# Meditation and vacation effects have an impact on disease-associated molecular phenotypes

**DOI:** 10.1038/tp.2016.164

**Published:** 2016-08-30

**Authors:** E S Epel, E Puterman, J Lin, E H Blackburn, P Y Lum, N D Beckmann, J Zhu, E Lee, A Gilbert, R A Rissman, R E Tanzi, E E Schadt

**Affiliations:** 1 Department of Psychiatry, University of California, San Francisco, San Francisco, CA, USA; 2 Departments of Biochemistry and Biophysics, University of California, San Francisco, San Francisco, CA, USA; 3 Capella Biosciences Inc., Palo Alto, CA, USA; 4 Institute for Genomics and Multiscale Biology, Mount Sinai School of Medicine, New York, NY, USA; 5 Department of Neurosciences, University of California, San Diego, La Jolla, CA, USA; 6 Genetics and Aging Research Unit, Department of Neurology, Massachusetts General Hospital/Harvard Medical School, Charlestown, MA, USA

## Abstract

Meditation is becoming increasingly practiced, especially for stress-related medical conditions. Meditation may improve cellular health; however, studies have not separated out effects of meditation from vacation-like effects in a residential randomized controlled trial. We recruited healthy women non-meditators to live at a resort for 6 days and randomized to either meditation retreat or relaxing on-site, with both groups compared with ‘regular meditators' already enrolled in the retreat. Blood drawn at baseline and post intervention was assessed for transcriptome-wide expression patterns and aging-related biomarkers. Highly significant gene expression changes were detected across all groups (the ‘vacation effect') that could accurately predict (96% accuracy) between baseline and post-intervention states and were characterized by improved regulation of stress response, immune function and amyloid beta (Aβ) metabolism. Although a smaller set of genes was affected, regular meditators showed post-intervention differences in a gene network characterized by lower regulation of protein synthesis and viral genome activity. Changes in well-being were assessed post intervention relative to baseline, as well as 1 and 10 months later. All groups showed equivalently large immediate post-intervention improvements in well-being, but novice meditators showed greater maintenance of lower distress over time compared with those in the vacation arm. Regular meditators showed a trend toward increased telomerase activity compared with randomized women, who showed increased plasma Aβ42/Aβ40 ratios and tumor necrosis factor alpha (TNF-α) levels. This highly controlled residential study showed large salutary changes in gene expression networks due to the vacation effect, common to all groups. For those already trained in the practice of meditation, a retreat appears to provide additional benefits to cellular health beyond the vacation effect.

## Introduction

Ancient practices such as yoga and meditation have long been thought to combat stress and promote longevity, although empirical evidence for effects on aging processes under highly controlled experimental conditions is lacking. Further, it is inherently difficult to assess effects of meditation apart from simple relaxation. Advances in the understanding of the biological bases of aging enable better assessment of acute effects of salutary interventions on biomarkers of aging. For example, impaired regulatory systems leading to systemic inflammation, and excessive stress responsivity, are related to biological aging^1^ and may partly underlie pathogenesis of cardiovascular^2, 3^ and Alzheimer's^4, 5^ diseases (AD). More recent systems biology approaches have identified gene regulatory networks associated with a diversity of biological processes, including immune and stress responses, and objectively linked them with disease or salutary states.^6, 7^

Integrated systems biology approaches can identify gene regulatory networks, such as immune, stress and other regulatory responses, and link them with physiologic states.^6^ This bioinformatics approach provides an unbiased view of the immune system profile, and can be linked to changes in environmental conditions.^7^ These network approaches, while often applied to identifying disease profiles, can be used to identify salutary states as well, such as that which might result from intensive meditation. In addition to high-dimensional molecular data such as gene networks, blood-based biomarkers can provide an integrated overview that indexes biological aging. Telomere length predicts both cellular health and disease in rodent models and humans.^8^ Shorter telomeres predict onset of cardiometabolic diseases of aging.^9^ Chronic stress is associated with higher inflammation, shorter telomeres, and lower activity levels of telomerase, the cellular enzyme that elongates telomeric DNA.^10, 11^ Levels of amyloid beta (Aβ) proteins circulating in the blood appear to be stress-related in rodent models^12^ and may be affected by stress reduction, and greater Aβ42/Aβ40 ratios are associated with lower risk of dementia.^13^

Various types of meditation have been shown to improve well-being among different populations such as physicians and the general public.^14, 15, 16^ Preliminary evidence suggests that meditation-based interventions may slow cellular aging rates by increasing telomerase activity, but many such studies lacked an active control group.^17, 18^ Recent randomized trials in breast cancer suggest that long-term intensive meditation interventions might have positive effects on telomerase activity. One found that mindfulness-based and supportive expressive therapies were associated with telomere maintenance, compared with a ‘treatment as usual' control group.^19^ A second study found that mindfulness-based stress reduction was associated with increases in telomerase after 3 months.^20^ Long-term mind–body interventions, including tai chi, yoga and meditation, have been associated with gene expression (GE) changes associated with inflammatory pathways^21, 22^ as reviewed elsewhere.^23, 24^

Short-term interventions have examined changes after one session of meditation or yoga. One study compared GE changes in experienced versus novice meditators after one session. They found changes in both groups in inflammation, energy metabolism, mitochondrial function and telomere maintenance, but experienced meditators had greater changes.^25^ Another study comparing experienced meditators to novices after 8  hours of meditation examined GE changes specific to epigenetic regulatory enzymes. Changes were found only in the experienced meditators.^26^ Another study found threefold changes in GE in the immune cells after yoga versus a control movement program.^27^ These studies suggest that there are greater changes in experienced meditators than in novices after one bout. However, none of these studies examined people in more controlled residential vacation settings, where larger changes can occur in periods as short as a week. One limitation to meditation studies is that short-term relaxation as a control condition may not lead to the powerful changes that prolonged vacation could. No studies we are aware of have examined how a meditation retreat may affect GE above and beyond a relaxing vacation. Further, none of these previous studies took a systems biology approach by examining covariation across GE patterns (versus specific gene pathways).

Here we examined how exposure to a short-term intensive residential meditation retreat affected biomarkers of aging and more general regulatory networks defining a wide array of biological processes. A residential retreat provides intensive daily exposure in a controlled environment but has the added ‘vacation' effect of taking people away from the demands of their daily lives, which alone might affect regulation of stress pathways. Therefore, it is critical to compare the effects of a meditation retreat with an active randomized control group. Because regular meditators may have differences in brain function and structure, as suggested by meta-analyses,^28^ and greater changes in GE than novices, after meditation,^25^ we also recruited a third comparison group of experienced meditators. Our design allowed us to study the effects of meditation independent of the vacation effect, as well as to compare the effects of acute intensive meditation in regular meditators versus those newly trained in meditation.

## Materials and methods

### Study design, recruitment and intervention overview

The Retreat and Relaxation Study, a randomized trial, compared the effects of vacation at a resort to training in a meditation/yoga retreat at the same resort (La Costa Resort and Spa). Healthy women aged 30–60 were recruited mostly from the greater San Diego area and San Francisco and randomized to a vacation arm or novice meditator arm. A comparison group of regulator meditators was recruited from women aged 30–60 who had already enrolled in the retreat. The retreat goal was to promote an intensive period of learning and psychological change. The vacation group was hosted at the same resort, but they did not participate in any retreat activities. They reported on well-being immediately after (on day 5), 1 month later, and 10 months later to assess maintenance of benefits.

### Phenotyping and computational analyses

Measures including psychological measures (depressive symptoms, perceived stress, vitality and mindfulness) and detailed methods for the blood draw, processing, biomarker assays and RNA sequencing of blood samples are described in the Supplementary Appendix, along with all statistical analyses carried out for group comparisons, construction of differential expression signatures, construction of coexpression networks, construction of Bayesian networks and pathway enrichment analysis.

### Code and data availability

All codes used in the analyses described herein are available upon request. The RNA sequencing data and appropriate descriptions are freely available on the Synapse platform by Sage Bionetworks: https://www.synapse.org/#!Synapse:syn7058059.

## Results

### Study design

To conduct our study, we chose a 1-week retreat offered regularly by the Chopra Center for Wellbeing (Carlsbad, CA, USA) at a vacation resort (OMNI La Costa Resort and Spa). Whereas the Chopra Center retreat reflects just one of the many different types of meditation that could be studied, the vacation resort environment at this center allowed us to attempt to distinguish between molecular/biomarker effects induced by a more relaxed, vacation-like environment, versus those induced by the practice of meditation and related activities specific to the meditation arm of the study. We randomized female volunteers who were ‘non-meditators'—having little to no previous experience with meditation—to either the resort only (herein referred to as the vacation arm of the study) or to the retreat (herein referred to as the novice meditation arm) during the same week. The vacation arm serves as a relaxation control for the meditation arms of the study, which take place at a vacation resort, given those in the vacation arm experience all of the amenities of the vacation resort and are at least partially removed from the demands of their daily lives. Although it was not possible to blind participants as to the arm in which they were enrolled, the vacation arm nevertheless affords the possibility of helping tease apart a vacation effect from a meditation effect. The scoring of a comprehensive set of molecular features in this setting provides a more objective assessment of the impact of the different interventions, and the biological processes that were perturbed as a result. In addition to the vacation and novice meditation arms, we recruited a third comparison group of female regular meditators who had already signed up for the retreat for the same week at the resort. This design allowed us to study the effects of meditation independent of the vacation effect, as well as to compare the effects of acute intensive meditation in regular meditators versus those newly trained in meditation. Regular meditators may have differences in brain function and structure, as suggested by meta-analyses^28^ and may therefore respond differently to the retreat.

### Study cohort

**Figure 1:**
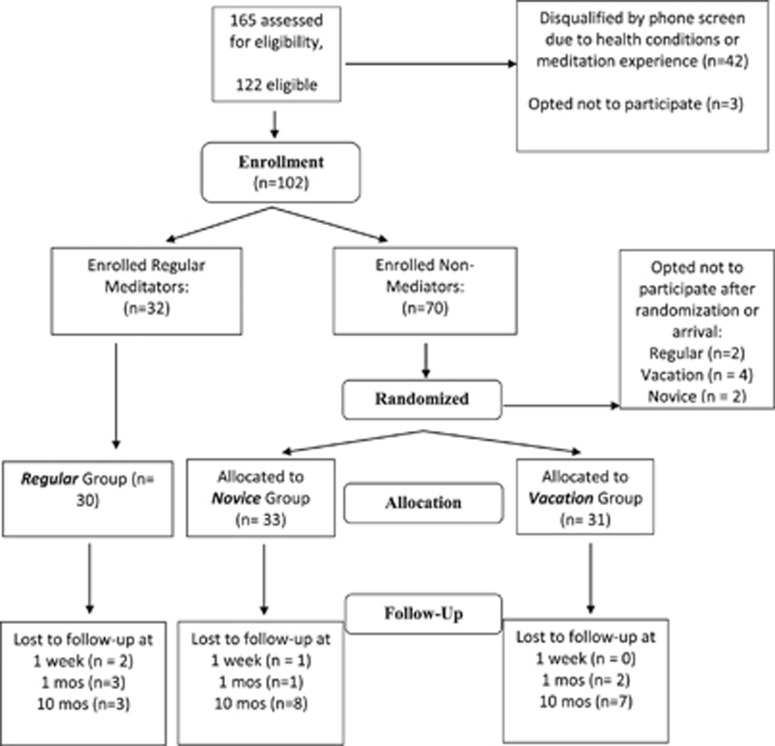
Study flow, including enrollment, randomization and retention, in our meditation study. Of the 165 women screened, 122 (73.9%) were eligible, and 102 (84%) of those eligible were interested and enrolled on a first come first serve basis. After the community women were randomly assigned to groups, and before arriving on site, there were six drop outs: four from Vacation and two from Meditation Groups. After arriving at the resort and before the retreat began, two from the Regular Meditator Group opted out of the study. After the retreat began, two participants dropped out, one from the Regular and one from Novice meditator groups.

Figure 1 (consort diagram) displays the study design, including enrollment, randomization and retention. Of 165 women screened, 122 (73.9%) were eligible, and 102 were interested and enrolled on a first come, first serve basis. Table 1 lists sociodemographic characteristics and body mass index of the three groups. Average age was 47 (s.d.=8.1, range 31–60 years). The sample was mostly white and college-educated (73% had at least a bachelor's degree), with 84% of the participants currently employed and 58% married or with a partner. The average body mass index was 24.5 (s.d.=4, range 17.9–36.2). Average annual household income was $90 ,000–99, 000. There were no significant differences by group on any sociodemographic variables.

#### Psychological changes

The changes in psychological well-being scores (Supplementary Table S1) indicated major improvements in all three groups from the first to fifth day and 1 month later on all measures (depressive symptoms, perceived stress, mindful awareness and vitality). Follow-up analyses showed that the novice group improved significantly more on mindfulness than the other two groups up to 1 month after the study (Supplementary Table S1). Ten months later depressive symptoms and perceived stress were measured again and compared with their pre-study baseline scores (Supplementary Table S2). Novice meditators showed significantly greater maintenance of improvements compared with those in the vacation arm, with greater decreases in depressive symptoms and stress levels at 10 months.

### Gene expression changes induced by vacation and meditation effects

To characterize the impact of vacation versus meditation on regulatory networks in blood, we computed three different GE signatures: (1) changes across all treatment groups between the baseline and follow-up time points, and (2 and 3) differences between the regular meditators versus the vacation and novice meditator groups at the follow-up time points. We also compared changes in the vacation versus novice arms at the follow-up time point, and, notably, there was not a significant differential GE signature beyond what would be expected by chance, suggesting that these groups changed in similar ways (see Supplementary Information for more explanation of the group comparisons made).

**Figure 2:**
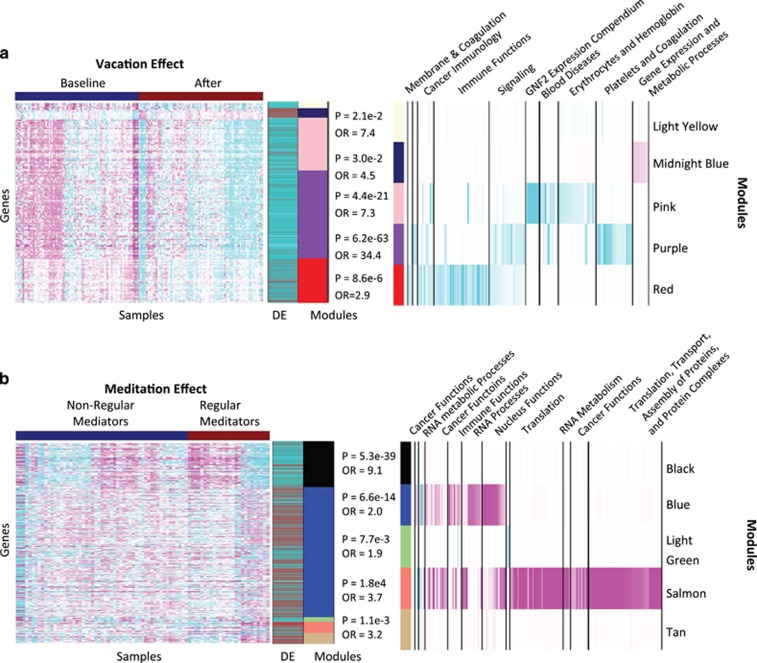
Characterizing molecular changes in response to vacation and meditation. (**a**) Heatmap of the top differentially expressed genes between baseline and follow-up (‘vacation' effect) after two-way hierarchical clustering. The expression values depicted in the heatmap were converted to *Z-*scores and ordered in the sample and gene dimensions according to the dendograms produced by the clustering procedure. Cyan (magenta) indicates genes that are down- (up) regulated at the baseline time point relative to the follow-up time point, with the intensity indicating the significance of the *Z-*score. The color band to the right of the heatmap indicates the mean difference in expression (DE) between the baseline and post-intervention time points for each of the differentially expressed genes. Red (cyan) indicates down- (up) regulation at the post-intervention time point compared with baseline. The module colors indicate the coexpression modules to which the genes belong, with the odds ratio and *P-*value given for each module reflecting the overall enrichment for differentially regulated genes in the module. The pathway enrichment heatmap indicates the –log10 of the *P*-value for pathways enriched in the indicated coexpression module. Brighter cyan (magenta) indicates larger –log10 *P-*values for pathways that are down- (up) regulated. (**b**) Similar to **a** but with genes differentially expressed between regular meditators with novices and the vacation group at the follow-up time point.

As shown in Supplementary Table S3, we identified significant GE signatures for all three group comparisons at a 5% false discovery rate. The comparison between all participants at baseline and post-intervention follow-up (presumably reflecting changes driven by a relaxation or effect unrelated to meditation) resulted in a signature comprising 390 genes (*P*-value threshold=7.9e−4). This signature was highly conserved among the vacation, novice and regular meditator groups (Supplementary Table S3). For those in the vacation and novice meditator arms versus the regular meditator arm, 479 and 855 genes, respectively, were significantly differentially regulated. Figure 2 depicts the top affected genes for each signature. In addition to significant suppression observed in pathways related to defense response, wounding and inflammation, one other notable change due to the relaxation effect was suppression of the polyamine synthesis pathway (DAVID enrichment score 1.78), where three genes (*ODC1*, *OAZ1* and *OAZ2*) involved in this pathway were significantly decreased (paired *t-*test statistics and *P*-values are *ODC1*: 6.87, 1.28E−09, *OAZ1*: 4.44, 2.83E−05 and *OAZ2*: 4.20, 6.94E−05). Other notable genes that were suppressed included stress-responsive genes, such as *MME* (membrane metallo proteinase; paired *t-*test statistic=4.18 and *P=*7.37E−05) and *FOXO3* (paired *t-*test=4.89 and *P=*5.21E−06).

#### Molecular profiles accurately predict vacation and meditation states

To assess the predictive power of the GE changes between the groups, we employed machine-learning procedures to construct classifiers to predict group membership based on pre- versus post-intervention GE (vacation effect) and regular versus non-regular post-intervention GE data (meditation effect). We constructed classifiers to distinguish between the baseline and post-intervention states (Supplementary Methods) and achieved prediction accuracies of 96% for the vacation effect (area under the receiver operating characteristic curve, or AUROC, equal to 0.98) and 85% for the meditation effect (AUROC=0.93; Supplementary Figure S1), supporting the potential for blood-based wellness biomarkers.

### The molecular architecture of response to resort and meditation interventions

To understand the molecular architecture of the relaxation and meditation GE signatures, we employed an integrative analysis strategy. GE traits from our study were used to construct a weighted coexpression network from which coherent modules of interconnected genes were identified (Supplementary Table S4). A broad range of biological processes, molecular functions, cell cycle pathways (Supplementary Table S5) and gene sets from the MSigDB (Supplementary Table S6) was enriched across these different modules. Five modules (red, pink, purple, midnight-blue and light-yellow) specific to the vacation effect (enriched by 2- to 35-fold for the relaxation effect signature) were highly enriched for pathways related to stress and immune processes, such as acute inflammatory response, defense response and IgG binding (red module), hydrogen peroxide catabolic process (pink module) and response to stress and wound healing (purple module). These pathways were all downregulated (Figure 2).

Whereas five modules (black, blue, salmon, tan and light-green) were enriched for the meditation signature, one module in particular (salmon) stood out as it was >50-fold enriched for pathways related to translation, transport and assembly of proteins and protein complexes, as well as processes relating to viral infection (Figure 2). The GO category ‘translation termination' comprised 31% of the salmon module, a 58.2-fold enrichment over what would have been expected by chance (Fisher exact test *P=*2.1e−60). Translational initiation, protein targeting to the endoplasmic reticulum, translational elongation, viral GE and viral infectious cycle are all closely related processes that are similarly significantly enriched. These pathways were all downregulated in the regular meditator group versus the resort group post-intervention. To identify the hierarchical themes of the most highly enriched pathways, Revigo was used to map out meta-pathways spatially^29^ (Supplementary Figure S2; Supplementary Tables S7 and S8).

**Figure 3:**
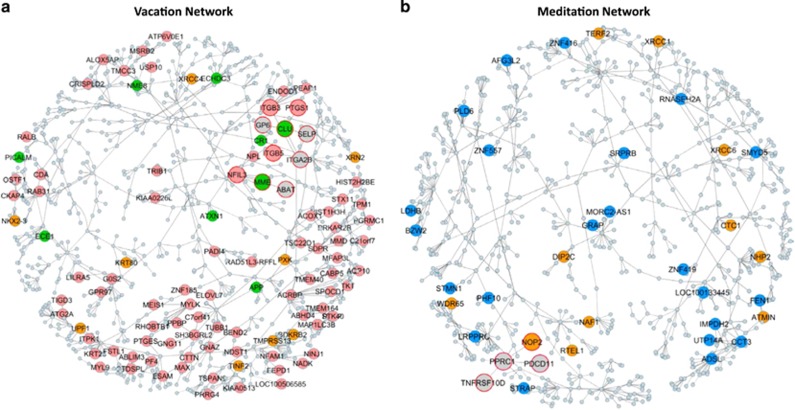
Identifying network structures in a blood Bayesian network reflecting vacation and meditation effects. (**a**) The red, brown, pink and purple coexpression network modules enriched for the vacation signature were projected on a blood Bayesian network. The subnetwork depicted is the largest connected subgraph comprising nodes within a path length of one of the projected nodes. The pink nodes represent genes in the vacation signature (2.7-fold enriched; Fisher exact test (FET) *P=*5.6e−19), green nodes are genes annotated as Alzheimer's disease genes (2.3-fold enriched; FET=0.012) and orange nodes are genes annotated as telomere genes. (**b**) Network constructed as in **a** using genes from the salmon, tan, blue and black modules all enriched for genes in the meditation signature. The blue nodes represent meditation signature genes (2.4-fold enriched; FET *P=*6.5e−5) and the orange nodes represent genes annotated as telomere genes (1.7-fold enriched; FET *P=*0.045). Oversized nodes with red borders are discussed in the main text.

To further characterize the structure of the molecular networks associated with the vacation and meditation effects, we projected the modules indicated in Figure 2 on a probabilistic causal network (Bayesian network)^30, 31, 32, 33^ constructed from a larger independent blood GE study in which hundreds of individuals were GE-profiled and genotyped (Supplementary Appendix). As we have done to elucidate complex diseases,^6^ for each projection on the blood causal network structure, we identified the largest connected subnetworks comprising genes directly connected to the projected module genes, resulting in the identification of relaxation- and meditation-specific subnetworks (Figure 3).

Of the 914 genes in the brown, pink, purple and red modules that map to the Bayesian network, 648 are in the 924-gene vacation subnetwork, a 6.1-fold enrichment over what would be expected by chance (Fisher exact test *P*<1e−300). The vacation subnetwork (Figure 3a) was enriched for blood coagulation, innate immune response, beta-amyloid metabolism, platelet degranulation, lipoxin metabolism and autophagy, in addition to being enriched for genes associated with AD processes. For example, the gene *MME* (a membrane metallo-endopeptoidase) was downregulated in the post-intervention time point relative to baseline. In the vacation subnetwork MME is linked to *NFIL3*, a gene found on activated T cells, natural killer cells and mast cells. Another interesting local network structure is centered around *CLU*, a gene shown to be involved in AD based on genome-wide association studies, with genes in the integrin family, involved in cell adhesion, clustered around CLU.

Similarly, of the 669 genes in the black, blue, salmon and tan modules that map to the Bayesian network, 508 are in the 836-gene meditation subnetwork, a 7.3-fold enrichment over what would be expected by chance (Fisher exact test *P*<1e−300). For the meditation subnetwork (Figure 3b), the genes *NOP2* and *PPRC1* formed a local network structure, with NOP2 observed as significantly upregulated after meditation. NOP2 and PPRC1 are involved in ribosomal biogenesis and are coexpressed with *DKC1*, a gene involved in ribosome biogenesis and also a component of the telomerase ribonucleoprotein enzyme complex. A linked node contains telomere-protective component RTEL1 and NAF1. NOP2 and NAF1 both interact with DKC1, and all three proteins are required for the stability, correct assembly and intranuclear trafficking of TERC, the RNA component of telomerase. The NOP2-centered subnetwork also contains PDCD11, an NF-kappa-B (NFKB1; 164011)-binding protein that colocalizes with U3 RNA (MIM 180710) in the nucleolus and is required for rRNA maturation and generation of 18 S rRNA.^34, 35^ TNFRSF10D is also linked to this subnetwork and is involved in apoptosis and cell senescence.

### Biomarker results

We scored several blood-based biomarkers to provide an integrated overview of biological aging as a short-term surrogate of the impacts vacation and meditation can have on wellness. Biomarkers such as telomerase activity and Aβ levels reflect cellular health, aging and stress.

#### Telomerase activity

Baseline telomerase was lower in the regular meditators. The analysis of variance of group differences in change scores was marginally significant (*P*<0.08; Supplementary Table S9). Day-5 follow-up *t*-tests showed a significant increase in telomerase activity only in the regular meditation group (*P*=0.004; Supplementary Figure S3).

#### Aβ peptide levels

An analysis of variance of group differences in pre- to post-intervention changes in plasma Aβ40 levels was significant (*P*<0.05; Supplementary Table S9). There were significant reductions in plasma Aβ40 levels in the vacation and novice meditator groups, but not in the regular meditator group. The analysis of variance for group differences in the ratio of Aβ42/Aβ40 was significant (*P*<0.001; Supplementary Table S9). *t*-tests showed a significantly higher Aβ42/Aβ40 ratio in the regular meditator group at baseline, but no increase in this ratio pre- to post intervention. In contrast, the vacation and novice meditator groups revealed significant post-intervention increases in Aβ42/Aβ40 ratio (*P*-value<0.01; Supplementary Table S9) driven by the decrease in plasma Aβ40 levels.

#### Tumor necrosis factor alpha

Analyses of variance of group comparisons showed significant group differences for change in cytokine tumor necrosis factor alpha (TNF-α *P*<0.001; Supplementary Table S9). *t*-tests showed no changes in the regular meditator group, and significant increases in the other groups, with the highest increase in the controls.

The complete blood count panel showed no group changes in percentage of each type for lymphocytes, neutrophils, eosinophils and basophils. There was a significant decrease in relative numbers of monocytes for the regular meditator group (Supplementary Table S10). To be conservative, all biomarker changes were re-analyzed with changes in monocytes as a covariate and none of the results changed in a meaningful way.

## Discussion

After 1 week at a resort, participants felt greater vitality and decreased distress, regardless of whether they were in the resort group or in an intensive meditation/yoga retreat. The randomized group immersed in meditation and yoga for the first time exhibited more sustained well-being up to 1 month later, compared with the resort group on mindfulness. Ten months later; the novices maintained a clinically meaningful improvement in depressive symptoms compared with the vacation group. Thus, we found both a short-term vacation effect for everyone and a significant benefit of learning meditation on longer-term mood.

A thematic examination of the coexpression modules enriched for relaxation effect genes showed a suppression of correlated networks regulating stress responses, inflammation and wound healing. A parsimonious interpretation of these data is that, while on vacation, genes that are normally needed for dealing with stress, wound healing and injury are downregulated. Among the genes that were downregulated after resort/treat were *MME* and *FOXO3*, both known stress-related genes. Psychological stress and glucorticoids have been shown to increase FOXO3 in animal studies,^36, 37, 38^ whereas low levels of FOXO3 in mice actually prevent the behavioral stress response in mice, serving an antidepressant effect.^39^ We believe our study is the first documentation that a stress reduction intervention can reduce FOXO3 expression.

A number of downregulated pathways in the regular meditator group versus the other groups defined the meditation effect, including regulation of protein synthesis and trafficking, viral expression and viral infectious cycle. Following a period of intensive meditation, genes involved with the infectious cycle may get downregulated along with related host genes regulating protein synthesis, an explanation that is supported by other stress reduction interventions that have shown increased innate antiviral activity, such as upregulated Type 1 interferon activity.^40^ Shorter telomeres in population-based studies have been predictive of earlier onset of chronic diseases of aging, including diabetes,^41^ cardiovascular disease^9^ and certain cancers.^42^

Changes in biomarkers during the retreat suggested salutary improvements in regular meditators compared with other groups. Regular meditators had lower telomerase at baseline, but also showed a significant increase in peripheral blood mononuclear cell telomerase activity post treatment that was not observed in the other two groups. Theoretically, a sustained increase could stabilize or even lengthen telomeres over time, as shown in a previous study where intensive lifestyle intervention led to higher telomerase activity after 4 months, and longer telomeres after 5 years, with such changes associated with better adherence.^43, 44^

There were also changes in the levels of plasma Aβ levels by study group. The regular meditator group started off with a higher plasma Aβ42/Aβ40 ratio, and this ratio did not change from pre- to post-intervention. Over the four days, plasma Aβ40 levels significantly decreased in the novice meditator group, accompanied by a significant increase in the Aβ42/Aβ40 ratio. A growing number of human studies suggest that a low plasma Aβ42/Aβ40 ratio is a risk factor for major depression,^45, 46^ dementia^47^ and higher mortality.^48^ The Framingham Study also showed that increased plasma Aβ42:Aβ40 ratios are associated with decreased risk of AD and dementia.^13^ Thus, the higher Aβ42/Aβ40 ratio observed in regular meditators at baseline and the increase in this ratio from pre- to post intervention in the novice meditator and vacation groups may be salutary to brain health.

The increase in the Aβ42/Aβ40 ratio (driven by a decrease in plasma Aβ40 levels) could also be related to the observed decrease in expression of *CLU* as part of the relaxation effect (*P=*0.00045). *CLU* is a confirmed AD gene from genome-wide association study, and encodes apolipoprotein J, which has been shown to chaperone re-entry of Aβ into the brain following export of the peptide from the brain into the blood.^49^ Interestingly, genes encoding two peptidases known to degrade Aβ were also decreased in expression in blood pre–post intervention as part of the vacation effect: *MME* (*P=*0.00019) and *ECE1* (*P=*0.0037). These data may reflect that there was less need for these peptidases, perhaps owing to lower plasma Aβ40 levels. Finally, expression of the known early-onset familial AD gene, *PSEN1*, was also decreased as part of the relaxation effect. *PSEN1* encodes the γ-secretase needed to produce the Aβ peptide.

The vacation group showed a statistically significant increase in circulating TNF-α compared with the regular meditators, and a marginally significant increase when compared with novice meditators. Reasons for this increase could include those in the vacation group having an acute stress-related inflammation response to the blood draw, overexposure to sun or some other exposure that can lead to acute physical stress such as exercise. All participants ate a healthy ayurvedic diet, which is thought to be anti-inflammatory.

One of the most comprehensive reviews on the effects of mindfulness meditation on immune parameters was recently conducted across 20 randomized clinical trials.^23^ Three of these studies found decreased NF-KB and no strong evidence for changes in interleukin inflammatory markers (interleukin-6) or TNF-α, but some evidence for a decrease in CRP and increases in telomerase. Our findings are consistent with this review. We also did not find decreases in circulating inflammatory cytokines but rather slight increases, particularly in the vacation group. We did not find a decrease in NF-KB specifically; however, we did find suppression in large gene networks known to be related to inflammatory processes. We found a trend for increases in telomerase activity in the regular meditator group.

Our study has several limitations. The changes in biomarkers and GE patterns appear salutary; however, no studies have tracked these markers to see how well they predict disease or longevity in a healthy sample. The sample size is relatively small and powered only to detect medium-to-large effects. Replication of these findings with larger controlled studies will be necessary. In addition, comparing regular meditators to non-meditators is somewhat problematic, given regular meditators likely differ on many lifestyle factors that make such comparisons complicated. For example, diet, exercise regimen and stress-reduction activities such as meditation likely differ between these groups and may not only lead to baseline differences in molecular and higher-order systems, but may prime such systems to respond differentially to resort and meditation interventions. In addition, the meditation arm of this study was not strictly sitting meditation, but also included yoga postures and self-reflection exercises and lectures. Further, whereas our design did attempt to control for the vacation effect, we did not randomize regular meditators to the vacation and meditation arms, making it difficult to interpret the changes observed in the regular meditators, given they were compared with a control group comprising non-meditators. A balanced design that included randomization of regular meditators as well as non-meditators would be useful in future studies to better localize the effects driving the expression changes. Despite these limitations, our study provides a strong distinction between beneficial effects of short-term relaxation typical of a vacation versus acute intensive meditation.

In summary, our results point to both a significant ‘vacation effect' that benefitted all groups, and a suppression of stress-related responses and immune function related to acute-phase wound healing and inflammation. We also identified a ‘meditation effect' within the regular meditator group, characterized by a distinct network of genes with cellular functions that may be relevant to healthy aging, and this network was associated with increased expression of a number of telomere maintenance pathway genes and an increase in measured telomerase enzymatic activity. This study provides a strong distinction between beneficial effects of short-term relaxation typical of a vacation versus acute intensive meditation for regular meditators. Future studies expanding upon these results will be critical for further understanding lifestyle acute adaptations capable of promoting stress reduction and overall health and well-being.

## Figures and Tables

**Table 1:** Sociodemographics and body mass index of study sample

	*Resort (*n=*31)*	*Retreat novice (*n=*33)*	*Retreat regular (*n=*30)*
Age, mean (s.d.)	45.87 (7.8)	47.52 (9.0)	49.27 (7.4)
			
*Racial/ethnic group, N (%)*			
European white	20 (64.5)	26 (78.8)	22 (73.3)
Asian	6 (19.4)	4 (12.1)	2 (6.7)
Black	2 (6.5)	2 (6.1)	1 (3.3)
Hispanic	2 (6.5)	1 (3.0)	5 (16.7)
Other	1 (3.2)	0	0
			
Marital status, *N* (%) married or with partner	22 (71)	19 (61.3)	12 (41.4)
Employed, *N* (%)	21 (75)	22 (88)	20 (90.9)
Education, *N* (%), with BA+	27 (87%)	24 (77%)	22 (76%)
Body mass index, mean (s.d.)	24.69 (4.04)	24.08 (3.92)	25.06 (4.08)

*Note*: There are no significant differences by group. Percentages are based on the total participants who completed the question in each group.
