# Supplementary material for: Meditation and vacation effects have an impact on disease-associated molecular phenotypes
Source: Transl Psychiatry. 2016 Aug 30;6(8):e880–. doi: 10.1038/tp.2016.164 (PMC5022094; doi:10.1038/tp.2016.164)
Supplement: Supplementary Information [file tp2016164x1.doc]

**Supplementary Appendix**

Supplement to: **“Meditation and vacation effects impact disease-associated molecular phenotypes”**

**Table of Contents**

Acknowledgements 2

Conflicts of Interest 2

Supplementary Methods 2

Blood collection methods 4

Assay Methods (Telomerase, Inflammation, Abeta proteins) 4

Transcriptome Profiling and Analysis 5

Statistical analyses of survey and biomarker data 7

Supplementary References 9

Supplementary Tables 11

Table S1. Psychological wellbeing during Retreat Week and one month later 11

Table S2. Psychological wellbeing ten months later 13

Table S3. Individual Gene Differential expression signatures for baseline vs. follow up (relaxation

effect) and regular meditators vs. others at follow-up (meditation effect) see excel file

Table S4. The blood weighted coexpression network for the study described in the main text see excel file

Table S5. Pathway enrichments for coexpression network modules identified in Table S4 see excel file

Table S6. Gene sets from the MsigDB database enriched for the coexpression network modules

identified in Table S4 see excel file

Table S7. Meta-themes from Gene Ontology Tree Maps for vacation network modules 14

Table S8. Meta-themes from Gene Ontology Tree Maps for meditation network modules

related to Meditation Signature 15

Table S9. Biomarker Means (Standard Errors) at baseline, follow up, and change scores 16

Table S10. Mean Cell type percentage values at baseline and follow up 17

Supplementary Figures 18

Figure S1. ROC curves for the prediction of vacation and meditation effects 18

Figure S2. REVIGO Gene Ontology treemap for the meditation-specific, salmon coexpression 19

Figure S3. Telomerase Activity at baseline and day 5 of retreat network module 20

**Acknowledgements:** This study was supported by The Chopra Foundation, and Marc and Lynn Benioff. The authors gratefully acknowledge the critical cooperation and assistance of The Chopra Center for Well-being, and The Mind Body Medical Group, Drs. Deepak Chopra, Sheila Patel, Valencia Porter and Tim Brieske, and Carolyn Rangel, and the OMNI La Costa Resort and Spa. We are particularly grateful for Dr. Chopra who opened up the center to research and supported all aspects of the endeavor. We are also grateful for the hard work of our research and technical team, Amanda Gilbert, Zoe Evans, Samantha Schilf, and Rylie Williams from UCSF and Setareh Moghadam and Louise Monte from UCSD and Lisa Pham from Capella Biosciences Inc.

**Conflicts of Interest:** Dr. Lin is a minor shareholder (< $10K) in Telomere Diagnostics, a telomere measurement company which had no role in the current research. Dr. Pek Lum and Lisa Pham are employees of Capella Biosciences, Inc. (a company focused on monoclonal antibody medicines, with no relation to outcomes studied here). Dr. Chopra assisted with study design. The funders (Chopra Foundation, Benioff Foundation) had no role in data collection, analysis, decision to publish, or preparation of the manuscript. Dr.’s Epel, Schadt, and Tanzi were past awardees of the Chopra Foundation’s Rustrum Roy Award.

**Supplementary Methods**

**Study Design and Intervention Protocols**

The Retreat and Relaxation Study (RARS), a randomized clinical trial funded by private foundations (The Chopra Foundation and Marc and Lynn Benioff Foundation), compared the effects of relaxation at a resort to training in a meditation/yoga retreat at the same resort (La Costa). Women stayed at the resort for six days total, all rooms were similar in size and with similar amenities, and all participants ate the same ayurvedic diet. The sample included women who were non-meditators and lived in California who were randomized (computer generated randomization scheme) to a “vacation” arm or a meditation arm. They did not pay for their stay at the resort. In addition, a comparison group of non-randomized women was recruited from the pool of those already registered for the retreat and thus they paid for their own expenses. While they could live outside of California, all had to be acclimated to the current time zone by the first day of retreat (using the one hour per time zone per day criterion). All participants were asked to not exercise any more than they typically would do each day. Exercise was recorded in daily diaries. The eligibility criteria for each group were identical except that prior regular meditation practice was required in the regular meditator arm. The study was approved by the UCSF and UCSD Investigational Review Boards, and all participants provided informed consent. In order to optimize the methods and accurate timing of the data collection methods, blood draw procedures, sample transport, and assays, an IRB-approved feasibility pilot study of ten participants was conducted at an earlier retreat at the same resort (data not included in this study).

*Power Analysis.* Using data from two previous publications^1,2^ and G Power, we calculated required sample size at 31 participants per group with small effect size of .29 at power = .80 and alpha set at .05 for a total of 93 participants. In greater detail, it was previously demonstrated that PBMC telomerase activity expressed as natural logarithms increased from 2·00 (SD 0·44) to 2·22 (SD 0·49; p=0·031)^2^. Daubenmier's publication demonstrated that pre-treatment experimental group telomerase levels were 1.67 (SD 0.3) and changed to 1.98 (SD 0.4) post-treatment, whereas control levels at baseline were 1.84 (SD 0.4) and follow-up were 1.98 (SD 0.3)^1^. Assuming the three group model we employed, (vacation, novice meditator and regular meditator arms), we assumed effect size from t1 to t2 in two groups of meditators to be between .2 and .3 (small effect size) compared to the vacation arm participants, providing a sample size calculation was 93 for full sample, so 31 per group. In order to account for participant attrition throughout the study, we recruited participants across the groups. Given the many outcomes examined in this study, including gene expression patterns, we view it as an exploratory trial. Further, the gene expression methods are inherently exploratory in nature, a bottom up approach to identifying group differences in genes and gene networks.

**Recruitment**

*Randomized women.* Women were recruited mostly from the greater San Diego area, as well as San Francisco, through many public venues including flyers, radio and media advertisements. The flyer stated “Participate in a University of California, San Francisco study examining the effects of a short meditation retreat on health and wellbeing. This study requires spending up to six days at La Costa Resort and Spa in Carlsbad, CA in April 2013” and stated basic eligibility criteria. Eligible participants were healthy women, aged 30-60, English speaking, with no history of chronic medical conditions and/or diseases. Exclusion criteria included pregnancy, antidepressants, estrogen or hormone replacement therapy use, smoking, or a diagnosis of post-traumatic stress disorder. They also could not have a current or past regular meditation practice.

Women first were screened for eligibility, learned details of study involvement, and were asked to consider their commitment and availability before they were enrolled into the study. It was emphasized that this included a commitment to stay in the study even if they were not randomized into their preferred group.

The randomization pattern was employed using a permuted block randomization, allocating a small but equal size group to one then the other condition. The study coordinator was given the randomization schedule to follow and this was based on timing of the completion of their questionnaire. In this way, there was no bias assigned to, for example, those who enrolled the earliest. Novice meditators were informed of the group they have been assigned to approximately 2-3 weeks prior to their arrival at La Costa Resort.

*Comparison group of regular meditators*. An inclusion criterion for “regular meditators” was to have meditated regularly for at least the past six months, at least 4 times a week, for at least 15 minutes each time. All of the women enrolled in the retreat who were within the age range 30-60 years were invited to learn more about the study. Interested women were screened on a first come first serve basis until we identified 31 eligible and interested women (of 44 total screened; 70.5% eligibility). Of the enrolled women, the average meditation practice was 2.3 years (range from 6 months to 7 years) and more than half (58%) engaged in meditation on a daily basis.

The intervention groups were not blinded, and site investigators and study personnel knew to which group participants had been randomized. The two groups (vacation arm and then novice/regular meditator arms) had minimal contact with each other during the week and did not know the details of the daily schedule of the other group. The teachers of the retreat delivered their standard meditation intervention program to a larger group of participants, without knowing who among them was enrolled in this intervention study.

**Intervention Overview**

*Meditation Groups program*. The Seduction of Spirit Retreat, led by Dr. Deepak Chopra and colleagues, at the Chopra Center, Carlsbad, CA, over the past decade, is a meditation retreat attended by several hundred participants per event. Its goal is to promote an intensive period of learning and psychological change. The retreat provides training in meditation (primordial sound meditation, which is similar to mantra meditation), foundations of yoga, and sutra, with the aim of promoting inner calm, expanded awareness of one’s body, breath, and self, and life-transforming skills. The retreat group had lectures and activities for the full day. Over the four days, this added up to 12 hours of meditation (4 times a day), up to 9 hours of yoga (2 times a day), and several lectures and, interactive self-reflection exercises each day.

*Vacation (Control) Group program*. The Vacation Group had an afternoon lecture on health behaviors each day for 1.5 hours and an optional activity in the morning, such as a leisurely walk. While dietary intake was not strictly monitored, both groups were served the same meals at the same dining center.

*Psychological measures.* As part of our original design, we assessed self-report measures of wellbeing and distress at baseline (the week before the retreat), day 5 (post retreat), and 1 month later. Standardized psychometric measures with extensive validation were used, for depression, stress, mindfulness, and vitality, as follows: The Center for Epidemiological Studies Depression (CES-D) Scale assesses 20 symptoms over the past week (from 1, rarely, to 4, most of the time) ^3^. The Perceived Stress Scale is a 10 item measure that assesses feelings of stress, overwhelm, and worry (from 0, never to 4, very often) ^4^. The Mindful Attention and Awareness Scale (MAAS) is a 15 item scale assessing frequency of experience of mindful presence (paying attention) or mindlessness (running on automatic, unaware of feelings or behavior) on a 1 (never) to 6 (almost always) scale, where higher scores represent greater mindfulness ^5^. The Vitality subscale of the SF-36 scale consists of four items that measure feelings of vitality (full of life, energetic) to feeling fatigued and worn out (from 1, none, to 5, all the time) ^6^. Ten months after the initial study was completed, we re-administered the depression and stress measures to assess any maintenance in the reduction in distress (83% of participants completed the surveys).

**Blood collection methods**

Blood (23 mls) was drawn by venipuncture in a fasting state at the same time of day on Day 1 and on Day 5. A pre-blood draw form asked about health behaviors and ensured they did not exercise or have any physical sickness symptoms that morning. Blood was drawn into a BD Vacutainer Cell Preparation Tube (CPT) to separate mononuclear cells, EDTA tubes kept at room temperature, and two PaxGene RNA tube to preserve RNA. These tubes were inverted 10 times and stored at room temperature for at least 2 hours for stabilization reagent to work, and then frozen. All tubes were placed on a rocker and blood was transported in batches to the laboratory site (UCSD, lab of Dr. Rissman) for processing within one hour of being drawn. A CBC panel was performed by Quest Diagnostics.

**Assay Methods (Telomerase, Inflammation, Abeta proteins)**

*Telomerase activity assays.* Four days after PBMCs were frozen, at each time point, the cell pellet was suspended in CHAPS buffer and a lysate was prepared. Samples were stored at -80 degrees C and shipped on dry ice to UCSF for assessment of telomerase activity in batch. Telomerase activity was assessed by Gel-TRAP assays performed by the Telomerase Repeat Amplification Protocol (TRAP) using a commercial kit (TRAPeze Telomerase Detection Kit, Millipore) with modifications^7,8^. PBMC were purified from whole blood collected in CPT tubes (BD). Cells were washed with DPBS and stained by Trypan blue, live cells were counted using a hemocytometer. 5X10^5^ - 1X10^6^ cells per sample were pelleted and lysed with 1XCHAPS buffer as directed by the manual for the TRAPeze kit. An extract corresponding to 5000 cells/µl was made and stored at -80°C and assayed in batches. The reaction was carried out according to the TRAPeze kit manual and run on an 8% polyacrylamide-8M urea sequencing gel. The gel was exposed to a phosphorimager plate overnight and scanned on a Typhoon 8600 Imager (GE Healthcare, Piscataway, NJ). The 293T cancer cell line was used as a positive telomerase activity control and standard. Telomerase activity was expressed as equivalent of number of 293T cells. Telomerase activity was quantified using the software ImageQuant 5.2 (GE Healthcare, Piscataway, NJ). Briefly, signals from the product ladders on the gels were added and normalized against the signal from internal control band for the same lane to get the product/internal control value. For each telomerase activity assay reaction, the product/internal value was divided by the product/internal control value from twenty 293T cells and then multiplied by 20 to obtain the final telomerase activity units, defined as 1 unit = the amount of product from one 293T cell/10,000 immune cells. The average intra assay variability of PBMC samples (N=6, assayed in triplicates) was 8% and the inter-assay variability of PBMC samples (N=24, assayed on 2 different days) was 6.7%.

*Aβ and inflammatory cytokine assays.*  Blood was collected in EDTA polypropylene tubes for plasma, and centrifuged immediately after each collection at 2200 rpm for 15 minutes at 4°C. Plasma was divided into 0.25 ml aliquots, and stored at -80ºC until analysis. Levels of inflammatory cytokines and Aβ40 and Aβ42 levels were measured in plasma using validated multiplex bioassays (Meso Scale Discovery, Gaithersburg, MD), by the Alzheimer’s Disease Cooperative Study (ADCS) Biomarker Core using established standard operating procedures.   Plasma was assayed, quantified, and quality controlled as previously described ^9^. Each assay plate also included a plasma sample derived from blood drawn by venipuncture of a 56-year-old healthy volunteer in a single afternoon. This internal standard provided a means for adjusting plate-to-plate variation and assessing freezer storage effects. Samples were not freeze thawed prior to analysis. All samples were run in duplicate, and internal standards were used to control for plate-to-plate variation.  Samples with CVs of greater than 20% were excluded from the analysis.  The intra-assay CV was 4.6%  and  for TNF-a, 3.75% for abeta-40,  and 3.6% for abeta-42.

**Transcriptome Profiling and Analyses**

*RNA Sequencing.* RNA isolated from 200 paired blood samples was quality controlled using the Agilent Bio-analyzer, Qubit 2.0 at the Icahn Institute Genomics Core at Mount Sinai (IGCMS). 1ug of RNA with RIN ≥ 9 were used for sequencing on an Illumina 2000/2500 V3 Instrument at IGCMS. RNA was fragmented and Illumina sequencing libraries were prepared according to the manufacturer's instructions. Briefly, total RNA was first degraded and converted to a library of cDNA fragments. Fragmentation sizes and final library sizes were analyzed by a Bioanalyzer. Subsequently, each fragment was extended with an 'A' base on the 3′ end, ligated with paired-end adaptors and amplified to enrich the targeted regions of the RNA (coding regions). cDNA Libraries were then amplified, denaturated and loaded onto an Illumina cBot for cluster generation according to the manufacturer's recommended protocols. The primer-hybridized flow cells were subjected to HiSeq 2500 Illumina sequencer (single- end, 100bp).

*RNA Seq Processing and QC.* Sample QC was performed to ensure sample quality and integrity. Samples were excluded if RIN < 9 or sex-specific expression values were inconsistent with subject descriptors such as sex. Of the 200 samples sent for sequencing, 4 samples were removed because they yielded < 20 million total reads or had > 5% of reads aligning to rRNA, based on two attempts to produce quality sequence (all samples failing either QC criterion on the first attempt were re-prepped and/or resequenced, and those failing twice were removed).

All RNA seq data were entered into a data processing and QC pipeline. Reads were mapped to human reference genome hg19 using TopHat^10^ version 2.0.9 and Bowtie version 2.1.0, with the following parameters: 0 mismatches in a 20 bp seed, reference guided against Ensembl genes and isoforms (version 70). For each sample, this produced a coordinate-sorted BAM file of mapped paired end reads including those spanning splice junctions, as well as a BAM file of unmapped reads. Overall quality control metrics were calculated using RNA-SeQCb for each sample, including total number of reads (counting twice each fragment sequenced, once for each end in pair), number of mapped reads (again, separately counting each end of a paired end since one may map and not the other), the rates of reads mapping to rRNA, intergenic regions, intragenic regions, introns, exons, and the number of genes and transcripts detected (defined here simply as those with at least 5 exon-mapping reads). UCSC Genome Browser transcripts were used for this quality control (QC) analysis.

*Genes***.** Known Ensembl gene levels were quantified by HTSeq version 0.6.0 in intersection-strict mode (the BAM file was streamed to HTSeq through novosort version 1.0.1, as HTSeq accepts read-name-sorted alignments). This provides an integral count of reads for each gene in each sample to be used in downstream analyses (a sample-by-gene "read count matrix").

*Isoforms***.** Relative isoform abundances (PSI = percent spliced in) of Ensembl genes were estimated using MISO (http://genes.mit.edu/burgelab/miso/; version 0.5.2, run with default parameters^11^). We processed the persample, per-gene MISO output files to extract the estimated PSI, as well as the standard deviations of the estimated sampled PSI values. We constructed corresponding sample-by-isoform matrices for all subsequent data processing and analysis (see “Isoform-level normalization and analyses” below). In addition, Cufflinks^12^ version 2.1.1 was applied to the BAM files to estimate both gene- and isoform-level FPKM (Fragments Per Kilobase per Million reads) values for all Ensembl genes and isoforms. Separately, Cufflinks was applied to the BAM files to assemble isoforms for each sample. These assembled isoforms were unified across samples using Cuffmerge, resulting in a single GTF file of "merged" genes and isoforms annotated by Ensembl annotations. Cufflinks was then applied to this GTF file to estimate both gene- and isoform-level FPKM values for all merged genes and isoforms.

*Differential Expression Analysis.*

*Comparisons made:* We tested for differential gene expression over the week overall, between baseline and follow up, to test the vacation effect.  For identifying the meditation effect, we examined differences among the different arms of the study at the follow-up time point, conditional on baseline expression values (equivalent to a change score). We found marginally significant differences weakly enriched for various GO pathways. We next examined expression differences among the different study arms at the follow up time point without conditioning on the baseline expression values and found a more significant differential expression signature that was more significantly enriched for a subset of GO pathways also identified as enriched in the conditional analysis. To assess whether these same enrichments were present at baseline, we carried out the same differential expression analysis among the study arm groups at the baseline time point and did not see the same pathway enrichments. Given the lack of baseline enrichments, the marginal change effect, and the larger post effect, we focused on the post-intervention time point as the strongest signal to identify retreat related differences between regular meditators and non-regular meditators. This comparison allows us to identify a meditation effect among practitioners, which may be present in non-significant ways at baseline but is observable after a retreat. This fits in with the literature cited showing greater benefits of meditation practice among regular practitioners.

*Methods for Differential Expression Analysis.* RNA sequencing data was normalized using the VOOM method ^13,14^, and differential gene expression analysis was carried out using the R statistical programming package LIMMA^15^. Differential expression analysis was carried out between the vacation and novice meditation group, the control and regular meditator group, and the novice and regular meditation groups, using the lmFit and eBayes functions in the LIMMA package, adjusting for age, RIN, and in cases where measures were matched (with respect to individual) over time, subject id. Multiple testing across all genes identified in the RNAseq data was accounted for by controlling the family-wise error rate using permutation methods to empirically estimate the null distribution of no difference between any of the two groups tested. From the permuted data a p value threshold was selected to control the false discovery rate^16^.

*Weighted interaction network analysis.* The blood coexpression network was constructed using a previously described weighted gene coexpression network analysis^17^. The weighted network analysis begins with a matrix of the Pearson correlations between all gene pairs, then converts the correlation matrix into an adjacency matrix using a power function f(x) = xb. The parameter b of the power function is determined in such a way that the resulting adjacency matrix, i.e., the weighted coexpression network, is approximately scale-free. To measure how well a network satisfies a scale-free topology, we use the model fitting index R2 of the linear model that regresses log(p(k)) on log(k), where k is connectivity and p(k) is the frequency distribution of connectivity^17^. The fitting index of a perfect scale-free network is 1. The connectivity between genes or kij is a transformed correlation between the expression profiles of two genes, jr(i,j)jb, with r as the Pearson correlation coefficient. The parameter b (>0) of the power function is determined in such a way that the global probability distribution of the resulted connectivity values for all the gene pairs is scale free. Thus, kij is a continuous value ranging from 0 to 1.

To explore the modular structures of the co-expression network, the adjacency matrix is further transformed into a topological overlap matrix (TOM)^18^. As the topological overlap between two genes reflects not only their direct interaction but also their indirect interactions through all the other genes in the network, previous studies^17,18^ have shown that topological overlap leads to more cohesive and biologically more meaningful modules. To identify modules of highly coregulated genes, we used average linkage hierarchical clustering to group genes based on the topological overlap of their connectivity, followed by a dynamic cut-tree algorithm to dynamically cut clustering dendrogram branches into gene modules ^19^. To distinguish between modules, each module was assigned a unique color identifier, with the remaining, less well connected genes colored gray. To compare and contrast two multitissue networks, we combined their TOM heatmaps into a single large network. In the combined heatmap, the upper panel shows the hierarchical clustering on the TOM of the LOAD network, whereas the color bar below represents the gene modules. Similarly, the lower panel represents the TOM from normal multitissue network. The color intensity in the map represents the interaction strength between genes. This connectivity map highlights how genes in the multitissue transcriptional networks fall into distinct network modules, where genes within a given module are more highly interconnected with each other (blocks along the diagonal of the matrix) than with genes in other modules.

For the coexpression network we constructed, all genes were entered into the reconstruction pipeline. If a gene was not observed to be expressed (at least one read detected) across at least 50% of the samples, it was filtered out of the analysis. The parameter b (beta) was set to 6 to ensure the network followed a scale free distribution. There were no samples detected as outliers, so all samples were retained in the construction.

*Reconstruction of the Bayesian networks:* Bayesian networks are directed acyclic graphs in which the edges of the graph are defined by conditional probabilities that characterize the distribution of states of each node given the state of its parents. The network topology defines a partitioned joint probability distribution over all nodes in a network, such that the probability distribution of states of a node depends only on the states of its parent nodes: formally, a joint probability distribution $p(X)$ on a set of nodes $X$ can be decomposed as$p(X)=\prod_{i} p(X^{i}|\text{Pa}(X^{i}))$, where $\text{Pa}(X^{i})$ represents the parent set of $X^{i}$. In our networks, each node represents transcription expression of a gene. These conditional probabilities reflect not only relationships between genes, but also the stochastic nature of these relationships, as well as noise in the data used to reconstruct the network.

Bayes formula allows us to determine the likelihood of a network model $M$ given observed data $D$ as a function of our prior belief that the model is correct and the probability of the observed data given the model: $P(M|D)\sim P(D|M)*P(M)$. The number of possible network structures grows super-exponentially with the number of nodes, so an exhaustive search of all possible structures to find the one best supported by the data is not feasible, even for a relatively small number of nodes. We employed Monte Carlo Markov Chain (MCMC) ^20^ simulation to identify potentially thousands of different plausible networks, which are then combined to obtain a consensus network (see below). Each reconstruction begins with a null network. Small random changes are then made to the network by flipping, adding, or deleting individual edges, ultimately accepting those changes that lead to an overall improvement in the fit of the network to the data. We assess whether a change improves the network model using the Bayesian Information Criterion (BIC)^21^, which avoids over-fitting by imposing a cost on the addition of new parameters. This is equivalent to imposing a lower prior probability $P(M)$ on models with larger numbers of parameters.

Although edges in Bayesian networks are directed, we can’t infer causal relationships from the structure directly in general. For example, in a network with two nodes, $X^{1}$and $X^{2}$, the two models $X^{1}\to X^{2}$ and $X^{2}\to X^{1}$ have equal probability distributions as $p(X^{1}\text{,}X^{2})=p(X^{2}\text{|}X^{1})p(X^{1})=p(X^{1}\text{|}X^{2})p(X^{2})$. Thus, by the data itself, we can’t infer whether $X^{1}$ is causal to$X^{2}$, or vice versa. In a more general case, a network with three nodes,$X^{1}$, $X^{2}$, and $X^{3}$, there are multiple groups of structures that are mathematically equivalent. For example, the following three different models,$\text{M}1: X^{1}\to X^{2}, X^{2}\to X^{3}$, $\text{M2}: X^{2}\to X^{1}, X^{2}\to X^{3}$, and $\text{M3}: X^{2}\to X^{1}, X^{3}\to X^{2}$, are Markov equivalent (which means that they all encode for the same conditional independent relationships). In the above case, all three structures encode the same conditional independent relationship, , $X^{1}$ and $X^{3}$ are independent conditioning on $X^{2}$, and they are mathematically equal

$\begin{matrix} p(X) =p(\text{M}1|D)=p(X^{2}|X^{1})p(X^{1})p(X^{3}|X^{2}) \\ =p(\text{M2}|D)=p(X^{1}|X^{2})p(X^{2})p(X^{3}|X^{2}) \\ =p(\text{M3}|D)=p(X^{2}|X^{3})p(X^{3})p(X^{1}|X^{2}) \end{matrix}$ .

Thus, we can’t infer whether $X^{1}$ is causal to $X^{2}$ or vice versa from these types of structures. However, there is a class of structures, V-shape structure (e.g. $\text{Mv}: X^{1}\to X^{2}, X^{3}\to X^{2}$), which has no Markov equivalent structure. In this case, we can infer causal relationships. There are more parameters to estimate in the Mv model than M1, M2, or M3, which means a large penalty in BIC score for the Mv model. In practice, a large sample size is needed to differentiate the Mv model from the M1, M2, or M3 models.

*Classification analysis.* To discover a parsimonious set of gene expression biomarkers that are capable of predicting the vacation or meditation status of a study participant, we employed a rigorous machine learning pipeline that combined feature (gene) selection and classification techniques in a wrapper setting ^22^. Briefly, we used the Recursive Feature Elimination (RFE) algorithm ^23^, wrapped within the Logistic Regression (LR) ^24^ and SVM-Linear (kernel) ^25^ classification algorithms (referred to as LR-RFE and SVM-RFE respectively). These methods were applied within a nested (outer and inner) cross-validation (CV) setup ^26^ to 150 randomly selected samples (training set) from the development set to select sets of predictive genes. The inner CV round was used to determine the optimal number of genes to be selected by each feature selection strategy, and the outer one was used to select the set of predictive genes based on this number. Four outer classification algorithms, namely Logistic Regression ^24^, SVM-Linear ^25^, AdaBoost ^27^ and Random Forest (RF) ^28^, were used to learn the final classification models over the training based on these predictive genes. These models were evaluated on the corresponding remaining 50 samples (holdout set) in terms of their F-measure, a harmonic mean of precision and recall ^29^. To obtain a more realistic estimate of the performance of these models on different test sets, which may not be determined by the default probability thresholds assigned by the algorithm implementations used, the optimal threshold for classifying samples as post-vacation or pre-vacation (or post-meditation versus pre-meditation) was determined for each model by maximizing the F-measure of the intervention class (again, either vacation or meditation) on the respective holdout set. This optimization closely mirrors the F-measure maximization-based evaluation strategies in other large computational biology studies, such as those on protein function prediction^26,30^. AUC curves were plotted using the Scikit-learn python library.

To obtain a comprehensive view of the performance of these proposed models, the above two components (feature selection and global classification) were executed on 100 random training-holdout splits of the development set. To determine the best performing combination of feature selection and global classification algorithms, we conducted a statistical analysis of the classification performance of all the models resulting from all the considered combinations using the Friedman followed by the Nemenyi test.^31,32^ These tests, which account for multiple hypothesis testing, assessed the statistical significance of the relative difference of performance of the combinations in terms of their relative ranks across the 100 splits, and allowed the ordering of the overall performance of each combination in terms of the significance of their pairwise comparison. This statistical comparison was a novel aspect of our pipeline, as in typical studies, this task, generally referred to as “model selection,” is based on a single training-holdout split. Even if multiple such splits are employed, models are generally selected based on absolute performance scores (F-measure here), and not based on the statistical significance of performance comparisons, as was done in our study.

**Statistical analyses of survey and biomarker data**

A series of group comparisons were performed to test several independent hypotheses. First, we were interested in whether groups differed at study entry, and thus, we compared baseline values in survey and biomarker data with ANOVAs (see Tables S1 and S3 for ANOVA Baseline F statistics and significance values).

*Survey data analysis.* For the survey data (Table S1), we completed 4 mixed ANOVAs to examine the differences in psychological wellbeing scores between groups at baseline, study follow-up, and 1 month. Significant within-subject effects were followed up with t-tests for difference scores with baseline subtracted from (1) follow-up and (2) from one month for all participants. Significant between-subject effects were followed up with contrasts between the three groups on average scores across all three-time points. Finally, significant mixed effects were followed up with two separate ANOVAs comparing all three groups and their difference scores of (1) follow-up and (2) 1 month from baseline. Additionally, we conducted ANOVAs on the difference scores between baseline and 10 month stress and depression surveys for those who completed follow up (83%) to examine whether participants returned to their baseline states of wellbeing 10 months after intervention exposure (see Table S2). Significant ANOVAs were followed up with comparisons between the groups.

*Biomarker data processing.* For the biomarker data, we calculated difference scores, subtracting study entry (day 1) from post-study (day 5) to test whether change scores were different among the three groups (see Table S3 for ANOVA Change F statistics and significance values). Given the exploratory nature of this study and the fact that this is the first of its kind to utilize a vacation-based control group, we followed up these ANOVAs with paired t-tests within each group for pre to post changes if F tests for any omnibus or follow-up ANOVA was at least marginally significant, p < .10 (see Tables S1, S2, and S3 for paired t-tests within each group).

Supplementary References

1. Daubenmier J, Lin J, Blackburn E, et al. Changes in stress, eating, and metabolic factors are related to changes in telomerase activity in a randomized mindfulness intervention pilot study. Psychoneuroendocrinology 2012;37:917-28.

2. Ornish D, Lin J, Daubenmier J, et al. Increased telomerase activity and comprehensive lifestyle changes: a pilot study. Lancet Oncol 2008;9:1048-57.

3. Radloff LS. The use of the Center for Epidemiologic Studies Depression Scale in adolescents and young adults. J Youth Adolesc 1991;20:149-66.

4. Cohen S, Kamarck T, Mermelstein R. A global measure of perceived stress. J Health Soc Behav 1983;24:385-96.

5. Brown KW, Ryan RM. The benefits of being present: mindfulness and its role in psychological well-being. J Pers Soc Psychol 2003;84:822-48.

6. Ware JE, Jr., Sherbourne CD. The MOS 36-item short-form health survey (SF-36). I. Conceptual framework and item selection. Med Care 1992;30:473-83.

7. Cawthon RM. Telomere measurement by quantitative PCR. Nucleic Acids Res 2002;30:e47.

8. Lin J, Epel E, Cheon J, et al. Analyses and comparisons of telomerase activity and telomere length in human T and B cells: insights for epidemiology of telomere maintenance. J Immunol Methods 2010;352:71-80.

9. Donohue MC, Moghadam SH, Roe AD, et al. Longitudinal plasma amyloid beta in Alzheimer's disease clinical trials. Alzheimer's & dementia : the journal of the Alzheimer's Association 2014.

10. Kim D, Pertea G, Trapnell C, Pimentel H, Kelley R, Salzberg SL. TopHat2: accurate alignment of transcriptomes in the presence of insertions, deletions and gene fusions. Genome Biol 2013;14:R36.

11. Katz Y, Wang ET, Airoldi EM, Burge CB. Analysis and design of RNA sequencing experiments for identifying isoform regulation. Nat Methods 2010;7:1009-15.

12. Trapnell C, Hendrickson DG, Sauvageau M, Goff L, Rinn JL, Pachter L. Differential analysis of gene regulation at transcript resolution with RNA-seq. Nat Biotechnol 2013;31:46-53.

13. Law CW, Chen Y, Shi W, Smyth GK. voom: Precision weights unlock linear model analysis tools for RNA-seq read counts. Genome Biol 2014;15:R29.

14. Smyth GK. Linear models and empirical bayes methods for assessing differential expression in microarray experiments. Statistical applications in genetics and molecular biology 2004;3:Article3.

15. Ritchie ME, Phipson B, Wu D, et al. limma powers differential expression analyses for RNA-sequencing and microarray studies. Nucleic Acids Res 2015;43:e47.

16. Millstein J, Volfson D. Computationally efficient permutation-based confidence interval estimation for tail-area FDR. Front Genet 2013;4:179.

17. Zhang B, Horvath S. A general framework for weighted gene co-expression network analysis. Statistical applications in genetics and molecular biology 2005;4:Article17.

18. Ravasz E, Somera AL, Mongru DA, Oltvai ZN, Barabasi AL. Hierarchical organization of modularity in metabolic networks. Science 2002;297:1551-5.

19. Langfelder P, Castellani LW, Zhou Z, et al. A systems genetic analysis of high density lipoprotein metabolism and network preservation across mouse models. Biochimica et biophysica acta 2012;1821:435-47.

20. Madigan DaY, J. Bayesian graphical models for discrete data. International Statistical Review 1995;63:215-32.

21. Schwarz G. Estimating the dimension of a model. Annals of Statistics 461-464 1978;6:461-4.

22. Saeys Y, Inza I, Larranaga P. A review of feature selection techniques in bioinformatics. Bioinformatics 2007;23:2507-17.

23. Guyon I, Weston, J, Barnhill, S, Vapnik, V. Gene selection for cancer classification using support vector machines. Machine Learning 2002;46:389-422.

24. Bewick V, Cheek L, Ball J. Statistics review 14: Logistic regression. Crit Care 2005;9:112-8.

25. Burges CJ. A tutorial on support vector machines for pattern recognition. Data mining and knowledge discovery 1998;2:121-67.

26. Whalen S, Pandey OP, Pandey G. Predicting protein function and other biomedical characteristics with heterogeneous ensembles. Methods 2016;93:92-102.

27. Freund Y, Schapire RE. A Decision-Theoretic Generalization of On-Line Learning and an Application to Boosting. J Comput Syst Sci 1997;55:119-39.

28. Breiman L. Random Forests. Machine Learning 2001;45:5-32.

29. Powers DM. Evaluation: from precision, recall and F-measure to ROC, informedness, markedness and correlation. 2011.

30. Radivojac P, Clark WT, Oron TR, et al. A large-scale evaluation of computational protein function prediction. Nature methods 2013;10:221-7.

31. Hollander M, Wolfe DA, Chicken E. Nonparametric statistical methods: John Wiley & Sons; 2013.

32. Demsar J. Statistical Comparisons of Classifiers over Multiple Data Sets. J Mach Learn Res 2006;7:1-30.

**Supplementary Tables**

**Table S1. Psychological wellbeing during retreat week and one month later**

| **Scores on Psychological Measures** | | | | | | | |
| --- | --- | --- | --- | --- | --- | --- | --- |
|  | Baseline  Mean (SE) | | Follow up  Mean (SE) | One month later, Mean  (SE) | F test for psych measure | F test for Group | F test  measure*Group |
| **Mindful Attention and Awareness Scale^Ŧ^** | | | | | | | |
| Full | | 3.96 (0.08) | 5.05 (0.06) | 4.82 (0.08) | F(1.79, 146.96) = 153.50, p = .000 | F(2, 82) = 0.93, p = .40 | **F(3.58, 146.96) = 3.23, p = .018** |
| Vacation Arm (V) | | 3.96 (0.15) | 4.91 (0.10) | 4.62 (0.13) | Follow-up t-tests:  FU-BL: t(87) = 14.99, p = .000;  1M-BL: t(84) = 12.71, p = .000 |  | **Follow up contrasts from ANOVAS**  FU-BL; F(2,85) = 2.98, p = .056  N > V*; V=R; N>R*  1M-BL:F(2,82) = 5.35, p = .01  N>R*; N>V^*^; V=R |
| Novice Meditator Arm (N) | | 3.80 (0.14) | 5.13 (0.10) | 4.96 (0.13) |
| Regular Meditator Arm (R) | | 4.13 (0.15) | 5.11 (0.11) | 4.89 (0.14) |
| Depressive Symptoms | | | | | | | |
| Full | 12.01 (1.00) | | 5.45 (0.50) | 7.26 (.78) | F(1.74, 142.74) = 43.25, p = .000 | F(2, 82) = 0.56, p = .58 | F(3.48, 142.74) = 1.73, p =.16 |
| Vacation Arm (V) | 10.14 (1.71) | | 5.48 (0.86) | 7.66 (1.33) | Follow-up t-tests:  FU-BL: t(87) = 7.98, p = .000;  1M-BL: t(84) = 6.77, p = .000 |  |  |
| Novice Meditator Arm (N) | 14.20 (1.69) | | 6.03 (0.85) | 7.43 (1.31) |
| Regular Meditator Arm (R) | 11.69 (1.81) | | 4.85 (0.91) | 6.69 (1.41) |
| Perceived Stress | | | | | | | |
| Full | 14.92 (1.20) | | 5.86 (0.82) | 10.76 (0.68) | F(2, 164) = 122.54, p = .000 | F(2, 82) = 3.00, p = .06 | F(4, 164) = 2.00, p =.10 |
| Vacation Arm (V) | 16.17 (1.20) | | 6.45 (0.82) | 13.03 (1.16) | Follow-up t-tests:  FU-BL: t(87) = 15.19, p = .000;  1M-BL: t(84) = 7.71, p=.000 | V >R*; V=N; N=R |  |
| Novice Meditator Arm (N) | 15.97 (1.18) | | 6.10 (0.80) | 10.50 (1.14) |
| Regular Meditator Arm (R) | 12.31 (1.27) | | 5.04 (0.86) | 8.73 (1.22) |
| **Vitality** | | | | | | | |
| Full | 12.77 (0.29) | | 15.61 (0.25) | 15.19 (0.23) | F(2, 166) = 71.34, p = .000 | F(2, 83) = 0.25, p = .78 | F(4, 166) = 2.22, p =.07 |
| Vacation Arm (V) | 12.97 (0.49) | | 16.14 (0.43) | 14.86 (0.40) | Follow-up t-tests:  FU-BL: t(87) = 10.98, p = .000;  1M-BL: t(85) = 9.54, p = .000 |  | Follow up contrasts  FU-BL; F(2,85) = .68, p=.51  1M-BL: F(2,83) = 2.54, p=.08  N > V*; V=R; N=R |
| Novice Meditator Arm (N) | 12.33 (0.48) | | 15.10 (0.42) | 15.53 (0.39) |
| Regular Meditator Arm (R) | 13.00 (0.51) | | 15.59 (0.44) | 15.19 (0.41) |

Notes. **^Ŧ^** Sphericity assumption violated. Greenhouse-Geisser correction applied to degrees of freedom; * p < 0.05.

There were no significant differences in baseline levels of any psychological measures (not shown).

**Table S2. Psychological wellbeing ten months later (change scores)**

| **Psychological Questionnaires 10 months later** | | | |
| --- | --- | --- | --- |
| **Depression Scale** |  |  |  |
|  | **Mean (SE)** | **Difference** | **Paired t tests, p** |
| *Full sample* | 9.15 (1.10) | -2.40 (1.19) | t(71)=2.02, p=.047 |
| Vacation Arm (V) | 11.46 (2.07) | 1.87 (2.33) | t(23)=.80, p=.430 |
| Novice Meditator Arm (N) | 7.61 (2.26) | -4.78 (2.00) | t(22)=2.38, p=.026 |
| Regular Meditator Arm (R) | 8.36 (1.34) | -4.32 (1.61) | t(24)=2.69, p=.013 |
| ANOVA between group differences | Difference score (9month – Baseline)  **F(2,69)=3.47, p=.037**  **N < V*, R < V*; N=R** | | |
| **Perceived Stress** |  |  |  |
|  | **Mean (SE)** | **Difference** | **Paired t tests, p** |
| *Full sample* | 11.71 (.81) | -2.97 (.74) | t(69)=4.00, p=.000 |
| Vacation Arm (V) | 15.45 (1.49) | -1.18 (1.07) | t(21)=1.10, p=.280 |
| Novice Meditator Arm (N) | 9.48 (1.43) | -5.43 (1.56) | t(22)=3.49, p=.002 |
| Regular Meditator Arm (R) | 10.48 (1.07) | -2.28 (1.06) | t(24)=2.14, p=.043 |
| ANOVA between group differences | Difference score (9month – Baseline)  **F(2,67)=3.05, p=.054**  **N < V*; N < R^M^ ; R=V** | | |

Notes: * = p < 0.05; ^m^ = 0.10

**Table S7. Meta-themes from Gene Ontology Tree Maps for modules of the co-expression network related to vacation signature.** When possible, examples of pathways related to stress and aging within each of the large meta-paths are also listed.

| **Module** | Highest significance | Medium significance | Lowest significance |
| --- | --- | --- | --- |
| Red  (**Suppressed** in Regular Meditator group relative to Novices) | **Immune response** (response to wounding, cytokine signaling, inflammatory response) | Organic Cation Transport  Hyaluranon biosynthesis  Lipoxin metabolism  Membrane fusion | Glycogen metabolism |
|  | **Positive regulation of chemokine production**  (epigenetic regulation of gene expression) |  |  |
| **Module** | Highest significance | Medium significance | Lowest significance |
| Pink  (**Suppressed** in post relative to baseline) | **Erythrocyte differentiation** | Porphyrin-containing compound metabolism | Plasma membrane organization |
|  | **Oxygen transport** |  |  |
|  | **Hydrogen peroxide metabolism**  (DNA damage response, mitochondrion degradation) |  |  |
| **Module** | Highest significance | Medium significance | Lowest significance |
| Purple  (**Suppressed** in post relative to baseline) | **Blood coagulation**  (acute inflammatory response, response to wounding, beta amyloid metabolic process, senescence) | Platelet degranulation  Arachidonic acid metabolism | Extracellular matrix organization |
| **Module** | Highest significance | Medium significance | Lowest significance |
| Brown | **Blood coagulation**  (macroautophagy, response to cytokines) |  |  |
| **Module** | Highest significance | Medium significance | Lowest significance |
| Midnight Blue | **Sphingosine-1-pohsphate signaling pathway** (response to stress) | GPI anchor biosynthesis | Glutamate metabolism |

**Table S8**. **Meta-themes from Gene Ontology Tree Maps for modules of the co-expression network**

**enriched for genes in the meditation signature.**

| **Module** | Highest significance | Medium significance | Lowest significance |
| --- | --- | --- | --- |
| Salmon  (**Suppressed** in Regular Meditator Arm relative to Novice Meditator and Vacation Arms) | **Translational Termination**  (represents 50 paths, largest include protein targeting to membrame which is ER stress, RNA and mRNA metabolic process, small include TOR signaling, mitochondrion morphogenesis, regulation of RNA, DNA) | **Viral life cycle** (positive regulation of viral genome replication) | Regulation of sodium ion transmembrane transport  Optic nerve development |
| **Module** | Highest significance | Medium significance | Lowest significance |
| Light-Yellow (**Suppressed** in Novice Relative to Vacation arm, and Suppressed in Follow Up Relative to Baseline | **Regulation of protein kinase C signaling** (inflammatory response, response to nutrients, regulation of cell death) | Nucleic acid phosphodiester bond hydrolysis  Embryonic placenta development | Long chain fatty acid import |
| **Module** | Highest significance | Medium significance | Lowest significance |
| Green-Yellow (**Activated** in Regular Meditator Relative to Vacation arm) (Activated in post Relative to Baseline) | **Chemokine mediated signaling** (cellular defense, response to wounding, sleep) | Carbohydrate biosynthesis  Cytidine deamination | Adherens junction assembly |
| Module | Highest significance | Medium significance | Lowest significance |
| Light Green (**Activated** in Regular Meditator Relative to Vacation and Novice arms) | **Positive regulation of chemokine biosynthesis** (immune response, DNA damage response, regulation of G1/S transition, senescence) | Glutamate secretion |  |
| Module | Highest significance | Medium significance | Lowest significance |
| Tan  (**Suppressed** in Regular Meditator Relative to Novice arm) (Activated in Follow Up Relative to Baseline | **RNA phophodiester bond dydrolysis exonucleolytic** (regulation of DNA, RNA methylation) | Fatty acid alpha-oxidation  Cellular response to granulocyte-macrophage colony stimulating factor stimulus (response to toxic substance, UV damage repair) | ATP transport  Chromatin organization  Salivary gland morphogenesis |
|  | **Positive regulation of viral genome replication** |  |  |

†The most significant meta-paths represent the largest space and typically have the most clustered pathways. Medium significance typically represents around 10 pathways, and small are typically 3 pathways. Meta-paths that represent one or two pathways are not represented here.

**Table S9. Biomarker Means (Standard Errors) at baseline, follow up, and change scores**

| **A Beta Protein 40** | Baseline | Post | Difference | | Paired t tests, p |
| --- | --- | --- | --- | --- | --- |
| *Full sample* | 369.49 (9.20) | 342.01 (7.43) | -27.48 (11.17) | | **t(85)=2.46, p=.016** |
| Vacation Arm (V) | 390.63 (18.97) | 340.48 (14.87) | -50.15 (22.49) | | **t(27)=2.23, p = .03** |
| Novice Meditator Arm (N) | 383.67 (14.97) | 324.28 (11.54) | -59.40 (16.41) | | **t(28)=3.62, p=.001** |
| Regular Meditator Arm (R) | 334.91 (11.76) | 361.22 (11.56) | +26.31 (15.02) | | **t(28)=1.75, p=.09** |
| ANOVA | Baseline:  F(2,83)=2.15, p = .123 | | **Change:**  **F(2,83) = 6.77, p = .002**  V=N; V<R*; N<R* | | |
| **A Beta Protein 42** | Baseline | Post | Difference | | Paired t tests, p |
| *Full sample* | 47.80 (1.35) | 49.84 (1.27) | 2.04 (1.43) | | t(86)=1.43, p=.16 |
| Vacation Arm (V) | 47.98 (2.22) | 49.70 (2.36) | 1.72 (3.13) | | t(28)=.55, p=.59 |
| Novice Meditator Arm (N) | 49.66 (2.36) | 49.97 (1.94) | 0.30 (2.26) | | t(28)=.14, p=.89 |
| Regular Meditator Arm (R) | 45.77 (2.07) | 49.85 (1.99) | 4.07 (1.90) | | **t(28)=2.15, p=.04** |
| ANOVA | Baseline:  F(2,84)=0.77, p = .46 | | Change:  F(2,84) = 0.59, p = .56 | | |
| **A Beta 42/40 Ratio** | Baseline | Post | Difference | | Paired t tests, p |
| *Full sample* | .13 (.002) | .15 (.002) | .02 (.002) | | **t(70)=8.05, p=.000** |
| Vacation Arm (V) | .12 (.003) | .15 (.004) | .02 (.003) | | **t(28)=7.52, p=.000** |
| Novice Meditator Arm (N) | .13 (.003) | .15 (.004) | .02 (.003) | | **t(28)=8.23, p=.000** |
| Regular Meditator Arm (R) | .14 (.004) | .14 (.003) | .00 (.002) | | t(28)=0.40, p=.70 |
| ANOVA | **Baseline:**  **F(2,83)=3.32, p = .04**  V=N; V<R*; N=R | | **Change:**  **F(2,83) = 22.30, p = .0001**  V=N; V>R*; N>R* | | |
| **Telomerase (logged)** | Baseline | Post | Difference | | Paired t tests, p |
| *Full sample* | *.64 (.02)* | .69 (.02) | *.06 (.03)* | | **t(86)=2.10, p=.039** |
| Vacation Arm (V) | .70 (.04) | .76 (.04) | .06 (.05) | | t(28)=1.17, p=.25 |
| Novice Meditator Arm (N) | .66 (.03) | .64 (.04) | -.02 (.05) | | t(28)=0.37, p=.72 |
| Regular Meditator Arm (R) | .56 (.04) | .69 (.04) | .13 (.04) | | **t(28)=3.15, p=.004** |
| ANOVA between group differences | **Baseline:**  **F(2,87)= 4.14, p= .02**  V=N; C>R*; N>R* | | **Change:**  **F(2,84) = 2.62, p = .08**  V=N; V=R; N<R* | | |
| **TNF-a** | Baseline | Post | Difference | | Paired t tests, p |
| *Full* | *2.08 (.08)* | 2.37 (.08) | 29 (.06) | **t(88)=5.23, p=.000** | |
| Vacation Arm (V) | 1.97 (.13) | 2.51 (.16) | .53 (.08) | **t(30)=7.01, p=.000** | |
| Novice Meditator Arm (N) | 2.07 (.11) | 2.32 (.11) | .26 (.11) | **t(29)=2.32, p=.03** | |
| Regular Meditator Arm (R) | 2.22 (.15) | 2.28 (.17) | .06 (.08) | t(27)=0.74, p=.46 | |
| ANOVA between group differences | Baseline:  F(2,86) = 0.89, p= .41 | | **Change:**  **F(2,86) = 6.92, p = .002**  V>N^*^; V>R*; N=R | | |

Notes: * = p < .05

**Table S10. Mean Cell type percentage values at baseline and follow up**

| **Cell raw values** | | | | | |
| --- | --- | --- | --- | --- | --- |
|  | **Baseline Mean (SE)**  **(Day 1)** | **Follow up**  **Mean (SE)**  **(Day 5)** | | **Difference Mean (SE)** | **T test**  **T(df), p value** |
| **Neutrophils** |  |  | |  |  |
| *Full sample* | 55.31 (0.96) | 53.47 (0.95) | | -1.83 (0.67) | T(90) = -2.72, p = .008 |
| Vacation Arm (V) | 56.96 (1.64) | 55.02 (1.83) | | -1.94 (1.58) | T(30) = -1.23, p = .23 |
| Novice Meditator Arm (N) | 55.59 (1.67) | 54.16 (1.41) | | 1.43 (0.85) | T(30) = -1.69, p = .10 |
| Regular Meditator Arm (R) | 53.23 (1.65) | 51.09 (1.67) | | -2.15 (0.95) | T(28) = -2.26, p = .03 |
| ANOVA | F (2, 90) = 1,80 p = .17 | | **F (2, 88) = 0.10 p = .91** | | |
| **Lymphocytes** |  |  | |  |  |
| *Full* | 34.97 (0.89) | 36.81 (0.87) | | 1.84 (0.61) | T(90) = 3.04, p = .003 |
| Vacation Arm (V) | 34.18 (1.58) | 35.83 (1.66) | | 1.65 (1.34) | T(30) = 1.18, p = .25 |
| Novice Meditator Arm (N) | 34.71 (1.63) | 36.12 (1.41) | | 1.41 (0.78) | T(30) = 1.81, p = .08 |
| Regular Meditator Arm (R) | 36.08 (1.44) | 38.61 (1.43) | | 2.52 (0.87) | T(28) = 2.89, p = .007 |
| ANOVA | F (2, 90) = 0.53, p = .59 | | **F (2, 88) = 0.30, p = .74** | | |
| **Monocytes** |  |  | |  |  |
| *Full sample* | 6.54 (0.21) | 6.50 (0,21) | | -0.04 (0.10) | T(90) = -.25, p = .73 |
| Vacation Arm (V) | 6.13 (0.25) | 6.36 (0.31) | | 0.23 (0.20) | T(30) = 1.15, p = .26 |
| Novice Meditator Arm (N) | 6.37 (0.34) | 6.42 (0.35) | | 0.04 (0.13) | T(30) = 0.32, p = .75 |
| Regular Meditator Arm (R) | 7.15 (0.48) | 6.74 (0.45) | | -0.41 (0.19) | T(28) = -2.10, p = .045 |
| ANOVA | F (2, 90) = 1.92, p = .15 | | **F (2, 88) = 3.32, p = .041**  **V= N; V>R*; N= R** | | |
| **Eosinophils** |  |  | |  |  |
| *Full sample* | 2.73 (0.21) | 2.72 (0.20) | | -0.01 (0.11) | T(90) =- 0.16, p = .88 |
| Vacation Arm (V) | 2.53 (0.32) | 2.32 (0.28) | | -0.21 (0.27) | T(30) =- 0.78, p = .44 |
| Novice Meditator Arm (N) | 2.75 (0.29) | 2.82 (0.31) | | 0.06 (0.12) | T(30) = 0.51, p = .61 |
| Regular Meditator Arm (R) | 2.93 (0.49) | 3.03 (0.45) | | 0.11 (0.11) | T(28) = 0.93, p = .36 |
| ANOVA | F (2, 90) = 1.02, p = .37 | | **F (2, 88) = 0.86, p = .43** | | |
| **Basophils** |  |  | |  |  |
| *Full sample* | 0.56 (0.05) | 0.49 (0.03) | | -0.07 (0.05) | T(90) = -1.56, p = .12 |
| Vacation Arm (V) | 0.53 (0.12) | 0.47 (0.03) | | 0.05 (0.12) | T(30) = 0.47, p = .64 |
| Novice Meditator Arm (N) | 0.57 (0.04) | 0.49 (0.06) | | -0.08 (0.04) | T(30) = -1.81, p = .08 |
| Regular Meditator Arm (R) | 0.60 (0.04) | 0.53 (0.06) | | -0.08 (0.05) | T(28) = -1.43, p = .16 |
| ANOVA | F (2, 90) = 0.27, p = .76 | | **F (2, 88) = 0.03, p = .97** | | |

**Supplementary Figures**


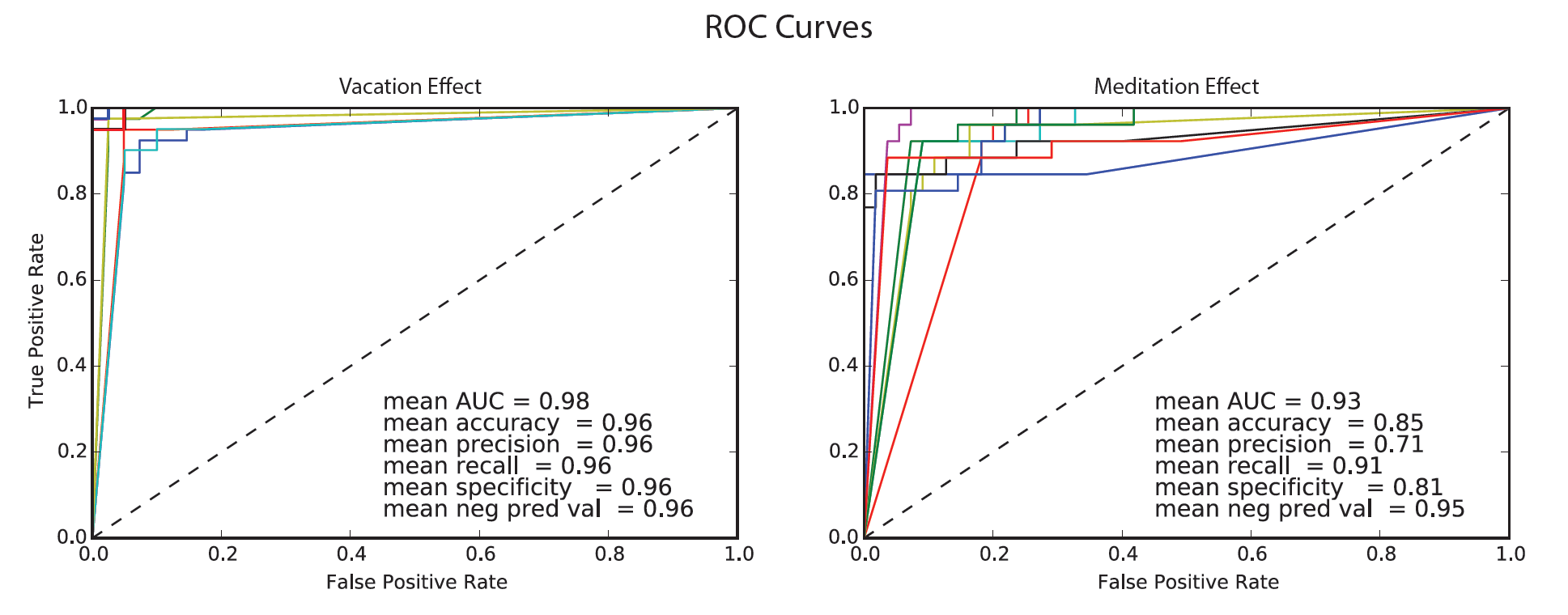


**Figure S1. Receiver operating characteristic (ROC) curves for the prediction of vacation (left panel) and meditation (right panel) effects.** For each curve a percentage of the data were randomly sampled to build the classifier and then the remaining data were used to assess the accuracy of the classifier, plotted here as the false positive rate versus the true positive rate. This process was repeated multiple times, with a sampling of those results depicted here as different colored ROC curves. The mean measures noted such as area under the curve (AUC) represent the mean measure across all runs.


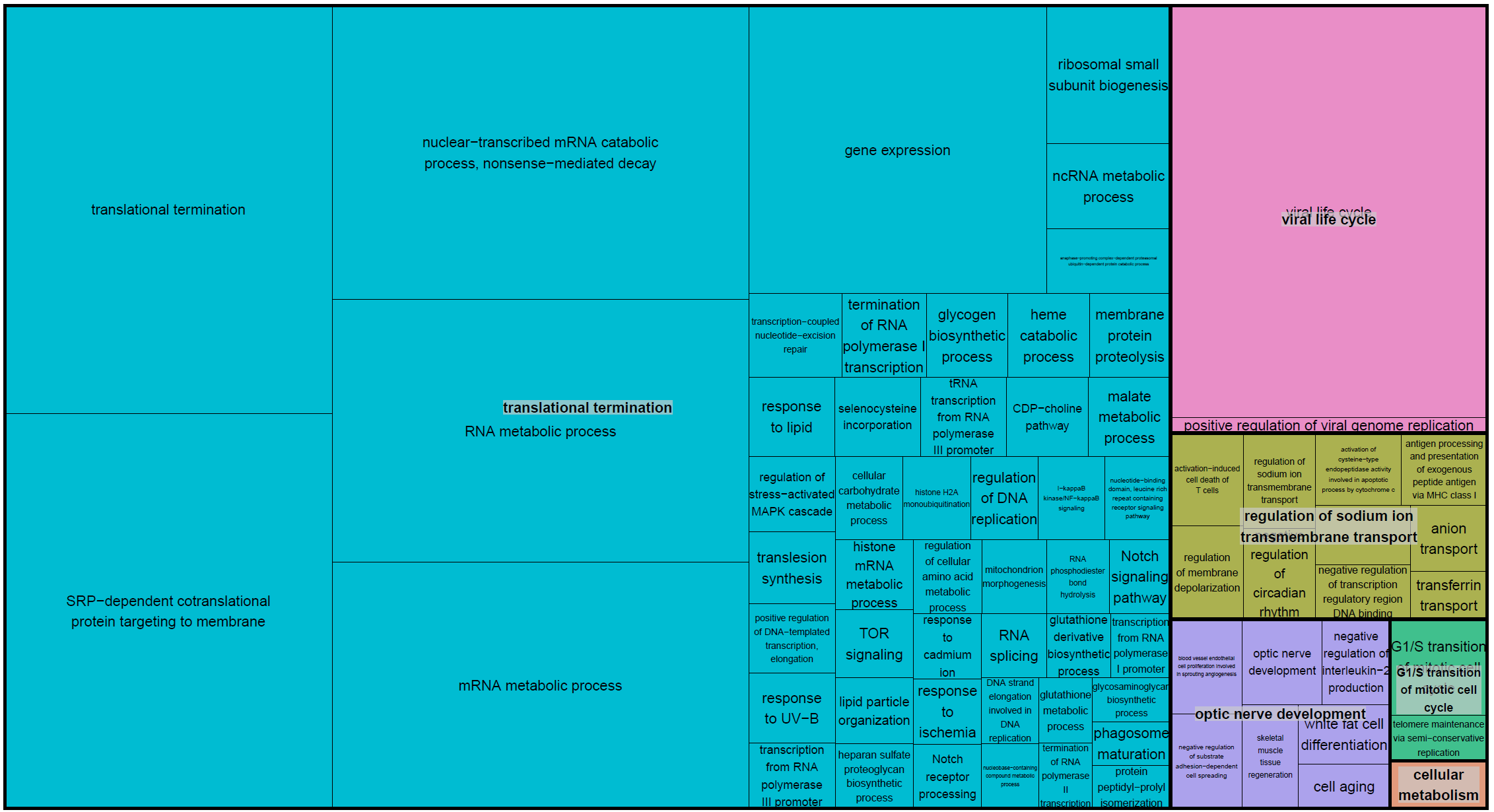


**Figure S2. REVIGO Gene Ontology treemap for the meditation-specific, salmon coexpression network module.** The GO biological pathway plot for the salmon module from the blood coexpression network was generated using the software Revigo. Each rectangle is a representative of a single GO cluster which may contain several other similar GO terms. The representatives are then joined into superclusters of loosely related terms as represented by different colors. Size of the rectangles are proportional to the -log10 p-value such that larger boxes reflect more significant terms.


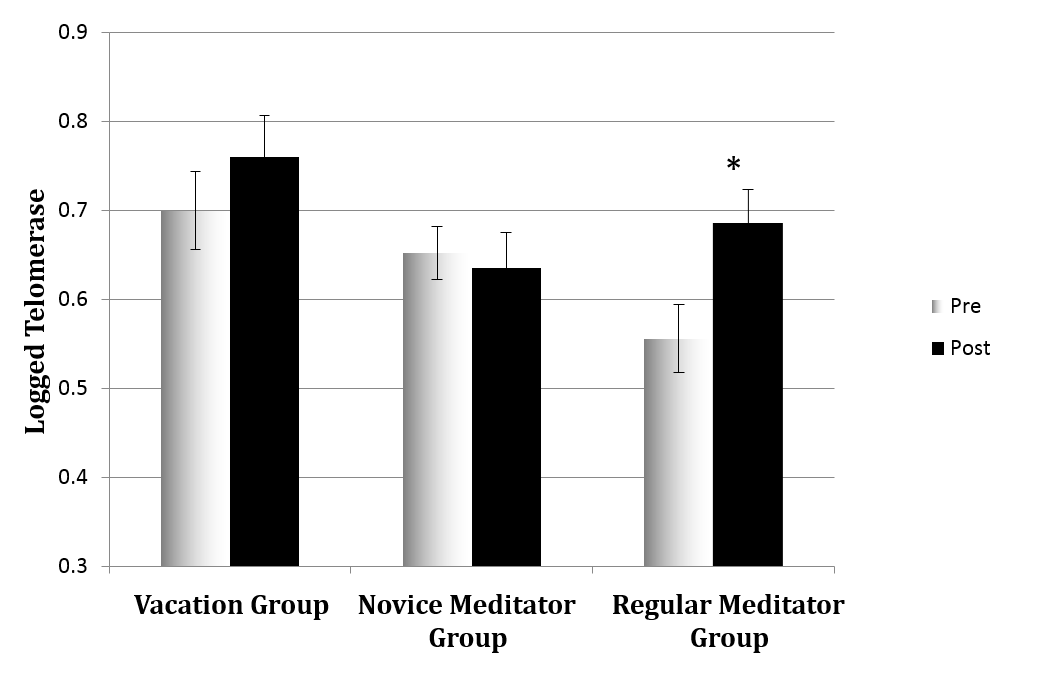


**Figure S3. Telomerase Activity at baseline and day 5 of retreat.** Mean levels of logged telomerase by natural log (in activity per 10,000 cells), at baseline and post, in vacation, novice, and regular meditator groups. Regular meditators had lower telomerase at baseline and a significant increase from baseline at post. There were no significant changes in telomerase in the other groups. This differential difference was marginally significantly different compared to the other groups.
