# Supplementary material for: Meditation and vacation effects have an impact on disease-associated molecular phenotypes
Source: Transl Psychiatry. 2016 Aug 30;6(8):e880–. doi: 10.1038/tp.2016.164 (PMC5022094; doi:10.1038/tp.2016.164)
Supplement: Supplementary Table 3 [file tp2016164x2.pdf]

Table S3. Individual Gene Differential expression signatures for baseline vs. followup for all group comparisons all samples (columns 2 and 3), vacation arm versus regular meditator arm (column 4 and 5), and novice vs. regular meditators (columns 6 and 7). The test statistics (columns 2, 4, and 6) and p values (columns 3, 5, and 7) for each gene tested are provided. The p value thresholds corresponding to a 5% FDR for columns 3, 5, and 7 are 0.0008, 0.0013, 0.0020, respectively.

gene_symbol	baseline versus followup test stat	baseline versus followup pvalue	follow up regular versus vacation test stat	follow up regular versus vacation_pvalue	follow up regular versus novices_test	follow up regular versus novices_pvalue
1/2-SBSRNA4	0.668193707	0.50577502	-1.525323838	0.132651089	-1.212065312	0.230555243
A1BG	0.279596098	0.780449607	-0.594984143	0.554184511	-0.108864675	0.913697344
A1BG-AS1	1.354105113	0.179202149	-0.540621067	0.590853435	-0.302437195	0.7634346
A2LD1	-0.199519956	0.842320617	-0.631304846	0.530338731	-1.17930584	0.243241602
A2M	1.18232864	0.240289683	0.48038963	0.632769964	0.879644729	0.382795263
A4GALT	-0.258017147	0.797001721	1.449006111	0.152758765	0.651969795	0.517076549
AAAS	-0.523703738	0.60181327	0.169118768	0.866295773	1.577854063	0.120208219
AACS	1.566373419	0.120883624	2.078225455	0.042154907	1.991401459	0.05130235
AAGAB	-0.554828523	0.580432533	1.860485939	0.067924581	2.98797082	0.004157018
AAK1	1.678403359	0.096849335	0.340115444	0.735006888	1.262032957	0.212145982
AAMP	-0.086534672	0.931239816	2.460985758	0.016874612	2.549305801	0.013547773
AANAT	-0.213098009	0.831747992	-0.600077688	0.550808209	0.143174177	0.886664402
AARS	0.394307379	0.69431692	2.203366572	0.0315824	2.879653692	0.005622415
AARS2	2.364612138	0.020268837	1.571062126	0.121646042	3.057380213	0.003414162
AARSD1	0.264456459	0.792052346	2.213690244	0.030824603	3.406268187	0.001222124
AASDH	0.295199476	0.768543317	1.237070461	0.221081023	1.642597278	0.106046912
AASDHPPT	-0.336399744	0.737377738	-0.328376669	0.743817752	-0.858859374	0.39406421
AASS	0.238533208	0.812027302	-1.31976653	0.192134741	0.174749919	0.861903764
AATF	-1.61237196	0.110495094	2.596084175	0.011944757	2.938699337	0.004772923
AATK	-1.883590544	0.062953706	-0.606639682	0.546473894	-0.90182687	0.370995143
ABAT	-2.765195864	0.006942419	-0.274136528	0.784960501	-0.874461094	0.385586466
ABCA1	-1.901265168	0.060570293	-0.302426428	0.763416482	-1.735486482	0.088131762
ABCA10	0.426732091	0.67062632	0.226044665	0.821966925	0.580186005	0.564106731
ABCA11P	1.355189286	0.178857858	0.47254963	0.638320252	1.499285212	0.139392811
ABCA13	-2.298153176	0.0239459	1.456000701	0.150822041	0.111536298	0.911588226
ABCA2	0.903282539	0.368864836	-2.611281449	0.011481327	-2.017427476	0.048432927
ABCA3	1.299517775	0.197191844	-0.433885138	0.665992782	0.444430974	0.658436694
ABCA5	1.874271201	0.064241855	-0.941959133	0.350147804	-1.330862	0.188605292
ABCA6	1.335885202	0.185063321	0.861093323	0.392757256	0.0177211	0.985924071
ABCA7	0.280603859	0.779679008	-0.309746357	0.757871752	0.372278166	0.711085013
ABCA9	1.271748254	0.206844391	0.008010429	0.993636352	-0.476888003	0.635289842
ABCB1	1.286946824	0.201519079	0.377156143	0.707443674	0.726899455	0.470302678
ABCB10	0.936416881	0.351645128	0.353191803	0.725234077	-1.499324347	0.139382686
ABCB4	0.733858661	0.465003732	2.148227425	0.035913718	1.76298179	0.083336584
ABCB6	1.474651065	0.143908216	1.326420504	0.189936047	1.771219666	0.081942864
ABCB7	0.398661332	0.691117418	1.134737248	0.26118585	1.87310648	0.066254459
ABCB8	0.019544353	0.984451512	0.381108912	0.704524629	1.346406586	0.183573339
ABCB9	1.256298835	0.212363371	1.272422234	0.208336047	2.175359301	0.033822527
ABCC1	2.780114977	0.00665646	1.424067365	0.159822356	1.147141896	0.256180063
ABCC10	1.943352229	0.055199561	1.628406999	0.108896918	1.589848217	0.11747563
ABCC13	-3.619660505	0.000494249	1.000885267	0.321068779	0.53623341	0.593912097
ABCC2	0.783841195	0.435257547	-0.309267525	0.758234073	-1.093320294	0.278914443
ABCC3	-2.716559774	0.007954319	1.795936045	0.077750305	1.641688592	0.106235838
ABCC4	-0.079403057	0.936894	0.920316288	0.36124611	1.619152255	0.111009701
ABCC5	0.274441772	0.784394351	-0.230782547	0.818301551	0.017797061	0.985863742
ABCC6	-1.105708359	0.271894469	1.955186513	0.055419302	1.958698052	0.055114108
ABCC9	-1.332342342	0.186219596	2.14896078	0.035852859	0.74504571	0.459346572
ABCD1	-1.015562556	0.312647654	-0.411444873	0.682273394	-0.599122418	0.551497265
ABCD2	1.833327604	0.070166288	-0.278423562	0.781684379	-0.27002827	0.788126961
ABCD3	1.038155469	0.302068224	1.405130485	0.165353319	0.63111636	0.530521267
ABCD4	0.76794676	0.444593704	2.111038974	0.039120732	3.034562208	0.003643471
ABCE1	1.134601907	0.259651855	0.096345397	0.923580713	-0.354429067	0.724342955
ABCF1	-0.532943562	0.595428449	2.739693616	0.008172788	2.822476734	0.006576586
ABCF2	-0.263711213	0.792624724	1.924550781	0.059230181	2.62920692	0.011016744
ABCF3	-0.254124848	0.799997461	1.878688843	0.065350935	2.243397171	0.028827837
ABCG1	-3.629692894	0.000477873	0.553801653	0.581858305	0.525669148	0.601184846
ABCG2	-1.590268658	0.11539387	0.108417165	0.914041601	-0.019586531	0.984442537
ABHD1	0.106247431	0.915630115	-0.547621575	0.58606774	-0.354158325	0.724544724
ABHD10	1.36403319	0.17606805	-0.137515134	0.891103237	0.176918073	0.860208379
ABHD11	-0.188266325	0.851105377	-1.393659025	0.168775039	-0.896021625	0.374060717
ABHD12	-0.504364458	0.61527754	1.282429957	0.204829279	1.611364739	0.112699215
ABHD12B	-0.499341065	0.618796823	-1.254687586	0.214659744	-1.550228281	0.126696588

ABHD13	0.201507207	0.840771377	-0.677875803	0.500564661	-1.392496857	0.169253587
ABHD14A	0.47615649	0.635153916	-0.422102103	0.674521959	0.635253628	0.527839445
ABHD14B	1.070986723	0.287131133	0.990352714	0.326144129	1.953658462	0.05572255
ABHD15	2.745242957	0.007342409	-1.800155516	0.077073568	-1.710237663	0.092734218
ABHD16A	-3.173454496	0.002081284	-0.510651043	0.61154715	-1.876682572	0.06575346
ABHD16B	0.720039742	0.473426386	-2.649684737	0.010382311	-2.137592836	0.036910789
ABHD2	-3.112646137	0.002507631	0.54633506	0.586945843	0.561942193	0.57638834
ABHD3	-0.515628698	0.607418797	-1.896981369	0.062848229	-2.525971587	0.014380674
ABHD4	-3.586647964	0.000551978	-0.193545715	0.847212552	-0.398759834	0.691580282
ABHD5	-4.35135743	3.66E-05	-0.961487245	0.340326476	-1.432864353	0.157432501
ABHD6	0.577249884	0.565258269	-0.421961897	0.674623709	0.393007395	0.695799889
ABHD8	0.447218428	0.655826394	-1.807315618	0.075936474	-0.299357413	0.765770936
ABI1	-0.107242795	0.914842753	-1.439819902	0.155331756	-2.786804158	0.00724544
ABI2	1.996278596	0.049023767	0.187274039	0.852103927	0.13440078	0.893565522
ABI3	-0.433143774	0.665979992	-0.025014389	0.980129952	0.363126719	0.717871614
ABL1	0.064231578	0.94893287	0.55376321	0.581884445	0.932210451	0.355212818
ABL2	0.164562456	0.8696693	1.046484585	0.299709269	0.569830701	0.571061969
ABLIM1	1.299291299	0.197269186	1.268201382	0.209828379	2.189799279	0.032703082
ABLIM2	1.186972998	0.238462274	1.61676414	0.111393769	2.059634949	0.044073626
ABLIM3	-5.266199015	9.91E-07	1.488794304	0.141996893	0.341356512	0.734107398
ABO	2.528257391	0.013263901	-0.38767033	0.699689008	0.046988035	0.962689232
ABP1	-0.637823194	0.525260355	-0.939657511	0.351317405	-0.820137194	0.415600583
ABR	-1.691777062	0.094259781	0.57914803	0.564747559	0.351236569	0.726723393
ABT1	-0.097236587	0.922761689	-2.062403941	0.043689514	-1.673572784	0.099768702
ABTB1	-2.39832147	0.018602663	-0.173023609	0.863239483	0.075345856	0.940206931
ABTB2	-0.405549021	0.686067462	-0.860306921	0.393186878	-1.965922003	0.054251868
ACAA1	1.531501243	0.129266016	-0.029073717	0.976906306	-0.276511735	0.783168775
ACAA2	-0.056247455	0.955273375	4.187235486	9.79E-05	3.94773127	0.000221786
ACACA	2.012464294	0.047257038	1.053886405	0.296336011	1.089573194	0.28054816
ACACB	3.896040431	0.000191301	1.190096639	0.23889077	1.551876688	0.126301732
ACAD10	1.616005228	0.109706074	2.684191212	0.009477657	3.46319857	0.001027642
ACAD11	1.218456182	0.226337388	-0.475441747	0.636270349	-1.881555919	0.065075884
ACAD8	-0.7021556	0.48445241	0.411065375	0.682550059	-0.343350338	0.732615222
ACAD9	0.286127905	0.775458899	1.309515267	0.195559602	4.100833176	0.000133945
ACADM	1.379136328	0.171380263	0.29312805	0.770477818	-0.060493376	0.951977161
ACADS	-0.092560167	0.926465339	0.680481893	0.498925791	-0.076945799	0.938939768
ACADSB	0.900471306	0.370349986	-0.887535467	0.378481962	-1.943215551	0.057001652
ACADVL	0.60677732	0.545575507	2.601732499	0.011770564	2.242608777	0.028881764
ACAP1	-0.423473444	0.672992686	1.266944665	0.210274237	2.440537201	0.017840394
ACAP2	0.198670764	0.842982829	-0.802689679	0.425453993	-2.193378801	0.032430673
ACAP3	0.460836243	0.646063541	-0.836074073	0.406568457	-0.094725975	0.924869202
ACAT1	1.789205572	0.077054258	0.612004638	0.542943173	2.103869288	0.039874047
ACAT2	3.530832732	0.000664362	2.445553261	0.01754125	2.778848937	0.007402904
ACBD3	0.240138339	0.810786712	-0.987315599	0.32761752	-1.50170309	0.138768287
ACBD4	2.732839874	0.007601537	0.383989687	0.702400038	1.22561772	0.225450608
ACBD5	0.065614612	0.947834871	-2.240641935	0.028921252	-3.203648439	0.002236093
ACBD6	0.825073206	0.411581729	1.226901201	0.224851371	2.074756355	0.042596759
ACCN2	0.498054922	0.619699304	-1.294159868	0.200775336	0.208705981	0.835431877
ACCN3	2.692482889	0.008503439	-1.513288494	0.135674719	-0.026072925	0.979291473
ACCS	1.634126847	0.105838096	1.67188891	0.099971	0.993615149	0.324666417
ACD	-0.964758806	0.337333929	-0.381018513	0.704591337	-0.972470478	0.334980914
ACE	1.240849676	0.217989707	-0.250620485	0.802999419	0.458743795	0.648186247
ACER1	-1.001736715	0.31924283	2.038104484	0.046140671	1.757729392	0.084235458
ACER2	0.418740443	0.676435567	1.931631497	0.058330098	0.486651504	0.628396612
ACER3	0.921608143	0.359276154	0.009589145	0.992382224	0.090444783	0.928255144
ACHE	-2.029841942	0.04542112	0.12637791	0.899872927	0.375908653	0.708399153
ACIN1	-0.386745333	0.69988705	-0.635678274	0.527503897	-0.236031217	0.814265972
ACLY	-0.100902763	0.919859322	1.290207373	0.202134583	0.85826094	0.394391682
ACN9	-0.410313562	0.682582477	-1.147159945	0.256058518	-1.55325813	0.125971583
ACO1	-0.44831546	0.655037664	1.842080498	0.070613312	1.472776981	0.146386297
ACO2	-0.892103512	0.374792938	1.807834611	0.075854602	1.611997272	0.112561214
ACOT1	0.255021718	0.79930691	0.893867391	0.375112797	0.269275304	0.788703363
ACOT11	0.169109211	0.866102461	1.103514126	0.274393198	0.861225715	0.392770972
ACOT13	-0.691310429	0.491207102	-1.956857827	0.055217574	-1.658101862	0.102865288
ACOT2	1.793241851	0.076401684	-2.37652821	0.020821882	-1.897737786	0.062868277
ACOT4	-0.854896626	0.394951408	-0.13217194	0.895308946	-0.293986906	0.769850255
ACOT7	-2.184244187	0.03162844	-0.108487084	0.913986387	0.697136682	0.488589266
ACOT8	-1.259155784	0.211334712	0.518347646	0.606201402	0.550027175	0.58447877
ACOT9	-1.63588628	0.105468486	2.129460965	0.03750214	1.379878949	0.173085556
ACOX1	-4.056502633	0.000108151	0.405001913	0.686976426	0.773565217	0.442427636
ACOX3	-0.21402689	0.831025821	1.740973523	0.087027882	1.940631059	0.057322058
ACP1	-0.899060447	0.371096753	-1.546034869	0.127573483	-0.754992918	0.453403596

ACP2	-1.208643304	0.23006759	0.603874938	0.548297942	0.169045468	0.866367454
ACP5	-1.386805315	0.169036606	-0.321890646	0.74870091	0.192971192	0.84767676
ACP6	0.933291937	0.353246674	-0.085036203	0.932527508	0.380329768	0.705133405
ACPL2	1.696401638	0.09337757	-0.076625665	0.939186896	-0.071495435	0.943257116
ACPP	-1.170709018	0.244905559	-0.399989804	0.69064364	-1.161247442	0.250446641
ACRBP	-4.213418248	6.11E-05	0.227103823	0.821147178	0.351354199	0.726635635
ACRC	3.300965767	0.001397378	0.651515978	0.517304737	0.831086188	0.409439717
ACSBG1	-1.025822388	0.307812949	0.232834565	0.816715305	-0.949090723	0.346635383
ACSF2	0.308157132	0.758697773	1.110097612	0.271570132	0.414809593	0.679859497
ACSF3	1.326205919	0.188235165	2.442686296	0.017667673	2.15472859	0.035480229
ACSL1	-0.121111266	0.903881584	-0.334046705	0.739557566	-1.072403018	0.288119551
ACSL3	-0.730331453	0.467145481	0.156194359	0.876426003	-1.042123898	0.301813661
ACSL4	0.093647861	0.925603755	-0.359865683	0.72026378	-1.916475547	0.06039173
ACSL5	0.17103274	0.864594321	-0.631178516	0.530420735	-0.303701608	0.762476049
ACSL6	1.876104246	0.063986751	0.129956957	0.897053286	0.87785868	0.383755548
ACSM1	1.091467362	0.278074266	0.793737235	0.43060643	0.476117182	0.635835453
ACSM3	1.489633431	0.139929254	0.809848377	0.421360618	-0.006532709	0.994810809
ACSS1	0.606660571	0.545652645	2.442092085	0.017693978	2.102654796	0.039984524
ACSS2	-1.672515565	0.098007596	2.16474389	0.034564763	2.43146387	0.018248692
ACTA1	-0.829917601	0.408851966	-0.584626385	0.561082146	-0.655894084	0.514566867
ACTA2	-1.231960235	0.221276103	1.454879258	0.151131257	-0.095104713	0.924569729
ACTB	-2.183077908	0.031717118	1.455356542	0.150999595	1.552240217	0.126214786
ACTG1	-1.578482349	0.118076244	2.21997775	0.030370964	2.60850582	0.011627424
ACTL6A	1.729134508	0.087324242	0.50533464	0.615252171	1.53817247	0.129614482
ACTN1	-1.85311719	0.067248141	0.594208797	0.554699367	0.427171285	0.670885689
ACTN3	-0.156886537	0.875696995	-1.693212342	0.095817644	-0.013872653	0.988980678
ACTN4	-1.881080944	0.063298436	-0.559880878	0.577731763	-0.665811473	0.508253538
ACTR10	-0.49006099	0.625321645	0.602655711	0.54910331	1.038702067	0.303388763
ACTR1A	-1.894443286	0.061481051	1.165380137	0.248668791	1.803652046	0.076643166
ACTR1B	2.682663797	0.008737117	-0.495547386	0.622099289	-0.601189298	0.550129617
ACTR2	-0.549425885	0.584117739	-0.154479599	0.877771643	-1.445596836	0.153840292
ACTR3	-0.263764767	0.792583589	0.438082232	0.662965309	-0.204098078	0.839013671
ACTR3B	-2.444423999	0.016521562	1.525989324	0.132485474	1.511410981	0.136283044
ACTR3C	-0.646528747	0.51963529	-2.084071896	0.041599791	-3.15852707	0.002550953
ACTR5	2.748025333	0.007285397	1.551865285	0.126172404	2.701407853	0.009108873
ACTR6	0.486615897	0.627751549	0.042816719	0.965995726	0.115271067	0.908640879
ACTR8	1.10861122	0.270646616	0.895153004	0.374431063	0.692385435	0.491544507
ACVR1	-1.781585343	0.078298871	2.402828775	0.019512096	1.241581295	0.219544674
ACVR1B	0.30642306	0.760013099	1.040459395	0.302474485	0.585375882	0.560636726
ACVR1C	1.692168722	0.094184803	0.078367853	0.937807073	-0.23839849	0.812438597
ACVR2A	2.788587576	0.0064989	-0.142814181	0.886935378	0.642084844	0.523426959
ACVR2B	2.47606404	0.015216274	0.36228371	0.718465958	0.654763557	0.515289201
ACVRL1	-2.836486468	0.005670565	0.883021673	0.38089535	0.88407435	0.380420171
ACY1	1.471667268	0.14471108	-0.857099695	0.394942053	-1.798202038	0.077513148
ACY3	0.731855091	0.466219634	0.469271639	0.640647094	0.68218633	0.497921422
ACYP1	2.175725509	0.032281169	-1.123268915	0.26598357	-0.3632096	0.717810047
ACYP2	-2.240755362	0.027582905	-0.301110254	0.764414795	-0.328807333	0.743522656
ADA	1.605071375	0.112094311	-0.077676206	0.938354839	0.175784255	0.861094885
ADAL	1.514676782	0.133471223	0.655280389	0.514896031	1.560047522	0.124359017
ADAM1	1.627406618	0.107259464	0.221292242	0.825647559	0.509574116	0.612343794
ADAM10	0.406097139	0.685666198	-1.196838827	0.236272483	-2.752000646	0.007957888
ADAM11	2.897800595	0.004751674	0.943525955	0.349353053	0.438409543	0.662769001
ADAM12	0.45565145	0.649773423	-2.611249649	0.011482279	-1.263377383	0.211666173
ADAM15	1.01902724	0.311009344	2.567656834	0.012857568	2.403368679	0.019566771
ADAM17	0.991877226	0.324002162	0.542460515	0.589594163	-0.345570275	0.730955045
ADAM19	-0.832450527	0.407429064	0.190635421	0.849481595	0.261862363	0.794384324
ADAM22	2.023232264	0.046112087	-2.023598274	0.047659895	-1.458496864	0.150266385
ADAM23	1.992056481	0.049493795	-0.394706243	0.694517549	0.454597409	0.65114885
ADAM28	2.266510406	0.0258949	0.191758008	0.848606205	0.783553201	0.436589983
ADAM8	-0.555552134	0.579939791	-0.045686701	0.963718	-0.746284071	0.458604278
ADAM9	0.098047164	0.922119895	1.534237795	0.130446349	-0.340388339	0.73483235
ADAMTS1	1.436610012	0.154407505	-0.995183125	0.323809877	-0.155150757	0.877258003
ADAMTS10	2.373345719	0.019824903	-4.082532191	0.00013906	-2.058245057	0.044211574
ADAMTS13	1.444055464	0.152307251	-0.11090034	0.912080925	0.823664214	0.413609858
ADAMTS14	-0.669956443	0.504656072	-1.92812712	0.058774098	-1.953114286	0.055788592
ADAMTS17	0.798799174	0.426577475	2.357723277	0.021806033	0.944631048	0.348888228
ADAMTS4	1.296075663	0.198369767	0.738709306	0.463083231	-0.020429011	0.983773453
ADAMTS5	-0.900691261	0.37023365	-0.251435704	0.802372205	-1.40351356	0.165961614
ADAMTS6	0.101239052	0.919593149	-1.15615259	0.252391944	-1.067222243	0.290431614
ADAMTSL2	1.305133011	0.195281449	0.527110927	0.600141036	1.897432149	0.062909377
ADAMTSL4	-1.096010762	0.276092204	-0.461752514	0.645998135	-1.2353766	0.221826493
ADAMTSL5	1.681632689	0.096218797	-0.283002833	0.778189318	0.711069982	0.479979836

ADAP1	-0.547950108	0.585126307	-0.558162792	0.578896556	-0.678606147	0.50017058
ADAP2	1.061557307	0.291368341	1.621430765	0.110387483	1.710470925	0.092690808
ADAR	-2.162313796	0.033332614	0.671527342	0.504569196	-0.722200029	0.473164015
ADARB1	2.515492005	0.013719388	0.927105869	0.357740331	1.642772096	0.106010596
ADARB2	-1.483565615	0.141530223	1.281000361	0.205327505	-0.347059765	0.729841856
ADAT1	0.756683558	0.451279334	-0.442445254	0.659824126	-0.391638415	0.696805513
ADAT2	1.753980678	0.08294766	-0.818544317	0.416420264	1.045262862	0.300373689
ADAT3	1.295755119	0.198479726	-3.154472743	0.002555277	-2.463780629	0.016831682
ADC	0.578170922	0.564639101	1.476933224	0.145140685	1.701164595	0.094435754
ADCK1	-1.493022311	0.1390413	-1.302657805	0.19787613	0.579392608	0.564638139
ADCK2	0.877137704	0.382822329	1.932268699	0.058249673	2.258478484	0.027813271
ADCK3	-0.896522881	0.372442275	1.629927303	0.108574263	1.84765538	0.069914143
ADCK4	-0.626988948	0.53230488	-0.766982586	0.446224437	-0.978481335	0.332026959
ADCK5	1.258164004	0.211691392	0.367751956	0.714406182	0.495481265	0.622191123
ADCY3	-0.669646921	0.504852454	1.140260666	0.258897192	0.553449973	0.582149104
ADCY4	-1.887264804	0.062451843	0.001838968	0.998539072	0.059806491	0.952521782
ADCY5	1.075369421	0.285176193	0.365870018	0.715802456	0.505010583	0.615524801
ADCY6	-0.335109101	0.738347705	0.719213931	0.474917179	1.516037753	0.135111058
ADCY7	1.849484523	0.067776065	-1.42272492	0.160209661	-1.904839131	0.061919735
ADCY9	0.981663619	0.328981763	-1.312638472	0.194511335	-0.301225944	0.764353198
ADD1	-1.219648223	0.225887261	1.375345843	0.174350154	1.666227057	0.101229338
ADD2	1.23658584	0.219561552	-0.236553309	0.813842615	0.2577321	0.797554469
ADD3	1.670157764	0.098474574	0.291370155	0.77181501	-0.394194062	0.694928631
ADH5	2.321961102	0.022565964	0.167925204	0.867230379	1.350899808	0.182138025
ADHFE1	2.713534701	0.008021491	-2.220455059	0.030336769	-2.580994523	0.012486718
ADI1	-1.014433568	0.313182754	1.763279518	0.083157563	1.530520309	0.131494182
ADIPOR1	-4.00691708	0.000129183	-0.798501966	0.427859553	-0.989148901	0.326827209
ADIPOR2	-1.500270696	0.137156901	1.425096708	0.159525876	1.915788443	0.060481055
ADK	1.965648391	0.052521763	0.872363038	0.386632576	0.734622431	0.465621777
ADM	-1.56922646	0.120217425	0.37135078	0.711738826	-0.914741679	0.364232853
ADM2	0.551191641	0.582912076	2.582896388	0.012360631	1.959194477	0.055054483
ADNP	0.050279703	0.960014505	0.507378511	0.613826591	0.101613035	0.919425269
ADNP2	-0.325911175	0.745272483	0.2700741	0.788068594	-0.486299047	0.62864488
ADO	1.001309506	0.319448081	-1.735449987	0.088008988	-2.222486168	0.030288668
ADORA1	0.431575551	0.667115234	1.160415589	0.250666972	0.348390212	0.728848024
ADORA2A	-0.103477066	0.917821994	1.454990152	0.151100658	2.884978873	0.005540408
ADORA2B	0.129479084	0.897276809	0.286486651	0.775533422	-0.436488058	0.664153924
ADORA3	3.267815336	0.001551392	-0.086533464	0.931342485	-0.734518466	0.465684613
ADPGK	-0.691997094	0.490777907	-0.177601058	0.859659423	-0.50300105	0.616927905
ADPRH	-1.734652842	0.08633617	1.951094089	0.055915923	1.396618552	0.168016116
ADPRHL1	-1.221380295	0.225234371	0.028191478	0.97760689	-0.507507538	0.613783376
ADPRHL2	-1.144378475	0.255598651	0.648401739	0.519301938	0.310325263	0.757460802
ADRA2A	-2.53128783	0.013157788	1.290979323	0.201868573	-0.105344759	0.916477099
ADRB1	0.33853523	0.735773775	-2.670440134	0.009829157	-2.042044907	0.045847253
ADRB2	1.013523224	0.313614671	-3.997643505	0.000184293	-4.072093905	0.000147342
ADRBK1	-0.82250305	0.41303444	0.245055649	0.807284358	0.461027449	0.646557006
ADRBK2	0.774567573	0.440690684	1.668611822	0.100622183	0.749193861	0.456862821
ADRM1	-1.119806678	0.26587145	-0.111425284	0.911666507	-0.015974851	0.987310996
ADSL	1.015505599	0.312674635	2.246380008	0.02852969	3.306921876	0.001647622
ADSS	0.865865049	0.388940545	1.182736368	0.241772999	1.661261684	0.102226533
ADSSL1	-0.061798366	0.95086485	-0.087610056	0.930490501	1.496873267	0.140018015
AEBP1	1.977466486	0.051147746	0.972592791	0.334822714	1.507497788	0.137280554
AEBP2	0.102484583	0.91860739	-1.351023338	0.181971499	-1.83780248	0.071375965
AEN	3.053053125	0.003003133	1.915683275	0.060374054	2.457832474	0.017084804
AES	0.771204693	0.442670626	-0.9264388	0.358083794	0.260827486	0.795178309
AFAP1	0.78692351	0.433460454	0.204397815	0.838763193	0.06540144	0.94808632
AFAP1L2	-1.526673675	0.130461797	1.008068921	0.317637702	-0.108667337	0.913853157
AFF1	-0.541959144	0.589229056	-0.091494702	0.92741699	-2.097445164	0.040461455
AFF2	-0.832134731	0.407606302	0.171460536	0.864462639	0.975147184	0.333663341
AFF3	1.340651007	0.18351645	1.303705843	0.197520764	0.700140411	0.486726054
AFF4	0.669291279	0.505078148	-1.326384536	0.189947881	-2.400061954	0.019727394
AFG3L1P	3.438853612	0.000898013	1.498724607	0.139406345	2.446203938	0.017589579
AFG3L2	0.358693487	0.720691498	3.543290585	0.00079146	3.977357834	0.000201305
AFMID	-1.059848459	0.292140785	2.432571898	0.01812025	1.553984242	0.125798328
AFTPH	-1.960606113	0.053117482	1.330400503	0.188630045	0.695549132	0.489575615
AGA	1.888821848	0.062240185	1.153750402	0.253367694	0.634166095	0.528543708
AGAP1	0.513696005	0.608763941	-3.089649146	0.003083615	-1.046718419	0.299707563
AGAP2	-1.299349682	0.197249246	-0.595830559	0.553622738	-0.023120119	0.981636299
AGAP3	0.607712571	0.544957771	1.396809533	0.167829915	0.195683445	0.845563288
AGAP4	-0.241137684	0.810014573	0.217780087	0.82837017	-0.142798114	0.886960035
AGAP5	1.398163985	0.165610424	-0.47694856	0.635203465	1.555577744	0.125418775
AGAP6	2.127823305	0.036174511	-3.291150054	0.001706973	-1.686159953	0.097306006

AGAP7	-1.347394972	0.18134419	-1.005937429	0.318653169	0.875901937	0.384809345
AGAP8	0.041337245	0.967121555	-0.358595849	0.721208548	-0.173897646	0.862570377
AGBL2	1.788419492	0.077181885	-0.785125891	0.435597446	0.361436228	0.719127787
AGBL3	0.428105286	0.669630126	0.383435317	0.702808704	-0.237373072	0.813230022
AGBL5	1.13218205	0.26066204	-1.22353924	0.226108161	-0.151674099	0.879986797
AGER	-0.48597425	0.628204573	0.006698427	0.994678616	1.670238884	0.100429467
AGFG1	0.675286811	0.501280555	-0.252468798	0.80157755	-0.815480956	0.418237543
AGFG2	0.080258256	0.936215792	-0.1124341	0.910870164	0.673788121	0.503206096
AGGF1	1.196228502	0.234850327	0.148917843	0.882138648	-0.61555102	0.540674143
AGK	4.239158149	5.56E-05	-0.898495546	0.372662263	0.957495995	0.342415298
AGL	1.010761059	0.314927633	-0.503136424	0.616787071	-1.400230527	0.166937425
AGMAT	-0.331604775	0.740983472	1.007489221	0.317913662	2.441407425	0.017801669
AGPAT1	-2.66829866	0.009089508	0.212850068	0.83219547	0.408139657	0.684720965
AGPAT2	-1.234157084	0.220460594	0.565946068	0.573628886	-0.183262151	0.855251464
AGPAT3	-0.333501213	0.739556687	1.408319597	0.164411647	1.014246565	0.314809027
AGPAT4	-1.733785463	0.086490865	0.105426977	0.91640332	0.132761951	0.894855553
AGPAT5	0.952821569	0.343314562	0.733147479	0.466442035	0.130474058	0.896656982
AGPAT6	1.503023539	0.136446503	2.410550917	0.01914181	3.389396766	0.001286158
AGPAT9	-3.295266533	0.001422792	1.969665469	0.053692433	1.007819685	0.317857807
AGPHD1	1.152489211	0.252270183	-0.927198767	0.357692517	0.106990335	0.915177418
AGPS	0.679328911	0.498728983	-0.417745316	0.677686604	-0.957804602	0.342260999
AGRN	0.582091418	0.562007268	0.391328744	0.696998239	-1.514858422	0.135409026
AGTPBP1	0.778241112	0.438533752	-0.096195733	0.92369905	-0.269500192	0.788531196
AGTRAP	-2.953894568	0.00403317	-0.358875855	0.721000184	-0.530459636	0.597881814
AGXT2L2	1.657414076	0.101029919	-0.560581778	0.577256906	0.368824899	0.713643187
AHCTF1	-0.39212673	0.695921459	0.576762883	0.56634708	-0.381033122	0.70461437
AHCY	-0.351399158	0.726136679	2.101988777	0.039937968	2.116781513	0.038715819
AHCYL1	-0.656377954	0.513309587	-0.034027241	0.972973078	-0.503861283	0.616327094
AHCYL2	0.071161929	0.943431857	-0.32901265	0.743339504	0.042220863	0.966472179
AHDC1	0.88001293	0.381271458	-0.589767415	0.557653208	-0.216639989	0.829272895
AHI1	2.44730819	0.016398563	0.080190973	0.936363357	0.225898113	0.822099659
AHNAK	-0.088707463	0.929517842	2.648406737	0.010417283	1.940844683	0.057295517
AHNAK2	1.086550423	0.280230361	1.458203764	0.15021603	1.188690399	0.239556908
AHR	0.020889133	0.983381827	-0.14146426	0.887996831	-1.374341261	0.174788196
AHRR	2.348918609	0.02108876	0.510624815	0.611565403	0.8799101	0.382652713
AHSA1	-0.829485561	0.409094969	2.700562325	0.009074202	2.744988543	0.008109008
AHSA2	2.996184534	0.003559333	-0.873069854	0.386250451	-0.813216404	0.419523671
AHSP	-4.023569603	0.000121717	-0.244957236	0.80736019	-0.898327385	0.372841193
AIDA	0.18008974	0.857500024	-0.663593775	0.509597864	-0.653399666	0.516161357
AIF1	-0.333683402	0.739419664	0.199702868	0.842416362	0.337181859	0.737235038
AIFM1	0.103226077	0.918020605	2.120138158	0.038313803	2.384877579	0.020480245
AIFM2	2.057975609	0.042578509	-0.338309736	0.736359919	0.247398527	0.805500829
AIFM3	0.863537901	0.390211101	-0.564540388	0.574578514	-0.299443683	0.765705462
AIG1	-0.855673165	0.394523982	1.902581572	0.062098479	1.372278818	0.175425594
AIM1	2.058075137	0.042568731	1.255060872	0.214525193	1.292970763	0.201307793
AIM1L	-0.777223309	0.43913074	-1.531945783	0.13101044	-0.596728059	0.553083746
AIM2	-0.607287053	0.545238783	-0.597625022	0.552432684	-1.841405525	0.070838444
AIMP1	1.175723338	0.242905921	0.308924204	0.758493889	0.482275954	0.631481774
AIMP2	-1.492190995	0.139258712	0.330365573	0.742322464	0.628491207	0.532226596
AIP	-0.201680375	0.840636407	1.167057401	0.2479963	1.267993268	0.210024953
AIRE	-0.203031606	0.839583396	-0.565579134	0.5738767	-2.007752549	0.049483031
AJAP1	1.980094335	0.050846418	-0.279985593	0.780491669	-1.309520137	0.195683119
AK1	-0.584696742	0.560261653	-0.344922257	0.731409224	1.070795856	0.288835423
AK2	1.548021909	0.125239294	0.239776353	0.811354925	1.522107038	0.133585817
AK3	1.716428456	0.089634609	-0.168437051	0.86682956	-0.615945164	0.540415827
AK4	0.686324978	0.49432937	0.20034658	0.841915278	0.555115545	0.581017075
AK5	2.652661513	0.009487734	1.77849968	0.080599694	2.365000378	0.021504611
AKAP1	3.10823485	0.002541526	2.034975833	0.046464746	2.448760175	0.01747748
AKAP10	0.584751027	0.560225309	-0.853112389	0.3971309	-1.628700448	0.108966287
AKAP11	1.3335863	0.185812991	-1.177576018	0.243808676	-1.976171854	0.053048308
AKAP12	-3.304580748	0.001381479	2.357418394	0.021822326	-0.393848562	0.695182256
AKAP13	-0.577687031	0.564964356	0.570790707	0.57036188	0.288031087	0.774381819
AKAP17A	2.115620928	0.037229099	-3.265877773	0.001840504	-2.3994759	0.019755984
AKAP3	1.332810667	0.186066439	1.525105966	0.132705345	2.201546111	0.031816584
AKAP5	-0.56192862	0.575606386	-1.350753573	0.182057428	-1.64188782	0.106194393
AKAP6	1.211301395	0.229052796	1.13549523	0.260870929	2.009837682	0.04925507
AKAP7	1.514036606	0.133633334	0.634866356	0.528029578	-0.128853818	0.897933049
AKAP8	0.495661698	0.62138017	-0.10733003	0.914900156	0.280131512	0.78040449
AKAP8L	-0.545135116	0.587052396	-1.325484603	0.190244146	0.020073841	0.984055522
AKAP9	0.184250294	0.85424497	-0.613503216	0.541959035	-0.18158018	0.856565101
AKD1	1.483380611	0.14157926	-1.550532093	0.126491687	-1.55110397	0.126486705
AKIP1	-1.023568868	0.308870531	0.177092156	0.860057295	-0.504476826	0.615897342

AKIRIN1	-0.042433629	0.966250046	-1.174998946	0.244829904	-2.29531553	0.025466908
AKIRIN2	-1.650592049	0.102419792	-1.182164642	0.241997929	-2.389593829	0.0202437
AKIRIN2-AS1	0.229395919	0.819098478	-2.003350036	0.049852508	-2.686206086	0.009483422
AKNA	-1.427414586	0.157032214	-1.172847794	0.245684707	-0.965861088	0.338249019
AKR1A1	0.32585588	0.745314176	2.061848224	0.043744284	2.730678168	0.008425629
AKR1B1	0.765205805	0.446215371	2.549696472	0.013466469	3.894808801	0.000263472
AKR1C1	-0.081912093	0.934904367	2.770342556	0.007525022	2.694457235	0.009278411
AKR1C2	1.087677315	0.279735197	1.234801923	0.221918049	0.957274093	0.342526274
AKR1C3	0.788324246	0.432645223	0.068737245	0.945436853	0.928475011	0.357129355
AKR1E2	0.80125114	0.425164463	0.811857924	0.420215825	0.024413255	0.980609397
AKR7A2	-0.38713249	0.699601472	2.052234225	0.044701289	2.583662921	0.012400913
AKR7A3	0.009843691	0.992168486	-0.39694983	0.692871543	-0.425962621	0.671761007
AKR7L	0.909041513	0.365834207	-0.192951355	0.847675846	1.117284707	0.268623261
AKT1	-1.296247896	0.198310703	-1.492621939	0.140993907	-1.791912823	0.078527343
AKT1S1	-1.12007829	0.265756338	-3.11739146	0.00284608	-3.09504775	0.003064908
AKT2	-0.852827043	0.396091946	-1.20593114	0.23277461	-0.553106546	0.58238265
AKT3	2.299726952	0.023852435	-0.225919805	0.822063575	0.421564916	0.674949686
AKTIP	-0.630323754	0.530131385	-0.231778625	0.817531469	0.870065633	0.387963242
ALAD	-0.413941313	0.679933592	-0.356151789	0.723028179	0.062878258	0.950086406
ALAS1	-1.067282952	0.288790389	0.415133217	0.679586764	0.095205404	0.924490113
ALAS2	-2.867858164	0.005181777	0.594478709	0.554520109	0.824495799	0.41314134
ALB	1.337445916	0.184555672	0.278224326	0.781836546	-0.761095171	0.449779951
ALCAM	1.040275002	0.301088305	0.929019989	0.356755965	-0.676284772	0.501631877
ALDH16A1	-0.075411212	0.9400603	1.693301579	0.095800565	1.918842998	0.060084814
ALDH18A1	1.007567374	0.316450288	2.461501599	0.016852729	2.664768829	0.010035592
ALDH1A1	-0.853434954	0.395756719	1.084789496	0.282534722	1.271335682	0.208842444
ALDH1B1	1.784803149	0.07777129	0.963474381	0.339337332	2.09562092	0.040629628
ALDH1L2	0.349666808	0.727431953	2.864168772	0.005824346	1.189305462	0.239316832
ALDH2	-2.577790857	0.011622503	1.619077573	0.110893988	0.812889263	0.419709665
ALDH3A2	2.131652603	0.035848949	2.289509647	0.025733565	2.886632662	0.005515167
ALDH3B1	-1.308802743	0.194040427	0.489852899	0.626098677	-0.493111378	0.623853982
ALDH4A1	-0.186438792	0.852533784	2.03241942	0.046731004	2.280883811	0.026364351
ALDH5A1	0.750772145	0.454811273	1.557448718	0.12484223	2.31475511	0.024300984
ALDH6A1	0.632136521	0.528951822	2.459293751	0.016946572	1.756713631	0.084410218
ALDH7A1	1.566277614	0.120906046	1.747356981	0.085905336	1.946527997	0.056593237
ALDH8A1	0.423699813	0.672828194	0.478555094	0.63406683	1.150751405	0.254704083
ALDH9A1	-2.010741573	0.047442452	0.365047868	0.716412743	0.736750361	0.464336723
ALDOA	-2.011361804	0.047375626	1.219814974	0.227506387	1.290863069	0.202032748
ALDOB	0.532242598	0.595911719	0.135369311	0.892791876	-1.843193986	0.070572901
ALDOC	-0.115701546	0.908155146	2.31860603	0.023987289	2.25206575	0.028240748
ALG1	1.764676572	0.08112009	2.287548369	0.025855236	2.406509352	0.019415302
ALG10	-0.241709537	0.809572817	-1.176937668	0.244061351	-1.877591392	0.065626649
ALG10B	1.378553219	0.171559469	-0.902141142	0.370739162	-1.744031853	0.086617561
ALG11	0.180756023	0.856978584	-1.97823589	0.052692139	-2.426465765	0.018477176
ALG12	0.763799922	0.44704848	-1.943017641	0.056907176	-1.271705508	0.208711908
ALG13	2.145786745	0.034669192	-1.039058885	0.303119726	-1.708642431	0.093031538
ALG14	-0.473097957	0.637325586	-0.599582456	0.551136021	0.108905479	0.913665126
ALG2	1.753637142	0.083006916	0.113032423	0.910397902	-0.368968267	0.713536914
ALG3	-0.104536084	0.916984036	-0.651045545	0.517606169	0.238918788	0.812037102
ALG5	0.83304842	0.407093629	-0.253238019	0.800986002	0.630182953	0.531127293
ALG6	1.277349042	0.204870007	-0.785537014	0.435358388	-1.491588567	0.141395614
ALG8	0.314995186	0.753517937	0.269773052	0.788299059	1.386874363	0.170952953
ALG9	0.52443004	0.601310251	-1.59110738	0.117059657	0.311914612	0.75625894
ALKBH1	0.488585846	0.626361593	-0.299553024	0.765596467	0.66435633	0.509177251
ALKBH2	1.975743089	0.051346188	-1.921263755	0.059652023	-0.207165902	0.836628618
ALKBH3	1.560443227	0.122277753	2.055547544	0.044369445	3.218941618	0.002137942
ALKBH4	-1.739642023	0.085450768	0.426639775	0.671232194	1.314895096	0.193881999
ALKBH5	0.361906911	0.718297238	-0.885714019	0.37945467	-0.847743279	0.400174653
ALKBH6	-0.23404264	0.815500559	-2.147223884	0.035997145	-1.741501133	0.087063729
ALKBH7	0.773611086	0.441253303	-2.68113636	0.009554735	-1.935859453	0.057917649
ALKBH8	3.17448652	0.002074669	0.515841237	0.607939896	0.66786913	0.506948894
ALMS1	0.036174995	0.971225519	0.889140368	0.377626203	1.558300534	0.124772361
ALOX12	-3.225386843	0.001771748	0.75301664	0.454506869	-1.224037959	0.226041337
ALOX12B	0.254741504	0.799522645	-0.290663748	0.772352554	-0.591823373	0.556340706
ALOX12P2	1.535511704	0.12827923	-0.391162877	0.69712015	1.066034476	0.290963489
ALOX15	4.038619194	0.000115327	0.520810059	0.60449565	0.473565288	0.63764321
ALOX15B	-0.809198207	0.420603905	0.33052009	0.742206337	1.340742336	0.185394964
ALOX5	-1.318058735	0.190936429	-1.055421123	0.295639863	-1.438366964	0.155872122
ALOX5AP	-5.031377542	2.59E-06	-1.328752265	0.189170073	-0.509387339	0.61247384
ALPK1	0.227413274	0.82063481	-0.122046585	0.903286958	-0.791214619	0.432143173
ALPK2	-0.878611475	0.3820269	0.145165735	0.885086831	0.415657935	0.679242146
ALPK3	-0.851408514	0.396874859	1.761588114	0.083445939	0.173172855	0.863137358

ALPL	-0.80972869	0.420300522	-0.833268195	0.408135711	-0.955988876	0.343169494
ALS2	1.017412721	0.311772067	1.175148033	0.244770741	0.226703748	0.821476162
ALS2CL	1.29181967	0.199833435	1.135317371	0.2609448	2.309502578	0.024611237
ALS2CR11	-0.607037838	0.545403399	-0.032794648	0.97395173	-0.53547783	0.594430886
ALS2CR12	-0.493721164	0.62274457	-0.997163177	0.322856271	0.232666409	0.816865161
ALS2CR8	1.819354777	0.072289759	-2.12924161	0.037521063	-2.252466558	0.028213861
ALX3	1.400920965	0.164786891	0.170976006	0.864841867	0.72818011	0.469524631
AMACR	-1.393632956	0.166970718	-1.892964198	0.063390763	-2.461193287	0.016941367
AMBP	0.329934602	0.742240778	-1.490604626	0.141521821	-2.393295031	0.020059783
AMBRA1	-1.429171032	0.15652822	1.557379345	0.124858688	1.462181895	0.149257487
AMD1	-2.461043745	0.015823952	-0.227443269	0.820884502	-0.907215982	0.368163691
AMDHD1	0.657094372	0.512851061	-0.000171976	0.999863377	0.201420927	0.841096241
AMDHD2	0.598996102	0.550728688	0.262671466	0.7937411	0.24703638	0.805779694
AMFR	-0.685927418	0.494578816	4.136802421	0.000115983	4.146217001	0.000115156
AMH	2.84102012	0.005597405	-2.846698992	0.006111248	-2.281421881	0.026330394
AMICA1	-1.225921472	0.223529125	1.902058723	0.062168155	2.016403578	0.048543143
AMIGO1	1.381943883	0.170519414	1.358976443	0.179452002	1.069942621	0.289215977
AMIGO2	1.342809979	0.182818912	0.120688899	0.904357492	0.130877628	0.896339181
AMIGO3	2.20576759	0.030030303	0.497112578	0.621002009	1.388377081	0.170497485
AMMECR1	1.627742852	0.107187984	-0.970738453	0.335737584	-0.507858553	0.61353875
AMMECR1L	1.118783704	0.266305311	-0.050255408	0.960092736	0.409753142	0.683543717
AMN	1.565859604	0.121003914	-0.992196475	0.325251827	-0.775874481	0.441073885
AMN1	0.281182942	0.779236304	-0.900636118	0.371532314	-1.047896941	0.299168962
AMOT	-0.610780299	0.54293401	-1.339385646	0.185706655	-0.569844969	0.571052357
AMOTL1	0.042057999	0.966548628	1.954186604	0.055540293	1.562570903	0.123763914
AMPD2	-2.576157534	0.011673562	-1.052629402	0.296907024	-0.763849627	0.448149835
AMPD3	-0.321142946	0.748870605	-0.438193565	0.662885078	-0.938697741	0.351900263
AMPH	1.087470459	0.279826046	-1.927264238	0.058883865	-1.859666197	0.068166367
AMT	1.256972117	0.212120622	-0.22364933	0.823821556	0.747215113	0.458046651
AMY2B	2.323187668	0.022496815	0.956136613	0.342999299	0.651340638	0.517479515
AMZ1	0.411734679	0.681544339	0.519307296	0.605536375	-0.136608559	0.891828091
AMZ2	-0.06054193	0.95186258	-2.04593245	0.045338372	-0.236512217	0.813894588
AMZ2P1	0.843247406	0.401397553	0.391814954	0.696640924	0.375633676	0.708602453
ANAPC1	1.205102526	0.231424417	0.06800162	0.946019867	0.552951031	0.582488423
ANAPC10	0.209568282	0.834493539	-2.241562229	0.028858134	-1.42350324	0.16011493
ANAPC11	-0.596488509	0.552394541	0.299167292	0.765889258	0.542572855	0.589567772
ANAPC13	-1.141925401	0.256611427	0.109305766	0.913339914	-0.933855053	0.354371142
ANAPC16	-0.650636869	0.516991863	-1.430290227	0.1580365	-0.580429476	0.563943707
ANAPC2	0.727463429	0.468891061	-0.58353779	0.561809553	0.336674344	0.737615574
ANAPC4	1.515999702	0.13313671	1.304847122	0.197134326	1.09032591	0.280219445
ANAPC5	0.375499331	0.708201131	1.732356268	0.08856249	2.759617014	0.007796683
ANAPC7	0.703510785	0.483611975	1.334367431	0.187335106	2.873294352	0.005721821
ANG	-2.503020697	0.014177902	1.352580614	0.181476054	0.883095933	0.380943981
ANGEL1	2.867067381	0.005193607	-0.550001296	0.584445125	0.537634945	0.592950353
ANGEL2	1.392493582	0.167314118	-1.537348222	0.129683932	-2.035958186	0.046475202
ANGPT1	-1.406415864	0.163154879	-1.914803409	0.060488571	-1.805398808	0.076366066
ANGPT2	-1.062762486	0.29082441	1.629082138	0.108753537	0.795677789	0.42956514
ANGPTL1	-0.617334237	0.538623217	1.602148001	0.114593664	0.574706474	0.567781891
ANGPTL6	0.914227836	0.363118501	-0.69708148	0.488555965	-2.031067463	0.046985136
ANK1	-2.677071901	0.008872789	0.680121479	0.499152267	-0.041094377	0.967366209
ANK3	2.058161266	0.04256027	0.18161799	0.856520182	-0.287902604	0.774479665
ANKAR	1.29441956	0.198938363	-0.946380312	0.347908239	-0.862453398	0.392101068
ANKDD1A	-0.800198291	0.425770855	-0.451451154	0.653359754	-0.700506386	0.48649931
ANKFY1	-0.809003705	0.420715174	1.722054615	0.090426366	0.432248144	0.667214035
ANKH	0.483035974	0.630280908	0.182043768	0.856187572	0.411302304	0.682414142
ANKHD1	-2.526115066	0.013339379	0.18080362	0.857156428	2.114984482	0.038875234
ANKHD1-EIF4EI	-1.085859414	0.280534294	0.2089235	0.835245098	0.647212829	0.520127455
ANKIB1	0.815724557	0.416880593	-1.074031286	0.28728772	-2.16616441	0.034552761
ANKK1	1.452576135	0.149930911	1.215499727	0.229134386	1.943867042	0.056921126
ANKLE1	-0.056093224	0.955395886	0.828236658	0.410955365	1.131813821	0.262515864
ANKLE2	2.754722312	0.007149827	2.496032371	0.015444707	3.27446646	0.001814634
ANKMY1	0.694950957	0.488933953	-1.175996764	0.244434129	-1.466193891	0.148165116
ANKMY2	2.22178416	0.02888731	0.781749708	0.437563542	0.404325472	0.687507039
ANKRA2	0.463559569	0.64411849	-1.217000217	0.228567339	-1.940933481	0.057284487
ANKRD10	3.058346288	0.002955693	1.46524665	0.148291476	1.393113069	0.169068135
ANKRD11	1.131814952	0.260815529	-1.703846389	0.093799934	-1.641500967	0.106274881
ANKRD12	0.603994968	0.547415347	-0.339415404	0.735531333	-1.32869626	0.189314579
ANKRD13A	-2.752995108	0.007184569	-0.599703483	0.5510559	-0.454052572	0.651538559
ANKRD13B	-0.199235111	0.842542731	-2.95828488	0.004481801	-1.690052143	0.096554672
ANKRD13C	1.354163742	0.179183518	0.07170789	0.9430828	-0.947299402	0.34753914
ANKRD13D	-1.527228028	0.130324043	-1.007114958	0.318091912	-1.076990746	0.286082817
ANKRD16	1.988952207	0.049841828	-1.557722775	0.12477723	-0.281333047	0.779487551

ANKRD17	-0.042013629	0.966583897	0.108544705	0.913940885	-0.26673648	0.790647727
ANKRD18A	0.656573506	0.513184407	-0.335960079	0.738121793	0.403188061	0.688338708
ANKRD19P	-0.925622376	0.35719721	-0.463868942	0.644490046	-1.967525642	0.054062035
ANKRD20A5P	-0.587667111	0.558274717	0.734872693	0.46539869	-0.347303137	0.729660024
ANKRD20A9P	0.331287499	0.741222264	1.237807476	0.220809586	1.250861105	0.216164318
ANKRD22	-2.422714052	0.017474167	-0.083895855	0.933430153	-1.052663576	0.296997313
ANKRD23	2.265272228	0.025973924	-1.193229198	0.237671658	-1.101032828	0.275572762
ANKRD24	1.248238186	0.215285474	-0.785331903	0.435477645	0.346619929	0.730170512
ANKRD26	1.243548738	0.216998971	-0.132370889	0.895152295	-1.545330384	0.127875642
ANKRD27	1.420602177	0.158998811	2.855643516	0.005962761	2.363024393	0.021608906
ANKRD28	1.1542781	0.251540219	-0.484608845	0.629791727	-1.121194284	0.266970076
ANKRD31	-0.572632163	0.568367527	1.647721396	0.104855226	0.95109279	0.345627121
ANKRD32	0.047531626	0.962198232	-1.398465308	0.167334836	-1.429871546	0.158286262
ANKRD33B	-1.376121578	0.172308323	0.744287087	0.459728731	-1.221443283	0.22701404
ANKRD34A	1.819116977	0.072326356	-2.823592426	0.006510794	-1.826340913	0.073108734
ANKRD34B	0.290177516	0.772369461	0.421456222	0.674990737	-0.129918572	0.89709444
ANKRD35	-0.201030594	0.841142883	1.443954683	0.154169485	1.597827208	0.115685692
ANKRD36	1.363777254	0.176148318	0.489543096	0.626316585	0.407316834	0.685321627
ANKRD36B	0.546748899	0.58594784	-0.685058107	0.496055079	-1.186093636	0.240572417
ANKRD36BP1	-0.300612552	0.764425605	-0.853999502	0.396643267	-1.076614158	0.286249627
ANKRD36BP2	1.316136164	0.191578085	1.080635461	0.28436346	0.772279721	0.443182283
ANKRD37	1.62396609	0.107993087	-0.532050695	0.596737314	-0.48376443	0.630431519
ANKRD39	1.95660779	0.053593936	-2.250830787	0.028229207	-1.463207119	0.148977745
ANKRD40	2.622529751	0.010300015	-0.170069688	0.865551304	1.137731572	0.260056736
ANKRD42	1.318526967	0.190780401	-1.517615121	0.134581515	0.299963737	0.765310803
ANKRD44	0.982668445	0.328489638	-0.789856948	0.432851163	-2.047832801	0.045256954
ANKRD46	0.981184035	0.329216817	-0.540815381	0.590720349	-0.321753122	0.74883266
ANKRD49	0.477643418	0.634099295	-1.354623994	0.180827515	-1.499756345	0.139270946
ANKRD5	1.575796067	0.118694509	-0.15123997	0.880314894	0.98155767	0.330521842
ANKRD50	0.123771371	0.901781189	0.761902589	0.449226853	0.300547691	0.764867725
ANKRD52	2.452736802	0.016169271	-1.227487981	0.224632543	-0.091811778	0.927173856
ANKRD54	-0.964084851	0.33766976	1.49276056	0.140957688	1.221327697	0.227057442
ANKRD55	1.957420287	0.053496823	-0.066705539	0.947047138	-0.117370429	0.906984703
ANKRD57	0.371810555	0.710935996	0.213191593	0.831930342	-0.752782279	0.454720482
ANKRD58	-1.805123646	0.074507237	-2.71490536	0.008733723	-2.771938411	0.007542242
ANKRD6	0.631221799	0.52954686	0.086389754	0.93145622	-0.038669789	0.969290611
ANKRD9	-2.318245667	0.022776576	-0.679538604	0.499518651	-2.068003883	0.043250861
ANKS1A	-1.341309038	0.183303635	1.149265457	0.25519665	0.603607121	0.548531932
ANKS3	1.864254378	0.065651021	0.679657358	0.499443993	1.689967417	0.096570976
ANKS6	2.585527698	0.011383369	1.016290548	0.313741237	2.057006054	0.044334862
ANKUB1	-2.227058083	0.028519376	-2.141901065	0.036442488	-2.413458492	0.019083889
ANKZF1	1.060976653	0.291630656	1.267222452	0.210175623	2.649682666	0.010441632
ANLN	-0.260912025	0.794775623	1.393505987	0.168821053	0.464538428	0.644055541
ANO10	-2.197858912	0.030609135	1.62765534	0.109056731	2.82154981	0.006593219
ANO5	2.189346546	0.031243019	-0.072045099	0.942815614	-0.333144252	0.740264261
ANO6	-2.059091347	0.042469002	-0.128936032	0.897857455	-1.207821723	0.232170845
ANO8	2.169286326	0.032782322	-3.524447626	0.00083913	-2.374884295	0.020989655
ANO9	-0.006578977	0.994765803	0.591534705	0.556476891	1.354117049	0.181115573
ANP32A	-1.965255233	0.052568007	0.494334743	0.622950006	0.478763012	0.633963502
ANP32B	0.151032433	0.880299021	3.416007683	0.00117111	3.884374933	0.000272536
ANP32E	0.789868162	0.431747707	0.428774419	0.669686828	0.47511778	0.636543163
ANPEP	-1.970674889	0.051933573	-0.057336397	0.95447568	-0.010459662	0.991691567
ANTXR2	-2.967486996	0.003874869	0.251823989	0.802073512	-0.723425483	0.472416926
ANUBL1	0.031385414	0.975033908	-1.144087333	0.257319977	-2.474069522	0.01640181
ANXA1	-0.179020837	0.858336693	-0.018535341	0.985275845	-0.13142319	0.895909592
ANXA11	-2.270418162	0.025646885	0.823484588	0.413629278	0.883017419	0.380986035
ANXA2	-0.237212391	0.813048509	1.859912532	0.068007023	1.790131152	0.078816662
ANXA3	0.525245605	0.600745641	-0.645603425	0.52110001	-0.979518242	0.331519138
ANXA4	-0.580118302	0.563331069	0.451578816	0.653268309	-0.075847123	0.939809908
ANXA5	-1.74895304	0.083818368	1.761209333	0.083510634	0.912283476	0.365513854
ANXA6	-1.071777053	0.286777921	2.12636189	0.03777027	2.396341701	0.019909515
ANXA7	-1.099858656	0.274421241	1.929593531	0.058587957	1.853454949	0.069065548
ANXA9	-2.553346646	0.012408113	0.946237455	0.347980458	1.933103184	0.058264086
AOAH	-2.408023732	0.018146251	1.094893422	0.278120857	0.491404836	0.625052617
AOC2	-2.187265325	0.031399732	-1.935381514	0.057858146	-1.693208172	0.095948947
AOC3	-2.650343882	0.009548085	-1.578475584	0.119933302	-1.186188575	0.240535235
AOC4	-0.508670791	0.61226776	0.865880047	0.390148507	0.658479391	0.512917058
AP1AR	1.402235637	0.164395293	-0.481337003	0.632100703	-1.387232735	0.170844247
AP1B1	-1.19111409	0.236841317	2.602957417	0.011733094	2.232585416	0.029575177
AP1G1	0.056757777	0.954868019	0.326615609	0.745142573	0.753593109	0.454237211
AP1G2	-0.062811154	0.950060656	0.794187299	0.430346519	2.090554277	0.041099904
AP1M1	-1.194932648	0.235353646	1.223815443	0.226004715	1.220284023	0.227449617

AP1M2	-3.444528665	0.000881599	1.361439032	0.178677289	1.022193934	0.311066336
AP1S1	0.302148769	0.763258237	-1.067168958	0.290348282	-0.691528536	0.492078539
AP1S2	-0.256559842	0.798122994	0.212447043	0.832508367	-0.07249344	0.942466443
AP1S3	-0.430357033	0.66799786	-1.640399759	0.10637272	0.456765373	0.649599125
AP2A1	-1.813421093	0.073207567	0.021922618	0.982585455	-0.489443801	0.626431261
AP2A2	0.376206788	0.707677057	1.313141307	0.19434296	1.909777221	0.061267335
AP2B1	-1.39323512	0.167090562	1.243408448	0.2187548	1.885341277	0.064553668
AP2M1	-2.344586543	0.021320211	1.523268427	0.133163643	1.708387026	0.093079214
AP2S1	-1.432633564	0.155538337	0.25452294	0.799998132	0.575978856	0.566927447
AP3B1	-0.175292499	0.861256255	1.284310286	0.204175344	1.046404572	0.299851107
AP3B2	-0.911430866	0.364581476	1.950006445	0.056048548	1.470189945	0.14708333
AP3D1	-0.264456363	0.792052421	0.144075783	0.885943559	0.549145732	0.585079429
AP3M1	0.021664135	0.982765376	-1.957073585	0.055191578	-1.877804853	0.065596894
AP3M2	2.217271433	0.029205433	2.237426506	0.029142739	1.85472922	0.068880265
AP3S1	-0.698043762	0.48700734	-0.044316544	0.964805372	-0.504974417	0.61555004
AP3S2	-1.29871318	0.197466716	-2.194061991	0.032279391	-1.785458174	0.079579722
AP4B1	0.631396084	0.529433458	1.512619966	0.135844262	2.314551163	0.024312965
AP4E1	-0.676169389	0.500722829	0.218369258	0.827913298	-0.13902153	0.889929791
AP4M1	0.003084434	0.997546028	1.145515396	0.256733137	1.574893344	0.120890555
AP4S1	2.183596011	0.031677697	-1.271202004	0.208766661	-0.666220321	0.507994167
APAF1	-1.481368778	0.142113367	-1.264804013	0.211035316	-1.313579751	0.194321605
APBA1	2.191917051	0.031050406	0.289216916	0.773453879	1.092766728	0.279155376
APBA2	2.781593728	0.006628711	-0.938795631	0.351756034	0.169280946	0.866183106
APBA3	-0.431215631	0.667375891	-2.083536362	0.041650374	-2.066855006	0.043363014
APBB1	1.40418623	0.16381559	1.926326591	0.05900334	3.038635451	0.00360151
APBB1IP	-1.831226985	0.070482158	0.083011051	0.934130581	-0.12716548	0.899263037
APBB2	0.224249696	0.823087682	0.567271926	0.572733883	0.443662983	0.658988598
APBB3	1.131403366	0.260987695	0.322248016	0.748431584	0.913814183	0.364715844
APC	-1.03472127	0.303660528	-0.742165721	0.461002882	-2.098321424	0.040380891
APC2	0.91574642	0.362325763	-1.230925458	0.223353741	-0.639682752	0.524976324
APCDD1	1.836069928	0.069755705	1.258873365	0.213154551	0.601967709	0.54961499
APEH	-0.427159568	0.670316141	2.416668585	0.018852948	2.224223505	0.030164858
APEX1	0.577174216	0.565309152	-1.723933965	0.09008394	-0.510831712	0.611468501
APEX2	-0.235312202	0.814518231	2.423484148	0.018535746	3.122996125	0.002827701
APH1A	-1.385589667	0.169406471	0.464758126	0.643856893	0.573180335	0.568807574
APH1B	0.276909078	0.782505345	-0.414215999	0.680254488	-0.332694865	0.740601671
API5	1.621296432	0.108565117	-0.136185376	0.89214962	0.028906504	0.977041498
APIP	1.502522934	0.136575474	-0.861325895	0.392630255	0.467301819	0.642089615
APITD1	0.18881522	0.850676455	-0.687400608	0.494589107	0.185744392	0.853313559
APLF	1.198199251	0.23408636	0.284507206	0.777042128	-1.025624497	0.309460116
APLP2	-2.533351247	0.013085973	2.479448644	0.016107103	1.984749213	0.05205881
APOA1BP	-0.173887163	0.86235724	0.871925768	0.386869096	1.429778429	0.158312883
APOBEC2	0.22990518	0.818703971	-1.197526894	0.236006452	-0.481352733	0.632133575
APOBEC3A	-2.892356774	0.00482734	-1.059940464	0.293596423	-2.818594873	0.006646503
APOBEC3B	-1.633949386	0.105875433	-0.173265884	0.863049925	-1.066428148	0.290787131
APOBEC3C	-0.822098004	0.413263663	-1.545596218	0.127679393	-0.934968832	0.353801866
APOBEC3D	1.199008766	0.233773069	0.061485348	0.951185548	1.780212059	0.080443708
APOBEC3F	-0.793940987	0.429385349	0.256691406	0.79833172	1.232028869	0.223064867
APOBEC3G	-1.833897108	0.070080856	-4.171315871	0.000103283	-3.447521017	0.001078045
APOBEC3H	0.888392489	0.376774021	-3.424457213	0.001141314	-2.260186912	0.027700357
APOBR	-2.19445386	0.030861335	0.796001253	0.429299912	0.632862533	0.529388513
APOD	0.976213991	0.331659245	-0.178414358	0.859023642	0.045105754	0.964182802
APOL1	-2.474778035	0.015267472	1.508406803	0.136916609	0.255935293	0.798934665
APOL2	-2.069423249	0.041466418	0.866073724	0.390043181	0.371319545	0.711794824
APOL3	-0.878239373	0.382227634	0.616222356	0.540175669	0.9749061	0.333781871
APOL4	-1.373513049	0.173114414	-0.620804383	0.537177348	-1.259642119	0.213001234
APOL6	-0.600632612	0.549642874	-0.475361022	0.636327527	-2.609220696	0.011605832
APOLD1	0.150949597	0.88036417	-1.937456332	0.057598426	-0.739519221	0.462667651
APOM	0.088838378	0.929414101	0.322268475	0.748416166	1.128127112	0.264056192
APOO	0.541112243	0.58981012	-0.164472514	0.869935033	-0.694391962	0.490295261
APOOL	0.11351602	0.909882439	-1.002013883	0.320528087	0.701997634	0.485575995
APP	-0.28066789	0.779630054	2.19215023	0.032424262	1.31303615	0.194503503
APPBP2	0.778232419	0.438538849	-1.540137056	0.129003364	-2.510610384	0.014954015
APPL1	1.068499404	0.288244706	-0.499776561	0.61913641	-1.270038141	0.20930091
APPL2	0.984985229	0.327356818	-0.263179153	0.793351708	-0.874287623	0.385680095
APRT	0.183570828	0.854776387	1.119891473	0.267408302	1.504884768	0.13794985
APTX	1.26710145	0.208493113	1.124300977	0.26554928	1.8881771	0.064164777
AQP1	-1.115700051	0.26761614	2.114349444	0.038825457	1.821122326	0.073909317
AQP10	-5.11468607	1.85E-06	0.779502646	0.438875003	0.423933509	0.673231522
AQP11	0.76714462	0.44506793	-0.753465482	0.45423931	-0.291129149	0.772023621
AQP3	1.197589386	0.234322584	1.106291789	0.273199609	1.235545848	0.221764021
AQP9	-2.164945945	0.03312394	-0.219745296	0.826846483	-0.943619309	0.349400643

AQR	0.796927855	0.427657745	0.434426877	0.665601696	0.352301203	0.725929263
ARAF	-1.026912709	0.307302135	0.478511365	0.634097757	1.134421173	0.261430351
ARAP1	-1.977572437	0.051135567	0.605458973	0.547252493	0.028621539	0.977267764
ARAP2	1.003504605	0.318394387	-1.062553545	0.292419353	-1.975061594	0.053177561
ARAP3	-1.50798798	0.135172687	-0.008064893	0.993593086	0.166991329	0.867975882
ARCN1	-1.145758095	0.255030305	0.789352852	0.433143291	0.791605021	0.431917301
ARF1	-2.409615591	0.018072327	-0.293133187	0.770473912	-0.592247128	0.556058932
ARF3	-2.59223332	0.011179713	-0.083877983	0.9334443	0.072135765	0.942749806
ARF4	-1.332606682	0.186133137	-1.37406278	0.174745988	-1.080607078	0.284484394
ARF5	-1.916916295	0.058523694	-0.916236	0.363363554	-0.892299165	0.376034877
ARF6	-1.337141372	0.184654648	0.048295677	0.961647681	-0.465464873	0.643396165
ARFGAP1	-0.309595605	0.757607203	0.848996958	0.399397938	1.65979521	0.102522575
ARFGAP2	-0.748624385	0.456098425	2.498237565	0.015358504	3.322460245	0.001572905
ARFGAP3	-0.34480896	0.731068373	1.089144018	0.280626523	0.243551048	0.808464808
ARFGEF1	0.545732227	0.586643588	1.197157152	0.23614938	0.348097654	0.729066522
ARFGEF2	1.915341255	0.058726973	0.35708831	0.722330738	-0.488216669	0.62729464
ARFIP1	-1.169955105	0.245207221	0.296437407	0.767962367	-0.896710643	0.373696028
ARFIP2	-0.655661629	0.513768271	0.278077927	0.781948364	2.046685301	0.04537346
ARFRP1	-0.706232781	0.481926329	1.697633246	0.094974529	2.566238272	0.012971043
ARG1	-3.187098244	0.001995406	-1.186969758	0.24011218	-0.818130177	0.416735975
ARG2	-1.398810646	0.165416979	-0.832148018	0.408762428	-0.840558132	0.404155228
ARGFXP2	-0.130747012	0.896276645	0.667373308	0.507198865	1.601694645	0.114826059
ARGLU1	1.103955145	0.272650062	-0.513655089	0.609458111	-1.245954705	0.217946748
ARHGAP1	-1.898138373	0.060986315	0.312976204	0.755429234	0.9565083	0.34290944
ARHGAP10	1.387994784	0.168675304	0.125465382	0.900592043	1.01472511	0.314582807
ARHGAP11A	-0.128122582	0.898347026	0.490361394	0.625741085	0.644266743	0.522021711
ARHGAP11B	1.479997321	0.142478367	-2.023933303	0.047624327	-1.164690218	0.249061344
ARHGAP12	0.284348993	0.776817175	-0.764340151	0.447784722	-1.133172152	0.261949955
ARHGAP15	-0.019598663	0.984408312	1.00325519	0.319934114	1.427024547	0.159101765
ARHGAP17	1.273693606	0.206157031	1.306943188	0.196426077	1.423972601	0.159979595
ARHGAP18	-0.722275381	0.472057998	1.240371023	0.219867361	-0.248663807	0.804526721
ARHGAP19	-2.330169125	0.022106781	0.466706323	0.642470585	0.864882552	0.390777663
ARHGAP21	-2.294662976	0.024154332	1.011065035	0.316214004	0.011671148	0.990729288
ARHGAP22	1.194968383	0.235339756	0.73322052	0.466397835	1.128957748	0.26370859
ARHGAP23	-1.09517816	0.276454692	0.358772399	0.721077167	-0.032570119	0.974132722
ARHGAP24	-0.255365904	0.799041944	2.029209757	0.047067166	0.894595593	0.374816215
ARHGAP25	-2.267480785	0.025833117	1.176735727	0.244141324	1.129967125	0.263286626
ARHGAP26	-1.820813068	0.072065668	0.310911017	0.75699071	0.019584516	0.984444137
ARHGAP27	-0.5583266	0.578052376	-1.84448676	0.070256783	-2.389085466	0.020269079
ARHGAP29	-1.221317746	0.225257924	0.756734792	0.452293195	0.023241336	0.981540037
ARHGAP30	-2.073965779	0.041032114	1.481693052	0.143872576	1.800609673	0.077127805
ARHGAP31	-0.847507679	0.399032696	0.547124135	0.58640719	0.853219671	0.397157039
ARHGAP32	1.65489368	0.101541616	0.256679307	0.798341015	0.962067669	0.340134193
ARHGAP33	1.370844739	0.173941942	-1.374002525	0.174764594	-0.096338403	0.923594311
ARHGAP35	0.75471728	0.452452386	0.422887706	0.673951945	0.631715678	0.530132343
ARHGAP39	-0.337101132	0.736850797	-2.26729694	0.027141724	-2.332573562	0.02327427
ARHGAP4	-0.043025815	0.965779337	0.295649262	0.768561212	0.920993184	0.360988116
ARHGAP42	0.1241783	0.901459943	1.631136641	0.108318159	0.89967773	0.372128168
ARHGAP44	-0.946364701	0.346578039	-0.619301794	0.538159645	-1.168242643	0.247637702
ARHGAP5	0.829470123	0.409103654	0.146474934	0.884057953	-1.065758196	0.291087302
ARHGAP6	-2.538127051	0.012921109	1.520829033	0.133773991	-0.02724218	0.978363017
ARHGAP9	-2.382107168	0.019388251	-0.942019666	0.350117077	-0.983876265	0.329390455
ARHGDIA	-0.990915653	0.324468833	-0.605885683	0.546971042	-0.577954705	0.565601859
ARHGDIB	-2.569430865	0.011885985	-1.322697857	0.191163778	-1.147188221	0.256161082
ARHGEF1	-0.116879675	0.907224214	0.639800251	0.52483933	1.490879839	0.141581174
ARHGEF10	1.737537474	0.085823336	-0.496062064	0.621738378	-1.216062915	0.229040813
ARHGEF10L	1.168649773	0.24573015	1.597654723	0.115592157	1.351966688	0.181798481
ARHGEF11	-1.481482948	0.142083015	0.511809297	0.610741305	0.317416332	0.752103245
ARHGEF12	-3.853688827	0.000221864	0.302835194	0.763106517	0.312249867	0.756005499
ARHGEF17	-0.342347544	0.732913261	-0.051599561	0.959026309	-0.392294663	0.696323378
ARHGEF18	0.415851663	0.678540317	0.277423035	0.782448622	0.691267812	0.49224109
ARHGEF19	-1.681516924	0.096241342	2.191673851	0.03246045	0.605855558	0.54704829
ARHGEF2	-0.928337785	0.355795297	-0.362972886	0.717953843	-0.065097652	0.94832711
ARHGEF25	1.129540466	0.261767941	-0.99639448	0.323226258	-0.905091023	0.369278496
ARHGEF26	-0.755613908	0.451917254	2.131596719	0.037318324	2.782354991	0.007333122
ARHGEF26-AS1	-1.228199839	0.222677128	2.455499006	0.017108964	1.991353719	0.051307745
ARHGEF3	0.335593116	0.7379839	1.255346909	0.214422133	0.839794271	0.404579833
ARHGEF35	-1.308830877	0.194030935	-1.136727519	0.260359522	-1.893791713	0.063400674
ARHGEF37	-2.893507438	0.004811255	1.694304947	0.095608702	0.944234122	0.349089201
ARHGEF4	0.223676102	0.823532606	-1.19800043	0.235823493	-0.281283839	0.779525098
ARHGEF40	-1.969649461	0.052053109	-0.009758937	0.992247343	-1.564513746	0.123307278
ARHGEF5	1.444245869	0.152253832	1.951660925	0.055846911	2.137026777	0.036958895

ARHGEF6	0.837055926	0.404849633	-0.157058726	0.875747839	0.287920439	0.774466083
ARHGEF7	0.256642885	0.798059088	0.203976631	0.839090775	0.605692482	0.547155828
ARHGEF9	2.37429743	0.019777053	-0.90739985	0.367976281	0.471441231	0.639149578
ARID1A	-0.798867936	0.426537811	-0.848209922	0.399832396	-1.100110411	0.275970942
ARID1B	0.131766578	0.895472514	0.222479588	0.824727617	-0.356128945	0.723076581
ARID2	-0.047676558	0.962083056	-0.948217959	0.346980129	-1.739608008	0.087398732
ARID3A	-0.818214258	0.415465432	-1.699445138	0.094630748	-1.565421304	0.123094434
ARID3B	-1.35834284	0.177859257	-1.535399104	0.130161276	-1.374169029	0.174841356
ARID4A	0.139521682	0.889359708	-0.049197912	0.960931785	-1.381828152	0.172489285
ARID4B	0.255408662	0.799009029	0.352169818	0.725996245	-1.290998367	0.201986153
ARID5A	-0.364251752	0.716551915	0.331792681	0.741250156	0.065819439	0.94775501
ARID5B	0.960625716	0.339396879	1.071998519	0.288191985	0.551194495	0.583683754
ARIH1	-1.145990859	0.254934503	-0.842009939	0.403265082	-1.725445275	0.089938956
ARIH2	0.793339708	0.429733627	1.844750354	0.07021782	2.54402626	0.013732298
ARL1	0.934018299	0.352873993	-0.769566477	0.444701797	-0.295159902	0.76895871
ARL10	1.858044292	0.066537605	-0.657021419	0.513784036	-0.737248784	0.464036019
ARL11	-1.825849365	0.0712962	-1.354520214	0.180860411	-2.19912183	0.031997785
ARL13B	0.377959725	0.706379113	-0.047733398	0.962093849	-1.967279931	0.054091084
ARL15	-0.244617375	0.807327475	-1.116045233	0.269037337	-1.691117106	0.096349927
ARL16	-0.006517629	0.994814611	-0.281528529	0.77931406	0.387495303	0.699852288
ARL17A	1.85576475	0.066865552	-0.7784411	0.439495362	-0.6171017	0.539658217
ARL2	-0.308227689	0.758644269	1.165401169	0.24866035	1.245303423	0.218184163
ARL2BP	0.744186562	0.458764603	-0.171798138	0.864198426	0.442970393	0.659486481
ARL3	-0.495815097	0.621272371	-1.700982381	0.094339881	-0.630284689	0.531061221
ARL4A	-0.374534672	0.708915963	0.567134594	0.572826555	0.993544982	0.32470029
ARL4C	1.70141593	0.092428641	-1.912863985	0.060741645	-1.020216475	0.311994761
ARL4D	0.150893787	0.880408064	0.304863093	0.761569348	0.410294277	0.683149064
ARL5A	0.279355519	0.780633602	-0.454529827	0.651155981	-1.242225993	0.219308577
ARL5B	-0.030234014	0.975949522	-1.352806099	0.181404401	-3.494108772	0.000934752
ARL6	1.59778501	0.113708876	-0.145054429	0.885174314	-0.961273444	0.340529764
ARL6IP1	1.303164942	0.195949441	1.312865768	0.194435211	1.259711056	0.212976538
ARL6IP4	0.84894796	0.398235136	1.635138794	0.10747411	2.028811907	0.047221937
ARL6IP5	1.142921143	0.256199984	0.373650079	0.710036536	0.172129303	0.863953823
ARL6IP6	1.520655812	0.131964619	-2.210113301	0.031085331	-2.619469586	0.011300255
ARL8A	-1.784059335	0.077892981	-2.404415325	0.019435499	-2.930099864	0.004888746
ARL8B	-0.635764689	0.52659507	-0.162688755	0.871332949	-0.317206235	0.752261806
ARMC1	1.521133876	0.131844737	0.003176727	0.99747632	-0.172343202	0.863786458
ARMC10	-0.255663978	0.798812497	-0.397746853	0.692287165	-0.580112766	0.564155775
ARMC2	2.446585155	0.01642932	-3.158108023	0.002528321	-1.625697478	0.109605636
ARMC5	0.154501193	0.877571653	-0.425812367	0.671831577	0.219428177	0.827111027
ARMC6	-0.662345976	0.509496534	2.418319303	0.018775679	1.747537866	0.086002582
ARMC7	-0.30669492	0.759806841	0.690566008	0.492611936	0.297874117	0.766896955
ARMC8	0.015852893	0.987387971	1.762487881	0.08329243	1.787005865	0.079326316
ARMCX1	2.582809941	0.01146686	1.34665807	0.183365789	0.539019758	0.592000803
ARMCX2	3.307825201	0.001367354	0.107976636	0.914389493	0.789564759	0.433098496
ARMCX3	-0.160108412	0.873166028	3.147340353	0.002608951	2.182104899	0.03329546
ARMCX4	3.31583575	0.001333058	-0.421117579	0.675236575	0.604472927	0.547960384
ARMCX5	0.703148622	0.483836496	-0.014454229	0.988517548	1.089962018	0.280378325
ARMCX6	1.282255742	0.203151789	0.654571984	0.515348856	1.668315201	0.10081236
ARNT	-0.844471871	0.400716983	0.57497119	0.567550085	0.266653799	0.790711072
ARNTL	-1.829286658	0.070774978	-0.248181189	0.804876944	-1.101676672	0.275295071
ARNTL2	-0.454555833	0.650558508	0.031992364	0.974588747	0.111065283	0.911960023
ARPC1A	-1.952820073	0.054048637	1.274388974	0.207643386	0.419962847	0.676112805
ARPC1B	-2.198135336	0.03058874	-0.585351993	0.56059755	-1.462477032	0.149176914
ARPC2	-0.563385735	0.574618331	-1.421720516	0.160499914	-1.225175081	0.225616012
ARPC3	-1.023361174	0.308968125	0.20685773	0.836850534	0.305660536	0.760991725
ARPC4	-3.0292967	0.003224878	0.423532558	0.673484201	0.963108346	0.339616332
ARPC5	-3.881075961	0.000201609	1.113056231	0.270308117	0.148663379	0.882351066
ARPC5L	1.943103365	0.055230092	-1.322535441	0.191217479	0.201826889	0.840780368
ARPP19	0.447934801	0.6553113	-1.920759927	0.059716908	-1.505391329	0.137819899
ARRB1	-2.029965378	0.045408302	0.499534616	0.619305742	0.987718816	0.327521114
ARRB2	-2.70555442	0.008201181	-2.330758204	0.023289725	-2.834010365	0.006372836
ARRDC1	-0.973386371	0.333054127	-2.672535588	0.009774835	-2.658853721	0.010193043
ARRDC2	1.521181187	0.131832877	0.408254574	0.684600581	0.403672344	0.687984556
ARRDC3	-3.733284558	0.000336306	-0.453473112	0.651912047	-1.789960909	0.078844354
ARRDC4	-1.480231157	0.142416083	0.722734984	0.472767325	-0.098769272	0.921672689
ARRDC5	2.132969557	0.035737572	0.577566733	0.565807755	2.13521896	0.037112896
ARSA	-0.684083169	0.495736872	0.210548986	0.833982329	0.600182564	0.550795555
ARSB	-1.843442053	0.068661879	-0.545468198	0.587537868	-1.1433459	0.257738872
ARSD	-1.515554687	0.133249163	1.054043361	0.296264764	0.950313585	0.346019308
ARSG	-0.997022907	0.321512424	-0.742155115	0.461009257	-0.58040309	0.563961373
ARSK	2.005383726	0.048023092	-0.609263734	0.544745527	-2.131436002	0.037436968

ARV1	1.848291369	0.067950218	-0.580225749	0.564025555	-0.577723504	0.565756892
ARVCF	1.395291011	0.166471953	-0.161130349	0.872554596	-0.20026794	0.841993509
ASAH1	1.757766441	0.082296956	-2.471796516	0.016421302	-3.052665039	0.003460412
ASAP1	-0.362527248	0.717835361	-0.894677678	0.374683027	-1.288795483	0.202745805
ASAP1-IT1	-0.229546287	0.818981989	1.696285246	0.095230959	2.215368911	0.030800568
ASAP2	-2.668259953	0.009090474	-0.424530816	0.672760368	-2.533017946	0.014124398
ASAP3	-1.464721332	0.146593578	1.579342849	0.119734209	1.678879485	0.098724284
ASB1	3.83383466	0.000237747	1.149241102	0.255206608	2.471606205	0.016503817
ASB13	0.360438085	0.719391285	2.506294006	0.015047252	3.516217616	0.000873271
ASB14	1.829845824	0.070690488	-0.299360041	0.765742947	-0.379387834	0.705828718
ASB16	1.064774162	0.289918033	0.524366725	0.602035801	1.201145645	0.234729209
ASB2	0.431521079	0.667154681	-0.126219905	0.899997436	0.352221648	0.725988594
ASB3	1.441428248	0.153045811	-3.708813014	0.000470217	-3.671432781	0.000538291
ASB6	-0.128050826	0.898403644	-0.77738152	0.440115087	0.046374925	0.963175714
ASB7	0.39017637	0.697357723	-0.048280997	0.961659329	-0.57951791	0.564554197
ASB8	-1.881652485	0.063219787	-0.471036876	0.63939361	-0.147991309	0.882878979
ASB9P1	-0.075545018	0.93995415	0.216165432	0.829622558	0.581329632	0.563341179
ASCC1	-0.143984182	0.885845238	0.783376499	0.436615538	1.9820203	0.052371889
ASCC2	-2.818759634	0.005965122	0.084192881	0.933195033	-0.156292628	0.876362084
ASCC3	0.203052565	0.839567065	1.936085964	0.057769853	1.883541611	0.0648015
ASCL2	-0.484591276	0.629181481	-3.600346601	0.000662347	-4.854311337	1.00E-05
ASF1A	1.318733633	0.190711564	-1.043058895	0.301279335	-0.637270017	0.526534977
ASF1B	-2.376105172	0.019686445	-1.978621394	0.052647524	-0.649005845	0.518976377
ASGR1	-1.234848096	0.220204532	1.024096194	0.310071903	0.041948625	0.966688236
ASGR2	1.025015776	0.308191214	1.534936304	0.130274823	1.785171681	0.079626704
ASH1L	-0.041734148	0.966806055	-0.31273722	0.755609876	-0.288207728	0.774247305
ASH2L	-1.551031354	0.1245166	0.901371332	0.37114472	1.133458109	0.261830929
ASL	-1.440736338	0.15324078	-0.172283208	0.86381883	0.282227176	0.778805411
ASMT	-1.099463182	0.274592653	1.125004061	0.26525371	0.804646184	0.424412611
ASMTL	0.202589309	0.839928045	0.832965654	0.408304919	0.992702934	0.325106973
ASMTL-AS1	0.642648851	0.522138362	0.448020997	0.655818784	1.10221089	0.275064811
ASNA1	-1.987787047	0.049972997	-0.268026487	0.789636504	-0.683742309	0.496945644
ASNS	0.979308529	0.3301371	1.657150605	0.102926943	2.734176238	0.008347202
ASNSD1	-0.082866366	0.934147749	-0.802518038	0.42555243	-1.149682314	0.255140615
ASPDH	1.860433214	0.06619537	-1.098878607	0.276393257	1.29277851	0.201373839
ASPH	0.26476635	0.791814372	1.261134519	0.212344707	-0.390850673	0.697384421
ASPHD2	-1.848358117	0.067940465	3.601788132	0.000659361	1.705608374	0.093599197
ASPM	0.240936744	0.810169813	1.037093845	0.304026637	0.310722656	0.757160238
ASPRV1	-3.485245957	0.000771872	-0.089336103	0.929124728	-0.054798017	0.956493595
ASPSCR1	0.63555838	0.526728936	1.267488232	0.210081304	1.195990195	0.236718831
ASRGL1	0.934378122	0.352689468	3.040733128	0.003548191	2.291761632	0.025685354
ASTE1	0.8499814	0.397663468	-0.155813806	0.876724607	0.329182233	0.743240801
ASTL	-1.307684166	0.194418079	0.104963562	0.916769404	1.297732518	0.199677133
ASTN2	-2.081122379	0.040355837	2.39416461	0.019935186	0.341597029	0.733927341
ASXL1	3.546977197	0.000629807	0.544881559	0.587938675	1.95193183	0.055932327
ASXL2	0.211883095	0.832692762	-0.18373384	0.854867575	-0.426172665	0.671608859
ATAD1	1.088634877	0.279314914	-0.303359251	0.762709184	-0.875305795	0.385130756
ATAD2	2.061631943	0.042220554	-0.54812576	0.585723782	-0.603583363	0.54854762
ATAD2B	0.462569044	0.644825656	0.233855567	0.815926338	-1.69271027	0.0960443
ATAD3A	-0.08972047	0.928715131	2.650907526	0.010348951	2.927776157	0.004920488
ATAD3B	0.948513764	0.34548962	0.607149897	0.546137617	1.725184508	0.089986294
ATAD3C	0.107544663	0.914603984	-0.198820144	0.843103607	0.053346547	0.957644849
ATAD5	1.944310223	0.055082169	0.205385655	0.837994999	-0.019848501	0.984234483
ATAT1	1.350740486	0.180273816	-1.797900873	0.077434562	-0.554378493	0.581517892
ATE1	-0.650185914	0.517281689	-0.217217134	0.828806768	-1.193423254	0.237714033
ATF1	1.438323343	0.153922227	-0.810483516	0.420998591	-1.850507002	0.069495801
ATF2	0.789315597	0.4320688	-1.196188488	0.236524127	-3.448365229	0.001075272
ATF3	-2.089386537	0.039586882	-0.835462475	0.406909757	-1.953694711	0.055718153
ATF4	-0.121934697	0.903231336	-0.170614908	0.86512451	0.098333797	0.922016902
ATF5	-1.023415183	0.308942745	-0.702087217	0.485452415	-0.885665428	0.379569334
ATF6	-0.115584832	0.908247377	-0.730078545	0.468301298	-1.508399394	0.137050215
ATF6B	-0.908385514	0.366178623	2.377649791	0.02076446	2.164353309	0.034698213
ATF7	-0.566161407	0.572738439	-1.231920823	0.222984447	-0.89979239	0.372067663
ATF7IP	-0.391176961	0.69662074	0.14498415	0.885229552	-0.086360893	0.931486287
ATF7IP2	1.126668177	0.262974165	-0.317802281	0.751784271	1.264006434	0.21144195
ATG10	-0.388366462	0.698691546	-0.812190116	0.420026764	-0.930944061	0.355861815
ATG12	-0.443814112	0.658276468	-1.22006768	0.227411312	-1.596243981	0.116039098
ATG13	-2.020618349	0.046387816	1.449818911	0.152532718	1.989018499	0.051572235
ATG14	2.215216375	0.029351318	-0.707694912	0.481988697	0.636055703	0.527320354
ATG16L1	2.207144807	0.029930489	-0.753689153	0.454106012	0.604083198	0.54821762
ATG16L2	-1.105652309	0.271918603	-0.159213835	0.874057394	0.168127743	0.86708598
ATG2A	-4.314402107	4.21E-05	-1.047692019	0.299157214	-0.875543041	0.385002824

ATG2B	2.197613139	0.030627278	-0.363313226	0.71770099	-0.777297259	0.440241031
ATG3	-0.647768174	0.518837017	0.197502745	0.844129496	-0.048778097	0.961268963
ATG4A	0.144517207	0.885425602	-1.196760321	0.23630285	-0.87663569	0.384413972
ATG4B	0.131709322	0.895517669	0.039221266	0.968849613	1.03313433	0.305963617
ATG4C	0.283095931	0.777774357	0.776255419	0.440774282	0.807472768	0.422796399
ATG4D	1.355981728	0.178606526	-0.998548132	0.322190386	-0.677714393	0.500731662
ATG5	0.564942633	0.573563518	0.187642807	0.851816156	0.237274805	0.813305876
ATG7	-1.522948845	0.131390386	2.974872665	0.004277336	2.600024202	0.011886418
ATG9A	-0.886486183	0.377794229	0.883759596	0.380500143	1.856950366	0.068558306
ATG9B	0.973046218	0.333222186	0.35716104	0.722276585	0.662748367	0.510199024
ATHL1	-0.491668129	0.624189506	-0.178863531	0.85867255	0.936531053	0.353004382
ATIC	0.322795396	0.747623026	2.555989346	0.013250211	3.48235432	0.000969094
ATL1	-0.253641602	0.800369605	-0.073930179	0.941322104	-0.762814241	0.448762183
ATL2	0.909064928	0.365821917	-1.22226762	0.226584864	-1.798889793	0.077402909
ATL3	-0.354140558	0.724088568	1.007135144	0.318082296	-0.003611114	0.997131534
ATM	1.655202265	0.101478854	-1.153339727	0.253534776	-1.036359539	0.30447028
ATMIN	-0.529438702	0.597846645	0.393607332	0.695324306	0.382027273	0.703880984
ATN1	1.106934897	0.271366729	-0.896368316	0.373787331	0.213431045	0.831762648
ATOH8	-2.850249097	0.005451147	1.15867448	0.251370468	0.593137294	0.555467254
ATOX1	-2.357056437	0.020659993	-0.943368667	0.349432782	-0.603971286	0.548291497
ATP10A	4.007675374	0.000128834	1.043902671	0.300892094	0.685607602	0.49577727
ATP10D	-0.188066206	0.851261767	-0.12244512	0.902972748	-2.476653423	0.016295422
ATP11A	-0.120952644	0.904006853	-1.518235644	0.134425302	-3.343370209	0.001477403
ATP11B	0.328228654	0.743525738	-0.601654743	0.549764953	-1.132543229	0.262211869
ATP11C	0.903692466	0.36864859	-0.483864653	0.630316587	-1.156467018	0.252379327
ATP13A1	0.826192488	0.410950051	0.356570675	0.722716199	0.582807902	0.562352377
ATP13A2	0.323883834	0.746801636	0.903203701	0.370179839	0.910887519	0.366242589
ATP13A3	1.474977772	0.143820518	-0.522000114	0.603672073	-2.200772313	0.031874322
ATP13A4	2.145560392	0.034687816	2.052960283	0.044628387	1.159824086	0.251020979
ATP1A1	0.543193576	0.588382585	2.748585133	0.007979779	2.990755876	0.004124516
ATP1A1OS	0.991679668	0.324098005	1.282467093	0.204816349	0.596668619	0.55312316
ATP1A3	1.277485974	0.204821911	-0.709308898	0.480994345	0.276302825	0.783328398
ATP1A4	-1.56629793	0.120901291	0.028759257	0.977156015	-2.064800456	0.043564208
ATP1B1	0.578171399	0.564638781	0.104634646	0.917029249	-0.09823064	0.922098442
ATP1B3	-0.288616194	0.773560158	2.071049636	0.042845029	1.835883308	0.071663675
ATP2A1	-0.366380618	0.71496865	-1.488287873	0.142130017	-1.688541091	0.096845792
ATP2A2	2.386690278	0.019163262	0.368839952	0.713599408	0.2120978	0.832797596
ATP2A3	-1.180440844	0.241035335	-0.182425595	0.855889317	0.48449883	0.629913615
ATP2B1	-1.408095824	0.162658405	0.429381455	0.669247629	-1.145401215	0.256894029
ATP2B4	1.063457739	0.290510939	-0.331084006	0.74178258	-0.177246639	0.859951515
ATP2C1	1.418424875	0.159631333	0.801704113	0.426019404	-0.211372802	0.83336051
ATP2C2	-1.619100945	0.109037371	-0.183758697	0.854848163	0.631684619	0.530152495
ATP5A1	-0.007446952	0.99407526	3.0487148	0.003468172	3.485447631	0.000959942
ATP5B	-0.176237548	0.860516028	1.461919036	0.149198365	2.185870773	0.033004364
ATP5C1	0.779238655	0.437949109	-1.287132486	0.203196774	-1.01551624	0.314209062
ATP5D	-0.130177659	0.89672574	2.279721331	0.026345898	1.908473565	0.061438999
ATP5E	-2.214588862	0.02939599	-3.43854571	0.001093224	-2.32931742	0.023458954
ATP5F1	0.467781564	0.641107958	0.053775744	0.957299927	0.893298682	0.375504148
ATP5G1	-2.082657609	0.040212022	-1.228871292	0.224117283	-0.343159308	0.732758143
ATP5G2	-1.141999154	0.256580936	-1.256251679	0.214096383	-0.725908068	0.470905485
ATP5G3	-0.403080341	0.687875839	-1.002240194	0.320419741	-0.640601141	0.524383672
ATP5H	-0.294493889	0.769080548	-0.321906822	0.748688719	0.942758774	0.349836865
ATP5I	-0.045913775	0.963483974	-2.715661511	0.008716102	-1.99285232	0.051138626
ATP5J	-0.285414385	0.776003619	-2.334539095	0.023076409	-1.715417288	0.091774231
ATP5J2	0.053285353	0.957626451	-5.687520932	4.52E-07	-4.351838305	5.75E-05
ATP5L	0.434657134	0.664885203	-0.305888616	0.760792357	0.528614368	0.599153121
ATP5O	0.262047159	0.793903193	-0.275358385	0.784026365	0.614889694	0.541107708
ATP5S	1.623637298	0.108063406	-2.502014346	0.015211875	-1.53997811	0.129174078
ATP5SL	-0.239750809	0.811086186	0.192127623	0.848318022	0.587911	0.558945587
ATP6AP1	-1.88862507	0.062266901	0.298365319	0.766498109	0.495454914	0.622209602
ATP6AP1L	-0.347196292	0.729280523	0.277439359	0.782436151	0.204411263	0.838770118
ATP6AP2	-0.188859749	0.850641661	2.341775327	0.022672994	1.767090808	0.082638957
ATP6V0A1	-1.759542668	0.08199311	1.417762907	0.161647558	1.93820105	0.057624715
ATP6V0A2	2.48018893	0.015053092	-0.977419304	0.332449206	-0.967370514	0.337500816
ATP6V0B	-2.910996533	0.004572766	-1.894397132	0.063196786	-2.051108317	0.044925812
ATP6V0C	-1.644151847	0.103746036	-1.815383953	0.074672008	-2.149397011	0.035920036
ATP6V0D1	-2.605433616	0.010788399	0.443811568	0.658841701	0.660691798	0.511507464
ATP6V0E1	-2.828220201	0.00580622	-1.603296209	0.11433963	-1.106091639	0.273396175
ATP6V0E2	-0.068325066	0.94568332	0.69387068	0.49055242	1.185384709	0.240850196
ATP6V1A	-2.286452955	0.024650948	-0.235127468	0.814943761	-1.471892474	0.14662432
ATP6V1B2	-3.539631345	0.000645313	0.852813203	0.397295441	0.269477988	0.788548195
ATP6V1C1	1.022506594	0.309369905	2.114715606	0.038792917	0.883456063	0.380751127

ATP6V1C2	-0.018349171	0.985402227	0.329319105	0.743109091	-0.928821217	0.356951447
ATP6V1D	-2.082834638	0.040195467	-0.662134934	0.510525467	-0.776945973	0.440446578
ATP6V1E1	-1.816143812	0.072785226	2.483483522	0.015943623	2.67088148	0.009875222
ATP6V1E2	0.023535986	0.981276516	2.159078285	0.035022403	3.112148068	0.002917652
ATP6V1F	-1.450168571	0.150599425	-4.41290256	4.53E-05	-4.141478865	0.000116991
ATP6V1G1	0.027980669	0.977741515	-1.361239484	0.17873997	-1.2666599	0.210498069
ATP6V1G2	-0.147275504	0.883254596	-1.165220977	0.248732674	0.364383922	0.716937918
ATP6V1H	-0.453946955	0.650994981	3.224415221	0.002081119	3.57132152	0.000736349
ATP7A	-0.074487438	0.94079317	-2.254972476	0.027952108	-2.758023173	0.007830167
ATP7B	0.185192289	0.853508339	-0.324781424	0.746523227	0.195813422	0.845462035
ATP8A1	1.58003693	0.117719623	-0.580303319	0.563973605	-1.620385172	0.11074411
ATP8A2	-0.530690811	0.596982225	-2.281568697	0.026229352	-1.551070438	0.126494736
ATP8B1	1.919565381	0.058183137	0.203544289	0.839427064	1.087551883	0.281432207
ATP8B2	2.699807895	0.008332835	0.08187889	0.935026898	0.843200428	0.402688574
ATP8B3	1.732141018	0.086784773	-1.415003532	0.162451488	-1.024433103	0.310017298
ATP8B4	0.577660394	0.564982263	0.808996611	0.421846415	-1.265217528	0.211010755
ATP9A	-1.824019747	0.071574941	-0.461351303	0.646284192	-0.309554688	0.758043726
ATP9B	1.50647325	0.135560353	0.377938687	0.706865428	1.076431491	0.286330564
ATPAF1	0.652485597	0.515804589	2.463379418	0.016773282	4.342987115	5.93E-05
ATPAF2	-0.636156936	0.526340605	2.11419954	0.038838785	2.436579577	0.018017465
ATPBD4	0.186789722	0.852259457	-0.400491945	0.6902759	0.506133303	0.614741518
ATPIF1	0.971150466	0.334159836	-1.560477877	0.124125285	-1.009423055	0.317095347
ATR	1.108486232	0.270700262	-0.165333538	0.869260405	-0.524553266	0.601955456
ATRIP	-0.341979737	0.733189076	2.015973533	0.048475592	3.349728788	0.001449467
ATRN	1.729657507	0.087230201	0.97185673	0.335185665	0.910707191	0.366336794
ATRNL1	0.871395573	0.38593133	1.183035256	0.241655469	1.90618539	0.06174129
ATRX	0.22164052	0.825112026	-0.012335109	0.990200884	-1.114089386	0.269979768
ATXN1	-0.113154402	0.910168279	-1.025977558	0.309191865	-2.208512451	0.031300907
ATXN10	2.273126584	0.025476216	4.368600671	5.28E-05	5.001206971	5.92E-06
ATXN1L	-0.50372532	0.615724809	-0.542194135	0.589776446	-0.717347856	0.476128647
ATXN2	-0.693693589	0.489718402	-1.947596605	0.056343356	-1.926419189	0.059111549
ATXN2L	0.574611685	0.567033634	-1.514586103	0.135346117	-1.073654815	0.287562818
ATXN3	1.588865112	0.115710715	-1.222974782	0.226319673	-1.017094589	0.313464314
ATXN7	-0.434540246	0.664969736	-0.252007893	0.801932052	-1.862082945	0.067819184
ATXN7L1	2.172386639	0.032540189	-1.285450631	0.203779518	-1.635818869	0.107462832
ATXN7L2	-1.663222326	0.099858669	-0.175044535	0.861658541	0.991883626	0.325503001
ATXN7L3	-0.674981147	0.501473792	0.836378851	0.406398442	1.786475338	0.079413104
ATXN7L3B	1.358074975	0.177943915	-0.4881221	0.627316509	0.005862188	0.995343423
AUH	0.483279438	0.630108751	-2.573239696	0.012673452	-2.184540907	0.033106904
AUP1	-0.84858951	0.398433537	-1.988991598	0.051459444	-0.728177937	0.46952595
AURKA	-0.191344709	0.848700425	-0.246787618	0.805950095	0.295640796	0.768593294
AURKAIP1	-0.577034548	0.565403077	0.028207705	0.977594003	-0.099753256	0.920894975
AURKAPS1	-0.031138459	0.975230288	-0.925483989	0.35857578	-0.640576333	0.524399677
AURKB	0.118021791	0.906321864	1.898936315	0.062585635	3.140556475	0.002687582
AURKC	-0.937342547	0.351171619	0.580471795	0.563860783	1.184044323	0.241376033
AUTS2	-0.966066953	0.336682705	-0.683003372	0.497342918	-1.290460428	0.202171461
AVEN	-1.592537529	0.114883149	3.750119128	0.000412144	2.98738072	0.004163934
AVIL	-0.63562044	0.526688666	-1.647175089	0.104967842	-1.516102357	0.13509475
AVL9	-0.596944348	0.552091529	-0.151463826	0.880139116	-0.994195274	0.324386452
AVPI1	0.781494062	0.436628933	2.394520549	0.019917644	0.402569571	0.688791107
AVPR2	1.08798698	0.279599235	-1.183816688	0.241348388	-1.474937592	0.145806149
AXIN1	0.299075375	0.765594249	-0.699778337	0.486882555	-0.08820485	0.930027215
AXIN2	3.532883823	0.000659875	1.060430806	0.293375299	1.643043349	0.105954269
AXL	-0.461081389	0.645888352	1.299304919	0.199016245	1.160566443	0.250721314
AZI1	1.226735469	0.223224458	0.770191758	0.444333788	1.58503457	0.118566234
AZI2	-0.598492255	0.551063204	-0.78007622	0.438540026	-1.191447092	0.238482255
AZIN1	-1.770646565	0.08011456	-0.052852011	0.958032706	-1.2052909	0.233138289
AZU1	-4.48506199	2.21E-05	2.977980577	0.004240006	1.021058667	0.31159912
B2M	-0.472157607	0.637993907	-1.032767399	0.306029906	-1.088044181	0.281216716
B3GALNT1	-0.293878443	0.769549238	0.44276723	0.65959256	0.161253369	0.872471762
B3GALNT2	0.693803938	0.48964953	-0.840723098	0.403979819	-1.017893821	0.31308765
B3GALT2	0.318636397	0.750764294	-0.7822371	0.43727939	-2.098927997	0.040325203
B3GALT4	-0.74258535	0.459728764	0.502135862	0.617486281	0.463397984	0.64486762
B3GALT6	0.51930703	0.604862434	0.452885623	0.652332546	1.256390822	0.214168388
B3GALTL	-0.427278318	0.670229985	-0.312283804	0.755952639	0.256027519	0.798863807
B3GAT1	1.810038779	0.07373506	-3.093843803	0.003046547	-2.944638439	0.004694426
B3GAT3	0.810610632	0.41979643	1.764826014	0.082894619	2.863014788	0.005885952
B3GNT1	1.062175898	0.291089067	1.142039025	0.258163363	1.672542666	0.099972486
B3GNT2	-0.870675387	0.38632237	0.993242257	0.324746438	-1.268339301	0.2099023
B3GNT3	-0.556171161	0.579518425	2.722748168	0.00855254	0.465359218	0.643471348
B3GNT5	-2.0299977	0.045404945	-0.462071294	0.645770889	-1.884354786	0.064689418
B3GNT7	0.509460657	0.611716428	0.29866087	0.766273712	0.496930404	0.621175292

B3GNT8	-2.785837539	0.006549663	0.243130132	0.808768402	0.263290227	0.793289189
B3GNT9	0.665180902	0.507690559	-1.544823456	0.127866144	-1.465578186	0.14833235
B3GNTL1	-1.495041397	0.138514362	0.23740913	0.813181866	-0.223093022	0.824271474
B4GALNT3	0.430190915	0.668118222	-0.030588241	0.975703668	-0.611884374	0.54308024
B4GALNT4	-0.541250813	0.589715028	-1.075343427	0.286705066	-1.078886844	0.285243964
B4GALT1	-2.345970853	0.021246007	0.018221126	0.985525423	0.017940668	0.985749686
B4GALT2	-0.587195262	0.558590112	-1.767773088	0.082395455	-2.017553503	0.048419376
B4GALT3	0.479704633	0.632638602	1.365629782	0.177364781	1.798691805	0.077434631
B4GALT4	3.355407065	0.001175169	1.633476481	0.107824039	2.012414866	0.048974566
B4GALT5	-3.341650396	0.001227951	-0.911505805	0.365828218	-1.411755511	0.163531286
B4GALT6	0.453888029	0.651037229	-1.52197994	0.133485752	-2.392186386	0.020114715
B4GALT7	-1.018773414	0.311129172	-0.34928237	0.728151126	-1.096084222	0.27771365
B7H6	0.949761629	0.344858642	-1.3290381	0.189076339	-1.01620106	0.313885781
B9D2	-3.10796625	0.002543604	-0.530486936	0.597813839	-0.850392794	0.398712948
BAALC	0.909039556	0.365835234	-1.327073739	0.189721226	0.130456734	0.896670624
BABAM1	-0.771446367	0.442528164	1.082293355	0.283632619	2.054861135	0.044548998
BACE1	1.449488049	0.150788806	1.177225641	0.243947341	0.856653475	0.395272145
BACE1-AS	3.790714128	0.000276063	1.758172767	0.084030773	2.937139362	0.004793743
BACE2	-0.540985369	0.589897192	2.156153107	0.035260755	0.919632205	0.361692919
BACH1	-2.256195643	0.026559713	-0.114741201	0.909049326	-1.584545178	0.118677568
BACH2	2.614120214	0.010537692	1.493249997	0.140829867	1.845475321	0.070235395
BAD	-0.972367088	0.333557888	-0.37644224	0.707971351	-0.595459827	0.553924996
BAG1	-3.766011701	0.000300594	1.663661755	0.101612372	1.066038504	0.290961684
BAG2	1.773854699	0.079578481	-0.330523807	0.742203544	-0.195315766	0.845849729
BAG3	1.692868398	0.094050981	2.28872579	0.025782132	2.012328369	0.048983958
BAG4	0.089609335	0.928803191	-1.742718401	0.086719841	-2.621512145	0.011240239
BAG5	-0.036216235	0.97119273	1.444858425	0.153916352	0.952339673	0.345000148
BAG6	-2.740173532	0.00744733	-0.265344331	0.791691622	-0.46244557	0.645546142
BAHCC1	2.451218401	0.016233114	-0.137723807	0.89093905	-0.51239036	0.610384465
BAHD1	0.385790129	0.700591822	0.368087019	0.71415769	0.678585054	0.500183847
BAI1	1.698409811	0.092996583	-0.553869747	0.581812005	0.064727098	0.948620827
BAI2	1.404643791	0.163679833	-2.2506125	0.028243879	-0.941745173	0.350351133
BAIAP2	-1.811563347	0.073496904	0.26622551	0.791016282	-0.56468351	0.574534659
BAIAP2L1	0.04262756	0.966095896	0.405608104	0.686533404	0.688500583	0.493968161
BAIAP2L2	1.011935712	0.314368828	-1.122749833	0.266202189	0.217345876	0.828725448
BAIAP3	-1.010740664	0.314937342	-0.21605714	0.82970657	-1.237310226	0.221113525
BAK1	-1.832466298	0.070295659	-2.093418591	0.040725518	-2.041752043	0.045877298
BAMBI	-1.79138666	0.076701055	1.618517825	0.111014746	1.013028144	0.3153855
BANF1	-0.575534183	0.566412534	1.249031561	0.216706103	2.046747368	0.045367152
BANK1	0.816681358	0.4163364	0.667343049	0.507218047	0.591427099	0.55660427
BANP	-2.163353207	0.033250075	-1.599001568	0.115292128	-2.264961842	0.027386919
BAP1	-0.849264847	0.39805979	1.633959274	0.107722312	2.595621363	0.012022924
BARD1	0.93465929	0.352545323	1.144552759	0.257128613	0.2563809	0.798592317
BASP1	-1.674981598	0.097521109	-1.261765343	0.212119182	-1.850105785	0.069554533
BATF	-0.086499454	0.931267729	-0.04224712	0.966447816	-0.409116136	0.684008402
BATF2	-2.70902589	0.008122569	0.143157229	0.88666567	-1.539307101	0.1293376
BATF3	-0.188321971	0.851061892	-2.199209626	0.031892137	-1.83983279	0.071072652
BAX	-1.978610236	0.051016409	-1.839644475	0.0709758	-1.459844751	0.149896739
BAZ1A	-0.473406836	0.637106126	0.065822915	0.947746756	-2.030178364	0.047078356
BAZ1B	-0.414234428	0.679719742	1.84393563	0.070338309	2.045038045	0.045541161
BAZ2A	-0.786420572	0.433753385	-0.847421231	0.400268059	-0.826861117	0.411810476
BAZ2B	-0.393542387	0.694879649	-0.786794917	0.434627434	-2.437706819	0.01796687
BBC3	-0.650901747	0.516821667	-2.52281995	0.014426567	-3.180905584	0.002389922
BBIP1	-0.550044603	0.583695142	-1.793901213	0.078078431	-1.608234951	0.113384065
BBS1	2.727958819	0.00770578	-0.582167534	0.562725832	-0.079590156	0.936845773
BBS10	0.192500354	0.847797959	-2.850333616	0.006050502	-3.020190757	0.003795193
BBS12	-1.269489081	0.207644757	-1.261371288	0.212260039	0.043567755	0.965403283
BBS2	3.239988438	0.001692805	1.054395807	0.296104821	2.231094942	0.029679534
BBS4	2.259220708	0.026363203	-1.439060253	0.155546031	-1.029289037	0.307750572
BBS5	0.66434554	0.508222367	-0.938625531	0.351842644	-1.514415739	0.135521009
BBS7	1.053282111	0.295121956	-0.06113183	0.951465856	-0.624586981	0.534768087
BBS9	2.212569955	0.02954012	0.881564716	0.381676406	1.738838838	0.087535147
BBX	-0.075976408	0.939611931	-1.524051052	0.132968296	-2.473884414	0.016409456
BCAM	-1.437570998	0.154135173	0.160477738	0.873066276	-0.386682339	0.700450713
BCAP29	-0.182551936	0.855573397	-1.598679798	0.11536375	-1.042958922	0.301430141
BCAP31	-2.307803475	0.023377828	2.260639319	0.027576869	2.836622654	0.006327506
BCAR3	-0.724036285	0.470981748	0.320047971	0.750090111	-1.484251734	0.14332586
BCAS1	2.074117395	0.041017686	-1.094987344	0.278080055	0.07692291	0.938957895
BCAS2	-0.831029197	0.408227143	-2.069325523	0.043012296	-0.851030048	0.398361875
BCAS3	-1.402807445	0.164225193	1.34686011	0.183301077	1.121144158	0.266991227
BCAS4	1.617823648	0.10931288	0.846125741	0.400984307	2.405420252	0.019467708
BCAT1	-1.73332813	0.086572521	0.057840727	0.954075701	-0.984919585	0.328882195

BCAT2	-0.735654481	0.463915434	0.930825862	0.355828874	2.008497014	0.049401537
BCCIP	2.019909203	0.046462864	0.498839034	0.619792678	1.757608653	0.084256216
BCDIN3D	1.206859369	0.230750473	-0.198561889	0.843304694	0.405651509	0.686537938
BCKDHA	-1.123398789	0.264351895	-1.172737053	0.24572877	-1.205753527	0.232961224
BCKDHB	1.260988071	0.21067692	-1.406206906	0.165035012	-1.523496756	0.133238506
BCKDK	-2.001033207	0.048499025	-0.881303793	0.38181639	-1.105770551	0.273533965
BCL10	-3.200106864	0.001916617	-0.186642769	0.852596591	-0.595518686	0.553885939
BCL11A	-0.438478607	0.662123926	0.631522574	0.530197413	0.862310009	0.392179274
BCL11B	1.249054369	0.214988262	-0.411151555	0.682487228	0.709873188	0.480715989
BCL2	1.549351785	0.124919526	1.397177751	0.16771972	1.214217407	0.229739049
BCL2A1	-0.421500681	0.674426872	0.114986297	0.908855916	0.341912229	0.733691396
BCL2L1	-3.616528941	0.00049947	0.276848299	0.782887725	-0.332418548	0.740809163
BCL2L11	0.699075597	0.486365504	-1.497207611	0.139799655	-1.954534993	0.055616313
BCL2L12	0.757391546	0.450857388	-2.612832428	0.011434964	-2.549370356	0.013545531
BCL2L13	-1.862328146	0.065924954	3.103221275	0.00296518	2.307718829	0.024717397
BCL2L15	1.626329711	0.107488659	0.316380677	0.752857371	1.032315698	0.306343452
BCL2L2	-0.018104762	0.985596646	-1.019272704	0.312335917	-1.269493197	0.20949368
BCL3	-1.347541019	0.181297363	-1.483003824	0.143524897	-2.326419559	0.023624414
BCL6	-1.181160149	0.240751025	-0.342737685	0.733043523	-0.634459092	0.528353921
BCL7A	2.137821571	0.035329817	-0.443207231	0.659276165	0.598080317	0.552187469
BCL7B	-0.044132677	0.964899564	-1.450390816	0.152373822	-0.972570989	0.334931376
BCL7C	-0.26182877	0.79407102	0.496210189	0.621634524	0.21031027	0.834185656
BCL9	-0.579174865	0.563964579	-0.055643718	0.955818211	0.59037221	0.557306189
BCL9L	0.469588184	0.63982156	-1.608777039	0.113133292	-0.58844434	0.558590129
BCLAF1	0.95469496	0.342371449	-0.262295159	0.79402976	-1.367970858	0.17676271
BCO2	1.543065151	0.126436866	0.798902639	0.427629042	0.110955041	0.912047046
BCOR	-0.135681887	0.892385537	0.603654425	0.548443559	-0.036710223	0.970846059
BCORL1	-2.031173696	0.04528298	-0.217578557	0.82852646	-0.320661719	0.749655306
BCR	-0.908849168	0.365935172	2.550651634	0.01343344	1.540594352	0.129024047
BCRP2	-0.517806826	0.605904451	1.160774435	0.250522156	1.69936719	0.094775865
BCRP3	-0.148346863	0.882411588	-0.140217221	0.888977571	-0.329045785	0.74334338
BCS1L	-0.163056039	0.870851653	1.081221488	0.284104975	2.294652521	0.025507535
BDH1	2.053308493	0.043039209	1.683456128	0.097700024	3.480382103	0.000974972
BDH2	1.224304172	0.224135353	-0.698466647	0.487696066	-0.06275081	0.950187441
BDNF-AS1	0.654931352	0.514236112	-1.680589554	0.098258835	-0.458466247	0.648384378
BDP1	0.948715125	0.345387752	-0.267358295	0.790148345	-1.600013026	0.115199204
BEAN1	-0.15415973	0.877840069	0.171794629	0.864201172	0.123248447	0.902349793
BECN1	-0.633044302	0.528361643	2.266637008	0.027184584	1.422771474	0.160326104
BEGAIN	0.534711379	0.594210459	0.085127963	0.93245488	1.415016275	0.162577415
BEND2	-4.391302764	3.15E-05	0.880701423	0.382139682	-1.166471908	0.248346595
BEND3	-1.075050597	0.285318098	1.110091694	0.271572661	0.040332311	0.967971042
BEND4	0.474169755	0.636564209	0.951735751	0.345207969	-0.191388969	0.848910198
BEND5	1.30641203	0.194848242	0.974412421	0.333926572	1.200905712	0.234821535
BEND7	-1.407751163	0.162760168	0.263676186	0.792970537	-0.230054186	0.818884436
BEST1	-0.774633489	0.440651927	-1.449844326	0.152525654	-1.284652917	0.204180129
BEST4	0.693850936	0.489620198	-0.590731542	0.557011326	-0.420743291	0.675546094
BET1	0.109258306	0.913248687	0.129230196	0.897625735	-0.1627091	0.871330743
BET1L	-0.180127902	0.857470156	2.806510995	0.006821507	3.370467058	0.001361786
BEX2	2.425317964	0.017357382	-0.275033833	0.78427446	0.720325929	0.474307834
BEX4	1.474008066	0.144080936	-2.345829152	0.022449754	-1.24000734	0.220121863
BEX5	1.321681046	0.18973186	-0.369085774	0.713417171	2.103764307	0.039883586
BFAR	-0.868459673	0.387526978	0.193200008	0.847482018	0.8857466	0.37952596
BFSP1	0.39144542	0.696423056	-1.457519402	0.150404078	-1.239869743	0.220172374
BHLHA15	0.293405236	0.769909665	-1.272251778	0.208396161	-1.815794549	0.074734229
BHLHB9	1.013429317	0.313659248	0.007838238	0.993773141	-0.228302688	0.820239052
BHLHE40	-1.859855545	0.066277991	-1.493077758	0.140874839	-2.129030378	0.037644333
BHLHE41	0.164716116	0.869548712	-1.303797434	0.19748973	-0.263754416	0.792933258
BICD1	0.495801732	0.621281762	-1.199136707	0.235384891	-0.58292899	0.56227142
BICD2	-0.111770132	0.911262583	0.917548878	0.362681375	0.200431096	0.841866526
BID	-2.373238756	0.019830288	-2.049053042	0.045021918	-1.996346812	0.050746129
BIK	-0.858894391	0.392753984	0.286575917	0.775465405	0.744853495	0.45946185
BIN1	0.304315081	0.761612991	2.545624797	0.013608095	2.873360528	0.005720778
BIN2	-1.291483837	0.199949272	-0.902852408	0.370364697	-0.204156511	0.838968229
BIN3	-3.030314829	0.003215071	-0.438790883	0.662454696	-0.847105335	0.400527092
BIRC2	-0.220829918	0.825741178	-0.636892118	0.526718498	-1.058674086	0.294274426
BIRC3	1.381110843	0.170774495	0.366744289	0.715153683	-0.346014181	0.730623225
BIRC5	0.492803592	0.623390179	2.000747235	0.050140558	2.709536189	0.008914213
BIRC6	0.613397038	0.541210779	0.363232589	0.717760895	0.003437981	0.99726906
BIRC7	1.725178403	0.08803829	-1.30382569	0.197480156	-0.535548832	0.594382127
BIVM	1.305299814	0.195224912	0.988547099	0.327019551	1.52753599	0.132233114
BLCAP	-0.86283056	0.390597797	-0.554369153	0.581472491	0.442828813	0.659588278
BLK	1.095319598	0.276393091	-0.291194118	0.771948956	-0.525415979	0.60135964

BLM	1.882328741	0.063126835	-0.211220678	0.833460649	1.505284001	0.137847424
BLMH	0.690088293	0.491971498	3.392440271	0.001258165	4.385848809	5.12E-05
BLNK	0.160130059	0.873149027	0.210701782	0.833863651	0.757139217	0.452127154
BLOC1S1	-2.136226436	0.035463422	-0.242070174	0.809585639	-0.123249755	0.902348762
BLOC1S2	-0.03652686	0.970945762	-0.467277753	0.642064207	-0.995425911	0.323793089
BLOC1S3	-1.012293617	0.314198697	-1.592094182	0.116837523	-1.170008072	0.246932382
BLVRA	0.321791018	0.748381239	1.938661455	0.057448028	1.533710818	0.130707834
BLVRB	-1.941420663	0.0554369	0.650038465	0.518251775	-0.118128156	0.906387037
BLZF1	-0.082982107	0.934055986	-0.782399487	0.437184741	-1.808558128	0.075867015
BMF	0.861877622	0.391119133	0.056607662	0.955053653	0.065519411	0.947992814
BMI1	0.800346588	0.425685412	-4.089805601	0.000135728	-4.378995192	5.24E-05
BMP1	1.004613296	0.317863073	-0.194708249	0.846306531	-0.940250028	0.351110618
BMP2	-0.866886353	0.388383752	0.344578932	0.731665987	1.562751809	0.123721337
BMP2K	-0.776425103	0.439599256	-0.638178663	0.525886728	-2.39911977	0.019773376
BMP6	-3.958496535	0.000153469	0.943791552	0.349218449	-1.053600778	0.296571606
BMP8A	3.306861543	0.001371535	1.473449401	0.146074397	2.548202725	0.01358614
BMP8B	2.748551722	0.007274657	-0.652695315	0.51654948	0.883266319	0.38085273
BMPR1A	0.516253764	0.606984043	-2.17340336	0.033875422	-1.933997582	0.058151475
BMPR2	0.478476277	0.633508909	-1.233630607	0.222351142	-1.922630146	0.059596611
BMS1	1.485919125	0.140907567	3.091658923	0.003065802	3.090854675	0.003102063
BMS1P4	1.709875273	0.090845619	-0.232576187	0.816914993	0.110503723	0.912403319
BMX	-3.218909648	0.001807858	-0.234780156	0.815212039	-0.232836881	0.816733428
BNC2	0.021727255	0.98271517	-2.165467843	0.034506665	-2.006438208	0.049627191
BNIP1	1.212729778	0.228508815	-0.792364745	0.431399618	-0.204394336	0.838783281
BNIP2	-0.523097784	0.602233087	0.670812399	0.505021257	-0.468095918	0.641525154
BNIP3	3.289123655	0.00145067	-0.971645262	0.335289988	1.094691325	0.278318344
BNIP3L	-4.582353327	1.52E-05	0.372493351	0.710892737	-0.951714448	0.345314438
BNIPL	1.770804529	0.080088094	0.860447547	0.393110031	1.46086409	0.149617668
BOD1	0.413735407	0.680083831	-0.728433354	0.469299741	-0.566663836	0.573197364
BOD1L	-0.884039131	0.379106365	-0.771599572	0.443505872	-1.842934737	0.070611341
BOK	-0.339211304	0.735266219	0.175054274	0.861650923	-0.280660445	0.780000802
BOLA1	0.019272404	0.984667834	1.308468559	0.195911867	1.242598993	0.219172066
BOLA3	-0.101718485	0.919213693	-3.386473634	0.001281159	-1.089225839	0.280699943
BOP1	0.324835359	0.746083807	-0.268658987	0.789152089	-1.125118227	0.265318056
BORA	0.617795766	0.538320309	0.112138789	0.911103269	0.151781134	0.879902764
BPGM	-4.457925481	2.45E-05	0.684454523	0.496433196	-0.647904328	0.519683367
BPHL	0.439954513	0.661058729	-0.05717611	0.954602804	0.799294912	0.427482551
BPI	-3.751726649	0.000315714	2.194102609	0.032276319	0.884233859	0.380334818
BPNT1	-0.675725939	0.501003016	0.596153874	0.553408226	2.199831709	0.031944632
BPTF	0.605010259	0.546743621	0.594698274	0.55437431	-0.19774405	0.843958362
BRAF	0.0377753	0.969953196	-1.505814653	0.137579693	-2.76269138	0.007732467
BRAP	-1.694872072	0.09366861	1.020705632	0.311662176	1.066176443	0.290899882
BRAT1	0.444395212	0.657857987	1.065325831	0.291174129	1.603780479	0.114364578
BRCA1	-1.607414865	0.11157895	0.830489858	0.409691215	0.782106884	0.437432477
BRCA2	1.235089713	0.22011505	-2.719937865	0.008617063	-2.828453089	0.00647027
BRCC3	-0.36789543	0.713842824	-1.264074927	0.211294999	-0.702034959	0.485552897
BRD1	2.498144762	0.014360877	0.032899476	0.973868497	-0.547543952	0.586171714
BRD2	0.293514564	0.769826389	-1.629556671	0.108652851	-1.346172937	0.183648211
BRD3	-1.273820239	0.206112346	0.174818912	0.861835014	-0.248754814	0.804456668
BRD4	-1.622261327	0.108358087	-1.111665068	0.27090101	-0.752003117	0.455185159
BRD7	-0.315086399	0.753448918	2.440157417	0.017779866	2.377284061	0.020866303
BRD7P3	-0.145573988	0.884593725	-0.90862151	0.367336319	-0.511334463	0.611118744
BRD8	-0.241174421	0.809986191	1.391341021	0.169473032	1.928820981	0.058805825
BRD9	1.119468531	0.266014809	1.420638175	0.160813147	2.688750137	0.009419773
BRE	-1.289510353	0.200630977	1.125589518	0.265007766	1.88977348	0.063946732
BREA2	0.614034937	0.540791118	0.456772164	0.64955284	1.088877104	0.280852386
BRF1	0.18514323	0.853546699	0.479766816	0.633210113	1.040251812	0.302674707
BRF2	-0.976102495	0.331714174	1.930083267	0.058525903	1.612385982	0.112476476
BRI3	-2.515342228	0.013724814	-0.89669636	0.373613692	-1.278076506	0.206472697
BRI3BP	-0.243178841	0.808438066	-2.532193954	0.014084884	-3.668660356	0.000543014
BRIP1	-0.090024163	0.928474498	-0.591305761	0.556629207	-0.408301826	0.684602606
BRIX1	1.767727135	0.080604987	-1.651315545	0.104116775	-0.261098133	0.794970641
BRK1	-1.92757384	0.057163766	-2.996451862	0.004024325	-2.111169609	0.039215552
BRMS1	-1.566338822	0.120891721	1.473323619	0.146108197	1.807915118	0.075968364
BRMS1L	0.992275923	0.323808798	-1.526859236	0.132269233	-1.620981204	0.110615899
BROX	-0.24572815	0.806470195	-0.238175133	0.812590577	-1.366480766	0.17722702
BRP44	-1.090209397	0.278624791	0.610608509	0.543860854	0.032204257	0.974423188
BRP44L	-0.856338607	0.394157933	-0.867077249	0.389497729	-0.224553885	0.823140241
BRPF1	0.521963465	0.603019324	-0.090451485	0.928242271	-0.162751367	0.871297617
BRPF3	0.680540236	0.497965705	-0.853137581	0.397117046	-0.223810864	0.82371556
BRSK1	2.01708407	0.046762874	-3.215639151	0.00213571	-1.88415735	0.064716616
BRSK2	0.478360403	0.633591034	-2.289794197	0.025715954	-1.906865063	0.061651367

BRWD1	1.275344937	0.205574884	-0.383338594	0.702880015	-1.275938515	0.207222131
BRWD3	-0.153060966	0.878703878	-2.093245981	0.040741518	-2.739929445	0.008219671
BSCL2	-0.150173382	0.880974688	-1.494777753	0.140431467	-1.343192528	0.184605303
BSDC1	-0.385019603	0.701160525	-0.177373908	0.859837009	-0.216568376	0.829328439
BSG	-2.969005369	0.003857547	-0.43137659	0.667804938	-0.934857283	0.353858854
BSN	-0.754996259	0.452285845	-0.12986911	0.897122478	1.368018982	0.176747731
BSPRY	0.13418992	0.893561667	0.658329049	0.512949698	0.342983895	0.73288939
BST1	-1.580234953	0.117674259	-0.143957816	0.886036292	-1.209946397	0.231360919
BST2	-2.144523239	0.034773265	1.657617994	0.102832119	-0.273273308	0.785644225
BTAF1	1.372653416	0.173380686	-1.597721647	0.115577234	-2.770244427	0.007576764
BTBD1	0.906077545	0.367392	-0.208826305	0.835320619	-1.506060996	0.137648253
BTBD10	-2.119365079	0.036902716	-0.28168809	0.779192309	-1.042287053	0.301738699
BTBD11	-0.259505269	0.795857177	-0.82310063	0.413845785	0.174100863	0.86241142
BTBD18	-0.221661357	0.825095855	-0.568490029	0.571912218	0.529982214	0.598210616
BTBD19	0.957069527	0.341178456	-1.134764637	0.261174466	-0.203298093	0.839635863
BTBD2	-0.124066398	0.901548282	-1.532363862	0.130907402	-0.836807779	0.406242556
BTBD3	0.923095035	0.358505203	0.359022308	0.72089121	-2.037289125	0.046337261
BTBD6	0.341559783	0.733504038	-0.779241101	0.439027799	-0.430984589	0.668127096
BTBD7	-0.459776463	0.646821123	-0.704637417	0.483875515	-1.082185567	0.283788648
BTBD9	-0.392798074	0.69542733	1.007268464	0.318018794	0.728352724	0.469419817
BTD	-1.068257354	0.28835323	1.035720662	0.30466149	0.346919835	0.729946409
BTF3	-0.94532919	0.347103278	0.668796357	0.506297187	0.876703565	0.384377412
BTF3L4	0.252026247	0.801613912	-0.881140293	0.381904124	-0.885098912	0.379872144
BTG1	0.508983066	0.612049763	-0.103137207	0.918212345	-0.661992566	0.510679674
BTG2	-0.844572922	0.40066085	-0.970803288	0.335705568	-2.152528527	0.035661139
BTG3	1.995789566	0.049078012	-0.389555162	0.698302225	-0.318393762	0.751365715
BTK	-1.079083441	0.283526706	2.445986813	0.017522203	1.927953022	0.058916151
BTLA	1.195835255	0.235002984	-0.63330375	0.529042067	-0.779984544	0.438670498
BTN1A1	1.833940445	0.070074358	0.558211498	0.57886352	0.487831313	0.627565875
BTN2A1	-1.061676909	0.291314331	-0.342082525	0.733533897	-0.882643746	0.38118622
BTN2A2	0.024533752	0.980482925	0.044418987	0.964724069	0.531274431	0.597320858
BTN2A3	0.22513717	0.822399403	-1.012285672	0.315635214	-1.075337383	0.286815678
BTN3A1	0.002744276	0.997816657	-1.019659503	0.312153953	-0.617416992	0.539451774
BTN3A2	-1.008785938	0.315868734	-0.342280209	0.733385923	1.66479634	0.10151585
BTN3A3	-0.003358526	0.997327962	0.093743802	0.925638019	0.109495477	0.913199299
BTNL3	-1.956200762	0.053642641	0.287807538	0.774527143	-0.024563161	0.980490356
BTNL8	-3.273751995	0.001522691	-1.491481973	0.141292036	-1.483852646	0.143431449
BTNL9	1.938725265	0.055769539	-0.586955698	0.559527256	-0.296923525	0.767618846
BTRC	0.330901116	0.741513101	-0.562528027	0.575939322	0.930396359	0.356142738
BUB1	1.018168791	0.311414732	1.515887378	0.135017221	2.469580161	0.016588144
BUB1B	0.105188591	0.91646778	1.59293386	0.116648774	1.852241008	0.06924245
BUB3	1.714625986	0.089966375	-0.436444176	0.664146213	-0.478246577	0.634328696
BUD13	-0.740951739	0.460713623	1.214289066	0.229592653	2.183939797	0.033153345
BUD31	-1.745410184	0.084436446	0.337198384	0.737193082	0.569907335	0.571010343
BYSL	1.399843026	0.165108506	1.306019521	0.196737943	2.26858567	0.027151144
BZRAP1	2.569816052	0.011873727	-2.230006343	0.029659585	-0.964831102	0.338760197
BZW1	0.29715384	0.767055865	0.342755571	0.733030137	0.291440493	0.77178675
BZW2	0.981279281	0.329170126	2.793401279	0.007069185	3.746459146	0.000424431
C10orf10	0.761058903	0.44867536	0.473478452	0.637661602	0.64262784	0.523077057
C10orf103	0.092107426	0.92682399	2.492771034	0.015572997	3.345635012	0.001467395
C10orf105	-1.095591388	0.276274745	-0.660184427	0.511767112	-0.473793583	0.637481396
C10orf11	-0.187553484	0.851662477	2.197000661	0.032057813	1.447794179	0.15322688
C10orf116	1.765648562	0.08095567	-2.444220613	0.017599914	-0.945424303	0.348486812
C10orf118	-0.203587154	0.839150544	-2.034657373	0.046497843	-2.788709995	0.007208178
C10orf12	-2.338383559	0.021655536	1.472302114	0.146382917	0.921145899	0.360909085
C10orf125	0.642256636	0.522391745	-0.039242668	0.968832624	0.919550291	0.361735368
C10orf128	-1.793267873	0.076397492	-2.034753112	0.046487891	-2.640705593	0.010690313
C10orf137	1.094013417	0.276962337	-1.321617384	0.191521238	-1.985772924	0.051941779
C10orf18	0.633726278	0.527918491	-1.413666216	0.162842221	-2.004237327	0.049869397
C10orf2	1.095610709	0.276266333	1.900368561	0.062393843	2.689906323	0.009390977
C10orf25	0.635489604	0.526773566	-0.342415473	0.733284678	-1.315507885	0.193677453
C10orf26	-2.099311379	0.038680153	2.080640607	0.041924815	2.168289813	0.034382749
C10orf28	-0.402034915	0.68864219	2.599518077	0.011838579	2.862961094	0.005886821
C10orf32	0.217584607	0.828261174	0.218587495	0.827744081	-0.256384265	0.798589732
C10orf35	1.111183749	0.269544105	0.320538209	0.749720437	1.127299398	0.264402894
C10orf46	-0.732988895	0.465531345	-0.022353642	0.982243123	-0.144411359	0.885691936
C10orf47	-3.483115124	0.000777279	1.159770131	0.250927605	0.457568095	0.649025709
C10orf54	-2.736955188	0.007514648	0.876190481	0.384566177	0.928674068	0.357027057
C10orf57	-0.060291037	0.952061823	-2.711354176	0.008816913	-2.816763106	0.006679733
C10orf58	3.331123119	0.001269837	1.373630195	0.174879599	2.380877873	0.020682787
C10orf68	1.608855773	0.111263023	-1.552044318	0.126129577	-0.344650312	0.731642882
C10orf76	-0.31960584	0.750031701	0.407940671	0.684829726	1.592254822	0.116933417

C10orf88	1.684716011	0.09561989	-0.445987531	0.657278358	-0.565308562	0.574112403
C10orf91	1.899417509	0.060815835	-0.552401782	0.582810527	0.350341464	0.727391303
C10orf95	0.83737719	0.404670068	-2.267673711	0.027117281	-2.71465679	0.00879355
C11orf1	-0.693639259	0.489752313	-0.441080032	0.660806367	0.52327112	0.602841451
C11orf10	-1.569608559	0.120128426	-6.643845633	1.19E-08	-5.603437439	6.58E-07
C11orf2	0.949986445	0.344745044	-0.303727587	0.762429955	0.417431387	0.67795229
C11orf21	2.03201561	0.045195836	-5.735669169	3.77E-07	-4.190586525	9.93E-05
C11orf24	-1.073677505	0.285929799	-0.922644946	0.360041232	-0.016133406	0.987185065
C11orf30	-1.513019229	0.133891284	-1.506667002	0.137361378	-2.953939082	0.004573907
C11orf31	0.768394984	0.44432884	0.047700582	0.962119888	0.329841751	0.742745051
C11orf35	0.624063189	0.534215547	-2.008423221	0.049295153	-0.963032907	0.339653854
C11orf42	0.663759588	0.508595572	-1.083817053	0.282962087	-0.307159385	0.759856624
C11orf45	2.658170544	0.009345666	0.56140891	0.576696771	1.574145845	0.121063317
C11orf46	1.568579976	0.120368126	-0.288412933	0.77406607	-0.341783746	0.73378757
C11orf48	-0.560443223	0.576614458	0.762845241	0.448668833	1.309240042	0.195777321
C11orf49	0.058221925	0.953705088	-0.28961824	0.773148345	1.113844229	0.270084044
C11orf51	0.934171911	0.35279521	-0.312215492	0.756004285	1.003061755	0.32012763
C11orf54	0.030725064	0.975559026	-2.444998455	0.017565652	-2.268936627	0.027128406
C11orf57	0.399423967	0.690557571	-0.2014158	0.84108311	-0.122658894	0.902814514
C11orf58	1.151436128	0.252700601	-0.880732242	0.382123138	-0.545209795	0.587765158
C11orf61	2.240082127	0.027628295	-2.65734859	0.010174833	-1.651930191	0.104122285
C11orf63	1.659585848	0.100590679	-1.43186722	0.157586402	-0.093145545	0.926118985
C11orf66	0.079640541	0.936705661	1.087646431	0.281281766	0.957811614	0.342257493
C11orf67	0.50106713	0.617586569	0.5177323	0.606628007	0.511184205	0.611223266
C11orf68	-1.962007267	0.052951369	0.511185831	0.611175015	-0.327167843	0.744755664
C11orf71	0.507779771	0.612889968	-0.352723303	0.725583437	-1.052733421	0.296965573
C11orf73	-0.144265471	0.885623783	-0.695393157	0.489605193	-0.238893758	0.812056416
C11orf75	-1.523471357	0.131259812	-0.265859186	0.791297015	-0.34619548	0.730487719
C11orf80	1.501694171	0.136789198	0.801456618	0.42616146	2.089807407	0.041169625
C11orf82	-2.374274395	0.01977821	1.727518035	0.089433876	1.334322804	0.187476047
C11orf83	-0.029525193	0.976513206	-0.305523951	0.761068619	0.124068291	0.901703601
C11orf84	0.471635621	0.638365019	-2.511458809	0.014850726	-2.919906367	0.005029417
C11orf9	-0.072066373	0.942714146	-1.37286798	0.175115212	-1.817704562	0.074437614
C11orf92	-0.489312043	0.625849544	-0.241371486	0.810124451	-0.131894589	0.895538426
C11orf95	2.602003048	0.010888887	-0.576433502	0.566568143	-0.509805854	0.61218246
C12orf10	-0.77101239	0.442784003	1.903236526	0.062011293	2.233593222	0.029504799
C12orf11	2.633129409	0.010007357	1.919722459	0.059850705	2.781494104	0.0073502
C12orf23	1.700291107	0.092640819	0.501691906	0.61779664	0.434722684	0.665427369
C12orf24	3.018278865	0.003332788	-1.917579076	0.060127936	-1.770759967	0.082020123
C12orf26	0.451366372	0.652846218	-0.180032493	0.857758977	0.077175011	0.938758244
C12orf29	1.373777267	0.173032635	-0.978046923	0.332141386	-1.474536229	0.145913782
C12orf32	-1.119796607	0.265875719	-0.886124528	0.379235307	-0.221478895	0.825521824
C12orf33	1.500458557	0.13710833	-2.569763687	0.012787803	-1.845935073	0.070167543
C12orf34	-0.59209659	0.555318243	-1.327638017	0.189535807	-0.959189203	0.341569278
C12orf35	-0.774334788	0.440827573	-0.989372513	0.326619168	-2.871147568	0.005755744
C12orf4	0.131443782	0.895727092	-1.233310572	0.222469582	-0.950675065	0.345837333
C12orf41	1.874374949	0.064227394	0.331830246	0.741221937	-0.074624963	0.940777933
C12orf42	1.194049066	0.235697281	-0.762264443	0.449012599	-0.722243715	0.47313737
C12orf43	-0.74429202	0.458701142	2.807113474	0.006810319	3.237088772	0.002026738
C12orf44	1.040306407	0.301073802	3.054868667	0.00340763	3.964033626	0.00021028
C12orf45	-0.059202319	0.952926444	1.330410478	0.188626781	0.968132998	0.337123278
C12orf47	2.193875321	0.030904366	1.05728397	0.294796388	1.061326993	0.293078087
C12orf48	0.356794067	0.722108032	1.002315669	0.320383613	0.341969082	0.733648841
C12orf49	-0.927931563	0.356004797	-0.768940639	0.445070312	-0.672493015	0.504023757
C12orf5	-0.857166088	0.393703041	-1.564955687	0.123071513	-1.582884831	0.119055915
C12orf51	0.511869958	0.610036098	-1.049885945	0.298155904	-0.525668261	0.601185458
C12orf52	0.086500939	0.931266553	-0.018015742	0.985688559	-0.648253176	0.519459409
C12orf53	-0.185241824	0.853469606	0.506635247	0.614344837	0.016023849	0.98727208
C12orf57	1.439345579	0.153633255	0.650454199	0.51798521	1.621251632	0.110557768
C12orf60	-0.061553796	0.951059055	-2.005410515	0.049625487	-1.303550102	0.197698376
C12orf61	0.984624288	0.327533135	-1.875193012	0.065838697	-1.498342217	0.139636983
C12orf62	0.788385702	0.432609476	0.590029666	0.557478574	1.170231568	0.246843195
C12orf65	1.833784532	0.070097737	-0.19801213	0.843732794	0.081714089	0.935164211
C12orf66	1.004642779	0.317848952	-0.42639488	0.671409576	-1.217890989	0.228350703
C12orf73	1.219043483	0.226115536	-0.501422844	0.617984768	-0.12060043	0.904437383
C12orf75	-0.460182202	0.646531037	-3.078846424	0.003181031	-2.803516168	0.006924649
C12orf76	-0.50595255	0.614166821	0.296727006	0.767742361	1.101440452	0.27539693
C13orf15	0.901556787	0.369776089	0.710648174	0.480170108	0.722206099	0.473160312
C13orf27	0.135674897	0.892391047	0.389412012	0.698407513	-0.256952392	0.798153313
C14orf1	0.353093939	0.724870265	-2.792510767	0.007086306	-1.6501487	0.104487447
C14orf101	0.401454922	0.689067496	-1.195207017	0.236904268	-1.868431409	0.066914289
C14orf102	0.333840957	0.739301176	2.72226846	0.008563523	3.013442385	0.003868452

C14orf109	-1.775380437	0.079324573	-3.454728675	0.001040359	-2.654934649	0.010298603
C14orf118	0.036126651	0.971263956	0.162856274	0.871201648	-0.514010668	0.609258475
C14orf119	-2.21910618	0.029075725	-0.772053693	0.443239003	-0.424592864	0.672753541
C14orf126	0.678089322	0.499510725	-1.149989278	0.254900841	-0.987554015	0.327601142
C14orf128	0.886680409	0.377690204	-1.546517088	0.127457134	-0.998033321	0.3225383
C14orf129	-0.458603439	0.647660092	-0.651129754	0.517552205	-0.906630668	0.368470546
C14orf132	3.946392449	0.000160191	0.521392493	0.604092512	1.537402441	0.129802659
C14orf135	0.312751122	0.755216588	-0.392602319	0.696062437	-0.851226932	0.398253448
C14orf142	0.866223255	0.388745202	-0.134581722	0.893411788	-0.20837689	0.835687569
C14orf148	-1.369832185	0.174256754	-1.186262625	0.240389023	-1.371575906	0.175643235
C14orf149	2.291508255	0.024344104	-2.556426078	0.01323532	-2.566978617	0.012946343
C14orf153	-1.102783065	0.273156014	-0.572003232	0.569545634	0.606130121	0.546867258
C14orf159	-3.231925138	0.001735983	-1.157442844	0.251868965	-0.364242731	0.717042755
C14orf166	0.250016864	0.803162452	0.343064695	0.732798805	0.931629342	0.355510529
C14orf167	-0.483113123	0.630226353	-4.164230478	0.000105776	-3.409219656	0.001211237
C14orf169	1.645781366	0.10340916	-0.937876328	0.35222428	-1.604542922	0.114196265
C14orf182	1.001471577	0.319370204	-0.728065692	0.469523035	0.362533651	0.718312223
C14orf2	0.649010896	0.51803727	-1.646204183	0.105168229	-0.786761236	0.434724702
C14orf21	3.443331395	0.000885038	-0.152466155	0.879352135	0.065558585	0.947961764
C14orf28	0.359679094	0.719956844	-0.683813529	0.49683492	-0.590752892	0.557052834
C14orf43	0.370260839	0.712086092	-1.24861276	0.216858197	-1.584465891	0.118695613
C14orf45	-2.538094007	0.012922243	0.231906064	0.817432958	0.03306601	0.97373903
C14orf49	1.170243582	0.245091762	0.51734561	0.60689616	-0.698880575	0.48750705
C14orf55	0.319316681	0.75025019	-0.709170884	0.481079328	0.575278322	0.567397798
C14orf64	-0.191276447	0.848753738	-3.414181749	0.001177646	-2.239652594	0.029084762
C14orf79	0.443456872	0.658533789	0.871847635	0.386911367	1.015442937	0.314243678
C14orf80	0.319341875	0.750231153	1.296026045	0.20013595	1.675278498	0.099432016
C14orf93	-0.071893814	0.942851075	0.853552446	0.396888961	1.332416227	0.188097519
C15orf17	3.551400875	0.000620639	0.898437196	0.372693095	2.217518123	0.03064519
C15orf23	-1.818292839	0.072453309	-0.609603409	0.544521999	1.11062023	0.271457982
C15orf24	-2.52557296	0.01335854	2.05176042	0.044748918	2.015749953	0.048613614
C15orf26	-3.505401053	0.000722456	2.401011722	0.019600154	1.430000553	0.158249386
C15orf27	1.868288885	0.06508036	1.139111281	0.259372268	1.376574324	0.174100071
C15orf29	-0.751706157	0.454252169	-1.398220886	0.167407847	-1.572618374	0.121416963
C15orf33	1.393083722	0.167136187	-0.914235012	0.364404863	-1.134158546	0.261539545
C15orf34	-0.837676497	0.40450282	-1.825967395	0.07304018	-2.423314326	0.01862256
C15orf37	-0.257196916	0.797632767	-3.491231209	0.000929887	-3.486345896	0.0009573
C15orf38	-1.271223734	0.207030011	-0.066292919	0.947374199	1.138323318	0.259811739
C15orf39	-2.428912231	0.017197323	0.232180453	0.817220861	-0.169875911	0.865717363
C15orf40	0.084778412	0.932631922	-2.491058764	0.015640736	-1.835141297	0.071775174
C15orf41	0.116850157	0.907247538	0.116076411	0.907995757	-0.899520151	0.37221133
C15orf42	-0.919470003	0.360386633	1.715442235	0.091639721	1.492860546	0.141063065
C15orf44	1.000902373	0.319643769	1.93749035	0.057594176	3.151127953	0.002606399
C15orf50	-0.32095764	0.74901055	-1.347014457	0.183251653	-1.555763743	0.125374532
C15orf52	-0.102817904	0.918343609	0.60102995	0.550178148	0.657151947	0.513763811
C15orf54	-2.571641905	0.01181578	0.279566458	0.780811653	0.516623076	0.60744505
C15orf57	1.543208197	0.126402179	1.560985949	0.124005357	2.370000582	0.021242704
C15orf58	-1.032920895	0.304497555	0.05330625	0.957672363	0.540100361	0.591260345
C15orf61	0.688329276	0.49307283	-3.482635109	0.000954851	-4.028622254	0.000170097
C15orf62	3.738453112	0.000330409	0.135775965	0.892471824	1.541067489	0.128908951
C16orf13	0.020502747	0.983689169	-2.436804999	0.017929584	-1.587765136	0.117946587
C16orf42	-0.474278138	0.636487237	0.879208266	0.382941802	0.677425379	0.500913579
C16orf45	-0.273885568	0.784820367	-0.712344116	0.479127502	-0.192697451	0.84789013
C16orf46	-0.433332456	0.665843457	-1.153144623	0.253614181	-1.467357083	0.147849582
C16orf48	0.21846929	0.827574035	-1.885669121	0.064386144	-1.121243462	0.266949327
C16orf5	-1.094325999	0.276826037	1.730875504	0.088828433	2.186718071	0.032939178
C16orf52	1.422242005	0.158523703	-2.212294197	0.030926132	-2.664865858	0.010033028
C16orf53	0.28765073	0.774296713	2.695187841	0.009204884	2.804600935	0.006904286
C16orf54	-2.546950687	0.01262142	-0.490940307	0.625334084	-0.199934943	0.84225269
C16orf55	-0.145517794	0.884637956	0.209922904	0.834468653	-1.067878452	0.290138055
C16orf57	-2.04258477	0.044114033	-0.37108552	0.711935307	-0.47720488	0.635065606
C16orf58	1.813767765	0.073153679	-0.544147458	0.588440413	0.611811206	0.543128309
C16orf61	0.915448688	0.362481099	-0.174296877	0.862243357	0.114381066	0.909343121
C16orf62	-0.64495599	0.520649177	1.688514561	0.09672029	2.029077933	0.047193954
C16orf7	-0.361586774	0.718535641	-1.260318458	0.21263672	-1.53798635	0.129659945
C16orf70	0.941688544	0.348954	0.458268926	0.648483666	1.014457447	0.314709324
C16orf71	0.72490124	0.4704536	-0.61854566	0.538654306	0.004792472	0.996193135
C16orf72	-0.328038253	0.743669198	0.035692971	0.971650592	-1.05981479	0.29375961
C16orf74	-0.524609213	0.60118619	2.22056346	0.030329007	1.212677143	0.230322988
C16orf79	0.208150713	0.835596754	1.322638856	0.191183285	1.770591643	0.082048428
C16orf80	1.734834231	0.086303848	-1.568948668	0.122137896	-0.756836731	0.452306922
C16orf86	-0.61341857	0.541196611	0.497505625	0.620726599	0.028499838	0.977364397

C16orf87	-0.023969569	0.980931656	-0.432177901	0.66722586	-0.486758127	0.628321516
C16orf88	1.561270993	0.12208239	0.901416177	0.371121086	1.775399716	0.081243122
C16orf91	0.623296233	0.53471699	1.235494956	0.221662093	1.327485342	0.189712038
C16orf93	1.232491669	0.221078624	0.63626924	0.527121445	-1.014722299	0.314584136
C16orf95	0.902962395	0.369033775	1.006958637	0.318166382	0.986487348	0.328119432
C17orf100	2.363351533	0.020333638	-0.743895685	0.459963666	-0.52325215	0.602854565
C17orf101	0.572051625	0.568759009	1.596488451	0.11585247	1.551910203	0.126293714
C17orf103	-1.677415079	0.09704297	0.006808018	0.994591555	-0.02009219	0.984040949
C17orf104	0.866483954	0.388603072	-1.054391014	0.296106996	-1.510057564	0.136627391
C17orf106-CDK	2.426969986	0.01728365	-4.018829577	0.000171829	-3.067146516	0.003320199
C17orf107	0.31898417	0.750501461	-2.433236423	0.018090199	-2.502278133	0.015273583
C17orf108	0.963023738	0.338198954	-0.499647352	0.619226838	-0.052576415	0.958255728
C17orf109	-2.232636518	0.028134672	0.665893497	0.508137426	0.054858877	0.956445325
C17orf110	-0.64694637	0.519366241	-1.560292586	0.124169045	-1.750221606	0.085534287
C17orf28	2.149900783	0.0343322	2.160426987	0.034912982	2.529902948	0.014237176
C17orf37	-1.931104909	0.056719131	-1.119030125	0.267772511	-1.16330184	0.249619334
C17orf39	-1.757527146	0.082337962	-0.310523969	0.757283469	0.101682296	0.91937054
C17orf42	0.621221584	0.536074624	-0.536461834	0.593705483	-0.301752269	0.763953997
C17orf46	1.747089757	0.084142966	-1.025873942	0.309240289	-1.393609454	0.168918859
C17orf48	1.034362521	0.303827193	-2.306203325	0.024718345	-1.568495732	0.122375596
C17orf49	-0.69259166	0.490406443	1.386847733	0.170832366	1.570355933	0.121942296
C17orf51	1.084033854	0.281338345	-0.537337711	0.593104344	0.685943682	0.495566919
C17orf53	0.627952367	0.531676491	2.088703784	0.041164515	3.238386662	0.002018997
C17orf54	0.40897079	0.683563944	0.338828739	0.73597094	0.290413402	0.772568246
C17orf55	1.813423474	0.073207196	0.05627446	0.95531793	1.872280347	0.066370656
C17orf56	-0.348636064	0.728203014	-0.857894337	0.394506728	-0.16152726	0.872257062
C17orf57	0.889434276	0.376217214	0.054432315	0.956779104	-0.907802097	0.36785658
C17orf58	0.435794982	0.664062543	0.409190073	0.683917855	-1.165982437	0.248542806
C17orf59	-0.241150815	0.810004428	-0.404561333	0.687298485	-0.804678033	0.424394379
C17orf61	1.272138025	0.206706536	-0.932282015	0.355082457	0.052047873	0.958674989
C17orf62	-0.625661629	0.533171249	1.476628666	0.145222123	1.472869305	0.14636147
C17orf63	-0.53698417	0.592646257	-1.001486597	0.320780621	-0.990501477	0.326171816
C17orf65	-0.877117529	0.382833225	-1.387838857	0.170531807	0.362243974	0.718527468
C17orf66	1.692554613	0.094110977	-1.046382818	0.29975583	-0.336200031	0.737971273
C17orf67	2.041968627	0.044176482	0.110032721	0.912765921	1.192127514	0.238217542
C17orf69	2.975528246	0.003783942	-0.033715465	0.973220618	1.295950368	0.200286266
C17orf70	1.227280748	0.223020538	0.61802178	0.538997164	1.356792706	0.180268575
C17orf72	0.673457752	0.502437455	-1.041173642	0.30214578	0.179798664	0.857956929
C17orf75	2.452687967	0.016171321	0.816310959	0.417685725	3.204717854	0.002229094
C17orf76	-4.79334021	6.69E-06	-0.940489277	0.350894439	0.003599408	0.997140832
C17orf79	0.151872308	0.879638522	1.391094049	0.16954753	1.16699261	0.248137987
C17orf80	2.148814219	0.034420925	0.692357568	0.491494822	0.393696081	0.695294201
C17orf81	0.326229889	0.74503218	1.059496454	0.293796752	0.505401033	0.615252346
C17orf85	1.61217297	0.110538439	0.333529597	0.739945759	-0.639045497	0.525387762
C17orf87	-1.360935383	0.177041473	-0.394863287	0.694402286	-0.251923922	0.802018276
C17orf89	0.527719597	0.599034407	-0.3434489	0.73251132	0.14149243	0.887986591
C17orf90	-0.465161468	0.642975538	-1.434519913	0.156831532	-0.717530566	0.476016824
C17orf91	-0.407315096	0.684774883	-0.907837181	0.367747105	-1.210343343	0.231209832
C17orf96	-0.466255163	0.642195683	-1.251161121	0.21593394	-0.663631107	0.509637954
C17orf97	0.342688031	0.732657964	0.118769727	0.905871062	-1.435932995	0.156560832
C18orf1	0.570567457	0.569760445	-0.77687022	0.440414319	-1.462050935	0.14929325
C18orf10	-2.378037827	0.019589984	-0.037949003	0.969859571	-0.802514525	0.425633921
C18orf18	1.577898728	0.118210349	-0.77522101	0.441380314	-0.291313071	0.771883691
C18orf19	2.529799671	0.013209801	0.679300948	0.499668079	0.77471573	0.441752871
C18orf21	-1.513509046	0.133767045	-2.060040983	0.043922815	-1.119337664	0.267754259
C18orf25	-0.110722354	0.912090994	-0.306469173	0.760352607	-2.61558175	0.011415299
C18orf32	0.170054761	0.865361041	-3.306186545	0.001631919	-3.312521777	0.001620316
C18orf45	-0.388417033	0.698654264	-1.110610652	0.271350996	-0.040733558	0.96765258
C18orf54	1.649182676	0.102708848	-1.936265316	0.057747392	-1.592170736	0.116952328
C18orf55	1.687542202	0.095073599	-1.720315446	0.090744206	-0.689880112	0.493106759
C18orf8	0.136536853	0.891711669	-2.063189266	0.043612215	-0.821343315	0.41491917
C19orf10	1.154906071	0.251284328	-1.150306411	0.254771313	-1.075347176	0.286811333
C19orf12	2.171201358	0.032632574	-2.075253421	0.042439558	-1.551990138	0.126274593
C19orf2	2.782679805	0.006608399	0.432812757	0.666767218	1.162318336	0.250015147
C19orf20	0.365413963	0.71568741	-2.321251011	0.023833897	-2.422797411	0.018646504
C19orf22	-2.53208719	0.013129925	-0.405214763	0.686820857	-0.947406006	0.347485313
C19orf23	1.465543108	0.14636987	-0.416462187	0.678619749	-0.297238051	0.767379968
C19orf24	-0.372813937	0.710191711	0.733454635	0.466256181	0.664600471	0.509022209
C19orf25	-0.013985847	0.988873226	1.032074474	0.306351582	0.775157139	0.441494149
C19orf28	-0.254848332	0.799440397	-0.202767386	0.840031441	-0.55546259	0.580781335
C19orf29	0.85360401	0.395663526	-2.092581119	0.040803197	-1.694868965	0.095631454
C19orf29OS	2.436052948	0.016883236	-2.071735833	0.042778614	-2.967139653	0.004407785

C19orf34	1.841678262	0.068922265	0.45225013	0.652787532	0.672762491	0.503853565
C19orf35	-1.815128912	0.072942417	0.023935187	0.98098704	-0.981442309	0.330578201
C19orf38	-2.440493934	0.016690491	0.056467506	0.955164816	-0.4145708	0.680033311
C19orf39	0.221188453	0.825462885	0.814529305	0.418696908	0.821572917	0.41478953
C19orf40	-0.785609484	0.434226041	-1.425034724	0.159543716	-0.055199725	0.956174991
C19orf42	-0.25610043	0.79847656	1.841863611	0.070645522	2.917395365	0.005064641
C19orf43	0.29251839	0.770585283	-4.404570428	4.67E-05	-3.654914211	0.000567023
C19orf44	0.617941096	0.538224946	1.069709382	0.289212651	1.709180138	0.092931233
C19orf46	0.950655534	0.344407102	1.603672471	0.114256484	1.253081128	0.215361363
C19orf47	-1.184901017	0.239276297	-2.111910713	0.039042789	-1.803978413	0.076591328
C19orf48	0.647799373	0.518816931	1.20809125	0.231949171	1.503310303	0.138354378
C19orf50	-1.546450704	0.125617926	-2.881503077	0.005552028	-2.025357134	0.047586636
C19orf52	0.269730554	0.788004911	1.317308598	0.192951763	1.310502509	0.195352994
C19orf53	-0.212756578	0.832013479	-2.543716465	0.013674936	-1.030892756	0.307004444
C19orf54	-0.93049839	0.354682345	-0.76218006	0.449062557	0.006184323	0.995087542
C19orf55	0.464812362	0.643224552	-0.475749637	0.636052287	0.768170373	0.445599728
C19orf56	-1.452993417	0.149815278	-1.936275548	0.057746111	-1.293073795	0.201272405
C19orf57	0.202374422	0.840095502	0.450463023	0.654067736	0.720097121	0.47444759
C19orf59	-1.578449556	0.118083776	1.408199866	0.164446926	0.822548281	0.414239086
C19orf6	1.271573676	0.206906158	-2.431642593	0.018162351	-2.014970638	0.048697752
C19orf60	0.977367993	0.331091074	-3.342528095	0.001463184	-1.644750405	0.105600344
C19orf63	-0.77885294	0.438175115	-0.097391659	0.922753489	-0.089724672	0.928824804
C19orf66	-0.908099776	0.366328707	-3.440600848	0.001086372	-4.185322729	0.000101034
C19orf70	-1.136702314	0.258777265	-2.909764221	0.005133207	-2.424352402	0.018574558
C19orf71	3.129026778	0.002385411	-1.828738581	0.072617879	-1.071859367	0.288361569
C19orf73	1.412161606	0.161461656	-1.986635358	0.05172735	-2.140519204	0.036662969
C19orf76	1.37248057	0.173434263	-0.871406478	0.387150097	0.427570453	0.670596712
C19orf77	-3.243399737	0.001674842	0.890134354	0.377096809	0.332973083	0.740392773
C19orf79	0.091845382	0.927031583	-3.365754747	0.001364131	-2.663002944	0.010082363
C1D	-0.337227196	0.736756101	0.377666086	0.707066841	0.195045714	0.846060127
C1GALT1	-0.32798672	0.743708028	-1.66111328	0.102125254	-2.4233246	0.018622084
C1GALT1C1	0.392335019	0.695768138	0.804357301	0.424498316	-0.125627829	0.900474575
C1QA	0.01925186	0.984684176	-0.392216226	0.696346082	-1.264516311	0.211260335
C1QB	-0.127351862	0.898955173	0.601754733	0.549698841	-1.255382933	0.214531161
C1QBP	1.210697735	0.229282974	2.063120369	0.043618991	1.848639929	0.069769467
C1QC	0.380876579	0.704221285	1.385209001	0.171330204	0.037411038	0.970289759
C1QL3	0.818161396	0.41549545	-1.325649257	0.190189914	-1.013500132	0.315162103
C1QTNF3	1.710352826	0.090756919	0.948158154	0.347010308	0.448014271	0.655864144
C1QTNF4	0.130778243	0.896252011	-1.820872163	0.073822023	-2.338884812	0.022919991
C1QTNF6	1.6619134	0.100121652	0.314088156	0.754588917	2.05357936	0.04467739
C1R	0.344857721	0.731031841	0.493609236	0.623459226	0.193914024	0.846941948
C1RL	-0.181379821	0.856490449	0.338442883	0.736260123	0.330664578	0.742126698
C1S	2.065984843	0.041797786	1.144725489	0.257057619	1.014341456	0.314764161
C1orf109	3.679212246	0.000404305	0.930713352	0.355886588	0.951516447	0.345414009
C1orf112	0.549993875	0.583729785	-0.923033252	0.359840569	-1.243710939	0.218765484
C1orf115	0.177964415	0.859163751	1.247784713	0.217159146	1.291158382	0.201931056
C1orf116	-4.122015225	8.54E-05	0.963294173	0.339426956	0.812223709	0.420088214
C1orf122	-0.408368554	0.684004311	0.294512803	0.769424961	0.300538206	0.764874922
C1orf123	-0.12540119	0.900494643	-0.731416337	0.467490301	0.135489924	0.892708345
C1orf124	-0.24799201	0.804723712	-1.671908058	0.099967206	-2.38922371	0.020262175
C1orf127	0.72882582	0.468061405	3.761575798	0.000397295	4.366876258	5.47E-05
C1orf131	0.634781812	0.527232979	-0.72812336	0.469488008	-0.54515224	0.587804475
C1orf135	0.049607565	0.960548584	0.020996365	0.983321125	1.156346638	0.252428133
C1orf144	-2.023679666	0.046065034	-0.290514505	0.772466135	-0.199351734	0.842706661
C1orf150	-2.658939886	0.009325981	2.198905042	0.031914936	-1.975906003	0.053079233
C1orf151	-0.76175974	0.448259066	-0.255794405	0.799020929	0.505353036	0.615285836
C1orf152	1.567011153	0.120734456	0.388006847	0.699441337	0.151098342	0.880438845
C1orf159	1.773612402	0.079618865	-0.238780177	0.812123611	0.813442749	0.419395013
C1orf162	-3.495367642	0.00074667	0.262657582	0.79375175	0.170434876	0.865279844
C1orf172	0.735971987	0.46372317	0.569848421	0.570996602	1.480000442	0.144453795
C1orf174	1.157180741	0.250358973	0.516141689	0.607731377	-0.047422307	0.962344662
C1orf177	0.403405189	0.687637774	-1.991455563	0.051180565	-1.305157773	0.197154159
C1orf183	-3.537597634	0.00064967	-1.119997451	0.267363515	-1.005911427	0.318766858
C1orf186	-2.089213148	0.039602884	1.218224963	0.228105258	-1.074246482	0.287299937
C1orf187	1.272669656	0.206518617	-1.793823335	0.078091012	-1.83531592	0.071748921
C1orf192	0.606012282	0.54608108	0.057422694	0.954407237	0.033727135	0.973214165
C1orf198	-2.20840979	0.029839066	0.776960643	0.440361392	-0.046097225	0.963396064
C1orf200	-1.431730451	0.155796056	-0.977770443	0.332276965	-0.491207462	0.625191314
C1orf201	-0.163681394	0.87036079	-1.925985398	0.059046866	0.0865376	0.931346454
C1orf204	0.830751187	0.408383357	-0.579599546	0.564445016	0.752877751	0.454663564
C1orf21	-1.669721795	0.098561118	-2.08680256	0.041342699	-1.173758844	0.245438681
C1orf212	-1.573459711	0.119234333	-1.118205173	0.268121658	-1.036789104	0.304271759

C1orf213	-0.302869845	0.762710482	-1.035312252	0.304850481	-1.350370674	0.182306607
C1orf216	1.843493639	0.068654276	1.615224601	0.111727372	2.568482239	0.012896309
C1orf220	1.066970132	0.288930831	0.468842381	0.640952067	0.327232997	0.74470665
C1orf226	0.925214199	0.35740825	2.4353791	0.017993606	0.527333499	0.60003632
C1orf228	0.467507093	0.64130349	-0.692787197	0.491227137	-0.123461766	0.902181651
C1orf27	0.177345223	0.85964858	-1.175170963	0.244761642	-1.509437843	0.136785295
C1orf31	-0.826736557	0.410643211	0.152403047	0.87940168	-0.071616454	0.943161235
C1orf35	1.007157825	0.316645904	-0.531408769	0.59717912	0.217161518	0.828868418
C1orf38	-3.13336069	0.002354016	0.079959929	0.936546307	-0.15033504	0.881038204
C1orf43	-0.673601876	0.502346243	-0.782630943	0.437049856	-0.691809412	0.491903458
C1orf50	-0.463607861	0.644084021	-2.453445186	0.017197438	-1.789447048	0.078927987
C1orf52	-0.433539417	0.665693707	-1.247560489	0.217240693	-1.526265032	0.132548811
C1orf55	-1.930736834	0.056765342	-0.871334934	0.387188822	-1.544022867	0.128191873
C1orf56	2.012750552	0.047226288	1.943477687	0.056850312	2.622099922	0.011223022
C1orf63	2.434143598	0.016966714	-1.326165574	0.190019933	-2.28026537	0.026403429
C1orf74	-1.689562678	0.09468461	0.717057331	0.476236652	-0.160046011	0.873418311
C1orf85	-1.979844975	0.050874946	-0.955832661	0.343151545	-1.910225527	0.061208398
C1orf86	0.062834427	0.950042177	0.04630036	0.963231016	0.33064094	0.742144459
C1orf87	-5.454321491	4.53E-07	0.223420052	0.823999131	-0.872686139	0.386545144
C1orf9	-0.318777495	0.750657654	-0.419735885	0.676239983	-1.528266914	0.132051829
C1orf93	0.14216298	0.887279266	-0.140335737	0.888884356	0.918747604	0.362151497
C1orf95	-0.370792185	0.711691688	0.640573645	0.524340175	0.477406358	0.634923049
C1orf96	0.131589178	0.895612422	-0.902717611	0.370435646	-1.686448805	0.097250083
C1orf97	-1.574876768	0.118906683	-1.734257208	0.08822205	0.331067858	0.741823695
C2	-1.622736923	0.108256159	1.326096079	0.190042805	0.333486656	0.740007211
C20orf103	0.376141973	0.707725065	0.506358023	0.614538184	1.002418583	0.320435295
C20orf106	1.273403785	0.206259328	1.727948478	0.089356065	3.400087472	0.001245223
C20orf107	-0.772335581	0.442004223	1.275310228	0.207319522	2.147034188	0.036116469
C20orf108	-3.446700894	0.000875392	-0.04686148	0.962785737	0.191955538	0.84846848
C20orf11	-2.38848914	0.01907559	2.148991805	0.035850286	2.882199256	0.005583075
C20orf111	-3.665283461	0.000423831	-1.120444982	0.267174443	-1.369866912	0.17617325
C20orf112	1.895343845	0.061360164	-1.38412348	0.171660593	0.003843107	0.996947252
C20orf118	1.04351315	0.299595383	1.419782918	0.161060995	1.595577306	0.116188173
C20orf12	0.525883705	0.600304059	-1.718369815	0.091100871	-1.058760757	0.294235288
C20orf132	-0.990271189	0.324781853	-0.057662944	0.954216697	0.653368874	0.516181057
C20orf134	-0.092756281	0.926309987	-2.945386904	0.004647025	-2.335021835	0.023136262
C20orf165	2.818777557	0.005964817	1.460666304	0.149540901	1.570252622	0.121966328
C20orf177	-0.267242244	0.789913767	0.09995248	0.920729149	-0.419325545	0.676575714
C20orf194	2.893765256	0.004807657	0.70549896	0.483343431	0.873587091	0.386058341
C20orf195	0.025663032	0.979584756	1.199365627	0.2352966	0.348487242	0.728775561
C20orf196	0.784008893	0.435159662	-1.132144666	0.262265037	-0.843043961	0.402775332
C20orf197	0.116457651	0.907557674	-2.207970002	0.031242488	-2.364935732	0.021508016
C20orf20	1.195161208	0.235264815	-0.249176283	0.804110881	-0.582223878	0.562742922
C20orf201	1.610940227	0.110807266	-2.184837266	0.032983715	-2.513821766	0.014832471
C20orf24	-0.880887058	0.38080074	-3.557166826	0.000758016	-3.318048658	0.001593786
C20orf27	-0.516030765	0.607139131	0.378632306	0.706353036	0.395420832	0.694028365
C20orf29	-1.417016754	0.160041433	1.253102964	0.215231616	2.813003746	0.006748413
C20orf3	-2.241407687	0.027538987	-0.123903628	0.901822974	0.297495933	0.767184127
C20orf30	0.265776766	0.791038579	-2.678884982	0.009611907	-2.371676174	0.021155577
C20orf4	-0.212423943	0.832272145	1.838984513	0.071074274	2.682903729	0.009566629
C20orf43	-3.068678929	0.002865085	1.425079703	0.15953077	1.44334081	0.154472078
C20orf7	0.443941755	0.658184537	-1.002975168	0.320068042	-0.48146548	0.632053958
C20orf72	-0.615447744	0.539862248	1.030020945	0.307306242	-0.447953948	0.655907417
C20orf94	0.131448625	0.895723271	1.20410405	0.23347446	0.263578732	0.793067964
C20orf96	-0.095386595	0.924226654	-0.015568031	0.987632812	0.625319699	0.534290639
C21orf119	-0.059757062	0.95248588	-0.298162703	0.766651957	-0.162195124	0.871733572
C21orf122	-1.409674953	0.162192788	-0.866602969	0.389755458	1.298772713	0.199322241
C21orf128	0.946870069	0.346321889	-0.381878028	0.703957168	0.527878164	0.599660682
C21orf15	-2.148004026	0.034487213	-1.778872878	0.080537808	-1.674321749	0.099620751
C21orf2	0.984193453	0.327743676	0.274552421	0.784642506	1.431641992	0.157780771
C21orf33	0.178676846	0.858605981	2.445629514	0.017537899	3.701297365	0.000489846
C21orf49	2.150840902	0.034255593	-2.029689754	0.047016761	-0.276767822	0.782973119
C21orf56	0.689608679	0.492271655	1.915084152	0.060452011	2.982232065	0.004224742
C21orf58	0.041910378	0.96666597	0.705202496	0.483526489	1.683664952	0.097790152
C21orf59	-0.852995871	0.39599883	-0.790272867	0.432610224	-0.010959914	0.991294218
C21orf62	-1.119492083	0.266004822	2.353844611	0.022014116	1.900000221	0.062564748
C21orf63	-1.381989328	0.170505506	0.569654297	0.571127407	1.06641708	0.290792088
C21orf67	3.699690895	0.000377142	-0.602241685	0.549376934	1.698660986	0.094909771
C21orf7	-5.682138501	1.73E-07	-0.174340014	0.862209613	-0.766743993	0.446440638
C21orf70	0.546975895	0.58579255	-1.300335364	0.198665329	-0.483286298	0.630768801
C21orf90	-0.808518041	0.420993083	-0.858617048	0.394111067	-1.90260367	0.06221701
C21orf91	0.01043973	0.991694303	-1.196295133	0.236482848	-2.060153579	0.044022247

C21orf96	0.138164307	0.89042916	-1.107505139	0.272679365	0.320256887	0.749960523
C22orf13	-2.254827964	0.026648981	1.506443737	0.137418538	1.635323182	0.107566975
C22orf23	0.039756194	0.968378394	1.103811752	0.274265131	1.444944402	0.154022794
C22orf25	-2.245197318	0.027285061	1.088796249	0.280778588	1.722017754	0.090562803
C22orf26	0.13945113	0.88941529	0.699715697	0.486921387	-0.012437299	0.990120745
C22orf28	0.533922493	0.594753842	3.506464021	0.000887165	3.730148185	0.000447027
C22orf29	3.492090124	0.000754744	1.737189486	0.087699031	2.92118257	0.005011602
C22orf32	-0.101438911	0.919434964	0.798479658	0.427872389	0.894189785	0.375031386
C22orf34	0.464764136	0.643258954	1.776871539	0.080870147	1.487101078	0.142573776
C22orf36	-0.905069099	0.367922973	-0.42185373	0.674702212	-0.602472129	0.549281636
C22orf39	2.66482022	0.009176756	-0.427009119	0.670964707	0.310919278	0.757011539
C22orf40	0.832281846	0.407523729	0.381801713	0.704013466	1.280266778	0.205707036
C22orf43	1.302709894	0.196104133	1.016529244	0.313628596	0.970313414	0.336045201
C22orf45	0.651280491	0.516578358	0.557465684	0.579369489	1.315357045	0.193727787
C22orf46	-0.244844839	0.807151903	0.305528604	0.761065093	1.137718088	0.26006232
C2CD2	1.661761098	0.100152288	-0.004574113	0.996366205	1.260716977	0.212616419
C2CD2L	0.711622743	0.47859811	-0.231332784	0.817876132	0.674306514	0.502879012
C2CD3	0.129383113	0.89735252	-0.06181996	0.950920235	-0.368454423	0.713917832
C2CD4D	-0.342760529	0.732603609	-2.455535263	0.017107406	-2.730118277	0.008438245
C2orf15	1.389447051	0.168234975	-1.324608716	0.190532829	0.178473515	0.85899251
C2orf16	0.791642755	0.43071745	1.764111015	0.083016102	0.620807694	0.537234222
C2orf18	-1.501113981	0.136938976	1.196710019	0.236322309	1.746850717	0.086122827
C2orf28	-0.285777155	0.775726657	-2.410707704	0.019134358	-1.665117184	0.101451541
C2orf29	0.161739765	0.871885008	0.728658169	0.469163233	1.537775362	0.129711499
C2orf3	2.300746892	0.023792034	-0.44053459	0.661198967	0.869673075	0.388175957
C2orf40	-0.294013137	0.769446655	0.017522956	0.98607998	-0.224044247	0.823534841
C2orf42	0.063395692	0.949596533	1.453786786	0.151432961	2.566016645	0.012978446
C2orf43	2.094791514	0.039090836	0.69851879	0.487663713	-0.602519654	0.549250233
C2orf44	0.94243828	0.348572351	-0.232635356	0.816869263	1.322742343	0.19127491
C2orf47	0.054739007	0.956471629	-0.876997656	0.384131279	-0.209534939	0.834787884
C2orf49	1.034673325	0.303682798	-2.270022642	0.026965332	-3.614487143	0.000643651
C2orf55	-1.86560861	0.065459006	-0.792119675	0.43154134	-1.46651295	0.148078514
C2orf56	2.261044069	0.026245374	-1.170206654	0.246737151	0.141821022	0.887728227
C2orf62	-0.741907487	0.460137283	-0.263124152	0.793393891	-0.724360419	0.471847399
C2orf63	1.641661837	0.104262516	-0.963305179	0.339421482	0.585556717	0.56051601
C2orf65	-0.121540076	0.903542953	0.030996483	0.975379506	-0.024916994	0.980209378
C2orf67	0.221511334	0.825212287	-1.58390902	0.118690359	-2.493358439	0.015622512
C2orf68	0.465470561	0.6427551	-0.005936286	0.995284071	1.575637701	0.120718716
C2orf69	0.511675117	0.61017191	-1.035561783	0.304735002	-2.017876066	0.048384707
C2orf74	0.919212765	0.360520381	0.802656508	0.425473016	1.015646079	0.314147752
C2orf76	2.189421269	0.031237405	-1.853829583	0.068886822	-1.331468524	0.188407014
C2orf81	1.121167591	0.265295031	1.492666997	0.140982134	2.156414861	0.035342113
C2orf85	-0.783211676	0.435625115	-1.846520774	0.069956593	0.028840352	0.977094024
C2orf88	-5.668837577	1.83E-07	0.315599665	0.753447129	-0.701795237	0.485701253
C2orf89	1.949668575	0.054429454	1.629854817	0.108589629	3.109607149	0.002939105
C3	1.427765898	0.156931309	0.660794279	0.511378721	-0.369228018	0.713344386
C3AR1	-1.444619305	0.152149105	-0.463676985	0.644626766	-0.801008176	0.426498236
C3orf14	-0.901362924	0.369878543	0.766286574	0.446635105	0.684687761	0.496353248
C3orf17	1.696107062	0.093433563	0.546517881	0.586821022	1.410611249	0.163867042
C3orf18	1.128754447	0.262097644	0.144126899	0.885903377	1.028679765	0.308034357
C3orf19	0.702782418	0.484063581	1.619331309	0.110839283	2.787511106	0.007231598
C3orf21	-2.199593787	0.030481332	-0.228333809	0.820195468	-0.631459859	0.530298337
C3orf23	-1.247830673	0.215433983	-1.132390136	0.262162722	-1.376438065	0.174142
C3orf25	-0.131477888	0.895700192	-1.701451125	0.094251335	-1.37584904	0.174323341
C3orf26	0.28050312	0.77975603	1.039702357	0.30282315	1.851073168	0.069412994
C3orf33	1.727630948	0.087595061	1.339914209	0.18553576	1.769487865	0.082234235
C3orf35	1.000504169	0.319835241	-0.916884128	0.363026681	-0.679147615	0.499830062
C3orf37	-0.523683343	0.601827397	2.315282354	0.024181286	3.421182009	0.001168048
C3orf38	-0.036134922	0.97125738	-0.398833636	0.691490637	-0.76976326	0.444661751
C3orf39	-1.988940788	0.049843112	-0.758913843	0.450998765	0.238580035	0.8122985
C3orf47	0.911245546	0.364678541	-0.43513308	0.665092023	-0.798102171	0.428168614
C3orf52	0.448042592	0.65523381	-1.008143483	0.31760222	0.064387617	0.948889921
C3orf54	-1.701593302	0.092395219	0.882026919	0.381428516	0.485935027	0.628901339
C3orf58	0.200969349	0.841190624	-0.646863755	0.520289771	-2.282052373	0.026290653
C3orf62	-4.037362067	0.000115848	0.594825205	0.554290032	0.899395636	0.372277051
C3orf63	1.23603036	0.219766935	0.138429781	0.890383616	-0.536969668	0.593406779
C3orf64	1.955167033	0.053766509	0.101940714	0.919157804	-1.037917801	0.303750557
C3orf71	-0.982734903	0.328457107	-1.554997055	0.125424916	-2.167095905	0.03447816
C3orf75	1.402804414	0.164226095	1.069492788	0.289309354	2.354947105	0.022039948
C3orf78	-0.415884245	0.678516564	-2.992961187	0.004064284	-2.304323565	0.024920592
C4BPA	-0.685669081	0.494740945	-0.833579004	0.407961923	-1.412875329	0.163203218
C4orf10	-0.465666343	0.642615488	-0.386266118	0.700722839	0.334267976	0.739420767

C4orf14	1.219152631	0.226074322	1.10010242	0.275864238	0.408742937	0.684280701
C4orf19	-0.471055118	0.638777844	-0.036755619	0.970806957	0.702551344	0.48523341
C4orf21	2.128821377	0.03608941	0.271412024	0.787044586	0.691187075	0.492291432
C4orf27	0.563305814	0.574672504	1.095705448	0.277768228	1.944224859	0.056876941
C4orf29	0.08004263	0.936386788	-0.673667768	0.503217107	-1.637619204	0.107085275
C4orf3	-1.495911885	0.138287666	-0.666214976	0.50793345	-1.207988389	0.232107237
C4orf32	-0.527722585	0.599032341	-1.141992493	0.258182546	-2.333419282	0.023226514
C4orf33	-0.968237271	0.335604089	-1.058473839	0.294258496	-1.753406377	0.084981313
C4orf34	-0.331262525	0.74124106	0.895813778	0.374080974	-0.849087124	0.399432857
C4orf41	1.806268187	0.07432683	1.006618643	0.318328394	-0.115284901	0.908629965
C4orf42	2.794563824	0.006389823	-3.19570849	0.002264732	-2.664953579	0.01003071
C4orf43	1.940495487	0.055550886	-1.602082373	0.114608197	-1.017630082	0.313211911
C4orf44	0.635000743	0.527090853	-0.908568704	0.367363967	-0.806875014	0.42313788
C4orf46	0.478696858	0.633352587	-1.387882268	0.170518652	-1.680234691	0.098459006
C4orf48	-0.938760125	0.350447279	-1.465176335	0.148310594	-1.885263469	0.064564366
C4orf52	-0.621728433	0.535742783	-1.936015659	0.05777866	-1.307091389	0.196501099
C5	-0.684781853	0.495297976	1.205440233	0.232962497	0.332541119	0.74071712
C5AR1	-4.353484618	3.63E-05	-0.334651924	0.739103316	-0.626116182	0.533771891
C5orf13	-0.689256737	0.492491974	-0.524630121	0.601853817	-0.095199705	0.924494619
C5orf15	0.157764236	0.875007382	-1.254811388	0.214615113	-1.922214345	0.059650046
C5orf20	1.615426192	0.109831516	-0.104721151	0.916960909	0.517400173	0.606906099
C5orf22	-1.062878454	0.290772108	-1.498806771	0.139385067	-0.641995951	0.523484253
C5orf24	1.3690104	0.174512571	-1.891673274	0.063565948	-2.55070946	0.013499094
C5orf25	-0.875477405	0.383719668	0.390535985	0.697580984	1.493606057	0.140868445
C5orf28	0.599566654	0.550350009	-0.691032302	0.492321047	-1.202075223	0.234371758
C5orf30	1.114219095	0.26824728	0.00649711	0.994838544	-0.959078278	0.34162466
C5orf32	-1.232193034	0.22118958	-1.065394284	0.291143429	-1.611599931	0.112647887
C5orf34	1.446181882	0.151711498	-1.608821812	0.11312348	-0.86292216	0.391845469
C5orf35	-0.110416602	0.912332752	-0.548830037	0.585243481	-0.383062622	0.703117508
C5orf39	0.192677273	0.847659818	-0.414618394	0.679961517	0.016127042	0.98719012
C5orf4	-3.184508124	0.00201145	1.840323482	0.070874605	1.014986779	0.314459156
C5orf41	-0.237435552	0.812875946	-1.058007491	0.294469232	-2.174547753	0.033886429
C5orf42	-0.497754895	0.619909915	-0.646708653	0.520389447	-0.578123511	0.565488679
C5orf43	-0.222105724	0.824751008	0.530308134	0.597936987	0.044737705	0.96447486
C5orf44	0.313178788	0.754892772	-1.059758721	0.293678411	-1.188157023	0.23976524
C5orf45	3.106185197	0.00255742	0.118896121	0.90577137	1.129715099	0.263391939
C5orf51	0.124636915	0.901097913	-2.447818009	0.017441959	-1.764190546	0.083130855
C5orf53	3.599724837	0.000528385	-1.016359461	0.313708714	-1.074954103	0.286985754
C5orf54	0.165300873	0.869089841	0.299816746	0.765396307	0.169843265	0.865742917
C5orf55	1.916767238	0.058542907	1.100491482	0.275696206	0.760119866	0.450357973
C5orf56	-0.859160346	0.392608066	-4.271958455	7.35E-05	-6.169539094	7.93E-08
C5orf62	-3.953605205	0.000156152	0.512079133	0.610553638	-0.223593517	0.823883868
C5orf63	2.336160993	0.021776817	0.252044227	0.801904104	0.851463736	0.39812306
C6orf1	-1.525241928	0.130818113	-2.049640347	0.044962575	-1.612693028	0.112409577
C6orf105	0.676686194	0.500396401	2.355078226	0.021947743	2.845496452	0.006175736
C6orf106	-1.157545356	0.25021087	3.088985198	0.003089521	2.604275381	0.011755955
C6orf108	1.488248517	0.140293404	-1.598389068	0.115428493	-0.502465601	0.617302011
C6orf115	0.708604592	0.480460197	-0.318556875	0.751214864	0.131716898	0.895678332
C6orf120	1.110333315	0.269908229	-1.597578758	0.115609098	-1.571145492	0.121758756
C6orf125	-0.490130021	0.625272998	-3.957992556	0.000210002	-2.650246709	0.010426184
C6orf130	1.025602317	0.307916122	-1.021265958	0.311398987	-0.197046642	0.844501473
C6orf136	0.828856967	0.409448682	-3.865124706	0.000284444	-3.19230969	0.002311584
C6orf138	-1.152287403	0.252352627	-0.705828025	0.483140288	-0.018320762	0.985447811
C6orf145	-1.331296728	0.186561887	-1.084107677	0.282834318	-1.425825898	0.159446085
C6orf147	0.262812079	0.793315446	-0.725700537	0.470960917	0.026166954	0.979216808
C6orf162	0.790090688	0.431618438	-2.147439441	0.035979211	-1.958099246	0.055186105
C6orf163	0.606532626	0.545737187	-0.891578563	0.376328463	-0.121495331	0.903731802
C6orf164	0.777083085	0.439213025	-0.657319224	0.513593957	0.069143912	0.945120343
C6orf170	1.446334511	0.151668806	-0.373265978	0.710320803	-0.926144012	0.3583287
C6orf174	0.583635963	0.560972072	-0.579046662	0.564815493	-1.840089074	0.071034443
C6orf192	2.993269849	0.003590261	2.07361402	0.042597287	3.495156966	0.000931747
C6orf203	-0.928837206	0.355537841	-1.098863572	0.276399761	1.10118869	0.27550552
C6orf204	1.120958443	0.265383559	-1.247341811	0.217320244	-1.864762881	0.067435939
C6orf211	0.274116733	0.784643302	-0.438120619	0.662937646	-1.023059836	0.310660381
C6orf226	0.198809721	0.84287446	-1.781290082	0.08013793	-0.345974632	0.730652786
C6orf25	-3.906683441	0.000184279	0.294876787	0.769148288	-1.03207174	0.306456708
C6orf27	0.287412139	0.774478766	-0.6494275	0.518643653	-0.421044425	0.675327481
C6orf35	0.114688024	0.908956111	-0.05120227	0.959341505	0.176891631	0.860229051
C6orf47	-1.511735284	0.134217378	1.448391113	0.152929976	1.638587773	0.106882598
C6orf48	0.305393749	0.760794185	-0.880574167	0.382208003	-0.140825034	0.888511387
C6orf57	1.135855922	0.259129444	0.331601275	0.741393946	0.482546366	0.631290917
C6orf62	-0.433233803	0.665914844	1.177044127	0.244019199	0.608589537	0.545247013

C6orf64	0.628108964	0.531574386	-0.274324147	0.784817042	0.553010171	0.582448198
C6orf70	1.940645359	0.055532408	0.41530897	0.679458848	0.893719598	0.37528079
C6orf72	-0.368388052	0.713476838	0.628870375	0.531920176	0.085906008	0.931846261
C6orf81	0.340848083	0.734037912	-0.126875762	0.899480631	1.484492863	0.143262093
C6orf89	-1.106135467	0.271710617	0.400408112	0.69033729	0.853452152	0.39702925
C6orf97	-0.206313182	0.837027292	1.087360131	0.281407153	-1.882850916	0.064896829
C7orf11	0.261222605	0.794536895	-1.812955106	0.075050786	-1.626036646	0.109533273
C7orf13	2.583755339	0.011437754	-0.644524258	0.521794313	-1.407272368	0.164849805
C7orf16	-0.51945875	0.604757097	-0.324698779	0.746585457	-0.0896095	0.928915917
C7orf23	1.280972089	0.203600261	-0.960012596	0.341061743	-0.98117061	0.330710963
C7orf25	-0.482811348	0.630439763	0.004437538	0.996474703	-0.554580641	0.581380515
C7orf26	1.18731596	0.238327726	1.405190996	0.165335412	2.204129649	0.031624473
C7orf28B	-1.46224676	0.147268826	-0.847768179	0.400076373	-0.664919787	0.508819466
C7orf29	0.780883694	0.436985975	0.362131799	0.718578858	1.747327071	0.086039455
C7orf30	-0.374131594	0.70921473	-1.26427518	0.21122365	-0.294496035	0.769463249
C7orf31	-1.069902768	0.287616056	-1.187894472	0.239750502	-1.195674518	0.236841056
C7orf34	0.163871833	0.870211318	-0.045519878	0.963850389	-0.938822877	0.351836564
C7orf36	0.022490962	0.982107714	-0.803188851	0.425167794	-1.041832255	0.301947689
C7orf40	0.996325062	0.32184933	-0.213501487	0.831689785	0.606703853	0.546489069
C7orf41	-5.756205242	1.26E-07	0.466679186	0.642489887	-1.139403824	0.259364806
C7orf42	-1.043583796	0.299562868	1.433321643	0.157172171	1.916726812	0.060359093
C7orf43	-1.949264033	0.054478503	-4.664599685	1.88E-05	-3.128409917	0.002783789
C7orf44	-0.977775359	0.330890662	-2.728744121	0.008416356	-1.994576325	0.050944663
C7orf46	0.771765889	0.442339854	-1.770555275	0.081926515	-0.518749921	0.605970513
C7orf47	0.611518154	0.542447821	-2.839190576	0.006238524	-2.536894297	0.013985185
C7orf49	-1.892029687	0.061806029	0.720556737	0.474096651	0.256417176	0.798564449
C7orf50	-0.033949995	0.972994628	0.549809911	0.584575542	0.839724087	0.40461886
C7orf51	0.43787874	0.662557063	-1.412269866	0.163250977	-1.624521271	0.109856885
C7orf53	-1.929616599	0.056906182	0.235268726	0.814834654	-0.366684179	0.715230693
C7orf54	0.900787191	0.370182918	-0.10313138	0.918216949	-0.286011279	0.77592042
C7orf55	-1.497502919	0.137874075	-2.748380086	0.007984183	-1.720402251	0.09085808
C7orf58	1.93135426	0.056687844	-0.349420426	0.728048045	-0.345198988	0.731232621
C7orf59	-1.347956781	0.181164109	0.649577906	0.518547167	0.491094416	0.625270758
C7orf60	-1.262586969	0.210104146	-1.343478957	0.184386315	-1.706889874	0.093359087
C7orf61	-0.23840785	0.81212421	1.75317686	0.084892386	1.917294148	0.060285456
C7orf64	0.589961548	0.556742314	-1.662289281	0.101888321	-2.090414352	0.041112958
C7orf68	1.442778236	0.152665958	-0.454622952	0.651089369	0.476120076	0.635833404
C7orf70	1.554104299	0.123782075	-0.448001954	0.655832446	-0.516274597	0.607686807
C7orf73	-3.271786073	0.001532139	-0.992928221	0.324898145	-0.827525354	0.411437209
C7orf74	-0.540737399	0.590067389	-0.998929141	0.322007358	-3.132883057	0.00274799
C8G	-0.541925766	0.589251951	-1.121539849	0.266712284	-1.320065611	0.192161208
C8ORFK29	-0.077442819	0.938448721	-1.597532387	0.115619441	-0.726406832	0.47060216
C8orf31	0.141361682	0.887910332	0.76475225	0.44754118	0.551281333	0.583624633
C8orf33	1.389301855	0.168278959	0.935721109	0.353323623	2.018342576	0.048334605
C8orf37	0.101178064	0.91964142	-1.103749622	0.274291862	-2.01498162	0.048696566
C8orf38	0.815332513	0.417103695	-0.987654526	0.327452877	-0.224659074	0.823058802
C8orf39	-0.084666601	0.932720556	-1.747769491	0.085833211	-3.449450401	0.001071718
C8orf40	1.269800035	0.207534458	-1.520978799	0.133736455	-0.752802629	0.45470835
C8orf41	-0.857479352	0.393530915	0.909730035	0.366756239	1.647765749	0.104977524
C8orf44	0.854801158	0.395003975	0.938900424	0.351702684	0.925248576	0.35879011
C8orf45	0.999438758	0.32034791	-1.007299503	0.31800401	0.423136907	0.673809178
C8orf46	2.453588228	0.01613357	-0.889973924	0.377182221	0.132899456	0.894747303
C8orf55	-0.175264093	0.861278507	0.010414235	0.991726781	0.494451525	0.622913409
C8orf58	1.051181155	0.296080169	0.85937538	0.39369617	1.700739909	0.094516024
C8orf59	1.261541189	0.210478646	-0.958679589	0.341727284	0.382245047	0.703720371
C8orf73	0.811896393	0.419062175	0.548907057	0.585190967	0.022704845	0.981966082
C8orf76	0.237771213	0.812616409	-0.48005561	0.633006003	-0.272141104	0.786510206
C8orf77	1.317987964	0.19096002	-1.991870114	0.051133773	-1.060959198	0.293243745
C8orf80	1.397748746	0.165734732	0.51923437	0.6055869	1.227551782	0.224728935
C8orf82	-0.005037244	0.995992386	-3.311889748	0.001604275	-3.637031621	0.000599777
C8orf83	-1.464058093	0.146774323	-0.618878452	0.538436565	-1.076127984	0.286465078
C8orf84	1.473554437	0.144202886	-2.257084123	0.027811757	-0.973886192	0.33428362
C9orf100	-0.627414992	0.532026946	0.178452593	0.858993755	-0.309462795	0.758113251
C9orf102	1.166860746	0.246448143	0.409986237	0.683337023	-0.5667968	0.57310763
C9orf103	-0.35335278	0.724676915	0.537501235	0.592992145	1.424390058	0.159859301
C9orf106	0.174302356	0.862031936	-0.234598695	0.815352216	0.60854092	0.545279018
C9orf114	0.019360536	0.984597729	0.148509967	0.882459052	-0.012447893	0.99011233
C9orf117	0.035367693	0.971867393	-0.817470556	0.417028387	0.506540918	0.614457251
C9orf123	1.396387487	0.166142745	-1.547038241	0.127331486	-1.474235275	0.14599453
C9orf130	0.796206251	0.428074743	-0.288224102	0.774209877	0.742656118	0.460780884
C9orf131	0.85856544	0.39293451	0.248783961	0.804412883	0.946724555	0.347829487
C9orf139	0.898069189	0.371621993	-0.321639178	0.748890444	-0.185088339	0.853825657

C9orf140	-1.869491004	0.064911133	-0.296030189	0.76827176	-0.466292299	0.642807507
C9orf142	0.597417609	0.551777025	-0.031503499	0.974976917	0.726041561	0.47082429
C9orf156	0.5770516	0.565391609	0.099942651	0.920736918	1.306789399	0.196602986
C9orf16	-1.599526421	0.113321317	-0.226658205	0.821492045	-0.10214624	0.919003951
C9orf163	0.631205956	0.529557169	-1.487643602	0.142299518	-1.466435708	0.148099476
C9orf167	0.648754041	0.518202514	-0.511034243	0.611280488	-0.658207042	0.513090723
C9orf172	0.710918015	0.479032542	-4.71335955	1.59E-05	-3.825227132	0.000329871
C9orf21	-2.412479652	0.017939999	-0.850120442	0.398778261	-1.18850852	0.239627933
C9orf23	-1.326720008	0.188065681	0.904208318	0.369651512	0.880502559	0.382334581
C9orf25	0.530777639	0.596922303	-2.77422501	0.007446425	-1.860352448	0.06806763
C9orf3	-0.158810503	0.874185451	-0.619087051	0.538300105	-0.868384259	0.388874838
C9orf30	-0.125230064	0.900629714	-0.056134282	0.955429112	-0.127613667	0.89890995
C9orf37	1.413361626	0.161109729	0.141143039	0.88824944	1.143663867	0.257608041
C9orf40	0.539787108	0.590719843	-0.977395129	0.332461066	-1.287101133	0.203331543
C9orf41	1.22426831	0.224148809	-1.510153524	0.136471217	-1.819507651	0.07415851
C9orf43	-0.252039367	0.801603803	0.30950219	0.758056501	0.787925976	0.434048646
C9orf46	-1.591469944	0.115123236	-3.167366588	0.002460866	-1.544160064	0.128158662
C9orf47	0.239878601	0.810987428	-0.050179799	0.960152725	-0.345969701	0.730656472
C9orf5	1.917921023	0.058394332	0.143288594	0.886562393	-0.606457924	0.546651163
C9orf6	0.31267601	0.755273465	-1.897662814	0.06275659	-1.900338339	0.062519494
C9orf64	-0.203052186	0.83956736	0.070279533	0.944214622	-0.443587832	0.659042615
C9orf66	-1.896610453	0.06119048	-3.534731764	0.000812784	-5.259675415	2.32E-06
C9orf68	2.140718106	0.035088327	-0.404172449	0.687582802	-0.409906757	0.683431675
C9orf69	-0.502992115	0.616238085	-1.04362641	0.301018843	-1.461051508	0.149566402
C9orf7	0.901341834	0.36988969	1.951553287	0.05586001	1.881646937	0.065063285
C9orf71	-2.016174655	0.046859801	-1.086578778	0.281749549	-3.248498528	0.001959634
C9orf72	0.003712152	0.997046618	-0.922989055	0.359863405	-1.648395276	0.104847874
C9orf78	-3.872469602	0.000207776	0.863220567	0.391596578	0.031104541	0.975296297
C9orf80	0.009475593	0.992461331	-0.703445364	0.484612258	-0.606709462	0.546485372
C9orf82	1.090178318	0.278638402	0.912755362	0.365176099	-0.124200093	0.901599723
C9orf84	-0.795914415	0.428243456	-0.375072568	0.708984137	-0.479539755	0.633414404
C9orf85	0.249221314	0.803775765	-0.923823631	0.359432353	0.101030581	0.919885528
C9orf86	-0.293339687	0.769959595	0.579698697	0.56437859	1.564992835	0.123194884
C9orf89	-2.320483247	0.02264953	-0.609990053	0.54426762	-1.341159974	0.185260185
C9orf9	0.426460489	0.670823426	-2.156202484	0.03525672	-1.741136748	0.087128127
C9orf91	-0.969077651	0.335187041	1.609116187	0.113058985	1.179549239	0.243145522
C9orf93	2.000265469	0.04858343	1.169265434	0.247112991	0.116083276	0.908000083
C9orf95	1.261847783	0.210368802	-1.51300791	0.135745856	-0.137510794	0.891118222
C9orf96	0.296201254	0.767780763	0.564301401	0.574740041	0.431399249	0.667827402
CA1	-2.709722911	0.008106868	-0.445358573	0.657730082	-0.925923852	0.358442111
CA11	0.922781746	0.358667555	-0.367140909	0.714859431	0.81472761	0.418665133
CA13	-0.067150021	0.946616019	-0.631763563	0.530041021	-2.261705242	0.027600347
CA14	0.607029505	0.545408904	-0.757412608	0.451890318	0.860030928	0.39342361
CA2	-4.064993643	0.000104897	1.090260068	0.280138907	0.517351129	0.606940107
CA4	-2.223148508	0.02879173	1.047212319	0.299376456	1.040929595	0.302362774
CA5B	0.685539736	0.49482213	-1.37673257	0.173923111	-0.558671615	0.578603691
CA5BP1	0.801735952	0.424885407	0.395941732	0.69361095	1.471685936	0.146679944
CA6	1.52102807	0.131871262	1.502277795	0.138488535	1.184728204	0.241107641
CA8	-2.852165279	0.005421224	0.85377164	0.396768484	-2.913652266	0.005117574
CAB39	-1.567395491	0.120644628	0.997830668	0.322535227	-0.135947262	0.892348449
CAB39L	0.135964334	0.892162909	-1.559199511	0.124427447	0.106329244	0.915699522
CABIN1	0.639074218	0.524450064	-1.816248514	0.074537568	-0.404517416	0.687366729
CABLES1	-1.07874923	0.283674868	2.233905031	0.029387025	1.060896191	0.293272131
CABLES2	0.115875143	0.908017965	-1.088321366	0.280986328	-0.32800989	0.744122303
CABP4	1.541979551	0.126700359	0.684680819	0.496291413	1.611343556	0.112703839
CABP5	-3.658056062	0.000434312	1.154057241	0.253242908	0.378378662	0.706573946
CABP7	-1.370457421	0.174062312	-1.645474165	0.105319103	-0.328930078	0.74343037
CABYR	0.69024892	0.491870996	0.372647058	0.710778943	1.525296215	0.132789861
CACHD1	0.696695365	0.487846789	-0.927929444	0.35731658	0.28468316	0.776932615
CACNA1A	-2.366164212	0.020189305	-1.791092154	0.078533305	-1.784776443	0.079691557
CACNA1C	1.901944869	0.060480176	-1.293489832	0.201005276	-0.887788193	0.378436049
CACNA1D	0.848746297	0.398346749	-0.35247559	0.725768179	-1.391392358	0.169586385
CACNA1E	-2.467644063	0.015554297	-0.106427007	0.915613388	-1.688045972	0.096941339
CACNA1F	2.358540504	0.020582639	0.871634241	0.387026833	1.875582325	0.065907258
CACNA1H	1.777432788	0.078984086	-0.245402239	0.807017308	-0.083503761	0.933747521
CACNA1I	4.00259195	0.000131193	1.723092881	0.090237057	2.034949469	0.046579983
CACNA2D2	1.308619635	0.19410221	-3.551661713	0.00077112	-1.624484627	0.10986472
CACNA2D3	-1.258973774	0.211400136	0.007729565	0.993859471	0.502136909	0.617531712
CACNA2D4	-0.045302676	0.963969653	0.2459348	0.806607011	-0.047544864	0.96224742
CACNB1	1.493388483	0.138945621	-2.144722798	0.036205806	-0.349427285	0.728073663
CACNB2	0.6903786	0.491789865	0.008100164	0.993565066	0.157702254	0.875256308
CACNB3	1.595887694	0.114132339	-0.573174306	0.568757835	-0.525950909	0.600990338

CACNB4	0.697194731	0.487535814	-1.787644334	0.079094636	-1.699242627	0.094799473
CACNG6	-1.972027109	0.051776299	-0.115133619	0.908739664	-1.174447266	0.245165234
CACNG8	0.263179475	0.79303319	0.239627893	0.81146947	-0.385606907	0.701242634
CACYBP	0.885233777	0.378465427	1.038652469	0.303307144	1.454888579	0.151259436
CAD	1.194525361	0.235512	0.813958545	0.419021157	1.621888851	0.11042089
CADM1	-0.17662582	0.860211943	1.418393851	0.16146417	1.276589569	0.206993702
CADM2	-0.958978902	0.340221143	-0.542716693	0.589418887	-0.662832303	0.510145659
CADM4	-2.647832329	0.009613878	0.239977507	0.81119973	-0.531499461	0.597165977
CALCOCO1	-1.0485761	0.297271231	-0.22937017	0.819393787	0.129874234	0.897129359
CALCOCO2	-2.086171315	0.039884532	1.828301808	0.072684303	1.494699097	0.140583482
CALCRL	0.749560773	0.455536991	-2.084186057	0.041589015	-2.111906091	0.03914965
CALD1	-1.131957419	0.260755954	1.856085017	0.068559497	1.83899822	0.071197199
CALHM1	0.703744238	0.483467278	-0.429193459	0.669383634	-0.075608696	0.93999875
CALHM2	1.67417732	0.097679557	-1.527584695	0.132089114	-2.576285674	0.012639454
CALM1	0.393020665	0.695263526	-0.444160347	0.658591014	0.841356513	0.403711726
CALM2	-1.820575162	0.072102186	-0.090724408	0.928026356	0.099421231	0.92115739
CALM3	-2.808194347	0.006147284	-0.282336633	0.778697501	0.398792964	0.691556009
CALML4	-0.241191702	0.809972841	-1.371027837	0.17568503	-1.032909602	0.306067856
CALML6	-0.432196976	0.66666529	1.785829533	0.079391443	1.317985034	0.19285225
CALR	-0.389958006	0.697518598	1.145989803	0.256538398	1.740680751	0.087208771
CALU	1.279885064	0.20398061	-1.719151997	0.090957346	-1.622674698	0.110252274
CALY	-0.349845335	0.727298432	-2.536172535	0.013942093	-2.040288185	0.046027731
CAMK1	0.189190235	0.850383434	0.236240789	0.814083936	0.268549025	0.789259448
CAMK1D	-0.399854573	0.690241541	-2.187862919	0.032751227	-3.425132477	0.001154108
CAMK2D	0.875148162	0.383897768	-0.233113602	0.816499663	-0.149880826	0.881394894
CAMK2G	-0.224825543	0.822641069	-0.686649059	0.495059179	-0.786120131	0.435097088
CAMK2N1	1.510765958	0.13446398	-1.161917216	0.250061373	-0.808578675	0.422165062
CAMK4	2.664089669	0.009195176	-0.857214154	0.394879332	0.479974394	0.633107238
CAMKK1	-4.087140482	9.68E-05	-0.102458933	0.918748297	1.309121038	0.195817356
CAMKK2	-1.910452137	0.059361779	0.588740749	0.558337131	0.739505006	0.462676211
CAMKMT	2.033027741	0.045091264	0.198506632	0.843347721	0.996333874	0.323355771
CAMLG	-0.258930012	0.796299566	-0.193963501	0.846886926	-0.513276988	0.609768209
CAMP	-2.908734951	0.00460298	0.288730124	0.773824528	0.443979943	0.658760797
CAMSAP1	1.706494911	0.091475515	-0.020541902	0.983682085	0.178041305	0.859330329
CAMSAP1L1	0.862790966	0.39061945	1.045040678	0.300370358	0.268166152	0.789552644
CAMTA1	0.697999809	0.487034691	-2.194928318	0.03221393	-1.295605755	0.200404214
CAMTA2	-0.745813342	0.457786227	-0.937760306	0.352283404	0.092911467	0.926304107
CAND1	2.339631185	0.021587718	1.409498821	0.164064501	1.736250399	0.087995514
CAND2	1.899353888	0.060824305	-0.327721144	0.744310804	1.302724244	0.197978377
CANT1	-2.238216825	0.027754398	-0.109251114	0.913383068	-0.242247461	0.809469694
CANX	1.57739599	0.118325966	1.759449657	0.083811725	1.245869777	0.217977696
CAP1	-1.418208867	0.159694191	0.325495118	0.745985906	0.618362998	0.538832603
CAPG	-1.520828612	0.131921276	1.662581738	0.101829469	1.637546864	0.107100424
CAPN1	-2.020464307	0.046404109	1.790955748	0.078555449	1.30110644	0.198527746
CAPN10	1.319147306	0.190573833	-1.023045542	0.310564101	-1.078615197	0.285364039
CAPN12	0.71012145	0.479523851	-0.522012306	0.603663638	-0.049928736	0.960356091
CAPN14	0.55671518	0.579148236	-1.024413614	0.309923306	0.455350253	0.650610518
CAPN2	0.55574839	0.579806185	2.349724122	0.022237112	1.332714491	0.188000193
CAPN3	0.42620695	0.671007444	0.706277394	0.482862955	1.006385286	0.31854096
CAPN5	-0.496234645	0.62097758	-0.645828173	0.520955475	0.5449807	0.587921663
CAPN7	1.243458533	0.217032029	-1.411030532	0.163614434	-2.030032651	0.047093648
CAPNS1	-2.387395913	0.019128828	0.552050417	0.583049651	1.059901797	0.293720368
CAPRIN1	-0.031178524	0.975198428	-0.326632546	0.745129828	-1.063068303	0.292294661
CAPRIN2	1.677161818	0.097092642	-2.095986589	0.040488129	-1.759118424	0.083996965
CAPS	0.933867117	0.35295154	1.30274712	0.197845827	2.288715434	0.025873915
CAPZA1	-0.310240907	0.757118131	-0.631684399	0.530092392	-0.754932919	0.453439308
CAPZA2	-0.581229356	0.562585454	-0.423399824	0.673580469	-0.936766465	0.35288431
CAPZB	-2.23568499	0.027926369	1.317980008	0.192728325	1.587811158	0.117936166
CARD11	1.602239196	0.112719687	0.109567249	0.913133446	0.759010411	0.451016025
CARD14	2.430171687	0.017141549	0.121220088	0.903938629	-0.56092464	0.577077147
CARD16	-0.815548045	0.416981032	-1.367184292	0.1768798	-2.29514045	0.025477631
CARD17	-1.272786242	0.206477424	-0.783380352	0.436613294	-1.934733487	0.058058959
CARD6	-2.132381751	0.035787247	1.927438862	0.058861637	1.071788459	0.288393146
CARD8	0.008399506	0.993317432	-1.106118742	0.273273862	-2.016594904	0.048522531
CARD9	0.180341219	0.857303207	0.842475947	0.403006444	0.223237616	0.82415949
CARHSP1	-1.793171062	0.07641309	-1.065494582	0.29109845	-1.103531131	0.274496337
CARKD	1.705389476	0.091682275	0.815897872	0.417920043	2.516482449	0.014732448
CARM1	-1.169428225	0.245418199	-0.235647602	0.814542029	-0.668661533	0.506446959
CARNS1	1.190881267	0.236932241	-0.453785076	0.651688803	1.159125091	0.251303376
CARS	-0.94707326	0.346218935	2.106401947	0.039537618	2.360376781	0.021749358
CARS2	-3.439955683	0.000894803	-0.036309337	0.971161256	0.392897806	0.69588037
CASC1	-1.774039001	0.079547775	0.139021833	0.889917853	0.176359048	0.860645445

CASC3	-2.65537723	0.009417458	-0.783164945	0.436738752	-1.139911241	0.25915511
CASC4	-0.102768313	0.918382853	-0.747056011	0.458068688	-1.828442369	0.072788414
CASC5	1.016234274	0.312329574	1.346253075	0.183495557	-0.212594441	0.832412037
CASD1	1.70132089	0.092446553	-1.089917046	0.280288715	-1.879968148	0.06529599
CASK	1.151356118	0.252733324	-0.537608447	0.592918589	-0.953133359	0.344601446
CASKIN2	-0.198484436	0.843128145	0.543587083	0.588823552	-0.53509044	0.594696954
CASP1	0.344960824	0.730954599	0.244964277	0.807354764	-0.834665967	0.40743758
CASP10	-0.667834369	0.506003284	1.769287083	0.082139994	1.867420442	0.067057703
CASP2	1.907872005	0.059699114	0.242358254	0.809363506	1.749152734	0.085720544
CASP3	0.42968314	0.66848619	-0.271103266	0.787280867	-0.95496868	0.343680641
CASP4	-1.398273408	0.165577678	-1.043438709	0.301104982	-1.934962482	0.058030196
CASP5	0.016574343	0.986814062	-1.283336297	0.20451388	-2.142916268	0.03646106
CASP6	1.831842908	0.070389419	1.59523646	0.116132441	2.005259567	0.049756774
CASP7	0.288810905	0.773411638	0.230829218	0.818265465	-0.475600926	0.636200988
CASP8	-2.224025986	0.028730404	-0.765198509	0.447277537	-1.072270365	0.288178591
CASP8AP2	1.449607789	0.15075547	-0.147180828	0.883503285	-1.222628164	0.226569467
CASP9	-3.214092172	0.00183516	1.707740946	0.093069781	1.927386417	0.058988268
CASQ1	1.507884393	0.13519917	0.265760464	0.791372675	-1.847620981	0.069919202
CASS4	-1.965161238	0.052579068	-0.589407537	0.557892896	-1.759838999	0.083873465
CAST	-0.433641326	0.665619974	2.327458937	0.023477301	1.454196601	0.151450464
CASZ1	-1.061909947	0.291209114	-1.358950446	0.179460194	-1.610575756	0.112871541
CAT	0.901690947	0.369705197	-0.098233946	0.922087601	-0.365586902	0.716044897
CATSPER1	-0.580560749	0.56303409	-1.285632091	0.203716584	-0.602416379	0.549318474
CATSPER2	0.979886824	0.329853159	-1.434112873	0.156947179	-0.016026352	0.987270092
CATSPER2P1	0.180613631	0.857090015	-1.582628132	0.118982435	-0.17437647	0.862195849
CATSPERB	0.181620558	0.856302083	-0.926104244	0.358256131	-2.006674026	0.049601299
CATSPERG	0.938222948	0.350721647	-0.586040667	0.560137812	-0.804103183	0.424723518
CAV1	0.897077318	0.372148027	0.536913584	0.593395399	0.849288224	0.399321924
CAV2	-3.04177361	0.003106591	0.684665317	0.496301125	-1.530774812	0.131431318
CBFA2T2	2.518187543	0.013622053	0.988264364	0.327156771	1.003141953	0.32008928
CBFA2T3	0.266981276	0.790114037	-1.129835094	0.263229076	-1.030127788	0.30736019
CBFB	0.614144351	0.540719153	-1.230072855	0.223670425	-1.843641524	0.070506583
CBL	-0.773398945	0.441378144	-0.240636541	0.810691322	-0.38707724	0.700160001
CBLB	1.100925306	0.273959291	-0.432203898	0.667207076	0.101228479	0.919729145
CBLL1	-0.712208776	0.478237015	-1.812149192	0.075176823	-3.123869256	0.002820575
CBLN3	2.480166691	0.015053968	0.007907708	0.993717954	0.31164988	0.756459089
CBR1	0.075984964	0.939605144	2.101322002	0.039998761	2.663520279	0.01006864
CBR3	2.213020375	0.029507911	1.000040545	0.321473864	1.278134128	0.206452527
CBR4	1.246799744	0.215810015	-0.335882819	0.73817975	-0.626621423	0.533442964
CBS	-1.781073035	0.07838314	-1.193494524	0.237568608	-1.141495317	0.25850125
CBWD1	0.933193712	0.353297091	-0.324751756	0.746545566	-0.416239222	0.678819261
CBWD2	1.080036097	0.28310467	-1.058315826	0.294329888	-1.512275825	0.136063364
CBWD6	2.071903203	0.041228825	0.796999683	0.42872449	1.368457	0.176611431
CBX1	-0.855819249	0.394443606	-0.277860616	0.782114353	0.258989456	0.796589031
CBX2	-0.251370547	0.802119144	1.035641421	0.304698152	0.938570193	0.351965198
CBX3	1.310370494	0.193512047	0.293170575	0.77044548	0.163001845	0.871101319
CBX4	0.055439608	0.955915086	-4.433349949	4.22E-05	-4.666641457	1.94E-05
CBX5	1.642416256	0.104105815	0.921637148	0.360562362	1.550406258	0.126653908
CBX6	0.729875272	0.467422883	-0.81391997	0.419043077	-0.818156795	0.416720905
CBX7	2.545589957	0.012667224	-0.478863515	0.633848721	0.565371726	0.574069741
CBX8	-2.579356578	0.011573746	-1.266077099	0.210582442	-1.768630622	0.082378783
CBY1	1.608617492	0.111315218	2.14522725	0.036163636	3.406908881	0.001219753
CBY3	2.122264793	0.036651649	-1.812319386	0.075150192	-3.053279405	0.003454353
CC2D1A	-0.015866525	0.987377127	1.151442914	0.254307512	1.549570276	0.12685448
CC2D1B	0.27142983	0.786702089	2.154305536	0.035412032	3.045145749	0.003535379
CC2D2A	1.332911065	0.186033618	0.128267363	0.898384216	-1.348399171	0.18293577
CC2D2B	-0.425359881	0.67162239	1.439993336	0.155282868	0.635525782	0.527663282
CCAR1	2.85773069	0.005335166	0.733738794	0.46608428	0.620268583	0.537586491
CCBL1	1.289675292	0.200573936	2.377560467	0.020769028	2.954260933	0.004569788
CCBL2	1.450651352	0.150465185	-0.159452145	0.873870503	0.491411769	0.625047745
CCDC101	-0.710539305	0.479266089	-0.202475038	0.840258892	0.460136277	0.647192594
CCDC102A	1.309898057	0.193671159	0.268014735	0.789645505	0.375806805	0.70847445
CCDC103	-1.105095458	0.272158448	1.93058557	0.058462315	1.423127859	0.160223231
CCDC104	3.250470391	0.001638177	0.768275523	0.445462152	1.560040892	0.124360584
CCDC106	-0.291440846	0.771406418	-0.39391764	0.69509646	0.539579163	0.591617429
CCDC107	0.451029318	0.653088172	-2.410774088	0.019131203	-1.685341236	0.097464658
CCDC109B	2.043388616	0.044032673	-0.11795483	0.906513844	0.172645142	0.863550217
CCDC111	1.767621935	0.080622705	-0.256532884	0.798453508	-1.204564624	0.233416459
CCDC112	1.390635594	0.167875262	-0.274221875	0.784895241	-0.154370423	0.87787035
CCDC114	1.508199589	0.135118598	-0.733690011	0.466113788	1.050631857	0.297921625
CCDC115	0.330858438	0.741545228	0.38290388	0.703200548	0.410305603	0.683140805
CCDC117	1.621380545	0.108547057	0.307265133	0.759749825	-0.320458576	0.749808458

CCDC12	-1.105994618	0.271771237	1.151140531	0.254430854	1.625865529	0.109569777
CCDC120	1.105724438	0.271887546	-0.688417435	0.493953503	0.425269598	0.672263102
CCDC121	0.011681046	0.990706773	-1.131279848	0.262625727	-1.261081733	0.212485948
CCDC122	0.865611279	0.389078971	-1.426205122	0.159207098	-0.273656879	0.785350906
CCDC124	-1.37237009	0.173468515	-0.863998095	0.391172873	-0.462219169	0.64570748
CCDC125	-2.045287145	0.043841028	-1.432079408	0.157525916	-0.875561142	0.384993064
CCDC126	-0.745828632	0.457777037	-1.002416511	0.320335346	-1.888744245	0.064087241
CCDC127	0.499421871	0.618740141	2.893968918	0.005363532	3.950226717	0.000219986
CCDC13	-1.204303632	0.231731351	0.944384802	0.348917913	1.198730937	0.235659588
CCDC130	0.864829946	0.389505365	-1.281857915	0.205028532	-0.155046041	0.877340171
CCDC132	1.249306442	0.21489653	1.310990845	0.195063813	1.213198121	0.230125356
CCDC134	1.48189067	0.141974663	1.449327936	0.152669231	0.757375039	0.451987034
CCDC135	0.287033665	0.774767582	1.525520116	0.132602225	2.233060463	0.029541985
CCDC136	3.181266534	0.0020317	1.751470716	0.085188308	1.726053824	0.089828566
CCDC137	-0.051331714	0.959178617	1.37002599	0.175995859	2.298843205	0.025251708
CCDC138	-0.384209408	0.70175869	-0.042335747	0.966377472	-0.675416	0.502179362
CCDC14	1.983122446	0.05050107	-0.119906415	0.904974562	-0.922100346	0.360415409
CCDC141	0.48767866	0.627001518	1.461871922	0.149211236	1.826390824	0.073101113
CCDC142	1.783709703	0.077950237	-1.049562929	0.298303185	0.31818702	0.751521696
CCDC144A	0.039547947	0.968543944	0.510954775	0.611335784	-0.448801178	0.655299761
CCDC144B	-0.488800424	0.626210273	-0.0470637	0.962625268	0.337448971	0.737034784
CCDC146	0.375649413	0.708089941	-1.19409375	0.237335992	-1.274169601	0.207843721
CCDC147	-1.550247331	0.124704558	-0.433322719	0.666398896	-1.740198647	0.087294099
CCDC149	-2.773342041	0.006784917	2.409691004	0.01918273	1.431420014	0.157844081
CCDC15	1.524595808	0.130979162	0.473234491	0.637834573	1.391268221	0.16962382
CCDC151	-0.745380292	0.458046555	-1.519484308	0.134111396	-1.966508586	0.054182364
CCDC152	1.560577114	0.122246137	-1.259947592	0.212769526	-1.302464172	0.198066615
CCDC153	-2.456248452	0.016022478	-0.997217198	0.322830281	0.800529085	0.426773349
CCDC154	0.778635654	0.438302463	0.324789618	0.746517058	0.28307467	0.77815901
CCDC157	0.502427168	0.616633704	1.287168606	0.203184273	0.388410321	0.69917897
CCDC159	-0.604506055	0.547077155	2.20975361	0.031111657	3.196495057	0.002283444
CCDC163P	3.51133646	0.000708483	1.208013855	0.231978709	0.541453792	0.590333554
CCDC165	-0.400440329	0.689811732	0.069909808	0.944507609	-0.286901598	0.775242103
CCDC167	0.690348366	0.491808779	-0.051603636	0.959023077	0.679114018	0.499851187
CCDC17	-0.000885466	0.999295523	0.032415227	0.97425299	-0.523087852	0.602968144
CCDC18	0.284884652	0.776408103	0.386422005	0.700608041	0.668233565	0.506718015
CCDC19	-1.003083708	0.318596247	-0.90793616	0.367695249	-1.526968164	0.132374084
CCDC22	-0.573295803	0.567920165	3.037940086	0.003576598	3.669773434	0.000541113
CCDC23	-1.899584782	0.060793571	-0.691971432	0.491735476	0.212888992	0.832183387
CCDC24	-1.697810664	0.093110119	1.340395262	0.185380329	-0.257176523	0.797981159
CCDC25	0.555595009	0.579910602	0.641419938	0.523794256	1.710003676	0.092777779
CCDC28A	-2.026304461	0.045789812	1.03589503	0.304580826	0.15052499	0.880889045
CCDC28B	-0.384656443	0.701428622	-0.180295078	0.857553787	0.436502479	0.664143526
CCDC30	-0.080061192	0.936372068	-1.307882847	0.196109194	-1.020153105	0.312024545
CCDC34	1.613966294	0.1101483	0.611907257	0.543007156	0.958398678	0.341964097
CCDC36	-0.209558312	0.834501297	1.348403676	0.182807261	1.921352928	0.059760879
CCDC39	1.644207783	0.103734457	-2.585312907	0.012283455	-3.555582392	0.000773214
CCDC41	0.567229516	0.572015828	-0.523819938	0.602413667	-0.805561892	0.423888613
CCDC43	0.955053017	0.342191386	-0.109972977	0.912813093	0.246709726	0.80603125
CCDC47	0.757572192	0.450749763	2.584861427	0.012297841	2.075745004	0.042501715
CCDC50	1.814229249	0.073081995	0.777486242	0.440053814	0.274819524	0.78446202
CCDC51	-0.080220455	0.936245769	2.110471772	0.039171519	2.34119881	0.022791306
CCDC53	1.152461989	0.252281303	-0.058258891	0.953744068	0.8098712	0.421427908
CCDC56	-0.545712939	0.586656792	1.426741643	0.159052974	2.253588059	0.028138747
CCDC57	2.102620773	0.038381826	-0.452349365	0.652716476	0.867955244	0.389107653
CCDC58	0.040527855	0.967764961	-1.126375028	0.264678037	-0.461539159	0.646192172
CCDC59	-0.292196597	0.770830476	-1.905209854	0.061749234	-0.620364663	0.537523701
CCDC6	0.144200147	0.885675211	0.148972554	0.882095671	0.714711262	0.477743955
CCDC61	-0.306749079	0.759765753	0.724093218	0.471939498	0.582442317	0.562596833
CCDC62	0.53491826	0.594067998	-0.647104175	0.520135286	-1.096410253	0.277572245
CCDC64	1.580531824	0.117606275	-9.69E-05	0.999923051	1.380464298	0.172906329
CCDC64B	-0.069752412	0.944550457	-0.240864891	0.810515183	0.270076622	0.788089951
CCDC65	1.943713314	0.055155289	-1.224053898	0.225915435	0.444129743	0.658653146
CCDC66	1.80254393	0.074915198	-1.199157973	0.235376689	-0.856803534	0.395189901
CCDC69	-4.032085258	0.000118061	-0.431365524	0.667812936	-0.62566696	0.534064437
CCDC7	-0.444567174	0.657734169	0.10697233	0.915182669	-1.089169082	0.280724749
CCDC71	-0.920318467	0.359945707	2.077983169	0.04217805	2.323583078	0.023787375
CCDC72	0.66989327	0.50469615	-1.156018341	0.252446404	-0.817010347	0.417370291
CCDC73	0.12860891	0.897963314	0.654679289	0.515280251	0.330882258	0.74196314
CCDC75	1.05843554	0.292780517	-0.090425564	0.928262778	0.177419717	0.859816213
CCDC76	1.472257032	0.144552114	-1.437228433	0.156063681	-1.724862076	0.090044854
CCDC77	0.407786169	0.684430267	1.94098453	0.057159058	2.559477684	0.013198585

CCDC78	0.793659137	0.429548584	-0.135669463	0.892555643	-0.252862628	0.801296391
CCDC82	0.852607002	0.396213329	-0.793836599	0.43054904	-0.977571694	0.332472876
CCDC84	1.984449429	0.050350362	-0.263534757	0.793078993	1.959751052	0.054987698
CCDC85B	0.663907947	0.508501065	-3.63906523	0.000586455	-2.544653972	0.01371024
CCDC85C	1.96071374	0.053104706	2.679225103	0.00960325	2.137674509	0.036903853
CCDC86	-0.181280667	0.856568035	2.361690312	0.021595028	2.503390323	0.015230573
CCDC88A	2.052177213	0.043151523	-0.267218899	0.790255136	-0.425943191	0.671775082
CCDC88B	-0.937118816	0.351286027	0.574135047	0.56811193	1.147966448	0.255842356
CCDC88C	3.69044124	0.000389189	-0.227998806	0.820454652	0.479020194	0.633781671
CCDC9	-0.760759485	0.448853281	-0.118704345	0.905922632	-0.452994821	0.652295423
CCDC90A	-1.236354313	0.219647139	-0.494136598	0.623089062	-0.932370347	0.35513093
CCDC90B	-1.1110662	0.269594414	-0.926383017	0.358112525	-0.826956626	0.411756793
CCDC91	1.074891778	0.285388804	-0.218019221	0.828184727	-0.72771278	0.469808467
CCDC92	-0.922463055	0.358832754	-1.369302306	0.176220647	-0.643188645	0.522715809
CCDC93	2.160667081	0.033463746	1.718655196	0.091048484	1.870875258	0.066568679
CCDC94	-1.067315641	0.288775716	1.191715915	0.238260025	1.372054385	0.175495062
CCDC96	-1.092747657	0.277514741	1.719101221	0.090966657	0.630909241	0.53065571
CCDC97	-1.250643227	0.214410543	0.248816224	0.804388047	-0.262136051	0.794174381
CCDC99	2.556992919	0.012287968	-0.771188004	0.443747815	-0.371148808	0.711921273
CCHCR1	1.327756911	0.187724187	1.998220786	0.050421526	1.673921315	0.099699831
CCL2	-1.102457305	0.273296751	-0.896870975	0.373521286	-1.311028015	0.195176571
CCL23	-0.889766037	0.376040005	0.356404649	0.722839848	-1.128642335	0.263840545
CCL28	1.300769587	0.196764756	-0.727774904	0.469699684	0.343954328	0.732163401
CCL3	-0.403712719	0.687412431	-0.567923594	0.572294233	-0.623097087	0.535739601
CCL4	1.547284153	0.125416967	-3.660105904	0.000548772	-2.96645051	0.004416317
CCL5	-1.035312358	0.30338606	-1.898889982	0.062591848	-1.381009585	0.172739496
CCM2	-1.379719766	0.171201098	1.466924638	0.147835808	1.730552385	0.089015998
CCNA1	-1.575547982	0.118751737	0.686827416	0.494947599	-2.184300712	0.033125454
CCNA2	1.634704068	0.105716722	-0.405554204	0.686572791	-0.362172723	0.718580414
CCNB1	0.377439755	0.706764029	1.528901047	0.131762785	1.840908405	0.070912404
CCNB1IP1	0.094368312	0.92503312	-0.701990607	0.48551221	0.493054644	0.623893814
CCNB2	-0.218319477	0.827690386	1.154965303	0.252873874	2.971881239	0.004349498
CCNC	1.073968679	0.285800008	-0.337523005	0.736949686	-0.195569308	0.845652204
CCND1	0.631417521	0.529419511	-1.62775704	0.109035097	0.364395056	0.71692965
CCND2	0.447572852	0.655571531	0.679247502	0.499701688	2.115274378	0.038849478
CCND3	-1.10594105	0.271794295	-1.113832263	0.26997778	-1.82125323	0.073889146
CCNDBP1	-1.795643913	0.076015507	0.110032288	0.912766263	-0.097597855	0.922598646
CCNE1	2.328944085	0.022174785	0.981212614	0.330591636	1.669177976	0.100640484
CCNE2	-0.614990944	0.540162489	-1.918479479	0.060011343	-1.982064832	0.052366767
CCNF	1.640285175	0.104548955	1.802321666	0.07672807	2.931021844	0.004876204
CCNG1	0.933784371	0.352993989	1.344760596	0.183974379	1.137862167	0.260002652
CCNG2	-1.067066325	0.28888764	-1.773873006	0.081370215	-2.495839194	0.015524752
CCNH	0.521732434	0.603179518	-0.743069827	0.460459603	-0.502416663	0.617336208
CCNI	-1.435687131	0.154669389	0.782297445	0.437244216	0.284732865	0.776894726
CCNI2	-0.571481447	0.569143633	0.562706858	0.575818329	2.076130299	0.042464724
CCNJ	-0.467096342	0.641596155	-1.436430868	0.156289482	-1.671196168	0.100239372
CCNJL	-1.554716079	0.123636253	-1.022118847	0.310998667	-0.853832977	0.396819974
CCNK	-1.520863786	0.131912455	-2.161474358	0.034828216	-2.127495007	0.037777206
CCNL1	0.119810613	0.904908818	-1.080633603	0.28436428	-1.778837097	0.080671441
CCNL2	2.450738426	0.016253342	-0.189230289	0.850577582	0.271883517	0.786707263
CCNT1	-0.077923348	0.938067578	-0.799886231	0.427063487	-1.709972638	0.092783559
CCNT2	0.913642033	0.3634246	-0.973593762	0.334329553	-1.919022833	0.060061555
CCNY	-2.359181664	0.0205493	-1.032571517	0.306120817	-1.377722641	0.173747024
CCNYL1	0.449887545	0.653908069	-1.004334022	0.319418486	-0.504672775	0.615760565
CCP110	0.442049062	0.659548242	-0.609220407	0.544774042	-1.60040858	0.115111344
CCPG1	-1.675613443	0.097396778	-0.885801853	0.379407727	-1.442287224	0.154767821
CCR1	-0.825404176	0.411394882	-0.259022334	0.796541512	-1.901790254	0.06232548
CCR10	-1.279554567	0.204096355	-0.553583296	0.582006787	-1.727649324	0.089539674
CCR2	-1.607561637	0.111546737	2.681004813	0.009558067	1.164026977	0.249327789
CCR3	-2.095111037	0.03906168	-0.827301124	0.41148094	-1.178481953	0.243567031
CCR4	0.494487956	0.622205276	1.70337871	0.09388793	1.123260701	0.26609919
CCR5	-0.418306156	0.676751823	-0.94663718	0.34777841	-1.098007535	0.2768802
CCR6	-0.036874496	0.970669371	1.858353725	0.068231571	1.536834553	0.129941578
CCR7	2.200413868	0.030421082	1.587042086	0.117978368	2.484449267	0.015978201
CCR8	1.031417467	0.305197718	0.704277373	0.484097973	0.050838928	0.959634017
CCR9	-0.215078739	0.830208222	1.625235575	0.109572498	1.896335766	0.063056999
CCRL2	-2.095744777	0.039003908	-0.144610556	0.885523198	0.258627248	0.796867113
CCRN4L	-0.334230323	0.739008382	1.043887571	0.300899021	1.010803353	0.316439953
CCS	-0.961510191	0.33895472	1.550120847	0.126590306	1.738732461	0.087554028
CCT2	2.685701311	0.008664213	0.824863014	0.412852574	1.710818705	0.092626117
CCT3	-1.114302539	0.268211691	3.24328938	0.001968132	4.310420152	6.62E-05
CCT4	0.969701347	0.334877744	4.255455549	7.77E-05	4.461646172	3.95E-05

CCT5	-0.8175843	0.415823231	2.627269562	0.011011507	2.533805543	0.014096011
CCT6A	0.841846848	0.402176862	2.480579648	0.016061127	2.506078842	0.015127056
CCT6B	1.133274725	0.260205554	1.584208797	0.118622085	1.288640974	0.202799166
CCT6P1	1.907415106	0.059759018	-0.583434012	0.561878923	1.144361552	0.257321138
CCT6P3	0.981182637	0.329217502	-0.149720854	0.881507905	-0.498718025	0.619923209
CCT7	-0.285421651	0.775998071	3.303783612	0.0016437	3.81746106	0.00033821
CCT8	1.108945607	0.27050313	2.098273096	0.040277775	3.023464236	0.003760124
CCZ1	0.028120922	0.977629974	-1.043468188	0.301091452	-1.388121431	0.170574906
CD101	1.839073304	0.06930834	1.857257149	0.068389908	1.749421305	0.085673713
CD109	0.216210489	0.829328726	-0.001826398	0.998549057	-0.613329369	0.542131363
CD14	-1.607758913	0.11150345	2.783139095	0.007268818	2.278777111	0.026497678
CD151	-1.386242615	0.169207733	0.82674715	0.411792352	-0.017717977	0.985926551
CD160	2.960518926	0.00395529	-2.14270354	0.03637504	-1.439179693	0.155642681
CD163	-0.080305479	0.936178344	2.787172288	0.007189748	1.933144471	0.058258884
CD164	0.25722511	0.797611074	-1.200502087	0.234858641	-2.097866788	0.040422673
CD177	-0.809745507	0.420290907	1.403188578	0.165928756	1.35144059	0.181965856
CD180	-0.316687895	0.752237437	0.36561512	0.715991648	0.976805561	0.332848753
CD19	0.314676384	0.753759181	2.48242682	0.015986292	2.785592056	0.007269232
CD1A	-1.662391921	0.100025445	1.154571551	0.253033847	0.586056081	0.560182726
CD1C	0.561434659	0.575941521	0.845045605	0.401582095	1.182951617	0.241805317
CD1D	-3.432588763	0.000916466	2.534296643	0.014009254	1.370597106	0.175946644
CD1E	0.866697697	0.388486566	-0.081653344	0.93520547	-1.497842512	0.13976651
CD2	1.364633127	0.175880002	-0.613378889	0.542040647	0.349437704	0.728065885
CD200	1.624767349	0.107821874	0.32483673	0.746481584	0.087535054	0.930557178
CD200R1	0.439351074	0.661494161	-0.310098123	0.757605615	-1.968377251	0.053961456
CD209	-2.785586358	0.006554317	1.151142245	0.254430155	0.635675175	0.527566593
CD22	0.229947432	0.818671242	1.263803003	0.211391913	1.731600564	0.088827543
CD226	-0.536247089	0.593153328	-1.724982492	0.08989336	-2.511919334	0.014904365
CD24	-1.760262783	0.081870189	0.999648385	0.321662041	-0.158544138	0.874596015
CD244	0.112769687	0.91047239	0.206023501	0.837499062	1.339245833	0.185878525
CD247	0.24838541	0.80442032	-1.175124099	0.244780238	-0.20098393	0.841436293
CD248	-0.836596742	0.40510637	0.012104992	0.990383682	0.10856291	0.913935612
CD27	1.559089688	0.122597742	2.827783698	0.006436576	3.377810534	0.001331961
CD274	-1.432037587	0.155708372	-1.754029943	0.084744743	-2.407232158	0.019380591
CD28	2.007081424	0.047838455	-0.468280055	0.641351674	0.315292517	0.753706591
CD2AP	0.753994746	0.452883879	-0.423866554	0.673241987	-1.602944232	0.114549413
CD2BP2	-0.73850235	0.462192537	1.283722695	0.204379527	1.926282996	0.059128926
CD300A	-1.5340621	0.12863522	1.611676201	0.112499357	1.629827119	0.108727196
CD300C	-0.816447134	0.416469578	2.949379423	0.004595286	1.152345102	0.254054333
CD300E	0.715569307	0.47616929	1.947832848	0.056314397	0.721100141	0.473835121
CD300LB	-2.748184778	0.007282142	0.692074871	0.491671002	1.034939951	0.305126965
CD300LD	-2.281694082	0.024942909	-1.696326101	0.095223179	0.139436416	0.889603463
CD300LF	-3.753783129	0.000313493	1.307307019	0.196303336	0.672608905	0.503950561
CD302	-1.746438212	0.084256714	-4.237780705	8.25E-05	-4.852323826	1.01E-05
CD320	1.232209327	0.221183525	0.826614406	0.411866994	1.330378393	0.1887635
CD33	-2.517685568	0.013640132	-0.428703066	0.669738461	0.046154624	0.963350519
CD34	0.982378014	0.32863183	1.183769768	0.241366818	2.061788837	0.043860587
CD36	2.745126276	0.007344808	0.442170384	0.660021839	1.033242785	0.30591332
CD37	-1.851978182	0.067413298	-2.362982083	0.021526714	-2.444031974	0.017685334
CD38	-0.328322676	0.7434549	-0.511506053	0.610952237	-1.135881586	0.260823734
CD3D	1.805512784	0.074445859	-0.89625004	0.373849949	0.832635542	0.408572442
CD3E	0.717526591	0.474967279	-0.721586085	0.473468205	0.835699269	0.406860781
CD3EAP	-0.900360932	0.370408372	-0.083297394	0.933903901	0.960213187	0.341058306
CD3G	1.611603353	0.110662592	-0.177669288	0.859606082	0.00381331	0.996970921
CD4	-0.870590101	0.386368694	2.091655877	0.040889166	1.804986846	0.076431343
CD40	-0.523987174	0.601616946	0.657934579	0.513201315	1.31671522	0.193274926
CD40LG	-0.688024103	0.493264038	-0.992335055	0.325184826	0.139371679	0.889654381
CD44	-0.652834269	0.51558083	1.344833187	0.183951068	1.422816032	0.160313239
CD46	-0.754001942	0.45287958	-0.864767688	0.390753773	-1.876375886	0.065796299
CD47	1.970454849	0.051959204	-0.665098178	0.508642237	-1.130888717	0.262901777
CD48	0.739631168	0.461510635	0.48972672	0.626187423	0.464649024	0.643976812
CD5	-0.563718092	0.574393078	0.929427103	0.356546826	1.830866221	0.072420417
CD52	0.429125446	0.668890426	-1.690943738	0.096252673	-1.205151822	0.233191538
CD53	-2.398408468	0.018598526	-1.386771036	0.170855641	-1.492939734	0.141042383
CD55	-2.532444825	0.013117477	0.267705269	0.789882548	-0.467414215	0.642009709
CD58	-1.490115707	0.139802618	-2.536616744	0.013926232	-2.702500675	0.009082477
CD59	-0.407934058	0.684322091	0.929695366	0.35640906	0.660751654	0.511469356
CD6	0.892276231	0.374700894	-1.048317388	0.298871561	0.352038343	0.726125306
CD63	-2.176504395	0.032221004	0.387991889	0.699452345	-0.091943729	0.927069491
CD68	-2.160489009	0.033477953	0.333718908	0.739803636	0.139564072	0.889503059
CD69	1.717627365	0.089414491	-0.761805645	0.449284264	-0.838249295	0.405439473
CD7	-0.254054803	0.800051399	-0.408295037	0.684571046	0.393089408	0.695739661

CD70	-0.529158295	0.598040309	-1.510149063	0.136472353	-0.464996986	0.643729137
CD72	-0.981161132	0.329228045	1.838742882	0.071110356	2.211808822	0.031059473
CD74	-1.150901962	0.252919125	0.667921348	0.506851511	0.690526017	0.492703729
CD79A	-1.472297297	0.144541265	-0.062817901	0.950129007	0.199788165	0.842366938
CD79B	-0.222053587	0.824791467	1.564221412	0.123243818	1.701667186	0.094340832
CD80	1.00041766	0.319876849	-0.616334426	0.540102232	-1.179013838	0.243356905
CD81	-0.492936005	0.623296994	1.114514673	0.269687531	1.745628011	0.086337133
CD82	-3.169200607	0.002108756	0.467405383	0.641973456	0.435241028	0.665053361
CD83	-0.986750427	0.326495437	0.090713865	0.928034697	0.674289217	0.502889924
CD84	1.437997696	0.154014372	-1.334128151	0.187413024	-1.444094544	0.154260775
CD86	-2.69623156	0.008415737	2.062518271	0.043678253	0.781146049	0.437992703
CD8A	0.172619642	0.863350489	-2.556112442	0.013246012	-0.548266035	0.585679191
CD8B	1.519690541	0.132206938	-1.31971861	0.192150645	0.465501112	0.643370379
CD9	-2.872018176	0.005119953	-0.54890859	0.585189921	-1.410238723	0.163976466
CD93	-0.882259994	0.380062147	0.650826011	0.517746869	-0.0612627	0.951367204
CD96	2.936433643	0.004245288	0.561767694	0.576453884	1.726133265	0.089814164
CD97	-3.265878761	0.001560863	0.590100154	0.55743164	0.225100452	0.822717099
CD99	-0.581526867	0.56238588	0.612398784	0.542684243	0.721134903	0.473813902
CD99L2	-0.438631034	0.662013883	2.905998132	0.005187276	3.001907255	0.003996738
CD99P1	1.329014835	0.187310526	0.681748225	0.498130501	-0.941019988	0.350719369
CDA	-3.795093234	0.000271918	-1.151862473	0.254136445	-0.796565582	0.429053426
CDADC1	-1.185534338	0.239027272	-1.524476083	0.132862302	-0.954207199	0.34406249
CDAN1	0.441487124	0.659953346	0.566756411	0.573081794	1.046302199	0.29989794
CDC123	-1.746620081	0.08422495	-0.624109544	0.535019906	-0.228160657	0.820348924
CDC14A	1.320009657	0.190286956	-1.118976223	0.267795315	-2.484982154	0.015956722
CDC14B	-1.006760731	0.316835648	0.357046534	0.722361844	-0.982855948	0.329888013
CDC16	1.842639247	0.068780294	1.691668182	0.096113577	3.163211341	0.002516425
CDC20	0.042595085	0.966121709	1.736792055	0.087769768	2.573015375	0.012746527
CDC23	1.501448263	0.136852664	0.326825185	0.744984872	0.755875581	0.452878408
CDC25A	-0.770358904	0.443169408	0.335197932	0.738693587	1.745826014	0.086302399
CDC25B	1.854422226	0.067059326	1.435233609	0.156628918	2.27325928	0.026849724
CDC26	-1.582857752	0.117074723	0.311482589	0.756558446	0.689685732	0.493228083
CDC27	-0.172570375	0.8633891	-0.530079766	0.598094292	-1.447613848	0.153277149
CDC34	-2.353804736	0.020830383	0.573015394	0.568864707	0.402061919	0.689162518
CDC37	-0.715140848	0.476432642	-0.040222538	0.968054812	0.757494213	0.451916233
CDC37L1	0.488787284	0.626219539	0.696872212	0.488685949	0.250572877	0.803057564
CDC40	1.038169568	0.302061699	-0.666596774	0.50769126	-0.814529613	0.418777557
CDC42	-1.777494491	0.078973868	-1.409596449	0.164035786	-1.171387027	0.246382471
CDC42BPA	-1.053244643	0.295139026	0.394856481	0.694407282	0.14963325	0.881589324
CDC42BPB	-0.164285173	0.869886911	1.251079497	0.215963499	1.132193786	0.262357475
CDC42BPG	2.169029529	0.032802448	0.119239295	0.905500701	1.249486683	0.216662537
CDC42EP1	-0.931992938	0.353913795	-0.867755206	0.389129504	-0.577650756	0.565805678
CDC42EP2	-2.694882817	0.008447196	0.393390539	0.695483504	-0.053487177	0.957533303
CDC42EP3	0.32401624	0.746701735	-1.23471891	0.221948723	-1.985558547	0.051966268
CDC42EP4	-1.035567868	0.303267468	0.722851003	0.472696581	0.937751567	0.352382146
CDC42SE1	-0.061992336	0.950710826	-1.269486208	0.209373275	-0.72645432	0.470573285
CDC42SE2	-0.00815434	0.993512479	-1.044545727	0.3005972	-1.40586565	0.165265218
CDC45	-0.091314764	0.927451956	0.201598806	0.840940696	1.437309465	0.156171059
CDC5L	-0.353523326	0.72454953	1.249521589	0.216528242	1.372132053	0.175471019
CDC6	0.619825692	0.536989077	0.215493448	0.830143906	1.136992308	0.26036304
CDC7	0.775799539	0.439966642	-0.866793428	0.389651948	-0.782572961	0.437160878
CDC73	-0.383944834	0.701954065	-0.561916238	0.576353338	-2.333090752	0.023245055
CDCA2	1.605977352	0.11189485	1.986910531	0.051696001	0.958094159	0.342116265
CDCA3	1.490539043	0.139691532	0.134504912	0.893472249	0.920566046	0.36120922
CDCA4	2.513383601	0.013795956	-0.953507066	0.344317879	-1.405221265	0.16545578
CDCA5	0.431458413	0.667200062	1.575660514	0.120581375	1.897682195	0.062875751
CDCA7	1.212533886	0.228583363	0.750393952	0.456072099	-0.873303285	0.386211645
CDCA7L	1.826909063	0.07113517	1.662992819	0.101746792	1.453360203	0.151681612
CDCA8	-2.409198116	0.018091688	0.458217345	0.6485205	0.113604416	0.909955984
CDCP1	-2.497488321	0.014385672	1.287946702	0.202915106	1.796885587	0.077724527
CDH1	0.080621213	0.935927967	1.946973998	0.056419738	-0.37392516	0.709866096
CDH13	0.417456019	0.677371076	0.872336098	0.386647145	-0.283008995	0.778209095
CDH2	-3.035530102	0.00316527	0.838337254	0.405307021	-0.438710583	0.662552131
CDH23	-0.237515143	0.812814404	-0.875444471	0.384968396	-0.39393169	0.695121231
CDH24	-1.023729582	0.308795027	-1.25937829	0.212973512	-0.393681194	0.695305131
CDH26	-0.450659806	0.653353469	-0.075619494	0.939983875	0.420611208	0.675641992
CDHR1	0.885244636	0.378459604	-1.390820445	0.169630092	-0.287850331	0.774519474
CDHR2	-0.270357335	0.787524292	-2.245687552	0.028576693	-1.730492771	0.089026726
CDHR3	3.038257196	0.003139515	0.145346569	0.884944705	2.138021184	0.036874423
CDHR5	1.441555172	0.153010066	-0.422254255	0.674411546	-0.441329522	0.660666672
CDIPT	-2.288091941	0.024551095	-0.970885106	0.33566517	-1.19292183	0.237908789
CDK1	1.012265248	0.31421218	1.036888707	0.30412142	0.960057374	0.341136025

CDK10	-0.17845229	0.85878178	-0.966172509	0.337997305	0.609355237	0.544743075
CDK11A	-2.523100593	0.01344624	-0.556283067	0.580172229	-0.386025534	0.700934328
CDK11B	-1.620617909	0.108710893	0.291924288	0.771393418	-0.213392079	0.831792892
CDK12	-0.967308829	0.336065234	-0.524186648	0.602160234	-0.767233103	0.446152183
CDK13	0.517510148	0.606110616	-1.004464742	0.319356046	-1.940516606	0.057336283
CDK14	-2.153727627	0.034021292	0.698833395	0.487468533	-1.259987312	0.212877594
CDK16	0.32033625	0.749479893	-2.587606614	0.012210607	-2.244556631	0.028748692
CDK17	-0.213517604	0.831421754	-0.846714471	0.400658714	-2.749174492	0.008018481
CDK18	0.447378771	0.655711088	-0.918705443	0.3620811	-0.606271304	0.546774182
CDK19	-3.246298045	0.001659721	-0.794756866	0.430017729	-1.633966327	0.107852467
CDK2	1.997614733	0.048875817	-0.67713037	0.501033974	-0.93807303	0.352218378
CDK20	0.876187692	0.38333562	-0.017438252	0.986147261	0.163792349	0.870481857
CDK2AP1	-1.948578093	0.054561754	-0.711909757	0.47939441	-0.384915346	0.701752058
CDK2AP2	-1.127433145	0.262652535	-2.992650596	0.004067857	-3.03169867	0.003673242
CDK4	0.870649094	0.386336651	0.768584453	0.445280127	2.483324226	0.016023633
CDK5	-1.223882341	0.224293668	2.137895628	0.036780779	2.536277195	0.014007264
CDK5R1	0.851118346	0.397035125	-0.930716386	0.355885032	-0.92330578	0.359792534
CDK5RAP1	1.459397719	0.148049249	2.383667972	0.020458755	3.012956949	0.003873773
CDK5RAP2	-0.09931338	0.921117442	3.409857713	0.00119326	3.472203665	0.000999712
CDK5RAP3	-0.119817793	0.904903147	2.378726834	0.020709451	3.117113036	0.002876155
CDK6	2.370746758	0.019956099	0.292518345	0.770941529	-0.121104824	0.904039686
CDK7	-0.422203022	0.673916136	-2.534548187	0.014000231	-2.337772082	0.022982102
CDK8	-0.050470087	0.95986323	-1.298914761	0.199149233	-2.407504165	0.019367544
CDK9	-0.582236474	0.561910008	-1.115609097	0.269222499	0.188579491	0.85110129
CDKAL1	0.055552137	0.955825697	0.935378445	0.353498616	2.524973691	0.014417306
CDKL1	-1.542153328	0.126658151	-1.218997006	0.227814328	-1.40164634	0.166516063
CDKL5	-1.169371904	0.245440759	-0.208431073	0.83562773	-1.813417871	0.075104699
CDKN1A	-2.71965582	0.007886101	1.063480247	0.2920027	-0.388937894	0.698790865
CDKN1B	-1.230414646	0.221851169	-1.789053451	0.078864818	-2.634653674	0.010861001
CDKN1C	0.141066422	0.888142885	-0.555426459	0.580754013	-1.270675718	0.209075538
CDKN2A	0.948311191	0.345592121	-3.411202292	0.001188384	-3.69972376	0.000492291
CDKN2AIP	0.502357468	0.616682521	-0.736811413	0.464227816	-1.798426647	0.077477132
CDKN2AIPNL	-0.48004078	0.632400528	-2.278945995	0.026394949	-1.670757389	0.100326467
CDKN2B	-0.565750204	0.573016749	1.334136483	0.18741031	0.200924374	0.841482639
CDKN2C	-1.551723673	0.124350814	-0.961039303	0.340549712	-0.336360397	0.737851004
CDKN2D	-2.460273796	0.015855679	-1.206973114	0.232376176	-1.854195376	0.068957837
CDNF	1.293169564	0.19936833	-1.356540771	0.180220766	0.201307999	0.841184114
CDO1	0.561761051	0.575720065	-0.244717064	0.807545262	-0.28401279	0.77744367
CDON	-1.016018548	0.312431704	-0.188030564	0.851513587	1.018250143	0.31291982
CDR2	1.443093597	0.152577329	2.121904764	0.038158832	3.302719798	0.001668397
CDRT15P	2.346150993	0.021236368	0.261533193	0.794614341	0.022995654	0.98173514
CDS1	-2.708588576	0.008132434	-0.800923529	0.426467538	0.119598412	0.905227511
CDS2	-0.287081417	0.77473114	-0.362910327	0.718000324	-0.121423012	0.903788819
CDT1	0.351655197	0.725945306	0.640119504	0.524633251	1.558147318	0.124808665
CDV3	-0.692840853	0.490250801	1.854384903	0.068806108	1.357107704	0.18016906
CDYL	0.287060357	0.774747212	0.905083901	0.369191436	0.350441263	0.727316824
CDYL2	2.256175082	0.026561053	1.563590479	0.123392028	-0.375063911	0.709023768
CEACAM1	-3.874242551	0.000206491	-0.192144218	0.848305083	-0.55975371	0.577870271
CEACAM19	-0.287424646	0.774469223	-0.773901853	0.442153883	0.80790919	0.422547188
CEACAM21	-0.22793789	0.820228222	2.707970766	0.008896848	1.471931801	0.146613731
CEACAM22P	-1.737364299	0.085854052	2.304271039	0.024834006	1.988171991	0.051668401
CEACAM3	-2.712454258	0.008045607	-1.498246209	0.139530284	-1.528305437	0.13204228
CEACAM4	-2.275959489	0.025298774	-2.411131403	0.019114231	-1.430879377	0.157998356
CEACAM6	-2.654291306	0.009445502	2.08941116	0.04109839	-0.08453912	0.932928037
CEACAM8	-3.738914701	0.000329887	1.800589648	0.077004221	-0.363146739	0.717856743
CEBPA	-0.656491924	0.513236628	-0.319100094	0.750805044	-0.802879374	0.425424736
CEBPB	-1.291703389	0.199873537	-1.938606135	0.057454925	-3.253934599	0.001928404
CEBPD	-0.121333613	0.903705995	-1.790928321	0.078559903	-3.054357621	0.003443743
CEBPE	0.80119084	0.42519918	-0.594767928	0.554328062	-1.258904258	0.213265698
CEBPG	1.362669462	0.176496072	-0.113578469	0.909966931	-0.112458047	0.910860697
CEBPZ	1.658970515	0.100714972	1.170286046	0.246705468	2.038310778	0.046231616
CECR1	-0.672025776	0.503344197	0.952234299	0.344957295	0.80120407	0.426385776
CECR5	0.589541969	0.557022385	1.389761357	0.169949969	0.834230244	0.407680956
CECR6	-1.28243868	0.203087935	0.31765161	0.751897981	-1.579745782	0.119773871
CECR7	-1.25996749	0.211043123	1.814109479	0.074870562	1.415786947	0.162352601
CEL	-0.238666893	0.81192396	-1.379372565	0.173112341	0.151362019	0.880231818
CELA1	0.595871356	0.552804915	-1.52695024	0.132246627	0.148791605	0.88225035
CELF1	2.125330157	0.036387851	0.136452339	0.891939532	0.123128926	0.902444004
CELF2	1.213553651	0.22819548	-2.252601678	0.02811043	-2.83135142	0.006419284
CELF3	-0.439213275	0.661593611	0.939399286	0.351448784	1.213316992	0.230080279
CELF6	0.381649013	0.703650259	-1.538936042	0.129296101	-1.447710772	0.153250129
CELSR1	1.945804583	0.054899473	1.668027276	0.1007387	1.208463671	0.231925916

CELSR2	0.59853606	0.551034117	-0.101281779	0.919678539	0.62269362	0.536002847
CELSR3	0.871754712	0.385736421	-0.076556177	0.939241935	-0.500632689	0.618583398
CEMP1	0.717012443	0.475282865	1.965924601	0.054134122	2.802192061	0.00694958
CEND1	0.435570221	0.664225012	-0.128121299	0.898499287	0.155609369	0.876898154
CENPB	-0.780524854	0.437195962	-1.184274813	0.241168488	-0.275553819	0.783900771
CENPBD1	-0.061047635	0.951460993	2.060083331	0.043918625	1.35213779	0.181744071
CENPC1	1.886331873	0.062578951	0.389812764	0.698112772	0.569634704	0.571194015
CENPE	-0.056533243	0.955046368	0.527134221	0.600124965	-0.678112299	0.500481261
CENPF	0.520550606	0.603999286	2.055467699	0.044377416	2.905828584	0.005229875
CENPH	1.578386949	0.118098157	1.362470829	0.178353453	3.071712205	0.003277106
CENPJ	0.770370217	0.443162735	-0.111233911	0.911817584	-0.332607485	0.740667285
CENPK	2.5938858	0.011130034	-0.728992213	0.468960443	-1.736000247	0.08804011
CENPL	0.899331672	0.370953119	0.364253667	0.71700246	0.084953777	0.932599858
CENPM	-0.149138227	0.881788983	1.01946293	0.312246419	1.563702438	0.123497799
CENPN	-0.95594581	0.341742678	0.099752927	0.920886878	0.180340938	0.857533223
CENPO	0.249343627	0.803681462	0.160995189	0.872660564	0.847517349	0.400299449
CENPP	-0.567587979	0.571773414	0.128328934	0.89833571	0.216501321	0.829380449
CENPQ	0.779407409	0.43785025	0.094664211	0.924910113	-0.204984863	0.838324091
CENPT	1.439606414	0.153559588	-0.45995851	0.647277648	0.967337344	0.337517246
CENPV	-0.744838023	0.458372659	0.431062991	0.66803162	0.031135312	0.975271867
CENPW	1.501492291	0.136841299	-0.640813765	0.52418525	-0.297831968	0.766928958
CEP104	1.005559646	0.317410025	1.239933184	0.220028078	0.593112245	0.5554839
CEP120	2.253651747	0.026725962	-0.30336973	0.76270124	-1.180819613	0.242644493
CEP128	0.677508542	0.499877219	0.168145281	0.867058036	0.191718997	0.84865289
CEP135	0.48066165	0.631960901	-0.664438359	0.509061249	-1.219073419	0.227905138
CEP152	-1.145267602	0.255232265	0.821381537	0.414815997	-0.27620383	0.783404041
CEP164	0.054162429	0.956929667	1.318000815	0.192721404	1.484626079	0.143226874
CEP170	-0.052248472	0.958450233	-0.209807363	0.834558409	-1.631771121	0.10831566
CEP19	-2.349419218	0.021062158	-0.814565126	0.418676563	-1.631135329	0.108450114
CEP192	0.494585433	0.622136735	0.870125046	0.387844061	-0.089923262	0.928667701
CEP250	0.921846088	0.359152708	2.549559274	0.013471219	3.040129305	0.003586234
CEP290	0.553504127	0.581334894	-0.407088596	0.685451881	-1.308759136	0.195939141
CEP350	-0.308305791	0.758585046	-0.758369165	0.45132212	-1.806250909	0.076231197
CEP44	1.633694543	0.105929071	-0.716828051	0.476377053	-0.904361639	0.369661645
CEP55	0.987261617	0.326246266	0.666894792	0.507502258	-0.505064476	0.615487192
CEP57	1.321516664	0.1897864	-0.02261637	0.982034458	-0.372029096	0.711269412
CEP57L1	0.698125082	0.486956739	-0.134519484	0.893460779	-1.320888837	0.191888298
CEP63	-2.361271745	0.02044095	-0.460964991	0.646559678	-1.293981834	0.200960721
CEP68	3.602967597	0.000522685	1.713361882	0.092024226	1.044963579	0.300510779
CEP70	0.509257818	0.611857989	-0.681334058	0.498390532	0.035629505	0.971703951
CEP72	0.195070133	0.845791898	1.831969792	0.072128068	2.185041437	0.033068278
CEP76	1.782425688	0.078160805	1.96499154	0.054244773	1.539517563	0.129286294
CEP78	0.791873747	0.430583453	-2.335228945	0.023037676	-0.902982151	0.37038699
CEP85	-0.09896041	0.921396873	-0.209622507	0.834702016	-0.416128628	0.67889971
CEP89	-0.535630805	0.593577452	0.044115192	0.964965172	0.714808981	0.477684033
CEP95	2.328306803	0.022210234	0.869436677	0.388217171	1.669276998	0.100620773
CEP97	-0.443756544	0.658317932	-0.698239677	0.48783691	-1.740080514	0.087315019
CEPT1	1.157667727	0.250161178	-0.471882148	0.638793761	-0.853684736	0.396901429
CERCAM	0.268288491	0.789111003	0.3496327	0.727889559	-0.273387629	0.7855568
CERK	2.305997625	0.023483216	-2.329237752	0.023376002	-1.04941176	0.298477643
CERKL	0.885635043	0.378250296	-0.536729413	0.593521806	-0.638878471	0.525495629
CERS2	-1.944720294	0.055031984	-2.041451636	0.045796128	-2.413805822	0.019067459
CERS3	1.981923654	0.050637549	0.294069505	0.769761962	0.281315695	0.779500791
CERS4	-0.605333499	0.54652985	0.256124102	0.798767588	0.843356236	0.402602192
CERS5	0.024875134	0.980211405	0.196698134	0.844756198	1.334086446	0.187553006
CERS6	1.187800322	0.238137797	-0.273614081	0.78536002	-0.031265755	0.975168301
CES1	-1.530606631	0.129486954	1.558615078	0.124565783	1.286856413	0.203416248
CES1P1	1.271849982	0.206808405	0.53900762	0.591959032	-0.199844847	0.842322818
CES2	-1.434978182	0.154870798	0.171580294	0.864368913	0.329848837	0.742739724
CES4A	0.548372259	0.584837719	0.54269141	0.589436185	1.433957121	0.157121661
CETN2	-1.434997201	0.154865392	2.875796565	0.005640346	3.004122695	0.003971796
CETN3	0.400086808	0.690071122	-0.199082462	0.842899366	-0.544671871	0.588132671
CETP	-0.69755729	0.487310103	1.53445863	0.130392101	1.187556806	0.239999836
CFB	-0.217378012	0.828421656	1.600697566	0.114915215	0.346030178	0.730611268
CFD	0.254858814	0.799432327	-0.906068744	0.368674386	-1.143050149	0.257860604
CFDP1	-0.678308816	0.499372254	1.304330191	0.197309288	1.196292248	0.236601924
CFH	1.020708673	0.310216342	-0.581294322	0.563310128	-1.001340849	0.320951281
CFL1	-1.947292195	0.054718113	0.938544913	0.351883697	1.065448004	0.291226356
CFL2	0.465404073	0.642802514	-1.474097829	0.145900253	-0.734614769	0.465626408
CFLAR	-2.432933607	0.017019806	-0.679999435	0.499228969	-0.633777215	0.528795657
CFLAR-AS1	-1.532011811	0.129140057	0.160610975	0.872961807	0.922595626	0.360159402
CFP	-3.029870788	0.003219345	0.944037811	0.349093675	0.706719996	0.482658552

CG030	2.444557359	0.016515857	-1.273280565	0.208033543	0.70869451	0.481441616
CGGBP1	0.380981335	0.704143834	-0.74488638	0.459369147	-1.640794435	0.106422011
CGN	1.196718376	0.234660258	0.81406057	0.418963186	1.683937592	0.097737151
CGRRF1	-0.973499255	0.332998367	-1.081622963	0.283927987	-1.204268841	0.233529816
CHAC2	0.924161066	0.357953119	-1.361517024	0.178652795	-2.027581618	0.047351533
CHAD	1.836362016	0.069712091	-0.558401306	0.578734785	-0.188874775	0.850870944
CHADL	-0.252172402	0.801501307	1.35891586	0.179471093	1.167314548	0.248009073
CHAF1A	-0.813996931	0.417864282	1.597991543	0.115517067	1.249967875	0.216488012
CHAF1B	1.109573912	0.270233666	-0.099920878	0.920754128	2.37187739	0.021145136
CHCHD1	-0.424003979	0.672607197	-1.044596833	0.300573772	-0.312430654	0.755868841
CHCHD10	-0.020992976	0.983299227	-4.276032125	7.25E-05	-4.40516956	4.80E-05
CHCHD2	0.457401828	0.648519979	-0.867613406	0.389206503	-0.189235659	0.850589442
CHCHD3	0.830758728	0.408379119	2.008666253	0.049268588	3.831971392	0.000322791
CHCHD4	0.828539226	0.409627547	0.661948662	0.510643974	0.540611441	0.590910292
CHCHD5	-1.008646847	0.315935078	0.29760678	0.767074121	0.557498385	0.579399387
CHCHD6	0.930175827	0.354848359	-1.128621418	0.263736683	-0.3089652	0.758489757
CHCHD7	1.918143534	0.058365716	1.84607724	0.07002196	1.944524536	0.056839958
CHCHD8	0.61685433	0.538938278	0.607019628	0.546223465	0.749389872	0.45674565
CHD1	-0.067525666	0.94631784	-0.144517872	0.88559605	-2.408846192	0.019303283
CHD1L	2.079264727	0.040530449	2.552459678	0.013371119	3.534633595	0.000825017
CHD2	0.218729809	0.827371713	0.307589638	0.759504119	-0.196003855	0.84531369
CHD3	1.083768598	0.281455306	0.677615163	0.500728729	1.682743557	0.097969444
CHD4	-1.151967389	0.252483399	1.893418543	0.063329203	2.014572072	0.048740832
CHD5	2.201357717	0.030351867	-0.670161662	0.505432911	-0.102778596	0.918504318
CHD6	2.510858029	0.01388818	0.62124769	0.53688772	0.4051396	0.686911991
CHD7	1.598587993	0.113530036	1.971492311	0.05347786	1.10665989	0.273152437
CHD8	-0.493240125	0.623082995	1.278160544	0.206319884	1.405453239	0.165387159
CHD9	0.21415747	0.830924312	-1.887665148	0.064112488	-2.886416217	0.005518464
CHDH	-0.069953901	0.944390548	0.891919012	0.376147482	-0.545002396	0.587906841
CHEK1	0.409882717	0.682897334	-1.559457238	0.124366482	-0.776714374	0.440582123
CHEK2	0.415465661	0.67882175	1.624017295	0.109832919	2.818370174	0.006650571
CHERP	-0.632617797	0.528638885	-0.002167077	0.998278413	-0.183816329	0.854818735
CHFR	-1.446850315	0.151524598	-0.872771053	0.386411962	-0.598582381	0.551854888
CHI3L1	-0.110028088	0.91263996	-0.253625608	0.800687982	0.65344175	0.516134435
CHI3L2	0.706441423	0.481797259	0.323646163	0.747378196	0.694918074	0.489967999
CHIC1	1.907234649	0.059782692	-1.604975654	0.113968883	-1.21895534	0.227949605
CHIC2	-0.75173732	0.454233522	-1.440779335	0.155061457	-1.909700148	0.061277473
CHID1	-0.726645737	0.469389409	1.800971616	0.076943249	2.25985221	0.027722446
CHIT1	0.702561512	0.484200594	0.608114609	0.545502071	-0.219095676	0.827368766
CHKA	-1.115882582	0.267538423	-0.594898303	0.5542415	0.246004808	0.806574178
CHKB	0.595781177	0.552864892	2.145446923	0.036145285	2.719155355	0.008688782
CHKB-CPT1B	-0.013855159	0.988977191	3.745807081	0.000417869	4.272308912	7.54E-05
CHL1	0.583950455	0.560761406	1.435740929	0.156485017	0.00666474	0.994705933
CHM	1.165193013	0.247118803	-1.65902843	0.102546402	-2.472268853	0.01647632
CHML	0.440521928	0.660649398	-1.06655259	0.290624277	-1.56645614	0.1228521
CHMP1A	-1.793849705	0.076303807	1.287867657	0.202942438	1.320301571	0.192082954
CHMP1B	-1.028374426	0.306618219	-1.38599027	0.17109272	-1.828420079	0.072791806
CHMP2A	-2.567237113	0.011956012	1.516023828	0.13498277	1.519299546	0.134289644
CHMP2B	-0.40669808	0.685226367	0.374292303	0.709561329	-0.10972441	0.913018555
CHMP4A	-0.203941814	0.838874238	1.92308426	0.059418073	2.435728292	0.018055759
CHMP4B	-2.092625058	0.039289015	0.603735844	0.548389791	0.780561536	0.438333718
CHMP5	-1.177053953	0.242377258	-0.37210207	0.711182445	-1.181855349	0.242236556
CHMP6	-1.378079139	0.171705274	1.021795013	0.311150624	1.434658995	0.156922263
CHMP7	1.529577299	0.129741532	1.4985641	0.139447918	2.987930452	0.00415749
CHN1	0.285754761	0.775743753	0.440286686	0.661377435	1.002230497	0.320525304
CHN2	1.234573642	0.220306208	0.245390799	0.807026122	-0.690516304	0.492709788
CHORDC1	1.171450754	0.244609027	0.565566267	0.573885391	-0.776865263	0.440493811
CHP	-0.160815517	0.872610732	0.140470923	0.888778031	0.231958657	0.817412138
CHPF	-0.849254545	0.39806549	0.950595473	0.345781758	0.600207545	0.550779026
CHPF2	-0.537974509	0.591965278	-0.406157017	0.686132337	-0.478367761	0.634242993
CHPT1	-3.363975523	0.001143379	-0.325663177	0.745859397	-0.944849979	0.348777411
CHRAC1	-1.44727805	0.151405093	-2.06788188	0.043152788	-1.768842305	0.082343069
CHRM5	1.318238853	0.190876397	-0.668126756	0.506721354	0.564217606	0.574849499
CHRNA10	-0.357258663	0.721761461	-1.842317468	0.070578133	-2.16452013	0.034684793
CHRNA2	-1.065295463	0.289683472	-0.181041999	0.856970179	0.386468076	0.700608464
CHRNA5	1.669805388	0.09854452	1.031564605	0.306588426	1.219046603	0.227915236
CHRNA7	0.326269232	0.745002518	-0.296838618	0.767657575	0.068451449	0.945669075
CHRNB1	-1.026693199	0.30740493	-1.652039006	0.103968648	-1.837614991	0.071404029
CHRNB2	-1.672402184	0.098030011	-0.250891964	0.802790534	-1.319826606	0.192240496
CHRNE	-0.034074766	0.972895418	-2.413741333	0.018990675	-3.145072844	0.002652613
CHST10	0.35636759	0.722426221	-0.064066483	0.949139125	0.791349034	0.432065397
CHST11	-2.847405877	0.005495827	-0.82364163	0.413540744	-1.867520729	0.067043465

CHST12	2.691313746	0.008530962	-4.179761912	0.000100387	-3.72293818	0.000457379
CHST13	0.567014663	0.572161148	-0.605139225	0.547463442	-0.868073278	0.38904359
CHST14	-0.808327338	0.421102238	-0.515202839	0.608383066	0.037966727	0.969848668
CHST15	-2.958675715	0.003976819	-0.868197993	0.388889127	-0.871237894	0.387328466
CHST2	-1.079494317	0.283344631	-0.404499473	0.687343708	-0.894243607	0.375002844
CHST7	-1.507794028	0.135222276	-1.744142944	0.086469023	-1.878417099	0.065511615
CHST8	-3.012767644	0.003388013	0.130640159	0.896515197	0.039095847	0.968952434
CHSY1	-1.76676352	0.080767405	0.604210544	0.54807636	-1.061015687	0.293218298
CHSY3	-0.720195955	0.473330699	0.944522544	0.348848158	-1.414632297	0.162689515
CHTF18	1.160355178	0.249071636	-1.165259778	0.248717099	-0.445603637	0.657594347
CHTF8	-0.346820076	0.729562168	1.160557492	0.250609699	1.77481881	0.081340067
CHTOP	-1.609368729	0.111150728	-2.82458758	0.006493101	-1.971201659	0.053629028
CHUK	-0.47744244	0.634241796	-0.504161698	0.616070961	-1.413696925	0.162962844
CHURC1	0.278756007	0.781092162	-0.503382236	0.616615347	-0.822809761	0.414091597
CIAO1	0.16535238	0.869049424	0.874479626	0.385488993	1.548836381	0.127030768
CIAPIN1	1.143004691	0.256165483	2.476009838	0.016247625	3.113999249	0.002902115
CIB1	-0.858856419	0.39277482	-0.790190409	0.432657985	-0.069932748	0.944495275
CIB2	2.796101698	0.006362027	0.152276765	0.879500825	0.811763282	0.420350212
CIC	0.338168636	0.73604904	-1.161822747	0.250099441	-0.787808796	0.434116633
CIDEB	-2.952896671	0.004045024	1.216967171	0.228579816	1.916348163	0.060408281
CIDECP	-1.795957555	0.075965203	-0.080805809	0.935876521	0.483136896	0.630874208
CIITA	0.920062058	0.36007892	0.573408085	0.568600632	0.428174399	0.67015958
CILP	-0.503435285	0.615927823	-0.40030093	0.69041578	0.535733732	0.594255158
CINP	-2.407820659	0.018155701	-1.02912977	0.307721167	-0.360575921	0.719767364
CIR1	-1.443084157	0.152579981	-0.06958348	0.944766213	-0.166734999	0.868176633
CIRBP	0.112356449	0.910799062	0.209514466	0.834785952	1.747437218	0.086020186
CIRH1A	0.61919414	0.53740307	0.415318778	0.67945171	1.761265798	0.08362937
CISD1	1.243013561	0.217195154	0.198118352	0.843650074	-0.173756584	0.86268072
CISD2	-0.261177442	0.794571609	-0.626139799	0.533696885	-0.672474571	0.504035407
CISD3	-0.105887573	0.915914794	-1.031843703	0.306458764	-0.798899977	0.427709644
CISH	-3.308093779	0.001366191	1.18135703	0.24231592	0.155880992	0.876685037
CIT	0.253400921	0.800554969	0.792459723	0.4313447	0.682956286	0.49743844
CITED2	-0.492210814	0.623807418	-0.703318058	0.484690976	-1.54397369	0.128203779
CITED4	-1.488010547	0.14035605	-2.030226586	0.046960444	-2.253731963	0.028129122
CIZ1	-0.928272762	0.355828826	0.106019689	0.915935121	0.520607211	0.604684204
CKAP2	0.621029591	0.536200352	-0.010196926	0.991899409	-0.952679125	0.344829589
CKAP2L	-0.689501403	0.492338805	0.390142348	0.697870408	-0.458202151	0.648572929
CKAP4	-3.751642996	0.000315804	2.709181594	0.008868166	2.604968994	0.011734793
CKAP5	0.121232149	0.903786121	1.583087906	0.118877528	1.931060689	0.058521947
CKB	-0.22679245	0.821116024	-0.32999794	0.742598781	-1.499210676	0.139412099
CKLF	-0.821437941	0.413637369	-0.862067773	0.392225306	-1.343733492	0.184431304
CKM	-1.550418261	0.124663561	-0.565016281	0.574256932	-1.951435476	0.055992757
CKS1B	1.056796591	0.293523787	0.184620818	0.854174984	0.930517666	0.356080506
CKS2	0.760103338	0.44924332	-2.608242396	0.01157267	-3.109367393	0.002941137
CLASP1	-2.126094696	0.036322313	0.222026695	0.825078484	0.432992466	0.666676416
CLASP2	2.875002957	0.005076014	0.347301094	0.729631015	-0.707945245	0.481903202
CLASRP	0.112799306	0.910448976	-2.723542855	0.008534375	-2.721793727	0.008627871
CLC	1.036023023	0.30305629	0.358650521	0.721167862	-0.605294105	0.547418576
CLCC1	2.877968003	0.005032708	0.720821111	0.473935198	1.239870827	0.220171976
CLCF1	2.249309685	0.027011837	-1.880058973	0.065160601	-1.304648741	0.197326351
CLCN1	-2.064657074	0.041926355	-0.258364857	0.797046359	-1.167582192	0.247901936
CLCN2	1.300455145	0.196871971	-1.000559915	0.321224761	-0.272615351	0.786147439
CLCN3	-0.767838445	0.444657722	-0.164117275	0.870213398	-0.758161445	0.451519951
CLCN4	-3.085192178	0.002725617	-1.674056192	0.099542242	-1.792374094	0.078452584
CLCN5	1.19558008	0.235102082	1.178734347	0.243350661	1.410098822	0.164017574
CLCN6	2.663416964	0.009212168	-1.042567162	0.301505167	-0.774863566	0.44166621
CLCN7	-0.728381041	0.468332171	1.367632446	0.176740172	1.851380112	0.069368136
CLDN12	-0.431310264	0.667307354	0.061807194	0.950930357	-1.695657299	0.095481051
CLDN15	0.532506714	0.595729606	-2.197090834	0.032051035	-1.449118452	0.152858119
CLDN16	-0.006684345	0.994681975	1.863071123	0.067553938	1.162978313	0.249749488
CLDN20	2.285115279	0.02473271	0.216836462	0.829102028	0.147096363	0.883582045
CLDN23	1.09762874	0.275388733	0.140793513	0.888524322	-0.666192652	0.508011717
CLDN5	-3.162640067	0.002151789	0.525619718	0.601170315	-0.100198063	0.920543437
CLDN7	0.161900959	0.871758449	-0.986186329	0.32816649	1.050848345	0.297823041
CLDN9	-1.257904385	0.211784833	-0.367592937	0.714524126	0.198386044	0.843458474
CLDND1	1.73531673	0.08621792	-1.50914154	0.13672912	-1.43618911	0.156488251
CLDND2	0.540187028	0.590445223	-3.129741324	0.002745949	-1.632678443	0.108124018
CLEC10A	1.018502562	0.311257072	1.476783703	0.145180662	1.576769055	0.120457912
CLEC11A	-0.999612453	0.320264291	-2.704120269	0.008988626	-1.663258683	0.101824515
CLEC12A	0.929180341	0.355361021	-0.883122272	0.380841457	-0.348062585	0.729092715
CLEC12B	0.981683812	0.328971869	-0.172065743	0.863989005	0.119002347	0.905697576
CLEC16A	-1.442640959	0.152704551	0.352074186	0.726067578	0.905081571	0.36928346

CLEC17A	-1.560155058	0.122345823	1.882579826	0.064811638	2.486407397	0.015899404
CLEC18A	-0.596881298	0.552133436	-0.316324702	0.752899634	1.929684499	0.058696237
CLEC1A	-0.466914672	0.641725616	0.189680469	0.850226414	-1.039223738	0.303148271
CLEC1B	-0.758949321	0.449929787	2.157108588	0.035182744	1.109594093	0.27189631
CLEC2B	-0.062117543	0.950611405	-1.416678372	0.161963164	-1.180128211	0.242917086
CLEC2D	2.655407968	0.009416665	-1.744874289	0.086340489	-0.526112176	0.600879023
CLEC2L	-2.251449442	0.026870624	1.351175448	0.18192306	0.221179494	0.825753799
CLEC3B	-0.360925558	0.719028129	0.389611636	0.698260689	-0.32500579	0.746382701
CLEC4A	-1.751372733	0.08339838	0.17648499	0.860532039	-0.039947738	0.968276276
CLEC4C	1.672050047	0.098099652	0.426858323	0.671073912	-0.058470409	0.953581206
CLEC4D	-1.225724512	0.22360289	0.307052404	0.75991091	-0.518964027	0.605822164
CLEC4E	-1.899078519	0.060860976	-0.158487652	0.874626939	-1.275470702	0.207386384
CLEC4F	-0.333464463	0.739584327	-0.406653016	0.68577001	-0.087941344	0.930235706
CLEC4G	-1.777427893	0.078984897	2.328454881	0.023420537	1.37446047	0.174751409
CLEC5A	0.675234468	0.501313643	-0.829241865	0.410391104	-0.595483131	0.553909532
CLEC6A	-0.721130734	0.472758335	-1.573129785	0.121166386	-2.998652806	0.004033643
CLEC7A	-0.295585972	0.768249089	0.044063305	0.965006352	-1.430922756	0.157985973
CLEC9A	0.595310453	0.553178018	-2.674981972	0.009711763	-1.155796811	0.252651136
CLECL1	-9.31E-05	0.999925914	-1.910482341	0.061053656	-2.071627317	0.042898789
CLIC1	-0.924461253	0.357797755	-0.280021348	0.780464374	-0.374361938	0.709542971
CLIC2	0.746918694	0.457122128	0.207263599	0.836535053	-0.435076646	0.66517196
CLIC3	-0.551660686	0.58259201	-1.722360563	0.090370548	-1.375006399	0.174583014
CLIC4	-0.042913477	0.96586863	0.169307723	0.866147832	-1.556420317	0.125218455
CLIC5	1.352488458	0.179716467	-0.191828756	0.848551042	0.724891626	0.471523981
CLINT1	2.044377937	0.043932717	-0.134941316	0.893128743	-1.59786398	0.115677494
CLIP1	-0.736032637	0.463686449	0.244321077	0.807850427	-0.609163616	0.544869166
CLIP2	-1.168003447	0.245989369	1.63157567	0.108225306	1.306582885	0.196672683
CLIP3	1.311057394	0.19328088	0.321335209	0.749119568	0.825805899	0.41240388
CLIP4	1.043816829	0.299455632	-0.747135716	0.458020954	-0.650000903	0.518338153
CLK1	2.19385855	0.030905614	-0.96062686	0.340755342	-1.679587842	0.098585552
CLK2	1.538429373	0.12756508	-2.618569875	0.011264933	-1.537226715	0.129845633
CLK3	-1.110348735	0.269901623	0.835798838	0.406722029	0.995761977	0.323631177
CLK4	1.947799499	0.054656382	-1.194143647	0.23731663	-1.347315292	0.18328237
CLMN	-0.349458209	0.727587976	1.648389253	0.104717686	0.992663385	0.325126083
CLMP	0.704403976	0.483058492	0.03622639	0.971227107	1.262554167	0.211959873
CLN3	-0.924529058	0.357762667	-0.369321385	0.713242519	-0.349249958	0.72820605
CLN5	0.374211916	0.70915519	0.151616041	0.880019597	0.370928269	0.712084618
CLN6	-0.957930863	0.340746386	-1.41097514	0.163630694	-0.70381761	0.484450466
CLN8	4.452486147	2.50E-05	-0.211405579	0.833317056	0.489021998	0.626727972
CLNS1A	1.519797563	0.132180054	0.500559154	0.618588835	0.777100724	0.440356022
CLOCK	-0.180628969	0.857078012	-2.029224282	0.04706564	-1.735412378	0.088144988
CLP1	-1.336119589	0.184987016	1.809850636	0.07553727	1.695664356	0.095479706
CLPB	-1.499484632	0.137360284	1.658375838	0.102678519	2.004622023	0.049826988
CLPP	-0.206862811	0.836599342	-1.850421184	0.069383972	-1.575449378	0.120762173
CLPTM1	-0.899766918	0.370722699	-0.655103071	0.515009356	-0.857261364	0.39493904
CLPTM1L	-0.95262176	0.343415251	2.795392079	0.007031048	2.94979844	0.004627202
CLPX	0.515400886	0.607577283	2.64137894	0.010611525	2.186423974	0.032961791
CLSPN	-0.475666107	0.635501892	0.764269725	0.447826351	1.01504319	0.314432503
CLSTN1	1.15692125	0.250464413	1.524497982	0.132856842	1.568709708	0.122325691
CLSTN3	0.060520161	0.951879868	-0.980715554	0.330834651	0.675840039	0.501912099
CLTA	-0.76582281	0.445850026	-0.069748509	0.944635432	0.156076658	0.876531523
CLTB	-1.739196489	0.08552953	1.229715991	0.223803073	1.089984338	0.280368578
CLTC	-0.600542902	0.549702369	2.120799237	0.038255747	1.770907553	0.081995313
CLTCL1	-1.31485325	0.192007153	2.588353436	0.012186973	2.918550468	0.005048409
CLU	-3.64679649	0.000451133	2.643596451	0.010549881	1.052899909	0.296889924
CLUAP1	2.177734402	0.032126192	2.226448548	0.029910257	3.347958778	0.001457193
CLYBL	1.8962158	0.061243308	1.471403275	0.146624982	1.733132569	0.088552694
CMAHP	-0.715614313	0.476141632	0.394200084	0.694889098	-0.774431472	0.441919529
CMAS	2.555013664	0.012353054	0.732556361	0.466799826	1.234225077	0.222251887
CMBL	-3.219029869	0.001807182	2.18490259	0.03297868	0.556327274	0.580194172
CMC1	0.730207158	0.467221055	-1.797522698	0.077495251	-0.831555921	0.409176658
CMIP	-2.463614689	0.015718423	-1.104524703	0.273958521	-1.384599944	0.171644098
CMKLR1	-1.54040194	0.127084044	0.441435487	0.660550569	0.625907336	0.533907887
CMPK1	0.450246527	0.653650241	1.284013988	0.204278286	1.004083619	0.319639223
CMPK2	-1.931419781	0.056679625	0.748470059	0.457222262	-2.470828677	0.016536133
CMTM1	-0.041919316	0.966658865	-2.386456342	0.020318485	-4.069909406	0.000148412
CMTM2	-2.413868656	0.017876134	-0.002940972	0.99766361	0.089578273	0.928940621
CMTM3	-2.735564882	0.007543901	-0.048702679	0.961324733	-0.71687054	0.476420847
CMTM4	1.239904953	0.218337263	0.406153744	0.686134729	1.079816038	0.284833503
CMTM5	-4.198035877	6.47E-05	0.825171562	0.412678837	-0.253912629	0.800489124
CMTM6	-1.491510767	0.139436808	-0.268875523	0.788986269	-1.060180148	0.29359485
CMTM7	-1.607609804	0.111536167	0.10551533	0.916333526	0.484668829	0.629793757

CMTM8	1.298115451	0.197671102	0.206001455	0.837516202	-0.347122315	0.729795121
CN5H6.4	-0.470665046	0.639055307	1.415193048	0.162396175	0.730445508	0.468150106
CNBP	-1.08088466	0.282729112	0.777530939	0.440027664	0.877182096	0.384119715
CNDP2	-0.746545285	0.457346412	2.135661417	0.036970666	1.645860681	0.105370667
CNFN	0.736574745	0.463358298	0.694110523	0.490403132	0.676076703	0.501762968
CNGA4	-0.199455716	0.842370709	-1.297120409	0.199761711	-1.174268734	0.245236127
CNIH	-0.171286298	0.864395556	-0.259992158	0.795796987	0.081205339	0.935566972
CNIH2	-1.159791036	0.249300069	-2.126772931	0.037734611	-0.708117413	0.481797116
CNIH4	0.195032233	0.845821477	-0.68677927	0.494977718	-1.088878497	0.280851777
CNKSR1	-0.44139277	0.660021376	1.286696533	0.203347708	2.313050539	0.024401285
CNKSR2	1.781174155	0.078366501	0.031503738	0.974976727	-0.653031278	0.516397062
CNN2	-2.471232118	0.01540944	0.551642143	0.583327562	0.531150836	0.597405933
CNN3	1.823112534	0.071713491	0.133664556	0.894133775	1.554442783	0.125689015
CNNM1	-2.443796806	0.016548419	0.953640145	0.344251067	1.248648165	0.216966911
CNNM2	0.773882799	0.441093435	-0.373053612	0.710477989	-1.069949361	0.289212969
CNNM3	-0.406485902	0.685381648	-1.861106052	0.067835518	-1.019939213	0.312125086
CNNM4	-1.31822183	0.19088207	-0.797980074	0.428159913	-1.155621801	0.252722148
CNO	-1.12405298	0.264075813	-2.799889507	0.006945588	-3.09406159	0.003073609
CNOT1	-1.038225768	0.302035688	1.414091155	0.162717985	1.192341305	0.238134412
CNOT10	-1.379907986	0.171143329	1.843729066	0.070368885	2.32262532	0.023842626
CNOT2	0.483179907	0.630179129	-1.430343915	0.15802116	-0.655509461	0.514812556
CNOT3	-1.087155515	0.279964404	-1.329799871	0.188826701	-1.051031604	0.297739607
CNOT4	-1.310424337	0.193493919	-1.373126451	0.175035287	-0.746990178	0.458181335
CNOT6	-0.462832222	0.644637733	-0.833453078	0.408032329	-2.013476428	0.048859425
CNOT6L	0.118874356	0.905648358	-0.0254745	0.979764545	-0.387904139	0.699551415
CNOT7	0.740866328	0.460765148	-0.32497645	0.746376385	-0.544027859	0.588572807
CNOT8	-0.535356745	0.593766104	0.7097167	0.480743286	0.242676484	0.809138941
CNP	-0.623642121	0.534490816	3.72390476	0.000448145	2.954572196	0.004565809
CNPPD1	-2.217203959	0.029210213	-0.484638561	0.629770773	-0.688908738	0.493713216
CNPY2	0.525830488	0.600340881	1.189187016	0.239245618	1.537071691	0.129883554
CNPY3	-2.298523221	0.023923894	1.155958209	0.252470801	1.841455106	0.070831071
CNPY4	1.920276928	0.058091949	1.120139473	0.267303504	2.523458914	0.014473074
CNR1	-0.315224562	0.753344378	0.064511036	0.948786701	-1.578476152	0.120065244
CNR2	1.099955527	0.274379266	2.275998374	0.026582168	2.268259016	0.027172323
CNST	1.695819849	0.093488183	1.922293437	0.059519604	1.788466544	0.079087775
CNTD1	0.128216989	0.898272537	-0.738370768	0.463287279	0.671134907	0.504881967
CNTLN	-0.378453777	0.706013454	0.760576972	0.45001226	0.501467654	0.617999527
CNTNAP1	-0.894321187	0.373612192	-0.523239752	0.602814733	-0.321451676	0.749059846
CNTNAP2	2.202106225	0.030297076	0.147286807	0.883420015	0.959980003	0.341174622
CNTNAP3	-2.210191644	0.029710701	-1.622473494	0.110163644	-2.273877976	0.026810045
CNTNAP3B	-2.117379659	0.037075478	-0.699281646	0.487190515	-1.893638858	0.063421374
CNTRL	2.205247636	0.030068062	2.734759532	0.008281733	2.226043068	0.030035666
CNTROB	0.183650464	0.854714099	2.118289234	0.038476583	2.104821585	0.039787606
COA5	0.550940106	0.583083752	-2.579261292	0.012477554	-1.898689926	0.062740386
COASY	-1.155882767	0.250886702	1.276625223	0.206857891	1.803255722	0.076706154
COBLL1	2.686981977	0.008633642	1.715439349	0.091640254	0.749512683	0.456672245
COBRA1	-0.259925521	0.795534034	0.46428655	0.64419265	1.368263227	0.176671718
COCH	1.052817815	0.295333532	-0.299740252	0.765454363	0.078637074	0.937600442
COG1	1.290940444	0.200136805	0.402699322	0.688660233	1.615442204	0.111812025
COG2	0.987357441	0.326199572	2.441820453	0.017706014	2.92111399	0.005012558
COG3	0.778891854	0.438152311	0.845371817	0.401401499	-0.135856619	0.892419777
COG4	-0.284856365	0.776429704	1.125055849	0.265231947	4.054469393	0.000156189
COG5	0.706139442	0.481984078	-1.324657892	0.190516613	-0.281218952	0.779574608
COG6	1.372149312	0.173536978	-0.940170807	0.351056346	-1.085066169	0.282522027
COG7	0.840188366	0.403100879	0.78536237	0.43545993	1.034517817	0.305322425
COG8	-0.341757094	0.733356051	-1.98256689	0.052192757	-2.018858052	0.048279296
COIL	0.488076957	0.626720526	-0.944298061	0.348961845	-0.758819945	0.451129053
COL11A2	0.896410759	0.372501797	1.688708847	0.096682821	1.039916003	0.302829335
COL13A1	-0.479917899	0.632487553	-0.032329861	0.974320771	0.306280862	0.760521879
COL17A1	-2.451153915	0.016235831	0.183769812	0.854839484	0.342098047	0.733552314
COL18A1	0.161474166	0.872093546	-1.224797013	0.225637371	-0.650718146	0.517878377
COL19A1	1.338159334	0.18432397	0.066297182	0.94737082	-0.272004976	0.786614344
COL1A1	1.152150027	0.252408759	2.715335371	0.008723698	3.587179719	0.000700904
COL1A2	1.26801407	0.208168546	3.047526394	0.003479978	3.364779971	0.001385318
COL24A1	2.599083843	0.010975061	-0.369769944	0.712910058	-1.031086714	0.306914288
COL27A1	1.029071153	0.306292592	0.84434379	0.401970799	1.097441645	0.277125242
COL4A1	0.581111699	0.562664389	2.938909352	0.004732122	2.921403485	0.005008524
COL4A3	0.657583938	0.51253785	-0.665496384	0.508389451	-1.511790229	0.136186676
COL4A3BP	-0.694371837	0.48929517	-1.147685163	0.255843332	-2.349496801	0.022335125
COL4A4	1.693828274	0.093867644	0.111237341	0.911814876	0.35488461	0.724003509
COL5A1	1.688634313	0.094863181	-1.383438887	0.171869207	0.012299611	0.990230109
COL5A2	2.701030862	0.008304656	0.943571346	0.349330046	0.859174911	0.393891612

COL5A3	2.917517066	0.004486672	1.313196726	0.19432441	0.811225825	0.420656169
COL6A1	3.324338612	0.001297539	-0.81315584	0.41947743	0.300469127	0.764927332
COL6A2	2.38665007	0.019165225	-3.129672161	0.0027465	-1.097234248	0.277215086
COL6A3	2.530115753	0.013198738	0.83192674	0.408886299	1.426258637	0.159321711
COL6A4P2	0.241974938	0.809367816	-0.229356236	0.819404565	-0.520830113	0.604529912
COL7A1	-0.902589642	0.369230537	1.875918206	0.065737261	2.276983905	0.026611636
COL8A2	1.236507408	0.219590543	1.093521131	0.278717495	1.303505832	0.197713378
COL9A2	0.923259849	0.358419812	0.396617658	0.693115147	0.266682685	0.790688941
COL9A3	-2.573656935	0.011752125	-1.044370191	0.300677678	0.055980667	0.955555628
COLEC12	1.962443724	0.052899716	1.751596378	0.085166483	1.754272414	0.084831457
COLQ	2.260069763	0.026308278	-0.984433158	0.329019968	0.487758531	0.627617109
COMMD1	0.673993501	0.502098439	0.612933213	0.542333257	1.700785538	0.094507397
COMMD10	0.175987536	0.860711843	-1.661491183	0.102049068	-1.487983596	0.142341467
COMMD2	-0.387473181	0.699350204	-0.587070752	0.559450509	-0.987259133	0.32774437
COMMD3	0.568808775	0.570948215	-0.397242141	0.692657198	0.180854023	0.857132364
COMMD4	-0.380617707	0.704412696	3.118942964	0.002833315	3.23511446	0.002038566
COMMD5	-0.772071269	0.442159923	1.268122775	0.209856247	1.163579186	0.249507796
COMMD6	0.330808939	0.74158249	-0.010340227	0.991785573	0.773261688	0.442605753
COMMD7	0.782781505	0.435876391	0.148305413	0.882619745	1.299544911	0.19905909
COMMD8	0.783935996	0.43520221	0.31702029	0.752374497	0.134213457	0.893712962
COMMD9	-1.284447268	0.202387822	-0.01720521	0.986332368	1.292156954	0.201587477
COMT	-0.390832002	0.696874786	2.448742643	0.017401567	2.77014698	0.007578754
COMTD1	0.333515683	0.739545803	-2.920470792	0.004982338	-1.865021965	0.067398986
COPA	-0.47568971	0.635485141	0.303615827	0.762514675	0.467322794	0.642074702
COPB1	1.051847425	0.295776064	1.778853873	0.080540959	1.732038837	0.088748843
COPB2	0.661086076	0.510300243	2.420053423	0.018694811	2.373672601	0.021052186
COPE	-1.9309066	0.056744025	0.386625448	0.700458233	0.828533443	0.410871108
COPG	-1.30558975	0.195126668	1.796162943	0.077713789	1.818650818	0.074291031
COPG2	1.105141528	0.2721386	1.829465096	0.072507506	2.245736249	0.028668367
COPS2	0.41202622	0.681331442	-0.284692031	0.77690122	-0.559492196	0.578047478
COPS3	-2.224116885	0.028724058	-1.34231359	0.184761488	-0.143520747	0.886391969
COPS4	0.689869828	0.492108208	-0.40737848	0.685240194	0.782959365	0.436935782
COPS5	-0.073636262	0.94146849	-0.117516765	0.90685941	-0.282913835	0.77828167
COPS6	-1.487725666	0.140431075	-0.257614127	0.797622918	0.525574275	0.601250345
COPS7A	-2.199731119	0.030471235	-1.346036961	0.183564832	-1.038174675	0.303632024
COPS7B	0.86209206	0.39100178	1.025202636	0.309554142	2.625940839	0.011111108
COPS8	1.781072198	0.078383278	-0.431170924	0.667953598	-0.390467251	0.697666261
COPZ1	-1.842988839	0.068728708	0.500459212	0.618658751	1.972909268	0.053428899
COPZ2	-0.404962071	0.686497254	0.809897878	0.421332396	0.156239588	0.876403696
COQ10A	0.584779237	0.560206423	0.137071563	0.89145226	1.180755309	0.242669836
COQ10B	-1.642557667	0.104076463	-1.190139112	0.23887421	-1.952982404	0.055804607
COQ2	-0.613595182	0.541080406	-0.742939154	0.460538102	-0.759642583	0.450640996
COQ3	1.400940973	0.164780926	0.989164465	0.326720055	1.487343699	0.14250988
COQ4	0.899976198	0.370611938	0.794187638	0.430346324	1.463805425	0.148814681
COQ5	0.822062684	0.413283655	-0.164261133	0.870100668	0.70422852	0.484196549
COQ6	1.901379024	0.06055519	0.177177759	0.859990365	0.174236043	0.862305685
COQ7	0.619400998	0.537267453	-1.050810129	0.297734795	1.783608542	0.079883451
COQ9	-0.020838971	0.983421727	1.417258155	0.161794385	0.10846301	0.914014493
CORIN	1.408542032	0.162526734	-0.062315132	0.950527626	-1.511555087	0.13624642
CORO1A	-1.881774331	0.063203031	0.790240896	0.432628741	0.918446122	0.362307871
CORO1B	-1.165024237	0.247186746	-0.706959153	0.482442368	-0.745825115	0.458879303
CORO1C	-4.02036435	0.000123121	2.125577164	0.037838429	1.63874543	0.106849637
CORO2A	-0.016724602	0.986694532	0.175364391	0.861408373	0.304746542	0.761684167
CORO2B	-0.120264167	0.90455059	0.981335303	0.330531671	0.893309338	0.375498492
CORO6	-0.068667915	0.945411196	-0.782913132	0.436885439	-0.706459319	0.482819341
CORO7	-2.022768403	0.046160915	0.935734308	0.353316884	1.80790284	0.075970301
COTL1	-3.415373113	0.000969034	0.555535236	0.580680119	0.510142263	0.61194829
COX10	0.369461741	0.71267939	-0.070363942	0.944147734	0.533184825	0.596006588
COX11	1.399802478	0.165120613	-2.777029032	0.007390131	-2.238842757	0.029140593
COX15	-0.866405438	0.388645875	3.34544038	0.001450401	3.517740968	0.00086918
COX16	0.81883845	0.415111092	-0.733510245	0.466222537	-0.599269949	0.551399587
COX17	-0.067185379	0.946587952	-2.201899535	0.031691407	-1.594759359	0.116371285
COX18	-0.329127595	0.742848544	-0.515483995	0.608187872	-0.133173418	0.894531633
COX19	2.245919778	0.027236886	0.400323052	0.69039958	1.358419637	0.179755038
COX4I1	0.17733112	0.859659624	1.150342581	0.254756543	1.53539014	0.130295448
COX4NB	-0.998850882	0.320631026	0.904519458	0.369487981	0.244063506	0.808069862
COX5A	0.29326876	0.770013623	-3.37458035	0.001328184	-2.219256634	0.030520011
COX5B	0.410950619	0.682117026	-2.58796845	0.012199152	-1.127152489	0.264464462
COX6A1	-0.306355194	0.760064591	-1.408204463	0.164445571	-0.485001937	0.629558929
COX6B1	-2.499713752	0.014301769	-1.743802384	0.08652893	-0.478107561	0.634427017
COX6C	0.919131419	0.360562683	0.61714972	0.539568142	1.533209505	0.130831142
COX7A2	0.556389047	0.579370146	-1.818478743	0.0741917	-0.963290771	0.339525608

COX7A2L	0.568536476	0.571132226	0.702944998	0.484921693	0.700912829	0.486247562
COX7B	-0.495542768	0.621463752	-1.305196025	0.197016301	-0.488362827	0.627191779
COX7C	0.602251622	0.548569729	-0.410958711	0.682627829	0.042272999	0.966430803
COX8A	-1.687821291	0.09501979	0.325000249	0.746358466	0.402379165	0.688930403
CPA3	-2.974140407	0.003799493	1.311318798	0.194953751	-2.00001701	0.05033669
CPA5	-1.138787794	0.257910946	0.905135612	0.369164276	-0.834730876	0.407401333
CPAMD8	-0.071031812	0.943535115	2.337582345	0.022905977	1.390342016	0.169903331
CPD	-2.008284289	0.047708003	0.09560872	0.924163215	-0.79469069	0.43013452
CPEB2	-1.130383116	0.261414808	-1.537960834	0.12953419	-3.690191043	0.000507353
CPEB3	-0.442738054	0.659051682	-0.236431204	0.8139369	-2.931285765	0.00487262
CPEB4	-1.275780829	0.205421422	-0.935686123	0.353341487	-3.109670754	0.002938566
CPLX1	0.30472156	0.761304405	-1.815943926	0.074584908	-1.83786851	0.071366083
CPM	-3.302430159	0.001390916	0.791270263	0.432032762	1.094488723	0.278406376
CPNE1	-1.471118441	0.144859135	2.763131423	0.007673033	3.516892278	0.000871457
CPNE2	-2.248932132	0.02703682	-0.161041391	0.87262434	-0.637193032	0.52658475
CPNE3	-0.432655859	0.666333112	-0.781327444	0.437809813	-1.334864245	0.187299841
CPNE5	-2.586664142	0.01134862	1.590270215	0.117248374	0.950309476	0.346021377
CPNE7	1.633695003	0.105928974	-1.817339623	0.074368188	0.116311109	0.907820344
CPNE8	-0.34610686	0.730096203	-0.010578813	0.991596044	-1.151453862	0.254417545
CPOX	2.466735989	0.015591151	1.045579576	0.300123508	-0.759523075	0.450711879
CPPED1	-4.156699029	7.52E-05	-1.026156452	0.309108273	-1.236868545	0.221276233
CPSF1	1.063786032	0.290363001	0.137791804	0.89088555	0.986124232	0.328295994
CPSF2	0.051963904	0.958676324	1.679385136	0.098494405	1.910535721	0.061167645
CPSF3	0.505997313	0.614135526	2.565182018	0.012939959	3.518548416	0.000867019
CPSF3L	-0.079430512	0.936872226	-0.348545329	0.728701529	0.331363473	0.741601613
CPSF4	0.712485725	0.478066421	0.78569471	0.435266713	2.059851242	0.044052192
CPSF6	2.621058084	0.010341255	-0.492823052	0.624011242	-1.167782318	0.247821848
CPSF7	-1.531200878	0.129340163	-1.965495715	0.054184959	-1.499288392	0.139391989
CPT1A	-1.280931094	0.203614596	4.215595576	8.89E-05	2.382775923	0.020586449
CPT1B	0.695944309	0.488314705	1.398840226	0.167222891	1.963325358	0.05456046
CPT2	-2.748173769	0.007282367	1.430460605	0.157987823	1.998020076	0.05055911
CPVL	-1.689342406	0.094726954	1.646153589	0.105178679	1.01602293	0.313969848
CPXM1	0.948381595	0.345556495	-1.001143151	0.32094518	0.763780735	0.448190564
CR1	-1.016803332	0.312060277	0.486354923	0.628561015	-0.522301565	0.603511839
CR1L	-2.972922141	0.003813193	-1.08009084	0.284603828	-1.183860369	0.241448263
CR2	-0.362006085	0.71822339	1.287314233	0.203133876	1.402903721	0.166142542
CRABP2	-1.356169984	0.178546858	-0.646555827	0.52048767	-0.777639912	0.44004059
CRADD	-1.844830589	0.068457471	-0.904141075	0.36968686	-0.985334807	0.328680063
CRAMP1L	1.698582425	0.092963894	0.477663825	0.6346973	0.688854377	0.493747167
CRAT	-1.93588182	0.056122281	2.063914787	0.043540907	0.88731685	0.378687501
CRB2	1.516916076	0.132905384	0.673245809	0.503483499	1.494503684	0.140634394
CRB3	0.800989667	0.425315012	0.382222919	0.703702759	0.481334974	0.632146116
CRBN	0.640391928	0.523597285	-0.231082536	0.818069607	-0.594604218	0.554492905
CRCP	-2.241648253	0.027522806	2.170649414	0.034093331	2.309406828	0.024616925
CREB1	-1.14742441	0.254345044	-0.831850225	0.408929137	-2.160418621	0.035016064
CREB3	-0.492889938	0.623329413	2.367025578	0.021314123	3.232144778	0.002056482
CREB3L2	0.890825885	0.375474243	0.263084236	0.793424504	0.116677541	0.907531273
CREB3L4	0.993837628	0.323052119	-0.664007132	0.509335195	-0.268412075	0.789364318
CREB5	-0.580588941	0.56301517	-1.098731682	0.276456817	-2.506836751	0.015097989
CREBBP	-2.262743479	0.026135973	-0.864802237	0.390734965	-1.05872147	0.294253029
CREBL2	0.932483295	0.35366187	0.422912442	0.673934001	0.335078665	0.738812444
CREBZF	2.116659944	0.037138277	-0.583795223	0.561637492	-1.156915995	0.252197356
CREG1	-2.4898192	0.014678205	1.388264832	0.170402755	0.466482323	0.642672351
CRELD1	0.737815342	0.462607829	0.917639302	0.362634421	2.324894559	0.023711904
CRELD2	-0.07431417	0.940930637	2.493380903	0.015548934	2.090482224	0.041106625
CREM	-2.511291334	0.013872318	0.061710154	0.9510073	-0.411509633	0.682263023
CRHBP	-0.161093956	0.872392088	-1.940678091	0.057197105	-0.745376774	0.459148059
CRIM1	1.588833832	0.115717785	-0.678978021	0.499871161	-1.125318694	0.265233852
CRIP1	-0.65407333	0.51478608	0.238637648	0.812233608	0.852818164	0.397377798
CRIP2	1.262385603	0.210176218	-0.00101551	0.99919325	0.665406489	0.508510528
CRIP3	1.816802068	0.072683424	0.490611827	0.625565006	1.628347767	0.109041218
CRIPAK	0.557148307	0.578853587	1.594253277	0.116352683	2.28788515	0.025925523
CRIPT	0.138276269	0.890340939	-1.220865331	0.227111405	-0.991348721	0.32576173
CRISP3	-2.213417294	0.029479553	0.841208293	0.403710241	0.409370494	0.683822837
CRISPLD2	-4.704763672	9.47E-06	0.690106568	0.492898641	-0.505924807	0.614886945
CRK	-1.596036962	0.114098978	-0.7268875	0.470238999	-3.260375117	0.001892012
CRKL	-1.643632921	0.103853501	1.073921468	0.287336521	0.532244651	0.596653219
CRLF3	-0.33971315	0.734889539	0.24117552	0.810275592	0.112803679	0.910587912
CRLS1	0.209146737	0.834821569	-0.052228694	0.958527193	0.041522829	0.967026165
CRNKL1	-1.796444472	0.075887162	2.022079491	0.047821424	1.936426832	0.057846553
CROCC	2.101424444	0.038489439	0.261482625	0.794653142	1.06703251	0.29051653
CROCCP2	2.243088816	0.027426087	0.810691527	0.420880066	-0.335048853	0.738834811

CROCCP3	3.808341108	0.000259738	-0.718522283	0.475340125	0.711446495	0.479748372
CROT	1.184452772	0.239452662	0.03858585	0.969354018	0.661720497	0.510852755
CRTAM	2.260496542	0.026280708	-2.388915056	0.020195513	-1.10923089	0.272051576
CRTAP	-0.631021552	0.529677169	-1.038075916	0.303573158	-1.257291947	0.213844428
CRTC1	0.556261592	0.579456881	-1.399230274	0.16710649	-0.515952613	0.607910222
CRTC2	-1.467403367	0.145864443	-1.265953225	0.210626476	-0.804141878	0.424701357
CRTC3	0.658210916	0.512136876	0.363760191	0.717368969	0.998362681	0.322380031
CRX	-0.522098788	0.602925503	2.740799345	0.008148554	3.053538187	0.003451803
CRY1	-0.212773705	0.832000161	-0.027143594	0.978439034	-1.107256258	0.272896804
CRY2	1.113695733	0.26847057	1.328020009	0.189410364	2.197078477	0.032151219
CRYBB2P1	0.464492917	0.643452444	-0.968061314	0.337061308	-0.951117628	0.345614624
CRYGS	0.777591577	0.438914679	0.877727282	0.383738428	2.131915876	0.037395722
CRYL1	-0.100889696	0.919869664	0.195654351	0.84556934	2.029428233	0.047157129
CRYM	-1.223694338	0.224364253	0.174138525	0.86236723	0.282525345	0.778577973
CRYZ	2.151443183	0.034206593	-0.720083655	0.474385639	-1.653090477	0.103885015
CRYZL1	2.290608678	0.024398459	-0.905862081	0.368782847	0.339380816	0.735587024
CS	0.250020349	0.803159766	0.623054297	0.535708231	0.792302315	0.431514047
CSAD	0.091793012	0.927073071	0.466753148	0.642437281	0.795647533	0.429582585
CSDA	-3.465784046	0.000822604	0.218510011	0.82780416	0.341821872	0.733759031
CSDC2	0.503978459	0.615547645	-0.636082076	0.527242555	0.311113979	0.756864302
CSDE1	-0.987213177	0.326269872	-0.127622499	0.898892267	-0.249612179	0.803796796
CSE1L	1.445124707	0.152007458	2.268133723	0.027087464	3.730424393	0.000446635
CSF1	-0.852906083	0.39604835	-0.293574119	0.770138615	-1.037010245	0.304169594
CSF1R	-0.073222265	0.941796969	-0.002897505	0.997698141	-0.273053328	0.785812457
CSF2RA	-0.945894084	0.346816684	0.461679583	0.64605013	0.925061817	0.358886394
CSF2RB	-0.931409379	0.354213755	-0.524770595	0.601756771	-2.278087412	0.026541457
CSF3R	-1.784926812	0.077751074	0.007659338	0.99391526	-0.272101025	0.786540866
CSGALNACT1	1.078598298	0.283741796	-0.485889682	0.628888834	-0.620590795	0.537375935
CSGALNACT2	-0.166298858	0.868306804	0.32759944	0.744402355	-0.903076059	0.370337584
CSK	-0.781074568	0.436874302	-0.133802015	0.894025563	-0.166066063	0.868700566
CSMD1	-0.911677924	0.3644521	0.19675559	0.844711444	0.464555809	0.644043167
CSNK1A1	0.059992166	0.95229917	0.664455875	0.509050123	-1.165909416	0.248572087
CSNK1A1L	-0.315362969	0.753239657	-0.326441351	0.745273707	-1.359171113	0.179518214
CSNK1D	-0.976190594	0.331670771	0.205477295	0.837923743	-0.084257811	0.933150686
CSNK1E	1.227556358	0.222917518	-2.521940591	0.014459002	-2.053078058	0.044727691
CSNK1G1	-0.899639623	0.37079008	0.358790496	0.7210637	0.612198284	0.542874034
CSNK1G2	-0.439927363	0.661078318	-2.734866904	0.008279348	-3.254193274	0.00192693
CSNK1G3	0.65534629	0.51397026	-0.980664086	0.330859821	-2.003211271	0.049982662
CSNK2A1	-1.161978223	0.24841526	0.49407614	0.623131494	0.498134898	0.620331518
CSNK2A2	1.893734958	0.061576274	2.686639369	0.009416298	2.846381766	0.006160781
CSNK2B	-1.944630328	0.05504299	0.81152666	0.42040441	1.433955532	0.157122112
CSPG4	-0.02645579	0.978954254	2.623453286	0.01112203	1.078572106	0.285383089
CSPP1	2.013582286	0.047137041	-1.473948361	0.14594038	-0.313657782	0.754941456
CSRNP1	-0.490962938	0.624686162	0.820087608	0.415547166	-1.110312032	0.271589581
CSRNP2	0.750559726	0.454938482	-0.952970305	0.344587444	0.75013996	0.456297422
CSRP1	-1.593610749	0.114642199	3.153475941	0.002562716	2.715979212	0.008762632
CSRP2BP	0.128500404	0.898048923	2.408838534	0.019223373	3.165806675	0.002497485
CST3	-0.909695214	0.365491202	0.662153416	0.51051371	0.522223154	0.60356607
CST7	-0.793070815	0.429889431	-0.223101904	0.824245552	-0.180939533	0.857065561
CSTA	-0.986815597	0.326463663	-2.389829167	0.020149964	-1.624493832	0.109862752
CSTB	-1.817296397	0.072607053	-2.878970328	0.005591067	-2.815056324	0.006710834
CSTF1	0.539667468	0.59080201	0.257988443	0.79733543	0.791099786	0.432209624
CSTF2	1.215704628	0.227378889	2.117947265	0.038506756	2.223508107	0.030215786
CSTF2T	0.318039557	0.75121543	0.158966683	0.874251228	-0.605023597	0.547597025
CSTF3	1.307569165	0.194456936	0.338909974	0.735910062	1.127896899	0.264152588
CTAG2	-0.539544876	0.590886209	-0.943817529	0.349205285	0.167407956	0.86764961
CTAGE5	-0.651884965	0.516190164	1.476255101	0.145322063	2.080948658	0.042004487
CTAGE7P	1.600205057	0.11317057	-1.327173269	0.189688511	-0.253198295	0.801038298
CTBP1	0.597658277	0.551617123	-2.2457184	0.028574598	-1.241113675	0.219716039
CTBP2	-1.548403356	0.125147509	-1.252458151	0.215464644	-3.330184657	0.001536965
CTBS	-1.448208119	0.151145494	-1.155503302	0.252655415	-1.994589405	0.050943194
CTC1	0.842037239	0.402070869	0.930190469	0.356154891	1.67372725	0.099738174
CTCF	-0.764418131	0.446682026	1.607575134	0.113396949	1.610778312	0.11282728
CTDNEP1	-1.904066747	0.060199576	0.063350619	0.949706656	-0.000360072	0.999713978
CTDP1	-0.993594838	0.323169679	0.872499238	0.386558924	0.310576863	0.757270503
CTDSP1	-1.375427375	0.172522567	-0.581594194	0.56310944	0.101081197	0.919845529
CTDSP2	-1.327830763	0.187699882	-0.085876148	0.931862706	-0.026466034	0.978979319
CTDSPL	-4.94794162	3.62E-06	2.795399643	0.007030903	1.690371513	0.096493234
CTDSPL2	0.312509696	0.755399408	-1.296729896	0.199895194	-2.594238024	0.012066106
CTGF	-0.088161259	0.929950687	-0.707504973	0.482105791	0.035169497	0.972069123
CTH	-0.887523956	0.377238624	0.53405465	0.595359075	0.786905431	0.434640972
CTIF	-1.243573855	0.216989767	2.502287051	0.015201337	1.801962846	0.076911934

CTLA4	0.974510016	0.332499365	1.207085198	0.232333346	0.881555171	0.381769772
CTNNA1	-1.604719267	0.112171908	1.898017072	0.062708995	1.589265003	0.117607334
CTNNAL1	-3.105771627	0.002560638	1.290784064	0.201935833	2.490425077	0.015738826
CTNNB1	1.713674909	0.090141837	3.46184691	0.001017882	2.493313396	0.015624292
CTNNBIP1	0.783314194	0.435565244	0.799093659	0.427519172	1.589435765	0.11756876
CTNNBL1	-1.144979335	0.255351011	2.434372676	0.018038918	2.414947483	0.019013541
CTNND1	0.226638534	0.821235339	1.316791066	0.193124125	0.702690573	0.485147289
CTNS	-0.572619397	0.568376134	-0.855560948	0.395785866	-0.806170105	0.423540788
CTPS	1.508534594	0.135033005	1.536759401	0.129827988	1.920567306	0.059862112
CTPS2	1.476735648	0.143349371	-0.544341918	0.588307485	-0.466988972	0.64231205
CTR9	-0.026916296	0.978588008	1.819955866	0.073963367	1.659323275	0.102617995
CTRC	-0.228546009	0.819756979	1.511490075	0.13613119	1.440016673	0.155406668
CTRL	-0.214584331	0.830592501	0.237642392	0.813001796	1.03624375	0.304523807
CTSA	-2.573278805	0.011764047	0.167933938	0.867223539	-0.545810311	0.587355011
CTSB	-2.535157748	0.01302339	2.073716666	0.042587396	1.400417006	0.16688188
CTSC	-0.217643037	0.828215787	0.561017638	0.576961708	-0.723186402	0.472562628
CTSD	-2.840576519	0.005604525	2.087019324	0.04132235	2.273025212	0.026864749
CTSE	-2.339753331	0.021581089	0.941976411	0.350139033	0.64574549	0.52107046
CTSF	2.421324916	0.017536756	1.881681997	0.064935743	1.861251651	0.067938437
CTSG	-3.752756546	0.0003146	1.156456094	0.252268855	-0.582146289	0.562794817
CTSH	0.135663044	0.892400389	2.528383302	0.014222889	1.797599092	0.077609902
CTSK	1.657970143	0.100917307	0.668764707	0.506317232	1.795205263	0.077995032
CTSL1	-0.980304933	0.329647968	-0.019713751	0.984339856	-0.098532823	0.921859584
CTSO	1.729891736	0.087188111	-0.825200656	0.412662457	-0.463341636	0.644907755
CTSS	-2.03659115	0.044724747	0.116567279	0.907608472	-0.074093457	0.941198946
CTSW	0.277253777	0.782241542	-1.857607799	0.068339243	-0.144229364	0.885834979
CTSZ	-2.350939291	0.020981565	0.659582532	0.51215059	-0.023621614	0.981238049
CTTN	-3.543009977	0.000638137	2.269942409	0.02697051	1.02518963	0.309663412
CTTNBP2NL	0.151034346	0.880297517	0.511927279	0.610659247	-0.797793385	0.428346334
CTU1	-0.26921111	0.788403285	-2.443617149	0.017626536	-2.763909895	0.00770715
CTU2	-0.019005719	0.984879969	1.695387504	0.095402052	0.970009559	0.336195301
CUBN	3.150910753	0.002230774	-0.145849839	0.88454918	1.112185052	0.270790505
CUEDC1	-0.661519566	0.510023638	-0.747352715	0.457891012	-1.395089618	0.168474337
CUEDC2	-1.020787353	0.310179268	1.009573829	0.31692206	1.370838859	0.175871668
CUL1	0.585411838	0.559782993	1.767960747	0.082363754	1.499281564	0.139393755
CUL2	0.708392597	0.480591141	0.019899969	0.984191948	0.063804645	0.949352035
CUL3	-1.423526464	0.158152321	0.282231657	0.778777586	-0.15612467	0.876493855
CUL4A	2.107206404	0.037971741	2.628005012	0.010990323	2.377878013	0.020835873
CUL4B	0.312236264	0.755606482	0.554065979	0.581678588	0.06436991	0.948903957
CUL5	1.025915071	0.307769505	-0.544837806	0.587968572	-1.578188145	0.12013142
CUL7	0.994414916	0.322772708	0.841474994	0.403562106	1.972377668	0.053491133
CUL9	1.569978513	0.120042305	1.148999525	0.255305392	1.863781034	0.067576136
CUTA	0.267673466	0.789582872	1.230367337	0.223561008	1.809256577	0.075757057
CUTC	0.149160667	0.881771329	0.89380878	0.375143896	1.120623217	0.267211106
CUX1	-1.489405041	0.139989256	0.523228292	0.602822656	-0.231102244	0.818074127
CUX2	0.630574295	0.529968278	0.099013373	0.921471457	0.125718477	0.900403145
CWC15	0.2778658	0.781773215	-0.932418003	0.355012802	-0.082586822	0.934473336
CWC22	0.64328992	0.521724349	1.143122685	0.257716924	1.559371553	0.124518824
CWC25	-0.343962882	0.73170235	1.007775889	0.317777177	0.393153641	0.695692492
CWC27	1.227273974	0.223023071	1.994661603	0.050819641	3.098180699	0.003037419
CWF19L1	-0.008743192	0.993044005	2.545848395	0.013600282	3.635904689	0.000601901
CWF19L2	1.27789834	0.204677122	0.676507082	0.50142657	1.800543105	0.077138438
CX3CR1	0.760085673	0.449253824	-2.010121293	0.049109801	-1.073326632	0.287708705
CXCL1	-1.380428174	0.170983748	-1.701517421	0.094238817	-1.591073474	0.117199325
CXCL10	-0.529924662	0.597511083	-0.564689756	0.57447757	-1.662485124	0.101980087
CXCL16	-3.238514693	0.001700621	-1.304527012	0.197242658	-2.615827789	0.011407987
CXCL5	-3.728227117	0.000342172	1.366746864	0.177016167	-1.335221881	0.187183522
CXCL6	-0.049386429	0.960724302	-0.744706136	0.459477279	-0.811434836	0.42053717
CXCL9	-0.002954564	0.997649352	-2.33161966	0.023240968	-2.340633671	0.022822675
CXCR1	-2.563758553	0.012067814	-0.034111606	0.972906096	-0.590919772	0.556941788
CXCR2	-2.563860418	0.012064527	-0.799648787	0.427199974	-1.686247893	0.097288978
CXCR2P1	-5.013473671	2.78E-06	-0.150856634	0.880615914	-1.275206442	0.207479211
CXCR3	-1.274249551	0.205960907	0.192807708	0.847787825	0.724205189	0.471941932
CXCR4	1.614752982	0.109977505	-0.133445387	0.894306318	-0.427601521	0.670574222
CXCR5	-0.536116951	0.593242876	1.673586868	0.099634962	1.914553128	0.060641932
CXCR6	0.486330844	0.627952788	-1.656987244	0.102960102	-0.970814657	0.33579769
CXCR7	-0.341368429	0.733647567	-2.568955966	0.012814509	-2.031041314	0.046987875
CXXC1	0.543613202	0.588094971	0.717066311	0.476231153	1.749543408	0.085652428
CXXC5	0.250092867	0.803103866	0.41495845	0.679713973	-0.102691513	0.918573121
CXorf21	-1.466309498	0.146161479	0.696526551	0.488900694	-1.135167642	0.261120163
CXorf23	0.949525238	0.344978115	-2.124361867	0.037944198	-2.280328777	0.02639942
CXorf26	0.888296741	0.376825222	2.390720319	0.020105648	3.019061837	0.003807357

CXorf38	-2.472195589	0.015370749	0.454649672	0.651070257	-0.607296379	0.546098631
CXorf40A	0.170781831	0.864791017	1.054163312	0.296210322	1.849208839	0.069685983
CXorf40B	-0.048661669	0.961300221	2.279297021	0.026372732	2.262217756	0.027566661
CXorf56	-0.706049721	0.482039591	1.061391015	0.292942616	2.96198623	0.004471959
CXorf57	2.747858805	0.007288798	0.234154794	0.815695151	-0.335574602	0.738440387
CXorf65	0.656763286	0.513062937	-0.137604901	0.891032606	1.54886122	0.127024798
CYB561	-0.047527368	0.962201616	0.200424692	0.841854477	0.190600337	0.849525126
CYB561D1	0.758546329	0.450169649	0.839077536	0.40489493	2.144367537	0.036339289
CYB561D2	-1.075051511	0.285317691	-1.042583738	0.301497553	-0.478301992	0.634289505
CYB5A	0.28309674	0.77777374	-0.574262455	0.5680263	-0.57533764	0.567357964
CYB5B	-0.054123757	0.956960389	1.104118682	0.274133103	1.935226883	0.057997001
CYB5D1	0.628401342	0.531383778	-0.510623371	0.611566407	0.641900947	0.523545489
CYB5D2	1.708881096	0.091030505	-2.547500052	0.0135427	-2.20305606	0.03170418
CYB5R1	-1.979882534	0.050870648	4.449521252	3.99E-05	3.47427479	0.000993391
CYB5R2	-0.737591228	0.46274335	1.822109542	0.073631509	1.136352698	0.26062826
CYB5R3	-1.930666992	0.056774114	-0.505221514	0.615331119	-0.764052205	0.448030084
CYB5R4	-2.488385474	0.014733481	-0.481638247	0.631887957	-1.075644415	0.286679486
CYB5RL	4.652107138	1.16E-05	-1.302349279	0.197980836	-0.436162461	0.664388717
CYBA	-1.923760384	0.057647269	-1.634046864	0.107703865	-1.928153052	0.05889071
CYBASC3	0.10316006	0.918072846	1.956194374	0.055297578	1.880729959	0.065190305
CYBB	0.793042783	0.429905676	1.247746327	0.217173105	0.496291936	0.62162276
CYBRD1	-1.926453648	0.057305436	-1.158442444	0.25146433	-1.953026595	0.05579924
CYC1	-0.183085558	0.855155962	-0.452249389	0.652788063	-0.628811522	0.532018363
CYCS	0.342639255	0.732694534	-0.976668029	0.332817922	-0.558902244	0.578447338
CYFIP1	0.38789952	0.699035815	1.758720757	0.083936708	1.333579366	0.187718194
CYFIP2	-0.800472092	0.425613109	0.936457148	0.352947931	1.537754099	0.129716695
CYHR1	-0.526698243	0.599740594	-0.112745356	0.910624483	0.728331563	0.469432666
CYLD	0.548047728	0.585059567	1.547527369	0.12721365	0.630074349	0.531197828
CYP1B1	-1.5249291	0.130896068	2.102353808	0.03990472	0.419956354	0.67611752
CYP1B1-AS1	-2.442989586	0.016583041	0.199986518	0.842195552	-0.34308403	0.732814467
CYP20A1	-0.203415976	0.839283911	1.919537464	0.05987459	2.869110745	0.005788101
CYP26B1	-0.496335153	0.620906969	-0.91488336	0.364067255	-0.474736374	0.636813339
CYP27A1	-2.968902131	0.003858723	2.118687325	0.038441484	1.422456749	0.160416994
CYP2B7P1	0.734238821	0.464773229	-0.812752334	0.419706905	-0.162254138	0.871687318
CYP2D6	0.910188668	0.365232416	-1.273094817	0.208098979	-1.344375878	0.184224844
CYP2D7P1	0.168775004	0.866364545	-0.898006843	0.372920542	-0.540570959	0.590938015
CYP2E1	0.233793899	0.815693057	-1.14709902	0.256083488	0.114847459	0.908975112
CYP2J2	-0.666531027	0.506831672	0.535779089	0.594174269	1.171349928	0.246397255
CYP2R1	0.825629053	0.411267958	0.049212326	0.960920348	-0.031005898	0.975374616
CYP2S1	0.842640446	0.401735169	-0.728778349	0.469090269	-0.306951803	0.760013797
CYP2U1	1.330510454	0.186819592	-1.113439706	0.270144846	0.308558478	0.758797548
CYP3A5	-1.194869516	0.235378187	-1.300213648	0.198706755	-1.085068532	0.282520989
CYP4F12	1.115939871	0.267514033	0.563848191	0.575046418	0.869607425	0.388211538
CYP4F22	-0.665217242	0.507667431	-2.488405719	0.015746218	-1.362565679	0.178451398
CYP4F3	-2.515417785	0.013722077	-0.629475458	0.531526882	0.006931724	0.99449386
CYP4V2	2.153717304	0.034022127	-0.433422291	0.666326989	-0.578048646	0.565538873
CYP51A1	4.390752411	3.16E-05	0.820722747	0.415188166	1.42134561	0.160738203
CYSLTR1	-0.562910086	0.574940774	-0.500005298	0.618976342	-2.161515236	0.034927222
CYSLTR2	-0.888494791	0.376719321	-0.524846181	0.601704556	-0.450013241	0.654430844
CYTH1	0.777295435	0.439088419	1.753161247	0.08489509	2.490654232	0.015729711
CYTH2	-1.293894333	0.199118944	-0.402855406	0.688546044	0.497770876	0.620586469
CYTH3	1.001355726	0.319425871	-1.52295993	0.133240708	-1.511833843	0.136175597
CYTH4	-0.51959792	0.604660481	-0.29353083	0.770171531	-0.430672402	0.668352764
CYTIP	0.938314932	0.350674656	-0.148808254	0.882224733	0.016329843	0.987029047
CYTL1	0.946864108	0.34632491	1.417825173	0.161629453	1.05024144	0.298099467
CYYR1	-0.747250235	0.456923044	-1.511812104	0.136049364	-1.124264742	0.265676766
D2HGDH	1.901147316	0.06058593	-1.701988836	0.094149845	-0.252898645	0.801268696
D4S234E	2.344899062	0.021303439	-0.264627689	0.792240979	-1.133230251	0.261925769
DAAM1	1.8923784	0.061758987	-0.432926779	0.666684858	-0.799926567	0.42711949
DAAM2	-6.012719339	4.15E-08	-1.144730625	0.257055508	-0.251904011	0.80203359
DAB2	-0.729119623	0.467882595	0.293386134	0.770281559	0.865509872	0.390436351
DAB2IP	-1.08768414	0.2797322	-0.433630835	0.666176398	-0.401367081	0.689671004
DACH1	0.462836232	0.644634869	1.113122709	0.270279807	-1.404411812	0.165695397
DACT1	-1.035826379	0.303147514	2.951507426	0.004567928	0.957547002	0.342389792
DAD1	-2.086508517	0.039853225	-4.358506634	5.47E-05	-3.371599001	0.001357148
DAG1	-2.076101673	0.040829263	-1.442294855	0.154635239	-0.434860659	0.665327805
DAGLA	0.554014452	0.580987111	-0.512019318	0.610595236	-0.837824505	0.405676027
DAGLB	0.563610657	0.574465887	1.741803089	0.086881318	3.065563246	0.003335266
DAK	-0.183348668	0.854950155	3.773989851	0.000381785	4.182308682	0.00010206
DALRD3	1.565979858	0.120975753	-0.487514807	0.627744063	-0.819907504	0.415730426
DAP	-3.571490844	0.000580572	1.257187541	0.213759825	1.470835621	0.14690912
DAP3	-0.761557243	0.448379326	1.433397811	0.157150501	2.173396945	0.033977225

DAPK1	-2.175904137	0.032267362	0.429949403	0.668836815	-1.201982054	0.234407567
DAPK2	-2.971995138	0.003823647	0.127834758	0.898725036	0.374392388	0.709520445
DAPK3	-1.400734375	0.164842528	-1.622943053	0.110062966	-2.018152365	0.048355028
DAPP1	-0.37010708	0.712200238	-0.826561779	0.411896588	-2.601797872	0.011831827
DARC	-1.758186068	0.08222509	0.813596254	0.419227054	0.537799134	0.592837733
DARS	2.134079434	0.035643942	1.419509227	0.161140372	1.766486913	0.08274118
DARS2	0.018444886	0.985326089	1.399008863	0.167172557	1.912593068	0.060897941
DAXX	-0.764602649	0.446572684	0.608130124	0.545491853	1.19066316	0.238787502
DAZAP1	1.706905481	0.09139882	-0.471169805	0.63929926	0.795729273	0.429535455
DAZAP2	-3.775922396	0.000290513	-0.921230898	0.36077257	-1.279313032	0.20604018
DBF4	0.246133689	0.806157265	1.071071598	0.288604975	1.146731204	0.256348387
DBF4B	-0.878297274	0.382196394	-0.64729935	0.520009891	-0.688986495	0.493664655
DBH	2.126687284	0.036271586	0.153235061	0.878748509	1.222060034	0.226782552
DBI	-0.093217928	0.925944303	-2.236318699	0.029219394	-1.62257073	0.11027457
DBN1	-1.063721752	0.290391963	-0.166182846	0.868595052	0.193763795	0.847059022
DBNDD1	-0.730361592	0.467127156	-0.584338323	0.561274586	0.443806268	0.658885614
DBNDD2	0.461022067	0.645930744	-1.089729853	0.280370491	-1.071652115	0.288453869
DBNL	-2.024768595	0.045950683	1.283390237	0.204495121	1.701854139	0.094305543
DBP	0.468639308	0.640497067	-0.170302421	0.865369117	-0.068782592	0.945406663
DBR1	1.08084166	0.282748135	0.214738779	0.830729495	-0.462071811	0.6458125
DBT	1.165077651	0.247165242	-0.006057389	0.995187864	-0.169134973	0.866297383
DCAF10	0.848440898	0.398515811	-1.564484685	0.123182016	-2.305025773	0.024878446
DCAF11	-2.657911932	0.009352292	1.041473238	0.302007975	0.286958611	0.775198671
DCAF12	-3.983465057	0.000140446	0.037131711	0.970508386	-0.588865259	0.558309676
DCAF13	1.716987977	0.089531827	-0.20178182	0.840798281	0.310113646	0.757620872
DCAF15	-1.058923095	0.292559657	-2.747014562	0.008013564	-2.318086332	0.024106026
DCAF16	1.594187521	0.114512875	-0.756074301	0.452685973	0.479474674	0.633460404
DCAF17	0.941774666	0.348910146	-2.663192341	0.010019181	-2.065739683	0.043472133
DCAF4	1.865968082	0.065408116	1.524597149	0.132832122	2.66093742	0.010137323
DCAF4L1	-0.63133182	0.529475272	-0.855550239	0.395791742	-0.702294386	0.485392375
DCAF5	1.181151773	0.240754334	-1.377817274	0.173589635	-0.284594148	0.777000467
DCAF6	-2.931853966	0.004302602	0.795918768	0.429347471	0.996226857	0.323407295
DCAF7	-0.289200983	0.773114122	1.256076191	0.214159537	0.964633598	0.338858276
DCAF8	1.714652629	0.089961464	1.065080373	0.291284234	1.533451495	0.130771608
DCAKD	1.374299356	0.172871127	-0.935507498	0.353432704	0.931112664	0.355775366
DCBLD1	0.897909513	0.371706644	-0.100587889	0.920226936	-0.22565945	0.822284387
DCBLD2	0.910346079	0.365149888	-1.695059653	0.095464597	-0.809872266	0.4214273
DCDC2B	1.157415753	0.250263506	-0.620792764	0.537184941	0.006729132	0.994654784
DCHS1	3.553250038	0.000616845	-0.265978784	0.791205358	1.340481476	0.185479186
DCK	1.351604912	0.179998027	-0.225580432	0.822326286	-1.749395282	0.085678249
DCLK2	1.624591317	0.10785947	0.808132615	0.422339532	1.082463592	0.283666227
DCLRE1A	0.168982453	0.866201862	-1.274662081	0.207547337	-2.278158515	0.026536941
DCLRE1B	-0.176762245	0.860105104	0.463007232	0.645103889	0.487785516	0.627598113
DCLRE1C	-0.647584615	0.518955201	0.878342984	0.383407114	0.640580722	0.524396846
DCP1A	1.122028435	0.26493087	-1.03088212	0.306905647	-1.664492878	0.101576705
DCP1B	-0.316771337	0.752174333	-0.54297796	0.589240154	0.450336018	0.654199529
DCP2	-0.03361124	0.973263986	0.206203897	0.837358813	-0.692564557	0.491432916
DCPS	-1.278808717	0.204357741	2.745011919	0.008056834	2.3624566	0.021638958
DCST2	0.283467437	0.777490537	-0.306380315	0.760419908	0.776872221	0.440489739
DCTD	1.010897739	0.314862578	-0.303237005	0.762801863	0.193746369	0.847072603
DCTN1	-0.972113778	0.33368316	1.843209667	0.070445817	2.176011574	0.033771244
DCTN2	-0.948028513	0.345735187	2.564874105	0.012950243	3.721003737	0.000460195
DCTN3	-2.663672304	0.009205715	-1.654204474	0.103526297	-1.445151983	0.15396471
DCTN4	-0.545600814	0.586733547	-0.731329641	0.467542834	-1.30804768	0.196178722
DCTN5	-1.14681837	0.254594123	0.437371907	0.66347729	1.447816527	0.153220651
DCTN6	0.283371159	0.777564088	-1.756285099	0.084355469	-1.067018735	0.290522696
DCTPP1	-0.54028591	0.590377331	-1.101955116	0.275064719	0.224451181	0.823219759
DCUN1D1	-1.066204299	0.28927485	-0.145456512	0.884858297	-0.970399352	0.336002757
DCUN1D2	1.129062761	0.261968284	0.801660799	0.426044263	1.755654192	0.084592813
DCUN1D3	-1.417697017	0.159843213	-0.735398666	0.465080867	-2.394040768	0.020022908
DCUN1D4	1.485332643	0.141062529	-0.822204795	0.414351199	-1.192289937	0.238154384
DCUN1D5	1.611571033	0.110669639	-0.607990391	0.545583884	-0.847744115	0.400174191
DCXR	-0.953689914	0.342877206	-0.498434479	0.620075963	0.173978916	0.862506807
DDA1	0.099174713	0.921227218	-1.747554565	0.085870783	-0.255635836	0.799164752
DDAH2	-0.807688306	0.421468132	1.000016958	0.321485181	0.502076335	0.617574047
DDB1	-0.607082043	0.545374198	1.974517225	0.053124182	1.915190021	0.060558943
DDB2	0.976440056	0.331547892	0.277491642	0.78239621	1.42894245	0.158552037
DDHD1	1.855829552	0.06685621	-1.49413073	0.140600086	-1.623387521	0.110099508
DDHD2	3.070151214	0.002852386	-0.334765955	0.73901774	0.723347722	0.472464313
DDI2	-0.364709273	0.716211546	-0.018631257	0.98519966	-0.241388083	0.810132333
DDIT3	-2.593998332	0.011126658	-1.180155425	0.242789601	-1.015709474	0.314117819
DDIT4	6.190867122	1.90E-08	-1.077048109	0.285949328	0.270727697	0.787591651

DDO	-0.191097149	0.848893776	-0.067986636	0.946031743	-0.904294762	0.369696788
DDOST	-0.233856126	0.8156449	1.268109386	0.209860994	1.417508249	0.161851347
DDR1	3.976620242	0.000143907	0.62368453	0.535297083	1.223856676	0.226109198
DDRGK1	0.238045713	0.812404177	1.057334313	0.294773616	1.558181972	0.124800453
DDT	0.259138355	0.796139336	1.1147511	0.269587023	0.559279344	0.57819173
DDTL	0.905538511	0.367675755	-0.389460236	0.698372043	-0.107790424	0.91454559
DDX1	1.755426828	0.082698594	1.770358213	0.081959657	3.058298261	0.003405225
DDX10	-0.902530129	0.369261958	2.253648893	0.0280404	2.38220949	0.020615157
DDX11	1.858995135	0.066401211	0.7978242	0.428249646	1.725081227	0.090005048
DDX11L2	-1.704525057	0.091844221	1.173472881	0.245436095	1.152494006	0.253993686
DDX12	2.477903722	0.015143301	0.185643958	0.853376216	1.199863395	0.235222923
DDX17	2.028734761	0.045536241	-1.985182055	0.051893188	-1.754748603	0.084749153
DDX18	1.622958984	0.108208594	1.503564572	0.138157333	1.866162241	0.067236552
DDX19A	-0.852221843	0.396425851	2.215160899	0.030717965	2.370547457	0.021214232
DDX19B	1.099695478	0.274491959	2.500648277	0.015264764	2.507630556	0.015067599
DDX20	0.841879837	0.402158495	1.000571688	0.321219116	1.086189701	0.28202907
DDX21	2.187615227	0.031373337	0.298639399	0.766290013	-0.352750602	0.725594137
DDX23	-0.411863258	0.681450442	1.550397372	0.126523987	1.719410115	0.091039813
DDX24	0.498719804	0.619232686	1.929807787	0.058560802	2.492830371	0.015643393
DDX26B	2.051614313	0.043207502	-0.69725459	0.488448454	-1.163990022	0.249342641
DDX27	-1.01668544	0.312116055	2.394948414	0.019896575	2.735644031	0.008314494
DDX28	1.196284018	0.234828781	0.891549478	0.376343927	-0.12134937	0.90384688
DDX31	2.117190139	0.037092006	0.733862958	0.466009178	1.357423916	0.180069203
DDX39A	0.360495611	0.719348426	1.421403431	0.160591631	1.683053618	0.09790908
DDX3X	0.281456221	0.77902741	-0.809433021	0.42159747	-2.301696617	0.025078824
DDX41	-0.26244688	0.793596041	1.784660848	0.07958307	2.247865451	0.028523884
DDX42	0.695016175	0.488893283	2.270379469	0.026942316	1.966479223	0.054185841
DDX43	0.192730264	0.847618442	-1.673424331	0.099667089	-1.411136028	0.163712992
DDX46	1.662545981	0.099994487	2.009718586	0.049153704	1.674199141	0.099644959
DDX47	1.357151731	0.178235935	2.292822278	0.025529213	3.173698467	0.00244071
DDX49	-0.633693145	0.527940016	1.411117852	0.163588806	1.480876987	0.144220664
DDX5	2.277514695	0.025201825	-0.213150683	0.8319621	0.204488498	0.838710058
DDX50	0.572458248	0.568484792	1.429331895	0.15831051	2.15763446	0.035242513
DDX51	1.199511484	0.233578664	0.939912072	0.351187922	2.142190464	0.036522093
DDX52	1.270914811	0.207139392	0.227660446	0.820716453	1.977920066	0.052845332
DDX54	0.314399711	0.753968565	0.833479244	0.408017699	1.020087287	0.312055481
DDX55	2.317933926	0.022794325	1.090095171	0.280210916	2.653668602	0.010332917
DDX56	0.096441629	0.923391165	1.814351929	0.074832756	3.322329672	0.00157352
DDX58	-1.935311086	0.05619331	1.559092295	0.124452816	-1.975071291	0.053176431
DDX59	0.348445569	0.728345547	-2.498723821	0.015339554	-3.165452712	0.00250006
DDX6	-0.09204133	0.926876351	-1.995829356	0.050688724	-1.513487983	0.135755935
DDX60	-0.423699404	0.672828492	2.117656413	0.038532435	-0.942038897	0.350202056
DDX60L	-0.425525147	0.671502394	0.664087227	0.509284307	-1.633684355	0.107911874
DEAF1	-1.212125157	0.228738963	-0.341001878	0.734342983	-0.372186978	0.711152522
DECR1	-2.050939792	0.043274664	0.919278373	0.361783976	1.090424051	0.280176607
DECR2	-0.850007298	0.397649148	0.384227611	0.702224673	0.442676483	0.65969781
DEDD	-2.014725748	0.047014581	-0.872388336	0.386618895	-0.674327676	0.502865662
DEDD2	-1.288495557	0.200982192	-1.921113917	0.059671314	-2.462461187	0.016887537
DEF6	0.072093337	0.94269275	0.666179047	0.507956245	1.733459074	0.088494209
DEF8	-1.557916998	0.122875514	0.72276587	0.472748491	1.573797026	0.121144004
DEFA4	-2.839574256	0.005620643	1.228392722	0.224295444	-0.33339517	0.740075888
DEGS1	-2.574560834	0.011723672	-1.866937085	0.067002854	-2.574877049	0.012685473
DEGS2	1.184318661	0.239505447	0.975141757	0.33356783	0.329266499	0.743177453
DEK	0.438451371	0.662143589	-0.014475323	0.988500792	-0.719134926	0.475035549
DEM1	2.12680319	0.036261671	-0.970135598	0.336035368	-0.109467404	0.913221463
DENND1A	-1.732303857	0.086755633	1.020133967	0.311930846	0.951333633	0.345505959
DENND1B	0.474299099	0.636472352	-0.580190732	0.564049007	-1.463192846	0.148981636
DENND1C	-0.998931004	0.32059243	0.216700482	0.829207504	1.398274153	0.167521017
DENND2C	-0.944700345	0.347422496	0.871919459	0.386872509	0.096333604	0.923598105
DENND2D	1.80098163	0.075163164	0.808174272	0.422315748	2.74155476	0.008183968
DENND3	-0.354538838	0.723791178	-0.850395528	0.398626623	-1.201008584	0.234781946
DENND4A	0.887647839	0.377172333	-0.385969185	0.700941525	-1.637406838	0.107129755
DENND4B	0.531882383	0.596160135	0.241210319	0.810248752	0.789535178	0.433115636
DENND4C	0.825937225	0.411094059	-0.599126817	0.551437713	-1.772878395	0.081664594
DENND5A	-1.619456454	0.108960788	0.295486145	0.768685168	-0.37426123	0.709617469
DENND5B	0.590652549	0.556281219	1.865756052	0.067170803	1.04130466	0.302190254
DENR	0.32594789	0.745244799	0.972585374	0.33482637	0.842353274	0.403158445
DEPDC1	-0.391946664	0.696054014	1.955667188	0.05536122	1.460475995	0.149723871
DEPDC1B	-0.59610706	0.552648166	0.785149329	0.435583815	-0.215951379	0.829807026
DEPDC5	0.494634098	0.622102516	1.079134527	0.285026236	0.634609618	0.528256433
DEPDC7	1.500991281	0.136970667	-0.528768584	0.598997831	0.53772048	0.592891681
DEPTOR	3.071055718	0.00284461	0.359937468	0.720210384	2.065175114	0.043527459

DERA	-0.270885788	0.787119136	-0.339576961	0.735410289	-0.736019362	0.464777944
DERL1	-0.905229868	0.367838291	-1.178771557	0.243335959	-1.353216919	0.181401196
DERL2	-1.405547848	0.163411856	-2.875865536	0.005639271	-2.124634269	0.038025871
DERL3	2.47315744	0.01533221	-0.538364019	0.592400324	0.681645795	0.498260645
DES	0.070817431	0.943705242	-0.886670534	0.378943665	0.314943473	0.753970202
DET1	1.404423365	0.163745222	-1.503197849	0.138251659	-0.459118155	0.647919046
DEXI	1.19905699	0.233754415	0.411787271	0.682023812	0.719780282	0.474641152
DFFA	0.981898326	0.32886677	2.523043467	0.014418334	2.180909401	0.033388341
DFFB	1.80240125	0.074937816	0.120318332	0.904649716	0.480795756	0.632526946
DFNA5	0.719302283	0.473878255	2.345640032	0.022460125	2.052022675	0.04483375
DFNB31	0.613125421	0.541389521	-1.08360823	0.283053918	-0.458368327	0.648454285
DFNB59	2.125708336	0.03635542	-0.307512949	0.759562184	0.503882625	0.616312191
DGAT1	-1.752319157	0.083234581	-1.042868245	0.301366879	-0.999320422	0.321920099
DGAT2	-2.374403408	0.019771731	-0.518905774	0.605814584	-0.469756958	0.640345142
DGCR10	-0.189979968	0.849766438	-0.822485168	0.414192977	-1.101802461	0.275240841
DGCR11	-0.683691111	0.495983246	0.351465293	0.726521822	0.388805883	0.69888797
DGCR14	-1.682146839	0.096118716	0.754395204	0.453685384	0.991458097	0.325708814
DGCR2	-2.057250937	0.042649763	0.809769629	0.421405517	0.595287481	0.554039367
DGCR6	-1.474757898	0.143879534	1.975291712	0.053033951	2.158755333	0.035151193
DGCR6L	0.635037495	0.527066996	0.331780914	0.741258996	0.506588235	0.614424256
DGCR8	0.34215365	0.733058655	2.004542299	0.049721037	2.839916283	0.006270777
DGCR9	-0.389741468	0.69767814	-0.04442888	0.964716218	-1.72566812	0.089898519
DGKA	1.226961698	0.223139838	2.18325325	0.033106006	4.133223617	0.000120257
DGKD	2.16577327	0.033058586	1.534159154	0.130465672	2.201430555	0.0318252
DGKE	2.756994461	0.007104357	-0.977792311	0.332266239	-1.372478847	0.175363697
DGKG	-0.999726089	0.320209594	1.524544462	0.132845255	1.127503481	0.26431738
DGKH	-0.087330476	0.930609089	-0.636434178	0.527014729	-1.235194025	0.2218939
DGKK	-0.715934964	0.475944603	-0.959805392	0.34116514	-0.417147761	0.678158511
DGKQ	0.806895532	0.421922318	-0.582768324	0.562323998	-0.766809571	0.446401957
DGKZ	-0.325791404	0.745362793	-0.305511391	0.761078134	0.658567942	0.512860599
DGUOK	-2.517383161	0.013651034	-0.470154163	0.640020287	0.820064158	0.415641867
DHCR24	3.643759818	0.000455775	-0.158752777	0.874418994	0.856566449	0.395319847
DHCR7	0.614052497	0.540779567	0.26459635	0.792265005	-0.165696281	0.868990217
DHDDS	-0.801867596	0.424809652	0.386480459	0.700564996	0.843601417	0.402466284
DHFR	2.24279351	0.02744589	0.215740239	0.829952429	1.579339019	0.119867159
DHFRL1	0.970442405	0.33451049	-1.69932916	0.094652723	-0.661978988	0.510688311
DHODH	1.872795357	0.064447867	0.4511397	0.653582872	2.059916043	0.044045773
DHPS	-0.283728106	0.777291412	-1.408366128	0.164397939	-0.509299312	0.612535134
DHRS1	0.155443525	0.876830984	0.371010241	0.711991071	1.456564525	0.150797555
DHRS11	-1.175707921	0.242912052	1.219081736	0.227782415	1.252762543	0.215476455
DHRS12	-0.822944177	0.412784886	-1.351985979	0.181665114	-0.953678326	0.34432786
DHRS13	0.065362483	0.948035031	1.146358885	0.256386967	0.390742135	0.697464199
DHRS3	0.733556334	0.46518709	0.447984675	0.655844844	1.387204292	0.170852873
DHRS4	0.108252784	0.914043909	0.264925723	0.792012502	1.214866476	0.229493301
DHRS4L2	0.038792529	0.969144488	2.292854046	0.025527261	2.627468422	0.011066881
DHRS7	-2.007215513	0.047823898	0.490535444	0.625618708	0.473334524	0.637806792
DHRS7B	0.129211557	0.897487861	1.853847729	0.068884183	1.183085438	0.241752714
DHRS9	-3.364556756	0.001141253	-0.532844838	0.596190956	-0.837934683	0.405614663
DHRSX	-2.971431438	0.003830018	0.232350076	0.817089754	-0.664319225	0.509200817
DHTKD1	-0.82454967	0.411877393	0.705451687	0.483372618	0.50121396	0.618176903
DHX15	1.451909175	0.150115877	0.694502717	0.490159068	0.378151066	0.706742055
DHX16	0.890443015	0.375678563	-0.108152396	0.914250691	0.339569783	0.73544546
DHX29	0.614936623	0.540198198	1.553955314	0.125673164	1.719915594	0.090947185
DHX30	1.06813398	0.288408556	0.636030716	0.527275792	1.155483468	0.252778288
DHX32	3.095647289	0.002640605	2.493224528	0.0155551	2.912513043	0.005133786
DHX33	2.181848541	0.031810828	0.137552953	0.89107348	-0.871183368	0.387357977
DHX34	-1.137162416	0.25858596	-1.396361358	0.167964114	-1.182156557	0.242118014
DHX35	0.652220056	0.515975034	4.032748636	0.000164087	3.84667075	0.00030786
DHX36	1.267887269	0.208213619	1.256943013	0.213847725	1.224009567	0.226051964
DHX37	1.169282653	0.245476513	0.884033181	0.380353686	0.90421856	0.369736835
DHX38	-0.638846885	0.524597259	1.887211081	0.064174654	1.391605643	0.169522081
DHX40	-0.105122057	0.916520419	-0.59317594	0.555385591	-1.511558722	0.136245496
DHX57	0.793407665	0.429694256	2.260679691	0.027574211	2.604609365	0.011745761
DHX58	-1.479816213	0.142526622	0.511092561	0.61123991	-1.536536095	0.130014635
DHX8	-0.841878583	0.402159193	1.83237949	0.072066161	1.809950012	0.07564802
DHX9	0.217022955	0.828697482	1.785863009	0.079385959	1.772472301	0.081732648
DIABLO	-0.719965132	0.473472091	0.567711007	0.572437638	1.119811269	0.267554068
DIAPH1	-1.621393909	0.108544187	1.495934121	0.140130508	1.770473724	0.082068261
DIAPH2	-0.174777687	0.861659544	2.007379149	0.049409418	2.203524184	0.031669404
DICER1	0.118761718	0.905737336	-0.819086493	0.416113409	-1.836681398	0.071543912
DIDO1	4.643901185	1.20E-05	-0.771709137	0.443441476	0.621701711	0.53665031
DIEXF	1.759711435	0.081964288	0.446042985	0.657238537	1.231353683	0.223315242

DIMT1L	1.70053633	0.092594528	-0.876463456	0.384419066	-0.735755154	0.464937475
DIP2A	3.35487513	0.00117717	-1.223312736	0.226193018	-1.248623271	0.216975952
DIP2B	-1.129468959	0.261797923	-0.03426173	0.972786904	-2.316067602	0.024224004
DIP2C	1.745845244	0.084360345	0.414222942	0.680249432	0.640079704	0.524720121
DIRAS1	-0.770243271	0.443237625	0.966818363	0.33767706	1.614518509	0.112012509
DIRC2	-2.692329524	0.008507045	-1.440568436	0.155120842	-2.394195597	0.02001526
DIRC3	-0.411680009	0.681584265	-0.102622475	0.918619068	0.28995632	0.772916108
DIS3	1.318625546	0.190747563	0.128123717	0.898497383	-0.526451038	0.600645156
DIS3L	2.164984029	0.033120929	0.170949115	0.864862915	0.346646196	0.730150883
DIS3L2	0.567784077	0.571640822	0.930108247	0.356197093	2.022051741	0.047937839
DISC1	0.443555565	0.658462697	-0.988243528	0.327166885	-1.822471821	0.073701589
DISP1	-0.540936009	0.58993107	-0.986516595	0.328005874	-0.169398664	0.866090952
DISP2	2.156463539	0.033800519	-1.827411564	0.072819847	-1.290093709	0.202297861
DIXDC1	0.022382372	0.982194087	-0.475391679	0.636305812	0.116099069	0.907987624
DKC1	1.239033101	0.21865837	2.583252261	0.012349238	2.74735018	0.00805782
DKFZP434I0714	1.385612798	0.169399427	-1.988518725	0.051513114	-2.207916328	0.031344744
DKFZP586I1420	0.170297632	0.865170622	-0.323284716	0.74765047	-0.95043319	0.34595909
DKFZP686I1521	-1.185565424	0.239015053	-1.68970183	0.096491508	0.028274094	0.977543641
DKFZp761E198	-1.166490574	0.246596891	-0.080522336	0.936100975	-0.859581158	0.393669465
DKK3	1.871236072	0.06466613	0.144412147	0.885679154	-0.667337158	0.507286014
DLAT	2.306892536	0.023430937	0.848470657	0.399688433	0.823217832	0.413861485
DLC1	-2.090623526	0.039472879	1.742913491	0.086685456	1.592437887	0.116892255
DLD	0.651633353	0.516351731	1.287828625	0.202955935	0.699383079	0.487195455
DLEC1	2.290799989	0.024386891	-1.667262232	0.100891363	-0.921824525	0.360558029
DLEU1	-0.126758988	0.899423028	-0.769705079	0.444620207	-1.901677561	0.06234052
DLEU2	-0.295761795	0.768115251	-1.695958085	0.09529328	-1.941209231	0.057250248
DLEU7	-2.358293029	0.02059552	-0.519952195	0.605089655	-2.324206972	0.023751445
DLG1	1.462776759	0.147123999	-1.148012587	0.255709249	-2.119561551	0.038470324
DLG3	1.583850105	0.116848521	0.434570445	0.665498069	1.756817939	0.084392258
DLG4	-1.318865117	0.190667779	0.273025224	0.785810393	-0.163317954	0.870853597
DLG5	1.995732452	0.049084351	-1.207193143	0.232292104	-0.927396538	0.357683928
DLGAP3	0.055199984	0.956105436	-1.87245918	0.066222282	-2.145963459	0.036205793
DLGAP4	-2.045627203	0.043806777	-0.194101838	0.846779111	-0.404217868	0.687585702
DLGAP5	1.180458141	0.241028495	1.504303658	0.137967384	2.0605402	0.04398398
DLK2	1.773972733	0.079558814	0.297246501	0.767347753	0.246001261	0.80657691
DLL1	0.11664105	0.90741276	-0.25893678	0.7966072	-1.167925017	0.247764753
DLST	-0.460864384	0.646043429	1.341909106	0.184891842	2.056630625	0.044372278
DLX4	-0.321940031	0.748268732	0.524653571	0.601837616	-0.349889962	0.727728284
DMAP1	-0.300419437	0.764572392	2.212718327	0.030895256	3.373760405	0.001348333
DMC1	-1.373946894	0.172980148	-0.168312373	0.86692719	-1.158263289	0.251651861
DMD	1.903831373	0.060230648	1.03245718	0.30617389	1.260098073	0.212837933
DMKN	0.02528481	0.979885571	-1.015561049	0.314085658	-0.619561088	0.538048967
DMPK	1.26927264	0.207721556	-0.234540993	0.815396791	1.018291537	0.312900328
DMRTC2	0.435023529	0.664620256	-0.235033075	0.815016671	-1.730135136	0.089091109
DMTF1	0.621008422	0.536214215	-1.611222965	0.112598272	-2.268476144	0.027158244
DMWD	0.814858508	0.417373536	-2.126234546	0.037781324	-1.205797567	0.232944373
DMXL1	1.21075091	0.229262691	-0.921443491	0.360662557	-2.664752584	0.010036021
DMXL2	-0.970728351	0.334368851	-1.484446685	0.143142946	-2.356475979	0.021957776
DNA2	1.133381405	0.260161017	-0.985183046	0.328654727	-1.814136262	0.074992556
DNAAF2	1.415693444	0.16042757	-1.891987257	0.063523301	-1.99053023	0.051400881
DNAH1	1.161614095	0.24856241	-1.174396971	0.245068895	-0.119980775	0.904925993
DNAH10	-0.795073522	0.428729805	0.923027571	0.359843504	0.934113698	0.35423889
DNAH14	-0.349197021	0.727783348	1.048377687	0.298844028	0.715477442	0.477274239
DNAH17	0.719017475	0.474052833	1.200047406	0.235033791	0.626765923	0.53334891
DNAH6	1.462792906	0.147119589	-0.613648526	0.541863656	0.344569699	0.731703165
DNAH8	2.015636421	0.046917247	-0.32693827	0.744899782	-1.089397556	0.280624901
DNAI2	1.387722834	0.168757857	1.755734795	0.084450323	1.39446486	0.168661854
DNAJA1	-0.076771005	0.938981612	0.814082343	0.418950815	-0.500111061	0.618948285
DNAJA2	-0.003902215	0.996895405	1.573536872	0.121072129	1.085636102	0.28227189
DNAJA3	2.012054667	0.047301069	0.435081016	0.665129592	1.670401618	0.100397131
DNAJA4	-0.697790725	0.487164808	0.973643875	0.334304876	0.934885398	0.35384449
DNAJB1	1.293013897	0.199421924	1.270524382	0.209006075	2.23757718	0.029228031
DNAJB11	1.402131472	0.164426295	0.229474712	0.81931293	0.353348089	0.725148665
DNAJB12	-1.481036586	0.14220171	-0.190475093	0.849606634	0.030223718	0.975995641
DNAJB13	1.382170726	0.170450003	0.556385571	0.580102629	1.473018266	0.14632142
DNAJB14	0.161551254	0.872033019	-0.631747171	0.530051658	-2.130433052	0.037523301
DNAJB2	-1.91731326	0.058472555	0.159802002	0.873596145	0.159593526	0.8737731
DNAJB4	1.606719293	0.111731716	-0.715458439	0.477216231	-1.049464986	0.298453373
DNAJB5	-3.282273651	0.00148236	-3.059703736	0.003360757	-2.758086567	0.007828833
DNAJB6	-3.439038951	0.000897472	-1.182310415	0.241940564	-2.040939685	0.045960727
DNAJB7	-0.367329543	0.714263324	-1.320073225	0.192032979	-1.587153612	0.118085133
DNAJB9	1.254195654	0.21312298	0.149331387	0.881813811	0.160502196	0.873060648

DNAJC1	-0.270120874	0.787705602	1.516775658	0.134793073	1.782849949	0.080008299
DNAJC10	1.080918915	0.282713959	0.743979183	0.459913542	0.510605487	0.611625913
DNAJC11	2.159144032	0.033585428	0.679998031	0.499229852	1.326579145	0.190009892
DNAJC13	0.243297583	0.808346379	1.911043536	0.060980013	0.683652727	0.497001793
DNAJC14	-2.522833349	0.01345575	0.334016128	0.739580519	0.675698911	0.502001041
DNAJC15	0.259951004	0.79551444	-1.217472707	0.228388994	-0.488495719	0.627098262
DNAJC16	1.691160196	0.09437797	-0.06749371	0.946422424	1.092080966	0.279454047
DNAJC17	1.413733705	0.161000731	0.595242	0.554013339	2.084532409	0.041664994
DNAJC18	2.533977384	0.013064251	-0.120844624	0.904234695	0.25121432	0.802564091
DNAJC19	1.993492847	0.049333461	-1.635687555	0.107358795	-0.818758543	0.416380299
DNAJC2	1.38190869	0.170530184	1.268518519	0.209715976	2.379231538	0.020766676
DNAJC21	0.945900067	0.34681365	0.938777419	0.351765307	0.162567072	0.871442054
DNAJC24	1.896573649	0.061195405	-0.884742464	0.379974153	-1.395187777	0.16844489
DNAJC25	0.775666461	0.44004482	-0.959745828	0.341194866	-1.458308037	0.150318226
DNAJC27	0.426304223	0.670936841	-0.522263234	0.603490051	-1.542461405	0.12857034
DNAJC3	-1.902280067	0.060435776	0.819353319	0.415962442	0.654390081	0.515527947
DNAJC30	-1.990651657	0.049651038	-3.933213877	0.000227788	-2.525315143	0.014404762
DNAJC4	-0.455279841	0.650039662	-3.254195371	0.001905504	-3.128612898	0.002782155
DNAJC5	-1.153561922	0.251832279	1.820224001	0.073921982	1.714250228	0.091989814
DNAJC6	-0.769669211	0.443576379	-0.431364528	0.667813656	0.275393764	0.784023098
DNAJC7	0.453806187	0.651095908	3.152431492	0.002570532	3.456427452	0.00104913
DNAJC8	-1.148585253	0.253868426	1.888693107	0.063971939	2.919267633	0.005038355
DNAJC9	0.873976537	0.384531965	1.597977376	0.115520224	2.234215223	0.029461436
DNAL1	1.744693485	0.084561935	-0.429277099	0.669323124	1.059574647	0.293867938
DNAL4	-1.833023048	0.070212011	-1.215626885	0.229086292	-0.571208286	0.570134288
DNASE1	0.819283467	0.414858575	-1.968599372	0.053817993	-1.705393301	0.093639544
DNASE1L1	-0.190922241	0.849030389	-0.987258652	0.327645188	-0.978497802	0.33201889
DNASE1L2	-1.356400479	0.178473823	-0.084354315	0.933067247	-0.820372882	0.415467374
DNASE1L3	0.246253868	0.806064535	-0.62072782	0.537227378	0.34180339	0.733772865
DNASE2	-0.136056387	0.892090353	0.044332178	0.964792964	0.180363865	0.857515311
DND1	1.451171548	0.150320646	-0.497739344	0.620562856	0.518262188	0.606308511
DNHD1	3.358074749	0.001165184	0.762090375	0.449115658	0.987542022	0.327606966
DNLZ	-0.389919183	0.697547201	2.099786709	0.040139049	0.632666835	0.529515401
DNM1	-0.839462548	0.403505673	-1.178000459	0.243640776	0.006248261	0.995036754
DNM1L	0.534629024	0.594267174	-0.800848159	0.426510823	-0.802717024	0.425517811
DNM1P35	1.906419379	0.059889745	-1.158105023	0.251600865	-0.782353285	0.437288878
DNM1P46	1.19683858	0.234613636	-2.25458997	0.027977599	-1.700352515	0.094589294
DNM2	-0.637778901	0.525289055	-1.526432471	0.132375282	-1.456109707	0.150922791
DNM3	-3.280232309	0.001491929	1.658897758	0.102572846	-0.201067045	0.841371614
DNMBP	-1.437564127	0.154137119	0.420128503	0.675954798	0.070448359	0.944086727
DNMT1	1.659145238	0.100679667	1.253282361	0.215166817	1.354189708	0.181092532
DNMT3A	0.750194206	0.455157427	0.804211303	0.424581933	1.065566185	0.291173372
DNMT3B	1.030561823	0.305596685	-1.312000603	0.194725084	-0.314916658	0.753990454
DNPEP	-0.043864515	0.965112704	-0.248166695	0.804888104	0.316038258	0.753143479
DNTTIP1	-2.967323567	0.003876738	-0.011616817	0.990771474	-0.351078168	0.726841573
DNTTIP2	1.143778059	0.255846277	0.038229307	0.969637053	0.955960375	0.343183767
DOC2GP	1.441037678	0.153155844	1.120042926	0.267344299	0.758228436	0.451480175
DOCK1	-0.805456564	0.42274746	1.760178589	0.083686891	-0.762687991	0.448836883
DOCK10	3.406064421	0.000998629	1.508063101	0.137004384	1.17990581	0.243004817
DOCK11	0.282582605	0.778166574	0.357196483	0.722250195	-0.894992392	0.374605898
DOCK2	-1.29476047	0.198821218	1.456514993	0.150680401	1.30206588	0.198201805
DOCK3	1.320550545	0.190107185	0.14528375	0.884994077	-0.591241563	0.556727693
DOCK4	-1.853042097	0.067259019	-0.289679979	0.773101345	-2.333111454	0.023243886
DOCK5	-1.161190216	0.248733784	-0.896421241	0.373759314	-1.366516812	0.177215777
DOCK6	-1.427643479	0.156966465	0.783659485	0.436450754	-0.993275131	0.324830583
DOCK7	1.569338758	0.120191263	1.289130073	0.202506253	1.392636494	0.169211549
DOCK8	-0.255152806	0.799205991	0.709577704	0.48082885	-0.133094095	0.894594077
DOCK9	2.799121069	0.006307774	0.943436374	0.34939846	1.020829265	0.311706853
DOHH	-0.020870641	0.983396535	-2.970076195	0.004335549	-1.566371903	0.122871812
DOK1	-0.957060708	0.341182881	0.544112612	0.588464235	0.090149992	0.928488341
DOK2	-0.758262968	0.450338351	-0.456976561	0.64940679	-0.227106979	0.821164136
DOK3	-1.163805244	0.247677868	-0.889442923	0.377465013	-1.285870974	0.203757603
DOK4	-0.408123448	0.684183568	0.497997169	0.620382248	0.409151023	0.683982949
DOK6	1.678544904	0.096821627	-2.167469674	0.034346461	-1.90794012	0.06150936
DOK7	-1.055088756	0.294299666	-1.360189515	0.179070058	-1.539457014	0.129301053
DOLK	-0.591585127	0.555659223	-1.484681441	0.143080878	-2.155437482	0.035422109
DOLPP1	1.161591906	0.248571379	0.652182996	0.516877501	1.959831371	0.054978066
DOM3Z	1.735551755	0.08617609	-0.092718455	0.926448992	1.136368075	0.260621882
DONSON	0.155724489	0.876610169	-1.019250715	0.312346264	0.546257112	0.587049938
DOPEY1	1.333945116	0.185695831	-0.412394414	0.681581341	-0.874447301	0.38559391
DOPEY2	-0.07784073	0.938133107	1.054966286	0.29584606	1.410501531	0.163899264
DOT1L	2.145170309	0.034719933	-1.776965317	0.080854549	-0.908904807	0.367279229

DPAGT1	-0.309745418	0.757493652	-1.372331659	0.175281142	-0.31482001	0.754063453
DPCD	-1.555341409	0.123487342	0.994098892	0.324332849	0.784901286	0.435805576
DPEP2	-1.932217294	0.056579667	0.592584928	0.555778446	1.795537847	0.077941429
DPEP3	-1.245377942	0.216329407	0.653100307	0.516290255	0.297374055	0.767276682
DPF2	-2.377373545	0.019623092	-0.97123458	0.335492649	-0.667327656	0.507292037
DPF3	0.025781402	0.979490612	-2.196940947	0.032062302	-2.218031474	0.03060818
DPH1	0.945664868	0.346932957	-1.932866463	0.058174312	-0.896502392	0.373806228
DPH2	1.039786724	0.301313859	0.833887281	0.407789596	2.145669988	0.036230309
DPH3	-1.589473812	0.115573218	-0.731414249	0.467491567	-0.936325093	0.353109453
DPH3P1	-0.91758953	0.361365097	-2.546850643	0.013565314	-2.602493472	0.01181048
DPH5	1.281387922	0.2034549	0.407508367	0.685145352	1.460134484	0.149817375
DPM1	0.907315954	0.36674061	-1.405744861	0.165171584	-1.397052642	0.167886195
DPM2	-2.68164493	0.008761695	-0.679228213	0.499713817	-0.57379071	0.568397245
DPM3	-0.071444448	0.943207662	-1.384866728	0.171434326	-0.703508084	0.484641784
DPP3	-1.334036441	0.185666021	1.811399251	0.075294266	1.899954207	0.062570909
DPP4	-0.23324038	0.816121462	0.95910944	0.341512576	1.21426797	0.229719898
DPP7	0.752420254	0.453824974	-0.097328206	0.922803656	0.235836219	0.814416544
DPP8	0.342115112	0.733087555	-2.366015928	0.02136703	-3.338815634	0.001497726
DPP9	0.609687469	0.543654507	-0.636376105	0.527052301	-0.435823581	0.664633125
DPPA4	1.746934679	0.084170028	-0.862246048	0.392128035	-0.719230333	0.474977232
DPRXP4	-0.619787612	0.537014034	-1.355889019	0.180426901	-0.940018102	0.351228525
DPY19L1	0.811914692	0.41905173	-0.8635372	0.391423998	-1.936617969	0.05782262
DPY19L1P1	0.2368413	0.813335481	0.052724026	0.958134238	1.726331933	0.089778154
DPY19L2	0.287434586	0.774461638	-1.80079408	0.076971583	0.168903777	0.866478382
DPY19L2P2	1.858921277	0.066411797	-0.739544785	0.462579881	-1.202849305	0.234074403
DPY19L3	-1.9279404	0.057117472	-1.427691798	0.15878031	-1.689625085	0.096636876
DPY19L4	1.700029081	0.092690303	-1.120626433	0.267097811	-1.56376588	0.123482893
DPY30	0.299861908	0.764996217	-1.738907001	0.087393878	-1.448757764	0.152958488
DPYD	-0.661467531	0.510056837	2.311834218	0.024384022	1.049425768	0.298471256
DPYSL2	-0.279717756	0.780356567	2.155882308	0.035282893	1.572573972	0.121427256
DPYSL4	-0.700588662	0.485425169	0.443967037	0.658729952	-0.649569713	0.518614664
DR1	-0.967164611	0.336136902	-1.181597054	0.242221381	-3.161018709	0.002532532
DRAM1	-1.652345481	0.10206109	-0.214591075	0.830844117	-1.342644026	0.184781855
DRAM2	1.301569647	0.196492156	-0.396403697	0.693272077	0.206037981	0.837505333
DRAP1	-2.169341125	0.032778029	-1.960890113	0.054733459	-3.301722292	0.001673364
DRD4	0.694339533	0.489315323	-2.806740336	0.006817246	-2.515366251	0.014774335
DRG1	0.465955359	0.642409417	2.110479894	0.039170792	3.200843047	0.002254551
DRG2	-0.948554418	0.345469051	1.479715652	0.14439833	2.369756848	0.021255404
DROSHA	0.229189151	0.819258668	0.663829023	0.509448366	0.988411714	0.327184784
DRP2	0.266872621	0.790197425	-1.425807743	0.159321326	-0.84528013	0.401536505
DSC1	2.027478076	0.045667209	0.372942932	0.710559916	0.527855078	0.599676602
DSC2	-0.460175315	0.646535961	0.203663085	0.839334658	-0.262506654	0.793890118
DSCAML1	0.226379399	0.821436228	-0.512592678	0.610196549	-0.170938958	0.864885319
DSCC1	0.130203233	0.896705567	0.634949718	0.527975593	1.044417311	0.300761114
DSCR3	-0.622809075	0.535035624	-0.127988479	0.898603927	0.027038042	0.978525112
DSE	-1.986970278	0.05006512	0.667701149	0.50699106	-2.132527135	0.037343242
DSEL	2.706086516	0.008189087	1.157867847	0.251696868	0.64373739	0.522362457
DSG3	-0.910308611	0.365169531	1.970035925	0.053648861	0.881880063	0.381595548
DSN1	0.225091257	0.822435007	3.469328214	0.000994756	3.439136652	0.001105955
DSP	-0.702037212	0.484525868	0.151645702	0.879996307	0.97857729	0.331979943
DST	-0.36757733	0.714079187	2.216589511	0.030614686	1.069478303	0.289423214
DSTN	-0.246305976	0.80602433	1.888661678	0.063976232	2.534188273	0.014082236
DSTNP2	-0.60405669	0.547374499	3.160969378	0.002507291	3.650873002	0.000574272
DSTYK	0.913045939	0.363736244	1.348960427	0.182629394	1.704798837	0.093751139
DTD1	-0.938062681	0.350803532	1.516839702	0.134776923	1.182650043	0.241923892
DTHD1	-0.237660203	0.812702241	-1.811520673	0.07527524	-3.155012709	0.002577148
DTL	0.029839793	0.976263021	1.091092714	0.279775499	0.887009902	0.378851308
DTNA	-0.341693996	0.733403374	1.199305905	0.235319632	-0.003437311	0.997269592
DTNB	1.547926757	0.125262199	2.453122561	0.017211374	2.678571567	0.009676797
DTNBP1	-1.226308898	0.22338408	1.874944722	0.065873457	2.001296743	0.050194596
DTWD1	0.737108117	0.463035563	-1.808027693	0.075824162	-1.532391513	0.131032541
DTWD2	0.336376146	0.737395469	-1.426206963	0.159206569	-0.955006136	0.343661866
DTX1	0.138671979	0.89002915	0.765458598	0.447123922	0.824294808	0.41325455
DTX2	-1.396933879	0.165978882	0.89260991	0.375780375	1.0269954	0.308819823
DTX2P1-UPK3B	-0.366035816	0.715225	-2.520299987	0.014519691	-2.031555546	0.046934029
DTX3	1.680260945	0.096486225	-2.490883609	0.01564768	-0.526525609	0.600593697
DTX3L	-1.174687394	0.243318081	1.184530725	0.241068037	-0.60823718	0.545478993
DTX4	-2.256556063	0.026536233	1.313700921	0.194155703	2.079642874	0.042128782
DTYMK	0.785943155	0.43403156	2.127060479	0.037709683	2.186480021	0.032957481
DUOX1	1.968926215	0.05213756	0.215912067	0.829819118	0.709736321	0.480800216
DUS1L	0.657216263	0.512773068	0.653345719	0.516133207	1.191639924	0.238407214
DUS2L	-0.819368676	0.414810236	3.520788258	0.000848699	4.185012242	0.00010114

DUS3L	1.170501037	0.244988752	-0.692817818	0.491208062	0.362941014	0.71800957
DUS4L	2.138198427	0.035298316	-1.439725285	0.155358432	-1.021264173	0.311502629
DUSP1	0.945892929	0.34681727	-2.389030463	0.020189757	-2.640164049	0.010705487
DUSP10	-0.011674316	0.990712126	-1.130968641	0.262755609	-1.871369541	0.066498962
DUSP11	-0.118349794	0.906062741	-1.139931714	0.259033095	-1.001363988	0.320940196
DUSP12	0.408068091	0.684224057	-0.616345	0.540095304	-0.073052893	0.94202324
DUSP13	-2.209170933	0.029784174	-0.004136901	0.996713536	0.856765977	0.395210484
DUSP14	0.988361305	0.325710669	-1.847644883	0.069791155	-0.942111867	0.350165027
DUSP16	0.959462134	0.33997914	0.62604583	0.533758083	0.768037338	0.445678118
DUSP18	-0.137772586	0.890737828	-1.993823149	0.050913819	-0.88248564	0.381270941
DUSP19	1.585176699	0.116546678	-0.148602119	0.882386661	0.37004523	0.712738788
DUSP2	3.107777651	0.002545064	-1.502478693	0.138436784	-1.0085992	0.317486966
DUSP22	0.365216344	0.715834382	0.21469943	0.83076003	-0.992302319	0.325300577
DUSP23	0.277632884	0.781951435	-0.575112533	0.567455137	-0.485731494	0.629044752
DUSP28	1.290552424	0.200270797	-3.136937519	0.002689135	-2.338797527	0.022924858
DUSP3	0.622360444	0.535329145	1.898018007	0.062708869	0.695801769	0.489418578
DUSP4	-0.504508663	0.615176645	0.423396372	0.673582973	0.980476085	0.331050496
DUSP5	-1.202644102	0.232369881	-1.280022305	0.205668885	-1.758429349	0.084115207
DUSP6	-1.294164978	0.199025877	-0.649306312	0.518721403	-1.45269691	0.151865117
DUSP7	0.670429465	0.504356033	-0.57026614	0.570715185	0.008071791	0.993588278
DUSP8	0.285239559	0.776137102	-2.601986487	0.011762786	-1.286731755	0.203459406
DUT	1.663354999	0.099832044	-0.853235705	0.397063092	-0.917546733	0.362774628
DVL1	1.449260561	0.150852154	-1.720667937	0.090679713	-1.735904931	0.088057108
DVL2	-0.221184448	0.825465994	-2.222967306	0.030157342	-1.49637487	0.140147481
DVL3	-0.508615708	0.612306217	-2.30263078	0.024932564	-2.085250716	0.041597235
DYM	-0.467089065	0.64160134	-0.21796684	0.828225347	0.691538385	0.492072399
DYNC1H1	0.22756443	0.820517656	1.885709484	0.064380601	1.700162342	0.09462528
DYNC1I2	1.773862599	0.079577165	3.343332393	0.001459643	3.831269807	0.00032352
DYNC1LI1	-3.709074035	0.000365285	0.11630198	0.907817785	0.106271801	0.91574489
DYNC1LI2	1.303540371	0.195821883	0.263978226	0.79273893	-0.068062644	0.945977189
DYNC2H1	-1.260293461	0.210926107	0.218783466	0.827592137	1.067860189	0.290146223
DYNC2LI1	0.165305268	0.869086392	0.314079737	0.754595278	1.670378386	0.100401747
DYNLL1	-1.610193198	0.110970428	-1.877895053	0.065461421	-1.368009296	0.176750746
DYNLL2	-0.892909023	0.374363793	-0.817678942	0.416910326	-0.262652497	0.79377826
DYNLRB1	-2.512035815	0.013845103	-1.423378716	0.160020945	-1.009114893	0.317241793
DYNLT1	-2.437368542	0.016825931	-1.936365411	0.05773486	-2.321343869	0.023916728
DYNLT3	0.597925398	0.551439674	-1.067982264	0.28998438	-1.445975284	0.153734508
DYRK1A	-1.012148092	0.314267865	-0.619698442	0.537900252	-2.414636294	0.019028224
DYRK1B	-1.305167876	0.19526963	-2.289001897	0.025765015	-2.230501894	0.029721147
DYRK2	1.248264551	0.215275869	-1.976225238	0.052925366	-2.132493137	0.037346159
DYRK3	-0.639689409	0.524051842	1.57693631	0.120287319	1.342708671	0.18476104
DYRK4	0.635259137	0.526923134	-1.691943198	0.096060816	-0.533245895	0.595964597
DYSF	-1.707668463	0.091256432	0.439593109	0.661876852	0.047272732	0.96246334
DZIP1L	0.196776105	0.844460715	0.63235432	0.529657744	-0.322655749	0.748152525
DZIP3	1.845717406	0.068327187	-0.458696547	0.648178342	0.602520796	0.549249478
E2F1	-1.728088449	0.087512584	0.977254189	0.332530219	-0.391859892	0.696642784
E2F2	-2.767250862	0.006902375	1.951230326	0.055899329	1.976173938	0.053048066
E2F3	-0.824799186	0.411736465	0.987928447	0.327319853	-0.971207277	0.335603901
E2F4	-1.094435794	0.276778172	-0.128309842	0.898350751	0.134598914	0.893409578
E2F5	0.074637181	0.940674369	-1.05020898	0.298008665	-0.17899367	0.858585988
E2F6	1.064382433	0.290094378	-0.592379965	0.555914721	0.244905105	0.80742136
E2F8	-1.468387963	0.145597481	0.075394438	0.940162148	0.078746204	0.937514027
E4F1	0.7526828	0.453667969	-1.052915062	0.296777192	-0.324530108	0.746740829
EAF1	-1.061805403	0.291256312	0.732209612	0.46700978	-0.374765055	0.709244795
EAF2	0.141685448	0.88765534	-1.097470198	0.277002951	-1.386883387	0.170950215
EAPP	-1.894731124	0.061442391	-0.834524488	0.407433538	-0.141035823	0.88834563
EARS2	0.522285591	0.602796	0.892249804	0.375971688	2.149347085	0.035924177
EBAG9	1.723509449	0.088340957	-0.284445194	0.777089406	-0.309306953	0.758231162
EBF1	-0.823677127	0.412370442	-0.336138386	0.737988041	0.861718018	0.392502254
EBF4	1.177988119	0.242006598	0.272268836	0.786389006	-0.037194445	0.970461686
EBI3	-3.111242047	0.002518374	-0.838125912	0.405424716	-0.75052726	0.456066083
EBLN2	0.596885645	0.552130546	-0.084383038	0.933044512	-1.573825483	0.12113742
EBNA1BP2	0.925407561	0.357308266	2.469655154	0.016510211	3.423245509	0.001160747
EBP	1.018782511	0.311124877	1.238918386	0.22040091	2.299514343	0.02521095
EBPL	-0.517756495	0.605939425	1.83393373	0.071831718	0.265700676	0.791441386
ECD	0.724307463	0.470816129	1.432517141	0.157401195	0.985919083	0.328395774
ECE1	-2.979701542	0.003737533	0.32845967	0.743755331	0.454285364	0.651372036
ECE2	-0.032656027	0.974023532	0.868172727	0.38890284	-0.394491863	0.694710049
ECH1	-1.38865244	0.168475793	2.187343015	0.032791073	2.030649906	0.047028896
ECHDC1	0.930777243	0.354538867	-0.583752669	0.561665933	-0.611504648	0.543329733
ECHDC2	0.662603659	0.509332237	0.49830796	0.620164568	2.11801074	0.038607102
ECHDC3	-1.56948196	0.120157908	0.553620699	0.581981351	0.12153001	0.903704462

ECHS1	-0.740704762	0.460862623	2.922963319	0.00494781	3.545394475	0.000798008
ECI1	-0.836864371	0.404956723	0.870683241	0.387541673	0.283228172	0.778041947
ECI2	0.882199015	0.380094933	1.761499792	0.083461021	2.760692847	0.007774156
ECM1	0.662395769	0.509464784	-0.419339627	0.676527861	-1.292315525	0.201532958
ECRP	-0.099810352	0.920724028	-1.28392288	0.204309947	-0.971449019	0.335484619
ECSIT	-1.481849091	0.14198571	1.536390427	0.129918324	0.897183224	0.373446027
ECT2	-1.116060492	0.267462688	-0.879507721	0.382780851	-2.109225784	0.039389954
ECT2L	-0.789292022	0.432082503	-1.62175631	0.11031756	-2.01088315	0.049141114
EDA	3.753959774	0.000313303	4.3016439	6.64E-05	3.323118686	0.001569811
EDAR	3.124774691	0.002416592	0.374488929	0.709415861	0.487208329	0.628004475
EDARADD	0.412196724	0.681206944	0.219214879	0.827257666	-0.173019452	0.86325737
EDC3	0.822513518	0.413028518	2.248209361	0.028405846	2.534940434	0.014055199
EDC4	0.063231994	0.949726508	1.018215603	0.312833581	2.020026642	0.048154112
EDEM1	0.553161308	0.581568578	0.266001066	0.791188282	0.295580701	0.768638955
EDEM2	-2.470147292	0.015453109	1.86883634	0.066733514	1.060281165	0.293549307
EDEM3	0.85857765	0.392927809	-0.167988648	0.867180695	-1.575329387	0.120789868
EDF1	-1.292209855	0.199698914	-1.148672956	0.255438975	-0.618446691	0.538777843
EDN1	0.573442206	0.567821498	-0.82159407	0.414695974	-0.028365028	0.977471439
EEA1	1.090139325	0.278655479	-0.932030399	0.355211362	-1.806743169	0.076153374
EED	1.313147134	0.192578873	-0.244032386	0.808072924	-0.34733494	0.729636264
EEF1A1	0.387744497	0.699150125	-0.081607344	0.935241891	0.352741727	0.725600755
EEF1A2	-1.023508397	0.308898944	2.660751011	0.010083941	2.702523056	0.009081938
EEF1B2	0.526077076	0.60017027	-0.079010603	0.93729806	1.008707956	0.31743525
EEF1D	0.326349436	0.744942051	1.65636597	0.10308629	1.974576358	0.053234136
EEF1E1	1.245390147	0.216324945	-1.215789516	0.229024792	-0.463397182	0.644868191
EEF1G	0.448509392	0.654898274	0.954309683	0.343915057	1.430787732	0.158024519
EEF2	0.389566107	0.697807354	0.803101818	0.425217685	0.879062748	0.383108004
EEF2K	1.182478766	0.240230457	1.069434322	0.289335461	1.222183489	0.226736236
EEFSEC	0.015214449	0.987895855	0.650031988	0.518255929	1.66725876	0.101023143
EEPD1	-3.432208453	0.000917598	0.959372509	0.341381218	0.959695613	0.341316514
EFCAB11	1.562321063	0.121834916	-0.019353885	0.984625688	0.420404864	0.675791817
EFCAB2	-1.655566875	0.101404738	-1.930497703	0.058473434	-3.430753887	0.001134545
EFCAB4A	-0.623862251	0.534346899	-0.502412689	0.617292794	-0.177049612	0.860105544
EFCAB4B	0.890214557	0.375800513	-0.428048326	0.670212319	-1.056931405	0.295062123
EFCAB5	0.700157767	0.485692858	0.443002938	0.65942306	0.291984053	0.771373261
EFCAB7	1.398574363	0.165487641	-0.295897083	0.768372899	-0.869668537	0.388178417
EFEMP2	-0.00495066	0.99606127	-2.036889573	0.046266282	-1.369750974	0.176209251
EFHA1	1.438108394	0.153983044	0.801990502	0.425855059	0.810962842	0.420805926
EFHA2	0.73870612	0.462069401	-0.307421522	0.759631409	-0.796379122	0.429160869
EFHC1	3.323343666	0.001301649	-1.357202106	0.180011783	-0.919993284	0.361505843
EFHC2	1.368207706	0.174762721	-1.922742057	0.059461989	-2.111647558	0.039172773
EFHD1	-2.462943072	0.01574593	0.708537786	0.481469273	1.224224041	0.225971695
EFHD2	-1.38660994	0.169096008	0.177808337	0.859497378	-0.199797224	0.842359886
EFNA1	1.851580516	0.067471039	0.718730945	0.475212505	2.079810414	0.042112817
EFNA3	-0.352963402	0.724967781	-1.662758416	0.101793929	-0.523769275	0.602497142
EFNA4	-0.7835955	0.435400984	1.103040881	0.27459692	0.585032366	0.560866076
EFNA5	-0.591218679	0.55590359	-1.689336143	0.096561927	-0.964747195	0.338801863
EFNB1	0.619483032	0.537213676	0.016798422	0.986655484	-0.193397762	0.847344288
EFR3A	0.73010807	0.467281307	-0.25316865	0.801039344	-2.022791106	0.047859087
EFR3B	1.609686124	0.111081291	1.195337826	0.236853578	0.597538187	0.552546705
EFTUD1	0.314040168	0.754240693	2.811376219	0.006731644	3.098652216	0.003033301
EFTUD2	-0.233319187	0.816060465	2.211703768	0.030969161	2.355555086	0.022007238
EGF	-3.708017345	0.000366602	0.606968336	0.54625727	-0.632341583	0.529726325
EGFL7	-4.128983799	8.32E-05	0.810716995	0.420865556	-0.078480811	0.937724179
EGLN1	-1.031446874	0.305184012	-0.994682269	0.324051391	-2.239691325	0.029082094
EGLN2	0.749186188	0.455761535	-5.24217462	2.36E-06	-5.091037296	4.28E-06
EGLN3	-0.960641217	0.339389127	2.863005685	0.005843053	1.689139915	0.096730336
EGR1	-1.815731406	0.072849067	0.933729446	0.354341517	0.162734838	0.871310572
EGR2	0.662042367	0.509690149	0.032063504	0.974532262	-1.097640036	0.277039317
EGR3	-0.768473393	0.444282517	1.014705353	0.314489985	0.405403114	0.686719431
EHBP1	1.204473473	0.231666074	0.553042776	0.582374417	0.374924658	0.709126753
EHBP1L1	-0.378273663	0.706146753	-0.152684635	0.879180611	0.08860066	0.929714053
EHD1	-2.235348954	0.027949264	0.29027213	0.772650606	0.222365795	0.824834749
EHD3	-3.417874709	0.000961223	0.80553144	0.423826217	0.102588484	0.918654524
EHD4	-1.934643762	0.056276457	1.737948988	0.08756398	1.069592504	0.289372233
EHHADH	1.555799822	0.12337827	1.555992253	0.125188126	-0.901520146	0.371156713
EHMT1	1.126649479	0.26298203	-0.719729506	0.47460204	-0.636583741	0.526978762
EHMT2	0.296343804	0.767672272	1.270272342	0.209095178	1.626269836	0.109483544
EI24	2.487439241	0.014770065	1.689667105	0.096498193	2.246833352	0.028593839
EID1	0.573752339	0.567612513	-0.366160787	0.71558666	-0.433778614	0.666108776
EID2	0.081853353	0.934950942	-1.135467312	0.260882523	-1.865886001	0.067275872
EID2B	1.232255257	0.221166458	-1.107697102	0.272597122	-1.131377752	0.262697723

EID3	2.54605759	0.012651466	-0.601635472	0.549777695	-0.161557374	0.872233457
EIF1	-2.870793498	0.005138083	-0.122283276	0.903100346	0.137579958	0.891063808
EIF1AD	0.054284031	0.956833064	0.920112351	0.361351754	1.241333835	0.219635346
EIF1AX	0.924903474	0.357568957	-0.239665386	0.811440542	-0.354086291	0.724598411
EIF1B	-3.504898065	0.000723652	-0.858072582	0.394409122	-0.951984927	0.34517845
EIF2A	1.162931862	0.248030174	0.085251043	0.932357462	0.498438857	0.62011867
EIF2AK1	-2.475951358	0.015220754	0.224400599	0.823239763	1.008110927	0.317719219
EIF2AK2	-1.814215573	0.073084119	-0.33276308	0.740521307	-2.823233208	0.00656304
EIF2AK3	1.313172339	0.192570418	0.100454633	0.920332256	1.105001874	0.27386403
EIF2AK4	1.113269493	0.26865252	1.634050407	0.107703119	1.480221846	0.144394881
EIF2B1	1.451000723	0.150368099	2.115867072	0.038690743	3.231008391	0.002063376
EIF2B2	-0.339532194	0.735025355	-0.997982399	0.322462279	-0.312869314	0.755537288
EIF2B3	0.604732039	0.546927653	3.476302974	0.000973647	5.089636299	4.31E-06
EIF2B4	0.87593172	0.383473994	1.656098052	0.103140746	2.364657662	0.021522668
EIF2B5	1.959504317	0.053248415	2.672520997	0.009775212	3.905769444	0.000254262
EIF2C1	-1.310111936	0.193599114	0.526108436	0.600832896	0.917222536	0.362942972
EIF2C2	-3.156303887	0.002194127	-0.489065294	0.626652725	-1.42929868	0.158450094
EIF2C3	-1.028999053	0.306326278	-3.17986851	0.002372461	-3.676981028	0.000528958
EIF2C4	-0.874764088	0.384105595	-0.876012285	0.38466223	-1.672995033	0.099882954
EIF2D	2.071932576	0.041226018	1.022079085	0.311017323	1.311705574	0.194949277
EIF2S1	1.977090357	0.051191	1.140771359	0.258686306	1.737071232	0.087849308
EIF2S2	0.706667564	0.481657384	2.639203547	0.010672317	3.12266569	0.002830402
EIF2S3	0.09119505	0.9275468	1.748608702	0.085686632	2.652618567	0.010361456
EIF3A	1.126779111	0.262927506	2.66874149	0.009873394	1.614393492	0.112039665
EIF3B	-0.317536031	0.7515961	2.967956556	0.004361508	2.901969005	0.005286113
EIF3D	-0.494753222	0.62201876	2.269466328	0.027001251	3.01806212	0.00381816
EIF3E	0.787542625	0.433100016	-0.262474022	0.793892553	0.129030439	0.897793933
EIF3F	0.769912487	0.443432803	1.267170979	0.210193893	1.524566972	0.132971532
EIF3G	-0.235009303	0.814752573	-0.72594693	0.470811007	-0.280631836	0.780022635
EIF3H	0.167514544	0.867353133	1.267657439	0.210021273	2.139761084	0.036727031
EIF3I	0.501741463	0.617114039	1.227844037	0.224499835	2.022618641	0.047877447
EIF3J	0.108867432	0.913557802	-0.369423448	0.713166868	0.043877317	0.965157623
EIF3K	-0.139411441	0.889446557	2.551660488	0.013398633	3.079936173	0.003200802
EIF3L	-0.38404408	0.701880775	1.120080968	0.267328224	2.129143478	0.037634561
EIF3M	1.13013182	0.261520085	3.002603295	0.003954796	3.441463135	0.001098143
EIF4A2	1.490560731	0.139685843	0.214470382	0.830937782	-0.261194233	0.794896906
EIF4A3	-0.779392775	0.437858822	3.788958791	0.000363855	3.529590553	0.000837972
EIF4B	1.148496834	0.253904707	2.236265601	0.029223073	3.087678999	0.003130483
EIF4E	1.578894384	0.11798164	-1.473734483	0.145997815	-1.290385753	0.202197195
EIF4E2	-2.748211874	0.007281589	-0.942990788	0.349624377	-0.828495454	0.410892432
EIF4E3	-0.350692157	0.726665206	-0.070980511	0.943659156	-0.230422605	0.818599568
EIF4EBP1	-1.587536867	0.116011202	-1.095918134	0.27767592	-1.288457782	0.202862447
EIF4EBP2	-2.738778519	0.007476442	-0.894668766	0.374687752	-0.898474667	0.372763382
EIF4EBP3	-0.986285961	0.326721941	-2.830917365	0.006381598	-2.76531404	0.007678071
EIF4ENIF1	4.83E-05	0.999961536	1.35731425	0.179976363	1.980842143	0.052507553
EIF4G1	-0.279039438	0.780875359	1.883755813	0.064649387	1.50935753	0.136805769
EIF4G2	-0.093254673	0.925915197	1.693491541	0.095764216	1.555460077	0.12544677
EIF4G3	-2.202661814	0.030256462	-0.039231437	0.96884154	-0.689873549	0.493110855
EIF4H	-1.615619025	0.109789728	-1.503207589	0.138249153	-0.900870546	0.371499045
EIF5	0.84489774	0.400480446	0.139718823	0.889369587	-0.304085636	0.762184991
EIF5A	-1.247742647	0.215466071	0.372104109	0.711180935	1.215982481	0.229071212
EIF5A2	0.489879836	0.625449314	-2.372096181	0.021050184	-2.68589895	0.009491132
EIF5AL1	0.25499844	0.799324831	1.77223768	0.081644024	2.825551387	0.00652169
EIF5B	0.697227195	0.487515601	2.564418992	0.012965458	2.776218263	0.007455665
EIF6	-1.102378922	0.273330621	1.089367837	0.280528686	1.44308479	0.154543903
ELAC1	0.856883822	0.393858175	-0.392418308	0.696197616	0.430169669	0.668716234
ELAC2	1.287390288	0.201365238	1.483469647	0.143401498	2.512858683	0.014868827
ELANE	-2.285013251	0.024738956	1.265704407	0.210714945	-0.366553326	0.715327771
ELAVL1	0.263941249	0.792448036	-0.960176139	0.340980148	-0.351886931	0.72623824
ELF1	-0.4164233	0.678123622	0.468402102	0.641264934	-1.749870792	0.085595382
ELF2	-0.505287593	0.614631786	-2.267277562	0.027142981	-2.628385982	0.011040393
ELF3	0.49022128	0.625208689	2.378783165	0.020706577	1.181706046	0.24229533
ELF4	-1.84312842	0.06870812	-1.151491084	0.254287867	-1.566912997	0.122745237
ELFN2	1.550861155	0.124557384	-0.442991252	0.659431463	0.35771412	0.721896359
ELK1	-0.483201791	0.630163655	-3.169033248	0.002448905	-3.320642013	0.00158148
ELK3	0.020936541	0.983344117	-1.070915095	0.288674745	-2.238548659	0.029160891
ELK4	1.880587867	0.063366353	-0.448570676	0.655424469	-1.118939432	0.267922673
ELL	-2.711962958	0.008056595	-1.44871816	0.15283891	-1.418030639	0.161699461
ELL2	0.736025942	0.463690503	-1.135241544	0.260976299	-1.722213084	0.090527155
ELL3	-0.102957501	0.918233138	0.137241936	0.891318199	1.027848918	0.308421634
ELMO1	-1.31928821	0.190526937	-0.411858213	0.681972106	-0.382993584	0.703168408
ELMO2	-0.76146423	0.44843457	1.17570836	0.244548474	1.136531878	0.260553942

ELMO3	-0.067729343	0.94615617	0.239103281	0.811874272	0.752805836	0.454706438
ELMOD2	0.365974435	0.715270638	-0.235344312	0.814776272	-0.502030935	0.617605778
ELMOD3	0.605767335	0.546243002	1.21723734	0.228477822	1.250250944	0.216385393
ELOF1	-2.279931481	0.025051816	-0.595167776	0.554062609	-0.825500281	0.412575842
ELOVL1	-1.867074912	0.065251631	-1.832872758	0.071991687	-2.279734878	0.02643699
ELOVL3	-0.923873044	0.358102228	-0.124448061	0.901393839	-0.457863737	0.648814575
ELOVL4	0.403320376	0.687699926	-1.411681309	0.163423504	-1.662574059	0.101962192
ELOVL5	-1.384185266	0.169834532	-1.677923329	0.098780938	-3.369041375	0.00136765
ELOVL6	0.674668684	0.501671367	-3.748609987	0.000414139	-1.987751533	0.051716223
ELOVL7	-5.210850997	1.25E-06	0.824443034	0.413089126	-0.361110446	0.719369958
ELP2	1.831109256	0.070499896	2.83639769	0.006286489	3.694363575	0.000500707
ELP3	-0.655610924	0.513800747	1.511885206	0.136030794	1.781430471	0.080242353
ELP4	0.184497774	0.854051431	-0.81444477	0.418744923	0.518175378	0.60636868
EMB	1.000848385	0.319669724	-2.321605379	0.023813413	-2.792353517	0.007137431
EMBP1	1.157911413	0.250062244	-1.570855339	0.121694096	-1.384150572	0.171780906
EMD	-1.600673658	0.113066573	-3.630004994	0.000603432	-2.581250104	0.012478476
EME1	0.659898125	0.511058673	-0.992962571	0.324881549	-1.265325617	0.210972303
EME2	1.913021589	0.059027442	-1.759349154	0.083828949	-1.845407046	0.070245476
EMG1	1.200756386	0.233097752	-0.06503874	0.948368372	-0.027102114	0.978474236
EMID1	-1.774699389	0.079437828	0.794266093	0.430301026	0.288796292	0.773799157
EMID2	2.702212643	0.008277509	1.212167	0.230397524	0.043735746	0.96526997
EMILIN1	0.507685839	0.612955578	1.329559305	0.188905509	0.181645622	0.856513982
EMILIN2	-1.193047995	0.236087044	2.722897109	0.008549133	1.403661868	0.165917636
EML2	1.083841878	0.281422991	-0.769148835	0.444947699	0.456203838	0.650000377
EML3	-0.060776214	0.95167653	0.241920175	0.809701307	1.321595483	0.19165427
EML4	2.184665962	0.031596423	0.268092842	0.78958568	-0.32283832	0.748014981
EML5	1.814708048	0.073007684	-0.063554317	0.949545163	-0.627292611	0.533006164
EML6	0.869605979	0.38690348	-0.67220406	0.504141507	0.357971667	0.72170467
EMP1	0.870258908	0.386548618	0.278343124	0.781745812	0.080023179	0.936502915
EMP3	-2.081701324	0.040301552	-2.823162662	0.006518449	-2.231918449	0.029621835
EMR1	-0.17212008	0.863742015	1.897565592	0.062769657	1.715802162	0.091703227
EMR2	-1.190231721	0.237186039	-0.90454491	0.369474606	-0.590208708	0.557415022
EMR3	-2.80116407	0.006271306	-0.529312808	0.598622728	-0.275018235	0.784310127
EMR4P	2.281642459	0.024946093	-0.766843997	0.446306191	-1.927354263	0.058992363
ENAH	-0.705760247	0.482218722	-0.056460773	0.955170156	-0.044726976	0.964483374
ENAM	1.500518433	0.137092851	0.183846618	0.854779506	2.145054055	0.03628181
ENC1	-1.038630091	0.301848606	1.721583758	0.090512326	0.971848671	0.335287482
ENDOD1	-4.346862293	3.73E-05	2.745494874	0.00804638	0.967077076	0.337646184
ENDOG	-0.384571243	0.701491524	-1.035553153	0.304738995	-1.549423881	0.12688963
ENDOV	2.948209125	0.004101139	-0.786913593	0.43455851	-0.601226091	0.550105287
ENG	0.235037994	0.814730376	0.811446371	0.420450125	0.857172945	0.39498748
ENGASE	2.281962746	0.024926345	0.576996746	0.566190149	2.03534716	0.046538648
ENHO	0.464711439	0.643296546	0.424253749	0.672961237	-0.132314344	0.895207944
ENKUR	-4.238887575	5.56E-05	0.760032036	0.450335357	-0.440767825	0.66107087
ENO1	-1.453955679	0.149548889	1.628064329	0.108969751	1.769067904	0.082305022
ENO2	2.937334568	0.004234097	0.776412477	0.440682309	2.391539906	0.02014681
ENO3	0.181650339	0.856278781	0.569182938	0.571445078	1.749818539	0.085604485
ENO4	1.484534515	0.141273626	1.099658413	0.276056088	1.704439284	0.093818688
ENOPH1	0.855624414	0.394550808	-0.893693944	0.375204833	-0.365511825	0.716100618
ENOSF1	2.798287388	0.006322712	0.052438347	0.95836087	0.469332892	0.640646311
ENOX2	-0.148798701	0.882056096	0.377322502	0.707320731	1.029405711	0.307696248
ENPP2	-0.051322781	0.959185715	2.115245334	0.038745883	0.840634094	0.404113018
ENPP3	-2.259795335	0.026326019	0.654652046	0.515297668	-1.706265522	0.093476006
ENPP4	1.650900298	0.102356659	-1.222708237	0.226419603	-1.95787996	0.05521249
ENPP5	0.908921587	0.365897156	-1.524771643	0.132788634	-1.523000144	0.133362534
ENSA	-0.465577046	0.642679164	-1.110302463	0.271482619	-0.24119753	0.810279281
ENTPD1	0.194129161	0.846526337	-1.22611018	0.225146614	-1.551297973	0.126440244
ENTPD2	-2.263391828	0.02609434	-0.788607607	0.43357538	-1.486639819	0.142695313
ENTPD3-AS1	0.847577924	0.398993775	-0.603742092	0.548385665	0.70954493	0.480918012
ENTPD4	1.508294158	0.135094432	0.160177522	0.873301679	-0.34013647	0.735020985
ENTPD5	-0.947879281	0.345810731	0.242012211	0.809630335	0.256257755	0.798686922
ENTPD6	1.070101825	0.287526963	2.574923636	0.01261839	3.213259636	0.002173926
ENTPD7	0.027481088	0.978138827	-0.638960541	0.525381569	-1.194745574	0.237200992
ENY2	-0.731659634	0.466338346	-1.331298593	0.188336287	-0.837949713	0.405606293
EOMES	-0.34970537	0.727403112	-2.108155418	0.039379522	-0.995453607	0.323779743
EP300	-0.866044525	0.388842663	-0.5320774	0.596718938	-1.082075691	0.28383704
EP400	1.852507622	0.067336486	0.432575008	0.666938961	0.499223211	0.619569572
EP400NL	0.060056056	0.952248432	-1.644416432	0.10553802	-1.66774089	0.100926903
EPAS1	-3.104411599	0.002571248	0.687051757	0.494807273	-1.997844327	0.050578725
EPB41	-2.847816414	0.005489355	1.228829088	0.22413299	1.042443357	0.301666897
EPB41L2	0.793602374	0.429581463	1.497311222	0.139772764	1.442393912	0.154737853
EPB41L3	-0.643665036	0.521482172	2.013580251	0.048734095	0.764147287	0.447973884

EPB41L4A	-0.080183804	0.936274834	-0.248628227	0.804532773	-1.500189708	0.139158925
EPB41L5	0.089147194	0.929169389	0.183314454	0.855195089	-0.312737478	0.755636929
EPB42	-3.281591504	0.001485552	0.92399544	0.359343655	-0.108920656	0.913653143
EPB49	-3.137738494	0.002322693	0.818582473	0.416398665	-0.144220735	0.885841761
EPC1	0.359908227	0.719786091	-1.839837688	0.070946992	-2.364693226	0.021520794
EPC2	-0.088670868	0.929546842	-0.969206298	0.336494744	-2.697933625	0.009193258
EPDR1	1.174748438	0.24329378	-1.432299425	0.157463218	-1.223905163	0.226091046
EPG5	0.039863383	0.968293183	-0.096215871	0.923683128	-0.6480255	0.51960557
EPHA1	1.308910783	0.194003979	1.084626902	0.282606146	1.879137076	0.06541145
EPHA2	1.640765984	0.104448842	-0.015687456	0.987537949	-0.847189737	0.400480452
EPHA4	1.009875488	0.315349356	0.856955475	0.395021093	0.828887517	0.410672387
EPHB1	-2.602235138	0.010882062	0.613291568	0.542097972	-1.369101792	0.176410935
EPHB2	-2.026755365	0.045742674	1.94927481	0.056137913	0.490034013	0.62601619
EPHB3	-0.439718086	0.661229317	1.595426675	0.11608987	0.451299829	0.653509027
EPHB4	-2.729876768	0.007664664	1.945493132	0.056601767	1.985123028	0.052016049
EPHB6	1.418924465	0.159486028	2.17767019	0.033540216	3.229341854	0.002073527
EPHX1	0.381026059	0.704110768	-0.255259053	0.799432342	0.396675006	0.693108446
EPHX2	4.298478266	4.46E-05	2.625036359	0.01107606	3.2151257	0.002162046
EPHX4	1.379223257	0.171353559	-2.066762653	0.043261982	-2.253738271	0.0281287
EPM2A	2.010118608	0.047509654	0.18365938	0.854925721	0.996071888	0.323481915
EPM2AIP1	1.357596492	0.178095213	-2.501377177	0.015236523	-2.629612576	0.011005075
EPN1	-0.679538027	0.49859717	-0.491623151	0.624854165	-0.798320488	0.428042989
EPN2	-0.449113369	0.654464239	-2.606828036	0.011615405	-2.765672991	0.007670654
EPOR	-2.674508945	0.008935609	-0.654331907	0.515502365	-0.802620451	0.425573182
EPPK1	1.203832947	0.231912326	-0.437089998	0.663680526	-0.034649267	0.972482109
EPRS	1.629860623	0.106738654	2.469843296	0.016502382	2.687312066	0.009455703
EPS15	0.841800195	0.402202836	1.063122634	0.292163437	0.176657254	0.860412291
EPS15L1	-1.958468195	0.053371794	-0.723857074	0.472083366	-1.313095011	0.194483801
EPS8	3.600696464	0.000526671	0.058733027	0.953368056	0.868517216	0.388802704
EPS8L1	1.016709974	0.312104447	-1.82906369	0.072568471	-2.835706871	0.006343363
EPS8L2	-0.32329563	0.747245487	-2.105607258	0.039609451	-1.37516813	0.174533151
EPSTI1	-1.417798751	0.159813585	1.352081391	0.181634769	-0.726922491	0.470288676
EPT1	0.902438135	0.369310531	0.316462225	0.752795802	-0.428581562	0.669864943
ERAL1	-0.846303839	0.399700075	1.987606026	0.05161684	3.126873108	0.002796188
ERAP1	1.272005347	0.206753454	-0.832724378	0.408439893	-1.358379751	0.179767615
ERAP2	0.992649495	0.323627687	-1.224151368	0.225878949	-1.68033956	0.098438502
ERBB2	0.876964361	0.382915954	-0.726184736	0.470666349	0.671358296	0.504740749
ERBB2IP	0.446255317	0.656519161	-0.731920491	0.46718488	-1.964472723	0.054423919
ERBB3	1.067110509	0.288867802	1.093666103	0.278654422	1.054147277	0.296323562
ERC1	-0.080234407	0.936234705	1.678953615	0.098578918	1.198031397	0.235929619
ERCC1	0.496248176	0.620968075	0.516128685	0.607740401	1.329862907	0.188932248
ERCC2	0.623372813	0.53466691	1.199061037	0.235414081	1.532599568	0.130981292
ERCC3	1.932550096	0.056537998	-0.291772841	0.771508634	1.031948616	0.306513878
ERCC4	1.248664202	0.215130303	-0.114132108	0.909529994	0.419674022	0.67632258
ERCC5	2.421017416	0.017550638	-1.24873551	0.21681361	-1.697349833	0.095158797
ERCC6	0.55582415	0.579754614	0.545761551	0.587337491	-0.015538116	0.987657871
ERCC6L	1.121387222	0.265202087	-0.080693782	0.935965223	-0.274738842	0.784523695
ERCC8	0.274779131	0.784135987	-1.065794693	0.290963893	-1.510048423	0.136629719
EREG	2.048879449	0.043480366	-0.216183241	0.829608742	-1.702111673	0.094256949
ERF	-1.684613133	0.095639824	-3.447123533	0.001064893	-3.710070378	0.000476426
ERG	0.899546884	0.370839175	1.312533053	0.194546648	1.1935994	0.237645644
ERGIC1	-3.876590186	0.000204801	-2.209468291	0.031132553	-2.762356538	0.007739438
ERGIC2	0.2743754	0.784445184	-1.91437502	0.060544394	-2.32745215	0.023565337
ERGIC3	-0.44171933	0.659785935	1.949862496	0.056066121	1.947050182	0.056529081
ERH	0.994030163	0.322958913	-3.11548893	0.002861806	-2.655957929	0.010270945
ERI1	-0.062735347	0.950120848	-1.058569012	0.294215501	-1.510442023	0.136529504
ERI2	0.56368128	0.574418025	-0.48069524	0.632554036	-1.442485746	0.154712062
ERI3	-0.046227297	0.963234803	1.459225701	0.149935567	2.880033049	0.005616536
ERICH1	-3.069151738	0.002861001	0.043637756	0.96534409	-0.651842904	0.517157808
ERLEC1	0.615742634	0.539668472	-0.956584969	0.342774803	-0.991863383	0.325512789
ERLIN1	0.066841573	0.946860864	0.311746405	0.756358955	-0.058568933	0.95350308
ERLIN2	0.186221904	0.852703337	-0.331861686	0.74119832	0.087841856	0.930314423
ERMAP	-2.239150076	0.027691243	1.463910674	0.148655055	1.030536451	0.307170108
ERMN	3.192518229	0.001962218	-1.425788134	0.159326965	-1.550866491	0.126543596
ERMP1	0.560162512	0.576805059	0.040625417	0.96773502	0.061467207	0.951205066
ERN1	-0.737915753	0.462547119	-0.940209237	0.351036806	-1.559474454	0.124494486
ERO1L	-0.211935142	0.832652283	-0.72906388	0.468916941	-0.824867844	0.412931833
ERO1LB	0.859662476	0.39233266	-1.101527866	0.275248952	-1.837903796	0.071360803
ERP27	2.525264943	0.013369438	-2.254418033	0.027989063	-0.637956625	0.526091174
ERP29	-0.507032128	0.613412273	-1.245628494	0.21794426	-0.539523296	0.591655711
ERP44	-1.989462565	0.049784467	-0.225525196	0.822369047	-0.791521184	0.4319658
ERRFI1	0.272848879	0.785614581	-0.046347941	0.963193257	-1.586436519	0.118247763

ERV3-1	-0.297268119	0.766968915	-2.230476933	0.029626568	-4.294635755	6.99E-05
ESAM	-4.612422827	1.36E-05	1.881488508	0.064962515	0.5974355	0.552614763
ESCO1	0.413505471	0.68025162	-0.179664787	0.858046327	-0.818478214	0.416538952
ESCO2	-0.411214845	0.681924011	-1.1063026	0.27319497	-1.929042768	0.058777661
ESD	0.406715079	0.685213927	1.178973925	0.243256007	2.345192891	0.022570705
ESF1	1.070852081	0.287191336	0.253737928	0.800601624	-0.432511409	0.66702386
ESM1	-1.777259363	0.079012811	-0.810399636	0.421046392	-1.458514994	0.150261408
ESPL1	0.314806981	0.753660352	1.1048831	0.27380448	0.822314835	0.414370791
ESPN	-1.850334406	0.067652245	-0.512971478	0.609933214	0.234993015	0.815067722
ESPNL	0.078912825	0.937282794	1.307534259	0.196226704	-0.414918197	0.679780452
ESPNP	0.96233411	0.338543172	1.669984879	0.100348925	2.210963401	0.031121236
ESR1	0.627837422	0.531751443	0.692632437	0.491323553	0.624948012	0.534532807
ESR2	0.357441357	0.721625193	0.913787419	0.364638051	0.064453217	0.948837922
ESRG	-2.848768405	0.005474374	1.564835694	0.123099658	1.93547715	0.057965596
ESRP2	0.23876367	0.811849151	0.415806625	0.679096696	0.47393637	0.637380198
ESRRA	-1.009815197	0.315378081	-1.607144622	0.113491509	-1.903870536	0.062048392
ESYT1	0.519868579	0.604472601	3.015062162	0.00381739	3.0844523	0.00315961
ESYT2	3.565083001	0.000593078	-0.793149188	0.430946167	0.294346886	0.769576616
ETAA1	0.792591151	0.430167445	-0.281149045	0.779603644	-0.13606018	0.892259593
ETF1	-2.710040153	0.008099731	2.396582712	0.019816285	1.896963121	0.062972493
ETFA	-0.219873661	0.826483526	0.435876117	0.664555937	1.318420587	0.192707431
ETFB	-0.597169116	0.551942149	3.428584995	0.00112702	3.434958851	0.001120117
ETFDH	0.547336834	0.58554567	3.158646273	0.002524352	2.238075032	0.029193607
ETHE1	-0.205842684	0.837393668	-0.41580012	0.679101429	0.029159134	0.976840909
ETNK1	0.96007155	0.339674105	-0.408349805	0.684531069	-0.987091575	0.327825773
ETNK2	0.417127411	0.677610498	0.794658715	0.430074377	0.10870593	0.913822685
ETS1	2.863793483	0.005242846	2.239645322	0.028989742	1.987940414	0.051694735
ETS2	-1.002372924	0.318937328	0.716411913	0.476631938	-1.053427799	0.296650147
ETV3	-0.772284568	0.442034271	0.220961959	0.825903502	-0.648131485	0.519537528
ETV5	-0.544486218	0.587496812	2.244505801	0.028657067	1.703189109	0.094053872
ETV6	-1.09731523	0.275524945	-0.072267321	0.942639541	-1.292634394	0.201423359
ETV7	-2.370077621	0.019990002	-0.644068059	0.522087964	-2.672072663	0.009844244
EVC	0.016116509	0.987178265	0.091733407	0.927228164	-0.744064705	0.459935093
EVC2	0.4480606	0.655220865	0.941897309	0.350179187	0.791060515	0.432232351
EVI2A	-0.316761566	0.752181722	-0.499801653	0.61911885	-0.682036586	0.498015384
EVI2B	-1.883480739	0.062968756	-1.914006322	0.060592474	-2.230990589	0.029686852
EVI5	-1.280957091	0.203605505	0.486851498	0.6282112	0.906059874	0.368769946
EVI5L	0.668587291	0.505525066	-1.198411381	0.235664798	-0.298321081	0.766557596
EVL	0.861262356	0.391455962	-0.95866885	0.341732649	0.113957738	0.909677167
EVPL	-0.748311701	0.45628599	1.676346437	0.099090795	1.526400695	0.132515085
EWSR1	-0.545557416	0.586763258	0.873744069	0.38588617	1.498091578	0.139701939
EXD2	2.228045797	0.028450926	-0.054493371	0.956730672	0.917467916	0.362815551
EXD3	2.268055818	0.025796566	0.128851537	0.897924016	-0.163804727	0.870472158
EXO1	-0.90088552	0.370130923	1.254932314	0.214571525	2.885923969	0.00552597
EXOC1	1.552448977	0.124177318	0.51706295	0.607092207	-0.57486753	0.567673701
EXOC2	2.347788216	0.021148938	0.840715621	0.403983974	1.24297297	0.219035259
EXOC3	-0.774059559	0.440989453	1.072267951	0.288072017	1.483121457	0.143625061
EXOC3L1	-1.804492449	0.074606885	-1.255855335	0.214239036	-2.258620306	0.027803882
EXOC3L2	-2.317459912	0.022821338	2.161763122	0.034804877	1.378626963	0.173469379
EXOC4	-1.073208419	0.286138979	1.568857249	0.122159207	1.382585272	0.172258104
EXOC5	1.002636827	0.318810662	-0.338333556	0.736342065	-1.219578467	0.22771502
EXOC6	-0.531739595	0.59625862	0.669512489	0.505843756	-0.790832575	0.432364277
EXOC6B	0.792242023	0.430369868	-0.792281297	0.431447872	-0.653422223	0.516146927
EXOC7	0.240787105	0.810285425	0.76305204	0.448546469	1.205580154	0.233027568
EXOC8	0.162821958	0.871035405	-1.065594335	0.29105372	-2.980529293	0.004245033
EXOG	1.531753057	0.12920388	0.391627944	0.696778349	1.868186036	0.066949073
EXOSC1	-0.086363914	0.931375159	-3.312757025	0.00160011	-1.596214431	0.116045702
EXOSC10	0.489512663	0.625708117	0.208598228	0.835497841	0.633452441	0.529006122
EXOSC2	1.349435387	0.180690804	0.434625467	0.665458356	2.563677702	0.013056802
EXOSC3	1.09722035	0.275566177	0.037856352	0.969933121	0.399817489	0.690805522
EXOSC4	-0.037073414	0.970511221	1.543863131	0.128098526	1.847032369	0.070005823
EXOSC5	0.405334344	0.686224646	1.500735027	0.138886453	1.857639067	0.068458735
EXOSC6	0.760972355	0.448726785	-0.638667479	0.525570882	-0.748669044	0.457176633
EXOSC7	0.437615548	0.662747138	0.124272701	0.901532059	1.201787298	0.234482432
EXOSC8	2.631843428	0.010042457	0.69875105	0.487519615	1.085187455	0.282468783
EXOSC9	1.308804064	0.194039981	1.084567186	0.282632382	2.102229855	0.040023243
EXPH5	1.743346695	0.084798163	1.647203892	0.104961902	1.763914035	0.08317788
EXT1	-1.703762766	0.091987228	0.438449027	0.662700997	-0.625328017	0.53428522
EXT2	-0.450941141	0.653151476	1.338601399	0.185960439	1.062023186	0.292764692
EXTL2	-0.142222108	0.887232702	-1.033446319	0.305714954	-0.52932553	0.598663013
EXTL3	-3.000794635	0.003510919	0.431595956	0.66764639	0.515222334	0.608417079
EYA3	-0.016400721	0.986952176	0.465781414	0.643128577	1.963578039	0.054530365

EYS	1.247448765	0.215573228	-2.333587425	0.023129937	-2.044214868	0.045625166
EZH1	1.186593462	0.238611234	1.114749958	0.269587508	2.256316278	0.027956761
EZH2	0.005597625	0.995546553	0.516479941	0.607496662	1.330897467	0.188593693
EZR	-0.287838249	0.774153638	2.327727818	0.023461964	1.94785367	0.056430487
F11R	-4.1089707	8.95E-05	1.353356557	0.181229573	2.185581905	0.033026614
F12	-0.623922591	0.534307453	-1.062227052	0.292566245	-0.80489295	0.424271365
F13A1	-3.165215172	0.002134801	2.07054695	0.04289374	0.520903177	0.604479342
F2R	-1.299522448	0.197190248	-1.912919266	0.060734419	-1.672724785	0.099936434
F2RL1	-3.374381387	0.00110586	-0.135981722	0.892309892	-2.337257299	0.023010888
F2RL2	0.322949071	0.747507037	0.229075018	0.819622084	0.763677988	0.448251312
F2RL3	-2.539473964	0.012874952	1.671479703	0.100052124	0.513318778	0.609739169
F5	-1.323207671	0.189225903	0.481064969	0.632292847	0.189474747	0.850402957
F8	0.389057666	0.698182047	-1.312951845	0.194406389	-1.34986774	0.182466951
F8A1	-1.906298442	0.059905639	-0.79690624	0.428778325	-1.290465903	0.202169574
FA2H	0.311424121	0.756221633	0.289329578	0.773368103	-0.923380803	0.359753791
FAAH	0.662759168	0.509233099	1.813058823	0.075034579	1.64146996	0.106281335
FAAH2	3.494061626	0.000749877	2.49382772	0.015531325	3.623882051	0.000625008
FABP5	3.110128275	0.002526926	-0.251826111	0.80207188	-0.55317435	0.582336537
FADD	-1.464937777	0.146534631	-2.423519969	0.018534091	-1.901773933	0.062327658
FADS1	2.16690897	0.032969054	-1.182890196	0.241712505	-0.62474644	0.534664162
FADS2	1.75385554	0.082969241	-0.947403773	0.347391137	-2.134367724	0.037185603
FADS3	0.541492095	0.589549468	-0.30258324	0.763297568	-0.579751416	0.564397783
FAF1	1.05185491	0.295772649	2.169857868	0.034156191	3.151774066	0.002601512
FAF2	-0.847740179	0.398903882	0.263624947	0.793009829	0.61963238	0.538002355
FAH	-1.716273312	0.089663126	0.435200033	0.66504371	1.733079619	0.088562182
FAHD1	0.175533029	0.861067844	0.579046254	0.564815766	0.645636472	0.521140558
FAHD2A	2.12252301	0.036629364	1.997380597	0.050515263	1.847739043	0.069901839
FAHD2B	1.105243431	0.272094699	0.529945885	0.59818652	0.224051684	0.823529083
FAIM	-0.326408955	0.74489718	-0.614641903	0.541211853	-0.00991161	0.992126887
FAIM3	1.788441863	0.077178251	-0.535811626	0.594151925	0.387380087	0.699937087
FAM100A	-1.691504536	0.094311981	-2.290656384	0.02566266	-2.585797233	0.012332668
FAM100B	-1.189092889	0.237631488	-3.786147326	0.00036716	-4.418075277	4.59E-05
FAM101B	-2.191896721	0.031051925	1.350622798	0.182099096	1.896205071	0.063074616
FAM102A	1.795340607	0.07606418	1.407464294	0.164663791	2.400919655	0.019685618
FAM102B	0.209490821	0.834553814	-1.32749681	0.189582194	-2.236196463	0.02932369
FAM103A1	-2.243825461	0.027376744	0.531327879	0.597234803	-0.203759721	0.839276818
FAM104A	-3.568951423	0.000585498	0.05166636	0.958973315	0.073653051	0.941547811
FAM104B	0.16145432	0.872109129	-1.771931003	0.081695457	0.000207	0.99983557
FAM105A	0.334095507	0.739109756	1.278566105	0.206177942	0.349184235	0.728255119
FAM105B	0.544766308	0.587304965	-0.091565867	0.927360695	-0.444642285	0.658284872
FAM106A	0.860515818	0.391864898	-0.605515609	0.547215132	0.340197977	0.734974918
FAM107B	-0.054122565	0.956961336	1.444994638	0.153878228	1.791071709	0.078663818
FAM108A1	0.729796505	0.46747079	-4.510240146	3.24E-05	-3.932088107	0.000233396
FAM108B1	-0.501856812	0.617033225	-1.176750687	0.244135399	-1.606355237	0.113796993
FAM108C1	-1.0042193	0.318051818	-0.364569208	0.716768141	-1.068964089	0.289652841
FAM109A	1.922442145	0.057815208	-0.136639185	0.891792497	-0.137519259	0.891111562
FAM109B	1.515389032	0.133291042	1.203044019	0.233881196	0.063771094	0.949378631
FAM110A	-0.981937511	0.328847573	-0.152550815	0.879285669	-0.823396999	0.413760477
FAM110B	-0.078035944	0.937978271	1.672472554	0.099855388	0.880965068	0.382086344
FAM110C	-0.772355993	0.4419922	-1.020929948	0.311556795	-0.989533165	0.326640924
FAM111A	0.50536528	0.614577455	-0.390531445	0.697584322	-0.627040502	0.533170212
FAM111B	-0.417443367	0.677380293	0.580748317	0.563675631	1.258321333	0.213474801
FAM113A	1.35170797	0.179965168	-2.219126203	0.030432056	-0.592412677	0.555948871
FAM113B	1.585950304	0.116370945	-0.259318536	0.796314101	0.854053165	0.396699004
FAM114A1	-0.639865173	0.523938097	-1.003439227	0.319846114	-0.858893999	0.394045268
FAM114A2	1.281254497	0.203501533	-0.147335644	0.883381644	0.948281792	0.347043314
FAM115A	1.353939107	0.179254911	0.500039729	0.618952249	0.574582174	0.567865396
FAM115C	0.423723069	0.672811296	1.122540026	0.266290588	1.998580177	0.05049664
FAM116A	0.120209184	0.904594016	-0.771599057	0.443506174	-1.087032458	0.281659698
FAM116B	-1.272198809	0.206685044	1.279398467	0.205886851	1.820642787	0.073983251
FAM117A	-3.303060645	0.001388143	-1.129206798	0.263491767	-0.591009806	0.556881882
FAM117B	1.787107869	0.077395229	0.447131232	0.656457271	0.163263162	0.870896534
FAM118A	1.930621641	0.056779811	0.131948621	0.89548479	0.619807114	0.537888121
FAM118B	0.974611914	0.332449087	-0.481714191	0.631834329	-0.383804723	0.702570464
FAM120A	-0.947966658	0.345766498	-0.918898851	0.36198078	-2.283370631	0.026207734
FAM120AOS	-3.627955433	0.000480671	-1.483303428	0.14344552	-1.452540964	0.151908286
FAM120B	-1.045089571	0.298870407	1.658608489	0.102631403	1.511227864	0.136329594
FAM120C	1.909710334	0.0594586	-0.674697159	0.502567549	0.230634077	0.818436067
FAM122A	0.077499756	0.938403559	-2.700184238	0.009083339	-2.463322383	0.016851062
FAM122B	1.42741702	0.157031515	-2.267288858	0.027142248	-2.581092977	0.012483543
FAM122C	0.088781754	0.929458971	-3.89321434	0.000259596	-3.69254149	0.000503599
FAM123B	0.731172099	0.466634529	-0.596195162	0.553380836	0.067759194	0.946217668

FAM124B	-1.133251241	0.260215359	0.083628057	0.933642142	-1.073578512	0.287596732
FAM125A	-1.01954004	0.310767352	1.696342735	0.095220011	1.442772883	0.154631441
FAM125B	0.528898466	0.598219786	1.041066844	0.302194914	0.515697553	0.608087226
FAM126A	0.782479725	0.436052721	-1.070205109	0.288991408	-1.867031928	0.067112886
FAM126B	-0.632348332	0.528814086	-1.405881066	0.165131315	-2.562083514	0.013110455
FAM127A	-0.782693257	0.43592795	-2.823883898	0.006505607	-3.252118467	0.001938785
FAM127B	0.75771671	0.450663673	-4.29690552	6.75E-05	-4.056137473	0.00015533
FAM127C	-1.084928535	0.280944092	-0.372415265	0.710950549	0.129924541	0.89708974
FAM129A	-0.439744223	0.661210458	-0.1511118	0.880415539	-0.802411326	0.425693101
FAM129B	-0.556504674	0.579291466	1.490367613	0.141583948	1.260145408	0.212820985
FAM129C	1.118900429	0.266255781	0.725765353	0.470921479	1.167200407	0.248054773
FAM131A	-0.721310191	0.472648499	-0.120644421	0.904392567	-0.388804598	0.698888916
FAM131B	1.310394493	0.193503967	-2.446896881	0.017482282	-1.466314784	0.148132298
FAM132A	-0.759440722	0.449637403	-1.041512775	0.301989792	0.029803429	0.976329343
FAM133B	1.966041561	0.052475551	-0.057980183	0.953965101	-0.375124455	0.708978994
FAM134A	-2.847346748	0.00549676	0.334978596	0.73885817	0.974063358	0.334196426
FAM134B	1.845095668	0.068418506	1.212200519	0.230384795	1.341759751	0.185066755
FAM134C	-1.12413106	0.264042875	1.630439098	0.108465819	2.665529839	0.010015497
FAM135A	1.17013926	0.245133511	-1.41546866	0.162315759	-2.010181363	0.049217584
FAM136A	1.830994981	0.070517117	-0.722680922	0.472800292	0.966297257	0.338032703
FAM13A	-1.370258836	0.174124052	2.606399809	0.011628373	1.473861538	0.146094855
FAM13AOS	0.237578934	0.812765079	-2.11848379	0.038459426	-1.16455418	0.249115978
FAM13B	-0.055031093	0.956239599	-0.937767252	0.352279864	-2.539068504	0.013907648
FAM149B1	0.08324758	0.933845511	-2.360120295	0.021678317	-1.880979413	0.06515573
FAM151B	-0.243638312	0.808083298	2.063968015	0.04353568	0.781316594	0.437893234
FAM153A	2.48571868	0.014836794	1.140325486	0.258870419	1.50181427	0.138739623
FAM153B	3.733917794	0.000335578	0.426054479	0.671656166	0.359884882	0.720281249
FAM154B	1.209329275	0.229805392	0.728723216	0.469123741	1.614365377	0.112045773
FAM157A	0.604572477	0.547033211	-0.305321171	0.761222254	0.135488576	0.892709405
FAM157B	-1.155373723	0.251093885	-3.755388673	0.000405249	-3.056192473	0.003425757
FAM158A	-2.138224535	0.035296135	0.409589754	0.683626249	-1.015369841	0.314278201
FAM159A	2.496213306	0.014433941	0.625051498	0.534405868	0.937554972	0.352482324
FAM160A1	1.143545712	0.255942148	0.244586905	0.807645565	-1.077516927	0.285849858
FAM160A2	-0.567469388	0.571853607	1.10625252	0.273216458	0.789624071	0.433064131
FAM160B1	-0.841860652	0.402169176	-1.074061524	0.287274283	-2.099760745	0.040248861
FAM160B2	0.965433151	0.336998123	0.486580868	0.628401836	1.898989217	0.062700231
FAM161A	1.493810815	0.138835331	-1.139194424	0.259337881	-0.912435397	0.365434603
FAM161B	-2.425097253	0.017367253	-0.357334694	0.722147291	-1.590708233	0.117281635
FAM162A	1.181701693	0.240537135	0.302091073	0.763670809	0.784231443	0.436195232
FAM164A	1.661423441	0.100220236	-1.206232127	0.232659466	-1.810177389	0.075612295
FAM165B	1.621218507	0.10858185	-0.435283241	0.664983671	-0.138994265	0.889951237
FAM167A	-1.6206087	0.108712872	0.402461736	0.688834062	0.644186567	0.522073313
FAM167B	-0.428437908	0.669388912	-1.730711609	0.088857909	-0.391336188	0.697027597
FAM168A	0.217319204	0.82846734	-1.204525089	0.233313049	-0.941448462	0.350501767
FAM168B	1.046547257	0.298201097	0.673977787	0.503021433	1.174825966	0.245014905
FAM169A	0.858747949	0.392834344	-1.372501621	0.175228546	-1.609315435	0.113147257
FAM171A1	2.29480785	0.024145648	1.743194975	0.086635864	2.178667734	0.033563115
FAM172A	0.059648918	0.952571764	-1.647730282	0.104853395	-2.659163374	0.010184745
FAM173A	-0.07890456	0.937289349	-2.74481418	0.008061118	-2.600678873	0.011866242
FAM173B	2.006011533	0.047954743	-1.882447042	0.06482998	-1.727008039	0.089655697
FAM174A	-1.463083258	0.147040297	-0.61943384	0.538073284	-1.267598323	0.210165008
FAM174B	0.240421505	0.810567906	3.037060159	0.003585591	1.594612849	0.116404108
FAM175A	0.911269904	0.364665782	-0.336947011	0.737381576	-0.659341701	0.512367406
FAM175B	0.706700441	0.48163705	2.056544512	0.044270012	2.356615763	0.021950276
FAM176B	-1.376917928	0.172062804	-1.898497121	0.062644548	-1.358045002	0.179873192
FAM177A1	-2.254736728	0.026654945	-1.864608005	0.067334402	-2.65533738	0.01028771
FAM177B	1.684594363	0.095643461	-0.928733417	0.356903228	0.479151557	0.633688805
FAM178A	2.196665052	0.030697354	-0.695459015	0.489564241	-0.03475618	0.972397236
FAM178B	0.623466769	0.534605471	-2.019191126	0.048129917	-0.174605973	0.862016346
FAM179A	-0.548229884	0.584935041	-2.405968475	0.019360776	-1.245343093	0.218169696
FAM179B	0.847909816	0.398809913	-1.548897511	0.126884031	-2.750848694	0.007982535
FAM182A	0.132232288	0.895105246	-0.892973638	0.3755872	0.224882916	0.822885504
FAM184A	1.573461718	0.119233868	0.900073297	0.3718292	0.335208591	0.738714966
FAM184B	2.986607318	0.003661893	0.438803482	0.662445619	1.50073467	0.139018158
FAM185A	0.249109778	0.803861761	-1.374642034	0.174567199	-1.701106696	0.094446694
FAM186B	0.247431882	0.805155738	-0.488046135	0.627369984	0.144922259	0.885290404
FAM188A	0.210466286	0.833794843	-0.782937082	0.436871486	-0.73332015	0.466409223
FAM189B	-0.204059827	0.838782302	-0.124602928	0.901271774	0.347251722	0.729698437
FAM18A	1.421125475	0.158847077	0.555414379	0.580762219	1.110813647	0.271375417
FAM18B1	-0.618730478	0.537707113	-1.801383304	0.076877578	-2.323034849	0.023818987
FAM18B2	2.051533708	0.043215523	-0.270913107	0.787426398	0.571855262	0.569698862
FAM190A	-0.40805032	0.684237054	0.837697836	0.405663172	-0.948988733	0.346686798

FAM190B	1.174829519	0.243261506	0.145561527	0.884775763	0.322934857	0.747942256
FAM192A	-0.894751635	0.373383283	1.680995166	0.098179606	3.042075636	0.003566422
FAM193A	-2.12562242	0.036362785	-0.171496772	0.86443428	-0.030129006	0.97607084
FAM193B	0.548502644	0.584748599	-1.516099433	0.134963684	-0.144796536	0.88538921
FAM195A	0.598245186	0.551227275	0.501068498	0.618232566	0.515095565	0.608505084
FAM195B	-0.327084943	0.744387616	1.137690005	0.25996058	1.506186876	0.137616007
FAM196B	-1.42134457	0.158783582	2.468391232	0.016562892	2.264134584	0.027440996
FAM198B	2.677476338	0.008862912	-0.562871804	0.575706741	-0.841536462	0.403611806
FAM199X	0.763251617	0.447373643	-1.05016881	0.298026972	-1.468372553	0.147574551
FAM19A1	-0.155666418	0.876655807	-0.403559947	0.688030701	-0.702079999	0.485525027
FAM19A2	-0.885927786	0.378093397	0.31498033	0.753914905	-0.772975435	0.442773772
FAM200A	1.047255074	0.297876464	-0.965530432	0.338315876	-1.084011302	0.282985402
FAM200B	-0.768096239	0.444505363	-0.78317805	0.436731119	-1.258875828	0.213275892
FAM201A	0.967921842	0.335760712	-1.347309445	0.183157222	-1.335569933	0.187070373
FAM202A	-1.084279103	0.281230234	-0.6285144	0.532151625	0.30983127	0.757834482
FAM203A	0.153022677	0.878733982	3.072916921	0.003235718	2.789421846	0.007194305
FAM204A	0.640628769	0.523444086	0.768386635	0.445396679	1.343295713	0.184572104
FAM20A	0.026526346	0.97889814	-1.094114425	0.278459436	-1.797633927	0.077604309
FAM20B	-0.486467638	0.627856212	1.605648199	0.113820687	2.327517761	0.023561587
FAM20C	1.57347666	0.11923041	1.820924861	0.0738139	1.852225478	0.069244715
FAM21A	-0.922409702	0.358860415	-0.692149892	0.491624245	-0.859467826	0.39373143
FAM21B	-2.20180355	0.030319221	-0.478296805	0.634249512	0.249556256	0.803839832
FAM21C	0.37988968	0.704951102	2.015901302	0.048483377	1.830014825	0.0725495
FAM22A	-0.892033118	0.374830456	0.316790347	0.752548081	0.739237461	0.462837339
FAM22D	1.11365856	0.268486435	0.169411816	0.866066335	-0.566142045	0.573549577
FAM22G	-0.062674825	0.950168903	0.737911307	0.463564293	1.257499751	0.213769772
FAM24B	0.143930242	0.885887706	1.057326235	0.29477727	3.259544464	0.001896669
FAM26F	-1.747160551	0.084130614	-1.252273507	0.215531406	-2.34829972	0.022400427
FAM32A	-0.678571952	0.499206278	-1.578833529	0.119851099	-0.424751549	0.672638526
FAM35A	1.15605088	0.250818307	0.522721096	0.60317337	0.679557779	0.499572202
FAM35B	2.377477415	0.019617912	1.559399737	0.124380082	2.940697999	0.004746372
FAM36A	0.280119553	0.780049313	-5.149855205	3.30E-06	-5.772731778	3.51E-07
FAM38A	1.100479797	0.274152168	-1.755756972	0.084446499	-1.455478311	0.151096784
FAM3A	0.572165497	0.56868221	0.311989066	0.756175476	0.416998099	0.678267337
FAM3B	0.120808845	0.904120417	0.561268756	0.576791665	-0.1428361	0.886930172
FAM3C	1.861401955	0.06605701	-1.259608326	0.212891071	-1.219065593	0.227908085
FAM3D	-0.949469449	0.345006314	0.770882658	0.443927366	-1.337843673	0.18633247
FAM40A	0.057033083	0.954649343	1.178280008	0.243530237	1.319489536	0.192352358
FAM40B	-1.45314875	0.149772251	-1.390003342	0.169876841	0.208698095	0.835438004
FAM41C	0.505384887	0.614563744	1.161491005	0.250233155	0.411116461	0.682549611
FAM43A	-0.020477405	0.983709327	-2.944839242	0.004654165	-3.12007732	0.002851644
FAM45B	-1.19376931	0.235806156	0.826186033	0.412107924	0.989801015	0.326511117
FAM46A	-2.572604396	0.011785336	0.73818114	0.463401596	-1.359007109	0.179569879
FAM46C	-4.985923253	3.11E-06	1.080286592	0.284517416	0.942177664	0.35013164
FAM48A	-0.088299974	0.929840758	-2.117605222	0.038536956	-2.702143579	0.009091095
FAM49A	-4.054697172	0.000108856	1.304427889	0.197276213	0.58680318	0.559684284
FAM49B	-1.671563117	0.098196018	-0.756956539	0.452161371	-1.172999376	0.245740604
FAM50A	-0.578269179	0.564573068	2.421060001	0.018648015	3.050461427	0.003482227
FAM50B	-0.219599716	0.826696221	1.778579825	0.080586401	1.645608927	0.10542271
FAM53A	0.622140655	0.535472973	0.488367477	0.627143792	-0.212135028	0.832768694
FAM53B	-2.190540492	0.031153425	0.236089445	0.814200807	0.60314998	0.548833827
FAM53C	-2.188582448	0.031300475	0.101508244	0.919499567	-0.057029092	0.954724168
FAM54A	1.044349657	0.299210535	-2.195797361	0.032148381	-1.030512973	0.307181026
FAM54B	0.779500733	0.437795585	0.629817273	0.531304774	2.071480658	0.042912991
FAM55C	1.367244346	0.1750633	1.216879152	0.228613052	2.019606471	0.04819909
FAM57A	-0.470535326	0.63914759	-1.247917994	0.217110686	0.095747474	0.924061515
FAM58A	1.146975352	0.254529587	-1.745769322	0.086183402	-0.792290293	0.431520997
FAM59B	0.449709999	0.654035601	1.712486247	0.092186464	2.095325119	0.040656954
FAM60A	1.389018907	0.168364697	-0.852021794	0.397730891	-0.935132383	0.353718321
FAM63A	-3.757944059	0.000309046	0.086006674	0.931759401	0.576464768	0.566601309
FAM63B	0.167901434	0.86704967	-1.653996865	0.10356864	-3.681983458	0.000520675
FAM65A	0.158428746	0.874485338	-0.086294019	0.931531986	0.009325467	0.992592465
FAM65B	1.528069136	0.13011525	0.177647593	0.859623043	-0.985360094	0.328667756
FAM65C	-1.485396697	0.141045598	1.3113721	0.194935867	1.04925732	0.298548075
FAM66B	1.422093669	0.158566636	-2.290366967	0.025680539	-1.362128786	0.178588429
FAM66C	1.672576596	0.097995532	0.196685707	0.844765878	-1.226327735	0.225185477
FAM69A	1.143502403	0.255960021	-0.238893575	0.8120361	0.110415178	0.912473219
FAM69B	1.494682458	0.138607923	-0.061990952	0.950784659	0.41989783	0.676160025
FAM70A	1.10829701	0.270781492	-2.257444525	0.027787865	-3.733750654	0.000441939
FAM71F2	-1.49194001	0.139324403	-0.618326242	0.538797893	-0.562340312	0.576118951
FAM73A	1.527976441	0.130138247	-1.382410115	0.172183068	-1.918280162	0.060157659
FAM73B	0.787851652	0.432920173	0.375679367	0.708535382	1.229108887	0.224149152

FAM76A	2.071336229	0.04128304	-0.30205182	0.76370058	0.564332914	0.57477157
FAM76B	1.223022778	0.224616518	-1.573802641	0.121010625	-2.05532046	0.044503066
FAM78A	0.071577927	0.943101741	-1.33511782	0.187090913	-0.867113782	0.389564544
FAM81A	1.577064033	0.118402357	-0.663689557	0.509536992	-0.205186686	0.838167168
FAM82A1	0.720446108	0.473177493	-2.095092525	0.04057064	-1.841187357	0.070870894
FAM82A2	0.494510378	0.622189509	0.764711016	0.447565545	1.632391452	0.108184605
FAM82B	0.196467601	0.84470141	-0.098985574	0.921493432	0.503696866	0.616441907
FAM83A	-1.943240945	0.055213212	2.597649683	0.011896244	1.264058896	0.211423258
FAM83D	1.027845668	0.306865499	0.188139775	0.851428374	-0.799142383	0.427570249
FAM83G	0.969781654	0.334837933	-0.534225446	0.595241677	-0.21631958	0.829521415
FAM83H	2.659368531	0.009315029	-0.96290469	0.339620715	-0.075651665	0.939964716
FAM84B	2.216208148	0.029280834	0.455343928	0.650573754	-0.242976745	0.808907477
FAM86A	0.216278516	0.829275868	-1.237066857	0.221082351	-0.697330264	0.488469068
FAM86B1	0.459984244	0.646672562	-0.249044264	0.804212504	0.793344832	0.430911565
FAM86C1	-0.759491774	0.449607033	-0.418536298	0.677111621	0.706516906	0.482783818
FAM86C2P	-0.600526543	0.549713218	0.371703904	0.711477294	0.795668993	0.429570212
FAM86DP	-0.397219276	0.692176494	-2.624407817	0.011094291	-1.757716745	0.084237632
FAM86EP	-1.119194771	0.266130911	2.454612944	0.017147084	1.35098834	0.182109831
FAM86FP	-0.211772331	0.832778909	-0.518514339	0.606085862	2.364081769	0.02155304
FAM86HP	0.374882277	0.708658351	-0.466570029	0.642567529	-0.531649939	0.597062418
FAM89A	-0.110564975	0.912215432	-0.076605635	0.939202761	-1.105601073	0.273606714
FAM89B	-1.528665356	0.129967407	-1.615824479	0.111597288	-2.209875767	0.031200854
FAM8A1	0.033280114	0.973527281	-1.97556641	0.053001979	-3.134377582	0.002736125
FAM90A1	-0.721519482	0.47252042	1.516213083	0.134934998	0.762669996	0.448847531
FAM91A1	0.084802519	0.932612811	-1.174266917	0.24512055	-1.969907273	0.053781157
FAM92A1	1.047934664	0.297565003	-2.350899895	0.022173276	-1.491034771	0.141540593
FAM95B1	1.539172987	0.127383571	2.303946345	0.024853488	1.284881689	0.204100721
FAM96A	0.884970704	0.37860651	0.003357479	0.997332726	-0.170678672	0.86508903
FAM96B	-0.438517272	0.662096012	-4.211611104	9.02E-05	-3.357375553	0.001416532
FAM98A	2.688144756	0.00860597	0.068308486	0.945776659	1.015528193	0.314203417
FAM98B	1.193016874	0.236099169	-2.77907384	0.007349326	-1.87011347	0.066676249
FAM98C	-0.390324826	0.69724836	-2.108539197	0.039344993	-2.187375388	0.032888686
FAN1	1.231039914	0.221618395	1.227232909	0.224727648	1.564805741	0.123238766
FANCA	-0.996912923	0.321565507	-1.645247852	0.105365912	-1.045310431	0.300351903
FANCB	1.660886804	0.100328303	-1.345367696	0.183779492	-1.244362333	0.218527561
FANCC	2.344086674	0.021347061	-1.509267774	0.136696929	-0.408488456	0.684466404
FANCD2	0.960771807	0.339323821	1.617459312	0.111243397	1.937518948	0.057709915
FANCE	0.682593259	0.496673503	0.765885852	0.446871642	1.313437763	0.194369104
FANCF	0.060886949	0.951588595	-0.443076143	0.659370421	-0.94602632	0.34818237
FANCG	2.161966481	0.033360235	1.21627633	0.228840773	1.783764202	0.079857853
FANCI	2.068614783	0.041544127	2.967260736	0.004370061	2.713908387	0.008811091
FANCL	1.425358176	0.157623869	-0.148288702	0.882632873	-1.275581791	0.207347371
FANCM	1.286817852	0.201563836	0.045930932	0.963524183	-0.854334398	0.39654453
FAR1	-0.298899324	0.765728127	-0.082535957	0.934506696	-1.032849639	0.306095674
FAR2	-3.551314094	0.000620818	-0.874735927	0.385350659	-2.404794351	0.019497883
FARP1	-0.113305362	0.910048952	-0.389823043	0.698105213	0.60820929	0.545497357
FARP2	1.237621262	0.219179089	1.999377823	0.050292683	1.701993065	0.094279326
FARS2	2.686906474	0.008635441	-0.123531663	0.902116182	1.685102248	0.097511009
FARSA	-0.464871906	0.643182076	0.932570186	0.354934862	0.516098266	0.607809152
FARSB	0.895677045	0.372891454	-1.932633398	0.058203685	0.821699525	0.414718054
FAS	-1.104277598	0.272510983	-0.846325797	0.400873649	-2.062058086	0.043834019
FAS-AS1	-1.826699182	0.071167039	-1.917443552	0.060145502	-2.705179209	0.009018076
FASLG	0.161874813	0.871778977	-2.14952782	0.035805863	-1.554888203	0.125582903
FASN	1.330839519	0.186711706	-0.193974738	0.846878168	0.458935598	0.648049341
FASTK	0.195899963	0.845144319	0.761053968	0.449729555	1.536132935	0.130113373
FASTKD1	0.638340046	0.52492551	0.541266808	0.590411221	0.082221723	0.934762351
FASTKD2	1.434858193	0.154904907	0.97841864	0.331959165	2.878395761	0.005641951
FASTKD3	0.130929064	0.896133052	-0.889958871	0.377190236	-0.57154506	0.569907613
FASTKD5	0.562647627	0.575118733	0.796582315	0.428964974	0.659331883	0.512373662
FAT1	-0.720083151	0.473399795	4.718127259	1.56E-05	3.063606535	0.003353975
FAT2	0.224755432	0.822695443	1.927497902	0.058854123	0.542599626	0.589549459
FAT4	-1.392157732	0.167415444	1.673708052	0.099611014	1.473426401	0.14621173
FAU	0.067695808	0.946182788	-0.836900157	0.406107742	-0.080022043	0.936503815
FBF1	2.02062165	0.046387467	-0.244655443	0.807592748	0.705126164	0.483642115
FBL	0.24622714	0.806085158	0.731010465	0.467736265	1.603670234	0.114388931
FBLIM1	-0.094436703	0.924978953	3.498050641	0.000910524	1.640905162	0.106398942
FBLN2	1.334174677	0.185620905	-0.460797713	0.646678982	0.821306014	0.414940233
FBLN5	0.666192069	0.507047228	0.04612727	0.963368374	0.126882515	0.899485972
FBLN7	0.338447499	0.735839646	1.544221565	0.128011752	2.07235562	0.042828324
FBN1	0.604770139	0.546902449	2.076547056	0.042315454	1.155374839	0.252822379
FBN2	-0.967315259	0.336062038	2.291015874	0.025640469	1.233639476	0.222468448
FBP1	-2.486243521	0.014816411	2.040431229	0.045900928	2.521086959	0.014560792

FBRS	-2.255120017	0.026629896	-1.908363808	0.061332341	-1.594667652	0.116391829
FBRSL1	1.220741977	0.22547482	-2.135445024	0.036989102	-1.823852963	0.073489497
FBXL12	1.621198955	0.108586049	0.088995128	0.929394516	-0.247076999	0.805748415
FBXL13	-1.193779955	0.235802013	-2.063384175	0.043593048	-3.115932033	0.002885975
FBXL14	1.692178044	0.094183019	-3.03354034	0.003621775	-3.09911332	0.00302928
FBXL15	0.548983145	0.584420227	-1.691918831	0.09606549	-1.047868876	0.299181781
FBXL16	1.667374696	0.099028103	1.516640551	0.134827147	1.431843667	0.15772327
FBXL17	0.506724806	0.613627027	-1.875373306	0.065813466	-2.567461091	0.012930269
FBXL18	0.735589839	0.463954584	0.611377022	0.54335561	1.129208026	0.263603918
FBXL19	0.187945603	0.851356018	-2.090562913	0.040990922	-1.806859535	0.076134987
FBXL19-AS1	0.938824376	0.350414472	-2.421750461	0.018615975	-2.487350422	0.015861581
FBXL2	0.84705171	0.399285393	-0.594267746	0.554660214	1.844021572	0.070450308
FBXL20	-1.382343159	0.170397256	0.423189839	0.673732777	0.850344358	0.398739639
FBXL3	0.260204433	0.795319591	-0.547050064	0.586457744	-1.941652534	0.05719524
FBXL4	0.706697749	0.481638715	0.334346846	0.739332282	-0.418623776	0.677085594
FBXL5	-0.818386868	0.415367427	-0.600005335	0.550856096	-1.319964736	0.192194669
FBXL6	0.477371723	0.634291942	-0.449865045	0.654496335	0.519076783	0.605744046
FBXL8	1.377700178	0.171821891	-1.563190825	0.123485982	-1.096762868	0.277419365
FBXO10	1.93322879	0.056453103	-0.248962744	0.804275256	0.749991673	0.456386013
FBXO11	0.433673442	0.665596739	-0.494383075	0.622916089	-1.72686108	0.089682303
FBXO15	0.686204054	0.494405236	-0.841544628	0.403523435	0.391472068	0.696927746
FBXO18	1.045915654	0.298490978	3.045371005	0.003501487	2.469207758	0.016603686
FBXO2	1.039451862	0.301468609	0.304359487	0.761950997	0.392956229	0.695837464
FBXO21	1.728204758	0.087491626	4.038057573	0.000161224	3.005762463	0.003953428
FBXO22	1.388729237	0.168452507	-2.18084765	0.033292487	-2.371843992	0.021146869
FBXO24	-1.2031385	0.232179521	-0.129830824	0.897152633	0.693787059	0.490671684
FBXO25	1.096507783	0.275875975	1.389404319	0.170057909	2.105513138	0.039724936
FBXO27	-0.858696062	0.392862819	1.753529869	0.084831264	0.781091819	0.438024335
FBXO28	0.127205229	0.899070882	-0.589036601	0.558140004	-0.986953439	0.327892893
FBXO3	1.122586135	0.264695135	-1.060103802	0.293522752	-1.701084881	0.094450816
FBXO30	-0.049169576	0.960896619	-0.768501257	0.445329143	-1.793929352	0.078200958
FBXO31	0.554192662	0.580865685	3.467158704	0.001001411	3.26221889	0.001881713
FBXO32	3.26426356	0.001568805	0.512219369	0.610456117	0.896938066	0.373575705
FBXO33	-0.506302433	0.613922232	-0.375295255	0.708819437	-1.932045692	0.058397471
FBXO34	-0.378370718	0.706074923	-0.202304898	0.84039127	-1.441913919	0.154872714
FBXO36	0.405355075	0.686209466	0.194248379	0.846664905	-0.422851851	0.674015933
FBXO38	0.292952157	0.770254808	1.660667707	0.102215143	1.105354966	0.273712381
FBXO39	-1.484954561	0.141162497	0.710615478	0.480190222	0.210929555	0.833704706
FBXO4	1.666848835	0.099132976	-0.557917473	0.579062965	-0.220759976	0.826078867
FBXO40	-0.74380861	0.45899208	0.570702721	0.570421133	0.230603428	0.818459763
FBXO41	2.473152261	0.015332417	-0.027929281	0.977815103	0.205462349	0.837952842
FBXO42	-1.429021868	0.156570972	2.279574378	0.026355189	1.814589504	0.074921876
FBXO44	1.240518752	0.218111405	-0.551772645	0.583238723	0.149224635	0.881910239
FBXO45	-0.68803408	0.493257786	0.837031833	0.406034334	0.185576412	0.853444674
FBXO46	0.359315353	0.72022794	-1.178190658	0.243565564	0.279076307	0.781210017
FBXO48	-0.164008774	0.870103838	-1.303846049	0.197473259	-0.827481017	0.411462118
FBXO5	1.841062177	0.069013411	-1.293584904	0.200972638	-1.236972692	0.221237859
FBXO6	-1.927464924	0.057177528	-1.015426612	0.314149157	-2.806886943	0.006861555
FBXO7	-3.574872295	0.000574074	0.115514431	0.908439176	-0.644545214	0.521842506
FBXO8	0.410776679	0.682244099	-1.154077784	0.253234555	-1.14764805	0.255972722
FBXO9	-1.124452182	0.263907441	1.405005604	0.165390278	-0.034359604	0.972712063
FBXW11	-1.267776579	0.208252972	-0.666201374	0.507942079	-0.632779272	0.529442496
FBXW2	-0.644098754	0.521202235	1.511235834	0.136195818	1.997763689	0.050587727
FBXW4	0.096426413	0.923403214	0.814258995	0.418850453	2.330934733	0.023367059
FBXW4P1	-0.558082211	0.578218511	-0.143477842	0.886413612	-0.216237267	0.829585263
FBXW5	0.112874161	0.910389804	-1.008935426	0.317225513	-0.832119144	0.408861379
FBXW7	-0.299254246	0.765458233	-1.177206894	0.243954762	-1.818012626	0.074389866
FBXW8	2.068164863	0.041587428	-1.742302433	0.086793194	-1.101473358	0.275382739
FBXW9	0.84268569	0.401709996	-0.89306273	0.375539893	0.262745835	0.793706674
FCAR	-1.448095918	0.151176793	0.282588133	0.778505643	0.019409473	0.984583154
FCER1A	-0.932876695	0.35345984	0.042820773	0.965992509	-1.96804773	0.054000355
FCER1G	-1.22223377	0.224913166	-0.858353118	0.394255532	-0.897690617	0.373177728
FCER2	0.179072205	0.858296481	2.437112295	0.017915813	3.04419164	0.003545
FCF1	-0.900293217	0.370444196	-1.071278891	0.28851258	0.061483034	0.951192517
FCGBP	1.936421455	0.056055192	0.210509471	0.834013021	0.444416867	0.65844683
FCGR1A	0.214282808	0.83082688	-0.29729982	0.767307255	-1.243673396	0.218779202
FCGR1B	-0.457571067	0.648398841	-0.55630708	0.580155924	-2.208068495	0.031333549
FCGR1C	-0.323536729	0.747063547	-0.752504889	0.45481204	-1.569595668	0.122119237
FCGR2A	-2.160321082	0.033491355	-1.10850894	0.272249495	-2.267605036	0.027214768
FCGR2B	-1.832971486	0.070219754	0.289825941	0.772990233	-0.111260313	0.911806073
FCGR2C	0.151176458	0.88018575	-1.344273831	0.184130749	-1.777658107	0.080867143
FCGR3A	-2.312129773	0.023127043	-1.538013806	0.129521248	-2.880573016	0.005608178

FCGR3B	-3.966019992	0.000149428	-0.785998481	0.43509015	-2.621858978	0.011230077
FCGRT	-2.491918189	0.014597615	-0.305850218	0.760821445	-0.464572104	0.644031567
FCHO1	-1.251253399	0.214188984	-0.937012133	0.352664825	-0.434910409	0.665291907
FCHO2	-0.207755678	0.835904247	-2.162764506	0.034724049	-3.145534153	0.002649065
FCHSD1	0.497503158	0.620086653	-0.371575702	0.711572239	-0.019683944	0.984365172
FCHSD2	0.734182006	0.464807673	0.602189637	0.549411336	-0.198540052	0.843338565
FCN1	-2.108892934	0.037821874	1.526430221	0.132375841	0.452830417	0.652413094
FCRL1	1.9854668	0.050235077	0.18763459	0.851822567	-0.111172439	0.911875438
FCRL2	2.057720314	0.042603599	0.507240567	0.613922758	0.579012836	0.564892593
FCRL3	4.10159275	9.19E-05	-0.474926417	0.636635401	0.045634721	0.963763059
FCRL5	0.220182734	0.826243574	1.46483248	0.148404115	0.863185898	0.391701708
FCRL6	1.67954186	0.096626656	-2.562820515	0.013019024	-1.519735174	0.134180241
FCRLA	-0.380813772	0.704267723	1.133047128	0.26188902	1.305519664	0.19703181
FCRLB	-1.822571371	0.071796244	-2.303043432	0.024907736	-2.544337857	0.013721345
FDFT1	-0.833758953	0.406695218	-0.072033905	0.942824483	0.492650821	0.624177365
FDPS	-1.089231413	0.279053309	1.220218342	0.227354643	2.704294392	0.009039304
FDPSL2A	0.323392273	0.747172556	0.910514016	0.366346343	1.552358856	0.126186421
FDX1	1.91144416	0.059232508	-0.209119323	0.835092949	0.326982372	0.744895193
FDX1L	0.533880027	0.594783099	1.156214344	0.252366896	2.323464976	0.023794182
FDXACB1	-2.051797889	0.043189239	-1.500703836	0.138894507	-1.168875981	0.247384506
FDXR	0.101692346	0.919234381	1.58371878	0.118733702	2.157971803	0.035215007
FECH	-3.937058501	0.000165566	0.262281093	0.79404055	-0.187504797	0.851939749
FEM1A	-0.47592508	0.635318115	-0.290601721	0.772399759	-0.498277964	0.620231331
FEM1B	0.267526599	0.789695565	-1.450419592	0.15236583	-3.240405722	0.00200701
FEM1C	-0.329624964	0.742473951	-1.301132859	0.198394063	-1.935229359	0.05799669
FEN1	1.012984479	0.313870469	2.998264121	0.004003724	4.188940741	9.98E-05
FER	1.092919004	0.277439917	-0.570512821	0.570549027	-0.29935011	0.76577648
FER1L4	1.030168688	0.305780114	0.992582519	0.325065205	1.468606472	0.147511253
FERMT3	-1.651538748	0.102225998	0.782176756	0.437314565	0.330489583	0.742258191
FES	-1.904185895	0.060183852	1.311341356	0.194946182	1.200256256	0.235071577
FEZ1	3.619086947	0.000495202	-1.458479594	0.15014029	0.327512919	0.744496087
FEZ2	0.111497376	0.911478224	0.543280992	0.589032881	-0.97517972	0.333647347
FFAR2	-0.975891028	0.33181837	0.171097816	0.864746528	-0.852241976	0.397694735
FFAR3	-1.575911609	0.118667863	0.312027091	0.756146726	-0.617899759	0.539135752
FGD1	2.110921093	0.037642328	1.039279281	0.303018123	2.165619395	0.034596475
FGD2	0.309772118	0.757473415	0.39795829	0.69213217	0.280356724	0.780232598
FGD3	-1.756990641	0.082429958	1.284223958	0.204205332	1.384985534	0.171526775
FGD4	-1.708525182	0.091096768	-0.662136126	0.51052471	-2.032556977	0.046829321
FGD6	1.621082031	0.108611162	-1.545303152	0.127750192	-0.91677221	0.363176894
FGF11	0.067289598	0.946505225	-1.592699409	0.116701451	-2.19810287	0.032074217
FGF13	-1.307050948	0.194632108	-0.274355323	0.784793204	-1.572581033	0.121425619
FGF5	-1.134645454	0.259633701	2.596973	0.011917191	1.417207645	0.161938798
FGF7	-0.097859327	0.922268615	0.109797279	0.912951818	-1.005827986	0.318806648
FGF9	0.566206687	0.572707797	0.215033596	0.830500719	0.060328721	0.952107712
FGFBP2	0.76593998	0.445780666	-2.022668251	0.047758751	-1.668882659	0.100699288
FGFBP3	-0.304422394	0.761531519	-2.496530972	0.015425178	-2.772446505	0.007531915
FGFR1	-0.400587156	0.689704011	-0.757958964	0.45156573	-0.718450545	0.475453998
FGFR1OP	1.878542425	0.063648751	-0.523673805	0.602514673	-1.98023048	0.052578105
FGFR1OP2	-0.032259029	0.974339215	-0.69821419	0.487852727	-1.318681198	0.192620818
FGFR2	2.146484059	0.034611872	-0.0670728	0.946756038	-0.545237232	0.587746416
FGFR4	1.530728776	0.129456771	-0.721617931	0.47344877	-0.741975946	0.461189615
FGFRL1	3.325459837	0.001292922	-1.440198348	0.155225093	-1.495035516	0.140495867
FGGY	0.735976816	0.463720246	0.769601727	0.444681046	-1.187624583	0.239973337
FGL2	-1.723400049	0.088360827	-0.863935518	0.391206963	-1.892654179	0.063554856
FGR	-2.463478882	0.015723982	-1.174322779	0.245098361	-1.551368361	0.126423325
FH	0.602969212	0.548094418	3.043984782	0.003515386	4.340011619	5.99E-05
FHAD1	0.594326433	0.553832875	0.238764644	0.812135599	-0.215497364	0.830159234
FHDC1	-0.99286955	0.323521035	-0.384256041	0.702203719	-2.053636994	0.04467161
FHIT	1.068156732	0.288398352	1.318410911	0.192585028	0.699936719	0.486852279
FHL1	-0.246080975	0.806197938	1.346148034	0.183529225	1.197964746	0.235955359
FHL2	-2.534269166	0.01305414	-0.20817654	0.835825525	-0.878268431	0.383535108
FHL3	-1.980706747	0.050776412	-0.191565822	0.848756057	0.149098093	0.882009625
FHOD1	0.308557239	0.758394386	-0.161707584	0.872102061	0.079700028	0.936758777
FHOD3	0.132142354	0.895176167	-2.148701894	0.035874332	-1.126109587	0.26490183
FIBCD1	0.393682625	0.694776477	-1.768748792	0.082230745	-1.737001775	0.087861672
FIBP	-1.345356049	0.181998878	2.432890478	0.018105838	2.751459936	0.007969449
FICD	-0.696545869	0.487939907	-0.313395737	0.755112152	-0.667945285	0.506900643
FIG4	-0.553523704	0.58132155	1.275478561	0.207260386	1.349003239	0.182742819
FIGNL1	0.487456529	0.627158251	-0.338089851	0.736524738	-0.177737861	0.859567519
FIGNL2	1.066696254	0.289053827	-3.257947424	0.001884396	-3.268077191	0.001849341
FILIP1L	0.359577751	0.720032372	1.772434288	0.081611065	1.885829562	0.064486565
FIP1L1	-0.470771927	0.638979276	0.561696843	0.576501844	1.054618115	0.296109974

FIS1	-1.385080709	0.169561506	-1.207767342	0.232072809	-1.231102209	0.223408548
FITM1	0.612479887	0.541814445	-0.743117675	0.460430862	-0.584771663	0.561040167
FITM2	1.909531312	0.059481986	-1.877805701	0.065473868	-1.508340103	0.137065353
FIZ1	-0.121311326	0.903723595	-0.660774524	0.5113913	0.629702823	0.531439163
FKBP10	1.096712771	0.275786829	-0.924527771	0.359068928	0.657831176	0.513330449
FKBP11	2.12529356	0.03639099	-0.287311759	0.774904792	1.357906235	0.179916972
FKBP14	0.267803952	0.789482752	-0.724357707	0.471778391	-0.830441535	0.409800903
FKBP15	-1.428117757	0.156830296	1.439085304	0.155538961	1.098213346	0.276791118
FKBP1A	-2.052086377	0.043160553	-2.50430451	0.01512358	-2.153238382	0.03560268
FKBP1AP1	0.217240039	0.828528839	0.460282297	0.647046639	0.578072063	0.565523173
FKBP1B	-2.150555918	0.034278799	0.708684678	0.481378782	-0.394752952	0.694518434
FKBP2	-0.743574966	0.459132736	0.680530487	0.49889526	0.515847346	0.607983271
FKBP3	1.174296933	0.243473559	-0.702934798	0.484928002	0.113686504	0.909891204
FKBP4	-0.687406405	0.493651182	2.084086085	0.041598451	2.182711352	0.033248431
FKBP5	1.279530692	0.204104718	0.730016002	0.468339232	0.143920129	0.886078038
FKBP7	1.240471454	0.218128803	-0.721508112	0.473515793	-1.131559179	0.26262205
FKBP8	-2.973266941	0.003809311	0.31382186	0.754790134	-0.329883292	0.742713828
FKBP9	-0.634278021	0.527560106	0.332921986	0.740401979	1.255222969	0.214588779
FKBPL	-1.690988141	0.094410957	2.919741806	0.004992478	2.255700833	0.027997723
FKRP	-0.002565866	0.997958599	-2.595054889	0.011976752	-1.64132841	0.1063108
FKTN	1.741323877	0.085153987	-1.874447457	0.06594312	-2.214138489	0.030889833
FLAD1	-0.810039788	0.420122667	-1.186119539	0.240445069	-0.648175075	0.519509545
FLCN	-0.889001872	0.37644826	1.09122783	0.279716558	1.049965711	0.298225111
FLI1	-1.808134886	0.074033372	-1.237087576	0.221074717	-2.873438866	0.005719544
FLII	-1.139098363	0.257782109	1.208900255	0.231640577	1.207317264	0.232363447
FLJ10038	1.652819647	0.101964264	-0.893574421	0.375268264	-0.49781783	0.620553582
FLJ10661	-1.579150376	0.117922893	-1.050512654	0.297870296	-1.32965433	0.18900056
FLJ12334	0.582653991	0.561630108	-1.497023138	0.139847543	-1.47012533	0.147100773
FLJ12825	0.247174539	0.805354247	2.011069925	0.049006515	1.105788154	0.27352641
FLJ13197	4.079285238	9.96E-05	-1.712695855	0.092147607	-0.74019068	0.462263413
FLJ14107	1.50974675	0.134723656	-1.070795279	0.288728167	0.054248445	0.956929485
FLJ14186	-1.062645277	0.29087728	0.679911585	0.499284185	0.722123429	0.473210736
FLJ20021	-0.688284309	0.493101002	0.706592611	0.482668468	-0.307792519	0.759377301
FLJ22184	-1.346592328	0.181601703	-2.152202957	0.03558488	-1.516608788	0.134966967
FLJ22447	-0.083813626	0.933396752	-1.546755913	0.127399542	0.999891606	0.321646011
FLJ23867	-1.667338602	0.099035299	1.886815235	0.064228891	1.670600499	0.100357624
FLJ27354	2.271924881	0.025551817	-1.859975132	0.067998019	-3.094748887	0.003067542
FLJ30403	-3.18492082	0.002008886	0.543433716	0.588928432	2.079541756	0.042138421
FLJ31306	0.772151737	0.442112517	-0.257068437	0.798042077	-0.52759087	0.599858806
FLJ31813	0.560888586	0.576312122	-0.646577427	0.520473786	-0.904843146	0.369408678
FLJ32224	1.444804756	0.152097117	0.708648942	0.481400796	0.035785022	0.971580497
FLJ33065	-1.485871879	0.140920046	-0.208975755	0.835204497	0.983078647	0.329779371
FLJ33360	0.191091353	0.848898303	1.185144927	0.240827072	0.974282863	0.334088416
FLJ33630	1.266531386	0.208696041	-2.417326293	0.018822127	-2.813669059	0.006736211
FLJ35390	0.290118878	0.77241417	-1.551954252	0.12615112	-1.598790384	0.115471118
FLJ35776	0.647201094	0.519202174	-1.94464546	0.056706189	-0.502044064	0.617596602
FLJ36644	-0.064575773	0.948659603	-1.352401677	0.18153293	-2.773771531	0.007505047
FLJ37453	0.845158864	0.400335455	-0.897131322	0.373383538	-1.282826605	0.204814874
FLJ38109	0.432089837	0.666742856	-1.758588502	0.083959402	-1.074911679	0.287004583
FLJ39051	-0.231607933	0.817385245	-0.280670163	0.779969123	-1.427134929	0.159070086
FLJ39534	-0.602602097	0.548337559	0.129205703	0.897645028	0.089542961	0.928968557
FLJ39582	1.718874033	0.089186075	0.888790683	0.377812556	-0.367232568	0.7148239
FLJ39639	-0.152123698	0.879440839	-3.649030396	0.00056831	-3.507399394	0.000897317
FLJ39653	-0.754647047	0.452494318	-2.039414145	0.046005593	-1.500540324	0.139068346
FLJ40292	1.314462658	0.192137928	0.475958102	0.635904662	0.40107522	0.689884633
FLJ40852	1.411434098	0.161675297	0.647003902	0.520199714	0.48782539	0.627570044
FLJ41484	-0.159500785	0.873643253	-0.500897278	0.618352318	0.902279013	0.370757054
FLJ42289	0.346015019	0.730164981	-0.41563424	0.679222135	-0.411681693	0.682137621
FLJ42351	1.49162673	0.139406434	2.375202279	0.02088995	3.108428302	0.002949108
FLJ42418	-1.107158266	0.271270697	-0.241450788	0.81006329	-1.024617316	0.309931102
FLJ42627	0.230232624	0.818450336	-1.785679049	0.079416096	-2.694285782	0.009282629
FLJ43663	1.919697237	0.05816623	-1.183931546	0.241303275	-1.310148673	0.195471852
FLJ44511	-3.340659972	0.001231836	0.145531959	0.884799002	-0.029063545	0.976916807
FLJ44635	-2.058240831	0.042552456	-3.927515806	0.000232078	-3.945612941	0.000223325
FLJ45244	0.792576811	0.430175759	1.473070743	0.146176166	1.148462341	0.255639408
FLJ45340	-0.369268425	0.712822945	0.821412145	0.41479871	0.838785552	0.405140967
FLJ45445	-0.049745643	0.960438866	0.457901432	0.648746107	-0.004793855	0.996192037
FLJ45513	0.090042184	0.928460219	0.907101046	0.368132917	2.197123309	0.032147846
FLJ46906	0.257142841	0.797674374	2.551332303	0.013409947	2.736313592	0.008299613
FLJ90757	-0.435846741	0.664025132	0.087628197	0.930476145	-0.020023536	0.984095473
FLNA	-0.703563769	0.483579133	0.520560191	0.604668637	0.782371262	0.437278402
FLNB	1.411893286	0.161540426	1.621744769	0.110320038	1.683231652	0.097874433

FLNC	-0.752368469	0.453855946	1.421176091	0.160657413	1.636417252	0.107337222
FLOT1	-1.75734581	0.082369047	0.428636303	0.669786773	-0.128446421	0.898253949
FLOT2	-1.031488244	0.305164732	0.753033975	0.454496534	0.719594992	0.47475437
FLRT1	0.454103899	0.650882465	1.254257892	0.214814704	0.477381405	0.634940703
FLT1	0.541218348	0.589737306	-0.698112696	0.487915716	-0.648290298	0.51943558
FLT3	0.878715831	0.381970615	0.32865489	0.743608522	0.716230707	0.476812693
FLT3LG	0.193681502	0.846875788	-0.05862289	0.953455398	0.772227819	0.443212768
FLT4	2.287343419	0.024596653	0.203046121	0.839814593	-0.595627063	0.553814027
FLVCR1	0.588034292	0.558029344	-3.934793189	0.000226612	-4.037722891	0.000165069
FLVCR2	-0.086486404	0.931278072	1.138738832	0.259526345	1.092096309	0.279447362
FLYWCH1	2.691956268	0.008515826	0.666544536	0.507724393	2.17137437	0.03413732
FLYWCH2	0.567571172	0.571784779	0.306393297	0.760410075	0.858117586	0.394470152
FMN1	0.148130802	0.882581586	-1.42810835	0.158660888	-1.754241354	0.084836828
FMNL1	-1.999866884	0.0486273	-0.064785865	0.948568833	-0.299586045	0.76559742
FMNL2	-0.560398202	0.576645025	-2.044063601	0.045528809	-3.798244986	0.000359722
FMNL3	3.054720858	0.00298811	-1.291526423	0.201680204	0.13324665	0.894473984
FMO4	1.090580875	0.278462142	-1.175634778	0.244577654	-0.316859551	0.752523473
FMO5	-0.267316886	0.789856488	0.61685986	0.539757994	-0.5744523	0.567952652
FMOD	0.794264925	0.429197784	2.272613819	0.026798589	1.966961235	0.054128782
FMR1	0.271151512	0.786915432	-0.190150698	0.849859639	-1.521679917	0.133692705
FN1	1.304250452	0.195580792	2.633036788	0.010846381	2.717747856	0.008721438
FN3K	0.549097681	0.584341967	1.032671127	0.306074584	1.053069109	0.296813056
FN3KRP	1.126526842	0.26303362	1.247567062	0.217238302	0.799654587	0.427275795
FNBP1	-1.524524491	0.130996948	0.587924598	0.558881116	0.655128917	0.515055701
FNBP1L	-0.670919434	0.504045346	1.284334711	0.20416686	-0.609285608	0.544788891
FNBP4	3.330525131	0.001272257	-0.222082804	0.825035013	0.831931569	0.408966362
FNDC3A	0.579645627	0.563648423	-0.852635925	0.397392958	-1.95534138	0.055518731
FNDC3B	-0.477521071	0.634186042	-0.775743013	0.441074426	-2.524480345	0.014435448
FNDC5	-0.022248067	0.982300913	-0.601390939	0.549939395	1.003778114	0.319785189
FNDC8	0.63752869	0.525451201	0.159283897	0.874002448	1.227017135	0.224928262
FNDC9	-0.331944014	0.740728179	0.254286658	0.800179765	0.135317856	0.892843757
FNIP1	-1.018541435	0.311238714	-1.365867749	0.177290474	-2.924674364	0.004963157
FNIP2	0.387892737	0.699040816	-1.010106014	0.316669245	-1.991805425	0.051256719
FNTA	0.055278831	0.956042802	-1.653681987	0.103632887	-1.736330667	0.087981207
FNTB	-0.328882831	0.743032911	-2.213381324	0.030847044	-1.404046538	0.165803614
FOLR2	-0.550046936	0.583693549	0.702388889	0.485265729	1.692186401	0.096144709
FOLR3	-2.394446879	0.018787767	-1.201066235	0.234641454	-2.206712969	0.031433402
FOPNL	0.59629852	0.552520857	-0.215589693	0.830069231	-0.57839776	0.565304826
FOS	-0.167844697	0.867094171	-1.327950641	0.18943314	-2.244410084	0.028758684
FOSB	2.075123418	0.040922063	-0.652789969	0.516488888	-0.19688634	0.844626319
FOSL2	-2.124384274	0.036469075	-0.201046933	0.841370176	-1.478962993	0.144730104
FOXC1	-1.893135463	0.061656963	1.231636649	0.223089834	0.386097302	0.700881478
FOXD2	0.036844003	0.970693614	-2.200142761	0.031822377	-3.282282178	0.001773013
FOXJ1	-0.153889101	0.878052814	-2.281616144	0.026226365	-1.942904941	0.057040078
FOXJ2	-1.271052366	0.207090683	0.456083604	0.650044943	0.903965694	0.369869743
FOXJ3	0.467486694	0.641318023	2.227009757	0.029870592	1.632548769	0.10815139
FOXK1	1.41631704	0.160245518	1.993398795	0.050961541	1.456665	0.1507699
FOXK2	0.807624656	0.421504587	0.610963115	0.543627695	0.205180542	0.838171945
FOXM1	1.183270118	0.239918432	1.395637036	0.168181176	1.961113826	0.054824469
FOXN2	0.447298646	0.655768706	-1.037194585	0.303980098	-1.95865294	0.05511953
FOXN3	-0.952579688	0.343436454	0.122988462	0.902544395	-0.148068761	0.882818137
FOXO1	0.610234599	0.543293727	0.181500266	0.856612151	0.390466009	0.697667174
FOXO3	-4.987601245	3.09E-06	0.286014274	0.775893383	-0.706044749	0.483075115
FOXO3B	1.488943675	0.140110526	-1.550260385	0.126556837	-0.778229197	0.439696002
FOXO4	-3.475453991	0.000797016	-0.760907241	0.449816506	-1.095762995	0.277853022
FOXP1	0.665393038	0.507555557	1.663227766	0.101699564	2.162546112	0.034843886
FOXP3	-0.886351566	0.377866338	-0.825050019	0.41274727	-0.232238086	0.817196174
FOXP4	0.049464932	0.960661922	-0.094037329	0.925405877	0.403476343	0.688127881
FOXRED1	1.38114255	0.170764781	1.892925842	0.063395962	2.584767874	0.012365539
FOXRED2	0.47624911	0.635088202	0.260299882	0.79556079	0.791166966	0.432170748
FP588	1.503831408	0.136238574	-2.394651034	0.019911216	-0.658265845	0.513053225
FPGS	2.244651404	0.027321513	-0.510957441	0.611333929	0.221271677	0.825682374
FPGT	0.359320802	0.720223878	-0.785543897	0.435354387	-0.987087079	0.327827958
FPR1	-2.760666459	0.007031431	-0.363679616	0.717428818	-1.530225644	0.131566996
FPR2	-2.240894685	0.02757352	-0.217364232	0.828692681	-1.934550718	0.058081925
FPR3	-0.575139999	0.566677889	1.936272472	0.057746496	-0.158554076	0.874588221
FRA10AC1	0.743291515	0.459303408	-0.037394063	0.970300113	0.82730419	0.411561469
FRAT1	-2.569325612	0.011889336	-1.862630697	0.067616961	-2.061513241	0.043887796
FRAT2	-3.191880315	0.001966098	-0.718961241	0.475071675	-0.958029621	0.342148521
FRG1	-0.574772282	0.566925483	0.580500894	0.563841298	1.319147586	0.192465889
FRG1B	1.810436836	0.073672816	0.921066211	0.360857807	0.737214792	0.464056523
FRMD3	-4.180084173	6.91E-05	3.24648371	0.00194959	0.583990781	0.56156178

FRMD4A	1.110029523	0.270038384	-0.374194704	0.709633539	-0.132653483	0.894940945
FRMD4B	-0.121346069	0.903696158	0.849460293	0.399142306	0.608520593	0.5452924
FRMD6	-0.750622227	0.454901051	0.494167207	0.62306758	-0.274585052	0.784641259
FRMD8	-0.407910945	0.684338997	0.478113471	0.634379193	0.538101091	0.592630642
FRRS1	2.08240188	0.040235947	-0.159996435	0.873443676	-0.970952729	0.335729533
FRS2	-0.018767245	0.985069665	-2.256291639	0.027864357	-2.612963253	0.011493383
FRS3	0.782640802	0.435958598	-2.074660467	0.042496548	-2.879364626	0.005626899
FRY	-2.451930997	0.016203124	-0.89531522	0.374345099	-1.967628943	0.054049826
FRYL	0.022861513	0.981812981	0.082392514	0.934620258	-0.914052942	0.364591472
FSCN1	-0.927945746	0.355997481	0.717865522	0.475741934	0.525195524	0.601511867
FSCN2	0.609656692	0.543674804	-0.718402069	0.475413659	0.340730183	0.734576355
FSD1	-0.389867207	0.697585496	-1.025654138	0.309343029	-0.58973175	0.557732565
FSD1L	-0.054768699	0.956448042	-0.414512235	0.680038803	-1.90381643	0.062055585
FSIP2	-0.808556051	0.420971328	1.51198091	0.136006486	0.470342014	0.639929737
FSTL1	-4.705470315	9.45E-06	1.742247032	0.086802968	0.616399571	0.540118094
FSTL3	-0.426452371	0.670829318	-0.773369543	0.442466261	0.327151093	0.744768264
FSTL4	-0.852915998	0.396042882	1.145251192	0.256841636	1.6792744	0.09864692
FTH1	-2.555556269	0.012335181	-0.182897263	0.855520914	-0.224763688	0.822977809
FTH1P3	-0.257721261	0.797229346	0.635250368	0.527780913	0.298340271	0.766543027
FTL	-3.517647761	0.000693906	-0.277951932	0.782044601	-0.142141283	0.887476425
FTO	-0.244085816	0.807737809	1.099371874	0.276179947	1.644649928	0.105621149
FTSJ1	1.112863995	0.268825695	1.011256917	0.316122972	2.358013119	0.021875435
FTSJ2	-0.206628333	0.836781904	3.667686815	0.000535771	4.218318669	9.04E-05
FTSJ3	0.74585998	0.457758196	2.007798748	0.049363469	2.583093581	0.012419176
FTSJD1	1.341255419	0.183320969	0.018516944	0.985290458	-0.496212532	0.62167842
FTSJD2	-0.517278248	0.606271788	0.547125373	0.586406346	-0.478492908	0.634154493
FTX	-0.664722373	0.507982431	-1.179332358	0.243114445	-1.501782156	0.138747902
FUBP1	0.772196842	0.442085946	0.522571983	0.603276496	0.659847421	0.512045197
FUBP3	2.107331885	0.037960573	-1.576083199	0.120483887	-2.410052013	0.019245708
FUCA1	0.700016276	0.485780776	3.91188907	0.000244247	3.173345209	0.002443225
FUCA2	-0.965672228	0.336879121	2.270405592	0.026940632	1.672331035	0.100014394
FUK	2.313707231	0.023036195	0.909544748	0.366853157	1.491880505	0.141319235
FUNDC1	-0.148190206	0.882534846	-1.133519986	0.261692154	-0.302767078	0.76318448
FUNDC2	-1.443573749	0.152442463	-0.305771849	0.760880814	-0.170699518	0.865072714
FURIN	-2.308156355	0.023357282	-0.422504013	0.674230319	-1.187819763	0.239897039
FUS	-0.730575418	0.466997164	-1.403093684	0.165956915	-0.430323503	0.668605006
FUT10	1.102493711	0.27328102	-1.13808226	0.259798118	-0.701077022	0.486145882
FUT11	0.77388335	0.441093111	-3.340826403	0.001470702	-2.17052989	0.034204359
FUT2	1.058527612	0.2927388	-0.315183652	0.753761329	1.562363592	0.123812719
FUT4	0.25645262	0.798205509	-2.860814904	0.00587844	-3.815516944	0.000340329
FUT7	-1.282800596	0.202961653	0.420322796	0.675813687	-0.198736738	0.843185433
FUT8	0.408173266	0.684147133	1.639768546	0.106504379	2.31276767	0.024417965
FUZ	-0.785022363	0.434568371	-1.602267509	0.114567202	-1.429168531	0.158487333
FXC1	0.960817041	0.339301202	-1.661072155	0.102133548	0.082400014	0.934621213
FXN	2.257376682	0.02648284	-1.536478545	0.129896745	1.00926453	0.317170677
FXR1	0.949049855	0.345218456	0.483279072	0.630729717	0.706901668	0.482546512
FXR2	-0.639573306	0.524126985	-0.187188902	0.852170367	-0.120458436	0.904549345
FXYD1	-1.444352017	0.152224058	-4.435591537	4.19E-05	-4.499926457	3.46E-05
FXYD2	1.744712488	0.084558606	-1.496149106	0.140074611	-1.017982678	0.313045792
FXYD5	-1.293642111	0.199205705	-1.645918172	0.105227318	-1.549281921	0.126923722
FXYD6	-2.088352031	0.039682442	2.037646753	0.046187962	2.025551201	0.047566085
FXYD7	1.152684214	0.252190539	-2.168990349	0.0342252	-0.083947908	0.933395971
FYB	0.34984498	0.727298698	-1.313187331	0.194327555	-2.460661454	0.016963993
FYCO1	1.385521793	0.16942714	0.265272486	0.791746691	0.033495483	0.973398072
FYN	0.864005516	0.389955591	-0.134528433	0.893453734	0.832213417	0.408808621
FYTTD1	-0.419341715	0.675997806	-0.571972116	0.569566573	-1.248707455	0.216945379
FZD1	-1.236791623	0.219485501	-0.143238699	0.88660162	-0.606484067	0.546633931
FZD2	-0.315524819	0.753117205	1.064663269	0.291471398	0.362712279	0.718179504
FZD3	1.103078739	0.273028319	-1.615165264	0.111740246	-1.466187081	0.148166965
FZD4	-1.743734007	0.084730173	-0.398892502	0.691447503	-1.923327802	0.059507045
FZD5	-2.098073935	0.038792217	0.339951233	0.735129897	-1.014929463	0.314486238
FZD6	0.556522975	0.579279013	-0.204671121	0.83855064	-0.529859101	0.598295418
FZR1	-0.68046708	0.498011784	-0.389031341	0.698687528	-0.373561799	0.710134948
G0S2	-3.878105871	0.000203717	-1.634380546	0.107633612	-1.962255325	0.054688062
G2E3	0.322549701	0.74780848	-1.238878407	0.220415608	-1.88467616	0.064645167
G3BP1	0.933035997	0.353378052	1.812729364	0.075086072	1.103570935	0.274479211
G3BP2	0.265078647	0.791574569	0.169556225	0.865953276	-0.055129215	0.956230914
G6PC3	-0.696628418	0.487888487	-2.149808572	0.035782615	-0.942189277	0.350125748
G6PD	-2.21208511	0.029574825	-0.348776722	0.728528715	-0.515850987	0.607980745
GAA	-1.788406458	0.077184003	0.412214754	0.681712261	-0.000662262	0.999473935
GAB1	-1.544747568	0.126029375	-1.155305688	0.252735642	-2.386970372	0.020374977
GAB2	-2.40923854	0.018089812	0.006336076	0.994966471	-0.796819431	0.428907177

GAB3	1.608938993	0.111244799	-0.858009844	0.394443475	-0.647917012	0.519675223
GABARAP	-3.615183481	0.000501728	-0.633025062	0.529222749	-0.622739004	0.535973232
GABARAPL1	-5.975372056	4.89E-08	-2.233670877	0.029403332	-2.715403108	0.008776089
GABARAPL2	-2.459677762	0.015880279	-0.241564739	0.80997541	-0.466454412	0.642692202
GABBR1	2.222352675	0.028847449	-1.011993986	0.315773458	0.255781082	0.799053151
GABPA	0.405475985	0.686120936	-1.014659384	0.314511716	-2.073699355	0.042698579
GABPB1	-0.082325332	0.934576714	0.467904051	0.641618934	-0.006461523	0.994867354
GABPB2	1.48267009	0.141767712	-0.51894887	0.605784721	0.35358532	0.724971818
GABRR2	2.277672369	0.025192015	-0.655628829	0.51467338	-1.375994952	0.174278406
GADD45A	-1.477017993	0.143273809	-0.049838825	0.96042326	-0.782916099	0.436960983
GADD45B	-2.456363164	0.016017703	0.453373642	0.651983235	-1.706736687	0.093387762
GADD45G	-1.703508143	0.092035036	-0.365260134	0.716255159	-1.374219706	0.174825713
GADD45GIP1	0.734212192	0.464789372	0.006266905	0.995021422	0.822906817	0.41403686
GAK	-0.524901353	0.600983935	0.389355288	0.698449236	1.0457962	0.300129493
GAL3ST4	0.942691588	0.348443467	0.314481693	0.754291588	1.578140014	0.120142482
GALC	0.55738427	0.578693095	-1.440394911	0.155169716	-2.324848344	0.02371456
GALE	-0.32454201	0.746305085	0.15854468	0.87458221	0.292176748	0.771226692
GALK1	-0.094950694	0.924571872	0.430557866	0.668396809	-0.044158214	0.964934714
GALK2	0.274873806	0.784063486	1.221094832	0.22702517	0.989892534	0.326466772
GALM	-2.181134016	0.031865405	1.257128826	0.213780929	0.573689031	0.56846559
GALNS	-1.15705363	0.250410619	-0.16290469	0.871163701	0.010454027	0.991696043
GALNT1	-0.556935386	0.578998424	-0.131980535	0.895459661	-0.573770905	0.568410557
GALNT10	-0.608367698	0.544525268	1.479359571	0.144493166	1.39705865	0.167884397
GALNT11	1.010049036	0.31526668	-0.321877982	0.748710455	-0.136231344	0.892124906
GALNT12	2.924942849	0.004390446	1.253015852	0.215263086	1.805522141	0.076346533
GALNT14	-0.048466861	0.961455025	0.965638926	0.338262032	-0.024718239	0.980367209
GALNT2	-0.740516105	0.460976457	0.816679484	0.417476752	0.31451549	0.75429347
GALNT3	-0.909648805	0.365515547	-0.683543862	0.49700398	-2.044567448	0.045589169
GALNT4	1.155517072	0.251035529	-1.089441171	0.280496635	-3.125270359	0.002809175
GALNT6	-1.044268535	0.299247842	2.890552988	0.005414586	3.470871092	0.001003799
GALNT7	-0.672527514	0.503026391	-1.025959703	0.309200209	-2.422935227	0.018640118
GALNT9	-0.284786526	0.776483035	-0.013559915	0.989227946	-0.532960761	0.596160664
GALT	1.807497363	0.074133486	-0.492229121	0.624428411	-0.451848351	0.653116185
GAMT	1.137562205	0.258419813	-1.05884607	0.294090364	-0.832653506	0.408562393
GAN	0.99624172	0.321889581	-0.296657421	0.767795222	-1.352499213	0.18162918
GANAB	-0.08992895	0.92854994	1.595422851	0.116090725	1.88107622	0.065142316
GANC	1.513235871	0.133836323	0.928190325	0.357182418	0.8713332	0.387276887
GAPDH	-1.697991311	0.093075875	0.572426696	0.569260702	0.51895668	0.605827254
GAPDHS	1.387790265	0.168737384	1.438503622	0.155703186	2.021320155	0.048015873
GAPT	-0.315716657	0.752972074	-1.099789593	0.275999396	-1.668227807	0.100829783
GAPVD1	-0.024782238	0.980285291	0.406349516	0.685991709	0.63374829	0.5288144
GAR1	-0.084577728	0.932791007	-0.170069897	0.865551141	1.182134057	0.242126868
GARNL3	1.521119713	0.131848287	-0.342201848	0.733444578	-0.539156398	0.591907149
GARS	-0.046350477	0.963136907	3.398254441	0.001236136	4.120389302	0.000125511
GART	2.007656274	0.047776074	3.305652125	0.001634532	2.847455872	0.006142681
GAS1	-0.184271461	0.854228416	-1.378953169	0.173240948	-1.805958437	0.076277467
GAS2	-0.081103212	0.935545754	-1.287103459	0.203206821	-1.177105337	0.244111483
GAS2L1	-2.364647613	0.020267016	0.446919381	0.656609331	-1.217728096	0.228412134
GAS5	0.635013329	0.527082683	-0.02348091	0.981347828	0.462565494	0.645460689
GAS6	4.250326539	5.33E-05	1.04441143	0.30065877	1.203254464	0.233918875
GAS7	-0.612711858	0.54166173	-0.001726695	0.998628264	0.202726006	0.84008087
GAS8	0.552445144	0.582056899	1.675969597	0.099164962	0.71863989	0.475338207
GATA1	-3.008418346	0.003432191	0.674263748	0.502840981	-0.128189284	0.898456501
GATA2	-4.229826252	5.75E-05	1.010179361	0.316634412	-2.524851675	0.014421791
GATA3	-0.035857734	0.971477766	1.220230917	0.227349914	0.707265395	0.482322239
GATAD1	2.413335431	0.017900627	-1.97815744	0.052701223	-1.596485566	0.115985115
GATAD2A	-1.482412595	0.141836055	-0.060465946	0.951993859	-0.177638519	0.859645173
GATAD2B	-0.740205191	0.461164095	-0.181649167	0.856495827	0.490553985	0.625650616
GATC	2.023836985	0.046048498	-0.833485676	0.408014102	0.111540853	0.91158463
GATM	0.831446626	0.407992658	-0.292555405	0.77091334	-0.368682623	0.713748656
GATS	-2.275724778	0.025313434	-1.343110886	0.184504748	-0.555892368	0.580489459
GATSL3	-0.74528456	0.458104115	-0.318623205	0.751164819	-0.018261639	0.985494767
GBA	-2.164016254	0.033197515	1.116923638	0.268664683	1.369340392	0.176336788
GBA2	-1.014687646	0.313062277	0.258248479	0.79713573	1.201842313	0.234461282
GBAP1	0.31042094	0.756981702	-0.697228851	0.488464439	0.56077054	0.577181495
GBAS	0.563075238	0.574828807	-1.818325803	0.074215375	-1.725194189	0.089984536
GBE1	-1.495397907	0.138421483	0.989648575	0.326485332	0.484952812	0.629593557
GBF1	-0.748379735	0.456245175	0.842950087	0.402743397	1.22166485	0.226930858
GBGT1	-1.013279437	0.313730405	0.287890583	0.774463891	-0.556609022	0.580002914
GBP1	-1.153959153	0.251670257	0.426394454	0.671409885	-1.731077337	0.088921575
GBP1P1	-0.876289989	0.383280328	-0.446527596	0.656890581	-2.434877476	0.018094105
GBP2	-1.341541878	0.183228377	1.172780132	0.245711629	0.06030632	0.952125473

GBP3	-0.825147517	0.411539773	0.437869354	0.663118728	-0.610789472	0.543799788
GBP4	-1.250919512	0.214310201	-1.487911312	0.142229067	-1.802477624	0.076829944
GBP5	-0.740461698	0.461009289	-1.665409611	0.101261833	-3.134394558	0.002735991
GBP6	-2.498799885	0.01433617	-0.192594247	0.847954232	-1.568639487	0.122342067
GCA	-0.369015807	0.713010554	-0.059696892	0.952603698	-0.678567753	0.50019473
GCAT	-2.223951553	0.028735602	0.009665564	0.992321517	-0.821633233	0.414755478
GCC1	-1.563714537	0.121507128	2.579872778	0.012457816	2.1958561	0.032243316
GCC2	0.97085575	0.334305759	-0.701709787	0.485686039	-1.005789658	0.318824926
GCDH	0.330164899	0.742067369	1.217496404	0.228380052	2.229141757	0.029816779
GCET2	2.691494029	0.008526712	-0.389950186	0.698011714	0.954339876	0.343995938
GCFC1	1.84724031	0.06810394	0.038120501	0.969723428	-0.522500695	0.603374124
GCFC1-AS1	3.130892907	0.002371845	0.049601584	0.960611494	-0.937527469	0.352496341
GCH1	-0.247410806	0.805171995	-0.153937838	0.87819686	-2.172619048	0.034038721
GCHFR	1.389200411	0.168309695	-0.136485582	0.891913372	0.281385339	0.779447652
GCLC	0.444257367	0.657957247	0.6066763	0.546449757	0.906740313	0.368413051
GCLM	-0.688609827	0.492897086	-0.688895479	0.493654839	-1.716806824	0.091518095
GCM1	-0.502900283	0.616302385	0.745094957	0.459244036	0.975271591	0.333602187
GCN1L1	1.630115426	0.106684695	2.527263431	0.014263678	2.537424627	0.013966236
GCNT1	0.904723597	0.368105	1.377194413	0.173781064	0.029228351	0.976785951
GCNT2	0.180064815	0.857519532	1.022823613	0.310668136	0.537155734	0.593279109
GCNT4	1.628564237	0.107013529	-0.408643784	0.684316504	-0.751335421	0.455583578
GCNT7	0.500054481	0.618296474	0.260775078	0.795196086	0.554888854	0.581171087
GCOM1	-2.386904081	0.019152823	0.795063925	0.429840537	0.599249823	0.551412911
GCSH	0.324233177	0.746538066	0.244912197	0.807394895	0.478785649	0.633947496
GDAP1	0.51203278	0.609922616	1.776593284	0.080916444	0.209250251	0.835009037
GDAP2	0.206491514	0.836888435	-0.915787483	0.363596795	-1.746764716	0.086137886
GDE1	-3.295853593	0.001420154	0.429291881	0.66931243	-0.195985618	0.845327896
GDF11	0.749790103	0.455399552	-1.844643423	0.070233624	-0.98843833	0.327171869
GDF15	-0.199029854	0.842702792	-1.910288283	0.061079139	-1.292916677	0.201326372
GDF9	-0.355894238	0.722779439	-0.411064594	0.682550629	-0.126269457	0.899968998
GDI1	-1.103873624	0.272685231	-0.510623482	0.61156633	-0.159589518	0.873776243
GDI2	-0.36767318	0.714007964	1.923168642	0.059407248	2.041927197	0.045859327
GDPD1	0.226641783	0.82123282	-1.607068425	0.113508252	-1.834663165	0.071847098
GDPD3	0.165799246	0.868698792	-0.177326283	0.859874244	0.713187643	0.478678802
GDPD5	0.394599478	0.694102097	1.376087934	0.174121527	1.188034104	0.23981327
GEMIN2	0.521626427	0.603253029	-0.016841374	0.986621367	0.41569963	0.679211809
GEMIN4	0.788432039	0.432582525	3.315788212	0.001585634	4.675610876	1.88E-05
GEMIN5	1.305819706	0.195048775	2.889987856	0.005423075	2.714467325	0.008797987
GEMIN6	0.226554338	0.821300608	-0.246421278	0.806232265	0.445251176	0.65784748
GEMIN7	0.212269759	0.832392048	0.683308329	0.497151666	2.103653597	0.039893648
GEMIN8	0.745615929	0.457904891	-1.958386064	0.055033665	-0.579275426	0.564716647
GEN1	0.902721437	0.36916096	0.049423862	0.960752506	-0.027249763	0.978356996
GET4	0.025909017	0.979389116	0.873607505	0.385959939	0.952175971	0.34508242
GFER	0.965827172	0.336802012	-1.203961918	0.233528967	-0.734411354	0.465749356
GFI1	1.704388365	0.091869851	-2.867309664	0.005774104	-1.865991295	0.067260882
GFI1B	-3.587076561	0.000551189	1.334299407	0.187357255	0.689322378	0.493454918
GFM1	0.341987056	0.733183587	0.840889398	0.403887409	1.25973734	0.212967123
GFM2	0.509005672	0.612033983	-0.676744926	0.501276738	-0.270414575	0.787831288
GFOD1	0.117529357	0.906710906	-2.399329678	0.019681986	-3.58760368	0.000699979
GFOD2	-2.286286743	0.024661095	0.970453236	0.335878447	1.710150737	0.092750399
GFPT1	1.58176232	0.117324826	-0.442740157	0.65961203	-0.417341735	0.678017472
GFPT2	-1.796113808	0.075940152	-0.117086587	0.907198773	-0.156913071	0.875875349
GFRA2	-0.192883073	0.847499131	0.387151593	0.700070855	0.739109227	0.462914579
GGA1	0.232158617	0.816958871	-0.167053843	0.867912808	1.470701428	0.146945313
GGA2	1.668672956	0.098769577	1.500230161	0.139016866	2.309166131	0.02463123
GGA3	-0.581936661	0.562111042	-0.441468338	0.660526931	0.07441103	0.94094739
GGCT	2.368473179	0.020071501	-0.479431658	0.633447028	0.763487943	0.448363687
GGCX	0.698250294	0.486878833	-0.448452353	0.65550934	-0.062563002	0.950336328
GGH	-0.593353953	0.55448043	1.071426862	0.288446639	0.962042913	0.340146518
GGN	1.450058779	0.150629966	-2.683184203	0.009503002	-0.798650847	0.427852934
GGNBP2	-0.015259433	0.98786007	2.946685469	0.004630138	2.632963036	0.010909127
GGPS1	-0.063243791	0.94971714	-0.756767256	0.452273894	-0.912930925	0.36517618
GGT1	-0.316652945	0.752263869	1.871312281	0.066383766	0.652374333	0.516817536
GGT5	-0.291068559	0.771690177	0.175236091	0.861508718	-0.088966201	0.929424849
GGT7	3.534301745	0.000656789	-1.10872905	0.272155299	0.396201989	0.693455342
GGTA1P	-2.966282499	0.003888662	0.21332351	0.831827939	-0.078067224	0.938051688
GHDC	0.334506158	0.738800984	2.115557282	0.038718209	2.46730726	0.016683208
GHITM	-1.860573479	0.066175322	0.620982986	0.537060651	1.245777205	0.218011434
GHRL	-2.160518159	0.033475627	-1.273406152	0.207989309	-1.818259432	0.074351631
GHRLOS	0.082611793	0.934349588	0.426411876	0.671397265	1.400366767	0.166896843
GHRLOS2	1.176498348	0.242597904	-0.942251427	0.349999452	1.188722604	0.239544333
GIGYF1	0.896878617	0.372253464	0.253514283	0.800773578	1.630092614	0.108670917

GIGYF2	-0.329834364	0.74231626	-0.075529013	0.940055547	-0.033269683	0.973577333
GIMAP1	2.715923232	0.00796841	-0.98534803	0.328574406	-0.124879471	0.901064304
GIMAP2	1.221917938	0.22503199	-0.259033014	0.796533312	-0.345467758	0.731031683
GIMAP4	-0.757663398	0.45069543	1.57561761	0.120591274	0.767294983	0.446115697
GIMAP5	1.822439613	0.071816404	-3.241994782	0.001975693	-2.815856243	0.006696242
GIMAP6	2.526294011	0.01333306	-1.164875084	0.248871546	-0.272225339	0.786445768
GIMAP7	1.751512555	0.083374164	0.017242438	0.986302798	0.437305398	0.663564677
GIMAP8	-0.018426797	0.985340478	2.139165251	0.036673253	1.460732945	0.14965355
GIN1	1.281343553	0.203470407	-0.129074969	0.89774801	-0.081803124	0.935093727
GINS1	0.090120428	0.928398223	1.20022495	0.234965387	1.24617641	0.217865972
GINS2	0.562296854	0.575356613	0.834796957	0.407281346	0.940696167	0.350883882
GINS3	-0.894194525	0.373679567	0.010612431	0.991569338	1.876137065	0.065829676
GINS4	2.002375592	0.048351744	0.679295323	0.499671616	1.669852858	0.100506207
GIPC1	1.433943913	0.155164993	0.990041796	0.326294761	1.817914916	0.074405007
GIPC3	0.245840704	0.80638334	1.431997881	0.157549154	1.944738964	0.056813508
GIPR	4.093527839	9.46E-05	-0.064447839	0.948836801	-0.108172399	0.914243965
GIT1	0.295455106	0.768348709	-0.335108674	0.738760562	0.382397074	0.703608255
GIT2	-1.614380059	0.110058443	0.281694167	0.779187671	0.273499375	0.785471346
GJA3	0.612324811	0.541916549	-0.903317567	0.370119933	-0.253989307	0.800430179
GJB6	0.80044318	0.425629764	-0.150059149	0.881242208	2.413545855	0.019079755
GJC2	0.053620764	0.957359983	-0.546998323	0.586493059	-0.952887129	0.344725105
GJD3	1.340380993	0.183603829	-0.168104754	0.867089772	-2.445782075	0.017608141
GK	-0.152142724	0.879425879	-1.257638015	0.213597964	-2.653848745	0.010328028
GK3P	-0.567773213	0.571648168	0.546163351	0.58706309	-0.635616605	0.527604499
GK5	1.849852056	0.067722496	-2.713635218	0.008763394	-1.711377863	0.092522186
GKAP1	0.844280498	0.400823304	0.488544944	0.627018889	-0.137169037	0.891387102
GLA	-2.066611918	0.041737183	1.064226568	0.291667445	0.691765572	0.491930783
GLB1	-2.061760128	0.042208052	0.963847815	0.339151657	0.429266196	0.669369637
GLB1L	-0.299522173	0.765254514	1.43270354	0.157348108	0.735792867	0.464914702
GLB1L2	-0.515917485	0.607217919	-0.586958233	0.559525565	-0.554827957	0.581212463
GLB1L3	0.327389644	0.744157968	0.885228543	0.379714194	0.838493493	0.405303525
GLCCI1	0.548886007	0.584486604	-2.804477626	0.006859391	-3.134905097	0.002731949
GLCE	1.905081039	0.060065833	-0.264627261	0.792241307	-1.512390494	0.136034258
GLDC	0.078468242	0.937635399	0.557059252	0.579645308	0.959427455	0.341450344
GLE1	0.229653954	0.818898581	2.396244766	0.019832864	2.839179684	0.006283423
GLG1	-0.423052606	0.673298532	1.920564658	0.059742071	2.259862468	0.027721769
GLI1	-0.02471785	0.980336502	-0.003931659	0.996876584	0.473378689	0.637775483
GLI4	-0.561987979	0.575566119	-2.375232429	0.0208884	-1.934138792	0.058133713
GLIPR1	0.335247021	0.738244033	-2.299784223	0.025104431	-2.309123677	0.024633753
GLIPR2	-3.214036429	0.001835478	1.617927607	0.111142193	1.561359734	0.124049265
GLIS2	-0.439504218	0.661383644	-0.58121664	0.563362122	-1.523113208	0.133334288
GLIS3	0.344758681	0.731106042	-1.127233203	0.264318137	-1.37685499	0.17401373
GLMN	0.545410667	0.586863725	1.015490455	0.314119001	0.05437072	0.956832502
GLO1	1.350403257	0.180381493	-1.300018457	0.198773201	-0.277834464	0.78215833
GLOD4	1.379179605	0.171366968	1.130444683	0.262974384	1.884893535	0.064615251
GLRX	0.027376343	0.97822213	-1.09688505	0.277256535	-1.02736214	0.308648686
GLRX2	0.397364973	0.692069464	-2.635739286	0.010769784	-2.252539043	0.028209001
GLRX3	0.909102197	0.365802357	1.46003669	0.149713289	2.942991128	0.004716078
GLRX5	-3.257710583	0.001601412	-1.056228518	0.295274081	-1.132666941	0.262160335
GLS	1.472900147	0.144378921	-0.619529865	0.538010487	-1.83728048	0.071454123
GLS2	2.553769984	0.01239411	1.298506898	0.199288328	1.885185596	0.064575075
GLT1D1	-2.853817619	0.005395541	-0.473732173	0.637481733	-0.996686976	0.323185807
GLT25D1	0.212003473	0.832599139	3.111023106	0.002899041	2.793022917	0.007124503
GLT25D2	-1.502658808	0.136540459	-0.285237203	0.776485633	-1.31555874	0.193660485
GLT8D1	0.408196434	0.684130189	0.312698838	0.755638889	0.803055227	0.425323933
GLTP	-3.498457633	0.000739132	-0.437449981	0.663421008	-0.721205337	0.473770911
GLTPD1	-0.351981056	0.725701773	-0.566954417	0.572948151	-0.204604346	0.838619972
GLTPD2	-0.085103341	0.932374349	-3.39714146	0.001240325	-2.191096698	0.032604114
GLTSCR1	-0.266406324	0.790555314	-2.010014625	0.049121426	-1.53403543	0.130628039
GLTSCR2	1.103559806	0.272820645	0.707519086	0.482097091	1.081334265	0.284163727
GLUD1	1.340840312	0.183455207	1.712550906	0.092174476	1.261837169	0.212215923
GLUL	-4.188724805	6.69E-05	0.347261091	0.729660906	-0.812551293	0.419901867
GLYCTK	1.475608955	0.14365121	0.06108996	0.951499055	0.527745921	0.599751876
GLYR1	-2.880669573	0.004993547	0.350570975	0.727189175	0.426989277	0.671017471
GM2A	-3.179259449	0.002044332	0.71620859	0.476756502	1.108757722	0.272253944
GMCL1	0.075768037	0.939777229	-1.926695678	0.058956286	-3.308123618	0.001641726
GMDS	-1.782993168	0.078067685	2.783370021	0.007264269	3.333526018	0.00152166
GMEB1	-0.506150471	0.614028457	1.213097253	0.230044438	1.215602621	0.229214817
GMEB2	0.708233318	0.480689536	1.07763536	0.285689302	1.855562415	0.068759343
GMFB	0.069785624	0.944524099	-0.746889583	0.458168369	-1.333899008	0.187614054
GMFG	-0.221184692	0.825465805	-0.94631706	0.347940214	-0.640083835	0.524717455
GMIP	-0.720055948	0.473416458	-0.836976706	0.406065066	-0.883159841	0.380909753

GMNN	-0.53528832	0.59381321	0.196361532	0.845018404	0.106708062	0.915400342
GMPPA	-0.288782568	0.773433252	-0.221601024	0.825408294	0.768195938	0.445584665
GMPPB	1.734486385	0.086365839	-0.669213241	0.506033204	-0.484592368	0.629847665
GMPR	-3.999281287	0.000132752	1.819920478	0.07396883	0.901456666	0.371190157
GMPR2	-1.438901177	0.15375883	0.866066829	0.39004693	0.018028823	0.985679672
GMPS	1.905087504	0.060064981	2.261338018	0.027530913	2.970714839	0.00436377
GNA11	1.632729344	0.106132417	-0.116311791	0.907810044	0.752291408	0.455013196
GNA12	-1.618658723	0.109132694	1.467350785	0.14772026	1.493217766	0.140969784
GNA13	-0.250906138	0.802477032	-0.546443945	0.5868715	-1.89967164	0.062608754
GNA15	-1.108888207	0.270527756	0.101320189	0.919648185	-0.415849772	0.679102573
GNAI1	2.062251757	0.042160132	0.70269332	0.485077377	-0.039730604	0.968448616
GNAI2	-2.51940566	0.013578271	0.224571419	0.823107492	0.140040159	0.889128624
GNAI3	-2.458673281	0.015921813	0.034745773	0.972402599	-0.472091398	0.638688321
GNAL	-0.599896761	0.550130974	-0.018319423	0.985447347	0.637002528	0.526707927
GNAO1	-1.07014541	0.287507458	-0.77635908	0.440713577	-0.188895101	0.850855088
GNAQ	-0.170624447	0.8649144	-2.227025486	0.029869481	-3.906944567	0.000253293
GNAS	-2.319207673	0.022721878	0.356969309	0.722419347	0.895127014	0.37453456
GNAT2	-0.427312578	0.67020513	-1.396919125	0.167797112	-1.033432842	0.305825191
GNAZ	-4.358464619	3.57E-05	1.672699412	0.09981048	0.911313605	0.36602006
GNB1	-2.447676925	0.016382897	-0.442729562	0.65961965	-0.722439139	0.473018191
GNB1L	0.163115547	0.870804941	0.729486427	0.468660507	0.49564472	0.622076506
GNB2	-1.914904079	0.058783502	-0.572529317	0.569191663	-1.165747939	0.248636847
GNB2L1	0.48261472	0.630578832	1.509159799	0.136724463	1.857360339	0.068499018
GNB3	-0.034530358	0.972533165	-1.47660496	0.145228463	0.470172697	0.640049945
GNB4	-1.40995429	0.16211053	-0.496626189	0.6213429	-2.388303787	0.020308158
GNB5	0.353251789	0.724752351	1.029634981	0.307485898	1.237945768	0.220879556
GNE	0.27044894	0.787454056	0.878021234	0.383580228	1.373996789	0.174894531
GNG10	-0.588844954	0.557487801	-0.593793078	0.554975517	-1.461474533	0.149450738
GNG11	-5.484206149	4.00E-07	1.601889142	0.114650998	0.794309367	0.430354596
GNG2	-2.157994518	0.033677523	-1.593145715	0.116601191	-0.88412628	0.380392382
GNG5	-2.578382694	0.011604051	-2.702060821	0.009038069	-2.750662323	0.00798653
GNG7	0.062650943	0.950187866	-0.883972217	0.380386318	-0.114290281	0.909414758
GNGT2	0.02217513	0.982358927	0.290552505	0.772437215	0.520706995	0.604615132
GNL1	0.244840069	0.807155585	1.876283708	0.065686187	2.446095285	0.017594358
GNL2	2.022481569	0.04619113	2.959746341	0.004463429	3.285270475	0.00175734
GNL3	2.193921073	0.030900961	0.930334365	0.356081042	0.91975407	0.361629774
GNL3L	-2.636639176	0.009912126	1.452252918	0.151857359	1.926870698	0.059053973
GNLY	0.773246601	0.441467808	-0.237925564	0.812783211	0.624216101	0.535009841
GNMT	1.619403708	0.108972148	-0.487562095	0.627710766	0.141390817	0.88806649
GNN	1.694802377	0.093681889	-0.248750052	0.804438987	0.77400077	0.442172117
GNPAT	1.036860792	0.30266785	1.34265524	0.184651439	2.047950948	0.045244973
GNPDA1	0.029893452	0.976220349	2.775132509	0.007428163	2.451144398	0.017373504
GNPDA2	1.320576507	0.19009856	-0.781993081	0.43742164	-0.664162828	0.509300153
GNPNAT1	1.988321853	0.049912754	0.554394944	0.58145496	2.637185757	0.010789286
GNPTAB	1.539999619	0.127182039	-0.845015403	0.401598818	-1.018088068	0.312996151
GNPTG	-1.080012992	0.2831149	0.9815004	0.33045099	0.803757659	0.424921425
GNRH1	3.036105118	0.003159823	-0.149373346	0.881780853	1.128600417	0.263858085
GNRHR	0.159021711	0.874019547	-1.65903735	0.102544597	-1.666968205	0.101081178
GNRHR2	-0.893196095	0.374210926	-2.19151368	0.032472625	-0.574498847	0.567921378
GNS	-1.771683049	0.079941038	1.885867134	0.064358952	1.456006891	0.150951114
GOLGA1	1.113691716	0.268472285	-0.147410719	0.883322657	0.87549056	0.385031121
GOLGA2	-1.271464346	0.206944847	0.220002447	0.826647156	0.284569477	0.777019274
GOLGA2B	2.105143263	0.038155773	0.491159294	0.625180157	0.750887575	0.455850924
GOLGA3	1.370220269	0.174136044	2.222796489	0.030169512	2.293543519	0.02557562
GOLGA4	0.996854272	0.321593817	1.035461539	0.304781389	-0.036166583	0.971277605
GOLGA5	-0.90845259	0.366143397	1.038644409	0.303310862	0.902564255	0.370606902
GOLGA6L5	2.722772702	0.007817964	-1.264818341	0.211030215	-1.300117529	0.198864121
GOLGA7	-0.95439439	0.34252265	-2.944483592	0.004658806	-3.083151457	0.003171424
GOLGA7B	2.979891916	0.003735429	0.518531761	0.606073786	1.261349594	0.212390174
GOLGA8A	2.583351961	0.011450165	-0.977177833	0.332567687	0.576517184	0.566566134
GOLGA8B	1.967095943	0.052351795	0.276660123	0.783031509	0.592959494	0.555585409
GOLGB1	-2.202659923	0.0302566	1.670437572	0.100258966	2.136292529	0.037021375
GOLIM4	0.191799823	0.848344994	0.354680834	0.724124099	0.042175774	0.966507963
GOLM1	-1.019624546	0.310727485	-0.333529271	0.739946004	-0.970846616	0.335781913
GOLPH3	-2.520210983	0.013549396	-0.312386918	0.755874686	-1.585550583	0.118448935
GOLPH3L	0.409649705	0.683067639	0.121176188	0.903973245	-0.711215318	0.479890482
GOLT1B	0.561338697	0.576006638	-0.750879571	0.455782046	-1.175617465	0.244700924
GON4L	0.071007212	0.943554636	1.395439712	0.168240346	1.777056051	0.080967232
GOPC	1.104758815	0.272303518	-0.880563042	0.382213976	-1.845560389	0.070222836
GORAB	1.352211864	0.179804573	-0.495585301	0.622072698	-0.906027619	0.36878687
GORASP1	-0.691241713	0.491250065	0.314672699	0.75414729	-0.344004461	0.732125903
GORASP2	-0.556136689	0.579541885	0.676836813	0.501218859	1.075170026	0.286889932

GOSR1	-1.030028748	0.305845424	-0.462187122	0.645688328	-0.300502563	0.764901963
GOSR2	1.565416073	0.121107827	-0.433243784	0.666455901	-0.464599666	0.644011947
GOT1	1.284271027	0.202449181	3.707043153	0.000472873	4.124834998	0.000123666
GOT2	-0.755700725	0.451865458	1.459081229	0.149975192	2.032860415	0.046797634
GP1BA	0.780805278	0.437031857	1.055688752	0.295518582	1.46151028	0.149440967
GP5	-0.327709437	0.74391697	1.337157212	0.186428469	0.80954517	0.421613776
GP6	-3.111696193	0.002514894	0.599820812	0.550978233	0.284271205	0.777246656
GP9	-3.689122958	0.000390935	0.402590377	0.68873994	-0.925046638	0.35889422
GPA33	4.983310154	3.14E-06	1.275779035	0.207154859	1.889673434	0.063960379
GPAA1	-0.056024835	0.95545021	-0.024081566	0.980870786	-0.011829867	0.99060322
GPAM	0.112135605	0.91097365	0.075006457	0.940469487	-0.405189043	0.68687586
GPANK1	-2.195504438	0.03078333	-1.092523785	0.279151676	-1.146962729	0.256253486
GPAT2	1.781388412	0.078331255	0.222662172	0.824586175	1.254820434	0.214733821
GPATCH1	0.805233123	0.422875673	0.81510988	0.418367239	1.825173828	0.073287142
GPATCH2	1.184022579	0.239622012	-0.687106043	0.49477332	-0.960795061	0.340768173
GPATCH3	-1.166946875	0.246413543	3.225454863	0.00207474	2.806998214	0.006859481
GPATCH4	0.960546483	0.339436507	2.615657193	0.011350961	3.093712777	0.003076692
GPATCH8	0.352402452	0.725386882	-1.228297675	0.22433084	-1.032361887	0.306322013
GPBAR1	-1.536950802	0.127926592	0.335134311	0.738741325	-0.0506188	0.959808645
GPBP1	0.014541841	0.988430922	0.672526901	0.503937538	-0.149329005	0.881828267
GPBP1L1	-0.923508285	0.358291121	-0.169300393	0.86615357	-0.904991188	0.369330925
GPC1	0.371521241	0.711150655	0.545949776	0.587208939	0.950487461	0.345931768
GPC2	2.389716632	0.01901597	-1.240934956	0.219660487	-1.482502416	0.143789139
GPC4	-0.622627226	0.53515459	1.759441642	0.083813098	1.810906313	0.075497865
GPCPD1	-0.582515145	0.561723181	-0.274036934	0.785036657	-0.197293718	0.844309052
GPD1	1.423134864	0.158265476	-0.244790088	0.80748899	-0.46593808	0.643059482
GPD1L	1.019284571	0.310887893	0.043845417	0.965179279	0.063993467	0.949202357
GPD2	-0.383286637	0.702440199	-0.157728022	0.875222788	-1.398344371	0.167500044
GPER	1.192205124	0.236415573	0.218472638	0.827833138	1.478809032	0.144771145
GPHN	0.886472951	0.377801316	0.831367129	0.409199668	1.045721204	0.300163822
GPI	-1.278202852	0.204570252	1.364664094	0.177666571	1.376636492	0.174080944
GPKOW	-0.442848817	0.658971869	2.360694512	0.021647822	3.206030902	0.002220528
GPLD1	-0.403175587	0.687806034	0.570032752	0.57087241	-0.460433836	0.646980344
GPM6A	-0.364118021	0.716651414	2.012680589	0.048831575	0.437363311	0.663522934
GPM6B	1.580358659	0.117645926	-0.293338953	0.770317437	-0.965299301	0.338527769
GPN1	0.648785249	0.518182436	0.3288423	0.743467595	1.997303643	0.050639112
GPN2	-0.124967566	0.900836912	-2.044198954	0.045514993	-1.329092593	0.189184628
GPN3	0.348141936	0.728572753	-0.429332284	0.669283201	-0.482194746	0.631539095
GPNMB	0.482319576	0.630787604	2.318936945	0.02396805	1.952235318	0.055895406
GPR107	-1.726044691	0.087881523	0.790450997	0.432507058	0.87742747	0.383987619
GPR108	-1.572177362	0.119531455	-1.599663605	0.115144881	-1.749743104	0.085617627
GPR113	1.095730374	0.276214239	-0.529360581	0.598589806	-0.296231909	0.768144197
GPR114	0.165691692	0.868783182	-2.780850772	0.007314036	-1.726025662	0.089833672
GPR124	1.072934963	0.28626097	-0.545388729	0.587592156	-0.509294744	0.612538316
GPR125	1.758415527	0.082185814	0.497041741	0.621051651	-0.081710907	0.93516673
GPR126	1.138622492	0.257979538	0.279562725	0.780814504	-0.718129153	0.475650577
GPR128	-1.240700793	0.218044453	-0.807166316	0.422891447	-1.177059453	0.244129646
GPR132	0.591378844	0.555796777	-1.237703591	0.220847831	-0.307182758	0.759838927
GPR133	2.896059314	0.004775757	1.155334876	0.252723791	0.742464112	0.460896244
GPR137	-1.247672023	0.215491819	-1.15071915	0.254602807	-0.653854889	0.515870172
GPR137B	0.356535608	0.722300859	-0.91569026	0.363647366	-2.852790778	0.006053508
GPR141	-2.026643187	0.045754397	-1.215605086	0.229094536	-1.680201476	0.098465501
GPR146	-2.819696143	0.005949216	-0.101240536	0.919711134	-0.50636715	0.614578428
GPR15	0.601745164	0.548905317	-0.333327277	0.740097659	-2.017784425	0.048394554
GPR150	0.84678761	0.399431802	-3.437878771	0.001095457	-3.249278726	0.001955121
GPR152	2.336471575	0.021759833	0.217078153	0.828914562	0.10500109	0.916748559
GPR153	0.816022743	0.41671095	-0.85941573	0.393674102	-0.470419353	0.639874833
GPR155	1.417070469	0.160025774	-0.229847827	0.81902436	-1.405103289	0.165490687
GPR157	-0.556342056	0.579402123	-2.051784025	0.044746544	-1.538997399	0.12941313
GPR160	-2.51930677	0.013581821	-0.564239971	0.574781564	-0.988663711	0.327062523
GPR162	-1.383569406	0.170022506	-2.356453555	0.021873957	-1.361557846	0.178767625
GPR171	0.548929574	0.584456833	-1.464074528	0.148610425	-1.446306418	0.153641995
GPR172A	0.220272984	0.82617351	0.401019475	0.689889648	0.028334264	0.977495865
GPR172B	0.614934789	0.540199404	0.858947461	0.393930259	0.293237487	0.770420021
GPR174	0.667530899	0.506196101	-1.538930316	0.129297498	-2.055226519	0.044512457
GPR18	0.171729865	0.864047864	-4.426156494	4.33E-05	-3.405359341	0.001225495
GPR180	1.993052897	0.049382523	-0.059873122	0.95246395	-1.369492834	0.176289427
GPR183	1.988643072	0.0498766	-0.973304319	0.334472107	-0.516640688	0.607432833
GPR19	1.56901191	0.120267422	-1.706513265	0.09329944	-0.438817993	0.66247476
GPR20	0.524244146	0.601438978	-0.880465284	0.382266465	-0.854081919	0.396683209
GPR25	0.29542715	0.768369992	-2.867425382	0.005772261	-2.807041886	0.006858667
GPR27	0.3305757	0.741758076	-1.611405141	0.112558505	-2.823834327	0.006552294

GPR34	0.063653366	0.949391945	-1.397013908	0.167768746	-2.108893405	0.039419843
GPR35	0.652046353	0.516086546	1.855961549	0.068577382	2.108717693	0.039435651
GPR44	0.184555982	0.854005911	3.891093743	0.000261397	2.340553766	0.022827113
GPR55	0.241003209	0.810118463	2.190475591	0.032551633	2.568169142	0.012906713
GPR56	0.904486355	0.368230024	-1.853538447	0.068929169	-0.909374501	0.367033485
GPR65	-0.995919563	0.322045204	-1.273023776	0.208124009	-1.674762392	0.099533791
GPR68	0.516401995	0.606880965	-3.26711679	0.001833734	-1.941178971	0.057254004
GPR75	0.242986343	0.80858671	-2.359514548	0.021710529	-1.194807634	0.237176933
GPR77	-1.344593689	0.182244126	-1.996091443	0.050659381	-2.692211493	0.009333804
GPR82	1.094016131	0.276961153	-1.192605295	0.237914105	-1.164793204	0.24901999
GPR84	-1.70751536	0.09128499	-0.214292776	0.831075618	-1.192841008	0.237940191
GPR89A	1.06400408	0.290264771	-0.897110535	0.373394535	-1.426426915	0.159273366
GPR89B	0.515658541	0.607398037	-2.486682506	0.015815075	-3.911830155	0.000249304
GPR97	-4.141750363	7.94E-05	-0.163089151	0.871019126	0.116019985	0.908050015
GPRASP1	3.611747428	0.000507541	1.437176213	0.156078457	2.608838322	0.011617377
GPRC5B	0.68022637	0.498163417	1.990909374	0.051242272	1.146957503	0.256255628
GPRC5C	0.36040089	0.719418997	-0.364088285	0.717125284	0.701640168	0.485797233
GPRC5D	0.483444887	0.629991771	-0.39337815	0.695492602	0.859019888	0.393976404
GPRIN1	0.061718669	0.950928134	1.528960983	0.131747942	1.166250998	0.248435136
GPRIN3	0.413909357	0.679956908	-1.190000594	0.238928219	-2.237414349	0.029239298
GPS1	-0.82322025	0.412628752	0.65031217	0.51807627	1.048066117	0.299091701
GPS2	-2.305215213	0.023529008	-1.745924678	0.08615616	-1.010018126	0.316812683
GPSM1	1.864598011	0.065602253	0.058454443	0.953588984	0.491758843	0.624803887
GPSM2	-1.420693463	0.158972334	0.70775544	0.481951387	0.439466908	0.662007402
GPSM3	-2.388077562	0.019095618	0.335738357	0.738288123	0.2553591	0.799377398
GPT	1.938060594	0.055851826	-1.127822426	0.26407123	-1.381616646	0.172553909
GPT2	-0.51831402	0.605552071	2.968526891	0.004354509	1.838593451	0.071257671
GPX1	-2.00662339	0.04788821	-0.430230386	0.668633609	-0.562258074	0.576174593
GPX2	0.190058991	0.849704705	1.224895401	0.225600574	1.508293923	0.137077144
GPX3	-5.065023962	2.26E-06	0.042463323	0.966276215	1.479859255	0.144491374
GPX4	-0.445829999	0.656825188	-0.147646735	0.883137223	0.085698681	0.932010335
GPX7	0.775884804	0.439916557	-1.525402997	0.13263138	-0.97798268	0.332271357
GRAMD1A	0.087018581	0.930856281	-0.987266754	0.327641252	-0.423382708	0.673630914
GRAMD1B	-1.653828591	0.101758483	-0.9400218	0.351132118	-1.173247835	0.245641801
GRAMD1C	0.468197849	0.640811446	-1.175328059	0.244699314	-1.424891551	0.159714883
GRAMD3	1.798922081	0.075491094	-2.156624317	0.035222264	-1.478750888	0.144786646
GRAMD4	-0.893917076	0.373827176	1.051904972	0.297236451	-0.053406451	0.957597334
GRAP	1.071360287	0.286964143	2.882219609	0.005541029	3.783604561	0.000376987
GRAP2	-1.912779065	0.059058931	-0.179620943	0.858080591	-0.036744204	0.970819085
GRAPL	1.885490534	0.062693766	1.174214819	0.245141244	1.292515026	0.201464382
GRASP	1.354155402	0.179186169	0.356501563	0.72276767	0.500121154	0.618941224
GRB10	-3.010091477	0.003415133	1.929621661	0.058584391	0.820267093	0.415527162
GRB14	-1.602416676	0.112680415	-0.084516834	0.932938605	-1.74773802	0.085967583
GRB2	-2.540388683	0.01284369	1.532240772	0.130937731	1.236268099	0.22149757
GREB1	-0.667749608	0.506057135	2.350258202	0.022208095	1.486038429	0.142853897
GREM2	0.445543266	0.657031533	1.070407829	0.288900968	-0.233671593	0.816088476
GRHL1	-0.377772118	0.706517983	0.080617704	0.936025463	-0.005409979	0.995702628
GRHPR	-0.756411295	0.451441658	0.500723615	0.61847379	0.274766927	0.784502226
GRID2IP	1.661474671	0.100209924	-1.109976533	0.271621867	0.318803539	0.751056584
GRIK4	0.641263001	0.523033953	-1.756166054	0.084375981	-1.192521882	0.238064214
GRIK5	-1.140935149	0.257021064	1.027241273	0.308601694	0.39651064	0.693228979
GRIN2C	-0.453661566	0.651199604	-0.769220772	0.444905338	0.985667617	0.328518109
GRIN2D	-0.541528935	0.589524191	0.057142161	0.954629729	-0.167403263	0.867653285
GRIN3A	-0.502694943	0.616446173	0.31952098	0.750487565	0.318361024	0.751390415
GRIN3B	1.401776643	0.164531931	-3.869300351	0.000280611	-3.700664959	0.000490827
GRINA	-1.996258027	0.049026047	-0.773909704	0.442149276	-1.087986307	0.281242043
GRINL1A	0.141831286	0.887540484	-2.761649507	0.007703779	-3.570892021	0.000737333
GRIP1	0.856932799	0.393831255	-0.396375727	0.693292592	0.4613216	0.646347274
GRIP2	-1.816239402	0.072770436	1.403284056	0.165900428	0.639257304	0.525250992
GRIPAP1	-0.942474957	0.348553687	1.86916692	0.066686727	1.914201524	0.060687789
GRK4	0.994520092	0.32272182	-2.458962412	0.016960695	-2.385401579	0.020453842
GRK5	-3.429863737	0.000924604	1.116665477	0.268774167	-0.14875193	0.882281512
GRK6	-1.348649453	0.180942266	0.604054384	0.548179458	1.173276453	0.245630423
GRM2	2.486712152	0.014798231	-1.57680234	0.120318171	-0.597056733	0.552865834
GRN	-2.278476244	0.025142048	2.085690704	0.041447213	1.665060014	0.101462997
GRPEL1	0.096220063	0.923566617	0.626742291	0.533304596	2.321292398	0.023919709
GRPEL2	0.620729128	0.536397142	0.090560006	0.928156418	-0.25600094	0.798884228
GRSF1	0.79382835	0.429450579	2.959881966	0.004461727	3.749991575	0.000419684
GRTP1	-0.180968601	0.85681223	-0.268931351	0.788943518	0.544785334	0.588055143
GRWD1	0.832620277	0.407333812	3.398519415	0.001235141	2.58342228	0.012408629
GSDMA	-0.198895392	0.84280765	0.588073725	0.5587817	-0.279035494	0.781241178
GSDMB	2.416562051	0.017752873	0.601682278	0.549746747	1.28750466	0.203191929

GSDMD	-1.141507757	0.256784138	0.664191861	0.509217832	0.541888078	0.590036314
GSG2	0.127734588	0.89865317	0.367506193	0.714588467	0.663708539	0.509588755
GSK3A	-1.6021076	0.112748812	-1.237464476	0.22093588	-0.999910657	0.321636872
GSK3B	-2.189113012	0.031260569	-1.130439339	0.262976616	-1.853582596	0.069046969
GSN	-2.37840255	0.019571828	1.554202329	0.125614264	1.364093702	0.177972768
GSPT1	-3.245414631	0.001664316	1.760504787	0.083631077	0.99528645	0.323860295
GSPT2	1.386919297	0.169001958	1.725322419	0.089831646	1.670603691	0.10035699
GSR	-1.902512278	0.060405033	2.044539118	0.045480288	1.361997724	0.178629552
GSS	0.586182745	0.559267201	1.890799781	0.063684718	2.937757802	0.004785479
GSTA4	1.421650372	0.158694991	0.952548303	0.344799473	0.882810172	0.381097053
GSTCD	0.110519439	0.912251438	-1.891584982	0.063577944	-1.321415294	0.191713925
GSTK1	0.212410755	0.8322824	0.427473361	0.670628553	0.682399342	0.497787777
GSTM1	-0.739480051	0.461601889	0.318053857	0.751594419	0.348876031	0.728485238
GSTM2	1.450292206	0.150565039	0.202195723	0.840476216	0.997525301	0.322782524
GSTM3	1.805389357	0.074465322	0.527139313	0.600121452	2.184034524	0.033146023
GSTM4	1.454718201	0.149338057	2.275285585	0.026627617	1.12298073	0.266217066
GSTO1	-0.866991069	0.388326691	0.764868665	0.447472395	0.227339058	0.820984564
GSTO2	-0.915423923	0.362494022	1.098812811	0.276421719	-0.046824185	0.962819241
GSTP1	-1.043450385	0.299624272	1.107374503	0.272735344	1.573546815	0.121201908
GSTT1	-0.203792478	0.838990579	-1.384661238	0.17149686	-1.261790605	0.21223256
GSTZ1	-0.306365591	0.760056703	2.431587806	0.018164836	2.049427514	0.045095472
GTDC1	0.022319352	0.982244213	-1.346923337	0.18328083	-1.640824975	0.106415648
GTF2A1	-0.126092659	0.899948891	-0.535070623	0.594660899	-1.449998231	0.152613516
GTF2A2	-0.115519176	0.908299263	-2.833025325	0.006344859	-1.778723672	0.080690251
GTF2B	-0.281868593	0.778712224	-1.253213129	0.215191822	-1.243718636	0.218762671
GTF2E1	-0.36044637	0.719385112	-0.118960616	0.905720501	-1.085841411	0.28218182
GTF2E2	0.645630122	0.520214467	0.481262027	0.632153658	0.547926021	0.585911085
GTF2F1	-1.722483277	0.088527478	0.947710632	0.347236194	1.347710557	0.183155915
GTF2F2	0.378185501	0.706212002	0.260171207	0.795659554	0.399090879	0.691337748
GTF2H1	1.155610014	0.250997699	1.223515132	0.226117191	1.955732603	0.055471442
GTF2H2	0.132774365	0.894677781	0.304647212	0.761732942	0.506630579	0.61439473
GTF2H2B	1.084509515	0.281128691	0.929267878	0.356628613	1.020352346	0.31193091
GTF2H3	1.049431419	0.296879811	-0.10888803	0.913669772	-0.544004599	0.588588706
GTF2H4	1.064076224	0.290232275	-0.544406468	0.588263363	0.998935847	0.322104729
GTF2H5	0.790482494	0.431390887	-2.296431427	0.025308211	-1.137631559	0.26009816
GTF2I	-1.788374244	0.077189237	1.089779198	0.280348932	2.032273532	0.046858937
GTF2IP1	-0.580573311	0.56302566	-0.850663217	0.398479097	-2.925186918	0.004956082
GTF2IRD1	1.509853084	0.134696545	-0.520370373	0.604800065	0.154381168	0.877861917
GTF2IRD2	1.143014181	0.256161564	2.261480345	0.02752156	3.577144125	0.000723141
GTF2IRD2B	0.59617786	0.552601087	1.178948413	0.243266085	3.648610511	0.000578369
GTF3A	0.322212664	0.748062905	-0.144191921	0.885852265	1.04708825	0.299538473
GTF3C1	-0.768797903	0.444090827	1.851243993	0.069263681	1.878291526	0.065529098
GTF3C2	0.796628858	0.427830498	0.464652866	0.64393183	1.332597729	0.188038289
GTF3C3	1.894490811	0.061474666	-0.922665586	0.360030564	-0.668092299	0.506807504
GTF3C4	1.099477071	0.274586631	1.196083995	0.236564578	0.924535056	0.359158055
GTF3C5	-0.167413495	0.867432396	0.923343115	0.359680495	1.409694074	0.164136549
GTF3C6	-2.496109491	0.014437877	-0.2574308	0.79776373	0.720494193	0.474205074
GTPBP1	-1.868682873	0.065024856	-0.196590121	0.844840337	-0.252583345	0.801511147
GTPBP10	0.912327787	0.364111928	-1.448054276	0.153023812	-0.831572719	0.409167253
GTPBP2	-1.806319363	0.074318772	-1.018865699	0.312527464	-2.493102406	0.015632633
GTPBP3	1.99286329	0.049403681	1.120950567	0.266960958	0.955557494	0.343385568
GTPBP4	0.977851642	0.330853141	3.363788726	0.001372263	3.535031866	0.000824002
GTPBP5	-0.341226622	0.733753939	0.044033084	0.965030337	0.020442309	0.983762892
GTPBP6	-0.387123511	0.699608095	0.107028606	0.915138222	0.784160411	0.436236564
GTPBP8	-0.602783899	0.548217145	2.090921029	0.040957557	2.218498131	0.03057457
GTSE1	-0.530222089	0.597305749	0.312255028	0.755974395	0.743555542	0.460240718
GTSF1	-0.104883113	0.916709466	-1.627216839	0.10915005	-0.281969754	0.779001783
GUCA1B	2.147486521	0.034529612	1.020232344	0.3118846	2.14032188	0.036679634
GUCY1A3	0.357169428	0.721828022	-0.245370644	0.807041651	-1.150507326	0.254803698
GUCY1B3	-3.463003022	0.000830105	0.13231882	0.895193293	-0.401606139	0.689496044
GUCY2C	-1.127512274	0.262619281	-1.228798884	0.224144232	-0.563861667	0.575090085
GUCY2D	1.165363137	0.24705033	0.209098545	0.835109093	-0.07625736	0.939484996
GUF1	1.048701331	0.2972139	-0.467065203	0.642215351	-0.310336462	0.757452332
GUK1	-2.863453166	0.005247989	-0.153963999	0.878176326	-0.744311046	0.459787267
GUSB	-1.043326066	0.299681499	1.366283116	0.177160828	2.071149466	0.042945077
GUSBP1	1.772618225	0.079784743	-0.437382136	0.663469916	-0.534698067	0.594966502
GUSBP11	2.653203583	0.009473669	-0.482562298	0.631235565	0.433185693	0.666536878
GUSBP2	1.12434212	0.263953854	-0.193529554	0.847225148	-1.218075761	0.228281035
GUSBP3	0.918078647	0.361110433	0.913453966	0.364811836	1.167093451	0.248097602
GUSBP4	0.54610101	0.58639117	-1.970595636	0.053583088	-3.053637904	0.003450822
GUSBP5	-0.030041498	0.976102617	-0.726157959	0.470682636	0.069806026	0.944595686
GVINP1	0.19091945	0.849032569	-1.254105361	0.214869732	-1.52483825	0.132903927

GXYLT1	0.258977991	0.796262666	-0.196196411	0.845147037	-0.931311951	0.355673201
GYG1	-2.013115883	0.04718707	0.650742654	0.517800298	0.440234052	0.661455068
GYLTL1B	1.302606121	0.196139423	-0.86534842	0.390437707	-0.038430307	0.969480699
GYPA	-1.790965302	0.076769184	0.569012464	0.57155999	0.135864531	0.892413551
GYPB	-1.254953766	0.212848942	-0.094317786	0.925184076	-0.06172377	0.951001659
GYPC	-3.552708135	0.000617954	-0.902408963	0.370598132	-1.495150462	0.140465941
GYPE	-1.76903373	0.08038519	0.560223802	0.57749941	1.704893362	0.093733387
GYS1	0.117775038	0.906516805	0.341035692	0.734317662	1.241272378	0.219657869
GZF1	0.733642174	0.465135025	-0.779651912	0.438787814	-0.362060661	0.718663691
GZMA	1.352546263	0.179698058	-2.386730024	0.020304763	-1.587656452	0.117971201
GZMB	-0.598583516	0.551002606	-1.734917582	0.088104037	-1.176522854	0.24434212
GZMH	0.590391888	0.556455132	-2.413834508	0.018986277	-0.838986545	0.40502912
GZMK	1.574072232	0.119092618	-0.899292331	0.372241408	-0.705926317	0.483148196
GZMM	0.472359532	0.63785037	-2.539362128	0.013828566	-1.552679084	0.126109885
H19	-1.616287773	0.109644905	-1.008756939	0.317310388	-1.638946009	0.106807715
H1F0	-3.748436767	0.000319297	0.293419942	0.77025585	-0.690417247	0.492771586
H1FNT	0.847658866	0.39894893	-3.859134042	0.000290031	-1.183216435	0.241701229
H1FX	-1.335971258	0.185035302	-2.075325813	0.042432605	-2.417065515	0.018913876
H2AFJ	-1.566256271	0.120911041	0.316424578	0.752824225	0.385400357	0.70139477
H2AFV	-1.24943759	0.214848816	-1.148556605	0.255486579	0.008294525	0.993411357
H2AFX	0.299784823	0.765054821	-1.989359118	0.051417764	-1.763009249	0.083331906
H2AFY	-2.028987848	0.045509904	2.018159904	0.048240472	1.714072757	0.092022633
H2AFY2	0.696791156	0.487787127	0.006065486	0.995181432	1.160730535	0.250655109
H2AFZ	-1.050313943	0.296476307	-0.42833053	0.670008062	-0.393949446	0.695108196
H3F3B	-3.985288578	0.000139538	1.060672935	0.293266151	-0.428221932	0.670125181
H3F3C	0.195244832	0.845655558	2.047480638	0.045181133	0.778612219	0.439472114
H6PD	-1.115369862	0.267756767	0.989306082	0.32665138	1.736597143	0.087933727
HAAO	-2.056442903	0.042729335	0.080217228	0.936342567	-0.826824983	0.411830788
HABP4	3.179497069	0.002042833	0.885356794	0.379645623	2.691208656	0.009358637
HACE1	1.811615926	0.073488702	-1.595557351	0.116060631	-1.593723124	0.116603598
HACL1	1.38487057	0.169625548	1.407735519	0.164583801	2.553537355	0.013401504
HADH	0.700713446	0.485347664	1.505800993	0.137583194	2.52768588	0.014317941
HADHA	-0.835732276	0.405589975	3.153153109	0.002565129	3.609592292	0.000653574
HADHB	-0.70496342	0.482712	3.343285274	0.00145985	3.110133468	0.002934649
HAGH	-2.463440475	0.015725554	-0.055213184	0.956159706	-0.736157696	0.464694429
HAGHL	1.198026296	0.234153335	-1.720982029	0.090622277	-1.76613827	0.082800244
HAL	-3.055667672	0.002979612	-0.217524392	0.828568467	-1.362498696	0.178472402
HAPLN3	2.565572674	0.012009391	1.662736852	0.101798266	1.663486415	0.101778752
HAR1A	1.453388413	0.149705884	0.131154093	0.896110455	0.815167839	0.418415232
HARBI1	-2.019946026	0.046458964	-0.291797381	0.771489965	-0.357968993	0.72170666
HARS	0.062149317	0.950586175	2.161462678	0.03482916	3.201019925	0.002253383
HARS2	-0.60734462	0.545200762	2.561143213	0.013075447	2.149186542	0.035937495
HAS1	0.050020604	0.960220382	-0.893592038	0.375258914	-2.315448408	0.024260294
HAS3	-0.374727543	0.708773021	-1.29198702	0.20152172	1.339890728	0.185670025
HAT1	-0.032414682	0.974215444	-0.216812775	0.8291204	-0.27008691	0.788082077
HAUS1	0.25463496	0.799604677	-0.341802707	0.73374337	0.937174443	0.352676283
HAUS2	0.124320219	0.90134791	0.514528231	0.608851532	1.726894906	0.089676178
HAUS3	0.547286761	0.585579917	-0.448587164	0.655412643	-0.510234216	0.61188429
HAUS4	-1.137001816	0.258652724	1.185566148	0.24066192	0.541690595	0.59017147
HAUS5	0.98037973	0.32961127	-0.725752935	0.470929035	0.644476856	0.521886493
HAUS6	0.664676587	0.50801158	-0.002908371	0.997689509	-1.120936833	0.267078719
HAUS7	1.278236661	0.204558389	1.243666613	0.218660429	1.954806917	0.055583391
HAUS8	-1.41242427	0.161384574	0.827363527	0.41144587	1.822335754	0.073722512
HAVCR1	0.067027637	0.946713166	0.524024054	0.602272597	-0.027199968	0.978396535
HAVCR2	-0.340750835	0.734110871	2.98926946	0.004106947	1.291128864	0.201941219
HAX1	0.834763596	0.406132297	-0.791487507	0.431907045	-0.025201921	0.97998312
HBA1	-3.238621306	0.001700054	-1.29278943	0.201245847	-1.926261104	0.059131719
HBA2	-3.450660568	0.000864183	-0.155509017	0.876963774	-1.248205197	0.217127831
HBB	-3.280756089	0.001489469	0.457564847	0.648986514	0.234354349	0.81556103
HBD	-3.027649498	0.003240802	0.981604885	0.330399936	0.68335878	0.497186063
HBEGF	-1.233077746	0.220860989	-0.476965336	0.635191591	-0.486636338	0.628407294
HBG1	-2.747487033	0.007296395	0.607931216	0.54562286	0.56721363	0.572826363
HBG2	-1.87150097	0.064629007	1.520661042	0.133816105	1.588248799	0.117837102
HBM	-2.174407745	0.032383182	-1.360288508	0.179038917	-2.685936205	0.009490197
HBP1	-0.929389369	0.355253335	0.422647766	0.67412602	-0.660535697	0.511606852
HBQ1	-2.760531236	0.007034104	-0.156915173	0.875860461	-0.551678665	0.583354157
HBS1L	1.366095231	0.175422349	1.343469621	0.184389319	1.551632227	0.126360228
HBXIP	-1.884885845	0.062776396	-1.847126297	0.069867436	-1.242175767	0.219326964
HBZ	-0.751567525	0.45433513	-0.045117826	0.964169458	-1.111146536	0.271233356
HCAR2	1.118018249	0.266630278	-0.965818147	0.3381731	-1.426427669	0.15927315
HCAR3	1.000699795	0.319741167	-0.078998837	0.937307377	-0.935052203	0.353759277
HCCS	-2.779155708	0.006674518	-2.101711933	0.039963199	-1.382767619	0.172202461

HCFC1	-0.371046586	0.71150288	0.29122051	0.771928874	0.232943173	0.816651293
HCFC1R1	0.123633589	0.901889963	-1.435897215	0.156440707	-1.190114751	0.23900121
HCFC2	1.934557478	0.056287215	-1.775778534	0.081052131	-1.381832938	0.172487823
HCG11	0.518417456	0.605480219	-1.186386397	0.240340549	-2.799114741	0.00700784
HCG18	1.072353532	0.286520471	-2.412792856	0.019035493	-1.74305127	0.086790213
HCG25	-1.430438883	0.156165199	0.341676464	0.733837882	0.35597175	0.723193656
HCG26	-0.478533686	0.633468222	0.810632402	0.420913754	1.618406029	0.111170701
HCG27	-0.640108299	0.523780779	-2.630013446	0.010932661	-2.104179066	0.03984591
HCG4	1.588356874	0.115825619	0.129083231	0.897741502	-0.520666979	0.604642831
HCK	-1.380013965	0.171110808	-0.042352499	0.966364176	-0.174926966	0.861765298
HCLS1	-2.455135678	0.016068865	1.241618269	0.219410011	1.383459586	0.171991435
HCN2	0.380810017	0.704270499	-2.291687792	0.025599035	-2.355887603	0.021989367
HCN3	1.746426988	0.084258675	-2.167848823	0.034316192	-1.621405463	0.110524712
HCP5	-0.267786458	0.789496174	0.138851117	0.89005215	0.261519034	0.794647712
HCST	-0.683258104	0.49625543	-2.269816338	0.026978648	-0.867432842	0.389391264
HDAC1	-1.135107592	0.259441102	1.553874242	0.1256925	2.397728344	0.019841458
HDAC10	-0.662641218	0.509308292	0.157024455	0.875774725	0.756376933	0.452580259
HDAC11	0.711888206	0.478434522	1.150896158	0.254530565	1.514392884	0.135526792
HDAC2	2.51627804	0.01369094	2.050339061	0.044892062	3.049737443	0.003489422
HDAC3	-1.097064335	0.275633986	2.081321644	0.04186013	3.068310069	0.003309167
HDAC4	0.465518357	0.642721016	-1.240208701	0.219926934	-2.759510694	0.007798913
HDAC5	0.154396243	0.877654151	-0.081132431	0.935617907	0.252834277	0.801318191
HDAC6	1.030391668	0.305676067	1.041631	0.301935426	1.643888737	0.105778873
HDAC7	1.800952277	0.07516783	-0.888674078	0.37787471	-0.369538971	0.713113931
HDAC8	0.096153228	0.923619543	2.174567797	0.033783652	1.227014381	0.224929289
HDAC9	2.867137749	0.005192553	-0.380424677	0.705029605	-1.796305625	0.077817803
HDC	-4.368515227	3.44E-05	1.514295421	0.135419674	-1.907111805	0.061618749
HDDC2	-0.175553952	0.861051455	-0.612155556	0.542844022	-0.203789927	0.839253325
HDDC3	0.25273825	0.801065393	0.400919566	0.689962794	0.80710451	0.423006755
HDGF	-2.037940224	0.044586655	1.356128701	0.180351074	1.451302363	0.152251494
HDGFRP2	-0.1860253	0.852857039	1.299790743	0.198850741	1.349574426	0.182560514
HDGFRP3	1.524814895	0.130924536	-0.33870744	0.736061844	-1.947893216	0.056425638
HDHD1	0.163571361	0.870447155	3.16035652	0.002511781	2.799637168	0.006997918
HDHD2	-0.190140476	0.849641049	1.157377236	0.251895539	0.550804034	0.583949624
HDHD3	0.518673894	0.6053021	0.982534447	0.329945962	1.461627269	0.149408994
HDLBP	-2.258392511	0.026416876	1.345053815	0.183880232	1.483384119	0.143555487
HDX	-0.597100634	0.551987659	-0.445303235	0.657769832	-1.276593609	0.206992284
HEATR1	1.124246945	0.263993995	1.22541402	0.225406686	1.349979925	0.182431175
HEATR2	1.770448391	0.080147772	1.219963309	0.227450576	0.907467266	0.368032004
HEATR3	2.32858353	0.022194835	2.819133705	0.006590614	3.107028915	0.002961024
HEATR4	1.312748701	0.192712572	1.124467017	0.265479457	0.857423646	0.394850144
HEATR5A	-0.261164696	0.794581405	-1.545217967	0.127770776	-3.059673832	0.003391874
HEATR5B	1.581456493	0.117394727	0.606805196	0.546364794	-0.130409871	0.896707529
HEATR6	0.28879314	0.773425188	0.595246839	0.554010128	0.940133554	0.351169828
HEATR7A	2.521483668	0.013503875	0.063440141	0.949635682	0.411974511	0.681924228
HEBP1	-1.755137712	0.082748338	1.892230606	0.063490265	1.825111312	0.073296708
HEBP2	-2.12439894	0.036467814	-0.740358908	0.462089698	-0.184381363	0.854377575
HECA	0.330209157	0.742034045	1.675136195	0.099329146	0.834003413	0.407807688
HECTD1	1.00323272	0.318524772	2.012383784	0.048863772	1.365182124	0.177632435
HECTD2	1.007611013	0.316429449	-1.516762367	0.134796424	-0.886628678	0.379054816
HECTD3	-0.096184799	0.923594542	-0.318860571	0.750985738	0.475731325	0.636108651
HECW2	-2.341195112	0.021502973	-1.25939165	0.212968723	-2.428321236	0.018392057
HEG1	1.90639267	0.059893255	-0.018915429	0.984973946	0.061484857	0.951191072
HELB	1.389814146	0.168123811	1.122758829	0.2661984	1.047992202	0.299125455
HELLS	2.529055036	0.013235896	-0.551807954	0.583214687	0.250287901	0.803283752
HELQ	1.872542048	0.064483282	0.649024737	0.518902075	0.33552054	0.738480942
HELZ	0.561328222	0.576013747	-1.806210544	0.076111049	-2.907691973	0.005202922
HEMGN	-4.793956257	6.68E-06	0.657402405	0.513540872	0.198324485	0.843506404
HEMK1	2.587508065	0.011322878	-0.531030841	0.597439301	0.151406298	0.880197053
HENMT1	1.611484948	0.110688413	-0.033404491	0.973467523	0.515463155	0.608249913
HERC1	0.771940927	0.442236716	0.960170949	0.340982737	0.956247231	0.34304013
HERC2	2.364941886	0.020251917	-0.327360736	0.74458193	0.348649691	0.72865425
HERC2P2	3.16194366	0.002156405	-0.97254062	0.334848431	0.765300449	0.447292611
HERC2P3	0.912755112	0.363888353	-1.484343805	0.143170154	-0.765178897	0.447364394
HERC2P9	1.038937578	0.301706382	-0.908149861	0.367583306	-1.542704632	0.128511329
HERC3	0.279278819	0.780692264	-0.821922487	0.41451055	-1.297921128	0.199612749
HERC4	1.210642439	0.229304067	0.407564908	0.685104068	-0.78899351	0.43342956
HERC5	-1.358686902	0.177750563	1.828240147	0.072693684	-1.658926173	0.102698341
HERC6	-1.427689936	0.156953123	1.186661588	0.2402328	-0.472260863	0.638568118
HERPUD1	-1.43798913	0.154016796	0.296866116	0.767636687	0.408914336	0.684155637
HERPUD2	-2.434257569	0.016961721	-1.285160537	0.203880158	-1.874264733	0.066091839
HES1	0.848293838	0.398597236	0.708298551	0.48161667	-0.729181548	0.468916728

HES4	0.140944261	0.888239103	-1.120569908	0.267121681	-2.855938126	0.006001464
HES6	0.83074505	0.408386805	-1.427254446	0.158905771	-1.752445898	0.085147768
HEXA	0.27034132	0.787536571	2.229576048	0.029689803	2.237705879	0.029219129
HEXB	-0.408153106	0.684161877	1.050382018	0.297929815	0.16761685	0.867486028
HEXDC	0.530592379	0.597050158	0.274863381	0.784404768	1.170747959	0.246637214
HEXIM1	-0.818873865	0.415090993	-3.058147586	0.003375777	-2.50923943	0.015006176
HEXIM2	-1.765034858	0.081059451	2.881073597	0.00555863	1.480312759	0.144370696
HEY1	-2.045600437	0.043809472	1.860860193	0.067870817	0.416854236	0.678371954
HFE	0.041348875	0.967112311	0.709589359	0.480821675	0.412115023	0.681821839
HGD	-2.167642217	0.032911362	1.35151631	0.181814549	1.325829479	0.190256562
HGF	0.164702795	0.869559166	1.837799727	0.071251345	0.827538982	0.411429553
HGS	-1.068161659	0.288396143	-0.930771069	0.355856981	-0.64165247	0.523705665
HGSNAT	0.457795472	0.64823823	-0.454630186	0.651084195	0.362421183	0.71839579
HHAT	1.094188477	0.276885997	-0.351384718	0.726581939	0.088553896	0.929751052
HHEX	-2.189557791	0.03122715	1.226053411	0.225167813	-0.536081421	0.594016438
HHLA2	0.94280307	0.348386755	0.056684725	0.954992531	1.243765756	0.218745455
HHLA3	0.150998578	0.880325647	0.562305811	0.576089685	1.613854103	0.112156895
HIAT1	-0.108340412	0.913974605	-1.376024424	0.174141084	-2.406730281	0.019404687
HIATL1	-1.275637707	0.205471801	-2.298675779	0.025171639	-3.188017821	0.002340778
HIATL2	-0.022669524	0.981965687	-3.641477131	0.000582013	-3.18968702	0.002329383
HIBADH	-0.597722112	0.551574715	0.904410738	0.369545117	1.144770549	0.257153056
HIBCH	1.889864936	0.062098731	1.862129283	0.067688773	1.748155419	0.085894635
HIC1	-1.720084873	0.088964681	-3.446510865	0.001066893	-3.049861826	0.003488185
HIC2	2.408999551	0.018100903	-0.369833378	0.712863047	0.112888141	0.910521253
HIF1A	-1.952941243	0.05403404	0.667506604	0.507114368	-1.595703282	0.116159991
HIF1AN	0.694181973	0.489413627	0.81472388	0.418586405	1.446315139	0.153639559
HIGD1A	-0.187586299	0.851636831	-1.047786796	0.29911391	-0.946563075	0.347911077
HIGD2A	-1.467488627	0.145841311	-1.754541371	0.084656332	-1.667287221	0.10101746
HINFP	0.663106875	0.509011472	0.813688851	0.419174423	1.630447885	0.108595645
HINT1	0.666570679	0.506806459	-1.217494633	0.22838072	-0.659435166	0.512307848
HINT2	1.320818213	0.19001827	0.156629726	0.876084412	1.93469964	0.058063212
HINT3	-1.426238271	0.157370445	-0.967123359	0.337525897	-1.708429344	0.093071313
HIP1	-1.188313486	0.237936694	1.515044183	0.135230265	1.718219471	0.091258304
HIP1R	1.952555433	0.054080528	0.323209933	0.747706807	1.099508963	0.276230787
HIPK1	-0.349309902	0.727698909	0.562063152	0.576253903	-0.366488889	0.715375578
HIPK2	-1.074475834	0.285574041	0.387210671	0.700027362	0.190691184	0.849454284
HIPK3	0.577785985	0.564897835	-0.865843365	0.390168457	-3.271187875	0.001832366
HIRA	-1.747300043	0.084106281	1.020151657	0.31192253	0.558379765	0.578801577
HIRIP3	-0.725886129	0.469852625	1.081755986	0.283869362	2.094631228	0.040721119
HIST1H1C	-2.695846908	0.008424698	-1.485658533	0.142822767	0.040150438	0.968115393
HIST1H1D	-2.440289507	0.01669932	2.47745391	0.016188481	2.038800744	0.046181024
HIST1H1E	-2.854514806	0.005384739	0.839069954	0.404899149	-0.198646197	0.843255924
HIST1H2AC	-3.77418886	0.000292252	-1.454610746	0.151205367	-2.837203199	0.006317472
HIST1H2AE	-3.949698368	0.000158328	-2.515905007	0.014683406	-2.267583638	0.027216158
HIST1H2AG	-1.106183177	0.271690085	-0.264168721	0.792592866	-1.015450945	0.314239897
HIST1H2AK	-1.404886729	0.16360779	-1.585505415	0.118327146	-3.018007355	0.003818753
HIST1H2BC	-2.847964332	0.005487025	-4.2613524	7.62E-05	-4.634865395	2.17E-05
HIST1H2BD	-3.53486804	0.000655561	-2.496923946	0.015409802	-1.986440342	0.051865601
HIST1H2BE	-2.374084296	0.01978776	-1.252334297	0.215509425	-1.048391765	0.298943019
HIST1H2BG	-3.090173097	0.002684804	-0.513509692	0.609559146	-1.088209995	0.281144161
HIST1H2BH	-2.544437795	0.012706124	-1.582256174	0.119067359	-1.551752567	0.12633143
HIST1H2BJ	-2.903076021	0.004679391	-0.716258018	0.476726219	-1.646805102	0.10517562
HIST1H2BK	-3.641055359	0.000459946	-1.446394327	0.153486899	-3.123434381	0.002824122
HIST1H2BO	-1.946734445	0.054786051	-0.607403228	0.545970687	-0.561717468	0.576540428
HIST1H3D	-2.13891896	0.035238156	-0.904417535	0.369541545	-2.15813592	0.035201633
HIST1H3H	-4.599503732	1.43E-05	-0.078474571	0.937722558	-1.256907458	0.213982609
HIST1H4D	-0.99402623	0.322960817	-0.646235189	0.520693779	-0.460739536	0.646762318
HIST1H4E	-2.503920058	0.014144382	0.233446191	0.816242655	-1.536503265	0.130022673
HIST1H4H	-3.273857498	0.001522185	-0.756763208	0.452276301	-0.971553646	0.335433003
HIST2H2AC	0.151440453	0.879978134	0.680617901	0.498840341	0.168105375	0.867103494
HIST2H2BE	-4.864877547	5.04E-06	-0.925551655	0.358540899	-1.828115258	0.072838197
HIST2H2BF	-1.031064939	0.305362051	-1.816304554	0.07452886	-3.233755697	0.002046745
HIST3H2A	-0.782692832	0.435928199	-1.826422735	0.07297065	-1.078394831	0.285461471
HIST4H4	-0.953278461	0.343084396	-1.886437959	0.064280619	-2.285318562	0.026085633
HIVEP1	-2.17783217	0.032118666	-0.499456391	0.619360494	-1.920202609	0.059909156
HIVEP2	0.33442844	0.738859419	1.241450108	0.219471633	0.732451752	0.466934735
HIVEP3	1.53509521	0.128381432	-0.79647775	0.429025237	-0.758758681	0.451165412
HJURP	-0.347419343	0.729113559	0.772937835	0.442719697	1.921285302	0.059769587
HK1	-2.131461143	0.035865166	2.375389276	0.020880338	1.747467631	0.086014866
HK2	-1.370527296	0.174040591	1.00649921	0.318385318	-0.327226654	0.744711422
HK3	-0.875398222	0.383762496	1.607396863	0.113436097	0.911955859	0.365684798
HKDC1	1.109158519	0.270411796	2.572458188	0.01269908	2.616731837	0.011381157

HKR1	1.986398443	0.050129704	1.204468449	0.233334758	3.159564368	0.002543269
HLA-A	-2.56582598	0.012001253	2.462699505	0.016802009	1.046286202	0.299905258
HLA-B	-2.926656059	0.004368517	2.92817006	0.0048764	1.588219773	0.11784367
HLA-C	-3.062567825	0.002918357	2.162847521	0.034717355	0.044433331	0.964716394
HLA-DMA	-1.628197649	0.107091361	0.209004695	0.835182011	0.603766606	0.548426627
HLA-DMB	-0.891856353	0.374924676	-0.570607929	0.570484972	-0.479978314	0.633104468
HLA-DOA	0.662447387	0.509431872	1.494895905	0.140400693	1.944454262	0.056848629
HLA-DOB	-0.849041934	0.398183131	0.583792562	0.561639271	1.553876733	0.125823968
HLA-DPA1	-1.046041436	0.298433234	-0.983999427	0.329231344	0.053269638	0.957705854
HLA-DPB1	-0.621370728	0.535976967	0.232644981	0.816861824	0.83821388	0.405459191
HLA-DQA1	-0.837585859	0.404553462	0.144201571	0.88584468	1.056914959	0.295069563
HLA-DQA2	-0.731688523	0.466320799	1.315301832	0.193620756	1.394298646	0.168711769
HLA-DQB1	-2.322936348	0.022510968	0.158462421	0.874646729	0.674023642	0.503057478
HLA-DQB2	0.234574703	0.815088838	0.822454279	0.414210407	-0.205270433	0.838102053
HLA-DRA	0.653687775	0.51503331	-0.56277364	0.575773149	-0.028112234	0.977672163
HLA-DRB1	-1.220445791	0.225586454	1.421099185	0.160679671	1.33420126	0.187515619
HLA-DRB5	-0.119158615	0.905423817	0.316965433	0.752415908	-0.547373609	0.586287931
HLA-DRB6	-0.673558602	0.502373629	-0.131649066	0.895720672	0.652911335	0.516473817
HLA-E	-2.558740503	0.012230758	1.497029479	0.139845897	1.330762381	0.188637873
HLA-F	-1.572331191	0.119495781	-1.45774344	0.150342497	-1.019642488	0.312264602
HLA-F-AS1	0.020227972	0.983907734	-0.896251136	0.373849369	-0.642041901	0.523454636
HLA-G	-2.027547937	0.04565992	1.659006958	0.102550747	1.155497203	0.252772713
HLA-H	-0.860102194	0.392091584	2.658384741	0.010147074	0.429532475	0.669177034
HLA-J	0.626510951	0.532616796	-0.084448649	0.932992577	-1.925587317	0.059217754
HLA-L	-0.188695715	0.850769836	1.367112335	0.176902227	1.157871014	0.251810599
HLCS	2.156161719	0.033824813	-0.888836996	0.377787872	0.961084075	0.340624125
HLF	0.886134566	0.377982595	-0.178889692	0.858652102	-0.983393638	0.329625746
HLTF	1.771943486	0.079897486	-0.042592424	0.966173748	-0.184437364	0.854333853
HLX	-1.39378137	0.166926026	0.15715139	0.875675143	-1.050683286	0.297898203
HM13	-3.104755218	0.002568564	-0.199159424	0.842839445	-0.014589662	0.988411184
HMBOX1	0.125908417	0.900094303	-1.689346276	0.096559975	-2.349620579	0.022328383
HMBS	-0.970273292	0.334594276	1.8030623	0.076610237	0.99092665	0.325965979
HMG20A	0.986432521	0.326650458	0.083329762	0.933878277	0.712709484	0.478972397
HMG20B	-0.067941024	0.945988147	-1.220083915	0.227405205	-1.139990766	0.259122257
HMGA1	0.118820683	0.905690757	0.370745588	0.712187127	0.678818224	0.500037194
HMGB1	0.067346244	0.946460261	-1.112144756	0.270696471	-1.031339369	0.306796877
HMGB2	-1.051958852	0.295725227	-0.250964094	0.802735037	-0.710630843	0.480249879
HMGB3	-0.350393515	0.726888499	2.252084384	0.02814508	1.930028607	0.058652616
HMGCL	-0.224838218	0.82263124	2.739794661	0.008170571	3.529025453	0.000839436
HMGCR	-2.96076926	0.003952374	1.572318693	0.121354362	0.719792426	0.474633732
HMGCS1	2.023907755	0.046041061	-0.486000597	0.628810674	-1.090513316	0.280137646
HMGN1	0.195013141	0.845836377	-0.033513494	0.973380977	0.734551922	0.465664392
HMGN2	-3.274784372	0.00151775	1.273885298	0.207820611	1.68337007	0.097847503
HMGN3	2.074807132	0.040952105	0.228289098	0.820230059	0.950755101	0.34579705
HMGN4	0.36423608	0.716563575	-0.492474314	0.624256176	-0.09682545	0.923209259
HMGN5	0.249878425	0.80326917	0.751108844	0.455645141	0.372688983	0.710780901
HMGXB3	3.265069739	0.001564836	2.219414427	0.030411366	2.386219716	0.020412679
HMGXB4	0.064640641	0.948608102	-0.477204159	0.635022567	-1.448081595	0.153146786
HMHA1	-0.613138747	0.541380751	0.54854084	0.585440684	1.14469035	0.257186009
HMMR	-0.058083736	0.953814843	0.187360671	0.852036321	0.204839055	0.838437465
HMOX1	-1.850178301	0.067674974	-0.307852665	0.759304982	-0.812730306	0.419800056
HMOX2	-0.924535096	0.357759543	0.790080511	0.432721644	1.785357684	0.079596199
HN1	-2.22993455	0.028320435	0.330375563	0.742314955	0.000990211	0.999213431
HN1L	1.518498415	0.132506689	2.334584268	0.023073871	3.49553312	0.00093067
HNF1A	0.586845731	0.558823804	1.650899122	0.104202115	2.30791951	0.024705433
HNMT	-0.18788903	0.851400232	-0.050823268	0.959642197	-0.360899286	0.719526942
HNRNPA0	-0.473401248	0.637110096	-1.033329975	0.30576891	-1.457280188	0.150600659
HNRNPA1	-0.17720473	0.859758594	1.187757281	0.239804136	1.813759235	0.075051393
HNRNPA1L2	2.065408588	0.041853543	1.282200314	0.204909251	1.905174498	0.061875242
HNRNPA2B1	1.711544547	0.090535879	1.643614659	0.105704208	1.768319707	0.082431262
HNRNPA3	1.275189343	0.205629684	0.481385014	0.632066795	1.503216839	0.138378421
HNRNPAB	-0.009308678	0.992594123	-0.524345257	0.602050635	-0.33304829	0.740336308
HNRNPC	-1.653241566	0.10187817	-1.370485232	0.175853324	-1.402696153	0.166204158
HNRNPD	0.297536553	0.766764687	0.704153221	0.484174695	0.8818824	0.381594296
HNRNPF	-0.690963385	0.491424099	0.706260576	0.482873332	0.466286271	0.642811795
HNRNPH1	2.542662565	0.012766272	0.694305267	0.490281933	1.872413477	0.066351919
HNRNPH2	-0.532908419	0.595452673	-1.208648926	0.231736414	-1.76953252	0.082226711
HNRNPH3	-0.981996214	0.328818817	-1.857248434	0.068391167	-2.282901682	0.026237205
HNRNPK	-1.530260807	0.12957244	-0.174791783	0.861856234	-0.445525189	0.657650684
HNRNPL	0.10672526	0.915252127	0.041619241	0.966946175	0.197994931	0.843763006
HNRNPM	-0.501243214	0.617463165	1.870669436	0.066474424	1.833648926	0.071999868
HNRNPR	-0.122348838	0.902904322	1.245758903	0.217896717	1.259374911	0.213096978

HNRNPU	-0.295810874	0.768077893	1.426296565	0.159180821	1.659598934	0.102562251
HNRNPUL1	-0.530378127	0.597198039	-0.361235539	0.719245085	-0.010220826	0.991881275
HNRNPUL2	-2.76146089	0.007015744	-0.995843651	0.323491555	-0.064860904	0.948514766
HNRPDL	1.644220824	0.103731758	-0.274248121	0.784875172	0.759429987	0.450767096
HNRPLL	-0.181455653	0.856431113	-0.765364028	0.447179774	-2.19566312	0.032257876
HOMER1	-0.274257488	0.784535493	-1.25086271	0.216042019	-1.791220083	0.078639729
HOMER2	-2.774091694	0.006770588	0.251002086	0.802705807	0.045878805	0.963569378
HOMER3	-1.797315657	0.075747699	0.934519807	0.353937354	0.847694154	0.400201786
HOMEZ	0.547327223	0.585552244	0.2158515	0.829866108	0.28527561	0.776481045
HOOK1	2.440452721	0.016692271	-0.196377831	0.845005707	-0.298007665	0.766795553
HOOK2	1.0922034	0.277752501	1.021848387	0.311125575	1.878077904	0.065558849
HOOK3	0.542714372	0.588711115	-1.838166796	0.071196445	-1.662970403	0.101882469
HOPX	1.752756732	0.083158939	-2.386524995	0.020315042	-1.689268666	0.096705527
HORMAD1	-0.531797107	0.596218952	-0.13613435	0.892189776	-3.11868088	0.002863166
HOTAIRM1	-0.343037999	0.73239559	-1.950594429	0.055976817	0.306491598	0.760362284
HOXA1	-1.751509958	0.083374614	-2.800342126	0.00693704	-1.257927034	0.213616328
HOXA10	-1.734272417	0.08640399	0.129765944	0.897203736	-1.379128164	0.173315647
HOXA2	-0.404251982	0.687017352	-1.24469194	0.218285926	0.512589322	0.610246151
HOXA3	0.264681844	0.791879264	-1.554831183	0.125464417	-0.403421038	0.688168325
HOXA4	0.724201323	0.470880949	0.201710442	0.840853825	0.840658837	0.40409927
HOXA5	-0.9223184	0.358907754	0.084708697	0.932786737	0.38077105	0.704807747
HOXB2	-0.423551357	0.672936069	-4.891622894	8.40E-06	-3.474159766	0.000993741
HOXB3	1.545711617	0.125796346	-3.576665979	0.000713288	-2.275495479	0.026706554
HOXB4	-0.694575462	0.489168145	-5.027349455	5.15E-06	-5.389899279	1.44E-06
HOXC4	-0.40631127	0.685509462	-1.319033014	0.19237829	-1.114215371	0.269926193
HOXC5	-2.593553997	0.011139993	-0.963895074	0.339128164	0.217644672	0.828493743
HP	-0.145397309	0.884732794	1.888404818	0.064011329	1.300512189	0.198729827
HP1BP3	0.070368105	0.944061827	0.299462828	0.765664927	0.296183395	0.768181053
HPCAL1	-2.317610815	0.022812736	0.710409614	0.480316869	1.040255829	0.302672857
HPCAL4	3.008585365	0.003430485	0.535516614	0.594354536	0.94027569	0.351097574
HPD	-2.828300952	0.00580488	-0.427675631	0.670482113	0.499493652	0.619380298
HPDL	-0.089469325	0.928914132	-1.644311464	0.105559766	-1.291911085	0.201672033
HPGD	0.33228006	0.740475317	-0.300223748	0.765087433	-0.793381923	0.430890139
HPRT1	0.761006473	0.448706512	1.463607553	0.148737645	1.893598713	0.063426811
HPS1	-1.931612991	0.056655395	0.212163089	0.832728837	0.014674314	0.988343948
HPS3	0.440620515	0.660578288	-0.132002597	0.895442289	0.230563715	0.818490467
HPS4	3.547457368	0.000628805	1.818420217	0.074200759	2.636255893	0.010815572
HPS5	1.050734503	0.296284152	3.065059428	0.003309541	2.597987118	0.0119494
HPS6	-1.158449091	0.24984405	2.104970131	0.039667124	1.485437926	0.143012386
HPSE	-2.045759518	0.043793456	0.798904226	0.427628129	0.579763023	0.564390009
HPX	1.09368916	0.277103777	-1.261982352	0.21204164	0.082418631	0.934606476
HRAS	-0.088898557	0.929366413	0.585897891	0.56023311	0.695112028	0.489847382
HRASLS	-2.422103023	0.017501673	-0.019241077	0.98471529	-1.053210206	0.296748966
HRASLS5	-0.572066734	0.568748819	0.37065566	0.712253751	0.585560209	0.560513679
HRH2	-0.471041831	0.638787294	-0.897522143	0.373176818	-1.546813991	0.127517577
HRH4	-0.937822155	0.350926446	0.895227966	0.374391336	-1.800675782	0.077117247
HRK	-2.5367465	0.012968573	-1.315723999	0.193479874	-1.107826955	0.272652331
HRSP12	-0.826790695	0.410612687	0.07892753	0.937363846	-0.704839887	0.483818897
HS1BP3	-1.92969279	0.056896594	2.233797449	0.029394516	2.053595302	0.044675791
HS2ST1	1.383887582	0.169925372	-0.180166888	0.857653956	-0.835747553	0.406833841
HS3ST1	0.640774282	0.523349973	0.044287737	0.964828234	-0.455258198	0.650676333
HS3ST3B1	1.716235732	0.089670034	-3.117813659	0.002842601	-3.300564311	0.001679149
HS6ST1	-2.552826573	0.012425336	-0.156106264	0.876495126	-0.432315549	0.667165342
HSBP1	-1.605298825	0.112044208	-1.506236083	0.137471717	-0.965084977	0.338634153
HSBP1L1	2.870514674	0.005142219	0.39290298	0.695841586	1.435975372	0.156548821
HSCB	-0.601410108	0.549127388	1.324409071	0.190598677	0.247328858	0.805554474
HSD11B1L	1.671698664	0.098169185	-1.018824273	0.312546965	-0.452295743	0.652795844
HSD17B1	1.345929797	0.181814471	-1.197675642	0.23594897	-0.73426391	0.465838487
HSD17B10	-0.929023687	0.355441739	0.820818528	0.415134044	1.52021328	0.134060252
HSD17B11	-1.964408415	0.05266773	-0.034720912	0.972422337	-0.614417437	0.541417428
HSD17B12	0.97147663	0.33399839	3.556021238	0.000760725	2.753630612	0.007923134
HSD17B13	-0.163979562	0.870126765	0.081523336	0.935308403	-0.801889307	0.425992532
HSD17B14	0.453510548	0.651307894	-0.927464052	0.357555997	0.14065825	0.888642542
HSD17B4	-0.488227807	0.626614118	2.273004064	0.026773556	2.038013722	0.046262312
HSD17B7	1.608175748	0.111412033	0.212158404	0.832732475	1.440884355	0.155162293
HSD17B7P2	0.492397007	0.623676349	-0.419854594	0.676153752	1.931793205	0.058429357
HSD17B8	1.43295439	0.155446863	1.438601801	0.155675458	1.627320693	0.109259668
HSD3B7	2.040795503	0.044295594	1.828458122	0.072660525	1.001509924	0.320870296
HSDL1	1.380594875	0.170932633	1.334647909	0.187243804	0.890298519	0.377098619
HSDL2	-1.869352904	0.064930555	-0.639232036	0.525206221	-1.528782034	0.131924187
HSF1	0.739651488	0.461498365	-0.092668362	0.926488614	0.490456388	0.625719226
HSF2	3.532186638	0.000661397	1.369116153	0.176278505	1.783284503	0.079936761

HSF4	0.631110029	0.529619591	-0.843419809	0.402482905	-0.331808599	0.741267251
HSF5	0.58693053	0.558767105	-0.366238147	0.715529251	-0.394871362	0.694431539
HSH2D	-5.055947504	2.34E-06	-0.591355407	0.556596177	-1.372236025	0.175438838
HSP90AA1	1.258074421	0.21172363	2.529260492	0.014191013	2.557292716	0.013272896
HSP90AB1	-0.691503253	0.491086559	2.61809065	0.011279046	2.721332468	0.008638492
HSP90B1	1.516005663	0.133135204	3.241619529	0.00197789	3.203500994	0.002237059
HSPA12B	0.366402375	0.714952476	0.110896076	0.912084291	-0.451698994	0.653223142
HSPA13	0.583746033	0.560898336	0.069868498	0.944540345	-1.161879526	0.250191891
HSPA14	0.010761272	0.991438499	-0.313423781	0.755090959	-0.742260258	0.46101874
HSPA1A	-2.490065922	0.014668711	0.93551508	0.353428832	0.483389789	0.63069579
HSPA1B	-0.194713524	0.846070219	1.507565383	0.13713157	0.600022172	0.550901689
HSPA1L	-1.811554409	0.073498298	1.224225857	0.225851068	2.565926326	0.012981463
HSPA4	0.743752851	0.459025645	3.876576751	0.000274049	3.667986743	0.000544168
HSPA5	-1.477889484	0.143040775	2.110894868	0.03913363	1.71646247	0.091581515
HSPA6	-2.051443943	0.043224457	-0.178246001	0.859155244	-1.176758179	0.244248923
HSPA7	1.650243454	0.102491226	0.252530455	0.80153013	-0.197843877	0.843880628
HSPA8	1.572346713	0.119492182	3.557234831	0.000757856	3.397977936	0.001253202
HSPA9	0.304398721	0.761549491	2.417369031	0.018820126	2.476758528	0.016291107
HSPB1	-0.896459189	0.372476086	-1.981007991	0.052372035	-1.520289322	0.134041176
HSPB11	1.723762536	0.088295004	-1.084110903	0.2828329	-0.293748958	0.770031148
HSPBAP1	1.135152924	0.259422215	1.379272162	0.173143122	1.735303866	0.088164359
HSPBP1	-0.854728376	0.395044054	1.502625256	0.13839904	2.545778181	0.013670816
HSPC159	-4.142546068	7.92E-05	-0.142330084	0.887316003	-1.82922747	0.072669046
HSPD1	1.606685421	0.111739159	3.539658683	0.000800443	3.834262772	0.000320418
HSPE1	0.881920161	0.380244882	-1.080466537	0.284437999	0.029410936	0.976640978
HSPG2	1.306061444	0.194966915	1.281818426	0.205042292	0.703067265	0.484914327
HSPH1	0.149317488	0.88164796	3.553837571	0.000765915	3.029407521	0.003697226
HTATIP2	-2.420084297	0.017592822	0.226578642	0.821553622	-0.768857693	0.445194855
HTATSF1	0.790287435	0.431504163	3.952222917	0.00021402	3.671293011	0.000538529
HTR2B	-0.139895341	0.889065345	-0.651144792	0.517542568	-0.253628787	0.800707327
HTR3A	-1.86882294	0.065005133	-0.507516084	0.613730688	-0.378678785	0.706352289
HTR6	0.48656488	0.627787563	-0.053157595	0.957790289	-0.30382344	0.762383708
HTR7	-0.096165879	0.923609525	0.529382607	0.598574627	-0.270496853	0.787768317
HTR7P1	-0.830593721	0.408471853	-1.385889609	0.171123304	-1.349928799	0.182447478
HTRA1	0.658845127	0.511731445	1.174322108	0.245098628	0.902431078	0.370677002
HTRA2	0.567763579	0.571654681	-1.760662732	0.083604064	-1.193848092	0.237549114
HTRA3	-1.428077281	0.156841914	1.818449952	0.074196156	0.206305149	0.837297649
HTT	0.492914983	0.623311788	1.199462668	0.23525918	1.529192927	0.131822441
HUS1	0.763346546	0.447317337	-2.037661276	0.046186461	-1.233408231	0.222554007
HUWE1	0.589142047	0.557289401	0.706854771	0.482506749	1.094560473	0.278375198
HVCN1	-0.564096451	0.574136699	-2.081337957	0.041858581	-1.222494319	0.226619654
HYAL1	-0.326660189	0.744707785	1.004784927	0.319203141	2.705546256	0.009009284
HYAL2	-0.102010581	0.918982518	-1.19344974	0.237585999	-2.379799626	0.020737695
HYAL3	0.929425919	0.355234507	-0.117207355	0.907103499	0.006705132	0.994673848
HYDIN	-0.462337775	0.644990813	1.038257946	0.303489155	0.253170163	0.801059927
HYLS1	0.714038237	0.477110734	-0.673133802	0.503554224	-0.204189125	0.838942866
HYOU1	-0.442324384	0.659349797	1.297640093	0.199584179	1.450549729	0.152460341
IAH1	1.702460267	0.092232	-1.727015945	0.089524709	-1.075427903	0.286775521
IARS	3.529824073	0.00066658	2.859962875	0.005892256	3.685526069	0.000514884
IARS2	0.929626429	0.355131232	2.125869941	0.037812987	2.239174696	0.029117697
IBA57	0.461858743	0.645332962	0.552867909	0.582493375	1.174113803	0.245297661
IBTK	1.15757379	0.250199323	1.16634175	0.248283077	1.186648374	0.240355216
ICA1	-0.622234556	0.535411523	-0.085289437	0.932327073	-0.68135974	0.498440215
ICA1L	1.525917589	0.130649867	-1.330042485	0.188747247	-0.370479187	0.712417278
ICAM1	-2.218672415	0.029106345	-0.135062922	0.893033027	-0.571077374	0.570222414
ICAM2	0.11046592	0.912293755	2.178027594	0.033512271	2.501329409	0.015310358
ICAM3	-3.031737424	0.003201415	-0.995761929	0.323530927	-1.280108014	0.205762464
ICAM4	-0.702686598	0.484123009	-0.106972014	0.915182918	0.542789062	0.589419874
ICAM5	1.321618234	0.189752699	0.231914357	0.817426547	-0.527603792	0.599849894
ICK	1.018371856	0.311318806	-0.531332763	0.597231442	-0.531522004	0.597150463
ICMT	-0.281599778	0.778917682	0.739116318	0.46283798	1.276113908	0.207160574
ICOS	1.311538362	0.193119139	-0.80226811	0.425695789	-0.682701537	0.497598212
ICOSLG	0.945578425	0.346976812	1.782767607	0.07989432	2.042355892	0.045815368
ICT1	0.733082251	0.465474697	1.530499366	0.131367418	1.758671211	0.084073689
ID2	-0.649079903	0.517992879	-4.519885702	3.13E-05	-3.764105032	0.00040122
ID3	-1.433064845	0.15541538	1.439260664	0.155489478	0.594863959	0.554320471
IDE	1.759464357	0.082006487	0.241527486	0.810004139	0.298033842	0.766775678
IDH1	1.134640795	0.259635644	1.277264827	0.206633634	0.212027484	0.832852188
IDH2	-0.76207539	0.448071645	0.983481289	0.329483975	1.386709567	0.171002958
IDH3A	1.574617903	0.118966484	0.394001121	0.695035168	0.792380997	0.431468558
IDH3B	-0.41262362	0.680895273	1.276016336	0.207071547	2.515378157	0.014773887
IDH3G	-1.48610553	0.140858343	0.007528238	0.994019407	0.250136601	0.803393244

IDI1	0.41154316	0.68168421	-0.250711134	0.802929669	-1.013464058	0.315179173
IDI2	1.623894922	0.108008304	2.455435745	0.017111683	3.79846884	0.000359464
IDI2-AS1	0.925576211	0.357221075	1.555922497	0.125204712	0.176691707	0.860385355
IDO1	-0.189546129	0.850105372	0.001419451	0.998872347	-1.125824886	0.265021315
IDS	-2.199457246	0.030491374	-2.35277007	0.022072076	-2.614701729	0.011441488
IDUA	0.45533053	0.650003344	-1.991434766	0.051182914	-1.733962647	0.088404068
IER2	-3.193525665	0.001956107	-3.041234499	0.003543115	-3.298105184	0.001691496
IER3	-2.46046018	0.015847994	-2.053975505	0.044526625	-2.978319157	0.004271506
IER3IP1	1.127152239	0.262770609	-1.140756536	0.258692426	-1.18072383	0.242682243
IER5	-0.948186692	0.345655127	-2.689199784	0.009352514	-3.691264227	0.000505636
IER5L	-0.339701621	0.734898192	-3.186098804	0.002329526	-3.422825833	0.001162228
IFFO1	1.23834398	0.218912421	0.237853056	0.812839179	0.794349026	0.430331704
IFFO2	0.745013966	0.458266837	-1.078438462	0.285333965	-0.065799428	0.947770871
IFI16	-0.457857434	0.648193886	1.336036679	0.186792225	-0.342986901	0.73288714
IFI27	-3.125505264	0.002411208	1.741251172	0.086978805	0.219351223	0.827170676
IFI27L1	1.773247926	0.079679645	-1.377475668	0.173694604	-1.4132599	0.16309067
IFI27L2	0.158409311	0.874500606	-1.403555714	0.165819846	-0.911420027	0.365964493
IFI30	-2.186947405	0.031423731	0.815100793	0.418372398	-0.047981357	0.961901097
IFI35	-1.819036161	0.072338797	2.150774793	0.035702707	-0.271813731	0.786760653
IFI44	-1.053463888	0.29503915	1.100912358	0.275514515	-1.296034115	0.20025761
IFI44L	-1.288539154	0.200967094	0.958213852	0.341960017	-1.549900496	0.126775222
IFI6	-1.143970638	0.255766834	-0.37363386	0.710048538	-2.590562442	0.012181531
IFIH1	-1.072089515	0.286638359	1.47934741	0.144496406	-1.499175107	0.139421304
IFIT1	-1.325958661	0.188316721	0.985347493	0.328574668	-2.152078192	0.03569827
IFIT1B	-3.957450761	0.000154039	1.396211873	0.168008893	0.895728414	0.374215978
IFIT2	-2.269147022	0.02572733	0.22741583	0.820905734	-3.120533875	0.002847886
IFIT3	-1.676988521	0.097126643	0.375066752	0.708988438	-2.682516928	0.009576418
IFIT5	-1.364708978	0.175856237	0.025266326	0.97992987	-2.545137435	0.013693273
IFITM1	-1.551824586	0.124326664	-3.070108425	0.003261926	-3.213991872	0.002169257
IFITM10	-1.2891405	0.200758928	1.002506023	0.320292507	0.846179316	0.401039022
IFITM2	-2.918701112	0.004471199	-2.657100133	0.010181499	-3.814391005	0.000341562
IFITM3	-2.491249661	0.014623239	-1.66140337	0.102066767	-0.316793616	0.752573243
IFITM5	1.090376637	0.278551558	-1.737730321	0.087602845	-1.98183186	0.052393567
IFNAR1	-1.262986068	0.209961356	0.257104954	0.798014026	-0.053043322	0.957885369
IFNAR2	-0.175454822	0.861129105	0.238568514	0.812286962	0.190722638	0.849429757
IFNG	0.050281776	0.960012858	-0.659789944	0.512018427	-0.614912869	0.541092511
IFNGR1	0.318851479	0.75060174	-0.601178585	0.550079836	-1.136086822	0.260738565
IFNGR2	-2.722749394	0.007818472	-1.100080889	0.275873539	-1.369367082	0.176328495
IFRD1	-1.097183024	0.275582399	-0.499044268	0.619648988	-1.351155815	0.182056505
IFRD2	1.986544825	0.050113165	0.191889056	0.848504026	0.314715294	0.754142547
IFT122	-0.52352773	0.601935198	1.728082074	0.089331927	1.708527131	0.093053059
IFT140	1.769827784	0.080251855	0.007625141	0.993942426	0.451706668	0.653217646
IFT172	1.765775668	0.080934189	0.232692142	0.816825375	1.836278048	0.071604418
IFT20	1.840636485	0.069076448	-1.329872775	0.188802823	-0.558841478	0.578488532
IFT27	1.554652891	0.123651308	0.470757296	0.639592068	0.068263171	0.945818278
IFT43	0.374510768	0.70893368	0.238640689	0.81223126	0.362545517	0.718303406
IFT46	0.569618015	0.570401526	0.790869371	0.432264811	2.359539242	0.021793957
IFT52	-0.103158953	0.918073723	-0.590477112	0.557180681	1.276332077	0.207084023
IFT57	1.592995771	0.114780219	0.359676672	0.720404378	0.485456775	0.629238346
IFT74	1.935873758	0.056123284	0.200295988	0.841954658	-0.407718747	0.685028204
IFT80	1.718083281	0.089330902	-0.917428119	0.362744088	-1.356773944	0.180274504
IFT81	-0.970125374	0.334667573	-0.754200286	0.453801483	-0.27431005	0.784851497
IFT88	2.366452022	0.020174588	0.325227176	0.746187618	1.189946544	0.239066786
IGBP1	-0.273818024	0.784872107	1.265451532	0.210804884	1.604239076	0.114263317
IGF1R	0.292137563	0.77087546	-0.523896132	0.602361005	-0.575791727	0.56705307
IGF2BP2	-3.489252305	0.000761802	1.089576376	0.28043755	0.714984284	0.477576546
IGF2BP3	-2.526945673	0.01331007	0.087198772	0.93081597	-1.527371707	0.132273887
IGF2R	-2.536669091	0.01297124	-0.16493229	0.869574778	-0.182896311	0.855537153
IGFALS	0.81912819	0.414946674	-0.295091023	0.768985456	-0.234793645	0.815221708
IGFBP2	-1.822731539	0.071771743	1.235174445	0.22178044	1.490408348	0.141704726
IGFBP3	0.366101116	0.715176449	-0.791401229	0.431956971	0.492303408	0.624421354
IGFBP4	0.826053067	0.411028702	0.968789819	0.336700755	1.26393761	0.211466473
IGFBP6	1.843063601	0.06871768	0.682051096	0.497940392	-0.403182549	0.688342739
IGFBP7	0.571887383	0.568869789	-1.776166822	0.080987443	-2.841423742	0.00624497
IGFLR1	0.146320471	0.884006186	2.185799963	0.032909586	1.520923184	0.133882246
IGHMBP2	1.148820593	0.253771877	0.135657855	0.892564779	1.523873161	0.13314456
IGJ	-0.21650974	0.82909621	0.62803611	0.532462683	0.268822039	0.789050398
IGLL5	-1.027128182	0.307201254	1.538051135	0.129512129	0.559477103	0.578057706
IGSF22	-0.22445773	0.822926329	-0.339336762	0.735590257	-0.012076384	0.990407414
IGSF23	-2.249953359	0.026969289	0.470960825	0.639447592	0.96625804	0.338052149
IGSF6	-2.164403891	0.03316682	-2.036027626	0.046355579	-2.448428031	0.017492009
IGSF8	1.029350002	0.306162333	1.19623534	0.236505991	2.751819376	0.007961762

IGSF9B	1.161985512	0.248412315	-0.314057516	0.754612068	0.064348213	0.948921156
IK	-1.571879004	0.11960067	-0.031553839	0.974936945	0.456694777	0.649649564
IKBIP	-2.841013028	0.005597519	-0.501819839	0.617707198	-0.914650066	0.364280542
IKBKAP	2.530512867	0.013184851	2.17238248	0.033956057	2.430980516	0.018270677
IKBKB	1.158255365	0.24992265	0.866307558	0.389916041	0.872341202	0.386731622
IKBKE	0.170256548	0.865202832	1.636253389	0.107239998	2.097634978	0.040443991
IKBKG	-1.891859476	0.061829002	-0.533881405	0.595478166	-0.611446842	0.543367719
IKZF1	0.64034271	0.523629124	0.114123441	0.909536834	0.060147033	0.952251768
IKZF2	1.340673714	0.183509103	-2.49288588	0.015568463	-2.067805018	0.043270256
IKZF3	0.143001956	0.886618603	-0.880731207	0.382123693	-0.275839506	0.783682442
IKZF4	0.170766697	0.864802881	-0.691178584	0.492229811	-1.178774658	0.243451379
IKZF5	-0.586970062	0.558740673	-1.979767358	0.052515088	-3.031782419	0.003672368
IL10	-3.027325735	0.003243941	0.77925713	0.439018435	0.963583699	0.33937996
IL10RA	0.603937955	0.54745308	0.206066153	0.837465902	0.064851213	0.948522447
IL10RB	-1.81105906	0.073575608	0.339829751	0.735220903	-0.012987753	0.98968353
IL11RA	1.155002855	0.251244906	1.501896333	0.138586839	2.877795111	0.005651301
IL12A	-0.62247788	0.535252303	-1.462222792	0.149115401	-2.261726768	0.027598932
IL12RB1	-1.350226351	0.180437999	-1.977781189	0.052744805	-0.721561971	0.473553266
IL12RB2	-0.381855982	0.703497285	-0.929402157	0.356559639	-0.097145466	0.92295627
IL13RA1	-0.747032263	0.457053927	-1.045147687	0.30032133	-1.796278925	0.077822099
IL15	0.485765942	0.628351675	-0.736407032	0.4644719	-1.694110058	0.095776427
IL15RA	-0.787089369	0.433363877	-0.018362659	0.985413005	-1.251146875	0.216060834
IL16	-2.488059855	0.014746061	0.567511544	0.572572205	0.949540611	0.346408647
IL17RA	-0.833189112	0.407014721	0.636962779	0.526672796	0.452963533	0.652317817
IL17RB	-1.109120714	0.270428012	-0.318663643	0.751134309	-0.953020469	0.344658136
IL17RC	0.596998877	0.552055287	0.224291206	0.823324472	-0.961092636	0.340619859
IL17RD	0.761567627	0.448373158	2.177165338	0.033579725	2.32527726	0.023689921
IL17RE	-0.457266756	0.648616668	-0.727195687	0.47005166	-0.984215899	0.329224943
IL18	-2.068721059	0.041533905	0.981978352	0.330217495	0.325374228	0.746105353
IL18BP	0.359805089	0.719862949	-1.354394151	0.180900376	-0.347633816	0.729412988
IL18R1	-1.359877365	0.177374866	0.611165097	0.543494912	-0.867626253	0.389286246
IL18RAP	-0.147510097	0.883069993	1.185046091	0.240865836	0.900289315	0.371805517
IL1B	-2.13150802	0.035861195	-0.996627796	0.323113929	-2.255187464	0.028031932
IL1R1	-2.445054126	0.01649462	-0.743525938	0.460185665	-2.132115279	0.037378595
IL1R2	-2.276359943	0.025273779	-0.327803449	0.744248893	-1.079552968	0.28494967
IL1RAP	-1.272322428	0.20664134	-1.47313917	0.146157772	-3.059440252	0.003394138
IL1RL1	-1.043361312	0.299665274	-0.709574768	0.480830657	-1.477535472	0.145110983
IL1RN	-3.024010852	0.003276238	-0.175419084	0.861365598	-1.38504682	0.171508133
IL20RB	1.453982553	0.149541455	0.497677682	0.620606055	1.96370564	0.054515172
IL21R	1.170664202	0.244923483	0.265526498	0.791551996	1.247421448	0.217412766
IL23A	1.837357452	0.069563628	0.406137302	0.68614674	0.458764885	0.648171193
IL23R	0.038266372	0.969562785	-1.898443411	0.062651756	-1.882228312	0.064982862
IL24	2.197906186	0.030605646	1.136792448	0.260332595	2.971623383	0.004352649
IL27	-0.879278	0.381667499	0.154199366	0.877991587	-1.299117657	0.199204658
IL27RA	0.565910706	0.57290811	2.147553533	0.035969722	2.659513092	0.010175381
IL28RA	2.590784173	0.011223444	0.307755439	0.75937859	0.13823199	0.890550857
IL2RA	-0.933508364	0.353135603	0.709493465	0.480880709	-0.13156895	0.895794823
IL2RB	1.255549158	0.212633904	-1.403383803	0.165870836	-0.535822165	0.594194436
IL2RG	-1.61834818	0.109199673	0.012849183	0.989792522	0.809716398	0.421516153
IL31RA	-4.244687373	5.45E-05	-1.397019676	0.167767019	-0.813644516	0.419280347
IL32	-0.321071237	0.748924759	-0.551506058	0.58342021	-0.122583783	0.902873723
IL34	1.072725182	0.28635458	0.210566924	0.833968396	1.034678737	0.305247904
IL3RA	-1.071529842	0.286888372	1.299695059	0.198883329	0.911444519	0.365951706
IL4I1	0.207008537	0.836485886	-0.120144632	0.904786697	-0.058773028	0.953341241
IL4R	1.190337989	0.237144503	1.412187573	0.163275092	1.566136365	0.122926943
IL5RA	0.63990207	0.523914221	-0.412707583	0.681353155	-1.194408508	0.237331692
IL6R	-2.758801733	0.007068379	0.916538338	0.363206385	0.68663586	0.495133839
IL6ST	2.11436822	0.037338857	-1.182039991	0.24204699	-1.394270708	0.16872016
IL7	-0.022962758	0.981732451	-0.435246076	0.665010487	-1.074099516	0.287365219
IL7R	2.916596586	0.004498734	0.793492961	0.430747537	0.570097668	0.570882133
IL8	-0.011638682	0.990740475	-0.955253433	0.343441798	-0.852856642	0.397356639
IL9R	1.275506452	0.205518011	-1.154914405	0.252894549	-0.467640859	0.641848592
ILDR1	0.188062232	0.851264872	-1.184914061	0.240917626	0.43199061	0.667400092
ILF2	1.784223904	0.077866043	2.903615203	0.005221759	4.830832668	1.09E-05
ILF3	3.243084588	0.001676494	0.678969461	0.499876545	1.574540762	0.120972019
ILK	-2.324251359	0.022436999	2.247113108	0.028480003	2.153890002	0.035549091
ILKAP	1.181619605	0.240569548	-0.424412894	0.672845857	2.404891369	0.019493203
ILVBL	-0.03626095	0.971157178	2.393013154	0.019992028	2.88447351	0.005548143
IMMP1L	0.394509235	0.694168463	-0.24465741	0.807591233	0.091195109	0.927661623
IMMP2L	1.235880342	0.219822426	1.420402426	0.160881436	1.335303552	0.187156967
IMMT	0.235781941	0.814154844	1.861207592	0.067820944	2.471891605	0.016491969
IMP3	0.600110038	0.549989482	0.353510361	0.724996562	0.917308573	0.362898291

IMP4	0.93852251	0.350568626	1.397282828	0.167688284	1.770167974	0.082119706
IMPA1	0.482447814	0.630696891	-1.52884555	0.13177653	-2.10467764	0.039800661
IMPA2	-0.842983645	0.401544246	0.05416035	0.956994837	-0.175878038	0.861021551
IMPACT	1.407727321	0.162767209	-0.960013087	0.341061498	-0.727034834	0.470220395
IMPAD1	1.769390731	0.080325221	-1.437746323	0.155917195	-1.840362151	0.070993748
IMPDH1	-1.484327559	0.141328405	0.406506774	0.685876833	0.608658781	0.545201431
IMPDH2	-0.439518	0.661373698	3.755896395	0.000404591	4.52695125	3.15E-05
IMPG2	-1.337057141	0.184682029	0.215373891	0.83023667	-0.100609128	0.92021858
INADL	2.294211751	0.024181395	1.586921087	0.118005801	2.188137378	0.032830239
INCA1	1.484457682	0.141293961	-1.198446883	0.235651092	-0.474194903	0.637196985
INCENP	0.62826245	0.53147432	-0.126367629	0.899881028	-0.920488977	0.361249124
INE1	-0.448512633	0.654895944	-0.16656517	0.868295569	0.415265756	0.679527513
INF2	0.619156335	0.537427857	0.960853755	0.34064221	2.491361023	0.015701629
ING1	-0.939325622	0.350158597	-3.077138465	0.003196694	-3.636663194	0.000600471
ING2	-0.55231309	0.582146962	0.903373019	0.370090761	1.320338085	0.192070847
ING3	-0.995509333	0.322243445	-1.759382635	0.083823211	-1.787832527	0.079191242
ING4	0.23004193	0.818598043	-0.052556936	0.958266792	0.09701935	0.92305597
ING5	2.831460805	0.005752688	1.40447287	0.165548014	1.767444129	0.082579198
INHBB	-2.076144254	0.040825228	-0.278542581	0.781593482	-1.329362235	0.189096256
INMT	0.369511252	0.712642624	1.770183967	0.081988971	1.309651959	0.195638796
INO80	-0.602685052	0.548282613	0.852013759	0.397735313	0.9243853	0.359235312
INO80B	-0.579856351	0.563506932	-2.268832102	0.027042252	-1.472348397	0.146501592
INO80C	-1.962162142	0.052933035	-1.005991629	0.31862732	0.799117132	0.427584768
INO80D	0.718886238	0.474133289	-1.517596544	0.134586194	-1.808036336	0.075949249
INO80E	-1.264350235	0.209473821	-4.320045326	6.24E-05	-3.475638862	0.000989249
INPP1	-0.092003668	0.926906188	-1.442543631	0.154565362	-0.962637414	0.339850612
INPP4A	1.42845938	0.156732271	-0.935585074	0.353393087	-1.223861524	0.226107383
INPP4B	1.061252833	0.291505869	0.095086655	0.924576045	0.494790964	0.622675277
INPP5A	-0.753967845	0.452899949	-0.148462865	0.882496054	-0.290579509	0.772441842
INPP5B	0.71646811	0.475617105	1.950695728	0.055964467	1.81942154	0.074171819
INPP5D	-1.887672214	0.062396403	0.898005753	0.372921118	1.011215132	0.316244609
INPP5E	1.294325201	0.198970796	-1.809738887	0.07555483	-1.203813206	0.233704514
INPP5F	0.169692058	0.865645427	0.507380994	0.613824859	0.571706847	0.569798733
INPP5K	-2.417094564	0.017728592	1.069010581	0.289524726	1.654729258	0.103550649
INPPL1	-0.73426447	0.464757679	-0.105394458	0.916429008	0.218451142	0.827868435
INSC	-0.459785226	0.646814858	0.404128458	0.687614967	-0.322629214	0.748172516
INSIG1	1.583120311	0.11701484	-0.861907291	0.392312882	-1.039440547	0.30304836
INSIG2	1.000269183	0.319948268	-0.162209647	0.871708492	-1.003410476	0.3199609
INSL3	-0.293177098	0.770083448	-2.272851599	0.026783334	-1.436244173	0.156472649
INSR	0.606634557	0.545669834	1.713144509	0.092064479	1.941483746	0.057216179
INTS1	0.040238304	0.967995138	0.197412744	0.844199591	1.111346983	0.271147841
INTS10	2.485240106	0.014855403	2.784562466	0.007240822	3.855230719	0.000299474
INTS12	-0.748283581	0.45630286	-1.000660963	0.32117631	0.077603974	0.938418536
INTS2	1.810427524	0.073674272	0.682646251	0.497566936	0.248456495	0.804686303
INTS3	-0.194503693	0.846233994	1.240616711	0.219777215	1.769053478	0.082307455
INTS4	0.118378609	0.906039978	0.775006473	0.441506067	2.433293386	0.018165694
INTS4L1	1.167203125	0.24631062	-2.244075348	0.028686392	-1.181242894	0.242477718
INTS5	-0.081915831	0.934901404	2.025989894	0.047406491	2.701221209	0.009113389
INTS6	-0.657332149	0.512698925	-1.230338775	0.223571618	-2.398160341	0.019820298
INTS7	-1.198627896	0.233920432	1.293623325	0.200959449	2.107861046	0.039512803
INTS8	1.32062128	0.190083685	-0.153290498	0.87870499	-0.29188616	0.771447723
INTS9	-1.698421377	0.092994392	0.844292163	0.401999403	2.007523588	0.049508118
INVS	1.19532438	0.235201413	-1.202626861	0.234041402	0.920452079	0.361268229
IP6K1	-2.781435964	0.006631667	0.721418919	0.473570232	0.642666629	0.523052066
IP6K2	-1.936149711	0.056088967	-0.54467632	0.58807893	-0.008479728	0.993264247
IPCEF1	1.97060774	0.051941393	0.623269096	0.535568083	0.829189243	0.410503093
IPMK	-0.987003399	0.326372114	-1.315170293	0.193664668	-3.010371988	0.003902219
IPO11	1.012801871	0.313957204	0.283346022	0.777927568	0.554162686	0.581664569
IPO13	-0.188333827	0.851052626	1.764869518	0.082887232	1.978232258	0.052809156
IPO4	-0.149943009	0.881155898	2.00624329	0.049533986	2.26129422	0.027627389
IPO5	2.335378782	0.021819644	3.564079027	0.000741863	4.231501234	8.65E-05
IPO7	0.777393621	0.439030811	0.056042896	0.955501596	-0.312844565	0.755555993
IPO8	1.227940211	0.222774097	2.13361265	0.037145544	1.521455026	0.133749012
IPO9	1.587942814	0.115919299	0.772040505	0.443246752	1.623504767	0.110074397
IPP	1.757585213	0.08232801	0.425296123	0.672205658	2.386639098	0.020391607
IPPK	0.57600722	0.566094176	0.199373564	0.842672727	1.217931364	0.228335478
IPW	1.338980639	0.1840575	-1.669844568	0.100376821	-1.022928798	0.310721791
IQCB1	1.543338504	0.126370588	-0.173052391	0.863216963	-0.067710475	0.946256277
IQCC	1.440187894	0.153395459	0.676427547	0.501476679	0.835085837	0.407203145
IQCE	-0.552868661	0.581768097	0.122446278	0.902971835	0.158633484	0.874525946
IQCG	0.909994194	0.365334392	0.244766258	0.807507354	1.34942079	0.182609536
IQCK	-0.287072349	0.77473806	-1.061349389	0.292961364	0.133369653	0.894377157

IQGAP1	-1.271006867	0.207106793	-0.229351962	0.819407871	-1.045813842	0.300121417
IQGAP2	-1.375428911	0.172522093	0.069496696	0.944834987	-1.445697947	0.153812023
IQSEC1	-1.582909929	0.117062821	-0.933667031	0.354373446	-1.233740313	0.222431146
IQSEC2	0.332219575	0.740520828	-0.29869682	0.766246419	-0.547051499	0.586507722
IQSEC3	2.487853047	0.014754056	1.234293111	0.222106106	0.252500329	0.801574986
IRAK1	0.024596683	0.980432873	0.591422226	0.556551721	0.283198033	0.778064931
IRAK1BP1	1.102773264	0.273160247	-1.013675295	0.314977165	-1.459351842	0.150031832
IRAK2	-0.365933746	0.715300892	-0.193597995	0.847171803	-0.558771352	0.578536073
IRAK3	0.116139178	0.907809324	0.217315119	0.828730771	-0.958762818	0.341782194
IRAK4	0.152723063	0.878969554	-0.39424877	0.694853356	-0.411578152	0.682213084
IREB2	0.499523643	0.618668756	-1.069608258	0.289257797	-2.237184901	0.029255181
IRF1	-2.230318736	0.028293956	-0.346864466	0.729957289	-1.446092545	0.153701742
IRF2	-1.905167237	0.060054479	-1.196369169	0.236454194	-2.451093742	0.017375707
IRF2BP1	0.44788355	0.655348146	-0.93668575	0.3528313	-0.809233615	0.421791439
IRF2BP2	-0.73852702	0.462177629	0.412324769	0.681632091	-1.070712575	0.288872552
IRF2BPL	0.628067907	0.531601155	-2.644700705	0.010519306	-3.38968714	0.00128503
IRF3	-0.444805116	0.657562859	1.390062583	0.169858942	2.023828687	0.047748759
IRF4	0.780652105	0.43712149	1.110251009	0.271504598	0.682004455	0.498035547
IRF5	-0.199564114	0.842286186	1.269358844	0.209418357	0.206194751	0.837383465
IRF6	-1.192992261	0.236108758	1.983592783	0.052075063	1.568827027	0.122298336
IRF7	-0.814905146	0.417346982	-0.537653694	0.592887547	-2.361696805	0.021679231
IRF8	1.721013728	0.088795151	0.72350128	0.472300176	-0.011369104	0.9909692
IRF9	-1.322011017	0.189622415	-1.281821341	0.205041276	-3.21921037	0.002136254
IRGM	0.58144268	0.56244235	-0.383187106	0.702991707	0.397277523	0.692666673
IRGQ	1.940060239	0.055604579	1.344490809	0.184061034	1.7049001	0.093732121
IRS1	-1.835325902	0.069866902	-0.537993171	0.592654671	-1.018607328	0.312751645
IRS2	0.605011126	0.546743047	-0.484669014	0.629749299	-2.105724757	0.039705775
ISCA1	-1.403204219	0.164107241	-2.235185042	0.029298022	-1.933477724	0.058216906
ISCA2	-0.053854476	0.957174313	-2.803603278	0.006875741	-2.037032378	0.046363843
ISCU	-1.791386436	0.076701091	-0.438514841	0.662653577	0.165669206	0.869011426
ISG15	-1.795203552	0.076086182	1.440551914	0.155125495	-1.123572711	0.265967869
ISG20	-3.472192874	0.00080556	0.35978302	0.720325269	-0.58815977	0.558779774
ISG20L2	-1.322883098	0.189333389	0.236615852	0.813794323	0.621120167	0.537030098
ISL2	-2.904949594	0.004653964	-2.294687306	0.025414798	-3.493649666	0.000936072
ISM1	1.287605413	0.201290641	2.565949409	0.01291436	1.994982103	0.050899102
ISOC1	0.692491032	0.490469301	-0.055404623	0.956007858	-0.172351544	0.863779932
ISOC2	0.01116706	0.991115673	1.410409796	0.163796712	2.34186385	0.022754442
ISPD	-2.303942281	0.023603676	0.82065155	0.415228399	-1.140004255	0.259116684
ISY1	-1.258136429	0.211701314	2.828455005	0.006424762	3.070628859	0.003287283
ISYNA1	0.587952414	0.558084056	-1.628696029	0.108835518	-0.947289842	0.347543967
ITCH	-0.136318317	0.891883907	-1.189085422	0.239285275	-1.821074349	0.073916711
ITFG1	-0.38788774	0.699044501	0.265965394	0.791215619	-0.760317153	0.450241015
ITFG2	1.358634157	0.177767223	-0.181879438	0.85631594	0.871033227	0.387439246
ITFG3	-0.95537558	0.342029225	2.651176417	0.010341628	3.235673609	0.00203521
ITGA1	-0.44182792	0.659707652	-0.170058558	0.865560017	-1.318914912	0.192543169
ITGA10	2.158350238	0.033649	-0.360569471	0.719740344	0.642339258	0.523263002
ITGA2	-0.942883112	0.348346039	-0.851374515	0.398087257	-1.475893666	0.145550009
ITGA2B	-3.106591728	0.00255426	2.727314529	0.008448644	1.210542968	0.231133877
ITGA3	0.36370234	0.716960721	0.53330345	0.595875545	1.494148661	0.140726927
ITGA4	1.739912776	0.085402934	-0.752041783	0.455088305	-1.989292223	0.051541172
ITGA5	-1.139110448	0.257777097	0.348096755	0.729036582	0.706277646	0.482931417
ITGA6	2.850101619	0.005453457	1.12838236	0.263836747	2.340689511	0.022819574
ITGA7	-1.200913468	0.233037121	1.355962544	0.180403638	1.432444804	0.15755197
ITGA9	-2.049201096	0.043448198	2.406167881	0.019351201	0.345560864	0.73096208
ITGAD	-0.338345022	0.735916592	-0.439823613	0.661710858	-1.849263621	0.069677949
ITGAE	0.678008786	0.499561538	1.174795795	0.244910538	1.186655495	0.240352429
ITGAL	0.115225968	0.908530974	-0.94877553	0.346698845	0.555305191	0.580888247
ITGAM	-1.445137975	0.152003741	0.741805694	0.461219324	0.15444745	0.877809901
ITGAV	1.144805046	0.255422826	-0.760089295	0.450301401	-2.31614085	0.024219715
ITGAX	-1.008544116	0.315984085	-1.09150807	0.27959434	-1.240883689	0.219800357
ITGB1	0.291437564	0.77140892	0.668077577	0.506752515	-0.450636921	0.653983921
ITGB1BP1	2.685212494	0.008675908	-1.14444336	0.257173584	-0.057482554	0.954364563
ITGB1BP2	0.173763156	0.862454404	1.827739	0.072769968	2.360589271	0.021738055
ITGB2	-1.674214134	0.0976723	1.063991123	0.291773181	1.042206187	0.301775851
ITGB3	-5.291579054	8.93E-07	2.250840594	0.028228548	1.070802151	0.288832617
ITGB3BP	-0.393052621	0.695240011	-0.648129265	0.519476873	0.128023589	0.898587025
ITGB4	-0.709719868	0.479771646	1.108367858	0.272309884	0.531069825	0.597461699
ITGB5	-4.021088056	0.000122803	3.003375783	0.003946144	1.347045165	0.183368828
ITGB7	1.031012014	0.305386728	1.02884692	0.307852939	1.311316553	0.195079754
ITGB8	-0.409064218	0.683495637	-0.219909517	0.826719188	-3.244594343	0.001982356
ITIH1	-0.287850045	0.774144638	0.237638738	0.813004616	0.798853695	0.427736262
ITIH2	1.172962148	0.244005594	-2.6255561	0.011061006	-0.545897117	0.587295735

ITIH4	0.894486742	0.373524141	-0.232856367	0.816698455	0.717664212	0.475935039
ITK	2.464127921	0.015697432	-0.109952891	0.912828952	0.211230526	0.833470989
ITLN1	-3.164960331	0.002136477	1.236257423	0.221380744	1.805318977	0.076378712
ITM2A	1.581981305	0.117274795	-0.479956204	0.633076256	-0.324301539	0.746912931
ITM2B	-2.551021437	0.012485281	-1.661686345	0.102009741	-2.177336671	0.033667272
ITM2C	0.997286725	0.321385119	0.262613244	0.79378576	0.198423025	0.843429681
ITPA	0.697271314	0.487488133	1.100231644	0.275808419	1.514475675	0.135505843
ITPK1	-3.6156624	0.000500923	0.015298229	0.987847125	-0.247310299	0.805568765
ITPK1-AS1	-1.186403706	0.238685734	1.281766203	0.20506049	0.956681945	0.342822532
ITPKB	1.280984084	0.203596067	1.469527054	0.147131279	2.016310445	0.048553178
ITPKC	-0.943024128	0.348274316	3.203432347	0.002213885	2.744110002	0.008128126
ITPR1	1.373022331	0.173266377	1.743343951	0.086609627	0.084908832	0.932635429
ITPR2	-0.005389409	0.995712206	0.259105312	0.796477803	-1.566845818	0.122760946
ITPR3	2.96529341	0.003900022	-0.449608251	0.654680427	0.471605874	0.639032759
ITPRIP	-2.466817145	0.015587854	-0.236997032	0.813500015	-1.448738907	0.152963737
ITPRIPL1	3.926603782	0.000171792	-2.428832975	0.018290171	-1.273367859	0.208125908
ITPRIPL2	0.088765869	0.929471559	0.038971927	0.969047541	-0.181448632	0.856667858
ITSN1	-2.624981126	0.010231652	0.584902135	0.560897962	0.40631648	0.68605216
ITSN2	0.865710662	0.389024756	-0.232811916	0.816732808	-1.050589387	0.297940967
IVD	2.462959659	0.01574525	1.355371355	0.180590756	2.607352712	0.011662331
IVNS1ABP	-1.13475647	0.259587426	1.008249101	0.317551962	1.045006256	0.300491228
IWS1	-0.128765435	0.897839822	3.061705945	0.003341524	3.029935875	0.003691682
IZUMO4	0.033038819	0.973719149	-1.88375678	0.064649254	-1.215897154	0.229103464
JAG1	-1.224980619	0.223881648	0.356857895	0.72250231	-0.512504565	0.61030507
JAG2	2.166860943	0.032972836	-1.982059613	0.052251037	-0.081118376	0.93563582
JAGN1	-0.214446135	0.830699921	0.737907749	0.463566439	1.211109663	0.230918356
JAK1	-0.155551427	0.87674618	0.887382233	0.378563732	0.646000715	0.520906372
JAK2	1.225288016	0.223766427	0.082612375	0.934446197	-2.018900944	0.048274696
JAK3	0.667320592	0.506329748	-1.263784634	0.21139846	-0.690389349	0.492788992
JAKMIP1	-0.114960409	0.908740841	-0.591781905	0.556312453	-0.500125317	0.618938311
JAKMIP2	0.206113276	0.837182955	-0.209817099	0.834550846	0.526309457	0.600742864
JAKMIP3	0.57868641	0.56429271	-0.161941722	0.871918515	-0.101683677	0.919369448
JAM2	0.645375792	0.520378448	-1.407393036	0.164684811	-0.897396542	0.373333214
JAM3	-0.94281885	0.348378727	2.495312909	0.015472926	1.884939722	0.064608896
JARID2	-2.403480064	0.018358737	0.340607817	0.734638094	-0.104680993	0.917001408
JAZF1	-1.71584821	0.089741301	-1.811606746	0.075261756	-2.379951004	0.020729978
JDP2	-2.29276363	0.02426843	-0.488544135	0.627019459	-1.401665715	0.166510303
JHDM1D	-0.125047667	0.900773685	-0.809610697	0.421496143	-1.256750014	0.214039213
JKAMP	-1.166475042	0.246603134	-2.184751896	0.032990295	-2.521539279	0.014544028
JMJD1C	-0.102284849	0.918765459	-0.376757145	0.707738572	-2.435850619	0.018050251
JMJD4	0.261615466	0.794234948	1.91233712	0.060810551	2.045108611	0.045533966
JMJD5	2.24249365	0.02746601	-0.176910599	0.860199249	0.683766136	0.49693071
JMJD6	1.119319327	0.266078082	1.198571839	0.235602856	2.37595417	0.020934581
JMJD7	1.690698171	0.094466571	1.349018113	0.182610973	1.802350826	0.076850133
JMJD8	-0.13864675	0.890049028	1.969665038	0.053692484	2.321771493	0.023891977
JMY	1.565467434	0.12109579	-0.926802733	0.357896385	-1.322300404	0.191421028
JOSD1	-1.539355927	0.127338949	0.326355456	0.745338349	1.216457993	0.228891539
JOSD2	-0.182224089	0.855829882	-0.515358525	0.608274977	-0.039010528	0.969020154
JPH3	-0.833314595	0.406944351	-2.275035682	0.026643568	-0.237552413	0.813091592
JPH4	-0.85172978	0.396697463	2.513303334	0.014781105	1.42313584	0.160220928
JPX	-0.975128963	0.332194041	-2.550539508	0.013437313	-2.653435752	0.010339239
JRK	3.516891769	0.000695637	1.005184203	0.319012535	1.59054575	0.117318267
JRKL	0.75574444	0.451839379	0.18034837	0.857512144	-1.202228039	0.234313034
JSRP1	-1.527131435	0.130348038	-0.762214593	0.449042112	-0.581262076	0.563386387
JTB	0.487638285	0.627030005	-0.08195491	0.934966711	0.124128321	0.901656288
JUB	0.810796118	0.419690458	1.165253927	0.248719448	0.00868557	0.993100744
JUN	-0.598781944	0.55087086	-0.797987323	0.42815574	-1.54207948	0.128663047
JUNB	-1.11572573	0.267605205	-2.63118768	0.010899077	-3.666838426	0.00054614
JUND	-0.38351509	0.702271453	-2.764541331	0.007643885	-2.830158785	0.006440219
JUP	-0.879758631	0.381408466	1.897691871	0.062752685	0.673815967	0.503188524
KALRN	0.554138042	0.5809029	2.489245582	0.015712757	1.90588245	0.061781406
KANK1	4.865653975	5.03E-06	1.263755323	0.211408909	1.510194909	0.136592415
KANK2	-3.067427382	0.002875921	1.302253593	0.198013318	0.684809433	0.496277039
KANK3	0.970838403	0.33431435	-1.877309813	0.06554298	-0.873785285	0.385951304
KARS	-1.802658943	0.07489697	1.750818391	0.085301677	1.834663254	0.071847085
KAT2A	0.574212887	0.567302239	0.444246944	0.658528778	1.190902958	0.2386941
KAT2B	-1.350826165	0.180246466	-1.965480337	0.054186783	-2.990444272	0.004128141
KAT5	-1.029851076	0.305928359	0.721237829	0.473680772	1.403893407	0.165848997
KAT6A	-0.921392707	0.359387945	-0.046967429	0.962701663	-0.637761253	0.526217436
KAT6B	0.352225931	0.725518783	-0.026518892	0.978935134	-0.011349194	0.990985014
KAT7	-0.613314007	0.541265415	-0.795361142	0.429669067	-0.268193708	0.789531542
KAT8	-0.386415075	0.700130692	0.628686022	0.532040032	0.753355355	0.454378887

KATNA1	0.488903984	0.626137248	2.604656869	0.011681288	3.285263616	0.001757375
KATNAL1	0.386032419	0.70041303	-1.455542538	0.150948311	-1.25269838	0.21549964
KATNAL2	2.53277682	0.01310593	1.202164686	0.234218989	1.576985768	0.120408007
KATNB1	-1.283693656	0.202650291	0.335726325	0.738297149	-0.130284096	0.896806578
KAZN	-1.102584709	0.273241703	-0.467505501	0.641902272	-1.380233084	0.172977107
KBTBD11	-0.618781243	0.53767382	1.395552308	0.168206581	1.980257392	0.052574999
KBTBD12	-0.95653094	0.341448807	1.932600994	0.05820777	2.487006872	0.015875351
KBTBD2	-0.338341975	0.73591888	0.194322032	0.846607506	-0.888528149	0.37804151
KBTBD3	0.076437754	0.939245961	-0.782718344	0.436998929	-0.920483197	0.361252117
KBTBD4	-0.453487608	0.651324344	1.039137319	0.303083565	1.278663189	0.206267401
KBTBD6	1.072091611	0.286637423	-0.679666519	0.499438234	-1.579117581	0.119917968
KBTBD7	-1.885350243	0.062712929	-0.96578761	0.338188252	-3.434623274	0.001121262
KBTBD8	0.651735052	0.516286424	-0.20244169	0.840284838	-0.14571791	0.884665136
KCMF1	-1.716909166	0.089546298	-1.705086329	0.09356696	-1.784492596	0.079738159
KCNA2	0.917426848	0.361449825	-1.456187955	0.150770457	-1.586715986	0.118184361
KCNA3	0.362940018	0.717528086	-1.981797282	0.052281198	-2.209738172	0.031210939
KCNA5	2.008430719	0.047692143	0.628262597	0.532315374	-0.45859547	0.648292127
KCNA6	-0.877292274	0.382738855	-1.193118846	0.237714528	0.392707237	0.696020332
KCNAB1	1.601240993	0.112940766	-1.370506379	0.175846763	-2.123812892	0.038097531
KCNAB2	-0.579466256	0.563768876	0.707245123	0.48226601	1.008827906	0.317378218
KCNAB3	1.04837665	0.297362557	-0.448436588	0.655520648	0.934066652	0.354262943
KCNC3	2.052266027	0.043142697	-2.376496664	0.020823499	-1.769172526	0.082287383
KCNC4	2.709096251	0.008120983	-0.195804528	0.845452337	0.657811069	0.513343275
KCND1	-0.943916997	0.34782041	-1.202972913	0.233908499	-0.290154376	0.772765371
KCNE1	-2.706007292	0.008190886	0.517658336	0.606679293	-0.191470566	0.848846579
KCNE1L	0.532147596	0.595977231	-1.191800574	0.238227082	0.416320595	0.678760071
KCNE3	-4.866142415	5.02E-06	-1.028495626	0.308016651	-1.224479223	0.225876218
KCNG1	0.920341581	0.3599337	-0.912044939	0.365546763	-0.549726445	0.584683669
KCNH2	-0.981037068	0.32928887	0.739204185	0.462785044	-0.423895612	0.673258998
KCNH3	-0.133894502	0.893794576	2.258127269	0.027742655	1.765703238	0.082873992
KCNH7	-0.084406252	0.932926943	0.670550034	0.505187207	-1.302227508	0.198146936
KCNH8	1.734091339	0.086436287	-0.477560488	0.634770417	-0.338428751	0.736300399
KCNIP2	1.060483047	0.291853772	-0.770608745	0.444088469	0.726456776	0.470571792
KCNIP4	2.17092025	0.032654519	-2.299464402	0.025123806	-3.687839481	0.000511136
KCNJ13	0.583704	0.560926493	0.310667433	0.75717495	0.772136392	0.443266471
KCNJ14	1.595058845	0.114317727	0.386211043	0.700763399	1.789333307	0.078946509
KCNJ15	-1.388709875	0.168458378	-0.754685731	0.453512369	-1.553613295	0.125886815
KCNJ2	0.063446347	0.949556314	-1.722412735	0.090361032	-3.02703845	0.00372218
KCNK10	2.121632579	0.036706262	-0.191826166	0.848553062	0.757641151	0.451828946
KCNK13	0.53922017	0.591109254	1.113218414	0.270239056	0.623561597	0.53543661
KCNK17	1.363914333	0.176105323	-1.554270934	0.12559791	-0.272840517	0.785975219
KCNK5	-0.667662945	0.506112197	-0.82045752	0.415338058	-0.81344462	0.41939395
KCNK6	0.602705945	0.548268775	1.351485672	0.1818243	1.317096352	0.193147988
KCNK7	-0.14937187	0.881605179	0.366489004	0.715343101	0.854398444	0.396509357
KCNMA1	-1.521666081	0.131711379	2.145723687	0.036122177	2.271492272	0.026963335
KCNMB1	-0.965182448	0.337122941	-2.548174	0.013519268	-2.161429855	0.034934132
KCNMB3	0.208715161	0.835157436	-0.950937774	0.345609446	-0.287045052	0.775132824
KCNMB4	2.57805672	0.011614211	1.628804774	0.108812423	1.651066591	0.104299171
KCNN3	-0.033806549	0.973108688	1.431831029	0.15759672	0.483207787	0.630824192
KCNN4	1.370958429	0.173906623	0.670128288	0.505454028	0.505634921	0.615089167
KCNQ1	-1.137503324	0.258444279	1.760520826	0.083628334	1.278659094	0.206268833
KCNQ1OT1	-0.475897288	0.635337836	0.024196128	0.980779801	-0.932196284	0.355220074
KCNQ4	2.051210206	0.043247728	0.099291468	0.921251632	0.48107664	0.632328555
KCNQ5	1.534736164	0.128469588	-0.874283986	0.385594607	-0.157880779	0.875116282
KCNRG	-2.23019302	0.028302619	-0.543583997	0.588825662	-0.377301332	0.707369823
KCNS1	1.136553774	0.258839047	0.636988383	0.526656237	-0.257830563	0.797478854
KCNT1	0.301738859	0.763569674	-1.482333386	0.143702646	-0.292174817	0.771228161
KCTD1	0.237386746	0.812913685	0.130562825	0.896576103	-0.964927947	0.338712112
KCTD10	1.467450113	0.14585176	0.582118011	0.562758962	0.953877026	0.344228143
KCTD11	-0.201418319	0.84084066	1.62732187	0.109127692	1.7730785	0.081631077
KCTD12	-1.265542919	0.209048256	-0.388380097	0.699166669	-1.30405911	0.197525948
KCTD13	0.690971907	0.491418769	-3.412520476	0.001183622	-3.111464894	0.002923405
KCTD15	1.063693524	0.290404683	-1.537198356	0.129720585	-1.697448417	0.095140054
KCTD17	-0.781250441	0.436771421	0.123996614	0.901749678	0.836797288	0.406248404
KCTD18	-0.334769482	0.738603012	0.746143258	0.458615526	-0.028977195	0.97698537
KCTD2	-1.419596364	0.159290767	1.258530349	0.213277604	2.075170314	0.042556941
KCTD20	-0.994160128	0.322896007	0.874177414	0.385652147	0.423243858	0.673731611
KCTD21	-1.509577463	0.134766825	0.11075603	0.912194854	-0.227954906	0.820508095
KCTD3	0.233571557	0.815865136	1.131664788	0.262465136	-0.201342778	0.841157051
KCTD5	-0.059943081	0.952338151	0.318773538	0.751051398	-0.840224684	0.404340547
KCTD6	0.385810782	0.70057658	-1.505383655	0.137690191	-0.900201527	0.371851819
KCTD7	2.825011476	0.005859674	-1.447376965	0.153212634	-0.619056958	0.538378633

KCTD9	0.183741409	0.854642968	-1.376046868	0.174134173	-2.021941951	0.047949543
KDELC1	1.457500214	0.148570804	-0.14862628	0.882367681	0.660341426	0.511730558
KDELC2	1.063219123	0.290618499	-0.776857794	0.440421592	-0.816625598	0.417588363
KDELR1	-0.422391361	0.673779204	0.321999804	0.748618641	0.882902577	0.381047551
KDELR2	-0.352204152	0.725535057	-0.328066774	0.744050826	-0.642894717	0.522905129
KDELR3	0.571305666	0.569262235	-1.005567261	0.318829743	-0.899405666	0.372271756
KDM1A	2.110597213	0.03767095	2.363551083	0.021496684	2.683352598	0.00955528
KDM1B	-1.958260631	0.053396539	-0.731444131	0.467473461	-1.046944974	0.299603973
KDM2A	-0.757506192	0.450789083	0.487796058	0.627546038	0.674523367	0.502742222
KDM2B	1.987905913	0.049959602	0.025792412	0.979512071	0.562534028	0.575987895
KDM3A	-0.288969844	0.77329041	1.402056173	0.16626503	0.347974574	0.729158452
KDM3B	0.722932055	0.471656483	1.552748178	0.125961316	1.105144622	0.273802715
KDM4A	-0.558996054	0.5775974	2.371230369	0.021095044	2.162166144	0.034874582
KDM4B	-1.357856983	0.178012833	-0.178696998	0.858802715	-0.246785535	0.805972867
KDM4C	1.296277869	0.198300426	0.448805981	0.655255702	1.011440384	0.316137786
KDM5A	-1.645587491	0.103449194	2.238644357	0.029058675	1.719978982	0.090935575
KDM5B	-1.129469912	0.261797524	1.089442306	0.280496139	1.209745452	0.231437431
KDM5C	0.938247423	0.350709143	-0.144385117	0.885700401	0.015469995	0.987711976
KDM6A	-0.557798863	0.578411159	-2.121754932	0.038171954	-3.356786132	0.001419046
KDM6B	-1.582027356	0.117264275	-1.501155059	0.138778027	-1.7912117	0.07864109
KDSR	0.873703828	0.384679675	1.998713896	0.05036658	1.998984712	0.050451563
KEAP1	0.197679987	0.843755592	1.195225582	0.236897073	1.770450268	0.082072207
KEL	-2.756847971	0.00710728	0.503839538	0.616295936	-0.146377337	0.884146977
KGFLP2	0.047703598	0.962061568	1.459769278	0.149786554	1.118966481	0.267911231
KHDRBS1	-0.29144866	0.771400462	2.937585916	0.004749685	2.812872785	0.006750817
KHDRBS2	0.154113223	0.877876629	0.793549689	0.430714765	0.842333719	0.403169295
KHK	0.140206952	0.888819874	2.726942568	0.008457064	3.899403367	0.000259573
KHNYN	1.314774409	0.192033545	1.373687971	0.17486175	1.117397613	0.268575416
KHSRP	-0.291228871	0.771567982	-0.850926522	0.398334019	-0.789572237	0.433094163
KIAA0020	1.116583597	0.267240093	1.153792653	0.253350509	1.997077504	0.050664387
KIAA0040	-1.43420527	0.15509061	-0.678354378	0.500263485	-1.157239871	0.252066147
KIAA0090	2.529167529	0.013231951	2.788090179	0.007171864	2.305433008	0.024854033
KIAA0100	1.225229374	0.223788404	1.906674029	0.061555399	1.304755057	0.197290378
KIAA0101	0.771547267	0.442468694	1.298578493	0.199263906	0.742102754	0.461113397
KIAA0114	0.653812276	0.514953469	-1.513279058	0.135677111	-0.833359234	0.408167729
KIAA0125	3.095322254	0.002643211	1.059391558	0.293844093	1.653705816	0.103759362
KIAA0141	1.828715994	0.070861291	-1.005741496	0.318746623	-0.649721374	0.518517399
KIAA0146	-0.754286877	0.452709392	2.546728793	0.013569561	0.634243034	0.528493868
KIAA0174	-1.946590483	0.054803598	2.881727765	0.005548576	3.258271067	0.001903829
KIAA0182	-0.04158618	0.966923675	-2.072207688	0.042732997	-1.801180482	0.077036682
KIAA0195	-0.070851955	0.943677845	0.636668348	0.526863239	1.333047824	0.187891469
KIAA0196	1.197844272	0.234223836	1.041300222	0.302087552	0.793374708	0.430894307
KIAA0226	-1.206002193	0.231079118	1.186044562	0.240474441	1.42156299	0.160675323
KIAA0226L	-3.473573125	0.000801933	1.243172571	0.218841049	1.096448454	0.277555679
KIAA0232	-1.555607623	0.123423992	-0.815509041	0.418140673	-1.463108574	0.149004616
KIAA0240	-0.116375797	0.907622352	-1.347329987	0.183150647	-1.553010308	0.126030759
KIAA0247	-1.454655902	0.149355273	0.085334242	0.93229161	-0.649637883	0.518570943
KIAA0284	1.522113571	0.131599331	0.800276222	0.426839371	1.974533181	0.053239172
KIAA0317	-1.522985686	0.131381176	0.241020746	0.810394968	0.355994478	0.723176728
KIAA0319	-1.210578064	0.229328624	0.312213719	0.756005626	-0.887271222	0.378711848
KIAA0319L	-2.932740089	0.004291457	2.117900529	0.038510881	1.942415022	0.057100732
KIAA0355	0.86783034	0.38786955	-0.955400695	0.343367989	-0.841698617	0.403521779
KIAA0368	0.786305413	0.433820475	3.057813307	0.003379012	2.238772246	0.029145458
KIAA0391	-0.711876734	0.478441591	1.920872817	0.059702364	1.655888332	0.103314689
KIAA0415	-0.814430505	0.41761728	-0.427759527	0.670421376	-0.111939197	0.911270211
KIAA0430	-1.609513216	0.111119114	0.488946769	0.626736122	0.562699151	0.575876194
KIAA0494	0.100355196	0.920292741	0.164002831	0.870303079	0.44124983	0.660724013
KIAA0513	-4.337524465	3.86E-05	0.932738256	0.354848799	0.676146699	0.501718866
KIAA0528	0.940937194	0.349336741	2.620002197	0.011222846	1.443785978	0.154347251
KIAA0556	-0.224787781	0.822670355	-1.23690566	0.221141752	-1.654195544	0.103659448
KIAA0564	0.43813452	0.662372362	0.376370747	0.708024202	0.580965878	0.563584622
KIAA0586	0.994097409	0.322926364	1.001263585	0.320887468	0.606443653	0.54666057
KIAA0649	1.53638149	0.128066005	0.43858314	0.662604367	0.232199564	0.817225946
KIAA0664	0.870820534	0.38624354	-0.712235749	0.479194085	-0.211876107	0.832969717
KIAA0664L3	1.271346403	0.206986589	-1.579632873	0.11966769	-1.162012959	0.250138137
KIAA0748	3.161397913	0.002160029	2.261593053	0.027514156	3.36202182	0.001396868
KIAA0753	0.57541892	0.56649012	-0.227315749	0.820983179	-0.228140501	0.820364517
KIAA0754	0.838067333	0.404284489	0.839448012	0.404688794	0.716921677	0.476389537
KIAA0776	1.579449484	0.117854282	-1.033277917	0.305793055	-1.332271799	0.18814466
KIAA0825	-1.880709867	0.063349543	-1.231097934	0.223289718	-2.169779297	0.034264042
KIAA0889	1.198753152	0.233871961	1.532323423	0.130917365	1.043609729	0.301131462
KIAA0895	0.760622832	0.448934497	-1.387741629	0.170561274	-0.688735299	0.493821542

KIAA0895L	-1.036922108	0.302639434	-0.865284436	0.390472523	-0.442474455	0.659843091
KIAA0907	3.262633856	0.001576855	-3.893889107	0.000259026	-4.107609903	0.000130962
KIAA0913	-0.240587772	0.810439437	-1.477184789	0.145073444	-1.246169159	0.217868613
KIAA0922	-0.044263861	0.964795296	0.794107511	0.43039259	1.33899335	0.185960204
KIAA0930	-0.910887828	0.36486595	1.753250405	0.084879649	0.980411772	0.331081949
KIAA0947	0.585231406	0.559903749	1.266808358	0.210322638	1.069444304	0.289438392
KIAA1009	-0.937297389	0.351194709	0.007326751	0.994179469	-1.044725274	0.300619968
KIAA1024	3.137410914	0.002325024	-1.810071784	0.075502528	-1.453265273	0.151707865
KIAA1033	0.224467181	0.822919	-0.362232018	0.718504375	-1.766455042	0.082746578
KIAA1045	-2.515322767	0.01372552	-2.470565917	0.016472343	-2.221695868	0.030345137
KIAA1107	0.353995108	0.724197184	0.54010045	0.591210076	-1.495239348	0.140442803
KIAA1109	0.269006888	0.788559923	0.357427692	0.722078053	-1.717396999	0.091409485
KIAA1143	-0.22423253	0.823100997	-1.930656941	0.058453285	-1.228357322	0.224428857
KIAA1147	2.504988924	0.014104637	0.629063805	0.531794433	0.736055533	0.464756106
KIAA1161	-0.249133737	0.803843288	-0.010059578	0.992008516	0.335659897	0.738376404
KIAA1191	-0.268671102	0.78881749	-0.058250679	0.95375058	0.935903061	0.353324819
KIAA1199	-0.031836196	0.974675447	-0.023229787	0.981547271	-1.078665684	0.285341719
KIAA1211	0.336040453	0.737647717	0.937259339	0.352538769	-1.198460455	0.235763971
KIAA1257	-1.900032502	0.060734014	-0.328421454	0.743784072	-2.110033566	0.039317397
KIAA1267	-0.868841606	0.387319169	0.42404631	0.673111643	0.351914037	0.726218022
KIAA1274	1.345357363	0.181998456	1.730714616	0.088857368	1.531678439	0.131208309
KIAA1279	2.68492546	0.008682782	0.363898779	0.717266031	1.078411147	0.285454256
KIAA1310	0.487674211	0.627004657	2.20823693	0.031222878	2.195849817	0.03224379
KIAA1324	-3.193553729	0.001955937	1.048048435	0.298994389	1.854681856	0.068887144
KIAA1324L	1.479679933	0.142562941	0.450035956	0.654373823	0.203667896	0.839348235
KIAA1328	2.067997682	0.041603528	-0.690839135	0.49244154	-0.764434184	0.447804332
KIAA1370	-0.598497683	0.551059599	-2.264652256	0.027313848	-2.91067267	0.005160077
KIAA1377	1.150604191	0.253040999	-1.554319624	0.125586304	-0.60645921	0.546650315
KIAA1383	-0.679971469	0.498324016	-1.111986165	0.270764082	-2.156455052	0.035338826
KIAA1407	2.88557449	0.004923162	1.122130997	0.266462987	0.65754448	0.513513343
KIAA1429	0.482480162	0.630674009	1.50316229	0.138260808	2.249354524	0.028423222
KIAA1430	0.477017974	0.634542807	-0.913560572	0.364756271	-1.570270198	0.12196224
KIAA1432	-0.387816056	0.699097358	-0.755281275	0.453157829	-2.238555266	0.029160435
KIAA1467	-0.834495927	0.406282231	0.410476585	0.682979392	0.756860481	0.452292805
KIAA1468	1.288281149	0.201056456	-0.961522035	0.340309143	-1.509692135	0.136720484
KIAA1522	1.839515907	0.069242616	0.851658819	0.397930706	0.236101895	0.814211398
KIAA1524	1.391031445	0.167755588	0.406968341	0.685539705	-0.44886414	0.655254612
KIAA1530	1.381746842	0.170579722	0.092829413	0.926361228	0.123038486	0.902515293
KIAA1539	-3.853187566	0.000222252	-1.625947994	0.109420442	-1.549920091	0.12677052
KIAA1543	1.459127365	0.148123473	-0.452355397	0.652712156	0.106107962	0.91587429
KIAA1586	0.982063012	0.328786098	-1.504975212	0.13779497	-1.841103176	0.070883419
KIAA1598	1.119921454	0.265822803	0.678537132	0.5001485	-0.510099495	0.611978058
KIAA1609	2.413412357	0.017897092	0.911753503	0.36569889	2.439964114	0.017865937
KIAA1614	-2.19149566	0.03108191	1.974564217	0.053118704	1.71924078	0.091070861
KIAA1671	-0.562299103	0.575355088	-1.783662747	0.079747032	-1.188349503	0.239690044
KIAA1683	-0.715774999	0.47604289	0.26701078	0.790414582	-0.913560086	0.364848236
KIAA1704	1.379866699	0.171156	-0.606178317	0.546778067	-0.698105394	0.487987943
KIAA1715	0.681935411	0.497087364	-1.391066082	0.169555968	-0.941904471	0.350270277
KIAA1731	0.145461458	0.8846823	-0.747037483	0.458079785	-1.996903098	0.050683888
KIAA1737	-0.857439606	0.393552751	0.377397716	0.70726515	0.325309725	0.746153907
KIAA1797	1.037275753	0.302475575	1.572247987	0.12137076	1.739864683	0.087353249
KIAA1804	1.930610717	0.056781183	-0.207474551	0.836371091	0.098728536	0.921704888
KIAA1826	1.052257734	0.295588894	-0.176656478	0.860397947	0.087616274	0.930492913
KIAA1841	1.501166595	0.136925388	-0.465829436	0.643094407	-0.88640844	0.379172417
KIAA1875	2.704449214	0.008226353	-0.717048875	0.476241829	-0.239816251	0.811344683
KIAA1908	4.071901385	0.000102318	-0.128558505	0.898154856	-0.232583539	0.816929201
KIAA1919	2.382036429	0.019391742	-0.6508333	0.517742197	-0.008042847	0.99361127
KIAA1949	-1.972012691	0.051777974	-1.36610617	0.177216048	-1.972529912	0.053473303
KIAA1958	-1.288144586	0.201103767	-1.007943011	0.317697627	-2.023988906	0.047731742
KIAA1967	-0.154035289	0.877937893	2.218972132	0.030443121	2.775811839	0.007463847
KIAA1984	1.509967816	0.134667299	-0.515284699	0.608326232	0.388668418	0.698989093
KIAA2013	-1.517418581	0.132778668	0.195743205	0.845500113	-0.120659354	0.904390922
KIAA2018	-1.62439126	0.107902209	-1.118842649	0.267851829	-1.665149905	0.101444984
KIAA2022	-1.663031194	0.099897035	1.04607974	0.299894526	1.3497612	0.182500931
KIAA2026	0.026286198	0.979089134	-2.521053946	0.014491772	-2.292090553	0.025665066
KIDINS220	0.074772037	0.94056738	0.918293389	0.362294889	-0.536677537	0.593607254
KIF11	1.182105326	0.240377803	1.120472709	0.267162732	0.739889465	0.462444728
KIF13A	-2.313039108	0.023074635	0.446095129	0.657201093	-0.313173515	0.755307391
KIF13B	0.863776755	0.390080576	0.731625484	0.467363584	0.57396354	0.568281086
KIF14	0.007085019	0.994363206	0.881307608	0.381814343	0.093209984	0.926068024
KIF15	-0.193608142	0.846933056	1.018573341	0.312665105	1.081524552	0.284079857
KIF16B	1.265958485	0.208900127	1.440031729	0.155272047	0.256807113	0.798264906

KIF18A	-0.76499335	0.446341212	0.768275574	0.445462122	1.114676738	0.269730059
KIF18B	-0.306979576	0.759590894	0.080911273	0.935793015	-0.345191134	0.731238493
KIF19	-1.163459	0.247817494	-1.729462985	0.089082735	-0.795852183	0.429464592
KIF1B	-1.914030338	0.058896618	0.876245138	0.384536719	0.29989278	0.765364647
KIF1C	-1.243311372	0.217085968	1.124416327	0.265500772	1.146509452	0.256439306
KIF20A	0.352079743	0.725628024	0.870618945	0.387576496	0.54121101	0.590499754
KIF20B	1.043603172	0.29955395	-0.111228663	0.911821726	-0.712905468	0.478852048
KIF21A	0.936363971	0.351672205	-0.9802082	0.331082822	-1.18784791	0.239886037
KIF21B	0.978880112	0.330347556	-0.379796531	0.705493304	0.210215518	0.834259248
KIF22	0.74578588	0.457802733	0.870763559	0.387498175	1.817991262	0.074393176
KIF23	0.536352071	0.593081094	1.156550391	0.252230621	0.562362875	0.576103686
KIF24	-0.912601253	0.363968842	0.471975857	0.638727275	-0.209962975	0.8344554
KIF26A	-1.292095703	0.199738263	-0.696960332	0.488631212	-0.106647415	0.915448238
KIF26B	2.18794254	0.031348663	-0.005843421	0.995357844	0.146011174	0.884434691
KIF27	-1.874565118	0.064200893	-0.893541949	0.375285498	-1.841295529	0.070854803
KIF2A	0.320523627	0.749338355	0.83762205	0.405705397	-0.649949692	0.518370989
KIF2C	0.464902531	0.643160231	1.833859144	0.071842954	0.880579142	0.382293471
KIF3A	1.407778851	0.162751991	-0.884797122	0.379944916	-1.664451657	0.101584974
KIF3B	-0.410798043	0.682228491	1.411038938	0.163611967	1.52243078	0.133504844
KIF3C	-2.319311993	0.022715953	1.271986008	0.208489914	0.78881011	0.43353588
KIF4A	0.602024147	0.548720445	2.79058574	0.007123448	3.317514627	0.001596332
KIF5A	-0.912469571	0.364037737	-0.92342803	0.359636636	-1.795797878	0.077899541
KIF5B	0.365457146	0.715655295	0.278128471	0.781909758	-1.032951826	0.306048269
KIF5C	-0.01254274	0.990021259	-0.136454878	0.891937534	0.035983653	0.971422819
KIF7	-0.312123373	0.755691981	1.277540967	0.206536871	0.577503461	0.565904462
KIF9	0.314574536	0.753836257	-0.561701143	0.576498933	-0.567087621	0.572911384
KIFAP3	1.647215056	0.103113501	1.04030991	0.302543311	1.805861999	0.076292728
KIFC1	0.124738005	0.901018116	1.048122586	0.298960522	1.777109283	0.080958378
KIFC2	1.393825341	0.166912787	-2.16182881	0.03479957	-0.983021551	0.329807223
KIFC3	-1.691692396	0.094275995	0.577436352	0.565895214	-0.669677814	0.505803604
KIN	-1.475310381	0.14373128	-1.216045387	0.228928058	-1.045293739	0.300359547
KIR2DL1	-1.158200654	0.249944851	-1.776423877	0.080944641	-0.486219069	0.628701222
KIR2DL3	0.12143962	0.903622281	-0.853989201	0.396648927	-0.355715338	0.723384638
KIR2DL4	-2.291210397	0.02436209	0.1594559	0.873867557	-2.134168711	0.03720262
KIR2DS4	-1.623237646	0.10814893	-1.385882693	0.171125405	0.439007343	0.662338374
KIR3DL1	-0.160186969	0.873104333	-0.506335142	0.614554144	0.023757014	0.981130526
KIR3DL2	0.693886564	0.489597964	-0.753820296	0.454027867	-1.814219056	0.074979641
KIR3DX1	0.289915452	0.772569278	-2.16557322	0.034498216	-1.617113739	0.111449964
KIRREL3	2.064769831	0.041915424	-0.090233292	0.928414891	0.117000009	0.907276896
KISS1R	-0.047917444	0.961891628	-2.749966045	0.007950182	-2.356527167	0.021955029
KIT	-1.521142603	0.131842549	-0.79810248	0.428089454	-1.342899911	0.184699475
KL	0.601894505	0.548806351	0.001310667	0.998958768	-0.261183434	0.794905192
KLB	1.902451536	0.060413073	-0.643452808	0.522484133	0.490344079	0.625798182
KLC1	0.287955779	0.774063968	1.738864053	0.087401498	1.581540635	0.119362931
KLC2	-0.318090192	0.751177153	-2.345284266	0.022479646	-0.681582172	0.498300581
KLC3	-1.569347229	0.12018929	1.706160847	0.093365452	0.776473637	0.440723043
KLC4	0.250591256	0.802719714	-0.215192401	0.830377493	0.083194363	0.933992423
KLF1	-2.007381542	0.047805879	0.422012124	0.674587257	-1.024290647	0.310083966
KLF10	0.951324145	0.344069617	0.460448641	0.646927972	-1.961365807	0.054794332
KLF11	0.174023719	0.862250246	1.76487734	0.082885904	-0.180741738	0.857220085
KLF12	2.013692418	0.047125235	-1.382742199	0.172081707	-1.514931678	0.135390502
KLF13	-0.302983788	0.762623937	-0.438004167	0.663021568	-0.436011809	0.664497366
KLF16	0.737364724	0.46288034	-1.130298833	0.263035306	-1.010281284	0.316687735
KLF2	-0.016502173	0.986871472	-2.421598259	0.018623034	-2.668324445	0.00994202
KLF3	-0.5080546	0.612698022	-0.284731403	0.776871204	-1.397156339	0.16785517
KLF4	-0.624673925	0.533816416	-0.855201003	0.395983412	-1.83344081	0.072031249
KLF5	-3.405849595	0.000999322	0.970696243	0.335758428	-1.588073314	0.117876816
KLF6	-0.386639953	0.699964789	-1.190390005	0.238776407	-2.20068052	0.031881178
KLF7	0.039967788	0.968210185	-2.785702346	0.007218474	-4.288175332	7.14E-05
KLF8	1.860150896	0.066235738	-0.57550274	0.567193053	0.411643123	0.682165732
KLF9	3.412070916	0.000979437	-0.316376347	0.752860641	-0.727270873	0.470076952
KLHDC1	1.86228281	0.065931413	-2.689748144	0.009338905	-2.554874378	0.013355586
KLHDC10	1.249671537	0.21476372	-1.823492683	0.073419042	-1.897199967	0.062940614
KLHDC2	0.654213474	0.51469623	-1.956567565	0.055252564	-1.547642702	0.127317918
KLHDC3	-0.740311749	0.461099782	1.69642117	0.095205077	2.502920291	0.015248736
KLHDC4	0.873121001	0.384995475	0.654845666	0.515173888	1.212597572	0.230353184
KLHDC5	1.746098086	0.084316144	-1.090932315	0.279845479	-1.231838609	0.223135399
KLHDC7A	-2.201194651	0.030363816	2.187337887	0.032791467	0.470727733	0.63965593
KLHDC7B	-1.105507066	0.271981147	0.962253243	0.339944958	-0.881985874	0.381538818
KLHDC8A	-3.288041051	0.001455636	0.745678014	0.458894402	-0.939981985	0.351246889
KLHDC8B	-4.054399189	0.000108973	2.210645472	0.031046418	1.965117256	0.054347346
KLHL11	0.835266746	0.40585055	-0.101202627	0.919741093	-1.94148858	0.057215579

KLHL12	0.261309052	0.794470451	1.291353657	0.201739674	-0.14448494	0.885634105
KLHL13	0.26492583	0.791691909	0.868076499	0.388955073	0.270043133	0.788115585
KLHL14	2.309290084	0.023291383	0.718864532	0.475130811	0.338983446	0.735884742
KLHL15	0.598811682	0.550851117	-0.200098829	0.842108126	-1.141383564	0.25854734
KLHL17	2.838471814	0.00563842	-1.827199032	0.072852237	-1.373608624	0.175014414
KLHL18	-0.495169366	0.621726206	0.845174101	0.401510952	0.624919119	0.534551634
KLHL2	-0.122564582	0.902733973	-0.137504523	0.891111586	-1.657575271	0.102972054
KLHL20	0.976274677	0.33162935	-1.818164304	0.074240382	-2.49672459	0.015489994
KLHL21	-1.706553767	0.091464517	0.269332812	0.788636116	-0.406467677	0.685941725
KLHL22	0.537758023	0.592114108	-0.094312878	0.925187958	1.227796773	0.22463764
KLHL24	1.090602382	0.278452727	-0.6447056	0.521677609	-1.44893068	0.152910364
KLHL25	1.589511588	0.11556469	1.016987426	0.313412457	1.817014485	0.074544665
KLHL26	-0.240161024	0.810769183	-0.659993753	0.511888577	-0.563280977	0.575482691
KLHL28	0.348488078	0.728313741	-0.785728665	0.435246975	-1.897811261	0.0628584
KLHL29	1.96920865	0.052104567	0.222761909	0.824508914	0.465600986	0.643299314
KLHL3	2.001591232	0.048437754	-0.241485256	0.810036708	0.796576606	0.429047074
KLHL31	0.187470694	0.851727185	-0.236543309	0.813850337	-0.819546555	0.415934519
KLHL32	3.189786274	0.001978882	1.140252046	0.258900753	0.452914163	0.652353152
KLHL34	0.383596624	0.702211233	-0.412302209	0.681648531	-0.720308039	0.474318761
KLHL35	-0.874252153	0.384382716	1.525696568	0.13255831	0.528356001	0.599331224
KLHL36	2.904809405	0.004655862	-3.715728965	0.000459976	-4.014409807	0.000178247
KLHL5	1.567829364	0.120543288	-0.937934293	0.352194743	-1.699788978	0.094695963
KLHL6	1.994067506	0.049269439	0.718247102	0.475508459	0.707566912	0.482136369
KLHL7	1.246483093	0.21592561	0.268672058	0.789142079	0.074184811	0.941126582
KLHL8	-0.825836098	0.41115112	-0.882339124	0.38126113	-2.443232787	0.017720686
KLHL9	0.541791415	0.589344114	-0.675345627	0.502158591	-1.548129504	0.127200751
KLK2	-0.979429679	0.330077602	2.127583447	0.037664383	1.842572957	0.070665013
KLK4	-1.719273425	0.089112999	0.841170817	0.403731059	0.886340279	0.379208818
KLKB1	2.381905298	0.019398215	1.480580625	0.144168164	2.297887706	0.025309836
KLLN	-0.809448403	0.420460802	-0.53997065	0.591299009	-1.540938191	0.128940396
KLRAP1	1.667033819	0.099096074	-2.393239822	0.019980827	-2.265147926	0.027374768
KLRAQ1	-0.727657522	0.468772814	1.584772438	0.118493802	1.415320146	0.162488743
KLRB1	0.514396515	0.608276234	-2.301828072	0.024980923	-0.872858295	0.386452095
KLRC1	0.973137772	0.333176946	-1.605082601	0.113945307	-1.534610464	0.130486782
KLRC2	0.085910013	0.93173493	-3.303445627	0.001645364	-2.37856216	0.020800871
KLRC3	0.438521239	0.662093148	-3.516709239	0.000859488	-3.479949991	0.000976265
KLRC4	1.66679267	0.099144182	-5.951454835	1.67E-07	-5.542275042	8.25E-07
KLRC4-KLRK1	1.680216033	0.096494991	-0.555214206	0.580898212	-0.569948889	0.570982351
KLRD1	1.933367723	0.056435738	-2.25213765	0.028141511	-2.359872058	0.021776225
KLRF1	0.927928878	0.356006182	-2.536529496	0.013929346	-1.991126498	0.051333429
KLRG1	1.580827079	0.117538693	-1.6979199	0.094920073	-0.457172052	0.649308591
KLRK1	1.930105163	0.056844722	-2.747623858	0.008000442	-1.926788575	0.059064442
KMO	-0.4927956	0.623395803	0.87041092	0.387689178	0.793525052	0.430807465
KNDC1	1.070502151	0.287347844	-3.032403728	0.003633533	-2.498586926	0.015417116
KNTC1	2.358817206	0.020568245	1.283782786	0.204358639	0.997373101	0.322855716
KPNA1	-0.215618433	0.829788792	-0.033900851	0.973073427	0.570411954	0.570670457
KPNA2	-0.187437549	0.85175309	1.943324095	0.056869292	2.768806427	0.007606184
KPNA3	-0.188304969	0.851075178	0.445787626	0.657421918	-0.004959397	0.996060541
KPNA4	-1.773660308	0.079610879	-0.933171035	0.354627249	-1.259771944	0.212954728
KPNA5	1.526504903	0.13050376	-0.901024251	0.371327664	-1.146825532	0.256309719
KPNA6	0.652429744	0.515840438	0.708739141	0.481345233	1.447684149	0.15325755
KPNB1	-1.451588227	0.150204947	0.974937823	0.333668114	0.243308576	0.808651696
KPTN	-2.746967671	0.007307021	1.581259974	0.119295049	0.602771316	0.549083962
KRAS	-0.254066949	0.800042045	-1.317678484	0.192828645	-1.807883229	0.075973393
KRBA1	1.757546596	0.082334628	1.056294466	0.295244217	1.966676356	0.054162499
KRBA2	1.534556589	0.128513697	-0.031986138	0.974593691	0.335911963	0.738187331
KRCC1	-0.006724207	0.994650261	-0.644047433	0.522101243	-0.825115924	0.41279217
KREMEN1	-0.745057314	0.458240768	0.307977173	0.759210722	-0.674284408	0.502892958
KREMEN2	-0.578498549	0.564418934	0.532574597	0.596376851	0.946366201	0.348010567
KRI1	1.122402595	0.264772699	1.920969438	0.059689919	2.719639202	0.008677582
KRIT1	1.089778507	0.278813536	-0.259515423	0.796162949	-0.038402447	0.969502812
KRR1	0.224167931	0.823151102	-1.082525493	0.283530391	-1.377058796	0.173951054
KRT1	-3.05746314	0.002963559	0.997012027	0.322929	0.052253148	0.958512155
KRT10	-2.798901928	0.006311698	-4.192049765	9.63E-05	-3.717766592	0.000464945
KRT18	0.411235688	0.681908787	2.963558166	0.004415837	2.873240792	0.005722665
KRT23	-3.433838613	0.000912756	-0.980900423	0.330744254	-0.750028982	0.456363723
KRT5	1.239783094	0.218382124	-0.369719914	0.712947136	0.682450952	0.4977554
KRT72	1.27978005	0.204017382	2.374200749	0.020941496	2.062218334	0.043818213
KRT73	3.249182505	0.001644799	2.02242816	0.0477843	1.19865525	0.235688794
KRT74	-0.619602492	0.53713537	1.190870668	0.238589117	0.418345218	0.677288027
KRT81	-0.713384249	0.477513183	0.572328666	0.569326656	-0.058911886	0.953231135
KRT86	0.085736595	0.931872388	-0.245949555	0.806595644	-0.272508733	0.78622899

KRTAP5-1	1.087156867	0.27996381	-1.33274048	0.187865381	-0.33973193	0.735323996
KRTCAP2	-0.921873935	0.359138263	-2.99251474	0.004069421	-1.826700399	0.073053855
KRTCAP3	0.325456012	0.745615707	0.819729199	0.415749832	1.068693314	0.289773809
KSR1	2.929914521	0.004327088	-2.78694054	0.00719427	-2.579192469	0.01254497
KSR2	-0.120860051	0.904079977	3.187629376	0.002319091	2.487847198	0.015841689
KTI12	-0.256895164	0.797864955	-2.128969163	0.037544579	-2.042914642	0.045758127
KTN1	1.318831409	0.190679003	2.04135099	0.045806455	1.751858825	0.085249644
KY	-2.521643652	0.013498162	-3.497198718	0.000912922	-3.843667739	0.000310855
KYNU	-3.175339505	0.002069217	1.220634521	0.227198157	0.352969922	0.725430606
L1CAM	-0.568587949	0.57109744	0.717671348	0.475860767	1.43766575	0.156070293
L1TD1	-0.386888463	0.699781468	1.276613902	0.206861862	1.746648028	0.086158322
L2HGDH	2.061555492	0.042228012	-1.195485998	0.236796169	-1.045593985	0.300222064
L3MBTL1	3.522228389	0.000683504	-0.772866842	0.442761382	-1.469743972	0.147203753
L3MBTL2	-0.516764638	0.60662882	3.442084185	0.001081452	3.801957702	0.000355466
L3MBTL3	1.939585515	0.055663193	1.366010104	0.177246033	0.268177027	0.789544316
L3MBTL4	1.706113303	0.091546848	0.683511604	0.497024205	1.238456079	0.220691822
LACC1	0.312464578	0.755433576	-1.438559532	0.155687396	-1.234357808	0.222202823
LACE1	-0.323334676	0.747216021	0.26999198	0.788131459	-0.06625345	0.94741102
LACTB	0.127506491	0.898833156	2.234233833	0.02936414	0.972845369	0.334796172
LACTB2	-0.05845071	0.953523379	-0.821456985	0.414773387	-1.407205421	0.164869556
LAG3	0.078952654	0.937251206	-1.049601449	0.298285619	-0.236521131	0.813887706
LAGE3	0.660590137	0.510616795	-0.057014997	0.954730585	0.457902021	0.648787236
LAIR1	2.541098685	0.012819471	-0.922585587	0.360071913	0.920708391	0.361135527
LAIR2	0.805809628	0.422544915	-0.921650482	0.360555463	-1.551394677	0.12641709
LAMA2	1.802553272	0.074913717	0.237587964	0.813043811	-0.020459011	0.983749627
LAMA5	0.57531072	0.566562957	-0.526243644	0.600739563	0.211612354	0.833174504
LAMB2	0.057634586	0.954171582	2.643442596	0.010554147	2.923341623	0.004981596
LAMB2P1	0.931992569	0.353913985	0.407264812	0.685323197	1.771445458	0.081904939
LAMB3	-1.722545589	0.088516143	2.939328896	0.004726566	2.283141715	0.026222116
LAMC1	-0.363248096	0.717298777	1.521232983	0.133672768	1.662346805	0.102007925
LAMC3	1.896882298	0.061154113	1.873898129	0.066020148	0.730200018	0.468298946
LAMP1	-1.965854313	0.052497555	1.026701589	0.308853639	0.66328377	0.509858682
LAMP2	-2.008375353	0.047698139	-0.602351061	0.549304642	-1.520069204	0.134096402
LAMP3	-3.157919395	0.002183259	0.313579072	0.754973602	-1.81758243	0.07445655
LAMTOR1	-3.023610511	0.003280158	0.326925813	0.744909155	-0.005098602	0.995949965
LAMTOR2	-1.028237937	0.306682037	-0.177975092	0.859367018	-0.173584065	0.862815673
LAMTOR3	-0.613788496	0.540953227	-1.822570618	0.073560625	-2.191144472	0.032600475
LANCL1	1.218889477	0.226173696	-2.238370253	0.029077577	-1.85616919	0.068671394
LANCL2	1.568178947	0.120461684	1.117792479	0.268296445	1.833416213	0.072034959
LANCL3	0.027097589	0.978443824	0.383823012	0.702522896	-0.57314986	0.568828065
LAP3	-1.181514532	0.240611041	2.712393376	0.008792494	-0.049334921	0.960827194
LAPTM4A	-0.453092417	0.651607761	-2.779119574	0.007348416	-2.956474937	0.00454155
LAPTM4B	0.165855604	0.868654573	2.013549361	0.048737439	2.176815439	0.033708136
LAPTM5	-3.546788301	0.000630201	-0.800948094	0.426453431	-0.43246987	0.667053866
LARGE	-0.566320286	0.572630924	0.226114873	0.821912581	1.092064367	0.279461279
LARP1	0.512665128	0.609481977	1.361308763	0.178718206	1.248353759	0.217073852
LARP1B	1.143885783	0.255801836	-0.680027249	0.499211488	0.326853348	0.744992262
LARP4	1.662877762	0.099927843	-0.117080572	0.907203518	-0.750833065	0.455883471
LARP4B	-1.008455728	0.316026254	0.475764547	0.636041728	-0.195862445	0.845423846
LARP7	0.394869106	0.693903821	-0.152538407	0.87929541	-0.057360189	0.9544616
LARS	1.641091653	0.104381076	3.608519275	0.000645586	3.524555763	0.000851099
LARS2	0.43361624	0.665638125	2.806040025	0.006830265	2.471725897	0.016498847
LAS1L	1.318284575	0.19086116	2.157073119	0.035185637	2.507132074	0.015086676
LASP1	-2.777578718	0.006704301	0.086856577	0.931086774	0.008957916	0.992884415
LAT	-0.089685578	0.928742779	0.706250061	0.482879821	1.653919332	0.103715791
LAT2	-2.210967946	0.029654927	0.333707451	0.739812237	0.407106858	0.685474941
LATS1	-0.242206614	0.809188876	-1.235015036	0.221839318	-2.053745807	0.044660699
LATS2	-1.193174173	0.236037892	-0.487069411	0.628057718	-2.381417024	0.020655381
LAX1	2.247382556	0.027139574	-0.360581022	0.719731754	0.548350957	0.58562128
LAYN	0.021088094	0.983223568	1.943315623	0.056870339	1.213184639	0.230130469
LBH	0.447272161	0.655787752	-0.400767634	0.690074033	0.49607905	0.621771992
LBR	-1.43163647	0.155822894	0.037528889	0.970193079	-1.019086254	0.312526246
LCA5L	-2.698440609	0.008364441	-1.224884284	0.225604732	0.972820004	0.334808669
LCAT	-0.960210745	0.339604458	-1.320450077	0.191907993	-0.017903088	0.985779533
LCK	0.335146374	0.738319687	-1.447139137	0.153278979	-0.098611271	0.921797577
LCLAT1	0.219131006	0.827060163	-1.159324515	0.251107656	-0.738895991	0.463043035
LCMT1	0.064762381	0.94851145	2.133429064	0.03716125	2.912700785	0.005131111
LCMT2	1.329999398	0.186987236	-1.088074395	0.281094409	-0.524731858	0.601832093
LCN10	-1.435624908	0.154687058	-0.845217321	0.401487024	-0.007071733	0.994382647
LCN2	-4.375748128	3.34E-05	0.200980434	0.841421931	-0.224277882	0.823353938
LCN8	0.424684216	0.672113061	0.219057426	0.827379734	0.567420719	0.57268665
LCNL1	0.337424304	0.736608045	-0.194390444	0.846554191	-1.343458304	0.184519802

LCOR	-0.031976713	0.974563709	-1.985763977	0.051826729	-2.834473922	0.00636477
LCORL	-0.548946991	0.584444932	-0.823531335	0.413602923	-1.935437112	0.057970619
LCP1	-2.257822845	0.02645385	1.15027066	0.254785913	1.108304905	0.272447708
LCP2	-1.03621031	0.302969423	-2.675280922	0.009704081	-2.897798067	0.005347518
LDB1	0.81290074	0.418489161	-3.424630938	0.001140709	-3.331139136	0.001532578
LDB2	-0.466106753	0.642301482	-2.757078486	0.007799333	-0.958853797	0.341736756
LDHA	0.318696793	0.750718647	2.616402372	0.011328895	2.687590488	0.009448737
LDHAL6A	-0.3163892	0.752463343	-0.045074312	0.96420399	0.199117749	0.842888811
LDHB	1.637793476	0.105069014	3.373510347	0.001332494	4.164608748	0.000108291
LDHC	-0.88870668	0.37660604	1.442698476	0.154521881	1.500947421	0.138963233
LDHD	0.671620513	0.503600973	1.854548275	0.068782377	0.0965959	0.923390736
LDLR	3.395822482	0.001032173	-0.882564119	0.38114053	-0.780538475	0.438347175
LDLRAD2	0.305339935	0.760835028	0.557381953	0.579426307	0.586908253	0.559614201
LDLRAD3	-0.73349853	0.465222152	0.047123505	0.962577811	0.296703777	0.767785754
LDLRAP1	1.577715909	0.118252383	2.104242767	0.039733054	3.047609571	0.003510649
LDOC1	0.843494287	0.401260277	-0.789607901	0.432995473	0.30572973	0.760939312
LDOC1L	0.891615961	0.375052836	-1.10328779	0.274490618	-0.320311284	0.749919509
LEAP2	0.195342102	0.845579648	1.341177086	0.185127928	1.939949594	0.057406797
LEF1	2.367225004	0.020135107	0.857106727	0.3949382	1.811667536	0.075378519
LEFTY1	-3.361106247	0.001153934	0.068005174	0.94601705	-0.482486085	0.631333461
LEMD2	2.059464531	0.04243243	0.550626686	0.584019058	1.164909375	0.248973347
LEMD3	0.872133517	0.385530904	-0.347769454	0.729281086	-1.761641208	0.083565244
LENG1	-1.107414116	0.27116073	1.099486717	0.2761303	1.681849234	0.098143726
LENG8	2.323904958	0.022456463	-1.87880126	0.065335301	-0.749857627	0.456466105
LENG9	-0.646370416	0.519737312	-0.225096955	0.822700584	-1.543928352	0.128214756
LEO1	0.219750388	0.826579236	1.616465329	0.111458455	2.280205485	0.026407215
LEP	0.062398713	0.950388144	0.665403371	0.508448491	1.104569814	0.274049677
LEPR	-0.607258172	0.545257859	-0.822466035	0.414203774	-2.240526919	0.029024592
LEPRE1	2.789684754	0.006478747	0.410058535	0.683284289	0.209330629	0.834946596
LEPREL1	2.259958346	0.02631548	-0.163568831	0.870643189	0.747690773	0.457761915
LEPREL2	0.959674368	0.339872889	0.978654018	0.331843813	1.956928018	0.055327158
LEPREL4	-0.58443622	0.560436087	0.126613754	0.899687084	0.796132336	0.429303098
LEPROT	-1.262664993	0.210076225	-1.386748706	0.170862418	-1.591664519	0.117066227
LEPROTL1	0.970239839	0.334610852	-0.857500248	0.394722583	-1.289035523	0.202662924
LETM1	0.879196303	0.38171154	2.741780496	0.008127107	2.578122553	0.012579673
LETM2	-1.178934716	0.241631421	-2.697121971	0.009157658	-1.008738285	0.317420829
LETMD1	2.479250016	0.015090098	1.402603279	0.166102499	2.756717196	0.007857702
LFNG	-0.994241239	0.322856752	-0.92185826	0.360447983	-0.29241162	0.771048055
LGALS1	-0.690098987	0.491964807	-0.934373683	0.354012054	-1.015186368	0.314364863
LGALS12	-3.353105906	0.001183847	1.527271154	0.132166937	0.845493288	0.401418539
LGALS2	-1.055006397	0.294337117	-2.655146443	0.010234059	-2.364773497	0.021516564
LGALS3	-0.757489053	0.450799293	-0.432135327	0.667256622	-0.18841588	0.851228925
LGALS3BP	-0.79421736	0.429225321	3.21661765	0.002129558	2.210239724	0.031174192
LGALS4	1.283768733	0.202624132	-2.803712645	0.006873694	-0.500661634	0.618563153
LGALS8	0.326167551	0.745079179	-0.470659139	0.63966175	-1.141996044	0.258294809
LGALS9	-2.761481359	0.00701534	0.584195494	0.561370015	0.365946441	0.715778074
LGALS9B	0.710769816	0.479123928	-1.851014167	0.069297262	-0.986099219	0.328308159
LGALS9C	-1.715017393	0.089894247	-0.739407419	0.462662618	0.09051646	0.928198444
LGI2	-0.663253931	0.508917754	1.73799597	0.087555632	0.971225603	0.335594858
LGI4	0.797945893	0.427069853	-0.784329162	0.436060943	-0.868767636	0.388666864
LGMN	1.557134091	0.123061237	3.05493515	0.003406982	2.488467841	0.015816868
LGR4	1.438310632	0.153925823	-0.882971903	0.380922014	-1.248033538	0.217190215
LGR6	1.229751107	0.222098386	-1.498270534	0.13952398	-0.091188423	0.927666911
LGSN	-1.453055967	0.149797951	-0.540671308	0.590819023	1.015635402	0.314152793
LHFPL2	-2.463643993	0.015717224	2.698472024	0.009124825	0.690685262	0.492604392
LHPP	-0.527058188	0.599491676	0.118049412	0.906439235	0.793704656	0.430703735
LHX4	1.496911395	0.138027729	0.963433216	0.339357803	1.047746535	0.299237663
LIAS	1.045081297	0.298874209	-0.495041911	0.622453836	0.831451216	0.409235286
LIF	-2.80953626	0.006123869	-2.102238145	0.039915252	-1.919166416	0.06004299
LIG1	2.138772384	0.035250387	1.897835733	0.062733354	2.305182298	0.02486906
LIG3	1.104554386	0.272391639	1.066000287	0.290871739	1.855547738	0.068761471
LIG4	-0.152550418	0.879105302	-0.432866477	0.666728414	-1.461620203	0.149410925
LILRA1	-1.107768943	0.271008273	-1.638504745	0.106768381	-2.796048856	0.007066332
LILRA2	-3.375627323	0.001101446	0.120529216	0.904483414	-0.407809758	0.684961768
LILRA3	-2.732290305	0.007613209	1.119874337	0.267415544	1.019389022	0.31238381
LILRA4	1.370952615	0.173908429	0.386016229	0.700906876	0.098590073	0.921814332
LILRA5	-4.039207501	0.000115084	1.925769822	0.059074382	0.804247076	0.424641115
LILRA6	-1.899890104	0.060752951	-0.127285269	0.899157967	-0.213583554	0.831644279
LILRB1	-0.128114475	0.898353423	-0.25736083	0.797817475	-0.317205135	0.752262636
LILRB2	-2.679967765	0.008802291	-0.018616614	0.985211291	-1.127396186	0.264362336
LILRB3	-2.79013531	0.006470489	-0.115931613	0.908110005	-0.542499641	0.589617859
LILRB4	-1.339309822	0.183950779	1.350806769	0.182040481	1.079899544	0.284796636

LILRB5	-1.011144717	0.314745047	-0.79136425	0.43197837	-0.544866679	0.587999564
LIM2	-0.14306921	0.886565646	-3.397185569	0.001240158	-2.322659739	0.023840638
LIMA1	3.091879479	0.002670953	0.028361371	0.977471976	1.958838124	0.055097279
LIMD1	2.610836596	0.010631835	1.509368178	0.136671328	1.760664606	0.083732149
LIMD2	-0.827338965	0.410303632	-0.601128068	0.550113249	-0.406945216	0.685592975
LIME1	1.186987708	0.238456502	-0.367613038	0.714509216	0.398304002	0.691914294
LIMK1	-1.003165354	0.318557083	-0.366767798	0.71513624	-0.838732363	0.405170569
LIMK2	-0.416512006	0.678058969	0.577868724	0.565605207	-0.216585438	0.829315205
LIMS1	-2.199671366	0.030475628	-0.012920982	0.989735488	-1.043269529	0.301287567
LIMS2	-1.589028801	0.115673727	1.273933852	0.207803522	2.337149885	0.023016898
LIN37	-1.890693426	0.061986571	0.68348251	0.497042447	0.876621266	0.384421742
LIN52	0.454803401	0.650381074	-0.249328856	0.803993441	-0.085670645	0.932032522
LIN54	-0.763666311	0.447127703	-1.580026968	0.119577348	-2.624991239	0.011138681
LIN7A	-2.084221528	0.040065974	-1.073055916	0.287721363	-2.6578602	0.01021971
LIN7B	0.271673006	0.786515697	0.192625434	0.847929919	0.318836974	0.751031363
LIN7C	0.527030662	0.59951071	-0.427580432	0.670551034	-0.917981488	0.362548955
LIN9	0.129127497	0.897554177	-1.230724481	0.223428361	-0.957606452	0.342360066
LINGO2	1.286923151	0.201527293	-0.944137059	0.349043397	-1.296113311	0.200230515
LINGO3	0.698311733	0.486840609	-2.704613115	0.008976831	-2.650965442	0.010406531
LINS	0.881979802	0.380212808	-0.056180286	0.955392624	-0.333569718	0.739944858
LIPA	1.584420205	0.116718728	0.804090045	0.424651388	0.833442827	0.408120996
LIPC	-0.752284799	0.45390599	-0.392065809	0.696456598	-1.683008547	0.097917853
LIPE	0.586048247	0.559357173	0.316445204	0.752808652	0.807578919	0.422735775
LIPN	-2.93715265	0.004236354	0.281714775	0.779171947	-0.764783824	0.447597751
LIPT1	2.138496975	0.035273379	-0.134521094	0.893459512	0.281157825	0.77962125
LIPT2	0.218917052	0.827226306	-1.838606341	0.071130753	-2.645757808	0.0105497
LITAF	-3.532739843	0.000660189	-0.129480425	0.89742863	-0.669111899	0.506161801
LIX1	0.879996621	0.381280244	-0.169136281	0.866282061	0.470714588	0.63966526
LIX1L	1.658920783	0.100725023	-1.387218479	0.170719889	-1.929578986	0.058709618
LLGL1	0.533268944	0.59520418	0.04230795	0.966399534	0.294803112	0.769229858
LLGL2	1.217782123	0.226592208	-2.148293162	0.035908259	-1.014662066	0.314612603
LLPH	-0.942428391	0.348577383	-1.163790755	0.249307254	-0.362074943	0.718653078
LMAN1	1.949445785	0.054456462	-0.622255193	0.536229781	-1.076738772	0.286194422
LMAN2	-1.005339773	0.317515247	-0.552680768	0.582620696	-0.882296325	0.3813724
LMAN2L	-0.688059897	0.493241609	1.989410726	0.051411914	3.852540154	0.000302086
LMBR1	-0.434252878	0.665177577	-2.557129244	0.013211376	-3.714745483	0.00046942
LMBR1L	0.869200742	0.387123826	-2.445932141	0.017524604	-0.663129699	0.509956608
LMBRD1	-0.560334834	0.57668805	-1.575729386	0.120565486	-1.949366567	0.056245241
LMBRD2	0.157028415	0.875585514	-0.354375843	0.724351401	-3.03420111	0.003647212
LMF1	2.02053785	0.04639633	0.472413505	0.638416807	1.230719854	0.223550469
LMF2	-0.180560426	0.857131653	-0.7359003	0.464777866	-0.209443962	0.834858556
LMLN	1.884783683	0.062790366	0.470857445	0.639520975	0.246871236	0.805906869
LMNA	-2.269675871	0.025693835	1.868990482	0.066711695	1.007925358	0.317807517
LMNB1	-1.438605528	0.153842416	0.98982655	0.326399069	-0.391626866	0.696813999
LMNB2	-0.05240303	0.958327437	0.668268347	0.506631646	1.194408325	0.237331763
LMO2	-1.215873237	0.227314968	-2.392257818	0.020029394	-2.919465467	0.005035585
LMO4	-1.526917926	0.130401088	-2.262759676	0.027437616	-2.840347692	0.006263381
LMO7	3.284738522	0.001470883	0.756021624	0.452717307	1.573627213	0.1211833
LMOD1	-0.949468084	0.345007005	0.157820781	0.875150024	0.461201069	0.64643321
LMOD3	1.359080954	0.177626138	-0.520997695	0.604365762	-0.55574317	0.580590776
LMTK2	-2.26090462	0.026254369	0.450815123	0.653815424	-0.528255987	0.599400175
LMTK3	1.415440397	0.16050149	-1.960027006	0.054836776	-0.367149927	0.714885197
LNP1	2.334361058	0.021875476	-0.00971721	0.99228049	0.866325021	0.389993124
LNPEP	-0.158553944	0.874386987	0.409821458	0.683457221	-1.674756507	0.099534951
LNX1	0.183128074	0.855122705	-1.312524932	0.194549369	-2.422159114	0.01867611
LNX2	0.914450213	0.363002346	0.239944825	0.811224944	0.501290423	0.618123439
LOC100009676	0.945501242	0.347015972	-0.940763546	0.350755041	-2.230645705	0.029711051
LOC100124692	-2.895474154	0.004783875	-0.848205907	0.399834613	-3.098967373	0.003030552
LOC100125556	0.457325192	0.648574837	-0.14811469	0.882769578	1.375014801	0.174580424
LOC100128071	0.565744256	0.573020775	-3.402967165	0.001218549	-2.38381513	0.020533873
LOC100128164	-0.451780223	0.652549186	2.090571447	0.040990127	2.244607527	0.028745222
LOC100128191	0.916456609	0.361955406	-0.772371625	0.443052222	0.897044661	0.373519317
LOC100128239	1.469048269	0.14541866	-0.00908916	0.992779409	0.972008573	0.335208628
LOC100128252	-0.003723226	0.997037808	-1.473216307	0.146137038	0.695347946	0.489700691
LOC100128288	2.016227114	0.046854205	-0.32253618	0.748214437	0.883483597	0.380736385
LOC100128361	0.891561391	0.375081933	-0.042123988	0.966545547	-1.561750503	0.123957142
LOC100128398	-0.149857295	0.881223321	-2.088682185	0.041166536	-0.84138651	0.403695069
LOC100128420	1.909084561	0.059540379	-1.538045337	0.129513545	-0.020524338	0.983697746
LOC100128542	0.544462688	0.58751293	-1.284567359	0.204086061	0.658687921	0.512784108
LOC100128573	0.922597075	0.358763276	0.504967822	0.615508181	0.898005893	0.373011079
LOC100128682	-0.606556455	0.545721441	-0.239856009	0.811293468	-0.790819654	0.432371756
LOC100128822	-0.987001478	0.326373051	-1.453650885	0.151470526	-2.366386518	0.021431719

LOC100128881	0.752950256	0.45350806	-1.630572808	0.108437502	-1.708369282	0.093082527
LOC100129034	-0.944658864	0.34744356	-0.229340857	0.81941646	-1.297923559	0.199611919
LOC100129083	1.946199957	0.054851222	-3.128169768	0.002758505	-1.579101381	0.119921686
LOC100129196	1.919592911	0.058179607	-0.781495619	0.437711721	-0.667148487	0.507405607
LOC100129250	-2.077318459	0.040714089	-3.995591711	0.000185546	-2.801413509	0.006964277
LOC100129269	-0.937790158	0.350942799	-1.835840858	0.07154492	-1.268328096	0.20990627
LOC100129316	1.058703543	0.292659099	-0.371791976	0.711412071	0.196768198	0.844718334
LOC100129361	0.033382739	0.973445679	-1.093825596	0.278585044	-0.862027917	0.392333158
LOC100129387	3.782589438	0.000283913	2.149405642	0.035815985	0.808499408	0.422210294
LOC100129534	0.547528988	0.585414258	-0.938841025	0.351732924	-0.457529473	0.649053293
LOC100129550	0.876624574	0.383099519	-0.777340931	0.440138837	-0.081340712	0.9354598
LOC100129716	-0.850341208	0.397464551	-1.369965974	0.176014492	-2.541807746	0.013810515
LOC100129722	0.42605665	0.67111654	1.119815305	0.267440495	1.910755705	0.061138758
LOC100129726	1.791619672	0.0766634	-1.979485057	0.052547686	-1.515636095	0.135212482
LOC100129827	-1.858248118	0.066508348	-1.68611245	0.097184529	-0.245291818	0.80712342
LOC100129845	-0.378014164	0.706338818	0.409984305	0.683338433	-0.226204021	0.821862896
LOC100129917	2.704482753	0.008225588	-1.783474248	0.079778029	-2.004164189	0.049877463
LOC100129931	1.365935149	0.175472412	-0.550996531	0.58376716	-1.800214341	0.077190968
LOC100129961	1.034032996	0.303980335	-1.219185718	0.227743256	0.176814446	0.860289395
LOC100130000	0.670546544	0.504281784	-1.175746924	0.244533183	-1.936198647	0.057875137
LOC100130015	0.580388545	0.563149667	0.525798396	0.601046943	0.248049718	0.804999453
LOC100130093	0.075625431	0.939890359	-0.107545344	0.914730106	-0.488972985	0.626762453
LOC100130231	2.154373878	0.03396903	-2.244864753	0.028632632	-0.867091775	0.389576497
LOC100130298	-0.859224581	0.392572828	1.12249527	0.266309448	0.483495201	0.630621428
LOC100130357	-0.540982926	0.589898869	-0.120950718	0.904151035	0.944982369	0.34871041
LOC100130451	-0.782334987	0.436137306	0.28686747	0.775243267	-0.794706985	0.430125117
LOC100130557	0.914404483	0.363026231	-2.62545533	0.011063923	-2.533619785	0.014102702
LOC100130581	0.104682184	0.91686844	0.266963517	0.790450793	1.292919231	0.201325495
LOC100130691	1.649026793	0.102740859	-0.42038534	0.675768265	-1.503226479	0.138375941
LOC100130705	1.085656276	0.280623686	-0.616542425	0.539965948	-1.038341184	0.303555207
LOC100130776	0.521401327	0.603409137	0.263134633	0.793385852	1.065392009	0.291251463
LOC100130855	0.540602609	0.590159913	-1.153482137	0.253476828	-1.521840021	0.13365263
LOC100130872	1.372874848	0.173312068	-2.21527581	0.030709646	-1.015787337	0.314081059
LOC100130890	1.151739847	0.252576411	0.36470523	0.716667141	0.426229331	0.671567815
LOC100130932	1.019103442	0.310973376	0.063154315	0.949862289	0.8900102	0.377252074
LOC100130950	1.944210622	0.055094364	1.754889208	0.084596245	2.705663349	0.009006481
LOC100130987	1.600018084	0.113212087	-0.184934466	0.853930102	-0.6361907	0.527233012
LOC100130992	0.579695526	0.563614917	-2.021483974	0.047884889	-2.379634382	0.020746121
LOC100131067	1.253970847	0.213204292	-0.194870398	0.846180176	1.067605265	0.290260242
LOC100131089	1.837427569	0.069553181	-0.138766911	0.890118393	-0.342753252	0.733061972
LOC100131094	1.350499214	0.180350849	-3.186130335	0.002329311	-2.268046634	0.027186101
LOC100131096	0.125554058	0.900373986	0.520575874	0.604657778	2.740521194	0.008206656
LOC100131176	-0.083156759	0.933917516	-0.341602642	0.733893151	-0.412289505	0.681694704
LOC100131193	2.334480571	0.021868913	-2.055585936	0.044365612	-0.454784573	0.651014998
LOC100131289	1.173856659	0.243648957	-0.421859914	0.674697723	0.241598541	0.809970042
LOC100131347	0.017449109	0.9861182	-0.054060174	0.957074301	2.965136207	0.004432631
LOC100131434	0.764040202	0.44690603	-0.146212655	0.884264058	-0.041246982	0.967245092
LOC100131564	3.661747817	0.000428928	-0.1704999	0.865214535	0.052593182	0.958242428
LOC100131655	-0.037301354	0.970329999	-2.968953214	0.004349284	-2.284012032	0.026167474
LOC100131691	0.563155503	0.574774395	-0.356943166	0.722438813	-0.822299766	0.414379293
LOC100131733	0.530966885	0.596791709	-1.794402476	0.077997493	0.038516592	0.96941221
LOC100132077	2.897573412	0.004754809	-1.837041475	0.071364864	-1.824403858	0.073405044
LOC100132111	-0.835523397	0.40570688	0.24021079	0.811019756	-0.053551925	0.957481946
LOC100132163	0.605566831	0.546375563	-0.071995464	0.942854941	-0.793644668	0.43073838
LOC100132215	0.919249956	0.360501042	-0.316006975	0.753139542	-0.226592195	0.821562488
LOC100132247	-0.880751429	0.380873753	-2.254035516	0.028014584	-2.087626293	0.041373826
LOC100132273	1.06935667	0.287860574	-0.147553447	0.883210517	-1.397296973	0.167813102
LOC100132352	-0.447240641	0.655810419	-0.778151856	0.439664484	0.132083506	0.895389686
LOC100132356	1.626673842	0.107415376	-4.199216653	9.40E-05	-4.456222405	4.02E-05
LOC100132618	1.338710319	0.184145173	-0.911199529	0.365988171	-2.324512822	0.023733849
LOC100132707	-0.277397286	0.78213172	-1.447432891	0.153197036	-2.164442924	0.034691003
LOC100132774	-0.525263598	0.600733187	0.978083672	0.332123368	1.325914188	0.190228678
LOC100132832	1.40847123	0.162547622	0.840680655	0.404003406	-0.025757244	0.979542148
LOC100132891	-0.702734797	0.484093115	-1.377673226	0.173633892	-1.698048477	0.095026037
LOC100133091	0.917710572	0.361302064	-3.276811543	0.001781567	-2.293345186	0.025587813
LOC100133161	0.28227467	0.778401885	-0.672518993	0.503942534	-0.356991992	0.722433926
LOC100133315	1.084779693	0.281009655	-0.366717343	0.715173676	0.453813803	0.651709376
LOC100133331	1.158657367	0.249759566	1.87140819	0.066370249	2.017896078	0.048382557
LOC100133445	1.659602395	0.100587338	-4.499396612	3.36E-05	-4.898100484	8.56E-06
LOC100133545	1.270359377	0.207336163	1.556921221	0.124967417	1.566958799	0.122734527
LOC100133612	-1.653613989	0.101802225	1.057587709	0.294659015	0.921647088	0.360649797
LOC100133991	0.389738769	0.697680129	-2.073350157	0.042622721	-0.416876654	0.678355651

LOC100134229	-4.316717648	4.17E-05	0.537453044	0.59302521	-0.113136352	0.910325365
LOC100134368	1.012553716	0.314075097	-1.120921523	0.266973219	-1.175827376	0.244617703
LOC100134713	0.096972962	0.922970431	-4.162536849	0.00010638	-2.86953857	0.005781291
LOC100144603	1.129576391	0.261752879	-2.554515839	0.013300564	-3.155916144	0.00257039
LOC100170939	-0.369594828	0.712580565	-0.573727186	0.568386091	0.231089212	0.818084202
LOC100188949	-1.501781102	0.136766768	-2.380215388	0.020633639	-0.760317381	0.45024088
LOC100190939	2.028498794	0.045560808	-1.936514614	0.057716184	-0.302963169	0.763035814
LOC100216545	0.131068606	0.896022992	-2.962661475	0.004426989	-2.125317079	0.037966389
LOC100216546	1.609104973	0.111208457	-1.757451273	0.084154754	-0.963297478	0.339522272
LOC100233209	1.34966842	0.180616295	-0.848574285	0.399631225	0.560308959	0.57749411
LOC100240735	1.300224074	0.196950787	1.40031904	0.166781903	0.173319695	0.863022484
LOC100270804	-0.110338872	0.912394214	-1.002027313	0.320521657	-0.689107276	0.49358923
LOC100271722	-0.237697794	0.812673175	2.064994455	0.04343498	1.104974637	0.273875731
LOC100271836	1.576057994	0.118634111	-1.302365402	0.197975363	-1.354339869	0.181044922
LOC100272216	0.619775821	0.537021762	1.512481993	0.135879273	0.946624159	0.347880213
LOC100272217	2.330668038	0.022079139	-0.337282873	0.737129731	0.148214216	0.882703879
LOC100272228	2.701828802	0.008286318	-1.838090528	0.071207849	-0.973350261	0.334547473
LOC100286793	1.395001128	0.166559072	-2.010012474	0.049121661	-2.004455094	0.049845387
LOC100286844	0.847001159	0.399313415	-0.680306007	0.499036306	-0.424710852	0.672668022
LOC100286979	0.399660526	0.690383949	-0.767932156	0.445664519	-0.288375768	0.774119347
LOC100287015	1.413956165	0.160935589	-1.838672435	0.071120879	-0.836136908	0.406616637
LOC100287036	-3.896712252	0.000190851	2.376515131	0.020822553	1.578621395	0.120031883
LOC100287042	0.28950666	0.772881003	-3.728959489	0.000440977	-3.942202528	0.000225824
LOC100287177	0.831035778	0.408223446	-0.4834477	0.630610737	-1.757254821	0.08431707
LOC100287216	1.774063876	0.079543631	-0.397716588	0.692309352	-1.671046429	0.100269087
LOC100287482	-0.541238024	0.589723804	-3.194582904	0.002272233	-1.937087212	0.057763898
LOC100287559	-1.214802104	0.227721262	-3.168609447	0.002451941	-2.560518129	0.013163332
LOC100287616	2.24875582	0.027048495	-3.087498261	0.003102786	-2.259438403	0.027749778
LOC100287722	0.768384351	0.444335123	-0.992402526	0.325152209	-0.34772763	0.729342908
LOC100288069	0.479646258	0.63267995	-0.585827773	0.560279913	0.175392542	0.861401198
LOC100288123	2.242488344	0.027466367	0.685344428	0.495875767	1.140228986	0.25902386
LOC100288432	-0.816634685	0.416362936	0.115921279	0.908118158	0.308690362	0.758697739
LOC100288615	2.852403675	0.005417511	-0.526621799	0.600478557	-0.831464892	0.409227628
LOC100288637	1.487885832	0.140388891	0.160758166	0.872846399	-0.843187574	0.402695701
LOC100288778	0.242078457	0.80928786	0.516377397	0.607567813	-0.653549039	0.516065801
LOC100288842	2.097112486	0.03887948	-1.332612599	0.187907109	-1.043752607	0.301065917
LOC100289019	2.185134383	0.031560899	0.748411385	0.457257366	1.764096839	0.083146789
LOC100289187	-0.110650834	0.912147544	-1.784543996	0.079602251	-0.513889687	0.609342514
LOC100289230	-0.180479921	0.857194656	-2.282467717	0.0261728	-2.009879738	0.049250482
LOC100289341	-0.010767267	0.991433729	-2.307310853	0.024652267	0.323571426	0.747462763
LOC100289473	-1.6033935	0.112464467	-0.213727116	0.83151465	-3.041162428	0.003575705
LOC100289495	1.037890699	0.302190786	0.705859037	0.483121146	0.011975302	0.990487702
LOC100289511	1.182719994	0.240135312	-1.989203262	0.051435436	-2.043067093	0.04574252
LOC100289561	-0.903999081	0.368486897	0.134586194	0.893408269	-0.43105304	0.668077619
LOC100293516	-0.43965889	0.661272031	1.834090525	0.071808102	3.680589996	0.00052297
LOC100294145	0.950126548	0.344674264	-1.378677551	0.173325505	-1.526276042	0.132546074
LOC100294362	-0.260451052	0.795129989	0.531159349	0.597350825	0.631410064	0.530330651
LOC100302650	-0.469303827	0.640023963	-1.628383827	0.108901842	-1.135122924	0.261138737
LOC100303728	0.80156291	0.424984997	-3.245555874	0.001954959	-2.737631443	0.008270394
LOC100306951	2.568250325	0.011923623	-1.227080992	0.224784305	0.2567254	0.798327674
LOC100329109	0.243105715	0.808494532	0.363140061	0.717829638	0.947724095	0.347324735
LOC100499177	0.465362385	0.642832245	-0.944784745	0.3487154	-1.105264151	0.273751379
LOC100499194	-1.446831194	0.151529942	0.639999668	0.524710601	0.120394458	0.904599792
LOC100499405	3.169579223	0.002106297	-3.625338791	0.000612357	-2.364070812	0.021553619
LOC100499466	2.350965646	0.02098017	-0.645396444	0.521233137	0.123875175	0.901855807
LOC100499484	0.643909236	0.521324547	-1.847695349	0.069783736	-1.23471567	0.22207058
LOC100499489	-0.303165982	0.76248556	0.012265474	0.9902562	-0.568876183	0.571705185
LOC100505483	1.864333973	0.065639723	-1.838179194	0.071194592	-0.853590758	0.396953073
LOC100505549	-0.507903824	0.612803324	0.052991482	0.957922065	-0.020483544	0.983730144
LOC100505576	0.73184919	0.466223217	-2.225939451	0.029946279	-2.558640887	0.013227
LOC100505622	1.191637901	0.236636847	-2.187205285	0.032801636	-1.408281443	0.164552315
LOC100505624	1.157020251	0.250424182	0.803410377	0.425040819	1.080019094	0.28474386
LOC100505648	3.140297715	0.002304563	-0.806251032	0.423414624	-0.149388472	0.881781563
LOC100505658	0.830181176	0.408703759	1.041200947	0.302133218	1.048021731	0.29911197
LOC100505666	0.480760074	0.631891221	-0.51993453	0.605101889	-0.01185852	0.990580461
LOC100505678	1.522699917	0.131452628	-0.599992104	0.550864853	-0.824861361	0.412935483
LOC100505681	0.334449386	0.738843669	-0.426541341	0.67130349	-0.713358155	0.47857413
LOC100505687	1.458006709	0.148431448	-0.610110071	0.544188671	-0.696782219	0.4888094
LOC100505702	-0.51623679	0.606995848	-0.08046072	0.936149764	-1.586267132	0.118286205
LOC100505715	-0.060664429	0.951765301	-1.628530094	0.108870765	-1.236754841	0.221318135
LOC100505716	0.159015292	0.874024589	-1.359011857	0.179440843	-0.275614064	0.783854729
LOC100505738	0.814959788	0.417315871	-0.470944561	0.639459136	-1.561115211	0.124106939

LOC100505746	1.290336584	0.20034536	-0.761225565	0.449627879	-0.180109749	0.857713858
LOC100505761	1.197338671	0.234419746	0.254355713	0.80012668	1.109546867	0.271916495
LOC100505783	-2.10032347	0.038588705	0.828171316	0.41099206	1.513907675	0.135649621
LOC100505812	-2.428552909	0.017213264	-2.009916614	0.04913211	-2.167107409	0.034477239
LOC100505854	-1.508442127	0.135056626	-2.746778272	0.008018659	-2.384808349	0.020483735
LOC100505876	0.473162591	0.63727966	-1.359202588	0.179380752	-0.012178516	0.990326292
LOC100505881	-1.907466716	0.059752249	-0.222861558	0.824431723	-0.418511057	0.677167507
LOC100506023	0.721778186	0.472362129	-4.37682326	5.13E-05	-4.75855367	1.40E-05
LOC100506033	-1.687336625	0.09511325	0.302983113	0.76299436	-0.757567865	0.45187248
LOC100506035	-6.700020786	1.96E-09	0.721785429	0.473346554	-0.401149269	0.689830429
LOC100506046	-0.092685218	0.92636628	-0.793868612	0.430530551	0.71961168	0.474744173
LOC100506054	0.368349602	0.713505402	-1.354287917	0.18093406	-0.906472726	0.368553376
LOC100506068	1.090324853	0.278574233	-0.921141282	0.360818951	0.266472526	0.790849955
LOC100506083	1.031822939	0.305008778	0.594604569	0.554436531	0.248407612	0.804723933
LOC100506123	3.377736353	0.001094012	0.302751002	0.763170356	1.45902516	0.150121419
LOC100506178	0.255724403	0.798765985	1.365350386	0.177452056	2.651623106	0.010388577
LOC100506190	-1.565581201	0.121069131	-0.841527692	0.40353284	-1.161681455	0.2502717
LOC100506229	-0.374078094	0.709254388	0.902164772	0.370726717	-0.062804816	0.950144627
LOC100506233	1.305894433	0.195023467	-0.539962404	0.591304659	-0.324277016	0.746931397
LOC100506305	0.104916594	0.916682977	1.626439256	0.109315689	2.413122194	0.01909981
LOC100506314	-0.408531324	0.683885279	0.234855655	0.815153718	0.474329082	0.637101906
LOC100506334	1.903240004	0.060308775	0.004784336	0.996199199	-0.452404711	0.652717831
LOC100506343	-4.251089955	5.32E-05	0.622544578	0.536040878	0.432732417	0.666864228
LOC100506428	0.401012358	0.689392093	0.19338802	0.847335467	-0.625980442	0.53386028
LOC100506436	0.589028382	0.557365302	1.539389589	0.129185491	0.441198718	0.66076079
LOC100506469	0.893400976	0.374101851	0.382177223	0.703736465	1.988259112	0.051658497
LOC100506472	2.405875668	0.018246431	-0.181305254	0.856764504	1.782629263	0.08004465
LOC100506548	1.307672408	0.194422052	1.738369186	0.087489337	3.269582119	0.00184111
LOC100506585	-5.102395372	1.94E-06	1.463890595	0.148660524	0.913264809	0.365002123
LOC100506649	-0.199889691	0.842032329	-1.384359906	0.171588593	-1.509449855	0.136782233
LOC100506655	-0.17370195	0.862502362	-0.0371161	0.970520779	-0.12151569	0.903715751
LOC100506668	1.272791442	0.206475587	0.32013184	0.750026863	1.056157528	0.295412381
LOC100506686	2.309534406	0.023277203	-1.404578936	0.1655166	-2.469541214	0.016589769
LOC100506710	1.368395614	0.174704137	-2.032524022	0.046720084	-2.175248556	0.033831241
LOC100506713	-0.173743806	0.862469566	-3.49161188	0.000928796	-3.723772778	0.000456169
LOC100506714	-2.177358474	0.032155144	0.652964566	0.516377131	0.150358321	0.881019922
LOC100506730	0.766752996	0.445299565	-0.637031852	0.526628124	1.065310041	0.291288217
LOC100506746	2.623603576	0.010270018	0.66970961	0.505718984	1.952776141	0.055829663
LOC100506776	1.134108253	0.25985771	-2.800777294	0.006928831	-2.128575584	0.037683649
LOC100506779	-0.184623457	0.853953144	-1.998479275	0.050392717	-1.558538233	0.124716056
LOC100506801	0.972852647	0.333317848	0.046326317	0.963210418	1.441242037	0.155061642
LOC100506844	-0.324632666	0.746236699	-0.853374827	0.396986603	-0.599859809	0.551009139
LOC100506866	0.363229968	0.71731227	-0.777398236	0.440105306	-1.697221467	0.095183206
LOC100506930	0.840809056	0.402754912	-1.678503224	0.098667189	-1.073045299	0.287833805
LOC100506963	0.735701128	0.463887184	-1.773711549	0.081397215	-0.788501162	0.433715018
LOC100506990	1.340102437	0.183694005	0.838321789	0.405315633	1.433738711	0.15718375
LOC100507032	2.040556355	0.04431991	-1.328974045	0.189097342	-0.366323511	0.71549828
LOC100507034	2.804767487	0.006207455	0.45440926	0.651242227	1.435474061	0.156690956
LOC100507043	0.875944861	0.38346689	1.285834585	0.203646372	0.616203023	0.540246864
LOC100507053	1.968052765	0.052239705	-0.197719353	0.843960799	-0.583372662	0.561974841
LOC100507062	0.691323075	0.491199197	-0.286204321	0.775748557	0.045551628	0.963828994
LOC100507173	1.160392647	0.249056469	0.387612724	0.699731408	1.418455748	0.161575942
LOC100507178	1.188730161	0.237773493	0.791172379	0.432089414	1.956049423	0.055433171
LOC100507206	0.7391345	0.461810594	-2.215197805	0.030715293	-1.596984233	0.115873751
LOC100507217	-2.082725478	0.040205674	0.144309373	0.88575994	-0.479536057	0.633417018
LOC100507218	3.144134278	0.00227763	0.016383464	0.986985093	0.60832298	0.545422501
LOC100507246	-0.980224271	0.329687548	1.242246408	0.219179946	1.846260543	0.070119542
LOC100507250	-0.065769768	0.947711698	-1.42232534	0.160325083	0.139216931	0.889776097
LOC100507254	1.239388568	0.218527409	-0.21589039	0.829835935	1.107317896	0.272870392
LOC100507266	-0.254263036	0.799891052	0.590788119	0.556973671	1.187101908	0.240177745
LOC100507321	3.030349134	0.003214741	-3.057516155	0.003381889	-3.32312182	0.001569796
LOC100507331	0.979554443	0.330016337	0.00171519	0.998637404	1.723853741	0.09022819
LOC100507387	0.711662874	0.478573378	-0.686924959	0.494886583	-0.493239338	0.623764147
LOC100507392	-2.790071222	0.006471663	-2.199198347	0.031892981	-3.127212905	0.002793442
LOC100507421	-0.261457317	0.794356496	1.918718004	0.059980489	1.688464956	0.096860479
LOC100507423	-0.494215541	0.622396845	-0.037361731	0.97032578	-0.050723741	0.959725395
LOC100507424	0.495980437	0.621156189	-0.085753403	0.931959854	0.646243951	0.520750016
LOC100507433	1.564649384	0.121287617	-2.244879199	0.028631649	-0.315889457	0.753255828
LOC100507463	-1.917211779	0.058485625	-3.089007816	0.00308932	-2.502196865	0.01527673
LOC100507495	-0.106968714	0.91505955	-1.417906769	0.16160573	-1.932067687	0.058394694
LOC100507501	0.572675014	0.568338635	1.088704012	0.280818929	1.515970283	0.13512809
LOC100507557	0.814148804	0.417777751	-1.039541633	0.302897209	-2.000602582	0.050271628

LOC100507567	0.135882681	0.892227268	0.360218803	0.720001133	-1.111328733	0.271155626
LOC100507577	2.588916552	0.011280032	-0.062434275	0.950433163	0.4475746	0.656179572
LOC100507589	1.456135341	0.14894684	-2.144278179	0.03624301	-2.317574848	0.02413587
LOC100507632	-1.809085707	0.073884267	-0.293332703	0.770322189	-0.305750711	0.76092342
LOC100507634	-1.138898945	0.257864831	-3.139736878	0.002667334	-2.193130148	0.032449531
LOC100526820	1.178578192	0.241772678	-1.938402091	0.057480368	-1.153272933	0.253676603
LOC100527964	1.424947496	0.157742233	0.683904834	0.496777687	2.502171248	0.015277722
LOC100616668	1.694640325	0.09371277	-1.928265749	0.058756479	-0.242193295	0.809511455
LOC100630917	1.477001291	0.143278278	3.912123313	0.00024406	4.440690719	4.25E-05
LOC100630918	-0.527064343	0.59948742	-0.129293219	0.897576091	-0.450031261	0.654417929
LOC100630923	0.626038541	0.532925157	0.194126132	0.846760177	1.516924155	0.134887443
LOC113230	-2.95287834	0.004045242	-0.966402531	0.337883226	-1.315819725	0.193573425
LOC115110	0.677358329	0.499972033	-0.126355396	0.899890668	0.311221152	0.756783259
LOC121952	-2.170825356	0.032661929	-1.341190858	0.185123484	-0.978062152	0.332232398
LOC143188	-2.159391818	0.033565605	0.467497553	0.641907923	1.123324377	0.266072386
LOC143666	0.340440499	0.734343715	-1.870662949	0.066475339	-1.826168585	0.073135055
LOC144438	0.15885581	0.874149862	1.173072146	0.245595456	2.318166634	0.024101344
LOC144571	0.28396866	0.777107665	-1.32877869	0.189161406	-1.650974425	0.104318064
LOC145783	1.933546077	0.056413452	-0.243169947	0.808737708	-0.046201397	0.963313405
LOC146880	0.228513119	0.819782465	-3.033360434	0.003623634	-2.510238616	0.014968143
LOC147646	3.429188127	0.000926632	-2.188574773	0.032696739	-0.495053676	0.622491
LOC147727	2.213542412	0.029470619	0.591145652	0.55673574	0.570889938	0.570348601
LOC147804	1.996283246	0.049023251	0.48877932	0.626853951	0.832636388	0.408571969
LOC148189	-0.957683449	0.340870459	-0.759078925	0.450900788	-0.088974364	0.929418391
LOC148413	0.652455016	0.515824217	-1.232231465	0.222869287	-0.752439701	0.454924756
LOC148696	-0.19915799	0.84260287	-0.805864682	0.423635579	0.044761256	0.964456171
LOC149837	-1.617170888	0.109453895	0.384791212	0.70180933	-1.14027674	0.259004139
LOC150197	0.23861178	0.811966564	-1.803592701	0.076525945	-2.667191894	0.009971738
LOC150381	-1.956828879	0.053567495	-1.207842366	0.232044168	-1.400968794	0.166717606
LOC150776	3.673753867	0.000411853	-0.053440777	0.957565645	0.373481695	0.710194223
LOC151162	-0.930455081	0.354704632	2.187975558	0.0327426	2.229198805	0.029812763
LOC151174	0.46386111	0.643903275	-1.112130362	0.270702607	0.577034663	0.566218921
LOC151534	0.456992214	0.648813213	-2.927754857	0.004882059	-2.192377175	0.032506697
LOC152217	1.403920692	0.163894414	-1.791165899	0.078521335	-1.813469428	0.075096646
LOC153684	1.590544466	0.115331689	-1.13296461	0.261923386	-2.535007868	0.014052777
LOC154761	0.403948258	0.687239857	-0.863072143	0.391677493	0.001443914	0.998853033
LOC154822	-0.258778354	0.796416206	1.19838605	0.235674578	0.984086684	0.329287906
LOC155060	3.260161064	0.001589145	0.184699871	0.854113262	2.136748055	0.036982601
LOC158572	-0.473267915	0.637204826	-0.44322886	0.659260613	0.107978769	0.914396863
LOC162632	2.276411097	0.025270588	0.126647031	0.899660862	-0.741342978	0.461570168
LOC200772	1.00938772	0.315581799	-0.493148696	0.623782566	-0.185643071	0.853392644
LOC202181	2.631919183	0.010040387	-1.146120667	0.256484699	-0.824822278	0.412957489
LOC202781	2.990540334	0.003619449	-2.281017019	0.026264108	-0.812602556	0.419872711
LOC219347	2.970054755	0.003845617	-1.47226258	0.146393557	0.218022575	0.828200717
LOC220594	0.929555073	0.355167983	-0.476531222	0.635498879	-0.629844594	0.531347065
LOC220729	2.443283982	0.016570406	-2.654649988	0.010247454	-2.230111706	0.029748553
LOC220906	0.699918497	0.485841538	1.432464424	0.157416211	1.315006618	0.193844762
LOC221442	2.072621365	0.041160241	2.677744549	0.009640987	2.723433131	0.00859022
LOC221710	0.100952214	0.91982018	-1.068706644	0.289660532	-2.957880725	0.004523704
LOC253039	1.725944313	0.087899676	-0.245311976	0.807086854	-1.379122635	0.173317342
LOC254099	0.016133221	0.987164971	0.98706703	0.327738304	1.503111327	0.138405567
LOC254100	1.535196198	0.128356645	-2.554251208	0.013309626	-2.477593456	0.016256872
LOC254128	1.188146257	0.238002216	-1.325953002	0.190089902	-0.28026528	0.780302391
LOC254559	1.452767734	0.149877809	-0.62040264	0.537439891	-0.201067487	0.84137127
LOC255512	-0.370950623	0.711574098	-1.589702241	0.117376548	-2.073200366	0.042746719
LOC256880	0.892676195	0.374487802	-2.353222983	0.022047629	-1.995879846	0.050798428
LOC257358	-0.678922941	0.498984934	0.580953339	0.563538373	1.365621742	0.177495114
LOC257396	0.627133997	0.532210247	-0.463030291	0.645087459	-1.335933561	0.186952215
LOC282997	-1.643911445	0.10379581	-2.416388503	0.018866087	-1.56450586	0.123309129
LOC283050	0.787577907	0.433079481	0.931274576	0.355598757	1.234851087	0.222020553
LOC283070	-1.088568171	0.279344177	0.102400392	0.918794556	0.058887647	0.953250354
LOC283089	-2.553461473	0.012404314	1.219824124	0.227502944	0.872904844	0.386426938
LOC283104	1.302291743	0.196246362	-0.162000376	0.871872536	0.452118328	0.652922868
LOC283174	1.139424445	0.257646886	1.977032712	0.052831596	2.477040745	0.016279528
LOC283177	1.475171195	0.143768618	-2.204360842	0.03150871	-0.245511411	0.806954249
LOC283624	0.777735007	0.438830546	-1.882346873	0.064843819	-1.506470905	0.137543271
LOC283663	1.7078862	0.091215832	-0.565283834	0.574076173	0.427529137	0.67062662
LOC283693	1.155553394	0.251020744	0.686402788	0.495213267	1.755839439	0.084560862
LOC283875	-1.39631341	0.16616497	1.325420948	0.190265115	1.890671377	0.063824368
LOC283888	2.035790987	0.044806825	0.425504904	0.672054361	0.240326414	0.810951144
LOC283922	1.687782106	0.095027344	-0.617221174	0.539521346	0.981912338	0.33034861
LOC284009	0.394660216	0.69405743	-0.689529078	0.493259144	0.639798947	0.524901322

LOC284023	-0.186583803	0.852420425	-2.593783295	0.012016386	-1.881833726	0.065037437
LOC284233	0.609254819	0.543939884	0.396199274	0.693422023	0.691079871	0.492358281
LOC284385	-0.391368407	0.696479764	0.503903106	0.616251541	0.931086097	0.355788987
LOC284408	0.866034195	0.388848296	-0.450960408	0.653711327	0.13005521	0.896986832
LOC284440	2.010938162	0.047421262	2.911352342	0.005110564	3.891253327	0.000266528
LOC284454	0.720938914	0.472875755	-1.627498133	0.109090179	-0.681306926	0.498473372
LOC284551	3.481637735	0.000781049	-2.327440008	0.023478381	-0.385689826	0.701181563
LOC284749	0.990980037	0.324437572	0.876302814	0.384505635	1.084606408	0.282723922
LOC284751	-1.536084985	0.128138661	-1.082650326	0.283475428	-0.632596262	0.529561164
LOC284837	1.006899915	0.316769132	-1.019268897	0.312337709	-0.574165379	0.568145444
LOC284889	2.125622473	0.036362781	0.08536334	0.93226858	-0.937123312	0.35270235
LOC285033	-0.08123571	0.935440689	0.889061837	0.377668049	1.100656417	0.275735199
LOC285074	-1.532817188	0.128941565	-1.364896946	0.177593766	-2.479642367	0.016173133
LOC285359	1.335696046	0.185124919	2.27136654	0.026878738	3.6283504	0.000616322
LOC285540	0.801400268	0.425078614	-0.5165592	0.60744167	-1.003037481	0.320139238
LOC285696	0.123785161	0.901770302	-1.851122308	0.069281459	-1.67313312	0.099855638
LOC285740	-0.550660618	0.583274536	0.360568694	0.719740921	0.830514771	0.409759861
LOC285819	-1.200544327	0.233179621	-1.789579177	0.078779218	-1.129192303	0.263610493
LOC285965	0.410601947	0.68237176	-0.839186338	0.404834385	-0.114792195	0.909018717
LOC285972	0.527696173	0.599050598	-0.223385309	0.82402604	-0.012354574	0.990186452
LOC286059	-0.121048076	0.903931487	-2.769901846	0.007533992	-2.135082785	0.037124519
LOC286367	0.786606973	0.433644804	-0.382262697	0.703673419	0.227038542	0.821217091
LOC286437	1.127210715	0.262746027	-1.968462908	0.053834083	-1.501501353	0.138820309
LOC286442	1.393560506	0.166992538	1.420819116	0.160760749	1.197120828	0.236281446
LOC286467	-0.527991212	0.59884667	1.508949913	0.136778	-0.194599729	0.846407618
LOC338651	0.355166466	0.723322624	-4.416132539	4.48E-05	-3.839543353	0.000315015
LOC338758	0.212973848	0.831844534	-3.349616224	0.001432257	-3.185941682	0.002355025
LOC338799	1.229743158	0.222101348	-0.088689371	0.929636445	1.34723205	0.18330901
LOC338817	2.607181113	0.010737534	0.663949934	0.509371537	0.179358401	0.858300959
LOC339290	2.584306027	0.011420831	-0.16863254	0.866676485	-1.274915495	0.207581448
LOC339666	0.916078309	0.362152656	0.594080831	0.554784364	0.719147846	0.475027652
LOC339803	-0.006679928	0.994685488	0.869449584	0.388210173	0.816046818	0.41791654
LOC339874	1.24211678	0.217524184	-1.773105322	0.081498656	-1.196442677	0.236543718
LOC339894	0.975369906	0.332075234	-0.079567332	0.93685719	0.882332489	0.381353018
LOC340037	0.309804034	0.757449225	-0.334910579	0.73890921	0.112337181	0.910956091
LOC340544	1.078658488	0.283715104	-1.071285917	0.288509448	0.160556858	0.873017793
LOC344595	1.133063711	0.260293666	-0.245029167	0.807304764	-0.870556407	0.387697411
LOC348761	-0.175561237	0.861045749	-1.276154907	0.207022908	-1.590879902	0.117242942
LOC374443	0.388233173	0.698789811	-1.53493719	0.130274605	-1.712911832	0.092237561
LOC375190	1.923055528	0.057737014	0.5762425	0.566696353	2.159222985	0.035113154
LOC386597	-0.029133957	0.976824338	0.296542723	0.767882357	-1.065182341	0.291345485
LOC386758	-0.78495653	0.434606765	-1.209070965	0.231575498	0.6051471	0.547515549
LOC387646	0.888135615	0.376911393	1.915092577	0.060450915	0.744806143	0.459490252
LOC387647	2.21148516	0.029617819	0.559331524	0.578104079	0.183941939	0.854720658
LOC387723	0.965674922	0.33687778	-1.228509599	0.224251924	-1.246970535	0.217576822
LOC387895	0.699111387	0.48634325	-2.202158499	0.031672141	-0.964885713	0.338733081
LOC388152	0.621355854	0.535986705	0.951714457	0.345218679	1.436297132	0.156457646
LOC388387	-0.5819785	0.562082986	-1.085922123	0.282037526	-0.247359432	0.805530931
LOC388564	-1.621834743	0.108449576	-2.157872384	0.035120492	-2.768817778	0.007605951
LOC388588	-1.812797526	0.073304579	-0.561143462	0.576876504	-1.379730044	0.173131172
LOC388692	1.22762631	0.222891377	-0.459041943	0.647931772	-0.264143008	0.792635327
LOC388789	-0.652967376	0.515495422	-0.404850296	0.68708725	-0.039071825	0.968971501
LOC388796	1.550042262	0.124753757	0.907541287	0.367902153	1.915227604	0.060554049
LOC389333	1.156832786	0.250500367	-2.129670774	0.037484047	-2.552794382	0.013427081
LOC389634	-0.519241338	0.604908045	-0.427991218	0.670253657	0.448874927	0.655246877
LOC389641	0.284882203	0.776409973	-1.541784258	0.128602732	-0.941820263	0.350313017
LOC389765	-0.007995157	0.993639121	0.335256034	0.738649991	0.840044356	0.404440789
LOC389791	1.001245651	0.319478768	-1.040921992	0.302261564	-1.176359773	0.24440672
LOC389906	0.49087314	0.624749419	1.419811391	0.161052739	2.901427835	0.005294043
LOC390595	2.082676742	0.040210232	0.467107894	0.642184992	0.306454116	0.760390669
LOC391322	-0.815200798	0.417178667	-1.805688006	0.076193714	-1.77677665	0.081013716
LOC399715	0.480680731	0.631947392	-0.093621716	0.925734576	0.463065614	0.645104374
LOC399744	0.943872989	0.347842773	0.656220906	0.514295164	1.007200549	0.318152558
LOC399753	2.132235149	0.035799645	-1.188993631	0.239321108	0.084917757	0.932628366
LOC399815	1.640660376	0.104470825	-0.495864865	0.621876649	-1.223259448	0.226332867
LOC400027	0.736495153	0.463406468	-1.882649487	0.064802017	-1.753226613	0.085012447
LOC400236	0.380238928	0.7046928	-0.558151359	0.578904311	-0.498186362	0.620295478
LOC400604	0.507969414	0.612757514	-0.925213418	0.358715277	-1.397967606	0.167612603
LOC400657	-0.129629512	0.89715814	-1.094634033	0.278233565	-1.33078153	0.18863161
LOC400680	-0.87886464	0.381890364	-0.686192059	0.495345138	-0.246144365	0.806466683
LOC400685	-0.21008824	0.834088967	0.910663471	0.366268235	-0.049168357	0.96095934
LOC400752	-0.1167406	0.907334102	-1.066524121	0.290637029	-0.650349858	0.518114435

LOC400927	-1.479404813	0.142636284	-6.012621457	1.33E-07	-4.884779914	8.97E-06
LOC400960	-1.903653346	0.060254158	-1.929318454	0.058622836	-2.019588185	0.048201049
LOC401052	-0.631283084	0.529506982	-0.166500084	0.868346551	2.053363044	0.044699089
LOC401093	-0.892152751	0.374766696	-0.536687781	0.593550382	-2.869164289	0.005787249
LOC401127	-0.463414352	0.644222144	-0.6323426	0.529665346	0.878055722	0.383649532
LOC401233	-0.385453677	0.700840126	-1.377745105	0.173611807	-0.919132985	0.361951669
LOC401320	1.278687663	0.204400189	-0.482249414	0.631456433	1.731728516	0.088804561
LOC401321	1.497370604	0.137908434	-1.09317715	0.278867189	1.150566179	0.254779676
LOC401397	0.61133347	0.542569492	-1.27765071	0.206498424	-0.223497681	0.823958085
LOC401431	0.155036036	0.877151256	-0.020834272	0.983449868	1.028534504	0.308102043
LOC401588	0.32504664	0.745924447	-3.252021787	0.001917834	-1.98164546	0.052415019
LOC439949	2.13035607	0.035958893	0.393126202	0.695677635	1.473224276	0.146266045
LOC439994	0.015027613	0.988044485	-2.732768173	0.008326078	-2.986025317	0.004179863
LOC440104	2.508131201	0.013988374	-0.01082141	0.991403329	0.945654005	0.34837063
LOC440288	1.209772294	0.229636172	1.803574747	0.076528797	1.897448259	0.06290721
LOC440354	-0.641613822	0.522807162	1.21468967	0.22944094	-0.345065464	0.731332454
LOC440434	-0.152050273	0.879498577	0.692193355	0.491597157	0.05522753	0.956152939
LOC440461	1.051280931	0.296034615	-0.716347092	0.476671648	-0.777919108	0.439877309
LOC440600	0.33769022	0.736408322	-1.158752862	0.251338768	-0.366328582	0.715494517
LOC440944	1.879420382	0.063527409	-0.79765806	0.428345302	0.111842531	0.91134651
LOC441208	0.040471858	0.967809475	-0.676239163	0.501595378	-0.244142886	0.808008689
LOC441242	1.782618637	0.078129133	-3.057782476	0.00337931	-2.778418232	0.007411519
LOC441454	0.504549036	0.615148399	0.626602688	0.53339548	0.865791419	0.390283228
LOC493754	-0.433270395	0.665888365	0.337166516	0.737216978	0.728625886	0.469253976
LOC541471	-1.550767865	0.124579743	1.575939794	0.120516955	0.845301238	0.401524822
LOC550112	1.570277445	0.119972753	-1.375865839	0.174189927	-0.981729339	0.330437985
LOC550643	-0.107656344	0.914515649	1.120046608	0.267342742	1.674192733	0.099646225
LOC553103	-0.989987413	0.324919748	-0.240725804	0.810622467	0.046456726	0.963110807
LOC554206	1.065246671	0.289705421	-0.86646306	0.389831507	-0.403280781	0.688270897
LOC595101	0.565452063	0.573218577	-4.230477104	8.46E-05	-3.990223087	0.00019299
LOC606724	-0.367326851	0.714265325	-1.090102528	0.280207702	-0.236505686	0.81389963
LOC613037	-1.081367189	0.282515708	-0.689521948	0.493263595	-0.116124787	0.907967334
LOC619207	1.97479124	0.05145607	-3.150271407	0.002586767	-1.696847546	0.095254337
LOC641298	1.575045409	0.118867738	0.16148793	0.872274258	-1.686070511	0.097323328
LOC641367	0.82933272	0.409180956	-0.098982286	0.921496031	-0.951202982	0.345571682
LOC641467	1.041320739	0.300605626	-0.763527515	0.448265201	-0.851171579	0.39828393
LOC641518	4.42058819	2.82E-05	0.46478732	0.643836109	0.9718861	0.335269023
LOC642361	0.593125116	0.554632863	-2.848087005	0.006087984	-3.263521661	0.001874468
LOC642846	0.159292179	0.873807101	0.374154778	0.709663079	0.988589671	0.327098442
LOC642852	2.560462034	0.012174635	-0.28558546	0.77622019	-0.901620379	0.371103909
LOC643529	2.010226868	0.04749797	-1.646399193	0.105127955	-0.752971469	0.454607695
LOC643669	1.362962621	0.176403994	1.325890089	0.190110613	2.044265503	0.045619995
LOC643723	-2.320407015	0.022653848	-0.031523193	0.974961279	0.90063522	0.371623108
LOC643733	0.759052591	0.449868332	-1.0468465	0.299543724	0.231765062	0.817561771
LOC643770	-0.808683271	0.420898522	-1.602449251	0.11452697	-0.348106041	0.729060258
LOC643802	-0.309817652	0.757438904	-0.946501808	0.347846828	-1.383595698	0.171949949
LOC643837	0.60335547	0.547838658	1.928129173	0.058773837	2.845001557	0.006184111
LOC643988	-0.019057312	0.984838929	1.468618057	0.147377063	0.750142834	0.456295705
LOC644172	-0.62891786	0.531047132	1.718319165	0.091110171	0.467524322	0.641931433
LOC644242	-0.549585496	0.584008707	0.295883144	0.76838349	0.368598242	0.71381121
LOC644246	-1.711280774	0.090584765	0.571846076	0.569651396	-0.319181554	0.750771449
LOC644656	0.393709067	0.694757024	-1.130764832	0.262840692	0.176312954	0.860681484
LOC644714	-1.387269499	0.16889554	0.886408363	0.379083683	-0.750240162	0.456237564
LOC644961	0.086018928	0.9316486	-1.128892876	0.263623088	-1.325013461	0.190525342
LOC645158	1.755740966	0.082644573	-0.54733033	0.586266473	-0.027474478	0.978178562
LOC645212	1.955842323	0.053685564	-0.94993744	0.346113164	-0.979559155	0.331499111
LOC645431	0.477708517	0.634053139	-0.262366075	0.793975359	-1.662009887	0.10207576
LOC645513	0.28680536	0.774941818	-2.781188737	0.007307341	-2.410424376	0.01922796
LOC645638	-0.396506037	0.692700539	1.828053232	0.072722128	0.835087832	0.407202031
LOC645676	1.842520781	0.068797782	-1.219900371	0.227474255	-4.365533097	5.49E-05
LOC646214	2.03621002	0.044763826	-0.043155092	0.965727165	-1.918062166	0.060185894
LOC646278	0.351009064	0.726428282	1.838540338	0.071140614	2.135567216	0.037083186
LOC646329	-0.4277789	0.669866852	-2.190204973	0.032572257	-1.16263297	0.249888472
LOC646471	2.314745822	0.022976553	-1.36209483	0.17847141	-1.197694427	0.236059774
LOC646762	0.103924374	0.917468046	0.521395973	0.604090104	0.825006547	0.412853743
LOC647979	-0.034438711	0.972606036	-0.222376724	0.824807305	-1.298375321	0.199457767
LOC648740	1.076554226	0.284649277	0.001705896	0.998644787	0.026813817	0.978703159
LOC648987	0.691666999	0.490984205	1.8034583	0.076547297	-0.024039417	0.980906266
LOC650623	1.387761242	0.168746196	0.898701344	0.372553533	0.396228735	0.693435726
LOC650794	1.551377703	0.12443364	0.605994344	0.546899382	-0.685235834	0.496010016
LOC652276	1.316763585	0.191368507	-3.333030436	0.001505615	-1.34029094	0.185540722
LOC653160	1.294636954	0.198863655	-3.642745304	0.000579691	-2.529613336	0.014247702

LOC653566	-0.757592767	0.450737506	0.139403597	0.889617544	0.750699427	0.455963268
LOC653712	1.577893778	0.118211487	-0.098632777	0.921772315	0.903058658	0.370346739
LOC654433	0.022582541	0.982034873	-1.160862232	0.250486734	1.002999679	0.320157315
LOC678655	-0.857996151	0.393247055	-1.449144454	0.152720272	-1.327769808	0.189618611
LOC727896	0.667919783	0.505949021	-0.324050911	0.747073343	-0.429852528	0.668945565
LOC728024	-0.538104166	0.59187615	-0.895238739	0.374385627	-1.984456949	0.052092264
LOC728084	-3.531448378	0.000663012	0.707259128	0.482257374	-1.070300492	0.289056319
LOC728175	-1.427543834	0.156995085	0.134446383	0.893518321	-1.93041167	0.058604088
LOC728190	-1.356312681	0.17850164	-2.490837063	0.015649526	-3.692875062	0.000503068
LOC728323	0.543509289	0.588166187	-1.2588416	0.213165944	-1.28836017	0.202896172
LOC728377	1.531728899	0.129209841	-0.081183845	0.935577198	0.024889052	0.980231567
LOC728392	-0.190563889	0.849310297	0.073215602	0.941888224	-0.116815973	0.907422071
LOC728431	-0.489117272	0.625986861	-0.357612138	0.721940739	-0.514496115	0.60892131
LOC728537	1.310692631	0.19340361	-1.715875777	0.091559758	-1.080813812	0.284393205
LOC728554	0.345922539	0.730234239	-1.743391778	0.086601205	-2.697561534	0.009202338
LOC728558	0.590136614	0.556625477	-0.286478245	0.775539827	0.863148037	0.391722344
LOC728613	-0.214700641	0.830502095	-0.6143202	0.541422894	-1.123895094	0.26583223
LOC728730	0.809776992	0.420272905	2.353155617	0.022051264	2.334009729	0.023193225
LOC728743	1.977048712	0.051195791	1.323620061	0.190859078	2.081836087	0.041920197
LOC728752	0.120438604	0.904412821	2.881857736	0.005546581	2.451260792	0.017368442
LOC728855	-3.057781172	0.002960724	-0.441935749	0.660190631	-1.527973636	0.132124545
LOC728989	3.014719073	0.003368363	0.001833014	0.998543801	1.183448265	0.241610134
LOC729013	0.035891926	0.971450581	-2.569142205	0.012808347	-1.736018218	0.088036906
LOC729082	0.376600366	0.707385561	-1.385456867	0.171254832	-1.13237966	0.262280018
LOC729178	0.716369185	0.475677863	0.217095073	0.828901439	-0.232626861	0.816895723
LOC729234	1.650446747	0.102449562	1.296340714	0.200028289	2.373984724	0.021036063
LOC729513	2.534169631	0.013057588	0.947607958	0.347288033	1.921736029	0.059711566
LOC729603	0.030524722	0.975718343	-1.057415798	0.29473676	-0.340399004	0.734824363
LOC729678	-2.042500017	0.044122619	0.088144148	0.930067866	0.316247683	0.752985366
LOC729723	-0.137429361	0.891008295	-0.694088601	0.490416776	0.310097628	0.757632989
LOC729737	-0.486126895	0.628096787	-1.249797782	0.216428043	-0.392037162	0.696512545
LOC729799	1.381969361	0.170511617	-1.22537751	0.225420331	-0.330065909	0.742576579
LOC729852	-0.954512937	0.34246301	-0.258421057	0.797003202	-1.422609452	0.16037289
LOC730091	2.006905464	0.047857564	-0.66636835	0.507836151	-1.16555302	0.248715035
LOC730101	1.320596533	0.190091907	0.126393953	0.899860285	-0.866202443	0.390059754
LOC730102	-0.266250299	0.790675075	0.568344623	0.572010271	1.357959875	0.179900048
LOC730227	-1.736486753	0.086009844	-1.769415099	0.082118423	-0.741083436	0.461726261
LOC731275	0.212594211	0.832139737	-0.271870867	0.786693488	-0.674052968	0.503038974
LOC731424	-1.752682267	0.083171807	-0.117945551	0.906521163	-1.543358073	0.128352897
LOC731789	1.057096512	0.293387675	-1.023625311	0.310292432	0.354848496	0.724030417
LOC79015	0.198074447	0.843447912	-0.702508177	0.485191921	1.203164959	0.233953226
LOC80054	1.483085911	0.141657399	-0.930594228	0.355947702	0.573785371	0.568400834
LOC81691	0.459570678	0.646968272	2.000612708	0.050155484	0.906988594	0.368282881
LOC90784	1.042074543	0.30025802	2.084543069	0.041555331	2.736655336	0.008292027
LOC90834	3.735453686	0.000333819	0.094794988	0.924806694	-0.278695313	0.781500921
LOC91450	0.767246658	0.445007589	-0.178660123	0.858831539	-0.382088218	0.703836035
LOC92249	-0.820836482	0.413978072	-3.435593237	0.00110314	-2.655988602	0.010270117
LOC93432	-0.800888545	0.425373243	1.74589561	0.086161257	-1.459476734	0.149997594
LOC93622	0.075589664	0.939918733	-0.567531323	0.57255886	-0.842999782	0.402799831
LOC96610	0.644360032	0.521033636	1.004574075	0.319303828	1.185607768	0.24076277
LOH12CR1	2.061184276	0.042264241	1.235984691	0.221481351	2.096264368	0.04057024
LOH12CR2	-0.80820604	0.421171676	1.462263966	0.149104158	0.558577504	0.578667498
LONP1	0.301023214	0.76411349	1.438583438	0.155680644	2.207187447	0.031398418
LONP2	1.064149827	0.290199125	0.338090011	0.736524618	0.586918202	0.559607565
LONRF1	-0.265162087	0.791510502	-0.915706151	0.3636391	-2.009772084	0.049262228
LONRF2	-0.011309707	0.99100219	-0.228506049	0.820062218	-0.345203011	0.731229614
LONRF3	0.54689506	0.585847848	-0.865171069	0.390534214	-0.659470728	0.512285189
LOX	-0.122701247	0.902626067	-1.112786282	0.270423094	-0.305561899	0.761066443
LOXHD1	-2.712989915	0.008033643	-0.844861012	0.40168431	-1.295504865	0.200438754
LOXL1	-0.389731151	0.697685742	-2.069855054	0.042960863	-2.794392257	0.007098124
LOXL2	1.675302843	0.09745788	1.187514272	0.23989916	-0.598577123	0.55185837
LOXL3	-0.445326445	0.657187584	1.715599194	0.091610765	1.693497361	0.095893601
LOXL4	0.012895408	0.9897407	-0.213677638	0.831553055	0.37262804	0.710826012
LPAL2	1.686177472	0.095337077	-1.563024412	0.123525121	-0.413676487	0.68068442
LPAR1	3.661720257	0.000428968	-1.539808729	0.129083339	-1.829232147	0.072668335
LPAR2	-1.635962032	0.105452596	-0.780709227	0.438170514	-0.119939089	0.904958864
LPAR5	-0.281096155	0.779302647	-3.454837566	0.001040011	-2.311969458	0.024465089
LPAR6	0.288776075	0.773438205	-0.299075209	0.76595916	-0.79613235	0.429303091
LPCAT1	-2.378533545	0.01956531	0.076976007	0.938909408	1.396008207	0.168198921
LPCAT2	-0.059740451	0.952499072	-1.045090573	0.300347498	-2.538070638	0.013943186
LPCAT3	-1.065117351	0.2897636	-0.523529647	0.602614321	-0.281787109	0.779141121
LPCAT4	0.281335828	0.779119436	-1.439776511	0.155343989	-1.41394943	0.162889023

LPGAT1	-1.701602905	0.092393409	-0.405774728	0.68641165	-1.324619498	0.190655207
LPHN1	2.684759929	0.008686748	-0.902395239	0.370605358	0.145284702	0.885005566
LPIN1	2.896456538	0.004770253	-0.42231268	0.67436915	0.174065058	0.862439427
LPIN2	-1.232263953	0.221163227	0.493851606	0.62328909	-0.110297996	0.912565727
LPL	0.218183546	0.827795959	-1.58446355	0.11856409	-1.815038284	0.074851947
LPP	-0.342731286	0.732625533	-2.059260735	0.044000088	-3.007441891	0.003934699
LPP-AS2	-0.533081812	0.595333156	-0.998831678	0.32205417	-1.190297342	0.238930041
LPPR2	-2.139674146	0.035175198	-0.575832062	0.566971909	-0.539497105	0.591673658
LPPR3	0.939612896	0.350012004	-3.360989438	0.001383921	-2.155044513	0.035454317
LPXN	0.914578882	0.362935149	-0.404089674	0.687643326	0.461561783	0.646176044
LRBA	0.149462812	0.881533638	1.162779092	0.249714256	1.364201977	0.17793889
LRCH1	1.901318768	0.060563183	0.030730951	0.975590349	-0.366707174	0.715213633
LRCH3	0.295280229	0.76848184	-0.076830415	0.939024723	0.010613755	0.991569171
LRCH4	-1.022801968	0.309230995	-1.288894565	0.202587571	-0.4754374	0.636316793
LRFN1	0.337720356	0.736385688	-1.530036103	0.131481916	-1.17880344	0.243440009
LRFN2	0.915654296	0.362373823	0.487075109	0.628053705	-1.043442551	0.301208167
LRFN3	-0.013496754	0.989262311	0.523005146	0.602976944	1.280404709	0.20565889
LRFN4	1.137175664	0.258580453	-1.44605152	0.15358267	-1.762931557	0.083345142
LRG1	-2.957658798	0.003988744	-0.021204478	0.983155832	-0.730962297	0.467836866
LRGUK	-1.318912605	0.190651967	-0.559839322	0.577759923	-0.47124325	0.639290063
LRIF1	-1.415706156	0.160423857	-1.611992578	0.112430351	-2.366739767	0.021413178
LRIG1	1.242847009	0.217256234	1.634023476	0.10770879	1.396796901	0.167962728
LRIG2	2.067104441	0.041689638	0.279957984	0.780512746	0.794868675	0.430031821
LRIT3	-0.303745692	0.762045318	-0.395521531	0.693919242	0.060485896	0.951983092
LRMP	-1.749570241	0.083711075	-0.058353186	0.953669286	-1.176210372	0.244465913
LRP1	-1.283512943	0.202713267	1.097183667	0.277127104	0.803865575	0.424859608
LRP10	-1.491692769	0.139389139	-0.666294568	0.507882957	-1.435731256	0.156618021
LRP11	-1.03869994	0.301816294	1.307643238	0.196189961	-0.599552244	0.551212709
LRP12	-0.93540019	0.352165672	-0.836358685	0.40640969	-4.081796521	0.00014268
LRP2BP	2.159728285	0.033538705	0.281885084	0.779042002	-0.149275969	0.881869921
LRP3	-0.722679304	0.471811002	-1.517234282	0.13467746	-1.561306582	0.1240618
LRP5	1.32319301	0.189230757	0.618918059	0.538410654	0.471370518	0.639199753
LRP5L	4.018905919	0.000123765	0.988274195	0.327151999	0.128431086	0.898266029
LRP6	0.89782607	0.371750886	1.117699122	0.268335995	0.213846421	0.831440268
LRP8	2.925029151	0.004389339	-0.17187156	0.864140967	-0.314139694	0.754577357
LRPAP1	-1.298593006	0.197507796	2.197342505	0.032032125	2.345646632	0.022545764
LRPPRC	1.8546982	0.067019455	3.780272424	0.000374159	4.467684862	3.87E-05
LRR1	-1.254018051	0.213187216	0.186184606	0.852954193	0.420550874	0.675685799
LRRC1	0.052008011	0.958641281	1.445228307	0.153812844	0.958688407	0.34181936
LRRC14	2.13586931	0.035493394	0.549436311	0.584830166	0.760817627	0.449944396
LRRC16A	0.517882103	0.605852146	1.035702816	0.304669747	1.984890821	0.052042608
LRRC16B	-0.451324474	0.652876292	-1.835259191	0.07163229	-1.640747429	0.106431805
LRRC17	0.088424121	0.929742377	-0.905999306	0.368710826	-1.122627906	0.266365666
LRRC20	0.597135061	0.55196478	1.237098154	0.22107082	2.381270238	0.020662839
LRRC23	0.214612501	0.830570604	0.148492646	0.882472659	-0.29254531	0.770946379
LRRC25	-2.824295641	0.005871661	0.544624261	0.588114507	0.27259725	0.786161284
LRRC26	0.322646801	0.747735186	-2.076240595	0.042344826	-1.996691319	0.050707575
LRRC27	0.147477837	0.883095378	2.793851923	0.007060536	3.212410972	0.002179349
LRRC28	-0.765840448	0.445839584	1.021680905	0.31120418	1.74791662	0.085936364
LRRC29	0.461400843	0.64566009	0.782924941	0.436878559	0.740034552	0.462357389
LRRC3	-0.332355557	0.740418513	-0.030487437	0.975783712	0.430669168	0.668355101
LRRC32	-2.075158843	0.040918699	-0.364083944	0.717128508	-1.511874398	0.136165296
LRRC33	-0.326273514	0.74499929	-0.21623867	0.829565742	-0.852602239	0.397496551
LRRC34	0.372124989	0.710702726	-1.290930102	0.201885526	-1.438751388	0.155763562
LRRC36	0.541987828	0.58920938	0.567256713	0.572744149	-0.171150161	0.86472003
LRRC37A	0.7783363	0.438477944	-0.759447058	0.450682345	0.839104399	0.404963546
LRRC37A2	0.791333297	0.430897005	-0.13418753	0.893722083	0.441966161	0.660208668
LRRC37A3	0.671456567	0.50370487	0.264735155	0.792158592	-0.809678344	0.421537848
LRRC37A4	0.791153201	0.431001521	0.21169703	0.833090728	0.20288475	0.839957383
LRRC37B	0.358367213	0.720934755	0.10080163	0.920058008	-0.929755637	0.356471554
LRRC37BP1	0.454561935	0.650554134	-3.373440575	0.001332775	-2.323652963	0.023783348
LRRC39	2.358082138	0.020606503	-0.213135803	0.831973651	-1.711208652	0.092553628
LRRC4	-3.460557451	0.000836755	-1.530560005	0.131352437	-1.705618144	0.093597364
LRRC40	1.942596801	0.055292281	-0.35757048	0.721971751	-0.858979037	0.393998749
LRRC41	-0.720450004	0.473175107	1.538715231	0.12934998	2.183433324	0.033192519
LRRC42	0.864661067	0.389597564	2.124078918	0.037968861	2.770536459	0.007570802
LRRC43	1.204674653	0.231588769	0.437544484	0.663352887	1.086998578	0.281674541
LRRC45	0.582509604	0.561726896	0.103342733	0.918049952	0.936547378	0.352996054
LRRC46	-0.419374086	0.675974242	-0.005709561	0.995464184	0.523807113	0.602470994
LRRC47	0.239393697	0.811362179	4.144924449	0.000112865	3.246735248	0.001969865
LRRC48	-0.915262828	0.36257809	-1.064670096	0.291468334	-2.325306115	0.023688264
LRRC4B	1.200982696	0.233010404	1.17795518	0.243658683	1.097199163	0.277230287

LRRC56	0.404115718	0.687117174	-1.142553044	0.257951533	-0.023113924	0.981641219
LRRC57	-0.188266913	0.851104918	-1.146743084	0.256229401	-0.69791403	0.488106699
LRRC58	1.597759545	0.113714551	-0.257290305	0.797871647	-0.751219137	0.455652987
LRRC59	-1.276997199	0.204993627	1.435780271	0.156473862	1.59200671	0.116989224
LRRC6	0.224477504	0.822910993	-0.858746785	0.394040067	-0.160993245	0.87267568
LRRC61	-1.103657646	0.272778421	0.466219603	0.642816808	0.346834315	0.730010312
LRRC68	0.784842608	0.434673211	-2.100420014	0.040081129	-0.992040549	0.325427124
LRRC70	0.094420835	0.924991521	-1.383233857	0.171931723	-1.520261643	0.134048119
LRRC8A	0.562189681	0.575429303	0.828643746	0.410726794	1.004707435	0.319341312
LRRC8B	0.829517225	0.409077157	-0.971441005	0.335390773	-1.403082456	0.166089498
LRRC8C	3.331272928	0.001269232	-0.268422066	0.789333531	0.327967653	0.744154068
LRRC8D	2.868668056	0.005169688	-0.670386574	0.505290612	-0.442600357	0.659752553
LRRCC1	-0.196680739	0.844535118	0.153890928	0.87823368	-1.419253474	0.161344353
LRRFIP1	-1.408290619	0.162600913	0.023528955	0.98130967	-2.09834194	0.040379006
LRRFIP2	-0.835867896	0.405514082	1.605066515	0.113948853	1.338513031	0.186115662
LRRK1	0.074562631	0.940733515	-0.689280012	0.493414669	-1.541922907	0.128701068
LRRK2	-0.872332924	0.385422745	-0.06461786	0.948702017	-1.804297944	0.076540605
LRRN1	-1.098223606	0.275130408	0.064693243	0.948642258	-0.210825668	0.833785383
LRRN2	0.779076839	0.438043915	0.566460767	0.573281364	0.375219244	0.708908897
LRRN3	1.605266823	0.112051257	-0.592676819	0.555717356	-0.03553899	0.971775805
LRRTM2	-0.467427489	0.641360205	0.963659046	0.339245506	1.267984276	0.210028141
LRSAM1	0.927730735	0.356108399	1.753893118	0.084768408	2.674047777	0.009793076
LRTOMT	-0.194763921	0.846030884	-0.814963961	0.418450082	-1.279351903	0.206026594
LRWD1	-0.555567562	0.579929288	-1.020452551	0.311781099	-0.819784355	0.415800052
LSG1	1.104626749	0.272360444	0.903340571	0.370107831	2.185839766	0.033006752
LSM1	0.220860605	0.825717358	-0.830600715	0.409629081	-0.368282603	0.714045221
LSM10	-2.48902602	0.014708762	-2.211130716	0.031010973	-1.537605597	0.129752992
LSM11	2.550345413	0.012507797	-0.22960957	0.819208626	-1.682146814	0.098085707
LSM12	-1.324888156	0.188670125	-1.600281308	0.11500763	-0.932178104	0.355229386
LSM14A	-0.211238028	0.833194493	-0.919140664	0.361855379	-1.55885353	0.124641401
LSM14B	-1.055151991	0.294270913	-0.13336553	0.894369186	0.064479056	0.94881744
LSM2	1.3891672	0.168319758	-0.670840821	0.505003282	-0.153575593	0.87849415
LSM3	0.017890231	0.985767299	-1.677547602	0.098854696	-1.100200635	0.275931978
LSM4	0.702994409	0.483932117	0.409403503	0.683762131	0.847451962	0.400335571
LSM5	1.523072494	0.131359477	-0.386691983	0.700409242	0.228144787	0.820361201
LSM6	-1.459892664	0.147913441	-2.193163633	0.032347396	-1.54976781	0.126807064
LSM7	0.968316593	0.33556471	-0.625223394	0.534293853	-0.284126349	0.777357091
LSMD1	-0.131949743	0.895328064	-0.932367587	0.355038625	-0.744324519	0.459779184
LSP1	-2.646020948	0.009661585	0.7442561	0.459747329	0.916115164	0.363518369
LSR	2.372638903	0.019860507	1.062567595	0.292413033	0.788289224	0.433837932
LSS	2.650879682	0.009534102	0.626096437	0.533725124	0.717163328	0.476241598
LST1	-2.196399901	0.030716977	-0.086948118	0.93101433	0.209046684	0.835167181
LTA	-0.195725068	0.845280794	-0.755248768	0.453177177	-0.271646954	0.786888248
LTA4H	0.016867316	0.986581005	3.088445717	0.003094328	1.705264334	0.093663745
LTB	-0.320693536	0.749210019	-2.02579317	0.047427291	-1.276953904	0.206865953
LTB4R	-2.61821216	0.010421429	-1.474522028	0.145786417	-1.348529498	0.182894128
LTB4R2	1.402645488	0.164273358	-3.940599276	0.00022234	-3.726781636	0.000451832
LTBP1	-2.788085657	0.006508138	4.03200587	0.000164492	1.886499643	0.064394575
LTBP2	0.230707574	0.818082478	0.070377633	0.944136885	-1.250166597	0.216415966
LTBP3	1.248674705	0.215126479	-0.832352851	0.408647784	0.341281499	0.734163558
LTBP4	1.431898693	0.15574802	-0.827595603	0.41131546	-0.403470616	0.688132069
LTBR	-0.545224178	0.586991412	0.350595412	0.727170938	-0.899479631	0.372232716
LTC4S	-0.462691049	0.644738535	-1.625618637	0.109490717	-2.400831262	0.01968992
LTF	-3.934248473	0.000167218	2.625882607	0.011051558	1.272286686	0.208506896
LTK	1.989569336	0.049772474	-1.792304808	0.078336668	-0.430353791	0.668583107
LTN1	1.01805319	0.31146935	-0.750114137	0.456239278	-1.986301325	0.05188146
LTV1	2.052149957	0.043154233	1.02134343	0.311362609	2.3076067	0.024724084
LUC7L	1.742441732	0.084957199	1.635147876	0.107472201	2.963967324	0.004447187
LUC7L2	2.137826469	0.035329407	2.266567065	0.02718913	2.703176921	0.009066178
LUC7L3	2.539279625	0.012881602	-0.384723118	0.701859507	-0.497670695	0.620656641
LUZP1	-1.302710202	0.196104028	1.286277035	0.203493022	1.006632645	0.318423081
LXN	-0.91596229	0.362213163	-1.590485215	0.117199885	-2.057615649	0.044274167
LY6E	-0.91559031	0.362407205	1.711847833	0.092304899	-0.421036804	0.675333013
LY6G5B	0.744027208	0.458860505	0.826774578	0.41177693	2.090443705	0.041110219
LY6G5C	-0.604023794	0.547396269	1.100085718	0.275871453	1.402799335	0.166173526
LY6G6E	-2.961963342	0.003938494	1.035006906	0.304991832	0.986716506	0.328008039
LY6G6F	-4.87075044	4.93E-06	1.738097314	0.087537626	0.394462919	0.694731292
LY75	-2.094912509	0.039079793	-0.134449196	0.893516106	0.281732973	0.779182421
LY86	-1.026196601	0.307637567	0.800266384	0.426845024	1.074763472	0.28707037
LY9	2.799016623	0.006309644	-0.955675952	0.343230057	0.507583977	0.613730101
LY96	-0.898765592	0.371252939	-1.278386734	0.206240711	-1.484940599	0.143143749
LYAR	1.126660322	0.262977469	-0.561729231	0.57647992	0.861310834	0.392724503

LYG1	1.022931036	0.30917031	1.069889324	0.28913233	2.248290211	0.028495138
LYL1	-2.520058937	0.013554843	-0.099617468	0.920993948	-1.110671236	0.271436207
LYN	-2.079602315	0.040498669	0.298931981	0.766067889	-0.921117679	0.360923688
LYNX1	0.193876462	0.846723595	0.314790233	0.754058502	0.625048289	0.534467467
LYPD2	1.292519761	0.199592119	-2.200318993	0.031809217	-1.743926082	0.086636171
LYPD3	-0.024351646	0.980627765	-0.489807375	0.626130695	0.389559862	0.698333422
LYPD6B	-1.563747654	0.121499347	-0.28622909	0.775729683	-1.660122697	0.102456403
LYPLA1	-0.140538209	0.888558939	-1.706980365	0.093212006	-2.495406296	0.015541771
LYPLA2	-1.49740321	0.137899966	0.884612866	0.380043483	1.524890575	0.132890891
LYPLAL1	1.516768922	0.13294251	-0.765027776	0.447378393	-0.879075828	0.383100973
LYRM1	-1.283161827	0.202835668	1.65021886	0.104341648	1.202134845	0.234348845
LYRM2	0.564909307	0.573586086	-0.103272262	0.918105633	-0.161996985	0.871888873
LYRM4	-0.122984619	0.90240233	-0.237195364	0.813346894	0.172222723	0.863880726
LYRM5	-0.007578241	0.99397081	-1.526952201	0.13224614	-1.199272959	0.235450516
LYRM7	0.502795482	0.61637577	-1.356517041	0.180228268	-0.381422026	0.704327442
LYSMD1	-1.743300563	0.084806264	0.680704745	0.498785783	1.994014425	0.051007811
LYSMD2	-1.150126068	0.253236777	-0.295279466	0.768842238	-1.247203587	0.217492019
LYSMD3	0.020521359	0.983674364	-1.003782511	0.319682011	-1.806811227	0.07614262
LYSMD4	3.177045593	0.002058352	0.286905004	0.77521467	1.699113451	0.09482396
LYST	0.254203039	0.79993725	-1.029011461	0.307776279	-2.618098356	0.01134071
LYVE1	-1.002677843	0.318790978	-0.537259644	0.593157912	-0.668557169	0.506513051
LYZ	-0.3934026	0.694982494	0.94989872	0.346132671	0.483580793	0.63056105
LZIC	-0.731538565	0.466411887	0.582724814	0.562353094	2.028510198	0.04725369
LZTFL1	0.56949692	0.570483316	-1.958876238	0.054974788	-1.172510161	0.245935231
LZTR1	1.238472316	0.218865092	-2.044939457	0.045439473	-1.155664874	0.25270467
LZTS1	-2.331543847	0.022030689	1.947683112	0.056332751	0.05046866	0.959927753
LZTS2	1.472676141	0.144439228	0.785962104	0.435111292	1.207479443	0.232301515
M6PR	2.052401936	0.043129193	0.600426802	0.550577176	-0.154538321	0.87773859
MAB21L3	-0.541097564	0.589820194	2.075953466	0.042372361	1.26088283	0.212557088
MACC1	0.49094524	0.624698629	1.483432436	0.143411352	1.652236668	0.10405957
MACF1	1.58152524	0.117379011	1.112231192	0.270659626	1.215846351	0.229122668
MACROD1	1.553362174	0.123959149	-0.703697809	0.484456184	-0.336229711	0.737949013
MACROD2	-0.796604788	0.427844407	0.248589619	0.804562495	0.132496729	0.895064354
MAD1L1	-0.435855013	0.664019153	1.916770374	0.060232819	1.60509985	0.114073448
MAD2L1	1.43888846	0.153762424	-0.268001457	0.789655675	0.169391825	0.866096306
MAD2L1BP	-1.266789392	0.20860418	1.525000347	0.132731653	1.099742736	0.27612977
MAD2L2	-0.411222656	0.681918306	-0.232167291	0.817231035	-0.88828846	0.378169281
MADCAM1	-1.362046657	0.176691809	-2.286061151	0.025947838	-1.775844393	0.081168977
MADD	-0.684086891	0.495734534	2.202296651	0.031661867	1.988243813	0.051660236
MAEA	-1.496007538	0.138262774	0.392487703	0.696146635	0.586317704	0.560008154
MAF	-0.981103422	0.329256338	-1.277650262	0.206498581	-3.311285239	0.001626309
MAF1	-1.846368077	0.068231728	-0.731894255	0.467200771	-1.051476003	0.297537349
MAFB	-1.504481466	0.136071443	1.3508958	0.18201212	-0.265113807	0.791891158
MAFF	-1.068933734	0.288050043	-2.436992206	0.017921193	-3.022945409	0.003765662
MAFG	1.924190118	0.057592611	0.585664605	0.560388837	0.487183451	0.628021993
MAFK	0.698017058	0.487023957	-1.801994539	0.076780163	-1.209320722	0.231599212
MAGED1	0.251331035	0.802149591	2.181876814	0.033212594	3.092884339	0.003084026
MAGED2	1.278445152	0.204485244	4.241287725	8.15E-05	5.160332619	3.33E-06
MAGEE1	2.184153872	0.031635299	-0.45800821	0.648669848	-0.459467294	0.647669889
MAGEF1	2.538066039	0.012923203	-0.540555148	0.590898586	-0.569864682	0.571039077
MAGEH1	2.629380962	0.01010998	0.119856256	0.90501412	-0.244368955	0.80783448
MAGI3	0.066185765	0.947381458	-0.186616529	0.85261707	-0.060506142	0.95196704
MAGIX	-0.485564201	0.628494155	0.97336172	0.334443833	1.453187678	0.151729326
MAGOH	-0.791104003	0.431030074	-2.830812298	0.006383434	-2.836212153	0.006334609
MAGOHB	0.354143776	0.724086165	-2.478838488	0.016131956	-1.912737841	0.060879
MAGT1	1.388830299	0.168421868	-1.782040427	0.080014138	-2.759701036	0.007794922
MAK	-0.867669933	0.387956896	-0.974305687	0.333979093	-2.572305203	0.012769887
MAK16	0.340553956	0.734258586	-1.543817977	0.128109461	-0.424977921	0.672474466
MAL	1.024208469	0.308570117	3.174746323	0.002408315	3.245424216	0.001977505
MALAT1	-0.654119601	0.514756413	-1.35280504	0.181404738	-1.624151186	0.109936036
MALT1	1.903777548	0.060237755	-0.945098664	0.348556499	-1.012188618	0.315783119
MAMDC4	1.403365862	0.164059207	-1.341090775	0.185155779	-0.870897571	0.387512683
MAML1	-0.961760979	0.338829417	-2.029669699	0.047018866	-2.288100165	0.02591215
MAML2	-0.123591578	0.90192313	-1.239849923	0.22005865	-1.412252688	0.163385568
MAML3	-2.143367922	0.034868663	1.020923063	0.311560029	-0.146792988	0.883820397
MAMLD1	0.668883555	0.505336961	0.352321961	0.725882763	1.346467004	0.183553982
MAMSTR	2.474601619	0.015274507	1.417907155	0.161605617	1.095794466	0.277839366
MAN1A1	-1.289796213	0.200532125	-1.433743094	0.157052297	-3.690276357	0.000507217
MAN1A2	1.253528535	0.213364341	-1.183516611	0.241466276	-2.485709985	0.015927428
MAN1B1	-0.198613546	0.843027452	2.512293986	0.014819166	2.62521665	0.01113213
MAN1C1	2.104351249	0.038226625	0.178665501	0.858827335	0.930663358	0.356005773
MAN2A1	0.437451933	0.662865311	-0.493062684	0.623842962	-1.245751053	0.218020966

MAN2A2	-1.556577935	0.123193306	-1.187482063	0.239911757	-1.914233846	0.060683572
MAN2B1	-0.166467129	0.868174789	0.149628407	0.881580516	0.208967397	0.835228778
MAN2B2	-0.83125093	0.408102577	0.017941924	0.985747193	-0.036101592	0.971329196
MAN2C1	1.201148235	0.232946527	0.924239875	0.35921749	0.428402452	0.669994547
MANBA	-0.54931441	0.584193893	1.579862901	0.119614952	0.611633215	0.543245253
MANBAL	-0.315817062	0.752896118	-0.813977203	0.419010555	-1.138877649	0.259582381
MANEA	0.309771429	0.757473937	-0.794694103	0.430053952	-1.961744961	0.054749013
MANEAL	-0.509960857	0.611367399	-0.759485735	0.450659398	-0.242953734	0.808925215
MANF	0.35420206	0.724042642	-3.280377502	0.001762734	-2.779436336	0.007391171
MANSC1	-3.482370302	0.000779177	0.327191336	0.744709377	-0.164321418	0.870067309
MAP1A	-1.294580305	0.198883121	2.184236862	0.033030021	1.092237792	0.279385725
MAP1B	-0.453974346	0.650975343	2.406744456	0.019323539	2.066575467	0.043390341
MAP1LC3A	-2.173807449	0.032429746	-1.046956002	0.299493648	-1.540972803	0.128931978
MAP1LC3B	-4.561438432	1.65E-05	-0.172522584	0.863631515	-0.324360738	0.746868355
MAP1LC3B2	-1.670373946	0.098431683	0.793811567	0.430563498	0.193410818	0.847334113
MAP1LC3C	-1.668875457	0.098729301	4.476740662	3.64E-05	3.486278791	0.000957497
MAP1S	-0.88852709	0.376702052	0.397967573	0.692125365	0.099256185	0.921287837
MAP2	0.666486031	0.506860284	0.669697505	0.505726646	0.9190919	0.361972969
MAP2K1	-1.38609561	0.169252461	0.584689771	0.561039806	-0.116268195	0.907854198
MAP2K2	-1.07071359	0.28725327	0.799242568	0.427433536	0.900892145	0.371487659
MAP2K3	-2.810048273	0.006114956	0.740484489	0.462014112	0.816229142	0.417813143
MAP2K4	-3.510671341	0.000710036	-0.590866912	0.556921231	-0.976566509	0.332966093
MAP2K5	-0.166725855	0.867971815	2.687088764	0.009405074	2.382055524	0.020622966
MAP2K6	-1.581431525	0.117400435	2.116240153	0.038657689	1.364013372	0.177997906
MAP2K7	-0.621525519	0.535875621	-4.037805006	0.000161359	-3.800157667	0.000357523
MAP3K1	0.246309358	0.806021721	-0.267438337	0.790087028	-1.960964146	0.054842376
MAP3K10	0.879188351	0.381715827	-3.633157723	0.000597472	-3.125574521	0.002806706
MAP3K11	-1.046755847	0.298105404	-2.192115604	0.032426891	-2.586014508	0.01232574
MAP3K12	3.106892288	0.002551927	0.99954435	0.321711974	1.548179154	0.127188806
MAP3K13	-0.838017157	0.404312514	-1.587777625	0.117811715	-1.543860821	0.128231109
MAP3K14	-0.453325068	0.651440906	0.609143282	0.544824803	1.052939687	0.296871851
MAP3K15	-0.935564482	0.352081521	-1.814852896	0.074754689	-0.482567365	0.631276097
MAP3K2	0.051028289	0.959419702	-0.239440142	0.811614337	-1.638069847	0.106990937
MAP3K3	-1.810547148	0.073655575	0.012966767	0.989699118	-0.371737535	0.711485291
MAP3K4	1.846727341	0.068179069	2.060714669	0.043856191	1.972852498	0.053435542
MAP3K5	-2.01502007	0.046983105	0.731870855	0.467214945	-0.493237649	0.623765332
MAP3K6	1.633760663	0.105915152	-0.408780862	0.684216464	1.060797401	0.293316641
MAP3K7	0.77240329	0.441964342	-1.388124073	0.170445391	-2.262828924	0.027526539
MAP3K8	-0.09253098	0.92648846	-1.217384943	0.228422113	-2.884126791	0.005553455
MAP3K9	1.053321339	0.295104085	-0.467216194	0.64210798	0.173221635	0.863099197
MAP4	0.033870008	0.973058229	1.411441111	0.163493955	1.517539448	0.134732394
MAP4K1	-0.042350028	0.966316498	1.328724138	0.189179299	2.251895637	0.028252167
MAP4K2	-0.674822833	0.501573891	-0.214115844	0.831212936	0.168398403	0.866874056
MAP4K3	1.008357199	0.316073266	-0.024026193	0.980914763	-1.600648261	0.115058133
MAP4K4	-1.374351342	0.172855052	1.064019126	0.291760603	1.230348791	0.223688263
MAP4K5	0.977323222	0.331113105	-0.898720309	0.372543515	-2.515537567	0.014767899
MAP6D1	1.622834006	0.108235362	1.609819539	0.112905006	1.815841401	0.074726941
MAP7	-2.393718932	0.018822727	-0.606032636	0.546874131	-0.880494256	0.382339039
MAP7D1	-1.300037325	0.197014502	0.502588833	0.617169693	0.146020167	0.884427625
MAP7D2	1.342011027	0.18307681	-0.759054168	0.45091548	-0.627174345	0.533083117
MAP7D3	-1.037853573	0.302207974	1.197141856	0.236155295	1.768715077	0.082364533
MAP9	1.566646187	0.120819805	-0.862147866	0.392181604	-1.347311121	0.183283705
MAPK1	-1.865811349	0.0654303	-0.441934426	0.660191583	-1.015563763	0.31418662
MAPK11	1.121065965	0.265338045	0.969703977	0.336248677	1.324779625	0.190602415
MAPK13	2.167375352	0.032932349	0.397787923	0.692257058	0.855896348	0.395687272
MAPK14	-1.058674754	0.29267214	0.286587221	0.775456792	-0.700233014	0.486668676
MAPK1IP1L	-0.379606316	0.705160703	-0.34278034	0.733011601	-0.189234697	0.850590193
MAPK3	-1.669410144	0.098623022	-1.545381043	0.127731372	-1.856912502	0.068563783
MAPK6	1.18688289	0.238497633	-0.236410927	0.813952557	-0.575277104	0.567398617
MAPK7	0.219485935	0.826784566	-2.421238562	0.018639724	-2.714167343	0.008805018
MAPK8	0.043106984	0.96571482	-1.932784274	0.058184669	-3.566917277	0.000746493
MAPK8IP1	1.438070879	0.15399366	1.367405589	0.176810841	2.607096329	0.011670105
MAPK8IP2	0.851401381	0.396878798	-0.950610987	0.345773947	-0.887230251	0.378733711
MAPK8IP3	1.523854083	0.131164235	-2.090369986	0.041008907	-1.211319137	0.230838727
MAPK9	1.304374323	0.195538757	0.340168515	0.734967134	1.177213752	0.244068573
MAPKAP1	-0.332648238	0.74019831	1.411314763	0.163531023	1.402293346	0.166323781
MAPKAPK2	-2.54756966	0.012600633	-1.207844668	0.232043289	-1.18133078	0.242443102
MAPKAPK3	-2.528687339	0.013248799	-0.572760146	0.569036386	-0.80282876	0.425453752
MAPKAPK5	1.83435813	0.070011761	2.56001951	0.013113372	2.002793571	0.050028834
MAPKBP1	0.838714311	0.403923229	-0.346784349	0.730017162	0.653224936	0.516273148
MAPRE1	-2.052522276	0.043117239	0.442677752	0.659656909	0.611660147	0.543227558
MAPRE2	-1.91458428	0.058824883	-1.71170526	0.092331365	-1.495034198	0.14049621

MAPRE3	-0.765082197	0.446288584	-1.678531087	0.098661727	-0.597539398	0.552545903
MAPT	-0.883025678	0.379650625	-0.353658877	0.724885838	-0.218148499	0.828103081
MARCH1	1.309886352	0.193675102	-0.109964296	0.912819947	-0.787585806	0.434246028
MARCH2	-2.842402665	0.005575268	0.156477685	0.876203701	-0.532747452	0.596307363
MARCH3	0.309292808	0.757836726	0.014548615	0.988442572	0.123798983	0.901915859
MARCH5	0.354015451	0.724181992	-1.408323948	0.164410365	-1.748660083	0.085806505
MARCH6	1.69861243	0.092958213	-1.262656668	0.211800827	-2.020581888	0.048094731
MARCH7	-0.317694284	0.751476453	-0.501848029	0.61768749	-1.490533644	0.141671885
MARCH8	-3.094391008	0.002650688	0.613871166	0.541717536	-0.324087702	0.747073953
MARCH9	1.574198575	0.119063404	0.338178392	0.736458369	0.498988683	0.619733734
MARCKS	-2.567823189	0.011937267	-0.427451158	0.670644629	-1.54544026	0.127849096
MARCKSL1	-2.644853665	0.009692441	1.595817538	0.116002432	2.369933561	0.021246195
MARCO	0.134524453	0.893297931	2.209383929	0.031138734	2.355787984	0.021994719
MARK2	-2.119919958	0.036854557	-1.261667381	0.212154192	-1.13847115	0.259750558
MARK3	-4.042922045	0.00011356	-0.007968086	0.99366999	0.593833549	0.555004686
MARK4	-0.838799654	0.40387559	-1.050462351	0.297893214	-0.77730998	0.440233589
MARS	-0.416900257	0.677776021	1.621614346	0.110348047	2.343414025	0.022668719
Mar-02	1.141609957	0.256741866	-1.625239128	0.109571739	-2.509683505	0.014989262
MARVELD1	0.473033438	0.63737143	1.750531951	0.085351497	0.737963686	0.463604906
MASP2	1.446674217	0.151573819	-0.811822511	0.420235983	1.029123424	0.307827693
MAST1	0.671421948	0.50372681	0.118082016	0.906413517	0.037558625	0.970172607
MAST2	0.132035793	0.895260203	-0.27516791	0.784171966	0.448778077	0.655316326
MAST3	-1.644371182	0.10370064	-0.888489266	0.377973233	-0.322531178	0.748246378
MAST4	2.752030719	0.007204035	-0.58265197	0.562401809	-1.139802237	0.259200147
MASTL	0.382816818	0.702787276	-0.721041084	0.473800884	-1.244031903	0.218648227
MAT2A	2.6615591	0.009259243	-0.429106032	0.669446887	-0.913841601	0.364701561
MAT2B	-0.305410767	0.760781269	0.34802553	0.729089787	-0.360193493	0.720051737
MATK	0.868466314	0.387523365	-1.954230109	0.055535024	-0.843504842	0.402519814
MATL2963	1.677436167	0.097038835	0.337717432	0.736803921	0.635283733	0.527819958
MATN2	2.630412378	0.010081648	0.996478925	0.323185599	0.769539612	0.444793376
MATR3	1.456841006	0.14875233	1.431758085	0.157617518	1.307399824	0.196397078
MAU2	-0.219899046	0.826463818	-0.478946215	0.633790243	-0.580416728	0.563952242
MAVS	-0.448474209	0.654923561	0.98333774	0.329553988	1.411673767	0.163555254
MAX	-3.476070978	0.000795409	-2.945041829	0.004651523	-3.050812689	0.003478741
MAZ	-0.142272206	0.88719325	-2.314865552	0.024205712	-1.484127861	0.143358628
MB21D1	-2.080166476	0.040445607	0.266897983	0.790501003	-0.453568048	0.65188521
MB21D2	-1.09317783	0.277326919	1.103023055	0.274604596	0.005689693	0.995480442
MBD1	-0.313624116	0.754555629	-0.575233295	0.56737402	-0.217550773	0.828566557
MBD2	-1.924189027	0.05759275	0.259603706	0.796095175	0.196394063	0.845009741
MBD3	0.206597028	0.836806279	1.216378254	0.228802259	1.779185217	0.080613732
MBD4	-0.406393626	0.685449184	2.421120602	0.0186452	3.01672505	0.003832653
MBD5	0.287063429	0.774744868	-1.635649202	0.107366851	-0.955935477	0.343196236
MBD6	-0.941783447	0.348905675	-3.301628706	0.001654333	-2.927406121	0.00492556
MBIP	1.386785236	0.16904271	-0.387466788	0.699838827	0.142679361	0.887053393
MBLAC1	-0.609781618	0.543592416	-1.006095768	0.318577659	-0.637218562	0.526568244
MBLAC2	1.664273036	0.099647972	-1.519481242	0.134112166	-2.263827156	0.027461117
MBNL1	0.50122538	0.617475663	-0.829522375	0.410233727	-1.750064333	0.085561672
MBNL2	0.873237949	0.384932095	-1.158982335	0.251245977	-1.750204814	0.085537211
MBNL3	-2.778115198	0.006694155	-1.700484985	0.094433915	-1.159857865	0.251007339
MBOAT1	-0.25634391	0.798289172	-1.856811927	0.068454283	-2.150899511	0.035795613
MBOAT2	-0.976221215	0.331655686	0.833885633	0.407790517	-0.052549616	0.958276986
MBOAT4	2.559270019	0.012213471	2.099258437	0.040187419	1.96730782	0.054087786
MBOAT7	-2.178150902	0.032094142	-0.464812606	0.643818108	-0.563104728	0.575601878
MBP	-2.488857474	0.014715263	-0.63491594	0.527997467	-1.162554388	0.249920106
MBTD1	0.1524546	0.879180643	-1.379633163	0.173032466	-2.184547615	0.033106386
MBTPS1	2.44806146	0.016366574	1.81135114	0.075301806	1.915198585	0.060557828
MBTPS2	0.815555363	0.416976868	0.126306423	0.899929259	0.242551746	0.809235103
MC1R	1.845627335	0.06834041	-1.800570177	0.07700733	0.023485828	0.98134588
MCAM	-0.099458349	0.92100268	0.202122819	0.840532942	-0.363504198	0.717591223
MCART1	-0.897726992	0.371803422	-2.067927755	0.043148317	-1.804029563	0.076583207
MCART6	1.618887946	0.109083276	-1.28737189	0.203113925	-1.618522294	0.111145605
MCAT	-0.009109603	0.992752501	0.810932841	0.420742591	0.417761375	0.677712393
MCC	0.171716921	0.86405801	0.717850413	0.475751181	-0.24665566	0.806072888
MCCC1	2.470303589	0.01544681	0.85594813	0.395573439	2.022345066	0.047906583
MCCC2	1.46737319	0.145872631	-0.833368884	0.408079406	-0.047361833	0.962392644
MCEE	-0.392102688	0.695939156	-1.216152812	0.228887454	-1.884908386	0.064613208
MCF2	1.291217896	0.200041036	0.509539913	0.612320661	0.201189154	0.841276593
MCF2L	3.530638257	0.000664789	0.457135763	0.649293044	1.016868546	0.3135709
MCF2L-AS1	-0.40724471	0.68482638	-1.056494122	0.29515382	-0.076798157	0.939056694
MCF2L2	-0.398910367	0.690934584	-0.669479643	0.505864549	-1.457375609	0.150574422
MCFD2	0.060745468	0.951700946	-0.159680723	0.87369125	-0.277876246	0.782126418
MCHR1	0.753187064	0.453366501	-1.161478591	0.25023816	0.250273164	0.803288166

MCL1	-1.160492987	0.249015856	-0.880105505	0.382459681	-2.395624485	0.019944799
MCM10	1.383757579	0.169965055	1.836476606	0.07144953	1.207457229	0.232309997
MCM2	1.319980009	0.190296814	2.645192676	0.010505711	3.39093802	0.001280179
MCM3	-0.264505999	0.792014302	2.939934797	0.004718555	3.215869613	0.002157327
MCM3AP	1.700021681	0.092691701	1.956794927	0.055225155	2.249074355	0.028442138
MCM3AP-AS1	1.270958312	0.207123987	-1.510400928	0.136408225	-0.312135272	0.756092126
MCM4	0.852538514	0.396251114	0.854416799	0.396414014	2.554976906	0.013352071
MCM5	-0.417538586	0.677310923	1.805640284	0.076201267	1.819480505	0.074162705
MCM6	2.182861137	0.031733624	2.088891264	0.04114698	3.404826888	0.001227474
MCM7	0.102315324	0.91874134	0.862183952	0.392161915	1.838360983	0.07129242
MCM8	2.759270373	0.007059076	1.232075313	0.22292717	1.769398389	0.082249313
MCM9	0.687711973	0.493459646	-1.36123984	0.178739858	-2.541554548	0.013819467
MCMBP	-0.767837722	0.44465815	0.539716539	0.591473133	-1.000639656	0.321287289
MCOLN1	-2.245203668	0.027284637	0.094458237	0.925073002	-0.740352124	0.462166249
MCOLN2	3.835129799	0.000236679	0.059810625	0.952513509	0.878806111	0.383245965
MCOLN3	1.114178043	0.26826479	1.957033072	0.055196458	2.298372843	0.025280308
MCPH1	0.308538325	0.758408726	1.997686507	0.050481116	2.456930794	0.017123472
MCRS1	-0.237975089	0.81245878	1.022010837	0.311049345	1.229581701	0.223973318
MCTP1	-0.268641835	0.788839941	0.419034383	0.676749651	-1.187809789	0.239900937
MCTP2	0.184742129	0.853860343	-0.633376451	0.528994938	-0.787890052	0.434069488
MCTS1	1.331636207	0.186450704	-1.069087199	0.289490498	-0.102064682	0.919068393
MCU	-1.42338278	0.158193832	-2.724208349	0.008519191	-2.80444135	0.006907278
MDC1	2.27015031	0.025663818	-0.730343825	0.468140416	-0.1780905	0.859291877
MDFIC	1.980241236	0.050829618	-0.093454017	0.92586721	-1.039831136	0.302868423
MDGA1	1.612558505	0.110454472	1.288648008	0.202672731	0.481595025	0.631962486
MDH1	2.253140555	0.026759479	2.023073245	0.047715681	2.516440496	0.014734021
MDH2	-0.752855619	0.453564639	2.235904304	0.029248114	2.916431596	0.005078221
MDK	-1.894930076	0.061415681	-0.715829157	0.476989006	0.730618512	0.46804523
MDM1	0.584050627	0.560694313	-0.727963659	0.469585014	-1.713416558	0.092144068
MDM2	-0.216441325	0.829149366	-1.489375389	0.141844266	-0.872073556	0.386876355
MDM4	1.805013943	0.074524548	-0.2831885	0.778047707	1.243762004	0.218746825
MDN1	2.020223294	0.046429611	0.630159231	0.531082623	0.9442971	0.349057309
MDP1	0.366550499	0.714842362	0.037295587	0.97037829	-0.89906178	0.372453301
MDS2	1.999488152	0.048669016	1.64078143	0.106293175	3.335407922	0.001513104
ME1	0.838676237	0.403944483	1.744711894	0.086369016	1.64411007	0.105732992
ME2	-0.780708396	0.437088548	0.21032434	0.834156821	-0.101492895	0.919520202
ME3	0.980316684	0.329642203	-0.379155256	0.705966812	0.285579136	0.776249725
MEA1	-0.918741291	0.360765601	-1.690278888	0.096380472	-0.827680581	0.411350009
MEAF6	-0.724704122	0.470573933	-0.277365794	0.782492351	0.578680618	0.565115232
MECP2	0.29238986	0.770683215	-0.237585182	0.813045959	0.133906634	0.893954467
MECR	0.517254281	0.606288446	0.963389957	0.339379317	2.395418216	0.019954956
MED1	-0.615098247	0.540091954	-0.541631943	0.59016124	-0.455155656	0.65074965
MED10	1.059188907	0.292439294	0.889464821	0.377453349	1.285765047	0.203794322
MED11	0.653455786	0.5151821	-0.100724464	0.920118995	0.177714197	0.859586017
MED12	-0.772344408	0.441999023	-0.321758046	0.74880085	-0.012885035	0.989765117
MED12L	-2.721483164	0.007846089	-1.960907783	0.054731345	-2.899373411	0.005324248
MED13	-0.161573162	0.872015817	-0.962873522	0.339636224	-2.560030474	0.013179845
MED13L	-0.356951442	0.72199063	-0.534035267	0.595372398	-2.113106436	0.039042448
MED14	0.98839109	0.32569617	-2.205857648	0.03139806	-2.444380932	0.017669918
MED15	-0.966627946	0.336403683	0.135641966	0.892577284	0.859959182	0.393462821
MED16	-0.180293938	0.85734021	0.836269661	0.406459347	1.007623352	0.317951255
MED17	0.937667683	0.351005399	-0.529007273	0.598833303	-0.89835924	0.372824363
MED18	-3.119687178	0.002454399	2.892241817	0.005389289	3.189094189	0.002333424
MED19	-0.026557014	0.978873749	-2.427607086	0.018346195	-2.516108944	0.014746452
MED20	0.564872034	0.573611329	0.377656897	0.707073631	-0.711723466	0.479578142
MED21	0.549335167	0.584179713	-1.958818985	0.054981662	-2.320109186	0.02398832
MED22	-0.219800755	0.82654013	0.823853785	0.413421159	1.677867808	0.098922699
MED23	0.807509118	0.421570765	-0.643867753	0.522216926	-1.68647846	0.097244343
MED24	0.363325792	0.717240951	1.718237778	0.091125117	2.160723563	0.034991339
MED25	-2.677147011	0.008870954	-1.105777285	0.273420421	-1.832336973	0.072197887
MED26	0.726357032	0.469565434	-0.847534955	0.400205221	-0.917584847	0.362754841
MED27	0.105960637	0.915856992	1.896141762	0.062961295	2.709460071	0.008916018
MED28	-0.735220168	0.464178503	-1.962506051	0.054540471	-2.398520509	0.019802672
MED29	-1.195162971	0.23526413	-0.72382569	0.472102488	0.27936657	0.780988411
MED30	-0.551210401	0.582899273	-2.144939955	0.036187647	-1.884325501	0.064693451
MED31	-0.262826339	0.79330449	-2.539978935	0.013806709	-2.334492599	0.023166033
MED4	-0.350826909	0.72656446	-1.246178313	0.217743864	-0.658939353	0.51262383
MED6	-1.476349704	0.143452709	0.979847782	0.331259194	2.001308074	0.050193339
MED7	0.683890384	0.495858012	-0.867165145	0.389449977	-0.924749116	0.359047644
MED8	-1.31655511	0.191438125	2.208559847	0.031199168	1.260582049	0.212664697
MED9	-1.384167461	0.169839964	-1.481480626	0.143928984	-0.283410307	0.777903056
MEF2A	1.319933189	0.190312382	-0.672863175	0.503725131	-1.695638619	0.095484613

MEF2BNB	-2.281821084	0.024935078	-0.718496022	0.475356188	-0.388466439	0.699137684
MEF2BNB-MEF	-1.429404995	0.156461181	-0.253900295	0.800476791	-0.277725084	0.782241872
MEF2C	0.414556641	0.679484693	-0.250290482	0.803253355	-0.699623335	0.487046515
MEF2D	-1.605028886	0.112103672	-1.405131419	0.165353042	-1.303339805	0.197769647
MEFV	-2.740545924	0.007439577	1.131380276	0.262583824	0.027846567	0.97788311
MEG3	2.048178652	0.043550524	1.278497918	0.206201801	0.627672976	0.53275871
MEGF11	0.448866636	0.654641534	-0.670264119	0.505368085	-0.900827535	0.371521718
MEGF6	2.152288695	0.034137908	0.334881439	0.738931077	0.599362073	0.551338597
MEGF8	1.732706377	0.086683635	-0.240197995	0.811029627	0.470119797	0.640087503
MEGF9	-2.737690884	0.007499211	-0.652919277	0.516406119	-1.265688463	0.210843261
MEI1	1.356104013	0.178567766	0.772975388	0.442697648	1.172331626	0.246006286
MEIS1	-5.149295771	1.60E-06	0.549906872	0.584509467	0.172111395	0.863967835
MELK	2.201649954	0.030330465	-0.976705021	0.33279976	0.551250614	0.583645546
MEMO1	-0.409881155	0.682898475	-3.566669542	0.000735894	-3.392519157	0.001274073
MEN1	0.496904628	0.620506954	-2.152980321	0.035520889	-1.171677028	0.246266935
MEOX1	-1.5441072	0.126184353	2.689500211	0.009345056	1.703392108	0.094015651
MEPCE	-1.070096312	0.28752943	-0.164775005	0.869698014	0.631690352	0.530148776
MERTK	0.03392556	0.973014057	1.053814817	0.29636851	0.240740541	0.810631723
MESDC1	0.358941542	0.720506578	-3.524763483	0.000838309	-4.00226684	0.000185508
MESDC2	-0.441573515	0.65989106	1.247593406	0.21722872	2.538970297	0.013911142
MEST	-1.343327774	0.182651916	-0.767350971	0.446007169	-1.090341098	0.280212816
METAP1	2.745726373	0.007332474	1.16018	0.25076208	1.114387555	0.269852983
METAP1D	-0.09613468	0.923634231	-2.171313964	0.034040636	-0.981008149	0.330790365
METAP2	0.902655111	0.369195973	1.654045594	0.1035587	2.272848913	0.026876071
METRN	1.75152185	0.083372555	-2.049017433	0.045025518	-2.179657512	0.033485847
METRNL	-1.037839413	0.302214531	-1.022766839	0.310694754	-2.766811995	0.007647161
METTL1	-0.377948923	0.706387109	-0.049461345	0.960722765	1.178911094	0.243397484
METTL10	0.551679256	0.58257934	-0.49368593	0.623405386	-0.879671844	0.382780696
METTL11A	-0.045543651	0.963778133	0.311141614	0.756816306	0.84251836	0.403066854
METTL12	1.257669355	0.21186945	-2.162264542	0.034764384	-1.107465971	0.27280695
METTL13	-0.616896757	0.53891042	3.613655209	0.000635261	3.207684778	0.002209784
METTL14	0.836798836	0.404993364	-0.743440419	0.460237021	-0.894174615	0.375039431
METTL15	0.145335051	0.8847818	-0.481200986	0.632196772	-0.34485954	0.731486427
METTL16	1.373365786	0.173160007	2.149365065	0.035819347	2.412523273	0.019128193
METTL17	2.223790258	0.028746868	0.11124123	0.911811805	1.623420928	0.110092352
METTL18	0.463001959	0.644516544	-0.551752129	0.583252689	0.188622065	0.851068078
METTL19	1.187151715	0.238392154	2.175976883	0.033672894	1.62917446	0.108865645
METTL20	-0.551220512	0.582892373	0.833330575	0.408100828	0.129732576	0.897240924
METTL21A	-0.910846607	0.364887549	-2.210737458	0.031039696	-1.725100592	0.090001531
METTL21B	0.137678604	0.890811886	-3.027941961	0.003680031	-1.531522383	0.131246801
METTL21D	-0.10017802	0.920432987	-0.386076181	0.700862721	-0.726088624	0.470795667
METTL22	0.306202179	0.760180692	-1.015766888	0.313988448	-0.9780351	0.332245659
METTL23	1.819666271	0.072241844	-1.71825229	0.091122452	-0.627656513	0.532769419
METTL2A	-0.654489853	0.514519061	1.90913953	0.061230173	2.4049979	0.019488065
METTL2B	0.159150117	0.873918686	1.352242144	0.18158365	1.810603724	0.075545349
METTL3	5.045717919	2.44E-06	1.051475813	0.297431724	1.477902329	0.145013026
METTL4	0.298179779	0.766275379	-1.042910092	0.301347662	-1.428812035	0.158589371
METTL5	0.733279712	0.465354893	-0.678789439	0.499989778	0.886069478	0.379353458
METTL6	1.124995006	0.263678614	-0.098014418	0.922261148	0.165948649	0.868792535
METTL7A	1.204406061	0.231691982	2.586093928	0.012258606	2.470627768	0.016544492
METTL8	0.864819013	0.389511333	0.781090485	0.437948047	0.801896467	0.425988424
METTL9	-0.345476026	0.730568663	0.305574217	0.761030537	0.121473328	0.90374915
MEX3B	1.390885129	0.167799815	-2.454301852	0.017160485	-3.019999839	0.003797247
MEX3C	1.632214005	0.106241117	-2.180662164	0.033306904	-2.856583082	0.00599085
MEX3D	-1.464355968	0.146693125	-3.094923448	0.003037074	-3.580963954	0.000714599
MFAP1	-0.511688897	0.610162304	1.711456406	0.092377575	1.919700101	0.059974027
MFAP3	-0.217861983	0.82804572	-1.346283218	0.183485896	-2.621451879	0.011242005
MFAP3L	-6.124016741	2.55E-08	2.650563453	0.010358328	0.618803531	0.538544395
MFAP4	0.592550302	0.55501585	-0.055834747	0.955666692	0.664344684	0.509184648
MFF	0.893738486	0.37392221	0.890641986	0.376826626	1.28677903	0.203443038
MFGE8	2.44413282	0.016534026	-0.922930747	0.359893533	0.526041285	0.600927955
MFHAS1	2.045020207	0.04386793	0.788243604	0.43378652	0.502803439	0.61706596
MFI2	0.392500171	0.695646579	-1.773782752	0.081385307	-0.132443364	0.895106367
MFI2-AS1	1.401548615	0.164599845	-1.431040224	0.157822315	-0.655383068	0.514893307
MFN1	0.933223999	0.353281545	-0.427109125	0.670892288	-1.057568763	0.294773867
MFN2	-3.228355384	0.001755425	2.3809532	0.020596155	2.33424557	0.02317994
MFNG	-0.605198658	0.54661902	0.355872698	0.723236068	1.057603052	0.294758365
MFSD1	-1.321110659	0.189921158	2.373836025	0.020960296	0.682483528	0.497734964
MFSD10	-0.792311171	0.430329772	-0.703003122	0.484885743	-0.888549808	0.378029965
MFSD11	-1.047276524	0.29786663	1.842126274	0.070606515	1.85779109	0.068436773
MFSD2A	-0.173764002	0.862453741	0.94683234	0.347679791	-0.171965992	0.864081609
MFSD2B	-2.271366658	0.025587002	1.282269421	0.204885182	-0.551477561	0.583491047

MFSD3	0.195349195	0.845574113	-1.807042064	0.075979658	-1.783194416	0.079951587
MFSD4	1.352415928	0.179739567	-0.356825297	0.722526584	-0.173178137	0.863133227
MFSD5	-2.590130541	0.01124322	0.801507304	0.426132366	0.764136344	0.447980352
MFSD6	1.504166095	0.136152505	-0.171145487	0.864709216	-0.539284848	0.591819116
MFSD6L	1.377775296	0.17179877	-0.883098059	0.380854428	-0.906984041	0.368285268
MFSD7	-0.536162115	0.593211798	-1.880347828	0.065120534	-2.554757309	0.013359601
MFSD8	2.513793417	0.013781043	-2.2226394	0.030180708	-1.740693966	0.087206433
MFSD9	-0.319073863	0.75043368	-1.451917147	0.151950385	-2.482639989	0.016051322
MGA	1.747199668	0.08412379	0.872010806	0.386823091	0.52971715	0.598393203
MGAM	-3.231095488	0.001740483	-0.384166817	0.70226948	-1.038445528	0.303507076
MGAT1	-1.963132589	0.052818278	1.561187991	0.123957692	1.503444327	0.138319907
MGAT2	1.145922485	0.254962642	-1.253841781	0.214964845	-2.059465488	0.044090425
MGAT3	-0.622572458	0.535190422	0.55608933	0.580303785	-0.870827763	0.387550477
MGAT4A	2.125569665	0.036367308	-0.150429556	0.880951304	-0.004638563	0.996315391
MGAT4B	-1.029365219	0.306155225	1.041615336	0.301942629	0.727228339	0.470102798
MGAT5	0.164216955	0.86994045	0.630964332	0.530559783	-0.377199861	0.707444803
MGC12916	0.220510921	0.8259888	-1.104381818	0.27401995	-1.109442427	0.271961138
MGC12982	2.553820051	0.012392455	-1.692435558	0.095966418	-0.612215397	0.542862794
MGC16142	-0.190297442	0.849518431	-1.968948295	0.053776871	0.138599311	0.890261907
MGC16275	-0.036787467	0.970738564	-2.16399303	0.034625111	-2.06563584	0.043482305
MGC16703	1.113540446	0.268536848	-0.463578196	0.644697132	0.250039182	0.803468206
MGC23270	1.202950715	0.232251812	0.532575773	0.596376043	-0.292722362	0.770811732
MGC23284	-0.847124324	0.399245144	0.089510621	0.928986649	0.625167474	0.534389813
MGC2752	-1.485742546	0.140954209	-0.578300763	0.565315495	-2.346590928	0.022493939
MGC3771	-0.406070512	0.685685689	1.07515684	0.286787869	2.320466572	0.023967578
MGC39372	-0.362925617	0.717538806	1.247811809	0.217149294	0.443550093	0.659069741
MGC57346	1.147663325	0.254246899	-1.019130013	0.312403062	0.582030569	0.562872221
MGC72080	0.842114373	0.402027932	-2.477952752	0.016168095	-1.414206471	0.162813903
MGEA5	0.140383968	0.888680435	0.570484148	0.57056834	0.504292826	0.616025791
MGLL	-2.839910624	0.005615229	1.006264231	0.318497335	0.019231962	0.984724133
MGMT	-0.211256645	0.833180012	0.565646723	0.573831049	2.067532848	0.043296813
MGP	2.382432035	0.019372226	2.014497267	0.048634906	2.160489892	0.035010284
MGRN1	-2.05399245	0.042971428	0.048427341	0.961543207	0.069971807	0.944464325
MGST1	1.692157495	0.094186951	1.743901943	0.086511413	1.864342924	0.067495874
MGST2	-1.469504697	0.145295153	-0.490542944	0.625613435	-0.468720306	0.641081475
MGST3	1.629705126	0.106771595	-1.470360954	0.146906083	-0.32571568	0.74584835
MIA3	-0.955362446	0.342035828	1.375105916	0.174424121	0.923926209	0.359472215
MIAT	3.749484093	0.000318152	-2.395227329	0.019882852	-2.304878214	0.024887297
MIB1	0.007270578	0.994215579	-0.320257911	0.749931793	-1.593682448	0.116612725
MIB2	0.143693673	0.886073962	-2.532409332	0.01407712	-2.201498253	0.031820152
MICA	0.626402327	0.532687691	2.82699393	0.006450501	-0.308768366	0.758638708
MICAL1	-0.153537061	0.878329571	1.133606175	0.261656282	2.010244925	0.049210654
MICAL2	-2.316714206	0.022863892	-0.107934764	0.914422561	-0.520485479	0.604768473
MICAL3	1.046549398	0.298200115	0.161689127	0.872116529	0.3587015	0.721161561
MICALCL	-3.167737861	0.002118281	0.487707069	0.627608691	0.229583064	0.819248749
MICALL1	-0.566953607	0.572202447	1.016634356	0.313579003	1.438941544	0.155709885
MICALL2	-0.397033031	0.692313321	1.386948145	0.170801897	0.176951829	0.860181989
MICB	-1.886154013	0.062603208	-1.823045629	0.073487658	-2.301946675	0.025063724
MICU1	-1.330523878	0.18681519	1.847470738	0.069816763	1.919818089	0.05995879
MID1IP1	-1.826395825	0.071213123	-2.586629318	0.012241598	-3.281739147	0.001775876
MID2	0.449315073	0.654319315	1.000835556	0.321092609	2.00073	0.050257481
MIDN	-1.389838956	0.1681163	-1.41681366	0.161923768	-1.940172977	0.057379008
MIER1	0.054239495	0.956868445	-0.530678736	0.597681751	-1.173967322	0.245355849
MIER2	0.281521251	0.778977703	-0.065880099	0.947701427	-0.336905056	0.737442577
MIER3	0.849233538	0.398077112	-0.061103391	0.951488405	-0.789243503	0.43328466
MIF4GD	0.366565878	0.714830929	0.247395397	0.805482014	0.71841992	0.475472728
MIIP	-1.721155091	0.088769374	0.818545593	0.416419542	0.671485691	0.504660224
MILR1	-0.997238699	0.321408291	0.396078868	0.693510347	0.891952502	0.376219061
MINA	0.600659835	0.549624821	-0.016881595	0.986589419	0.125222595	0.900793904
MINK1	-0.081530571	0.935206883	0.765756921	0.446947763	0.837625013	0.405787147
MINPP1	-0.414941236	0.679204179	-1.345020493	0.183890929	-1.456569194	0.15079627
MIOS	1.388625602	0.168483931	2.142716354	0.036373964	3.565178289	0.000750534
MIPEP	0.364559908	0.716322658	1.913499844	0.060658573	2.557816809	0.013255037
MIR155HG	1.631857364	0.106316395	-0.55346466	0.582087467	-1.360010671	0.179253913
MIR17HG	0.183344208	0.854953644	-0.008338361	0.993375842	-0.120180043	0.904768862
MIR210HG	-0.124955489	0.900846444	0.566002381	0.573590859	0.957058459	0.342634139
MIR223	-0.882011174	0.380195937	2.251353204	0.028194122	1.509835482	0.13668396
MIR3648	-0.728957467	0.467981279	0.60269804	0.549075339	-0.956788243	0.342769337
MIR497HG	0.585399806	0.559791045	-0.855004631	0.396091212	-0.48952017	0.626377547
MIRLET7BHG	-0.818092	0.415534857	-0.484429743	0.629918025	-0.363255148	0.717776213
MIS12	1.712989605	0.090268441	-0.380751361	0.70478849	-0.453346008	0.652044093
MIS18A	1.434719518	0.154944334	-1.320980506	0.191732177	-0.546410563	0.58694518

MIS18BP1	-0.020718169	0.983517815	0.232109814	0.817275462	-1.31503675	0.193834701
MITD1	-0.652134623	0.516029878	-2.017116083	0.048352601	-1.416385254	0.162178234
MITF	0.737806489	0.462613182	-1.410275747	0.163836095	-0.8399247	0.404507313
MKI67	0.431891075	0.666886763	1.546690188	0.127415389	2.06528401	0.043516783
MKI67IP	0.991971224	0.323956567	-0.641325933	0.523854881	-0.457944199	0.648757117
MKKS	1.412737663	0.161292643	-0.586276893	0.559980158	-0.232136157	0.817274951
MKL1	-2.032778918	0.045116953	0.786982805	0.434518317	0.729281266	0.468856221
MKL2	1.988398707	0.049904102	-1.248064716	0.217057347	-1.155023363	0.252965077
MKLN1	0.501728852	0.617122874	-0.348370499	0.728832107	-1.668973673	0.100681163
MKNK1	0.097982055	0.922171445	-0.008832748	0.992983101	0.402584184	0.688780417
MKNK2	-1.77212344	0.079867405	-1.368497937	0.176470755	-2.047726749	0.04526771
MKRN1	-3.312772199	0.001346078	1.585853314	0.118248111	1.056542519	0.295238097
MKRN2	-0.842692254	0.401706344	-1.589025216	0.11752948	-1.149068973	0.255391296
MKRN3	0.752162298	0.453979265	-1.217462469	0.228392857	0.006086106	0.995165559
MKS1	0.278825632	0.781038903	0.859565295	0.393592306	2.299356234	0.025220546
MLANA	1.207595217	0.230468616	-1.966098974	0.054113464	-0.090687904	0.928062826
MLC1	0.059825539	0.952431498	0.405595224	0.686542816	0.482185506	0.631545618
MLEC	1.224973305	0.223884391	2.13703561	0.036853771	2.078067234	0.042279189
MLF1	-0.284046562	0.777048162	0.778943524	0.439201685	2.057564514	0.044279255
MLF1IP	1.511770806	0.134208348	-0.459698768	0.64746299	-0.008285568	0.993418471
MLF2	-3.453995432	0.000854847	-0.477112114	0.635087709	-0.696532083	0.488964775
MLH1	0.736354139	0.46349182	2.270732206	0.026919581	3.605256109	0.000662486
MLH3	1.591095677	0.115207499	-0.511075845	0.611251541	0.253242051	0.801004655
MLKL	-1.936530729	0.056041615	0.611849247	0.543045274	-0.766877381	0.446361961
MLL	1.335132361	0.185308571	0.256285909	0.798643263	0.501374714	0.618064506
MLL2	-0.026569629	0.978863717	-1.655495088	0.103263388	-1.48725961	0.142532023
MLL3	0.271082467	0.78696836	-0.311980792	0.756181732	-1.847390099	0.069953168
MLL4	-0.39809253	0.691535085	-1.164409285	0.249058648	-0.825171472	0.412760901
MLL5	-0.921316016	0.359427746	-1.8047846	0.076336809	-2.484622996	0.015971196
MLLT1	-0.385793373	0.700589428	-0.690051142	0.492933235	-0.315394059	0.753629908
MLLT10	-0.04286506	0.965907115	-2.030327801	0.046949832	-2.958878676	0.004511075
MLLT11	1.58279242	0.117089627	-1.283213873	0.204556461	0.340216603	0.734960968
MLLT3	0.808748324	0.420861295	0.084863589	0.932664135	1.032724344	0.306153806
MLLT4	-0.590854122	0.556146748	-1.432453201	0.157419408	0.147893666	0.882955682
MLLT6	0.633979595	0.527753933	-0.180106414	0.857701212	0.699174466	0.487324799
MLST8	-0.16888917	0.866275015	0.096403948	0.923534417	-0.021300175	0.983081601
MLX	-0.343168336	0.732297883	-2.962945629	0.004423452	-2.792624932	0.007132187
MLXIP	1.19658516	0.234711934	-0.009900548	0.992134848	0.235870693	0.814389924
MLYCD	1.055703341	0.294020296	-0.611064881	0.543560792	-0.078680831	0.937565793
MMAA	0.417337787	0.677457215	-1.423843042	0.159887024	0.508897628	0.612814868
MMAB	0.435268546	0.664443103	-1.560778685	0.124054269	-0.194783893	0.846264122
MMACHC	-0.526519111	0.59986449	1.102651433	0.274764649	1.198999213	0.23555609
MMADHC	-1.985564453	0.050224023	-0.048786338	0.961258353	0.047616645	0.962190468
MMD	-4.830410382	5.78E-06	0.897261745	0.373314544	-1.524982344	0.132868029
MME	-3.895963119	0.000191353	-0.396087959	0.693503679	-0.467304071	0.642088014
MMGT1	-0.736714247	0.463273875	0.243924859	0.8081558	0.511548571	0.610969819
MMP11	-0.039382151	0.968675747	-0.343877275	0.73219083	0.222150569	0.825001471
MMP14	0.506384764	0.613864684	0.585192915	0.560703772	1.22941198	0.224036424
MMP15	0.803265531	0.4240057	0.44457386	0.65829385	-0.121171989	0.903986731
MMP17	1.632636587	0.106151976	1.376209542	0.174084083	0.633731036	0.528825581
MMP19	-1.458802095	0.148212811	1.541258253	0.128730559	0.899768538	0.372080249
MMP23B	0.769383057	0.443745295	-3.43335089	0.001110728	-2.351308796	0.022236602
MMP24	-0.304376868	0.761566082	2.638915585	0.010680388	3.276831924	0.001801941
MMP25	-0.766156267	0.445652649	-1.044060431	0.30081973	-1.108733139	0.272264461
MMP28	2.696508576	0.008409289	0.309681437	0.757920872	0.719883235	0.474578252
MMP8	-3.716195937	0.000356522	1.360499604	0.178972525	-0.19190391	0.848508729
MMP9	-3.531707416	0.000662445	1.022735216	0.31070958	1.23017968	0.223751083
MMRN1	-0.605278951	0.546565921	-0.712904042	0.478783557	-0.93052958	0.356074394
MMS19	2.587628167	0.011319219	1.573644162	0.121047297	3.402930175	0.001234548
MMS22L	0.161575462	0.872014011	-1.499284411	0.139261426	-0.093199514	0.926076304
MN1	0.184226967	0.854263213	1.2403484	0.219875663	0.962352682	0.339992313
MNAT1	0.054399603	0.956741252	0.25986601	0.795893819	1.285333257	0.203944049
MNDA	-3.094884484	0.002646723	-0.670419036	0.505270075	-0.988849484	0.326972411
MNT	-1.143276582	0.256053228	-0.261860506	0.794363212	-0.050840479	0.959632786
MOAP1	2.083147224	0.040166249	-1.233612773	0.222357741	-0.259356942	0.796306924
MOB2	-2.506559886	0.014046404	-1.247421195	0.217291363	-1.125649603	0.265094898
MOBKL1A	0.808891212	0.420779535	-0.17754796	0.859700935	-0.840026392	0.404450776
MOBKL1B	-0.996210715	0.321904557	0.182442853	0.855875837	-1.571286759	0.12172594
MOBKL2A	-3.20611745	0.001881203	0.939018	0.351642832	0.964269252	0.339039256
MOBKL2B	0.955305417	0.342064494	0.129076008	0.897747192	-0.429443925	0.66924108
MOBKL2C	0.123610929	0.901907853	2.327932815	0.023450277	1.666067136	0.101261331
MOCS1	-1.457711723	0.148512597	0.342333806	0.733345805	1.597485567	0.115761879

MOCS2	0.725201177	0.470270534	0.200261248	0.841981698	0.633444872	0.529011027
MOCS3	-2.137570659	0.035350804	-1.564917373	0.123080499	-2.621240636	0.0112482
MOGS	0.454341831	0.650711901	-0.777060141	0.440303156	0.13913485	0.889840658
MON1A	0.724386834	0.47076766	-0.139876221	0.889245782	0.33292999	0.740425128
MON1B	-0.967969005	0.33573729	0.252521614	0.80153693	-0.00157093	0.998752139
MON2	1.616183394	0.109667499	-1.815835158	0.07460182	-2.718152501	0.008712038
MORC2	1.614558108	0.110019794	2.783152342	0.007268557	3.593285308	0.000687696
MORC2-AS1	-0.148502431	0.882289189	2.291458428	0.025613172	4.209389102	9.32E-05
MORC3	0.632850401	0.528487675	-0.435377084	0.664915959	-1.473664401	0.146147796
MORC4	2.59258743	0.011169051	3.083425843	0.003139389	4.704517261	1.70E-05
MORF4L1	-1.086499887	0.280252581	0.386455495	0.70058338	0.298767543	0.766218666
MORF4L2	-0.178426814	0.858801725	-0.444895359	0.658062849	-0.808935868	0.421961269
MORN1	1.054081897	0.294757743	-3.23203574	0.002034784	-2.928963589	0.004904244
MORN2	-1.068105863	0.288421166	-0.867488371	0.389274406	0.625787018	0.533986243
MORN3	1.806423348	0.074302401	-0.261602847	0.794560898	1.231771508	0.223160279
MORN4	1.014068921	0.313355715	-1.190498297	0.238734202	-0.250343329	0.803234179
MOSC1	-3.724351952	0.000346733	1.437553565	0.155971705	1.299882243	0.198944216
MOSC2	-0.665282821	0.507625696	1.199580655	0.235213689	0.610350967	0.544088101
MOSPD1	0.028735042	0.97714158	-1.831044205	0.072268091	-2.28233777	0.026272682
MOSPD2	-0.985908024	0.326906326	-1.287667658	0.203011605	-1.230946765	0.223466237
MOSPD3	-1.08747415	0.279824425	-2.524531276	0.014363635	-2.192472663	0.032499443
MOV10	-0.465757499	0.642550491	1.63488359	0.107527773	0.849739963	0.3990728
MOXD1	0.874816415	0.384077276	1.993859694	0.050909711	2.609313202	0.01160304
MPDU1	-1.525939067	0.130644522	1.214529835	0.229501462	1.327567805	0.189684952
MPEG1	-1.566303068	0.120900089	0.353412391	0.725069605	-0.072431138	0.942515801
MPG	0.461134388	0.64585048	-0.419665557	0.676291072	-0.968742562	0.336821658
MPHOSPH10	2.422109438	0.017501384	1.362859732	0.178231509	2.388685565	0.020289063
MPHOSPH6	0.248440085	0.804378156	-0.30316084	0.762859609	0.861838552	0.39243648
MPHOSPH8	0.837943284	0.404353778	2.087164772	0.041308701	2.510807827	0.014946516
MPHOSPH9	2.444599877	0.016514038	-1.05412237	0.296228904	-1.159946763	0.250971441
MPI	1.156293818	0.250719492	1.569456209	0.122019633	3.141520206	0.002680084
MPL	-1.063941983	0.290292743	0.716078927	0.476835948	0.494701459	0.622738065
MPND	-0.325173558	0.745828724	1.181943809	0.24208485	0.668319158	0.506663798
MPO	-2.936652009	0.004242573	3.398079041	0.001236795	1.749770226	0.085612902
MPP1	-3.081353026	0.002757467	1.062341459	0.292514767	0.718809962	0.475234215
MPP2	1.303256542	0.195918313	0.956543697	0.342795464	1.986902685	0.051812886
MPP5	0.65005185	0.517367868	-1.972901795	0.053312811	-1.723259719	0.09033634
MPP6	0.706720304	0.481624766	0.835138203	0.407090787	1.357218733	0.180133993
MPP7	-0.640158935	0.523748018	-0.303481164	0.762616761	-1.100883463	0.275637211
MPPE1	-0.850268243	0.397504884	-0.858278191	0.39429655	-1.39048386	0.169860503
MPPED2	-1.688949838	0.094802459	-0.487697295	0.627615572	0.646105439	0.52083905
MPRIP	3.220688886	0.001797871	-0.021460251	0.982952685	0.371739668	0.711483712
MPST	-0.37250025	0.710424366	0.936362362	0.352996298	-0.197612521	0.844060785
MPV17	-0.407670794	0.684514664	-2.172879733	0.03391676	-2.65624028	0.010263326
MPV17L2	0.521530803	0.603319342	-0.559019731	0.578315444	-0.547777812	0.58601218
MPZ	-1.470072717	0.145141563	-3.048706989	0.003468249	-3.510649114	0.000888383
MPZL1	-4.218923938	5.99E-05	-0.193082042	0.847573973	-1.127266645	0.26441662
MPZL2	-1.364784834	0.175832474	-2.036933405	0.046261745	-2.308965603	0.024643152
MPZL3	-1.622457004	0.108316141	-2.273749491	0.026725797	-3.288764823	0.001739177
MR1	-1.06865013	0.288177142	-0.07776725	0.938282732	-0.902225802	0.370785069
MRAS	0.641317104	0.522998974	0.528206624	0.599385275	-0.050072658	0.960241912
MRC2	-1.49111215	0.139541256	1.245271186	0.218074563	0.59815724	0.552136506
MRE11A	2.436197681	0.016876924	-0.453429149	0.65194351	-1.308233268	0.196116205
MREG	0.190670404	0.849227096	-0.591267522	0.55665465	-0.567055889	0.572932795
MRFAP1	-2.06027856	0.042352747	3.074661132	0.003219541	2.946457452	0.004670626
MRFAP1L1	0.040428184	0.967844193	-0.287723498	0.774591155	-0.1084276	0.914042453
MRGPRE	0.06185056	0.950823404	1.514131067	0.135461277	2.84903421	0.006116173
MRGPRX3	0.426351473	0.670902547	3.319633045	0.00156745	2.743409864	0.008143392
MRI1	2.374049839	0.019789492	-0.37474772	0.709224418	0.207439681	0.836415846
MRM1	1.893373897	0.06162486	1.804015162	0.076458862	2.523378059	0.014476056
MRP63	1.255062504	0.212809657	-1.840144887	0.07090121	-0.715358604	0.477347077
MRPL1	1.180356927	0.241068519	-0.107994296	0.914375546	0.223667754	0.82382638
MRPL10	-2.021618862	0.04628211	-2.993801873	0.004054627	-2.883273529	0.005566549
MRPL11	1.567654768	0.12058406	1.271387224	0.208701255	1.813761035	0.075051112
MRPL12	0.987053785	0.326347555	0.588453557	0.558528522	1.181901573	0.242218361
MRPL13	1.298378309	0.197581202	-0.024695106	0.980383519	0.252763228	0.801372823
MRPL14	-0.10766714	0.91450711	-1.641951524	0.106049615	-1.742027118	0.086970841
MRPL15	1.355534648	0.17874829	-0.390184354	0.697839521	0.244937515	0.807396388
MRPL16	-0.577124701	0.565342449	1.675022966	0.099351469	2.498449933	0.015422466
MRPL17	2.184561691	0.031604336	0.595240872	0.554014088	0.736832067	0.464287421
MRPL18	1.091346947	0.27812693	-0.14266507	0.887052615	-0.56431186	0.574785799
MRPL19	1.534917258	0.128425118	-0.903219361	0.3701716	0.369252024	0.713326593

MRPL2	-1.488324646	0.140273367	-0.444294701	0.658494457	0.886737431	0.378996754
MRPL20	0.720440769	0.473180762	-0.387541511	0.699783825	0.32916398	0.743254523
MRPL21	-1.251285848	0.214177206	0.190987795	0.849206794	0.871334832	0.387276004
MRPL22	1.480919128	0.142232957	-1.955019852	0.055439453	-0.644715839	0.521732719
MRPL23	-0.516320345	0.606937743	-1.436010831	0.156408502	-0.879773944	0.382725848
MRPL24	0.665027916	0.507787931	0.750979517	0.455722363	1.872047439	0.066403446
MRPL27	-0.34420425	0.731521471	0.255056481	0.799588032	1.377756134	0.173736735
MRPL28	-1.607313726	0.111601153	1.530647936	0.131330715	1.371811731	0.175570194
MRPL3	1.541379223	0.126846254	-0.404917992	0.687037767	0.375241176	0.708892679
MRPL30	0.510441917	0.611031811	-0.342506375	0.733216642	0.472023006	0.638736834
MRPL32	1.257272259	0.212012472	-1.586837321	0.118024795	-1.373167901	0.175150605
MRPL33	0.123301928	0.902151807	-2.89905592	0.005288329	-2.117314202	0.038668673
MRPL34	1.47323065	0.144289979	-2.12080724	0.038255045	-2.031483835	0.046941535
MRPL35	1.409422984	0.162267014	-0.948191806	0.346993326	-1.149751238	0.255112456
MRPL36	-0.030758666	0.975532305	-3.265979521	0.001839947	-2.507930919	0.015056115
MRPL37	-0.372411605	0.710490118	0.486605623	0.628384397	1.702181588	0.09424376
MRPL38	0.915449932	0.362480451	2.003151784	0.049874398	2.47453672	0.016382528
MRPL39	0.999392554	0.320370155	-1.20225695	0.23418353	-1.219750623	0.227650241
MRPL4	0.770840308	0.442885473	-0.810264218	0.421123569	-0.43524059	0.665053677
MRPL40	1.108903196	0.270521325	-0.30296266	0.763009868	1.023245934	0.310573181
MRPL41	0.37915801	0.705492355	-1.953166735	0.055663932	-1.178967335	0.243375271
MRPL42	1.276916172	0.205022103	-1.454723298	0.151174299	-1.407995802	0.164636484
MRPL42P5	1.859712126	0.066298517	-1.17715491	0.24397534	1.046760677	0.299688239
MRPL43	0.106157937	0.915700911	-1.702739444	0.094008322	-0.855803606	0.39573814
MRPL44	-2.567630766	0.011943418	2.726045021	0.008477412	1.91597668	0.060456572
MRPL45	0.90927709	0.365710574	-0.501133783	0.618186908	0.859427812	0.39375331
MRPL45P2	0.771912717	0.442253337	-0.085872973	0.931865219	-1.557571858	0.124945093
MRPL46	0.111924834	0.911140279	-0.889507007	0.377430877	0.397371795	0.692597562
MRPL47	1.282963966	0.202904668	-0.155562107	0.876922114	-0.020065217	0.984062371
MRPL48	0.772818617	0.44171976	-0.155714624	0.876802433	0.714664746	0.477772481
MRPL49	-0.48744305	0.627167763	-0.282047742	0.7789179	0.665266122	0.508599616
MRPL50	1.044980819	0.298920382	-2.261225603	0.027538302	-1.894617855	0.063288897
MRPL51	-1.626286806	0.107497799	-0.759283639	0.450779307	-0.004194551	0.996668087
MRPL52	0.85919925	0.392586724	-1.48816394	0.14216261	-0.25790401	0.797422452
MRPL53	-0.327761666	0.743877612	1.24111991	0.219592669	1.607161839	0.113619653
MRPL54	-1.126661782	0.262976855	-1.39562165	0.168185789	0.220995782	0.825896147
MRPL55	0.777014619	0.439253205	1.708268056	0.092971319	2.350682075	0.022270635
MRPL9	0.024114381	0.980816477	-0.421122011	0.675233357	1.258665738	0.21335124
MRPS10	0.294614579	0.768988647	-0.978604422	0.331868116	-0.059567613	0.95271119
MRPS11	-1.360997709	0.177021848	-0.046046113	0.963432778	-0.020068669	0.984059629
MRPS12	-0.707777484	0.480971192	0.026454389	0.97898636	0.792448143	0.431429741
MRPS14	0.438446887	0.662146826	-1.627459378	0.109098426	-0.975812392	0.333336435
MRPS15	0.017534209	0.986050505	1.322831848	0.191119484	2.655092208	0.01029434
MRPS16	-1.396112702	0.1662252	3.097787276	0.003012079	4.668166246	1.93E-05
MRPS17	1.842179544	0.068848177	-0.239489973	0.811575887	1.469372281	0.147304177
MRPS18A	-1.479051369	0.14273055	-0.543224386	0.589071597	-0.359578198	0.720509354
MRPS18B	-0.366885627	0.714593252	1.087127588	0.281509025	1.035156473	0.305026742
MRPS18C	0.157068232	0.875554228	-1.422907934	0.160156817	-1.561595071	0.123993779
MRPS2	-0.610219176	0.543303895	-0.98632261	0.328100207	-0.396209666	0.693449712
MRPS21	2.129031796	0.036071491	-1.227218157	0.224733149	-0.529751028	0.598369865
MRPS22	1.188303695	0.23794053	-0.594340499	0.554611896	0.085112557	0.932474195
MRPS23	1.611405104	0.110705828	0.174316516	0.862227994	0.724735329	0.471619128
MRPS24	0.925262697	0.357383171	-4.5550026	2.77E-05	-4.33388677	6.12E-05
MRPS25	3.206551387	0.00187867	0.357777585	0.721817575	0.900389295	0.371752787
MRPS26	0.977113442	0.331216347	-2.176297621	0.033647727	-1.341927026	0.185012836
MRPS27	1.632908923	0.10609456	3.087222836	0.003105249	4.863630653	9.67E-06
MRPS28	1.845863891	0.068305687	-0.328918234	0.743410497	0.235493694	0.814681049
MRPS30	0.88371489	0.379280441	-0.134882192	0.89317528	0.350044516	0.727612926
MRPS31	-0.901716495	0.369691698	-0.391085738	0.697176849	-1.068263408	0.289965939
MRPS33	0.150391094	0.880803444	-1.451267504	0.152130498	-0.587137313	0.559461433
MRPS34	0.179469772	0.857985275	-1.937626408	0.05757718	-1.33990353	0.185665887
MRPS35	0.180513752	0.85716818	0.070833285	0.943775818	1.140676206	0.258839208
MRPS36	-1.30522972	0.195248668	-4.681874573	1.77E-05	-3.659885267	0.000558226
MRPS5	0.381578205	0.703702597	1.278337984	0.206257773	2.128942596	0.037651919
MRPS6	-1.177443955	0.242222463	0.391763997	0.696678369	0.241837534	0.809785758
MRPS7	0.050887518	0.959531553	-0.177102972	0.860048838	-0.013913296	0.988948397
MRPS9	0.535631972	0.593576648	1.437276965	0.156049949	3.29206619	0.001722181
MRRF	2.212522924	0.029543485	-0.14470603	0.885448153	0.655428632	0.514864196
MRS2	2.716737029	0.007950399	0.182607904	0.855746918	0.749979434	0.456393326
MRS2P2	-0.767977546	0.444575509	2.597134367	0.011912193	1.215988088	0.229069093
MRTO4	0.411314338	0.681851338	2.330468516	0.023306141	2.964203839	0.004444238
MRVI1	-3.825255678	0.000244941	-0.07361263	0.941573677	-0.431415608	0.667815579

MS4A1	0.667149046	0.506438776	-0.223984609	0.823561899	0.083042551	0.934112591
MS4A14	0.174600889	0.861798051	0.298872572	0.76611299	0.610009491	0.544312673
MS4A2	-2.410977378	0.0180093	1.643359496	0.105757142	-2.833752407	0.006377329
MS4A3	-2.469067358	0.015496691	1.432402008	0.157433992	-2.073166043	0.042750032
MS4A4A	0.31948145	0.750125688	0.35715704	0.722279563	-0.617140928	0.539632529
MS4A6A	-0.900582206	0.370291327	-0.300091555	0.76518775	-0.917176494	0.362966884
MS4A7	1.487653808	0.140450004	-1.685973365	0.097211465	-2.606943397	0.011674745
MSC	-1.460204828	0.147827837	-2.32710864	0.023497296	-2.767808986	0.007626652
MSH2	1.884671354	0.062805728	0.262409205	0.793942274	0.150088377	0.881231903
MSH3	0.617549903	0.538481662	0.699846094	0.486840552	-0.523964678	0.602362111
MSH5-C6orf26	0.354565104	0.723771567	0.296912179	0.767601696	1.69642669	0.09533445
MSH6	2.224532988	0.028695023	3.508505613	0.000881583	2.584350572	0.012378888
MSI2	-0.327821624	0.743832431	-2.872447262	0.005692787	-3.070992201	0.003283867
MSL1	-1.385949647	0.169296881	-0.266598346	0.790730586	-0.669063906	0.506192185
MSL2	0.393575992	0.694854925	0.105315086	0.916491708	-0.194645646	0.846371839
MSL3	1.191085131	0.236852625	0.804672369	0.424317903	1.129525017	0.263471388
MSL3P1	-0.396032895	0.693048257	1.839425694	0.071008432	0.135924453	0.892366398
MSLN	0.567439438	0.57187386	1.093489994	0.278731043	1.437576435	0.156095549
MSMP	0.682433149	0.496774213	1.012841812	0.315371746	0.732018889	0.467196809
MSN	-1.853767326	0.067154023	0.507043804	0.614059943	0.429088708	0.669498028
MSR1	-0.490102003	0.625292743	-0.931880348	0.355288248	-1.724868934	0.090043608
MSRA	-1.489048769	0.140082895	1.086168016	0.281929665	0.777795789	0.439949424
MSRB2	-4.334317208	3.90E-05	0.49186183	0.624686454	0.548124419	0.58577577
MSRB3	-3.278835227	0.001498511	1.176646701	0.244176586	-0.53441987	0.59515765
MST1	0.264109622	0.792318716	0.395390016	0.694015742	0.322995075	0.747896893
MST1P2	3.127490106	0.002396636	0.117861295	0.906587627	-0.936207603	0.353169401
MST1P9	1.080102012	0.283075485	-0.053158296	0.957789733	0.187008826	0.852326756
MST1R	2.0349519	0.044893034	-0.568330341	0.572019902	-0.230460917	0.818569946
MST4	1.206002876	0.231078856	-0.691505044	0.492026233	-1.173993127	0.245345597
MSTO1	1.414214161	0.160860067	-0.102463544	0.918744654	1.83300032	0.072097708
MSTO2P	1.747887963	0.084003786	-0.610380949	0.544010505	0.764325205	0.447868733
MSX2P1	1.101459035	0.273728344	-2.181114615	0.033271747	-1.51634471	0.135033587
MT1E	-2.266552095	0.025892243	0.450717111	0.653885655	1.308838212	0.195912526
MT1F	0.3565728	0.722273111	-0.564400461	0.574673086	1.212249995	0.230485118
MT1L	-2.773266911	0.006786354	0.386368929	0.700647127	1.484104	0.14336494
MT1X	-1.840435333	0.069106252	0.265207036	0.791796861	1.740655076	0.087213313
MT2A	-3.838856669	0.00023363	0.151237344	0.880316956	-0.825671442	0.412479529
MTA1	2.63788301	0.009878574	-3.692644694	0.000495017	-3.099326888	0.003027419
MTA2	0.44567238	0.656938614	1.017798372	0.313030153	1.771067597	0.081968415
MTA3	0.337046334	0.736891961	0.098017703	0.922258551	0.507298567	0.613929031
MTAP	2.013531012	0.047142539	0.448834127	0.655235516	1.210742695	0.231057902
MTBP	0.958417213	0.340502578	-0.415400998	0.679391872	-0.30352165	0.762612452
MTCH1	-1.276539115	0.205154656	-0.643490429	0.522459904	-0.056942512	0.954792829
MTCH2	0.213778903	0.831218608	2.236913063	0.029178245	3.387569628	0.001293281
MTCP1	1.227753795	0.22284374	-0.193406016	0.84732144	-0.27222733	0.786444246
MTCP1NB	1.317022817	0.191281965	0.431275893	0.667877722	0.812597309	0.419875695
MTDH	1.850180769	0.067674614	0.623997927	0.535092691	-0.116548156	0.907633341
MTERF	1.243731953	0.216931839	-0.199231555	0.842783287	-0.310733191	0.75715227
MTERFD1	2.186422519	0.031463389	0.238738291	0.812155936	1.107588313	0.272754541
MTERFD2	3.077591032	0.002789012	0.248995388	0.804250127	1.057033915	0.295015747
MTERFD3	2.437185367	0.0168339	-1.147005639	0.256121763	0.921334273	0.360811617
MTF1	-3.644727374	0.000454291	0.832433034	0.408602911	0.0686106	0.945542956
MTF2	0.314470068	0.753915318	-0.215903467	0.82982579	-0.963498325	0.339422405
MTFMT	-2.592968659	0.011157582	-0.876698808	0.384292259	-0.063518225	0.949579083
MTFP1	-0.230984274	0.817868188	2.055901261	0.044334145	1.553194921	0.125986674
MTFR1	0.333904097	0.739253693	-0.398272875	0.691901588	-1.204029274	0.233621659
MTG1	1.036539888	0.302816601	1.653091876	0.103753381	2.595501951	0.012026646
MTHFD1	-0.18784122	0.851437597	1.165704229	0.248538746	2.027178231	0.047394092
MTHFD1L	1.194104954	0.235675535	4.388526986	4.93E-05	3.780178471	0.00038114
MTHFD2	0.330955921	0.741471846	0.156643859	0.876073323	-0.747464513	0.457897345
MTHFD2L	0.100768298	0.919965753	-1.493216973	0.140838489	-1.729705092	0.089168577
MTHFR	1.436415834	0.154462578	1.658035056	0.102747566	1.897970038	0.06283706
MTHFS	-1.72631607	0.08783246	-2.937989826	0.004744318	-2.785941521	0.007262365
MTHFSD	1.488201417	0.140305801	1.971522304	0.053474343	1.97312739	0.053403381
MTIF2	2.61394754	0.010542623	2.665051424	0.009970122	3.627452474	0.000618058
MTIF3	-0.129331236	0.897393445	0.35430502	0.724404188	1.319202843	0.19244754
MTL5	-0.253917189	0.800157371	-1.775632832	0.081076416	-1.410569361	0.163879343
MTM1	-0.530945107	0.596806737	-0.000364916	0.9997101	-0.923388606	0.359749762
MTMR1	1.312613894	0.192757823	0.662118886	0.510535677	0.211211941	0.83348542
MTMR10	-0.683028263	0.496399939	0.169766975	0.865788283	-0.836705743	0.406299438
MTMR11	-0.431451026	0.667205412	-0.159252446	0.874027113	-0.139034335	0.88991972
MTMR12	-1.106615903	0.271503913	-0.365419409	0.716136923	-0.516234245	0.607714803

MTMR14	-2.047238661	0.043644781	1.235991898	0.221478692	1.311629176	0.194974896
MTMR2	0.439230173	0.661581415	-1.648992913	0.104593495	-1.193863278	0.237543221
MTMR3	-2.003266075	0.048254255	1.004246945	0.319460084	0.626049201	0.533815506
MTMR4	1.1106313	0.269780603	1.587119546	0.117960808	1.836065268	0.071636355
MTMR6	-1.103929428	0.272661156	-0.056527508	0.955117226	-1.381243806	0.172667874
MTMR7	1.554466573	0.123695708	0.015550019	0.98764712	0.686210985	0.495399649
MTMR8	-0.06787621	0.946039594	-0.128023971	0.898575965	-0.817060693	0.417341761
MTMR9	0.888737605	0.376589509	-0.928446156	0.357050884	-0.994136139	0.324414983
MTMR9LP	0.841572128	0.402329831	-1.02255321	0.310794925	-0.35306827	0.72535728
MTO1	0.611261007	0.542617236	1.313292988	0.194292192	1.460415835	0.149740339
MTOR	1.481442747	0.142093702	0.538544834	0.592276331	0.5596566	0.577936071
MTPAP	1.47426962	0.144010659	-0.138912973	0.89000349	1.692344204	0.096114454
MTR	1.78173382	0.078274462	-0.076415548	0.939353323	-0.07564427	0.939970573
MTRF1	1.627441192	0.107252112	-1.726184315	0.089675326	-1.188034847	0.23981298
MTRF1L	0.181471952	0.856418359	-1.159421022	0.251068655	-0.747281134	0.458007123
MTRNR2L4	1.358515164	0.177804811	-0.481382171	0.632068803	0.95010646	0.346123607
MTRNR2L8	-1.114838473	0.267983193	-0.264615501	0.792250323	-0.172384193	0.863754386
MTRNR2L9	-4.296704972	4.49E-05	0.813668017	0.419186264	1.125211152	0.265279021
MTRR	0.959282091	0.340069293	-0.920868645	0.360960079	-1.86259764	0.067745438
MTSS1	4.093999814	9.45E-05	-1.33234305	0.187995088	-1.419818486	0.161180479
MTSS1L	1.385082773	0.169560877	-1.629067017	0.108756747	-0.256810006	0.798262684
MTUS1	1.924120727	0.057601434	0.106745577	0.915361765	0.129094952	0.89774312
MTVR2	-0.743939816	0.458913104	0.667140455	0.507346488	-0.159762139	0.87364089
MTX1	-3.301795312	0.001393714	-1.860439935	0.067931192	-1.51264909	0.135968637
MTX2	0.770960047	0.442814865	-0.823223621	0.413776425	-0.964543625	0.338902962
MTX3	1.695813159	0.093489456	-0.469652573	0.640376506	-0.170163453	0.865492289
MUC1	-0.043553469	0.965359934	1.180869147	0.242508166	1.128469875	0.263912714
MUC12	-0.079546384	0.936780332	1.448879654	0.152793958	0.262938577	0.793558857
MUC16	-0.615516473	0.539817083	3.436284066	0.001100812	2.45619556	0.017155061
MUC20	1.622091794	0.108394439	2.079361551	0.042046535	1.820743187	0.073967766
MUC4	0.980362963	0.329619496	0.633833275	0.528698849	0.095056074	0.924608188
MUC5B	0.397803972	0.691747007	0.431272896	0.667879888	-0.22470603	0.823022448
MUC6	1.51186757	0.134183751	0.447020144	0.656537005	0.414017568	0.680436065
MUDENG	0.274408954	0.784419485	-0.564469824	0.574626205	-0.574814885	0.567709064
MUL1	-2.107508803	0.037944832	2.54866471	0.01350223	2.789167602	0.007199257
MUM1	1.719271268	0.089113393	0.624630737	0.534680108	1.636804045	0.107256092
MUS81	1.967609091	0.052291655	-2.305151662	0.024781235	-1.936849539	0.057793634
MUSTN1	0.1558233	0.876532513	-0.537267463	0.593152547	1.028038709	0.308333139
MUT	1.75428731	0.082894798	1.913541231	0.06065317	1.866303929	0.067216391
MUTYH	1.516651875	0.132972046	-1.051643993	0.297355189	0.048452125	0.961527586
MVD	-0.084040093	0.933217215	-0.005350532	0.995749403	0.933721292	0.354439549
MVK	1.7305288	0.087073718	2.089173972	0.041120552	2.73245041	0.008385811
MVP	-1.5568201	0.123135786	0.275648345	0.783804731	-0.044949045	0.964307155
MX1	-1.773078065	0.079707984	1.494974793	0.140380149	-1.091982232	0.279497067
MX2	-2.904797267	0.004656027	-0.416628624	0.67849868	-1.99306624	0.051114524
MXD1	-2.104026922	0.038255671	-0.893201297	0.375466323	-1.918296036	0.060155604
MXD3	0.184147706	0.854325201	0.654467569	0.515415617	-0.226350159	0.821749797
MXD4	-1.289234612	0.200726365	-1.071082739	0.288600008	-0.873905665	0.3858863
MXI1	-3.777798428	0.000288641	-0.830456972	0.409709649	-0.904307653	0.369690014
MXRA7	-1.047760087	0.297644991	-1.966156006	0.05410671	0.042993102	0.965859322
MXRA8	2.463342364	0.015729571	-1.945466518	0.056605043	-2.005169849	0.049766649
MYADM	-3.870130567	0.000209483	-0.359678115	0.720403305	-0.171948821	0.864095046
MYB	0.131142897	0.895964397	0.290703651	0.772322187	-1.256944037	0.213969461
MYBBP1A	1.602822735	0.112590607	0.890249655	0.37703543	0.886928322	0.378894852
MYBL1	0.512928472	0.609298514	-2.362463626	0.021554108	-2.6056151	0.011715231
MYBL2	-0.285651949	0.775822244	1.199190913	0.235363983	1.092156608	0.279421092
MYBPC2	-1.569465349	0.120161776	0.508304209	0.613181417	0.420869793	0.675454255
MYBPC3	-0.422501265	0.673699303	0.375472167	0.708688604	0.213921914	0.83138168
MYBPH	1.234274317	0.220417137	-0.329917535	0.742659219	0.758293472	0.451441561
MYC	-0.874691136	0.384145078	0.859357753	0.393705811	0.273492547	0.785476567
MYCBP	1.197997996	0.234164295	-2.691679409	0.00929112	-2.496572076	0.015495976
MYCBP2	0.365661405	0.715503399	0.715235352	0.477352998	-0.055747884	0.955740244
MYCBPAP	2.896831069	0.004765069	0.444516109	0.658335349	1.21550796	0.229250613
MYCL1	-1.385872455	0.169320376	-0.212551912	0.832426948	-0.942115737	0.350163063
MYCT1	-0.451511074	0.652742355	-0.841354648	0.403628946	-1.116726736	0.268859788
MYD88	-0.43736503	0.662928081	-1.155396719	0.252698683	-2.081193094	0.041981255
MYEF2	2.596757348	0.01104418	-0.595140673	0.5540806	-0.500557894	0.618635712
MYEOV	-2.955208747	0.004017608	1.0768963	0.286016573	-0.369298873	0.713291871
MYEOV2	0.082198042	0.934677639	-0.475666046	0.636111487	0.366706471	0.715214155
MYH10	-0.857423998	0.393561326	0.416501381	0.678591238	0.159719459	0.873674355
MYH11	-0.282135431	0.778508292	0.222571479	0.824656431	0.545435645	0.587610888
MYH3	1.759865417	0.081937999	2.514273834	0.014744592	3.329475071	0.001540234

MYH7	-2.140099386	0.03513979	-0.291133117	0.771995373	-0.370892418	0.712111173
MYH7B	2.445888388	0.016459009	-1.295002963	0.200486288	-0.786391621	0.43493937
MYH9	-2.103481199	0.038304589	1.594294885	0.116343356	1.5086201	0.136993876
MYL12A	-2.053561319	0.043014143	-0.659670782	0.512094354	0.036724447	0.970834768
MYL12B	-0.719839012	0.473549357	-1.150428236	0.254721568	-0.519929751	0.605153249
MYL4	-3.060713135	0.002934706	1.276013889	0.207072405	0.606552692	0.546588698
MYL5	0.634859431	0.527182588	0.345982945	0.730616164	0.878766015	0.383267522
MYL6	-0.2968102	0.767317344	-0.772264488	0.443115159	-0.253824526	0.80055685
MYL6B	-0.613445498	0.541178892	0.665332955	0.50849319	1.487576641	0.142448554
MYL9	-3.655430713	0.00043818	0.801807187	0.42596025	-0.031583654	0.974915906
MYLIP	-1.365734638	0.175535134	-0.452325405	0.652733632	-1.582413392	0.11916352
MYLK	-4.611295442	1.36E-05	0.596239553	0.553351387	-0.745414667	0.459125341
MYLK3	0.173593656	0.862587217	0.756025681	0.452714895	0.387033639	0.700192096
MYLK4	0.924000671	0.35803615	0.240712167	0.810632986	-0.187470327	0.851966644
MYNN	-0.189636854	0.850034491	-0.514058836	0.609177591	-0.925678685	0.358568431
MYO10	-1.209074653	0.22990269	3.461773017	0.001018113	1.566911588	0.122745566
MYO15A	1.673920381	0.097730219	0.266160775	0.79106589	0.381218802	0.704477373
MYO15B	0.640737255	0.523373921	0.293495377	0.77019849	0.667358287	0.507272622
MYO16	0.602955006	0.548103826	-0.480534215	0.632667804	1.482882148	0.143688473
MYO18A	1.522692098	0.131454584	1.299463852	0.19896209	1.754543016	0.084784678
MYO19	1.575553708	0.118750416	-2.412981278	0.019026582	-1.300210324	0.198832539
MYO1A	-0.011918531	0.990517843	0.265548955	0.791534783	1.207628024	0.232244785
MYO1B	-1.046157013	0.298380181	0.14515449	0.88509567	1.034121222	0.305506138
MYO1C	0.053160091	0.957725967	1.553952034	0.125673946	1.578141942	0.120142038
MYO1D	-0.269398567	0.788259513	1.960449879	0.054786136	2.559973555	0.013181773
MYO1E	2.560664688	0.012168044	0.856046863	0.395519281	1.612383836	0.112476944
MYO1F	-2.10157491	0.03847589	0.319388732	0.750587316	0.312170046	0.756065839
MYO1G	-0.152983177	0.878765038	-0.629718052	0.531369243	0.168653435	0.866674379
MYO3B	-1.450749338	0.150437951	-1.512263767	0.135934664	0.425163021	0.67234033
MYO5A	-0.436196297	0.663772493	-0.29293774	0.770622549	-1.230111017	0.223776593
MYO5B	-0.012009755	0.990445271	0.475940802	0.635916912	0.645075253	0.521501498
MYO5C	-2.135339561	0.035537894	-1.774618338	0.081245676	-0.303882818	0.762338704
MYO6	0.143409248	0.886297904	-1.947472565	0.056358567	-1.5193253	0.134283174
MYO7A	-0.761269881	0.448550016	3.255708919	0.001896963	2.754630864	0.007901875
MYO7B	-2.79965517	0.006298222	1.448061417	0.153021822	1.560949324	0.124146077
MYO9A	0.455273987	0.650043857	0.244291874	0.807872933	-0.952793651	0.344772058
MYO9B	-1.304377243	0.195537766	0.014348911	0.988601207	0.129231564	0.89763552
MYOF	0.528230023	0.59868163	2.160508466	0.034906382	1.555139305	0.125523114
MYOM1	0.227341698	0.820690287	0.213037224	0.832050178	0.092553622	0.926587119
MYOM2	0.115594853	0.908239459	0.993230692	0.324752024	0.686309545	0.495337981
MYPOP	-0.045900408	0.963494598	-4.281837829	7.10E-05	-4.701280534	1.72E-05
MYSM1	1.124642655	0.263827131	-1.98486353	0.051929596	-2.975552901	0.004304857
MYT1L	0.557617858	0.57853424	-1.059294464	0.293887918	0.480491699	0.632741736
MZB1	0.277061091	0.782389004	0.310181603	0.757542459	0.477279758	0.635012624
MZF1	1.926303084	0.057324501	-1.365494772	0.17740695	-1.053443501	0.296643017
MZT1	0.459726331	0.646856969	-1.293855211	0.200879863	-1.56182643	0.123939249
MZT2A	0.878134844	0.382284036	-1.673720792	0.099608496	-1.196159552	0.236653278
MZT2B	0.660341474	0.510775554	-0.269281909	0.788675091	-0.003262508	0.997408446
N4BP1	-2.901356049	0.004702846	-0.651482716	0.517326047	-0.359473674	0.720587101
N4BP2	1.403650768	0.16397457	-0.449638936	0.654658428	-1.780595659	0.080380269
N4BP2L1	0.198244039	0.843315637	-0.391394224	0.696950114	-1.268875114	0.209712483
N4BP2L2	0.364752488	0.716179399	-0.846870806	0.400572281	-1.285021468	0.204052215
N4BP3	0.730051374	0.467315785	0.515810113	0.607961499	0.271566706	0.786949646
N6AMT1	2.361754819	0.02041598	-0.729207522	0.468829759	-0.282541677	0.778565515
N6AMT2	1.635635847	0.105521032	-0.493918947	0.623241823	1.721975967	0.090570431
NAA10	-0.855999103	0.394344663	-1.422207745	0.160359064	0.021055526	0.983275892
NAA15	2.315307847	0.022944335	1.697643103	0.094972657	2.519593228	0.014616279
NAA16	1.75195284	0.083297948	-1.599430724	0.11519666	-0.803326939	0.425168209
NAA20	1.086676441	0.280174958	-2.038913767	0.046057162	-1.523136774	0.133328401
NAA25	2.307595062	0.023389969	0.888509875	0.377962246	0.818706928	0.416409508
NAA30	0.678800306	0.499062265	-0.266075811	0.791131	-0.702331495	0.485369416
NAA35	0.55941684	0.577311512	0.990412795	0.326115027	0.740467788	0.462096645
NAA38	0.931829081	0.353998004	-1.256901199	0.213862759	-1.72885785	0.089321363
NAA40	3.100167385	0.002604621	-0.696854219	0.488697126	0.1179526	0.906525504
NAA50	-0.29897311	0.765672015	-0.24595045	0.806594955	-1.001398036	0.320923887
NAA60	-2.128215083	0.036141085	-0.693516756	0.490772763	-0.262918117	0.793574548
NAAA	-0.600685276	0.54960795	1.218629564	0.227952757	0.800544305	0.426764608
NAALAD2	0.240769756	0.810298829	-1.785174517	0.079498797	-1.585517124	0.118456538
NAALADL1	0.651896162	0.516182975	-1.273033882	0.208120449	-0.6097127	0.544507896
NAB1	-0.085513454	0.932049262	-1.076141123	0.28635125	-2.367467618	0.02137502
NAB2	3.139911957	0.002307287	-1.905043076	0.061771346	-1.330458834	0.188737178
NACA	0.33433824	0.738927238	0.766775447	0.446346632	1.597861694	0.115678003

NACC1	-1.44902488	0.150917805	-1.330272238	0.188672028	-0.832479831	0.408659553
NACC2	-1.781560263	0.078302994	0.119002585	0.905687398	-1.144153596	0.257406631
NADK	-3.989990905	0.000137221	1.095150255	0.278009292	0.563977106	0.575012052
NADKD1	0.81396794	0.417880801	0.958340164	0.341896888	0.862094465	0.392296852
NADSYN1	0.229523311	0.818999788	2.013193204	0.048776012	2.931100605	0.004875134
NAE1	0.338683314	0.735662592	-0.08214986	0.934812367	1.244843746	0.218351847
NAF1	2.700925149	0.008307089	-1.394287434	0.168586195	-0.498749037	0.619901498
NAGA	0.608731632	0.544285081	1.709244368	0.092789175	1.202850823	0.23407382
NAGK	-2.063097152	0.042077839	1.11872848	0.26790014	0.042336305	0.966380562
NAGLU	1.080621869	0.282845382	-0.679706081	0.499413363	-0.434758523	0.665401507
NAGPA	0.085069441	0.932401221	2.878386768	0.005600098	2.039456396	0.046113399
NAGS	0.504810738	0.614965318	-2.173205444	0.033891041	-0.736427002	0.464531868
NAIF1	-3.040292278	0.003120421	1.619975475	0.1107005	1.674755892	0.099535073
NAIP	-0.126854046	0.899348012	0.501668884	0.617812735	0.375236121	0.708896416
NAMPT	-1.261508765	0.210490265	-0.443259811	0.65923836	-1.623981828	0.109972272
NANOS1	-0.549405243	0.58413184	0.642289398	0.523233704	1.801902324	0.076921578
NANP	-0.790536572	0.431359485	-1.171910789	0.246057715	-1.998366362	0.05052048
NANS	-1.250559232	0.214441056	2.134533357	0.037066865	1.960976641	0.054840881
NAP1L1	0.958437917	0.340492201	2.220586139	0.030327384	2.107159527	0.039576081
NAP1L2	0.723362728	0.471393258	0.58913255	0.55807608	0.838387184	0.405362705
NAP1L3	0.324162814	0.74659115	0.685234755	0.495944447	0.699259432	0.487272116
NAP1L4	0.105398203	0.916301945	1.471963743	0.146474006	3.417363762	0.001181672
NAP1L5	0.146067213	0.884205511	-1.387458987	0.170646955	-1.680676958	0.09837256
NAPA	-2.901324489	0.004703277	0.607971705	0.545596191	-0.525357566	0.601399973
NAPB	0.781377475	0.436697119	-0.781574857	0.437665508	-2.554049798	0.013383888
NAPEPLD	3.707523409	0.00036722	-1.037041728	0.304050716	0.225568895	0.82235448
NAPG	1.392802416	0.167220984	-1.605572972	0.113837256	-1.472399417	0.146487863
NAPRT1	0.272587957	0.78581451	1.58511637	0.118415579	1.29529056	0.200512139
NAPSA	-1.160937825	0.248835865	0.639674636	0.524920426	0.840931802	0.403947617
NAPSB	1.37453131	0.172799409	0.349244288	0.728179561	-0.516932215	0.607230623
NARF	-0.833453059	0.40686671	0.902300509	0.370655238	0.68875134	0.493811523
NARFL	-0.589765365	0.556873258	0.696483356	0.488927533	1.572631534	0.121413913
NARG2	1.357967509	0.177977887	-0.231660022	0.817623154	-0.426825178	0.671136294
NARS	2.009051278	0.047624981	2.242930732	0.028764501	1.727514136	0.089564123
NARS2	1.937926096	0.055868489	-0.26594779	0.791229111	-1.15636313	0.252421446
NASP	-0.254302409	0.799860734	3.240714449	0.001983198	3.388774341	0.001288581
NAT1	0.11867776	0.905803658	-1.435603306	0.156524043	-1.440884332	0.155162299
NAT10	0.649819199	0.517517438	2.362658759	0.021543794	2.579301748	0.012541431
NAT14	0.001088934	0.999133644	-0.416622094	0.67850343	0.516069636	0.607829018
NAT6	-0.149225238	0.881720532	-2.961238991	0.004444736	-1.870976257	0.066554429
NAT8B	-3.876391785	0.000204944	0.190478024	0.849604348	0.859744616	0.393580104
NAT8L	0.967173033	0.336132716	0.025930318	0.979402551	0.748196854	0.457459082
NAT9	1.285114727	0.202155569	1.178336415	0.243507937	1.382481509	0.172289773
NAV1	1.521784055	0.131681832	0.341121332	0.734253533	0.5766938	0.566447617
NAV2	-0.429425755	0.668672739	1.079923504	0.284677709	0.665665872	0.508345923
NAV3	-2.965613276	0.003896345	-1.15999616	0.250836314	-1.257531465	0.21375838
NBAS	-0.079018077	0.937199319	3.000034201	0.003983697	3.477338966	0.000984109
NBEA	1.71653733	0.089614602	0.445674607	0.657503088	0.255560776	0.799222428
NBEAL1	0.227608653	0.820483382	0.105957957	0.915983884	-0.532094053	0.596756827
NBEAL2	-1.12239063	0.264777756	-1.189969272	0.238940433	-1.334305512	0.187481676
NBN	-0.673348098	0.502506858	-0.678525285	0.500155953	-1.711693017	0.092463651
NBPF1	1.111419841	0.26944308	0.159569132	0.873778759	0.556437802	0.580119139
NBPF10	-0.299832962	0.765018223	-0.584295671	0.561303083	-1.953005955	0.055801747
NBPF14	0.310719135	0.756755747	-0.420331924	0.675807058	-0.693428801	0.490894698
NBPF15	1.672825044	0.097946435	1.554760249	0.125481313	1.878257901	0.06553378
NBPF16	2.411066378	0.018005188	-0.539836366	0.591391021	1.163413836	0.249574289
NBPF3	1.979671874	0.050894758	0.957341495	0.342396222	2.698013138	0.009191319
NBPF9	3.094626192	0.002648798	0.132773103	0.894835608	-0.134785003	0.893263117
NBR1	-1.795435604	0.076048932	1.573584025	0.121061215	0.622668559	0.536019201
NBR2	1.174113418	0.243546657	-0.970981981	0.335617341	-1.263231546	0.211718182
NCALD	0.002740642	0.997819548	-1.404558134	0.165522761	0.165151032	0.869417345
NCAM1	1.023746327	0.308787161	-1.836575143	0.071434754	-1.216904907	0.228722766
NCAPD2	-0.183126656	0.855123815	1.540509123	0.128912783	1.954825504	0.055581141
NCAPD3	-0.296871226	0.767270906	1.067368916	0.290258784	0.961822589	0.340256225
NCAPG	1.074873874	0.285396776	0.955830804	0.343152476	0.737299567	0.464005388
NCAPG2	1.638946316	0.104828138	1.731393079	0.088735403	0.59682527	0.55301929
NCAPH	0.379588597	0.70517381	0.872570683	0.386520292	0.945540257	0.34842816
NCAPH2	-0.491247618	0.624485645	2.713605148	0.008764098	1.740871073	0.087175104
NCBP1	2.829623858	0.005782977	-0.088945892	0.929433472	0.623228325	0.535653988
NCBP2	0.730900466	0.466799595	1.48217201	0.143745457	1.712869166	0.092245468
NCDN	1.20902633	0.229921159	0.776890514	0.44040244	1.097838753	0.27695327
NCEH1	-0.140311693	0.888737367	0.54012094	0.591196037	0.010073454	0.991998333

NCF1	-3.121091921	0.002443905	-0.83214295	0.408765265	-0.851557909	0.398071214
NCF1B	-2.180370898	0.031923783	-3.077878049	0.003189903	-1.844311542	0.070407397
NCF1C	-3.367486554	0.001130588	-0.749657908	0.456511932	-1.120928274	0.267082332
NCF2	-2.844249334	0.005545825	0.385543063	0.7012554	0.063877916	0.949293953
NCF4	-1.520576601	0.13198449	0.516891116	0.607211402	0.28631203	0.775691264
NCK1	1.174493336	0.243395344	-0.606248762	0.546731618	-0.786024367	0.435152729
NCK2	-0.462089714	0.645167981	-2.588790739	0.012173154	-3.523433703	0.000854052
NCKAP1	-1.212943591	0.228427468	0.062492465	0.950387026	-2.058996714	0.044136925
NCKAP1L	-0.168035305	0.866944671	2.047895459	0.045139083	1.632564607	0.108148046
NCKAP5L	0.182062096	0.85595662	0.996921647	0.322972493	0.69159764	0.492035461
NCKIPSD	-0.02097235	0.983315633	-0.121827346	0.903459816	0.396708836	0.693083638
NCL	-0.181440434	0.856443021	3.1326044	0.002723211	3.116996542	0.002877122
NCLN	0.783860811	0.435246097	0.946776422	0.347708046	1.71530236	0.091795442
NCOA1	-1.717955778	0.089354272	-0.50202444	0.617564167	-1.488629061	0.142171748
NCOA2	-0.943024941	0.348273903	-0.602322853	0.549323285	-2.784946922	0.007281924
NCOA3	0.435451401	0.664310907	0.206795225	0.836899122	-0.216124536	0.829672707
NCOA4	-4.988298794	3.08E-06	0.511733947	0.610793714	-0.343926748	0.732184031
NCOA5	0.395986719	0.693082196	0.863365122	0.391517783	1.384387589	0.171708738
NCOA6	-1.224657698	0.224002735	0.472587564	0.638293347	0.229595495	0.819239136
NCOA7	-0.266305292	0.790632863	-0.552307483	0.582874698	-2.141491322	0.036580968
NCOR1	0.260130197	0.795376665	0.462925228	0.645162317	0.088791514	0.929563054
NCOR2	-0.227558199	0.820522485	-1.148109359	0.25566963	-0.797620226	0.428446014
NCR1	0.222799675	0.824212542	0.487161666	0.627992745	1.022354011	0.310991261
NCR3	1.424044551	0.158002714	0.368881245	0.713568795	0.231203496	0.817995854
NCRNA00085	-1.771310897	0.080003306	-2.211740646	0.030966472	-2.366113052	0.021446082
NCRNA00086	1.984480536	0.050346834	0.283929499	0.777482611	-0.005190333	0.995877101
NCRNA00092	0.292100874	0.770903417	-0.610249146	0.544097192	0.077771462	0.9382859
NCRNA00094	1.31338397	0.192499433	0.102811837	0.918469438	0.573791139	0.568396957
NCRNA00115	3.025765987	0.0032591	-1.421185548	0.160654676	-1.101606807	0.275325194
NCRNA00116	1.139281276	0.257706251	-0.784952187	0.435698473	-0.141421635	0.888042257
NCRNA00152	-0.916712263	0.361822144	-0.396041715	0.693537602	-0.414394379	0.680161735
NCRNA00173	1.977722618	0.051118309	-1.968190581	0.053866205	-0.951176988	0.34558476
NCRNA00174	-0.043100032	0.965720345	-0.905289463	0.369083476	-0.240024928	0.811183704
NCRNA00176	1.04162744	0.300464162	0.470423605	0.63982897	0.84275987	0.402932886
NCRNA00188	-0.152526999	0.879123716	-0.226416819	0.82167887	0.511285742	0.611152634
NCRNA00189	-2.392537663	0.01887958	1.984203885	0.052005065	1.32420535	0.190791799
NCRNA00201	3.271893074	0.001531624	-0.669583641	0.505798717	-0.008039635	0.993613821
NCRNA00202	1.377584116	0.171857619	1.138608773	0.259580163	1.180223981	0.242879314
NCRNA00219	1.06535682	0.289655873	0.078664304	0.937572301	0.328953758	0.743412567
NCRNA00239	1.245444986	0.216304895	-1.118678877	0.267921132	-1.63113991	0.108449145
NCRNA00241	-0.584740037	0.560232667	-1.798934649	0.077268868	-0.670177565	0.5054874
NCRNA00247	-0.097721622	0.922377645	-1.011566203	0.315976279	-1.64356043	0.10584696
NCRNA00256A	-1.128012515	0.262409122	0.5040546	0.616145747	0.484306209	0.630049434
NCRNA00263	1.510844592	0.134443962	-1.595944351	0.115974075	-1.026947936	0.308841977
NCRNA00264	-0.792690316	0.43010996	1.296879289	0.199844122	2.484447025	0.015978292
NCRNA00265	-0.261788816	0.794101725	-0.050728098	0.959717704	0.137562684	0.891077398
NCRNA00282	0.872045425	0.385578692	-1.440495917	0.155141265	-0.983293825	0.329674421
NCRNA00287	2.847917502	0.005487763	0.952073638	0.345038064	1.901490736	0.062365462
NCRNA00292	-0.525706374	0.600426762	-0.791556264	0.431867261	0.171222612	0.86466333
NCRNA00294	-0.100051548	0.920533099	-1.065083996	0.291282608	0.228075025	0.820415168
NCRNA00299	2.394859776	0.018767964	-0.285013427	0.77665621	1.294943778	0.20063093
NCRNA00304	-0.287303331	0.774561795	0.358710447	0.721123268	0.295408841	0.768769543
NCRNA00321	-0.355966227	0.722725716	-0.036693875	0.970855974	-0.386448938	0.700622555
NCRNA00324	-0.02468958	0.980358986	-0.593406981	0.555232052	-0.966460618	0.337951709
NCRNA00338	-1.064019803	0.290257688	-0.390820147	0.697372079	0.909533047	0.366950557
NCRNA00339	0.443176615	0.658735689	0.39635529	0.693307582	1.161364594	0.250399411
NCRNA00341	2.573501869	0.011757013	3.201096348	0.002229149	2.944684091	0.004693828
NCRNA00346	-0.808601056	0.420945571	0.104424696	0.917195115	0.345163248	0.731259342
NCS1	1.120812311	0.265445426	-0.881694103	0.381607003	0.085916893	0.931837648
NCSTN	-1.530892759	0.129416258	-0.457680239	0.648904091	-0.766205559	0.446758309
NDC80	-0.545757122	0.586626547	1.695491305	0.095382256	0.264072028	0.792689744
NDE1	-1.144333828	0.255617059	-0.669606069	0.505784521	-0.574546204	0.567889561
NDEL1	-2.852366561	0.005418089	-0.435829076	0.664589871	-0.811291307	0.420618885
NDFIP1	2.25923844	0.026362055	0.58889423	0.558234862	-0.221660568	0.825381072
NDFIP2	1.291154402	0.200062949	-1.641491432	0.106145331	-1.476268087	0.145449795
NDN	-0.763494812	0.447229404	1.192390548	0.237997597	0.869827181	0.388092443
NDNL2	1.022475893	0.309384346	-0.318223951	0.751466066	-1.103669384	0.274436855
NDOR1	-0.219633159	0.826670254	0.315754597	0.753330124	1.01280803	0.315489718
NDRG1	-2.330544935	0.022085957	0.628306287	0.53228696	0.739761046	0.462522042
NDRG2	1.944486846	0.055060549	1.398229036	0.167405412	2.924149184	0.004970415
NDRG3	-1.165824576	0.246864674	0.236604606	0.813803007	0.941851058	0.350297387
NDRG4	1.53275366	0.128957213	0.172950578	0.863296624	0.88146421	0.38181856

NDST1	-3.424355528	0.000941262	0.907650347	0.367845001	0.430536543	0.66845098
NDST2	0.362751135	0.717668688	-1.100474394	0.275703585	-0.931085193	0.35578945
NDUFA1	-0.510416353	0.611049643	-1.308878358	0.195773894	-0.380767645	0.70481026
NDUFA10	0.11707711	0.907068219	2.000875855	0.05012629	3.60035417	0.000672701
NDUFA11	-0.798260823	0.426888086	-1.669101526	0.100524655	-0.906339313	0.368623352
NDUFA12	-0.317480748	0.751637898	-2.52545136	0.014329903	-1.784900649	0.079671172
NDUFA13	-0.879544472	0.381523872	-0.873600699	0.385963616	0.002383723	0.998106502
NDUFA2	-1.297681884	0.197819453	-2.01745127	0.04831657	-0.725065974	0.471417859
NDUFA3	-1.436911423	0.154322049	-3.852266215	0.000296566	-2.621417586	0.011243011
NDUFA4	0.669810123	0.504748902	-1.71504856	0.091712381	-1.155985663	0.252574524
NDUFA5	0.67435398	0.501870402	-1.205576174	0.232910457	-1.128745355	0.263797441
NDUFA6	0.678851948	0.4990297	-2.222656615	0.030179481	-1.098180992	0.27680512
NDUFA7	-1.293662243	0.199198778	-3.203618914	0.00221267	-2.843002667	0.006218044
NDUFA8	-0.070149403	0.944235392	-0.220426966	0.82631812	0.629598198	0.531507135
NDUFA9	-0.771620883	0.442425308	2.737617441	0.008218468	3.612545543	0.00064757
NDUFAB1	1.692585304	0.094105108	-0.597870162	0.552270212	0.693498129	0.490851537
NDUFAF1	-0.387892875	0.699040715	1.738887014	0.087397424	1.451968296	0.152066893
NDUFAF2	0.568771479	0.570973417	-1.575996533	0.120503871	-1.272706496	0.2083589
NDUFAF3	-0.017138389	0.986365371	-3.680946106	0.000513736	-3.070843004	0.003285269
NDUFAF4	2.109915686	0.037731241	-0.347032563	0.729831671	-0.265060171	0.791932267
NDUFB1	-0.002630158	0.997907449	-1.458373396	0.150169448	-0.911024351	0.366171118
NDUFB10	0.112876879	0.910387655	-3.508953415	0.000880363	-2.215059543	0.03082299
NDUFB11	0.423666351	0.672852509	2.018453122	0.048209015	2.353417802	0.022122418
NDUFB2	0.221838649	0.824958265	-0.361635161	0.718948003	-0.033155462	0.973668013
NDUFB3	0.161610747	0.871986307	-0.847618959	0.400158809	-0.42474924	0.6726402
NDUFB4	-0.539739645	0.590752439	-3.877209112	0.000273486	-2.607256912	0.011665235
NDUFB5	0.521255336	0.603510393	1.038993425	0.303149907	1.046907144	0.299621269
NDUFB6	-2.300275299	0.023819945	-2.279502902	0.026359709	-2.022846811	0.047853158
NDUFB7	0.242162125	0.809223238	-1.00544329	0.318888893	-0.455522449	0.650487414
NDUFB8	1.142901094	0.256208263	0.479413617	0.633459781	1.295329881	0.200498673
NDUFB9	0.015150138	0.987947015	0.960670181	0.340733739	1.778861866	0.080667334
NDUFC1	-1.269861623	0.207512618	-2.975579219	0.004268823	-2.279826983	0.02643116
NDUFC2	-0.508610254	0.612310025	1.233819385	0.2222813	2.53930502	0.013899237
NDUFS1	0.481892719	0.631089596	1.459510146	0.149857576	1.732286753	0.088704351
NDUFS2	0.09218103	0.926765682	2.634341747	0.010809333	3.167027643	0.00248862
NDUFS3	2.004558975	0.048113009	1.038622383	0.303321022	2.01898585	0.048265592
NDUFS4	0.637042099	0.525766602	0.230688864	0.818373988	0.046638991	0.962966185
NDUFS5	0.858211784	0.393128653	-0.743658116	0.460106297	0.409268881	0.683896966
NDUFS6	-1.614417802	0.110050249	-1.455962726	0.150832504	-0.849161112	0.39939204
NDUFS7	-1.619133908	0.109030269	-1.841490101	0.070701022	-1.725303297	0.089964727
NDUFS8	-0.927872615	0.356035205	2.543760863	0.013673377	3.24255105	0.001994347
NDUFV1	-0.67084638	0.504091663	1.454626358	0.151201058	2.048451193	0.045194276
NDUFV2	-0.103524937	0.917784114	-1.708738742	0.09288347	-0.972291439	0.335069166
NDUFV3	-0.943271439	0.348148553	-0.069183282	0.945083364	1.489060802	0.142058314
NEAT1	2.41172636	0.017974719	-0.763837523	0.448081871	-1.02093981	0.311654935
NEB	-0.27738932	0.782137816	-2.359412069	0.021715983	-2.735002085	0.008328784
NEBL	-0.717323466	0.475091944	0.005458982	0.995663248	0.928344327	0.357196526
NECAB1	-0.828270277	0.409778982	-0.412142254	0.681765096	-1.261831356	0.212218
NECAB2	-1.157947912	0.250047428	0.929542617	0.356487501	0.106070824	0.915903622
NECAB3	0.89346946	0.374065396	0.700807159	0.486245005	1.028382197	0.308173023
NECAP1	0.04032639	0.967925114	-2.913031365	0.005086726	-3.263284764	0.001875784
NECAP2	-0.555608135	0.579901666	2.437350175	0.01790516	2.467925942	0.016657283
NEDD1	0.969197811	0.335127438	-0.040550621	0.96779439	-0.235433447	0.814727575
NEDD4	1.554449142	0.123699863	-1.817133413	0.074400174	-1.379973775	0.173056512
NEDD4L	-2.462356346	0.015769995	2.788997446	0.007154227	2.53559471	0.014031738
NEDD8	-2.121117457	0.036750812	-1.646228095	0.10516329	-1.379640082	0.173158736
NEDD9	-3.064534807	0.002901111	-1.24454059	0.218341177	-2.67602433	0.009742115
NEFH	0.280493266	0.779763564	1.861479383	0.067781946	1.775206049	0.081275432
NEFL	1.223125434	0.224577943	2.885206098	0.005495403	2.106790132	0.039609437
NEFM	0.676660193	0.500412821	2.663390258	0.010013948	0.447768232	0.656040649
NEGR1	0.131928104	0.895345129	0.122515452	0.902917299	-0.006140215	0.995122578
NEIL1	1.289761484	0.200544133	-0.91246602	0.365327034	0.847551401	0.400280638
NEIL2	0.770653903	0.442995403	2.735020069	0.008275947	3.955789879	0.000216025
NEIL3	-1.413686477	0.161014563	2.007354725	0.049412093	2.401613457	0.019651884
NEK1	0.33007667	0.742133802	0.50890985	0.612759477	-0.081100674	0.935649834
NEK11	0.446529498	0.656321911	0.954199067	0.343970555	1.541538548	0.128794441
NEK2	1.490148205	0.139794088	1.26040911	0.212604267	1.638782932	0.106841798
NEK3	1.380835654	0.170858823	1.703438714	0.093876636	2.14350445	0.036411665
NEK4	1.713949426	0.090091163	1.094370032	0.278348309	0.870163898	0.387910007
NEK5	-0.269253192	0.788371009	1.181400102	0.242298953	0.944147622	0.349133008
NEK6	-1.05972812	0.292195234	-0.18252258	0.855813563	-2.120955719	0.038347722
NEK7	0.321288912	0.748760375	-0.426646731	0.671227156	-1.767374663	0.082590944

NEK8	-1.177687623	0.242125785	0.419512235	0.676402457	1.049699536	0.298346435
NEK9	1.787782719	0.077285399	2.10473765	0.039688186	1.943439903	0.056973911
NELF	-0.156508659	0.875993925	-2.64570559	0.010491553	-0.36545577	0.716142222
NELL2	5.498909986	3.76E-07	2.349828697	0.022231428	3.501651346	0.000913329
NEMF	1.360920683	0.177046102	0.843998642	0.402162047	-0.391382558	0.696993522
NENF	-0.646127886	0.519893609	0.910391598	0.366410328	0.724449068	0.471793418
NEO1	1.307737266	0.194400139	-0.917425951	0.362745214	0.042403139	0.966327521
NET1	2.734193587	0.007572855	1.828482092	0.072656879	2.570118706	0.012842054
NETO1	0.220253183	0.826188882	0.700257081	0.486585825	-0.54196377	0.589984515
NETO2	-1.444570581	0.152162766	-0.525789477	0.601053101	-0.827565409	0.411414707
NEU1	-1.802447727	0.074930448	1.207818824	0.232053155	0.702474948	0.485280669
NEU3	0.737147117	0.46301197	-0.88589464	0.379358142	-0.055379115	0.956032715
NEURL	1.380662391	0.170911933	-0.53524919	0.594538227	-1.218723851	0.228036797
NEURL1B	-1.251101741	0.214244037	0.363152528	0.717820375	-0.181406506	0.856700765
NEURL2	2.69296288	0.008492163	-1.180377315	0.24270208	-0.930581551	0.356047735
NEURL4	1.126165608	0.263185621	0.488680504	0.626923488	1.473007895	0.146324208
NEXN	-3.537964738	0.000648881	1.432113838	0.157516103	0.397804445	0.692280416
NF1	0.777149394	0.439174113	-0.914468147	0.364283442	-1.400184435	0.166951156
NF2	-0.563945544	0.574238948	1.484937112	0.143013304	1.638762234	0.106846124
NFAM1	-3.276887758	0.001507732	-0.465842033	0.643085443	-0.365718962	0.715946888
NFAT5	-0.252423099	0.801308169	-2.77578142	0.00741513	-2.383153586	0.020567328
NFATC1	-0.44766867	0.655502637	0.837965685	0.405513959	0.032203456	0.974423824
NFATC2	0.732098172	0.46607202	-1.326387204	0.189947003	-1.465459	0.148364739
NFATC2IP	3.666116581	0.000422638	-0.054950072	0.956368408	0.981739555	0.330432995
NFATC3	-1.266525289	0.208698213	1.889484569	0.063863904	1.898317099	0.062790437
NFE2	-2.601975228	0.010889705	1.2864464	0.203434345	0.679780912	0.499431954
NFE2L1	-0.916125889	0.362127843	1.78184967	0.080045594	1.418759217	0.161487811
NFE2L2	-0.72183248	0.472328912	0.590659416	0.557059332	-0.672401761	0.504081397
NFE2L3	1.763689157	0.081287401	0.258956354	0.796592172	-1.550549626	0.126619536
NFIA	2.218699837	0.029104408	0.258048927	0.797288979	1.196719438	0.236436656
NFIC	0.177259781	0.859715486	-1.906919828	0.06152291	-2.057440094	0.044291639
NFIL3	-3.866890291	0.000211869	-0.710568259	0.480219269	-1.448930541	0.152910403
NFIX	-2.628919577	0.010122677	-0.592508074	0.555829543	-0.738907853	0.463035889
NFKB1	-0.139588971	0.889306698	1.204047691	0.233496072	0.61602113	0.540366047
NFKB2	-0.368154428	0.713650397	0.767590541	0.445865906	0.464431199	0.644131877
NFKBIA	2.22065393	0.028966699	-0.710519791	0.480249086	-1.442502086	0.154707473
NFKBIB	-0.731239771	0.466593411	-1.660812982	0.102185828	-1.233215136	0.22262547
NFKBID	2.056508118	0.042722908	-1.425855895	0.159307481	-0.955207616	0.343560883
NFKBIE	0.688157439	0.49318049	2.021780857	0.047853241	1.658155332	0.102854452
NFKBIL1	-0.297775413	0.766582972	2.325889987	0.023566974	2.364489389	0.021531539
NFKBIZ	0.482351326	0.630765144	-0.873297837	0.386127247	-2.153500076	0.03558115
NFRKB	1.115335944	0.267771215	1.115830531	0.269128478	2.062134722	0.043826459
NFS1	1.001790824	0.31921684	0.436984974	0.663756247	1.519525624	0.134232859
NFU1	1.073986514	0.285792059	0.62897081	0.531854884	0.845224151	0.401567488
NFX1	2.421507622	0.017528513	0.932764482	0.35483537	1.673801614	0.09972348
NFXL1	-0.984404499	0.327640531	1.071051256	0.288614042	0.47144981	0.63914349
NFYA	-1.749732057	0.083682964	-0.006388746	0.99492463	-0.399488548	0.691046444
NFYB	0.263761317	0.792586239	-0.58766136	0.559056628	-0.764465739	0.447785686
NFYC	-2.223751607	0.028749568	-1.363189057	0.178128297	-1.576205696	0.120587723
NGDN	0.86987082	0.386759516	-0.27409351	0.784993395	1.495052395	0.140491472
NGFR	0.251755766	0.801822314	-0.779679357	0.438771785	0.329189748	0.74323515
NGFRAP1	-1.435087684	0.154839676	0.878175446	0.383497249	1.128934297	0.263718399
NGLY1	1.053465283	0.295038515	0.697020211	0.488594019	1.216755008	0.228779364
NGRN	0.565412743	0.573245198	0.164447928	0.869954298	0.866533517	0.389879807
NHEJ1	-1.563325472	0.121598578	1.212080658	0.230430316	1.225605258	0.225455263
NHLH1	0.02190168	0.98257643	0.027765017	0.977945548	-0.259803624	0.795964058
NHLRC1	0.141046076	0.88815891	0.271471577	0.786999015	0.277434933	0.782463496
NHLRC2	0.930215605	0.354827883	-1.979043304	0.052598732	-3.481429644	0.000971846
NHLRC3	1.645400131	0.103487895	1.642309259	0.105975242	1.278093258	0.206466834
NHLRC4	1.349233471	0.180755383	1.23910882	0.22033091	1.32819652	0.189478532
NHP2	-1.1063502	0.271618216	-0.832679052	0.408465252	0.786872694	0.434659981
NHP2L1	-0.9078655	0.36645179	-0.145274057	0.885001695	1.421564644	0.160674845
NHS	-1.880829293	0.063333091	0.017847522	0.985822176	-0.807936974	0.422531325
NHSL1	0.219671691	0.826640337	1.988285604	0.051539591	1.115022354	0.269583198
NHSL2	-3.096803405	0.002631358	-0.876325972	0.384493155	-1.661185154	0.102241965
NICN1	0.898957819	0.37115111	-0.952622919	0.344761977	0.851639684	0.398026197
NID1	2.618521916	0.010412675	0.512196043	0.610472337	-0.056295372	0.955306044
NID2	-0.902304368	0.369381167	-2.177026018	0.033590635	-1.640868031	0.106406677
NIF3L1	2.864190445	0.005236853	0.095226623	0.924465361	1.016323322	0.313828089
NIN	-0.181565062	0.856345505	0.497697691	0.620592037	-1.031579688	0.306685226
NINJ1	-4.35532871	3.61E-05	-0.705050243	0.483620515	-0.518593995	0.606078559
NINJ2	-4.563917063	1.64E-05	-1.643458984	0.105736501	-1.893572071	0.06343042

NINL	0.176118388	0.860609356	-1.256687413	0.213939634	-0.547955903	0.585890704
NIP7	1.280587073	0.203734918	-0.023941384	0.980982118	-0.265636131	0.791490849
NIPA1	0.677593377	0.499823676	-1.245777356	0.21788999	-1.770295457	0.082098253
NIPA2	-1.150758683	0.252977762	-0.584592138	0.561105023	-0.343287379	0.732662324
NIPAL2	1.40802276	0.162679974	-1.979632203	0.052530693	-2.31482161	0.024297079
NIPAL3	0.775589633	0.440089957	1.451253138	0.152134482	2.12157829	0.038293084
NIPAL4	0.716470274	0.475615776	0.53083211	0.597576136	0.049808069	0.96045182
NIPBL	-1.223132948	0.22457512	0.609018789	0.544906745	-0.396142363	0.693499075
NIPSNAP1	0.828678198	0.40954931	1.10818132	0.272389744	1.426992577	0.159110941
NIPSNAP3A	0.969856709	0.334800728	-0.355361642	0.723616798	-0.262785047	0.793676602
NIPSNAP3B	0.790394053	0.431442245	-1.180577231	0.242623246	-0.47765694	0.634745767
NISCH	1.972995652	0.051663899	-0.875681279	0.38484069	0.005147134	0.995911414
NIT1	-1.065601979	0.289545615	1.436225227	0.156347742	1.207832176	0.232166855
NIT2	2.478467367	0.015121006	-1.356700129	0.180170391	0.081415225	0.93540081
NKAP	-0.462836757	0.644634495	2.547469951	0.013543748	2.375099721	0.020978555
NKAPP1	1.506316328	0.135600565	-0.859086684	0.393854089	-1.851899781	0.069292244
NKD1	1.65547257	0.101423903	-1.305238568	0.197001913	-0.503360128	0.616677083
NKG7	0.405103093	0.686393982	-2.662229275	0.010044682	-1.621013429	0.110608971
NKIRAS1	-1.546586286	0.125585218	-1.804474517	0.076385977	-0.534253227	0.595272163
NKIRAS2	-1.679380556	0.09665818	0.093243048	0.926034068	0.005406487	0.995705402
NKPD1	0.767621271	0.444786098	0.530332138	0.597920454	0.091182092	0.927671919
NKRF	2.060988217	0.042283386	1.193864581	0.237424935	1.72134678	0.090685346
NKTR	2.115535354	0.037236588	-1.805266034	0.076260524	-2.307907199	0.024706167
NKX3-1	-2.375454508	0.019719015	0.638200366	0.525872702	0.094129242	0.925341068
NLE1	1.951860529	0.054164345	0.249831846	0.803606307	0.406848017	0.685663954
NLGN2	0.82044945	0.4141974	0.40343629	0.68812114	0.770211164	0.444398209
NLGN3	-0.397826114	0.691730745	-0.6467664	0.520352335	0.669310624	0.506036002
NLK	-1.413487526	0.161072841	-0.241095911	0.810336993	-1.667825632	0.100909995
NLN	1.565328777	0.121128287	2.627882135	0.01099386	2.29991603	0.025186583
NLRC3	2.031486059	0.045250632	-0.194447815	0.846509482	0.540347001	0.591091402
NLRC4	-2.804475768	0.006212602	2.377492738	0.020772492	1.257155382	0.2138935
NLRC5	0.554095518	0.580931874	-0.118136471	0.906370562	-0.327436706	0.744553414
NLRP1	1.654380296	0.101646102	-0.066034665	0.947578907	0.320904694	0.749472138
NLRP12	-2.227297242	0.028502789	1.935305611	0.057867666	2.185952063	0.032998105
NLRP2	3.731011269	0.000338931	0.40294339	0.688481679	1.654186775	0.103661236
NLRP3	-1.588318272	0.11583435	2.186601179	0.032848002	1.402812379	0.166169654
NLRP6	0.60535368	0.546516504	-1.976372246	0.052908284	-3.480447664	0.000974776
NLRP7	1.534467597	0.12853556	0.718035303	0.475638044	2.172609335	0.034039489
NLRP9	1.210887011	0.229210784	0.697364856	0.48837998	0.505000122	0.615532101
NLRX1	-1.686614983	0.095252545	0.283831583	0.777557276	1.440088082	0.155386545
NMB	0.135324259	0.892667436	-3.551136534	0.000772381	-1.766125208	0.082802457
NMD3	0.94443027	0.347559652	0.315041231	0.753868904	0.498479876	0.620089948
NME1	1.075758924	0.285002896	0.654317053	0.515511864	1.843671433	0.070502153
NME2	0.088134683	0.929971748	-5.143380477	3.38E-06	-3.51111957	0.000887097
NME3	1.109856623	0.27011248	-0.952749173	0.344698538	-0.771531989	0.443621585
NME4	-0.391732293	0.696211837	-0.724114196	0.471926719	-0.863679548	0.391432712
NME6	-1.543494922	0.126332674	-0.601983588	0.549547541	-0.75308852	0.454537922
NME7	1.273652375	0.206171582	0.269139788	0.788783912	0.7665732	0.44654139
NMI	-2.416496259	0.017755875	0.488525498	0.627032575	-0.727519969	0.4699256
NMNAT1	-4.193539643	6.57E-05	1.877279527	0.065547203	2.582461641	0.012439475
NMNAT3	-1.127861252	0.262472658	1.362690934	0.178284429	0.847341424	0.40039664
NMRAL1	0.420859715	0.67489311	1.879997208	0.065169171	2.591945741	0.012137974
NMT1	-0.511688035	0.610162905	0.718651693	0.475260974	1.20117869	0.234716495
NMT2	1.654285806	0.101665342	0.515076976	0.608470456	1.114380615	0.269855934
NMUR1	1.46433832	0.146697935	-2.860119812	0.005889709	-1.152713717	0.253904218
NNAT	1.076396885	0.284719212	-0.65771452	0.513341711	-1.231010991	0.2234424
NNT	0.37674378	0.707279355	1.501495522	0.138690189	1.253947007	0.215048784
NOB1	0.328038847	0.74366875	3.433892799	0.00110889	3.659562324	0.000558793
NOC2L	0.139408882	0.889448574	2.312108714	0.024367827	2.452058106	0.017333805
NOC3L	1.24555084	0.216266197	-0.585762555	0.560323449	-0.418262528	0.677348124
NOC4L	-0.223787434	0.823446244	-0.626816444	0.533256324	-0.863645311	0.391451365
NOD1	-1.299833515	0.197084056	1.005924028	0.31865956	1.609288254	0.11315321
NOD2	-1.730552819	0.087069407	-0.359730599	0.720364263	-0.263840001	0.792867637
NODAL	0.652070469	0.516071064	-0.042445232	0.966290573	0.45430762	0.651356117
NOG	2.390481086	0.018978924	-1.55323369	0.125845357	-0.622684749	0.536008635
NOL10	0.623227419	0.534761993	4.152923892	0.000109873	4.49507033	3.52E-05
NOL11	1.595739845	0.114165391	0.989372033	0.326619401	1.874051498	0.066121752
NOL12	0.808178191	0.421187619	0.364774915	0.716615399	0.992473231	0.325217972
NOL3	1.062450132	0.290965318	2.077475845	0.042226545	1.099481492	0.27624266
NOL6	1.727289907	0.087656585	1.803080069	0.076607412	2.53585056	0.014022546
NOL7	0.290735771	0.771943856	-2.078113855	0.042165566	-1.005602833	0.31891403
NOL8	0.766065046	0.445706639	1.416475515	0.162022249	2.071926731	0.042869808

NOL9	0.473658147	0.636927593	1.473986939	0.145930023	2.540693534	0.01384995
NOLC1	1.634094403	0.105844921	1.98497515	0.051916835	1.938925529	0.057534339
NOM1	1.719806525	0.089015535	1.302898017	0.197794637	1.966614649	0.054169805
NOMO1	-1.085951935	0.280493587	0.622363838	0.536158856	2.662170525	0.010104479
NOMO2	1.413776015	0.16098834	1.805385864	0.076241547	0.757010396	0.452203707
NOMO3	-0.683836743	0.495891721	1.173531745	0.245412693	0.801827735	0.426027858
NONO	-0.003655399	0.997091771	1.184754445	0.240980247	1.897402769	0.062913329
NOP10	-3.185450278	0.0020056	-3.251845725	0.001918836	-3.077909761	0.003219447
NOP14	2.628972856	0.01012121	2.856162089	0.005954255	2.92444862	0.004966276
NOP16	1.303971723	0.195675402	1.303662903	0.197535314	1.74748634	0.086011594
NOP2	2.12779213	0.036177172	0.760035265	0.450333442	2.498341273	0.015426711
NOP56	0.775357315	0.440226463	1.375410855	0.174330115	2.648938348	0.010462048
NOP58	1.630499555	0.10660339	0.197329505	0.844264422	0.842725633	0.402951876
NOS3	0.870979373	0.386157285	0.925679739	0.35847488	0.878183735	0.383580667
NOSIP	0.17197089	0.863858947	-0.161161617	0.872530082	0.127300269	0.899156847
NOTCH1	-0.985516429	0.327097445	-1.596601244	0.115827274	-1.749470206	0.085665188
NOTCH2	-0.713216068	0.477616708	0.535981173	0.594035497	-0.10544577	0.916397314
NOTCH2NL	-0.60425747	0.547241633	-1.564443984	0.123191569	-2.880013345	0.005616842
NOTCH3	-0.217512032	0.828317549	0.976883325	0.332712229	0.734855789	0.465480753
NOTCH4	3.083681475	0.002738109	1.417923195	0.161600954	0.981916706	0.330346477
NOV	-2.61089374	0.01063019	-1.157481107	0.251853467	-1.574460575	0.120990552
NOXA1	1.154189818	0.251576207	-0.859438078	0.393661879	0.861754452	0.392482372
NPAS2	0.871394051	0.385932157	-0.975745486	0.333271064	-0.618242727	0.538911301
NPAT	0.818695488	0.415192232	-1.46713627	0.147778416	-1.567093771	0.122702972
NPC1	0.949881352	0.344798144	-1.621191619	0.110438871	-1.334613491	0.187381431
NPC1L1	-1.80583016	0.074395831	-0.294723952	0.769264458	-0.025744372	0.979552369
NPC2	-3.307736651	0.001367738	0.954260361	0.343939802	0.562402751	0.576076707
NPDC1	-0.078964338	0.937241939	0.396158078	0.693452242	-0.383446401	0.702834583
NPEPL1	1.117604341	0.266806114	-3.32833199	0.001527033	-3.669547405	0.000541499
NPEPPS	-0.892682759	0.374484306	1.090529512	0.280021273	0.598863609	0.551668639
NPFF	1.245003817	0.216466229	1.002982634	0.32006447	2.867638543	0.005811594
NPHP3	1.765539961	0.080974027	-0.486127432	0.628721301	-0.542708149	0.589475221
NPHP3-ACAD1:	2.346957028	0.021193285	-1.846528611	0.069955439	-1.203753777	0.233727307
NPHP4	1.069331788	0.287871718	0.277433814	0.782440387	-0.631413971	0.530328116
NPIP	-0.1485613	0.882242872	-3.571808116	0.00072419	-1.144973143	0.257069827
NPIPL3	0.136345947	0.891862131	2.293063091	0.025514414	2.862200559	0.005899137
NPL	-3.749441429	0.000318198	0.568553419	0.571869475	0.37919258	0.705972882
NPLOC4	-0.442175493	0.659457111	1.815440185	0.074663258	2.472653135	0.016460393
NPM1	1.658441341	0.100821962	1.472929679	0.146214094	2.110175296	0.039304678
NPM3	0.25789063	0.797099048	-0.716318334	0.476689266	-0.345822499	0.730766501
NPPA	-0.375025808	0.708551989	0.553297259	0.582201319	1.282680524	0.204865709
NPPA-AS1	0.112951227	0.910328884	0.057041591	0.954709493	1.160280719	0.250836621
NPR2	2.738529491	0.00748165	-1.792884907	0.078242748	-0.18473369	0.854102513
NPR3	-0.946469257	0.346525034	-0.026713227	0.978780804	0.067133306	0.94671369
NPRL2	1.546127693	0.125695878	-0.993124469	0.324803335	-0.397411017	0.692568808
NPRL3	-2.731763903	0.007624405	0.879771025	0.382639366	0.763254115	0.448501973
NPTN	-1.969858895	0.052028676	-1.890682317	0.063700704	-3.071568411	0.003278455
NPTX1	-0.383799639	0.702061293	-0.970206532	0.336000321	-0.256315682	0.79864242
NPTXR	0.868621001	0.387439191	1.091399768	0.279641568	1.755454152	0.084627327
NPY6R	1.327852438	0.187692749	1.607501108	0.113413204	-0.411589473	0.682204832
NQO1	-0.483799979	0.629740737	0.865268237	0.390481337	0.401199603	0.689793587
NQO2	-2.324107051	0.022445106	-0.313151661	0.755296619	-0.537353655	0.593143317
NR1D1	1.081984746	0.282242747	-0.623560795	0.535377791	0.340725007	0.734580231
NR1D2	2.400010229	0.018522494	-0.457356206	0.649135555	-0.511555672	0.61096488
NR1H2	-1.66450511	0.099601483	-0.244409329	0.807782414	-0.773806448	0.442286106
NR1H3	-1.787110878	0.077394739	0.494419622	0.622890443	0.639407636	0.525153929
NR2C1	2.625604767	0.010214326	-1.123939035	0.265701527	-0.116841498	0.907401935
NR2C2	1.343226328	0.182684625	-1.02699298	0.308717589	-1.569906364	0.122046902
NR2C2AP	1.998184462	0.048812847	0.618615626	0.538608524	1.886107705	0.064448367
NR2F6	-0.106701353	0.915271039	0.278823223	0.781379162	0.864084206	0.391212295
NR3C1	-1.060319737	0.291927616	-0.416334314	0.678712771	-1.871268708	0.066513179
NR3C2	3.379947083	0.00108627	1.474498213	0.145792806	1.855262546	0.068802842
NR4A1	-0.46839014	0.640674501	-1.278742648	0.206116176	-0.965880637	0.338239322
NR4A2	-1.718363175	0.089279617	-1.2528047	0.215339382	-1.954457029	0.055625755
NR6A1	-0.976279398	0.331627025	-1.433571537	0.157101085	-1.576765269	0.120458785
NRADDP	-0.608044502	0.544738614	0.183037555	0.855411344	-0.57780445	0.565702611
NRARP	1.569085975	0.12025016	-0.319023712	0.750862664	0.76975698	0.444665446
NRAS	0.369692657	0.712507926	-0.565327854	0.574046435	-1.026716282	0.308950115
NRBF2	-2.602112973	0.010885654	-1.386808123	0.170844386	-2.029462541	0.047153524
NRBP1	-2.024766677	0.045950884	1.442802533	0.154492666	1.94401166	0.056903265
NRBP2	2.052452502	0.043124169	-2.960142647	0.004458458	-2.327700406	0.023551153
NRCAM	4.742165519	8.18E-06	-1.143188259	0.257689927	-0.361606024	0.719001579

NRD1	-1.404213775	0.163807416	1.029829843	0.307395186	0.899543764	0.372198867
NRF1	-0.409079159	0.683484714	1.50374567	0.138110771	1.622549957	0.110279025
NRG1	-0.392985272	0.69528957	-0.136222964	0.892120039	-0.282775679	0.778387038
NRG4	1.450335398	0.150553027	-4.419035641	4.44E-05	-2.307369645	0.024738226
NRGN	-3.15840812	0.002179981	-0.644792023	0.521621995	-1.69819752	0.094997735
NRIP1	-0.202578056	0.839936814	-0.373882459	0.709864576	-0.990157688	0.326338317
NRIP2	1.823369838	0.071674173	0.721144525	0.473737732	1.805063073	0.076419261
NRIP3	-1.304409126	0.195526948	2.216582969	0.030615159	1.020824626	0.311709032
NRL	-1.137355322	0.258505781	0.639516652	0.52502243	-1.367855673	0.176798569
NRM	-1.096103444	0.276051874	-0.617157674	0.539562932	-0.820891113	0.415174567
NRN1	-1.419518767	0.159313308	-1.283906114	0.204315774	0.199145419	0.84286727
NRN1L	1.584510398	0.116698205	-1.990737473	0.051261706	-0.8164138	0.417708437
NRP1	1.038178937	0.302057362	0.982236494	0.33009143	-0.050057012	0.960254325
NRP2	-1.997988315	0.048834518	2.017638583	0.048296445	-0.002971456	0.99763964
NRSN2	0.821580203	0.413556808	0.840567773	0.404066142	1.028744477	0.308004207
NRXN1	-1.009948494	0.315314575	-0.214297535	0.831071925	-1.547316626	0.127396449
NRXN2	0.558743688	0.577768893	-2.624442184	0.011093294	-2.278310637	0.026527281
NSA2	0.150568157	0.880664176	-0.193254126	0.847439834	0.758706175	0.451196575
NSD1	-1.253582503	0.213344808	1.059111504	0.293970511	1.050055751	0.298184077
NSDHL	0.728992104	0.467960199	-0.195001831	0.84607776	1.057929579	0.294610768
NSF	-1.365322582	0.175664083	1.301749811	0.198184399	0.668555527	0.506514091
NSFL1C	-2.396345785	0.018696846	1.234331743	0.222091823	0.836487491	0.406421124
NSL1	-0.482665101	0.630543199	-1.194275743	0.237265377	-1.178943371	0.243384736
NSMAF	0.202113976	0.840298471	0.007648081	0.993924203	-0.475442364	0.636313277
NSMCE1	-0.09337307	0.925821415	2.395568359	0.019866084	3.023870815	0.00375579
NSMCE2	0.620799399	0.536351114	-0.252743806	0.80136605	0.232583204	0.81692946
NSMCE4A	1.764692665	0.081117365	-0.867729396	0.389143519	-0.424590404	0.672755323
NSRP1	-1.316770833	0.191366086	0.897009111	0.373448195	1.030627536	0.307127752
NSUN2	2.713668358	0.008018512	1.827062523	0.072873048	2.470171892	0.016563475
NSUN3	-1.768138887	0.080535668	-0.300755917	0.764683627	-0.362514335	0.718326575
NSUN4	0.006282035	0.995002046	1.733461928	0.088364345	2.873796409	0.005713914
NSUN5	-0.642874957	0.521992318	1.399623983	0.16698906	1.534245179	0.1305765
NSUN5P1	0.911647622	0.364467967	1.063070819	0.292186732	2.466134233	0.016732462
NSUN5P2	1.486814277	0.140671305	1.814808973	0.074761531	2.433921689	0.018137269
NSUN6	1.910864858	0.059307968	0.704653606	0.483865514	2.039976991	0.046059766
NSUN7	0.16208878	0.871610988	-0.481661089	0.631871827	-0.406661669	0.685800043
NT5C	0.339409132	0.735117724	-2.094341203	0.040640092	-1.018135533	0.312973795
NT5C2	-3.105501368	0.002562743	-1.901263891	0.062274203	-2.655163934	0.0102924
NT5C3	-3.186536365	0.001998876	-0.065658485	0.947877097	-1.411089842	0.163726545
NT5C3L	1.442233152	0.152819243	-1.17873547	0.243350218	-0.516440272	0.607571864
NT5DC1	0.287120299	0.774701468	-0.348243706	0.728926814	-0.837344864	0.405943227
NT5DC2	0.729234854	0.467812476	1.081643523	0.283918925	1.088356236	0.281080181
NT5DC3	0.112027303	0.911059269	2.123574058	0.0380129	1.107366416	0.272849603
NT5E	1.914885542	0.0587859	-0.020438381	0.983764307	0.371519897	0.711646452
NT5M	-2.566640054	0.011975135	1.609885579	0.112890557	0.670816557	0.505083251
NTAN1	-2.702130806	0.008279386	-0.821030293	0.415014399	-0.458567185	0.648312319
NTHL1	1.092841561	0.277473733	2.359032861	0.021736174	2.070880925	0.042971108
NTN1	0.189232209	0.850350638	-1.574491339	0.120851363	-0.968989837	0.336699353
NTN4	1.234505234	0.220331556	-1.003352454	0.319887604	-0.120767713	0.904305484
NTN5	1.655034677	0.101512936	-0.940717928	0.350778224	0.237138167	0.813411351
NTNG2	-1.53542721	0.128299959	-1.184349915	0.241139006	-2.778719975	0.007405483
NTPCR	0.054024915	0.957038912	1.052367531	0.297026078	1.456976079	0.150684302
NTRK1	-3.593053629	0.000540299	1.759071563	0.083876537	-1.603055338	0.114524842
NTSR1	1.20645778	0.230904402	0.850269881	0.39869588	0.491448371	0.625022026
NUAK1	-1.448772356	0.150988173	-0.701963709	0.485528858	-0.271190481	0.787237513
NUAK2	-3.292531234	0.001435143	-0.500891055	0.618356671	-1.071490366	0.288525919
NUB1	-2.098508255	0.038752853	0.252473043	0.801574285	-0.136297261	0.892073037
NUBP1	0.316025339	0.752738565	-0.201004484	0.841403214	0.391230274	0.697105432
NUBP2	-0.32837099	0.743418499	0.227613846	0.820752511	0.243543699	0.808470472
NUBPL	1.423940651	0.158032708	-0.71050075	0.4802608	-1.050626973	0.297923849
NUCB1	-2.20282871	0.030244271	1.505123423	0.137756942	1.070017804	0.28918243
NUCB2	1.756624761	0.082492745	0.465109701	0.643606623	0.506243563	0.614664618
NUCKS1	2.18428701	0.031625188	0.458175207	0.64855059	1.72193837	0.090577294
NUDC	-0.919505065	0.360368405	2.840481667	0.006216466	3.167672088	0.002483954
NUDCD1	0.447180184	0.655853897	-0.55505878	0.581003816	-0.670054831	0.505565047
NUDCD2	1.553109128	0.124019573	-0.4116386	0.682132177	-0.34946273	0.728047203
NUDCD3	1.702745402	0.09217837	2.527166256	0.014267222	3.3736745	0.001348682
NUDT1	-0.358767744	0.72063614	0.032386096	0.97427612	0.848798542	0.399592081
NUDT11	0.893242798	0.37418606	-0.644523663	0.521794696	-0.716709079	0.476519711
NUDT12	-0.278642832	0.781178737	0.201503605	0.841014781	-0.112730019	0.910646046
NUDT13	2.075917748	0.040846697	0.778923021	0.439213667	1.505830088	0.137707419
NUDT14	0.672832106	0.502833513	-0.466647362	0.642512522	0.216205117	0.829610201

NUDT15	1.010296449	0.315148841	-1.016593161	0.313598439	0.66836168	0.506636865
NUDT16	-0.173866397	0.862373511	-0.165255839	0.86932128	-0.942080898	0.350180742
NUDT16L1	-0.509471412	0.611708922	-1.626141575	0.109379155	-0.692987144	0.491169704
NUDT16P1	-1.597852815	0.113693765	-1.495373453	0.140276365	-0.540539568	0.590959513
NUDT17	0.994431542	0.322764664	0.561029695	0.576953543	0.565174779	0.574202768
NUDT18	-0.374240678	0.709133871	1.11284262	0.270399095	0.462786594	0.645303154
NUDT19	-0.166133349	0.868436656	0.284066305	0.777378294	-0.364725002	0.71668468
NUDT2	-0.123768602	0.901783375	0.369969437	0.712762216	0.635966307	0.527378197
NUDT21	0.762485785	0.447828035	-0.36191028	0.718743502	-1.334939926	0.187275222
NUDT22	0.138311587	0.89031311	0.993829894	0.324462685	1.404264215	0.165739118
NUDT3	-0.042839364	0.96592754	-1.118230927	0.268110754	-2.165374619	0.034616124
NUDT4	-0.578490022	0.564424664	0.736262445	0.464559191	0.029706919	0.97640597
NUDT5	-2.05593341	0.042779574	0.511265144	0.611119834	-0.627180439	0.533079151
NUDT6	0.838802765	0.403873853	-0.074132704	0.94116166	-1.012927609	0.315433098
NUDT7	2.37596657	0.019693379	0.339400219	0.735542711	1.542616044	0.128532819
NUDT8	0.780182598	0.437396299	-0.647816339	0.519677818	-0.692171586	0.491677751
NUDT9	2.070137809	0.041397838	1.832064304	0.072113783	1.761271238	0.083628441
NUF2	0.117315625	0.906879769	-0.195948332	0.845340302	-0.067855157	0.946141619
NUFIP1	0.840637476	0.402850531	1.003895211	0.319628148	2.758362064	0.007823037
NUFIP2	0.701721137	0.484722018	-0.625390395	0.534185038	-1.870494771	0.066622389
NUMA1	0.822725196	0.412908757	1.37326249	0.174993232	1.717635476	0.091365629
NUMB	-1.201287106	0.232892949	-1.249066521	0.21669341	-2.123689416	0.038108314
NUMBL	1.060718107	0.291747507	-0.778690009	0.439349855	-0.292694118	0.770833211
NUP107	3.300520675	0.001399347	2.095837425	0.040501885	2.366898274	0.021404863
NUP133	1.647345129	0.103086711	2.951413369	0.004569134	3.223965045	0.002106597
NUP153	-0.394392109	0.694254602	0.455568855	0.65041293	-0.451516174	0.653354072
NUP155	0.101581326	0.919322248	-0.021977881	0.982541563	0.709725894	0.480806634
NUP160	0.971679897	0.333897802	1.706603106	0.093282618	0.647070088	0.520219149
NUP188	1.005473673	0.317451165	1.203931856	0.233540496	1.943895006	0.056917672
NUP205	2.095453959	0.03903041	0.60980479	0.5443895	1.56097329	0.124140422
NUP210	1.360264568	0.177252799	-0.464667739	0.643921242	0.371637436	0.711559413
NUP210L	0.842315309	0.401916094	-0.537664826	0.592879909	0.178017282	0.859349106
NUP214	-2.960484085	0.003955696	0.413940751	0.680454915	-0.474885473	0.636707716
NUP35	1.418259947	0.159679325	-1.496610788	0.139954633	-0.4510096	0.653716923
NUP37	1.114126102	0.268286946	-0.007018828	0.994424086	1.823250601	0.073581934
NUP43	2.367733918	0.020109152	0.275971757	0.783557549	2.413667917	0.019073981
NUP50	-1.205966978	0.231092626	0.353345007	0.725119845	-0.490325436	0.625811289
NUP54	0.976882238	0.331330157	-0.577060209	0.566147566	-0.404425631	0.687433822
NUP62	-0.372468044	0.710448255	1.480450602	0.144202745	1.113646476	0.270168177
NUP85	2.200585277	0.030408502	2.386794109	0.020301552	3.549264756	0.000788501
NUP88	3.01750958	0.003340447	1.600975685	0.114853501	1.861963811	0.067836264
NUP93	-0.599423193	0.550445212	2.3609688	0.021633268	2.110556214	0.039270513
NUP98	-2.237115716	0.027829075	1.1225153	0.266301008	1.03998839	0.302795999
NUPL1	-0.35369667	0.724420062	-1.474710379	0.145735895	-2.201474896	0.031821894
NUPL2	1.202067528	0.232592024	-2.300599259	0.025055114	-1.81793939	0.074401214
NUS1	1.302757954	0.196087791	0.051068292	0.9594478	-0.36127309	0.719249053
NUSAP1	-0.206053019	0.837229876	-0.471375545	0.639153244	-0.757730678	0.451775769
NUTF2	-1.67006082	0.098493813	-0.901547807	0.371051722	-0.329967091	0.742650847
NVL	1.671907304	0.098127894	0.700995665	0.486128241	2.477572725	0.016257721
NXF1	1.250343939	0.214519279	-1.179814233	0.242924223	-0.781794912	0.437614329
NXN	1.245781533	0.21618188	1.367486206	0.176785725	2.109182731	0.039393824
NXPH4	0.267257018	0.789902429	-0.848198506	0.3998387	0.138212871	0.890565897
NXT1	1.230223111	0.221922509	-1.130219987	0.263068244	-0.719898875	0.474568697
NXT2	-0.557527346	0.578595792	-1.591930331	0.116874383	-1.512195821	0.136083674
NYNRIN	0.648357285	0.518457817	2.346401895	0.022418373	2.284780625	0.026119301
O3FAR1	-1.357340647	0.178176152	0.554330328	0.581498883	1.060412562	0.293490074
OAF	-2.193941495	0.030899441	0.402901136	0.68851259	-0.68338961	0.497166735
OAS1	-1.40066396	0.164863528	-0.305294743	0.761242277	-1.881218104	0.065122661
OAS2	-1.293913996	0.199112181	2.143803217	0.036282791	-0.564794244	0.574459841
OAS3	-0.970770099	0.334348176	1.20277697	0.233983745	-1.874308852	0.066085651
OASL	-3.628020622	0.000480566	0.051891647	0.958794584	-2.257011661	0.027910542
OAT	0.947291619	0.346108318	0.913686394	0.364690697	0.455031479	0.650838439
OAZ1	-4.000319595	0.000132261	-0.687390802	0.494595239	-1.280656072	0.205571171
OAZ2	-3.716518867	0.00035613	-0.646203466	0.520714173	-0.8925617	0.375895429
OBFC1	-0.242718662	0.80879342	-2.196939653	0.032062399	-1.171581197	0.246305109
OBFC2A	0.327246062	0.74426618	-1.386460997	0.170949754	-2.95362611	0.004577915
OBFC2B	-0.563480457	0.57455413	-0.548620094	0.585386637	0.006573802	0.994778167
OBSCN	2.4540711	0.016113354	0.26344137	0.793150609	1.03780798	0.303801243
OBSL1	-0.299971502	0.764912899	-0.388854517	0.698817611	-0.739214925	0.462850913
OCEL1	-0.46225065	0.645053036	0.979459713	0.331449167	1.898303445	0.062792271
OCIAD1	0.657426457	0.51263859	-0.841566491	0.403511294	-0.368062416	0.71420848
OCIAD2	1.017217872	0.311864202	-2.356784605	0.021856229	-0.9217362	0.360603708

OCLM	1.58399626	0.116815236	0.184910119	0.853949111	-0.50731639	0.613916607
OCLN	1.953528117	0.053963391	0.286990341	0.775149655	1.449266485	0.15281694
OCM	1.641059301	0.104387806	1.473780897	0.145985349	2.953636303	0.004577785
OCRL	1.426358686	0.157335795	-0.358917736	0.72096902	0.373220896	0.710387219
ODC1	-7.02356042	4.51E-10	1.785604605	0.079428294	1.128565597	0.263872656
ODF2	0.176713353	0.860143392	1.891795768	0.063549307	2.011539197	0.04906972
ODF2L	1.457526471	0.148563577	-1.367558984	0.176763054	-1.742577977	0.086873648
ODF3B	-1.077630266	0.284171312	-1.714820198	0.091754551	-3.08295545	0.003173207
ODZ1	1.591316699	0.115157732	-0.530719231	0.597653865	-0.701758435	0.485724031
ODZ4	-0.700570713	0.485436319	-0.136752483	0.891703341	0.680819319	0.498779559
OFD1	2.701452196	0.008294968	0.342434172	0.733270683	2.053033879	0.044732126
OGDH	-1.335451193	0.185204676	0.366204493	0.715554226	0.406394789	0.685994962
OGFOD1	-0.44300084	0.658862331	1.63269096	0.107989718	2.191662145	0.032561064
OGFOD2	0.105011859	0.916607604	-0.053678346	0.957377189	0.56230294	0.576144237
OGFR	-1.664731197	0.09955621	0.196005871	0.845295475	0.157259533	0.875603573
OGFRL1	-1.703407705	0.0920539	-1.557352519	0.124865053	-2.517831976	0.014681949
OGG1	0.748339042	0.456269587	-1.690733549	0.096293061	-1.001477723	0.320885719
OGT	2.791499485	0.006445541	-1.042111396	0.301714584	-0.933225746	0.354693056
OIP5	-0.684589407	0.495418844	-0.914100546	0.364474908	-1.929941815	0.058663615
OLA1	0.849983216	0.397662464	0.736865295	0.464195299	1.73807571	0.087670665
OLAH	-2.347528007	0.021162813	0.093981527	0.925450009	-0.633448259	0.529008832
OLFM1	-0.037118473	0.970475398	0.420087717	0.675984421	0.249628028	0.803784599
OLFM2	0.389924543	0.697543252	-0.307473307	0.759592199	0.387841367	0.699597608
OLFM4	-4.112428118	8.84E-05	2.070617089	0.04288694	0.132957292	0.894701772
OLFML2A	2.013201604	0.047177871	-0.017463186	0.986127456	0.92803995	0.357353006
OLFML2B	-0.933904078	0.35293258	0.112627619	0.910717415	-0.791583166	0.431929943
OLIG1	3.304676188	0.001381061	-2.274187879	0.026697744	-1.647172476	0.105099828
OLIG2	3.303037904	0.001388243	-0.934644458	0.353873639	-1.562224304	0.123845519
OLR1	-0.737872999	0.462572968	0.279751746	0.780670192	-0.983013472	0.329811164
OMA1	-0.32204338	0.748190705	-0.486484351	0.62846983	-1.175539559	0.244731816
OMG	0.155178169	0.877039542	-0.698895206	0.487430191	-0.850101162	0.398873676
OPA1	1.806702302	0.074258498	1.139969482	0.259017489	1.183147825	0.241728194
OPA3	0.468807231	0.6403775	1.181939177	0.242086674	1.671774428	0.100124685
OPHN1	-0.213522647	0.831417833	-0.00574654	0.995434807	-0.011832067	0.990601473
OPLAH	-1.161840128	0.248471059	-0.357647627	0.721914319	-1.349251235	0.182663649
OPN3	0.619175706	0.537415156	-0.652012879	0.516986445	-1.179525716	0.243154806
OPRL1	-1.473951747	0.144096072	1.277735817	0.206468613	0.786690501	0.434765779
OPTN	-2.679614692	0.008810859	1.699309202	0.094656505	2.078707251	0.042218039
OR13A1	-0.555557694	0.579936006	2.189542623	0.032622783	1.965509376	0.054300805
OR2W3	-3.54633322	0.000631152	0.462062559	0.645777115	0.178825819	0.858717166
OR52N4	0.953769422	0.342837178	-2.628904843	0.010964455	-2.370698517	0.021206374
OR7D2	-0.269016751	0.788552358	-0.526423024	0.600615747	-0.775602727	0.441233068
ORAI1	-1.629796719	0.106752191	-2.277179297	0.026507021	-2.213969816	0.030902088
ORAI2	-2.73565625	0.007541975	-0.0061733	0.995095783	0.254768358	0.799831379
ORAI3	-1.887485134	0.062421855	-1.382517082	0.172150414	-1.564797604	0.123240675
ORAOV1	1.804498509	0.074605928	-0.731865216	0.467218361	0.134923834	0.893153853
ORC1	0.084511949	0.932843153	1.670464374	0.100253642	2.902543057	0.005277713
ORC2	1.318868282	0.190666725	-0.433062584	0.666586769	-0.958203278	0.342061733
ORC3	0.446053801	0.656664149	-1.92571443	0.059081454	-0.639531257	0.52507412
ORC4	-0.424724179	0.672084036	-1.41500281	0.162451699	-1.62993061	0.108705255
ORC5	1.105317715	0.272062701	0.099736524	0.920899843	2.028612366	0.047242935
ORC6	1.133802022	0.259985467	1.569935658	0.121908001	1.069637283	0.289352245
ORM1	-2.001237546	0.048476581	-0.010062146	0.992006476	0.434685388	0.665454282
ORM2	-0.455995422	0.649527026	0.874351294	0.38555827	0.510653983	0.611592167
ORMDL1	2.281927188	0.024928537	-1.465717746	0.148163436	-1.474870591	0.145824112
ORMDL2	-2.150278694	0.034301387	-3.320170197	0.001564926	-1.895297715	0.063197037
ORMDL3	-0.218763134	0.827345834	-0.10700925	0.915153509	0.308952078	0.758499686
OS9	-2.05560956	0.042811533	1.950765459	0.055955967	2.014213558	0.04877961
OSBP	-0.047246655	0.962424698	1.141762889	0.258277212	0.860743009	0.393034563
OSBP2	-3.260817143	0.001585876	1.207924255	0.232012908	-0.129194117	0.897665014
OSBPL10	1.405368314	0.163465047	1.650872842	0.104207503	1.401565616	0.166540065
OSBPL11	-0.091322592	0.927445754	-0.668587827	0.506429262	-2.209669657	0.031215962
OSBPL1A	-1.746521309	0.0842422	1.228202678	0.224366222	-0.297214258	0.767398038
OSBPL2	-2.512916226	0.013812981	0.160676098	0.872910746	0.252127646	0.801861593
OSBPL3	2.230938576	0.028251282	-1.941573035	0.057086051	-1.32644909	0.190052668
OSBPL5	0.161805613	0.871833308	-2.334351577	0.023086948	-1.617796566	0.111302335
OSBPL6	-3.550158069	0.000623202	1.623315627	0.109983136	1.982038864	0.052369753
OSBPL7	-0.532651289	0.59562993	-0.073320429	0.941805173	0.977854837	0.332334033
OSBPL8	0.90066001	0.370250177	-1.661012817	0.102145516	-2.419946613	0.018779056
OSBPL9	-0.384706979	0.701391312	1.080383994	0.284474427	0.995267158	0.323869592
OSCAR	-1.744621404	0.084574564	0.811273335	0.420548659	0.420843139	0.675473605
OSCP1	-1.329023591	0.187307649	-1.189675883	0.239054861	-1.012925216	0.315434231

OSGEP	1.84615968	0.06826229	1.894515369	0.063180803	2.99096814	0.004122049
OSGEPL1	2.881976801	0.004974698	-0.827741886	0.411233273	0.644870085	0.521633482
OSGIN1	-1.831183937	0.070488643	1.722247561	0.090391161	0.574291192	0.568060902
OSGIN2	-1.921391985	0.057949292	-1.433032659	0.157254408	-2.109717915	0.039345735
OSM	-2.44270934	0.016595076	-0.008053618	0.993602043	-1.766657164	0.082712351
OSMR	0.39775742	0.691781198	-0.067703638	0.946256039	0.711665265	0.47961391
OST4	-2.941169799	0.004186758	-2.786295201	0.007206876	-1.965497041	0.054302269
OSTC	0.221009621	0.825601691	-0.599987518	0.550867888	-0.312268654	0.755991298
OSTCL	0.270496574	0.787417534	-2.104009956	0.039754176	-1.165218789	0.248849147
OSTF1	-3.62945166	0.00047826	-0.386984791	0.700193655	-0.505157909	0.615421991
OSTM1	-0.346871138	0.72952394	0.135879233	0.892390551	-0.945428547	0.348484665
OSTalpha	-1.866069368	0.065393783	0.996631924	0.323111941	1.094956239	0.278203267
OTOF	-2.074823899	0.040950512	1.086215061	0.281909032	0.010331585	0.991793299
OTUB1	-1.091828536	0.277916344	-2.398077129	0.019743122	-2.068148036	0.043236807
OTUB2	-0.009504045	0.992438696	1.720034431	0.09079565	0.59536283	0.553989362
OTUD1	-1.92844818	0.057053395	0.535673484	0.594246795	-1.411658462	0.163559742
OTUD3	1.746138341	0.084309109	-1.900645805	0.062356775	-0.74884176	0.457073345
OTUD4	0.891143025	0.375305053	-0.503434688	0.616578708	-1.569594612	0.122119483
OTUD5	-2.101674323	0.03846694	-0.043257	0.965646284	0.300607399	0.764822426
OTUD6B	1.403632384	0.16398003	-0.747750881	0.457652638	-1.426512673	0.159248733
OTUD7A	-0.244390151	0.807502873	-1.086213234	0.281909834	-2.805778422	0.006882245
OTUD7B	0.627462898	0.531995698	0.257552865	0.797669972	1.195100732	0.237063332
OTX1	-3.040579679	0.003117734	-2.254545724	0.027980548	-2.304534856	0.024907904
OVCA2	0.378553573	0.705939601	-4.648146423	2.00E-05	-3.956790321	0.000215319
OVGP1	3.586022307	0.000553132	2.531308195	0.014116854	3.395492297	0.001262665
OXA1L	-0.214516865	0.830644942	-2.217885879	0.030521234	-1.470987212	0.146868243
OXCT1	1.543432977	0.126347688	-1.007013609	0.318140192	0.001924701	0.998471123
OXER1	-1.961449166	0.053017481	-0.186310054	0.852856276	-0.337361743	0.737100177
OXNAD1	1.853804276	0.067148677	0.302556828	0.763317596	0.086679607	0.931234081
OXR1	1.123059577	0.26449513	-0.058602066	0.953471912	-1.166872075	0.248186266
OXSM	1.394349125	0.166755145	0.076961973	0.938920523	-0.107329559	0.914909527
OXSR1	-2.179180047	0.032015069	1.003721681	0.319711086	0.911388465	0.365980972
OXTR	-0.680840867	0.497776369	1.054217696	0.296185642	0.166962202	0.867998693
P2RX1	-1.953627168	0.053951474	0.579297299	0.564647531	1.042581148	0.301603608
P2RX4	1.531606713	0.129239988	-0.630325533	0.530974603	0.39627765	0.693399851
P2RX5	0.081461543	0.935261617	1.052123038	0.297137261	1.710195664	0.092742035
P2RX5-TAX1BP:	0.393095003	0.695208824	-0.038169299	0.96968469	-0.262827359	0.793644151
P2RX6	-1.823046027	0.071723657	1.77954477	0.080426491	1.582944142	0.119042383
P2RX7	3.898112823	0.000189914	-0.889848491	0.37724901	-0.88142296	0.381840685
P2RY1	0.294781758	0.768861352	-0.943925164	0.349150747	-2.909340439	0.005179186
P2RY10	-0.820079923	0.414406873	-1.784280284	0.079645553	-2.270277031	0.027041717
P2RY11	1.539112526	0.127398321	-0.562091564	0.576234675	-0.080354078	0.936240926
P2RY12	-2.449401591	0.0163098	0.414905333	0.679752637	-1.870621501	0.066604496
P2RY13	-2.159773498	0.033535091	-2.077586511	0.042215963	-2.480602944	0.016134008
P2RY14	-1.092095129	0.277799817	-2.822237666	0.006534952	-3.804677928	0.000352378
P2RY2	-0.864958038	0.389435442	0.132165448	0.895314057	-0.956616666	0.342855201
P2RY6	-0.729595388	0.467593126	-0.882194605	0.381338607	-1.091406226	0.279748135
P2RY8	-0.845562303	0.400111504	1.923378302	0.059380359	1.883244442	0.0648425
P4HA1	-0.670235754	0.504478892	0.358506831	0.721274795	-0.125577213	0.900514461
P4HB	-0.899905345	0.370649435	2.044704129	0.045463461	2.150159243	0.035856868
P4HTM	2.478787636	0.015108351	-0.337203394	0.737189325	-0.201644298	0.840922436
PA2G4	-0.880569811	0.380971535	1.952425235	0.055753971	3.198002789	0.002273386
PA2G4P4	0.745153113	0.458183157	-1.48464429	0.143090699	-0.130283294	0.89680721
PAAF1	1.432852803	0.155475823	1.928733332	0.058697086	2.931208794	0.004873665
PABPC1	-0.035763809	0.971552445	1.289148112	0.202500025	1.255731193	0.214405759
PABPC1L	2.258657354	0.026399702	-0.268456948	0.789306816	-0.000497715	0.999604643
PABPC4	0.446552361	0.656305464	0.644560592	0.521770929	0.51972617	0.605294232
PABPN1	0.974465857	0.332521156	-3.402398944	0.001220657	-2.989321578	0.004141225
PABPN1L	-0.189870854	0.84985168	-0.348496396	0.728738076	0.18995748	0.850026458
PACRGL	1.372477437	0.173435234	-1.236806056	0.221178461	-2.716126613	0.008759192
PACS1	-1.248695813	0.215118793	0.091247098	0.927612861	0.734449897	0.465726059
PACS2	2.053588212	0.043011477	0.253078582	0.801108603	0.699847631	0.486907492
PACSIN1	1.959612576	0.053235537	-0.264816532	0.792096207	-0.520455074	0.604789522
PACSIN2	-0.739211079	0.461764338	-0.647811069	0.519681202	-0.783057788	0.436878458
PADI2	-2.630549698	0.010077881	0.87935618	0.382862296	-0.138958475	0.889979389
PADI4	-3.713451107	0.000359876	0.517589125	0.606727286	0.892363827	0.376000528
PADI6	-0.43251058	0.66643827	1.382722231	0.1720878	0.90317197	0.370287128
PAF1	-1.847400301	0.068080522	1.715359233	0.091655037	2.004476299	0.049843049
PAFAH1B1	0.101611326	0.919298504	1.836419733	0.071458059	2.061380283	0.043900928
PAFAH1B2	-0.939200507	0.350222453	0.05182502	0.958847442	0.692614889	0.491401562
PAFAH1B3	-0.296205593	0.767777461	2.394781264	0.019904804	2.640753371	0.010688975
PAFAH2	-0.189970584	0.849773769	-0.710555814	0.480226925	0.680007635	0.499289473

PAG1	2.033674812	0.045024518	-0.166116585	0.868646958	-0.762574117	0.448904267
PAICS	1.790299845	0.076876884	2.235518022	0.029274908	4.231905948	8.64E-05
PAIP1	0.78786231	0.432913971	-1.028877654	0.307838619	-1.134536526	0.2613824
PAIP2	-1.425487416	0.157586635	0.268601246	0.789196308	0.044343557	0.964787634
PAIP2B	2.350565857	0.021001339	1.472035384	0.146454717	1.776742278	0.081019436
PAK1	-2.899234022	0.004731932	1.461560741	0.149296271	1.326554907	0.190017863
PAK1IP1	1.560155253	0.122345777	0.803684081	0.424883968	1.330690458	0.188661399
PAK2	-2.694564591	0.008454635	0.447903987	0.655902735	-0.461308371	0.646356706
PAK4	0.79317187	0.429830873	-0.928129083	0.35721391	-1.127089626	0.264490811
PAK6	1.30917252	0.193915703	-1.774745119	0.081224507	-0.896258005	0.373935578
PALB2	0.382900528	0.702725431	-0.017862726	0.9858101	1.000354145	0.321424172
PALLD	-2.58061272	0.011534763	-1.006136465	0.318558254	-1.428920492	0.158558322
PALM	0.159946297	0.873293346	0.356658272	0.722650964	-0.426976328	0.671026847
PAM	0.670870356	0.504076461	1.484399331	0.143155469	1.099974917	0.276029465
PAM16	0.372781245	0.710215957	-3.103036048	0.002966768	-2.128714004	0.037671679
PAN2	0.855156324	0.394808432	0.317344274	0.752129944	0.589094416	0.55815702
PAN3	0.11415948	0.909373846	-1.865984368	0.067138308	-2.296735593	0.025380084
PAN3-AS1	1.293546834	0.199238486	-3.510438153	0.00087633	-1.974223814	0.053275272
PANK1	0.289236548	0.773086998	0.601751055	0.549701273	0.743855501	0.460060653
PANK2	-3.536345843	0.000652365	0.059197498	0.952999719	-1.149109096	0.255374892
PANK3	-0.161671942	0.871938259	-0.917143443	0.362891954	-1.42659175	0.159226022
PANK4	0.387163573	0.699578546	0.165842466	0.868861696	-0.643063946	0.522796124
PANX1	-1.751559193	0.083366088	0.361712601	0.718890438	0.367362545	0.714727496
PANX2	-1.409921562	0.162120166	-1.549223506	0.126805706	-1.56524029	0.123136863
PAOX	-0.360081106	0.719657267	0.90665026	0.368369303	1.85127607	0.069383338
PAPD4	0.958598156	0.3404119	-0.855691124	0.395714437	-1.445667774	0.153820459
PAPD5	0.349114441	0.727845124	-1.315105217	0.193686396	-2.114143445	0.038950039
PAPD7	3.724985055	0.000345984	-0.144345072	0.885731878	0.743826858	0.460077846
PAPLN	-0.15385703	0.878078026	-0.175681807	0.861160129	-0.560524201	0.577348323
PAPOLA	0.510427052	0.61104218	-1.275792482	0.207150137	-1.829928638	0.072562578
PAPOLG	0.193800057	0.846783239	-0.870808079	0.387474066	-1.785708559	0.07953868
PAPSS1	0.965741677	0.336844557	0.354203676	0.724479725	-0.047773928	0.962065675
PAPSS2	-2.030695994	0.04533249	0.23405192	0.815774631	-0.515712368	0.608076945
PAQR3	1.15485662	0.251304472	-1.214062389	0.22967853	-1.916911474	0.060335116
PAQR4	0.349113329	0.727845955	-0.061489008	0.951182645	0.454165033	0.65145811
PAQR5	-2.608789464	0.010690912	-1.074387554	0.287129438	0.735005586	0.46539024
PAQR6	-1.152178317	0.252397198	-1.236546072	0.221274301	-1.878115906	0.065553556
PAQR7	0.492147551	0.623851954	-0.89542198	0.37428853	1.108302931	0.272448553
PAQR8	-0.557444118	0.578652393	-0.587234528	0.559341272	-0.991517021	0.32568031
PAR-SN	1.862549097	0.065893484	-0.356272826	0.722938028	-0.338778744	0.736038125
PAR5	2.528478106	0.013256146	0.655650337	0.514659638	0.705598382	0.48335059
PARD3	-1.558658044	0.122699926	2.086258621	0.0413938	2.005449708	0.049735849
PARD6A	-1.503311065	0.136372471	-1.05321882	0.296639178	-0.882813419	0.381095314
PARD6B	0.37077429	0.711704969	-1.185792385	0.24057325	0.649849582	0.518435183
PARD6G	1.01735903	0.311797453	0.010158569	0.991929879	-1.600729991	0.115039992
PARG	0.195337943	0.845582894	1.62551586	0.109512654	2.652818902	0.010356005
PARK2	0.797683841	0.427221136	-0.159996516	0.873443613	-0.364404187	0.716922871
PARK7	-0.77454519	0.440703846	-0.901865503	0.370884343	0.02571748	0.979573723
PARL	1.174428201	0.243421281	0.771504146	0.443561962	1.945826907	0.05667947
PARM1	1.287675075	0.201266489	0.23391359	0.815881508	1.158767188	0.251448059
PARN	-0.708721322	0.480388104	1.842035661	0.07061997	2.593844718	0.01207841
PARP1	-0.629294811	0.53080152	2.147897574	0.03594112	2.039320554	0.046127403
PARP10	-1.209280474	0.229824038	0.215873245	0.829849237	-0.697269316	0.48850691
PARP11	0.820402338	0.414224103	0.224874637	0.822872713	-0.472918612	0.638101666
PARP12	-0.770617558	0.443016839	0.572259387	0.569373269	-1.126423231	0.264770242
PARP14	-0.817869268	0.415661354	0.140505783	0.888750615	-1.138898916	0.259573584
PARP15	2.126444082	0.036292397	-1.114674193	0.269619714	-1.35741374	0.180072416
PARP16	-1.307064526	0.194627517	-0.073738192	0.941474202	0.15274835	0.87914347
PARP2	1.725431691	0.08799243	0.628515487	0.532150917	1.017404603	0.313318173
PARP3	-1.106272138	0.271651804	0.000352319	0.999720108	-0.393803742	0.695215161
PARP4	-0.288947476	0.77330747	0.801709328	0.426016411	-0.073008202	0.942058644
PARP6	1.049525677	0.296836696	0.595398267	0.553909618	2.553008252	0.013419714
PARP8	-0.108083917	0.914177467	-1.644563518	0.105507556	-2.618534799	0.011327819
PARP9	-0.903467994	0.368766994	-0.508316658	0.613172743	-2.650326968	0.010423988
PARS2	1.014939184	0.312943035	2.343765426	0.022563155	2.075237026	0.042550527
PARVA	0.156098744	0.876316049	0.799239379	0.42743537	1.522465011	0.133496285
PARVB	-1.259470558	0.2112216	1.582017344	0.119121914	1.657631689	0.102960611
PARVG	-0.775925955	0.439892385	1.86017951	0.067968628	2.456041431	0.01716169
PASK	3.85927522	0.000217581	1.577634469	0.120126646	2.498468375	0.015421746
PATL1	-1.458891721	0.14818819	-2.120098906	0.038317253	-2.11681358	0.038712979
PATL2	2.016335914	0.046842601	-1.679777687	0.098417575	-0.750341529	0.456177014
PATZ1	2.799876187	0.006294273	-0.517632187	0.606697425	0.116633027	0.907566389

PAWR	0.151855088	0.879652064	0.260461978	0.79543638	0.42450747	0.672815437
PAX5	0.651597111	0.516375005	1.172851443	0.245683255	0.772280798	0.443181651
PAX6	-0.827935988	0.409967255	-0.978116748	0.332107152	-0.291745556	0.771554678
PAX8	0.351144682	0.7263269	-0.745269251	0.459139503	0.932406217	0.355112561
PAXIP1	0.830727431	0.408396707	0.140746965	0.88856093	0.763988937	0.448067482
PBLD	-0.023194826	0.981547868	1.217037366	0.228553313	0.410046964	0.683329419
PBRM1	1.563693221	0.121512137	-0.071387989	0.943336277	-0.694381685	0.490301655
PBX1	-3.167341316	0.00212087	0.398187459	0.691964193	0.0998471	0.920820807
PBX2	-1.598021453	0.113656192	-2.972875556	0.004301485	-2.328418605	0.023510163
PBX3	-0.150762849	0.880511047	-0.75688835	0.452201905	-1.077161718	0.286007107
PBX4	1.422064635	0.15857504	0.966431363	0.337868929	1.137621348	0.260102389
PBXIP1	-1.258298099	0.21164314	1.984704349	0.051947799	2.595582425	0.012024137
PC	-0.603064073	0.5480316	1.342866053	0.184583559	1.593769761	0.116593134
PCBD1	-0.159045405	0.874000935	-1.950821183	0.055949175	-2.155112113	0.035448775
PCBD2	0.323031373	0.747444921	-1.343922948	0.184243531	-0.4078065	0.684964146
PCBP1	-2.417014165	0.017732256	-1.564619553	0.123150367	-2.765812747	0.007667768
PCBP2	-2.30919177	0.023297091	-1.161405738	0.250267532	-1.235622943	0.221735568
PCBP3	-1.408830867	0.162441545	-0.782442285	0.437159798	-0.353360431	0.725139464
PCBP4	1.369029933	0.174506487	-0.247978241	0.805033205	0.787038656	0.434563621
PCCA	-0.489410141	0.625780388	2.176337647	0.033644588	2.3934069	0.020054247
PCCB	0.200707214	0.841394968	2.598104792	0.011882174	2.503203162	0.015237803
PCDH1	-2.170952838	0.032651974	-0.376085969	0.708234739	0.54951195	0.584829833
PCDH11Y	-0.125599781	0.900337897	1.463154817	0.148861066	1.187408811	0.240057706
PCDH12	0.80959869	0.420374857	0.774011663	0.442089459	0.110957079	0.912045437
PCDH7	-1.077478741	0.284238585	2.292612743	0.025542096	2.376379637	0.020912715
PCDH9	0.29915426	0.765534263	1.188389736	0.239556955	0.496974381	0.621144476
PCDHGB6	-0.986894853	0.326425026	-0.22294945	0.824363642	0.081461621	0.93536408
PCDHGC3	0.94772463	0.345889029	0.384496093	0.702026805	-0.184746837	0.85409225
PCF11	1.458977757	0.148164559	-0.240402941	0.810871523	-1.938414789	0.057598039
PCGF1	-2.499841608	0.014296962	-1.91221062	0.060827105	-2.043837041	0.045663768
PCGF3	2.021316066	0.04631408	-2.258819566	0.027696878	-2.773885781	0.007502734
PCGF5	-1.128269723	0.262301112	-0.329849088	0.742710669	-1.699967815	0.094662101
PCGF6	-1.084258573	0.281239283	-0.91034408	0.366435167	-1.012089325	0.31583017
PCID2	2.890813098	0.004848997	0.273572135	0.785392099	0.946363497	0.348011934
PCIF1	-1.142091113	0.256542922	0.518881852	0.605831161	0.659180472	0.512470152
PCK2	0.364908621	0.716063261	2.550738353	0.013430445	1.756253697	0.084489448
PCM1	1.993635213	0.049317594	2.929226413	0.004862029	2.933953635	0.004836522
PCMT1	-0.373938979	0.709357515	1.309672406	0.195506759	1.023697947	0.310361449
PCMTD1	-0.122488918	0.902793716	-0.16025205	0.87324324	-2.002455822	0.050066196
PCMTD2	1.169351105	0.245449091	-1.518890828	0.13426052	-2.372271611	0.021124693
PCNA	0.536631015	0.592889183	1.181677236	0.242189805	2.472009442	0.016487079
PCNP	0.37813785	0.706247271	-0.313246745	0.755224756	-0.786221291	0.435038317
PCNT	1.554966976	0.123576489	1.216027239	0.228934918	1.498365032	0.139631072
PCNX	-1.413373618	0.161106216	-0.281829861	0.779084136	-0.347848149	0.729252884
PCNXL2	2.399637681	0.018540153	-1.742674007	0.086727667	-0.81303864	0.419624731
PCNXL3	-0.35486777	0.723545601	0.131294882	0.895999583	0.922821634	0.36004262
PCOLCE	-0.363872087	0.716834408	-0.619812492	0.537825679	0.151930388	0.879785588
PCP2	-1.246832914	0.215797909	-0.611967912	0.542967303	-1.337417535	0.186470597
PCP4L1	0.950543582	0.344463631	0.150120954	0.881193667	0.7899905	0.432851858
PCSK1N	-0.754100396	0.452820771	-1.462414824	0.149062971	-1.203111889	0.233973596
PCSK4	0.848336037	0.39857387	-0.600026566	0.550842044	-1.029241396	0.307772755
PCSK5	1.028577291	0.306523383	0.842079632	0.403226395	0.69206194	0.491746076
PCSK6	-4.102284402	9.17E-05	0.767415742	0.445968974	-0.191718684	0.848653134
PCSK7	2.424396527	0.017398628	-0.301796675	0.763894098	1.136848612	0.260422608
PCTP	-4.413820517	2.90E-05	-0.345216817	0.731188956	-0.545788088	0.587370187
PCYOX1	0.783611566	0.435391603	1.833894426	0.071837638	2.328043855	0.023531543
PCYOX1L	2.964125446	0.003913475	0.238475315	0.812358891	1.073427843	0.287663708
PCYT1A	-1.046998377	0.297994167	-0.338048422	0.736555794	-0.503044005	0.616897898
PCYT1B	-2.662069156	0.009246297	1.477845283	0.144897017	1.005540924	0.31894356
PCYT2	-0.017594272	0.986002726	-1.460570436	0.149567139	-0.276780665	0.782963307
PDAP1	-1.902176637	0.060449473	-1.085714705	0.282128532	-0.737055424	0.464152662
PDCD1	2.089132683	0.039610313	-0.475891462	0.635951851	-0.817605496	0.417033102
PDCD10	-0.159531822	0.873618876	-0.725583367	0.471032215	-1.076299769	0.286388938
PDCD11	0.857208947	0.393679489	2.29271251	0.025535962	2.410371157	0.019230496
PDCD1LG2	-2.415228089	0.017813826	-1.827665808	0.072781115	-2.075838051	0.042492779
PDCD2	1.165016421	0.247189893	-0.041477518	0.967058666	-0.294175702	0.769706738
PDCD2L	1.021309422	0.309933344	-0.120088902	0.904830647	0.7389778	0.46299375
PDCD4	0.457813978	0.648224986	3.003841804	0.003940933	1.920245932	0.059903566
PDCD5	-0.010403921	0.991722791	-3.070501582	0.003258245	-1.583867707	0.118831828
PDCD6	-0.374287059	0.709099492	-0.017436539	0.986148622	0.152083221	0.879665605
PDCD6IP	1.519766462	0.132187866	-0.353154101	0.725262188	-0.692106881	0.491718071
PDCD7	0.49937391	0.618773783	1.850373818	0.069390902	0.706173956	0.482995391

PDCL	-0.276673745	0.782685463	0.436892613	0.663822841	-0.037412876	0.9702883
PDCL3	1.081363973	0.28251713	3.299760501	0.001663605	4.020725099	0.00017458
PDDC1	0.360030229	0.719695178	1.849559869	0.069510081	2.929006585	0.004903656
PDE12	-0.1782431	0.858945557	-0.49337413	0.623624281	-0.194917533	0.846159996
PDE1B	1.219374709	0.225990485	1.04074101	0.302344854	2.652360751	0.010368474
PDE2A	-1.446827248	0.151531045	0.491376567	0.625027451	0.112547192	0.910790339
PDE3B	1.402631086	0.164277642	-0.987668955	0.327445869	-1.618336881	0.11118563
PDE4A	-1.492140101	0.139272031	-0.683977299	0.496732265	0.166396003	0.868442138
PDE4B	-2.370311184	0.019978162	-0.825186978	0.412670158	-2.785394946	0.007273107
PDE4D	-0.397419798	0.69202919	-1.001926822	0.320569775	-1.497709501	0.139801004
PDE4DIP	3.576031614	0.000571862	2.696522417	0.009172273	3.508014964	0.000895618
PDE5A	-4.759817875	7.64E-06	0.892201722	0.375997237	-0.255941934	0.798929563
PDE6B	2.961939312	0.003938773	-1.594092169	0.116388805	-0.461815312	0.645995319
PDE6D	-0.781340967	0.436718472	-3.329065707	0.00152367	-2.449415624	0.01744884
PDE6G	-1.03259612	0.304648714	-0.889286582	0.377548301	0.440748956	0.66108445
PDE7A	1.844688741	0.068478329	0.249520281	0.803846102	0.818456036	0.416551505
PDE7B	1.939300438	0.055698416	0.952186584	0.344981282	0.693869084	0.490620631
PDE8A	0.731060752	0.466702189	-1.415479168	0.162312694	-1.40342308	0.165988448
PDE8B	0.981960618	0.328836254	-0.890105144	0.377112359	-0.857299552	0.39491812
PDE9A	0.887530732	0.377234998	1.844457693	0.070261081	1.908683595	0.061411315
PDF	0.728334158	0.468360717	-1.708773107	0.092877058	-1.916818253	0.060347219
PDGFA	-2.384460808	0.019272417	2.573834809	0.012653968	1.618445496	0.111162182
PDGFB	1.284404526	0.202402701	-1.528565023	0.131846026	-1.361060971	0.178923686
PDGFC	1.944152646	0.055101464	-0.124078269	0.901685314	-0.266004451	0.791208603
PDGFD	0.700220448	0.485653914	-2.711190356	0.008820768	-2.667679024	0.009958946
PDGFRB	0.946462542	0.346528438	-0.546579642	0.586778858	-0.939537619	0.351472876
PDHA1	1.456475162	0.148853147	1.049870444	0.298162971	2.077924913	0.042292798
PDHB	0.361873843	0.718321863	-0.228918716	0.819742987	1.147997085	0.255829814
PDHX	0.864830082	0.389505291	-0.085276464	0.932337341	0.49064218	0.625588619
PDIA3	-0.287211352	0.774631984	2.408964015	0.019217385	2.28653837	0.026009429
PDIA3P	0.401398627	0.689108782	-0.273494005	0.785451851	0.690034272	0.49301055
PDIA4	-0.060314021	0.95204357	1.42492189	0.159576198	1.722389283	0.090495009
PDIA5	1.006738768	0.316846144	1.852149925	0.06913144	2.651486401	0.010392307
PDIA6	0.731105362	0.466675081	2.025724617	0.047434541	2.080041326	0.042090821
PDIK1L	1.621307558	0.108562728	0.202057987	0.840583388	-0.524714713	0.601843936
PDK1	1.560638661	0.122231606	-0.891085763	0.37659053	-1.29526691	0.200520238
PDK2	-0.037857189	0.969888093	1.098111109	0.276725387	1.540922659	0.128944174
PDK3	-0.398369382	0.691331782	-0.194576363	0.846409306	0.013166584	0.989541489
PDK4	-1.520726883	0.131946791	2.216496513	0.0306214	-0.861487806	0.392627898
PDLIM1	-2.930084705	0.004324935	1.454374262	0.151270662	1.368448387	0.176614111
PDLIM2	-2.093039745	0.039251014	-1.684266417	0.097542539	-1.822524675	0.073693463
PDLIM5	-2.319876765	0.022683903	-0.589137665	0.558072673	-1.956256265	0.055408197
PDLIM7	-1.752473548	0.083207885	-1.04616926	0.299853555	-1.199896718	0.235210082
PDP1	0.3842184	0.70175205	-0.421600058	0.67488633	-1.628860547	0.108932287
PDP2	0.862864244	0.390579377	-0.193273861	0.847424451	0.812477137	0.419944047
PDPK1	0.051498855	0.959045817	-0.376970696	0.707580732	-1.047953019	0.29914335
PDPR	1.209105382	0.229890946	0.424853392	0.672526536	1.789500323	0.078919313
PDRG1	0.37507768	0.708513551	0.618232931	0.538858961	1.771957778	0.08181894
PDS5A	1.710690381	0.090694265	0.507958399	0.613422397	-0.361788896	0.718865661
PDS5B	0.454952061	0.650274538	1.154229519	0.253172865	0.019021756	0.984891078
PDSS1	1.162097886	0.248366916	0.302619329	0.763270201	0.022934743	0.981783512
PDSS2	2.360235888	0.020494585	0.701004431	0.486122811	1.198722845	0.235662711
PDXDC1	1.950893999	0.054281108	1.588543008	0.117638502	0.81088498	0.42085027
PDXDC2P	2.546707189	0.012629605	1.056621374	0.295096213	3.17428054	0.002436571
PDXK	-0.937954253	0.350858938	2.749020142	0.007970445	1.649662811	0.104587223
PDXP	-1.124297047	0.263972863	-1.86230528	0.06766356	-1.77808777	0.080795777
PDZD11	0.270860399	0.7871386	-1.346213285	0.18350831	-1.041061821	0.302301946
PDZD2	0.151440959	0.879977736	-1.348762211	0.182692704	0.256487004	0.798510806
PDZD3	-1.708626551	0.091077891	0.203738234	0.839276204	0.598865465	0.551667409
PDZD4	1.045405001	0.298725488	-2.136281993	0.036917837	-0.686584745	0.495165814
PDZD7	1.194476747	0.235530906	-1.917037293	0.060198184	-1.369955265	0.176145819
PDZD8	-1.163352555	0.24786043	0.255611977	0.799161117	-0.812905403	0.419700487
PDZK1	1.828228965	0.070935024	-0.506607129	0.614364446	-1.137868768	0.259999919
PDZK1IP1	-3.817960848	0.000251221	1.468514345	0.147405127	-1.005901994	0.318771356
PEA15	0.832491766	0.407405922	-0.854538035	0.396347425	-1.176105091	0.244507631
PEAK1	0.640219093	0.523709097	-0.153298167	0.878698971	0.173312777	0.863027897
PEAR1	-4.038797981	0.000115253	1.361587809	0.178630567	-0.153992883	0.878166642
PEBP1	0.749583483	0.45552338	2.492873474	0.015568952	3.461638175	0.001032557
PECAM1	-3.292566716	0.001434982	0.845866167	0.401127916	1.890391167	0.063862533
PECR	0.882891874	0.379722518	1.438140047	0.155805902	2.349956994	0.022310066
PEF1	-1.535105154	0.128378991	2.315885793	0.024145961	2.650867268	0.010409213
PEG10	-0.573458019	0.567810841	0.312758036	0.755594141	-0.700238413	0.48666533

PELI1	-0.568879808	0.570900218	-1.791137879	0.078525883	-3.338461486	0.001499317
PELI2	-2.128659623	0.03610319	-0.088925871	0.929449314	-1.965767488	0.054270189
PELI3	-1.097481775	0.27545258	2.033648663	0.046602811	2.126621828	0.037852954
PELO	-1.840524758	0.069093001	-1.401799261	0.166341395	-0.973482976	0.334482121
PELP1	0.231145891	0.81774303	2.421465342	0.0186292	3.033900168	0.003650334
PEMT	-0.812275636	0.418845749	0.977385511	0.332465785	0.512537548	0.610282142
PEPD	-1.055032022	0.294325464	1.955915786	0.055331201	2.447797215	0.017519633
PER1	2.689832615	0.008565945	0.022743219	0.981933711	0.203711592	0.83931425
PER2	1.550708279	0.124594025	-0.408534472	0.684396284	-0.220549014	0.826242344
PER3	3.659575838	0.000432088	-2.322811386	0.023743815	-0.392829554	0.695930495
PERP	-0.628238478	0.531489948	-0.809860393	0.421353767	-0.051271394	0.959290945
PES1	-1.117930297	0.266667635	3.343488849	0.001458955	3.868733349	0.00028669
PET112	1.178079078	0.241970529	0.428601229	0.669812155	3.102702813	0.002998147
PET117	1.268280647	0.20807381	0.230921697	0.818193961	-0.058795917	0.953323092
PEX1	2.975924094	0.003779517	0.461793382	0.645969001	0.269435556	0.788580679
PEX10	1.801999553	0.075001524	-1.721849237	0.090463851	-1.165012701	0.248931867
PEX11A	0.559822263	0.577036126	-0.532665509	0.596314311	0.88616877	0.37930042
PEX11B	2.208673565	0.029820033	-1.678942454	0.098581104	0.390041965	0.697978924
PEX11G	-0.762224024	0.447983407	4.818970407	1.09E-05	4.434888142	4.33E-05
PEX12	0.416197583	0.678288147	-0.97746544	0.332426572	-0.71973302	0.474670029
PEX13	1.082512658	0.282009554	-2.551909877	0.013390042	-1.547264868	0.127408917
PEX14	-0.401012016	0.689392344	-0.70628839	0.48285617	-0.292072801	0.771305756
PEX16	-1.510063887	0.134642813	0.358331597	0.721405209	0.443369324	0.659199682
PEX19	-0.145499483	0.88465237	-1.308375811	0.195943104	0.099167747	0.921357736
PEX2	0.440671301	0.660541658	-0.273591245	0.785377484	-0.201707395	0.840873342
PEX26	1.416755796	0.160117523	-0.237660672	0.812987684	0.951453027	0.345445906
PEX3	3.160270302	0.002167534	-1.357680052	0.179860866	-1.244284327	0.218556043
PEX5	1.237835193	0.219100128	2.407606723	0.019282238	2.031492268	0.046940652
PEX6	0.182226848	0.855827723	1.875766482	0.065758472	2.249909363	0.028385795
PEX7	4.821678056	5.98E-06	-0.86205181	0.392234017	0.507741441	0.613620362
PF4	-5.207323622	1.26E-06	2.30870752	0.024569163	0.928821008	0.356951554
PF4V1	-2.988612719	0.003640194	0.484123735	0.630133841	0.508912889	0.612804239
PFAS	1.040682306	0.300900244	1.092233214	0.279278262	1.783549241	0.079893205
PFDN1	0.751295662	0.454497845	-0.669414636	0.5059057	0.264892256	0.792060972
PFDN2	0.911878988	0.364346831	-2.201582901	0.031714977	-1.359672829	0.179360233
PFDN4	0.123033413	0.902363805	-0.298856951	0.766124849	0.383105298	0.703086045
PFDN5	0.405587538	0.686039262	-0.120796065	0.904272986	0.72484361	0.47155321
PFDN6	-0.672976585	0.502742037	-1.214822528	0.229390641	-0.545070468	0.587860337
PFKFB2	-1.327777966	0.187717257	0.549413282	0.584845863	-0.50087378	0.618414783
PFKFB3	-0.246931476	0.805541751	0.736707159	0.464290737	0.261971242	0.794300802
PFKFB4	-1.563847875	0.1214758	0.568126603	0.572157305	-0.419784096	0.676242629
PFKL	-0.297475848	0.76681087	1.016052549	0.313853576	1.237366738	0.221092714
PFKM	2.63130459	0.010057198	2.583497675	0.012341387	2.6463162	0.010534263
PFKP	0.007864203	0.993743305	1.098693616	0.276473286	0.164651085	0.869809019
PFN1	-1.741928915	0.08504743	-1.018283518	0.312801592	-0.480829936	0.632502803
PFN2	1.361803925	0.17676814	-0.213453646	0.831726921	0.668735663	0.506400016
PGAM1	-2.205272346	0.030066267	1.398233516	0.167404074	1.997994901	0.050561919
PGAM2	0.872604469	0.385275489	-0.909760537	0.366740285	0.996403094	0.323322448
PGAM4	-1.480995144	0.142212734	-1.227071678	0.224787779	-1.301490444	0.198397244
PGAM5	-0.232236431	0.816898627	1.888607418	0.063983645	2.097192863	0.040484678
PGAP1	1.087722479	0.279715364	-0.621180348	0.536931712	-2.125783238	0.037925827
PGAP2	-0.127167864	0.899100367	-0.942429765	0.349908958	-0.509012784	0.612734667
PGAP3	0.212739769	0.832026549	1.147224043	0.256032249	2.232075065	0.029610873
PGBD1	1.582386481	0.117182269	0.37395045	0.709814266	1.565861583	0.122991284
PGBD2	2.485638927	0.014839894	-0.841079167	0.403781973	-0.930559039	0.356059283
PGBD3	0.45409901	0.650885969	1.365933692	0.177269887	2.245469122	0.028686539
PGBD4	1.131264649	0.261045738	-2.777381018	0.007383092	-3.539858045	0.000811797
PGBD5	1.332538605	0.186155401	1.288089909	0.202865595	0.61326849	0.542171323
PGCP	-2.468895508	0.015503636	1.21504246	0.229307395	0.85132726	0.398198202
PGD	-2.933289633	0.004284559	1.208564212	0.231768724	1.254467423	0.214861078
PGGT1B	-0.731665108	0.466335021	0.317701452	0.751860365	-0.903283826	0.370228289
PGK1	-1.206485958	0.230893599	1.165650792	0.248560185	1.201199614	0.234708445
PGLS	-0.961838667	0.338790607	0.902107636	0.370756807	1.080196628	0.2846655
PGLYRP1	-2.661246335	0.00926719	-0.463431696	0.644801489	-0.989583453	0.326616551
PGM1	-1.089929087	0.278747566	2.127291088	0.037689701	2.545820199	0.013669344
PGM2	1.282104182	0.203204701	0.084930014	0.932611559	-0.718533872	0.475403039
PGM2L1	1.052741307	0.295368406	-1.094592323	0.278251691	-1.272526348	0.208422398
PGM3	2.273273169	0.025467008	-1.576138332	0.120471176	-1.620081016	0.110809582
PGM5	-1.575731218	0.118709466	0.489358507	0.626446436	-0.096936398	0.923121548
PGP	1.521768825	0.131685646	0.050696652	0.959742653	-0.22149951	0.825505853
PGPEP1	-0.093735113	0.925534645	0.971178594	0.335520283	2.398812264	0.019788404
PGRMC1	-4.914846669	4.13E-06	-0.890897327	0.376690769	-2.312776275	0.024417458

PGRMC2	0.277366391	0.782155362	-1.096567622	0.277394165	-0.615102473	0.540968191
PGS1	1.04636692	0.298283845	-0.585479503	0.560512414	-1.658001602	0.102885608
PHACTR1	-2.800603673	0.00628129	1.084919911	0.282477442	0.79554036	0.429644385
PHACTR2	-0.118910179	0.905620061	-0.737079373	0.464066117	-1.492010963	0.141285114
PHACTR4	0.221805986	0.824983613	1.147055733	0.256101229	1.914229533	0.060684135
PHAX	0.525365551	0.600662624	1.410188177	0.163861827	2.154241347	0.035520225
PHB	0.412694057	0.680843853	-0.144067939	0.885949725	0.86421947	0.391138633
PHB2	0.60128497	0.549210339	-1.141140141	0.258534097	-0.384407718	0.70212608
PHC1	0.679896764	0.49837109	-2.088503692	0.041183236	-1.340898404	0.185344589
PHC2	-2.654439384	0.009441673	-0.347157923	0.729737995	-0.917074587	0.363019813
PHC3	1.632560748	0.106167969	-0.271141912	0.787251292	-0.705734081	0.483266835
PHEX	0.769839667	0.443475777	1.17312872	0.245572954	-0.065452801	0.94804561
PHF1	-0.12497021	0.900834824	-0.635966492	0.527317354	-0.198018098	0.843744967
PHF10	1.455723444	0.149060466	2.373352083	0.020985264	3.464849777	0.001022466
PHF11	-1.387863004	0.168715303	-1.719033	0.090979169	-2.624100073	0.011164615
PHF12	-1.135486903	0.259283097	-1.032594997	0.306109918	-1.01652609	0.313732424
PHF13	1.272604707	0.206541568	2.728324234	0.008425828	2.248704564	0.028467121
PHF14	2.25508754	0.026632018	1.79977562	0.077134295	1.762231872	0.083464431
PHF15	1.109094304	0.27043934	1.393048144	0.168958771	1.553136685	0.12600058
PHF16	-0.090396063	0.92817983	1.436189289	0.156357926	0.493157212	0.623821803
PHF17	-1.070421875	0.287383756	1.480033008	0.14431385	0.585577964	0.560501827
PHF19	2.575835177	0.011683663	-0.076409463	0.939358142	1.434266719	0.157033682
PHF2	0.472398044	0.637822995	-0.814720564	0.418588288	-1.495835615	0.140287667
PHF20	-1.386194444	0.169222388	0.493012208	0.623878407	-0.164624777	0.869829631
PHF20L1	-0.754208583	0.452756152	-1.136776636	0.260339152	-2.305783929	0.024833013
PHF21A	-1.205754666	0.231174083	-0.09363786	0.925721808	-0.168103252	0.867105156
PHF23	-1.45494544	0.149275271	0.292986911	0.770585154	0.080100642	0.936441583
PHF3	-0.615344	0.539930429	0.060250499	0.9521647	-0.171978094	0.86407214
PHF5A	-0.036768428	0.970753701	-0.795081776	0.429830238	-0.316647886	0.752683247
PHF6	0.299600834	0.765194706	-0.695359322	0.489626232	-1.082385879	0.283700442
PHF7	1.94605186	0.054869291	-1.07975539	0.284751948	0.093329171	0.925973767
PHF8	0.307515849	0.759184116	0.339085964	0.735778182	0.509698572	0.612257146
PHGDH	-0.124660386	0.901079386	1.774639826	0.081242088	3.534952388	0.000824204
PHIP	0.353528959	0.724545322	-0.700634573	0.486351923	-1.898265321	0.062797391
PHKA2	-1.205752224	0.231175021	1.912275744	0.060818582	2.392146683	0.020116685
PHKB	-0.738844674	0.461985684	1.116423131	0.268876974	0.300124736	0.765188637
PHKG1	-0.4253479	0.671631088	-1.482455984	0.143670129	-1.257338469	0.213827712
PHKG2	0.026497574	0.978921023	1.045500657	0.30015965	1.629578948	0.108779824
PHLDA1	0.417395064	0.677415485	-1.705885777	0.093417003	-1.871001591	0.066550855
PHLDA3	0.788938758	0.432287861	0.764160154	0.447891121	0.005512256	0.995621386
PHLDB1	2.808317907	0.006145125	-0.132516533	0.895037619	1.12100379	0.267050461
PHLDB2	0.355020378	0.723431676	-1.892825456	0.063409571	-0.651229959	0.517550421
PHLDB3	1.989064651	0.049829185	0.80955828	0.421526034	1.615873575	0.111718497
PHLPP1	-2.001986896	0.048394351	0.153026589	0.878912161	-0.45179343	0.653155514
PHLPP2	2.223873868	0.028741028	0.454142184	0.651433292	0.6754417	0.502163161
PHOCN	-1.449950771	0.150660016	-4.196507187	9.49E-05	-3.982010647	0.000198259
PHOSPHO1	-3.558397608	0.000606397	-2.286280302	0.025934174	-2.405138315	0.019481295
PHOSPHO2	1.608346621	0.111374575	-1.347755067	0.18301464	-0.799839003	0.427169809
PHPT1	-1.985398597	0.050242799	-2.173660809	0.033855113	-1.396727661	0.167983453
PHRF1	0.693889128	0.489596364	0.22507834	0.822714997	0.47317103	0.637922699
PHTF1	-0.031142594	0.975227	0.327315641	0.744615856	-0.577494812	0.565910263
PHTF2	1.107718558	0.271029919	-0.739202168	0.462786259	-1.55738452	0.124989532
PHYH	0.589316957	0.557172611	1.54947745	0.126744719	2.719322769	0.008684905
PHYHD1	0.239056445	0.811622844	0.421550636	0.674922204	0.274825284	0.784457617
PI16	-0.176355297	0.860423808	1.982834345	0.052162051	1.11281972	0.270520116
PI3	-0.749583467	0.45552339	-0.757467002	0.451857997	-0.173675709	0.862743984
PI4K2A	-0.531062289	0.596725878	1.397708856	0.167560876	1.260871078	0.212561291
PI4K2B	0.544121992	0.587746331	-0.2551038	0.799551663	-1.45785973	0.150441361
PI4KA	0.137656886	0.890829	0.290083191	0.772794416	0.637236363	0.526556735
PI4KAP1	2.024635162	0.045964682	-1.530541976	0.131356891	-1.467811997	0.147726323
PI4KAP2	2.105606885	0.038114351	-0.625127956	0.534356043	0.599982937	0.550927653
PI4KB	-0.038881293	0.969073922	0.716672888	0.476472082	1.09214712	0.279425225
PIAS1	-1.736333086	0.086037148	-1.32429993	0.190634681	-2.137706249	0.036901157
PIAS2	-0.436708593	0.663402305	-0.982064407	0.330175466	-1.065493542	0.291205939
PIAS3	0.711449533	0.478704866	-2.127392545	0.037680913	-1.537618193	0.129749913
PIAS4	0.192046058	0.848152704	-0.5657524	0.573759676	-0.445449605	0.657704966
PIBF1	0.605283533	0.546562891	-0.307097877	0.759876476	0.183057511	0.855411268
PICALM	-1.714129347	0.090057964	-1.14172427	0.258293137	-1.774231	0.081438262
PICK1	-0.358907951	0.720531619	-0.448687634	0.655340581	0.623793026	0.535285687
PID1	1.519924384	0.132148202	-0.570079718	0.570840769	-0.53547148	0.594435247
PIDD	1.074279086	0.285661689	0.725631817	0.471002732	1.76272815	0.083379807
PIF1	0.933438794	0.353171305	-0.996466007	0.323191818	-0.724187827	0.471952506

PIGA	0.211050096	0.833340679	-0.599299085	0.55132364	-0.896231868	0.373949414
PIGB	-2.270671297	0.025630891	-3.077496956	0.003193401	-2.683591537	0.009549244
PIGC	0.023533675	0.981278354	1.301535787	0.198257114	1.4593204	0.150040453
PIGF	1.156172774	0.250768723	-1.254679072	0.214662813	-0.93416658	0.354211853
PIGG	2.431825236	0.017068569	0.267964626	0.789683886	0.354553423	0.724250286
PIGH	0.361603574	0.718523129	-2.01526076	0.048552456	-1.518806315	0.134413599
PIGK	1.132604902	0.260485319	-0.671446421	0.504620352	-0.387568165	0.699798663
PIGL	1.654177109	0.101687479	-0.357654908	0.721908899	-0.301065644	0.764474793
PIGM	0.642024874	0.522541501	-2.37503901	0.020898345	-3.480326594	0.000975138
PIGN	1.510176589	0.134614092	0.70473212	0.483817011	0.109237433	0.913403031
PIGO	0.646603777	0.519586948	0.211053809	0.833590244	1.283997644	0.204407704
PIGP	-0.337223941	0.736758545	-2.99821134	0.004004322	-2.676193438	0.009737766
PIGQ	0.555628968	0.579887483	2.15733241	0.035164492	2.876013432	0.00567912
PIGR	0.829606583	0.409026891	0.988014474	0.327278083	0.626442495	0.53355944
PIGS	-0.990616392	0.32461416	0.195046328	0.846043088	0.288872041	0.773741485
PIGT	-2.233370103	0.028084422	0.540705698	0.590795469	0.809769197	0.421486054
PIGU	-0.268995729	0.788568482	0.741499205	0.461403627	2.285869111	0.026051215
PIGV	-0.712964834	0.47777138	0.957803649	0.342165085	1.020950372	0.311649975
PIGW	1.27336273	0.206273822	-1.598641299	0.115372322	-1.462931365	0.149052946
PIGX	-2.596286094	0.011058228	-0.510728772	0.611493055	-1.239480138	0.220315444
PIGY	0.478662726	0.633376774	-1.492151103	0.141116982	-1.27751317	0.206669968
PIGZ	0.905119245	0.367896558	-1.887753513	0.064100396	-0.608904274	0.545039843
PIH1D1	0.173231517	0.862870987	1.419683147	0.161089928	2.293827119	0.025558194
PIK3AP1	-1.177541684	0.242183684	-0.263480568	0.793120549	-2.66394559	0.010057371
PIK3C2A	1.144352905	0.255609193	-2.201552128	0.031717269	-2.67831717	0.009683302
PIK3C2B	1.021154959	0.310006092	0.512000551	0.610608288	0.653953419	0.515807158
PIK3C3	0.358328409	0.720963688	1.347606455	0.183062181	1.357154651	0.180154232
PIK3CA	-0.086714092	0.93109761	0.466552573	0.642579946	-0.776201581	0.440882328
PIK3CB	-0.050046321	0.960199948	2.097590364	0.040340485	1.821298726	0.073882136
PIK3CD	-0.938981186	0.350334411	0.713112927	0.478655282	0.406470165	0.685939908
PIK3CG	-1.184490683	0.239437742	0.802416013	0.425610948	-0.929101687	0.356807362
PIK3IP1	3.811320567	0.000257071	1.043037185	0.301289303	1.773624435	0.081539694
PIK3R1	0.931890924	0.35396622	1.624585922	0.109711306	2.121381063	0.038310386
PIK3R2	0.184077609	0.854380023	-0.938255034	0.352031338	-0.745176172	0.459268338
PIK3R3	-0.332056215	0.740643749	-0.783226571	0.436702857	0.806025862	0.423623262
PIK3R4	0.192823053	0.847545994	1.708778841	0.092875989	1.678503143	0.098798056
PIK3R5	-2.1107467	0.037657737	0.374538233	0.709379387	0.373756864	0.709990615
PIK3R6	1.064203819	0.290174809	-0.463865226	0.644492692	-0.832083729	0.408881199
PIKFYVE	0.979305788	0.330138446	-0.125013031	0.900948547	-1.298568307	0.199391943
PILRA	-4.126551559	8.40E-05	-1.015668071	0.314035113	-1.360005901	0.179255413
PILRB	0.968287827	0.33557899	0.11219346	0.911060114	0.752127519	0.45511095
PIM1	-2.816550048	0.006002805	-0.438207226	0.662875234	-1.491816186	0.14133606
PIM2	-1.117706225	0.266762824	-0.849642898	0.399041586	-0.993092048	0.324919001
PIM3	0.434292448	0.665148956	-0.950744059	0.345706954	-1.556687285	0.125155037
PIN1	-0.050959745	0.959474164	-1.536789388	0.129820649	-0.696057496	0.489259648
PIN4	1.435290516	0.15478204	-2.098129455	0.040290961	-2.388120079	0.020317352
PINK1	-3.124291746	0.002420158	1.639893725	0.106478258	1.16292037	0.249772804
PINX1	0.568729321	0.571001905	1.704159128	0.093741128	2.893749577	0.005407753
PION	1.950182929	0.054367146	-1.464839745	0.148402139	-1.802985187	0.076749175
PIP4K2A	-3.053163826	0.003002133	-0.416081408	0.678896764	0.441172167	0.660779895
PIP4K2B	0.902268819	0.36939994	2.368027675	0.021261728	2.534971985	0.014054066
PIP4K2C	-1.830586231	0.070578744	-0.387109468	0.700101866	0.266114641	0.791124169
PIP5K1A	-0.069390841	0.94483742	-0.511429632	0.6110054	-0.744734032	0.459533505
PIP5K1B	-2.78218738	0.006617601	-0.372713654	0.710729642	0.23830603	0.81250995
PIP5K1C	-0.385319614	0.700939075	0.343793864	0.73225323	0.510653199	0.611592712
PIP5KL1	0.38314917	0.702541746	-0.488141701	0.627302712	-0.833263414	0.4082213
PIPOX	-0.031955528	0.974580555	2.315482763	0.024169549	1.486054851	0.142849565
PISD	-3.060899576	0.002933058	-0.527710811	0.599727209	-0.62789283	0.532615707
PITHD1	-2.743285299	0.007382765	-0.716506135	0.47657422	-0.106054929	0.915916176
PITPNA	-3.560346675	0.000602485	-0.115151283	0.908725726	-0.025172434	0.980006536
PITPNB	-0.210274275	0.833944227	1.312237564	0.194645658	1.469005119	0.14740343
PITPNC1	-2.021904215	0.046252	0.350408678	0.727310307	1.373332083	0.17509986
PITPNM1	-1.051129337	0.296103829	-0.757611468	0.45177216	-0.643403294	0.522577576
PITPNM2	1.541667905	0.12677608	-0.419496928	0.676413577	0.601647888	0.549826402
PITPNM3	0.508417136	0.612444861	-1.687724704	0.096872739	0.145184211	0.885084539
PITRM1	0.11378964	0.909666164	2.756392833	0.00781376	3.076588832	0.003231656
PIWIL2	0.942736836	0.348420448	0.211059738	0.833585639	-0.296498289	0.767941842
PIWIL4	0.071301177	0.943321356	-0.923487641	0.359605849	-2.406551663	0.019413268
PJA1	1.291033906	0.200104541	2.291140094	0.025632804	3.160107647	0.002539253
PJA2	0.22400119	0.823280436	0.166960006	0.867986304	-1.425093858	0.159656652
PKD1	1.932403501	0.056556349	-0.595331482	0.553953945	-0.020608602	0.983630826
PKD1L1	0.442702149	0.659077555	-0.486349909	0.628564548	-0.074562572	0.940827353

PKD1P1	0.493439182	0.622942943	-0.497176884	0.620956946	-0.110273334	0.912585197
PKD2	1.461668125	0.147427069	-1.47337828	0.146093508	-2.694890105	0.009267769
PKDCC	-0.393480735	0.694925007	-0.632275106	0.529709129	0.196167941	0.845185874
PKDREJ	0.233465931	0.815946886	0.461400133	0.646249375	0.17761555	0.859663128
PKHD1L1	-1.097467658	0.275458713	0.923250146	0.359728517	0.779141451	0.439162871
PKI55	0.369531649	0.712627479	-0.073571807	0.941606018	2.002577418	0.050052742
PKIA	-0.009375921	0.992540627	-0.324328451	0.746864325	-0.198907318	0.843052631
PKIG	-1.077459586	0.28424709	0.83059779	0.40963072	1.399848804	0.167051171
PKM2	-1.717198336	0.08949321	2.614670851	0.011380229	2.628941715	0.011024379
PKMYT1	0.021509479	0.982888391	-0.093080617	0.92616254	1.027789812	0.308449198
PKN1	-1.051583335	0.295896577	0.736745446	0.464267629	1.309176168	0.195798809
PKN2	-0.46567961	0.642606028	-1.733681088	0.088325113	-2.46959728	0.01658743
PKN3	1.312176778	0.192904608	-0.580072143	0.564128433	0.738699186	0.463161611
PKNOX1	-1.403023653	0.164160911	0.91450888	0.36426223	0.995166599	0.323918058
PKP2	-0.831294127	0.408078312	1.656357328	0.103088046	0.23946036	0.811619246
PKP4	-1.838631795	0.069373954	0.85986846	0.393426542	1.890775762	0.063810155
PLA2G12A	-0.969767817	0.334844792	-0.801181577	0.426319361	-1.578355249	0.120093021
PLA2G15	1.161188478	0.248734487	2.706730296	0.008926321	3.63141563	0.000610431
PLA2G16	-0.801260356	0.425159158	-0.173854102	0.862589732	1.109840653	0.271790943
PLA2G2D	1.829033727	0.070813223	0.734138768	0.465842378	0.953213488	0.34456121
PLA2G4A	2.652234019	0.00949884	-0.038368793	0.969526325	0.114116055	0.909552238
PLA2G4C	-0.923069986	0.358518182	-0.222098739	0.825022668	-0.105511485	0.916345408
PLA2G6	1.338315587	0.184273252	-1.070578032	0.288825049	-0.210890889	0.833734733
PLA2G7	-1.833268613	0.070175142	1.283092455	0.204598699	0.736872128	0.46426325
PLAA	-1.212995852	0.228407588	2.202579488	0.031640843	1.879192447	0.065403752
PLAC2	0.2206186	0.825905212	-0.4149836	0.679695666	0.666615296	0.507743664
PLAC4	-0.396141863	0.69296817	-0.494514812	0.622823648	-0.338694432	0.736101303
PLAC8	1.860201702	0.066228472	-0.545667339	0.587401839	0.542223222	0.589806978
PLAC8L1	-0.590943022	0.556087447	1.295586725	0.200286332	1.675028298	0.099481344
PLAG1	0.974813455	0.332349657	-0.887240992	0.378639113	-0.961943743	0.340195895
PLAGL1	-2.529141523	0.013232863	-2.43826765	0.017864124	-3.707954864	0.00047963
PLAGL2	-0.918987298	0.360637637	-0.900969312	0.371356627	-0.490569328	0.62563983
PLAT	-1.128392639	0.262249506	-0.690602454	0.492589196	0.925778257	0.358517124
PLAU	-1.970074156	0.052003573	0.594739403	0.554347001	-0.143528762	0.886385669
PLAUR	-3.448151485	0.00087127	1.061896011	0.292715235	-0.11883417	0.90583021
PLB1	0.527409671	0.599248656	1.904351367	0.061863126	1.163855752	0.249396609
PLBD1	-2.996786604	0.003552976	-0.670115218	0.505462298	-0.722980156	0.472688339
PLBD2	0.804673433	0.423196931	0.261518279	0.794625785	0.589573511	0.557837935
PLCB1	-0.924427298	0.357815326	-0.745167052	0.459200795	-1.647302172	0.105073081
PLCB2	-2.172417474	0.032537789	-0.220990907	0.825881069	0.441716551	0.660388225
PLCB3	0.481421873	0.631422782	1.590284869	0.117245069	1.838815395	0.071224507
PLCD1	0.745446877	0.458006521	1.30294642	0.197778219	1.741968641	0.086981164
PLCD3	0.394854953	0.693914228	1.601828188	0.114664502	0.857732122	0.394681199
PLCD4	0.639212348	0.524360637	1.26018759	0.212683577	2.490803941	0.015723759
PLCG1	1.85618257	0.066805341	1.130691724	0.262871218	2.109598979	0.039356418
PLCG2	-2.42439086	0.017398882	0.841236633	0.403694498	0.247912725	0.805104922
PLCH2	1.526396923	0.130530612	-1.339388352	0.18570578	-0.460550196	0.646897352
PLCL1	2.647126636	0.009632439	0.355334021	0.723637377	1.091032576	0.279911085
PLCL2	-2.652933982	0.009480662	3.203738734	0.002211891	2.503980114	0.015207809
PLCXD1	1.39565941	0.166361289	1.923564935	0.059356432	2.652276074	0.01037078
PLCXD2	0.747866908	0.456552877	-1.521554283	0.133592298	-2.296994181	0.025364302
PLD1	-1.469680801	0.145247522	0.355551495	0.723475352	0.821887308	0.414612056
PLD2	0.389377879	0.697946059	0.29382901	0.769944809	1.054210736	0.296294769
PLD3	-0.959806436	0.339806782	1.313951543	0.194071883	0.624266547	0.534976955
PLD4	1.262489132	0.210139161	0.859575731	0.3935866	1.145952947	0.256667575
PLD6	3.300080014	0.001401299	2.015062196	0.048573888	3.265296435	0.001864641
PLDN	0.361395556	0.718678052	0.103361951	0.918034768	-0.568364592	0.572050073
PLEC	1.627797025	0.107176471	-0.951363299	0.345395318	0.138840526	0.890072167
PLEK	-2.056142661	0.042758934	-0.656465043	0.514139254	-1.144700538	0.257181822
PLEK2	-2.445633854	0.016469867	1.242396349	0.219125054	0.80721669	0.422942669
PLEKHA1	1.655510363	0.101416222	-1.471955969	0.1464761	-1.282741864	0.204844363
PLEKHA2	-1.588063477	0.115891993	2.072644069	0.042690846	1.755026353	0.084701177
PLEKHA3	-0.469615607	0.639802041	-1.192893958	0.237801909	-2.313554444	0.024371596
PLEKHA4	0.242872866	0.808674338	1.501801426	0.138611306	1.940433426	0.057346623
PLEKHA5	1.960752656	0.053100088	-0.674229619	0.502862516	-0.408240992	0.684647005
PLEKHA6	2.342383752	0.02143876	0.506693196	0.614304425	-0.328918052	0.743439412
PLEKHA7	1.279997068	0.203941396	-1.098032205	0.276759548	-0.023566162	0.981282085
PLEKHA8	0.973694668	0.332901855	-0.789959718	0.432791622	-2.190890993	0.032619788
PLEKHA8P1	-0.132637866	0.894785417	2.609075512	0.011547564	2.519281988	0.014627865
PLEKHB1	1.07821003	0.283914017	0.532498145	0.596429447	1.478756871	0.144785051
PLEKHB2	-1.903790309	0.06023607	1.475045633	0.145646001	0.72015284	0.474413555
PLEKHD1	3.804737522	0.000262999	-0.529071488	0.598789043	-0.550065614	0.584452582

PLEKHF1	-0.369663565	0.712529527	-2.597950668	0.011886937	-1.841652068	0.070801789
PLEKHF2	-0.345689179	0.730409012	0.961264877	0.340437283	-0.222603896	0.824650317
PLEKHG1	-0.959627637	0.339896283	0.780381302	0.438361914	1.188542431	0.23961469
PLEKHG2	1.449982207	0.15065127	-1.268716129	0.20964596	0.746626009	0.458399436
PLEKHG3	-2.396414685	0.018693554	-0.990309786	0.326164923	-0.314288774	0.754464733
PLEKHG4	3.284144992	0.001473639	-0.465952987	0.643006497	0.123028444	0.902523208
PLEKHG5	2.031622606	0.045236497	-0.585885041	0.560241687	-0.277368582	0.782514179
PLEKHG6	-0.709410083	0.479962846	-0.632163603	0.529781463	-0.448944752	0.655196809
PLEKHG7	0.625776839	0.533096021	-1.021267627	0.311398203	-0.595751764	0.55373129
PLEKHH1	-0.812337929	0.418810205	0.554393568	0.581455896	0.897278838	0.373395459
PLEKHH2	1.53678651	0.127966811	-0.004113944	0.996731773	0.190271343	0.849781684
PLEKHH3	-0.402142782	0.688563103	0.44933091	0.654879272	0.034261342	0.97279007
PLEKHJ1	-0.175232241	0.861303458	1.367304733	0.176842266	1.547428368	0.127369533
PLEKHM1	-2.762045238	0.007004225	-0.004408932	0.996497428	-0.294991559	0.769086642
PLEKHM1P	0.687677957	0.493480966	-2.083637082	0.041640856	-2.503997654	0.015207133
PLEKHM2	-1.247350352	0.21560912	-0.691547254	0.491999914	-0.391545464	0.696873813
PLEKHM3	0.046089503	0.963344314	0.280154167	0.780362983	-0.248717898	0.804485084
PLEKHN1	0.447733221	0.655456225	1.13348599	0.261706304	0.777826451	0.439931493
PLEKHO1	-2.418958821	0.017643823	0.506444996	0.614477523	0.30883015	0.758591953
PLEKHO2	-3.069980518	0.002853855	0.174726222	0.861907515	-0.038927145	0.969086338
PLIN2	-1.166489364	0.246597377	3.357531715	0.00139845	2.107444333	0.039550381
PLIN3	-1.6421352	0.104164171	1.588803789	0.117579532	0.741724392	0.461340833
PLIN4	-0.387559657	0.69928643	0.337120314	0.737251622	0.221690833	0.825357625
PLIN5	-0.696816938	0.487771071	-0.786909416	0.434560936	-1.170588511	0.246700803
PLK1	1.539217047	0.127372823	-0.151930196	0.879772929	1.440836242	0.155175836
PLK1S1	0.835962363	0.405461224	-0.082330143	0.934669636	-0.284100792	0.777376576
PLK2	-0.798206718	0.426919311	0.432316255	0.667125898	0.281023611	0.779723664
PLK3	0.177802235	0.859290733	-1.308353223	0.195950712	-0.737295927	0.464007583
PLK4	0.28288642	0.777934431	-1.296702286	0.199904635	-1.258029855	0.213579415
PLLP	2.120220968	0.036828455	1.044728942	0.300513217	1.426252981	0.159323336
PLN	0.430551075	0.667857275	0.388550502	0.699041285	-0.618233404	0.538917403
PLOD1	-2.755841828	0.00712739	1.359824096	0.179185048	1.378766534	0.173426558
PLOD3	-0.124563736	0.901155679	1.114302622	0.269777699	0.765605865	0.447112277
PLP2	-2.201743823	0.030323593	-1.112267507	0.270644147	-1.374638631	0.17469644
PLRG1	1.428229562	0.15679821	0.241225076	0.81023737	1.419032577	0.161408456
PLS1	0.610118934	0.543369987	0.126691179	0.899626075	-0.903822119	0.369945221
PLS3	-0.756115412	0.451618102	1.321296691	0.191627433	1.861934886	0.067840411
PLSCR1	-1.212408897	0.228630937	0.249062208	0.80419869	-2.570771619	0.012820465
PLSCR3	0.606811665	0.545552816	-3.950943095	0.000214921	-4.841311253	1.05E-05
PLTP	-0.817004564	0.416152669	-0.493181399	0.623759603	0.808164559	0.422401405
PLVAP	-1.983329852	0.050477489	1.095860699	0.277700846	-0.087747063	0.930389427
PLXDC1	2.062295969	0.042155825	-1.793838633	0.078088541	0.718984612	0.475127438
PLXDC2	-1.909039669	0.05954625	-0.170598175	0.865137608	-0.946217733	0.348085608
PLXNA1	2.847417474	0.005495645	0.70440269	0.484020538	1.492606568	0.141129416
PLXNA2	-3.248444791	0.001648603	-1.065071842	0.291288061	-1.343062817	0.184647043
PLXNA3	1.717278308	0.089478532	-0.47271771	0.638201041	0.432271127	0.667197432
PLXNA4	1.558453773	0.122748307	1.893991833	0.0632516	1.538986204	0.12941586
PLXNB1	1.126550315	0.263023745	0.054018106	0.957107672	-0.213028192	0.832075336
PLXNB2	0.093200204	0.925958342	0.521081374	0.604307841	0.418958627	0.676842284
PLXNB3	-1.427750516	0.156935726	0.411576532	0.68217742	-0.382894446	0.703241502
PLXNC1	-1.221143549	0.225323529	-0.604609968	0.547812703	-1.683566263	0.097809343
PLXND1	0.218128709	0.82783855	0.471701529	0.638921917	0.552672247	0.582678058
PM20D1	0.527291484	0.599330367	0.25009661	0.803402548	-1.069236961	0.289530972
PM20D2	1.596224824	0.114057003	-0.068304041	0.945780182	-1.934623957	0.058072721
PMAIP1	-0.656845457	0.513010348	-0.051160533	0.959374618	-0.558968938	0.578402127
PMEL	0.910072977	0.365293079	0.617025448	0.539649533	1.054571476	0.296131126
PMEPA1	0.662241419	0.509563207	1.715997498	0.091537318	1.734705512	0.088271233
PMF1	-2.511213116	0.01387518	0.820973658	0.415046395	1.949937654	0.05617545
PMFBP1	0.292623341	0.77050532	0.478708208	0.633958547	0.058279469	0.953732615
PML	-1.77043879	0.080149382	-0.351826268	0.726252517	-1.922493244	0.0596142
PMM1	0.198955647	0.84276066	2.341128506	0.022708796	2.536896298	0.013985114
PMM2	-0.667720134	0.506075861	-0.271605716	0.786896371	-1.521676991	0.133693437
PMP22	0.49313047	0.623160152	0.135089926	0.893011773	-0.928892449	0.35691485
PMPCA	0.48274204	0.630488781	1.764601137	0.082932811	2.339961435	0.022860038
PMPCB	0.900928807	0.370108035	0.139581631	0.889477501	0.617127456	0.539641351
PMS1	1.586839802	0.116169147	-0.726416495	0.470525392	-1.643551197	0.105848875
PMS2	1.458514001	0.148291974	0.183340383	0.855174839	1.451747642	0.152128041
PMS2CL	0.701374791	0.484937004	0.365979088	0.715721506	2.036197322	0.046450391
PMS2P1	1.299860693	0.19707478	-0.336157778	0.737973495	0.545825887	0.587344375
PMS2P3	2.702874142	0.008262349	-1.475096891	0.145632261	-0.840863203	0.403985725
PMS2P4	-0.299682858	0.765132343	-1.529395292	0.131640426	-0.964156213	0.339095418
PMS2P5	2.1341343	0.035639319	-0.336272151	0.737887706	-1.990914675	0.051357382

PMVK	-0.58259906	0.561666929	1.822274968	0.07360607	1.94298395	0.057030301
PNISR	2.40707394	0.018190486	-1.216713378	0.228675658	-1.827320123	0.072959329
PNKD	-1.511937513	0.134165975	0.443930547	0.65875618	0.274958308	0.784355933
PNKP	0.750029995	0.455255808	-0.700765672	0.486270705	-0.785054268	0.435716614
PNLDC1	0.13882376	0.889909563	0.970087907	0.336058933	-1.847570085	0.069926689
PNMA1	0.478236235	0.633679042	-0.164193146	0.870153944	-0.849184158	0.399379328
PNMA3	2.919842297	0.004456333	1.936808	0.057679475	2.824168019	0.006546336
PNMA5	1.013792091	0.313487065	0.536724802	0.593524971	0.412675645	0.681413378
PNMAL1	0.843477818	0.401269434	-0.169820575	0.865746321	1.061460317	0.293018053
PNMAL2	-1.262501576	0.210134707	0.111632675	0.911502788	0.676748741	0.501339625
PNN	2.235247572	0.027956175	0.301002233	0.764496746	0.607683201	0.545843816
PNO1	1.182055438	0.240397492	-0.523341568	0.602744341	0.602084864	0.549537557
PNOC	-1.757799365	0.082291316	0.97576255	0.333262679	2.278439301	0.026519113
PNP	0.68300497	0.496414585	0.501944807	0.617619834	1.217683422	0.228428984
PNPLA1	-2.982620383	0.003705388	0.860903654	0.392860848	-0.456853381	0.649536247
PNPLA2	-0.698051694	0.487002404	-1.171142247	0.246363965	-0.49781693	0.620554212
PNPLA4	0.129910452	0.89693652	-0.884279782	0.380221704	-0.434397677	0.66566192
PNPLA6	-0.498976757	0.619052397	0.862272756	0.392113464	0.470843219	0.63957396
PNPLA7	1.451316623	0.150280355	1.527664301	0.132069361	2.195289867	0.032286055
PNPLA8	-0.038312219	0.969526336	-0.215051506	0.830486821	-0.923828532	0.359522633
PNPO	0.957448068	0.340988526	2.600220487	0.011816966	5.183074389	3.07E-06
PNPT1	0.41796079	0.677003366	-0.124560832	0.901304954	-1.798541533	0.077458715
PNRC1	-1.674225537	0.097670052	0.046700792	0.962913251	-0.894663856	0.374780028
PNRC2	0.383632831	0.70218449	0.311526656	0.756525122	-0.326541081	0.745227209
POC1A	1.675787036	0.097362642	1.463697322	0.148713182	1.259507919	0.213049316
POC1B	-0.538321114	0.591727031	-0.785869007	0.4351654	-1.296592291	0.200066699
POC5	1.325425182	0.188492775	-0.141460447	0.88799983	0.392079598	0.696481369
PODN	0.356230756	0.722528321	-1.642117624	0.106015078	-1.053756942	0.296500712
PODNL1	1.016717061	0.312101094	0.23204656	0.817324356	0.269406639	0.788602816
PODXL	-0.801779073	0.424860592	-0.550016538	0.584434739	0.652063486	0.517016556
PODXL2	-1.337076528	0.184675727	0.014449893	0.988520992	-0.014221905	0.988703279
POFUT1	-0.312592527	0.755336683	1.468988096	0.147276969	1.94085345	0.057294428
POFUT2	1.0171523	0.311895211	-2.368537576	0.021235112	-1.364987403	0.177693284
POGK	1.777317039	0.079003257	-0.081316648	0.93547205	0.389385553	0.698461611
POGLUT1	0.604668075	0.546969966	-0.772714658	0.442850747	-1.086420773	0.28192776
POGZ	1.148733151	0.253807748	-0.827584098	0.411321925	0.014420153	0.988545818
POLA1	-0.051299454	0.959204249	1.664978722	0.101348158	2.22268257	0.030274649
POLA2	-0.14755638	0.883033573	2.466616487	0.01663712	3.2935367	0.001714661
POLB	-2.419790447	0.017606126	-0.657570549	0.513433574	-0.526173275	0.600836853
POLD1	0.322355142	0.747955346	0.729814222	0.468461631	1.36824929	0.176676055
POLD2	0.139474572	0.889396822	0.61466379	0.541197496	1.827731012	0.072896712
POLD3	-2.788959244	0.006492067	0.978511028	0.331913885	0.71312338	0.478718254
POLD4	-0.907170485	0.366817088	0.762163684	0.449072253	0.996882872	0.323091539
POLDIP2	-1.144579099	0.255515947	3.077230489	0.003195848	3.974262039	0.000203357
POLDIP3	-1.941121498	0.055473737	0.928012323	0.357273955	0.592347779	0.555992015
POLE	2.114421839	0.037334153	0.834787418	0.407286674	1.221633274	0.226942711
POLE2	1.142100887	0.256538882	-0.45149933	0.653325245	-0.817032255	0.417357876
POLE3	-1.242090164	0.217533955	-0.557424802	0.579397231	1.196164211	0.236651475
POLE4	-0.71806967	0.474634063	-0.146571357	0.883982184	0.144808253	0.885380002
POLG	1.315467869	0.191801505	-0.279151278	0.781128655	0.625195095	0.534371817
POLG2	2.217223999	0.029208793	0.690112249	0.492895095	1.170076043	0.246905256
POLH	-0.620067065	0.536830896	1.876496867	0.065656416	2.424671267	0.018559835
POLI	0.890524035	0.37563532	-1.807116067	0.075967974	-2.893593599	0.005410086
POLK	0.985981464	0.326870491	-1.265163727	0.210907282	-2.320031071	0.023992856
POLL	-1.835833411	0.069791037	0.833048346	0.408258666	0.360018407	0.720181944
POLM	1.796192174	0.075927591	-1.362927744	0.17821019	-0.218639944	0.827722061
POLN	0.562846871	0.574983634	-0.16601063	0.868729959	-0.913882018	0.364680505
POLQ	0.652676002	0.515682391	-0.488871411	0.626789148	0.279434991	0.780936176
POLR1A	0.988698314	0.325546647	0.539601615	0.59155189	1.257288011	0.213845842
POLR1B	1.976344731	0.051276837	1.941869586	0.057049292	2.113112515	0.039041905
POLR1C	1.39114923	0.167719992	-0.664908058	0.508762953	1.042836246	0.301486464
POLR1D	-3.479171011	0.000787382	-0.158007182	0.875003808	-0.109339111	0.913322753
POLR1E	2.088305668	0.039686729	2.703476376	0.009004058	4.110619236	0.000129658
POLR2A	-1.007551515	0.316457861	-0.267289846	0.790200783	-0.10701628	0.915156929
POLR2B	2.320099194	0.022671291	0.211395989	0.833324503	0.343202733	0.732725653
POLR2C	-1.30625569	0.194901156	-2.01490013	0.048591386	-0.79642553	0.429134126
POLR2D	0.613313884	0.541265497	-0.133098758	0.894579214	1.190718231	0.23876605
POLR2E	-2.120384742	0.036814261	-0.884173836	0.380278403	-1.059410718	0.293941902
POLR2F	-1.140016527	0.257401483	-2.35603948	0.021896149	-1.523618889	0.133208017
POLR2G	-0.935936179	0.351891184	-0.322349273	0.748355279	0.298545237	0.766387423
POLR2H	1.724602597	0.088142617	0.411858027	0.681972242	1.626804646	0.109369561
POLR2I	-1.056857889	0.293495965	-3.40524522	0.001210133	-1.629350347	0.10882832

POLR2J	-2.112218663	0.037527845	-2.223161368	0.030143521	-2.421997541	0.01868361
POLR2J2	-0.606636464	0.545668574	-2.717144786	0.008681632	-5.199588928	2.89E-06
POLR2J3	-0.302297029	0.763145603	-0.63325144	0.529075979	0.349786369	0.72780561
POLR2J4	0.026274432	0.979098492	-2.167501285	0.034343937	-0.970959587	0.335726147
POLR2K	0.362484061	0.717867512	-1.676280216	0.099103825	-1.27635135	0.207077261
POLR2L	-0.665949191	0.507201714	-0.754013187	0.453912942	-0.447806151	0.656013445
POLR3A	1.497184966	0.13795665	2.440449534	0.017766874	2.156772879	0.035312849
POLR3B	0.969869939	0.33479417	1.587121934	0.117960267	1.815065824	0.074847657
POLR3C	-0.655279364	0.514013135	1.407269875	0.164721147	1.353720715	0.181241293
POLR3D	2.82718046	0.005823492	2.090196155	0.041025117	1.806503446	0.076191264
POLR3E	3.117537471	0.002470538	-1.700315734	0.094465929	-1.777255462	0.080934069
POLR3F	2.464746985	0.015672146	-0.130705883	0.896463435	0.072847083	0.942186282
POLR3G	1.220109818	0.225713132	-0.794115086	0.430388216	-1.161418746	0.250377582
POLR3GL	0.742376626	0.459854531	-0.828489101	0.410813615	-0.643989069	0.522200436
POLR3H	0.416235701	0.678260361	2.212930512	0.030879819	2.266738403	0.027271106
POLR3K	0.124726863	0.901026911	0.620598929	0.537311607	0.979827703	0.331367681
POLRMT	1.895458895	0.061344735	-0.30959512	0.757986184	-0.217118184	0.828902024
POM121	0.229561099	0.818970514	0.208277779	0.835746852	0.717885113	0.475799875
POM121C	-0.45883081	0.647497437	0.527213327	0.600070387	0.984383409	0.329143331
POM121L10P	-0.374801403	0.708718284	1.422676262	0.160223713	0.646561742	0.520545773
POMC	0.269708783	0.788021606	0.243796094	0.808255048	-0.463502226	0.644793375
POMGNT1	2.199891208	0.030459469	0.063832855	0.949324341	1.712805275	0.092257309
POMP	-1.423393072	0.158190858	-0.51668802	0.607352296	0.344315138	0.731893539
POMT1	2.037062079	0.0446765	0.753980428	0.453932458	1.180910471	0.242608687
POMT2	3.437890026	0.000900828	-0.499475602	0.619347047	0.834827706	0.407347263
POMZP3	0.081019369	0.935612239	2.614863045	0.01137452	2.986647653	0.004172542
PON2	0.747844952	0.456566054	-1.555775936	0.125239564	-0.661530385	0.510973717
POP1	0.194186575	0.846481521	1.088076333	0.281093561	2.904541499	0.005248567
POP4	-0.265133757	0.791532254	0.713147516	0.478634043	2.379319004	0.020762212
POP5	1.715199505	0.089860703	-0.445510479	0.65762097	0.662014781	0.510665543
POP7	-0.545088777	0.587084127	-0.511738999	0.6107902	-0.117963957	0.906516546
POPDC2	1.581500309	0.11738471	-1.054165746	0.296209218	0.967950608	0.337213561
POR	-1.445743354	0.151834211	0.649325815	0.51870889	0.566700009	0.573172951
PORCN	-0.387004563	0.69969583	-0.644199431	0.522003392	-0.175562627	0.861268191
POT1	0.504144562	0.615431406	-0.16686915	0.868057467	-0.501799256	0.617767714
POU2AF1	0.697942783	0.487070177	0.994675177	0.324054812	1.49868947	0.139547029
POU2F1	0.356015881	0.722688662	-0.346388134	0.730313288	-0.404907576	0.687081558
POU2F2	1.231738915	0.221358383	-0.759372865	0.450726365	-0.38190664	0.703969961
POU5F1	0.024602964	0.980427877	1.922603115	0.059479828	1.543822756	0.128240326
POU5F1B	-0.014232434	0.988677061	1.468426892	0.147428794	2.014851711	0.048710603
POU5F1P3	-0.198173657	0.843370532	0.237837919	0.812850863	1.159998327	0.250950621
POU6F1	3.780362368	0.000286101	1.12731071	0.264285649	2.200502908	0.031894446
PP7080	1.343392172	0.182631155	1.344257926	0.18413586	1.96096106	0.054842746
PPA1	0.612169489	0.542018825	2.682115343	0.009529972	3.444040698	0.001089548
PPA2	1.399490371	0.165213829	0.675368119	0.50214441	1.620234159	0.110776613
PPAN	1.150721323	0.252993054	0.040054714	0.968188027	1.136096615	0.260734502
PPAN-P2RY11	1.473492466	0.144219552	1.662523117	0.101841263	1.852785466	0.069163061
PPAP2A	1.681341841	0.096275449	-0.770683045	0.444044766	-1.030170159	0.307340478
PPAP2B	0.3846397	0.701440983	3.002468774	0.003956304	0.945001928	0.348700512
PPAP2C	0.428627713	0.669251283	-1.835012141	0.071669426	-1.879307205	0.0653878
PPAPDC1B	1.224305417	0.224134886	0.183256172	0.855240605	0.886272743	0.379244887
PPAPDC2	1.335578168	0.185163313	-2.462205076	0.016822926	-1.774875791	0.081330553
PPAPDC3	-1.59584636	0.114141579	1.176847357	0.244097114	0.245379551	0.807055831
PPARA	-0.205143685	0.837938045	-0.918769695	0.362047771	-0.520974077	0.604430271
PPARD	0.16740933	0.867435663	0.37314778	0.710408288	1.39993108	0.167026649
PPARGC1A	-0.706229166	0.481928566	0.401155164	0.689790311	1.849473608	0.069647159
PPARGC1B	3.996091571	0.00013427	-1.039562934	0.302887393	-0.640002998	0.524769624
PPAT	2.342913713	0.021410186	-0.222970777	0.824347121	-0.529824343	0.598319361
PPBP	-3.97543668	0.000144513	1.783947528	0.079700221	0.773744624	0.442322375
PPCDC	-1.316492065	0.191459182	0.499667442	0.619212777	0.819889209	0.415740769
PPCS	-0.27675301	0.782624794	-1.249275687	0.216617482	-1.012385813	0.315689693
PPDPF	-1.124070166	0.264068563	-2.475733696	0.016258957	-2.159016671	0.035129931
PPEF1	1.098752762	0.274900759	-1.965158834	0.054224919	-0.716762418	0.47648705
PPFIA1	-0.849119693	0.398140104	-0.507635411	0.613647511	-0.540813579	0.590771868
PPFIA3	2.121684706	0.036701756	-2.172941873	0.033911852	0.027983216	0.977774606
PPFIA4	1.971413002	0.051847674	2.521778308	0.014464995	1.256960164	0.213963664
PPFIBP1	1.943325827	0.0552028	0.879829414	0.382607995	0.853274892	0.397126683
PPFIBP2	-1.068939451	0.288047482	2.33312495	0.02315599	1.789419068	0.078932543
PPHLN1	0.658123849	0.512192548	-0.988023265	0.327273815	-0.225219986	0.822624564
PPIA	1.34964469	0.180623881	-1.183379664	0.241520091	-0.283951768	0.777490195
PPIB	-0.530600982	0.597044221	0.064079181	0.949129059	0.901057021	0.371400754
PPID	-1.743018935	0.084855735	2.097319912	0.04036535	1.58239868	0.119166879

PPIE	1.070134133	0.287512504	1.563951227	0.123307269	1.899513787	0.062629903
PPIEL	2.232871347	0.028118578	-0.099255728	0.921279883	0.909772901	0.366825124
PPIF	-0.724592616	0.47064201	2.088132041	0.041218029	1.521178586	0.133818251
PPIG	0.491632685	0.624214464	-0.180470676	0.857416575	-0.099863916	0.920807517
PPIH	-0.278609928	0.781203908	-3.263166683	0.001855399	-0.806270691	0.423483281
PPIL1	-0.011783823	0.990625009	-1.161900003	0.250068309	-0.246582103	0.806129538
PPIL2	0.377595851	0.706648468	-1.427402701	0.158863233	-0.511780185	0.610808736
PPIL3	0.753149971	0.453388673	-2.369917393	0.021163236	-1.032152644	0.306419146
PPIL4	0.723590918	0.471253823	-0.140834415	0.888492154	-0.438445217	0.6627433
PPIP5K1	1.936090931	0.056096276	0.981661177	0.330372433	1.926899965	0.059050243
PPIP5K2	0.602924528	0.54812401	-0.635415579	0.527673951	-1.878578899	0.065489093
PPL	-0.260132971	0.795374533	-1.794054183	0.078053724	-1.066018424	0.290970682
PPM1A	-4.194198629	6.56E-05	-0.62921046	0.531699108	-1.084345477	0.282838551
PPM1B	0.522635366	0.602553549	0.19025464	0.849778571	-1.055347007	0.295779532
PPM1D	-1.595007537	0.114329211	-1.181937482	0.242087341	-2.890922056	0.005450193
PPM1F	-0.603763366	0.547568635	-0.935473563	0.353450035	0.07689261	0.938981891
PPM1G	-0.650676865	0.516966162	2.395110371	0.019888606	3.136492305	0.00271942
PPM1H	0.018119578	0.98558486	1.907358775	0.061464927	1.036675388	0.304324304
PPM1J	1.303141497	0.195957408	-1.044218826	0.300747086	0.462548263	0.645472967
PPM1K	1.135314465	0.259354919	1.136980237	0.26025473	1.052997309	0.296845673
PPM1L	-1.67257052	0.097996733	0.505263895	0.615301542	-0.139937958	0.889209001
PPM1M	-0.618170936	0.538074145	1.107760183	0.2725701	1.84030101	0.071002858
PPM1N	3.490312305	0.000759158	-1.281767425	0.205060065	-1.196558671	0.236498842
PPME1	-0.039684741	0.968435196	0.859395763	0.393685022	1.760161604	0.083818222
PPOX	0.818341126	0.415393398	1.033254876	0.305803742	1.021562772	0.311362465
PPP1CA	-2.062568834	0.042129251	0.179175399	0.858428798	0.251864326	0.802064112
PPP1CB	-0.034196714	0.972798454	0.279478761	0.78087861	-0.411089212	0.682569475
PPP1CC	1.13206922	0.260709209	-0.534676794	0.594931492	-0.601893655	0.549663939
PPP1R10	-1.490407948	0.139725925	1.113703677	0.270032496	1.035980158	0.304645683
PPP1R11	-2.7431288	0.007386	-0.673223741	0.503497433	-0.137122477	0.891423735
PPP1R12A	0.107026565	0.91501379	-0.532733449	0.596267576	-2.072244615	0.042839058
PPP1R12B	-1.2360564	0.219757304	-0.752503444	0.454812902	0.46568582	0.643238954
PPP1R12C	-0.227122785	0.820859966	0.292071238	0.771281628	0.195054375	0.846053378
PPP1R13B	1.618412389	0.109185821	-1.386964373	0.170796974	0.140232963	0.888976994
PPP1R13L	1.961686039	0.052989413	0.260133817	0.795688253	0.357603864	0.721978427
PPP1R14A	-0.960971268	0.339224089	0.133366652	0.894368304	-1.188100613	0.239787281
PPP1R14B	1.419704654	0.159259314	-0.090539236	0.928172849	-0.51312027	0.609877116
PPP1R15A	-1.776037708	0.079215399	-0.518045311	0.606410987	-1.237034095	0.221215237
PPP1R15B	-0.338414918	0.73586411	-1.183876453	0.241324913	-2.165810548	0.034581138
PPP1R16A	0.355634042	0.722973624	-0.410835202	0.682717884	-0.698178455	0.487942608
PPP1R16B	-0.105582313	0.916156289	0.030875458	0.975475605	0.721784564	0.473417451
PPP1R2	1.324971606	0.188642557	0.656737532	0.513965268	0.554687758	0.581307725
PPP1R3B	-1.063179165	0.290636513	-0.106528933	0.915532881	-1.359478419	0.179421437
PPP1R3D	-0.900308313	0.370436209	-1.546808223	0.12738693	-2.477369471	0.01626605
PPP1R3E	2.661100964	0.009270885	0.309693892	0.757911449	2.592321815	0.012126156
PPP1R3F	3.107226827	0.002549331	0.075888923	0.939770456	-0.102128962	0.919017602
PPP1R3G	-0.465532336	0.642711047	-0.107302174	0.914922157	-0.185632859	0.853400615
PPP1R7	-1.247651664	0.215499242	2.620531434	0.011207332	3.066039016	0.003330731
PPP1R8	0.051147679	0.959324841	-0.951251724	0.345451456	-0.207845373	0.836100577
PPP1R9A	1.389656761	0.168171464	-0.152156016	0.879595628	-0.803227045	0.425225457
PPP1R9B	-1.124510453	0.26388287	-0.218114915	0.828110521	-0.031563907	0.974931584
PPP2CA	0.561742538	0.575732625	-0.718410077	0.47540876	-0.189016753	0.850760194
PPP2CB	-0.732337093	0.465926959	-1.314732564	0.193810851	-1.015140591	0.314386488
PPP2R1A	-1.31435556	0.192173798	1.931405575	0.058358636	2.452384343	0.017319651
PPP2R1B	0.322074162	0.748167466	1.210114322	0.231178034	2.009306167	0.049313093
PPP2R2A	-2.304043527	0.023597729	-2.087990728	0.041231265	-2.118938873	0.038525192
PPP2R2B	-0.181793136	0.856167053	-2.604625412	0.011682245	-1.60095804	0.114989389
PPP2R2D	-0.487642639	0.627026933	-0.624902511	0.534502965	1.035680733	0.304784168
PPP2R3A	-0.636390838	0.526188895	-1.319126951	0.192347087	-1.455343815	0.151133867
PPP2R3B	1.992263565	0.049470652	0.706562313	0.482687159	-0.109686418	0.913048549
PPP2R3B-AS1	-0.045772736	0.963596065	0.736681825	0.464306027	1.014362695	0.314754119
PPP2R3C	-1.760222431	0.081877073	0.495662663	0.622018444	-0.968965139	0.336711568
PPP2R4	-1.061881775	0.291221832	2.127071421	0.037708735	2.588354032	0.012251364
PPP2R5A	-2.241500855	0.02753272	-0.857733653	0.394594731	-0.630675199	0.530807651
PPP2R5B	-2.540347051	0.012845111	-1.50125008	0.138753507	-1.746884025	0.086116995
PPP2R5C	-0.913768356	0.363358579	0.234002492	0.81581282	0.109073034	0.913532831
PPP2R5D	0.037197648	0.970412449	1.02532279	0.309497951	1.161275752	0.250435227
PPP2R5E	-0.869349729	0.387042806	-1.43322596	0.157199396	-2.128997338	0.037647188
PPP3CA	-0.437658435	0.662716164	0.137930077	0.890776758	-0.91911412	0.361961449
PPP3CB	2.199506134	0.030487778	0.404837883	0.687096323	0.43759263	0.663357652
PPP3CC	3.797289959	0.000269862	-1.179216478	0.243160205	-0.261593162	0.794590842
PPP3R1	-2.502974383	0.01417963	-0.405839006	0.686364683	-0.278688198	0.781506354

PPP4C	-1.871647299	0.064608508	0.251748301	0.802131734	0.268319256	0.789435397
PPP4R1	-0.983203371	0.32822785	-1.112363325	0.270603309	-2.059270864	0.044109726
PPP4R1L	-1.865895636	0.065418369	0.15782961	0.875143099	-1.330923028	0.188585334
PPP4R2	0.356189202	0.722559328	0.29188064	0.771426624	-1.044460703	0.300741224
PPP4R4	0.742779419	0.459611845	-0.682575107	0.49761157	-0.066413373	0.94728427
PPP5C	2.255364106	0.026613956	-2.051632402	0.044761794	-0.005834357	0.995365531
PPP6C	-0.726569335	0.469435988	-1.022806361	0.310676224	-0.230631781	0.818437842
PPP6R1	-1.238788996	0.218748337	0.615238183	0.540820797	0.846747719	0.400724745
PPP6R2	0.727281748	0.469001762	1.347722367	0.1830251	1.907694866	0.061541731
PPP6R3	0.831775716	0.407807853	0.79448618	0.430173967	0.576498768	0.566578492
PPPDE1	1.112458102	0.268999116	-1.72074711	0.090665232	-2.444624301	0.017659174
PPPDE2	-1.707905062	0.091212315	-2.171652262	0.034013838	-1.062933486	0.292355264
PPRC1	1.916610272	0.058563144	0.836730134	0.406202539	1.301098609	0.198530408
PPT1	-1.6067649	0.111721694	-0.093722676	0.925654728	0.166942694	0.868013971
PPT2	-0.256619722	0.798076913	-0.088244829	0.929988199	1.061141615	0.293161575
PPT2-EGFL8	-0.10003306	0.920547734	-2.951198061	0.004571896	-3.921615318	0.000241494
PPTC7	-0.589441123	0.557089711	-1.89307729	0.063375435	-3.583852624	0.000708203
PPWD1	2.087566125	0.039755171	-1.675623747	0.099233068	-1.608566648	0.113311325
PQBP1	-1.110276521	0.269932558	1.442753467	0.154506441	1.015744074	0.314101484
PQLC1	-1.787146595	0.077388923	1.318669209	0.19249917	0.506811374	0.61426867
PQLC2	-0.298400581	0.766107434	0.578563801	0.565139147	1.072337194	0.288148846
PQLC3	0.967430986	0.336004536	-1.364978771	0.177568188	-1.207716595	0.232210973
PRADC1	-0.225569126	0.822064451	0.2797816	0.780647401	0.243732931	0.808324627
PRAF2	-1.659764766	0.100554562	-2.976007106	0.004263674	-2.574180509	0.012708285
PRAM1	-1.760204135	0.081880194	0.646021084	0.520831432	1.45530456	0.151144691
PRAME	0.709858906	0.479685844	1.370936964	0.175713207	-0.252827105	0.801323705
PRC1	1.530990147	0.129392202	1.03385755	0.305524291	1.096425815	0.277565496
PRCC	-1.33148154	0.186501353	0.268016404	0.789644227	0.703031185	0.484936637
PRCD	-0.786578079	0.433661635	0.135747111	0.892494533	-1.211181085	0.230891204
PRCP	-2.577133589	0.011643025	1.231866194	0.223004704	0.841980378	0.403365377
PRDM1	0.925085421	0.357474848	-2.245368565	0.028598369	-3.317843752	0.001594763
PRDM10	-0.415342362	0.678911656	-1.033406305	0.30573351	-2.221999555	0.030323427
PRDM11	-0.294421739	0.769135489	0.096099526	0.923775122	0.163379396	0.870805449
PRDM15	1.629329438	0.106851214	0.355451188	0.723550082	0.294179202	0.769704078
PRDM2	0.614209771	0.540676126	-0.897385586	0.37324904	-1.073600379	0.287587013
PRDM4	1.427654552	0.156963285	-0.205791416	0.837679505	0.249209285	0.804106866
PRDM5	-2.314320442	0.023000964	-1.001486671	0.320780585	-2.507023564	0.015090832
PRDM8	-0.165954472	0.868577001	-3.820694345	0.000328474	-4.252765294	8.05E-05
PRDX1	0.686135135	0.494448477	-0.216976165	0.828993667	0.830993268	0.409491767
PRDX2	-0.636213862	0.52630368	1.785651369	0.079420631	2.404143523	0.019529304
PRDX3	-0.431099112	0.667460284	-0.476811035	0.635300806	-1.317534917	0.193001998
PRDX4	0.935028474	0.352356113	0.514969262	0.60854525	0.696503565	0.488982492
PRDX5	-2.709776602	0.008105659	-0.362781119	0.718096329	-0.730757518	0.467960974
PRDX6	-3.608490734	0.000513109	2.502254847	0.015202581	2.710362023	0.008894651
PREB	0.820499343	0.414169122	0.158368105	0.874720706	1.320361123	0.192063208
PRELID1	0.035239572	0.971969262	-1.640735005	0.106302848	-1.676226675	0.099245261
PRELID2	-0.478763816	0.633305138	0.150036019	0.881260373	-0.456141469	0.650044951
PREP	-0.636702794	0.525986593	1.747934152	0.085804434	2.205328149	0.031535701
PREPL	1.557188001	0.123048441	-1.041576395	0.301960536	-1.343146818	0.184620012
PREX1	-2.69448258	0.008456553	-0.05572737	0.955751861	-1.042802774	0.301501833
PRF1	-0.303618778	0.762141692	-0.828148951	0.411004621	-0.35701675	0.722415494
PRG2	-1.636167746	0.105409455	1.2109131	0.230874078	0.367668003	0.714500955
PRG4	0.151279628	0.880104612	1.782696985	0.07990595	0.600284509	0.550728101
PRH1-PRR4	0.056175634	0.955330425	-1.280896358	0.205363786	-3.108111336	0.002951803
PRH2	1.424510244	0.15786833	0.527278322	0.600025546	1.500438838	0.13909456
PRIC285	-1.253761741	0.213279945	-0.518281871	0.606246996	-2.104652972	0.039802899
PRICKLE1	0.106526874	0.915409059	3.085549711	0.00312025	2.115444438	0.038834377
PRICKLE2	0.509012876	0.612028955	-0.603388374	0.548619273	0.753082687	0.454541398
PRICKLE3	-0.772218967	0.442072914	-0.903619043	0.369961353	-0.956123899	0.343101881
PRICKLE4	0.505358699	0.614582058	-0.325863	0.745708986	-1.072691572	0.287991151
PRIM1	0.683031199	0.496398093	-0.003820143	0.996965175	1.310120018	0.19548148
PRIM2	1.467872261	0.14573726	2.10047599	0.040076013	2.367835773	0.021355743
PRKAA1	1.586450883	0.116257346	-2.553898552	0.01332171	-3.298171565	0.001691161
PRKAB1	-3.908605782	0.000183037	-0.714899518	0.477558928	0.128487716	0.898221421
PRKAB2	1.813486711	0.073197364	-0.483675671	0.630449901	-0.238454265	0.812395556
PRKACA	-1.775311308	0.079336063	-1.290036841	0.202193382	-1.037141449	0.30410899
PRKACB	1.124937697	0.263702765	-0.905298626	0.369078664	-0.957002277	0.342662245
PRKAG1	-2.198794463	0.030540158	-0.561327402	0.576751957	0.856891703	0.395141583
PRKAG2	-0.522049564	0.60295963	-1.647561568	0.104888162	-2.594935931	0.012044303
PRKAR1A	-2.661829559	0.009252377	-0.474245907	0.637117602	-2.063460536	0.043695857
PRKAR1B	0.197109145	0.844200894	1.9921237	0.051105168	2.823118899	0.006565085
PRKAR2A	-0.515666481	0.607392514	-0.060036795	0.95233416	-0.173382031	0.862973719

PRKAR2B	-5.047529125	2.42E-06	0.819934794	0.415633569	-0.64250358	0.523157118
PRKCA	2.528636952	0.013250568	1.500263774	0.13900818	1.850967146	0.069428494
PRKCB	-2.327189147	0.022272526	1.2432012	0.218830579	0.174943862	0.861752084
PRKCD	-2.543570558	0.012735476	0.554165988	0.581610598	-0.265510251	0.791587318
PRKCDBP	0.154597869	0.877495661	0.226880415	0.82132007	-0.055625852	0.955837027
PRKCE	-0.542299428	0.58899566	0.131523074	0.895819886	-0.801437409	0.426251843
PRKCH	1.979315024	0.050935621	-0.075939801	0.939730156	0.915410388	0.363884879
PRKCI	0.936807307	0.351445362	-0.997166245	0.322854795	-1.036609495	0.304354754
PRKCQ	3.358739454	0.001162708	1.030871535	0.306910569	2.033499413	0.046730966
PRKCSH	-1.381336281	0.170705436	0.70139549	0.485880632	1.047313953	0.299435313
PRKCZ	-1.007317084	0.316569826	-0.777106384	0.440276092	-0.276416743	0.783241356
PRKD2	-1.685374899	0.095492301	-1.575364359	0.120649718	-0.934077855	0.354257215
PRKD3	1.364284193	0.175989355	-0.554879744	0.581125473	-1.006207941	0.318625491
PRKDC	-2.381725975	0.01940707	0.683460114	0.49705649	0.39044372	0.697683559
PRKG2	0.57113625	0.569376554	2.901052978	0.005259074	1.405435709	0.165392344
PRKRA	1.05614445	0.293819893	-0.125319675	0.900706874	0.676439673	0.501534295
PRKRIP1	1.044049392	0.299348638	-2.920577165	0.00498086	-2.882125888	0.005584205
PRKRIR	0.643632296	0.521503307	-0.106081714	0.915886128	-0.117401465	0.906960222
PRKX	1.866478785	0.065335873	0.419742763	0.676234987	0.150329784	0.881042331
PRLR	0.036061204	0.971315991	-0.681297153	0.498413706	-1.094625995	0.278346728
PRMT1	0.40054943	0.689731688	-1.385317754	0.171297131	-0.807108562	0.42300444
PRMT10	1.547367853	0.1253968	0.235183735	0.8149003	0.847799595	0.40014355
PRMT2	2.439327631	0.016740919	0.083656647	0.933619509	0.909520557	0.366957089
PRMT3	2.400787018	0.018485721	-1.265194064	0.210896487	-0.652235094	0.516906679
PRMT5	-0.693667383	0.489734759	1.353234431	0.18126835	0.929455406	0.356625699
PRMT6	0.740367597	0.461066077	-1.539223767	0.129225923	-1.475935208	0.145538888
PRMT7	1.894396024	0.0614874	1.579045474	0.119802446	2.20004719	0.031928513
PRNP	-0.146523347	0.883846518	0.940180481	0.351051428	-0.43140266	0.667824936
PROC	-1.144910143	0.25537952	0.042379152	0.966343022	-0.028568807	0.977309634
PROCA1	1.470875504	0.144924709	0.986196016	0.328161778	1.472634608	0.14642459
PROCR	-0.687984592	0.493288797	-1.428649376	0.158505884	-1.670993723	0.100279548
PROK2	0.735828483	0.463810063	-0.465546992	0.643295394	-0.96527054	0.338542043
PROM2	-0.749321238	0.455680572	0.88455124	0.380076453	0.859869045	0.393512088
PRORSD1P	1.666722581	0.099158168	-2.176335882	0.033644726	-2.283449533	0.026202779
PROS1	-2.452883498	0.016163115	1.514984895	0.135245256	0.494056504	0.623190586
PROSC	-0.648100496	0.518623089	0.41783105	0.677624273	0.209430633	0.834868911
PROSER1	0.347497486	0.729055068	-0.720922846	0.473873077	-0.445248504	0.657849399
PROX2	3.148075769	0.002250266	1.023361122	0.310416206	1.304310421	0.197440858
PROZ	1.875378036	0.064087715	0.419341905	0.676526206	0.295550742	0.768661719
PRPF18	0.344089012	0.731607828	0.583057713	0.562130492	-0.056169824	0.955405612
PRPF19	0.138993105	0.88977614	1.715018826	0.091717871	1.960034289	0.054953738
PRPF3	0.817075816	0.416112171	1.406560753	0.164930479	2.976007613	0.004299358
PRPF31	-1.068682224	0.288162757	1.93074909	0.058441628	2.53618904	0.01401042
PRPF38A	-0.222607739	0.824361465	1.486525151	0.142594147	1.732569861	0.088653565
PRPF38B	1.990369935	0.049682623	-1.773910978	0.081363867	-3.077754338	0.003220882
PRPF39	1.784079543	0.077889673	-0.651874569	0.51707503	-1.020439536	0.311889939
PRPF4	-0.725012269	0.470385829	1.851775587	0.069186057	2.591487807	0.012152377
PRPF40A	1.234025418	0.220509409	0.257516645	0.797697792	-0.491005803	0.625333036
PRPF40B	0.409500091	0.683176999	-0.605532793	0.547203797	1.299288062	0.199146591
PRPF4B	2.232914207	0.028115641	-0.694275987	0.490300155	-1.465340993	0.148396814
PRPF6	-0.872011317	0.385597195	3.213589291	0.002148654	3.724653639	0.000454895
PRPF8	-0.276139649	0.783094292	0.975500421	0.333391506	1.162331122	0.250009998
PRPH2	0.21450799	0.83065184	-0.039109002	0.96893873	0.307230915	0.759802466
PRPS1	1.739379749	0.085497126	-0.379116175	0.705995672	0.641542519	0.523776551
PRPS2	0.709067626	0.480174262	0.890515719	0.376893819	0.692148549	0.491692106
PRPSAP1	0.189892775	0.849834555	0.159905506	0.873514979	1.02748643	0.308590702
PRPSAP2	1.160803308	0.248890284	3.413518939	0.001180027	4.392129147	5.01E-05
PRR11	-1.629884505	0.106733596	0.219865105	0.826753614	-0.377727816	0.707054719
PRR12	0.884275346	0.37897958	-1.385022062	0.171387066	-0.695493666	0.489610096
PRR13	-3.345204997	0.001214103	-0.458609624	0.648240401	-0.570653689	0.570507671
PRR14	-1.608689001	0.111299552	-1.450209954	0.152424058	-1.266917195	0.210406712
PRR14L	-1.435218789	0.15480242	0.696476257	0.488931945	0.767670536	0.445894297
PRR22	-0.641034007	0.523182015	-1.111008084	0.271181325	-1.125071755	0.265337579
PRR24	-1.624621577	0.107853006	-0.957938217	0.342097803	-1.790973444	0.078679775
PRR3	-0.1469053	0.883545925	0.229238958	0.819495276	1.081221137	0.284213596
PRR5	0.13058676	0.896403045	-2.219560867	0.030400859	-0.554780149	0.581244947
PRR5L	-1.487353459	0.140529145	-1.633684251	0.107780251	-1.184138308	0.241339135
PRR7	-0.435083126	0.664577164	-3.186033366	0.002329973	-2.819192024	0.006635703
PRRC1	0.797904139	0.427093956	-0.508781253	0.612849057	-0.800529986	0.426772832
PRRC2A	-1.139224599	0.257729754	-0.611501951	0.5432735	-0.332017276	0.741110519
PRRC2B	0.213943899	0.831090337	0.541811505	0.590038326	1.02597389	0.309296843
PRRC2C	0.003264601	0.997402688	0.183432804	0.855102662	-0.048588057	0.961419739

PRRG4	-5.150402342	1.60E-06	0.055655769	0.955808653	-1.296280829	0.20017321
PRRT1	0.033086284	0.973681407	-2.793724028	0.00706299	-2.181569218	0.03333705
PRRT2	0.6584776	0.511966372	-1.948722767	0.056205422	-0.985450618	0.3286237
PRRT3	0.37349342	0.709687845	-0.199544966	0.842539288	0.019950821	0.984153222
PRRT4	-0.175364318	0.861199997	1.442209826	0.154659128	0.245620099	0.80687052
PRSS16	-0.353438285	0.724613048	0.173170644	0.86312444	-0.033532009	0.973369074
PRSS21	0.731118142	0.466667315	0.542826602	0.589343695	1.144929135	0.257087904
PRSS22	1.55038132	0.12467242	-1.997704356	0.050479124	-0.286900352	0.775243052
PRSS23	-0.200321611	0.841695581	-2.349890359	0.022228077	-1.510274048	0.136572265
PRSS27	-0.258523439	0.796612272	-1.129921996	0.263192757	-1.15653128	0.252353275
PRSS30P	1.351853161	0.179918883	-3.283205868	0.00174793	-0.913094711	0.36509079
PRSS33	2.908506277	0.004606046	0.007304902	0.994196826	0.135107181	0.893009556
PRSS35	1.565001957	0.121204911	-0.811960895	0.420157216	-1.041141786	0.302265163
PRSS36	-2.124277442	0.036478259	1.344954372	0.183912157	0.901781604	0.371018985
PRSS41	0.672605425	0.502977052	0.25612303	0.798768412	-0.658536234	0.512880815
PRSS53	0.356957858	0.721985844	0.03882578	0.969163555	0.524690587	0.6018606
PRSS57	-0.306385457	0.76004163	-1.283204532	0.204559711	-1.127933561	0.264137235
PRSS8	-1.277710188	0.204743177	-0.235601805	0.814577399	-0.248919754	0.804329711
PRTFDC1	-1.024817236	0.308284368	-1.029291366	0.3076459	-0.488416023	0.627154344
PRTN3	-2.717259954	0.007938844	1.491566768	0.141269843	0.645193907	0.521425176
PRUNE	-2.768078157	0.006886313	0.401898239	0.689246409	0.021097106	0.983242871
PRUNE2	-1.636754118	0.105286561	0.137585456	0.891047906	0.099701309	0.920936031
PRX	2.843124857	0.005563737	2.871502642	0.005707658	3.28772431	0.001744567
PSAP	-2.344771436	0.021310286	2.211907122	0.030954335	2.05351553	0.044683792
PSAT1	0.156363624	0.876107895	1.137275503	0.260132334	1.71845597	0.09121487
PSD	-0.09504876	0.924494206	-1.018077905	0.312898446	-0.185008471	0.853888005
PSD3	-0.213429659	0.83149013	2.210726827	0.031040473	-0.037659771	0.97009232
PSD4	-1.285402182	0.202055606	0.583369365	0.561922137	1.353370613	0.181352403
PSEN1	-2.986907575	0.003658636	-0.280750669	0.779907678	-0.349304065	0.728165654
PSEN2	-0.055395276	0.955950301	1.388130382	0.17044348	1.17100619	0.246534256
PSENEN	-2.567964801	0.011932742	-0.915700636	0.363641969	-1.192417038	0.238104969
PSIMCT-1	0.515977003	0.607176522	-0.586150284	0.560064653	-0.964469239	0.338939909
PSIP1	1.844998938	0.068432722	1.142482171	0.257980733	0.885116814	0.379862573
PSKH1	0.469779939	0.639685086	1.475406619	0.145549257	1.621377772	0.110530662
PSMA1	-1.408685724	0.162484349	-1.358208944	0.179693975	-0.928283459	0.357227815
PSMA2	0.416322798	0.678196876	-0.481029257	0.632318073	0.267647193	0.789950101
PSMA3	1.06928008	0.287894879	-0.596703364	0.553043749	0.09895111	0.921528964
PSMA4	0.331972446	0.740706784	-0.070353751	0.944155809	-0.126324476	0.899925647
PSMA5	0.325022764	0.745942455	0.493589484	0.623473092	1.228543913	0.224359391
PSMA6	0.522716308	0.602497449	-0.301658987	0.763998535	-0.293850513	0.769953942
PSMA7	-0.122289386	0.902951266	-0.352219866	0.725958914	-0.02768969	0.978007676
PSMB1	-0.79084579	0.431179956	0.820138456	0.415518418	1.857388056	0.068495012
PSMB10	-2.025154986	0.045910166	0.641873024	0.523502106	0.429970185	0.668860481
PSMB2	-2.163244979	0.033258661	-1.658866939	0.102579083	-0.395312625	0.694107755
PSMB3	-1.630475789	0.106608419	0.935660126	0.353354762	0.941420515	0.350515957
PSMB4	-1.459035907	0.148148588	-0.065843878	0.947730139	0.445696522	0.657527645
PSMB5	1.074684261	0.285481209	-0.489630129	0.626255364	0.646371263	0.520668188
PSMB6	-0.659222304	0.511490408	-0.355938234	0.72318725	0.691725025	0.491956056
PSMB7	-1.234260427	0.220422285	-1.654464246	0.103473335	-0.50084443	0.618435309
PSMB8	-1.405271833	0.163493636	1.091712005	0.279505423	0.316963683	0.752444874
PSMB9	-2.51083432	0.013889048	-0.807214321	0.422864018	-2.013862579	0.048817599
PSMC1	-0.678272827	0.499394957	3.018951645	0.003775414	3.674344301	0.000533374
PSMC2	1.624037558	0.107977806	-0.32030371	0.749897258	0.374890402	0.709152088
PSMC3	-0.108606368	0.913764266	2.465043402	0.016703165	3.509814732	0.000890669
PSMC3IP	-1.618447907	0.10917816	-0.169497065	0.865999592	1.034409485	0.3053726
PSMC4	-1.600402804	0.113126675	0.065727044	0.947822751	0.722768056	0.472817638
PSMC5	-1.021117222	0.310023866	1.443459082	0.154308438	2.706424839	0.00898827
PSMC6	0.421828574	0.674188412	0.077761395	0.93828737	0.128004612	0.898601975
PSMD1	-0.619825117	0.536989454	2.369697193	0.021174692	2.607541539	0.011656608
PSMD10	0.802124534	0.424661819	-1.069821983	0.289162387	-1.198653408	0.235689504
PSMD11	-0.212426077	0.832270484	1.295478037	0.200323549	1.665985973	0.101277571
PSMD12	1.383301601	0.170104296	1.160816984	0.250504989	2.280848419	0.026366586
PSMD13	-0.85442529	0.395210981	3.220714407	0.002103978	3.868976339	0.000286465
PSMD14	0.815697136	0.416896195	0.090839198	0.927935545	0.703708334	0.484518005
PSMD2	-0.667388368	0.506286675	2.751770493	0.00791166	2.809991209	0.006803919
PSMD3	-0.348622111	0.728213454	-1.174466315	0.245041356	-1.106044284	0.273416493
PSMD4	-0.883020827	0.379653231	2.5447251	0.01363957	3.290762115	0.001728876
PSMD5	1.418392782	0.159640671	-1.214274398	0.229598209	-0.767482252	0.446005288
PSMD6	-1.927753181	0.057141112	-0.085143446	0.932442625	0.80316672	0.42526003
PSMD7	-1.308225817	0.194235139	1.562657998	0.123611334	2.896484358	0.005366995
PSMD8	-1.178003017	0.24200069	-1.713915344	0.091921802	-1.218246141	0.228216807
PSMD9	-0.746739778	0.457229584	1.702993999	0.093960367	1.240766916	0.219843177

PSME1	-2.341638812	0.021478983	1.799363138	0.077200276	1.380060559	0.173029934
PSME2	-1.957682242	0.053465545	-0.001720841	0.998632915	-0.314223348	0.754514159
PSME3	-1.71036149	0.09075531	0.207231253	0.836560195	0.205084655	0.838246499
PSME4	-0.912380234	0.364084483	0.87304417	0.386264332	1.402283023	0.166326847
PSMF1	-2.516992903	0.013665114	0.270877679	0.787453513	-0.101509046	0.91950744
PSMG1	0.833569344	0.406801513	-0.997969329	0.322468562	0.676123837	0.501733271
PSMG2	-2.243295958	0.027412204	-0.590154851	0.557395223	-0.650317698	0.51813505
PSMG3	0.64546497	0.520320947	0.564011633	0.57493592	0.752888275	0.45465729
PSMG4	1.389271548	0.168288141	-0.39094378	0.697281197	-0.48482757	0.629681846
PSPC1	-0.005540223	0.995592221	0.595251432	0.554007079	-0.216687215	0.829236267
PSPH	-0.228124878	0.820083314	1.207560725	0.232151702	1.631542323	0.108364029
PSRC1	-2.479435442	0.015082783	0.764081898	0.447937384	1.799602938	0.077288738
PSTK	-1.067368211	0.288752121	-0.044563515	0.964609368	-0.787962148	0.43402766
PSTPIP1	-0.736066687	0.463665834	0.396299431	0.693348555	0.914696841	0.364256193
PSTPIP2	-0.139746723	0.889182422	-0.643615182	0.522379563	-1.419976901	0.161134555
PTAFR	-3.729314941	0.000340902	-0.041061976	0.967388498	-0.315103937	0.75384901
PTAR1	0.907927491	0.366419219	-1.498129762	0.139560465	-2.350666106	0.022271502
PTBP1	-0.271168254	0.786902598	-1.258282381	0.213366592	-1.210730416	0.231062572
PTBP2	0.813687309	0.418040724	-0.914931992	0.36404194	-1.498192636	0.139675746
PTCD1	0.695342429	0.488689861	-2.135207255	0.03700937	-1.194734454	0.237205303
PTCD2	0.845682917	0.400044566	-0.016034467	0.987262309	-0.19724644	0.844345871
PTCD3	3.084157279	0.002734169	0.32242116	0.748301108	0.931063095	0.35580078
PTCH1	2.987994694	0.003646868	-1.710283352	0.092595661	-1.078329935	0.285490169
PTCH2	1.549889884	0.124790325	-1.446164781	0.153551023	-0.957077852	0.342624437
PTCHD2	0.746301963	0.457492594	0.551181541	0.58364117	0.088790045	0.929564217
PTCRA	-2.463890645	0.015707134	2.755968413	0.007822702	1.410935853	0.163771741
PTDSS1	1.513498076	0.133769826	1.812414066	0.07513538	1.814633418	0.074915031
PTDSS2	-0.167317747	0.867507502	1.934406472	0.057980544	2.085577129	0.041566476
PTEN	-1.116787869	0.267153206	-0.097401175	0.922745966	-0.893291176	0.375508132
PTENP1	-0.967066644	0.336185592	-2.953388546	0.00454387	-2.374683268	0.021000018
PTER	1.004812294	0.317767771	-0.857867342	0.394521512	-1.651885701	0.104131392
PTGDR	3.807822823	0.000260205	-3.176163952	0.002398341	-3.129915045	0.002771694
PTGDS	0.583690971	0.560935221	-1.087205913	0.28147471	-0.282056254	0.778935795
PTGER2	2.723125157	0.007810293	-1.518146791	0.134447662	-2.529212466	0.014262285
PTGER4	-0.592175019	0.555265965	-1.722453992	0.090353508	-3.556634514	0.000770696
PTGES	-5.755445635	1.26E-07	2.444010036	0.017609199	3.921851268	0.000241308
PTGES2	-0.072716983	0.942197891	-0.112959837	0.910455193	0.02224424	0.982331867
PTGES3	-0.255237522	0.799140774	2.005866228	0.049575398	1.929077562	0.058773244
PTGFRN	1.397456794	0.165822174	1.168934533	0.247245222	2.075636177	0.042512168
PTGIR	1.506245632	0.135618684	0.58694407	0.559535013	0.789730091	0.433002707
PTGR2	1.026596436	0.30745025	1.087920237	0.281161888	0.756382866	0.452576731
PTGS1	-4.267472481	5.01E-05	2.330601951	0.023298578	1.09725284	0.277207031
PTGS2	-1.307924065	0.194337038	-1.352906074	0.181372639	-2.605158517	0.011729017
PTK2	-1.09185715	0.277903835	1.997534354	0.050498098	2.588689652	0.012240727
PTK2B	-1.465133263	0.146481407	0.499580701	0.619273487	0.557605426	0.57932677
PTK6	-0.402970178	0.687956578	-0.267051391	0.790383468	1.040270202	0.30266624
PTK7	-1.043289992	0.299698106	-0.027768487	0.977942792	-0.263402979	0.793202729
PTMA	0.927389008	0.356284731	0.744725879	0.459465434	1.353268935	0.181384682
PTMS	-2.139323508	0.035204418	-1.367156257	0.176888537	-2.023744592	0.047757693
PTOV1	-0.738983973	0.461901526	-0.862092845	0.392211626	-0.384019214	0.702412381
PTP4A1	-3.018485876	0.003330731	0.199653099	0.842455106	-1.474163952	0.146013672
PTP4A2	-2.336177161	0.021775933	1.247067101	0.217420209	1.364523792	0.177838226
PTP4A3	-0.994822707	0.322575432	0.244589458	0.807643598	0.456075777	0.6500919
PTPDC1	2.889256019	0.004870933	-0.558918614	0.578383999	-1.655461797	0.103401471
PTPLA	0.317120442	0.751910335	0.675047106	0.502346831	0.396921435	0.692927748
PTPLAD1	0.374873071	0.708665173	-0.487672351	0.627633134	0.280664332	0.779997835
PTPLAD2	-1.588670832	0.115754628	-2.601779698	0.011769118	-2.979870794	0.004252905
PTPLB	0.462191399	0.645095354	-1.536091278	0.129991601	-2.539606562	0.01388852
PTPMT1	1.582202481	0.11722428	-2.132059397	0.037278607	-1.02659323	0.309007567
PTPN1	-0.258061414	0.796967668	-0.039688132	0.968479015	-0.241883757	0.809750117
PTPN11	1.012404733	0.31414589	2.087733568	0.041255361	1.109553507	0.271913657
PTPN12	-1.164307521	0.247475421	-0.17850023	0.858956519	-1.380371026	0.172934878
PTPN13	0.764665086	0.446535688	0.091849628	0.92713623	-0.864539399	0.390964443
PTPN14	-0.601134574	0.549310041	0.570662612	0.570448144	0.954857601	0.343736325
PTPN18	-1.870526066	0.064765719	0.485196184	0.629377627	0.850045365	0.398904432
PTPN2	1.588630284	0.115763795	-0.84323708	0.402584228	-0.729390062	0.468790211
PTPN22	0.075286866	0.940158946	-1.752217121	0.085058742	-2.276596142	0.026636335
PTPN23	-0.082640807	0.934326584	-0.526245209	0.600738482	0.217664743	0.82847818
PTPN4	1.450423517	0.150528524	-0.439421128	0.662000713	-0.331008206	0.741868512
PTPN6	-1.699326072	0.092823174	0.154878719	0.877458405	0.121551013	0.903687903
PTPN7	1.00418243	0.318069485	-0.250900311	0.802784112	1.787960433	0.07917036
PTPN9	-0.855296829	0.394731091	0.193315102	0.847392305	0.001537116	0.998778999

PTPRA	0.483562806	0.629908403	-0.457688304	0.64889833	0.409118006	0.684007037
PTPRB	0.92530283	0.357362418	-0.263786174	0.792886195	-0.755165778	0.453300716
PTPRC	0.269665029	0.788055161	-0.88818846	0.378133628	-2.066081251	0.04343869
PTPRCAP	1.072074446	0.286645088	-1.762876092	0.08322627	-0.760434827	0.450171262
PTPRE	-1.333813128	0.185738921	-0.15651545	0.876174071	-0.917184207	0.362962878
PTPRF	-2.784496948	0.00657454	1.333554068	0.187600065	0.279208851	0.781108822
PTPRJ	-0.503025424	0.616214764	-0.224936239	0.822825017	-0.771068172	0.443894211
PTPRK	1.077953672	0.284027767	0.752570226	0.454773071	0.834296815	0.407643766
PTPRM	2.928242367	0.004348303	-0.637425976	0.526373268	0.001260015	0.998999113
PTPRN	-2.423594695	0.017434592	-0.439324293	0.662070457	-0.564205881	0.574857423
PTPRN2	-2.659150901	0.009320588	-0.737280073	0.463945026	-2.018355579	0.048333209
PTPRO	5.233627802	1.13E-06	-0.326325659	0.745360773	-0.379750601	0.705560903
PTPRS	3.897884976	0.000190066	-0.588272007	0.558649527	-0.29652632	0.767920549
PTPRU	-0.139489012	0.889385446	0.856115567	0.395481597	0.340023126	0.735105879
PTPRVP	0.176560575	0.86026304	0.157118365	0.875701051	0.218404522	0.827904579
PTRF	-2.875930063	0.005062436	1.856881817	0.068444174	1.801375609	0.077005553
PTRH1	-0.472427436	0.637802104	-2.246333221	0.028532864	-1.448108067	0.153139411
PTRH2	2.400690834	0.018490271	0.537359842	0.593089159	0.710319771	0.48044122
PTRHD1	-0.802437419	0.424481837	-1.472853495	0.14623458	-0.696064734	0.48925515
PTS	0.11820395	0.906177957	-1.661917001	0.101963277	-0.087940447	0.930236415
PTTG1	0.993130672	0.323394509	1.044389905	0.300668639	2.808404121	0.00683333
PTTG1IP	-1.154436218	0.25147577	0.557884212	0.579085529	-0.233155285	0.816487394
PTX3	1.12547679	0.263475635	2.216257896	0.030638632	0.917922389	0.362579627
PTX4	0.5570287	0.578934946	-1.18093023	0.242484091	-0.72238529	0.473051029
PUF60	-0.659359904	0.511402489	1.518698675	0.134308831	2.21917881	0.030525605
PUM1	0.85713412	0.393720609	0.868434031	0.388761026	-0.079760586	0.936710829
PUM2	0.859393901	0.392479952	-0.866801976	0.389647302	-1.812490362	0.075249693
PURA	1.816881014	0.072671223	0.086628363	0.931267382	-0.690120679	0.49295663
PURB	0.701581042	0.484808972	-3.343376578	0.001459449	-3.853968258	0.000300697
PUS1	0.319506373	0.750106856	0.746396416	0.45846382	1.614268127	0.112066903
PUS10	0.118559636	0.905896971	-1.94321966	0.0568822	-2.122080561	0.038249054
PUS3	0.285039212	0.776290081	0.933142472	0.354641868	0.827357988	0.411531241
PUS7	-0.134014914	0.893699642	-0.646111061	0.520773582	-0.363809132	0.717364748
PUS7L	0.534614866	0.594276924	-1.003856415	0.319646689	-2.258067476	0.027840496
PUSL1	1.239686214	0.218417793	0.234388841	0.815514334	-0.001761391	0.998600848
PVALB	-3.493941627	0.000750173	1.050115582	0.298051231	0.806087519	0.423588008
PVR	0.589217065	0.557239309	0.238820609	0.812092409	1.248248806	0.217111986
PVRIG	-0.468439724	0.64063919	-1.395459069	0.168234541	-0.601860741	0.549685695
PVRL1	-2.311500856	0.023163352	0.377394117	0.707267809	0.339185758	0.735733161
PVRL2	-1.793067171	0.076429831	1.11161567	0.27092208	0.400725868	0.690140374
PVRL3	0.002084263	0.998341761	0.369315358	0.713246987	-0.093357598	0.925951286
PVT1	3.176100526	0.002064364	0.935346459	0.353514953	0.959647567	0.34134049
PWP1	1.796539517	0.075871937	0.681403583	0.498346876	2.347809197	0.022427235
PWP2	1.147922312	0.254140538	1.191261137	0.238437049	2.090856734	0.041071698
PWWP2A	0.089246819	0.929090445	-1.759811091	0.083749808	-2.947093625	0.004662328
PWWP2B	1.189421198	0.237503009	-0.636638561	0.526882507	-0.546974524	0.586560252
PXDN	0.149210899	0.881731812	-0.809414108	0.421608257	-0.759950935	0.450458136
PXK	-0.756357968	0.451473455	0.484773822	0.629675399	-0.217156675	0.828872173
PXMP2	1.058609663	0.292701627	0.13506526	0.893031187	-0.683919701	0.496834465
PXMP4	-0.187897098	0.851393926	0.616248384	0.540158613	2.16680771	0.034501225
PXN	-2.188643093	0.031295911	-0.410697746	0.682818115	-0.493294913	0.623725132
PYCARD	-1.071009307	0.287121035	-0.467248691	0.642084872	-0.789075904	0.4333818
PYCR1	-0.614536549	0.540461233	1.440586953	0.155115627	1.139487907	0.259330049
PYCR2	0.361666572	0.718476213	0.87327054	0.386141997	1.403434875	0.16598495
PYCRL	1.955307854	0.053749621	-0.94250821	0.349869157	-0.059129853	0.9530583
PYGB	-0.737701622	0.462676592	0.965496885	0.338332526	1.215612083	0.229211239
PYGL	-1.491075028	0.139550986	0.816686033	0.417473039	0.476394355	0.635639238
PYGM	0.220273202	0.826173341	-0.857742165	0.394590069	-0.944371147	0.349019813
PYGO2	0.219064292	0.827111968	0.272845471	0.785947887	1.328997168	0.18921591
PYHIN1	0.902853432	0.369091285	-3.269285012	0.001821943	-1.991700251	0.051268596
PYROXD1	-0.614881054	0.540234729	-1.217026199	0.228557529	-1.067068251	0.290500533
PYROXD2	1.569097357	0.120247508	-1.846845508	0.069908767	-0.783035249	0.436891585
PZP	-1.141600649	0.256745716	-2.845211172	0.006136276	0.42524303	0.672282354
ProSAPiP1	3.529936302	0.000666333	1.022663916	0.310743012	0.796125489	0.429307045
QARS	0.197794836	0.843666007	1.591389616	0.116996089	1.974379571	0.053257094
QDPR	0.276262482	0.783000263	3.566160048	0.000737065	3.084279101	0.00316118
QKI	-0.388858272	0.698329009	-0.578935832	0.564889773	-2.349749358	0.02232137
QPCT	-3.899082308	0.000189268	0.213357536	0.831801526	0.046649623	0.962957749
QPCTL	0.749847201	0.455365336	0.530843504	0.597568291	1.183643688	0.241533363
QPRT	-0.091012079	0.927691762	0.551453939	0.583455694	2.063815338	0.043660964
QRICH1	0.771569064	0.442455847	-0.626704504	0.533329195	0.617846562	0.53917057
QRICH2	2.483759011	0.014913127	2.114533409	0.038809105	1.451235901	0.152269928

QRSL1	1.078501375	0.283784781	0.906614575	0.36838802	1.366234216	0.177303935
QSER1	1.258915954	0.211420923	-0.816845298	0.417382748	-1.302967511	0.197895868
QSOX1	-2.000681203	0.048537709	2.495021059	0.015484386	1.99793466	0.050568642
QSOX2	1.840801121	0.069052063	0.772516441	0.442967161	0.62529037	0.534309745
QTRT1	0.694015298	0.489517628	-0.756303987	0.452549362	-0.412443668	0.681582382
QTRTD1	-0.075869451	0.939696779	-0.18061928	0.857300461	-0.239019328	0.811959526
R3HCC1	0.023613619	0.981214768	1.106794575	0.272983946	1.131197063	0.262773104
R3HDM1	0.470084954	0.639468029	-1.763725401	0.083081681	-1.340860166	0.185356931
R3HDM2	-0.700881767	0.485243128	-2.403782792	0.019466005	-1.866387739	0.067204469
RAB10	-0.246783435	0.80565596	-1.01273361	0.315422994	-1.978903788	0.052731411
RAB11A	-1.991595161	0.049545384	0.087558985	0.930530916	-0.319394937	0.750610511
RAB11B	-0.825253804	0.411479767	-1.563387992	0.123439623	-1.113214818	0.270351888
RAB11FIP1	-4.042888339	0.000113573	-0.250313659	0.803235519	-0.740276029	0.462212045
RAB11FIP2	0.646669024	0.51954491	-1.276835852	0.206784021	-2.082259875	0.041879996
RAB11FIP3	1.567825198	0.12054426	0.001437989	0.99885762	0.783670468	0.436521716
RAB11FIP4	-3.18527176	0.002006708	0.475675831	0.636104557	0.99445812	0.324259658
RAB11FIP5	-0.63855581	0.524785759	-0.114160136	0.909507875	0.557034412	0.579714204
RAB12	0.40927189	0.683343815	-1.108842223	0.272106875	-1.051807077	0.297386729
RAB13	-0.684519137	0.495462983	0.893034232	0.375555025	0.071579053	0.943190867
RAB14	-0.677114858	0.500125731	0.639483989	0.52504352	-0.808078423	0.422450574
RAB15	0.77553359	0.440122885	0.038743154	0.969229146	0.162847906	0.871221959
RAB18	-0.156609249	0.875914881	-1.31499425	0.193723449	-1.82020607	0.074050636
RAB19	-1.146113256	0.254884137	-0.18854998	0.851108321	0.150740909	0.880719499
RAB1A	-1.635697353	0.105508125	-0.99055171	0.326047745	-1.993531901	0.051062093
RAB1B	-2.51683806	0.013670704	-0.911705104	0.365724158	-0.449066985	0.655109165
RAB20	-3.091530769	0.002673779	1.620363177	0.110617039	0.390266243	0.697814033
RAB21	-2.621544139	0.010327618	-1.892393223	0.063468196	-3.570861031	0.000737404
RAB22A	-0.849076882	0.398163793	-0.882251332	0.381308195	-1.374546143	0.174724974
RAB23	0.85311324	0.395934104	-0.103134986	0.9182141	-1.197374518	0.236183386
RAB24	-0.871333119	0.385965232	0.028599093	0.977283201	-0.361764932	0.718883472
RAB25	0.824145245	0.412105878	2.385358319	0.020373618	1.926562153	0.059093313
RAB27A	-1.612344703	0.11050103	-0.170856236	0.864935614	-1.12785738	0.264169138
RAB27B	-2.031562154	0.045242754	-0.964637549	0.338759215	-1.468893272	0.147433675
RAB28	0.264521728	0.792002223	-0.212416617	0.832531991	-0.66349552	0.509724112
RAB2A	-0.906472354	0.367184255	-1.245763856	0.217894911	-1.598087272	0.115627723
RAB2B	0.787482913	0.433134772	-4.193286258	9.59E-05	-3.31318168	0.001617127
RAB30	0.347639063	0.728949101	-0.886537091	0.379014929	-0.55277081	0.58261101
RAB31	-3.388391812	0.00105717	0.767182675	0.44610642	-0.193675155	0.847128102
RAB32	-3.306956061	0.001371124	0.98036195	0.331007602	0.409051705	0.68405541
RAB33A	0.657583923	0.512537859	-0.117390063	0.906959362	1.003207886	0.320057755
RAB33B	-0.301394717	0.763831172	-0.185726077	0.853312112	-1.750270659	0.085525748
RAB34	1.226949283	0.223144481	2.501421119	0.015234822	2.231508568	0.029650541
RAB35	-2.208084128	0.029862579	-2.087295695	0.041296418	-2.01095789	0.049132976
RAB36	-2.556415201	0.012306934	0.222713603	0.824546334	0.05166543	0.958978366
RAB37	-3.976506129	0.000143965	-1.45521274	0.151039254	-0.13923337	0.889763167
RAB38	-1.375356142	0.172544563	-1.521990821	0.13348303	-0.573904292	0.568320905
RAB39	-0.87997735	0.381290626	-0.218717942	0.827642939	-0.660954533	0.511340205
RAB39B	0.099074331	0.921306686	-1.430408624	0.158002673	-1.785530441	0.079567874
RAB3A	0.813768002	0.417994735	-1.10187737	0.275098238	-1.319840464	0.192235898
RAB3D	-3.346941059	0.001207393	0.634000041	0.528590781	0.654301267	0.51558473
RAB3GAP1	0.959384605	0.340017959	3.129242628	0.002749927	2.614563006	0.011445621
RAB3GAP2	0.686866595	0.493989646	-0.225833081	0.822130707	-1.704936882	0.093725215
RAB3IL1	-1.380308425	0.171020474	1.626033027	0.109402305	0.530458144	0.597882842
RAB3IP	0.215364524	0.829986115	0.289256173	0.77342399	-0.089094932	0.929323004
RAB40B	0.992139175	0.32387511	-1.184309091	0.241155032	-1.774062864	0.081466368
RAB40C	0.063467697	0.949539362	-1.23362528	0.222353113	-0.814548193	0.418767007
RAB42	-0.64463879	0.520853787	0.148037461	0.88283025	-0.183058363	0.855410602
RAB43	0.854727755	0.395044396	-1.989136943	0.051442957	-2.505994895	0.015130278
RAB4A	0.677456865	0.499909836	0.223013122	0.824314321	0.193003022	0.847651951
RAB5A	-1.8773674	0.063811455	-1.462684284	0.148989425	-2.361217625	0.021704664
RAB5B	-2.035391817	0.044847819	-0.945565712	0.348320174	-0.55464043	0.581339886
RAB5C	-2.834613294	0.005701047	-1.520057096	0.133967595	-1.962881662	0.05461334
RAB6A	-0.446198136	0.6565603	-0.802806026	0.425387275	-1.660774471	0.10232481
RAB6B	-1.988889751	0.049848852	-0.505176142	0.615362784	-0.14942473	0.881753087
RAB7A	-2.275464415	0.025329705	-0.792855625	0.431115829	-0.796837868	0.428896556
RAB7L1	1.121555919	0.265130714	-1.196908597	0.236245497	0.301503868	0.764142393
RAB8A	-2.744971423	0.007347994	-0.720889958	0.473893159	-0.728918431	0.469076405
RAB8B	-0.331138179	0.741334655	-1.274503483	0.20760311	-2.168925094	0.034332075
RAB9A	-0.10543648	0.916271662	-0.218215648	0.828032409	-0.419891692	0.676164483
RAB9B	1.324315188	0.188859482	-1.21285596	0.230135985	0.092779252	0.926408672
RABAC1	-0.699584665	0.486049019	0.209910237	0.834478493	0.466388311	0.642739216
RABEP1	1.637987296	0.105028485	0.238254895	0.812529013	-0.69931926	0.487235022

RABEP2	0.113774851	0.909677854	1.234476082	0.222038466	2.023813335	0.04775039
RABEPK	1.2160583	0.227244825	-0.547365122	0.586242731	1.453759009	0.151571364
RABGAP1	0.312586941	0.755340912	2.029951101	0.046989337	1.567584079	0.122588399
RABGAP1L	1.269609574	0.207602011	1.70995866	0.0926561	2.242396561	0.028896294
RABGEF1	0.108507287	0.913842626	1.031886832	0.306438731	0.506862414	0.614233084
RABGGTA	-0.384925628	0.701229897	0.912598474	0.365257935	1.647335765	0.105066154
RABGGTB	1.014886945	0.312967796	-0.32652441	0.745211202	0.035740714	0.97161567
RABIF	-1.698617487	0.092957255	-3.741914698	0.000423102	-3.001512998	0.004001192
RABL2A	0.353075434	0.724884089	-2.114686842	0.038795472	-0.962277684	0.340029643
RABL2B	0.779624566	0.437723055	-1.190604631	0.238692766	-0.066122384	0.947514899
RABL3	1.16723619	0.246297341	-1.297625893	0.199589028	-0.77963161	0.438876575
RABL5	0.758248283	0.450347094	0.269965767	0.788151525	0.805127887	0.424136916
RAC1	-2.907525857	0.004619209	0.495766789	0.621945424	0.136500613	0.891913027
RAC2	-2.502774098	0.014187106	-0.585554329	0.560462457	-0.538955691	0.592044717
RAC3	-0.869492011	0.386965442	1.178899424	0.243285439	2.089707489	0.041178961
RACGAP1	0.283566344	0.777414981	1.43827057	0.155769021	1.958697678	0.055114153
RAD1	1.329991659	0.186989776	-0.394150396	0.694925576	0.206610963	0.837059937
RAD17	1.39465977	0.166661704	-0.053050008	0.957875636	0.264824021	0.792113273
RAD18	0.423134564	0.673238964	0.140912488	0.888430753	0.428805356	0.669703021
RAD21	-1.484039789	0.141404601	0.527053735	0.600180497	-0.253051404	0.801151239
RAD23A	-1.229894124	0.222045084	0.001760832	0.998601145	0.079438731	0.93696567
RAD23B	-1.442770759	0.15266806	-0.193770851	0.847037075	-0.467970156	0.641614533
RAD50	3.194853917	0.001948076	1.3031665	0.197703583	1.94363035	0.056950371
RAD51	1.876213531	0.063971569	1.400530353	0.166718962	2.430306491	0.018301373
RAD51AP1	0.81068512	0.419753871	-0.342840983	0.732966217	-0.798162168	0.428134088
RAD51B	0.277468766	0.782077021	0.592430963	0.555880813	1.3410203	0.185305251
RAD51C	-0.531754461	0.596248366	-1.580031218	0.119576374	-1.117330774	0.268603739
RAD51D	1.641979585	0.104196493	-0.893266678	0.375431613	-0.415263461	0.679529183
RAD52	0.633045824	0.528360653	0.767606212	0.445856667	-0.558430325	0.578767293
RAD54B	1.582797746	0.117088412	-1.067991592	0.289980208	-1.234894008	0.222004699
RAD54L	1.499828297	0.137271337	1.119019699	0.267776922	2.99717268	0.004050532
RAD54L2	1.322015818	0.189620822	0.281427225	0.779391362	0.191917865	0.84849785
RAD9A	0.02961668	0.976440451	-0.080179425	0.936372501	0.64038735	0.524521604
RAD9B	-0.855187605	0.394791212	-0.332336647	0.740841564	1.708735937	0.093014089
RAE1	-0.434927751	0.664689511	2.547166112	0.013554325	3.109036105	0.002943947
RAF1	-2.94875247	0.004094598	-1.518134998	0.13445063	-1.37322109	0.175134164
RAG1	0.105364605	0.916328525	-1.19399644	0.237373756	-1.67137928	0.100203044
RAGE	0.679269479	0.498766449	-1.380862018	0.172656195	-1.42453906	0.159816381
RAI1	1.950723548	0.054301722	-1.161729477	0.25013703	-0.742369965	0.460952815
RAI14	0.080920126	0.935690935	1.64116224	0.106213858	1.967611903	0.05405184
RAI2	-0.670616642	0.504237333	1.67376098	0.099600556	1.58409628	0.118779764
RALA	1.18781975	0.238130181	-0.420926551	0.675375267	0.329736467	0.742824184
RALB	-3.408173965	0.000991848	0.508581988	0.612987877	-0.231887998	0.817466751
RALBP1	-2.461798013	0.015792926	1.545954719	0.127592829	1.329494161	0.189053029
RALGAPA1	1.486419276	0.140775521	-1.292839623	0.2012286	-1.122120623	0.266579425
RALGAPA2	-1.862945279	0.065837087	0.024364838	0.980645814	0.208087841	0.835912165
RALGAPB	1.002992389	0.318640054	0.446207685	0.657120273	0.045410568	0.963940927
RALGDS	0.730425364	0.467088385	-2.271005782	0.026901959	-1.46560113	0.148326115
RALGPS1	1.015444593	0.312703536	-1.134519685	0.261276291	-1.642491104	0.106068972
RALGPS2	0.576393523	0.565834256	0.192270548	0.848206591	-0.586771884	0.55970516
RALY	-1.900385495	0.060687092	-0.206128889	0.837417128	-0.057440618	0.954397819
RAMP1	-1.74678068	0.084196909	0.094737083	0.924852485	0.202483788	0.840269299
RAN	1.634669271	0.105724036	0.573212952	0.568731846	1.320600704	0.191983785
RANBP1	2.275765129	0.025310913	0.011051145	0.991220832	1.540814774	0.128970416
RANBP10	-2.205010736	0.030085279	0.871006987	0.38736636	0.90818587	0.36765558
RANBP2	0.581452275	0.562435914	2.768686157	0.007558786	2.270586248	0.027021753
RANBP3	-0.654010651	0.514826267	-0.424355146	0.672887724	0.237336027	0.813258618
RANBP6	0.284650473	0.776586933	-0.518994674	0.605752981	-1.406113216	0.165192052
RANBP9	-0.013950242	0.988901551	-2.0801415	0.041972275	-2.691375976	0.009354489
RANGAP1	-0.813189677	0.418324399	3.104014749	0.00295839	3.177950968	0.002410622
RANGRF	0.395640381	0.693336771	1.894857612	0.063134558	2.23220616	0.0296017
RAP1A	-1.699572373	0.092776604	-1.574993437	0.120735358	-1.76372334	0.083210323
RAP1B	-0.105329214	0.916356525	-2.251699366	0.028170895	-2.434074762	0.01813035
RAP1GAP	-3.439846389	0.000895121	0.734087574	0.465873336	0.459964064	0.647315448
RAP1GAP2	-1.227017939	0.223118805	-0.767408231	0.445973403	-0.310504867	0.757324956
RAP1GDS1	2.06764857	0.041637164	0.384818389	0.701789304	1.35806433	0.179867095
RAP2A	0.788906893	0.432306387	-1.565983189	0.122830723	-2.124451691	0.038041789
RAP2B	-1.113996311	0.268342314	0.251614084	0.80223498	-0.157867845	0.875126427
RAP2C	-0.16059594	0.87278316	-1.012253051	0.315650673	-1.684466281	0.097634442
RAPGEF1	0.327854415	0.743807722	-0.702520702	0.485184171	-0.113340815	0.910164007
RAPGEF2	-2.193751624	0.030913573	-1.306999082	0.196407217	-2.32271155	0.023837647
RAPGEF3	-0.229924473	0.818689026	1.310203816	0.195328135	1.00634949	0.31855802

RAPGEF5	0.154242682	0.877774861	-1.022598001	0.310773921	0.002681524	0.997869946
RAPGEF6	1.229508314	0.222188894	-0.972274429	0.334979666	-0.945168124	0.348616415
RAPGEFL1	-0.53332142	0.595168015	0.542816469	0.589350627	1.054936626	0.295965546
RAPH1	4.235411689	5.64E-05	-0.573439	0.568579846	-0.377510826	0.707215034
RAPSN	-0.104506576	0.917007383	1.195774709	0.236684337	1.128900164	0.263732677
RARA	-2.56793349	0.011933742	-1.447286674	0.153237819	-2.070776214	0.042981262
RARB	-1.02229498	0.309469449	1.628688334	0.108837152	3.244957456	0.001980232
RARG	2.217333623	0.029201029	-1.320436201	0.191912594	0.132307946	0.895212981
RARRES3	-1.070849709	0.287192397	0.614249437	0.541469321	1.444128912	0.154251145
RARS	1.877392235	0.063808012	1.881209523	0.065001133	3.939230254	0.000228025
RARS2	0.129182123	0.897511081	-1.755461757	0.084497418	-1.514474013	0.135506264
RASA1	1.594641929	0.114411069	-1.303395472	0.197625954	-2.357768404	0.021888525
RASA2	1.00708103	0.316682593	-0.762868622	0.448654998	-1.75152375	0.085307835
RASA3	1.670763417	0.098354448	0.794184175	0.430348323	0.814704882	0.418678037
RASA4	1.821563127	0.071950634	1.98431086	0.051992819	1.34589379	0.183737692
RASA4P	-0.574804189	0.566903997	0.028841921	0.977090372	0.787847063	0.43409443
RASAL2	-0.474627421	0.636239213	2.414971365	0.018932692	2.450040289	0.017421585
RASAL3	-0.197996276	0.843508884	-0.500454061	0.618662355	0.845077136	0.401648865
RASD1	2.348347272	0.021119157	-0.798372484	0.427934061	-0.909858194	0.366780526
RASGEF1A	-2.395144777	0.018754306	-1.5125589	0.135859757	-0.789971335	0.432862959
RASGEF1B	-0.719434594	0.473797166	2.201196223	0.031743783	1.556167532	0.125278527
RASGRF1	0.157081962	0.87554344	-0.124146727	0.901631353	-0.133404691	0.894349576
RASGRF2	1.715906414	0.089730594	0.450656474	0.653929106	0.860728085	0.393042714
RASGRP1	1.541175544	0.126895784	-0.735635047	0.464938072	-0.930868813	0.355900402
RASGRP2	-0.488916541	0.626128394	-0.813559797	0.419247776	0.308941623	0.758507598
RASGRP3	1.181013442	0.240808992	1.103531835	0.274385577	1.079337611	0.285044792
RASGRP4	-2.224494335	0.028697719	-0.700198612	0.486622059	-1.159229045	0.251261364
RASIP1	-1.345757027	0.181869986	-0.206247947	0.837324567	0.012828182	0.989810274
RASL11A	-0.471718226	0.638306284	-1.544206108	0.128015493	-0.64086453	0.524213768
RASSF1	1.474479873	0.143954185	0.406529554	0.685860192	1.11720973	0.268655035
RASSF2	-3.196885437	0.001935853	0.08001717	0.936500981	-0.556564965	0.580032819
RASSF3	-1.42599049	0.157441762	0.126145311	0.900056217	-0.856512003	0.395349692
RASSF4	0.879113909	0.38175596	1.533185128	0.130705182	2.08668126	0.041462576
RASSF5	-1.657202948	0.101072703	0.468314198	0.641327408	0.673407264	0.503446474
RASSF6	2.465933368	0.01562379	0.026299656	0.97910924	-1.097415581	0.277136531
RASSF7	0.969683965	0.334886362	0.350012901	0.727605727	0.422541413	0.674241129
RAVER1	-1.009551358	0.315503806	-0.365473299	0.716096919	0.168761757	0.866589571
RAVER2	-0.491408384	0.62437242	0.319679289	0.750368161	0.588456223	0.55858221
RB1	0.293527322	0.769816671	-0.397262722	0.692642107	-1.546929999	0.127489612
RB1CC1	-0.295065789	0.768645097	-0.062190946	0.950626089	-1.190204044	0.238966404
RBAK	1.071026953	0.287113146	-0.681645664	0.498194885	-0.48229093	0.631471203
RBBP4	2.639353336	0.009839045	1.851090306	0.069286136	2.108147807	0.039486962
RBBP5	-0.648396273	0.518432726	-0.099750502	0.920888794	-0.358354667	0.721419641
RBBP6	-0.276955046	0.782470164	-1.320410499	0.191921117	-2.660008801	0.010162121
RBBP7	0.611233341	0.542635464	0.802415032	0.42561151	1.36663359	0.177179358
RBBP8	0.347838598	0.728799761	0.469750103	0.640307235	-0.853791942	0.39684252
RBBP9	-0.42857372	0.669290433	-0.567651724	0.572477631	-0.125737938	0.90038781
RBCK1	-2.165910156	0.033047783	-0.922625363	0.360051353	-0.948698307	0.346833233
RBFA	0.955610855	0.341910978	0.624490972	0.534771219	1.688507788	0.096852216
RBFOX2	0.846339617	0.399680231	0.196428521	0.84496622	0.861414852	0.39266772
RBFOX3	1.773918499	0.07956785	-0.180707234	0.857231738	0.957267399	0.342529622
RBKS	0.886416068	0.377831786	-1.720889198	0.090639249	1.156068367	0.252540978
RBL1	2.000303001	0.048579301	-1.0200273	0.311980995	-0.524547964	0.601959119
RBL2	2.099375903	0.038674317	0.116052951	0.908014267	0.339325367	0.735628565
RBM10	-1.640173628	0.104572192	0.247640483	0.805293282	0.802893656	0.425416549
RBM11	0.298665678	0.765905814	0.589621869	0.557750139	-0.228199936	0.820318538
RBM12	0.090935104	0.927752748	1.624881835	0.109648062	0.424427426	0.672873457
RBM12B	0.831945643	0.407712449	-0.7578357	0.451638949	-1.704880406	0.09373582
RBM14	0.738548608	0.462164583	-1.335731621	0.186891348	-0.178596929	0.858896054
RBM15	1.086171695	0.280396913	-1.022834816	0.310662883	-3.200308916	0.002258082
RBM15B	0.428953541	0.669015048	-1.050445778	0.297900765	-1.047682411	0.299266956
RBM17	1.972643624	0.051704728	2.647919158	0.010430654	3.373466203	0.00134953
RBM18	-0.959149469	0.34013571	-0.889800798	0.377274407	-1.537461089	0.129788319
RBM19	2.24609104	0.027225477	1.384356291	0.171589694	2.309525187	0.024609894
RBM20	1.441442296	0.153041854	1.042635442	0.301473802	0.676005372	0.501807914
RBM22	-2.931168739	0.004311239	0.160787596	0.872823325	0.201369153	0.841136528
RBM23	-3.068722474	0.002864708	0.967084802	0.337545004	0.72353394	0.472350839
RBM25	2.594171517	0.011121465	0.024722548	0.980361726	-0.960114766	0.341107397
RBM26	-0.451593987	0.652682846	1.507959935	0.137030739	0.803858004	0.424863945
RBM26-AS1	0.979750097	0.329920277	-1.691410184	0.096163095	-1.000781275	0.321219407
RBM27	0.286288068	0.775336643	-0.719908402	0.474492719	-1.908098049	0.061488522
RBM28	3.417260481	0.000963136	1.142755251	0.257868236	1.910937329	0.061114918

RBM3	0.926782326	0.356597918	-0.334679604	0.739082543	0.573652429	0.568490193
RBM33	1.288967577	0.200818772	-0.801643941	0.426053939	-0.829017426	0.410599492
RBM34	1.333435075	0.185862386	0.818476584	0.416458609	2.048241912	0.045215479
RBM38	-2.819145648	0.005958561	-0.126932384	0.899436016	-0.07339122	0.941755224
RBM39	0.903653382	0.368669204	-0.128738646	0.898012946	-0.770946511	0.443965738
RBM4	2.101376788	0.038493731	-3.236482173	0.002008199	-1.376454737	0.17413687
RBM41	1.036694952	0.302744717	-0.258345786	0.797061004	-0.402872429	0.688569565
RBM42	-0.793475176	0.429655145	0.292109199	0.77125275	0.237798277	0.812901822
RBM43	-1.109401657	0.270307523	-2.143016811	0.03634874	-2.182379641	0.033274147
RBM44	-0.518736429	0.605258668	-0.240508606	0.810790011	-1.439205777	0.155635321
RBM45	0.797164507	0.427521041	0.18595244	0.853135414	1.422323942	0.16045536
RBM47	-1.192591312	0.236265006	-0.695681186	0.489426106	-2.465620844	0.016754059
RBM4B	0.467081949	0.641606412	-1.49456015	0.140488158	-1.147531037	0.256020645
RBM5	-0.248764616	0.804127902	1.506692331	0.137354895	2.14428989	0.036345795
RBM6	0.535899909	0.593392238	1.84814964	0.069716976	2.884849163	0.005542393
RBM7	-0.653705352	0.515022038	-2.077815713	0.042194052	-2.029310603	0.047169492
RBM8A	0.567669039	0.571718604	-0.839073764	0.404897029	-0.893688688	0.375297189
RBMS1	0.152955129	0.87878709	-0.986180349	0.328169398	-1.854194329	0.068957989
RBMS2	-1.404919726	0.163598006	0.088727556	0.92960623	0.080422787	0.936186526
RBMX	2.543422689	0.012740487	1.983838345	0.052046926	2.828637594	0.006467013
RBMX2	0.699464623	0.486123638	-0.062212429	0.950609056	0.883307972	0.380830424
RBMXL1	-0.428783195	0.66913855	1.138080924	0.259798671	0.108228203	0.914199901
RBP5	0.024391611	0.980595978	-0.737028181	0.464097006	-0.29096731	0.772146757
RBP7	-3.160426975	0.00216649	-0.722641832	0.47282413	0.145595134	0.884761616
RBPJ	-2.320236902	0.022663486	0.443622505	0.658977608	-1.022619898	0.310866589
RBPMS	1.488795955	0.140149372	-0.900315602	0.371701367	0.349735386	0.727843666
RBPMS2	-2.928118518	0.004349878	-0.406242424	0.686069942	-1.230517227	0.223625707
RBX1	0.758996137	0.449901927	-1.505813711	0.137579935	-0.969054112	0.336667567
RC3H1	-0.806593789	0.422095266	-0.865749666	0.39021942	-1.520752141	0.133925117
RC3H2	0.584977688	0.560073574	0.094143024	0.925322286	-0.649334444	0.518765569
RCAN1	0.455036963	0.650213697	1.001723003	0.320667382	-0.077560466	0.93845299
RCAN2	0.264849943	0.791750182	-0.724014894	0.471987213	-0.818009371	0.416804376
RCAN3	2.347337233	0.02117299	0.919263881	0.36179149	0.619364713	0.53817737
RCBTB1	1.521041084	0.131867999	0.603452672	0.548576805	-2.003970726	0.049898806
RCBTB2	-1.111920397	0.269228977	0.347126417	0.729761538	-0.981170297	0.330711117
RCC1	0.888619648	0.376652567	0.522416782	0.60338384	0.055290541	0.956102964
RCC2	0.688204676	0.493150894	2.712101294	0.008799351	2.089442624	0.041203716
RCCD1	0.751823826	0.45418176	-2.364079193	0.021468845	-2.526895469	0.014346835
RCE1	0.79685434	0.427700216	0.689429113	0.493321562	1.174402766	0.245182903
RCHY1	-0.028479607	0.97734472	-0.705506091	0.483339028	-1.982228714	0.052347921
RCL1	2.039572101	0.044420105	1.466685149	0.147900775	2.616379873	0.011391596
RCN1	0.994134046	0.322908631	1.012733444	0.315423073	1.663224762	0.101831333
RCN2	1.612192988	0.110534078	0.37252934	0.710866093	0.166385496	0.868450368
RCN3	-1.063350874	0.290559107	-0.176978403	0.860146234	0.404040744	0.687715194
RCOR1	-2.235835245	0.027916138	0.770468628	0.444170893	0.068623909	0.945532409
RCOR3	-0.144846066	0.885166717	-1.692371376	0.095978719	-2.49174619	0.015686344
RCSD1	-4.260657117	5.13E-05	1.130326453	0.263023768	0.710650723	0.480237652
RCVRN	-0.216030619	0.82946849	0.594861516	0.554265924	1.556220931	0.125265836
RDBP	-1.587279529	0.116069491	0.905409983	0.369020189	1.995070608	0.050889169
RDH10	-0.223765321	0.823463398	-1.247406895	0.217296565	-1.173391871	0.245584536
RDH11	-0.378131342	0.706252087	-0.048537032	0.96145617	-0.876032812	0.384738806
RDH13	2.134301005	0.035625275	-0.138589327	0.890258098	0.838128198	0.4055069
RDH14	0.4854174	0.628597842	-1.89505681	0.063107655	-2.004010144	0.049894457
RDH16	1.125479415	0.263474529	0.559431569	0.578036267	1.600104075	0.115178975
RDH5	0.670706434	0.504180396	0.571780648	0.56969543	1.97196679	0.053539277
RDX	0.81025944	0.419997118	0.991101668	0.32578147	0.697348655	0.48845765
REC8	-0.577007071	0.565421556	0.833811238	0.4078321	0.635962895	0.527380405
RECK	2.230248763	0.028298777	-0.172531049	0.863624892	-0.519755874	0.605273661
RECQL	1.786126718	0.077555139	-0.251740205	0.802137962	-1.116187435	0.269088542
RECQL4	0.676686814	0.500396009	-0.059386772	0.952849622	0.142974641	0.88682126
RECQL5	0.030797593	0.97550135	1.859175327	0.06811314	3.276844336	0.001801875
REEP3	0.077690241	0.938252471	-1.519832228	0.134024034	-2.174510108	0.033889396
REEP4	-1.34750347	0.181309402	-0.353710001	0.724847726	-0.727577105	0.469890888
REEP5	-0.397173664	0.692210003	0.013647343	0.989158497	-0.362478752	0.718353014
REEP6	0.896621753	0.372389791	0.206723494	0.836954881	1.000356134	0.321423218
REG4	0.48558806	0.628477304	0.341832462	0.733721093	0.172323136	0.863802158
REL	-0.379282125	0.705400531	-1.882190537	0.064865424	-1.919076047	0.060054674
RELA	-1.77483011	0.079416079	-0.219122227	0.827329496	-0.069488916	0.94484696
RELB	-1.204862092	0.231516761	-0.111902987	0.911289405	-1.579252828	0.119886934
RELL1	-3.430486998	0.000922737	-1.429122775	0.158370352	-1.560330173	0.124292244
RELL2	1.68622951	0.09532702	-3.322053579	0.001556104	-2.74344362	0.008142656
RELT	-1.691479941	0.094316693	-1.547888414	0.127126726	-2.267631749	0.027213033

REM2	-1.794422397	0.076211686	-2.55614936	0.013244753	-2.10878106	0.03942995
RENBP	-1.123072166	0.264489813	0.764957077	0.44742016	0.711410855	0.479770279
REPIN1	0.899026649	0.371114653	-3.723059738	0.000449354	-3.078987627	0.003209517
REPS1	1.912195184	0.0591348	2.482797995	0.015971292	2.58605674	0.012324393
REPS2	-4.803487326	6.43E-06	-0.707129078	0.482337571	-1.454323725	0.151415356
RER1	0.094571789	0.924871963	-0.334225854	0.739423096	0.521538144	0.604039938
RERE	-1.918835302	0.058276826	1.28882029	0.202613223	0.344366399	0.731855202
REREP3	-0.717846025	0.474771269	-2.42515416	0.018458756	-2.223674613	0.030203926
REST	-0.253956031	0.80012746	-0.360275575	0.71995891	-0.890130084	0.377188262
RET	-0.090168621	0.928360038	0.119463962	0.905323508	-0.733837642	0.466096222
RETN	-1.385724277	0.169365485	-0.865159622	0.390540443	0.057865285	0.954061057
RETSAT	1.201430143	0.232837774	1.256056185	0.214166737	0.680321597	0.499092203
REV1	2.175108936	0.032328865	-0.624908836	0.534498843	-0.89504082	0.374580234
REV3L	0.526770258	0.599690789	-0.610366754	0.544019841	-2.408028462	0.019342416
REXO1	0.874033991	0.38450085	-2.386866012	0.020297949	-2.025261383	0.047596778
REXO1L1	-0.844156238	0.400892348	4.094656076	0.000133548	4.06666446	0.000150014
REXO2	0.133508325	0.894099054	0.364232976	0.717017827	-0.043349296	0.965576648
REXO4	1.872206561	0.064530212	0.937653023	0.352338081	1.558600048	0.124701417
RFC1	0.791433423	0.430838904	1.971736695	0.053449211	2.397052947	0.019874581
RFC2	-0.63774479	0.525311159	0.75341508	0.454269351	1.763018232	0.083330375
RFC3	2.350151565	0.021023295	0.65145147	0.517346065	0.433225238	0.666508322
RFC4	0.913977919	0.36324907	0.919049156	0.36190283	1.968571679	0.053938516
RFC5	0.888346455	0.376798637	-0.802590148	0.425511072	-1.01536496	0.314280506
RFESD	0.698732823	0.486578669	-0.441024884	0.660846058	-0.304664146	0.7617466
RFFL	-0.080629923	0.93592106	-1.933286849	0.058121364	-3.201583229	0.002249667
RFK	0.917587364	0.361366225	0.093627205	0.925730235	-0.629887204	0.531319386
RFNG	-0.649859838	0.51749131	0.346421795	0.730288129	0.489154356	0.62663486
RFPL1-AS1	-2.272698666	0.025503114	-0.526965381	0.600241461	0.756734941	0.452367425
RFPL2	-0.802881645	0.424226382	0.755236469	0.453184497	1.144975173	0.257068993
RFPL3-AS1	-0.236757913	0.813399969	0.842730763	0.402865062	-0.542275139	0.589771455
RFPL4A	-1.011443404	0.314602948	-0.357394206	0.722102984	0.161918674	0.871950254
RFT1	1.895160489	0.061384761	1.308512129	0.195897194	2.210003847	0.031191469
RFTN1	0.774745131	0.440586288	1.217737408	0.228289124	1.399363367	0.167195907
RFTN2	0.953137145	0.343155575	0.47650829	0.635515113	-0.876882916	0.384280816
RFWD2	-2.457562905	0.015967839	1.25495412	0.214563665	0.609620859	0.544568315
RFWD3	0.249644734	0.803449323	0.634160523	0.528486796	1.79221178	0.078478884
RFX1	-0.754861482	0.452366298	-2.004601527	0.049714514	-2.220210889	0.030451492
RFX2	-1.709816011	0.090856632	-0.638092427	0.525942459	-1.550318737	0.126674895
RFX3	-0.344260513	0.73147931	1.085881716	0.282055253	-0.902436377	0.370674212
RFX5	1.05975584	0.292182691	0.994682838	0.324051117	0.563117564	0.575593198
RFX7	1.168714787	0.245704086	-0.810687422	0.420882405	-1.982295155	0.052340283
RFXANK	-1.466152937	0.146204031	1.631669024	0.108205571	1.492048431	0.141275315
RFXAP	0.774551162	0.440700334	-2.315939182	0.024142838	-2.268116583	0.027181562
RG9MTD1	0.178922438	0.858413721	-0.877813271	0.383692146	-0.404015888	0.687733367
RG9MTD2	0.70512159	0.482614062	-0.321416819	0.749058051	-0.615379176	0.540786786
RG9MTD3	0.870590492	0.386368482	-0.673148424	0.503544991	1.152875623	0.253838303
RGAG4	-0.989999071	0.324914082	-0.505893159	0.614862461	-0.221989562	0.825126197
RGL1	0.730597223	0.46698391	3.282759951	0.001750256	1.614278545	0.112064639
RGL2	-0.882316529	0.380031752	-0.926460554	0.35807259	-0.295562618	0.768652695
RGL3	-2.018517866	0.046610408	-0.378708503	0.706296756	-0.627134879	0.533108797
RGL4	-0.023136017	0.981594643	-1.307384651	0.196277154	-0.765442094	0.447208971
RGMB	1.834114866	0.070048213	2.671408218	0.009804026	1.845298652	0.070261483
RGNEF	-0.01329459	0.989423138	-1.916865255	0.060220506	-1.0138089	0.315016018
RGP1	0.181489475	0.856404648	-1.869604677	0.066624813	-1.901022195	0.062428049
RGPD1	0.753813143	0.452992369	-0.918795743	0.362034259	0.862521154	0.392064117
RGS1	-0.037157754	0.970444167	-1.050861367	0.297711459	-0.816630735	0.417585451
RGS10	-1.808631727	0.073955427	0.193707289	0.847086616	0.510433668	0.61174548
RGS12	0.696378778	0.488043996	1.552054177	0.126127219	2.280954299	0.026359901
RGS14	-0.833924357	0.406602506	0.438337455	0.662781392	-0.233350059	0.816336899
RGS16	-0.024660975	0.980381737	2.489196294	0.015714719	0.794474817	0.4302591
RGS18	-1.292735735	0.199517718	-0.247189616	0.805640489	-0.959005806	0.341660847
RGS19	-1.42544675	0.15759835	-1.181071846	0.242428281	-1.739666986	0.087388279
RGS2	-3.883419765	0.00019996	0.250184586	0.803334845	0.127170169	0.899259343
RGS3	-1.155398115	0.251083955	-1.845397545	0.070122231	-2.005500979	0.049730208
RGS5	0.75178125	0.454207236	-0.314402135	0.754351693	-0.166281496	0.868531825
RGS6	-2.670724621	0.009029105	2.182750931	0.033144869	2.120880153	0.038354359
RGS9	-0.954793737	0.342321769	-2.290065786	0.025699156	-1.260705174	0.212620642
RHBDD1	0.065160706	0.948195219	0.324750517	0.7465465	0.979185385	0.331682097
RHBDD2	-2.759757369	0.007049421	0.720135397	0.474354026	0.659292218	0.512398938
RHBDD3	1.225776076	0.223583576	0.495200879	0.622342324	1.196103739	0.23667488
RHBDF2	-0.568601133	0.57108853	-0.737324472	0.46391824	-1.666527664	0.101169224
RHBDL1	0.128606198	0.897965454	0.38232898	0.70362453	-0.190043621	0.849959277

RHCE	-1.900767258	0.06063638	1.281618496	0.205111969	0.71715257	0.476248184
RHD	-0.417871514	0.677068395	-1.757388255	0.08416559	-1.290285067	0.202231897
RHEB	-0.348219285	0.728514871	-0.391179231	0.69710813	-0.911410217	0.365969615
RHEBL1	-0.11931421	0.905300912	-1.062669354	0.292367262	-1.838338849	0.07129573
RHOA	-2.530966413	0.013169006	1.36312837	0.178147313	1.536368278	0.130055728
RHOB	-3.489008923	0.00076241	-2.805099172	0.006847791	-2.379882268	0.020733482
RHOBTB1	-4.210092742	6.19E-05	1.297008174	0.199800068	-0.011078461	0.991200056
RHOBTB2	1.84000047	0.06917072	0.344864358	0.731452523	0.862237223	0.392218976
RHOBTB3	0.389639021	0.697753627	-1.485055689	0.142981972	-2.152788342	0.035639733
RHOC	0.165699529	0.868777033	-2.02575922	0.047430881	-1.088509279	0.281013236
RHOF	0.407852333	0.684381869	-3.670114407	0.00053167	-2.798202257	0.007025202
RHOG	-2.559156453	0.012217176	-0.537917035	0.592706895	-0.61698141	0.53973699
RHOH	-0.683437894	0.496142406	0.747773468	0.457639118	1.189382214	0.239286886
RHOQ	-0.415801021	0.678577237	-1.844247226	0.070292206	-2.065339227	0.04351137
RHOT1	0.380326329	0.704628164	-1.095279791	0.277953034	-2.3892528	0.020260722
RHOT2	0.261006068	0.794703335	-0.571424301	0.569935287	-0.288532377	0.774000099
RHOU	-1.137990094	0.258242072	0.314882046	0.753989147	-0.177059145	0.860098091
RHOXF1	0.687605994	0.493526071	-1.076883493	0.286022247	-0.462921365	0.645207137
RHPN1	-0.136151542	0.892015354	0.926901315	0.35784563	1.261592561	0.212303329
RHPN2	0.940775636	0.349419074	-0.687048592	0.494809252	-0.476451781	0.635598589
RIBC1	-0.549129214	0.584320422	-0.713746078	0.478266583	-0.356805516	0.722572766
RIC3	3.531497346	0.000662905	0.357505774	0.722019923	1.59678918	0.115917301
RIC8A	-0.901521417	0.36979478	2.050604472	0.044865303	1.902333705	0.062252993
RIC8B	0.980954507	0.329329352	-1.558416424	0.124612833	-1.03498252	0.305107259
RICTOR	0.181467551	0.856421803	-1.156436749	0.252276699	-2.808126061	0.006838495
RIF1	0.459924787	0.646715072	0.182340662	0.855955658	-1.284092295	0.20437482
RILP	-2.417168137	0.01772524	-0.400165282	0.690515122	-1.991779476	0.051259649
RILPL1	-0.558029396	0.578254418	-1.818162405	0.074240676	-0.237893912	0.81282801
RILPL2	-2.580690995	0.011532338	0.798977359	0.427586063	-0.052494407	0.958320779
RIMBP2	1.04985242	0.296687276	1.299778097	0.198855047	0.491691469	0.624851222
RIMBP3	-1.881619844	0.063224277	1.075486519	0.286641576	1.239350379	0.220363109
RIMKLA	-0.563631924	0.574451474	1.416999719	0.1618696	0.444587665	0.658324114
RIMKLB	0.603650288	0.547643485	-0.621669353	0.536612307	-1.119687487	0.26760638
RIMS3	3.262927247	0.001575403	0.600121232	0.55077939	1.642937658	0.105976213
RIN1	-0.433314651	0.665856341	0.932998708	0.354715456	0.264552402	0.792321481
RIN2	-0.567646094	0.571734118	1.516474687	0.134868987	0.486857015	0.628251871
RIN3	-1.10281663	0.273141516	-0.539062408	0.591921473	-0.561427268	0.576736856
RING1	-0.343610708	0.731966293	-2.578299014	0.012508674	-1.431908331	0.157704836
RINL	-1.150537343	0.253068365	-0.491212379	0.625142846	0.047529102	0.962259927
RINT1	1.979468888	0.050917999	-0.732896874	0.466593701	-0.278482859	0.781663152
RIOK1	2.452133075	0.016194629	3.117015758	0.002849179	4.259926483	7.86E-05
RIOK2	1.159445457	0.249440075	0.081725739	0.935148152	0.583520931	0.561875746
RIOK3	-1.195243642	0.235232783	2.294522225	0.025424907	1.886273017	0.064425674
RIPK1	-0.843993558	0.40098275	1.784209592	0.079657165	2.187183493	0.03290342
RIPK2	-2.45279265	0.016166927	-1.259904965	0.212784795	-2.616687822	0.011382462
RIPK3	-1.126360882	0.263103445	0.181227236	0.856825456	0.743074113	0.460529804
RIPK4	-1.972993507	0.051664148	-0.420726773	0.675520324	1.328158311	0.189491072
RIT1	-2.445303428	0.016483971	-0.186863321	0.852424458	-1.242289314	0.219285398
RLF	-0.74948989	0.455579477	-0.008400918	0.993326147	-0.747142281	0.458090258
RLIM	0.904717607	0.368108157	-0.173473403	0.862887566	-0.6997144	0.486990069
RLN2	0.006166427	0.995094021	-2.088889848	0.041147113	-0.957678356	0.342324114
RLTPR	0.286384138	0.775263313	0.678789133	0.49998997	1.654804457	0.103535327
RMI1	-1.391544735	0.167600507	-0.820752125	0.415171565	-1.912816039	0.060868772
RMI2	0.153314495	0.878504549	0.516487978	0.607491086	0.170605517	0.865146286
RMND1	-0.928398691	0.355763894	1.860218919	0.067962961	1.860400991	0.06806065
RMND5A	-2.899066656	0.004734233	-1.240996583	0.219637888	-1.417260119	0.16192353
RMND5B	-0.250534877	0.802763168	1.998744384	0.050363185	1.99403323	0.051005697
RMRP	-2.36772553	0.020109579	-1.599615576	0.115155558	-1.350462702	0.182277278
RNASE1	-0.350597517	0.726735965	1.948466288	0.056236811	1.283177453	0.204692821
RNASE2	-0.371411054	0.711232416	-2.306058634	0.024726989	-2.571928457	0.012782296
RNASE3	0.293213576	0.770055661	-0.592526199	0.555817492	-1.437802062	0.156031754
RNASE4	-2.44521019	0.016487953	2.324203346	0.023663712	2.010608977	0.049170977
RNASE6	-2.16031084	0.033492173	-0.117558661	0.90682636	0.986419084	0.32815262
RNASEH1	1.068132632	0.28840916	0.964283209	0.33893526	1.718723559	0.091165748
RNASEH2A	-0.354185631	0.724054911	3.189689935	0.002305111	3.949486776	0.000220519
RNASEH2B	0.240199784	0.810739232	-1.306067882	0.196721605	-0.663112126	0.509967779
RNASEH2C	1.165749365	0.246894928	0.105278214	0.916520836	0.846201122	0.401026963
RNASEK	-2.610459992	0.010642681	-3.295662221	0.001684115	-2.876615412	0.005669707
RNASEK-C17OR	-0.656992514	0.512916239	-2.561092638	0.013077152	-3.228983375	0.002075716
RNASEL	-1.433800114	0.15520593	0.645684497	0.521047871	-1.074483162	0.287194824
RNASET2	-1.478450108	0.142891022	-2.019801827	0.048064548	-1.63595915	0.107433374
RNF10	-3.410428129	0.000984651	1.70907539	0.092820679	1.659259883	0.102630817

RNF103	0.456591348	0.649100237	-2.128458335	0.037588704	-2.452344709	0.01732137
RNF11	-3.640164688	0.000461328	-0.68162156	0.498210018	-1.578245243	0.120118298
RNF111	-0.194177417	0.84648867	-2.067620258	0.043178291	-3.50493802	0.000904141
RNF112	0.313651837	0.754534644	0.106619622	0.91546125	0.043449483	0.965497142
RNF113A	0.291872055	0.771077786	1.597541022	0.115617515	1.163319225	0.249612341
RNF114	-2.616659292	0.010465413	0.25440076	0.800092052	-0.28972673	0.773090855
RNF115	-1.068000601	0.288468377	-2.401253962	0.019588394	-1.751445842	0.08532137
RNF121	-0.167257371	0.867554862	-0.306387463	0.760414494	-0.480489007	0.632743637
RNF122	-1.288337547	0.201036919	-0.393551287	0.69536546	0.909948352	0.366733389
RNF123	-1.29181974	0.199833411	0.409238791	0.683882307	-0.055667708	0.955803831
RNF125	0.299989597	0.764899143	-0.759033934	0.450927489	0.40370658	0.687959521
RNF126	1.219670961	0.225878681	-3.206641979	0.002193073	-2.101230231	0.040114451
RNF13	-1.352745478	0.179634625	-0.273747856	0.785257716	-1.407212519	0.164867462
RNF130	-3.630012161	0.00047736	-0.520009864	0.605049715	-1.20019453	0.235095351
RNF135	-1.951153006	0.054249797	1.06146827	0.292907823	1.304956965	0.197222073
RNF138	0.491036195	0.62463456	-0.481696436	0.631846867	-1.038003283	0.303711108
RNF138P1	-0.07469777	0.9406263	0.69058602	0.49259945	0.532861194	0.596229137
RNF139	-0.745994751	0.457677198	-1.731782884	0.088665391	-2.285476228	0.026075772
RNF14	-0.119416959	0.905219751	1.887350905	0.064155505	1.511720743	0.136204329
RNF141	-3.260206047	0.001588921	-1.592110224	0.116833914	-1.392861611	0.169143794
RNF144A	0.86391261	0.390006347	-0.692664003	0.491303886	-1.297533905	0.19974495
RNF144B	0.40633784	0.685490015	0.424067216	0.673096484	-0.661403725	0.511054315
RNF145	-1.0790183	0.28355558	-0.168325135	0.866917196	-0.403646631	0.688003357
RNF146	1.395120769	0.166523112	-1.256466097	0.214019239	-2.657080714	0.010240676
RNF149	-1.546863238	0.125518425	0.386891295	0.700262491	-0.848175889	0.399935761
RNF157	4.436027272	2.66E-05	2.458279958	0.016989819	3.255559654	0.00191916
RNF165	0.250389269	0.802875398	-2.282225404	0.026188032	-1.266522791	0.210546765
RNF166	-1.914495326	0.058836397	-2.973517005	0.004293715	-2.671894063	0.009848883
RNF167	-1.795780472	0.075993601	-2.780903441	0.007312992	-2.21885332	0.030549011
RNF168	0.785073894	0.434538319	2.829721684	0.006402524	4.166380308	0.000107651
RNF169	-0.782313546	0.436149837	-0.362469225	0.718328092	-0.716128441	0.476875339
RNF17	-0.915907496	0.362241742	0.187151342	0.852199679	0.653907704	0.515836395
RNF170	3.298344289	0.001409014	-1.329988315	0.188764985	-0.672548464	0.503988734
RNF175	1.159051507	0.249599746	0.125243173	0.900767166	-0.325233738	0.746211105
RNF181	-1.357643876	0.178080225	0.462731233	0.64530055	0.678277828	0.500377114
RNF182	-1.530818343	0.129434642	0.66131596	0.511046609	1.101269091	0.275470838
RNF185	-2.70638602	0.008182286	0.778959412	0.4391924	1.116363908	0.269013673
RNF187	-1.974086787	0.051537522	0.848638793	0.399595615	0.931642251	0.355503913
RNF19A	0.546707392	0.585976237	-1.13768796	0.259961427	-2.276933641	0.026614836
RNF19B	-3.918862283	0.000176545	-0.599162414	0.55141414	-1.625377374	0.109673967
RNF2	-0.087641988	0.930362208	-1.475754562	0.145456056	-2.107567381	0.039539281
RNF20	-0.756031397	0.451668209	2.775279138	0.007425216	2.255722741	0.027996264
RNF207	0.411575211	0.681660802	-1.272744755	0.208222342	-0.742834724	0.46067359
RNF208	-2.705503092	0.008202348	0.050068308	0.960241184	0.302228595	0.763592775
RNF212	1.491551185	0.139426221	-0.607642914	0.545812773	-0.619895267	0.537830495
RNF213	-0.752280646	0.453908474	0.031237078	0.975188464	-0.992422895	0.325242299
RNF214	1.984891335	0.050300259	-1.304674336	0.197192795	-0.476493325	0.635569182
RNF215	0.110958728	0.9119041	0.100939176	0.919949301	-0.442284421	0.659979759
RNF216	1.50054848	0.137085085	1.883913143	0.064627707	2.614176205	0.011457153
RNF216L	1.244663921	0.216590588	0.185764043	0.853282476	1.874940241	0.065997153
RNF217	-0.271750245	0.786456498	-1.009538806	0.316938702	-2.216882725	0.030691054
RNF219	1.022458532	0.309392512	0.58340611	0.561897574	0.911025153	0.366170699
RNF220	0.214352407	0.830772778	0.261161325	0.794899682	1.113345471	0.270296273
RNF222	0.523164609	0.602186782	-2.794719703	0.007043907	-1.473690282	0.146140845
RNF24	-0.586997794	0.558722131	-0.548143078	0.585711969	-1.462373559	0.149205158
RNF25	-0.786065196	0.433960441	0.252689809	0.801407575	1.757687074	0.084242733
RNF26	-0.042838162	0.965928495	0.153215303	0.878764019	1.354498901	0.18099451
RNF31	-1.073148493	0.28616571	0.91599602	0.363488338	-0.12125737	0.903919414
RNF32	0.668965289	0.505285072	-1.454545443	0.151223396	-1.365712785	0.177466686
RNF34	0.475795154	0.635410312	-0.125656587	0.900441356	0.297131869	0.767460609
RNF38	-0.294069895	0.76940343	-0.892215593	0.375989867	-1.370368147	0.176017674
RNF39	0.949547301	0.344966963	0.259184213	0.796417226	1.316595106	0.193314944
RNF4	-1.681376142	0.096268766	0.013732109	0.989091163	0.642423364	0.523208805
RNF40	-1.163842968	0.247662659	0.971792475	0.335217361	0.876636322	0.384413632
RNF41	-1.55971411	0.122450041	-1.738446891	0.08747554	-1.414264405	0.162796976
RNF43	2.471575699	0.015395632	-0.188332152	0.851278274	0.998318239	0.322401384
RNF44	0.405630973	0.686007461	-2.006304758	0.049527238	-1.660077212	0.102465592
RNF5P1	-1.033602999	0.304180249	1.765090526	0.082849715	0.882607847	0.381205455
RNF6	0.370413665	0.711972645	-0.563437512	0.575324112	-1.153046892	0.25376859
RNF7	-0.90880302	0.365959398	-1.968660748	0.053810757	-1.648687144	0.104787809
RNF8	0.050994559	0.959446503	2.276587797	0.026544637	3.139763004	0.00269377
RNFT1	1.120053865	0.265766688	-2.906866751	0.005174759	-2.766832761	0.007646733

RNFT2	0.391986774	0.696024486	1.06253511	0.292427646	1.85112481	0.069405445
RNGTT	0.991143727	0.324358103	0.390067691	0.697925305	0.101922851	0.919180461
RNH1	-0.91842234	0.360931553	2.829126781	0.006412959	3.10856058	0.002947984
RNLS	-0.278618261	0.781197533	-2.358943949	0.021740911	-0.327067764	0.744830951
RNMT	1.914831502	0.058792891	0.497063661	0.62103629	1.470722019	0.146939759
RNMTL1	0.428692475	0.669204326	2.694609344	0.009219053	3.373865856	0.001347904
RNPC3	1.552444877	0.124178298	-0.577206076	0.566049698	-2.167532624	0.034443232
RNPEP	-1.423517268	0.158154978	2.080772881	0.041912245	1.702148912	0.094249924
RNPEPL1	-0.944857119	0.347342896	1.021978395	0.311064567	2.154932249	0.035463523
RNPS1	-0.738342988	0.462288852	0.671346836	0.50468331	1.054189498	0.296304405
ROBO1	1.244332367	0.216711945	0.983985224	0.329238268	0.825620214	0.412508354
ROBO3	1.239555209	0.218466034	1.91008771	0.061105486	2.127352659	0.037789545
ROCK1	0.341423794	0.733606039	0.237110797	0.813412182	-1.325213785	0.190459333
ROCK2	-1.860784194	0.066145214	-0.403078031	0.688383187	-2.264081143	0.027444493
ROD1	-0.661905344	0.509777543	-1.404443415	0.165556739	-2.043040233	0.045745269
ROGDI	-2.501859559	0.014221284	-0.378106279	0.706741612	-1.339566678	0.185774771
ROM1	1.623890188	0.108009316	0.051269259	0.959288358	-1.364275698	0.177915826
ROMO1	-0.064378647	0.948816107	-0.479900891	0.63311535	-0.282206152	0.778821448
ROPN1L	-1.523618088	0.131223163	-0.150018637	0.881274025	0.102560555	0.918676591
ROR2	-0.676529855	0.500495137	0.993664645	0.324542462	1.147873945	0.255880226
RORA	1.351480696	0.180037637	-2.345212754	0.022483572	-2.530405418	0.014218929
RORC	-0.714181073	0.477022861	-1.075947416	0.286437141	-0.521435635	0.604110865
RP1L1	-3.354824917	0.001177359	0.81026641	0.421122319	1.717886959	0.091319399
RP2	-1.092491618	0.277626575	-0.988042661	0.327264398	-2.601458823	0.011842245
RP9	0.173634737	0.862555027	0.679596861	0.499482026	1.150465263	0.254820868
RP9P	-0.081279208	0.935406198	-0.934112784	0.354145454	-0.084089084	0.93328423
RPA1	-0.236082268	0.813922535	2.1902604	0.032568032	2.246164829	0.028639233
RPA2	-1.346656553	0.181581087	0.290468038	0.772501501	1.598355424	0.115567977
RPA3	-0.052310718	0.958400778	-0.46221251	0.645670232	-0.014281576	0.988655885
RPAIN	2.636124352	0.009926043	-0.625257145	0.53427186	-0.522412392	0.603435191
RPAP1	1.054359682	0.294631314	1.390419027	0.169751278	2.419982934	0.018777362
RPAP2	1.112050937	0.269173161	0.067683597	0.946271923	0.591837476	0.556331326
RPAP3	0.690620649	0.491638452	0.53140738	0.597180076	-0.241483696	0.810058602
RPE	0.526766275	0.599693544	-0.641718914	0.523601467	-0.814818639	0.418613452
RPF1	0.430426506	0.667947525	-2.0571208	0.044212624	-1.111924274	0.27090166
RPF2	0.782168467	0.436234632	-0.04288452	0.965941913	0.303060307	0.762962172
RPGR	-0.682278922	0.496871233	-1.688120526	0.096796317	-3.067245864	0.003319255
RPGRIP1	0.683861473	0.49587618	0.14475035	0.885413317	0.430989039	0.668123879
RPGRIP1L	0.935184447	0.352276195	-1.64131994	0.106181026	-1.197685333	0.236063286
RPH3A	-2.188701162	0.031291542	-0.074631305	0.94076667	-0.086814162	0.931127606
RPH3AL	-1.399019593	0.16535451	1.599232293	0.115240794	2.444617389	0.017659479
RPIA	-1.87871893	0.06362434	2.029070827	0.047081763	1.721451461	0.090666218
RPL10	0.331736844	0.74088408	-1.598877781	0.115319677	-0.382140027	0.703797824
RPL10A	0.740437626	0.461023815	-0.067824838	0.946159978	0.687536713	0.494570508
RPL11	0.170799504	0.864777162	0.494412905	0.622895157	1.091853966	0.279552962
RPL12	0.023357039	0.981418846	-0.45486207	0.650918342	0.217801682	0.828371995
RPL13	1.04983954	0.296693165	-1.360013911	0.17912531	-0.115945437	0.908108827
RPL13A	0.662335559	0.509503176	1.292873156	0.201217078	1.54895559	0.12700212
RPL13P5	-1.173139379	0.243934904	2.393238334	0.0199809	1.343892505	0.184380181
RPL14	-0.471792231	0.638253665	-1.22688651	0.224856852	-0.230055468	0.818883444
RPL15	0.593465287	0.554406276	-0.929854317	0.356327448	-0.227252499	0.821051538
RPL17	0.666938503	0.506572608	0.205842302	0.837639941	1.043669125	0.301104213
RPL18	-0.107558978	0.914592661	1.979838199	0.052506911	1.880396353	0.065236568
RPL18A	-0.564909512	0.573585948	-1.350779946	0.182049026	-0.424001077	0.673182534
RPL19	0.106808132	0.915186574	0.5749803	0.567543965	1.700469225	0.094567215
RPL21	-0.653496028	0.515156288	3.409501817	0.001194554	3.664049031	0.000550958
RPL21P44	-0.209780871	0.834328121	-0.755453977	0.453055046	0.370973523	0.712051099
RPL22	0.239542449	0.811247213	-0.174172987	0.862340271	0.750125478	0.456306073
RPL22L1	0.778878473	0.438160152	-0.998270287	0.3223239	-0.242155042	0.809540949
RPL23	0.850511196	0.397370596	0.460694018	0.646752942	1.046161523	0.299962303
RPL23A	-0.191164581	0.848841109	-0.073729974	0.941480712	0.474759469	0.636796978
RPL23AP53	0.515793172	0.607304386	1.167566176	0.247792567	0.657623045	0.51346322
RPL23AP64	1.922936202	0.057752219	0.890359975	0.376976709	1.298389573	0.199452905
RPL23AP7	-3.785410076	0.000281164	1.264088487	0.211290167	0.068980316	0.94524998
RPL23AP82	-0.666949316	0.506565734	3.496288907	0.000915489	2.472687473	0.016458971
RPL24	0.523082501	0.602243677	-0.590676856	0.557047724	0.286330707	0.775677033
RPL26	0.727839913	0.46866171	-0.367058553	0.714920527	0.213818395	0.831462018
RPL26L1	0.521051841	0.603651545	-1.293717462	0.200927137	-0.33580619	0.738266669
RPL27	0.027765363	0.977912746	-2.628402325	0.010978894	-1.56895407	0.12226872
RPL27A	0.366625176	0.714786849	-0.869093384	0.388403326	0.367395962	0.714702711
RPL28	-1.845789025	0.068316675	-1.180603614	0.242612844	-0.95727443	0.342526106
RPL29	-0.682996728	0.496419768	-2.79331532	0.007070836	-1.506724582	0.137478333

RPL3	0.809463076	0.420452411	0.382287653	0.703655012	1.147381287	0.256081985
RPL30	0.106496854	0.915432806	-1.177815693	0.243713855	-0.445270866	0.657833337
RPL31	0.633486544	0.528074249	-0.78140777	0.437762959	-0.006309654	0.994987987
RPL31P11	-1.030472282	0.305638457	-1.260397428	0.212608449	-2.079626601	0.042130333
RPL32	0.089204497	0.929123981	-1.357938321	0.179779355	0.064135404	0.949089845
RPL32P3	0.536021298	0.5933087	-0.67755711	0.500765277	-0.193157343	0.847531669
RPL34	0.790719573	0.431253231	-0.071246524	0.94344837	0.885169009	0.379834668
RPL35	0.579812216	0.563536565	-0.822609993	0.414122547	0.187120901	0.8522393
RPL35A	0.093882677	0.925417765	-1.935358838	0.05786099	-0.56631697	0.57343149
RPL36	-0.140747855	0.888393804	-1.904508971	0.061842204	-0.582378909	0.562639238
RPL36A	0.645951382	0.520007372	0.120301717	0.904662818	0.511482346	0.61101588
RPL36AL	-0.83439179	0.406340572	-0.660579906	0.511515229	-0.622197882	0.536326387
RPL37	0.825327163	0.411438355	-1.965692134	0.054161672	-0.900197275	0.371854062
RPL37A	-0.063373545	0.949614118	-0.028241918	0.977566835	0.579920111	0.564284796
RPL38	-1.354868407	0.178959705	-2.992359207	0.004071213	-1.606906923	0.113675675
RPL39	0.076069293	0.939538248	-0.351888895	0.726205798	0.604061479	0.548231957
RPL39L	0.598252614	0.551222342	-0.782730883	0.436991622	-0.686488524	0.495226006
RPL3L	-0.088315273	0.929828635	0.694826209	0.489957808	-0.480038689	0.633061805
RPL4	0.630808762	0.529815658	0.662572091	0.510247406	1.456007613	0.150950915
RPL41	0.45673603	0.648996637	-0.199752019	0.842378099	0.536906958	0.593449812
RPL5	0.454510515	0.65059099	-0.561738018	0.576473971	0.421196424	0.675217145
RPL6	0.642533871	0.522212635	0.616336597	0.54010081	1.311599517	0.194984842
RPL7	0.414800734	0.679306652	0.5638048	0.575075756	0.746658495	0.458379977
RPL7A	0.367188503	0.714368142	0.023478328	0.981349878	0.717053114	0.476309069
RPL7L1	1.35294344	0.179571608	0.210154353	0.834288862	0.482081902	0.631618752
RPL8	0.104916106	0.916683363	-2.704935598	0.00896912	-1.017619861	0.313216728
RPL9	0.29108975	0.771674024	0.576719239	0.566376369	1.01457511	0.314653704
RPLP0	-0.179944335	0.857613828	-0.398117326	0.692015598	0.001225251	0.999026727
RPLP0P2	1.823230068	0.071695528	1.628049509	0.108972902	0.594178311	0.55477571
RPLP1	-0.787526552	0.433109372	0.361364138	0.719149479	0.726155515	0.470754985
RPLP2	-0.349825778	0.727313058	0.438909674	0.662369118	0.866897488	0.38968204
RPN1	-1.810165896	0.073715178	1.780600798	0.08025179	2.608351099	0.011632103
RPN2	-0.572983497	0.568130671	2.742474512	0.008111967	2.786551496	0.007250394
RPP14	-1.614325556	0.110070276	1.769489929	0.082105817	2.306087309	0.024814853
RPP21	-0.36458077	0.716307139	-2.902743258	0.00523443	-3.327502803	0.001549354
RPP25	0.056769231	0.954858921	0.268829777	0.789021299	-1.230029332	0.223806944
RPP30	1.855239911	0.066941249	-1.859786586	0.068025143	-0.059559808	0.952717379
RPP38	1.128985318	0.262000773	-1.713547937	0.091989784	-0.681213862	0.498531802
RPP40	0.36002569	0.71969856	-1.534253262	0.130442549	-0.222512706	0.824720951
RPPH1	-3.246744964	0.001657401	2.776047048	0.007409801	2.662430656	0.010097563
RPRD1A	1.169497372	0.245390503	-2.205401936	0.031431712	-1.943979577	0.056907227
RPRD1B	-0.372185762	0.710657644	-1.502280261	0.138487899	-0.758435363	0.451357324
RPRD2	-0.766368093	0.445527293	0.290623772	0.772382978	0.497249919	0.620951415
RPRM	0.881196771	0.380634047	0.301456871	0.764151848	-0.671351263	0.504745195
RPS10	0.16853088	0.866555997	0.839498184	0.404660883	1.512266428	0.136065749
RPS10P7	-0.100084957	0.920506654	-0.738098827	0.463451224	0.883881802	0.380523218
RPS11	-0.613015849	0.541461635	-0.765453092	0.447127174	-0.00815165	0.993524845
RPS12	-1.020072068	0.310516419	0.530497122	0.597806824	1.510113721	0.136613089
RPS13	0.464957091	0.643121313	-2.057569081	0.044168028	-0.586355123	0.559983188
RPS14	0.771726744	0.442362922	0.264790137	0.792116442	1.025728787	0.309411375
RPS15	0.785431253	0.434329945	-0.117501984	0.906871071	0.661788457	0.510809518
RPS15A	-0.07362602	0.941476616	-0.787581124	0.434170948	-0.03814024	0.96971094
RPS15AP10	-0.951944562	0.34375665	-1.550307425	0.126545556	-2.141083855	0.03661532
RPS16	-0.105842841	0.915950181	-0.026627302	0.978849041	0.668987083	0.506240822
RPS18	0.595831576	0.552831372	-1.264771707	0.211046818	-0.076493057	0.939298326
RPS19	-0.181728669	0.856217493	0.845020519	0.401595985	1.347888146	0.183099122
RPS19BP1	0.091463542	0.927334087	1.297785805	0.199534423	1.401811649	0.166466919
RPS2	1.630812687	0.106537149	-1.342880359	0.184578953	-0.54463909	0.58815507
RPS20	0.260022693	0.79545932	0.730373767	0.46812226	1.407791732	0.164696637
RPS21	0.487664389	0.627011587	-1.774324982	0.081294674	-0.546189816	0.587095883
RPS23	0.511482018	0.61030652	-0.59323469	0.555346546	0.079236761	0.937125591
RPS24	0.760019739	0.449293029	0.333912434	0.739658358	1.234521123	0.222142465
RPS25	0.351716407	0.725899558	-1.818641056	0.074166581	-0.672577483	0.503970406
RPS26	-0.036032162	0.971339082	-3.400075026	0.001229314	-2.397359826	0.019859525
RPS27	0.363077532	0.717425729	-1.291546752	0.201673207	-0.797253241	0.428657317
RPS27A	0.858457443	0.39299379	0.715122048	0.477422469	1.617360346	0.111396629
RPS27L	1.444012764	0.152319233	-1.733822909	0.088299733	-1.255442645	0.214509656
RPS28	0.728922919	0.468002306	-1.711765339	0.092320211	-1.146653784	0.256380127
RPS29	-0.123241013	0.9021999	-2.259937472	0.027623099	-1.363350555	0.178205425
RPS2P32	0.036515735	0.970954607	-1.486855944	0.142506957	-0.076924054	0.938956989
RPS3	0.213058972	0.831778345	0.936077742	0.353141557	1.25910599	0.213193369
RPS3A	0.334730915	0.738632006	0.513189903	0.609781392	0.945768684	0.348312635

RPS4X	0.3565104	0.722319667	1.308571102	0.195877336	1.999421446	0.050402936
RPS5	0.39600801	0.693066548	-0.654459929	0.515420503	0.247543866	0.80538892
RPS6	0.664155336	0.508343496	-0.121811416	0.903472375	1.138333663	0.259807457
RPS6KA1	-2.153849766	0.034011409	0.931676351	0.355392794	0.788286781	0.433839349
RPS6KA2	2.932320355	0.004296733	0.62111796	0.536972469	0.81576942	0.418073884
RPS6KA3	1.349246643	0.180751169	-1.407701929	0.164593706	-2.551426754	0.013474279
RPS6KA4	-0.168762268	0.866374534	-1.764322227	0.0829802	-1.9848588	0.052046271
RPS6KA5	-0.722496553	0.471922744	-0.314026752	0.754635313	-2.860858235	0.005920934
RPS6KB1	0.098722688	0.921585072	-1.133309167	0.261779912	-1.547944279	0.127245322
RPS6KB2	-1.034439115	0.303791604	-1.118973849	0.267796319	-0.297109521	0.767477582
RPS6KC1	-0.581883951	0.56214639	0.511710975	0.610809693	-0.18745442	0.851979056
RPS6KL1	1.284807436	0.202262471	-1.319465781	0.19223457	0.31698086	0.75243191
RPS7	0.230239822	0.818444761	-0.599188338	0.551396974	-0.09226881	0.926812377
RPS8	0.806391756	0.422211087	0.105504053	0.916342434	0.989932602	0.326447359
RPS9	0.042524369	0.966177919	-0.697473234	0.488312682	-0.215183552	0.830402698
RPSA	-0.044937223	0.964260109	0.366519671	0.715320345	0.993075012	0.324927229
RPSAP58	0.854473633	0.395184353	0.478536357	0.634080081	-0.992682204	0.32511699
RPTOR	0.174513843	0.861866245	0.817116061	0.417229272	1.331521057	0.188389848
RPUSD1	0.437902315	0.662540038	-0.039023507	0.969006597	0.644082703	0.522140164
RPUSD2	0.688992844	0.492657209	2.221719503	0.030246344	3.098280964	0.003036543
RPUSD3	-0.326658844	0.744708799	1.812346818	0.0751459	2.338932381	0.022917339
RPUSD4	2.284418266	0.024775408	2.488217247	0.015753736	3.362266749	0.001395839
RQCD1	1.005030316	0.317663379	-1.373062083	0.175055188	-0.606214349	0.546811729
RRAGA	-1.199281478	0.233667594	-0.669687041	0.505733269	-0.947961811	0.347204762
RRAGB	0.636592603	0.526058046	-0.19771234	0.843966261	-0.316524545	0.752776354
RRAGC	-0.865707283	0.389026599	0.125317378	0.900708685	-0.419798472	0.676232188
RRAGD	-2.464548993	0.015680229	0.87854663	0.38329757	-0.525504271	0.601298678
RRAS	-0.496401295	0.620860504	-1.851637375	0.069206232	-1.658200238	0.102845352
RRAS2	1.662873026	0.099928794	-2.086209472	0.04139842	-1.471945617	0.146610011
RRBP1	0.247079902	0.80542725	1.621035342	0.110472463	0.87774745	0.383815402
RREB1	0.756465939	0.451409076	0.225116976	0.822685084	-0.113330985	0.910171765
RRM1	2.530215019	0.013195265	3.353936675	0.00141371	3.961636536	0.000211935
RRM2	0.746826839	0.457177294	0.339753368	0.735278127	0.34277621	0.733044792
RRM2B	-1.063510889	0.290486985	-0.855584243	0.395773083	-2.296364304	0.02540276
RRN3	1.99459227	0.049211038	2.237436082	0.029142077	2.138748006	0.036812789
RRN3P1	1.745350041	0.084446971	0.003056455	0.997571867	1.097237665	0.277213606
RRN3P2	1.047608129	0.297714629	-1.464272228	0.148556591	-1.026946101	0.308842833
RRN3P3	2.541854734	0.012793728	-2.078749961	0.042104844	-0.822592965	0.41421388
RRNAD1	0.854098193	0.395391181	0.547524849	0.586133738	0.459781897	0.647445413
RRP1	0.929466745	0.355213478	2.307484917	0.024641897	3.045227812	0.003534553
RRP12	-0.855515104	0.394610959	0.677817857	0.500601135	1.195029557	0.237090915
RRP15	2.517561412	0.013644607	-0.645663106	0.521061627	0.139142705	0.88983448
RRP1B	0.999416496	0.320358628	2.117083618	0.03858305	2.441187137	0.017811464
RRP36	-1.2163037	0.227151837	2.675735081	0.009692421	4.306400362	6.72E-05
RRP7A	0.805382996	0.422789672	1.192204988	0.238069758	1.497394505	0.139882717
RRP7B	3.359652099	0.001159317	-0.80610356	0.423498956	-0.653041629	0.516390439
RRP8	-0.483157161	0.630195213	2.335052016	0.023047605	3.085430721	0.003150751
RRP9	-0.015511466	0.987659577	1.345148441	0.183849858	2.419517159	0.018799097
RRS1	0.727823845	0.468671498	0.317784635	0.751797588	0.008088514	0.993574995
RSAD1	1.430435943	0.15616604	1.669781168	0.100389428	2.671446429	0.009860519
RSAD2	-1.904513187	0.060140678	0.839544795	0.404634954	-2.126502696	0.037863299
RSBN1	1.389804673	0.168126679	-0.508940766	0.612737942	-1.206735174	0.232585835
RSBN1L	-1.379939385	0.171133693	-1.973531841	0.053239174	-3.690746557	0.000506464
RSC1A1	-1.027671048	0.306947192	-1.979318818	0.052566891	-1.968134238	0.053990141
RSF1	0.216580723	0.829041059	1.00957779	0.316920177	0.470483309	0.639829431
RSG1	0.127622195	0.898741856	0.492854005	0.623989504	0.079585007	0.93684985
RSL1D1	0.866799719	0.388430963	2.66457295	0.009982727	4.039024669	0.000164362
RSL24D1	0.4147037	0.679377426	-0.085991497	0.931771413	-0.043719376	0.96528296
RSPH3	-0.829663044	0.408995132	-0.293796948	0.769969186	-0.511555851	0.610964755
RSPH4A	0.243586275	0.808123475	-0.714435588	0.477843486	0.096388272	0.923554884
RSPH9	-1.764059733	0.081224576	1.941536334	0.057090601	-1.247608825	0.21734462
RSPO4	-0.104304959	0.917166908	0.672023201	0.504255791	0.190174359	0.849857317
RSPRY1	-0.090972232	0.927723332	0.41932326	0.676539752	-0.171276644	0.864621046
RSRC1	-0.714342431	0.476923605	-0.526783261	0.600367132	-0.436764386	0.663954686
RSRC2	0.853462166	0.395741717	0.044952511	0.964300652	0.353551607	0.724996949
RSU1	-1.683809877	0.095795582	-1.699660901	0.094589879	-1.33421778	0.18751024
RTCD1	1.692469762	0.094127206	-1.402730591	0.166064695	-1.406607484	0.165046049
RTDR1	-3.065477396	0.00289288	-1.184636033	0.24102671	-1.42266069	0.160358093
RTEL1	1.029827182	0.305939513	0.981808077	0.330300667	1.163543578	0.249522115
RTEL1-TNFRSF6	0.652266999	0.5159449	0.707953627	0.48182923	0.703383927	0.484718537
RTF1	-3.072732249	0.00283025	1.756818369	0.084263637	1.238787126	0.220570099
RTKN	0.366713262	0.714721371	-0.429928369	0.668852028	0.40911886	0.684006415

RTKN2	1.477658854	0.143102416	-0.318598319	0.751183595	-1.306241285	0.196788012
RTN1	-0.613800669	0.540945219	0.632797563	0.529370268	0.009261412	0.992643345
RTN2	-1.869898757	0.064853815	0.329928105	0.742651274	0.446146798	0.657204335
RTN3	-3.452499957	0.000859022	0.885645575	0.379491252	0.785551414	0.435427586
RTN4	0.271108685	0.786948262	-0.11782729	0.906614451	-1.314291563	0.194083615
RTN4IP1	1.550634699	0.124611664	0.573061733	0.568833542	2.349103773	0.022356547
RTN4R	-0.387654727	0.699216323	0.06329967	0.949747049	-0.312009343	0.756187324
RTN4RL1	-1.011391836	0.314627477	0.385695529	0.70114309	1.400691436	0.166800163
RTP4	-2.60172913	0.010896947	1.271343885	0.208716557	-0.148531732	0.882454471
RTTN	1.86558112	0.065462899	-0.835791495	0.406726127	0.013360526	0.989387446
RUFY1	-2.366733108	0.020160223	2.473672796	0.016343754	1.367549094	0.176894037
RUFY2	2.862230867	0.005266499	-2.434482932	0.018033949	-3.337531257	0.001503504
RUFY3	0.257280624	0.79756836	-0.69651116	0.488910258	-0.840908054	0.403960809
RUFY4	0.062688813	0.950157797	2.576989459	0.012551137	0.902454926	0.370664448
RUNDC1	-2.368398455	0.020075304	1.258485656	0.213293641	0.035193598	0.972049991
RUNDC2A	-0.189065973	0.850480525	0.131975316	0.89546377	-0.566460741	0.573334443
RUNDC2C	-0.623467727	0.534604845	1.243865344	0.218587805	1.929958147	0.058661545
RUNDC3A	-3.328157558	0.001281877	0.662968896	0.509995082	-0.355503842	0.72354218
RUNDC3B	0.239192917	0.811517361	-0.225905108	0.822074952	-0.266091285	0.791142066
RUNX1	-1.529991436	0.129639059	-0.956281882	0.342926551	-1.213238165	0.23011017
RUNX2	0.658924164	0.511680931	-2.33557336	0.02301836	-3.094590294	0.003068941
RUNX3	-0.391246427	0.696569586	-1.24650917	0.21762334	-0.516489563	0.607537669
RUSC1	-0.002698633	0.99785297	-0.881083746	0.38193447	-0.342650943	0.73313853
RUSC1-AS1	0.157605551	0.875132054	-2.112641705	0.038977534	-0.094914332	0.924720265
RUSC2	0.679926054	0.498352633	0.959699116	0.34121818	0.523196213	0.602893233
RUVBL1	0.133984191	0.893723864	1.849245339	0.069556181	3.043984016	0.003547096
RUVBL2	-0.79236243	0.43030005	0.587027638	0.559479268	1.374492534	0.174741515
RWDD1	0.673961613	0.502118614	0.233036454	0.816559282	0.785609433	0.435393863
RWDD2A	0.689803797	0.492149532	-0.61085611	0.543698047	1.19945381	0.235380787
RWDD2B	0.703512165	0.48361112	-0.573839055	0.568310886	0.74533492	0.459173153
RWDD3	0.758817716	0.45000811	0.52199454	0.603675929	0.461976229	0.645880623
RWDD4	-0.201357161	0.84088833	-1.306271885	0.196652698	-1.356092897	0.180489812
RXFP4	-0.687819238	0.493392419	-0.043055019	0.965806591	0.335287956	0.738655424
RXRA	-2.666296502	0.009139635	0.003355295	0.997334461	-0.410039379	0.683334951
RXRB	-0.176665073	0.860181203	-2.09765929	0.04033415	-0.894130358	0.375062902
RYBP	-2.729700363	0.007668437	-1.567748773	0.12241785	-2.398339607	0.019811523
RYK	1.27962423	0.204071954	-1.249617307	0.216493513	-1.263536297	0.211609512
RYR1	0.084265393	0.933038607	-0.08084019	0.935849297	0.832076825	0.408885063
RYR3	-0.036030387	0.971340493	-1.083060457	0.283294903	-2.617750097	0.011351005
S100A10	-0.864965985	0.389431104	-0.979968764	0.331199984	-0.39425785	0.694881809
S100A11	-2.95991345	0.00396235	-2.171260033	0.03404491	-2.401598094	0.01965263
S100A12	-1.969190829	0.052106648	-1.139650985	0.259149115	-0.750822262	0.455889921
S100A13	1.070755272	0.287234629	1.600771429	0.114898822	2.433488202	0.018156876
S100A4	-1.616014691	0.109704025	1.158146822	0.251583948	2.11131767	0.039202295
S100A6	-1.91711489	0.058498105	-1.442269613	0.154642331	-0.632509343	0.529617528
S100A8	-0.343860462	0.731779108	-0.680343333	0.499012852	-0.665666709	0.508345392
S100A9	-1.50642008	0.135573977	-0.660275246	0.511709263	-0.393924767	0.695126313
S100B	0.68523983	0.4950104	-0.958516718	0.34180866	0.261804299	0.794428867
S100P	-2.375756004	0.019703917	-0.871413544	0.387146273	-0.896167537	0.373983468
S100PBP	3.538325527	0.000648107	-0.36598636	0.715716109	-0.161056647	0.872625976
S100Z	0.429539284	0.668590453	-0.082400986	0.934613551	0.28250828	0.778590989
S1PR1	0.824616194	0.411839817	-0.864485764	0.390907269	-1.003539414	0.319899267
S1PR2	0.037907429	0.969848151	1.152474315	0.253887125	1.097130099	0.277260212
S1PR3	-2.052834238	0.043086263	-0.88602534	0.379288303	-0.208762518	0.835387951
S1PR4	-0.240152719	0.8107756	-1.141661791	0.258318903	-1.149563949	0.255188979
S1PR5	0.65111308	0.516685897	-2.994260699	0.004049365	-1.98840747	0.051641634
SAAL1	0.185186535	0.853512838	1.157714203	0.251759073	2.632783568	0.010914247
SAC3D1	-1.011388153	0.31462923	0.987816906	0.327374016	0.121345785	0.903849706
SACM1L	0.935750826	0.35198609	-1.532108229	0.130970396	-2.041669894	0.045885729
SACS	1.101003817	0.27392531	0.02810783	0.977673315	-0.288502666	0.774022722
SAE1	-0.573746843	0.567616216	3.303795461	0.001643642	3.699439628	0.000492733
SAFB	0.015203974	0.987904188	1.297492687	0.199634523	1.330695607	0.188659715
SAFB2	0.642793477	0.522044945	0.226119779	0.821908783	0.702629148	0.485185283
SALL2	0.873856129	0.384597179	1.062955655	0.292238511	2.374822982	0.020992815
SALL4	0.625164334	0.533496031	-2.085205754	0.04149287	-0.862110738	0.392287974
SAMD1	1.733360536	0.086566733	-0.165572215	0.869073414	0.192445898	0.848086216
SAMD10	1.591595277	0.115095029	-1.167731289	0.247726475	0.505714231	0.615033837
SAMD12	-0.254487572	0.79971816	0.054510368	0.95671719	1.343136431	0.184623354
SAMD14	-3.307670414	0.001368025	0.310084444	0.757615963	-0.045326853	0.964007355
SAMD3	0.713610185	0.477374126	-1.313466814	0.194234023	-0.165479731	0.86915985
SAMD4A	-0.906865863	0.366977268	-0.199686573	0.842429047	-1.115199581	0.269507912
SAMD4B	0.504771987	0.614992426	-1.74763703	0.085856365	-1.385447739	0.171386221

SAMD8	-0.55193295	0.582406261	-1.259191744	0.213040384	-2.624678405	0.011147779
SAMD9	-0.759454331	0.449629307	-0.855903535	0.395597902	-2.523727788	0.014463161
SAMD9L	-0.456724501	0.649004892	-1.037098529	0.304024473	-3.301204533	0.001675948
SAMHD1	-1.906077656	0.059934665	2.50249151	0.01519344	1.643094897	0.105943567
SAMM50	-0.438660966	0.661992275	1.91613437	0.060315414	3.554360089	0.00077615
SAMSN1	-0.095162228	0.924404343	0.087468666	0.930602389	-0.448382489	0.655600027
SAP130	-1.678608015	0.096809275	-0.237255838	0.813300207	-0.07785909	0.938216507
SAP18	-3.213975219	0.001835827	-2.220536782	0.030330917	-1.865064244	0.067392958
SAP25	-0.992700884	0.323602779	-0.624410519	0.534823668	0.820885937	0.415177491
SAP30	-1.622981193	0.108203838	0.110271596	0.91257732	-1.744165199	0.086594105
SAP30BP	-0.206486205	0.836892568	1.909418067	0.061193522	2.960145021	0.004495097
SAP30L	0.057489853	0.954286539	-0.613027887	0.542271091	-1.23694076	0.221249625
SAR1A	0.129834022	0.896996811	0.540340477	0.591045636	0.998118117	0.322497548
SAR1B	0.618134363	0.538098139	-0.169854659	0.865719638	-0.292182386	0.771222404
SARDH	2.026576669	0.04576135	-0.289770054	0.773032776	0.916290377	0.363427288
SARM1	2.386663557	0.019164567	0.40715388	0.685404206	1.483053557	0.143643051
SARNP	1.09628371	0.275973444	0.242557555	0.809209837	0.516855548	0.607283798
SARS	-0.41021427	0.682655033	1.283084271	0.204601547	2.319400879	0.024029477
SARS2	0.273809643	0.784878526	0.652969186	0.516374174	1.778784791	0.080680115
SART1	-0.468522745	0.640580069	1.930834757	0.058430792	1.898805025	0.062724941
SART3	1.184562673	0.239409412	2.876990692	0.005621758	2.836462981	0.006330268
SASH1	1.315983631	0.191629062	0.610736589	0.543776634	-0.00315709	0.997492184
SASH3	-2.36762015	0.020114952	0.384573135	0.70197003	0.752372532	0.454964813
SASS6	0.799819318	0.425989251	-0.988392807	0.327094429	-1.288003775	0.203019342
SAT1	-2.104516973	0.03821179	0.252112175	0.80185184	-0.556394414	0.580148593
SAT2	-0.653634571	0.515067431	2.439877104	0.017792342	2.280060023	0.026416415
SATB1	2.78882513	0.006494532	0.16519212	0.869371202	-0.3140641	0.754634467
SATB2	-1.230312339	0.221889273	1.078950864	0.285107411	0.694525445	0.490212218
SAV1	-0.666050131	0.507137507	0.395333465	0.694057239	-0.480029917	0.633068004
SBDS	1.315358189	0.191838191	-1.237447195	0.220942244	-0.581275829	0.563377184
SBDSP1	0.164528932	0.869695609	-1.149314032	0.255176791	-0.656643973	0.514088036
SBF1	1.01620543	0.312343228	0.099283279	0.921258105	0.672096617	0.504274165
SBF2	-1.098391793	0.275057402	-0.296342022	0.768034834	-2.0526765	0.044768019
SBK1	1.269756851	0.207549773	-1.730378713	0.088917804	-0.600332475	0.550696365
SBNO1	-0.294063463	0.769408328	1.111655625	0.270905038	0.777696747	0.440007348
SBNO2	-0.442240847	0.659410006	-1.648301634	0.104735722	-2.404806006	0.01949732
SC4MOL	1.426127532	0.157402315	-1.812147419	0.075177101	-0.871574822	0.387146141
SC5DL	1.569928351	0.120053979	-1.687842697	0.096849952	-1.687161351	0.097112243
SCAF1	-0.796988734	0.427622575	-0.640134325	0.524623685	-0.487154232	0.628042568
SCAF11	0.993704247	0.323116699	0.248474694	0.804650972	-1.439299721	0.155608818
SCAF4	-0.368129943	0.713668588	0.275328677	0.784049073	0.529866815	0.598290104
SCAF8	-0.963875823	0.337773963	1.828719695	0.072620751	1.111593667	0.271042625
SCAI	1.169303924	0.245467991	-1.859812817	0.068021369	-1.953355674	0.055759288
SCAMP1	0.959165056	0.340127904	-0.553075443	0.582352196	-1.870361377	0.066641227
SCAMP2	-0.77716934	0.439162408	-0.686490752	0.495158226	-0.329032666	0.743353243
SCAMP3	-0.402412433	0.688365412	0.129551067	0.897372987	0.696180712	0.489183081
SCAMP4	0.43433251	0.66511998	-0.681544758	0.498258236	0.320154529	0.7500377
SCAMP5	3.189783222	0.001978901	1.284795681	0.204006788	0.836693656	0.406306177
SCAND1	-0.8473317	0.399130212	-0.40821458	0.684629774	-0.320049396	0.750116973
SCAND2	1.496409066	0.138158319	2.147237942	0.035995975	3.007278425	0.003936518
SCAP	-0.2469815	0.805503161	-0.466828691	0.642383553	0.356871424	0.722523694
SCAPER	1.34069506	0.183502197	1.017350434	0.313241285	0.992011916	0.325440968
SCARA5	0.42093427	0.674838872	-0.369048391	0.713444883	-0.572700442	0.569130287
SCARB1	0.552404742	0.582084452	-0.352069757	0.726070882	0.035733013	0.971621784
SCARB2	-0.978453801	0.330557064	0.251605627	0.802241486	-1.497318485	0.139902443
SCARF1	-1.736966724	0.085924605	0.205940083	0.837563917	-0.647391726	0.520012546
SCARF2	0.367479085	0.714152194	-1.698640188	0.09478335	-0.681462361	0.498375791
SCARNA10	0.035570367	0.971706248	0.68211592	0.497899708	0.336464814	0.737772698
SCARNA12	1.121949293	0.264964335	0.134566318	0.893423914	0.873148665	0.386295183
SCARNA16	-1.504825835	0.13598297	0.884309244	0.380205938	1.204336231	0.233503986
SCARNA17	-0.431446415	0.667208751	-3.198672111	0.002245094	-3.861792889	0.000293194
SCARNA2	0.711733368	0.478529935	-2.69160346	0.009292995	-3.342387543	0.001481766
SCARNA7	-2.137168739	0.035384444	-0.621198151	0.536920082	-1.767839975	0.082512289
SCARNA9	-0.015774942	0.987449981	-3.264690466	0.001847013	-2.93177669	0.004865959
SCCPDH	-1.646398035	0.103281906	1.686466679	0.097115955	1.251343322	0.215989718
SCD	2.402829162	0.018389357	0.527628377	0.599784068	0.414690301	0.679946325
SCD5	-0.861263182	0.39145551	-1.269755187	0.209278092	-0.878476113	0.383423409
SCFD1	2.062794712	0.042107263	0.146289852	0.884203394	0.390776533	0.697438915
SCFD2	-1.659312047	0.100645969	1.256309474	0.214075588	1.362667977	0.178419324
SCGB3A1	0.651800137	0.516244631	-0.319237521	0.750701376	-1.728493535	0.089387127
SCLT1	-1.079305401	0.283428337	0.305160486	0.761344002	-1.63345895	0.107959382
SCLY	-0.802018609	0.42472276	-2.885550917	0.005490158	-2.421356393	0.018713401

SCMH1	1.597590832	0.113752157	-1.013254658	0.315176258	-0.658244459	0.513066862
SCML1	1.990088981	0.049714138	-0.796429968	0.429052776	-1.098459837	0.276684453
SCML2	0.4859252	0.628239209	0.39230647	0.696279779	0.155416482	0.877049499
SCML4	2.929648374	0.004330459	-0.184021207	0.854643173	0.50642882	0.614535421
SCN11A	2.472801006	0.015346481	0.152154475	0.879596838	1.607525129	0.113539853
SCN1B	-2.064628449	0.041929131	-1.084397569	0.28270691	-1.937450215	0.057718506
SCN3A	0.593736714	0.554225512	0.204069914	0.839018219	-0.552536712	0.582770263
SCN3B	-1.405149708	0.16352983	0.563390956	0.575355597	-0.918059775	0.362508327
SCN4A	-0.189096117	0.850456972	-0.019482963	0.984523165	0.82279452	0.414100193
SCN5A	-0.283713366	0.777302672	-1.638138098	0.106845072	-0.328020771	0.74411412
SCN8A	1.087138388	0.27997193	-0.787394425	0.434279323	-1.112337855	0.270725389
SCN9A	-1.277811909	0.204707463	-2.038726428	0.046076481	-1.857693358	0.068450891
SCNM1	-1.885460939	0.062697808	-2.358176935	0.02178181	-1.653586002	0.103783819
SCNN1A	0.601866224	0.548825091	1.252261888	0.215535608	2.638055424	0.010764755
SCNN1D	1.080511228	0.282894344	-0.638131581	0.525917155	0.698406562	0.487801079
SCO1	0.432474834	0.666464145	0.403458474	0.688104915	0.222519627	0.82471559
SCO2	-0.08204598	0.934798208	-0.701190721	0.486007435	-2.399725982	0.019743779
SCOC	0.623786761	0.534396251	-0.307511318	0.759563419	-0.47097389	0.639481218
SCP2	0.238290288	0.812215095	0.409306001	0.683833269	0.777695207	0.440008249
SCPEP1	-2.106943778	0.037995125	3.218046391	0.002120604	2.61096945	0.011553165
SCRIB	0.753134449	0.453397951	-0.640608676	0.524317571	-0.187859623	0.8516629
SCRN1	1.783893586	0.07792012	0.559018259	0.578316442	0.856272599	0.395480942
SCRN2	1.184976619	0.23924656	0.786375694	0.434870959	1.109711572	0.271846102
SCRN3	1.012300657	0.314195351	-2.27962141	0.026352215	-2.835888264	0.006340219
SCRT2	-0.936223529	0.351744085	-2.181393838	0.033250066	-1.127867884	0.264164739
SCT	0.711895747	0.478429875	-0.443448599	0.659102629	-0.496703877	0.621334036
SCUBE3	2.96895044	0.003858172	-0.088015149	0.930169943	0.709196777	0.481132331
SCYL1	-0.664093705	0.508382748	1.987538778	0.051624489	2.162476394	0.034849516
SCYL2	-1.187516954	0.238248898	-1.256337571	0.214065478	-2.712521181	0.008843691
SCYL3	0.395473791	0.693459235	1.011443192	0.316034617	0.561888678	0.576424555
SDAD1	1.118481399	0.266433619	2.696928804	0.009162364	3.450142158	0.001069458
SDC1	-0.526723923	0.599722834	3.446848956	0.001065789	1.531033452	0.131367457
SDC2	-1.452913299	0.149837474	-1.223253308	0.226215287	-0.889943762	0.377287441
SDC3	-2.764866146	0.006948864	1.369001417	0.176314173	1.016268968	0.313853736
SDC4	-2.010482274	0.047470414	2.191467401	0.032476144	2.959614832	0.00450178
SDCBP	-2.895293879	0.004786379	0.576407891	0.566585334	-0.459308195	0.647783422
SDCCAG3	1.249823981	0.214708284	1.306184244	0.196682299	2.391433419	0.020152101
SDCCAG8	-0.365541158	0.715592819	0.657590129	0.51342108	2.175493341	0.033811983
SDF2	-1.3587917	0.177717466	1.444158749	0.154112299	0.685286695	0.495978171
SDF2L1	0.412703996	0.680836598	-2.818206101	0.006607333	-2.314230536	0.024331812
SDF4	-0.91963527	0.36030072	-0.045883807	0.963561581	-0.051364834	0.95921682
SDHA	-0.525453361	0.600601852	2.607238992	0.011602973	2.442545288	0.017751148
SDHAF1	1.200762666	0.233095327	-0.623675366	0.53530306	-0.663566874	0.50967877
SDHAF2	-1.500491516	0.137099809	1.652955668	0.103781209	1.437872645	0.156011802
SDHAP1	3.963067261	0.000151002	-2.369185166	0.021201351	-1.561387885	0.124042627
SDHAP2	2.250723567	0.026918455	-0.094509628	0.925032361	-0.966204764	0.338078567
SDHAP3	1.385689067	0.169376205	-1.278653654	0.20614731	-0.786909856	0.434638403
SDHB	-2.79830284	0.006322434	0.61650812	0.539988425	0.798953367	0.42767894
SDHC	-2.06738188	0.041662876	-0.272391518	0.78629515	0.250767412	0.802907896
SDHD	-1.124200644	0.264013524	-2.133598993	0.037146712	-1.58582045	0.118387626
SDK1	-1.744468194	0.084601414	-0.353841438	0.724749742	-0.125355547	0.900689135
SDK2	0.970242747	0.334609411	0.609884967	0.544336751	2.025633601	0.047557362
SDPR	-5.442724231	4.76E-07	1.634856712	0.107533425	-0.710684866	0.480216653
SDR39U1	2.42786014	0.017244037	-0.020499673	0.983715625	-0.069177767	0.945093516
SDR42E1	-0.301208564	0.763972632	1.023372682	0.310410789	0.524843421	0.601755036
SDSL	-2.350502914	0.021004673	1.815991378	0.074577531	0.885978977	0.379401805
SEC1	0.276242794	0.783015334	0.616928545	0.539713004	-0.289861661	0.772988154
SEC11A	-1.215386519	0.227499521	-1.550047293	0.126607951	-1.200377038	0.235025061
SEC11C	1.118382352	0.266475667	-1.154832501	0.252927821	-0.216345415	0.829501376
SEC13	-1.158027516	0.250015117	1.26910478	0.209508306	1.816227983	0.074666832
SEC14L1	-3.151509229	0.002226679	0.097662218	0.922539587	-0.461306988	0.646357692
SEC14L2	2.379252891	0.019529555	-0.652778657	0.516496129	0.755985172	0.452813225
SEC14L4	-0.782027802	0.436316857	2.366858015	0.021322895	0.55507555	0.581044246
SEC14L5	-3.359473547	0.00115998	1.608959294	0.113093355	0.56792804	0.572344454
SEC16A	0.965196482	0.337115953	0.535712841	0.594219766	1.075758739	0.286628786
SEC16B	0.616530035	0.539151232	0.249818092	0.803616892	-0.979620671	0.331469001
SEC22A	1.814119412	0.073099051	-0.924279442	0.35919707	-0.783512058	0.436613936
SEC22B	0.148481595	0.882305583	-0.211109575	0.833546934	-1.435569322	0.156663939
SEC22C	2.748896477	0.00726763	0.222728302	0.824534947	1.943595735	0.056954649
SEC23A	-0.18306658	0.855170807	0.084686727	0.932804127	-1.296325172	0.200158043
SEC23B	-0.990034623	0.324896804	1.727160832	0.08949849	1.463152814	0.148992552
SEC23IP	0.498341318	0.61949829	1.678451062	0.098677417	0.52100979	0.604405555

SEC24A	0.238328467	0.812185579	-0.382270058	0.703667989	-2.027551681	0.04735469
SEC24B	-0.45792355	0.64814657	-1.26288775	0.21171835	-1.661701114	0.102137959
SEC24C	-1.485573309	0.140998924	0.592849548	0.555602532	0.556390778	0.580151062
SEC24D	-2.283662859	0.024821756	0.312654924	0.755672085	-0.45053732	0.654055285
SEC31A	-0.191940145	0.848235412	0.583289526	0.561975511	0.539278487	0.591823475
SEC31B	2.299123226	0.023888251	0.683001402	0.497344153	2.110061345	0.039314904
SEC61A1	-0.251803363	0.801785639	1.338191184	0.186093291	2.095473627	0.040643233
SEC61A2	-0.321679694	0.748465293	-2.991233657	0.004084196	-1.968152334	0.053988004
SEC61B	-1.723574656	0.088329116	-3.693795713	0.000493211	-2.848687019	0.006121995
SEC61G	-0.82088309	0.413951664	-1.498487514	0.139467758	-0.203636345	0.839372774
SEC62	0.25887784	0.796339691	-0.09727426	0.922846306	-0.126492286	0.899793427
SEC63	2.124865612	0.036427722	0.41911143	0.676693666	0.633253389	0.529135136
SECISBP2	2.034131779	0.044977433	0.826276592	0.412056984	1.659085577	0.102666082
SECISBP2L	0.97941524	0.330084693	-0.660232666	0.511736384	-2.026900041	0.047423461
SECTM1	-1.567588796	0.120599469	-0.961869085	0.340136259	-2.279463246	0.026454189
SEH1L	3.583280688	0.000558213	1.523226229	0.133174183	3.126969077	0.002795413
SEL1L	-0.236550469	0.813560404	-0.484004619	0.630217858	-1.430508923	0.158104136
SEL1L3	1.424587952	0.157845914	0.650052634	0.518242689	0.690952807	0.492437521
SELENBP1	-2.728061447	0.007703574	1.192642524	0.237899633	0.326625342	0.745163809
SELK	-2.882507497	0.004967065	-0.947214364	0.347486798	-1.187585652	0.239988558
SELL	-2.277125472	0.025226058	-0.039968211	0.968256692	-0.58174186	0.563065356
SELM	2.857243656	0.005342646	0.676333646	0.501535843	1.486224855	0.142804723
SELO	0.495421142	0.621549234	-0.131362167	0.895946597	0.658902643	0.51264723
SELP	-2.453582281	0.016133819	2.325211265	0.02360586	1.422189875	0.160494097
SELPLG	-2.154012248	0.033998266	0.261186347	0.794880481	-0.188092363	0.851481318
SELRC1	1.05151481	0.295927853	-2.507140688	0.015014875	-0.129821864	0.897170604
SELS	1.404737835	0.163651942	-0.117768022	0.906661204	-0.539866974	0.591420231
SELT	-0.614317496	0.54060528	-0.598220682	0.552037938	-1.073329191	0.287707567
SEMA3B	0.581936595	0.562111086	-1.18229093	0.241948232	-1.047202597	0.299486207
SEMA3C	-0.568454362	0.571187722	-0.139777741	0.889323244	-1.873575666	0.066188544
SEMA3G	1.136674815	0.258788701	0.483674761	0.630450543	0.171328612	0.864580377
SEMA4A	-1.99634966	0.049015888	0.556117407	0.580284719	0.163861525	0.870427653
SEMA4B	-1.807959689	0.074060873	0.003953534	0.996859206	-0.448902041	0.655227435
SEMA4C	2.392114707	0.018899973	-0.370793101	0.712151928	0.218699826	0.827675636
SEMA4D	-2.061688047	0.042215082	0.23977277	0.81135769	0.698581194	0.487692743
SEMA4F	3.877612497	0.00020407	-0.830257908	0.40982124	0.899290318	0.372332645
SEMA4G	2.560779046	0.012164326	-2.104805643	0.039682025	-0.145501092	0.884835516
SEMA5A	1.499257088	0.137419202	2.475609709	0.016264047	0.56420205	0.574860012
SEMA6A	0.695991133	0.488285526	-0.641748738	0.523582237	0.338459824	0.736277112
SEMA6B	-0.625917085	0.533004451	-0.087327499	0.930714101	-0.849394244	0.399263448
SEMA6C	0.334814121	0.738569453	-0.130595179	0.896550621	-0.578395415	0.565306397
SEMA7A	-0.680694182	0.497868746	0.110836465	0.912131352	-1.96292849	0.054607757
SEMG1	-0.714395184	0.476891157	-1.6528009	0.103812836	-0.766901439	0.446347772
SENP1	-0.991599691	0.32413681	-2.452344428	0.017245026	-1.96153671	0.054773901
SENP2	-1.477862724	0.143047926	0.168669975	0.866647173	-0.55737225	0.579484965
SENP3	-2.75303732	0.007183719	-2.594295533	0.012000405	-1.684481234	0.097631538
SENP5	2.574448504	0.011727205	2.036136664	0.046344275	3.32734071	0.001550106
SENP6	1.089818703	0.278795925	-0.751132872	0.455630795	-1.90366415	0.062075835
SENP7	0.954744571	0.342346497	-1.410110366	0.163884694	-2.201727398	0.03180307
SENP8	0.521809875	0.60312582	-1.127193353	0.264334841	-2.099644165	0.040259541
Sep-15	0.276103758	0.783121767	-0.976480571	0.332909966	-1.006923013	0.318284744
SEPHS1	0.821731962	0.413470879	-0.493271231	0.623696528	-0.041493366	0.967049549
SEPHS1P	1.023608199	0.308852052	-0.492782337	0.624039835	0.452839245	0.652406775
SEPHS2	-2.331739652	0.022019869	-0.377750737	0.707004294	-0.823211822	0.413864874
SEPN1	0.358602672	0.720759204	0.412687857	0.681367527	0.728004074	0.469631536
SEPSECS	0.611022531	0.542774374	-1.510769267	0.136314485	-1.748957996	0.085754515
Sep-01	0.081548849	0.935192389	0.271734937	0.786797494	2.003170052	0.049987217
Sep-10	0.097038418	0.922918601	1.245420035	0.218020274	0.702153664	0.485479444
Sep-11	-0.238896167	0.811746733	0.050704177	0.959736683	0.110008075	0.912794607
Sep-14	-0.60431069	0.547206418	0.699677912	0.486944812	-1.003370805	0.319979865
Sep-02	1.561919321	0.121929549	2.480150889	0.016078542	1.79225886	0.078471255
Sep-04	-1.907616227	0.059732643	0.885655218	0.379486097	0.648512778	0.51929278
SEPT5-GP1BB	-0.742702948	0.459657914	-0.387769842	0.699615766	-0.795150423	0.42986928
Sep-06	1.858050126	0.066536768	-0.275142155	0.784191654	0.752335137	0.454987115
Sep-07	0.5966649	0.552277277	0.050266314	0.960084083	-0.500964893	0.618351065
SEPT7P2	2.73987324	0.007453588	-0.714629698	0.477724414	-0.342444404	0.733293093
Sep-08	0.572137261	0.568701253	-2.556378755	0.013236933	-0.270369965	0.78786543
Sep-09	-0.709549109	0.479877034	1.608852301	0.113116799	2.004860143	0.049800753
SEPW1	0.640123014	0.523771259	1.317606425	0.192852625	1.368231001	0.176681746
SEPX1	-2.691512897	0.008526268	-0.585097114	0.560767747	-0.971833451	0.335294988
SERAC1	-1.026227986	0.307622861	0.097847569	0.922393052	-0.611850271	0.543102645
SERBP1	1.276913885	0.205022907	1.708322382	0.092961176	1.414520521	0.162722159

SERF2	-2.106279692	0.038054308	-1.887336338	0.0641575	-1.54498477	0.12795917
SERGEF	0.666608537	0.506782388	1.987765491	0.051598704	2.23473436	0.029425288
SERINC1	-0.201414097	0.840843951	0.17868639	0.858811007	-0.588519478	0.55854006
SERINC2	-2.770393256	0.006841549	1.070853303	0.288702295	0.314795517	0.754081953
SERINC3	-0.949749906	0.344864566	0.446258728	0.657083622	-0.431359858	0.667855869
SERINC4	2.503794595	0.014149054	0.877070504	0.384092044	1.896200742	0.063075199
SERINC5	2.543279029	0.012745356	1.552672302	0.125979446	1.665174071	0.101440142
SERP1	-0.333064125	0.739885449	-1.592702326	0.116700795	-1.610840804	0.112813627
SERPINA1	-2.530095957	0.013199431	-0.301106677	0.764417509	0.049545399	0.960660209
SERPINB1	-2.222999412	0.028802162	0.498599195	0.619960616	0.114943227	0.908899548
SERPINB10	-1.401421607	0.164637682	0.707948976	0.481832097	0.046304221	0.963231816
SERPINB2	-1.282889294	0.202930713	1.459094763	0.149971479	0.434946915	0.665265565
SERPINB6	-0.435079909	0.66457949	0.982116471	0.330150039	2.942777247	0.004718896
SERPINB8	-0.232422089	0.816754894	0.973863887	0.33419655	0.339404501	0.73556928
SERPINB9	0.957357627	0.341033897	-0.590480558	0.557178387	-1.848376968	0.069808084
SERPIND1	0.255006046	0.799318975	1.725185418	0.089856515	1.113119433	0.270392495
SERPINE1	-1.953776175	0.053933552	0.688924845	0.493636495	0.526645416	0.600511025
SERPINE2	0.395969004	0.693095217	0.421172495	0.675196706	2.556252909	0.013308393
SERPINF1	3.752992766	0.000314345	0.963242269	0.339452773	1.663878034	0.101700096
SERPINF2	0.872995917	0.385063273	-0.549172963	0.585009681	-0.022069922	0.982470302
SERPING1	-2.207310369	0.02991851	0.688460378	0.49392667	-1.066922446	0.290565798
SERPINH1	1.543841137	0.126248789	0.417171749	0.678103663	0.774473156	0.441895087
SERPINI1	1.209228341	0.229843958	-0.856497917	0.395271921	-0.851058168	0.398346388
SERTAD1	0.005770912	0.995408687	0.954355988	0.343891827	1.086207399	0.28202131
SERTAD2	-0.482749672	0.630483384	0.881688791	0.381609852	1.079879424	0.284805518
SERTAD3	-2.764540626	0.006955232	0.067731811	0.946233709	-1.167334119	0.248001237
SESN1	3.588476733	0.00054862	-0.371690022	0.711487575	0.275488655	0.783950574
SESN2	-1.910962411	0.059295255	0.750956166	0.455736307	0.789843604	0.432936947
SESN3	-4.788206642	6.83E-06	0.244380455	0.807804666	-0.588347042	0.558654967
SESTD1	-1.138825236	0.257895411	-0.088701927	0.929626509	-1.385809148	0.171276381
SET	-0.030858743	0.975452722	2.141586215	0.03646898	2.456984219	0.017121179
SETBP1	1.333624655	0.185800465	-1.233718191	0.222318736	-1.71025211	0.092731528
SETD1A	1.339792607	0.183794345	-0.18205211	0.856181055	0.091958152	0.927058084
SETD1B	-0.50695867	0.613463602	-1.148215707	0.255626095	-1.153133406	0.25373338
SETD2	-1.678926333	0.096746994	0.388609219	0.698998083	0.177690841	0.859604273
SETD3	-0.18961634	0.850050518	1.631804482	0.108176939	1.650680886	0.104378252
SETD4	0.983863617	0.327904922	1.058690738	0.294160517	2.157680161	0.035238786
SETD5	1.578417477	0.118091145	-0.402670435	0.688681367	0.099542836	0.921061279
SETD6	1.770748108	0.080097546	-1.288034537	0.202884738	0.372847559	0.710663527
SETD7	0.564527865	0.573844436	-1.163454573	0.24944245	-1.060812379	0.293309892
SETD8	-0.814246124	0.417722308	0.858937873	0.393935505	1.385235229	0.171450833
SETDB1	-0.220128794	0.826285449	0.935166256	0.353607005	1.534582225	0.130493716
SETDB2	1.421093769	0.158856267	-0.408106809	0.684708444	-0.096684499	0.923320691
SETMAR	1.620897522	0.108650801	-1.039140413	0.303082139	0.954025338	0.344153726
SETX	-1.391246059	0.167690733	0.883461664	0.380659674	-0.213950019	0.831359869
SEZ6	0.468383674	0.640679105	0.394282872	0.694828322	-0.564415008	0.574716092
SEZ6L	-1.460559342	0.147730665	0.56147772	0.576650185	0.904680897	0.369493906
SF1	-0.259881069	0.795568212	-1.60899941	0.113084567	-0.877427956	0.383987357
SF3A1	-1.878857643	0.063605162	0.642594829	0.523036864	0.410121198	0.683275282
SF3A2	-0.464861513	0.64318949	-0.738205393	0.463386974	-0.609296587	0.544781667
SF3A3	0.735566444	0.463968753	2.323070921	0.023728862	3.22830995	0.002079835
SF3B1	1.414224995	0.160856897	-0.56311139	0.575544677	-1.361515391	0.178780955
SF3B14	-0.080456212	0.936058812	-0.765746243	0.446954068	-0.617815511	0.539190895
SF3B2	-0.999388193	0.320372255	2.382251078	0.020530364	2.587871736	0.012266663
SF3B3	0.014146367	0.988745529	2.651305051	0.010338126	3.075191697	0.003244617
SF3B4	-1.80740506	0.074147991	-1.307560913	0.196217717	-1.061281832	0.293098424
SF3B5	-1.049598985	0.296803168	-1.76947556	0.082108237	-1.124569981	0.265548438
SFI1	2.225830445	0.028604653	0.827807131	0.411196619	2.181061983	0.033376474
SFMBT1	1.655073854	0.101504967	2.120386118	0.038292018	2.411674139	0.019168499
SFMBT2	1.74184381	0.085062411	0.1869468	0.852359307	-0.017361986	0.986209287
SFN	-1.919805818	0.05815231	0.947962136	0.347109236	0.87321434	0.386259699
SFPQ	1.541103785	0.126913237	2.473505743	0.016350645	1.701426808	0.094386221
SFR1	1.447576291	0.151321811	0.423458707	0.673537762	0.794861225	0.43003612
SFRP1	-1.294382074	0.198951247	1.897685396	0.062753555	0.024181663	0.980793306
SFRP2	-1.684593008	0.095643724	2.033535872	0.046614561	0.672994486	0.50370707
SFRP5	1.765888757	0.080915082	0.682802865	0.497468687	0.940301429	0.351084491
SFSWAP	1.304722954	0.195420488	0.604783667	0.547698065	1.492969626	0.141034576
SFT2D1	-0.894419546	0.373559878	-0.6339235	0.528640379	-0.757930847	0.451656885
SFT2D2	-0.420337253	0.675273241	-2.352979633	0.022060761	-2.819895038	0.006623009
SFT2D3	0.985541507	0.327085204	-1.358092284	0.179730777	-1.52961533	0.131717911
SFTPB	-0.013864612	0.988969671	1.475546047	0.145511904	1.376415479	0.174148951
SFTPD	0.378659979	0.70586086	-0.350450231	0.727279293	0.609433595	0.544691518

SFXN1	2.331795251	0.022016798	0.421184895	0.675187704	1.558393449	0.124750349
SFXN2	1.220188752	0.225683366	0.065888237	0.947694977	0.728535065	0.469309111
SFXN3	1.263809057	0.209667131	1.334340384	0.187343912	1.402090278	0.166384111
SFXN4	-0.002869229	0.997717244	0.653837842	0.515818356	1.200870099	0.234835241
SFXN5	-1.616347105	0.109632064	1.875911566	0.065738189	1.513878982	0.135656887
SGCA	0.760157002	0.449211413	1.265302477	0.210857912	0.831646828	0.40912576
SGCB	1.371745846	0.173662145	-1.398629452	0.167285818	-1.283798681	0.20447684
SGCD	-1.058306733	0.292838885	0.46020508	0.647101727	0.379868385	0.705473955
SGCE	0.300945175	0.764172799	-1.017285807	0.313271755	-2.391331086	0.020157187
SGIP1	-3.166571988	0.002125901	2.354403404	0.021984029	1.497539165	0.139845186
SGK1	-3.130229023	0.002376663	-0.721320811	0.473630117	-0.922570652	0.360172308
SGK196	0.040954708	0.967425641	-0.369060443	0.713435949	-0.593500946	0.555225632
SGK2	1.126915791	0.262870026	-0.496915392	0.621140199	-0.470792005	0.63961031
SGK223	1.104940554	0.272225194	-0.49596757	0.621804633	-0.376204528	0.708180426
SGK494	1.593395327	0.114690532	-0.659446176	0.512237486	0.764640677	0.447682322
SGMS1	-0.488679708	0.6262954	-1.422114763	0.160385936	-2.141958857	0.036541587
SGMS2	-0.705819667	0.482181948	0.548213482	0.585663947	-0.762660699	0.448853032
SGOL1	-0.829232499	0.409237346	-0.318540392	0.7512273	-0.332693777	0.740602488
SGOL2	1.369000136	0.174515768	1.501393572	0.138716487	1.010917111	0.316385979
SGPL1	0.57165232	0.569028355	1.557942618	0.124725108	1.484751482	0.143193726
SGPP1	0.750137491	0.455191404	-1.581160929	0.119317706	-2.917507978	0.005063056
SGPP2	0.437425733	0.662884236	0.861314892	0.392636263	0.652566116	0.516694768
SGSH	2.980965707	0.00372358	0.612259623	0.542775656	0.193584586	0.847198685
SGSM1	2.697998371	0.008374688	-0.130489629	0.89663375	-0.440298951	0.661408351
SGSM2	2.001223008	0.048478178	0.552002148	0.583082504	1.78784786	0.079188738
SGSM3	0.097493058	0.922558616	1.022120239	0.310998015	1.857303295	0.068507265
SGTA	-0.828658545	0.409560374	1.763615456	0.083100386	1.945723634	0.056692182
SGTB	1.079986801	0.283126498	-0.452778812	0.652409009	-0.662536806	0.510333541
SH2B1	2.141410002	0.035030855	-2.084168936	0.041590631	-1.019204128	0.312470787
SH2B2	-1.875513176	0.064068917	-1.023122839	0.310527872	-2.049871432	0.045050609
SH2B3	-2.057698806	0.042605714	-1.618949475	0.110921614	-1.950836597	0.056065743
SH2D1A	1.594523444	0.114437608	-1.24168925	0.219384004	-1.050026153	0.298197565
SH2D1B	0.445359313	0.657163927	-1.695411313	0.095397511	-1.61754741	0.111356185
SH2D2A	-2.530334287	0.013191094	-0.736404568	0.464473388	-0.377918864	0.706913581
SH2D3A	1.927410433	0.057184414	-1.34959515	0.182426779	-0.934698227	0.353940123
SH2D3C	-1.054521448	0.294557706	-1.991123926	0.051218025	-1.621136218	0.110582575
SH3BGR	-0.794196464	0.429237419	0.313614135	0.754947105	0.65501837	0.515126345
SH3BGRL	0.135042701	0.892889383	-1.246198956	0.217736342	-1.688120116	0.096927026
SH3BGRL2	-5.74024367	1.35E-07	1.077259545	0.285855688	-0.271042022	0.787351114
SH3BGRL3	-3.052386008	0.003009161	-2.233858869	0.029390239	-2.106442593	0.039640841
SH3BP1	-0.568438797	0.571198242	-0.250853583	0.802820065	0.125482272	0.900589274
SH3BP2	-0.671885878	0.503432829	-1.33783625	0.186208297	-1.290253425	0.202242803
SH3BP4	0.991622246	0.324125866	2.194447399	0.032250255	0.829683411	0.410225913
SH3BP5	0.219870673	0.826485846	1.135950759	0.260681799	0.79847219	0.427955709
SH3BP5L	-2.109062912	0.037806798	0.853151482	0.397109403	0.378605134	0.706406682
SH3D21	-2.104217999	0.038238556	-0.22043318	0.826313304	0.262909225	0.793581367
SH3GL1	-1.468269988	0.145629449	-1.441051829	0.154984754	-1.76758005	0.082556218
SH3GL1P1	1.056150828	0.293816996	-1.6766023	0.099040464	-1.707293497	0.093283567
SH3GLB1	-0.268156199	0.789212495	-0.867543936	0.38924423	-1.090152321	0.280295228
SH3GLB2	-0.607419022	0.545151623	1.033713119	0.305591245	0.81579105	0.418061613
SH3KBP1	-1.671397411	0.098228829	0.522923478	0.603033417	0.648682471	0.519183876
SH3PXD2A	3.997476486	0.000133609	0.700595929	0.486375864	0.904840105	0.369410276
SH3PXD2B	-3.052078841	0.003011941	2.958936124	0.004473606	0.985375366	0.328660323
SH3RF1	5.558469654	2.92E-07	-0.303972318	0.762244447	-0.334561221	0.739200703
SH3RF2	0.446856015	0.656087042	-0.684818078	0.496205427	-1.475658819	0.145612894
SH3RF3	-0.563690068	0.574412069	0.373267083	0.710319985	-0.01132538	0.99100393
SH3TC1	0.657778086	0.512413668	1.195339778	0.236852821	1.557068458	0.125064535
SH3TC2	-4.268793521	4.98E-05	2.373481018	0.020978609	1.142934356	0.257908276
SH3YL1	1.656571749	0.101200698	0.087504311	0.930574181	-0.707441951	0.482213396
SHANK1	1.606280909	0.111828082	-2.5655171	0.012928776	-1.331160029	0.188507844
SHARPIN	-2.092279899	0.039320669	-0.652481586	0.51668631	-1.205678177	0.232990056
SHB	-1.772619952	0.079784455	0.539811985	0.591407728	-0.236431561	0.813956861
SHC1	-0.077616416	0.938311027	-0.084626078	0.932852133	-0.050096344	0.960223121
SHC2	0.837219616	0.404758136	1.428973333	0.158413127	-0.882084452	0.38148597
SHCBP1	1.88087354	0.063326996	0.545838593	0.587284871	0.304637118	0.761767079
SHD	2.365825063	0.02020666	-1.447255702	0.153246459	-0.337071239	0.737317976
SHE	-0.828636488	0.40957279	1.677298296	0.098903661	1.646593712	0.105219252
SHF	-1.825252541	0.071387026	-0.16742248	0.867624088	0.63227081	0.529772227
SHFM1	0.847316608	0.399138575	-1.768162565	0.082329674	-0.791405039	0.432032994
SHISA4	-3.360224288	0.001157196	-0.940019519	0.351133277	-2.144289813	0.036345802
SHISA5	-2.694267388	0.008461587	0.398855607	0.691474537	-1.147618069	0.255985001
SHISA7	-1.825385152	0.071366836	-0.363241117	0.71775456	-0.306713093	0.760194552

SHISA8	0.248452927	0.804368253	-2.337642667	0.02290261	-2.61018539	0.011576751
SHISA9	-0.138288539	0.890331271	1.076976853	0.28598089	0.315606269	0.753469659
SHKBP1	-1.847053919	0.068131231	0.403412564	0.688138493	-0.24860427	0.804572549
SHMT1	0.347033458	0.729402421	2.038107767	0.046140332	2.912300194	0.00513682
SHMT2	-0.581432934	0.562448888	2.31194928	0.024377232	2.237411011	0.029239529
SHOC2	-1.201378056	0.232857865	-0.064399874	0.948874825	-1.278517632	0.206318321
SHPK	0.697914741	0.487087628	1.915706673	0.060371011	1.72819373	0.089441277
SHPRH	1.080076365	0.28308684	-0.429951027	0.668835641	-1.383092498	0.17210336
SHQ1	2.942496076	0.004170501	2.597231617	0.011909182	3.327761868	0.001548153
SHROOM1	-1.693101729	0.094006388	-0.825199765	0.412662958	-0.744020724	0.459961488
SHROOM4	-3.350669647	0.0011931	1.888740259	0.063965498	-0.060007016	0.952362787
SIAE	0.958017592	0.340702901	-0.932815244	0.35480938	0.523332951	0.602798711
SIAH1	-0.447920565	0.655321535	-1.170877053	0.246469703	-1.090961442	0.279942114
SIAH2	-3.087971341	0.002702774	0.081588041	0.935257173	-0.586088046	0.560161395
SIDT1	1.189747103	0.237375522	-1.611388015	0.112562243	-0.006914806	0.994507298
SIDT2	-0.555611457	0.579899404	1.242344757	0.21914394	0.436909024	0.663850409
SIGIRR	-0.368895062	0.713100233	-0.51796482	0.60646679	0.273622604	0.785377116
SIGLEC1	-1.112816865	0.268845828	1.80981479	0.075542902	-0.732303569	0.467024442
SIGLEC10	-1.949012354	0.054509036	-0.919232251	0.36180789	-0.611821189	0.54312175
SIGLEC11	-0.628268094	0.531470641	0.189461913	0.850396897	-0.181239163	0.856831489
SIGLEC12	-4.912685335	4.17E-06	1.083492789	0.283104693	0.283844331	0.77757211
SIGLEC14	-2.655770277	0.009407326	0.582008527	0.562832207	0.092870563	0.926336457
SIGLEC15	0.075500537	0.939989437	-0.747414425	0.457854062	-1.348348518	0.182951957
SIGLEC16	-2.18871272	0.031290672	1.985105106	0.051901981	2.394655974	0.019992533
SIGLEC5	-3.175933137	0.00206543	1.156003564	0.252452399	0.956943463	0.342691671
SIGLEC6	0.928519774	0.355701467	-0.975132542	0.333572361	-0.269034727	0.788887551
SIGLEC7	-2.156947849	0.033761568	0.442499163	0.659785352	1.603601514	0.114404114
SIGLEC8	2.620401044	0.010359715	0.289236975	0.773438606	0.264307903	0.792508912
SIGLEC9	-0.989926638	0.324949285	0.436941833	0.663787353	0.336883712	0.737458581
SIGLECP3	0.142602166	0.886933413	-3.929616913	0.000230487	-2.508479408	0.015035164
SIGMAR1	-0.170308604	0.86516202	-2.142551406	0.036387819	-1.540839825	0.128964322
SIK1	0.560012921	0.576906643	-1.173095029	0.245586354	-0.513601923	0.609542432
SIK2	0.696405461	0.488027373	0.129227789	0.897627631	0.451805179	0.6531471
SIK3	-2.527913691	0.013275984	0.085518733	0.932145589	-0.052783433	0.958091516
SIKE1	0.508855801	0.612138602	-1.424295365	0.159756648	-1.787523764	0.079241671
SIL1	-0.50660339	0.613711881	4.78412149	1.23E-05	4.135534049	0.000119334
SIM2	1.493406285	0.13894097	0.051412939	0.959174368	1.369525446	0.176279297
SIN3A	-0.235912103	0.814054159	0.683056142	0.497309821	0.707920633	0.481918369
SIN3B	0.540092918	0.590509842	-0.786204756	0.43497028	-0.336012965	0.738111575
SIPA1	-1.9296615	0.056900531	-0.038032758	0.969793082	0.040097568	0.968157356
SIPA1L1	-2.636754399	0.009909013	-0.081771931	0.935111581	-0.300672888	0.764772742
SIPA1L2	-2.309548899	0.023276362	0.25279115	0.80132964	-0.865183559	0.390613868
SIPA1L3	0.486980157	0.627494433	-1.366367572	0.177134476	-0.488121136	0.627361877
SIRPA	-1.140445589	0.257223751	-0.21679709	0.829132567	-1.10226413	0.275041871
SIRPB1	-2.885880168	0.004918806	-0.063464233	0.949616582	-1.115219369	0.269499507
SIRPB2	-1.806057677	0.074359984	-0.126622368	0.899680296	-1.490780027	0.141607322
SIRPD	-2.409252347	0.018089172	-1.142148691	0.258118159	-2.45395261	0.017251753
SIRPG	0.225266583	0.822299049	0.707089231	0.482362144	1.75004719	0.085564657
SIRT1	0.394746741	0.693993802	-0.53102027	0.597446579	-0.823150014	0.413899722
SIRT2	-2.018074458	0.046657513	-0.956147975	0.342993609	-0.393799427	0.695218328
SIRT3	0.805543433	0.422697619	1.029066453	0.307750661	2.782897659	0.007322375
SIRT4	2.036242064	0.044760539	1.885717248	0.064379534	2.063719547	0.043670382
SIRT5	1.385758433	0.169355086	0.065613495	0.94791276	1.11832492	0.268182698
SIRT6	-1.111207962	0.269533743	0.303364907	0.762704896	-0.142845859	0.8869225
SIRT7	-1.042492837	0.300065247	-0.21944725	0.827077524	-0.380728004	0.704839512
SIT1	1.238499945	0.218854904	0.215281778	0.830308142	1.336570292	0.186745451
SIVA1	-0.039169428	0.968844858	-0.253206568	0.801010187	0.05634295	0.955268311
SIX4	-1.359181082	0.177594532	2.780868008	0.007313694	1.964389896	0.054433766
SIX5	0.993864692	0.323039017	-1.000422217	0.321290792	-0.956887329	0.342719757
SKA1	0.344175748	0.731542829	1.97072061	0.053568411	2.08850081	0.041291847
SKA2	-0.021446224	0.982938705	-0.900327282	0.371695206	-0.804560462	0.424461684
SKAP1	1.341325942	0.183298171	1.212239045	0.230370165	3.021214179	0.003784196
SKAP2	-2.130105583	0.035980168	-0.717687352	0.475850973	-0.826463784	0.412033856
SKI	-0.682266749	0.496878891	-0.219270326	0.827214681	-0.145709007	0.884672133
SKIL	-0.140147982	0.888866326	0.229161639	0.819555082	-2.244914336	0.028724313
SKIV2L	-0.237417399	0.812889983	0.92014493	0.361334876	1.340880969	0.185350216
SKIV2L2	1.792742268	0.076482206	1.021606562	0.311239076	0.853376798	0.397070667
SKOR1	0.604389145	0.547154506	-2.766649389	0.007600494	-0.875337761	0.385113517
SKP1	-0.426297999	0.670941359	1.924328837	0.059258584	2.485053252	0.015953859
SKP2	1.277881479	0.204683041	-1.099411248	0.276162925	-0.840935927	0.403945325
SLA	-1.541791602	0.126746021	1.845215921	0.070149046	1.916208251	0.060426465
SLA2	-3.739013761	0.000329776	0.425633174	0.671961415	0.797749935	0.428371345

SLAIN1	2.064750612	0.041917287	-0.938224901	0.352046687	-1.163968358	0.249351348
SLAIN2	0.441639262	0.659843659	-0.427821967	0.670376175	-1.082636351	0.283590175
SLAMF1	0.305086135	0.761027664	1.373399156	0.174950991	2.978723051	0.004266657
SLAMF6	1.487835932	0.140402032	-0.47234806	0.63846323	0.749527784	0.456663219
SLAMF7	0.406395772	0.685447613	-1.755869219	0.084427145	-0.60388673	0.54834732
SLAMF8	-0.197500889	0.843895298	-0.392275036	0.696302874	-0.628675304	0.532106911
SLBP	-1.278940894	0.204311401	0.947080404	0.347554465	0.097441187	0.922722494
SLC10A1	-0.274802097	0.7841184	1.240925856	0.219663824	1.456085125	0.150929562
SLC10A3	-2.17784431	0.032117732	-0.558481503	0.578680397	-1.558775701	0.124659826
SLC10A7	0.902663434	0.36919158	-2.179491861	0.033397993	-2.282593563	0.026256584
SLC11A1	-1.833068037	0.070205255	-1.353799021	0.181089137	-1.01554024	0.314197728
SLC11A2	3.640375946	0.000461	-0.949929129	0.346117351	0.045225491	0.964087788
SLC12A1	-1.757836331	0.082284983	-1.036918466	0.304107669	0.492910103	0.623995299
SLC12A2	0.319205476	0.750334222	-0.322212673	0.748458218	-0.548934689	0.585223288
SLC12A3	3.067456283	0.00287567	1.118484201	0.268003529	0.564060269	0.57495584
SLC12A4	-0.717502023	0.474982356	-1.038418926	0.303414879	-1.225233265	0.225594265
SLC12A6	-0.280426292	0.779814772	-1.483166338	0.143481836	-1.395385068	0.168385716
SLC12A7	0.560158581	0.576807728	1.493919429	0.140655187	1.663738071	0.101728202
SLC12A9	0.520986574	0.60369682	-0.417836131	0.677620578	-0.11086288	0.912119797
SLC13A4	-0.929319633	0.355289259	1.117258421	0.268522751	1.354033128	0.181142188
SLC14A1	-0.036051739	0.971323516	-1.166675643	0.248149249	-0.346662117	0.730138986
SLC14A2	-0.476183436	0.635134798	1.210800985	0.230916723	0.960218832	0.341055491
SLC15A2	0.787505111	0.433121852	-0.980703254	0.330840666	-0.234409606	0.815518346
SLC15A3	-2.617379695	0.010444987	-0.698049755	0.487954781	-0.997045729	0.323013185
SLC15A4	-1.642465763	0.104095538	0.321281008	0.749160426	-0.913301836	0.364982824
SLC16A1	0.357713963	0.721421878	2.864164431	0.005824415	3.811185017	0.000345096
SLC16A10	1.811088059	0.073571081	0.340166038	0.734968989	0.90424305	0.369723964
SLC16A11	0.001165674	0.99907259	-2.65644065	0.010199214	-0.930636474	0.356019563
SLC16A13	-0.490727746	0.624851843	-0.249592019	0.803790888	0.614860019	0.541127167
SLC16A14	-0.150193618	0.880958771	-0.339165303	0.735718731	-0.314025902	0.754663325
SLC16A3	-3.240118111	0.001692119	-0.839614854	0.404595983	-1.764773942	0.083031713
SLC16A4	1.801621155	0.075061577	-0.626555349	0.533426299	-0.704218476	0.484202754
SLC16A5	-1.724841795	0.088099265	-1.298804782	0.199186732	-0.810163639	0.421261231
SLC16A6	-1.903929052	0.060217751	-1.669874315	0.100370906	-2.166354359	0.034537537
SLC16A7	0.387839937	0.699079749	-0.955218761	0.343459177	-1.730672951	0.088994305
SLC17A5	-0.759848359	0.449394942	-0.665746535	0.508230686	-0.280699271	0.779971172
SLC17A9	1.753951933	0.082952617	-1.399360592	0.167067613	-1.121076329	0.267019849
SLC18A1	-0.134026526	0.893690487	-1.535776095	0.130068841	-1.795021753	0.078024622
SLC18A2	-1.666292143	0.099244095	-1.058810946	0.294106226	-1.752028405	0.085220206
SLC19A1	-1.428533713	0.156710947	-1.269185442	0.209479745	-1.572380027	0.121472221
SLC19A2	0.532112297	0.596001573	-2.421547286	0.018625398	-2.295114608	0.025479214
SLC1A2	1.206852312	0.230753177	2.220820892	0.030310582	1.801825702	0.076933789
SLC1A3	2.841484153	0.005589966	0.846566791	0.400740372	-0.188927126	0.850830107
SLC1A4	1.45648706	0.148849867	2.087989545	0.041231375	2.422954441	0.018639228
SLC1A5	0.004732071	0.996235178	-0.084439758	0.932999614	0.009479296	0.992470277
SLC1A7	0.214898584	0.830348243	-0.051578591	0.959042946	-0.210391128	0.834122856
SLC20A1	1.835204802	0.069885015	0.886525867	0.379020924	-0.119380353	0.905399472
SLC20A2	2.724942816	0.007770841	0.800590521	0.426658804	1.363012121	0.178311455
SLC22A1	-4.31145022	4.25E-05	0.643602198	0.522387924	-0.192703008	0.847885799
SLC22A15	-1.371704751	0.173674898	-0.386749139	0.700367158	-2.053245577	0.044710876
SLC22A16	-0.9343996	0.352678456	1.052951976	0.296760418	1.026939095	0.308846103
SLC22A17	3.420246995	0.000953871	0.380300101	0.705121558	0.594498163	0.554563319
SLC22A18	-1.769815176	0.080253971	-0.084750473	0.93275367	-0.052505608	0.958311894
SLC22A18AS	-2.398585322	0.018590117	1.450593943	0.152317417	0.840757727	0.404044324
SLC22A20	0.75601705	0.451676766	0.418973342	0.676794007	-0.474407705	0.637046198
SLC22A23	3.345079521	0.001214589	1.159357218	0.25109444	1.945786278	0.056684471
SLC22A3	2.320887987	0.022626617	-1.291920413	0.201544633	0.048892476	0.961178217
SLC22A31	-0.991230392	0.324316035	0.281667079	0.77920834	1.273537704	0.208066104
SLC22A4	-1.292776742	0.199503594	-0.292242559	0.771151305	-1.62224256	0.11034497
SLC22A5	2.500293443	0.014279986	-0.836691161	0.406224271	-2.02202745	0.047940428
SLC23A1	0.68654481	0.494191467	-1.269310082	0.209435618	0.218864735	0.827547792
SLC23A2	0.812754046	0.418572825	-0.508472727	0.613064	-0.676922889	0.501229954
SLC23A3	1.294396345	0.198946342	-0.238957089	0.811987086	-0.033746681	0.973198649
SLC24A1	1.641716636	0.104251127	-0.842390857	0.403053661	0.605619963	0.547203653
SLC24A3	-2.633795243	0.009989228	0.500780516	0.618433988	-0.208678622	0.835453133
SLC24A4	-1.349814427	0.180569623	0.801291441	0.426256284	2.04822871	0.045216817
SLC24A6	1.004965959	0.317694191	2.82029598	0.00656972	3.561464862	0.000759235
SLC25A1	0.643296104	0.521720356	2.344548005	0.022520093	2.210704715	0.031140157
SLC25A10	0.855225467	0.394770371	0.563810846	0.575071667	1.572301902	0.121490338
SLC25A11	-1.877981969	0.063726313	0.17447608	0.862103177	0.229227378	0.819523823
SLC25A12	2.129852404	0.036001682	0.090635342	0.928096818	0.613623104	0.541938582
SLC25A13	0.266335719	0.790609508	0.167009856	0.86794726	-0.460669581	0.646812207

SLC25A14	0.557564962	0.578570211	0.90155422	0.371048343	-0.472294455	0.638544293
SLC25A15	0.807524603	0.421561895	-0.204652772	0.83856491	1.144021153	0.25746109
SLC25A16	1.871140118	0.064679581	-1.827001454	0.072882359	-1.823342645	0.073567803
SLC25A17	2.400331922	0.018507257	0.857033024	0.394978591	2.239365022	0.029104577
SLC25A19	1.458040208	0.148422234	-0.209640353	0.834688153	-1.026850862	0.308887288
SLC25A20	-0.717528765	0.474965945	4.47594573	3.65E-05	2.807139826	0.006856843
SLC25A22	0.43625949	0.663726824	-0.957476121	0.342328881	-0.301127695	0.764427724
SLC25A23	0.489433407	0.625763987	0.295826942	0.768426196	0.835168174	0.407157181
SLC25A24	0.525263068	0.600733554	-0.321982541	0.748631652	-1.944527106	0.056839641
SLC25A25	1.467604999	0.145809742	-0.986299849	0.328111277	0.572918248	0.568983808
SLC25A26	1.80471214	0.07457219	1.768984993	0.082190913	2.745538364	0.008097064
SLC25A28	-1.811214627	0.073551321	-3.023847096	0.003723193	-1.564459486	0.123320013
SLC25A29	1.978750236	0.051000352	-2.454918504	0.017133929	-1.058456377	0.294372752
SLC25A3	-0.654582745	0.514459522	2.506365267	0.015044525	3.46911328	0.001009215
SLC25A30	0.540753884	0.590056074	0.392611192	0.696055919	-0.027941189	0.977807977
SLC25A32	-0.30392527	0.76190896	-0.673117713	0.503564384	-0.774303348	0.441994659
SLC25A33	-0.310032217	0.757276286	1.259958002	0.212765798	1.127910573	0.264146862
SLC25A34	-0.105484824	0.916233416	-0.596925806	0.552896238	0.211169114	0.833518677
SLC25A35	0.676059563	0.500792213	0.105035324	0.916712713	0.203337966	0.83960485
SLC25A36	1.629766163	0.106758664	-1.115378648	0.269320372	-1.717742747	0.091345907
SLC25A37	-2.273152647	0.025474579	-2.266893835	0.027167897	-3.052147002	0.003465529
SLC25A38	0.5567861	0.579099985	0.973553006	0.334349623	2.050984641	0.044938277
SLC25A39	-2.654084947	0.00945084	1.0675892	0.290160211	0.510969477	0.611372651
SLC25A4	1.093030714	0.277391143	-1.130619311	0.262901455	0.164941547	0.869581458
SLC25A40	0.197099733	0.844208237	-0.980679572	0.330852248	-2.111820535	0.039157301
SLC25A42	2.947364485	0.004111326	-0.175315581	0.861446548	1.374203945	0.174830578
SLC25A43	1.286734133	0.201592893	-1.438261667	0.155771537	-1.571321229	0.121717934
SLC25A44	-2.446295897	0.01644164	0.970014945	0.336094987	0.771729771	0.443505361
SLC25A45	0.672058888	0.50332322	-0.655408836	0.514813948	1.296141444	0.20022089
SLC25A46	1.363814981	0.176136484	-1.584557193	0.118542778	-2.187333804	0.032891878
SLC25A5	-1.05450775	0.294563939	1.434551749	0.156822489	1.615393265	0.111822639
SLC25A6	-0.743768233	0.459016386	-2.366357024	0.021349143	-1.885851574	0.064483541
SLC26A1	1.988487347	0.049894125	-1.513893871	0.135521337	-1.404561019	0.165651208
SLC26A11	2.959750769	0.003964249	0.067822482	0.946161846	0.116114091	0.907975772
SLC26A2	1.717909	0.089362848	0.164700056	0.86975674	-0.274014535	0.785077434
SLC26A5	-0.919153656	0.360551119	-0.136320915	0.892042956	0.850946208	0.398408053
SLC26A6	1.221685534	0.225119456	1.37770283	0.173624796	2.194480416	0.03234724
SLC26A8	-1.40070043	0.164852651	-0.344646612	0.731615368	-1.420442112	0.160999754
SLC27A1	0.258412141	0.79669788	0.94225533	0.349997471	1.891297174	0.063739202
SLC27A2	-0.582767074	0.561554309	2.975392056	0.004271076	-0.096817136	0.923215832
SLC27A3	1.119856231	0.265850446	-2.269572851	0.02699437	-2.636318082	0.010813812
SLC27A4	-0.770136994	0.443300329	1.364525567	0.177709895	1.581030848	0.119479534
SLC27A5	-1.532456362	0.129030464	-0.542309459	0.589697527	-0.615070397	0.540989222
SLC28A2	-0.038675413	0.969237595	2.574670275	0.01262666	1.382083256	0.172411364
SLC28A3	0.640203801	0.52371899	-1.08638229	0.281835698	-0.471713923	0.638956101
SLC29A1	1.023359593	0.308968868	-1.028725517	0.307909509	-1.464626914	0.148591018
SLC29A2	2.701519239	0.008293428	-0.60830939	0.545373796	1.152967242	0.253801009
SLC29A3	-1.160782825	0.248898571	0.345258392	0.73115787	-0.027853494	0.97787761
SLC29A4	0.99293226	0.323490646	0.301792102	0.763897566	0.592405785	0.555953453
SLC2A1	-1.715091363	0.089880621	-1.001707287	0.32067491	-1.142759392	0.25798032
SLC2A11	0.70110187	0.48510645	-0.516796723	0.607276884	0.954909803	0.343710156
SLC2A12	0.222442309	0.824489827	-0.355874757	0.723234535	-0.183827199	0.854810247
SLC2A13	0.92666155	0.356660287	-1.222536823	0.226483884	-2.813718484	0.006735306
SLC2A14	0.151291807	0.880095034	-0.048066876	0.961829233	-1.353376265	0.181350609
SLC2A3	-2.077377459	0.040708512	0.88893438	0.377735971	0.038934637	0.969080391
SLC2A4RG	1.493244467	0.138983245	-1.910067383	0.061108157	-1.013448928	0.315186333
SLC2A5	-1.145085916	0.255307103	1.296455677	0.199988966	1.900713101	0.062469367
SLC2A6	-0.065160237	0.948195591	-0.473852202	0.63739665	-0.885715437	0.379542612
SLC2A8	0.044039649	0.964973504	-1.471821666	0.146512267	-0.749046119	0.456951151
SLC2A9	-1.342101327	0.183047648	1.24803733	0.217067301	0.728900123	0.469087517
SLC30A1	-0.944651569	0.347447264	0.703511609	0.4845713	-1.454121343	0.151471251
SLC30A4	-0.442686801	0.659088615	-1.238996432	0.22037222	-1.194971387	0.23711346
SLC30A5	1.426870098	0.157188703	0.302793832	0.76313788	-0.26987582	0.788243654
SLC30A6	-0.117533498	0.906707634	-0.779631839	0.438799539	-1.357335333	0.180097172
SLC30A7	1.100090238	0.2743209	-1.038435156	0.303407391	-1.831472901	0.072328554
SLC30A9	0.399542051	0.690470901	-0.620979176	0.53706314	-1.196501158	0.236521092
SLC31A1	-4.453019697	2.50E-05	0.369710652	0.712954	0.450352384	0.654187801
SLC31A2	-2.773995991	0.006772416	-1.362906934	0.178216713	-2.230615446	0.029713175
SLC33A1	0.811232937	0.419440956	-1.128882724	0.263627336	-2.776604785	0.007447891
SLC34A3	0.053791471	0.957224367	-1.22397756	0.225944014	-1.080100139	0.284708087
SLC35A1	1.275239716	0.205611942	-1.205366297	0.232990805	-1.084736865	0.282666625
SLC35A2	-1.878210043	0.06369474	-0.673483692	0.503333309	-0.604234447	0.548117782

SLC35A3	0.974342245	0.332582158	-0.923526036	0.35958602	-2.62971721	0.011002067
SLC35A4	-1.145286185	0.255224611	0.506232149	0.614625983	0.909724547	0.366850409
SLC35A5	-1.553044344	0.124035046	-1.513220653	0.135691916	-2.329365633	0.02345621
SLC35B1	-0.916708444	0.361824134	-0.959173005	0.341480833	-0.464389216	0.644161766
SLC35B2	2.404097083	0.018329752	0.061847177	0.950898656	0.410689538	0.682860854
SLC35B3	-0.177275283	0.859703347	-1.302710509	0.197858248	-1.759775742	0.083884301
SLC35B4	1.069664571	0.287722692	-0.085519446	0.932145024	0.980170928	0.331199752
SLC35C1	-0.480362866	0.632172448	0.262870144	0.793588709	0.638965974	0.525439117
SLC35C2	-0.715202177	0.476394941	1.286226898	0.203510396	2.875106567	0.005693329
SLC35D1	1.059115053	0.292472732	0.351902264	0.726195825	0.343753334	0.732313746
SLC35D2	-1.466296305	0.146165065	1.808089377	0.075814439	1.838942275	0.071205554
SLC35E1	-1.320636353	0.190078678	1.443847478	0.154199534	1.446501325	0.153587562
SLC35E2	2.351856089	0.020933089	0.674841581	0.502476453	0.566977119	0.572985947
SLC35E2B	1.605580918	0.111982094	0.88542377	0.379609817	1.624227415	0.109919729
SLC35E3	-1.159297247	0.249500137	-1.87533919	0.06581824	-2.258387411	0.027819302
SLC35E4	-0.077770686	0.938188663	-0.713403316	0.478476987	-0.239598031	0.811513033
SLC35F2	1.24645754	0.21593494	0.701512085	0.485808438	2.126548843	0.037859292
SLC35F3	-0.197557884	0.843850838	-0.427381219	0.670695267	-2.041946169	0.045857381
SLC35F5	-0.417275624	0.677502507	-1.546980844	0.12734532	-2.891151863	0.005446733
SLC35G1	-0.025739211	0.979524168	-1.436530504	0.156261259	-1.872235396	0.066376983
SLC36A1	-0.74605571	0.457640565	1.252511692	0.215445288	0.383534804	0.702769418
SLC36A4	-0.130922183	0.896138479	-0.375205825	0.708885578	-0.826593665	0.411960829
SLC37A1	-1.888402065	0.062297189	-0.992286053	0.325208517	-2.443776185	0.017696642
SLC37A2	-2.712065356	0.008054304	2.263334573	0.027399967	1.73444422	0.088317937
SLC37A3	-0.468524181	0.640579046	-0.162351648	0.871597183	-0.539214036	0.591867646
SLC37A4	1.986497769	0.050118481	1.185371807	0.240738107	2.397615176	0.019847004
SLC38A1	1.896393925	0.06121946	-0.149520775	0.881665055	-0.335305778	0.738642054
SLC38A10	-0.498185918	0.619607358	1.510074282	0.136491398	1.214282717	0.229714313
SLC38A11	-0.728174902	0.468457691	2.28854974	0.025793051	2.534933656	0.014055442
SLC38A2	2.076329365	0.04080769	-0.399320199	0.691134138	-1.142441913	0.258111084
SLC38A5	-2.928769009	0.004341611	-1.031075878	0.306815565	-0.314898958	0.754003823
SLC38A6	1.902321351	0.060430309	-1.70885331	0.092862097	-0.590707188	0.557083248
SLC38A7	-0.319128687	0.75039225	0.278037558	0.781979198	1.290364575	0.202204494
SLC38A9	-0.602330229	0.548517652	-2.44782018	0.017441864	-3.071483877	0.003279248
SLC39A1	-0.121903037	0.903256337	-0.504486592	0.615844115	-0.745105596	0.459310659
SLC39A10	1.925435052	0.057434516	-0.772494803	0.442979869	-1.230244429	0.223727029
SLC39A11	-0.674035466	0.502071889	2.999268115	0.003992353	2.86716113	0.005819231
SLC39A13	1.721324363	0.088738515	1.140587656	0.258762151	1.344197251	0.184282236
SLC39A14	-0.06167453	0.950963184	2.045617006	0.045370468	1.572507488	0.121442668
SLC39A3	-0.255673903	0.798804857	-1.242739939	0.218999307	-1.023246815	0.310572768
SLC39A4	0.251007567	0.802398864	-0.445226128	0.657825222	0.670791248	0.505099255
SLC39A6	2.297104193	0.024008377	1.910313095	0.06107588	1.086763714	0.281777449
SLC39A7	-0.899187359	0.371029539	2.398337492	0.0197304	2.383320294	0.020558893
SLC39A8	0.431765244	0.666977874	1.63637254	0.107214996	1.302107718	0.198187601
SLC39A9	-0.677871474	0.499648178	-0.527188691	0.600087384	0.966392835	0.337985314
SLC3A2	-1.139505015	0.257613482	1.075036264	0.286841388	0.877481305	0.383958641
SLC40A1	-2.439243418	0.016744566	0.070181412	0.944292377	-1.184428245	0.241225334
SLC41A1	1.184022034	0.239622226	-1.105793117	0.273413624	0.379434264	0.705794439
SLC41A2	-0.110622485	0.91216996	-0.319712142	0.750343383	0.498679366	0.619950275
SLC41A3	1.693455452	0.093938819	1.249314331	0.216603456	1.303233089	0.197805822
SLC43A1	0.387946129	0.699001448	0.482484829	0.631290249	1.589275185	0.117605034
SLC43A2	-2.747296119	0.0073003	-0.462347325	0.645574144	-0.913979269	0.364629846
SLC43A3	-1.520309636	0.132051481	2.61212503	0.011456089	2.391565527	0.020145537
SLC44A1	-0.234553382	0.815105335	0.848933757	0.399432815	-0.370895013	0.712109251
SLC44A2	-2.631417786	0.0100541	-0.687825028	0.494323754	-0.536302404	0.593864736
SLC44A5	-1.053394296	0.29507085	0.621083013	0.5369953	0.37650003	0.707961999
SLC45A1	-0.469624976	0.639795373	1.004696767	0.319245237	0.576332895	0.566689811
SLC45A3	-3.563290603	0.000596622	1.789423411	0.078804572	-2.514949216	0.014790012
SLC45A4	-2.047229656	0.043645685	-2.073426728	0.042615339	-1.888508837	0.064119414
SLC46A1	1.023398553	0.308950559	0.140516579	0.888742123	0.165185367	0.869390447
SLC46A2	-1.244122434	0.216788812	0.024550442	0.980498409	-0.70953755	0.480922555
SLC46A3	2.626306888	0.010194851	-1.297883496	0.199501069	-0.667811786	0.506985228
SLC47A1	-1.585073726	0.116570085	-0.986225057	0.328147653	-0.500893597	0.618400924
SLC48A1	0.054676432	0.956521338	-1.334841109	0.187180933	-0.422121968	0.674545446
SLC4A1	-2.985391028	0.003675112	1.269212354	0.209470217	0.512327763	0.610427984
SLC4A10	1.245270345	0.21636875	-2.806560686	0.006820584	-1.774532282	0.08138792
SLC4A1AP	0.440424039	0.660720007	2.844507528	0.006148145	2.614449895	0.011448992
SLC4A2	0.020143266	0.983975112	0.693050036	0.491063413	0.541160457	0.590534364
SLC4A3	1.515252228	0.133325635	-0.400063099	0.690589959	0.331019227	0.741860232
SLC4A4	1.353744842	0.179316668	-2.375706551	0.020864039	-2.45227808	0.01732426
SLC4A5	1.43326421	0.155358567	2.048050871	0.045123338	1.333140027	0.187861404
SLC4A7	2.386920516	0.019152021	-1.239391608	0.220226993	-1.807795207	0.075987277

SLC4A8	0.605210136	0.546611429	-0.614229574	0.541482353	-0.214383856	0.8310232
SLC50A1	-2.225690328	0.0286144	1.533780388	0.130558768	1.136014785	0.260768457
SLC5A10	0.681823376	0.497157865	-0.196595575	0.844836088	0.919978763	0.361513365
SLC5A11	-0.45806215	0.648047386	1.266429567	0.210457186	1.286989041	0.203370338
SLC5A2	2.720349859	0.007870883	-2.106692426	0.03951139	-1.825774727	0.073195239
SLC5A3	0.344519932	0.731284925	0.28775851	0.774564487	-0.987063517	0.327839406
SLC5A5	2.598815285	0.01098302	0.387708351	0.699661024	0.358228167	0.721513778
SLC5A6	0.89177036	0.374970519	1.334108522	0.187419417	2.210626437	0.031145884
SLC5A9	-1.181913252	0.240453614	-0.996964302	0.322951966	-1.346986123	0.18338773
SLC6A12	-2.459597695	0.015883586	1.183565303	0.241447144	0.263739568	0.792944643
SLC6A16	0.738187565	0.462382797	-1.324854043	0.190451939	0.583627467	0.561804548
SLC6A4	-2.386861647	0.019154894	1.350746006	0.182059839	-0.118866185	0.90580496
SLC6A6	-0.931328049	0.354255572	-1.208214648	0.231902082	-1.698557602	0.094929387
SLC6A8	-3.121635232	0.002439858	1.233720286	0.222317961	0.449397853	0.65487195
SLC6A9	-1.108782484	0.270573119	1.023123158	0.310527722	1.073232847	0.287750404
SLC7A1	3.041443726	0.003109666	0.290342426	0.772597103	0.633680193	0.528858526
SLC7A11	-0.32490603	0.746030502	-0.180922468	0.857063569	-1.803022877	0.076743181
SLC7A5	-0.369126243	0.712928536	0.423532028	0.673484585	0.457779752	0.64887455
SLC7A5P1	0.692948623	0.490183499	-3.155752562	0.002545756	-2.503002196	0.01524557
SLC7A5P2	3.027007122	0.003247032	-1.809478433	0.075595772	-1.215565536	0.22922884
SLC7A6	2.547393579	0.012606543	-1.31500161	0.193720991	-0.382661282	0.703413424
SLC7A6OS	1.66295563	0.099912207	-1.297067702	0.199779723	-1.693091481	0.095971288
SLC7A7	-2.910547451	0.004578751	0.584379061	0.561247369	0.643571985	0.522468952
SLC7A8	-1.516379174	0.133040879	0.524106407	0.602215684	-0.443894102	0.658822488
SLC8A1	-0.916827743	0.361761959	-0.473368284	0.63773971	-1.158551966	0.251535091
SLC8A3	-4.048612678	0.000111263	0.108340937	0.914101799	0.158089411	0.874952648
SLC9A1	-1.15222841	0.25237673	0.579303792	0.56464318	0.520807837	0.604545331
SLC9A3	1.185141876	0.239181568	-1.205044927	0.233113875	0.274169568	0.7849589
SLC9A3R1	-1.944476224	0.055061849	-1.52864318	0.131826661	-0.83862393	0.405230919
SLC9A3R2	-0.590871069	0.556135443	0.691498591	0.492030256	-0.168914996	0.866469598
SLC9A4	-0.000722945	0.999424825	0.573004138	0.568872276	-1.148710058	0.255538071
SLC9A5	2.659221307	0.009318789	0.090501267	0.928202887	0.151202768	0.880356854
SLC9A6	1.902338797	0.060427999	-0.352094406	0.726052495	-0.404476341	0.687396754
SLC9A7	1.85080262	0.067584111	0.693169962	0.49098872	0.574884477	0.567662318
SLC9A7P1	-1.20475766	0.231556878	1.256940221	0.213848729	0.482701271	0.631181596
SLC9A8	-1.469644044	0.145257462	-0.614027037	0.541615249	-0.114017528	0.909629986
SLC9A9	0.574314629	0.567233706	1.626753969	0.109248625	2.286196733	0.026030752
SLC9B1	1.306256805	0.194900779	-0.189008355	0.850750714	0.093202872	0.926073648
SLC9B2	1.377069703	0.172016041	-1.624916807	0.109640589	-0.160272305	0.873240887
SLCO3A1	-1.910395732	0.059369137	0.296878403	0.767627354	-0.148071574	0.882815927
SLCO4A1	0.873202047	0.384951552	0.500897701	0.618352022	0.100302069	0.920461242
SLCO4C1	-1.516155505	0.133097358	-0.356233537	0.722967291	-0.594210506	0.554754329
SLED1	-1.808075065	0.074042761	0.666142723	0.50797929	0.056380382	0.955238625
SLFN11	1.167495665	0.246193159	-0.837442742	0.405805311	-1.068053718	0.290059684
SLFN12	-1.396769236	0.166028245	0.782507574	0.437121748	-0.939849088	0.351314465
SLFN12L	1.332674969	0.186110807	-2.092677566	0.040794245	-1.076398537	0.286345167
SLFN13	1.75266509	0.083174776	-0.080642922	0.936005495	0.690503156	0.49271799
SLFN14	-0.202080579	0.840324498	0.550616633	0.584025906	0.172677285	0.863525069
SLFN5	0.119499034	0.905154922	-0.636851551	0.526744736	-1.380353906	0.172940119
SLIRP	1.299940393	0.19704758	0.073313917	0.941810333	1.783708295	0.079867046
SLIT1	0.820164208	0.414359089	0.889984785	0.377176439	0.356850416	0.722539335
SLITRK4	1.884117222	0.06288156	0.050691678	0.959746599	-0.212768481	0.832276935
SLK	-0.63892864	0.524544321	-0.456677131	0.649620749	-2.037924085	0.046271578
SLMAP	-0.615795351	0.539633834	-0.017970303	0.985724651	-1.582081183	0.119239393
SLMO1	3.27613351	0.001511317	2.148131729	0.035921666	0.999631632	0.321770743
SLMO2	0.33416267	0.739059253	-0.221019975	0.825858543	-0.695182239	0.489803724
SLPI	-0.475068935	0.635925756	0.875907777	0.384718569	0.615406967	0.540768569
SLTM	2.630465853	0.010080181	2.035452761	0.046415218	2.101089172	0.040127337
SLU7	-1.114273669	0.268224004	0.210330361	0.834152144	-0.051808697	0.958864717
SLX4	1.501350353	0.13687794	-0.373172393	0.71039007	0.757631145	0.45183489
SMAD1	-0.67752616	0.4998661	0.778280574	0.439589217	-0.834350904	0.407613552
SMAD2	-0.582784393	0.561542701	-0.546033083	0.587152047	-1.40123012	0.16663985
SMAD3	4.192494486	6.60E-05	0.86472213	0.390778575	0.822695696	0.414155932
SMAD4	0.251226005	0.802230527	-0.9154457	0.363774596	-0.825480426	0.412587015
SMAD5	1.087147484	0.279967933	-1.354293621	0.180932251	-2.482013589	0.016076707
SMAD7	-0.346564251	0.729753708	-3.723974035	0.000448046	-3.872226066	0.000283469
SMAD9	1.821480876	0.071963241	-0.163548134	0.870659409	1.793577374	0.078257846
SMAGP	1.298635707	0.197493198	-0.34137098	0.734066601	1.024639668	0.309920645
SMAP1	-1.108165842	0.270837811	0.854246525	0.396507549	0.708148771	0.481777795
SMAP2	-1.978596261	0.051018012	-0.499781349	0.61913306	-0.917638919	0.362726769
SMARCA1	0.869719535	0.386841749	1.693148827	0.095829802	2.060800903	0.043958192
SMARCA2	0.323023421	0.747450923	1.887063199	0.064194911	1.324775685	0.190603714

SMARCA4	0.269369223	0.788282018	1.342676315	0.184644652	1.608095579	0.11341464
SMARCA5	1.565264737	0.121143298	1.853511213	0.068933132	1.060599432	0.29340585
SMARCAD1	1.476104608	0.143518364	-0.988847126	0.326873979	-1.525958956	0.132624928
SMARCAL1	-0.3595777	0.72003241	2.407030094	0.019309849	2.679716248	0.009647575
SMARCB1	-0.598695739	0.550928094	1.227755322	0.224532895	1.495459869	0.140385413
SMARCC1	-1.052578439	0.295442654	2.232091346	0.029513545	2.218422928	0.030579984
SMARCC2	-0.563824021	0.574321294	-0.096388783	0.923546408	0.543830672	0.588707601
SMARCD1	-0.211712462	0.832825472	0.363112934	0.717849791	1.299915225	0.198932986
SMARCD2	-1.239900705	0.218338827	1.184421422	0.241110937	1.706377157	0.093455092
SMARCD3	-2.470549491	0.015436906	-0.092177547	0.926876841	-0.832373316	0.408719148
SMARCE1	0.415485061	0.678807604	-0.424827603	0.672545229	0.526341463	0.600720776
SMC1A	0.878055605	0.382326795	1.831813141	0.07215175	1.315486377	0.193684629
SMC1B	0.406892837	0.685083846	0.767892452	0.445687922	1.421901879	0.160577334
SMC2	1.166258608	0.246690136	0.689734205	0.493131075	0.346900481	0.729960871
SMC3	1.787805553	0.077281685	2.310767801	0.024447028	2.257238355	0.027895489
SMC4	-0.23993248	0.810945792	-0.483625503	0.630485294	-1.530385395	0.131527516
SMC5	1.079404609	0.283384377	0.142418546	0.887246447	-0.526678643	0.600488098
SMC6	-0.13724765	0.891151492	-0.288801739	0.773769997	-0.728474361	0.469345965
SMCHD1	-0.44911869	0.654460416	-0.70984024	0.480667244	-2.699849691	0.009146631
SMCR7	-0.295351048	0.768427926	1.365363711	0.177447893	0.943550231	0.349435647
SMCR7L	0.926128342	0.356935722	1.434311933	0.156890614	1.517517703	0.134737871
SMCR8	0.333350437	0.739670089	-0.167557347	0.867518463	-0.157690541	0.875265495
SMEK1	0.741944647	0.460114883	1.031193097	0.306761076	0.850184013	0.39882801
SMEK2	0.572950495	0.568152917	-0.307237279	0.759770917	-1.459867066	0.149890625
SMG1	0.851471999	0.3968398	-1.145460127	0.256755831	-2.868659488	0.005795293
SMG5	-0.32336696	0.747191658	1.114007657	0.269903159	1.245913805	0.217961652
SMG6	0.460856139	0.646049322	1.675695732	0.09921889	2.212075391	0.03104002
SMG7	-1.725317545	0.088013095	0.346264448	0.730405738	0.239035818	0.811946802
SMG8	-0.692789868	0.490282643	1.279472399	0.205861011	0.653284303	0.516235164
SMG9	-0.080485677	0.936035447	1.911159585	0.060964794	2.41589088	0.018969091
SMNDC1	-0.617651962	0.53841468	0.17042117	0.865276162	-1.204555939	0.233419787
SMO	1.236716718	0.219513181	1.908080347	0.061369711	0.881347962	0.381880915
SMOC1	0.589182045	0.557262692	0.857689802	0.394618749	1.115660195	0.269312312
SMOX	-3.396459818	0.001030055	1.597135024	0.115708096	0.34261679	0.733164088
SMPD1	-0.475348658	0.635727197	0.571941696	0.569587045	1.730880385	0.088956991
SMPD2	0.419203726	0.67609826	0.332823697	0.740475787	0.094952919	0.924689753
SMPD3	3.718291047	0.000353983	-0.651457519	0.51734219	-0.400906909	0.690007839
SMPD4	1.494564972	0.138638557	0.280689987	0.779953993	1.186248101	0.240511924
SMPDL3A	-1.199712428	0.23350099	-0.415567551	0.679270665	-1.771855464	0.081836109
SMPDL3B	-1.816872469	0.072672544	-0.112456172	0.910852742	1.438276765	0.155897602
SMS	-0.942963554	0.348305124	1.622337066	0.11019291	1.707708635	0.093205944
SMTN	0.71719652	0.475169864	2.373934312	0.020955228	1.59316775	0.116728261
SMTNL1	-2.015598094	0.04692134	-0.601420649	0.549919748	-1.049872589	0.298267553
SMU1	-1.43889565	0.153760392	2.43318297	0.018092614	2.413164272	0.019097817
SMUG1	-1.232551517	0.221056392	-0.406215296	0.686089761	-0.147247661	0.883463179
SMURF1	1.076152738	0.284827755	-0.185951352	0.853136263	0.136558009	0.891867865
SMURF2	-0.089043571	0.9292515	-1.53594926	0.1300264	-2.965012735	0.004434166
SMYD2	-0.067060776	0.946686861	-0.095023323	0.924626127	-0.083020689	0.934129896
SMYD3	1.293596057	0.19922155	1.274542528	0.207589379	2.729971413	0.008441557
SMYD4	2.434296646	0.016960009	1.170014081	0.246814014	2.260279982	0.027694217
SMYD5	1.972749456	0.051692451	2.145104822	0.036173866	3.813904382	0.000342096
SNAI1	0.118658489	0.905818881	0.685280349	0.495915894	-0.594046597	0.554863183
SNAI3	-1.873239352	0.064385832	-0.890378803	0.376966687	-2.00063377	0.050268165
SNAP23	-1.29699574	0.198054397	-1.002122017	0.320476315	-2.263812171	0.027462098
SNAP29	-3.257370374	0.001603122	-0.760835696	0.449858907	-1.31267998	0.194622753
SNAP47	-0.449958227	0.6538573	3.229365969	0.002050906	4.4144856	4.65E-05
SNAP91	-0.67113457	0.503908961	-0.26892746	0.788946497	0.025052028	0.980102149
SNAPC1	0.952716341	0.343367587	-0.27018315	0.787985116	-0.317414653	0.752104513
SNAPC2	0.329794145	0.742346546	-0.705117044	0.48357926	-1.401920879	0.166434452
SNAPC3	1.917447944	0.058455213	-1.896772789	0.062876302	-2.028748712	0.047228586
SNAPC4	0.167767602	0.867154641	-0.147585328	0.883185469	0.480106948	0.633013573
SNAPC5	0.586219979	0.559242295	-1.864559842	0.067341273	-1.788478447	0.079085834
SNAPIN	-0.101658203	0.919261403	-2.43931746	0.017817273	-2.029847936	0.047113041
SNCA	-3.747953477	0.000319826	0.37617368	0.708169892	-0.165431429	0.869197688
SND1	-1.074995449	0.285342648	2.599923922	0.011826087	2.420746042	0.0187418
SNED1	4.18392375	6.81E-05	0.382546311	0.70346424	0.272525853	0.786215895
SNF8	-1.180813104	0.240888167	1.984585334	0.051961412	2.984792177	0.004194403
SNHG1	1.367013816	0.175135286	-1.591758541	0.116913039	-0.172105332	0.86397258
SNHG10	2.371708689	0.019907451	0.443367635	0.659160837	0.350942849	0.726942537
SNHG11	-0.565861193	0.572941623	0.746095872	0.458643926	1.558880152	0.124635099
SNHG12	3.520859381	0.000686597	0.225629791	0.822288075	0.637864392	0.526150779
SNHG3	1.388959285	0.168382768	-0.395509438	0.693928116	0.721615334	0.473520705

SNHG4	1.013316202	0.31371295	2.01835756	0.048219265	3.319290603	0.001587882
SNHG5	1.022040299	0.309589281	0.360000958	0.72016316	0.434016478	0.665937065
SNHG6	-1.006128345	0.317137978	-1.294402857	0.200691998	-0.949120061	0.346620594
SNHG7	2.041404172	0.044233759	0.220791721	0.826035431	0.628595925	0.532158515
SNHG8	1.159240172	0.24952327	1.033457298	0.305709863	1.563700708	0.123498206
SNHG9	-1.488470808	0.140234904	-4.794520078	1.19E-05	-4.095388048	0.000136389
SNIP1	-1.358004206	0.177966286	-1.708143601	0.092994559	-1.485687434	0.142946517
SNN	-4.190898626	6.64E-05	-1.543953277	0.128076698	-1.737476934	0.087777119
SNORA67	0.517367826	0.606209528	1.65874213	0.102604346	2.409569847	0.019268712
SNORA70	1.715577941	0.089791031	1.543834704	0.12810541	1.967400107	0.054076875
SNORD10	1.099631001	0.274519905	1.755664423	0.084462459	0.95729099	0.342517823
SNORD17	-1.919164415	0.058234576	2.090355152	0.04101029	1.847360684	0.069957497
SNPH	1.463974583	0.146797093	2.020525771	0.047987157	3.018599285	0.003812352
SNRK	-0.925997824	0.357003163	-0.703206232	0.484760128	-2.030014431	0.047095561
SNRNP200	0.629922871	0.530392422	1.523938908	0.132996274	1.725683372	0.089895752
SNRNP25	0.206738123	0.836696422	0.423874706	0.673236076	0.365869613	0.715835087
SNRNP27	-0.304628801	0.761374821	-1.484624656	0.14309589	-1.346438348	0.183563163
SNRNP35	-1.150881858	0.252927352	0.421922097	0.674652593	1.582354656	0.119176932
SNRNP40	2.792250219	0.00643185	1.386491482	0.170940498	3.387978093	0.001291685
SNRNP48	1.683417772	0.09587169	-0.464712279	0.643889532	0.081503411	0.935330996
SNRNP70	0.526714658	0.599729241	-1.902618806	0.06209352	-0.894529424	0.374851295
SNRPA	0.231297483	0.817625639	-3.038661742	0.003569238	-2.177328732	0.033667894
SNRPA1	1.452281791	0.150012519	-1.569958081	0.121902782	0.832761178	0.408502164
SNRPB	-1.286841431	0.201555653	3.733051612	0.000435254	4.080269777	0.000143404
SNRPB2	-0.083594889	0.933570163	-0.254393816	0.800097389	1.088153992	0.281168664
SNRPC	-0.67470477	0.501648547	-1.681851898	0.098012431	-0.731886799	0.467276798
SNRPD1	0.882294885	0.380043389	0.323687458	0.747347091	1.20159806	0.234555192
SNRPD2	0.781314959	0.436733684	-2.197444007	0.032024501	-1.055983322	0.295491267
SNRPD3	-0.220550575	0.825958017	-1.303165395	0.197703958	0.198812976	0.843126079
SNRPE	0.204345533	0.838559736	-0.205052644	0.838253947	0.081466525	0.935360198
SNRPF	-0.252398716	0.801326954	-0.901511701	0.371070748	-0.04841244	0.961559073
SNRPG	1.418323515	0.159660826	-1.910566705	0.061042581	-1.494328152	0.140680139
SNTA1	0.29829524	0.766187557	1.085318765	0.282302309	1.585419581	0.118478705
SNTB1	0.705204515	0.48256272	-1.167782742	0.247705882	-3.014858637	0.003852969
SNTB2	0.536674561	0.592859227	-3.78221206	0.000371834	-4.369189744	5.42E-05
SNUPN	1.254607747	0.212973987	2.829252528	0.006410752	3.068721113	0.003305278
SNW1	-0.52671209	0.599731018	2.660213216	0.010098258	2.034819498	0.046593498
SNX1	-0.079303372	0.936973057	1.58394374	0.118682449	1.459687833	0.149939736
SNX10	-1.257477838	0.21193842	-1.49914829	0.139296653	-2.833525682	0.00638128
SNX11	-2.444872958	0.016502362	0.863477938	0.391456295	0.382741865	0.703354005
SNX12	-0.356654972	0.722211804	-0.170485366	0.865225911	-1.078664251	0.285342353
SNX13	-0.676619846	0.500438301	-1.085287513	0.282316029	-2.142457297	0.036499644
SNX14	1.180576676	0.240981628	0.740947602	0.461735432	0.185928485	0.853169873
SNX16	0.190586838	0.849292371	-0.856871769	0.395066972	-1.816342591	0.07464902
SNX17	-0.611028656	0.542770338	3.171756238	0.002429479	3.806247139	0.000350609
SNX18	-1.140037793	0.257392672	-1.394753803	0.168446152	-3.110515981	0.002931415
SNX19	1.717332272	0.089468629	2.815473037	0.006656821	2.32915819	0.023468019
SNX2	-0.707331063	0.48124712	0.677804969	0.500609248	0.210031847	0.834401906
SNX20	-1.433959641	0.155160516	-1.514128463	0.135461936	-2.345373654	0.022560766
SNX21	0.662092834	0.509657963	1.184331797	0.241146118	1.768673925	0.082371476
SNX22	0.20063755	0.841449276	0.245771383	0.806732905	0.795700949	0.429551786
SNX24	-0.477801907	0.633986929	0.25546421	0.799274674	0.37061556	0.712316253
SNX25	0.037861082	0.969884998	-0.127592375	0.898916001	0.501318127	0.618104069
SNX27	-2.626107135	0.010200388	-0.689612387	0.493207128	-0.740911219	0.461829853
SNX29	-0.256342108	0.798290559	2.387799392	0.02025123	1.240463197	0.219954579
SNX3	-2.158715109	0.033619766	-1.801872982	0.076799528	-2.047557841	0.045284847
SNX30	0.31055038	0.756883617	2.052047562	0.044720048	0.579063897	0.564858378
SNX32	1.115791687	0.267577122	0.432467677	0.6670165	-0.139678971	0.889412691
SNX33	-1.031350197	0.305229071	0.592883749	0.555579797	0.594033612	0.554871807
SNX4	1.266105981	0.20884757	-0.149304367	0.881835034	-0.726293457	0.470671099
SNX5	1.111146447	0.269560069	-0.065141246	0.948287113	-0.004976305	0.99604711
SNX6	-1.537339335	0.127831516	0.612127371	0.542862539	-0.162006888	0.871881111
SNX7	1.000358651	0.319905231	0.431718954	0.667557499	0.670326221	0.505393363
SNX8	0.419552636	0.67584427	1.76347073	0.083125015	1.840336718	0.070997538
SNX9	2.999910571	0.003520155	0.677353958	0.500893182	1.327756159	0.189623093
SOAT1	-0.800879843	0.425378254	1.256633477	0.213959033	0.876773059	0.384339982
SOAT2	-1.82285441	0.071752952	-0.117014328	0.907255779	0.000579294	0.99953984
SOBP	-0.094855329	0.9246474	1.229735709	0.223795743	0.570597746	0.570545342
SOCS1	-0.486160898	0.628072778	-1.533631961	0.130595264	-2.781112447	0.007357783
SOCS2	-1.09807227	0.275196111	0.727342422	0.469962478	-0.413709546	0.680660347
SOCS3	-0.100536427	0.920149287	0.239076762	0.811894736	-1.404458325	0.16568162
SOCS4	0.945239774	0.347148656	-1.161479228	0.250237903	-2.064665687	0.043577434

SOCS5	1.763420783	0.081332924	0.313501261	0.755032404	-0.708282099	0.481695652
SOCS6	-0.004418567	0.996484599	0.061571639	0.951117127	-1.34829827	0.182968015
SOCS7	2.350831053	0.020987294	-0.078574146	0.9376437	0.558132565	0.578969215
SOD1	1.15254414	0.252247747	0.015187781	0.987934858	0.931723277	0.355462394
SOD2	-3.027978179	0.003237619	-0.465136609	0.64358747	-1.445870462	0.153763802
SOLH	1.218294523	0.226398482	-1.71234808	0.092212086	-0.580085362	0.564174127
SON	0.675218309	0.501323858	2.41200975	0.019072569	1.555410468	0.125458575
SORBS1	0.374795915	0.708722351	-0.506070105	0.614739019	-1.277520811	0.206667291
SORBS3	1.017569473	0.31169796	0.671500649	0.50458607	1.648518111	0.104822592
SORCS2	-0.691704709	0.490960635	-1.221129914	0.22701199	-0.662265054	0.510506358
SORCS3	1.099219443	0.274698334	1.86758644	0.066910663	2.354174987	0.022081552
SORD	-0.258910142	0.796314847	0.166582499	0.868281995	0.302290569	0.763545781
SORL1	-1.51618779	0.133089204	0.490014903	0.62598474	-0.178147791	0.859247096
SORT1	0.011997104	0.990455335	1.286878231	0.203284791	1.083913257	0.283028497
SOS1	0.16623189	0.868359344	-2.020990372	0.047937547	-2.826885319	0.006498005
SOS2	-0.456227279	0.649360961	-0.491724078	0.624783245	-1.43153104	0.157812413
SOX10	0.645573056	0.520251258	-0.038991494	0.969032009	0.91040795	0.366493155
SOX12	1.80968237	0.073790827	0.574285748	0.568010647	0.521349845	0.604170227
SOX13	-1.096171881	0.276022097	-1.758686354	0.083942611	-1.316031945	0.193502653
SOX15	0.33321714	0.73977035	-0.916936432	0.362999504	-0.758127564	0.451540068
SOX4	-0.945998208	0.346763875	2.079339093	0.042048675	0.996724351	0.323167821
SOX6	-2.82053995	0.005934918	-0.158549151	0.874578703	0.563401942	0.575400895
SOX7	0.194644819	0.846123844	0.35267937	0.7256162	1.019009694	0.312562271
SOX8	1.722938859	0.08844463	1.414502638	0.162597753	0.649122416	0.518901587
SP1	-0.317747981	0.751435857	-1.078848226	0.285152781	-1.110617383	0.271459197
SP100	-1.048404967	0.297349589	0.157035306	0.875766213	-1.435418253	0.156706785
SP110	-1.983618059	0.050444737	2.026804948	0.047320397	0.052334712	0.958447455
SP140	-2.164763949	0.033138332	1.583228773	0.118845401	1.157371697	0.252012756
SP140L	-0.276981743	0.782449732	-0.229858111	0.819016406	1.755250359	0.084662501
SP2	-1.326432234	0.18816054	-1.711367001	0.092394182	-1.696096469	0.095397348
SP3	0.261054143	0.794666381	-0.625015476	0.534429343	-1.693502316	0.095892653
SP4	1.017127418	0.311906979	-1.30367123	0.197532492	-2.077495676	0.042333864
SPAG1	-1.204828425	0.231529693	-0.628851757	0.53193228	-2.343992925	0.02263678
SPAG16	1.099740305	0.274472531	-0.099209234	0.921316635	0.508979251	0.612758021
SPAG5	1.389310594	0.168276312	1.604879675	0.113990045	2.532047683	0.014159439
SPAG7	-0.045293322	0.963977087	1.465240361	0.148293185	2.405841137	0.01944744
SPAG8	1.431199199	0.155947811	-0.280939943	0.779763223	-0.170891269	0.864922642
SPAG9	-1.033745514	0.304113981	-1.506648301	0.137366166	-2.489740176	0.015766096
SPARC	-4.679809316	1.04E-05	1.23657182	0.221264808	-0.045599906	0.963790685
SPARCL1	1.543072372	0.126435115	2.852499104	0.006014576	1.687916305	0.096966374
SPAST	-1.251229114	0.214197799	-0.15894434	0.874268751	-1.007651743	0.317937741
SPATA13	1.091324281	0.278136844	-1.694574688	0.095557177	-2.74928751	0.00801605
SPATA2	-1.526935632	0.130396688	-0.921865543	0.360444217	-1.317945479	0.192865406
SPATA20	1.637720411	0.105084295	0.280912438	0.779784215	0.996660241	0.323198674
SPATA24	0.87245033	0.385359073	-1.781537101	0.080097159	-1.363997559	0.178002854
SPATA2L	-0.561261153	0.576059261	1.40394328	0.165704935	0.452695475	0.652509685
SPATA5	1.46636829	0.146145503	-0.249119161	0.804154851	1.200594436	0.234941352
SPATA5L1	0.096334223	0.923476217	-0.629427942	0.531557761	-0.680728716	0.498836463
SPATA6	-1.248988195	0.215012348	-0.527170803	0.600099725	-1.080521147	0.284522303
SPATA7	-0.2514763	0.802037653	-1.86828458	0.066811666	-1.792519748	0.07842899
SPATC1	0.302727674	0.76281847	-0.747369579	0.457880914	-1.72245156	0.090483649
SPATS2	1.067348324	0.288761047	0.09052328	0.928185472	1.278058903	0.20647886
SPATS2L	-2.258725378	0.026395292	0.64114958	0.523968623	-1.461308782	0.149496049
SPC25	-0.575910109	0.566159525	2.027565031	0.047240232	2.763719465	0.007711102
SPCS1	-1.007228317	0.316612228	-2.32843902	0.02342144	-1.405549218	0.165358774
SPCS2	-1.428733938	0.156653523	-1.032404966	0.306198129	-0.914959744	0.364119356
SPCS3	0.619972233	0.53689304	-1.086846225	0.281632319	-0.959778144	0.341275333
SPDYA	1.951778311	0.054174269	-1.026422186	0.30898413	-0.454837945	0.650976831
SPDYC	-1.03415296	0.303924577	-0.917409765	0.36275362	-0.748926058	0.457022937
SPDYE1	1.828523244	0.070890464	-2.456218523	0.017078066	-1.707195308	0.093301934
SPDYE3	1.298067156	0.197687623	0.748761164	0.457048123	0.821879258	0.4146166
SPDYE5	0.905635643	0.367624613	-2.774381973	0.007443264	-1.834238772	0.071910989
SPDYE6	2.60658035	0.010754996	0.034119301	0.972899987	0.431476679	0.667771445
SPECC1	-4.014634287	0.00012567	2.573948227	0.012650258	2.289328446	0.025835871
SPECC1L	-0.757663774	0.450695206	1.324158007	0.190681508	1.179814333	0.243040909
SPEF2	1.179874316	0.241259428	-1.078339197	0.285377869	0.039235277	0.968841765
SPEG	2.558849253	0.012227206	-1.828647084	0.07263179	-0.457230909	0.649266548
SPEN	-0.679842533	0.498405263	-0.609415806	0.544645448	-0.856115165	0.395567268
SPG11	0.128120771	0.898348455	1.027008733	0.308710235	0.048930725	0.961147871
SPG20	-0.581943537	0.562106431	3.241700252	0.001977417	1.756889154	0.084379998
SPG21	-3.473286199	0.000802686	-0.506439444	0.614481396	-0.269620386	0.788439185
SPG7	0.94166828	0.348964319	1.359358506	0.17933164	2.491688512	0.015688632

SPHAR	-0.457431998	0.648498383	-0.628319944	0.532278078	-0.29853389	0.766396037
SPHK1	0.089723049	0.928713088	1.425009302	0.159551034	0.678445615	0.500271559
SPHK2	-0.0993037	0.921125105	-0.929721986	0.356395392	-0.103168757	0.918196063
SPI1	-1.78915178	0.077062986	-0.51461028	0.608794547	-1.215760484	0.229155129
SPIB	-1.164414099	0.247432479	1.14055602	0.258775214	1.335428811	0.187116244
SPICE1	1.886872128	0.062505317	0.579856209	0.564273072	0.390514122	0.697631805
SPIN1	1.098201733	0.275139903	-0.231589138	0.81767795	-0.348337517	0.728887377
SPIN2B	0.669107448	0.505194831	0.908360969	0.367472743	1.163664769	0.249473386
SPIN3	1.358382229	0.177846811	-0.180776657	0.857177495	-0.63892692	0.525464339
SPIN4	1.22153696	0.225175385	-1.292665828	0.201288324	-2.338150929	0.022960939
SPINK2	0.10202989	0.918967236	-1.516409869	0.13488534	-1.547652342	0.127315597
SPINT1	-1.206131896	0.231029367	1.041844897	0.301837082	1.260087628	0.212841673
SPINT2	-0.758245308	0.450348866	0.455476578	0.650478906	0.765931539	0.446920028
SPIRE1	1.801792959	0.075034306	0.328077054	0.744043094	-0.619409841	0.538147861
SPIRE2	1.448408572	0.151089589	-1.599386312	0.115206537	0.708909764	0.481309054
SPN	-0.309524196	0.75766133	0.515839397	0.607941173	1.027004239	0.308815697
SPNS1	-0.212004795	0.832598111	-0.25996613	0.795816966	-0.033921322	0.973060004
SPNS2	-1.553651065	0.123890195	-1.230537763	0.223497702	-0.922640486	0.36013622
SPNS3	1.653200158	0.101886617	0.669953784	0.505564452	0.934249089	0.354169674
SPOCD1	-3.65006243	0.000446192	0.556884873	0.579763667	-1.20257101	0.234181275
SPOCK1	-1.166524717	0.246583169	0.362320915	0.718438308	-0.081843815	0.935061514
SPOCK2	0.696360512	0.488055376	1.696682997	0.095155236	2.380159219	0.020719369
SPON1	2.893577966	0.00481027	-0.901659644	0.370992795	-0.436684475	0.664012301
SPON2	1.169794454	0.245271537	-2.849203089	0.006069337	-1.607619639	0.1135191
SPOP	-2.192234255	0.031026709	2.430561584	0.018211435	2.046984827	0.045343024
SPOPL	-0.448920803	0.65460261	-0.992732665	0.32499264	-2.17233238	0.034061407
SPP1	-2.074839124	0.040949066	-0.508373072	0.613133435	-0.239957806	0.811235483
SPPL2A	0.855574178	0.394578451	1.028325581	0.308095918	0.292958588	0.770632093
SPPL2B	0.699392153	0.486168689	0.033019195	0.973773442	1.215874304	0.229112101
SPPL3	-3.214500968	0.001832828	1.867086054	0.066981695	1.678587396	0.098781536
SPR	-1.43125399	0.155932155	0.692457681	0.491432438	-0.516774382	0.607340096
SPRED1	3.371346287	0.001116682	-1.755920653	0.084418277	-1.443219407	0.154506133
SPRED2	-0.530368962	0.597204365	-1.602078682	0.114609015	-2.385993758	0.02042404
SPRN	0.013683981	0.989113367	1.111702001	0.270885258	1.273776249	0.207982133
SPRY1	0.536370193	0.593068625	-1.313477527	0.194230438	-0.648220544	0.519480357
SPRY2	-0.213460311	0.831466298	-2.404044514	0.019453377	-1.114849627	0.269656587
SPRY3	-1.563125506	0.121645601	-0.996151628	0.323343205	-2.601308273	0.011846874
SPRYD3	-1.129422158	0.261817548	0.227391048	0.820924912	0.713296629	0.478611897
SPRYD4	0.562240666	0.575394722	-1.457981536	0.150277073	-0.446132801	0.657214384
SPRYD7	0.864647795	0.389604811	-1.287592605	0.203037566	-1.070197739	0.289102155
SPSB1	-0.955980099	0.341725453	-1.264097199	0.211287063	-1.533703717	0.13070958
SPSB2	-1.123549303	0.264288357	1.054839366	0.295903615	-0.829248937	0.410469604
SPSB3	-0.360667869	0.719220093	-0.595928472	0.553557771	-0.236907293	0.813589579
SPTA1	-3.608947766	0.000512324	1.633971561	0.107719724	-0.001060041	0.999157962
SPTAN1	0.034531336	0.972532387	1.996808379	0.050579189	2.386010378	0.020423204
SPTB	-2.680302922	0.008794165	-2.22354712	0.030116065	-0.655868679	0.514583094
SPTBN1	2.081876264	0.04028516	1.505984605	0.137536141	1.60358901	0.114406877
SPTBN2	1.393635306	0.16697001	1.667119171	0.100919932	2.019105838	0.048252729
SPTBN4	1.192872256	0.236155516	0.248300848	0.804784816	1.562118169	0.123870516
SPTBN5	2.301268486	0.023761197	-0.176922849	0.860189671	0.579195801	0.564769997
SPTLC1	-0.003914551	0.996885591	0.431790473	0.667505814	-0.337801071	0.736770842
SPTLC2	-2.112986918	0.037460206	2.079906228	0.041994664	1.518511516	0.134487729
SPTLC3	0.183938178	0.854489071	0.201383023	0.841108618	-0.325998207	0.74563572
SPTSSA	-0.764387466	0.446700199	-1.515562507	0.135099272	-1.945365812	0.056736245
SPTSSB	0.678225989	0.499424504	-2.547055012	0.013558194	-3.330431369	0.00153583
SPTY2D1	-0.397704847	0.691819812	0.349537951	0.727960298	-0.954722726	0.343803946
SQLE	6.678372741	2.16E-09	-0.742435377	0.460840807	-0.025549757	0.979706909
SQRDL	-2.599923346	0.010950216	1.677693761	0.098825999	1.07259184	0.288035525
SQSTM1	-2.220704209	0.028963163	1.541842406	0.128588608	1.548749501	0.12705165
SRA1	-1.90002374	0.060735179	0.414796087	0.679832161	0.975096219	0.333688396
SRBD1	-1.900044842	0.060732373	1.621947338	0.110276546	0.570025129	0.570930994
SRC	-0.951335278	0.344063999	1.030211747	0.307217456	0.880836791	0.382155182
SRCAP	-0.864289582	0.389800425	-0.235543541	0.814622398	0.004660476	0.996297985
SRCRB4D	-0.850166874	0.397560923	0.473335959	0.637762629	0.641491931	0.523809168
SRD5A1	0.411453961	0.681749358	-0.494002634	0.623183084	0.178752325	0.858774604
SRD5A3	0.134846955	0.893043691	0.053597738	0.957441133	0.569943143	0.570986222
SREBF1	-0.651442498	0.516474302	-0.211120234	0.833538656	0.273290347	0.785631194
SREBF2	2.653728854	0.009460057	0.072003166	0.942848839	-0.084814102	0.932710402
SREK1	1.598048291	0.113650213	-0.118678708	0.905942854	-1.109842631	0.271790098
SREK1IP1	1.42488709	0.157759648	-1.055783429	0.295475685	-1.156075651	0.252538024
SRF	-0.419687408	0.675746172	-0.676505795	0.501427381	-0.941187941	0.350634063
SRFBP1	0.172989574	0.863060582	0.502767243	0.617045019	0.006407451	0.994910304

SRGAP2	-2.542564363	0.012769607	0.445357535	0.657730828	0.134397945	0.893567754
SRGAP2P2	-0.319756326	0.749918002	0.78122257	0.43787099	-0.381026035	0.704619599
SRGAP3	0.383522287	0.702266137	0.49532185	0.622257472	0.229017412	0.819686215
SRGN	-2.434497161	0.016951228	0.058626572	0.953452478	-0.964276505	0.339035653
SRI	-2.036099446	0.044775169	-1.423330811	0.160034767	-2.245226734	0.028703037
SRM	0.181254414	0.856588578	0.840258026	0.40423832	0.766010895	0.44687319
SRP14	0.589388047	0.557125146	-1.034799709	0.305087772	-0.704898253	0.483782852
SRP19	0.182295748	0.85577382	-1.933868024	0.05804823	-1.987932935	0.051695586
SRP54	0.935261854	0.352236538	0.30256522	0.763311233	1.042749994	0.301526069
SRP68	-0.442287529	0.65937636	1.420717558	0.160790157	1.982155474	0.052356343
SRP72	1.550932588	0.124540266	-0.124564629	0.901301961	0.483460156	0.630646149
SRP9	0.457805168	0.648231291	-0.221144766	0.825761839	-0.269418448	0.788593776
SRPK1	-0.921620084	0.359269958	-0.358172816	0.721523385	-0.508952095	0.612776934
SRPK2	-1.875463515	0.064075825	-0.592398104	0.55590266	-1.765217537	0.082956395
SRPK3	0.171432576	0.864280892	-0.276064013	0.783487042	-0.954478421	0.343926452
SRPR	-0.836411586	0.405209922	0.734138101	0.465842781	0.915009543	0.36409344
SRPRB	0.158439233	0.874477099	3.599038844	0.000665067	4.288070818	7.15E-05
SRR	0.092879528	0.926212358	0.64458604	0.521754551	1.348413299	0.182931255
SRRD	-3.591830164	0.000542512	0.1377708	0.890902076	1.082596907	0.283607538
SRRM1	0.594414572	0.553774203	-0.306650379	0.760215366	-0.010461591	0.991690035
SRRM2	0.521400667	0.603409595	-0.353936864	0.724678607	0.328222361	0.743962517
SRRM5	1.51613014	0.133103764	0.088455622	0.929821401	1.047788426	0.299218527
SRRT	-0.052856487	0.957967172	0.98662064	0.327955286	2.012557182	0.048959116
SRSF1	2.856206373	0.005358611	-0.322535551	0.748214911	-0.604098283	0.548207662
SRSF10	0.818373626	0.415374945	-0.663913293	0.509394819	-1.521935618	0.133628707
SRSF11	2.314646998	0.022982222	-0.773083384	0.442634242	-1.0656603	0.291131183
SRSF12	0.121831055	0.903313177	0.989161454	0.326721515	0.972955507	0.33474191
SRSF2	2.611815163	0.0106037	-0.058792012	0.953321278	-0.286466608	0.775573491
SRSF3	-1.067587385	0.288653759	1.40541075	0.165270396	1.03645327	0.304426955
SRSF4	-1.340510462	0.183561928	0.702790461	0.485017284	0.6328022	0.52942763
SRSF5	0.098991203	0.921372495	-0.558571517	0.578619354	0.524600712	0.601922682
SRSF6	1.631422215	0.106408303	0.509075498	0.612644095	0.760261552	0.450273975
SRSF7	0.140429657	0.888644445	-0.35769584	0.721878428	-0.545420056	0.587621536
SRSF8	1.002174513	0.319032583	-1.133972197	0.261503981	-1.177641687	0.243899252
SRSF9	-1.860231032	0.066224277	-0.872559582	0.386526295	-1.519882638	0.134143224
SRXN1	-3.660329251	0.000430989	1.070495183	0.288862002	0.440073579	0.661570591
SS18	1.507146362	0.135387975	0.97188991	0.335169298	1.439962236	0.15542201
SS18L1	1.606550648	0.11176878	-1.483983311	0.143265522	-3.304771012	0.001658225
SS18L2	0.179700712	0.857804511	-0.8945805	0.374734553	-0.513947243	0.609302532
SSB	0.773590123	0.441265638	0.76023642	0.45021416	1.487205668	0.142546228
SSBP1	0.771493501	0.442500383	0.786287911	0.434921963	2.138861561	0.036803168
SSBP2	-0.58029198	0.563214484	-2.068980288	0.043045857	-2.189943876	0.032692039
SSBP3	-1.810037356	0.073735282	-0.95432334	0.343908205	-0.510123986	0.611961012
SSBP4	0.050255797	0.9600335	-3.401827227	0.001222781	-3.033487074	0.003654622
SSC5D	0.658081502	0.512219626	0.760041707	0.450329621	1.04269786	0.301550009
SSFA2	-0.725103065	0.470330412	-0.310567182	0.757250781	-1.497524052	0.139849107
SSH1	-1.075182984	0.285259168	0.158985162	0.874236735	0.374392477	0.70952038
SSH2	-1.754737543	0.08281723	0.216616031	0.829273011	-0.366176266	0.715607534
SSH3	0.013236369	0.989469455	-1.970745414	0.053565498	-1.4318665	0.157716761
SSNA1	-1.172529101	0.244178382	-0.744384488	0.459670278	-0.861486532	0.392628594
SSPN	-0.007046264	0.994394039	-2.002097837	0.049990909	-2.519061673	0.014636071
SSPO	1.995870234	0.04906906	0.274829744	0.784430483	0.952927251	0.344704953
SSR1	0.36031181	0.719485367	0.339033346	0.735817611	0.180222778	0.857625544
SSR2	0.2694356	0.788231111	0.523733914	0.602473125	0.950285069	0.346033666
SSR3	-0.713825472	0.477241644	0.031958587	0.974615567	1.614325866	0.112054358
SSR4	-0.97379526	0.332852181	-2.119036209	0.038410747	-1.123668614	0.265927514
SSRP1	0.651069479	0.516713907	2.101219623	0.040008103	2.812547598	0.006756791
SSSCA1	0.84574111	0.400012272	-1.159729632	0.250943965	-0.340612466	0.734664505
SSTR3	2.045963346	0.043772943	0.123199042	0.902378388	-0.466459456	0.642688615
SSU72	-2.561090325	0.01215421	-0.173773264	0.862652972	-0.366637565	0.715265274
SSX2IP	0.255730196	0.798761526	-0.923611341	0.359541967	-0.955044051	0.343642861
ST13	-0.272794814	0.785656006	3.433075101	0.001111665	3.883600771	0.000273221
ST14	-0.793223146	0.429801162	4.209545099	9.08E-05	2.76030624	0.007782244
ST20	-1.461946034	0.147351051	-1.242873415	0.218950472	-0.023551028	0.981294104
ST3GAL1	-0.501983212	0.616944675	1.341484739	0.185028678	1.364433334	0.177866517
ST3GAL2	-1.440991677	0.153168807	-0.895932335	0.374018183	-1.607027433	0.113649188
ST3GAL3	0.178310883	0.858892488	0.839742533	0.404524967	-0.158375359	0.874728382
ST3GAL4	-1.270735878	0.207202767	0.318261325	0.751437865	-0.253721206	0.800636278
ST3GAL5	1.26560714	0.209025359	0.019118159	0.984812921	1.322018348	0.191514328
ST3GAL6	1.169784564	0.245275496	-0.506602492	0.61436768	-0.516573776	0.607479249
ST5	1.284093269	0.202511082	0.610054703	0.544225092	0.061862016	0.950892057
ST6GAL1	0.077648526	0.938285558	1.639725667	0.106513327	1.833461987	0.072028055

ST6GALNAC1	1.725686992	0.087946226	2.570037276	0.012778769	1.8309351	0.072409983
ST6GALNAC2	-3.00202709	0.003498079	-1.064940037	0.291347196	-1.580830659	0.119525348
ST6GALNAC3	-0.694218591	0.489390779	-0.444772793	0.65815091	-1.868976684	0.066837045
ST6GALNAC4	-2.36621838	0.020186535	0.220787968	0.826038339	-0.648487068	0.519309281
ST6GALNAC6	0.94413763	0.347708306	-0.699427419	0.487100121	0.295259847	0.768882761
ST7	-2.020553955	0.046394627	-0.363094856	0.717863222	-0.265807707	0.791359366
ST7L	-0.44798168	0.655277599	0.576466144	0.566546234	0.398936877	0.69145057
ST8SIA1	1.298703886	0.197469893	-0.104344434	0.917258525	0.291145055	0.77201152
ST8SIA4	-0.202488912	0.840006281	-0.903167074	0.37019911	-2.303215547	0.024987224
ST8SIA6	1.116019935	0.267479951	-2.515494373	0.014698787	-3.637623053	0.000598665
STAB1	0.458234566	0.647924011	1.459354356	0.149900288	1.449942641	0.152628962
STAC3	-1.789737341	0.07696802	2.018821669	0.048169501	1.783829978	0.079847038
STAG1	1.076242249	0.284787957	0.994682196	0.324051427	0.603587885	0.548544633
STAG2	0.312728643	0.75523361	-0.087054374	0.930930242	-1.320026044	0.192174332
STAG3	0.175327919	0.86122851	0.0416714	0.966904775	0.360805646	0.71959656
STAG3L1	1.00880106	0.315861521	0.523881209	0.602371319	0.668106237	0.506798675
STAG3L2	0.695504092	0.488589079	0.377588731	0.707124	0.909486011	0.366975158
STAG3L3	1.111413125	0.269445953	-0.67933254	0.499648215	1.707328459	0.093277027
STAG3L4	1.952406277	0.054098509	-2.014453445	0.048639642	-1.764524207	0.083074141
STAM	-1.85465499	0.067025696	-0.933533192	0.354441921	-1.630819676	0.108516918
STAM2	0.341286388	0.733709107	-0.986832344	0.327852369	-2.247258509	0.028565004
STAMBP	-1.892115134	0.061794499	-2.313955827	0.024259102	-1.670825215	0.100313
STAMBPL1	2.621390321	0.010331932	-0.644483686	0.521820425	-1.069527844	0.289401098
STAP1	1.960621672	0.053115635	-0.084661399	0.932824176	0.278465427	0.781676464
STAP2	0.410090518	0.682745468	1.442735588	0.154511461	2.351067719	0.022249688
STAR	-1.32046912	0.19013424	0.717056402	0.47623722	1.555347392	0.125473585
STARD10	-1.221341296	0.225249056	-1.260873135	0.212438206	-0.660232431	0.51179997
STARD13	-0.721450592	0.472562576	1.731642657	0.088690571	1.970927081	0.053661268
STARD3	-1.763841725	0.081261531	-0.476307366	0.635657361	0.470066277	0.640125503
STARD3NL	-0.512602634	0.609525519	-1.483903816	0.143286559	-1.34763874	0.183178887
STARD4	2.138359078	0.035284895	-0.624354531	0.53486017	-2.383272893	0.020561291
STARD5	-1.979812549	0.050878657	0.14336007	0.886506201	0.447641218	0.656131775
STARD7	2.114464575	0.037330405	2.114391482	0.03882172	2.829828251	0.006446033
STARD8	-0.356220668	0.722535848	1.299007659	0.199117562	0.524294485	0.602134232
STARD9	3.647420617	0.000450185	-1.254607115	0.214688758	0.151218495	0.880344505
STAT1	-0.653663915	0.515048612	0.723354143	0.472389853	-1.540747092	0.128986882
STAT2	-1.27831324	0.20453152	1.31099939	0.195060945	-0.717036681	0.476319129
STAT3	-2.28775171	0.024571794	0.410779844	0.682758249	-0.257418783	0.797795093
STAT4	2.175429808	0.032304036	-1.492793647	0.140949045	-0.226524298	0.821615031
STAT5A	-1.364952612	0.175779923	2.176103523	0.033662955	1.486198875	0.142811575
STAT5B	-0.194038828	0.84659685	0.374593603	0.709338426	0.102494441	0.918728828
STAT6	-2.116190472	0.037179291	0.552026567	0.583065884	0.822261856	0.414400683
STAU1	-2.515401328	0.013722673	1.74977162	0.085483858	1.022718276	0.310820469
STAU2	-1.041651879	0.300452892	-0.638201266	0.52587212	-2.435341101	0.0180732
STBD1	1.259483671	0.211216889	-2.772509938	0.007481052	-2.793582996	0.007113703
STEAP2	-0.258852975	0.796358814	1.622664508	0.11012268	0.725299728	0.4712756
STEAP3	0.529380139	0.597887089	0.379611115	0.7056302	0.271896474	0.78669735
STEAP4	-0.629456126	0.530696429	-0.347657621	0.729364636	-0.792925334	0.431153937
STIL	1.265628145	0.209017871	0.256455741	0.798512777	0.271952128	0.786654774
STIM1	-1.109081856	0.27044468	0.895365298	0.374318563	1.272592522	0.208399072
STIM2	-0.172383598	0.863535482	-0.769466213	0.444760824	-0.921431674	0.360761226
STIP1	-1.196113767	0.234894859	0.887827711	0.378326041	1.017590114	0.313230745
STK10	-1.778582463	0.078793883	0.894476785	0.37478955	1.129447271	0.263503888
STK11	0.212459341	0.832244617	-0.506296572	0.614581046	-0.781062845	0.438041237
STK11IP	-0.294553764	0.769034955	1.933380218	0.058109609	2.842339268	0.006229344
STK16	-2.216645924	0.02924977	-1.376005546	0.174146898	-0.668527214	0.506532023
STK17A	-0.344159416	0.731555068	0.882738864	0.381046881	1.215880689	0.229109688
STK17B	0.804074006	0.423541159	-0.91250526	0.365306563	-1.327388286	0.189743922
STK19	-1.517102336	0.132858404	0.974402286	0.333931558	1.097118755	0.277265127
STK24	-2.337371008	0.021710715	2.117632756	0.038534524	2.073680954	0.042700353
STK25	0.540078149	0.590519983	-1.379833294	0.172971144	-0.685528545	0.495826759
STK3	-0.29974315	0.765086504	0.601954047	0.54956707	0.202434771	0.840307432
STK32B	-0.771369738	0.442573332	-1.085769332	0.282104562	0.310447957	0.757368
STK32C	0.180149281	0.857453424	1.376210641	0.174083745	2.057526676	0.044283021
STK33	2.589177905	0.011272097	2.529116868	0.014196228	1.7893197	0.078948725
STK35	0.822309051	0.413144218	-0.702678034	0.485086833	-0.639778884	0.524914272
STK36	0.722426186	0.471965774	1.269329961	0.209428581	1.653575493	0.103785964
STK38	-0.322990703	0.747475615	1.889605259	0.063847443	2.740968591	0.008196828
STK38L	-0.333743951	0.739374128	-0.721997506	0.473217152	-1.756813003	0.084393108
STK39	1.687093118	0.095160234	0.094730141	0.924857975	1.251315822	0.215999672
STK4	-1.433821927	0.15519972	0.71384137	0.478208097	0.81475939	0.418647089
STK40	-3.620986541	0.000492055	0.532293578	0.59657019	0.754901311	0.453458123

STMN1	1.46850886	0.145564728	2.690580906	0.009318273	4.959118566	6.88E-06
STMN3	1.575172155	0.118838475	-0.107974805	0.914390939	0.949447794	0.346455418
STOM	-1.0812103	0.282585082	-0.784243737	0.436110656	-1.636955526	0.107224332
STOML1	0.268142384	0.789223094	0.437224761	0.663583368	0.914783849	0.364210903
STOML2	-1.554443382	0.123701236	2.624302315	0.011097354	2.796358431	0.007060406
STON1	-1.980600418	0.050788561	-1.542563069	0.128413656	-1.074997088	0.286966676
STOX1	0.814218854	0.417737844	-1.308273961	0.19597741	-2.400766942	0.01969305
STRA13	0.120668046	0.904231613	0.687622028	0.494450662	0.799565285	0.427327124
STRADA	0.402084484	0.688605846	1.077357083	0.285812498	1.900849216	0.062451169
STRADB	-3.465790367	0.000822587	0.208966647	0.835211574	0.126713932	0.899618794
STRAP	0.079214781	0.937043316	3.953489971	0.000213131	3.777151469	0.000384846
STRBP	0.580646327	0.562976657	0.879724505	0.38266436	0.886889914	0.378915354
STRC	-0.245366814	0.806749043	0.596033423	0.553488138	0.952238735	0.345050875
STRN	-0.14577438	0.884435994	-1.177882631	0.243687378	-1.83968753	0.071094317
STRN3	0.583369989	0.561150268	-0.747197474	0.45798397	-1.874924245	0.065999394
STRN4	-1.417431048	0.15992069	-1.136098235	0.260620589	-0.776073574	0.440957286
STS	-3.028767578	0.003229985	0.084088356	0.933277772	-0.382645585	0.703424998
STT3A	0.266369002	0.790583961	0.35955726	0.720493209	0.330717289	0.742087091
STT3B	1.947115246	0.054739659	0.867713254	0.389152284	0.220780353	0.826063076
STUB1	-0.403806267	0.687343889	0.690455019	0.492681188	0.665486969	0.508459452
STX10	-1.575740443	0.118707338	-1.5757338	0.120564468	-1.536192441	0.130098796
STX11	-3.569104263	0.000585201	-1.385781321	0.17115621	-2.68210739	0.009586793
STX12	-0.008865202	0.992946938	0.799403344	0.427341086	0.250430718	0.80316694
STX16	0.460825637	0.646071121	1.006606628	0.31833412	1.269314486	0.209556927
STX16-NPEPL1	0.77608544	0.439798714	-0.904986846	0.369242415	-0.666066835	0.508091529
STX17	1.014062122	0.31335894	-1.109346042	0.271891377	-0.586207909	0.560081413
STX18	1.445044888	0.152029822	0.663355759	0.509749144	2.532145651	0.014155897
STX1A	-0.849816367	0.397754726	-1.510975747	0.136261959	0.473577782	0.637634354
STX1B	3.187402677	0.001993528	1.508073236	0.137001795	2.133933547	0.037222736
STX2	1.846851701	0.068160849	-0.544040575	0.588513482	0.080539651	0.936094002
STX3	-1.069563164	0.287768098	-0.588770549	0.558317274	-1.356896958	0.180235635
STX4	-1.010473462	0.31506455	0.66533802	0.508489975	1.197987357	0.235946626
STX5	-1.994505979	0.049220638	0.032663941	0.974055511	-0.022196866	0.98236949
STX6	-1.287531325	0.20131633	1.120485783	0.26715721	0.180663845	0.85728094
STX7	-0.077280497	0.938577474	-0.705554441	0.483309178	-1.957938598	0.055205434
STX8	0.821644958	0.413520141	1.001944681	0.320561223	1.837647102	0.071399222
STXBP1	2.789947116	0.006473937	1.06625452	0.290757811	1.656205284	0.103250242
STXBP2	-2.427523012	0.01725903	0.309595564	0.757985848	0.289897071	0.772961203
STXBP3	0.505555763	0.614444252	0.502938482	0.616925368	-0.532496159	0.596480205
STXBP4	1.390110175	0.168034208	-0.860441313	0.393113438	-0.842855879	0.402879636
STXBP5	0.090115235	0.928402338	-0.451493591	0.653329356	-1.438545776	0.155821619
STYK1	1.83883214	0.069344174	-0.836260517	0.406464447	-1.380749022	0.172819201
STYX	0.790870632	0.431165535	-0.776402362	0.440688232	-1.385335678	0.17142029
STYXL1	-1.5021421	0.136673652	1.766525541	0.082606453	1.159929093	0.250978576
SUB1	0.641441098	0.522918814	0.098676282	0.921737924	-0.014709536	0.988315973
SUCLA2	1.669147361	0.098675244	0.355221548	0.723721178	1.047787981	0.299218731
SUCLG1	-0.279748285	0.780333221	0.3569445	0.72243782	1.198913	0.235589347
SUCLG2	0.360817532	0.7191086	0.623204191	0.535610429	0.770607992	0.444164797
SUDS3	-0.00324124	0.997421274	-1.556769796	0.125003372	-0.662613418	0.510284826
SUFU	-0.589756881	0.556878921	2.019205475	0.04812838	1.693399178	0.095912389
SUGP1	-1.026288555	0.307594481	0.544095194	0.588476142	1.367650936	0.176862319
SUGP2	3.188218113	0.001988506	0.355330227	0.723640204	0.954687631	0.343821542
SUGT1	-0.010525004	0.991626462	1.660844968	0.102179375	2.267256586	0.027237408
SUGT1P3	-1.528872036	0.129916189	0.02284219	0.981855107	-1.157207248	0.252079361
SULF2	-2.892660935	0.004823083	0.637421059	0.526376446	-0.450590215	0.654017385
SULT1A1	0.118484455	0.905956362	0.678973469	0.499874024	1.271008873	0.208957846
SULT1A2	-0.864151713	0.389875728	0.924992149	0.358829381	1.055655135	0.295639919
SULT1B1	-0.922281018	0.358927138	0.529927279	0.598199339	-1.515315423	0.135293499
SUMF1	-0.062792952	0.950075109	-0.311402113	0.756619303	-1.436824955	0.15630817
SUMF2	-0.184054857	0.854397816	2.925971183	0.00490644	3.362627027	0.001394326
SUMO1	-1.005470637	0.317452618	0.134492319	0.893482162	0.844619802	0.401902079
SUMO1P1	-0.325207057	0.745803459	-1.543870248	0.128096803	-0.645215661	0.521411184
SUMO2	-0.167314643	0.867509937	-1.422421451	0.160297315	-0.871870699	0.386986075
SUMO3	0.569312345	0.570607994	-1.80988017	0.075532629	-0.380608902	0.704927403
SUN1	3.601601033	0.00052508	0.946885649	0.347652856	1.165552991	0.248715047
SUN2	0.058010316	0.953873156	0.676704259	0.501302355	1.379752029	0.173124436
SUOX	0.151973016	0.879559329	2.118955339	0.038417869	2.240952835	0.028995321
SUPT16H	0.919511817	0.360364895	2.553803612	0.013324965	2.140435121	0.03667007
SUPT3H	1.612552212	0.110455842	-1.1679195	0.247651153	-0.177971511	0.859384883
SUPT4H1	-3.173009361	0.002084143	-3.67849172	0.000517749	-3.491341675	0.000942731
SUPT5H	-0.530168615	0.597342663	1.523312879	0.133152542	1.935777892	0.057927875
SUPT6H	-0.442769719	0.659028865	1.167428099	0.247847846	1.532087588	0.131107434

SUPT7L	1.629302277	0.106856971	0.315389453	0.753605889	1.215255476	0.229346111
SUPV3L1	2.298961154	0.023897875	0.754933351	0.453364936	2.249600453	0.028406627
SURF1	-0.245814416	0.806403626	1.191787492	0.238232172	1.298564444	0.19939326
SURF2	-0.238275618	0.812226436	1.203992643	0.233517183	1.325252078	0.190446716
SURF4	-0.562384153	0.575297406	0.924550625	0.359057137	1.43597475	0.156548997
SURF6	0.795249168	0.42862819	2.45639685	0.017070416	2.545244303	0.013689525
SUSD1	-2.225324133	0.028639889	1.262595313	0.21182273	0.111374169	0.9117162
SUSD2	1.604982447	0.112113905	-1.302527105	0.197920481	-0.789567504	0.433096905
SUSD3	0.606701853	0.545625369	-0.233878256	0.815908808	0.779699867	0.438836715
SUSD4	0.032939133	0.973798416	1.04457598	0.300583331	1.089010745	0.28079396
SUV39H1	-1.042827346	0.299911147	2.355983376	0.021899157	1.861242754	0.067939714
SUV39H2	1.046873605	0.298051391	0.253351058	0.800899082	-0.114399215	0.9093288
SUV420H1	0.390291553	0.69727287	1.651467489	0.104085651	1.329367816	0.189094427
SUV420H2	-0.940620612	0.34949809	-0.895848576	0.374062543	-1.193605926	0.237643111
SUZ12	0.401271731	0.68920185	-0.798558818	0.427826841	-1.242675853	0.219143944
SUZ12P	1.959386925	0.053262381	-0.36987305	0.712833646	1.617081878	0.111456857
SV2A	1.143304033	0.256041897	1.543297961	0.128235445	2.114733766	0.038897521
SV2B	0.201743898	0.840586897	1.160095713	0.250796113	0.149563263	0.881644288
SVIL	-0.852633666	0.396198619	0.509570908	0.612299078	-0.154922033	0.87743748
SVIP	0.376603108	0.70738353	-1.385468482	0.171251301	-1.533119663	0.13085325
SWAP70	0.600426069	0.549779856	2.343816511	0.022560342	1.755569528	0.08460742
SWI5	-1.809914547	0.073754494	0.526952554	0.600250312	1.479528897	0.144579332
SWT1	0.098767613	0.921549506	-0.4442832	0.658502722	-0.477393288	0.634932296
SYAP1	-3.116234717	0.002480367	2.741592011	0.008131223	3.142545399	0.002672129
SYBU	-0.219672224	0.826639924	-1.318566233	0.192533396	-0.392447869	0.696210839
SYCE1	0.279205259	0.780748527	-0.781643737	0.437625338	0.817525356	0.417078497
SYCE1L	1.411569546	0.161635505	-0.97947741	0.331440502	0.336239978	0.737941314
SYCE2	1.339414527	0.183916844	0.146069702	0.884376397	0.582225154	0.562742069
SYCP2	1.383923518	0.169914404	-0.343090186	0.73277973	-1.092817258	0.279133377
SYCP2L	0.633586831	0.528009089	0.94535802	0.348425253	-0.85819138	0.394429757
SYCP3	0.722262555	0.472065842	0.014538432	0.988450661	-0.628571695	0.532174268
SYDE2	0.756197776	0.451568982	2.035537402	0.046406433	1.173887363	0.245387616
SYF2	-2.008045195	0.047733909	0.158756084	0.8744164	0.002129982	0.998308059
SYK	-1.832708579	0.070259248	1.094854827	0.278137625	1.019219906	0.312463365
SYMPK	-0.158305228	0.87458237	1.430600089	0.157947982	2.118071683	0.038601719
SYN1	0.369200242	0.712873581	0.116165789	0.907925238	-0.149099561	0.882008472
SYN2	-0.624527443	0.533912131	0.472763926	0.638168264	-0.506930458	0.614185645
SYNCRIP	1.875211158	0.064110935	0.445611879	0.65754814	0.195082891	0.846031161
SYNE1	2.663939353	0.009198971	0.159911899	0.873509966	0.311359352	0.756678758
SYNE2	0.428883725	0.669065665	0.013367426	0.989380851	-0.229556271	0.819269468
SYNGAP1	0.979501267	0.330042448	-1.657560613	0.102843757	-0.717524763	0.476020376
SYNGR1	2.689644085	0.008570407	0.103551989	0.917884615	1.059524797	0.293890429
SYNGR2	-1.291352261	0.199994668	-0.50363064	0.616441835	-0.639273959	0.525240238
SYNGR3	1.289720966	0.200558142	-1.216793271	0.228645484	-0.892311182	0.376028493
SYNJ1	-1.250888931	0.214321306	-1.352778222	0.181413259	-2.890741039	0.005452921
SYNJ2	-0.070744896	0.943762806	0.784261942	0.436100062	0.764946474	0.447501671
SYNJ2BP	1.424204966	0.157956413	-0.855221143	0.395972357	-0.118231951	0.906305172
SYNM	-2.125808018	0.036346875	0.189833154	0.850107317	0.520148255	0.605001947
SYNPO	0.262097417	0.793864572	1.430710119	0.157916559	1.897668352	0.062877612
SYNPO2	-0.879509183	0.381542891	0.885444096	0.379598951	1.721420479	0.090671879
SYNRG	0.148890342	0.881983998	0.906530701	0.368432015	1.651946912	0.104118863
SYP	-0.120915069	0.904036527	0.150312529	0.881043211	0.459442603	0.647687508
SYPL1	0.694985521	0.488912398	0.841604185	0.403490361	0.837222854	0.406011214
SYS1	-1.041811824	0.300379138	0.251049017	0.802669699	1.500924439	0.138969166
SYT11	0.112023028	0.911062649	0.193287899	0.847413509	0.239147194	0.811860866
SYT15	0.027927824	0.977783542	-0.302971126	0.763003449	0.965202005	0.338576061
SYT17	1.571576176	0.119670954	-1.137100863	0.260204722	-0.553086855	0.582396042
SYT2	0.046239347	0.963225227	-1.098382543	0.276607894	-0.758369723	0.451396292
SYTL1	-0.519800178	0.60452008	-0.069943413	0.944480979	1.094484993	0.278407996
SYTL2	1.751594271	0.083360014	-0.639908178	0.524769658	-0.32008151	0.750092758
SYTL3	-1.582384201	0.117182789	-0.19934903	0.842691828	0.522471242	0.603394492
SYTL4	-2.202674739	0.030255517	1.458940224	0.150013873	1.074232047	0.287306348
SYVN1	-0.071496318	0.943166502	-1.687563465	0.096903883	-1.210622055	0.231103791
SZT2	1.085076604	0.280878881	-0.477223441	0.635008921	0.176574916	0.860476666
TAB1	1.547811442	0.125289961	1.401235306	0.16650912	1.936885343	0.057789154
TAB2	-1.776177491	0.079192197	-0.016844978	0.986618505	-1.581167511	0.119448266
TAB3	-0.234397121	0.815226249	-1.425182329	0.159501233	-2.830626325	0.006432005
TACC1	-1.046027611	0.29843958	-0.406558887	0.685838766	-0.757175433	0.452105634
TACC3	-1.513121309	0.133865384	2.447164592	0.017470554	1.999028144	0.050446725
TACO1	-0.389186909	0.698086794	1.742643027	0.086733129	2.141451626	0.036584314
TACR2	-0.163638285	0.870394626	-1.479251539	0.144521948	-0.941649481	0.350399709
TACSTD2	-1.171968924	0.244402025	-0.604965593	0.547578011	-3.37208561	0.001355159

TADA1	0.364642537	0.71626119	-1.383834635	0.171748589	-0.40469178	0.687239279
TADA2A	1.386885274	0.1690123	-1.269669432	0.209308435	0.172625518	0.863565571
TADA2B	-1.307453331	0.194496081	0.633769106	0.528740434	0.415061648	0.67967605
TADA3	-1.081912526	0.282274659	1.458905109	0.150023507	1.46096678	0.149589576
TAF1	0.631646214	0.52927073	0.511872804	0.610697134	-0.102104866	0.919036642
TAF10	-2.002531926	0.048334617	-3.314295124	0.001592749	-2.802737581	0.006939299
TAF11	1.962723089	0.052866676	0.802924054	0.4253196	1.378123757	0.173623831
TAF12	-2.163548885	0.033234556	-3.088414092	0.00309461	-3.138992168	0.002699795
TAF13	-0.02101585	0.983281033	0.567074395	0.57286718	0.309564881	0.758036014
TAF15	-1.252788513	0.213632312	-0.796603023	0.428953041	-0.247017297	0.805794389
TAF1A	1.115139099	0.267855079	-0.431165851	0.667957265	0.049346635	0.9608179
TAF1B	2.051302627	0.043238525	3.026149584	0.003698866	3.049557976	0.003491207
TAF1C	1.16345885	0.247817554	-0.26849774	0.789275576	0.203027301	0.839846496
TAF1D	0.626147247	0.532854192	0.381708909	0.704081931	-0.047834405	0.962017691
TAF2	1.063232622	0.290612413	-1.905869972	0.06166178	-2.521644846	0.014540118
TAF3	0.032857422	0.973863389	1.728308365	0.089291052	1.691881416	0.096203205
TAF4	-0.885951179	0.37808086	-2.100370799	0.040085627	-2.882796132	0.005573887
TAF4B	1.309186121	0.193911117	-1.057549176	0.29467644	-1.520361218	0.134023142
TAF5	2.200932419	0.030383038	0.272504984	0.786208347	0.245971356	0.806599945
TAF5L	1.016844386	0.312040855	-2.168533119	0.034261621	-2.679673291	0.00964867
TAF6	-0.986453251	0.326640348	-0.545070302	0.587809706	0.349351268	0.728130414
TAF6L	1.11015788	0.269983386	-1.060281574	0.293442584	-1.317170298	0.193123367
TAF7	0.630604818	0.529948409	-0.056130136	0.955432401	-0.500355294	0.618777428
TAF8	0.407949737	0.684310623	0.831422884	0.40916844	1.235276187	0.221863564
TAF9	0.823535052	0.412450758	-0.837451879	0.405800219	-0.430469565	0.668499403
TAF9B	1.217912417	0.226542935	-1.120108707	0.267316503	-1.099070521	0.276420316
TAGAP	-0.017990462	0.985687567	-1.049959579	0.298122338	-2.569247087	0.012870926
TAGLN	0.894465843	0.373535255	2.638514272	0.010691645	1.844071241	0.070442956
TAGLN2	-3.678322927	0.000405526	-1.685713582	0.097261792	-2.295913908	0.025430291
TAL1	-4.095839176	9.38E-05	1.760764429	0.083586674	0.737924872	0.463628307
TALDO1	-2.596957905	0.011038206	1.276352503	0.206953567	1.226569324	0.225095316
TAMM41	-0.338638774	0.735696032	-0.696762128	0.488754334	0.243725795	0.808330127
TANC1	0.657231803	0.512763126	0.788721015	0.43350961	0.038945025	0.969072146
TANC2	-0.61160837	0.54238839	-0.508208708	0.613247964	1.226524624	0.225111995
TANK	-1.261972604	0.210324094	-1.654731037	0.103418966	-2.685037777	0.009512783
TAOK1	0.081987805	0.934844335	-1.624567694	0.109715203	-2.727949678	0.008487271
TAOK2	0.37239816	0.71050009	-1.145036616	0.256929778	-0.646725987	0.52044023
TAOK3	0.057175678	0.954536082	-0.354351713	0.724369386	-0.600374591	0.550668501
TAP1	-1.399006126	0.165358536	0.084466775	0.932978229	-1.700963943	0.094473672
TAP2	-1.623951563	0.107996193	-0.836296789	0.406444214	-1.807377679	0.07605316
TAPBP	-2.275936668	0.025300199	-1.068369226	0.28981135	-0.741848956	0.46126595
TAPBPL	-0.615281214	0.539971694	0.708294134	0.481619392	1.359767059	0.179330574
TAPT1	1.182025085	0.240409472	0.746853413	0.458190034	0.893352843	0.375475403
TARBP1	3.495361893	0.000746684	-0.128520402	0.898184873	0.154373639	0.877867826
TARBP2	0.388339231	0.698711621	0.821645124	0.414667145	1.390755479	0.169778512
TARDBP	2.491139009	0.014627485	2.783598707	0.007259767	3.123207925	0.00282597
TARM1	-0.739117614	0.461820795	1.345708463	0.183670171	-0.597855875	0.552336179
TARP	-0.038593004	0.96930311	-2.604817222	0.011676411	-1.658112431	0.102863146
TARS	-0.506857661	0.613534185	2.70585557	0.008947158	2.520236909	0.014592345
TARS2	0.558478845	0.577948892	2.773457505	0.007461902	3.256253319	0.001915227
TARSL2	3.00335032	0.003484343	2.183478923	0.033088559	3.394889369	0.00126497
TAS1R3	2.383753879	0.019307144	-2.083286959	0.041673949	-1.427871865	0.158858716
TAS2R14	1.229258869	0.222281909	-2.291774743	0.025593678	-2.746467881	0.008076909
TAS2R20	2.552118182	0.012448829	0.907367679	0.367993143	0.939448428	0.351518246
TAS2R4	1.606266592	0.111831231	-0.29318239	0.770436495	-0.202180188	0.840505491
TAS2R40	0.266954871	0.790134302	-1.614627871	0.111856895	-3.204667463	0.002229423
TAS2R5	0.034807168	0.972313069	-1.335931841	0.186826286	-0.315806014	0.753318833
TASP1	1.586482554	0.116250161	0.781143602	0.437917058	0.121591672	0.903655847
TATDN1	0.520393875	0.60410804	-0.677788437	0.500619654	0.004095828	0.996746506
TATDN2	0.187807224	0.851464166	2.544301988	0.013654396	2.864208844	0.005866666
TATDN3	-3.361457378	0.001152637	-1.045394513	0.300208264	-1.187386915	0.240066269
TAX1BP1	-0.051936846	0.958697823	0.484482177	0.629881049	-0.157098128	0.875730183
TAX1BP3	-2.856576059	0.005352916	-4.308143461	6.49E-05	-5.340226268	1.73E-06
TAZ	0.324168009	0.746587231	-0.102605293	0.918632645	1.152144334	0.254136121
TBC1D1	-1.534226438	0.128594823	0.981090298	0.330651426	0.334577351	0.739188599
TBC1D10A	0.375687903	0.708061426	-0.008777973	0.993026615	0.538599812	0.592288679
TBC1D10B	-0.924458052	0.357799411	0.99774008	0.322578785	1.164752201	0.249036454
TBC1D10C	-0.424140729	0.672507848	-0.798334099	0.42795615	0.197808542	0.843908143
TBC1D12	1.142417076	0.256408207	0.041049271	0.967398583	-0.647612646	0.519870664
TBC1D13	-0.117222403	0.906953423	2.843335809	0.006167958	3.449827935	0.001070484
TBC1D14	-0.043835654	0.965135644	-0.458373908	0.648408702	-1.293582787	0.201097649
TBC1D15	1.166400532	0.246633083	-0.285356296	0.776394857	-0.537843555	0.592807265

TBC1D16	1.060130437	0.292013228	0.971401469	0.335410284	0.699960584	0.48683749
TBC1D17	-1.598086457	0.113641711	-0.963025748	0.339560484	-0.05303345	0.957893199
TBC1D19	0.768164359	0.444465109	-1.48822359	0.142146922	0.119142716	0.905586876
TBC1D2	-1.447621225	0.151309267	0.63894333	0.525392686	0.847489489	0.400314839
TBC1D20	-0.554269525	0.580813318	-1.420499803	0.160853226	-0.920546611	0.361219283
TBC1D22A	-1.348796695	0.180895136	1.454767641	0.15116206	1.912542168	0.060904601
TBC1D22B	-2.623597094	0.010270199	1.151148354	0.254427662	1.368269738	0.176669692
TBC1D23	0.09029682	0.928258462	-0.739367948	0.462686394	-1.535643637	0.130233288
TBC1D24	2.538025719	0.012924587	1.245694604	0.217920157	1.343885466	0.184382444
TBC1D25	-1.095193868	0.27644785	-0.503509641	0.616526351	0.207924447	0.836039131
TBC1D2B	0.741488325	0.460389998	0.834984226	0.407176765	0.462791963	0.645299329
TBC1D30	-2.156819276	0.033771905	-0.002534335	0.997986653	-0.153792758	0.878323706
TBC1D4	2.335495875	0.021813228	0.837992662	0.405498932	0.131546917	0.895812171
TBC1D5	-0.423337309	0.673091616	0.041477153	0.967058955	-0.401430036	0.689624927
TBC1D7	0.848754892	0.398341991	2.24061887	0.028922835	1.888143885	0.064169321
TBC1D8	-1.671448798	0.098218653	1.072967104	0.28776087	0.760315913	0.45024175
TBC1D8B	-0.500593859	0.617918305	-0.259156229	0.796438711	-0.621827315	0.536568301
TBC1D9	0.899154056	0.371047176	1.811856602	0.075222625	1.004582766	0.319400834
TBC1D9B	0.749525345	0.455558226	0.544874441	0.587943538	0.845233387	0.401562376
TBCA	-0.657883046	0.512346539	-0.315133078	0.753799528	0.234814389	0.815205686
TBCB	0.259381873	0.795952066	-2.084050021	0.041601856	-1.147926535	0.255858695
TBCC	-0.858502399	0.392969113	-1.578324235	0.119968074	-1.350525393	0.182257301
TBCCD1	0.104192496	0.917255893	-3.559422492	0.000752708	-2.432611933	0.018196569
TBCD	0.685406386	0.494905838	2.507006794	0.015019991	2.527053823	0.014341042
TBCE	0.160261816	0.873045553	-1.154517806	0.253055688	-1.099920829	0.276052829
TBCEL	0.679973172	0.498322943	-0.089192954	0.92923799	-0.289369907	0.773362466
TBCK	-0.503121679	0.616147371	-0.600858633	0.550291472	-1.539729418	0.129234664
TBK1	-0.43838817	0.662189219	-0.695909018	0.489284473	-1.788514321	0.079079983
TBKBP1	-0.39927364	0.690667912	-1.35068133	0.182080445	-2.674005589	0.009794167
TBL1X	-0.98059638	0.329504988	0.809950928	0.421302151	0.589492512	0.557891875
TBL1XR1	0.46813864	0.640853616	-0.495978204	0.621797177	-0.524032167	0.602315476
TBL2	0.37726675	0.706892115	3.025002097	0.003710971	3.26164882	0.001884892
TBL3	-0.506300424	0.613923636	1.893665175	0.063295808	1.95782892	0.055218633
TBP	0.155766924	0.876576819	-0.975170123	0.333553883	-0.604481747	0.547954563
TBPL1	-0.842865605	0.401609906	-1.161660573	0.250164801	-0.999987102	0.321600202
TBRG1	1.848040147	0.067986933	-0.702221398	0.485369374	-0.485587814	0.629146
TBRG4	0.717216562	0.475157562	1.739194125	0.08734295	2.244786756	0.028733006
TBX19	1.19063789	0.237027313	0.510204689	0.611857825	2.03959426	0.046099191
TBX21	-0.798051051	0.427009154	-2.306482598	0.024701668	-1.451658239	0.152152821
TBX6	-0.076971729	0.938822393	-0.892610431	0.375780098	-1.154824491	0.253045844
TBXA2R	-0.233162892	0.81618144	-0.110585462	0.912329518	-0.093980706	0.925458527
TBXAS1	-3.04219558	0.003102662	-0.174367622	0.862188017	-0.53507272	0.594709127
TC2N	1.91675862	0.058544017	-0.681043244	0.498573162	-0.563406483	0.575397825
TCAP	0.966881761	0.336277491	0.22791718	0.820517807	1.351431007	0.181968906
TCEA1	1.121638295	0.265095867	0.684354612	0.4964958	0.758455374	0.451345445
TCEA2	0.82958652	0.409038176	1.377565632	0.173666955	2.584899537	0.01236133
TCEA3	1.182681606	0.240150451	1.356005615	0.180390011	2.389856339	0.020230606
TCEAL1	1.454288283	0.149456898	-1.057570742	0.294666688	-0.738557168	0.463247188
TCEAL3	0.390030517	0.697465175	-0.80567284	0.42374532	-0.139869286	0.88926301
TCEAL4	0.459423234	0.647073711	0.514386975	0.608949646	2.152764097	0.03564173
TCEAL8	1.271927538	0.206780973	-1.179396244	0.243089219	-0.031871328	0.974687511
TCEANC	-0.570766256	0.569626257	-1.209830194	0.231286222	-1.423767034	0.160038857
TCEANC2	0.467590557	0.641244028	-0.806222498	0.423430941	0.611194906	0.543533288
TCEB1	-0.621183972	0.536099253	-2.700634481	0.009072459	-1.431014135	0.157959891
TCEB2	-0.95601845	0.341706188	-0.687904434	0.494274117	-0.133825261	0.894018518
TCEB3	1.201091521	0.232968409	2.439030196	0.017830082	1.614624643	0.111989458
TCERG1	3.486754766	0.000768065	-0.12444454	0.901396614	0.086064656	0.931720713
TCF12	1.306762628	0.194729619	-1.631924326	0.108151613	-2.454260064	0.01723847
TCF19	2.050054076	0.04336299	0.002917434	0.997682309	0.930255792	0.35621486
TCF20	-0.822765833	0.412885768	0.383521239	0.702745359	0.292354647	0.771091386
TCF25	-0.490214804	0.625213253	0.114062479	0.909584945	1.553000294	0.126033151
TCF3	1.586775199	0.116183794	-0.724822952	0.471495073	-0.478631355	0.634056594
TCF4	2.264240557	0.026039929	1.07713084	0.285912686	0.504874947	0.61561946
TCF7	1.174746112	0.243294706	-0.200920279	0.841468749	0.478640144	0.634050379
TCF7L1	-0.75429405	0.452705108	-1.287557401	0.203049744	0.310083661	0.757643554
TCF7L2	0.541895632	0.589272622	-2.024258352	0.04758984	-3.498112118	0.000923323
TCFL5	-0.681291308	0.497492758	-1.755417426	0.084505067	-1.394243198	0.168728423
TCHP	1.581955073	0.117280787	1.679573662	0.098457501	2.273384662	0.026841678
TCIRG1	-1.308701273	0.194074662	-0.975078034	0.333599163	-0.997403555	0.32284107
TCL1A	-0.254089793	0.800024454	0.887614721	0.378439673	1.266320574	0.210618598
TCL1B	-0.347557115	0.729010436	0.580928736	0.563554843	0.599342464	0.551351579
TCL6	1.310667358	0.193412116	0.579730439	0.564357325	0.083291502	0.933915533

TCN1	-4.661106841	1.12E-05	0.222493845	0.824716572	-1.460956261	0.149592454
TCN2	-3.227879542	0.001758032	2.591860566	0.012076541	2.185785581	0.033010924
TCOF1	0.766874612	0.445227625	2.045247354	0.045408104	2.172034384	0.034085005
TCP1	0.843035691	0.401515297	3.089408845	0.003085752	3.794498787	0.000364066
TCP11L1	0.74284375	0.459573092	-0.420770093	0.675488869	-0.275558884	0.783896901
TCP11L2	-1.498043947	0.137733656	-0.611645948	0.543178866	-1.98193193	0.052382054
TCTA	-0.654394984	0.514579872	-2.208519407	0.031202137	-0.6250974	0.534435468
TCTEX1D2	3.605961281	0.000517474	-2.013893185	0.048700227	-1.373632785	0.17500695
TCTEX1D4	1.213558023	0.228193818	-2.103005902	0.039845385	-1.477507529	0.145118446
TCTN1	1.03908149	0.301639834	-0.637315937	0.526444416	1.169363321	0.247189804
TCTN2	2.295654962	0.024094929	0.596734892	0.55302284	1.355983507	0.180524414
TCTN3	2.2567364	0.026524491	1.475632427	0.145488766	3.061400423	0.003375186
TDG	1.142617385	0.256325448	-0.842323357	0.403091121	-1.343755997	0.184424068
TDP1	0.793329965	0.429739272	1.457575908	0.150388544	2.835618785	0.00634489
TDP2	-2.25178432	0.026848583	0.597333827	0.552625711	-0.06377808	0.949373094
TDRD3	2.675866631	0.008902281	0.915451306	0.363771679	2.091930043	0.040971742
TDRD6	1.428792404	0.156636758	0.445109683	0.657908872	-0.818796296	0.416358936
TDRD7	-1.881052393	0.063302367	1.299150221	0.199068967	-1.521124481	0.133831806
TDRD9	-0.934869177	0.352437746	0.994097706	0.324333421	1.131342057	0.262712613
TDRKH	0.708370747	0.480604638	0.094368725	0.925143792	1.37981774	0.173104306
TEAD2	0.3524868	0.725323858	0.125231033	0.900776734	0.38230192	0.703678427
TEAD3	2.674694174	0.008931055	-0.814709856	0.418594369	-0.487923407	0.627501049
TEC	1.93605382	0.05610089	0.12127512	0.903895235	-1.033078893	0.305989329
TECPR1	1.065946312	0.289390804	0.295606119	0.768593997	0.824644879	0.413057382
TECPR2	-0.828456105	0.409674346	0.278455057	0.781660325	-0.757746649	0.451766283
TECR	-0.345913738	0.73024083	-0.537610312	0.592917309	0.26648203	0.790842672
TECTA	-0.151644627	0.879817568	-0.540296065	0.59107606	0.380856778	0.704744488
TEF	2.581962338	0.011493011	1.270489625	0.209018361	1.829560568	0.072618451
TEK	-1.59313906	0.114748049	-0.079966517	0.93654109	0.367642865	0.714519598
TEKT2	-2.069318464	0.041476483	-2.002712739	0.049922905	-0.772696786	0.442937363
TEKT4P2	0.832387932	0.407464191	-0.101189866	0.919751178	-1.462033198	0.149298095
TELO2	1.24130958	0.217820658	0.680642347	0.498824983	0.824058135	0.413387882
TENC1	0.385158859	0.701057731	0.099729184	0.920905644	0.662588491	0.510300676
TEP1	1.72319346	0.088398358	-0.329767673	0.74277187	-0.571529968	0.56991777
TEPP	1.156877816	0.250482066	-0.898672086	0.37256899	0.790865424	0.432345264
TERF1	0.215096826	0.830194165	0.615029617	0.540957564	0.431241926	0.6679411
TERF2	-0.788342288	0.432634728	1.421023623	0.160701543	1.832982477	0.072100401
TERF2IP	-0.637232568	0.525643131	1.078138745	0.285466541	0.837787294	0.405696752
TES	0.774578206	0.440684432	-0.474701795	0.636794548	0.207282454	0.836538036
TESC	-2.240208191	0.027619791	1.020344644	0.311831814	0.76757881	0.445948366
TESK1	0.074640063	0.940672082	-1.012780171	0.315400941	-0.314540012	0.754274947
TESK2	-0.674128229	0.502013204	-2.189137022	0.032653758	-2.292547914	0.025636881
TET1	0.02467127	0.980373549	-2.507036818	0.015018844	-1.492096208	0.141262821
TET2	0.239218153	0.811497856	-0.616470741	0.540012915	-2.641002097	0.010682014
TET3	-0.991433959	0.324217233	-0.055552356	0.955890678	-0.912951046	0.365165689
TEX10	0.593988883	0.5540576	1.952083379	0.055795524	3.545622462	0.000797445
TEX101	-1.370780531	0.173961892	1.311394293	0.194928421	0.000690542	0.999451471
TEX14	0.37529261	0.708354293	-0.381961024	0.703895943	1.352872799	0.181510481
TEX2	3.118898207	0.002460311	1.084845214	0.282510249	1.753162632	0.08502353
TEX21P	1.488765082	0.140157491	-0.55038273	0.584185243	-0.243922381	0.80817862
TEX22	0.725786259	0.469913545	-0.077180661	0.938747315	0.035544538	0.971771401
TEX261	-0.046335176	0.963149068	-0.040414731	0.967902255	0.786345547	0.434966134
TEX264	-0.498363495	0.619482727	1.460386451	0.149617505	2.606565796	0.011686207
TF	1.854796646	0.067005237	-0.14118152	0.888219178	-0.798653835	0.427851215
TFAM	0.70622027	0.48193407	-0.185056923	0.853834497	-0.697035921	0.488651836
TFAP2E	3.316411321	0.001330625	1.315367198	0.193598938	1.455963329	0.150963114
TFAP4	2.411959501	0.017963967	-0.096048921	0.923815135	0.393237288	0.695631068
TFB1M	0.988361731	0.325710461	-0.008806659	0.993003826	2.178314787	0.033590706
TFB2M	1.188469836	0.237875446	-0.187627812	0.851827857	-0.123107475	0.902460913
TFCP2	-1.155703043	0.250959837	0.874006738	0.385744308	0.783638837	0.43654013
TFCP2L1	-2.332773674	0.021962813	-0.970150013	0.336028246	-0.397067048	0.692820984
TFDP1	-3.126948597	0.002400604	-0.177855296	0.859460667	-0.694585657	0.490174761
TFDP2	1.613602357	0.110227385	0.00724827	0.994241815	0.944953459	0.34872504
TFE3	-1.948908989	0.05452158	-0.270234895	0.787945506	-0.514603968	0.608846414
TFEB	-2.290102899	0.024429066	0.358686723	0.721140923	-0.154727029	0.877590503
TFEC	-0.723955572	0.47103105	-0.555270705	0.580859827	-1.837602752	0.071405861
TFF3	1.77562199	0.079284436	1.452976777	0.151656963	1.252119605	0.215708863
TFG	-1.912864166	0.05904788	0.234777345	0.81521421	-0.235764365	0.814472029
TFIP11	-0.900035809	0.370580393	1.175096104	0.244791347	2.273745079	0.026818563
TFPT	-0.35391461	0.724257299	1.512412329	0.135896954	1.788074756	0.079151699
TFR2	-1.453834337	0.149582461	-2.279964349	0.026330541	-2.634728881	0.010858865
TFRC	0.848700472	0.398372113	-0.87610831	0.384610468	-0.810911387	0.42083523

TG	0.087563075	0.930424747	-0.247484627	0.8054133	-2.001389627	0.050184296
TGDS	0.487761882	0.626942802	-0.748792092	0.457029625	-1.519327909	0.134282519
TGFA	-2.499884706	0.014295342	-0.196290489	0.845073747	-1.243017518	0.219018967
TGFB1	-1.818162148	0.072473459	-1.647582607	0.104883826	-1.848069243	0.069853297
TGFB1I1	-1.433403311	0.155318937	-0.007148302	0.994321231	-0.128367892	0.898315807
TGFB3	0.612308032	0.541927597	-2.24044137	0.028935024	-0.09497705	0.924670673
TGFBI	-2.589467129	0.011263323	2.991456316	0.004081625	1.700899317	0.094485888
TGFBR1	0.077195794	0.938644662	-0.252717802	0.801386048	-0.965039623	0.338656668
TGFBR2	0.03985467	0.968300109	-2.071945092	0.042758379	-2.139184146	0.036775848
TGFBR3	1.271020531	0.207101955	-2.402101948	0.019547277	-1.765230611	0.082954176
TGFBRAP1	0.557019198	0.57894141	-0.825705505	0.412378291	-0.427624667	0.670557468
TGIF1	2.33042092	0.022092827	-0.320522931	0.749731957	-0.599130284	0.551492056
TGIF2	-0.511687342	0.610163388	2.455317733	0.017116756	2.597892391	0.011952336
TGM1	0.860250131	0.392010498	0.378143988	0.706713753	-0.464732863	0.643917132
TGM2	-3.266702163	0.00155683	1.870707608	0.066469038	0.586446006	0.559922553
TGM3	-2.656114646	0.009398457	-0.430414267	0.668500641	0.526554708	0.600573616
TGOLN2	-1.38636802	0.169169583	1.234486018	0.222034794	1.23306636	0.222680542
TGS1	1.632664593	0.10614607	1.136892152	0.260291252	1.861399584	0.067917202
TH1L	1.290559536	0.200268341	0.571245592	0.570055594	0.893108036	0.375605342
THADA	1.151575414	0.252643642	3.786285364	0.000366997	4.111903757	0.000129105
THAP1	-0.705521547	0.48236646	-0.384435764	0.702071265	-0.332431391	0.740799518
THAP11	0.177004031	0.859915758	2.681698706	0.009540503	1.860416145	0.068058472
THAP2	1.275456482	0.205535605	-1.88724552	0.064169937	-1.82277133	0.073655552
THAP3	0.298335167	0.766157188	2.233193776	0.029436584	2.155538011	0.035413873
THAP4	-0.889612577	0.376121969	1.059223491	0.293919955	1.272792352	0.208328643
THAP5	-0.457922318	0.648147452	-1.140420864	0.258831027	-1.499206218	0.139413253
THAP6	0.264065516	0.792352592	-1.124212785	0.265586371	-1.45805317	0.15038822
THAP7	0.07036114	0.944067354	0.913037405	0.36502901	1.422264904	0.160472417
THAP8	-1.740828494	0.085241315	1.102399686	0.27487311	0.60359669	0.54853882
THAP9	0.682754088	0.496572352	-0.508253335	0.613216866	-1.023585662	0.310414037
THBD	-1.278944062	0.204310291	-1.213986762	0.229707187	-2.035829161	0.046488593
THBS1	-2.809171279	0.006130229	1.952257421	0.055774366	3.394939668	0.001264778
THBS2	1.887562502	0.062411328	-0.177686192	0.859592867	0.461785425	0.646016623
THBS3	0.832417592	0.407447546	0.713908943	0.478166626	1.611714427	0.112622906
THBS4	2.039574552	0.044419855	0.123337961	0.902268876	0.410631369	0.682903266
THEM4	1.688372793	0.094913533	0.53483033	0.594825994	2.37620884	0.020921491
THEM5	-0.820969744	0.41390257	0.242264784	0.809435577	0.448366787	0.655611289
THEMIS	1.576573871	0.118515227	-1.28048772	0.205506384	-0.957782858	0.342271869
THG1L	-1.15769697	0.250149304	0.149885102	0.881378903	-0.194172819	0.846740275
THNSL1	1.819897771	0.072206251	0.92857115	0.356986631	1.587641956	0.117974484
THNSL2	0.271008764	0.787024861	1.023144735	0.31051761	0.312130796	0.75609551
THOC1	1.76386908	0.081256893	0.919889047	0.361467452	2.35874324	0.021836421
THOC2	1.734667337	0.086333586	1.074723939	0.286980046	0.746121367	0.458701766
THOC3	1.386410679	0.169156608	-0.058050153	0.95390961	-1.425178024	0.159632431
THOC4	-1.340192329	0.183664901	2.26937654	0.027007052	1.566328739	0.122881914
THOC5	-1.914986848	0.058772796	1.138117244	0.259783632	0.749130652	0.456900611
THOC6	-0.353561458	0.724521049	3.050361385	0.003451875	3.721098968	0.000460056
THOC7	-1.65207629	0.102116093	0.388004283	0.699443224	0.837928187	0.405618281
THOP1	0.823637126	0.412393054	0.696133	0.489145256	1.706358122	0.093458658
THRA	2.097807974	0.038816339	-0.0641842	0.949045803	1.404738458	0.16559867
THRAP3	-1.965824583	0.05250105	1.363305271	0.178091886	1.299485623	0.199079285
THSD1	1.320020365	0.190283396	-1.698908657	0.094732431	-1.0863509	0.281958392
THSD1P1	1.135869269	0.259123888	-0.436689202	0.663969515	0.424746323	0.672642314
THSD7A	-2.521812859	0.013492123	2.776972962	0.007391253	-1.668314829	0.100812434
THTPA	2.513343553	0.013797414	-0.000639596	0.999491887	1.29893832	0.199265784
THUMPD1	1.551044918	0.12451335	-0.465314276	0.643461015	-1.086246468	0.282004179
THUMPD2	0.88422925	0.37900432	0.514247974	0.609046199	0.528684116	0.599105045
THUMPD3	1.31819094	0.190892365	0.715465776	0.477211733	1.765734764	0.082868646
THYN1	1.857056376	0.066679566	1.380713591	0.17270161	2.631231533	0.010958617
TIA1	1.235875383	0.219824261	-0.708244461	0.48165	-1.231366211	0.223310595
TIAF1	1.735473119	0.086190084	0.475540791	0.636200198	2.12557183	0.037944218
TIAL1	0.878142387	0.382279966	-0.945697889	0.348253312	-0.31426735	0.754480918
TIAM1	4.088583851	9.63E-05	1.336691612	0.186579551	1.527574835	0.132223475
TIAM2	-1.951267759	0.05423593	0.339349261	0.735580892	1.197726501	0.236047383
TICAM1	0.565915436	0.572904909	-1.200079632	0.235021374	-1.25015488	0.216420214
TICAM2	-0.685213514	0.495026922	-3.360261229	0.001386969	-3.596708386	0.000680396
TIE1	0.53775329	0.592117362	0.087102481	0.930892172	0.081173026	0.935592554
TIFA	0.271266956	0.786826937	-1.066201838	0.290781416	-1.773686076	0.081529382
TIFAB	2.316122904	0.022897684	-0.845921345	0.401097386	-0.965873476	0.338242874
TIGD1	0.311850071	0.755898981	-1.632148376	0.108104279	-1.243300223	0.218915597
TIGD2	-0.639292704	0.524308616	1.934018425	0.058029317	2.387165128	0.020365205
TIGD3	-3.565918227	0.000591434	-0.170162319	0.86547879	-0.419371877	0.676542056

TIGD5	0.58787094	0.5581385	-0.974911416	0.333681101	-2.246004504	0.028650128
TIGD6	-0.073716171	0.941405088	1.985253713	0.051885	1.620769606	0.110661402
TIGD7	-0.033129047	0.973647403	-0.943249548	0.349493172	-0.651467955	0.517397957
TIGIT	0.420554151	0.675115421	-0.018933512	0.984959583	0.248745119	0.804464131
TIMD4	2.843338897	0.005560323	0.928103269	0.357227184	0.357060288	0.722383079
TIMELESS	-0.018378084	0.985379227	2.873199189	0.005680974	2.399356465	0.019761815
TIMM10	-1.330385953	0.186860422	-0.309429929	0.75811118	-0.031379616	0.975077902
TIMM13	0.234547249	0.815110081	0.554509257	0.581377261	0.582313618	0.562682903
TIMM17A	1.130458502	0.261383231	-0.363084843	0.717870662	-0.004156047	0.996698672
TIMM17B	-1.969265726	0.052097902	-2.424245002	0.018500634	-2.2009521	0.031860899
TIMM22	-1.117630964	0.266794802	0.699674392	0.486946994	2.423829148	0.01859874
TIMM23	-0.161332815	0.872204533	1.769774565	0.08205788	1.48991408	0.141834338
TIMM44	0.704008575	0.483303467	-0.818505149	0.416442438	0.568267454	0.57211557
TIMM50	-0.561772186	0.57571251	1.214112973	0.229659364	1.961594562	0.054766986
TIMM8A	-0.102126377	0.918890875	0.360801829	0.719567559	0.269422826	0.788590424
TIMM8B	0.880173014	0.381185226	-1.412920594	0.163060389	-0.992462757	0.325223034
TIMM9	-0.972881312	0.33330368	0.402094974	0.689102435	0.821330951	0.414926151
TIMMDC1	1.466687133	0.146058881	2.467869865	0.016584667	2.670197007	0.009893062
TIMP1	-2.406922419	0.018197552	0.727775143	0.469699539	-0.01448573	0.988493733
TIMP2	-2.392911821	0.018861555	0.824102605	0.413280933	0.522822498	0.603151603
TINF2	-0.909364081	0.365664926	0.362305736	0.718449589	0.457886945	0.648798001
TIPARP	-0.2948013	0.768846473	-0.792998258	0.431033391	-1.975726099	0.053100169
TIPIN	0.839053273	0.403734037	0.181118319	0.85691055	0.77137941	0.443711258
TIPRL	0.368047621	0.713729749	-0.804164786	0.424608577	-1.43133356	0.157868743
TIRAP	-2.040300276	0.044345959	-0.202726054	0.840063596	0.144123521	0.885918171
TJAP1	1.011489014	0.314581253	-0.308286052	0.758976901	0.486322617	0.628628277
TJP2	-2.287680212	0.024576145	1.254381976	0.214769948	0.741468982	0.461494397
TJP3	2.20982753	0.029736892	1.004642611	0.319271099	1.322246285	0.191438927
TK1	-0.271529477	0.786625709	1.600686851	0.114917593	2.62077141	0.01126197
TK2	-0.599086267	0.550668837	1.037547994	0.303816873	1.3825528	0.172268014
TKT	-3.300282537	0.001400402	-0.150965141	0.880530706	-0.487467504	0.627821992
TKTL1	-1.017075776	0.311931403	0.738223086	0.463376308	2.444231461	0.01767652
TLCD2	0.014683029	0.988318605	0.052038943	0.958677729	1.9117901	0.061003084
TLE1	-0.196025266	0.845046545	0.669237389	0.506017914	0.191482219	0.848837494
TLE2	1.355785142	0.178668851	0.89655701	0.373687446	1.486966125	0.142609326
TLE3	-1.919054154	0.058248728	0.172108376	0.863955642	-0.41346015	0.680841962
TLE4	-3.98775009	0.00013832	0.398805697	0.69151111	-1.346990309	0.18338639
TLK1	0.334388723	0.738889281	0.113784843	0.909804056	-0.733368439	0.46638001
TLK2	-0.528442234	0.59853499	0.067294342	0.946580443	0.79938562	0.427430403
TLN1	-2.223969088	0.028734378	1.343931326	0.184240838	1.369680197	0.176231231
TLN2	-1.903132251	0.060323019	1.24339503	0.218759705	1.726060163	0.089827417
TLR1	-2.120195094	0.036830698	-0.625534691	0.534091028	-1.762132658	0.083481358
TLR10	-0.6837597	0.495940139	-0.285357665	0.776393813	-1.024989194	0.309757145
TLR2	1.721110135	0.088777571	-0.032847354	0.973909882	-0.812504014	0.419928758
TLR3	-0.706636011	0.481676899	-1.978900879	0.052615198	-1.467713363	0.147753041
TLR4	-1.913711191	0.058937982	-0.910851233	0.366170123	-1.835278284	0.071754578
TLR5	-0.799410727	0.42622479	0.387015823	0.700170808	-0.503902768	0.616298126
TLR6	0.612919929	0.541524768	-1.564604249	0.123153958	-2.496548975	0.015496883
TLR7	-1.143095938	0.256127806	1.831755393	0.072160482	1.623119116	0.11015701
TLR8	-2.010322677	0.047487631	-0.790706133	0.43235932	-1.865732529	0.067297726
TLR9	-1.832953324	0.070222482	-0.014936286	0.98813463	-0.007724602	0.993864058
TM2D1	1.730812919	0.08702274	1.795400461	0.077836559	1.03742009	0.303980311
TM2D2	-0.384145564	0.701805834	1.45358915	0.151487592	2.397101961	0.019872175
TM2D3	-0.017991655	0.985686619	0.090852773	0.927924806	-0.130036125	0.897001862
TM4SF19	1.04152258	0.300512523	-1.198655397	0.235570604	-1.025283191	0.309619666
TM6SF1	-2.996480512	0.003556207	0.105141086	0.916629163	-1.704765937	0.093757318
TM7SF2	1.037299706	0.302464478	-1.948309089	0.056256057	-0.318891262	0.750990412
TM7SF3	-0.251911741	0.801702135	-0.741482097	0.461413916	-0.876712356	0.384372677
TM9SF1	-1.721784351	0.088654702	0.404941249	0.687020768	-0.692867866	0.49124399
TM9SF2	0.269276575	0.788353075	-0.362845158	0.718048745	-1.136779645	0.260451201
TM9SF3	0.941455074	0.349072901	-0.019084036	0.984840024	-0.874505923	0.385562273
TM9SF4	0.04203158	0.966569627	1.1514245	0.254315022	1.473706669	0.146136444
TMBIM1	-1.798829034	0.075505937	0.605668833	0.547114063	0.193766128	0.847057204
TMBIM4	-0.016035871	0.987242412	-0.395841487	0.693684493	-1.511739593	0.13619954
TMBIM6	-2.300155588	0.023827035	1.469454128	0.147150985	1.84666572	0.070059825
TMC2	0.451864397	0.652488779	-0.140373653	0.888854534	1.425428444	0.159560384
TMC4	-0.716725526	0.475459026	-1.61955044	0.110792056	-1.196929058	0.236355591
TMC5	-3.784233603	0.000282307	0.315801348	0.75329482	0.132944872	0.89471155
TMC6	0.347620041	0.728963338	-1.226538988	0.224986529	-0.329054836	0.743336575
TMC8	0.549013531	0.584399465	-2.817975196	0.006611501	-1.302627487	0.198011202
TMCC1	-3.853380431	0.000222103	-1.628212908	0.108938167	-2.633757018	0.010886501
TMCC2	-3.246367128	0.001659362	0.966910594	0.337631343	0.260246297	0.795624309

TMCC3	-5.098623193	1.97E-06	0.758461882	0.451267068	0.340871097	0.734470838
TMCO1	0.927244473	0.356359328	-0.655390229	0.514825838	-0.886398419	0.379177769
TMCO3	-0.940468287	0.349575741	-0.16515842	0.869397606	-1.328145836	0.189495166
TMCO4	1.46355657	0.146911112	3.080038398	0.003170143	3.409789626	0.001209145
TMCO6	-0.602317454	0.548526116	-1.832723122	0.072014272	-1.191996723	0.238268409
TMCO7	1.179356275	0.241464472	2.270173664	0.026955589	2.555318291	0.013340373
TMED1	0.278434789	0.78133789	0.709708361	0.480748419	0.649784461	0.518476942
TMED10	1.128347499	0.262268457	-0.80264189	0.425481399	0.031634738	0.974875349
TMED10P1	0.850483394	0.397385962	-0.333490814	0.739974876	-0.845084037	0.401645045
TMED2	-0.199321915	0.842475042	-0.970800422	0.335706984	-1.232124419	0.223029451
TMED3	0.420850456	0.674899846	1.020304667	0.311850605	1.749141362	0.085722528
TMED4	2.498085383	0.014363118	0.04822132	0.961706683	-0.013904257	0.988955576
TMED5	0.411749218	0.681533722	-0.968500104	0.336844111	-1.461689122	0.149392092
TMED6	-0.912735902	0.363898402	1.378661243	0.173330509	3.294984468	0.001707288
TMED7	-0.663656259	0.5086614	-1.548166352	0.127059843	-2.25450092	0.028077738
TMED8	1.135214158	0.259396705	-0.052716968	0.958139837	-0.95489672	0.343716714
TMED9	0.241896782	0.809428185	2.153572694	0.035472194	2.427631174	0.018423672
TMEM101	0.39069632	0.696974718	2.089452791	0.041094501	1.173123487	0.245691246
TMEM102	-0.159119638	0.873942626	1.635908895	0.107312312	0.79408172	0.430486012
TMEM104	-2.153152574	0.034067855	1.366631978	0.177051996	2.103432106	0.039913785
TMEM106A	0.25745521	0.797434034	1.842811816	0.070504794	1.711398154	0.092518417
TMEM106B	0.2940958	0.769383701	-0.992744236	0.324987049	-1.652186023	0.104069931
TMEM106C	1.069647172	0.287730483	-0.004505222	0.996420934	0.779841512	0.438754007
TMEM107	0.561435881	0.575940692	-0.530405735	0.597869764	-1.04518359	0.300409996
TMEM109	0.710558339	0.47925435	0.737418153	0.463861727	0.702838242	0.485055957
TMEM11	-1.511591922	0.134253828	-2.404194254	0.019446156	-1.896306654	0.063060922
TMEM110	1.492340139	0.139219687	1.278361847	0.206249421	1.804956769	0.07643611
TMEM111	-4.086045	9.72E-05	-0.245312645	0.807086339	0.166742072	0.868171093
TMEM115	-0.35088295	0.726522563	1.347503077	0.183095257	1.440872884	0.155165522
TMEM116	0.76023768	0.449163446	-1.692171677	0.096017002	-0.184205618	0.854514786
TMEM117	-0.021064353	0.983242453	-0.408929153	0.684108247	-0.125761566	0.900369192
TMEM119	-1.786514611	0.077491887	1.048362573	0.298850929	-1.621115199	0.110587093
TMEM120A	-2.684342915	0.008696748	-1.101543815	0.275242073	-1.294986419	0.20061632
TMEM120B	0.835841598	0.405528798	-1.66988252	0.100369275	-0.023633529	0.981228587
TMEM121	1.458522702	0.148289582	-0.69378676	0.490604661	-0.463871673	0.644530266
TMEM123	0.448751894	0.654723991	-0.061299282	0.95133308	-1.580470288	0.119607855
TMEM126A	0.71673876	0.475450899	-1.379210044	0.173162169	-0.270636347	0.78766156
TMEM126B	0.746823621	0.457179226	-1.368202492	0.176562688	-1.061424188	0.29303432
TMEM127	-2.507359896	0.014016832	1.00117322	0.32093077	0.750324734	0.456187046
TMEM128	1.908464834	0.059621463	-0.284190733	0.777283419	-0.35651826	0.722786659
TMEM129	1.49566034	0.138353145	-0.051125294	0.959402576	0.322737644	0.748090827
TMEM131	-0.273234075	0.785319453	0.260726638	0.795233261	0.593934759	0.554937461
TMEM132A	1.111631151	0.269352682	-0.389275433	0.698507973	-0.707304049	0.482298409
TMEM132D	-0.721608513	0.472465942	1.021303728	0.311381251	-0.313541012	0.755029688
TMEM132E	0.379877004	0.704960478	-0.686158879	0.495365903	-0.231254784	0.817956207
TMEM134	1.028497948	0.306560472	-2.590686757	0.0121134	-1.840158288	0.071024126
TMEM135	0.562793886	0.57501956	-0.960005871	0.341065099	-2.373226993	0.021075224
TMEM138	0.686555521	0.494184749	-2.626606107	0.011030649	-1.573294753	0.121260263
TMEM140	-3.494962005	0.000747664	-0.139359098	0.889652548	-2.502079039	0.015281294
TMEM141	0.442772972	0.659026521	-1.21453842	0.229498211	-0.94151459	0.350468191
TMEM143	0.314268304	0.75406802	-1.192403966	0.237992379	0.263760559	0.792928548
TMEM144	2.397120045	0.018659886	1.048268021	0.298894104	0.330599667	0.742175471
TMEM147	0.60921172	0.543968316	-3.20617525	0.002196088	-2.656125777	0.010266415
TMEM14A	0.384215127	0.701754467	-0.555584495	0.580646658	-1.265909621	0.210764638
TMEM14B	1.008991092	0.315770895	-0.709146698	0.481094223	-0.340728501	0.734577614
TMEM14C	1.019392031	0.310837185	-0.986733659	0.327900341	-1.289953871	0.202346075
TMEM14E	1.012767506	0.313973528	-0.053540941	0.957486188	0.028702823	0.977203223
TMEM150A	-0.605532243	0.546398433	-0.805984209	0.423567214	-1.289487392	0.202506974
TMEM150B	-1.994901797	0.049176619	2.280164391	0.026317905	1.162729544	0.2498496
TMEM154	-2.258578798	0.026404795	-2.234583635	0.02933981	-3.562301855	0.000757265
TMEM156	2.194281595	0.030874142	-0.655873345	0.514517166	-0.857545459	0.394783424
TMEM158	-3.500984956	0.00073302	1.613023514	0.112205731	-0.022063251	0.9824756
TMEM159	-2.04418262	0.043952436	1.218759491	0.227903802	0.998132003	0.322490875
TMEM160	0.381942886	0.703433056	-2.682575555	0.009518351	-2.228269955	0.029878219
TMEM161A	1.140598949	0.257160245	1.247123839	0.217399559	1.38946843	0.170167288
TMEM161B	1.06928191	0.287894059	-1.031512807	0.306612494	-0.804625297	0.424424568
TMEM163	-1.457020257	0.148702953	0.741787324	0.46123037	-0.458476481	0.648377072
TMEM164	-3.767813768	0.000298737	-0.011514172	0.990853013	-0.377276511	0.707388164
TMEM165	0.946394531	0.346562916	-1.314674911	0.19383011	-2.85895004	0.005952047
TMEM167A	0.186078641	0.852815338	-0.05044841	0.959939608	-0.363955171	0.717256292
TMEM167B	-1.004159314	0.318080561	0.058952329	0.953194142	-0.6566642	0.514075123
TMEM168	1.952385486	0.054101016	-1.137450336	0.26005988	-1.637715563	0.107065097

TMEM169	0.254205392	0.799935439	0.132339443	0.895177055	-0.296881469	0.767650788
TMEM170A	0.089070562	0.929230113	-2.029518104	0.047034781	-3.629755301	0.000613616
TMEM170B	-0.063531907	0.949488381	0.056818018	0.954886813	-1.56242165	0.123799049
TMEM171	0.562087725	0.575498459	-1.774113822	0.081329959	-1.227205171	0.224858144
TMEM173	1.074090896	0.285745542	-0.869292329	0.388295439	-0.129126906	0.897717952
TMEM175	-0.321537814	0.748572424	-1.336940576	0.186498754	-0.854926425	0.396219468
TMEM176A	0.836584131	0.405113423	-1.645890895	0.105232955	-2.066930244	0.043355662
TMEM176B	-0.002125115	0.998309259	-1.4294498	0.158276778	-1.951021206	0.056043235
TMEM177	-0.291745304	0.771174381	-1.131120186	0.262692356	-0.221308578	0.825653784
TMEM179B	-2.37406669	0.019788645	-0.963420216	0.339364268	-0.39316953	0.695680824
TMEM18	0.851094544	0.397048273	0.34107878	0.734285397	1.042503541	0.301639253
TMEM180	0.169847442	0.865523593	-0.640453437	0.524417742	-0.336612961	0.737661603
TMEM181	2.094098318	0.039154153	-1.116363345	0.26890234	-1.886557021	0.064386703
TMEM182	-1.827679262	0.071018322	-1.013683837	0.314973123	-0.737100831	0.464125269
TMEM183A	0.071630366	0.943060129	0.310501485	0.757300476	1.350561276	0.182245868
TMEM184A	-0.044073122	0.964946899	0.076804749	0.939045052	0.550910676	0.583877004
TMEM184B	-2.736489565	0.007524434	-1.682677758	0.097851501	-1.797702861	0.077593243
TMEM184C	-1.728976373	0.087352692	-1.186791987	0.240181756	-1.275339418	0.207432496
TMEM185A	-0.294741922	0.768891684	-0.213546906	0.83165453	-0.273340153	0.785593105
TMEM185B	0.096743509	0.92315212	0.382237817	0.703691771	-1.294799555	0.200680349
TMEM186	0.506474053	0.613802276	1.530850808	0.131280611	2.062388468	0.043801437
TMEM187	-0.164817145	0.869469429	2.118164068	0.038487624	1.603683853	0.114385923
TMEM188	-0.62818866	0.531522427	-1.523386339	0.133134197	-1.724496204	0.090111341
TMEM189	0.325971763	0.745226798	3.695803531	0.000490076	3.776485917	0.000385666
TMEM19	1.559233606	0.122563687	0.097411292	0.922737967	0.446882494	0.656676227
TMEM191A	1.142221072	0.256489206	-1.54996354	0.126628045	-0.74229496	0.460997886
TMEM191B	0.899513015	0.370857105	-1.398179796	0.167420124	-0.665516883	0.508440468
TMEM192	1.392020456	0.167456874	0.484442987	0.629908685	1.932851356	0.058295826
TMEM194A	1.847340776	0.068089234	-0.465697444	0.643188329	-0.859556776	0.393682796
TMEM194B	1.190879065	0.236933101	0.385148344	0.701546191	-0.240422129	0.810877315
TMEM198	-1.267665737	0.208292384	0.568266766	0.572062777	-1.149735903	0.255118721
TMEM199	-2.551580568	0.012466685	0.263036089	0.793461432	-0.324393846	0.746843426
TMEM2	-0.310714462	0.756759288	0.869621366	0.388117044	0.358348877	0.721423949
TMEM200A	-1.163075049	0.24797239	2.190629745	0.032539889	0.789787692	0.432969337
TMEM201	0.402897441	0.68800989	1.061288085	0.292988977	1.246308807	0.217817745
TMEM203	-1.193152152	0.23604647	1.057894681	0.294520225	1.328577118	0.189353657
TMEM204	1.233639023	0.220652709	1.043165602	0.301230344	0.461216077	0.64642251
TMEM205	-1.566638521	0.120821598	0.516649417	0.607379078	0.886707443	0.379012763
TMEM206	0.62016721	0.536765273	1.154956746	0.25287735	-1.021625721	0.311332922
TMEM208	-1.06959326	0.287754622	-1.241812108	0.219338996	-0.715788312	0.47708373
TMEM209	1.681919226	0.096163011	0.778073978	0.439710025	0.03053266	0.975750349
TMEM212	-0.502498395	0.616583819	2.013663504	0.048725082	-0.649641874	0.518568384
TMEM214	-0.702368969	0.484320033	0.466453474	0.642650437	1.041381136	0.302155086
TMEM216	-1.171499968	0.244589361	-1.698219986	0.094863092	-1.526244861	0.132553826
TMEM218	1.085726336	0.280592854	-1.294400302	0.200692874	-0.650273963	0.518163087
TMEM219	-0.972637628	0.333424131	1.565626708	0.12291422	1.411904962	0.163487472
TMEM220	2.071310822	0.041285471	-0.836261048	0.406464151	0.434341968	0.665702127
TMEM221	1.289866132	0.200507952	2.127920221	0.037635236	3.258031903	0.001905177
TMEM222	-1.849896417	0.067716032	-0.18019564	0.857631488	0.223918276	0.823632385
TMEM223	0.910208367	0.365222088	-1.382722383	0.172087754	-0.473793549	0.637481419
TMEM229B	-0.635364229	0.526854929	0.805997957	0.423559351	0.953073973	0.344631267
TMEM231	1.008898187	0.315815199	-0.93414359	0.354129701	-1.17263038	0.245887393
TMEM234	-0.417050602	0.677666466	-1.112419754	0.270579261	-1.190282809	0.238935705
TMEM237	1.215975989	0.227276021	0.056612776	0.955049596	0.63388225	0.528727601
TMEM25	2.735183385	0.007551946	0.298926883	0.766071759	1.077117037	0.286026892
TMEM30A	-0.116833767	0.907260487	-0.164301408	0.870069109	-1.484079795	0.143371343
TMEM30B	0.4964269	0.620842516	0.414225516	0.680247558	-0.155748944	0.876788642
TMEM33	-1.81843273	0.072431747	-0.576160674	0.566751283	-1.509897071	0.13666827
TMEM38A	-0.743250562	0.45932807	2.570233274	0.012772301	2.488449383	0.015817606
TMEM38B	1.258278141	0.211650321	-1.075049641	0.28683545	-1.764167389	0.083134792
TMEM39A	0.97549334	0.332014381	-1.888529287	0.06399432	-1.681136956	0.098282714
TMEM39B	0.086877132	0.930968388	-0.111843114	0.911336668	1.291631441	0.201768236
TMEM40	-4.988974728	3.07E-06	1.638355621	0.106799568	0.053895045	0.95720979
TMEM41A	2.866300013	0.00520511	2.028178498	0.047175616	3.015855846	0.003842102
TMEM41B	2.543116031	0.012750884	-1.341270193	0.185097887	-0.734595871	0.465637829
TMEM42	1.569350233	0.12018859	-0.436222353	0.664306195	-0.242623991	0.809179408
TMEM43	-1.611736847	0.110633485	0.395505051	0.693931335	-0.234670664	0.815316698
TMEM44	2.274545026	0.025387234	1.067176261	0.290345013	0.5244081	0.60205574
TMEM45B	-0.431080926	0.667473456	-0.474695524	0.636798991	1.118631009	0.268053157
TMEM48	1.722223077	0.088574824	0.210262816	0.834204611	0.260006255	0.795808534
TMEM5	1.297480809	0.197888282	0.08111538	0.935631407	0.87408994	0.385786808
TMEM50A	-2.226870345	0.028532402	-1.922024283	0.059554194	-2.009150423	0.049330106

TMEM50B	1.447665915	0.151296791	-1.416059353	0.162143515	-0.455023266	0.650844311
TMEM51	0.92947354	0.355209978	2.777492183	0.00738087	1.566740512	0.122785574
TMEM53	-0.255097618	0.799248478	-1.745728415	0.086190577	-0.863651354	0.391448072
TMEM55A	-3.209704827	0.001860359	-1.658247203	0.102704578	-2.591596153	0.012148968
TMEM55B	-0.162157707	0.871556874	-1.607068409	0.113508255	-1.367006442	0.177063114
TMEM56	-2.050718688	0.043296698	0.444739758	0.658174646	0.34754149	0.729481957
TMEM56-RWD	0.73914736	0.461802826	0.248966123	0.804272654	-0.134863787	0.893201111
TMEM57	0.004607576	0.996334225	0.773234451	0.442545558	2.157739724	0.035233928
TMEM59	-1.248264754	0.215275795	0.378874932	0.706173835	0.244729184	0.807556906
TMEM60	-2.422294536	0.017493048	-1.773502521	0.08143218	-1.643013123	0.105960544
TMEM62	0.173302809	0.862815122	0.056956136	0.954777269	1.244359668	0.218528534
TMEM63A	1.98073519	0.050773163	-0.780183114	0.438477615	-0.119677501	0.905165143
TMEM63B	-2.199325893	0.030501037	-1.468680672	0.147360123	-1.202368296	0.234259145
TMEM63C	0.634984783	0.527101214	0.357100484	0.722321673	-0.802722269	0.425514804
TMEM64	0.416369935	0.678162518	-1.519099627	0.13420804	-2.399397592	0.019759807
TMEM65	0.298244766	0.766225948	-1.718647237	0.091049945	-3.095011267	0.003065229
TMEM66	0.23639819	0.81367818	1.269191045	0.209477761	0.545259444	0.587731243
TMEM67	0.190229434	0.849571556	-1.0226374	0.310755445	-0.681585745	0.498298338
TMEM68	1.324908664	0.18866335	-1.14370766	0.257476158	-1.458576587	0.150244501
TMEM69	-1.592507422	0.114889915	-2.337716706	0.022898478	-1.011737392	0.31599697
TMEM70	0.540135643	0.590480505	-1.215309364	0.229206399	-1.906272836	0.061729715
TMEM71	-3.110354025	0.00252519	-1.238742469	0.220465588	-1.299770149	0.198982382
TMEM79	-1.996127776	0.049040491	1.188985153	0.239324418	-0.101139033	0.919799826
TMEM80	0.699596721	0.486041524	-0.500972768	0.618299519	-0.412274047	0.681705967
TMEM81	-1.439629445	0.153553084	1.505572014	0.137641891	1.410858583	0.163794422
TMEM85	0.455406209	0.649949121	-0.813588773	0.419231306	-0.476324566	0.63568864
TMEM86A	-1.623890059	0.108009344	0.046529628	0.963049078	0.489209166	0.626596304
TMEM86B	-0.551207008	0.582901589	-0.05892559	0.953215347	0.163960881	0.870349801
TMEM87A	1.867466643	0.065196323	2.980075757	0.004215011	3.227605413	0.002084153
TMEM87B	1.885283015	0.062722113	0.076419472	0.939350214	-0.54721605	0.586395436
TMEM88	-2.054963934	0.042875309	-1.552510015	0.12601823	-1.552459117	0.126162454
TMEM8A	-0.350558975	0.726764782	-1.229916024	0.223728713	-1.588201709	0.117847758
TMEM8B	2.011725224	0.047336507	0.750459158	0.456033147	1.607288635	0.113591796
TMEM9	1.674654588	0.097585507	0.645130313	0.521404335	1.266890085	0.210416337
TMEM91	-0.943371969	0.34809744	-0.419278007	0.676572632	-0.778306173	0.439651002
TMEM92	-1.58133391	0.117422754	-0.292208446	0.771177254	-0.306409006	0.760424831
TMEM93	1.618773787	0.109107885	-3.147447995	0.002608133	-3.150726946	0.002609436
TMEM97	0.864530988	0.38966859	-1.205799185	0.232825103	-0.60327214	0.548753145
TMEM99	-1.407359271	0.162875934	-1.407350458	0.164697372	0.096018634	0.923847125
TMEM9B	-0.237634135	0.812722397	-2.140352681	0.036572937	-2.034650849	0.046611041
TMF1	0.4075417	0.684609101	-0.046094571	0.963394323	-1.508041061	0.137141724
TMIE	0.619922992	0.536925309	1.479135478	0.144552874	2.411976737	0.019154127
TMIGD2	2.369356825	0.020026579	-0.689164328	0.493486916	-0.281442409	0.779404108
TMLHE	-0.984896325	0.327400242	-1.915465806	0.060402341	-2.905146975	0.005239766
TMOD1	-3.07378167	0.002821296	1.519323959	0.134151674	1.199266601	0.235452968
TMOD2	1.751523233	0.083372315	-0.359048216	0.720871933	-2.852726706	0.006054572
TMOD3	-0.745877959	0.457747389	-1.235548127	0.221642464	-2.103350379	0.039921217
TMOD4	1.985926281	0.050183084	-1.061465976	0.292908856	1.083275288	0.283309026
TMPO	1.227185005	0.223056334	-0.066721178	0.947034742	-0.313178054	0.755303961
TMPPE	-1.282333067	0.203124797	0.433203708	0.666484844	-1.130193753	0.263191951
TMPRSS13	3.068658539	0.002865261	1.986244343	0.051771924	2.257006526	0.027910883
TMPRSS3	0.951501683	0.34398004	1.27670831	0.206828749	1.282692811	0.204861433
TMPRSS5	2.657533292	0.009362	1.032789653	0.306019579	0.51813296	0.606398081
TMPRSS6	0.634624386	0.527335189	0.414219969	0.680251597	1.047842378	0.299193883
TMPRSS9	-0.48615191	0.628079124	-1.034616236	0.305172745	-1.912544532	0.060904292
TMSB10	-0.243724163	0.808017015	-0.179017676	0.858552071	0.235810707	0.814436244
TMSB15B	0.273438844	0.785162577	-0.712056061	0.479304499	-1.397274042	0.16781996
TMSB4X	-1.102467498	0.273292346	-1.299817831	0.198841516	-0.840473677	0.40420216
TMTC1	-1.592765767	0.114831873	1.994881325	0.050794986	-2.020197393	0.048135844
TMTC2	1.438417925	0.153895473	-1.326530675	0.189899804	-1.974210854	0.053276785
TMTC3	1.130383841	0.261414504	-0.202819443	0.839990941	-1.018760038	0.312679763
TMTC4	1.95169097	0.054184813	0.843195338	0.402607376	0.934282437	0.354152626
TMUB1	-0.289963239	0.772532841	-2.031552559	0.046821589	-1.512214715	0.136078877
TMUB2	-1.429676279	0.156383476	-1.735633677	0.087976214	-0.86437821	0.391052199
TMX1	0.741951889	0.460110518	-0.481712483	0.631835535	-0.931024429	0.355820606
TMX2	-0.546595025	0.586053117	1.820340615	0.073903989	2.486010207	0.015915359
TMX3	1.543420145	0.126350798	-0.690351554	0.49274575	-1.178604592	0.243518569
TMX4	-3.689312278	0.000390684	-0.375687024	0.708529719	-1.160653575	0.250686158
TNF	-1.102279774	0.27337347	-2.077146821	0.042258023	-2.266557499	0.027282879
TNFAIP1	1.184915788	0.239270487	-1.312960184	0.194403597	-1.443597834	0.154399998
TNFAIP2	-2.450359734	0.016269318	-1.157587366	0.251810434	-1.486273306	0.142791945
TNFAIP3	1.911222365	0.05926139	0.101088826	0.919831031	-2.224109294	0.030172983

TNFAIP6	-2.285850881	0.024687719	-1.676710274	0.099019231	-2.342930646	0.022695418
TNFAIP8	1.190976765	0.236894943	0.665829473	0.508178054	0.627122995	0.533116531
TNFAIP8L1	0.271479109	0.786664316	1.339279953	0.185740842	1.92421792	0.059392938
TNFAIP8L2	-1.622546301	0.108297003	1.768632542	0.082250355	1.604230173	0.114265282
TNFAIP8L2-SCN	0.969096766	0.335177558	0.811458587	0.420443169	1.23614267	0.221543826
TNFRSF10A	2.438431099	0.016779775	3.455212974	0.001038815	3.141575131	0.002679657
TNFRSF10B	1.453383279	0.149707306	-1.147765302	0.255810509	-1.323322902	0.191083089
TNFRSF10C	-2.326902487	0.022288528	-0.100285784	0.920465709	-0.853450857	0.397029961
TNFRSF10D	2.092749434	0.039277614	0.750697065	0.455891042	1.502563341	0.138546623
TNFRSF11A	-0.881176983	0.380644696	0.447586387	0.656130624	-0.197810212	0.843906842
TNFRSF12A	-1.632875076	0.106101695	-2.881710995	0.005548834	-2.548688413	0.013569235
TNFRSF13B	-0.172858164	0.863163563	0.5600854	0.577593181	0.492999486	0.623932541
TNFRSF13C	0.785554116	0.434258317	-0.059884345	0.95245505	-0.311217355	0.75678613
TNFRSF14	-1.431746339	0.155791519	-0.694914824	0.489902685	-0.771482351	0.443650757
TNFRSF17	1.172275443	0.244279633	0.455944296	0.650144524	0.864625674	0.390917478
TNFRSF18	-0.266735657	0.790302542	-1.093599799	0.278683268	-0.456865126	0.649527856
TNFRSF1A	-1.708652192	0.091073117	-0.472504949	0.638351944	-1.310247613	0.195438611
TNFRSF1B	-2.13422381	0.035631778	1.545303535	0.127750099	1.532072995	0.131111031
TNFRSF21	2.340581494	0.021536188	0.400308633	0.690410139	0.029178491	0.976825539
TNFRSF25	2.030068942	0.045397549	-1.83229194	0.072079387	-0.628131683	0.532460369
TNFRSF4	-0.731120501	0.466665882	-0.131760756	0.895632722	-0.541127865	0.590556677
TNFRSF8	-0.6909963	0.491403516	0.241127722	0.810312458	0.328517436	0.743740629
TNFRSF9	-2.120315703	0.036820244	0.12220099	0.903165221	0.417185784	0.678130863
TNFSF10	-0.979197103	0.330191829	0.174477741	0.862101879	-1.760717719	0.083723065
TNFSF12	-1.996499878	0.048999238	-0.709191547	0.481066605	-1.820681726	0.073977245
TNFSF13	-0.532129524	0.595989693	2.171682655	0.034011431	1.36306751	0.178294099
TNFSF13B	-1.239834625	0.218363153	-0.708451287	0.481522564	-2.797642644	0.00703587
TNFSF14	-1.338179845	0.184317312	-2.150442628	0.03573016	-2.663960502	0.010056977
TNFSF15	0.642582875	0.522180979	0.056964269	0.954770818	-1.134017178	0.261598336
TNFSF4	-3.082752628	0.002745816	0.944353192	0.348933922	0.069627793	0.944736915
TNFSF8	0.572612515	0.568380774	0.109630264	0.913083689	-0.976111338	0.333189592
TNFSF9	-1.330188452	0.186925207	-1.472304607	0.146382246	-0.282807695	0.77836262
TNIK	1.417652795	0.159856093	0.050756909	0.959694846	0.552274599	0.582948599
TNIP1	-1.954860922	0.053803235	0.990482733	0.326081152	1.07083318	0.288818784
TNIP2	0.546188697	0.58633116	0.857465859	0.394741422	0.520259401	0.604924992
TNIP3	1.827762435	0.071005713	-0.232556109	0.816930511	-0.668220844	0.506726073
TNK1	2.001587776	0.048438134	-0.18773942	0.851740766	0.379185278	0.705978274
TNK2	-0.226110045	0.821645051	0.03302655	0.973767602	0.512292898	0.610452224
TNKS	0.936800672	0.351448756	-1.217168194	0.228503923	-1.432834235	0.157441075
TNKS1BP1	1.356304779	0.178504144	2.869491325	0.005739442	2.633844212	0.010884019
TNKS2	1.180878739	0.240862225	-0.836479909	0.406342078	-1.469369074	0.147305043
TNNC1	0.403743972	0.687389531	0.743399658	0.460261499	1.366622363	0.177182859
TNNC2	-1.870487742	0.064771098	-1.151467846	0.254297344	-0.879565768	0.382837685
TNNI2	-2.398399481	0.018598953	1.491029749	0.141410441	0.628789682	0.53203256
TNNT1	-1.033282317	0.304329398	0.855988633	0.395551221	1.103591132	0.274470521
TNNT3	0.443771903	0.658306869	1.555653888	0.125268594	2.212407097	0.03101583
TNPO1	1.395995717	0.166260314	-0.523180584	0.602855641	-1.777383944	0.080912709
TNPO2	2.53952323	0.012873266	-1.478795818	0.144643412	-0.877271292	0.384071693
TNPO3	-1.51562511	0.133231362	1.298248238	0.199376577	1.2766165	0.206984256
TNRC18	-0.385664753	0.700684347	-0.533144656	0.595984747	-0.564255947	0.574823586
TNRC6A	2.844616593	0.005539987	0.683613382	0.496960393	1.12905239	0.263669005
TNRC6B	-0.464221037	0.64364643	-0.849364298	0.39919526	-0.752077801	0.455140607
TNRC6C	1.703494858	0.092037531	0.387969188	0.699469052	1.031799903	0.30658294
TNS1	-3.261586552	0.00158205	1.807013342	0.075984193	0.39721476	0.692712687
TNS3	-1.198077001	0.234133698	1.799040421	0.077251931	1.489743927	0.141878979
TNS4	1.119163543	0.266144157	-0.041406679	0.967114893	1.235723742	0.221698371
TNXB	1.075007258	0.285337391	-0.058172203	0.953812817	-0.104229192	0.917358308
TOB1	-0.488884905	0.626150702	1.047214335	0.299375534	0.044530132	0.964639578
TOB2	-0.077276326	0.938580783	0.519087087	0.605688948	0.63525234	0.52784028
TOE1	0.017086555	0.986406605	1.945868995	0.056555518	1.458188202	0.150351133
TOLLIP	-0.862722258	0.390657026	1.744769951	0.086358816	1.544978641	0.127960652
TOM1	-2.151161195	0.034229527	1.936472207	0.057721492	2.131639361	0.037419484
TOM1L2	1.522633236	0.131469305	1.370020473	0.175997572	2.176200485	0.033756404
TOMM20	0.646799194	0.51946105	1.032625798	0.306095622	1.186669834	0.240346816
TOMM22	-0.767788623	0.444687171	0.215054477	0.830484516	0.680260098	0.49913084
TOMM34	2.178015515	0.032104557	2.578050395	0.012516725	2.609216042	0.011605972
TOMM40	-0.014840427	0.988193393	0.736045969	0.464689899	0.448285134	0.655669854
TOMM40L	0.47266271	0.637634885	1.125657334	0.264979288	2.669127649	0.009920994
TOMM5	1.357971101	0.177976752	-2.336390484	0.022972591	-1.702654575	0.094154577
TOMM6	-0.383690101	0.702142192	-1.956193597	0.055297672	-1.141385387	0.258546588
TOMM7	0.685303579	0.494970378	0.372988795	0.710525967	1.172772776	0.24583074
TOMM70A	2.032051865	0.045192087	0.939016948	0.351643367	2.062661308	0.043774546

TONSL	-0.650323906	0.517192994	0.144549092	0.88557151	0.608763878	0.545132251
TOP1	-2.003820696	0.048193621	0.534298308	0.595191598	-0.29564429	0.768590639
TOP1MT	-0.176068832	0.86064817	3.554892105	0.000763405	3.651080298	0.000573898
TOP1P1	0.49715386	0.620331923	-0.127076108	0.89932277	-0.477523976	0.634839834
TOP2A	0.362167926	0.718102884	1.633878955	0.10773923	0.804624332	0.42442512
TOP2B	2.052487577	0.043120685	3.185020812	0.002336902	3.602686636	0.000667822
TOP3A	-1.657483438	0.101015867	2.442963429	0.017655417	2.766411358	0.007655417
TOP3B	1.980205804	0.050833669	-1.50419052	0.137996448	-0.923492225	0.359696256
TOPBP1	0.448628694	0.65481253	0.047535849	0.962250606	-0.825416168	0.412623177
TOPORS	-1.565745912	0.121030544	-1.463563111	0.148749756	-2.233967186	0.029478721
TOR1A	-1.739443222	0.085485905	0.054069319	0.957067047	-2.099360963	0.040285495
TOR1AIP1	-1.139216095	0.257733281	0.427802217	0.670390472	-0.788416289	0.433764238
TOR1AIP2	-2.214496152	0.029402595	0.65071377	0.517818813	1.033089962	0.305984195
TOR1B	-2.245059031	0.02729429	0.594515653	0.554495576	-2.058579251	0.044178371
TOR2A	-1.081512326	0.282451541	-0.714521915	0.477790529	-0.95057605	0.345887173
TOR3A	0.69729974	0.487470435	1.440143834	0.155240454	1.118856124	0.267957913
TOX	-0.84685885	0.399392306	-2.5828993	0.012360538	-1.733852611	0.088423758
TOX2	-0.161613422	0.871984207	-0.328709483	0.743567469	0.649827099	0.5184496
TOX4	-2.008492668	0.047685435	0.925388826	0.358624839	0.937861059	0.35232636
TP53	-0.547214501	0.58562934	0.850950183	0.398320984	1.141891249	0.258338005
TP53BP1	0.15141785	0.879995909	1.720715221	0.090671064	1.650285474	0.104459374
TP53BP2	1.061377663	0.29144948	1.735488851	0.088002053	0.85865897	0.394173855
TP53I11	-1.622389025	0.108330712	-0.299802519	0.765407105	-0.122864363	0.902652547
TP53I13	2.05972278	0.042407137	-1.414828691	0.162502531	-0.379058184	0.706072118
TP53I3	0.643670728	0.521478497	0.701414129	0.48586909	-0.196774029	0.844713792
TP53INP1	-0.155770461	0.876574039	-1.301169537	0.198381594	-2.140442326	0.036669461
TP53INP2	-3.373600851	0.001108633	-2.074397679	0.042521827	-1.814970416	0.074862518
TP53RK	0.946154308	0.346684714	-1.176574923	0.244205019	-0.144860098	0.885339256
TP53TG1	-0.494485176	0.622207231	-0.818785782	0.416283585	-0.732256529	0.467052921
TP63	-0.662757441	0.5092342	-0.035809437	0.971558128	0.102535231	0.9186966
TP73	2.345945827	0.021247347	1.095801915	0.277726358	1.337218596	0.186535107
TP73-AS1	2.895584364	0.004782345	1.19175373	0.23824531	1.404613461	0.165635679
TPBG	-0.614285479	0.540626336	-0.876524582	0.384386129	-1.031229245	0.306848048
TPCN1	0.363488928	0.717119539	1.412560488	0.163165837	2.224626127	0.030136229
TPCN2	-1.69644144	0.093370006	1.171163859	0.246355349	0.347704852	0.729359924
TPD52	1.820505122	0.072112941	-0.615908215	0.540381547	-0.932474655	0.355077516
TPD52L2	-2.836695937	0.005667165	-0.111519704	0.911591969	-0.452972552	0.652311362
TPI1	-1.071188641	0.287040864	2.114077937	0.0388496	2.9693805	0.004380149
TPK1	-0.8011366	0.425230408	-0.001253532	0.999004158	-0.117545551	0.906846569
TPM1	-4.558726113	1.67E-05	1.006684773	0.318296877	0.392403672	0.696243304
TPM2	1.297008325	0.198050086	0.84422489	0.402036676	2.717262054	0.008732735
TPM3	-2.395092815	0.018756795	1.120266981	0.267249633	1.311982924	0.194856295
TPM4	-2.060995526	0.042282673	-0.13766237	0.890987388	0.127591791	0.898927183
TPMT	0.261888588	0.794025051	1.45053748	0.152333094	0.787656717	0.434204878
TPP1	-1.31133676	0.193186921	0.781563321	0.437672236	0.748722619	0.457144592
TPP2	3.481642261	0.000781037	0.561633282	0.576544871	0.624479341	0.534838245
TPPP	-0.084391834	0.932938372	-0.65572167	0.514614064	0.202163297	0.840518633
TPPP3	-3.591548578	0.000543022	0.282701657	0.778419045	0.790482464	0.43256696
TPR	1.001185806	0.319507529	1.475716762	0.145466179	1.086853964	0.281737902
TPRA1	-0.866435737	0.388629357	0.254434458	0.800066148	-0.586720629	0.559739349
TPRG1	-0.115095686	0.908633933	-0.122063773	0.903273407	0.001859058	0.998523266
TPRG1L	-3.464299592	0.0008266	0.313500691	0.755032835	0.448034167	0.655849871
TPRKB	-0.199965593	0.841973149	-0.138490373	0.890335946	-0.592092037	0.556162051
TPRN	0.158578232	0.874367908	0.407464157	0.685177633	0.728818467	0.469137078
TPSAB1	-1.741654436	0.085095756	0.177513735	0.859727691	0.911695079	0.365820904
TPST1	-4.431810417	2.71E-05	-1.112445381	0.27056834	-1.353374112	0.181351292
TPST2	1.582486442	0.11715945	-1.573370561	0.12111063	-1.837809566	0.071374904
TPT1	0.201926712	0.840444416	-0.569510025	0.571224629	0.055948088	0.955581465
TPX2	-0.33674697	0.737116857	2.583765459	0.012332825	2.600635005	0.011867593
TRA2A	-0.580474593	0.563091914	-0.087365387	0.930684118	-0.800511524	0.426783435
TRA2B	1.059896858	0.292118888	-0.392381873	0.696224383	-0.789502295	0.433134689
TRABD	0.672737934	0.502893141	-0.881066197	0.381943888	-0.14646933	0.884074696
TRADD	0.574495449	0.567111917	-0.924831369	0.358912307	-0.699849645	0.486906244
TRAF1	3.905846979	0.000184822	0.369307154	0.713253067	1.81588931	0.07471949
TRAF2	0.067667347	0.946205379	2.753736656	0.00786988	2.8306452	0.006431673
TRAF3	1.598825639	0.113477151	0.311301898	0.756695089	0.018243192	0.985509418
TRAF3IP1	2.115796239	0.037213762	1.324641216	0.190522112	1.464016117	0.148757292
TRAF3IP2	-1.495213858	0.138469426	2.137754991	0.036792706	2.60686244	0.011677201
TRAF3IP2-AS1	1.02312625	0.309078539	-2.318148752	0.024013897	-0.641601853	0.523738298
TRAF3IP3	2.202026967	0.030302873	1.785691868	0.079413995	3.401783966	0.001238842
TRAF4	0.918074809	0.36111243	0.025269921	0.979927015	-0.213411716	0.83177765
TRAF5	1.796970112	0.07580299	0.478895781	0.633825905	1.370157979	0.176082895

TRAF6	-0.482719935	0.630504416	-0.732909647	0.466585971	-0.25286078	0.801297812
TRAF7	-0.610077378	0.543397386	-0.354387777	0.724342507	-1.294696396	0.200715703
TRAFD1	-2.45846586	0.015930402	0.876298725	0.384507839	-0.571739449	0.569776794
TRAIP	1.193129314	0.236055366	1.860684402	0.067896066	2.096051823	0.040589849
TRAK1	0.149821449	0.881251519	-0.033389355	0.973479541	-0.232351841	0.81710826
TRAK2	0.386085877	0.700373584	0.684534132	0.496383315	0.681444659	0.498386903
TRAM1	1.388695886	0.168462619	-0.954751325	0.343693535	-1.64258702	0.106049043
TRAM2	0.507130303	0.613343677	0.495009589	0.622476511	1.037791318	0.303808933
TRANK1	-2.67206725	0.008995833	0.774411553	0.441854895	-0.327685024	0.744366635
TRAP1	1.235385952	0.220005374	1.62424626	0.109783937	2.582632746	0.012433976
TRAPPC1	-1.894554418	0.061466122	-2.862284157	0.005854686	-2.516876872	0.014717673
TRAPPC10	2.218205134	0.029139362	-1.18200085	0.242062397	-1.479246842	0.144654464
TRAPPC2	0.473329926	0.637160768	-0.782804361	0.43694881	0.667069205	0.507455866
TRAPPC2L	-0.110400187	0.912345731	-3.110716175	0.002901616	-2.282304724	0.026274762
TRAPPC2P1	0.89818131	0.371562559	0.271637304	0.786872201	1.153018833	0.25378001
TRAPPC3	-1.425341991	0.157628533	0.797723895	0.428307396	2.283305246	0.026211841
TRAPPC4	1.012222774	0.314232368	0.574440307	0.567906779	0.790263258	0.432693889
TRAPPC5	-0.771548383	0.442468036	-3.922085389	0.000236239	-3.460336063	0.001036675
TRAPPC6A	-0.132756891	0.89469156	-0.869763633	0.388039926	0.156934104	0.87585885
TRAPPC6B	1.151348825	0.252736307	0.462381726	0.645549626	0.154486185	0.877779504
TRAPPC8	-0.058275634	0.95366243	-0.802788258	0.425397464	-1.609270401	0.113157119
TRAPPC9	-1.202094827	0.232581502	2.054142807	0.044509874	2.002855046	0.050022037
TRAT1	0.739815697	0.461399218	-1.303286651	0.197662845	-1.155457012	0.252789026
TRDMT1	0.977050655	0.331247251	-2.345188205	0.02248492	-1.68552712	0.097428619
TREM1	-4.631466587	1.26E-05	0.011992095	0.990473364	-0.742230817	0.461036433
TREML1	-3.757961723	0.000309027	1.486431216	0.142618914	0.810411561	0.421119958
TREML2	-3.213122124	0.001840704	0.489107423	0.626623084	0.845469665	0.401431611
TREML3	-4.473560684	2.31E-05	0.584513186	0.561157765	-0.545720467	0.587416365
TREML4	-2.288772452	0.02450974	-0.828952143	0.410553687	-1.512601061	0.135980823
TRERF1	0.254496128	0.799711572	-1.015708251	0.314016138	-1.821043681	0.073921438
TREX1	0.232564407	0.816644718	-0.40471917	0.687183101	-1.776448729	0.081068301
TREX2	0.930221091	0.354825059	-0.409968976	0.683349614	0.963918541	0.339213523
TRIAP1	0.734875418	0.464387383	-0.049537574	0.960662282	0.150248365	0.881106267
TRIB1	-5.389482568	5.94E-07	-0.977053047	0.332628926	-1.675959017	0.099297951
TRIB2	1.946008547	0.054874577	0.189596271	0.850292091	0.744646316	0.459586122
TRIB3	0.359133275	0.720363656	2.393928857	0.019946812	4.192297827	9.87E-05
TRIM10	-3.340349065	0.001233057	2.428572945	0.018302042	1.395794285	0.168263029
TRIM11	-0.643887685	0.521338457	1.425912899	0.159291093	1.778201967	0.080776818
TRIM13	0.941642994	0.348977195	-1.300549826	0.198592353	-1.041761565	0.301980181
TRIM14	1.17110374	0.244747725	-1.749875235	0.08546581	-1.978239124	0.05280836
TRIM16	-0.710371465	0.479369615	2.105041008	0.039660704	2.814919708	0.006713329
TRIM16L	-0.551767684	0.582519008	0.882851883	0.38098632	1.760394546	0.083778352
TRIM17	1.055369325	0.294172106	-2.676426206	0.009674703	-2.304016715	0.024939029
TRIM2	0.591923728	0.555433474	0.729899076	0.468410157	0.034273235	0.972780629
TRIM21	-2.534941336	0.013030873	1.177241011	0.243941257	0.020783539	0.983491896
TRIM22	-0.109425297	0.91311663	0.577836782	0.565626628	-2.143436543	0.036417364
TRIM23	0.187987132	0.851323564	-0.710866102	0.480036063	-1.604403333	0.114227065
TRIM24	-0.475989399	0.635272475	0.465234257	0.643517968	1.124460861	0.265594309
TRIM25	0.623915944	0.534311799	0.25305279	0.801128437	-1.059436988	0.293930048
TRIM26	-1.508882204	0.134944236	-0.918982351	0.361937474	-0.404774427	0.687178872
TRIM27	-1.258119331	0.211707468	-0.835978302	0.40662189	-1.0936251	0.278781842
TRIM28	-0.218344494	0.827670956	1.614955233	0.111785825	1.794294618	0.07814196
TRIM3	0.8276502	0.410128254	-1.546974233	0.127346913	-0.446448164	0.656987983
TRIM32	2.272869646	0.025492364	0.397949415	0.692138676	1.233412527	0.222552418
TRIM33	0.9950531	0.32246401	-1.792541925	0.078298266	-2.920994497	0.005014223
TRIM34	-0.058960487	0.953118507	-1.085490184	0.282227065	-1.847222406	0.069977847
TRIM35	2.050054796	0.043362918	-0.35935248	0.720645555	-1.276844399	0.206904343
TRIM36	2.173170782	0.032479195	1.442261019	0.154644745	-0.188573551	0.851105923
TRIM37	1.331920601	0.1863576	0.523235236	0.602817855	0.735188833	0.46527953
TRIM38	-1.575614258	0.118736446	0.377277547	0.707353954	-0.401775837	0.689371857
TRIM39	-1.370460265	0.174061427	0.822789893	0.414021055	1.752542081	0.085131087
TRIM4	1.042093477	0.300249292	1.359066205	0.179423719	0.256713951	0.798336469
TRIM41	-0.138294341	0.890326699	-1.520853509	0.133767856	-1.526255699	0.132551132
TRIM44	0.504460542	0.615210312	-0.0115588	0.990817562	-0.574894367	0.567655674
TRIM45	-0.37964577	0.705131518	-0.42612137	0.671607707	-1.775212955	0.081274279
TRIM46	0.438946619	0.661786075	0.104357289	0.917248369	0.309340436	0.758205828
TRIM47	-0.755253709	0.452132187	0.401070197	0.689852514	0.344381235	0.731844106
TRIM5	-2.510793169	0.013890555	1.366131106	0.177208265	-0.446805406	0.656731556
TRIM52	1.553527247	0.123919745	-1.489439238	0.141827503	-1.743281676	0.08674962
TRIM56	1.246479522	0.215926914	-1.373491223	0.174922539	-1.701438715	0.094383972
TRIM58	-3.650056818	0.0004462	1.162698155	0.249746838	0.185876134	0.853210733
TRIM59	0.439228441	0.661582666	-0.947787238	0.347197521	-0.991812827	0.325537238

TRIM6	-1.860367157	0.066204814	-0.834390815	0.407508216	-2.293286031	0.025591451
TRIM61	1.867750248	0.065156306	-1.085272929	0.282322432	-0.893771995	0.375252991
TRIM62	1.54537326	0.125878094	-1.692650622	0.095925209	-2.193927727	0.032389075
TRIM64B	-1.221977683	0.225009509	1.995248082	0.050753854	-0.999188092	0.321983621
TRIM65	0.519646347	0.604626863	0.624252026	0.534927002	1.643504202	0.105858624
TRIM66	1.943811634	0.055143239	1.186439885	0.240319604	1.567418974	0.12262697
TRIM68	0.655251102	0.514031241	-0.392882705	0.695856478	0.084909134	0.93263519
TRIM69	0.381589039	0.703694589	2.250389116	0.0282589	1.348239024	0.18298695
TRIM7	0.49686191	0.620536957	1.044320864	0.300700296	0.495858782	0.621926416
TRIM78P	-0.174929452	0.861540652	-0.110109345	0.912705423	-0.582373603	0.562642786
TRIM8	-0.729154269	0.467861513	-0.828639896	0.410728955	-0.999567341	0.321801594
TRIM9	1.452967498	0.149822459	0.447544643	0.65616058	0.103018343	0.918314899
TRIO	2.95479012	0.00402256	0.058672111	0.953416364	-0.478360029	0.634248462
TRIOBP	-1.133680339	0.260036244	0.235784936	0.814435966	0.089062804	0.929348422
TRIP10	0.150537663	0.88068816	-0.365721649	0.715912577	1.126394309	0.264782374
TRIP11	0.357606251	0.72150221	0.182167221	0.856091137	0.384280377	0.702219917
TRIP12	-1.028764073	0.306436082	2.446800663	0.017486499	1.103799796	0.274380755
TRIP13	0.800706495	0.425478089	1.382691542	0.172097166	0.913109294	0.365083188
TRIP4	-0.30690967	0.759643925	0.582856515	0.562265024	0.792263461	0.431536511
TRIP6	0.318350132	0.750980663	1.884991885	0.064479218	1.28960592	0.202466082
TRIT1	0.267934463	0.789382615	2.587051665	0.012228197	3.61322278	0.000646201
TRMT1	1.43535713	0.154763115	-0.611401628	0.543339437	0.330746151	0.742065405
TRMT11	2.55830517	0.012244987	-0.77385454	0.442181642	-0.889624015	0.377457681
TRMT112	-0.585274224	0.559875091	-1.111495117	0.270973504	-0.473648167	0.637584463
TRMT12	0.634990933	0.527097222	1.997457914	0.050506631	1.817195208	0.074516617
TRMT1L	0.270871881	0.787129797	0.31331175	0.755175626	-0.927215581	0.357777034
TRMT2A	0.602077981	0.548684775	-0.402514906	0.688795159	-0.033161076	0.973663556
TRMT2B	0.905223498	0.367841647	2.835854852	0.006295851	4.040712338	0.000163449
TRMT5	0.533710538	0.594899875	-0.161018303	0.872642442	0.370278016	0.712566315
TRMT6	0.237454388	0.812861381	-0.413105018	0.681063613	-0.519965539	0.605128466
TRMT61A	0.724066028	0.470963582	0.04349975	0.96545362	0.144534431	0.885595208
TRMT61B	2.536872124	0.012964248	-0.967660841	0.337259619	-0.511165107	0.611236552
TRMU	0.953351595	0.343047562	0.544495485	0.588202521	1.476351333	0.145427522
TRNAU1AP	0.201790149	0.84055085	-0.943307418	0.349463832	-0.167730247	0.867397231
TRNP1	0.20030164	0.84171115	-2.035907896	0.046367995	-1.431072096	0.157943348
TRNT1	1.75116767	0.083433906	-0.435743127	0.664651874	-1.00667435	0.318403209
TRO	1.317474591	0.191131216	-0.906557043	0.368418197	1.541618814	0.128774938
TROAP	0.83864867	0.403959872	0.201322057	0.841156063	1.452118841	0.152025185
TROVE2	-1.165687234	0.246919922	-0.271403444	0.787051152	-1.664839684	0.10150716
TRPC1	1.502886978	0.136481676	-0.41274495	0.68132593	-0.920470458	0.361258713
TRPC2	-0.114899876	0.908788681	-1.489244689	0.141878584	-1.626385088	0.109458972
TRPC4AP	-1.095269707	0.276414819	1.61814772	0.111094649	2.165208201	0.034629488
TRPC6	1.252998885	0.213556109	0.077916327	0.938164666	0.160875715	0.872767817
TRPM2	-0.360334105	0.719468756	1.702609542	0.094032802	0.912688781	0.365302446
TRPM4	-0.101900347	0.91906976	0.894031383	0.375025792	0.793719436	0.430695199
TRPM5	0.97925791	0.330161962	0.952958942	0.344593152	0.729247679	0.468876601
TRPM6	0.723750737	0.471156179	-0.076354351	0.939401795	-0.116311616	0.907819944
TRPM7	0.909391111	0.365650743	-0.193610874	0.847161764	-0.570508133	0.570605687
TRPS1	1.42650951	0.157292405	0.434006649	0.665905055	0.215469245	0.830181048
TRPT1	0.836498539	0.40516129	0.418108893	0.677422287	0.380246826	0.705194621
TRPV1	0.932439037	0.353684603	-0.070730591	0.943857193	0.316802261	0.752566717
TRPV2	-0.657426699	0.512638436	1.348201058	0.182872024	2.19328849	0.032437521
TRPV4	-3.084376692	0.002732354	0.585237157	0.560674229	0.844263211	0.402099582
TRPV5	0.538156218	0.591840371	-1.15679424	0.252131767	-0.537485604	0.593052797
TRPV6	-0.002102295	0.998327415	-0.329447029	0.743012916	1.046451004	0.299829868
TRRAP	1.468234859	0.145638969	0.368590119	0.713784636	0.420050876	0.676048874
TRUB1	1.273792575	0.206122107	-1.709242673	0.092789491	-1.813151414	0.075146329
TRUB2	1.027169396	0.307181961	0.90826079	0.367525207	2.637666629	0.010775716
TSC1	1.867043833	0.065256021	1.366409945	0.177121256	1.746719264	0.086145846
TSC2	-0.325818111	0.745342655	0.558357344	0.5787646	1.214275741	0.229716955
TSC22D1	-5.394999603	5.81E-07	1.325967855	0.190085012	-0.140124675	0.889062156
TSC22D2	-0.484589795	0.629182528	-1.466721593	0.147890887	-3.512345621	0.000883753
TSC22D3	0.338904888	0.735496243	0.285627742	0.776187964	-0.350714806	0.727112696
TSC22D4	-1.904126251	0.060191723	-1.139574453	0.25918075	-1.682901804	0.097938632
TSEN15	1.628585704	0.107008973	-2.157949846	0.035114184	-1.708220467	0.093110316
TSEN2	1.885706244	0.062664312	-0.891675725	0.376276806	0.181101435	0.856939081
TSEN34	-1.85017284	0.067675769	-0.709604254	0.480812505	-1.472490686	0.146463307
TSEN54	0.136343617	0.891863967	0.129662242	0.897285418	0.815800217	0.418056414
TSFM	0.569977783	0.570158563	4.124777922	0.000120752	4.446081097	4.17E-05
TSG101	-2.513993073	0.013773783	0.881441916	0.381742284	0.990388987	0.32622629
TSGA10	0.664353795	0.50821711	0.046849681	0.962795101	-1.306625005	0.196658466
TSGA14	1.301702658	0.196446864	0.008785347	0.993020757	1.157340068	0.252025565

TSHR	-0.780376023	0.437283072	-0.872313939	0.386659129	-0.087936143	0.930239821
TSHZ1	0.606267153	0.545912623	-0.654334532	0.515500686	-1.132982024	0.262029114
TSHZ2	2.888525671	0.004881254	2.363514021	0.021498639	2.189799452	0.032703069
TSHZ3	-2.419521791	0.017618296	-0.645009665	0.521481956	-1.3766502	0.174076726
TSIX	-0.065177596	0.94818181	2.291692993	0.025598715	2.870963026	0.005758669
TSKS	0.223354173	0.823782345	0.194836855	0.846206314	0.703181962	0.484843406
TSN	-0.54296442	0.588539678	0.899914517	0.371912984	1.339531124	0.185786266
TSNARE1	4.559430951	1.66E-05	-1.716093448	0.091519632	-1.764811186	0.083025387
TSNAX	0.542079621	0.589146417	0.34027026	0.734890922	-0.472416462	0.638457759
TSPAN13	0.735909006	0.463761304	0.718856225	0.475135891	0.807129725	0.42299235
TSPAN14	0.059738485	0.952500633	0.861577841	0.392492703	1.07231395	0.288159191
TSPAN15	-1.180168014	0.241143235	-1.864268056	0.06738291	-1.202098804	0.234362696
TSPAN16	-0.07321115	0.941805788	-0.60844322	0.54528567	-0.665314202	0.5085691
TSPAN17	-0.690609421	0.491645475	0.275125824	0.784204138	0.357385663	0.722140852
TSPAN18	-0.720547541	0.473115378	1.077248893	0.285860405	1.749935034	0.085584191
TSPAN2	-3.686045856	0.000395041	-1.897825498	0.062734729	-1.854811091	0.068868375
TSPAN3	1.706033537	0.091561764	1.786902254	0.07921589	1.486776439	0.142659307
TSPAN31	-0.313070562	0.754974713	1.70816606	0.092990365	2.851296536	0.006078363
TSPAN32	0.612923552	0.541522383	-2.314830246	0.024207783	-0.654284756	0.515595286
TSPAN33	-2.200949283	0.030381802	3.198770156	0.002244447	3.327111781	0.001551169
TSPAN4	1.644320486	0.103711131	-0.489878874	0.626080408	0.238521149	0.812343941
TSPAN5	-3.450265641	0.000865294	0.221579702	0.825424815	0.754158088	0.453900647
TSPAN6	-0.3705639	0.711861128	-0.31932346	0.750636551	2.184264799	0.033128229
TSPAN7	-1.588798909	0.115725678	0.330379683	0.742311859	0.897565909	0.37324366
TSPAN9	-4.582845912	1.52E-05	1.824609882	0.073247802	0.809506066	0.421636072
TSPEAR	-0.711049412	0.478951525	-1.833636582	0.07187649	-1.065046009	0.291406632
TSPO	-0.793521063	0.429628563	-0.092208514	0.926852346	-0.142420638	0.887256794
TSPO2	-2.520981336	0.013521826	0.466744969	0.642443099	0.379868272	0.705474039
TSPY26P	1.602900939	0.112573317	-1.763909901	0.083050299	-1.298359571	0.19946314
TSPYL1	-0.676672698	0.500404924	1.29036544	0.202080092	1.009445721	0.317084577
TSPYL2	1.750998571	0.08346321	-0.642378536	0.523176253	1.425027927	0.159675628
TSPYL4	0.710176705	0.479489761	-2.182602601	0.033156353	-2.354673926	0.02205466
TSPYL5	2.046507506	0.043718218	-0.226329243	0.821746653	0.153512794	0.878543439
TSR1	3.3324428	0.001264514	2.790975962	0.007115905	2.970625043	0.00436487
TSR2	-1.202132633	0.232566932	1.484385493	0.143159128	1.697851784	0.095063398
TSSC1	-0.488141498	0.626674999	1.276483687	0.206907541	2.204392032	0.03160502
TSSC4	-1.548812943	0.125049012	1.15604842	0.252434202	0.975234432	0.333620453
TSSK3	1.726399301	0.087817417	-2.070640497	0.042884671	0.204954978	0.838347328
TSSK4	1.571463968	0.119697005	0.152514179	0.879314431	0.65471622	0.515319458
TSSK6	0.296678856	0.767417292	-1.465692301	0.148170349	-1.164835175	0.249003137
TST	-2.398103255	0.018613045	1.377686542	0.173629801	0.153391388	0.878638729
TSTA3	-2.810950294	0.006099283	-0.390557508	0.69756516	-0.367370206	0.714721813
TSTD1	0.255115331	0.799234842	0.736059127	0.464681953	0.839021634	0.405009596
TSTD2	1.253082695	0.213525756	-0.44953064	0.654736069	-0.783289016	0.436743802
TTBK2	0.273940279	0.784778459	-0.908378758	0.367463427	-2.031708335	0.046918041
TTC1	-2.566286194	0.011986482	1.557138323	0.124915882	1.889403293	0.063997239
TTC12	2.797651658	0.006334124	0.439651794	0.66183459	1.362210625	0.178562754
TTC13	1.657371153	0.101038616	-2.322654836	0.023752839	-1.640684485	0.106444922
TTC14	1.650288605	0.102481971	-1.998397217	0.050401861	-2.437939802	0.017956428
TTC15	-0.816033017	0.416705105	-1.715059274	0.091710403	-2.225312832	0.030087455
TTC16	1.439315028	0.153641885	-3.103738379	0.002960753	-1.554353201	0.125710365
TTC17	1.171074462	0.244759429	-1.19437223	0.237227945	-1.005546197	0.318941045
TTC18	2.010063807	0.047515569	-0.921202569	0.360787231	-0.834783837	0.407371759
TTC19	0.645437877	0.520338416	1.088219828	0.281030761	0.984228592	0.329218758
TTC21A	0.476500028	0.634910191	1.734416744	0.088193528	1.528241011	0.13205825
TTC21B	1.773268555	0.079676204	0.621595431	0.536660584	0.532767769	0.596293389
TTC22	1.161888139	0.248451659	-4.042984303	0.00015861	-3.116077685	0.002884762
TTC23	0.279492064	0.78052917	1.270954057	0.20885424	0.788935757	0.433463039
TTC24	1.568523934	0.120381196	-1.155785567	0.252540852	-0.802921915	0.425400349
TTC25	-1.539169737	0.127384364	0.468453553	0.641228369	1.448752879	0.152959848
TTC26	-0.6850687	0.495117846	-1.639937002	0.106469229	-2.291364391	0.025709874
TTC27	0.76424614	0.44678396	2.556819619	0.013221914	3.080184483	0.003198524
TTC28	-0.730261355	0.467188101	0.960792668	0.340672666	1.735672601	0.088098551
TTC28-AS1	2.438518775	0.016775972	-1.635880083	0.107318362	0.335414676	0.738560358
TTC3	2.897441569	0.00475663	1.628697652	0.108835173	2.11123515	0.039209683
TTC30A	-0.776527261	0.439539277	-1.155754404	0.252553498	-1.734264686	0.088350039
TTC30B	-0.590076936	0.556665304	-0.752135391	0.455032455	-1.421020784	0.160832198
TTC31	1.516351588	0.133047844	-1.744228505	0.086453977	0.04457138	0.964606846
TTC32	0.351045924	0.726400727	0.1764932	0.860525619	-0.439394351	0.662059652
TTC33	0.750832185	0.454775321	-0.00211525	0.998319585	-0.576148999	0.566813239
TTC35	0.19498766	0.845856263	-1.575497339	0.120619027	-1.729352519	0.089232131
TTC37	1.709189982	0.090973029	0.584560097	0.561126427	0.568287269	0.572102209

TTC38	-0.490288032	0.625161652	-2.113799611	0.038874364	-1.641689942	0.106235557
TTC39A	-0.195333847	0.845586091	-0.427747572	0.670430031	-0.043242486	0.965661411
TTC39B	0.822140989	0.413239333	-1.467997647	0.147545002	-2.242342707	0.028899983
TTC39C	1.224483949	0.224067906	1.618577214	0.111001928	1.98303028	0.052255829
TTC4	0.383951796	0.701948924	-3.170395202	0.00243917	-2.543700371	0.013743763
TTC5	0.92694024	0.356516382	-1.456669293	0.150637925	-1.7177114	0.09135167
TTC7A	-1.110136202	0.269992674	2.420458452	0.018675968	1.65442684	0.103612286
TTC7B	-2.434558706	0.016948534	1.435272037	0.156618014	-0.194441721	0.846530737
TTC8	0.420747276	0.674974911	-0.757956117	0.451567421	-0.334236802	0.739444162
TTC9	-0.593407631	0.554444678	-2.517175359	0.014635914	-1.296392537	0.200135004
TTC9C	0.318948071	0.750528742	-2.39621286	0.01983443	-2.854000442	0.006033456
TTF1	-0.524391414	0.601336998	2.179939989	0.033363088	1.828614844	0.072762177
TTF2	2.111533467	0.037588261	1.684892805	0.09742094	-0.196044778	0.845281812
TTI1	-0.792727425	0.43008845	-0.565726538	0.573777142	0.163053476	0.871060857
TTK	0.218896589	0.827242197	2.640736708	0.010629439	2.811149627	0.006782525
TTL	0.902951795	0.369039369	0.670086234	0.505480638	0.342857508	0.732983959
TTLL1	0.002295755	0.998173499	2.095025966	0.040576789	2.45050173	0.017401476
TTLL11	0.376113908	0.707745853	-2.352723511	0.02207459	-3.459061382	0.001040721
TTLL12	-0.758093424	0.450439308	0.491023179	0.625275831	0.723996923	0.472068781
TTLL3	0.259207865	0.796085881	-0.684445446	0.496438883	0.617197779	0.539595303
TTLL4	-4.232015085	5.71E-05	0.913299433	0.364892392	1.047073113	0.299545393
TTLL5	0.337683773	0.736413164	-1.213272124	0.229978109	-0.437882156	0.663149001
TTN	-0.295596787	0.768240856	-0.396160918	0.693450159	-0.106785077	0.915339519
TTPAL	-1.448484459	0.151068429	1.268358854	0.209772561	1.84913086	0.069697421
TTYH2	0.034542087	0.972523839	0.265967957	0.791213655	-0.483934017	0.630311909
TTYH3	-1.133710844	0.260023514	-0.30881394	0.75857734	-0.421229335	0.675193255
TUB	1.46862475	0.145533336	-1.202909736	0.233932758	-0.612940899	0.542386374
TUBA1A	-2.147148747	0.03455731	-1.286032192	0.203577873	-1.016619503	0.313688359
TUBA1B	-0.689956688	0.492053851	-0.534854086	0.594809671	-0.080735941	0.935938595
TUBA1C	-2.708764347	0.008128468	0.243454353	0.808518467	-0.331772802	0.741294139
TUBA3D	0.762740877	0.447676651	-0.834410891	0.407497	-0.341195147	0.734228209
TUBA4A	-2.123454503	0.036549069	0.437791801	0.663174624	0.62996928	0.531266072
TUBA8	-4.560962099	1.65E-05	1.085315746	0.282303635	-0.335305593	0.738642193
TUBB	-0.25976978	0.795653783	-0.686206341	0.4953362	-0.147442991	0.883309724
TUBB1	-4.975605223	3.24E-06	0.772865605	0.442762108	-0.441391208	0.660622289
TUBB2A	-2.701552392	0.008292666	1.464917602	0.14838096	-0.115839188	0.908192651
TUBB2C	-1.40398441	0.163875497	0.922920639	0.359898756	1.672378319	0.10000503
TUBB3	0.745886769	0.457742095	-0.835249831	0.407028463	-1.285302081	0.203954862
TUBB4	-0.282325383	0.778363131	-1.463001722	0.14890282	-0.341320844	0.734134101
TUBB6	-0.303728536	0.762058346	1.857221335	0.068395084	-0.27666369	0.783052677
TUBBP5	0.8019085	0.424786115	0.808905909	0.421898166	0.927432121	0.357665621
TUBD1	1.940909166	0.055499894	0.550550406	0.584071019	2.090481353	0.041106707
TUBE1	1.737185904	0.085885704	-1.226537409	0.224987118	-1.279119316	0.206107894
TUBG1	0.816857337	0.416236356	-2.704836146	0.008971498	-1.481020178	0.144182608
TUBG2	1.92238538	0.057822449	-0.650091651	0.518217669	-0.030192967	0.976020056
TUBGCP2	0.158187202	0.87467509	0.56963599	0.571139743	1.319548527	0.192332777
TUBGCP3	1.91162068	0.059209531	-0.303706384	0.762446028	-0.648717159	0.519161615
TUBGCP4	1.009741501	0.315413195	-0.778946573	0.439199903	-0.851982727	0.397837388
TUBGCP5	3.50209034	0.000730362	0.550535091	0.584081451	0.991148698	0.325858515
TUBGCP6	2.372886838	0.019848012	-0.166762421	0.868141065	0.842020067	0.403343349
TUFM	-0.336621002	0.737211497	2.364669822	0.02143775	1.797319179	0.077654853
TUFT1	-2.919947846	0.004454961	-0.233092011	0.816516348	0.088467404	0.929819483
TUG1	0.847524521	0.399023364	-0.448910183	0.65518097	1.110534759	0.271494473
TULP3	-1.136739197	0.258761925	-1.4115472	0.163462836	-0.095519778	0.924241545
TULP4	1.979660548	0.050896055	1.555343057	0.12534255	1.230944393	0.223467118
TUSC1	0.768278589	0.444397612	-0.311329026	0.756674573	-2.339251669	0.022899547
TUSC2	-0.105837939	0.915954059	-2.674224532	0.009731251	-2.812416528	0.0067592
TUSC3	-2.052191276	0.043150126	1.291772853	0.2015954	0.984654191	0.329011434
TUT1	-0.400016202	0.690122933	0.965069919	0.338544485	1.839347857	0.071144999
TWF1	-0.309770746	0.757474455	-0.673648369	0.503229352	-1.17345257	0.245560407
TWF2	-1.471726875	0.144695008	0.965190565	0.338484584	0.952242043	0.345049212
TWISTNB	1.618429659	0.109182096	-0.174700074	0.861927967	1.072730072	0.287974023
TWSG1	-0.13967584	0.889238263	-0.980794467	0.330796063	-1.18266804	0.241916815
TXK	3.847937793	0.000226358	0.46735919	0.642006301	0.047376446	0.96238105
TXLNA	-0.630085903	0.530286255	3.13694125	0.002689106	2.761364956	0.007760113
TXLNB	0.322717213	0.747682038	-1.581460545	0.119249179	-2.327132436	0.023583614
TXLNG	1.270352255	0.207338687	0.801190927	0.426313992	1.16484734	0.248998253
TXN	-1.47959293	0.142586132	-0.341624442	0.73387683	-0.014769657	0.988268221
TXN2	-0.178551077	0.858704441	0.315748984	0.753334363	0.695198962	0.489793325
TXNDC11	-0.262853026	0.793283986	1.629897981	0.108580479	2.160295485	0.035026052
TXNDC12	-1.110303928	0.269920817	-1.038791198	0.303243161	-1.024499469	0.309986243
TXNDC15	0.350858662	0.726540721	0.953897368	0.344121953	1.152747274	0.253890555

TXNDC16	-0.167768744	0.867153746	-2.562009256	0.013046286	-2.136367681	0.037014976
TXNDC17	0.07713958	0.938689251	0.123816236	0.901891861	-0.000210812	0.999832542
TXNDC3	0.355481091	0.723087781	0.719681537	0.474631355	0.185414619	0.853570964
TXNDC5	0.949094273	0.345195995	-0.73043602	0.468084511	-1.730231126	0.089073824
TXNDC6	0.714207894	0.477006362	0.417183262	0.67809529	0.045821158	0.963615121
TXNDC9	0.051838971	0.958775586	-0.068287757	0.945793088	0.190178705	0.849853928
TXNIP	-0.816923945	0.416198493	0.979773096	0.33129575	-0.596350385	0.553334199
TXNL1	-0.764496644	0.446635499	-0.54885211	0.585228431	-0.139679478	0.889412292
TXNL4A	0.477820863	0.63397349	0.166645415	0.868232714	0.442886625	0.65954671
TXNL4B	-1.453263677	0.149740423	-1.239200232	0.220297314	-0.599495718	0.551250126
TXNRD1	-1.542123727	0.12666534	0.376274854	0.708095093	-0.320755515	0.749584596
TXNRD2	-0.143961115	0.885863399	2.37097103	0.021108498	2.219751578	0.030484455
TXNRD3	1.764035693	0.08122865	2.64201245	0.010593881	2.602747602	0.01180269
TYK2	-0.673422655	0.502459668	-0.664571693	0.508976561	-0.206334612	0.837274746
TYMP	-0.812122621	0.418933063	0.734293064	0.46574908	-0.838883937	0.405086217
TYMS	1.012174189	0.314255461	0.453006017	0.652246364	0.6652693	0.508597599
TYROBP	-3.053825626	0.002996165	-2.156261971	0.03525186	-2.621862833	0.011229964
TYSND1	2.51240119	0.013831764	-1.24853455	0.216886609	-1.058048165	0.294557177
TYW1	2.571088321	0.011833322	0.929957397	0.356274528	1.657850556	0.102916229
TYW1B	1.912645157	0.059076323	0.455453138	0.650495666	1.81199579	0.075327104
TYW3	1.491780251	0.139366231	-0.2083449	0.835694693	-0.038414849	0.969492969
TYW5	0.800770443	0.425441259	-1.414299284	0.162657162	-1.564225988	0.123374826
U2AF1	-1.751247706	0.083420039	1.210062463	0.231197778	1.183197266	0.241708763
U2AF1L4	-0.891870656	0.374917052	-3.349591067	0.001432365	-2.761812033	0.007750785
U2AF2	-0.497532584	0.620065993	-2.157312581	0.035166109	-2.267454872	0.027224523
U2SURP	1.734710092	0.086325967	0.825244582	0.412637728	0.437700151	0.663280163
UACA	0.435358377	0.664378159	1.751601709	0.085165558	1.891154021	0.063758676
UAP1	1.945254927	0.054966612	0.50445283	0.615867686	0.538902894	0.592080907
UAP1L1	1.654190946	0.101684661	1.194255608	0.237273189	1.549463739	0.126880059
UBA1	-1.525064665	0.130862281	1.640904236	0.106267591	1.945995722	0.056658695
UBA2	1.666190423	0.09926441	0.552147965	0.582983259	0.628549789	0.532188509
UBA3	0.569130003	0.570731176	-0.863204147	0.391605529	-1.669162021	0.100643661
UBA5	3.090395317	0.002682997	0.484208996	0.630073706	1.031697081	0.306630696
UBA52	-3.575092279	0.000573654	-0.853584043	0.396871592	-0.44771017	0.656082305
UBA6	1.294557878	0.198890827	0.749397222	0.456667767	-0.372065055	0.711242788
UBA7	0.103519966	0.917788047	1.964620035	0.054288884	2.115289611	0.038848125
UBAC1	-1.271341516	0.206988319	1.781433813	0.080114205	1.304733039	0.197297827
UBAC2	-0.982641851	0.328502657	1.866183044	0.067110042	2.453613046	0.017266434
UBAC2-AS1	1.560321607	0.122306478	0.266944872	0.790465078	0.726569546	0.470503228
UBAP1	-3.484997086	0.000772502	0.222130078	0.824998387	-0.209908496	0.834497716
UBAP2	-1.175905175	0.242833628	-1.071397112	0.288459895	-0.703833926	0.484440383
UBAP2L	-0.519541117	0.604699914	-0.832521494	0.40855341	-0.875112596	0.385234956
UBASH3A	2.173908526	0.032421901	1.655066224	0.103350691	2.514074858	0.01482293
UBASH3B	0.586881417	0.558799943	0.876117285	0.38460563	0.108510119	0.913977296
UBB	-3.17627325	0.002063264	0.994601547	0.324090327	0.45508531	0.650799948
UBC	-1.51920737	0.132328363	2.728147107	0.008429826	3.125125501	0.002810352
UBE2A	-0.284184093	0.776943118	-1.800084464	0.077084923	-1.653666767	0.103767332
UBE2B	-2.711104508	0.008075827	-0.600923965	0.550248254	-1.120999344	0.267052337
UBE2C	0.400062496	0.690088962	0.50523024	0.615325029	0.715866391	0.477035888
UBE2CBP	1.298489952	0.197543028	0.090807301	0.927960779	1.380320739	0.172950272
UBE2D1	-1.468456302	0.145578966	-1.293535358	0.200989646	-2.370213697	0.021231604
UBE2D2	0.360265574	0.719519817	-4.993292618	5.83E-06	-4.101176566	0.000133792
UBE2D3	-2.96111609	0.003948338	-1.306749874	0.196491317	-2.475457887	0.016344569
UBE2D4	1.215555708	0.227435356	-0.103331497	0.91805883	1.026733853	0.308941912
UBE2E1	0.218355251	0.827662602	-2.793918808	0.007059253	-2.337382835	0.023003865
UBE2E2	-0.32729177	0.744231731	-0.867438239	0.389301634	-0.749015158	0.456969662
UBE2E3	-0.720578608	0.473096354	-2.227908764	0.029807149	-1.221377618	0.227038696
UBE2F	-3.451022652	0.000863164	1.428961145	0.158416616	1.266730363	0.210473048
UBE2G1	-1.61224289	0.110523207	-1.66649037	0.101045578	-1.587338225	0.118043293
UBE2G2	1.186588142	0.238613323	-0.795771782	0.429432227	0.309504364	0.7580818
UBE2H	-3.462234933	0.000832188	1.790428707	0.07864106	1.543059539	0.12842526
UBE2I	-1.307690687	0.194415876	-1.197722469	0.235930876	-1.019775982	0.312201829
UBE2J1	3.675639896	0.00040923	-1.203806733	0.233588489	-0.996301005	0.323371595
UBE2J2	-1.287237821	0.20141812	0.746924585	0.458147404	1.141652986	0.258436233
UBE2K	-0.507950575	0.612770672	-1.353646274	0.181137609	-1.429390808	0.158423737
UBE2L3	-1.877070566	0.063852612	0.195367251	0.84579303	1.024983068	0.30976001
UBE2L6	-2.708623928	0.008131636	-0.76786276	0.445705425	-2.629719356	0.011002005
UBE2M	-1.538744483	0.12748814	-1.547020475	0.127335768	-2.186481831	0.032957342
UBE2N	1.332423308	0.186193111	-2.896625264	0.005324139	-2.602947173	0.011796575
UBE2O	-1.347335619	0.181363223	1.867128782	0.066975627	1.155703302	0.252689077
UBE2Q1	-0.84474896	0.400563072	0.05396624	0.957148815	0.323948244	0.747178974
UBE2Q2	1.322499789	0.189460385	-1.09682783	0.277281341	-1.475252105	0.145721849

UBE2Q2P1	1.503481	0.136328731	-1.345201069	0.183832966	0.335760599	0.738300866
UBE2R2	-2.490277971	0.014660556	-0.000999852	0.999205688	-0.511336551	0.611117291
UBE2S	-1.180729479	0.240921221	-0.167431176	0.867617277	-0.847805703	0.400140176
UBE2T	0.203622306	0.839123157	-1.140931576	0.258620172	-1.081338549	0.284161838
UBE2V1	0.969391945	0.335031156	-3.251414276	0.001921294	-2.827895535	0.006480121
UBE2V2	-0.061532873	0.95107567	-0.894631033	0.374707758	-0.952925529	0.344705818
UBE2W	-0.435749004	0.664095777	-1.018907579	0.312507751	-2.002659045	0.050043713
UBE2Z	-1.051409585	0.295975883	0.786575604	0.434754822	0.719650051	0.474720726
UBE3A	1.711932496	0.090464017	0.445329855	0.657750711	0.036617619	0.970919568
UBE3B	0.011452924	0.990888254	1.287302946	0.203137782	0.779718095	0.438826072
UBE3C	0.173908796	0.86234029	0.591611646	0.556425707	0.302742468	0.763203138
UBE4A	1.169255018	0.245487584	-0.319436621	0.750551194	-1.244585259	0.218446181
UBE4B	-2.30907478	0.023303885	0.561581109	0.57658019	0.300675307	0.764770907
UBFD1	0.962607419	0.338406727	1.432983199	0.157268487	1.421964368	0.160559271
UBIAD1	0.158021787	0.874805042	-0.620322499	0.537492273	1.132535407	0.262215128
UBL3	-0.282234104	0.778432886	-1.113115505	0.270282875	-2.054492001	0.04458594
UBL4A	-0.287495505	0.774415154	2.542116363	0.01373121	1.845243783	0.070269587
UBL5	-0.824664868	0.411812325	-1.336392067	0.186676798	-0.717154359	0.476247089
UBL7	-1.907046835	0.05980734	0.730361894	0.468129459	0.478444289	0.634188875
UBLCP1	-0.260490357	0.795099772	-0.047494921	0.962283083	0.152425538	0.879396875
UBN1	-2.767276361	0.006901879	0.416672567	0.678466717	0.492095584	0.624567329
UBN2	0.909243108	0.365728406	-2.36394784	0.021475767	-2.994688191	0.00407903
UBOX5	-2.056940063	0.042680362	1.561451841	0.123895467	2.154752934	0.035478232
UBP1	2.188135517	0.031334124	-1.687136348	0.096986424	-2.224820406	0.030122423
UBQLN1	1.027618656	0.306971705	-0.786990583	0.434513801	-2.110894412	0.039240202
UBQLN2	0.186312627	0.852632413	0.176811873	0.860276442	-1.136493468	0.260569872
UBQLN4	0.286221264	0.775387635	1.019783645	0.312095568	1.864535331	0.067468409
UBQLNL	-1.353130566	0.179512055	-1.035013388	0.304988831	-0.444980238	0.658042091
UBR1	0.545612018	0.586725878	0.315116694	0.753811903	-1.441273461	0.155052802
UBR2	-2.343671727	0.021369373	-0.076537143	0.939257011	-1.452791946	0.151838814
UBR3	-0.243962561	0.807832963	0.850338285	0.398658174	-0.741054344	0.46174376
UBR4	-0.582046286	0.562037531	1.099877852	0.275961259	1.252054137	0.215732538
UBR5	0.896643185	0.372378415	1.094294373	0.278381199	-0.999325534	0.321917646
UBR7	0.104468615	0.917037418	0.712412895	0.479085246	0.925526015	0.358647107
UBTD1	0.144107161	0.885748418	0.47735239	0.634917669	-0.428524794	0.66990602
UBTD2	0.576471211	0.565781991	0.623357491	0.535510414	-0.231504648	0.817763061
UBTF	-1.331917289	0.186358684	2.110246396	0.039191715	2.632241051	0.010929738
UBXN1	-0.439280079	0.661545398	2.520024934	0.014529888	2.626894506	0.011083478
UBXN10	-2.915970217	0.004506959	-0.379525942	0.705693089	0.027401598	0.978236432
UBXN11	0.595136708	0.553293616	0.408893879	0.684133988	0.653393631	0.516165218
UBXN2A	-0.901869034	0.369611107	0.289189132	0.773475032	0.706248945	0.482949124
UBXN2B	-1.874055377	0.064271947	-2.064284921	0.043504568	-2.674068991	0.009792528
UBXN4	1.302970029	0.196015689	-0.545820494	0.587297232	-1.676959834	0.099101053
UBXN6	-3.339602601	0.001235996	0.621732514	0.536571059	0.19857414	0.843312025
UBXN7	-0.890974853	0.375394764	-0.677267497	0.500947624	-0.674392035	0.502825064
UBXN8	1.297135744	0.198006441	-0.209289854	0.834960456	-0.429215338	0.669406425
UCHL3	-0.07624222	0.93940107	-1.232077018	0.222926538	-0.559018017	0.578368858
UCHL5	0.476905493	0.634622583	-0.585382291	0.56057732	-0.967166604	0.337601827
UCK1	-0.773678396	0.441213696	1.12457209	0.265435279	1.893223283	0.06347768
UCK2	1.031499832	0.305159332	0.969780654	0.336210777	0.529273158	0.5986991
UCKL1	1.413146469	0.161172785	-1.418198373	0.16152097	-1.438381231	0.155868092
UCKL1-AS1	2.034031288	0.044987784	-0.666287599	0.507887377	0.459856187	0.64739241
UCN	0.223898879	0.823359797	-0.619217543	0.53821475	-0.571021874	0.570259777
UCP2	-1.511569442	0.134259544	0.757283757	0.451966888	1.322601834	0.191321358
UCP3	1.911377538	0.059241182	-0.831919264	0.408890484	-0.48271141	0.631174441
UEVLD	-0.901216927	0.369955713	-1.517741445	0.134549702	-3.556482264	0.00077106
UFC1	-0.997051757	0.321498501	-1.354067628	0.181003923	-0.55995376	0.57773473
UFD1L	-1.8511036	0.067540343	-1.779083213	0.080502947	-1.286578822	0.203512362
UFM1	1.053486302	0.295028941	-1.009761938	0.316832683	-0.900748323	0.371563477
UFSP1	1.282834718	0.20294975	-3.802584822	0.000348237	-3.440078125	0.001102787
UFSP2	1.161414962	0.248642909	0.310031374	0.757656113	2.505293632	0.015157223
UGCG	1.724337195	0.088190738	-0.741245357	0.461556307	-2.07049967	0.04300809
UGDH	1.088623019	0.279320116	-0.605713939	0.547084313	-1.240949119	0.219776366
UGGT1	-1.553532344	0.123918528	1.18703733	0.240085738	1.818876631	0.074256087
UGGT2	2.102727094	0.038372275	-0.929935395	0.356285823	-0.43711961	0.663698599
UGP2	-1.931695398	0.056645063	2.634133637	0.010815233	2.446581665	0.017572974
UGT2B11	1.84719234	0.068110962	-0.53896268	0.59198984	-3.059108761	0.003397352
UGT8	1.649541098	0.102635274	1.471090069	0.146709405	1.936759823	0.057804862
UHMK1	0.829559983	0.409053104	-1.435872798	0.156447629	-1.976137544	0.053052298
UHRF1	2.293326532	0.024234566	-1.022558516	0.310792437	-0.573225852	0.56877697
UHRF1BP1	1.277748016	0.204729895	-1.554599893	0.125519514	-1.030766162	0.307063296
UHRF1BP1L	-1.827611696	0.071028566	0.29245781	0.770987573	-1.46308367	0.149011407

UHRF2	1.539391894	0.127330177	1.409580662	0.164040429	1.497308499	0.139905035
UIMC1	-0.91880052	0.36073479	1.12620925	0.2647476	0.811199075	0.420671401
ULK1	-1.288920026	0.20083523	-0.801651904	0.426049369	-0.67286855	0.50378659
ULK2	3.970039634	0.000147311	0.677178901	0.501003413	0.027553681	0.978115672
ULK3	3.101692826	0.00259258	-1.400463796	0.166738785	0.38748744	0.699858075
ULK4	2.996399685	0.00355706	0.374323617	0.709538162	-0.114600972	0.909169601
UMPS	2.350912335	0.020982992	1.221603446	0.226834141	2.355785536	0.021994851
UNC119	-1.275408379	0.205552543	-3.129027127	0.002751648	-2.706683823	0.008982084
UNC119B	1.012824788	0.313946318	1.498530026	0.139456745	2.005839244	0.049693006
UNC13B	0.214585366	0.830591696	2.271758822	0.026853507	2.549583535	0.013538129
UNC13D	-1.640454309	0.104513729	0.5219147	0.603731167	0.494895646	0.622601846
UNC45A	-0.211853297	0.832715937	3.113663725	0.00287697	3.288397122	0.00174108
UNC45B	-0.339511784	0.735040675	-0.831869907	0.408918117	-0.713715888	0.47835457
UNC50	1.185614426	0.238995794	-1.465592309	0.14819752	-0.83298957	0.408374426
UNC5A	0.438320798	0.662237861	-0.892036352	0.376085118	-2.016243345	0.04856041
UNC5B	-0.884134714	0.37905506	1.803952643	0.076468787	0.688846866	0.493751858
UNC5CL	1.518405158	0.132530161	-1.572898586	0.121219944	-0.644113989	0.522120027
UNC93B1	-0.987718768	0.326023542	-1.873897996	0.066020167	-3.389857991	0.001284366
UNG	2.174631165	0.032365866	0.531556926	0.597077137	1.569228004	0.122204879
UNK	0.863108747	0.390445687	-3.272345928	0.00180542	-2.708667526	0.008934833
UNKL	2.175200122	0.032321807	-1.794531524	0.077976667	-1.991931326	0.051242504
UPB1	0.95394281	0.342749898	1.16343635	0.24944978	1.03098864	0.306959873
UPF1	-1.541652584	0.126779804	-0.566342259	0.573361371	-0.62425226	0.534986269
UPF2	-1.707908052	0.091211758	1.316950404	0.193071046	0.899337132	0.372307932
UPF3A	1.171981106	0.24439716	0.600238291	0.550701921	0.516185565	0.60774858
UPF3B	2.659263971	0.009317699	-0.92850185	0.357022253	-1.149961233	0.255026675
UPK3A	-1.187489006	0.238259858	-0.12254706	0.90289238	0.594969654	0.554250311
UPK3B	0.434686423	0.664864022	-1.103898953	0.274227616	1.020153681	0.312024274
UPP1	0.198572624	0.843059367	2.151382466	0.035652531	1.656992194	0.103090378
UPRT	-0.948678068	0.345406498	-0.195561185	0.845641928	-1.655089128	0.103477341
UQCC	3.821526381	0.000248133	0.806264383	0.42340699	2.106697853	0.039617773
UQCR10	-1.289684831	0.200570637	-4.744741095	1.42E-05	-3.217531332	0.002146821
UQCR11	-0.824505279	0.411902469	-1.500315204	0.138994891	-1.058426032	0.294386459
UQCRB	0.025098506	0.980033747	-0.658528021	0.512822807	-0.340066025	0.735073747
UQCRC1	-0.909251341	0.365724085	2.267029998	0.027159053	3.017725855	0.0038218
UQCRC2	-0.010775462	0.991427209	-0.006186406	0.995085372	1.28190611	0.205135356
UQCRFS1	-0.142685934	0.88686745	2.466469626	0.016643276	2.293283327	0.025591617
UQCRH	0.16409405	0.87003691	-1.514534539	0.135359163	-0.489128062	0.626653357
UQCRQ	-0.081471781	0.935253499	-1.746811637	0.086000765	-1.040833301	0.302407077
URB1	2.020697982	0.046379395	1.469095775	0.147247853	1.634934271	0.107648742
URB2	1.908210721	0.059654737	2.265798698	0.027239116	2.406121718	0.01943394
URGCP	1.231882653	0.221304943	2.92544785	0.004913615	3.567847692	0.000744339
URM1	-0.338266918	0.73597524	0.549832825	0.584559926	1.410199476	0.163987997
UROD	0.697642658	0.487256965	0.880139781	0.382441271	1.660241046	0.102432498
UROS	0.65224356	0.515959946	0.739105993	0.4628442	1.734546482	0.088299656
USE1	-0.812729465	0.418586845	1.41229256	0.163244327	2.070146047	0.043042415
USF1	-2.321484743	0.02259287	1.034981599	0.305003549	0.500197201	0.618888022
USF2	-0.918577468	0.360850834	-2.982553099	0.004185635	-3.288872371	0.001738621
USMG5	-0.249071294	0.803891433	-1.02491423	0.309689046	-0.515437593	0.608267656
USO1	0.713715699	0.477309193	-0.204338911	0.838809004	-1.104852943	0.273928013
USP1	0.290982114	0.77175607	0.770856202	0.443942924	0.077446685	0.938543096
USP10	-4.503017933	2.06E-05	0.006293439	0.995000343	-0.211334086	0.833390573
USP11	0.12341922	0.902059205	0.92852498	0.357010363	1.44129371	0.155047106
USP12	-1.004804374	0.317771563	-1.455988444	0.150825418	-2.267449108	0.027224897
USP13	1.120651503	0.265513518	0.516354732	0.60758354	1.254257693	0.21493671
USP14	2.018958955	0.046563589	1.27475367	0.207515133	1.42305422	0.160244484
USP15	-0.36334147	0.717229282	-0.00943449	0.992505081	-0.915745546	0.363710555
USP16	1.870652969	0.064747909	-0.464170737	0.64427512	0.014815054	0.988232165
USP18	-1.730660436	0.087050096	1.107361834	0.272740773	-1.057783037	0.294677002
USP19	-1.312096597	0.192931541	1.207549789	0.232155878	0.91254858	0.365375566
USP20	0.319209704	0.750331027	-0.193235029	0.84745472	0.691624821	0.492018517
USP21	-0.231152296	0.81773807	1.50844558	0.136906708	2.106604706	0.03962619
USP22	-1.70527778	0.091703188	1.656269183	0.10310596	1.269976423	0.209322735
USP24	1.79326335	0.076398221	0.489074427	0.626646299	0.516801861	0.607321035
USP25	-1.249490497	0.21482957	-0.494161466	0.623071609	-2.591613938	0.012148408
USP27X	0.814232478	0.417730082	0.258190622	0.797180161	0.815838402	0.418034753
USP28	0.001071425	0.999147574	-1.724684492	0.089947491	-1.240538917	0.219926802
USP3	-1.698624132	0.092955997	-1.354980809	0.180714449	-2.079149435	0.042175835
USP30	0.367067167	0.714458321	-1.898617606	0.062628382	-0.334566941	0.73919641
USP31	-2.503143982	0.014173303	-0.586762508	0.559656135	-0.719658168	0.474715766
USP32	-2.276809989	0.025245715	-0.33822641	0.736422376	-2.022290133	0.047912435
USP33	0.627460859	0.531997028	0.555557599	0.580664928	-1.115702806	0.269294222

USP34	0.909040099	0.365834949	-0.242537597	0.809225226	-0.024608403	0.98045443
USP35	0.632638273	0.528625574	-2.185444031	0.032936976	-1.943662779	0.056946363
USP36	2.508056542	0.013991126	0.347101053	0.729780492	0.561389655	0.576762317
USP37	0.530740071	0.596948229	-0.558285393	0.5788134	-0.231516626	0.817753801
USP38	-0.159320414	0.873784924	0.431053678	0.668038352	-1.364811281	0.177748336
USP39	-0.947866538	0.345817182	0.029982497	0.976184664	0.427888364	0.670366596
USP4	-2.188075895	0.031338616	0.473512525	0.637637446	0.450751611	0.653901748
USP40	2.561188444	0.012151024	0.405333063	0.686734399	2.980932703	0.004240218
USP42	0.226722789	0.821170025	-2.429731848	0.018249191	-3.026627894	0.00372652
USP43	0.208779387	0.835107451	-1.891984961	0.063523613	-1.376586212	0.174096413
USP44	1.253312221	0.213442645	0.05574854	0.955735069	0.191434728	0.848874521
USP45	2.46990767	0.015462769	-1.012268893	0.315643165	-1.172674581	0.245869806
USP46	2.272361112	0.02552435	1.377873529	0.173572354	0.45735411	0.649178546
USP47	1.243006102	0.217197889	1.808884379	0.075689224	1.543154061	0.128402345
USP48	1.286249332	0.20176122	2.886590156	0.005474377	2.773274972	0.007515106
USP49	-0.445766997	0.656870525	-0.639411817	0.525090123	-2.545771031	0.013671066
USP5	-0.272994657	0.785502885	2.305265862	0.024774399	2.820467694	0.006612686
USP51	1.12224191	0.264840619	0.791714111	0.431775935	0.88436454	0.380264901
USP53	2.141205592	0.035047826	-0.785788787	0.435212028	-1.470105166	0.147106216
USP54	2.769766586	0.00685364	-0.724261333	0.471837091	0.52892814	0.598936857
USP6	-1.177928691	0.242030166	0.278325455	0.781759307	-1.433696199	0.157195837
USP6NL	-1.095300738	0.276401305	-1.147430281	0.255947743	-1.901460041	0.06236956
USP7	-1.253759788	0.213280652	1.583162856	0.118860433	1.66213526	0.102050513
USP8	0.185670262	0.853134618	0.146066193	0.884379154	-0.506694512	0.614350151
USP9X	-1.362388562	0.176584334	-0.097683732	0.922522578	-0.358512777	0.721301986
USPL1	0.471607301	0.638385157	-1.600073774	0.115053728	-1.85126849	0.069384446
UST	0.648971659	0.518062511	-2.4754128	0.016272134	-0.697852947	0.488144609
UTF1	-0.785518529	0.434279064	-1.267105672	0.210217076	-1.946484064	0.056598637
UTP11L	-0.043750723	0.96520315	-0.657903515	0.513221133	1.67783376	0.098929382
UTP14A	0.41732107	0.677469395	3.031829195	0.003639489	3.89385963	0.000264284
UTP14C	0.490321104	0.625138348	0.055807065	0.955688649	-0.465729894	0.643207596
UTP15	0.786610196	0.433642927	-1.676584228	0.099044019	-1.023715328	0.31035331
UTP18	3.464364665	0.000826424	2.24604152	0.028552658	2.509809378	0.014984471
UTP20	1.539967337	0.127189904	1.701456125	0.094250391	2.311513264	0.024492057
UTP23	0.042222244	0.966418071	-0.55436574	0.581474812	-1.837427089	0.071432164
UTP3	0.423335104	0.673093219	2.004158208	0.049763358	2.146641542	0.036149202
UTP6	1.197014834	0.234545287	1.295089131	0.200456763	1.794703171	0.078076013
UTRN	1.494438636	0.138671506	-1.690232282	0.096389436	-2.518618675	0.014652583
UTS2	-0.449950984	0.653862503	-1.044541056	0.300599341	-0.40013852	0.690570424
UTS2D	0.521647244	0.603238593	-0.843205657	0.402601653	0.842800972	0.402910088
UVRAG	-0.088349105	0.929801824	0.684408665	0.49646193	0.50763026	0.613697845
UXS1	0.924977105	0.357530871	1.788755879	0.078913304	2.864018734	0.005869732
UXT	-0.703953126	0.483337826	0.934654696	0.353868407	1.804782483	0.076463742
VAC14	0.589222238	0.557235855	1.22244803	0.226517188	1.953239668	0.055773369
VAMP1	2.41065894	0.018024021	-1.28699589	0.203244057	-0.501144597	0.618225404
VAMP2	-1.348571301	0.180967286	-2.667506732	0.009905665	-1.398260801	0.167525005
VAMP3	-2.383043042	0.019342119	-1.48600473	0.142731403	-1.269373767	0.209535946
VAMP4	1.706685259	0.091439951	-0.890154766	0.377085942	-1.276474165	0.207034179
VAMP5	-2.925681591	0.004380978	0.333314919	0.740106938	-0.470880514	0.63954749
VAMP7	-0.734303469	0.464734037	-0.130012213	0.897009764	-0.48891176	0.626805527
VAMP8	0.232214056	0.81691595	0.114819958	0.908987176	0.725914902	0.470901328
VANGL1	-0.103495381	0.917807501	-0.006718425	0.994662729	0.178868482	0.858683824
VAPA	0.268249408	0.789140986	-0.856608522	0.39521128	-0.91808189	0.362496851
VAPB	-0.366481848	0.714893396	1.32883541	0.189142804	0.938196878	0.352155297
VARS	-0.348304427	0.72845116	1.930249352	0.058504871	2.147784116	0.036054022
VARS2	1.030814882	0.305478653	0.523184922	0.602852642	1.730503422	0.08902481
VASH1	1.174250073	0.243492223	0.380604256	0.70489706	0.824115565	0.413355526
VASH2	0.267050837	0.790060654	0.376368068	0.708026182	-0.944243256	0.349084575
VASN	1.915209721	0.058743976	-1.380244477	0.172845206	-0.779746621	0.438809415
VASP	-2.34630146	0.02122832	-0.196815069	0.844665113	-0.47405434	0.637296595
VAT1	-0.733722312	0.465086421	2.800242883	0.006938913	2.709756442	0.008908992
VAV1	-2.120954988	0.036764872	0.861168641	0.392716125	0.837700227	0.405745249
VAV2	0.252762689	0.801046567	1.550136422	0.12658657	1.681024927	0.098304589
VAV3	-0.868921645	0.387275628	-0.764154687	0.447894354	-1.303967482	0.197556979
VBP1	0.120629562	0.904262007	0.043248303	0.965653186	0.179707039	0.858028525
VCAN	0.127478471	0.898855266	2.234913683	0.02931687	1.212537621	0.230375936
VCL	-1.060409141	0.291887188	0.362504533	0.718301854	0.103778815	0.917714099
VCP	-1.516787824	0.13293774	2.456370683	0.017071538	2.29213901	0.025662079
VCPIP1	-0.03670054	0.970807676	-0.478676889	0.633980695	-2.081980124	0.04190653
VDAC1	-0.640282297	0.523668207	1.719133249	0.090960784	2.174019957	0.033928044
VDAC2	-0.089850031	0.928612472	3.633851134	0.000596168	4.904085589	8.37E-06
VDAC3	1.111834183	0.269265845	2.107820438	0.039409682	2.778122556	0.007417438

VDR	-3.519972296	0.000688609	0.835181791	0.407066451	0.118020576	0.906471889
VEGFA	-0.062144695	0.950589845	-1.202354941	0.234145873	-1.240692217	0.219870573
VEGFB	0.792724104	0.430090375	1.38584076	0.171138147	2.225834636	0.030050441
VENTX	1.677438632	0.097038352	0.379333315	0.705835324	0.962632199	0.339853207
VEPH1	-1.100651339	0.27407789	1.956255047	0.055290257	1.88370251	0.064779309
VEZF1	-0.797362003	0.427406977	-1.19604401	0.236580058	-2.478523864	0.016218798
VEZT	1.23056978	0.221793399	-0.58501949	0.560819585	-1.071005246	0.288742086
VGLL4	0.220415026	0.826063241	0.286759678	0.775325393	0.109978318	0.912818099
VHL	-2.443682291	0.016553326	1.139133935	0.259362898	2.403685253	0.019551456
VIL1	-2.893761641	0.004807708	1.787573527	0.079106199	1.52402906	0.133105665
VILL	1.045161724	0.298837253	0.305555154	0.761044979	0.993230699	0.32485204
VIM	-1.677665876	0.096993801	2.218012364	0.030512129	2.600918086	0.011858877
VIPAR	0.117679131	0.906592576	1.832301973	0.072077871	1.991999973	0.051234755
VIPR1	0.434561831	0.664954125	0.151855052	0.87983193	1.105865592	0.273493175
VIPR2	0.937227621	0.351230385	-1.257806275	0.213537529	-0.740241806	0.462232642
VIT	-0.389703715	0.697705957	-2.813631723	0.006690355	-2.881882475	0.005587957
VKORC1	-0.875895569	0.38349354	-0.269907072	0.788196458	0.438091036	0.662998486
VKORC1L1	-1.127927402	0.262444871	-3.100262329	0.002990632	-4.896950153	8.59E-06
VLDLR	0.151706358	0.879769023	-1.023263201	0.310462091	-1.158359019	0.251613134
VMA21	0.763516039	0.447216815	-0.569878912	0.570976058	-0.397980577	0.692151321
VMAC	3.189910022	0.001978124	-1.09011306	0.280203103	0.058705673	0.953394651
VMO1	-0.706379458	0.48183559	-0.944748868	0.348733563	-0.734487548	0.465703301
VMP1	-1.931088777	0.056721156	-2.189423857	0.032631851	-2.545798391	0.013670108
VN1R1	0.203076677	0.839548278	-0.652292486	0.516807389	0.689086006	0.493602512
VNN1	-0.658235239	0.512121324	0.721238123	0.473680593	-0.41047338	0.683018463
VNN2	-1.471996466	0.14462233	-0.091150362	0.927689386	-0.395083557	0.69427583
VNN3	-3.661028929	0.000429972	-0.406371756	0.685975462	-0.602202488	0.54945982
VOPP1	-0.136189161	0.891985704	1.82474513	0.073227095	2.113524008	0.039005215
VPRBP	3.047515491	0.003053519	1.249767073	0.216439182	1.631259514	0.108423842
VPREB3	-2.843358021	0.005560018	0.60526283	0.547381891	0.058684137	0.953411728
VPS11	-0.589521301	0.557036183	-0.076897664	0.938971459	0.641152782	0.524027859
VPS13A	1.356471835	0.178451217	-1.101850604	0.275109778	-2.319086534	0.024047762
VPS13B	0.083030707	0.934017453	-1.17897146	0.243256981	-1.264209131	0.211369737
VPS13C	0.938424888	0.350618489	-0.308572499	0.75876008	-1.89708925	0.062955515
VPS13D	1.256615432	0.212249198	0.728506417	0.469255375	0.976933582	0.332785924
VPS16	-0.2099025	0.834233484	-0.143985029	0.8860149	0.601825889	0.549708733
VPS18	-1.408344965	0.162584876	1.985682047	0.051836082	1.882899923	0.064890061
VPS24	0.621341704	0.53599597	-2.35483201	0.021960976	-3.466319851	0.001017879
VPS25	-0.046164822	0.963284455	-2.426804902	0.018382938	-0.869678262	0.388173146
VPS26A	-0.02393779	0.980956932	-0.764363453	0.447770949	-0.563987417	0.575005082
VPS26B	-1.856848523	0.066709466	1.582325615	0.119051501	1.611594669	0.112649035
VPS28	-1.595806178	0.114150561	-0.182009125	0.856214633	0.242812899	0.809033779
VPS29	1.202420559	0.232455989	0.078427177	0.937760091	-0.05038354	0.959995281
VPS33A	-0.716329227	0.475702405	3.676868189	0.000520419	3.308176378	0.001641467
VPS33B	1.689082227	0.09477699	1.903826498	0.061932846	3.101173672	0.003011373
VPS35	-0.625046985	0.533572686	2.231889257	0.029527672	1.996725286	0.050703775
VPS36	1.845194999	0.06840391	-0.867178056	0.389442963	-1.263031714	0.211789461
VPS37A	-0.246682735	0.805733648	-0.989545825	0.326535142	-1.597537919	0.115750201
VPS37B	-1.571908608	0.119593801	-0.065684411	0.947856546	1.372765015	0.175275175
VPS37C	-1.132861116	0.260378281	-1.00028595	0.321356146	-0.681989374	0.49804501
VPS39	-0.935844333	0.35193821	1.609188925	0.113043053	2.165647503	0.034594219
VPS41	-1.513416977	0.13379039	0.027420369	0.97821924	0.493627001	0.623492021
VPS45	1.439505947	0.153587959	1.809754751	0.075552337	2.617990214	0.011343906
VPS4A	0.030695834	0.97558227	1.616948521	0.11135387	2.42984311	0.018322504
VPS4B	-0.915643169	0.362379628	-0.156996286	0.875796825	-1.414914723	0.162607056
VPS52	0.728730488	0.468119432	-0.295810907	0.76843838	1.855993594	0.068696836
VPS53	-0.183979297	0.854456911	2.388183919	0.020232011	2.618515774	0.011328381
VPS54	0.435819789	0.664044612	0.048249261	0.961684512	-0.418709823	0.677023068
VPS72	-0.516301686	0.606950718	-1.049040109	0.298541672	0.134162452	0.893753108
VPS8	-1.671397277	0.098228856	0.071464924	0.943275316	-0.493406847	0.623646555
VRK1	1.127312462	0.262703257	-0.289039747	0.773588771	0.863785142	0.391375187
VRK2	-0.457211551	0.648656187	0.132564896	0.89499954	-1.177750881	0.243856061
VRK3	-2.475774237	0.015227798	2.18313177	0.033115401	2.193305453	0.032436235
VSIG1	1.037345434	0.302443295	-0.133049294	0.894618156	1.315389704	0.193716889
VSIG10	-0.645339865	0.520401614	1.689706634	0.096490583	1.357490226	0.180048268
VSIG10L	-0.414483108	0.679538332	1.148891684	0.255349499	-0.623464498	0.535499939
VSIG2	-3.113887365	0.002498169	-0.0360314	0.97138191	-0.330833838	0.741999519
VSIG4	-2.566614815	0.011975944	0.892096898	0.376052941	-0.138116302	0.890641864
VSIG8	-1.798241043	0.075599793	-0.803261383	0.425126217	-1.235641326	0.221728784
VSTM1	-1.089749759	0.278826132	0.023119524	0.981634843	0.125000578	0.900968864
VSTM4	-1.428204089	0.15680552	0.81358401	0.419234013	-0.095394185	0.924340847
VTA1	-0.266506741	0.790478239	-1.307140672	0.196359448	-1.541874149	0.12871291

VTI1A	-0.39889523	0.690945696	-1.272243978	0.208398912	0.11442084	0.909311736
VTI1B	-2.759329407	0.007057905	-0.908266263	0.36752234	-0.454544048	0.651187013
VTN	0.194560083	0.84618998	-0.175157428	0.861570242	-1.22691791	0.22496527
VWA5A	-0.449061441	0.654501551	1.463656349	0.148724347	2.167539711	0.034442666
VWCE	-2.497981404	0.014367044	2.264506497	0.027323363	0.365147022	0.716371391
VWDE	-0.116754437	0.907323169	2.388521117	0.020215171	2.723373098	0.008591596
VWF	-2.698642522	0.008359767	1.172005489	0.246019998	1.28340732	0.204612884
WAC	-1.058837233	0.292598544	-1.612722903	0.11227119	-2.371524134	0.02116347
WAPAL	-0.087966058	0.930105381	-0.263618338	0.793014898	-1.446983069	0.153453087
WARS	-2.73171528	0.00762544	-0.326588988	0.745162606	-0.327674603	0.744374474
WARS2	1.717463572	0.089444537	-0.398657028	0.691620053	-0.193434366	0.84731576
WAS	-2.163837508	0.033211677	-1.165828778	0.248488782	-1.574549455	0.12097001
WASF1	1.859048332	0.066393587	1.481446191	0.143938129	1.429177404	0.158484794
WASF2	-1.737680764	0.085797927	-0.315675383	0.753389946	0.062778655	0.950165366
WASF3	-3.291815535	0.001438391	0.588185037	0.558707499	-0.923164362	0.359865572
WASH1	0.331486987	0.74107212	-1.473823409	0.145973933	0.683614801	0.497025566
WASH2P	0.535910409	0.593385012	-0.212366452	0.832570939	0.781977723	0.437507761
WASH3P	0.471775368	0.638265655	0.652036077	0.516971589	0.602483377	0.549274203
WASH7P	0.044776369	0.964387953	0.921083674	0.360848768	0.751458037	0.455510398
WASL	0.557863399	0.578367278	-0.67283718	0.503741549	-0.983071306	0.329782952
WBP1	-0.562012028	0.575549806	-1.717910873	0.091185171	-1.858881266	0.068279451
WBP11	-1.354876938	0.178956997	0.176018871	0.860896533	0.526291123	0.600755517
WBP2	-3.013002207	0.003385645	-0.873878339	0.38581365	-1.47243822	0.146477423
WBP2NL	0.906727932	0.367049812	-0.331684889	0.741331131	-1.857568141	0.068468984
WBP4	-0.574073581	0.567396082	0.660305766	0.511689823	-0.392633998	0.696074124
WBSCR16	-0.468020935	0.640937451	2.759667924	0.00774507	2.829840663	0.006445814
WBSCR22	1.064984458	0.289823394	1.983347407	0.052103193	3.017369168	0.003825665
WBSCR27	0.616100613	0.539433286	0.23797923	0.812741786	0.583367029	0.561978606
WDFY1	-0.871897087	0.385659168	-0.395036081	0.694275471	-1.919858593	0.05995356
WDFY2	3.804896571	0.000262854	-1.145975922	0.256544095	-1.908931057	0.06137871
WDFY3	-0.685079066	0.495111338	-0.565838905	0.573701254	-1.789132644	0.078979195
WDFY4	-1.315663129	0.191736207	1.264829181	0.211026356	1.256845162	0.214005004
WDHD1	1.642456608	0.104097438	-0.904042177	0.369738852	-0.979049841	0.331748471
WDPCP	1.443548747	0.152449483	-0.206181445	0.837376268	-0.475275037	0.636431781
WDR1	-1.489884623	0.139863285	0.414438863	0.680092222	0.463647527	0.64468989
WDR11	2.605047438	0.010799669	2.940122569	0.004716074	1.639053505	0.106785253
WDR12	2.155416846	0.033884834	0.467656356	0.641795019	0.912940102	0.365171396
WDR13	-1.378719756	0.171508273	-0.980901848	0.330743558	-0.759291413	0.450849301
WDR17	0.258684474	0.796488412	-0.869971581	0.387927223	-1.918750338	0.060096801
WDR18	0.137821394	0.890699367	-0.517605967	0.606715607	0.81552477	0.418212682
WDR19	3.143433443	0.002282528	0.468469894	0.641216756	1.311864597	0.19489596
WDR20	-1.406799146	0.163041507	-5.04541738	4.83E-06	-5.93046851	1.95E-07
WDR24	1.385043419	0.169572869	0.276629646	0.783054797	0.824514321	0.413130909
WDR25	-1.281588125	0.203384944	0.60323397	0.548721263	0.665713249	0.508315861
WDR26	0.163253297	0.870696813	-1.104138724	0.274124484	-2.00702643	0.049562629
WDR27	2.611490694	0.010613021	-0.831811881	0.408950605	-0.710316048	0.480443511
WDR3	2.177516574	0.032142965	2.146919788	0.036022459	1.971574163	0.053585317
WDR33	1.533237453	0.128838083	0.820772033	0.415160316	1.580535072	0.119593019
WDR34	0.382712158	0.702864602	1.53435979	0.130416379	1.954435766	0.055628331
WDR35	0.972049496	0.333714954	-0.368214163	0.714063405	-0.417643922	0.677797776
WDR36	1.478567808	0.142859597	-0.892706885	0.375728865	-1.305387324	0.197076545
WDR37	2.539908834	0.012860081	1.492747484	0.140961105	2.167993145	0.034406435
WDR4	1.028179405	0.306709407	0.295301366	0.768825594	-0.748132612	0.457497518
WDR41	0.539976944	0.590589478	0.844319067	0.401984497	0.248673174	0.80451951
WDR43	1.884987454	0.062762505	0.421544496	0.67492666	0.381647566	0.704161069
WDR44	0.395441234	0.69348317	0.586394035	0.559901987	-0.820405248	0.415449084
WDR45	-2.513620867	0.013787321	-0.679736104	0.49939449	-0.595620891	0.553818122
WDR45L	-0.403322762	0.687698178	-1.262753391	0.211766302	-2.395976046	0.019927497
WDR46	-0.49481994	0.621971852	2.585349995	0.012282274	2.966261114	0.004418664
WDR47	0.350417575	0.726870508	0.650963101	0.517659006	-0.832991345	0.408373434
WDR48	0.235645898	0.814260082	-0.227974272	0.820473634	0.624198167	0.535021533
WDR5	1.131795708	0.260823577	2.283336005	0.026118284	2.682742301	0.009570713
WDR52	2.651751021	0.009511402	0.151036792	0.880474441	1.017765709	0.313148006
WDR53	-0.578432985	0.56446299	1.27918011	0.205963184	1.299633766	0.199028827
WDR54	-0.40008527	0.690072251	0.936651046	0.352849004	1.834865065	0.071816719
WDR55	0.752439347	0.453813556	1.231688563	0.223070579	2.31883655	0.024062313
WDR59	0.728553502	0.468227172	2.096206601	0.040467847	2.369105203	0.021289392
WDR5B	0.38366587	0.702160089	-2.130436277	0.037418101	-1.860364538	0.068065892
WDR6	1.992352327	0.049460735	1.923534266	0.059360364	2.675836398	0.00974695
WDR60	3.310469563	0.001355942	-0.138444195	0.890372276	1.309054755	0.195839657
WDR61	0.997171542	0.321440697	0.036616615	0.97091731	1.485927561	0.142883148
WDR62	0.648942238	0.518081437	0.184047001	0.854623032	0.944630298	0.348888608

WDR66	-0.199258314	0.842524637	1.432422799	0.157428069	2.243320416	0.028833083
WDR67	1.037807685	0.30222922	-0.71689677	0.47633497	0.586556024	0.559849155
WDR7	0.763057947	0.447488528	0.922477412	0.360127829	-0.09789044	0.92236736
WDR70	0.770516239	0.443076599	1.50016445	0.139033847	3.031140858	0.003679068
WDR73	1.64521494	0.103526159	0.90506168	0.369203107	1.813934791	0.075023992
WDR74	1.193660282	0.235848597	2.28552465	0.025981316	3.110637135	0.002930391
WDR75	2.511783812	0.01385431	0.13403644	0.89384102	0.479729282	0.633280454
WDR76	2.830230668	0.005772955	0.268069061	0.789603895	0.273200409	0.785699974
WDR77	1.901567011	0.06053026	2.478254779	0.016155764	2.887207504	0.005506418
WDR78	0.923391124	0.358351808	-1.636728091	0.107140416	-0.882814606	0.381094678
WDR81	0.584203044	0.560592235	0.984694501	0.328892647	1.024838249	0.309827746
WDR82	-1.150398964	0.253125021	-0.175966122	0.860937784	0.150057075	0.881256484
WDR83	-0.223093672	0.823984444	1.149819448	0.254970225	1.252036822	0.2157388
WDR85	1.881302391	0.063267953	1.335985113	0.186808978	1.617210774	0.111428975
WDR86	1.786952249	0.077420574	-1.224250201	0.225841957	-0.558370424	0.578807911
WDR88	1.052317636	0.295561575	-0.594543559	0.554477045	-0.510511578	0.611691262
WDR89	1.683884465	0.09578111	0.378751546	0.706264964	0.938946399	0.351773693
WDR90	2.460465149	0.015847789	-0.032863163	0.97389733	-0.07532281	0.940225184
WDR91	1.641367761	0.10432365	2.88871062	0.005442308	2.333740249	0.023208413
WDR92	0.268026572	0.789311947	1.660647261	0.102219269	2.182018558	0.033302161
WDSUB1	0.495602594	0.621421707	0.233517282	0.816187722	0.202927094	0.839924445
WDTC1	-1.872712918	0.064459391	-0.455901121	0.650175388	-0.194082082	0.846810983
WDYHV1	1.455468576	0.149130808	0.012981036	0.989687783	0.486833216	0.628268631
WEE1	0.51852526	0.605405337	-0.100995591	0.919904715	-0.880462805	0.382355923
WFIKKN1	1.932694038	0.056519984	-1.692751922	0.095905804	-2.149206756	0.035935818
WFS1	0.574603302	0.567039279	0.91983127	0.361497391	1.194202862	0.237411459
WHAMM	0.865339141	0.389227451	-0.610981132	0.54361585	-0.737858061	0.463668587
WHAMMP2	1.163616613	0.247753928	-1.135358367	0.260927772	-1.368525328	0.176590177
WHAMMP3	0.531324405	0.59654503	0.490496631	0.625645997	-1.641118833	0.106354437
WHSC1	3.12246424	0.002433694	0.505106026	0.61541172	0.832849059	0.40845301
WHSC1L1	-0.629002642	0.530991885	0.775694732	0.441102713	0.057212987	0.954578334
WHSC2	0.304598362	0.761397929	-0.869898444	0.387966859	-1.511048744	0.13637514
WIBG	-0.099830097	0.920708398	-0.584796655	0.560968413	0.366555982	0.7153258
WIPF1	-1.65355671	0.101813902	-1.717290021	0.091299313	-3.379342189	0.001325819
WIPF2	-1.369853982	0.174249972	-0.919233646	0.361807166	-0.892475414	0.375941257
WIPI1	-2.956357027	0.004004056	-2.211063863	0.031015854	-2.814847902	0.006714641
WIPI2	-1.700142463	0.092668888	1.894087659	0.063238637	2.403094999	0.01958002
WIZ	-0.746791376	0.457198593	-2.339843101	0.022780094	-1.27310421	0.208218766
WLS	-2.925637801	0.004381538	-0.271456741	0.787010367	-0.014249731	0.988681178
WNK1	-2.815275976	0.006024632	-0.703865021	0.48435282	-0.850846152	0.398463167
WNK2	-0.600777628	0.549546709	-0.554961114	0.581070179	1.161632689	0.250291352
WNK3	0.710904787	0.479040699	0.578690069	0.565054503	-0.378599705	0.706410691
WNT1	0.583537583	0.561037981	-3.366175577	0.001362396	-2.297404155	0.025339299
WNT10A	0.527085203	0.599472996	0.822079557	0.414421886	0.619981959	0.537773827
WNT10B	3.585318479	0.000554432	-0.938449977	0.351932045	0.064365116	0.948907757
WNT11	0.106259324	0.915620706	1.029733189	0.307440178	1.477401438	0.145146785
WNT16	-0.517588964	0.606055843	0.681664554	0.498183027	0.393430105	0.695489485
WNT3	0.69733046	0.48745131	0.360377498	0.719883108	-0.066095938	0.94753586
WNT4	-0.870712685	0.386302112	0.525590812	0.601190275	1.336573767	0.186744323
WNT5B	-0.506274305	0.613941893	0.95126522	0.345444665	1.749586121	0.085644984
WNT6	-0.429164981	0.668861767	-1.535369568	0.130168521	-0.948304006	0.347032108
WNT7A	1.637508519	0.105128622	0.757645517	0.451751931	1.858854949	0.068283245
WRAP53	-0.693773614	0.489668455	2.666685408	0.009927184	3.112129892	0.002917805
WRAP73	0.141435614	0.887852104	1.778498027	0.080599969	1.145396329	0.256896036
WRB	-1.405439873	0.163443844	-0.192181261	0.848276203	-0.317626746	0.751944455
WRN	1.050275329	0.296493954	0.739964426	0.462327178	1.124753132	0.265471459
WRNIP1	1.119060093	0.266188041	1.428014492	0.15868779	0.445745803	0.657492257
WSB1	1.863022354	0.06582612	-0.009414443	0.992521005	-0.356931545	0.722478931
WSB2	-0.986944459	0.326400844	1.370265628	0.175921472	0.244510281	0.807725578
WTAP	0.633784954	0.527880371	-0.110751279	0.912198605	-0.245165853	0.807220465
WWC1	-0.749925209	0.455318593	0.604826741	0.547669639	0.759465485	0.45074604
WWC2	0.124196934	0.901445233	0.950210947	0.345975392	-1.166897201	0.248176201
WWC3	-0.250185868	0.803032179	-0.880852851	0.382058395	-1.434950502	0.156839506
WWOX	1.595081049	0.114312758	-0.172701836	0.863491254	-0.544268704	0.588408188
WWP1	2.109845638	0.037737442	0.497750474	0.620555059	0.547960601	0.585887499
WWP2	-2.436320931	0.016871549	1.253387615	0.215128806	1.091445792	0.279730885
XAB2	-0.691283199	0.491224127	1.000520241	0.321243785	1.126640837	0.264678973
XAF1	-1.779133787	0.078702806	0.544509531	0.58819292	-1.334983388	0.187261085
XBP1	-0.267854198	0.7894442	1.003239797	0.319941475	2.01474469	0.04872217
XCL1	1.118900052	0.266255941	-2.11405149	0.038851953	-1.598167444	0.115609858
XCL2	1.357890287	0.178002302	-3.401292718	0.00122477	-2.787676625	0.00722836
XIAP	-2.061196786	0.04226302	0.071351505	0.943365186	0.385009987	0.701682335

XIST	0.440470789	0.660686285	-1.638942085	0.106676963	-2.364033495	0.021555588
XK	-3.22117351	0.00179516	0.516312161	0.60761308	-0.291550647	0.77170295
XKR3	-2.197600787	0.03062819	-0.067953197	0.946058245	0.517873202	0.606578141
XKR6	0.376559061	0.707416151	-2.489232877	0.015713263	-2.051386533	0.044897783
XKR8	-4.006021062	0.000129597	-0.700492512	0.486439939	-0.77441235	0.441930741
XKR9	0.293019289	0.770203665	0.325657492	0.745863676	-0.214064749	0.831270832
XKRX	1.968484214	0.052189228	-0.644447696	0.521843589	1.14010433	0.259075346
XPA	1.651661781	0.102200834	-1.129901118	0.263201482	-0.079234166	0.937127646
XPC	-1.271703215	0.206860325	1.543696669	0.128138841	1.252009834	0.215748561
XPNPEP1	-1.162868643	0.248055689	0.797225857	0.428594204	0.847782383	0.400153056
XPNPEP2	1.577628991	0.118272371	-2.379316337	0.020679398	-0.815359173	0.418306647
XPNPEP3	0.084759549	0.932646874	-1.75029006	0.085393588	-0.399846983	0.690783921
XPO1	-0.190928491	0.849025507	-0.23274784	0.816782329	-1.030220476	0.307317071
XPO4	1.572681551	0.119414563	-0.341313218	0.73410985	-0.590266953	0.557376251
XPO5	0.727911399	0.468618169	2.600763419	0.011800285	2.868716893	0.005794378
XPO6	-1.124108824	0.264052255	-0.407521268	0.685135932	-0.755197022	0.453282122
XPO7	-2.087014509	0.039806287	0.17041589	0.865280295	0.100709019	0.92013964
XPOT	1.621869512	0.108442117	-0.763139579	0.448494677	-0.742382815	0.460945093
XPR1	0.411930162	0.681401586	-2.398634201	0.01971591	-2.674596373	0.009778907
XRCC1	-1.065644518	0.289526487	0.776741967	0.440489396	1.023947218	0.310244729
XRCC2	-0.562508646	0.575212979	1.372483942	0.175234016	0.806346788	0.423439779
XRCC3	2.763430925	0.00697698	2.179239029	0.033417701	2.329529165	0.023446904
XRCC4	-0.532114338	0.596000166	-0.349287714	0.728147136	-0.537564393	0.592998748
XRCC5	-0.367166368	0.714384593	2.625183403	0.011071799	2.628870974	0.011026416
XRCC6	-1.284955975	0.202210792	2.478608751	0.016141323	2.871858851	0.005744484
XRCC6BP1	0.107350463	0.91475759	-1.096554355	0.277399919	-0.155284917	0.877152732
XRN1	0.080160844	0.936293041	-1.979611507	0.052533083	-3.711956528	0.000473588
XRN2	0.151622577	0.879834908	1.185697685	0.240610363	0.429060046	0.669518762
XRRA1	-0.05945497	0.952725793	0.792422929	0.431365975	1.370546499	0.175962342
XYLB	2.477156873	0.015172888	-0.236340167	0.814007197	-0.521256943	0.604234514
XYLT1	-0.564179596	0.574080366	0.171645055	0.86431823	0.411884627	0.681989729
XYLT2	2.485845418	0.01483187	-0.556922552	0.579738091	1.108782283	0.272243437
YAF2	0.003644026	0.997100819	-1.530907534	0.131266603	-1.257934032	0.213613816
YARS	-0.000128687	0.999897617	2.500620582	0.015265838	3.023827229	0.003756254
YARS2	1.080913521	0.282716345	-1.352738139	0.181425995	0.110434262	0.912458154
YBEY	0.632978259	0.528404567	-1.448125218	0.153004045	0.482248722	0.631500996
YBX1	-3.829868005	0.000241048	-1.98476176	0.051941233	-1.998257875	0.050532579
YDJC	1.235978216	0.219786222	-2.562082059	0.013043838	-1.701399815	0.094391319
YEATS2	0.280371889	0.779856368	1.844342689	0.070278087	1.33916922	0.185903307
YEATS4	0.807478748	0.421588161	1.114768947	0.269579437	1.360899943	0.178974285
YES1	1.180319839	0.241083187	-0.770322343	0.444256954	-0.603528645	0.548583751
YIF1A	0.17100449	0.864616466	-1.149685727	0.255024866	-0.166156919	0.868629401
YIF1B	-1.460947191	0.147624412	-1.9763122	0.05291526	-1.637371652	0.107137126
YIPF1	-1.197169197	0.23448544	1.32622701	0.189999715	0.901448353	0.371194537
YIPF2	-0.660053519	0.51095943	-0.090804643	0.927962881	0.075422886	0.940145919
YIPF3	-3.226470627	0.001765773	-0.257544559	0.797676352	-0.218883273	0.827533422
YIPF4	0.194891431	0.845931366	-0.504067643	0.616136638	-1.178206704	0.243675822
YIPF5	-0.177076847	0.859858736	0.329498033	0.742974572	0.947689592	0.34734215
YIPF6	-0.039580036	0.968518434	-1.652148746	0.103946193	-3.034685499	0.003642194
YJEFN3	0.077202491	0.938639349	0.081037743	0.935692878	1.39286675	0.169142247
YKT6	-1.800282375	0.075274369	-1.01139343	0.316058219	0.28377915	0.777621808
YLPM1	0.817170368	0.416058434	1.771287982	0.081803387	1.499841739	0.139248867
YME1L1	-0.076373942	0.93929658	0.380573235	0.704919956	-0.364911508	0.71654622
YOD1	-1.060091998	0.292030614	-0.767240071	0.446072569	-1.591867621	0.117020518
YPEL1	1.396238479	0.166187455	-3.031519313	0.003642706	-1.913499702	0.060779409
YPEL2	2.089265162	0.039598083	1.321131944	0.191682004	0.25499827	0.799654685
YPEL3	-1.99114374	0.04959591	-2.31699743	0.024081007	-2.738206565	0.008257673
YPEL4	-3.036438813	0.003156666	0.308373862	0.758910433	-1.405288132	0.165435997
YPEL5	-1.346637548	0.181587187	-0.744087489	0.459848529	-1.017321619	0.313357288
YRDC	0.411255708	0.681894163	-1.812707458	0.075089497	-1.870668322	0.066597886
YTHDC1	0.864599827	0.389631002	0.087765628	0.930367393	-0.516703982	0.607388928
YTHDC2	2.085123938	0.03998191	0.596544214	0.553149301	0.436702299	0.66399945
YTHDF1	0.510278409	0.611145866	0.880176283	0.382421665	0.894554705	0.374837892
YTHDF2	-0.015939054	0.98731943	1.544950698	0.127835379	1.459194313	0.150075027
YTHDF3	-0.057510807	0.954269896	-0.774620254	0.441732506	-1.811895613	0.075342792
YWHAB	-2.089585707	0.039568507	1.717352986	0.091287732	1.939818338	0.057423131
YWHAE	-0.087934897	0.930130076	1.89274108	0.063421012	1.80530665	0.076380665
YWHAG	1.47562681	0.143646422	0.734214471	0.465796601	-0.004341565	0.996551308
YWHAH	-2.090709478	0.039464968	1.868085544	0.066839877	1.481955465	0.143934232
YWHAQ	-0.472033767	0.638081944	-1.455110265	0.151067521	-1.172514256	0.245933601
YWHAZ	-1.195558236	0.235110566	-0.125727121	0.900385771	-0.007088889	0.99436902
YY1	-1.110301322	0.269921933	-0.994226608	0.324271217	-2.823728783	0.00655418

YY1AP1	-2.763851685	0.006968727	0.808014497	0.422406973	1.210106839	0.231299842
YY2	0.591250828	0.555882149	-0.161813888	0.872018726	-0.776836839	0.440510447
ZADH2	1.765925781	0.080908827	-1.261688113	0.212146783	-1.145856508	0.256707147
ZAK	-0.120072256	0.904702164	1.747321083	0.085911616	0.945783957	0.348304912
ZAP70	1.618652805	0.10913397	-2.017690324	0.048290887	-0.872423914	0.386686902
ZBED1	-1.722609794	0.088504464	1.442020152	0.154712427	1.292822968	0.201358565
ZBED2	-0.890479443	0.37565912	-1.401150902	0.166534233	-0.232229925	0.817202481
ZBED3	2.252396398	0.026808338	-0.645215276	0.521349677	-0.516743187	0.607361733
ZBED4	1.263946792	0.209617919	-0.309207574	0.758279441	-0.348362994	0.728868351
ZBED5	1.093490455	0.277190476	-0.295299922	0.768826691	-0.565108205	0.574247738
ZBED6	0.137254243	0.891146297	0.383669583	0.702635999	-0.758023255	0.451602008
ZBP1	-3.58137964	0.000561762	-0.417916953	0.67756182	-1.816281669	0.074658488
ZBTB1	1.120099852	0.265747201	-0.243041842	0.808836466	-1.306033453	0.196858205
ZBTB10	2.054047367	0.042965989	-0.301624315	0.764024834	-1.428060886	0.158804536
ZBTB11	0.848066482	0.398723141	-0.815821922	0.417963133	-1.810150041	0.075616591
ZBTB12	0.394757585	0.693985828	-2.183234186	0.03310748	-1.818664056	0.074288982
ZBTB16	1.271565975	0.206908883	-0.244519147	0.807697782	-1.627008495	0.10932614
ZBTB17	0.124382066	0.901299088	0.225312199	0.822533942	0.385282215	0.701481795
ZBTB2	-1.234616431	0.220290354	-1.291026796	0.201852223	-1.531473007	0.131258982
ZBTB20	-0.077464155	0.938431798	-0.589364066	0.557921853	-1.337372978	0.186485044
ZBTB22	-0.001794735	0.99857211	-4.123695868	0.000121191	-3.992083115	0.000191816
ZBTB24	0.507863366	0.612831581	-0.536316484	0.593805269	1.472959918	0.146337107
ZBTB25	2.667963187	0.009097889	-1.078201617	0.285438727	-1.250650151	0.216240732
ZBTB26	1.422916102	0.158328715	-1.741491433	0.086936356	-2.456055869	0.017161069
ZBTB3	-1.224055902	0.22422852	2.220247096	0.030351663	3.269601703	0.001841003
ZBTB32	0.556638866	0.579200159	0.859506017	0.393624724	1.168113735	0.24768926
ZBTB33	0.599303257	0.550524811	-1.264958587	0.210980291	-2.030192761	0.047076845
ZBTB34	-1.06006782	0.29204155	-0.861853048	0.392342486	-2.586227319	0.012318957
ZBTB37	-1.198715037	0.23388671	-2.977006445	0.004251674	-3.248243445	0.001961111
ZBTB38	1.141748604	0.256684529	-0.619588997	0.537971818	-1.729519174	0.089202086
ZBTB39	1.089186828	0.279072856	-0.969617511	0.33629142	-0.763072154	0.448609603
ZBTB4	1.971280681	0.051863064	-0.861035577	0.392788794	-0.413494586	0.680816884
ZBTB40	3.895899202	0.000191396	1.007292692	0.318007254	2.168438229	0.034370905
ZBTB41	0.491551256	0.624271806	-0.114178708	0.909493218	-1.060519359	0.293441937
ZBTB42	0.117091803	0.90705661	1.06297309	0.292230671	-0.505225264	0.615374991
ZBTB43	0.073613388	0.941486639	-1.778629835	0.080578107	-2.344440004	0.022612142
ZBTB44	-0.553964259	0.581021313	-0.643330399	0.522562973	-1.585325557	0.118500076
ZBTB45	-0.243276538	0.808362629	-1.266280576	0.210510126	-1.054383027	0.296216605
ZBTB46	1.463170021	0.147016609	-1.520461047	0.133866255	0.32233396	0.748394972
ZBTB47	-1.02300634	0.309134907	0.310929594	0.75697666	0.625621672	0.534093934
ZBTB48	-0.579632614	0.563657161	0.538742506	0.592140791	1.409944755	0.164062854
ZBTB49	-0.775084569	0.440386755	-2.750013629	0.007949164	-3.493987947	0.000935099
ZBTB5	1.751329839	0.08340581	-0.907992315	0.367665831	-1.150627465	0.254754662
ZBTB6	0.036817004	0.970715081	-1.137300134	0.260122125	-1.78276811	0.080021778
ZBTB7A	0.214384286	0.830747997	-2.757768987	0.007784829	-2.642339035	0.010644665
ZBTB7B	-0.972144781	0.333667826	-2.207422161	0.031282771	-2.652963469	0.010352074
ZBTB7C	0.537086171	0.592576103	-0.042307832	0.966399629	0.855382831	0.395968983
ZBTB8A	0.137009941	0.891338824	2.47787611	0.016171226	1.904416488	0.061975846
ZBTB8OS	1.465226455	0.14645604	-1.485126888	0.142963162	-0.839394068	0.404802403
ZBTB9	0.150321925	0.880857849	-0.208644245	0.835462083	0.786445971	0.434907801
ZC3H10	-1.438459677	0.153883663	-1.314933905	0.193743601	-2.020048569	0.048151766
ZC3H11A	0.556734727	0.579134937	-0.259297764	0.796330047	-1.137115325	0.260312052
ZC3H12A	0.662916949	0.509132523	-2.727056253	0.00845449	-3.122992963	0.002827726
ZC3H12B	1.709557193	0.090904739	0.434073069	0.665857103	0.656179765	0.514384422
ZC3H12C	1.923359703	0.05769827	-1.325831967	0.19012975	-0.372110476	0.71120916
ZC3H12D	1.919169923	0.058233869	0.902653189	0.370469557	1.394319734	0.168705435
ZC3H13	0.14265346	0.886893021	1.631661002	0.108207266	1.020259107	0.311974725
ZC3H14	1.809218636	0.073863441	0.429919168	0.668858682	0.730729306	0.467978074
ZC3H15	0.915740394	0.362328907	0.056005978	0.955530878	0.045758742	0.963664648
ZC3H18	-1.044951756	0.298933739	0.605981584	0.546907797	0.445047074	0.657994081
ZC3H3	-1.441105872	0.153136627	-1.036679878	0.304217928	-0.294558454	0.769415807
ZC3H4	-0.468843606	0.640351601	0.68726678	0.494672795	0.662720035	0.510217037
ZC3H6	1.785817143	0.077605651	-1.34536406	0.183780659	-1.930570817	0.058583937
ZC3H7A	0.711333558	0.478776353	2.580729301	0.012430215	2.265860113	0.027328307
ZC3H7B	2.515374828	0.013723633	-0.850045714	0.39881946	-0.309704291	0.757930544
ZC3H8	0.77044983	0.443115771	-1.380855747	0.172658114	-1.613583056	0.112215841
ZC3HAV1	0.010741241	0.991454434	-1.656772532	0.103003698	-2.726060318	0.008530198
ZC3HAV1L	1.221992427	0.225003961	-0.690264783	0.492799899	-1.716915258	0.091498132
ZC3HC1	0.205699078	0.837505501	2.606828204	0.0116154	3.270443243	0.001836416
ZC4H2	-0.191353033	0.848693924	1.195202948	0.236905845	2.802972951	0.006934867
ZCCHC10	1.133876655	0.259954326	-0.225155292	0.822655419	-0.399163234	0.691284742
ZCCHC11	1.896041048	0.061266713	-0.121700748	0.903559633	0.016765062	0.986683381

ZCCHC14	1.539067573	0.127409289	-0.935287808	0.353544912	-1.553937692	0.125809429
ZCCHC17	-0.753673742	0.453075657	-0.349123765	0.728269556	-0.184197923	0.854520793
ZCCHC18	1.235915893	0.219809275	-1.153820499	0.253339183	-0.010521411	0.99164252
ZCCHC2	-1.911803965	0.05918568	-0.666058557	0.508032691	-3.417367439	0.001181659
ZCCHC24	-3.233270091	0.00172871	2.526569413	0.014289009	0.664157772	0.509303365
ZCCHC3	1.263915491	0.209629102	-4.200564944	9.36E-05	-4.224011991	8.87E-05
ZCCHC4	2.113340581	0.037429104	-0.90516952	0.369146467	-0.789463207	0.433157339
ZCCHC6	0.290535115	0.772096825	-0.911479931	0.365841729	-2.533935344	0.014091338
ZCCHC7	0.416433767	0.678115993	-0.600467412	0.550550305	-0.613826885	0.541804859
ZCCHC8	1.521012017	0.131875287	-2.14559008	0.036133331	-2.570196355	0.012839485
ZCCHC9	-0.017585637	0.986009595	-2.040172724	0.045927511	-2.260575011	0.027674763
ZCRB1	1.016437737	0.312233271	-1.356308068	0.180294344	-0.749676883	0.456574111
ZCWPW1	-0.200033142	0.841920483	1.667498693	0.100844158	1.815973702	0.074706365
ZDBF2	1.062433295	0.290972915	-0.01524538	0.987889105	-1.142300702	0.258169262
ZDHHC1	-2.042021896	0.04417108	-2.002983793	0.049892953	-1.869424223	0.066773703
ZDHHC11	0.432118694	0.666721963	0.866211931	0.389968032	1.904624904	0.061948171
ZDHHC12	-1.605721808	0.111951082	0.478515091	0.634095122	0.143220163	0.886628252
ZDHHC13	0.767187109	0.445042803	-1.682751463	0.09783715	-1.691028971	0.096366858
ZDHHC14	0.558095296	0.578209615	-2.325777651	0.023573406	-1.772707432	0.081693238
ZDHHC15	0.430238144	0.668084001	-0.487098409	0.628037295	-1.05161573	0.297473775
ZDHHC16	1.553824261	0.12384887	-1.188448512	0.239533993	-1.19764416	0.236079193
ZDHHC17	0.454436942	0.650643725	-1.530782902	0.13129738	-2.698314616	0.009183969
ZDHHC18	-2.70656234	0.008178285	-0.254564245	0.799966381	0.015085866	0.988017071
ZDHHC19	-0.071254737	0.943358209	0.26405759	0.792678076	-1.570461303	0.121917789
ZDHHC2	-0.815875413	0.416794763	-0.923492774	0.359603198	-1.476292449	0.145443277
ZDHHC20	0.618456439	0.537886853	-0.997908317	0.322497894	-1.816691879	0.074594755
ZDHHC21	1.617266468	0.109433238	-0.210379213	0.834114198	-1.76713443	0.082631577
ZDHHC23	1.502467658	0.13658972	-0.521863058	0.603766896	-1.057333165	0.294880397
ZDHHC24	1.216618593	0.227032556	-1.273044493	0.20811671	-1.549482937	0.126875449
ZDHHC3	-1.219353813	0.225998373	-0.91051612	0.366345243	-0.111328334	0.91175238
ZDHHC4	0.450540891	0.653438855	0.683263756	0.497179617	2.797992593	0.007029197
ZDHHC5	-1.685996001	0.095372157	0.128655883	0.898078144	0.508021735	0.613425042
ZDHHC6	1.249060769	0.214985933	-1.621583891	0.110354589	-0.739654757	0.462586038
ZDHHC7	-2.536351283	0.01298219	1.110908218	0.271223952	0.847741585	0.400175588
ZDHHC8	1.662933916	0.099916567	-1.829576977	0.072490521	-0.92500359	0.358916416
ZDHHC9	0.97178138	0.33384759	0.316646558	0.752656633	0.549679801	0.584715453
ZEB1	0.09468531	0.924782054	-0.624260686	0.534921356	-2.312402541	0.024439511
ZEB1-AS1	0.718004021	0.474674336	-1.9645496	0.05429725	-2.435305507	0.018074804
ZEB2	0.484609288	0.629168754	-1.230688549	0.223441704	-1.851010841	0.069422106
ZER1	-2.895529175	0.004783112	0.683797956	0.496844682	0.649538102	0.518634939
ZFAND1	0.73901002	0.461885791	-0.895049749	0.374485788	-0.68185251	0.498130902
ZFAND2A	-1.595333409	0.114256289	-1.568613444	0.122216057	-0.882475252	0.381276507
ZFAND2B	-1.522615288	0.131473794	0.754123647	0.453847137	0.741681123	0.461366846
ZFAND3	-2.185697896	0.031518209	-1.696360146	0.095216696	-2.043152196	0.04573381
ZFAND5	-1.010376148	0.315110888	0.710051438	0.480537263	-1.049943134	0.2982354
ZFAND6	-0.768004137	0.444559793	-0.464146677	0.644292253	-0.436335536	0.664263906
ZFAT	-1.325434921	0.18848956	0.546869377	0.586581072	0.096841581	0.923196507
ZFC3H1	1.781864269	0.078253022	-2.191154862	0.032499915	-2.690720038	0.009370758
ZFHX2	0.79759187	0.427274239	0.696332228	0.489021443	0.640737486	0.524295717
ZFHX3	1.675052236	0.097507203	-0.305388773	0.761171034	0.678942161	0.499959253
ZFP1	0.809778579	0.420271998	-1.183634149	0.241420095	-1.531965393	0.131137554
ZFP106	-2.656168087	0.009397081	1.858096457	0.06826869	0.793364853	0.4309
ZFP112	0.111086361	0.911803186	0.581309852	0.563299734	0.061518095	0.951164721
ZFP14	2.139000886	0.035231322	-0.089517369	0.92898131	-0.2062838	0.837314244
ZFP161	1.676705238	0.097182245	0.509947274	0.612037026	-0.376076391	0.708275149
ZFP2	-0.156625984	0.87590173	-0.030678521	0.975631982	0.110621163	0.912310609
ZFP28	2.154635637	0.033947882	-0.981220356	0.330587852	0.740324937	0.46218261
ZFP3	0.121362389	0.90368327	-3.942212827	0.000221167	-3.233654203	0.002047357
ZFP30	0.949101575	0.345192302	-0.246933753	0.805837542	-0.916253517	0.363446448
ZFP36	-1.308094806	0.194279375	-2.611645429	0.011470431	-3.418414887	0.001177906
ZFP36L1	-1.427013771	0.157147399	-2.132722173	0.037221778	-3.011104667	0.003894136
ZFP36L2	0.834187653	0.406454951	-1.437263591	0.156053733	-3.095149538	0.003064011
ZFP37	1.459429173	0.148040616	0.767630934	0.445842091	1.058825682	0.294205973
ZFP41	2.26776407	0.025815105	-0.612619705	0.542539139	-0.049311047	0.960846135
ZFP57	1.696356655	0.093386118	0.343315239	0.732611328	-1.064606513	0.291603813
ZFP62	1.882975104	0.063038099	-0.298511974	0.766386759	6.00E-05	0.999952327
ZFP64	1.873763338	0.064312685	0.121410862	0.903788203	0.23880688	0.812123454
ZFP82	0.411432553	0.681764994	0.336490142	0.737724205	1.780459468	0.080402787
ZFP90	1.255291836	0.212726822	-1.224886358	0.225603956	0.446085771	0.65724815
ZFP91	-0.371352304	0.71127601	-0.905660667	0.368888574	-0.047547007	0.96224572
ZFPL1	-1.595835734	0.114143954	-1.381536415	0.172449965	-1.37725737	0.173890004
ZFPM1	0.560394796	0.576647337	-2.877415391	0.005615161	-3.131929474	0.002755585

ZFR	0.589877537	0.556798386	-0.882857781	0.38098316	-2.041644965	0.045888287
ZFR2	-0.155920756	0.876455924	-1.011928083	0.315804698	1.717038858	0.091475381
ZFX	0.455166431	0.650120924	0.465777971	0.643131028	-0.207287715	0.836533948
ZFYVE1	-2.591868158	0.011190718	1.456887206	0.150577955	1.292809865	0.201363066
ZFYVE16	0.097232506	0.92276492	-1.100087691	0.275870601	-2.563255862	0.01307098
ZFYVE19	0.007838809	0.993763508	2.504026129	0.015134288	2.802919057	0.006935881
ZFYVE20	1.476257569	0.143477387	0.53870131	0.592169037	2.769608378	0.007589764
ZFYVE21	0.026920108	0.978584976	-2.044503368	0.045483934	-2.697582656	0.009201822
ZFYVE26	0.52590075	0.600292265	2.776136763	0.007408002	2.055602066	0.044474927
ZFYVE27	0.84185499	0.402172328	0.160835766	0.872785557	0.89094408	0.376755166
ZFYVE28	1.843878953	0.068597507	-2.835253361	0.006306239	-2.077027587	0.042378687
ZFYVE9	0.457293288	0.648597675	1.653791604	0.103610517	2.557073748	0.013280364
ZG16B	-0.929683305	0.355101942	-0.603091039	0.548815683	0.669733802	0.505768174
ZGLP1	0.252386771	0.801336156	1.358568942	0.179580446	2.220793584	0.03040972
ZGPAT	0.101974144	0.919011354	2.077495677	0.042224649	2.794801042	0.007090267
ZHX1	0.653714452	0.515016202	-0.54718707	0.586364239	-1.209626687	0.23148266
ZHX1-C8ORF76	0.545189086	0.58701544	1.041942949	0.301792008	1.903808243	0.062056674
ZHX2	-1.263447845	0.209796229	1.755811106	0.084437164	1.659464866	0.102589359
ZHX3	1.338632938	0.184170275	1.727892683	0.089366148	2.576774251	0.012623528
ZIK1	1.799871894	0.075339714	0.525945547	0.600945348	1.916637871	0.060370643
ZKSCAN1	0.185689461	0.853119607	0.55829763	0.5788051	-0.217695612	0.828454243
ZKSCAN2	0.731587487	0.46638217	1.949459984	0.056115284	2.981474558	0.004233758
ZKSCAN3	0.401063077	0.68935489	-0.42333564	0.673627022	-0.365917344	0.715799667
ZKSCAN4	-0.964946231	0.337240574	1.569309967	0.122053699	1.988693533	0.051609134
ZKSCAN5	-2.234491403	0.028007766	1.590713108	0.117148505	2.638684511	0.010747041
ZMAT1	1.495102471	0.138498447	-1.062145478	0.292602954	-1.61174058	0.112617201
ZMAT2	-2.909315348	0.004595209	-1.102648341	0.274765981	-0.774846976	0.441675934
ZMAT3	0.00317005	0.997477912	-0.689780057	0.493102451	-0.64344582	0.522550191
ZMAT4	-0.150161462	0.880984065	-0.369372714	0.713204472	0.529860439	0.598294496
ZMAT5	-1.163233378	0.247908507	1.300553478	0.198591111	0.923091762	0.359903071
ZMIZ1	-1.918354468	0.058338599	-0.613769291	0.541784395	-1.47860793	0.144824765
ZMIZ2	0.356925444	0.722010024	-0.477990358	0.634466284	0.386698715	0.700438656
ZMPSTE24	-0.952974309	0.343237606	-0.784181062	0.436147132	-1.667786594	0.100917784
ZMYM1	0.418526142	0.676591618	-0.260116515	0.795701533	-0.754648954	0.453608351
ZMYM2	2.201643422	0.030330943	-0.53069114	0.597673209	-1.156034834	0.252554579
ZMYM3	1.21252347	0.228587327	0.6381039	0.525935044	0.930861879	0.355903958
ZMYM4	1.004005287	0.318154374	2.022681736	0.047757317	2.00367073	0.049931916
ZMYM5	-0.490555045	0.624973515	-1.994083032	0.050884612	-2.983271038	0.004212405
ZMYM6	1.929549663	0.056914607	-0.97649	0.332905336	-1.118447439	0.268130841
ZMYM6NB	-0.019731512	0.984302638	-4.188984751	9.73E-05	-3.180595253	0.002392089
ZMYND10	0.079970012	0.936444376	0.114536003	0.909211254	-0.256606951	0.798418664
ZMYND11	2.26599124	0.025928009	-1.17705254	0.244015868	-1.860252133	0.068082056
ZMYND15	-2.590140925	0.011242905	-0.344710415	0.731567651	-0.719339615	0.474910438
ZMYND17	3.113278568	0.002502806	1.138874829	0.259470077	1.354266101	0.18106831
ZMYND19	1.374074101	0.172940796	-0.494229727	0.623023703	0.0133307	0.989411135
ZMYND8	1.653533001	0.101818736	1.110834411	0.27125546	0.954435638	0.343947909
ZNF10	0.513913448	0.608612535	-0.66236555	0.51037877	-0.50462328	0.615795113
ZNF100	-0.516188853	0.607029185	-1.125547922	0.265025235	-1.222457622	0.226633416
ZNF101	1.024330176	0.308512974	2.012365964	0.048865705	3.248858063	0.001957553
ZNF107	0.006921817	0.994493046	-0.401116569	0.689818566	-1.646792166	0.10517829
ZNF117	0.299363494	0.765375164	-1.356414918	0.180260557	-2.96636453	0.004417383
ZNF12	0.694171443	0.489420196	-0.909500118	0.366876504	-1.545378175	0.127864095
ZNF121	0.028673793	0.977190289	0.100024179	0.920672478	-0.08368984	0.933600235
ZNF124	1.955446517	0.053732995	-0.985729178	0.328388898	-1.346055102	0.183685979
ZNF131	1.885906654	0.062636957	-0.313298367	0.75518574	-0.602374905	0.549345879
ZNF132	-0.188142909	0.851201823	2.555452276	0.013268544	2.253172268	0.028166575
ZNF133	1.12756772	0.262595981	0.404756754	0.687155627	1.645088219	0.105530419
ZNF134	0.941746966	0.348924251	0.261289939	0.794800991	1.735648571	0.088102838
ZNF135	0.951099825	0.34418282	0.228694984	0.819916058	0.989994325	0.326417455
ZNF136	0.300983342	0.764143793	0.322654348	0.748125397	0.580935799	0.563604756
ZNF137P	-0.337950039	0.736213195	-1.64828341	0.104739474	-0.553690783	0.581985368
ZNF138	1.110179284	0.269974215	-0.171171001	0.864689247	-0.99339076	0.324774749
ZNF14	0.568942811	0.570857648	0.127566081	0.898936718	0.213770342	0.831499312
ZNF140	1.037452991	0.302393475	-0.979703498	0.331329818	-0.637278393	0.526529562
ZNF141	0.756453989	0.451416201	-0.256733223	0.798299594	-0.140266273	0.888950797
ZNF142	1.042337768	0.300136701	-0.474956773	0.636613895	0.160181807	0.87331184
ZNF143	-0.353801065	0.724342096	-1.237711531	0.220844908	-1.12294568	0.266231825
ZNF146	1.509143349	0.134877578	0.424522375	0.672766488	0.663000614	0.510038662
ZNF148	-0.198192261	0.843356021	-0.766077963	0.446758234	-1.919968639	0.059939353
ZNF154	2.650519686	0.009543495	0.328436988	0.743772389	0.914505438	0.364355836
ZNF155	0.3900316	0.697464378	-0.294263412	0.769614546	-0.265269277	0.791772
ZNF157	0.438337361	0.662225903	0.790648345	0.43239278	1.229341972	0.224062458

ZNF16	0.707013074	0.481443719	1.031264011	0.306728115	0.946578853	0.347903105
ZNF160	0.090334631	0.928228504	2.034222522	0.046543069	2.335242976	0.023123832
ZNF165	-1.137655693	0.258380972	1.642615946	0.105911515	2.292610892	0.025633002
ZNF167	-1.22519537	0.223801149	-3.056399731	0.003392722	-2.587226722	0.012287151
ZNF169	2.694730684	0.008450752	-2.368553154	0.021234299	-2.677666081	0.00969997
ZNF17	3.224945323	0.001774188	0.266796215	0.790578976	1.17759155	0.243919086
ZNF174	-0.610119317	0.543369734	1.097601556	0.276946047	0.890709982	0.376879688
ZNF175	0.537819199	0.592072049	-0.466235537	0.642805473	-0.659918453	0.51199995
ZNF18	-0.408389755	0.683988806	2.54999062	0.01345629	3.898944621	0.00025996
ZNF180	-0.262362809	0.793660641	-0.218140799	0.828090449	-0.505777847	0.614989459
ZNF181	1.429648003	0.156391574	-0.627372091	0.532894686	-0.529090047	0.598825279
ZNF182	-0.581056182	0.562701637	2.102394498	0.039901015	3.086321559	0.003142706
ZNF184	1.224611507	0.22402006	-1.337486404	0.186321707	-0.66875962	0.506384846
ZNF185	-4.012969897	0.00012642	1.117248037	0.268527152	-0.654260341	0.515610897
ZNF187	1.37230455	0.173488837	-1.149344043	0.255164522	-0.508603446	0.613019773
ZNF189	-0.283528803	0.777443658	0.558072656	0.578957695	0.761589689	0.449487036
ZNF19	0.405420562	0.686161517	1.770559853	0.081925745	1.55133013	0.126432544
ZNF192	0.8335405	0.406817684	-1.639609238	0.106537628	-1.535498907	0.130268774
ZNF193	0.543051421	0.588480034	-0.135203176	0.892922635	0.523309076	0.602815214
ZNF195	1.117538241	0.266834202	-0.086927096	0.931030967	0.529736564	0.598379829
ZNF197	0.650831173	0.516867011	-0.464201619	0.644253129	-0.895363909	0.374409048
ZNF2	1.806557933	0.074281216	1.660198135	0.102309943	2.248674379	0.028469161
ZNF200	-2.467095182	0.015576564	0.169617853	0.865905028	-0.494267385	0.62304261
ZNF202	1.317270055	0.191199455	0.069481691	0.944846878	1.467117092	0.14791464
ZNF204P	1.255508466	0.212648596	-0.889297215	0.377542636	-0.407280608	0.685348076
ZNF205	0.115128154	0.908608274	1.9199183	0.059825429	1.986473973	0.051861765
ZNF207	0.597648997	0.551623289	-1.4829194	0.14354727	-1.898009962	0.062831696
ZNF208	0.569025557	0.570801741	0.496580987	0.621374585	-1.72333407	0.090322798
ZNF211	1.679239519	0.09668575	1.006153565	0.3185501	2.69230974	0.009331374
ZNF212	0.428950427	0.669017306	3.165544924	0.002474003	4.233847016	8.58E-05
ZNF213	-1.61796095	0.109283238	-0.893184696	0.375475136	-1.716877514	0.09150508
ZNF217	-0.703307017	0.483738293	-0.066376772	0.947307732	-2.602169609	0.011820415
ZNF219	-0.428624023	0.669253959	-1.16030343	0.250712247	-0.642846981	0.52293588
ZNF22	1.132411122	0.260566294	-1.157213315	0.251961943	-0.980512257	0.331032807
ZNF221	0.512511702	0.609588876	-0.222276399	0.824885028	-0.075072839	0.940423177
ZNF222	-0.680865964	0.497760566	0.685139834	0.496003893	0.184467886	0.854310024
ZNF223	2.068099249	0.041593746	0.000972321	0.99922756	0.164461789	0.869957328
ZNF224	1.271521172	0.206924737	-0.290595865	0.772404216	-0.166796197	0.868128703
ZNF225	1.263347694	0.209832034	-0.9453746	0.348416864	0.068429402	0.945686546
ZNF226	1.218318924	0.22638926	-1.242350363	0.219141888	-0.589073186	0.558171162
ZNF227	-0.134884227	0.89301431	-1.531098593	0.131219434	-1.008264484	0.317646165
ZNF229	0.342512946	0.732789239	-0.093685572	0.925684073	0.269998225	0.788149958
ZNF23	1.382735804	0.170277193	-0.12508121	0.900894813	-0.804887763	0.424274333
ZNF230	-0.380836173	0.704251161	-0.017077872	0.986433514	-0.878192482	0.383575962
ZNF232	2.225154554	0.028651699	0.358186031	0.72151355	1.509095964	0.136872467
ZNF233	1.041015833	0.300746307	0.09060192	0.928123258	1.610441939	0.11290079
ZNF234	0.720478998	0.473157352	-0.136822605	0.891648162	1.109398334	0.271979987
ZNF235	0.622530588	0.535217817	-0.694245019	0.490319427	-0.342616206	0.733164525
ZNF236	1.63309143	0.106056097	-1.14048572	0.258804243	-1.579392808	0.119854819
ZNF238	-0.303447004	0.762272137	-1.27699995	0.206726483	-1.859524584	0.068186758
ZNF239	1.398077442	0.165636326	0.502236359	0.617416036	0.425540798	0.672066599
ZNF24	-0.140607067	0.8885047	-1.026452886	0.308969791	-1.827530981	0.07292719
ZNF248	2.423031374	0.017459898	-1.226228661	0.225102374	-0.167607547	0.867493313
ZNF25	1.361914832	0.176733261	-0.958016673	0.342058581	-0.918615572	0.362219975
ZNF250	1.223551496	0.224417893	1.794154004	0.078037605	2.63803951	0.010765203
ZNF251	2.207686149	0.029891335	0.111573557	0.911549457	0.326595978	0.745185903
ZNF252	-0.46800518	0.640948673	-0.342357136	0.733328343	-0.584201274	0.561421151
ZNF253	1.24111367	0.217892658	-0.91964648	0.361593158	0.345103497	0.731304017
ZNF254	0.472190418	0.637970582	-0.988303913	0.327137574	-1.680678907	0.098372179
ZNF256	1.309507432	0.193802792	0.320110143	0.750043225	1.902102847	0.062283776
ZNF257	1.142777479	0.256259317	-0.392569202	0.696086765	-2.155463438	0.035419982
ZNF259	2.198676906	0.030548818	2.05606611	0.044317701	2.320120243	0.023987678
ZNF26	1.847877871	0.068010659	0.302097088	0.763666248	0.151890484	0.879816916
ZNF260	0.203679748	0.839078404	-0.812476618	0.419863748	-0.778427459	0.439580104
ZNF263	2.476589304	0.015195407	1.647652557	0.104869411	2.604896191	0.011737013
ZNF264	-0.22201306	0.824822916	-0.767417662	0.445967842	1.291935233	0.201663727
ZNF266	2.611415462	0.010615184	1.42481916	0.159605775	2.38786058	0.020330346
ZNF267	-0.528593248	0.598430649	0.065378382	0.948099133	-1.19694745	0.236348479
ZNF268	0.245370817	0.806745954	0.541126493	0.590507298	0.758822461	0.45112756
ZNF271	-0.562071088	0.575509744	-0.928791016	0.356873626	-1.127343384	0.264384461
ZNF273	0.44426096	0.657954659	-1.399656579	0.16697934	-1.403392023	0.165997659
ZNF274	-0.216250066	0.829297974	-0.004332298	0.996558308	1.55544605	0.125450108

ZNF275	1.504014245	0.13619155	-1.169025497	0.247208867	-0.121826893	0.903470404
ZNF276	0.139908285	0.889055148	-1.959968687	0.054843763	-1.025104765	0.309703097
ZNF277	0.631326023	0.529479043	1.293848346	0.200882219	1.014819603	0.314538151
ZNF28	0.361992478	0.718233523	-1.721140789	0.090593257	-1.128044558	0.264090757
ZNF280B	-0.810466796	0.419878618	0.181487836	0.856621862	0.784294549	0.436158514
ZNF280C	0.831789259	0.407800249	-2.131446222	0.037331251	-2.688463114	0.009426935
ZNF280D	0.684537906	0.495451194	-0.648112836	0.519487422	-1.722322838	0.09050713
ZNF281	-1.492455011	0.139189636	-1.099892304	0.275955015	-2.108174389	0.039484567
ZNF282	0.320707094	0.749199779	-0.071479901	0.943263449	0.425202012	0.672312076
ZNF283	1.189644635	0.2374156	1.471849087	0.146504882	0.962319664	0.340008748
ZNF284	0.388646282	0.698485269	0.870939108	0.387403113	0.18393734	0.854724249
ZNF285	2.549653025	0.012530897	0.475581699	0.636171224	1.339545728	0.185781544
ZNF286A	1.449994428	0.150647869	-0.052101053	0.958628455	1.074567214	0.287157502
ZNF286B	0.651671641	0.516327143	-0.336692774	0.737572235	0.352820459	0.725542049
ZNF287	1.168888816	0.245634328	-0.109170191	0.913446966	0.217514598	0.828594609
ZNF292	-0.04288851	0.965888476	-0.684205724	0.496589102	-2.129477867	0.037605683
ZNF295	-0.654970811	0.514210828	-1.714878464	0.09174379	-2.697178091	0.009211703
ZNF296	-0.257839398	0.797138462	-1.907878122	0.061396383	-2.009443886	0.049298054
ZNF3	0.328161628	0.743576239	2.196608924	0.032087273	3.37927039	0.001326106
ZNF30	0.97234591	0.333568361	0.960545799	0.340795765	1.850321169	0.069522999
ZNF300	1.747650356	0.084045197	-0.660389229	0.511636664	-0.547863478	0.585953745
ZNF302	1.58290536	0.117063863	0.081011801	0.935713418	0.072805369	0.942219328
ZNF304	1.469215451	0.145373412	1.158606009	0.251398163	1.956801057	0.055342466
ZNF317	-0.926175301	0.35691146	1.017078533	0.313369491	0.975633943	0.333424111
ZNF318	3.311657269	0.001350846	0.931654207	0.355404144	1.213969124	0.229833104
ZNF319	-1.241480911	0.217757705	-0.591176314	0.556715338	-1.115268075	0.26947882
ZNF32	1.180401818	0.241050767	0.633626268	0.528833009	1.500890657	0.138977886
ZNF320	0.894314706	0.37361564	-1.106001992	0.273323967	0.008928523	0.992907763
ZNF322	1.130130439	0.261520663	-0.010744029	0.991464798	-1.501428812	0.138839019
ZNF323	-0.563568452	0.57449449	-0.15295579	0.87896774	0.289974566	0.772902221
ZNF324	-0.227674599	0.820432271	-0.98833908	0.327120505	-1.139820267	0.259192697
ZNF324B	2.664861807	0.009175708	-0.519107248	0.605674978	1.244662517	0.218417983
ZNF326	1.259142368	0.211339534	0.060555896	0.951922534	-1.23546163	0.221795106
ZNF329	-1.347469971	0.181320142	1.675737523	0.099210659	2.976514214	0.00429324
ZNF330	1.298590121	0.197508782	3.379505299	0.001308516	3.338751157	0.001498015
ZNF331	2.839267395	0.005625586	-0.750668902	0.455907863	-0.051662596	0.958980614
ZNF333	0.432563816	0.666399735	-0.599824692	0.550975664	-1.566375356	0.122871004
ZNF335	1.356347278	0.178490678	-2.106158395	0.039559621	-0.892046164	0.376169292
ZNF337	3.530915622	0.00066418	1.069045542	0.289509107	2.786326947	0.007254799
ZNF33A	-0.584718411	0.560247145	-1.238064816	0.220714867	-1.85882065	0.06828819
ZNF33B	1.367500859	0.174983226	-0.22900219	0.819678418	0.025948222	0.979390496
ZNF34	0.382001926	0.703389423	1.157098004	0.252008663	1.410744135	0.163828022
ZNF341	-1.920882305	0.058014462	0.48926745	0.626510496	0.290471581	0.772523972
ZNF343	1.289826664	0.200521597	2.168009718	0.034303354	3.106130121	0.002968701
ZNF345	1.583944707	0.116826976	-0.39454232	0.69463787	-0.31137139	0.756669656
ZNF346	1.312223831	0.192888803	-0.980390806	0.330993486	-0.783247192	0.436768156
ZNF347	-0.305293738	0.760870091	-0.206026392	0.837496814	0.563014076	0.575663186
ZNF35	0.44719019	0.655846701	0.235651278	0.81453919	0.606165734	0.546843779
ZNF350	-0.200302565	0.841710429	0.086265334	0.931554689	0.555512834	0.580747209
ZNF354A	-0.333275065	0.739726781	-1.631566876	0.108227165	-1.201794772	0.234479558
ZNF354B	2.267148727	0.025854244	-1.112324062	0.270620042	-0.308632316	0.758741667
ZNF354C	0.079978259	0.936437836	0.769726604	0.444607537	0.40583644	0.686402828
ZNF358	-0.782542362	0.436016119	-0.809683771	0.421454473	-1.108306387	0.272447074
ZNF362	-1.040336719	0.301059804	0.310977864	0.756940152	0.623154139	0.535702382
ZNF365	1.126191861	0.263174572	-1.81851283	0.074186424	-1.637724159	0.107063297
ZNF366	0.253103887	0.80078375	0.797630157	0.428361369	-0.176557914	0.860489959
ZNF367	-0.253298223	0.800634066	-0.653624301	0.515954963	-1.387961629	0.170623314
ZNF37A	0.163063304	0.870845951	-1.712993526	0.092092447	-1.15340382	0.25362335
ZNF37BP	1.957680332	0.053465773	-2.493923274	0.015527562	-1.127658639	0.26425238
ZNF382	-0.522417712	0.602704413	-1.472890335	0.146224673	-1.289162099	0.202619231
ZNF383	1.596211543	0.11405997	0.726166857	0.470677224	1.45138084	0.152229731
ZNF384	-1.90886975	0.059568474	-1.186091131	0.240456198	-0.938327957	0.352088541
ZNF385A	-1.86728166	0.065222436	-0.35045382	0.727276614	-0.474484243	0.636991968
ZNF385C	0.305057048	0.761049742	-2.004231716	0.049755256	-1.163514443	0.24953383
ZNF391	-0.387522519	0.699313818	-1.155202032	0.252777732	-2.260898652	0.027653436
ZNF394	-1.753280407	0.083068487	1.484868979	0.143031309	1.551385684	0.126419243
ZNF395	2.085321903	0.039963489	0.795976271	0.429314316	1.291216539	0.201911034
ZNF396	-1.381134317	0.170767303	0.66348714	0.509665637	0.396622688	0.693146811
ZNF397	-0.124010214	0.901592635	0.522593955	0.6032613	1.015285563	0.314318007
ZNF398	0.196978767	0.844302607	1.075373678	0.286691642	1.836864675	0.071516433
ZNF404	1.377286884	0.171949143	0.139039179	0.889904208	0.286716227	0.775383319
ZNF407	-1.844690312	0.068478098	0.591298523	0.556634023	0.986603594	0.328062923

ZNF408	-1.441528663	0.153017531	1.687647484	0.096887653	1.140736373	0.258814373
ZNF41	0.878918781	0.381861169	0.073462491	0.941692624	-0.285197073	0.776540902
ZNF410	-0.956607317	0.34141046	3.274565082	0.001793529	3.198785964	0.002268178
ZNF414	0.46703853	0.641637352	-1.837600351	0.071281179	-1.053750753	0.296503522
ZNF415	0.491076501	0.62460617	-0.107056253	0.915116386	-0.221292791	0.825666015
ZNF416	-0.527850101	0.598944201	3.958441257	0.000209693	4.461054227	3.96E-05
ZNF417	0.099502406	0.920967803	-0.756968719	0.45215413	0.58067798	0.563777336
ZNF418	1.665994551	0.099303538	1.355730789	0.180476974	3.29938154	0.001685077
ZNF419	1.118040233	0.266620941	1.475448125	0.145538137	3.527661374	0.000842979
ZNF420	1.538750522	0.127486665	1.158346693	0.251503069	1.219783822	0.22763775
ZNF425	0.318604933	0.750788075	-0.109922176	0.912853203	-0.322443681	0.748312301
ZNF426	2.088878395	0.039633795	3.294391119	0.001690525	3.381389468	0.00131765
ZNF428	0.660045107	0.510964802	-3.236719323	0.00200679	-1.683235969	0.097873593
ZNF429	-0.179181672	0.858210791	1.512283922	0.135929547	-0.148079185	0.882809949
ZNF43	-0.733061073	0.465487547	-0.251685344	0.802180163	0.864683713	0.390885885
ZNF430	0.505248417	0.614659184	-0.83240584	0.40861813	-1.751745191	0.085269375
ZNF431	1.469001329	0.145431367	-2.868054608	0.005762247	-1.540362202	0.12908055
ZNF432	0.506280646	0.613937462	-0.283936155	0.777477536	0.172345174	0.863784916
ZNF433	0.12824874	0.898247485	1.069865081	0.28914315	0.056667759	0.95501072
ZNF434	1.317326207	0.191180719	-0.047855146	0.961997241	0.7551748	0.453295346
ZNF436	0.719726312	0.473618407	0.698986644	0.487373473	2.147976936	0.036037981
ZNF438	-1.178038795	0.241986502	1.059218848	0.293922051	-0.186497041	0.85272614
ZNF439	1.341537668	0.183229738	0.734272773	0.465761348	1.031865987	0.306552249
ZNF44	2.234912905	0.027978998	2.795368864	0.007031491	4.007924994	0.00018209
ZNF440	0.353197158	0.72479316	1.399780209	0.166942481	2.276405864	0.026648462
ZNF441	-0.351071328	0.726381736	-0.393251646	0.695585505	-1.024371271	0.310046234
ZNF442	-0.665256706	0.507642315	1.308518166	0.195895161	-0.359876647	0.720287374
ZNF443	0.348503442	0.728302245	0.109498853	0.913187451	0.452509198	0.65264303
ZNF444	0.284951372	0.776357155	-1.400661053	0.166680041	0.071807027	0.94301025
ZNF445	1.395193307	0.166501312	0.815609859	0.41808346	1.49544817	0.140388457
ZNF446	0.153815276	0.878110851	0.553817464	0.581847554	1.225662241	0.225433976
ZNF449	2.716534861	0.00795487	-1.893207914	0.063357735	-0.975556062	0.33346238
ZNF45	1.066660246	0.289070001	0.851029315	0.398277391	0.352278043	0.725946535
ZNF451	1.791169332	0.076736188	-0.398560786	0.691690582	-0.828604825	0.41083104
ZNF460	-0.380274581	0.704666433	-0.242544443	0.809219947	-1.413439322	0.163038181
ZNF461	1.782292883	0.078182611	-0.96686795	0.33765248	-0.362672792	0.718208842
ZNF467	-0.914725112	0.362858789	-1.740310094	0.087145241	-2.123902067	0.038089745
ZNF468	-0.026721407	0.978743005	-1.356531931	0.18022356	-1.933436743	0.058222067
ZNF469	1.454922277	0.14928167	-0.616178376	0.54020449	-0.902004508	0.370901591
ZNF470	0.71756864	0.474941474	0.68488951	0.496160681	-0.018256053	0.985499203
ZNF471	1.068127869	0.288411297	-0.580310577	0.563968744	0.787863823	0.434084706
ZNF473	0.618128538	0.538101961	0.300218258	0.7650916	0.315633326	0.753449228
ZNF48	1.000313158	0.319927114	1.726875743	0.089550086	2.099864096	0.040239395
ZNF480	0.388367511	0.698690773	-2.482314843	0.015990819	-1.851395495	0.069365888
ZNF483	1.158772171	0.249713007	-0.166263745	0.868531681	0.243560802	0.80845729
ZNF484	1.276879798	0.205034888	-0.97750995	0.332404736	-1.816844463	0.07457106
ZNF485	-0.322245795	0.748037893	-1.737364084	0.087667969	-0.963430272	0.339456241
ZNF486	-1.210253521	0.229452461	-0.75086712	0.455789481	-1.692871286	0.096013455
ZNF487P	-1.194851614	0.235385146	-1.197506683	0.236014263	-1.225108533	0.225640887
ZNF490	-1.690313223	0.094540444	1.555959559	0.125195899	2.174937828	0.033855701
ZNF491	1.000453586	0.319859569	-0.148597946	0.882389939	-0.00744134	0.99408906
ZNF493	0.647553779	0.518975055	-1.463665983	0.148721722	-1.81462383	0.074916525
ZNF496	1.503997965	0.136195736	0.731263541	0.467582889	0.316229647	0.752998982
ZNF497	0.641275146	0.5230261	-0.147396603	0.883333748	0.723340496	0.472468717
ZNF498	-0.057816231	0.954027309	-1.630956651	0.108356245	-0.404276939	0.687542518
ZNF500	0.308456451	0.758470806	1.899168144	0.062554557	2.785238904	0.007276177
ZNF501	0.220798866	0.825765281	-0.133508813	0.894256384	-0.340785518	0.73453492
ZNF502	0.864283758	0.389803605	-1.545253798	0.127762118	-0.095470647	0.924280391
ZNF503	0.148006563	0.882679341	-1.231990169	0.222958736	-2.041953658	0.045856612
ZNF506	1.55121575	0.124472427	-0.629543202	0.531482859	-0.537491536	0.593048727
ZNF507	0.197794371	0.84366637	-1.471179972	0.146685169	-2.102684279	0.039981839
ZNF510	0.192690695	0.847649337	-0.486547393	0.628425418	-0.548686335	0.585392601
ZNF511	0.554347705	0.580760055	-0.990336075	0.326152189	-0.862361426	0.39215123
ZNF512	1.969613902	0.052057258	0.966410589	0.33787923	3.007254913	0.00393678
ZNF512B	2.844552622	0.005541003	-1.056883138	0.29497774	-0.665559366	0.508413508
ZNF513	0.600787648	0.549540065	-3.331742591	0.001511458	-2.777481616	0.007430284
ZNF514	3.584867715	0.000555266	0.875756651	0.384800049	0.654489034	0.515464685
ZNF516	-0.00762718	0.993931875	-1.447261697	0.153244787	-2.169967252	0.034249089
ZNF517	1.890894783	0.061959337	0.536530647	0.593658245	1.091468673	0.279720908
ZNF518A	0.442678855	0.659094341	-0.582573619	0.562454209	-1.163305009	0.249618059
ZNF518B	-0.385016403	0.701162887	-0.340321114	0.734852831	-1.015450258	0.314240221
ZNF519	2.144270516	0.034794113	-1.956096465	0.055309393	-2.014561671	0.048741956

ZNF524	-0.75780708	0.450609844	-1.687487303	0.096918597	-1.541216823	0.128872641
ZNF525	0.315996734	0.752760202	-1.6249077	0.109642535	-0.370022595	0.712755559
ZNF526	0.718217216	0.474543556	1.360469752	0.178981912	1.519256905	0.134300357
ZNF527	-0.133444025	0.894149752	-0.828360258	0.410885958	-0.558664145	0.578608755
ZNF528	2.205000582	0.030086018	-1.587305621	0.117918636	-0.049632601	0.960591028
ZNF529	2.141901491	0.034990079	-1.088393314	0.280954847	-0.375313228	0.708839398
ZNF530	2.263468665	0.02608941	0.933759445	0.354326171	2.161464061	0.034931363
ZNF532	0.977521607	0.331015491	-0.677712192	0.500667648	-0.447242261	0.656418039
ZNF540	1.430024274	0.156283841	-0.345655037	0.730861304	-0.24542172	0.807023345
ZNF541	1.995558057	0.04910371	-1.427703403	0.158776982	-0.007040377	0.994407554
ZNF542	-0.706778811	0.481588583	-0.487460222	0.627782499	1.452593294	0.151893799
ZNF543	-0.876740622	0.38303682	0.277829994	0.782137744	1.575599812	0.120727458
ZNF544	2.009890127	0.047534322	1.973159607	0.053282668	2.796427829	0.007059078
ZNF546	2.670133171	0.009043798	0.899565725	0.372097073	1.220427268	0.227395761
ZNF547	0.41837359	0.676702712	2.459263516	0.01694786	3.198819773	0.002267953
ZNF548	1.259629819	0.211164388	-0.733898021	0.465987972	0.676717602	0.501359236
ZNF549	1.130617717	0.261316552	0.635529904	0.52759994	1.947060834	0.056527773
ZNF550	0.775307072	0.440255988	0.398721404	0.691572878	-0.090945184	0.927859313
ZNF551	1.853962818	0.067125743	0.785152203	0.435582143	0.99558977	0.323714137
ZNF552	-4.290868241	4.59E-05	-0.168158088	0.867048007	0.750280947	0.456213201
ZNF554	2.26547286	0.025961104	2.077892155	0.042186747	3.556294012	0.00077151
ZNF555	-0.35703778	0.721926224	0.217459725	0.82861862	0.71193287	0.479449462
ZNF557	0.121573261	0.903516748	2.07542367	0.042423207	3.813888381	0.000342114
ZNF558	2.326250292	0.022324972	0.52147491	0.604035477	0.275218817	0.784156811
ZNF559	2.199435522	0.030492972	2.109757114	0.039235591	2.906239117	0.005223926
ZNF561	-0.667983743	0.50590839	-2.479506702	0.01610474	-1.595929381	0.116109426
ZNF562	-0.411018629	0.682067343	-1.222519122	0.226490523	0.595608393	0.553826414
ZNF563	2.916885206	0.004494948	1.65380923	0.103606921	2.388980992	0.020274298
ZNF564	-1.663243449	0.09985443	3.06979897	0.003264826	3.302004543	0.001671957
ZNF565	1.521091171	0.131855442	0.629411932	0.531568165	0.598984126	0.551588833
ZNF566	1.851564653	0.067473344	-0.581856502	0.56293392	0.147856415	0.882984945
ZNF567	0.880373448	0.381077275	-1.128304628	0.26386929	-0.999991337	0.321598171
ZNF568	-0.650307311	0.51720366	-0.20616481	0.837389201	1.232939293	0.222727587
ZNF569	0.861468961	0.391342836	0.820940691	0.415065021	0.590160454	0.557447144
ZNF57	0.436025613	0.663895848	0.256032762	0.798837772	1.280186423	0.205735088
ZNF570	0.89525052	0.373118089	0.489254138	0.626519861	-0.605759911	0.547111361
ZNF571	0.805596594	0.422667121	-1.010407015	0.316526314	-0.048208148	0.961721158
ZNF572	-0.730928443	0.466782593	-0.654927065	0.515121855	-0.087821733	0.930330346
ZNF573	1.632229941	0.106237754	-0.558785898	0.578473984	-0.074317557	0.941021432
ZNF574	-0.16381246	0.870257918	-0.250783511	0.80287398	0.32171836	0.748858857
ZNF575	0.177979363	0.859152047	-2.528649335	0.014213215	-2.48984519	0.015761912
ZNF576	0.288712447	0.773486738	-1.76767406	0.082412187	-2.378007344	0.020829252
ZNF577	0.599096656	0.550661941	-0.252876517	0.801263991	0.438572752	0.662651421
ZNF578	1.421527269	0.158730649	0.541131083	0.590504155	1.96196117	0.054723185
ZNF579	-0.321600933	0.748524763	-2.031364389	0.046841273	-2.414618494	0.019029065
ZNF580	1.494597089	0.138630182	-2.614299233	0.011391273	-1.259523559	0.213043712
ZNF581	-0.424172045	0.672485097	0.960490832	0.340823178	0.534444584	0.595140667
ZNF582	0.896607302	0.372397462	-0.345678993	0.730843394	0.437385294	0.663507088
ZNF583	0.156947696	0.875648938	-1.627711298	0.109044827	-0.20160467	0.84095327
ZNF584	2.127535902	0.036199049	0.338959552	0.735872909	-0.132441554	0.895107792
ZNF585A	0.084146709	0.933132694	-2.671360513	0.009805263	-3.017718038	0.003821885
ZNF585B	-0.905146505	0.367882199	-1.86787687	0.066869465	-0.346796656	0.730038451
ZNF586	-0.641941259	0.522595535	-0.016762634	0.986683911	-0.002350886	0.998132585
ZNF587	-1.870050483	0.064832498	0.482559051	0.631237858	1.77675528	0.081017272
ZNF589	2.429441288	0.017173874	0.489371486	0.626437306	2.598167992	0.011943795
ZNF592	-2.142762053	0.034918783	0.454064605	0.651488796	0.196229694	0.845137772
ZNF593	1.333360112	0.185886874	0.547126051	0.586405883	0.312259888	0.755997924
ZNF594	1.269294665	0.20771374	-0.948788254	0.346692428	-1.554273548	0.12572935
ZNF595	-0.376313003	0.707598386	-0.258308281	0.797089806	0.000571242	0.999546236
ZNF596	0.334781188	0.738594211	-0.459854051	0.647352183	-0.896490306	0.373812625
ZNF597	0.242714523	0.808796617	-2.799218631	0.006958275	-2.721665637	0.008630819
ZNF598	0.856031049	0.39432709	-1.837075878	0.07135971	-1.225217624	0.225600111
ZNF599	0.379671704	0.705112334	-0.656470234	0.514135939	-1.354561291	0.180974736
ZNF600	2.784300277	0.006578197	-1.736997033	0.087733279	0.029056532	0.976922376
ZNF605	0.959010978	0.340205076	-0.879622308	0.382719274	0.070531343	0.944020976
ZNF606	0.300689507	0.764367115	-0.083831408	0.933481168	-0.284366541	0.777173976
ZNF607	1.259150477	0.21133662	-0.14463621	0.885503033	-0.218996015	0.827446023
ZNF608	-0.398137076	0.691502371	-0.062931803	0.950038701	-0.64416738	0.522085662
ZNF609	0.850887075	0.39716289	-0.451134202	0.653586811	0.05741579	0.954417508
ZNF610	-0.395804653	0.693216019	-0.012703811	0.989908	0.620328406	0.537547395
ZNF611	0.379213622	0.705451211	-1.461726489	0.149250973	0.11483407	0.908985676
ZNF613	0.530705366	0.59697218	-1.2957333	0.200236149	-0.818554998	0.416495492

ZNF614	0.235064861	0.814709589	-1.78943452	0.078802764	-1.70688815	0.09335941
ZNF615	1.057292715	0.293298657	-0.67425433	0.502846923	-0.059069424	0.953106216
ZNF616	-0.249824046	0.80331109	0.393298567	0.695551046	1.485004662	0.143126822
ZNF618	-0.937029915	0.351331494	0.166657285	0.868223416	0.01984689	0.984235762
ZNF619	0.262732965	0.79337623	-0.685443604	0.495813666	-0.147507057	0.883259394
ZNF620	1.100363543	0.274202515	2.246650616	0.028511341	2.084565713	0.04166185
ZNF621	1.480895226	0.142239316	-0.974893529	0.333689898	-0.294823893	0.769214065
ZNF622	-0.039271061	0.968764062	3.42639466	0.001134583	3.524263307	0.000851868
ZNF623	1.625010998	0.107769855	-0.213258297	0.831878561	-0.170939842	0.864884627
ZNF624	0.599446553	0.55042971	-1.14395305	0.257375208	-1.803506586	0.076666279
ZNF625	0.464808559	0.643227264	0.287723625	0.774591058	0.613307917	0.542145444
ZNF626	1.919289396	0.058218538	-0.887922427	0.378275517	-1.544128991	0.128166183
ZNF627	-0.54134873	0.589647838	-0.28893456	0.773668862	0.123525504	0.902131413
ZNF628	0.051054777	0.959398657	-2.703419393	0.009005425	-2.719289901	0.008685666
ZNF629	0.419479233	0.675897701	1.778630651	0.080577971	2.544664896	0.013709857
ZNF630	1.304033546	0.195654414	0.149946476	0.881330699	0.087472692	0.930606523
ZNF638	0.758041586	0.450470178	2.843110711	0.006171771	1.954725467	0.05559325
ZNF639	1.788606393	0.077151524	-0.893636664	0.375235231	-0.209079278	0.83514186
ZNF641	-0.048723564	0.961251036	0.739344141	0.462700734	0.55066355	0.584045295
ZNF642	1.831408479	0.07045482	-1.64796554	0.104804929	-0.281548421	0.779323224
ZNF643	1.17033252	0.245056173	-0.814188481	0.418890512	-0.746984368	0.458184814
ZNF644	0.665659715	0.507385872	-0.53270524	0.596286981	-1.112165033	0.270799037
ZNF646	-0.71566109	0.476112886	-0.599256812	0.551351632	-0.661285504	0.511129549
ZNF649	2.084902365	0.040002536	-1.224120304	0.225890577	-0.641458305	0.523830848
ZNF652	-1.365549959	0.175592919	0.741220907	0.461571014	0.906968646	0.368293339
ZNF653	-1.128418733	0.262238552	-0.885318328	0.379666189	-0.584486608	0.56123055
ZNF654	0.03934632	0.968704232	-0.306615731	0.760241607	-1.228588295	0.22434287
ZNF655	1.137308498	0.258525242	-2.857347946	0.005934847	-2.908971892	0.005184484
ZNF658	-1.145142228	0.255283906	0.468411735	0.641258088	0.634277842	0.52847132
ZNF662	0.88891131	0.376496661	-0.296349929	0.768028827	1.132805403	0.262102665
ZNF664	0.8164998	0.41643963	-0.742173866	0.460997986	-0.460533661	0.646909145
ZNF665	1.209418268	0.229771392	0.153753396	0.878341633	2.558574579	0.013229254
ZNF667	1.246715292	0.21584084	-0.2108254	0.833767639	1.259058641	0.213210344
ZNF668	-0.240863279	0.810226571	-1.633582323	0.107801731	-1.081249603	0.284201047
ZNF669	1.129989817	0.261579588	2.009413204	0.049187019	1.987505299	0.051744247
ZNF670	-0.548442005	0.584790046	-0.901186822	0.371241967	-0.817097767	0.417320752
ZNF671	1.876467616	0.063936283	1.571132893	0.1216296	2.846956316	0.006151093
ZNF672	-0.106099922	0.915746806	0.284100172	0.777352471	0.479150404	0.63368962
ZNF673	1.571697846	0.119642711	0.265982365	0.791202613	0.533861976	0.595541061
ZNF674	-1.056067785	0.293854717	-1.768126461	0.08233577	-1.743613552	0.086691177
ZNF675	-0.00055007	0.999562364	-0.313318678	0.75517039	-0.331727037	0.741328514
ZNF677	2.140223772	0.035129439	0.539372521	0.591708902	0.689951095	0.493062459
ZNF678	1.271872206	0.206800544	-0.875936253	0.384703217	-1.498291372	0.139650159
ZNF680	-0.017686892	0.985929049	0.113463484	0.910057681	-1.02132649	0.311473374
ZNF681	1.636880656	0.105260056	-0.705618835	0.483269423	-0.652333236	0.516843846
ZNF682	1.74655727	0.084235919	0.751706927	0.455288122	1.11069916	0.271424287
ZNF683	-0.607125145	0.545345726	-2.374588624	0.02092152	-0.6143059	0.541490591
ZNF684	-0.317430919	0.751675573	-1.502272186	0.13848998	-1.740018514	0.087325999
ZNF687	-2.024805754	0.045946785	-0.320311336	0.749891508	0.309118347	0.758373872
ZNF688	0.223482619	0.8236827	4.891163624	8.42E-06	4.27831415	7.39E-05
ZNF689	-0.610457692	0.543146653	-0.588400018	0.558564205	-1.142313265	0.258164085
ZNF69	2.242218749	0.027484468	1.504251953	0.137980666	2.634718063	0.010859172
ZNF691	0.528109667	0.598764805	1.604500416	0.114073696	3.349951235	0.001448498
ZNF692	0.708520466	0.480512157	0.375191822	0.708895934	1.383443054	0.171996474
ZNF696	2.302055411	0.023714741	-1.633016897	0.107920947	-1.500382737	0.139109052
ZNF697	-1.437311095	0.15420879	0.631225616	0.530390161	-0.218421559	0.82789137
ZNF699	0.847638632	0.39896014	0.555607805	0.580630824	0.097556987	0.922630953
ZNF7	2.350177939	0.021021897	-0.601209203	0.550059586	0.65063076	0.517934383
ZNF70	3.362790791	0.001147726	1.318116342	0.192682979	1.688736611	0.096808083
ZNF700	1.646139111	0.103335321	-0.261325909	0.794773391	-0.331337648	0.741621013
ZNF701	-0.485462433	0.628566034	-2.412325561	0.019057609	-2.877661478	0.005653383
ZNF702P	-0.063528141	0.949491371	-1.23076884	0.223411889	-1.009953697	0.316843279
ZNF703	-0.44818661	0.655130282	-3.207627668	0.002186719	-2.996970325	0.004052846
ZNF704	-1.2623287	0.210196588	1.775678198	0.081068854	1.084039884	0.28297284
ZNF706	-0.900145017	0.370522606	-2.015252267	0.048553373	-1.476764533	0.145317006
ZNF707	1.1057182	0.271890232	0.022380874	0.982221495	0.65671636	0.514041827
ZNF708	0.271257714	0.786834021	-0.519963476	0.605081842	-0.189854335	0.8501069
ZNF709	0.735245491	0.464163162	-0.404315625	0.687478119	-1.041764321	0.301978914
ZNF71	1.150054718	0.253266002	-0.496047261	0.621748757	-0.768950289	0.445140327
ZNF710	-0.963125169	0.338148345	-0.6971564	0.488509434	-0.781523737	0.437772437
ZNF711	-0.499216271	0.618884365	-0.560377998	0.577394947	-1.056861994	0.295093526
ZNF713	-0.560416074	0.57663289	-1.277842871	0.206431117	0.133374959	0.89437298

ZNF714	0.979273417	0.330154345	-1.938838722	0.057425934	-3.045214263	0.003534689
ZNF717	1.23350119	0.220703843	0.226735697	0.821432071	-0.655578058	0.514768733
ZNF718	0.412336373	0.681104982	-0.346887553	0.729940036	-0.702384007	0.485336929
ZNF720	1.835590753	0.069827302	-0.720451495	0.474160931	-1.087550165	0.28143296
ZNF721	0.824382682	0.411971726	0.363539508	0.717532893	-0.053243117	0.95772689
ZNF736	1.60339855	0.112463351	-0.007392824	0.99412698	-1.260687331	0.212627026
ZNF737	0.533137036	0.595295093	-0.151147038	0.880387869	-1.33384495	0.187631663
ZNF738	1.660605385	0.100385012	-1.946652345	0.056459233	-1.326198621	0.190135069
ZNF74	1.1042569	0.272519909	0.117009096	0.907259907	1.788037622	0.07915776
ZNF740	0.668853785	0.505355861	0.896957993	0.373475242	1.906418681	0.061710412
ZNF746	-1.183314901	0.239900784	-0.653178553	0.516240179	-1.236110811	0.221555576
ZNF747	0.803850941	0.423669299	-1.462404062	0.149065909	-0.950245542	0.346053569
ZNF749	0.788355361	0.432627124	0.191704355	0.848648039	0.953146749	0.344594722
ZNF750	0.432208041	0.666657279	0.617574109	0.539290236	0.206458893	0.837178141
ZNF75A	2.001978938	0.048395224	-1.373494399	0.174921558	-0.40311422	0.688392713
ZNF75D	1.475749488	0.143613534	-0.61215066	0.542847238	-0.981765543	0.330420302
ZNF76	3.06162677	0.002926642	-0.909687581	0.366778444	0.380474446	0.705026629
ZNF761	1.242314422	0.217451638	0.323956773	0.747144243	0.803732246	0.424935984
ZNF763	0.797369712	0.427402525	0.106343523	0.915679329	0.221646228	0.825392182
ZNF764	1.554890553	0.123594691	-3.253111387	0.001911644	-2.709054719	0.008925637
ZNF765	-0.109184683	0.913306909	-1.514605306	0.135341259	-1.576388737	0.120545534
ZNF766	0.371772952	0.710963896	0.425961549	0.671723493	1.558165731	0.124804301
ZNF767	2.949895156	0.004080873	-0.814122657	0.41892791	1.823926201	0.073478265
ZNF768	-0.090500231	0.928097296	0.76968361	0.444632844	0.366295473	0.715519083
ZNF77	0.685749014	0.494690777	1.845635206	0.070087157	2.043933577	0.045653902
ZNF770	0.084742425	0.932660449	0.115689413	0.908301107	-0.637781959	0.526204054
ZNF771	0.598335856	0.551167061	-1.821384529	0.073743086	-1.638781263	0.106842147
ZNF772	0.61084445	0.542891731	-0.076422387	0.939347906	-0.272475925	0.786254085
ZNF773	0.456906635	0.648874484	1.764042924	0.083027678	2.89239726	0.005428012
ZNF775	0.076312027	0.939345695	-0.372721981	0.710723477	-1.122623057	0.266367709
ZNF776	-0.532049612	0.596044803	-2.975933778	0.004264556	-1.918142474	0.060175491
ZNF777	0.197904766	0.843580261	0.918255853	0.362314368	1.162824335	0.24981145
ZNF778	1.327243086	0.187893352	1.22753268	0.22461588	1.369185538	0.176384908
ZNF780A	0.438158401	0.662355118	-1.278364493	0.206248495	-1.144093452	0.25743136
ZNF780B	1.149085201	0.253663353	-1.088579394	0.280873439	-0.723985369	0.472075819
ZNF781	0.796914378	0.42766553	-0.580860296	0.563600661	-0.804016046	0.424773422
ZNF782	0.425717656	0.671362629	-2.477196369	0.016199015	-3.106888796	0.00296222
ZNF783	2.5411097	0.012819096	0.463503117	0.644750612	1.714157445	0.092006971
ZNF784	-0.964591661	0.337417197	-1.341070554	0.185162305	-0.67204438	0.504307168
ZNF785	2.424857952	0.017377962	-0.351742938	0.726314682	0.402982726	0.688488889
ZNF786	1.208944153	0.22995257	-0.497855182	0.620481709	-0.674527662	0.502739512
ZNF787	-0.936324087	0.351692617	-2.436240788	0.017954892	-2.950780114	0.004614515
ZNF788	-0.743857446	0.458962684	0.210219636	0.834238152	1.217575348	0.228469751
ZNF789	2.521180731	0.013514698	1.051448418	0.297444193	3.25544989	0.001919783
ZNF79	0.178144597	0.859022677	0.248151126	0.804900091	1.471441213	0.146745873
ZNF790	1.072700181	0.286365737	0.137702305	0.890955968	0.458674242	0.648235896
ZNF791	0.254551995	0.799668557	-0.320493982	0.749753785	-1.156697493	0.252285903
ZNF792	1.77716953	0.079027693	1.146317443	0.256403967	1.464920252	0.148511217
ZNF793	0.204442396	0.838484283	0.367071502	0.714910921	1.228965279	0.224202577
ZNF799	1.318490285	0.190792621	-0.252442937	0.80159744	0.174352644	0.862214484
ZNF8	0.687724371	0.493451875	0.122447093	0.902971192	0.791704189	0.431859937
ZNF80	1.482381622	0.141844278	-1.794785383	0.077935713	-1.508956569	0.136908023
ZNF800	0.621882841	0.535641711	-1.33896657	0.185842236	-1.571649993	0.121641596
ZNF804A	-0.357993814	0.721213182	-2.115377468	0.038734159	-3.136085354	0.002722627
ZNF805	-0.908413736	0.366163802	-1.74245744	0.086765854	-0.439934549	0.661670684
ZNF808	1.019090756	0.310979364	-1.593455755	0.116531583	-1.312481502	0.19468923
ZNF81	-1.802781309	0.074877581	-1.413568066	0.162870926	-1.678391127	0.098820022
ZNF813	-0.119955954	0.904794023	0.382388142	0.703580894	0.870198968	0.38789101
ZNF814	0.627633278	0.531884574	1.140535136	0.258783838	2.453110016	0.017288203
ZNF815	3.446392806	0.00087627	0.565960511	0.573619133	1.298704554	0.199345481
ZNF816	0.591587249	0.555657808	-1.359711947	0.17922035	-1.250214483	0.216398608
ZNF821	1.099944305	0.274384128	1.531596906	0.131096473	2.334013294	0.023193024
ZNF823	1.359650006	0.177446572	0.554831423	0.581158309	-0.523966475	0.602360869
ZNF827	0.407513389	0.684629813	-0.711802322	0.47946044	-1.15028091	0.25489613
ZNF828	-0.038216096	0.969602755	0.912318095	0.365404215	1.102258982	0.275044089
ZNF829	-0.614396243	0.540553495	-2.006848934	0.049467532	-0.920543526	0.36122088
ZNF83	1.776392616	0.0791565	-0.870672094	0.387547711	0.026005398	0.979345094
ZNF830	0.116732691	0.907340351	-0.966079906	0.338043239	-0.661171812	0.511201906
ZNF831	2.102937111	0.038353414	-1.321364147	0.191605091	-0.914783958	0.364210846
ZNF835	-0.22794349	0.820223882	-0.517785429	0.606591168	0.090185187	0.928460499
ZNF836	0.572572284	0.5684079	-2.295710187	0.025352239	-0.319345599	0.750647722
ZNF837	0.656034375	0.513529563	-1.417955891	0.161591449	-1.34968144	0.182526373

ZNF839	0.744729652	0.458437845	0.704861951	0.483736813	2.41930101	0.018809191
ZNF84	2.065848843	0.041810939	-0.100490413	0.920303977	0.269946488	0.78818956
ZNF841	2.257472021	0.026476642	-1.341966113	0.184873466	0.287147832	0.775054533
ZNF844	-2.813312448	0.006058413	-0.720024342	0.474421877	-0.130998725	0.896243823
ZNF845	-0.8786828	0.38198843	0.040291799	0.967999834	-0.049400537	0.960775137
ZNF846	2.7041109	0.008234073	0.77927212	0.439009677	1.134809246	0.261269058
ZNF85	0.77899371	0.438092624	-1.057326344	0.29477722	-1.542691646	0.128514479
ZNF850	1.425216071	0.157664818	-0.685795183	0.495593549	0.832712537	0.408529372
ZNF853	0.400733452	0.689596685	1.128107279	0.263951924	1.856862833	0.06857097
ZNF860	0.094111633	0.925236419	0.236460075	0.813914606	0.346621443	0.730169381
ZNF862	3.508659646	0.000714753	0.406033622	0.686222489	2.336179311	0.02307127
ZNF865	-0.561256732	0.576062261	-3.137194354	0.002687128	-2.655429247	0.010285227
ZNF876P	1.592158319	0.114968383	-2.177394802	0.033561763	-2.352236448	0.022186313
ZNF879	1.979471023	0.050917754	-0.723491111	0.472306374	-0.869884032	0.388061637
ZNF880	1.83099728	0.07051677	0.019915539	0.984179582	-0.841640293	0.403554159
ZNF883	1.278724681	0.204387208	-0.593015044	0.555492527	-2.332874076	0.023257291
ZNF890P	2.922618789	0.004420355	-0.425432208	0.67210704	-0.729901506	0.468479969
ZNF90	0.468752909	0.640416178	-1.505806428	0.137581801	-1.456012134	0.150949669
ZNF91	2.141636518	0.035012057	-0.948657408	0.346758423	-1.570402087	0.121931561
ZNF92	0.439854317	0.661131021	-0.497385736	0.6208106	-1.118414985	0.268144577
ZNF93	-0.166295826	0.868309183	-0.730748884	0.467894825	-0.445081945	0.657969034
ZNFX1	-2.137997832	0.035315081	1.010827543	0.316326699	0.208471085	0.835614381
ZNFX1-AS1	0.504024882	0.615515157	-2.337497896	0.022910691	-2.294943212	0.025489715
ZNHIT1	-0.9311585	0.354342761	1.206090046	0.232713815	1.123545002	0.26597953
ZNHIT2	0.314731226	0.753717679	0.279925523	0.780537527	0.385627253	0.701227648
ZNHIT3	1.015942199	0.312467855	0.003897702	0.99690356	0.084695485	0.932804282
ZNHIT6	1.577026551	0.118410986	0.052160932	0.95858095	1.904425646	0.06197463
ZNRD1	0.519428855	0.604777852	0.319490208	0.750510774	0.775697283	0.441177677
ZNRD1-AS1	1.047023127	0.297982817	0.832111637	0.408782793	0.549949912	0.584531409
ZNRF1	-1.570942568	0.119818116	2.286144326	0.025942652	0.686625865	0.495140092
ZNRF2	0.857216866	0.393675138	-0.617681058	0.539220213	-2.703776456	0.009051751
ZNRF3	1.087845519	0.279661339	-0.263381372	0.793196621	-1.444861005	0.154046135
ZP3	0.334744953	0.738621453	1.887883445	0.064082619	2.248360053	0.028490414
ZRANB1	-1.824434661	0.071511649	0.35491052	0.723952936	0.905423638	0.369103856
ZRANB2	1.589324328	0.115606972	-0.343567741	0.732422404	-0.544213847	0.588445681
ZRANB3	0.886946556	0.37754769	0.052014636	0.958697012	0.915821041	0.363671295
ZRSR2	-0.133932496	0.893764621	0.012537702	0.990039951	1.980619763	0.052533194
ZSCAN10	0.75422714	0.452745069	-2.274525233	0.026676175	-1.551037604	0.126502601
ZSCAN12	0.294169462	0.769327603	-1.407605395	0.164622174	-1.931574861	0.058456943
ZSCAN16	0.664169368	0.508334559	-0.143188472	0.886641107	-0.023034511	0.981704283
ZSCAN18	1.507586511	0.13527535	0.036683779	0.970863989	2.014457025	0.048753273
ZSCAN2	-0.288264907	0.773828132	0.436553658	0.664067259	1.397957478	0.16761563
ZSCAN20	0.120896991	0.904050803	1.311235073	0.194981845	1.529342796	0.131785346
ZSCAN21	-0.808709885	0.420883292	0.30414643	0.762112476	1.668608854	0.100753834
ZSCAN22	1.317803424	0.191021546	-0.958464718	0.341834644	-0.62400831	0.535145312
ZSCAN29	2.415128643	0.017818377	1.277426472	0.206576987	0.998627961	0.322252593
ZSCAN30	1.847016022	0.068136781	-1.64998006	0.104390666	-1.858950361	0.06826949
ZSCAN5A	-1.35291808	0.179579679	1.114381421	0.26974419	2.68290232	0.009566665
ZSWIM1	-0.199910217	0.842016325	-0.964466526	0.338844176	-0.861311834	0.392723957
ZSWIM3	-0.621280118	0.536036295	1.599920324	0.115087823	0.355855405	0.72328031
ZSWIM4	-0.763397205	0.447287291	-0.078602136	0.937621534	-0.259314887	0.796339207
ZSWIM5	1.235267906	0.220049073	-1.319826082	0.192114978	-1.554110562	0.125768206
ZSWIM6	-1.736324347	0.086038701	-1.826357226	0.072980649	-3.290964735	0.001727834
ZSWIM7	2.058408504	0.042535993	1.263379751	0.211542824	2.073001204	0.042765947
ZUFSP	1.113462154	0.268570268	-0.280459078	0.780130238	0.074865968	0.940587035
ZW10	-0.892022776	0.374835968	2.673395275	0.009752628	2.960426056	0.004491558
ZWILCH	1.871675849	0.064604509	0.356761155	0.722574349	0.256802982	0.798268079
ZWINT	0.355407593	0.723142639	0.301424707	0.764176247	0.626431165	0.533566816
ZXDA	0.576911385	0.565485909	-2.312155766	0.024365052	-2.548542769	0.013574302
ZXDB	0.496714428	0.620640543	-1.032181421	0.30630192	-1.143483344	0.257682314
ZXDC	1.234275339	0.220416758	-0.643671574	0.522343248	-0.39420052	0.694923891
ZYG11B	-1.609286806	0.111168656	-1.543099872	0.128283462	-2.684771345	0.00951949
ZYX	-1.926078708	0.057352921	-0.127195724	0.899228522	-0.272748174	0.786045847
ZZEF1	-0.288074492	0.773973399	-0.173497022	0.862869088	-0.002714729	0.99784357
ZZZ3	1.885572929	0.062682514	0.812279097	0.419976131	0.325842842	0.745752645
psiTPTE22	-3.388469974	0.001056904	1.305139396	0.197035453	-0.170293393	0.865390583
