# Supplementary material for: Meditation and vacation effects have an impact on disease-associated molecular phenotypes
Source: Transl Psychiatry. 2016 Aug 30;6(8):e880–. doi: 10.1038/tp.2016.164 (PMC5022094; doi:10.1038/tp.2016.164)
Supplement: Supplementary Table 4 [file tp2016164x3.pdf]

Supplementary Table 4. The blood weighted coexpression network for the study described in the main text.

Gene_symbol	Module Colors
ABHD16B	black
ABHD8	black
ACOT2	black
ACY1	black
ADAT3	black
AKT1S1	black
ALKBH6	black
AMH	black
AMN	black
ANGPTL6	black
ANKRD16	black
ANKRD9	black
APBA3	black
ARMC8	black
ARRDC1	black
ASCL2	black
ATP6V1E1	black
BBC3	black
BBS5	black
BOP1	black
BRSK1	black
C10orf28	black
C10orf95	black
C11orf2	black
C11orf35	black
C17orf106-CDK3	black
C17orf107	black
C17orf76	black
C19orf20	black
C19orf43	black
C19orf6	black
C1orf85	black
C1QTNF4	black
C20orf134	black
C20orf201	black
C20orf24	black
C2CD4D	black
C4orf48	black
C5	black
C8orf82	black
C9orf172	black
C9orf69	black
CACNA1A	black
CALY	black
CASKIN1	black
CBX4	black
CBY3	black
CCDC124	black
CCDC85B	black
CDK16	black
CDK2AP2	black
CEBPB	black
CEBPD	black
CENPB	black
CFD	black
CHCHD10	black
CLASRP	black
CLEC11A	black
COPB1	black
CORO1B	black
CPLX1	black
CSNK1G2	black
CTU1	black
CWC22	black
CYLD	black

CYP51A1	black
CYTIP	black
DBNDD2	black
DCAF15	black
DLGAP3	black
DNAJC4	black
DOK2	black
EGLN2	black
EID2	black
EPS8L2	black
EXOSC6	black
EXOSC9	black
FAM108A1	black
FAM109A	black
FAM173A	black
FAM43A	black
FAM89B	black
FBXL14	black
FBXL8	black
FGFBP3	black
FLJ22184	black
FLJ35390	black
GAS2L1	black
GDAP1	black
GFOD1	black
GJD3	black
GLI4	black
GLTPD2	black
GMPPB	black
GPAA1	black
GPN1	black
GPR135	black
GPR150	black
GPR25	black
GPR27	black
GRIN1	black
GRIN3B	black
H1FX	black
H2AFX	black
HCFC1R1	black
HCN2	black
HECA	black
HES4	black
HIATL2	black
HIC1	black
HIGD2A	black
HMG20B	black
HSPB1	black
IDUA	black
IER5	black
IER5L	black
INO80B	black
IRF2BP1	black
JUNB	black
JUND	black
KCNK12	black
KIFC2	black
KISS1R	black
KLF2	black
LINGO3	black
LOC100128071	black
LOC100131655	black
LOC100133091	black
LOC100134368	black
LOC100286793	black
LOC100506023	black
LOC100630917	black
LOC143666	black

LOC144438	black
LOC221442	black
LOC254100	black
LOC388564	black
LOC389333	black
LOC439994	black
LOC441208	black
LOC595101	black
LOC642361	black
LOC643988	black
LOC645781	black
LOXL1	black
LPPR3	black
LRFN4	black
LRRC26	black
LTB	black
LTB4R2	black
LTC4S	black
MAB21L3	black
MAST1	black
MAT2B	black
MBD4	black
MESDC1	black
METTL21A	black
MEX3D	black
MGAT4B	black
MGC72080	black
MIB2	black
MMP23B	black
MOSPD3	black
MRPL10	black
MRPL4	black
MRPL41	black
MTIF2	black
MYPOP	black
NCK2	black
NDUFAF3	black
NEURL	black
NPAS1	black
NPB	black
NPEPL1	black
NR2F6	black
NUDT3	black
NUTF2	black
OAT	black
OTUD7A	black
OVCA2	black
OXT	black
PABPN1	black
PCSK1N	black
PDF	black
PDXP	black
PDZD7	black
PEX10	black
PGLYRP2	black
PHOSPHO1	black
PKP3	black
PLSCR3	black
PODXL2	black
PPT2-EGFL8	black
PRDM8	black
PRR7	black
PSD3	black
PSMD12	black
PWWP2B	black
RASL10A	black
RCE1	black
RDH14	black

REPIN1	black
RNF126	black
RPP25	black
RRAS	black
RTN4RL2	black
SAMD1	black
SCARF2	black
SCGB3A1	black
SDF2L1	black
SHISA8	black
SKOR1	black
SLC25A28	black
SLC39A3	black
SLC7A5P1	black
SNAPC2	black
SNRPA	black
SOCS1	black
SQLE	black
SSBP4	black
STBD1	black
STK11	black
SUV420H2	black
TAF6L	black
TBX6	black
TCTEX1D4	black
TFR2	black
TIGD5	black
TMEM102	black
TMEM121	black
TMEM160	black
TMEM175	black
TMEM55B	black
TMEM93	black
TMUB1	black
TOR2A	black
TPRA1	black
TPRN	black
TPST2	black
TRADD	black
TRAPPC5	black
TTC22	black
TUSC1	black
UACA	black
UCN	black
UFSP1	black
UFSP2	black
UGP2	black
USF2	black
USP33	black
UTF1	black
VAPB	black
WDR86	black
WFIKKN1	black
WNT6	black
XKR6	black
YIF1A	black
YIF1B	black
ZDHHC1	black
ZDHHC24	black
ZFPM1	black
ZNF219	black
ZNF35	black
ZNF358	black
ZNF414	black
ZNF428	black
ZNF444	black
ZNF564	black
ZNF575	black

ZNF579		black
ZNF628		black
ZNF638		black
ZNF668		black
ZNF747		black
ZNF764		black
ZNF771		black
ZNF784		black
ZNF787		black
ZNF804A		black
ZNF837		black
ZNF865		black
ZSCAN10		black
ZSWIM1		black
	6-Sep	blue
	11-Sep	blue
A1BG-AS1		blue
AAK1		blue
AARS		blue
AARS2		blue
ABCA2		blue
ABCB1		blue
ABCC1		blue
ABCC10		blue
ABCF1		blue
ABCF2		blue
ABHD11-AS1		blue
ABHD15		blue
ABHD6		blue
ABL1		blue
ABL2		blue
ABLIM1		blue
ACAA2		blue
ACACA		blue
ACACB		blue
ACAD8		blue
ACE		blue
ACER2		blue
ACLY		blue
ACN9		blue
ACO2		blue
ACOX2		blue
ACOX3		blue
ACSL5		blue
ACTR10		blue
ACTR8		blue
ACVR1		blue
ADAM11		blue
ADAM23		blue
ADAMTS10		blue
ADAMTS17		blue
ADAMTS4		blue
ADAT1		blue
ADCY3		blue
ADCY6		blue
ADCY7		blue
ADCY9		blue
ADD1		blue
ADIPOR2		blue
ADPGK		blue
ADSS		blue
AFG3L1P		blue
AFG3L2		blue
AGAP5		blue
AGAP6		blue
AGBL5		blue
AGK		blue
AGMAT		blue

AGPAT3	blue
AGPAT6	blue
AGXT2L2	blue
AHCYL2	blue
AHDC1	blue
AHNAK	blue
AHSA1	blue
AIF1	blue
AIFM1	blue
AIFM2	blue
AIG1	blue
AIM1	blue
AK2	blue
AK5	blue
AKAP1	blue
AKAP17A	blue
AKAP3	blue
AKAP8	blue
AKAP9	blue
AKNA	blue
AKT2	blue
AKT3	blue
ALAD	blue
ALDH18A1	blue
ALDH1B1	blue
ALDH3A2	blue
ALDH5A1	blue
ALDH6A1	blue
ALDH7A1	blue
ALG8	blue
ALG9	blue
ALMS1	blue
ALS2CL	blue
ALX3	blue
AMACR	blue
AMBRA1	blue
AMFR	blue
AMIGO1	blue
AMIGO3	blue
AMMECR1L	blue
AMOTL1	blue
AMZ1	blue
ANAPC1	blue
ANAPC13	blue
ANAPC5	blue
ANAPC7	blue
ANGEL1	blue
ANK3	blue
ANKH	blue
ANKLE2	blue
ANKRD11	blue
ANKRD20A5P	blue
ANKRD27	blue
ANKRD36	blue
ANKRD36B	blue
ANKRD40	blue
ANKRD52	blue
ANKS1B	blue
ANKS6	blue
ANKZF1	blue
ANO8	blue
ANP32B	blue
ANXA2	blue
ANXA6	blue
AP2B1	blue
AP3D1	blue
AP3S1	blue
APBA1	blue

APBA2	blue
APBB1	blue
APOBEC3F	blue
APOL3	blue
APOLD1	blue
APOOL	blue
AQP3	blue
AQR	blue
ARG1	blue
ARHGAP15	blue
ARHGAP17	blue
ARHGAP18	blue
ARHGAP29	blue
ARHGAP32	blue
ARHGAP33	blue
ARHGAP35	blue
ARHGAP39	blue
ARHGEF1	blue
ARHGEF16	blue
ARHGEF18	blue
ARHGEF3	blue
ARHGEF4	blue
ARHGEF5	blue
ARHGEF6	blue
ARHGEF7	blue
ARHGEF9	blue
ARID1B	blue
ARIH2	blue
ARL10	blue
ARL13A	blue
ARL17A	blue
ARL3	blue
ARMCX1	blue
ARMCX4	blue
ARNTL2	blue
ARPC2	blue
ARPC3	blue
ARPM1	blue
ARRDC5	blue
ASB1	blue
ASB16	blue
ASCC1	blue
ASMT	blue
ASPDH	blue
ASXL1	blue
ATAD3C	blue
ATAT1	blue
ATF6B	blue
ATG10	blue
ATG13	blue
ATG14	blue
ATG3	blue
ATG9B	blue
ATIC	blue
ATM	blue
ATN1	blue
ATOX1	blue
ATP10A	blue
ATP13A1	blue
ATP1A1	blue
ATP1A3	blue
ATP1B3	blue
ATP2A2	blue
ATP2A3	blue
ATP5A1	blue
ATP5B	blue
ATP5J	blue
ATP5J2	blue

ATP6V0E1	blue
ATP6V1D	blue
ATP6V1F	blue
ATP6V1H	blue
ATP8B1	blue
ATP8B2	blue
ATP9B	blue
ATRN	blue
ATXN1L	blue
ATXN2L	blue
ATXN7L1	blue
AURKAPS1	blue
AVPR2	blue
AXIN1	blue
AXIN2	blue
B3GALT4	blue
B3GNTL1	blue
B7H6	blue
BACE1	blue
BACE1-AS	blue
BACH2	blue
BAD	blue
BAG3	blue
BAHCC1	blue
BAHD1	blue
BAIAP2L1	blue
BATF	blue
BATF3	blue
BAZ1B	blue
BBS1	blue
BBS2	blue
BBS9	blue
BCL10	blue
BCL11B	blue
BCL2	blue
BCL9L	blue
BDH1	blue
BET1L	blue
BEX1	blue
BICD1	blue
BID	blue
BIK	blue
BIRC6	blue
BLCAP	blue
BLM	blue
BLMH	blue
BLOC1S1	blue
BMP2	blue
BMP8A	blue
BMP8B	blue
BMS1	blue
BMS1P4	blue
BPTF	blue
BRD1	blue
BRD2	blue
BRD7	blue
BRI3	blue
BRI3BP	blue
BRK1	blue
BRPF1	blue
BRSK2	blue
BSDC1	blue
BSN	blue
BTBD11	blue
BTBD18	blue
BTBD2	blue
BTN3A2	blue
BTN3A3	blue

BTNL3	blue
BTRC	blue
C10orf111	blue
C10orf128	blue
C10orf76	blue
C11orf10	blue
C11orf21	blue
C11orf71	blue
C11orf74	blue
C11orf75	blue
C11orf82	blue
C11orf9	blue
C11orf95	blue
C12orf33	blue
C12orf39	blue
C12orf43	blue
C12orf49	blue
C12orf51	blue
C12orf60	blue
C12orf61	blue
C14orf102	blue
C14orf109	blue
C14orf169	blue
C14orf182	blue
C14orf21	blue
C14orf49	blue
C15orf23	blue
C15orf27	blue
C15orf29	blue
C15orf37	blue
C15orf44	blue
C15orf48	blue
C15orf52	blue
C15orf62	blue
C16orf48	blue
C16orf58	blue
C16orf62	blue
C16orf80	blue
C16orf88	blue
C16orf93	blue
C16orf96	blue
C17orf28	blue
C17orf37	blue
C17orf46	blue
C17orf50	blue
C17orf65	blue
C17orf69	blue
C17orf91	blue
C17orf97	blue
C18orf21	blue
C18orf32	blue
C18orf45	blue
C19orf23	blue
C19orf29	blue
C19orf29OS	blue
C19orf34	blue
C19orf50	blue
C19orf55	blue
C19orf56	blue
C19orf71	blue
C1D	blue
C1GALT1C1	blue
C1orf109	blue
C1orf114	blue
C1orf144	blue
C1orf152	blue
C1orf192	blue
C1orf194	blue

C1orf201	blue
C1orf227	blue
C1orf52	blue
C1orf53	blue
C1orf61	blue
C1orf63	blue
C20orf112	blue
C20orf118	blue
C20orf12	blue
C20orf132	blue
C20orf165	blue
C20orf194	blue
C20orf4	blue
C21orf62	blue
C21orf96	blue
C22orf13	blue
C22orf23	blue
C22orf28	blue
C22orf29	blue
C22orf36	blue
C22orf41	blue
C22orf43	blue
C22orf45	blue
C2CD2	blue
C2CD3	blue
C2orf27A	blue
C2orf42	blue
C2orf44	blue
C2orf50	blue
C2orf62	blue
C3orf19	blue
C3orf37	blue
C3orf54	blue
C4orf3	blue
C4orf42	blue
C5orf13	blue
C5orf20	blue
C5orf25	blue
C5orf45	blue
C5orf62	blue
C6orf1	blue
C6orf106	blue
C6orf136	blue
C6orf165	blue
C6orf52	blue
C7orf25	blue
C7orf42	blue
C7orf44	blue
C7orf73	blue
C8orf44	blue
C9orf150	blue
C9orf46	blue
C9orf6	blue
C9orf66	blue
C9orf89	blue
C9orf9	blue
C9orf91	blue
C9orf96	blue
CABP4	blue
CACNA1F	blue
CACNA1I	blue
CACNA2D3	blue
CACNB1	blue
CAD	blue
CADM1	blue
CADM2	blue
CALM1	blue
CALM3	blue

CALR	blue
CAMP	blue
CAMSAP1	blue
CAMTA2	blue
CAND2	blue
CAPN2	blue
CAPN3	blue
CAPN5	blue
CAPNS2	blue
CARD11	blue
CARD14	blue
CARD16	blue
CARNS1	blue
CASKIN2	blue
CASP1	blue
CASP10	blue
CASP2	blue
CATSPER1	blue
CATSPER2	blue
CBFA2T2	blue
CBLB	blue
CBWD3	blue
CBWD6	blue
CBX2	blue
CBX5	blue
CBX7	blue
CC2D1B	blue
CC2D2B	blue
CCAR1	blue
CCDC120	blue
CCDC127	blue
CCDC134	blue
CCDC141	blue
CCDC142	blue
CCDC148	blue
CCDC153	blue
CCDC164	blue
CCDC23	blue
CCDC24	blue
CCDC28A	blue
CCDC64	blue
CCDC68	blue
CCDC88C	blue
CCDC93	blue
CCL8	blue
CCND1	blue
CCND2	blue
CCNDBP1	blue
CCNF	blue
CCNT1	blue
CCR4	blue
CCR7	blue
CCR8	blue
CCRL2	blue
CCT3	blue
CCT5	blue
CCT6A	blue
CCT7	blue
CCT8	blue
CD1A	blue
CD247	blue
CD3E	blue
CD3EAP	blue
CD4	blue
CD40LG	blue
CD6	blue
CD74	blue
CD84	blue

CD8A	blue
CD96	blue
CD99L2	blue
CDAN1	blue
CDC16	blue
CDC25B	blue
CDC42BPA	blue
CDC42BPB	blue
CDC42BPG	blue
CDH13	blue
CDH20	blue
CDH23	blue
CDH24	blue
CDHR2	blue
CDK5RAP2	blue
CDKAL1	blue
CDKN2A	blue
CDKN2B	blue
CDON	blue
CDR2	blue
CECR1	blue
CELF1	blue
CELSR2	blue
CELSR3	blue
CEND1	blue
CENPN	blue
CENPO	blue
CEP104	blue
CEP164	blue
CEP72	blue
CEP85	blue
CEP89	blue
CERK	blue
CES2	blue
CG030	blue
CHAD	blue
CHCHD3	blue
CHD1L	blue
CHD2	blue
CHD3	blue
CHD4	blue
CHD6	blue
CHEK2	blue
CHMP4C	blue
CHMP5	blue
CHMP7	blue
CHRNA2	blue
CHRNA5	blue
CHST12	blue
CHTF8	blue
CIAO1	blue
CIC	blue
CIDECP	blue
CIITA	blue
CINP	blue
CIRBP	blue
CIRH1A	blue
CIT	blue
CIZ1	blue
CKAP5	blue
CKLF	blue
CKM	blue
CLCC1	blue
CLCN3	blue
CLCN6	blue
CLDN15	blue
CLDN20	blue
CLEC12A	blue

CLEC16A	blue
CLEC18C	blue
CLEC3B	blue
CLEC4A	blue
CLEC4D	blue
CLEC4E	blue
CLEC9A	blue
CLIC1	blue
CLK2	blue
CLN8	blue
CLPB	blue
CLSTN1	blue
CLUAP1	blue
CMBL	blue
CMKLR1	blue
CMTM2	blue
CMTM4	blue
CNDP2	blue
CNIH3	blue
CNIH4	blue
CNNM2	blue
CNNM3	blue
CNOT10	blue
CNTNAP1	blue
CNTRL	blue
COG1	blue
COG2	blue
COG5	blue
COG7	blue
COL17A1	blue
COL27A1	blue
COL5A1	blue
COL6A3	blue
COPG2	blue
COPS7B	blue
CORO2A	blue
COX10	blue
COX15	blue
COX5B	blue
COX8A	blue
CPSF3	blue
CRADD	blue
CRAMP1L	blue
CRB2	blue
CREB3L2	blue
CREG1	blue
CREM	blue
CRISP2	blue
CROCC	blue
CROCCP3	blue
CRTC1	blue
CRTC3	blue
CRY2	blue
CRYBB1	blue
CSNK1E	blue
CSNK2A2	blue
CSRNP2	blue
CSRP2BP	blue
CSTB	blue
CSTF1	blue
CSTF2	blue
CSTF3	blue
CTC1	blue
CTCF	blue
CTNNBL1	blue
CTPS	blue
CTPS2	blue
CTR9	blue

CUBN	blue
CUL1	blue
CUL4A	blue
CUL7	blue
CUL9	blue
CXCL1	blue
CXCL11	blue
CXCR3	blue
CXXC5	blue
CYB561D1	blue
CYB5B	blue
CYB5RL	blue
CYFIP2	blue
CYP20A1	blue
CYP2D6	blue
CYTH1	blue
CYTH3	blue
CYTL1	blue
D2HGDH	blue
DAD1	blue
DAGLA	blue
DARS2	blue
DBF4B	blue
DBH	blue
DBR1	blue
DCAF16	blue
DCAF4	blue
DCAF4L1	blue
DCAF5	blue
DCAF7	blue
DCAF8	blue
DCHS1	blue
DCLK2	blue
DCLRE1C	blue
DCN	blue
DCP1B	blue
DCTD	blue
DCTN1	blue
DCTN5	blue
DDB1	blue
DDHD2	blue
DDI2	blue
DDIT3	blue
DDIT4	blue
DDIT4L	blue
DDR1	blue
DDX10	blue
DDX17	blue
DDX18	blue
DDX19A	blue
DDX19B	blue
DDX20	blue
DDX23	blue
DDX24	blue
DDX27	blue
DDX31	blue
DDX42	blue
DDX46	blue
DDX47	blue
DDX51	blue
DDX52	blue
DDX55	blue
DDX56	blue
DDX6	blue
DENND2D	blue
DENND4B	blue
DEPDC5	blue
DERA	blue

DERL2	blue
DET1	blue
DFFA	blue
DFFB	blue
DFNB31	blue
DGKA	blue
DGKD	blue
DGKH	blue
DGKQ	blue
DHCR24	blue
DHCR7	blue
DHDDS	blue
DHODH	blue
DHX15	blue
DHX16	blue
DHX32	blue
DHX33	blue
DHX35	blue
DHX37	blue
DHX38	blue
DHX57	blue
DHX9	blue
DIAPH1	blue
DIDO1	blue
DIEXF	blue
DIP2A	blue
DIS3L	blue
DKC1	blue
DKFZp779M0652	blue
DLD	blue
DLEC1	blue
DLG3	blue
DLK2	blue
DLST	blue
DMWD	blue
DNAH1	blue
DNAJA3	blue
DNAJA4	blue
DNAJB1	blue
DNAJB13	blue
DNAJC1	blue
DNAJC11	blue
DNAJC16	blue
DNAJC7	blue
DNAL1	blue
DNASE1	blue
DNHD1	blue
DNM1	blue
DNMT1	blue
DNMT3A	blue
DOCK10	blue
DOCK7	blue
DOCK9	blue
DOPEY2	blue
DPH1	blue
DPP4	blue
DPP9	blue
DPYSL2	blue
DRAM1	blue
DRD4	blue
DRG1	blue
DROSHA	blue
DSCAML1	blue
DST	blue
DSTNP2	blue
DSTYK	blue
DTL	blue
DTX4	blue

DUOX1	blue
DUSP16	blue
DUSP28	blue
DUSP7	blue
DVL2	blue
DYNC1H1	blue
DYNC1I2	blue
DYNC1LI2	blue
DYNLT1	blue
DYTN	blue
DYX1C1	blue
EARS2	blue
ECD	blue
ECHDC2	blue
ECI2	blue
ECRP	blue
EDAR	blue
EDC3	blue
EDC4	blue
EDEM1	blue
EEF2	blue
EEF2K	blue
EFCAB1	blue
EFCAB4B	blue
EFEMP2	blue
EFHC2	blue
EFHD1	blue
EFNA4	blue
EFTUD1	blue
EFTUD2	blue
EGFL7	blue
EHBP1	blue
EHD4	blue
EHMT1	blue
EIF1AD	blue
EIF2AK3	blue
EIF2AK4	blue
EIF2B1	blue
EIF2S3	blue
EIF3A	blue
EIF3B	blue
EIF4A3	blue
EIF4B	blue
EIF4ENIF1	blue
EIF4G1	blue
EIF4G2	blue
EIF5A	blue
EIF5B	blue
ELAVL1	blue
ELFN2	blue
ELP3	blue
EMD	blue
EME2	blue
EML3	blue
ENO1-AS1	blue
ENO2	blue
ENOSF1	blue
ENOX2	blue
ENTPD4	blue
ENTPD5	blue
ENTPD8	blue
EP400	blue
EP400NL	blue
EPB41L2	blue
EPHA1	blue
EPHA2	blue
EPHX1	blue
EPHX2	blue

EPPK1	blue
EPRS	blue
EPS8	blue
ERBB3	blue
ERCC3	blue
ERCC5	blue
ESYT1	blue
ESYT2	blue
ETFDH	blue
ETHE1	blue
ETS1	blue
EVI5L	blue
EVL	blue
EXD2	blue
EXOC2	blue
EXOC4	blue
EXOC7	blue
EXOG	blue
EXOSC10	blue
EXT2	blue
EZH1	blue
EZR	blue
F12	blue
F2	blue
FADS1	blue
FADS2	blue
FAF1	blue
FAIM3	blue
FAM102A	blue
FAM103A1	blue
FAM105B	blue
FAM106A	blue
FAM110B	blue
FAM115A	blue
FAM117A	blue
FAM118A	blue
FAM120C	blue
FAM122A	blue
FAM123B	blue
FAM125B	blue
FAM132A	blue
FAM134C	blue
FAM150B	blue
FAM153A	blue
FAM153B	blue
FAM160B2	blue
FAM161B	blue
FAM168B	blue
FAM171A1	blue
FAM174A	blue
FAM175B	blue
FAM176B	blue
FAM184B	blue
FAM186B	blue
FAM18B2	blue
FAM193B	blue
FAM198A	blue
FAM201A	blue
FAM20B	blue
FAM22D	blue
FAM22G	blue
FAM26F	blue
FAM38A	blue
FAM40A	blue
FAM57A	blue
FAM70B	blue
FAM72B	blue
FAM78A	blue

FAM81B	blue
FAM83G	blue
FAM86A	blue
FAM89A	blue
FAM98A	blue
FAN1	blue
FANCI	blue
FARSB	blue
FAS-AS1	blue
FASN	blue
FASTKD5	blue
FAT2	blue
FBF1	blue
FBLN2	blue
FBRSL1	blue
FBXL12	blue
FBXL18	blue
FBXL19	blue
FBXL19-AS1	blue
FBXO18	blue
FBXO21	blue
FBXO41	blue
FBXW2	blue
FBXW4P1	blue
FBXW8	blue
FCER1G	blue
FCGBP	blue
FEN1	blue
FER1L4	blue
FEZ2	blue
FGD1	blue
FGF2	blue
FGFR1	blue
FGFRL1	blue
FGGY	blue
FH	blue
FHL1	blue
FIP1L1	blue
FITM2	blue
FKBP1AP1	blue
FKBP4	blue
FKRP	blue
FLJ12334	blue
FLJ13224	blue
FLJ27352	blue
FLJ30403	blue
FLJ33065	blue
FLJ35776	blue
FLJ36777	blue
FLJ37201	blue
FLJ38109	blue
FLJ39639	blue
FLJ39653	blue
FLJ42289	blue
FLJ42627	blue
FLJ43390	blue
FLJ43663	blue
FLNA	blue
FLNB	blue
FLNC	blue
FLT4	blue
FLVCR1	blue
FLYWCH1	blue
FMNL3	blue
FN3KRP	blue
FNBP1	blue
FNBP4	blue
FNDC5	blue

FNDC9	blue
FOSB	blue
FOXC1	blue
FOXI1	blue
FOXJ1	blue
FOXJ3	blue
FOXK1	blue
FOXK2	blue
FOXM1	blue
FOXP1	blue
FOXP4	blue
FOXRED2	blue
FP588	blue
FRMD4A	blue
FSCN2	blue
FSIP1	blue
FTSJ2	blue
FTSJ3	blue
FTX	blue
FUBP1	blue
FUT11	blue
FUT2	blue
FXN	blue
FXYD1	blue
FYCO1	blue
FYN	blue
FZR1	blue
G0S2	blue
G3BP1	blue
GABARAPL2	blue
GABBR1	blue
GABPB2	blue
GADD45A	blue
GAL3ST4	blue
GALK2	blue
GALM	blue
GALNT10	blue
GALNT6	blue
GALNT9	blue
GALT	blue
GANAB	blue
GANC	blue
GAR1	blue
GARS	blue
GART	blue
GATA3	blue
GBF1	blue
GCN1L1	blue
GDF11	blue
GEMIN4	blue
GEMIN5	blue
GFM1	blue
GGA3	blue
GGCX	blue
GGH	blue
GGN	blue
GGNBP2	blue
GGT7	blue
GGTA1P	blue
GHRLOS	blue
GIGYF1	blue
GIMAP6	blue
GINS4	blue
GIPC2	blue
GIPC3	blue
GIPR	blue
GK5	blue
GLE1	blue

GLG1	blue
GLIPR1	blue
GLOD4	blue
GLS2	blue
GLTP	blue
GLUD1	blue
GMEB2	blue
GMFG	blue
GMPS	blue
GNE	blue
GNG10	blue
GNL2	blue
GNL3L	blue
GNPDA1	blue
GOLGA1	blue
GOLGA2B	blue
GOLGA3	blue
GOLGA7	blue
GON4L	blue
GORASP2	blue
GOSR2	blue
GOT1	blue
GOT2	blue
GP1BA	blue
GP5	blue
GPA33	blue
GPANK1	blue
GPATCH1	blue
GPATCH4	blue
GPATCH8	blue
GPC2	blue
GPHN	blue
GPN2	blue
GPN3	blue
GPR133	blue
GPR137C	blue
GPR152	blue
GPR157	blue
GPR20	blue
GPR55	blue
GPR61	blue
GPR75	blue
GPR88	blue
GPR89B	blue
GPRASP1	blue
GPRC5C	blue
GPT	blue
GPT2	blue
GPX2	blue
GRAMD4	blue
GRAP2	blue
GRID2IP	blue
GRIN2C	blue
GRIPAP1	blue
GRM2	blue
GRPEL1	blue
GSDMB	blue
GSTM4	blue
GSTO1	blue
GTF2A2	blue
GTF3C1	blue
GTF3C2	blue
GTF3C4	blue
GTPBP4	blue
GUCY2D	blue
GUSBP11	blue
GUSBP3	blue
GYS1	blue

GYS2	blue
H3F3A	blue
H3F3C	blue
H6PD	blue
HABP4	blue
HACL1	blue
HADH	blue
HADHA	blue
HADHB	blue
HARS	blue
HAUS5	blue
HBXIP	blue
HCFC1	blue
HCG18	blue
HCG26	blue
HCN3	blue
HDAC1	blue
HEATR1	blue
HEATR2	blue
HEATR4	blue
HEATR5B	blue
HEATR6	blue
HEATR7A	blue
HEBP2	blue
HECTD1	blue
HEG1	blue
HELB	blue
HEMK1	blue
HERC1	blue
HERC2	blue
HEXIM1	blue
HGF	blue
HHAT	blue
HHEX	blue
HHLA2	blue
HIC2	blue
HIF1AN	blue
HIST1H2AC	blue
HIST1H2AK	blue
HIST1H2AL	blue
HIST1H2BF	blue
HIST1H2BN	blue
HIST1H4I	blue
HIST2H2AC	blue
HIST2H2BC	blue
HIST2H2BE	blue
HIST2H3D	blue
HIST4H4	blue
HKDC1	blue
HLA-DRB1	blue
HLCS	blue
HMG20A	blue
HMGA1	blue
HMGXB3	blue
HMHA1	blue
HN1L	blue
HNRNPA0	blue
HNRNPA2B1	blue
HNRNPA3	blue
HNRNPAB	blue
HNRNPH1	blue
HNRNPM	blue
HNRNPR	blue
HNRNPU	blue
HOMEZ	blue
HOTAIRM1	blue
HOXA9	blue
HOXB2	blue

HP1BP3	blue
HPD	blue
HPS4	blue
HPS5	blue
HRASLS2	blue
HSBP1	blue
HSD17B13	blue
HSP90AB1	blue
HSP90B1	blue
HSPA1L	blue
HSPA4	blue
HSPA8	blue
HSPA9	blue
HSPD1	blue
HSPG2	blue
HSPH1	blue
HTATIP2	blue
HTATSF1	blue
HTRA2	blue
HTT	blue
HUWE1	blue
HYAL1	blue
HYAL2	blue
HYOU1	blue
IARS	blue
IARS2	blue
IBA57	blue
ICMT	blue
ID1	blue
IDH3A	blue
IDI2	blue
IFFO2	blue
IFITM5	blue
IFT172	blue
IGFBP7	blue
IGLL1	blue
IGSF9B	blue
IK	blue
IKBIP	blue
IKBKAP	blue
IKBKB	blue
IKBKE	blue
IKZF3	blue
IL10	blue
IL10RA	blue
IL11RA	blue
IL12RB2	blue
IL16	blue
IL18	blue
IL18BP	blue
IL21R	blue
IL26	blue
IL27	blue
IL29	blue
IL2RA	blue
IL2RB	blue
IL2RG	blue
IL31RA	blue
IL4	blue
IL7R	blue
ILF2	blue
ILF3	blue
IMMT	blue
INADL	blue
INCA1	blue
INCENP	blue
INE1	blue
ING1	blue

ING2	blue
ING5	blue
INO80	blue
INPP4A	blue
INPP5B	blue
INPP5E	blue
INPPL1	blue
INTS1	blue
INTS4	blue
INTS7	blue
INTS9	blue
INVS	blue
IP6K2	blue
IPCEF1	blue
IPO5	blue
IPO9	blue
IPP	blue
IRAK1	blue
IRF8	blue
IRGM	blue
IRGQ	blue
IRS1	blue
ISCA2	blue
ITFG2	blue
ITGA6	blue
ITGAL	blue
ITM2C	blue
ITPKB	blue
ITPR3	blue
IVD	blue
IWS1	blue
JAM3	blue
JAZF1	blue
JMJD5	blue
JOSD1	blue
JPH3	blue
JRK	blue
JTB	blue
JUB	blue
KALRN	blue
KANK1	blue
KARS	blue
KAT6B	blue
KAZALD1	blue
KCMF1	blue
KCNA6	blue
KCNAB3	blue
KCNC4	blue
KCNE3	blue
KCNJ13	blue
KCNJ14	blue
KCNJ9	blue
KCNK10	blue
KCTD1	blue
KCTD10	blue
KCTD13	blue
KCTD5	blue
KDELR2	blue
KDELR3	blue
KDM1A	blue
KDM2B	blue
KDM4A	blue
KDM4C	blue
KDM5C	blue
KHDRBS1	blue
KHNYN	blue
KHSRP	blue
KIAA0090	blue

KIAA0100	blue
KIAA0141	blue
KIAA0174	blue
KIAA0182	blue
KIAA0226	blue
KIAA0355	blue
KIAA0368	blue
KIAA0494	blue
KIAA0586	blue
KIAA0664	blue
KIAA0748	blue
KIAA0753	blue
KIAA0754	blue
KIAA0889	blue
KIAA0947	blue
KIAA1147	blue
KIAA1191	blue
KIAA1274	blue
KIAA1310	blue
KIAA1324L	blue
KIAA1429	blue
KIAA1530	blue
KIAA1609	blue
KIAA1797	blue
KIAA1908	blue
KIAA1919	blue
KIF13B	blue
KIF16B	blue
KIF21B	blue
KIF3B	blue
KIF4A	blue
KIF5C	blue
KIR2DL3	blue
KIR3DL2	blue
KITLG	blue
KLB	blue
KLC1	blue
KLC2	blue
KLF11	blue
KLHDC8B	blue
KLHL29	blue
KLHL3	blue
KLHL31	blue
KLHL34	blue
KLHL36	blue
KLHL6	blue
KNTC1	blue
KPNA6	blue
KRBA1	blue
KRBA2	blue
KRT10	blue
KRT5	blue
KRT7	blue
KRTAP5-1	blue
KY	blue
KYNU	blue
L3MBTL1	blue
L3MBTL2	blue
LAIR1	blue
LAMA2	blue
LAMB2P1	blue
LAMTOR1	blue
LANCL2	blue
LAPTM4B	blue
LARP1	blue
LARP7	blue
LARS	blue
LARS2	blue

LAS1L	blue
LAX1	blue
LBH	blue
LCK	blue
LDB1	blue
LDHA	blue
LDHAL6A	blue
LDLRAP1	blue
LEF1	blue
LENG8	blue
LEO1	blue
LEPREL1	blue
LETM1	blue
LGALS17A	blue
LGALS2	blue
LGALS8-AS1	blue
LGMN	blue
LGSN	blue
LHX4	blue
LIG1	blue
LIG3	blue
LILRA5	blue
LIPT2	blue
LIX1L	blue
LLPH	blue
LMAN2L	blue
LMLN	blue
LMO2	blue
LMO7	blue
LMOD3	blue
LMTK3	blue
LNPEP	blue
LOC100128288	blue
LOC100128361	blue
LOC100128573	blue
LOC100128822	blue
LOC100129250	blue
LOC100129480	blue
LOC100129716	blue
LOC100129827	blue
LOC100129845	blue
LOC100129917	blue
LOC100130932	blue
LOC100130950	blue
LOC100131094	blue
LOC100131096	blue
LOC100131193	blue
LOC100131347	blue
LOC100131434	blue
LOC100131564	blue
LOC100132273	blue
LOC100132352	blue
LOC100132356	blue
LOC100132774	blue
LOC100133286	blue
LOC100133315	blue
LOC100133445	blue
LOC100133545	blue
LOC100133957	blue
LOC100133991	blue
LOC100144603	blue
LOC100216545	blue
LOC100216546	blue
LOC100271722	blue
LOC100271836	blue
LOC100272228	blue
LOC100287042	blue
LOC100287722	blue

LOC100288123	blue
LOC100288615	blue
LOC100289019	blue
LOC100289230	blue
LOC100289361	blue
LOC100289511	blue
LOC100293516	blue
LOC100329109	blue
LOC100499466	blue
LOC100505495	blue
LOC100505648	blue
LOC100505678	blue
LOC100505681	blue
LOC100505716	blue
LOC100505839	blue
LOC100505854	blue
LOC100505933	blue
LOC100506033	blue
LOC100506046	blue
LOC100506123	blue
LOC100506334	blue
LOC100506436	blue
LOC100506472	blue
LOC100506655	blue
LOC100506713	blue
LOC100506714	blue
LOC100506730	blue
LOC100506779	blue
LOC100506801	blue
LOC100506810	blue
LOC100506939	blue
LOC100507034	blue
LOC100507178	blue
LOC100507217	blue
LOC100507218	blue
LOC100507321	blue
LOC100507424	blue
LOC100507433	blue
LOC100507463	blue
LOC100507634	blue
LOC100526820	blue
LOC100527964	blue
LOC121952	blue
LOC147670	blue
LOC147804	blue
LOC150381	blue
LOC151162	blue
LOC202181	blue
LOC283174	blue
LOC283547	blue
LOC283888	blue
LOC283922	blue
LOC284551	blue
LOC284801	blue
LOC285074	blue
LOC285758	blue
LOC285965	blue
LOC285972	blue
LOC286437	blue
LOC338758	blue
LOC339529	blue
LOC339666	blue
LOC340037	blue
LOC344887	blue
LOC375190	blue
LOC388387	blue
LOC388692	blue
LOC389791	blue

LOC389906	blue
LOC400236	blue
LOC401097	blue
LOC401320	blue
LOC401588	blue
LOC440104	blue
LOC441454	blue
LOC493754	blue
LOC494141	blue
LOC541471	blue
LOC619207	blue
LOC641298	blue
LOC642236	blue
LOC644246	blue
LOC644961	blue
LOC645513	blue
LOC646329	blue
LOC646471	blue
LOC646903	blue
LOC652276	blue
LOC653160	blue
LOC653566	blue
LOC728190	blue
LOC728377	blue
LOC729013	blue
LOC730091	blue
LOC730227	blue
LOC90784	blue
LOC90834	blue
LOC92249	blue
LOH12CR1	blue
LOH12CR2	blue
LONP2	blue
LPAR5	blue
LPHN1	blue
LPIN1	blue
LPP-AS2	blue
LPXN	blue
LRBA	blue
LRCH1	blue
LRCH3	blue
LRIG1	blue
LRIG2	blue
LRP5	blue
LRP5L	blue
LRP8	blue
LRRC16A	blue
LRRC28	blue
LRRC37A2	blue
LRRC37A4	blue
LRRC41	blue
LRRC42	blue
LRRC48	blue
LRRC4B	blue
LRRC8A	blue
LRRC8D	blue
LRRIQ3	blue
LRRN2	blue
LSG1	blue
LSM10	blue
LSM14B	blue
LSMD1	blue
LSS	blue
LST1	blue
LTBP3	blue
LUC7L2	blue
LUZP1	blue
LY6G5B	blue

LY86-AS1	blue
LY9	blue
LY96	blue
LYPD6B	blue
LYRM1	blue
LYSMD2	blue
LZTR1	blue
LZTS2	blue
M6PR	blue
MACF1	blue
MAD2L1BP	blue
MAD2L2	blue
MADD	blue
MAFK	blue
MAGED1	blue
MAGED2	blue
MAGOH	blue
MAL2	blue
MALAT1	blue
MAMLD1	blue
MAN1C1	blue
MANBAL	blue
MAP1A	blue
MAP1LC3B2	blue
MAP2K6	blue
MAP2K7	blue
MAP3K12	blue
MAP3K4	blue
MAP3K9	blue
MAP4	blue
MAP7D3	blue
MAPK7	blue
MAPK8IP3	blue
MAPK9	blue
MAPKAP1	blue
MAPRE2	blue
MAPT	blue
MARS	blue
MASP2	blue
MATN2	blue
MAVS	blue
MBD1	blue
MBD2	blue
MBOAT4	blue
MBTPS1	blue
MCAM	blue
MCCC1	blue
MCCC2	blue
MCCD1	blue
MCF2L	blue
MCM2	blue
MCM3	blue
MCM3AP	blue
MCM3AP-AS1	blue
MCM4	blue
MCM6	blue
MDC1	blue
MDM4	blue
MDN1	blue
MDS2	blue
MECP2	blue
MED11	blue
MED24	blue
MED27	blue
MED29	blue
MED30	blue
MED31	blue
MED6	blue

MEGF6	blue
MEIS2	blue
MEMO1	blue
MESDC2	blue
METAP1	blue
METTL13	blue
METTL16	blue
METTL2A	blue
METTL2B	blue
METTL7A	blue
METTL7B	blue
METTL9	blue
MFAP1	blue
MFAP4	blue
MFGE8	blue
MFHAS1	blue
MFSD1	blue
MGA	blue
MGAT5	blue
MGC16121	blue
MGC21881	blue
MGC3771	blue
MGC57346	blue
MGST1	blue
MIAT	blue
MID2	blue
MILR1	blue
MINK1	blue
MIOX	blue
MIPEP	blue
MIR181A2HG	blue
MIR223	blue
MIR5047	blue
MIR7-1	blue
MKL1	blue
MLANA	blue
MLC1	blue
MLEC	blue
MLH1	blue
MLL	blue
MLL4	blue
MLLT6	blue
MLXIP	blue
MMACHC	blue
MMADHC	blue
MMEL1	blue
MMGT1	blue
MMP1	blue
MMS19	blue
MND1	blue
MNS1	blue
MNT	blue
MORC4	blue
MORN1	blue
MPHOSPH8	blue
MPP2	blue
MPRIP	blue
MPZ	blue
MRPL16	blue
MRPL33	blue
MRPL44	blue
MRPL49	blue
MRPS24	blue
MRPS27	blue
MRPS36	blue
MRPS5	blue
MRPS9	blue
MS4A3	blue

MS4A4A	blue
MS4A6A	blue
MSH5-C6orf26	blue
MSH6	blue
MSI2	blue
MSRB2	blue
MSRB3	blue
MTA1	blue
MTA2	blue
MTA3	blue
MTCH2	blue
MTHFD1	blue
MTHFD1L	blue
MTHFR	blue
MTHFS	blue
MTMR4	blue
MTMR9LP	blue
MTOR	blue
MTPAP	blue
MTR	blue
MTRNR2L9	blue
MTSS1L	blue
MUL1	blue
MXD3	blue
MXRA8	blue
MYBBP1A	blue
MYC	blue
MYCBP	blue
MYCBP2	blue
MYCT1	blue
MYH3	blue
MYH7B	blue
MYO15A	blue
MYO15B	blue
MYO18A	blue
MYO19	blue
MYO1C	blue
MYO1D	blue
MYO1E	blue
MYO5B	blue
MYO9B	blue
MYOM1	blue
N4BP3	blue
NAA40	blue
NAB2	blue
NACC1	blue
NARS	blue
NASP	blue
NAT10	blue
NAT8L	blue
NAV1	blue
NBAS	blue
NBPF1	blue
NBPF15	blue
NBPF9	blue
NCAPD2	blue
NCAPD3	blue
NCAPG	blue
NCAPG2	blue
NCKAP1L	blue
NCL	blue
NCOA5	blue
NCOR2	blue
NCR1	blue
NCRNA00094	blue
NCRNA00152	blue
NCRNA00174	blue
NCRNA00176	blue

NCRNA00202	blue
NCRNA00241	blue
NCRNA00255	blue
NCRNA00258	blue
NCRNA00292	blue
NCRNA00294	blue
NCRNA00299	blue
NCRNA00324	blue
NCRNA00341	blue
NDC80	blue
NDUFA10	blue
NDUFA2	blue
NDUFA9	blue
NDUFB4	blue
NDUFB6	blue
NDUFS1	blue
NDUFV3	blue
NEAT1	blue
NECAB1	blue
NEDD8	blue
NEFH	blue
NEIL2	blue
NEK9	blue
NELL2	blue
NEO1	blue
NET1	blue
NEU3	blue
NEURL4	blue
NEXN	blue
NF2	blue
NFATC2	blue
NFATC2IP	blue
NFATC3	blue
NFE2L1	blue
NFRKB	blue
NFS1	blue
NFX1	blue
NICN1	blue
NINL	blue
NIPAL3	blue
NIPSNAP3A	blue
NISCH	blue
NKAP	blue
NKAPL	blue
NKD1	blue
NLGN2	blue
NLRC3	blue
NLRC5	blue
NLRP1	blue
NLRP7	blue
NME1-NME2	blue
NME6	blue
NMI	blue
NMT1	blue
NMT2	blue
NNAT	blue
NNT	blue
NOD1	blue
NODAL	blue
NOL10	blue
NOL11	blue
NOL9	blue
NOLC1	blue
NOM1	blue
NONO	blue
NOP10	blue
NOP14	blue
NPAS2	blue

NPHP4	blue
NPY6R	blue
NR1D1	blue
NRBP2	blue
NRCAM	blue
NRF1	blue
NRG1	blue
NRG2	blue
NRIP2	blue
NRXN2	blue
NSDHL	blue
NSUN2	blue
NSUN4	blue
NT5E	blue
NTSR1	blue
NUCKS1	blue
NUDT16P1	blue
NUDT9	blue
NUMA1	blue
NUMBL	blue
NUP133	blue
NUP155	blue
NUP188	blue
NUP205	blue
NUP210	blue
NUP210L	blue
NUP214	blue
NUP62	blue
NUP93	blue
NXF1	blue
NXN	blue
NYNRIN	blue
OBSCN	blue
OCLM	blue
OCRL	blue
ODF2	blue
OGDH	blue
OGFOD1	blue
OMG	blue
OPA3	blue
OR2B6	blue
OR6K3	blue
ORAOV1	blue
ORMDL2	blue
ORMDL3	blue
OTOA	blue
OTUD1	blue
OTUD7B	blue
P2RY8	blue
PA2G4	blue
PAAF1	blue
PABPC1	blue
PABPC1L	blue
PABPC4	blue
PACSIN1	blue
PAFAH2	blue
PAICS	blue
PALB2	blue
PALM3	blue
PAM	blue
PAM16	blue
PAN2	blue
PAN3-AS1	blue
PAPD7	blue
PAR5	blue
PARD6A	blue
PARN	blue
PARP1	blue

PARP15	blue
PARP6	blue
PAR-SN	blue
PARVA	blue
PASK	blue
PATZ1	blue
PAXIP1	blue
PCBD1	blue
PCCA	blue
PCCB	blue
PCGF1	blue
PCIF1	blue
PCM1	blue
PCNT	blue
PCNXL2	blue
PCNXL3	blue
PCSK5	blue
PCSK7	blue
PCYOX1	blue
PCYOX1L	blue
PDCD11	blue
PDCD4	blue
PDDC1	blue
PDE4DIP	blue
PDE6H	blue
PDE9A	blue
PDGFC	blue
PDHA1	blue
PDHB	blue
PDIA3	blue
PDIA4	blue
PDIA5	blue
PDIA6	blue
PDPK1	blue
PDPR	blue
PDXDC1	blue
PDXDC2P	blue
PEA15	blue
PEAK1	blue
PEBP1	blue
PER1	blue
PET112	blue
PEX26	blue
PEX5	blue
PFAS	blue
PFKP	blue
PFN4	blue
PGAM2	blue
PGPEP1	blue
PHACTR4	blue
PHC1	blue
PHF10	blue
PHF13	blue
PHF15	blue
PHF19	blue
PHLPP2	blue
PHOCN	blue
PHRF1	blue
PI3	blue
PI4K2A	blue
PI4KA	blue
PI4KAP2	blue
PI4KB	blue
PIGO	blue
PIGP	blue
PIGT	blue
PIGU	blue
PIK3IP1	blue

PIK3R2	blue
PIK3R4	blue
PILRB	blue
PIM3	blue
PINX1	blue
PIP4K2A	blue
PIP4K2B	blue
PIP5K1C	blue
PITPNB	blue
PITPNC1	blue
PITPNM2	blue
PITPNM3	blue
PITRM1	blue
PJA1	blue
PKD1	blue
PKD1P1	blue
PKI55	blue
PKM2	blue
PKN3	blue
PLA2G7	blue
PLAC4	blue
PLAT	blue
PLCD4	blue
PLCG1	blue
PLCH2	blue
PLCXD1	blue
PLD6	blue
PLEC	blue
PLEKHA2	blue
PLEKHA7	blue
PLEKHG2	blue
PLIN2	blue
PLLP	blue
PLSCR4	blue
PLXDC1	blue
PLXNA1	blue
PLXNA3	blue
PLXNA4	blue
PLXND1	blue
PMEL	blue
PMS2	blue
PMS2CL	blue
PMS2P3	blue
PMS2P5	blue
PNN	blue
PNO1	blue
PNPO	blue
PNRC1	blue
POFUT1	blue
POFUT2	blue
POGK	blue
POGZ	blue
POLA1	blue
POLA2	blue
POLB	blue
POLDIP3	blue
POLE	blue
POLE3	blue
POLG	blue
POLH	blue
POLR1A	blue
POLR1B	blue
POLR2B	blue
POLR2C	blue
POLR2J	blue
POLR3A	blue
POLR3B	blue
POLR3C	blue

POLR3E	blue
POM121	blue
POM121C	blue
POMGNT1	blue
POP1	blue
POU5F1P4	blue
POU6F1	blue
PPARA	blue
PPARD	blue
PPARGC1B	blue
PPCS	blue
PPFIBP1	blue
PPFIBP2	blue
PPIEL	blue
PPIL2	blue
PPIP5K1	blue
PPM1K	blue
PPME1	blue
PPP1R13B	blue
PPP1R16B	blue
PPP1R3E	blue
PPP1R9B	blue
PPP2R1B	blue
PPP2R3B-AS1	blue
PPP2R5C	blue
PPP6R2	blue
PPRC1	blue
PPT2	blue
PRAP1	blue
PRC1	blue
PRDM15	blue
PRDM2	blue
PRDM4	blue
PREP	blue
PRF1	blue
PRICKLE4	blue
PRKAG1	blue
PRKCA	blue
PRKCH	blue
PRKCQ	blue
PRKX	blue
PRLR	blue
PRMT2	blue
PRO1768	blue
ProSAPiP1	blue
PROSER1	blue
PROX2	blue
PRPF19	blue
PRPF3	blue
PRPF4	blue
PRPF40B	blue
PRPF8	blue
PRPS1	blue
PRR12	blue
PRR15L	blue
PRR16	blue
PRR25	blue
PRRC2B	blue
PRRG1	blue
PRRT1	blue
PRRT2	blue
PRRT3	blue
PRSS36	blue
PSAT1	blue
PSD4	blue
PSENEN	blue
PSMC4	blue
PSMD1	blue

PSMD11	blue
PSMD2	blue
PSMD6	blue
PSMD7	blue
PSME4	blue
PSPC1	blue
PTBP1	blue
PTCH2	blue
PTDSS1	blue
PTENP1	blue
PTGFR	blue
PTGFRN	blue
PTK2	blue
PTMA	blue
PTPN13	blue
PTPN18	blue
PTPRA	blue
PTPRN2	blue
PTPRS	blue
PTTG2	blue
PUM1	blue
PUS3	blue
PWP1	blue
PXMP4	blue
PYGB	blue
PYGO2	blue
QRICH1	blue
QTRTD1	blue
R3HDM1	blue
RAB11A	blue
RAB11FIP3	blue
RAB15	blue
RAB24	blue
RAB32	blue
RAB3GAP1	blue
RAB41	blue
RABGAP1	blue
RABIF	blue
RABL2A	blue
RABL2B	blue
RACGAP1	blue
RAD18	blue
RAD51D	blue
RAD54L2	blue
RAI1	blue
RAI14	blue
RALGDS	blue
RALGPS1	blue
RAMP3	blue
RANBP10	blue
RAP1GDS1	blue
RAPGEF1	blue
RASA3	blue
RASA4	blue
RASAL3	blue
RASGRF2	blue
RASGRP2	blue
RAVER1	blue
RBBP4	blue
RBBP7	blue
RBFOX2	blue
RBL2	blue
RBM14	blue
RBM15B	blue
RBM17	blue
RBM19	blue
RBM26	blue
RBM28	blue

RBM33	blue
RBM4B	blue
RBM6	blue
RBP7	blue
RC3H2	blue
RCAN1	blue
RCAN3	blue
RCC2	blue
RCCD1	blue
RCL1	blue
RCN1	blue
RDH11	blue
RDH13	blue
RDH5	blue
REL	blue
RELA	blue
REPS1	blue
RETN	blue
RETSAT	blue
REXO1	blue
RFC1	blue
RFT1	blue
RFTN1	blue
RFWD3	blue
RFX5	blue
RG9MTD3	blue
RGMB	blue
RGS13	blue
RGS17	blue
RHBDD1	blue
RHOBTB2	blue
RHOF	blue
RIMBP3	blue
RIMS3	blue
RIOK1	blue
RLTPR	blue
RMRP	blue
RNASE2	blue
RNASE3	blue
RNASE4	blue
RNASEH1	blue
RNASEK-C17ORF49	blue
RNF125	blue
RNF144A	blue
RNF157	blue
RNF175	blue
RNF20	blue
RNF214	blue
RNF216	blue
RNF224	blue
RNF4	blue
RNF43	blue
RNF44	blue
RNF8	blue
RNFT2	blue
RNMT	blue
RNPS1	blue
RP1L1	blue
RPA1	blue
RPA2	blue
RPAP1	blue
RPL13P5	blue
RPL21	blue
RPL21P44	blue
RPL23AP64	blue
RPN2	blue
RPP14	blue
RPP21	blue

RPRD1B	blue
RPS15AP10	blue
RPS6KA2	blue
RRAGA	blue
RREB1	blue
RRM1	blue
RRN3	blue
RRN3P3	blue
RRP1B	blue
RSL1D1	blue
RTN4IP1	blue
RTTN	blue
RUNX1	blue
RUNX2	blue
RUNX3	blue
RUSC1-AS1	blue
RUVBL1	blue
RXFP4	blue
RYR2	blue
S100A12	blue
S100A6	blue
S100A8	blue
S100A9	blue
SAE1	blue
SALL2	blue
SAMD10	blue
SAMD12	blue
SAMD13	blue
SAMM50	blue
SAP30	blue
SARM1	blue
SARS	blue
SART3	blue
SATB1	blue
SBF1	blue
SBK1	blue
SCAF1	blue
SCAF4	blue
SCAF8	blue
SCAMP4	blue
SCAMP5	blue
SCAND2	blue
SCAPER	blue
SCARNA10	blue
SCARNA17	blue
SCARNA2	blue
SCCPDH	blue
SCD	blue
SCFD2	blue
SCMH1	blue
SCN7A	blue
SCO1	blue
SCO2	blue
SCRG1	blue
SCRIB	blue
SCRN1	blue
SCUBE3	blue
SDAD1	blue
SDF2	blue
SDHA	blue
SDHAP1	blue
SDHB	blue
SDK2	blue
SEC14L2	blue
SEC24C	blue
SEC31B	blue
SEC61B	blue
SECISBP2	blue

SEH1L	blue
SELRC1	blue
SEMA4C	blue
SEMA4G	blue
SENP5	blue
SENP8	blue
SEPHS2	blue
SEPN1	blue
SERINC4	blue
SERINC5	blue
SERPINB2	blue
SERPINB6	blue
SERPINC1	blue
SERPIND1	blue
SERPINE2	blue
SERPINI2	blue
SETD1A	blue
SETD5	blue
SETD6	blue
SEZ6	blue
SF1	blue
SF3A3	blue
SF3B2	blue
SF3B3	blue
SFI1	blue
SFMBT1	blue
SFMBT2	blue
SFPQ	blue
SFRP5	blue
SFT2D1	blue
SFXN1	blue
SFXN2	blue
SFXN3	blue
SGK1	blue
SGK196	blue
SGK223	blue
SGK494	blue
SGPP2	blue
SGSH	blue
SGSM2	blue
SH2B1	blue
SH2D4B	blue
SH3BP4	blue
SH3BP5	blue
SH3GL1P1	blue
SH3PXD2A	blue
SHANK1	blue
SHC1	blue
SHMT1	blue
SHPK	blue
SIDT1	blue
SIDT2	blue
SIGMAR1	blue
SIK1	blue
SIK2	blue
SIN3B	blue
SIPA1L3	blue
SIRPG	blue
SKAP1	blue
SLA2	blue
SLAMF1	blue
SLC10A1	blue
SLC12A3	blue
SLC12A4	blue
SLC13A4	blue
SLC16A1	blue
SLC1A4	blue
SLC20A2	blue

SLC22A23	blue
SLC23A2	blue
SLC23A3	blue
SLC24A1	blue
SLC25A12	blue
SLC25A15	blue
SLC25A23	blue
SLC25A27	blue
SLC25A42	blue
SLC25A45	blue
SLC26A1	blue
SLC26A7	blue
SLC2A1	blue
SLC34A3	blue
SLC35B1	blue
SLC35D1	blue
SLC35E2	blue
SLC35E2B	blue
SLC35F2	blue
SLC35G6	blue
SLC37A1	blue
SLC39A14	blue
SLC41A1	blue
SLC46A1	blue
SLC4A1AP	blue
SLC4A3	blue
SLC4A5	blue
SLC6A16	blue
SLC7A1	blue
SLC7A5P2	blue
SLC7A6	blue
SLC9A3	blue
SLC9A5	blue
SLC9A9	blue
SLFN12L	blue
SLFN13	blue
SLFN5	blue
SLFNL1	blue
SLITRK4	blue
SLPI	blue
SLTM	blue
SLX4	blue
SMAD3	blue
SMARCA4	blue
SMARCAL1	blue
SMARCC1	blue
SMARCC2	blue
SMARCD1	blue
SMARCD3	blue
SMARCE1	blue
SMC1A	blue
SMCR7L	blue
SMG6	blue
SMS	blue
SMU1	blue
SMYD3	blue
SMYD4	blue
SNAP47	blue
SNAPC4	blue
SNAPIN	blue
SND1	blue
SNED1	blue
SNHG9	blue
SNORA24	blue
SNORA25	blue
SNORA48	blue
SNORA57	blue
SNORA72	blue

SNPH	blue
SNRNP200	blue
SNRNP40	blue
SNRNP70	blue
SNW1	blue
SNX29	blue
SNX6	blue
SNX9	blue
SOCS7	blue
SOLH	blue
SON	blue
SORCS2	blue
SOX10	blue
SOX18	blue
SP140L	blue
SPAG1	blue
SPATA5	blue
SPATS2	blue
SPDYE1	blue
SPDYE3	blue
SPDYE5	blue
SPDYE6	blue
SPECC1L	blue
SPG11	blue
SPG7	blue
SPINK8	blue
SPN	blue
SPOCK2	blue
SPON1	blue
SPRNP1	blue
SPTAN1	blue
SPTBN1	blue
SRBD1	blue
SRCAP	blue
SREBF2	blue
SRGAP1	blue
SRGN	blue
SRP68	blue
SRPK3	blue
SRRM1	blue
SRRM2	blue
SRRM5	blue
SRRT	blue
SRSF5	blue
SSBP2	blue
SSRP1	blue
SSTR3	blue
ST13	blue
ST20	blue
ST3GAL1	blue
ST3GAL6	blue
ST6GAL1	blue
ST6GALNAC3	blue
STAG3L1	blue
STAG3L2	blue
STAG3L3	blue
STAR	blue
STARD7	blue
STARD9	blue
STIM1	blue
STIP1	blue
STK16	blue
STK36	blue
STK38	blue
STK39	blue
STX17	blue
STX1B	blue
STXBP1	blue

STXBP6	blue
SUFU	blue
SUGP2	blue
SUN2	blue
SUPT16H	blue
SUPT4H1	blue
SUPT6H	blue
SUPV3L1	blue
SURF4	blue
SUSD4	blue
SV2A	blue
SVOPL	blue
SYCE2	blue
SYDE1	blue
SYF2	blue
SYNE1	blue
SYNE2	blue
SYNGAP1	blue
SYNJ2	blue
SYNRG	blue
SYT15	blue
SYVN1	blue
SZT2	blue
TAC4	blue
TADA2A	blue
TAF1	blue
TAF10	blue
TAF1C	blue
TAF3	blue
TAF6	blue
TAF8	blue
TAMM41	blue
TAOK2	blue
TARDBP	blue
TARS	blue
TAS2R3	blue
TAS2R4	blue
TATDN2	blue
TAX1BP3	blue
TBC1D16	blue
TBC1D22B	blue
TBC1D24	blue
TBC1D7	blue
TBC1D9B	blue
TBCC	blue
TBCCD1	blue
TBP	blue
TBX19	blue
TCEAL4	blue
TCEANC2	blue
TCEB1	blue
TCEB3	blue
TCF3	blue
TCF7	blue
TCF7L2	blue
TCFL5	blue
TCHP	blue
TCOF1	blue
TCP1	blue
TCP11L1	blue
TCTN2	blue
TCTN3	blue
TDP1	blue
TDRKH	blue
TEAD2	blue
TECPR1	blue
TEF	blue
TEP1	blue

TERF2	blue
TERF2IP	blue
TES	blue
TEX10	blue
TEX12	blue
TEX2	blue
TFCP2L1	blue
TFDP2	blue
TFF3	blue
TFIP11	blue
TGFBRAP1	blue
TGIF2	blue
TGS1	blue
TH1L	blue
THADA	blue
THEM4	blue
THOC1	blue
THOC2	blue
THRA	blue
THTPA	blue
THUMPD3	blue
TIAF1	blue
TIAM1	blue
TIGD4	blue
TIMD4	blue
TIMELESS	blue
TIMM17B	blue
TKTL1	blue
TLCD2	blue
TLE1	blue
TLR8-AS1	blue
TM4SF1	blue
TMC6	blue
TMC8	blue
TMCO7	blue
TMED11P	blue
TMED4	blue
TMEM106A	blue
TMEM109	blue
TMEM110	blue
TMEM120B	blue
TMEM14E	blue
TMEM155	blue
TMEM191B	blue
TMEM192	blue
TMEM208	blue
TMEM214	blue
TMEM216	blue
TMEM229B	blue
TMEM25	blue
TMEM44	blue
TMEM57	blue
TMEM59	blue
TMEM60	blue
TMEM63A	blue
TMOD4	blue
TNF	blue
TNFAIP1	blue
TNFAIP8L1	blue
TNFAIP8L2-SCNM1	blue
TNFRSF10A	blue
TNFRSF11A	blue
TNFRSF14	blue
TNFRSF17	blue
TNFRSF21	blue
TNIK	blue
TNK2	blue
TNKS1BP1	blue

TNNC2	blue
TNNI2	blue
TNNT1	blue
TNPO2	blue
TNRC18	blue
TNRC6A	blue
TNRC6B	blue
TNRC6C	blue
TNS4	blue
TNXB	blue
TOB2	blue
TOM1L2	blue
TOMM34	blue
TOP1P1	blue
TOP2B	blue
TP53	blue
TP53BP1	blue
TP53I3	blue
TP73-AS1	blue
TPCN1	blue
TPI1P2	blue
TPMT	blue
TPPP	blue
TRAF1	blue
TRAF3	blue
TRAF3IP1	blue
TRAF3IP2	blue
TRAF3IP3	blue
TRAM2	blue
TRAPPC10	blue
TREML3	blue
TRIM14	blue
TRIM16	blue
TRIM17	blue
TRIM26	blue
TRIM35	blue
TRIM4	blue
TRIM44	blue
TRIM56	blue
TRIM62	blue
TRIM65	blue
TRIM66	blue
TRIM68	blue
TRIO	blue
TRIP4	blue
TRIT1	blue
TRMT2B	blue
TRPC2	blue
TRPC3	blue
TRPV1	blue
TRRAP	blue
TSC1	blue
TSFM	blue
TSGA14	blue
TSHZ1	blue
TSHZ2	blue
TSNARE1	blue
TSPAN14	blue
TSPAN18	blue
TSPYL2	blue
TSR1	blue
TSR2	blue
TSSK3	blue
TSSK4	blue
TSTD2	blue
TTC1	blue
TTC17	blue
TTC21A	blue

TTC24	blue
TTC27	blue
TTC28	blue
TTC3	blue
TTC31	blue
TTC9	blue
TTF1	blue
TTL	blue
TTLL3	blue
TTLL5	blue
TTPAL	blue
TTYH2	blue
TTYH3	blue
TUB	blue
TUBA3D	blue
TUBB	blue
TUBB4	blue
TUBGCP4	blue
TUBGCP5	blue
TUBGCP6	blue
TUG1	blue
TULP3	blue
TULP4	blue
TUSC2	blue
TXK	blue
TXLNA	blue
TXLNG	blue
TXN	blue
TXNDC11	blue
TXNRD3	blue
TYK2	blue
TYW1	blue
U2AF1L4	blue
U2AF2	blue
UBE2B	blue
UBE2D2	blue
UBE2F	blue
UBE2G2	blue
UBE2I	blue
UBE2L3	blue
UBE2Q1	blue
UBE2Q2P1	blue
UBE2Z	blue
UBE3C	blue
UBFD1	blue
UBIAD1	blue
UBQLN4	blue
UBR4	blue
UBR7	blue
UBTF	blue
UBXN7	blue
UCK2	blue
UCKL1-AS1	blue
UFD1L	blue
UGT2B28	blue
UHRF1	blue
UHRF1BP1	blue
ULK2	blue
UNC119B	blue
UNC13B	blue
UNC5CL	blue
UNG	blue
UNK	blue
UNKL	blue
URB1	blue
URB2	blue
USP11	blue
USP13	blue

USP20	blue
USP24	blue
USP27X	blue
USP35	blue
USP36	blue
USP39	blue
USP40	blue
USP48	blue
USP54	blue
USP7	blue
UTP14A	blue
UTP20	blue
UTRN	blue
UXS1	blue
VAMP1	blue
VANGL2	blue
VASH1	blue
VASN	blue
VAT1	blue
VAV2	blue
VCP	blue
VDAC1	blue
VDAC2	blue
VGLL4	blue
VHL	blue
VIPR1	blue
VMAC	blue
VNN3	blue
VOPP1	blue
VPRBP	blue
VPS11	blue
VPS13D	blue
VPS24	blue
VPS26B	blue
VPS33A	blue
VPS39	blue
VPS45	blue
VPS52	blue
VPS53	blue
VWA3A	blue
VWA5A	blue
WBP1	blue
WBP11	blue
WDFY2	blue
WDR11	blue
WDR27	blue
WDR3	blue
WDR33	blue
WDR37	blue
WDR4	blue
WDR45L	blue
WDR52	blue
WDR55	blue
WDR59	blue
WDR6	blue
WDR60	blue
WDR67	blue
WDR70	blue
WHAMM	blue
WHSC1	blue
WIZ	blue
WRNIP1	blue
WSB2	blue
XBP1	blue
XKRX	blue
XPC	blue
XPNPEP1	blue
XPNPEP3	blue

XPO5	blue
XRCC3	blue
XRCC5	blue
XRCC6	blue
XYLT2	blue
YARS	blue
YEATS2	blue
YLPM1	blue
YWHAE	blue
ZBED2	blue
ZBED3	blue
ZBED4	blue
ZBED6	blue
ZBTB16	blue
ZBTB22	blue
ZBTB3	blue
ZBTB39	blue
ZBTB4	blue
ZBTB40	blue
ZBTB46	blue
ZBTB7A	blue
ZBTB9	blue
ZC3H12B	blue
ZC3H12D	blue
ZC3H14	blue
ZC3H18	blue
ZC3H7B	blue
ZC4H2	blue
ZCCHC11	blue
ZCCHC14	blue
ZCWPW2	blue
ZDHHC11	blue
ZDHHC12	blue
ZDHHC8	blue
ZDHHC9	blue
ZFHX2	blue
ZFP41	blue
ZFP64	blue
ZFYVE20	blue
ZFYVE21	blue
ZFYVE26	blue
ZFYVE9	blue
ZHX2	blue
ZHX3	blue
ZKSCAN1	blue
ZKSCAN2	blue
ZKSCAN5	blue
ZMIZ2	blue
ZMYM3	blue
ZMYM4	blue
ZMYND12	blue
ZMYND8	blue
ZNF101	blue
ZNF135	blue
ZNF137P	blue
ZNF142	blue
ZNF154	blue
ZNF207	blue
ZNF212	blue
ZNF233	blue
ZNF236	blue
ZNF263	blue
ZNF264	blue
ZNF275	blue
ZNF276	blue
ZNF3	blue
ZNF317	blue
ZNF318	blue

ZNF320	blue
ZNF324	blue
ZNF330	blue
ZNF335	blue
ZNF337	blue
ZNF346	blue
ZNF362	blue
ZNF395	blue
ZNF397	blue
ZNF417	blue
ZNF445	blue
ZNF460	blue
ZNF469	blue
ZNF490	blue
ZNF496	blue
ZNF498	blue
ZNF512	blue
ZNF512B	blue
ZNF513	blue
ZNF541	blue
ZNF543	blue
ZNF549	blue
ZNF557	blue
ZNF562	blue
ZNF580	blue
ZNF584	blue
ZNF587	blue
ZNF589	blue
ZNF598	blue
ZNF611	blue
ZNF619	blue
ZNF621	blue
ZNF623	blue
ZNF629	blue
ZNF689	blue
ZNF696	blue
ZNF70	blue
ZNF732	blue
ZNF74	blue
ZNF740	blue
ZNF76	blue
ZNF763	blue
ZNF767	blue
ZNF783	blue
ZNF785	blue
ZNF786	blue
ZNF792	blue
ZNF793	blue
ZNF8	blue
ZNF808	blue
ZNF814	blue
ZNF815	blue
ZNF827	blue
ZNF828	blue
ZNF835	blue
ZNF862	blue
ZNF878	blue
ZNFX1-AS1	blue
ZNHIT6	blue
ZP1	blue
ZRSR2	blue
ZSCAN16	blue
ZSCAN2	blue
ZSCAN21	blue
ZSCAN22	blue
ZSCAN29	blue
ZW10	blue
ZXDC	blue

	8-Mar	brown
1/2-SBSRNA4		brown
AATF		brown
AATK		brown
ABAT		brown
ABCA11P		brown
ABCA17P		brown
ABCC5		brown
ABCC6		brown
ABCG1		brown
ABHD2		brown
ABHD4		brown
ACIN1		brown
ACO1		brown
ACOX1		brown
ACP1		brown
ACRC		brown
ACSM1		brown
ACSM3		brown
ACSS2		brown
ACTA1		brown
ACTB		brown
ACTG1		brown
ACTN1		brown
ACVR1B		brown
ACYP1		brown
ACYP2		brown
ADAL		brown
ADAM19		brown
ADAMTSL4		brown
ADAP2		brown
ADAR		brown
ADCY4		brown
ADH5		brown
ADK		brown
ADO		brown
ADPRH		brown
ADRBK2		brown
ADSL		brown
AFAP1		brown
AFF2		brown
AGA		brown
AGBL2		brown
AGPAT1		brown
AHI1		brown
AKAP13		brown
AKR1C3		brown
ALAS1		brown
ALB		brown
ALDH1A1		brown
ALDH2		brown
ALDH3B1		brown
ALDH9A1		brown
ALDOC		brown
ALG14		brown
ALG1L2		brown
ALG5		brown
ALKBH2		brown
ALKBH3		brown
ALOX15B		brown
ALPK1		brown
ALPK3		brown
ALPL		brown
AMICA1		brown
AMIGO2		brown
AMPD3		brown
AMY2B		brown
AMZ2		brown

AMZ2P1	brown
ANAPC16	brown
ANAPC4	brown
ANKFY1	brown
ANKRA2	brown
ANKRD13A	brown
ANKRD29	brown
ANKRD31	brown
ANKRD36BP2	brown
ANKRD37	brown
ANKRD39	brown
ANKS1A	brown
ANO10	brown
ANO7	brown
ANP32A	brown
ANPEP	brown
ANXA11	brown
ANXA4	brown
ANXA5	brown
ANXA7	brown
ANXA9	brown
AOAH	brown
AOC2	brown
AP1M1	brown
AP2A1	brown
AP2M1	brown
AP3B1	brown
APBB1IP	brown
APC2	brown
APITD1	brown
APLP2	brown
APOA1	brown
APOBEC3C	brown
APOBR	brown
APP	brown
ARAP1	brown
ARAP3	brown
ARCN1	brown
ARF1	brown
ARF3	brown
ARF6	brown
ARFGAP3	brown
ARGFXP2	brown
ARHGAP1	brown
ARHGAP21	brown
ARHGAP23	brown
ARHGAP24	brown
ARHGAP25	brown
ARHGAP26	brown
ARHGAP27	brown
ARHGAP30	brown
ARHGAP31	brown
ARHGDIB	brown
ARHGEF10	brown
ARHGEF11	brown
ARHGEF12	brown
ARID1A	brown
ARID3A	brown
ARID3B	brown
ARL1	brown
ARL6	brown
ARNT	brown
ARSD	brown
ARSG	brown
ASAH1	brown
ASAP1-IT1	brown
ASB8	brown
ASB9P1	brown

ASCC2	brown
ASH2L	brown
ASPRV1	brown
ATF5	brown
ATF7	brown
ATG2A	brown
ATG7	brown
ATP2B4	brown
ATP5S	brown
ATP6V0A1	brown
ATP6V0C	brown
ATP6V0D1	brown
ATP6V1B2	brown
ATPIF1	brown
ATXN2	brown
B3GNT9	brown
B4GALT1	brown
B4GALT4	brown
B4GALT5	brown
BASP1	brown
BAZ2A	brown
BBS4	brown
BCAS3	brown
BCCIP	brown
BCDIN3D	brown
BCKDHA	brown
BCL2L13	brown
BCL6	brown
BCL9	brown
BCO2	brown
BCOR	brown
BCORL1	brown
BDNF-AS1	brown
BEAN1	brown
BECN1	brown
BEND3	brown
BEX2	brown
BEX5	brown
BHLHE40	brown
BICD2	brown
BIN2	brown
BIVM	brown
BLOC1S3	brown
BNIP3	brown
BOD1	brown
BOLA3	brown
BPNT1	brown
BRAP	brown
BRCA1	brown
BRD3	brown
BRD4	brown
BRD8	brown
BRE	brown
BRIX1	brown
BRP44	brown
BRPF3	brown
BSCL2	brown
BSG	brown
BTBD19	brown
BTBD9	brown
BTG2	brown
BTG3	brown
BTK	brown
BTN3A1	brown
BUB3	brown
BZW2	brown
C10orf10	brown
C10orf105	brown

C10orf12	brown
C10orf26	brown
C10orf54	brown
C10orf88	brown
C11orf31	brown
C11orf42	brown
C11orf51	brown
C11orf73	brown
C12orf24	brown
C12orf32	brown
C12orf41	brown
C12orf47	brown
C12orf57	brown
C12orf62	brown
C12orf73	brown
C12orf76	brown
C13orf27	brown
C14orf128	brown
C14orf149	brown
C14orf159	brown
C14orf64	brown
C14orf79	brown
C15orf33	brown
C15orf38	brown
C15orf39	brown
C15orf40	brown
C15orf57	brown
C15orf61	brown
C16orf54	brown
C16orf57	brown
C16orf70	brown
C16orf91	brown
C16orf95	brown
C17orf100	brown
C17orf103	brown
C17orf108	brown
C17orf48	brown
C17orf63	brown
C17orf85	brown
C17orf87	brown
C17orf89	brown
C18orf18	brown
C18orf55	brown
C18orf8	brown
C19orf12	brown
C19orf22	brown
C19orf42	brown
C19orf73	brown
C19orf79	brown
C1orf101	brown
C1orf135	brown
C1orf151	brown
C1orf183	brown
C1orf213	brown
C1orf216	brown
C1orf228	brown
C1orf31	brown
C1orf38	brown
C1orf54	brown
C1QTNF3	brown
C1RL	brown
C20orf11	brown
C20orf20	brown
C20orf3	brown
C20orf30	brown
C20orf43	brown
C20orf7	brown
C21orf119	brown

C21orf122	brown
C21orf49	brown
C22orf32	brown
C22orf39	brown
C2orf18	brown
C2orf28	brown
C2orf29	brown
C2orf3	brown
C2orf56	brown
C2orf63	brown
C2orf84	brown
C3	brown
C3orf25	brown
C3orf26	brown
C3orf33	brown
C3orf52	brown
C3orf62	brown
C3orf71	brown
C3orf78	brown
C4BPB	brown
C4orf43	brown
C5AR1	brown
C5orf28	brown
C5orf34	brown
C5orf39	brown
C5orf55	brown
C6orf115	brown
C6orf125	brown
C6orf130	brown
C6orf170	brown
C6orf225	brown
C6orf27	brown
C6orf48	brown
C6orf57	brown
C6orf64	brown
C6orf70	brown
C6orf89	brown
C7orf11	brown
C7orf13	brown
C7orf23	brown
C7orf30	brown
C7orf46	brown
C7orf47	brown
C7orf54	brown
C7orf55	brown
C7orf57	brown
C7orf68	brown
C7orf70	brown
C8orf38	brown
C8orf41	brown
C8orf76	brown
C8orf77	brown
C9orf103	brown
C9orf123	brown
C9orf131	brown
C9orf163	brown
C9orf25	brown
C9orf30	brown
C9orf40	brown
C9orf84	brown
C9orf95	brown
CA13	brown
CA14	brown
CA3	brown
CA6	brown
CABLES2	brown
CACNA2D4	brown
CACNG8	brown

CADM4	brown
CALCOCO1	brown
CALCOCO2	brown
CAMK1D	brown
CAMK2G	brown
CAMKK2	brown
CAMTA1	brown
CANT1	brown
CAP1	brown
CAPN1	brown
CAPNS1	brown
CAPZB	brown
CARM1	brown
CARS	brown
CASC3	brown
CASP6	brown
CASP9	brown
CASS4	brown
CAST	brown
CASZ1	brown
CBFA2T3	brown
CBL	brown
CBR3	brown
CBX1	brown
CBX6	brown
CBY1	brown
CC2D2A	brown
CCBL1	brown
CCDC102B	brown
CCDC109B	brown
CCDC111	brown
CCDC135	brown
CCDC149	brown
CCDC152	brown
CCDC167	brown
CCDC36	brown
CCDC69	brown
CCDC99	brown
CCL3	brown
CCNB1IP1	brown
CCNK	brown
CCRN4L	brown
CCT6P1	brown
CCT6P3	brown
CD101	brown
CD14	brown
CD163	brown
CD1C	brown
CD1D	brown
CD209	brown
CD300C	brown
CD300E	brown
CD300LB	brown
CD300LF	brown
CD3D	brown
CD44	brown
CD53	brown
CD68	brown
CD82	brown
CD86	brown
CD93	brown
CD97	brown
CDC42	brown
CDC42EP4	brown
CDC42SE1	brown
CDC5L	brown
CDCA4	brown
CDCP1	brown

CDH1	brown
CDKL3	brown
CDKN2AIPNL	brown
CDNF	brown
CDV3	brown
CECR6	brown
CELA1	brown
CELF3	brown
CENPH	brown
CERKL	brown
CERS2	brown
CES1	brown
CFL1	brown
CFL2	brown
CFLAR	brown
CFLAR-AS1	brown
CHCHD1	brown
CHCHD2	brown
CHCHD6	brown
CHCHD7	brown
CHCHD8	brown
CHD8	brown
CHEK1	brown
CHGA	brown
CHI3L2	brown
CHKA	brown
CHMP4B	brown
CHP	brown
CHRNB1	brown
CHRNB2	brown
CHST11	brown
CHST15	brown
CHST7	brown
CITED2	brown
CKAP4	brown
CLC	brown
CLCN5	brown
CLDN16	brown
CLDN9	brown
CLEC4C	brown
CLIP2	brown
CLPX	brown
CLTA	brown
CLTC	brown
CLTCL1	brown
CMIP	brown
CMTM1	brown
CNFN	brown
CNN2	brown
CNNM4	brown
CNOT1	brown
CNOT4	brown
CNPY2	brown
CNTD1	brown
COA5	brown
COG8	brown
COL15A1	brown
COL5A2	brown
COL8A2	brown
COLEC12	brown
COPA	brown
COPB2	brown
COPG	brown
COPS7A	brown
COPS8	brown
COPZ2	brown
COQ3	brown
COQ5	brown

CORO1C	brown
COTL1	brown
COX11	brown
COX17	brown
COX18	brown
COX5A	brown
COX6A1	brown
CPM	brown
CPNE2	brown
CPNE7	brown
CPOX	brown
CPPED1	brown
CPSF7	brown
CPT1A	brown
CPT2	brown
CPVL	brown
CREBBP	brown
CRISPLD2	brown
CRKL	brown
CRNKL1	brown
CRTAM	brown
CRYGS	brown
CRYZ	brown
CS	brown
CSAD	brown
CSF1	brown
CSF1R	brown
CSF2RA	brown
CSF2RB	brown
CSF3R	brown
CSNK1D	brown
CSNK2A1	brown
CTDP1	brown
CTDSP2	brown
CTIF	brown
CTNNA1	brown
CTNNAL1	brown
CTNNB1	brown
CTNND1	brown
CTSA	brown
CTSB	brown
CTSD	brown
CTSZ	brown
CUX1	brown
CUX2	brown
CWC15	brown
CXCR2	brown
CXCR7	brown
CXorf56	brown
CYB5A	brown
CYB5D1	brown
CYB5R1	brown
CYB5R3	brown
CYFIP1	brown
CYP27A1	brown
CYP2R1	brown
CYP4F3	brown
DAB2	brown
DAG1	brown
DAP	brown
DAP3	brown
DAPK1	brown
DAPK2	brown
DAZAP2	brown
DBNL	brown
DCLRE1B	brown
DCUN1D2	brown
DDTL	brown

DEDD	brown
DENND1A	brown
DENND3	brown
DENND5A	brown
DGCR11	brown
DGCR2	brown
DGKG	brown
DHTKD1	brown
DHX34	brown
DHX8	brown
DIRC3	brown
DISC1	brown
DKFZP686I15217	brown
DKFZp761E198	brown
DLEU1	brown
DMKN	brown
DNAAF2	brown
DNAH17	brown
DNAJB12	brown
DNAJB5	brown
DNAJB6	brown
DNAJC13	brown
DNAJC14	brown
DNAJC18	brown
DNAJC19	brown
DNAJC5	brown
DNAJC5B	brown
DNM2	brown
DNMBP	brown
DNMT3B	brown
DOCK2	brown
DOCK5	brown
DOCK6	brown
DOCK8	brown
DOK3	brown
DPAGT1	brown
DPF2	brown
DPH5	brown
DPRXP4	brown
DSCC1	brown
DSCR3	brown
DTX2P1-UPK3BP1-PMS2P11	brown
DUS4L	brown
DUSP12	brown
DUSP18	brown
DUT	brown
DVL3	brown
DYNLL1	brown
DYNLL2	brown
DYRK4	brown
DYSF	brown
ECE1	brown
EEPD1	brown
EFHA2	brown
EFHC1	brown
EGR1	brown
EHBP1L1	brown
EHD1	brown
EHD3	brown
EID2B	brown
EID3	brown
EIF2AK1	brown
EIF2B3	brown
EIF2C1	brown
EIF2C2	brown
EIF2D	brown
EIF4A2	brown
EIF4EBP2	brown

EIF4H	brown
ELAC1	brown
ELF4	brown
ELK1	brown
ELL	brown
ELL3	brown
ELMO1	brown
ELMO2	brown
ELOVL1	brown
ELOVL3	brown
EMG1	brown
EMILIN2	brown
EMR2	brown
ENDOU	brown
ENO1	brown
ENOPH1	brown
ENSA	brown
ENTPD3-AS1	brown
EP300	brown
EPB41	brown
EPB41L3	brown
EPHB1	brown
EPHB2	brown
EPS15L1	brown
ERC1	brown
ERF	brown
ERGIC1	brown
ERICH1	brown
ERMAP	brown
ERMN	brown
ESD	brown
ESR1	brown
ESRP2	brown
ETV6	brown
EVC	brown
EXOSC1	brown
EXOSC3	brown
EXOSC8	brown
EXTL3	brown
EYA3	brown
F11R	brown
F13A1	brown
F2RL2	brown
FABP6	brown
FAHD1	brown
FAHD2A	brown
FAIM	brown
FAM101B	brown
FAM104B	brown
FAM110C	brown
FAM120B	brown
FAM129A	brown
FAM129B	brown
FAM136A	brown
FAM157A	brown
FAM160A2	brown
FAM162A	brown
FAM165B	brown
FAM168A	brown
FAM192A	brown
FAM193A	brown
FAM19A1	brown
FAM202A	brown
FAM20A	brown
FAM21A	brown
FAM21C	brown
FAM24B	brown
FAM35B2	brown

FAM36A	brown
FAM53C	brown
FAM58A	brown
FAM63A	brown
FAM66C	brown
FAM71F2	brown
FAM76A	brown
FAM96B	brown
FAM98C	brown
FANCF	brown
FANK1	brown
FBN2	brown
FBP1	brown
FBRS	brown
FBXL20	brown
FBXO22	brown
FBXO25	brown
FBXO40	brown
FCAR	brown
FCN1	brown
FDPSL2A	brown
FDX1	brown
FGF9	brown
FHDC1	brown
FIG4	brown
FIS1	brown
FKBP15	brown
FKBP1A	brown
FKBP5	brown
FKBP9	brown
FLCN	brown
FLJ10038	brown
FLJ14107	brown
FLJ14186	brown
FLJ23867	brown
FLJ31813	brown
FLJ37453	brown
FLJ39582	brown
FLJ40292	brown
FLJ44635	brown
FLJ45340	brown
FLJ45445	brown
FLOT2	brown
FLT3	brown
FLVCR2	brown
FNTB	brown
FOSL2	brown
FOXJ2	brown
FOXN3	brown
FOXO4	brown
FOXP3	brown
FRS3	brown
FSCN1	brown
FTH1P3	brown
FTO	brown
FUCA2	brown
FUNDC1	brown
FURIN	brown
FUS	brown
FXYD6	brown
FZD1	brown
G6PD	brown
GAB2	brown
GAB3	brown
GABRR2	brown
GALNS	brown
GALNT11	brown
GALNT2	brown

GAPDHS	brown
GAS7	brown
GATAD2B	brown
GATS	brown
GBA	brown
GCC1	brown
GCK	brown
GCNT7	brown
GDF15	brown
GDI2	brown
GDPD1	brown
GFOD2	brown
GHITM	brown
GHRLOS2	brown
GIGYF2	brown
GIMAP4	brown
GIMAP8	brown
GIT2	brown
GK3P	brown
GLB1	brown
GLB1L	brown
GLI1	brown
GLMN	brown
GLO1	brown
GLUL	brown
GLYR1	brown
GM2A	brown
GMDS	brown
GMEB1	brown
GMIP	brown
GNA11	brown
GNA12	brown
GNA15	brown
GNAI2	brown
GNAL	brown
GNAS	brown
GNAT2	brown
GNB1	brown
GNB5	brown
GNRH1	brown
GNS	brown
GOLGA2	brown
GOLGA5	brown
GOLGA6L5	brown
GOLGA6L9	brown
GOLGB1	brown
GOLIM4	brown
GOLM1	brown
GOSR1	brown
GPR107	brown
GPR18	brown
GPR183	brown
GPR64	brown
GPRIN1	brown
GPX7	brown
GRAMD1B	brown
GRB10	brown
GRB2	brown
GRIN2D	brown
GRINA	brown
GRN	brown
GSK3A	brown
GSN	brown
GSR	brown
GSTA4	brown
GTF2F1	brown
GTF2H5	brown
GTF2I	brown

GTF2IP1	brown
GTF3A	brown
GTPBP1	brown
GTPBP5	brown
GUSBP1	brown
GUSBP4	brown
GUSBP5	brown
H1F0	brown
H2AFY	brown
HAAO	brown
HAR1A	brown
HAR1B	brown
HAX1	brown
HCG4	brown
HCK	brown
HCLS1	brown
HDAC4	brown
HDDC2	brown
HDDC3	brown
HDGF	brown
HDLBP	brown
HENMT1	brown
HERPUD1	brown
HESX1	brown
HEXB	brown
HFE	brown
HGD	brown
HGSNAT	brown
HIBADH	brown
HIP1	brown
HIPK2	brown
HIST3H2A	brown
HIVEP3	brown
HK1	brown
HK2	brown
HLA-B	brown
HLA-C	brown
HLA-DPA1	brown
HLA-E	brown
HM13	brown
HMGN3	brown
HMGN4	brown
HMOX1	brown
HNRNPD	brown
HNRNPF	brown
HNRNPK	brown
HNRNPUL1	brown
HNRNPUL2	brown
HRH2	brown
HRK	brown
HSBP1L1	brown
HSCB	brown
HSD17B14	brown
HSD17B3	brown
HSD17B4	brown
HSD17B6	brown
HSPA1A	brown
HSPA1B	brown
HSPA5	brown
HYLS1	brown
ICOS	brown
IFI27L1	brown
IFI27L2	brown
IFNAR2	brown
IFT43	brown
IFT46	brown
IGF1R	brown
IGF2R	brown

IKZF1	brown
IL17RA	brown
IL23A	brown
IL4R	brown
IL6R	brown
IMMP2L	brown
IMPDH1	brown
ING4	brown
INSR	brown
INTS3	brown
IP6K1	brown
IQCB1	brown
IQCE	brown
IQSEC1	brown
IRAK2	brown
IRF2	brown
IRF2BPL	brown
IRS2	brown
ISCU	brown
ISG20L2	brown
ITGA5	brown
ITGAE	brown
ITGAM	brown
ITGAX	brown
ITGB2	brown
ITGB4	brown
ITM2A	brown
ITPK1	brown
ITPK1-AS1	brown
ITSN1	brown
JAK1	brown
JAK3	brown
JARID2	brown
JPH4	brown
KAT5	brown
KAT6A	brown
KAT7	brown
KATNA1	brown
KBTBD11	brown
KCNA5	brown
KCNC3	brown
KCND1	brown
KCNG2	brown
KCNK13	brown
KCNK6	brown
KCNMB3	brown
KCNMB4	brown
KCNQ1OT1	brown
KCNRG	brown
KCTD21	brown
KDELR1	brown
KDM2A	brown
KDM3B	brown
KDM4B	brown
KDM5B	brown
KDM6B	brown
KGFLP2	brown
KHDRBS2	brown
KIAA0040	brown
KIAA0196	brown
KIAA0226L	brown
KIAA0247	brown
KIAA0317	brown
KIAA0319L	brown
KIAA0430	brown
KIAA0513	brown
KIAA0556	brown
KIAA0913	brown

KIAA0922	brown
KIAA1267	brown
KIAA1324	brown
KIAA1598	brown
KIAA1614	brown
KIAA1949	brown
KIAA1958	brown
KIF13A	brown
KIF1B	brown
KIF1C	brown
KIF3C	brown
KIR3DL1	brown
KLF13	brown
KLF4	brown
KLF7	brown
KLF9	brown
KLHDC2	brown
KLHL18	brown
KLHL21	brown
KLHL32	brown
KLRC4-KLRK1	brown
KPNB1	brown
KREMEN1	brown
KRT2	brown
KSR1	brown
KTI12	brown
LAIR2	brown
LAMP1	brown
LAPTM5	brown
LASP1	brown
LAT2	brown
LCMT2	brown
LCP1	brown
LCP2	brown
LDLRAD3	brown
LEFTY2	brown
LEKR1	brown
LILRA1	brown
LILRA4	brown
LILRB1	brown
LILRB2	brown
LIMK1	brown
LIMK2	brown
LIN7B	brown
LIPA	brown
LIPT1	brown
LMTK2	brown
LNP1	brown
LOC100128682	brown
LOC100128881	brown
LOC100129034	brown
LOC100129196	brown
LOC100129518	brown
LOC100129931	brown
LOC100129961	brown
LOC100130093	brown
LOC100130298	brown
LOC100131733	brown
LOC100132247	brown
LOC100133161	brown
LOC100270804	brown
LOC100272217	brown
LOC100287015	brown
LOC100287482	brown
LOC100288069	brown
LOC100289473	brown
LOC100289495	brown
LOC100294362	brown

LOC100303728	brown
LOC100303749	brown
LOC100306951	brown
LOC100499177	brown
LOC100499194	brown
LOC100505483	brown
LOC100505549	brown
LOC100505622	brown
LOC100505687	brown
LOC100505738	brown
LOC100505761	brown
LOC100505881	brown
LOC100506054	brown
LOC100506068	brown
LOC100506207	brown
LOC100506233	brown
LOC100506428	brown
LOC100506585	brown
LOC100506686	brown
LOC100506844	brown
LOC100506963	brown
LOC100507032	brown
LOC100507250	brown
LOC100507423	brown
LOC100507547	brown
LOC100630918	brown
LOC139201	brown
LOC144486	brown
LOC145474	brown
LOC145783	brown
LOC147727	brown
LOC148189	brown
LOC148696	brown
LOC150197	brown
LOC152217	brown
LOC152225	brown
LOC202781	brown
LOC254128	brown
LOC255512	brown
LOC256880	brown
LOC257358	brown
LOC257396	brown
LOC283070	brown
LOC283104	brown
LOC283481	brown
LOC284385	brown
LOC284408	brown
LOC286442	brown
LOC339290	brown
LOC374443	brown
LOC387723	brown
LOC388152	brown
LOC388789	brown
LOC389765	brown
LOC399744	brown
LOC399815	brown
LOC400027	brown
LOC400604	brown
LOC400657	brown
LOC400752	brown
LOC400960	brown
LOC440288	brown
LOC440434	brown
LOC440944	brown
LOC550112	brown
LOC554206	brown
LOC613037	brown
LOC643733	brown

LOC643770	brown
LOC644656	brown
LOC644714	brown
LOC645212	brown
LOC645431	brown
LOC645638	brown
LOC645676	brown
LOC646762	brown
LOC678655	brown
LOC727896	brown
LOC728175	brown
LOC728558	brown
LOC728730	brown
LOC729234	brown
LOC729603	brown
LOC729737	brown
LOC731275	brown
LOC79015	brown
LOC93622	brown
LOXHD1	brown
LOXL3	brown
LPCAT3	brown
LPIN2	brown
LPPR2	brown
LRFN1	brown
LRIT3	brown
LRP1	brown
LRP10	brown
LRRC23	brown
LRRC25	brown
LRRC3	brown
LRRC37A	brown
LRRC37A3	brown
LRRC39	brown
LRRC59	brown
LRRFIP2	brown
LRRK1	brown
LSM5	brown
LSM7	brown
LSP1	brown
LTA4H	brown
LTB4R	brown
LTBP2	brown
LYG1	brown
LYRM4	brown
LYSMD1	brown
LZTS1	brown
MAEA	brown
MAFB	brown
MAFG	brown
MAGEH1	brown
MAGI2	brown
MAGOHB	brown
MAML1	brown
MAML3	brown
MAN2A2	brown
MAN2B2	brown
MANBA	brown
MANF	brown
MAP1S	brown
MAP2K1	brown
MAP2K3	brown
MAP3K3	brown
MAP3K5	brown
MAP4K4	brown
MAP7D1	brown
MAPK13	brown
MAPK14	brown

MAPKAPK5	brown
MAPRE1	brown
MAPRE3	brown
MARK2	brown
MARK3	brown
MARVELD1	brown
MAST3	brown
MATL2963	brown
MAU2	brown
MAX	brown
MBD6	brown
MBOAT7	brown
MBP	brown
MCF2L-AS1	brown
MDP1	brown
ME3	brown
MED12	brown
MED25	brown
MEFV	brown
MERTK	brown
MEST	brown
METTL10	brown
METTL23	brown
METTL5	brown
METTL6	brown
MFN2	brown
MFSD11	brown
MFSD2A	brown
MFSD6L	brown
MGAM	brown
MGRN1	brown
MGST3	brown
MICAL2	brown
MICALCL	brown
MICU1	brown
MIDN	brown
MIR17HG	brown
MIR210HG	brown
MIR3648	brown
MIS12	brown
MIS18A	brown
MKNK1	brown
MKNK2	brown
MLF1	brown
MLF2	brown
MLH3	brown
MLL2	brown
MLLT1	brown
MMP14	brown
MMP25	brown
MMP9	brown
MOBKL2A	brown
MON1B	brown
MOSC1	brown
MPEG1	brown
MPP1	brown
MREG	brown
MRFAP1L1	brown
MRP63	brown
MRPL11	brown
MRPL14	brown
MRPL17	brown
MRPL21	brown
MRPL27	brown
MRPL30	brown
MRPL34	brown
MRPL35	brown
MRPL36	brown

MRPL43	brown
MRPL45	brown
MRPL45P2	brown
MRPL46	brown
MRPL51	brown
MRPL53	brown
MRPS14	brown
MRPS21	brown
MRPS23	brown
MRPS30	brown
MRPS35	brown
MRPS6	brown
MRS2P2	brown
MRTO4	brown
MS4A14	brown
MSL3P1	brown
MSMP	brown
MSN	brown
MSRA	brown
MST1P9	brown
MSX2P1	brown
MT1E	brown
MTCP1	brown
MTCP1NB	brown
MTERFD3	brown
MTF1	brown
MTMR12	brown
MTMR14	brown
MTMR3	brown
MTVR2	brown
MX2	brown
MYADM	brown
MYB	brown
MYCL1	brown
MYD88	brown
MYH11	brown
MYH9	brown
MYL6	brown
MYL6B	brown
MYLK-AS1	brown
MYO1F	brown
MYO7A	brown
MYOF	brown
N4BP1	brown
N6AMT1	brown
N6AMT2	brown
NAAA	brown
NACC2	brown
NADK	brown
NAE1	brown
NAF1	brown
NAGA	brown
NAGK	brown
NANOS1	brown
NAP1L3	brown
NAP1L5	brown
NAPA	brown
NBEAL2	brown
NBR1	brown
NCF2	brown
NCOA6	brown
NCOR1	brown
NCRNA00086	brown
NCRNA00115	brown
NCRNA00116	brown
NCRNA00219	brown
NCRNA00282	brown
NCRNA00321	brown

NCRNA00339	brown
NCSTN	brown
NDEL1	brown
NDNL2	brown
NDRG1	brown
NDRG3	brown
NDST1	brown
NDST2	brown
NDUFA12	brown
NDUFA7	brown
NDUFAB1	brown
NDUFAF4	brown
NDUFB2	brown
NDUFC1	brown
NDUFS3	brown
NDUFV2	brown
NEK3	brown
NEK6	brown
NEU1	brown
NFAM1	brown
NFIC	brown
NFKB1	brown
NFKBIA	brown
NFYC	brown
NGDN	brown
NGRN	brown
NHEJ1	brown
NHLH1	brown
NHSL1	brown
NHSL2	brown
NID1	brown
NIT2	brown
NKAPP1	brown
NKIRAS2	brown
NLRC4	brown
NLRP12	brown
NLRP3	brown
NLRP6	brown
NLRX1	brown
NMB	brown
NME1	brown
NME2	brown
NMNAT1	brown
NOD2	brown
NOL7	brown
NOMO2	brown
NOMO3	brown
NOTCH1	brown
NOTCH2	brown
NPM3	brown
NR2C1	brown
NRBP1	brown
NSD1	brown
NSFL1C	brown
NSMCE4A	brown
NT5C3L	brown
NTAN1	brown
NTNG2	brown
NTPCR	brown
NUAK2	brown
NUB1	brown
NUCB1	brown
NUDT15	brown
NUDT2	brown
NUDT7	brown
NUMB	brown
NUP35	brown
NUP98	brown

NUPL2	brown
NXT1	brown
OAZ2	brown
OCIAD2	brown
OLFML2B	brown
OPTN	brown
ORAI2	brown
ORMDL1	brown
OS9	brown
OSBP	brown
OSBPL2	brown
OSMR	brown
OST4	brown
OSTalpha	brown
OSTF1	brown
OXNAD1	brown
P2RX1	brown
PACS1	brown
PACSIN2	brown
PADI2	brown
PADI4	brown
PAF1	brown
PAFAH1B1	brown
PAK1	brown
PAPLN	brown
PAQR6	brown
PARP4	brown
PBX2	brown
PCBP1	brown
PCBP2	brown
PCDHGC3	brown
PCP4L1	brown
PCYT1A	brown
PDCD5	brown
PDCD6	brown
PDE7B	brown
PDZD11	brown
PECAM1	brown
PET117	brown
PEX7	brown
PFDN1	brown
PFDN2	brown
PFDN6	brown
PFKFB3	brown
PGAM1	brown
PGBD1	brown
PGBD2	brown
PGCP	brown
PGD	brown
PGK1	brown
PGM1	brown
PHB2	brown
PHF12	brown
PHF2	brown
PHF21A	brown
PHF23	brown
PHF8	brown
PHKA2	brown
PHLPP1	brown
PHOSPHO2	brown
PHYH	brown
PIAS3	brown
PID1	brown
PIGC	brown
PIGH	brown
PIK3CD	brown
PIK3R5	brown
PILRA	brown

PIM1	brown
PIM2	brown
PINK1	brown
PIP4K2C	brown
PITPNA	brown
PKNOX1	brown
PLA2G12A	brown
PLA2G16	brown
PLAC8	brown
PLAGL2	brown
PLAUR	brown
PLBD2	brown
PLCG2	brown
PLCL2	brown
PLEK	brown
PLEKHB2	brown
PLEKHG6	brown
PLEKHM1P	brown
PLEKHO2	brown
PLIN1	brown
PLIN3	brown
PLIN4	brown
PLOD1	brown
PLXNA2	brown
PMPCB	brown
PNMA1	brown
POC5	brown
POLE4	brown
POLR2A	brown
POLR3F	brown
POLR3GL	brown
POMC	brown
POP5	brown
POU2F2	brown
POU5F1	brown
POU5F1B	brown
POU5F1P3	brown
PP7080	brown
PPA2	brown
PPAPDC2	brown
PPFIA1	brown
PPIAL4G	brown
PPID	brown
PPIL1	brown
PPL	brown
PPM1F	brown
PPM1H	brown
PPP1R10	brown
PPP1R11	brown
PPP1R12B	brown
PPP2R2D	brown
PPT1	brown
PRAF2	brown
PRAM1	brown
PRCP	brown
PRDX4	brown
PREX1	brown
PRG2	brown
PRH1-PRR4	brown
PRIC285	brown
PRIM1	brown
PRKAB1	brown
PRKACA	brown
PRKCB	brown
PRKCD	brown
PRKD2	brown
PRKDC	brown
PRKRA	brown

PRMT5	brown
PRMT6	brown
PRORSD1P	brown
PROSC	brown
PRPH2	brown
PRR14L	brown
PRRC2A	brown
PRSS27	brown
PRSS57	brown
PSAP	brown
PSEN1	brown
PSMB2	brown
PSMD3	brown
PSME3	brown
PSMF1	brown
PSTK	brown
PTAFR	brown
PTGER2	brown
PTK2B	brown
PTPLA	brown
PTPMT1	brown
PTPN1	brown
PTPN6	brown
PTPN9	brown
PTPRJ	brown
PTRF	brown
PTRHD1	brown
PTTG1	brown
PTTG1IP	brown
PTX3	brown
PTX4	brown
PVR	brown
PVRL1	brown
PXMP2	brown
PXN	brown
PYGL	brown
QSOX1	brown
R3HDM2	brown
RAB11FIP1	brown
RAB11FIP4	brown
RAB19	brown
RAB23	brown
RAB35	brown
RAB36	brown
RAB3D	brown
RAB40B	brown
RAB42	brown
RAB43	brown
RAB5B	brown
RAB5C	brown
RAB7A	brown
RAB8A	brown
RABGGTB	brown
RABL5	brown
RAC1	brown
RAC2	brown
RAD1	brown
RAD23B	brown
RAD54B	brown
RAF1	brown
RAGE	brown
RALB	brown
RALBP1	brown
RALGAPA2	brown
RAMP1	brown
RAP1GAP2	brown
RAPGEFL1	brown
RARS	brown

RASSF2	brown
RASSF5	brown
RASSF8	brown
RBM20	brown
RBM22	brown
RBM23	brown
RBM8A	brown
RBMS2	brown
RBMX2	brown
RBP5	brown
RBPJ	brown
RCC1	brown
RCOR1	brown
RCSD1	brown
RERE	brown
REXO2	brown
RFC4	brown
RFESD	brown
RFFL	brown
RFX1	brown
RFX2	brown
RGAG4	brown
RGL3	brown
RGL4	brown
RGP1	brown
RGS10	brown
RGS16	brown
RGS3	brown
RHBDF2	brown
RHEBL1	brown
RHOA	brown
RILPL1	brown
RIN2	brown
RIN3	brown
RIPK1	brown
RNASEH2B	brown
RNASET2	brown
RNF114	brown
RNF122	brown
RNF14	brown
RNF167	brown
RNF170	brown
RNF185	brown
RNF213	brown
RNF24	brown
RNF34	brown
RNF39	brown
RNF41	brown
RNPEP	brown
ROMO1	brown
RP9	brown
RP9P	brown
RPA3	brown
RPAIN	brown
RPH3A	brown
RPL6	brown
RPLP0	brown
RPLP0P2	brown
RPN1	brown
RPP30	brown
RPP40	brown
RPRD2	brown
RPS10	brown
RPS2P32	brown
RPS6KA1	brown
RPS9	brown
RPSAP9	brown
RPUSD4	brown

RRAGB	brown
RRAGC	brown
RRAS2	brown
RRBP1	brown
RRP12	brown
RRP15	brown
RSPH3	brown
RSU1	brown
RTCD1	brown
RTF1	brown
RTN1	brown
RTN4	brown
RUFY1	brown
RUNDC1	brown
RUNDC2A	brown
RUSC2	brown
RXRA	brown
SAAL1	brown
SAMD4B	brown
SAMHD1	brown
SAP130	brown
SASH1	brown
SBDS	brown
SBDSP1	brown
SC4MOL	brown
SCAMP2	brown
SCAND3	brown
SCLY	brown
SCPEP1	brown
SCRT2	brown
SDHC	brown
SEC14L1	brown
SEC16A	brown
SEC22A	brown
SEC23B	brown
SEC31A	brown
SEC61G	brown
SELL	brown
SELPLG	brown
SELS	brown
SEMA4A	brown
SEMA4B	brown
SEMA4D	brown
SEPHS1	brown
SERF2	brown
SERPINA1	brown
SERPINB8	brown
SESN2	brown
SETD1B	brown
SETDB1	brown
SETMAR	brown
SF3B4	brown
SFT2D3	brown
SFTPB	brown
SGCE	brown
SGPL1	brown
SH2B3	brown
SH2D1A	brown
SH2D3C	brown
SH3BGR	brown
SH3BP5L	brown
SH3KBP1	brown
SH3PXD2B	brown
SH3RF3	brown
SH3YL1	brown
SHISA2	brown
SHKBP1	brown
SIAE	brown

SIGLEC10	brown
SIGLEC5	brown
SIGLEC7	brown
SIGLEC9	brown
SIK3	brown
SIN3A	brown
SIPA1L1	brown
SIPA1L2	brown
SIRPA	brown
SIRPB1	brown
SIRPB2	brown
SIRT4	brown
SKI	brown
SLA	brown
SLC11A1	brown
SLC1A5	brown
SLC24A4	brown
SLC25A14	brown
SLC25A26	brown
SLC25A33	brown
SLC25A44	brown
SLC27A5	brown
SLC2A3	brown
SLC31A1	brown
SLC35A2	brown
SLC35E1	brown
SLC35E3	brown
SLC36A1	brown
SLC37A2	brown
SLC38A6	brown
SLC38A7	brown
SLC39A11	brown
SLC43A2	brown
SLC43A3	brown
SLC44A2	brown
SLC46A2	brown
SLC48A1	brown
SLC5A2	brown
SLC6A12	brown
SLC6A6	brown
SLC7A5	brown
SLC7A6OS	brown
SLC7A7	brown
SLC9A1	brown
SLC9A7P1	brown
SLC9A8	brown
SLCO3A1	brown
SLED1	brown
SLIRP	brown
SMAD9	brown
SMAP2	brown
SMARCA2	brown
SMARCD2	brown
SMCR8	brown
SMG7	brown
SMG8	brown
SMUG1	brown
SMURF1	brown
SNAP29	brown
SNAPC5	brown
SNHG1	brown
SNHG10	brown
SNHG12	brown
SNHG3	brown
SNHG5	brown
SNHG7	brown
SNHG8	brown
SNIP1	brown

SNN	brown
SNORA10	brown
SNORA52	brown
SNORA67	brown
SNORA70	brown
SNORD17	brown
SNORD22	brown
SNRPA1	brown
SNRPF	brown
SNURF	brown
SNX1	brown
SNX12	brown
SNX19	brown
SNX24	brown
SNX27	brown
SNX30	brown
SNX33	brown
SOCS2	brown
SOD1	brown
SORL1	brown
SOX4	brown
SP140	brown
SP2	brown
SPATA2	brown
SPATA24	brown
SPATA7	brown
SPCS1	brown
SPDYA	brown
SPECC1	brown
SPEN	brown
SPG21	brown
SPINK2	brown
SPINT1	brown
SPOP	brown
SPPL3	brown
SPRED1	brown
SPRY2	brown
SPRYD4	brown
SPRYD7	brown
SPTB	brown
SPTLC2	brown
SQSTM1	brown
SRC	brown
SRF	brown
SRGAP2	brown
SRI	brown
SRP54	brown
SRPR	brown
SRSF2	brown
SRSF4	brown
SRSF7	brown
SRXN1	brown
SSBP3	brown
SSH1	brown
SSH2	brown
SSR2	brown
ST14	brown
ST3GAL2	brown
STAG3L4	brown
STAMBPL1	brown
STARD8	brown
STAT3	brown
STAT5A	brown
STAT5B	brown
STAT6	brown
STAU1	brown
STEAP3	brown
STK24	brown

STK32B	brown
STK35	brown
STK4	brown
STK40	brown
STRAP	brown
STT3A	brown
STX16-NPEPL1	brown
STX18	brown
SULF2	brown
SUMF1	brown
SUMO1P3	brown
SUPT3H	brown
SUSD1	brown
SUZ12P	brown
SV2B	brown
SVIL	brown
SYAP1	brown
SYK	brown
SYT2	brown
TACC3	brown
TADA2B	brown
TAF11	brown
TAF15	brown
TAF4	brown
TAGLN2	brown
TALDO1	brown
TANC2	brown
TAP2	brown
TAPBP	brown
TASP1	brown
TBC1D1	brown
TBC1D14	brown
TBC1D19	brown
TBC1D2	brown
TBC1D20	brown
TBC1D2B	brown
TBC1D5	brown
TBC1D8	brown
TBC1D9	brown
TBCE	brown
TBL1X	brown
TBXAS1	brown
TCEAL1	brown
TCF20	brown
TCN2	brown
TCTN1	brown
TECPR2	brown
TEPP	brown
TET3	brown
TEX261	brown
TFB2M	brown
TFCP2	brown
TFE3	brown
TFG	brown
TGFB1	brown
TGFBI	brown
TGOLN2	brown
THG1L	brown
THOC5	brown
THRAP3	brown
THUMPD2	brown
TIAL1	brown
TIMM8A	brown
TIMM9	brown
TIMP2	brown
TINF2	brown
TIPARP-AS1	brown
TIRAP	brown

TJP2	brown
TK2	brown
TKT	brown
TLE3	brown
TLN1	brown
TLR9	brown
TM2D1	brown
TM2D2	brown
TM9SF1	brown
TM9SF4	brown
TMBIM1	brown
TMBIM6	brown
TMEM106C	brown
TMEM107	brown
TMEM116	brown
TMEM117	brown
TMEM119	brown
TMEM127	brown
TMEM131	brown
TMEM132A	brown
TMEM132E	brown
TMEM140	brown
TMEM14A	brown
TMEM14C	brown
TMEM156	brown
TMEM164	brown
TMEM177	brown
TMEM18	brown
TMEM184B	brown
TMEM185A	brown
TMEM19	brown
TMEM191A	brown
TMEM198	brown
TMEM218	brown
TMEM223	brown
TMEM237	brown
TMEM27	brown
TMEM38A	brown
TMEM41B	brown
TMEM42	brown
TMEM43	brown
TMEM5	brown
TMEM50B	brown
TMEM62	brown
TMEM67	brown
TMEM68	brown
TMEM86A	brown
TMEM92	brown
TMPPE	brown
TMSB10	brown
TMSB15B	brown
TMTC4	brown
TMUB2	brown
TMX2	brown
TNFAIP2	brown
TNFRSF10C	brown
TNFRSF1B	brown
TNFRSF9	brown
TNFSF13	brown
TNIP1	brown
TNNC1	brown
TNPO3	brown
TNS3	brown
TOE1	brown
TOMM22	brown
TOMM7	brown
TOP3A	brown
TOR3A	brown

TOX4	brown
TP53BP2	brown
TP53I11	brown
TP53INP2	brown
TP53RK	brown
TP53TG1	brown
TPCN2	brown
TPD52L2	brown
TPM3	brown
TPM4	brown
TPP1	brown
TPRG1	brown
TPRG1L	brown
TRAIP	brown
TRAK1	brown
TRANK1	brown
TRAPPC2	brown
TRAT1	brown
TRERF1	brown
TRIM16L	brown
TRIM25	brown
TRIM27	brown
TRIM67	brown
TRIM8	brown
TRIOBP	brown
TRMT11	brown
TRMT61B	brown
TRPC4AP	brown
TSEN15	brown
TSG101	brown
TSIX	brown
TSPAN31	brown
TTC13	brown
TTC19	brown
TTC39B	brown
TTC5	brown
TTI1	brown
TTLL4	brown
TUFT1	brown
TXNDC15	brown
TXNDC5	brown
TXNIP	brown
UAP1	brown
UBA1	brown
UBAC2-AS1	brown
UBAP1	brown
UBAP2	brown
UBAP2L	brown
UBE2E3	brown
UBE2H	brown
UBE2V1	brown
UBE3B	brown
UBE4B	brown
UBL5	brown
UBN1	brown
UFC1	brown
UGGT1	brown
UGT8	brown
ULK1	brown
UNC45B	brown
UNC5B	brown
UQCR10	brown
UQCR11	brown
USMG5	brown
USP22	brown
USP4	brown
UTP18	brown
UTP3	brown

UTS2	brown
UVRAG	brown
VAMP2	brown
VAMP8	brown
VASP	brown
VAV1	brown
VCAN	brown
VCL	brown
VDR	brown
VIM	brown
VIPAR	brown
VPS25	brown
VPS35	brown
VRK3	brown
WAS	brown
WASF2	brown
WBP2	brown
WBP5	brown
WDFY4	brown
WDR12	brown
WDR61	brown
WDR78	brown
WDR82	brown
WDYHV1	brown
WHAMMP3	brown
WIPF2	brown
WIPI1	brown
WIPI2	brown
WNK1	brown
WWC3	brown
WWP2	brown
XIAP	brown
XKR7	brown
XPA	brown
XPO6	brown
XPO7	brown
XRCC6BP1	brown
XRN2	brown
XYLT1	brown
YBEY	brown
YEATS4	brown
YIPF3	brown
YRDC	brown
YTHDF1	brown
YTHDF2	brown
YWHAB	brown
YWHAG	brown
YWHAH	brown
YY1	brown
YY1AP1	brown
ZBED1	brown
ZBTB37	brown
ZBTB47	brown
ZBTB5	brown
ZC3H13	brown
ZC3H3	brown
ZC3H7A	brown
ZC3HC1	brown
ZCCHC24	brown
ZCCHC3	brown
ZDHHC18	brown
ZDHHC3	brown
ZDHHC5	brown
ZDHHC7	brown
ZER1	brown
ZFAND2A	brown
ZFAND3	brown
ZFHX3	brown

ZFP106	brown
ZFP30	brown
ZFP36L2	brown
ZFP37	brown
ZFYVE1	brown
ZMIZ1	brown
ZMYM6NB	brown
ZNF10	brown
ZNF14	brown
ZNF140	brown
ZNF16	brown
ZNF165	brown
ZNF174	brown
ZNF193	brown
ZNF195	brown
ZNF2	brown
ZNF204P	brown
ZNF223	brown
ZNF23	brown
ZNF235	brown
ZNF239	brown
ZNF26	brown
ZNF287	brown
ZNF30	brown
ZNF300	brown
ZNF319	brown
ZNF32	brown
ZNF341	brown
ZNF345	brown
ZNF367	brown
ZNF383	brown
ZNF385A	brown
ZNF398	brown
ZNF404	brown
ZNF407	brown
ZNF416	brown
ZNF440	brown
ZNF461	brown
ZNF485	brown
ZNF491	brown
ZNF501	brown
ZNF502	brown
ZNF503	brown
ZNF516	brown
ZNF527	brown
ZNF540	brown
ZNF559	brown
ZNF565	brown
ZNF568	brown
ZNF569	brown
ZNF57	brown
ZNF576	brown
ZNF582	brown
ZNF596	brown
ZNF599	brown
ZNF607	brown
ZNF609	brown
ZNF618	brown
ZNF626	brown
ZNF630	brown
ZNF646	brown
ZNF652	brown
ZNF673	brown
ZNF677	brown
ZNF678	brown
ZNF682	brown
ZNF687	brown
ZNF706	brown

ZNF710		brown
ZNF713		brown
ZNF720		brown
ZNF749		brown
ZNF766		brown
ZNF77		brown
ZNF790		brown
ZNF823		brown
ZNF83		brown
ZNFX1		brown
ZNRD1-AS1		brown
ZSWIM7		brown
ZYX		brown
ZZEF1		brown
	4-Sep	cyan
ACOT9		cyan
ACP6		cyan
ACTA2		cyan
APOL1		cyan
APOL2		cyan
APOL4		cyan
APOL6		cyan
ASPHD2		cyan
ATF3		cyan
BATF2		cyan
BTN2A2		cyan
C2		cyan
C7orf49		cyan
CARD6		cyan
CEACAM1		cyan
CETP		cyan
DTX3L		cyan
EMR1		cyan
ERP27		cyan
ETV7		cyan
FANCA		cyan
FBXO6		cyan
FTSJD2		cyan
FZD5		cyan
GAS8		cyan
GBP1		cyan
GBP2		cyan
GBP4		cyan
GBP5		cyan
GPR19		cyan
HCAR1		cyan
ICAM1		cyan
IFI16		cyan
IFI35		cyan
IRF1		cyan
IRF9		cyan
LACTB		cyan
LAP3		cyan
LHFPL2		cyan
LOC100128191		cyan
LOC100616668		cyan
LOC641467		cyan
LRRC18		cyan
MB21D1		cyan
MEX3B		cyan
MLKL		cyan
NRARP		cyan
NUDT11		cyan
OR52K2		cyan
PARP14		cyan
PARP9		cyan
PML		cyan
PSTPIP2		cyan

RAB20	cyan
RMI2	cyan
RNASEL	cyan
RNF222	cyan
SDC3	cyan
SERPING1	cyan
SHISA5	cyan
SLAMF8	cyan
SMTNL1	cyan
SORT1	cyan
SP100	cyan
SP110	cyan
STAT1	cyan
STAT2	cyan
TAP1	cyan
TGIF1	cyan
TRAFD1	cyan
TRIM21	cyan
TRIM38	cyan
TRIM5	cyan
UBE2L6	cyan
WARS	cyan
ZNF883	cyan
10-Mar	green
2-Sep	green
5-Sep	green
A2ML1	green
AAGAB	green
AASDH	green
AASS	green
ABCB7	green
ABCC11	green
ABHD11	green
ABHD16A	green
ABHD5	green
ABTB1	green
ACAA1	green
ACAD11	green
ACCN4	green
ACOXL	green
ACPT	green
ACSS3	green
ACTG2	green
ACVR2B	green
ADAD2	green
ADAM8	green
ADAMTS2	green
ADAMTS7	green
ADCK4	green
ADNP2	green
ADPRHL1	green
ADRA1A	green
AFAP1L2	green
AGAP7	green
AGPAT2	green
AGTRAP	green
AIPL1	green
AMOT	green
AMOTL2	green
AMPD2	green
AMPH	green
ANGPTL1	green
ANK2	green
ANKRD13D	green
ANKRD2	green
ANKRD42	green
ANKRD5	green
ANKRD58	green

ANO2	green
ANTXRL	green
ANXA2P2	green
AOC3	green
AP1M2	green
AP1S3	green
AP2S1	green
AP4E1	green
AP4M1	green
APCDD1L	green
APLP1	green
APOBEC2	green
AQP8	green
AR	green
ARF5	green
ARHGAP9	green
ARHGEF40	green
ARL2BP	green
ARL8A	green
ARMCX3	green
ARPC1B	green
ARRB2	green
ARSJ	green
ART1	green
ARTN	green
AS3MT	green
ASB12	green
ASB14	green
ASCC3	green
ASF1B	green
ASL	green
ASPHD1	green
ASS1	green
ATAD5	green
ATE1	green
ATG2B	green
ATL1	green
ATMIN	green
ATP2A1	green
ATP2B2	green
ATP6V0A2	green
ATP6V0B	green
ATP6V1G2	green
ATPAF1	green
ATR	green
ATXN10	green
ATXN3	green
ATXN7L3B	green
AURKAIP1	green
AZU1	green
B3GNT8	green
B9D2	green
BAG5	green
BAI3	green
BAIAP3	green
BAK1	green
BANP	green
BARD1	green
BAX	green
BCAN	green
BCAS1	green
BCKDK	green
BCL3	green
BEND4	green
BEX4	green
BFAR	green
BIN3	green
BLOC1S1-RDH5	green

BMPR1B	green
BTN2A3	green
BTNL8	green
C10orf103	green
C10orf137	green
C10orf18	green
C10orf55	green
C10orf67	green
C11orf63	green
C11orf92	green
C12orf11	green
C12orf66	green
C12orf69	green
C13orf33	green
C13orf35	green
C14orf178	green
C15orf38-AP3S2	green
C15orf41	green
C15orf43	green
C15orf56	green
C17orf47	green
C17orf51	green
C17orf57	green
C17orf61-PLSCR3	green
C17orf80	green
C19orf2	green
C19orf35	green
C19orf40	green
C1orf226	green
C1orf70	green
C20orf144	green
C20orf160	green
C21orf71	green
C2orf15	green
C2orf55	green
C2orf65	green
C2orf82	green
C3orf70	green
C4orf38	green
C4orf44	green
C5orf42	green
C5orf47	green
C6orf192	green
C6orf222	green
C7orf43	green
C7orf74	green
C8orf73	green
C9orf102	green
C9orf125	green
C9orf129	green
C9orf5	green
C9orf93	green
CA7	green
CABP1	green
CACNB2	green
CACNG1	green
CAMK2B	green
CAMKK1	green
CAND1	green
CAPN11	green
CARD9	green
CASK	green
CASP7	green
CASR	green
CBX8	green
CCDC144B	green
CCDC15	green
CCDC151	green

CCDC168	green
CCDC17	green
CCDC19	green
CCDC50	green
CCDC74A	green
CCDC96	green
CCIN	green
CCL25	green
CCL27	green
CCL28	green
CCNJ	green
CD151	green
CD164L2	green
CD200R1L	green
CD300A	green
CD3G	green
CDC14B	green
CDC23	green
CDH5	green
CDK15	green
CDK5	green
CDK6	green
CDKN2D	green
CDYL	green
CELA2B	green
CENPC1	green
CENPJ	green
CEP128	green
CEP192	green
CEP68	green
CEP76	green
CERS1	green
CERS4	green
CERS6	green
CHIC2	green
CHRM4	green
CHRM5	green
CHRNA3	green
CHST1	green
CHST9-AS1	green
CIB2	green
CITED4	green
CLASP2	green
CLCN1	green
CLDN10	green
CLDN11	green
CLEC10A	green
CLEC18B	green
CLEC4G	green
CLIC5	green
CLINT1	green
CLLU1	green
CLOCK	green
CLTB	green
CMAS	green
CNIH2	green
CNTN2	green
COL16A1	green
COL18A1	green
COL23A1	green
COL4A2	green
COL9A1	green
CORO7-PAM16	green
COX6B1	green
CP	green
CPE	green
CPNE4	green
CPT1B	green

CPXM2	green
CRISPLD1	green
CRTAP	green
CRYZL1	green
CSE1L	green
CSPG5	green
CSPP1	green
CTAGE7P	green
CTLA4	green
CTNNBIP1	green
CTNND2	green
CTRL	green
CUEDC1	green
CWC27	green
CXCL2	green
CXCL3	green
CYP11B1	green
CYP1A1	green
CYP2A7	green
CYP2D7P1	green
CYP4V2	green
DAAM2	green
DARS	green
DBT	green
DCAF17	green
DCTN3	green
DDAH2	green
DDHD1	green
DDN	green
DDX1	green
DEDD2	green
DGAT1	green
DHRS7B	green
DHX29	green
DIP2C	green
DISP1	green
DKFZp566F0947	green
DLEU2L	green
DLL3	green
DNAH2	green
DNAH9	green
DNAJA2	green
DNAJB7	green
DNAJC2	green
DNAJC8	green
DNER	green
DNM1L	green
DNM1P46	green
DNTTIP1	green
DOCK3	green
DOK1	green
DOK6	green
DOPEY1	green
DPEP3	green
DPPA4	green
DRD3	green
DSTN	green
DTWD2	green
DUSP1	green
DYNC1I1	green
DYNC2H1	green
DYSFIP1	green
EBPL	green
ECE2	green
EDNRB	green
EEF1DP3	green
EFCAB3	green
EFCAB4A	green

EGFEM1P	green
EGFL8	green
EI24	green
EIF1B	green
EIF2C3	green
EIF2S1	green
EIF4EBP1	green
EIF4EBP3	green
ELANE	green
ELF3	green
ELFN1	green
ELP2	green
EML4	green
EMP2	green
ENPP7	green
EPCAM	green
EPHA4	green
EPM2A	green
EPO	green
EPOR	green
ERAP1	green
ERCC4	green
ERCC6	green
ERMP1	green
ERRFI1	green
ESRRA	green
ESRRB	green
ETV3L	green
EXD1	green
EXOSC4	green
F3	green
FAAH	green
FAAH2	green
FADD	green
FAH	green
FAM100B	green
FAM107B	green
FAM114A2	green
FAM115C	green
FAM116B	green
FAM124A	green
FAM131A	green
FAM151A	green
FAM166B	green
FAM170B	green
FAM176A	green
FAM178A	green
FAM180B	green
FAM182B	green
FAM18A	green
FAM190B	green
FAM27A	green
FAM55C	green
FAM74A1	green
FAM83E	green
FAM83F	green
FANCC	green
FANCD2	green
FASTKD2	green
FAT1	green
FBLN1	green
FBXO15	green
FBXO32	green
FBXO36	green
FBXO43	green
FCGRT	green
FCN2	green
FER1L5	green

FGF17	green
FGF18	green
FHOD3	green
FKBP14	green
FKBPL	green
FKTN	green
FLJ20021	green
FLJ22447	green
FLJ34208	green
FLJ40194	green
FLJ43860	green
FMO4	green
FOS	green
FOXD4L1	green
FOXO1	green
FOXO3B	green
FRAT1	green
FRAT2	green
FRMD5	green
FRYL	green
FSTL3	green
FTH1	green
FUBP3	green
FUT1	green
FUT3	green
FUT7	green
FUT8	green
FUZ	green
FZD9	green
G3BP2	green
GABARAP	green
GABRD	green
GADD45G	green
GALK1	green
GALNTL4	green
GALR2	green
GAN	green
GARNL3	green
GATAD1	green
GATM	green
GATSL2	green
GCLC	green
GEMIN8P4	green
GGT5	green
GJA4	green
GJB3	green
GJB5	green
GJB7	green
GJC1	green
GJC2	green
GJD4	green
GLDN	green
GLIS2	green
GNB2	green
GNB3	green
GNG4	green
GNG5	green
GNN	green
GNPAT	green
GNPNAT1	green
GNPTAB	green
GNRHR	green
GOLGA6A	green
GOLGA8B	green
GOLT1A	green
GPD1L	green
GPLD1	green
GPM6B	green

GPR108	green
GPR125	green
GPR161	green
GPR162	green
GPR176	green
GPR37L1	green
GPR62	green
GPR77	green
GPR85	green
GPSM3	green
GPX3	green
GRB7	green
GRIA4	green
GRID1	green
GRIK3	green
GRM4	green
GRSF1	green
GSDMD	green
GSG1L	green
GSPT2	green
GSTCD	green
GSTK1	green
GSTZ1	green
GTF2H3	green
GTF3C3	green
GUSBP2	green
GVINP1	green
HAPLN2	green
HAS3	green
HAUS2	green
HAVCR1	green
HDAC2	green
HELQ	green
HERC2P2	green
HERC2P9	green
HES1	green
HEXIM2	green
HHATL	green
HIBCH	green
HIST1H1C	green
HIST1H2AD	green
HIST1H2AE	green
HIST1H2BK	green
HIST1H2BO	green
HIST1H3A	green
HIST1H3D	green
HIST1H3H	green
HIST2H2AB	green
HLA-F	green
HLX	green
HMBOX1	green
HMGA2	green
HMP19	green
HN1	green
HNF1A-AS1	green
HNRNPA1L2	green
HOMER3	green
HOXB6	green
HPCA	green
HPN	green
HRH3	green
HSD3B7	green
HSP90AA1	green
HTR3B	green
HTRA3	green
HUS1	green
HUS1B	green
HYAL3	green

ICAM3	green
ICAM5	green
IER2	green
IER3	green
IFITM10	green
IFITM2	green
IFITM4P	green
IGFBP2	green
IGFLR1	green
IGLON5	green
IGSF23	green
IHH	green
IL15RA	green
IL17RC	green
IMPA2	green
INPP4B	green
INPP5F	green
INPP5J	green
INSL3	green
INSRR	green
INTS10	green
INTU	green
IPO7	green
IPPK	green
IRGC	green
ISG20	green
ISL2	green
ITGA7	green
ITGB1	green
ITK	green
ITLN2	green
ITPKA	green
ITPR1	green
JDP2	green
KATNAL2	green
KCNA3	green
KCNB1	green
KCNE1L	green
KCNH3	green
KCNJ12	green
KCNQ5	green
KCTD4	green
KDSR	green
KIAA0146	green
KIAA0528	green
KIAA0564	green
KIAA0907	green
KIAA1045	green
KIAA1161	green
KIAA1199	green
KIAA1244	green
KIAA1279	green
KIAA1328	green
KIAA1407	green
KIAA1462	green
KIAA1737	green
KIFAP3	green
KLF12	green
KLHL11	green
KPTN	green
KRIT1	green
KRT36	green
KRTAP10-2	green
KRTAP16-1	green
KSR2	green
L3MBTL3	green
LAD1	green
LAMA3	green

LCA5L	green
LCTL	green
LDHD	green
LEFTY1	green
LENG9	green
LETM2	green
LILRA2	green
LIMA1	green
LIN37	green
LMOD1	green
LOC100128126	green
LOC100128252	green
LOC100128398	green
LOC100128542	green
LOC100128590	green
LOC100129924	green
LOC100130231	green
LOC100130691	green
LOC100130890	green
LOC100130987	green
LOC100131067	green
LOC100131089	green
LOC100132111	green
LOC100132707	green
LOC100133920	green
LOC100133985	green
LOC100271702	green
LOC100286844	green
LOC100287216	green
LOC100288181	green
LOC100288637	green
LOC100302650	green
LOC100505658	green
LOC100505746	green
LOC100505817	green
LOC100506190	green
LOC100506274	green
LOC100506649	green
LOC100506757	green
LOC100506994	green
LOC100507173	green
LOC100507299	green
LOC100507351	green
LOC100507387	green
LOC100507392	green
LOC100507564	green
LOC100507567	green
LOC100507577	green
LOC100507589	green
LOC113230	green
LOC200261	green
LOC219347	green
LOC219731	green
LOC220729	green
LOC282997	green
LOC283392	green
LOC283440	green
LOC283624	green
LOC284276	green
LOC284454	green
LOC284648	green
LOC284950	green
LOC286367	green
LOC339902	green
LOC340544	green
LOC400685	green
LOC400891	green
LOC401074	green

LOC402160	green
LOC440900	green
LOC642852	green
LOC643406	green
LOC645166	green
LOC648740	green
LOC650623	green
LOC653786	green
LOC728723	green
LOC729082	green
LOC729799	green
LOC731223	green
LOC80054	green
LOC92659	green
LPPR4	green
LRP1B	green
LRP2BP	green
LRP3	green
LRPPRC	green
LRRC37B	green
LRRC37BP1	green
LRRC49	green
LTBR	green
LTV1	green
LUM	green
LY6G5C	green
LYPD1	green
LYPD4	green
LYRM7	green
MADCAM1	green
MAFF	green
MAGEE1	green
MAGIX	green
MAP1LC3A	green
MAP1LC3B	green
MAP3K6	green
MAP6	green
MAP7	green
MAPK10	green
MAPK3	green
MASTL	green
MAT2A	green
MCART1	green
MCART6	green
MCFD2	green
MCM8	green
MCOLN2	green
MCPH1	green
MED17	green
MEF2BNB	green
MEF2BNB-MEF2B	green
MEGF11	green
MEIS3	green
MEP1B	green
MESP2	green
MET	green
METRNL	green
METTL8	green
MFAP2	green
MFSD6	green
MGC23284	green
MGC2752	green
MIIP	green
MIOS	green
MKL2	green
MLK7-AS1	green
MLLT10	green
MLX	green

MMP2	green
MOCOS	green
MORF4L1	green
MOV10L1	green
MPHOSPH10	green
MPHOSPH9	green
MPPE1	green
MPST	green
MPZL1	green
MRE11A	green
MRS2	green
MSTN	green
MT1DP	green
MT1G	green
MTAP	green
MTCH1	green
MTDH	green
MTFMT	green
MTL5	green
MTMR11	green
MTMR8	green
MTRNR2L2	green
MTRNR2L4	green
MTX1	green
MVP	green
MYBPC3	green
MYEF2	green
MYEOV	green
MYLPF	green
MYO1B	green
MYO7B	green
MYO9A	green
NAA15	green
NAA35	green
NANOG	green
NANOS3	green
NAPRT1	green
NBEAL1	green
NBPF3	green
NCBP1	green
NCBP2	green
NCF1	green
NCF1B	green
NCF1C	green
NCRNA00085	green
NCRNA00222	green
NCRNA00242	green
NCRNA00256B	green
NCRNA00285	green
NCRNA00323	green
NDUFA4L2	green
NEK4	green
NEURL3	green
NFIB	green
NFKBID	green
NHLRC3	green
NINJ1	green
NINJ2	green
NIP7	green
NIPAL2	green
NIT1	green
NKAIN2	green
NKTR	green
NLRP4	green
NOL3	green
NOL8	green
NOS1AP	green
NOV	green

NPAT	green
NPC2	green
NPHP3-ACAD11	green
NPM2	green
NR1H3	green
NR2C2	green
NRTN	green
NRXN1	green
NSUN6	green
NUDT13	green
NUFIP1	green
NUP107	green
NUP160	green
NUP88	green
NXF3	green
NXNL2	green
NYX	green
OBSL1	green
OGT	green
OIT3	green
OLFML3	green
OR52W1	green
ORAI3	green
ORC5	green
OSGIN1	green
OSTCL	green
OTUB1	green
OTUD3	green
OXGR1	green
P4HA2	green
PABPN1L	green
PACSIN3	green
PANX2	green
PARG	green
PARP2	green
PAX9	green
PBRM1	green
PCBD2	green
PCBP3	green
PCDH17	green
PCDHGB7	green
PCDHGC5	green
PCGF2	green
PCTP	green
PDCL	green
PDE12	green
PDE2A	green
PDE4C	green
PDE8A	green
PDIA2	green
PDLIM7	green
PDP2	green
PDS5A	green
PDSS2	green
PDZD2	green
PECR	green
PENK	green
PEX19	green
PGA5	green
PGAP2	green
PGLS	green
PGPEP1L	green
PGRMC2	green
PHACTR2	green
PHF14	green
PHLDA2	green
PIAS2	green
PIGN	green

PIGX	green
PIH1D2	green
PIK3R1	green
PISD	green
PIWIL2	green
PKD2L1	green
PLAA	green
PLEKHA5	green
PLEKHG7	green
PLEKHO1	green
PLK3	green
PLP2	green
PMF1-BGLAP	green
PMFBP1	green
PNMA6C	green
POC1A	green
POLR2D	green
PON2	green
POR	green
PPAP2A	green
PPCDC	green
PPHLN1	green
PPM1M	green
PPP1R8	green
PPP2CA	green
PQLC1	green
PRDM7	green
PRELID1	green
PRIM2	green
PRKCE	green
PRKG1	green
PRNP	green
PRO0611	green
PRPF38A	green
PRR13	green
PRR15	green
PRR24	green
PRRG2	green
PRRT4	green
PRSS1	green
PRTN3	green
PSCA	green
PSIMCT-1	green
PSMB10	green
PSMB8	green
PSMB9	green
PSMD5	green
PSME2	green
PSRC1	green
PTCD2	green
PTGER1	green
PTGES3	green
PTP4A2	green
PTP4A3	green
PTPN11	green
PTPN14	green
PTPN21	green
PUS7	green
PVRL4	green
PYCARD	green
PYGM	green
RAB13	green
RAB30	green
RAB34	green
RAB40C	green
RAB4B-EGLN2	green
RABAC1	green
RABGAP1L	green

RAD50	green
RAD52	green
RADIL	green
RALGAPA1	green
RANBP2	green
RARA	green
RASGEF1B	green
RASGRP1	green
RASSF6	green
RBL1	green
RBM12	green
RBM14-RBM4	green
RBM45	green
RBMS3	green
RBMX	green
RECK	green
REEP2	green
RELL1	green
RELT	green
REM2	green
RFXANK	green
RGL2	green
RGS11	green
RGS14	green
RGS19	green
RGS2	green
RHBDL1	green
RHOB	green
RHOD	green
RHOG	green
RILPL2	green
RNASEK	green
RNF103-VPS24	green
RNF168	green
RNF208	green
ROBO4	green
ROGDI	green
ROM1	green
RPGRIP1	green
RPGRIP1L	green
RPL7L1	green
RPPH1	green
RPRML	green
RRAGD	green
RRN3P2	green
RSPH1	green
RTDR1	green
RTN2	green
S100A2	green
S100A3	green
S100A5	green
S100Z	green
S1PR4	green
SAV1	green
SBNO1	green
SCAND1	green
SCGB3A2	green
SCML2	green
SCN1B	green
SCNM1	green
SCRT1	green
SCT	green
SDC2	green
SDCCAG8	green
SDSL	green
SEC23IP	green
SEC63	green
SEMA3B	green

SEMA3D	green
SEMG1	green
SEPHS1P	green
SEPX1	green
SERBP1	green
SERTAD1	green
SET	green
SFN	green
SFT2D2	green
SFXN5	green
SGK2	green
SGOL1	green
SH2B2	green
SH3D21	green
SHOX2	green
SHPRH	green
SHQ1	green
SHROOM1	green
SHROOM2	green
SIRT6	green
SIRT7	green
SLC15A3	green
SLC16A3	green
SLC16A5	green
SLC19A1	green
SLC22A31	green
SLC24A2	green
SLC25A16	green
SLC25A30	green
SLC30A4	green
SLC31A2	green
SLC35F1	green
SLC38A1	green
SLC39A1	green
SLC39A4	green
SLC39A6	green
SLC44A4	green
SLC45A2	green
SLC4A8	green
SLC7A10	green
SLC9B2	green
SLCO2B1	green
SLFN11	green
SLFN14	green
SLIT3	green
SMAD6	green
SMC1B	green
SMC3	green
SMPDL3B	green
SNAI3	green
SNAPC3	green
SNCG	green
SNORA12	green
SNORA45	green
SNORA74B	green
SNORA78	green
SNX11	green
SNX5	green
SPAG6	green
SPATA2L	green
SPATA5L1	green
SPEF1	green
SPERT	green
SPESP1	green
SPHK1	green
SPI1	green
SPICE1	green
SPIN1	green

SPNS2	green
SPOCK3	green
SRA1	green
SRCIN1	green
SRCRB4D	green
SRFBP1	green
SRP72	green
SRR	green
SRSF9	green
SSB	green
SSH3	green
SSTR2	green
ST7-AS1	green
ST8SIA1	green
STARD10	green
STIM2	green
STK19	green
STX10	green
SULT1A2	green
SUN3	green
SVEP1	green
SYNCRIP	green
SYNDIG1L	green
SYNJ2BP	green
SYNPO2L	green
SYPL1	green
SYPL2	green
SYT6	green
SYT9	green
TAF1B	green
TAF4B	green
TAF5L	green
TAPT1	green
TARSL2	green
TAS1R1	green
TAS2R20	green
TAS2R5	green
TBCK	green
TCAM1P	green
TCERG1	green
TCF12	green
TCIRG1	green
TCN1	green
TDRD3	green
TERC	green
TERF1	green
TEX21P	green
TFB1M	green
TFEB	green
THBD	green
THBS1	green
THRSP	green
TIGD3	green
TIPIN	green
TLL2	green
TM7SF3	green
TMCO6	green
TMEM105	green
TMEM11	green
TMEM120A	green
TMEM125	green
TMEM130	green
TMEM132B	green
TMEM191C	green
TMEM194A	green
TMEM205	green
TMEM213	green
TMEM232	green

TMEM234	green
TMEM39A	green
TMEM41A	green
TMEM54	green
TMEM87A	green
TMEM88	green
TMEM8A	green
TMEM91	green
TMEM97	green
TMPRSS4	green
TNC	green
TNFAIP8L2	green
TNFRSF12A	green
TNFSF12-TNFSF13	green
TNFSF8	green
TNKS	green
TOMM70A	green
TPD52	green
TPH1	green
TPO	green
TPP2	green
TPST1	green
TRAK2	green
TREML2P1	green
TRIM31	green
TRIM32	green
TRIM37	green
TRIM7	green
TRIP11	green
TRIP6	green
TRMT5	green
TRMT6	green
TRPM4	green
TSC22D3	green
TSEN34	green
TSLP	green
TSN	green
TSPAN4	green
TSPEAR	green
TSPO	green
TSPYL1	green
TSPYL5	green
TST	green
TTBK2	green
TTC21B	green
TTC36	green
TTC37	green
TTF2	green
TUBA1A	green
TUBA1C	green
TUBA4A	green
TXNDC6	green
TYROBP	green
UBAC1	green
UBD	green
UBE2C	green
UBE2CBP	green
UBE2S	green
UBN2	green
UBP1	green
UBTD1	green
UBXN2A	green
UHRF2	green
UMPS	green
UNC119	green
UNC5C	green
UNC93B1	green
UPF3B	green

UPK2	green
UPP1	green
UPRT	green
USF1	green
USHBP1	green
USP10	green
USP47	green
UST	green
UTP6	green
VAMP5	green
VGF	green
VPREB1	green
VPS37C	green
VSIG8	green
VWA5B1	green
WDR13	green
WDR19	green
WDR25	green
WDR48	green
WDR7	green
WDR76	green
WDR92	green
WDSUB1	green
WFDC2	green
WFDC3	green
WFIKKN2	green
WISP3	green
WNK2	green
WNK3	green
WNT11	green
WRN	green
XIRP2	green
XKR8	green
XPO4	green
XPOT	green
XYLB	green
YAP1	green
YARS2	green
YPEL2	green
YPEL3	green
YSK4	green
ZACN	green
ZBTB44	green
ZC3H12A	green
ZC3H6	green
ZC3H8	green
ZCCHC4	green
ZDHHC23	green
ZFP90	green
ZFPL1	green
ZIK1	green
ZKSCAN3	green
ZMAT5	green
ZNF134	green
ZNF160	green
ZNF19	green
ZNF192	green
ZNF20	green
ZNF205	green
ZNF229	green
ZNF250	green
ZNF280B	green
ZNF284	green
ZNF285	green
ZNF286B	green
ZNF304	green
ZNF37A	green
ZNF37BP	green

ZNF385D	green
ZNF41	green
ZNF426	green
ZNF431	green
ZNF436	green
ZNF45	green
ZNF467	green
ZNF471	green
ZNF473	green
ZNF483	green
ZNF506	green
ZNF550	green
ZNF551	green
ZNF573	green
ZNF577	green
ZNF616	green
ZNF625	green
ZNF660	green
ZNF664	green
ZNF665	green
ZNF667	green
ZNF669	green
ZNF695	green
ZNF699	green
ZNF7	green
ZNF714	green
ZNF737	green
ZNF746	green
ZNF768	green
ZNF775	green
ZNF778	green
ZNF805	green
ZNF81	green
ZNF846	green
ZSCAN20	green
ZSCAN30	green
ADAMTS1	greenyellow
AGAP1	greenyellow
APOBEC3G	greenyellow
APOBEC3H	greenyellow
ARL4C	greenyellow
AUTS2	greenyellow
B3GAT1	greenyellow
B3GNT7	greenyellow
BAI2	greenyellow
BFSP1	greenyellow
BNC2	greenyellow
BZRAP1	greenyellow
C14orf1	greenyellow
C17orf66	greenyellow
C1orf174	greenyellow
C1orf177	greenyellow
C1orf187	greenyellow
C1orf21	greenyellow
C8orf80	greenyellow
CACNA2D2	greenyellow
CAMK2N1	greenyellow
CCL4	greenyellow
CCL5	greenyellow
CCR5	greenyellow
CD160	greenyellow
CD2	greenyellow
CEP78	greenyellow
CHST10	greenyellow
CLDND2	greenyellow
CLIC3	greenyellow
CX3CR1	greenyellow
CXCR6	greenyellow

DLG5	greenyellow
DTHD1	greenyellow
DUSP8	greenyellow
EDARADD	greenyellow
EFNA5	greenyellow
EOMES	greenyellow
EPB41L4A	greenyellow
EPDR1	greenyellow
EPHX4	greenyellow
ERBB2	greenyellow
F2R	greenyellow
FAM131B	greenyellow
FAM179A	greenyellow
FASLG	greenyellow
FCRL6	greenyellow
FEZ1	greenyellow
FGFBP2	greenyellow
FZD4	greenyellow
GFI1	greenyellow
GFPT2	greenyellow
GLB1L2	greenyellow
GNLY	greenyellow
GPR114	greenyellow
GPR153	greenyellow
GPR56	greenyellow
GPR68	greenyellow
GZMB	greenyellow
GZMH	greenyellow
HOXC4	greenyellow
HOXC5	greenyellow
ITPRIPL1	greenyellow
JAKMIP1	greenyellow
JAKMIP2	greenyellow
KIAA1671	greenyellow
KIF19	greenyellow
KIR2DL1	greenyellow
KIR3DX1	greenyellow
KLRD1	greenyellow
KLRG1	greenyellow
LAG3	greenyellow
LDB2	greenyellow
LDOC1L	greenyellow
LGR6	greenyellow
LIM2	greenyellow
LLGL2	greenyellow
LOC100129083	greenyellow
LOC100129316	greenyellow
LOC100130872	greenyellow
LOC100188949	greenyellow
LOC100499405	greenyellow
LOC100507206	greenyellow
LOC283177	greenyellow
LRRC16B	greenyellow
MANEAL	greenyellow
MGC12982	greenyellow
MSC	greenyellow
MTSS1	greenyellow
MYO3B	greenyellow
NCALD	greenyellow
NCAM1	greenyellow
NEB	greenyellow
NKG7	greenyellow
NMUR1	greenyellow
NPC1	greenyellow
NUAK1	greenyellow
PAK6	greenyellow
PATL2	greenyellow
PCDH1	greenyellow

PDGFRB		greenyellow
PDZD4		greenyellow
PIK3R3		greenyellow
PODN		greenyellow
PPP2R2B		greenyellow
PRR5L		greenyellow
PRSS23		greenyellow
PTCH1		greenyellow
PTGDR		greenyellow
PYHIN1		greenyellow
RAB11FIP5		greenyellow
RAB7L1		greenyellow
RASGEF1A		greenyellow
RCAN2		greenyellow
RGNEF		greenyellow
RGS9		greenyellow
RNF165		greenyellow
S1PR5		greenyellow
SAMD3		greenyellow
SCD5		greenyellow
SGCD		greenyellow
SGSM1		greenyellow
SH2D1B		greenyellow
SH2D2A		greenyellow
SH3RF2		greenyellow
SIGLECP3		greenyellow
SLAMF6		greenyellow
SLAMF7		greenyellow
SLC1A7		greenyellow
SMAD7		greenyellow
SOX13		greenyellow
SPON2		greenyellow
SYT11		greenyellow
SYTL2		greenyellow
TARP		greenyellow
TBX21		greenyellow
TIGIT		greenyellow
TM4SF19		greenyellow
TOX		greenyellow
TTC16		greenyellow
TTC38		greenyellow
USP28		greenyellow
VANGL1		greenyellow
VIT		greenyellow
WNT1		greenyellow
XPNPEP2		greenyellow
YPEL1		greenyellow
YWHAQ		greenyellow
ZFYVE28		greenyellow
ZNF683		greenyellow
	3-Sep	grey
A2M		grey
A2MP1		grey
ABCA13		grey
ABCA6		grey
ABCA9		grey
ABCC9		grey
ABI3BP		grey
ABP1		grey
ABRA		grey
ACAN		grey
ACBD7		grey
ACER1		grey
ACOT1		grey
ACOT4		grey
ACSBG2		grey
ACTN3		grey
ACTR3B		grey

ACTR3C	grey
ADAM20	grey
ADAM21	grey
ADAM33	grey
ADAMTS14	grey
ADAMTS16	grey
ADAMTS8	grey
ADARB2	grey
ADCY1	grey
ADCY10	grey
ADCY5	grey
ADORA2B	grey
ADRB1	grey
AFAP1-AS1	grey
AFAP1L1	grey
AGAP11	grey
AGAP4	grey
AGAP8	grey
AGAP9	grey
AGPAT4	grey
AGPHD1	grey
AGRP	grey
AHRR	grey
AICDA	grey
AIF1L	grey
AIRE	grey
AJAP1	grey
AK7	grey
AK8	grey
AKAP2	grey
AKR1C1	grey
AKR1CL1	grey
AKR1E2	grey
AKR7A2P1	grey
AKR7L	grey
ALDH1A3	grey
ALDH1L2	grey
ALDOB	grey
ALG1L	grey
ALK	grey
ALKBH1	grey
ALMS1P	grey
ALOX12B	grey
ALOX12P2	grey
ALOXE3	grey
ALPK2	grey
ALS2CR12	grey
AMDHD1	grey
ANGPT2	grey
ANGPT4	grey
ANGPTL2	grey
ANKHD1	grey
ANKRD18A	grey
ANKRD19P	grey
ANKRD20A9P	grey
ANKRD35	grey
ANKRD53	grey
ANKRD55	grey
AOC4	grey
APCDD1	grey
APOA2	grey
APOBEC3B	grey
APOF	grey
AQP7	grey
ARHGAP11B	grey
ARHGAP20	grey
ARHGAP44	grey
ARHGEF17	grey

ARHGEF26-AS1	grey
ARHGEF33	grey
ARHGEF35	grey
ARL5C	grey
ARMC2	grey
ARMC9	grey
ARNT2	grey
ARPP21	grey
ARR3	grey
ASB9	grey
ASIP	grey
ATOH8	grey
ATP13A4	grey
ATP1A2	grey
ATP1A4	grey
ATP1B1	grey
ATP1B2	grey
ATP5L2	grey
ATP6AP1L	grey
ATP6V1C2	grey
ATP8B3	grey
ATRNL1	grey
AURKA	grey
AURKB	grey
AXL	grey
B3GALNT2	grey
B3GNT3	grey
B3GNT4	grey
B4GALNT3	grey
B9D1	grey
BAALC	grey
BCAR1	grey
BCAR3	grey
BCL2L14	grey
BCL2L2	grey
BCL6B	grey
BCRP3	grey
BDAG1	grey
BDNF	grey
BEGAIN	grey
BEND6	grey
BFSP2	grey
BHLHA15	grey
BICC1	grey
BIRC5	grey
BIRC7	grey
BMP3	grey
BMP4	grey
BPI	grey
BREA2	grey
BSPRY	grey
BTBD17	grey
BTBD8	grey
BTG1	grey
BTNL9	grey
BVES	grey
C10orf140	grey
C10orf25	grey
C10orf57	grey
C10orf91	grey
C10orf92	grey
C11orf34	grey
C11orf45	grey
C11orf65	grey
C11orf85	grey
C12orf42	grey
C12orf59	grey
C12orf68	grey

C12orf71	grey
C12orf74	grey
C13orf16	grey
C14orf119	grey
C14orf132	grey
C14orf183	grey
C14orf184	grey
C14orf19	grey
C14orf33	grey
C14orf50	grey
C14orf55	grey
C14orf82	grey
C15orf42	grey
C15orf53	grey
C15orf55	grey
C15orf63	grey
C16orf3	grey
C16orf45	grey
C16orf46	grey
C16orf59	grey
C16orf89	grey
C16orf90	grey
C17orf110	grey
C17orf53	grey
C17orf77	grey
C17orf78	grey
C17orf88	grey
C19orf18	grey
C1orf106	grey
C1orf115	grey
C1orf170	grey
C1orf173	grey
C1orf182	grey
C1orf190	grey
C1orf204	grey
C1orf229	grey
C1orf51	grey
C1orf56	grey
C1orf74	grey
C1orf88	grey
C1orf95	grey
C1S	grey
C20orf103	grey
C20orf106	grey
C20orf152	grey
C20orf173	grey
C20orf202	grey
C20orf203	grey
C20orf46	grey
C20orf54	grey
C21orf128	grey
C21orf15	grey
C21orf56	grey
C21orf67	grey
C21orf81	grey
C21orf90	grey
C22orf15	grey
C22orf24	grey
C2orf16	grey
C2orf40	grey
C2orf48	grey
C2orf52	grey
C2orf61	grey
C3orf15	grey
C3orf20	grey
C3orf35	grey
C3orf49	grey
C3orf67	grey

C4BPA	grey
C4orf26	grey
C4orf36	grey
C4orf49	grey
C5orf27	grey
C5orf58	grey
C5orf64	grey
C6orf124	grey
C6orf132	grey
C6orf138	grey
C6orf163	grey
C6orf164	grey
C6orf174	grey
C6orf201	grey
C6orf81	grey
C7orf10	grey
C7orf16	grey
C7orf28B	grey
C7orf31	grey
C8orf31	grey
C8orf45	grey
C8orf46	grey
C8orf51	grey
C9orf106	grey
C9orf116	grey
C9orf117	grey
C9orf128	grey
C9orf140	grey
C9orf169	grey
C9orf24	grey
C9orf43	grey
CABLES1	grey
CABP7	grey
CACHD1	grey
CACNA1C	grey
CACNA1D	grey
CACNG4	grey
CALHM1	grey
CALN1	grey
CAMK2N2	grey
CAMKMT	grey
CARD10	grey
CASC1	grey
CASQ1	grey
CATSPER2P1	grey
CATSPER3	grey
CAV1	grey
CCBP2	grey
CCDC11	grey
CCDC110	grey
CCDC113	grey
CCDC116	grey
CCDC122	grey
CCDC144A	grey
CCDC144C	grey
CCDC150	grey
CCDC163P	grey
CCDC37	grey
CCDC40	grey
CCDC42	grey
CCDC62	grey
CCDC81	grey
CCL16	grey
CCL20	grey
CCL22	grey
CCNB1	grey
CCNB2	grey
CCNB3	grey

CCNE1	grey
CCNI2	grey
CCR10	grey
CCR3	grey
CCR9	grey
CCRL1	grey
CD163L1	grey
CD1B	grey
CD1E	grey
CD207	grey
CD276	grey
CD34	grey
CD70	grey
CD99P1	grey
CDC20	grey
CDC45	grey
CDCA2	grey
CDCA3	grey
CDCA5	grey
CDCP2	grey
CDH3	grey
CDH4	grey
CDKN2B-AS1	grey
CDO1	grey
CDRT1	grey
CDRT15	grey
CDRT15P	grey
CEACAM21	grey
CEACAM22P	grey
CEACAM6	grey
CEACAM8	grey
CECR2	grey
CECR5-AS1	grey
CECR7	grey
CELF4	grey
CELF5	grey
CENPA	grey
CENPBD1	grey
CENPI	grey
CENPP	grey
CEP112	grey
CERS3	grey
CES3	grey
CFB	grey
CGREF1	grey
CHAC1	grey
CHDH	grey
CHGB	grey
CHI3L1	grey
CHIT1	grey
CHL1	grey
CHN1	grey
CHODL	grey
CHRFAM7A	grey
CHRM3	grey
CHRNA6	grey
CHRNA7	grey
CHRNA9	grey
CHRNE	grey
CHRNG	grey
CHST5	grey
CHST6	grey
CHST8	grey
CHTOP	grey
CIB3	grey
CILP	grey
CILP2	grey
CKAP2L	grey

CKMT2	grey
CLDN23	grey
CLDN4	grey
CLEC12B	grey
CLEC18A	grey
CLEC4F	grey
CLGN	grey
CLIC6	grey
CLMP	grey
CLNK	grey
CMTM8	grey
CMYA5	grey
CNDP1	grey
CNGA1	grey
CNKSR3	grey
CNNM1	grey
CNRIP1	grey
CNTD2	grey
CNTN4	grey
COBL	grey
COL10A1	grey
COL13A1	grey
COL1A1	grey
COL28A1	grey
COL4A1	grey
COL5A3	grey
COL6A4P2	grey
COL6A6	grey
COL9A3	grey
COPS3	grey
CORIN	grey
CORT	grey
COX6B2	grey
COX7A1	grey
CPA5	grey
CPEB1	grey
CPLX2	grey
CPLX3	grey
CPNE9	grey
CPS1	grey
CPXM1	grey
CRB1	grey
CREB3L3	grey
CREG2	grey
CRHBP	grey
CRHR2	grey
CRISP3	grey
CRLF1	grey
CRLF2	grey
CRMP1	grey
CRX	grey
CRYBA1	grey
CRYGN	grey
CRYM	grey
CSDC2	grey
CSMD1	grey
CSPG4	grey
CTAGE4	grey
CTF1	grey
CTH	grey
CTHRC1	grey
CTRC	grey
CTSG	grey
CTSK	grey
CTSL2	grey
CTTNBP2	grey
CXCR4	grey
CXorf36	grey

CXorf58	grey
CXorf65	grey
CYB5R2	grey
CYCSP52	grey
CYGB	grey
CYP17A1	grey
CYP21A1P	grey
CYP21A2	grey
CYP24A1	grey
CYP27B1	grey
CYP2A6	grey
CYP2B7P1	grey
CYP2C8	grey
CYP2F1	grey
CYP2G1P	grey
CYP2J2	grey
CYP3A4	grey
CYP3A5	grey
CYP46A1	grey
CYP4F2	grey
CYP7B1	grey
DAB1	grey
DACT1	grey
DACT3	grey
DAZL	grey
DBIL5P	grey
DCDC5	grey
DCHS2	grey
DCST1	grey
DCT	grey
DDAH1	grey
DDO	grey
DDR2	grey
DDX11L2	grey
DDX39B	grey
DEFA4	grey
DEPDC4	grey
DEPTOR	grey
DES	grey
DFNA5	grey
DGCR10	grey
DGCR9	grey
DGKK	grey
DIO1	grey
DISP2	grey
DKFZP434L187	grey
DKK2	grey
DLC1	grey
DLG2	grey
DLGAP1	grey
DLL1	grey
DLL4	grey
DLX4	grey
DMC1	grey
DMD	grey
DNAAF1	grey
DNAH10	grey
DNAH11	grey
DNAH12	grey
DNAH7	grey
DNAH8	grey
DNAJC12	grey
DNAJC28	grey
DNAJC6	grey
DNALI1	grey
DNASE1L3	grey
DNM1P35	grey
DNMT3L	grey

DNTT	grey
DOC2A	grey
DPPA3	grey
DPY19L1P1	grey
DPY19L2	grey
DPY19L2P1	grey
DPYSL3	grey
DPYSL4	grey
DQX1	grey
DRP2	grey
DSC3	grey
DSG2	grey
DSP	grey
DTNA	grey
DUOX2	grey
DUSP14	grey
DUSP22	grey
DUSP4	grey
DUSP5	grey
DZIP1L	grey
E2F8	grey
EBF3	grey
EBI3	grey
EBLN2	grey
ECM1	grey
ECM2	grey
EDA	grey
EDA2R	grey
EDN1	grey
EDN3	grey
EFCAB10	grey
EFCAB5	grey
EFCAB6	grey
EFHB	grey
EFNA1	grey
EFNB2	grey
EFR3B	grey
EGLN3	grey
EGOT	grey
EGR2	grey
EHF	grey
EIF3C	grey
EIF5AL1	grey
ELOVL2	grey
EME1	grey
EMID2	grey
EMP1	grey
ENAM	grey
ENC1	grey
ENO4	grey
ENPEP	grey
ENPP1	grey
ENPP2	grey
ENTHD1	grey
ENTPD2	grey
ENTPD3	grey
EPB41L1	grey
EPHA10	grey
EPHX3	grey
EPS8L1	grey
EPX	grey
ERAP2	grey
ERCC6L	grey
ERG	grey
ERVFRD-1	grey
ESPL1	grey
ESPNP	grey
ESRG	grey

ETV1	grey
ETV4	grey
ETV5	grey
EVPL	grey
EXO1	grey
EYA2	grey
EZH2	grey
F10	grey
F7	grey
FA2H	grey
FABP3	grey
FAIM2	grey
FAM106CP	grey
FAM111B	grey
FAM127C	grey
FAM149A	grey
FAM154B	grey
FAM155B	grey
FAM156A	grey
FAM160A1	grey
FAM164C	grey
FAM166A	grey
FAM167A	grey
FAM171B	grey
FAM172BP	grey
FAM174B	grey
FAM181B	grey
FAM182A	grey
FAM183B	grey
FAM186A	grey
FAM189A1	grey
FAM190A	grey
FAM194A	grey
FAM196A	grey
FAM196B	grey
FAM22A	grey
FAM3B	grey
FAM3D	grey
FAM40B	grey
FAM46B	grey
FAM54A	grey
FAM59A	grey
FAM64A	grey
FAM66A	grey
FAM66B	grey
FAM66D	grey
FAM71E1	grey
FAM72A	grey
FAM78B	grey
FAM81A	grey
FAM83D	grey
FAM86C1	grey
FAM86C2P	grey
FAM86DP	grey
FAM86FP	grey
FAM90A1	grey
FAM95B1	grey
FARP1	grey
FAT4	grey
FBLIM1	grey
FBLN5	grey
FBN1	grey
FBXL2	grey
FBXL22	grey
FBXO27	grey
FERMT2	grey
FGD5	grey
FGF11	grey

FGF22	grey
FGF5	grey
FGFR2	grey
FGFR3	grey
FHAD1	grey
FHIT	grey
FIBCD1	grey
FILIP1L	grey
FKBP6	grey
FKBP9L	grey
FKSG29	grey
FLG	grey
FLJ11235	grey
FLJ12825	grey
FLJ26484	grey
FLJ26850	grey
FLJ32224	grey
FLJ33360	grey
FLJ35946	grey
FLJ37035	grey
FLJ39534	grey
FLJ40852	grey
FLJ41649	grey
FLJ43681	grey
FLJ44054	grey
FLJ45079	grey
FLJ45983	grey
FLT1	grey
FMN1	grey
FMR1-AS1	grey
FN1	grey
FNDC7	grey
FNDC8	grey
FOLR3	grey
FONG	grey
FOSL1	grey
FOXD1	grey
FOXD2	grey
FOXD4	grey
FOXE1	grey
FPR3	grey
FREM1	grey
FRG1B	grey
FRMD6	grey
FSCN3	grey
FSD2	grey
FSIP2	grey
FST	grey
FTCD	grey
FUT10	grey
FZD7	grey
FZD8	grey
GAB4	grey
GAL3ST1	grey
GALNT8	grey
GAS1	grey
GATA6	grey
GCOM1	grey
GDF10	grey
GDF7	grey
GEM	grey
GGNBP1	grey
GGT8P	grey
GH1	grey
GINS1	grey
GINS2	grey
GINS3	grey
GJA3	grey

GJA9-MYCBP	grey
GJB6	grey
GJC3	grey
GLDC	grey
GLI3	grey
GLIPR1L2	grey
GLIS3	grey
GLP1R	grey
GLT25D2	grey
GNA14	grey
GNAS-AS1	grey
GNG3	grey
GPC3	grey
GPC4	grey
GPER	grey
GPHA2	grey
GPM6A	grey
GPNMB	grey
GPR113	grey
GPR126	grey
GPR137B	grey
GPR142	grey
GPR172B	grey
GPR173	grey
GPR179	grey
GPR182	grey
GPR3	grey
GPR45	grey
GPR52	grey
GPR63	grey
GPR83	grey
GPR98	grey
GPRC5B	grey
GPRC5D	grey
GRAPL	grey
GREB1	grey
GREB1L	grey
GREM2	grey
GRIK1	grey
GRIK1-AS1	grey
GRIK4	grey
GRK4	grey
GRK7	grey
GRPR	grey
GRTP1	grey
GSDMA	grey
GSG1	grey
GSG2	grey
GSTM1	grey
GSTM5	grey
GSTO2	grey
GTF2H2	grey
GTF2H2B	grey
GTF2H2C	grey
GTF2IRD1	grey
GTSF1	grey
GTSF1L	grey
GUCY1B2	grey
GYPE	grey
H19	grey
H1FNT	grey
H2BFM	grey
H2BFXP	grey
HAP1	grey
HARBI1	grey
HAVCR2	grey
HBEGF	grey
HCG4B	grey

HCG9	grey
HCRTR1	grey
HEATR3	grey
HEATR8	grey
HERC2P3	grey
HERC2P7	grey
HEY2	grey
HEYL	grey
HFE2	grey
HHIPL1	grey
HHIPL2	grey
HIGD1B	grey
HILS1	grey
HIPK4	grey
HIST1H1D	grey
HIST1H1E	grey
HIST1H2AH	grey
HIST1H2AM	grey
HIST1H4C	grey
HIST1H4E	grey
HIST3H2BB	grey
HIST3H3	grey
HJURP	grey
HLA-DMB	grey
HLA-DPB2	grey
HLA-DQA1	grey
HLA-DQA2	grey
HLA-DQB1	grey
HLA-DQB2	grey
HLA-DRB5	grey
HLA-DRB6	grey
HLA-F-AS1	grey
HLA-G	grey
HLA-H	grey
HLA-J	grey
HLA-L	grey
HMCN1	grey
HMGB3	grey
HMGN2	grey
HMGN2P46	grey
HNRNPC	grey
HOXA1	grey
HOXA10	grey
HOXA2	grey
HOXA3	grey
HOXA4	grey
HOXA5	grey
HOXB3	grey
HOXB4	grey
HOXB7	grey
HOXB9	grey
HPDL	grey
HPSE2	grey
HRH1	grey
HRNR	grey
HS3ST3A1	grey
HSD11B1	grey
HSD11B2	grey
HSD3BP4	grey
HSF2BP	grey
HSP90AB2P	grey
HSP90AB4P	grey
HSPA12B	grey
HSPA2	grey
HSPA7	grey
HSPB6	grey
HSPB9	grey
HSPBAP1	grey

HTR2B	grey
HTR6	grey
HTR7	grey
HTRA1	grey
HTRA4	grey
HUNK	grey
HYDIN	grey
HYMAI	grey
ICA1	grey
IDO2	grey
IFNK	grey
IGDCC4	grey
IGF1	grey
IGFBP3	grey
IGFL4	grey
IGLL3P	grey
IGLL5	grey
IGSF10	grey
IGSF11	grey
IGSF22	grey
IGSF3	grey
IL11	grey
IL17B	grey
IL17RB	grey
IL17RD	grey
IL23R	grey
IL36A	grey
IL6	grey
IL9R	grey
ILDR1	grey
ILDR2	grey
INGX	grey
INHBE	grey
INMT	grey
INPP1	grey
INTS4L1	grey
IQCA1	grey
IQCD	grey
IQCH	grey
IQCK	grey
IQGAP3	grey
IQSEC3	grey
IQUB	grey
IRF6	grey
ISM1	grey
ITGA9	grey
ITIH1	grey
ITIH2	grey
ITIH3	grey
JAKMIP3	grey
JAM2	grey
JMJD7-PLA2G4B	grey
KBTBD10	grey
KBTBD12	grey
KBTBD4	grey
KCNA7	grey
KCNC1	grey
KCNE2	grey
KCNH1	grey
KCNJ1	grey
KCNJ10	grey
KCNJ11	grey
KCNK1	grey
KCNK17	grey
KCNK5	grey
KCNMA1	grey
KCNMB1	grey
KCNN1	grey

KCNN3	grey
KCNQ3	grey
KCP	grey
KCTD19	grey
KDELC1	grey
KDM4D	grey
KDM4DL	grey
KGFLP1	grey
KHDC1	grey
KHDRBS3	grey
KIAA0087	grey
KIAA0895	grey
KIAA0895L	grey
KIAA1217	grey
KIAA1409	grey
KIAA1456	grey
KIAA1549	grey
KIAA2022	grey
KIF14	grey
KIF17	grey
KIF18B	grey
KIF26A	grey
KIF26B	grey
KIF2C	grey
KIF5A	grey
KIF9	grey
KIR2DL4	grey
KIR2DS4	grey
KIRREL2	grey
KIRREL3	grey
KIT	grey
KLF15	grey
KLHDC9	grey
KLHL10	grey
KLHL13	grey
KLHL30	grey
KLHL33	grey
KLHL4	grey
KLK1	grey
KLK14	grey
KNG1	grey
KRT73	grey
KRT74	grey
KRT8	grey
KRT80	grey
KRT86	grey
KRTAP5-9	grey
L1TD1	grey
L3MBTL4	grey
LAMC3	grey
LANCL3	grey
LAYN	grey
LBX2	grey
LCA5	grey
LCN12	grey
LCN2	grey
LCN8	grey
LCT	grey
LDB3	grey
LDLRAD2	grey
LEAP2	grey
LENEP	grey
LEP	grey
LGI2	grey
LHFP	grey
LHFPL1	grey
LHFPL4	grey
LIF	grey

LILRA3	grey
LIMCH1	grey
LINGO2	grey
LINGO4	grey
LIPC	grey
LIPH	grey
LMAN1L	grey
LMCD1	grey
LOC100009676	grey
LOC100101266	grey
LOC100125556	grey
LOC100128081	grey
LOC100128164	grey
LOC100128239	grey
LOC100128338	grey
LOC100128593	grey
LOC100128640	grey
LOC100128788	grey
LOC100129046	grey
LOC100129148	grey
LOC100129269	grey
LOC100129387	grey
LOC100129534	grey
LOC100129726	grey
LOC100129858	grey
LOC100130000	grey
LOC100130015	grey
LOC100130197	grey
LOC100130348	grey
LOC100130357	grey
LOC100130451	grey
LOC100130522	grey
LOC100130894	grey
LOC100130954	grey
LOC100131060	grey
LOC100131176	grey
LOC100131691	grey
LOC100132724	grey
LOC100132781	grey
LOC100132832	grey
LOC100133612	grey
LOC100134259	grey
LOC100134868	grey
LOC100170939	grey
LOC100190938	grey
LOC100192204	grey
LOC100240735	grey
LOC100268168	grey
LOC100286979	grey
LOC100288122	grey
LOC100288778	grey
LOC100288842	grey
LOC100289187	grey
LOC100289561	grey
LOC100306975	grey
LOC100335030	grey
LOC100499467	grey
LOC100505666	grey
LOC100505676	grey
LOC100505826	grey
LOC100505894	grey
LOC100506012	grey
LOC100506071	grey
LOC100506083	grey
LOC100506134	grey
LOC100506136	grey
LOC100506305	grey
LOC100506314	grey

LOC100506388	grey
LOC100506462	grey
LOC100506497	grey
LOC100506540	grey
LOC100506599	grey
LOC100506660	grey
LOC100506668	grey
LOC100506746	grey
LOC100506874	grey
LOC100507091	grey
LOC100507127	grey
LOC100507156	grey
LOC100507246	grey
LOC100507300	grey
LOC100507341	grey
LOC100507346	grey
LOC100507421	grey
LOC100507462	grey
LOC143188	grey
LOC144481	grey
LOC144571	grey
LOC148709	grey
LOC149086	grey
LOC149134	grey
LOC151009	grey
LOC151484	grey
LOC151534	grey
LOC154761	grey
LOC154822	grey
LOC220594	grey
LOC220980	grey
LOC253039	grey
LOC283050	grey
LOC283143	grey
LOC283194	grey
LOC283335	grey
LOC283432	grey
LOC283683	grey
LOC284009	grey
LOC284100	grey
LOC284798	grey
LOC285033	grey
LOC285456	grey
LOC285484	grey
LOC285540	grey
LOC285593	grey
LOC285740	grey
LOC285847	grey
LOC286190	grey
LOC286467	grey
LOC338651	grey
LOC338739	grey
LOC338817	grey
LOC339524	grey
LOC339685	grey
LOC339788	grey
LOC339803	grey
LOC339894	grey
LOC340508	grey
LOC348761	grey
LOC348840	grey
LOC386597	grey
LOC387647	grey
LOC388906	grey
LOC388948	grey
LOC389247	grey
LOC389641	grey
LOC390940	grey

LOC391322	grey
LOC399753	grey
LOC400043	grey
LOC400548	grey
LOC400680	grey
LOC400958	grey
LOC401052	grey
LOC401127	grey
LOC401321	grey
LOC440028	grey
LOC440131	grey
LOC440600	grey
LOC441204	grey
LOC441242	grey
LOC441455	grey
LOC441461	grey
LOC441617	grey
LOC441666	grey
LOC441869	grey
LOC442028	grey
LOC442421	grey
LOC442454	grey
LOC553103	grey
LOC554202	grey
LOC641367	grey
LOC642846	grey
LOC643201	grey
LOC643719	grey
LOC644242	grey
LOC644538	grey
LOC646278	grey
LOC646324	grey
LOC646576	grey
LOC646851	grey
LOC646999	grey
LOC647859	grey
LOC648987	grey
LOC650794	grey
LOC653712	grey
LOC654342	grey
LOC654433	grey
LOC727849	grey
LOC728554	grey
LOC728613	grey
LOC728752	grey
LOC728758	grey
LOC728989	grey
LOC729156	grey
LOC729444	grey
LOC729609	grey
LOC729723	grey
LOC729970	grey
LOC730101	grey
LOC730102	grey
LOC730668	grey
LOC731789	grey
LOC84989	grey
LOC91450	grey
LOC91948	grey
LONRF2	grey
LOXL2	grey
LOXL4	grey
LPAR3	grey
LPIN3	grey
LPL	grey
LRFN2	grey
LRGUK	grey
LRP11	grey

LRRC1	grey
LRRC17	grey
LRRC19	grey
LRRC2	grey
LRRC36	grey
LRRC46	grey
LRRC66	grey
LRRC69	grey
LRRC7	grey
LRRC8E	grey
LRRN4	grey
LRRN4CL	grey
LRRTM2	grey
LRTOMT	grey
LTA	grey
LTF	grey
LYPD5	grey
MAB21L2	grey
MACC1	grey
MAFA	grey
MAG	grey
MALL	grey
MAMDC2	grey
MANSC4	grey
MAOA	grey
MAP1LC3C	grey
MAP3K15	grey
MAPK12	grey
MARCO	grey
MARVELD2	grey
MARVELD3	grey
MATN1	grey
MCF2L2	grey
MCHR1	grey
MCM10	grey
MDGA1	grey
ME1	grey
MECOM	grey
MED19	grey
MED20	grey
MEG3	grey
MEIS3P1	grey
MELK	grey
MEOX1	grey
MESTIT1	grey
METTL20	grey
METTL21B	grey
MEX3A	grey
MFAP5	grey
MGAT5B	grey
MGC12916	grey
MGC16025	grey
MGC16142	grey
MGC16275	grey
MGC16703	grey
MGC23270	grey
MGC45922	grey
MGP	grey
MGST2	grey
MIA	grey
MICA	grey
MID1	grey
MIR497HG	grey
MKX	grey
MMP19	grey
MMP21	grey
MMP8	grey
MMRN2	grey

MN1	grey
MOCS1	grey
MOG	grey
MORN4	grey
MOSC2	grey
MPL	grey
MPO	grey
MPP3	grey
MPPED2	grey
MPV17L	grey
MRAS	grey
MRC1	grey
MRGPRD	grey
MRGPRE	grey
MRRF	grey
MS4A7	grey
MSH4	grey
MSH5	grey
MSLN	grey
MST1R	grey
MSX2	grey
MT3	grey
MTRNR2L3	grey
MTUS1	grey
MTUS2	grey
MUC12	grey
MUC16	grey
MUC4	grey
MURC	grey
MXRA7	grey
MYBL2	grey
MYBPH	grey
MYCN	grey
MYH15	grey
MYO16	grey
MYO18B	grey
MYO1A	grey
MYO1H	grey
MYOM2	grey
MYOT	grey
MYOZ2	grey
MYOZ3	grey
MYRIP	grey
MYT1	grey
MYT1L	grey
MZB1	grey
NAT8	grey
NAV2	grey
NAV3	grey
NBPF11	grey
NBPF16	grey
NBPF7	grey
NBR2	grey
NCKAP5	grey
NCRNA00092	grey
NCRNA00102	grey
NCRNA00161	grey
NCRNA00167	grey
NCRNA00169	grey
NCRNA00235	grey
NCRNA00245	grey
NCRNA00257	grey
NCRNA00266-1	grey
NCRNA00277	grey
NCRNA00304	grey
NCRNA00310	grey
NCRNA00336	grey
NCRNA00346	grey

NDRG4	grey
NEBL	grey
NEFL	grey
NEFM	grey
NEIL3	grey
NEK10	grey
NEK11	grey
NEK2	grey
NEK5	grey
NEURL1B	grey
NFATC4	grey
NGFR	grey
NHLRC1	grey
NID2	grey
NIM1	grey
NIPAL1	grey
NKX3-1	grey
NLRP2	grey
NLRP9	grey
NMNAT3	grey
NOS2	grey
NOTCH2NL	grey
NOTCH4	grey
NOX1	grey
NPC1L1	grey
NPHP1	grey
NPIP	grey
NPR3	grey
NPTX1	grey
NPTX2	grey
NR1I2	grey
NR1I3	grey
NR4A2	grey
NR5A1	grey
NRIP3	grey
NRN1L	grey
NRP2	grey
NT5C1B	grey
NTN1	grey
NTN4	grey
NTNG1	grey
NTRK2	grey
NUDT10	grey
NUDT19	grey
NUDT6	grey
NWD1	grey
NXPH3	grey
O3FAR1	grey
OAZ3	grey
OCLN	grey
ODF3	grey
ODZ4	grey
OGN	grey
OLFM1	grey
OLFM4	grey
OLFML1	grey
OLR1	grey
OMD	grey
OPHN1	grey
OR10AD1	grey
OR10G2	grey
OR13A1	grey
OR2A7	grey
OR2B11	grey
OR2C1	grey
OR2H2	grey
OR2L13	grey
OR4D1	grey

OR52K1	grey
OR56B1	grey
OR56B4	grey
OR5K2	grey
OR7D2	grey
ORC1	grey
ORC6	grey
OSBPL6	grey
OSCP1	grey
OTUB2	grey
OXA1L	grey
OXCT2	grey
OXTR	grey
P2RX2	grey
P2RY1	grey
P2RY4	grey
P2RY6	grey
PACRG	grey
PADI6	grey
PAK3	grey
PALLD	grey
PALM	grey
PAPL	grey
PAPPA	grey
PAQR5	grey
PAR1	grey
PARD3B	grey
PAX8	grey
PCDH11X	grey
PCDH18	grey
PCDHB9	grey
PCDHGA10	grey
PCDHGA11	grey
PCDHGA12	grey
PCDHGA5	grey
PCDHGA6	grey
PCDHGA7	grey
PCDHGA9	grey
PCDHGB2	grey
PCDHGB3	grey
PCDHGB4	grey
PCDHGB5	grey
PCDHGB6	grey
PCGF3	grey
PCOLCE2	grey
PDC	grey
PDE6A	grey
PDE6C	grey
PDE6D	grey
PDE8B	grey
PDGFB	grey
PDZD9	grey
PDZK1	grey
PEG3	grey
PERP	grey
PEX5L	grey
PFKFB1	grey
PGAM4	grey
PGBD3	grey
PGBD5	grey
PGF	grey
PGM5	grey
PGM5P2	grey
PHACTR3	grey
PHKA1	grey
PHYHD1	grey
PI4KAP1	grey
PIGR	grey

PIGV	grey
PIP5K1P1	grey
PIPOX	grey
PIPSL	grey
PKD1L1	grey
PKD1L3	grey
PKD2L2	grey
PKDCC	grey
PKDREJ	grey
PKMYT1	grey
PKP2	grey
PKP4	grey
PLA2G10	grey
PLA2G2D	grey
PLA2G4B	grey
PLA2G4C	grey
PLAC2	grey
PLAC9	grey
PLCB4	grey
PLCE1	grey
PLCH1	grey
PLEKHA8P1	grey
PLEKHD1	grey
PLEKHG4B	grey
PLEKHH1	grey
PLEKHN1	grey
PLK1	grey
PLK2	grey
PLN	grey
PLS3	grey
PLTP	grey
PM20D1	grey
PMM2	grey
PMS2P4	grey
PNLDC1	grey
PNMA2	grey
PNMA5	grey
PNMAL1	grey
PNMAL2	grey
PNPLA4	grey
POF1B	grey
POLE2	grey
POLN	grey
POLR2J2	grey
POLR2J3	grey
POM121L10P	grey
POM121L9P	grey
POU3F1	grey
POU4F1	grey
POU5F2	grey
PP2D1	grey
PPAPDC3	grey
PPARG	grey
PPARGC1A	grey
PPEF2	grey
PPFIA4	grey
PPIL6	grey
PPM1E	grey
PPP1R14C	grey
PPP1R1B	grey
PPP1R3G	grey
PPP1R9A	grey
PRB3	grey
PRCD	grey
PRDM11	grey
PRDM16	grey
PRELID2	grey
PRG4	grey

PRH2	grey
PRICKLE2	grey
PRKAG3	grey
PRKCDBP	grey
PRKCG	grey
PRKD1	grey
PRKG2	grey
PRKRIP1	grey
PRL	grey
PRODH	grey
PROM1	grey
PRR19	grey
PRSS12	grey
PRSS16	grey
PRSS21	grey
PRSS35	grey
PRUNE2	grey
PSD2	grey
PSMB11	grey
PSMC3IP	grey
PSMG2	grey
PSORS1C3	grey
PSPH	grey
PSPN	grey
PTCD1	grey
PTPN3	grey
PTPRB	grey
PTPRG	grey
PTPRH	grey
PTPRM	grey
PTPRU	grey
PXDN	grey
PXDNL	grey
PYCR1	grey
PYY2	grey
PZP	grey
QRFP	grey
RAB26	grey
RAB38	grey
RAB3A	grey
RAB3B	grey
RAB9A	grey
RAD51	grey
RAD54L	grey
RAD9B	grey
RAET1K	grey
RAI2	grey
RANBP17	grey
RAP2B	grey
RAPGEF3	grey
RAPGEF4	grey
RAPH1	grey
RARB	grey
RARRES1	grey
RASAL1	grey
RASAL2	grey
RASD1	grey
RASGRF1	grey
RASIP1	grey
RASL10B	grey
RASL11B	grey
RAVER2	grey
RAX2	grey
RCOR2	grey
RDH12	grey
RDM1	grey
REEP1	grey
REG4	grey

REREP3	grey
RFPL1-AS1	grey
RFPL2	grey
RFPL3-AS1	grey
RFPL4A	grey
RFX8	grey
RGMA	grey
RGPD1	grey
RGPD2	grey
RGPD3	grey
RGS20	grey
RGS7	grey
RGS9BP	grey
RHBDF1	grey
RHBDL2	grey
RHD	grey
RHOXF1	grey
RIBC1	grey
RIBC2	grey
RIMBP2	grey
RIPK4	grey
RLN3	grey
RNASE1	grey
RNASE6	grey
RND1	grey
RND2	grey
RNF112	grey
RNF138P1	grey
RNF182	grey
RNF207	grey
RNF212	grey
RNF32	grey
RNF5	grey
RNF5P1	grey
ROBO1	grey
ROCK1P1	grey
ROR1	grey
ROR2	grey
RPA4	grey
RPL13AP20	grey
RPL13AP6	grey
RPL19P12	grey
RPL23AP7	grey
RPS26	grey
RPS6KA6	grey
RPSAP58	grey
RRAD	grey
RRH	grey
RRM2	grey
RSPO4	grey
RTN4RL1	grey
RWDD2B	grey
S100A1	grey
S100B	grey
SALL4	grey
SAMD11	grey
SAP18	grey
SATB2	grey
SCARA5	grey
SCARNA15	grey
SCARNA16	grey
SCARNA21	grey
SCARNA5	grey
SCARNA6	grey
SCARNA7	grey
SCG3	grey
SCG5	grey
SCIN	grey

SCN11A	grey
SCN2A	grey
SCN2B	grey
SCN3B	grey
SCN4B	grey
SCN8A	grey
SCNN1A	grey
SCUBE2	grey
SDC1	grey
SDHAP2	grey
SDK1	grey
SDR16C5	grey
SDS	grey
SEBOX	grey
SEC16B	grey
SEC61A2	grey
SEMA5A	grey
SENP3	grey
SENP3-EIF4A1	grey
SEPP1	grey
SEPT7L	grey
SERHL2	grey
SERINC2	grey
SERP2	grey
SERPINE3	grey
SETD3	grey
SETD8	grey
SEZ6L	grey
SFRP1	grey
SFRP4	grey
SH2D4A	grey
SH2D7	grey
SH3D19	grey
SH3GL1P2	grey
SHANK3	grey
SHC2	grey
SHC3	grey
SHD	grey
SHISA9	grey
SIAH3	grey
SIGLEC11	grey
SIGLEC12	grey
SIGLEC15	grey
SIX4	grey
SKA1	grey
SKA3	grey
SLBP	grey
SLC10A4	grey
SLC10A5	grey
SLC12A1	grey
SLC12A8	grey
SLC13A1	grey
SLC13A3	grey
SLC13A5	grey
SLC14A2	grey
SLC16A2	grey
SLC16A8	grey
SLC17A3	grey
SLC17A7	grey
SLC18A1	grey
SLC1A1	grey
SLC1A2	grey
SLC22A13	grey
SLC22A16	grey
SLC22A20	grey
SLC22A3	grey
SLC22A5	grey
SLC22A7	grey

SLC23A1	grey
SLC25A13	grey
SLC25A18	grey
SLC25A20	grey
SLC25A41	grey
SLC26A4	grey
SLC26A5	grey
SLC28A2	grey
SLC28A3	grey
SLC2A14	grey
SLC34A1	grey
SLC35F3	grey
SLC35G3	grey
SLC35G5	grey
SLC38A11	grey
SLC39A5	grey
SLC3A1	grey
SLC44A5	grey
SLC45A1	grey
SLC47A1	grey
SLC47A2	grey
SLC4A9	grey
SLC5A10	grey
SLC5A11	grey
SLC6A13	grey
SLC6A20	grey
SLC7A3	grey
SLC7A9	grey
SLC9A2	grey
SLC9A4	grey
SLCO4A1	grey
SLCO5A1	grey
SLITRK5	grey
SMAD1	grey
SMAD5-AS1	grey
SMCR5	grey
SMO	grey
SMOC1	grey
SNAI1	grey
SNORA16A	grey
SNORA16B	grey
SNORA18	grey
SNORA21	grey
SNORA27	grey
SNORA29	grey
SNORA2A	grey
SNORA3	grey
SNORA33	grey
SNORA38	grey
SNORA4	grey
SNORA40	grey
SNORA44	grey
SNORA53	grey
SNORA56	grey
SNORA5A	grey
SNORA5C	grey
SNORA6	grey
SNORA61	grey
SNORA62	grey
SNORA63	grey
SNORA64	grey
SNORA65	grey
SNORA70G	grey
SNORA71B	grey
SNORA75	grey
SNORA81	grey
SNORA9	grey
SNORD10	grey

SNORD94	grey
SNORD97	grey
SNX7	grey
SORBS1	grey
SORBS2	grey
SORCS3	grey
SOX15	grey
SOX30	grey
SOX5	grey
SOX7	grey
SP6	grey
SP7	grey
SPAG17	grey
SPARCL1	grey
SPATA18	grey
SPATA21	grey
SPATA9	grey
SPC24	grey
SPC25	grey
SPDYE4	grey
SPDYE7P	grey
SPIRE1	grey
SPOCK1	grey
SPP1	grey
SPRED3	grey
SPSB1	grey
SPTLC3	grey
SPTSSB	grey
SRD5A3	grey
SRMS	grey
SRPX	grey
SRSF12	grey
SSC5D	grey
ST6GAL2	grey
ST6GALNAC1	grey
ST8SIA6	grey
STAB2	grey
STAC	grey
STAC2	grey
STARD13	grey
STARD5	grey
STEAP1B	grey
STIL	grey
STK31	grey
STK33	grey
STL	grey
STOX1	grey
STX19	grey
SUGT1P1	grey
SULT1C4	grey
SULT2B1	grey
SUMO4	grey
SUSD5	grey
SYCE1	grey
SYCP2L	grey
SYN3	grey
SYNC	grey
SYNM	grey
SYNPO2	grey
SYS1	grey
SYT1	grey
SYT3	grey
SYTL5	grey
TAC3	grey
TACR2	grey
TACSTD2	grey
TANC1	grey
TARM1	grey

TAS2R10	grey
TAS2R13	grey
TAS2R19	grey
TAS2R31	grey
TAS2R50	grey
TAS2R60	grey
TBC1D3P1-DHX40P1	grey
TBX1	grey
TCEAL2	grey
TCP10L	grey
TCTE1	grey
TCTEX1D1	grey
TDH	grey
TDRD1	grey
TEAD1	grey
TECTA	grey
TEK	grey
TEKT2	grey
TEKT3	grey
TEKT4P2	grey
TEKT5	grey
TERT	grey
TEX14	grey
TF	grey
TGFB2	grey
THAP10	grey
THEM5	grey
THNSL2	grey
THRB	grey
THSD1	grey
TIFAB	grey
TIGD6	grey
TIMP3	grey
TLCD1	grey
TLE6	grey
TLN2	grey
TLX2	grey
TM4SF20	grey
TMC2	grey
TMC3	grey
TMED6	grey
TMED7-TICAM2	grey
TMEM108	grey
TMEM132C	grey
TMEM133	grey
TMEM136	grey
TMEM139	grey
TMEM150C	grey
TMEM151B	grey
TMEM159	grey
TMEM163	grey
TMEM17	grey
TMEM171	grey
TMEM176A	grey
TMEM176B	grey
TMEM178	grey
TMEM200B	grey
TMEM212	grey
TMEM217	grey
TMEM231	grey
TMEM236	grey
TMEM37	grey
TMEM45A	grey
TMEM45B	grey
TMEM69	grey
TMEM81	grey
TMPRSS3	grey
TMTC1	grey

TNFRSF13B	grey
TNFRSF19	grey
TNFSF11	grey
TNFSF9	grey
TNIP3	grey
TNR	grey
TOB2P1	grey
TOMM20L	grey
TP53TG5	grey
TPBG	grey
TPD52L1	grey
TPSAB1	grey
TREM2	grey
TREML4	grey
TRIM36	grey
TRIM45	grey
TRIP13	grey
TRNP1	grey
TRO	grey
TROAP	grey
TRPC5	grey
TRPC6	grey
TRPM8	grey
TRPV3	grey
TSHR	grey
TSKS	grey
TSNAXIP1	grey
TSPAN1	grey
TSPAN10	grey
TSPAN15	grey
TSPY26P	grey
TSPYL6	grey
TTC23	grey
TTC39A	grey
TTC8	grey
TTLL10	grey
TTLL11	grey
TTLL13	grey
TTLL2	grey
TTLL7	grey
TUBA3E	grey
TUBA4B	grey
TUBB3	grey
TUBBP5	grey
TULP2	grey
TUSC5	grey
TXNDC2	grey
TYMS	grey
TYRO3	grey
TYRO3P	grey
TYW1B	grey
UBE2D4	grey
UBE2E2	grey
UBQLNL	grey
UBXN10	grey
UCHL1	grey
ULBP2	grey
ULBP3	grey
ULK4	grey
UMODL1	grey
UNC13A	grey
UPB1	grey
UPK1A	grey
UPK1B	grey
UQCRHL	grey
USP2	grey
USP43	grey
USP49	grey

UTS2D	grey
VCAM1	grey
VLDLR	grey
VN1R1	grey
VN1R2	grey
VPS37D	grey
VSIG10L	grey
VSIG4	grey
VSNL1	grey
VSTM2L	grey
VSTM4	grey
VTN	grey
VWA5B2	grey
VWDE	grey
WASF1	grey
WASH1	grey
WASH5P	grey
WBP2NL	grey
WDR31	grey
WDR49	grey
WDR63	grey
WDR64	grey
WDR66	grey
WDR88	grey
WDR96	grey
WEE2	grey
WNK4	grey
WNT16	grey
WNT2B	grey
WNT3	grey
WNT4	grey
WNT5B	grey
WNT7B	grey
WNT8B	grey
WSCD2	grey
WTIP	grey
WWC1	grey
WWOX	grey
WWTR1	grey
XCR1	grey
XG	grey
XKR3	grey
XKR9	grey
XRCC2	grey
YBX2	grey
YY2	grey
ZBTB20-AS1	grey
ZBTB32	grey
ZBTB49	grey
ZBTB7C	grey
ZBTB8A	grey
ZC3HAV1L	grey
ZCCHC12	grey
ZDHHC15	grey
ZFP112	grey
ZFP57	grey
ZHX1-C8ORF76	grey
ZMAT4	grey
ZNF114	grey
ZNF132	grey
ZNF155	grey
ZNF311	grey
ZNF321P	grey
ZNF334	grey
ZNF389	grey
ZNF425	grey
ZNF433	grey
ZNF462	grey

ZNF474	grey
ZNF521	grey
ZNF556	grey
ZNF704	grey
ZNF726	grey
ZNF727	grey
ZNF774	grey
ZNF79	grey
ZNF826P	grey
ZNF843	grey
ZNRF3	grey
ZPBP2	grey
ZSCAN12P1	grey
ZSCAN23	grey
ZSWIM3	grey
ZWINT	grey
ZYG11A	grey
ABO	grey60
ABTB2	grey60
ACOT11	grey60
ADORA3	grey60
ALOX15	grey60
ASB2	grey60
ASRGL1	grey60
BACE2	grey60
CACNG6	grey60
CAT	grey60
CCL23	grey60
CEBPE	grey60
CYP4F12	grey60
CYSLTR2	grey60
EMR4P	grey60
EPN2	grey60
FAM124B	grey60
GPR44	grey60
HRASLS5	grey60
IDO1	grey60
IL1RL1	grey60
IL34	grey60
IL5RA	grey60
KCTD15	grey60
LGALS12	grey60
MGAT3	grey60
OLIG1	grey60
OLIG2	grey60
P2RY2	grey60
PIK3R6	grey60
PMP22	grey60
PRSS33	grey60
PRSS41	grey60
SEMA7A	grey60
SIGLEC8	grey60
SLC29A1	grey60
SMPD3	grey60
SORD	grey60
SPNS3	grey60
SRGAP3	grey60
TEC	grey60
THBS4	grey60
VSTM1	grey60
ZBTB42	grey60
AGRN	lightcyan
BST2	lightcyan
CCL2	lightcyan
CMPK2	lightcyan
CTSL1	lightcyan
DDX58	lightcyan
DDX60	lightcyan

DDX60L	lightcyan
DHX58	lightcyan
EIF2AK2	lightcyan
EIF3L	lightcyan
EPSTI1	lightcyan
EXOC3L1	lightcyan
FBXO39	lightcyan
FLJ42418	lightcyan
HERC5	lightcyan
HERC6	lightcyan
IFI44	lightcyan
IFI44L	lightcyan
IFI6	lightcyan
IFIH1	lightcyan
IFIT1	lightcyan
IFIT2	lightcyan
IFIT3	lightcyan
IFIT5	lightcyan
IFITM3	lightcyan
IRF7	lightcyan
ISG15	lightcyan
KLHDC7B	lightcyan
LAMP3	lightcyan
LILRB5	lightcyan
LOC100133669	lightcyan
LY6E	lightcyan
MT2A	lightcyan
MX1	lightcyan
NCRNA00256A	lightcyan
OAS1	lightcyan
OAS2	lightcyan
OAS3	lightcyan
OASL	lightcyan
ODF3B	lightcyan
OTOF	lightcyan
PARP12	lightcyan
PHF11	lightcyan
PLSCR1	lightcyan
PNPT1	lightcyan
RSAD2	lightcyan
RSPH9	lightcyan
RTP4	lightcyan
RUFY4	lightcyan
SAMD4A	lightcyan
SAMD9L	lightcyan
SIGLEC1	lightcyan
SPATS2L	lightcyan
TDRD7	lightcyan
TOR1B	lightcyan
TRIM22	lightcyan
TRIM69	lightcyan
USP18	lightcyan
XAF1	lightcyan
ZBP1	lightcyan
ZCCHC2	lightcyan
ADRB2	lightgreen
C12orf75	lightgreen
C17orf54	lightgreen
CD226	lightgreen
DSG3	lightgreen
ELOVL6	lightgreen
ENPP4	lightgreen
ENPP5	lightgreen
EPHB4	lightgreen
ESM1	lightgreen
FAM108C1	lightgreen
FAM70A	lightgreen
GZMA	lightgreen

GZMK	lightgreen
HOPX	lightgreen
ID2	lightgreen
IFNG	lightgreen
KATNAL1	lightgreen
KIF21A	lightgreen
KLRB1	lightgreen
KLRC1	lightgreen
KLRC2	lightgreen
KLRC3	lightgreen
KLRC4	lightgreen
KLRF1	lightgreen
KLRK1	lightgreen
LOC387895	lightgreen
LPAL2	lightgreen
MYBL1	lightgreen
MYO6	lightgreen
PDGFD	lightgreen
PHLDA1	lightgreen
PHLDB2	lightgreen
SLC4A4	lightgreen
STYK1	lightgreen
TGFBR3	lightgreen
TLR3	lightgreen
TMEM22	lightgreen
XCL1	lightgreen
XCL2	lightgreen
ZNF365	lightgreen
ZNF600	lightgreen
AKAP12	lightyellow
C10orf82	lightyellow
C1orf150	lightyellow
C1orf186	lightyellow
CA8	lightyellow
CCNA1	lightyellow
CPA3	lightyellow
CTNNA2	lightyellow
CYP11A1	lightyellow
ENPP3	lightyellow
EPAS1	lightyellow
GATA2	lightyellow
HDC	lightyellow
HPGDS	lightyellow
HRH4	lightyellow
ITGB8	lightyellow
KLHDC7A	lightyellow
KRT81	lightyellow
LOC100130899	lightyellow
LOC201477	lightyellow
LOC399940	lightyellow
LOC728084	lightyellow
LOC81691	lightyellow
MS4A2	lightyellow
NTRK1	lightyellow
PRSS3	lightyellow
PTGER3	lightyellow
SLC27A2	lightyellow
SLC2A10	lightyellow
SLC45A3	lightyellow
TEX101	lightyellow
THSD7A	lightyellow
TRIM64B	lightyellow
3-Mar	magenta
ABCB4	magenta
ADAM28	magenta
ADARB1	magenta
ADD2	magenta
AFF3	magenta

AHNAK2	magenta
AKAP6	magenta
BANK1	magenta
BCL11A	magenta
BCL7A	magenta
BLK	magenta
BLNK	magenta
C11orf80	magenta
C1orf220	magenta
CABYR	magenta
CBLN3	magenta
CCDC165	magenta
CCR6	magenta
CD180	magenta
CD19	magenta
CD200	magenta
CD22	magenta
CD24	magenta
CD40	magenta
CD72	magenta
CD79A	magenta
CD79B	magenta
CD83	magenta
CDCA7L	magenta
CDHR3	magenta
CELSR1	magenta
CLEC17A	magenta
CNR2	magenta
CNTNAP2	magenta
COBLL1	magenta
COCH	magenta
COL19A1	magenta
COL4A3	magenta
COL4A4	magenta
CORO2B	magenta
CR2	magenta
CTGF	magenta
CXCR5	magenta
DBNDD1	magenta
DENND5B	magenta
DIRAS1	magenta
DPF3	magenta
DTX1	magenta
E2F5	magenta
EBF1	magenta
EGR3	magenta
EML6	magenta
ENAH	magenta
EVC2	magenta
FAM129C	magenta
FAM177B	magenta
FARP2	magenta
FBXO10	magenta
FCER2	magenta
FCRL1	magenta
FCRL2	magenta
FCRL3	magenta
FCRL5	magenta
FCRLA	magenta
FLJ41484	magenta
GGA2	magenta
GHRL	magenta
GNG7	magenta
GYLTL1B	magenta
HLA-DOA	magenta
HLA-DOB	magenta
HS3ST1	magenta
HTR3A	magenta

ICOSLG	magenta
IL28RA	magenta
IRF4	magenta
KCNG1	magenta
KCNH8	magenta
KCNIP2	magenta
KIAA0125	magenta
KLF8	magenta
KLHL14	magenta
L1CAM	magenta
LAMA5	magenta
LAMC1	magenta
LARGE	magenta
LCN10	magenta
LIX1	magenta
LOC100506178	magenta
LOC100506930	magenta
LOC100507043	magenta
LOC254099	magenta
LOC283663	magenta
LOC284749	magenta
LOC375196	magenta
LOC96610	magenta
MACROD2	magenta
MAP2	magenta
MGC39372	magenta
MICAL3	magenta
MMP11	magenta
MOBKL2B	magenta
MOXD1	magenta
MRPL42P5	magenta
MS4A1	magenta
MYBPC2	magenta
NCRNA00287	magenta
NEK8	magenta
NETO1	magenta
NIPAL4	magenta
NXPH4	magenta
OLFML2A	magenta
OSBPL10	magenta
P2RX5	magenta
P2RX5-TAX1BP3	magenta
PARM1	magenta
PAWR	magenta
PAX5	magenta
PCDH9	magenta
PEG10	magenta
PHACTR1	magenta
PIK3C2B	magenta
PKIG	magenta
PLEKHG1	magenta
PMEPA1	magenta
PNOC	magenta
POU2AF1	magenta
PPAPDC1B	magenta
PRICKLE1	magenta
PTPRK	magenta
QRSL1	magenta
QSOX2	magenta
RAPGEF5	magenta
RASGRP3	magenta
RUNDC2C	magenta
SCN4A	magenta
SEL1L3	magenta
SEMA3G	magenta
SETBP1	magenta
SIGLEC6	magenta
SLC15A2	magenta

SLC2A5		magenta
SLC5A9		magenta
SLC9A7		magenta
SNX22		magenta
SOBP		magenta
SPIB		magenta
SPRY1		magenta
STAG3		magenta
STAP1		magenta
STRBP		magenta
SWAP70		magenta
SYBU		magenta
SYNPO		magenta
TCF4		magenta
TCL1A		magenta
TCL1B		magenta
TCL6		magenta
TNFRSF13C		magenta
TPSB2		magenta
TSPAN13		magenta
TSPAN3		magenta
VPREB3		magenta
ZNF418		magenta
ZNF532		magenta
ZNF860		magenta
	8-Sep	midnightblue
ADAM1		midnightblue
ADAT2		midnightblue
ATF7IP2		midnightblue
C16orf7		midnightblue
C19orf38		midnightblue
C5orf63		midnightblue
CA5B		midnightblue
CAPRIN2		midnightblue
CARS2		midnightblue
CNST		midnightblue
DDX5		midnightblue
FES		midnightblue
FXC1		midnightblue
GADD45B		midnightblue
GATC		midnightblue
GBGT1		midnightblue
GGT1		midnightblue
GOLGA8A		midnightblue
HAUS4		midnightblue
HK3		midnightblue
HNRPDL		midnightblue
ICA1L		midnightblue
KCTD7		midnightblue
LOC100506776		midnightblue
MFSD7		midnightblue
MOAP1		midnightblue
MRPS25		midnightblue
MTERFD2		midnightblue
NARF		midnightblue
NR3C2		midnightblue
OFD1		midnightblue
OSBPL3		midnightblue
PAIP2B		midnightblue
PER3		midnightblue
PIGG		midnightblue
PIGL		midnightblue
PTCD3		midnightblue
RIC3		midnightblue
S100PBP		midnightblue
S1PR1		midnightblue
SCML4		midnightblue
SEC22C		midnightblue

SEMA6B		midnightblue
SEPT7P2		midnightblue
SERTAD3		midnightblue
SLC11A2		midnightblue
SRSF1		midnightblue
STAT4		midnightblue
SUN1		midnightblue
TARBP1		midnightblue
TBRG1		midnightblue
THSD1P1		midnightblue
TRAF5		midnightblue
TSEN2		midnightblue
UPF3A		midnightblue
WDR75		midnightblue
ZBTB24		midnightblue
ZFP28		midnightblue
ZMYND15		midnightblue
ZNF234		midnightblue
ZNF251		midnightblue
ZNF331		midnightblue
ZNF514		midnightblue
ZNF528		midnightblue
ZNF548		midnightblue
ZNF585B		midnightblue
ZNF831		midnightblue
ZNF841		midnightblue
ZNF850		midnightblue
ZNF880		midnightblue
	2-Mar	pink
A4GALT		pink
ABCC13		pink
ABCC4		pink
ABCG2		pink
ACHE		pink
ADIPOR1		pink
AHSP		pink
ALAS2		pink
ANK1		pink
AQP1		pink
ARHGEF37		pink
BAG1		pink
BAMBI		pink
BCAM		pink
BCL2L1		pink
BEST2		pink
BLVRB		pink
BPGM		pink
C14orf45		pink
C17orf99		pink
C18orf10		pink
C19orf51		pink
C19orf69		pink
C19orf77		pink
C1orf116		pink
C20orf108		pink
C2orf66		pink
C5orf4		pink
C6orf147		pink
C6orf154		pink
C7orf29		pink
C9orf3		pink
C9orf78		pink
CA1		pink
CDC34		pink
CHPT1		pink
CLRN1-AS1		pink
CNN1		pink
CR1L		pink

CSDA	pink
CTSE	pink
DARC	pink
DCAF12	pink
DCAF6	pink
DDX11L9	pink
DNMBP-AS1	pink
DPCD	pink
DPM2	pink
DYRK3	pink
E2F2	pink
ECSIT	pink
EHD2	pink
EMID1	pink
EPB42	pink
EPB49	pink
ESPN	pink
FAM104A	pink
FAM107A	pink
FAM46C	pink
FAM65C	pink
FAM83A	pink
FBXO17	pink
FBXO7	pink
FBXO9	pink
FECH	pink
FHL2	pink
FKBP1B	pink
FKBP8	pink
FTL	pink
GALNT5	pink
GATA1	pink
GCAT	pink
GFAP	pink
GLRX5	pink
GMPR	pink
GPR146	pink
GPX1	pink
GSPT1	pink
GUK1	pink
GYPA	pink
GYPB	pink
GYPC	pink
HAGH	pink
HBA1	pink
HBA2	pink
HBB	pink
HBD	pink
HBG1	pink
HBG2	pink
HBM	pink
HBQ1	pink
HBZ	pink
HEMGN	pink
HEPACAM2	pink
HERC2P4	pink
HIST1H2AI	pink
HIST1H3B	pink
HMBS	pink
HPS1	pink
HSD17B7P2	pink
IFI27	pink
IFIT1B	pink
IGF2BP2	pink
ITLN1	pink
KANK2	pink
KCNH2	pink
KEL	pink

KLC3	pink
KLF1	pink
KLHDC8A	pink
KRT1	pink
KRT79	pink
LGALS3	pink
LOC100132163	pink
LOC100134229	pink
LOC100505536	pink
LOC388588	pink
LOC401109	pink
LY75-CD302	pink
LYL1	pink
MBNL3	pink
MEF2B	pink
MFSD2B	pink
MKRN1	pink
MRC2	pink
MT1L	pink
MXI1	pink
MYL4	pink
NEDD4L	pink
NFIX	pink
NPRL3	pink
NT5M	pink
NUDT4	pink
OAZ1	pink
ODC1	pink
OR2W3	pink
OSBP2	pink
PAGE2B	pink
PAQR9	pink
PBX1	pink
PDE3A	pink
PDZK1IP1	pink
PITHD1	pink
PLEK2	pink
PLVAP	pink
POLL	pink
POLR1D	pink
PPM1A	pink
PRDX5	pink
PRDX6	pink
PTPRF	pink
PTPRN	pink
RAB3IL1	pink
RAG1	pink
RAP1GAP	pink
RBM38	pink
RHAG	pink
RHCE	pink
RHOV	pink
RILP	pink
RIOK3	pink
RNF10	pink
RPL3L	pink
RUNDC3A	pink
SEC14L4	pink
SELENBP1	pink
SESN3	pink
SFRP2	pink
SGIP1	pink
SHARPIN	pink
SHISA4	pink
SHISA7	pink
SIAH2	pink
SIM2	pink
SLC14A1	pink

SLC25A37	pink
SLC25A39	pink
SLC2A4	pink
SLC38A5	pink
SLC4A1	pink
SLC6A19	pink
SLC6A8	pink
SLC6A9	pink
SNCA	pink
SNORA66	pink
SOX6	pink
SPDYC	pink
SPTA1	pink
SRRD	pink
ST6GALNAC4	pink
STRADB	pink
STX2	pink
TAL1	pink
TESC	pink
TFDP1	pink
TGM2	pink
TMC5	pink
TMCC2	pink
TMEM111	pink
TMEM158	pink
TMEM56	pink
TMEM98	pink
TMOD1	pink
TMPRSS9	pink
TNS1	pink
TRIM10	pink
TRIM50	pink
TRIM58	pink
TSPAN5	pink
TSPAN7	pink
TSPO2	pink
TSTA3	pink
TTC25	pink
TUBB2A	pink
TUBB2B	pink
UBA52	pink
UBB	pink
UBXN6	pink
UTS2R	pink
VIPR2	pink
VTI1B	pink
VWCE	pink
WDR45	pink
WNT9A	pink
XK	pink
YBX1	pink
YPEL4	pink
ABCC3	purple
ABLIM3	purple
ACRBP	purple
ACSBG1	purple
ADRA2A	purple
ALOX12	purple
AQP10	purple
ARHGAP6	purple
ARMC3	purple
ASAP2	purple
AVPR1A	purple
BEND2	purple
BET3L	purple
BMP6	purple
C10orf47	purple
C15orf26	purple

C15orf54	purple
C21orf7	purple
C2orf88	purple
C6orf145	purple
C6orf25	purple
C7orf41	purple
C8orf42	purple
CA2	purple
CABP5	purple
CALD1	purple
CAV2	purple
CCDC3	purple
CD9	purple
CDK2AP1	purple
CDKN1A	purple
CDR2L	purple
CLDN5	purple
CLEC1B	purple
CLEC2L	purple
CLU	purple
CMTM5	purple
CTDSPL	purple
CTTN	purple
CXCL5	purple
CXCR2P1	purple
DNM3	purple
EGF	purple
ELOVL7	purple
ENDOD1	purple
ENKUR	purple
ESAM	purple
F2RL3	purple
FAM26E	purple
FLJ44511	purple
FSTL1	purple
FSTL4	purple
GFI1B	purple
GNAZ	purple
GNG11	purple
GP6	purple
GP9	purple
GRB14	purple
GRK5	purple
GUCY1A3	purple
GUCY1B3	purple
HIST1H2AG	purple
HIST1H2BG	purple
HIST1H2BH	purple
HIST1H2BJ	purple
HOMER2	purple
HRASLS	purple
ITGA2B	purple
ITGB3	purple
ITGB5	purple
KCND3	purple
KIAA1211	purple
LOC100287036	purple
LOC100506035	purple
LOC100506343	purple
LOC151174	purple
LOC283089	purple
LTBP1	purple
LY6G6E	purple
LY6G6F	purple
MAOB	purple
MAP1B	purple
MEIS1	purple
MFAP3L	purple

MGLL	purple
MMD	purple
MMRN1	purple
MYL9	purple
MYLK	purple
NAT8B	purple
NRGN	purple
PARD3	purple
PCSK6	purple
PCYT1B	purple
PDE5A	purple
PDGFA	purple
PDGFRA	purple
PDLIM1	purple
PEAR1	purple
PF4	purple
PF4V1	purple
PGRMC1	purple
PKHD1L1	purple
PLOD2	purple
PLXNB3	purple
PPBP	purple
PRKAR2B	purple
PROS1	purple
psiTPTE22	purple
PTCRA	purple
PTGES	purple
PTGS1	purple
PVALB	purple
RAB6B	purple
RBPMS2	purple
RGS6	purple
RHOBTB1	purple
SAMD14	purple
SDC4	purple
SDPR	purple
SEC14L5	purple
SELP	purple
SERPINE1	purple
SH3BGRL2	purple
SH3TC2	purple
SLC24A3	purple
SLC35D3	purple
SLC6A4	purple
SLC8A3	purple
SMOX	purple
SPARC	purple
SPOCD1	purple
ST7	purple
STON2	purple
SYTL4	purple
TFPI	purple
TGFB1I1	purple
TMEM40	purple
TNFSF4	purple
TOM1L1	purple
TPM1	purple
TREML1	purple
TRIM40	purple
TSC22D1	purple
TSPAN33	purple
TSPAN9	purple
TTC18	purple
TTC7B	purple
TUBA8	purple
TUBB1	purple
VEGFC	purple
VEPH1	purple

VIL1	purple
VSIG2	purple
VWF	purple
WASF3	purple
ZNF492	purple
A2LD1	red
ABHD12B	red
ACPP	red
ACSL1	red
ACTR2	red
ADAM17	red
ADAMTSL5	red
ADM	red
AGFG1	red
AGPAT9	red
AGTPBP1	red
AHCTF1	red
AKR7A3	red
ALDH1A2	red
ALOX5	red
ALOX5AP	red
ANKRD6	red
ANTXR2	red
ANXA3	red
AP3B2	red
APAF1	red
APBB2	red
APH1B	red
APOA1BP	red
APOBEC3A	red
AQP9	red
ARHGAP19	red
ARHGEF19	red
ARL11	red
ARL4D	red
ARMCX6	red
ARPC5	red
ARPC5L	red
ARSB	red
ASAP1	red
ASTN2	red
ATF6	red
ATP11A	red
ATP2C2	red
ATP5G1	red
ATP6V1A	red
ATP7B	red
ATP9A	red
ATXN1	red
ATXN7	red
AVIL	red
AVL9	red
BCL2L11	red
BEND5	red
BEND7	red
BEST1	red
BMX	red
BOD1L	red
BRWD3	red
BST1	red
C10orf116	red
C10orf46	red
C15orf50	red
C17orf81	red
C19orf59	red
C1orf98	red
C21orf59	red
C22orf46	red

C3AR1	red
C3orf75	red
C5orf32	red
C7orf34	red
C7orf53	red
C7orf71	red
C9orf152	red
C9orf68	red
CA4	red
CACNA1E	red
CAPN12	red
CAPN13	red
CAPN8	red
CASP4	red
CASP5	red
CBS	red
CCDC125	red
CCDC147	red
CCDC64B	red
CCNI	red
CCNJL	red
CCR1	red
CCR2	red
CCT4	red
CD177	red
CD300LD	red
CD55	red
CD59	red
CDA	red
CDC123	red
CDC42EP3	red
CDH2	red
CDK14	red
CDK19	red
CDK4	red
CDKL5	red
CEACAM3	red
CEACAM4	red
CENPV	red
CEP63	red
CHRNA10	red
CIR1	red
CLDN6	red
CLEC1A	red
CLMN	red
CNGA4	red
CNTNAP3	red
COMMD7	red
COQ4	red
CR1	red
CSRNP1	red
CST7	red
CTBP2	red
CUL3	red
CWC25	red
CXCR1	red
CYBB	red
CYP1B1-AS1	red
CYTH4	red
DCTPP1	red
DGAT2	red
DHRS12	red
DHRS13	red
DHRS7	red
DHRSX	red
DISC2	red
DLEU2	red
DMRTC2	red

DMXL2	red
DNAH14	red
DNAJB11	red
DNAJC3	red
DOCK1	red
DOCK4	red
DOK4	red
DPYD	red
DUSP13	red
E2F3	red
EBP	red
ECHDC3	red
EDEM2	red
EFCAB2	red
EIF2C4	red
ELMOD3	red
ELOVL5	red
EMR3	red
ENO3	red
ENTPD1	red
ETS2	red
EVI5	red
EXT1	red
F5	red
F8A1	red
FAM120A	red
FAM157B	red
FAM169B	red
FAM49A	red
FAM65B	red
FCGR1A	red
FCGR1B	red
FCGR1C	red
FCGR2A	red
FCGR3B	red
FFAR2	red
FFAR3	red
FGF13	red
FLI1	red
FLJ10661	red
FLJ39051	red
FLOT1	red
FOXO3	red
FPR1	red
FPR2	red
FRMD4B	red
FRY	red
FYB	red
GABARAPL1	red
GALNT14	red
GBE1	red
GCM1	red
GDE1	red
GIMAP1	red
GLIPR2	red
GLT1D1	red
GMPR2	red
GNAQ	red
GOLGA7B	red
GPR141	red
GPR17	red
GPR84	red
GPR89A	red
GPR97	red
GPSM2	red
GRIP2	red
GSDMC	red
GSK3B	red

GTSE1	red
H3F3B	red
HAL	red
HAS1	red
HCAR2	red
HCAR3	red
HCG27	red
HEY1	red
HIF1A	red
HIST1H1T	red
HIST1H2BE	red
HIST1H4D	red
HIVEP1	red
HMGCR	red
HP	red
HPSE	red
HSPA6	red
IDH1	red
IDS	red
IFNAR1	red
IFNGR2	red
IGSF6	red
IL10RB	red
IL13RA1	red
IL18RAP	red
IL1B	red
IL1R1	red
IL1R2	red
IL1RN	red
IMP3	red
INHBB	red
INPP5A	red
INSC	red
IQGAP1	red
IRAK3	red
ITGAD	red
ITM2B	red
ITPRIP	red
JHDM1D	red
JMJD1C	red
KAL1	red
KAZN	red
KCNE1	red
KCNH7	red
KCNJ15	red
KCNS1	red
KDM1B	red
KIAA0232	red
KIAA0319	red
KIAA1257	red
KIAA1539	red
KLF6	red
KPNA1	red
KPNA4	red
KRT23	red
LAMP2	red
LBR	red
LILRA6	red
LILRB3	red
LIMD1	red
LITAF	red
LMNB1	red
LMO4	red
LOC100124692	red
LOC100131289	red
LOC100233209	red
LOC100288432	red
LOC100289178	red

LOC100505702	red
LOC100506229	red
LOC100507404	red
LOC149837	red
LOC151475	red
LOC284751	red
LOC285084	red
LOC285696	red
LOC338799	red
LOC399715	red
LOC401233	red
LOC440461	red
LOC731424	red
LRG1	red
LRRC4	red
LRRC6	red
LRRFIP1	red
LYN	red
LYVE1	red
MAK	red
MANSC1	red
MARCKS	red
MBOAT2	red
MCMBP	red
MCTP2	red
ME2	red
MEGF9	red
MLLT11	red
MME	red
MPZL3	red
MRPL9	red
MRVI1	red
MSL1	red
MTMR10	red
MXD1	red
MYO10	red
MYO5A	red
MZT2A	red
NAIP	red
NCF4	red
NCOA1	red
NCOA2	red
NCRNA00173	red
NECAB2	red
NEDD9	red
NFE2	red
NFE2L2	red
NFIL3	red
NFYA	red
NHP2L1	red
NLN	red
NMNAT2	red
NPEPPS	red
NPL	red
NPTN	red
NQO2	red
NR6A1	red
NRADDP	red
NRBF2	red
NRD1	red
NSUN7	red
NT5C2	red
NUDT5	red
NUP50	red
NUPL1	red
OLAH	red
OPLAH	red
ORM1	red

ORM2	red
OSBPL11	red
OSBPL1A	red
OSGEP	red
OSM	red
OTX1	red
OXER1	red
PAK2	red
PANK2	red
PAPSS2	red
PCBP4	red
PCNX	red
PDCD2L	red
PDCD7	red
PDE4B	red
PDK3	red
PDRG1	red
PDZD3	red
PDZD8	red
PELI2	red
PELO	red
PFKFB4	red
PGLYRP1	red
PGS1	red
PHB	red
PHC2	red
PIAS1	red
PICALM	red
PIF1	red
PLAU	red
PLB1	red
PLBD1	red
PLD1	red
PLIN5	red
PLXDC2	red
PLXNC1	red
PNP	red
PNPLA1	red
POLD3	red
PPAP2B	red
PPP1R3B	red
PPP3CC	red
PPP3R1	red
PPP3R2	red
PPP4R1	red
PROK2	red
PRR11	red
PRRG4	red
PRUNE	red
PTPRE	red
PVRL2	red
QPCT	red
RAB1A	red
RAB27A	red
RAB31	red
RANBP1	red
RAPGEF2	red
RASGRP4	red
RASSF3	red
REPS2	red
RHOH	red
RMND5A	red
RNF111	red
RNF130	red
RNF145	red
RNF149	red
RNF19B	red
ROD1	red

ROPN1L	red
RPL32P3	red
RS1	red
RTN3	red
S100A11	red
SAMD15	red
SBF2	red
SBNO2	red
SCARF1	red
SCARNA3	red
SDCBP	red
SDHAP3	red
SEC24D	red
SECTM1	red
SERPINB1	red
SH3GLB1	red
SHROOM4	red
SIRPD	red
SIT1	red
SLC12A6	red
SLC15A4	red
SLC22A1	red
SLC22A14	red
SLC22A4	red
SLC26A8	red
SLC2A11	red
SLC45A4	red
SLC8A1	red
SMAGP	red
SNAP23	red
SNORD89	red
SNTA1	red
SNTG2	red
SOCS3	red
SOD2	red
SOS2	red
SPATC1	red
SPIN2B	red
SQRDL	red
SRPK1	red
SRPK2	red
ST3GAL4	red
ST3GAL5	red
ST6GALNAC2	red
STK3	red
STX11	red
STX12	red
STX3	red
STX6	red
SURF2	red
SUSD3	red
SYN2	red
SYNGR3	red
TARBP2	red
TBC1D30	red
TCF19	red
TCP11L2	red
TDRD9	red
TEAD3	red
TEX264	red
TG	red
TGM3	red
TIAM2	red
TIMM44	red
TLE4	red
TLR2	red
TLR4	red
TLR5	red

TM9SF2	red
TMCC1	red
TMCC3	red
TMCO3	red
TMEM132D	red
TMEM154	red
TMLHE	red
TMX4	red
TNFRSF1A	red
TNFSF14	red
TOR1AIP2	red
TPX2	red
TREM1	red
TREML2	red
TRIB1	red
TRIM9	red
TRPM6	red
TRPV5	red
TSEN54	red
TSHZ3	red
TSPAN16	red
UBE2R2	red
UBR2	red
UHRF1BP1L	red
UIMC1	red
UNQ6494	red
URM1	red
USP3	red
USP32	red
VAMP3	red
VPS8	red
VSIG10	red
WAC	red
WDFY3	red
WDR26	red
WIPF1	red
WLS	red
WNT10B	red
YIPF1	red
ZAK	red
ZCCHC18	red
ZDHHC19	red
ZFP36	red
ZFP36L1	red
ZNF143	red
ZNF200	red
ZNF217	red
ZNF259	red
ZNF438	red
ZNF552	red
ZNF578	red
ZNF608	red
ZSWIM6	red
ARPC1A	salmon
ATP6V1E2	salmon
BLVRA	salmon
BTF3	salmon
BUD31	salmon
C11orf67	salmon
C14orf43	salmon
C20orf107	salmon
C4orf27	salmon
C6orf203	salmon
C9orf156	salmon
CACYBP	salmon
CCDC56	salmon
CCDC77	salmon
CETN2	salmon

CHKB	salmon
CHKB-CPT1B	salmon
COMMD1	salmon
CWF19L1	salmon
CXorf26	salmon
DIABLO	salmon
EBNA1BP2	salmon
EIF3H	salmon
EIF3M	salmon
FAU	salmon
GSTM3	salmon
GTF3C6	salmon
HDHD1	salmon
HMGN1	salmon
HNRNPA1	salmon
HS3ST3B1	salmon
ICT1	salmon
IL20RB	salmon
ISY1	salmon
ITGB1BP1	salmon
LOC100505783	salmon
LOC400927	salmon
MDH1	salmon
MED10	salmon
MRPS16	salmon
MRPS7	salmon
MYEOV2	salmon
MYL12A	salmon
MYL5	salmon
NACA	salmon
NAP1L1	salmon
NBPF10	salmon
NDUFC2	salmon
NOP16	salmon
NPM1	salmon
PARL	salmon
PCNA	salmon
PDCL3	salmon
PER2	salmon
PLAC8L1	salmon
POLR2H	salmon
POMP	salmon
POP4	salmon
PPIA	salmon
PRDX1	salmon
PSMA5	salmon
PSMB6	salmon
RAN	salmon
RANGRF	salmon
RARRES3	salmon
RPIA	salmon
RPL10	salmon
RPL10A	salmon
RPL12	salmon
RPL13A	salmon
RPL14	salmon
RPL19	salmon
RPL22	salmon
RPL23A	salmon
RPL24	salmon
RPL27A	salmon
RPL3	salmon
RPL32	salmon
RPL35A	salmon
RPL36	salmon
RPL37	salmon
RPL37A	salmon
RPL4	salmon

RPL5	salmon
RPL7A	salmon
RPS11	salmon
RPS12	salmon
RPS13	salmon
RPS14	salmon
RPS16	salmon
RPS18	salmon
RPS19	salmon
RPS20	salmon
RPS21	salmon
RPS23	salmon
RPS25	salmon
RPS27A	salmon
RPS3	salmon
RPS4X	salmon
RPS5	salmon
RPS6	salmon
RPS8	salmon
RPSA	salmon
SKP1	salmon
SNHG6	salmon
SNRPD2	salmon
SNRPD3	salmon
SNUPN	salmon
SPAG7	salmon
SRPRB	salmon
SSBP1	salmon
STX8	salmon
SUCLG1	salmon
SUGT1	salmon
THYN1	salmon
TICAM2	salmon
TOMM6	salmon
TRAPPC3	salmon
TRNAU1AP	salmon
UBAC2	salmon
UQCRFS1	salmon
USP42	salmon
UXT	salmon
VDAC3	salmon
ZGLP1	salmon
ZMYND17	salmon
ZNRD1	salmon
AARSD1	tan
ABCD4	tan
ACTR5	tan
AEN	tan
AKIRIN2	tan
ALDH8A1	tan
AP3M2	tan
AP4B1	tan
ARMCX2	tan
ASNS	tan
ATG16L1	tan
ATP1A1OS	tan
BPHL	tan
C10orf58	tan
C12orf65	tan
C15orf17	tan
C17orf67	tan
C17orf75	tan
C2orf89	tan
C3orf18	tan
C8orf33	tan
CARKD	tan
CCDC84	tan
CD302	tan

CDK20	tan
CDK5RAP1	tan
CIAPIN1	tan
CLYBL	tan
CNPY4	tan
COQ10A	tan
COQ6	tan
CRIP3	tan
CXCL16	tan
DEM1	tan
DEPDC7	tan
DFNB59	tan
DNAH3	tan
DNAJC9	tan
DPH2	tan
DSN1	tan
DUSP6	tan
EXOSC2	tan
EXOSC7	tan
FAM86B1	tan
FAM86HP	tan
FANCE	tan
FGR	tan
FLJ42351	tan
GALNT12	tan
GCET2	tan
GEMIN8	tan
GPD1	tan
GTF2IRD2	tan
GTF2IRD2B	tan
HIST1H2BC	tan
HIST1H2BD	tan
HIST2H2BF	tan
HKR1	tan
HPCAL4	tan
HSD17B7	tan
IL24	tan
ILKAP	tan
ITGA10	tan
KLKB1	tan
LDHB	tan
LETMD1	tan
LIAS	tan
LOC100129722	tan
LOC100506469	tan
LOC100506548	tan
LOC100506990	tan
LOC100507501	tan
LOC283693	tan
LOC284440	tan
LOC285359	tan
LOC386758	tan
LOC439949	tan
LOC728537	tan
LOC93432	tan
LUC7L	tan
MAGEF1	tan
MEAF6	tan
METTL17	tan
METTL3	tan
MSTO2P	tan
NAP1L4	tan
NPR2	tan
NUP43	tan
NVL	tan
OCM	tan
OVGP1	tan
PCID2	tan

PDE6B		tan
PEX11B		tan
PMS2P1		tan
POLR1C		tan
POPDC2		tan
PPIE		tan
PPIH		tan
PRPSAP2		tan
PRR3		tan
PVT1		tan
RBFA		tan
RDH16		tan
RNF216L		tan
RPP38		tan
RPS10P7		tan
RRN3P1		tan
RSAD1		tan
SDCCAG3		tan
SETD4		tan
SIRT5		tan
SLC25A17		tan
SLC25A4		tan
SMYD5		tan
SNHG4		tan
STMN1		tan
SUPT7L		tan
TCEA3		tan
TGFB3		tan
TSC22D2		tan
TTC12		tan
TTC28-AS1		tan
TTC39C		tan
UQCC		tan
VSIG1		tan
WDR77		tan
ZNF133		tan
ZNF185		tan
ZNF202		tan
ZNF211		tan
ZNF232		tan
ZNF266		tan
ZNF329		tan
ZNF343		tan
ZNF419		tan
ZNF44		tan
ZNF530		tan
ZNF542		tan
ZNF544		tan
ZNF563		tan
ZNF671		tan
ZNF69		tan
ZNF773		tan
ZNF789		tan
	1-Mar	turquoise
	5-Mar	turquoise
	6-Mar	turquoise
	7-Mar	turquoise
	7-Sep	turquoise
	9-Sep	turquoise
	10-Sep	turquoise
	14-Sep	turquoise
	15-Sep	turquoise
AAAS		turquoise
AACS		turquoise
AAMP		turquoise
AANAT		turquoise
AASDHPPT		turquoise
ABCA1		turquoise

ABCA10	turquoise
ABCA3	turquoise
ABCA5	turquoise
ABCA7	turquoise
ABCB10	turquoise
ABCB6	turquoise
ABCC2	turquoise
ABCD1	turquoise
ABCD2	turquoise
ABCD3	turquoise
ABCE1	turquoise
ABCF3	turquoise
ABHD10	turquoise
ABHD12	turquoise
ABHD13	turquoise
ABHD14B	turquoise
ABHD3	turquoise
ABI1	turquoise
ABI2	turquoise
ABI3	turquoise
ABLIM2	turquoise
ABR	turquoise
ACAD10	turquoise
ACAD9	turquoise
ACADM	turquoise
ACADSB	turquoise
ACADVL	turquoise
ACAP1	turquoise
ACAP2	turquoise
ACAT1	turquoise
ACAT2	turquoise
ACBD3	turquoise
ACBD5	turquoise
ACBD6	turquoise
ACCN2	turquoise
ACD	turquoise
ACOT13	turquoise
ACOT7	turquoise
ACP2	turquoise
ACPL2	turquoise
ACSF2	turquoise
ACSF3	turquoise
ACSL4	turquoise
ACSL6	turquoise
ACSS1	turquoise
ACTL6A	turquoise
ACTL9	turquoise
ACTN4	turquoise
ACTR1A	turquoise
ACTR6	turquoise
ACVR1C	turquoise
ACVR2A	turquoise
ACVRL1	turquoise
ACY3	turquoise
ADA	turquoise
ADAM10	turquoise
ADAM15	turquoise
ADAM22	turquoise
ADAMTS5	turquoise
ADAMTS6	turquoise
ADAP1	turquoise
ADC	turquoise
ADCK1	turquoise
ADCK2	turquoise
ADCK3	turquoise
ADD3	turquoise
ADI1	turquoise
ADM2	turquoise

ADORA1	turquoise
ADRBK1	turquoise
ADRM1	turquoise
ADSSL1	turquoise
AEBP1	turquoise
AEBP2	turquoise
AFF4	turquoise
AGAP2	turquoise
AGAP3	turquoise
AGBL3	turquoise
AGER	turquoise
AGFG2	turquoise
AGGF1	turquoise
AGL	turquoise
AGPAT5	turquoise
AGPS	turquoise
AHR	turquoise
AIDA	turquoise
AIFM3	turquoise
AIM2	turquoise
AIMP1	turquoise
AIMP2	turquoise
AK3	turquoise
AKAP11	turquoise
AKAP5	turquoise
AKAP7	turquoise
AKAP8L	turquoise
AKD1	turquoise
AKIP1	turquoise
AKIRIN1	turquoise
AKIRIN2-AS1	turquoise
AKR1B1	turquoise
AKR7A2	turquoise
AKT1	turquoise
AKTIP	turquoise
ALCAM	turquoise
ALDH16A1	turquoise
ALDH4A1	turquoise
ALDOA	turquoise
ALG1	turquoise
ALG10	turquoise
ALG10B	turquoise
ALG11	turquoise
ALG13	turquoise
ALG2	turquoise
ALG3	turquoise
ALG6	turquoise
ALKBH4	turquoise
ALKBH5	turquoise
ALKBH8	turquoise
ALS2CR11	turquoise
ALS2CR8	turquoise
AMD1	turquoise
AMMECR1	turquoise
AMN1	turquoise
ANAPC10	turquoise
ANG	turquoise
ANGEL2	turquoise
ANGPT1	turquoise
ANGPTL4	turquoise
ANGPTL7	turquoise
ANKAR	turquoise
ANKHD1-EIF4EBP3	turquoise
ANKIB1	turquoise
ANKK1	turquoise
ANKLE1	turquoise
ANKMY2	turquoise
ANKRD10	turquoise

ANKRD12	turquoise
ANKRD13C	turquoise
ANKRD22	turquoise
ANKRD24	turquoise
ANKRD26	turquoise
ANKRD28	turquoise
ANKRD32	turquoise
ANKRD34B	turquoise
ANKRD44	turquoise
ANKRD46	turquoise
ANKRD49	turquoise
ANKRD50	turquoise
ANKRD57	turquoise
ANKUB1	turquoise
ANLN	turquoise
ANO5	turquoise
ANP32E	turquoise
ANUBL1	turquoise
ANXA1	turquoise
AP1AR	turquoise
AP1B1	turquoise
AP1S2	turquoise
AP2A2	turquoise
AP3M1	turquoise
AP3S2	turquoise
AP4S1	turquoise
APC	turquoise
APEH	turquoise
APEX1	turquoise
APEX2	turquoise
APH1A	turquoise
API5	turquoise
APIP	turquoise
APLF	turquoise
APOBEC3D	turquoise
APOO	turquoise
APPBP2	turquoise
APPL1	turquoise
APTX	turquoise
AQP11	turquoise
ARAF	turquoise
ARAP2	turquoise
ARF4	turquoise
ARFGAP1	turquoise
ARFGAP2	turquoise
ARFGEF2	turquoise
ARFIP1	turquoise
ARG2	turquoise
ARGLU1	turquoise
ARHGAP10	turquoise
ARHGAP11A	turquoise
ARHGAP12	turquoise
ARHGAP22	turquoise
ARHGAP42	turquoise
ARHGAP5	turquoise
ARHGDIA	turquoise
ARHGEF10L	turquoise
ARHGEF2	turquoise
ARID2	turquoise
ARID4A	turquoise
ARID4B	turquoise
ARID5A	turquoise
ARID5B	turquoise
ARL13B	turquoise
ARL15	turquoise
ARL2	turquoise
ARL4A	turquoise
ARL5A	turquoise

ARL5B	turquoise
ARL6IP1	turquoise
ARL6IP5	turquoise
ARL6IP6	turquoise
ARL8B	turquoise
ARMC1	turquoise
ARMC10	turquoise
ARMC7	turquoise
ARMCX5	turquoise
ARMCX5-GPRASP2	turquoise
ARPC4	turquoise
ARPP19	turquoise
ARRB1	turquoise
ARRDC3	turquoise
ARRDC4	turquoise
ARSA	turquoise
ARSK	turquoise
ARV1	turquoise
ARVCF	turquoise
ASAP3	turquoise
ASB13	turquoise
ASB7	turquoise
ASF1A	turquoise
ASGR1	turquoise
ASGR2	turquoise
ASMTL	turquoise
ASMTL-AS1	turquoise
ASNA1	turquoise
ASNSD1	turquoise
ASPH	turquoise
ASPM	turquoise
ASTE1	turquoise
ASTL	turquoise
ATAD1	turquoise
ATAD2	turquoise
ATAD2B	turquoise
ATAD3A	turquoise
ATF1	turquoise
ATF2	turquoise
ATG12	turquoise
ATG16L2	turquoise
ATG4A	turquoise
ATG4B	turquoise
ATG4C	turquoise
ATG5	turquoise
ATG9A	turquoise
ATL2	turquoise
ATP10D	turquoise
ATP11B	turquoise
ATP11C	turquoise
ATP13A2	turquoise
ATP13A3	turquoise
ATP2B1	turquoise
ATP2C1	turquoise
ATP5C1	turquoise
ATP5E	turquoise
ATP5F1	turquoise
ATP5I	turquoise
ATP5L	turquoise
ATP5O	turquoise
ATP6AP1	turquoise
ATP6V1G1	turquoise
ATP7A	turquoise
ATP8A1	turquoise
ATP8A2	turquoise
ATP8B4	turquoise
ATPAF2	turquoise
ATPBD4	turquoise

ATRX	turquoise
ATXN7L2	turquoise
ATXN7L3	turquoise
AUH	turquoise
AURKC	turquoise
AZI1	turquoise
AZI2	turquoise
AZIN1	turquoise
B2M	turquoise
B3GALNT1	turquoise
B3GALT2	turquoise
B3GALTL	turquoise
B3GNT1	turquoise
B3GNT2	turquoise
B3GNT5	turquoise
B4GALT3	turquoise
B4GALT6	turquoise
BACH1	turquoise
BAG2	turquoise
BAG4	turquoise
BAG6	turquoise
BAIAP2	turquoise
BANF1	turquoise
BAP1	turquoise
BAZ1A	turquoise
BAZ2B	turquoise
BBIP1	turquoise
BBS10	turquoise
BBS12	turquoise
BBS7	turquoise
BBX	turquoise
BCAP29	turquoise
BCAP31	turquoise
BCAS2	turquoise
BCAS4	turquoise
BCAT1	turquoise
BCAT2	turquoise
BCKDHB	turquoise
BCL2A1	turquoise
BCL2L15	turquoise
BCLAF1	turquoise
BCR	turquoise
BCRP2	turquoise
BDH2	turquoise
BDP1	turquoise
BET1	turquoise
BGLAP	turquoise
BHLHB9	turquoise
BIN1	turquoise
BIRC2	turquoise
BIRC3	turquoise
BLOC1S2	turquoise
BLZF1	turquoise
BMF	turquoise
BMI1	turquoise
BMP1	turquoise
BMPR1A	turquoise
BMPR2	turquoise
BNIP1	turquoise
BNIP2	turquoise
BNIP3L	turquoise
BNIPL	turquoise
BORA	turquoise
BRCA2	turquoise
BRCC3	turquoise
BRD9	turquoise
BRIP1	turquoise
BRMS1	turquoise

BRMS1L	turquoise
BRP44L	turquoise
BRWD1	turquoise
BTAF1	turquoise
BTBD1	turquoise
BTBD10	turquoise
BTBD3	turquoise
BTD	turquoise
BTF3L4	turquoise
BTLA	turquoise
BUD13	turquoise
BYSL	turquoise
BZW1	turquoise
C10orf11	turquoise
C10orf118	turquoise
C10orf131	turquoise
C10orf2	turquoise
C10orf32	turquoise
C10orf68	turquoise
C11orf1	turquoise
C11orf24	turquoise
C11orf46	turquoise
C11orf49	turquoise
C11orf54	turquoise
C11orf57	turquoise
C11orf58	turquoise
C11orf61	turquoise
C11orf66	turquoise
C11orf68	turquoise
C11orf84	turquoise
C12orf23	turquoise
C12orf26	turquoise
C12orf29	turquoise
C12orf34	turquoise
C12orf35	turquoise
C12orf4	turquoise
C12orf45	turquoise
C12orf48	turquoise
C12orf52	turquoise
C12orf53	turquoise
C14orf101	turquoise
C14orf126	turquoise
C14orf129	turquoise
C14orf135	turquoise
C14orf142	turquoise
C14orf148	turquoise
C14orf153	turquoise
C14orf166	turquoise
C14orf167	turquoise
C14orf176	turquoise
C14orf2	turquoise
C14orf28	turquoise
C14orf93	turquoise
C15orf24	turquoise
C15orf34	turquoise
C15orf58	turquoise
C16orf5	turquoise
C16orf52	turquoise
C16orf61	turquoise
C16orf71	turquoise
C16orf72	turquoise
C16orf87	turquoise
C17orf101	turquoise
C17orf104	turquoise
C17orf42	turquoise
C17orf55	turquoise
C17orf58	turquoise
C17orf61	turquoise

C17orf62	turquoise
C17orf72	turquoise
C17orf96	turquoise
C18orf1	turquoise
C18orf19	turquoise
C18orf25	turquoise
C18orf54	turquoise
C19orf44	turquoise
C19orf47	turquoise
C19orf52	turquoise
C19orf54	turquoise
C19orf63	turquoise
C19orf76	turquoise
C1GALT1	turquoise
C1orf112	turquoise
C1orf124	turquoise
C1orf127	turquoise
C1orf131	turquoise
C1orf162	turquoise
C1orf172	turquoise
C1orf200	turquoise
C1orf27	turquoise
C1orf43	turquoise
C1orf9	turquoise
C1orf93	turquoise
C1orf96	turquoise
C1orf97	turquoise
C1QA	turquoise
C1QB	turquoise
C1QC	turquoise
C1QL3	turquoise
C1QTNF7	turquoise
C1R	turquoise
C20orf111	turquoise
C20orf177	turquoise
C20orf197	turquoise
C20orf29	turquoise
C20orf72	turquoise
C20orf94	turquoise
C20orf96	turquoise
C21orf33	turquoise
C21orf70	turquoise
C21orf91	turquoise
C22orf25	turquoise
C2CD2L	turquoise
C2orf43	turquoise
C2orf47	turquoise
C2orf49	turquoise
C2orf67	turquoise
C2orf69	turquoise
C2orf74	turquoise
C2orf76	turquoise
C2orf85	turquoise
C3orf14	turquoise
C3orf17	turquoise
C3orf21	turquoise
C3orf23	turquoise
C3orf38	turquoise
C3orf39	turquoise
C3orf45	turquoise
C3orf58	turquoise
C3orf63	turquoise
C3orf64	turquoise
C4orf10	turquoise
C4orf21	turquoise
C4orf29	turquoise
C4orf32	turquoise
C4orf33	turquoise

C4orf34	turquoise
C4orf39	turquoise
C4orf46	turquoise
C4orf52	turquoise
C5orf15	turquoise
C5orf22	turquoise
C5orf24	turquoise
C5orf30	turquoise
C5orf35	turquoise
C5orf41	turquoise
C5orf43	turquoise
C5orf44	turquoise
C5orf53	turquoise
C5orf54	turquoise
C5orf56	turquoise
C6orf105	turquoise
C6orf120	turquoise
C6orf162	turquoise
C6orf204	turquoise
C6orf211	turquoise
C6orf35	turquoise
C6orf47	turquoise
C6orf62	turquoise
C6orf72	turquoise
C6orf97	turquoise
C7orf26	turquoise
C7orf36	turquoise
C7orf58	turquoise
C7orf60	turquoise
C7orf61	turquoise
C7orf64	turquoise
C8orf37	turquoise
C8orf39	turquoise
C8orf40	turquoise
C8orf58	turquoise
C8orf59	turquoise
C8orf83	turquoise
C8orf84	turquoise
C9orf130	turquoise
C9orf139	turquoise
C9orf167	turquoise
C9orf21	turquoise
C9orf41	turquoise
C9orf71	turquoise
C9orf72	turquoise
C9orf80	turquoise
C9orf82	turquoise
C9orf85	turquoise
C9orf86	turquoise
CA11	turquoise
CA12	turquoise
CA5BP1	turquoise
CAB39	turquoise
CAB39L	turquoise
CABIN1	turquoise
CACNA1H	turquoise
CACNB3	turquoise
CACNB4	turquoise
CALB1	turquoise
CALCRL	turquoise
CALHM2	turquoise
CALM2	turquoise
CALML4	turquoise
CALML6	turquoise
CALU	turquoise
CAMK1	turquoise
CAMK2D	turquoise
CAMK4	turquoise

CAMLG	turquoise
CAMSAP1L1	turquoise
CAPG	turquoise
CAPN14	turquoise
CAPN7	turquoise
CAPS2	turquoise
CAPZA1	turquoise
CAPZA2	turquoise
CARD17	turquoise
CARD8	turquoise
CARHSP1	turquoise
CASC4	turquoise
CASC5	turquoise
CASD1	turquoise
CASP3	turquoise
CASP8	turquoise
CASP8AP2	turquoise
CATSPERB	turquoise
CATSPERG	turquoise
CBFB	turquoise
CBLL1	turquoise
CBR4	turquoise
CBWD1	turquoise
CBWD2	turquoise
CBX3	turquoise
CC2D1A	turquoise
CCBL2	turquoise
CCDC102A	turquoise
CCDC103	turquoise
CCDC104	turquoise
CCDC112	turquoise
CCDC115	turquoise
CCDC117	turquoise
CCDC121	turquoise
CCDC126	turquoise
CCDC13	turquoise
CCDC132	turquoise
CCDC136	turquoise
CCDC137	turquoise
CCDC138	turquoise
CCDC14	turquoise
CCDC146	turquoise
CCDC154	turquoise
CCDC159	turquoise
CCDC18	turquoise
CCDC25	turquoise
CCDC30	turquoise
CCDC34	turquoise
CCDC39	turquoise
CCDC41	turquoise
CCDC42B	turquoise
CCDC43	turquoise
CCDC53	turquoise
CCDC58	turquoise
CCDC59	turquoise
CCDC6	turquoise
CCDC65	turquoise
CCDC66	turquoise
CCDC7	turquoise
CCDC71	turquoise
CCDC72	turquoise
CCDC73	turquoise
CCDC75	turquoise
CCDC76	turquoise
CCDC82	turquoise
CCDC85C	turquoise
CCDC86	turquoise
CCDC88A	turquoise

CCDC88B	turquoise
CCDC9	turquoise
CCDC90A	turquoise
CCDC90B	turquoise
CCDC91	turquoise
CCDC92	turquoise
CCDC97	turquoise
CCM2	turquoise
CCNA2	turquoise
CCNC	turquoise
CCND3	turquoise
CCNE2	turquoise
CCNG1	turquoise
CCNG2	turquoise
CCNH	turquoise
CCNL1	turquoise
CCNT2	turquoise
CCNY	turquoise
CCNYL1	turquoise
CCP110	turquoise
CCPG1	turquoise
CCT2	turquoise
CCT6B	turquoise
CCZ1	turquoise
CD109	turquoise
CD164	turquoise
CD200R1	turquoise
CD244	turquoise
CD248	turquoise
CD27	turquoise
CD274	turquoise
CD28	turquoise
CD2AP	turquoise
CD2BP2	turquoise
CD33	turquoise
CD37	turquoise
CD46	turquoise
CD47	turquoise
CD48	turquoise
CD5	turquoise
CD52	turquoise
CD58	turquoise
CD63	turquoise
CD69	turquoise
CD80	turquoise
CD81	turquoise
CDADC1	turquoise
CDC14A	turquoise
CDC25C	turquoise
CDC26	turquoise
CDC27	turquoise
CDC37	turquoise
CDC37L1	turquoise
CDC40	turquoise
CDC42EP1	turquoise
CDC42EP2	turquoise
CDC42SE2	turquoise
CDC7	turquoise
CDC73	turquoise
CDCA7	turquoise
CDH26	turquoise
CDHR1	turquoise
CDHR5	turquoise
CDIPT	turquoise
CDK1	turquoise
CDK11A	turquoise
CDK11B	turquoise
CDK17	turquoise

CDK18	turquoise
CDK2	turquoise
CDK5R1	turquoise
CDK5RAP3	turquoise
CDK7	turquoise
CDK8	turquoise
CDK9	turquoise
CDKL1	turquoise
CDKL2	turquoise
CDKN1B	turquoise
CDKN1C	turquoise
CDKN2AIP	turquoise
CDKN2C	turquoise
CDKN3	turquoise
CDS1	turquoise
CDT1	turquoise
CEACAM19	turquoise
CEBPA	turquoise
CEBPG	turquoise
CEBPZ	turquoise
CENPE	turquoise
CENPK	turquoise
CENPL	turquoise
CENPQ	turquoise
CENPW	turquoise
CEP120	turquoise
CEP135	turquoise
CEP152	turquoise
CEP170	turquoise
CEP19	turquoise
CEP250	turquoise
CEP290	turquoise
CEP350	turquoise
CEP44	turquoise
CEP57	turquoise
CEP57L1	turquoise
CEP70	turquoise
CEP95	turquoise
CEP97	turquoise
CEPT1	turquoise
CERCAM	turquoise
CERS5	turquoise
CES4A	turquoise
CETN3	turquoise
CFDP1	turquoise
CFH	turquoise
CFL1P1	turquoise
CFP	turquoise
CGGBP1	turquoise
CGN	turquoise
CGRRF1	turquoise
CHAC2	turquoise
CHAF1A	turquoise
CHAF1B	turquoise
CHCHD4	turquoise
CHD1	turquoise
CHD5	turquoise
CHD9	turquoise
CHERP	turquoise
CHFR	turquoise
CHIC1	turquoise
CHID1	turquoise
CHM	turquoise
CHML	turquoise
CHMP1A	turquoise
CHMP1B	turquoise
CHMP2A	turquoise
CHMP2B	turquoise

CHN2	turquoise
CHORDC1	turquoise
CHPF	turquoise
CHPF2	turquoise
CHST13	turquoise
CHST14	turquoise
CHST2	turquoise
CHSY1	turquoise
CHUK	turquoise
CHURC1	turquoise
CIDEB	turquoise
CISD1	turquoise
CISD2	turquoise
CISH	turquoise
CKAP2	turquoise
CKB	turquoise
CKS1B	turquoise
CKS2	turquoise
CLCN2	turquoise
CLCN7	turquoise
CLDN12	turquoise
CLDND1	turquoise
CLEC2B	turquoise
CLEC2D	turquoise
CLEC5A	turquoise
CLEC6A	turquoise
CLEC7A	turquoise
CLECL1	turquoise
CLIC2	turquoise
CLIC4	turquoise
CLIP3	turquoise
CLIP4	turquoise
CLK1	turquoise
CLK3	turquoise
CLK4	turquoise
CLN3	turquoise
CLN5	turquoise
CLN6	turquoise
CLNS1A	turquoise
CLP1	turquoise
CLPTM1	turquoise
CLPTM1L	turquoise
CLSTN3	turquoise
CMAHP	turquoise
CMC1	turquoise
CMPK1	turquoise
CMTM3	turquoise
CMTM6	turquoise
CMTM7	turquoise
CNBP	turquoise
CNIH	turquoise
CNKSR1	turquoise
CNKSR2	turquoise
CNN3	turquoise
CNOT2	turquoise
CNOT3	turquoise
CNOT6	turquoise
CNOT6L	turquoise
CNOT7	turquoise
CNOT8	turquoise
CNP	turquoise
CNPPD1	turquoise
CNPY3	turquoise
CNR1	turquoise
CNTROB	turquoise
COASY	turquoise
COBRA1	turquoise
COG3	turquoise

COG4	turquoise
COG6	turquoise
COIL	turquoise
COL24A1	turquoise
COL4A3BP	turquoise
COL7A1	turquoise
COLQ	turquoise
COMMD10	turquoise
COMMD2	turquoise
COMMD3	turquoise
COMMD6	turquoise
COMMD8	turquoise
COMT	turquoise
COPE	turquoise
COPS2	turquoise
COPS4	turquoise
COPS5	turquoise
COPS6	turquoise
COPZ1	turquoise
COQ10B	turquoise
COQ2	turquoise
CORO1A	turquoise
CORO7	turquoise
COX16	turquoise
COX19	turquoise
COX4NB	turquoise
COX6C	turquoise
COX7A2	turquoise
COX7A2L	turquoise
COX7B	turquoise
COX7C	turquoise
CPAMD8	turquoise
CPD	turquoise
CPEB2	turquoise
CPEB3	turquoise
CPEB4	turquoise
CPNE1	turquoise
CPNE3	turquoise
CPNE5	turquoise
CPNE8	turquoise
CPSF1	turquoise
CPSF6	turquoise
CRABP2	turquoise
CRAT	turquoise
CRBN	turquoise
CRCP	turquoise
CREB1	turquoise
CREB5	turquoise
CREBL2	turquoise
CREBZF	turquoise
CRIM1	turquoise
CRIPAK	turquoise
CRIPT	turquoise
CRK	turquoise
CRLF3	turquoise
CRLS1	turquoise
CROCCP2	turquoise
CROT	turquoise
CRTC2	turquoise
CRY1	turquoise
CSGALNACT1	turquoise
CSGALNACT2	turquoise
CSK	turquoise
CSNK1A1	turquoise
CSNK1A1L	turquoise
CSNK1G3	turquoise
CSNK2B	turquoise
CSRP1	turquoise

CST3	turquoise
CSTA	turquoise
CSTF2T	turquoise
CTBP1	turquoise
CTBS	turquoise
CTDNEP1	turquoise
CTDSP1	turquoise
CTDSPL2	turquoise
CTNS	turquoise
CTSH	turquoise
CTSO	turquoise
CTSS	turquoise
CTSW	turquoise
CUL2	turquoise
CUL4B	turquoise
CUL5	turquoise
CUTC	turquoise
CWF19L2	turquoise
CXADR	turquoise
CXCL10	turquoise
CXCL6	turquoise
CXCL9	turquoise
CXorf21	turquoise
CXorf23	turquoise
CXorf57	turquoise
CXXC1	turquoise
CYB561	turquoise
CYB5R4	turquoise
CYBA	turquoise
CYBASC3	turquoise
CYBRD1	turquoise
CYCS	turquoise
CYHR1	turquoise
CYP2S1	turquoise
CYP2U1	turquoise
CYP2W1	turquoise
CYP4F22	turquoise
CYSLTR1	turquoise
CYTH2	turquoise
CYYR1	turquoise
D4S234E	turquoise
DAB2IP	turquoise
DACH1	turquoise
DAGLB	turquoise
DAK	turquoise
DAPK3	turquoise
DAPP1	turquoise
DAXX	turquoise
DAZAP1	turquoise
DBF4	turquoise
DBI	turquoise
DBN1	turquoise
DBP	turquoise
DCAF10	turquoise
DCAF11	turquoise
DCAF13	turquoise
DCAKD	turquoise
DCBLD2	turquoise
DCDC2B	turquoise
DCK	turquoise
DCLRE1A	turquoise
DCP1A	turquoise
DCP2	turquoise
DCPS	turquoise
DCST2	turquoise
DCTN2	turquoise
DCTN4	turquoise
DCTN6	turquoise

DCUN1D1	turquoise
DCUN1D4	turquoise
DCUN1D5	turquoise
DDA1	turquoise
DDB2	turquoise
DDOST	turquoise
DDRGK1	turquoise
DDX11	turquoise
DDX12	turquoise
DDX26B	turquoise
DDX3X	turquoise
DDX41	turquoise
DDX50	turquoise
DDX54	turquoise
DDX59	turquoise
DECR1	turquoise
DEF6	turquoise
DEF8	turquoise
DEGS1	turquoise
DEGS2	turquoise
DEK	turquoise
DENND1B	turquoise
DENND1C	turquoise
DENND4A	turquoise
DENND4C	turquoise
DENR	turquoise
DEPDC1	turquoise
DEPDC1B	turquoise
DERL1	turquoise
DEXI	turquoise
DGCR14	turquoise
DGCR8	turquoise
DGKE	turquoise
DGKZ	turquoise
DHFR	turquoise
DHFRL1	turquoise
DHPS	turquoise
DHRS1	turquoise
DHRS11	turquoise
DHRS9	turquoise
DHX30	turquoise
DHX36	turquoise
DHX40	turquoise
DIAPH3	turquoise
DICER1	turquoise
DIMT1L	turquoise
DIP2B	turquoise
DIRC2	turquoise
DIS3	turquoise
DIS3L2	turquoise
DIXDC1	turquoise
DKFZP434I0714	turquoise
DKFZP564C196	turquoise
DKFZP586I1420	turquoise
DKK3	turquoise
DLAT	turquoise
DLEU7	turquoise
DLG1	turquoise
DLG4	turquoise
DLGAP4	turquoise
DMAP1	turquoise
DMPK	turquoise
DMTF1	turquoise
DMXL1	turquoise
DNA2	turquoise
DNAH6	turquoise
DNAI2	turquoise
DNAJA1	turquoise

DNAJB14	turquoise
DNAJB2	turquoise
DNAJB4	turquoise
DNAJB9	turquoise
DNAJC10	turquoise
DNAJC15	turquoise
DNAJC21	turquoise
DNAJC24	turquoise
DNAJC25	turquoise
DNAJC27	turquoise
DNASE1L1	turquoise
DNASE2	turquoise
DNPEP	turquoise
DNTTIP2	turquoise
DOCK11	turquoise
DOHH	turquoise
DOLPP1	turquoise
DONSON	turquoise
DOT1L	turquoise
DPEP2	turquoise
DPH3	turquoise
DPM1	turquoise
DPP3	turquoise
DPY19L1	turquoise
DPY19L2P2	turquoise
DPY19L3	turquoise
DPY19L4	turquoise
DPY30	turquoise
DR1	turquoise
DRAM2	turquoise
DRAP1	turquoise
DSC1	turquoise
DSC2	turquoise
DSEL	turquoise
DTNB	turquoise
DTNBP1	turquoise
DTWD1	turquoise
DTX2	turquoise
DUSP11	turquoise
DUSP19	turquoise
DYNC2LI1	turquoise
DYNLRB1	turquoise
DYNLT3	turquoise
DYRK1A	turquoise
DYRK1B	turquoise
DYRK2	turquoise
DZIP3	turquoise
E2F4	turquoise
E2F6	turquoise
E2F7	turquoise
EAF2	turquoise
EAPP	turquoise
EBAG9	turquoise
EBF4	turquoise
ECH1	turquoise
ECHDC1	turquoise
ECHS1	turquoise
ECT2	turquoise
ECT2L	turquoise
EDEM3	turquoise
EEA1	turquoise
EED	turquoise
EEF1A1	turquoise
EEF1B2	turquoise
EEF1E1	turquoise
EEFSEC	turquoise
EFCAB11	turquoise
EFCAB7	turquoise

EFHA1	turquoise
EFHD2	turquoise
EFNA3	turquoise
EFNB1	turquoise
EFR3A	turquoise
EGLN1	turquoise
EHMT2	turquoise
EID1	turquoise
EIF1AX	turquoise
EIF2A	turquoise
EIF2B5	turquoise
EIF2S2	turquoise
EIF3D	turquoise
EIF3E	turquoise
EIF3J	turquoise
EIF4E	turquoise
EIF4E2	turquoise
EIF4E3	turquoise
EIF5	turquoise
EIF5A2	turquoise
EIF6	turquoise
ELAC2	turquoise
ELF2	turquoise
ELK4	turquoise
ELL2	turquoise
ELMOD2	turquoise
ELOF1	turquoise
ELOVL4	turquoise
EMB	turquoise
EMBP1	turquoise
EMILIN1	turquoise
EML2	turquoise
EML5	turquoise
EMP3	turquoise
ENDOG	turquoise
ENDOV	turquoise
ENG	turquoise
ENTPD6	turquoise
ENY2	turquoise
EPB41L5	turquoise
EPC2	turquoise
EPHB3	turquoise
EPHB6	turquoise
EPM2AIP1	turquoise
EPN1	turquoise
EPS15	turquoise
EPT1	turquoise
ERAL1	turquoise
ERBB2IP	turquoise
ERCC2	turquoise
ERCC8	turquoise
EREG	turquoise
ERGIC2	turquoise
ERH	turquoise
ERI1	turquoise
ERI2	turquoise
ERLEC1	turquoise
ERLIN1	turquoise
ERN1	turquoise
ERO1L	turquoise
ERO1LB	turquoise
ESCO1	turquoise
ESCO2	turquoise
ESF1	turquoise
ESR2	turquoise
ETAA1	turquoise
ETFA	turquoise
ETNK1	turquoise

EVI2A	turquoise
EVI2B	turquoise
EWSR1	turquoise
EXOC1	turquoise
EXOC3	turquoise
EXOC3L2	turquoise
EXOC5	turquoise
EXOC6	turquoise
EXOC8	turquoise
EXPH5	turquoise
EXTL2	turquoise
EYS	turquoise
F2RL1	turquoise
F8	turquoise
FABP5	turquoise
FAM100A	turquoise
FAM102B	turquoise
FAM105A	turquoise
FAM108B1	turquoise
FAM109B	turquoise
FAM110A	turquoise
FAM111A	turquoise
FAM113B	turquoise
FAM114A1	turquoise
FAM116A	turquoise
FAM117B	turquoise
FAM120AOS	turquoise
FAM122B	turquoise
FAM125A	turquoise
FAM126A	turquoise
FAM126B	turquoise
FAM127A	turquoise
FAM133B	turquoise
FAM134A	turquoise
FAM134B	turquoise
FAM135A	turquoise
FAM13AOS	turquoise
FAM13B	turquoise
FAM149B1	turquoise
FAM151B	turquoise
FAM160B1	turquoise
FAM161A	turquoise
FAM164A	turquoise
FAM169A	turquoise
FAM171A2	turquoise
FAM172A	turquoise
FAM173B	turquoise
FAM175A	turquoise
FAM177A1	turquoise
FAM179B	turquoise
FAM184A	turquoise
FAM185A	turquoise
FAM188A	turquoise
FAM189B	turquoise
FAM18B1	turquoise
FAM198B	turquoise
FAM199X	turquoise
FAM19A2	turquoise
FAM200A	turquoise
FAM200B	turquoise
FAM203A	turquoise
FAM204A	turquoise
FAM20C	turquoise
FAM32A	turquoise
FAM35A	turquoise
FAM35B	turquoise
FAM3C	turquoise
FAM45B	turquoise

FAM46A	turquoise
FAM49B	turquoise
FAM50A	turquoise
FAM50B	turquoise
FAM53B	turquoise
FAM59B	turquoise
FAM60A	turquoise
FAM63B	turquoise
FAM65A	turquoise
FAM69A	turquoise
FAM72D	turquoise
FAM73A	turquoise
FAM76B	turquoise
FAM82A1	turquoise
FAM82A2	turquoise
FAM82B	turquoise
FAM83H	turquoise
FAM84B	turquoise
FAM86EP	turquoise
FAM8A1	turquoise
FAM91A1	turquoise
FAM92A1	turquoise
FAM96A	turquoise
FANCB	turquoise
FANCL	turquoise
FANCM	turquoise
FAR1	turquoise
FAR2	turquoise
FARS2	turquoise
FARSA	turquoise
FAS	turquoise
FASTKD1	turquoise
FASTKD3	turquoise
FBXL13	turquoise
FBXL16	turquoise
FBXL17	turquoise
FBXL3	turquoise
FBXL4	turquoise
FBXL5	turquoise
FBXO11	turquoise
FBXO28	turquoise
FBXO3	turquoise
FBXO30	turquoise
FBXO31	turquoise
FBXO33	turquoise
FBXO4	turquoise
FBXO42	turquoise
FBXO45	turquoise
FBXO46	turquoise
FBXO48	turquoise
FBXO5	turquoise
FBXO8	turquoise
FBXW4	turquoise
FBXW7	turquoise
FCER1A	turquoise
FCF1	turquoise
FCGR2B	turquoise
FCGR2C	turquoise
FCHO1	turquoise
FCHO2	turquoise
FDFT1	turquoise
FDPS	turquoise
FDXACB1	turquoise
FEM1A	turquoise
FEM1C	turquoise
FER	turquoise
FERMT3	turquoise
FGD2	turquoise

FGD3	turquoise
FGD4	turquoise
FGF7	turquoise
FGFR1OP	turquoise
FGFR1OP2	turquoise
FGFR4	turquoise
FGL2	turquoise
FHL3	turquoise
FHOD1	turquoise
FIGNL1	turquoise
FITM1	turquoise
FIZ1	turquoise
FKBP10	turquoise
FKBP3	turquoise
FKBP7	turquoise
FLII	turquoise
FLJ21408	turquoise
FLJ27354	turquoise
FLJ31306	turquoise
FLJ33630	turquoise
FLJ36644	turquoise
FLJ42393	turquoise
FLJ45513	turquoise
FLJ90757	turquoise
FLRT1	turquoise
FMNL1	turquoise
FMNL2	turquoise
FMO5	turquoise
FMR1	turquoise
FN3K	turquoise
FNBP1L	turquoise
FNDC3A	turquoise
FNDC3B	turquoise
FNIP1	turquoise
FNTA	turquoise
FOLR2	turquoise
FOPNL	turquoise
FOXH1	turquoise
FOXN2	turquoise
FOXRED1	turquoise
FPGT	turquoise
FRA10AC1	turquoise
FRG1	turquoise
FRMD8	turquoise
FRRS1	turquoise
FRS2	turquoise
FSD1L	turquoise
FTSJ1	turquoise
FTSJD1	turquoise
FUCA1	turquoise
FUNDC2	turquoise
FUT4	turquoise
FXR1	turquoise
FXR2	turquoise
FXYD5	turquoise
FXYD7	turquoise
FYTTD1	turquoise
FZD2	turquoise
FZD3	turquoise
FZD6	turquoise
G2E3	turquoise
G6PC3	turquoise
GAA	turquoise
GAB1	turquoise
GABPA	turquoise
GABPB1	turquoise
GAK	turquoise
GALC	turquoise

GALE	turquoise
GALNT1	turquoise
GALNT3	turquoise
GALNT4	turquoise
GALNT7	turquoise
GAPDH	turquoise
GAPT	turquoise
GAS2	turquoise
GAS2L2	turquoise
GAS5	turquoise
GAS6	turquoise
GATAD2A	turquoise
GBA2	turquoise
GBAS	turquoise
GBP1P1	turquoise
GBP3	turquoise
GBP6	turquoise
GCA	turquoise
GCC2	turquoise
GCFC1	turquoise
GCFC1-AS1	turquoise
GCH1	turquoise
GCLM	turquoise
GCNT1	turquoise
GCNT2	turquoise
GCNT4	turquoise
GCSH	turquoise
GDAP1L1	turquoise
GDF9	turquoise
GDI1	turquoise
GDPD3	turquoise
GDPD5	turquoise
GEMIN2	turquoise
GEMIN6	turquoise
GEN1	turquoise
GFM2	turquoise
GFPT1	turquoise
GFRA2	turquoise
GGCT	turquoise
GGPS1	turquoise
GIF	turquoise
GIMAP2	turquoise
GIMAP7	turquoise
GIN1	turquoise
GIPC1	turquoise
GIT1	turquoise
GK	turquoise
GKAP1	turquoise
GLA	turquoise
GLB1L3	turquoise
GLCCI1	turquoise
GLCE	turquoise
GLRX	turquoise
GLRX2	turquoise
GLRX3	turquoise
GLS	turquoise
GLT25D1	turquoise
GLT8D1	turquoise
GLT8D2	turquoise
GLTPD1	turquoise
GLTSCR1	turquoise
GMCL1	turquoise
GMFB	turquoise
GMNN	turquoise
GMPPA	turquoise
GNA13	turquoise
GNAI1	turquoise
GNAO1	turquoise

GNB4	turquoise
GNG2	turquoise
GNL1	turquoise
GNL3	turquoise
GNMT	turquoise
GNPDA2	turquoise
GNRHR2	turquoise
GOLGA4	turquoise
GOLPH3L	turquoise
GOLT1B	turquoise
GOPC	turquoise
GORAB	turquoise
GORASP1	turquoise
GPAM	turquoise
GPAT2	turquoise
GPATCH2	turquoise
GPATCH3	turquoise
GPBAR1	turquoise
GPBP1	turquoise
GPC1	turquoise
GPCPD1	turquoise
GPD2	turquoise
GPI	turquoise
GPKOW	turquoise
GPR124	turquoise
GPR132	turquoise
GPR137	turquoise
GPR15	turquoise
GPR160	turquoise
GPR171	turquoise
GPR174	turquoise
GPR180	turquoise
GPR34	turquoise
GPR35	turquoise
GPR65	turquoise
GPR82	turquoise
GPRIN3	turquoise
GPS1	turquoise
GPS2	turquoise
GPSM1	turquoise
GRAMD1A	turquoise
GRAMD1C	turquoise
GRAMD3	turquoise
GRAP	turquoise
GRHL1	turquoise
GRHPR	turquoise
GRIK5	turquoise
GRINL1A	turquoise
GRIP1	turquoise
GRK6	turquoise
GRPEL2	turquoise
GRWD1	turquoise
GSS	turquoise
GSTM2	turquoise
GSTP1	turquoise
GTDC1	turquoise
GTF2B	turquoise
GTF2E1	turquoise
GTF2E2	turquoise
GTF2F2	turquoise
GTF2H1	turquoise
GTF2H4	turquoise
GTPBP10	turquoise
GTPBP2	turquoise
GTPBP8	turquoise
GUCA1B	turquoise
GUCY2C	turquoise
GUF1	turquoise

GUSB	turquoise
GXYLT1	turquoise
GZF1	turquoise
H2AFV	turquoise
H2AFZ	turquoise
HACE1	turquoise
HARS2	turquoise
HAT1	turquoise
HAUS1	turquoise
HAUS3	turquoise
HAUS6	turquoise
HAUS7	turquoise
HBS1L	turquoise
HCFC2	turquoise
HCG11	turquoise
HCG25	turquoise
HCP5	turquoise
HDAC3	turquoise
HDAC5	turquoise
HDAC6	turquoise
HDAC7	turquoise
HDAC8	turquoise
HDAC9	turquoise
HDGFRP2	turquoise
HDGFRP3	turquoise
HDHD2	turquoise
HDX	turquoise
HEATR5A	turquoise
HEBP1	turquoise
HECTD2	turquoise
HECTD3	turquoise
HECW2	turquoise
HELLS	turquoise
HEPH	turquoise
HERC4	turquoise
HEXA	turquoise
HGS	turquoise
HHLA3	turquoise
HIGD1A	turquoise
HINT1	turquoise
HINT3	turquoise
HIRA	turquoise
HIRIP3	turquoise
HIST1H3E	turquoise
HIST1H4H	turquoise
HLA-A	turquoise
HLA-DMA	turquoise
HLA-DPB1	turquoise
HLA-DRA	turquoise
HLF	turquoise
HLTF	turquoise
HMGB1	turquoise
HMGB2	turquoise
HMGCL	turquoise
HMGCS1	turquoise
HMGN5	turquoise
HMGXB4	turquoise
HMMR	turquoise
HMOX2	turquoise
HNF1A	turquoise
HNMT	turquoise
HNRNPH2	turquoise
HNRNPL	turquoise
HNRPLL	turquoise
HOMER1	turquoise
HOOK1	turquoise
HOOK2	turquoise
HORMAD1	turquoise

HPCAL1	turquoise
HPGD	turquoise
HPRT1	turquoise
HPS6	turquoise
HRSP12	turquoise
HS1BP3	turquoise
HS2ST1	turquoise
HS6ST1	turquoise
HSD17B10	turquoise
HSD17B11	turquoise
HSDL1	turquoise
HSDL2	turquoise
HSF1	turquoise
HSF2	turquoise
HSF4	turquoise
HSF5	turquoise
HSH2D	turquoise
HSPA13	turquoise
HSPA14	turquoise
HSPB11	turquoise
HSPBP1	turquoise
HSPC159	turquoise
HSPE1	turquoise
HTR7P1	turquoise
HVCN1	turquoise
IAH1	turquoise
IBTK	turquoise
ICAM2	turquoise
ICAM4	turquoise
ICK	turquoise
IDE	turquoise
IDH2	turquoise
IDH3B	turquoise
IDH3G	turquoise
IDI1	turquoise
IDI2-AS1	turquoise
IER3IP1	turquoise
IFI30	turquoise
IFITM1	turquoise
IFNGR1	turquoise
IFRD1	turquoise
IFT122	turquoise
IFT140	turquoise
IFT20	turquoise
IFT52	turquoise
IFT57	turquoise
IFT74	turquoise
IFT80	turquoise
IFT81	turquoise
IFT88	turquoise
IGBP1	turquoise
IGF2BP3	turquoise
IGFALS	turquoise
IGFBP4	turquoise
IGHMBP2	turquoise
IGJ	turquoise
IGSF9	turquoise
IKBKG	turquoise
IKZF2	turquoise
IKZF5	turquoise
IL12A	turquoise
IL12RB1	turquoise
IL15	turquoise
IL17RE	turquoise
IL17REL	turquoise
IL18R1	turquoise
IL1RAP	turquoise
IL27RA	turquoise

IL32	turquoise
IL3RA	turquoise
IL4I1	turquoise
IL6ST	turquoise
IL7	turquoise
IL8	turquoise
ILK	turquoise
IMMP1L	turquoise
IMPA1	turquoise
IMPACT	turquoise
IMPAD1	turquoise
IMPDH2	turquoise
IMPG2	turquoise
INF2	turquoise
ING3	turquoise
INO80C	turquoise
INO80D	turquoise
INPP5D	turquoise
INPP5K	turquoise
INSIG1	turquoise
INSIG2	turquoise
INTS12	turquoise
INTS2	turquoise
INTS5	turquoise
INTS6	turquoise
INTS8	turquoise
IPMK	turquoise
IPO11	turquoise
IPO13	turquoise
IPO4	turquoise
IPW	turquoise
IQCG	turquoise
IQSEC2	turquoise
IRAK1BP1	turquoise
IREB2	turquoise
IRF2BP2	turquoise
IRF5	turquoise
ISCA1	turquoise
ISPD	turquoise
ITCH	turquoise
ITFG3	turquoise
ITGA1	turquoise
ITGA2	turquoise
ITGA3	turquoise
ITGA4	turquoise
ITGAV	turquoise
ITGB3BP	turquoise
ITGB7	turquoise
ITIH4	turquoise
ITPKC	turquoise
ITPR2	turquoise
IVNS1ABP	turquoise
JAG1	turquoise
JAK2	turquoise
JKAMP	turquoise
JMJD6	turquoise
JMY	turquoise
JPX	turquoise
JRKL	turquoise
JUN	turquoise
JUP	turquoise
KAT2B	turquoise
KAT8	turquoise
KATNB1	turquoise
KBTBD2	turquoise
KBTBD3	turquoise
KBTBD6	turquoise
KBTBD7	turquoise

KBTBD8	turquoise
KCNA2	turquoise
KCNAB1	turquoise
KCNAB2	turquoise
KCNIP4	turquoise
KCNJ2	turquoise
KCNK7	turquoise
KCNQ1	turquoise
KCNT1	turquoise
KCTD11	turquoise
KCTD12	turquoise
KCTD18	turquoise
KCTD2	turquoise
KCTD3	turquoise
KCTD6	turquoise
KCTD9	turquoise
KDELC2	turquoise
KEAP1	turquoise
KIAA0020	turquoise
KIAA0101	turquoise
KIAA0195	turquoise
KIAA0284	turquoise
KIAA0391	turquoise
KIAA0649	turquoise
KIAA0664L3	turquoise
KIAA0776	turquoise
KIAA0825	turquoise
KIAA0930	turquoise
KIAA1009	turquoise
KIAA1024	turquoise
KIAA1033	turquoise
KIAA1107	turquoise
KIAA1109	turquoise
KIAA1143	turquoise
KIAA1370	turquoise
KIAA1377	turquoise
KIAA1383	turquoise
KIAA1430	turquoise
KIAA1467	turquoise
KIAA1468	turquoise
KIAA1522	turquoise
KIAA1524	turquoise
KIAA1543	turquoise
KIAA1586	turquoise
KIAA1683	turquoise
KIAA1704	turquoise
KIAA1715	turquoise
KIAA1731	turquoise
KIAA1804	turquoise
KIAA1826	turquoise
KIAA1841	turquoise
KIAA1967	turquoise
KIAA2013	turquoise
KIF11	turquoise
KIF18A	turquoise
KIF20B	turquoise
KIF22	turquoise
KIF23	turquoise
KIF27	turquoise
KIF2A	turquoise
KIF3A	turquoise
KIF5B	turquoise
KIFC1	turquoise
KIFC3	turquoise
KIN	turquoise
KL	turquoise
KLC4	turquoise
KLF16	turquoise

KLF3	turquoise
KLF5	turquoise
KLHDC1	turquoise
KLHDC10	turquoise
KLHDC3	turquoise
KLHDC4	turquoise
KLHDC5	turquoise
KLHL15	turquoise
KLHL2	turquoise
KLHL20	turquoise
KLHL22	turquoise
KLHL24	turquoise
KLHL25	turquoise
KLHL26	turquoise
KLHL28	turquoise
KLHL5	turquoise
KLHL7	turquoise
KLHL9	turquoise
KLLN	turquoise
KLRAP1	turquoise
KMO	turquoise
KNDC1	turquoise
KPNA2	turquoise
KPNA3	turquoise
KPNA5	turquoise
KRAS	turquoise
KRCC1	turquoise
KRI1	turquoise
KRR1	turquoise
KRT72	turquoise
KTN1	turquoise
L2HGDH	turquoise
LACC1	turquoise
LACE1	turquoise
LACTB2	turquoise
LAMB2	turquoise
LAMB3	turquoise
LAMTOR3	turquoise
LANCL1	turquoise
LAPTM4A	turquoise
LARP1B	turquoise
LARP4	turquoise
LCAT	turquoise
LCLAT1	turquoise
LCOR	turquoise
LCORL	turquoise
LDLR	turquoise
LDOC1	turquoise
LEMD3	turquoise
LENG1	turquoise
LEPR	turquoise
LEPREL4	turquoise
LEPROT	turquoise
LEPROTL1	turquoise
LFNG	turquoise
LGALS3BP	turquoise
LGALS9	turquoise
LGALS9C	turquoise
LGI4	turquoise
LGR4	turquoise
LHPP	turquoise
LIG4	turquoise
LILRB4	turquoise
LIMS1	turquoise
LIN28A	turquoise
LIN52	turquoise
LIN54	turquoise
LIN7A	turquoise

LIN7C	turquoise
LIN9	turquoise
LINS	turquoise
LIPE	turquoise
LIPN	turquoise
LLGL1	turquoise
LMAN1	turquoise
LMAN2	turquoise
LMBR1	turquoise
LMBRD1	turquoise
LMBRD2	turquoise
LMNA	turquoise
LMNB2	turquoise
LNX1	turquoise
LNX2	turquoise
LOC100127983	turquoise
LOC100128420	turquoise
LOC100129361	turquoise
LOC100130557	turquoise
LOC100130581	turquoise
LOC100130776	turquoise
LOC100130855	turquoise
LOC100130992	turquoise
LOC100131496	turquoise
LOC100132215	turquoise
LOC100132618	turquoise
LOC100132891	turquoise
LOC100133331	turquoise
LOC100134015	turquoise
LOC100190939	turquoise
LOC100287616	turquoise
LOC100287765	turquoise
LOC100288198	turquoise
LOC100288846	turquoise
LOC100294145	turquoise
LOC100499484	turquoise
LOC100505576	turquoise
LOC100505624	turquoise
LOC100505715	turquoise
LOC100505812	turquoise
LOC100505876	turquoise
LOC100506710	turquoise
LOC100506804	turquoise
LOC100506866	turquoise
LOC100507053	turquoise
LOC100507254	turquoise
LOC100507266	turquoise
LOC100507331	turquoise
LOC100507489	turquoise
LOC100507557	turquoise
LOC100507582	turquoise
LOC100507632	turquoise
LOC115110	turquoise
LOC146880	turquoise
LOC147646	turquoise
LOC148824	turquoise
LOC153684	turquoise
LOC158572	turquoise
LOC162632	turquoise
LOC200772	turquoise
LOC220906	turquoise
LOC221710	turquoise
LOC254559	turquoise
LOC283038	turquoise
LOC283875	turquoise
LOC284023	turquoise
LOC284233	turquoise
LOC284576	turquoise

LOC284837	turquoise
LOC339874	turquoise
LOC344595	turquoise
LOC387646	turquoise
LOC389634	turquoise
LOC401093	turquoise
LOC401397	turquoise
LOC440354	turquoise
LOC606724	turquoise
LOC643529	turquoise
LOC643723	turquoise
LOC643802	turquoise
LOC643837	turquoise
LOC645158	turquoise
LOC646214	turquoise
LOC647979	turquoise
LOC728024	turquoise
LOC728392	turquoise
LOC729178	turquoise
LOC729852	turquoise
LONP1	turquoise
LONRF1	turquoise
LOX	turquoise
LPAR2	turquoise
LPAR6	turquoise
LPCAT1	turquoise
LPCAT2	turquoise
LPCAT4	turquoise
LPGAT1	turquoise
LRCH4	turquoise
LRFN3	turquoise
LRIF1	turquoise
LRMP	turquoise
LRP12	turquoise
LRP6	turquoise
LRPAP1	turquoise
LRR1	turquoise
LRRC20	turquoise
LRRC33	turquoise
LRRC34	turquoise
LRRC40	turquoise
LRRC43	turquoise
LRRC47	turquoise
LRRC58	turquoise
LRRC61	turquoise
LRRC70	turquoise
LRRC8B	turquoise
LRRC8C	turquoise
LRRCC1	turquoise
LRRK2	turquoise
LRRN1	turquoise
LRRN3	turquoise
LRSAM1	turquoise
LRWD1	turquoise
LSM1	turquoise
LSM11	turquoise
LSM3	turquoise
LSM6	turquoise
LSR	turquoise
LTN1	turquoise
LUC7L3	turquoise
LXN	turquoise
LY6G6C	turquoise
LY75	turquoise
LYAR	turquoise
LYNX1	turquoise
LYPD3	turquoise
LYPLA1	turquoise

LYPLA2	turquoise
LYPLAL1	turquoise
LYRM2	turquoise
LYRM5	turquoise
LYSMD3	turquoise
LYST	turquoise
LZIC	turquoise
LZTFL1	turquoise
MAD2L1	turquoise
MAF	turquoise
MAF1	turquoise
MAGT1	turquoise
MAK16	turquoise
MALT1	turquoise
MAMSTR	turquoise
MAN1A1	turquoise
MAN1A2	turquoise
MAN2A1	turquoise
MAN2B1	turquoise
MAN2C1	turquoise
MANEA	turquoise
MAP2K2	turquoise
MAP2K4	turquoise
MAP2K5	turquoise
MAP3K1	turquoise
MAP3K11	turquoise
MAP3K13	turquoise
MAP3K14	turquoise
MAP3K2	turquoise
MAP3K7	turquoise
MAP3K8	turquoise
MAP4K1	turquoise
MAP4K3	turquoise
MAP4K5	turquoise
MAP6D1	turquoise
MAP7D2	turquoise
MAP9	turquoise
MAPK6	turquoise
MAPK8	turquoise
MAPK8IP1	turquoise
MAPK8IP2	turquoise
MAPKAPK2	turquoise
MAPKAPK3	turquoise
MAPKBP1	turquoise
MARCKSL1	turquoise
MARK4	turquoise
MARS2	turquoise
MAST2	turquoise
MAST4	turquoise
MATR3	turquoise
MB21D2	turquoise
MBIP	turquoise
MBLAC2	turquoise
MBNL1	turquoise
MBNL2	turquoise
MBOAT1	turquoise
MBTD1	turquoise
MBTPS2	turquoise
MCAT	turquoise
MCEE	turquoise
MCF2	turquoise
MCL1	turquoise
MCM5	turquoise
MCM9	turquoise
MCOLN1	turquoise
MCOLN3	turquoise
MCTP1	turquoise
MCTS1	turquoise

MCU	turquoise
MDFIC	turquoise
MDH2	turquoise
MDK	turquoise
MDM1	turquoise
MDM2	turquoise
MED12L	turquoise
MED13	turquoise
MED14	turquoise
MED15	turquoise
MED16	turquoise
MED21	turquoise
MED22	turquoise
MED23	turquoise
MED26	turquoise
MED28	turquoise
MED4	turquoise
MED7	turquoise
MED8	turquoise
MED9	turquoise
MEF2A	turquoise
MEF2C	turquoise
MEF2D	turquoise
MEGF8	turquoise
MEN1	turquoise
MEPCE	turquoise
METAP2	turquoise
METTL1	turquoise
METTL14	turquoise
METTL15	turquoise
METTL18	turquoise
METTL19	turquoise
METTL21D	turquoise
METTL4	turquoise
MEX3C	turquoise
MFAP3	turquoise
MFF	turquoise
MFN1	turquoise
MFSD4	turquoise
MFSD5	turquoise
MFSD8	turquoise
MGAT1	turquoise
MGAT2	turquoise
MGAT4A	turquoise
MGEA5	turquoise
MIB1	turquoise
MICAL1	turquoise
MICALL1	turquoise
MICALL2	turquoise
MID1IP1	turquoise
MIER1	turquoise
MIER2	turquoise
MIER3	turquoise
MINA	turquoise
MINPP1	turquoise
MIR155HG	turquoise
MIS18BP1	turquoise
MITD1	turquoise
MITF	turquoise
MKI67IP	turquoise
MKKS	turquoise
MKLN1	turquoise
MKRN2	turquoise
MKRN3	turquoise
MKS1	turquoise
MLF1IP	turquoise
MLLT3	turquoise
MLLT4	turquoise

MLYCD	turquoise
MMAA	turquoise
MMP15	turquoise
MMP17	turquoise
MMP24	turquoise
MMS22L	turquoise
MNAT1	turquoise
MNDA	turquoise
MOBKL1A	turquoise
MOBKL1B	turquoise
MOBKL2C	turquoise
MOCS2	turquoise
MOCS3	turquoise
MON1A	turquoise
MON2	turquoise
MORC2	turquoise
MORC3	turquoise
MORF4L2	turquoise
MORN2	turquoise
MORN3	turquoise
MOSPD1	turquoise
MOSPD2	turquoise
MOV10	turquoise
MPHOSPH6	turquoise
MPI	turquoise
MPND	turquoise
MPP5	turquoise
MPP6	turquoise
MPP7	turquoise
MPV17	turquoise
MPZL2	turquoise
MRFAP1	turquoise
MRGPRX3	turquoise
MRM1	turquoise
MRPL1	turquoise
MRPL13	turquoise
MRPL15	turquoise
MRPL18	turquoise
MRPL19	turquoise
MRPL2	turquoise
MRPL22	turquoise
MRPL28	turquoise
MRPL3	turquoise
MRPL32	turquoise
MRPL37	turquoise
MRPL39	turquoise
MRPL40	turquoise
MRPL42	turquoise
MRPL47	turquoise
MRPL48	turquoise
MRPL50	turquoise
MRPS10	turquoise
MRPS17	turquoise
MRPS18B	turquoise
MRPS18C	turquoise
MRPS2	turquoise
MRPS22	turquoise
MRPS28	turquoise
MRPS31	turquoise
MRPS33	turquoise
MSH2	turquoise
MSL2	turquoise
MSL3	turquoise
MSR1	turquoise
MST4	turquoise
MSTO1	turquoise
MTBP	turquoise
MTERF	turquoise

MTERFD1	turquoise
MTF2	turquoise
MTFR1	turquoise
MTHFD2	turquoise
MTHFD2L	turquoise
MTHFSD	turquoise
MTIF3	turquoise
MTMR2	turquoise
MTMR6	turquoise
MTMR7	turquoise
MTMR9	turquoise
MTO1	turquoise
MTRF1	turquoise
MTRF1L	turquoise
MTRR	turquoise
MTX2	turquoise
MTX3	turquoise
MUC1	turquoise
MUC20	turquoise
MUC5B	turquoise
MUC6	turquoise
MUDENG	turquoise
MUM1	turquoise
MUSTN1	turquoise
MYCBPAP	turquoise
MYL12B	turquoise
MYLK4	turquoise
MYNN	turquoise
MYO1G	turquoise
MYO5C	turquoise
MYSM1	turquoise
MZT1	turquoise
N4BP2	turquoise
N4BP2L1	turquoise
N4BP2L2	turquoise
NAA16	turquoise
NAA20	turquoise
NAA25	turquoise
NAA30	turquoise
NAA38	turquoise
NAA50	turquoise
NAA60	turquoise
NAALAD2	turquoise
NAB1	turquoise
NACAD	turquoise
NADKD1	turquoise
NADSYN1	turquoise
NAGPA	turquoise
NAIF1	turquoise
NAMPT	turquoise
NANP	turquoise
NANS	turquoise
NAP1L2	turquoise
NAPB	turquoise
NAPEPLD	turquoise
NAPG	turquoise
NARG2	turquoise
NARS2	turquoise
NAT1	turquoise
NBEA	turquoise
NBL1	turquoise
NBN	turquoise
NBPF14	turquoise
NCDN	turquoise
NCK1	turquoise
NCKAP1	turquoise
NCKAP5L	turquoise
NCKIPSD	turquoise

NCLN	turquoise
NCOA4	turquoise
NCOA7	turquoise
NCRNA00188	turquoise
NCRNA00189	turquoise
NCRNA00201	turquoise
NCRNA00239	turquoise
NCRNA00247	turquoise
NCRNA00264	turquoise
NCRNA00265	turquoise
NCS1	turquoise
NDE1	turquoise
NDFIP1	turquoise
NDFIP2	turquoise
NDN	turquoise
NDRG2	turquoise
NDUFA1	turquoise
NDUFA4	turquoise
NDUFA5	turquoise
NDUFA6	turquoise
NDUFAF1	turquoise
NDUFAF2	turquoise
NDUFB1	turquoise
NDUFB3	turquoise
NDUFB5	turquoise
NDUFS2	turquoise
NDUFS4	turquoise
NDUFS5	turquoise
NECAP1	turquoise
NECAP2	turquoise
NEDD1	turquoise
NEDD4	turquoise
NEGR1	turquoise
NEK1	turquoise
NEK7	turquoise
NEMF	turquoise
NETO2	turquoise
NFASC	turquoise
NFATC1	turquoise
NFIA	turquoise
NFKB2	turquoise
NFKBIB	turquoise
NFKBIE	turquoise
NFKBIZ	turquoise
NFU1	turquoise
NFXL1	turquoise
NFYB	turquoise
NGLY1	turquoise
NHLRC2	turquoise
NHS	turquoise
NIF3L1	turquoise
NIN	turquoise
NIPA1	turquoise
NIPSNAP1	turquoise
NIPSNAP3B	turquoise
NKIRAS1	turquoise
NKRF	turquoise
NLGN3	turquoise
NLK	turquoise
NMD3	turquoise
NME7	turquoise
NOC2L	turquoise
NOC3L	turquoise
NOC4L	turquoise
NOL6	turquoise
NOMO1	turquoise
NOP2	turquoise
NOP56	turquoise

NOP58	turquoise
NOS3	turquoise
NOTCH3	turquoise
NPFF	turquoise
NPHP3	turquoise
NPIPL3	turquoise
NPLOC4	turquoise
NPPA	turquoise
NPPA-AS1	turquoise
NPTXR	turquoise
NQO1	turquoise
NR1D2	turquoise
NR1H2	turquoise
NR3C1	turquoise
NR4A1	turquoise
NR4A3	turquoise
NRAS	turquoise
NRG4	turquoise
NRIP1	turquoise
NRM	turquoise
NRN1	turquoise
NRSN2	turquoise
NSA2	turquoise
NSL1	turquoise
NSMAF	turquoise
NSMCE2	turquoise
NSUN3	turquoise
NT5C3	turquoise
NT5DC1	turquoise
NT5DC2	turquoise
NT5DC3	turquoise
NTN3	turquoise
NTN5	turquoise
NUBP1	turquoise
NUBPL	turquoise
NUCB2	turquoise
NUDC	turquoise
NUDCD1	turquoise
NUDCD2	turquoise
NUDCD3	turquoise
NUDT12	turquoise
NUDT16	turquoise
NUDT21	turquoise
NUDT9P1	turquoise
NUF2	turquoise
NUFIP2	turquoise
NUP37	turquoise
NUP54	turquoise
NUP62CL	turquoise
NUP85	turquoise
NUS1	turquoise
NXT2	turquoise
OAF	turquoise
OBFC2A	turquoise
OCEL1	turquoise
OCIAD1	turquoise
ODF2L	turquoise
ODZ1	turquoise
OGFR	turquoise
OGFRL1	turquoise
OIP5	turquoise
OLA1	turquoise
OLFM2	turquoise
OMA1	turquoise
OPA1	turquoise
OPN3	turquoise
OPRL1	turquoise
OR52N4	turquoise

ORAI1	turquoise
ORC2	turquoise
ORC3	turquoise
ORC4	turquoise
OSBPL5	turquoise
OSBPL7	turquoise
OSBPL8	turquoise
OSCAR	turquoise
OSGEPL1	turquoise
OSGIN2	turquoise
OSTC	turquoise
OSTM1	turquoise
OTUD4	turquoise
OTUD5	turquoise
OTUD6B	turquoise
OXCT1	turquoise
OXR1	turquoise
OXSM	turquoise
P2RX4	turquoise
P2RY10	turquoise
P2RY11	turquoise
P2RY12	turquoise
P2RY13	turquoise
P2RY14	turquoise
P4HA1	turquoise
P4HB	turquoise
PA2G4P4	turquoise
PACRGL	turquoise
PACS2	turquoise
PAG1	turquoise
PAIP1	turquoise
PAIP2	turquoise
PAK1IP1	turquoise
PAK4	turquoise
PAN3	turquoise
PANK1	turquoise
PANK3	turquoise
PAPD4	turquoise
PAPD5	turquoise
PAPOLA	turquoise
PAPOLG	turquoise
PAQR3	turquoise
PAQR4	turquoise
PAQR7	turquoise
PAQR8	turquoise
PARD6B	turquoise
PARK2	turquoise
PARP11	turquoise
PARP16	turquoise
PARP8	turquoise
PART1	turquoise
PARVB	turquoise
PARVG	turquoise
PAX2	turquoise
PAX6	turquoise
PBK	turquoise
PBX3	turquoise
PBXIP1	turquoise
PC	turquoise
PCDH11Y	turquoise
PCDH12	turquoise
PCF11	turquoise
PCGF5	turquoise
PCGF6	turquoise
PCK2	turquoise
PCMT1	turquoise
PCMTD1	turquoise
PCMTD2	turquoise

PCNP	turquoise
PDAP1	turquoise
PDCD10	turquoise
PDCD1LG2	turquoise
PDCD2	turquoise
PDCD6IP	turquoise
PDE1B	turquoise
PDE3B	turquoise
PDE4A	turquoise
PDE4D	turquoise
PDE7A	turquoise
PDHX	turquoise
PDIA3P	turquoise
PDIK1L	turquoise
PDK1	turquoise
PDK2	turquoise
PDLIM2	turquoise
PDLIM5	turquoise
PDP1	turquoise
PDS5B	turquoise
PDSS1	turquoise
PDXK	turquoise
PEF1	turquoise
PELI1	turquoise
PELI3	turquoise
PELP1	turquoise
PEPD	turquoise
PES1	turquoise
PEX1	turquoise
PEX11A	turquoise
PEX11G	turquoise
PEX12	turquoise
PEX13	turquoise
PEX14	turquoise
PEX2	turquoise
PEX3	turquoise
PFDN4	turquoise
PFDN5	turquoise
PFKL	turquoise
PFKM	turquoise
PFN1	turquoise
PFN2	turquoise
PGAM5	turquoise
PGAP1	turquoise
PGAP3	turquoise
PGBD4	turquoise
PGGT1B	turquoise
PGM2L1	turquoise
PGM3	turquoise
PHAX	turquoise
PHC3	turquoise
PHF17	turquoise
PHF20L1	turquoise
PHF5A	turquoise
PHF6	turquoise
PHF7	turquoise
PHGDH	turquoise
PHIP	turquoise
PHKG2	turquoise
PHLDB1	turquoise
PHLDB3	turquoise
PHOX2A	turquoise
PHTF1	turquoise
PHTF2	turquoise
PI4K2B	turquoise
PIAS4	turquoise
PIBF1	turquoise
PIGA	turquoise

PIGB	turquoise
PIGF	turquoise
PIGK	turquoise
PIGM	turquoise
PIGS	turquoise
PIGW	turquoise
PIGY	turquoise
PIGZ	turquoise
PIK3AP1	turquoise
PIK3C2A	turquoise
PIK3CA	turquoise
PIKFYVE	turquoise
PIN4	turquoise
PION	turquoise
PITPNM1	turquoise
PIWIL4	turquoise
PJA2	turquoise
PKD2	turquoise
PKIA	turquoise
PKIB	turquoise
PKN1	turquoise
PKN2	turquoise
PLA2G15	turquoise
PLA2G4A	turquoise
PLAG1	turquoise
PLAGL1	turquoise
PLCB1	turquoise
PLCB2	turquoise
PLCB3	turquoise
PLCD3	turquoise
PLCL1	turquoise
PLCXD2	turquoise
PLD2	turquoise
PLD3	turquoise
PLD4	turquoise
PLDN	turquoise
PLEKHA1	turquoise
PLEKHA3	turquoise
PLEKHA6	turquoise
PLEKHA8	turquoise
PLEKHB1	turquoise
PLEKHF2	turquoise
PLEKHG3	turquoise
PLEKHH2	turquoise
PLEKHH3	turquoise
PLEKHM1	turquoise
PLEKHM2	turquoise
PLK1S1	turquoise
PLK4	turquoise
PLOD3	turquoise
PLRG1	turquoise
PLS1	turquoise
PLXNB1	turquoise
PLXNB2	turquoise
PM20D2	turquoise
PMAIP1	turquoise
PMF1	turquoise
PMPCA	turquoise
PMS1	turquoise
PNISR	turquoise
PNKD	turquoise
PNMA3	turquoise
PNPLA2	turquoise
PNPLA6	turquoise
PNPLA7	turquoise
PNPLA8	turquoise
PNRC2	turquoise
POC1B	turquoise

PODNL1	turquoise
PODXL	turquoise
POGLUT1	turquoise
POLD2	turquoise
POLDIP2	turquoise
POLG2	turquoise
POLI	turquoise
POLK	turquoise
POLQ	turquoise
POLR1E	turquoise
POLR2E	turquoise
POLR2K	turquoise
POLR3D	turquoise
POLR3G	turquoise
POLR3H	turquoise
POMT2	turquoise
PORCN	turquoise
POT1	turquoise
PPA1	turquoise
PPAN-P2RY11	turquoise
PPAT	turquoise
PPDPF	turquoise
PPEF1	turquoise
PPFIA3	turquoise
PPIB	turquoise
PPIF	turquoise
PPIG	turquoise
PPIL3	turquoise
PPIL4	turquoise
PPIP5K2	turquoise
PPM1B	turquoise
PPM1G	turquoise
PPP1CA	turquoise
PPP1CB	turquoise
PPP1CC	turquoise
PPP1R12A	turquoise
PPP1R13L	turquoise
PPP1R15A	turquoise
PPP1R15B	turquoise
PPP1R2	turquoise
PPP1R3D	turquoise
PPP1R7	turquoise
PPP2CB	turquoise
PPP2R1A	turquoise
PPP2R2A	turquoise
PPP2R3A	turquoise
PPP2R3C	turquoise
PPP2R4	turquoise
PPP2R5A	turquoise
PPP2R5B	turquoise
PPP2R5D	turquoise
PPP2R5E	turquoise
PPP3CA	turquoise
PPP3CB	turquoise
PPP4C	turquoise
PPP4R1L	turquoise
PPP4R2	turquoise
PPP4R4	turquoise
PPP5C	turquoise
PPP6C	turquoise
PPP6R1	turquoise
PPP6R3	turquoise
PPPDE1	turquoise
PPPDE2	turquoise
PPWD1	turquoise
PQBP1	turquoise
PQLC2	turquoise
PQLC3	turquoise

PRCC	turquoise
PRDM10	turquoise
PRDM5	turquoise
PRDX2	turquoise
PRDX3	turquoise
PREPL	turquoise
PRICKLE3	turquoise
PRKAA1	turquoise
PRKAB2	turquoise
PRKACB	turquoise
PRKAG2	turquoise
PRKAR1A	turquoise
PRKAR1B	turquoise
PRKCI	turquoise
PRKCSH	turquoise
PRKCZ	turquoise
PRKD3	turquoise
PRKRIR	turquoise
PRMT1	turquoise
PRMT10	turquoise
PRMT3	turquoise
PROC	turquoise
PROCA1	turquoise
PROM2	turquoise
PROZ	turquoise
PRPF18	turquoise
PRPF31	turquoise
PRPF38B	turquoise
PRPF39	turquoise
PRPF40A	turquoise
PRPF4B	turquoise
PRPF6	turquoise
PRPS2	turquoise
PRPSAP1	turquoise
PRR14	turquoise
PRRC1	turquoise
PRSS50	turquoise
PRSS8	turquoise
PRTFDC1	turquoise
PRX	turquoise
PSEN2	turquoise
PSIP1	turquoise
PSKH1	turquoise
PSMA1	turquoise
PSMA2	turquoise
PSMA3	turquoise
PSMA4	turquoise
PSMA6	turquoise
PSMA8	turquoise
PSMB1	turquoise
PSMB3	turquoise
PSMC1	turquoise
PSMC2	turquoise
PSMC3	turquoise
PSMC5	turquoise
PSMC6	turquoise
PSMD10	turquoise
PSMD13	turquoise
PSMD14	turquoise
PSMD4	turquoise
PSMD8	turquoise
PSME1	turquoise
PSMG1	turquoise
PSMG4	turquoise
PSTPIP1	turquoise
PTAR1	turquoise
PTBP2	turquoise
PTCHD2	turquoise

PTEN	turquoise
PTER	turquoise
PTGDS	turquoise
PTGER4	turquoise
PTGIR	turquoise
PTGR1	turquoise
PTGR2	turquoise
PTGS2	turquoise
PTH2R	turquoise
PTK6	turquoise
PTK7	turquoise
PTMS	turquoise
PTOV1	turquoise
PTP4A1	turquoise
PTPDC1	turquoise
PTPLAD1	turquoise
PTPLAD2	turquoise
PTPN12	turquoise
PTPN2	turquoise
PTPN22	turquoise
PTPN23	turquoise
PTPN4	turquoise
PTPN7	turquoise
PTPRO	turquoise
PTPRVP	turquoise
PTRH2	turquoise
PTS	turquoise
PUF60	turquoise
PUM2	turquoise
PURA	turquoise
PURB	turquoise
PUS10	turquoise
PUS7L	turquoise
PVRL3	turquoise
PWP2	turquoise
PWWP2A	turquoise
PXT1	turquoise
PYCR2	turquoise
PYROXD1	turquoise
QDPR	turquoise
QKI	turquoise
QPRT	turquoise
QRICH2	turquoise
QSER1	turquoise
RAB10	turquoise
RAB11B	turquoise
RAB11FIP2	turquoise
RAB12	turquoise
RAB14	turquoise
RAB18	turquoise
RAB1B	turquoise
RAB22A	turquoise
RAB27B	turquoise
RAB28	turquoise
RAB2A	turquoise
RAB2B	turquoise
RAB33B	turquoise
RAB37	turquoise
RAB39B	turquoise
RAB3GAP2	turquoise
RAB3IP	turquoise
RAB4A	turquoise
RAB5A	turquoise
RAB6A	turquoise
RAB8B	turquoise
RAB9B	turquoise
RABEP1	turquoise
RABL3	turquoise

RAD17	turquoise
RAD23A	turquoise
RAD51AP1	turquoise
RAD51B	turquoise
RAD51C	turquoise
RAE1	turquoise
RALA	turquoise
RALGPS2	turquoise
RALY	turquoise
RANBP3	turquoise
RANBP6	turquoise
RANGAP1	turquoise
RAP1A	turquoise
RAP1B	turquoise
RAP2A	turquoise
RAP2C	turquoise
RAPGEF6	turquoise
RAPSN	turquoise
RARG	turquoise
RARS2	turquoise
RASA1	turquoise
RASL11A	turquoise
RASSF1	turquoise
RASSF4	turquoise
RB1	turquoise
RB1CC1	turquoise
RBAK	turquoise
RBBP6	turquoise
RBBP8	turquoise
RBBP9	turquoise
RBCK1	turquoise
RBFOX3	turquoise
RBKS	turquoise
RBM10	turquoise
RBM11	turquoise
RBM12B	turquoise
RBM15	turquoise
RBM18	turquoise
RBM25	turquoise
RBM26-AS1	turquoise
RBM27	turquoise
RBM3	turquoise
RBM34	turquoise
RBM39	turquoise
RBM4	turquoise
RBM41	turquoise
RBM42	turquoise
RBM43	turquoise
RBM44	turquoise
RBM47	turquoise
RBM5	turquoise
RBM7	turquoise
RBMS1	turquoise
RBX1	turquoise
RCBTB1	turquoise
RCBTB2	turquoise
RCHY1	turquoise
RCN2	turquoise
RCN3	turquoise
RCOR3	turquoise
RCVRN	turquoise
RDBP	turquoise
RDH10	turquoise
RDX	turquoise
RECQL	turquoise
RECQL5	turquoise
REEP3	turquoise
REEP4	turquoise

REEP5	turquoise
RELB	turquoise
RENBP	turquoise
RER1	turquoise
REST	turquoise
RET	turquoise
REV1	turquoise
REV3L	turquoise
REXO4	turquoise
RFC2	turquoise
RFC3	turquoise
RFC5	turquoise
RFK	turquoise
RFNG	turquoise
RFWD2	turquoise
RFX7	turquoise
RFXAP	turquoise
RG9MTD1	turquoise
RG9MTD2	turquoise
RGS1	turquoise
RGS12	turquoise
RGS18	turquoise
RGS5	turquoise
RHBDD2	turquoise
RHEB	turquoise
RHOBTB3	turquoise
RHOQ	turquoise
RHOT1	turquoise
RHOU	turquoise
RHPN2	turquoise
RIC8A	turquoise
RIC8B	turquoise
RICTOR	turquoise
RIF1	turquoise
RIMKLA	turquoise
RIMKLB	turquoise
RIN1	turquoise
RING1	turquoise
RINT1	turquoise
RIOK2	turquoise
RIPK2	turquoise
RIPK3	turquoise
RIT1	turquoise
RLF	turquoise
RLN1	turquoise
RLN2	turquoise
RMI1	turquoise
RMND1	turquoise
RMND5B	turquoise
RNASEH2A	turquoise
RNF103	turquoise
RNF11	turquoise
RNF121	turquoise
RNF123	turquoise
RNF13	turquoise
RNF135	turquoise
RNF138	turquoise
RNF139	turquoise
RNF141	turquoise
RNF144B	turquoise
RNF146	turquoise
RNF187	turquoise
RNF19A	turquoise
RNF2	turquoise
RNF217	turquoise
RNF219	turquoise
RNF220	turquoise
RNF26	turquoise

RNF31	turquoise
RNF38	turquoise
RNF40	turquoise
RNF6	turquoise
RNF7	turquoise
RNFT1	turquoise
RNGTT	turquoise
RNH1	turquoise
RNLS	turquoise
RNPC3	turquoise
RNPEPL1	turquoise
ROCK1	turquoise
RORA	turquoise
RORC	turquoise
RP2	turquoise
RPAP2	turquoise
RPAP3	turquoise
RPE	turquoise
RPF1	turquoise
RPF2	turquoise
RPGR	turquoise
RPH3AL	turquoise
RPL11	turquoise
RPL15	turquoise
RPL17	turquoise
RPL22L1	turquoise
RPL23	turquoise
RPL23AP53	turquoise
RPL23AP82	turquoise
RPL26	turquoise
RPL26L1	turquoise
RPL27	turquoise
RPL30	turquoise
RPL31	turquoise
RPL31P11	turquoise
RPL34	turquoise
RPL35	turquoise
RPL36A	turquoise
RPL36AL	turquoise
RPL39	turquoise
RPL39L	turquoise
RPL41	turquoise
RPL7	turquoise
RPL9	turquoise
RPRD1A	turquoise
RPS15A	turquoise
RPS24	turquoise
RPS27	turquoise
RPS27L	turquoise
RPS29	turquoise
RPS3A	turquoise
RPS6KA3	turquoise
RPS6KA4	turquoise
RPS6KA5	turquoise
RPS6KB1	turquoise
RPS7	turquoise
RPTOR	turquoise
RPUSD2	turquoise
RRM2B	turquoise
RRNAD1	turquoise
RRP1	turquoise
RRP36	turquoise
RRP7A	turquoise
RRP8	turquoise
RRS1	turquoise
RSBN1	turquoise
RSBN1L	turquoise
RSG1	turquoise

RSL24D1	turquoise
RSPH4A	turquoise
RSPH6A	turquoise
RSPRY1	turquoise
RSRC2	turquoise
RTKN	turquoise
RTKN2	turquoise
RUFY2	turquoise
RUFY3	turquoise
RUNDC3B	turquoise
RUVBL2	turquoise
RWDD1	turquoise
RWDD2A	turquoise
RWDD3	turquoise
RWDD4	turquoise
RXFP2	turquoise
RXRB	turquoise
RYBP	turquoise
RYK	turquoise
RYR1	turquoise
RYR3	turquoise
S100A10	turquoise
S100A4	turquoise
S100P	turquoise
S1PR2	turquoise
SACM1L	turquoise
SACS	turquoise
SAFB	turquoise
SAFB2	turquoise
SAMD8	turquoise
SAMD9	turquoise
SAMSN1	turquoise
SAP25	turquoise
SAP30BP	turquoise
SAR1A	turquoise
SAR1B	turquoise
SARNP	turquoise
SART1	turquoise
SASH3	turquoise
SASS6	turquoise
SAT1	turquoise
SC5DL	turquoise
SCAF11	turquoise
SCAI	turquoise
SCAMP1	turquoise
SCAMP3	turquoise
SCAP	turquoise
SCARB1	turquoise
SCFD1	turquoise
SCLT1	turquoise
SCML1	turquoise
SCN3A	turquoise
SCN5A	turquoise
SCN9A	turquoise
SCOC	turquoise
SCP2	turquoise
SCRN3	turquoise
SCUBE1	turquoise
SCYL1	turquoise
SCYL2	turquoise
SCYL3	turquoise
SDF4	turquoise
SDHD	turquoise
SDR42E1	turquoise
SEC1	turquoise
SEC11A	turquoise
SEC11C	turquoise
SEC13	turquoise

SEC23A	turquoise
SEC24A	turquoise
SEC61A1	turquoise
SEC62	turquoise
SECISBP2L	turquoise
SEL1L	turquoise
SELK	turquoise
SELT	turquoise
SEMA3A	turquoise
SEMA3C	turquoise
SEMA4F	turquoise
SEMA6C	turquoise
SENP1	turquoise
SENP6	turquoise
SENP7	turquoise
SEPSECS	turquoise
SEPT5-GP1BB	turquoise
SERAC1	turquoise
SERINC1	turquoise
SERP1	turquoise
SERPINB10	turquoise
SERPINH1	turquoise
SERPINI1	turquoise
SESN1	turquoise
SESTD1	turquoise
SETDB2	turquoise
SF3A1	turquoise
SF3A2	turquoise
SF3B1	turquoise
SF3B14	turquoise
SFR1	turquoise
SFSWAP	turquoise
SFTPD	turquoise
SFXN4	turquoise
SGCB	turquoise
SGMS1	turquoise
SGMS2	turquoise
SGOL2	turquoise
SGPP1	turquoise
SGTA	turquoise
SGTB	turquoise
SH3BGRL	turquoise
SH3BGRL3	turquoise
SH3BP1	turquoise
SH3BP2	turquoise
SH3GL1	turquoise
SH3TC1	turquoise
SHB	turquoise
SHFM1	turquoise
SHMT2	turquoise
SHOC2	turquoise
SIAH1	turquoise
SIGLEC16	turquoise
SIKE1	turquoise
SIL1	turquoise
SIPA1	turquoise
SIRT1	turquoise
SIRT2	turquoise
SKA2	turquoise
SKAP2	turquoise
SKIL	turquoise
SKIV2L	turquoise
SKIV2L2	turquoise
SKP2	turquoise
SLAIN1	turquoise
SLAIN2	turquoise
SLC10A3	turquoise
SLC10A7	turquoise

SLC12A2	turquoise
SLC12A5	turquoise
SLC12A7	turquoise
SLC12A9	turquoise
SLC16A10	turquoise
SLC16A13	turquoise
SLC16A14	turquoise
SLC16A6	turquoise
SLC16A7	turquoise
SLC17A5	turquoise
SLC18A2	turquoise
SLC19A2	turquoise
SLC20A1	turquoise
SLC22A15	turquoise
SLC22A18	turquoise
SLC22A18AS	turquoise
SLC24A6	turquoise
SLC25A1	turquoise
SLC25A10	turquoise
SLC25A11	turquoise
SLC25A22	turquoise
SLC25A24	turquoise
SLC25A3	turquoise
SLC25A32	turquoise
SLC25A34	turquoise
SLC25A36	turquoise
SLC25A38	turquoise
SLC25A40	turquoise
SLC25A43	turquoise
SLC25A46	turquoise
SLC26A2	turquoise
SLC26A6	turquoise
SLC27A1	turquoise
SLC27A3	turquoise
SLC27A4	turquoise
SLC29A3	turquoise
SLC29A4	turquoise
SLC2A12	turquoise
SLC2A13	turquoise
SLC2A6	turquoise
SLC2A9	turquoise
SLC30A1	turquoise
SLC30A5	turquoise
SLC30A6	turquoise
SLC30A7	turquoise
SLC30A9	turquoise
SLC33A1	turquoise
SLC35A1	turquoise
SLC35A3	turquoise
SLC35A4	turquoise
SLC35A5	turquoise
SLC35B3	turquoise
SLC35B4	turquoise
SLC35C1	turquoise
SLC35C2	turquoise
SLC35E4	turquoise
SLC35F5	turquoise
SLC35G1	turquoise
SLC36A4	turquoise
SLC37A3	turquoise
SLC38A10	turquoise
SLC38A2	turquoise
SLC38A9	turquoise
SLC39A10	turquoise
SLC39A7	turquoise
SLC39A8	turquoise
SLC3A2	turquoise
SLC40A1	turquoise

SLC41A2	turquoise
SLC43A1	turquoise
SLC44A3	turquoise
SLC46A3	turquoise
SLC4A10	turquoise
SLC4A11	turquoise
SLC4A2	turquoise
SLC4A7	turquoise
SLC5A3	turquoise
SLC5A6	turquoise
SLC7A11	turquoise
SLC7A8	turquoise
SLC9A3R1	turquoise
SLC9A3R2	turquoise
SLC9A6	turquoise
SLC9B1	turquoise
SLCO4C1	turquoise
SLFN12	turquoise
SLIT1	turquoise
SLK	turquoise
SLMAP	turquoise
SLMO2	turquoise
SLU7	turquoise
SMAD2	turquoise
SMAD4	turquoise
SMAD5	turquoise
SMARCA1	turquoise
SMARCA5	turquoise
SMARCAD1	turquoise
SMARCB1	turquoise
SMC2	turquoise
SMC4	turquoise
SMC5	turquoise
SMC6	turquoise
SMCHD1	turquoise
SMEK1	turquoise
SMEK2	turquoise
SMG1	turquoise
SMG5	turquoise
SMG9	turquoise
SMNDC1	turquoise
SMPD1	turquoise
SMPD4	turquoise
SMPDL3A	turquoise
SMTN	turquoise
SMURF2	turquoise
SMYD2	turquoise
SNAPC1	turquoise
SNORA31	turquoise
SNORA32	turquoise
SNORA8	turquoise
SNRK	turquoise
SNRNP27	turquoise
SNRNP35	turquoise
SNRNP48	turquoise
SNRPB	turquoise
SNRPB2	turquoise
SNRPD1	turquoise
SNRPE	turquoise
SNRPG	turquoise
SNTB1	turquoise
SNX10	turquoise
SNX13	turquoise
SNX14	turquoise
SNX16	turquoise
SNX17	turquoise
SNX18	turquoise
SNX2	turquoise

SNX21	turquoise
SNX25	turquoise
SNX3	turquoise
SNX32	turquoise
SNX4	turquoise
SNX8	turquoise
SOCS4	turquoise
SOCS5	turquoise
SOCS6	turquoise
SORBS3	turquoise
SP3	turquoise
SP4	turquoise
SPA17	turquoise
SPAG16	turquoise
SPAG5	turquoise
SPAG9	turquoise
SPAST	turquoise
SPATA6	turquoise
SPCS2	turquoise
SPCS3	turquoise
SPEF2	turquoise
SPHAR	turquoise
SPIN3	turquoise
SPIN4	turquoise
SPINT2	turquoise
SPIRE2	turquoise
SPNS1	turquoise
SPOPL	turquoise
SPR	turquoise
SPRYD3	turquoise
SPTBN2	turquoise
SPTBN5	turquoise
SPTLC1	turquoise
SPTSSA	turquoise
SPTY2D1	turquoise
SRD5A1	turquoise
SREBF1	turquoise
SREK1	turquoise
SREK1IP1	turquoise
SRM	turquoise
SRP14	turquoise
SRP19	turquoise
SRP9	turquoise
SRSF10	turquoise
SRSF11	turquoise
SRSF3	turquoise
SRSF6	turquoise
SRSF8	turquoise
SS18	turquoise
SS18L1	turquoise
SS18L2	turquoise
SSFA2	turquoise
SSPN	turquoise
SSPO	turquoise
SSR3	turquoise
SSSCA1	turquoise
SSU72	turquoise
SSX2IP	turquoise
ST18	turquoise
ST5	turquoise
ST7L	turquoise
ST8SIA4	turquoise
STAB1	turquoise
STAC3	turquoise
STAG1	turquoise
STAG2	turquoise
STAM2	turquoise
STARD3	turquoise

STARD3NL	turquoise
STARD4	turquoise
STAU2	turquoise
STEAP2	turquoise
STEAP4	turquoise
STK10	turquoise
STK11IP	turquoise
STK17A	turquoise
STK17B	turquoise
STK32C	turquoise
STK38L	turquoise
STOM	turquoise
STON1	turquoise
STRADA	turquoise
STRN3	turquoise
STRN4	turquoise
STT3B	turquoise
STX16	turquoise
STX4	turquoise
STX5	turquoise
STX7	turquoise
STXBP2	turquoise
STXBP3	turquoise
STXBP4	turquoise
STXBP5	turquoise
STYX	turquoise
STYXL1	turquoise
SUB1	turquoise
SUCLA2	turquoise
SUCLG2	turquoise
SUCNR1	turquoise
SUGP1	turquoise
SUGT1P3	turquoise
SULT1A1	turquoise
SULT1B1	turquoise
SUMF2	turquoise
SUMO1	turquoise
SUMO1P1	turquoise
SUMO2	turquoise
SUMO3	turquoise
SUOX	turquoise
SUPT5H	turquoise
SURF6	turquoise
SUV39H1	turquoise
SUV39H2	turquoise
SUZ12	turquoise
SVIP	turquoise
SWT1	turquoise
SYCP2	turquoise
SYCP3	turquoise
SYDE2	turquoise
SYMPK	turquoise
SYN1	turquoise
SYNGR2	turquoise
SYNJ1	turquoise
SYP	turquoise
SYTL3	turquoise
TAB1	turquoise
TAB2	turquoise
TAB3	turquoise
TACO1	turquoise
TADA1	turquoise
TAF12	turquoise
TAF13	turquoise
TAF1A	turquoise
TAF1D	turquoise
TAF2	turquoise
TAF5	turquoise

TAF7	turquoise
TAF9	turquoise
TAF9B	turquoise
TAGLN	turquoise
TANK	turquoise
TAOK1	turquoise
TAPBPL	turquoise
TARS2	turquoise
TAS1R3	turquoise
TAS2R14	turquoise
TAS2R40	turquoise
TATDN1	turquoise
TATDN3	turquoise
TAX1BP1	turquoise
TBC1D10B	turquoise
TBC1D12	turquoise
TBC1D13	turquoise
TBC1D15	turquoise
TBC1D22A	turquoise
TBC1D23	turquoise
TBC1D25	turquoise
TBC1D4	turquoise
TBC1D8B	turquoise
TBCA	turquoise
TBCB	turquoise
TBCD	turquoise
TBCEL	turquoise
TBK1	turquoise
TBKBP1	turquoise
TBL1XR1	turquoise
TBPL1	turquoise
TBXA2R	turquoise
TC2N	turquoise
TCAP	turquoise
TCEA1	turquoise
TCEAL3	turquoise
TCEAL8	turquoise
TCEANC	turquoise
TCF25	turquoise
TCF7L1	turquoise
TCTE3	turquoise
TCTEX1D2	turquoise
TDG	turquoise
TDP2	turquoise
TDRD6	turquoise
TECR	turquoise
TENC1	turquoise
TESK1	turquoise
TESK2	turquoise
TET1	turquoise
TET2	turquoise
TEX9	turquoise
TFAM	turquoise
TFAP2E	turquoise
TFAP4	turquoise
TFEC	turquoise
TFPT	turquoise
TFRC	turquoise
TGDS	turquoise
TGFA	turquoise
TGFBR1	turquoise
TGFBR2	turquoise
TGM4	turquoise
THAP1	turquoise
THAP2	turquoise
THAP4	turquoise
THAP5	turquoise
THAP6	turquoise

THAP8	turquoise
THAP9	turquoise
THBS2	turquoise
THBS3	turquoise
THEMIS	turquoise
THNSL1	turquoise
THOC3	turquoise
THOC4	turquoise
THOC7	turquoise
THUMPD1	turquoise
TIA1	turquoise
TICAM1	turquoise
TIE1	turquoise
TIFA	turquoise
TIGD1	turquoise
TIGD2	turquoise
TIGD7	turquoise
TIMM17A	turquoise
TIMM22	turquoise
TIMM23	turquoise
TIMM8B	turquoise
TIMMDC1	turquoise
TIMP1	turquoise
TIPRL	turquoise
TJAP1	turquoise
TJP3	turquoise
TK1	turquoise
TLK1	turquoise
TLR1	turquoise
TLR10	turquoise
TLR6	turquoise
TLR8	turquoise
TM2D3	turquoise
TM6SF1	turquoise
TM9SF3	turquoise
TMBIM4	turquoise
TMC4	turquoise
TMCO1	turquoise
TMCO4	turquoise
TMED10	turquoise
TMED10P1	turquoise
TMED2	turquoise
TMED5	turquoise
TMED7	turquoise
TMEM104	turquoise
TMEM106B	turquoise
TMEM115	turquoise
TMEM123	turquoise
TMEM126A	turquoise
TMEM126B	turquoise
TMEM128	turquoise
TMEM135	turquoise
TMEM144	turquoise
TMEM14B	turquoise
TMEM150B	turquoise
TMEM161B	turquoise
TMEM165	turquoise
TMEM167A	turquoise
TMEM167B	turquoise
TMEM168	turquoise
TMEM169	turquoise
TMEM170A	turquoise
TMEM170B	turquoise
TMEM173	turquoise
TMEM180	turquoise
TMEM181	turquoise
TMEM182	turquoise
TMEM183A	turquoise

TMEM184A	turquoise
TMEM185B	turquoise
TMEM188	turquoise
TMEM194B	turquoise
TMEM199	turquoise
TMEM200A	turquoise
TMEM201	turquoise
TMEM204	turquoise
TMEM206	turquoise
TMEM209	turquoise
TMEM220	turquoise
TMEM221	turquoise
TMEM30A	turquoise
TMEM30B	turquoise
TMEM33	turquoise
TMEM38B	turquoise
TMEM39B	turquoise
TMEM48	turquoise
TMEM50A	turquoise
TMEM51	turquoise
TMEM55A	turquoise
TMEM56-RWDD3	turquoise
TMEM63B	turquoise
TMEM63C	turquoise
TMEM64	turquoise
TMEM65	turquoise
TMEM66	turquoise
TMEM70	turquoise
TMEM71	turquoise
TMEM86B	turquoise
TMEM87B	turquoise
TMEM8B	turquoise
TMEM9B	turquoise
TMF1	turquoise
TMOD2	turquoise
TMPO	turquoise
TMPRSS13	turquoise
TMPRSS6	turquoise
TMTC3	turquoise
TMX1	turquoise
TMX3	turquoise
TNFAIP6	turquoise
TNFAIP8	turquoise
TNFRSF10B	turquoise
TNFRSF10D	turquoise
TNFRSF8	turquoise
TNFSF10	turquoise
TNFSF13B	turquoise
TNKS2	turquoise
TNPO1	turquoise
TOB1	turquoise
TOLLIP	turquoise
TOM1	turquoise
TOMM20	turquoise
TOMM40	turquoise
TOMM5	turquoise
TONSL	turquoise
TOP1MT	turquoise
TOPORS	turquoise
TOR1A	turquoise
TOX2	turquoise
TP53INP1	turquoise
TP63	turquoise
TPI1	turquoise
TPK1	turquoise
TPPP3	turquoise
TPRKB	turquoise
TPT1	turquoise

TRA2A	turquoise
TRA2B	turquoise
TRAF3IP2-AS1	turquoise
TRAF7	turquoise
TRAM1	turquoise
TRAP1	turquoise
TRAPPC1	turquoise
TRAPPC2P1	turquoise
TRAPPC6B	turquoise
TRAPPC8	turquoise
TRAPPC9	turquoise
TRDMT1	turquoise
TREX1	turquoise
TRIAP1	turquoise
TRIB2	turquoise
TRIM11	turquoise
TRIM13	turquoise
TRIM2	turquoise
TRIM23	turquoise
TRIM28	turquoise
TRIM3	turquoise
TRIM33	turquoise
TRIM39	turquoise
TRIM41	turquoise
TRIM47	turquoise
TRIM52	turquoise
TRIM59	turquoise
TRIM6	turquoise
TRIM61	turquoise
TRIM72	turquoise
TRIP10	turquoise
TRMT12	turquoise
TRMT1L	turquoise
TRNT1	turquoise
TROVE2	turquoise
TRPC1	turquoise
TRPM2	turquoise
TRPM5	turquoise
TRPM7	turquoise
TRPV2	turquoise
TRPV4	turquoise
TRPV6	turquoise
TRUB1	turquoise
TSC2	turquoise
TSC22D4	turquoise
TSGA10	turquoise
TSGA10IP	turquoise
TSNAX	turquoise
TSPAN2	turquoise
TSPAN6	turquoise
TSPYL4	turquoise
TTC14	turquoise
TTC15	turquoise
TTC26	turquoise
TTC30A	turquoise
TTC30B	turquoise
TTC32	turquoise
TTC33	turquoise
TTC35	turquoise
TTC3P1	turquoise
TTC7A	turquoise
TTK	turquoise
TTLL1	turquoise
TTLL12	turquoise
TTYH1	turquoise
TUBA1B	turquoise
TUBB2C	turquoise
TUBD1	turquoise

TUBE1	turquoise
TUBG1	turquoise
TUBG2	turquoise
TUBGCP2	turquoise
TWF1	turquoise
TWF2	turquoise
TWISTNB	turquoise
TWSG1	turquoise
TXLNB	turquoise
TXNDC12	turquoise
TXNDC16	turquoise
TXNDC17	turquoise
TXNDC3	turquoise
TXNDC9	turquoise
TXNL1	turquoise
TXNL4B	turquoise
TXNRD2	turquoise
TYMP	turquoise
TYW3	turquoise
TYW5	turquoise
U2AF1	turquoise
U2SURP	turquoise
UAP1L1	turquoise
UBA2	turquoise
UBA3	turquoise
UBA5	turquoise
UBA6	turquoise
UBASH3A	turquoise
UBC	turquoise
UBE2A	turquoise
UBE2D1	turquoise
UBE2D3	turquoise
UBE2E1	turquoise
UBE2G1	turquoise
UBE2J1	turquoise
UBE2J2	turquoise
UBE2K	turquoise
UBE2M	turquoise
UBE2N	turquoise
UBE2O	turquoise
UBE2Q2	turquoise
UBE2T	turquoise
UBE2V2	turquoise
UBE2W	turquoise
UBE3A	turquoise
UBE4A	turquoise
UBL3	turquoise
UBL7	turquoise
UBLCP1	turquoise
UBOX5	turquoise
UBR1	turquoise
UBR3	turquoise
UBR5	turquoise
UBTD2	turquoise
UBXN2B	turquoise
UBXN4	turquoise
UBXN8	turquoise
UCHL3	turquoise
UCHL5	turquoise
UCP2	turquoise
UCP3	turquoise
UFM1	turquoise
UGCG	turquoise
UGDH	turquoise
UGGT2	turquoise
UGT2B11	turquoise
UHMK1	turquoise
UNC13D	turquoise

UNC45A	turquoise
UNC50	turquoise
UNC5A	turquoise
UPF1	turquoise
UPK3A	turquoise
UPK3B	turquoise
UQCRB	turquoise
UQCRC1	turquoise
UQCRC2	turquoise
UQCRH	turquoise
UQCRQ	turquoise
URGCP	turquoise
USO1	turquoise
USP1	turquoise
USP12	turquoise
USP14	turquoise
USP15	turquoise
USP16	turquoise
USP19	turquoise
USP21	turquoise
USP25	turquoise
USP31	turquoise
USP37	turquoise
USP38	turquoise
USP44	turquoise
USP45	turquoise
USP46	turquoise
USP5	turquoise
USP51	turquoise
USP53	turquoise
USP6	turquoise
USPL1	turquoise
UTP11L	turquoise
UTP14C	turquoise
UTP15	turquoise
UTP23	turquoise
VAC14	turquoise
VAMP4	turquoise
VAMP7	turquoise
VAPA	turquoise
VARS	turquoise
VASH2	turquoise
VBP1	turquoise
VCPIP1	turquoise
VEGFA	turquoise
VEGFB	turquoise
VENTX	turquoise
VEZF1	turquoise
VEZT	turquoise
VMA21	turquoise
VMP1	turquoise
VNN1	turquoise
VNN2	turquoise
VPS13A	turquoise
VPS13C	turquoise
VPS18	turquoise
VPS26A	turquoise
VPS28	turquoise
VPS29	turquoise
VPS33B	turquoise
VPS36	turquoise
VPS37A	turquoise
VPS37B	turquoise
VPS4A	turquoise
VPS4B	turquoise
VPS54	turquoise
VPS72	turquoise
VRK1	turquoise

VRK2	turquoise
VTA1	turquoise
WAPAL	turquoise
WARS2	turquoise
WASL	turquoise
WBP4	turquoise
WBSCR16	turquoise
WBSCR22	turquoise
WDFY1	turquoise
WDHD1	turquoise
WDPCP	turquoise
WDR1	turquoise
WDR17	turquoise
WDR35	turquoise
WDR36	turquoise
WDR41	turquoise
WDR43	turquoise
WDR44	turquoise
WDR46	turquoise
WDR47	turquoise
WDR5	turquoise
WDR53	turquoise
WDR5B	turquoise
WDR62	turquoise
WDR81	turquoise
WDR89	turquoise
WDR91	turquoise
WDTC1	turquoise
WEE1	turquoise
WFS1	turquoise
WHAMMP2	turquoise
WHSC2	turquoise
WNT10A	turquoise
WRAP73	turquoise
WRB	turquoise
WSB1	turquoise
WSCD1	turquoise
WTAP	turquoise
WWC2	turquoise
WWP1	turquoise
XAB2	turquoise
XIST	turquoise
XPO1	turquoise
XRCC1	turquoise
XRCC4	turquoise
XRN1	turquoise
XRRA1	turquoise
YAF2	turquoise
YES1	turquoise
YIPF2	turquoise
YIPF4	turquoise
YIPF6	turquoise
YJEFN3	turquoise
YKT6	turquoise
YOD1	turquoise
YPEL5	turquoise
YTHDC2	turquoise
YTHDF3	turquoise
ZADH2	turquoise
ZBED5	turquoise
ZBTB1	turquoise
ZBTB10	turquoise
ZBTB11	turquoise
ZBTB12	turquoise
ZBTB2	turquoise
ZBTB25	turquoise
ZBTB26	turquoise
ZBTB33	turquoise

ZBTB34	turquoise
ZBTB38	turquoise
ZBTB41	turquoise
ZBTB43	turquoise
ZBTB45	turquoise
ZBTB48	turquoise
ZBTB6	turquoise
ZBTB7B	turquoise
ZBTB8OS	turquoise
ZC3H10	turquoise
ZC3H11A	turquoise
ZC3H12C	turquoise
ZC3H15	turquoise
ZC3H4	turquoise
ZCCHC10	turquoise
ZCCHC17	turquoise
ZCCHC6	turquoise
ZCCHC7	turquoise
ZCCHC8	turquoise
ZCCHC9	turquoise
ZCRB1	turquoise
ZCWPW1	turquoise
ZDBF2	turquoise
ZDHHC13	turquoise
ZDHHC14	turquoise
ZDHHC16	turquoise
ZDHHC17	turquoise
ZDHHC2	turquoise
ZDHHC20	turquoise
ZDHHC21	turquoise
ZDHHC6	turquoise
ZEB1	turquoise
ZEB1-AS1	turquoise
ZFAND1	turquoise
ZFAND5	turquoise
ZFAND6	turquoise
ZFAT	turquoise
ZFC3H1	turquoise
ZFP1	turquoise
ZFP14	turquoise
ZFP161	turquoise
ZFP2	turquoise
ZFP3	turquoise
ZFP62	turquoise
ZFP82	turquoise
ZFP92	turquoise
ZFR	turquoise
ZFX	turquoise
ZFYVE16	turquoise
ZGPAT	turquoise
ZHX1	turquoise
ZKSCAN4	turquoise
ZMAT1	turquoise
ZMAT2	turquoise
ZMAT3	turquoise
ZMPSTE24	turquoise
ZMYM1	turquoise
ZMYM2	turquoise
ZMYM5	turquoise
ZMYM6	turquoise
ZMYND11	turquoise
ZNF100	turquoise
ZNF107	turquoise
ZNF117	turquoise
ZNF12	turquoise
ZNF121	turquoise
ZNF124	turquoise
ZNF131	turquoise

ZNF136	turquoise
ZNF138	turquoise
ZNF141	turquoise
ZNF146	turquoise
ZNF148	turquoise
ZNF157	turquoise
ZNF167	turquoise
ZNF169	turquoise
ZNF17	turquoise
ZNF175	turquoise
ZNF18	turquoise
ZNF180	turquoise
ZNF181	turquoise
ZNF182	turquoise
ZNF184	turquoise
ZNF187	turquoise
ZNF189	turquoise
ZNF197	turquoise
ZNF208	turquoise
ZNF213	turquoise
ZNF215	turquoise
ZNF22	turquoise
ZNF221	turquoise
ZNF222	turquoise
ZNF224	turquoise
ZNF225	turquoise
ZNF226	turquoise
ZNF227	turquoise
ZNF230	turquoise
ZNF238	turquoise
ZNF24	turquoise
ZNF248	turquoise
ZNF25	turquoise
ZNF252	turquoise
ZNF253	turquoise
ZNF254	turquoise
ZNF256	turquoise
ZNF257	turquoise
ZNF260	turquoise
ZNF267	turquoise
ZNF268	turquoise
ZNF271	turquoise
ZNF273	turquoise
ZNF277	turquoise
ZNF28	turquoise
ZNF280C	turquoise
ZNF280D	turquoise
ZNF281	turquoise
ZNF282	turquoise
ZNF283	turquoise
ZNF286A	turquoise
ZNF292	turquoise
ZNF295	turquoise
ZNF296	turquoise
ZNF302	turquoise
ZNF322	turquoise
ZNF323	turquoise
ZNF324B	turquoise
ZNF326	turquoise
ZNF33A	turquoise
ZNF33B	turquoise
ZNF34	turquoise
ZNF347	turquoise
ZNF350	turquoise
ZNF354A	turquoise
ZNF354B	turquoise
ZNF354C	turquoise
ZNF382	turquoise

ZNF384	turquoise
ZNF385C	turquoise
ZNF391	turquoise
ZNF396	turquoise
ZNF408	turquoise
ZNF410	turquoise
ZNF415	turquoise
ZNF420	turquoise
ZNF429	turquoise
ZNF43	turquoise
ZNF430	turquoise
ZNF432	turquoise
ZNF434	turquoise
ZNF439	turquoise
ZNF441	turquoise
ZNF442	turquoise
ZNF443	turquoise
ZNF449	turquoise
ZNF451	turquoise
ZNF468	turquoise
ZNF470	turquoise
ZNF480	turquoise
ZNF484	turquoise
ZNF487P	turquoise
ZNF493	turquoise
ZNF497	turquoise
ZNF507	turquoise
ZNF510	turquoise
ZNF518A	turquoise
ZNF518B	turquoise
ZNF519	turquoise
ZNF525	turquoise
ZNF526	turquoise
ZNF529	turquoise
ZNF546	turquoise
ZNF547	turquoise
ZNF554	turquoise
ZNF555	turquoise
ZNF558	turquoise
ZNF561	turquoise
ZNF566	turquoise
ZNF567	turquoise
ZNF570	turquoise
ZNF571	turquoise
ZNF572	turquoise
ZNF574	turquoise
ZNF583	turquoise
ZNF585A	turquoise
ZNF586	turquoise
ZNF592	turquoise
ZNF594	turquoise
ZNF595	turquoise
ZNF597	turquoise
ZNF605	turquoise
ZNF606	turquoise
ZNF610	turquoise
ZNF613	turquoise
ZNF614	turquoise
ZNF615	turquoise
ZNF620	turquoise
ZNF622	turquoise
ZNF624	turquoise
ZNF627	turquoise
ZNF639	turquoise
ZNF642	turquoise
ZNF643	turquoise
ZNF644	turquoise
ZNF649	turquoise

ZNF654	turquoise
ZNF655	turquoise
ZNF658	turquoise
ZNF662	turquoise
ZNF670	turquoise
ZNF672	turquoise
ZNF674	turquoise
ZNF675	turquoise
ZNF680	turquoise
ZNF681	turquoise
ZNF684	turquoise
ZNF697	turquoise
ZNF700	turquoise
ZNF701	turquoise
ZNF702P	turquoise
ZNF703	turquoise
ZNF708	turquoise
ZNF709	turquoise
ZNF711	turquoise
ZNF717	turquoise
ZNF718	turquoise
ZNF721	turquoise
ZNF736	turquoise
ZNF738	turquoise
ZNF750	turquoise
ZNF75A	turquoise
ZNF75D	turquoise
ZNF761	turquoise
ZNF765	turquoise
ZNF770	turquoise
ZNF772	turquoise
ZNF776	turquoise
ZNF777	turquoise
ZNF780A	turquoise
ZNF780B	turquoise
ZNF781	turquoise
ZNF782	turquoise
ZNF788	turquoise
ZNF791	turquoise
ZNF799	turquoise
ZNF80	turquoise
ZNF800	turquoise
ZNF813	turquoise
ZNF816	turquoise
ZNF829	turquoise
ZNF830	turquoise
ZNF836	turquoise
ZNF839	turquoise
ZNF84	turquoise
ZNF844	turquoise
ZNF845	turquoise
ZNF85	turquoise
ZNF853	turquoise
ZNF876P	turquoise
ZNF879	turquoise
ZNF90	turquoise
ZNF91	turquoise
ZNF92	turquoise
ZNF93	turquoise
ZNHIT3	turquoise
ZNRF1	turquoise
ZNRF2	turquoise
ZRANB1	turquoise
ZRANB2	turquoise
ZRANB3	turquoise
ZSCAN12	turquoise
ZSCAN5A	turquoise
ZSWIM4	turquoise

ZSWIM5		turquoise
ZUFSP		turquoise
ZWILCH		turquoise
ZXDA		turquoise
ZXDB		turquoise
ZZZ3		turquoise
	9-Mar	yellow
	1-Sep	yellow
A1BG		yellow
ABCB8		yellow
ABCB9		yellow
ABHD1		yellow
ABHD14A		yellow
ABT1		yellow
ACADS		yellow
ACAP3		yellow
ACBD4		yellow
ACCN3		yellow
ACCS		yellow
ACER3		yellow
ACOT8		yellow
ACP5		yellow
ACSL3		yellow
ACTR1B		yellow
ACTR3		yellow
ADAM12		yellow
ADAM9		yellow
ADAMDEC1		yellow
ADAMTS13		yellow
ADAMTSL2		yellow
ADCK5		yellow
ADHFE1		yellow
ADNP		yellow
ADORA2A		yellow
ADPRHL2		yellow
AES		yellow
AFF1		yellow
AFMID		yellow
AFTPH		yellow
AHCY		yellow
AHCYL1		yellow
AHSA2		yellow
AIM1L		yellow
AIP		yellow
AK1		yellow
AK4		yellow
AKAP10		yellow
AKR1A1		yellow
ALG12		yellow
ALKBH7		yellow
ALS2		yellow
AMDHD2		yellow
AMT		yellow
ANAPC11		yellow
ANAPC2		yellow
ANKDD1A		yellow
ANKMY1		yellow
ANKRD13B		yellow
ANKRD17		yellow
ANKRD23		yellow
ANKRD33B		yellow
ANKRD34A		yellow
ANKRD36BP1		yellow
ANKRD54		yellow
ANKS3		yellow
ANO6		yellow
ANO9		yellow
AP1G1		yellow

AP1G2	yellow
AP1S1	yellow
APBB3	yellow
APOE	yellow
APOM	yellow
APPL2	yellow
APRT	yellow
ARC	yellow
ARFGEF1	yellow
ARFIP2	yellow
ARFRP1	yellow
ARHGAP4	yellow
ARHGEF25	yellow
ARHGEF26	yellow
ARIH1	yellow
ARL16	yellow
ARL6IP4	yellow
ARMC5	yellow
ARMC6	yellow
ARNTL	yellow
ARRDC2	yellow
ASB3	yellow
ASB6	yellow
ASH1L	yellow
ASPSCR1	yellow
ASXL2	yellow
ATAD3B	yellow
ATF4	yellow
ATF7IP	yellow
ATG4D	yellow
ATHL1	yellow
ATL3	yellow
ATP5D	yellow
ATP5G2	yellow
ATP5G3	yellow
ATP5H	yellow
ATP5SL	yellow
ATP6AP2	yellow
ATP6V0E2	yellow
ATP6V1C1	yellow
ATRIP	yellow
AUP1	yellow
AVEN	yellow
AVPI1	yellow
B3GALT6	yellow
B3GAT3	yellow
B4GALNT4	yellow
B4GALT2	yellow
B4GALT7	yellow
BABAM1	yellow
BAI1	yellow
BAIAP2L2	yellow
BCL2L12	yellow
BCL7B	yellow
BCL7C	yellow
BCS1L	yellow
BEST4	yellow
BHLHE41	yellow
BMP2K	yellow
BOK	yellow
BOLA1	yellow
BRAF	yellow
BRAT1	yellow
BRD7P3	yellow
BRF1	yellow
BRF2	yellow
BROX	yellow
BTBD6	yellow

BTBD7	yellow
BTN1A1	yellow
BTN2A1	yellow
BUB1	yellow
BUB1B	yellow
C10orf125	yellow
C10orf35	yellow
C11orf30	yellow
C11orf48	yellow
C11orf83	yellow
C12orf10	yellow
C12orf44	yellow
C12orf5	yellow
C13orf15	yellow
C14orf118	yellow
C14orf80	yellow
C16orf13	yellow
C16orf42	yellow
C16orf53	yellow
C16orf55	yellow
C16orf74	yellow
C16orf79	yellow
C16orf86	yellow
C17orf109	yellow
C17orf39	yellow
C17orf49	yellow
C17orf56	yellow
C17orf59	yellow
C17orf70	yellow
C17orf79	yellow
C17orf90	yellow
C19orf10	yellow
C19orf24	yellow
C19orf25	yellow
C19orf28	yellow
C19orf39	yellow
C19orf46	yellow
C19orf48	yellow
C19orf53	yellow
C19orf57	yellow
C19orf60	yellow
C19orf66	yellow
C19orf70	yellow
C1orf122	yellow
C1orf123	yellow
C1orf159	yellow
C1orf198	yellow
C1orf212	yellow
C1orf35	yellow
C1orf50	yellow
C1orf55	yellow
C1orf86	yellow
C1QBP	yellow
C1QTNF6	yellow
C20orf195	yellow
C20orf196	yellow
C20orf27	yellow
C21orf2	yellow
C21orf58	yellow
C21orf63	yellow
C22orf26	yellow
C22orf34	yellow
C22orf40	yellow
C2orf68	yellow
C2orf81	yellow
C3orf47	yellow
C4orf14	yellow
C4orf19	yellow

C4orf41	yellow
C5orf51	yellow
C6orf108	yellow
C6orf226	yellow
C7orf40	yellow
C7orf50	yellow
C7orf51	yellow
C7orf59	yellow
C8G	yellow
C8orf55	yellow
C8ORFK29	yellow
C9orf100	yellow
C9orf114	yellow
C9orf142	yellow
C9orf16	yellow
C9orf173	yellow
C9orf23	yellow
C9orf37	yellow
C9orf47	yellow
C9orf64	yellow
C9orf7	yellow
CANX	yellow
CAPN10	yellow
CAPRIN1	yellow
CAPS	yellow
CBR1	yellow
CCDC101	yellow
CCDC106	yellow
CCDC107	yellow
CCDC114	yellow
CCDC12	yellow
CCDC130	yellow
CCDC157	yellow
CCDC22	yellow
CCDC28B	yellow
CCDC47	yellow
CCDC48	yellow
CCDC51	yellow
CCDC57	yellow
CCDC61	yellow
CCDC78	yellow
CCDC94	yellow
CCHCR1	yellow
CCNL2	yellow
CCS	yellow
CD320	yellow
CD36	yellow
CD38	yellow
CD7	yellow
CD8B	yellow
CD99	yellow
CDC25A	yellow
CDC6	yellow
CDCA8	yellow
CDK10	yellow
CDK12	yellow
CDK13	yellow
CDS2	yellow
CDYL2	yellow
CECR5	yellow
CEL	yellow
CELF2	yellow
CELF6	yellow
CEMP1	yellow
CENPF	yellow
CENPM	yellow
CENPT	yellow
CEP55	yellow

CHADL	yellow
CHCHD5	yellow
CHD7	yellow
CHMP4A	yellow
CHMP6	yellow
CHRAC1	yellow
CHTF18	yellow
CIB1	yellow
CISD3	yellow
CLASP1	yellow
CLCF1	yellow
CLCN4	yellow
CLDN7	yellow
CLIP1	yellow
CLPP	yellow
CLSPN	yellow
CN5H6.4	yellow
CNO	yellow
CNTLN	yellow
COL11A2	yellow
COL6A1	yellow
COL6A2	yellow
COL9A2	yellow
COMMD4	yellow
COMMD5	yellow
COMMD9	yellow
COMTD1	yellow
COQ7	yellow
COQ9	yellow
CORO6	yellow
COX4I1	yellow
CPSF2	yellow
CPSF3L	yellow
CPSF4	yellow
CPT1C	yellow
CRB3	yellow
CREB3	yellow
CREB3L4	yellow
CRELD1	yellow
CRELD2	yellow
CRIP1	yellow
CRIP2	yellow
CRYBB2P1	yellow
CRYL1	yellow
CSDE1	yellow
CSNK1G1	yellow
CTAGE5	yellow
CTSC	yellow
CTSF	yellow
CTTNBP2NL	yellow
CTU2	yellow
CUEDC2	yellow
CUTA	yellow
CXorf38	yellow
CXorf40A	yellow
CXorf40B	yellow
CYB561D2	yellow
CYB5D2	yellow
CYC1	yellow
CYP1B1	yellow
CYP2E1	yellow
DAAM1	yellow
DALRD3	yellow
DCBLD1	yellow
DCUN1D3	yellow
DCXR	yellow
DDT	yellow
DDX21	yellow

DDX28	yellow
DDX39A	yellow
DDX43	yellow
DDX49	yellow
DEAF1	yellow
DECR2	yellow
DENND2C	yellow
DERL3	yellow
DGCR6	yellow
DGCR6L	yellow
DGUOK	yellow
DHDH	yellow
DHRS3	yellow
DHRS4	yellow
DHRS4L2	yellow
DIAPH2	yellow
DLGAP5	yellow
DNAJC17	yellow
DNAJC30	yellow
DNAL4	yellow
DNASE1L2	yellow
DND1	yellow
DNLZ	yellow
DOC2GP	yellow
DOK7	yellow
DOLK	yellow
DOM3Z	yellow
DPH3P1	yellow
DPM3	yellow
DPP7	yellow
DPP8	yellow
DRG2	yellow
DSE	yellow
DTD1	yellow
DTX3	yellow
DTYMK	yellow
DUS1L	yellow
DUS2L	yellow
DUS3L	yellow
DUSP10	yellow
DUSP2	yellow
DUSP23	yellow
DUSP3	yellow
DVL1	yellow
DYM	yellow
DYNC1LI1	yellow
E2F1	yellow
E4F1	yellow
EAF1	yellow
ECI1	yellow
EDF1	yellow
EEF1A2	yellow
EEF1D	yellow
EEF1G	yellow
EHHADH	yellow
EIF1	yellow
EIF2B2	yellow
EIF2B4	yellow
EIF3F	yellow
EIF3G	yellow
EIF3I	yellow
EIF3K	yellow
EIF4G3	yellow
ELF1	yellow
ELK3	yellow
ELMO3	yellow
ELP4	yellow
ENGASE	yellow

ENHO	yellow
ENTPD7	yellow
EPC1	yellow
EPG5	yellow
ERCC1	yellow
ERGIC3	yellow
ERI3	yellow
ERLIN2	yellow
ERP29	yellow
ERP44	yellow
ERV3-1	yellow
ESPNL	yellow
ETF1	yellow
ETFB	yellow
ETNK2	yellow
ETV2	yellow
ETV3	yellow
EXD3	yellow
EXOC6B	yellow
EXOSC5	yellow
FADS3	yellow
FAF2	yellow
FAHD2B	yellow
FAM113A	yellow
FAM118B	yellow
FAM122C	yellow
FAM127B	yellow
FAM13A	yellow
FAM158A	yellow
FAM159A	yellow
FAM167B	yellow
FAM178B	yellow
FAM195A	yellow
FAM195B	yellow
FAM21B	yellow
FAM3A	yellow
FAM41C	yellow
FAM48A	yellow
FAM53A	yellow
FAM54B	yellow
FAM69B	yellow
FAM73B	yellow
FAM98B	yellow
FANCG	yellow
FASTK	yellow
FBL	yellow
FBLN7	yellow
FBXL15	yellow
FBXL6	yellow
FBXO2	yellow
FBXO24	yellow
FBXO34	yellow
FBXO38	yellow
FBXO44	yellow
FBXW11	yellow
FBXW5	yellow
FBXW9	yellow
FCGR3A	yellow
FCHSD1	yellow
FCHSD2	yellow
FCRLB	yellow
FDX1L	yellow
FDXR	yellow
FEM1B	yellow
FGD6	yellow
FIBP	yellow
FICD	yellow
FIGNL2	yellow

FKBP11	yellow
FKBP2	yellow
FLAD1	yellow
FLJ13197	yellow
FLJ45244	yellow
FLJ46906	yellow
FLT3LG	yellow
FLYWCH2	yellow
FNIP2	yellow
FPGS	yellow
FRMD3	yellow
FSD1	yellow
FUK	yellow
FXYD2	yellow
GADD45GIP1	yellow
GAMT	yellow
GAPVD1	yellow
GAS2L3	yellow
GATSL3	yellow
GBAP1	yellow
GCDH	yellow
GCHFR	yellow
GDAP2	yellow
GEMIN7	yellow
GET4	yellow
GFER	yellow
GGA1	yellow
GHDC	yellow
GIMAP5	yellow
GLTSCR2	yellow
GLYCTK	yellow
GNAI3	yellow
GNB1L	yellow
GNB2L1	yellow
GNG8	yellow
GNGT2	yellow
GNPTG	yellow
GOLPH3	yellow
GPBP1L1	yellow
GPR155	yellow
GPR172A	yellow
GPX4	yellow
GRASP	yellow
GRIN3A	yellow
GSTT1	yellow
GTF2A1	yellow
GTF2H2D	yellow
GTF3C5	yellow
GTPBP3	yellow
GTPBP6	yellow
GYG1	yellow
GZMM	yellow
H2AFJ	yellow
H2AFY2	yellow
HAGHL	yellow
HAPLN3	yellow
HAUS8	yellow
HBP1	yellow
HCCS	yellow
HCST	yellow
HDAC10	yellow
HDAC11	yellow
HDHD3	yellow
HELZ	yellow
HERC3	yellow
HERPUD2	yellow
HES6	yellow
HES7	yellow

HEXDC	yellow
HIAT1	yellow
HIATL1	yellow
HINFP	yellow
HINT2	yellow
HIP1R	yellow
HIPK1	yellow
HIPK3	yellow
HIVEP2	yellow
HNRNPH3	yellow
HOOK3	yellow
HPS3	yellow
HPX	yellow
HRAS	yellow
HSD11B1L	yellow
HSD17B1	yellow
HSD17B12	yellow
HSD17B8	yellow
ID3	yellow
IFFO1	yellow
IFRD2	yellow
IFT27	yellow
IGFBP6	yellow
IGSF8	yellow
IKZF4	yellow
IL17C	yellow
IL17D	yellow
ILVBL	yellow
IMP4	yellow
INO80E	yellow
IPO8	yellow
IQCC	yellow
IQGAP2	yellow
IRAK4	yellow
IRF3	yellow
ISOC1	yellow
ISOC2	yellow
ISYNA1	yellow
ITFG1	yellow
ITGB1BP2	yellow
ITPA	yellow
ITPRIPL2	yellow
ITSN2	yellow
IZUMO4	yellow
JAG2	yellow
JAGN1	yellow
JMJD4	yellow
JMJD7	yellow
JMJD8	yellow
JOSD2	yellow
JSRP1	yellow
KANK3	yellow
KAT2A	yellow
KCNH4	yellow
KCNN4	yellow
KCNQ4	yellow
KCTD17	yellow
KCTD20	yellow
KDM3A	yellow
KDM5A	yellow
KDM6A	yellow
KHK	yellow
KIAA0114	yellow
KIAA0240	yellow
KIAA0415	yellow
KIAA1432	yellow
KIAA1875	yellow
KIAA1984	yellow

KIAA2018	yellow
KIAA2026	yellow
KIDINS220	yellow
KIF15	yellow
KIF20A	yellow
KIF24	yellow
KIF7	yellow
KLF10	yellow
KLHL12	yellow
KLHL17	yellow
KLHL35	yellow
KLHL8	yellow
KLRAQ1	yellow
KREMEN2	yellow
KRT18	yellow
KRTCAP2	yellow
KRTCAP3	yellow
LAGE3	yellow
LAMB1	yellow
LAMTOR2	yellow
LARP4B	yellow
LAT	yellow
LATS1	yellow
LATS2	yellow
LCMT1	yellow
LCNL1	yellow
LEMD2	yellow
LEPRE1	yellow
LEPREL2	yellow
LGALS1	yellow
LGALS4	yellow
LGALS8	yellow
LGALS9B	yellow
LHB	yellow
LIMD2	yellow
LIME1	yellow
LIMS2	yellow
LMBR1L	yellow
LMF1	yellow
LMF2	yellow
LOC100129550	yellow
LOC100130705	yellow
LOC100131320	yellow
LOC100132077	yellow
LOC100134713	yellow
LOC100272216	yellow
LOC100287177	yellow
LOC100287559	yellow
LOC100289341	yellow
LOC100379224	yellow
LOC100499489	yellow
LOC100507062	yellow
LOC100507495	yellow
LOC100630923	yellow
LOC148413	yellow
LOC150776	yellow
LOC155060	yellow
LOC284889	yellow
LOC285819	yellow
LOC286059	yellow
LOC388796	yellow
LOC390595	yellow
LOC401431	yellow
LOC550643	yellow
LOC641518	yellow
LOC643669	yellow
LOC728323	yellow
LOC728431	yellow

LOC728743	yellow
LOC729513	yellow
LOC729678	yellow
LONRF3	yellow
LPAR1	yellow
LPP	yellow
LRRC14	yellow
LRRC27	yellow
LRRC29	yellow
LRRC32	yellow
LRRC45	yellow
LRRC56	yellow
LRRC57	yellow
LRRC68	yellow
LSM12	yellow
LSM14A	yellow
LSM2	yellow
LSM4	yellow
LTBP4	yellow
LTK	yellow
LY86	yellow
LYPD2	yellow
LYSMD4	yellow
LYZ	yellow
MACROD1	yellow
MAD1L1	yellow
MAGI3	yellow
MAL	yellow
MAMDC4	yellow
MAML2	yellow
MAN1B1	yellow
MAP3K10	yellow
MAP4K2	yellow
MAPK1	yellow
MAPK11	yellow
MAPK1IP1L	yellow
MATK	yellow
MAZ	yellow
MBD3	yellow
MBD5	yellow
MBLAC1	yellow
MC1R	yellow
MCC	yellow
MCM7	yellow
MCRS1	yellow
MEA1	yellow
MECR	yellow
MED1	yellow
MED13L	yellow
MED18	yellow
MEI1	yellow
MESP1	yellow
METAP1D	yellow
METRN	yellow
METTL11A	yellow
METTL12	yellow
METTL22	yellow
MFI2	yellow
MFI2-AS1	yellow
MFNG	yellow
MFSD10	yellow
MFSD3	yellow
MFSD9	yellow
MGMT	yellow
MIA3	yellow
MICB	yellow
MIF4GD	yellow
MIRLET7BHG	yellow

MKI67	yellow
MLL3	yellow
MLL5	yellow
MLST8	yellow
MMAB	yellow
MMP28	yellow
MOB2	yellow
MOGS	yellow
MORC2-AS1	yellow
MPDU1	yellow
MPG	yellow
MPV17L2	yellow
MR1	yellow
MRI1	yellow
MRPL12	yellow
MRPL20	yellow
MRPL23	yellow
MRPL24	yellow
MRPL38	yellow
MRPL52	yellow
MRPL54	yellow
MRPL55	yellow
MRPS11	yellow
MRPS12	yellow
MRPS15	yellow
MRPS18A	yellow
MRPS26	yellow
MRPS34	yellow
MSH3	yellow
MST1	yellow
MST1P2	yellow
MT1F	yellow
MT1X	yellow
MTFP1	yellow
MTG1	yellow
MTM1	yellow
MTMR1	yellow
MTRNR2L8	yellow
MUS81	yellow
MUT	yellow
MUTYH	yellow
MVD	yellow
MVK	yellow
MXD4	yellow
MYH10	yellow
MYLIP	yellow
MYLK3	yellow
MZF1	yellow
MZT2B	yellow
NAA10	yellow
NAALADL1	yellow
NAGLU	yellow
NAGS	yellow
NAPSA	yellow
NAPSB	yellow
NARFL	yellow
NAT14	yellow
NAT6	yellow
NAT9	yellow
NCAPH	yellow
NCAPH2	yellow
NCEH1	yellow
NCOA3	yellow
NCR3	yellow
NCRNA00087	yellow
NCRNA00263	yellow
NCRNA00338	yellow
NDOR1	yellow

NDUFA11	yellow
NDUFA13	yellow
NDUFA3	yellow
NDUFA8	yellow
NDUFB10	yellow
NDUFB11	yellow
NDUFB7	yellow
NDUFB8	yellow
NDUFB9	yellow
NDUFS6	yellow
NDUFS7	yellow
NDUFS8	yellow
NDUFV1	yellow
NECAB3	yellow
NEIL1	yellow
NELF	yellow
NENF	yellow
NEURL2	yellow
NF1	yellow
NFAT5	yellow
NFE2L3	yellow
NFKBIL1	yellow
NGFRAP1	yellow
NHLRC4	yellow
NHP2	yellow
NIPA2	yellow
NIPBL	yellow
NKPD1	yellow
NLE1	yellow
NME3	yellow
NME4	yellow
NMRAL1	yellow
NOB1	yellow
NOG	yellow
NOL12	yellow
NOSIP	yellow
NOXA1	yellow
NOXO1	yellow
NPDC1	yellow
NPRL2	yellow
NR2C2AP	yellow
NRL	yellow
NRP1	yellow
NSF	yellow
NSMCE1	yellow
NSRP1	yellow
NSUN5	yellow
NSUN5P1	yellow
NSUN5P2	yellow
NT5C	yellow
NTHL1	yellow
NUBP2	yellow
NUDT1	yellow
NUDT14	yellow
NUDT16L1	yellow
NUDT17	yellow
NUDT18	yellow
NUDT22	yellow
NUDT8	yellow
NUP153	yellow
NUSAP1	yellow
OBFC1	yellow
OBFC2B	yellow
OGFOD2	yellow
OGG1	yellow
OSBPL9	yellow
OXSR1	yellow
P2RX6	yellow

P2RX7	yellow
P4HTM	yellow
PAFAH1B2	yellow
PAFAH1B3	yellow
PANK4	yellow
PANX1	yellow
PAOX	yellow
PAPSS1	yellow
PARD6G	yellow
PARK7	yellow
PARP10	yellow
PARP3	yellow
PARS2	yellow
PATL1	yellow
PBLD	yellow
PBX4	yellow
PCOLCE	yellow
PCP2	yellow
PCSK4	yellow
PCYT2	yellow
PDCD1	yellow
PDE6G	yellow
PDK4	yellow
PEMT	yellow
PEX16	yellow
PEX6	yellow
PFKFB2	yellow
PGM2	yellow
PGP	yellow
PHEX	yellow
PHF1	yellow
PHF16	yellow
PHF20	yellow
PHF3	yellow
PHKB	yellow
PHKG1	yellow
PHLDA3	yellow
PHPT1	yellow
PI16	yellow
PICK1	yellow
PIDD	yellow
PIGQ	yellow
PIH1D1	yellow
PIK3C3	yellow
PIK3CB	yellow
PIK3CG	yellow
PIN1	yellow
PIP5K1A	yellow
PIP5K1B	yellow
PIP5KL1	yellow
PLA2G6	yellow
PLCD1	yellow
PLEKHA4	yellow
PLEKHF1	yellow
PLEKHG4	yellow
PLEKHG5	yellow
PLEKHJ1	yellow
PLEKHM3	yellow
PMM1	yellow
PMVK	yellow
PNKP	yellow
PNMT	yellow
POLD1	yellow
POLD4	yellow
POLM	yellow
POLR2F	yellow
POLR2G	yellow
POLR2I	yellow

POLR2J4	yellow
POLR2L	yellow
POLR3K	yellow
POLRMT	yellow
POMT1	yellow
POMZP3	yellow
POP7	yellow
POU2F1	yellow
PPAN	yellow
PPAP2C	yellow
PPM1D	yellow
PPM1J	yellow
PPM1L	yellow
PPM1N	yellow
PPOX	yellow
PPP1R12C	yellow
PPP1R14A	yellow
PPP1R14B	yellow
PPP1R16A	yellow
PPP1R3F	yellow
PPP2R3B	yellow
PPTC7	yellow
PRADC1	yellow
PRDM1	yellow
PREB	yellow
PRKAR2A	yellow
PRMT7	yellow
PROCR	yellow
PRR22	yellow
PRR5	yellow
PRRC2C	yellow
PRSS22	yellow
PRSS30P	yellow
PRSS53	yellow
PSD	yellow
PSMA7	yellow
PSMB4	yellow
PSMB5	yellow
PSMB7	yellow
PSMD9	yellow
PSMG3	yellow
PTDSS2	yellow
PTGES2	yellow
PTPLB	yellow
PTPRC	yellow
PTPRCAP	yellow
PTRH1	yellow
PUS1	yellow
PUSL1	yellow
PVRIG	yellow
PXK	yellow
PYCRL	yellow
PYROXD2	yellow
QARS	yellow
QPCTL	yellow
QTRT1	yellow
R3HCC1	yellow
RAB21	yellow
RAB25	yellow
RAB33A	yellow
RAB39	yellow
RABEP2	yellow
RABEPK	yellow
RABGEF1	yellow
RABGGTA	yellow
RAC3	yellow
RAD21	yellow
RAD9A	yellow

RALGAPB	yellow
RANBP9	yellow
RASA2	yellow
RASA4P	yellow
RASSF7	yellow
RBBP5	yellow
RBMXL1	yellow
RBPMS	yellow
RC3H1	yellow
REC8	yellow
RECQL4	yellow
REEP6	yellow
RELL2	yellow
REXO1L1	yellow
RFTN2	yellow
RFX3	yellow
RGL1	yellow
RHBDD3	yellow
RHOC	yellow
RHOT2	yellow
RHPN1	yellow
RINL	yellow
RLIM	yellow
RNASEH2C	yellow
RNF113A	yellow
RNF115	yellow
RNF166	yellow
RNF169	yellow
RNF181	yellow
RNF215	yellow
RNF25	yellow
RNMTL1	yellow
ROBO3	yellow
ROCK2	yellow
RPL13	yellow
RPL18	yellow
RPL18A	yellow
RPL28	yellow
RPL29	yellow
RPL38	yellow
RPL8	yellow
RPLP1	yellow
RPLP2	yellow
RPS15	yellow
RPS19BP1	yellow
RPS2	yellow
RPS28	yellow
RPS6KB2	yellow
RPS6KC1	yellow
RPS6KL1	yellow
RPUSD1	yellow
RPUSD3	yellow
RQCD1	yellow
RRP7B	yellow
RRP9	yellow
RSC1A1	yellow
RSF1	yellow
RSRC1	yellow
RTEL1	yellow
RTEL1-TNFRSF6B	yellow
RTN4R	yellow
RUSC1	yellow
S100A13	yellow
S1PR3	yellow
SAC3D1	yellow
SAP30L	yellow
SARDH	yellow
SARS2	yellow

SAT2	yellow
SCARB2	yellow
SCARNA12	yellow
SCARNA9	yellow
SCNN1D	yellow
SCRN2	yellow
SDHAF1	yellow
SDHAF2	yellow
SDR39U1	yellow
SEC22B	yellow
SEC24B	yellow
SELM	yellow
SELO	yellow
SEMA6A	yellow
SENP2	yellow
SEPW1	yellow
SERGEF	yellow
SERINC3	yellow
SERPINB9	yellow
SERPINF1	yellow
SERPINF2	yellow
SERTAD2	yellow
SETD2	yellow
SETD7	yellow
SETX	yellow
SF3B5	yellow
SGCA	yellow
SGK3	yellow
SGSM3	yellow
SH2D3A	yellow
SH3GLB2	yellow
SH3RF1	yellow
SHCBP1	yellow
SHE	yellow
SHF	yellow
SIGIRR	yellow
SIGLEC14	yellow
SIRT3	yellow
SIVA1	yellow
SIX5	yellow
SLC16A11	yellow
SLC16A4	yellow
SLC17A9	yellow
SLC1A3	yellow
SLC22A17	yellow
SLC25A19	yellow
SLC25A25	yellow
SLC25A29	yellow
SLC25A35	yellow
SLC25A5	yellow
SLC25A6	yellow
SLC26A11	yellow
SLC29A2	yellow
SLC2A4RG	yellow
SLC2A8	yellow
SLC35B2	yellow
SLC35D2	yellow
SLC37A4	yellow
SLC39A13	yellow
SLC39A9	yellow
SLC41A3	yellow
SLC44A1	yellow
SLC50A1	yellow
SLC5A5	yellow
SLC7A4	yellow
SLMO1	yellow
SMAP1	yellow
SMCR7	yellow

SMPD2	yellow
SNF8	yellow
SNHG11	yellow
SNRNP25	yellow
SNRPC	yellow
SNTB2	yellow
SNX20	yellow
SOAT1	yellow
SOAT2	yellow
SOS1	yellow
SOX12	yellow
SOX8	yellow
SP1	yellow
SPAG4	yellow
SPAG8	yellow
SPATA13	yellow
SPATA20	yellow
SPEG	yellow
SPG20	yellow
SPHK2	yellow
SPPL2A	yellow
SPPL2B	yellow
SPRED2	yellow
SPRN	yellow
SPRY3	yellow
SPSB2	yellow
SPSB3	yellow
SPTBN4	yellow
SRGAP2P2	yellow
SSNA1	yellow
SSR1	yellow
SSR4	yellow
ST3GAL3	yellow
ST6GALNAC6	yellow
STAM	yellow
STAMBP	yellow
STAP2	yellow
STK25	yellow
STMN3	yellow
STOML1	yellow
STOML2	yellow
STRA13	yellow
STRC	yellow
STRN	yellow
STS	yellow
STUB1	yellow
STX1A	yellow
SUDS3	yellow
SURF1	yellow
SUSD2	yellow
SUV420H1	yellow
SWI5	yellow
SYCE1L	yellow
SYNGR1	yellow
SYT17	yellow
SYTL1	yellow
TACC1	yellow
TADA3	yellow
TAGAP	yellow
TAOK3	yellow
TAZ	yellow
TBC1D10A	yellow
TBC1D10C	yellow
TBC1D17	yellow
TBL2	yellow
TBL3	yellow
TBRG4	yellow
TCEA2	yellow

TCEB2	yellow
TCTA	yellow
TEDDM1	yellow
TELO2	yellow
TEX22	yellow
TGM1	yellow
THAP11	yellow
THAP3	yellow
THAP7	yellow
THOC6	yellow
THOP1	yellow
TIMM10	yellow
TIMM13	yellow
TIMM50	yellow
TIPARP	yellow
TLE2	yellow
TLK2	yellow
TLR7	yellow
TM7SF2	yellow
TMED1	yellow
TMED3	yellow
TMED8	yellow
TMED9	yellow
TMEM101	yellow
TMEM129	yellow
TMEM134	yellow
TMEM138	yellow
TMEM141	yellow
TMEM143	yellow
TMEM147	yellow
TMEM150A	yellow
TMEM161A	yellow
TMEM179B	yellow
TMEM184C	yellow
TMEM186	yellow
TMEM187	yellow
TMEM189	yellow
TMEM2	yellow
TMEM203	yellow
TMEM219	yellow
TMEM222	yellow
TMEM53	yellow
TMEM79	yellow
TMEM80	yellow
TMEM85	yellow
TMEM9	yellow
TMEM99	yellow
TMIE	yellow
TMIGD2	yellow
TMOD3	yellow
TMPRSS5	yellow
TMSB4X	yellow
TMTC2	yellow
TNFAIP3	yellow
TNFRSF18	yellow
TNFRSF25	yellow
TNFRSF4	yellow
TNFRSF6B	yellow
TNFSF12	yellow
TNFSF15	yellow
TNIP2	yellow
TNK1	yellow
TNNT3	yellow
TOMM40L	yellow
TOP1	yellow
TOP2A	yellow
TOP3B	yellow
TOPBP1	yellow

TOR1AIP1	yellow
TP53I13	yellow
TP73	yellow
TPM2	yellow
TPR	yellow
TRABD	yellow
TRAF2	yellow
TRAF4	yellow
TRAF6	yellow
TRAPPC2L	yellow
TRAPPC4	yellow
TRAPPC6A	yellow
TREX2	yellow
TRIB3	yellow
TRIM24	yellow
TRIM34	yellow
TRIM46	yellow
TRIM78P	yellow
TRIP12	yellow
TRMT1	yellow
TRMT112	yellow
TRMT2A	yellow
TRMT61A	yellow
TRMU	yellow
TRPS1	yellow
TRPT1	yellow
TRUB2	yellow
TSPAN17	yellow
TSPAN32	yellow
TSSC1	yellow
TSSC4	yellow
TSSK6	yellow
TSTD1	yellow
TTC34	yellow
TTC4	yellow
TTC9C	yellow
TTN	yellow
TUBB6	yellow
TUBGCP3	yellow
TUFM	yellow
TUT1	yellow
TXN2	yellow
TXNL4A	yellow
TXNRD1	yellow
TYSND1	yellow
UBA7	yellow
UBASH3B	yellow
UBL4A	yellow
UBQLN1	yellow
UBQLN2	yellow
UBXN1	yellow
UBXN11	yellow
UCK1	yellow
UCKL1	yellow
UEVLD	yellow
ULK3	yellow
UPF2	yellow
UROD	yellow
UROS	yellow
USE1	yellow
USP30	yellow
USP34	yellow
USP6NL	yellow
USP8	yellow
USP9X	yellow
VARS2	yellow
VAV3	yellow
VILL	yellow

VKORC1	yellow
VKORC1L1	yellow
VMO1	yellow
VPS13B	yellow
VPS16	yellow
VPS41	yellow
VTI1A	yellow
WASH2P	yellow
WASH3P	yellow
WASH7P	yellow
WBSCR27	yellow
WDR18	yellow
WDR20	yellow
WDR24	yellow
WDR34	yellow
WDR54	yellow
WDR73	yellow
WDR74	yellow
WDR83	yellow
WDR85	yellow
WDR90	yellow
WHSC1L1	yellow
WIBG	yellow
WNT7A	yellow
WRAP53	yellow
XPR1	yellow
YDJC	yellow
YIPF5	yellow
YME1L1	yellow
YTHDC1	yellow
YWHAZ	yellow
ZAP70	yellow
ZBTB17	yellow
ZBTB20	yellow
ZC3HAV1	yellow
ZDHHC4	yellow
ZEB2	yellow
ZFAND2B	yellow
ZFP91	yellow
ZFR2	yellow
ZFYVE19	yellow
ZFYVE27	yellow
ZG16B	yellow
ZMYND10	yellow
ZMYND19	yellow
ZNF274	yellow
ZNF333	yellow
ZNF366	yellow
ZNF394	yellow
ZNF446	yellow
ZNF48	yellow
ZNF486	yellow
ZNF500	yellow
ZNF511	yellow
ZNF517	yellow
ZNF524	yellow
ZNF581	yellow
ZNF593	yellow
ZNF641	yellow
ZNF653	yellow
ZNF688	yellow
ZNF691	yellow
ZNF692	yellow
ZNF707	yellow
ZNF71	yellow
ZNF821	yellow
ZNF890P	yellow
ZNHIT1	yellow

ZNHIT2	yellow
ZP3	yellow
ZSCAN18	yellow
ZYG11B	yellow
