# Supplementary material for: Meditation and vacation effects have an impact on disease-associated molecular phenotypes
Source: Transl Psychiatry. 2016 Aug 30;6(8):e880–. doi: 10.1038/tp.2016.164 (PMC5022094; doi:10.1038/tp.2016.164)
Supplement: Supplementary Table 5 [file tp2016164x4.pdf]

Supplementary Table 5. Pathway enrichments for the coexpression network modules identified in Supplementary Table 4

module	GO Type	GO.ID	Term	Total Gene	Annotatec	Genes in Sig	Significant	Expected	classic	fold_enric	gene_list
yellow	CC	GO:0005622	intracellular	17735	12344	1225	958	837.77	8.70E-19	1.143512	ABCB8;ABCB9;ABHD14A;ABT1;ACADS;ACER3;ACOT8;ACP5;ACSL3;ACTR1B;ACTR3;ADAM12;ADAM9;ADCK5;
yellow	CC	GO:0044444	cytoplasmic part	17735	6562	1225	581	445.35	2.10E-17	1.304592	ABCB8;ABCB9;ACADS;ACER3;ACOT8;ACP5;ACSL3;ACTR1B;ACTR3;ADAM12;ADCK5;ADHFE1;ADPRHL2;AFMI
yellow	CC	GO:0044424	intracellular part	17735	12103	1225	937	821.41	9.00E-17	1.140721	ABCB8;ABCB9;ABHD14A;ABT1;ACADS;ACER3;ACOT8;ACP5;ACSL3;ACTR1B;ACTR3;ADAM12;ADAM9;ADCK5;
yellow	CC	GO:0005737	cytoplasm	17735	9016	1225	744	611.9	1.30E-16	1.215885	ABCB8;ABCB9;ABHD14A;ACADS;ACER3;ACOT8;ACP5;ACSL3;ACTR1B;ACTR3;ADAM12;ADAM9;ADCK5;ADHF
yellow	MF	GO:0003824	catalytic activity	17735	5264	1225	480	368.81	1.70E-13	1.301483	ABCB8;ABCB9;ABHD1;ABHD14A;ACADS;ACER3;ACOT8;ACP5;ACSL3;ADAM12;ADAM9;ADAMDEC1;ADAMTS
yellow	CC	GO:0005739	mitochondrion	17735	1488	1225	174	100.99	2.30E-13	1.722943	ABCB8;ACADS;ACOT8;ACSL3;ADAM12;ADCK5;ADHFE1;ADPRHL2;AK4;AKAP10;ALKBH7;AMT;ATAD3B;ATP5D
yellow	CC	GO:0043227	membrane-bounded organelle	17735	9590	1225	749	650.86	4.50E-10	1.150785	ABCB8;ABCB9;ABT1;ACADS;ACER3;ACOT8;ACP5;ACSL3;ACTR3;ADAM12;ADCK5;ADHFE1;ADNP;ADPRHL2;AE
yellow	CC	GO:0043226	organelle	17735	10552	1225	811	716.15	5.20E-10	1.132444	ABCB8;ABCB9;ABT1;ACADS;ACER3;ACOT8;ACP5;ACSL3;ACTR1B;ACTR3;ADAM12;ADCK5;ADHFE1;ADNP;ADC
yellow	CC	GO:0043231	intracellular membrane-bounded organelle	17735	9560	1225	746	648.82	6.90E-10	1.14978	ABCB8;ABCB9;ABT1;ACADS;ACER3;ACOT8;ACP5;ACSL3;ACTR3;ADAM12;ADCK5;ADHFE1;ADNP;ADPRHL2;AE
yellow	CC	GO:0043229	intracellular organelle	17735	10522	1225	808	714.11	8.00E-10	1.131478	ABCB8;ABCB9;ABT1;ACADS;ACER3;ACOT8;ACP5;ACSL3;ACTR1B;ACTR3;ADAM12;ADCK5;ADHFE1;ADNP;ADC
yellow	CC	GO:0044429	mitochondrial part	17735	740	1225	94	50.22	2.10E-09	1.871764	ABCB8;ACADS;ACSL3;ADHFE1;ADPRHL2;AK4;ATAD3B;ATP5D;ATP5G2;ATP5G3;ATP5H;BCS1L;BOK;C1QBP;CC
yellow	CC	GO:0044422	organelle part	17735	6321	1225	516	429	2.80E-08	1.202797	ABCB8;ABCB9;ABT1;ACADS;ACER3;ACOT8;ACSL3;ACTR1B;ACTR3;ADHFE1;ADORA2A;ADPRHL2;AFTPH;AK1;
yellow	CC	GO:0044446	intracellular organelle part	17735	6219	1225	508	422.07	3.80E-08	1.203592	ABCB8;ABCB9;ABT1;ACADS;ACER3;ACOT8;ACSL3;ACTR1B;ACTR3;ADHFE1;ADORA2A;ADPRHL2;AFTPH;AK4;
yellow	CC	GO:0031974	membrane-enclosed lumen	17735	2770	1225	248	188	9.70E-07	1.319149	ABCB8;ABT1;ACADS;ACOT8;ADHFE1;ADPRHL2;AK4;ANAPC11;ANAPC2;APPL2;ARFGEF1;ARNTL;ATF4;ATF7IP;
yellow	CC	GO:0005740	mitochondrial envelope	17735	520	1225	64	35.29	2.50E-06	1.813545	ABCB8;ACADS;ACSL3;ATAD3B;ATP5D;ATP5G2;ATP5G3;ATP5H;BCS1L;BOK;CCS;CDS2;CHCHD5;COQ7;COX4I1
yellow	CC	GO:0019866	organelle inner membrane	17735	377	1225	50	25.59	4.00E-06	1.953888	ABCB8;ATAD3B;ATP5D;ATP5G2;ATP5G3;ATP5H;BCS1L;CCS;CDS2;COQ7;COX4I1;CYC1;ECI1;FDXR;FPGS;GCDH
yellow	CC	GO:0005743	mitochondrial inner membrane	17735	347	1225	47	23.55	4.40E-06	1.995754	ABCB8;ATAD3B;ATP5D;ATP5G2;ATP5G3;ATP5H;BCS1L;CCS;CDS2;COQ7;COX4I1;CYC1;ECI1;FDXR;FPGS;GCDH
yellow	BP	GO:0022900	electron transport chain	17735	151	1225	27	10.62	6.00E-06	2.542373	ATP5D;ATP5H;COQ7;COX4I1;CYB561D2;CYC1;ETFB;FADS3;FDX1L;FDXR;NDUFA11;NDUFA13;NDUFA3;NDUF
yellow	CC	GO:0070013	intracellular organelle lumen	17735	2677	1225	236	181.68	6.20E-06	1.298987	ABCB8;ABT1;ACADS;ACOT8;ADHFE1;ADPRHL2;AK4;ANAPC11;ANAPC2;APPL2;ARFGEF1;ARNTL;ATF4;ATF7IP;
yellow	BP	GO:0043412	macromolecule modification	17735	2584	1225	235	181.79	7.10E-06	1.2927	ADAM9;ADORA2A;ALG12;ALS2;ANAPC11;ANAPC2;APOE;ARFGEF1;ARIH1;ASB3;ASB6;ATF4;ATF7IP;AVPI1;B3
yellow	CC	GO:0005747	mitochondrial respiratory chain complex ...	17735	46	1225	13	3.12	7.50E-06	4.166667	NDUFA11;NDUFA13;NDUFA3;NDUFA8;NDUFB10;NDUFB11;NDUFB7;NDUFB8;NDUFB9;NDUFS6;NDUFS7;ND
yellow	CC	GO:0030964	NADH dehydrogenase complex	17735	46	1225	13	3.12	7.50E-06	4.166667	NDUFA11;NDUFA13;NDUFA3;NDUFA8;NDUFB10;NDUFB11;NDUFB7;NDUFB8;NDUFB9;NDUFS6;NDUFS7;ND
yellow	CC	GO:0045271	respiratory chain complex I	17735	46	1225	13	3.12	7.50E-06	4.166667	NDUFA11;NDUFA13;NDUFA3;NDUFA8;NDUFB10;NDUFB11;NDUFB7;NDUFB8;NDUFB9;NDUFS6;NDUFS7;ND
yellow	BP	GO:0044267	cellular protein metabolic process	17735	3210	1225	283	225.83	7.60E-06	1.253155	ADAM9;ADORA2A;AIP;ALG12;ALS2;ANAPC11;ANAPC2;APOE;ARFGEF1;ARIH1;ASB3;ASB6;ATF4;AVPI1;B3GAI
yellow	BP	GO:0046386	deoxyribose phosphate catabolic process	17735	17	1225	8	1.2	8.00E-06	6.666667	MPG;MUTYH;NT5C;NTHL1;NUDT1;NUDT18;OGG1;PGM2
yellow	MF	GO:0016853	isomerase activity	17735	138	1225	25	9.67	9.80E-06	2.585315	DDT;DSE;ECI1;EHHADH;ERP29;ERP44;FKBP11;FKBP2;ISYNA1;MRI1;MUT;PBLD;PGM2;PIN1;PMM1;PTGES2;P
yellow	CC	GO:0005759	mitochondrial matrix	17735	286	1225	40	19.41	1.00E-05	2.060793	ACADS;ADHFE1;ADPRHL2;AK4;ATP5D;C1QBP;CLPP;DDX28;DGUOK;DTYMK;ECI1;ETFB;FDX1L;FDXR;FPGS;GCI
yellow	CC	GO:0043233	organelle lumen	17735	2722	1225	238	184.74	1.00E-05	1.288297	ABCB8;ABT1;ACADS;ACOT8;ADHFE1;ADPRHL2;AK4;ANAPC11;ANAPC2;APPL2;ARFGEF1;ARNTL;ATF4;ATF7IP;
yellow	CC	GO:0032991	macromolecular complex	17735	3893	1225	324	264.21	1.20E-05	1.226297	ABCB8;ABCB9;ABT1;ACTR1B;ACTR3;AFTPH;ALS2;ANAPC11;ANAPC2;ANO6;AP1G1;AP1G2;AP1S1;APOE;APOI
yellow	CC	GO:0031966	mitochondrial membrane	17735	496	1225	59	33.66	1.70E-05	1.752822	ABCB8;ACADS;ACSL3;ATAD3B;ATP5D;ATP5G2;ATP5G3;ATP5H;BCS1L;BOK;CCS;CDS2;COQ7;COX4I1;CPT1C;C
yellow	BP	GO:0019692	deoxyribose phosphate metabolic process	17735	30	1225	10	2.11	2.30E-05	4.739336	DGUOK;DTYMK;MPG;MUTYH;NT5C;NTHL1;NUDT1;NUDT18;OGG1;PGM2
yellow	CC	GO:0005840	ribosome	17735	206	1225	31	13.98	2.40E-05	2.217454	CANX;GNB2L1;LARP4B;MRPL12;MRPL20;MRPL23;MRPL24;MRPL38;MRPL52;MRPL54;MRPL55;MRPS11;MRI
yellow	BP	GO:0022904	respiratory electron transport chain	17735	111	1225	21	7.81	2.70E-05	2.68886	ATP5D;ATP5H;COQ7;COX4I1;CYC1;ETFB;NDUFA11;NDUFA13;NDUFA3;NDUFA8;NDUFB10;NDUFB11;NDUFB
yellow	BP	GO:0006974	response to DNA damage stimulus	17735	608	1225	70	42.77	2.80E-05	1.636661	ATRIP;BABAM1;C11orf30;C17orf70;C1orf86;CIB1;CLSPN;E2F1;ERCC1;FANCG;FEM1B;FNIP2;GTF2H2D;HINFP
yellow	MF	GO:0016655	oxidoreductase activity, acting on NAD(P...	17735	58	1225	14	4.06	3.50E-05	3.448276	CBR1;DCXR;DHRS4;NDUFA13;NDUFA3;NDUFA8;NDUFB10;NDUFB7;NDUFB8;NDUFB9;NDUFS6;NDUFS7;NDU
yellow	MF	GO:0016740	transferase activity	17735	1705	1225	161	119.46	3.50E-05	1.347731	ADCK5;AK1;AK4;ALG12;AMT;APRT;ASH1L;B3GALT6;B3GAT3;B4GALNT4;B4GALT2;B4GALT7;BMP2K;BRAF;BU
yellow	MF	GO:0008168	methyltransferase activity	17735	177	1225	28	12.4	4.00E-05	2.258065	AMT;ASH1L;COMTD1;FBL;GAMT;LCMT1;METTL12;METTL22;MGMT;MLL3;MLL5;NSUN5;NSUN5P2;PEMT;PN
yellow	BP	GO:0006281	DNA repair	17735	388	1225	49	27.3	4.60E-05	1.794872	ATRIP;BABAM1;C11orf30;C17orf70;C1orf86;CIB1;CLSPN;ERCC1;FANCG;GTF2H2D;HINFP;INO80E;MC1R;MCF
yellow	BP	GO:0009264	deoxyribonucleotide catabolic process	17735	16	1225	7	1.13	5.40E-05	6.19469	MPG;MUTYH;NT5C;NTHL1;NUDT1;NUDT18;OGG1
yellow	CC	GO:0005746	mitochondrial respiratory chain	17735	70	1225	15	4.75	5.70E-05	3.157895	BCS1L;NDUFA11;NDUFA13;NDUFA3;NDUFA8;NDUFB10;NDUFB11;NDUFB7;NDUFB8;NDUFB9;NDUFS6;NDU
yellow	CC	GO:0044455	mitochondrial membrane part	17735	148	1225	24	10.04	5.80E-05	2.390438	ATP5D;ATP5G2;ATP5G3;ATP5H;BCS1L;NDUFA11;NDUFA13;NDUFA3;NDUFA8;NDUFB10;NDUFB11;NDUFB7;
yellow	CC	GO:0031090	organelle membrane	17735	2366	1225	206	160.58	6.10E-05	1.28285	ABCB8;ABCB9;ACADS;ACER3;ACSL3;ACTR3;AFTPH;ALG12;AP1G1;AP1G2;AP1S1;APPL2;ARFGEF1;ASPSCR1;A
yellow	CC	GO:0070469	respiratory chain	17735	79	1225	16	5.36	6.70E-05	2.985075	BCS1L;CYC1;NDUFA11;NDUFA13;NDUFA3;NDUFA8;NDUFB10;NDUFB11;NDUFB7;NDUFB8;NDUFB9;NDUFS6
yellow	MF	GO:0016741	transferase activity, transferring one-c...	17735	183	1225	28	12.82	7.40E-05	2.184087	AMT;ASH1L;COMTD1;FBL;GAMT;LCMT1;METTL12;METTL22;MGMT;MLL3;MLL5;NSUN5;NSUN5P2;PEMT;PN
yellow	BP	GO:0002832	negative regulation of response to bioti...	17735	12	1225	6	0.84	7.60E-05	7.142857	C1QBP;MICB;NFKBIL1;PRDM1;SIGIRR;TNFAIP3
yellow	BP	GO:0009394	2'-deoxyribonucleotide metabolic process	17735	28	1225	9	1.97	8.30E-05	4.568528	DGUOK;DTYMK;MPG;MUTYH;NT5C;NTHL1;NUDT1;NUDT18;OGG1
yellow	CC	GO:0044464	cell part	17735	14451	1225	1022	980.77	8.30E-05	1.042038	ABCB8;ABCB9;ABHD14A;ABT1;ACADS;ACER3;ACOT8;ACP5;ACSL3;ACTR1B;ACTR3;ADAM12;ADAM9;ADAMT
yellow	CC	GO:0005623	cell	17735	14452	1225	1022	980.83	8.50E-05	1.041975	ABCB8;ABCB9;ABHD14A;ABT1;ACADS;ACER3;ACOT8;ACP5;ACSL3;ACTR1B;ACTR3;ADAM12;ADAM9;ADAMT
yellow	CC	GO:0044391	ribosomal subunit	17735	134	1225	22	9.09	9.60E-05	2.420242	GNB2L1;MRPL12;MRPL23;MRPL52;MRPL55;MRPS11;MRPS12;MRPS15;MRPS18A;MRPS26;RPL13;RPL18;RPI
yellow	BP	GO:0019538	protein metabolic process	17735	3895	1225	326	274.02	1.00E-04	1.189694	ADAM12;ADAM9;ADAMDEC1;ADAMTS13;ADORA2A;AIP;ALG12;ALS2;ANAPC11;ANAPC2;APOE;APOM;ARFG
yellow	BP	GO:0006464	cellular protein modification process	17735	2482	1225	219	174.62	0.00011	1.254152	ADAM9;ADORA2A;ALG12;ALS2;ANAPC11;ANAPC2;APOE;ARFGEF1;ARIH1;ASB3;ASB6;ATF4;AVPI1;B3GALT6;
yellow	BP	GO:0036211	protein modification process	17735	2482	1225	219	174.62	0.00011	1.254152	ADAM9;ADORA2A;ALG12;ALS2;ANAPC11;ANAPC2;APOE;ARFGEF1;ARIH1;ASB3;ASB6;ATF4;AVPI1;B3GALT6;
yellow	BP	GO:0009151	purine deoxyribonucleotide metabolic pro...	17735	13	1225	6	0.91	0.00013	6.593407	DGUOK;MPG;MUTYH;NUDT1;NUDT18;OGG1
yellow	BP	GO:0006119	oxidative phosphorylation	17735	73	1225	15	5.14	0.00014	2.918288	ATP5D;COQ7;NDUFA3;NDUFA8;NDUFB10;NDUFB7;NDUFB8;NDUFB9;NDUFS6;NDUFS7;NDUFS8;NDUFV1;SD
yellow	MF	GO:0003954	NADH dehydrogenase activity	17735	44	1225	11	3.08	0.00017	3.571429	NDUFA13;NDUFA3;NDUFA8;NDUFB10;NDUFB7;NDUFB8;NDUFB9;NDUFS6;NDUFS7;NDUFS8;NDUFV1
yellow	MF	GO:0008137	NADH dehydrogenase (ubiquinone) activity	17735	44	1225	11	3.08	0.00017	3.571429	NDUFA13;NDUFA3;NDUFA8;NDUFB10;NDUFB7;NDUFB8;NDUFB9;NDUFS6;NDUFS7;NDUFS8;NDUFV1
yellow	MF	GO:0050136	NADH dehydrogenase (quinone) activity	17735	44	1225	11	3.08	0.00017	3.571429	NDUFA13;NDUFA3;NDUFA8;NDUFB10;NDUFB7;NDUFB8;NDUFB9;NDUFS6;NDUFS7;NDUFS8;NDUFV1
yellow	BP	GO:0042773	ATP synthesis coupled electron transport	17735	60	1225	13	4.22	0.00022	3.080569	COQ7;NDUFA3;NDUFA8;NDUFB10;NDUFB7;NDUFB8;NDUFB9;NDUFS6;NDUFS7;NDUFS8;NDUFV1;SDHAF2;
yellow	BP	GO:0042775	mitochondrial ATP synthesis coupled elec...	17735	60	1225	13	4.22	0.00022	3.080569	COQ7;NDUFA3;NDUFA8;NDUFB10;NDUFB7;NDUFB8;NDUFB9;NDUFS6;NDUFS7;NDUFS8;NDUFV1;SDHAF2;
yellow	MF	GO:0016772	transferase activity, transferring phosp...	17735	889	1225	90	62.29	0.00023	1.444855	ADCK5;AK1;AK4;BMP2K;BRAF;BUB1;BUB1B;CDK10;CDK12;CDK13;CDS2;CHRAC1;CNTLN;CSNK1G1;DGUOK;D
yellow	CC	GO:0031981	nuclear lumen	17735	2101	1225	182	142.59	0.00023	1.276387	ABCB8;ABT1;ANAPC11;ANAPC2;APPL2;ARFGEF1;ARNTL;ATF4;ATF7IP;ATRIP;BRF1;BRF2;BUB1;BUB1B;C17or
yellow	BP	GO:0044237	cellular metabolic process	17735	8597	1225	658	604.82	0.00024	1.087927	ABT1;ACADS;ACAP3;ACER3;ACOT8;ACP5;ACSL3;ADAM9;ADAMTS13;ADHFE1;ADNP;ADORA2A;AES;AFF1;AFI
yellow	BP	GO:0008152	metabolic process	17735	9452	1225	716	664.97	0.00026	1.07674	ABT1;ACADS;ACAP3;ACER3;ACOT8;ACP5;ACSL3;ADAM12;ADAM9;ADAMDEC1;ADAMTS13;ADHFE1;ADNP;AI
yellow	CC	GO:0005829	cytosol	17735	2362	1225	201	160.31	0.00028	1.253821	ACP5;ACTR1B;AFMID;AFTPH;AHCY;AIP;AK1;AKAP10;AKR1A1;ALS2;ANAPC11;ANAPC2;AP1G1;AP1S1;APRT;A
yellow	CC	GO:0005654	nucleoplasm	17735	1481	1225	134	100.51	0.00029	1.333201	ABT1;ANAPC11;ANAPC2;APPL2;ARNTL;ATF4;ATF7IP;ATRIP;BRF1;BRF2;C17orf49;C17orf70;C1orf86;CCDC101
yellow	BP	GO:0031664	regulation of lipopolysaccharide-mediate...	17735	10	1225	5	0.7	0.00032	7.142857	NFKBIL1;PRDM1;SIGIRR;TNFAIP3;TRAF6

yellow	BP	GO:0009262	deoxyribonucleotide metabolic process	17735	33	1225	9	2.32	0.00034	3.87931	DGUOK;DTYMK;MPG;MUTYH;NT5C;NTHL1;NUDT1;NUDT18;OGG1
yellow	CC	GO:0005758	mitochondrial intermembrane space	17735	49	1225	11	3.33	0.00036	3.303303	CHCHD5;DTYMK;GFER;GOLPH3;NDUFA8;NDUFB7;NME4;PPOX;STOML2;TIMM10;TIMM13
yellow	BP	GO:0016310	phosphorylation	17735	1280	1225	121	90.05	4.00E-04	1.343698	ADAM9;ADORA2A;ALS2;ANAPC2;APOE;ATF4;ATP5D;AVPI1;BRAF;C19orf10;CCNL2;CD36;CDC25A;CDC6;CDK
yellow	BP	GO:0045333	cellular respiration	17735	162	1225	24	11.4	0.00041	2.105263	ATP5D;ATP5H;COQ7;COX4I1;CYC1;ETFB;NDUFA11;NDUFA13;NDUFA3;NDUFA8;NDUFB10;NDUFB11;NDUFB
yellow	MF	GO:0016651	oxidoreductase activity, acting on NAD(P...	17735	98	1225	17	6.87	0.00043	2.474527	CBR1;DCXR;DHDH;DHRS4;NDOR1;NDUFA13;NDUFA3;NDUFA8;NDUFB10;NDUFB7;NDUFB8;NDUFB9;NDUFS
yellow	MF	GO:0016866	intramolecular transferase activity	17735	28	1225	8	1.96	5.00E-04	4.081633	MUT;PGM2;PMM1;PUS1;PUSL1;RPUSD1;RPUSD3;TRUB2
yellow	BP	GO:0045184	establishment of protein localization	17735	1288	1225	121	90.61	0.00051	1.335393	ABCB9;ACSL3;ADAM9;ADORA2A;AFTPH;AHCYL1;AIP;AP1G1;AP1G2;AP1S1;ARFGEF1;ARFRP1;ARNTL;ASPSCR
yellow	MF	GO:0005085	guanyl-nucleotide exchange factor activi...	17735	176	1225	25	12.33	0.00056	2.027575	ALS2;ARFGEF1;ARHGEF25;ARHGEF26;DENND2C;EIF2B2;EIF2B4;FGD6;GAPVD1;ITSN2;LAMTOR2;PCP2;PLEKH
yellow	BP	GO:0006412	translation	17735	464	1225	52	32.64	0.00056	1.593137	C1QBP;CAPRIN1;DALRD3;EEF1D;EEF1G;EIF1;EIF2B2;EIF2B4;EIF3F;EIF3G;EIF3I;EIF3K;EIF4G3;ETF1;GNB2L1;LA
yellow	BP	GO:0008104	protein localization	17735	1586	1225	144	111.58	0.00065	1.290554	ABCB9;ACSL3;ADAM9;ADORA2A;AFTPH;AHCYL1;AIP;AKAP10;ALS2;AP1G1;AP1G2;AP1S1;ARFGEF1;ARFRP1;
yellow	MF	GO:0016301	kinase activity	17735	762	1225	77	53.39	0.00068	1.442218	ADCK5;AK1;AK4;BMP2K;BRAF;BUB1;BUB1B;CDK10;CDK12;CDK13;CNTLN;CSNK1G1;DGUOK;DOLK;DTYMK;E
yellow	CC	GO:0031967	organelle envelope	17735	825	1225	80	55.99	0.00074	1.428827	ABCB8;ACADS;ACSL3;ATAD3B;ATP5D;ATP5G2;ATP5G3;ATP5H;BCS1L;BOK;CCS;CDS2;CENPF;CHCHD5;COQ7;
yellow	BP	GO:0001522	pseudouridine synthesis	17735	17	1225	6	1.2	0.00075	5	NHP2;PUS1;PUSL1;RPUSD1;RPUSD3;TRUB2
yellow	BP	GO:0006120	mitochondrial electron transport, NADH t...	17735	44	1225	10	3.1	0.00078	3.225806	NDUFA3;NDUFA8;NDUFB10;NDUFB7;NDUFB8;NDUFB9;NDUFS6;NDUFS7;NDUFS8;NDUFV1
yellow	CC	GO:0031975	envelope	17735	839	1225	81	56.94	0.00078	1.42255	ABCB8;ACADS;ACSL3;ATAD3B;ATP5D;ATP5G2;ATP5G3;ATP5H;BCS1L;BOK;CCS;CDS2;CENPF;CHCHD5;COQ7;
yellow	MF	GO:0051539	4 iron, 4 sulfur cluster binding	17735	37	1225	9	2.59	0.00082	3.474903	MUTYH;NARFL;NDUFS7;NDUFS8;NDUFV1;NTHL1;NUBP2;POLD1;RTEL1
yellow	BP	GO:0015031	protein transport	17735	1245	1225	116	87.59	9.00E-04	1.324352	ABCB9;ACSL3;ADAM9;ADORA2A;AFTPH;AHCYL1;AIP;AP1G1;AP1G2;AP1S1;ARFGEF1;ARFRP1;ARNTL;ASPSCR
yellow	BP	GO:0006413	translational initiation	17735	152	1225	22	10.69	0.00096	2.057998	EIF1;EIF2B2;EIF2B4;EIF3F;EIF3G;EIF3I;EIF3K;EIF4G3;RPL13;RPL18;RPL18A;RPL28;RPL29;RPL38;RPL8;RPLP1;R
yellow	BP	GO:0006259	DNA metabolic process	17735	866	1225	85	60.93	0.001	1.395043	ASH1L;ATF7IP;ATRIP;BABAM1;C11orf30;C17orf70;C1orf86;CDC25A;CDC6;CENPF;CHTF18;CIB1;CLCF1;CLSPN
yellow	CC	GO:0031970	organelle envelope lumen	17735	56	1225	11	3.8	0.00118	2.894737	CHCHD5;DTYMK;GFER;GOLPH3;NDUFA8;NDUFB7;NME4;PPOX;STOML2;TIMM10;TIMM13
yellow	BP	GO:0055114	oxidation-reduction process	17735	561	1225	59	39.47	0.00121	1.494806	ACADS;ACOT8;APOM;ATP5D;ATP5H;CBR1;COQ7;COX4I1;CPT1C;CYB561D2;CYC1;CYP1B1;CYP2E1;DCXR;DH
yellow	MF	GO:0003723	RNA binding	17735	851	1225	83	59.62	0.00123	1.39215	ABT1;ANKRD17;C1QBP;CAPRIN1;CELF2;CELF6;CPSF2;CPSF4;CSDE1;DDX21;DDX28;DDX43;DDX49;DNAJC17;
yellow	MF	GO:0016773	phosphotransferase activity, alcohol gro...	17735	706	1225	71	49.46	0.00124	1.435503	ADCK5;BMP2K;BRAF;BUB1;BUB1B;CDK10;CDK12;CDK13;CNTLN;CSNK1G1;DGUOK;DOLK;ETNK2;FASTK;FUK;
yellow	MF	GO:0019205	nucleobase-containing compound kinase ac...	17735	47	1225	10	3.29	0.0013	3.039514	AK1;AK4;DGUOK;DTYMK;MAGI3;NME3;NME4;PNKP;UCK1;UCKL1
yellow	BP	GO:0007094	mitotic cell cycle spindle assembly chec...	17735	32	1225	8	2.25	0.00134	3.555556	ANAPC11;ANAPC2;BUB1;BUB1B;CENPF;DYNC1LI1;MAD1L1;TPR
yellow	BP	GO:0007091	mitotic metaphase/anaphase transition	17735	47	1225	10	3.31	0.00134	3.021148	ANAPC11;ANAPC2;BUB1;BUB1B;CENPF;DLGAP5;DYNC1LI1;MAD1L1;RAD21;TPR
yellow	BP	GO:0033554	cellular response to stress	17735	1209	1225	112	85.06	0.00135	1.316718	APOE;ATF4;ATRIP;BABAM1;C11orf30;C12orf44;C17orf70;C19orf10;C1orf86;CCDC47;CCS;CD36;CIB1;CLSPN;
yellow	MF	GO:0003735	structural constituent of ribosome	17735	157	1225	22	11	0.0014	2	MRPL12;MRPL20;MRPL23;MRPL24;MRPL52;MRPL55;MRPS11;MRPS12;MRPS15;MRPS18A;RPL13;RPL18;RP
yellow	CC	GO:0030529	ribonucleoprotein complex	17735	573	1225	58	38.89	0.00145	1.491386	ARFGEF1;BTBD6;CANX;CAPRIN1;FBL;GEMIN7;GNB2L1;HNRNPH3;IMP4;LARP4B;LSM14A;LSM2;LSM4;MRPL1
yellow	CC	GO:0015934	large ribosomal subunit	17735	75	1225	13	5.09	0.00149	2.554028	MRPL12;MRPL23;MRPL52;MRPL55;RPL13;RPL18;RPL18A;RPL28;RPL29;RPL38;RPL8;RPLP1;RPLP2
yellow	BP	GO:0007005	mitochondrion organization	17735	230	1225	29	16.18	0.00159	1.792336	AIP;BCS1L;C1QBP;COQ7;FANCG;GFER;GIMAP5;GOLPH3;MRPL12;MTFP1;MTM1;NDUFS7;NDUFS8;P2RX7;PA
yellow	BP	GO:0045841	negative regulation of mitotic metaphase...	17735	33	1225	8	2.32	0.00166	3.448276	ANAPC11;ANAPC2;BUB1;BUB1B;CENPF;DYNC1LI1;MAD1L1;TPR
yellow	BP	GO:0071173	spindle assembly checkpoint	17735	33	1225	8	2.32	0.00166	3.448276	ANAPC11;ANAPC2;BUB1;BUB1B;CENPF;DYNC1LI1;MAD1L1;TPR
yellow	BP	GO:0071704	organic substance metabolic process	17735	8996	1225	677	632.89	0.00169	1.069696	ABT1;ACADS;ACAP3;ACER3;ACOT8;ACP5;ACSL3;ADAM12;ADAM9;ADAMDEC1;ADAMTS13;ADHFE1;ADNP;A
yellow	BP	GO:0030071	regulation of mitotic metaphase/anaphase...	17735	41	1225	9	2.88	0.00184	3.125	ANAPC11;ANAPC2;BUB1;BUB1B;CENPF;DLGAP5;DYNC1LI1;MAD1L1;TPR
yellow	MF	GO:0009982	pseudouridine synthase activity	17735	14	1225	5	0.98	0.00196	5.102041	PUS1;PUSL1;RPUSD1;RPUSD3;TRUB2
yellow	CC	GO:0043234	protein complex	17735	3294	1225	262	223.56	0.00196	1.171945	ABCB8;ABCB9;ABT1;ACTR1B;ACTR3;AFTPH;ALS2;ANAPC11;ANAPC2;ANO6;AP1G1;AP1G2;AP1S1;APPL2;ARI
yellow	CC	GO:0044428	nuclear part	17735	2482	1225	203	168.45	0.00197	1.205105	ABCB8;ABT1;ANAPC11;ANAPC2;APPL2;ARFGEF1;ARNTL;ATF4;ATF7IP;ATRIP;BABAM1;BRF1;BRF2;BUB1;BUB
yellow	MF	GO:0016798	hydrolase activity, acting on glycosyl b...	17735	112	1225	17	7.85	0.00199	2.165605	ACER3;ADPRHL2;ATHL1;CD38;ENGASE;HEXDC;KIAA2018;LYZ;MAN1B1;MOGS;MPG;MUTYH;NAGLU;NEIL1;N
yellow	MF	GO:0008757	S-adenosylmethionine-dependent methyltra...	17735	103	1225	16	7.22	0.00209	2.216066	ASH1L;GAMT;LCMT1;MLL3;MLL5;PEMT;PNMT;PRMT7;RBBP5;SETD2;SETD7;SUV420H1;TRMT1;TRMT61A;T
yellow	MF	GO:0008170	N-methyltransferase activity	17735	67	1225	12	4.69	0.00217	2.558635	ASH1L;MLL3;MLL5;PEMT;PNMT;PRMT7;RBBP5;SETD2;SETD7;SUV420H1;TRMT1;WHSC1L
yellow	CC	GO:0000313	organellar ribosome	17735	52	1225	10	3.53	0.0023	2.832861	MRPL12;MRPL20;MRPL23;MRPL52;MRPL55;MRPS11;MRPS12;MRPS15;MRPS18A;MRPS2
yellow	CC	GO:0005761	mitochondrial ribosome	17735	52	1225	10	3.53	0.0023	2.832861	MRPL12;MRPL20;MRPL23;MRPL52;MRPL55;MRPS11;MRPS12;MRPS15;MRPS18A;MRPS2
yellow	BP	GO:0071174	mitotic cell cycle spindle checkpoint	17735	35	1225	8	2.46	0.00249	3.252033	ANAPC11;ANAPC2;BUB1;BUB1B;CENPF;DYNC1LI1;MAD1L1;TPR
yellow	MF	GO:0008080	N-acetyltransferase activity	17735	77	1225	13	5.39	0.00251	2.411874	ELP4;EPC1;KAT2A;MCRS1;NAA10;NAGS;NAT14;NAT6;NAT9;NCOA3;PHF20;SAT2;TADA3
yellow	MF	GO:0051536	iron-sulfur cluster binding	17735	61	1225	11	4.27	0.0031	2.576112	CISD3;FDX1L;MUTYH;NARFL;NDUFS7;NDUFS8;NDUFV1;NTHL1;NUBP2;POLD1;RTEL1
yellow	MF	GO:0051540	metal cluster binding	17735	61	1225	11	4.27	0.0031	2.576112	CISD3;FDX1L;MUTYH;NARFL;NDUFS7;NDUFS8;NDUFV1;NTHL1;NUBP2;POLD1;RTEL1
yellow	MF	GO:0008135	translation factor activity, nucleic aci...	17735	79	1225	13	5.53	0.00317	2.350814	EEF1A2;EEF1D;EEF1G;EIF1;EIF2B2;EIF2B4;EIF3F;EIF3G;EIF3I;EIF3K;EIF4G3;ETF1;TUF
yellow	BP	GO:0006479	protein methylation	17735	79	1225	13	5.56	0.00328	2.338129	ETF1;KDM3A;KDM6A;LCMT1;MLL3;MLL5;PRMT7;RBBP5;SETD2;SETD7;SUV420H1;TRMT112;WHSC1L
yellow	BP	GO:0008213	protein alkylation	17735	79	1225	13	5.56	0.00328	2.338129	ETF1;KDM3A;KDM6A;LCMT1;MLL3;MLL5;PRMT7;RBBP5;SETD2;SETD7;SUV420H1;TRMT112;WHSC1L
yellow	BP	GO:0072594	establishment of protein localization to...	17735	210	1225	26	14.77	0.00347	1.760325	AIP;CCDC101;GFER;PEX16;PEX6;RPL13;RPL18;RPL18A;RPL28;RPL29;RPL38;RPL8;RPLP1;RPLP2;RPS15;RPS2;R
yellow	BP	GO:0006996	organelle organization	17735	2139	1225	181	150.48	0.00355	1.202818	ACOT8;ACTR3;AIP;ALS2;ANAPC11;ANAPC2;APOE;ARC;ARFGEF1;ARFIP2;ARHGAP4;ATL3;ATP6V0E2;ATP6V1C
yellow	BP	GO:0000075	cell cycle checkpoint	17735	243	1225	29	17.1	0.00361	1.695906	ANAPC11;ANAPC2;ATRIP;BABAM1;BUB1;BUB1B;CDC25A;CDC6;CENPF;CLSPN;DYNC1LI1;E2F1;FANCG;FEM1
yellow	BP	GO:0000281	cytokinesis after mitosis	17735	10	1225	4	0.7	0.00363	5.714286	MYH10;NUSAP1;STAMBP;USP8
yellow	BP	GO:0006793	phosphorus metabolic process	17735	2513	1225	209	176.8	0.00377	1.182127	ACAP3;ACER3;ACOT8;ACSL3;ADAM9;ADORA2A;AK1;AK4;ALG12;ALS2;ANAPC2;APOE;APRT;ARFGEF1;ARFRP
yellow	BP	GO:0044238	primary metabolic process	17735	8755	1225	656	615.94	0.00422	1.065039	ABT1;ACADS;ACAP3;ACER3;ACOT8;ACSL3;ADAM12;ADAM9;ADAMDEC1;ADAMTS13;ADNP;ADORA2A;AES;A
yellow	BP	GO:0006796	phosphate-containing compound metabolic ...	17735	2466	1225	205	173.49	0.00423	1.181624	ACAP3;ACER3;ACSL3;ADAM9;ADORA2A;AK1;AK4;ALG12;ALS2;ANAPC2;APOE;APRT;ARFGEF1;ARFRP1;ARHG
yellow	MF	GO:0016627	oxidoreductase activity, acting on the C...	17735	55	1225	10	3.85	0.00443	2.597403	ACADS;DECR2;DHDH;DUS1L;DUS2L;DUS3L;GCDH;MECR;PPOX;TM7SF2
yellow	CC	GO:0000793	condensed chromosome	17735	167	1225	21	11.33	0.00457	1.853486	BUB1;BUB1B;CENPF;CENPM;CENPT;CLASP1;DYNC1LI1;MAD1L1;MKI67;NCAPH;NSMCE1;RAD21;RAD9A;REC
yellow	MF	GO:0016787	hydrolase activity	17735	2319	1225	193	162.48	0.00458	1.187839	ABCB8;ABCB9;ABHD1;ABHD14A;ACER3;ACOT8;ACP5;ADAM12;ADAM9;ADAMDEC1;ADAMTS13;ADAMTSL2;
yellow	CC	GO:0005815	microtubule organizing center	17735	482	1225	48	32.71	0.00487	1.467441	ACTR1B;ALS2;ATF4;BUB1B;CCDC28B;CCDC61;CDCA8;CEP55;CLASP1;CLIP1;CNTLN;DLGAP5;DYNC1LI1;FBXW
yellow	BP	GO:0007059	chromosome segregation	17735	142	1225	19	9.99	0.00503	1.901902	BUB1;CDC6;CENPF;CENPT;DLGAP5;LATS1;NCAPH;NIPBL;NUSAP1;RAD21;REC8;SLC25A5;STRA13;TLK2;TOP1;
yellow	MF	GO:0036094	small molecule binding	17735	2525	1225	208	176.91	0.00509	1.175739	ABCB8;ABCB9;ABT1;ACADS;ACSL3;ACTR1B;ACTR3;AHCY;AHCYL1;AK1;AK4;APRT;ARFIP2;ARFRP1;ARL16;ATA
yellow	BP	GO:0051338	regulation of transferase activity	17735	659	1225	64	46.36	0.00516	1.3805	ADAM9;ADORA2A;ALS2;ANAPC2;APOE;ATF4;AVPI1;BRAF;C19orf10;CCNL2;CDC25A;CDC6;CDK12;CLSPN;DO
yellow	BP	GO:0033108	mitochondrial respiratory chain complex ...	17735	17	1225	5	1.2	0.00517	4.166667	BCS1L;NDUFS7;NDUFS8;SDHAF1;TAZ
yellow	BP	GO:0034377	plasma lipoprotein particle assembly	17735	17	1225	5	1.2	0.00517	4.166667	ACSL3;APOE;APOM;SOAT1;SOAT2
yellow	BP	GO:0065005	protein-lipid complex assembly	17735	17	1225	5	1.2	0.00517	4.166667	ACSL3;APOE;APOM;SOAT1;SOAT2
yellow	CC	GO:0044451	nucleoplasm part	17735	823	1225	75	55.86	0.00534	1.342642	ABT1;APPL2;ARNTL;ATF4;ATF7IP;BRF1;C17orf49;C1orf86;CCDC101;CCNL2;CDK12;CDK13;CPSF2;CPSF4;CREE
yellow	MF	GO:0000166	nucleotide binding	17735	2385	1225	197	167.1	0.00577	1.178935	ABCB8;ABCB9;ABT1;ACADS;ACSL3;ACTR1B;ACTR3;AHCY;AHCYL1;AK1;AK4;APRT;ARFIP2;ARFRP1;ARL16;ATA

yellow	MF	GO:0018024	histone-lysine N-methyltransferase activ...	17735	40	1225	8	2.8	0.00581	2.857143	ASH1L;MLL3;MLL5;RBBP5;SETD2;SETD7;SUV420H1;WHSC1L1
yellow	MF	GO:1901265	nucleoside phosphate binding	17735	2386	1225	197	167.17	0.00588	1.178441	ABCB8;ABCB9;ABT1;ACADS;ACSL3;ACTR1B;ACTR3;AHCY;AHCYL1;AK1;AK4;APRT;ARFIP2;ARFRP1;ARL16;ATA
yellow	BP	GO:0045839	negative regulation of mitosis	17735	40	1225	8	2.81	0.00595	2.846975	ANAPC11;ANAPC2;BUB1;BUB1B;CENPF;DYNC1LI1;MAD1L1;TPR
yellow	BP	GO:0051784	negative regulation of nuclear division	17735	40	1225	8	2.81	0.00595	2.846975	ANAPC11;ANAPC2;BUB1;BUB1B;CENPF;DYNC1LI1;MAD1L1;TPR
yellow	MF	GO:0042054	histone methyltransferase activity	17735	49	1225	9	3.43	0.00634	2.623907	ASH1L;MLL3;MLL5;PRMT7;RBBP5;SETD2;SETD7;SUV420H1;WHSC1L1
yellow	MF	GO:0008276	protein methyltransferase activity	17735	67	1225	11	4.69	0.0065	2.345416	ASH1L;LCMT1;MLL3;MLL5;PRMT7;RBBP5;SETD2;SETD7;SUV420H1;TRMT112;WHSC1L
yellow	BP	GO:0050854	regulation of antigen receptor-mediated ...	17735	25	1225	6	1.76	0.00664	3.409091	DUSP3;ELF1;KCNN4;PHPT1;PTPRC;RC3H1
yellow	BP	GO:0009148	pyrimidine nucleoside triphosphate biosy...	17735	18	1225	5	1.27	0.00675	3.937008	DTYMK;NME3;NME4;UCK1;UCKL1
yellow	BP	GO:0070849	response to epidermal growth factor stim...	17735	18	1225	5	1.27	0.00675	3.937008	BRAF;MAPK1;MCM7;MED1;TPR
yellow	BP	GO:0031577	spindle checkpoint	17735	41	1225	8	2.88	0.00695	2.777778	ANAPC11;ANAPC2;BUB1;BUB1B;CENPF;DYNC1LI1;MAD1L1;TPR
yellow	BP	GO:0006402	mRNA catabolic process	17735	179	1225	22	12.59	0.00742	1.747419	ETF1;EXOSC5;LSM2;LSM4;PATL1;RC3H1;RPL13;RPL18;RPL18A;RPL28;RPL29;RPL38;RPL8;RPLP1;RPLP2;RPS1
yellow	MF	GO:0005123	death receptor binding	17735	12	1225	4	0.84	0.00752	4.761905	FEM1B;NGFRAP1;PIDD;TNFSF15
yellow	MF	GO:0019104	DNA N-glycosylase activity	17735	12	1225	4	0.84	0.00752	4.761905	MPG;NEIL1;NTHL1;OGG1
yellow	BP	GO:0010257	NADH dehydrogenase complex assembly	17735	12	1225	4	0.84	0.00763	4.761905	BCS1L;NDUFS7;NDUFS8;TAZ
yellow	BP	GO:0032981	mitochondrial respiratory chain complex ...	17735	12	1225	4	0.84	0.00763	4.761905	BCS1L;NDUFS7;NDUFS8;TAZ
yellow	BP	GO:0097031	mitochondrial respiratory chain complex ...	17735	12	1225	4	0.84	0.00763	4.761905	BCS1L;NDUFS7;NDUFS8;TAZ
yellow	CC	GO:0022625	cytosolic large ribosomal subunit	17735	52	1225	9	3.53	0.00772	2.549575	RPL13;RPL18;RPL18A;RPL28;RPL29;RPL38;RPL8;RPLP1;RPLP2
yellow	BP	GO:0000956	nuclear-transcribed mRNA catabolic proce...	17735	169	1225	21	11.89	0.00776	1.76619	ETF1;EXOSC5;LSM2;LSM4;PATL1;RC3H1;RPL13;RPL18;RPL18A;RPL28;RPL29;RPL38;RPL8;RPLP1;RPLP2;RPS1
yellow	BP	GO:0019080	viral genome expression	17735	148	1225	19	10.41	0.00784	1.825168	POLR2F;POLR2G;POLR2I;POLR2L;RPL13;RPL18;RPL18A;RPL28;RPL29;RPL38;RPL8;RPLP1;RPLP2;RPS15;RPS2;
yellow	BP	GO:0019083	viral transcription	17735	148	1225	19	10.41	0.00784	1.825168	POLR2F;POLR2G;POLR2I;POLR2L;RPL13;RPL18;RPL18A;RPL28;RPL29;RPL38;RPL8;RPLP1;RPLP2;RPS15;RPS2;
yellow	BP	GO:0007093	mitotic cell cycle checkpoint	17735	138	1225	18	9.71	0.00808	1.853759	ANAPC11;ANAPC2;BUB1;BUB1B;CENPF;CLSPN;DYNC1LI1;HRAS;MAD1L1;PIDD;PSMA7;PSMB4;PSMB5;PSMB
yellow	CC	GO:0005819	spindle	17735	232	1225	26	15.75	0.00812	1.650794	BUB1B;CDC6;CDCA8;CENPF;CLASP1;DLGAP5;DYNC1LI1;E4F1;HAUS8;KAT2A;KIF15;KIF20A;LATS1;LATS2;MA
yellow	BP	GO:0002702	positive regulation of production of mol...	17735	26	1225	6	1.83	0.00813	3.278689	CD36;CLCF1;GIMAP5;HPX;TRAF2;TRAF6
yellow	BP	GO:0044281	small molecule metabolic process	17735	2545	1225	208	179.05	0.00838	1.161687	ACADS;ACAP3;ACER3;ACOT8;ACP5;ACSL3;ADHFE1;ADORA2A;AFMID;AHCY;AHCYL1;AK1;AK4;AKR1A1;ALS2;
yellow	MF	GO:0000062	fatty-acyl-CoA binding	17735	19	1225	5	1.33	0.0085	3.759398	ACADS;ACBD4;GCDH;SOAT1;SOAT2
yellow	BP	GO:0000910	cytokinesis	17735	108	1225	15	7.6	0.00851	1.973684	ACTR3;CDC6;CEP55;DIAPH2;KIF20A;MYH10;NUSAP1;PIK3C3;PIN1;RABEP2;RHOC;ROCK2;SPG20;STAMBP;US
yellow	BP	GO:0006414	translational elongation	17735	108	1225	15	7.6	0.00851	1.973684	EEF1D;EEF1G;RPL13;RPL18;RPL18A;RPL28;RPL29;RPL38;RPL8;RPLP1;RPLP2;RPS15;RPS2;RPS28;TUF
yellow	BP	GO:0043549	regulation of kinase activity	17735	638	1225	61	44.89	0.00863	1.358877	ADAM9;ADORA2A;ALS2;ANAPC2;APOE;ATF4;AVPI1;BRAF;C19orf10;CCNL2;CDC25A;CDC6;CDK12;CLSPN;DO
yellow	BP	GO:0050856	regulation of T cell receptor signaling ...	17735	19	1225	5	1.34	0.00864	3.731343	DUSP3;ELF1;KCNN4;PHPT1;RC3H1
yellow	BP	GO:0008654	phospholipid biosynthetic process	17735	215	1225	25	15.13	0.00907	1.652346	ACSL3;ALG12;CDS2;DOLK;DPM3;ETNK2;ISYNA1;MTM1;MTMR1;MVK;PCYT2;PEMT;PIGQ;PIK3C3;PIK3CB;PIK
yellow	BP	GO:0022415	viral reproductive process	17735	542	1225	53	38.13	0.00909	1.389982	ACOT8;ANKRD17;AP1G2;ATF7IP;BUB1;C1QBP;CDK13;CPSF4;CREB3;DYNC1LI1;E4F1;EIF4G3;GADD45GIP1;G
yellow	BP	GO:0016032	viral reproduction	17735	689	1225	65	48.47	0.00912	1.341036	ACOT8;ANKRD17;AP1G1;AP1G2;AP1S1;ATF7IP;BUB1;C1QBP;CD8B;CDK13;CPSF4;CREB3;DYNC1LI1;E4F1;EIF
yellow	CC	GO:0005849	mRNA cleavage factor complex	17735	13	1225	4	0.88	0.00919	4.545455	CPSF2;CPSF4;PIP5K1A;TUT1
yellow	MF	GO:0016779	nucleotidyltransferase activity	17735	120	1225	16	8.41	0.00952	1.902497	CDS2;CHRAC1;FICD;FLAD1;PAPSS1;PCYT2;POLD1;POLD4;POLM;POLR2F;POLR2G;POLR2I;POLR2L;POLR3K;PC
yellow	BP	GO:0044764	multi-organism cellular process	17735	691	1225	65	48.61	0.00969	1.337173	ACOT8;ANKRD17;AP1G1;AP1G2;AP1S1;ATF7IP;BUB1;C1QBP;CD8B;CDK13;CPSF4;CREB3;DYNC1LI1;E4F1;EIF
yellow	MF	GO:0016407	acetyltransferase activity	17735	90	1225	13	6.31	0.00971	2.060222	ELP4;EPC1;KAT2A;MCRS1;NAA10;NAGS;NAT14;NAT6;NAT9;NCOA3;PHF20;SAT2;TADA3
yellow	BP	GO:0033205	cell cycle cytokinesis	17735	35	1225	7	2.46	0.00987	2.845528	ACTR3;CDC6;MYH10;NUSAP1;RHOC;STAMBP;USP8
yellow	BP	GO:0006401	RNA catabolic process	17735	206	1225	24	14.49	0.01019	1.656315	ETF1;EXOSC5;LSM2;LSM4;PATL1;POLD1;RC3H1;RNASEH2C;RPL13;RPL18;RPL18A;RPL28;RPL29;RPL38;RPL8;
yellow	BP	GO:0033036	macromolecule localization	17735	1852	1225	155	130.29	0.01041	1.189654	ABCB9;ACSL3;ADAM9;ADORA2A;AFTPH;AHCYL1;AIP;AKAP10;ALS2;AP1G1;AP1G2;AP1S1;APOE;APOM;ARFG
yellow	MF	GO:0016860	intramolecular oxidoreductase activity	17735	44	1225	8	3.08	0.01046	2.597403	DDT;ECI1;EHHADH;ERP29;ERP44;MRI1;PTGES2;SPATA20
yellow	MF	GO:0016491	oxidoreductase activity	17735	709	1225	66	49.67	0.01058	1.32877	ACADS;ADHFE1;AKR1A1;ALKBH7;CBR1;CCS;COQ7;COX4I1;CRYL1;CYP1B1;CYP2E1;DCXR;DECR2;DHDH;DHRS
yellow	BP	GO:0006399	tRNA metabolic process	17735	121	1225	16	8.51	0.01066	1.880141	CTU2;DALRD3;FBL;GTPBP3;PARS2;POP7;PUS1;PUSL1;QARS;QTRT1;SARS2;TPR;TRMU;TRPT1;TRUB2;VARS2
yellow	MF	GO:0016799	hydrolase activity, hydrolyzing N-glycos...	17735	20	1225	5	1.4	0.01069	3.571429	CD38;MPG;NEIL1;NTHL1;OGG1
yellow	BP	GO:0045859	regulation of protein kinase activity	17735	608	1225	58	42.77	0.01073	1.356091	ADAM9;ADORA2A;ALS2;ANAPC2;APOE;ATF4;AVPI1;BRAF;C19orf10;CCNL2;CDC25A;CDC6;CDK12;CLSPN;DO
yellow	BP	GO:0015985	energy coupled proton transport, down el...	17735	20	1225	5	1.41	0.01087	3.546099	ATP5D;ATP5G2;ATP5G3;ATP5H;STOML2
yellow	BP	GO:0015986	ATP synthesis coupled proton transport	17735	20	1225	5	1.41	0.01087	3.546099	ATP5D;ATP5G2;ATP5G3;ATP5H;STOML2
yellow	BP	GO:0006415	translational termination	17735	91	1225	13	6.4	0.01097	2.03125	ETF1;RPL13;RPL18;RPL18A;RPL28;RPL29;RPL38;RPL8;RPLP1;RPLP2;RPS15;RPS2;RPS2
yellow	BP	GO:0007088	regulation of mitosis	17735	91	1225	13	6.4	0.01097	2.03125	ANAPC11;ANAPC2;BUB1;BUB1B;CDC6;CDK13;CENPF;DLGAP5;DYNC1LI1;MAD1L1;NUSAP1;PIN1;TPR
yellow	BP	GO:0051783	regulation of nuclear division	17735	91	1225	13	6.4	0.01097	2.03125	ANAPC11;ANAPC2;BUB1;BUB1B;CDC6;CDK13;CENPF;DLGAP5;DYNC1LI1;MAD1L1;NUSAP1;PIN1;TPR
yellow	BP	GO:0071900	regulation of protein serine/threonine k...	17735	368	1225	38	25.89	0.01111	1.467748	ALS2;ANAPC2;APOE;AVPI1;CCNL2;CDC25A;CDC6;CDK12;DUSP10;DUSP2;DUSP3;HIPK3;HRAS;LATS1;LATS2;L
yellow	MF	GO:0032813	tumor necrosis factor receptor superfami...	17735	36	1225	7	2.52	0.01129	2.777778	FEM1B;NGFRAP1;PIDD;SIVA1;TNFSF12;TNFSF15;TRAF2
yellow	BP	GO:0009142	nucleoside triphosphate biosynthetic pro...	17735	72	1225	11	5.07	0.01145	2.169625	ATP5D;ATP5G2;ATP5G3;ATP5H;DTYMK;NME3;NME4;STOML2;SURF1;UCK1;UCKL1
yellow	BP	GO:0043506	regulation of JUN kinase activity	17735	72	1225	11	5.07	0.01145	2.169625	DUSP10;HIPK3;MAGI3;MAP3K10;MAP4K2;TAOK3;TP73;TRAF2;TRAF6;WNT7A;ZEB2
yellow	BP	GO:0043405	regulation of MAP kinase activity	17735	253	1225	28	17.8	0.01147	1.573034	APOE;AVPI1;CDK12;DUSP10;DUSP2;DUSP3;HIPK3;HRAS;LIME1;LPAR1;MAGI3;MAP3K10;MAP4K2;MAPK1;M
yellow	MF	GO:0016410	N-acyltransferase activity	17735	92	1225	13	6.45	0.01161	2.015504	ELP4;EPC1;KAT2A;MCRS1;NAA10;NAGS;NAT14;NAT6;NAT9;NCOA3;PHF20;SAT2;TADA3
yellow	MF	GO:0016278	lysine N-methyltransferase activity	17735	45	1225	8	3.15	0.01196	2.539683	ASH1L;MLL3;MLL5;RBBP5;SETD2;SETD7;SUV420H1;WHSC1L1
yellow	MF	GO:0016279	protein-lysine N-methyltransferase activ...	17735	45	1225	8	3.15	0.01196	2.539683	ASH1L;MLL3;MLL5;RBBP5;SETD2;SETD7;SUV420H1;WHSC1L1
yellow	CC	GO:0000780	condensed nuclear chromosome, centromeri...	17735	14	1225	4	0.95	0.01219	4.210526	BUB1;BUB1B;REC8;SUV420H1
yellow	CC	GO:0005665	DNA-directed RNA polymerase II, core com...	17735	14	1225	4	0.95	0.01219	4.210526	POLR2F;POLR2G;POLR2I;POLR2L
yellow	BP	GO:0071156	regulation of cell cycle arrest	17735	266	1225	29	18.71	0.01241	1.549973	ANAPC11;ANAPC2;ATRIP;BABAM1;BUB1;BUB1B;CDC25A;CDC6;CENPF;CLSPN;DYNC1LI1;E2F1;FANCG;FEM1
yellow	BP	GO:0035023	regulation of Rho protein signal transdu...	17735	166	1225	20	11.68	0.01275	1.712329	ALS2;APOE;ARFGEF1;ARHGAP4;ARHGEF25;ARHGEF26;FGD6;FICD;HRAS;ITSN2;LPAR1;MLST8;PIN1;PIP5K1A;
yellow	MF	GO:0042826	histone deacetylase binding	17735	64	1225	10	4.48	0.013	2.232143	BHLHE41;HDAC10;KAT2A;NIPBL;PRDM1;RAD9A;SP1;SUDS3;TOP2A;TRAF6
yellow	MF	GO:0005515	protein binding	17735	7306	1225	547	511.88	0.01365	1.06861	ABCB9;ABT1;ACAP3;ACCS;ACOT8;ACSL3;ACTR1B;ACTR3;ADAM12;ADAM9;ADAMTS13;ADAMTSL2;ADNP;AD
yellow	BP	GO:0006241	CTP biosynthetic process	17735	14	1225	4	0.98	0.01379	4.081633	NME3;NME4;UCK1;UCKL1
yellow	BP	GO:0046036	CTP metabolic process	17735	14	1225	4	0.98	0.01379	4.081633	NME3;NME4;UCK1;UCKL1
yellow	BP	GO:0002705	positive regulation of leukocyte mediate...	17735	46	1225	8	3.24	0.01393	2.469136	ABCB9;GIMAP5;HPX;NCR3;P2RX7;TRAF2;TRAF6;ZP3
yellow	BP	GO:0002708	positive regulation of lymphocyte mediat...	17735	46	1225	8	3.24	0.01393	2.469136	ABCB9;GIMAP5;HPX;NCR3;P2RX7;TRAF2;TRAF6;ZP3
yellow	BP	GO:0034968	histone lysine methylation	17735	46	1225	8	3.24	0.01393	2.469136	KDM3A;KDM6A;MLL3;MLL5;RBBP5;SETD2;SUV420H1;WHSC1L1
yellow	BP	GO:0034613	cellular protein localization	17735	956	1225	85	67.26	0.01402	1.263753	ACSL3;AHCYL1;AIP;AP1G1;AP1G2;AP1S1;ARFRP1;ARNTL;ASPSCR1;BUB1B;CCDC101;CCHCR1;CD36;CIB1;CLA

yellow	BP	GO:0002831	regulation of response to biotic stimulu...	17735	84	1225	12	5.91	0.0142	2.030457	AP1G1;AP1S1;C1QBP;CD8B;CREB3;MICB;NFKBIL1;PRDM1;SIGIRR;TNFAIP3;TRAF6;ZC3HAV1
yellow	BP	GO:0042325	regulation of phosphorylation	17735	869	1225	78	61.14	0.01474	1.275761	ADAM9;ADORA2A;ALS2;ANAPC2;APOE;ATF4;AVPI1;BRAF;C19orf10;CCNL2;CD36;CDC25A;CDC6;CDK12;CLCF
yellow	MF	GO:0016776	phosphotransferase activity, phosphate g...	17735	38	1225	7	2.66	0.01512	2.631579	AK1;AK4;DTYMK;MAGI3;NME3;NME4;PMVK
yellow	BP	GO:0006614	SRP-dependent cotranslational protein ta...	17735	105	1225	14	7.39	0.01518	1.894452	RPL13;RPL18;RPL18A;RPL28;RPL29;RPL38;RPL8;RPLP1;RPLP2;RPS15;RPS2;RPS28;SSR1;SSR4
yellow	BP	GO:0070727	cellular macromolecule localization	17735	960	1225	85	67.54	0.01545	1.258513	ACSL3;AHCYL1;AIP;AP1G1;AP1G2;AP1S1;ARFRP1;ARNTL;ASPSCR1;BUB1B;CCDC101;CCHCR1;CD36;CIB1;CLA
yellow	BP	GO:0006888	ER to Golgi vesicle-mediated transport	17735	56	1225	9	3.94	0.01548	2.284264	KLHL12;LMF1;PREB;SEC22B;SEC24B;TRAPPC2L;TRAPPC4;USE1;YIPF5
yellow	BP	GO:0034655	nucleobase-containing compound catabolic...	17735	770	1225	70	54.17	0.01556	1.292228	ACAP3;ALS2;ARFGEF1;ARFRP1;ARHGAP4;ATL3;ATP5D;ATP5H;DNASE1L2;ERCC1;ETF1;EXOSC5;FGD6;FICD;GN
yellow	CC	GO:0000315	organellar large ribosomal subunit	17735	15	1225	4	1.02	0.01575	3.921569	MRPL12;MRPL23;MRPL52;MRPL55
yellow	CC	GO:0005762	mitochondrial large ribosomal subunit	17735	15	1225	4	1.02	0.01575	3.921569	MRPL12;MRPL23;MRPL52;MRPL55
yellow	CC	GO:0005852	eukaryotic translation initiation factor...	17735	15	1225	4	1.02	0.01575	3.921569	EIF3F;EIF3G;EIF3I;EIF3K
yellow	BP	GO:0033365	protein localization to organelle	17735	510	1225	49	35.88	0.01597	1.365663	AIP;ARFRP1;ARNTL;BUB1B;CCDC101;CD36;CREB3;DVL1;FBXW11;GET4;GFER;HOOK3;LIME1;MAPK1;MED1;N
yellow	MF	GO:0019201	nucleotide kinase activity	17735	22	1225	5	1.54	0.01617	3.246753	AK1;AK4;DTYMK;MAGI3;PNKP
yellow	BP	GO:0051301	cell division	17735	474	1225	46	33.35	0.01628	1.37931	ACTR3;ANAPC11;ANAPC2;BUB1;BUB1B;CDC25A;CDC6;CDCA8;CENPF;CENPT;CEP55;CLASP1;DIAPH2;DYNC1L
yellow	BP	GO:0009147	pyrimidine nucleoside triphosphate metab...	17735	22	1225	5	1.55	0.01643	3.225806	DTYMK;NME3;NME4;UCK1;UCKL1
yellow	BP	GO:0044260	cellular macromolecule metabolic process	17735	6621	1225	499	465.81	0.01673	1.071252	ABT1;ADAM9;ADAMTS13;ADNP;ADORA2A;AES;AFF1;AHCYL1;AIP;ALG12;ALS2;ANAPC11;ANAPC2;APOE;ARF
yellow	BP	GO:0006289	nucleotide-excision repair	17735	76	1225	11	5.35	0.01683	2.056075	ERCC1;NEIL1;NTHL1;OGG1;PNKP;POLD1;POLD4;POLR2F;POLR2G;POLR2I;POLR2L
yellow	BP	GO:0015980	energy derivation by oxidation of organi...	17735	331	1225	34	23.29	0.01684	1.459854	ATP5D;ATP5H;COQ7;COX4I1;CYC1;ETFB;GNG8;GYG1;NDUFA11;NDUFA13;NDUFA3;NDUFA8;NDUFB10;NDU
yellow	CC	GO:0043025	neuronal cell body	17735	259	1225	27	17.58	0.01738	1.535836	ADORA2A;ALS2;APOE;CANX;CREB3;DVL1;EEF1A2;GNB2L1;GRIN3A;KLHL17;MAPK1;MYH10;NDUFS7;NRP1;P
yellow	BP	GO:0043414	macromolecule methylation	17735	128	1225	16	9.01	0.01763	1.775805	ATF7IP;ETF1;KDM3A;KDM6A;LCMT1;MGMT;MLL3;MLL5;PICK1;PRMT7;RBBP5;SETD2;SETD7;SUV420H1;TRM
yellow	BP	GO:0006613	cotranslational protein targeting to mem...	17735	107	1225	14	7.53	0.01768	1.85923	RPL13;RPL18;RPL18A;RPL28;RPL29;RPL38;RPL8;RPLP1;RPLP2;RPS15;RPS2;RPS28;SSR1;SSR4
yellow	BP	GO:0045047	protein targeting to ER	17735	107	1225	14	7.53	0.01768	1.85923	RPL13;RPL18;RPL18A;RPL28;RPL29;RPL38;RPL8;RPLP1;RPLP2;RPS15;RPS2;RPS28;SSR1;SSR4
yellow	BP	GO:0006284	base-excision repair	17735	39	1225	7	2.74	0.0177	2.554745	MPG;MUTYH;NEIL1;NTHL1;OGG1;POLD1;POLD4
yellow	BP	GO:0009209	pyrimidine ribonucleoside triphosphate b...	17735	15	1225	4	1.06	0.01777	3.773585	NME3;NME4;UCK1;UCKL1
yellow	BP	GO:0032259	methylation	17735	139	1225	17	9.78	0.01797	1.738241	AHCY;ATF7IP;ETF1;KDM3A;KDM6A;LCMT1;MGMT;MLL3;MLL5;PICK1;PRMT7;RBBP5;SETD2;SETD7;SUV420H
yellow	BP	GO:0000184	nuclear-transcribed mRNA catabolic proce...	17735	118	1225	15	8.3	0.01837	1.807229	ETF1;RPL13;RPL18;RPL18A;RPL28;RPL29;RPL38;RPL8;RPLP1;RPLP2;RPS15;RPS2;RPS28;UPF2;WIBG
yellow	MF	GO:0005088	Ras guanyl-nucleotide exchange factor ac...	17735	108	1225	14	7.57	0.01846	1.849406	ALS2;ARHGEF25;ARHGEF26;DENND2C;FGD6;ITSN2;PLEKHG4;PLEKHG5;RABGEF1;RGL1;SERGEF;SOS1;SPATA
yellow	BP	GO:0016044	cellular membrane organization	17735	417	1225	41	29.34	0.01846	1.39741	ANO6;AP1G1;AP1S1;ARFGEF1;BAIAP2L2;CD36;CHMP4A;CHMP6;CIB1;CRB3;DOK7;DVL1;GET4;GIMAP5;GNA
yellow	BP	GO:0016567	protein ubiquitination	17735	540	1225	51	37.99	0.01902	1.342459	ANAPC11;ANAPC2;ARIH1;ASB3;ASB6;BUB1B;DTX3;E4F1;FBXL15;FBXL6;FBXO2;FBXO24;FBXW11;FBXW5;FEM
yellow	BP	GO:0072599	establishment of protein localization to...	17735	108	1225	14	7.6	0.01905	1.842105	RPL13;RPL18;RPL18A;RPL28;RPL29;RPL38;RPL8;RPLP1;RPLP2;RPS15;RPS2;RPS28;SSR1;SSR4
yellow	CC	GO:0015935	small ribosomal subunit	17735	60	1225	9	4.07	0.01915	2.211302	GNB2L1;MRPS11;MRPS12;MRPS15;MRPS18A;MRPS26;RPS15;RPS2;RPS28
yellow	BP	GO:0044710	single-organism metabolic process	17735	3059	1225	242	215.21	0.01919	1.124483	ACADS;ACAP3;ACER3;ACOT8;ACP5;ACSL3;ADHFE1;ADORA2A;AFMID;AHCY;AHCYL1;AK1;AK4;AKR1A1;ALG12
yellow	CC	GO:0000794	condensed nuclear chromosome	17735	70	1225	10	4.75	0.01922	2.105263	BUB1;BUB1B;RAD21;RAD9A;REC8;SUV420H1;SYCE1L;TOP2A;TOPBP1;TTN
yellow	BP	GO:0070647	protein modification by small protein co...	17735	640	1225	59	45.03	0.0194	1.310238	ANAPC11;ANAPC2;ARIH1;ASB3;ASB6;BABAM1;BUB1B;DTX3;E4F1;FBXL15;FBXL6;FBXO2;FBXO24;FBXW11;FE
yellow	MF	GO:0043425	bHLH transcription factor binding	17735	23	1225	5	1.61	0.0195	3.10559	ARNTL;BHLHE41;MAP3K10;PSMD9;SP1
yellow	MF	GO:0003743	translation initiation factor activity	17735	49	1225	8	3.43	0.01957	2.332362	EIF1;EIF2B2;EIF2B4;EIF3F;EIF3G;EIF3I;EIF3K;EIF4G3
yellow	BP	GO:0046578	regulation of Ras protein signal transdu...	17735	276	1225	29	19.42	0.01968	1.493306	ACAP3;ALS2;APOE;ARFGEF1;ARHGAP4;ARHGEF25;ARHGEF26;FGD6;FICD;HRAS;ITSN2;LPAR1;MLST8;NF1;PIN
yellow	CC	GO:0022626	cytosolic ribosome	17735	91	1225	12	6.18	0.01983	1.941748	RPL13;RPL18;RPL18A;RPL28;RPL29;RPL38;RPL8;RPLP1;RPLP2;RPS15;RPS2;RPS28
yellow	CC	GO:0005865	striated muscle thin filament	17735	16	1225	4	1.09	0.01989	3.669725	TMOD3;TNNT3;TPM2;TTN
yellow	BP	GO:0009201	ribonucleoside triphosphate biosynthetic...	17735	68	1225	10	4.78	0.01993	2.09205	ATP5D;ATP5G2;ATP5G3;ATP5H;NME3;NME4;STOML2;SURF1;UCK1;UCKL1
yellow	BP	GO:0051641	cellular localization	17735	2049	1225	167	144.15	0.02003	1.158515	ACSL3;ACTR3;ADAM9;ADORA2A;AHCYL1;AIP;ALS2;ANKRD54;AP1G1;AP1G2;AP1S1;APOE;ARFGEF1;ARFRP1;
yellow	BP	GO:0006468	protein phosphorylation	17735	1126	1225	97	79.22	0.0201	1.224438	ADAM9;ADORA2A;ALS2;ANAPC2;APOE;ATF4;AVPI1;BRAF;C19orf10;CCNL2;CD36;CDC25A;CDC6;CDK12;CDK
yellow	CC	GO:0005789	endoplasmic reticulum membrane	17735	757	1225	66	51.38	0.02112	1.284547	ABCB9;ACER3;ACSL3;ALG12;ATL3;AUP1;CANX;CREB3;CREB3L4;CYP1B1;CYP2E1;DERL3;DOLK;DPM3;ERGIC3;
yellow	BP	GO:0061024	membrane organization	17735	421	1225	41	29.62	0.02126	1.3842	ANO6;AP1G1;AP1S1;ARFGEF1;BAIAP2L2;CD36;CHMP4A;CHMP6;CIB1;CRB3;DOK7;DVL1;GET4;GIMAP5;GNA
yellow	BP	GO:0009451	RNA modification	17735	59	1225	9	4.15	0.02131	2.168675	CTU2;GTPBP3;NHP2;PUS1;PUSL1;QTRT1;RPUSD1;RPUSD3;TRUB2
yellow	BP	GO:0016482	cytoplasmic transport	17735	719	1225	65	50.58	0.02144	1.285093	ACSL3;AHCYL1;AIP;ARFRP1;ARNTL;CCHCR1;CD36;CPSF2;CREB3;DDX39A;FBXW11;GFER;GIMAP5;HOOK3;KLH
yellow	CC	GO:0016604	nuclear body	17735	276	1225	28	18.73	0.02166	1.494928	ARNTL;CCNL2;CDK12;CDK13;CREB3;EAF1;FBL;GEMIN7;HINFP;HIPK1;HIPK3;MAML2;MLL5;NHP2;NSRP1;OGG
yellow	BP	GO:0031399	regulation of protein modification proce...	17735	1026	1225	89	72.18	0.02167	1.233029	ADAM9;ADORA2A;ALS2;ANAPC11;ANAPC2;APOE;ARFGEF1;ATF4;AVPI1;BRAF;BUB1B;C19orf10;CCNL2;CD36
yellow	MF	GO:0005524	ATP binding	17735	1462	1225	122	102.43	0.02175	1.191057	ABCB8;ABCB9;ACSL3;ACTR1B;ACTR3;AK1;AK4;ATAD3B;ATP5D;BCS1L;BMP2K;BRAF;BUB1;BUB1B;CDC6;CDK
yellow	BP	GO:0042455	ribonucleoside biosynthetic process	17735	110	1225	14	7.74	0.02202	1.808786	APRT;ATP5D;ATP5G2;ATP5G3;ATP5H;NME3;NME4;PANK4;PAPSS1;QTRT1;STOML2;SURF1;UCK1;UCKL1
yellow	BP	GO:0016071	mRNA metabolic process	17735	607	1225	56	42.7	0.02204	1.311475	AHCYL1;C1QBP;CDK12;CDK13;CELF2;CELF6;CPSF2;CPSF4;DDX39A;ETF1;EXOSC5;GEMIN7;HNRNPH3;LSM2;L
yellow	BP	GO:0009208	pyrimidine ribonucleoside triphosphate m...	17735	16	1225	4	1.13	0.02241	3.539823	NME3;NME4;UCK1;UCKL1
yellow	BP	GO:0031146	SCF-dependent proteasomal ubiquitin-depe...	17735	16	1225	4	1.13	0.02241	3.539823	FBXL15;FBXO2;FBXW11;FBXW5
yellow	BP	GO:0070972	protein localization to endoplasmic reti...	17735	121	1225	15	8.51	0.02259	1.762632	PEX16;RPL13;RPL18;RPL18A;RPL28;RPL29;RPL38;RPL8;RPLP1;RPLP2;RPS15;RPS2;RPS28;SSR1;SSR4
yellow	CC	GO:0043228	non-membrane-bounded organelle	17735	3122	1225	238	211.89	0.02275	1.123224	ABCB8;ABT1;ACTR1B;ACTR3;ADORA2A;ALS2;ANKRD23;APBB3;ARC;ARFGEF1;ARHGEF25;ASH1L;ATF4;BTBD6
yellow	CC	GO:0043232	intracellular non-membrane-bounded organ...	17735	3122	1225	238	211.89	0.02275	1.123224	ABCB8;ABT1;ACTR1B;ACTR3;ADORA2A;ALS2;ANKRD23;APBB3;ARC;ARFGEF1;ARHGEF25;ASH1L;ATF4;BTBD6
yellow	MF	GO:0032559	adenyl ribonucleotide binding	17735	1491	1225	124	104.46	0.0228	1.187057	ABCB8;ABCB9;ACSL3;ACTR1B;ACTR3;AK1;AK4;APRT;ATAD3B;ATP5D;BCS1L;BMP2K;BRAF;BUB1;BUB1B;CDC6
yellow	BP	GO:0016571	histone methylation	17735	60	1225	9	4.22	0.02356	2.132701	KDM3A;KDM6A;MLL3;MLL5;PRMT7;RBBP5;SETD2;SUV420H1;WHSC1L1
yellow	BP	GO:0048709	oligodendrocyte differentiation	17735	60	1225	9	4.22	0.02356	2.132701	APOE;BOK;EIF2B2;EIF2B4;HDAC10;HDAC11;NF1;SOX8;TP73
yellow	BP	GO:0035020	regulation of Rac protein signal transdu...	17735	24	1225	5	1.69	0.02361	2.95858	ALS2;HRAS;MLST8;NF1;STMN3
yellow	BP	GO:0043170	macromolecule metabolic process	17735	7334	1225	547	515.97	0.0238	1.060139	ABT1;ADAM12;ADAM9;ADAMDEC1;ADAMTS13;ADNP;ADORA2A;AES;AFF1;AHCYL1;AIP;ALG12;ALS2;ANAPC
yellow	BP	GO:0044265	cellular macromolecule catabolic process	17735	698	1225	63	49.11	0.02399	1.282834	ANAPC11;ANAPC2;APOE;ARIH1;BUB1B;DERL3;DNASE1L2;DVL1;ERCC1;ERLIN2;ETF1;EXOSC5;FBXL15;FBXO2;
yellow	BP	GO:0046328	regulation of JNK cascade	17735	133	1225	16	9.36	0.02444	1.709402	DUSP10;DUSP3;HIPK3;HRAS;MAGI3;MAP3K10;MAP4K2;SERPINF2;SH3RF1;TAOK3;TP73;TRAF2;TRAF4;TRAF6
yellow	BP	GO:0001932	regulation of protein phosphorylation	17735	813	1225	72	57.2	0.02444	1.258741	ADAM9;ADORA2A;ALS2;ANAPC2;APOE;ATF4;AVPI1;BRAF;C19orf10;CCNL2;CD36;CDC25A;CDC6;CDK12;CLCF
yellow	MF	GO:0030554	adenyl nucleotide binding	17735	1495	1225	124	104.74	0.02452	1.183884	ABCB8;ABCB9;ACSL3;ACTR1B;ACTR3;AK1;AK4;APRT;ATAD3B;ATP5D;BCS1L;BMP2K;BRAF;BUB1;BUB1B;CDC6
yellow	CC	GO:0000922	spindle pole	17735	94	1225	12	6.38	0.02497	1.880878	CDC6;CDCA8;CENPF;DLGAP5;DYNC1LI1;HAUS8;LATS1;LATS2;ROCK2;THAP11;TOPBP1;TUBGCP3
yellow	CC	GO:0044450	microtubule organizing center part	17735	94	1225	12	6.38	0.02497	1.880878	CDCA8;CEP55;CLASP1;CNTLN;HOOK3;KIF24;KRT18;MZT2B;PARP3;THAP11;TOP2A;TUBGCP3
yellow	BP	GO:0097006	regulation of plasma lipoprotein particl...	17735	51	1225	8	3.59	0.02498	2.228412	ACSL3;APOE;APOM;CD36;LMF1;MYLIP;SOAT1;SOAT2
yellow	BP	GO:0002819	regulation of adaptive immune response	17735	91	1225	12	6.4	0.02538	1.875	ABCB9;CLCF1;DUSP10;GIMAP5;HPX;P2RX7;PTPRC;RC3H1;TNFAIP3;TRAF2;TRAF6;ZP3
yellow	MF	GO:0005083	small GTPase regulator activity	17735	295	1225	30	20.67	0.02546	1.451379	ACAP3;ALS2;ARFGEF1;ARHGAP4;ARHGEF25;ARHGEF26;DENND2C;FGD6;IQGAP2;ITSN2;MAP4K2;NF1;PLEKH

yellow	CC	GO:0005744	mitochondrial inner membrane presequence...	17735	10	1225	3	0.68	0.02609	4.411765	SLC25A6;TIMM10;TIMM50
yellow	CC	GO:0031010	ISWI-type complex	17735	10	1225	3	0.68	0.02609	4.411765	C17orf49;CHRAC1;RSF1
yellow	BP	GO:0006304	DNA modification	17735	71	1225	10	5	0.02622	2	ATF7IP;EXOSC5;MGMT;MLL5;MPG;MUTYH;NTHL1;OGG1;PICK1;PRMT7
yellow	MF	GO:0004721	phosphoprotein phosphatase activity	17735	168	1225	19	11.77	0.02622	1.614274	CDC25A;DLGAP5;DUSP10;DUSP2;DUSP23;DUSP3;MTM1;MTMR1;PHPT1;PPAP2C;PPM1D;PPM1J;PPM1L;PPI
yellow	BP	GO:0006886	intracellular protein transport	17735	702	1225	63	49.39	0.02662	1.275562	ACSL3;AHCYL1;AIP;AP1G1;AP1G2;AP1S1;ARFRP1;ARNTL;ASPSCR1;CCHCR1;CD36;CREB3;ERP29;FBXW11;GFI
yellow	BP	GO:0051056	regulation of small GTPase mediated sign...	17735	367	1225	36	25.82	0.0268	1.394268	ACAP3;ALS2;APOE;ARFGEF1;ARHGAP4;ARHGEF25;ARHGEF26;FAM13A;FGD6;FICD;GAPVD1;HRAS;ITSN2;LPA
yellow	BP	GO:0097190	apoptotic signaling pathway	17735	202	1225	22	14.21	0.02714	1.548205	ADORA2A;ATF4;BOK;CD38;FASTK;FNIP2;GNB2L1;HIPK1;HRAS;NDUFA13;NGFRAP1;PARK7;PHLDA3;PLA2G6;S
yellow	BP	GO:1900117	regulation of execution phase of apoptos...	17735	202	1225	22	14.21	0.02714	1.548205	ADORA2A;ATF4;BOK;CD38;FASTK;FNIP2;GNB2L1;HIPK1;HRAS;NDUFA13;NGFRAP1;PARK7;PHLDA3;PLA2G6;S
yellow	BP	GO:0009116	nucleoside metabolic process	17735	602	1225	55	42.35	0.02725	1.298701	ACAP3;AK1;AK4;ALS2;APRT;ARFGEF1;ARFRP1;ARHGAP4;ATL3;ATP5D;ATP5G2;ATP5G3;ATP5H;DGUOK;FGD6
yellow	MF	GO:0003746	translation elongation factor activity	17735	17	1225	4	1.19	0.02734	3.361345	EEF1A2;EEF1D;EEF1G;TUFM
yellow	BP	GO:0045786	negative regulation of cell cycle	17735	453	1225	43	31.87	0.02737	1.349231	AK1;ANAPC11;ANAPC2;ATRIP;BABAM1;BUB1;BUB1B;CDC25A;CDC6;CENPF;CLSPN;CREB3;DCUN1D3;DYNC1I
yellow	BP	GO:0007250	activation of NF-kappaB-inducing kinase ...	17735	17	1225	4	1.2	0.02771	3.333333	TNFSF15;TRAF2;TRAF6;ZFP91
yellow	BP	GO:0015949	nucleobase-containing small molecule int...	17735	17	1225	4	1.2	0.02771	3.333333	AK1;DTYMK;NME4;TXNRD1
yellow	BP	GO:1901222	regulation of NIK/NF-kappaB cascade	17735	17	1225	4	1.2	0.02771	3.333333	TNFSF15;TRAF2;TRAF6;ZFP91
yellow	BP	GO:0002711	positive regulation of T cell mediated i...	17735	25	1225	5	1.76	0.02786	2.840909	ABCB9;P2RX7;TRAF2;TRAF6;ZP3
yellow	BP	GO:0007032	endosome organization	17735	25	1225	5	1.76	0.02786	2.840909	ALS2;HOOK3;PIK3C3;PLEKHF1;USP8
yellow	BP	GO:0009057	macromolecule catabolic process	17735	883	1225	77	62.12	0.02811	1.239536	ADAM9;ANAPC11;ANAPC2;APOE;ARIH1;ARNTL;BUB1B;DERL3;DNASE1L2;DVL1;ERCC1;ERLIN2;ETF1;EXOSC5
yellow	MF	GO:0016303	1-phosphatidylinositol-3-kinase activity	17735	10	1225	3	0.7	0.02836	4.285714	PIK3C3;PIK3CB;PIK3CG
yellow	MF	GO:0019206	nucleoside kinase activity	17735	10	1225	3	0.7	0.02836	4.285714	DGUOK;UCK1;UCKL1
yellow	CC	GO:0000779	condensed chromosome, centromeric region	17735	85	1225	11	5.77	0.02838	1.906412	BUB1;BUB1B;CENPF;CENPM;CENPT;CLASP1;DYNC1LI1;MAD1L1;REC8;STRA13;SUV420H1
yellow	CC	GO:0019005	SCF ubiquitin ligase complex	17735	26	1225	5	1.76	0.02844	2.840909	FBXL15;FBXO2;FBXO44;FBXW11;FBXW5
yellow	CC	GO:0033116	endoplasmic reticulum-Golgi intermediate...	17735	26	1225	5	1.76	0.02844	2.840909	ERGIC3;SEC22B;TMED1;TMED3;TMED9
yellow	BP	GO:0000076	DNA replication checkpoint	17735	10	1225	3	0.7	0.02867	4.285714	CDC6;CLSPN;RAD9A
yellow	BP	GO:0032482	Rab protein signal transduction	17735	10	1225	3	0.7	0.02867	4.285714	ALS2;SGSM3;USP6NL
yellow	BP	GO:0032483	regulation of Rab protein signal transdu...	17735	10	1225	3	0.7	0.02867	4.285714	ALS2;SGSM3;USP6NL
yellow	BP	GO:0034261	negative regulation of Ras GTPase activi...	17735	10	1225	3	0.7	0.02867	4.285714	ARFGEF1;FICD;HRAS
yellow	BP	GO:0050860	negative regulation of T cell receptor s...	17735	10	1225	3	0.7	0.02867	4.285714	DUSP3;ELF1;PHPT1
yellow	BP	GO:0070208	protein heterotrimerization	17735	10	1225	3	0.7	0.02867	4.285714	C1QTNF6;COL6A1;COL6A2
yellow	BP	GO:2000114	regulation of establishment of cell pola...	17735	10	1225	3	0.7	0.02867	4.285714	ARFGEF1;GNB2L1;ROCK2
yellow	BP	GO:0006220	pyrimidine nucleotide metabolic process	17735	43	1225	7	3.03	0.02911	2.310231	DTYMK;NME3;NME4;NT5C;NTHL1;UCK1;UCKL1
yellow	BP	GO:0000087	M phase of mitotic cell cycle	17735	370	1225	36	26.03	0.02981	1.38302	ANAPC11;ANAPC2;BUB1;BUB1B;CDC25A;CDC6;CDCA8;CDK13;CENPF;CENPM;CENPT;CEP55;CLASP1;CLIP1;D
yellow	BP	GO:0051348	negative regulation of transferase activ...	17735	170	1225	19	11.96	0.03026	1.588629	ADORA2A;APOE;DUSP10;DUSP2;DUSP3;DVL1;GNB2L1;HIPK3;LATS1;LATS2;NF1;NPRL2;PHPT1;PTPRC;SPRED
yellow	MF	GO:0005089	Rho guanyl-nucleotide exchange factor ac...	17735	73	1225	10	5.11	0.03039	1.956947	ALS2;ARHGEF25;ARHGEF26;FGD6;ITSN2;PLEKHG4;PLEKHG5;SOS1;SPATA13;VAV3
yellow	BP	GO:0032268	regulation of cellular protein metabolic...	17735	1264	1225	106	88.93	0.0304	1.191949	ADAM9;ADORA2A;ALS2;ANAPC11;ANAPC2;APOE;ARFGEF1;ATF4;AVPI1;BRAF;BUB1B;C19orf10;C1QBP;CAPI
yellow	BP	GO:0002822	regulation of adaptive immune response b...	17735	83	1225	11	5.84	0.03048	1.883562	ABCB9;CLCF1;GIMAP5;HPX;P2RX7;PTPRC;RC3H1;TNFAIP3;TRAF2;TRAF6;ZP3
yellow	BP	GO:0048285	organelle fission	17735	383	1225	37	26.95	0.03068	1.372913	ACOT8;ANAPC11;ANAPC2;BUB1;BUB1B;CDC25A;CDC6;CDCA8;CDK13;CENPF;CENPM;CENPT;CEP55;CLASP1;
yellow	BP	GO:0033673	negative regulation of kinase activity	17735	159	1225	18	11.19	0.03069	1.608579	ADORA2A;APOE;DUSP10;DUSP2;DUSP3;DVL1;GNB2L1;HIPK3;LATS1;LATS2;NF1;NPRL2;PTPRC;SPRED2;TAOK
yellow	BP	GO:0006469	negative regulation of protein kinase ac...	17735	148	1225	17	10.41	0.03101	1.633045	ADORA2A;APOE;DUSP10;DUSP2;DUSP3;DVL1;GNB2L1;HIPK3;LATS1;LATS2;NF1;PTPRC;SPRED2;TAOK3;TNFA
yellow	BP	GO:0000280	nuclear division	17735	359	1225	35	25.26	0.03102	1.38559	ANAPC11;ANAPC2;BUB1;BUB1B;CDC25A;CDC6;CDCA8;CDK13;CENPF;CENPM;CENPT;CEP55;CLASP1;CLIP1;D
yellow	BP	GO:0007067	mitosis	17735	359	1225	35	25.26	0.03102	1.38559	ANAPC11;ANAPC2;BUB1;BUB1B;CDC25A;CDC6;CDCA8;CDK13;CENPF;CENPM;CENPT;CEP55;CLASP1;CLIP1;D
yellow	BP	GO:0008033	tRNA processing	17735	73	1225	10	5.14	0.03113	1.945525	CTU2;FBL;GTPBP3;POP7;PUS1;PUSL1;QTRT1;TRMU;TRPT1;TRUB2
yellow	CC	GO:0042175	nuclear outer membrane-endoplasmic retic...	17735	773	1225	66	52.46	0.03116	1.258101	ABCB9;ACER3;ACSL3;ALG12;ATL3;AUP1;CANX;CREB3;CREB3L4;CYP1B1;CYP2E1;DERL3;DOLK;DPM3;ERGIC3;
yellow	BP	GO:1901361	organic cyclic compound catabolic proces...	17735	849	1225	74	59.73	0.03121	1.238908	ACAP3;AFMID;ALS2;APOE;ARFGEF1;ARFRP1;ARHGAP4;ATL3;ATP5D;ATP5H;CEL;DNASE1L2;ERCC1;ETF1;EXO
yellow	BP	GO:0043900	regulation of multi-organism process	17735	205	1225	22	14.42	0.03137	1.525659	AP1G1;AP1S1;C1QBP;CD36;CD8B;CREB3;MICB;NFKBIL1;P2RX7;POLR2F;POLR2G;POLR2I;POLR2L;PRDM1;RSF
yellow	BP	GO:0007050	cell cycle arrest	17735	384	1225	37	27.02	0.03175	1.369356	AK1;ANAPC11;ANAPC2;ATRIP;BABAM1;BUB1;BUB1B;CDC25A;CDC6;CENPF;CLSPN;DYNC1LI1;E2F1;E4F1;FAN
yellow	BP	GO:0009141	nucleoside triphosphate metabolic proces...	17735	495	1225	46	34.82	0.03184	1.32108	ACAP3;AK1;AK4;ALS2;ARFGEF1;ARFRP1;ARHGAP4;ATL3;ATP5D;ATP5G2;ATP5G3;ATP5H;DGUOK;DTYMK;FGI
yellow	BP	GO:0051640	organelle localization	17735	171	1225	19	12.03	0.03189	1.579385	ACTR3;CENPF;CLASP1;DLGAP5;HOOK3;KLHL12;MAP4K2;MTM1;MYH10;NUSAP1;PIK3CG;PREB;RHOT2;RPS1!
yellow	BP	GO:0046907	intracellular transport	17735	1214	1225	102	85.41	0.03189	1.19424	ACSL3;AHCYL1;AIP;ALS2;ANKRD54;AP1G1;AP1G2;AP1S1;APOE;ARFRP1;ARNTL;ASPSCR1;ATP5D;ATP5H;CAPI
yellow	MF	GO:0005488	binding	17735	11799	1225	851	826.67	0.03213	1.029431	ABCB8;ABCB9;ABT1;ACADS;ACAP3;ACBD4;ACCS;ACOT8;ACP5;ACSL3;ACTR1B;ACTR3;ADAM12;ADAM9;ADAI
yellow	CC	GO:0044297	cell body	17735	286	1225	28	19.41	0.0325	1.442555	ADORA2A;ALS2;APOE;ATP6AP2;CANX;CREB3;DVL1;EEF1A2;GNB2L1;GRIN3A;KLHL17;MAPK1;MYH10;NDUFS
yellow	BP	GO:0002448	mast cell mediated immunity	17735	26	1225	5	1.83	0.03256	2.73224	LAT;PIK3CG;S100A13;SERPINB9;YWHAZ
yellow	MF	GO:0032553	ribonucleotide binding	17735	1846	1225	149	129.34	0.03283	1.152002	ABCB8;ABCB9;ACSL3;ACTR1B;ACTR3;AK1;AK4;APRT;ARFIP2;ARFRP1;ARL16;ATAD3B;ATL3;ATP5D;BCS1L;BM
yellow	BP	GO:0044270	cellular nitrogen compound catabolic pro...	17735	813	1225	71	57.2	0.0331	1.241259	ACAP3;AFMID;ALS2;ARFGEF1;ARFRP1;ARHGAP4;ATL3;ATP5D;ATP5H;DNASE1L2;ERCC1;ETF1;EXOSC5;FGD6;
yellow	BP	GO:0046700	heterocycle catabolic process	17735	813	1225	71	57.2	0.0331	1.241259	ACAP3;AFMID;ALS2;ARFGEF1;ARFRP1;ARHGAP4;ATL3;ATP5D;ATP5H;DNASE1L2;ERCC1;ETF1;EXOSC5;FGD6;
yellow	BP	GO:0010627	regulation of intracellular protein kina...	17735	698	1225	62	49.11	0.03318	1.262472	ADAM9;AIP;ANKRD54;APOE;ATP6AP2;AVPI1;BRAF;C1QBP;CD36;CDK12;CLCF1;DUSP10;DUSP2;DUSP3;EEF1I
yellow	CC	GO:0005694	chromosome	17735	631	1225	55	42.82	0.0332	1.284447	ASH1L;BUB1;BUB1B;C1orf86;CDCA8;CENPF;CENPM;CENPT;CHRAC1;CLASP1;CLIP1;DYNC1LI1;ERCC1;EXOSC5
yellow	CC	GO:0005783	endoplasmic reticulum	17735	1273	1225	103	86.4	0.03327	1.19213	ABCB9;ACER3;ACSL3;AHCYL1;ALG12;ATL3;AUP1;C19orf10;CANX;CCDC47;CCDC78;CD320;CDS2;CIB1;COL11/
yellow	BP	GO:0002709	regulation of T cell mediated immunity	17735	35	1225	6	2.46	0.03351	2.439024	ABCB9;P2RX7;PTPRC;TRAF2;TRAF6;ZP3
yellow	BP	GO:0019637	organophosphate metabolic process	17735	1204	1225	101	84.7	0.03384	1.192444	ACAP3;ACER3;ACSL3;ADORA2A;AK1;AK4;ALG12;ALS2;APOE;APRT;ARFGEF1;ARFRP1;ARHGAP4;ATL3;ATP5D;
yellow	CC	GO:0030130	clathrin coat of trans-Golgi network ves...	17735	11	1225	3	0.75	0.0341	4	AFTPH;AP1G2;AP1S1
yellow	CC	GO:0030897	HOPS complex	17735	11	1225	3	0.75	0.0341	4	HOOK3;VPS16;VPS41
yellow	MF	GO:0016791	phosphatase activity	17735	255	1225	26	17.87	0.03463	1.454952	ACP5;C12orf5;CDC25A;DLGAP5;DUSP10;DUSP2;DUSP23;DUSP3;HDHD3;MTM1;MTMR1;NT5C;PFKFB2;PGP;I
yellow	BP	GO:0019439	aromatic compound catabolic process	17735	815	1225	71	57.34	0.03465	1.238228	ACAP3;AFMID;ALS2;ARFGEF1;ARFRP1;ARHGAP4;ATL3;ATP5D;ATP5H;DNASE1L2;ERCC1;ETF1;EXOSC5;FGD6;
yellow	CC	GO:0000314	organellar small ribosomal subunit	17735	19	1225	4	1.29	0.03605	3.100775	MRPS11;MRPS15;MRPS18A;MRPS26
yellow	CC	GO:0005763	mitochondrial small ribosomal subunit	17735	19	1225	4	1.29	0.03605	3.100775	MRPS11;MRPS15;MRPS18A;MRPS26
yellow	BP	GO:0033619	membrane protein proteolysis	17735	45	1225	7	3.17	0.03634	2.208202	ADAM9;APOE;NAPSA;P2RX7;SPPL2A;SPPL2B;TRAF6
yellow	BP	GO:0043393	regulation of protein binding	17735	96	1225	12	6.75	0.0366	1.777778	AES;DVL1;MFNG;NOG;NRP1;PARK7;PRMT7;SENP2;SPAG8;TRAF2;TRAF4;TRIB3
yellow	BP	GO:0032872	regulation of stress-activated MAPK casc...	17735	151	1225	17	10.62	0.03662	1.600753	DUSP10;DUSP3;HIPK3;HRAS;MAGI3;MAP3K10;MAP4K2;MAPK1;SERPINF2;SH3RF1;TAOK3;TP73;TRAF2;TRAF
yellow	BP	GO:0002706	regulation of lymphocyte mediated immuni...	17735	75	1225	10	5.28	0.03666	1.893939	ABCB9;CLCF1;GIMAP5;HPX;NCR3;P2RX7;PTPRC;TRAF2;TRAF6;ZP3

yellow	CC	GO:0030660	Golgi-associated vesicle membrane	17735	37	1225	6	2.51	0.03676	2.390438	AFTPH;AP1G2;AP1S1;SPPL2A;SPPL2B;TMED3
yellow	MF	GO:0005031	tumor necrosis factor-activated receptor...	17735	11	1225	3	0.77	0.03701	3.896104	TNFRSF18;TNFRSF25;TNFRSF4
yellow	MF	GO:0008175	tRNA methyltransferase activity	17735	11	1225	3	0.77	0.03701	3.896104	TRMT1;TRMT61A;TRMU
yellow	BP	GO:0031570	DNA integrity checkpoint	17735	140	1225	16	9.85	0.03708	1.624365	ATRIP;BABAM1;CDC6;CLSPN;E2F1;FEM1B;HINFP;PIDD;PSMA7;PSMB4;PSMB5;PSMB7;PSMD9;RAD9A;TAOK
yellow	MF	GO:0015932	nucleobase-containing compound transmemb...	17735	27	1225	5	1.89	0.03715	2.645503	SLC25A19;SLC25A5;SLC29A2;SLC35B2;SLC35D2
yellow	BP	GO:0002922	positive regulation of humoral immune re...	17735	11	1225	3	0.77	0.0374	3.896104	GIMAP5;HPX;ZP3
yellow	BP	GO:0039528	cytoplasmic pattern recognition receptor...	17735	11	1225	3	0.77	0.0374	3.896104	C1QBP;IRF3;ZC3HAV1
yellow	BP	GO:0046501	protoporphyrinogen IX metabolic process	17735	11	1225	3	0.77	0.0374	3.896104	PPOX;UROD;UROS
yellow	CC	GO:0015030	Cajal body	17735	47	1225	7	3.19	0.03792	2.194357	EAF1;FBL;GEMIN7;HINFP;NHP2;SNRPC;WRAP53
yellow	CC	GO:0005768	endosome	17735	546	1225	48	37.06	0.03914	1.295197	ALS2;AP1G1;AP1G2;APOE;APPL2;ARC;ATP6V0E2;CD8B;CHMP4A;CHMP6;CLCN4;CLIP1;DIAPH2;FAM21B;GAI
yellow	CC	GO:0000775	chromosome, centromeric region	17735	146	1225	16	9.91	0.03916	1.614531	BUB1;BUB1B;CDCA8;CENPF;CENPM;CENPT;CLASP1;CLIP1;DYNC1LI1;MAD1L1;MKI67;RAD21;REC8;STRA13;S
yellow	MF	GO:0032555	purine ribonucleotide binding	17735	1831	1225	147	128.29	0.03941	1.145841	ABCB8;ABCB9;ACSL3;ACTR1B;ACTR3;AK1;AK4;APRT;ARFIP2;ARFRP1;ARL16;ATAD3B;ATL3;ATP5D;BCS1L;BM
yellow	MF	GO:0003684	damaged DNA binding	17735	46	1225	7	3.22	0.03961	2.173913	ERCC1;FANCG;MPG;NEIL1;OGG1;PNKP;TP73
yellow	MF	GO:0043168	anion binding	17735	2346	1225	185	164.37	0.03969	1.12551	ABCB8;ABCB9;ACADS;ACBD4;ACCS;ACSL3;ACTR1B;ACTR3;AK1;AK4;APOE;APRT;ARFIP2;ARFRP1;ARL16;ATAD
yellow	MF	GO:0070566	adenylyltransferase activity	17735	19	1225	4	1.33	0.03986	3.007519	FICD;FLAD1;PAPSS1;TUT1
yellow	MF	GO:1901677	phosphate transmembrane transporter acti...	17735	19	1225	4	1.33	0.03986	3.007519	SLC25A19;SLC35B2;SLC35D2;SLC37A4
yellow	MF	GO:0032550	purine ribonucleoside binding	17735	1805	1225	145	126.46	0.03997	1.146608	ABCB8;ABCB9;ACSL3;ACTR1B;ACTR3;AK1;AK4;APRT;ARFIP2;ARFRP1;ARL16;ATAD3B;ATL3;ATP5D;BCS1L;BM
yellow	CC	GO:0031300	intrinsic to organelle membrane	17735	218	1225	22	14.8	0.04036	1.486486	ABCB9;ACER3;ALG12;CANX;CREB3;CYP2E1;DERL3;DOLK;DPM3;LEMD2;MFNG;P2RX7;PEX16;PPOX;RHOT2;SL
yellow	MF	GO:0001883	purine nucleoside binding	17735	1807	1225	145	126.6	0.04119	1.14534	ABCB8;ABCB9;ACSL3;ACTR1B;ACTR3;AK1;AK4;APRT;ARFIP2;ARFRP1;ARL16;ATAD3B;ATL3;ATP5D;BCS1L;BM
yellow	MF	GO:0032549	ribonucleoside binding	17735	1809	1225	145	126.74	0.04245	1.144074	ABCB8;ABCB9;ACSL3;ACTR1B;ACTR3;AK1;AK4;APRT;ARFIP2;ARFRP1;ARL16;ATAD3B;ATL3;ATP5D;BCS1L;BM
yellow	MF	GO:0017076	purine nucleotide binding	17735	1836	1225	147	128.64	0.04248	1.142724	ABCB8;ABCB9;ACSL3;ACTR1B;ACTR3;AK1;AK4;APRT;ARFIP2;ARFRP1;ARL16;ATAD3B;ATL3;ATP5D;BCS1L;BM
yellow	CC	GO:0005839	proteasome core complex	17735	20	1225	4	1.36	0.04272	2.941176	PSMA7;PSMB4;PSMB5;PSMB7
yellow	MF	GO:0035639	purine ribonucleoside triphosphate bindi...	17735	1796	1225	144	125.83	0.04275	1.144401	ABCB8;ABCB9;ACSL3;ACTR1B;ACTR3;AK1;AK4;ARFIP2;ARFRP1;ARL16;ATAD3B;ATL3;ATP5D;BCS1L;BMP2K;B
yellow	CC	GO:0012510	trans-Golgi network transport vesicle me...	17735	12	1225	3	0.81	0.04322	3.703704	AFTPH;AP1G2;AP1S1
yellow	CC	GO:0032839	dendrite cytoplasm	17735	12	1225	3	0.81	0.04322	3.703704	CANX;KLHL17;MAPK1
yellow	CC	GO:0043240	Fanconi anaemia nuclear complex	17735	12	1225	3	0.81	0.04322	3.703704	C1orf86;FANCG;STRA13
yellow	CC	GO:0030131	clathrin adaptor complex	17735	29	1225	5	1.97	0.04346	2.538071	AFTPH;AP1G1;AP1G2;AP1S1;GGA1
yellow	CC	GO:0031301	integral to organelle membrane	17735	196	1225	20	13.3	0.04439	1.503759	ABCB9;ACER3;CANX;CREB3;DERL3;DOLK;DPM3;LEMD2;MFNG;P2RX7;PEX16;PPOX;RHOT2;SLC22A17;SLC37
yellow	CC	GO:0016607	nuclear speck	17735	149	1225	16	10.11	0.04581	1.582591	CCNL2;CDK12;CDK13;EAF1;HIPK1;MAML2;MLL5;NSRP1;OGG1;PATL1;PIN1;PIP5K1A;RSRC1;THAP7;TIMM50;
yellow	CC	GO:0032154	cleavage furrow	17735	39	1225	6	2.65	0.04605	2.264151	CEP55;FSD1;MYH10;RAB21;RHOC;STAMBP
yellow	MF	GO:0060589	nucleoside-triphosphatase regulator acti...	17735	447	1225	41	31.32	0.04611	1.309068	ACAP3;AHSA2;ALS2;ARFGEF1;ARHGAP4;ARHGEF25;ARHGEF26;DENND2C;EIF2B2;EIF2B4;FAM13A;FGD6;GAI
yellow	CC	GO:0000776	kinetochore	17735	103	1225	12	6.99	0.04617	1.716738	BUB1;BUB1B;CENPF;CENPM;CENPT;CLASP1;CLIP1;DYNC1LI1;MAD1L1;REC8;STRA13;TPR
yellow	MF	GO:0003906	DNA-(apurinic or apyrimidinic site) lyas...	17735	12	1225	3	0.84	0.04683	3.571429	NEIL1;NTHL1;OGG1
yellow	MF	GO:0015215	nucleotide transmembrane transporter act...	17735	12	1225	3	0.84	0.04683	3.571429	SLC25A19;SLC35B2;SLC35D2
yellow	MF	GO:0016307	phosphatidylinositol phosphate kinase ac...	17735	12	1225	3	0.84	0.04683	3.571429	PIP5K1A;PIP5K1B;PIP5KL1
yellow	MF	GO:0031996	thioesterase binding	17735	12	1225	3	0.84	0.04683	3.571429	TRAF2;TRAF4;TRAF6
yellow	MF	GO:0035004	phosphatidylinositol 3-kinase activity	17735	12	1225	3	0.84	0.04683	3.571429	PIK3C3;PIK3CB;PIK3CG
yellow	CC	GO:0000777	condensed chromosome kinetochore	17735	81	1225	10	5.5	0.04694	1.818182	BUB1;BUB1B;CENPF;CENPM;CENPT;CLASP1;DYNC1LI1;MAD1L1;REC8;STRA13
yellow	MF	GO:0016922	ligand-dependent nuclear receptor bindin...	17735	20	1225	4	1.4	0.04715	2.857143	MED1;NCOA3;TADA3;TRIM24
yellow	MF	GO:0031418	L-ascorbic acid binding	17735	20	1225	4	1.4	0.04715	2.857143	LEPRE1;LEPREL2;OGFOD2;P4HTM
yellow	MF	GO:1901363	heterocyclic compound binding	17735	5302	1225	397	371.47	0.04782	1.068727	ABCB8;ABCB9;ABT1;ACADS;ACSL3;ACTR1B;ACTR3;ADNP;AHCY;AHCYL1;AK1;AK4;ANKRD17;APRT;ARFIP2;AR
yellow	CC	GO:0005634	nucleus	17735	5873	1225	425	398.59	0.04813	1.066259	ABCB8;ABT1;ADAM12;ADNP;ADPRHL2;AES;AFF1;AFMID;AFTPH;AIP;ALKBH7;ANAPC11;ANAPC2;ANKRD17;A
yellow	MF	GO:0030695	GTPase regulator activity	17735	436	1225	40	30.55	0.04823	1.309329	ACAP3;ALS2;ARFGEF1;ARHGAP4;ARHGEF25;ARHGEF26;DENND2C;EIF2B2;EIF2B4;FAM13A;FGD6;GAPVD1;IC
yellow	MF	GO:0001882	nucleoside binding	17735	1818	1225	145	127.37	0.04849	1.138416	ABCB8;ABCB9;ACSL3;ACTR1B;ACTR3;AK1;AK4;APRT;ARFIP2;ARFRP1;ARL16;ATAD3B;ATL3;ATP5D;BCS1L;BM
yellow	MF	GO:0008173	RNA methyltransferase activity	17735	29	1225	5	2.03	0.04874	2.463054	RNMTL1;TRMT1;TRMT2A;TRMT61A;TRMU
yellow	MF	GO:0043022	ribosome binding	17735	29	1225	5	2.03	0.04874	2.463054	C1QBP;EIF3K;ETF1;NAA10;WIBG
yellow	MF	GO:0019899	enzyme binding	17735	1084	1225	90	75.95	0.04959	1.18499	ACSL3;ADAM9;ADORA2A;ALS2;ANAPC2;ARFIP2;ARIH1;ATP6AP2;BHLHE41;C1QBP;CCNL2;CDC25A;CDC6;CUT
turquoise	CC	GO:0005634	nucleus	17735	5873	3682	1527	1221.37	1.00E-30	1.250235	AAAS;ABHD14B;ABI1;ABLIM2;ACAD9;ACAT2;ACBD6;ACD;ACSL6;ACTL6A;ACTN4;ADAM10;ADAMTS5;ADAP1
turquoise	CC	GO:0043231	intracellular membrane-bounded organelle	17735	9560	3682	2438	1988.12	1.00E-30	1.226284	AAAS;ABCA1;ABCA3;ABCA5;ABCA7;ABCB10;ABCB6;ABCD1;ABCD2;ABCD3;ABCE1;ABHD10;ABHD14B;ABI1;A
turquoise	CC	GO:0043227	membrane-bounded organelle	17735	9590	3682	2444	1994.36	1.00E-30	1.225456	AAAS;ABCA1;ABCA3;ABCA5;ABCA7;ABCB10;ABCB6;ABCD1;ABCD2;ABCD3;ABCE1;ABHD10;ABHD14B;ABI1;A
turquoise	CC	GO:0043229	intracellular organelle	17735	10522	3682	2596	2188.18	1.00E-30	1.186374	AAAS;ABCA1;ABCA3;ABCA5;ABCA7;ABCB10;ABCB6;ABCD1;ABCD2;ABCD3;ABCE1;ABHD10;ABHD14B;ABI1;A
turquoise	CC	GO:0043226	organelle	17735	10552	3682	2603	2194.42	1.00E-30	1.18619	AAAS;ABCA1;ABCA3;ABCA5;ABCA7;ABCB10;ABCB6;ABCD1;ABCD2;ABCD3;ABCE1;ABHD10;ABHD14B;ABI1;A
turquoise	CC	GO:0044424	intracellular part	17735	12103	3682	2943	2516.97	1.00E-30	1.169263	AAAS;AACS;AAMP;AANAT;AASDHPPT;ABCA1;ABCA3;ABCA5;ABCA7;ABCB10;ABCB6;ABCD1;ABCD2;ABCD3;A
turquoise	CC	GO:0005737	cytoplasm	17735	9016	3682	2189	1874.99	1.00E-30	1.167473	AAAS;AACS;AAMP;AANAT;AASDHPPT;ABCA1;ABCA3;ABCA5;ABCA7;ABCB10;ABCB6;ABCD1;ABCD2;ABCD3;A
turquoise	CC	GO:0005622	intracellular	17735	12344	3682	2986	2567.09	1.00E-30	1.163185	AAAS;AACS;AAMP;AANAT;AASDHPPT;ABCA1;ABCA3;ABCA5;ABCA7;ABCB10;ABCB6;ABCD1;ABCD2;ABCD3;A
turquoise	CC	GO:0044464	cell part	17735	14451	3682	3193	3005.27	7.70E-28	1.062467	AAAS;AACS;AAMP;AANAT;AASDHPPT;ABCA1;ABCA3;ABCA5;ABCA7;ABCB10;ABCB6;ABCC2;ABCD1;ABCD2;A
turquoise	CC	GO:0005623	cell	17735	14452	3682	3193	3005.48	8.60E-28	1.062393	AAAS;AACS;AAMP;AANAT;AASDHPPT;ABCA1;ABCA3;ABCA5;ABCA7;ABCB10;ABCB6;ABCC2;ABCD1;ABCD2;A
turquoise	CC	GO:0044444	cytoplasmic part	17735	6562	3682	1643	1364.65	2.60E-27	1.203972	AACS;AANAT;AASDHPPT;ABCA1;ABCA3;ABCA5;ABCA7;ABCB10;ABCB6;ABCD1;ABCD2;ABCD3;ABCE1;ABHD1
turquoise	CC	GO:0044446	intracellular organelle part	17735	6219	3682	1568	1293.32	4.50E-27	1.212384	AAAS;ABCA3;ABCA5;ABCA7;ABCB10;ABCB6;ABCD1;ABCD2;ABCD3;ABHD14B;ACADM;ACADSB;ACADVL;ACA
turquoise	BP	GO:0044260	cellular macromolecule metabolic process	17735	6621	3682	1724	1458.89	1.30E-26	1.18172	AANAT;ABCA1;ABCE1;ABI1;ABI2;ACADM;ACADVL;ACD;ACTL6A;ACVR1C;ACVR2A;ACVRL1;ADAM10;ADM2;A
turquoise	CC	GO:0044422	organelle part	17735	6321	3682	1584	1314.53	5.30E-26	1.204993	AAAS;ABCA3;ABCA5;ABCA7;ABCB10;ABCB6;ABCD1;ABCD2;ABCD3;ABHD14B;ACADM;ACADSB;ACADVL;ACA
turquoise	BP	GO:0044237	cellular metabolic process	17735	8597	3682	2142	1894.28	1.70E-24	1.130773	AACS;AANAT;AASDHPPT;ABCA1;ABCB6;ABCD1;ABCD2;ABCD3;ABCE1;ABHD10;ABI1;ABI2;ACADM;ACADSB;A
turquoise	BP	GO:0044267	cellular protein metabolic process	17735	3210	3682	890	707.3	3.30E-18	1.258306	AANAT;ABCA1;ABI1;ABI2;ACADVL;ACTL6A;ACVR1C;ACVR2A;ACVRL1;ADAM10;ADM2;ADORA1;ADRBK1;AGA
turquoise	BP	GO:0044238	primary metabolic process	17735	8755	3682	2138	1929.1	4.50E-18	1.108289	AAAS;AACS;AANAT;AASDHPPT;ABCA1;ABCD1;ABCD2;ABCD3;ABCE1;ABHD10;ABI1;ABI2;ACADM;ACADSB;AC
turquoise	BP	GO:0043170	macromolecule metabolic process	17735	7334	3682	1828	1615.99	1.20E-17	1.131195	AANAT;AASDHPPT;ABCA1;ABCE1;ABI1;ABI2;ACADM;ACADVL;ACD;ACTL6A;ACVR1C;ACVR2A;ACVRL1;ADAM
turquoise	BP	GO:0071704	organic substance metabolic process	17735	8996	3682	2178	1982.2	2.50E-16	1.098779	AAAS;AACS;AANAT;AASDHPPT;ABCA1;ABCB6;ABCD1;ABCD2;ABCD3;ABCE1;ABHD10;ABI1;ABI2;ACADM;ACA
turquoise	MF	GO:0005488	binding	17735	11799	3682	2671	2505.78	2.70E-16	1.065936	AACS;AAMP;AASDHPPT;ABCA1;ABCA10;ABCA3;ABCA5;ABCA7;ABCB10;ABCB6;ABCC2;ABCD1;ABCD2;ABCD3
turquoise	CC	GO:0044428	nuclear part	17735	2482	3682	667	516.16	2.00E-15	1.292235	AAAS;ABHD14B;ACAT2;ACD;ACTL6A;ADD3;AEBP2;AFF4;AGAP2;AGPAT5;AHR;AKAP11;AKAP8L;AKIRIN1;AKT
turquoise	BP	GO:0008152	metabolic process	17735	9452	3682	2260	2082.68	3.40E-14	1.08514	AAAS;AACS;AANAT;AASDHPPT;ABCA1;ABCB6;ABCD1;ABCD2;ABCD3;ABCE1;ABHD10;ABI1;ABI2;ACADM;ACA

turquoise	MF	GO:0008270	zinc ion binding	17735	1977	3682	550	419.86	5.10E-14	1.30996	ABLIM2;ACAP1;ACAP2;ADA;ADAM10;ADAM15;ADAM22;ADAMTS5;ADAMTS6;ADAP1;AEBP1;AEBP2;AGAP2
turquoise	CC	GO:0031090	organelle membrane	17735	2366	3682	632	492.04	5.30E-14	1.284448	AAAS;ABCA3;ABCA5;ABCA7;ABCB10;ABCB6;ABCD1;ABCD2;ABCD3;ACADVL;ACAT1;ACBD3;ACBD5;ACP2;ACS
turquoise	BP	GO:0019941	modification-dependent protein catabolic...	17735	392	3682	150	86.37	1.20E-13	1.736714	AGAP3;AKT1;ANAPC10;ARRB1;BAG6;BAP1;BIRC2;CDC26;CDC27;CDK1;CDK2;CHFR;CRBN;CUL2;CUL4B;CUL5;
turquoise	BP	GO:0051603	proteolysis involved in cellular protein...	17735	413	3682	156	91	1.40E-13	1.714286	AGAP3;AKT1;ANAPC10;ARRB1;BAG6;BAP1;BIRC2;CASP8;CDC26;CDC27;CDK1;CDK2;CHFR;CRBN;CUL2;CUL4
turquoise	CC	GO:0070013	intracellular organelle lumen	17735	2677	3682	701	556.72	1.40E-13	1.259161	ABCD3;ABHD14B;ACADM;ACADSB;ACADVL;ACAT1;ACAT2;ACD;ACP2;ACSS1;ACTL6A;ADD3;AEBP2;AFF4;AG
turquoise	BP	GO:0006511	ubiquitin-dependent protein catabolic pr...	17735	386	3682	148	85.05	1.50E-13	1.740153	AGAP3;AKT1;ANAPC10;ARRB1;BAG6;BAP1;BIRC2;CDC26;CDC27;CDK1;CDK2;CHFR;CRBN;CUL2;CUL4B;CUL5;
turquoise	BP	GO:0044265	cellular macromolecule catabolic process	17735	698	3682	236	153.8	1.60E-13	1.53446	ABCE1;AGAP3;AKT1;ANAPC10;ARRB1;AUH;BAG6;BAP1;BIRC2;BNIP3L;CASP3;CASP8;CCNH;CDC26;CDC27;CD
turquoise	BP	GO:0043632	modification-dependent macromolecule cat...	17735	395	3682	150	87.04	2.50E-13	1.723346	AGAP3;AKT1;ANAPC10;ARRB1;BAG6;BAP1;BIRC2;CDC26;CDC27;CDK1;CDK2;CHFR;CRBN;CUL2;CUL4B;CUL5;
turquoise	CC	GO:0043233	organelle lumen	17735	2722	3682	708	566.07	4.60E-13	1.250729	ABCD3;ABHD14B;ACADM;ACADSB;ACADVL;ACAT1;ACAT2;ACD;ACP2;ACSS1;ACTL6A;ACTN4;ADA;ADD3;AEB
turquoise	BP	GO:0070647	protein modification by small protein co...	17735	640	3682	218	141.02	6.10E-13	1.54588	AIMP2;AKT1;ANAPC10;ANGPT1;ARRB1;ASB13;ASB7;ATG5;ATXN7L3;BAP1;BIRC2;BMI1;BRCC3;BTBD1;CBLL1
turquoise	BP	GO:0044257	cellular protein catabolic process	17735	428	3682	158	94.31	8.70E-13	1.675326	AGAP3;AKT1;ANAPC10;ARRB1;BAG6;BAP1;BIRC2;BNIP3L;CASP8;CDC26;CDC27;CDK1;CDK2;CHFR;CRBN;CUL
turquoise	CC	GO:0031974	membrane-enclosed lumen	17735	2770	3682	715	576.06	1.90E-12	1.24119	ABCD3;ABHD14B;ACADM;ACADSB;ACADVL;ACAT1;ACAT2;ACD;ACP2;ACSS1;ACTL6A;ACTN4;ADA;ADD3;AEB
turquoise	BP	GO:0007049	cell cycle	17735	1388	3682	411	305.84	2.40E-12	1.34384	ACTR1A;AHR;AKT1;ANAPC10;ANLN;ANXA1;APC;APEX1;APEX2;APPL1;ARHGEF2;ARL2;ARL8B;ARPP19;ASPM;
turquoise	BP	GO:0006464	cellular protein modification process	17735	2482	3682	680	546.89	2.40E-12	1.243394	AANAT;ABCA1;ABI1;ABI2;ACADVL;ACTL6A;ACVR1C;ACVR2A;ACVRL1;ADAM10;ADM2;ADORA1;ADRBK1;AID
turquoise	BP	GO:0036211	protein modification process	17735	2482	3682	680	546.89	2.40E-12	1.243394	AANAT;ABCA1;ABI1;ABI2;ACADVL;ACTL6A;ACVR1C;ACVR2A;ACVRL1;ADAM10;ADM2;ADORA1;ADRBK1;AID
turquoise	MF	GO:0043167	ion binding	17735	5761	3682	1393	1223.47	2.90E-12	1.138565	AACS;AAMP;AASDHPPT;ABCA1;ABCA10;ABCA3;ABCA5;ABCA7;ABCB10;ABCB6;ABCC2;ABCD1;ABCD2;ABCD3
turquoise	CC	GO:0031981	nuclear lumen	17735	2101	3682	560	436.93	3.30E-12	1.28167	ABHD14B;ACAT2;ACD;ACTL6A;ADD3;AEBP2;AFF4;AGAP2;AHR;AKAP11;AKAP8L;AKT1;AKTIP;ALKBH5;ALS2CR
turquoise	BP	GO:0043412	macromolecule modification	17735	2584	3682	703	569.36	4.10E-12	1.23472	AANAT;ABCA1;ABI1;ABI2;ACADVL;ACTL6A;ACVR1C;ACVR2A;ACVRL1;ADAM10;ADM2;ADORA1;ADRBK1;AID
turquoise	CC	GO:0005829	cytosol	17735	2362	3682	619	491.21	5.10E-12	1.260153	AACS;AANAT;AASDHPPT;ABCD1;ABCD2;ABCD3;ABHD10;ABI1;ABI2;ABR;ACOT7;ACTR1A;ADA;ADC;ADI1;ADR
turquoise	MF	GO:0003824	catalytic activity	17735	5264	3682	1278	1117.93	1.90E-11	1.143184	AACS;AANAT;AASDHPPT;ABCA1;ABCA10;ABCA3;ABCA5;ABCA7;ABCB10;ABCB6;ABCC2;ABCD1;ABCD2;ABCD
turquoise	BP	GO:0016070	RNA metabolic process	17735	3911	3682	1009	861.76	2.90E-11	1.17086	ABCE1;ACTL6A;ACVR2A;ACVRL1;ADRM1;AEBP1;AEBP2;AFF4;AGER;AGGF1;AHR;AIM2;AIMP1;AIMP2;AKAP7;
turquoise	MF	GO:1901363	heterocyclic compound binding	17735	5302	3682	1284	1126	3.50E-11	1.14032	AACS;ABCA1;ABCA10;ABCA3;ABCA5;ABCA7;ABCB10;ABCB6;ABCC2;ABCD1;ABCD2;ABCD3;ABCE1;ABCF3;AB
turquoise	BP	GO:0090304	nucleic acid metabolic process	17735	4431	3682	1128	976.34	3.70E-11	1.155335	ABCE1;ACD;ACTL6A;ACVR2A;ACVRL1;ADRM1;AEBP1;AEBP2;AFF4;AGER;AGGF1;AHR;AIM2;AIMP1;AIMP2;A
turquoise	BP	GO:0010498	proteasomal protein catabolic process	17735	234	3682	96	51.56	3.80E-11	1.861908	AGAP3;AKT1;ANAPC10;ARRB1;BAG6;BIRC2;CDC26;CDC27;CDK1;CDK2;CRBN;DERL1;DNAJB2;DNAJB9;DNAJC
turquoise	MF	GO:0046914	transition metal ion binding	17735	2217	3682	588	470.83	6.90E-11	1.248858	ABLIM2;ACAP1;ACAP2;ADA;ADAM10;ADAM15;ADAM22;ADAMTS5;ADAMTS6;ADAP1;AEBP1;AEBP2;AGAP2
turquoise	MF	GO:0097159	organic cyclic compound binding	17735	5372	3682	1295	1140.86	1.10E-10	1.135109	AACS;ABCA1;ABCA10;ABCA3;ABCA5;ABCA7;ABCB10;ABCB6;ABCC2;ABCD1;ABCD2;ABCD3;ABCE1;ABCF3;AB
turquoise	BP	GO:0032446	protein modification by small protein co...	17735	573	3682	191	126.26	1.40E-10	1.512751	AIMP2;AKT1;ANAPC10;ANGPT1;ARRB1;ASB13;ASB7;ATG5;BIRC2;BMI1;BTBD1;CBLL1;CDC26;CDC27;CDC73;
turquoise	BP	GO:0034641	cellular nitrogen compound metabolic pro...	17735	5595	3682	1387	1232.82	1.60E-10	1.125063	AANAT;AASDHPPT;ABCA1;ABCB6;ABCD1;ABCD3;ABCE1;ACADM;ACADSB;ACADVL;ACAP1;ACAP2;ACAT1;AC
turquoise	MF	GO:0046872	metal ion binding	17735	3936	3682	976	835.9	2.00E-10	1.167604	AASDHPPT;ABLIM2;ACAP1;ACAP2;ACAT1;ACTN4;ACVR1C;ACVR2A;ACVRL1;ACY3;ADA;ADAM10;ADAM15;A
turquoise	BP	GO:0044249	cellular biosynthetic process	17735	5073	3682	1268	1117.8	2.10E-10	1.134371	AANAT;ABCA1;ABCB6;ACADM;ACADVL;ACAT1;ACD;ACSF3;ACSL4;ACSL6;ACSS1;ACTL6A;ACVR2A;ACVRL1;A
turquoise	BP	GO:0043161	proteasomal ubiquitin-dependent protein ...	17735	224	3682	91	49.36	2.30E-10	1.843598	AGAP3;AKT1;ANAPC10;ARRB1;BAG6;BIRC2;CDC26;CDC27;CDK1;CDK2;CRBN;DERL1;DNAJB2;DNAJB9;DNAJC
turquoise	BP	GO:0010467	gene expression	17735	4403	3682	1115	970.17	2.30E-10	1.149283	ACTL6A;ACVR2A;ACVRL1;ADM2;ADRM1;AEBP1;AEBP2;AFF4;AGER;AGGF1;AHR;AIM2;AIMP1;AIMP2;AKT1;A
turquoise	BP	GO:0016567	protein ubiquitination	17735	540	3682	181	118.98	2.70E-10	1.521264	AIMP2;AKT1;ANAPC10;ANGPT1;ARRB1;ASB13;ASB7;ATG5;BIRC2;BMI1;BTBD1;CBLL1;CDC26;CDC27;CDC73;
turquoise	BP	GO:0034645	cellular macromolecule biosynthetic proc...	17735	4170	3682	1061	918.83	2.70E-10	1.154729	ABCA1;ACADM;ACD;ACTL6A;ACVR2A;ACVRL1;ADRM1;AEBP1;AEBP2;AFF4;AGER;AGL;AHR;AIM2;AIMP1;AI
turquoise	BP	GO:0030163	protein catabolic process	17735	531	3682	178	117	3.70E-10	1.521368	AGAP2;AGAP3;AKT1;ANAPC10;APC;ARRB1;BAG6;BAP1;BIRC2;BNIP3L;CASP8;CDC26;CDC27;CDK1;CDK2;CDK
turquoise	BP	GO:0009059	macromolecule biosynthetic process	17735	4294	3682	1088	946.15	4.10E-10	1.149923	AASDHPPT;ABCA1;ACADM;ACD;ACTL6A;ACVR2A;ACVRL1;ADC;ADRM1;AEBP1;AEBP2;AFF4;AGER;AGL;AHR;
turquoise	BP	GO:0006139	nucleobase-containing compound metabolic...	17735	5209	3682	1295	1147.76	5.70E-10	1.128285	ABCA1;ABCD1;ABCD3;ABCE1;ACADVL;ACAP1;ACAP2;ACD;ACTL6A;ACVR2A;ACVRL1;ADA;ADAP1;ADRM1;AD
turquoise	MF	GO:0005515	protein binding	17735	7306	3682	1705	1551.59	5.80E-10	1.098873	AASDHPPT;ABCA1;ABCB10;ABCC2;ABCD1;ABCD2;ABCD3;ABI1;ABI2;ABI3;ABLIM2;ACADM;ACAP1;ACAP2;AC
turquoise	CC	GO:0005815	microtubule organizing center	17735	482	3682	157	100.24	6.10E-10	1.566241	AAAS;ACTR1A;AKAP11;ANKRD26;ANKRD32;APC;APEX1;ARFGEF2;ARL2;AZI1;BBS7;BLOC1S2;BRCA2;C12orf5
turquoise	BP	GO:1901576	organic substance biosynthetic process	17735	5154	3682	1282	1135.65	6.60E-10	1.128869	AANAT;AASDHPPT;ABCA1;ABCB6;ACADM;ACADVL;ACBD3;ACD;ACSF3;ACSL4;ACSL6;ACSS1;ACTL6A;ACVR2A
turquoise	BP	GO:0009058	biosynthetic process	17735	5219	3682	1296	1149.97	7.90E-10	1.126986	AANAT;AASDHPPT;ABCA1;ABCB6;ACADM;ACADVL;ACAT1;ACBD3;ACD;ACSF3;ACSL4;ACSL6;ACSS1;ACTL6A;
turquoise	CC	GO:0005654	nucleoplasm	17735	1481	3682	401	307.99	8.30E-10	1.30199	ACD;ACTL6A;AEBP2;AHR;AKT1;ALKBH5;ALS2CR8;ANAPC10;ANKRD28;APEX1;APPL1;APTX;ARID4A;ARID5A;A
turquoise	MF	GO:0043169	cation binding	17735	4026	3682	989	855.01	1.40E-09	1.156712	AASDHPPT;ABLIM2;ACAP1;ACAP2;ACAT1;ACTN4;ACVR1C;ACVR2A;ACVRL1;ACY3;ADA;ADAM10;ADAM15;A
turquoise	CC	GO:0012505	endomembrane system	17735	1768	3682	466	367.68	1.70E-09	1.267406	AAAS;ABCA3;ABCA5;ABCA7;ACBD3;ACSL4;ACSL6;ADAM10;ADAM15;ADORA1;AGPAT5;AKIRIN1;ALG1;ALG1
turquoise	BP	GO:0006396	RNA processing	17735	646	3682	206	142.34	2.00E-09	1.447239	AGGF1;ALKBH5;ALKBH8;BCAS2;BUD13;CCNH;CCNL1;CD2BP2;CDC40;CDC73;CDK11A;CDK11B;CDK7;CHERP;
turquoise	CC	GO:0030529	ribonucleoprotein complex	17735	573	3682	178	119.16	2.70E-09	1.49379	ACTN4;APEX1;API5;APOBEC3D;BCAS2;BTBD1;CARHSP1;CASP8;CD2BP2;CDC40;CNOT3;CNOT7;CPSF6;CSNK1
turquoise	BP	GO:0006807	nitrogen compound metabolic process	17735	5942	3682	1453	1309.28	3.10E-09	1.10977	AANAT;AASDHPPT;ABCA1;ABCB6;ABCD1;ABCD3;ABCE1;ACADM;ACADSB;ACADVL;ACAP1;ACAP2;ACAT1;AC
turquoise	BP	GO:0046483	heterocycle metabolic process	17735	5361	3682	1322	1181.26	3.60E-09	1.119144	AANAT;ABCA1;ABCB6;ABCD1;ABCD3;ABCE1;ACADVL;ACAP1;ACAP2;ACD;ACTL6A;ACVR2A;ACVRL1;ADA;AD
turquoise	MF	GO:0016881	acid-amino acid ligase activity	17735	299	3682	106	63.5	8.00E-09	1.669291	ANAPC10;ATPBD4;BIRC2;BIRC3;BMI1;CBLL1;CHFR;DDB2;DZIP3;ERCC8;FANCL;FBXL3;FBXL5;FBXO11;FBXO3;
turquoise	CC	GO:0008287	protein serine/threonine phosphatase com...	17735	42	3682	26	8.73	8.30E-09	2.978236	CTDNEP1;CYCS;PPP1CA;PPP1CB;PPP1CC;PPP1R12A;PPP1R15B;PPP2CB;PPP2R1A;PPP2R2A;PPP2R3A;PPP2R
turquoise	BP	GO:0009987	cellular process	17735	12861	3682	2922	2833.82	8.70E-09	1.031117	AAAS;AACS;AAMP;AANAT;AASDHPPT;ABCA1;ABCA3;ABCA5;ABCA7;ABCB10;ABCB6;ABCC2;ABCD1;ABCD2;A
turquoise	BP	GO:0000278	mitotic cell cycle	17735	777	3682	237	171.21	1.00E-08	1.384265	ACTR1A;AKT1;ANAPC10;ANLN;APC;APEX1;ARHGEF2;ARPP19;ASPM;AURKC;AZI1;BACH1;BCAT1;BORA;BRCA
turquoise	BP	GO:0009057	macromolecule catabolic process	17735	883	3682	264	194.56	1.20E-08	1.356908	ABCE1;AGAP2;AGAP3;AGL;AKT1;ANAPC10;APC;ARRB1;AUH;BAG6;BAP1;BIRC2;BNIP3L;CASP3;CASP8;CCNH;
turquoise	MF	GO:0019787	small conjugating protein ligase activit...	17735	269	3682	97	57.13	1.30E-08	1.697882	ANAPC10;BIRC2;BIRC3;BMI1;CBLL1;CHFR;DDB2;DZIP3;ERCC8;FANCL;FBXL3;FBXL5;FBXO11;FBXO3;FBXO30;
turquoise	BP	GO:0016071	mRNA metabolic process	17735	607	3682	192	133.75	1.40E-08	1.435514	AKT1;ALKBH5;AUH;BCAS2;BUD13;CCNH;CD2BP2;CDC40;CDC73;CDK11A;CDK11B;CDK7;CEBPG;CLNS1A;CLP1
turquoise	BP	GO:0006725	cellular aromatic compound metabolic pro...	17735	5371	3682	1318	1183.46	1.60E-08	1.113684	AANAT;ABCA1;ABCB6;ABCD1;ABCD3;ABCE1;ACADVL;ACAP1;ACAP2;ACD;ACTL6A;ACVR2A;ACVRL1;ADA;AD
turquoise	MF	GO:0004842	ubiquitin-protein ligase activity	17735	253	3682	92	53.73	1.90E-08	1.712265	ANAPC10;BIRC2;BIRC3;BMI1;CBLL1;CHFR;DDB2;DZIP3;ERCC8;FANCL;FBXL3;FBXL5;FBXO11;FBXO3;FBXO30;
turquoise	MF	GO:0016874	ligase activity	17735	483	3682	154	102.58	1.90E-08	1.501267	AACS;ACSF2;ACSF3;ACSL4;ACSL6;ACSS1;ADSSL1;ANAPC10;ASNSD1;ATPBD4;BIRC2;BIRC3;BMI1;BTD;CBLL1;
turquoise	BP	GO:0019538	protein metabolic process	17735	3895	3682	981	858.23	2.20E-08	1.14305	AANAT;ABCA1;ABI1;ABI2;ACADVL;ACTL6A;ACVR1C;ACVR2A;ACVRL1;ADAM10;ADAM15;ADAM22;ADAMTS5
turquoise	CC	GO:0005813	centrosome	17735	374	3682	123	77.78	2.40E-08	1.581383	AAAS;ACTR1A;ANKRD26;ANKRD32;APC;APEX1;ARL2;AZI1;BBS7;BLOC1S2;BRCA2;C12orf52;CASP8;CCDC14;
turquoise	MF	GO:1901265	nucleoside phosphate binding	17735	2386	3682	607	506.72	4.40E-08	1.1979	AACS;ABCA1;ABCA10;ABCA3;ABCA5;ABCA7;ABCB10;ABCB6;ABCC2;ABCD1;ABCD2;ABCD3;ABCE1;ABCF3;AC
turquoise	MF	GO:0016879	ligase activity, forming carbon-nitrogen...	17735	330	3682	112	70.08	4.70E-08	1.598174	ADSSL1;ANAPC10;ASNSD1;ATPBD4;BIRC2;BIRC3;BMI1;BTD;CBLL1;CHFR;DDB2;DZIP3;ERCC8;FANCL;FBXL3;
turquoise	BP	GO:0022402	cell cycle process	17735	1059	3682	305	233.34	5.10E-08	1.307106	ACTR1A;AKT1;ANAPC10;ANLN;APC;APEX1;ARHGEF2;ARPP19;ASPM;AURKC;AZI1;BACH1;BAG6;BCAT1;BORA
turquoise	MF	GO:0000166	nucleotide binding	17735	2385	3682	606	506.51	5.50E-08	1.196423	AACS;ABCA1;ABCA10;ABCA3;ABCA5;ABCA7;ABCB10;ABCB6;ABCC2;ABCD1;ABCD2;ABCD3;ABCE1;ABCF3;AC
turquoise	CC	GO:0015630	microtubule cytoskeleton	17735	853	3682	241	177.39	6.50E-08	1.358588	AAAS;ACTR1A;AKAP11;AKT1;ALKBH8;ANKRD26;ANKRD32;APC;APEX1;APPBP2;ARFGEF2;ARHGEF2;ARL2;AR
turquoise	CC	GO:0032991	macromolecular complex	17735	3893	3682	928	809.6	7.40E-08	1.146245	AAAS;ABCA7;ABCB6;ACD;ACTL6A;ACTN4;ACTR1A;ACVR1C;ACVR2A;ADRM1;AEBP2;AGL;AHR;AIM2;AIMP1;A
turquoise	CC	GO:0005783	endoplasmic reticulum	17735	1273	3682	339	264.74	1.20E-07	1.280502	ABCB6;ABI1;ACSL4;ACSL6;ADORA1;AGL;AGPAT5;AIFM3;AIMP1;AKAP7;ALG1;ALG10;ALG11;ALG13;ALG2;AL
turquoise	BP	GO:0031323	regulation of cellular metabolic process	17735	4397	3682	1088	968.85	1.40E-07	1.122981	ABCA1;ABCD2;ACADM;ACADVL;ACAP1;ACAP2;ACD;ACSL6;ACTL6A;ACVR2A;ACVRL1;ADAP1;ADORA1;AEBP2
turquoise	CC	GO:0000139	Golgi membrane	17735	540	3682	162	112.3	1.90E-07	1.442565	ABCA5;ABCA7;ACBD3;AP1B1;AP1S2;APH1A;ARFGAP1;ARFGAP2;ARFGEF2;ARFIP1;ARRB1;ATP2C1;B2M;B3G

turquoise	BP	GO:0080090	regulation of primary metabolic process	17735	4337	3682	1073	955.63	2.00E-07	1.12282	ABCA1;ABCD2;ACADM;ACADVL;ACAP1;ACAP2;ACD;ACSL6;ACTL6A;ACVR2A;ACVRL1;ADAP1;ADORA1;AEBP2
turquoise	MF	GO:0004674	protein serine/threonine kinase activity	17735	434	3682	137	92.17	2.30E-07	1.486384	ACVR1C;ACVR2A;ACVRL1;ADCK1;ADCK2;ADCK3;ADRBK1;AKT1;ANKK1;ARAF;AURKC;BCR;BMPR1A;BMPR2;C
turquoise	BP	GO:0022403	cell cycle phase	17735	852	3682	249	187.73	2.60E-07	1.326373	ACTR1A;AKT1;ANAPC10;ANLN;APC;APEX1;ARHGEF2;ARPP19;ASPM;AURKC;AZI1;BACH1;BAG6;BCAT1;BORA
turquoise	BP	GO:1901360	organic cyclic compound metabolic proces...	17735	5578	3682	1352	1229.07	2.60E-07	1.100019	AANAT;ABCA1;ABCB6;ABCD1;ABCD3;ABCE1;ACADVL;ACAP1;ACAP2;ACBD3;ACD;ACTL6A;ACVR2A;ACVRL1;A
turquoise	MF	GO:0016740	transferase activity	17735	1705	3682	444	362.09	2.70E-07	1.226214	AANAT;AASDHPPT;ACAD10;ACAT1;ACAT2;ACVR1C;ACVR2A;ACVRL1;ADCK1;ADCK2;ADCK3;ADRBK1;AGL;AG
turquoise	MF	GO:0036094	small molecule binding	17735	2525	3682	632	536.24	2.80E-07	1.178577	AACS;ABCA1;ABCA10;ABCA3;ABCA5;ABCA7;ABCB10;ABCB6;ABCC2;ABCD1;ABCD2;ABCD3;ABCE1;ABCF3;AC
turquoise	MF	GO:0035639	purine ribonucleoside triphosphate bindi...	17735	1796	3682	463	381.42	5.10E-07	1.213885	AACS;ABCA1;ABCA10;ABCA3;ABCA5;ABCA7;ABCB10;ABCB6;ABCC2;ABCD1;ABCD2;ABCD3;ABCE1;ABCF3;AC
turquoise	BP	GO:0000209	protein polyubiquitination	17735	167	3682	65	36.8	5.30E-07	1.766304	ANAPC10;BIRC2;CDC26;CDC27;CHFR;DDB2;DZIP3;ERCC8;FBXO4;G2E3;HACE1;HDAC6;ITCH;LRSAM1;MARCH
turquoise	BP	GO:0019222	regulation of metabolic process	17735	4834	3682	1181	1065.14	5.50E-07	1.108774	ABCA1;ABCD2;ACADM;ACADVL;ACAP1;ACAP2;ACD;ACSL6;ACTL6A;ACVR1C;ACVR2A;ACVRL1;ADAP1;ADM2;
turquoise	MF	GO:0001883	purine nucleoside binding	17735	1807	3682	465	383.76	6.00E-07	1.211695	AACS;ABCA1;ABCA10;ABCA3;ABCA5;ABCA7;ABCB10;ABCB6;ABCC2;ABCD1;ABCD2;ABCD3;ABCE1;ABCF3;AC
turquoise	MF	GO:0032550	purine ribonucleoside binding	17735	1805	3682	463	383.33	9.50E-07	1.207837	AACS;ABCA1;ABCA10;ABCA3;ABCA5;ABCA7;ABCB10;ABCB6;ABCC2;ABCD1;ABCD2;ABCD3;ABCE1;ABCF3;AC
turquoise	MF	GO:0001882	nucleoside binding	17735	1818	3682	466	386.09	9.50E-07	1.206972	AACS;ABCA1;ABCA10;ABCA3;ABCA5;ABCA7;ABCB10;ABCB6;ABCC2;ABCD1;ABCD2;ABCD3;ABCE1;ABCF3;AC
turquoise	BP	GO:0006996	organelle organization	17735	2139	3682	557	471.31	1.10E-06	1.181812	ABCA1;ABCD1;ABCD3;ABI1;ABI2;ABLIM2;ABR;ACD;ACP2;ACTL6A;ACTN4;AEBP2;AKT1;AKTIP;ALDOA;ANAPC
turquoise	MF	GO:0032549	ribonucleoside binding	17735	1809	3682	463	384.18	1.20E-06	1.205164	AACS;ABCA1;ABCA10;ABCA3;ABCA5;ABCA7;ABCB10;ABCB6;ABCC2;ABCD1;ABCD2;ABCD3;ABCE1;ABCF3;AC
turquoise	MF	GO:0017076	purine nucleotide binding	17735	1836	3682	469	389.91	1.30E-06	1.202842	AACS;ABCA1;ABCA10;ABCA3;ABCA5;ABCA7;ABCB10;ABCB6;ABCC2;ABCD1;ABCD2;ABCD3;ABCE1;ABCF3;AC
turquoise	BP	GO:0060255	regulation of macromolecule metabolic pr...	17735	4178	3682	1028	920.59	1.40E-06	1.116675	ACADVL;ACD;ACTL6A;ACVR2A;ACVRL1;ADM2;ADORA1;AEBP2;AGAP2;AGER;AHR;AIDA;AIM2;AIMP2;AKT1;A
turquoise	BP	GO:0046907	intracellular transport	17735	1214	3682	334	267.5	1.50E-06	1.248598	AAAS;ABCA1;ABCD1;ACD;ADORA1;AGAP3;AKAP5;AKT1;AKTIP;ALKBH5;AP1B1;AP1S2;AP2A2;AP3M1;AP3S2;
turquoise	MF	GO:0032555	purine ribonucleotide binding	17735	1831	3682	467	388.85	1.70E-06	1.200977	AACS;ABCA1;ABCA10;ABCA3;ABCA5;ABCA7;ABCB10;ABCB6;ABCC2;ABCD1;ABCD2;ABCD3;ABCE1;ABCF3;AC
turquoise	BP	GO:0006397	mRNA processing	17735	405	3682	129	89.24	2.30E-06	1.44554	ALKBH5;BCAS2;BUD13;CCNH;CD2BP2;CDC40;CDC73;CDK11A;CDK11B;CDK7;CLNS1A;CLP1;CNOT2;CNOT3;C
turquoise	MF	GO:0032553	ribonucleotide binding	17735	1846	3682	469	392.04	2.50E-06	1.196306	AACS;ABCA1;ABCA10;ABCA3;ABCA5;ABCA7;ABCB10;ABCB6;ABCC2;ABCD1;ABCD2;ABCD3;ABCE1;ABCF3;AC
turquoise	BP	GO:2000112	regulation of cellular macromolecule bio...	17735	3117	3682	782	686.81	2.50E-06	1.138597	ACD;ACTL6A;ACVR2A;ACVRL1;AEBP2;AGER;AHR;AIM2;AKT1;ANG;ANKRD49;APEX1;ARGLU1;ARHGAP22;AR
turquoise	CC	GO:0016604	nuclear body	17735	276	3682	90	57.4	2.60E-06	1.567944	ALKBH5;APEX1;ATPAF2;BMI1;CCNL1;CD2BP2;CDK2;CDK9;CHFR;CLK3;COIL;CPSF6;CSNK1A1;CXXC1;DAPK3;D
turquoise	MF	GO:0003676	nucleic acid binding	17735	3299	3682	796	700.61	3.10E-06	1.136153	ABI2;ACD;AEBP1;AEBP2;AGGF1;AHR;AIM2;AIMP1;AKAP8L;ALKBH8;ANG;APEX1;APEX2;APTX;ARID2;ARID4A;
turquoise	BP	GO:0006974	response to DNA damage stimulus	17735	608	3682	181	133.97	3.40E-06	1.351049	ACD;ACTL6A;ALKBH8;APC;APEX1;APEX2;APLF;APTX;ASF1A;ASTE1;ATRX;BAG6;BCLAF1;BRCA2;BRCC3;BRIP1;
turquoise	BP	GO:0010556	regulation of macromolecule biosynthetic...	17735	3197	3682	798	704.43	4.30E-06	1.132831	ACD;ACTL6A;ACVR2A;ACVRL1;AEBP2;AGER;AHR;AIM2;AKT1;ANG;ANKRD13C;ANKRD49;APEX1;ARGLU1;AR
turquoise	CC	GO:0031902	late endosome membrane	17735	78	3682	34	16.22	4.60E-06	2.096178	ABCA5;ARL8B;ATG9A;CD63;CHMP1B;CHMP2A;CHMP2B;CYBASC3;HGS;HLA-DMA;HLA-DRA;LAMTOR3;MAR
turquoise	BP	GO:0031326	regulation of cellular biosynthetic proc...	17735	3328	3682	827	733.3	5.50E-06	1.127779	ABCA1;ACADM;ACADVL;ACD;ACSL6;ACTL6A;ACVR2A;ACVRL1;AEBP2;AGER;AHR;AIM2;AKAP5;AKT1;ANG;AN
turquoise	CC	GO:0044451	nucleoplasm part	17735	823	3682	223	171.15	5.60E-06	1.302951	ACTL6A;AEBP2;AHR;ALKBH5;APEX1;APPL1;ARID4A;ARID5A;ATF1;ATPAF2;ATXN7L3;BMI1;CCNC;CCNH;CCNL
turquoise	BP	GO:0045184	establishment of protein localization	17735	1288	3682	348	283.8	5.60E-06	1.226216	ABCA1;ACAP1;ACD;ACTN4;ADORA1;AGAP2;AGAP3;AIM2;AKAP5;AKT1;AKTIP;ANG;ANXA1;AP1AR;AP1B1;AP
turquoise	BP	GO:0000280	nuclear division	17735	359	3682	115	79.1	6.00E-06	1.453856	ANAPC10;ANLN;APC;ARHGEF2;ARPP19;ASPM;AURKC;BORA;CASC5;CCNA2;CCNG1;CCNG2;CD28;CD2AP;CD
turquoise	BP	GO:0007067	mitosis	17735	359	3682	115	79.1	6.00E-06	1.453856	ANAPC10;ANLN;APC;ARHGEF2;ARPP19;ASPM;AURKC;BORA;CASC5;CCNA2;CCNG1;CCNG2;CD28;CD2AP;CD
turquoise	CC	GO:0005794	Golgi apparatus	17735	1158	3682	301	240.82	6.20E-06	1.249896	ABCA1;ABCA5;ABCA7;ABCB6;ACBD3;ADAM10;ADI1;AIMP1;AKR7A2;AKTIP;AP1AR;AP1B1;AP1S2;AP3M1;AP
turquoise	BP	GO:0006355	regulation of transcription, DNA-depende...	17735	2869	3682	720	632.16	7.00E-06	1.138952	ACTL6A;ACVR2A;ACVRL1;AEBP2;AGER;AHR;AIM2;AKT1;ANKRD49;APEX1;ARGLU1;ARHGAP22;ARHGEF2;ARI
turquoise	BP	GO:0048285	organelle fission	17735	383	3682	121	84.39	7.30E-06	1.433819	ANAPC10;ANLN;APC;ARHGEF2;ARPP19;ASPM;AURKC;BORA;CASC5;CCNA2;CCNG1;CCNG2;CD28;CD2AP;CD
turquoise	BP	GO:0007050	cell cycle arrest	17735	384	3682	121	84.61	8.30E-06	1.430091	AKT1;ANAPC10;APC;BRCC3;BRIP1;CAB39;CAB39L;CASC5;CCNA2;CCNE2;CCNG1;CCNG2;CDC25C;CDC26;CDC
turquoise	BP	GO:0009889	regulation of biosynthetic process	17735	3356	3682	831	739.47	9.20E-06	1.123778	ABCA1;ACADM;ACADVL;ACD;ACSL6;ACTL6A;ACVR2A;ACVRL1;AEBP2;AGER;AHR;AIM2;AKAP5;AKT1;ANG;AN
turquoise	BP	GO:0000082	G1/S transition of mitotic cell cycle	17735	215	3682	75	47.37	9.60E-06	1.583281	AKT1;BACH1;BCAT1;CAMK2D;CCNE2;CCNH;CDC7;CDC73;CDK1;CDK2;CDK7;CDKN1B;CDKN2C;CDKN3;CDT1;
turquoise	BP	GO:0051252	regulation of RNA metabolic process	17735	2953	3682	738	650.67	9.60E-06	1.134216	ACTL6A;ACVR2A;ACVRL1;AEBP2;AGER;AHR;AIM2;AKT1;ANKRD49;APEX1;ARGLU1;ARHGAP22;ARHGEF2;ARI
turquoise	MF	GO:0004722	protein serine/threonine phosphatase act...	17735	51	3682	25	10.83	9.80E-06	2.308403	CDKN3;CTDNEP1;CTDSP1;CYCS;MTMR6;PDP1;PPEF1;PPM1B;PPM1G;PPP1CA;PPP1CB;PPP1CC;PPP1R15B;PP
turquoise	BP	GO:0000087	M phase of mitotic cell cycle	17735	370	3682	117	81.53	9.80E-06	1.435055	ANAPC10;ANLN;APC;ARHGEF2;ARPP19;ASPM;AURKC;BORA;CASC5;CCNA2;CCNG1;CCNG2;CD28;CD2AP;CD
turquoise	CC	GO:0005739	mitochondrion	17735	1488	3682	375	309.45	9.90E-06	1.211827	ABCB10;ABCB6;ABCD1;ABCD2;ABCD3;ABCE1;ABHD10;ACAD10;ACAD9;ACADM;ACADSB;ACADVL;ACAT1;AC
turquoise	CC	GO:0000775	chromosome, centromeric region	17735	146	3682	53	30.36	1.10E-05	1.745718	APC;AURKC;CASC5;CBX3;CENPE;CENPK;CENPL;CENPQ;CENPW;CSNK1A1;DAPK3;DAXX;DCTN2;DYNLT3;HELL
turquoise	CC	GO:0044431	Golgi apparatus part	17735	654	3682	181	136.01	1.10E-05	1.330785	ABCA5;ABCA7;ACBD3;ADAM10;AP1B1;AP1S2;APH1A;ARFGAP1;ARFGAP2;ARFGEF2;ARFIP1;ARRB1;ATP2C1;
turquoise	BP	GO:0015031	protein transport	17735	1245	3682	335	274.33	1.20E-05	1.221157	ABCA1;ACAP1;ACD;ACTN4;ADORA1;AGAP2;AGAP3;AIM2;AKAP5;AKT1;AKTIP;ANG;ANXA1;AP1AR;AP1B1;AP
turquoise	CC	GO:0000502	proteasome complex	17735	65	3682	29	13.52	1.30E-05	2.14497	ADRM1;DNAJB2;IDE;PSMA1;PSMA2;PSMA3;PSMA4;PSMA6;PSMA8;PSMB1;PSMB3;PSMC1;PSMC2;PSMC3;
turquoise	CC	GO:0042175	nuclear outer membrane-endoplasmic retic...	17735	773	3682	209	160.76	1.30E-05	1.300075	ACSL4;ACSL6;AGPAT5;ALG1;ALG10;ALG11;ALG13;ALG2;ALG3;ALG6;ANKRD13C;ANO5;APH1A;ARL6IP1;ARL6
turquoise	BP	GO:2001141	regulation of RNA biosynthetic process	17735	2889	3682	722	636.57	1.30E-05	1.134204	ACTL6A;ACVR2A;ACVRL1;AEBP2;AGER;AHR;AIM2;AKT1;ANKRD49;APEX1;ARGLU1;ARHGAP22;ARHGEF2;ARI
turquoise	BP	GO:0033554	cellular response to stress	17735	1209	3682	326	266.39	1.40E-05	1.22377	ACADVL;ACD;ACTL6A;AGAP3;AIDA;AKT1;ALKBH8;ANXA1;APC;APEX1;APEX2;APLF;APTX;ARFGAP1;ARHGEF2;
turquoise	BP	GO:0051171	regulation of nitrogen compound metaboli...	17735	3496	3682	861	770.32	1.40E-05	1.117717	ABCA1;ACADVL;ACAP1;ACAP2;ACD;ACTL6A;ACVR2A;ACVRL1;ADAP1;AEBP2;AGAP2;AGAP3;AGER;AGFG2;A
turquoise	MF	GO:0016772	transferase activity, transferring phosp...	17735	889	3682	240	188.8	1.50E-05	1.271186	AASDHPPT;ACAD10;ACVR1C;ACVR2A;ACVRL1;ADCK1;ADCK2;ADCK3;ADRBK1;AK3;AKD1;AKT1;ANKK1;ARAF;
turquoise	MF	GO:0032182	small conjugating protein binding	17735	71	3682	31	15.08	1.80E-05	2.055703	AGAP3;AGL;BAG6;BRCC3;CASP8AP2;DNAJB2;DYRK2;DZIP3;FAM175A;HDAC6;IDE;KIF18A;MARK4;NEDD4;RA
turquoise	BP	GO:0032774	RNA biosynthetic process	17735	3293	3682	813	725.59	1.90E-05	1.120467	ACTL6A;ACVR2A;ACVRL1;ADRM1;AEBP1;AEBP2;AFF4;AGER;AHR;AIM2;AKT1;ANG;ANKRD49;APEX1;ARGLU1
turquoise	CC	GO:0005789	endoplasmic reticulum membrane	17735	757	3682	204	157.43	2.00E-05	1.295814	ACSL4;ACSL6;AGPAT5;ALG1;ALG10;ALG11;ALG13;ALG2;ALG3;ALG6;ANKRD13C;ANO5;APH1A;ARL6IP1;ARL6
turquoise	MF	GO:0003723	RNA binding	17735	851	3682	230	180.73	2.10E-05	1.272617	AIMP1;ALKBH8;ANG;APEX1;APTX;AUH;CARHSP1;CDK9;CDKN2AIP;CHERP;CNOT6;CNP;COPS5;CPEB2;CPEB3;
turquoise	CC	GO:0043234	protein complex	17735	3294	3682	771	685.03	2.50E-05	1.125498	AAAS;ABCA7;ABCB6;ACTL6A;ACTN4;ACTR1A;ACVR1C;ACVR2A;ADRM1;AEBP2;AGL;AHR;AIM2;AIMP1;AKAP
turquoise	CC	GO:0044432	endoplasmic reticulum part	17735	905	3682	238	188.21	2.60E-05	1.264545	ACSL4;ACSL6;AGPAT5;ALG1;ALG10;ALG11;ALG13;ALG2;ALG3;ALG6;ANKRD13C;ANO5;APH1A;ARL6IP1;ARL6
turquoise	BP	GO:0044248	cellular catabolic process	17735	1692	3682	439	372.82	2.70E-05	1.177512	ABCD1;ABCD2;ABCD3;ABCE1;ABHD10;ACADM;ACADSB;ACADVL;ACAP1;ACAP2;ACAT1;ACOT7;ACSF3;ADA;A
turquoise	BP	GO:0006351	transcription, DNA-dependent	17735	3208	3682	791	706.86	3.20E-05	1.119033	ACTL6A;ACVR2A;ACVRL1;ADRM1;AEBP1;AEBP2;AFF4;AGER;AHR;AIM2;AKT1;ANG;ANKRD49;APEX1;ARGLU1
turquoise	BP	GO:0006281	DNA repair	17735	388	3682	119	85.49	3.90E-05	1.391976	ACTL6A;APEX1;APEX2;APLF;APTX;ASF1A;ASTE1;ATRX;BRCA2;BRCC3;BRIP1;CCNH;CDK1;CDK2;CDK7;CDK9;CE
turquoise	CC	GO:0044429	mitochondrial part	17735	740	3682	198	153.89	4.30E-05	1.286633	ABCB10;ABCB6;ABCD3;ACADM;ACADSB;ACADVL;ACAT1;ACSL4;ACSL6;ACSS1;AGPAT5;AIFM3;AK3;ALDH4A1
turquoise	BP	GO:0008380	RNA splicing	17735	328	3682	103	72.27	4.50E-05	1.425211	BCAS2;BUD13;CD2BP2;CDC40;CLK1;CLK3;CLK4;CLNS1A;CLP1;CPSF1;DCPS;DDX41;DGCR14;FRG1;GEMIN2;GE
turquoise	BP	GO:0010468	regulation of gene expression	17735	3347	3682	821	737.49	4.60E-05	1.113235	ACTL6A;ACVR2A;ACVRL1;ADM2;AEBP2;AGER;AHR;AIM2;AKT1;ANG;ANKRD49;APEX1;APTX;ARGLU1;ARHGA
turquoise	MF	GO:0005524	ATP binding	17735	1462	3682	370	310.49	4.80E-05	1.191665	AACS;ABCA1;ABCA10;ABCA3;ABCA5;ABCA7;ABCB10;ABCB6;ABCC2;ABCD1;ABCD2;ABCD3;ABCE1;ABCF3;AC
turquoise	BP	GO:0006793	phosphorus metabolic process	17735	2513	3682	628	553.72	5.60E-05	1.134147	ABCA1;ABCD1;ABCD3;ABI1;ABI2;ACADVL;ACAP1;ACAP2;ACSL4;ACSL6;ACSS1;ACVR1C;ACVR2A;ACVRL1;ADA
turquoise	BP	GO:0051726	regulation of cell cycle	17735	736	3682	206	162.17	5.80E-05	1.270272	AKT1;ANAPC10;ANLN;APC;APEX1;AURKC;BAP1;BCR;BIRC2;BORA;BRCA2;BRCC3;BRIP1;CAB39;CAB39L;CASC
turquoise	BP	GO:0008104	protein localization	17735	1586	3682	411	349.46	5.80E-05	1.1761	ABCA1;ACAP1;ACD;ACTN4;ADORA1;AGAP2;AGAP3;AIM2;AKAP5;AKAP7;AKT1;AKTIP;ANG;ANGPT1;ANKRD1
turquoise	BP	GO:0044271	cellular nitrogen compound biosynthetic ...	17735	3726	3682	906	821	6.00E-05	1.103532	AANAT;ABCA1;ABCB6;ACADM;ACTL6A;ACVR2A;ACVRL1;ADA;ADC;ADI1;ADRM1;ADSSL1;AEBP1;AEBP2;AFF4
turquoise	CC	GO:0043228	non-membrane-bounded organelle	17735	3122	3682	729	649.26	6.30E-05	1.122817	AAAS;ABHD14B;ABI1;ABI2;ABLIM2;ACADVL;ACAT2;ACD;ACTL6A;ACTL9;ACTN4;ACTR1A;ACTR6;ADAM10;AD
turquoise	CC	GO:0043232	intracellular non-membrane-bounded organ...	17735	3122	3682	729	649.26	6.30E-05	1.122817	AAAS;ABHD14B;ABI1;ABI2;ABLIM2;ACADVL;ACAT2;ACD;ACTL6A;ACTL9;ACTN4;ACTR1A;ACTR6;ADAM10;AD

turquoise	MF	GO:0030554	adenyl nucleotide binding	17735	1495	3682	376	317.5	7.30E-05	1.184252	AACS;ABCA1;ABCA10;ABCA3;ABCA5;ABCA7;ABCB10;ABCB6;ABCC2;ABCD1;ABCD2;ABCD3;ABCE1;ABCF3;AC
turquoise	BP	GO:0000226	microtubule cytoskeleton organization	17735	279	3682	89	61.48	7.70E-05	1.447625	APC;ARHGEF2;ARL2;AURKC;BLOC1S2;BORA;BRCA2;CASC5;CCDC88A;CCDC88B;CCP110;CDK1;CDK2;CDK5R1;
turquoise	BP	GO:0018130	heterocycle biosynthetic process	17735	3650	3682	887	804.25	8.20E-05	1.102891	AANAT;ABCA1;ABCB6;ACTL6A;ACVR2A;ACVRL1;ADA;ADRM1;ADSSL1;AEBP1;AEBP2;AFF4;AGER;AHR;AIM2;
turquoise	CC	GO:0010008	endosome membrane	17735	311	3682	93	64.68	8.30E-05	1.437848	ABCA5;ABCA7;APPL1;ARL8B;ATG9A;ATP11B;B2M;CD164;CD63;CHMP1A;CHMP1B;CHMP2A;CHMP2B;CLIP3;
turquoise	BP	GO:0070936	protein K48-linked ubiquitination	17735	37	3682	19	8.15	8.60E-05	2.331288	HACE1;ITCH;MARCH6;PARK2;PELI1;RNF146;RNF187;RNF6;TOPORS;UBE2A;UBE2D1;UBE2D3;UBE2E1;UBE2
turquoise	BP	GO:0070646	protein modification by small protein re...	17735	77	3682	32	16.97	9.20E-05	1.885681	ATXN7L3;BAP1;BRCC3;COPS2;COPS4;COPS5;COPS6;DNAJB2;ENY2;GPS1;ITCH;MYSM1;OTUD5;PSMD14;SEN
turquoise	BP	GO:0043687	post-translational protein modification	17735	195	3682	66	42.97	9.20E-05	1.535955	ALG1;ALG10;ALG10B;ALG11;ALG13;ALG2;ALG3;ALG6;ARSA;ARSK;ATG5;B3GNT2;B3GNT5;B4GALT3;B4GALT
turquoise	CC	GO:0044440	endosomal part	17735	320	3682	95	66.55	9.40E-05	1.427498	ABCA5;ABCA7;APPL1;ARL8B;ATG9A;ATP11B;B2M;CD164;CD63;CHMP1A;CHMP1B;CHMP2A;CHMP2B;CLIP3;
turquoise	MF	GO:0043130	ubiquitin binding	17735	60	3682	26	12.74	9.60E-05	2.040816	AGAP3;AGL;BAG6;BRCC3;DNAJB2;DYRK2;DZIP3;FAM175A;HDAC6;IDE;KIF18A;MARK4;NEDD4;RAD23A;RBC
turquoise	MF	GO:0032559	adenyl ribonucleotide binding	17735	1491	3682	374	316.65	9.60E-05	1.181115	AACS;ABCA1;ABCA10;ABCA3;ABCA5;ABCA7;ABCB10;ABCB6;ABCC2;ABCD1;ABCD2;ABCD3;ABCE1;ABCF3;AC
turquoise	BP	GO:0048193	Golgi vesicle transport	17735	181	3682	62	39.88	1.00E-04	1.554664	AP1B1;AP1S2;ARFGAP1;ARFGEF2;ARRB1;BET1;BLZF1;COG3;COG4;COG6;COPE;COPZ1;CSK;DTNBP1;EXOC5;
turquoise	BP	GO:0019219	regulation of nucleobase-containing comp...	17735	3414	3682	832	752.25	1.00E-04	1.106015	ABCA1;ACADVL;ACAP1;ACAP2;ACD;ACTL6A;ACVR2A;ACVRL1;ADAP1;AEBP2;AGAP2;AGAP3;AGER;AGFG2;A
turquoise	BP	GO:0019438	aromatic compound biosynthetic process	17735	3638	3682	883	801.61	1.00E-04	1.101533	AANAT;ABCA1;ABCB6;ACTL6A;ACVR2A;ACVRL1;ADA;ADRM1;ADSSL1;AEBP1;AEBP2;AFF4;AGER;AHR;AIM2;
turquoise	CC	GO:0000151	ubiquitin ligase complex	17735	150	3682	51	31.19	0.00011	1.635139	ANAPC10;BMI1;BRCC3;CBLL1;CDC26;CDC27;CRBN;CUL2;CUL4B;CUL5;DCAF10;DCAF11;DCAF13;DCUN1D1;D
turquoise	BP	GO:0022613	ribonucleoprotein complex biogenesis	17735	233	3682	76	51.34	0.00011	1.480327	BYSL;CD2BP2;CLNS1A;CLP1;CNOT7;DCAF13;DDX3X;DICER1;DIS3;EIF2A;EIF3D;EIF6;ERAL1;ERI1;FCF1;FRG1;G
turquoise	BP	GO:0051329	interphase of mitotic cell cycle	17735	408	3682	122	89.9	0.00011	1.357063	ACTR1A;AKT1;ANAPC10;APEX1;ARPP19;AZI1;BACH1;BCAT1;BRCA2;CAMK2D;CCNA2;CCNE2;CCNG1;CCNH;
turquoise	BP	GO:0033036	macromolecule localization	17735	1852	3682	471	408.07	0.00011	1.154214	ABCA1;ABCA5;ABCA7;ABCD1;ACAP1;ACD;ACSL4;ACSL6;ACTN4;ACVR1C;ADORA1;AGAP2;AGAP3;AIM2;AKAP
turquoise	BP	GO:0006796	phosphate-containing compound metabolic ...	17735	2466	3682	614	543.36	0.00011	1.130006	ABCA1;ABCD1;ABCD3;ABI1;ABI2;ACADVL;ACAP1;ACAP2;ACSL6;ACVR1C;ACVR2A;ACVRL1;ADA;ADAM10;AD
turquoise	BP	GO:0034654	nucleobase-containing compound biosynthe...	17735	3583	3682	870	789.49	0.00011	1.101977	ABCA1;ACTL6A;ACVR2A;ACVRL1;ADA;ADRM1;ADSSL1;AEBP1;AEBP2;AFF4;AGER;AHR;AIM2;AKAP5;AKT1;AL
turquoise	CC	GO:0005681	spliceosomal complex	17735	147	3682	50	30.57	0.00013	1.63559	API5;BCAS2;CDC40;DDX41;DGCR14;FRG1;GEMIN2;GEMIN6;IVNS1ABP;KIAA1967;LSM3;PHF5A;PLRG1;PPIL3;
turquoise	BP	GO:0051325	interphase	17735	414	3682	123	91.22	0.00013	1.348389	ACTR1A;AKT1;ANAPC10;APEX1;ARPP19;AZI1;BACH1;BCAT1;BRCA2;CAMK2D;CCNA2;CCNE2;CCNG1;CCNH;
turquoise	BP	GO:0045786	negative regulation of cell cycle	17735	453	3682	133	99.82	0.00013	1.332398	AKT1;ANAPC10;APC;BRCC3;BRIP1;CAB39;CAB39L;CASC5;CASP3;CCNA2;CCNE2;CCNG1;CCNG2;CDC25C;CDC
turquoise	BP	GO:0031398	positive regulation of protein ubiquitin...	17735	133	3682	48	29.31	0.00014	1.637666	AIMP2;ANAPC10;ANGPT1;BMI1;CDC26;CDC27;CDK1;DNAJB2;FBXO4;FBXO5;FBXW7;HSPBP1;LRRK2;MALT1;
turquoise	BP	GO:0016197	endosomal transport	17735	140	3682	50	30.85	0.00014	1.620746	ABCA1;AKTIP;CHMP2A;CHMP2B;DPY30;EEA1;EPS15;FAM109B;GCC2;HGS;HMGXB4;HOOK1;HOOK2;KIAA10
turquoise	CC	GO:0005768	endosome	17735	546	3682	149	113.55	0.00014	1.312197	ABCA5;ABCA7;AP1AR;APPL1;ARFGEF2;ARL8B;ATG9A;ATP11B;ATP7A;B2M;BLOC1S2;CALCRL;CCDC115;CD16
turquoise	CC	GO:0000159	protein phosphatase type 2A complex	17735	20	3682	12	4.16	0.00015	2.884615	CYCS;PPP2CB;PPP2R1A;PPP2R2A;PPP2R3A;PPP2R4;PPP2R5A;PPP2R5B;PPP2R5D;PPP2R5E;STRN3;STRN4
turquoise	BP	GO:0031575	mitotic cell cycle G1/S transition check...	17735	82	3682	33	18.07	0.00015	1.826231	CDK2;CDKN1B;DGKZ;DLG1;FBXO31;MDM2;MUC1;NBN;PSMA1;PSMA2;PSMA3;PSMA4;PSMA6;PSMA8;PSM
turquoise	MF	GO:0019783	small conjugating protein-specific prote...	17735	52	3682	23	11.04	0.00016	2.083333	BAP1;BRCC3;MYSM1;OTUD5;SENP1;SENP6;UCHL5;USP1;USP12;USP14;USP15;USP16;USP19;USP21;USP25;
turquoise	BP	GO:0000075	cell cycle checkpoint	17735	243	3682	78	53.54	0.00017	1.456855	AKT1;ANAPC10;APC;BRCC3;BRIP1;CASC5;CCNA2;CCNE2;CCNG1;CCNG2;CDC25C;CDC26;CDC27;CDC7;CDK1;
turquoise	BP	GO:0051351	positive regulation of ligase activity	17735	86	3682	34	18.95	0.00018	1.794195	ANAPC10;BMI1;CDC26;CDC27;CDK1;FBXO5;GCLM;MID1IP1;PSMA1;PSMA2;PSMA3;PSMA4;PSMA6;PSMA8;
turquoise	BP	GO:0071779	G1/S transition checkpoint	17735	86	3682	34	18.95	0.00018	1.794195	AKT1;CDK2;CDKN1B;DGKZ;DLG1;FBXO31;MDM2;MUC1;NBN;PSMA1;PSMA2;PSMA3;PSMA4;PSMA6;PSMA8
turquoise	BP	GO:0071156	regulation of cell cycle arrest	17735	266	3682	84	58.61	0.00018	1.433203	AKT1;ANAPC10;APC;BRCC3;BRIP1;CASC5;CCNA2;CCNE2;CCNG1;CCNG2;CDC25C;CDC26;CDC27;CDC7;CDK1;
turquoise	BP	GO:0034660	ncRNA metabolic process	17735	285	3682	89	62.8	0.00018	1.417197	AIMP1;AIMP2;ALKBH8;CLNS1A;CLP1;DCAF13;DGCR8;DICER1;DIS3;EEF1E1;ELAC2;ERI1;FARS2;FARSA;FCF1;F
turquoise	BP	GO:0031571	mitotic cell cycle G1/S transition DNA d...	17735	73	3682	30	16.09	0.00019	1.864512	CDK2;CDKN1B;DGKZ;FBXO31;MDM2;MUC1;PSMA1;PSMA2;PSMA3;PSMA4;PSMA6;PSMA8;PSMB1;PSMB3;
turquoise	BP	GO:0000279	M phase	17735	528	3682	151	116.34	0.00019	1.29792	ANAPC10;ANLN;APC;ARHGEF2;ARPP19;ASPM;AURKC;BAG6;BORA;BRCA2;CASC5;CCNA2;CCNG1;CCNG2;CD
turquoise	BP	GO:0007093	mitotic cell cycle checkpoint	17735	138	3682	49	30.41	2.00E-04	1.611312	ANAPC10;APC;CCNA2;CCNG1;CDC26;CDC27;CDK1;CDK2;CDKN1B;CHFR;DGKZ;DLG1;FBXO31;MAD2L1;MDM
turquoise	BP	GO:0051340	regulation of ligase activity	17735	100	3682	38	22.03	0.00021	1.724921	ANAPC10;BMI1;CDC26;CDC27;CDK1;CDK2;FBXO5;FEM1A;GCLM;MAD2L1;MID1IP1;PSMA1;PSMA2;PSMA3;
turquoise	MF	GO:0016301	kinase activity	17735	762	3682	202	161.83	0.00021	1.248223	ACVR1C;ACVR2A;ACVRL1;ADCK1;ADCK2;ADCK3;ADRBK1;AK3;AKD1;AKT1;ANKK1;ARAF;AURKC;BCR;BMPR1
turquoise	BP	GO:0051301	cell division	17735	474	3682	137	104.44	0.00022	1.311758	ANAPC10;ANLN;APC;ARHGEF2;ARL8B;ARPP19;ASPM;AURKC;BMI1;BORA;BRCA2;CASC5;CCNA2;CCND3;CCN
turquoise	BP	GO:2000045	regulation of G1/S transition of mitotic...	17735	104	3682	39	22.92	0.00024	1.701571	CDC73;CDK2;CDKN1B;DDX3X;DGKZ;DLG1;E2F7;FBXO31;MDM2;MUC1;NBN;PKD2;PSMA1;PSMA2;PSMA3;PS
turquoise	BP	GO:1901575	organic substance catabolic process	17735	1842	3682	465	405.87	0.00025	1.145687	ABCD1;ABCD2;ABCD3;ABCE1;ABHD10;ACADM;ACADSB;ACADVL;ACAP1;ACAP2;ACAT1;ACOT7;ACSF3;ADA;A
turquoise	BP	GO:0006487	protein N-linked glycosylation	17735	98	3682	37	21.59	0.00029	1.713756	ALG1;ALG10;ALG10B;ALG11;ALG13;ALG2;ALG3;ALG6;B4GALT3;B4GALT6;DDOST;DOLPP1;DPM1;EDEM3;GF
turquoise	BP	GO:0034130	toll-like receptor 1 signaling pathway	17735	68	3682	28	14.98	3.00E-04	1.869159	ATF1;ATF2;CDK1;CHUK;CREB1;IKBKG;JUN;MAP2K2;MAP2K4;MAP3K1;MAP3K7;MAPK8;MAPKAPK2;MAPKA
turquoise	BP	GO:0051439	regulation of ubiquitin-protein ligase a...	17735	78	3682	31	17.19	0.00031	1.803374	ANAPC10;CDC26;CDC27;CDK1;CDK2;FBXO5;MAD2L1;PSMA1;PSMA2;PSMA3;PSMA4;PSMA6;PSMA8;PSMB1
turquoise	CC	GO:0031300	intrinsic to organelle membrane	17735	218	3682	67	45.34	0.00033	1.477724	ABCB10;ABCB6;ABCD1;ASPH;B3GNT1;BCAP31;BNIP1;CCDC90A;CHST2;CLN3;CSGALNACT1;CSGALNACT2;DE
turquoise	MF	GO:0004672	protein kinase activity	17735	595	3682	161	126.36	0.00033	1.274137	ACVR1C;ACVR2A;ACVRL1;ADCK1;ADCK2;ADCK3;ADRBK1;AKT1;ANKK1;ARAF;AURKC;BCR;BMPR1A;BMPR2;C
turquoise	CC	GO:0005759	mitochondrial matrix	17735	286	3682	84	59.48	0.00034	1.412239	ACADM;ACADSB;ACADVL;ACAT1;ACSS1;AK3;ALDH4A1;ARG2;ATAD3A;ATP5C1;ATP5E;ATP5F1;AUH;BCAT2;B
turquoise	BP	GO:1901362	organic cyclic compound biosynthetic pro...	17735	3752	3682	902	826.72	0.00034	1.091059	AANAT;ABCA1;ABCB6;ACBD3;ACTL6A;ACVR2A;ACVRL1;ADA;ADRM1;ADSSL1;AEBP1;AEBP2;AFF4;AGER;AHR
turquoise	BP	GO:0018279	protein N-linked glycosylation via aspar...	17735	92	3682	35	20.27	0.00035	1.72669	ALG1;ALG10;ALG10B;ALG11;ALG13;ALG2;ALG3;ALG6;B4GALT3;B4GALT6;DDOST;DOLPP1;DPM1;EDEM3;GF
turquoise	BP	GO:0010565	regulation of cellular ketone metabolic ...	17735	170	3682	57	37.46	0.00035	1.521623	ABCD2;ACADM;ACADVL;AKT1;ANXA1;ARPP19;AZIN1;CAB39;CAB39L;CNR1;COMT;DKK3;DLAT;FGFR4;GNMT
turquoise	BP	GO:0034134	toll-like receptor 2 signaling pathway	17735	72	3682	29	15.86	0.00036	1.828499	ATF1;ATF2;CDK1;CHUK;CREB1;F2RL1;IKBKG;JUN;MAP2K2;MAP2K4;MAP3K1;MAP3K7;MAPK8;MAPKAPK2;
turquoise	BP	GO:0000375	RNA splicing, via transesterification re...	17735	215	3682	69	47.37	0.00038	1.456618	BCAS2;BUD13;CD2BP2;CDC40;CLNS1A;CLP1;CPSF1;DCPS;DDX41;DGCR14;FRG1;GEMIN2;GEMIN6;GTF2F2;H
turquoise	MF	GO:0019003	GDP binding	17735	45	3682	20	9.56	4.00E-04	2.09205	ARL2;ARL8B;KRAS;PRPS2;RAB10;RAB11B;RAB12;RAB14;RAB18;RAB22A;RAB27B;RAB28;RAB2A;RAB4A;RAB
turquoise	BP	GO:0000079	regulation of cyclin-dependent protein k...	17735	86	3682	33	18.95	0.00043	1.741425	AKT1;APC;CASP3;CCNA2;CCNC;CCND3;CCNE2;CCNG1;CCNH;CCNL1;CCNT2;CCNY;CCNYL1;CDC25C;CDC37;C
turquoise	CC	GO:0005770	late endosome	17735	150	3682	49	31.19	0.00044	1.571016	ABCA5;AP1AR;ARL8B;ATG9A;ATP7A;CD63;CHMP1B;CHMP2A;CHMP2B;CLN3;CST3;CTNS;CYBASC3;DERL1;H
turquoise	BP	GO:0018196	peptidyl-asparagine modification	17735	93	3682	35	20.49	0.00045	1.70815	ALG1;ALG10;ALG10B;ALG11;ALG13;ALG2;ALG3;ALG6;B4GALT3;B4GALT6;DDOST;DOLPP1;DPM1;EDEM3;GF
turquoise	BP	GO:0031145	anaphase-promoting complex-dependent pro...	17735	83	3682	32	18.29	0.00047	1.74959	ANAPC10;CDC26;CDC27;CDK1;CDK2;FBXO5;MAD2L1;PSMA1;PSMA2;PSMA3;PSMA4;PSMA6;PSMA8;PSMB1
turquoise	BP	GO:0042787	protein ubiquitination involved in ubiqu...	17735	60	3682	25	13.22	5.00E-04	1.891074	FBXO45;FBXW7;HACE1;HECTD2;HECTD3;HECW2;HERC4;ITCH;LNX1;MDM2;NEDD4;PARK2;PTEN;RBBP6;RCH
turquoise	MF	GO:0019900	kinase binding	17735	397	3682	112	84.31	0.00052	1.328431	ABI2;ACVRL1;ADAM10;AKAP7;AKT1;APC;APPL1;ARRB1;ATF2;BORA;CAB39;CCDC88A;CCNA2;CCNC;CCND3;C
turquoise	MF	GO:0016773	phosphotransferase activity, alcohol gro...	17735	706	3682	186	149.93	0.00052	1.240579	ACVR1C;ACVR2A;ACVRL1;ADCK1;ADCK2;ADCK3;ADRBK1;AKT1;ANKK1;ARAF;AURKC;BCR;BMPR1A;BMPR2;C
turquoise	BP	GO:0006886	intracellular protein transport	17735	702	3682	191	154.68	0.00053	1.234807	ACD;ADORA1;AGAP3;AKAP5;AKT1;AP1B1;AP1S2;AP2A2;AP3M1;AP3S2;APPBP2;ARFIP1;ARHGEF2;ARL6IP1;
turquoise	BP	GO:0031396	regulation of protein ubiquitination	17735	180	3682	59	39.66	0.00054	1.487645	AIMP2;ANAPC10;ANGPT1;ARRB1;ATG5;BMI1;CDC26;CDC27;CDK1;CDK2;DAXX;DNAJB2;FBXO4;FBXO5;FBX
turquoise	CC	GO:0031967	organelle envelope	17735	825	3682	210	171.57	0.00054	1.22399	AAAS;ABCB10;ABCB6;ABCD3;ACADVL;ACAT1;ACSL4;ACSL6;AGPAT5;AIFM3;AKIRIN1;ANKLE1;ANXA1;APEH;
turquoise	BP	GO:0016482	cytoplasmic transport	17735	719	3682	195	158.43	0.00056	1.230827	AGAP3;AKT1;AKTIP;ALKBH5;AP3M1;ARFGEF2;ASPH;BET1;BLZF1;BMPR1A;BUD13;C12orf52;CAMK1;CAMK2
turquoise	MF	GO:0004843	ubiquitin-specific protease activity	17735	46	3682	20	9.77	0.00057	2.047083	BAP1;BRCC3;MYSM1;OTUD5;UCHL5;USP1;USP12;USP14;USP15;USP16;USP19;USP21;USP25;USP37;USP44;
turquoise	BP	GO:0051436	negative regulation of ubiquitin-protein...	17735	67	3682	27	14.76	0.00057	1.829268	ANAPC10;CDC26;CDC27;FBXO5;MAD2L1;PSMA1;PSMA2;PSMA3;PSMA4;PSMA6;PSMB1;PSMB3;PSMC1;PS
turquoise	BP	GO:0000084	S phase of mitotic cell cycle	17735	140	3682	48	30.85	0.00057	1.555916	APEX1;BRCA2;CCNH;CDC73;CDK2;CDK7;CDKN1B;CDT1;CHMP1A;CKS1B;CRLF3;CUL4B;DDX11;DNA2;LSM11;
turquoise	CC	GO:0005840	ribosome	17735	206	3682	63	42.84	0.00057	1.470588	APEX1;DDX3X;DNAJC21;FMR1;FXR2;HSPA14;MRPL1;MRPL13;MRPL15;MRPL18;MRPL19;MRPL2;MRPL22;M
turquoise	CC	GO:0005730	nucleolus	17735	622	3682	163	129.35	0.00057	1.260147	ABHD14B;ACAT2;AFF4;AGAP2;AKAP11;AKTIP;ALS2CR8;ANG;AP1AR;APEX1;APTX;ARFGAP2;ARID5A;ARL4A;A

turquoise	BP	GO:0006412	translation	17735	464	3682	132	102.24	0.00059	1.29108	AIMP1;AIMP2;AKT1;ANG;CD28;COPS5;CPEB2;CPEB3;DAPK3;DDOST;DDX3X;DHPS;EEF1A1;EEF1B2;EEF1E1;EI
turquoise	MF	GO:0004659	prenyltransferase activity	17735	14	3682	9	2.97	0.00061	3.030303	CHM;CHML;COQ2;FDFT1;FDPS;FNTA;GGPS1;PGGT1B;PTAR1
turquoise	MF	GO:0043021	ribonucleoprotein complex binding	17735	56	3682	23	11.89	0.00061	1.934399	BAG6;CD2BP2;DDX3X;EIF2A;EIF5A2;EIF6;ERAL1;ERI1;GUF1;IGHMBP2;ITCH;MTIF3;NAA16;PPP1CA;PRMT1;PI
turquoise	BP	GO:0032268	regulation of cellular protein metabolic...	17735	1264	3682	325	278.51	0.00065	1.166924	ACADVL;ACVR2A;ACVRL1;ADORA1;AIDA;AIMP2;AKT1;AKTIP;ANAPC10;ANG;ANGPT1;APC;ARAF;ARFGAP1;AI
turquoise	CC	GO:0016592	mediator complex	17735	35	3682	16	7.28	0.00081	2.197802	CCNC;CDK8;MED12L;MED13;MED14;MED15;MED16;MED21;MED22;MED23;MED26;MED28;MED4;MED7;N
turquoise	BP	GO:0051443	positive regulation of ubiquitin-protein...	17735	82	3682	31	18.07	0.00085	1.715551	ANAPC10;BMI1;CDC26;CDC27;CDK1;FBXO5;PSMA1;PSMA2;PSMA3;PSMA4;PSMA6;PSMA8;PSMB1;PSMB3;F
turquoise	BP	GO:2000602	regulation of interphase of mitotic cell...	17735	157	3682	52	34.59	0.00086	1.503325	APEX1;BRCA2;CCNA2;CCNG1;CDC73;CDK1;CDK2;CDKN1B;CDT1;CHMP1A;CRLF3;CUL4B;DDX3X;DGKZ;DLG1;I
turquoise	BP	GO:0034470	ncRNA processing	17735	198	3682	63	43.63	0.00086	1.443961	ALKBH8;CLP1;DCAF13;DGCR8;DICER1;DIS3;ELAC2;ERI1;FARS2;FCF1;FDXACB1;FRG1;FTSJ1;HSD17B10;INTS12
turquoise	BP	GO:0051438	regulation of ubiquitin-protein ligase a...	17735	96	3682	35	21.15	0.00088	1.654846	ANAPC10;BMI1;CDC26;CDC27;CDK1;CDK2;FBXO5;FEM1A;MAD2L1;PSMA1;PSMA2;PSMA3;PSMA4;PSMA6;P
turquoise	BP	GO:0051437	positive regulation of ubiquitin-protein...	17735	72	3682	28	15.86	0.00089	1.765448	ANAPC10;CDC26;CDC27;CDK1;FBXO5;PSMA1;PSMA2;PSMA3;PSMA4;PSMA6;PSMA8;PSMB1;PSMB3;PSMC1
turquoise	CC	GO:0019866	organelle inner membrane	17735	377	3682	104	78.4	0.00089	1.326531	ABCB10;ABCD3;ACADVL;ACAT1;AIFM3;APEX2;ATAD3A;ATP11B;ATP5C1;ATP5E;ATP5F1;ATP5I;ATP5L;ATP5O
turquoise	CC	GO:0005778	peroxisomal membrane	17735	55	3682	22	11.44	9.00E-04	1.923077	ABCD1;ABCD2;ABCD3;ACBD5;ACSL4;ACSL6;AGPS;ATAD1;FAR1;FAR2;IMPDH2;MAP2K2;PEX1;PEX11A;PEX11
turquoise	CC	GO:0031903	microbody membrane	17735	55	3682	22	11.44	9.00E-04	1.923077	ABCD1;ABCD2;ABCD3;ACBD5;ACSL4;ACSL6;AGPS;ATAD1;FAR1;FAR2;IMPDH2;MAP2K2;PEX1;PEX11A;PEX11
turquoise	BP	GO:0000377	RNA splicing, via transesterification re...	17735	210	3682	66	46.27	0.00094	1.42641	BUD13;CD2BP2;CDC40;CLNS1A;CLP1;CPSF1;DCPS;DDX41;DGCR14;FRG1;GEMIN2;GEMIN6;GTF2F2;HNRNPH
turquoise	BP	GO:0000398	mRNA splicing, via spliceosome	17735	210	3682	66	46.27	0.00094	1.42641	BUD13;CD2BP2;CDC40;CLNS1A;CLP1;CPSF1;DCPS;DDX41;DGCR14;FRG1;GEMIN2;GEMIN6;GTF2F2;HNRNPH
turquoise	BP	GO:0072594	establishment of protein localization to...	17735	210	3682	66	46.27	0.00094	1.42641	AP3M1;CHCHD4;DDOST;GCC2;GOLGA4;GRPEL2;LRWD1;MFF;MTX2;MTX3;NAGPA;NEDD4;PEX1;PEX12;PEX1
turquoise	MF	GO:0004712	protein serine/threonine/tyrosine kinase...	17735	35	3682	16	7.43	0.00103	2.153432	AURKC;CLK1;CLK3;CLK4;DYRK1A;DYRK1B;DYRK2;LRRK2;MAP2K2;MAP3K1;MAP3K7;MAPKAPK3;TESK1;TESK
turquoise	BP	GO:0002755	MyD88-dependent toll-like receptor signa...	17735	76	3682	29	16.75	0.00103	1.731343	ATF1;ATF2;CDK1;CHUK;CREB1;IKBKG;JUN;MAP2K2;MAP2K4;MAP3K1;MAP3K7;MAPK8;MAPKAPK2;MAPKAF
turquoise	CC	GO:0031975	envelope	17735	839	3682	211	174.48	0.00103	1.209308	AAAS;ABCB10;ABCB6;ABCD3;ACADVL;ACAT1;ACSL4;ACSL6;AGPAT5;AIFM3;AKIRIN1;ANKLE1;ANXA1;APEH;A
turquoise	BP	GO:0006888	ER to Golgi vesicle-mediated transport	17735	56	3682	23	12.34	0.00104	1.863857	BET1;COG3;GOPC;INSIG1;LMAN1;RAB2A;RNF139;SAR1A;SAR1B;SCFD1;SEC13;SEC23A;SEC24A;SPAST;STX5;
turquoise	BP	GO:0000245	spliceosomal complex assembly	17735	37	3682	17	8.15	0.00105	2.08589	GEMIN2;GEMIN6;MBNL1;PRPF6;PSIP1;PTBP2;RBM5;SCAF11;SF3A1;SF3A2;SFSWAP;SLU7;SNRPD1;SNRPE;SN
turquoise	BP	GO:0006977	DNA damage response, signal transduction...	17735	66	3682	26	14.54	0.00107	1.788171	CDK2;CDKN1B;MDM2;MUC1;PSMA1;PSMA2;PSMA3;PSMA4;PSMA6;PSMA8;PSMB1;PSMB3;PSMC1;PSMC2;
turquoise	BP	GO:0072413	signal transduction involved in mitotic ...	17735	66	3682	26	14.54	0.00107	1.788171	CDK2;CDKN1B;MDM2;MUC1;PSMA1;PSMA2;PSMA3;PSMA4;PSMA6;PSMA8;PSMB1;PSMB3;PSMC1;PSMC2;
turquoise	BP	GO:0072431	signal transduction involved in mitotic ...	17735	66	3682	26	14.54	0.00107	1.788171	CDK2;CDKN1B;MDM2;MUC1;PSMA1;PSMA2;PSMA3;PSMA4;PSMA6;PSMA8;PSMB1;PSMB3;PSMC1;PSMC2;
turquoise	BP	GO:0072474	signal transduction involved in mitotic ...	17735	66	3682	26	14.54	0.00107	1.788171	CDK2;CDKN1B;MDM2;MUC1;PSMA1;PSMA2;PSMA3;PSMA4;PSMA6;PSMA8;PSMB1;PSMB3;PSMC1;PSMC2;
turquoise	MF	GO:0043168	anion binding	17735	2346	3682	555	498.22	0.0011	1.113966	AACS;AAMP;ABCA1;ABCA10;ABCA3;ABCA5;ABCA7;ABCB10;ABCB6;ABCC2;ABCD1;ABCD2;ABCD3;ABCE1;AB(
turquoise	BP	GO:0034138	toll-like receptor 3 signaling pathway	17735	73	3682	28	16.09	0.00114	1.740211	ATF1;ATF2;CDK1;CHUK;CREB1;F2RL1;IKBKG;JUN;MAP2K2;MAP2K4;MAP3K7;MAPK8;MAPKAPK2;MAPKAPK3
turquoise	BP	GO:0007017	microtubule-based process	17735	423	3682	120	93.2	0.00114	1.287554	AP3M1;AP3S2;APC;ARHGEF2;ARL2;AURKC;BLOC1S2;BORA;BRCA2;CASC5;CCDC103;CCDC39;CCDC88A;CCDC
turquoise	MF	GO:0000287	magnesium ion binding	17735	180	3682	56	38.23	0.00118	1.464818	AASDHPPT;ADSSL1;ATP10D;ATP11B;ATP11C;ATP8A1;ATP8A2;ATP8B4;COMT;DYRK2;ENDOV;ERN1;FARS2;FD
turquoise	BP	GO:0051320	S phase	17735	148	3682	49	32.61	0.0012	1.502607	APEX1;BRCA2;CCNH;CDC7;CDC73;CDK2;CDK7;CDKN1B;CDT1;CHMP1A;CKS1B;CRLF3;CUL4B;DDX11;DNA2;LS
turquoise	CC	GO:0000776	kinetochore	17735	103	3682	35	21.42	0.00126	1.633987	APC;CASC5;CENPE;CENPK;CENPW;CSNK1A1;DCTN2;DYNLT3;ITGB3BP;KAT2B;KAT8;KIF22;MAD2L1;MLF1IP;N
turquoise	CC	GO:0031301	integral to organelle membrane	17735	196	3682	59	40.76	0.00127	1.447498	ABCB10;ABCB6;ABCD1;ASPH;B3GNT1;BCAP31;BNIP1;CCDC90A;CLN3;CSGALNACT1;CSGALNACT2;DERL1;DH
turquoise	BP	GO:0034613	cellular protein localization	17735	956	3682	249	210.65	0.00132	1.182056	ACD;ADORA1;AGAP3;AKAP5;AKT1;ANGPT1;ANKRD13C;AP1AR;AP1B1;AP1S2;AP2A2;AP3M1;AP3S2;APC;APF
turquoise	CC	GO:0005637	nuclear inner membrane	17735	30	3682	14	6.24	0.00134	2.24359	ATP11B;CBX3;ERN1;FAM169A;LEMD3;LMNB2;MATR3;NRM;PSEN2;RNF13;SIRT1;TMEM201;TMPO;UNC5(
turquoise	CC	GO:0031966	mitochondrial membrane	17735	496	3682	131	103.15	0.00137	1.269995	ABCB10;ABCB6;ABCD3;ACADVL;ACAT1;ACSL4;ACSL6;AGPAT5;AIFM3;ANXA1;APEX2;ATAD3A;ATP5C1;ATP5E
turquoise	BP	GO:0035666	TRIF-dependent toll-like receptor signal...	17735	67	3682	26	14.76	0.00138	1.761518	ATF1;ATF2;CDK1;CHUK;CREB1;IKBKG;JUN;MAP2K2;MAP2K4;MAP3K7;MAPK8;MAPKAPK2;MAPKAPK3;MEF2
turquoise	BP	GO:0072401	signal transduction involved in DNA inte...	17735	67	3682	26	14.76	0.00138	1.761518	CDK2;CDKN1B;MDM2;MUC1;PSMA1;PSMA2;PSMA3;PSMA4;PSMA6;PSMA8;PSMB1;PSMB3;PSMC1;PSMC2;
turquoise	BP	GO:0072404	signal transduction involved in G1/S tra...	17735	67	3682	26	14.76	0.00138	1.761518	CDK2;CDKN1B;MDM2;MUC1;PSMA1;PSMA2;PSMA3;PSMA4;PSMA6;PSMA8;PSMB1;PSMB3;PSMC1;PSMC2;
turquoise	BP	GO:0072422	signal transduction involved in DNA dama...	17735	67	3682	26	14.76	0.00138	1.761518	CDK2;CDKN1B;MDM2;MUC1;PSMA1;PSMA2;PSMA3;PSMA4;PSMA6;PSMA8;PSMB1;PSMB3;PSMC1;PSMC2;
turquoise	BP	GO:0008063	Toll signaling pathway	17735	81	3682	30	17.85	0.00149	1.680672	ATF1;ATF2;CDK1;CHUK;CREB1;IKBKG;JUN;MAP2K2;MAP2K4;MAP3K1;MAP3K7;MAPK8;MAPKAPK2;MAPKAF
turquoise	BP	GO:0016568	chromatin modification	17735	434	3682	122	95.63	0.0015	1.27575	ACTL6A;AEBP2;ARID2;ARID5A;ARRB1;ASF1A;ATXN7L3;AURKC;BAG6;BAP1;BAZ1A;BMI1;BRCA2;BRCC3;BRD9
turquoise	BP	GO:0032873	negative regulation of stress-activated ...	17735	26	3682	13	5.73	0.00156	2.268761	AIDA;AKT1;DUSP19;F2RL1;GPS2;GSTP1;HDAC3;IGBP1;ITCH;MAPK8IP1;MEN1;ZMYND11;ZNF675
turquoise	BP	GO:0070303	negative regulation of stress-activated ...	17735	26	3682	13	5.73	0.00156	2.268761	AIDA;AKT1;DUSP19;F2RL1;GPS2;GSTP1;HDAC3;IGBP1;ITCH;MAPK8IP1;MEN1;ZMYND11;ZNF675
turquoise	BP	GO:0071702	organic substance transport	17735	1950	3682	481	429.67	0.00156	1.119464	AAAS;AACS;ABCA1;ABCA5;ABCA7;ABCB6;ABCC2;ABCD1;ACAP1;ACD;ACSL4;ACSL6;ACTN4;ACVR1C;ADA;ADC
turquoise	BP	GO:0009056	catabolic process	17735	1989	3682	490	438.26	0.00157	1.118058	ABCD1;ABCD2;ABCD3;ABCE1;ABHD10;ACADM;ACADSB;ACADVL;ACAP1;ACAP2;ACAT1;ACOT7;ACSF3;ADA;A
turquoise	BP	GO:0006623	protein targeting to vacuole	17735	10	3682	7	2.2	0.00159	3.181818	AP3M1;GCC2;NAGPA;NEDD4;SNX16;VAMP7;ZFYVE16
turquoise	BP	GO:0006991	response to sterol depletion	17735	10	3682	7	2.2	0.00159	3.181818	EIF2A;FBXW7;INSIG1;INSIG2;LMNA;SCAP;SREBF1
turquoise	BP	GO:0072665	protein localization to vacuole	17735	10	3682	7	2.2	0.00159	3.181818	AP3M1;GCC2;NAGPA;NEDD4;SNX16;VAMP7;ZFYVE16
turquoise	BP	GO:0072666	establishment of protein localization to...	17735	10	3682	7	2.2	0.00159	3.181818	AP3M1;GCC2;NAGPA;NEDD4;SNX16;VAMP7;ZFYVE16
turquoise	BP	GO:0044106	cellular amine metabolic process	17735	191	3682	60	42.09	0.00159	1.425517	AANAT;ADC;ADI1;AMD1;APIP;ATP7A;AZIN1;CCBL2;CDS1;CEPT1;CLN3;COMT;DHPS;FABP5;HPRT1;KMO;LCAT
turquoise	MF	GO:0017111	nucleoside-triphosphatase activity	17735	756	3682	194	160.55	0.0016	1.208346	ABCA1;ABCA10;ABCA3;ABCA5;ABCA7;ABCB10;ABCB6;ABCC2;ABCD1;ABCD2;ABCD3;ABCE1;ABCF3;AGAP3;A
turquoise	MF	GO:0016462	pyrophosphatase activity	17735	791	3682	202	167.99	0.00165	1.202453	ABCA1;ABCA10;ABCA3;ABCA5;ABCA7;ABCB10;ABCB6;ABCC2;ABCD1;ABCD2;ABCD3;ABCE1;ABCF3;AGAP3;A
turquoise	BP	GO:0070727	cellular macromolecule localization	17735	960	3682	249	211.53	0.00168	1.177138	ACD;ADORA1;AGAP3;AKAP5;AKT1;ANGPT1;ANKRD13C;AP1AR;AP1B1;AP1S2;AP2A2;AP3M1;AP3S2;APC;APF
turquoise	BP	GO:0072395	signal transduction involved in cell cyc...	17735	68	3682	26	14.98	0.00178	1.735648	CDK2;CDKN1B;MDM2;MUC1;PSMA1;PSMA2;PSMA3;PSMA4;PSMA6;PSMA8;PSMB1;PSMB3;PSMC1;PSMC2;
turquoise	BP	GO:0009108	coenzyme biosynthetic process	17735	103	3682	36	22.7	0.00179	1.585903	ACSL4;ACSL6;ACSS1;COASY;COQ2;DCAKD;DHFRL1;DLAT;ELOVL4;GCH1;KMO;MLYCD;MOCS2;MOCS3;MTHFD
turquoise	BP	GO:0009308	amine metabolic process	17735	211	3682	65	46.49	0.0018	1.39815	AANAT;ADC;ADI1;AMD1;APIP;ATP7A;AZIN1;CCBL2;CDS1;CEPT1;CLN3;COMT;DHPS;FABP5;GNMT;HPRT1;KM
turquoise	BP	GO:0008654	phospholipid biosynthetic process	17735	215	3682	66	47.37	0.00184	1.393287	ACSL6;AGPAT5;CD81;CDIPT;CDS1;CEPT1;CRLS1;DGKE;DOLPP1;DPM1;EPT1;ETNK1;FABP5;FAR1;FDPS;GGPS1
turquoise	MF	GO:0016817	hydrolase activity, acting on acid anhyd...	17735	797	3682	203	169.26	0.00184	1.199338	ABCA1;ABCA10;ABCA3;ABCA5;ABCA7;ABCB10;ABCB6;ABCC2;ABCD1;ABCD2;ABCD3;ABCE1;ABCF3;AGAP3;A
turquoise	MF	GO:0016818	hydrolase activity, acting on acid anhyd...	17735	793	3682	202	168.41	0.00188	1.199454	ABCA1;ABCA10;ABCA3;ABCA5;ABCA7;ABCB10;ABCB6;ABCC2;ABCD1;ABCD2;ABCD3;ABCE1;ABCF3;AGAP3;A
turquoise	BP	GO:0042180	cellular ketone metabolic process	17735	223	3682	68	49.14	0.00191	1.383801	ABCD2;ACADM;ACADVL;AKT1;ANXA1;ARPP19;AZIN1;CAB39;CAB39L;CACNA1H;CCBL2;CNR1;COMT;COQ2;D
turquoise	MF	GO:0019901	protein kinase binding	17735	357	3682	99	75.82	0.00193	1.305724	ACVRL1;ADAM10;AKAP7;AKT1;APC;APPL1;ARRB1;ATF2;BORA;CCDC88A;CCNA2;CCNC;CCND3;CCNE2;CCNH;
turquoise	BP	GO:0061136	regulation of proteasomal protein catabo...	17735	65	3682	25	14.32	0.00196	1.74581	AKT1;BAG6;DNAJB2;FBXO5;GIPC1;HSPBP1;LRRK2;MAD2L1;MDM2;PSMD10;PSMD14;PTEN;RAD23A;RCHY1;
turquoise	BP	GO:0031570	DNA integrity checkpoint	17735	140	3682	46	30.85	0.00198	1.491086	BRCC3;BRIP1;CCNA2;CCNG1;CDK1;CDK2;CDKN1B;CDT1;DGKZ;DNA2;FAM175A;FBXO31;MAPKAPK2;MDM2;I
turquoise	BP	GO:0051352	negative regulation of ligase activity	17735	72	3682	27	15.86	0.00204	1.702396	ANAPC10;CDC26;CDC27;FBXO5;MAD2L1;PSMA1;PSMA2;PSMA3;PSMA4;PSMA6;PSMB1;PSMB3;PSMC1;PSN
turquoise	BP	GO:0051444	negative regulation of ubiquitin-protein...	17735	72	3682	27	15.86	0.00204	1.702396	ANAPC10;CDC26;CDC27;FBXO5;MAD2L1;PSMA1;PSMA2;PSMA3;PSMA4;PSMA6;PSMB1;PSMB3;PSMC1;PSN
turquoise	BP	GO:0010564	regulation of cell cycle process	17735	434	3682	121	95.63	0.00215	1.265293	AKT1;ANAPC10;ANLN;APC;APEX1;AURKC;BORA;BRCA2;BRCC3;BRIP1;CASC5;CCNA2;CCNE2;CCNG1;CCNG2;C
turquoise	BP	GO:0046329	negative regulation of JNK cascade	17735	21	3682	11	4.63	0.00223	2.37581	AIDA;AKT1;DUSP19;F2RL1;GPS2;HDAC3;ITCH;MAPK8IP1;MEN1;ZMYND11;ZNF675
turquoise	BP	GO:0002756	MyD88-independent toll-like receptor sig...	17735	69	3682	26	15.2	0.00226	1.710526	ATF1;ATF2;CDK1;CHUK;CREB1;IKBKG;JUN;MAP2K2;MAP2K4;MAP3K7;MAPK8;MAPKAPK2;MAPKAPK3;MEF2
turquoise	MF	GO:0008353	RNA polymerase II carboxy-terminal domai...	17735	16	3682	9	3.4	0.00229	2.647059	CCNH;CDK1;CDK7;CDK8;CDK9;ERCC2;GTF2H1;GTF2H4;MNAT1

turquoise	MF	GO:0016780	phosphotransferase activity, for other s...	17735	16	3682	9	3.4	0.00229	2.647059	AASDHPPT;CDIPT;CDS1;CEPT1;CRLS1;EPT1;PIGF;SGMS1;SGMS2
turquoise	BP	GO:0051403	stress-activated MAPK cascade	17735	213	3682	65	46.93	0.00233	1.385042	AIDA;AKT1;ATF1;ATF2;CCM2;CD27;CDK1;COPS5;CREB1;DAB2IP;DAXX;DIXDC1;DUSP19;F2RL1;FCER1A;GAB1
turquoise	CC	GO:0016607	nuclear speck	17735	149	3682	46	30.99	0.00238	1.48435	ALKBH5;APEX1;ATPAF2;CCNL1;CD2BP2;CLK3;CSNK1A1;CXXC1;DDX3X;DYRK1A;EAF2;FAM76B;FRG1;FYTTD1;
turquoise	CC	GO:0016605	PML body	17735	73	3682	26	15.18	0.00241	1.71278	CDK9;CHFR;DAPK3;DAXX;EIF3E;HIRA;KLHL20;LRCH4;MORC3;NSMCE2;PIAS4;PTEN;RB1;RNF6;SIRT1;SKIL;SM
turquoise	BP	GO:0000077	DNA damage checkpoint	17735	134	3682	44	29.53	0.00248	1.49001	BRCC3;BRIP1;CCNA2;CCNG1;CDK1;CDK2;CDKN1B;DGKZ;FAM175A;FBXO31;MAPKAPK2;MDM2;MSH2;MUC1
turquoise	MF	GO:0031593	polyubiquitin binding	17735	28	3682	13	5.95	0.00255	2.184874	AGAP3;AGL;BAG6;BRCC3;DNAJB2;DZIP3;FAM175A;HDAC6;RAD23A;TAB2;ZFAND6;ZRANB1;ZRANB3
turquoise	BP	GO:0000216	M/G1 transition of mitotic cell cycle	17735	80	3682	29	17.63	0.00258	1.644923	CDC7;CDK2;CDT1;DBF4;GMNN;MCM5;ORC2;ORC3;ORC4;PSMA1;PSMA2;PSMA3;PSMA4;PSMA6;PSMA8;PS
turquoise	BP	GO:0071158	positive regulation of cell cycle arrest	17735	80	3682	29	17.63	0.00258	1.644923	CDK2;CDKN1B;CRLF3;MDM2;MUC1;PKD2;PSMA1;PSMA2;PSMA3;PSMA4;PSMA6;PSMA8;PSMB1;PSMB3;PS
turquoise	BP	GO:0032434	regulation of proteasomal ubiquitin-depe...	17735	56	3682	22	12.34	0.00262	1.78282	AKT1;BAG6;DNAJB2;FBXO5;GIPC1;HSPBP1;LRRK2;MAD2L1;MDM2;PSMD10;PTEN;RAD23A;RCHY1;SENP1;SC
turquoise	CC	GO:0044391	ribosomal subunit	17735	134	3682	42	27.87	0.00263	1.506997	DDX3X;FXR2;MRPL1;MRPL13;MRPL15;MRPL22;MRPL3;MRPL32;MRPL42;MRPS17;MRPS18B;MRPS18C;MRP
turquoise	BP	GO:0051297	centrosome organization	17735	63	3682	24	13.88	0.00277	1.729107	ARL2;BRCA2;CCP110;CDK1;CDK2;CEP135;CEP152;CEP250;CETN3;CHORDC1;CNTROB;HAUS1;HAUS3;HAUS6;
turquoise	MF	GO:0008565	protein transporter activity	17735	87	3682	30	18.48	0.00292	1.623377	AP1B1;AP1S2;AP2A2;AP3S2;AP4S1;CALCRL;CCT6B;COG3;IPO11;IPO13;IPO4;KPNA2;KPNA3;KPNA5;MCL1;RA
turquoise	BP	GO:0031023	microtubule organizing center organizati...	17735	67	3682	25	14.76	0.00318	1.693767	ARL2;BRCA2;CCP110;CDK1;CDK2;CEP135;CEP152;CEP250;CETN3;CHORDC1;CNTROB;GCC2;HAUS1;HAUS3;H
turquoise	CC	GO:0000793	condensed chromosome	17735	167	3682	50	34.73	0.00322	1.439678	ADD3;AURKC;CASC5;CBX3;CDK2;CENPE;CENPK;CENPW;CHMP1A;CSNK1A1;DYNLT3;HMGB1;HMGB2;ITGB3B
turquoise	CC	GO:0005740	mitochondrial envelope	17735	520	3682	134	108.14	0.00323	1.239134	ABCB10;ABCB6;ABCD3;ACADVL;ACAT1;ACSL4;ACSL6;AGPAT5;AIFM3;ANXA1;APEX2;ARL2;ATAD3A;ATP5C1;
turquoise	BP	GO:0002474	antigen processing and presentation of p...	17735	99	3682	34	21.81	0.00327	1.558918	B2M;BCAP31;CTSS;CYBA;HLA-A;IFI30;ITGAV;PSMA1;PSMA2;PSMA3;PSMA4;PSMA6;PSMA8;PSMB1;PSMB3;
turquoise	CC	GO:0033116	endoplasmic reticulum-Golgi intermediate...	17735	26	3682	12	5.41	0.00328	2.218115	BCAP31;ERGIC2;LMAN1;LMAN2;RAB2A;STX5;TMED10;TMED2;TMED5;TMED7;VMA21;VMP
turquoise	BP	GO:0007031	peroxisome organization	17735	34	3682	15	7.49	0.00334	2.00267	ABCD1;ABCD3;MFF;PEX1;PEX11A;PEX11G;PEX12;PEX13;PEX14;PEX2;PEX3;RAB8B;SCP2;TMEM135;ZFAND
turquoise	BP	GO:0031098	stress-activated protein kinase signalin...	17735	216	3682	65	47.59	0.00336	1.365833	AIDA;AKT1;ATF1;ATF2;CCM2;CD27;CDK1;COPS5;CREB1;DAB2IP;DAXX;DIXDC1;DUSP19;F2RL1;FCER1A;GAB1
turquoise	CC	GO:0022624	proteasome accessory complex	17735	23	3682	11	4.78	0.00344	2.301255	PSMC1;PSMC2;PSMC3;PSMC5;PSMC6;PSMD10;PSMD13;PSMD14;PSMD4;PSMD8;PSME
turquoise	BP	GO:0006461	protein complex assembly	17735	783	3682	204	172.53	0.00345	1.182403	ACAT1;ACOT13;ACVRL1;ADRM1;AHR;AIM2;ALDOA;ANG;ANGPT1;ANGPTL4;ANLN;AP1AR;APC;AQP11;ARL2;
turquoise	MF	GO:0004693	cyclin-dependent protein kinase activity	17735	32	3682	14	6.8	0.00349	2.058824	CCND3;CDK1;CDK11A;CDK11B;CDK17;CDK18;CDK2;CDK5R1;CDK7;CDK8;CDK9;CDKL1;CDKL2;ICK
turquoise	BP	GO:0016050	vesicle organization	17735	103	3682	35	22.7	0.0035	1.54185	ABCA1;ANXA1;ARFGAP1;BET1;BLOC1S2;COPE;COPZ1;DLG4;DTNBP1;EEA1;EPS15;FBXO5;FCHO1;FCHO2;FNB
turquoise	BP	GO:0016579	protein deubiquitination	17735	64	3682	24	14.1	0.00353	1.702128	ATXN7L3;BAP1;BRCC3;DNAJB2;ENY2;ITCH;MYSM1;OTUD5;PSMD14;UCHL5;USP1;USP12;USP15;USP16;USP
turquoise	BP	GO:0043244	regulation of protein complex disassembl...	17735	64	3682	24	14.1	0.00353	1.702128	APC;ARHGEF2;CAPG;CAPZA1;CAPZA2;CKAP2;EIF5A2;F2RL1;GAS2L2;HDAC6;HRSP12;KATNB1;MAP6D1;MGE
turquoise	BP	GO:0006470	protein dephosphorylation	17735	144	3682	46	31.73	0.00367	1.449732	ADORA1;CDC25C;CDKN3;CTDNEP1;CTDSP1;DAPP1;DUSP11;DUSP19;IGBP1;LHPP;MTMR2;MTMR6;MTMR7;
turquoise	CC	GO:0015934	large ribosomal subunit	17735	75	3682	26	15.6	0.00369	1.666667	FXR2;MRPL1;MRPL13;MRPL15;MRPL22;MRPL3;MRPL32;RBM3;RPL11;RPL15;RPL17;RPL23;RPL26;RPL26L1;
turquoise	MF	GO:0003735	structural constituent of ribosome	17735	157	3682	48	33.34	0.00373	1.439712	MRPL1;MRPL13;MRPL15;MRPL18;MRPL19;MRPL2;MRPL22;MRPL28;MRPL3;MRPL32;MRPL37;MRPL42;MRP
turquoise	BP	GO:0071840	cellular component organization or bioge...	17735	4237	3682	995	933.59	0.00377	1.065778	ABCA1;ABCA5;ABCD1;ABCD3;ABI1;ABI2;ABLIM2;ABR;ACAT1;ACD;ACOT13;ACP2;ACSL4;ACSL6;ACTL6A;ACT
turquoise	BP	GO:0034142	toll-like receptor 4 signaling pathway	17735	89	3682	31	19.61	0.0038	1.580826	ATF1;ATF2;CDK1;CHUK;CREB1;F2RL1;IKBKG;JUN;MAP2K2;MAP2K4;MAP3K1;MAP3K7;MAPK8;MAPKAPK2;
turquoise	BP	GO:0070271	protein complex biogenesis	17735	785	3682	204	172.97	0.0039	1.179395	ACAT1;ACOT13;ACVRL1;ADRM1;AHR;AIM2;ALDOA;ANG;ANGPT1;ANGPTL4;ANLN;AP1AR;APC;AQP11;ARL2;
turquoise	CC	GO:0044438	microbody part	17735	79	3682	27	16.43	0.00392	1.643335	ABCD1;ABCD2;ABCD3;ACBD5;ACSL4;ACSL6;AGPS;ATAD1;CRAT;CROT;FAR1;FAR2;GRHPR;IDE;IMPDH2;MAP2
turquoise	CC	GO:0044439	peroxisomal part	17735	79	3682	27	16.43	0.00392	1.643335	ABCD1;ABCD2;ABCD3;ACBD5;ACSL4;ACSL6;AGPS;ATAD1;CRAT;CROT;FAR1;FAR2;GRHPR;IDE;IMPDH2;MAP2
turquoise	MF	GO:0000030	mannosyltransferase activity	17735	17	3682	9	3.61	0.00396	2.493075	ALG1;ALG11;ALG2;ALG3;DPM1;PIGB;PIGM;PIGZ;POMT2
turquoise	BP	GO:0006892	post-Golgi vesicle-mediated transport	17735	75	3682	27	16.53	0.00398	1.633394	AP1B1;AP1S2;ARFGEF2;ARRB1;BLZF1;CSK;DTNBP1;EXOC5;GOLGA4;GOPC;LLGL1;MON2;OSBPL5;RAB10;RAB
turquoise	BP	GO:0033238	regulation of cellular amine metabolic p...	17735	75	3682	27	16.53	0.00398	1.633394	AZIN1;COMT;HPRT1;LDLR;LPCAT1;NQO1;PDE1B;PSMA1;PSMA2;PSMA3;PSMA4;PSMA6;PSMA8;PSMB1;PSM
turquoise	MF	GO:0008601	protein phosphatase type 2A regulator ac...	17735	20	3682	10	4.25	0.00411	2.352941	ARPP19;IGBP1;PPP2R1A;PPP2R2A;PPP2R3A;PPP2R4;PPP2R5A;PPP2R5B;PPP2R5D;PPP2R5E
turquoise	BP	GO:0051289	protein homotetramerization	17735	51	3682	20	11.24	0.00412	1.779359	ACOT13;ALDOA;CBR4;CRTC2;DECR1;DHPS;GFPT1;GLS;GNMT;HPRT1;IDE;IMPDH2;PEX14;PFKL;PPAT;RYR3;S
turquoise	CC	GO:0030880	RNA polymerase complex	17735	102	3682	33	21.21	0.00415	1.55587	CCNC;CCNH;CDC73;CDK7;ERCC2;GTF2E2;GTF2F2;GTF2H1;GTF2H4;INTS12;INTS2;INTS5;INTS6;INTS8;MNAT1
turquoise	BP	GO:0072331	signal transduction by p53 class mediato...	17735	130	3682	42	28.64	0.00426	1.46648	BAG6;BRCA2;CDK2;CDKN1B;DYRK1A;DYRK2;E2F7;EEF1E1;MDM2;MSH2;MUC1;NBN;PDK2;PMAIP1;PSMA1;
turquoise	BP	GO:0006521	regulation of cellular amino acid metabo...	17735	58	3682	22	12.78	0.00434	1.72144	AZIN1;COMT;NQO1;PSMA1;PSMA2;PSMA3;PSMA4;PSMA6;PSMA8;PSMB1;PSMB3;PSMC1;PSMC2;PSMC3;P
turquoise	MF	GO:0005525	GTP binding	17735	366	3682	99	77.73	0.00436	1.27364	ADSSL1;AGAP2;AGAP3;AK3;ARF4;ARHGAP5;ARL13B;ARL15;ARL2;ARL4A;ARL5A;ARL5B;ARL8B;ATL2;DNAJC2
turquoise	CC	GO:0000932	cytoplasmic mRNA processing body	17735	51	3682	19	10.61	0.00506	1.790763	APOBEC3D;BTBD1;CARHSP1;CNOT3;CNOT7;DCP1A;DCP2;EIF4E;LIN28A;MOV10;PAN3;PNRC2;PSMA2;PSMA
turquoise	CC	GO:0005819	spindle	17735	232	3682	65	48.25	0.00511	1.34715	AKT1;ARHGEF2;ARL8B;ASPM;AURKC;CBX3;CDC14A;CDC27;CDK1;CENPE;CEP170;CEP19;CEP250;CEP350;CEP
turquoise	CC	GO:0005689	U12-type spliceosomal complex	17735	24	3682	11	4.99	0.00517	2.204409	PHF5A;RNPC3;SF3B1;SF3B14;SNRNP35;SNRNP48;SNRPB;SNRPD1;SNRPE;SNRPG;ZCRB1
turquoise	CC	GO:0035770	ribonucleoprotein granule	17735	88	3682	29	18.3	0.00518	1.584699	APOBEC3D;BTBD1;CARHSP1;CNOT3;CNOT7;DCP1A;DCP2;DDX3X;EIF4E;FMR1;LIN28A;MBNL1;MOV10;PAN3
turquoise	CC	GO:0005777	peroxisome	17735	123	3682	38	25.58	0.00533	1.485536	ABCD1;ABCD2;ABCD3;ACBD5;ACSL4;ACSL6;AGPS;ATAD1;CRAT;CROT;ECH1;FAR1;FAR2;GRHPR;HMGCL;HSD
turquoise	CC	GO:0042579	microbody	17735	123	3682	38	25.58	0.00533	1.485536	ABCD1;ABCD2;ABCD3;ACBD5;ACSL4;ACSL6;AGPS;ATAD1;CRAT;CROT;ECH1;FAR1;FAR2;GRHPR;HMGCL;HSD
turquoise	MF	GO:0004559	alpha-mannosidase activity	17735	12	3682	7	2.55	0.00555	2.745098	EDEM3;MAN1A1;MAN1A2;MAN2A1;MAN2B1;MAN2C1;MANEA
turquoise	MF	GO:0016667	oxidoreductase activity, acting on a sul...	17735	54	3682	20	11.47	0.00564	1.743679	CHCHD4;COIL;DNAJC10;ERO1L;ERO1LB;GLRX;GLRX2;GLRX3;IFI30;P4HB;PRDX3;SH3BGRL3;STAB1;SUOX;TM
turquoise	MF	GO:0019899	enzyme binding	17735	1084	3682	264	230.21	0.00565	1.146779	ABCA1;ABCD1;ABI2;ACAT1;ACD;ACSL6;ACVRL1;ADAM10;ADRM1;AKAP11;AKAP5;AKAP7;AKAP8L;AKT1;APC;
turquoise	MF	GO:0003677	DNA binding	17735	2332	3682	542	495.25	0.00575	1.094397	ABI2;ACD;AEBP1;AEBP2;AHR;AIM2;AKAP8L;ANG;APEX1;APEX2;APTX;ARID2;ARID4A;ARID4B;ARID5A;ARID5
turquoise	CC	GO:0035097	histone methyltransferase complex	17735	59	3682	21	12.27	0.0061	1.711491	AEBP2;CXXC1;DPY30;E2F6;EED;HDAC9;INO80C;KAT8;MEN1;PELP1;PPP1CA;PPP1CB;PPP1CC;PRPF31;RNF2;R
turquoise	CC	GO:0016591	DNA-directed RNA polymerase II, holoenzy...	17735	89	3682	29	18.51	0.00619	1.566721	CCNC;CCNH;CDC73;CDK7;ERCC2;GTF2E2;GTF2F2;GTF2H1;GTF2H4;INTS12;INTS2;INTS5;INTS6;INTS8;MNAT1
turquoise	MF	GO:0017136	NAD-dependent histone deacetylase activi...	17735	15	3682	8	3.19	0.00622	2.507837	HDAC3;HDAC5;HDAC6;HDAC7;HDAC8;HDAC9;SIRT1;SIRT2
turquoise	MF	GO:0034979	NAD-dependent protein deacetylase activi...	17735	15	3682	8	3.19	0.00622	2.507837	HDAC3;HDAC5;HDAC6;HDAC7;HDAC8;HDAC9;SIRT1;SIRT2
turquoise	CC	GO:0005694	chromosome	17735	631	3682	157	131.22	0.00651	1.196464	ACD;ACTL6A;ADD3;APC;APTX;ARRB1;ATRX;AURKC;BANF1;BAZ1A;CASC5;CBX3;CDK2;CENPE;CENPK;CENPL;C
turquoise	CC	GO:0000428	DNA-directed RNA polymerase complex	17735	101	3682	32	21	0.00667	1.52381	CCNC;CCNH;CDC73;CDK7;ERCC2;GTF2E2;GTF2F2;GTF2H1;GTF2H4;INTS12;INTS2;INTS5;INTS6;INTS8;MNAT1
turquoise	CC	GO:0055029	nuclear DNA-directed RNA polymerase comp...	17735	101	3682	32	21	0.00667	1.52381	CCNC;CCNH;CDC73;CDK7;ERCC2;GTF2E2;GTF2F2;GTF2H1;GTF2H4;INTS12;INTS2;INTS5;INTS6;INTS8;MNAT1
turquoise	MF	GO:0004221	ubiquitin thiolesterase activity	17735	73	3682	25	15.5	0.0069	1.612903	BAP1;BRCC3;MYSM1;PSMD14;UCHL3;UCHL5;USP1;USP12;USP14;USP15;USP16;USP19;USP21;USP25;USP31
turquoise	MF	GO:0019001	guanyl nucleotide binding	17735	380	3682	101	80.7	0.00694	1.251549	ADSSL1;AGAP2;AGAP3;AK3;ARF4;ARHGAP5;ARL13B;ARL15;ARL2;ARL4A;ARL5A;ARL5B;ARL8B;ATL2;DNAJC2
turquoise	MF	GO:0032561	guanyl ribonucleotide binding	17735	380	3682	101	80.7	0.00694	1.251549	ADSSL1;AGAP2;AGAP3;AK3;ARF4;ARHGAP5;ARL13B;ARL15;ARL2;ARL4A;ARL5A;ARL5B;ARL8B;ATL2;DNAJC2
turquoise	MF	GO:0008234	cysteine-type peptidase activity	17735	147	3682	44	31.22	0.00805	1.409353	ATG4A;ATG4B;ATG4C;BAP1;BRCC3;CAPN14;CAPN7;CARD17;CASP3;CASP8;CTSH;CTSO;CTSS;CTSW;MALT1;
turquoise	CC	GO:0032391	photoreceptor connecting cilium	17735	22	3682	10	4.58	0.00814	2.183406	CEP290;CETN3;FAM161A;IFT122;IFT140;IFT52;IFT57;IFT88;KIF3A;TOPORS
turquoise	CC	GO:0005743	mitochondrial inner membrane	17735	347	3682	91	72.16	0.00829	1.261086	ABCB10;ABCD3;ACADVL;ACAT1;AIFM3;APEX2;ATAD3A;ATP5C1;ATP5E;ATP5F1;ATP5I;ATP5L;ATP5O;C14orf2
turquoise	MF	GO:0001608	G-protein coupled nucleotide receptor ac...	17735	25	3682	11	5.31	0.00884	2.071563	GPR15;GPR171;GPR174;GPR34;LPAR6;P2RY10;P2RY11;P2RY12;P2RY13;P2RY14;SUCNR1
turquoise	MF	GO:0045028	G-protein coupled purinergic nucleotide ...	17735	25	3682	11	5.31	0.00884	2.071563	GPR15;GPR171;GPR174;GPR34;LPAR6;P2RY10;P2RY11;P2RY12;P2RY13;P2RY14;SUCNR1
turquoise	CC	GO:0030684	preribosome	17735	16	3682	8	3.33	0.00892	2.402402	CASP8;PES1;PIN4;RPS7;RRP1;UTP11L;UTP14C;WDR36
turquoise	MF	GO:0004702	receptor signaling protein serine/threon...	17735	49	3682	18	10.41	0.00925	1.729107	ACVR2A;ACVRL1;KIAA1804;MAP3K1;MAP3K11;MAP3K13;MAP3K14;MAP3K2;MAP3K7;MAP3K8;MAP4K1;M

turquoise	CC	GO:0005793	endoplasmic reticulum-Golgi intermediate...	17735	61	3682	21	12.69	0.00934	1.654846	BCAP31;ERGIC2;LMAN1;LMAN2;MAN1A1;NUCB2;P4HB;RAB2A;RAB37;SCYL1;SERPINH1;SLC35C2;STK17B;ST
turquoise	CC	GO:0034708	methyltransferase complex	17735	61	3682	21	12.69	0.00934	1.654846	AEBP2;CXXC1;DPY30;E2F6;EED;HDAC9;INO80C;KAT8;MEN1;PELP1;PPP1CA;PPP1CB;PPP1CC;PRPF31;RNF2;R
turquoise	CC	GO:0005575	cellular_component	17735	16733	3682	3484	3479.84	0.00941	1.001195	AAAS;AACS;AAMP;AANAT;AASDHPPT;ABCA1;ABCA10;ABCA3;ABCA5;ABCA7;ABCB10;ABCB6;ABCC2;ABCD1;
turquoise	CC	GO:0031901	early endosome membrane	17735	84	3682	27	17.47	0.00982	1.545507	APPL1;B2M;CLIP3;EEA1;EPS15;FGD2;HGS;HLA-A;HPS6;LLGL1;MARCH1;MTMR2;PIKFYVE;RAB14;RAB5A;SH3
turquoise	MF	GO:0015923	mannosidase activity	17735	13	3682	7	2.76	0.00984	2.536232	EDEM3;MAN1A1;MAN1A2;MAN2A1;MAN2B1;MAN2C1;MANEA
turquoise	MF	GO:0032129	histone deacetylase activity (H3-K9 spec...	17735	13	3682	7	2.76	0.00984	2.536232	HDAC3;HDAC5;HDAC6;HDAC7;HDAC8;HDAC9;SIRT1
turquoise	MF	GO:0046969	NAD-dependent histone deacetylase activi...	17735	13	3682	7	2.76	0.00984	2.536232	HDAC3;HDAC5;HDAC6;HDAC7;HDAC8;HDAC9;SIRT1
turquoise	CC	GO:0044455	mitochondrial membrane part	17735	148	3682	43	30.78	0.01048	1.397011	ABCB10;ABCB6;ATP5C1;ATP5E;ATP5F1;ATP5I;ATP5L;ATP5O;C14orf2;CCDC90A;COX7A2;COX7A2L;COX7B;D
turquoise	MF	GO:0003684	damaged DNA binding	17735	46	3682	17	9.77	0.01054	1.74002	APEX1;APTX;DDB2;HMGB1;HMGB2;MSH2;NBN;POLI;POLK;POLQ;RAD23A;RBBP8;REV1;RUVBL2;TDG;TP63;
turquoise	CC	GO:0031227	intrinsic to endoplasmic reticulum membr...	17735	112	3682	34	23.29	0.01075	1.459854	ASPH;BCAP31;BNIP1;CLN3;DERL1;DHRS9;DOLPP1;ELOVL4;ERN1;EXTL2;FITM1;FMO5;HLA-A;HLA-DPB1;HLA-
turquoise	CC	GO:0030532	small nuclear ribonucleoprotein complex	17735	33	3682	13	6.86	0.01133	1.895044	CD2BP2;LSM11;LSM6;PHF5A;PRPF31;PRPF6;SF3A2;SLU7;SNRPB;SNRPB2;SNRPD1;SNRPE;SNRPG
turquoise	CC	GO:0005769	early endosome	17735	190	3682	53	39.51	0.01161	1.341433	AP1AR;APPL1;ATP11B;B2M;CHMP1A;CLIP3;CLN3;DERL1;EEA1;EPS15;FAM109B;FGD2;HGS;HLA-A;HPS6;KCN
turquoise	MF	GO:0016887	ATPase activity	17735	353	3682	93	74.97	0.01184	1.240496	ABCA1;ABCA10;ABCA3;ABCA5;ABCA7;ABCB10;ABCB6;ABCC2;ABCD1;ABCD2;ABCD3;ABCE1;ABCF3;ASNA1;A
turquoise	CC	GO:0000123	histone acetyltransferase complex	17735	74	3682	24	15.39	0.01279	1.559454	ACTL6A;ATXN7L3;DMAP1;DR1;ENY2;EPC2;ING3;KAT2B;KAT8;MAP3K7;MBIP;MSL2;MSL3;PHF17;RUVBL2;TA
turquoise	MF	GO:0051082	unfolded protein binding	17735	127	3682	38	26.97	0.01316	1.408973	CCT2;CCT6B;CDC37;CHAF1A;CHAF1B;CLN3;DNAJA1;DNAJB14;DNAJB2;DNAJB4;DNAJB9;DNAJC10;DNAJC21;
turquoise	MF	GO:0003674	molecular_function	17735	15097	3682	3210	3206.18	0.01357	1.001191	AAAS;AACS;AAMP;AANAT;AASDHPPT;ABCA1;ABCA10;ABCA3;ABCA5;ABCA7;ABCB10;ABCB6;ABCC2;ABCD1;
turquoise	CC	GO:0044450	microtubule organizing center part	17735	94	3682	29	19.55	0.01396	1.483376	BLOC1S2;CCDC92;CCP110;CEP135;CEP170;CEP19;CEP250;CEP290;CETN3;CNTROB;FOPNL;IFT20;IFT88;KATN
turquoise	MF	GO:0042578	phosphoric ester hydrolase activity	17735	351	3682	92	74.54	0.01404	1.234237	ACP2;ACPL2;ADORA1;APEX1;APTX;CDC14A;CDC25C;CDKN3;CNP;CTDNEP1;CTDSP1;CTDSPL2;CYCS;DAPP1;D
turquoise	CC	GO:0005675	holo TFIIH complex	17735	11	3682	6	2.29	0.01412	2.620087	CCNH;CDK7;ERCC2;GTF2H1;GTF2H4;MNAT1
turquoise	CC	GO:0005779	integral to peroxisomal membrane	17735	14	3682	7	2.91	0.01437	2.405498	ABCD1;PEX11A;PEX11G;PEX12;PEX13;PEX2;PEX3
turquoise	CC	GO:0031231	intrinsic to peroxisomal membrane	17735	14	3682	7	2.91	0.01437	2.405498	ABCD1;PEX11A;PEX11G;PEX12;PEX13;PEX2;PEX3
turquoise	CC	GO:0000313	organellar ribosome	17735	52	3682	18	10.81	0.01454	1.665125	MRPL13;MRPL18;MRPL28;MRPL3;MRPL32;MRPL37;MRPL39;MRPL40;MRPL42;MRPL47;MRPL48;MRPS17;
turquoise	CC	GO:0005761	mitochondrial ribosome	17735	52	3682	18	10.81	0.01454	1.665125	MRPL13;MRPL18;MRPL28;MRPL3;MRPL32;MRPL37;MRPL39;MRPL40;MRPL42;MRPL47;MRPL48;MRPS17;
turquoise	CC	GO:0022625	cytosolic large ribosomal subunit	17735	52	3682	18	10.81	0.01454	1.665125	FXR2;RPL11;RPL15;RPL17;RPL23;RPL26;RPL27;RPL30;RPL31;RPL34;RPL35;RPL36A;RPL39;RPL39L;RPL41;RPL
turquoise	MF	GO:0016538	cyclin-dependent protein kinase regulato...	17735	20	3682	9	4.25	0.01475	2.117647	CASP3;CCNE2;CCNY;CDKN1B;CDKN1C;CDKN2C;CKS1B;CKS2;KAT2B
turquoise	CC	GO:0071013	catalytic step 2 spliceosome	17735	79	3682	25	16.43	0.0155	1.521607	CDC40;DDX41;DGCR14;FRG1;LSM3;PLRG1;PPIL3;PPWD1;PRPF4B;PRPF6;RALY;SART1;SF3A1;SF3A2;SF3B1;S
turquoise	CC	GO:0005765	lysosomal membrane	17735	139	3682	40	28.91	0.01551	1.383604	ABCA5;ACP2;AP1B1;AP1S2;ARL8B;ARRB1;CCZ1;CD164;CD63;CLCN7;CLN3;CLN5;CTNS;CYBASC3;DIRC2;DRA
turquoise	MF	GO:0035586	purinergic receptor activity	17735	37	3682	14	7.86	0.01556	1.78117	ADORA1;DLG4;GPR15;GPR171;GPR174;GPR34;LPAR6;P2RX4;P2RY10;P2RY11;P2RY12;P2RY13;P2RY14;SUCN
turquoise	MF	GO:0031078	histone deacetylase activity (H3-K14 spe...	17735	11	3682	6	2.34	0.01565	2.564103	HDAC3;HDAC5;HDAC6;HDAC7;HDAC8;HDAC9
turquoise	MF	GO:0032041	NAD-dependent histone deacetylase activi...	17735	11	3682	6	2.34	0.01565	2.564103	HDAC3;HDAC5;HDAC6;HDAC7;HDAC8;HDAC9
turquoise	MF	GO:0046970	NAD-dependent histone deacetylase activi...	17735	11	3682	6	2.34	0.01565	2.564103	HDAC3;HDAC5;HDAC6;HDAC7;HDAC8;HDAC9
turquoise	MF	GO:0004675	transmembrane receptor protein serine/th...	17735	17	3682	8	3.61	0.01566	2.216066	ACVR1C;ACVR2A;ACVRL1;BMPR1A;BMPR2;ENG;TGFBR1;TGFBR2
turquoise	MF	GO:0005024	transforming growth factor beta-activate...	17735	17	3682	8	3.61	0.01566	2.216066	ACVR1C;ACVR2A;ACVRL1;BMPR1A;BMPR2;ENG;TGFBR1;TGFBR2
turquoise	MF	GO:0015295	solute:hydrogen symporter activity	17735	14	3682	7	2.97	0.01611	2.356902	SLC17A5;SLC2A9;SLC35A1;SLC35A3;SLC35A4;SLC35A5;SLC4A11
turquoise	MF	GO:0019887	protein kinase regulator activity	17735	113	3682	34	24	0.01678	1.416667	ABI1;APC;CAB39;CASP3;CCNE2;CCNY;CDK5R1;CDKN1B;CDKN1C;CDKN2C;CKS1B;CKS2;CSNK2B;CXCL10;DUS
turquoise	MF	GO:0019207	kinase regulator activity	17735	133	3682	39	28.25	0.01698	1.380531	ABI1;APC;CAB39;CASP3;CCNE2;CCNY;CDK5R1;CDKN1B;CDKN1C;CDKN2C;CKS1B;CKS2;CSNK2B;CXCL10;DUS
turquoise	CC	GO:0032592	integral to mitochondrial membrane	17735	31	3682	12	6.45	0.01713	1.860465	ABCB10;ABCB6;CCDC90A;DMPK;L2HGDH;MFF;RHOT1;SLC24A6;TIMM17A;TIMM23;TMEM70;TOMM4
turquoise	CC	GO:0031965	nuclear membrane	17735	194	3682	53	40.34	0.01746	1.313832	AAAS;AKIRIN1;APEH;ARFGAP1;ATP11B;BRIP1;CAPG;CBX3;CEP70;CEPT1;CERS5;CST3;CTDNEP1;DMPK;DTX2;
turquoise	CC	GO:0005635	nuclear envelope	17735	314	3682	81	65.3	0.01822	1.240429	AAAS;AGPAT5;AKIRIN1;ANKLE1;APEH;ARFGAP1;ATP11B;BNIP1;BNIP2;BNIP3L;BRIP1;CAPG;CBX3;CEP70;CEP
turquoise	CC	GO:0032153	cell division site	17735	42	3682	15	8.73	0.01827	1.718213	ANLN;ECT2;MEN1;NDE1;PITPNM1;PKN1;PKN2;PLCD3;PLK4;PPP1CC;PSTPIP1;RALA;RDX;SEPT7;SPIRE2
turquoise	CC	GO:0032155	cell division site part	17735	42	3682	15	8.73	0.01827	1.718213	ANLN;ECT2;MEN1;NDE1;PITPNM1;PKN1;PKN2;PLCD3;PLK4;PPP1CC;PSTPIP1;RALA;RDX;SEPT7;SPIRE2
turquoise	CC	GO:0030496	midbody	17735	88	3682	27	18.3	0.01856	1.47541	ARL8B;ASPM;AURKC;CDK1;CEP44;CLIC4;DDX11;ECT2;ERH;GDI1;GNAI1;KATNB1;KEAP1;KIF23;KLHL9;MITD1;
turquoise	MF	GO:0016758	transferase activity, transferring hexos...	17735	183	3682	51	38.86	0.01946	1.312403	AGL;ALG1;ALG10;ALG10B;ALG11;ALG13;ALG2;ALG3;ALG6;B3GALNT1;B3GALT2;B3GNT1;B3GNT2;B3GNT5;B
turquoise	MF	GO:0016788	hydrolase activity, acting on ester bond...	17735	730	3682	178	155.03	0.01973	1.148165	ABHD12;ABHD3;ACOT13;ACOT7;ACP2;ACPL2;ACY3;ADORA1;ANG;APEX1;APEX2;APLF;APTX;ARSA;ARSK;AST
turquoise	CC	GO:0005932	microtubule basal body	17735	65	3682	21	13.52	0.01992	1.553254	CEP250;CEP290;CETN3;DYNC2LI1;FAM161A;FOPNL;IFT122;IFT140;IFT20;IFT57;IFT80;IFT88;MKS1;PKD2;POC
turquoise	CC	GO:0005774	vacuolar membrane	17735	183	3682	50	38.06	0.02044	1.313715	ABCA5;ABCB6;ACP2;AKTIP;AP1B1;AP1S2;ARL8B;ARRB1;ATG9A;ATP6AP1;ATP6V1G1;CCZ1;CD164;CD63;CLC
turquoise	MF	GO:0004298	threonine-type endopeptidase activity	17735	21	3682	9	4.46	0.02104	2.017937	PRSS50;PSMA1;PSMA2;PSMA3;PSMA4;PSMA6;PSMA8;PSMB1;PSMB3
turquoise	MF	GO:0070003	threonine-type peptidase activity	17735	21	3682	9	4.46	0.02104	2.017937	PRSS50;PSMA1;PSMA2;PSMA3;PSMA4;PSMA6;PSMA8;PSMB1;PSMB3
turquoise	CC	GO:0032154	cleavage furrow	17735	39	3682	14	8.11	0.0212	1.726264	ECT2;MEN1;NDE1;PITPNM1;PKN1;PKN2;PLCD3;PLK4;PPP1CC;PSTPIP1;RALA;RDX;SEPT7;SPIRE2
turquoise	CC	GO:0000777	condensed chromosome kinetochore	17735	81	3682	25	16.84	0.02126	1.484561	CASC5;CENPE;CENPK;CENPW;CSNK1A1;DYNLT3;ITGB3BP;MAD2L1;MLF1IP;NDE1;NSL1;NUDCD2;NUF2;NUP
turquoise	CC	GO:0000779	condensed chromosome, centromeric region	17735	85	3682	26	17.68	0.02141	1.470588	CASC5;CBX3;CENPE;CENPK;CENPW;CSNK1A1;DYNLT3;ITGB3BP;MAD2L1;MLF1IP;NDE1;NSL1;NUDCD2;NUF2
turquoise	CC	GO:0044447	axoneme part	17735	32	3682	12	6.65	0.02234	1.804511	ARFGEF2;CTSH;DNAH6;DNAI2;DYNC2LI1;HSPB11;IFT20;IFT52;IFT74;IFT81;IFT88;RSPH4A
turquoise	MF	GO:0004721	phosphoprotein phosphatase activity	17735	168	3682	47	35.68	0.02254	1.317265	CDC14A;CDC25C;CDKN3;CTDNEP1;CTDSP1;CTDSPL2;CYCS;DAPP1;DLG1;DUSP11;DUSP19;MTMR2;MTMR6;
turquoise	MF	GO:0070412	R-SMAD binding	17735	18	3682	8	3.82	0.02304	2.094241	FOXH1;JUN;MEN1;PAX6;RANBP3;SMAD2;SMAD4;TRIM33
turquoise	CC	GO:0032039	integrator complex	17735	12	3682	6	2.5	0.02333	2.4	INTS12;INTS2;INTS5;INTS6;INTS8;SHFM1
turquoise	CC	GO:0005741	mitochondrial outer membrane	17735	118	3682	34	24.54	0.02373	1.385493	ABCB6;ACSL4;ACSL6;AGPAT5;BMF;BNIP3L;CASP8;CISD1;CISD2;CNP;COASY;DDX3X;DMPK;GK;GPAM;GPAT2;
turquoise	CC	GO:0005795	Golgi stack	17735	110	3682	32	22.88	0.02419	1.398601	APH1A;B4GALT3;B4GALT6;BET1;CHPF;CHPF2;CHSY1;CLIP3;CLN3;COG3;CSGALNACT1;CSGALNACT2;FUT4;GA
turquoise	MF	GO:0016854	racemase and epimerase activity	17735	12	3682	6	2.55	0.02576	2.352941	DHRS9;GALE;GLCE;MCEE;RENBP;RPE
turquoise	MF	GO:0034739	histone deacetylase activity (H4-K16 spe...	17735	12	3682	6	2.55	0.02576	2.352941	HDAC3;HDAC5;HDAC6;HDAC7;HDAC8;HDAC9
turquoise	MF	GO:0097372	NAD-dependent histone deacetylase activi...	17735	12	3682	6	2.55	0.02576	2.352941	HDAC3;HDAC5;HDAC6;HDAC7;HDAC8;HDAC9
turquoise	MF	GO:0001614	purinergic nucleotide receptor activity	17735	32	3682	12	6.8	0.02609	1.764706	GPR15;GPR171;GPR174;GPR34;LPAR6;P2RX4;P2RY10;P2RY11;P2RY12;P2RY13;P2RY14;SUCNR1
turquoise	MF	GO:0016502	nucleotide receptor activity	17735	32	3682	12	6.8	0.02609	1.764706	GPR15;GPR171;GPR174;GPR34;LPAR6;P2RX4;P2RY10;P2RY11;P2RY12;P2RY13;P2RY14;SUCNR1
turquoise	MF	GO:0008022	protein C-terminus binding	17735	158	3682	44	33.55	0.02866	1.311475	AKAP7;ARL6IP5;BAIAP2;BLOC1S2;CDK7;CEP120;CEP135;CEP250;COIL;CORO1A;CSK;CTBP1;DGKZ;DLG1;DLG4
turquoise	CC	GO:0000314	organellar small ribosomal subunit	17735	19	3682	8	3.95	0.02896	2.025316	MRPL42;MRPS17;MRPS18B;MRPS18C;MRPS22;MRPS28;MRPS31;MRPS33
turquoise	CC	GO:0005763	mitochondrial small ribosomal subunit	17735	19	3682	8	3.95	0.02896	2.025316	MRPL42;MRPS17;MRPS18B;MRPS18C;MRPS22;MRPS28;MRPS31;MRPS33
turquoise	CC	GO:0005720	nuclear heterochromatin	17735	26	3682	10	5.41	0.02984	1.848429	ATRX;CBX3;EED;PSIP1;RING1;RNF2;SIRT1;SUV39H2;SUZ12;TRIM28
turquoise	CC	GO:0070461	SAGA-type complex	17735	26	3682	10	5.41	0.02984	1.848429	ATXN7L3;ENY2;KAT2B;TADA1;TAF12;TAF2;TAF5;TAF7;TAF9;TAF9B
turquoise	MF	GO:0004520	endodeoxyribonuclease activity	17735	29	3682	11	6.16	0.02984	1.785714	APEX1;APLF;DNA2;DNASE1L1;DNASE2;ENDOV;RAD51C;RBBP8;TATDN1;TATDN3;ZRANB3
turquoise	MF	GO:0008094	DNA-dependent ATPase activity	17735	70	3682	22	14.87	0.02995	1.479489	BRIP1;CCNH;CDK7;CHD1;DDX11;DDX3X;DNA2;ERCC2;GTF2H1;GTF2H4;IGHMBP2;MNAT1;MSH2;NBN;POLQ;

turquoise	MF	GO:0003712	transcription cofactor activity	17735	475	3682	118	100.88	0.0307	1.169707	ACTL6A;AEBP1;AEBP2;APEX1;ARID5B;ATF2;ATXN7L3;BIRC2;CASP8AP2;CBFB;CDK7;CNOT2;COPS2;COPS5;CR
turquoise	CC	GO:0012507	ER to Golgi transport vesicle membrane	17735	37	3682	13	7.69	0.03093	1.690507	B2M;HLA-A;HLA-DPB1;HLA-DRA;LMAN1;SAR1B;SCAP;SEC13;SEC23A;SEC24A;SREBF1;TMED7;VMA21
turquoise	CC	GO:0031519	PcG protein complex	17735	37	3682	13	7.69	0.03093	1.690507	AEBP2;BAP1;BMI1;CSNK2B;EED;PCGF5;PCGF6;PHC3;RING1;RNF2;RYBP;SIRT1;SUZ12
turquoise	CC	GO:0000792	heterochromatin	17735	60	3682	19	12.48	0.03186	1.522436	ATRX;CBX3;DAXX;EED;HELLS;LRWD1;ORC2;PSIP1;RING1;RNF2;RNF40;RRP8;SIRT1;SMARCAD1;SUV39H1;SU\
turquoise	MF	GO:0004407	histone deacetylase activity	17735	19	3682	8	4.04	0.03255	1.980198	HDAC3;HDAC5;HDAC6;HDAC7;HDAC8;HDAC9;SIRT1;SIRT2
turquoise	MF	GO:0004709	MAP kinase kinase kinase activity	17735	19	3682	8	4.04	0.03255	1.980198	KIAA1804;MAP3K1;MAP3K11;MAP3K13;MAP3K14;MAP3K2;MAP3K7;MAP3K8
turquoise	CC	GO:0005801	cis-Golgi network	17735	30	3682	11	6.24	0.03332	1.762821	BET1;COG3;FAM134B;MAP6D1;SCFD1;SCYL1;SLC35C2;TMED10;TMED5;TRAPPC1;TRAPPC8
turquoise	MF	GO:0019210	kinase inhibitor activity	17735	48	3682	16	10.19	0.03513	1.570167	CASP3;CDKN1B;CDKN1C;CDKN2C;DUSP19;FGFR1OP;GMFB;IBTK;KAT2B;LRP6;MAPK8IP1;MBIP;PKIA;PKIB;PR
turquoise	MF	GO:0052745	inositol phosphate phosphatase activity	17735	16	3682	7	3.4	0.03611	2.058824	IMPA1;IMPAD1;INPP5D;INPP5K;MINPP1;PTEN;SYNJ1
turquoise	MF	GO:0070063	RNA polymerase binding	17735	16	3682	7	3.4	0.03611	2.058824	C14orf166;CDC73;KIAA1967;NEDD4;SMYD2;WDHD1;ZNF326
turquoise	MF	GO:0005057	receptor signaling protein activity	17735	103	3682	30	21.87	0.03611	1.371742	ACVR2A;ACVRL1;ARAF;BAG4;CDKN1B;DAXX;ENG;FLRT1;IFITM1;KIAA1804;MAP3K1;MAP3K11;MAP3K13;MA
turquoise	MF	GO:0016765	transferase activity, transferring alkyl...	17735	52	3682	17	11.04	0.03656	1.539855	AGPS;CHM;CHML;COQ2;DHPS;FDFT1;FDPS;FNTA;GGPS1;GSTM2;GSTP1;NANS;NUS1;PDSS1;PGGT1B;PTAR1;
turquoise	MF	GO:0008047	enzyme activator activity	17735	404	3682	101	85.8	0.03677	1.177156	ABI1;ABR;ACAP1;ACAP2;ADAP1;ADC;ADRM1;AGAP2;AGAP3;AGFG2;AIFM3;ARAP2;ARFGAP1;ARFGAP2;ARH
turquoise	CC	GO:0044427	chromosomal part	17735	538	3682	129	111.88	0.03803	1.153021	ACD;ACTL6A;APC;APTX;ARRB1;ATRX;AURKC;CASC5;CBX3;CDK2;CENPE;CENPK;CENPL;CENPQ;CENPW;CREB1
turquoise	CC	GO:0008385	IkappaB kinase complex	17735	10	3682	5	2.08	0.0383	2.403846	CHUK;IKBKG;MAP3K13;MAP3K7;PRDX3
turquoise	MF	GO:0030145	manganese ion binding	17735	41	3682	14	8.71	0.03858	1.607348	ATP2C1;CDIPT;DCP2;DYRK2;GALNT1;GALNT3;HMGCL;IDI1;NUDT16;PAPOLA;PEPD;PPEF1;PPM1B;TDP2
turquoise	CC	GO:0044665	MLL1/2 complex	17735	27	3682	10	5.61	0.0388	1.782531	E2F6;INO80C;KAT8;PELP1;PRPF31;RNF2;RUVBL2;TAF7;TAF9;WDR5
turquoise	CC	GO:0071339	MLL1 complex	17735	27	3682	10	5.61	0.0388	1.782531	E2F6;INO80C;KAT8;PELP1;PRPF31;RNF2;RUVBL2;TAF7;TAF9;WDR5
turquoise	CC	GO:0000803	sex chromosome	17735	20	3682	8	4.16	0.03968	1.923077	CDK2;EED;RING1;RNF2;SMARCB1;SMCHD1;SUZ12;UBE2A
turquoise	CC	GO:0005839	proteasome core complex	17735	20	3682	8	4.16	0.03968	1.923077	PSMA1;PSMA2;PSMA3;PSMA4;PSMA6;PSMA8;PSMB1;PSMB3
turquoise	MF	GO:0030695	GTPase regulator activity	17735	436	3682	108	92.59	0.04004	1.166433	ABR;ACAP1;ACAP2;ADAP1;AGAP2;AGAP3;AGFG2;ARAP2;ARFGAP1;ARFGAP2;ARFGEF2;ARHGAP10;ARHGAP
turquoise	MF	GO:0004860	protein kinase inhibitor activity	17735	45	3682	15	9.56	0.04057	1.569038	CASP3;CDKN1B;CDKN1C;CDKN2C;DUSP19;FGFR1OP;GMFB;IBTK;KAT2B;MAPK8IP1;MBIP;PKIA;PKIB;PRKAG2
turquoise	MF	GO:0005072	transforming growth factor beta receptor...	17735	10	3682	5	2.12	0.04162	2.358491	CDKN1B;ENG;SMAD2;SMAD4;SMAD5
turquoise	MF	GO:0008641	small protein activating enzyme activity	17735	10	3682	5	2.12	0.04162	2.358491	MOCS3;UBA2;UBA3;UBA5;UBA6
turquoise	MF	GO:0043175	RNA polymerase core enzyme binding	17735	10	3682	5	2.12	0.04162	2.358491	C14orf166;CDC73;KIAA1967;SMYD2;ZNF326
turquoise	MF	GO:0015036	disulfide oxidoreductase activity	17735	34	3682	12	7.22	0.04179	1.66205	CHCHD4;COIL;DNAJC10;GLRX;GLRX2;GLRX3;P4HB;SH3BGRL3;STAB1;TMX1;TMX3;TXNL1
turquoise	MF	GO:0016791	phosphatase activity	17735	255	3682	66	54.15	0.04221	1.218837	ACP2;ACPL2;APTX;CDC14A;CDC25C;CDKN3;CTDNEP1;CTDSP1;CTDSPL2;CYCS;DAPP1;DLG1;DUSP11;DUSP19;
turquoise	MF	GO:0050662	coenzyme binding	17735	179	3682	48	38.01	0.04334	1.262826	ACAD10;ACAD9;ACADM;ACADSB;ACADVL;ACAT1;ACBD3;ACBD5;ACBD6;ACOT7;AGPS;AIFM3;BDH2;C1orf43
turquoise	MF	GO:0005096	GTPase activator activity	17735	247	3682	64	52.46	0.04396	1.219977	ABR;ACAP1;ACAP2;ADAP1;AGAP2;AGAP3;AGFG2;ARAP2;ARFGAP1;ARFGAP2;ARHGAP10;ARHGAP11A;ARHG
turquoise	CC	GO:0032838	cell projection cytoplasm	17735	66	3682	20	13.73	0.04402	1.456664	ADA;CCDC103;CCDC39;DLG4;DNAH6;DNAI2;DYNC2LI1;FMR1;IFT140;IFT80;IFT88;LRRK2;PQBP1;RPGR;RSPH4
turquoise	MF	GO:0016787	hydrolase activity	17735	2319	3682	524	492.49	0.04415	1.063981	ABCA1;ABCA10;ABCA3;ABCA5;ABCA7;ABCB10;ABCB6;ABCC2;ABCD1;ABCD2;ABCD3;ABCE1;ABCF3;ABHD10;
turquoise	MF	GO:0033558	protein deacetylase activity	17735	20	3682	8	4.25	0.04441	1.882353	HDAC3;HDAC5;HDAC6;HDAC7;HDAC8;HDAC9;SIRT1;SIRT2
turquoise	MF	GO:0004536	deoxyribonuclease activity	17735	38	3682	13	8.07	0.04462	1.610905	APEX1;APLF;APTX;DNA2;DNASE1L1;DNASE2;ENDOV;RAD51C;RBBP8;TATDN1;TATDN3;TREX1;ZRANB3
turquoise	MF	GO:0008375	acetylglucosaminyltransferase activity	17735	38	3682	13	8.07	0.04462	1.610905	ALG13;B3GNT1;B3GNT5;EXTL2;GCNT1;GCNT2;GCNT4;LFNG;MGAT1;MGAT2;MGAT4A;PIGA;RFNG
turquoise	MF	GO:0060589	nucleoside-triphosphatase regulator acti...	17735	447	3682	110	94.93	0.04535	1.158749	ABR;ACAP1;ACAP2;ADAP1;AGAP2;AGAP3;AGFG2;ARAP2;ARFGAP1;ARFGAP2;ARFGEF2;ARHGAP10;ARHGAP
turquoise	MF	GO:0051536	iron-sulfur cluster binding	17735	61	3682	19	12.95	0.04539	1.467181	ABCE1;AIFM3;BRIP1;CISD1;CISD2;CMAHP;DDX11;DNA2;ERCC2;GLRX2;GLRX3;IREB2;ISCA1;NDUFS2;NFU1;NI
turquoise	MF	GO:0051540	metal cluster binding	17735	61	3682	19	12.95	0.04539	1.467181	ABCE1;AIFM3;BRIP1;CISD1;CISD2;CMAHP;DDX11;DNA2;ERCC2;GLRX2;GLRX3;IREB2;ISCA1;NDUFS2;NFU1;NI
turquoise	CC	GO:0030176	integral to endoplasmic reticulum membra...	17735	95	3682	27	19.76	0.0474	1.366397	ASPH;BCAP31;BNIP1;CLN3;DERL1;DHRS9;DOLPP1;ELOVL4;ERN1;FITM1;HLA-A;HLA-DPB1;HLA-DRA;INSIG1;II
turquoise	MF	GO:0016746	transferase activity, transferring acyl ...	17735	227	3682	59	48.21	0.04864	1.223812	AANAT;ACAT1;ACAT2;AGPAT5;BRCA2;CERS5;CRAT;CROT;DLAT;ESCO1;ESCO2;GGCT;GPAM;GPAT2;HAT1;HM
turquoise	CC	GO:0031984	organelle subcompartment	17735	83	3682	24	17.26	0.04931	1.390498	APH1A;B4GALT3;B4GALT6;BET1;CHPF;CHPF2;CHSY1;COG3;CSGALNACT1;CSGALNACT2;FUT4;GALNT1;GALN
turquoise	CC	GO:0015030	Cajal body	17735	47	3682	15	9.77	0.0495	1.535312	CDK2;COIL;FRG1;GEMIN2;GEMIN6;NOP58;OIP5;PHAX;PRPF31;SART1;SMNDC1;TAF9;U2AF1;USPL1;XPO1
turquoise	MF	GO:0016757	transferase activity, transferring glyco...	17735	253	3682	65	53.73	0.04981	1.209752	AGL;ALG1;ALG10;ALG10B;ALG11;ALG13;ALG2;ALG3;ALG6;B3GALNT1;B3GALT2;B3GALTL;B3GNT1;B3GNT2;E
tan	BP	GO:0046483	heterocycle metabolic process	17735	5361	111	56	35.76	2.20E-05	1.565996	AARSD1;ABCD4;ACTR5;AKIRIN2;ASNS;CARKD;CDK5RAP1;EXOSC2;EXOSC7;FANCE;GEMIN8;GPD1;GTF2IRD2;
tan	BP	GO:0006725	cellular aromatic compound metabolic pro...	17735	5371	111	56	35.83	2.40E-05	1.562936	AARSD1;ABCD4;ACTR5;AKIRIN2;ASNS;CARKD;CDK5RAP1;CLYBL;EXOSC2;EXOSC7;FANCE;GEMIN8;GPD1;GTF
tan	BP	GO:1901360	organic cyclic compound metabolic proces...	17735	5578	111	57	37.21	3.50E-05	1.531846	AARSD1;ABCD4;ACTR5;AKIRIN2;ASNS;CARKD;CDK5RAP1;EXOSC2;EXOSC7;FANCE;GEMIN8;GPD1;GTF2IRD2;
tan	BP	GO:0006139	nucleobase-containing compound metabolic...	17735	5209	111	53	34.75	0.00011	1.52518	AARSD1;ACTR5;AKIRIN2;ASNS;CARKD;CDK5RAP1;EXOSC2;EXOSC7;FANCE;GEMIN8;GPD1;GTF2IRD2;GTF2IRD
tan	BP	GO:0006807	nitrogen compound metabolic process	17735	5942	111	57	39.64	0.00027	1.437941	AARSD1;ABCD4;ACTR5;AKIRIN2;ASNS;BPHL;CARKD;CDK5RAP1;EXOSC2;EXOSC7;FANCE;GEMIN8;GPD1;GTF2
tan	BP	GO:0090304	nucleic acid metabolic process	17735	4431	111	46	29.56	0.00032	1.556157	AARSD1;ACTR5;AKIRIN2;CDK5RAP1;EXOSC2;EXOSC7;FANCE;GEMIN8;GTF2IRD2;GTF2IRD2B;HIST1H2BC;HIST
tan	BP	GO:0034641	cellular nitrogen compound metabolic pro...	17735	5595	111	54	37.32	0.00042	1.446945	AARSD1;ACTR5;AKIRIN2;ASNS;CARKD;CDK5RAP1;EXOSC2;EXOSC7;FANCE;GEMIN8;GPD1;GTF2IRD2;GTF2IRD
tan	BP	GO:0006733	oxidoreduction coenzyme metabolic proces...	17735	57	111	4	0.38	0.00056	10.52632	CARKD;COQ6;GPD1;LDHB
tan	MF	GO:0008270	zinc ion binding	17735	1977	111	25	12.82	0.00065	1.950078	CRIP3;GTF2IRD2;GTF2IRD2B;HKR1;PRR3;SIRT5;TCEA3;ZNF133;ZNF185;ZNF202;ZNF211;ZNF232;ZNF266;ZNF
tan	CC	GO:0043231	intracellular membrane-bounded organelle	17735	9560	111	77	61.06	0.001	1.261055	ABCD4;ACTR5;AEN;AKIRIN2;AP3M2;AP4B1;ATG16L1;BPHL;C12orf65;CARKD;CDK20;CIAPIN1;CLYBL;COQ10A
tan	CC	GO:0043227	membrane-bounded organelle	17735	9590	111	77	61.25	0.0011	1.257143	ABCD4;ACTR5;AEN;AKIRIN2;AP3M2;AP4B1;ATG16L1;BPHL;C12orf65;CARKD;CDK20;CIAPIN1;CLYBL;COQ10A
tan	BP	GO:0044237	cellular metabolic process	17735	8597	111	72	57.35	0.00133	1.255449	AARSD1;ABCD4;ACTR5;AKIRIN2;ALDH8A1;ASNS;ATG16L1;BPHL;CARKD;CDK5RAP1;CLYBL;COQ6;DNAJC9;DPI
tan	BP	GO:0016070	RNA metabolic process	17735	3911	111	40	26.09	0.0015	1.533154	AARSD1;ACTR5;AKIRIN2;CDK5RAP1;EXOSC2;EXOSC7;GEMIN8;GTF2IRD2;GTF2IRD2B;HKR1;MEAF6;METTL3;I
tan	MF	GO:0046914	transition metal ion binding	17735	2217	111	26	14.37	0.00153	1.809325	CRIP3;GTF2IRD2;GTF2IRD2B;HKR1;METTL17;PRR3;SIRT5;TCEA3;ZNF133;ZNF185;ZNF202;ZNF211;ZNF232;ZN
tan	MF	GO:1901363	heterocyclic compound binding	17735	5302	111	49	34.38	0.00164	1.425247	AARSD1;ABCD4;ACTR5;AEN;ASNS;C12orf65;CARKD;CDK20;COQ6;DNAH3;EXOSC2;EXOSC7;FGR;GPD1;GTF2II
tan	MF	GO:0051539	4 iron, 4 sulfur cluster binding	17735	37	111	3	0.24	0.00175	12.5	CDK5RAP1;LIAS;RSAD1
tan	MF	GO:0097159	organic cyclic compound binding	17735	5372	111	49	34.83	0.00225	1.406833	AARSD1;ABCD4;ACTR5;AEN;ASNS;C12orf65;CARKD;CDK20;COQ6;DNAH3;EXOSC2;EXOSC7;FGR;GPD1;GTF2II
tan	MF	GO:0003676	nucleic acid binding	17735	3299	111	34	21.39	0.0023	1.589528	AARSD1;AEN;C12orf65;EXOSC2;EXOSC7;GTF2IRD2;GTF2IRD2B;HIST1H2BC;HIST1H2BD;HIST2H2BF;HKR1;ME
tan	CC	GO:0005739	mitochondrion	17735	1488	111	19	9.5	0.0026	2	BPHL;C12orf65;CARKD;CLYBL;COQ10A;COQ6;FGR;GPD1;LDHB;LETMD1;LIAS;METTL17;PEX11B;RBFA;RSAD1;
tan	CC	GO:0044424	intracellular part	17735	12103	111	90	77.3	0.0028	1.164295	AARSD1;ABCD4;ACTR5;AEN;AKIRIN2;ALDH8A1;AP3M2;AP4B1;ASNS;ATG16L1;BPHL;C12orf65;CARKD;CD302
tan	MF	GO:0051287	NAD binding	17735	44	111	3	0.29	0.00289	10.34483	GPD1;LDHB;SIRT5
tan	BP	GO:0034660	ncRNA metabolic process	17735	285	111	7	1.9	0.00295	3.684211	AARSD1;CDK5RAP1;EXOSC2;EXOSC7;RBFA;RPP38;WDR77
tan	BP	GO:0008152	metabolic process	17735	9452	111	76	63.05	0.00302	1.205393	AARSD1;ABCD4;ACTR5;AKIRIN2;ALDH8A1;ASNS;ATG16L1;BPHL;CARKD;CDK5RAP1;CLYBL;COQ6;DNAJC9;DPI
tan	MF	GO:0043169	cation binding	17735	4026	111	39	26.1	0.00303	1.494253	AARSD1;CDK5RAP1;CLYBL;CRIP3;GTF2IRD2;GTF2IRD2B;HKR1;HPCAL4;ILKAP;ITGA10;LIAS;METTL17;OCM;OV
tan	MF	GO:0003677	DNA binding	17735	2332	111	26	15.12	0.00316	1.719577	GTF2IRD2;GTF2IRD2B;HIST1H2BC;HIST1H2BD;HIST2H2BF;HKR1;PMS2P1;POLR1C;TCEA3;ZNF133;ZNF202;ZN
tan	CC	GO:0005779	integral to peroxisomal membrane	17735	14	111	2	0.09	0.0035	22.22222	PEX11B;SLC25A17
tan	CC	GO:0031231	intrinsic to peroxisomal membrane	17735	14	111	2	0.09	0.0035	22.22222	PEX11B;SLC25A17

tan	CC	GO:0005622	intracellular	17735	12344	111	91	78.84	0.0035	1.154236	AARSD1;ABCD4;ACTR5;AEN;AKIRIN2;ALDH8A1;AP3M2;AP4B1;ASNS;ATG16L1;BPHL;C12orf65;CARKD;CD302
tan	BP	GO:0071704	organic substance metabolic process	17735	8996	111	73	60.01	0.00361	1.216464	AARSD1;ABCD4;ACTR5;AKIRIN2;ALDH8A1;ASNS;BPHL;CARKD;CDK5RAP1;COQ6;DNAJC9;DPH2;DUSP6;EXOS
tan	BP	GO:0019362	pyridine nucleotide metabolic process	17735	47	111	3	0.31	0.00377	9.677419	CARKD;GPD1;LDHB
tan	BP	GO:0046496	nicotinamide nucleotide metabolic proces...	17735	47	111	3	0.31	0.00377	9.677419	CARKD;GPD1;LDHB
tan	MF	GO:0046872	metal ion binding	17735	3936	111	38	25.52	0.00377	1.489028	AARSD1;CDK5RAP1;CLYBL;CRIP3;GTF2IRD2;GTF2IRD2B;HKR1;HPCAL4;ILKAP;ITGA10;LIAS;METTL17;OCM;PD
tan	MF	GO:0000175	3'-5'-exoribonuclease activity	17735	15	111	2	0.1	0.00414	20	EXOSC2;EXOSC7
tan	BP	GO:0010467	gene expression	17735	4403	111	42	29.37	0.0044	1.430031	AARSD1;ACTR5;AKIRIN2;CDK5RAP1;EXOSC2;EXOSC7;GEMIN8;GTF2IRD2;GTF2IRD2B;HKR1;KLKB1;MEAF6;MI
tan	MF	GO:0005488	binding	17735	11799	111	87	76.5	0.00466	1.137255	AARSD1;ABCD4;ACTR5;AEN;ASNS;ATG16L1;C12orf65;CARKD;CD302;CDK20;CDK5RAP1;CLYBL;CNPY4;COQ6;
tan	BP	GO:0051186	cofactor metabolic process	17735	233	111	6	1.55	0.00467	3.870968	CARKD;COQ6;GPD1;LDHB;LIAS;RSAD1
tan	BP	GO:0045070	positive regulation of viral genome repl...	17735	16	111	2	0.11	0.00497	18.18182	PPIE;PPIH
tan	BP	GO:0072524	pyridine-containing compound metabolic p...	17735	52	111	3	0.35	0.00502	8.571429	CARKD;GPD1;LDHB
tan	CC	GO:0005778	peroxisomal membrane	17735	55	111	3	0.35	0.0052	8.571429	ABCD4;PEX11B;SLC25A17
tan	CC	GO:0031903	microbody membrane	17735	55	111	3	0.35	0.0052	8.571429	ABCD4;PEX11B;SLC25A17
tan	MF	GO:0016896	exoribonuclease activity, producing 5'-p...	17735	17	111	2	0.11	0.00531	18.18182	EXOSC2;EXOSC7
tan	BP	GO:0006334	nucleosome assembly	17735	108	111	4	0.72	0.00588	5.555556	HIST1H2BC;HIST1H2BD;HIST2H2BF;NAP1L4
tan	MF	GO:0004532	exoribonuclease activity	17735	18	111	2	0.12	0.00595	16.66667	EXOSC2;EXOSC7
tan	MF	GO:0043167	ion binding	17735	5761	111	50	37.35	0.00615	1.338688	AARSD1;ABCD4;ACTR5;ASNS;CARKD;CDK20;CDK5RAP1;CLYBL;COQ6;CRIP3;DNAH3;FGR;GTF2IRD2;GTF2IRD
tan	MF	GO:0016616	oxidoreductase activity, acting on the C...	17735	115	111	4	0.75	0.00664	5.333333	GPD1;HSD17B7;LDHB;RDH16
tan	MF	GO:0004527	exonuclease activity	17735	60	111	3	0.39	0.00692	7.692308	AEN;EXOSC2;EXOSC7
tan	BP	GO:0000413	protein peptidyl-prolyl isomerization	17735	19	111	2	0.13	0.00699	15.38462	PPIE;PPIH
tan	CC	GO:0000178	exosome (RNase complex)	17735	20	111	2	0.13	0.0071	15.38462	EXOSC2;EXOSC7
tan	MF	GO:0051536	iron-sulfur cluster binding	17735	61	111	3	0.4	0.00725	7.5	CDK5RAP1;LIAS;RSAD1
tan	MF	GO:0051540	metal cluster binding	17735	61	111	3	0.4	0.00725	7.5	CDK5RAP1;LIAS;RSAD1
tan	BP	GO:0031497	chromatin assembly	17735	119	111	4	0.79	0.00824	5.063291	HIST1H2BC;HIST1H2BD;HIST2H2BF;NAP1L4
tan	BP	GO:0006732	coenzyme metabolic process	17735	190	111	5	1.27	0.00884	3.937008	CARKD;COQ6;GPD1;LDHB;LIAS
tan	CC	GO:0000786	nucleosome	17735	67	111	3	0.43	0.009	6.976744	HIST1H2BC;HIST1H2BD;HIST2H2BF
tan	BP	GO:1901362	organic cyclic compound biosynthetic pro...	17735	3752	111	36	25.03	0.00903	1.438274	ACTR5;AKIRIN2;GTF2IRD2;GTF2IRD2B;HKR1;HSD17B7;LIAS;MEAF6;NPR2;PCID2;POLR1C;PPIE;PRPSAP2;RSAI
tan	BP	GO:0019674	NAD metabolic process	17735	22	111	2	0.15	0.00933	13.33333	GPD1;LDHB
tan	BP	GO:0044238	primary metabolic process	17735	8755	111	70	58.4	0.00936	1.19863	AARSD1;ACTR5;AKIRIN2;ALDH8A1;ASNS;BPHL;CARKD;CDK5RAP1;DNAJC9;DPH2;DUSP6;EXOSC2;EXOSC7;FAI
tan	MF	GO:0016614	oxidoreductase activity, acting on CH-OH...	17735	128	111	4	0.83	0.00961	4.819277	GPD1;HSD17B7;LDHB;RDH16
tan	MF	GO:0004540	ribonuclease activity	17735	68	111	3	0.44	0.00977	6.818182	EXOSC2;EXOSC7;RPP38
tan	BP	GO:0044260	cellular macromolecule metabolic process	17735	6621	111	56	44.17	0.01034	1.267829	AARSD1;ACTR5;AKIRIN2;ASNS;CDK5RAP1;DNAJC9;DPH2;DUSP6;EXOSC2;EXOSC7;FANCE;FGR;GALNT12;GEM
tan	BP	GO:0018130	heterocycle biosynthetic process	17735	3650	111	35	24.35	0.01038	1.437372	ACTR5;AKIRIN2;GTF2IRD2;GTF2IRD2B;HKR1;LIAS;MEAF6;NPR2;PCID2;POLR1C;PPIE;PRPSAP2;RSAD1;SIRT5;S
tan	CC	GO:0043229	intracellular organelle	17735	10522	111	79	67.2	0.0104	1.175595	ABCD4;ACTR5;AEN;AKIRIN2;AP3M2;AP4B1;ATG16L1;BPHL;C12orf65;CARKD;CDK20;CIAPIN1;CLYBL;COQ10A
tan	BP	GO:0034470	ncRNA processing	17735	198	111	5	1.32	0.01045	3.787879	CDK5RAP1;EXOSC2;EXOSC7;RBFA;RPP38
tan	MF	GO:0016796	exonuclease activity, active with either...	17735	24	111	2	0.16	0.01047	12.5	EXOSC2;EXOSC7
tan	BP	GO:0034728	nucleosome organization	17735	128	111	4	0.85	0.01058	4.705882	HIST1H2BC;HIST1H2BD;HIST2H2BF;NAP1L4
tan	CC	GO:0043226	organelle	17735	10552	111	79	67.39	0.0115	1.172281	ABCD4;ACTR5;AEN;AKIRIN2;AP3M2;AP4B1;ATG16L1;BPHL;C12orf65;CARKD;CDK20;CIAPIN1;CLYBL;COQ10A
tan	BP	GO:0000291	nuclear-transcribed mRNA catabolic proce...	17735	26	111	2	0.17	0.0129	11.76471	EXOSC2;EXOSC7
tan	BP	GO:0043928	exonucleolytic nuclear-transcribed mRNA ...	17735	26	111	2	0.17	0.0129	11.76471	EXOSC2;EXOSC7
tan	MF	GO:0015932	nucleobase-containing compound transmemb...	17735	27	111	2	0.18	0.01314	11.11111	SLC25A17;SLC25A4
tan	BP	GO:0065004	protein-DNA complex assembly	17735	137	111	4	0.91	0.01331	4.395604	HIST1H2BC;HIST1H2BD;HIST2H2BF;NAP1L4
tan	CC	GO:0044452	nucleolar part	17735	28	111	2	0.18	0.0137	11.11111	POLR1C;RPP38
tan	CC	GO:0005634	nucleus	17735	5873	111	49	37.51	0.0138	1.306318	ACTR5;AEN;AKIRIN2;CDK20;CIAPIN1;DSN1;DUSP6;EXOSC2;EXOSC7;FANCE;GEMIN8;GTF2IRD2;GTF2IRD2B;H
tan	CC	GO:0044438	microbody part	17735	79	111	3	0.5	0.0141	6	ABCD4;PEX11B;SLC25A17
tan	CC	GO:0044439	peroxisomal part	17735	79	111	3	0.5	0.0141	6	ABCD4;PEX11B;SLC25A17
tan	BP	GO:0006333	chromatin assembly or disassembly	17735	140	111	4	0.93	0.01431	4.301075	HIST1H2BC;HIST1H2BD;HIST2H2BF;NAP1L4
tan	BP	GO:0044271	cellular nitrogen compound biosynthetic ...	17735	3726	111	35	24.86	0.01431	1.407884	ACTR5;AKIRIN2;ASNS;GTF2IRD2;GTF2IRD2B;HKR1;MEAF6;NPR2;PCID2;POLR1C;PPIE;PRPSAP2;RSAD1;SIRT5;
tan	CC	GO:0030131	clathrin adaptor complex	17735	29	111	2	0.19	0.0147	10.52632	AP3M2;AP4B1
tan	BP	GO:0018208	peptidyl-proline modification	17735	28	111	2	0.19	0.01487	10.52632	PPIE;PPIH
tan	BP	GO:0006351	transcription, DNA-dependent	17735	3208	111	31	21.4	0.01529	1.448598	ACTR5;AKIRIN2;GTF2IRD2;GTF2IRD2B;HKR1;MEAF6;PCID2;POLR1C;PPIE;SIRT5;SUPT7L;TCEA3;TGFB3;WDR7
tan	BP	GO:0019438	aromatic compound biosynthetic process	17735	3638	111	34	24.27	0.01731	1.400906	ACTR5;AKIRIN2;GTF2IRD2;GTF2IRD2B;HKR1;MEAF6;NPR2;PCID2;POLR1C;PPIE;PRPSAP2;RSAD1;SIRT5;SUPT
tan	BP	GO:0000387	spliceosomal snRNP assembly	17735	31	111	2	0.21	0.01806	9.52381	GEMIN8;WDR77
tan	BP	GO:0006323	DNA packaging	17735	153	111	4	1.02	0.0192	3.921569	HIST1H2BC;HIST1H2BD;HIST2H2BF;NAP1L4
tan	MF	GO:0008408	3'-5' exonuclease activity	17735	33	111	2	0.21	0.01928	9.52381	EXOSC2;EXOSC7
tan	BP	GO:0006355	regulation of transcription, DNA-depende...	17735	2869	111	28	19.14	0.0194	1.462905	ACTR5;AKIRIN2;GTF2IRD2;GTF2IRD2B;HKR1;MEAF6;PCID2;PPIE;SIRT5;SUPT7L;TCEA3;TGFB3;WDR77;ZNF20
tan	BP	GO:0022613	ribonucleoprotein complex biogenesis	17735	233	111	5	1.55	0.01983	3.225806	EXOSC2;EXOSC7;GEMIN8;RBFA;WDR77
tan	MF	GO:0004518	nuclease activity	17735	161	111	4	1.04	0.02068	3.846154	AEN;EXOSC2;EXOSC7;RPP38
tan	BP	GO:0044249	cellular biosynthetic process	17735	5073	111	44	33.84	0.02082	1.300236	AARSD1;ACTR5;AKIRIN2;ALDH8A1;ASNS;COQ6;DPH2;GALNT12;GPD1;GTF2IRD2;GTF2IRD2B;HKR1;HSD17B7
tan	BP	GO:0071824	protein-DNA complex subunit organization	17735	157	111	4	1.05	0.02089	3.809524	HIST1H2BC;HIST1H2BD;HIST2H2BF;NAP1L4
tan	BP	GO:2001141	regulation of RNA biosynthetic process	17735	2889	111	28	19.27	0.02115	1.453036	ACTR5;AKIRIN2;GTF2IRD2;GTF2IRD2B;HKR1;MEAF6;PCID2;PPIE;SIRT5;SUPT7L;TCEA3;TGFB3;WDR77;ZNF20
tan	BP	GO:0032774	RNA biosynthetic process	17735	3293	111	31	21.97	0.02183	1.411015	ACTR5;AKIRIN2;GTF2IRD2;GTF2IRD2B;HKR1;MEAF6;PCID2;POLR1C;PPIE;SIRT5;SUPT7L;TCEA3;TGFB3;WDR7
tan	CC	GO:0030119	AP-type membrane coat adaptor complex	17735	36	111	2	0.23	0.0221	8.695652	AP3M2;AP4B1
tan	BP	GO:0034654	nucleobase-containing compound biosynthe...	17735	3583	111	33	23.9	0.02375	1.380753	ACTR5;AKIRIN2;GTF2IRD2;GTF2IRD2B;HKR1;MEAF6;NPR2;PCID2;POLR1C;PPIE;PRPSAP2;SIRT5;SUPT7L;TCEA
tan	MF	GO:0003755	peptidyl-prolyl cis-trans isomerase acti...	17735	37	111	2	0.24	0.02392	8.333333	PPIE;PPIH
tan	MF	GO:0048037	cofactor binding	17735	255	111	5	1.65	0.02511	3.030303	ASNS;COQ6;GPD1;LDHB;SIRT5
tan	MF	GO:0016859	cis-trans isomerase activity	17735	39	111	2	0.25	0.02639	8	PPIE;PPIH
tan	BP	GO:0051252	regulation of RNA metabolic process	17735	2953	111	28	19.7	0.02765	1.42132	ACTR5;AKIRIN2;GTF2IRD2;GTF2IRD2B;HKR1;MEAF6;PCID2;PPIE;SIRT5;SUPT7L;TCEA3;TGFB3;WDR77;ZNF20
tan	CC	GO:0030118	clathrin coat	17735	41	111	2	0.26	0.0282	7.692308	AP3M2;AP4B1

tan	BP	GO:0006396	RNA processing	17735	646	111	9	4.31	0.02834	2.088167	CDK5RAP1;EXOSC2;EXOSC7;GEMIN8;PPIE;PPIH;RBFA;RPP38;WDR77
tan	MF	GO:0050662	coenzyme binding	17735	179	111	4	1.16	0.02908	3.448276	COQ6;GPD1;LDHB;SIRT5
tan	BP	GO:0006364	rRNA processing	17735	105	111	3	0.7	0.03309	4.285714	EXOSC2;EXOSC7;RBFA
tan	CC	GO:0032993	protein-DNA complex	17735	110	111	3	0.7	0.0334	4.285714	HIST1H2BC;HIST1H2BD;HIST2H2BF
tan	BP	GO:0009058	biosynthetic process	17735	5219	111	44	34.81	0.03396	1.264005	AARSD1;ACTR5;AKIRIN2;ALDH8A1;ASNS;COQ6;DPH2;GALNT12;GPD1;GTF2IRD2;GTF2IRD2B;HKR1;HSD17B7
tan	BP	GO:0071103	DNA conformation change	17735	189	111	4	1.26	0.03766	3.174603	HIST1H2BC;HIST1H2BD;HIST2H2BF;NAP1L4
tan	BP	GO:0043966	histone H3 acetylation	17735	46	111	2	0.31	0.0377	6.451613	MEAF6;SUPT7L
tan	BP	GO:0016072	rRNA metabolic process	17735	111	111	3	0.74	0.03807	4.054054	EXOSC2;EXOSC7;RBFA
tan	CC	GO:0005758	mitochondrial intermembrane space	17735	49	111	2	0.31	0.0391	6.451613	FGR;SIRT5
tan	BP	GO:0051704	multi-organism process	17735	1380	111	15	9.21	0.0396	1.628664	AKIRIN2;DNAJC9;FGR;HIST1H2BC;IL24;LIAS;NUP43;OVGP1;PPIE;PPIH;SLC25A4;STMN1;TGFB3;ZNF202;ZNF2
tan	BP	GO:0043170	macromolecule metabolic process	17735	7334	111	58	48.92	0.03996	1.185609	AARSD1;ACTR5;AKIRIN2;ASNS;CDK5RAP1;DNAJC9;DPH2;DUSP6;EXOSC2;EXOSC7;FANCE;FGR;GALNT12;GEM
tan	BP	GO:0033013	tetrapyrrole metabolic process	17735	48	111	2	0.32	0.04074	6.25	ABCD4;RSAD1
tan	BP	GO:0045069	regulation of viral genome replication	17735	48	111	2	0.32	0.04074	6.25	PPIE;PPIH
tan	BP	GO:1901576	organic substance biosynthetic process	17735	5154	111	43	34.38	0.0434	1.250727	AARSD1;ACTR5;AKIRIN2;ALDH8A1;ASNS;COQ6;GALNT12;GPD1;GTF2IRD2;GTF2IRD2B;HKR1;HSD17B7;LIAS;
tan	CC	GO:0005777	peroxisome	17735	123	111	3	0.79	0.0442	3.797468	ABCD4;PEX11B;SLC25A17
tan	CC	GO:0042579	microbody	17735	123	111	3	0.79	0.0442	3.797468	ABCD4;PEX11B;SLC25A17
tan	CC	GO:0005730	nucleolus	17735	622	111	8	3.97	0.0457	2.015113	AEN;CIAPIN1;EXOSC2;EXOSC7;MEAF6;NVL;POLR1C;RPP38
tan	BP	GO:0006399	tRNA metabolic process	17735	121	111	3	0.81	0.04715	3.703704	AARSD1;CDK5RAP1;RPP38
tan	CC	GO:0031970	organelle envelope lumen	17735	56	111	2	0.36	0.0498	5.555556	FGR;SIRT5
salmon	CC	GO:0022627	cytosolic small ribosomal subunit	17735	36	114	20	0.24	1.00E-30	83.33333	FAU;MRPS7;RPS11;RPS12;RPS13;RPS14;RPS16;RPS18;RPS19;RPS20;RPS21;RPS23;RPS25;RPS27A;RPS3;RPS4
salmon	CC	GO:0022626	cytosolic ribosome	17735	91	114	39	0.61	1.00E-30	63.93443	FAU;MRPS7;RPL10;RPL10A;RPL12;RPL13A;RPL14;RPL19;RPL22;RPL23A;RPL24;RPL27A;RPL3;RPL32;RPL35A;
salmon	BP	GO:0006415	translational termination	17735	91	114	39	0.67	1.00E-30	58.20896	FAU;ICT1;RPL10;RPL10A;RPL12;RPL13A;RPL14;RPL19;RPL22;RPL23A;RPL24;RPL27A;RPL3;RPL32;RPL35A;RPL
salmon	CC	GO:0015935	small ribosomal subunit	17735	60	114	21	0.4	1.00E-30	52.5	FAU;MRPS16;MRPS7;RPS11;RPS12;RPS13;RPS14;RPS16;RPS18;RPS19;RPS20;RPS21;RPS23;RPS25;RPS27A;R
salmon	BP	GO:0006614	SRP-dependent cotranslational protein ta...	17735	105	114	39	0.77	1.00E-30	50.64935	FAU;RPL10;RPL10A;RPL12;RPL13A;RPL14;RPL19;RPL22;RPL23A;RPL24;RPL27A;RPL3;RPL32;RPL35A;RPL36;R
salmon	BP	GO:0006613	cotranslational protein targeting to mem...	17735	107	114	39	0.79	1.00E-30	49.36709	FAU;RPL10;RPL10A;RPL12;RPL13A;RPL14;RPL19;RPL22;RPL23A;RPL24;RPL27A;RPL3;RPL32;RPL35A;RPL36;R
salmon	BP	GO:0045047	protein targeting to ER	17735	107	114	39	0.79	1.00E-30	49.36709	FAU;RPL10;RPL10A;RPL12;RPL13A;RPL14;RPL19;RPL22;RPL23A;RPL24;RPL27A;RPL3;RPL32;RPL35A;RPL36;R
salmon	BP	GO:0006414	translational elongation	17735	108	114	39	0.79	1.00E-30	49.36709	FAU;RPL10;RPL10A;RPL12;RPL13A;RPL14;RPL19;RPL22;RPL23A;RPL24;RPL27A;RPL3;RPL32;RPL35A;RPL36;R
salmon	BP	GO:0072599	establishment of protein localization to...	17735	108	114	39	0.79	1.00E-30	49.36709	FAU;RPL10;RPL10A;RPL12;RPL13A;RPL14;RPL19;RPL22;RPL23A;RPL24;RPL27A;RPL3;RPL32;RPL35A;RPL36;R
salmon	CC	GO:0044391	ribosomal subunit	17735	134	114	41	0.9	1.00E-30	45.55556	FAU;ICT1;MRPS16;MRPS7;RPL10;RPL10A;RPL12;RPL13A;RPL14;RPL19;RPL22;RPL23A;RPL24;RPL27A;RPL3;R
salmon	BP	GO:0070972	protein localization to endoplasmic reti...	17735	121	114	39	0.89	1.00E-30	43.82022	FAU;RPL10;RPL10A;RPL12;RPL13A;RPL14;RPL19;RPL22;RPL23A;RPL24;RPL27A;RPL3;RPL32;RPL35A;RPL36;R
salmon	BP	GO:0000184	nuclear-transcribed mRNA catabolic proce...	17735	118	114	38	0.87	1.00E-30	43.67816	FAU;RPL10;RPL10A;RPL12;RPL13A;RPL14;RPL19;RPL22;RPL23A;RPL24;RPL27A;RPL3;RPL32;RPL35A;RPL36;R
salmon	MF	GO:0003735	structural constituent of ribosome	17735	157	114	40	1.05	1.00E-30	38.09524	FAU;MRPS16;MRPS7;RPL10;RPL10A;RPL12;RPL13A;RPL14;RPL19;RPL22;RPL23A;RPL24;RPL27A;RPL3;RPL32;
salmon	BP	GO:0006612	protein targeting to membrane	17735	151	114	40	1.11	1.00E-30	36.03604	FAU;MYL12A;RPL10;RPL10A;RPL12;RPL13A;RPL14;RPL19;RPL22;RPL23A;RPL24;RPL27A;RPL3;RPL32;RPL35A
salmon	BP	GO:0019080	viral genome expression	17735	148	114	39	1.09	1.00E-30	35.77982	FAU;POLR2H;RPL10;RPL10A;RPL12;RPL13A;RPL14;RPL19;RPL22;RPL23A;RPL24;RPL27A;RPL3;RPL32;RPL35A
salmon	BP	GO:0019083	viral transcription	17735	148	114	39	1.09	1.00E-30	35.77982	FAU;POLR2H;RPL10;RPL10A;RPL12;RPL13A;RPL14;RPL19;RPL22;RPL23A;RPL24;RPL27A;RPL3;RPL32;RPL35A
salmon	BP	GO:0006413	translational initiation	17735	152	114	40	1.12	1.00E-30	35.71429	EIF3H;EIF3M;FAU;RPL10;RPL10A;RPL12;RPL13A;RPL14;RPL19;RPL22;RPL23A;RPL24;RPL27A;RPL3;RPL32;RPL
salmon	BP	GO:0043624	cellular protein complex disassembly	17735	158	114	39	1.16	1.00E-30	33.62069	FAU;ICT1;RPL10;RPL10A;RPL12;RPL13A;RPL14;RPL19;RPL22;RPL23A;RPL24;RPL27A;RPL3;RPL32;RPL35A;RPL
salmon	CC	GO:0044445	cytosolic part	17735	181	114	40	1.21	1.00E-30	33.05785	FAU;MRPS7;RPL10;RPL10A;RPL12;RPL13A;RPL14;RPL19;RPL22;RPL23A;RPL24;RPL27A;RPL3;RPL32;RPL35A;
salmon	BP	GO:0043241	protein complex disassembly	17735	163	114	39	1.2	1.00E-30	32.5	FAU;ICT1;RPL10;RPL10A;RPL12;RPL13A;RPL14;RPL19;RPL22;RPL23A;RPL24;RPL27A;RPL3;RPL32;RPL35A;RPL
salmon	BP	GO:0000956	nuclear-transcribed mRNA catabolic proce...	17735	169	114	38	1.24	1.00E-30	30.64516	FAU;RPL10;RPL10A;RPL12;RPL13A;RPL14;RPL19;RPL22;RPL23A;RPL24;RPL27A;RPL3;RPL32;RPL35A;RPL36;R
salmon	CC	GO:0005840	ribosome	17735	206	114	41	1.38	1.00E-30	29.71014	FAU;ICT1;MRPS16;MRPS7;RPL10;RPL10A;RPL12;RPL13A;RPL14;RPL19;RPL22;RPL23A;RPL24;RPL27A;RPL3;R
salmon	BP	GO:0032984	macromolecular complex disassembly	17735	184	114	39	1.35	1.00E-30	28.88889	FAU;ICT1;RPL10;RPL10A;RPL12;RPL13A;RPL14;RPL19;RPL22;RPL23A;RPL24;RPL27A;RPL3;RPL32;RPL35A;RPL
salmon	BP	GO:0006402	mRNA catabolic process	17735	179	114	38	1.32	1.00E-30	28.78788	FAU;RPL10;RPL10A;RPL12;RPL13A;RPL14;RPL19;RPL22;RPL23A;RPL24;RPL27A;RPL3;RPL32;RPL35A;RPL36;R
salmon	BP	GO:0072594	establishment of protein localization to...	17735	210	114	40	1.55	1.00E-30	25.80645	FAU;RPL10;RPL10A;RPL12;RPL13A;RPL14;RPL19;RPL22;RPL23A;RPL24;RPL27A;RPL3;RPL32;RPL35A;RPL36;R
salmon	BP	GO:0019058	viral infectious cycle	17735	223	114	41	1.64	1.00E-30	25	FAU;POLR2H;PPIA;RAN;RPL10;RPL10A;RPL12;RPL13A;RPL14;RPL19;RPL22;RPL23A;RPL24;RPL27A;RPL3;RPL
salmon	BP	GO:0006401	RNA catabolic process	17735	206	114	38	1.52	1.00E-30	25	FAU;RPL10;RPL10A;RPL12;RPL13A;RPL14;RPL19;RPL22;RPL23A;RPL24;RPL27A;RPL3;RPL32;RPL35A;RPL36;R
salmon	BP	GO:0022411	cellular component disassembly	17735	296	114	39	2.18	1.00E-30	17.88991	FAU;ICT1;RPL10;RPL10A;RPL12;RPL13A;RPL14;RPL19;RPL22;RPL23A;RPL24;RPL27A;RPL3;RPL32;RPL35A;RPL
salmon	BP	GO:0006412	translation	17735	464	114	46	3.41	1.00E-30	13.48974	EIF3H;EIF3M;FAU;ICT1;MRPS16;MRPS7;NACA;RPL10;RPL10A;RPL12;RPL13A;RPL14;RPL19;RPL22;RPL23A;RP
salmon	CC	GO:0030529	ribonucleoprotein complex	17735	573	114	47	3.83	1.00E-30	12.27154	FAU;HNRNPA1;ICT1;ISY1;MRPS16;MRPS7;NPM1;POP4;RPL10;RPL10A;RPL12;RPL13A;RPL14;RPL19;RPL22;R
salmon	BP	GO:0006605	protein targeting	17735	479	114	43	3.52	1.00E-30	12.21591	FAU;MYL12A;PRDX1;RPL10;RPL10A;RPL12;RPL13A;RPL14;RPL19;RPL22;RPL23A;RPL24;RPL27A;RPL3;RPL32;
salmon	BP	GO:0022415	viral reproductive process	17735	542	114	47	3.99	1.00E-30	11.77945	C9orf156;FAU;HNRNPA1;NACA;NPM1;PDCL3;POLR2H;PPIA;PSMB6;RAN;RPL10;RPL10A;RPL12;RPL13A;RPL1
salmon	BP	GO:0033365	protein localization to organelle	17735	510	114	42	3.75	1.00E-30	11.2	FAU;PRDX1;RPL10;RPL10A;RPL12;RPL13A;RPL14;RPL19;RPL22;RPL23A;RPL24;RPL27A;RPL3;RPL32;RPL35A;R
salmon	BP	GO:0016071	mRNA metabolic process	17735	607	114	47	4.47	1.00E-30	10.51454	FAU;HNRNPA1;ISY1;POLR2H;POP4;PSMA5;PSMB6;RPL10;RPL10A;RPL12;RPL13A;RPL14;RPL19;RPL22;RPL23
salmon	BP	GO:0016032	viral reproduction	17735	689	114	49	5.07	1.00E-30	9.664694	C9orf156;FAU;HNRNPA1;NACA;NPM1;PDCL3;POLR2H;PPIA;PSMA5;PSMB6;RAN;RPL10;RPL10A;RPL12;RPL1
salmon	BP	GO:0044764	multi-organism cellular process	17735	691	114	49	5.08	1.00E-30	9.645669	C9orf156;FAU;HNRNPA1;NACA;NPM1;PDCL3;POLR2H;PPIA;PSMA5;PSMB6;RAN;RPL10;RPL10A;RPL12;RPL1
salmon	BP	GO:0044703	multi-organism reproductive process	17735	730	114	47	5.37	1.00E-30	8.752328	C9orf156;FAU;HNRNPA1;NACA;NPM1;PDCL3;POLR2H;PPIA;PSMB6;RAN;RPL10;RPL10A;RPL12;RPL13A;RPL1
salmon	BP	GO:0006886	intracellular protein transport	17735	702	114	45	5.17	1.00E-30	8.704062	FAU;MYL12A;NPM1;PRDX1;RAN;RPL10;RPL10A;RPL12;RPL13A;RPL14;RPL19;RPL22;RPL23A;RPL24;RPL27A;
salmon	BP	GO:0016482	cytoplasmic transport	17735	719	114	46	5.29	1.00E-30	8.695652	FAU;HNRNPA1;PRDX1;RAN;RPL10;RPL10A;RPL12;RPL13A;RPL14;RPL19;RPL22;RPL23A;RPL24;RPL27A;RPL3;
salmon	MF	GO:0005198	structural molecule activity	17735	620	114	41	4.14	1.30E-30	9.903382	FAU;MRPS16;MRPS7;MYL5;RPL10;RPL10A;RPL12;RPL13A;RPL14;RPL19;RPL22;RPL23A;RPL24;RPL27A;RPL3;
salmon	BP	GO:0048610	cellular process involved in reproductio...	17735	544	114	40	4	8.20E-30	10	FAU;POLR2H;RPL10;RPL10A;RPL12;RPL13A;RPL14;RPL19;RPL22;RPL23A;RPL24;RPL27A;RPL3;RPL32;RPL35A
salmon	BP	GO:0044265	cellular macromolecule catabolic process	17735	698	114	43	5.14	5.00E-29	8.365759	COMMD1;FAU;PSMA5;PSMB6;RPL10;RPL10A;RPL12;RPL13A;RPL14;RPL19;RPL22;RPL23A;RPL24;RPL27A;RP
salmon	CC	GO:0022625	cytosolic large ribosomal subunit	17735	52	114	19	0.35	6.10E-29	54.28571	RPL10;RPL10A;RPL12;RPL13A;RPL14;RPL19;RPL22;RPL23A;RPL24;RPL27A;RPL3;RPL32;RPL35A;RPL36;RPL37
salmon	CC	GO:0015934	large ribosomal subunit	17735	75	114	20	0.5	3.20E-27	40	ICT1;RPL10;RPL10A;RPL12;RPL13A;RPL14;RPL19;RPL22;RPL23A;RPL24;RPL27A;RPL3;RPL32;RPL35A;RPL36;R
salmon	BP	GO:0034613	cellular protein localization	17735	956	114	46	7.03	1.40E-26	6.543385	FAU;MYL12A;NPM1;PRDX1;RAN;RANGRF;RPL10;RPL10A;RPL12;RPL13A;RPL14;RPL19;RPL22;RPL23A;RPL24;
salmon	BP	GO:0070727	cellular macromolecule localization	17735	960	114	46	7.06	1.60E-26	6.515581	FAU;MYL12A;NPM1;PRDX1;RAN;RANGRF;RPL10;RPL10A;RPL12;RPL13A;RPL14;RPL19;RPL22;RPL23A;RPL24;
salmon	BP	GO:0046907	intracellular transport	17735	1214	114	49	8.93	4.30E-25	5.487122	FAU;HNRNPA1;MYL12A;NPM1;PRDX1;RAN;RPL10;RPL10A;RPL12;RPL13A;RPL14;RPL19;RPL22;RPL23A;RPL2
salmon	BP	GO:0009057	macromolecule catabolic process	17735	883	114	43	6.5	7.00E-25	6.615385	COMMD1;FAU;PSMA5;PSMB6;RPL10;RPL10A;RPL12;RPL13A;RPL14;RPL19;RPL22;RPL23A;RPL24;RPL27A;RP
salmon	BP	GO:0015031	protein transport	17735	1245	114	49	9.16	1.30E-24	5.349345	ATP6V1E2;FAU;MYL12A;NACA;NPM1;PPIA;PRDX1;RAN;RANGRF;RPL10;RPL10A;RPL12;RPL13A;RPL14;RPL19
salmon	BP	GO:0045184	establishment of protein localization	17735	1288	114	49	9.48	6.00E-24	5.168776	ATP6V1E2;FAU;MYL12A;NACA;NPM1;PPIA;PRDX1;RAN;RANGRF;RPL10;RPL10A;RPL12;RPL13A;RPL14;RPL19

salmon	CC	GO:0005829	cytosol	17735	2362	114	61	15.79	9.50E-24	3.863205	ATP6V1E2;BLVRA;CETN2;CHKB;DIABLO;EIF3H;FAU;ITGB1BP1;MDH1;MRPS7;POMP;PPIA;PRDX1;PSMA5;PSM
salmon	BP	GO:0051704	multi-organism process	17735	1380	114	50	10.15	1.50E-23	4.926108	ATP6V1E2;C9orf156;FAU;HNRNPA1;NACA;NPM1;PDCL3;POLR2H;PPIA;PSMA5;PSMB6;RAN;RPL10;RPL10A;R
salmon	CC	GO:0032991	macromolecular complex	17735	3893	114	75	26.03	7.40E-23	2.881291	ATP6V1E2;CACYBP;CETN2;COMMD1;DIABLO;EIF3H;EIF3M;FAU;GTF3C6;HNRNPA1;ICT1;ISY1;MED10;MRPS1
salmon	BP	GO:0044270	cellular nitrogen compound catabolic pro...	17735	813	114	39	5.98	3.80E-22	6.521739	BLVRA;FAU;RPL10;RPL10A;RPL12;RPL13A;RPL14;RPL19;RPL22;RPL23A;RPL24;RPL27A;RPL3;RPL32;RPL35A;R
salmon	BP	GO:0046700	heterocycle catabolic process	17735	813	114	39	5.98	3.80E-22	6.521739	BLVRA;FAU;RPL10;RPL10A;RPL12;RPL13A;RPL14;RPL19;RPL22;RPL23A;RPL24;RPL27A;RPL3;RPL32;RPL35A;R
salmon	BP	GO:0019439	aromatic compound catabolic process	17735	815	114	39	6	4.10E-22	6.5	BLVRA;FAU;RPL10;RPL10A;RPL12;RPL13A;RPL14;RPL19;RPL22;RPL23A;RPL24;RPL27A;RPL3;RPL32;RPL35A;R
salmon	BP	GO:0034655	nucleobase-containing compound catabolic...	17735	770	114	38	5.67	5.60E-22	6.70194	FAU;RPL10;RPL10A;RPL12;RPL13A;RPL14;RPL19;RPL22;RPL23A;RPL24;RPL27A;RPL3;RPL32;RPL35A;RPL36;R
salmon	BP	GO:0022414	reproductive process	17735	1510	114	50	11.11	8.30E-22	4.50045	C9orf156;CETN2;FAU;HNRNPA1;NACA;NPM1;PDCL3;POLR2H;PPIA;PSMB6;RAN;RPL10;RPL10A;RPL12;RPL13
salmon	BP	GO:1901361	organic cyclic compound catabolic proces...	17735	849	114	39	6.25	1.80E-21	6.24	BLVRA;FAU;RPL10;RPL10A;RPL12;RPL13A;RPL14;RPL19;RPL22;RPL23A;RPL24;RPL27A;RPL3;RPL32;RPL35A;R
salmon	BP	GO:0071822	protein complex subunit organization	17735	1027	114	42	7.56	2.50E-21	5.555556	ARPC1A;FAU;ICT1;NPM1;POMP;RPL10;RPL10A;RPL12;RPL13A;RPL14;RPL19;RPL22;RPL23A;RPL24;RPL27A;R
salmon	BP	GO:0043933	macromolecular complex subunit organizat...	17735	1289	114	46	9.49	3.90E-21	4.847208	ARPC1A;FAU;ICT1;NAP1L1;NPM1;POMP;RPL10;RPL10A;RPL12;RPL13A;RPL14;RPL19;RPL22;RPL23A;RPL24;R
salmon	BP	GO:0000003	reproduction	17735	1591	114	50	11.71	8.40E-21	4.269855	C9orf156;CETN2;FAU;HNRNPA1;NACA;NPM1;PDCL3;POLR2H;PPIA;PSMB6;RAN;RPL10;RPL10A;RPL12;RPL13
salmon	BP	GO:0008104	protein localization	17735	1586	114	49	11.67	5.30E-20	4.1988	ATP6V1E2;FAU;MYL12A;NACA;NPM1;PPIA;PRDX1;RAN;RANGRF;RPL10;RPL10A;RPL12;RPL13A;RPL14;RPL19
salmon	MF	GO:0003723	RNA binding	17735	851	114	35	5.69	4.50E-19	6.151142	EIF3H;EIF3M;FAU;HNRNPA1;ICT1;MRPS7;NPM1;POP4;RPL10A;RPL12;RPL14;RPL19;RPL22;RPL23A;RPL24;RP
salmon	BP	GO:0051649	establishment of localization in cell	17735	1820	114	50	13.39	2.90E-18	3.73413	FAU;HNRNPA1;MYL12A;NPM1;PPIA;PRDX1;RAN;RPL10;RPL10A;RPL12;RPL13A;RPL14;RPL19;RPL22;RPL23A;
salmon	CC	GO:0043228	non-membrane-bounded organelle	17735	3122	114	62	20.87	3.50E-18	2.970771	ARPC1A;CCDC77;CETN2;COMMD1;EBNA1BP2;FAU;HMGN1;HNRNPA1;ICT1;MDH1;MRPS16;MRPS7;MYL12A
salmon	CC	GO:0043232	intracellular non-membrane-bounded organ...	17735	3122	114	62	20.87	3.50E-18	2.970771	ARPC1A;CCDC77;CETN2;COMMD1;EBNA1BP2;FAU;HMGN1;HNRNPA1;ICT1;MDH1;MRPS16;MRPS7;MYL12A
salmon	BP	GO:0033036	macromolecule localization	17735	1852	114	50	13.63	6.00E-18	3.668379	ATP6V1E2;FAU;HNRNPA1;MYL12A;NACA;NPM1;PPIA;PRDX1;RAN;RANGRF;RPL10;RPL10A;RPL12;RPL13A;RP
salmon	BP	GO:0071702	organic substance transport	17735	1950	114	51	14.35	8.90E-18	3.554007	ATP6V1E2;FAU;HNRNPA1;MYL12A;NACA;NPM1;PPIA;PRDX1;RAN;RANGRF;RPL10;RPL10A;RPL12;RPL13A;RP
salmon	BP	GO:0051641	cellular localization	17735	2049	114	51	15.08	7.50E-17	3.381963	FAU;HNRNPA1;MYL12A;NPM1;PPIA;PRDX1;RAN;RANGRF;RPL10;RPL10A;RPL12;RPL13A;RPL14;RPL19;RPL22
salmon	BP	GO:0044248	cellular catabolic process	17735	1692	114	46	12.45	2.00E-16	3.694779	BLVRA;COMMD1;FAU;GSTM3;PRDX1;PSMA5;PSMB6;RPL10;RPL10A;RPL12;RPL13A;RPL14;RPL19;RPL22;RPL
salmon	BP	GO:0022613	ribonucleoprotein complex biogenesis	17735	233	114	20	1.71	3.80E-16	11.69591	EBNA1BP2;NPM1;POP4;RPL13A;RPL14;RPL35A;RPL5;RPL7A;RPS14;RPS16;RPS18;RPS19;RPS21;RPS25;RPS6;
salmon	CC	GO:0044422	organelle part	17735	6321	114	84	42.26	9.40E-16	1.987695	CACYBP;CCDC77;CETN2;COMMD1;DIABLO;EBNA1BP2;FAU;GTF3C6;HMGN1;HNRNPA1;HS3ST3B1;ICT1;ISY1;
salmon	CC	GO:0044446	intracellular organelle part	17735	6219	114	83	41.58	1.60E-15	1.996152	CACYBP;CCDC77;CETN2;COMMD1;DIABLO;EBNA1BP2;FAU;GTF3C6;HMGN1;HNRNPA1;HS3ST3B1;ICT1;ISY1;
salmon	BP	GO:0009056	catabolic process	17735	1989	114	48	14.64	3.70E-15	3.278689	BLVRA;COMMD1;FAU;GSTM3;PRDX1;PSMA5;PSMB6;RARRES3;RPIA;RPL10;RPL10A;RPL12;RPL13A;RPL14;RP
salmon	BP	GO:1901575	organic substance catabolic process	17735	1842	114	46	13.55	5.30E-15	3.394834	BLVRA;COMMD1;FAU;PSMA5;PSMB6;RARRES3;RPIA;RPL10;RPL10A;RPL12;RPL13A;RPL14;RPL19;RPL22;RPL
salmon	BP	GO:0042254	ribosome biogenesis	17735	151	114	16	1.11	1.60E-14	14.41441	EBNA1BP2;NPM1;POP4;RPL14;RPL35A;RPL5;RPL7A;RPS14;RPS16;RPS18;RPS19;RPS21;RPS25;RPS6;RPS8;RP
salmon	BP	GO:0042274	ribosomal small subunit biogenesis	17735	18	114	8	0.13	2.70E-13	61.53846	RPS14;RPS16;RPS19;RPS21;RPS25;RPS6;RPS8;RPSA
salmon	BP	GO:0034645	cellular macromolecule biosynthetic proc...	17735	4170	114	66	30.68	1.10E-12	2.151239	BTF3;BUD31;CACYBP;COMMD1;EIF3H;EIF3M;FAU;GTF3C6;HMGN1;HS3ST3B1;ICT1;MED10;MRPS16;MRPS7
salmon	BP	GO:0010467	gene expression	17735	4403	114	68	32.4	1.10E-12	2.098765	BTF3;BUD31;COMMD1;EIF3H;EIF3M;FAU;GTF3C6;HMGN1;HNRNPA1;ICT1;ISY1;MED10;MRPS16;MRPS7;NA
salmon	BP	GO:0044765	single-organism transport	17735	2753	114	52	20.26	3.20E-12	2.566634	ATP6V1E2;FAU;HNRNPA1;MYL12A;PPIA;PRDX1;RAN;RANGRF;RPL10;RPL10A;RPL12;RPL13A;RPL14;RPL19;RP
salmon	CC	GO:0044444	cytoplasmic part	17735	6562	114	80	43.87	4.00E-12	1.82357	ATP6V1E2;BLVRA;CACYBP;CETN2;CHKB;DIABLO;EIF3H;EIF3M;FAU;HS3ST3B1;ICT1;ITGB1BP1;MDH1;MRPS16
salmon	BP	GO:0009059	macromolecule biosynthetic process	17735	4294	114	66	31.6	4.70E-12	2.088608	BTF3;BUD31;CACYBP;COMMD1;EIF3H;EIF3M;FAU;GTF3C6;HMGN1;HS3ST3B1;ICT1;MED10;MRPS16;MRPS7
salmon	BP	GO:0044267	cellular protein metabolic process	17735	3210	114	56	23.62	6.50E-12	2.370872	COMMD1;EIF3H;EIF3M;FAU;ICT1;MRPS16;MRPS7;NACA;POLR2H;PPIA;PSMA5;PSMB6;RPL10;RPL10A;RPL12
salmon	BP	GO:0090304	nucleic acid metabolic process	17735	4431	114	66	32.61	2.20E-11	2.023919	BTF3;BUD31;CACYBP;CETN2;COMMD1;FAU;GTF3C6;HMGN1;HNRNPA1;ISY1;MED10;NACA;NAP1L1;NPM1;F
salmon	BP	GO:0016070	RNA metabolic process	17735	3911	114	61	28.78	4.50E-11	2.119527	BTF3;BUD31;COMMD1;FAU;GTF3C6;HMGN1;HNRNPA1;ISY1;MED10;NACA;NPM1;PCNA;PER2;POLR2H;POP
salmon	CC	GO:0005737	cytoplasm	17735	9016	114	93	60.28	6.40E-11	1.5428	ARPC1A;ATP6V1E2;BLVRA;CACYBP;CETN2;CHKB;COMMD1;DIABLO;EIF3H;EIF3M;FAU;GSTM3;HMGN1;HNRN
salmon	CC	GO:0044424	intracellular part	17735	12103	114	107	80.91	1.80E-10	1.322457	ARPC1A;ATP6V1E2;BLVRA;BTF3;BUD31;C4orf27;CACYBP;CCDC77;CETN2;CHKB;COMMD1;DIABLO;EBNA1BP
salmon	BP	GO:0032774	RNA biosynthetic process	17735	3293	114	54	24.23	2.60E-10	2.228642	BTF3;BUD31;COMMD1;FAU;GTF3C6;HMGN1;MED10;NACA;NPM1;PCNA;PER2;POLR2H;RAN;RPL10;RPL10A;
salmon	BP	GO:0006364	rRNA processing	17735	105	114	11	0.77	2.80E-10	14.28571	POP4;RPL14;RPL35A;RPL5;RPS14;RPS16;RPS19;RPS21;RPS6;RPS8;RPSA
salmon	BP	GO:0071840	cellular component organization or bioge...	17735	4237	114	62	31.18	4.60E-10	1.988454	ARPC1A;ATP6V1E2;CETN2;EBNA1BP2;FAU;HMGN1;ICT1;MYL12A;NAP1L1;NPM1;PARL;PCNA;POMP;POP4;P
salmon	BP	GO:0016072	rRNA metabolic process	17735	111	114	11	0.82	5.20E-10	13.41463	POP4;RPL14;RPL35A;RPL5;RPS14;RPS16;RPS19;RPS21;RPS6;RPS8;RPSA
salmon	BP	GO:0006810	transport	17735	3358	114	54	24.71	5.70E-10	2.18535	ATP6V1E2;FAU;HNRNPA1;MYL12A;NACA;NPM1;PPIA;PRDX1;RAN;RANGRF;RPL10;RPL10A;RPL12;RPL13A;RP
salmon	BP	GO:0006725	cellular aromatic compound metabolic pro...	17735	5371	114	71	39.52	5.70E-10	1.796559	BLVRA;BTF3;BUD31;CACYBP;CETN2;COMMD1;FAU;GSTM3;GTF3C6;HDHD1;HMGN1;HNRNPA1;ISY1;MED10
salmon	BP	GO:0051234	establishment of localization	17735	3408	114	54	25.08	1.00E-09	2.15311	ATP6V1E2;FAU;HNRNPA1;MYL12A;NACA;NPM1;PPIA;PRDX1;RAN;RANGRF;RPL10;RPL10A;RPL12;RPL13A;RP
salmon	CC	GO:0043229	intracellular organelle	17735	10522	114	99	70.34	1.00E-09	1.40745	ARPC1A;ATP6V1E2;BTF3;BUD31;C4orf27;CACYBP;CCDC77;CETN2;COMMD1;DIABLO;EBNA1BP2;FAU;GTF3C
salmon	CC	GO:0005622	intracellular	17735	12344	114	107	82.52	1.10E-09	1.296655	ARPC1A;ATP6V1E2;BLVRA;BTF3;BUD31;C4orf27;CACYBP;CCDC77;CETN2;CHKB;COMMD1;DIABLO;EBNA1BP
salmon	BP	GO:0034641	cellular nitrogen compound metabolic pro...	17735	5595	114	72	41.17	1.30E-09	1.748846	BLVRA;BTF3;BUD31;CACYBP;CETN2;CHKB;COMMD1;FAU;GSTM3;GTF3C6;HDHD1;HMGN1;HNRNPA1;ISY1;M
salmon	CC	GO:0043226	organelle	17735	10552	114	99	70.54	1.30E-09	1.403459	ARPC1A;ATP6V1E2;BTF3;BUD31;C4orf27;CACYBP;CCDC77;CETN2;COMMD1;DIABLO;EBNA1BP2;FAU;GTF3C
salmon	BP	GO:0006139	nucleobase-containing compound metabolic...	17735	5209	114	69	38.33	1.40E-09	1.800157	BTF3;BUD31;CACYBP;CETN2;COMMD1;FAU;GTF3C6;HDHD1;HMGN1;HNRNPA1;ISY1;MED10;NACA;NAP1L1;
salmon	BP	GO:0019538	protein metabolic process	17735	3895	114	58	28.66	1.60E-09	2.023726	COMMD1;EIF3H;EIF3M;FAU;HS3ST3B1;ICT1;MRPS16;MRPS7;NACA;PARL;POLR2H;PPIA;PSMA5;PSMB6;RPL1
salmon	BP	GO:0016043	cellular component organization	17735	4124	114	60	30.35	1.60E-09	1.976936	ARPC1A;ATP6V1E2;CETN2;FAU;HMGN1;ICT1;MYL12A;NAP1L1;NPM1;PARL;PCNA;POMP;PPIA;RAN;RPL10;RI
salmon	BP	GO:0046483	heterocycle metabolic process	17735	5361	114	70	39.45	1.70E-09	1.774398	BLVRA;BTF3;BUD31;CACYBP;CETN2;COMMD1;FAU;GTF3C6;HDHD1;HMGN1;HNRNPA1;ISY1;MED10;NACA;N
salmon	BP	GO:1901576	organic substance biosynthetic process	17735	5154	114	68	37.93	2.80E-09	1.792776	BTF3;BUD31;CACYBP;CHKB;COMMD1;EIF3H;EIF3M;FAU;GTF3C6;HMGN1;HS3ST3B1;ICT1;MDH1;MED10;MF
salmon	BP	GO:0042255	ribosome assembly	17735	19	114	6	0.14	3.50E-09	42.85714	NPM1;RPS14;RPS19;RPS25;RPS6;RPSA
salmon	BP	GO:1901360	organic cyclic compound metabolic proces...	17735	5578	114	71	41.05	3.80E-09	1.729598	BLVRA;BTF3;BUD31;CACYBP;CETN2;COMMD1;FAU;GSTM3;GTF3C6;HDHD1;HMGN1;HNRNPA1;ISY1;MED10
salmon	BP	GO:0044249	cellular biosynthetic process	17735	5073	114	67	37.33	4.20E-09	1.794803	BTF3;BUD31;CACYBP;CHKB;COMMD1;EIF3H;EIF3M;FAU;GTF3C6;HMGN1;HS3ST3B1;ICT1;MED10;MRPS16;N
salmon	BP	GO:0022618	ribonucleoprotein complex assembly	17735	104	114	10	0.77	4.50E-09	12.98701	NPM1;RPL13A;RPS14;RPS19;RPS25;RPS6;RPSA;SNRPD2;SNRPD3;SNUPN
salmon	BP	GO:0009058	biosynthetic process	17735	5219	114	68	38.4	5.00E-09	1.770833	BTF3;BUD31;CACYBP;CHKB;COMMD1;EIF3H;EIF3M;FAU;GTF3C6;HMGN1;HS3ST3B1;ICT1;MDH1;MED10;MF
salmon	BP	GO:0034654	nucleobase-containing compound biosynthe...	17735	3583	114	54	26.37	6.80E-09	2.047782	BTF3;BUD31;COMMD1;FAU;GTF3C6;HMGN1;MED10;NACA;NPM1;PCNA;PER2;POLR2H;RAN;RPL10;RPL10A;
salmon	BP	GO:0071826	ribonucleoprotein complex subunit organi...	17735	111	114	10	0.82	8.40E-09	12.19512	NPM1;RPL13A;RPS14;RPS19;RPS25;RPS6;RPSA;SNRPD2;SNRPD3;SNUPN
salmon	BP	GO:0006807	nitrogen compound metabolic process	17735	5942	114	73	43.72	8.90E-09	1.669716	BLVRA;BTF3;BUD31;CACYBP;CETN2;CHKB;COMMD1;FAU;GSTM3;GTF3C6;HDHD1;HMGN1;HNRNPA1;HS3ST
salmon	BP	GO:0019438	aromatic compound biosynthetic process	17735	3638	114	54	26.77	1.20E-08	2.017183	BTF3;BUD31;COMMD1;FAU;GTF3C6;HMGN1;MED10;NACA;NPM1;PCNA;PER2;POLR2H;RAN;RPL10;RPL10A;
salmon	BP	GO:0018130	heterocycle biosynthetic process	17735	3650	114	54	26.86	1.40E-08	2.010424	BTF3;BUD31;COMMD1;FAU;GTF3C6;HMGN1;MED10;NACA;NPM1;PCNA;PER2;POLR2H;RAN;RPL10;RPL10A;
salmon	BP	GO:0034660	ncRNA metabolic process	17735	285	114	14	2.1	2.10E-08	6.666667	POP4;RPL14;RPL35A;RPL5;RPS14;RPS16;RPS19;RPS21;RPS6;RPS8;RPSA;SNRPD2;SNRPD3;SNUPN
salmon	BP	GO:0044260	cellular macromolecule metabolic process	17735	6621	114	77	48.72	2.50E-08	1.58046	BTF3;BUD31;CACYBP;CETN2;COMMD1;EIF3H;EIF3M;FAU;GTF3C6;HMGN1;HNRNPA1;HS3ST3B1;ICT1;ISY1;M
salmon	BP	GO:0044271	cellular nitrogen compound biosynthetic ...	17735	3726	114	54	27.42	2.90E-08	1.969365	BTF3;BUD31;COMMD1;FAU;GTF3C6;HMGN1;MED10;NACA;NPM1;PCNA;PER2;POLR2H;RAN;RPL10;RPL10A;
salmon	BP	GO:1901362	organic cyclic compound biosynthetic pro...	17735	3752	114	54	27.61	3.80E-08	1.955813	BTF3;BUD31;COMMD1;FAU;GTF3C6;HMGN1;MED10;NACA;NPM1;PCNA;PER2;POLR2H;RAN;RPL10;RPL10A;
salmon	MF	GO:0019843	rRNA binding	17735	31	114	6	0.21	4.90E-08	28.57143	RPL23A;RPL37;RPL5;RPS11;RPS18;RPS4X

salmon	CC	GO:0005730	nucleolus	17735	622	114	18	4.16	1.50E-07	4.326923	COMMD1;EBNA1BP2;HNRNPA1;NOP16;NPM1;POLR2H;POP4;PRDX1;RPL3;RPL36;RPL4;RPL5;RPS13;RPS14;F
salmon	BP	GO:0034470	ncRNA processing	17735	198	114	11	1.46	2.20E-07	7.534247	POP4;RPL14;RPL35A;RPL5;RPS14;RPS16;RPS19;RPS21;RPS6;RPS8;RPSA
salmon	BP	GO:0044237	cellular metabolic process	17735	8597	114	88	63.26	2.40E-07	1.391084	BLVRA;BTF3;BUD31;CACYBP;CETN2;CHKB;COMMD1;EIF3H;EIF3M;FAU;GSTM3;GTF3C6;HDHD1;HMGN1;HNI
salmon	MF	GO:0003676	nucleic acid binding	17735	3299	114	45	22.04	2.80E-07	2.041742	CETN2;EIF3H;EIF3M;FAU;GTF3C6;HMGN1;HNRNPA1;ICT1;MRPS7;NACA;NPM1;PCNA;POLR2H;POP4;RPL10A
salmon	BP	GO:0051179	localization	17735	4195	114	55	30.87	7.20E-07	1.781665	ATP6V1E2;FAU;HNRNPA1;ITGB1BP1;MYL12A;NACA;NPM1;PPIA;PRDX1;RAN;RANGRF;RPL10;RPL10A;RPL12;
salmon	BP	GO:0006396	RNA processing	17735	646	114	18	4.75	1.00E-06	3.789474	HNRNPA1;ISY1;POLR2H;POP4;RPL14;RPL35A;RPL5;RPS13;RPS14;RPS16;RPS19;RPS21;RPS6;RPS8;RPSA;SNRF
salmon	BP	GO:0043170	macromolecule metabolic process	17735	7334	114	78	53.97	1.70E-06	1.445247	BTF3;BUD31;CACYBP;CETN2;COMMD1;EIF3H;EIF3M;FAU;GTF3C6;HMGN1;HNRNPA1;HS3ST3B1;ICT1;ISY1;N
salmon	BP	GO:0008152	metabolic process	17735	9452	114	90	69.55	8.50E-06	1.294033	BLVRA;BTF3;BUD31;CACYBP;CETN2;CHKB;COMMD1;DIABLO;EIF3H;EIF3M;FAU;GSTM3;GTF3C6;HDHD1;HM
salmon	BP	GO:0044238	primary metabolic process	17735	8755	114	85	64.42	1.70E-05	1.319466	BTF3;BUD31;CACYBP;CETN2;CHKB;COMMD1;EIF3H;EIF3M;FAU;GSTM3;GTF3C6;HDHD1;HMGN1;HNRNPA1;
salmon	BP	GO:0071704	organic substance metabolic process	17735	8996	114	86	66.2	2.80E-05	1.299094	BLVRA;BTF3;BUD31;CACYBP;CETN2;CHKB;COMMD1;EIF3H;EIF3M;FAU;GSTM3;GTF3C6;HDHD1;HMGN1;HNI
salmon	BP	GO:0042273	ribosomal large subunit biogenesis	17735	13	114	3	0.1	0.00011	30	RPL14;RPL35A;RPL5
salmon	BP	GO:0070925	organelle assembly	17735	106	114	6	0.78	0.00013	7.692308	NPM1;RPS14;RPS19;RPS25;RPS6;RPSA
salmon	CC	GO:0044428	nuclear part	17735	2482	114	32	16.59	0.00013	1.928873	CACYBP;CETN2;COMMD1;EBNA1BP2;GTF3C6;HNRNPA1;ISY1;MED10;NOP16;NPM1;PCNA;POLR2H;POP4;PR
salmon	BP	GO:0034622	cellular macromolecular complex assembly	17735	547	114	13	4.03	0.00019	3.225806	ARPC1A;NAP1L1;NPM1;POMP;RPL13A;RPS14;RPS19;RPS25;RPS6;RPSA;SNRPD2;SNRPD3;SNUPN
salmon	CC	GO:0031974	membrane-enclosed lumen	17735	2770	114	34	18.52	2.00E-04	1.835853	CACYBP;COMMD1;DIABLO;EBNA1BP2;GTF3C6;HNRNPA1;ICT1;MED10;MRPS16;NOP16;NPM1;PCNA;POLR2I
salmon	CC	GO:0031981	nuclear lumen	17735	2101	114	28	14.05	0.00023	1.992883	COMMD1;EBNA1BP2;GTF3C6;HNRNPA1;MED10;NOP16;NPM1;PCNA;POLR2H;POP4;PRDX1;PSMA5;PSMB6;
salmon	MF	GO:1901363	heterocyclic compound binding	17735	5302	114	53	35.43	0.00024	1.495907	BLVRA;CETN2;CHKB;EIF3H;EIF3M;FAU;GTF3C6;HMGN1;HNRNPA1;ICT1;MDH1;MRPS7;NACA;NPM1;PCNA;P
salmon	MF	GO:0097159	organic cyclic compound binding	17735	5372	114	53	35.9	0.00035	1.476323	BLVRA;CETN2;CHKB;EIF3H;EIF3M;FAU;GTF3C6;HMGN1;HNRNPA1;ICT1;MDH1;MRPS7;NACA;NPM1;PCNA;P
salmon	CC	GO:0070013	intracellular organelle lumen	17735	2677	114	32	17.9	0.00053	1.787709	COMMD1;EBNA1BP2;GTF3C6;HNRNPA1;ICT1;MED10;MRPS16;NOP16;NPM1;PCNA;POLR2H;POP4;PRDX1;P
salmon	CC	GO:0043233	organelle lumen	17735	2722	114	32	18.2	0.00071	1.758242	COMMD1;EBNA1BP2;GTF3C6;HNRNPA1;ICT1;MED10;MRPS16;NOP16;NPM1;PCNA;POLR2H;POP4;PRDX1;P
salmon	BP	GO:0051701	interaction with host	17735	411	114	10	3.02	0.00089	3.311258	ATP6V1E2;C9orf156;HNRNPA1;NACA;NPM1;PDCL3;PPIA;PSMB6;RAN;RPS27A
salmon	CC	GO:0044464	cell part	17735	14451	114	107	96.61	0.00118	1.107546	ARPC1A;ATP6V1E2;BLVRA;BTF3;BUD31;C4orf27;CACYBP;CCDC77;CETN2;CHKB;COMMD1;DIABLO;EBNA1BP
salmon	CC	GO:0005623	cell	17735	14452	114	107	96.62	0.00119	1.107431	ARPC1A;ATP6V1E2;BLVRA;BTF3;BUD31;C4orf27;CACYBP;CCDC77;CETN2;CHKB;COMMD1;DIABLO;EBNA1BP
salmon	BP	GO:0000387	spliceosomal snRNP assembly	17735	31	114	3	0.23	0.0015	13.04348	SNRPD2;SNRPD3;SNUPN
salmon	BP	GO:0044403	symbiosis, encompassing mutualism throug...	17735	445	114	10	3.27	0.00162	3.058104	ATP6V1E2;C9orf156;HNRNPA1;NACA;NPM1;PDCL3;PPIA;PSMB6;RAN;RPS27A
salmon	BP	GO:0044419	interspecies interaction between organis...	17735	445	114	10	3.27	0.00162	3.058104	ATP6V1E2;C9orf156;HNRNPA1;NACA;NPM1;PDCL3;PPIA;PSMB6;RAN;RPS27A
salmon	BP	GO:0019048	virus-host interaction	17735	371	114	9	2.73	0.00165	3.296703	C9orf156;HNRNPA1;NACA;NPM1;PDCL3;PPIA;PSMB6;RAN;RPS27A
salmon	CC	GO:0071013	catalytic step 2 spliceosome	17735	79	114	4	0.53	0.00193	7.54717	HNRNPA1;ISY1;SNRPD2;SNRPD3
salmon	BP	GO:0051437	positive regulation of ubiquitin-protein...	17735	72	114	4	0.53	0.00194	7.54717	PSMA5;PSMB6;RPS27A;SKP1
salmon	BP	GO:0006289	nucleotide-excision repair	17735	76	114	4	0.56	0.00237	7.142857	CETN2;HMGN1;PCNA;POLR2H
salmon	BP	GO:0044085	cellular component biogenesis	17735	1686	114	23	12.41	0.00237	1.853344	ARPC1A;EBNA1BP2;NAP1L1;NPM1;POMP;POP4;RPL13A;RPL14;RPL35A;RPL5;RPL7A;RPS14;RPS16;RPS18;RP
salmon	BP	GO:0051439	regulation of ubiquitin-protein ligase a...	17735	78	114	4	0.57	0.0026	7.017544	PSMA5;PSMB6;RPS27A;SKP1
salmon	BP	GO:0031398	positive regulation of protein ubiquitin...	17735	133	114	5	0.98	0.003	5.102041	COMMD1;PSMA5;PSMB6;RPS27A;SKP1
salmon	BP	GO:0051443	positive regulation of ubiquitin-protein...	17735	82	114	4	0.6	0.00312	6.666667	PSMA5;PSMB6;RPS27A;SKP1
salmon	BP	GO:0031145	anaphase-promoting complex-dependent pro...	17735	83	114	4	0.61	0.00326	6.557377	PSMA5;PSMB6;RPS27A;SKP1
salmon	BP	GO:0000460	maturation of 5.8S rRNA	17735	12	114	2	0.09	0.00337	22.22222	RPS21;RPSA
salmon	BP	GO:0006417	regulation of translation	17735	200	114	6	1.47	0.00359	4.081633	EIF3H;RPL13A;RPS14;RPS4X;RPS5;TRNAU1AP
salmon	MF	GO:0003729	mRNA binding	17735	94	114	4	0.63	0.00362	6.349206	RPS13;RPS14;RPS3;RPS5
salmon	BP	GO:0051351	positive regulation of ligase activity	17735	86	114	4	0.63	0.00371	6.349206	PSMA5;PSMB6;RPS27A;SKP1
salmon	BP	GO:0000084	S phase of mitotic cell cycle	17735	140	114	5	1.03	0.00373	4.854369	PCNA;PSMA5;PSMB6;RPS27A;SKP1
salmon	BP	GO:0007099	centriole replication	17735	13	114	2	0.1	0.00397	20	CETN2;NPM1
salmon	CC	GO:0005852	eukaryotic translation initiation factor...	17735	15	114	2	0.1	0.00439	20	EIF3H;EIF3M
salmon	BP	GO:0000377	RNA splicing, via transesterification re...	17735	210	114	6	1.55	0.00456	3.870968	HNRNPA1;ISY1;POLR2H;SNRPD2;SNRPD3;SNUPN
salmon	BP	GO:0000398	mRNA splicing, via spliceosome	17735	210	114	6	1.55	0.00456	3.870968	HNRNPA1;ISY1;POLR2H;SNRPD2;SNRPD3;SNUPN
salmon	BP	GO:0006283	transcription-coupled nucleotide-excisio...	17735	46	114	3	0.34	0.00467	8.823529	HMGN1;PCNA;POLR2H
salmon	BP	GO:0051320	S phase	17735	148	114	5	1.09	0.00473	4.587156	PCNA;PSMA5;PSMB6;RPS27A;SKP1
salmon	BP	GO:0000375	RNA splicing, via transesterification re...	17735	215	114	6	1.58	0.00511	3.797468	HNRNPA1;ISY1;POLR2H;SNRPD2;SNRPD3;SNUPN
salmon	BP	GO:0051438	regulation of ubiquitin-protein ligase a...	17735	96	114	4	0.71	0.00548	5.633803	PSMA5;PSMB6;RPS27A;SKP1
salmon	BP	GO:0051340	regulation of ligase activity	17735	100	114	4	0.74	0.00633	5.405405	PSMA5;PSMB6;RPS27A;SKP1
salmon	BP	GO:0019067	viral assembly, maturation, egress, and ...	17735	18	114	2	0.13	0.0076	15.38462	PPIA;RPS27A
salmon	CC	GO:0005839	proteasome core complex	17735	20	114	2	0.13	0.00778	15.38462	PSMA5;PSMB6
salmon	CC	GO:0005634	nucleus	17735	5873	114	52	39.26	0.00833	1.324503	BTF3;BUD31;C4orf27;CACYBP;CETN2;COMMD1;EBNA1BP2;GTF3C6;HMGN1;HNRNPA1;ISY1;MED10;NACA;N
salmon	MF	GO:0004298	threonine-type endopeptidase activity	17735	21	114	2	0.14	0.00855	14.28571	PSMA5;PSMB6
salmon	MF	GO:0070003	threonine-type peptidase activity	17735	21	114	2	0.14	0.00855	14.28571	PSMA5;PSMB6
salmon	CC	GO:0000502	proteasome complex	17735	65	114	3	0.43	0.00941	6.976744	POMP;PSMA5;PSMB6
salmon	MF	GO:0051059	NF-kappaB binding	17735	23	114	2	0.15	0.01021	13.33333	NPM1;RPS3
salmon	CC	GO:0005844	polysome	17735	23	114	2	0.15	0.01023	13.33333	RPL7A;RPS4X
salmon	BP	GO:0031396	regulation of protein ubiquitination	17735	180	114	5	1.32	0.01059	3.787879	COMMD1;PSMA5;PSMB6;RPS27A;SKP1
salmon	CC	GO:0044429	mitochondrial part	17735	740	114	11	4.95	0.01062	2.222222	DIABLO;ICT1;MRPS16;NDUFC2;PARL;PRDX1;SSBP1;SUCLG1;TOMM6;UQCRFS1;VDAC3
salmon	BP	GO:0008380	RNA splicing	17735	328	114	7	2.41	0.01064	2.904564	HNRNPA1;ISY1;POLR2H;RPS13;SNRPD2;SNRPD3;SNUPN
salmon	CC	GO:0005689	U12-type spliceosomal complex	17735	24	114	2	0.16	0.01111	12.5	SNRPD2;SNRPD3
salmon	CC	GO:0016460	myosin II complex	17735	24	114	2	0.16	0.01111	12.5	MYL12A;MYL5
salmon	BP	GO:0051297	centrosome organization	17735	63	114	3	0.46	0.01117	6.521739	CETN2;NPM1;UXT
salmon	BP	GO:0009987	cellular process	17735	12861	114	102	94.64	0.0118	1.077768	ARPC1A;ATP6V1E2;BLVRA;BTF3;BUD31;C9orf156;CACYBP;CETN2;CHKB;COMMD1;DIABLO;EIF3H;EIF3M;FAU
salmon	BP	GO:0090305	nucleic acid phosphodiester bond hydroly...	17735	65	114	3	0.48	0.01216	6.25	POP4;RPS21;RPSA
salmon	BP	GO:0006977	DNA damage response, signal transduction...	17735	66	114	3	0.49	0.01267	6.122449	PSMA5;PSMB6;RPS27A
salmon	BP	GO:0072413	signal transduction involved in mitotic ...	17735	66	114	3	0.49	0.01267	6.122449	PSMA5;PSMB6;RPS27A
salmon	BP	GO:0072431	signal transduction involved in mitotic ...	17735	66	114	3	0.49	0.01267	6.122449	PSMA5;PSMB6;RPS27A
salmon	BP	GO:0072474	signal transduction involved in mitotic ...	17735	66	114	3	0.49	0.01267	6.122449	PSMA5;PSMB6;RPS27A

salmon	BP	GO:0031023	microtubule organizing center organizati...	17735	67	114	3	0.49	0.0132	6.122449	CETN2;NPM1;UXT
salmon	BP	GO:0051436	negative regulation of ubiquitin-protein...	17735	67	114	3	0.49	0.0132	6.122449	PSMA5;PSMB6;RPS27A
salmon	BP	GO:0072401	signal transduction involved in DNA inte...	17735	67	114	3	0.49	0.0132	6.122449	PSMA5;PSMB6;RPS27A
salmon	BP	GO:0072404	signal transduction involved in G1/S tra...	17735	67	114	3	0.49	0.0132	6.122449	PSMA5;PSMB6;RPS27A
salmon	BP	GO:0072422	signal transduction involved in DNA dama...	17735	67	114	3	0.49	0.0132	6.122449	PSMA5;PSMB6;RPS27A
salmon	BP	GO:0072395	signal transduction involved in cell cyc...	17735	68	114	3	0.5	0.01374	6	PSMA5;PSMB6;RPS27A
salmon	MF	GO:0008135	translation factor activity, nucleic aci...	17735	79	114	3	0.53	0.01587	5.660377	EIF3H;EIF3M;ICT1
salmon	BP	GO:0032069	regulation of nuclease activity	17735	72	114	3	0.53	0.016	5.660377	NPM1;PCNA;SRPRB
salmon	BP	GO:0051352	negative regulation of ligase activity	17735	72	114	3	0.53	0.016	5.660377	PSMA5;PSMB6;RPS27A
salmon	BP	GO:0051444	negative regulation of ubiquitin-protein...	17735	72	114	3	0.53	0.016	5.660377	PSMA5;PSMB6;RPS27A
salmon	BP	GO:0050657	nucleic acid transport	17735	132	114	4	0.97	0.01632	4.123711	HNRNPA1;RAN;RPSA;SNUPN
salmon	BP	GO:0050658	RNA transport	17735	132	114	4	0.97	0.01632	4.123711	HNRNPA1;RAN;RPSA;SNUPN
salmon	BP	GO:0051236	establishment of RNA localization	17735	132	114	4	0.97	0.01632	4.123711	HNRNPA1;RAN;RPSA;SNUPN
salmon	BP	GO:0006405	RNA export from nucleus	17735	73	114	3	0.54	0.0166	5.555556	HNRNPA1;RAN;RPSA
salmon	BP	GO:0031571	mitotic cell cycle G1/S transition DNA d...	17735	73	114	3	0.54	0.0166	5.555556	PSMA5;PSMB6;RPS27A
salmon	CC	GO:0005681	spliceosomal complex	17735	147	114	4	0.98	0.01703	4.081633	HNRNPA1;ISY1;SNRPD2;SNRPD3
salmon	BP	GO:0002479	antigen processing and presentation of e...	17735	75	114	3	0.55	0.01784	5.454545	PSMA5;PSMB6;RPS27A
salmon	BP	GO:0006099	tricarboxylic acid cycle	17735	29	114	2	0.21	0.01914	9.52381	MDH1;SUCLG1
salmon	BP	GO:0006403	RNA localization	17735	139	114	4	1.02	0.01935	3.921569	HNRNPA1;RAN;RPSA;SNUPN
salmon	BP	GO:0051298	centrosome duplication	17735	30	114	2	0.22	0.02041	9.090909	CETN2;NPM1
salmon	CC	GO:0030532	small nuclear ribonucleoprotein complex	17735	33	114	2	0.22	0.02044	9.090909	SNRPD2;SNRPD3
salmon	BP	GO:0042590	antigen processing and presentation of e...	17735	79	114	3	0.58	0.02046	5.172414	PSMA5;PSMB6;RPS27A
salmon	BP	GO:0000216	M/G1 transition of mitotic cell cycle	17735	80	114	3	0.59	0.02114	5.084746	PSMA5;PSMB6;RPS27A
salmon	BP	GO:0071158	positive regulation of cell cycle arrest	17735	80	114	3	0.59	0.02114	5.084746	PSMA5;PSMB6;RPS27A
salmon	BP	GO:0000082	G1/S transition of mitotic cell cycle	17735	215	114	5	1.58	0.02128	3.164557	PCNA;PSMA5;PSMB6;RPS27A;SKP1
salmon	BP	GO:0031575	mitotic cell cycle G1/S transition check...	17735	82	114	3	0.6	0.02255	5	PSMA5;PSMB6;RPS27A
salmon	CC	GO:0046930	pore complex	17735	91	114	3	0.61	0.0231	4.918033	RAN;SNUPN;VDAC3
salmon	BP	GO:0006281	DNA repair	17735	388	114	7	2.86	0.02442	2.447552	CETN2;HMGN1;NPM1;PCNA;POLR2H;RPS27A;RPS3
salmon	BP	GO:0043161	proteasomal ubiquitin-dependent protein ...	17735	224	114	5	1.65	0.02487	3.030303	COMMD1;PSMA5;PSMB6;RPS27A;SKP1
salmon	BP	GO:0006259	DNA metabolic process	17735	866	114	12	6.37	0.02524	1.88383	CACYBP;CETN2;HMGN1;NAP1L1;NPM1;PCNA;POLR2H;PPIA;RAN;RPS27A;RPS3;SSBP1
salmon	BP	GO:0071779	G1/S transition checkpoint	17735	86	114	3	0.63	0.02552	4.761905	PSMA5;PSMB6;RPS27A
salmon	CC	GO:0042470	melanosome	17735	95	114	3	0.64	0.02583	4.6875	NAP1L1;PRDX1;RAN
salmon	CC	GO:0048770	pigment granule	17735	95	114	3	0.64	0.02583	4.6875	NAP1L1;PRDX1;RAN
salmon	BP	GO:0015931	nucleobase-containing compound transport	17735	154	114	4	1.13	0.02696	3.539823	HNRNPA1;RAN;RPSA;SNUPN
salmon	BP	GO:0010498	proteasomal protein catabolic process	17735	234	114	5	1.72	0.02928	2.906977	COMMD1;PSMA5;PSMB6;RPS27A;SKP1
salmon	BP	GO:0006397	mRNA processing	17735	405	114	7	2.98	0.0299	2.348993	HNRNPA1;ISY1;POLR2H;POP4;SNRPD2;SNRPD3;SNUPN
salmon	BP	GO:0045333	cellular respiration	17735	162	114	4	1.19	0.03165	3.361345	MDH1;NDUFC2;SUCLG1;UQCRFS1
salmon	MF	GO:0003899	DNA-directed RNA polymerase activity	17735	42	114	2	0.28	0.03201	7.142857	POLR2H;ZNRD1
salmon	MF	GO:0034062	RNA polymerase activity	17735	42	114	2	0.28	0.03201	7.142857	POLR2H;ZNRD1
salmon	BP	GO:0034101	erythrocyte homeostasis	17735	95	114	3	0.7	0.03293	4.285714	PRDX1;RPS14;RPS19
salmon	BP	GO:0031397	negative regulation of protein ubiquitin...	17735	96	114	3	0.71	0.03381	4.225352	PSMA5;PSMB6;RPS27A
salmon	BP	GO:0065003	macromolecular complex assembly	17735	1014	114	13	7.46	0.03501	1.742627	ARPC1A;NAP1L1;NPM1;POMP;RPL13A;RPS14;RPS19;RPS25;RPS6;RPSA;SNRPD2;SNRPD3;SNUPN
salmon	BP	GO:0006974	response to DNA damage stimulus	17735	608	114	9	4.47	0.03513	2.013423	CETN2;HMGN1;NPM1;PCNA;POLR2H;PSMA5;PSMB6;RPS27A;RPS3
salmon	BP	GO:0032092	positive regulation of protein binding	17735	41	114	2	0.3	0.03652	6.666667	RAN;ZGLP1
salmon	BP	GO:0002474	antigen processing and presentation of p...	17735	99	114	3	0.73	0.03654	4.109589	PSMA5;PSMB6;RPS27A
salmon	BP	GO:0007098	centrosome cycle	17735	42	114	2	0.31	0.03816	6.451613	CETN2;NPM1
salmon	BP	GO:0006913	nucleocytoplasmic transport	17735	342	114	6	2.52	0.04053	2.380952	HNRNPA1;NPM1;PRDX1;RAN;RPSA;SNUPN
salmon	CC	GO:0043234	protein complex	17735	3294	114	30	22.02	0.04089	1.362398	ATP6V1E2;CACYBP;CETN2;COMMD1;DIABLO;EIF3H;EIF3M;GTF3C6;MED10;MYL12A;MYL5;NACA;NDUFC2;N
salmon	BP	GO:0048872	homeostasis of number of cells	17735	176	114	4	1.3	0.04096	3.076923	IL20RB;PRDX1;RPS14;RPS19
salmon	BP	GO:2000045	regulation of G1/S transition of mitotic...	17735	104	114	3	0.77	0.04134	3.896104	PSMA5;PSMB6;RPS27A
salmon	MF	GO:0003743	translation initiation factor activity	17735	49	114	2	0.33	0.04243	6.060606	EIF3H;EIF3M
salmon	BP	GO:0051169	nuclear transport	17735	346	114	6	2.55	0.04249	2.352941	HNRNPA1;NPM1;PRDX1;RAN;RPSA;SNUPN
salmon	CC	GO:0005759	mitochondrial matrix	17735	286	114	5	1.91	0.04302	2.617801	ICT1;MRPS16;PRDX1;SSBP1;SUCLG1
salmon	BP	GO:0000080	G1 phase of mitotic cell cycle	17735	46	114	2	0.34	0.04502	5.882353	RPS27A;SKP1
salmon	BP	GO:0030330	DNA damage response, signal transduction...	17735	108	114	3	0.79	0.04539	3.797468	PSMA5;PSMB6;RPS27A
salmon	BP	GO:0009060	aerobic respiration	17735	47	114	2	0.35	0.0468	5.714286	MDH1;SUCLG1
salmon	BP	GO:0010608	posttranscriptional regulation of gene e...	17735	355	114	6	2.61	0.04711	2.298851	EIF3H;RPL13A;RPS14;RPS4X;RPS5;TRNAU1AP
salmon	CC	GO:0000313	organellar ribosome	17735	52	114	2	0.35	0.0473	5.714286	ICT1;MRPS16
salmon	CC	GO:0005761	mitochondrial ribosome	17735	52	114	2	0.35	0.0473	5.714286	ICT1;MRPS16
salmon	BP	GO:0035872	nucleotide-binding domain, leucine rich ...	17735	48	114	2	0.35	0.0486	5.714286	RPS27A;SUGT1
salmon	BP	GO:0051318	G1 phase	17735	48	114	2	0.35	0.0486	5.714286	RPS27A;SKP1
salmon	CC	GO:0031967	organelle envelope	17735	825	114	10	5.52	0.04943	1.811594	CACYBP;DIABLO;NDUFC2;PARL;RAN;SNUPN;SUCLG1;TOMM6;UQCRFS1;VDAC3
red	BP	GO:0023052	signaling	17735	4666	466	176	127.07	1.30E-07	1.385063	ACSL1;ADAM17;ADM;AGFG1;AGPAT9;AGTPBP1;ALDH1A2;ANKRD6;AP3B2;APAF1;APBB2;APH1B;ARHGAP19
red	BP	GO:0044700	single organism signaling	17735	4666	466	176	127.07	1.30E-07	1.385063	ACSL1;ADAM17;ADM;AGFG1;AGPAT9;AGTPBP1;ALDH1A2;ANKRD6;AP3B2;APAF1;APBB2;APH1B;ARHGAP19
red	BP	GO:0007165	signal transduction	17735	4165	466	159	113.43	4.10E-07	1.401746	ACSL1;ADAM17;ADM;AGFG1;AGPAT9;ALDH1A2;ANKRD6;APAF1;APBB2;APH1B;ARHGAP19;ARHGEF19;ARL1
red	BP	GO:0007154	cell communication	17735	4775	466	177	130.04	4.10E-07	1.36112	ACSL1;ADAM17;ADM;AGFG1;AGPAT9;AGTPBP1;ALDH1A2;ANKRD6;AP3B2;APAF1;APBB2;APH1B;ARHGAP19
red	BP	GO:0050896	response to stimulus	17735	6581	466	225	179.22	1.90E-06	1.25544	ACSL1;ADAM17;ADM;AGFG1;AGPAT9;AGTPBP1;ALDH1A2;ALOX5;ALOX5AP;ANKRD6;ANXA3;APAF1;APBB2;
red	BP	GO:0009611	response to wounding	17735	1090	466	56	29.68	2.90E-06	1.886792	ADAM17;ADM;ALOX5;ALOX5AP;ARHGEF19;C3AR1;CCR1;CCR2;CD177;CD55;CD59;CR1;CXCR1;CYBB;DOCK1;
red	MF	GO:0019864	IgG binding	17735	10	466	5	0.27	3.00E-06	18.51852	FCGR1A;FCGR1B;FCGR1C;FCGR2A;FCGR3B

red	BP	GO:0002376	immune system process	17735	1816	466	80	49.46	7.30E-06	1.617469	ADAM17;ANXA3;APOBEC3A;AQP9;BCL2L11;BST1;C3AR1;CCR1;CCR2;CD177;CD55;CD59;CR1;CST7;CXCR1;CY
red	BP	GO:0051716	cellular response to stimulus	17735	5052	466	179	137.58	8.40E-06	1.301061	ACSL1;ADAM17;ADM;AGFG1;AGPAT9;ALDH1A2;ALOX5AP;ANKRD6;APAF1;APBB2;APH1B;APOBEC3A;AQP9;
red	BP	GO:0006952	defense response	17735	1119	466	54	30.47	2.60E-05	1.772235	ALOX5;ALOX5AP;ANXA3;APAF1;APOBEC3A;C3AR1;CACNA1E;CCR1;CCR2;CD55;CD59;CLEC1A;CR1;CXCR1;CY
red	CC	GO:0016020	membrane	17735	7690	466	245	202.89	2.90E-05	1.207551	ACPP;ACSL1;ADAM17;AGFG1;AGPAT9;AHCTF1;ALOX5;ALOX5AP;ANTXR2;ANXA3;AP3B2;APBB2;APH1B;AQP
red	BP	GO:0006954	inflammatory response	17735	489	466	30	13.32	3.00E-05	2.252252	ALOX5;ALOX5AP;C3AR1;CCR1;CCR2;CD55;CD59;CR1;CXCR1;CYBB;FCGR1A;FPR2;HIF1A;HP;IL10RB;IL18RAP;I
red	MF	GO:0005515	protein binding	17735	7306	466	235	195.28	3.70E-05	1.2034	ACPP;ACTR2;ADAM17;ADM;AGFG1;AGTPBP1;ALOX5;ALOX5AP;ANTXR2;APAF1;APBB2;APH1B;APOA1BP;AP
red	BP	GO:0019221	cytokine-mediated signaling pathway	17735	335	466	23	9.12	4.70E-05	2.52193	ACSL1;CCR1;CCR2;COMMD7;CXCR1;FCGR1A;FCGR1B;GPR17;HIF1A;IFNAR1;IFNGR2;IL1B;IL1R1;IL1RN;IRAK3;
red	MF	GO:0004896	cytokine receptor activity	17735	81	466	10	2.17	5.80E-05	4.608295	CCR1;CCR2;CXCR1;GPR17;IFNAR1;IFNGR2;IL10RB;IL13RA1;IL1R1;IL1R2
red	BP	GO:0010563	negative regulation of phosphorus metabo...	17735	254	466	19	6.92	7.00E-05	2.745665	ATXN1;ATXN7;CCR2;CDA;DNAJC3;GNAQ;GSK3B;HMGCR;IL1B;IQGAP1;IRAK3;LYN;PAK2;PDZD3;PPAP2B;RHO
red	BP	GO:0045936	negative regulation of phosphate metabol...	17735	254	466	19	6.92	7.00E-05	2.745665	ATXN1;ATXN7;CCR2;CDA;DNAJC3;GNAQ;GSK3B;HMGCR;IL1B;IQGAP1;IRAK3;LYN;PAK2;PDZD3;PPAP2B;RHO
red	MF	GO:0019865	immunoglobulin binding	17735	18	466	5	0.48	8.60E-05	10.41667	FCGR1A;FCGR1B;FCGR1C;FCGR2A;FCGR3B
red	BP	GO:0009605	response to external stimulus	17735	1238	466	56	33.71	0.00011	1.661228	ACSL1;ADAM17;ADM;ALDH1A2;ALOX5AP;ARSB;BEST1;C3AR1;CASP5;CBS;CCR1;CCR2;CD55;CD59;CHRNA10;
red	BP	GO:0080134	regulation of response to stress	17735	737	466	38	20.07	0.00012	1.893373	ALOX5AP;CBS;CCR2;CD55;CD59;CR1;FCGR1A;HMGCR;HP;HPSE;IFNAR1;IFNGR2;IL1B;IL1RN;IRAK3;LYN;NFE2
red	BP	GO:0071345	cellular response to cytokine stimulus	17735	412	466	25	11.22	0.00016	2.228164	ACSL1;CCR1;CCR2;COMMD7;CXCR1;FCGR1A;FCGR1B;GPR17;HIF1A;IFNAR1;IFNGR2;IL1B;IL1R1;IL1RN;IRAK3;
red	BP	GO:0002526	acute inflammatory response	17735	109	466	11	2.97	0.00019	3.703704	ALOX5AP;CD55;CD59;CR1;FCGR1A;HP;IL1B;IL1RN;ORM1;ORM2;OSM
red	CC	GO:0044459	plasma membrane part	17735	1895	466	75	50	0.00021	1.5	ADAM17;AQP9;ATP6V1A;ATP7B;BEST1;BMX;C3AR1;CA4;CACNA1E;CCR1;CCR2;CD55;CD59;CDH2;CDKL5;CE
red	BP	GO:0035556	intracellular signal transduction	17735	1936	466	78	52.72	0.00021	1.479514	ADAM17;ADM;AGFG1;AGPAT9;APAF1;APBB2;ARHGAP19;ARHGEF19;ARL11;ARL4D;ASAP1;BCL2L11;BMX;CB
red	CC	GO:0071944	cell periphery	17735	4360	466	148	115.03	0.00025	1.286621	ACPP;ACSL1;ADAM17;ANTXR2;ANXA3;APH1B;AQP9;ARL4D;ATP11A;ATP6V1A;ATP7B;BCL2L11;BEST1;BMX;B
red	BP	GO:0032103	positive regulation of response to exter...	17735	153	466	13	4.17	0.00028	3.117506	ADAM17;ALOX5AP;C3AR1;CCR1;CCR2;FCGR1A;IL1B;OSM;SCARF1;TLR2;TLR4;TNFRSF1A;WAC
red	BP	GO:0006955	immune response	17735	1081	466	49	29.44	0.00029	1.664402	ADAM17;ANXA3;APOBEC3A;AQP9;BST1;CCR1;CCR2;CD55;CD59;CR1;CST7;CYBB;FCGR1A;FCGR1B;FCGR3B;F
red	CC	GO:0005886	plasma membrane	17735	4273	466	145	112.74	3.00E-04	1.286145	ACPP;ACSL1;ADAM17;ANTXR2;ANXA3;APH1B;AQP9;ARL4D;ATP11A;ATP6V1A;ATP7B;BCL2L11;BEST1;BMX;B
red	BP	GO:0032101	regulation of response to external stimu...	17735	380	466	23	10.35	0.00031	2.222222	ADAM17;ALOX5AP;C3AR1;CCR1;CCR2;CD55;CD59;CR1;FCGR1A;HMGCR;HPSE;IL1B;LYN;NFE2L2;OSM;PLAU;
red	BP	GO:0006950	response to stress	17735	2926	466	108	79.68	0.00032	1.355422	ADAM17;ADM;ALOX5;ALOX5AP;ANXA3;APAF1;APOBEC3A;AQP9;ARHGEF19;ATF6;AVIL;BCL2L11;BMX;C3AR
red	BP	GO:0010573	vascular endothelial growth factor produ...	17735	23	466	5	0.63	0.00033	7.936508	C3AR1;CCR2;HIF1A;HPSE;IL1B
red	BP	GO:0010574	regulation of vascular endothelial growt...	17735	23	466	5	0.63	0.00033	7.936508	C3AR1;CCR2;HIF1A;HPSE;IL1B
red	BP	GO:0048583	regulation of response to stimulus	17735	2347	466	90	63.92	0.00034	1.40801	ADAM17;ADM;AGFG1;AGPAT9;ALOX5AP;ANKRD6;ARHGAP19;ARHGEF19;ASAP1;ATXN1;ATXN7;BCL2L11;C3
red	BP	GO:0044763	single-organism cellular process	17735	9577	466	292	260.81	0.00039	1.119589	ACSL1;ACTR2;ADAM17;ADM;AGFG1;AGPAT9;AGTPBP1;AHCTF1;ALDH1A2;ALOX5AP;ANKRD6;ANXA3;AP3B2
red	BP	GO:0071310	cellular response to organic substance	17735	1333	466	57	36.3	4.00E-04	1.570248	ACSL1;ADAM17;ALDH1A2;AQP9;ATF6;ATP6V1A;CACNA1E;CCR1;CCR2;COMMD7;CXCR1;DGAT2;DNAJB11;DI
red	CC	GO:0044425	membrane part	17735	6026	466	193	158.99	0.00044	1.213913	ACPP;ACSL1;ADAM17;AGFG1;AGPAT9;AHCTF1;ALOX5AP;ANTXR2;AP3B2;APH1B;AQP9;ARMCX6;ASTN2;ATF
red	BP	GO:0070887	cellular response to chemical stimulus	17735	1709	466	69	46.54	0.00048	1.482596	ACSL1;ADAM17;ALDH1A2;ALOX5AP;APOBEC3A;AQP9;ATF6;ATP6V1A;C3AR1;CACNA1E;CBS;CCR1;CCR2;COM
red	CC	GO:0016023	cytoplasmic membrane-bounded vesicle	17735	848	466	39	22.37	0.00054	1.743406	ACPP;AGFG1;ANXA3;AP3B2;APH1B;ATP7B;CA4;CCT4;CYBB;DMXL2;F5;FCGR1A;FCGR1B;FLOT1;GABARAPL1;
red	BP	GO:0002673	regulation of acute inflammatory respons...	17735	53	466	7	1.44	0.00055	4.861111	ALOX5AP;CD55;CD59;CR1;FCGR1A;IL1B;OSM
red	BP	GO:0002698	negative regulation of immune effector p...	17735	53	466	7	1.44	0.00055	4.861111	CCR2;CD55;CD59;CR1;IRAK3;PGLYRP1;TARBP2
red	BP	GO:0050729	positive regulation of inflammatory resp...	17735	70	466	8	1.91	0.00061	4.188482	ALOX5AP;CCR2;FCGR1A;IL1B;OSM;TLR2;TLR4;TNFRSF1A
red	BP	GO:0031347	regulation of defense response	17735	428	466	24	11.66	0.00069	2.058319	ALOX5AP;CCR2;CD55;CD59;CR1;FCGR1A;IFNAR1;IFNGR2;IL1B;IRAK3;LYN;OSM;PAK2;PELI2;PIAS1;PVRL2;SBN
red	BP	GO:0042326	negative regulation of phosphorylation	17735	212	466	15	5.77	0.00071	2.599653	ATXN1;ATXN7;DNAJC3;GNAQ;GSK3B;HMGCR;IL1B;IRAK3;LYN;PAK2;PPAP2B;RHOH;SOCS3;TARBP2;TRIB1
red	BP	GO:0050727	regulation of inflammatory response	17735	191	466	14	5.2	0.00075	2.692308	ALOX5AP;CCR2;CD55;CD59;CR1;FCGR1A;IL1B;LYN;OSM;SBNO2;TLR2;TLR4;TNFRSF1A;ZFP36
red	BP	GO:0070098	chemokine-mediated signaling pathway	17735	29	466	5	0.79	0.00101	6.329114	CCR1;CCR2;CXCR1;GPR17;HIF1A
red	MF	GO:0000287	magnesium ion binding	17735	180	466	13	4.81	0.0011	2.702703	ATP11A;ATP9A;DCTPP1;ENO3;IDH1;IRAK3;NUDT5;PIF1;SRPK1;SRPK2;STK3;TGM3;ZAK
red	BP	GO:0034097	response to cytokine stimulus	17735	497	466	26	13.53	0.00116	1.921656	ACSL1;ALDH1A2;CCR1;CCR2;COMMD7;CXCR1;FCGR1A;FCGR1B;GPR17;HIF1A;IFNAR1;IFNGR2;IL1B;IL1R1;IL1
red	BP	GO:0032722	positive regulation of chemokine product...	17735	30	466	5	0.82	0.00119	6.097561	ADAM17;HIF1A;IL1B;TLR2;TLR4
red	CC	GO:0045177	apical part of cell	17735	279	466	17	7.36	0.00124	2.309783	ACPP;ADAM17;ATP6V1A;CA4;CD55;GPSM2;INSC;KCNE1;PDZD3;PLB1;RAB27A;SHROOM4;SLC22A4;STX3;TR
red	CC	GO:0031226	intrinsic to plasma membrane	17735	1252	466	51	33.03	0.00128	1.544051	ADAM17;AQP9;ATP6V1A;ATP7B;C3AR1;CA4;CCR1;CCR2;CD55;CD59;CEACAM4;CLEC1A;CR1;CYBB;EBP;ENTP
red	BP	GO:0023051	regulation of signaling	17735	2036	466	77	55.45	0.00149	1.388638	ADAM17;ADM;AGFG1;AGPAT9;ANKRD6;ARHGAP19;ARHGEF19;ASAP1;ATXN1;ATXN7;BCL2L11;CACNA1E;CE
red	MF	GO:0004693	cyclin-dependent protein kinase activity	17735	32	466	5	0.86	0.0015	5.813953	CDK14;CDK19;CDK4;CDKL5;MAK
red	BP	GO:0002262	myeloid cell homeostasis	17735	19	466	4	0.52	0.00152	7.692308	ADAM17;BCL2L11;HCAR2;PDE4B
red	BP	GO:0010575	positive regulation vascular endothelial...	17735	19	466	4	0.52	0.00152	7.692308	C3AR1;HIF1A;HPSE;IL1B
red	BP	GO:0007166	cell surface receptor signaling pathway	17735	2331	466	86	63.48	0.00158	1.354757	ACSL1;ADAM17;ADM;ANKRD6;APH1B;ATP6V1A;ATXN1;ATXN7;BCL2L11;C3AR1;CCR1;CCR2;CD59;CDA;CDH2
red	BP	GO:0051262	protein tetramerization	17735	81	466	8	2.21	0.0016	3.61991	APOA1BP;CDA;DCTPP1;HMGCR;SBF2;SOD2;TGM3;TRPV5
red	BP	GO:0010646	regulation of cell communication	17735	2042	466	77	55.61	0.00162	1.384643	ADAM17;ADM;AGFG1;AGPAT9;ANKRD6;ARHGAP19;ARHGEF19;ASAP1;ATXN1;ATXN7;BCL2L11;CACNA1E;CE
red	CC	GO:0005887	integral to plasma membrane	17735	1208	466	49	31.87	0.00174	1.537496	ADAM17;AQP9;ATP6V1A;ATP7B;C3AR1;CCR1;CCR2;CD55;CEACAM4;CLEC1A;CR1;CYBB;EBP;ENTPD1;FFAR2;
red	BP	GO:0065007	biological regulation	17735	8616	466	263	234.64	0.00177	1.120866	ACSL1;ADAM17;ADM;AGFG1;AGPAT9;AGTPBP1;ALDH1A2;ALOX5AP;ANKRD6;ANXA3;APAF1;APBB2;APH1B;
red	CC	GO:0031988	membrane-bounded vesicle	17735	905	466	39	23.88	0.0018	1.633166	ACPP;AGFG1;ANXA3;AP3B2;APH1B;ATP7B;CA4;CCT4;CYBB;DMXL2;F5;FCGR1A;FCGR1B;FLOT1;GABARAPL1;
red	MF	GO:0005488	binding	17735	11799	466	339	315.37	0.0018	1.074928	ACPP;ACSL1;ACTR2;ADAM17;ADAMTSL5;ADM;AGFG1;AGTPBP1;AHCTF1;ALDH1A2;ALOX5;ALOX5AP;ANTXR
red	BP	GO:0007243	intracellular protein kinase cascade	17735	879	466	39	23.94	0.00181	1.629073	ADAM17;CBS;CCR1;CCR2;CDH2;DOK4;FGF13;FPR1;FYB;GPR89A;GSK3B;HMGCR;HPSE;IFNAR1;IL1B;IL1RN;IR
red	BP	GO:0009966	regulation of signal transduction	17735	1795	466	69	48.88	0.00181	1.41162	ADAM17;ADM;AGFG1;AGPAT9;ANKRD6;ARHGAP19;ARHGEF19;ASAP1;ATXN1;ATXN7;BCL2L11;CBS;CCR1;CD
red	BP	GO:0032757	positive regulation of interleukin-8 pro...	17735	20	466	4	0.54	0.00186	7.407407	IL1B;TLR2;TLR4;TLR5
red	CC	GO:0042581	specific granule	17735	10	466	3	0.26	0.00191	11.53846	ANXA3;SNAP23;STX3
red	CC	GO:0031224	intrinsic to membrane	17735	5319	466	169	140.33	0.002	1.204304	ACPP;ACSL1;ADAM17;AGFG1;AGPAT9;AHCTF1;ALOX5AP;ANTXR2;APH1B;AQP9;ARMCX6;ASTN2;ATF6;ATP1
red	BP	GO:0065008	regulation of biological quality	17735	2547	466	92	69.36	0.00202	1.326413	ADAM17;ADM;AGTPBP1;ALDH1A2;AQP9;ARPC5;ARPC5L;ATP11A;ATP6V1A;ATP7B;ATP9A;ATXN1;AVIL;BCL2
red	BP	GO:0010935	regulation of macrophage cytokine produc...	17735	10	466	3	0.27	0.00209	11.11111	IRAK3;TLR2;TLR4
red	BP	GO:0042535	positive regulation of tumor necrosis fa...	17735	10	466	3	0.27	0.00209	11.11111	CCR2;TLR2;TLR4
red	MF	GO:0005484	SNAP receptor activity	17735	21	466	4	0.56	0.0021	7.142857	STX11;STX12;STX3;STX6
red	CC	GO:0005794	Golgi apparatus	17735	1158	466	47	30.55	0.00213	1.538462	ACPP;AGFG1;AP3B2;APAF1;ARSB;ATF6;ATP2C2;ATP7B;ATP9A;BEND5;CA4;CAPN8;CUL3;EXT1;GABARAPL1;G
red	CC	GO:0031410	cytoplasmic vesicle	17735	914	466	39	24.11	0.00215	1.617586	ACPP;AGFG1;ANXA3;AP3B2;APH1B;ATP7B;CA4;CCT4;CYBB;DMXL2;F5;FCGR1A;FCGR1B;FLOT1;GABARAPL1;
red	BP	GO:1901701	cellular response to oxygen-containing c...	17735	387	466	21	10.54	0.00218	1.99241	ALDH1A2;AQP9;CACNA1E;DGAT2;HIF1A;HMGCR;HP;IL1B;LITAF;LYN;NFE2L2;PDE4B;PDK3;SLC8A1;SOD2;TLR
red	BP	GO:0042346	positive regulation of NF-kappaB import ...	17735	21	466	4	0.57	0.00224	7.017544	IL1B;TLR2;TLR4;TNFSF14
red	CC	GO:0012505	endomembrane system	17735	1768	466	66	46.65	0.00233	1.414791	ACSL1;AGFG1;AGPAT9;AHCTF1;ALOX5;ALOX5AP;ANTXR2;ANXA3;AP3B2;ATF6;ATP2C2;ATP7B;BCL2L11;CA4;
red	BP	GO:0006469	negative regulation of protein kinase ac...	17735	148	466	11	4.03	0.00242	2.729529	DNAJC3;GNAQ;GSK3B;HMGCR;IL1B;IRAK3;LYN;PAK2;SOCS3;TARBP2;TRIB1

red	BP	GO:0051348	negative regulation of transferase activ...	17735	170	466	12	4.63	0.00242	2.591793	DNAJC3;GNAQ;GSK3B;HMGCR;IL1B;IRAK3;LYN;PAK2;PIF1;SOCS3;TARBP2;TRIB1
red	BP	GO:0002697	regulation of immune effector process	17735	217	466	14	5.91	0.00253	2.368866	CCR2;CD55;CD59;CR1;FCGR1A;IL1B;IRAK3;LYN;PAK2;PGLYRP1;PVRL2;TARBP2;TLR2;TLR4
red	BP	GO:0002675	positive regulation of acute inflammator...	17735	22	466	4	0.6	0.00269	6.666667	ALOX5AP;FCGR1A;IL1B;OSM
red	MF	GO:0019956	chemokine binding	17735	11	466	3	0.29	0.0027	10.34483	CCR1;CXCR1;ZFP36
red	MF	GO:0036041	long-chain fatty acid binding	17735	11	466	3	0.29	0.0027	10.34483	ALOX5AP;OXER1;STX3
red	CC	GO:0005737	cytoplasm	17735	9016	466	267	237.87	0.00272	1.122462	ACPP;ACSL1;ACTR2;ADAM17;ADM;AGFG1;AGPAT9;AGTPBP1;AHCTF1;AKR7A3;ALDH1A2;ALOX5;ALOX5AP;A
red	BP	GO:0031663	lipopolysaccharide-mediated signaling pa...	17735	36	466	5	0.98	0.00275	5.102041	IL1B;LYN;TLR2;TLR4;TRIB1
red	BP	GO:0042345	regulation of NF-kappaB import into nucl...	17735	36	466	5	0.98	0.00275	5.102041	IL1B;LITAF;TLR2;TLR4;TNFSF14
red	BP	GO:0042348	NF-kappaB import into nucleus	17735	36	466	5	0.98	0.00275	5.102041	IL1B;LITAF;TLR2;TLR4;TNFSF14
red	CC	GO:0030426	growth cone	17735	91	466	8	2.4	0.00277	3.333333	APBB2;CDKL5;FGF13;GSK3B;MYO5A;STX3;TIAM2;TSHZ3
red	BP	GO:0050777	negative regulation of immune response	17735	52	466	6	1.42	0.00278	4.225352	CCR2;CD55;CD59;CR1;IRAK3;LYN
red	BP	GO:0010934	macrophage cytokine production	17735	11	466	3	0.3	0.00281	10	IRAK3;TLR2;TLR4
red	BP	GO:0044273	sulfur compound catabolic process	17735	37	466	5	1.01	0.00311	4.950495	ARSB;CBS;HPSE;IDS;SQRDL
red	BP	GO:0044710	single-organism metabolic process	17735	3059	466	106	83.31	0.00339	1.272356	ACPP;ACSL1;ADM;AGFG1;AGPAT9;AKR7A3;ALDH1A2;ALOX5;ALOX5AP;APOBEC3A;ARHGEF19;ARL4D;ARSB;A
red	CC	GO:0016021	integral to membrane	17735	5202	466	164	137.25	0.00347	1.1949	ACPP;ACSL1;ADAM17;AGFG1;AGPAT9;AHCTF1;ALOX5AP;ANTXR2;APH1B;AQP9;ARMCX6;ASTN2;ATF6;ATP1
red	MF	GO:0008289	lipid binding	17735	721	466	32	19.27	0.0035	1.660612	ALDH1A2;ALOX5AP;ANXA3;ARHGEF19;ASAP1;ATP5G1;BMX;CYTH4;DOK4;FFAR2;FFAR3;LYN;MYO10;NCF4;C
red	CC	GO:0030427	site of polarized growth	17735	95	466	8	2.51	0.00362	3.187251	APBB2;CDKL5;FGF13;GSK3B;MYO5A;STX3;TIAM2;TSHZ3
red	BP	GO:0006796	phosphate-containing compound metabolic ...	17735	2466	466	88	67.16	0.00368	1.310304	ACPP;ACSL1;ADAM17;ADM;AGFG1;AGPAT9;ARHGEF19;ARL4D;ARSB;ASAP1;ATF6;ATP2C2;ATP5G1;ATP7B;A
red	BP	GO:0042221	response to chemical stimulus	17735	2766	466	97	75.33	0.00373	1.287668	ACSL1;ADAM17;ADM;ALDH1A2;ALOX5AP;APOBEC3A;AQP9;ARSB;ATF6;ATP6V1A;ATP7B;C3AR1;CA4;CACNA
red	BP	GO:0015893	drug transport	17735	24	466	4	0.65	0.00374	6.153846	EBP;LYN;SLC22A1;TLR2
red	BP	GO:0006144	purine nucleobase metabolic process	17735	39	466	5	1.06	0.00392	4.716981	ACPP;DPYD;GMPR2;NT5C2;PNP
red	BP	GO:0001776	leukocyte homeostasis	17735	56	466	6	1.53	0.00404	3.921569	BCL2L11;HCAR2;HIF1A;LYN;PDE4B;TNFSF14
red	BP	GO:0071260	cellular response to mechanical stimulus	17735	56	466	6	1.53	0.00404	3.921569	CASP5;IL1B;TLR4;TLR5;TNFRSF1A;TNFSF14
red	BP	GO:0090316	positive regulation of intracellular pro...	17735	94	466	8	2.56	0.00408	3.125	GSK3B;IL1B;KPNA1;TLR2;TLR4;TNFRSF1A;TNFSF14;WIPF1
red	MF	GO:0001637	G-protein coupled chemoattractant recept...	17735	25	466	4	0.67	0.0041	5.970149	CCR1;CCR2;CXCR1;GPR17
red	MF	GO:0004950	chemokine receptor activity	17735	25	466	4	0.67	0.0041	5.970149	CCR1;CCR2;CXCR1;GPR17
red	BP	GO:0033673	negative regulation of kinase activity	17735	159	466	11	4.33	0.00421	2.540416	DNAJC3;GNAQ;GSK3B;HMGCR;IL1B;IRAK3;LYN;PAK2;SOCS3;TARBP2;TRIB1
red	CC	GO:0016324	apical plasma membrane	17735	212	466	13	5.59	0.00423	2.325581	ADAM17;ATP6V1A;CA4;CD55;KCNE1;PLB1;RAB27A;SHROOM4;SLC22A4;STX3;TRPM6;TRPV5;VAMP3
red	BP	GO:0006793	phosphorus metabolic process	17735	2513	466	89	68.44	0.00431	1.300409	ACPP;ACSL1;ADAM17;ADM;AGFG1;AGPAT9;ARHGEF19;ARL4D;ARSB;ASAP1;ATF6;ATP2C2;ATP5G1;ATP7B;A
red	BP	GO:0002532	production of molecular mediator involve...	17735	25	466	4	0.68	0.00435	5.882353	ALOX5;ALOX5AP;LYN;TLR4
red	BP	GO:0044699	single-organism process	17735	10656	466	313	290.2	0.00437	1.078567	ACSL1;ACTR2;ADAM17;ADM;AGFG1;AGPAT9;AGTPBP1;AHCTF1;ALDH1A2;ALOX5;ALOX5AP;ANKRD6;ANXA3
red	BP	GO:0032677	regulation of interleukin-8 production	17735	40	466	5	1.09	0.00439	4.587156	ADAM17;IL1B;TLR2;TLR4;TLR5
red	MF	GO:0005149	interleukin-1 receptor binding	17735	13	466	3	0.35	0.0044	8.571429	IL1B;IL1RN;TLR5
red	MF	GO:0070851	growth factor receptor binding	17735	97	466	8	2.59	0.0044	3.088803	ADAM17;IL1B;IL1R1;IL1RN;LYN;NPTN;SDCBP;TLR5
red	BP	GO:0070304	positive regulation of stress-activated ...	17735	57	466	6	1.55	0.00442	3.870968	HMGCR;IL1B;LYN;SDCBP;STK3;TLR4
red	BP	GO:0009914	hormone transport	17735	232	466	14	6.32	0.00461	2.21519	ADM;CACNA1E;HCAR2;HIF1A;HMGCR;IL1B;IL1RN;INHBB;IQGAP1;LYN;MARCKS;MYO5A;OSM;SLC22A1
red	BP	GO:0046827	positive regulation of protein export fr...	17735	13	466	3	0.35	0.00468	8.571429	GSK3B;IL1B;WIPF1
red	MF	GO:0005504	fatty acid binding	17735	26	466	4	0.69	0.0047	5.797101	ALOX5AP;OXER1;SH3GLB1;STX3
red	CC	GO:0031982	vesicle	17735	993	466	40	26.2	0.00512	1.526718	ACPP;AGFG1;ANXA3;AP3B2;APH1B;ATP7B;CA4;CCT4;CYBB;DMXL2;F5;FCGR1A;FCGR1B;FLOT1;GABARAPL1;(
red	BP	GO:0046824	positive regulation of nucleocytoplasmic...	17735	79	466	7	2.15	0.00568	3.255814	GSK3B;IL1B;KPNA1;TLR2;TLR4;TNFSF14;WIPF1
red	BP	GO:0034121	regulation of toll-like receptor signali...	17735	27	466	4	0.74	0.00579	5.405405	IRAK3;LYN;TLR2;TLR5
red	BP	GO:0006607	NLS-bearing substrate import into nucleu...	17735	14	466	3	0.38	0.00583	7.894737	FYB;KPNA1;KPNA4
red	BP	GO:0070498	interleukin-1-mediated signaling pathway	17735	14	466	3	0.38	0.00583	7.894737	IL1R1;IL1RN;IRAK3
red	BP	GO:0090025	regulation of monocyte chemotaxis	17735	14	466	3	0.38	0.00583	7.894737	CCR1;CCR2;LYN
red	BP	GO:0032637	interleukin-8 production	17735	43	466	5	1.17	0.00601	4.273504	ADAM17;IL1B;TLR2;TLR4;TLR5
red	BP	GO:0044281	small molecule metabolic process	17735	2545	466	89	69.31	0.00612	1.284086	ACPP;ACSL1;ADM;AGFG1;AGPAT9;ALDH1A2;ALOX5;ALOX5AP;APOBEC3A;ARHGEF19;ARL4D;ARSB;ASAP1;AT
red	BP	GO:0001892	embryonic placenta development	17735	81	466	7	2.21	0.0065	3.167421	ADM;GCM1;HEY1;HIF1A;SLC8A1;SOCS3;STK3
red	CC	GO:0031201	SNARE complex	17735	29	466	4	0.77	0.00673	5.194805	STX12;STX3;STX6;VAMP3
red	MF	GO:0016411	acylglycerol O-acyltransferase activity	17735	15	466	3	0.4	0.0068	7.5	DGAT2;MBOAT2;SH3GLB1
red	MF	GO:0030332	cyclin binding	17735	15	466	3	0.4	0.0068	7.5	CDK14;CDK4;CUL3
red	BP	GO:0061041	regulation of wound healing	17735	82	466	7	2.23	0.00695	3.139013	HMGCR;HPSE;LYN;NFE2L2;PLAU;PPAP2B;TLR4
red	BP	GO:0060713	labyrinthine layer morphogenesis	17735	15	466	3	0.41	0.00715	7.317073	ADM;GCM1;SOCS3
red	BP	GO:0006953	acute-phase response	17735	45	466	5	1.23	0.00729	4.065041	HP;IL1B;IL1RN;ORM1;ORM2
red	BP	GO:0006753	nucleoside phosphate metabolic process	17735	834	466	35	22.71	0.0073	1.541171	ACPP;ADM;AGFG1;ARHGEF19;ARL4D;ASAP1;ATP2C2;ATP5G1;ATP7B;CBS;CCR2;CDA;CDKL5;DCTPP1;DOCK1;
red	BP	GO:0061025	membrane fusion	17735	83	466	7	2.26	0.00741	3.097345	PVRL2;SNAP23;STX11;STX3;STX6;TRIM9;VAMP3
red	CC	GO:0030659	cytoplasmic vesicle membrane	17735	360	466	18	9.5	0.00741	1.894737	ANXA3;AP3B2;CA4;CYBB;DMXL2;FCGR1A;FCGR1B;GABARAPL1;GPR89A;ITM2B;LAMP2;PICALM;RAB27A;RAB
red	BP	GO:0019637	organophosphate metabolic process	17735	1204	466	47	32.79	0.00767	1.433364	ACPP;ADM;AGFG1;AGPAT9;ARHGEF19;ARL4D;ARSB;ASAP1;ATP2C2;ATP5G1;ATP7B;CBS;CCR2;CDA;CDKL5;D
red	BP	GO:0001933	negative regulation of protein phosphory...	17735	197	466	12	5.36	0.00782	2.238806	DNAJC3;GNAQ;GSK3B;HMGCR;IL1B;IRAK3;LYN;PAK2;PPAP2B;SOCS3;TARBP2;TRIB1
red	BP	GO:0001959	regulation of cytokine-mediated signalin...	17735	84	466	7	2.29	0.0079	3.056769	HIF1A;IFNAR1;IFNGR2;IL1RN;IRAK3;PIAS1;SOCS3
red	BP	GO:0007259	JAK-STAT cascade	17735	105	466	8	2.86	0.00791	2.797203	ADAM17;CCR2;IFNAR1;LYN;OSM;PIAS1;SOCS3;TNFRSF1A
red	BP	GO:0045055	regulated secretory pathway	17735	46	466	5	1.25	0.008	4	ANXA3;CCR2;LYN;RAB27A;RAB31
red	BP	GO:0048066	developmental pigmentation	17735	46	466	5	1.25	0.008	4	BCL2L11;GNAQ;MYO5A;RAB27A;SOD2
red	MF	GO:0004198	calcium-dependent cysteine-type endopept...	17735	16	466	3	0.43	0.0082	6.976744	CAPN12;CAPN13;CAPN8
red	MF	GO:0008329	pattern recognition receptor activity	17735	16	466	3	0.43	0.0082	6.976744	PGLYRP1;TLR2;TLR4
red	BP	GO:0009112	nucleobase metabolic process	17735	65	466	6	1.77	0.00839	3.389831	ACPP;CDA;DPYD;GMPR2;NT5C2;PNP
red	BP	GO:0032729	positive regulation of interferon-gamma ...	17735	30	466	4	0.82	0.00848	4.878049	CCR2;IFNAR1;IL1B;TLR4
red	BP	GO:0045429	positive regulation of nitric oxide bios...	17735	30	466	4	0.82	0.00848	4.878049	IL1B;SOD2;TLR2;TLR4
red	BP	GO:0055086	nucleobase-containing small molecule met...	17735	873	466	36	23.77	0.00853	1.514514	ACPP;ADM;AGFG1;APOBEC3A;ARHGEF19;ARL4D;ASAP1;ATP2C2;ATP5G1;ATP7B;CBS;CCR2;CDA;CDKL5;DCTI
red	BP	GO:0010955	negative regulation of protein processin...	17735	16	466	3	0.44	0.00862	6.818182	CD55;CD59;CR1

red	BP	GO:0016578	histone deubiquitination	17735	16	466	3	0.44	0.00862	6.818182	ATXN7;UIMC1;USP3
red	BP	GO:0030502	negative regulation of bone mineralizati...	17735	16	466	3	0.44	0.00862	6.818182	CCR1;HIF1A;NFE2
red	BP	GO:0042533	tumor necrosis factor biosynthetic proce...	17735	16	466	3	0.44	0.00862	6.818182	CCR2;TLR2;TLR4
red	BP	GO:0042534	regulation of tumor necrosis factor bios...	17735	16	466	3	0.44	0.00862	6.818182	CCR2;TLR2;TLR4
red	BP	GO:0045070	positive regulation of viral genome repl...	17735	16	466	3	0.44	0.00862	6.818182	SRPK1;SRPK2;TARBP2
red	BP	GO:0045087	innate immune response	17735	550	466	25	14.98	0.00872	1.668892	APOBEC3A;CCR2;CD55;CR1;CYBB;FCGR1A;FCGR1B;IFNAR1;IFNGR2;IL18RAP;IL1R1;IRAK3;LYN;NAIP;PELI2;PG
red	BP	GO:0042306	regulation of protein import into nucleu...	17735	129	466	9	3.51	0.00875	2.564103	GNAQ;GSK3B;IL1B;KPNA1;LITAF;TLR2;TLR4;TNFRSF1A;TNFSF14
red	BP	GO:0002444	myeloid leukocyte mediated immunity	17735	47	466	5	1.28	0.00876	3.90625	ADAM17;ANXA3;CCR2;FCGR1A;LYN
red	BP	GO:0032496	response to lipopolysaccharide	17735	200	466	12	5.45	0.00877	2.201835	ADAM17;ADM;IL1B;IRAK3;LITAF;LYN;PDE4B;SOD2;TLR2;TLR4;TNFRSF1A;TRIB1
red	BP	GO:0050789	regulation of biological process	17735	8134	466	245	221.52	0.0089	1.105995	ACSL1;ADAM17;ADM;AGFG1;AGPAT9;ALDH1A2;ALOX5AP;ANKRD6;ANXA3;APAF1;APBB2;APH1B;APOBEC3
red	CC	GO:0045335	phagocytic vesicle	17735	68	466	6	1.79	0.00899	3.351955	ANXA3;CYBB;LAMP2;NCF4;RAB31;STX12
red	BP	GO:0032640	tumor necrosis factor production	17735	66	466	6	1.8	0.00902	3.333333	CCR2;IRAK3;TLR2;TLR4;TNFRSF1A;ZFP36
red	BP	GO:0032680	regulation of tumor necrosis factor prod...	17735	66	466	6	1.8	0.00902	3.333333	CCR2;IRAK3;TLR2;TLR4;TNFRSF1A;ZFP36
red	BP	GO:0046879	hormone secretion	17735	226	466	13	6.15	0.00913	2.113821	ADM;CACNA1E;HCAR2;HIF1A;HMGCR;IL1B;IL1RN;INHBB;IQGAP1;LYN;MARCKS;MYO5A;OSM
red	BP	GO:0071216	cellular response to biotic stimulus	17735	108	466	8	2.94	0.00932	2.721088	GSK3B;IL1B;LITAF;LYN;PDE4B;TLR2;TLR4;TRIB1
red	CC	GO:0005635	nuclear envelope	17735	314	466	16	8.28	0.00947	1.932367	AGFG1;AHCTF1;ALOX5;ALOX5AP;ATF6;CDK4;GNAQ;KAZN;KPNA1;LBR;LMNB1;NUP50;NUPL1;RANBP1;TOR1
red	BP	GO:0042990	regulation of transcription factor impor...	17735	67	466	6	1.82	0.00969	3.296703	GSK3B;IL1B;LITAF;TLR2;TLR4;TNFSF14
red	CC	GO:0012506	vesicle membrane	17735	371	466	18	9.79	0.00993	1.838611	ANXA3;AP3B2;CA4;CYBB;DMXL2;FCGR1A;FCGR1B;GABARAPL1;GPR89A;ITM2B;LAMP2;PICALM;RAB27A;RAB
red	BP	GO:0031349	positive regulation of defense response	17735	229	466	13	6.24	0.01013	2.083333	ALOX5AP;CCR2;FCGR1A;IL1B;IRAK3;LYN;OSM;PELI2;PVRL2;TLR2;TLR4;TLR5;TNFRSF1A
red	BP	GO:0032715	negative regulation of interleukin-6 pro...	17735	17	466	3	0.46	0.01026	6.521739	IRAK3;TLR4;TNFRSF1A
red	BP	GO:0060669	embryonic placenta morphogenesis	17735	17	466	3	0.46	0.01026	6.521739	ADM;GCM1;SOCS3
red	BP	GO:0061082	myeloid leukocyte cytokine production	17735	17	466	3	0.46	0.01026	6.521739	IRAK3;TLR2;TLR4
red	BP	GO:0042991	transcription factor import into nucleus	17735	68	466	6	1.85	0.01039	3.243243	GSK3B;IL1B;LITAF;TLR2;TLR4;TNFSF14
red	BP	GO:0032642	regulation of chemokine production	17735	49	466	5	1.33	0.01042	3.759398	ADAM17;HIF1A;IL1B;TLR2;TLR4
red	BP	GO:0009117	nucleotide metabolic process	17735	825	466	34	22.47	0.01054	1.513129	ACPP;ADM;AGFG1;ARHGEF19;ARL4D;ASAP1;ATP2C2;ATP5G1;ATP7B;CBS;CCR2;CDA;CDKL5;DOCK1;DOCK4;D
red	BP	GO:1900180	regulation of protein localization to nu...	17735	133	466	9	3.62	0.01058	2.486188	GNAQ;GSK3B;IL1B;KPNA1;LITAF;TLR2;TLR4;TNFRSF1A;TNFSF14
red	BP	GO:0048584	positive regulation of response to stimu...	17735	1164	466	45	31.7	0.01063	1.419558	ADAM17;ALOX5AP;ANKRD6;BCL2L11;C3AR1;CCR1;CCR2;CD55;CD59;CDH2;CR1;FCGR1A;FPR1;FYB;GPR89A;I
red	BP	GO:0042993	positive regulation of transcription fac...	17735	32	466	4	0.87	0.01066	4.597701	IL1B;TLR2;TLR4;TNFSF14
red	BP	GO:0010033	response to organic substance	17735	1870	466	67	50.93	0.01076	1.315531	ACSL1;ADAM17;ADM;ALDH1A2;AQP9;ARSB;ATF6;ATP6V1A;CA4;CACNA1E;CBS;CCR1;CCR2;CD55;CDK4;COM
red	BP	GO:0046822	regulation of nucleocytoplasmic transpor...	17735	158	466	10	4.3	0.01132	2.325581	GNAQ;GSK3B;IL1B;KPNA1;LITAF;TLR2;TLR4;TNFRSF1A;TNFSF14;WIPF1
red	BP	GO:0060759	regulation of response to cytokine stimu...	17735	90	466	7	2.45	0.01136	2.857143	HIF1A;IFNAR1;IFNGR2;IL1RN;IRAK3;PIAS1;SOCS3
red	MF	GO:0030234	enzyme regulator activity	17735	970	466	38	25.93	0.0115	1.465484	AGFG1;ALOX5AP;ANXA3;APAF1;ARHGAP19;ARHGEF19;ASAP1;CD55;CST7;CYTH4;DNAJC3;DOCK1;DOCK4;EV
red	BP	GO:0071706	tumor necrosis factor superfamily cytoki...	17735	70	466	6	1.91	0.0119	3.141361	CCR2;IRAK3;TLR2;TLR4;TNFRSF1A;ZFP36
red	BP	GO:0051656	establishment of organelle localization	17735	113	466	8	3.08	0.01206	2.597403	CUL3;GPSM2;INSC;MYO5A;PVRL2;RAB27A;SEC24D;SNAP23
red	BP	GO:0002407	dendritic cell chemotaxis	17735	18	466	3	0.49	0.01207	6.122449	CCR1;CCR2;CXCR1
red	BP	GO:0070168	negative regulation of biomineral tissue...	17735	18	466	3	0.49	0.01207	6.122449	CCR1;HIF1A;NFE2
red	BP	GO:0048518	positive regulation of biological proces...	17735	3464	466	114	94.34	0.01209	1.208395	ACSL1;ADAM17;ADM;ALDH1A2;ALOX5AP;ANKRD6;ANXA3;ATF6;ATXN1;ATXN7;AVIL;BCL2L11;C3AR1;CASP4
red	BP	GO:0009617	response to bacterium	17735	341	466	17	9.29	0.01238	1.829925	ADAM17;ADM;ANXA3;FCGR1A;HP;IL1B;IRAK3;LITAF;LYN;PDE4B;PGLYRP1;PLD1;SOD2;TLR2;TLR4;TNFRSF1A;
red	BP	GO:0002237	response to molecule of bacterial origin	17735	211	466	12	5.75	0.01303	2.086957	ADAM17;ADM;IL1B;IRAK3;LITAF;LYN;PDE4B;SOD2;TLR2;TLR4;TNFRSF1A;TRIB1
red	MF	GO:0005083	small GTPase regulator activity	17735	295	466	15	7.88	0.0133	1.903553	AGFG1;ARHGEF19;ASAP1;CYTH4;DOCK4;EVI5;GPSM2;IQGAP1;RANBP1;RAPGEF2;RASGRP4;SBF2;SOS2;TBC1
red	BP	GO:0071222	cellular response to lipopolysaccharide	17735	93	466	7	2.53	0.01345	2.766798	IL1B;LITAF;LYN;PDE4B;TLR2;TLR4;TRIB1
red	MF	GO:0042578	phosphoric ester hydrolase activity	17735	351	466	17	9.38	0.0136	1.812367	ACPP;C3AR1;CCR1;DUSP13;GDE1;INPP5A;MTMR10;NT5C2;PDE4B;PFKFB4;PLD1;PPAP2B;PPP3CC;PPP3R1;PF
red	BP	GO:0051174	regulation of phosphorus metabolic proce...	17735	1246	466	47	33.93	0.01383	1.385205	ACSL1;ADAM17;ADM;AGFG1;ARHGEF19;ASAP1;ATF6;ATXN1;ATXN7;CBS;CCR2;CDA;CDKL5;DNAJB11;DNAJC
red	BP	GO:0071396	cellular response to lipid	17735	213	466	12	5.8	0.01395	2.068966	ALDH1A2;DGAT2;IL1B;LITAF;LYN;PDE4B;PDK3;TLR2;TLR4;TNFRSF1A;TRIB1;WNT10B
red	BP	GO:0036336	dendritic cell migration	17735	19	466	3	0.52	0.01405	5.769231	CCR1;CCR2;CXCR1
red	BP	GO:0060706	cell differentiation involved in embryon...	17735	19	466	3	0.52	0.01405	5.769231	GCM1;SOCS3;STK3
red	CC	GO:0031965	nuclear membrane	17735	194	466	11	5.12	0.01413	2.148438	AHCTF1;ALOX5;ALOX5AP;CDK4;GNAQ;KAZN;LBR;LMNB1;NUP50;NUPL1;TOR1AIP2
red	BP	GO:0032602	chemokine production	17735	53	466	5	1.44	0.01436	3.472222	ADAM17;HIF1A;IL1B;TLR2;TLR4
red	BP	GO:0050931	pigment cell differentiation	17735	35	466	4	0.95	0.01457	4.210526	GNAQ;MYO5A;RAB27A;SOD2
red	CC	GO:0030136	clathrin-coated vesicle	17735	195	466	11	5.14	0.01463	2.140078	AP3B2;DMXL2;FCGR1A;FCGR1B;MME;PICALM;STX6;SYN2;SYNGR3;TRIM9;VAMP3
red	BP	GO:0002274	myeloid leukocyte activation	17735	117	466	8	3.19	0.01465	2.507837	ANXA3;CCR2;LYN;PTPRE;PVRL2;RHOH;SBNO2;TLR4
red	BP	GO:0048585	negative regulation of response to stimu...	17735	784	466	32	21.35	0.01465	1.498829	ADM;ANKRD6;ATXN1;ATXN7;CCR2;CD55;CD59;CDH2;CR1;CUL3;GSK3B;HEY1;HIF1A;HMGCR;HP;IL1B;IL1RN;
red	BP	GO:0009628	response to abiotic stimulus	17735	754	466	31	20.53	0.01471	1.509985	ADAM17;ADM;AQP9;ARSB;ATXN1;BEST1;CACNA1E;CASP5;CBS;CDK4;CHRNA10;HIF1A;HMGCR;IL1B;LIMD1;I
red	BP	GO:0032940	secretion by cell	17735	694	466	29	18.9	0.01475	1.534392	ADM;ANXA3;ARL4D;CACNA1E;CCR1;CCR2;F5;HCAR2;HIF1A;HMGCR;IL1B;IL1RN;INHBB;IQGAP1;LAMP2;LYN;
red	MF	GO:0032403	protein complex binding	17735	327	466	16	8.74	0.015	1.830664	ADAM17;CDK4;DOK4;FCGR1A;FCGR1B;FCGR1C;FCGR2A;FCGR3B;FYB;GNAQ;GSK3B;IQGAP1;LYN;PPAP2B;TN
red	BP	GO:0072521	purine-containing compound metabolic pro...	17735	725	466	30	19.74	0.01501	1.519757	ACPP;ADM;AGFG1;ARHGEF19;ARL4D;ASAP1;ATP2C2;ATP5G1;ATP7B;CBS;CCR2;CDKL5;DOCK1;DOCK4;DPYD
red	BP	GO:0009607	response to biotic stimulus	17735	606	466	26	16.5	0.01506	1.575758	ADAM17;ADM;ANXA3;APOBEC3A;BCL2L11;DNAJC3;FCGR1A;GSK3B;HP;IFNAR1;IFNGR2;IL1B;IRAK3;LITAF;LY
red	BP	GO:0040011	locomotion	17735	1253	466	47	34.12	0.01518	1.377491	ADAM17;ANXA3;AVL9;C3AR1;CACNA1E;CCR1;CCR2;CD177;CDH2;CDKL5;CENPV;CUL3;CXCR1;DOCK1;DOCK4
red	MF	GO:0017016	Ras GTPase binding	17735	120	466	8	3.21	0.0152	2.492212	CCDC64B;CDKL5;DMXL2;DOCK4;IQGAP1;MYO5A;RANBP1;RHOH
red	MF	GO:0008373	sialyltransferase activity	17735	20	466	3	0.53	0.0154	5.660377	ST3GAL4;ST3GAL5;ST6GALNAC2
red	MF	GO:0048365	Rac GTPase binding	17735	20	466	3	0.53	0.0154	5.660377	CDKL5;DOCK4;IQGAP1
red	BP	GO:0006997	nucleus organization	17735	54	466	5	1.47	0.01549	3.401361	AHCTF1;ATXN7;PVRL2;SRPK1;SRPK2
red	BP	GO:0043901	negative regulation of multi-organism pr...	17735	54	466	5	1.47	0.01549	3.401361	APOBEC3A;SRPK1;SRPK2;TARBP2;TLR2
red	CC	GO:0044433	cytoplasmic vesicle part	17735	418	466	19	11.03	0.01554	1.722575	ANXA3;AP3B2;CA4;CYBB;DMXL2;F5;FCGR1A;FCGR1B;GABARAPL1;GPR89A;ITM2B;LAMP2;PICALM;RAB27A;I
red	MF	GO:0005096	GTPase activator activity	17735	247	466	13	6.6	0.0157	1.969697	AGFG1;ARHGAP19;ARHGEF19;ASAP1;DOCK1;DOCK4;EVI5;GNAQ;IQGAP1;RANBP1;RAPGEF2;TBC1D30;TIAM
red	BP	GO:0030449	regulation of complement activation	17735	20	466	3	0.54	0.0162	5.555556	CD55;CD59;CR1
red	BP	GO:2000257	regulation of protein activation cascade	17735	20	466	3	0.54	0.0162	5.555556	CD55;CD59;CR1
red	BP	GO:0042060	wound healing	17735	610	466	26	16.61	0.01624	1.565322	ADAM17;ARHGEF19;CD177;CD59;DOCK1;ENTPD1;F5;GNAQ;H3F3B;HIF1A;HMGCR;HPSE;JMJD1C;LAMP2;LYI
red	BP	GO:0051650	establishment of vesicle localization	17735	55	466	5	1.5	0.01667	3.333333	CUL3;MYO5A;RAB27A;SEC24D;SNAP23

red	BP	GO:0006887	exocytosis	17735	271	466	14	7.38	0.01672	1.897019	ANXA3;CCR1;CCR2;F5;LAMP2;LYN;MYO5A;RAB27A;RAB31;SNAP23;STX12;STX3;TRIM9;VAMP3
red	BP	GO:0002682	regulation of immune system process	17735	884	466	35	24.07	0.01673	1.454092	ADAM17;C3AR1;CCR1;CCR2;CD55;CD59;CR1;FCGR1A;FCGR1B;FOXO3;FYB;HCAR2;HIF1A;IFNAR1;IFNGR2;IL1
red	BP	GO:0033157	regulation of intracellular protein tran...	17735	168	466	10	4.58	0.0168	2.183406	GNAQ;GSK3B;IL1B;KPNA1;LITAF;TLR2;TLR4;TNFRSF1A;TNFSF14;WIPF1
red	BP	GO:1901135	carbohydrate derivative metabolic proces...	17735	1327	466	49	36.14	0.0173	1.355838	ACPP;ADM;AGFG1;APOBEC3A;ARHGEF19;ARL4D;ARSB;ASAP1;ATP2C2;ATP5G1;ATP7B;CBS;CCR2;CDA;CDKL
red	BP	GO:0001817	regulation of cytokine production	17735	382	466	18	10.4	0.01734	1.730769	ADAM17;C3AR1;CCR2;HIF1A;HPSE;IFNAR1;IL1B;INHBB;IQGAP1;IRAK3;LITAF;LYN;PGLYRP1;TLR2;TLR4;TLR5;
red	BP	GO:0009892	negative regulation of metabolic process	17735	1456	466	53	39.65	0.01737	1.336696	ATXN1;ATXN7;CCR1;CCR2;CD55;CD59;CDA;CIR1;COMMD7;CR1;CTBP2;DNAJC3;FOXO3;GNAQ;GSK3B;HCAR
red	BP	GO:0043122	regulation of I-kappaB kinase/NF-kappaB ...	17735	194	466	11	5.28	0.01738	2.083333	GPR89A;IL1B;LITAF;PELI2;RHOH;SECTM1;TLR2;TLR4;TNFRSF1A;TNFSF14;WLS
red	BP	GO:0071219	cellular response to molecule of bacteri...	17735	98	466	7	2.67	0.01752	2.621723	IL1B;LITAF;LYN;PDE4B;TLR2;TLR4;TRIB1
red	BP	GO:0008542	visual learning	17735	37	466	4	1.01	0.01761	3.960396	ATXN1;CACNA1E;HIF1A;HMGCR
red	BP	GO:0045576	mast cell activation	17735	37	466	4	1.01	0.01761	3.960396	LYN;PTPRE;PVRL2;RHOH
red	BP	GO:0019220	regulation of phosphate metabolic proces...	17735	1233	466	46	33.58	0.01771	1.369863	ACSL1;ADAM17;ADM;AGFG1;ARHGEF19;ASAP1;ATF6;ATXN1;ATXN7;CBS;CCR2;CDA;CDKL5;DNAJB11;DNAJC
red	BP	GO:0009119	ribonucleoside metabolic process	17735	556	466	24	15.14	0.01787	1.585205	ACPP;AGFG1;APOBEC3A;ARHGEF19;ARL4D;ASAP1;ATP2C2;ATP5G1;ATP7B;CDA;CDKL5;DOCK1;DOCK4;DPYD
red	BP	GO:0032874	positive regulation of stress-activated ...	17735	56	466	5	1.53	0.0179	3.267974	HMGCR;IL1B;SDCBP;STK3;TLR4
red	BP	GO:0001782	B cell homeostasis	17735	21	466	3	0.57	0.01852	5.263158	BCL2L11;HIF1A;LYN
red	BP	GO:0046135	pyrimidine nucleoside catabolic process	17735	21	466	3	0.57	0.01852	5.263158	APOBEC3A;CDA;DPYD
red	BP	GO:0046627	negative regulation of insulin receptor ...	17735	21	466	3	0.57	0.01852	5.263158	IL1B;PTPRE;SOCS3
red	BP	GO:0002252	immune effector process	17735	471	466	21	12.83	0.01877	1.636789	ADAM17;ANXA3;APOBEC3A;CCR2;CD55;CD59;CR1;DNAJC3;FCGR1A;IFNAR1;IL1B;IRAK3;LYN;PAK2;PGLYRP1
red	BP	GO:0007204	elevation of cytosolic calcium ion conce...	17735	171	466	10	4.66	0.01878	2.145923	ADM;C3AR1;CCR1;CCR2;CD55;CHRNA10;LYN;NPTN;PROK2;SLC8A1
red	MF	GO:0046983	protein dimerization activity	17735	909	466	35	24.3	0.019	1.440329	ACPP;ALOX5AP;APOA1BP;BCL2L11;CBS;CCR2;CDA;CYBB;DGAT2;DPYD;EXT1;H3F3B;HEY1;HIF1A;HMGCR;HP
red	BP	GO:0006733	oxidoreduction coenzyme metabolic proces...	17735	57	466	5	1.55	0.0192	3.225806	COQ4;HMGCR;IDH1;NMNAT2;PNP
red	BP	GO:0030595	leukocyte chemotaxis	17735	123	466	8	3.35	0.01925	2.38806	ADAM17;C3AR1;CCR1;CCR2;CXCR1;IL1B;LYN;PDE4B
red	BP	GO:0048522	positive regulation of cellular process	17735	3140	466	103	85.51	0.01926	1.204537	ACSL1;ADAM17;ADM;ALDH1A2;ANKRD6;ANXA3;ATF6;ATXN1;ATXN7;AVIL;BCL2L11;C3AR1;CASP4;CCR1;CCR
red	BP	GO:0006928	cellular component movement	17735	1208	466	45	32.9	0.01934	1.367781	ACTR2;ADAM17;ANXA3;AP3B2;ARPC5;AVL9;C3AR1;CACNA1E;CCR1;CCR2;CD177;CDH2;CDKL5;CENPV;CUL3;
red	BP	GO:0002699	positive regulation of immune effector p...	17735	100	466	7	2.72	0.01937	2.573529	CCR2;FCGR1A;IL1B;LYN;PVRL2;TLR2;TLR4
red	BP	GO:0010942	positive regulation of cell death	17735	444	466	20	12.09	0.0195	1.65426	ALDH1A2;BCL2L11;CASP4;CDK4;FOXO3;GSK3B;HCAR2;HMGCR;HP;ITM2B;LYN;MLLT11;NCOA1;PDCD7;SRPK
red	MF	GO:0008374	O-acyltransferase activity	17735	39	466	4	1.04	0.0198	3.846154	AGPAT9;DGAT2;MBOAT2;SH3GLB1
red	CC	GO:0044464	cell part	17735	14451	466	396	381.27	0.02031	1.038634	ACPP;ACSL1;ACTR2;ADAM17;ADM;AGFG1;AGPAT9;AGTPBP1;AHCTF1;AKR7A3;ALDH1A2;ALOX5;ALOX5AP;A
red	CC	GO:0005623	cell	17735	14452	466	396	381.29	0.02049	1.03858	ACPP;ACSL1;ACTR2;ADAM17;ADM;AGFG1;AGPAT9;AGTPBP1;AHCTF1;AKR7A3;ALDH1A2;ALOX5;ALOX5AP;A
red	BP	GO:0045088	regulation of innate immune response	17735	225	466	12	6.13	0.02057	1.957586	CR1;IFNAR1;IFNGR2;IRAK3;LYN;PELI2;PIAS1;PVRL2;SOCS3;TLR2;TLR4;TLR5
red	BP	GO:0033993	response to lipid	17735	534	466	23	14.54	0.02062	1.581843	ACSL1;ADAM17;ADM;ALDH1A2;ARSB;CA4;CDK4;DGAT2;IDH1;IL1B;IL1RN;IRAK3;LITAF;LYN;PDCD7;PDE4B;PD
red	CC	GO:0031090	organelle membrane	17735	2366	466	78	62.42	0.02067	1.249599	ACPP;ACSL1;AGPAT9;AHCTF1;ALOX5;ALOX5AP;ANTXR2;ANXA3;AP3B2;ATF6;ATP2C2;ATP5G1;ATP7B;ATP9A
red	BP	GO:1901136	carbohydrate derivative catabolic proces...	17735	564	466	24	15.36	0.02081	1.5625	AGFG1;APOBEC3A;ARHGEF19;ARL4D;ARSB;ASAP1;CDA;CDKL5;DOCK1;DOCK4;DPYD;GNAQ;GSK3B;HPSE;IDS
red	CC	GO:0030175	filopodium	17735	60	466	5	1.58	0.02082	3.164557	ACPP;FAM65B;FGF13;MYO10;TIAM2
red	CC	GO:0031252	cell leading edge	17735	260	466	13	6.86	0.02097	1.895044	ADAM17;APBB2;ARPC5;BMX;CDH2;CDKL5;FRMD4B;IQGAP1;MYO10;MYO5A;NEDD9;S100A11;TIAM2
red	BP	GO:0015695	organic cation transport	17735	22	466	3	0.6	0.02102	5	SLC22A1;SLC22A14;SLC22A4
red	BP	GO:0071320	cellular response to cAMP	17735	22	466	3	0.6	0.02102	5	AQP9;SLC8A1;WNT10B
red	BP	GO:0043299	leukocyte degranulation	17735	39	466	4	1.06	0.02103	3.773585	ANXA3;CCR2;LYN;RAB27A
red	BP	GO:0042325	regulation of phosphorylation	17735	869	466	34	23.67	0.0213	1.436417	ACSL1;ADAM17;ATF6;ATXN1;ATXN7;CBS;DNAJB11;DNAJC3;FGF13;FPR1;GNAQ;GSK3B;HMGCR;IL1B;IL1RN;I
red	BP	GO:0016310	phosphorylation	17735	1280	466	47	34.86	0.02141	1.34825	ACSL1;ADAM17;ATF6;ATXN1;ATXN7;BMX;CBS;CDK4;CDKL5;DNAJB11;DNAJC3;FGF13;FPR1;FYB;GNAQ;GSK3
red	BP	GO:0042493	response to drug	17735	335	466	16	9.12	0.02149	1.754386	ACSL1;ADAM17;CA4;CDK4;EBP;GSK3B;IL1B;IL1RN;LYN;NCOA1;PDE4B;PNP;SLC22A1;SLC8A1;SOD2;TLR2
red	BP	GO:0030203	glycosaminoglycan metabolic process	17735	150	466	9	4.09	0.02166	2.200489	ARSB;EXT1;HAS1;HPSE;IDS;IL1B;LYVE1;PGLYRP1;ST3GAL4
red	BP	GO:0060326	cell chemotaxis	17735	150	466	9	4.09	0.02166	2.200489	ADAM17;C3AR1;CCR1;CCR2;CXCR1;DOCK4;IL1B;LYN;PDE4B
red	BP	GO:0006944	cellular membrane fusion	17735	80	466	6	2.18	0.02178	2.752294	SNAP23;STX11;STX3;STX6;TRIM9;VAMP3
red	BP	GO:0060828	regulation of canonical Wnt receptor sig...	17735	126	466	8	3.43	0.02191	2.332362	ANKRD6;CDH2;CDK14;GSK3B;LIMD1;STK3;WLS;WNT10B
red	BP	GO:0006027	glycosaminoglycan catabolic process	17735	59	466	5	1.61	0.02198	3.10559	ARSB;HPSE;IDS;LYVE1;PGLYRP1
red	BP	GO:0043065	positive regulation of apoptotic process	17735	421	466	19	11.47	0.02206	1.656495	ALDH1A2;BCL2L11;CASP4;CDK4;FOXO3;GSK3B;HCAR2;HMGCR;ITM2B;LYN;MLLT11;NCOA1;PDCD7;SRPK2;S
red	CC	GO:0042383	sarcolemma	17735	83	466	6	2.19	0.02237	2.739726	CA4;CD59;FLOT1;SLC8A1;SNTA1;SNTG2
red	BP	GO:0009108	coenzyme biosynthetic process	17735	103	466	7	2.81	0.02239	2.491103	ACSL1;COQ4;ELOVL5;NMNAT2;PANK2;PDK3;PNP
red	MF	GO:0015298	solute:cation antiporter activity	17735	23	466	3	0.61	0.0226	4.918033	SLC22A4;SLC8A1;TMCO3
red	BP	GO:0001819	positive regulation of cytokine producti...	17735	202	466	11	5.5	0.02268	2	ADAM17;C3AR1;CCR2;HIF1A;HPSE;IFNAR1;IL1B;TLR2;TLR4;TLR5;TNFRSF1A
red	BP	GO:0080135	regulation of cellular response to stres...	17735	282	466	14	7.68	0.0227	1.822917	CBS;HMGCR;HP;IL1B;IL1RN;LYN;NFE2L2;SCARF1;SDCBP;STK3;TLR4;UIMC1;WAC;ZAK
red	BP	GO:0032388	positive regulation of intracellular tra...	17735	127	466	8	3.46	0.02285	2.312139	GSK3B;IL1B;KPNA1;TLR2;TLR4;TNFRSF1A;TNFSF14;WIPF1
red	MF	GO:0008047	enzyme activator activity	17735	404	466	18	10.8	0.0242	1.666667	AGFG1;ALOX5AP;APAF1;ARHGAP19;ARHGEF19;ASAP1;DOCK1;DOCK4;EVI5;FGF13;GNAQ;IQGAP1;PAK2;RAN
red	MF	GO:0015101	organic cation transmembrane transporter...	17735	24	466	3	0.64	0.0253	4.6875	SLC22A1;SLC22A14;SLC22A4
red	CC	GO:0030666	endocytic vesicle membrane	17735	109	466	7	2.88	0.02537	2.430556	ANXA3;CYBB;FCGR1A;FCGR1B;LAMP2;PICALM;RAB31
red	MF	GO:0030695	GTPase regulator activity	17735	436	466	19	11.65	0.0256	1.630901	AGFG1;ARHGAP19;ARHGEF19;ASAP1;CYTH4;DOCK1;DOCK4;EVI5;GNAQ;GPSM2;IQGAP1;RANBP1;RAPGEF2;
red	MF	GO:0019207	kinase regulator activity	17735	133	466	8	3.55	0.0264	2.253521	DNAJC3;FGF13;PAK2;RHOH;ROPN1L;SOCS3;STK3;TRIB1
red	MF	GO:0031267	small GTPase binding	17735	133	466	8	3.55	0.0264	2.253521	CCDC64B;CDKL5;DMXL2;DOCK4;IQGAP1;MYO5A;RANBP1;RHOH
red	CC	GO:0043020	NADPH oxidase complex	17735	10	466	2	0.26	0.02717	7.692308	CYBB;NCF4
red	CC	GO:0030135	coated vesicle	17735	242	466	12	6.38	0.0274	1.880878	AP3B2;DMXL2;FCGR1A;FCGR1B;MME;PICALM;SEC24D;STX6;SYN2;SYNGR3;TRIM9;VAMP3
red	MF	GO:0008252	nucleotidase activity	17735	10	466	2	0.27	0.0278	7.407407	ACPP;NT5C2
red	MF	GO:0001608	G-protein coupled nucleotide receptor ac...	17735	25	466	3	0.67	0.0282	4.477612	GPR17;HCAR2;HCAR3
red	MF	GO:0045028	G-protein coupled purinergic nucleotide ...	17735	25	466	3	0.67	0.0282	4.477612	GPR17;HCAR2;HCAR3
red	CC	GO:0030139	endocytic vesicle	17735	163	466	9	4.3	0.02911	2.093023	ANXA3;CYBB;FCGR1A;FCGR1B;LAMP2;NCF4;PICALM;RAB31;STX12
red	CC	GO:0000139	Golgi membrane	17735	540	466	22	14.25	0.02967	1.54386	ATF6;ATP2C2;ATP7B;EXT1;GALNT14;GIMAP1;GLIPR2;GOLGA7B;GPR89A;ITM2B;NMNAT2;PLD1;RTN3;SEC24
red	MF	GO:0005516	calmodulin binding	17735	162	466	9	4.33	0.0301	2.078522	IQGAP1;MARCKS;MYO10;MYO5A;PPP3CC;PPP3R1;SLC8A1;SNTA1;TRPV5
red	MF	GO:0019887	protein kinase regulator activity	17735	113	466	7	3.02	0.0319	2.317881	DNAJC3;FGF13;PAK2;ROPN1L;SOCS3;STK3;TRIB1
red	MF	GO:0060589	nucleoside-triphosphatase regulator acti...	17735	447	466	19	11.95	0.0319	1.589958	AGFG1;ARHGAP19;ARHGEF19;ASAP1;CYTH4;DOCK1;DOCK4;EVI5;GNAQ;GPSM2;IQGAP1;RANBP1;RAPGEF2;
red	CC	GO:0005768	endosome	17735	546	466	22	14.41	0.03295	1.526718	ACPP;ATP11A;ATP7B;ATP9A;AVL9;FCGR1A;FCGR1B;FLOT1;FPR1;ITM2B;KIAA0319;LAMP2;NMNAT2;OSBPL1

red	MF	GO:0042625	ATPase activity, coupled to transmembran...	17735	67	466	5	1.79	0.0333	2.793296	ATP11A;ATP2C2;ATP6V1A;ATP7B;ATP9A
red	MF	GO:0008528	G-protein coupled peptide receptor activ...	17735	115	466	7	3.07	0.0346	2.28013	C3AR1;CCR1;CCR2;CXCR1;FPR1;FPR2;GPR17
red	CC	GO:0044444	cytoplasmic part	17735	6562	466	192	173.13	0.03534	1.108993	ACPP;ACSL1;AGFG1;AGPAT9;AGTPBP1;AHCTF1;AKR7A3;ALDH1A2;ALOX5;ALOX5AP;ANTXR2;ANXA3;AP3B2;
red	MF	GO:0001653	peptide receptor activity	17735	116	466	7	3.1	0.0361	2.258065	C3AR1;CCR1;CCR2;CXCR1;FPR1;FPR2;GPR17
red	MF	GO:0022804	active transmembrane transporter activit...	17735	306	466	14	8.18	0.0361	1.711491	AQP9;ATP11A;ATP2C2;ATP6V1A;ATP7B;ATP9A;SLC12A6;SLC15A4;SLC22A1;SLC22A4;SLC26A8;SLC8A1;TIMM
red	MF	GO:0042803	protein homodimerization activity	17735	545	466	22	14.57	0.0363	1.509952	ACPP;ALOX5AP;APOA1BP;CBS;CCR2;CDA;DGAT2;DPYD;EXT1;HMGCR;IDH1;INHBB;IRAK3;NR6A1;PTPRE;PVRI
red	MF	GO:0019210	kinase inhibitor activity	17735	48	466	4	1.28	0.0389	3.125	DNAJC3;RHOH;SOCS3;TRIB1
red	MF	GO:0004197	cysteine-type endopeptidase activity	17735	70	466	5	1.87	0.0391	2.673797	CAPN12;CAPN13;CAPN8;CASP4;CASP5
red	MF	GO:0016493	C-C chemokine receptor activity	17735	12	466	2	0.32	0.0394	6.25	CCR1;CCR2
red	MF	GO:0051020	GTPase binding	17735	145	466	8	3.88	0.041	2.061856	CCDC64B;CDKL5;DMXL2;DOCK4;IQGAP1;MYO5A;RANBP1;RHOH
red	MF	GO:0033293	monocarboxylic acid binding	17735	49	466	4	1.31	0.0415	3.053435	ALOX5AP;OXER1;SH3GLB1;STX3
red	MF	GO:0016791	phosphatase activity	17735	255	466	12	6.82	0.0418	1.759531	ACPP;DUSP13;INPP5A;MTMR10;NT5C2;PFKFB4;PPAP2B;PPP3CC;PPP3R1;PPP4R1;PTPRE;SBF2
red	MF	GO:0016746	transferase activity, transferring acyl ...	17735	227	466	11	6.07	0.0419	1.812191	AGPAT9;DGAT2;ELOVL5;MBOAT2;NCOA1;NCOA2;OLAH;QPCT;SH3GLB1;TGM3;ZDHHC19
red	CC	GO:0048471	perinuclear region of cytoplasm	17735	469	466	19	12.37	0.04325	1.535974	ALDH1A2;ATP7B;ATP9A;CA4;CCR2;CDK4;DGAT2;GSK3B;INHBB;LYN;MRVI1;PAK2;PDE4B;PICALM;PLD1;SEC2
red	MF	GO:0015662	ATPase activity, coupled to transmembran...	17735	50	466	4	1.34	0.0442	2.985075	ATP11A;ATP2C2;ATP7B;ATP9A
red	CC	GO:0030670	phagocytic vesicle membrane	17735	51	466	4	1.35	0.04517	2.962963	ANXA3;CYBB;LAMP2;RAB31
red	MF	GO:0005095	GTPase inhibitor activity	17735	13	466	2	0.35	0.0458	5.714286	IQGAP1;RHOH
red	MF	GO:0005161	platelet-derived growth factor receptor ...	17735	13	466	2	0.35	0.0458	5.714286	IL1R1;LYN
red	CC	GO:0001726	ruffle	17735	124	466	7	3.27	0.04611	2.140673	ADAM17;BMX;CDKL5;FRMD4B;MYO10;MYO5A;S100A11
red	MF	GO:0017048	Rho GTPase binding	17735	51	466	4	1.36	0.047	2.941176	CDKL5;DOCK4;IQGAP1;RHOH
red	MF	GO:0005085	guanyl-nucleotide exchange factor activi...	17735	176	466	9	4.7	0.0471	1.914894	ARHGEF19;CYTH4;DOCK1;DOCK4;RAPGEF2;RASGRP4;SBF2;SOS2;TIAM2
red	CC	GO:0030669	clathrin-coated endocytic vesicle membra...	17735	31	466	3	0.82	0.04752	3.658537	FCGR1A;FCGR1B;PICALM
red	CC	GO:0005643	nuclear pore	17735	75	466	5	1.98	0.04798	2.525253	AGFG1;AHCTF1;KPNA1;NUP50;NUPL1
red	CC	GO:0034399	nuclear periphery	17735	100	466	6	2.64	0.04898	2.272727	AHCTF1;ALOX5;ATXN1;ATXN7;LMNB1;SRPK1
red	CC	GO:0042995	cell projection	17735	1194	466	41	31.5	0.04992	1.301587	ACPP;ACTR2;ADAM17;APBB2;ARPC5;ATP6V1A;AVIL;BMX;CA4;CCR2;CDH2;CDKL5;CHRNA10;DNAH14;DOCK
purple	CC	GO:0031091	platelet alpha granule	17735	60	134	16	0.47	8.50E-21	34.04255	CD9;CLU;EGF;ITGA2B;ITGB3;MMRN1;PDGFA;PF4;PPBP;PROS1;SELP;SERPINE1;SPARC;TREML1;VEGFC;VWF
purple	BP	GO:0042060	wound healing	17735	610	134	34	4.66	1.20E-20	7.296137	ADRA2A;CD9;CLU;EGF;ESAM;F2RL3;GP6;GP9;GRB14;GUCY1A3;GUCY1B3;ITGA2B;ITGB3;MGLL;MMRN1;MYL
purple	BP	GO:0007596	blood coagulation	17735	498	134	30	3.8	4.10E-19	7.894737	ADRA2A;CD9;CLU;EGF;ESAM;F2RL3;GP6;GP9;GRB14;GUCY1A3;GUCY1B3;ITGA2B;ITGB3;MGLL;MMRN1;PDE
purple	BP	GO:0050817	coagulation	17735	501	134	30	3.82	4.80E-19	7.853403	ADRA2A;CD9;CLU;EGF;ESAM;F2RL3;GP6;GP9;GRB14;GUCY1A3;GUCY1B3;ITGA2B;ITGB3;MGLL;MMRN1;PDE
purple	BP	GO:0007599	hemostasis	17735	502	134	30	3.83	5.10E-19	7.832898	ADRA2A;CD9;CLU;EGF;ESAM;F2RL3;GP6;GP9;GRB14;GUCY1A3;GUCY1B3;ITGA2B;ITGB3;MGLL;MMRN1;PDE
purple	BP	GO:0030168	platelet activation	17735	219	134	22	1.67	8.30E-19	13.17365	ADRA2A;CD9;CLU;EGF;F2RL3;GP6;GP9;ITGA2B;ITGB3;MGLL;MMRN1;PDGFA;PDGFRA;PF4;PPBP;PROS1;SELF
purple	BP	GO:0002576	platelet degranulation	17735	83	134	15	0.63	5.20E-17	23.80952	CD9;CLU;EGF;ITGA2B;ITGB3;MMRN1;PDGFA;PF4;PPBP;PROS1;SELP;SERPINE1;SPARC;VEGFC;VWF
purple	BP	GO:0050878	regulation of body fluid levels	17735	594	134	30	4.53	5.40E-17	6.622517	ADRA2A;CD9;CLU;EGF;ESAM;F2RL3;GP6;GP9;GRB14;GUCY1A3;GUCY1B3;ITGA2B;ITGB3;MGLL;MMRN1;PDE
purple	BP	GO:0009611	response to wounding	17735	1090	134	37	8.32	2.20E-15	4.447115	ADRA2A;BMP6;CD9;CLU;EGF;ESAM;F2RL3;GP6;GP9;GRB14;GUCY1A3;GUCY1B3;ITGA2B;ITGB3;MGLL;MMRN
purple	CC	GO:0031093	platelet alpha granule lumen	17735	47	134	11	0.37	6.00E-14	29.72973	CLU;EGF;MMRN1;PDGFA;PF4;PPBP;PROS1;SERPINE1;SPARC;VEGFC;VWF
purple	CC	GO:0030141	secretory granule	17735	251	134	19	1.96	8.70E-14	9.693878	ACRBP;CAV2;CD9;CLU;EGF;ITGA2B;ITGB3;MMRN1;PDGFA;PF4;PPBP;PROS1;SELP;SERPINE1;SPARC;SYTL4;TR
purple	CC	GO:0034774	secretory granule lumen	17735	50	134	11	0.39	1.30E-13	28.20513	CLU;EGF;MMRN1;PDGFA;PF4;PPBP;PROS1;SERPINE1;SPARC;VEGFC;VWF
purple	CC	GO:0060205	cytoplasmic membrane-bounded vesicle lum...	17735	54	134	11	0.42	3.10E-13	26.19048	CLU;EGF;MMRN1;PDGFA;PF4;PPBP;PROS1;SERPINE1;SPARC;VEGFC;VWF
purple	CC	GO:0031983	vesicle lumen	17735	55	134	11	0.43	3.90E-13	25.5814	CLU;EGF;MMRN1;PDGFA;PF4;PPBP;PROS1;SERPINE1;SPARC;VEGFC;VWF
purple	BP	GO:0006887	exocytosis	17735	271	134	17	2.07	2.30E-11	8.21256	ADRA2A;CD9;CLU;EGF;ITGA2B;ITGB3;MMRN1;PDGFA;PF4;PPBP;PROS1;SELP;SERPINE1;SPARC;SYTL4;VEGFC
purple	BP	GO:0001775	cell activation	17735	758	134	25	5.79	3.80E-10	4.317789	ADRA2A;CD9;CDKN1A;CLU;EGF;F2RL3;GP6;GP9;ITGA2B;ITGB3;MGLL;MMRN1;PDE5A;PDGFA;PDGFRA;PF4;P
purple	BP	GO:0065008	regulation of biological quality	17735	2547	134	47	19.44	7.60E-10	2.417695	ACSBG1;ADRA2A;ALOX12;AVPR1A;BMP6;CA2;CD9;CLDN5;CLU;EGF;ESAM;F2RL3;GP6;GP9;GRB14;GUCY1A3
purple	BP	GO:0032940	secretion by cell	17735	694	134	23	5.3	2.00E-09	4.339623	ADRA2A;AVPR1A;BMP6;CD9;CLU;EGF;ITGA2B;ITGB3;MAOB;MMRN1;PCSK6;PDGFA;PF4;PPBP;PRKAR2B;PRC
purple	BP	GO:0046903	secretion	17735	792	134	24	6.05	4.80E-09	3.966942	ADRA2A;AVPR1A;BMP6;CA2;CD9;CLU;EGF;ITGA2B;ITGB3;MAOB;MMRN1;PCSK6;PDGFA;PF4;PPBP;PRKAR2B
purple	CC	GO:0044433	cytoplasmic vesicle part	17735	418	134	17	3.27	2.80E-08	5.198777	CAV2;CD9;CLU;EGF;ITGA2B;ITGB3;MMRN1;PDGFA;PF4;PPBP;PROS1;SELP;SERPINE1;SPARC;SYTL4;VEGFC;V
purple	BP	GO:0006950	response to stress	17735	2926	134	47	22.34	7.50E-08	2.10385	ADRA2A;AVPR1A;BMP6;CA2;CD9;CDKN1A;CLEC1B;CLU;EGF;ESAM;F2RL3;GP6;GP9;GRB14;GUCY1A3;GUCY1
purple	BP	GO:0061041	regulation of wound healing	17735	82	134	8	0.63	2.00E-07	12.69841	ADRA2A;MYLK;PDGFA;PDGFRA;PROS1;SELP;SERPINE1;VIL1
purple	BP	GO:0016192	vesicle-mediated transport	17735	894	134	23	6.82	2.20E-07	3.372434	ADRA2A;BET3L;CAV2;CD9;CLU;DNM3;EGF;ITGA2B;ITGB3;MMRN1;PDGFA;PEAR1;PF4;PPBP;PROS1;RAB6B;S
purple	CC	GO:0031988	membrane-bounded vesicle	17735	905	134	23	7.08	5.00E-07	3.248588	ACRBP;ACSBG1;BMP6;CAV2;CD9;CLU;EGF;ITGA2B;ITGB3;ITGB5;MMRN1;PDGFA;PF4;PPBP;PROS1;RAB6B;SE
purple	BP	GO:0044707	single-multicellular organism process	17735	5398	134	67	41.21	5.20E-07	1.625819	ABLIM3;ACSBG1;ADRA2A;ALOX12;AVPR1A;BMP6;CA2;CALD1;CAV2;CD9;CDKN1A;CLDN5;CLU;DNM3;EGF;ES
purple	CC	GO:0031410	cytoplasmic vesicle	17735	914	134	23	7.15	6.00E-07	3.216783	ACRBP;ACSBG1;AVPR1A;CAV2;CD9;CLU;EGF;ITGA2B;ITGB3;ITGB5;MMRN1;PDGFA;PF4;PPBP;PROS1;RAB6B;
purple	BP	GO:0050896	response to stimulus	17735	6581	134	76	50.24	6.20E-07	1.512739	ABLIM3;ACSBG1;ADRA2A;ALOX12;AQP10;ARHGAP6;ASAP2;AVPR1A;BMP6;CA2;CABP5;CAV2;CD9;CDKN1A;
purple	CC	GO:0016023	cytoplasmic membrane-bounded vesicle	17735	848	134	22	6.63	6.50E-07	3.31825	ACRBP;ACSBG1;CAV2;CD9;CLU;EGF;ITGA2B;ITGB3;ITGB5;MMRN1;PDGFA;PF4;PPBP;PROS1;RAB6B;SELP;SER
purple	CC	GO:0031982	vesicle	17735	993	134	24	7.76	6.80E-07	3.092784	ACRBP;ACSBG1;AVPR1A;BMP6;CAV2;CD9;CLU;EGF;ITGA2B;ITGB3;ITGB5;MMRN1;PDGFA;PF4;PPBP;PROS1;
purple	BP	GO:0044763	single-organism cellular process	17735	9577	134	96	73.11	6.80E-07	1.31309	ABCC3;ABLIM3;ACSBG1;ADRA2A;ALOX12;AQP10;ARHGAP6;ASAP2;AVPR1A;BMP6;CA2;CABP5;CALD1;CAV2;
purple	CC	GO:0071944	cell periphery	17735	4360	134	60	34.09	7.70E-07	1.760047	ABCC3;ADRA2A;ALOX12;AQP10;ASAP2;AVPR1A;C2orf88;C6orf25;CA2;CALD1;CAV2;CD9;CLDN5;CLEC1B;CTT
purple	CC	GO:0005886	plasma membrane	17735	4273	134	59	33.41	9.20E-07	1.765938	ABCC3;ADRA2A;ALOX12;AQP10;ASAP2;AVPR1A;C2orf88;C6orf25;CA2;CALD1;CAV2;CD9;CLDN5;CLEC1B;EGF
purple	BP	GO:0032501	multicellular organismal process	17735	5593	134	67	42.69	2.30E-06	1.569454	ABLIM3;ACSBG1;ADRA2A;ALOX12;AVPR1A;BMP6;CA2;CALD1;CAV2;CD9;CDKN1A;CLDN5;CLU;DNM3;EGF;ES
purple	BP	GO:0042221	response to chemical stimulus	17735	2766	134	42	21.11	2.70E-06	1.989578	ABLIM3;ACSBG1;ADRA2A;ALOX12;AQP10;AVPR1A;BMP6;CA2;CAV2;CD9;CDKN1A;CLU;CMTM5;CXCL5;GNG
purple	CC	GO:0016020	membrane	17735	7690	134	86	60.13	3.80E-06	1.430234	ABCC3;ADRA2A;ALOX12;AQP10;ASAP2;AVPR1A;C2orf88;C6orf25;CA2;CALD1;CAV2;CD9;CLDN5;CLEC1B;CLE
purple	BP	GO:0040011	locomotion	17735	1253	134	25	9.56	6.40E-06	2.615063	ABLIM3;ADRA2A;ALOX12;CMTM5;CXCL5;ESAM;GP6;GRB14;ITGA2B;ITGB3;MYL9;MYLK;PARD3;PDGFA;PDGI
purple	BP	GO:0030335	positive regulation of cell migration	17735	223	134	10	1.7	7.90E-06	5.882353	ADRA2A;ALOX12;ITGB3;MYLK;PDGFA;PDGFRA;SELP;SERPINE1;VEGFC;VIL1
purple	BP	GO:2000147	positive regulation of cell motility	17735	228	134	10	1.74	9.50E-06	5.747126	ADRA2A;ALOX12;ITGB3;MYLK;PDGFA;PDGFRA;SELP;SERPINE1;VEGFC;VIL1
purple	BP	GO:0044699	single-organism process	17735	10656	134	100	81.34	9.80E-06	1.229407	ABCC3;ABLIM3;ACSBG1;ADRA2A;ALOX12;AQP10;ARHGAP6;ASAP2;AVPR1A;BMP6;CA2;CABP5;CALD1;CAV2;
purple	BP	GO:0051649	establishment of localization in cell	17735	1820	134	31	13.89	1.00E-05	2.231821	ADRA2A;AVPR1A;BMP6;CD9;CDKN1A;CLU;EGF;F2RL3;ITGA2B;ITGB3;MAOB;MAP1B;MMRN1;PARD3;PCSK6;
purple	BP	GO:0051272	positive regulation of cellular componen...	17735	234	134	10	1.79	1.20E-05	5.586592	ADRA2A;ALOX12;ITGB3;MYLK;PDGFA;PDGFRA;SELP;SERPINE1;VEGFC;VIL1
purple	BP	GO:0040017	positive regulation of locomotion	17735	237	134	10	1.81	1.30E-05	5.524862	ADRA2A;ALOX12;ITGB3;MYLK;PDGFA;PDGFRA;SELP;SERPINE1;VEGFC;VIL1
purple	BP	GO:0009719	response to endogenous stimulus	17735	1063	134	22	8.11	1.50E-05	2.7127	ACSBG1;ADRA2A;AVPR1A;BMP6;CA2;CAV2;CDKN1A;GNG11;HOMER2;LTBP1;MAOB;PARD3;PDGFA;PDGFRA
purple	BP	GO:0007154	cell communication	17735	4775	134	58	36.45	1.70E-05	1.591221	ACSBG1;ADRA2A;ARHGAP6;ASAP2;AVPR1A;BMP6;CABP5;CAV2;CD9;CDKN1A;CLDN5;CLEC1B;CLU;CXCL5;EG

purple	MF	GO:0001664	G-protein coupled receptor binding	17735	195	134	9	1.5	1.90E-05	6	ADRA2A;AVPR1A;CAV2;CXCL5;GNAZ;HOMER2;PF4;PF4V1;PPBP
purple	BP	GO:0023052	signaling	17735	4666	134	57	35.62	1.90E-05	1.600225	ACSBG1;ADRA2A;ARHGAP6;ASAP2;AVPR1A;BMP6;CABP5;CAV2;CD9;CDKN1A;CLDN5;CLEC1B;CLU;CXCL5;EG
purple	BP	GO:0044700	single organism signaling	17735	4666	134	57	35.62	1.90E-05	1.600225	ACSBG1;ADRA2A;ARHGAP6;ASAP2;AVPR1A;BMP6;CABP5;CAV2;CD9;CDKN1A;CLDN5;CLEC1B;CLU;CXCL5;EG
purple	CC	GO:0030667	secretory granule membrane	17735	52	134	5	0.41	5.20E-05	12.19512	CAV2;CD9;ITGA2B;ITGB3;SELP
purple	BP	GO:0033993	response to lipid	17735	534	134	14	4.08	5.50E-05	3.431373	ACSBG1;ALOX12;AVPR1A;BMP6;CA2;CDKN1A;MAOB;PDGFA;PTGES;SELP;SERPINE1;SLC6A4;SPARC;TNFSF4
purple	BP	GO:1901700	response to oxygen-containing compound	17735	755	134	17	5.76	5.60E-05	2.951389	ADRA2A;AVPR1A;BMP6;CD9;CDKN1A;HOMER2;MAOB;PDGFA;PDGFRA;PTGES;SELP;SERPINE1;SLC6A4;SLC8/
purple	BP	GO:0042127	regulation of cell proliferation	17735	1174	134	22	8.96	6.60E-05	2.455357	ADRA2A;ALOX12;AVPR1A;BMP6;CAV2;CD9;CDKN1A;CXCL5;EGF;ITGB3;PDE5A;PDGFA;PDGFRA;PTGES;PTGS1
purple	BP	GO:0051241	negative regulation of multicellular org...	17735	359	134	11	2.74	9.30E-05	4.014599	ADRA2A;AVPR1A;GUCY1A3;MGLL;PDE5A;PDGFA;PDGFRA;PROS1;SERPINE1;SLC6A4;TNFSF4
purple	BP	GO:0044765	single-organism transport	17735	2753	134	38	21.02	9.30E-05	1.807802	ABCC3;ADRA2A;AQP10;AVPR1A;BMP6;CA2;CD9;CDKN1A;CLU;EGF;F2RL3;ITGA2B;ITGB3;KCND3;MAOB;MAP
purple	BP	GO:0051179	localization	17735	4195	134	51	32.02	9.30E-05	1.592755	ABCC3;ADRA2A;ALOX12;AQP10;AVPR1A;BET3L;BMP6;CA2;CAV2;CD9;CDKN1A;CLU;DNM3;EGF;ESAM;F2RL3
purple	CC	GO:0005615	extracellular space	17735	844	134	18	6.6	1.00E-04	2.727273	BMP6;CA2;CLU;CMTM5;CXCL5;EGF;FSTL1;PCSK6;PDGFA;PF4;PKHD1L1;PPBP;SELP;SERPINE1;SPARC;TFPI;TNI
purple	BP	GO:0006928	cellular component movement	17735	1208	134	22	9.22	1.00E-04	2.386117	ADRA2A;ALOX12;CALD1;CD9;ESAM;GP6;GRB14;ITGB3;MAP1B;MYLK;PARD3;PDGFA;PDGFRA;PF4;PROS1;SEI
purple	MF	GO:0005102	receptor binding	17735	1201	134	22	9.22	1.00E-04	2.386117	ADRA2A;AVPR1A;BMP6;CAV2;CD9;CMTM5;CXCL5;EGF;GNAZ;HOMER2;ITGB3;ITGB5;PDGFA;PDGFRA;PF4;PI
purple	BP	GO:0051641	cellular localization	17735	2049	134	31	15.64	1.00E-04	1.982097	ADRA2A;AVPR1A;BMP6;CD9;CDKN1A;CLU;EGF;F2RL3;ITGA2B;ITGB3;MAOB;MAP1B;MMRN1;PARD3;PCSK6;
purple	BP	GO:0030193	regulation of blood coagulation	17735	62	134	5	0.47	0.00011	10.6383	PDGFA;PDGFRA;PROS1;SELP;SERPINE1
purple	BP	GO:1900046	regulation of hemostasis	17735	62	134	5	0.47	0.00011	10.6383	PDGFA;PDGFRA;PROS1;SELP;SERPINE1
purple	BP	GO:0071363	cellular response to growth factor stimu...	17735	433	134	12	3.31	0.00011	3.625378	CAV2;ITGB3;LTBP1;PARD3;PDGFA;PDGFRA;PRKAR2B;SERPINE1;SPARC;TGFB1I1;VEGFC;VIL1
purple	MF	GO:0005161	platelet-derived growth factor receptor ...	17735	13	134	3	0.1	0.00012	30	ITGB3;PDGFA;PDGFRA
purple	BP	GO:0010033	response to organic substance	17735	1870	134	29	14.27	0.00012	2.032235	ACSBG1;ADRA2A;ALOX12;AVPR1A;BMP6;CA2;CAV2;CDKN1A;CLU;GNG11;HOMER2;ITGB3;LTBP1;MAOB;PAI
purple	BP	GO:0048545	response to steroid hormone stimulus	17735	254	134	9	1.94	0.00014	4.639175	ACSBG1;AVPR1A;BMP6;CA2;CDKN1A;MAOB;PDGFA;SLC6A4;SPARC
purple	BP	GO:0014070	response to organic cyclic compound	17735	513	134	13	3.92	0.00014	3.316327	ACSBG1;AVPR1A;BMP6;CA2;CDKN1A;HOMER2;MAOB;PDGFA;PRKAR2B;PTGES;SLC6A4;SLC8A3;SPARC
purple	BP	GO:0009605	response to external stimulus	17735	1238	134	22	9.45	0.00014	2.328042	ABLIM3;AVPR1A;CDKN1A;CMTM5;CXCL5;ITGA2B;ITGB3;MGLL;MYL9;PDGFA;PDGFRA;PF4;PGRMC1;PLXNB3;
purple	BP	GO:0050900	leukocyte migration	17735	257	134	9	1.96	0.00015	4.591837	ESAM;GP6;GRB14;ITGB3;PF4;PROS1;SELP;SERPINE1;VEGFC
purple	BP	GO:0070848	response to growth factor stimulus	17735	446	134	12	3.4	0.00015	3.529412	CAV2;ITGB3;LTBP1;PARD3;PDGFA;PDGFRA;PRKAR2B;SERPINE1;SPARC;TGFB1I1;VEGFC;VIL1
purple	BP	GO:0006810	transport	17735	3358	134	43	25.63	0.00015	1.677721	ABCC3;ADRA2A;AQP10;AVPR1A;BET3L;BMP6;CA2;CAV2;CD9;CDKN1A;CLU;DNM3;EGF;F2RL3;ITGA2B;ITGB3
purple	BP	GO:0050818	regulation of coagulation	17735	67	134	5	0.51	0.00016	9.803922	PDGFA;PDGFRA;PROS1;SELP;SERPINE1
purple	BP	GO:0008277	regulation of G-protein coupled receptor...	17735	106	134	6	0.81	0.00016	7.407407	ADRA2A;CAV2;GRK5;HOMER2;MGLL;RGS6
purple	BP	GO:0006935	chemotaxis	17735	596	134	14	4.55	0.00017	3.076923	ABLIM3;CMTM5;CXCL5;ITGA2B;ITGB3;MYL9;PDGFA;PDGFRA;PF4;PGRMC1;PLXNB3;PPBP;SERPINE1;VEGFC
purple	BP	GO:0042330	taxis	17735	596	134	14	4.55	0.00017	3.076923	ABLIM3;CMTM5;CXCL5;ITGA2B;ITGB3;MYL9;PDGFA;PDGFRA;PF4;PGRMC1;PLXNB3;PPBP;SERPINE1;VEGFC
purple	BP	GO:0051384	response to glucocorticoid stimulus	17735	108	134	6	0.82	0.00018	7.317073	ACSBG1;AVPR1A;BMP6;CDKN1A;MAOB;SPARC
purple	BP	GO:0030334	regulation of cell migration	17735	392	134	11	2.99	2.00E-04	3.67893	ADRA2A;ALOX12;ITGB3;MYLK;PDGFA;PDGFRA;SELP;SERPINE1;TPM1;VEGFC;VIL1
purple	BP	GO:0006690	icosanoid metabolic process	17735	71	134	5	0.54	0.00021	9.259259	ALOX12;AVPR1A;MGLL;PTGES;PTGS1
purple	BP	GO:1901568	fatty acid derivative metabolic process	17735	71	134	5	0.54	0.00021	9.259259	ALOX12;AVPR1A;MGLL;PTGES;PTGS1
purple	BP	GO:0032879	regulation of localization	17735	1361	134	23	10.39	0.00021	2.213667	ADRA2A;ALOX12;AVPR1A;BMP6;CDKN1A;EGF;F2RL3;ITGB3;MAOB;MAP1B;MYLK;PARD3;PDGFA;PDGFRA;PI
purple	BP	GO:0030195	negative regulation of blood coagulation	17735	39	134	4	0.3	0.00022	13.33333	PDGFA;PDGFRA;PROS1;SERPINE1
purple	BP	GO:1900047	negative regulation of hemostasis	17735	39	134	4	0.3	0.00022	13.33333	PDGFA;PDGFRA;PROS1;SERPINE1
purple	BP	GO:0016477	cell migration	17735	844	134	17	6.44	0.00022	2.639752	ADRA2A;ALOX12;ESAM;GP6;GRB14;ITGB3;MYLK;PARD3;PDGFA;PDGFRA;PF4;PROS1;SELP;SERPINE1;TPM1;\
purple	BP	GO:0051234	establishment of localization	17735	3408	134	43	26.02	0.00022	1.652575	ABCC3;ADRA2A;AQP10;AVPR1A;BET3L;BMP6;CA2;CAV2;CD9;CDKN1A;CLU;DNM3;EGF;F2RL3;ITGA2B;ITGB3
purple	BP	GO:0031960	response to corticosteroid stimulus	17735	114	134	6	0.87	0.00024	6.896552	ACSBG1;AVPR1A;BMP6;CDKN1A;MAOB;SPARC
purple	BP	GO:0007178	transmembrane receptor protein serine/th...	17735	273	134	9	2.08	0.00024	4.326923	BMP6;CAV2;FSTL1;LTBP1;PARD3;PCSK6;PDGFA;SERPINE1;TGFB1I1
purple	BP	GO:0051412	response to corticosterone stimulus	17735	17	134	3	0.13	0.00027	23.07692	AVPR1A;CDKN1A;MAOB
purple	CC	GO:0044459	plasma membrane part	17735	1895	134	29	14.82	0.00028	1.956815	ABCC3;ADRA2A;AVPR1A;CA2;CAV2;CD9;CLEC1B;F2RL3;GNAZ;GNG11;GP6;GP9;ITGA2B;ITGB3;ITGB5;KCND3
purple	BP	GO:0050819	negative regulation of coagulation	17735	42	134	4	0.32	0.00029	12.5	PDGFA;PDGFRA;PROS1;SERPINE1
purple	MF	GO:1901681	sulfur compound binding	17735	167	134	7	1.28	3.00E-04	5.46875	C6orf25;FSTL1;PCSK6;PF4;PF4V1;PTGES;SELP
purple	BP	GO:2000145	regulation of cell motility	17735	414	134	11	3.16	0.00032	3.481013	ADRA2A;ALOX12;ITGB3;MYLK;PDGFA;PDGFRA;SELP;SERPINE1;TPM1;VEGFC;VIL1
purple	BP	GO:0008015	blood circulation	17735	349	134	10	2.66	0.00034	3.759398	ADRA2A;ALOX12;AVPR1A;GUCY1A3;GUCY1B3;PDE5A;PTGS1;SLC6A4;TPM1;VEGFC
purple	BP	GO:0003013	circulatory system process	17735	350	134	10	2.67	0.00034	3.745318	ADRA2A;ALOX12;AVPR1A;GUCY1A3;GUCY1B3;PDE5A;PTGS1;SLC6A4;TPM1;VEGFC
purple	BP	GO:0032231	regulation of actin filament bundle asse...	17735	44	134	4	0.34	0.00035	11.76471	ARHGAP6;SDC4;TPM1;VIL1
purple	MF	GO:0005080	protein kinase C binding	17735	44	134	4	0.34	0.00035	11.76471	AVPR1A;GRK5;PARD3;SDC4
purple	BP	GO:0007167	enzyme linked receptor protein signaling...	17735	884	134	17	6.75	0.00037	2.518519	ADRA2A;BMP6;CAV2;EGF;FSTL1;GP6;ITGB3;LTBP1;PARD3;PCSK6;PDGFA;PDGFRA;PRKAR2B;SERPINE1;TGFB:
purple	BP	GO:0045932	negative regulation of muscle contractio...	17735	19	134	3	0.15	0.00038	20	ADRA2A;GUCY1A3;PDE5A
purple	CC	GO:0032432	actin filament bundle	17735	44	134	4	0.34	0.00038	11.76471	MYL9;MYLK;TPM1;VIL1
purple	BP	GO:0051239	regulation of multicellular organismal p...	17735	1804	134	27	13.77	0.00039	1.960784	ADRA2A;ALOX12;AVPR1A;BMP6;CA2;EGF;GUCY1A3;ITGB3;MAP1B;MEIS1;MGLL;MYL9;PDE5A;PDGFA;PDGFI
purple	MF	GO:0005518	collagen binding	17735	46	134	4	0.35	0.00042	11.42857	GP6;PDGFA;SPARC;VWF
purple	CC	GO:0009986	cell surface	17735	485	134	12	3.79	0.00042	3.166227	CAV2;CD9;ITGA2B;PCSK6;PDGFA;PDGFRA;PGRMC1;SDC4;SELP;TNFSF4;TREML1;VWF
purple	BP	GO:0033559	unsaturated fatty acid metabolic process	17735	83	134	5	0.63	0.00043	7.936508	ALOX12;AVPR1A;MGLL;PTGES;PTGS1
purple	CC	GO:0005887	integral to plasma membrane	17735	1208	134	21	9.45	0.00044	2.222222	ABCC3;ADRA2A;AVPR1A;CAV2;CD9;CLEC1B;F2RL3;GP6;GP9;ITGA2B;ITGB3;ITGB5;KCND3;MMD;PDGFRA;SD(
purple	BP	GO:0042552	myelination	17735	84	134	5	0.64	0.00045	7.8125	ACSBG1;CD9;CLDN5;SH3TC2;WASF3
purple	BP	GO:0072376	protein activation cascade	17735	84	134	5	0.64	0.00045	7.8125	CLU;GP9;PROS1;TFPI;VWF
purple	MF	GO:0008201	heparin binding	17735	128	134	6	0.98	0.00045	6.122449	C6orf25;FSTL1;PCSK6;PF4;PF4V1;SELP
purple	CC	GO:0044421	extracellular region part	17735	1123	134	20	8.78	0.00045	2.277904	BMP6;CA2;CLU;CMTM5;CXCL5;EGF;FSTL1;LTBP1;PCSK6;PDGFA;PF4;PKHD1L1;PPBP;SELP;SERPINE1;SPARC;T
purple	MF	GO:0008009	chemokine activity	17735	47	134	4	0.36	0.00046	11.11111	CXCL5;PF4;PF4V1;PPBP
purple	BP	GO:0051716	cellular response to stimulus	17735	5052	134	56	38.56	0.00046	1.452282	ADRA2A;ALOX12;ARHGAP6;ASAP2;AVPR1A;BMP6;CABP5;CAV2;CDKN1A;CLEC1B;CLU;CXCL5;EGF;F2RL3;FST
purple	BP	GO:0008284	positive regulation of cell proliferatio...	17735	662	134	14	5.05	0.00051	2.772277	ADRA2A;ALOX12;AVPR1A;BMP6;CAV2;CDKN1A;CXCL5;EGF;ITGB3;PDGFA;PDGFRA;TNFSF4;TSC22D1;VEGFC
purple	BP	GO:0001516	prostaglandin biosynthetic process	17735	21	134	3	0.16	0.00052	18.75	AVPR1A;PTGES;PTGS1
purple	BP	GO:0046457	prostanoid biosynthetic process	17735	21	134	3	0.16	0.00052	18.75	AVPR1A;PTGES;PTGS1
purple	BP	GO:0051385	response to mineralocorticoid stimulus	17735	21	134	3	0.16	0.00052	18.75	AVPR1A;CDKN1A;MAOB
purple	BP	GO:0072378	blood coagulation, fibrin clot formation	17735	21	134	3	0.16	0.00052	18.75	GP9;TFPI;VWF
purple	BP	GO:0007272	ensheathment of neurons	17735	87	134	5	0.66	0.00053	7.575758	ACSBG1;CD9;CLDN5;SH3TC2;WASF3

purple	BP	GO:0008366	axon ensheathment	17735	87	134	5	0.66	0.00053	7.575758	ACSBG1;CD9;CLDN5;SH3TC2;WASF3
purple	BP	GO:0006633	fatty acid biosynthetic process	17735	134	134	6	1.02	0.00056	5.882353	ALOX12;AVPR1A;ELOVL7;MGLL;PTGES;PTGS1
purple	BP	GO:0048870	cell motility	17735	916	134	17	6.99	0.00056	2.432046	ADRA2A;ALOX12;ESAM;GP6;GRB14;ITGB3;MYLK;PARD3;PDGFA;PDGFRA;PF4;PROS1;SELP;SERPINE1;TPM1;
purple	BP	GO:0051674	localization of cell	17735	916	134	17	6.99	0.00056	2.432046	ADRA2A;ALOX12;ESAM;GP6;GRB14;ITGB3;MYLK;PARD3;PDGFA;PDGFRA;PF4;PROS1;SELP;SERPINE1;TPM1;
purple	BP	GO:0046456	icosanoid biosynthetic process	17735	51	134	4	0.39	0.00061	10.25641	ALOX12;AVPR1A;PTGES;PTGS1
purple	BP	GO:1901570	fatty acid derivative biosynthetic proce...	17735	51	134	4	0.39	0.00061	10.25641	ALOX12;AVPR1A;PTGES;PTGS1
purple	BP	GO:0040012	regulation of locomotion	17735	448	134	11	3.42	0.00062	3.216374	ADRA2A;ALOX12;ITGB3;MYLK;PDGFA;PDGFRA;SELP;SERPINE1;TPM1;VEGFC;VIL1
purple	CC	GO:0031234	extrinsic to internal side of plasma mem...	17735	51	134	4	0.4	0.00067	10	CAV2;GNAZ;GNG11;RGS6
purple	BP	GO:0010543	regulation of platelet activation	17735	23	134	3	0.18	0.00069	16.66667	PDGFA;PDGFRA;SELP
purple	BP	GO:0007165	signal transduction	17735	4165	134	48	31.79	0.00069	1.509909	ADRA2A;ARHGAP6;ASAP2;AVPR1A;BMP6;CABP5;CAV2;CDKN1A;CLEC1B;CLU;CXCL5;EGF;F2RL3;FSTL1;GNAZ
purple	CC	GO:0031226	intrinsic to plasma membrane	17735	1252	134	21	9.79	7.00E-04	2.145046	ABCC3;ADRA2A;AVPR1A;CAV2;CD9;CLEC1B;F2RL3;GP6;GP9;ITGA2B;ITGB3;ITGB5;KCND3;MMD;PDGFRA;SD
purple	BP	GO:0006636	unsaturated fatty acid biosynthetic proc...	17735	55	134	4	0.42	0.00081	9.52381	ALOX12;AVPR1A;PTGES;PTGS1
purple	BP	GO:0051270	regulation of cellular component movemen...	17735	463	134	11	3.53	0.00082	3.116147	ADRA2A;ALOX12;ITGB3;MYLK;PDGFA;PDGFRA;SELP;SERPINE1;TPM1;VEGFC;VIL1
purple	MF	GO:0042379	chemokine receptor binding	17735	55	134	4	0.42	0.00083	9.52381	CXCL5;PF4;PF4V1;PPBP
purple	BP	GO:0006936	muscle contraction	17735	262	134	8	2	0.00089	4	ADRA2A;CALD1;GUCY1A3;ITGB5;MYL9;MYLK;PDE5A;TPM1
purple	MF	GO:0070851	growth factor receptor binding	17735	97	134	5	0.74	9.00E-04	6.756757	EGF;ITGB3;PDGFA;PDGFRA;VEGFC
purple	CC	GO:0044425	membrane part	17735	6026	134	65	47.12	9.00E-04	1.379457	ABCC3;ADRA2A;AQP10;AVPR1A;C6orf25;CA2;CAV2;CD9;CLDN5;CLEC1B;CLEC2L;CMTM5;EGF;ELOVL7;ESAM
purple	BP	GO:0007155	cell adhesion	17735	957	134	17	7.31	0.00091	2.325581	ALOX12;ARHGAP6;CD9;CLDN5;ESAM;GP9;ITGA2B;ITGB3;ITGB5;MMRN1;PDGFRA;SDC4;SELP;SERPINE1;TGF
purple	BP	GO:0022610	biological adhesion	17735	959	134	17	7.32	0.00093	2.322404	ALOX12;ARHGAP6;CD9;CLDN5;ESAM;GP9;ITGA2B;ITGB3;ITGB5;MMRN1;PDGFRA;SDC4;SELP;SERPINE1;TGF
purple	BP	GO:0007160	cell-matrix adhesion	17735	148	134	6	1.13	0.00095	5.309735	ARHGAP6;ITGA2B;ITGB3;ITGB5;SDC4;SERPINE1
purple	CC	GO:0005576	extracellular region	17735	2141	134	30	16.74	0.00095	1.792115	ACRBP;BMP6;C2orf88;CA2;CCDC3;CLU;CMTM5;CXCL5;EGF;ENDOD1;F2RL3;FSTL1;FSTL4;LTBP1;MMRN1;PCS
purple	MF	GO:0005125	cytokine activity	17735	204	134	7	1.57	0.00099	4.458599	BMP6;CMTM5;CXCL5;PF4;PF4V1;PPBP;TNFSF4
purple	BP	GO:0071495	cellular response to endogenous stimulus	17735	716	134	14	5.47	0.00109	2.559415	ADRA2A;CAV2;GNG11;LTBP1;PARD3;PDGFA;PDGFRA;PRKAR2B;SERPINE1;SLC6A4;SLC8A3;TGFB1I1;TNFSF4;
purple	BP	GO:0019228	regulation of action potential in neuron	17735	103	134	5	0.79	0.00114	6.329114	ACSBG1;CD9;CLDN5;SH3TC2;WASF3
purple	BP	GO:0023051	regulation of signaling	17735	2036	134	28	15.54	0.00115	1.801802	ADRA2A;ARHGAP6;ASAP2;AVPR1A;BMP6;CAV2;CLU;EGF;GRK5;HOMER2;ITGB3;LTBP1;MAOB;MGLL;PCSK6;
purple	BP	GO:0032970	regulation of actin filament-based proce...	17735	211	134	7	1.61	0.00116	4.347826	ARHGAP6;PARD3;PDGFA;PDGFRA;SDC4;TPM1;VIL1
purple	BP	GO:0009617	response to bacterium	17735	341	134	9	2.6	0.0012	3.461538	HIST1H2BJ;MAOB;PPBP;PTGES;SELP;SERPINE1;SPARC;TNFSF4;VIL1
purple	BP	GO:0010646	regulation of cell communication	17735	2042	134	28	15.59	0.00121	1.796023	ADRA2A;ARHGAP6;ASAP2;AVPR1A;BMP6;CAV2;CLU;EGF;GRK5;HOMER2;ITGB3;LTBP1;MAOB;MGLL;PCSK6;
purple	BP	GO:0008283	cell proliferation	17735	1542	134	23	11.77	0.00122	1.954121	ADRA2A;ALOX12;AVPR1A;BMP6;CAV2;CD9;CDKN1A;CXCL5;EGF;GFI1B;ITGB3;PDE5A;PDGFA;PDGFRA;PTGES
purple	BP	GO:0010863	positive regulation of phospholipase C a...	17735	62	134	4	0.47	0.00128	8.510638	ARHGAP6;AVPR1A;PDGFRA;PRKAR2B
purple	BP	GO:0009725	response to hormone stimulus	17735	647	134	13	4.94	0.00129	2.631579	ACSBG1;ADRA2A;AVPR1A;BMP6;CA2;CDKN1A;GNG11;MAOB;PDGFA;PRKAR2B;SLC6A4;SPARC;TNFSF4
purple	MF	GO:0005126	cytokine receptor binding	17735	215	134	7	1.65	0.00134	4.242424	CXCL5;ITGB3;PF4;PF4V1;PPBP;TNFSF4;VEGFC
purple	BP	GO:0032846	positive regulation of homeostatic proce...	17735	63	134	4	0.48	0.00135	8.333333	ALOX12;AVPR1A;CA2;F2RL3
purple	BP	GO:1900274	regulation of phospholipase C activity	17735	63	134	4	0.48	0.00135	8.333333	ARHGAP6;AVPR1A;PDGFRA;PRKAR2B
purple	BP	GO:0006692	prostanoid metabolic process	17735	29	134	3	0.22	0.00137	13.63636	AVPR1A;PTGES;PTGS1
purple	BP	GO:0006693	prostaglandin metabolic process	17735	29	134	3	0.22	0.00137	13.63636	AVPR1A;PTGES;PTGS1
purple	CC	GO:0008305	integrin complex	17735	29	134	3	0.23	0.00147	13.04348	ITGA2B;ITGB3;ITGB5
purple	BP	GO:0031589	cell-substrate adhesion	17735	221	134	7	1.69	0.00152	4.142012	ARHGAP6;ITGA2B;ITGB3;ITGB5;SDC4;SERPINE1;VWF
purple	BP	GO:0050880	regulation of blood vessel size	17735	110	134	5	0.84	0.00153	5.952381	ADRA2A;ALOX12;AVPR1A;GUCY1A3;SLC6A4
purple	MF	GO:0046983	protein dimerization activity	17735	909	134	16	6.98	0.00155	2.292264	ADRA2A;BMP6;CAV2;GUCY1B3;HIST1H2AG;HIST1H2BH;HIST1H2BJ;HOMER2;MAOB;MEIS1;MGLL;PDGFA;PD
purple	BP	GO:0035150	regulation of tube size	17735	111	134	5	0.85	0.0016	5.882353	ADRA2A;ALOX12;AVPR1A;GUCY1A3;SLC6A4
purple	MF	GO:0046982	protein heterodimerization activity	17735	354	134	9	2.72	0.00162	3.308824	ADRA2A;BMP6;GUCY1B3;HIST1H2AG;HIST1H2BH;HIST1H2BJ;HOMER2;MEIS1;PDGFA
purple	BP	GO:0006937	regulation of muscle contraction	17735	112	134	5	0.85	0.00166	5.882353	ADRA2A;GUCY1A3;MYL9;PDE5A;TPM1
purple	BP	GO:0014909	smooth muscle cell migration	17735	31	134	3	0.24	0.00167	12.5	ITGB3;PDGFA;SERPINE1
purple	BP	GO:0032233	positive regulation of actin filament bu...	17735	31	134	3	0.24	0.00167	12.5	SDC4;TPM1;VIL1
purple	BP	GO:0015837	amine transport	17735	67	134	4	0.51	0.0017	7.843137	ADRA2A;AVPR1A;MAOB;SLC6A4
purple	BP	GO:0065007	biological regulation	17735	8616	134	81	65.77	0.00176	1.231565	ABLIM3;ACSBG1;ADRA2A;ALOX12;ARHGAP6;ASAP2;AVPR1A;BMP6;CA2;CABP5;CAV2;CD9;CDKN1A;CLDN5;
purple	BP	GO:0060341	regulation of cellular localization	17735	672	134	13	5.13	0.00181	2.534113	ADRA2A;AVPR1A;BMP6;CDKN1A;EGF;F2RL3;MAOB;MAP1B;PARD3;PRKAR2B;SYTL4;TNFSF4;VEGFC
purple	BP	GO:0003012	muscle system process	17735	294	134	8	2.24	0.00185	3.571429	ADRA2A;CALD1;GUCY1A3;ITGB5;MYL9;MYLK;PDE5A;TPM1
purple	MF	GO:0005515	protein binding	17735	7306	134	72	56.07	0.00195	1.284109	ABLIM3;ADRA2A;ALOX12;ARHGAP6;ASAP2;AVPR1A;BMP6;CALD1;CAV2;CD9;CDKN1A;CLDN5;CLEC1B;CLU;C
purple	BP	GO:0007179	transforming growth factor beta receptor...	17735	171	134	6	1.31	0.00198	4.580153	CAV2;LTBP1;PARD3;PDGFA;SERPINE1;TGFB1I1
purple	BP	GO:0051970	negative regulation of transmission of n...	17735	33	134	3	0.25	0.002	12	AVPR1A;MGLL;SLC6A4
purple	CC	GO:0044444	cytoplasmic part	17735	6562	134	68	51.31	0.00201	1.325278	ACRBP;ACSBG1;ALOX12;ARHGAP6;ASAP2;AVPR1A;BET3L;C6orf25;CA2;CABP5;CALD1;CAV2;CCDC3;CD9;CDK
purple	MF	GO:0005539	glycosaminoglycan binding	17735	171	134	6	1.31	0.00204	4.580153	C6orf25;FSTL1;PCSK6;PF4;PF4V1;SELP
purple	BP	GO:0070887	cellular response to chemical stimulus	17735	1709	134	24	13.05	0.00213	1.83908	ADRA2A;ALOX12;AVPR1A;CAV2;GNG11;ITGB3;LTBP1;MAOB;PARD3;PDGFA;PDGFRA;PF4;PRKAR2B;PTGS1;S
purple	BP	GO:0051240	positive regulation of multicellular org...	17735	524	134	11	4	0.00219	2.75	ADRA2A;ALOX12;AVPR1A;BMP6;CA2;PDE5A;PF4;SERPINE1;TNFSF4;TPM1;WASF3
purple	BP	GO:0044057	regulation of system process	17735	448	134	10	3.42	0.00224	2.923977	ADRA2A;ALOX12;AVPR1A;GUCY1A3;MGLL;MYL9;PDE5A;SLC6A4;TPM1;WASF3
purple	BP	GO:0010518	positive regulation of phospholipase act...	17735	73	134	4	0.56	0.00234	7.142857	ARHGAP6;AVPR1A;PDGFRA;PRKAR2B
purple	BP	GO:0014812	muscle cell migration	17735	35	134	3	0.27	0.00237	11.11111	ITGB3;PDGFA;SERPINE1
purple	BP	GO:0006631	fatty acid metabolic process	17735	307	134	8	2.34	0.00243	3.418803	ACSBG1;ALOX12;AVPR1A;ELOVL7;MGLL;PRKAR2B;PTGES;PTGS1
purple	BP	GO:0021936	regulation of cerebellar granule cell pr...	17735	10	134	2	0.08	0.0025	25	EGF;SLC6A4
purple	BP	GO:0045741	positive regulation of epidermal growth ...	17735	10	134	2	0.08	0.0025	25	ADRA2A;EGF
purple	MF	GO:0019001	guanyl nucleotide binding	17735	380	134	9	2.92	0.00262	3.082192	DNM3;GNAZ;GUCY1A3;GUCY1B3;PDE5A;RAB6B;RHOBTB1;TUBA8;TUBB1
purple	MF	GO:0032561	guanyl ribonucleotide binding	17735	380	134	9	2.92	0.00262	3.082192	DNM3;GNAZ;GUCY1A3;GUCY1B3;PDE5A;RAB6B;RHOBTB1;TUBA8;TUBB1
purple	BP	GO:0032956	regulation of actin cytoskeleton organiz...	17735	182	134	6	1.39	0.00271	4.316547	ARHGAP6;PDGFA;PDGFRA;SDC4;TPM1;VIL1
purple	CC	GO:0005834	heterotrimeric G-protein complex	17735	36	134	3	0.28	0.00276	10.71429	GNAZ;GNG11;RGS6
purple	BP	GO:0051048	negative regulation of secretion	17735	126	134	5	0.96	0.00278	5.208333	ADRA2A;EGF;MAOB;SYTL4;TNFSF4
purple	BP	GO:0022607	cellular component assembly	17735	1545	134	22	11.79	0.00284	1.865988	ARHGAP6;CAV2;CD9;CLU;DNM3;EGF;HIST1H2AG;HIST1H2BH;HIST1H2BJ;KCND3;MYLK;PARD3;PDGFA;SDC4;
purple	BP	GO:0048146	positive regulation of fibroblast prolif...	17735	38	134	3	0.29	0.00301	10.34483	CDKN1A;PDGFA;PDGFRA

purple	BP	GO:0006837	serotonin transport	17735	11	134	2	0.08	0.00304	25	MAOB;SLC6A4
purple	BP	GO:0021924	cell proliferation in external granule l...	17735	11	134	2	0.08	0.00304	25	EGF;SLC6A4
purple	BP	GO:0021930	cerebellar granule cell precursor prolif...	17735	11	134	2	0.08	0.00304	25	EGF;SLC6A4
purple	BP	GO:0045601	regulation of endothelial cell different...	17735	11	134	2	0.08	0.00304	25	ALOX12;BMP6
purple	BP	GO:0090303	positive regulation of wound healing	17735	11	134	2	0.08	0.00304	25	ADRA2A;MYLK
purple	BP	GO:2000060	positive regulation of protein ubiquitin...	17735	11	134	2	0.08	0.00304	25	CLU;EGF
purple	MF	GO:0048407	platelet-derived growth factor binding	17735	11	134	2	0.08	0.00307	25	PDGFA;PDGFRA
purple	BP	GO:0003018	vascular process in circulatory system	17735	129	134	5	0.98	0.00308	5.102041	ADRA2A;ALOX12;AVPR1A;GUCY1A3;SLC6A4
purple	BP	GO:0090257	regulation of muscle system process	17735	129	134	5	0.98	0.00308	5.102041	ADRA2A;GUCY1A3;MYL9;PDE5A;TPM1
purple	CC	GO:0019898	extrinsic to membrane	17735	127	134	5	0.99	0.00322	5.050505	CAV2;GNAZ;GNG11;RGS6;SYTL4
purple	BP	GO:0051492	regulation of stress fiber assembly	17735	39	134	3	0.3	0.00324	10	ARHGAP6;SDC4;TPM1
purple	BP	GO:0010517	regulation of phospholipase activity	17735	80	134	4	0.61	0.00326	6.557377	ARHGAP6;AVPR1A;PDGFRA;PRKAR2B
purple	BP	GO:0051017	actin filament bundle assembly	17735	80	134	4	0.61	0.00326	6.557377	ARHGAP6;SDC4;TPM1;VIL1
purple	BP	GO:0072330	monocarboxylic acid biosynthetic process	17735	190	134	6	1.45	0.00335	4.137931	ALOX12;AVPR1A;ELOVL7;MGLL;PTGES;PTGS1
purple	BP	GO:0060193	positive regulation of lipase activity	17735	81	134	4	0.62	0.00341	6.451613	ARHGAP6;AVPR1A;PDGFRA;PRKAR2B
purple	MF	GO:0097367	carbohydrate derivative binding	17735	190	134	6	1.46	0.00345	4.109589	C6orf25;FSTL1;PCSK6;PF4;PF4V1;SELP
purple	BP	GO:0031645	negative regulation of neurological syst...	17735	40	134	3	0.31	0.00348	9.677419	AVPR1A;MGLL;SLC6A4
purple	MF	GO:0030295	protein kinase activator activity	17735	40	134	3	0.31	0.00354	9.677419	CDKN1A;EGF;TOM1L1
purple	BP	GO:0021534	cell proliferation in hindbrain	17735	12	134	2	0.09	0.00363	22.22222	EGF;SLC6A4
purple	BP	GO:0001676	long-chain fatty acid metabolic process	17735	41	134	3	0.31	0.00374	9.677419	ACSBG1;MGLL;PTGS1
purple	BP	GO:0051049	regulation of transport	17735	1000	134	16	7.63	0.00375	2.096986	ADRA2A;AVPR1A;BMP6;CDKN1A;EGF;F2RL3;ITGB3;MAOB;MAP1B;MYLK;PRKAR2B;SERPINE1;STON2;SYTL4;[illegible]
purple	BP	GO:0048468	cell development	17735	1482	134	21	11.31	0.0038	1.856764	ABLIM3;BMP6;CAV2;CD9;ITGA2B;ITGB3;MAP1B;MEIS1;MYL9;PARD3;PDE5A;PDGFRA;PGRMC1;PLXNB3;RGS
purple	BP	GO:0044767	single-organism developmental process	17735	3427	134	39	26.16	0.00383	1.490826	ABLIM3;ACSBG1;ALOX12;AVPR1A;BMP6;CA2;CAV2;CD9;CDKN1A;EGF;ITGA2B;ITGB3;MAP1B;MEIS1;MYL9;[illegible]
purple	CC	GO:0019897	extrinsic to plasma membrane	17735	82	134	4	0.64	0.0039	6.25	CAV2;GNAZ;GNG11;RGS6
purple	CC	GO:0001725	stress fiber	17735	41	134	3	0.32	0.00401	9.375	MYL9;MYLK;TPM1
purple	BP	GO:0050714	positive regulation of protein secretion	17735	85	134	4	0.65	0.00405	6.153846	BMP6;SYTL4;TNFSF4;VEGFC
purple	BP	GO:0048856	anatomical structure development	17735	3903	134	43	29.79	0.00405	1.443437	ABLIM3;ACSBG1;ALOX12;AVPR1A;BMP6;CA2;CAV2;CD9;CDKN1A;CLDN5;DNM3;EGF;ITGA2B;ITGB3;MAP1B;
purple	BP	GO:0071560	cellular response to transforming growth...	17735	198	134	6	1.51	0.0041	3.97351	CAV2;LTBP1;PARD3;PDGFA;SERPINE1;TGFB1I1
purple	BP	GO:0071559	response to transforming growth factor b...	17735	199	134	6	1.52	0.0042	3.947368	CAV2;LTBP1;PARD3;PDGFA;SERPINE1;TGFB1I1
purple	BP	GO:0007320	insemination	17735	13	134	2	0.1	0.00426	20	AVPR1A;SLC6A4
purple	BP	GO:0019369	arachidonic acid metabolic process	17735	13	134	2	0.1	0.00426	20	MGLL;PTGS1
purple	BP	GO:0031954	positive regulation of protein autophosp...	17735	13	134	2	0.1	0.00426	20	PDGFA;VEGFC
purple	BP	GO:0045986	negative regulation of smooth muscle con...	17735	13	134	2	0.1	0.00426	20	ADRA2A;GUCY1A3
purple	BP	GO:0060055	angiogenesis involved in wound healing	17735	13	134	2	0.1	0.00426	20	ITGB3;SERPINE1
purple	BP	GO:0010595	positive regulation of endothelial cell ...	17735	43	134	3	0.33	0.00428	9.090909	ALOX12;ITGB3;VEGFC
purple	BP	GO:0065003	macromolecular complex assembly	17735	1014	134	16	7.74	0.00429	2.067183	ARHGAP6;CAV2;CLU;EGF;HIST1H2AG;HIST1H2BH;HIST1H2BJ;KCND3;PARD3;SELP;SLC6A4;TUBA8;TUBB1;VIL
purple	BP	GO:0007166	cell surface receptor signaling pathway	17735	2331	134	29	17.79	0.00429	1.630129	ADRA2A;AVPR1A;BMP6;CAV2;CLEC1B;EGF;F2RL3;FSTL1;GNAZ;GNG11;GP6;GRK5;HOMER2;ITGA2B;ITGB3;IT
purple	BP	GO:0032496	response to lipopolysaccharide	17735	200	134	6	1.53	0.0043	3.921569	MAOB;PTGES;SELP;SERPINE1;SPARC;TNFSF4
purple	BP	GO:0008217	regulation of blood pressure	17735	140	134	5	1.07	0.00437	4.672897	AVPR1A;GUCY1A3;PTGS1;TPM1;VEGFC
purple	BP	GO:0045597	positive regulation of cell differentiat...	17735	492	134	10	3.76	0.00437	2.659574	ALOX12;BMP6;CA2;MAP1B;PDE5A;PF4;RGS6;TGFB1I1;TNFSF4;VEGFC
purple	BP	GO:0051050	positive regulation of transport	17735	492	134	10	3.76	0.00437	2.659574	ADRA2A;AVPR1A;BMP6;EGF;F2RL3;MYLK;SERPINE1;SYTL4;TNFSF4;VEGFC
purple	BP	GO:0003008	system process	17735	1703	134	23	13	0.00439	1.769231	ACSBG1;ADRA2A;ALOX12;AVPR1A;CALD1;CAV2;CD9;CLDN5;GUCY1A3;GUCY1B3;ITGB5;KCND3;MGLL;MYL9;
purple	CC	GO:0005912	adherens junction	17735	196	134	6	1.53	0.00443	3.921569	ESAM;ITGA2B;ITGB5;PARD3;SDC4;TGFB1I1
purple	BP	GO:0014068	positive regulation of phosphatidylinosi...	17735	44	134	3	0.34	0.00457	8.823529	PDGFA;PDGFRA;SELP
purple	BP	GO:0051707	response to other organism	17735	578	134	11	4.41	0.00463	2.494331	CLU;GUCY1A3;HIST1H2BJ;MAOB;PPBP;PTGES;SELP;SERPINE1;SPARC;TNFSF4;VIL1
purple	BP	GO:0055082	cellular chemical homeostasis	17735	665	134	12	5.08	0.00477	2.362205	ACSBG1;ADRA2A;ALOX12;AVPR1A;CA2;CD9;CLDN5;F2RL3;PDGFRA;SH3TC2;SLC8A3;WASF3
purple	BP	GO:0007399	nervous system development	17735	1716	134	23	13.1	0.00482	1.755725	ABLIM3;ACSBG1;AVPR1A;BMP6;CD9;CLDN5;DNM3;EGF;ITGA2B;ITGB3;MAP1B;MEIS1;MYL9;NRGN;PARD3;P
purple	BP	GO:0048010	vascular endothelial growth factor recep...	17735	45	134	3	0.34	0.00487	8.823529	ITGB3;PDGFRA;VEGFC
purple	BP	GO:0007275	multicellular organismal development	17735	3940	134	43	30.08	0.00487	1.429521	ABLIM3;ACSBG1;ALOX12;AVPR1A;BMP6;CA2;CAV2;CD9;CDKN1A;CLDN5;DNM3;EGF;GFI1B;ITGA2B;ITGB3;IT
purple	BP	GO:0090075	relaxation of muscle	17735	14	134	2	0.11	0.00495	18.18182	GUCY1A3;PDE5A
purple	BP	GO:2000058	regulation of protein ubiquitination inv...	17735	14	134	2	0.11	0.00495	18.18182	CLU;EGF
purple	BP	GO:0032844	regulation of homeostatic process	17735	275	134	7	2.1	0.00512	3.333333	ADRA2A;ALOX12;AVPR1A;CA2;F2RL3;ITGB3;WASF3
purple	BP	GO:0048522	positive regulation of cellular process	17735	3140	134	36	23.97	0.00517	1.501877	ABLIM3;ADRA2A;ALOX12;AVPR1A;BMP6;CA2;CAV2;CDKN1A;CLU;CXCL5;EGF;F2RL3;GUCY1A3;ITGB3;MAOB;
purple	BP	GO:0090287	regulation of cellular response to growt...	17735	146	134	5	1.11	0.00521	4.504505	CAV2;ITGB3;LTBP1;TGFB1I1;VEGFC
purple	BP	GO:2000021	regulation of ion homeostasis	17735	147	134	5	1.12	0.00536	4.464286	ALOX12;AVPR1A;CA2;F2RL3;WASF3
purple	BP	GO:0032526	response to retinoic acid	17735	92	134	4	0.7	0.00537	5.714286	BMP6;PDGFA;PTGES;SLC6A4
purple	CC	GO:0042995	cell projection	17735	1194	134	18	9.34	0.00541	1.927195	CA2;CLDN5;CTTN;DNM3;ENKUR;HOMER2;KCND3;MAP1B;MYLK;PARD3;PDGFA;PTGS1;PVALB;SLC6A4;SLC8A
purple	BP	GO:0048519	negative regulation of biological proces...	17735	3150	134	36	24.05	0.00545	1.496881	ADRA2A;ALOX12;ARHGAP6;AVPR1A;CAV2;CD9;CDKN1A;CLU;EGF;F2RL3;GFI1B;GRK5;GUCY1A3;ITGB3;LTBP1
purple	BP	GO:0043149	stress fiber assembly	17735	47	134	3	0.36	0.0055	8.333333	ARHGAP6;SDC4;TPM1
purple	BP	GO:0002237	response to molecule of bacterial origin	17735	211	134	6	1.61	0.00557	3.726708	MAOB;PTGES;SELP;SERPINE1;SPARC;TNFSF4
purple	MF	GO:0019209	kinase activator activity	17735	47	134	3	0.36	0.00559	8.333333	CDKN1A;EGF;TOM1L1
purple	BP	GO:0071310	cellular response to organic substance	17735	1333	134	19	10.18	0.00561	1.866405	ADRA2A;ALOX12;CAV2;GNG11;ITGB3;LTBP1;PARD3;PDGFA;PDGFRA;PF4;PRKAR2B;SERPINE1;SLC6A4;SLC8A
purple	BP	GO:0048731	system development	17735	3385	134	38	25.84	0.00562	1.470588	ABLIM3;ACSBG1;AVPR1A;BMP6;CA2;CAV2;CD9;CDKN1A;CLDN5;DNM3;EGF;ITGA2B;ITGB3;MAP1B;MEIS1;[illegible]
purple	BP	GO:0032787	monocarboxylic acid metabolic process	17735	430	134	9	3.28	0.00563	2.743902	ABCC3;ACSBG1;ALOX12;AVPR1A;ELOVL7;MGLL;PRKAR2B;PTGES;PTGS1
purple	MF	GO:0003779	actin binding	17735	351	134	8	2.69	0.00565	2.973978	ABLIM3;CALD1;HOMER2;MYLK;SLC6A4;TPM1;VIL1;WASF3
purple	BP	GO:0001508	regulation of action potential	17735	149	134	5	1.14	0.00567	4.385965	ACSBG1;CD9;CLDN5;SH3TC2;WASF3
purple	BP	GO:0009653	anatomical structure morphogenesis	17735	2058	134	26	15.71	0.00583	1.654997	ABLIM3;BMP6;CA2;CD9;CDKN1A;EGF;ITGA2B;ITGB3;MAP1B;MEIS1;MYL9;MYLK;NAT8B;PARD3;PDGFA;PDGI
purple	BP	GO:0051128	regulation of cellular component organiz...	17735	1240	134	18	9.47	0.00586	1.900739	ALOX12;ARHGAP6;AVPR1A;CAV2;CDKN1A;CLU;EGF;MAP1B;PDGFA;PDGFRA;SDC4;SELP;SERPINE1;STON2;T[illegible]
purple	BP	GO:0051051	negative regulation of transport	17735	284	134	7	2.17	0.00608	3.225806	ADRA2A;EGF;ITGB3;MAOB;MAP1B;SYTL4;TNFSF4

purple	BP	GO:0007186	G-protein coupled receptor signaling pat...	17735	601	134	11	4.59	0.00617	2.396514	ADRA2A;AVPR1A;CAV2;F2RL3;GNAZ;GNG11;GRK5;HOMER2;MGLL;PARD3;RGS6
purple	BP	GO:2000177	regulation of neural precursor cell prol...	17735	49	134	3	0.37	0.00618	8.108108	EGF;SLC6A4;VEGFC
purple	BP	GO:0048514	blood vessel morphogenesis	17735	438	134	9	3.34	0.00633	2.694611	EGF;ITGB3;MEIS1;MYLK;PDGFA;PDGFRA;PF4;SERPINE1;VEGFC
purple	BP	GO:0097305	response to alcohol	17735	217	134	6	1.66	0.00637	3.614458	AVPR1A;CDKN1A;MAOB;PDGFA;SLC6A4;SPARC
purple	BP	GO:0006873	cellular ion homeostasis	17735	604	134	11	4.61	0.0064	2.386117	ACSBG1;ALOX12;AVPR1A;CA2;CD9;CLDN5;F2RL3;PDGFRA;SH3TC2;SLC8A3;WASF3
purple	CC	GO:0005737	cytoplasm	17735	9016	134	85	70.5	0.00652	1.205674	ABLIM3;ACRBP;ACSBG1;ADRA2A;ALOX12;ARHGAP6;ASAP2;AVPR1A;BET3L;BMP6;C6orf25;C8orf42;CA2;CAB
purple	CC	GO:0070161	anchoring junction	17735	214	134	6	1.67	0.00675	3.592814	ESAM;ITGA2B;ITGB5;PARD3;SDC4;TGFB1I1
purple	MF	GO:0005525	GTP binding	17735	366	134	8	2.81	0.00721	2.846975	DNM3;GNAZ;GUCY1A3;GUCY1B3;RAB6B;RHOBTB1;TUBA8;TUBB1
purple	CC	GO:0042641	actomyosin	17735	51	134	3	0.4	0.0074	7.5	MYL9;MYLK;TPM1
purple	MF	GO:0005516	calmodulin binding	17735	162	134	5	1.24	0.00822	4.032258	CALD1;ENKUR;MYLK;NRGN;SLC8A3
purple	CC	GO:0009898	internal side of plasma membrane	17735	102	134	4	0.8	0.00841	5	CAV2;GNAZ;GNG11;RGS6
purple	MF	GO:0008083	growth factor activity	17735	164	134	5	1.26	0.00864	3.968254	BMP6;EGF;PDGFA;PPBP;VEGFC
purple	MF	GO:0019838	growth factor binding	17735	105	134	4	0.81	0.00869	4.938272	LTBP1;PCSK6;PDGFA;PDGFRA
purple	MF	GO:0003924	GTPase activity	17735	232	134	6	1.78	0.00897	3.370787	DNM3;GNAZ;GNG11;RAB6B;TUBA8;TUBB1
purple	MF	GO:0042803	protein homodimerization activity	17735	545	134	10	4.18	0.00916	2.392344	ADRA2A;CAV2;HOMER2;MAOB;MGLL;PDGFA;PDGFRA;SLC6A4;VIL1;VWF
purple	MF	GO:0015491	cation:cation antiporter activity	17735	19	134	2	0.15	0.00917	13.33333	SLC24A3;SLC8A3
purple	MF	GO:0008289	lipid binding	17735	721	134	12	5.53	0.00932	2.169982	ASAP2;DNM3;GRB14;GRK5;MGLL;PARD3;PGRMC1;SDPR;SELP;SYTL4;VEPH1;VIL1
purple	MF	GO:0005488	binding	17735	11799	134	101	90.55	0.00947	1.115406	ABCC3;ABLIM3;ACSBG1;ADRA2A;ALOX12;ARHGAP6;ASAP2;AVPR1A;BMP6;C6orf25;CA2;CABP5;CALD1;CAV
purple	CC	GO:0012505	endomembrane system	17735	1768	134	23	13.82	0.00992	1.664255	ASAP2;CAV2;CD9;ELOVL7;GNAZ;GRB14;HRASLS;ITGA2B;ITGB3;MGLL;PARD3;PCSK6;PCYT1B;PDGFA;PGRMC
purple	CC	GO:0005783	endoplasmic reticulum	17735	1273	134	18	9.95	0.01023	1.809045	ACSBG1;BET3L;C6orf25;CAV2;CCDC3;CLU;ELOVL7;GNAZ;MGLL;PCSK6;PCYT1B;PDGFA;PGRMC1;PLOD2;PROS
purple	MF	GO:0009975	cyclase activity	17735	21	134	2	0.16	0.01115	12.5	GUCY1A3;GUCY1B3
purple	MF	GO:0016849	phosphorus-oxygen lyase activity	17735	21	134	2	0.16	0.01115	12.5	GUCY1A3;GUCY1B3
purple	MF	GO:0019887	protein kinase regulator activity	17735	113	134	4	0.87	0.01117	4.597701	CDKN1A;EGF;PRKAR2B;TOM1L1
purple	MF	GO:0031406	carboxylic acid binding	17735	177	134	5	1.36	0.01175	3.676471	PLOD2;PTGES;SDPR;SELP;VIL1
purple	MF	GO:0017124	SH3 domain binding	17735	115	134	4	0.88	0.01186	4.545455	ARHGAP6;ENKUR;SH3BGRL2;TOM1L1
purple	MF	GO:0005543	phospholipid binding	17735	487	134	9	3.74	0.01266	2.406417	ASAP2;DNM3;GRB14;GRK5;PARD3;SDPR;SYTL4;VEPH1;VIL1
purple	CC	GO:0045121	membrane raft	17735	178	134	5	1.39	0.01301	3.597122	CAV2;PRKAR2B;SDC4;SDPR;SLC6A4
purple	MF	GO:0015298	solute:cation antiporter activity	17735	23	134	2	0.18	0.0133	11.11111	SLC24A3;SLC8A3
purple	MF	GO:0043499	eukaryotic cell surface binding	17735	23	134	2	0.18	0.0133	11.11111	PCSK6;PDGFA
purple	MF	GO:0042802	identical protein binding	17735	852	134	13	6.54	0.0136	1.987768	ADRA2A;CAV2;CLDN5;HOMER2;ITGA2B;ITGB3;MAOB;MGLL;PDGFA;PDGFRA;SLC6A4;VIL1;VWF
purple	CC	GO:0005902	microvillus	17735	66	134	3	0.52	0.01495	5.769231	CA2;PDGFA;VIL1
purple	CC	GO:0030027	lamellipodium	17735	122	134	4	0.95	0.01544	4.210526	CTTN;MYLK;VIL1;WASF3
purple	CC	GO:0000786	nucleosome	17735	67	134	3	0.52	0.01556	5.769231	HIST1H2AG;HIST1H2BH;HIST1H2BJ
purple	CC	GO:0031252	cell leading edge	17735	260	134	6	2.03	0.01649	2.955665	CTTN;ITGB5;MYLK;TPM1;VIL1;WASF3
purple	MF	GO:0005546	phosphatidylinositol-4,5-bisphosphate bi...	17735	26	134	2	0.2	0.01683	10	PARD3;VIL1
purple	CC	GO:0005925	focal adhesion	17735	127	134	4	0.99	0.01764	4.040404	ITGA2B;ITGB5;SDC4;TGFB1I1
purple	MF	GO:0043168	anion binding	17735	2346	134	27	18	0.01797	1.5	ABCC3;ACSBG1;C6orf25;DNM3;FSTL1;GNAZ;GRK5;GUCY1A3;GUCY1B3;MAOB;MYLK;PARD3;PCSK6;PDE5A;P
purple	MF	GO:0020037	heme binding	17735	131	134	4	1.01	0.01832	3.960396	GUCY1A3;GUCY1B3;PGRMC1;PTGS1
purple	MF	GO:0019207	kinase regulator activity	17735	133	134	4	1.02	0.01925	3.921569	CDKN1A;EGF;PRKAR2B;TOM1L1
purple	CC	GO:0005924	cell-substrate adherens junction	17735	132	134	4	1.03	0.02002	3.883495	ITGA2B;ITGB5;SDC4;TGFB1I1
purple	MF	GO:0017022	myosin binding	17735	29	134	2	0.22	0.02071	9.090909	CALD1;SLC6A4
purple	CC	GO:0009897	external side of plasma membrane	17735	202	134	5	1.58	0.02129	3.164557	CD9;ITGA2B;PDGFRA;SELP;VWF
purple	MF	GO:0030551	cyclic nucleotide binding	17735	30	134	2	0.23	0.02208	8.695652	PDE5A;PRKAR2B
purple	CC	GO:0005796	Golgi lumen	17735	77	134	3	0.6	0.02246	5	PCSK6;PROS1;SDC4
purple	CC	GO:0043197	dendritic spine	17735	137	134	4	1.07	0.02259	3.738318	DNM3;HOMER2;MAP1B;SLC8A3
purple	CC	GO:0044309	neuron spine	17735	137	134	4	1.07	0.02259	3.738318	DNM3;HOMER2;MAP1B;SLC8A3
purple	MF	GO:0046906	tetrapyrrole binding	17735	140	134	4	1.07	0.02275	3.738318	GUCY1A3;GUCY1B3;PGRMC1;PTGS1
purple	CC	GO:0030055	cell-substrate junction	17735	138	134	4	1.08	0.02313	3.703704	ITGA2B;ITGB5;SDC4;TGFB1I1
purple	MF	GO:0016702	oxidoreductase activity, acting on singl...	17735	80	134	3	0.61	0.0236	4.918033	ALOX12;PLOD2;PTGS1
purple	MF	GO:0005178	integrin binding	17735	81	134	3	0.62	0.02438	4.83871	CD9;ITGB5;VWF
purple	MF	GO:0016701	oxidoreductase activity, acting on singl...	17735	81	134	3	0.62	0.02438	4.83871	ALOX12;PLOD2;PTGS1
purple	MF	GO:0051213	dioxygenase activity	17735	82	134	3	0.63	0.02516	4.761905	ALOX12;PLOD2;PTGS1
purple	CC	GO:0043005	neuron projection	17735	627	134	10	4.9	0.02536	2.040816	CA2;CLDN5;DNM3;HOMER2;KCND3;MAP1B;PARD3;PVALB;SLC6A4;SLC8A3
purple	CC	GO:0042383	sarcolemma	17735	83	134	3	0.65	0.02728	4.615385	ALOX12;KCND3;SLC8A3
purple	MF	GO:0019905	syntaxin binding	17735	35	134	2	0.27	0.02947	7.407407	CAV2;SLC6A4
purple	MF	GO:0005509	calcium ion binding	17735	657	134	10	5.04	0.0296	1.984127	CABP5;EGF;FSTL1;FSTL4;LTBP1;MYL9;PROS1;PVALB;SPARC;VIL1
purple	MF	GO:0050840	extracellular matrix binding	17735	36	134	2	0.28	0.03105	7.142857	ITGA2B;SPARC
purple	MF	GO:0004866	endopeptidase inhibitor activity	17735	162	134	4	1.24	0.03615	3.225806	PROS1;SERPINE1;TFPI;VIL1
purple	MF	GO:0061135	endopeptidase regulator activity	17735	166	134	4	1.27	0.03899	3.149606	PROS1;SERPINE1;TFPI;VIL1
purple	MF	GO:0032403	protein complex binding	17735	327	134	6	2.51	0.0402	2.390438	ADRA2A;CD9;CDKN1A;GNAZ;ITGB5;VWF
purple	CC	GO:0030016	myofibril	17735	165	134	4	1.29	0.04064	3.100775	CALD1;MYL9;SDC4;TPM1
purple	MF	GO:0030414	peptidase inhibitor activity	17735	169	134	4	1.3	0.0412	3.076923	PROS1;SERPINE1;TFPI;VIL1
purple	CC	GO:0043235	receptor complex	17735	167	134	4	1.31	0.04216	3.053435	ADRA2A;ITGA2B;ITGB3;ITGB5
purple	CC	GO:0005923	tight junction	17735	101	134	3	0.79	0.04477	3.797468	CLDN5;ESAM;PARD3
purple	CC	GO:0070160	occluding junction	17735	101	134	3	0.79	0.04477	3.797468	CLDN5;ESAM;PARD3
purple	MF	GO:0016860	intramolecular oxidoreductase activity	17735	44	134	2	0.34	0.04481	5.882353	ITGB3;PTGES
purple	MF	GO:0072341	modified amino acid binding	17735	44	134	2	0.34	0.04481	5.882353	PTGES;SDPR
purple	CC	GO:0031012	extracellular matrix	17735	422	134	7	3.3	0.048	2.121212	CLU;LTBP1;PCSK6;SERPINE1;SPARC;TGFB1I1;VWF
purple	MF	GO:0000149	SNARE binding	17735	46	134	2	0.35	0.04855	5.714286	CAV2;SLC6A4

purple	MF	GO:0008307	structural constituent of muscle	17735	46	134	2	0.35	0.04855	5.714286	MYL9;TPM1
purple	MF	GO:0017137	Rab GTPase binding	17735	46	134	2	0.35	0.04855	5.714286	SLC6A4;SYTL4
purple	CC	GO:0043292	contractile fiber	17735	175	134	4	1.37	0.0486	2.919708	CALD1;MYL9;SDC4;TPM1
pink	CC	GO:0005833	hemoglobin complex	17735	12	196	9	0.14	5.50E-16	64.28571	AHSP;HBA2;HBB;HBD;HBG1;HBG2;HBM;HBQ1;HBZ
pink	MF	GO:0005344	oxygen transporter activity	17735	13	196	8	0.15	2.80E-13	53.33333	HBA2;HBB;HBD;HBG1;HBG2;HBM;HBQ1;HBZ
pink	BP	GO:0048821	erythrocyte development	17735	21	196	7	0.24	2.10E-09	29.16667	ANK1;EPB42;GATA1;HBZ;KLF1;SOX6;TAL1
pink	BP	GO:0030218	erythrocyte differentiation	17735	88	196	11	0.99	4.10E-09	11.11111	ALAS2;ANK1;DYRK3;EPB42;FECH;GATA1;HBZ;KLF1;SOX6;TAL1;TRIM1C
pink	MF	GO:0019825	oxygen binding	17735	36	196	8	0.41	5.30E-09	19.5122	HBA2;HBB;HBD;HBG1;HBG2;HBM;HBQ1;HBZ
pink	BP	GO:0034101	erythrocyte homeostasis	17735	95	196	11	1.07	9.30E-09	10.28037	ALAS2;ANK1;DYRK3;EPB42;FECH;GATA1;HBZ;KLF1;SOX6;TAL1;TRIM1C
pink	BP	GO:0048872	homeostasis of number of cells	17735	176	196	13	1.99	9.60E-08	6.532663	ALAS2;ANK1;DYRK3;EPB42;FECH;GATA1;HBZ;KLF1;PRDX5;SOX6;SPTA1;TAL1;TRIM1C
pink	BP	GO:0065008	regulation of biological quality	17735	2547	196	56	28.73	1.90E-07	1.949182	ABCC4;ABCG2;ACHE;AHSP;ALAS2;ANK1;AQP1;BAMBI;BCL2L1;BEST2;CSDA;DPM2;DYRK3;EHD2;EPB42;EPB4
pink	BP	GO:0015669	gas transport	17735	13	196	5	0.15	2.10E-07	33.33333	AQP1;HBA2;HBB;HBQ1;RHAG
pink	BP	GO:0006778	porphyrin-containing compound metabolic ...	17735	39	196	7	0.44	2.30E-07	15.90909	ALAS2;ANK1;BLVRB;FECH;HMBS;SLC25A39;SPTA1
pink	MF	GO:0005506	iron ion binding	17735	164	196	12	1.86	3.50E-07	6.451613	C5orf4;FECH;FTL;HBA2;HBB;HBD;HBG1;HBG2;HBM;HBQ1;HBZ;SNCA
pink	BP	GO:0043154	negative regulation of cysteine-type end...	17735	63	196	8	0.71	5.00E-07	11.26761	AQP1;GPX1;PRDX5;RAG1;SFRP2;SIAH2;SNCA;WNT9A
pink	BP	GO:0006779	porphyrin-containing compound biosynthet...	17735	28	196	6	0.32	5.80E-07	18.75	ALAS2;ANK1;FECH;HMBS;SLC25A39;SPTA1
pink	BP	GO:0015701	bicarbonate transport	17735	29	196	6	0.33	7.20E-07	18.18182	AQP1;CA1;HBA2;HBB;RHAG;SLC4A1
pink	BP	GO:2000117	negative regulation of cysteine-type end...	17735	66	196	8	0.74	7.30E-07	10.81081	AQP1;GPX1;PRDX5;RAG1;SFRP2;SIAH2;SNCA;WNT9A
pink	BP	GO:0033013	tetrapyrrole metabolic process	17735	48	196	7	0.54	1.00E-06	12.96296	ALAS2;ANK1;BLVRB;FECH;HMBS;SLC25A39;SPTA1
pink	BP	GO:0033014	tetrapyrrole biosynthetic process	17735	31	196	6	0.35	1.10E-06	17.14286	ALAS2;ANK1;FECH;HMBS;SLC25A39;SPTA1
pink	CC	GO:0044445	cytosolic part	17735	181	196	12	2.05	1.10E-06	5.853659	AHSP;HBA2;HBB;HBD;HBG1;HBG2;HBM;HBQ1;HBZ;PRDX5;RPL3L;UBA52
pink	CC	GO:0030863	cortical cytoskeleton	17735	55	196	7	0.62	2.80E-06	11.29032	ANK1;EPB42;EPB49;GYPC;SLC4A1;SPTA1;TMOD1
pink	BP	GO:0042744	hydrogen peroxide catabolic process	17735	21	196	5	0.24	3.00E-06	20.83333	GPX1;HBA2;HBB;PRDX5;PRDX6
pink	BP	GO:0030097	hemopoiesis	17735	509	196	19	5.74	4.70E-06	3.310105	AHSP;ALAS2;ANK1;DYRK3;EPB42;FECH;GATA1;GLRX5;HBZ;KLF1;LYL1;PBX1;RAG1;SFRP2;SOX6;SPTA1;TAL1;T
pink	BP	GO:0043249	erythrocyte maturation	17735	11	196	4	0.12	4.80E-06	33.33333	EPB42;HBZ;KLF1;TAL1
pink	BP	GO:0042592	homeostatic process	17735	1235	196	32	13.93	6.70E-06	2.2972	ABCG2;ALAS2;ANK1;AQP1;BCL2L1;BEST2;DYRK3;EPB42;FECH;FKBP1B;FTL;GATA1;GLRX5;GPX1;HBZ;KCNH2;
pink	BP	GO:0002520	immune system development	17735	572	196	20	6.45	7.00E-06	3.100775	AHSP;ALAS2;ANK1;DYRK3;EPB42;FECH;GATA1;GLRX5;HBZ;KLF1;LYL1;PBX1;POLL;RAG1;SFRP2;SOX6;SPTA1;T
pink	BP	GO:0048534	hematopoietic or lymphoid organ developm...	17735	542	196	19	6.11	1.20E-05	3.109656	AHSP;ALAS2;ANK1;DYRK3;EPB42;FECH;GATA1;GLRX5;HBZ;KLF1;LYL1;PBX1;RAG1;SFRP2;SOX6;SPTA1;TAL1;T
pink	BP	GO:0020027	hemoglobin metabolic process	17735	14	196	4	0.16	1.40E-05	25	AHSP;ALAS2;EPB42;FECH
pink	MF	GO:0020037	heme binding	17735	131	196	9	1.48	1.80E-05	6.081081	FECH;HBA2;HBB;HBD;HBG1;HBG2;HBM;HBQ1;HBZ
pink	BP	GO:0042168	heme metabolic process	17735	30	196	5	0.34	1.90E-05	14.70588	ALAS2;BLVRB;FECH;HMBS;SLC25A39
pink	BP	GO:0030099	myeloid cell differentiation	17735	247	196	12	2.79	2.40E-05	4.301075	ALAS2;ANK1;DYRK3;EPB42;FECH;GATA1;HBZ;KLF1;SOX6;TAL1;TESC;TRIM1C
pink	BP	GO:0070301	cellular response to hydrogen peroxide	17735	52	196	6	0.59	2.50E-05	10.16949	AQP1;GPX1;HBA2;HBB;PRDX5;PRDX6
pink	MF	GO:0046906	tetrapyrrole binding	17735	140	196	9	1.58	3.00E-05	5.696203	FECH;HBA2;HBB;HBD;HBG1;HBG2;HBM;HBQ1;HBZ
pink	BP	GO:0042542	response to hydrogen peroxide	17735	86	196	7	0.97	5.20E-05	7.216495	AQP1;FKBP1B;GPX1;HBA2;HBB;PRDX5;PRDX6
pink	BP	GO:0042743	hydrogen peroxide metabolic process	17735	37	196	5	0.42	5.60E-05	11.90476	GPX1;HBA2;HBB;PRDX5;PRDX6
pink	CC	GO:0044448	cell cortex part	17735	90	196	7	1.02	7.30E-05	6.862745	ANK1;EPB42;EPB49;GYPC;SLC4A1;SPTA1;TMOD1
pink	MF	GO:0004601	peroxidase activity	17735	40	196	5	0.45	8.30E-05	11.11111	GPX1;HBA2;HBB;PRDX5;PRDX6
pink	MF	GO:0016684	oxidoreductase activity, acting on perox...	17735	40	196	5	0.45	8.30E-05	11.11111	GPX1;HBA2;HBB;PRDX5;PRDX6
pink	BP	GO:0006783	heme biosynthetic process	17735	22	196	4	0.25	9.70E-05	16	ALAS2;FECH;HMBS;SLC25A39
pink	BP	GO:0010951	negative regulation of endopeptidase act...	17735	128	196	8	1.44	1.00E-04	5.555556	AQP1;GPX1;PRDX5;RAG1;SFRP2;SIAH2;SNCA;WNT9A
pink	BP	GO:0000302	response to reactive oxygen species	17735	130	196	8	1.47	0.00011	5.442177	AQP1;FKBP1B;GPX1;HBA2;HBB;PRDX5;PRDX6;PTPRN
pink	BP	GO:0010466	negative regulation of peptidase activit...	17735	130	196	8	1.47	0.00011	5.442177	AQP1;GPX1;PRDX5;RAG1;SFRP2;SIAH2;SNCA;WNT9A
pink	MF	GO:0015101	organic cation transmembrane transporter...	17735	24	196	4	0.27	0.00014	14.81481	RHAG;RHCE;SLC14A1;SLC6A8
pink	BP	GO:0043281	regulation of cysteine-type endopeptidas...	17735	172	196	9	1.94	0.00015	4.639175	AQP1;GPX1;IFI27;PRDX5;RAG1;SFRP2;SIAH2;SNCA;WNT9A
pink	BP	GO:0035162	embryonic hemopoiesis	17735	25	196	4	0.28	0.00016	14.28571	GATA1;KLF1;PBX1;TAL1
pink	BP	GO:2000116	regulation of cysteine-type endopeptidas...	17735	179	196	9	2.02	2.00E-04	4.455446	AQP1;GPX1;IFI27;PRDX5;RAG1;SFRP2;SIAH2;SNCA;WNT9A
pink	BP	GO:0046501	protoporphyrinogen IX metabolic process	17735	11	196	3	0.12	0.00022	25	ALAS2;FECH;HMBS
pink	BP	GO:0034599	cellular response to oxidative stress	17735	108	196	7	1.22	0.00022	5.737705	AQP1;GPX1;HBA2;HBB;PRDX5;PRDX6;SNCA
pink	BP	GO:0015711	organic anion transport	17735	266	196	11	3	0.00022	3.666667	AQP1;CA1;HBA2;HBB;RHAG;SLC38A5;SLC4A1;SLC6A19;SLC6A8;SNCA;XK
pink	BP	GO:0034614	cellular response to reactive oxygen spe...	17735	80	196	6	0.9	0.00028	6.666667	AQP1;GPX1;HBA2;HBB;PRDX5;PRDX6
pink	MF	GO:0005215	transporter activity	17735	1166	196	27	13.19	0.00028	2.047005	ABCC13;ABCC4;ABCG2;AQP1;BEST2;FKBP1B;HBA2;HBB;HBD;HBG1;HBG2;HBM;HBQ1;HBZ;KCNH2;RHAG;RH
pink	BP	GO:0006970	response to osmotic stress	17735	55	196	5	0.62	0.00038	8.064516	AQP1;CSDA;GYPA;NEDD4L;SLC2A4
pink	BP	GO:0071470	cellular response to osmotic stress	17735	14	196	3	0.16	0.00047	18.75	AQP1;CSDA;SLC2A4
pink	BP	GO:0055072	iron ion homeostasis	17735	88	196	6	0.99	0.00047	6.060606	ABCG2;ALAS2;EPB42;FECH;FTL;SLC25A37
pink	BP	GO:0042440	pigment metabolic process	17735	59	196	5	0.67	0.00053	7.462687	ALAS2;BLVRB;FECH;HMBS;SLC25A39
pink	BP	GO:0006979	response to oxidative stress	17735	253	196	10	2.85	6.00E-04	3.508772	AQP1;FKBP1B;GPX1;HBA2;HBB;KRT1;PRDX5;PRDX6;PTPRN;SNCA
pink	BP	GO:0090101	negative regulation of transmembrane rec...	17735	95	196	6	1.07	0.00071	5.607477	BAMBI;NEDD4L;PPM1A;SFRP2;UBA52;UBB
pink	BP	GO:0051188	cofactor biosynthetic process	17735	132	196	7	1.49	0.00074	4.697987	ALAS2;ANK1;FECH;HMBS;SLC25A39;SNCA;SPTA1
pink	MF	GO:0016209	antioxidant activity	17735	65	196	5	0.74	0.00083	6.756757	GPX1;HBA2;HBB;PRDX5;PRDX6
pink	BP	GO:0030512	negative regulation of transforming grow...	17735	66	196	5	0.74	0.00088	6.756757	BAMBI;NEDD4L;PPM1A;UBA52;UBB
pink	BP	GO:0006820	anion transport	17735	317	196	11	3.58	0.00095	3.072626	AQP1;CA1;HBA2;HBB;RHAG;SLC38A5;SLC4A1;SLC6A19;SLC6A8;SNCA;XK
pink	BP	GO:1901566	organonitrogen compound biosynthetic pro...	17735	647	196	17	7.3	0.00103	2.328767	A4GALT;ACHE;ALAS2;ANK1;AQP1;CHPT1;FECH;GALNT5;GMPR;HAGH;HMBS;OAZ1;ODC1;SLC25A39;SNCA;SF
pink	MF	GO:0022892	substrate-specific transporter activity	17735	948	196	22	10.72	0.00105	2.052239	AQP1;BEST2;FKBP1B;HBA2;HBB;HBD;HBG1;HBG2;HBM;HBQ1;HBZ;KCNH2;RHAG;RHCE;SLC14A1;SLC25A37;S
pink	MF	GO:0008509	anion transmembrane transporter activity	17735	227	196	9	2.57	0.00113	3.501946	BEST2;RHAG;RHCE;SLC14A1;SLC38A5;SLC4A1;SLC6A19;SLC6A8;SLC6A9
pink	BP	GO:0060216	definitive hemopoiesis	17735	19	196	3	0.21	0.0012	14.28571	GATA1;LYL1;TAL1
pink	MF	GO:0005328	neurotransmitter:sodium symporter activi...	17735	19	196	3	0.21	0.00121	14.28571	SLC6A19;SLC6A8;SLC6A9
pink	CC	GO:0005938	cell cortex	17735	186	196	8	2.11	0.0013	3.791469	ANK1;EPB42;EPB49;GYPC;SLC4A1;SNCA;SPTA1;TMOD1
pink	BP	GO:0051186	cofactor metabolic process	17735	233	196	9	2.63	0.00132	3.422053	ALAS2;ANK1;BLVRB;FECH;HMBS;PRDX5;SLC25A39;SNCA;SPTA1

pink	MF	GO:0046983	protein dimerization activity	17735	909	196	21	10.28	0.00144	2.042802	ABCG2;ACHE;ADIPOR1;BCL2L1;CTSE;GYPA;HPS1;LYL1;MEF2B;MXI1;PBX1;PLVAP;PRDX5;RAG1;RAP1GAP;SIM
pink	BP	GO:1901701	cellular response to oxygen-containing c...	17735	387	196	12	4.36	0.00148	2.752294	AQP1;BCL2L1;FKBP1B;GPX1;HBA2;HBB;PDE3A;PRDX5;PRDX6;SNCA;TESC;WNT9A
pink	BP	GO:0051346	negative regulation of hydrolase activit...	17735	238	196	9	2.68	0.00153	3.358209	AQP1;FKBP1B;GPX1;PRDX5;RAG1;SFRP2;SIAH2;SNCA;WNT9A
pink	MF	GO:0015175	neutral amino acid transmembrane transpo...	17735	21	196	3	0.24	0.00163	12.5	SLC38A5;SLC6A19;SLC6A9
pink	BP	GO:0050801	ion homeostasis	17735	679	196	17	7.66	0.00173	2.219321	ABCG2;ALAS2;BCL2L1;BEST2;EPB42;FECH;FKBP1B;FTL;KCNH2;NEDD4L;RHAG;RNF10;SLC25A37;SLC4A1;SNC
pink	BP	GO:0000080	G1 phase of mitotic cell cycle	17735	46	196	4	0.52	0.00176	7.692308	E2F2;TFDP1;UBA52;UBB
pink	BP	GO:0046148	pigment biosynthetic process	17735	46	196	4	0.52	0.00176	7.692308	ALAS2;FECH;HMBS;SLC25A39
pink	CC	GO:0005829	cytosol	17735	2362	196	42	26.79	0.0018	1.567749	AHSP;ANK1;BCL2L1;BLVRB;CA1;FKBP1B;FTL;GFAP;GMPR;GPX1;GUK1;HBA2;HBB;HBD;HBG1;HBG2;HBM;HB
pink	BP	GO:0000122	negative regulation of transcription fro...	17735	507	196	14	5.72	0.00182	2.447552	CSDA;FHL2;HBZ;KANK2;MXI1;NEDD4L;NFIX;PPM1A;SIM2;SOX6;TAL1;UBA52;UBB;YBX1
pink	BP	GO:0071560	cellular response to transforming growth...	17735	198	196	8	2.23	0.00183	3.587444	BAMBI;NEDD4L;PDE3A;PPM1A;SOX6;TFDP1;UBA52;UBB
pink	BP	GO:0071559	response to transforming growth factor b...	17735	199	196	8	2.24	0.00189	3.571429	BAMBI;NEDD4L;PDE3A;PPM1A;SOX6;TFDP1;UBA52;UBB
pink	MF	GO:0005200	structural constituent of cytoskeleton	17735	78	196	5	0.88	0.0019	5.681818	ANK1;EPB42;GFAP;KRT1;SPTA1
pink	BP	GO:0052548	regulation of endopeptidase activity	17735	248	196	9	2.8	0.00202	3.214286	AQP1;GPX1;IFI27;PRDX5;RAG1;SFRP2;SIAH2;SNCA;WNT9A
pink	BP	GO:0051318	G1 phase	17735	48	196	4	0.54	0.00206	7.407407	E2F2;TFDP1;UBA52;UBB
pink	BP	GO:0030219	megakaryocyte differentiation	17735	23	196	3	0.26	0.00211	11.53846	GATA1;TAL1;TESC
pink	BP	GO:0060548	negative regulation of cell death	17735	633	196	16	7.14	0.00214	2.240896	AQP1;BAG1;BCL2L1;CSDA;FHL2;GATA1;GPX1;KANK2;PDE3A;PRDX5;RAG1;SFRP2;SIAH2;SNCA;STRADB;WNT
pink	MF	GO:0003730	mRNA 3'-UTR binding	17735	24	196	3	0.27	0.00242	11.11111	CSDA;IGF2BP2;RBM38
pink	MF	GO:0005326	neurotransmitter transporter activity	17735	24	196	3	0.27	0.00242	11.11111	SLC6A19;SLC6A8;SLC6A9
pink	BP	GO:0052547	regulation of peptidase activity	17735	255	196	9	2.88	0.00244	3.125	AQP1;GPX1;IFI27;PRDX5;RAG1;SFRP2;SIAH2;SNCA;WNT9A
pink	BP	GO:0090288	negative regulation of cellular response...	17735	86	196	5	0.97	0.00287	5.154639	BAMBI;NEDD4L;PPM1A;UBA52;UBB
pink	BP	GO:0043489	RNA stabilization	17735	26	196	3	0.29	0.00303	10.34483	CSDA;RBM38;YBX1
pink	BP	GO:0048255	mRNA stabilization	17735	26	196	3	0.29	0.00303	10.34483	CSDA;RBM38;YBX1
pink	BP	GO:0000082	G1/S transition of mitotic cell cycle	17735	215	196	8	2.42	0.00306	3.305785	CDC34;GSPT1;KANK2;RBM38;TFDP1;UBA52;UBB;WNT9A
pink	CC	GO:0031672	A band	17735	26	196	3	0.29	0.0031	10.34483	ANK1;FHL2;MYL4
pink	BP	GO:0002376	immune system process	17735	1816	196	33	20.48	0.00356	1.611328	AHSP;ALAS2;ANK1;BCL2L1;CTSE;DYRK3;ECSIT;EPB42;FECH;FKBP1B;GATA1;GLRX5;GPX1;HBZ;HPS1;IFI27;KLF
pink	BP	GO:0030851	granulocyte differentiation	17735	28	196	3	0.32	0.00375	9.375	GATA1;TAL1;TESC
pink	BP	GO:0071804	cellular potassium ion transport	17735	28	196	3	0.32	0.00375	9.375	AQP1;KCNH2;NEDD4L
pink	BP	GO:0071805	potassium ion transmembrane transport	17735	28	196	3	0.32	0.00375	9.375	AQP1;KCNH2;NEDD4L
pink	BP	GO:0043069	negative regulation of programmed cell d...	17735	610	196	15	6.88	0.0038	2.180233	AQP1;BAG1;BCL2L1;CSDA;FHL2;GATA1;GPX1;KANK2;PDE3A;PRDX5;RAG1;SFRP2;SIAH2;SNCA;STRADB
pink	BP	GO:0017015	regulation of transforming growth factor...	17735	92	196	5	1.04	0.00384	4.807692	BAMBI;NEDD4L;PPM1A;UBA52;UBB
pink	BP	GO:0048878	chemical homeostasis	17735	872	196	19	9.83	0.00442	1.932859	ABCG2;ALAS2;AQP1;BCL2L1;BEST2;EPB42;FECH;FKBP1B;FTL;KCNH2;NEDD4L;RHAG;RNF10;SLC25A37;SLC2A
pink	BP	GO:0019725	cellular homeostasis	17735	749	196	17	8.45	0.00472	2.011834	ABCG2;ALAS2;AQP1;BCL2L1;BEST2;FKBP1B;FTL;GLRX5;GPX1;KCNH2;NEDD4L;RHAG;RNF10;SLC4A1;SNCA;TG
pink	BP	GO:0021670	lateral ventricle development	17735	10	196	2	0.11	0.00536	18.18182	AQP1;DPCD
pink	BP	GO:0035413	positive regulation of catenin import in...	17735	10	196	2	0.11	0.00536	18.18182	BAMBI;SFRP2
pink	MF	GO:0030507	spectrin binding	17735	10	196	2	0.11	0.00539	18.18182	ANK1;PTPRN
pink	BP	GO:0008584	male gonad development	17735	100	196	5	1.13	0.00547	4.424779	BCL2L1;CSDA;GATA1;SFRP2;TESC
pink	MF	GO:0015171	amino acid transmembrane transporter act...	17735	63	196	4	0.71	0.00561	5.633803	SLC38A5;SLC6A19;SLC6A8;SLC6A9
pink	BP	GO:0007009	plasma membrane organization	17735	102	196	5	1.15	0.00594	4.347826	A4GALT;ANK1;BCL2L1;SPTA1;TESC
pink	BP	GO:2000045	regulation of G1/S transition of mitotic...	17735	104	196	5	1.17	0.00644	4.273504	KANK2;RBM38;UBA52;UBB;WNT9A
pink	BP	GO:0006596	polyamine biosynthetic process	17735	11	196	2	0.12	0.0065	16.66667	OAZ1;ODC1
pink	BP	GO:0070935	3'-UTR-mediated mRNA stabilization	17735	11	196	2	0.12	0.0065	16.66667	CSDA;RBM38
pink	MF	GO:0005372	water transmembrane transporter activity	17735	11	196	2	0.12	0.00654	16.66667	AQP1;SLC14A1
pink	BP	GO:0031647	regulation of protein stability	17735	105	196	5	1.18	0.0067	4.237288	AHSP;DPM2;FBXO7;SNCA;TESC
pink	BP	GO:0006810	transport	17735	3358	196	52	37.87	0.00685	1.373119	ABCC4;ABCG2;ACHE;ANK1;AQP1;BAMBI;BCL2L1;CA1;EHD2;FKBP1B;FTL;GFAP;HBA2;HBB;HBQ1;HPS1;ITLN1;
pink	BP	GO:0010608	posttranscriptional regulation of gene e...	17735	355	196	10	4	0.00704	2.5	AHSP;CSDA;DPM2;FBXO7;FECH;IGF2BP2;RBM38;SNCA;TESC;YBX1
pink	CC	GO:0000151	ubiquitin ligase complex	17735	150	196	6	1.7	0.0073	3.529412	DCAF12;DCAF6;FBXO17;FBXO7;FBXO9;SHARPIN
pink	BP	GO:0019935	cyclic-nucleotide-mediated signaling	17735	36	196	3	0.41	0.00766	7.317073	CDC34;NUDT4;PDE3A
pink	BP	GO:0046788	egress of virus within host cell	17735	12	196	2	0.14	0.00775	14.28571	UBA52;UBB
pink	MF	GO:0008198	ferrous iron binding	17735	12	196	2	0.14	0.0078	14.28571	FECH;SNCA
pink	BP	GO:0031333	negative regulation of protein complex a...	17735	70	196	4	0.79	0.00803	5.063291	EPB49;PPM1A;SNCA;SPTA1
pink	BP	GO:0043066	negative regulation of apoptotic process	17735	602	196	14	6.79	0.00824	2.061856	AQP1;BAG1;BCL2L1;CSDA;FHL2;GATA1;GPX1;PDE3A;PRDX5;RAG1;SFRP2;SIAH2;SNCA;STRADB
pink	BP	GO:0043488	regulation of mRNA stability	17735	37	196	3	0.42	0.00826	7.142857	CSDA;RBM38;YBX1
pink	BP	GO:0006873	cellular ion homeostasis	17735	604	196	14	6.81	0.00847	2.0558	ABCG2;ALAS2;BCL2L1;BEST2;FKBP1B;FTL;KCNH2;NEDD4L;RHAG;RNF10;SLC4A1;SNCA;TGM2;UTS2R
pink	BP	GO:2000602	regulation of interphase of mitotic cell...	17735	157	196	6	1.77	0.00877	3.389831	BCL2L1;KANK2;RBM38;UBA52;UBB;WNT9A
pink	BP	GO:0010035	response to inorganic substance	17735	312	196	9	3.52	0.00897	2.556818	AQP1;FKBP1B;GPX1;HBA2;HBB;NEDD4L;PRDX5;PRDX6;SNCA
pink	BP	GO:0042026	protein refolding	17735	13	196	2	0.15	0.00909	13.33333	BAG1;FKBP1B
pink	BP	GO:0051234	establishment of localization	17735	3408	196	52	38.44	0.00924	1.352758	ABCC4;ABCG2;ACHE;ANK1;AQP1;BAMBI;BCL2L1;CA1;EHD2;FKBP1B;FTL;GFAP;HBA2;HBB;HBQ1;HPS1;ITLN1;
pink	BP	GO:0055080	cation homeostasis	17735	430	196	11	4.85	0.00959	2.268041	ABCG2;ALAS2;EPB42;FECH;FKBP1B;FTL;NEDD4L;SLC25A37;SNCA;TGM2;UTS2R
pink	BP	GO:0048469	cell maturation	17735	116	196	5	1.31	0.01009	3.816794	EPB42;HBZ;KLF1;PDE3A;TAL1
pink	BP	GO:0048513	organ development	17735	2404	196	39	27.11	0.01029	1.438584	ACHE;AHSP;ALAS2;ANK1;AQP1;BAMBI;BCL2L1;CSDA;DPCD;DYRK3;EPB42;FECH;FHL2;GATA1;GLRX5;GPX1;H
pink	BP	GO:0071702	organic substance transport	17735	1950	196	33	21.99	0.01037	1.500682	ABCG2;ACHE;ANK1;AQP1;BAMBI;CA1;FKBP1B;HBA2;HBB;HPS1;ITLN1;MXI1;NUDT4;OSBP2;RAB3IL1;RHAG;R
pink	BP	GO:0046546	development of primary male sexual chara...	17735	117	196	5	1.32	0.01045	3.787879	BCL2L1;CSDA;GATA1;SFRP2;TESC
pink	BP	GO:0021781	glial cell fate commitment	17735	14	196	2	0.16	0.01053	12.5	SOX6;TAL1
pink	MF	GO:0015665	alcohol transmembrane transporter activi...	17735	14	196	2	0.16	0.01059	12.5	AQP1;SLC6A8
pink	BP	GO:0051239	regulation of multicellular organismal p...	17735	1804	196	31	20.35	0.0106	1.523342	ACHE;AQP1;BAMBI;CNN1;CSDA;DARC;FKBP1B;GATA1;GFAP;GPX1;HEMGN;IGF2BP2;KCNH2;KRT1;MBNL3;M
pink	BP	GO:0051494	negative regulation of cytoskeleton orga...	17735	76	196	4	0.86	0.01067	4.651163	EPB49;ESPN;SNCA;SPTA1
pink	BP	GO:0043487	regulation of RNA stability	17735	41	196	3	0.46	0.01097	6.521739	CSDA;RBM38;YBX1
pink	BP	GO:0055085	transmembrane transport	17735	688	196	15	7.76	0.01107	1.93299	ABCC4;ABCG2;AQP1;FKBP1B;FTL;KCNH2;NEDD4L;RHAG;SLC14A1;SLC2A4;SLC38A5;SLC4A1;SLC6A19;SLC6A
pink	BP	GO:0090092	regulation of transmembrane receptor pro...	17735	167	196	6	1.88	0.01167	3.191489	BAMBI;NEDD4L;PPM1A;SFRP2;UBA52;UBB

pink	MF	GO:0008092	cytoskeletal protein binding	17735	627	196	14	7.09	0.01188	1.974612	ANK1;CNN1;EPB49;ESPN;KLC3;MYL4;PTPRN;RHAG;SGIP1;SLC4A1;SNCA;SPTA1;TMOD1;TNS1
pink	BP	GO:0001919	regulation of receptor recycling	17735	15	196	2	0.17	0.01206	11.76471	ACHE;SNCA
pink	MF	GO:0005527	macrolide binding	17735	15	196	2	0.17	0.01213	11.76471	FKBP1B;FKBP8
pink	MF	GO:0005528	FK506 binding	17735	15	196	2	0.17	0.01213	11.76471	FKBP1B;FKBP8
pink	BP	GO:0007178	transmembrane receptor protein serine/th...	17735	273	196	8	3.08	0.01232	2.597403	BAMBI;ECSIT;NEDD4L;PPM1A;SFRP2;TFDP1;UBA52;UBB
pink	BP	GO:0072593	reactive oxygen species metabolic proces...	17735	123	196	5	1.39	0.01278	3.597122	GPX1;HBA2;HBB;PRDX5;PRDX6
pink	BP	GO:0032844	regulation of homeostatic process	17735	275	196	8	3.1	0.01283	2.580645	FKBP1B;GATA1;KCNH2;RNF10;SGIP1;SNCA;TAL1;UTS2R
pink	BP	GO:0007179	transforming growth factor beta receptor...	17735	171	196	6	1.93	0.013	3.108808	BAMBI;NEDD4L;PPM1A;TFDP1;UBA52;UBB
pink	BP	GO:0044765	single-organism transport	17735	2753	196	43	31.05	0.01313	1.384863	ABCC4;ABCG2;ACHE;ANK1;AQP1;BAMBI;BCL2L1;CA1;EHD2;FKBP1B;FTL;GFAP;HBA2;HBB;HBQ1;HPS1;ITLN1;
pink	BP	GO:0006865	amino acid transport	17735	124	196	5	1.4	0.01319	3.571429	SLC38A5;SLC6A19;SLC6A8;SNCA;XK
pink	BP	GO:1901564	organonitrogen compound metabolic proces...	17735	1688	196	29	19.04	0.01352	1.523109	A4GALT;ABCG2;ACHE;ALAS2;ANK1;AQP1;BLVRB;CHPT1;FECH;GALNT5;GCAT;GMPR;GPX1;GSPT1;GUK1;HAG
pink	BP	GO:0046661	male sex differentiation	17735	125	196	5	1.41	0.01362	3.546099	BCL2L1;CSDA;GATA1;SFRP2;TESC
pink	BP	GO:0060251	regulation of glial cell proliferation	17735	16	196	2	0.18	0.01368	11.11111	GFAP;RNF10
pink	MF	GO:0016780	phosphotransferase activity, for other s...	17735	16	196	2	0.18	0.01376	11.11111	CHPT1;KLC3
pink	MF	GO:0051184	cofactor transporter activity	17735	16	196	2	0.18	0.01376	11.11111	ABCG2;SLC6A8
pink	CC	GO:0031430	M band	17735	16	196	2	0.18	0.0138	11.11111	ANK1;FHL2
pink	BP	GO:0031575	mitotic cell cycle G1/S transition check...	17735	82	196	4	0.92	0.01381	4.347826	RBM38;UBA52;UBB;WNT9A
pink	BP	GO:0021700	developmental maturation	17735	174	196	6	1.96	0.01406	3.061224	EPB42;HBZ;KLF1;LYL1;PDE3A;TAL1
pink	BP	GO:0032272	negative regulation of protein polymeriz...	17735	45	196	3	0.51	0.01414	5.882353	EPB49;SNCA;SPTA1
pink	BP	GO:0006352	DNA-dependent transcription, initiation	17735	228	196	7	2.57	0.01488	2.723735	E2F2;NEDD4L;POLR1D;PPM1A;TFDP1;UBA52;UBB
pink	BP	GO:0061061	muscle structure development	17735	460	196	11	5.19	0.01525	2.119461	ACHE;CSDA;FHL2;GPX1;MBNL3;MEF2B;MYL4;RBM38;SOX6;TMOD1;YBX1
pink	BP	GO:0072661	protein targeting to plasma membrane	17735	17	196	2	0.19	0.01539	10.52632	ANK1;TESC
pink	BP	GO:0003018	vascular process in circulatory system	17735	129	196	5	1.45	0.01543	3.448276	GPX1;HBB;KEL;PDE3A;UTS2R
pink	BP	GO:0071495	cellular response to endogenous stimulus	17735	716	196	15	8.08	0.01545	1.856436	ADIPOR1;AQP1;BAMBI;BCL2L1;GATA1;NEDD4L;PDE3A;PPM1A;SLC2A4;SNCA;SOX6;STRADB;TFDP1;UBA52;U
pink	BP	GO:0090002	establishment of protein localization to...	17735	47	196	3	0.53	0.01589	5.660377	ANK1;BCL2L1;TESC
pink	BP	GO:0071779	G1/S transition checkpoint	17735	86	196	4	0.97	0.0162	4.123711	RBM38;UBA52;UBB;WNT9A
pink	MF	GO:0005515	protein binding	17735	7306	196	97	82.65	0.01653	1.173624	ABCG2;ACHE;ADIPOR1;AHSP;ALAS2;ANK1;AQP1;ARHGEF37;BAG1;BAMBI;BCAM;BCL2L1;CDC34;CNN1;CSDA
pink	BP	GO:0035872	nucleotide-binding domain, leucine rich ...	17735	48	196	3	0.54	0.01681	5.555556	BCL2L1;UBA52;UBB
pink	BP	GO:0019067	viral assembly, maturation, egress, and ...	17735	18	196	2	0.2	0.01718	10	UBA52;UBB
pink	BP	GO:0042401	cellular biogenic amine biosynthetic pro...	17735	18	196	2	0.2	0.01718	10	OAZ1;ODC1
pink	BP	GO:2001234	negative regulation of apoptotic signali...	17735	18	196	2	0.2	0.01718	10	BCL2L1;SIAH2
pink	MF	GO:0004602	glutathione peroxidase activity	17735	18	196	2	0.2	0.01729	10	GPX1;PRDX6
pink	MF	GO:0005001	transmembrane receptor protein tyrosine ...	17735	18	196	2	0.2	0.01729	10	PTPRF;PTPRN
pink	MF	GO:0019198	transmembrane receptor protein phosphata...	17735	18	196	2	0.2	0.01729	10	PTPRF;PTPRN
pink	MF	GO:0030506	ankyrin binding	17735	18	196	2	0.2	0.01729	10	RHAG;SLC4A1
pink	MF	GO:1901618	organic hydroxy compound transmembrane t...	17735	18	196	2	0.2	0.01729	10	AQP1;SLC6A8
pink	BP	GO:0051329	interphase of mitotic cell cycle	17735	408	196	10	4.6	0.0174	2.173913	BCL2L1;CDC34;E2F2;GSPT1;KANK2;RBM38;TFDP1;UBA52;UBB;WNT9A
pink	BP	GO:0008015	blood circulation	17735	349	196	9	3.94	0.01756	2.284264	FKBP1B;GPX1;HBB;KCNH2;KEL;MYL4;NEDD4L;PDE3A;UTS2R
pink	BP	GO:0003013	circulatory system process	17735	350	196	9	3.95	0.01785	2.278481	FKBP1B;GPX1;HBB;KCNH2;KEL;MYL4;NEDD4L;PDE3A;UTS2R
pink	MF	GO:0015370	solute:sodium symporter activity	17735	49	196	3	0.55	0.01792	5.454545	SLC6A19;SLC6A8;SLC6A9
pink	CC	GO:0005737	cytoplasm	17735	9016	196	117	102.25	0.0181	1.144254	A4GALT;ABCC4;ACHE;AHSP;ALAS2;ANK1;AQP1;ARHGEF37;BAG1;BAMBI;BCL2L1;BLVRB;C1orf116;C9orf3;C9
pink	BP	GO:0055082	cellular chemical homeostasis	17735	665	196	14	7.5	0.01833	1.866667	ABCG2;ALAS2;BCL2L1;BEST2;FKBP1B;FTL;KCNH2;NEDD4L;RHAG;RNF10;SLC4A1;SNCA;TGM2;UTS2R
pink	CC	GO:0031461	cullin-RING ubiquitin ligase complex	17735	89	196	4	1.01	0.0185	3.960396	DCAF12;DCAF6;FBXO17;FBXO9
pink	BP	GO:0071300	cellular response to retinoic acid	17735	50	196	3	0.56	0.01875	5.357143	AQP1;TESC;WNT9A
pink	BP	GO:0090150	establishment of protein localization to...	17735	50	196	3	0.56	0.01875	5.357143	ANK1;BCL2L1;TESC
pink	BP	GO:0051325	interphase	17735	414	196	10	4.67	0.01905	2.141328	BCL2L1;CDC34;E2F2;GSPT1;KANK2;RBM38;TFDP1;UBA52;UBB;WNT9A
pink	BP	GO:0000413	protein peptidyl-prolyl isomerization	17735	19	196	2	0.21	0.01906	9.52381	FKBP1B;FKBP8
pink	BP	GO:0006972	hyperosmotic response	17735	19	196	2	0.21	0.01906	9.52381	AQP1;CSDA
pink	BP	GO:0021591	ventricular system development	17735	19	196	2	0.21	0.01906	9.52381	AQP1;DPCD
pink	BP	GO:0045648	positive regulation of erythrocyte diffe...	17735	19	196	2	0.21	0.01906	9.52381	GATA1;TAL1
pink	MF	GO:0051537	2 iron, 2 sulfur cluster binding	17735	19	196	2	0.21	0.01918	9.52381	FECH;GLRX5
pink	BP	GO:0016567	protein ubiquitination	17735	540	196	12	6.09	0.01938	1.970443	CDC34;DCAF12;DCAF6;FBXO9;MARCH2;MKRN1;NEDD4L;RAG1;SHARPIN;SIAH2;UBA52;UBB
pink	BP	GO:0016044	cellular membrane organization	17735	417	196	10	4.7	0.01992	2.12766	A4GALT;ANK1;BCL2L1;FTL;SNCA;SPTA1;TESC;UBA52;UBB;VTI1B
pink	BP	GO:0007093	mitotic cell cycle checkpoint	17735	138	196	5	1.56	0.02006	3.205128	BCL2L1;RBM38;UBA52;UBB;WNT9A
pink	BP	GO:0044707	single-multicellular organism process	17735	5398	196	74	60.88	0.02102	1.215506	ABCC4;ACHE;AHSP;ALAS2;ANK1;AQP1;BAMBI;BCL2L1;BEST2;BPGM;CNN1;CSDA;CTSE;DARC;DPCD;DYRK3;E(
pink	BP	GO:0001504	neurotransmitter uptake	17735	20	196	2	0.23	0.02103	8.695652	GFAP;SNCA
pink	BP	GO:0006595	polyamine metabolic process	17735	20	196	2	0.23	0.02103	8.695652	OAZ1;ODC1
pink	BP	GO:0007220	Notch receptor processing	17735	20	196	2	0.23	0.02103	8.695652	UBA52;UBB
pink	BP	GO:0014009	glial cell proliferation	17735	20	196	2	0.23	0.02103	8.695652	GFAP;RNF10
pink	BP	GO:0046688	response to copper ion	17735	20	196	2	0.23	0.02103	8.695652	AQP1;SNCA
pink	BP	GO:0075733	intracellular transport of viral materia...	17735	20	196	2	0.23	0.02103	8.695652	UBA52;UBB
pink	BP	GO:0061024	membrane organization	17735	421	196	10	4.75	0.02112	2.105263	A4GALT;ANK1;BCL2L1;FTL;SNCA;SPTA1;TESC;UBA52;UBB;VTI1B
pink	MF	GO:0005217	intracellular ligand-gated ion channel a...	17735	20	196	2	0.23	0.02115	8.695652	AQP1;FKBP1B
pink	MF	GO:0043236	laminin binding	17735	20	196	2	0.23	0.02115	8.695652	ACHE;BCAM
pink	BP	GO:0006458	'de novo' protein folding	17735	53	196	3	0.6	0.02187	5	FKBP1B;TUBB2A;TUBB2B
pink	MF	GO:0003729	mRNA binding	17735	94	196	4	1.06	0.02196	3.773585	CSDA;FECH;IGF2BP2;RBM38
pink	BP	GO:0010941	regulation of cell death	17735	1242	196	22	14.01	0.02266	1.570307	AQP1;BAG1;BCL2L1;CSDA;FHL2;GATA1;GPX1;HBA2;HBB;IFI27;KANK2;PDE3A;PRDX5;RAG1;SFRP2;SIAH2;SN(
pink	MF	GO:0046943	carboxylic acid transmembrane transporte...	17735	95	196	4	1.07	0.02273	3.738318	SLC38A5;SLC6A19;SLC6A8;SLC6A9
pink	BP	GO:0043123	positive regulation of I-kappaB kinase/N...	17735	143	196	5	1.61	0.02299	3.10559	PPM1A;SHARPIN;TGM2;UBA52;UBB

pink	BP	GO:0001881	receptor recycling	17735	21	196	2	0.24	0.02307	8.333333	ACHE;SNCA
pink	BP	GO:0009651	response to salt stress	17735	21	196	2	0.24	0.02307	8.333333	AQP1;NEDD4L
pink	BP	GO:0051354	negative regulation of oxidoreductase ac...	17735	21	196	2	0.24	0.02307	8.333333	PRDX5;SNCA
pink	BP	GO:2000736	regulation of stem cell differentiation	17735	21	196	2	0.24	0.02307	8.333333	SOX6;TAL1
pink	MF	GO:0008200	ion channel inhibitor activity	17735	21	196	2	0.24	0.02321	8.333333	FKBP1B;NEDD4L
pink	MF	GO:0016248	channel inhibitor activity	17735	21	196	2	0.24	0.02321	8.333333	FKBP1B;NEDD4L
pink	MF	GO:0019894	kinesin binding	17735	21	196	2	0.24	0.02321	8.333333	KLC3;SNCA
pink	MF	GO:0043027	cysteine-type endopeptidase inhibitor ac...	17735	21	196	2	0.24	0.02321	8.333333	PRDX5;SNCA
pink	CC	GO:0080008	Cul4-RING ubiquitin ligase complex	17735	21	196	2	0.24	0.0233	8.333333	DCAF12;DCAF6
pink	BP	GO:0045892	negative regulation of transcription, DN...	17735	823	196	16	9.28	0.02361	1.724138	CSDA;FHL2;HBZ;KANK2;MXI1;NEDD4L;NFIX;PPM1A;PRDX5;SFRP2;SIM2;SOX6;TAL1;UBA52;UBB;YBX1
pink	BP	GO:0006367	transcription initiation from RNA polyme...	17735	197	196	6	2.22	0.02429	2.702703	E2F2;NEDD4L;PPM1A;TFDP1;UBA52;UBB
pink	BP	GO:0090287	regulation of cellular response to growt...	17735	146	196	5	1.65	0.02487	3.030303	BAMBI;NEDD4L;PPM1A;UBA52;UBB
pink	MF	GO:0005342	organic acid transmembrane transporter a...	17735	98	196	4	1.11	0.02513	3.603604	SLC38A5;SLC6A19;SLC6A8;SLC6A9
pink	MF	GO:0004842	ubiquitin-protein ligase activity	17735	253	196	7	2.86	0.02513	2.447552	CDC34;FBXO7;FBXO9;MARCH2;NEDD4L;RAG1;SIAH2
pink	BP	GO:0035412	regulation of catenin import into nucleu...	17735	22	196	2	0.25	0.02519	8	BAMBI;SFRP2
pink	BP	GO:0060291	long-term synaptic potentiation	17735	22	196	2	0.25	0.02519	8	GFAP;SNCA
pink	BP	GO:0043271	negative regulation of ion transport	17735	56	196	3	0.63	0.02525	4.761905	FKBP1B;NEDD4L;SNCA
pink	BP	GO:0071705	nitrogen compound transport	17735	562	196	12	6.34	0.02547	1.892744	ABCG2;AQP1;FKBP1B;HBB;NUDT4;RHAG;SLC14A1;SLC38A5;SLC6A19;SLC6A8;SNCA;XK
pink	BP	GO:2000021	regulation of ion homeostasis	17735	147	196	5	1.66	0.02552	3.012048	FKBP1B;KCNH2;RNF10;SNCA;UTS2R
pink	CC	GO:0005903	brush border	17735	56	196	3	0.64	0.0257	4.6875	AQP1;ESPN;ITLN1
pink	BP	GO:0071310	cellular response to organic substance	17735	1333	196	23	15.03	0.02614	1.530273	ADIPOR1;AQP1;BAMBI;BCL2L1;CSDA;DARC;FKBP1B;GATA1;IFI27;NEDD4L;PDE3A;PPM1A;PTPRN;SHARPIN;S
pink	MF	GO:0022857	transmembrane transporter activity	17735	901	196	17	10.19	0.02655	1.668302	ABCC13;ABCC4;ABCG2;AQP1;BEST2;FKBP1B;KCNH2;RHAG;RHCE;SLC14A1;SLC25A37;SLC2A4;SLC38A5;SLC4
pink	BP	GO:0070887	cellular response to chemical stimulus	17735	1709	196	28	19.27	0.0268	1.453036	ADIPOR1;AQP1;BAMBI;BCL2L1;CSDA;DARC;FKBP1B;GATA1;GPX1;HBA2;HBB;IFI27;NEDD4L;PDE3A;PPM1A;P
pink	BP	GO:0032409	regulation of transporter activity	17735	101	196	4	1.14	0.02739	3.508772	FKBP1B;NEDD4L;SNCA;TESC
pink	BP	GO:0048523	negative regulation of cellular process	17735	2883	196	43	32.52	0.02749	1.322263	ACHE;AQP1;BAG1;BAMBI;BCL2L1;CDC34;CSDA;EPB49;ESPN;FHL2;FKBP1B;GATA1;GFAP;GPX1;HBZ;IGF2BP2;
pink	MF	GO:0016755	transferase activity, transferring amino...	17735	23	196	2	0.26	0.02755	7.692308	EPB42;TGM2
pink	BP	GO:0031646	positive regulation of neurological syst...	17735	59	196	3	0.67	0.0289	4.477612	GFAP;RNF10;SNCA
pink	BP	GO:0032446	protein modification by small protein co...	17735	573	196	12	6.46	0.029	1.857585	CDC34;DCAF12;DCAF6;FBXO9;MARCH2;MKRN1;NEDD4L;RAG1;SHARPIN;SIAH2;UBA52;UBB
pink	BP	GO:0071214	cellular response to abiotic stimulus	17735	153	196	5	1.73	0.02965	2.890173	AQP1;BCL2L1;CSDA;SFRP2;SLC2A4
pink	BP	GO:0030104	water homeostasis	17735	24	196	2	0.27	0.02966	7.407407	AQP1;NEDD4L
pink	BP	GO:0035411	catenin import into nucleus	17735	24	196	2	0.27	0.02966	7.407407	BAMBI;SFRP2
pink	BP	GO:0015698	inorganic anion transport	17735	60	196	3	0.68	0.03018	4.411765	AQP1;RHAG;SLC4A1
pink	BP	GO:0051591	response to cAMP	17735	60	196	3	0.68	0.03018	4.411765	AQP1;PDE3A;PTPRN
pink	MF	GO:0016491	oxidoreductase activity	17735	709	196	14	8.02	0.0305	1.745636	ABCC4;BLVRB;C5orf4;ECSIT;FTL;GLRX5;GMPR;GPX1;HBA2;HBB;PRDX5;PRDX6;SNCA;TSTA3
pink	BP	GO:0006875	cellular metal ion homeostasis	17735	324	196	8	3.65	0.03066	2.191781	ABCG2;ALAS2;FKBP1B;FTL;NEDD4L;SNCA;TGM2;UTS2R
pink	BP	GO:0044057	regulation of system process	17735	448	196	10	5.05	0.03066	1.980198	ACHE;AQP1;CNN1;FKBP1B;GFAP;KCNH2;MYL4;RNF10;SNCA;UTS2R
pink	BP	GO:0044699	single-organism process	17735	10656	196	131	120.18	0.03089	1.090032	A4GALT;ABCC4;ABCG2;ACHE;ADIPOR1;AHSP;ALAS2;ANK1;AQP1;ARHGEF37;BAG1;BAMBI;BCAM;BCL2L1;BE
pink	BP	GO:0051253	negative regulation of RNA metabolic pro...	17735	852	196	16	9.61	0.03119	1.664932	CSDA;FHL2;HBZ;KANK2;MXI1;NEDD4L;NFIX;PPM1A;PRDX5;SFRP2;SIM2;SOX6;TAL1;UBA52;UBB;YBX1
pink	BP	GO:0048519	negative regulation of biological proces...	17735	3150	196	46	35.53	0.03134	1.294681	ACHE;AQP1;BAG1;BAMBI;BCL2L1;CDC34;CSDA;EPB49;ESPN;FHL2;FKBP1B;GATA1;GFAP;GPX1;HBZ;IGF2BP2;
pink	BP	GO:2000113	negative regulation of cellular macromol...	17735	923	196	17	10.41	0.0314	1.633045	CSDA;FHL2;HBZ;IGF2BP2;KANK2;MXI1;NEDD4L;NFIX;PPM1A;PRDX5;SFRP2;SIM2;SOX6;TAL1;UBA52;UBB;YB
pink	BP	GO:0045639	positive regulation of myeloid cell diff...	17735	61	196	3	0.69	0.03149	4.347826	GATA1;TAL1;TESC
pink	BP	GO:0006811	ion transport	17735	995	196	18	11.22	0.03168	1.604278	ANK1;AQP1;CA1;FKBP1B;FTL;HBA2;HBB;KCNH2;NEDD4L;RHAG;SLC25A37;SLC38A5;SLC4A1;SLC6A19;SLC6A
pink	BP	GO:0051693	actin filament capping	17735	25	196	2	0.28	0.03201	7.142857	EPB49;SPTA1
pink	BP	GO:0055001	muscle cell development	17735	157	196	5	1.77	0.03262	2.824859	FHL2;GPX1;MBNL3;RBM38;TMOD1
pink	BP	GO:0008219	cell death	17735	1741	196	28	19.64	0.03308	1.425662	AQP1;BAG1;BCL2L1;CSDA;E2F2;FBXO7;FHL2;FKBP8;FTL;GATA1;GPX1;HBA2;HBB;IFI27;KANK2;PDE3A;PRDX5
pink	BP	GO:0043086	negative regulation of catalytic activit...	17735	519	196	11	5.85	0.03325	1.880342	AQP1;FKBP1B;GPX1;PRDX5;RAG1;SFRP2;SIAH2;SNCA;UBA52;UBB;WNT9A
pink	BP	GO:0051258	protein polymerization	17735	158	196	5	1.78	0.03339	2.808989	EPB49;SNCA;SPTA1;TUBB2A;TUBB2B
pink	MF	GO:0019787	small conjugating protein ligase activit...	17735	269	196	7	3.04	0.03348	2.302632	CDC34;FBXO7;FBXO9;MARCH2;NEDD4L;RAG1;SIAH2
pink	BP	GO:0016265	death	17735	1743	196	28	19.66	0.03351	1.424212	AQP1;BAG1;BCL2L1;CSDA;E2F2;FBXO7;FHL2;FKBP8;FTL;GATA1;GPX1;HBA2;HBB;IFI27;KANK2;PDE3A;PRDX5
pink	BP	GO:0048536	spleen development	17735	26	196	2	0.29	0.03442	6.896552	EPB42;PBX1
pink	MF	GO:0001158	enhancer sequence-specific DNA binding	17735	26	196	2	0.29	0.03462	6.896552	GATA1;TAL1
pink	CC	GO:0019005	SCF ubiquitin ligase complex	17735	26	196	2	0.29	0.0348	6.896552	FBXO17;FBXO9
pink	BP	GO:0009891	positive regulation of biosynthetic proc...	17735	1300	196	22	14.66	0.03541	1.500682	BAMBI;CSDA;DCAF6;FHL2;GATA1;HBB;KLF1;LYL1;NFIX;PBX1;PPM1A;PRDX5;RNF10;SFRP2;SNCA;SOX6;TAL1;
pink	BP	GO:0048641	regulation of skeletal muscle tissue dev...	17735	64	196	3	0.72	0.03558	4.166667	CSDA;MBNL3;RBM38
pink	CC	GO:0030666	endocytic vesicle membrane	17735	109	196	4	1.24	0.0356	3.225806	RILP;SGIP1;UBA52;UBB
pink	BP	GO:0051129	negative regulation of cellular componen...	17735	334	196	8	3.77	0.03573	2.122016	BCL2L1;EPB49;ESPN;GFAP;PPM1A;SFRP2;SNCA;SPTA1
pink	BP	GO:0051146	striated muscle cell differentiation	17735	216	196	6	2.44	0.0358	2.459016	FHL2;GPX1;MBNL3;RBM38;TMOD1;YBX1
pink	BP	GO:0050880	regulation of blood vessel size	17735	110	196	4	1.24	0.03587	3.225806	GPX1;HBB;KEL;UTS2R
pink	MF	GO:0022890	inorganic cation transmembrane transport...	17735	459	196	10	5.19	0.03605	1.926782	AQP1;FKBP1B;KCNH2;RHAG;RHCE;SLC14A1;SLC25A37;SLC6A19;SLC6A8;SLC6A9
pink	BP	GO:0050878	regulation of body fluid levels	17735	594	196	12	6.7	0.03671	1.791045	ABCC4;AQP1;EHD2;GATA1;HBB;HBD;HBG1;HBG2;HPS1;KRT1;PDE3A;UTS2R
pink	BP	GO:0044767	single-organism developmental process	17735	3427	196	49	38.65	0.03671	1.267788	ACHE;AHSP;ALAS2;ANK1;AQP1;BAMBI;BCL2L1;CSDA;DPCD;DYRK3;ECSIT;EPB42;FECH;FHL2;GATA1;GFAP;GL
pink	BP	GO:0035150	regulation of tube size	17735	111	196	4	1.25	0.0369	3.2	GPX1;HBB;KEL;UTS2R
pink	BP	GO:0006656	phosphatidylcholine biosynthetic process	17735	27	196	2	0.3	0.03691	6.666667	ACHE;CHPT1
pink	MF	GO:0008514	organic anion transmembrane transporter ...	17735	112	196	4	1.27	0.03833	3.149606	SLC38A5;SLC6A19;SLC6A8;SLC6A9
pink	MF	GO:0015144	carbohydrate transmembrane transporter a...	17735	28	196	2	0.32	0.03968	6.25	AQP1;SLC2A4
pink	MF	GO:1901476	carbohydrate transporter activity	17735	28	196	2	0.32	0.03968	6.25	AQP1;SLC2A4
pink	CC	GO:0030016	myofibril	17735	165	196	5	1.87	0.04	2.673797	ANK1;FHL2;MYL4;SLC4A1;TMOD1
pink	MF	GO:0019904	protein domain specific binding	17735	535	196	11	6.05	0.04089	1.818182	ARHGEF37;BCL2L1;EHD2;ESPN;GATA1;GPX1;SFRP2;SGIP1;SNCA;TFDP1;TGM2
pink	MF	GO:0051015	actin filament binding	17735	68	196	3	0.77	0.04177	3.896104	ESPN;MYL4;SPTA1

pink	MF	GO:0005488	binding	17735	11799	196	143	133.49	0.04353	1.071241	ABCC13;ABCC4;ABCG2;ACHE;ADIPOR1;AHSP;ALAS2;ANK1;AQP1;ARHGEF37;BAG1;BAMBI;BCAM;BCL2L1;BL
pink	MF	GO:0030551	cyclic nucleotide binding	17735	30	196	2	0.34	0.04501	5.882353	FKBP1B;PDE3A
pink	MF	GO:0035326	enhancer binding	17735	30	196	2	0.34	0.04501	5.882353	GATA1;TAL1
pink	CC	GO:0031226	intrinsic to plasma membrane	17735	1252	196	21	14.2	0.046	1.478873	AQP1;BCAM;GYPA;GYPB;GYPC;HPS1;KCNH2;PTPRF;PTPRN;RHAG;RHCE;SLC14A1;SLC2A4;SLC4A1;SLC6A19;S
pink	MF	GO:0003779	actin binding	17735	351	196	8	3.97	0.04637	2.015113	CNN1;EPB49;ESPN;MYL4;SLC4A1;SPTA1;TMOD1;TNS1
pink	MF	GO:0005198	structural molecule activity	17735	620	196	12	7.01	0.04923	1.71184	ANK1;EPB42;GFAP;KRT1;KRT79;MYL4;RPL3L;SPTA1;TUBB2A;TUBB2B;UBA52;WNT9A
pink	CC	GO:0043292	contractile fiber	17735	175	196	5	1.98	0.0493	2.525253	ANK1;FHL2;MYL4;SLC4A1;TMOD1
pink	CC	GO:0016020	membrane	17735	7690	196	99	87.21	0.0494	1.135191	A4GALT;ABCC13;ABCC4;ABCG2;ACHE;ADIPOR1;ALAS2;ANK1;AQP1;BAMBI;BCAM;BCL2L1;BEST2;BLVRB;C19
pink	CC	GO:0044444	cytoplasmic part	17735	6562	196	86	74.42	0.0497	1.155603	A4GALT;ABCC4;ACHE;AHSP;ALAS2;ANK1;BCL2L1;BLVRB;CA1;CHPT1;CSDA;CTSE;DPM2;ECSIT;EHD2;EMID1;E
midnightb	BP	GO:0034645	cellular macromolecule biosynthetic proc...	17735	4170	65	37	16.63	2.80E-08	2.224895	ATF7IP2;CAPRIN2;CARS2;DDX5;GATC;GBGT1;HNRPDL;MRPS25;MTERFD2;NR3C2;PAIP2B;PER3;PIGG;PIGL;PT
midnightb	BP	GO:0009059	macromolecule biosynthetic process	17735	4294	65	37	17.13	6.50E-08	2.159953	ATF7IP2;CAPRIN2;CARS2;DDX5;GATC;GBGT1;HNRPDL;MRPS25;MTERFD2;NR3C2;PAIP2B;PER3;PIGG;PIGL;PT
midnightb	BP	GO:0044249	cellular biosynthetic process	17735	5073	65	39	20.23	5.00E-07	1.92783	ATF7IP2;CAPRIN2;CARS2;DDX5;GATC;GBGT1;GGT1;HNRPDL;MRPS25;MTERFD2;NR3C2;PAIP2B;PER3;PIGG;P
midnightb	BP	GO:0010467	gene expression	17735	4403	65	36	17.56	5.30E-07	2.050114	ADAT2;ATF7IP2;CAPRIN2;CARS2;DDX5;GATC;HNRPDL;MRPS25;MTERFD2;NR3C2;PAIP2B;PER3;PTCD3;S1PR1
midnightb	BP	GO:1901576	organic substance biosynthetic process	17735	5154	65	39	20.56	8.00E-07	1.896887	ATF7IP2;CAPRIN2;CARS2;DDX5;GATC;GBGT1;GGT1;HNRPDL;MRPS25;MTERFD2;NR3C2;PAIP2B;PER3;PIGG;P
midnightb	BP	GO:0009058	biosynthetic process	17735	5219	65	39	20.82	1.20E-06	1.873199	ATF7IP2;CAPRIN2;CARS2;DDX5;GATC;GBGT1;GGT1;HNRPDL;MRPS25;MTERFD2;NR3C2;PAIP2B;PER3;PIGG;P
midnightb	BP	GO:0016070	RNA metabolic process	17735	3911	65	32	15.6	4.80E-06	2.051282	ADAT2;ATF7IP2;CAPRIN2;CARS2;DDX5;GATC;HNRPDL;MTERFD2;NR3C2;PER3;S1PR1;SCML4;SERTAD3;SRSF1
midnightb	BP	GO:2000112	regulation of cellular macromolecule bio...	17735	3117	65	28	12.43	5.10E-06	2.252615	ATF7IP2;CAPRIN2;DDX5;GATC;HNRPDL;PAIP2B;PER3;PTCD3;S1PR1;SCML4;SERTAD3;STAT4;TARBP1;TRAF5;U
midnightb	BP	GO:0010468	regulation of gene expression	17735	3347	65	29	13.35	6.40E-06	2.172285	ATF7IP2;CAPRIN2;DDX5;GATC;HNRPDL;PAIP2B;PER3;PTCD3;S1PR1;SCML4;SERTAD3;STAT4;TARBP1;TBRG1;
midnightb	BP	GO:0010556	regulation of macromolecule biosynthetic...	17735	3197	65	28	12.75	8.60E-06	2.196078	ATF7IP2;CAPRIN2;DDX5;GATC;HNRPDL;PAIP2B;PER3;PTCD3;S1PR1;SCML4;SERTAD3;STAT4;TARBP1;TRAF5;U
midnightb	BP	GO:0031326	regulation of cellular biosynthetic proc...	17735	3328	65	28	13.27	1.90E-05	2.110023	ATF7IP2;CAPRIN2;DDX5;GATC;HNRPDL;PAIP2B;PER3;PTCD3;S1PR1;SCML4;SERTAD3;STAT4;TARBP1;TRAF5;U
midnightb	BP	GO:0009889	regulation of biosynthetic process	17735	3356	65	28	13.39	2.30E-05	2.091113	ATF7IP2;CAPRIN2;DDX5;GATC;HNRPDL;PAIP2B;PER3;PTCD3;S1PR1;SCML4;SERTAD3;STAT4;TARBP1;TRAF5;U
midnightb	BP	GO:0090304	nucleic acid metabolic process	17735	4431	65	33	17.67	2.60E-05	1.867572	ADAT2;ATF7IP2;CAPRIN2;CARS2;DDX5;GATC;HNRPDL;MTERFD2;NR3C2;PER3;S1PR1;SCML4;SERTAD3;SRSF1
midnightb	BP	GO:0006351	transcription, DNA-dependent	17735	3208	65	27	12.8	3.00E-05	2.109375	ATF7IP2;CAPRIN2;DDX5;HNRPDL;MTERFD2;NR3C2;PER3;S1PR1;SCML4;SERTAD3;SRSF1;STAT4;TARBP1;TRA
midnightb	MF	GO:0003676	nucleic acid binding	17735	3299	65	26	12.22	3.70E-05	2.12766	CAPRIN2;DDX5;HNRPDL;MTERFD2;NR3C2;PAIP2B;PTCD3;SCML4;SRSF1;STAT4;TARBP1;TSEN2;UPF3A;ZBTB2
midnightb	BP	GO:0032774	RNA biosynthetic process	17735	3293	65	27	13.13	5.00E-05	2.056359	ATF7IP2;CAPRIN2;DDX5;HNRPDL;MTERFD2;NR3C2;PER3;S1PR1;SCML4;SERTAD3;SRSF1;STAT4;TARBP1;TRA
midnightb	BP	GO:0044271	cellular nitrogen compound biosynthetic ...	17735	3726	65	29	14.86	5.70E-05	1.951548	ATF7IP2;CAPRIN2;DDX5;GGT1;HNRPDL;MTERFD2;NR3C2;PER3;S1PR1;SCML4;SERTAD3;SLC11A2;SRSF1;STA
midnightb	BP	GO:0044260	cellular macromolecule metabolic process	17735	6621	65	41	26.41	9.10E-05	1.552442	ADAT2;ATF7IP2;CAPRIN2;CARS2;DDX5;FES;GADD45B;GATC;GBGT1;HNRPDL;MRPS25;MTERFD2;NR3C2;PAIP
midnightb	BP	GO:0019438	aromatic compound biosynthetic process	17735	3638	65	28	14.51	0.00011	1.929704	ATF7IP2;CAPRIN2;DDX5;HNRPDL;MTERFD2;NR3C2;PER3;S1PR1;SCML4;SERTAD3;SLC11A2;SRSF1;STAT4;TAR
midnightb	BP	GO:0018130	heterocycle biosynthetic process	17735	3650	65	28	14.56	0.00011	1.923077	ATF7IP2;CAPRIN2;DDX5;HNRPDL;MTERFD2;NR3C2;PER3;S1PR1;SCML4;SERTAD3;SLC11A2;SRSF1;STAT4;TAR
midnightb	BP	GO:0006355	regulation of transcription, DNA-depende...	17735	2869	65	24	11.44	0.00013	2.097902	ATF7IP2;CAPRIN2;DDX5;HNRPDL;PER3;S1PR1;SCML4;SERTAD3;STAT4;TARBP1;TRAF5;ZBTB24;ZFP28;ZMYND
midnightb	BP	GO:2001141	regulation of RNA biosynthetic process	17735	2889	65	24	11.52	0.00014	2.083333	ATF7IP2;CAPRIN2;DDX5;HNRPDL;PER3;S1PR1;SCML4;SERTAD3;STAT4;TARBP1;TRAF5;ZBTB24;ZFP28;ZMYND
midnightb	MF	GO:0008270	zinc ion binding	17735	1977	65	18	7.32	0.00018	2.459016	ADAT2;CA5B;NR3C2;TRAF5;ZBTB24;ZFP28;ZMYND15;ZNF234;ZNF251;ZNF331;ZNF514;ZNF528;ZNF548;ZNF
midnightb	BP	GO:0019222	regulation of metabolic process	17735	4834	65	33	19.28	0.00018	1.711618	ATF7IP2;CAPRIN2;CNST;DDX5;GADD45B;GATC;HNRPDL;MOAP1;PAIP2B;PER3;PTCD3;S1PR1;SCML4;SERTAD
midnightb	BP	GO:1901362	organic cyclic compound biosynthetic pro...	17735	3752	65	28	14.97	0.00019	1.870407	ATF7IP2;CAPRIN2;DDX5;HNRPDL;MTERFD2;NR3C2;PER3;S1PR1;SCML4;SERTAD3;SLC11A2;SRSF1;STAT4;TAR
midnightb	BP	GO:0060255	regulation of macromolecule metabolic pr...	17735	4178	65	30	16.66	0.00019	1.80072	ATF7IP2;CAPRIN2;DDX5;GADD45B;GATC;HNRPDL;PAIP2B;PER3;PTCD3;S1PR1;SCML4;SERTAD3;STAT4;TARB
midnightb	BP	GO:0051252	regulation of RNA metabolic process	17735	2953	65	24	11.78	2.00E-04	2.037351	ATF7IP2;CAPRIN2;DDX5;HNRPDL;PER3;S1PR1;SCML4;SERTAD3;STAT4;TARBP1;TRAF5;ZBTB24;ZFP28;ZMYND
midnightb	BP	GO:0034654	nucleobase-containing compound biosynthe...	17735	3583	65	27	14.29	0.00023	1.889433	ATF7IP2;CAPRIN2;DDX5;HNRPDL;MTERFD2;NR3C2;PER3;S1PR1;SCML4;SERTAD3;SRSF1;STAT4;TARBP1;TRA
midnightb	CC	GO:0043231	intracellular membrane-bounded organelle	17735	9560	65	49	35.38	0.00024	1.384963	ATF7IP2;CA5B;CAPRIN2;CARS2;CNST;DDX5;FES;GADD45B;GATC;GBGT1;GOLGA8A;HK3;HNRPDL;MRPS25;M
midnightb	CC	GO:0043229	intracellular organelle	17735	10522	65	52	38.94	0.00024	1.335388	ATF7IP2;CA5B;CAPRIN2;CARS2;CNST;DDX5;FES;GADD45B;GATC;GBGT1;GOLGA8A;HAUS4;HK3;HNRPDL;MR
midnightb	CC	GO:0043227	membrane-bounded organelle	17735	9590	65	49	35.49	0.00026	1.380671	ATF7IP2;CA5B;CAPRIN2;CARS2;CNST;DDX5;FES;GADD45B;GATC;GBGT1;GOLGA8A;HK3;HNRPDL;MRPS25;M
midnightb	CC	GO:0043226	organelle	17735	10552	65	52	39.05	0.00027	1.331626	ATF7IP2;CA5B;CAPRIN2;CARS2;CNST;DDX5;FES;GADD45B;GATC;GBGT1;GOLGA8A;HAUS4;HK3;HNRPDL;MR
midnightb	CC	GO:0005622	intracellular	17735	12344	65	57	45.68	0.00029	1.247811	ATF7IP2;CA5B;CAPRIN2;CARS2;CNST;DDX5;FES;GADD45B;GATC;GBGT1;GOLGA8A;HAUS4;HK3;HNRPDL;KCT
midnightb	BP	GO:0044237	cellular metabolic process	17735	8597	65	47	34.29	0.00032	1.370662	ADAT2;ATF7IP2;C5orf63;CA5B;CAPRIN2;CARS2;CNST;DDX5;FES;GADD45B;GATC;GBGT1;GGT1;HK3;HNRPDL
midnightb	BP	GO:0031323	regulation of cellular metabolic process	17735	4397	65	30	17.54	5.00E-04	1.710376	ATF7IP2;CAPRIN2;CNST;DDX5;GADD45B;GATC;HNRPDL;PAIP2B;PER3;PTCD3;S1PR1;SCML4;SERTAD3;STAT4
midnightb	BP	GO:0046483	heterocycle metabolic process	17735	5361	65	34	21.38	6.00E-04	1.590271	ADAT2;ATF7IP2;CAPRIN2;CARS2;DDX5;GATC;HNRPDL;MTERFD2;NR3C2;PER3;S1PR1;SCML4;SERTAD3;SLC11
midnightb	BP	GO:0034641	cellular nitrogen compound metabolic pro...	17735	5595	65	35	22.32	6.00E-04	1.5681	ADAT2;ATF7IP2;CAPRIN2;CARS2;DDX5;GATC;GGT1;HNRPDL;MTERFD2;NR3C2;PER3;S1PR1;SCML4;SERTAD3
midnightb	BP	GO:0006725	cellular aromatic compound metabolic pro...	17735	5371	65	34	21.42	0.00063	1.587302	ADAT2;ATF7IP2;CAPRIN2;CARS2;DDX5;GATC;HNRPDL;MTERFD2;NR3C2;PER3;S1PR1;SCML4;SERTAD3;SLC11
midnightb	MF	GO:0046914	transition metal ion binding	17735	2217	65	18	8.21	0.00074	2.192448	ADAT2;CA5B;NR3C2;TRAF5;ZBTB24;ZFP28;ZMYND15;ZNF234;ZNF251;ZNF331;ZNF514;ZNF528;ZNF548;ZNF
midnightb	MF	GO:0097159	organic cyclic compound binding	17735	5372	65	32	19.9	0.00077	1.60804	CAPRIN2;CARS2;DDX5;FES;GATC;HK3;HNRPDL;MTERFD2;NR3C2;OSBPL3;PAIP2B;PTCD3;SCML4;SEPT8;SRSF
midnightb	BP	GO:0006139	nucleobase-containing compound metabolic...	17735	5209	65	33	20.78	0.00083	1.588065	ADAT2;ATF7IP2;CAPRIN2;CARS2;DDX5;GATC;HNRPDL;MTERFD2;NR3C2;PER3;S1PR1;SCML4;SERTAD3;SRSF1
midnightb	BP	GO:0080090	regulation of primary metabolic process	17735	4337	65	29	17.3	0.00097	1.676301	ATF7IP2;CAPRIN2;DDX5;GADD45B;GATC;HNRPDL;PAIP2B;PER3;PTCD3;S1PR1;SCML4;SERTAD3;STAT4;TARB
midnightb	BP	GO:0009247	glycolipid biosynthetic process	17735	51	65	3	0.2	0.00109	15	GBGT1;PIGG;PIGL
midnightb	BP	GO:0006417	regulation of translation	17735	200	65	5	0.8	0.00119	6.25	CAPRIN2;GATC;PAIP2B;PTCD3;UPF3A
midnightb	BP	GO:0043170	macromolecule metabolic process	17735	7334	65	41	29.25	0.00136	1.401709	ADAT2;ATF7IP2;CAPRIN2;CARS2;DDX5;FES;GADD45B;GATC;GBGT1;HNRPDL;MRPS25;MTERFD2;NR3C2;PAIP
midnightb	BP	GO:0006399	tRNA metabolic process	17735	121	65	4	0.48	0.00137	8.333333	ADAT2;CARS2;GATC;TSEN2
midnightb	BP	GO:1901360	organic cyclic compound metabolic proces...	17735	5578	65	34	22.25	0.00137	1.52809	ADAT2;ATF7IP2;CAPRIN2;CARS2;DDX5;GATC;HNRPDL;MTERFD2;NR3C2;PER3;S1PR1;SCML4;SERTAD3;SLC11
midnightb	MF	GO:1901363	heterocyclic compound binding	17735	5302	65	31	19.64	0.00147	1.578411	CAPRIN2;CARS2;DDX5;FES;GATC;HK3;HNRPDL;MTERFD2;NR3C2;PAIP2B;PTCD3;SCML4;SEPT8;SRSF1;STAT4;
midnightb	CC	GO:0044424	intracellular part	17735	12103	65	55	44.79	0.00152	1.227953	ATF7IP2;CA5B;CAPRIN2;CARS2;CNST;DDX5;FES;GADD45B;GATC;GBGT1;GOLGA8A;HAUS4;HK3;HNRPDL;KCT
midnightb	BP	GO:0032543	mitochondrial translation	17735	15	65	2	0.06	0.00159	33.33333	GATC;PTCD3
midnightb	BP	GO:0016254	preassembly of GPI anchor in ER membrane	17735	16	65	2	0.06	0.00181	33.33333	PIGG;PIGL
midnightb	BP	GO:0019219	regulation of nucleobase-containing comp...	17735	3414	65	24	13.62	0.00185	1.762115	ATF7IP2;CAPRIN2;DDX5;HNRPDL;PER3;S1PR1;SCML4;SERTAD3;STAT4;TARBP1;TRAF5;ZBTB24;ZFP28;ZMYND
midnightb	BP	GO:0006807	nitrogen compound metabolic process	17735	5942	65	35	23.7	0.00211	1.476793	ADAT2;ATF7IP2;CAPRIN2;CARS2;DDX5;GATC;GGT1;HNRPDL;MTERFD2;NR3C2;PER3;S1PR1;SCML4;SERTAD3
midnightb	BP	GO:0008152	metabolic process	17735	9452	65	48	37.7	0.00231	1.27321	ADAT2;ATF7IP2;C5orf63;CA5B;CAPRIN2;CARS2;CNST;DDX5;FES;GADD45B;GATC;GBGT1;GGT1;HK3;HNRPDL
midnightb	BP	GO:0006412	translation	17735	464	65	7	1.85	0.00237	3.783784	CAPRIN2;CARS2;GATC;MRPS25;PAIP2B;PTCD3;UPF3A
midnightb	CC	GO:0005634	nucleus	17735	5873	65	33	21.73	0.0025	1.518638	ATF7IP2;DDX5;GADD45B;HNRPDL;NARF;NR3C2;OFD1;OSBPL3;PER3;S100PBP;SCML4;SERTAD3;SLC11A2;SRS
midnightb	BP	GO:0051171	regulation of nitrogen compound metaboli...	17735	3496	65	24	13.94	0.00259	1.721664	ATF7IP2;CAPRIN2;DDX5;HNRPDL;PER3;S1PR1;SCML4;SERTAD3;STAT4;TARBP1;TRAF5;ZBTB24;ZFP28;ZMYND
midnightb	BP	GO:0010608	posttranscriptional regulation of gene e...	17735	355	65	6	1.42	0.00282	4.225352	CAPRIN2;GATC;PAIP2B;PTCD3;TBRG1;UPF3A
midnightb	BP	GO:0006501	C-terminal protein lipidation	17735	25	65	2	0.1	0.00442	20	PIGG;PIGL
midnightb	BP	GO:0034660	ncRNA metabolic process	17735	285	65	5	1.14	0.00548	4.385965	ADAT2;CARS2;GATC;MTERFD2;TSEN2

midnightb	MF	GO:0019843	rRNA binding	17735	31	65	2	0.11	0.00585	18.18182	MTERFD2;PTCD3
midnightb	CC	GO:0044464	cell part	17735	14451	65	60	53.48	0.0061	1.121915	ATF7IP2;CA5B;CAPRIN2;CARS2;CNST;DDX5;FES;GADD45B;GATC;GBGT1;GGT1;GOLGA8A;HAUS4;HK3;HNRPD
midnightb	CC	GO:0005623	cell	17735	14452	65	60	53.48	0.00612	1.121915	ATF7IP2;CA5B;CAPRIN2;CARS2;CNST;DDX5;FES;GADD45B;GATC;GBGT1;GGT1;GOLGA8A;HAUS4;HK3;HNRPD
midnightb	BP	GO:0018410	C-terminal protein amino acid modificati...	17735	30	65	2	0.12	0.00633	16.66667	PIGG;PIGL
midnightb	BP	GO:0046467	membrane lipid biosynthetic process	17735	97	65	3	0.39	0.00681	7.692308	GBGT1;PIGG;PIGL
midnightb	BP	GO:0006664	glycolipid metabolic process	17735	101	65	3	0.4	0.00761	7.5	GBGT1;PIGG;PIGL
midnightb	BP	GO:0006506	GPI anchor biosynthetic process	17735	33	65	2	0.13	0.00763	15.38462	PIGG;PIGL
midnightb	MF	GO:0048306	calcium-dependent protein binding	17735	36	65	2	0.13	0.00783	15.38462	DDX5;S100PBP
midnightb	BP	GO:0006505	GPI anchor metabolic process	17735	35	65	2	0.14	0.00855	14.28571	PIGG;PIGL
midnightb	MF	GO:0003723	RNA binding	17735	851	65	8	3.15	0.01259	2.539683	CAPRIN2;DDX5;HNRPDL;MTERFD2;PTCD3;SRSF1;TARBP1;UPF3A
midnightb	BP	GO:0006396	RNA processing	17735	646	65	7	2.58	0.01392	2.713178	ADAT2;DDX5;HNRPDL;MTERFD2;SRSF1;TARBP1;TSEN2
midnightb	CC	GO:0000313	organellar ribosome	17735	52	65	2	0.19	0.01587	10.52632	MRPS25;MTERFD2
midnightb	CC	GO:0005761	mitochondrial ribosome	17735	52	65	2	0.19	0.01587	10.52632	MRPS25;MTERFD2
midnightb	CC	GO:0005681	spliceosomal complex	17735	147	65	3	0.54	0.01717	5.555556	DDX5;HNRPDL;SRSF1
midnightb	BP	GO:0071704	organic substance metabolic process	17735	8996	65	44	35.88	0.01735	1.22631	ADAT2;ATF7IP2;CAPRIN2;CARS2;DDX5;FES;GADD45B;GATC;GBGT1;GGT1;HK3;HNRPDL;MRPS25;MTERFD2;I
midnightb	BP	GO:0043038	amino acid activation	17735	51	65	2	0.2	0.01759	10	CARS2;GATC
midnightb	BP	GO:0043039	tRNA aminoacylation	17735	51	65	2	0.2	0.01759	10	CARS2;GATC
midnightb	BP	GO:0044238	primary metabolic process	17735	8755	65	43	34.92	0.01885	1.231386	ADAT2;ATF7IP2;CAPRIN2;CARS2;DDX5;FES;GADD45B;GATC;GBGT1;GGT1;HK3;HNRPDL;MRPS25;MTERFD2;I
midnightb	BP	GO:0006497	protein lipidation	17735	54	65	2	0.22	0.01959	9.090909	PIGG;PIGL
midnightb	CC	GO:0005759	mitochondrial matrix	17735	286	65	4	1.06	0.0214	3.773585	CA5B;CARS2;MRPS25;MTERFD2
midnightb	BP	GO:0017148	negative regulation of translation	17735	59	65	2	0.24	0.02313	8.333333	CAPRIN2;PAIP2B
midnightb	BP	GO:0042158	lipoprotein biosynthetic process	17735	59	65	2	0.24	0.02313	8.333333	PIGG;PIGL
midnightb	MF	GO:0005488	binding	17735	11799	65	50	43.71	0.02419	1.143903	ADAT2;CA5B;CAPRIN2;CARS2;CNST;DDX5;FES;GATC;GBGT1;GGT1;HK3;HNRPDL;ICA1L;MOAP1;MTERFD2;N/
midnightb	BP	GO:0006643	membrane lipid metabolic process	17735	164	65	3	0.65	0.02766	4.615385	GBGT1;PIGG;PIGL
midnightb	BP	GO:0009987	cellular process	17735	12861	65	56	51.3	0.0295	1.091618	ADAT2;ATF7IP2;C5orf63;CA5B;CAPRIN2;CARS2;CNST;DDX5;FES;GADD45B;GATC;GBGT1;GGT1;HAUS4;HK3;I
midnightb	BP	GO:0048813	dendrite morphogenesis	17735	68	65	2	0.27	0.03011	7.407407	CAPRIN2;SLC11A2
midnightb	MF	GO:0005496	steroid binding	17735	78	65	2	0.29	0.03383	6.896552	NR3C2;OSBPL3
midnightb	BP	GO:0008033	tRNA processing	17735	73	65	2	0.29	0.0343	6.896552	ADAT2;TSEN2
midnightb	CC	GO:0071013	catalytic step 2 spliceosome	17735	79	65	2	0.29	0.0346	6.896552	DDX5;SRSF1
midnightb	MF	GO:0003840	gamma-glutamyltransferase activity	17735	10	65	1	0.04	0.03645	25	GGT1
midnightb	MF	GO:0036002	pre-mRNA binding	17735	10	65	1	0.04	0.03645	25	DDX5
midnightb	MF	GO:0046872	metal ion binding	17735	3936	65	21	14.58	0.03905	1.440329	ADAT2;CA5B;CARS2;GBGT1;NR3C2;STAT4;TRAF5;ZBTB24;ZFP28;ZMYND15;ZNF234;ZNF251;ZNF331;ZNF514
midnightb	BP	GO:0006448	regulation of translational elongation	17735	10	65	1	0.04	0.03919	25	GATC
midnightb	BP	GO:0031116	positive regulation of microtubule polym...	17735	10	65	1	0.04	0.03919	25	FES
midnightb	CC	GO:0031105	septin complex	17735	11	65	1	0.04	0.03998	25	8-Sep
midnightb	CC	GO:0032156	septin cytoskeleton	17735	11	65	1	0.04	0.03998	25	8-Sep
midnightb	CC	GO:0035631	CD40 receptor complex	17735	11	65	1	0.04	0.03998	25	TRAF5
midnightb	MF	GO:0008143	poly(A) RNA binding	17735	11	65	1	0.04	0.04002	25	HNRPDL
midnightb	MF	GO:0016884	carbon-nitrogen ligase activity, with gl...	17735	11	65	1	0.04	0.04002	25	GATC
midnightb	MF	GO:0090079	translation regulator activity, nucleic ...	17735	11	65	1	0.04	0.04002	25	PAIP2B
midnightb	BP	GO:0007623	circadian rhythm	17735	81	65	2	0.32	0.04144	6.25	DDX5;PER3
midnightb	MF	GO:0003677	DNA binding	17735	2332	65	14	8.64	0.04195	1.62037	HNRPDL;NR3C2;SCML4;STAT4;ZBTB24;ZFP28;ZNF234;ZNF251;ZNF331;ZNF514;ZNF528;ZNF548;ZNF841;ZNF
midnightb	BP	GO:0035082	axoneme assembly	17735	11	65	1	0.04	0.04303	25	OFD1
midnightb	BP	GO:2000251	positive regulation of actin cytoskeleto...	17735	11	65	1	0.04	0.04303	25	FES
midnightb	CC	GO:0035145	exon-exon junction complex	17735	12	65	1	0.04	0.04353	25	UPF3A
midnightb	MF	GO:0004549	tRNA-specific ribonuclease activity	17735	12	65	1	0.04	0.04358	25	TSEN2
midnightb	MF	GO:0031996	thioesterase binding	17735	12	65	1	0.04	0.04358	25	TRAF5
midnightb	MF	GO:0046625	sphingolipid binding	17735	12	65	1	0.04	0.04358	25	S1PR1
midnightb	BP	GO:0006919	activation of cysteine-type endopeptidas...	17735	84	65	2	0.34	0.04425	5.882353	MOAP1;SLC11A2
midnightb	BP	GO:0034470	ncRNA processing	17735	198	65	3	0.79	0.04444	3.797468	ADAT2;MTERFD2;TSEN2
midnightb	BP	GO:0097202	activation of cysteine-type endopeptidas...	17735	85	65	2	0.34	0.0452	5.882353	MOAP1;SLC11A2
midnightb	BP	GO:0043517	positive regulation of DNA damage respon...	17735	12	65	1	0.05	0.04685	20	DDX5
midnightb	BP	GO:0060999	positive regulation of dendritic spine d...	17735	12	65	1	0.05	0.04685	20	CAPRIN2
midnightb	BP	GO:1901798	positive regulation of signal transducti...	17735	12	65	1	0.05	0.04685	20	DDX5
midnightb	MF	GO:0005487	nucleocytoplasmic transporter activity	17735	13	65	1	0.05	0.04713	20	UPF3A
midnightb	MF	GO:0043169	cation binding	17735	4026	65	21	14.92	0.04883	1.407507	ADAT2;CA5B;CARS2;GBGT1;NR3C2;STAT4;TRAF5;ZBTB24;ZFP28;ZMYND15;ZNF234;ZNF251;ZNF331;ZNF514
midnightb	CC	GO:0005813	centrosome	17735	374	65	4	1.38	0.0496	2.898551	HAUS4;OFD1;TRAF5;TSEN2
magenta	BP	GO:0042113	B cell activation	17735	169	144	12	1.43	1.90E-08	8.391608	BANK1;BCL11A;BLNK;CD40;CD79A;CR2;CXCR5;ICOSLG;MS4A1;PAWR;SWAP70;TNFRSF13C
magenta	CC	GO:0009897	external side of plasma membrane	17735	202	144	12	1.66	1.10E-07	7.228916	CD19;CD22;CD40;CD79A;CD79B;CD83;CR2;FCER2;ICOSLG;L1CAM;MS4A1;TNFRSF13C
magenta	BP	GO:0046649	lymphocyte activation	17735	468	144	17	3.96	4.10E-07	4.292929	BANK1;BCL11A;BLNK;CD24;CD40;CD79A;CD83;CR2;CXCR5;DTX1;HLA-DOA;ICOSLG;IRF4;MS4A1;PAWR;SWA
magenta	CC	GO:0071944	cell periphery	17735	4360	144	63	35.91	4.70E-07	1.754386	ABCB4;ADAM28;ADD2;BLK;BLNK;CCR6;CD180;CD19;CD200;CD22;CD24;CD40;CD72;CD79A;CD79B;CD83;CD
magenta	CC	GO:0005886	plasma membrane	17735	4273	144	62	35.2	5.40E-07	1.761364	ABCB4;ADAM28;ADD2;BLK;BLNK;CCR6;CD180;CD19;CD200;CD22;CD24;CD40;CD72;CD79A;CD79B;CD83;CD
magenta	BP	GO:0045321	leukocyte activation	17735	552	144	18	4.67	8.80E-07	3.85439	BANK1;BCL11A;BLNK;CD24;CD40;CD79A;CD83;CNR2;CR2;CXCR5;DTX1;HLA-DOA;ICOSLG;IRF4;MS4A1;PAWF
magenta	CC	GO:0009986	cell surface	17735	485	144	16	4	2.50E-06	4	CD19;CD22;CD24;CD40;CD79A;CD79B;CD83;CLEC17A;CNTNAP2;CR2;FCER2;ICOSLG;L1CAM;MS4A1;PTPRK;T
magenta	BP	GO:0006955	immune response	17735	1081	144	25	9.14	3.30E-06	2.73523	ADARB1;BLK;BLNK;CCR6;CD180;CD19;CD200;CD24;CD40;CD79A;CD79B;CD83;CNR2;CR2;ENAH;FCER2;HLA-I
magenta	CC	GO:0005887	integral to plasma membrane	17735	1208	144	26	9.95	5.30E-06	2.613065	ABCB4;CCR6;CD19;CD200;CD22;CD40;CD72;CD79B;CD83;CELSR1;CNR2;CNTNAP2;CXCR5;FCER2;HTR3A;ICO:
magenta	CC	GO:0044459	plasma membrane part	17735	1895	144	34	15.61	8.20E-06	2.178091	ABCB4;CCR6;CD19;CD200;CD22;CD40;CD72;CD79A;CD79B;CD83;CELSR1;CNR2;CNTNAP2;CR2;CXCR5;EVC2;I
magenta	CC	GO:0031226	intrinsic to plasma membrane	17735	1252	144	26	10.31	1.00E-05	2.521823	ABCB4;CCR6;CD19;CD200;CD22;CD40;CD72;CD79B;CD83;CELSR1;CNR2;CNTNAP2;CXCR5;FCER2;HTR3A;ICO:
magenta	BP	GO:0001775	cell activation	17735	758	144	19	6.41	1.90E-05	2.964119	BANK1;BCL11A;BLNK;CD24;CD40;CD79A;CD83;CNR2;CR2;CTGF;CXCR5;DTX1;HLA-DOA;ICOSLG;IRF4;MS4A1;

magenta	BP	GO:0002376	immune system process	17735	1816	144	32	15.36	3.30E-05	2.083333	ADARB1;ADD2;BANK1;BCL11A;BLK;BLNK;CCR6;CD180;CD19;CD200;CD24;CD40;CD79A;CD79B;CD83;CNR2;C
magenta	BP	GO:0006959	humoral immune response	17735	126	144	7	1.07	9.60E-05	6.542056	BLNK;CCR6;CD83;CR2;FCER2;PAX5;POU2AF1
magenta	BP	GO:0002768	immune response-regulating cell surface ...	17735	142	144	7	1.2	2.00E-04	5.833333	BLK;CD24;CD40;CD79A;CD79B;ENAH;PAWR
magenta	BP	GO:0050865	regulation of cell activation	17735	359	144	11	3.04	0.00023	3.618421	CD24;CD40;CD83;CNR2;CTGF;DTX1;HLA-DOA;ICOSLG;IRF4;PAWR;TNFRSF13C
magenta	CC	GO:0016021	integral to membrane	17735	5202	144	62	42.85	4.00E-04	1.446908	ABCB4;ADAM28;AKAP6;CCR6;CD180;CD19;CD200;CD22;CD40;CD72;CD79A;CD79B;CD83;CDHR3;CELSR1;CL
magenta	CC	GO:0031224	intrinsic to membrane	17735	5319	144	63	43.81	0.00042	1.438028	ABCB4;ADAM28;AKAP6;CCR6;CD180;CD19;CD200;CD22;CD24;CD40;CD72;CD79A;CD79B;CD83;CDHR3;CELS
magenta	MF	GO:0005178	integrin binding	17735	81	144	5	0.64	0.00044	7.8125	COL4A3;CTGF;FCER2;L1CAM;LAMA5
magenta	BP	GO:0002682	regulation of immune system process	17735	884	144	18	7.48	0.00045	2.406417	BLK;CD19;CD200;CD24;CD40;CD79A;CD79B;CD83;CNR2;CR2;DTX1;ENAH;FCER2;HLA-DOA;ICOSLG;IRF4;PAW
magenta	BP	GO:0002694	regulation of leukocyte activation	17735	334	144	10	2.83	0.00054	3.533569	CD24;CD40;CD83;CNR2;DTX1;HLA-DOA;ICOSLG;IRF4;PAWR;TNFRSF13C
magenta	CC	GO:0005605	basal lamina	17735	20	144	3	0.16	0.00056	18.75	COL4A4;LAMA5;LAMC1
magenta	BP	GO:0050863	regulation of T cell activation	17735	226	144	8	1.91	0.00067	4.188482	CD24;CD83;DTX1;HLA-DOA;ICOSLG;IRF4;PAWR;TNFRSF13C
magenta	BP	GO:0002684	positive regulation of immune system pro...	17735	554	144	13	4.69	0.00082	2.771855	BLK;CD24;CD40;CD79A;CD79B;CD83;CR2;ENAH;FCER2;ICOSLG;IRF4;PAWR;TNFRSF13C
magenta	BP	GO:0051249	regulation of lymphocyte activation	17735	293	144	9	2.48	0.00086	3.629032	CD24;CD40;CD83;DTX1;HLA-DOA;ICOSLG;IRF4;PAWR;TNFRSF13C
magenta	BP	GO:0050776	regulation of immune response	17735	560	144	13	4.74	0.00091	2.742616	BLK;CD19;CD200;CD24;CD40;CD79A;CD79B;CR2;ENAH;FCER2;IRF4;PAWR;TNFRSF13C
magenta	CC	GO:0016020	membrane	17735	7690	144	82	63.35	0.00093	1.294396	ABCB4;ADAM28;ADD2;AKAP6;BLK;BLNK;CCR6;CD180;CD19;CD200;CD22;CD24;CD40;CD72;CD79A;CD79B;C
magenta	BP	GO:0032673	regulation of interleukin-4 production	17735	24	144	3	0.2	0.00105	15	CD83;ICOSLG;IRF4
magenta	BP	GO:0002764	immune response-regulating signaling pat...	17735	250	144	8	2.11	0.00128	3.791469	BLK;CD24;CD40;CD79A;CD79B;ENAH;IRF4;PAWR
magenta	BP	GO:0007166	cell surface receptor signaling pathway	17735	2331	144	33	19.72	0.00151	1.673428	BLK;BLNK;CCR6;CD19;CD24;CD40;CD79A;CD79B;CELSR1;CNR2;COL4A3;CTGF;CXCR5;DTX1;E2F5;ENAH;EVC2
magenta	CC	GO:0044425	membrane part	17735	6026	144	67	49.64	0.00157	1.349718	ABCB4;ADAM28;AKAP6;CCR6;CD180;CD19;CD200;CD22;CD24;CD40;CD72;CD79A;CD79B;CD83;CDHR3;CELS
magenta	MF	GO:0005088	Ras guanyl-nucleotide exchange factor ac...	17735	108	144	5	0.85	0.00163	5.882353	DENND5B;FARP2;PLEKHG1;RAPGEF5;RASGRP3
magenta	BP	GO:0042100	B cell proliferation	17735	60	144	4	0.51	0.00165	7.843137	CD40;CD79A;PAWR;TNFRSF13C
magenta	BP	GO:0042089	cytokine biosynthetic process	17735	101	144	5	0.85	0.00166	5.882353	GHRL;ICOSLG;IRF4;PAWR;TNFRSF13C
magenta	MF	GO:0003779	actin binding	17735	351	144	9	2.76	0.00182	3.26087	ADD2;COBLL1;CORO2B;ENAH;MICAL3;MYBPC2;PAWR;PHACTR1;SYNPO
magenta	BP	GO:0032633	interleukin-4 production	17735	29	144	3	0.25	0.00184	12	CD83;ICOSLG;IRF4
magenta	BP	GO:0042107	cytokine metabolic process	17735	104	144	5	0.88	0.00188	5.681818	GHRL;ICOSLG;IRF4;PAWR;TNFRSF13C
magenta	BP	GO:0030198	extracellular matrix organization	17735	207	144	7	1.75	0.00189	4	COL19A1;COL4A3;COL4A4;CTGF;LAMC1;MMP11;OLFML2A
magenta	BP	GO:0043062	extracellular structure organization	17735	208	144	7	1.76	0.00194	3.977273	COL19A1;COL4A3;COL4A4;CTGF;LAMC1;MMP11;OLFML2A
magenta	BP	GO:0042551	neuron maturation	17735	30	144	3	0.25	0.00203	12	CNTNAP2;FARP2;KCNIP2
magenta	BP	GO:0050853	B cell receptor signaling pathway	17735	30	144	3	0.25	0.00203	12	BLK;CD79A;CD79B
magenta	BP	GO:0042110	T cell activation	17735	339	144	9	2.87	0.00235	3.135889	BCL11A;CD24;CD83;DTX1;HLA-DOA;ICOSLG;IRF4;PAWR;TNFRSF13C
magenta	BP	GO:0007015	actin filament organization	17735	223	144	7	1.89	0.00287	3.703704	ADD2;CTGF;ENAH;GHRL;MICAL3;PAWR;SYNPO
magenta	CC	GO:0043256	laminin complex	17735	10	144	2	0.08	0.0029	25	LAMA5;LAMC1
magenta	BP	GO:0032836	glomerular basement membrane development	17735	10	144	2	0.08	0.00306	25	COL4A3;COL4A4
magenta	BP	GO:0030098	lymphocyte differentiation	17735	226	144	7	1.91	0.00309	3.664921	BCL11A;BLNK;CD79A;CD83;DTX1;HLA-DOA;IRF4
magenta	MF	GO:0005201	extracellular matrix structural constitu...	17735	78	144	4	0.61	0.00333	6.557377	COL19A1;COL4A3;COL4A4;LAMC1
magenta	BP	GO:0050851	antigen receptor-mediated signaling path...	17735	122	144	5	1.03	0.00376	4.854369	BLK;CD79A;CD79B;ENAH;PAWR
magenta	MF	GO:0032395	MHC class II receptor activity	17735	12	144	2	0.09	0.00385	22.22222	HLA-DOA;HLA-DOB
magenta	MF	GO:0008092	cytoskeletal protein binding	17735	627	144	12	4.94	0.00387	2.42915	ADD2;COBLL1;CORO2B;ENAH;FARP2;MAP2;MICAL3;MYBPC2;PAWR;PHACTR1;SYBU;SYNPO
magenta	BP	GO:0044699	single-organism process	17735	10656	144	103	90.14	0.00418	1.142667	ABCB4;ADAM28;ADARB1;ADD2;AFF3;AKAP6;BANK1;BCL11A;BLK;BLNK;CABYR;CCR6;CD19;CD22;CD24;CD40
magenta	CC	GO:0034703	cation channel complex	17735	130	144	5	1.07	0.00444	4.672897	AKAP6;CNTNAP2;KCNG1;KCNIP2;SCN4A
magenta	BP	GO:0045580	regulation of T cell differentiation	17735	79	144	4	0.67	0.00449	5.970149	CD83;DTX1;HLA-DOA;IRF4
magenta	MF	GO:0030296	protein tyrosine kinase activator activi...	17735	13	144	2	0.1	0.00453	20	CD24;GHRL
magenta	CC	GO:0043204	perikaryon	17735	41	144	3	0.34	0.00464	8.823529	CNR2;CNTNAP2;SYNPO
magenta	BP	GO:0051017	actin filament bundle assembly	17735	80	144	4	0.68	0.0047	5.882353	ADD2;CTGF;PAWR;SYNPO
magenta	BP	GO:0050778	positive regulation of immune response	17735	377	144	9	3.19	0.00473	2.821317	BLK;CD79A;CD79B;CR2;ENAH;FCER2;IRF4;PAWR;TNFRSF13C
magenta	BP	GO:0046651	lymphocyte proliferation	17735	185	144	6	1.56	0.00487	3.846154	CD24;CD40;CD79A;ICOSLG;PAWR;TNFRSF13C
magenta	BP	GO:0002429	immune response-activating cell surface ...	17735	131	144	5	1.11	0.00509	4.504505	BLK;CD79A;CD79B;ENAH;PAWR
magenta	BP	GO:0032943	mononuclear cell proliferation	17735	187	144	6	1.58	0.00513	3.797468	CD24;CD40;CD79A;ICOSLG;PAWR;TNFRSF13C
magenta	CC	GO:0005604	basement membrane	17735	86	144	4	0.71	0.00556	5.633803	COL4A3;COL4A4;LAMA5;LAMC1
magenta	BP	GO:0002460	adaptive immune response based on somati...	17735	193	144	6	1.63	0.00596	3.680982	CD40;CR2;FCER2;IRF4;SWAP70;TNFRSF13C
magenta	BP	GO:0070661	leukocyte proliferation	17735	194	144	6	1.64	0.00611	3.658537	CD24;CD40;CD79A;ICOSLG;PAWR;TNFRSF13C
magenta	BP	GO:0007155	cell adhesion	17735	957	144	16	8.1	0.00676	1.975309	CD22;CD24;CD72;CDHR3;CELSR1;CNTNAP2;COL19A1;COL4A3;CTGF;L1CAM;LAMA5;LAMC1;MYBPC2;PCDH9
magenta	BP	GO:0032623	interleukin-2 production	17735	46	144	3	0.39	0.00688	7.692308	CD83;IRF4;PAWR
magenta	BP	GO:0022610	biological adhesion	17735	959	144	16	8.11	0.00689	1.972873	CD22;CD24;CD72;CDHR3;CELSR1;CNTNAP2;COL19A1;COL4A3;CTGF;L1CAM;LAMA5;LAMC1;MYBPC2;PCDH9
magenta	MF	GO:0005070	SH3/SH2 adaptor activity	17735	50	144	3	0.39	0.00713	7.692308	BLNK;FCRL2;STAP1
magenta	BP	GO:0044763	single-organism cellular process	17735	9577	144	94	81.01	0.00726	1.160351	ABCB4;ADARB1;ADD2;AKAP6;BANK1;BCL11A;BLK;BLNK;CABYR;CCR6;CD19;CD22;CD24;CD40;CD72;CD79A;C
magenta	BP	GO:0042035	regulation of cytokine biosynthetic proc...	17735	91	144	4	0.77	0.0074	5.194805	GHRL;ICOSLG;IRF4;TNFRSF13C
magenta	CC	GO:0042613	MHC class II protein complex	17735	16	144	2	0.13	0.00749	15.38462	HLA-DOA;HLA-DOB
magenta	BP	GO:0030029	actin filament-based process	17735	484	144	10	4.09	0.00799	2.444988	ADD2;CELSR1;CORO2B;CTGF;ENAH;GHRL;MICAL3;MYBPC2;PAWR;SYNPO
magenta	CC	GO:0031012	extracellular matrix	17735	422	144	9	3.48	0.00828	2.586207	COCH;COL19A1;COL4A3;COL4A4;CTGF;LAMA5;LAMC1;MMP11;OLFML2A
magenta	BP	GO:0050670	regulation of lymphocyte proliferation	17735	148	144	5	1.25	0.00844	4	CD24;CD40;ICOSLG;PAWR;TNFRSF13C
magenta	MF	GO:0090484	drug transporter activity	17735	18	144	2	0.14	0.00866	14.28571	ABCB4;SLC15A2
magenta	BP	GO:0032944	regulation of mononuclear cell prolifera...	17735	149	144	5	1.26	0.00868	3.968254	CD24;CD40;ICOSLG;PAWR;TNFRSF13C
magenta	BP	GO:0046716	muscle cell homeostasis	17735	17	144	2	0.14	0.00888	14.28571	GYLTL1B;LARGE
magenta	BP	GO:0002250	adaptive immune response	17735	211	144	6	1.78	0.00907	3.370787	CD40;CR2;FCER2;IRF4;SWAP70;TNFRSF13C
magenta	BP	GO:0030888	regulation of B cell proliferation	17735	51	144	3	0.43	0.00915	6.976744	CD40;PAWR;TNFRSF13C
magenta	BP	GO:0032835	glomerulus development	17735	51	144	3	0.43	0.00915	6.976744	CD24;COL4A3;COL4A4
magenta	BP	GO:0002520	immune system development	17735	572	144	11	4.84	0.00917	2.272727	ADD2;BCL11A;BLNK;CD40;CD79A;CD83;CXCR5;DTX1;HLA-DOA;IRF4;SWAP70
magenta	BP	GO:0030036	actin cytoskeleton organization	17735	419	144	9	3.54	0.0092	2.542373	ADD2;CELSR1;CORO2B;CTGF;ENAH;GHRL;MICAL3;PAWR;SYNPO

magenta	BP	GO:0050671	positive regulation of lymphocyte prolif...	17735	97	144	4	0.82	0.00923	4.878049	CD24;CD40;ICOSLG;TNFRSF13C
magenta	BP	GO:0032946	positive regulation of mononuclear cell ...	17735	98	144	4	0.83	0.00956	4.819277	CD24;CD40;ICOSLG;TNFRSF13C
magenta	BP	GO:0070663	regulation of leukocyte proliferation	17735	153	144	5	1.29	0.00967	3.875969	CD24;CD40;ICOSLG;PAWR;TNFRSF13C
magenta	BP	GO:0042104	positive regulation of activated T cell ...	17735	18	144	2	0.15	0.00994	13.33333	CD24;ICOSLG
magenta	CC	GO:0005578	proteinaceous extracellular matrix	17735	360	144	8	2.97	0.00997	2.693603	COCH;COL19A1;COL4A3;COL4A4;CTGF;LAMA5;LAMC1;MMP11
magenta	BP	GO:0070665	positive regulation of leukocyte prolife...	17735	100	144	4	0.85	0.01025	4.705882	CD24;CD40;ICOSLG;TNFRSF13C
magenta	MF	GO:0050699	WW domain binding	17735	20	144	2	0.16	0.01065	12.5	ENAH;PMEPA1
magenta	BP	GO:0045619	regulation of lymphocyte differentiation	17735	102	144	4	0.86	0.01096	4.651163	CD83;DTX1;HLA-DOA;IRF4
magenta	BP	GO:0007411	axon guidance	17735	358	144	8	3.03	0.01109	2.640264	CD24;CD72;COL4A3;COL4A4;ENAH;FARP2;L1CAM;LAMC1
magenta	BP	GO:0030217	T cell differentiation	17735	160	144	5	1.35	0.01158	3.703704	BCL11A;CD83;DTX1;HLA-DOA;IRF4
magenta	BP	GO:0007409	axonogenesis	17735	515	144	10	4.36	0.01202	2.293578	BCL11A;CD24;CD72;COL4A3;COL4A4;ENAH;FARP2;L1CAM;LAMC1;MAP2
magenta	MF	GO:0019887	protein kinase regulator activity	17735	113	144	4	0.89	0.01218	4.494382	CABYR;CD24;GHRL;PKIG
magenta	BP	GO:0032501	multicellular organismal process	17735	5593	144	60	47.31	0.01233	1.268231	ADAM28;ADD2;AFF3;BCL11A;BLNK;CABYR;CD24;CD40;CD72;CD79A;CD83;CELSR1;CNR2;CNTNAP2;COCH;CO
magenta	MF	GO:0005085	guanyl-nucleotide exchange factor activi...	17735	176	144	5	1.39	0.01273	3.597122	DENND5B;FARP2;PLEKHG1;RAPGEF5;RASGRP3
magenta	BP	GO:0042129	regulation of T cell proliferation	17735	107	144	4	0.91	0.01289	4.395604	CD24;ICOSLG;PAWR;TNFRSF13C
magenta	BP	GO:0007275	multicellular organismal development	17735	3940	144	45	33.33	0.0131	1.350135	ADD2;AFF3;BCL11A;BLNK;CD24;CD40;CD72;CD79A;CD83;CELSR1;CNTNAP2;COL19A1;COL4A3;COL4A4;CTGF
magenta	BP	GO:0007044	cell-substrate junction assembly	17735	59	144	3	0.5	0.01361	6	LAMA5;LAMC1;PTPRK
magenta	BP	GO:0042108	positive regulation of cytokine biosynth...	17735	59	144	3	0.5	0.01361	6	ICOSLG;IRF4;TNFRSF13C
magenta	BP	GO:0031175	neuron projection development	17735	689	144	12	5.83	0.01376	2.058319	BCL11A;CD24;CD72;CNTNAP2;COL4A3;COL4A4;ENAH;FARP2;GHRL;L1CAM;LAMC1;MAP2
magenta	BP	GO:0044707	single-multicellular organism process	17735	5398	144	58	45.66	0.01409	1.270258	ADD2;AFF3;BCL11A;BLNK;CD24;CD40;CD72;CD79A;CD83;CELSR1;CNR2;CNTNAP2;COCH;COL19A1;COL4A3;C
magenta	CC	GO:0010008	endosome membrane	17735	311	144	7	2.56	0.01461	2.734375	GGA2;HLA-DOA;HLA-DOB;MARCH3;PARM1;PMEPA1;SLC9A7
magenta	BP	GO:0042094	interleukin-2 biosynthetic process	17735	22	144	2	0.19	0.01468	10.52632	IRF4;PAWR
magenta	MF	GO:0035591	signaling adaptor activity	17735	66	144	3	0.52	0.01519	5.769231	BLNK;FCRL2;STAP1
magenta	BP	GO:0002757	immune response-activating signal transd...	17735	237	144	6	2	0.01541	3	BLK;CD79A;CD79B;ENAH;IRF4;PAWR
magenta	CC	GO:0044420	extracellular matrix part	17735	177	144	5	1.46	0.01563	3.424658	COL19A1;COL4A3;COL4A4;LAMA5;LAMC1
magenta	BP	GO:0050896	response to stimulus	17735	6581	144	68	55.67	0.01588	1.221484	ABCB4;ADARB1;AFF3;BLK;BLNK;CABYR;CCR6;CD180;CD19;CD200;CD24;CD40;CD72;CD79A;CD79B;CD83;CEI
magenta	BP	GO:0050867	positive regulation of cell activation	17735	239	144	6	2.02	0.01599	2.970297	CD24;CD40;CD83;CTGF;ICOSLG;TNFRSF13C
magenta	BP	GO:0002253	activation of immune response	17735	310	144	7	2.62	0.01626	2.671756	BLK;CD79A;CD79B;CR2;ENAH;IRF4;PAWR
magenta	MF	GO:0001637	G-protein coupled chemoattractant recept...	17735	25	144	2	0.2	0.01638	10	CCR6;CXCR5
magenta	MF	GO:0004950	chemokine receptor activity	17735	25	144	2	0.2	0.01638	10	CCR6;CXCR5
magenta	BP	GO:0007399	nervous system development	17735	1716	144	23	14.52	0.01662	1.584022	BCL11A;CD24;CD72;CELSR1;CNTNAP2;COL4A3;COL4A4;DPF3;DTX1;EGR3;ENAH;FARP2;GHRL;KCNIP2;L1CAM
magenta	BP	GO:0048666	neuron development	17735	793	144	13	6.71	0.01663	1.937407	BCL11A;CD24;CD72;CNTNAP2;COL4A3;COL4A4;ENAH;FARP2;GHRL;KCNIP2;L1CAM;LAMC1;MAP2
magenta	CC	GO:0044440	endosomal part	17735	320	144	7	2.64	0.01683	2.651515	GGA2;HLA-DOA;HLA-DOB;MARCH3;PARM1;PMEPA1;SLC9A7
magenta	BP	GO:0016064	immunoglobulin mediated immune response	17735	116	144	4	0.98	0.01689	4.081633	CD40;CR2;FCER2;SWAP70
magenta	BP	GO:0048469	cell maturation	17735	116	144	4	0.98	0.01689	4.081633	CABYR;CNTNAP2;FARP2;KCNIP2
magenta	BP	GO:0048699	generation of neurons	17735	1061	144	16	8.97	0.01699	1.783724	BCL11A;CD24;CD72;CELSR1;CNTNAP2;COL4A3;COL4A4;DTX1;ENAH;FARP2;GHRL;KCNIP2;L1CAM;LAMC1;MA
magenta	MF	GO:0015079	potassium ion transmembrane transporter ...	17735	125	144	4	0.98	0.01707	4.081633	KCNG1;KCNH8;KCNIP2;SLC9A7
magenta	BP	GO:0050850	positive regulation of calcium-mediated ...	17735	24	144	2	0.2	0.01735	10	L1CAM;P2RX5
magenta	BP	GO:0007154	cell communication	17735	4775	144	52	40.39	0.01743	1.287447	BLK;BLNK;CABYR;CCR6;CD19;CD24;CD40;CD79A;CD79B;CD83;CELSR1;CNR2;CNTNAP2;COL4A3;CTGF;CXCR5
magenta	BP	GO:0023052	signaling	17735	4666	144	51	39.47	0.0176	1.292121	BLK;BLNK;CABYR;CCR6;CD19;CD24;CD40;CD79A;CD79B;CD83;CELSR1;CNR2;CNTNAP2;COL4A3;CTGF;CXCR5
magenta	BP	GO:0044700	single organism signaling	17735	4666	144	51	39.47	0.0176	1.292121	BLK;BLNK;CABYR;CCR6;CD19;CD24;CD40;CD79A;CD79B;CD83;CELSR1;CNR2;CNTNAP2;COL4A3;CTGF;CXCR5
magenta	BP	GO:0030182	neuron differentiation	17735	977	144	15	8.26	0.01785	1.815981	BCL11A;CD24;CD72;CNTNAP2;COL4A3;COL4A4;DTX1;ENAH;FARP2;GHRL;KCNIP2;L1CAM;LAMC1;MAP2;TCF
magenta	BP	GO:0048856	anatomical structure development	17735	3903	144	44	33.01	0.01804	1.332929	ADD2;AFF3;BCL11A;BLNK;CABYR;CD24;CD40;CD72;CD79A;CD83;CELSR1;CNTNAP2;COCH;COL19A1;COL4A3
magenta	BP	GO:0019724	B cell mediated immunity	17735	119	144	4	1.01	0.01837	3.960396	CD40;CR2;FCER2;SWAP70
magenta	BP	GO:0046006	regulation of activated T cell prolifera...	17735	25	144	2	0.21	0.01875	9.52381	CD24;ICOSLG
magenta	MF	GO:0022843	voltage-gated cation channel activity	17735	129	144	4	1.02	0.01894	3.921569	KCNG1;KCNH8;KCNIP2;SCN4A
magenta	CC	GO:0008076	voltage-gated potassium channel complex	17735	69	144	3	0.57	0.0193	5.263158	CNTNAP2;KCNG1;KCNIP2
magenta	CC	GO:0034705	potassium channel complex	17735	69	144	3	0.57	0.0193	5.263158	CNTNAP2;KCNG1;KCNIP2
magenta	MF	GO:0005261	cation channel activity	17735	268	144	6	2.11	0.01932	2.843602	HTR3A;KCNG1;KCNH8;KCNIP2;P2RX5;SCN4A
magenta	MF	GO:0030674	protein binding, bridging	17735	130	144	4	1.02	0.01942	3.921569	BLNK;COL19A1;FCRL2;STAP1
magenta	BP	GO:0048731	system development	17735	3385	144	39	28.63	0.01972	1.362207	ADD2;BCL11A;BLNK;CD24;CD40;CD72;CD79A;CD83;CELSR1;CNTNAP2;COL19A1;COL4A3;COL4A4;CTGF;CXCI
magenta	BP	GO:0061098	positive regulation of protein tyrosine ...	17735	26	144	2	0.22	0.0202	9.090909	CD24;GHRL
magenta	CC	GO:0043025	neuronal cell body	17735	259	144	6	2.13	0.02041	2.816901	CNR2;CNTNAP2;KLHL14;MAP2;PTPRK;SYNPO
magenta	BP	GO:0042102	positive regulation of T cell proliferat...	17735	69	144	3	0.58	0.02065	5.172414	CD24;ICOSLG;TNFRSF13C
magenta	MF	GO:0019207	kinase regulator activity	17735	133	144	4	1.05	0.02093	3.809524	CABYR;CD24;GHRL;PKIG
magenta	BP	GO:0048667	cell morphogenesis involved in neuron di...	17735	563	144	10	4.76	0.02108	2.10084	BCL11A;CD24;CD72;COL4A3;COL4A4;ENAH;FARP2;L1CAM;LAMC1;MAP2
magenta	BP	GO:0002521	leukocyte differentiation	17735	329	144	7	2.78	0.02175	2.517986	BCL11A;BLNK;CD79A;CD83;DTX1;HLA-DOA;IRF4
magenta	BP	GO:0030154	cell differentiation	17735	2673	144	32	22.61	0.02214	1.415303	BCL11A;BLNK;CABYR;CD24;CD72;CD79A;CD83;CELSR1;CNTNAP2;COL19A1;COL4A3;COL4A4;CTGF;DTX1;ENA
magenta	BP	GO:0008154	actin polymerization or depolymerization	17735	126	144	4	1.07	0.02215	3.738318	ADD2;ENAH;GHRL;MICAL3
magenta	BP	GO:0048812	neuron projection morphogenesis	17735	568	144	10	4.8	0.02225	2.083333	BCL11A;CD24;CD72;COL4A3;COL4A4;ENAH;FARP2;L1CAM;LAMC1;MAP2
magenta	CC	GO:0034702	ion channel complex	17735	194	144	5	1.6	0.02227	3.125	AKAP6;CNTNAP2;KCNG1;KCNIP2;SCN4A
magenta	BP	GO:0031295	T cell costimulation	17735	72	144	3	0.61	0.02308	4.918033	CD24;ICOSLG;TNFRSF13C
magenta	BP	GO:0043370	regulation of CD4-positive, alpha-beta T...	17735	28	144	2	0.24	0.02324	8.333333	CD83;IRF4
magenta	MF	GO:0005249	voltage-gated potassium channel activity	17735	78	144	3	0.61	0.02361	4.918033	KCNG1;KCNH8;KCNIP2
magenta	BP	GO:0044767	single-organism developmental process	17735	3427	144	39	28.99	0.02381	1.345291	ADD2;AFF3;BCL11A;BLNK;CABYR;CD24;CD72;CD79A;CD83;CELSR1;CNTNAP2;COCH;COL19A1;COL4A3;COL4
magenta	BP	GO:0031294	lymphocyte costimulation	17735	73	144	3	0.62	0.02392	4.83871	CD24;ICOSLG;TNFRSF13C
magenta	BP	GO:0001843	neural tube closure	17735	74	144	3	0.63	0.02478	4.761905	CELSR1;ENAH;PRICKLE1
magenta	BP	GO:0044708	single-organism behavior	17735	340	144	7	2.88	0.02545	2.430556	CELSR1;CNTNAP2;GHRL;GNG7;NETO1;SOBP;STRBP
magenta	BP	GO:0060606	tube closure	17735	75	144	3	0.63	0.02566	4.761905	CELSR1;ENAH;PRICKLE1

magenta	MF	GO:0060090	binding, bridging	17735	142	144	4	1.12	0.02586	3.571429	BLNK;COL19A1;FCRL2;STAP1
magenta	BP	GO:0042098	T cell proliferation	17735	133	144	4	1.13	0.02636	3.539823	CD24;ICOSLG;PAWR;TNFRSF13C
magenta	BP	GO:2000514	regulation of CD4-positive, alpha-beta T...	17735	30	144	2	0.25	0.02646	8	CD83;IRF4
magenta	BP	GO:0032502	developmental process	17735	4437	144	48	37.53	0.02679	1.278977	ADD2;AFF3;BCL11A;BLNK;CABYR;CD24;CD40;CD72;CD79A;CD83;CELSR1;CNTNAP2;COCH;COL19A1;COL4A3;
magenta	BP	GO:0048858	cell projection morphogenesis	17735	673	144	11	5.69	0.02731	1.933216	BCL11A;CD24;CD72;COL4A3;COL4A4;ENAH;FARP2;L1CAM;LAMA5;LAMC1;MAP2
magenta	BP	GO:0030183	B cell differentiation	17735	77	144	3	0.65	0.02746	4.615385	BCL11A;BLNK;CD79A
magenta	BP	GO:0022008	neurogenesis	17735	1126	144	16	9.52	0.02785	1.680672	BCL11A;CD24;CD72;CELSR1;CNTNAP2;COL4A3;COL4A4;DTX1;ENAH;FARP2;GHRL;KCNIP2;L1CAM;LAMC1;MA
magenta	BP	GO:0032233	positive regulation of actin filament bu...	17735	31	144	2	0.26	0.02813	7.692308	CTGF;SYNPO
magenta	BP	GO:0050798	activated T cell proliferation	17735	31	144	2	0.26	0.02813	7.692308	CD24;ICOSLG
magenta	BP	GO:0014020	primary neural tube formation	17735	79	144	3	0.67	0.02933	4.477612	CELSR1;ENAH;PRICKLE1
magenta	BP	GO:0001816	cytokine production	17735	430	144	8	3.64	0.0296	2.197802	CD24;CD40;CD83;GHRL;ICOSLG;IRF4;PAWR;TNFRSF13C
magenta	BP	GO:0007156	homophilic cell adhesion	17735	138	144	4	1.17	0.02965	3.418803	CDHR3;CELSR1;L1CAM;PCDH9
magenta	BP	GO:0006935	chemotaxis	17735	596	144	10	5.04	0.02971	1.984127	CCR6;CD24;CD72;COL4A3;COL4A4;EGR3;ENAH;FARP2;L1CAM;LAMC1
magenta	BP	GO:0042330	taxis	17735	596	144	10	5.04	0.02971	1.984127	CCR6;CD24;CD72;COL4A3;COL4A4;EGR3;ENAH;FARP2;L1CAM;LAMC1
magenta	BP	GO:0002889	regulation of immunoglobulin mediated im...	17735	32	144	2	0.27	0.02984	7.407407	CD40;FCER2
magenta	BP	GO:0032653	regulation of interleukin-10 production	17735	32	144	2	0.27	0.02984	7.407407	CD83;IRF4
magenta	BP	GO:0048869	cellular developmental process	17735	2842	144	33	24.04	0.03008	1.372712	BCL11A;BLNK;CABYR;CD24;CD72;CD79A;CD83;CELSR1;CNTNAP2;COCH;COL19A1;COL4A3;COL4A4;CTGF;DT
magenta	BP	GO:0032990	cell part morphogenesis	17735	685	144	11	5.79	0.03053	1.899827	BCL11A;CD24;CD72;COL4A3;COL4A4;ENAH;FARP2;L1CAM;LAMA5;LAMC1;MAP2
magenta	BP	GO:0016331	morphogenesis of embryonic epithelium	17735	140	144	4	1.18	0.03103	3.389831	CELSR1;ENAH;LAMA5;PRICKLE1
magenta	CC	GO:0044297	cell body	17735	286	144	6	2.36	0.03117	2.542373	CNR2;CNTNAP2;KLHL14;MAP2;PTPRK;SYNPO
magenta	BP	GO:0002712	regulation of B cell mediated immunity	17735	33	144	2	0.28	0.03159	7.142857	CD40;FCER2
magenta	MF	GO:0022836	gated channel activity	17735	301	144	6	2.37	0.03176	2.531646	HTR3A;KCNG1;KCNH8;KCNIP2;P2RX5;SCN4A
magenta	MF	GO:0022839	ion gated channel activity	17735	301	144	6	2.37	0.03176	2.531646	HTR3A;KCNG1;KCNH8;KCNIP2;P2RX5;SCN4A
magenta	BP	GO:0002822	regulation of adaptive immune response b...	17735	83	144	3	0.7	0.03327	4.285714	CD40;FCER2;TNFRSF13C
magenta	BP	GO:0050864	regulation of B cell activation	17735	83	144	3	0.7	0.03327	4.285714	CD40;PAWR;TNFRSF13C
magenta	BP	GO:0002204	somatic recombination of immunoglobulin ...	17735	34	144	2	0.29	0.03338	6.896552	CD40;SWAP70
magenta	BP	GO:0002208	somatic diversification of immunoglobuli...	17735	34	144	2	0.29	0.03338	6.896552	CD40;SWAP70
magenta	BP	GO:0032613	interleukin-10 production	17735	34	144	2	0.29	0.03338	6.896552	CD83;IRF4
magenta	BP	GO:0034446	substrate adhesion-dependent cell spread...	17735	34	144	2	0.29	0.03338	6.896552	LAMA5;LAMC1
magenta	BP	GO:0045190	isotype switching	17735	34	144	2	0.29	0.03338	6.896552	CD40;SWAP70
magenta	BP	GO:0050848	regulation of calcium-mediated signaling	17735	34	144	2	0.29	0.03338	6.896552	L1CAM;P2RX5
magenta	BP	GO:0051251	positive regulation of lymphocyte activa...	17735	211	144	5	1.78	0.03352	2.808989	CD24;CD40;CD83;ICOSLG;TNFRSF13C
magenta	BP	GO:0007165	signal transduction	17735	4165	144	45	35.23	0.03382	1.27732	BLK;BLNK;CABYR;CCR6;CD19;CD24;CD40;CD79A;CD79B;CD83;CELSR1;CNR2;CNTNAP2;COL4A3;CTGF;CXCR5
magenta	BP	GO:0048583	regulation of response to stimulus	17735	2347	144	28	19.85	0.03403	1.410579	BLK;CD19;CD200;CD24;CD40;CD79A;CD79B;CNR2;CR2;CTGF;DTX1;ENAH;FARP2;FCER2;GHRL;GNG7;IRF4;L1
magenta	BP	GO:0000904	cell morphogenesis involved in different...	17735	699	144	11	5.91	0.03463	1.861252	BCL11A;CD24;CD72;COL4A3;COL4A4;ENAH;FARP2;L1CAM;LAMA5;LAMC1;MAP2
magenta	MF	GO:0008324	cation transmembrane transporter activit...	17735	566	144	9	4.46	0.03467	2.017937	HTR3A;KCNG1;KCNH8;KCNIP2;NIPAL4;P2RX5;SCN4A;SLC15A2;SLC9A7
magenta	BP	GO:0040011	locomotion	17735	1253	144	17	10.6	0.03478	1.603774	ADARB1;CABYR;CCR6;CD24;CD72;CELSR1;COL4A3;COL4A4;CTGF;EGR3;ENAH;FARP2;GHRL;L1CAM;LAMA5;L
magenta	CC	GO:0005581	collagen	17735	87	144	3	0.72	0.03511	4.166667	COL19A1;COL4A3;COL4A4
magenta	BP	GO:0030890	positive regulation of B cell proliferat...	17735	35	144	2	0.3	0.03521	6.666667	CD40;TNFRSF13C
magenta	BP	GO:0050999	regulation of nitric-oxide synthase acti...	17735	35	144	2	0.3	0.03521	6.666667	CNR2;FCER2
magenta	BP	GO:0090288	negative regulation of cellular response...	17735	86	144	3	0.73	0.0364	4.109589	PEG10;PMEPA1;SPRY1
magenta	CC	GO:0005768	endosome	17735	546	144	9	4.5	0.03684	2	CD79A;CNTNAP2;GGA2;HLA-DOA;HLA-DOB;MARCH3;PARM1;PMEPA1;SLC9A7
magenta	MF	GO:0005543	phospholipid binding	17735	487	144	8	3.83	0.03855	2.088773	FAM129C;FARP2;OSBPL10;PIK3C2B;PLEKHG1;SNX22;STAP1;SWAP70
magenta	BP	GO:0002285	lymphocyte activation involved in immune...	17735	88	144	3	0.74	0.03856	4.054054	CD40;IRF4;SWAP70
magenta	BP	GO:0022617	extracellular matrix disassembly	17735	37	144	2	0.31	0.03899	6.451613	LAMC1;MMP11
magenta	BP	GO:0030042	actin filament depolymerization	17735	37	144	2	0.31	0.03899	6.451613	ADD2;MICAL3
magenta	CC	GO:0042611	MHC protein complex	17735	38	144	2	0.31	0.03904	6.451613	HLA-DOA;HLA-DOB
magenta	MF	GO:0030295	protein kinase activator activity	17735	40	144	2	0.31	0.03947	6.451613	CD24;GHRL
magenta	BP	GO:0007218	neuropeptide signaling pathway	17735	89	144	3	0.75	0.03967	4	CELSR1;NXPH4;PNOC
magenta	BP	GO:0072006	nephron development	17735	89	144	3	0.75	0.03967	4	CD24;COL4A3;COL4A4
magenta	BP	GO:0048534	hematopoietic or lymphoid organ developm...	17735	542	144	9	4.58	0.04045	1.965066	ADD2;BCL11A;BLNK;CD79A;CD83;CXCR5;DTX1;HLA-DOA;IRF4
magenta	BP	GO:0002819	regulation of adaptive immune response	17735	91	144	3	0.77	0.04193	3.896104	CD40;FCER2;TNFRSF13C
magenta	BP	GO:0051492	regulation of stress fiber assembly	17735	39	144	2	0.33	0.04291	6.060606	CTGF;SYNPO
magenta	BP	GO:0048468	cell development	17735	1482	144	19	12.54	0.04299	1.515152	BCL11A;CABYR;CD24;CD72;CNTNAP2;COL4A3;COL4A4;DTX1;ENAH;FARP2;GHRL;KCNIP2;L1CAM;LAMA5;LAN
magenta	BP	GO:0001841	neural tube formation	17735	92	144	3	0.78	0.04308	3.846154	CELSR1;ENAH;PRICKLE1
magenta	BP	GO:0001817	regulation of cytokine production	17735	382	144	7	3.23	0.04354	2.167183	CD24;CD40;CD83;GHRL;ICOSLG;IRF4;TNFRSF13C
magenta	CC	GO:0001725	stress fiber	17735	41	144	2	0.34	0.04482	5.882353	ENAH;SYNPO
magenta	BP	GO:0016447	somatic recombination of immunoglobulin ...	17735	40	144	2	0.34	0.04493	5.882353	CD40;SWAP70
magenta	BP	GO:0032663	regulation of interleukin-2 production	17735	40	144	2	0.34	0.04493	5.882353	CD83;IRF4
magenta	MF	GO:0003725	double-stranded RNA binding	17735	43	144	2	0.34	0.04501	5.882353	ADARB1;STRBP
magenta	BP	GO:0002696	positive regulation of leukocyte activat...	17735	229	144	5	1.94	0.04504	2.57732	CD24;CD40;CD83;ICOSLG;TNFRSF13C
magenta	BP	GO:0001738	morphogenesis of a polarized epithelium	17735	41	144	2	0.35	0.04697	5.714286	CELSR1;LAMA5
magenta	BP	GO:0046637	regulation of alpha-beta T cell differen...	17735	41	144	2	0.35	0.04697	5.714286	CD83;IRF4
magenta	BP	GO:0019226	transmission of nerve impulse	17735	738	144	11	6.24	0.04807	1.762821	CNR2;CNTNAP2;EGR3;GHRL;GNG7;HTR3A;KCNG1;KCNH8;KCNIP2;NETO1;PNOC
magenta	CC	GO:0005788	endoplasmic reticulum lumen	17735	166	144	4	1.37	0.0485	2.919708	COL19A1;COL4A3;COL4A4;GHRL
magenta	BP	GO:2000026	regulation of multicellular organismal d...	17735	1113	144	15	9.41	0.04855	1.594049	BCL11A;CD24;CD40;CD83;CELSR1;COL4A3;CTGF;DTX1;GHRL;HLA-DOA;IRF4;LAMA5;PRICKLE1;SPRY1;TCF4
magenta	BP	GO:0002312	B cell activation involved in immune res...	17735	42	144	2	0.36	0.04906	5.555556	CD40;SWAP70
magenta	BP	GO:0007268	synaptic transmission	17735	651	144	10	5.51	0.04925	1.814882	CNR2;EGR3;GHRL;GNG7;HTR3A;KCNG1;KCNH8;KCNIP2;NETO1;PNOC
magenta	BP	GO:0030030	cell projection organization	17735	926	144	13	7.83	0.04938	1.660281	BCL11A;CD24;CD72;CNTNAP2;COL4A3;COL4A4;ENAH;FARP2;GHRL;L1CAM;LAMA5;LAMC1;MAP2

magenta	MF	GO:0005244	voltage-gated ion channel activity	17735	175	144	4	1.38	0.04951	2.898551	KCNG1;KCNH8;KCNIP2;SCN4A
magenta	MF	GO:0022832	voltage-gated channel activity	17735	175	144	4	1.38	0.04951	2.898551	KCNG1;KCNH8;KCNIP2;SCN4A
lightyellow	BP	GO:0018958	phenol-containing compound metabolic pro...	17735	65	27	3	0.09	0.00011	33.33333	CYP11A1;EPAS1;HDC
lightyellow	BP	GO:0030902	hindbrain development	17735	115	27	3	0.17	0.00058	17.64706	CTNNA2;CYP11A1;GATA2
lightyellow	BP	GO:0001568	blood vessel development	17735	498	27	5	0.72	6.00E-04	6.944444	EPAS1;GATA2;ITGB8;NTRK1;THSD7A
lightyellow	BP	GO:0001944	vasculature development	17735	523	27	5	0.76	0.00074	6.578947	EPAS1;GATA2;ITGB8;NTRK1;THSD7A
lightyellow	BP	GO:0001890	placenta development	17735	132	27	3	0.19	0.00086	15.78947	EPAS1;GATA2;ITGB8
lightyellow	BP	GO:0048871	multicellular organismal homeostasis	17735	155	27	3	0.22	0.00137	13.63636	EPAS1;GATA2;PTGER3
lightyellow	BP	GO:0006584	catecholamine metabolic process	17735	41	27	2	0.06	0.00157	33.33333	EPAS1;HDC
lightyellow	BP	GO:0009712	catechol-containing compound metabolic p...	17735	41	27	2	0.06	0.00157	33.33333	EPAS1;HDC
lightyellow	BP	GO:0001525	angiogenesis	17735	362	27	4	0.52	0.00162	7.692308	EPAS1;GATA2;NTRK1;THSD7A
lightyellow	BP	GO:0034311	diol metabolic process	17735	42	27	2	0.06	0.00165	33.33333	EPAS1;HDC
lightyellow	BP	GO:0007202	activation of phospholipase C activity	17735	56	27	2	0.08	0.00292	25	MS4A2;NTRK1
lightyellow	BP	GO:0048514	blood vessel morphogenesis	17735	438	27	4	0.63	0.00323	6.349206	EPAS1;GATA2;NTRK1;THSD7A
lightyellow	BP	GO:0030816	positive regulation of cAMP metabolic pr...	17735	60	27	2	0.09	0.00334	22.22222	AKAP12;NTRK1
lightyellow	BP	GO:0030819	positive regulation of cAMP biosynthetic...	17735	60	27	2	0.09	0.00334	22.22222	AKAP12;NTRK1
lightyellow	MF	GO:0004527	exonuclease activity	17735	60	27	2	0.09	0.0034	22.22222	ENPP3;LOC81691
lightyellow	BP	GO:0010863	positive regulation of phospholipase C a...	17735	62	27	2	0.09	0.00357	22.22222	MS4A2;NTRK1
lightyellow	BP	GO:1900274	regulation of phospholipase C activity	17735	63	27	2	0.09	0.00368	22.22222	MS4A2;NTRK1
lightyellow	BP	GO:0044283	small molecule biosynthetic process	17735	456	27	4	0.66	0.00374	6.060606	CYP11A1;HDC;HPGDS;SLC27A2
lightyellow	BP	GO:0072358	cardiovascular system development	17735	768	27	5	1.11	0.00407	4.504505	EPAS1;GATA2;ITGB8;NTRK1;THSD7A
lightyellow	BP	GO:0072359	circulatory system development	17735	768	27	5	1.11	0.00407	4.504505	EPAS1;GATA2;ITGB8;NTRK1;THSD7A
lightyellow	BP	GO:0044711	single-organism biosynthetic process	17735	468	27	4	0.68	0.0041	5.882353	CYP11A1;HDC;HPGDS;SLC27A2
lightyellow	BP	GO:0030804	positive regulation of cyclic nucleotide...	17735	67	27	2	0.1	0.00415	20	AKAP12;NTRK1
lightyellow	MF	GO:0001077	RNA polymerase II core promoter proximal...	17735	67	27	2	0.1	0.0042	20	EPAS1;GATA2
lightyellow	BP	GO:0030810	positive regulation of nucleotide biosyn...	17735	69	27	2	0.1	0.0044	20	AKAP12;NTRK1
lightyellow	BP	GO:1900373	positive regulation of purine nucleotide...	17735	69	27	2	0.1	0.0044	20	AKAP12;NTRK1
lightyellow	BP	GO:0030801	positive regulation of cyclic nucleotide...	17735	70	27	2	0.1	0.00452	20	AKAP12;NTRK1
lightyellow	BP	GO:1900544	positive regulation of purine nucleotide...	17735	72	27	2	0.1	0.00478	20	AKAP12;NTRK1
lightyellow	BP	GO:0010518	positive regulation of phospholipase act...	17735	73	27	2	0.11	0.00491	18.18182	MS4A2;NTRK1
lightyellow	BP	GO:0045981	positive regulation of nucleotide metabo...	17735	73	27	2	0.11	0.00491	18.18182	AKAP12;NTRK1
lightyellow	MF	GO:0001228	RNA polymerase II transcription regulato...	17735	73	27	2	0.11	0.005	18.18182	EPAS1;GATA2
lightyellow	MF	GO:0000981	sequence-specific DNA binding RNA polyme...	17735	253	27	3	0.37	0.0056	8.108108	EPAS1;GATA2;PTGER3
lightyellow	BP	GO:0010517	regulation of phospholipase activity	17735	80	27	2	0.12	0.00587	16.66667	MS4A2;NTRK1
lightyellow	BP	GO:0044281	small molecule metabolic process	17735	2545	27	9	3.68	0.00591	2.445652	AKAP12;CA8;CYP11A1;ENPP3;EPAS1;HDC;HPGDS;NTRK1;SLC27A2
lightyellow	BP	GO:0044710	single-organism metabolic process	17735	3059	27	10	4.42	0.00593	2.262443	AKAP12;CA8;CYP11A1;ENPP3;EPAS1;HDC;HPGDS;ITGB8;NTRK1;SLC27A2
lightyellow	BP	GO:0001892	embryonic placenta development	17735	81	27	2	0.12	0.00601	16.66667	EPAS1;GATA2
lightyellow	BP	GO:0060193	positive regulation of lipase activity	17735	81	27	2	0.12	0.00601	16.66667	MS4A2;NTRK1
lightyellow	BP	GO:0030817	regulation of cAMP biosynthetic process	17735	86	27	2	0.12	0.00675	16.66667	AKAP12;NTRK1
lightyellow	BP	GO:0032844	regulation of homeostatic process	17735	275	27	3	0.4	0.00692	7.5	GATA2;MS4A2;PTGER3
lightyellow	BP	GO:0030218	erythrocyte differentiation	17735	88	27	2	0.13	0.00706	15.38462	EPAS1;GATA2
lightyellow	BP	GO:0030814	regulation of cAMP metabolic process	17735	88	27	2	0.13	0.00706	15.38462	AKAP12;NTRK1
lightyellow	BP	GO:0045766	positive regulation of angiogenesis	17735	89	27	2	0.13	0.00721	15.38462	GATA2;NTRK1
lightyellow	BP	GO:0006171	cAMP biosynthetic process	17735	92	27	2	0.13	0.00769	15.38462	AKAP12;NTRK1
lightyellow	BP	GO:0043279	response to alkaloid	17735	92	27	2	0.13	0.00769	15.38462	CYP11A1;NTRK1
lightyellow	BP	GO:0034101	erythrocyte homeostasis	17735	95	27	2	0.14	0.00818	14.28571	EPAS1;GATA2
lightyellow	BP	GO:0030802	regulation of cyclic nucleotide biosynth...	17735	98	27	2	0.14	0.00869	14.28571	AKAP12;NTRK1
lightyellow	MF	GO:0000982	RNA polymerase II core promoter proximal...	17735	98	27	2	0.14	0.0088	14.28571	EPAS1;GATA2
lightyellow	BP	GO:0008584	male gonad development	17735	100	27	2	0.14	0.00903	14.28571	CYP11A1;NTRK1
lightyellow	BP	GO:0030808	regulation of nucleotide biosynthetic pr...	17735	101	27	2	0.15	0.0092	13.33333	AKAP12;NTRK1
lightyellow	BP	GO:1900371	regulation of purine nucleotide biosynth...	17735	101	27	2	0.15	0.0092	13.33333	AKAP12;NTRK1
lightyellow	BP	GO:0030799	regulation of cyclic nucleotide metaboli...	17735	105	27	2	0.15	0.00992	13.33333	AKAP12;NTRK1
lightyellow	BP	GO:0060191	regulation of lipase activity	17735	107	27	2	0.15	0.01028	13.33333	MS4A2;NTRK1
lightyellow	BP	GO:0046058	cAMP metabolic process	17735	108	27	2	0.16	0.01047	12.5	AKAP12;NTRK1
lightyellow	BP	GO:0001894	tissue homeostasis	17735	110	27	2	0.16	0.01084	12.5	EPAS1;GATA2
lightyellow	BP	GO:0052652	cyclic purine nucleotide metabolic proce...	17735	111	27	2	0.16	0.01103	12.5	AKAP12;NTRK1
lightyellow	BP	GO:0009190	cyclic nucleotide biosynthetic process	17735	112	27	2	0.16	0.01122	12.5	AKAP12;NTRK1
lightyellow	BP	GO:0048469	cell maturation	17735	116	27	2	0.17	0.012	11.76471	EPAS1;GATA2
lightyellow	BP	GO:0046546	development of primary male sexual chara...	17735	117	27	2	0.17	0.0122	11.76471	CYP11A1;NTRK1
lightyellow	BP	GO:0046661	male sex differentiation	17735	125	27	2	0.18	0.01384	11.11111	CYP11A1;NTRK1
lightyellow	BP	GO:0010738	regulation of protein kinase A signaling...	17735	10	27	1	0.01	0.01435	100	AKAP12
lightyellow	BP	GO:0021535	cell migration in hindbrain	17735	10	27	1	0.01	0.01435	100	CTNNA2
lightyellow	BP	GO:0031650	regulation of heat generation	17735	10	27	1	0.01	0.01435	100	PTGER3
lightyellow	BP	GO:0043302	positive regulation of leukocyte degranu...	17735	10	27	1	0.01	0.01435	100	MS4A2
lightyellow	BP	GO:0060872	semicircular canal development	17735	10	27	1	0.01	0.01435	100	GATA2
lightyellow	BP	GO:0071236	cellular response to antibiotic	17735	10	27	1	0.01	0.01435	100	CYP11A1
lightyellow	MF	GO:0004467	long-chain fatty acid-CoA ligase activit...	17735	10	27	1	0.01	0.0145	100	SLC27A2
lightyellow	BP	GO:0017085	response to insecticide	17735	11	27	1	0.02	0.01578	50	CYP11A1
lightyellow	BP	GO:0021514	ventral spinal cord interneuron differen...	17735	11	27	1	0.02	0.01578	50	GATA2

lightyellow	BP	GO:0032354	response to follicle-stimulating hormone...	17735	11	27	1	0.02	0.01578	50	CYP11A1
lightyellow	BP	GO:0033005	positive regulation of mast cell activat...	17735	11	27	1	0.02	0.01578	50	MS4A2
lightyellow	BP	GO:0033327	Leydig cell differentiation	17735	11	27	1	0.02	0.01578	50	CYP11A1
lightyellow	BP	GO:0048875	chemical homeostasis within a tissue	17735	11	27	1	0.02	0.01578	50	EPAS1
lightyellow	BP	GO:0050951	sensory perception of temperature stimul...	17735	11	27	1	0.02	0.01578	50	NTRK1
lightyellow	MF	GO:0004955	prostaglandin receptor activity	17735	11	27	1	0.02	0.0159	50	PTGER3
lightyellow	BP	GO:0009187	cyclic nucleotide metabolic process	17735	138	27	2	0.2	0.01669	10	AKAP12;NTRK1
lightyellow	BP	GO:0048015	phosphatidylinositol-mediated signaling	17735	138	27	2	0.2	0.01669	10	CA8;NTRK1
lightyellow	BP	GO:0048017	inositol lipid-mediated signaling	17735	138	27	2	0.2	0.01669	10	CA8;NTRK1
lightyellow	BP	GO:0006694	steroid biosynthetic process	17735	139	27	2	0.2	0.01692	10	CYP11A1;SLC27A2
lightyellow	BP	GO:0010737	protein kinase A signaling cascade	17735	12	27	1	0.02	0.0172	50	AKAP12
lightyellow	BP	GO:0045652	regulation of megakaryocyte differentiat...	17735	12	27	1	0.02	0.0172	50	GATA2
lightyellow	BP	GO:0060134	prepulse inhibition	17735	12	27	1	0.02	0.0172	50	CTNNA2
lightyellow	MF	GO:0004953	icosanoid receptor activity	17735	12	27	1	0.02	0.0173	50	PTGER3
lightyellow	MF	GO:0004954	prostanoid receptor activity	17735	12	27	1	0.02	0.0173	50	PTGER3
lightyellow	BP	GO:1901615	organic hydroxy compound metabolic proce...	17735	387	27	3	0.56	0.01743	5.357143	CYP11A1;EPAS1;HDC
lightyellow	MF	GO:0019899	enzyme binding	17735	1084	27	5	1.58	0.0177	3.164557	AKAP12;CCNA1;EPAS1;MS4A2;SLC27A2
lightyellow	BP	GO:0001573	ganglioside metabolic process	17735	13	27	1	0.02	0.01862	50	ITGB8
lightyellow	BP	GO:0042448	progesterone metabolic process	17735	13	27	1	0.02	0.01862	50	CYP11A1
lightyellow	MF	GO:0070742	C2H2 zinc finger domain binding	17735	13	27	1	0.02	0.0188	50	GATA2
lightyellow	BP	GO:0001991	regulation of systemic arterial blood pr...	17735	14	27	1	0.02	0.02004	50	CPA3
lightyellow	BP	GO:0006702	androgen biosynthetic process	17735	14	27	1	0.02	0.02004	50	CYP11A1
lightyellow	BP	GO:0031649	heat generation	17735	14	27	1	0.02	0.02004	50	PTGER3
lightyellow	BP	GO:0045649	regulation of macrophage differentiation	17735	14	27	1	0.02	0.02004	50	GATA2
lightyellow	BP	GO:0060008	Sertoli cell differentiation	17735	14	27	1	0.02	0.02004	50	NTRK1
lightyellow	BP	GO:0060384	innervation	17735	14	27	1	0.02	0.02004	50	NTRK1
lightyellow	BP	GO:0071276	cellular response to cadmium ion	17735	14	27	1	0.02	0.02004	50	CYP11A1
lightyellow	BP	GO:0071371	cellular response to gonadotropin stimul...	17735	14	27	1	0.02	0.02004	50	CYP11A1
lightyellow	BP	GO:0097237	cellular response to toxin	17735	14	27	1	0.02	0.02004	50	CYP11A1
lightyellow	BP	GO:2000178	negative regulation of neural precursor ...	17735	14	27	1	0.02	0.02004	50	GATA2
lightyellow	MF	GO:0016829	lyase activity	17735	151	27	2	0.22	0.0201	9.090909	CA8;HDC
lightyellow	MF	GO:0004089	carbonate dehydratase activity	17735	14	27	1	0.02	0.0202	50	CA8
lightyellow	MF	GO:0015295	solute:hydrogen symporter activity	17735	14	27	1	0.02	0.0202	50	SLC2A10
lightyellow	MF	GO:0015645	fatty acid ligase activity	17735	14	27	1	0.02	0.0202	50	SLC27A2
lightyellow	BP	GO:0045765	regulation of angiogenesis	17735	157	27	2	0.23	0.02127	8.695652	GATA2;NTRK1
lightyellow	BP	GO:0050755	chemokine metabolic process	17735	15	27	1	0.02	0.02146	50	CYP11A1
lightyellow	BP	GO:1901617	organic hydroxy compound biosynthetic pr...	17735	158	27	2	0.23	0.02152	8.695652	CYP11A1;HDC
lightyellow	CC	GO:0005779	integral to peroxisomal membrane	17735	14	27	1	0.02	0.022	50	SLC27A2
lightyellow	CC	GO:0031231	intrinsic to peroxisomal membrane	17735	14	27	1	0.02	0.022	50	SLC27A2
lightyellow	BP	GO:0071407	cellular response to organic cyclic comp...	17735	162	27	2	0.23	0.02255	8.695652	CYP11A1;NTRK1
lightyellow	MF	GO:0004518	nuclease activity	17735	161	27	2	0.23	0.0227	8.695652	ENPP3;LOC81691
lightyellow	BP	GO:0007141	male meiosis I	17735	16	27	1	0.02	0.02287	50	CCNA1
lightyellow	BP	GO:0007617	mating behavior	17735	16	27	1	0.02	0.02287	50	CYP11A1
lightyellow	BP	GO:0009713	catechol-containing compound biosyntheti...	17735	16	27	1	0.02	0.02287	50	HDC
lightyellow	BP	GO:0034312	diol biosynthetic process	17735	16	27	1	0.02	0.02287	50	HDC
lightyellow	BP	GO:0042423	catecholamine biosynthetic process	17735	16	27	1	0.02	0.02287	50	HDC
lightyellow	BP	GO:0043304	regulation of mast cell degranulation	17735	16	27	1	0.02	0.02287	50	MS4A2
lightyellow	BP	GO:0045746	negative regulation of Notch signaling p...	17735	16	27	1	0.02	0.02287	50	GATA2
lightyellow	BP	GO:0046189	phenol-containing compound biosynthetic ...	17735	16	27	1	0.02	0.02287	50	HDC
lightyellow	BP	GO:0051968	positive regulation of synaptic transmis...	17735	16	27	1	0.02	0.02287	50	NTRK1
lightyellow	MF	GO:0000980	RNA polymerase II distal enhancer sequen...	17735	16	27	1	0.02	0.023	50	GATA2
lightyellow	MF	GO:0001871	pattern binding	17735	16	27	1	0.02	0.023	50	ENPP3
lightyellow	MF	GO:0008395	steroid hydroxylase activity	17735	16	27	1	0.02	0.023	50	CYP11A1
lightyellow	MF	GO:0030247	polysaccharide binding	17735	16	27	1	0.02	0.023	50	ENPP3
lightyellow	BP	GO:0033006	regulation of mast cell activation invol...	17735	17	27	1	0.02	0.02428	50	MS4A2
lightyellow	BP	GO:0042359	vitamin D metabolic process	17735	17	27	1	0.02	0.02428	50	CYP11A1
lightyellow	BP	GO:0051412	response to corticosterone stimulus	17735	17	27	1	0.02	0.02428	50	CYP11A1
lightyellow	MF	GO:0035035	histone acetyltransferase binding	17735	17	27	1	0.02	0.0245	50	EPAS1
lightyellow	BP	GO:1901342	regulation of vasculature development	17735	170	27	2	0.25	0.02467	8	GATA2;NTRK1
lightyellow	BP	GO:0001964	startle response	17735	18	27	1	0.03	0.02569	33.33333	CTNNA2
lightyellow	BP	GO:0021533	cell differentiation in hindbrain	17735	18	27	1	0.03	0.02569	33.33333	GATA2
lightyellow	BP	GO:0031638	zymogen activation	17735	18	27	1	0.03	0.02569	33.33333	PRSS3
lightyellow	BP	GO:0042401	cellular biogenic amine biosynthetic pro...	17735	18	27	1	0.03	0.02569	33.33333	HDC
lightyellow	BP	GO:0021700	developmental maturation	17735	174	27	2	0.25	0.02576	8	EPAS1;GATA2
lightyellow	MF	GO:0019865	immunoglobulin binding	17735	18	27	1	0.03	0.0259	33.33333	MS4A2
lightyellow	BP	GO:0048872	homeostasis of number of cells	17735	176	27	2	0.25	0.02631	8	EPAS1;GATA2
lightyellow	BP	GO:0006700	C21-steroid hormone biosynthetic process	17735	19	27	1	0.03	0.0271	33.33333	CYP11A1
lightyellow	BP	GO:0045648	positive regulation of erythrocyte diffe...	17735	19	27	1	0.03	0.0271	33.33333	GATA2
lightyellow	BP	GO:0060216	definitive hemopoiesis	17735	19	27	1	0.03	0.0271	33.33333	GATA2

lightyellow	BP	GO:0008210	estrogen metabolic process	17735	20	27	1	0.03	0.02851	33.33333	CYP11A1
lightyellow	BP	GO:0050974	detection of mechanical stimulus involve...	17735	20	27	1	0.03	0.02851	33.33333	NTRK1
lightyellow	BP	GO:0071312	cellular response to alkaloid	17735	20	27	1	0.03	0.02851	33.33333	NTRK1
lightyellow	MF	GO:0016878	acid-thiol ligase activity	17735	20	27	1	0.03	0.0287	33.33333	SLC27A2
lightyellow	CC	GO:0044425	membrane part	17735	6026	27	15	9.71	0.029	1.544799	C1orf186;CTNNA2;CYP11A1;ENPP3;HRH4;ITGB8;KLHDC7A;MS4A2;NTRK1;PTGER3;SLC27A2;SLC2A10;SLC45
lightyellow	BP	GO:0001516	prostaglandin biosynthetic process	17735	21	27	1	0.03	0.02991	33.33333	HPGDS
lightyellow	BP	GO:0003081	regulation of systemic arterial blood pr...	17735	21	27	1	0.03	0.02991	33.33333	CPA3
lightyellow	BP	GO:0009651	response to salt stress	17735	21	27	1	0.03	0.02991	33.33333	CYP11A1
lightyellow	BP	GO:0034698	response to gonadotropin stimulus	17735	21	27	1	0.03	0.02991	33.33333	CYP11A1
lightyellow	BP	GO:0043300	regulation of leukocyte degranulation	17735	21	27	1	0.03	0.02991	33.33333	MS4A2
lightyellow	BP	GO:0046457	prostanoid biosynthetic process	17735	21	27	1	0.03	0.02991	33.33333	HPGDS
lightyellow	BP	GO:0051385	response to mineralocorticoid stimulus	17735	21	27	1	0.03	0.02991	33.33333	CYP11A1
lightyellow	BP	GO:0008406	gonad development	17735	189	27	2	0.27	0.03001	7.407407	CYP11A1;NTRK1
lightyellow	BP	GO:0072330	monocarboxylic acid biosynthetic process	17735	190	27	2	0.27	0.0303	7.407407	HPGDS;SLC27A2
lightyellow	BP	GO:0060249	anatomical structure homeostasis	17735	192	27	2	0.28	0.03089	7.142857	EPAS1;GATA2
lightyellow	BP	GO:0002675	positive regulation of acute inflammator...	17735	22	27	1	0.03	0.03132	33.33333	PTGER3
lightyellow	BP	GO:0071320	cellular response to cAMP	17735	22	27	1	0.03	0.03132	33.33333	CYP11A1
lightyellow	BP	GO:0016054	organic acid catabolic process	17735	198	27	2	0.29	0.03269	6.896552	HDC;SLC27A2
lightyellow	BP	GO:0046395	carboxylic acid catabolic process	17735	198	27	2	0.29	0.03269	6.896552	HDC;SLC27A2
lightyellow	BP	GO:0000083	regulation of transcription involved in ...	17735	23	27	1	0.03	0.03272	33.33333	CCNA1
lightyellow	BP	GO:0002886	regulation of myeloid leukocyte mediated...	17735	23	27	1	0.03	0.03272	33.33333	MS4A2
lightyellow	BP	GO:0030219	megakaryocyte differentiation	17735	23	27	1	0.03	0.03272	33.33333	GATA2
lightyellow	BP	GO:0042537	benzene-containing compound metabolic pr...	17735	23	27	1	0.03	0.03272	33.33333	CYP11A1
lightyellow	BP	GO:0048873	homeostasis of number of cells within a ...	17735	23	27	1	0.03	0.03272	33.33333	GATA2
lightyellow	MF	GO:0045296	cadherin binding	17735	23	27	1	0.03	0.033	33.33333	CTNNA2
lightyellow	MF	GO:0051018	protein kinase A binding	17735	23	27	1	0.03	0.033	33.33333	AKAP12
lightyellow	MF	GO:0051119	sugar transmembrane transporter activity	17735	23	27	1	0.03	0.033	33.33333	SLC2A10
lightyellow	BP	GO:0000038	very long-chain fatty acid metabolic pro...	17735	24	27	1	0.03	0.03412	33.33333	SLC27A2
lightyellow	BP	GO:0010623	developmental programmed cell death	17735	24	27	1	0.03	0.03412	33.33333	NTRK1
lightyellow	BP	GO:0030225	macrophage differentiation	17735	24	27	1	0.03	0.03412	33.33333	GATA2
lightyellow	BP	GO:0033003	regulation of mast cell activation	17735	24	27	1	0.03	0.03412	33.33333	MS4A2
lightyellow	MF	GO:0004181	metallocarboxypeptidase activity	17735	24	27	1	0.03	0.0344	33.33333	CPA3
lightyellow	MF	GO:0004364	glutathione transferase activity	17735	24	27	1	0.03	0.0344	33.33333	HPGDS
lightyellow	BP	GO:0019752	carboxylic acid metabolic process	17735	876	27	4	1.27	0.03446	3.149606	CYP11A1;HDC;HPGDS;SLC27A2
lightyellow	BP	GO:0007420	brain development	17735	504	27	3	0.73	0.03462	4.109589	CTNNA2;CYP11A1;GATA2
lightyellow	BP	GO:0009152	purine ribonucleotide biosynthetic proce...	17735	205	27	2	0.3	0.03484	6.666667	AKAP12;NTRK1
lightyellow	BP	GO:0030097	hemopoiesis	17735	509	27	3	0.74	0.03549	4.054054	EPAS1;GATA2;NTRK1
lightyellow	BP	GO:0043303	mast cell degranulation	17735	25	27	1	0.04	0.03551	25	MS4A2
lightyellow	BP	GO:0051602	response to electrical stimulus	17735	25	27	1	0.04	0.03551	25	NTRK1
lightyellow	BP	GO:0006164	purine nucleotide biosynthetic process	17735	211	27	2	0.3	0.03672	6.666667	AKAP12;NTRK1
lightyellow	BP	GO:0002279	mast cell activation involved in immune ...	17735	26	27	1	0.04	0.03691	25	MS4A2
lightyellow	BP	GO:0002448	mast cell mediated immunity	17735	26	27	1	0.04	0.03691	25	MS4A2
lightyellow	BP	GO:0014037	Schwann cell differentiation	17735	26	27	1	0.04	0.03691	25	CYP11A1
lightyellow	BP	GO:0046579	positive regulation of Ras protein signa...	17735	26	27	1	0.04	0.03691	25	NTRK1
lightyellow	BP	GO:0048485	sympathetic nervous system development	17735	26	27	1	0.04	0.03691	25	NTRK1
lightyellow	MF	GO:0001158	enhancer sequence-specific DNA binding	17735	26	27	1	0.04	0.0372	25	GATA2
lightyellow	BP	GO:0007610	behavior	17735	519	27	3	0.75	0.03729	4	CYP11A1;HPGDS;NTRK1
lightyellow	BP	GO:0006699	bile acid biosynthetic process	17735	27	27	1	0.04	0.0383	25	SLC27A2
lightyellow	BP	GO:0050982	detection of mechanical stimulus	17735	27	27	1	0.04	0.0383	25	NTRK1
lightyellow	BP	GO:0060627	regulation of vesicle-mediated transport	17735	216	27	2	0.31	0.03832	6.451613	GATA2;MS4A2
lightyellow	BP	GO:0097305	response to alcohol	17735	217	27	2	0.31	0.03865	6.451613	CYP11A1;NTRK1
lightyellow	BP	GO:0002376	immune system process	17735	1816	27	6	2.62	0.03888	2.290076	ENPP3;EPAS1;GATA2;MS4A2;NTRK1;PRSS3
lightyellow	BP	GO:0009260	ribonucleotide biosynthetic process	17735	218	27	2	0.31	0.03897	6.451613	AKAP12;NTRK1
lightyellow	BP	GO:0045137	development of primary sexual characteri...	17735	219	27	2	0.32	0.0393	6.25	CYP11A1;NTRK1
lightyellow	BP	GO:0008207	C21-steroid hormone metabolic process	17735	28	27	1	0.04	0.03969	25	CYP11A1
lightyellow	BP	GO:0030851	granulocyte differentiation	17735	28	27	1	0.04	0.03969	25	GATA2
lightyellow	BP	GO:0048854	brain morphogenesis	17735	28	27	1	0.04	0.03969	25	CTNNA2
lightyellow	BP	GO:0051057	positive regulation of small GTPase medi...	17735	28	27	1	0.04	0.03969	25	NTRK1
lightyellow	BP	GO:0060674	placenta blood vessel development	17735	28	27	1	0.04	0.03969	25	ITGB8
lightyellow	BP	GO:0061418	regulation of transcription from RNA pol...	17735	28	27	1	0.04	0.03969	25	EPAS1
lightyellow	MF	GO:0015144	carbohydrate transmembrane transporter a...	17735	28	27	1	0.04	0.04	25	SLC2A10
lightyellow	MF	GO:1901476	carbohydrate transporter activity	17735	28	27	1	0.04	0.04	25	SLC2A10
lightyellow	BP	GO:0045165	cell fate commitment	17735	222	27	2	0.32	0.04028	6.25	EPAS1;GATA2
lightyellow	BP	GO:0046390	ribose phosphate biosynthetic process	17735	222	27	2	0.32	0.04028	6.25	AKAP12;NTRK1
lightyellow	CC	GO:0005887	integral to plasma membrane	17735	1208	27	5	1.95	0.041	2.564103	ENPP3;ITGB8;MS4A2;NTRK1;PTGER3
lightyellow	BP	GO:0006692	prostanoid metabolic process	17735	29	27	1	0.04	0.04108	25	HPGDS
lightyellow	BP	GO:0006693	prostaglandin metabolic process	17735	29	27	1	0.04	0.04108	25	HPGDS
lightyellow	BP	GO:0007190	activation of adenylate cyclase activity	17735	29	27	1	0.04	0.04108	25	NTRK1
lightyellow	BP	GO:0008209	androgen metabolic process	17735	29	27	1	0.04	0.04108	25	CYP11A1

lightyellow	MF	GO:0016877	ligase activity, forming carbon-sulfur b...	17735	29	27	1	0.04	0.0414	25	SLC27A2
lightyellow	MF	GO:0046875	ephrin receptor binding	17735	29	27	1	0.04	0.0414	25	NTRK1
lightyellow	BP	GO:0048534	hematopoietic or lymphoid organ developm...	17735	542	27	3	0.78	0.04159	3.846154	EPAS1;GATA2;NTRK1
lightyellow	BP	GO:0072522	purine-containing compound biosynthetic ...	17735	227	27	2	0.33	0.04194	6.060606	AKAP12;NTRK1
lightyellow	BP	GO:0048610	cellular process involved in reproductio...	17735	544	27	3	0.79	0.04197	3.797468	CCNA1;CYP11A1;NTRK1
lightyellow	BP	GO:0035065	regulation of histone acetylation	17735	30	27	1	0.04	0.04247	25	GATA2
lightyellow	BP	GO:0042181	ketone biosynthetic process	17735	30	27	1	0.04	0.04247	25	CYP11A1
lightyellow	BP	GO:0045599	negative regulation of fat cell differen...	17735	30	27	1	0.04	0.04247	25	GATA2
lightyellow	BP	GO:0050766	positive regulation of phagocytosis	17735	30	27	1	0.04	0.04247	25	GATA2
lightyellow	BP	GO:0051966	regulation of synaptic transmission, glu...	17735	30	27	1	0.04	0.04247	25	NTRK1
lightyellow	MF	GO:0035326	enhancer binding	17735	30	27	1	0.04	0.0428	25	GATA2
lightyellow	BP	GO:0001659	temperature homeostasis	17735	31	27	1	0.04	0.04386	25	PTGER3
lightyellow	BP	GO:0035094	response to nicotine	17735	31	27	1	0.04	0.04386	25	NTRK1
lightyellow	MF	GO:0016831	carboxy-lyase activity	17735	31	27	1	0.05	0.0442	20	HDC
lightyellow	MF	GO:0042169	SH2 domain binding	17735	31	27	1	0.05	0.0442	20	MS4A2
lightyellow	BP	GO:0001990	regulation of systemic arterial blood pr...	17735	32	27	1	0.05	0.04524	20	CPA3
lightyellow	BP	GO:0002762	negative regulation of myeloid leukocyte...	17735	32	27	1	0.05	0.04524	20	GATA2
lightyellow	BP	GO:0006730	one-carbon metabolic process	17735	32	27	1	0.05	0.04524	20	CA8
lightyellow	BP	GO:0007140	male meiosis	17735	32	27	1	0.05	0.04524	20	CCNA1
lightyellow	BP	GO:0014823	response to activity	17735	32	27	1	0.05	0.04524	20	NTRK1
lightyellow	BP	GO:2000756	regulation of peptidyl-lysine acetylatio...	17735	32	27	1	0.05	0.04524	20	GATA2
lightyellow	BP	GO:0008610	lipid biosynthetic process	17735	564	27	3	0.81	0.04593	3.703704	CYP11A1;HPGDS;SLC27A2
lightyellow	CC	GO:0008305	integrin complex	17735	29	27	1	0.05	0.046	20	ITGB8
lightyellow	BP	GO:0006775	fat-soluble vitamin metabolic process	17735	33	27	1	0.05	0.04662	20	CYP11A1
lightyellow	BP	GO:0009309	amine biosynthetic process	17735	33	27	1	0.05	0.04662	20	HDC
lightyellow	BP	GO:0021517	ventral spinal cord development	17735	33	27	1	0.05	0.04662	20	GATA2
lightyellow	BP	GO:0045646	regulation of erythrocyte differentiatio...	17735	33	27	1	0.05	0.04662	20	GATA2
lightyellow	BP	GO:0045762	positive regulation of adenylate cyclase...	17735	33	27	1	0.05	0.04662	20	NTRK1
lightyellow	BP	GO:0046686	response to cadmium ion	17735	33	27	1	0.05	0.04662	20	CYP11A1
lightyellow	MF	GO:0015485	cholesterol binding	17735	33	27	1	0.05	0.047	20	CYP11A1
lightyellow	CC	GO:0031226	intrinsic to plasma membrane	17735	1252	27	5	2.02	0.047	2.475248	ENPP3;ITGB8;MS4A2;NTRK1;PTGER3
lightyellow	BP	GO:0002520	immune system development	17735	572	27	3	0.83	0.04756	3.614458	EPAS1;GATA2;NTRK1
lightyellow	BP	GO:0045921	positive regulation of exocytosis	17735	34	27	1	0.05	0.048	20	MS4A2
lightyellow	BP	GO:0030099	myeloid cell differentiation	17735	247	27	2	0.36	0.04882	5.555556	EPAS1;GATA2
lightyellow	BP	GO:0007205	protein kinase C-activating G-protein co...	17735	35	27	1	0.05	0.04938	20	MS4A2
lightyellow	BP	GO:0007618	mating	17735	35	27	1	0.05	0.04938	20	CYP11A1
lightyellow	BP	GO:0043436	oxoacid metabolic process	17735	985	27	4	1.42	0.04972	2.816901	CYP11A1;HDC;HPGDS;SLC27A2
lightgreen	BP	GO:0031343	positive regulation of cell killing	17735	29	36	3	0.06	2.80E-05	50	CD226;IFNG;XCL1
lightgreen	BP	GO:0002889	regulation of immunoglobulin mediated im...	17735	32	36	3	0.07	3.80E-05	42.85714	CD226;IFNG;XCL1
lightgreen	BP	GO:0002712	regulation of B cell mediated immunity	17735	33	36	3	0.07	4.10E-05	42.85714	CD226;IFNG;XCL1
lightgreen	BP	GO:0031341	regulation of cell killing	17735	36	36	3	0.07	5.40E-05	42.85714	CD226;IFNG;XCL1
lightgreen	MF	GO:0030246	carbohydrate binding	17735	215	36	5	0.5	0.00013	10	KLRB1;KLRC1;KLRC2;KLRC3;KLRF1
lightgreen	BP	GO:0036037	CD8-positive, alpha-beta T cell activati...	17735	10	36	2	0.02	0.00018	100	IFNG;XCL1
lightgreen	BP	GO:0006968	cellular defense response	17735	60	36	3	0.12	0.00025	25	KLRC2;KLRC3;KLRC4
lightgreen	BP	GO:0045073	regulation of chemokine biosynthetic pro...	17735	12	36	2	0.02	0.00027	100	IFNG;TLR3
lightgreen	CC	GO:0044459	plasma membrane part	17735	1895	36	12	3.96	0.00029	3.030303	ADRB2;CD226;EPHB4;GZMA;IFNG;KLRC1;KLRC2;KLRF1;MYO6;SLC4A4;TGFBR3;TLR3
lightgreen	BP	GO:0042033	chemokine biosynthetic process	17735	13	36	2	0.03	0.00032	66.66667	IFNG;TLR3
lightgreen	BP	GO:0001906	cell killing	17735	68	36	3	0.14	0.00036	21.42857	CD226;IFNG;XCL1
lightgreen	BP	GO:0050755	chemokine metabolic process	17735	15	36	2	0.03	0.00042	66.66667	IFNG;TLR3
lightgreen	BP	GO:0051098	regulation of binding	17735	178	36	4	0.37	0.00046	10.81081	ADRB2;GZMA;ID2;TGFBR3
lightgreen	BP	GO:0002706	regulation of lymphocyte mediated immuni...	17735	75	36	3	0.15	0.00048	20	CD226;IFNG;XCL1
lightgreen	BP	GO:0051100	negative regulation of binding	17735	75	36	3	0.15	0.00048	20	ADRB2;GZMA;ID2
lightgreen	BP	GO:0002761	regulation of myeloid leukocyte differen...	17735	77	36	3	0.16	0.00052	18.75	ID2;IFNG;TLR3
lightgreen	BP	GO:0002822	regulation of adaptive immune response b...	17735	83	36	3	0.17	0.00065	17.64706	CD226;IFNG;XCL1
lightgreen	CC	GO:0005887	integral to plasma membrane	17735	1208	36	9	2.52	0.00065	3.571429	ADRB2;CD226;EPHB4;KLRC1;KLRC2;KLRF1;SLC4A4;TGFBR3;TLR3
lightgreen	BP	GO:0010771	negative regulation of cell morphogenesi...	17735	21	36	2	0.04	0.00084	50	IFNG;TGFBR3
lightgreen	CC	GO:0031226	intrinsic to plasma membrane	17735	1252	36	9	2.62	0.00084	3.435115	ADRB2;CD226;EPHB4;KLRC1;KLRC2;KLRF1;SLC4A4;TGFBR3;TLR3
lightgreen	BP	GO:0002819	regulation of adaptive immune response	17735	91	36	3	0.19	0.00085	15.78947	CD226;IFNG;XCL1
lightgreen	CC	GO:0005886	plasma membrane	17735	4273	36	18	8.93	0.00089	2.015677	ADRB2;CD226;DSG3;ENPP5;EPHB4;GZMA;IFNG;KLRB1;KLRC1;KLRC2;KLRF1;MYO6;PHLDA1;PHLDB2;SLC4A4;
lightgreen	BP	GO:0002703	regulation of leukocyte mediated immunit...	17735	94	36	3	0.19	0.00094	15.78947	CD226;IFNG;XCL1
lightgreen	BP	GO:0032735	positive regulation of interleukin-12 pr...	17735	24	36	2	0.05	0.0011	40	IFNG;TLR3
lightgreen	BP	GO:0002699	positive regulation of immune effector p...	17735	100	36	3	0.21	0.00112	14.28571	CD226;IFNG;XCL1
lightgreen	CC	GO:0071944	cell periphery	17735	4360	36	18	9.11	0.00115	1.975851	ADRB2;CD226;DSG3;ENPP5;EPHB4;GZMA;IFNG;KLRB1;KLRC1;KLRC2;KLRF1;MYO6;PHLDA1;PHLDB2;SLC4A4;
lightgreen	BP	GO:0006955	immune response	17735	1081	36	8	2.23	0.00121	3.587444	CD226;GZMA;IFNG;KLRC1;TGFBR3;TLR3;XCL1;XCL2
lightgreen	BP	GO:0001912	positive regulation of leukocyte mediate...	17735	26	36	2	0.05	0.0013	40	CD226;XCL1
lightgreen	BP	GO:0033002	muscle cell proliferation	17735	106	36	3	0.22	0.00132	13.63636	ID2;IFNG;TGFBR3
lightgreen	MF	GO:0005126	cytokine receptor binding	17735	215	36	4	0.5	0.00148	8	IFNG;TGFBR3;XCL1;XCL2
lightgreen	MF	GO:0004888	transmembrane signaling receptor activit...	17735	1232	36	9	2.85	0.00156	3.157895	ADRB2;EPHB4;KLRB1;KLRC1;KLRC2;KLRC3;KLRF1;TGFBR3;TLR3
lightgreen	BP	GO:0030099	myeloid cell differentiation	17735	247	36	4	0.51	0.00157	7.843137	ID2;IFNG;TGFBR3;TLR3

lightgreen	BP	GO:0016064	immunoglobulin mediated immune response	17735	116	36	3	0.24	0.00172	12.5	CD226;IFNG;XCL1
lightgreen	BP	GO:0032760	positive regulation of tumor necrosis fa...	17735	31	36	2	0.06	0.00184	33.33333	IFNG;TLR3
lightgreen	BP	GO:0010721	negative regulation of cell development	17735	119	36	3	0.25	0.00185	12	ID2;IFNG;TGFBR3
lightgreen	BP	GO:0019724	B cell mediated immunity	17735	119	36	3	0.25	0.00185	12	CD226;IFNG;XCL1
lightgreen	BP	GO:0001910	regulation of leukocyte mediated cytotox...	17735	33	36	2	0.07	0.00209	28.57143	CD226;XCL1
lightgreen	BP	GO:0002637	regulation of immunoglobulin production	17735	33	36	2	0.07	0.00209	28.57143	IFNG;XCL1
lightgreen	BP	GO:0045596	negative regulation of cell differentiat...	17735	454	36	5	0.94	0.00217	5.319149	HOPX;ID2;IFNG;TGFBR3;TLR3
lightgreen	BP	GO:0002763	positive regulation of myeloid leukocyte...	17735	35	36	2	0.07	0.00235	28.57143	ID2;IFNG
lightgreen	BP	GO:0045637	regulation of myeloid cell differentiati...	17735	132	36	3	0.27	0.00248	11.11111	ID2;IFNG;TLR3
lightgreen	BP	GO:0002573	myeloid leukocyte differentiation	17735	133	36	3	0.27	0.00254	11.11111	ID2;IFNG;TLR3
lightgreen	BP	GO:0043392	negative regulation of DNA binding	17735	37	36	2	0.08	0.00262	25	GZMA;ID2
lightgreen	MF	GO:0038023	signaling receptor activity	17735	1328	36	9	3.08	0.00262	2.922078	ADRB2;EPHB4;KLRB1;KLRC1;KLRC2;KLRC3;KLRF1;TGFBR3;TLR3
lightgreen	BP	GO:0045670	regulation of osteoclast differentiation	17735	40	36	2	0.08	0.00306	25	IFNG;TLR3
lightgreen	BP	GO:0032655	regulation of interleukin-12 production	17735	42	36	2	0.09	0.00337	22.22222	IFNG;TLR3
lightgreen	BP	GO:0002381	immunoglobulin production involved in im...	17735	43	36	2	0.09	0.00353	22.22222	IFNG;XCL1
lightgreen	BP	GO:0032615	interleukin-12 production	17735	43	36	2	0.09	0.00353	22.22222	IFNG;TLR3
lightgreen	BP	GO:0002705	positive regulation of leukocyte mediate...	17735	46	36	2	0.09	0.00403	22.22222	CD226;XCL1
lightgreen	BP	GO:0002708	positive regulation of lymphocyte mediat...	17735	46	36	2	0.09	0.00403	22.22222	CD226;XCL1
lightgreen	BP	GO:0002824	positive regulation of adaptive immune r...	17735	46	36	2	0.09	0.00403	22.22222	CD226;XCL1
lightgreen	BP	GO:0001909	leukocyte mediated cytotoxicity	17735	49	36	2	0.1	0.00456	20	CD226;XCL1
lightgreen	BP	GO:0002821	positive regulation of adaptive immune r...	17735	49	36	2	0.1	0.00456	20	CD226;XCL1
lightgreen	BP	GO:0032642	regulation of chemokine production	17735	49	36	2	0.1	0.00456	20	IFNG;TLR3
lightgreen	BP	GO:0002694	regulation of leukocyte activation	17735	334	36	4	0.69	0.00467	5.797101	CD226;ID2;IFNG;XCL1
lightgreen	BP	GO:0051090	regulation of sequence-specific DNA bind...	17735	338	36	4	0.7	0.00488	5.714286	ID2;TGFBR3;TLR3;XCL1
lightgreen	MF	GO:0008009	chemokine activity	17735	47	36	2	0.11	0.00527	18.18182	XCL1;XCL2
lightgreen	BP	GO:0051093	negative regulation of developmental pro...	17735	559	36	5	1.15	0.0053	4.347826	HOPX;ID2;IFNG;TGFBR3;TLR3
lightgreen	BP	GO:0030316	osteoclast differentiation	17735	53	36	2	0.11	0.00531	18.18182	IFNG;TLR3
lightgreen	BP	GO:0030593	neutrophil chemotaxis	17735	53	36	2	0.11	0.00531	18.18182	IFNG;XCL1
lightgreen	BP	GO:0032602	chemokine production	17735	53	36	2	0.11	0.00531	18.18182	IFNG;TLR3
lightgreen	BP	GO:0050776	regulation of immune response	17735	560	36	5	1.16	0.00534	4.310345	CD226;IFNG;KLRC1;TLR3;XCL1
lightgreen	BP	GO:0008015	blood circulation	17735	349	36	4	0.72	0.00546	5.555556	ADRB2;ID2;IFNG;XCL2
lightgreen	BP	GO:0003013	circulatory system process	17735	350	36	4	0.72	0.00551	5.555556	ADRB2;ID2;IFNG;XCL2
lightgreen	BP	GO:0048872	homeostasis of number of cells	17735	176	36	3	0.36	0.00557	8.333333	ID2;IFNG;TGFBR3
lightgreen	BP	GO:0002449	lymphocyte mediated immunity	17735	179	36	3	0.37	0.00583	8.108108	CD226;IFNG;XCL1
lightgreen	BP	GO:0050865	regulation of cell activation	17735	359	36	4	0.74	0.00603	5.405405	CD226;ID2;IFNG;XCL1
lightgreen	BP	GO:0042108	positive regulation of cytokine biosynth...	17735	59	36	2	0.12	0.00654	16.66667	IFNG;TLR3
lightgreen	BP	GO:0006952	defense response	17735	1119	36	7	2.31	0.00664	3.030303	CD226;IFNG;KLRC2;KLRC3;KLRC4;TLR3;XCL1
lightgreen	MF	GO:0004872	receptor activity	17735	1528	36	9	3.54	0.0067	2.542373	ADRB2;EPHB4;KLRB1;KLRC1;KLRC2;KLRC3;KLRF1;TGFBR3;TLR3
lightgreen	BP	GO:0045639	positive regulation of myeloid cell diff...	17735	61	36	2	0.13	0.00698	15.38462	ID2;IFNG
lightgreen	MF	GO:0042379	chemokine receptor binding	17735	55	36	2	0.13	0.00716	15.38462	XCL1;XCL2
lightgreen	BP	GO:0002460	adaptive immune response based on somati...	17735	193	36	3	0.4	0.00718	7.5	CD226;IFNG;XCL1
lightgreen	BP	GO:0001824	blastocyst development	17735	62	36	2	0.13	0.00721	15.38462	HOPX;TGFBR3
lightgreen	BP	GO:0002700	regulation of production of molecular me...	17735	65	36	2	0.13	0.0079	15.38462	IFNG;XCL1
lightgreen	BP	GO:0032640	tumor necrosis factor production	17735	66	36	2	0.14	0.00813	14.28571	IFNG;TLR3
lightgreen	BP	GO:0032680	regulation of tumor necrosis factor prod...	17735	66	36	2	0.14	0.00813	14.28571	IFNG;TLR3
lightgreen	BP	GO:0001819	positive regulation of cytokine producti...	17735	202	36	3	0.42	0.00814	7.142857	IFNG;TLR3;XCL1
lightgreen	BP	GO:0048660	regulation of smooth muscle cell prolife...	17735	67	36	2	0.14	0.00837	14.28571	ID2;IFNG
lightgreen	BP	GO:0051101	regulation of DNA binding	17735	67	36	2	0.14	0.00837	14.28571	GZMA;ID2
lightgreen	BP	GO:0051341	regulation of oxidoreductase activity	17735	67	36	2	0.14	0.00837	14.28571	GZMA;IFNG
lightgreen	BP	GO:0002682	regulation of immune system process	17735	884	36	6	1.82	0.00838	3.296703	CD226;ID2;IFNG;KLRC1;TLR3;XCL1
lightgreen	CC	GO:0044425	membrane part	17735	6026	36	20	12.59	0.00849	1.588562	ADRB2;CD226;DSG3;ELOVL6;ENPP4;ENPP5;EPHB4;GZMA;IFNG;KLRB1;KLRC1;KLRC2;KLRC3;KLRC4;KLRF1;MY
lightgreen	BP	GO:0032649	regulation of interferon-gamma productio...	17735	68	36	2	0.14	0.00861	14.28571	TLR3;XCL1
lightgreen	BP	GO:0032675	regulation of interleukin-6 production	17735	69	36	2	0.14	0.00886	14.28571	IFNG;TLR3
lightgreen	BP	GO:0042102	positive regulation of T cell proliferat...	17735	69	36	2	0.14	0.00886	14.28571	IFNG;XCL1
lightgreen	BP	GO:0002376	immune system process	17735	1816	36	9	3.75	0.00891	2.4	CD226;GZMA;ID2;IFNG;KLRC1;TGFBR3;TLR3;XCL1;XCL2
lightgreen	BP	GO:0032635	interleukin-6 production	17735	70	36	2	0.14	0.00911	14.28571	IFNG;TLR3
lightgreen	BP	GO:0048659	smooth muscle cell proliferation	17735	70	36	2	0.14	0.00911	14.28571	ID2;IFNG
lightgreen	BP	GO:0071706	tumor necrosis factor superfamily cytoki...	17735	70	36	2	0.14	0.00911	14.28571	IFNG;TLR3
lightgreen	BP	GO:0002250	adaptive immune response	17735	211	36	3	0.44	0.00916	6.818182	CD226;IFNG;XCL1
lightgreen	BP	GO:0002697	regulation of immune effector process	17735	217	36	3	0.45	0.00989	6.666667	CD226;IFNG;XCL1
lightgreen	BP	GO:0006921	cellular component disassembly involved ...	17735	74	36	2	0.15	0.01014	13.33333	DSG3;GZMA
lightgreen	CC	GO:0005576	extracellular region	17735	2141	36	10	4.47	0.01015	2.237136	ENPP5;ESM1;GZMA;GZMK;IFNG;LPAL2;PDGFD;TGFBR3;XCL1;XCL2
lightgreen	MF	GO:0004871	signal transducer activity	17735	1632	36	9	3.78	0.01022	2.380952	ADRB2;EPHB4;KLRB1;KLRC1;KLRC2;KLRC3;KLRF1;TGFBR3;TLR3
lightgreen	MF	GO:0060089	molecular transducer activity	17735	1632	36	9	3.78	0.01022	2.380952	ADRB2;EPHB4;KLRB1;KLRC1;KLRC2;KLRC3;KLRF1;TGFBR3;TLR3
lightgreen	BP	GO:0002443	leukocyte mediated immunity	17735	221	36	3	0.46	0.01039	6.521739	CD226;IFNG;XCL1
lightgreen	BP	GO:0002377	immunoglobulin production	17735	75	36	2	0.15	0.0104	13.33333	IFNG;XCL1
lightgreen	BP	GO:0045088	regulation of innate immune response	17735	225	36	3	0.46	0.01091	6.521739	CD226;IFNG;TLR3
lightgreen	BP	GO:0032609	interferon-gamma production	17735	77	36	2	0.16	0.01094	12.5	TLR3;XCL1
lightgreen	BP	GO:0031347	regulation of defense response	17735	428	36	4	0.88	0.01107	4.545455	CD226;IFNG;TLR3;XCL1

lightgreen	BP	GO:0001816	cytokine production	17735	430	36	4	0.89	0.01124	4.494382	CD226;IFNG;TLR3;XCL1
lightgreen	BP	GO:0002696	positive regulation of leukocyte activat...	17735	229	36	3	0.47	0.01144	6.382979	CD226;IFNG;XCL1
lightgreen	MF	GO:0005125	cytokine activity	17735	204	36	3	0.47	0.01154	6.382979	IFNG;XCL1;XCL2
lightgreen	BP	GO:0050864	regulation of B cell activation	17735	83	36	2	0.17	0.01262	11.76471	ID2;IFNG
lightgreen	BP	GO:0050867	positive regulation of cell activation	17735	239	36	3	0.49	0.01283	6.122449	CD226;IFNG;XCL1
lightgreen	BP	GO:0001656	metanephros development	17735	85	36	2	0.18	0.01321	11.11111	ID2;IFNG
lightgreen	BP	GO:0030218	erythrocyte differentiation	17735	88	36	2	0.18	0.01411	11.11111	ID2;TGFBR3
lightgreen	BP	GO:0046631	alpha-beta T cell activation	17735	88	36	2	0.18	0.01411	11.11111	IFNG;XCL1
lightgreen	BP	GO:0045944	positive regulation of transcription fro...	17735	710	36	5	1.46	0.01413	3.424658	ADRB2;ID2;IFNG;MYO6;TLR3
lightgreen	CC	GO:0016020	membrane	17735	7690	36	23	16.07	0.01437	1.431238	ADRB2;CD226;DSG3;ELOVL6;ENPP4;ENPP5;EPHB4;GZMA;IFNG;KLRB1;KLRC1;KLRC2;KLRC3;KLRC4;KLRF1;MY
lightgreen	BP	GO:0042035	regulation of cytokine biosynthetic proc...	17735	91	36	2	0.19	0.01504	10.52632	IFNG;TLR3
lightgreen	BP	GO:0002252	immune effector process	17735	471	36	4	0.97	0.01528	4.123711	CD226;IFNG;TLR3;XCL1
lightgreen	MF	GO:0019199	transmembrane receptor protein kinase ac...	17735	82	36	2	0.19	0.01541	10.52632	EPHB4;TGFBR3
lightgreen	BP	GO:0045893	positive regulation of transcription, DN...	17735	1016	36	6	2.1	0.01599	2.857143	ADRB2;ID2;IFNG;MYBL1;MYO6;TLR3
lightgreen	BP	GO:0034101	erythrocyte homeostasis	17735	95	36	2	0.2	0.01631	10	ID2;TGFBR3
lightgreen	BP	GO:0009615	response to virus	17735	263	36	3	0.54	0.01657	5.555556	IFNG;TLR3;XCL1
lightgreen	BP	GO:0043393	regulation of protein binding	17735	96	36	2	0.2	0.01664	10	ADRB2;TGFBR3
lightgreen	BP	GO:0051250	negative regulation of lymphocyte activa...	17735	96	36	2	0.2	0.01664	10	ID2;XCL1
lightgreen	BP	GO:0050671	positive regulation of lymphocyte prolif...	17735	97	36	2	0.2	0.01697	10	IFNG;XCL1
lightgreen	BP	GO:0048522	positive regulation of cellular process	17735	3140	36	12	6.48	0.01728	1.851852	ADRB2;CD226;GZMA;ID2;IFNG;MYBL1;MYO6;PDGFD;PHLDA1;TGFBR3;TLR3;XCL1
lightgreen	BP	GO:0032946	positive regulation of mononuclear cell ...	17735	98	36	2	0.2	0.0173	10	IFNG;XCL1
lightgreen	CC	GO:0009986	cell surface	17735	485	36	4	1.01	0.01787	3.960396	CD226;EPHB4;IFNG;TGFBR3
lightgreen	BP	GO:0070665	positive regulation of leukocyte prolife...	17735	100	36	2	0.21	0.01797	9.52381	IFNG;XCL1
lightgreen	BP	GO:0042089	cytokine biosynthetic process	17735	101	36	2	0.21	0.01831	9.52381	IFNG;TLR3
lightgreen	MF	GO:0005102	receptor binding	17735	1201	36	7	2.78	0.01843	2.517986	CD226;ESM1;IFNG;PDGFD;TGFBR3;XCL1;XCL2
lightgreen	BP	GO:0032844	regulation of homeostatic process	17735	275	36	3	0.57	0.01864	5.263158	ID2;IFNG;XCL1
lightgreen	BP	GO:0042107	cytokine metabolic process	17735	104	36	2	0.21	0.01935	9.52381	IFNG;TLR3
lightgreen	BP	GO:0051092	positive regulation of NF-kappaB transcr...	17735	104	36	2	0.21	0.01935	9.52381	TGFBR3;TLR3
lightgreen	BP	GO:0051254	positive regulation of RNA metabolic pro...	17735	1063	36	6	2.19	0.0196	2.739726	ADRB2;ID2;IFNG;MYBL1;MYO6;TLR3
lightgreen	BP	GO:0030097	hemopoiesis	17735	509	36	4	1.05	0.01977	3.809524	ID2;IFNG;TGFBR3;TLR3
lightgreen	BP	GO:0042129	regulation of T cell proliferation	17735	107	36	2	0.22	0.02041	9.090909	IFNG;XCL1
lightgreen	BP	GO:0002385	mucosal immune response	17735	10	36	1	0.02	0.02045	50	XCL1
lightgreen	BP	GO:0002418	immune response to tumor cell	17735	10	36	1	0.02	0.02045	50	CD226
lightgreen	BP	GO:0007171	activation of transmembrane receptor pro...	17735	10	36	1	0.02	0.02045	50	ADRB2
lightgreen	BP	GO:0007252	I-kappaB phosphorylation	17735	10	36	1	0.02	0.02045	50	TLR3
lightgreen	BP	GO:0032703	negative regulation of interleukin-2 pro...	17735	10	36	1	0.02	0.02045	50	XCL1
lightgreen	BP	GO:0048291	isotype switching to IgG isotypes	17735	10	36	1	0.02	0.02045	50	IFNG
lightgreen	BP	GO:0048541	Peyer's patch development	17735	10	36	1	0.02	0.02045	50	ID2
lightgreen	BP	GO:0008285	negative regulation of cell proliferatio...	17735	522	36	4	1.08	0.02147	3.703704	ID2;IFNG;TGFBR3;XCL1
lightgreen	BP	GO:0002695	negative regulation of leukocyte activat...	17735	110	36	2	0.23	0.02149	8.695652	ID2;XCL1
lightgreen	BP	GO:0051240	positive regulation of multicellular org...	17735	524	36	4	1.08	0.02174	3.703704	ADRB2;IFNG;TLR3;XCL1
lightgreen	BP	GO:0002440	production of molecular mediator of immu...	17735	111	36	2	0.23	0.02186	8.695652	IFNG;XCL1
lightgreen	BP	GO:0051249	regulation of lymphocyte activation	17735	293	36	3	0.6	0.022	5	ID2;IFNG;XCL1
lightgreen	CC	GO:0016021	integral to membrane	17735	5202	36	17	10.87	0.02241	1.563937	ADRB2;CD226;DSG3;ELOVL6;ENPP4;ENPP5;EPHB4;KLRB1;KLRC1;KLRC2;KLRC3;KLRC4;KLRF1;SLC4A4;STYK1;T
lightgreen	BP	GO:0002710	negative regulation of T cell mediated i...	17735	11	36	1	0.02	0.02247	50	XCL1
lightgreen	BP	GO:0002833	positive regulation of response to bioti...	17735	11	36	1	0.02	0.02247	50	CD226
lightgreen	BP	GO:0003161	cardiac conduction system development	17735	11	36	1	0.02	0.02247	50	ID2
lightgreen	BP	GO:0010893	positive regulation of steroid biosynthe...	17735	11	36	1	0.02	0.02247	50	IFNG
lightgreen	BP	GO:0032354	response to follicle-stimulating hormone...	17735	11	36	1	0.02	0.02247	50	TGFBR3
lightgreen	BP	GO:0033005	positive regulation of mast cell activat...	17735	11	36	1	0.02	0.02247	50	CD226
lightgreen	BP	GO:0034123	positive regulation of toll-like recepto...	17735	11	36	1	0.02	0.02247	50	TLR3
lightgreen	BP	GO:0045351	type I interferon biosynthetic process	17735	11	36	1	0.02	0.02247	50	TLR3
lightgreen	BP	GO:0045830	positive regulation of isotype switching	17735	11	36	1	0.02	0.02247	50	IFNG
lightgreen	BP	GO:0048537	mucosal-associated lymphoid tissue devel...	17735	11	36	1	0.02	0.02247	50	ID2
lightgreen	BP	GO:0048715	negative regulation of oligodendrocyte d...	17735	11	36	1	0.02	0.02247	50	ID2
lightgreen	BP	GO:0071636	positive regulation of transforming grow...	17735	11	36	1	0.02	0.02247	50	XCL1
lightgreen	BP	GO:0072182	regulation of nephron tubule epithelial ...	17735	11	36	1	0.02	0.02247	50	IFNG
lightgreen	BP	GO:2000406	positive regulation of T cell migration	17735	11	36	1	0.02	0.02247	50	XCL1
lightgreen	BP	GO:0043433	negative regulation of sequence-specific...	17735	113	36	2	0.23	0.0226	8.695652	ID2;XCL1
lightgreen	BP	GO:0010628	positive regulation of gene expression	17735	1102	36	6	2.27	0.02301	2.643172	ADRB2;ID2;IFNG;MYBL1;MYO6;TLR3
lightgreen	BP	GO:0009887	organ morphogenesis	17735	808	36	5	1.67	0.02345	2.994012	EPHB4;ID2;IFNG;MYO6;TGFBR3
lightgreen	BP	GO:0009967	positive regulation of signal transducti...	17735	812	36	5	1.68	0.0239	2.97619	ADRB2;CD226;IFNG;TGFBR3;TLR3
lightgreen	BP	GO:0048534	hematopoietic or lymphoid organ developm...	17735	542	36	4	1.12	0.02426	3.571429	ID2;IFNG;TGFBR3;TLR3
lightgreen	BP	GO:0002251	organ or tissue specific immune response	17735	12	36	1	0.02	0.02449	50	XCL1
lightgreen	BP	GO:0002701	negative regulation of production of mol...	17735	12	36	1	0.02	0.02449	50	XCL1
lightgreen	BP	GO:0002719	negative regulation of cytokine producti...	17735	12	36	1	0.02	0.02449	50	XCL1
lightgreen	BP	GO:0003085	negative regulation of systemic arterial...	17735	12	36	1	0.02	0.02449	50	ADRB2
lightgreen	BP	GO:0010818	T cell chemotaxis	17735	12	36	1	0.02	0.02449	50	XCL1
lightgreen	BP	GO:0034390	smooth muscle cell apoptotic process	17735	12	36	1	0.02	0.02449	50	IFNG

lightgreen BP	GO:0034391	regulation of smooth muscle cell apoptot...	17735	12	36	1	0.02	0.02449	50	IFNG
lightgreen BP	GO:0040015	negative regulation of multicellular org...	17735	12	36	1	0.02	0.02449	50	ADRB2
lightgreen BP	GO:0042362	fat-soluble vitamin biosynthetic process	17735	12	36	1	0.02	0.02449	50	IFNG
lightgreen BP	GO:0042510	regulation of tyrosine phosphorylation o...	17735	12	36	1	0.02	0.02449	50	IFNG
lightgreen BP	GO:0045078	positive regulation of interferon-gamma ...	17735	12	36	1	0.02	0.02449	50	TLR3
lightgreen BP	GO:0045346	regulation of MHC class II biosynthetic ...	17735	12	36	1	0.02	0.02449	50	IFNG
lightgreen BP	GO:0070670	response to interleukin-4	17735	12	36	1	0.02	0.02449	50	XCL1
lightgreen MF	GO:0019838	growth factor binding	17735	105	36	2	0.24	0.0245	8.333333	ESM1;TGFBR3
lightgreen BP	GO:0050866	negative regulation of cell activation	17735	119	36	2	0.25	0.02488	8	ID2;XCL1
lightgreen BP	GO:0006468	protein phosphorylation	17735	1126	36	6	2.32	0.02529	2.586207	ADRB2;EPHB4;IFNG;PDGFD;TGFBR3;TLR3
lightgreen BP	GO:0045321	leukocyte activation	17735	552	36	4	1.14	0.02574	3.508772	CD226;ID2;IFNG;XCL1
lightgreen BP	GO:0002684	positive regulation of immune system pro...	17735	554	36	4	1.14	0.02604	3.508772	CD226;IFNG;TLR3;XCL1
lightgreen BP	GO:0030595	leukocyte chemotaxis	17735	123	36	2	0.25	0.02646	8	IFNG;XCL1
lightgreen BP	GO:0001916	positive regulation of T cell mediated c...	17735	13	36	1	0.03	0.0265	33.33333	XCL1
lightgreen BP	GO:0002347	response to tumor cell	17735	13	36	1	0.03	0.0265	33.33333	CD226
lightgreen BP	GO:0002704	negative regulation of leukocyte mediate...	17735	13	36	1	0.03	0.0265	33.33333	XCL1
lightgreen BP	GO:0002707	negative regulation of lymphocyte mediat...	17735	13	36	1	0.03	0.0265	33.33333	XCL1
lightgreen BP	GO:0002714	positive regulation of B cell mediated i...	17735	13	36	1	0.03	0.0265	33.33333	CD226
lightgreen BP	GO:0002724	regulation of T cell cytokine production	17735	13	36	1	0.03	0.0265	33.33333	XCL1
lightgreen BP	GO:0002891	positive regulation of immunoglobulin me...	17735	13	36	1	0.03	0.0265	33.33333	CD226
lightgreen BP	GO:0034695	response to prostaglandin E stimulus	17735	13	36	1	0.03	0.0265	33.33333	TGFBR3
lightgreen BP	GO:0042832	defense response to protozoan	17735	13	36	1	0.03	0.0265	33.33333	IFNG
lightgreen BP	GO:0045342	MHC class II biosynthetic process	17735	13	36	1	0.03	0.0265	33.33333	IFNG
lightgreen BP	GO:0045911	positive regulation of DNA recombination	17735	13	36	1	0.03	0.0265	33.33333	IFNG
lightgreen BP	GO:0045986	negative regulation of smooth muscle con...	17735	13	36	1	0.03	0.0265	33.33333	ADRB2
lightgreen BP	GO:0051044	positive regulation of membrane protein ...	17735	13	36	1	0.03	0.0265	33.33333	IFNG
lightgreen BP	GO:0072160	nephron tubule epithelial cell different...	17735	13	36	1	0.03	0.0265	33.33333	IFNG
lightgreen BP	GO:1901623	regulation of lymphocyte chemotaxis	17735	13	36	1	0.03	0.0265	33.33333	XCL1
lightgreen BP	GO:2000404	regulation of T cell migration	17735	13	36	1	0.03	0.0265	33.33333	XCL1
lightgreen BP	GO:2000696	regulation of epithelial cell differenti...	17735	13	36	1	0.03	0.0265	33.33333	IFNG
lightgreen BP	GO:0006950	response to stress	17735	2926	36	11	6.04	0.02659	1.821192	ADRB2;CD226;ID2;IFNG;KLRC2;KLRC3;KLRC4;MYO6;TGFBR3;TLR3;XCL1
lightgreen MF	GO:0048185	activin binding	17735	12	36	1	0.03	0.02745	33.33333	TGFBR3
lightgreen MF	GO:0050431	transforming growth factor beta binding	17735	12	36	1	0.03	0.02745	33.33333	TGFBR3
lightgreen CC	GO:0031224	intrinsic to membrane	17735	5319	36	17	11.11	0.02785	1.530153	ADRB2;CD226;DSG3;ELOVL6;ENPP4;ENPP5;EPHB4;KLRB1;KLRC1;KLRC2;KLRC3;KLRC4;KLRF1;SLC4A4;STYK1;T
lightgreen BP	GO:0032388	positive regulation of intracellular tra...	17735	127	36	2	0.26	0.02807	7.692308	TLR3;XCL1
lightgreen BP	GO:0003337	mesenchymal to epithelial transition inv...	17735	14	36	1	0.03	0.02851	33.33333	IFNG
lightgreen BP	GO:0006925	inflammatory cell apoptotic process	17735	14	36	1	0.03	0.02851	33.33333	IFNG
lightgreen BP	GO:0010719	negative regulation of epithelial to mes...	17735	14	36	1	0.03	0.02851	33.33333	TGFBR3
lightgreen BP	GO:0010922	positive regulation of phosphatase activ...	17735	14	36	1	0.03	0.02851	33.33333	IFNG
lightgreen BP	GO:0031649	heat generation	17735	14	36	1	0.03	0.02851	33.33333	ADRB2
lightgreen BP	GO:0042508	tyrosine phosphorylation of Stat1 protei...	17735	14	36	1	0.03	0.02851	33.33333	IFNG
lightgreen BP	GO:0045649	regulation of macrophage differentiation	17735	14	36	1	0.03	0.02851	33.33333	ID2
lightgreen BP	GO:0045672	positive regulation of osteoclast differ...	17735	14	36	1	0.03	0.02851	33.33333	IFNG
lightgreen BP	GO:0046641	positive regulation of alpha-beta T cell...	17735	14	36	1	0.03	0.02851	33.33333	XCL1
lightgreen BP	GO:0051281	positive regulation of release of seques...	17735	14	36	1	0.03	0.02851	33.33333	XCL1
lightgreen BP	GO:0060347	heart trabecula formation	17735	14	36	1	0.03	0.02851	33.33333	TGFBR3
lightgreen BP	GO:2000178	negative regulation of neural precursor ...	17735	14	36	1	0.03	0.02851	33.33333	ID2
lightgreen BP	GO:0002520	immune system development	17735	572	36	4	1.18	0.02886	3.389831	ID2;IFNG;TGFBR3;TLR3
lightgreen BP	GO:0023056	positive regulation of signaling	17735	856	36	5	1.77	0.02921	2.824859	ADRB2;CD226;IFNG;TGFBR3;TLR3
lightgreen BP	GO:0048584	positive regulation of response to stimu...	17735	1164	36	6	2.4	0.02922	2.5	ADRB2;CD226;IFNG;TGFBR3;TLR3;XCL1
lightgreen BP	GO:0010647	positive regulation of cell communicatio...	17735	858	36	5	1.77	0.02947	2.824859	ADRB2;CD226;IFNG;TGFBR3;TLR3
lightgreen BP	GO:0002521	leukocyte differentiation	17735	329	36	3	0.68	0.02968	4.411765	ID2;IFNG;TLR3
lightgreen MF	GO:0005452	inorganic anion exchanger activity	17735	13	36	1	0.03	0.0297	33.33333	SLC4A4
lightgreen BP	GO:0045444	fat cell differentiation	17735	132	36	2	0.27	0.03014	7.407407	ADRB2;ID2
lightgreen BP	GO:0002021	response to dietary excess	17735	15	36	1	0.03	0.03052	33.33333	ADRB2
lightgreen BP	GO:0002029	desensitization of G-protein coupled rec...	17735	15	36	1	0.03	0.03052	33.33333	ADRB2
lightgreen BP	GO:0002823	negative regulation of adaptive immune r...	17735	15	36	1	0.03	0.03052	33.33333	XCL1
lightgreen MF	GO:1901338	catecholamine binding	17735	14	36	1	0.03	0.03195	33.33333	ADRB2
lightgreen MF	GO:0003774	motor activity	17735	130	36	2	0.3	0.03631	6.666667	KIF21A;MYO6
lightgreen MF	GO:0004675	transmembrane receptor protein serine/th...	17735	17	36	1	0.04	0.03866	25	TGFBR3
lightgreen MF	GO:0005003	ephrin receptor activity	17735	17	36	1	0.04	0.03866	25	EPHB4
lightgreen MF	GO:0005024	transforming growth factor beta-activate...	17735	17	36	1	0.04	0.03866	25	TGFBR3
lightgreen CC	GO:0031941	filamentous actin	17735	20	36	1	0.04	0.04099	25	MYO6
lightgreen MF	GO:0015301	anion:anion antiporter activity	17735	19	36	1	0.04	0.04312	25	SLC4A4
lightgreen MF	GO:0032393	MHC class I receptor activity	17735	19	36	1	0.04	0.04312	25	KLRF1
lightgreen MF	GO:0004713	protein tyrosine kinase activity	17735	145	36	2	0.34	0.04425	5.882353	EPHB4;STYK1
lightgreen CC	GO:0030057	desmosome	17735	22	36	1	0.05	0.045	20	DSG3
lightgreen MF	GO:0000146	microfilament motor activity	17735	20	36	1	0.05	0.04533	20	MYO6
lightgreen MF	GO:0005160	transforming growth factor beta receptor...	17735	20	36	1	0.05	0.04533	20	TGFBR3

lightgreen	MF	GO:0017134	fibroblast growth factor binding	17735	20	36	1	0.05	0.04533	20	TGFBR3
lightgreen	CC	GO:0001772	immunological synapse	17735	23	36	1	0.05	0.04699	20	GZMA
lightgreen	MF	GO:0004252	serine-type endopeptidase activity	17735	151	36	2	0.35	0.04759	5.714286	GZMA;GZMK
lightcyan	BP	GO:0051607	defense response to virus	17735	185	54	23	0.64	1.00E-30	35.9375	BST2;DDX58;DDX60;DHX58;EIF2AK2;HERC5;IFI44L;IFIH1;IFIT1;IFIT2;IFIT3;IFIT5;IFITM3;IRF7;ISG15;MX1;OAS
lightcyan	BP	GO:0009615	response to virus	17735	263	54	24	0.9	4.20E-29	26.66667	BST2;DDX58;DDX60;DHX58;EIF2AK2;HERC5;IFI44;IFI44L;IFIH1;IFIT1;IFIT2;IFIT3;IFIT5;IFITM3;IRF7;ISG15;MX1
lightcyan	BP	GO:0045087	innate immune response	17735	550	54	27	1.89	1.00E-25	14.28571	BST2;CCL2;CTSL1;DDX58;DDX60;DHX58;HERC5;IFI6;IFIH1;IFIT1;IFIT2;IFIT3;IFIT5;IFITM3;IRF7;ISG15;MT2A;M
lightcyan	BP	GO:0006952	defense response	17735	1119	54	32	3.85	7.00E-24	8.311688	BST2;CCL2;CTSL1;DDX58;DDX60;DHX58;EIF2AK2;HERC5;IFI44L;IFI6;IFIH1;IFIT1;IFIT2;IFIT3;IFIT5;IFITM3;IRF7;
lightcyan	BP	GO:0060337	type I interferon-mediated signaling pat...	17735	72	54	15	0.25	1.10E-23	60	IFI6;IFIT1;IFIT2;IFIT3;IFITM3;IRF7;ISG15;MX1;OAS1;OAS2;OAS3;OASL;USP18;XAF1;ZBP1
lightcyan	BP	GO:0071357	cellular response to type I interferon	17735	72	54	15	0.25	1.10E-23	60	IFI6;IFIT1;IFIT2;IFIT3;IFITM3;IRF7;ISG15;MX1;OAS1;OAS2;OAS3;OASL;USP18;XAF1;ZBP1
lightcyan	BP	GO:0051707	response to other organism	17735	578	54	26	1.99	1.10E-23	13.06533	BST2;CCL2;CMPK2;DDX58;DDX60;DHX58;EIF2AK2;HERC5;IFI44;IFI44L;IFIH1;IFIT1;IFIT2;IFIT3;IFIT5;IFITM3;IR
lightcyan	BP	GO:0034340	response to type I interferon	17735	73	54	15	0.25	1.40E-23	60	IFI6;IFIT1;IFIT2;IFIT3;IFITM3;IRF7;ISG15;MX1;OAS1;OAS2;OAS3;OASL;USP18;XAF1;ZBP1
lightcyan	BP	GO:0009607	response to biotic stimulus	17735	606	54	26	2.08	3.70E-23	12.5	BST2;CCL2;CMPK2;DDX58;DDX60;DHX58;EIF2AK2;HERC5;IFI44;IFI44L;IFIH1;IFIT1;IFIT2;IFIT3;IFIT5;IFITM3;IR
lightcyan	BP	GO:0002252	immune effector process	17735	471	54	24	1.62	5.60E-23	14.81481	BST2;CCL2;DDX58;DDX60;DHX58;EIF2AK2;HERC5;IFI44L;IFIH1;IFIT1;IFIT2;IFIT3;IFIT5;IFITM3;IRF7;ISG15;MX1
lightcyan	BP	GO:0006955	immune response	17735	1081	54	29	3.72	1.90E-20	7.795699	BST2;CCL2;CTSL1;DDX58;DDX60;DHX58;HERC5;IFI44L;IFI6;IFIH1;IFIT1;IFIT2;IFIT3;IFIT5;IFITM3;IRF7;ISG15;M
lightcyan	BP	GO:0034097	response to cytokine stimulus	17735	497	54	21	1.71	2.90E-18	12.2807	BST2;CCL2;HERC5;IFI6;IFIT1;IFIT2;IFIT3;IFITM3;IRF7;ISG15;MT2A;MX1;OAS1;OAS2;OAS3;OASL;PLSCR1;PNPT
lightcyan	BP	GO:0019221	cytokine-mediated signaling pathway	17735	335	54	18	1.15	2.00E-17	15.65217	CCL2;HERC5;IFI6;IFIT1;IFIT2;IFIT3;IFITM3;IRF7;ISG15;MT2A;MX1;OAS1;OAS2;OAS3;OASL;USP18;XAF1;ZBP1
lightcyan	BP	GO:0071345	cellular response to cytokine stimulus	17735	412	54	19	1.42	3.50E-17	13.38028	CCL2;HERC5;IFI6;IFIT1;IFIT2;IFIT3;IFITM3;IRF7;ISG15;MT2A;MX1;OAS1;OAS2;OAS3;OASL;PNPT1;USP18;XAF
lightcyan	BP	GO:0045071	negative regulation of viral genome repl...	17735	32	54	9	0.11	8.30E-16	81.81818	BST2;IFIT1;IFITM3;MX1;OAS1;OAS3;OASL;PLSCR1;RSAD2
lightcyan	BP	GO:0048525	negative regulation of viral reproductio...	17735	33	54	9	0.11	1.10E-15	81.81818	BST2;IFIT1;IFITM3;MX1;OAS1;OAS3;OASL;PLSCR1;RSAD2
lightcyan	BP	GO:0002376	immune system process	17735	1816	54	30	6.24	2.40E-15	4.807692	BST2;CCL2;CTSL1;DDX58;DDX60;DHX58;EIF2AK2;HERC5;IFI44L;IFI6;IFIH1;IFIT1;IFIT2;IFIT3;IFIT5;IFITM3;IRF7;
lightcyan	MF	GO:0003725	double-stranded RNA binding	17735	43	54	9	0.13	6.30E-15	69.23077	DDX58;DDX60;DHX58;EIF2AK2;IFIH1;OAS1;OAS2;OAS3;OASL
lightcyan	BP	GO:0019079	viral genome replication	17735	61	54	10	0.21	7.00E-15	47.61905	BST2;CCL2;IFIT1;IFITM3;MX1;OAS1;OAS3;OASL;PLSCR1;RSAD2
lightcyan	BP	GO:0051704	multi-organism process	17735	1380	54	26	4.75	2.60E-14	5.473684	BST2;CCL2;CMPK2;DDX58;DDX60;DHX58;EIF2AK2;HERC5;IFI44;IFI44L;IFIH1;IFIT1;IFIT2;IFIT3;IFIT5;IFITM3;IR
lightcyan	BP	GO:0045069	regulation of viral genome replication	17735	48	54	9	0.17	4.80E-14	52.94118	BST2;IFIT1;IFITM3;MX1;OAS1;OAS3;OASL;PLSCR1;RSAD2
lightcyan	BP	GO:0043900	regulation of multi-organism process	17735	205	54	13	0.7	1.30E-13	18.57143	BST2;DDX58;DDX60;DHX58;HERC5;IFIT1;IFITM3;MX1;OAS1;OAS3;OASL;PLSCR1;RSAD2
lightcyan	BP	GO:0043901	negative regulation of multi-organism pr...	17735	54	54	9	0.19	1.50E-13	47.36842	BST2;IFIT1;IFITM3;MX1;OAS1;OAS3;OASL;PLSCR1;RSAD2
lightcyan	BP	GO:0006950	response to stress	17735	2926	54	34	10.06	2.90E-13	3.379722	BST2;CCL2;CTSL1;DDX58;DDX60;DHX58;EIF2AK2;HERC5;IFI44L;IFI6;IFIH1;IFIT1;IFIT2;IFIT3;IFIT5;IFITM3;IRF7;
lightcyan	BP	GO:0022415	viral reproductive process	17735	542	54	17	1.86	1.30E-12	9.139785	BST2;CCL2;DDX58;DHX58;EIF2AK2;IFIH1;IFIT1;IFITM3;IRF7;ISG15;MX1;OAS1;OAS3;OASL;PLSCR1;RSAD2;TRI
lightcyan	BP	GO:2000242	negative regulation of reproductive proc...	17735	81	54	9	0.28	6.80E-12	32.14286	BST2;IFIT1;IFITM3;MX1;OAS1;OAS3;OASL;PLSCR1;RSAD2
lightcyan	BP	GO:0010033	response to organic substance	17735	1870	54	26	6.43	3.20E-11	4.043546	BST2;CCL2;CMPK2;CTSL1;DDX58;EIF2AK2;HERC5;IFI6;IFIT1;IFIT2;IFIT3;IFITM3;IRF7;ISG15;MT2A;MX1;OAS1;
lightcyan	BP	GO:0016032	viral reproduction	17735	689	54	17	2.37	5.70E-11	7.172996	BST2;CCL2;DDX58;DHX58;EIF2AK2;IFIH1;IFIT1;IFITM3;IRF7;ISG15;MX1;OAS1;OAS3;OASL;PLSCR1;RSAD2;TRI
lightcyan	BP	GO:0044764	multi-organism cellular process	17735	691	54	17	2.38	6.00E-11	7.142857	BST2;CCL2;DDX58;DHX58;EIF2AK2;IFIH1;IFIT1;IFITM3;IRF7;ISG15;MX1;OAS1;OAS3;OASL;PLSCR1;RSAD2;TRI
lightcyan	BP	GO:0071310	cellular response to organic substance	17735	1333	54	22	4.58	9.00E-11	4.803493	CCL2;CMPK2;CTSL1;EIF2AK2;HERC5;IFI6;IFIT1;IFIT2;IFIT3;IFITM3;IRF7;ISG15;MT2A;MX1;OAS1;OAS2;OAS3;C
lightcyan	BP	GO:0050792	regulation of viral reproduction	17735	109	54	9	0.37	1.00E-10	24.32432	BST2;IFIT1;IFITM3;MX1;OAS1;OAS3;OASL;PLSCR1;RSAD2
lightcyan	BP	GO:0034341	response to interferon-gamma	17735	112	54	9	0.39	1.30E-10	23.07692	BST2;CCL2;IFITM3;IRF7;MT2A;OAS1;OAS2;OAS3;OASL
lightcyan	BP	GO:0044703	multi-organism reproductive process	17735	730	54	17	2.51	1.40E-10	6.772908	BST2;CCL2;DDX58;DHX58;EIF2AK2;IFIH1;IFIT1;IFITM3;IRF7;ISG15;MX1;OAS1;OAS3;OASL;PLSCR1;RSAD2;TRI
lightcyan	BP	GO:0039528	cytoplasmic pattern recognition receptor...	17735	11	54	5	0.04	1.80E-10	125	DDX58;DDX60;DHX58;IFIH1;IRF7
lightcyan	BP	GO:0019058	viral infectious cycle	17735	223	54	11	0.77	1.90E-10	14.28571	BST2;CCL2;EIF2AK2;IFIT1;IFITM3;MX1;OAS1;OAS3;OASL;PLSCR1;RSAD2
lightcyan	MF	GO:0003723	RNA binding	17735	851	54	17	2.65	2.70E-10	6.415094	DDX58;DDX60;DDX60L;DHX58;EIF2AK2;EIF3L;IFIH1;IFIT1;IFIT2;IFIT5;OAS1;OAS2;OAS3;OASL;PNPT1;TDRD7;
lightcyan	BP	GO:0035456	response to interferon-beta	17735	14	54	5	0.05	7.70E-10	100	BST2;IFITM3;PLSCR1;PNPT1;XAF1
lightcyan	BP	GO:0035455	response to interferon-alpha	17735	15	54	5	0.05	1.10E-09	100	BST2;IFIT2;IFIT3;IFITM3;OAS1
lightcyan	MF	GO:0003727	single-stranded RNA binding	17735	41	54	6	0.13	2.70E-09	46.15385	DDX58;DDX60;DHX58;IFIH1;IFIT5;PNPT1
lightcyan	BP	GO:0032479	regulation of type I interferon producti...	17735	67	54	7	0.23	2.70E-09	30.43478	DDX58;DHX58;HERC5;IFIH1;IRF7;ISG15;ZBP1
lightcyan	BP	GO:0032606	type I interferon production	17735	71	54	7	0.24	4.10E-09	29.16667	DDX58;DHX58;HERC5;IFIH1;IRF7;ISG15;ZBP1
lightcyan	BP	GO:0042221	response to chemical stimulus	17735	2766	54	28	9.51	6.40E-09	2.944269	AGRN;BST2;CCL2;CMPK2;CTSL1;DDX58;EIF2AK2;HERC5;IFI6;IFIT1;IFIT2;IFIT3;IFITM3;IRF7;ISG15;MT2A;MX1;
lightcyan	BP	GO:0070887	cellular response to chemical stimulus	17735	1709	54	22	5.88	1.00E-08	3.741497	CCL2;CMPK2;CTSL1;EIF2AK2;HERC5;IFI6;IFIT1;IFIT2;IFIT3;IFITM3;IRF7;ISG15;MT2A;MX1;OAS1;OAS2;OAS3;C
lightcyan	BP	GO:2000241	regulation of reproductive process	17735	201	54	9	0.69	2.40E-08	13.04348	BST2;IFIT1;IFITM3;MX1;OAS1;OAS3;OASL;PLSCR1;RSAD2
lightcyan	BP	GO:0071346	cellular response to interferon-gamma	17735	94	54	7	0.32	3.00E-08	21.875	CCL2;IRF7;MT2A;OAS1;OAS2;OAS3;OASL
lightcyan	BP	GO:0019048	virus-host interaction	17735	371	54	11	1.28	3.90E-08	8.59375	BST2;DDX58;DHX58;EIF2AK2;IFIH1;IFIT1;IFITM3;IRF7;ISG15;RSAD2;TRIM22
lightcyan	BP	GO:0001817	regulation of cytokine production	17735	382	54	11	1.31	5.30E-08	8.396947	BST2;CCL2;DDX58;DDX60;DHX58;HERC5;IFIH1;IRF7;ISG15;RSAD2;ZBP1
lightcyan	BP	GO:0045088	regulation of innate immune response	17735	225	54	9	0.77	6.30E-08	11.68831	DDX58;DDX60;DHX58;IFIH1;IRF7;PLSCR1;RSAD2;USP18;ZBP1
lightcyan	BP	GO:0032480	negative regulation of type I interferon...	17735	33	54	5	0.11	8.60E-08	45.45455	DDX58;DHX58;HERC5;IFIH1;ISG15
lightcyan	CC	GO:0005737	cytoplasm	17735	9016	54	46	27.98	9.30E-08	1.644031	AGRN;BST2;CCL2;CMPK2;CTSL1;DDX58;DDX60;DHX58;EIF2AK2;EIF3L;EXOC3L1;HERC5;HERC6;IFI44;IFI44L;IF
lightcyan	BP	GO:0051701	interaction with host	17735	411	54	11	1.41	1.10E-07	7.801418	BST2;DDX58;DHX58;EIF2AK2;IFIH1;IFIT1;IFITM3;IRF7;ISG15;RSAD2;TRIM22
lightcyan	BP	GO:0032481	positive regulation of type I interferon...	17735	36	54	5	0.12	1.40E-07	41.66667	DDX58;DHX58;IFIH1;IRF7;ZBP1
lightcyan	BP	GO:0031347	regulation of defense response	17735	428	54	11	1.47	1.70E-07	7.482993	DDX58;DDX60;DHX58;HERC5;IFIH1;IFIT1;IRF7;PLSCR1;RSAD2;USP18;ZBP1
lightcyan	BP	GO:0001816	cytokine production	17735	430	54	11	1.48	1.80E-07	7.432432	BST2;CCL2;DDX58;DDX60;DHX58;HERC5;IFIH1;IRF7;ISG15;RSAD2;ZBP1
lightcyan	BP	GO:0060333	interferon-gamma-mediated signaling path...	17735	75	54	6	0.26	2.00E-07	23.07692	IRF7;MT2A;OAS1;OAS2;OAS3;OASL
lightcyan	BP	GO:0002753	cytoplasmic pattern recognition receptor...	17735	40	54	5	0.14	2.40E-07	35.71429	DDX58;DDX60;DHX58;IFIH1;IRF7
lightcyan	BP	GO:0044403	symbiosis, encompassing mutualism throug...	17735	445	54	11	1.53	2.50E-07	7.189542	BST2;DDX58;DHX58;EIF2AK2;IFIH1;IFIT1;IFITM3;IRF7;ISG15;RSAD2;TRIM22
lightcyan	BP	GO:0044419	interspecies interaction between organis...	17735	445	54	11	1.53	2.50E-07	7.189542	BST2;DDX58;DHX58;EIF2AK2;IFIH1;IFIT1;IFITM3;IRF7;ISG15;RSAD2;TRIM22
lightcyan	BP	GO:0002697	regulation of immune effector process	17735	217	54	8	0.75	6.80E-07	10.66667	BST2;CCL2;DDX58;DDX60;DHX58;HERC5;IFIT1;RSAD2
lightcyan	BP	GO:0022414	reproductive process	17735	1510	54	18	5.19	1.20E-06	3.468208	BST2;CCL2;DDX58;DHX58;EIF2AK2;IFIH1;IFIT1;IFITM3;IRF7;ISG15;MX1;OAS1;OAS3;OASL;PLSCR1;RSAD2;TD
lightcyan	BP	GO:0045089	positive regulation of innate immune res...	17735	167	54	7	0.57	1.50E-06	12.2807	DDX58;DDX60;DHX58;IFIH1;IRF7;PLSCR1;RSAD2
lightcyan	BP	GO:0050896	response to stimulus	17735	6581	54	39	22.63	2.30E-06	1.723376	AGRN;BST2;CCL2;CMPK2;CTSL1;DDX58;DDX60;DHX58;EIF2AK2;HERC5;IFI44;IFI44L;IFI6;IFIH1;IFIT1;IFIT2;IFIT
lightcyan	BP	GO:0000003	reproduction	17735	1591	54	18	5.47	2.70E-06	3.290676	BST2;CCL2;DDX58;DHX58;EIF2AK2;IFIH1;IFIT1;IFITM3;IRF7;ISG15;MX1;OAS1;OAS3;OASL;PLSCR1;RSAD2;TD
lightcyan	BP	GO:0007166	cell surface receptor signaling pathway	17735	2331	54	22	8.02	2.70E-06	2.743142	AGRN;CCL2;HERC5;IFI6;IFIT1;IFIT2;IFIT3;IFITM3;IRF7;ISG15;LILRB5;LY6E;MT2A;MX1;OAS1;OAS2;OAS3;OASL
lightcyan	BP	GO:0050688	regulation of defense response to virus	17735	65	54	5	0.22	2.80E-06	22.72727	DDX58;DDX60;DHX58;HERC5;IFIT1
lightcyan	CC	GO:0005829	cytosol	17735	2362	54	21	7.33	2.90E-06	2.864939	DDX58;EIF2AK2;HERC5;HERC6;IFIH1;IFIT1;IFIT2;IFIT3;IRF7;ISG15;MT2A;MX1;OAS1;OAS2;OAS3;OASL;OTOF;

lightcyan	BP	GO:0002221	pattern recognition receptor signaling p...	17735	128	54	6	0.44	4.80E-06	13.63636	DDX58;DDX60;DHX58;IFIH1;IRF7;RSAD2
lightcyan	BP	GO:0001819	positive regulation of cytokine producti...	17735	202	54	7	0.69	5.40E-06	10.14493	DDX58;DDX60;DHX58;IFIH1;IRF7;RSAD2;ZBP1
lightcyan	BP	GO:0080134	regulation of response to stress	17735	737	54	12	2.53	5.40E-06	4.743083	DDX58;DDX60;DHX58;HERC5;IFIH1;IFIT1;IRF7;PLSCR1;PNPT1;RSAD2;USP18;ZBP1
lightcyan	BP	GO:0002758	innate immune response-activating signal...	17735	132	54	6	0.45	5.70E-06	13.33333	DDX58;DDX60;DHX58;IFIH1;IRF7;RSAD2
lightcyan	BP	GO:0002682	regulation of immune system process	17735	884	54	13	3.04	6.10E-06	4.276316	BST2;CCL2;DDX58;DDX60;DHX58;HERC5;IFIH1;IFIT1;IRF7;PLSCR1;RSAD2;USP18;ZBP1
lightcyan	BP	GO:0007154	cell communication	17735	4775	54	32	16.42	6.10E-06	1.948843	AGRN;BST2;CCL2;DDX58;DDX60;DHX58;EIF2AK2;EXOC3L1;HERC5;IFI6;IFIH1;IFIT1;IFIT2;IFIT3;IFITM3;IRF7;IS
lightcyan	BP	GO:0032727	positive regulation of interferon-alpha ...	17735	11	54	3	0.04	6.20E-06	75	DDX58;IFIH1;IRF7
lightcyan	BP	GO:0001818	negative regulation of cytokine producti...	17735	135	54	6	0.46	6.50E-06	13.04348	BST2;DDX58;DHX58;HERC5;IFIH1;ISG15
lightcyan	BP	GO:0002218	activation of innate immune response	17735	140	54	6	0.48	8.00E-06	12.5	DDX58;DDX60;DHX58;IFIH1;IRF7;RSAD2
lightcyan	BP	GO:0002831	regulation of response to biotic stimulu...	17735	84	54	5	0.29	9.90E-06	17.24138	DDX58;DDX60;DHX58;HERC5;IFIT1
lightcyan	CC	GO:0044444	cytoplasmic part	17735	6562	54	36	20.37	1.10E-05	1.767305	AGRN;BST2;CMPK2;CTSL1;DDX58;EIF2AK2;EIF3L;EXOC3L1;HERC5;HERC6;IFI6;IFIH1;IFIT1;IFIT2;IFIT3;IFITM3;
lightcyan	BP	GO:0031349	positive regulation of defense response	17735	229	54	7	0.79	1.20E-05	8.860759	DDX58;DDX60;DHX58;IFIH1;IRF7;PLSCR1;RSAD2
lightcyan	BP	GO:0007165	signal transduction	17735	4165	54	29	14.32	1.30E-05	2.02514	AGRN;BST2;CCL2;DDX58;DDX60;DHX58;EIF2AK2;HERC5;IFI6;IFIH1;IFIT1;IFIT2;IFIT3;IFITM3;IRF7;ISG15;LILRB
lightcyan	BP	GO:0023052	signaling	17735	4666	54	31	16.04	1.30E-05	1.932668	AGRN;BST2;CCL2;DDX58;DDX60;DHX58;EIF2AK2;EXOC3L1;HERC5;IFI6;IFIH1;IFIT1;IFIT2;IFIT3;IFITM3;IRF7;IS
lightcyan	BP	GO:0044700	single organism signaling	17735	4666	54	31	16.04	1.30E-05	1.932668	AGRN;BST2;CCL2;DDX58;DDX60;DHX58;EIF2AK2;EXOC3L1;HERC5;IFI6;IFIH1;IFIT1;IFIT2;IFIT3;IFITM3;IRF7;IS
lightcyan	BP	GO:0002757	immune response-activating signal transd...	17735	237	54	7	0.81	1.50E-05	8.641975	DDX58;DDX60;DHX58;IFIH1;IRF7;PLSCR1;RSAD2
lightcyan	BP	GO:0050776	regulation of immune response	17735	560	54	10	1.93	1.70E-05	5.181347	BST2;DDX58;DDX60;DHX58;IFIH1;IRF7;PLSCR1;RSAD2;USP18;ZBP1
lightcyan	BP	GO:0032607	interferon-alpha production	17735	16	54	3	0.06	2.10E-05	50	DDX58;IFIH1;IRF7
lightcyan	BP	GO:0032647	regulation of interferon-alpha productio...	17735	16	54	3	0.06	2.10E-05	50	DDX58;IFIH1;IRF7
lightcyan	BP	GO:0002764	immune response-regulating signaling pat...	17735	250	54	7	0.86	2.20E-05	8.139535	DDX58;DDX60;DHX58;IFIH1;IRF7;PLSCR1;RSAD2
lightcyan	BP	GO:0051716	cellular response to stimulus	17735	5052	54	32	17.37	2.30E-05	1.842257	AGRN;BST2;CCL2;CMPK2;CTSL1;DDX58;DDX60;DHX58;EIF2AK2;HERC5;IFI6;IFIH1;IFIT1;IFIT2;IFIT3;IFITM3;IR
lightcyan	MF	GO:0070566	adenylyltransferase activity	17735	19	54	3	0.06	2.60E-05	50	OAS1;OAS2;OAS3
lightcyan	BP	GO:0032728	positive regulation of interferon-beta p...	17735	22	54	3	0.08	5.60E-05	37.5	DDX58;IFIH1;IRF7
lightcyan	CC	GO:0044424	intracellular part	17735	12103	54	49	37.57	6.60E-05	1.304232	AGRN;BST2;CCL2;CMPK2;CTSL1;DDX58;DDX60;DHX58;EIF2AK2;EIF3L;EXOC3L1;HERC5;HERC6;IFI44;IFI44L;IF
lightcyan	MF	GO:0004386	helicase activity	17735	143	54	5	0.44	7.90E-05	11.36364	DDX58;DDX60;DDX60L;DHX58;IFIH1
lightcyan	BP	GO:0002253	activation of immune response	17735	310	54	7	1.07	8.50E-05	6.542056	DDX58;DDX60;DHX58;IFIH1;IRF7;PLSCR1;RSAD2
lightcyan	BP	GO:0032648	regulation of interferon-beta production	17735	30	54	3	0.1	0.00015	30	DDX58;IFIH1;IRF7
lightcyan	CC	GO:0005622	intracellular	17735	12344	54	49	38.31	0.00015	1.279039	AGRN;BST2;CCL2;CMPK2;CTSL1;DDX58;DDX60;DHX58;EIF2AK2;EIF3L;EXOC3L1;HERC5;HERC6;IFI44;IFI44L;IF
lightcyan	BP	GO:0032608	interferon-beta production	17735	32	54	3	0.11	0.00018	27.27273	DDX58;IFIH1;IRF7
lightcyan	BP	GO:0060338	regulation of type I interferon-mediated...	17735	33	54	3	0.11	0.00019	27.27273	IRF7;USP18;ZBP1
lightcyan	BP	GO:0050778	positive regulation of immune response	17735	377	54	7	1.3	0.00028	5.384615	DDX58;DDX60;DHX58;IFIH1;IRF7;PLSCR1;RSAD2
lightcyan	BP	GO:0048519	negative regulation of biological proces...	17735	3150	54	22	10.83	0.00034	2.031394	BST2;CCL2;DDX58;DHX58;EIF2AK2;HERC5;IFI6;IFIH1;IFIT1;IFIT3;IFITM3;IRF7;ISG15;MT2A;MX1;OAS1;OAS3;
lightcyan	BP	GO:0051240	positive regulation of multicellular org...	17735	524	54	8	1.8	0.00038	4.444444	CCL2;DDX58;DDX60;DHX58;IFIH1;IRF7;RSAD2;ZBP1
lightcyan	MF	GO:0003676	nucleic acid binding	17735	3299	54	21	10.26	0.00039	2.046784	DDX58;DDX60;DDX60L;DHX58;EIF2AK2;EIF3L;IFIH1;IFIT1;IFIT2;IFIT5;IRF7;OAS1;OAS2;OAS3;OASL;PARP12;P
lightcyan	MF	GO:0016779	nucleotidyltransferase activity	17735	120	54	4	0.37	0.00052	10.81081	OAS1;OAS2;OAS3;PNPT1
lightcyan	BP	GO:0002684	positive regulation of immune system pro...	17735	554	54	8	1.9	0.00055	4.210526	CCL2;DDX58;DDX60;DHX58;IFIH1;IRF7;PLSCR1;RSAD2
lightcyan	BP	GO:0006925	inflammatory cell apoptotic process	17735	14	54	2	0.05	0.00103	40	CTSL1;IRF7
lightcyan	MF	GO:0005524	ATP binding	17735	1462	54	12	4.55	0.00135	2.637363	CMPK2;DDX58;DDX60;DDX60L;DHX58;EIF2AK2;IFIH1;OAS1;OAS2;OAS3;OASL;TOR1B
lightcyan	BP	GO:0051241	negative regulation of multicellular org...	17735	359	54	6	1.23	0.00138	4.878049	BST2;DDX58;DHX58;HERC5;IFIH1;ISG15
lightcyan	BP	GO:0030522	intracellular receptor mediated signalin...	17735	242	54	5	0.83	0.00141	6.024096	DDX58;DDX60;DHX58;IFIH1;IRF7
lightcyan	MF	GO:0032559	adenyl ribonucleotide binding	17735	1491	54	12	4.64	0.0016	2.586207	CMPK2;DDX58;DDX60;DDX60L;DHX58;EIF2AK2;IFIH1;OAS1;OAS2;OAS3;OASL;TOR1B
lightcyan	MF	GO:0030554	adenyl nucleotide binding	17735	1495	54	12	4.65	0.00164	2.580645	CMPK2;DDX58;DDX60;DDX60L;DHX58;EIF2AK2;IFIH1;OAS1;OAS2;OAS3;OASL;TOR1B
lightcyan	BP	GO:0001961	positive regulation of cytokine-mediated...	17735	19	54	2	0.07	0.00191	28.57143	IRF7;ZBP1
lightcyan	BP	GO:0032069	regulation of nuclease activity	17735	72	54	3	0.25	0.00193	12	CCL2;OAS1;OAS3
lightcyan	BP	GO:0060760	positive regulation of response to cytok...	17735	20	54	2	0.07	0.00212	28.57143	IRF7;ZBP1
lightcyan	BP	GO:0043330	response to exogenous dsRNA	17735	21	54	2	0.07	0.00233	28.57143	DDX58;IFIT1
lightcyan	MF	GO:0035639	purine ribonucleoside triphosphate bindi...	17735	1796	54	13	5.58	0.00258	2.329749	CMPK2;DDX58;DDX60;DDX60L;DHX58;EIF2AK2;IFIH1;MX1;OAS1;OAS2;OAS3;OASL;TOR1B
lightcyan	MF	GO:0032550	purine ribonucleoside binding	17735	1805	54	13	5.61	0.00269	2.317291	CMPK2;DDX58;DDX60;DDX60L;DHX58;EIF2AK2;IFIH1;MX1;OAS1;OAS2;OAS3;OASL;TOR1B
lightcyan	MF	GO:0001883	purine nucleoside binding	17735	1807	54	13	5.62	0.00272	2.313167	CMPK2;DDX58;DDX60;DDX60L;DHX58;EIF2AK2;IFIH1;MX1;OAS1;OAS2;OAS3;OASL;TOR1B
lightcyan	MF	GO:0032549	ribonucleoside binding	17735	1809	54	13	5.63	0.00275	2.309059	CMPK2;DDX58;DDX60;DDX60L;DHX58;EIF2AK2;IFIH1;MX1;OAS1;OAS2;OAS3;OASL;TOR1B
lightcyan	BP	GO:0050691	regulation of defense response to virus ...	17735	23	54	2	0.08	0.0028	25	DDX58;IFIT1
lightcyan	MF	GO:0001882	nucleoside binding	17735	1818	54	13	5.65	0.00287	2.300885	CMPK2;DDX58;DDX60;DDX60L;DHX58;EIF2AK2;IFIH1;MX1;OAS1;OAS2;OAS3;OASL;TOR1B
lightcyan	BP	GO:0001959	regulation of cytokine-mediated signalin...	17735	84	54	3	0.29	0.003	10.34483	IRF7;USP18;ZBP1
lightcyan	BP	GO:0043903	regulation of symbiosis, encompassing mu...	17735	24	54	2	0.08	0.00305	25	BST2;IFITM3
lightcyan	MF	GO:0032555	purine ribonucleotide binding	17735	1831	54	13	5.69	0.00306	2.28471	CMPK2;DDX58;DDX60;DDX60L;DHX58;EIF2AK2;IFIH1;MX1;OAS1;OAS2;OAS3;OASL;TOR1B
lightcyan	BP	GO:0002683	negative regulation of immune system pro...	17735	176	54	4	0.61	0.0031	6.557377	BST2;CCL2;DHX58;IFIT1
lightcyan	MF	GO:0017076	purine nucleotide binding	17735	1836	54	13	5.71	0.00313	2.276708	CMPK2;DDX58;DDX60;DDX60L;DHX58;EIF2AK2;IFIH1;MX1;OAS1;OAS2;OAS3;OASL;TOR1B
lightcyan	MF	GO:0032553	ribonucleotide binding	17735	1846	54	13	5.74	0.00329	2.264808	CMPK2;DDX58;DDX60;DDX60L;DHX58;EIF2AK2;IFIH1;MX1;OAS1;OAS2;OAS3;OASL;TOR1B
lightcyan	BP	GO:0044763	single-organism cellular process	17735	9577	54	42	32.93	0.00355	1.275433	AGRN;BST2;CCL2;CMPK2;CTSL1;DDX58;DDX60;DHX58;EIF2AK2;EIF3L;EXOC3L1;HERC5;IFI6;IFIH1;IFIT1;IFIT2;
lightcyan	BP	GO:0060759	regulation of response to cytokine stimu...	17735	90	54	3	0.31	0.00364	9.677419	IRF7;USP18;ZBP1
lightcyan	MF	GO:0043168	anion binding	17735	2346	54	15	7.29	0.00378	2.057613	AGRN;CCL2;CMPK2;DDX58;DDX60;DDX60L;DHX58;EIF2AK2;IFIH1;MX1;OAS1;OAS2;OAS3;OASL;TOR1B
lightcyan	BP	GO:0034121	regulation of toll-like receptor signali...	17735	27	54	2	0.09	0.00385	22.22222	IRF7;RSAD2
lightcyan	BP	GO:0044766	multi-organism transport	17735	27	54	2	0.09	0.00385	22.22222	BST2;IFIT1
lightcyan	CC	GO:0044464	cell part	17735	14451	54	51	44.85	0.00422	1.137124	AGRN;BST2;CCL2;CMPK2;CTSL1;DDX58;DDX60;DHX58;EIF2AK2;EIF3L;EXOC3L1;HERC5;HERC6;IFI44;IFI44L;IF
lightcyan	CC	GO:0005623	cell	17735	14452	54	51	44.86	0.00423	1.13687	AGRN;BST2;CCL2;CMPK2;CTSL1;DDX58;DDX60;DHX58;EIF2AK2;EIF3L;EXOC3L1;HERC5;HERC6;IFI44;IFI44L;IF
lightcyan	MF	GO:0008026	ATP-dependent helicase activity	17735	106	54	3	0.33	0.00435	9.090909	DDX58;DDX60;DDX60L
lightcyan	MF	GO:0070035	purine NTP-dependent helicase activity	17735	106	54	3	0.33	0.00435	9.090909	DDX58;DDX60;DDX60L
lightcyan	BP	GO:0070206	protein trimerization	17735	30	54	2	0.1	0.00474	20	PNPT1;TRIM22
lightcyan	BP	GO:0048584	positive regulation of response to stimu...	17735	1164	54	10	4	0.00557	2.5	BST2;CCL2;DDX58;DDX60;DHX58;IFIH1;IRF7;PLSCR1;RSAD2;ZBP1

lightcyan	BP	GO:0006915	apoptotic process	17735	1562	54	12	5.37	0.00567	2.234637	CCL2;CTSL1;EIF2AK2;IFI6;IFIH1;IFIT2;IFIT3;IRF7;MX1;PLSCR1;TRIM69;XAF1
lightcyan	BP	GO:0032091	negative regulation of protein binding	17735	34	54	2	0.12	0.00606	16.66667	IFIT1;IFIT2
lightcyan	BP	GO:0012501	programmed cell death	17735	1575	54	12	5.42	0.00606	2.214022	CCL2;CTSL1;EIF2AK2;IFI6;IFIH1;IFIT2;IFIT3;IRF7;MX1;PLSCR1;TRIM69;XAF1
lightcyan	BP	GO:0043331	response to dsRNA	17735	38	54	2	0.13	0.00753	15.38462	DDX58;IFIT1
lightcyan	CC	GO:0044437	vacuolar part	17735	252	54	4	0.78	0.00769	5.128205	AGRN;CTSL1;IFITM3;LAMP3
lightcyan	MF	GO:0017111	nucleoside-triphosphatase activity	17735	756	54	7	2.35	0.00835	2.978723	DDX58;DDX60;DDX60L;DHX58;IFIH1;MX1;TOR1B
lightcyan	BP	GO:0002718	regulation of cytokine production involv...	17735	41	54	2	0.14	0.00872	14.28571	BST2;RSAD2
lightcyan	BP	GO:0048583	regulation of response to stimulus	17735	2347	54	15	8.07	0.01028	1.858736	AGRN;BST2;CCL2;DDX58;DDX60;DHX58;HERC5;IFIH1;IFIT1;IRF7;PLSCR1;PNPT1;RSAD2;USP18;ZBP1
lightcyan	MF	GO:0016462	pyrophosphatase activity	17735	791	54	7	2.46	0.01058	2.845528	DDX58;DDX60;DDX60L;DHX58;IFIH1;MX1;TOR1B
lightcyan	BP	GO:0006986	response to unfolded protein	17735	133	54	3	0.46	0.01072	6.521739	CCL2;EIF2AK2;TOR1B
lightcyan	MF	GO:0016818	hydrolase activity, acting on acid anhyd...	17735	793	54	7	2.47	0.01072	2.834008	DDX58;DDX60;DDX60L;DHX58;IFIH1;MX1;TOR1B
lightcyan	MF	GO:0016817	hydrolase activity, acting on acid anhyd...	17735	797	54	7	2.48	0.011	2.822581	DDX58;DDX60;DDX60L;DHX58;IFIH1;MX1;TOR1B
lightcyan	BP	GO:0043632	modification-dependent macromolecule cat...	17735	395	54	5	1.36	0.01121	3.676471	HERC5;HERC6;ISG15;PNPT1;USP18
lightcyan	BP	GO:0002367	cytokine production involved in immune r...	17735	47	54	2	0.16	0.01135	12.5	BST2;RSAD2
lightcyan	MF	GO:0043167	ion binding	17735	5761	54	26	17.91	0.01221	1.451703	AGRN;CCL2;CMPK2;DDX58;DDX60;DDX60L;DHX58;EIF2AK2;IFIH1;MT2A;MX1;OAS1;OAS2;OAS3;OASL;OTOF
lightcyan	BP	GO:0035966	response to topologically incorrect prot...	17735	140	54	3	0.48	0.01231	6.25	CCL2;EIF2AK2;TOR1B
lightcyan	BP	GO:0048523	negative regulation of cellular process	17735	2883	54	17	9.91	0.01314	1.715439	BST2;CCL2;DHX58;EIF2AK2;IFI6;IFIT1;IFIT3;IFITM3;IRF7;MX1;OAS1;OAS3;OASL;PLSCR1;PNPT1;RSAD2;SAMD
lightcyan	BP	GO:0008219	cell death	17735	1741	54	12	5.99	0.01315	2.003339	CCL2;CTSL1;EIF2AK2;IFI6;IFIH1;IFIT2;IFIT3;IRF7;MX1;PLSCR1;TRIM69;XAF1
lightcyan	BP	GO:0016265	death	17735	1743	54	12	5.99	0.01327	2.003339	CCL2;CTSL1;EIF2AK2;IFI6;IFIH1;IFIT2;IFIT3;IRF7;MX1;PLSCR1;TRIM69;XAF1
lightcyan	BP	GO:0051336	regulation of hydrolase activity	17735	755	54	7	2.6	0.01411	2.692308	AGRN;CCL2;IFI6;IFIT1;OAS1;OAS3;PLSCR1
lightcyan	BP	GO:0002698	negative regulation of immune effector p...	17735	53	54	2	0.18	0.01428	11.11111	BST2;IFIT1
lightcyan	BP	GO:0040013	negative regulation of locomotion	17735	148	54	3	0.51	0.01428	5.882353	BST2;CCL2;IFITM3
lightcyan	CC	GO:0032587	ruffle membrane	17735	59	54	2	0.18	0.01444	11.11111	DDX58;IFIT5
lightcyan	MF	GO:0008270	zinc ion binding	17735	1977	54	12	6.15	0.01558	1.95122	DDX58;DHX58;IFIH1;MT2A;OAS1;OAS2;PARP12;PHF11;TRIM22;TRIM69;XAF1;ZCCHC2
lightcyan	BP	GO:0071887	leukocyte apoptotic process	17735	57	54	2	0.2	0.0164	10	CTSL1;IRF7
lightcyan	BP	GO:0051239	regulation of multicellular organismal p...	17735	1804	54	12	6.2	0.01712	1.935484	AGRN;BST2;CCL2;DDX58;DDX60;DHX58;HERC5;IFIH1;IRF7;ISG15;RSAD2;ZBP1
lightcyan	MF	GO:1901363	heterocyclic compound binding	17735	5302	54	24	16.49	0.01749	1.455428	CMPK2;DDX58;DDX60;DDX60L;DHX58;EIF2AK2;EIF3L;IFIH1;IFIT1;IFIT2;IFIT5;IRF7;MX1;OAS1;OAS2;OAS3;OA
lightcyan	BP	GO:0042787	protein ubiquitination involved in ubiqu...	17735	60	54	2	0.21	0.01807	9.52381	HERC5;HERC6
lightcyan	MF	GO:0016772	transferase activity, transferring phosp...	17735	889	54	7	2.76	0.0191	2.536232	CCL2;CMPK2;EIF2AK2;OAS1;OAS2;OAS3;PNPT1
lightcyan	BP	GO:0008637	apoptotic mitochondrial changes	17735	64	54	2	0.22	0.0204	9.090909	IFI6;IFIT2
lightcyan	CC	GO:0000323	lytic vacuole	17735	337	54	4	1.05	0.02042	3.809524	AGRN;CTSL1;IFITM3;LAMP3
lightcyan	CC	GO:0005764	lysosome	17735	337	54	4	1.05	0.02042	3.809524	AGRN;CTSL1;IFITM3;LAMP3
lightcyan	CC	GO:0043202	lysosomal lumen	17735	71	54	2	0.22	0.02049	9.090909	AGRN;CTSL1
lightcyan	MF	GO:0097159	organic cyclic compound binding	17735	5372	54	24	16.7	0.02067	1.437126	CMPK2;DDX58;DDX60;DDX60L;DHX58;EIF2AK2;EIF3L;IFIH1;IFIT1;IFIT2;IFIT5;IRF7;MX1;OAS1;OAS2;OAS3;OA
lightcyan	BP	GO:0002700	regulation of production of molecular me...	17735	65	54	2	0.22	0.021	9.090909	BST2;RSAD2
lightcyan	CC	GO:0005775	vacuolar lumen	17735	75	54	2	0.23	0.0227	8.695652	AGRN;CTSL1
lightcyan	BP	GO:0051098	regulation of binding	17735	178	54	3	0.61	0.02322	4.918033	IFIT1;IFIT2;PLSCR1
lightcyan	BP	GO:0044699	single-organism process	17735	10656	54	43	36.64	0.02501	1.173581	AGRN;BST2;CCL2;CMPK2;CTSL1;DDX58;DDX60;DHX58;EIF2AK2;EIF3L;EXOC3L1;HERC5;IFI6;IFIH1;IFIT1;IFIT2;
lightcyan	BP	GO:0023056	positive regulation of signaling	17735	856	54	7	2.94	0.02619	2.380952	BST2;CCL2;DDX60;DHX58;IRF7;RSAD2;ZBP1
lightcyan	BP	GO:0010647	positive regulation of cell communicatio...	17735	858	54	7	2.95	0.02649	2.372881	BST2;CCL2;DDX60;DHX58;IRF7;RSAD2;ZBP1
lightcyan	MF	GO:0000166	nucleotide binding	17735	2385	54	13	7.42	0.02671	1.752022	CMPK2;DDX58;DDX60;DDX60L;DHX58;EIF2AK2;IFIH1;MX1;OAS1;OAS2;OAS3;OASL;TOR1B
lightcyan	MF	GO:1901265	nucleoside phosphate binding	17735	2386	54	13	7.42	0.02679	1.752022	CMPK2;DDX58;DDX60;DDX60L;DHX58;EIF2AK2;IFIH1;MX1;OAS1;OAS2;OAS3;OASL;TOR1B
lightcyan	BP	GO:0051100	negative regulation of binding	17735	75	54	2	0.26	0.02742	7.692308	IFIT1;IFIT2
lightcyan	BP	GO:0045926	negative regulation of growth	17735	190	54	3	0.65	0.02747	4.615385	BST2;MT2A;PNPT1
lightcyan	BP	GO:0051259	protein oligomerization	17735	338	54	4	1.16	0.02857	3.448276	OAS1;PNPT1;TOR1B;TRIM22
lightcyan	BP	GO:0048524	positive regulation of viral reproductio...	17735	79	54	2	0.27	0.03018	7.407407	IFIT1;IFITM3
lightcyan	BP	GO:0009057	macromolecule catabolic process	17735	883	54	7	3.04	0.03036	2.302632	AGRN;HERC5;HERC6;ISG15;OAS2;PNPT1;USP18
lightcyan	MF	GO:0043566	structure-specific DNA binding	17735	219	54	3	0.68	0.0305	4.411765	DDX58;DDX60;ZBP1
lightcyan	CC	GO:0000145	exocyst	17735	10	54	1	0.03	0.03062	33.33333	EXOC3L1
lightcyan	MF	GO:0004000	adenosine deaminase activity	17735	10	54	1	0.03	0.03067	33.33333	ZBP1
lightcyan	BP	GO:0044265	cellular macromolecule catabolic process	17735	698	54	6	2.4	0.03162	2.5	HERC5;HERC6;ISG15;OAS2;PNPT1;USP18
lightcyan	MF	GO:0003824	catalytic activity	17735	5264	54	23	16.37	0.03205	1.405009	CCL2;CMPK2;CTSL1;DDX58;DDX60;DDX60L;DHX58;EIF2AK2;HERC5;HERC6;IFIH1;MX1;OAS1;OAS2;OAS3;PAF
lightcyan	BP	GO:0031348	negative regulation of defense response	17735	82	54	2	0.28	0.03232	7.142857	DHX58;IFIT1
lightcyan	BP	GO:0043902	positive regulation of multi-organism pr...	17735	83	54	2	0.29	0.03304	6.896552	IFIT1;IFITM3
lightcyan	CC	GO:0031904	endosome lumen	17735	11	54	1	0.03	0.03363	33.33333	CTSL1
lightcyan	CC	GO:0042599	lamellar body	17735	11	54	1	0.03	0.03363	33.33333	LAMP3
lightcyan	BP	GO:0051085	chaperone mediated protein folding requi...	17735	10	54	1	0.03	0.03387	33.33333	TOR1B
lightcyan	BP	GO:0051770	positive regulation of nitric-oxide synt...	17735	10	54	1	0.03	0.03387	33.33333	CCL2
lightcyan	BP	GO:0051900	regulation of mitochondrial depolarizati...	17735	10	54	1	0.03	0.03387	33.33333	IFI6
lightcyan	BP	GO:0071622	regulation of granulocyte chemotaxis	17735	10	54	1	0.03	0.03387	33.33333	CCL2
lightcyan	BP	GO:0071675	regulation of mononuclear cell migration	17735	10	54	1	0.03	0.03387	33.33333	CCL2
lightcyan	BP	GO:0097067	cellular response to thyroid hormone sti...	17735	10	54	1	0.03	0.03387	33.33333	CTSL1
lightcyan	BP	GO:2000772	regulation of cellular senescence	17735	10	54	1	0.03	0.03387	33.33333	PNPT1
lightcyan	BP	GO:0030968	endoplasmic reticulum unfolded protein r...	17735	86	54	2	0.3	0.03526	6.666667	CCL2;EIF2AK2
lightcyan	MF	GO:0046914	transition metal ion binding	17735	2217	54	12	6.89	0.03526	1.741655	DDX58;DHX58;IFIH1;MT2A;OAS1;OAS2;PARP12;PHF11;TRIM22;TRIM69;XAF1;ZCCHC2
lightcyan	CC	GO:0005773	vacuole	17735	402	54	4	1.25	0.03584	3.2	AGRN;CTSL1;IFITM3;LAMP3
lightcyan	BP	GO:0002250	adaptive immune response	17735	211	54	3	0.73	0.03585	4.109589	CTSL1;IRF7;RSAD2
lightcyan	BP	GO:0034620	cellular response to unfolded protein	17735	87	54	2	0.3	0.03601	6.666667	CCL2;EIF2AK2
lightcyan	MF	GO:0008191	metalloendopeptidase inhibitor activity	17735	12	54	1	0.04	0.0367	25	BST2

lightcyan	MF	GO:0048551	metalloenzyme inhibitor activity	17735	12	54	1	0.04	0.0367	25	BST2
lightcyan	CC	GO:0031256	leading edge membrane	17735	98	54	2	0.3	0.03717	6.666667	DDX58;IFIT5
lightcyan	BP	GO:0006878	cellular copper ion homeostasis	17735	11	54	1	0.04	0.03719	25	MT2A
lightcyan	BP	GO:0007213	G-protein coupled acetylcholine receptor...	17735	11	54	1	0.04	0.03719	25	AGRN
lightcyan	BP	GO:0009263	deoxyribonucleotide biosynthetic process	17735	11	54	1	0.04	0.03719	25	CMPK2
lightcyan	BP	GO:0034123	positive regulation of toll-like recepto...	17735	11	54	1	0.04	0.03719	25	RSAD2
lightcyan	BP	GO:0035082	axoneme assembly	17735	11	54	1	0.04	0.03719	25	RSPH9
lightcyan	BP	GO:0045351	type I interferon biosynthetic process	17735	11	54	1	0.04	0.03719	25	IRF7
lightcyan	BP	GO:0051882	mitochondrial depolarization	17735	11	54	1	0.04	0.03719	25	IFI6
lightcyan	BP	GO:0070584	mitochondrion morphogenesis	17735	11	54	1	0.04	0.03719	25	PNPT1
lightcyan	BP	GO:0071294	cellular response to zinc ion	17735	11	54	1	0.04	0.03719	25	MT2A
lightcyan	MF	GO:0000982	RNA polymerase II core promoter proximal...	17735	98	54	2	0.3	0.03724	6.666667	IRF7;PLSCR1
lightcyan	CC	GO:0005739	mitochondrion	17735	1488	54	9	4.62	0.03798	1.948052	CMPK2;IFI6;IFIT3;OAS1;OAS2;PNPT1;RSAD2;TDRD7;XAF1
lightcyan	BP	GO:0002819	regulation of adaptive immune response	17735	91	54	2	0.31	0.03908	6.451613	IRF7;RSAD2
lightcyan	CC	GO:0043186	P granule	17735	13	54	1	0.04	0.03962	25	TDRD7
lightcyan	CC	GO:0045495	pole plasm	17735	13	54	1	0.04	0.03962	25	TDRD7
lightcyan	CC	GO:0060293	germ plasm	17735	13	54	1	0.04	0.03962	25	TDRD7
lightcyan	MF	GO:0010576	metalloenzyme regulator activity	17735	13	54	1	0.04	0.03969	25	BST2
lightcyan	BP	GO:0035967	cellular response to topologically incor...	17735	92	54	2	0.32	0.03986	6.25	CCL2;EIF2AK2
lightcyan	MF	GO:0036094	small molecule binding	17735	2525	54	13	7.85	0.04048	1.656051	CMPK2;DDX58;DDX60;DDX60L;DHX58;EIF2AK2;IFIH1;MX1;OAS1;OAS2;OAS3;OASL;TOR1E
lightcyan	BP	GO:0002701	negative regulation of production of mol...	17735	12	54	1	0.04	0.04051	25	BST2
lightcyan	BP	GO:0002719	negative regulation of cytokine producti...	17735	12	54	1	0.04	0.04051	25	BST2
lightcyan	BP	GO:0002832	negative regulation of response to bioti...	17735	12	54	1	0.04	0.04051	25	IFIT1
lightcyan	BP	GO:0045824	negative regulation of innate immune res...	17735	12	54	1	0.04	0.04051	25	DHX58
lightcyan	BP	GO:0046788	egress of virus within host cell	17735	12	54	1	0.04	0.04051	25	BST2
lightcyan	BP	GO:0050913	sensory perception of bitter taste	17735	12	54	1	0.04	0.04051	25	RTP4
lightcyan	BP	GO:0071621	granulocyte chemotaxis	17735	12	54	1	0.04	0.04051	25	CCL2
lightcyan	BP	GO:0071674	mononuclear cell migration	17735	12	54	1	0.04	0.04051	25	CCL2
lightcyan	BP	GO:0071222	cellular response to lipopolysaccharide	17735	93	54	2	0.32	0.04065	6.25	CCL2;CMPK2
lightcyan	BP	GO:0050794	regulation of cellular process	17735	7700	54	33	26.48	0.04266	1.246224	AGRN;BST2;CCL2;DDX58;DDX60;DHX58;EIF2AK2;HERC5;IFI6;IFIH1;IFIT1;IFIT2;IFIT3;IFITM3;IRF7;ISG15;LILRB
lightcyan	MF	GO:0043395	heparan sulfate proteoglycan binding	17735	14	54	1	0.04	0.04268	25	AGRN
lightcyan	MF	GO:0048020	CCR chemokine receptor binding	17735	14	54	1	0.04	0.04268	25	CCL2
lightcyan	MF	GO:0070717	poly-purine tract binding	17735	14	54	1	0.04	0.04268	25	PNPT1
lightcyan	BP	GO:0065007	biological regulation	17735	8616	54	36	29.63	0.04303	1.214985	AGRN;BST2;CCL2;DDX58;DDX60;DHX58;EIF2AK2;EXOC3L1;HERC5;IFI6;IFIH1;IFIT1;IFIT2;IFIT3;IFITM3;IRF7;IS
lightcyan	BP	GO:0043393	regulation of protein binding	17735	96	54	2	0.33	0.04305	6.060606	IFIT1;IFIT2
lightcyan	MF	GO:0046872	metal ion binding	17735	3936	54	18	12.24	0.04375	1.470588	AGRN;DDX58;DHX58;IFIH1;MT2A;OAS1;OAS2;OAS3;OTOF;PARP12;PHF11;PLSCR1;RSAD2;RUFY4;TRIM22;TR
lightcyan	BP	GO:0002724	regulation of T cell cytokine production	17735	13	54	1	0.04	0.04381	25	RSAD2
lightcyan	BP	GO:0008347	glial cell migration	17735	13	54	1	0.04	0.04381	25	CCL2
lightcyan	BP	GO:0051767	nitric-oxide synthase biosynthetic proce...	17735	13	54	1	0.04	0.04381	25	CCL2
lightcyan	BP	GO:0051769	regulation of nitric-oxide synthase bios...	17735	13	54	1	0.04	0.04381	25	CCL2
lightcyan	BP	GO:0070207	protein homotrimerization	17735	13	54	1	0.04	0.04381	25	PNPT1
lightcyan	BP	GO:0097066	response to thyroid hormone stimulus	17735	13	54	1	0.04	0.04381	25	CTSL1
lightcyan	BP	GO:1901623	regulation of lymphocyte chemotaxis	17735	13	54	1	0.04	0.04381	25	CCL2
lightcyan	MF	GO:0004842	ubiquitin-protein ligase activity	17735	253	54	3	0.79	0.04382	3.797468	HERC5;HERC6;TRIM69
lightcyan	BP	GO:0007005	mitochondrion organization	17735	230	54	3	0.79	0.04445	3.797468	IFI6;IFIT2;PNPT1
lightcyan	BP	GO:0071219	cellular response to molecule of bacteri...	17735	98	54	2	0.34	0.04468	5.882353	CCL2;CMPK2
lightcyan	BP	GO:0019941	modification-dependent protein catabolic...	17735	392	54	4	1.35	0.04533	2.962963	HERC5;HERC6;ISG15;USP18
lightcyan	BP	GO:0006984	ER-nucleus signaling pathway	17735	99	54	2	0.34	0.0455	5.882353	CCL2;EIF2AK2
lightcyan	CC	GO:0005852	eukaryotic translation initiation factor...	17735	15	54	1	0.05	0.04558	20	EIF3L
lightcyan	MF	GO:0000175	3'-5'-exoribonuclease activity	17735	15	54	1	0.05	0.04566	20	PNPT1
lightcyan	BP	GO:0002699	positive regulation of immune effector p...	17735	100	54	2	0.34	0.04633	5.882353	CCL2;RSAD2
lightcyan	BP	GO:0002686	negative regulation of leukocyte migrati...	17735	14	54	1	0.05	0.0471	20	CCL2
lightcyan	BP	GO:0055070	copper ion homeostasis	17735	14	54	1	0.05	0.0471	20	MT2A
lightcyan	CC	GO:0043231	intracellular membrane-bounded organelle	17735	9560	54	36	29.67	0.04937	1.213347	AGRN;BST2;CMPK2;CTSL1;EIF3L;EXOC3L1;HERC5;HERC6;IFI6;IFIH1;IFIT2;IFIT3;IFITM3;IRF7;LAMP3;MT2A;M
grey60	BP	GO:0006954	inflammatory response	17735	489	43	8	1.35	4.60E-05	5.925926	ADORA3;ALOX15;CCL23;IDO1;IL1RL1;IL34;IL5RA;SEMA7A
grey60	BP	GO:0032101	regulation of response to external stimu...	17735	380	43	7	1.05	7.00E-05	6.666667	ADORA3;CYP4F12;IDO1;IL1RL1;SEMA7A;TEC;THBS4
grey60	BP	GO:0009611	response to wounding	17735	1090	43	11	3	0.00012	3.666667	ADORA3;ALOX15;CCL23;CYP4F12;IDO1;IL1RL1;IL34;IL5RA;PIK3R6;SEMA7A;TEC
grey60	BP	GO:0032634	interleukin-5 production	17735	12	43	2	0.03	0.00048	66.66667	IL1RL1;IL5RA
grey60	BP	GO:0032674	regulation of interleukin-5 production	17735	12	43	2	0.03	0.00048	66.66667	IL1RL1;IL5RA
grey60	BP	GO:0032941	secretion by tissue	17735	62	43	3	0.17	0.00065	17.64706	ADORA3;P2RY2;SLC29A1
grey60	BP	GO:0044282	small molecule catabolic process	17735	257	43	5	0.71	0.00066	7.042254	ASRGL1;CYP4F12;IDO1;SMPD3;SORD
grey60	BP	GO:0044712	single-organism catabolic process	17735	257	43	5	0.71	0.00066	7.042254	ASRGL1;CYP4F12;IDO1;SMPD3;SORD
grey60	BP	GO:0051239	regulation of multicellular organismal p...	17735	1804	43	13	4.96	0.00074	2.620968	ADORA3;CYP4F12;CYSLTR2;IDO1;IL1RL1;IL5RA;OLIG2;P2RY2;PIK3R6;PMP22;SEMA7A;TEC;THBS
grey60	BP	GO:0032103	positive regulation of response to exter...	17735	153	43	4	0.42	8.00E-04	9.52381	ADORA3;IDO1;IL1RL1;THBS4
grey60	BP	GO:0050729	positive regulation of inflammatory resp...	17735	70	43	3	0.19	0.00093	15.78947	ADORA3;IDO1;IL1RL1
grey60	BP	GO:0007589	body fluid secretion	17735	72	43	3	0.2	0.00101	15	ADORA3;P2RY2;SLC29A1
grey60	BP	GO:0050727	regulation of inflammatory response	17735	191	43	4	0.53	0.00182	7.54717	ADORA3;IDO1;IL1RL1;SEMA7A
grey60	BP	GO:0002532	production of molecular mediator involve...	17735	25	43	2	0.07	0.00213	28.57143	ADORA3;IDO1
grey60	BP	GO:0006955	immune response	17735	1081	43	9	2.97	0.00224	3.030303	ADORA3;CCL23;CYSLTR2;IDO1;IL1RL1;IL34;IL5RA;SEMA7A;TEC

grey60	BP	GO:0006952	defense response	17735	1119	43	9	3.08	0.00284	2.922078	ADORA3;ALOX15;CCL23;CEBPE;IDO1;IL1RL1;IL34;IL5RA;SEMA7A
grey60	BP	GO:0048584	positive regulation of response to stimu...	17735	1164	43	9	3.2	0.0037	2.8125	ADORA3;CAT;CYSLTR2;IDO1;IL1RL1;PIK3R6;SEMA7A;TEC;THBS4
grey60	BP	GO:0002684	positive regulation of immune system pro...	17735	554	43	6	1.52	0.00379	3.947368	ADORA3;IDO1;IL1RL1;SEMA7A;TEC;THBS4
grey60	BP	GO:0006691	leukotriene metabolic process	17735	34	43	2	0.09	0.00392	22.22222	ALOX15;CYP4F12
grey60	MF	GO:0035586	purinergic receptor activity	17735	37	43	2	0.1	0.0041	20	ADORA3;P2RY2
grey60	MF	GO:0020037	heme binding	17735	131	43	3	0.34	0.0046	8.823529	CAT;CYP4F12;IDO1
grey60	BP	GO:0050878	regulation of body fluid levels	17735	594	43	6	1.63	0.00532	3.680982	ADORA3;CYP4F12;P2RY2;PIK3R6;SLC29A1;TEC
grey60	BP	GO:0009605	response to external stimulus	17735	1238	43	9	3.41	0.00556	2.639296	ADORA3;CAT;CCL23;CYP4F12;IDO1;IL1RL1;SEMA7A;TEC;THBS4
grey60	MF	GO:0046906	tetrapyrrole binding	17735	140	43	3	0.36	0.0056	8.333333	CAT;CYP4F12;IDO1
grey60	BP	GO:0001816	cytokine production	17735	430	43	5	1.18	0.0062	4.237288	CEBPE;IDO1;IL1RL1;IL5RA;SEMA7A
grey60	BP	GO:0014068	positive regulation of phosphatidylinosi...	17735	44	43	2	0.12	0.00649	16.66667	ADORA3;CAT
grey60	BP	GO:0002376	immune system process	17735	1816	43	11	5	0.0082	2.2	ADORA3;CCL23;CEBPE;CYSLTR2;IDO1;IL1RL1;IL34;IL5RA;SEMA7A;TEC;THBS4
grey60	BP	GO:0006950	response to stress	17735	2926	43	15	8.05	0.0083	1.863354	ACOT11;ADORA3;ALOX15;CAT;CCL23;CEBPE;CYP4F12;IDO1;IL1RL1;IL34;IL5RA;PIK3R6;SEMA7A;SLC29A1;TE
grey60	BP	GO:0046164	alcohol catabolic process	17735	51	43	2	0.14	0.00864	14.28571	SMPD3;SORD
grey60	BP	GO:0045765	regulation of angiogenesis	17735	157	43	3	0.43	0.00911	6.976744	CYSLTR2;PIK3R6;THBS4
grey60	MF	GO:0016811	hydrolase activity, acting on carbon-nit...	17735	57	43	2	0.15	0.0095	13.33333	ASRGL1;CAT
grey60	BP	GO:0014066	regulation of phosphatidylinositol 3-kin...	17735	55	43	2	0.15	0.00999	13.33333	ADORA3;CAT
grey60	BP	GO:0050801	ion homeostasis	17735	679	43	6	1.87	0.01003	3.208556	CCL23;CYP4F12;OLIG2;P2RY2;PMP22;SLC29A1
grey60	BP	GO:1901616	organic hydroxy compound catabolic proce...	17735	56	43	2	0.15	0.01035	13.33333	SMPD3;SORD
grey60	BP	GO:0048663	neuron fate commitment	17735	57	43	2	0.16	0.0107	12.5	OLIG1;OLIG2
grey60	BP	GO:1901342	regulation of vasculature development	17735	170	43	3	0.47	0.0113	6.382979	CYSLTR2;PIK3R6;THBS4
grey60	BP	GO:0051781	positive regulation of cell division	17735	59	43	2	0.16	0.01144	12.5	CAT;THBS4
grey60	BP	GO:0030193	regulation of blood coagulation	17735	62	43	2	0.17	0.01257	11.76471	CYP4F12;TEC
grey60	BP	GO:1900046	regulation of hemostasis	17735	62	43	2	0.17	0.01257	11.76471	CYP4F12;TEC
grey60	CC	GO:0005615	extracellular space	17735	844	43	6	1.96	0.013	3.061224	CCL23;IL34;IL5RA;PRSS33;SORD;THBS4
grey60	BP	GO:0035556	intracellular signal transduction	17735	1936	43	11	5.33	0.01307	2.06379	ACOT11;ADORA3;ASB2;CAT;CYSLTR2;IL1RL1;LGALS12;PIK3R6;SEMA7A;SRGAP3;TEC
grey60	BP	GO:0002687	positive regulation of leukocyte migrati...	17735	65	43	2	0.18	0.01376	11.11111	ADORA3;THBS4
grey60	BP	GO:0023052	signaling	17735	4666	43	20	12.84	0.01383	1.557632	ACOT11;ADORA3;ASB2;CAT;CCL23;CYSLTR2;EPN2;IL1RL1;IL5RA;LGALS12;OLIG2;P2RY2;PIK3R6;PMP22;SEM
grey60	BP	GO:0044700	single organism signaling	17735	4666	43	20	12.84	0.01383	1.557632	ACOT11;ADORA3;ASB2;CAT;CCL23;CYSLTR2;EPN2;IL1RL1;IL5RA;LGALS12;OLIG2;P2RY2;PIK3R6;PMP22;SEM
grey60	BP	GO:0051240	positive regulation of multicellular org...	17735	524	43	5	1.44	0.01386	3.472222	ADORA3;IDO1;IL1RL1;P2RY2;SEMA7A
grey60	BP	GO:0050818	regulation of coagulation	17735	67	43	2	0.18	0.01457	11.11111	CYP4F12;TEC
grey60	BP	GO:0080134	regulation of response to stress	17735	737	43	6	2.03	0.01462	2.955665	ADORA3;CYP4F12;IDO1;IL1RL1;SEMA7A;TEC
grey60	BP	GO:0007229	integrin-mediated signaling pathway	17735	68	43	2	0.19	0.01499	10.52632	SEMA7A;TEC
grey60	CC	GO:0031985	Golgi cisterna	17735	80	43	2	0.19	0.015	10.52632	ABO;SMPD3
grey60	CC	GO:0031984	organelle subcompartment	17735	83	43	2	0.19	0.016	10.52632	ABO;SMPD3
grey60	BP	GO:0006690	icosanoid metabolic process	17735	71	43	2	0.2	0.01627	10	ALOX15;CYP4F12
grey60	BP	GO:1901568	fatty acid derivative metabolic process	17735	71	43	2	0.2	0.01627	10	ALOX15;CYP4F12
grey60	BP	GO:0050865	regulation of cell activation	17735	359	43	4	0.99	0.01659	4.040404	ADORA3;IDO1;IL1RL1;TEC
grey60	BP	GO:0016054	organic acid catabolic process	17735	198	43	3	0.54	0.01698	5.555556	ASRGL1;CYP4F12;IDO1
grey60	BP	GO:0046395	carboxylic acid catabolic process	17735	198	43	3	0.54	0.01698	5.555556	ASRGL1;CYP4F12;IDO1
grey60	MF	GO:0030246	carbohydrate binding	17735	215	43	3	0.55	0.0178	5.454545	LGALS12;SIGLEC8;SORD
grey60	BP	GO:0001819	positive regulation of cytokine producti...	17735	202	43	3	0.56	0.0179	5.357143	IDO1;IL1RL1;SEMA7A
grey60	BP	GO:0050776	regulation of immune response	17735	560	43	5	1.54	0.018	3.246753	ADORA3;IDO1;IL1RL1;SEMA7A;TEC
grey60	BP	GO:0007154	cell communication	17735	4775	43	20	13.14	0.018	1.52207	ACOT11;ADORA3;ASB2;CAT;CCL23;CYSLTR2;EPN2;IL1RL1;IL5RA;LGALS12;OLIG2;P2RY2;PIK3R6;PMP22;SEM
grey60	BP	GO:0014065	phosphatidylinositol 3-kinase cascade	17735	75	43	2	0.21	0.01804	9.52381	ADORA3;CAT
grey60	MF	GO:0016702	oxidoreductase activity, acting on singl...	17735	80	43	2	0.21	0.0181	9.52381	ALOX15;IDO1
grey60	MF	GO:0004896	cytokine receptor activity	17735	81	43	2	0.21	0.0185	9.52381	IL1RL1;IL5RA
grey60	MF	GO:0005178	integrin binding	17735	81	43	2	0.21	0.0185	9.52381	SEMA7A;THBS4
grey60	MF	GO:0016701	oxidoreductase activity, acting on singl...	17735	81	43	2	0.21	0.0185	9.52381	ALOX15;IDO1
grey60	MF	GO:0051213	dioxygenase activity	17735	82	43	2	0.21	0.0189	9.52381	ALOX15;IDO1
grey60	BP	GO:0001817	regulation of cytokine production	17735	382	43	4	1.05	0.02035	3.809524	IDO1;IL1RL1;IL5RA;SEMA7A
grey60	BP	GO:0061041	regulation of wound healing	17735	82	43	2	0.23	0.02133	8.695652	CYP4F12;TEC
grey60	BP	GO:0033559	unsaturated fatty acid metabolic process	17735	83	43	2	0.23	0.02182	8.695652	ALOX15;CYP4F12
grey60	BP	GO:0042552	myelination	17735	84	43	2	0.23	0.02232	8.695652	OLIG2;PMP22
grey60	BP	GO:1901606	alpha-amino acid catabolic process	17735	84	43	2	0.23	0.02232	8.695652	ASRGL1;IDO1
grey60	BP	GO:0002685	regulation of leukocyte migration	17735	85	43	2	0.23	0.02281	8.695652	ADORA3;THBS4
grey60	BP	GO:0051302	regulation of cell division	17735	85	43	2	0.23	0.02281	8.695652	CAT;THBS4
grey60	BP	GO:0051047	positive regulation of secretion	17735	223	43	3	0.61	0.02318	4.918033	ADORA3;IL1RL1;P2RY2
grey60	BP	GO:0007272	ensheathment of neurons	17735	87	43	2	0.24	0.02383	8.333333	OLIG2;PMP22
grey60	BP	GO:0008366	axon ensheathment	17735	87	43	2	0.24	0.02383	8.333333	OLIG2;PMP22
grey60	BP	GO:0070374	positive regulation of ERK1 and ERK2 cas...	17735	87	43	2	0.24	0.02383	8.333333	CYSLTR2;SEMA7A
grey60	BP	GO:0006873	cellular ion homeostasis	17735	604	43	5	1.66	0.0241	3.012048	CCL23;OLIG2;P2RY2;PMP22;SLC29A1
grey60	BP	GO:0031349	positive regulation of defense response	17735	229	43	3	0.63	0.02483	4.761905	ADORA3;IDO1;IL1RL1
grey60	BP	GO:0045766	positive regulation of angiogenesis	17735	89	43	2	0.24	0.02485	8.333333	CYSLTR2;PIK3R6
grey60	BP	GO:0044255	cellular lipid metabolic process	17735	837	43	6	2.3	0.0257	2.608696	ACOT11;ALOX15;CAT;CYP4F12;PIK3R6;SMPD3
grey60	CC	GO:0005795	Golgi stack	17735	110	43	2	0.26	0.027	7.692308	ABO;SMPD3
grey60	BP	GO:0002517	T cell tolerance induction	17735	10	43	1	0.03	0.02718	33.33333	IDO1
grey60	BP	GO:0002645	positive regulation of tolerance inducti...	17735	10	43	1	0.03	0.02718	33.33333	IDO1
grey60	BP	GO:0010935	regulation of macrophage cytokine produc...	17735	10	43	1	0.03	0.02718	33.33333	SEMA7A

grey60	BP	GO:0043032	positive regulation of macrophage activa...	17735	10	43	1	0.03	0.02718	33.33333	IL1RL1
grey60	BP	GO:0043302	positive regulation of leukocyte degranu...	17735	10	43	1	0.03	0.02718	33.33333	ADORA3
grey60	MF	GO:0005337	nucleoside transmembrane transporter act...	17735	11	43	1	0.03	0.028	33.33333	SLC29A1
grey60	BP	GO:0031347	regulation of defense response	17735	428	43	4	1.18	0.02936	3.389831	ADORA3;IDO1;IL1RL1;SEMA7A
grey60	BP	GO:0032787	monocarboxylic acid metabolic process	17735	430	43	4	1.18	0.0298	3.389831	ACOT11;ALOX15;CYP4F12;IDO1
grey60	BP	GO:0002643	regulation of tolerance induction	17735	11	43	1	0.03	0.02986	33.33333	IDO1
grey60	BP	GO:0006000	fructose metabolic process	17735	11	43	1	0.03	0.02986	33.33333	SORD
grey60	BP	GO:0006569	tryptophan catabolic process	17735	11	43	1	0.03	0.02986	33.33333	IDO1
grey60	BP	GO:0009650	UV protection	17735	11	43	1	0.03	0.02986	33.33333	CAT
grey60	BP	GO:0010934	macrophage cytokine production	17735	11	43	1	0.03	0.02986	33.33333	SEMA7A
grey60	BP	GO:0033005	positive regulation of mast cell activat...	17735	11	43	1	0.03	0.02986	33.33333	ADORA3
grey60	BP	GO:0042436	indole-containing compound catabolic pro...	17735	11	43	1	0.03	0.02986	33.33333	IDO1
grey60	BP	GO:0045117	azole transport	17735	11	43	1	0.03	0.02986	33.33333	ADORA3
grey60	BP	GO:0046218	indolalkylamine catabolic process	17735	11	43	1	0.03	0.02986	33.33333	IDO1
grey60	BP	GO:2000026	regulation of multicellular organismal d...	17735	1113	43	7	3.06	0.0304	2.287582	CYSLTR2;IDO1;OLIG2;PIK3R6;PMP22;SEMA7A;THBS4
grey60	MF	GO:0035004	phosphatidylinositol 3-kinase activity	17735	12	43	1	0.03	0.0305	33.33333	PIK3R6
grey60	BP	GO:0048878	chemical homeostasis	17735	872	43	6	2.4	0.03064	2.5	CCL23;CYP4F12;OLIG2;P2RY2;PMP22;SLC29A1
grey60	BP	GO:0002699	positive regulation of immune effector p...	17735	100	43	2	0.28	0.03083	7.142857	ADORA3;SEMA7A
grey60	CC	GO:0043218	compact myelin	17735	14	43	1	0.03	0.032	33.33333	PMP22
grey60	BP	GO:0002682	regulation of immune system process	17735	884	43	6	2.43	0.03247	2.469136	ADORA3;IDO1;IL1RL1;SEMA7A;TEC;THBS4
grey60	BP	GO:0015858	nucleoside transport	17735	12	43	1	0.03	0.03253	33.33333	SLC29A1
grey60	BP	GO:0042362	fat-soluble vitamin biosynthetic process	17735	12	43	1	0.03	0.03253	33.33333	CYP4F12
grey60	BP	GO:0045136	development of secondary sexual characte...	17735	12	43	1	0.03	0.03253	33.33333	CAT
grey60	BP	GO:0045426	quinone cofactor biosynthetic process	17735	12	43	1	0.03	0.03253	33.33333	CYP4F12
grey60	BP	GO:0090136	epithelial cell-cell adhesion	17735	12	43	1	0.03	0.03253	33.33333	THBS4
grey60	BP	GO:1901663	quinone biosynthetic process	17735	12	43	1	0.03	0.03253	33.33333	CYP4F12
grey60	BP	GO:0019228	regulation of action potential in neuron	17735	103	43	2	0.28	0.03255	7.142857	OLIG2;PMP22
grey60	MF	GO:0008242	omega peptidase activity	17735	13	43	1	0.03	0.033	33.33333	ASRGL1
grey60	BP	GO:0050900	leukocyte migration	17735	257	43	3	0.71	0.03335	4.225352	ADORA3;CCL23;THBS4
grey60	BP	GO:0051716	cellular response to stimulus	17735	5052	43	20	13.9	0.03339	1.438849	ACOT11;ADORA3;ASB2;CAT;CCL23;CEBPE;CYP4F12;CYSLTR2;EPN2;IL1RL1;IL5RA;LGALS12;P2RY2;PIK3R6;SEM
grey60	MF	GO:0016491	oxidoreductase activity	17735	709	43	5	1.83	0.0345	2.73224	ALOX15;CAT;CYP4F12;IDO1;SORD
grey60	BP	GO:0055082	cellular chemical homeostasis	17735	665	43	5	1.83	0.03457	2.73224	CCL23;OLIG2;P2RY2;PMP22;SLC29A1
grey60	BP	GO:0019369	arachidonic acid metabolic process	17735	13	43	1	0.04	0.03519	25	CYP4F12
grey60	MF	GO:0016810	hydrolase activity, acting on carbon-nit...	17735	115	43	2	0.3	0.0354	6.666667	ASRGL1;CAT
grey60	MF	GO:0047617	acyl-CoA hydrolase activity	17735	14	43	1	0.04	0.0355	25	ACOT11
grey60	MF	GO:0048020	CCR chemokine receptor binding	17735	14	43	1	0.04	0.0355	25	CCL23
grey60	BP	GO:0009063	cellular amino acid catabolic process	17735	109	43	2	0.3	0.0361	6.666667	ASRGL1;IDO1
grey60	BP	GO:0065008	regulation of biological quality	17735	2547	43	12	7.01	0.03684	1.71184	ADORA3;BACE2;CCL23;CYP4F12;OLIG2;P2RY2;PIK3R6;PMP22;SEMA7A;SLC29A1;SMPD3;TEC
grey60	BP	GO:0001977	renal system process involved in regulat...	17735	14	43	1	0.04	0.03785	25	CYP4F12
grey60	BP	GO:0003091	renal water homeostasis	17735	14	43	1	0.04	0.03785	25	CYP4F12
grey60	BP	GO:0006568	tryptophan metabolic process	17735	14	43	1	0.04	0.03785	25	IDO1
grey60	BP	GO:0006684	sphingomyelin metabolic process	17735	14	43	1	0.04	0.03785	25	SMPD3
grey60	BP	GO:0020027	hemoglobin metabolic process	17735	14	43	1	0.04	0.03785	25	CAT
grey60	BP	GO:0021781	glial cell fate commitment	17735	14	43	1	0.04	0.03785	25	OLIG2
grey60	BP	GO:0042391	regulation of membrane potential	17735	271	43	3	0.75	0.03812	4	OLIG2;PMP22;SLC29A1
grey60	BP	GO:0070372	regulation of ERK1 and ERK2 cascade	17735	115	43	2	0.32	0.03979	6.25	CYSLTR2;SEMA7A
grey60	BP	GO:0002823	negative regulation of adaptive immune r...	17735	15	43	1	0.04	0.0405	25	IL1RL1
grey60	BP	GO:0009068	aspartate family amino acid catabolic pr...	17735	15	43	1	0.04	0.0405	25	ASRGL1
grey60	BP	GO:0046174	polyol catabolic process	17735	15	43	1	0.04	0.0405	25	SORD
grey60	BP	GO:1901661	quinone metabolic process	17735	15	43	1	0.04	0.0405	25	CYP4F12
grey60	MF	GO:0071837	HMG box domain binding	17735	16	43	1	0.04	0.0405	25	OLIG2
grey60	MF	GO:0008194	UDP-glycosyltransferase activity	17735	124	43	2	0.32	0.0406	6.25	ABO;MGAT3
grey60	BP	GO:0002274	myeloid leukocyte activation	17735	117	43	2	0.32	0.04105	6.25	ADORA3;IL1RL1
grey60	BP	GO:0050769	positive regulation of neurogenesis	17735	117	43	2	0.32	0.04105	6.25	OLIG2;SEMA7A
grey60	MF	GO:0008201	heparin binding	17735	128	43	2	0.33	0.043	6.060606	CCL23;THBS4
grey60	MF	GO:0016614	oxidoreductase activity, acting on CH-OH...	17735	128	43	2	0.33	0.043	6.060606	CYP4F12;SORD
grey60	BP	GO:0002507	tolerance induction	17735	16	43	1	0.04	0.04314	25	IDO1
grey60	BP	GO:0032288	myelin assembly	17735	16	43	1	0.04	0.04314	25	PMP22
grey60	BP	GO:0042402	cellular biogenic amine catabolic proces...	17735	16	43	1	0.04	0.04314	25	IDO1
grey60	BP	GO:0043304	regulation of mast cell degranulation	17735	16	43	1	0.04	0.04314	25	ADORA3
grey60	BP	GO:0070232	regulation of T cell apoptotic process	17735	16	43	1	0.04	0.04314	25	IDO1
grey60	BP	GO:0090023	positive regulation of neutrophil chemot...	17735	16	43	1	0.04	0.04314	25	THBS4
grey60	CC	GO:0044421	extracellular region part	17735	1123	43	6	2.61	0.044	2.298851	CCL23;IL34;IL5RA;PRSS33;SORD;THBS4
grey60	BP	GO:0030595	leukocyte chemotaxis	17735	123	43	2	0.34	0.04493	5.882353	CCL23;THBS4
grey60	CC	GO:0031225	anchored to membrane	17735	145	43	2	0.34	0.045	5.882353	PRSS41;SEMA7A
grey60	MF	GO:0016289	CoA hydrolase activity	17735	18	43	1	0.05	0.0455	20	ACOT11
grey60	BP	GO:0002820	negative regulation of adaptive immune r...	17735	17	43	1	0.05	0.04577	20	IL1RL1
grey60	BP	GO:0033006	regulation of mast cell activation invol...	17735	17	43	1	0.05	0.04577	20	ADORA3
grey60	BP	GO:0042375	quinone cofactor metabolic process	17735	17	43	1	0.05	0.04577	20	CYP4F12

grey60	BP	GO:0061082	myeloid leukocyte cytokine production	17735	17	43	1	0.05	0.04577	20	SEMA7A
grey60	BP	GO:0070229	negative regulation of lymphocyte apopto...	17735	17	43	1	0.05	0.04577	20	IDO1
grey60	BP	GO:0006576	cellular biogenic amine metabolic proces...	17735	127	43	2	0.35	0.04758	5.714286	IDO1;SMPD3
grey60	BP	GO:0048583	regulation of response to stimulus	17735	2347	43	11	6.46	0.04767	1.702786	ADORA3;CAT;CYP4F12;CYSLTR2;IDO1;IL1RL1;PIK3R6;SEMA7A;SRGAP3;TEC;THBS4
grey60	BP	GO:0009310	amine catabolic process	17735	18	43	1	0.05	0.0484	20	IDO1
grey60	BP	GO:0036296	response to increased oxygen levels	17735	18	43	1	0.05	0.0484	20	CAT
grey60	BP	GO:0046514	ceramide catabolic process	17735	18	43	1	0.05	0.0484	20	SMPD3
grey60	BP	GO:0050891	multicellular organismal water homeostas...	17735	18	43	1	0.05	0.0484	20	CYP4F12
grey60	BP	GO:0055093	response to hyperoxia	17735	18	43	1	0.05	0.0484	20	CAT
greenyello	BP	GO:0007155	cell adhesion	17735	957	126	21	6.91	3.90E-06	3.039074	CCL4;CCL5;CD2;CHST10;CX3CR1;DLG5;EFNA5;EPDR1;ERBB2;FEZ1;FZD4;GPR56;MTSS1;NCAM1;NUAK1;PCDH
greenyello	BP	GO:0022610	biological adhesion	17735	959	126	21	6.92	4.00E-06	3.034682	CCL4;CCL5;CD2;CHST10;CX3CR1;DLG5;EFNA5;EPDR1;ERBB2;FEZ1;FZD4;GPR56;MTSS1;NCAM1;NUAK1;PCDH
greenyello	MF	GO:0004872	receptor activity	17735	1528	126	26	11.12	2.90E-05	2.338129	BAI2;CCR5;CD160;CD2;CX3CR1;CXCR6;ERBB2;F2R;FZD4;GPR114;GPR153;GPR56;GPR68;KIR2DL1;KLRD1;KLR
greenyello	CC	GO:0044459	plasma membrane part	17735	1895	126	30	13.91	3.30E-05	2.156722	CACNA2D2;CCR5;CD160;CD2;CX3CR1;CXCR6;EFNA5;ERBB2;F2R;FASLG;FCRL6;FZD4;GPR56;GPR68;GZMB;KI
greenyello	CC	GO:0071944	cell periphery	17735	4360	126	52	32.01	6.20E-05	1.624492	ARL4C;BAI2;BFSP1;BNC2;CACNA2D2;CAMK2N1;CCR5;CD160;CD2;CX3CR1;CXCR6;DLG5;EFNA5;ERBB2;F2R;F
greenyello	BP	GO:0006968	cellular defense response	17735	60	126	5	0.43	7.10E-05	11.62791	CCR5;CD160;CX3CR1;GNLY;KLRG1
greenyello	CC	GO:0005886	plasma membrane	17735	4273	126	51	31.37	7.60E-05	1.625757	ARL4C;BAI2;BFSP1;BNC2;CACNA2D2;CAMK2N1;CCR5;CD160;CD2;CX3CR1;CXCR6;DLG5;EFNA5;ERBB2;F2R;F
greenyello	CC	GO:0009897	external side of plasma membrane	17735	202	126	8	1.48	0.00012	5.405405	CCR5;CD2;EFNA5;FASLG;FCRL6;KLRD1;LAG3;NCAM1
greenyello	BP	GO:0007165	signal transduction	17735	4165	126	48	30.08	0.00014	1.595745	ADAMTS1;AGAP1;ARL4C;BAI2;CCL4;CCL5;CCR5;CD160;CD2;CLIC3;CX3CR1;CXCR6;DLG5;DTHD1;DUSP8;EDAF
greenyello	BP	GO:0007166	cell surface receptor signaling pathway	17735	2331	126	32	16.83	0.00015	1.901367	ADAMTS1;BAI2;CCL5;CCR5;CD160;CD2;CX3CR1;CXCR6;EFNA5;ERBB2;F2R;FASLG;FZD4;GFI1;GPR114;GPR56;
greenyello	MF	GO:0004888	transmembrane signaling receptor activit...	17735	1232	126	21	8.97	0.00019	2.341137	BAI2;CCR5;CD160;CX3CR1;CXCR6;ERBB2;F2R;FZD4;GPR114;GPR153;GPR56;GPR68;KLRD1;LAG3;LGR6;NMUI
greenyello	BP	GO:0007154	cell communication	17735	4775	126	52	34.48	0.00028	1.508121	ADAMTS1;AGAP1;ARL4C;BAI2;CACNA2D2;CCL4;CCL5;CCR5;CD160;CD2;CLIC3;CX3CR1;CXCR6;DLG5;DTHD1;I
greenyello	BP	GO:0023052	signaling	17735	4666	126	51	33.69	0.00031	1.513802	ADAMTS1;AGAP1;ARL4C;BAI2;CACNA2D2;CCL4;CCL5;CCR5;CD160;CD2;CLIC3;CX3CR1;CXCR6;DLG5;DTHD1;I
greenyello	BP	GO:0044700	single organism signaling	17735	4666	126	51	33.69	0.00031	1.513802	ADAMTS1;AGAP1;ARL4C;BAI2;CACNA2D2;CCL4;CCL5;CCR5;CD160;CD2;CLIC3;CX3CR1;CXCR6;DLG5;DTHD1;I
greenyello	BP	GO:0050794	regulation of cellular process	17735	7700	126	73	55.6	0.00038	1.31295	ADAMTS1;AGAP1;APOBEC3G;APOBEC3H;ARL4C;BAI2;BNC2;CACNA2D2;CCL4;CCL5;CCR5;CD160;CD2;CLIC3;(
greenyello	BP	GO:0034405	response to fluid shear stress	17735	22	126	3	0.16	0.00051	18.75	MTSS1;PDGFRB;SMAD7
greenyello	BP	GO:0010921	regulation of phosphatase activity	17735	92	126	5	0.66	0.00053	7.575758	NUAK1;PDGFRB;PPP2R2B;SH3RF2;SYTL2
greenyello	MF	GO:0038023	signaling receptor activity	17735	1328	126	21	9.66	0.00053	2.173913	BAI2;CCR5;CD160;CX3CR1;CXCR6;ERBB2;F2R;FZD4;GPR114;GPR153;GPR56;GPR68;KLRD1;LAG3;LGR6;NMUI
greenyello	BP	GO:0002682	regulation of immune system process	17735	884	126	16	6.38	0.00058	2.507837	APOBEC3G;CCL4;CCL5;CD160;CD2;ERBB2;GFI1;KIR2DL1;KLRD1;KLRG1;LAG3;PIK3R3;SH2D1B;SLAMF7;TBX21
greenyello	BP	GO:0019722	calcium-mediated signaling	17735	97	126	5	0.7	0.00068	7.142857	CCL4;CCR5;NCAM1;NMUR1;RCAN2
greenyello	BP	GO:0030155	regulation of cell adhesion	17735	268	126	8	1.94	0.00072	4.123711	CCL5;EFNA5;ERBB2;FZD4;NUAK1;SMAD7;VIT;WNT1
greenyello	MF	GO:0001637	G-protein coupled chemoattractant recept...	17735	25	126	3	0.18	0.00077	16.66667	CCR5;CX3CR1;CXCR6
greenyello	MF	GO:0004950	chemokine receptor activity	17735	25	126	3	0.18	0.00077	16.66667	CCR5;CX3CR1;CXCR6
greenyello	BP	GO:0042110	T cell activation	17735	339	126	9	2.45	0.00077	3.673469	CCL5;CD2;EOMES;ERBB2;LAG3;PIK3R3;TBX21;TIGIT;WNT1
greenyello	BP	GO:0051928	positive regulation of calcium ion trans...	17735	58	126	4	0.42	0.00081	9.52381	CCL4;CCL5;F2R;PDGFRB
greenyello	CC	GO:0005901	caveola	17735	57	126	4	0.42	0.00081	9.52381	EFNA5;F2R;FASLG;PTCH1
greenyello	BP	GO:0071396	cellular response to lipid	17735	213	126	7	1.54	0.00089	4.545455	CCL5;CCR5;FZD4;GFI1;NPC1;PTCH1;PTGDR
greenyello	CC	GO:0009986	cell surface	17735	485	126	11	3.56	0.00089	3.089888	CCR5;CD2;EFNA5;FASLG;FCRL6;FZD4;KLRD1;LAG3;NCAM1;TIGIT;WNT1
greenyello	BP	GO:0019079	viral genome replication	17735	61	126	4	0.44	0.00098	9.090909	APOBEC3G;APOBEC3H;CCL5;CXCR6
greenyello	BP	GO:0006952	defense response	17735	1119	126	18	8.08	0.00102	2.227723	APOBEC3G;APOBEC3H;CCL4;CCL5;CCR5;CD160;CX3CR1;F2R;GFI1;GNLY;GPR68;KLRG1;LAG3;NCAM1;PTGDR,
greenyello	BP	GO:0043666	regulation of phosphoprotein phosphatase...	17735	28	126	3	0.2	0.00105	15	NUAK1;PDGFRB;PPP2R2B
greenyello	MF	GO:0015631	tubulin binding	17735	159	126	6	1.16	0.00107	5.172414	ARL4C;FEZ1;JAKMIP1;JAKMIP2;NCALD;RAB11FIP5
greenyello	BP	GO:0002228	natural killer cell mediated immunity	17735	29	126	3	0.21	0.00117	14.28571	LAG3;SH2D1B;SLAMF7
greenyello	BP	GO:0070098	chemokine-mediated signaling pathway	17735	29	126	3	0.21	0.00117	14.28571	CCL5;CCR5;CX3CR1
greenyello	BP	GO:0035303	regulation of dephosphorylation	17735	111	126	5	0.8	0.00125	6.25	NUAK1;PDGFRB;PPP2R2B;SH3RF2;SYTL2
greenyello	BP	GO:0051249	regulation of lymphocyte activation	17735	293	126	8	2.12	0.00127	3.773585	CCL5;CD2;ERBB2;LAG3;PIK3R3;SLAMF7;TBX21;TIGIT
greenyello	BP	GO:0007186	G-protein coupled receptor signaling pat...	17735	601	126	12	4.34	0.0013	2.764977	BAI2;CCL5;CCR5;CX3CR1;CXCR6;F2R;GPR114;GPR56;GPR68;NMUR1;PTGDR;RGS9
greenyello	MF	GO:0004930	G-protein coupled receptor activity	17735	858	126	15	6.24	0.00136	2.403846	BAI2;CCR5;CX3CR1;CXCR6;F2R;FZD4;GPR114;GPR153;GPR56;GPR68;LGR6;NMUR1;PDGFRB;PTGDR;S1PR5
greenyello	MF	GO:0004871	signal transducer activity	17735	1632	126	23	11.88	0.00136	1.936027	BAI2;CCR5;CD160;CX3CR1;CXCR6;ERBB2;F2R;FZD4;GPR114;GPR153;GPR56;GPR68;KLRD1;LAG3;LGR6;NMUI
greenyello	MF	GO:0060089	molecular transducer activity	17735	1632	126	23	11.88	0.00136	1.936027	BAI2;CCR5;CD160;CX3CR1;CXCR6;ERBB2;F2R;FZD4;GPR114;GPR153;GPR56;GPR68;KLRD1;LAG3;LGR6;NMUI
greenyello	MF	GO:0008528	G-protein coupled peptide receptor activ...	17735	115	126	5	0.84	0.00152	5.952381	CCR5;CX3CR1;CXCR6;F2R;NMUR1
greenyello	MF	GO:0019902	phosphatase binding	17735	115	126	5	0.84	0.00152	5.952381	ERBB2;FCRL6;RCAN2;SH3RF2;SYTL2
greenyello	BP	GO:0050789	regulation of biological process	17735	8134	126	74	58.74	0.00153	1.259789	ADAMTS1;AGAP1;APOBEC3G;APOBEC3H;ARL4C;BAI2;BNC2;CACNA2D2;CCL4;CCL5;CCR5;CD160;CD2;CLIC3;(
greenyello	BP	GO:0045071	negative regulation of viral genome repl...	17735	32	126	3	0.23	0.00156	13.04348	APOBEC3G;APOBEC3H;CCL5
greenyello	MF	GO:0001653	peptide receptor activity	17735	116	126	5	0.84	0.00157	5.952381	CCR5;CX3CR1;CXCR6;F2R;NMUR1
greenyello	BP	GO:0033993	response to lipid	17735	534	126	11	3.86	0.00163	2.849741	CCL5;CCR5;F2R;FASLG;FZD4;GFI1;NPC1;PDGFRB;PTCH1;PTGDR;RGS9
greenyello	CC	GO:0046658	anchored to plasma membrane	17735	32	126	3	0.23	0.00164	13.04348	CD160;CD2;EFNA5
greenyello	BP	GO:0048525	negative regulation of viral reproductio...	17735	33	126	3	0.24	0.00171	12.5	APOBEC3G;APOBEC3H;CCL5
greenyello	BP	GO:0006955	immune response	17735	1081	126	17	7.81	0.00183	2.176697	APOBEC3G;CCL4;CCL5;CCR5;CD160;EOMES;FASLG;GFI1;KIR2DL1;KLRD1;KLRG1;LAG3;NCAM1;SH2D1B;SLAM
greenyello	BP	GO:0072676	lymphocyte migration	17735	34	126	3	0.25	0.00186	12	CCL4;CCL5;TBX21
greenyello	BP	GO:0022407	regulation of cell-cell adhesion	17735	73	126	4	0.53	0.00191	7.54717	CCL5;EFNA5;SMAD7;WNT1
greenyello	BP	GO:0048583	regulation of response to stimulus	17735	2347	126	29	16.95	0.00196	1.710914	AGAP1;APOBEC3G;CCL4;CCL5;CD160;DUSP8;EFNA5;ERBB2;F2R;FASLG;FZD4;GFI1;KIR2DL1;KLRD1;KLRG1;LA
greenyello	CC	GO:0045121	membrane raft	17735	178	126	6	1.31	0.00201	4.580153	EFNA5;F2R;FASLG;NPC1;PTCH1;WNT1
greenyello	MF	GO:0008092	cytoskeletal protein binding	17735	627	126	12	4.56	0.00201	2.631579	ARL4C;CCR5;DLG5;EPB41L4A;FEZ1;JAKMIP1;JAKMIP2;MTSS1;MYO3B;NCALD;NEB;RAB11FIP5
greenyello	BP	GO:0046649	lymphocyte activation	17735	468	126	10	3.38	0.00204	2.95858	CCL5;CD2;EOMES;ERBB2;LAG3;PIK3R3;SLAMF7;TBX21;TIGIT;WNT1
greenyello	MF	GO:0019903	protein phosphatase binding	17735	74	126	4	0.54	0.00207	7.407407	ERBB2;FCRL6;RCAN2;SH3RF2
greenyello	BP	GO:0014065	phosphatidylinositol 3-kinase cascade	17735	75	126	4	0.54	0.00211	7.407407	CCL5;ERBB2;F2R;PDGFRB
greenyello	BP	GO:0045321	leukocyte activation	17735	552	126	11	3.99	0.00211	2.756892	CCL5;CD2;EOMES;ERBB2;LAG3;PIK3R3;SH2D1B;SLAMF7;TBX21;TIGIT;WNT1
greenyello	BP	GO:0044763	single-organism cellular process	17735	9577	126	83	69.16	0.00217	1.200116	ADAMTS1;AGAP1;APOBEC3G;APOBEC3H;ARL4C;BAI2;BFSP1;CACNA2D2;CCL4;CCL5;CCR5;CD160;CD2;CEP78
greenyello	BP	GO:0045090	retroviral genome replication	17735	10	126	2	0.07	0.00224	28.57143	APOBEC3G;APOBEC3H

greenyello	BP	GO:0072203	cell proliferation involved in metanephr...	17735	10	126	2	0.07	0.00224	28.57143	PDGFRB;PTCH1
greenyello	MF	GO:0008158	hedgehog receptor activity	17735	10	126	2	0.07	0.00227	28.57143	NPC1;PTCH1
greenyello	BP	GO:0051716	cellular response to stimulus	17735	5052	126	51	36.48	0.00234	1.398026	ADAMTS1;AGAP1;ARL4C;BAI2;CCL4;CCL5;CCR5;CD160;CD2;CLIC3;CX3CR1;CXCR6;DLG5;DTHD1;DUSP8;EDA
greenyello	BP	GO:0007528	neuromuscular junction development	17735	37	126	3	0.27	0.00238	11.11111	CACNA2D2;ERBB2;F2R
greenyello	CC	GO:0019898	extrinsic to membrane	17735	127	126	5	0.93	0.00245	5.376344	EPB41L4A;JAKMIP1;RGS9;SMAD7;SYTL2
greenyello	BP	GO:0007223	Wnt receptor signaling pathway, calcium ...	17735	11	126	2	0.08	0.00272	25	FZD4;WNT1
greenyello	BP	GO:2000242	negative regulation of reproductive proc...	17735	81	126	4	0.58	0.00279	6.896552	APOBEC3G;APOBEC3H;CCL4;CCL5
greenyello	BP	GO:0002694	regulation of leukocyte activation	17735	334	126	8	2.41	0.00288	3.319502	CCL5;CD2;ERBB2;LAG3;PIK3R3;SLAMF7;TBX21;TIGIT
greenyello	BP	GO:0007517	muscle organ development	17735	339	126	8	2.45	0.00315	3.265306	CACNA2D2;ERBB2;F2R;MSC;NEB;SGCD;SMAD7;WNT1
greenyello	BP	GO:0043922	negative regulation by host of viral tra...	17735	12	126	2	0.09	0.00325	22.22222	CCL4;CCL5
greenyello	BP	GO:0048731	system development	17735	3385	126	37	24.44	0.00363	1.513912	ADAMTS1;BAI2;BFSP1;CACNA2D2;CD2;EDARADD;EFNA5;EOMES;ERBB2;F2R;FASLG;FEZ1;FZD4;GFI1;GPR56;
greenyello	BP	GO:0014911	positive regulation of smooth muscle cel...	17735	13	126	2	0.09	0.00382	22.22222	CCL5;PDGFRB
greenyello	BP	GO:0061298	retina vasculature development in camera...	17735	13	126	2	0.09	0.00382	22.22222	FZD4;PDGFRB
greenyello	BP	GO:0072111	cell proliferation involved in kidney de...	17735	13	126	2	0.09	0.00382	22.22222	PDGFRB;PTCH1
greenyello	BP	GO:1901623	regulation of lymphocyte chemotaxis	17735	13	126	2	0.09	0.00382	22.22222	CCL4;CCL5
greenyello	BP	GO:0014068	positive regulation of phosphatidylinosi...	17735	44	126	3	0.32	0.00391	9.375	CCL5;F2R;PDGFRB
greenyello	BP	GO:0007218	neuropeptide signaling pathway	17735	89	126	4	0.64	0.00392	6.25	BAI2;GPR114;GPR56;NMUR1
greenyello	CC	GO:0044425	membrane part	17735	6026	126	59	44.24	0.00405	1.333635	B3GAT1;B3GNT7;BAI2;C14orf1;CACNA2D2;CCR5;CD160;CD2;CHST10;CLDND2;CLIC3;CX3CR1;CXCR6;EFNA5;
greenyello	CC	GO:0031225	anchored to membrane	17735	145	126	5	1.06	0.00433	4.716981	CD160;CD2;EFNA5;NCAM1;XPNPEP2
greenyello	BP	GO:0006925	inflammatory cell apoptotic process	17735	14	126	2	0.1	0.00444	20	CCL5;FASLG
greenyello	BP	GO:0050865	regulation of cell activation	17735	359	126	8	2.59	0.00446	3.088803	CCL5;CD2;ERBB2;LAG3;PIK3R3;SLAMF7;TBX21;TIGIT
greenyello	MF	GO:0048020	CCR chemokine receptor binding	17735	14	126	2	0.1	0.00451	20	CCL4;CCL5
greenyello	CC	GO:0031226	intrinsic to plasma membrane	17735	1252	126	18	9.19	0.00451	1.958651	CCR5;CD160;CD2;CX3CR1;CXCR6;EFNA5;F2R;FASLG;FZD4;GPR56;GPR68;KIR2DL1;NKG7;NMUR1;NPC1;PCDH
greenyello	BP	GO:0002376	immune system process	17735	1816	126	23	13.11	0.00473	1.754386	APOBEC3G;APOBEC3H;CCL4;CCL5;CCR5;CD160;CD2;EOMES;ERBB2;FASLG;GFI1;KIR2DL1;KLRD1;KLRG1;LAG
greenyello	BP	GO:0045069	regulation of viral genome replication	17735	48	126	3	0.35	0.005	8.571429	APOBEC3G;APOBEC3H;CCL5
greenyello	BP	GO:0035556	intracellular signal transduction	17735	1936	126	24	13.98	0.00506	1.716738	AGAP1;ARL4C;CCL4;CCL5;CCR5;DLG5;DUSP8;EFNA5;ERBB2;F2R;FASLG;FZD4;NCAM1;NMUR1;PDGFRB;RAB7
greenyello	BP	GO:0032897	negative regulation of viral transcripti...	17735	15	126	2	0.11	0.0051	18.18182	CCL4;CCL5
greenyello	BP	GO:2000403	positive regulation of lymphocyte migrat...	17735	15	126	2	0.11	0.0051	18.18182	CCL4;CCL5
greenyello	BP	GO:0019932	second-messenger-mediated signaling	17735	154	126	5	1.11	0.00516	4.504505	CCL4;CCR5;NCAM1;NMUR1;RCAN2
greenyello	BP	GO:0050896	response to stimulus	17735	6581	126	61	47.52	0.00542	1.28367	ADAMTS1;AGAP1;APOBEC3G;APOBEC3H;ARL4C;BAI2;CCL4;CCL5;CCR5;CD160;CD2;CHST10;CLIC3;CX3CR1;C
greenyello	BP	GO:0002717	positive regulation of natural killer ce...	17735	16	126	2	0.12	0.0058	16.66667	LAG3;SH2D1B
greenyello	BP	GO:0050863	regulation of T cell activation	17735	226	126	6	1.63	0.00592	3.680982	CCL5;CD2;ERBB2;LAG3;PIK3R3;TIGIT
greenyello	BP	GO:0061061	muscle structure development	17735	460	126	9	3.32	0.00604	2.710843	CACNA2D2;ERBB2;F2R;MSC;NEB;PDGFRB;SGCD;SMAD7;WNT1
greenyello	BP	GO:0030217	T cell differentiation	17735	160	126	5	1.16	0.00605	4.310345	CD2;EOMES;ERBB2;TBX21;WNT1
greenyello	BP	GO:0006816	calcium ion transport	17735	228	126	6	1.65	0.00617	3.636364	CCL4;CCL5;CCR5;F2R;NMUR1;PDGFRB
greenyello	CC	GO:0000932	cytoplasmic mRNA processing body	17735	51	126	3	0.37	0.00622	8.108108	APOBEC3G;APOBEC3H;PATL2
greenyello	BP	GO:0065007	biological regulation	17735	8616	126	75	62.22	0.00637	1.2054	ADAMTS1;AGAP1;APOBEC3G;APOBEC3H;ARL4C;BAI2;BNC2;CACNA2D2;CCL4;CCL5;CCR5;CD160;CD2;CLIC3;
greenyello	MF	GO:0043015	gamma-tubulin binding	17735	17	126	2	0.12	0.00665	16.66667	FEZ1;RAB11FIP5
greenyello	BP	GO:0014706	striated muscle tissue development	17735	306	126	7	2.21	0.00671	3.167421	CACNA2D2;ERBB2;F2R;MSC;PDGFRB;SMAD7;WNT1
greenyello	BP	GO:0043901	negative regulation of multi-organism pr...	17735	54	126	3	0.39	0.00695	7.692308	APOBEC3G;APOBEC3H;CCL5
greenyello	BP	GO:0014066	regulation of phosphatidylinositol 3-kin...	17735	55	126	3	0.4	0.00731	7.5	CCL5;F2R;PDGFRB
greenyello	BP	GO:0002407	dendritic cell chemotaxis	17735	18	126	2	0.13	0.00733	15.38462	CCL5;CCR5
greenyello	BP	GO:0036314	response to sterol	17735	18	126	2	0.13	0.00733	15.38462	CCR5;PTCH1
greenyello	BP	GO:0070723	response to cholesterol	17735	18	126	2	0.13	0.00733	15.38462	CCR5;PTCH1
greenyello	BP	GO:0090342	regulation of cell aging	17735	18	126	2	0.13	0.00733	15.38462	NUAK1;WNT1
greenyello	BP	GO:0010810	regulation of cell-substrate adhesion	17735	107	126	4	0.77	0.0075	5.194805	EFNA5;FZD4;VIT;WNT1
greenyello	BP	GO:0070838	divalent metal ion transport	17735	238	126	6	1.72	0.00756	3.488372	CCL4;CCL5;CCR5;F2R;NMUR1;PDGFRB
greenyello	BP	GO:0050792	regulation of viral reproduction	17735	109	126	4	0.79	0.008	5.063291	APOBEC3G;APOBEC3H;CCL4;CCL5
greenyello	BP	GO:0002715	regulation of natural killer cell mediat...	17735	19	126	2	0.14	0.00815	14.28571	LAG3;SH2D1B
greenyello	BP	GO:0010714	positive regulation of collagen metaboli...	17735	19	126	2	0.14	0.00815	14.28571	F2R;PDGFRB
greenyello	BP	GO:0032967	positive regulation of collagen biosynth...	17735	19	126	2	0.14	0.00815	14.28571	F2R;PDGFRB
greenyello	BP	GO:0036336	dendritic cell migration	17735	19	126	2	0.14	0.00815	14.28571	CCL5;CCR5
greenyello	BP	GO:0072511	divalent inorganic cation transport	17735	242	126	6	1.75	0.00818	3.428571	CCL4;CCL5;CCR5;F2R;NMUR1;PDGFRB
greenyello	BP	GO:0051336	regulation of hydrolase activity	17735	755	126	12	5.45	0.00818	2.201835	AGAP1;CCL5;EFNA5;ERBB2;F2R;FASLG;NMUR1;NUAK1;PDGFRB;PPP2R2B;SH3RF2;SYTL2
greenyello	BP	GO:0040011	locomotion	17735	1253	126	17	9.05	0.00818	1.878453	CCL4;CCL5;CCR5;CD2;CX3CR1;EFNA5;ERBB2;F2R;FEZ1;FZD4;NCAM1;PDGFRB;PODN;RASGEF1A;SMAD7;SPO
greenyello	BP	GO:0060537	muscle tissue development	17735	318	126	7	2.3	0.00821	3.043478	CACNA2D2;ERBB2;F2R;MSC;PDGFRB;SMAD7;WNT1
greenyello	MF	GO:0005212	structural constituent of eye lens	17735	19	126	2	0.14	0.00828	14.28571	BFSP1;LIM2
greenyello	BP	GO:0001775	cell activation	17735	758	126	12	5.47	0.00843	2.193784	CCL5;CD2;EOMES;ERBB2;F2R;LAG3;PIK3R3;SH2D1B;SLAMF7;TBX21;TIGIT;WNT1
greenyello	BP	GO:0072001	renal system development	17735	246	126	6	1.78	0.00883	3.370787	ADAMTS1;MTSS1;PDGFRB;PTCH1;SMAD7;WNT1
greenyello	BP	GO:0051707	response to other organism	17735	578	126	10	4.17	0.00891	2.398082	APOBEC3G;APOBEC3H;CCL4;CCL5;CCR5;F2R;FASLG;GFI1;GNLY;TBX21
greenyello	BP	GO:0016337	cell-cell adhesion	17735	405	126	8	2.92	0.009	2.739726	CCL5;CD2;DLG5;EFNA5;NCAM1;PCDH1;SMAD7;WNT1
greenyello	BP	GO:0043921	modulation by host of viral transcriptio...	17735	20	126	2	0.14	0.00902	14.28571	CCL4;CCL5
greenyello	BP	GO:0052472	modulation by host of symbiont transcrip...	17735	20	126	2	0.14	0.00902	14.28571	CCL4;CCL5
greenyello	BP	GO:2000401	regulation of lymphocyte migration	17735	20	126	2	0.14	0.00902	14.28571	CCL4;CCL5
greenyello	BP	GO:0007519	skeletal muscle tissue development	17735	179	126	5	1.29	0.00959	3.875969	CACNA2D2;ERBB2;F2R;MSC;WNT1
greenyello	BP	GO:0035821	modification of morphology or physiology...	17735	61	126	3	0.44	0.00972	6.818182	CCL4;CCL5;GNLY
greenyello	BP	GO:0046135	pyrimidine nucleoside catabolic process	17735	21	126	2	0.15	0.00992	13.33333	APOBEC3G;APOBEC3H
greenyello	BP	GO:0052312	modulation of transcription in other org...	17735	21	126	2	0.15	0.00992	13.33333	CCL4;CCL5
greenyello	BP	GO:0016311	dephosphorylation	17735	253	126	6	1.83	0.01005	3.278689	DUSP8;NUAK1;PDGFRB;PPP2R2B;SH3RF2;SYTL2

greenyello	BP	GO:0043270	positive regulation of ion transport	17735	118	126	4	0.85	0.01051	4.705882	CCL4;CCL5;F2R;PDGFRB
greenyello	BP	GO:0032965	regulation of collagen biosynthetic proc...	17735	22	126	2	0.16	0.01086	12.5	F2R;PDGFRB
greenyello	BP	GO:0048247	lymphocyte chemotaxis	17735	22	126	2	0.16	0.01086	12.5	CCL4;CCL5
greenyello	BP	GO:0006935	chemotaxis	17735	596	126	10	4.3	0.0109	2.325581	CCL4;CCL5;CCR5;CX3CR1;EFNA5;ERBB2;FEZ1;NCAM1;PDGFRB;SPON2
greenyello	BP	GO:0042330	taxis	17735	596	126	10	4.3	0.0109	2.325581	CCL4;CCL5;CCR5;CX3CR1;EFNA5;ERBB2;FEZ1;NCAM1;PDGFRB;SPON2
greenyello	BP	GO:0060538	skeletal muscle organ development	17735	186	126	5	1.34	0.0112	3.731343	CACNA2D2;ERBB2;F2R;MSC;WNT1
greenyello	BP	GO:0010712	regulation of collagen metabolic process	17735	23	126	2	0.17	0.01184	11.76471	F2R;PDGFRB
greenyello	MF	GO:0015026	coreceptor activity	17735	23	126	2	0.17	0.01202	11.76471	CCR5;CXCR6
greenyello	MF	GO:0043014	alpha-tubulin binding	17735	23	126	2	0.17	0.01202	11.76471	ARL4C;NCALD
greenyello	BP	GO:0009607	response to biotic stimulus	17735	606	126	10	4.38	0.01215	2.283105	APOBEC3G;APOBEC3H;CCL4;CCL5;CCR5;F2R;FASLG;GFI1;GNLY;TBX21
greenyello	BP	GO:0045785	positive regulation of cell adhesion	17735	124	126	4	0.9	0.01243	4.444444	CCL5;ERBB2;SMAD7;VIT
greenyello	BP	GO:0050850	positive regulation of calcium-mediated ...	17735	24	126	2	0.17	0.01285	11.76471	CCL4;NCAM1
greenyello	BP	GO:0001906	cell killing	17735	68	126	3	0.49	0.01306	6.122449	GNLY;LAG3;SLAMF7
greenyello	MF	GO:0005102	receptor binding	17735	1201	126	16	8.74	0.01317	1.830664	BZRAP1;CCL4;CCL5;CD160;EFNA5;ERBB2;F2R;FASLG;JAKMIP1;LAG3;MTSS1;PDGFRB;PTCH1;SMAD7;TIGIT;W
greenyello	BP	GO:0006950	response to stress	17735	2926	126	31	21.13	0.01366	1.467108	APOBEC3G;APOBEC3H;CCL4;CCL5;CCR5;CD160;CD2;CX3CR1;ERBB2;F2R;FASLG;FZD4;GFI1;GNLY;GPR68;KLR
greenyello	BP	GO:0044253	positive regulation of multicellular org...	17735	25	126	2	0.18	0.01391	11.11111	F2R;PDGFRB
greenyello	BP	GO:0050868	negative regulation of T cell activation	17735	70	126	3	0.51	0.01412	5.882353	ERBB2;LAG3;TIGIT
greenyello	BP	GO:0044699	single-organism process	17735	10656	126	87	76.95	0.01433	1.130604	ADAMTS1;AGAP1;APOBEC3G;APOBEC3H;ARL4C;BAI2;BFSP1;CACNA2D2;CCL4;CCL5;CCR5;CD160;CD2;CEP78
greenyello	CC	GO:0031224	intrinsic to membrane	17735	5319	126	51	39.05	0.01434	1.306018	B3GAT1;B3GNT7;BAI2;C14orf1;CACNA2D2;CCR5;CD160;CD2;CHST10;CLDND2;CLIC3;CX3CR1;CXCR6;EFNA5;
greenyello	CC	GO:0016020	membrane	17735	7690	126	69	56.46	0.01452	1.222104	ARL4C;B3GAT1;B3GNT7;BAI2;BFSP1;BNC2;C14orf1;CACNA2D2;CAMK2N1;CCR5;CD160;CD2;CHST10;CLDND
greenyello	BP	GO:0051924	regulation of calcium ion transport	17735	130	126	4	0.94	0.01457	4.255319	CCL4;CCL5;F2R;PDGFRB
greenyello	BP	GO:0018108	peptidyl-tyrosine phosphorylation	17735	200	126	5	1.44	0.01494	3.472222	CCL5;EFNA5;F2R;PDGFRB;ZFYVE28
greenyello	BP	GO:0032496	response to lipopolysaccharide	17735	200	126	5	1.44	0.01494	3.472222	CCL5;CCR5;F2R;FASLG;GFI1
greenyello	BP	GO:0006821	chloride transport	17735	26	126	2	0.19	0.01499	10.52632	CLIC3;NMUR1
greenyello	BP	GO:0006904	vesicle docking involved in exocytosis	17735	26	126	2	0.19	0.01499	10.52632	NCAM1;SYTL2
greenyello	BP	GO:0014910	regulation of smooth muscle cell migrati...	17735	26	126	2	0.19	0.01499	10.52632	CCL5;PDGFRB
greenyello	BP	GO:0051851	modification by host of symbiont morphol...	17735	26	126	2	0.19	0.01499	10.52632	CCL4;CCL5
greenyello	BP	GO:0055002	striated muscle cell development	17735	132	126	4	0.95	0.01533	4.210526	CACNA2D2;ERBB2;F2R;PDGFRB
greenyello	BP	GO:0007275	multicellular organismal development	17735	3940	126	39	28.45	0.01533	1.370826	ADAMTS1;BAI2;BFSP1;CACNA2D2;CD2;EDARADD;EFNA5;EOMES;ERBB2;F2R;FASLG;FEZ1;FZD4;GFI1;GPR56;
greenyello	CC	GO:0005887	integral to plasma membrane	17735	1208	126	16	8.87	0.0154	1.803833	CCR5;CD2;CX3CR1;CXCR6;F2R;FASLG;FZD4;GPR56;GPR68;KIR2DL1;NKG7;NMUR1;NPC1;PCDH1;PDGFRB;SYT
greenyello	BP	GO:0018212	peptidyl-tyrosine modification	17735	202	126	5	1.46	0.01553	3.424658	CCL5;EFNA5;F2R;PDGFRB;ZFYVE28
greenyello	BP	GO:0010518	positive regulation of phospholipase act...	17735	73	126	3	0.53	0.01579	5.660377	CCL5;NMUR1;PDGFRB
greenyello	BP	GO:0019835	cytolysis	17735	27	126	2	0.19	0.01612	10.52632	GZMB;GZMH
greenyello	MF	GO:0019239	deaminase activity	17735	27	126	2	0.2	0.01636	10	APOBEC3G;APOBEC3H
greenyello	BP	GO:0002706	regulation of lymphocyte mediated immuni...	17735	75	126	3	0.54	0.01697	5.555556	LAG3;SH2D1B;TBX21
greenyello	BP	GO:0001655	urogenital system development	17735	285	126	6	2.06	0.01722	2.912621	ADAMTS1;MTSS1;PDGFRB;PTCH1;SMAD7;WNT1
greenyello	BP	GO:0032964	collagen biosynthetic process	17735	28	126	2	0.2	0.01728	10	F2R;PDGFRB
greenyello	BP	GO:0042267	natural killer cell mediated cytotoxicit...	17735	28	126	2	0.2	0.01728	10	LAG3;SLAMF7
greenyello	BP	GO:0048278	vesicle docking	17735	28	126	2	0.2	0.01728	10	NCAM1;SYTL2
greenyello	BP	GO:0048015	phosphatidylinositol-mediated signaling	17735	138	126	4	1	0.01776	4	CCL5;ERBB2;F2R;PDGFRB
greenyello	BP	GO:0048017	inositol lipid-mediated signaling	17735	138	126	4	1	0.01776	4	CCL5;ERBB2;F2R;PDGFRB
greenyello	BP	GO:0045087	innate immune response	17735	550	126	9	3.97	0.01794	2.267003	APOBEC3G;CCL5;GFI1;KLRG1;LAG3;NCAM1;SH2D1B;SLAMF7;SPON2
greenyello	BP	GO:0050921	positive regulation of chemotaxis	17735	77	126	3	0.56	0.01819	5.357143	CCL4;CCL5;PDGFRB
greenyello	BP	GO:0002237	response to molecule of bacterial origin	17735	211	126	5	1.52	0.0184	3.289474	CCL5;CCR5;F2R;FASLG;GFI1
greenyello	BP	GO:0044246	regulation of multicellular organismal m...	17735	30	126	2	0.22	0.0197	9.090909	F2R;PDGFRB
greenyello	BP	GO:0050776	regulation of immune response	17735	560	126	9	4.04	0.01992	2.227723	CCL5;CD160;GFI1;KIR2DL1;KLRD1;KLRG1;LAG3;SH2D1B;TBX21
greenyello	MF	GO:0004435	phosphatidylinositol phospholipase C act...	17735	30	126	2	0.22	0.01999	9.090909	CCL5;CCR5
greenyello	BP	GO:0010517	regulation of phospholipase activity	17735	80	126	3	0.58	0.02012	5.172414	CCL5;NMUR1;PDGFRB
greenyello	BP	GO:0051146	striated muscle cell differentiation	17735	216	126	5	1.56	0.02014	3.205128	CACNA2D2;ERBB2;F2R;PDGFRB;WNT1
greenyello	BP	GO:0060193	positive regulation of lipase activity	17735	81	126	3	0.58	0.02079	5.172414	CCL5;NMUR1;PDGFRB
greenyello	BP	GO:0014909	smooth muscle cell migration	17735	31	126	2	0.22	0.02096	9.090909	CCL5;PDGFRB
greenyello	BP	GO:0022409	positive regulation of cell-cell adhesio...	17735	31	126	2	0.22	0.02096	9.090909	CCL5;SMAD7
greenyello	BP	GO:0051702	interaction with symbiont	17735	31	126	2	0.22	0.02096	9.090909	CCL4;CCL5
greenyello	BP	GO:0072529	pyrimidine-containing compound catabolic...	17735	31	126	2	0.22	0.02096	9.090909	APOBEC3G;APOBEC3H
greenyello	MF	GO:0004896	cytokine receptor activity	17735	81	126	3	0.59	0.02123	5.084746	CCR5;CX3CR1;CXCR6
greenyello	BP	GO:2000021	regulation of ion homeostasis	17735	147	126	4	1.06	0.02184	3.773585	CCR5;F2R;SMAD7;YPEL1
greenyello	BP	GO:0031589	cell-substrate adhesion	17735	221	126	5	1.6	0.02199	3.125	EFNA5;EPDR1;FZD4;VIT;WNT1
greenyello	BP	GO:0048856	anatomical structure development	17735	3903	126	38	28.18	0.022	1.348474	ADAMTS1;BAI2;BFSP1;CACNA2D2;CD2;EDARADD;EFNA5;EOMES;ERBB2;F2R;FASLG;FEZ1;FZD4;GFI1;GPR56;
greenyello	BP	GO:1901701	cellular response to oxygen-containing c...	17735	387	126	7	2.79	0.02201	2.508961	CCL5;CCR5;EFNA5;FZD4;GFI1;PTCH1;PTGDR
greenyello	BP	GO:0046131	pyrimidine ribonucleoside metabolic proc...	17735	32	126	2	0.23	0.02225	8.695652	APOBEC3G;APOBEC3H
greenyello	CC	GO:0019897	extrinsic to plasma membrane	17735	82	126	3	0.6	0.02247	5	RGS9;SMAD7;SYTL2
greenyello	MF	GO:0004629	phospholipase C activity	17735	32	126	2	0.23	0.02258	8.695652	CCL5;CCR5
greenyello	MF	GO:0016814	hydrolase activity, acting on carbon-nit...	17735	32	126	2	0.23	0.02258	8.695652	APOBEC3G;APOBEC3H
greenyello	BP	GO:0019058	viral infectious cycle	17735	223	126	5	1.61	0.02275	3.10559	APOBEC3G;APOBEC3H;CCL4;CCL5;CXCR6
greenyello	BP	GO:0060326	cell chemotaxis	17735	150	126	4	1.08	0.02332	3.703704	CCL4;CCL5;CCR5;PDGFRB
greenyello	BP	GO:0010812	negative regulation of cell-substrate ad...	17735	33	126	2	0.24	0.02358	8.333333	FZD4;WNT1
greenyello	BP	GO:0021532	neural tube patterning	17735	33	126	2	0.24	0.02358	8.333333	PTCH1;WNT1
greenyello	MF	GO:0008378	galactosyltransferase activity	17735	33	126	2	0.24	0.02393	8.333333	B3GAT1;B3GNT7
greenyello	MF	GO:0015485	cholesterol binding	17735	33	126	2	0.24	0.02393	8.333333	NPC1;PTCH1

greenyello	BP	GO:0030098	lymphocyte differentiation	17735	226	126	5	1.63	0.02394	3.067485	CD2;EOMES;ERBB2;TBX21;WNT1
greenyello	BP	GO:0044767	single-organism developmental process	17735	3427	126	34	24.75	0.02449	1.373737	ADAMTS1;BAI2;BFSP1;CACNA2D2;CD2;EDARADD;EFNA5;EOMES;ERBB2;F2R;FASLG;FEZ1;FZD4;GFI1;GPR56;
greenyello	BP	GO:0022406	membrane docking	17735	34	126	2	0.25	0.02493	8	NCAM1;SYTL2
greenyello	BP	GO:0050848	regulation of calcium-mediated signaling	17735	34	126	2	0.25	0.02493	8	CCL4;NCAM1
greenyello	BP	GO:0048520	positive regulation of behavior	17735	88	126	3	0.64	0.02579	4.6875	CCL4;CCL5;PDGFRB
greenyello	BP	GO:0000578	embryonic axis specification	17735	35	126	2	0.25	0.02632	8	PTCH1;WNT1
greenyello	BP	GO:0014812	muscle cell migration	17735	35	126	2	0.25	0.02632	8	CCL5;PDGFRB
greenyello	BP	GO:0048741	skeletal muscle fiber development	17735	89	126	3	0.64	0.02656	4.6875	CACNA2D2;ERBB2;F2R
greenyello	CC	GO:0035770	ribonucleoprotein granule	17735	88	126	3	0.65	0.02698	4.615385	APOBEC3G;APOBEC3H;PATL2
greenyello	BP	GO:0055001	muscle cell development	17735	157	126	4	1.13	0.02699	3.539823	CACNA2D2;ERBB2;F2R;PDGFRB
greenyello	BP	GO:0033344	cholesterol efflux	17735	36	126	2	0.26	0.02774	7.692308	NPC1;PTCH1
greenyello	BP	GO:0050730	regulation of peptidyl-tyrosine phosphor...	17735	159	126	4	1.15	0.0281	3.478261	CCL5;EFNA5;PDGFRB;ZFYVE28
greenyello	BP	GO:0050790	regulation of catalytic activity	17735	1441	126	17	10.41	0.02886	1.633045	AGAP1;CCL5;DUSP8;EFNA5;ERBB2;F2R;FASLG;FZD4;GFI1;NMUR1;NUAK1;PDGFRB;PPP2R2B;SH3RF2;SMAD7
greenyello	BP	GO:0071222	cellular response to lipopolysaccharide	17735	93	126	3	0.67	0.02973	4.477612	CCL5;CCR5;GFI1
greenyello	BP	GO:0002703	regulation of leukocyte mediated immunit...	17735	94	126	3	0.68	0.03055	4.411765	LAG3;SH2D1B;TBX21
greenyello	MF	GO:0032934	sterol binding	17735	38	126	2	0.28	0.03112	7.142857	NPC1;PTCH1
greenyello	BP	GO:0019220	regulation of phosphate metabolic proces...	17735	1233	126	15	8.9	0.03123	1.685393	AGAP1;CCL5;DUSP8;EFNA5;ERBB2;F2R;FZD4;NUAK1;PDGFRB;PPP2R2B;SH3RF2;SMAD7;SYTL2;WNT1;ZFYVE
greenyello	BP	GO:0035567	non-canonical Wnt receptor signaling pat...	17735	39	126	2	0.28	0.03218	7.142857	FZD4;WNT1
greenyello	BP	GO:0048661	positive regulation of smooth muscle cel...	17735	39	126	2	0.28	0.03218	7.142857	CCL5;PDGFRB
greenyello	BP	GO:0051250	negative regulation of lymphocyte activa...	17735	96	126	3	0.69	0.03223	4.347826	ERBB2;LAG3;TIGIT
greenyello	BP	GO:0045089	positive regulation of innate immune res...	17735	167	126	4	1.21	0.03282	3.305785	CCL5;GFI1;LAG3;SH2D1B
greenyello	BP	GO:0043525	positive regulation of neuron apoptotic ...	17735	40	126	2	0.29	0.03372	6.896552	FASLG;PPP2R2B
greenyello	BP	GO:0051174	regulation of phosphorus metabolic proce...	17735	1246	126	15	9	0.03384	1.666667	AGAP1;CCL5;DUSP8;EFNA5;ERBB2;F2R;FZD4;NUAK1;PDGFRB;PPP2R2B;SH3RF2;SMAD7;SYTL2;WNT1;ZFYVE
greenyello	BP	GO:0071219	cellular response to molecule of bacteri...	17735	98	126	3	0.71	0.03396	4.225352	CCL5;CCR5;GFI1
greenyello	BP	GO:0048513	organ development	17735	2404	126	25	17.36	0.0342	1.440092	ADAMTS1;BFSP1;CACNA2D2;CD2;EDARADD;EOMES;ERBB2;F2R;FASLG;FZD4;GFI1;GPR56;HOXC4;LDB2;LIM2
greenyello	BP	GO:0061005	cell differentiation involved in kidney ...	17735	41	126	2	0.3	0.03528	6.666667	MTSS1;PTCH1
greenyello	BP	GO:1901216	positive regulation of neuron death	17735	41	126	2	0.3	0.03528	6.666667	FASLG;PPP2R2B
greenyello	BP	GO:0002699	positive regulation of immune effector p...	17735	100	126	3	0.72	0.03574	4.166667	LAG3;SH2D1B;TBX21
greenyello	CC	GO:0016021	integral to membrane	17735	5202	126	48	38.19	0.03621	1.256874	B3GAT1;B3GNT7;BAI2;C14orf1;CACNA2D2;CCR5;CD2;CHST10;CLDND2;CLIC3;CX3CR1;CXCR6;EPHX4;ERBB2;
greenyello	BP	GO:0048468	cell development	17735	1482	126	17	10.7	0.03642	1.588785	BFSP1;CACNA2D2;EFNA5;EOMES;ERBB2;F2R;FASLG;FEZ1;FZD4;GFI1;LDB2;NCAM1;PDGFRB;PPP2R2B;S1PR5
greenyello	BP	GO:0042454	ribonucleoside catabolic process	17735	431	126	7	3.11	0.0365	2.250804	AGAP1;APOBEC3G;APOBEC3H;ARL4C;EFNA5;ERBB2;RAB7L1
greenyello	BP	GO:0007169	transmembrane receptor protein tyrosine ...	17735	625	126	9	4.51	0.03676	1.995565	EFNA5;ERBB2;FASLG;FZD4;MTSS1;PDGFRB;PIK3R3;WNT1;ZFYVE28
greenyello	BP	GO:0034109	homotypic cell-cell adhesion	17735	42	126	2	0.3	0.03687	6.666667	CCL5;NCAM1
greenyello	BP	GO:0009617	response to bacterium	17735	341	126	6	2.46	0.03705	2.439024	CCL5;CCR5;F2R;FASLG;GFI1;GNLY
greenyello	BP	GO:0032502	developmental process	17735	4437	126	41	32.04	0.03803	1.27965	ADAMTS1;BAI2;BFSP1;CACNA2D2;CD2;EDARADD;EFNA5;EOMES;ERBB2;F2R;FASLG;FEZ1;FZD4;GFI1;GPR56;
greenyello	BP	GO:0001657	ureteric bud development	17735	103	126	3	0.74	0.0385	4.054054	PTCH1;SMAD7;WNT1
greenyello	BP	GO:0050920	regulation of chemotaxis	17735	103	126	3	0.74	0.0385	4.054054	CCL4;CCL5;PDGFRB
greenyello	BP	GO:0050900	leukocyte migration	17735	257	126	5	1.86	0.03851	2.688172	CCL4;CCL5;CCR5;CD2;TBX21
greenyello	MF	GO:0030545	receptor regulator activity	17735	43	126	2	0.31	0.03905	6.451613	CCL5;WNT1
greenyello	BP	GO:0048514	blood vessel morphogenesis	17735	438	126	7	3.16	0.03927	2.21519	BAI2;ERBB2;FASLG;FZD4;PDGFRB;SH2D2A;SMAD7
greenyello	BP	GO:0048747	muscle fiber development	17735	104	126	3	0.75	0.03944	4	CACNA2D2;ERBB2;F2R
greenyello	CC	GO:0005913	cell-cell adherens junction	17735	43	126	2	0.32	0.0397	6.25	DLG5;SMAD7
greenyello	BP	GO:0080134	regulation of response to stress	17735	737	126	10	5.32	0.04012	1.879699	APOBEC3G;CCL5;F2R;FZD4;GFI1;LAG3;NPC1;NUAK1;SH2D1B;WNT1
greenyello	BP	GO:0030101	natural killer cell activation	17735	44	126	2	0.32	0.04014	6.25	CD2;SLAMF7
greenyello	BP	GO:0002449	lymphocyte mediated immunity	17735	179	126	4	1.29	0.0407	3.100775	LAG3;SH2D1B;SLAMF7;TBX21
greenyello	MF	GO:0019838	growth factor binding	17735	105	126	3	0.76	0.04121	3.947368	ERBB2;FGFBP2;PDGFRB
greenyello	BP	GO:0007423	sensory organ development	17735	443	126	7	3.2	0.04134	2.1875	BFSP1;FASLG;FZD4;GFI1;LIM2;PDGFRB;WNT1
greenyello	BP	GO:0009164	nucleoside catabolic process	17735	443	126	7	3.2	0.04134	2.1875	AGAP1;APOBEC3G;APOBEC3H;ARL4C;EFNA5;ERBB2;RAB7L1
greenyello	MF	GO:0019904	protein domain specific binding	17735	535	126	8	3.89	0.0414	2.056555	FZD4;HOXC4;LDB2;LLGL2;MTSS1;SH2D2A;WNT1;YWHAQ
greenyello	BP	GO:0044087	regulation of cellular component biogene...	17735	351	126	6	2.53	0.04169	2.371542	EFNA5;LDB2;MYO3B;NCAM1;PATL2;WNT1
greenyello	BP	GO:0048010	vascular endothelial growth factor recep...	17735	45	126	2	0.32	0.04181	6.25	FZD4;PDGFRB
greenyello	BP	GO:0051817	modification of morphology or physiology...	17735	45	126	2	0.32	0.04181	6.25	CCL4;CCL5
greenyello	BP	GO:0009615	response to virus	17735	263	126	5	1.9	0.04185	2.631579	APOBEC3G;APOBEC3H;CCL4;CCL5;TBX21
greenyello	BP	GO:0060191	regulation of lipase activity	17735	107	126	3	0.77	0.04233	3.896104	CCL5;NMUR1;PDGFRB
greenyello	MF	GO:0005518	collagen binding	17735	46	126	2	0.33	0.04414	6.060606	PODN;SMAD7
greenyello	MF	GO:0008009	chemokine activity	17735	47	126	2	0.34	0.04589	5.882353	CCL4;CCL5
green	BP	GO:0071214	cellular response to abiotic stimulus	17735	153	1060	23	9.5	7.00E-05	2.421053	ATR;BAK1;BAX;CLOCK;EPO;FADD;GPLD1;HMGA2;HUS1;HYAL3;INSRR;ITGB1;LTBR;MAPK3;PDE2A;PIK3R1;PK
green	BP	GO:0009411	response to UV	17735	99	1060	17	6.15	0.00012	2.764228	ATR;BAK1;BAX;BCL3;CASP7;CDKN2D;ELANE;ERCC4;ERCC6;HUS1;HYAL3;PIK3R1;TIPIN;TRIM32;USF1;USP47;
green	CC	GO:0005922	connexon complex	17735	21	1060	7	1.27	0.00016	5.511811	GJA4;GJB3;GJB5;GJB7;GJC1;GJC2;GJD4
green	CC	GO:0005921	gap junction	17735	31	1060	8	1.88	4.00E-04	4.255319	GJA4;GJB3;GJB5;GJB7;GJC1;GJC2;GJD4;PANX2
green	BP	GO:0071478	cellular response to radiation	17735	75	1060	13	4.66	0.00065	2.7897	ATR;BAK1;BAX;CLOCK;HMGA2;HUS1;HYAL3;ITGB1;PIK3R1;RHOB;TSPYL5;USP47;YAP1
green	BP	GO:0009314	response to radiation	17735	305	1060	34	18.94	0.00067	1.795143	AIPL1;ATR;BAK1;BAX;BCL3;CASP7;CDK5;CDKN2D;CLOCK;DRD3;DUSP1;ELANE;ERCC4;ERCC6;FANCD2;FKBPL;
green	BP	GO:0071479	cellular response to ionizing radiation	17735	33	1060	8	2.05	0.00074	3.902439	ATR;CLOCK;HMGA2;HUS1;ITGB1;RHOB;TSPYL5;YAP1
green	MF	GO:0008417	fucosyltransferase activity	17735	14	1060	5	0.83	0.00094	6.024096	CLASP2;FUT1;FUT3;FUT7;FUT8
green	BP	GO:0009416	response to light stimulus	17735	208	1060	25	12.92	0.00116	1.934985	AIPL1;ATR;BAK1;BAX;BCL3;CASP7;CDK5;CDKN2D;CLOCK;DRD3;DUSP1;ELANE;ERCC4;ERCC6;FOS;HUS1;HYAL
green	BP	GO:2001235	positive regulation of apoptotic signali...	17735	36	1060	8	2.24	0.00137	3.571429	BAX;CAMK2B;DEDD2;DNM1L;FADD;LTBR;PYCARD;TNFRSF12A
green	BP	GO:0051053	negative regulation of DNA metabolic pro...	17735	64	1060	11	3.97	0.00179	2.770781	ATR;ENPP7;ERCC4;HMGA2;HUS1;MRE11A;OTUB1;PDS5A;SMC3;TERF1;TIPIN
green	BP	GO:0006974	response to DNA damage stimulus	17735	608	1060	56	37.76	0.00194	1.483051	ASCC3;ATAD5;ATMIN;ATR;ATXN3;BARD1;BAX;BCL3;C19orf40;CDC14B;CDKN2D;CLOCK;CTLA4;DDX1;ERCC4;
green	BP	GO:0008156	negative regulation of DNA replication	17735	38	1060	8	2.36	0.00198	3.389831	ATR;ENPP7;HUS1;MRE11A;PDS5A;SMC3;TERF1;TIPIN

green	CC	GO:0005694	chromosome	17735	631	1060	56	38.19	0.00257	1.466352	AR;ASF1B;ATR;CBX8;CENPC1;CLASP2;CLOCK;DCTN3;DYNC1I1;ERCC4;EXOSC4;FANCD2;HDAC2;HIST1H1C;HIS
green	BP	GO:0051928	positive regulation of calcium ion trans...	17735	58	1060	10	3.6	0.00278	2.777778	ANK2;ARRB2;ATP2B2;BAK1;BAX;CACNB2;CASK;STIM2;TSPO;WNK3
green	CC	GO:0044427	chromosomal part	17735	538	1060	49	32.56	0.00279	1.504914	AR;ASF1B;CBX8;CENPC1;CLASP2;DCTN3;DYNC1I1;ERCC4;EXOSC4;HDAC2;HIST1H1C;HIST1H2AD;HIST1H2BK;
green	CC	GO:0097060	synaptic membrane	17735	215	1060	24	13.01	0.0028	1.844735	ANK2;ARRB2;CABP1;CASK;CDK5;CHRM4;CHRM5;CHRNA3;CNIH2;EPHA4;GABRD;GRIA4;GRID1;GRIK3;GRM4;
green	BP	GO:0051248	negative regulation of protein metabolic...	17735	473	1060	45	29.37	0.00283	1.532176	ARRB2;BAG5;BAK1;BANP;BAX;BCL3;CDC23;CDK5;DUSP1;EDNRB;EIF4EBP1;EIF4EBP3;ELANE;ERRFI1;FBXO43;
green	BP	GO:0009628	response to abiotic stimulus	17735	754	1060	66	46.82	0.00283	1.409654	ADAM8;AGTRAP;AIPL1;ARRB2;ATP2B2;ATR;ATXN3;BAK1;BAX;BCL3;CASP7;CDK5;CDKN2D;CLOCK;CYP1A1;DI
green	BP	GO:0046825	regulation of protein export from nucleu...	17735	24	1060	6	1.49	0.00289	4.026846	BARD1;CDK5;PTPN11;PTPN14;SFN;XPO4
green	BP	GO:0007224	smoothened signaling pathway	17735	88	1060	13	5.46	0.00295	2.380952	DISP1;FUZ;GLIS2;HES1;IHH;INTU;POR;RPGRIP1L;SEPT2;SHOX2;TTBK2;TTC21B;WDR19
green	BP	GO:2001238	positive regulation of extrinsic apoptot...	17735	17	1060	5	1.06	0.00302	4.716981	DEDD2;FADD;LTBR;PYCARD;TNFRSF12A
green	BP	GO:0034220	ion transmembrane transport	17735	296	1060	31	18.38	0.00303	1.686616	ANK2;ATP2A1;ATP2B2;ATP6V0A2;ATP6V0B;BAK1;BAX;CACNB2;CASK;CASR;CDK5;CNIH2;CPT1B;DRD3;EPO;G
green	CC	GO:0005911	cell-cell junction	17735	291	1060	30	17.61	0.00308	1.703578	AMOT;AMOTL2;ANK2;CASK;CDH5;CLDN10;CLDN11;EPCAM;FAT1;GJA4;GJB3;GJB5;GJB7;GJC1;GJC2;GJD4;HP
green	BP	GO:0032269	negative regulation of cellular protein ...	17735	411	1060	40	25.52	0.00319	1.567398	ARRB2;BAG5;BAK1;BAX;CDC23;CDK5;DUSP1;EDNRB;EIF4EBP1;EIF4EBP3;ERRFI1;GADD45G;GCLC;GPD1L;GRI
green	BP	GO:0070230	positive regulation of lymphocyte apopto...	17735	11	1060	4	0.68	0.00343	5.882353	ADAM8;BAX;NFKBID;ZC3H8
green	CC	GO:0000228	nuclear chromosome	17735	294	1060	30	17.79	0.00358	1.686341	AR;CBX8;CENPC1;ERCC4;HDAC2;HMGA2;HUS1;HUS1B;KIFAP3;LRPPRC;MORF4L1;NPM2;NR1H3;NUFIP1;ORC
green	BP	GO:0071706	tumor necrosis factor superfamily cytoki...	17735	70	1060	11	4.35	0.00371	2.528736	ADAM8;ARRB2;AZU1;BCL3;CARD9;FADD;PYCARD;RARA;THBS1;TSPO;ZC3H12A
green	BP	GO:0010458	exit from mitosis	17735	18	1060	5	1.12	0.00397	4.464286	CDC23;NPM2;SIRT7;UBE2C;UBE2S
green	BP	GO:2000649	regulation of sodium ion transmembrane t...	17735	18	1060	5	1.12	0.00397	4.464286	DRD3;GPD1L;SCN1B;WNK2;WNK3
green	MF	GO:0016763	transferase activity, transferring pento...	17735	44	1060	8	2.62	0.00398	3.053435	ART1;MTAP;NAPRT1;PARP2;SIRT6;TNKS;UMPS;UPP1
green	BP	GO:0022403	cell cycle phase	17735	852	1060	72	52.91	0.00445	1.360801	ARL8A;AURKAIP1;B9D2;CAMK2B;CDC23;CDK6;CDKN2D;CENPC1;CENPJ;CEP192;CEP76;CLASP2;CYP1A1;DCTI
green	BP	GO:0007193	adenylate cyclase-inhibiting G-protein c...	17735	43	1060	8	2.67	0.00446	2.996255	APLP1;CHRM4;CHRM5;DRD3;GRIK3;GRM4;INSL3;SSTR2
green	BP	GO:0003417	growth plate cartilage development	17735	12	1060	4	0.75	0.00489	5.333333	COL9A1;POR;RARA;THBS1
green	BP	GO:0006446	regulation of translational initiation	17735	53	1060	9	3.29	0.00495	2.735562	DDX1;EIF1B;EIF2S1;EIF4EBP1;EIF4EBP3;NCBP1;NCBP2;RARA;TRMT6
green	BP	GO:0010332	response to gamma radiation	17735	36	1060	7	2.24	0.00595	3.125	ATR;BAK1;BAX;ERCC6;FANCD2;TSPYL5;YAP1
green	CC	GO:0044454	nuclear chromosome part	17735	255	1060	26	15.43	0.00646	1.685029	AR;CBX8;CENPC1;ERCC4;HDAC2;HUS1;HUS1B;MORF4L1;NPM2;NR1H3;NUFIP1;ORC5;PCGF2;RAD50;RARA;R
green	BP	GO:0042026	protein refolding	17735	13	1060	4	0.81	0.00672	4.938272	BAG5;HSP90AA1;PDCL;PTGES3
green	BP	GO:2000104	negative regulation of DNA-dependent DNA...	17735	13	1060	4	0.81	0.00672	4.938272	MRE11A;SMC3;TERF1;TIPIN
green	BP	GO:0032640	tumor necrosis factor production	17735	66	1060	10	4.1	0.0072	2.439024	ARRB2;AZU1;BCL3;CARD9;FADD;PYCARD;RARA;THBS1;TSPO;ZC3H12A
green	BP	GO:0032680	regulation of tumor necrosis factor prod...	17735	66	1060	10	4.1	0.0072	2.439024	ARRB2;AZU1;BCL3;CARD9;FADD;PYCARD;RARA;THBS1;TSPO;ZC3H12A
green	CC	GO:0045211	postsynaptic membrane	17735	183	1060	20	11.08	0.00758	1.805054	ANK2;ARRB2;CABP1;CDK5;CHRM4;CHRM5;CHRNA3;CNIH2;EPHA4;GABRD;GRIA4;GRID1;GRIK3;HOMER3;HT
green	BP	GO:0007049	cell cycle	17735	1388	1060	108	86.2	0.00761	1.2529	AFAP1L2;ARL8A;ATR;AURKAIP1;B9D2;BAK1;BANP;BARD1;BAX;BIN3;CAMK2B;CDC14B;CDC23;CDK5;CDK6;CI
green	MF	GO:0005243	gap junction channel activity	17735	14	1060	4	0.83	0.0077	4.819277	GJB3;GJC1;GJC2;PANX2
green	MF	GO:0005385	zinc ion transmembrane transporter activ...	17735	14	1060	4	0.83	0.0077	4.819277	SLC30A4;SLC39A1;SLC39A4;SLC39A6
green	BP	GO:0050657	nucleic acid transport	17735	132	1060	16	8.2	0.00772	1.95122	G3BP2;HNRNPA1L2;LRPPRC;MVP;NCBP1;NCBP2;NUP107;NUP160;NUP88;NXF3;RANBP2;SRSF9;TNKS;TST;U
green	BP	GO:0050658	RNA transport	17735	132	1060	16	8.2	0.00772	1.95122	G3BP2;HNRNPA1L2;LRPPRC;MVP;NCBP1;NCBP2;NUP107;NUP160;NUP88;NXF3;RANBP2;SRSF9;TNKS;TST;U
green	BP	GO:0051236	establishment of RNA localization	17735	132	1060	16	8.2	0.00772	1.95122	G3BP2;HNRNPA1L2;LRPPRC;MVP;NCBP1;NCBP2;NUP107;NUP160;NUP88;NXF3;RANBP2;SRSF9;TNKS;TST;U
green	BP	GO:0008589	regulation of smoothened signaling pathw...	17735	47	1060	8	2.92	0.00777	2.739726	DISP1;FUZ;GLIS2;IHH;INTU;POR;RPGRIP1L;SHOX2
green	BP	GO:0033574	response to testosterone stimulus	17735	21	1060	5	1.3	0.00807	3.846154	DUSP1;EPO;MSTN;MTAP;TSPO
green	BP	GO:0022402	cell cycle process	17735	1059	1060	85	65.76	0.00814	1.292579	ARL8A;ATR;AURKAIP1;B9D2;BARD1;CAMK2B;CDC14B;CDC23;CDK5;CDK6;CDKN2D;CENPC1;CENPJ;CEP192;C
green	BP	GO:0031400	negative regulation of protein modificat...	17735	317	1060	31	19.69	0.00819	1.574403	ARRB2;BAG5;BAK1;BAX;CDC23;CDK5;DUSP1;ERRFI1;GADD45G;GCLC;GPD1L;HEXIM2;HHATL;INPP5J;OTUB1;
green	BP	GO:0000278	mitotic cell cycle	17735	777	1060	65	48.25	0.00834	1.34715	AFAP1L2;ARL8A;AURKAIP1;B9D2;CAMK2B;CDC23;CDK6;CDKN2D;CENPC1;CENPJ;CEP192;CEP76;CLASP2;CYF
green	CC	GO:0005814	centriole	17735	59	1060	9	3.57	0.00861	2.521008	CENPJ;CEP128;CEP192;CEP76;MPHOSPH9;POC1A;SDCCAG8;SPICE1;TTBK2
green	BP	GO:0016043	cellular component organization	17735	4124	1060	288	256.1	0.00884	1.124561	AASS;ADAM8;ADAMTS2;ADNP2;AMOT;ANK2;AP1M2;AP1S3;AP2S1;APLP1;AR;ARL8A;ARPC1B;ARRB2;ARTN;
green	BP	GO:0006525	arginine metabolic process	17735	14	1060	4	0.87	0.00895	4.597701	ASL;ASS1;DDAH2;FAH
green	BP	GO:0033137	negative regulation of peptidyl-serine p...	17735	14	1060	4	0.87	0.00895	4.597701	BAK1;BAX;GPD1L;INPP5J
green	BP	GO:0010212	response to ionizing radiation	17735	101	1060	13	6.27	0.00957	2.073365	ATR;BAK1;BAX;CLOCK;ERCC6;FANCD2;HMGA2;HUS1;ITGB1;RHOB;RNF168;TSPYL5;YAP1
green	BP	GO:0045581	negative regulation of T cell differenti...	17735	22	1060	5	1.37	0.00992	3.649635	CTLA4;HLX;IHH;NFKBID;ZC3H8
green	BP	GO:0060351	cartilage development involved in endoch...	17735	22	1060	5	1.37	0.00992	3.649635	COL9A1;POR;RARA;SHOX2;THBS1
green	BP	GO:0051028	mRNA transport	17735	114	1060	14	7.08	0.01096	1.977401	G3BP2;HNRNPA1L2;LRPPRC;MVP;NCBP1;NCBP2;NUP107;NUP160;NUP88;NXF3;RANBP2;SRSF9;TNKS;UPF3E
green	MF	GO:0008408	3'-5' exonuclease activity	17735	33	1060	6	1.96	0.01212	3.061224	EXD1;EXOSC4;ISG20;MRE11A;RAD50;WRN
green	BP	GO:0006403	RNA localization	17735	139	1060	16	8.63	0.01242	1.853998	G3BP2;HNRNPA1L2;LRPPRC;MVP;NCBP1;NCBP2;NUP107;NUP160;NUP88;NXF3;RANBP2;SRSF9;TNKS;TST;U
green	BP	GO:0000279	M phase	17735	528	1060	46	32.79	0.01251	1.402867	ARL8A;AURKAIP1;B9D2;CAMK2B;CDC23;CENPC1;CEP192;CLASP2;DCTN3;DPEP3;DRD3;EML4;ERCC4;FANCD2
green	BP	GO:0051726	regulation of cell cycle	17735	736	1060	61	45.71	0.01252	1.3345	AFAP1L2;ATR;AURKAIP1;BAK1;BARD1;BAX;CAMK2B;CDC14B;CDC23;CDK5;CDK6;CDKN2D;CENPJ;CEP76;CLO
green	CC	GO:0000784	nuclear chromosome, telomeric region	17735	24	1060	5	1.45	0.01304	3.448276	ERCC4;RAD50;SIRT6;TERF1;TNKS
green	BP	GO:0045862	positive regulation of proteolysis	17735	62	1060	9	3.85	0.0138	2.337662	ADAM8;AURKAIP1;GCLC;GPLD1;HDAC2;OGT;PACSIN3;PLK3;TRIM32
green	CC	GO:0005604	basement membrane	17735	86	1060	11	5.21	0.01442	2.111324	ANXA2P2;APLP1;CASK;COL18A1;COL4A2;FBLN1;ITGB1;LAD1;LAMA3;SMC3;TNC
green	BP	GO:0090003	regulation of establishment of protein l...	17735	24	1060	5	1.49	0.01444	3.355705	AR;NRXN1;PIK3R1;RHOG;WNK3
green	BP	GO:0007004	telomere maintenance via telomerase	17735	16	1060	4	0.99	0.01474	4.040404	MRE11A;RAD50;TERF1;TNKS
green	BP	GO:0042533	tumor necrosis factor biosynthetic proce...	17735	16	1060	4	0.99	0.01474	4.040404	AZU1;BCL3;CARD9;THBS1
green	BP	GO:0042534	regulation of tumor necrosis factor bios...	17735	16	1060	4	0.99	0.01474	4.040404	AZU1;BCL3;CARD9;THBS1
green	BP	GO:0090004	positive regulation of establishment of ...	17735	16	1060	4	0.99	0.01474	4.040404	NRXN1;PIK3R1;RHOG;WNK3
green	BP	GO:2000108	positive regulation of leukocyte apoptot...	17735	16	1060	4	0.99	0.01474	4.040404	ADAM8;BAX;NFKBID;ZC3H8
green	CC	GO:0044456	synapse part	17735	353	1060	32	21.37	0.01475	1.497426	AMPH;ANK2;ARRB2;CABP1;CASK;CDK5;CHRM4;CHRM5;CHRNA3;CNIH2;EPHA4;GABRD;GRIA4;GRID1;GRIK3
green	CC	GO:0000785	chromatin	17735	300	1060	28	18.16	0.01518	1.54185	AR;ASF1B;CBX8;CENPC1;EXOSC4;HDAC2;HIST1H1C;HIST1H2AD;HIST1H2BK;HIST1H2BO;HIST2H2AB;HMGA2
green	MF	GO:0016247	channel regulator activity	17735	77	1060	10	4.58	0.01551	2.183406	ANK2;GNB2;GPD1L;GPLD1;KCNJ12;NRXN1;PRKG1;SCN1B;SGK2;STIM2
green	BP	GO:0031397	negative regulation of protein ubiquitin...	17735	96	1060	12	5.96	0.01556	2.013423	ARRB2;BAG5;CDC23;CDK5;GCLC;OTUB1;PSMB10;PSMB8;PSMB9;PSMD5;PSME2;UBE2C
green	BP	GO:0000724	double-strand break repair via homologou...	17735	53	1060	8	3.29	0.0158	2.431611	ERCC4;HUS1;MCM8;MORF4L1;MRE11A;RAD50;RAD52;SIRT6
green	BP	GO:0000725	recombinational repair	17735	53	1060	8	3.29	0.0158	2.431611	ERCC4;HUS1;MCM8;MORF4L1;MRE11A;RAD50;RAD52;SIRT6
green	MF	GO:0001968	fibronectin binding	17735	17	1060	4	1.01	0.01588	3.960396	FSTL3;ITGB1;MFAP2;THBS1
green	BP	GO:0051168	nuclear export	17735	120	1060	14	7.45	0.01669	1.879195	BARD1;CDK5;NCBP1;NCBP2;NUP107;NUP160;NXF3;PTPN11;PTPN14;SFN;SRSF9;UPF3B;XPO4;XPOT

green	BP	GO:0008334	histone mRNA metabolic process	17735	25	1060	5	1.55	0.01715	3.225806	EXOSC4;NCBP1;NCBP2;SSB;ZNF473
green	MF	GO:0072509	divalent inorganic cation transmembrane ...	17735	138	1060	15	8.21	0.01724	1.82704	ATP2A1;ATP2B2;CACNB2;CACNG1;ITPR1;MRS2;NIPAL2;PDE2A;SLC24A2;SLC30A4;SLC39A1;SLC39A4;SLC39A
green	CC	GO:0044424	intracellular part	17735	12103	1060	762	732.55	0.01736	1.040202	AAGAB;AASS;ABCB7;ABHD11;ABHD5;ABTB1;ACAA1;ACAD11;ACOXL;ACSS3;ACTG2;ACVR2B;ADAM8;ADCK4;
green	BP	GO:0030001	metal ion transport	17735	512	1060	44	31.8	0.01781	1.383648	ADRA1A;ANK2;ARRB2;ATP2A1;ATP2B2;ATP6V0A2;ATP6V0B;ATP6V1G2;BAK1;BAX;CACNB2;CAMK2B;CASK;C
green	CC	GO:0048471	perinuclear region of cytoplasm	17735	469	1060	40	28.39	0.01802	1.408947	ANK2;APLP1;ARF5;ATP2A1;ATXN10;BAG5;BCL3;CABP1;CCIN;CLINT1;CTLA4;DCTN3;DNM1L;DOK1;DYNC1I1;F
green	BP	GO:0003416	endochondral bone growth	17735	17	1060	4	1.06	0.01835	3.773585	COL9A1;POR;RARA;THBS1
green	BP	GO:0061001	regulation of dendritic spine morphogene...	17735	17	1060	4	1.06	0.01835	3.773585	CAMK2B;CDK5;EPHA4;SRCIN1
green	MF	GO:0016175	superoxide-generating NADPH oxidase acti...	17735	10	1060	3	0.59	0.01836	5.084746	NCF1;NCF1B;NCF1C
green	CC	GO:0033179	proton-transporting V-type ATPase, V0 do...	17735	10	1060	3	0.61	0.01925	4.918033	ATP6V0A2;ATP6V0B;TCIRG1
green	BP	GO:0006275	regulation of DNA replication	17735	99	1060	12	6.15	0.01946	1.95122	ATR;ENPP7;EPO;HUS1;MRE11A;NPM2;PDS5A;PPP2CA;SMC3;TERF1;TIPIN;TNKS
green	BP	GO:0034644	cellular response to UV	17735	35	1060	6	2.17	0.01948	2.764977	ATR;BAK1;BAX;HYAL3;PIK3R1;USP47
green	BP	GO:0071840	cellular component organization or bioge...	17735	4237	1060	291	263.12	0.01987	1.105959	AASS;ADAM8;ADAMTS2;ADNP2;AMOT;ANK2;AP1M2;AP1S3;AP2S1;APLP1;AR;ARL8A;ARPC1B;ARRB2;ARTN;
green	MF	GO:0046915	transition metal ion transmembrane trans...	17735	27	1060	5	1.61	0.01991	3.10559	SLC30A4;SLC31A2;SLC39A1;SLC39A4;SLC39A6
green	BP	GO:0051897	positive regulation of protein kinase B ...	17735	66	1060	9	4.1	0.02023	2.195122	ADAM8;ARRB2;F3;KIAA1161;MEIS3;MTDH;RARA;THBS1;TSPYL5
green	BP	GO:0010564	regulation of cell cycle process	17735	434	1060	38	26.95	0.0203	1.410019	ATR;AURKAIP1;CAMK2B;CDC14B;CDC23;CDK5;CENPJ;CEP76;CLOCK;CSPP1;CYP1A1;DRD3;FBXO43;HMGA2;H
green	BP	GO:0008635	activation of cysteine-type endopeptidas...	17735	10	1060	3	0.62	0.02061	4.83871	BAK1;BAX;CASP7
green	BP	GO:0010225	response to UV-C	17735	10	1060	3	0.62	0.02061	4.83871	BAK1;BCL3;WRN
green	BP	GO:0036065	fucosylation	17735	10	1060	3	0.62	0.02061	4.83871	FUT3;FUT7;FUT8
green	BP	GO:0050665	hydrogen peroxide biosynthetic process	17735	10	1060	3	0.62	0.02061	4.83871	CYP1A1;NCF1;ZNF205
green	BP	GO:0051918	negative regulation of fibrinolysis	17735	10	1060	3	0.62	0.02061	4.83871	THBD;THBS1;USF1
green	BP	GO:0070841	inclusion body assembly	17735	10	1060	3	0.62	0.02061	4.83871	BAG5;TRIM37;UBD
green	CC	GO:0046930	pore complex	17735	91	1060	11	5.51	0.02127	1.99637	BAK1;BAX;CSE1L;IPO7;MVP;NUP107;NUP160;NUP88;RANBP2;TNKS;XPOT
green	CC	GO:0044420	extracellular matrix part	17735	177	1060	18	10.71	0.02148	1.680672	ANXA2P2;APLP1;CASK;COL16A1;COL18A1;COL23A1;COL4A2;COL9A1;FBLN1;FCN2;GLDN;ITGB1;LAD1;LAMA
green	BP	GO:2001233	regulation of apoptotic signaling pathwa...	17735	78	1060	10	4.84	0.02209	2.066116	BAK1;BAX;CAMK2B;DEDD2;DNM1L;FADD;LTBR;PYCARD;TNFRSF12A;ZNF205
green	BP	GO:0030194	positive regulation of blood coagulation	17735	18	1060	4	1.12	0.02246	3.571429	F3;THBD;THBS1;USF1
green	BP	GO:0045880	positive regulation of smoothened signal...	17735	18	1060	4	1.12	0.02246	3.571429	DISP1;IHH;POR;SHOX2
green	BP	GO:0070059	intrinsic apoptotic signaling pathway in...	17735	18	1060	4	1.12	0.02246	3.571429	ATP2A1;BAK1;BAX;ITPR1
green	BP	GO:1900048	positive regulation of hemostasis	17735	18	1060	4	1.12	0.02246	3.571429	F3;THBD;THBS1;USF1
green	MF	GO:0032947	protein complex scaffold	17735	48	1060	7	2.85	0.02246	2.45614	ARRB2;CD3G;DEDD2;G3BP2;KSR2;RGS14;SEPT2
green	BP	GO:0000087	M phase of mitotic cell cycle	17735	370	1060	33	22.98	0.02305	1.436031	ARL8A;AURKAIP1;B9D2;CDC23;CENPC1;CLASP2;DCTN3;DRD3;EML4;HAUS2;HMGA2;MASTL;MPHOSPH9;NEI
green	MF	GO:0005158	insulin receptor binding	17735	28	1060	5	1.67	0.02309	2.994012	DOK1;DOK6;INSL3;PIK3R1;PTPN11
green	BP	GO:0000041	transition metal ion transport	17735	90	1060	11	5.59	0.02331	1.9678	ATP6V0A2;ATP6V0B;ATP6V1G2;CP;FTH1;SLC30A4;SLC31A2;SLC39A1;SLC39A6;TCIRG1;TCN1
green	MF	GO:0017080	sodium channel regulator activity	17735	19	1060	4	1.13	0.02353	3.539823	GPD1L;GPLD1;SCN1B;SGK2
green	MF	GO:0030159	receptor signaling complex scaffold acti...	17735	19	1060	4	1.13	0.02353	3.539823	CD3G;DEDD2;G3BP2;RGS14
green	BP	GO:0045931	positive regulation of mitotic cell cycl...	17735	37	1060	6	2.3	0.02514	2.608696	CYP1A1;EIF4EBP1;HES1;SMAD6;SPHK1;TERF1
green	CC	GO:0016234	inclusion body	17735	38	1060	6	2.3	0.02539	2.608696	AFAP1L2;ATXN3;BAG5;RANBP2;TRIM37;UBD
green	BP	GO:0006942	regulation of striated muscle contractio...	17735	58	1060	8	3.6	0.02609	2.222222	ADRA1A;ANK2;ATP2A1;GJC1;GPD1L;MYBPC3;RGS2;SCN1B
green	BP	GO:0006643	membrane lipid metabolic process	17735	164	1060	17	10.18	0.02618	1.669941	ARSJ;BAX;CERS1;CERS4;CERS6;ENPP7;GPLD1;KDSR;MPPE1;PGAP2;PIGN;PIGX;PPAP2A;PPP2CA;SMPDL3B;SP
green	MF	GO:0016859	cis-trans isomerase activity	17735	39	1060	6	2.32	0.02643	2.586207	CWC27;FKBP14;FKBPL;GSTZ1;NKTR;RANBP2
green	CC	GO:0005930	axoneme	17735	71	1060	9	4.3	0.02689	2.093023	B9D2;DNAH2;DNAH9;DYNC2H1;RPGRIP1;RPGRIP1L;SPAG6;SPEF1;TTC21B
green	CC	GO:0043229	intracellular organelle	17735	10522	1060	666	636.86	0.02698	1.045756	AASS;ABCB7;ABHD11;ABTB1;ACAA1;ACAD11;ACOXL;ACSS3;ACTG2;ADAM8;ADCK4;ADNP2;ADRA1A;AFAP1L
green	BP	GO:0006020	inositol metabolic process	17735	11	1060	3	0.68	0.02705	4.411765	IMPA2;IPPK;ITPKA
green	BP	GO:0006222	UMP biosynthetic process	17735	11	1060	3	0.68	0.02705	4.411765	UMPS;UPP1;UPRT
green	BP	GO:0009173	pyrimidine ribonucleoside monophosphate ...	17735	11	1060	3	0.68	0.02705	4.411765	UMPS;UPP1;UPRT
green	BP	GO:0009174	pyrimidine ribonucleoside monophosphate ...	17735	11	1060	3	0.68	0.02705	4.411765	UMPS;UPP1;UPRT
green	BP	GO:0010954	positive regulation of protein processin...	17735	11	1060	3	0.68	0.02705	4.411765	ADAM8;CNTN2;HPN
green	BP	GO:0030205	dermatan sulfate metabolic process	17735	11	1060	3	0.68	0.02705	4.411765	BCAN;CSPG5;UST
green	BP	GO:0030208	dermatan sulfate biosynthetic process	17735	11	1060	3	0.68	0.02705	4.411765	BCAN;CSPG5;UST
green	BP	GO:0046049	UMP metabolic process	17735	11	1060	3	0.68	0.02705	4.411765	UMPS;UPP1;UPRT
green	BP	GO:0070243	regulation of thymocyte apoptotic proces...	17735	11	1060	3	0.68	0.02705	4.411765	ADAM8;NFKBID;ZC3H8
green	BP	GO:0009303	rRNA transcription	17735	19	1060	4	1.18	0.02709	3.389831	GTF3C3;RNASEK;SIRT7;TAF1B
green	BP	GO:0033081	regulation of T cell differentiation in ...	17735	19	1060	4	1.18	0.02709	3.389831	ADAM8;IHH;NFKBID;ZC3H8
green	BP	GO:0086002	regulation of cardiac muscle cell action...	17735	19	1060	4	1.18	0.02709	3.389831	ANK2;GJC1;GPD1L;SCN1B
green	BP	GO:0010822	positive regulation of mitochondrion org...	17735	28	1060	5	1.74	0.02723	2.873563	BAK1;BAX;DNM1L;PYCARD;ZNF205
green	BP	GO:2001236	regulation of extrinsic apoptotic signal...	17735	28	1060	5	1.74	0.02723	2.873563	DEDD2;FADD;LTBR;PYCARD;TNFRSF12A
green	MF	GO:0042578	phosphoric ester hydrolase activity	17735	351	1060	30	20.88	0.02919	1.436782	ACPT;CASR;CDC14B;CHRM5;DUSP1;ENPP7;EPM2A;GPLD1;IMPA2;INPP4B;INPP5F;INPP5J;LPPR4;MPPE1;MTI
green	BP	GO:0090257	regulation of muscle system process	17735	129	1060	14	8.01	0.02926	1.747815	ADRA1A;ANK2;ATP2A1;CAMK2B;CHRNA3;GJC1;GPD1L;MSTN;MYBPC3;NCF1;RGS2;SCN1B;SPHK1;SSTR2
green	CC	GO:0044441	cilium part	17735	120	1060	13	7.26	0.02957	1.790634	B9D2;CASK;CLTB;DNAH2;DNAH9;DYNC2H1;ROM1;RPGRIP1L;SEPT2;SPEF1;TSPEAR;TTBK2;TTC21E
green	BP	GO:0015931	nucleobase-containing compound transport	17735	154	1060	16	9.56	0.0298	1.67364	G3BP2;HNRNPA1L2;LRPPRC;MVP;NCBP1;NCBP2;NUP107;NUP160;NUP88;NXF3;RANBP2;SRSF9;TNKS;TST;U
green	MF	GO:0003707	steroid hormone receptor activity	17735	51	1060	7	3.03	0.03032	2.310231	AR;ESRRA;ESRRB;NR1H3;NR2C2;PGRMC2;RARA
green	CC	GO:0043226	organelle	17735	10552	1060	667	638.68	0.03047	1.044341	AASS;ABCB7;ABHD11;ABTB1;ACAA1;ACAD11;ACOXL;ACSS3;ACTG2;ADAM8;ADCK4;ADNP2;ADRA1A;AFAP1L
green	MF	GO:0015267	channel activity	17735	395	1060	33	23.49	0.03061	1.404853	ANO2;AOC3;AQP8;BAX;CACNB2;CACNG1;CHRNA3;CLCN1;CLIC5;GABRD;GJB3;GJC1;GJC2;GRIA4;GRID1;GRIK
green	MF	GO:0022803	passive transmembrane transporter activi...	17735	395	1060	33	23.49	0.03061	1.404853	ANO2;AOC3;AQP8;BAX;CACNB2;CACNG1;CHRNA3;CLCN1;CLIC5;GABRD;GJB3;GJC1;GJC2;GRIA4;GRID1;GRIK
green	MF	GO:0008191	metalloendopeptidase inhibitor activity	17735	12	1060	3	0.71	0.0308	4.225352	RECK;SPOCK3;WFIKKN2
green	MF	GO:0048551	metalloenzyme inhibitor activity	17735	12	1060	3	0.71	0.0308	4.225352	RECK;SPOCK3;WFIKKN2
green	BP	GO:0048814	regulation of dendrite morphogenesis	17735	39	1060	6	2.42	0.03179	2.479339	CAMK2B;CDK5;CHRNA3;EPHA4;SDC2;SRCIN1
green	BP	GO:0051216	cartilage development	17735	168	1060	17	10.43	0.03207	1.629914	BMPR1B;COL9A1;ESRRA;FGF18;FOXD4L1;GPLD1;HMGA2;HYAL3;IHH;LUM;MAPK3;NFIB;POR;RARA;SHOX2;T
green	CC	GO:0008278	cohesin complex	17735	12	1060	3	0.73	0.03224	4.109589	SGOL1;SMC1B;SMC3
green	BP	GO:0050820	positive regulation of coagulation	17735	20	1060	4	1.24	0.03224	3.225806	F3;THBD;THBS1;USF1

green	BP	GO:0051329	interphase of mitotic cell cycle	17735	408	1060	35	25.34	0.03268	1.381215	CAMK2B;CDC23;CDK6;CDKN2D;CENPJ;CEP192;CEP76;CYP1A1;DCTN3;DNAJC2;EIF4EBP1;HAUS2;HMGA2;HSI
green	BP	GO:1901654	response to ketone	17735	72	1060	9	4.47	0.03355	2.013423	DUSP1;EPO;FOS;MSTN;MTAP;NR1H3;SLIT3;THBS1;TSPO
green	CC	GO:0005634	nucleus	17735	5873	1060	383	355.47	0.03372	1.077447	ABTB1;ACAD11;ADNP2;ADRA1A;AFAP1L2;AIPL1;AMOT;ANKRD42;ANO2;AR;ARL2BP;ARRB2;ASCC3;ASF1B;A
green	BP	GO:0006907	pinocytosis	17735	12	1060	3	0.75	0.03444	4	NR1H3;PYCARD;SNX5
green	BP	GO:0007158	neuron cell-cell adhesion	17735	12	1060	3	0.75	0.03444	4	CTNND2;NINJ2;NRXN1
green	BP	GO:0032469	endoplasmic reticulum calcium ion homeos...	17735	12	1060	3	0.75	0.03444	4	ATP2A1;BAK1;BAX
green	BP	GO:0045346	regulation of MHC class II biosynthetic ...	17735	12	1060	3	0.75	0.03444	4	AZU1;HDAC2;SPI1
green	BP	GO:0070242	thymocyte apoptotic process	17735	12	1060	3	0.75	0.03444	4	ADAM8;NFKBID;ZC3H8
green	BP	GO:0022607	cellular component assembly	17735	1545	1060	113	95.94	0.03455	1.177819	AASS;AMOT;AP2S1;AR;ARPC1B;ASF1B;ATL1;ATPAF1;B9D2;BAG5;BAX;BIN3;CAND1;CD151;CD3G;CDH5;CDK5
green	BP	GO:0042116	macrophage activation	17735	40	1060	6	2.48	0.03551	2.419355	AZU1;NR1H3;PRKCE;THBS1;TYROBP;ZC3H12A
green	CC	GO:0005622	intracellular	17735	12344	1060	772	747.14	0.03559	1.033274	AAGAB;AASS;ABCB7;ABHD11;ABHD5;ABTB1;ACAA1;ACAD11;ACOXL;ACSS3;ACTG2;ACVR2B;ADAD2;ADAM8
green	BP	GO:0032508	DNA duplex unwinding	17735	30	1060	5	1.86	0.03568	2.688172	ASCC3;DDX1;MRE11A;RAD50;WRN
green	BP	GO:0070509	calcium ion import	17735	30	1060	5	1.86	0.03568	2.688172	ATP2A1;CACNB2;CASK;CASR;CDK5
green	BP	GO:0000086	G2/M transition of mitotic cell cycle	17735	145	1060	15	9	0.03578	1.666667	CENPJ;CEP192;CEP76;DCTN3;HAUS2;HMGA2;HSP90AA1;MASTL;PLK3;SDCCAG8;TERF1;TIPIN;TUBA1A;TUBA
green	BP	GO:0032844	regulation of homeostatic process	17735	275	1060	25	17.08	0.03594	1.4637	ADAM8;ANK2;BAK1;BAX;CDK6;DRD3;EDNRB;ERCC4;FOXO1;GCLC;GPD1L;GPLD1;ITPR1;NR1H3;PRKCE;SCN1E
green	CC	GO:0000781	chromosome, telomeric region	17735	52	1060	7	3.15	0.03616	2.222222	ERCC4;MRE11A;PTGES3;RAD50;SIRT6;TERF1;TNKS
green	CC	GO:0035085	cilium axoneme	17735	52	1060	7	3.15	0.03616	2.222222	B9D2;DNAH2;DNAH9;DYNC2H1;RPGRIP1L;SPEF1;TTC21B
green	CC	GO:0097014	microtubule-based flagellar cytoplasm	17735	52	1060	7	3.15	0.03616	2.222222	B9D2;DNAH2;DNAH9;DYNC2H1;RPGRIP1L;SPEF1;TTC21B
green	CC	GO:0005643	nuclear pore	17735	75	1060	9	4.54	0.0367	1.982379	CSE1L;IPO7;MVP;NUP107;NUP160;NUP88;RANBP2;TNKS;XPOT
green	BP	GO:0032412	regulation of ion transmembrane transpor...	17735	85	1060	10	5.28	0.0374	1.893939	ANK2;CNIH2;DRD3;EPO;GPD1L;NRXN1;SCN1B;STIM2;WNK2;WNK3
green	BP	GO:0046467	membrane lipid biosynthetic process	17735	97	1060	11	6.02	0.03784	1.827243	CERS1;CERS4;CERS6;KDSR;MPPE1;PGAP2;PIGN;PIGX;PPAP2A;SPHK1;ST8SIA1
green	BP	GO:0006278	RNA-dependent DNA replication	17735	21	1060	4	1.3	0.03793	3.076923	MRE11A;RAD50;TERF1;TNKS
green	BP	GO:0006471	protein ADP-ribosylation	17735	21	1060	4	1.3	0.03793	3.076923	ART1;PARP2;SIRT6;TNKS
green	CC	GO:0031514	motile cilium	17735	137	1060	14	8.29	0.03793	1.688782	B9D2;CASK;CLTB;DNAH2;DNAH9;DYNC2H1;ROM1;RPGRIP1L;SEPT2;SPEF1;TSPEAR;TTBK2;TTC21B;WDR1
green	CC	GO:0043227	membrane-bounded organelle	17735	9590	1060	608	580.45	0.03796	1.047463	AASS;ABCB7;ABHD11;ABTB1;ACAA1;ACAD11;ACOXL;ACSS3;ADAM8;ADCK4;ADNP2;ADRA1A;AFAP1L2;AGPA
green	MF	GO:0005487	nucleocytoplasmic transporter activity	17735	13	1060	3	0.77	0.03831	3.896104	NUP107;NUP160;UPF3B
green	MF	GO:0010576	metalloenzyme regulator activity	17735	13	1060	3	0.77	0.03831	3.896104	RECK;SPOCK3;WFIKKN2
green	MF	GO:0031369	translation initiation factor binding	17735	13	1060	3	0.77	0.03831	3.896104	EIF4EBP1;EIF4EBP3;TRIM32
green	MF	GO:0043560	insulin receptor substrate binding	17735	13	1060	3	0.77	0.03831	3.896104	INSRR;PIK3R1;PTPN11
green	MF	GO:0003950	NAD+ ADP-ribosyltransferase activity	17735	22	1060	4	1.31	0.03859	3.053435	ART1;PARP2;SIRT6;TNKS
green	MF	GO:0022829	wide pore channel activity	17735	22	1060	4	1.31	0.03859	3.053435	GJB3;GJC1;GJC2;PANX2
green	BP	GO:0043067	regulation of programmed cell death	17735	1209	1060	90	75.08	0.03885	1.198721	ADAM8;AIPL1;AR;ARRB2;ATP2A1;AZU1;BAK1;BARD1;BAX;BCL3;BFAR;CAMK2B;CARD9;CASP7;CD3G;CDK5;C
green	CC	GO:0043231	intracellular membrane-bounded organelle	17735	9560	1060	606	578.63	0.03906	1.047301	AASS;ABCB7;ABHD11;ABTB1;ACAA1;ACAD11;ACOXL;ACSS3;ADAM8;ADCK4;ADNP2;ADRA1A;AFAP1L2;AGPA
green	BP	GO:0051325	interphase	17735	414	1060	35	25.71	0.03914	1.361338	CAMK2B;CDC23;CDK6;CDKN2D;CENPJ;CEP192;CEP76;CYP1A1;DCTN3;DNAJC2;EIF4EBP1;HAUS2;HMGA2;HSI
green	BP	GO:0045620	negative regulation of lymphocyte differ...	17735	31	1060	5	1.93	0.04045	2.590674	CTLA4;HLX;IHH;NFKBID;ZC3H8
green	BP	GO:0042981	regulation of apoptotic process	17735	1197	1060	89	74.33	0.0408	1.197363	ADAM8;AIPL1;AR;ARRB2;ATP2A1;AZU1;BAK1;BARD1;BAX;BCL3;BFAR;CAMK2B;CARD9;CASP7;CD3G;CDK5;C
green	BP	GO:0010941	regulation of cell death	17735	1242	1060	92	77.13	0.04109	1.192791	ADAM8;ADNP2;AIPL1;AR;ARRB2;ATP2A1;AZU1;BAK1;BARD1;BAX;BCL3;BFAR;CAMK2B;CARD9;CASP7;CD3G;
green	BP	GO:0006281	DNA repair	17735	388	1060	33	24.09	0.04118	1.369863	ASCC3;ATR;ATXN3;BARD1;C19orf40;CDC14B;CDKN2D;DDX1;ERCC4;ERCC6;FANCC;FANCD2;GADD45G;GTF2I
green	MF	GO:0005496	steroid binding	17735	78	1060	9	4.64	0.04141	1.939655	AR;ESRRA;ESRRB;IHH;NPC2;NR1H3;PDIA2;PGRMC2;TSPO
green	CC	GO:0044297	cell body	17735	286	1060	25	17.31	0.04151	1.444252	ATP2B2;ATXN10;CABP1;CDK5;CHRNA3;CNTN2;CTNND2;DDN;DNER;EPHA4;FADD;GABARAP;GNB2;GNB3;GR
green	CC	GO:0005923	tight junction	17735	101	1060	11	6.11	0.04167	1.800327	AMOT;AMOTL2;CDH5;CLDN10;CLDN11;EPCAM;MTDH;RAB13;RPGRIP1L;SHROOM2;WNK3
green	CC	GO:0070160	occluding junction	17735	101	1060	11	6.11	0.04167	1.800327	AMOT;AMOTL2;CDH5;CLDN10;CLDN11;EPCAM;MTDH;RAB13;RPGRIP1L;SHROOM2;WNK3
green	CC	GO:0005635	nuclear envelope	17735	314	1060	27	19.01	0.0419	1.420305	ADRA1A;CDC14B;CERS4;CERS6;CSE1L;DNAJC2;EDNRB;EI24;HPN;IL15RA;IPO7;KIAA1161;LRPPRC;MTDH;MTM
green	BP	GO:0051100	negative regulation of binding	17735	75	1060	9	4.66	0.04208	1.93133	ARRB2;BAX;CTNNBIP1;HMGA2;MAPK3;NFIB;PEX19;TNKS;ZFP90
green	BP	GO:0009987	cellular process	17735	12861	1060	815	798.67	0.04214	1.020446	AASDH;AASS;ABCB7;ABCC11;ABHD5;ACAA1;ACAD11;ACOXL;ACVR2B;ADAD2;ADAM8;ADAMTS2;ADNP2;ADI
green	CC	GO:0000790	nuclear chromatin	17735	152	1060	15	9.2	0.04226	1.630435	AR;CBX8;HDAC2;MORF4L1;NPM2;NR1H3;NUFIP1;PCGF2;RARA;RBMX;SIRT6;SIRT7;SPI1;TIPIN;UHRF2
green	BP	GO:0051321	meiotic cell cycle	17735	161	1060	16	10	0.04231	1.6	CAMK2B;DPEP3;ERCC4;FANCD2;FBXO43;MRE11A;NPM2;NR2C2;PIWIL2;PPP2CA;RAD50;RAD52;RSPH1;SGOI
green	BP	GO:0050804	regulation of synaptic transmission	17735	174	1060	17	10.81	0.04264	1.572618	ADRA1A;ARRB2;ATP2B2;CAMK2B;CDK5;CHRNA3;CNTN2;CSPG5;CTNND2;DRD3;GPM6B;GRIK3;NRXN1;PRKC
green	CC	GO:0005819	spindle	17735	232	1060	21	14.04	0.04268	1.495726	ARL2BP;ARL8A;CAMK2B;CEP128;CLASP2;CSPP1;DCTN3;DYNC1I1;HAUS2;KIFAP3;PPP2CA;PSRC1;RGS14;SEPT
green	BP	GO:0006417	regulation of translation	17735	200	1060	19	12.42	0.04271	1.529791	APLP1;BCL3;DDX1;EIF1B;EIF2S1;EIF4EBP1;EIF4EBP3;GRB7;NANOS3;NCBP1;NCBP2;PIWIL2;RARA;RGS2;RNF1
green	BP	GO:0007096	regulation of exit from mitosis	17735	13	1060	3	0.81	0.04275	3.703704	CDC23;NPM2;UBE2C
green	BP	GO:0007099	centriole replication	17735	13	1060	3	0.81	0.04275	3.703704	CENPJ;CEP76;SPICE1
green	BP	GO:0008347	glial cell migration	17735	13	1060	3	0.81	0.04275	3.703704	AZU1;EPHA4;TSPO
green	BP	GO:0009130	pyrimidine nucleoside monophosphate bios...	17735	13	1060	3	0.81	0.04275	3.703704	UMPS;UPP1;UPRT
green	BP	GO:0043173	nucleotide salvage	17735	13	1060	3	0.81	0.04275	3.703704	AMPD2;NAPRT1;UPP1
green	BP	GO:0045064	T-helper 2 cell differentiation	17735	13	1060	3	0.81	0.04275	3.703704	BCL3;HLX;RARA
green	BP	GO:0045342	MHC class II biosynthetic process	17735	13	1060	3	0.81	0.04275	3.703704	AZU1;HDAC2;SPI1
green	BP	GO:0050686	negative regulation of mRNA processing	17735	13	1060	3	0.81	0.04275	3.703704	BARD1;RBMX;SRSF9
green	BP	GO:0051044	positive regulation of membrane protein ...	17735	13	1060	3	0.81	0.04275	3.703704	ADAM8;GPLD1;PACSIN3
green	BP	GO:0060004	reflex	17735	13	1060	3	0.81	0.04275	3.703704	ADRA1A;DRD3;HPN
green	BP	GO:0086019	cell-cell signaling involved in cardiac ...	17735	13	1060	3	0.81	0.04275	3.703704	ANK2;GJC1;SCN1B
green	BP	GO:0044092	negative regulation of molecular functio...	17735	673	1060	53	41.79	0.04347	1.268246	AMOT;ARRB2;BAG5;BAX;CABP1;CAND1;CDC23;CDKN2D;CEP192;CTNNBIP1;DRD3;DUSP1;EDNRB;ELFN1;EPC
green	BP	GO:0008637	apoptotic mitochondrial changes	17735	64	1060	8	3.97	0.04366	2.015113	ATP2A1;BAK1;BAX;CASP7;DNM1L;PYCARD;SFN;ZNF205
green	BP	GO:0045744	negative regulation of G-protein coupled...	17735	64	1060	8	3.97	0.04366	2.015113	ARRB2;CAMK2B;DRD3;RGS11;RGS14;RGS19;RGS2;RPGRIP1L
green	BP	GO:0055085	transmembrane transport	17735	688	1060	54	42.72	0.04405	1.264045	ABCB7;ABCC11;ANK2;AQP8;ATP2A1;ATP2B2;ATP6V0A2;ATP6V0B;ATP6V1G2;BAK1;BAX;CACNB2;CASK;CASI
green	BP	GO:0003254	regulation of membrane depolarization	17735	22	1060	4	1.37	0.04416	2.919708	GCLC;GPD1L;SCN1B;TSPO
green	BP	GO:0031952	regulation of protein autophosphorylatio...	17735	22	1060	4	1.37	0.04416	2.919708	ERRFI1;MRE11A;PPP2CA;RAD50
green	BP	GO:0090200	positive regulation of release of cytoch...	17735	22	1060	4	1.37	0.04416	2.919708	BAK1;BAX;DNM1L;PYCARD
green	MF	GO:0051059	NF-kappaB binding	17735	23	1060	4	1.37	0.04459	2.919708	ANKRD42;MTDH;NFKBID;TAF4B

green	MF	GO:0042393	histone binding	17735	104	1060	11	6.19	0.04482	1.77706	ASF1B;CBX8;DNAJC2;IPO7;NPM2;RNF168;SET;SPIN1;TDRD3;UHRF2;WDR92
green	CC	GO:0032838	cell projection cytoplasm	17735	66	1060	8	3.99	0.04498	2.005013	B9D2;DNAH2;DNAH9;DYNC2H1;GRIK3;RPGRIP1L;SPEF1;TTC21B
green	BP	GO:0006302	double-strand break repair	17735	112	1060	12	6.96	0.04508	1.724138	DDX1;ERCC4;HMGA2;HUS1;MCM8;MORF4L1;MRE11A;OTUB1;RAD50;RAD52;RNF168;SIRT6
green	BP	GO:0006937	regulation of muscle contraction	17735	112	1060	12	6.96	0.04508	1.724138	ADRA1A;ANK2;ATP2A1;CHRNA3;GJC1;GPD1L;MYBPC3;NCF1;RGS2;SCN1B;SPHK1;SSTR2
green	BP	GO:0034765	regulation of ion transmembrane transpor...	17735	112	1060	12	6.96	0.04508	1.724138	ANK2;CASK;CNIH2;DRD3;EPO;GPD1L;NRXN1;SCN1B;STIM2;THBS1;WNK2;WNK3
green	BP	GO:0065004	protein-DNA complex assembly	17735	137	1060	14	8.51	0.0453	1.645123	ASF1B;CAND1;CENPC1;HIST1H1C;HIST1H2AD;HIST1H2BK;HIST1H2BO;HIST2H2AB;RAD52;SET;SHPRH;TAF1B
green	BP	GO:0032392	DNA geometric change	17735	32	1060	5	1.99	0.04558	2.512563	ASCC3;DDX1;MRE11A;RAD50;WRN
green	CC	GO:0044444	cytoplasmic part	17735	6562	1060	423	397.17	0.04673	1.065035	AAGAB;AASS;ABCB7;ABHD11;ABHD5;ACAA1;ACAD11;ACOXL;ACSS3;ACTG2;ADAM8;ADCK4;ADRA1A;AGPAT
green	BP	GO:0000723	telomere maintenance	17735	65	1060	8	4.04	0.04719	1.980198	ERCC4;MRE11A;PRIM2;PTGES3;RAD50;TERF1;TNKS;WRN
green	BP	GO:0043065	positive regulation of apoptotic process	17735	421	1060	35	26.14	0.04785	1.338944	ADAM8;BAK1;BARD1;BAX;CASP7;CDK5;COL18A1;CTLA4;DUSP1;EI24;FADD;GPLD1;HMGA2;IER3;ITGB1;MTCI
green	BP	GO:0032409	regulation of transporter activity	17735	101	1060	11	6.27	0.04851	1.754386	ANK2;CNIH2;DRD3;EPO;GPD1L;NRXN1;SCN1B;SGK2;STIM2;WNK2;WNK3
green	BP	GO:0090068	positive regulation of cell cycle proces...	17735	164	1060	16	10.18	0.04869	1.571709	CAMK2B;CSPP1;CYP1A1;DRD3;HMGA2;NPM2;PRKCE;PSMB10;PSMB8;PSMB9;PSMD5;PSME2;SMAD6;TERF1
green	BP	GO:2000112	regulation of cellular macromolecule bio...	17735	3117	1060	214	193.57	0.04873	1.105543	ACVR2B;ADAM8;AFAP1L2;ANKRD42;APLP1;AR;ARMCX3;ARRB2;ASCC3;ASF1B;ATMIN;ATR;ATXN3;BANP;BCL
green	CC	GO:0016471	vacuolar proton-transporting V-type ATPa...	17735	14	1060	3	0.85	0.04876	3.529412	ATP6V0A2;ATP6V1G2;TCIRG1
green	CC	GO:0031307	integral to mitochondrial outer membrane	17735	14	1060	3	0.85	0.04876	3.529412	ARMCX3;BAK1;SYNJ2BP
green	BP	GO:0010556	regulation of macromolecule biosynthetic...	17735	3197	1060	219	198.53	0.04995	1.103108	ACVR2B;ADAM8;AFAP1L2;ANKRD42;APLP1;AR;ARMCX3;ARRB2;ASCC3;ASF1B;ATMIN;ATR;ATXN3;AZU1;BAN
cyan	BP	GO:0045087	innate immune response	17735	550	74	21	2.35	4.00E-15	8.93617	APOL1;C2;GBP1;GBP2;GBP5;ICAM1;IFI16;IFI35;IRF1;IRF9;MB21D1;PARP9;PML;RNASEL;SERPING1;SP100;ST/
cyan	BP	GO:0006952	defense response	17735	1119	74	24	4.77	8.30E-12	5.031447	APOL1;APOL2;BATF2;C2;GBP1;GBP2;GBP5;ICAM1;IFI16;IFI35;IRF1;IRF9;MB21D1;PARP9;PML;RNASEL;SERPII
cyan	BP	GO:0034341	response to interferon-gamma	17735	112	74	10	0.48	3.80E-11	20.83333	GBP1;GBP2;GBP5;ICAM1;IRF1;IRF9;PARP9;PML;SP100;STAT1
cyan	BP	GO:0071346	cellular response to interferon-gamma	17735	94	74	9	0.4	2.00E-10	22.5	GBP1;GBP2;GBP5;ICAM1;IRF1;IRF9;PML;SP100;STAT1
cyan	BP	GO:0006955	immune response	17735	1081	74	22	4.61	2.30E-10	4.772234	APOL1;C2;GBP1;GBP2;GBP5;ICAM1;IFI16;IFI35;IRF1;IRF9;MB21D1;PARP9;PML;RNASEL;SERPING1;SP100;ST/
cyan	BP	GO:0060337	type I interferon-mediated signaling pat...	17735	72	74	8	0.31	6.60E-10	25.80645	GBP2;IFI35;IRF1;IRF9;RNASEL;SP100;STAT1;STAT2
cyan	BP	GO:0071357	cellular response to type I interferon	17735	72	74	8	0.31	6.60E-10	25.80645	GBP2;IFI35;IRF1;IRF9;RNASEL;SP100;STAT1;STAT2
cyan	BP	GO:0034340	response to type I interferon	17735	73	74	8	0.31	7.40E-10	25.80645	GBP2;IFI35;IRF1;IRF9;RNASEL;SP100;STAT1;STAT2
cyan	BP	GO:0060333	interferon-gamma-mediated signaling path...	17735	75	74	8	0.32	9.20E-10	25	GBP1;GBP2;ICAM1;IRF1;IRF9;PML;SP100;STAT1
cyan	BP	GO:0009615	response to virus	17735	263	74	12	1.12	9.20E-10	10.71429	ACTA2;GBP1;IFI16;IFI35;IRF1;IRF9;MB21D1;PML;RNASEL;STAT1;STAT2;TRIM5
cyan	BP	GO:0034097	response to cytokine stimulus	17735	497	74	15	2.12	1.70E-09	7.075472	GBP1;GBP2;GBP5;ICAM1;IFI35;IRF1;IRF9;PARP9;PML;RNASEL;SP100;STAT1;STAT2;TRAFD1;UBE2L6
cyan	BP	GO:0051607	defense response to virus	17735	185	74	10	0.79	5.30E-09	12.65823	GBP1;IFI16;IRF1;IRF9;MB21D1;PML;RNASEL;STAT1;STAT2;TRIM5
cyan	BP	GO:0002376	immune system process	17735	1816	74	26	7.74	6.20E-09	3.359173	APOL1;BATF2;BTN2A2;C2;FZD5;GBP1;GBP2;GBP5;ICAM1;IFI16;IFI35;IRF1;IRF9;MB21D1;NRARP;PARP9;PML
cyan	BP	GO:0002252	immune effector process	17735	471	74	14	2.01	8.10E-09	6.965174	C2;GBP1;ICAM1;IFI16;IRF1;IRF9;MB21D1;PML;RNASEL;SERPING1;STAT1;STAT2;TAP1;TRIM5
cyan	BP	GO:0051707	response to other organism	17735	578	74	15	2.46	1.30E-08	6.097561	ACTA2;BATF2;FZD5;GBP1;ICAM1;IFI16;IFI35;IRF1;IRF9;MB21D1;PML;RNASEL;STAT1;STAT2;TRIM5
cyan	BP	GO:0019221	cytokine-mediated signaling pathway	17735	335	74	12	1.43	1.40E-08	8.391608	GBP1;GBP2;ICAM1;IFI35;IRF1;IRF9;PML;RNASEL;SP100;STAT1;STAT2;UBE2L6
cyan	BP	GO:0071345	cellular response to cytokine stimulus	17735	412	74	13	1.76	1.50E-08	7.386364	GBP1;GBP2;GBP5;ICAM1;IFI35;IRF1;IRF9;PML;RNASEL;SP100;STAT1;STAT2;UBE2L6
cyan	BP	GO:0009607	response to biotic stimulus	17735	606	74	15	2.58	2.50E-08	5.813953	ACTA2;BATF2;FZD5;GBP1;ICAM1;IFI16;IFI35;IRF1;IRF9;MB21D1;PML;RNASEL;STAT1;STAT2;TRIM5
cyan	BP	GO:0045088	regulation of innate immune response	17735	225	74	9	0.96	4.30E-07	9.375	IFI16;IRF1;MB21D1;PARP9;SERPING1;STAT1;STAT2;TRAFD1;TRIM5
cyan	BP	GO:0051704	multi-organism process	17735	1380	74	20	5.88	5.70E-07	3.401361	ACTA2;APOL1;APOL2;BATF2;FZD5;GBP1;ICAM1;IFI16;IFI35;IRF1;IRF9;MB21D1;PML;RNASEL;SP100;SP110;ST
cyan	BP	GO:0010033	response to organic substance	17735	1870	74	23	7.97	1.10E-06	2.885822	ATF3;FBXO6;FZD5;GBP1;GBP2;GBP5;HCAR1;ICAM1;IFI35;IRF1;IRF9;MB21D1;PARP9;PML;RNASEL;SORT1;SP:
cyan	BP	GO:0031347	regulation of defense response	17735	428	74	11	1.82	1.70E-06	6.043956	C2;IFI16;IRF1;MB21D1;PARP9;PML;SERPING1;STAT1;STAT2;TRAFD1;TRIM5
cyan	BP	GO:0006950	response to stress	17735	2926	74	29	12.48	2.10E-06	2.323718	APOL1;APOL2;ATF3;BATF2;C2;DTX3L;FANCA;FBXO6;FZD5;GBP1;GBP2;GBP5;ICAM1;IFI16;IFI35;IRF1;IRF9;ME
cyan	BP	GO:0002682	regulation of immune system process	17735	884	74	15	3.77	3.20E-06	3.97878	BTN2A2;C2;ICAM1;IFI16;IRF1;MB21D1;NRARP;PARP9;PML;SERPING1;STAT1;STAT2;TAP1;TRAFD1;TRIM5
cyan	BP	GO:0050776	regulation of immune response	17735	560	74	12	2.39	3.50E-06	5.020921	C2;ICAM1;IFI16;IRF1;MB21D1;PARP9;SERPING1;STAT1;STAT2;TAP1;TRAFD1;TRIM5
cyan	BP	GO:0045824	negative regulation of innate immune res...	17735	12	74	3	0.05	1.60E-05	60	IFI16;SERPING1;TRAFD1
cyan	BP	GO:0071310	cellular response to organic substance	17735	1333	74	17	5.68	2.80E-05	2.992958	ATF3;GBP1;GBP2;GBP5;ICAM1;IFI35;IRF1;IRF9;MB21D1;PML;RNASEL;SP100;STAT1;STAT2;TGIF1;TRIM5;UBE
cyan	BP	GO:0080134	regulation of response to stress	17735	737	74	12	3.14	5.50E-05	3.821656	C2;FZD5;IFI16;IRF1;MB21D1;PARP9;PML;SERPING1;STAT1;STAT2;TRAFD1;TRIM5
cyan	BP	GO:0042157	lipoprotein metabolic process	17735	98	74	5	0.42	6.00E-05	11.90476	APOL1;APOL2;APOL4;APOL6;CETP
cyan	BP	GO:0022415	viral reproductive process	17735	542	74	10	2.31	8.90E-05	4.329004	ICAM1;IFI16;PML;RNASEL;SP100;SP110;STAT1;STAT2;TAP1;TRIM5
cyan	BP	GO:0002683	negative regulation of immune system pro...	17735	176	74	6	0.75	1.00E-04	8	BTN2A2;IFI16;IRF1;NRARP;SERPING1;TRAFD1
cyan	BP	GO:0019048	virus-host interaction	17735	371	74	8	1.58	0.00017	5.063291	ICAM1;PML;SP100;SP110;STAT1;STAT2;TAP1;TRIM5
cyan	BP	GO:0043900	regulation of multi-organism process	17735	205	74	6	0.87	0.00023	6.896552	IFI16;MB21D1;PML;RNASEL;SP100;TRIM5
cyan	BP	GO:0044703	multi-organism reproductive process	17735	730	74	11	3.11	0.00023	3.536977	APOL2;ICAM1;IFI16;PML;RNASEL;SP100;SP110;STAT1;STAT2;TAP1;TRIM5
cyan	BP	GO:0050896	response to stimulus	17735	6581	74	42	28.06	0.00029	1.496793	ACTA2;APOL1;APOL2;ATF3;BATF2;C2;CEACAM1;DTX3L;EMR1;FANCA;FBXO6;FZD5;GBP1;GBP2;GBP5;GPR19
cyan	BP	GO:0002697	regulation of immune effector process	17735	217	74	6	0.93	0.00032	6.451613	C2;ICAM1;MB21D1;PML;SERPING1;TAP1
cyan	BP	GO:0051701	interaction with host	17735	411	74	8	1.75	0.00034	4.571429	ICAM1;PML;SP100;SP110;STAT1;STAT2;TAP1;TRIM5
cyan	BP	GO:0044403	symbiosis, encompassing mutualism throug...	17735	445	74	8	1.9	0.00057	4.210526	ICAM1;PML;SP100;SP110;STAT1;STAT2;TAP1;TRIM5
cyan	BP	GO:0044419	interspecies interaction between organis...	17735	445	74	8	1.9	0.00057	4.210526	ICAM1;PML;SP100;SP110;STAT1;STAT2;TAP1;TRIM5
cyan	BP	GO:0070887	cellular response to chemical stimulus	17735	1709	74	17	7.29	0.00059	2.331962	ATF3;GBP1;GBP2;GBP5;ICAM1;IFI35;IRF1;IRF9;MB21D1;PML;RNASEL;SP100;STAT1;STAT2;TGIF1;TRIM5;UBE
cyan	BP	GO:0016032	viral reproduction	17735	689	74	10	2.94	0.00062	3.401361	ICAM1;IFI16;PML;RNASEL;SP100;SP110;STAT1;STAT2;TAP1;TRIM5
cyan	BP	GO:0044764	multi-organism cellular process	17735	691	74	10	2.95	0.00063	3.389831	ICAM1;IFI16;PML;RNASEL;SP100;SP110;STAT1;STAT2;TAP1;TRIM5
cyan	BP	GO:0042221	response to chemical stimulus	17735	2766	74	23	11.79	0.00065	1.950806	ATF3;FBXO6;FZD5;GBP1;GBP2;GBP5;HCAR1;ICAM1;IFI35;IRF1;IRF9;MB21D1;PARP9;PML;RNASEL;SORT1;SP:
cyan	BP	GO:0071219	cellular response to molecule of bacteri...	17735	98	74	4	0.42	8.00E-04	9.52381	FZD5;ICAM1;STAT1;TRIM5
cyan	BP	GO:0048384	retinoic acid receptor signaling pathway	17735	47	74	3	0.2	0.00105	15	PML;SP100;TGIF1
cyan	BP	GO:0048584	positive regulation of response to stimu...	17735	1164	74	13	4.96	0.00106	2.620968	C2;FZD5;IFI16;IRF1;MB21D1;NRARP;PML;SEPT4;SERPING1;SHISA5;TAP1;TRIM38;TRIM5
cyan	BP	GO:0050778	positive regulation of immune response	17735	377	74	7	1.61	0.00107	4.347826	C2;IFI16;IRF1;MB21D1;SERPING1;TAP1;TRIM5
cyan	BP	GO:0071216	cellular response to biotic stimulus	17735	108	74	4	0.46	0.00115	8.695652	FZD5;ICAM1;STAT1;TRIM5
cyan	BP	GO:0007166	cell surface receptor signaling pathway	17735	2331	74	20	9.94	0.00116	2.012072	CEACAM1;EMR1;FZD5;GBP1;GBP2;GPR19;ICAM1;IFI35;IRF1;IRF9;NRARP;PML;RNASEL;SORT1;SP100;STAT1;
cyan	CC	GO:0000932	cytoplasmic mRNA processing body	17735	51	74	3	0.22	0.0014	13.63636	MEX3B;TRIM21;TRIM5
cyan	BP	GO:0050777	negative regulation of immune response	17735	52	74	3	0.22	0.00141	13.63636	IFI16;SERPING1;TRAFD1
cyan	BP	GO:0035456	response to interferon-beta	17735	14	74	2	0.06	0.00157	33.33333	IRF1;STAT1
cyan	BP	GO:0042310	vasoconstriction	17735	54	74	3	0.23	0.00157	13.04348	ACTA2;ICAM1;SMTNL1

cyan	BP	GO:0008285	negative regulation of cell proliferatio...	17735	522	74	8	2.23	0.00161	3.587444	BTN2A2;FZD5;GAS8;IRF1;PML;STAT1;TGIF1;WARS
cyan	BP	GO:0006869	lipid transport	17735	207	74	5	0.88	0.00187	5.681818	APOL1;APOL2;APOL4;APOL6;CETP
cyan	BP	GO:0002253	activation of immune response	17735	310	74	6	1.32	0.00202	4.545455	C2;IFI16;IRF1;MB21D1;SERPING1;TRIM5
cyan	BP	GO:0002250	adaptive immune response	17735	211	74	5	0.9	0.00203	5.555556	C2;ICAM1;IRF1;SERPING1;TAP1
cyan	BP	GO:0002230	positive regulation of defense response ...	17735	16	74	2	0.07	0.00207	28.57143	MB21D1;PML
cyan	BP	GO:0002684	positive regulation of immune system pro...	17735	554	74	8	2.36	0.00233	3.389831	C2;ICAM1;IFI16;IRF1;MB21D1;SERPING1;TAP1;TRIM5
cyan	BP	GO:0045907	positive regulation of vasoconstriction	17735	18	74	2	0.08	0.00262	25	ICAM1;SMTNL1
cyan	BP	GO:0002218	activation of innate immune response	17735	140	74	4	0.6	0.00298	6.666667	IFI16;IRF1;MB21D1;TRIM5
cyan	BP	GO:0001906	cell killing	17735	68	74	3	0.29	0.00304	10.34483	APOL1;ICAM1;TAP1
cyan	BP	GO:0048583	regulation of response to stimulus	17735	2347	74	19	10.01	0.0032	1.898102	C2;FZD5;ICAM1;IFI16;IRF1;MB21D1;NRARP;PARP9;PML;SEPT4;SERPING1;SHISA5;STAT1;STAT2;TAP1;TGIF1;
cyan	BP	GO:0030449	regulation of complement activation	17735	20	74	2	0.09	0.00323	22.22222	C2;SERPING1
cyan	BP	GO:2000257	regulation of protein activation cascade	17735	20	74	2	0.09	0.00323	22.22222	C2;SERPING1
cyan	BP	GO:0050868	negative regulation of T cell activation	17735	70	74	3	0.3	0.0033	10	BTN2A2;IRF1;NRARP
cyan	BP	GO:0010876	lipid localization	17735	237	74	5	1.01	0.00335	4.950495	APOL1;APOL2;APOL4;APOL6;CETP
cyan	MF	GO:0003924	GTPase activity	17735	232	74	5	1.01	0.0034	4.950495	GBP1;GBP2;GBP4;GBP5;SEPT4
cyan	BP	GO:0032606	type I interferon production	17735	71	74	3	0.3	0.00343	10	IRF1;IRF9;UBE2L6
cyan	BP	GO:0043330	response to exogenous dsRNA	17735	21	74	2	0.09	0.00357	22.22222	MB21D1;STAT1
cyan	BP	GO:0022414	reproductive process	17735	1510	74	14	6.44	0.00389	2.173913	APOL2;FANCA;FZD5;ICAM1;IFI16;PML;RNASEL;SEPT4;SP100;SP110;STAT1;STAT2;TAP1;TRIM5
cyan	BP	GO:0045581	negative regulation of T cell differenti...	17735	22	74	2	0.09	0.00391	22.22222	IRF1;NRARP
cyan	MF	GO:0003950	NAD+ ADP-ribosyltransferase activity	17735	22	74	2	0.1	0.0041	20	PARP14;PARP9
cyan	BP	GO:0050691	regulation of defense response to virus ...	17735	23	74	2	0.1	0.00427	20	MB21D1;PML
cyan	BP	GO:0001525	angiogenesis	17735	362	74	6	1.54	0.00434	3.896104	CEACAM1;FZD5;NRARP;PML;STAT1;WARS
cyan	BP	GO:0051716	cellular response to stimulus	17735	5052	74	32	21.54	0.00459	1.485608	ATF3;CEACAM1;DTX3L;EMR1;FANCA;FBXO6;FZD5;GBP1;GBP2;GBP5;GPR19;ICAM1;IFI16;IFI35;IRF1;IRF9;ME
cyan	MF	GO:0004842	ubiquitin-protein ligase activity	17735	253	74	5	1.1	0.0049	4.545455	DTX3L;FBXO6;TRIM21;TRIM5;UBE2L6
cyan	BP	GO:2000242	negative regulation of reproductive proc...	17735	81	74	3	0.35	0.00497	8.571429	IFI16;RNASEL;SP100
cyan	BP	GO:0042771	intrinsic apoptotic signaling pathway in...	17735	25	74	2	0.11	0.00504	18.18182	IFI16;PML
cyan	BP	GO:0060330	regulation of response to interferon-gam...	17735	25	74	2	0.11	0.00504	18.18182	PARP9;STAT1
cyan	CC	GO:0034364	high-density lipoprotein particle	17735	25	74	2	0.11	0.0051	18.18182	APOL1;CETP
cyan	BP	GO:0001568	blood vessel development	17735	498	74	7	2.12	0.00512	3.301887	ACTA2;CEACAM1;FZD5;NRARP;PML;STAT1;WARS
cyan	BP	GO:0031348	negative regulation of defense response	17735	82	74	3	0.35	0.00515	8.571429	IFI16;SERPING1;TRAFD1
cyan	MF	GO:0005525	GTP binding	17735	366	74	6	1.6	0.0052	3.75	GBP1;GBP2;GBP4;GBP5;RAB20;SEPT4
cyan	CC	GO:0019005	SCF ubiquitin ligase complex	17735	26	74	2	0.11	0.0055	18.18182	FBXO6;TRIM21
cyan	BP	GO:0002831	regulation of response to biotic stimulu...	17735	84	74	3	0.36	0.0055	8.333333	MB21D1;PML;TRIM5
cyan	BP	GO:0045089	positive regulation of innate immune res...	17735	167	74	4	0.71	0.00558	5.633803	IFI16;IRF1;MB21D1;TRIM5
cyan	MF	GO:0019001	guanyl nucleotide binding	17735	380	74	6	1.66	0.0062	3.614458	GBP1;GBP2;GBP4;GBP5;RAB20;SEPT4
cyan	MF	GO:0032561	guanyl ribonucleotide binding	17735	380	74	6	1.66	0.0062	3.614458	GBP1;GBP2;GBP4;GBP5;RAB20;SEPT4
cyan	BP	GO:0000003	reproduction	17735	1591	74	14	6.78	0.0062	2.064897	APOL2;FANCA;FZD5;ICAM1;IFI16;PML;RNASEL;SEPT4;SP100;SP110;STAT1;STAT2;TAP1;TRIM5
cyan	CC	GO:0035770	ribonucleoprotein granule	17735	88	74	3	0.38	0.0064	7.894737	MEX3B;TRIM21;TRIM5
cyan	MF	GO:0019787	small conjugating protein ligase activit...	17735	269	74	5	1.17	0.0064	4.273504	DTX3L;FBXO6;TRIM21;TRIM5;UBE2L6
cyan	BP	GO:0001944	vasculature development	17735	523	74	7	2.23	0.00665	3.139013	ACTA2;CEACAM1;FZD5;NRARP;PML;STAT1;WARS
cyan	BP	GO:0060759	regulation of response to cytokine stimu...	17735	90	74	3	0.38	0.00666	7.894737	PARP9;STAT1;STAT2
cyan	BP	GO:0002449	lymphocyte mediated immunity	17735	179	74	4	0.76	0.00711	5.263158	C2;ICAM1;SERPING1;TAP1
cyan	BP	GO:0070206	protein trimerization	17735	30	74	2	0.13	0.00721	15.38462	TRIM21;TRIM5
cyan	BP	GO:0071222	cellular response to lipopolysaccharide	17735	93	74	3	0.4	0.00729	7.5	ICAM1;STAT1;TRIM5
cyan	BP	GO:0045620	negative regulation of lymphocyte differ...	17735	31	74	2	0.13	0.00768	15.38462	IRF1;NRARP
cyan	BP	GO:0051250	negative regulation of lymphocyte activa...	17735	96	74	3	0.41	0.00796	7.317073	BTN2A2;IRF1;NRARP
cyan	BP	GO:0045071	negative regulation of viral genome repl...	17735	32	74	2	0.14	0.00817	14.28571	IFI16;RNASEL
cyan	BP	GO:0072332	intrinsic apoptotic signaling pathway by...	17735	32	74	2	0.14	0.00817	14.28571	IFI16;PML
cyan	BP	GO:0007165	signal transduction	17735	4165	74	27	17.76	0.00854	1.52027	ATF3;CEACAM1;EMR1;FZD5;GBP1;GBP2;GPR19;ICAM1;IFI16;IFI35;IRF1;IRF9;NRARP;PML;RAB20;RNASEL;SE
cyan	BP	GO:0001910	regulation of leukocyte mediated cytotox...	17735	33	74	2	0.14	0.00868	14.28571	ICAM1;TAP1
cyan	BP	GO:0002920	regulation of humoral immune response	17735	33	74	2	0.14	0.00868	14.28571	C2;SERPING1
cyan	BP	GO:0048525	negative regulation of viral reproductio...	17735	33	74	2	0.14	0.00868	14.28571	IFI16;RNASEL
cyan	BP	GO:0060338	regulation of type I interferon-mediated...	17735	33	74	2	0.14	0.00868	14.28571	STAT1;STAT2
cyan	BP	GO:0048519	negative regulation of biological proces...	17735	3150	74	22	13.43	0.00869	1.638124	ATF3;BTN2A2;CETP;FBXO6;FZD5;GAS8;ICAM1;IFI16;IRF1;NRARP;PML;RNASEL;SERPING1;SMTNL1;SORT1;SP
cyan	BP	GO:0010212	response to ionizing radiation	17735	101	74	3	0.43	0.00915	6.976744	ICAM1;IFI16;PML
cyan	BP	GO:0002460	adaptive immune response based on somati...	17735	193	74	4	0.82	0.00922	4.878049	C2;ICAM1;SERPING1;TAP1
cyan	BP	GO:0043122	regulation of I-kappaB kinase/NF-kappaB ...	17735	194	74	4	0.83	0.00938	4.819277	SHISA5;STAT1;TRIM38;TRIM5
cyan	BP	GO:0006917	induction of apoptosis	17735	195	74	4	0.83	0.00955	4.819277	IRF1;PML;SHISA5;STAT1
cyan	MF	GO:0000978	RNA polymerase II core promoter proximal...	17735	34	74	2	0.15	0.0096	13.33333	IRF1;STAT1
cyan	BP	GO:0012502	induction of programmed cell death	17735	196	74	4	0.84	0.00972	4.761905	IRF1;PML;SHISA5;STAT1
cyan	MF	GO:0016881	acid-amino acid ligase activity	17735	299	74	5	1.31	0.0098	3.816794	DTX3L;FBXO6;TRIM21;TRIM5;UBE2L6
cyan	CC	GO:0005737	cytoplasm	17735	9016	74	49	38.75	0.0098	1.264516	ACOT9;ACP6;ACTA2;APOL2;APOL6;ASPHD2;C7orf49;DTX3L;ERP27;FANCA;FBXO6;FTSJD2;FZD5;GAS8;GBP1;(
cyan	BP	GO:0001816	cytokine production	17735	430	74	6	1.83	0.00983	3.278689	BTN2A2;FZD5;IFI16;IRF1;IRF9;UBE2L6
cyan	BP	GO:0042127	regulation of cell proliferation	17735	1174	74	11	5.01	0.01009	2.195609	ATF3;BTN2A2;FANCA;FZD5;GAS8;IRF1;NRARP;PML;STAT1;TGIF1;WARS
cyan	BP	GO:0065007	biological regulation	17735	8616	74	46	36.74	0.01019	1.252041	ACTA2;APOL1;ATF3;BTN2A2;C2;CARD6;CEACAM1;CETP;EMR1;FANCA;FBXO6;FZD5;GAS8;GBP1;GBP2;GPR1!
cyan	BP	GO:0031341	regulation of cell killing	17735	36	74	2	0.15	0.01027	13.33333	ICAM1;TAP1
cyan	BP	GO:0031663	lipopolysaccharide-mediated signaling pa...	17735	36	74	2	0.15	0.01027	13.33333	STAT1;TRIM5
cyan	BP	GO:2001235	positive regulation of apoptotic signali...	17735	36	74	2	0.15	0.01027	13.33333	PML;SEPT4
cyan	CC	GO:0034358	plasma lipoprotein particle	17735	36	74	2	0.15	0.0104	13.33333	APOL1;CETP

cyan	BP	GO:0048514	blood vessel morphogenesis	17735	438	74	6	1.87	0.0107	3.208556	CEACAM1;FZD5;NRARP;PML;STAT1;WARS
cyan	BP	GO:0097190	apoptotic signaling pathway	17735	202	74	4	0.86	0.01077	4.651163	IFI16;PML;SEPT4;SORT1
cyan	BP	GO:1900117	regulation of execution phase of apoptos...	17735	202	74	4	0.86	0.01077	4.651163	IFI16;PML;SEPT4;SORT1
cyan	BP	GO:0006513	protein monoubiquitination	17735	37	74	2	0.16	0.01082	12.5	DTX3L;TRIM21
cyan	BP	GO:0043392	negative regulation of DNA binding	17735	37	74	2	0.16	0.01082	12.5	IFI16;SP100
cyan	BP	GO:0050789	regulation of biological process	17735	8134	74	44	34.68	0.01084	1.268743	ATF3;BTN2A2;C2;CARD6;CEACAM1;CETP;EMR1;FANCA;FBXO6;FZD5;GAS8;GBP1;GBP2;GPR19;ICAM1;IFI16;I
cyan	CC	GO:0032994	protein-lipid complex	17735	37	74	2	0.16	0.011	12.5	APOL1;CETP
cyan	BP	GO:0002526	acute inflammatory response	17735	109	74	3	0.46	0.01125	6.521739	APOL2;C2;SERPING1
cyan	BP	GO:0050792	regulation of viral reproduction	17735	109	74	3	0.46	0.01125	6.521739	IFI16;RNASEL;SP100
cyan	BP	GO:0043331	response to dsRNA	17735	38	74	2	0.16	0.01139	12.5	MB21D1;STAT1
cyan	BP	GO:0002695	negative regulation of leukocyte activat...	17735	110	74	3	0.47	0.01153	6.382979	BTN2A2;IRF1;NRARP
cyan	BP	GO:0050880	regulation of blood vessel size	17735	110	74	3	0.47	0.01153	6.382979	ACTA2;ICAM1;SMTNL1
cyan	BP	GO:0035150	regulation of tube size	17735	111	74	3	0.47	0.01181	6.382979	ACTA2;ICAM1;SMTNL1
cyan	BP	GO:0002237	response to molecule of bacterial origin	17735	211	74	4	0.9	0.01248	4.444444	FZD5;ICAM1;STAT1;TRIM5
cyan	BP	GO:0008203	cholesterol metabolic process	17735	115	74	3	0.49	0.01299	6.122449	APOL1;APOL2;CETP
cyan	BP	GO:0002521	leukocyte differentiation	17735	329	74	5	1.4	0.01299	3.571429	BATF2;FZD5;IFI16;IRF1;NRARP
cyan	BP	GO:0061005	cell differentiation involved in kidney ...	17735	41	74	2	0.17	0.01318	11.76471	ACTA2;STAT1
cyan	BP	GO:0097193	intrinsic apoptotic signaling pathway	17735	116	74	3	0.49	0.0133	6.122449	IFI16;PML;SEPT4
cyan	BP	GO:0007249	I-kappaB kinase/NF-kappaB cascade	17735	217	74	4	0.93	0.01371	4.301075	SHISA5;STAT1;TRIM38;TRIM5
cyan	BP	GO:0019229	regulation of vasoconstriction	17735	42	74	2	0.18	0.0138	11.11111	ICAM1;SMTNL1
cyan	BP	GO:0070613	regulation of protein processing	17735	42	74	2	0.18	0.0138	11.11111	C2;SERPING1
cyan	BP	GO:0050866	negative regulation of cell activation	17735	119	74	3	0.51	0.01424	5.882353	BTN2A2;IRF1;NRARP
cyan	MF	GO:0016879	ligase activity, forming carbon-nitrogen...	17735	330	74	5	1.44	0.0145	3.472222	DTX3L;FBXO6;TRIM21;TRIM5;UBE2L6
cyan	BP	GO:0002443	leukocyte mediated immunity	17735	221	74	4	0.94	0.01457	4.255319	C2;ICAM1;SERPING1;TAP1
cyan	BP	GO:0042110	T cell activation	17735	339	74	5	1.45	0.01463	3.448276	BTN2A2;FZD5;ICAM1;IRF1;NRARP
cyan	BP	GO:0016125	sterol metabolic process	17735	121	74	3	0.52	0.01488	5.769231	APOL1;APOL2;CETP
cyan	BP	GO:0008630	intrinsic apoptotic signaling pathway in...	17735	45	74	2	0.19	0.01574	10.52632	IFI16;PML
cyan	MF	GO:0016763	transferase activity, transferring pento...	17735	44	74	2	0.19	0.0158	10.52632	PARP14;PARP9
cyan	BP	GO:0031349	positive regulation of defense response	17735	229	74	4	0.98	0.0164	4.081633	IFI16;IRF1;MB21D1;TRIM5
cyan	BP	GO:0008015	blood circulation	17735	349	74	5	1.49	0.0164	3.355705	ACTA2;ICAM1;SERPING1;SMTNL1;STAT1
cyan	MF	GO:0000987	core promoter proximal region sequence-s...	17735	45	74	2	0.2	0.0165	10	IRF1;STAT1
cyan	BP	GO:0003013	circulatory system process	17735	350	74	5	1.49	0.01658	3.355705	ACTA2;ICAM1;SERPING1;SMTNL1;STAT1
cyan	MF	GO:0001159	core promoter proximal region DNA bindin...	17735	46	74	2	0.2	0.0172	10	IRF1;STAT1
cyan	BP	GO:0043170	macromolecule metabolic process	17735	7334	74	40	31.27	0.01761	1.279181	APOL1;APOL2;APOL4;APOL6;ASPHD2;ATF3;BATF2;C2;CETP;DTX3L;ETV7;FANCA;FBXO6;FTSJD2;FZD5;ICAM1;
cyan	BP	GO:0003018	vascular process in circulatory system	17735	129	74	3	0.55	0.01764	5.454545	ACTA2;ICAM1;SMTNL1
cyan	BP	GO:0002456	T cell mediated immunity	17735	48	74	2	0.2	0.01779	10	ICAM1;TAP1
cyan	BP	GO:0045069	regulation of viral genome replication	17735	48	74	2	0.2	0.01779	10	IFI16;RNASEL
cyan	BP	GO:0072331	signal transduction by p53 class mediato...	17735	130	74	3	0.55	0.018	5.454545	IFI16;PML;SP100
cyan	BP	GO:0001909	leukocyte mediated cytotoxicity	17735	49	74	2	0.21	0.0185	9.52381	ICAM1;TAP1
cyan	MF	GO:0016874	ligase activity	17735	483	74	6	2.11	0.0186	2.843602	DTX3L;FBXO6;TRIM21;TRIM5;UBE2L6;WARS
cyan	BP	GO:0006958	complement activation, classical pathway	17735	51	74	2	0.22	0.01995	9.090909	C2;SERPING1
cyan	BP	GO:0030097	hemopoiesis	17735	509	74	6	2.17	0.02099	2.764977	BATF2;FZD5;IFI16;IRF1;NRARP;PML
cyan	BP	GO:0002673	regulation of acute inflammatory respons...	17735	53	74	2	0.23	0.02144	8.695652	C2;SERPING1
cyan	BP	GO:0043901	negative regulation of multi-organism pr...	17735	54	74	2	0.23	0.02221	8.695652	IFI16;RNASEL
cyan	MF	GO:0004871	signal transducer activity	17735	1632	74	13	7.13	0.0227	1.823282	EMR1;FZD5;GPR19;HCAR1;ICAM1;OR52K2;SHISA5;SORT1;SP110;STAT1;STAT2;TRIM38;TRIM5
cyan	MF	GO:0060089	molecular transducer activity	17735	1632	74	13	7.13	0.0227	1.823282	EMR1;FZD5;GPR19;HCAR1;ICAM1;OR52K2;SHISA5;SORT1;SP110;STAT1;STAT2;TRIM38;TRIM5
cyan	BP	GO:0016525	negative regulation of angiogenesis	17735	55	74	2	0.23	0.02298	8.695652	PML;STAT1
cyan	BP	GO:0045892	negative regulation of transcription, DN...	17735	823	74	8	3.51	0.02298	2.279202	ATF3;IFI16;IRF1;NRARP;PML;SP100;STAT1;TGIF1
cyan	BP	GO:0043123	positive regulation of I-kappaB kinase/N...	17735	143	74	3	0.61	0.0231	4.918033	SHISA5;TRIM38;TRIM5
cyan	BP	GO:0001817	regulation of cytokine production	17735	382	74	5	1.63	0.02322	3.067485	BTN2A2;FZD5;IFI16;IRF1;UBE2L6
cyan	BP	GO:0097194	execution phase of apoptosis	17735	260	74	4	1.11	0.02481	3.603604	IFI16;PML;SEPT4;SORT1
cyan	BP	GO:0019538	protein metabolic process	17735	3895	74	24	16.61	0.02681	1.444913	APOL1;APOL2;APOL4;APOL6;ASPHD2;ATF3;C2;CETP;DTX3L;FBXO6;FZD5;IRF1;IRF9;LAP3;MEX3B;PML;RNASE
cyan	BP	GO:0048534	hematopoietic or lymphoid organ developm...	17735	542	74	6	2.31	0.02752	2.597403	BATF2;FZD5;IFI16;IRF1;NRARP;PML
cyan	BP	GO:0051253	negative regulation of RNA metabolic pro...	17735	852	74	8	3.63	0.02756	2.203857	ATF3;IFI16;IRF1;NRARP;PML;SP100;STAT1;TGIF1
cyan	BP	GO:0019079	viral genome replication	17735	61	74	2	0.26	0.02786	7.692308	IFI16;RNASEL
cyan	BP	GO:0008283	cell proliferation	17735	1542	74	12	6.57	0.02791	1.826484	ATF3;BTN2A2;FANCA;FZD5;GAS8;IFI16;IRF1;NRARP;PML;STAT1;TGIF1;WARS
cyan	BP	GO:0045765	regulation of angiogenesis	17735	157	74	3	0.67	0.02937	4.477612	PML;STAT1;WARS
cyan	BP	GO:0045321	leukocyte activation	17735	552	74	6	2.35	0.02975	2.553191	BATF2;BTN2A2;FZD5;ICAM1;IRF1;NRARP
cyan	BP	GO:0030217	T cell differentiation	17735	160	74	3	0.68	0.03083	4.411765	FZD5;IRF1;NRARP
cyan	MF	GO:0046332	SMAD binding	17735	63	74	2	0.28	0.0309	7.142857	PML;TGIF1
cyan	BP	GO:0050688	regulation of defense response to virus	17735	65	74	2	0.28	0.03133	7.142857	MB21D1;PML
cyan	BP	GO:0002455	humoral immune response mediated by circ...	17735	66	74	2	0.28	0.03222	7.142857	C2;SERPING1
cyan	BP	GO:0006956	complement activation	17735	66	74	2	0.28	0.03222	7.142857	C2;SERPING1
cyan	BP	GO:0032479	regulation of type I interferon producti...	17735	67	74	2	0.29	0.03312	6.896552	IRF1;UBE2L6
cyan	BP	GO:0051101	regulation of DNA binding	17735	67	74	2	0.29	0.03312	6.896552	IFI16;SP100
cyan	BP	GO:0043065	positive regulation of apoptotic process	17735	421	74	5	1.8	0.03341	2.777778	IRF1;PML;SEPT4;SHISA5;STAT1
cyan	BP	GO:0002520	immune system development	17735	572	74	6	2.44	0.03454	2.459016	BATF2;FZD5;IFI16;IRF1;NRARP;PML
cyan	BP	GO:0043068	positive regulation of programmed cell d...	17735	426	74	5	1.82	0.03489	2.747253	IRF1;PML;SEPT4;SHISA5;STAT1
cyan	BP	GO:0007569	cell aging	17735	70	74	2	0.3	0.03589	6.666667	ICAM1;PML

cyan	BP	GO:1901342	regulation of vasculature development	17735	170	74	3	0.72	0.03593	4.166667	PML;STAT1;WARS
cyan	BP	GO:0071356	cellular response to tumor necrosis fact...	17735	71	74	2	0.3	0.03683	6.666667	ICAM1;STAT1
cyan	BP	GO:0001936	regulation of endothelial cell prolifera...	17735	72	74	2	0.31	0.03778	6.451613	NRARP;STAT1
cyan	BP	GO:0023052	signaling	17735	4666	74	27	19.89	0.03818	1.357466	ATF3;CEACAM1;EMR1;FZD5;GBP1;GBP2;GPR19;ICAM1;IFI16;IFI35;IRF1;IRF9;NRARP;PML;RAB20;RNASEL;SE
cyan	BP	GO:0044700	single organism signaling	17735	4666	74	27	19.89	0.03818	1.357466	ATF3;CEACAM1;EMR1;FZD5;GBP1;GBP2;GPR19;ICAM1;IFI16;IFI35;IRF1;IRF9;NRARP;PML;RAB20;RNASEL;SE
cyan	BP	GO:0010629	negative regulation of gene expression	17735	909	74	8	3.88	0.03837	2.061856	ATF3;IFI16;IRF1;NRARP;PML;SP100;STAT1;TGIF1
cyan	BP	GO:0051239	regulation of multicellular organismal p...	17735	1804	74	13	7.69	0.03853	1.690507	BTN2A2;FZD5;ICAM1;IFI16;IRF1;NRARP;PML;SERPING1;SMTNL1;STAT1;TGIF1;UBE2L6;WARS
cyan	CC	GO:0016605	PML body	17735	73	74	2	0.31	0.0394	6.451613	PML;SP100
cyan	CC	GO:0044444	cytoplasmic part	17735	6562	74	36	28.2	0.0399	1.276596	ACOT9;ACP6;ACTA2;APOL2;ASPHD2;ERP27;FZD5;GAS8;GBP1;GBP2;GBP5;IFI35;IRF1;IRF9;LACTB;LAP3;MB21
cyan	BP	GO:0006366	transcription from RNA polymerase II pro...	17735	1444	74	11	6.16	0.04016	1.785714	ETV7;FZD5;IFI16;IRF1;IRF9;NRARP;RNASEL;SP100;STAT1;STAT2;TGIF1
cyan	MF	GO:0003677	DNA binding	17735	2332	74	16	10.18	0.0404	1.571709	ATF3;BATF2;ETV7;IFI16;IRF1;IRF9;MB21D1;PML;RMI2;SP100;SP110;STAT1;STAT2;TGIF1;TRIM21;ZNF883
cyan	BP	GO:0010942	positive regulation of cell death	17735	444	74	5	1.89	0.04055	2.645503	IRF1;PML;SEPT4;SHISA5;STAT1
cyan	CC	GO:0005829	cytosol	17735	2362	74	16	10.15	0.0406	1.576355	ACTA2;GBP1;GBP2;IFI35;IRF1;IRF9;MB21D1;PARP9;PML;PSTPIP2;RNASEL;STAT1;STAT2;TAP1;UBE2L6;WARS
cyan	BP	GO:0051100	negative regulation of binding	17735	75	74	2	0.32	0.04068	6.25	IFI16;SP100
cyan	BP	GO:0006351	transcription, DNA-dependent	17735	3208	74	20	13.68	0.04118	1.461988	ATF3;BATF2;ETV7;FZD5;ICAM1;IFI16;IRF1;IRF9;NRARP;PARP14;PML;RNASEL;SP100;SP110;STAT1;STAT2;TGI
cyan	BP	GO:2000113	negative regulation of cellular macromol...	17735	923	74	8	3.94	0.04142	2.030457	ATF3;IFI16;IRF1;NRARP;PML;SP100;STAT1;TGIF1
cyan	MF	GO:0005319	lipid transporter activity	17735	74	74	2	0.32	0.0415	6.25	APOL6;CETP
cyan	BP	GO:0001775	cell activation	17735	758	74	7	3.23	0.04165	2.167183	BATF2;BTN2A2;FZD5;ICAM1;IRF1;NRARP;SERPING1
cyan	BP	GO:0006895	Golgi to endosome transport	17735	10	74	1	0.04	0.04184	25	SORT1
cyan	BP	GO:0031664	regulation of lipopolysaccharide-mediate...	17735	10	74	1	0.04	0.04184	25	TRIM5
cyan	BP	GO:0036037	CD8-positive, alpha-beta T cell activati...	17735	10	74	1	0.04	0.04184	25	IRF1
cyan	BP	GO:0072109	glomerular mesangium development	17735	10	74	1	0.04	0.04184	25	ACTA2
cyan	BP	GO:0072203	cell proliferation involved in metanephr...	17735	10	74	1	0.04	0.04184	25	STAT1
cyan	BP	GO:0009892	negative regulation of metabolic process	17735	1456	74	11	6.21	0.04227	1.771337	ATF3;BTN2A2;IFI16;IRF1;NRARP;PML;SERPING1;SP100;STAT1;TGIF1;TRIM21
cyan	BP	GO:0051091	positive regulation of sequence-specific...	17735	182	74	3	0.78	0.0426	3.846154	ICAM1;SP100;TRIM5
cyan	BP	GO:0045934	negative regulation of nucleobase-contai...	17735	932	74	8	3.97	0.04346	2.015113	ATF3;IFI16;IRF1;NRARP;PML;SP100;STAT1;TGIF1
cyan	BP	GO:2001233	regulation of apoptotic signaling pathwa...	17735	78	74	2	0.33	0.04367	6.060606	PML;SEPT4
cyan	BP	GO:0072358	cardiovascular system development	17735	768	74	7	3.27	0.04419	2.140673	ACTA2;CEACAM1;FZD5;NRARP;PML;STAT1;WARS
cyan	BP	GO:0072359	circulatory system development	17735	768	74	7	3.27	0.04419	2.140673	ACTA2;CEACAM1;FZD5;NRARP;PML;STAT1;WARS
cyan	BP	GO:0006974	response to DNA damage stimulus	17735	608	74	6	2.59	0.04438	2.316602	DTX3L;FANCA;FBXO6;IFI16;PML;SP100
cyan	BP	GO:0045580	regulation of T cell differentiation	17735	79	74	2	0.34	0.04469	5.882353	IRF1;NRARP
cyan	BP	GO:0006516	glycoprotein catabolic process	17735	11	74	1	0.05	0.04593	20	FBXO6
cyan	BP	GO:0034370	triglyceride-rich lipoprotein particle r...	17735	11	74	1	0.05	0.04593	20	CETP
cyan	BP	GO:0034372	very-low-density lipoprotein particle re...	17735	11	74	1	0.05	0.04593	20	CETP
cyan	BP	GO:0034374	low-density lipoprotein particle remodel...	17735	11	74	1	0.05	0.04593	20	CETP
cyan	BP	GO:0045351	type I interferon biosynthetic process	17735	11	74	1	0.05	0.04593	20	IRF9
cyan	BP	GO:0051972	regulation of telomerase activity	17735	11	74	1	0.05	0.04593	20	PML
cyan	BP	GO:0060192	negative regulation of lipase activity	17735	11	74	1	0.05	0.04593	20	SORT1
cyan	MF	GO:0032183	SUMO binding	17735	11	74	1	0.05	0.047	20	PML
brown	CC	GO:0044424	intracellular part	17735	12103	1802	1408	1232.48	1.10E-25	1.142412	AATF;AATK;ABAT;ABCG1;ACIN1;ACO1;ACOX1;ACP1;ACRC;ACSM1;ACSM3;ACSS2;ACTA1;ACTB;ACTG1;ACTN1
brown	CC	GO:0005622	intracellular	17735	12344	1802	1424	1257.02	3.30E-24	1.132838	AATF;AATK;ABAT;ABCG1;ACIN1;ACO1;ACOX1;ACP1;ACRC;ACSM1;ACSM3;ACSS2;ACTA1;ACTB;ACTG1;ACTN1
brown	CC	GO:0005737	cytoplasm	17735	9016	1802	1112	918.12	7.60E-24	1.211171	AATF;AATK;ABAT;ABCG1;ACIN1;ACO1;ACOX1;ACP1;ACSM1;ACSM3;ACSS2;ACTA1;ACTB;ACTG1;ACTN1;ACYP
brown	CC	GO:0043227	membrane-bounded organelle	17735	9590	1802	1159	976.57	8.80E-22	1.186807	AATF;AATK;ABAT;ABCG1;ACIN1;ACO1;ACOX1;ACP1;ACRC;ACSM1;ACSM3;ACSS2;ACTA1;ACTB;ACTG1;ACTN1
brown	CC	GO:0043231	intracellular membrane-bounded organelle	17735	9560	1802	1151	973.52	1.10E-20	1.182308	AATF;AATK;ABAT;ABCG1;ACIN1;ACO1;ACOX1;ACP1;ACRC;ACSM1;ACSM3;ACSS2;ACTB;ACTN1;ACYP2;ADAP2
brown	CC	GO:0043226	organelle	17735	10552	1802	1238	1074.54	7.40E-19	1.152121	AATF;AATK;ABAT;ABCG1;ACIN1;ACO1;ACOX1;ACP1;ACRC;ACSM1;ACSM3;ACSS2;ACTA1;ACTB;ACTG1;ACTN1
brown	CC	GO:0043229	intracellular organelle	17735	10522	1802	1234	1071.48	1.30E-18	1.151678	AATF;AATK;ABAT;ABCG1;ACIN1;ACO1;ACOX1;ACP1;ACRC;ACSM1;ACSM3;ACSS2;ACTA1;ACTB;ACTG1;ACTN1
brown	CC	GO:0044444	cytoplasmic part	17735	6562	1802	835	668.22	3.40E-18	1.249588	AATF;AATK;ABAT;ABCG1;ACIN1;ACO1;ACOX1;ACSM1;ACSM3;ACSS2;ACTA1;ACTB;ACTG1;ACTN1;ACYP2;ADA
brown	MF	GO:0005515	protein binding	17735	7306	1802	895	757.91	1.50E-13	1.180879	AATF;AATK;ABAT;ABCG1;ACIN1;ACO1;ACOX1;ACP1;ACTA1;ACTB;ACTG1;ACTN1;ACVR1B;ADAM19;ADAMTS
brown	CC	GO:0044446	intracellular organelle part	17735	6219	1802	752	633.3	3.00E-10	1.187431	AATF;ABAT;ABCG1;ACIN1;ACOX1;ACSM1;ACSM3;ACTA1;ACTB;ACTG1;ACTN1;ADAP2;ADAR;AFF2;AGPAT1;A
brown	CC	GO:0044422	organelle part	17735	6321	1802	761	643.68	5.20E-10	1.182264	AATF;ABAT;ABCG1;ACIN1;ACOX1;ACSM1;ACSM3;ACTA1;ACTB;ACTG1;ACTN1;ADAP2;ADAR;AFF2;AGPAT1;A
brown	CC	GO:0044464	cell part	17735	14451	1802	1550	1471.58	6.90E-10	1.05329	AATF;AATK;ABAT;ABCC5;ABCC6;ABCG1;ACIN1;ACO1;ACOX1;ACP1;ACRC;ACSM1;ACSM3;ACSS2;ACTA1;ACTB
brown	CC	GO:0005623	cell	17735	14452	1802	1550	1471.68	7.20E-10	1.053218	AATF;AATK;ABAT;ABCC5;ABCC6;ABCG1;ACIN1;ACO1;ACOX1;ACP1;ACRC;ACSM1;ACSM3;ACSS2;ACTA1;ACTB
brown	BP	GO:0016192	vesicle-mediated transport	17735	894	1802	154	95.97	9.50E-10	1.604668	ACTN1;ADRBK2;AHI1;ALB;ANXA11;AP1M1;AP2A1;AP2M1;AP3B1;APOA1;APP;ARAP3;ARCN1;ARF1;ARF3;ARF
brown	MF	GO:0005488	binding	17735	11799	1802	1314	1224	1.40E-09	1.073529	AATF;AATK;ABAT;ABCC5;ABCC6;ABCG1;ACIN1;ACO1;ACOX1;ACP1;ACSM1;ACSM3;ACSS2;ACTA1;ACTB;ACTG
brown	CC	GO:0005829	cytosol	17735	2362	1802	324	240.53	2.10E-09	1.347025	ACIN1;ACO1;ACSS2;ACTA1;ACTB;ACTG1;ACTN1;ADAL;ADK;ADSL;AGBL2;AKAP13;ALDH1A1;ALDH9A1;ALDOC
brown	BP	GO:0007264	small GTPase mediated signal transductio...	17735	619	1802	111	66.45	2.80E-08	1.670429	ADAP2;AKAP13;ALDH1A1;APOA1;ARAP1;ARAP3;ARF1;ARF3;ARF6;ARFGAP3;ARHGAP1;ARHGAP24;ARHGAP2
brown	BP	GO:0006897	endocytosis	17735	362	1802	74	38.86	3.00E-08	1.904272	ADRBK2;AHI1;ANXA11;AP2A1;AP3B1;APP;ARF6;ARHGAP27;ATXN2;BIN2;C3;CAMK1D;CAP1;CBL;CD14;CD20
brown	BP	GO:0002376	immune system process	17735	1816	1802	264	194.95	4.40E-08	1.354193	ACIN1;ACVR1B;ADAR;ADK;AMICA1;AP1M1;AP2A1;AP2M1;AP3B1;APOA1;APOBEC3C;APP;ARF1;ARHGDIB;AI
brown	CC	GO:0005773	vacuole	17735	402	1802	76	40.94	7.00E-08	1.856375	AGA;ANXA11;AP1M1;AP2M1;ARSD;ARSG;ASAH1;ATP6V0A1;ATP6V0C;ATP6V0D1;CD1D;CD68;CTSA;CTSB;CT
brown	CC	GO:0031982	vesicle	17735	993	1802	152	101.12	1.30E-07	1.503165	ACTA1;ACTB;ACTG1;ACTN1;ALB;ANXA11;ANXA5;ANXA7;AP1M1;AP2A1;AP2M1;AP3B1;APOA1;APP;ARCN1;A
brown	CC	GO:0005739	mitochondrion	17735	1488	1802	212	151.53	1.30E-07	1.399063	AATK;ABAT;ABCG1;ACO1;ACOX1;ACSM1;ACSM3;ACYP2;ADAP2;ADH5;ADO;ADSL;ALAS1;ALDH2;ALDH9A1;AI
brown	CC	GO:0031090	organelle membrane	17735	2366	1802	314	240.94	1.30E-07	1.303229	ABCG1;ACOX1;AGPAT1;ALG14;ALG5;ANKFY1;ANXA7;AP1M1;AP2A1;AP2M1;AP3B1;APC2;ARAP1;ARCN1;AR
brown	CC	GO:0000323	lytic vacuole	17735	337	1802	65	34.32	2.90E-07	1.893939	AGA;ANXA11;AP1M1;AP2M1;ARSD;ARSG;ASAH1;CD1D;CD68;CTSA;CTSB;CTSD;CTSZ;CXCR2;GALNS;GBA;GLB
brown	CC	GO:0005764	lysosome	17735	337	1802	65	34.32	2.90E-07	1.893939	AGA;ANXA11;AP1M1;AP2M1;ARSD;ARSG;ASAH1;CD1D;CD68;CTSA;CTSB;CTSD;CTSZ;CXCR2;GALNS;GBA;GLB
brown	BP	GO:0030097	hemopoiesis	17735	509	1802	92	54.64	3.10E-07	1.683748	ACIN1;ACVR1B;AP3B1;ARNT;ASH2L;ATPIF1;BCL6;BTK;CBFA2T3;CCL3;CD1D;CD3D;CD86;CDC42;CITED2;CNN2
brown	MF	GO:0019899	enzyme binding	17735	1084	1802	163	112.45	4.30E-07	1.449533	ABAT;ACIN1;ACTB;ACVR1B;ADAMTSL4;ALB;ANAPC4;ANKRA2;AP3B1;APOA1;ARF6;ATF7;ATP6V0A1;ATP6V0
brown	BP	GO:0046907	intracellular transport	17735	1214	1802	184	130.32	4.30E-07	1.411909	ABCG1;ADAR;AKR1C3;ANP32A;AP1M1;AP2A1;AP2M1;AP3B1;APC2;APP;ARCN1;ARF1;ARFGAP3;ARHGAP21;
brown	CC	GO:0005634	nucleus	17735	5873	1802	691	598.06	4.70E-07	1.155402	AATF;ACIN1;ACP1;ACRC;ACSS2;ACTB;ACTN1;ADAR;ADH5;ADK;AFF2;AKAP13;AKR1C3;ALAS1;ALKBH2;ALKBH
brown	BP	GO:0009611	response to wounding	17735	1090	1802	168	117.01	4.90E-07	1.435775	ABHD2;ACTB;ACTG1;ACTN1;ALB;AMICA1;ANXA5;AOAH;AP3B1;APBB1IP;APOA1;APP;ATP2B4;B4GALT1;BCL6

brown	CC	GO:0030139	endocytic vesicle	17735	163	1802	38	16.6	8.00E-07	2.289157	ANXA11;AP2A1;AP2M1;APOA1;ATP6V0A1;ATP6V0C;ATP6V0D1;CACNG8;CAMK2G;CLTA;CLTC;EHD1;EHD3;F
brown	BP	GO:0006950	response to stress	17735	2926	1802	388	314.11	8.10E-07	1.235236	AATF;ABAT;ABHD2;ACTB;ACTG1;ACTN1;ADAR;ADH5;ADSL;AKR1C3;ALB;ALDH3B1;ALDOC;ALKBH2;ALKBH3;A
brown	BP	GO:0048534	hematopoietic or lymphoid organ developm...	17735	542	1802	95	58.18	8.30E-07	1.632864	ACIN1;ACVR1B;AP3B1;ARNT;ASH2L;ATPIF1;BCL6;BTK;CBFA2T3;CCL3;CD1D;CD3D;CD86;CDC42;CITED2;CNN2
brown	BP	GO:0050817	coagulation	17735	501	1802	89	53.78	1.00E-06	1.65489	ACTB;ACTG1;ACTN1;ALB;AMICA1;ANXA5;AP3B1;APBB1IP;APOA1;APP;ATP2B4;BLOC1S3;BRPF3;BSG;C4BPB;
brown	BP	GO:0007599	hemostasis	17735	502	1802	89	53.89	1.10E-06	1.651512	ACTB;ACTG1;ACTN1;ALB;AMICA1;ANXA5;ANXA7;AP3B1;APBB1IP;APOA1;APP;ATP2B4;BLOC1S3;BRPF3;BSG;
brown	BP	GO:0001775	cell activation	17735	758	1802	123	81.37	1.40E-06	1.511614	ACTN1;ADK;ALB;AP3B1;APBB1IP;APOA1;APP;BCL6;BLOC1S3;BRPF3;BTK;BTN3A1;CAP1;CCL3;CD1C;CD1D;CD
brown	BP	GO:0007596	blood coagulation	17735	498	1802	88	53.46	1.50E-06	1.646091	ACTB;ACTG1;ACTN1;ALB;AMICA1;ANXA5;AP3B1;APBB1IP;APOA1;APP;ATP2B4;BLOC1S3;BRPF3;BSG;C4BPB;
brown	BP	GO:0015031	protein transport	17735	1245	1802	185	133.65	1.50E-06	1.384212	ABCG1;ADAR;AKR1C3;AP1M1;AP2A1;AP2M1;AP3B1;APC2;APOA1;ARCN1;ARF1;ARF3;ARF6;ARFGAP3;ARL6;
brown	BP	GO:0002520	immune system development	17735	572	1802	98	61.41	1.60E-06	1.595831	ACIN1;ACVR1B;AP3B1;ARNT;ASH2L;ATPIF1;BCL6;BTK;CBFA2T3;CCL3;CD1D;CD3D;CD86;CDC42;CITED2;CNN2
brown	CC	GO:0031988	membrane-bounded vesicle	17735	905	1802	136	92.16	1.70E-06	1.475694	ACTA1;ACTB;ACTG1;ACTN1;ALB;ANXA11;ANXA5;ANXA7;AP1M1;AP2A1;AP2M1;AP3B1;APOA1;APP;ARCN1;A
brown	BP	GO:0002682	regulation of immune system process	17735	884	1802	139	94.9	1.70E-06	1.4647	ACIN1;ACVR1B;ADK;AMICA1;AP1M1;AP2A1;AP2M1;AP3B1;APOA1;ARF1;ARNT;BCL6;BLOC1S3;BTK;BTN3A1;
brown	BP	GO:0051649	establishment of localization in cell	17735	1820	1802	255	195.38	1.80E-06	1.305149	ABAT;ABCG1;ACTN1;ADAR;AKR1C3;ALB;ANP32A;AP1M1;AP2A1;AP2M1;AP3B1;APC2;APOA1;APP;ARCN1;AI
brown	BP	GO:0033036	macromolecule localization	17735	1852	1802	258	198.82	2.50E-06	1.297656	ABCG1;ADAR;AHI1;AKR1C3;AP1M1;AP2A1;AP2M1;AP3B1;APC2;APOA1;APOBR;ARCN1;ARF1;ARF3;ARF6;AR
brown	MF	GO:0008047	enzyme activator activity	17735	404	1802	72	41.91	3.00E-06	1.717967	ADAP2;ALDH1A1;APOA1;APP;ARAP1;ARAP3;ARFGAP3;ARHGAP1;ARHGAP21;ARHGAP23;ARHGAP24;ARHGA
brown	BP	GO:0030099	myeloid cell differentiation	17735	247	1802	51	26.52	3.10E-06	1.923077	ACIN1;ACVR1B;ARNT;ATPIF1;BCL6;CBFA2T3;CCL3;CDC42;CITED2;CSF1;CSF1R;CTNNB1;DAB2;G6PD;GAB2;GN
brown	CC	GO:0031252	cell leading edge	17735	260	1802	51	26.48	3.30E-06	1.925982	ACTN1;APBB1IP;APC2;ARAP3;ARF6;ARHGAP1;ARHGAP31;BRCA1;CAPZB;CFL1;CTNNA1;CTNNB1;CTNND1;CY
brown	BP	GO:0007265	Ras protein signal transduction	17735	378	1802	70	40.58	3.30E-06	1.724988	ADAP2;AKAP13;ALDH1A1;APOA1;ARAP1;ARAP3;ARF6;ARFGAP3;ARHGAP1;ARHGAP26;ARHGAP27;ARHGDIB
brown	CC	GO:0031974	membrane-enclosed lumen	17735	2770	1802	349	282.08	4.10E-06	1.237238	AATF;ABAT;ACIN1;ACOX1;ACSM1;ACSM3;ACTB;ACTN1;ADAR;AFF2;ALAS1;ALB;ALDH2;ALKBH2;ALKBH3;ANA
brown	CC	GO:0031410	cytoplasmic vesicle	17735	914	1802	135	93.07	4.70E-06	1.450521	ACTN1;ALB;ANXA11;ANXA7;AP1M1;AP2A1;AP2M1;AP3B1;APOA1;APP;ARCN1;ARHGAP21;ARHGAP30;ARHG
brown	MF	GO:0008013	beta-catenin binding	17735	60	1802	19	6.22	5.70E-06	3.054662	APC2;BCL9;CALCOCO1;CARM1;CBY1;CDH1;CHD8;CSNK2A1;CTNNA1;DVL3;EP300;ESR1;MED12;NUMB;PSEN
brown	BP	GO:0030168	platelet activation	17735	219	1802	46	23.51	5.80E-06	1.956614	ACTN1;ALB;APBB1IP;APOA1;APP;BLOC1S3;BRPF3;CAP1;CFL1;DGKG;F13A1;F2RL2;GNA11;GNA12;GNA15;GN
brown	BP	GO:0006909	phagocytosis	17735	96	1802	26	10.31	6.00E-06	2.521823	ANXA11;BIN2;C3;CAMK1D;CD14;CD93;CDC42SE1;CNN2;COLEC12;CORO1C;DOCK2;ELMO1;ELMO2;FCN1;HC
brown	BP	GO:0008219	cell death	17735	1741	1802	242	186.9	6.30E-06	1.29481	AATF;AATK;ACIN1;ACTN1;ACVR1B;ADAMTSL4;ADAR;AHI1;AKAP13;AKR1C3;ALB;ALDOC;ALOX15B;AMIGO2;A
brown	BP	GO:0016265	death	17735	1743	1802	242	187.11	6.90E-06	1.293357	AATF;AATK;ACIN1;ACTN1;ACVR1B;ADAMTSL4;ADAR;AHI1;AKAP13;AKR1C3;ALB;ALDOC;ALOX15B;AMIGO2;A
brown	CC	GO:0043233	organelle lumen	17735	2722	1802	342	277.19	6.90E-06	1.233811	AATF;ABAT;ACIN1;ACOX1;ACSM1;ACSM3;ACTB;ACTN1;ADAR;AFF2;ALAS1;ALB;ALDH2;ALKBH2;ALKBH3;ANA
brown	BP	GO:0006007	glucose catabolic process	17735	75	1802	22	8.05	7.70E-06	2.732919	ALDOC;ARNT;DHTKD1;ENO1;FBP1;G6PD;GAPDHS;GCK;GLYR1;HDAC4;HIBADH;HK1;HK2;INSR;NCOR1;PFKFB
brown	BP	GO:0051641	cellular localization	17735	2049	1802	278	219.96	8.20E-06	1.263866	ABAT;ABCG1;ACTN1;ADAR;AHI1;AKR1C3;ALB;ANP32A;AP1M1;AP2A1;AP2M1;AP3B1;APC2;APOA1;APP;ARC
brown	BP	GO:0048583	regulation of response to stimulus	17735	2347	1802	313	251.95	8.50E-06	1.24231	ACVR1B;ADAP2;ADRBK2;AGPAT1;AHI1;AKAP13;AKR1C3;ALB;ALDH1A1;AMICA1;AOAH;AP1M1;AP2A1;AP2M
brown	BP	GO:0042060	wound healing	17735	610	1802	100	65.48	9.00E-06	1.527184	ACTB;ACTG1;ACTN1;ALB;AMICA1;ANXA5;AP3B1;APBB1IP;APOA1;APP;ATP2B4;B4GALT1;BCL9;BLOC1S3;BRP
brown	BP	GO:0045184	establishment of protein localization	17735	1288	1802	186	138.27	9.00E-06	1.345194	ABCG1;ADAR;AKR1C3;AP1M1;AP2A1;AP2M1;AP3B1;APC2;APOA1;ARCN1;ARF1;ARF3;ARF6;ARFGAP3;ARL6;
brown	CC	GO:0070013	intracellular organelle lumen	17735	2677	1802	336	272.61	9.30E-06	1.23253	AATF;ABAT;ACIN1;ACOX1;ACSM1;ACSM3;ACTB;ADAR;AFF2;ALAS1;ALDH2;ALKBH2;ALKBH3;ANAPC4;ANP32
brown	BP	GO:0009267	cellular response to starvation	17735	87	1802	24	9.34	9.70E-06	2.569593	AKR1C3;ALB;ATG7;BECN1;CTSD;DAP;FBXO22;GCK;HFE;HSPA5;KIAA1324;LZTS1;MAX;NBR1;NUAK2;PRKCD;P
brown	MF	GO:0050839	cell adhesion molecule binding	17735	53	1802	17	5.5	1.40E-05	3.090909	AMICA1;CD1D;CDH1;CTNNA1;CTNNAL1;CTNNB1;CTNND1;LILRB2;MSN;NDRG1;NUMB;PSEN1;PVR;PVRL1;SE
brown	BP	GO:0050878	regulation of body fluid levels	17735	594	1802	97	63.77	1.40E-05	1.521091	ACTB;ACTG1;ACTN1;ALB;AMICA1;ANXA5;ANXA7;AP3B1;APBB1IP;APOA1;APP;ATP2B4;BLOC1S3;BRPF3;BSG;
brown	BP	GO:0010468	regulation of gene expression	17735	3347	1802	426	359.31	1.70E-05	1.185606	AATF;ABCG1;ACO1;ACVR1B;ADAR;ADK;AFF2;AHI1;ALKBH2;ALKBH3;ANP32A;APOA1;APOBEC3C;APP;ARHGE
brown	CC	GO:0030055	cell-substrate junction	17735	138	1802	31	14.05	1.80E-05	2.206406	AATF;ACTN1;AFAP1;APBB1IP;ARHGAP24;ARHGAP26;ARHGAP31;BRCA1;CASS4;CDH1;CTNNB1;DAG1;HCK;IR
brown	BP	GO:0050794	regulation of cellular process	17735	7700	1802	904	826.61	1.80E-05	1.093623	AATF;ABCG1;ABHD2;ACIN1;ACO1;ACTN1;ACVR1B;ADAMTSL4;ADAP2;ADAR;ADCY4;ADK;ADRBK2;AFF2;AGP
brown	BP	GO:0050789	regulation of biological process	17735	8134	1802	950	873.2	1.80E-05	1.087952	AATF;ABCG1;ABHD2;ACIN1;ACO1;ACOX1;ACTN1;ACVR1B;ADAMTSL4;ADAP2;ADAR;ADCY4;ADK;ADRBK2;AF
brown	CC	GO:0005924	cell-substrate adherens junction	17735	132	1802	30	13.44	1.90E-05	2.232143	AATF;ACTN1;AFAP1;APBB1IP;ARHGAP24;ARHGAP26;ARHGAP31;BRCA1;CASS4;CDH1;CTNNB1;DAG1;HCK;IR
brown	BP	GO:0008104	protein localization	17735	1586	1802	220	170.26	2.10E-05	1.292141	ABCG1;ADAR;AHI1;AKR1C3;AP1M1;AP2A1;AP2M1;AP3B1;APC2;APOA1;ARCN1;ARF1;ARF3;ARF6;ARFGAP3;
brown	CC	GO:0005925	focal adhesion	17735	127	1802	29	12.93	2.40E-05	2.242846	AATF;ACTN1;AFAP1;APBB1IP;ARHGAP24;ARHGAP26;ARHGAP31;BRCA1;CASS4;CDH1;DAG1;HCK;IRF2;ITGA5
brown	BP	GO:0002521	leukocyte differentiation	17735	329	1802	60	35.32	2.60E-05	1.698754	ACIN1;AP3B1;BCL6;BTK;CBFA2T3;CCL3;CD1D;CD3D;CD86;CDC42;CITED2;CSF1;CSF1R;CTNNB1;DOCK2;EGR1;
brown	CC	GO:0005654	nucleoplasm	17735	1481	1802	197	150.81	3.50E-05	1.306279	ACIN1;ACTB;ADAR;AFF2;ALKBH2;ALKBH3;ANAPC4;ANP32A;ANXA11;APITD1;ARNT;ASH2L;ATF5;ATF7;BASP1
brown	BP	GO:0006996	organelle organization	17735	2139	1802	284	229.63	3.50E-05	1.236772	ACIN1;ACOX1;ACTA1;ACTG1;ACTN1;ANAPC16;ANAPC4;AP1M1;AP3B1;APITD1;ARAP1;ARAP3;ARCN1;ARF1;
brown	BP	GO:0030100	regulation of endocytosis	17735	124	1802	29	13.31	3.90E-05	2.178813	AHI1;ARF6;ATXN2;C3;CAMK1D;CBL;CD14;CNN2;DAB2;DOCK2;HCK;HIP1;HNRNPK;LILRB1;MAGI2;MERTK;NO
brown	CC	GO:0044428	nuclear part	17735	2482	1802	309	252.75	4.50E-05	1.222552	AATF;ACIN1;ACTB;ADAR;AFF2;ALKBH2;ALKBH3;ANAPC16;ANAPC4;ANP32A;ANXA11;ANXA7;APITD1;ARID1A
brown	BP	GO:0019320	hexose catabolic process	17735	89	1802	23	9.55	4.60E-05	2.408377	ALDOC;ARNT;DHTKD1;ENO1;FBP1;G6PD;GAPDHS;GCK;GLB1;GLYR1;HDAC4;HIBADH;HK1;HK2;INSR;NCOR1;
brown	BP	GO:0012501	programmed cell death	17735	1575	1802	216	169.08	5.10E-05	1.277502	AATF;AATK;ACIN1;ACTN1;ACVR1B;ADAMTSL4;ADAR;AHI1;AKAP13;AKR1C3;ALB;ALDOC;ALOX15B;AMIGO2;A
brown	BP	GO:0006810	transport	17735	3358	1802	423	360.49	5.20E-05	1.173403	ABAT;ABCC5;ABCC6;ABCG1;ACTN1;ADAR;ADCY4;ADRBK2;AHI1;AKR1C3;ALB;AMICA1;ANP32A;ANXA11;AP1
brown	BP	GO:0019222	regulation of metabolic process	17735	4834	1802	588	518.94	5.50E-05	1.133079	AATF;ABCG1;ACO1;ACVR1B;ADAP2;ADAR;ADCY4;ADK;AFF2;AGPAT1;AHI1;AKR1C3;ALDH1A1;ALKBH2;ALKB
brown	BP	GO:0060255	regulation of macromolecule metabolic pr...	17735	4178	1802	515	448.52	5.60E-05	1.148221	AATF;ABCG1;ACO1;ACVR1B;ADAR;ADCY4;ADK;AFF2;AHI1;ALKBH2;ALKBH3;ANAPC4;ANP32A;APITD1;APLP2;
brown	BP	GO:0002263	cell activation involved in immune respo...	17735	133	1802	30	14.28	5.90E-05	2.10084	BCL6;CCL3;CD1C;CD86;DOCK2;EXOSC3;FOXP3;GAB2;GPR183;HMOX1;IL23A;IL4R;JAK3;LAT2;LCP1;LILRB1;MY
brown	BP	GO:0002366	leukocyte activation involved in immune ...	17735	133	1802	30	14.28	5.90E-05	2.10084	BCL6;CCL3;CD1C;CD86;DOCK2;EXOSC3;FOXP3;GAB2;GPR183;HMOX1;IL23A;IL4R;JAK3;LAT2;LCP1;LILRB1;MY
brown	BP	GO:0051701	interaction with host	17735	411	1802	70	44.12	5.90E-05	1.586582	ALB;ANPEP;AP1M1;APOBEC3C;ATF7;ATP6V0A1;ATP6V0C;ATP6V0D1;ATP6V1B2;BECN1;BNIP3;BRD4;CARM1
brown	MF	GO:0005096	GTPase activator activity	17735	247	1802	46	25.62	6.00E-05	1.795472	ADAP2;ALDH1A1;ARAP1;ARAP3;ARFGAP3;ARHGAP1;ARHGAP21;ARHGAP23;ARHGAP24;ARHGAP25;ARHGA
brown	BP	GO:0006915	apoptotic process	17735	1562	1802	214	167.68	6.00E-05	1.27624	AATF;AATK;ACIN1;ACTN1;ACVR1B;ADAMTSL4;ADAR;AHI1;AKAP13;AKR1C3;ALB;ALDOC;ALOX15B;AMIGO2;A
brown	CC	GO:0042470	melanosome	17735	95	1802	23	9.67	6.10E-05	2.37849	ANXA11;ATP6V0A1;ATP6V1B2;BSG;CLTC;CTSB;CTSD;DNAJC5;HSPA5;LAMP1;MMP14;MYH11;MYO7A;NCSTN
brown	CC	GO:0048770	pigment granule	17735	95	1802	23	9.67	6.10E-05	2.37849	ANXA11;ATP6V0A1;ATP6V1B2;BSG;CLTC;CTSB;CTSD;DNAJC5;HSPA5;LAMP1;MMP14;MYH11;MYO7A;NCSTN
brown	MF	GO:0005543	phospholipid binding	17735	487	1802	78	50.52	6.20E-05	1.543943	ABCG1;ADAP2;ADRBK2;AFAP1;AKAP13;ANXA11;ANXA4;ANXA5;ANXA7;ANXA9;APBB1IP;APOA1;ARAP1;ARA
brown	BP	GO:0007169	transmembrane receptor protein tyrosine ...	17735	625	1802	98	67.09	6.80E-05	1.460724	AATF;ADCY4;AHI1;AKAP13;ANKS1A;AP2A1;AP2M1;APLP2;APP;ARHGEF11;ARHGEF12;ARNT;ATP6V0A1;ATP6
brown	CC	GO:0016023	cytoplasmic membrane-bounded vesicle	17735	848	1802	121	86.35	7.10E-05	1.401274	ACTN1;ALB;ANXA11;ANXA7;AP1M1;AP2A1;AP2M1;AP3B1;APOA1;APP;ARCN1;ARHGAP21;ARHGAP30;ARHG
brown	CC	GO:0005912	adherens junction	17735	196	1802	38	19.96	7.30E-05	1.903808	AATF;ACTN1;AFAP1;AHI1;APBB1IP;ARHGAP24;ARHGAP26;ARHGAP31;BRCA1;CASS4;CDH1;CTNNA1;CTNNB
brown	CC	GO:0005768	endosome	17735	546	1802	84	55.6	7.50E-05	1.510791	ABCG1;ANKFY1;ARF6;ATP6V0A1;ATP6V0C;ATP6V0D1;CD14;CD1C;CD1D;CD68;CDH1;CHMP4B;CLCN5;CTSB;(
brown	BP	GO:0031323	regulation of cellular metabolic process	17735	4397	1802	538	472.03	7.80E-05	1.139758	AATF;ABCG1;ACO1;ACVR1B;ADAP2;ADAR;ADCY4;AFF2;AGPAT1;AHI1;AKR1C3;ALDH1A1;ANAPC4;ANP32A;A
brown	BP	GO:0016052	carbohydrate catabolic process	17735	135	1802	30	14.49	8.00E-05	2.070393	ALDOC;ARNT;DHTKD1;ENO1;FBP1;G6PD;GAPDHS;GCK;GLB1;GLYR1;GM2A;GSK3A;HDAC4;HEXB;HIBADH;HK
brown	BP	GO:0065007	biological regulation	17735	8616	1802	994	924.94	8.60E-05	1.074664	AATF;ABAT;ABCG1;ABHD2;ACIN1;ACO1;ACOX1;ACSM1;ACSM3;ACTB;ACTG1;ACTN1;ACVR1B;ADAMTSL4;AD
brown	BP	GO:0051179	localization	17735	4195	1802	515	450.34	8.70E-05	1.14358	ABAT;ABCC5;ABCC6;ABCG1;ABHD2;ACTN1;ACVR1B;ADAR;ADCY4;ADRBK2;AHI1;AKR1C3;ALB;ALOX15B;AMI
brown	MF	GO:0043169	cation binding	17735	4026	1802	481	417.65	9.00E-05	1.151682	ACO1;ACSM1;ACSM3;ACTN1;ACVR1B;ADAL;ADAM19;ADAP2;ADAR;ADCY4;ADH5;ADK;ADO;ADPRH;AGBL2;A
brown	BP	GO:0035556	intracellular signal transduction	17735	1936	1802	257	207.83	9.10E-05	1.236588	ADAP2;ADCY4;AKAP13;AKR1C3;ALDH1A1;ANP32A;APOA1;ARAP1;ARAP3;ARF1;ARF3;ARF6;ARFGAP3;ARHGA

brown	BP	GO:0044724	single-organism carbohydrate catabolic p...	17735	130	1802	29	13.96	9.70E-05	2.077364 ALDOC;ARNT;DHTKD1;ENO1;FBP1;G6PD;GAPDHS;GCK;GLB1;GLYR1;GM2A;GSK3A;HDAC4;HEXB;HIBADH;HK
brown	CC	GO:0031981	nuclear lumen	17735	2101	1802	264	213.95	9.70E-05	1.233933 AATF;ACIN1;ACTB;ADAR;AFF2;ALKBH2;ALKBH3;ANAPC4;ANP32A;ANXA11;APITD1;ARID1A;ARNT;ASH2L;ATF
brown	BP	GO:0016236	macroautophagy	17735	43	1802	14	4.62	9.90E-05	3.030303 ATG7;BECN1;CTSD;KIAA1324;LZTS1;NBR1;PSEN1;RAB23;SQSTM1;TBC1D14;TP53INP2;ULK1;WIPI1;WIPI2
brown	CC	GO:0070062	extracellular vesicular exosome	17735	62	1802	17	6.31	0.00011	2.694136 ACTA1;ACTB;ACTG1;ACTN1;ALB;ANXA5;C3;CLTC;ENO1;GSN;LTBP2;MSN;MYH9;MYOF;TGFBI;TLN1;VIM
brown	BP	GO:0046365	monosaccharide catabolic process	17735	94	1802	23	10.09	0.00011	2.279485 ALDOC;ARNT;DHTKD1;ENO1;FBP1;G6PD;GAPDHS;GCK;GLB1;GLYR1;HDAC4;HIBADH;HK1;HK2;INSR;NCOR1;
brown	CC	GO:0001726	ruffle	17735	124	1802	27	12.63	0.00011	2.137767 ACTN1;ARAP3;ARF6;ARHGAP1;BRCA1;CFL1;CYFIP1;DBNL;EMR2;GAS7;GNAS;GSN;ITGA5;LCP1;MTMR14;MY
brown	CC	GO:0070161	anchoring junction	17735	214	1802	40	21.79	0.00011	1.835704 AATF;ACTN1;AFAP1;AHI1;APBB1IP;ARHGAP24;ARHGAP26;ARHGAP31;B4GALT1;BRCA1;CASS4;CDH1;CTNNA
brown	CC	GO:0010008	endosome membrane	17735	311	1802	53	31.67	0.00012	1.673508 ANKFY1;ARF6;ATP6V0A1;ATP6V0C;ATP6V0D1;CD14;CD1C;CD1D;CD68;CHMP4B;CLCN5;ECE1;EHD1;EHD3;E
brown	MF	GO:0016301	kinase activity	17735	762	1802	111	79.05	0.00012	1.404175 AATK;ACVR1B;ADK;ADRBK2;AKAP13;ALPK1;ALPK3;BTK;CAMK1D;CAMK2G;CAMKK2;CASS4;CCL3;CCNK;CDKL
brown	BP	GO:0042059	negative regulation of epidermal growth ...	17735	39	1802	13	4.19	0.00013	3.102625 AP2A1;AP2M1;CBL;CDC42;CLTA;CLTC;EPS15L1;GRB2;PSEN1;PTPRJ;RAB7A;SH3KBP1;SPRY2
brown	BP	GO:1901185	negative regulation of ERBB signaling pa...	17735	39	1802	13	4.19	0.00013	3.102625 AP2A1;AP2M1;CBL;CDC42;CLTA;CLTC;EPS15L1;GRB2;PSEN1;PTPRJ;RAB7A;SH3KBP1;SPRY2
brown	BP	GO:0031669	cellular response to nutrient levels	17735	113	1802	26	12.13	0.00013	2.143446 AKR1C3;ALB;ATG7;BECN1;CTSD;DAP;FBXO22;GCK;HFE;HMOX1;HSPA5;KIAA1324;LZTS1;MAX;NBR1;NUAK2;
brown	CC	GO:0044437	vacuolar part	17735	252	1802	45	25.66	0.00013	1.753702 AP1M1;AP2M1;ASAH1;ATP6V0A1;ATP6V0C;ATP6V0D1;CD1D;CD68;CTSA;CTSB;CTSD;DAPK2;GALNS;GBA;GL
brown	MF	GO:0016773	phosphotransferase activity, alcohol gro...	17735	706	1802	104	73.24	0.00013	1.419989 AATK;ACVR1B;ADK;ADRBK2;AKAP13;ALPK1;ALPK3;BTK;CAMK1D;CAMK2G;CAMKK2;CASS4;CCL3;CCNK;CDKL
brown	MF	GO:0051015	actin filament binding	17735	68	1802	18	7.05	0.00014	2.553191 ACTN1;CORO1C;CTNNA1;CYFIP1;DBNL;ERMN;FSCN1;GAS7;LASP1;LCP1;MAP1S;MYH9;MYO7A;RCSD1;SPTB;
brown	CC	GO:0044440	endosomal part	17735	320	1802	54	32.59	0.00014	1.65695 ANKFY1;ARF6;ATP6V0A1;ATP6V0C;ATP6V0D1;CD14;CD1C;CD1D;CD68;CHMP4B;CLCN5;CTSB;ECE1;EHD1;EH
brown	BP	GO:0044403	symbiosis, encompassing mutualism throug...	17735	445	1802	73	47.77	0.00014	1.528156 ALB;ANPEP;AP1M1;APOBEC3C;ATF7;ATP6V0A1;ATP6V0C;ATP6V0D1;ATP6V1B2;BECN1;BNIP3;BRD4;CARM1
brown	BP	GO:0044419	interspecies interaction between organis...	17735	445	1802	73	47.77	0.00014	1.528156 ALB;ANPEP;AP1M1;APOBEC3C;ATF7;ATP6V0A1;ATP6V0C;ATP6V0D1;ATP6V1B2;BECN1;BNIP3;BRD4;CARM1
brown	BP	GO:0045321	leukocyte activation	17735	552	1802	87	59.26	0.00014	1.468107 ADK;AP3B1;BCL6;BLOC1S3;BTK;BTN3A1;CCL3;CD1C;CD1D;CD209;CD3D;CD86;CD93;CDC42;CHRNB2;CSF1;C
brown	BP	GO:0006098	pentose-phosphate shunt	17735	13	1802	7	1.4	0.00015	5 G6PD;GLYR1;HIBADH;PGAM1;PGD;TALDO1;TKT
brown	MF	GO:0004672	protein kinase activity	17735	595	1802	90	61.72	0.00015	1.458198 AATK;ACVR1B;ADRBK2;AKAP13;ALPK1;ALPK3;BTK;CAMK1D;CAMK2G;CAMKK2;CASS4;CCL3;CCNK;CDKL3;CH
brown	BP	GO:0009987	cellular process	17735	12861	1802	1423	1380.65	0.00015	1.030674 AATF;AATK;ABAT;ABCC5;ABCC6;ABCG1;ABHD2;ACIN1;ACO1;ACOX1;ACP1;ACSM1;ACSM3;ACSS2;ACTA1;AC
brown	BP	GO:0016197	endosomal transport	17735	140	1802	30	15.03	0.00016	1.996008 AP1M1;AP2A1;ARL1;BLOC1S3;CHMP4B;DAB2;DENND3;EHD1;EHD3;ERC1;FAM160A2;FAM21A;FAM21C;GO
brown	BP	GO:0048193	Golgi vesicle transport	17735	181	1802	36	19.43	0.00018	1.852805 AP1M1;AP2A1;ARCN1;ARF1;ARL1;BLOC1S3;CLTA;CLTC;COPA;COPB2;CUX1;DNM2;ERGIC1;GCC1;GOLGA5;G
brown	BP	GO:0016568	chromatin modification	17735	434	1802	71	46.59	0.00019	1.523932 ARID1A;ASH2L;BAZ2A;BCOR;BCORL1;BNIP3;BRCA1;BRD3;BRD4;BRD8;BRE;BRPF3;CARM1;CBX6;CENPH;CHD
brown	MF	GO:0046872	metal ion binding	17735	3936	1802	468	408.31	0.00019	1.146188 ACO1;ACSM1;ACSM3;ACTN1;ACVR1B;ADAL;ADAM19;ADAP2;ADAR;ADCY4;ADH5;ADK;ADO;ADPRH;AGBL2;
brown	CC	GO:0043230	extracellular organelle	17735	65	1802	17	6.62	2.00E-04	2.567976 ACTA1;ACTB;ACTG1;ACTN1;ALB;ANXA5;C3;CLTC;ENO1;GSN;LTBP2;MSN;MYH9;MYOF;TGFBI;TLN1;VIM
brown	CC	GO:0065010	extracellular membrane-bounded organelle	17735	65	1802	17	6.62	2.00E-04	2.567976 ACTA1;ACTB;ACTG1;ACTN1;ALB;ANXA5;C3;CLTC;ENO1;GSN;LTBP2;MSN;MYH9;MYOF;TGFBI;TLN1;VIM
brown	BP	GO:0007033	vacuole organization	17735	62	1802	17	6.66	2.00E-04	2.552553 BECN1;CTSD;FAM160A2;FIG4;GBA;HEXB;KIAA1324;MYO7A;PPT1;PSEN1;RAB23;TBC1D14;TP53INP2;TPP1;U
brown	CC	GO:0030027	lamellipodium	17735	122	1802	26	12.42	2.00E-04	2.093398 APBB1IP;APC2;ARAP3;ARHGAP31;CAPZB;CFL1;CTNNA1;CTNNB1;CTNND1;CYFIP1;DAG1;DBNL;DYSF;GSN;HA
brown	BP	GO:0048011	nerve growth factor receptor signaling p...	17735	223	1802	42	23.94	2.00E-04	1.754386 AATF;ADCY4;AKAP13;AP2A1;AP2M1;ARHGEF11;ARHGEF12;CASP9;CLTA;CLTC;ELK1;FOXO4;FURIN;GRB2;GS
brown	BP	GO:0051234	establishment of localization	17735	3408	1802	423	365.85	2.00E-04	1.156212 ABAT;ABCC5;ABCC6;ABCG1;ACTN1;ADAR;ADCY4;ADRBK2;AHI1;AKR1C3;ALB;AMICA1;ANP32A;ANXA11;AP1
brown	BP	GO:0000045	autophagic vacuole assembly	17735	26	1802	10	2.79	0.00021	3.584229 BECN1;CTSD;KIAA1324;PSEN1;RAB23;TBC1D14;TP53INP2;ULK1;WIPI1;WIPI2
brown	BP	GO:0042594	response to starvation	17735	110	1802	25	11.81	0.00021	2.11685 ADSL;AKR1C3;ALB;ATG7;BECN1;CTSD;DAP;FBXO22;GCK;HFE;HSPA5;KIAA1324;LZTS1;MAX;NBR1;NUAK2;PR
brown	BP	GO:0046649	lymphocyte activation	17735	468	1802	75	50.24	0.00024	1.492834 ADK;AP3B1;BCL6;BLOC1S3;BTK;BTN3A1;CD1C;CD1D;CD209;CD3D;CD86;CDC42;CHRNB2;CTNNB1;DOCK2;E
brown	BP	GO:0050776	regulation of immune response	17735	560	1802	87	60.12	0.00024	1.447106 AMICA1;APOA1;BCL6;BTK;BTN3A1;C1RL;C3;C4BPB;C5AR1;CD14;CD1D;CD3D;CD86;CDH1;COLEC12;CRTAM;
brown	BP	GO:0044267	cellular protein metabolic process	17735	3210	1802	400	344.6	0.00024	1.160766 AATK;ACO1;ACTB;ACVR1B;ADAR;ADCY4;ADH5;ADPRH;AGA;ALG14;ALG5;ANAPC16;ANAPC4;APITD1;APLP2;
brown	BP	GO:0019219	regulation of nucleobase-containing comp...	17735	3414	1802	423	366.5	0.00024	1.154161 AATF;ABCG1;ACVR1B;ADAP2;AFF2;AHI1;ALDH1A1;ANP32A;APP;ARAP1;ARAP3;ARFGAP3;ARHGAP26;ARHG
brown	BP	GO:0030029	actin filament-based process	17735	484	1802	77	51.96	0.00025	1.481909 ACTA1;ACTG1;ACTN1;ARAP1;ARF6;ARHGAP26;ARHGDIB;ARHGEF10;ARHGEF11;BCL6;CAP1;CAPZB;CDC42;C
brown	BP	GO:0051171	regulation of nitrogen compound metaboli...	17735	3496	1802	432	375.3	0.00025	1.151079 AATF;ABCG1;ACVR1B;ADAP2;AFF2;AHI1;ALDH1A1;ANP32A;APP;ARAP1;ARAP3;ARFGAP3;ARHGAP26;ARHG
brown	BP	GO:0080090	regulation of primary metabolic process	17735	4337	1802	526	465.58	0.00025	1.129774 AATF;ABCG1;ACO1;ACVR1B;ADAP2;ADAR;ADCY4;AFF2;AHI1;AKR1C3;ALDH1A1;ANAPC4;ANP32A;APITD1;A
brown	BP	GO:0019048	virus-host interaction	17735	371	1802	62	39.83	0.00026	1.556616 ANPEP;AP1M1;APOBEC3C;ATF7;ATP6V0C;BECN1;BNIP3;BRD4;CARM1;CD1D;CD209;CD86;CD93;CFLAR;CRE
brown	BP	GO:0071702	organic substance transport	17735	1950	1802	255	209.34	0.00026	1.218114 ABCC5;ABCG1;ADAR;AKR1C3;ALB;AP1M1;AP2A1;AP2M1;AP3B1;APC2;APOA1;APOBR;ARCN1;ARF1;ARF3;A
brown	BP	GO:0006914	autophagy	17735	118	1802	26	12.67	0.00027	2.052092 ATG2A;ATG7;BECN1;CTSD;DAP;DAPK1;DAPK2;FIS1;FUNDC1;KIAA1324;LAMP1;LZTS1;NBR1;PIM2;PINK1;PSE
brown	BP	GO:0006740	NADPH regeneration	17735	14	1802	7	1.5	0.00028	4.666667 G6PD;GLYR1;HIBADH;PGAM1;PGD;TALDO1;TKT
brown	BP	GO:0032786	positive regulation of DNA-dependent tra...	17735	18	1802	8	1.93	0.00028	4.145078 BRD4;DAB2;ELL;ELL3;MAP2K1;PAF1;RTF1;WDR61
brown	MF	GO:0045296	cadherin binding	17735	23	1802	9	2.39	0.00029	3.76569 CTNNA1;CTNNAL1;CTNNB1;CTNND1;NDRG1;NUMB;PSEN1;TBC1D2;VCL
brown	MF	GO:0060589	nucleoside-triphosphatase regulator acti...	17735	447	1802	70	46.37	0.00029	1.509597 ADAP2;AKAP13;ALDH1A1;ARAP1;ARAP3;ARFGAP3;ARHGAP1;ARHGAP21;ARHGAP23;ARHGAP24;ARHGAP25
brown	CC	GO:0012505	endomembrane system	17735	1768	1802	223	180.04	0.00029	1.238614 ABCG1;AGPAT1;ALG14;ALG5;ANXA11;ANXA7;AP1M1;AP2A1;AP2M1;AP3B1;APC2;APP;ARAP1;ARCN1;ARF1;
brown	BP	GO:0044237	cellular metabolic process	17735	8597	1802	986	922.9	0.00031	1.068371 AATF;AATK;ABAT;ABCG1;ACIN1;ACO1;ACOX1;ACSM1;ACSM3;ACSS2;ACTB;ACVR1B;ACYP1;ACYP2;ADAL;AD
brown	CC	GO:0045335	phagocytic vesicle	17735	68	1802	17	6.92	0.00036	2.456647 ANXA11;ATP6V0A1;ATP6V0C;ATP6V0D1;HLA-B;HLA-C;HLA-E;NCF2;RAB11FIP1;RAB23;RAB43;RAB7A;RAB8A
brown	BP	GO:0045730	respiratory burst	17735	23	1802	9	2.47	0.00037	3.643725 CAMK1D;HCK;INSR;NCF2;PGAM1;PIK3CD;RAC1;RAC2;SLC11A1
brown	BP	GO:0006886	intracellular protein transport	17735	702	1802	104	75.36	0.00037	1.380042 ADAR;AKR1C3;AP1M1;AP2A1;AP2M1;AP3B1;APC2;ARCN1;ARFGAP3;ARL6;BCL6;C11orf73;C1QTNF3;CDC42;
brown	BP	GO:0051252	regulation of RNA metabolic process	17735	2953	1802	369	317.01	0.00037	1.164001 AATF;ABCG1;ACVR1B;AFF2;AHI1;ANP32A;APP;ARHGEF11;ARID1A;ARID3A;ARID3B;ARNT;ASCC2;ASH2L;ATF
brown	BP	GO:0044260	cellular macromolecule metabolic process	17735	6621	1802	774	710.78	0.00038	1.088945 AATF;AATK;ABCG1;ACIN1;ACO1;ACTB;ACVR1B;ADAR;ADCY4;ADH5;ADPRH;ADRBK2;AFF2;AGA;AHI1;ALG14;
brown	BP	GO:0002576	platelet degranulation	17735	83	1802	20	8.91	0.00039	2.244669 ACTN1;ALB;APOA1;APP;BRPF3;CAP1;CFL1;F13A1;HSPA5;LEFTY2;P2RX1;PECAM1;PLEK;PSAP;SERPINA1;SOD1
brown	MF	GO:0030695	GTPase regulator activity	17735	436	1802	68	45.23	0.00039	1.503427 ADAP2;AKAP13;ALDH1A1;ARAP1;ARAP3;ARFGAP3;ARHGAP1;ARHGAP21;ARHGAP23;ARHGAP24;ARHGAP25
brown	MF	GO:0003824	catalytic activity	17735	5264	1802	607	546.08	0.00039	1.111559 AATK;ABAT;ABCC5;ABCC6;ABCG1;ABHD2;ABHD4;ACIN1;ACO1;ACOX1;ACP1;ACSM1;ACSM3;ACSS2;ACVR1B;
brown	CC	GO:0005775	vacuolar lumen	17735	75	1802	18	7.64	0.00042	2.356021 ASAH1;CTSA;CTSB;CTSD;DAPK2;GALNS;GBA;GLB1;GM2A;GNS;HEXB;NEU1;PLBD2;PSAP;RNASET2;TPP1;TXN
brown	BP	GO:0002684	positive regulation of immune system pro...	17735	554	1802	85	59.47	0.00042	1.429292 ADK;AP3B1;BCL6;BLOC1S3;BTK;BTN3A1;C1RL;C3;C4BPB;C5AR1;CAMK1D;CCL3;CD14;CD1D;CD3D;CD86;CDC
brown	BP	GO:2000112	regulation of cellular macromolecule bio...	17735	3117	1802	387	334.62	0.00042	1.156536 AATF;ABCG1;ACO1;ACVR1B;AHI1;ANP32A;APP;ARHGEF11;ARID1A;ARID3A;ARID3B;ARNT;ASCC2;ASH2L;ATF
brown	BP	GO:0010556	regulation of macromolecule biosynthetic...	17735	3197	1802	396	343.2	0.00042	1.153846 AATF;ABCG1;ACO1;ACVR1B;AHI1;ANP32A;APP;ARHGEF11;ARID1A;ARID3A;ARID3B;ARNT;ASCC2;ASH2L;ATF
brown	BP	GO:0016569	covalent chromatin modification	17735	274	1802	48	29.41	0.00043	1.632098 ASH2L;BAZ2A;BCOR;BRCA1;BRD4;BRD8;BRPF3;CARM1;CHEK1;CREBBP;DNMT3B;EP300;EYA3;FLCN;FOXP3;H
brown	BP	GO:0016571	histone methylation	17735	60	1802	16	6.44	0.00044	2.484472 ASH2L;BAZ2A;BCOR;BRCA1;CARM1;DNMT3B;JARID2;MLL2;MYB;NSD1;PRMT5;PRMT6;RTF1;SETD1B;WDR61
brown	MF	GO:0008289	lipid binding	17735	721	1802	103	74.8	0.00044	1.377005 ABCG1;ACOX1;ADAP2;ADH5;ADRBK2;AFAP1;AKAP13;ALB;ALDH1A1;ANXA11;ANXA4;ANXA5;ANXA7;ANXA9;
brown	BP	GO:0031331	positive regulation of cellular cataboli...	17735	103	1802	23	11.06	0.00048	2.079566 ARHGEF10;ARNT;ATG7;BECN1;BTG2;CPT1A;CSNK1D;DAB2;FBXO22;FURIN;GAPDHS;GCK;GTPBP1;INSR;IRS2;
brown	BP	GO:0016570	histone modification	17735	268	1802	47	28.77	0.00048	1.633646 ASH2L;BAZ2A;BCOR;BRCA1;BRD4;BRD8;BRPF3;CARM1;CHEK1;CREBBP;DNMT3B;EP300;EYA3;FLCN;FOXP3;H
brown	BP	GO:0006096	glycolysis	17735	61	1802	16	6.55	0.00053	2.442748 ALDOC;ARNT;DHTKD1;ENO1;FBP1;GAPDHS;GCK;HDAC4;HK1;HK2;INSR;NCOR1;PFKFB3;PGAM1;PGK1;PGM1
brown	BP	GO:0030036	actin cytoskeleton organization	17735	419	1802	67	44.98	0.00054	1.489551 ACTA1;ACTG1;ACTN1;ARAP1;ARF6;ARHGAP26;ARHGDIB;ARHGEF10;ARHGEF11;BCL6;CAP1;CAPZB;CDC42;C

brown	BP	GO:0006479	protein methylation	17735	79	1802	19	8.48	0.00055	2.240566	ASH2L;BAZ2A;BCOR;BRCA1;BTG2;CARM1;DNMT3B;JARID2;MLL2;MYB;N6AMT1;NSD1;PRMT5;PRMT6;RAB3
brown	BP	GO:0008213	protein alkylation	17735	79	1802	19	8.48	0.00055	2.240566	ASH2L;BAZ2A;BCOR;BRCA1;BTG2;CARM1;DNMT3B;JARID2;MLL2;MYB;N6AMT1;NSD1;PRMT5;PRMT6;RAB3
brown	MF	GO:0004674	protein serine/threonine kinase activity	17735	434	1802	67	45.02	0.00057	1.488227	AATK;ACVR1B;ADRBK2;AKAP13;ALPK1;ALPK3;CAMK1D;CAMK2G;CAMKK2;CCL3;CCNK;CDKL3;CHEK1;CSNK1
brown	BP	GO:0051716	cellular response to stimulus	17735	5052	1802	601	542.34	0.00057	1.108161	AATF;ACSM1;ACSS2;ACVR1B;ADAP2;ADAR;ADCY4;ADRBK2;AGPAT1;AHI1;AKAP13;AKR1C3;ALB;ALDH1A1;AL
brown	BP	GO:0051568	histone H3-K4 methylation	17735	29	1802	10	3.11	0.00058	3.215434	ASH2L;BCOR;BRCA1;DNMT3B;MLL2;MYB;RTF1;SETD1B;WDR61;WDR82
brown	BP	GO:0031347	regulation of defense response	17735	428	1802	68	45.95	0.00059	1.479869	AOAH;AP1M1;AP2A1;AP2M1;APOA1;ARF1;BCL6;BTK;C1QTNF3;C3;C4BPB;CCL3;CD14;CD1D;CD86;COLEC12;
brown	CC	GO:0043202	lysosomal lumen	17735	71	1802	17	7.23	0.00062	2.351314	ASAH1;CTSA;CTSB;CTSD;GALNS;GBA;GLB1;GM2A;GNS;HEXB;NEU1;PLBD2;PSAP;RNASET2;TPP1;TXNDC5;VC
brown	BP	GO:0044765	single-organism transport	17735	2753	1802	344	295.54	0.00062	1.163971	ABAT;ABCC5;ABCC6;ABCG1;ACTN1;ADAR;ADCY4;ADRBK2;AHI1;AKR1C3;ALB;AMICA1;ANXA11;AP1M1;AP2A
brown	MF	GO:0051019	mitogen-activated protein kinase binding	17735	16	1802	7	1.66	0.00063	4.216867	ATF7;GTF2I;MAPKAPK5;MICALCL;NBR1;PTPRJ;TNIP1
brown	MF	GO:0019901	protein kinase binding	17735	357	1802	57	37.03	0.00063	1.539292	ACTB;ATF7;CASP9;CCNK;CDC42;CTNNB1;FAM58A;FOXO4;GRB2;GSK3A;GTF2I;HCLS1;HDAC4;IFNAR2;IRS2;IT
brown	BP	GO:0002764	immune response-regulating signaling pat...	17735	250	1802	44	26.84	0.00066	1.639344	BTK;BTN3A1;C5AR1;CD14;CD3D;CD86;COLEC12;ELK1;FOXP3;HCK;HLA-DPA1;IRAK2;KLRC4-KLRK1;LAT2;LCP2
brown	BP	GO:0006955	immune response	17735	1081	1802	149	116.05	0.00066	1.283929	ADAR;AMICA1;APOA1;APP;ARHGDIB;BCL6;BTK;BTN3A1;C1RL;C3;C4BPB;C5AR1;CALCOCO2;CAMK2G;CCL3;C
brown	BP	GO:0031325	positive regulation of cellular metaboli...	17735	1911	1802	247	205.15	0.00066	1.203997	AATF;ACVR1B;ADCY4;AGPAT1;AHI1;AKR1C3;ANAPC4;APITD1;APOA1;APP;ARHGEF10;ARHGEF11;ARID1A;AR
brown	BP	GO:0042110	T cell activation	17735	339	1802	56	36.39	0.00068	1.538884	ADK;AP3B1;BCL6;BTN3A1;CD1C;CD1D;CD209;CD3D;CD86;CDC42;CTNNB1;DOCK2;EGR1;ELF4;FKBP1A;FLT3;
brown	MF	GO:0016772	transferase activity, transferring phosp...	17735	889	1802	122	92.22	0.00068	1.322923	AATK;ACVR1B;ADK;ADRBK2;AKAP13;ALPK1;ALPK3;BTK;CAMK1D;CAMK2G;CAMKK2;CASS4;CCDC111;CCL3;C
brown	BP	GO:0045637	regulation of myeloid cell differentiati...	17735	132	1802	27	14.17	0.00072	1.905434	ACIN1;ACVR1B;ARNT;CCL3;CSF1;CTNNB1;GNAS;HAX1;HCLS1;HSPA1B;IKZF1;IL23A;KLF13;LILRB1;MAFB;MAP
brown	BP	GO:0033554	cellular response to stress	17735	1209	1802	164	129.79	0.00074	1.26358	AATF;AKR1C3;ALB;ALDH3B1;ALKBH2;ALKBH3;APITD1;APOA1;ARNT;ASH2L;ATG7;ATP6V0D1;BCCIP;BCL6;BE
brown	BP	GO:0002757	immune response-activating signal transd...	17735	237	1802	42	25.44	0.00075	1.650943	BTK;BTN3A1;C5AR1;CD14;CD3D;CD86;COLEC12;ELK1;FOXP3;HCK;HLA-DPA1;IRAK2;KLRC4-KLRK1;LAT2;LCP2
brown	BP	GO:2000377	regulation of reactive oxygen species me...	17735	57	1802	15	6.12	0.00076	2.45098	AATF;AKR1C3;BCO2;GRB2;MAPK14;PID1;PRKCD;PTK2B;RAC1;RAC2;RIPK1;RNF41;ROMO1;SYK;TGFB1
brown	MF	GO:0019904	protein domain specific binding	17735	535	1802	79	55.5	0.00076	1.423423	AATF;ACP1;ACTN1;ADAM19;APP;ARHGAP1;ARHGAP26;ARHGAP27;ARHGAP31;ARL1;BASP1;BHLHE40;BIN2;
brown	BP	GO:1901184	regulation of ERBB signaling pathway	17735	63	1802	16	6.76	0.00078	2.366864	AP2A1;AP2M1;APLP2;APP;CBL;CDC42;CLTA;CLTC;EPS15L1;GRB2;PSEN1;PTPRJ;RAB7A;RBPJ;SH3KBP1;SPRY2
brown	BP	GO:0032968	positive regulation of transcription elo...	17735	12	1802	6	1.29	0.00079	4.651163	BRD4;DAB2;MAP2K1;PAF1;RTF1;WDR61
brown	BP	GO:0018193	peptidyl-amino acid modification	17735	653	1802	96	70.1	8.00E-04	1.369472	ACVR1B;ADH5;ALG14;ALG5;APOA1;B4GALT1;B4GALT4;B4GALT5;BRCA1;BRD4;BRD8;BRPF3;BTK;CD44;CDC4
brown	BP	GO:0034968	histone lysine methylation	17735	46	1802	13	4.94	0.00081	2.631579	ASH2L;BAZ2A;BCOR;BRCA1;DNMT3B;JARID2;MLL2;MYB;NSD1;RTF1;SETD1B;WDR61;WDR82
brown	BP	GO:0006355	regulation of transcription, DNA-depende...	17735	2869	1802	356	307.99	0.00081	1.155882	AATF;ABCG1;ACVR1B;AHI1;ANP32A;APP;ARHGEF11;ARID1A;ARID3A;ARID3B;ARNT;ASCC2;ASH2L;ATF5;ATF
brown	BP	GO:0002703	regulation of leukocyte mediated immunit...	17735	94	1802	21	10.09	0.00083	2.081269	BCL6;BTK;C3;CRTAM;FOXP3;GAB2;HMOX1;IL23A;KLRC4-KLRK1;LILRB1;NOD2;PRAM1;PVR;SH2D1A;STAT5A;
brown	MF	GO:0030234	enzyme regulator activity	17735	970	1802	131	100.63	0.00083	1.301799	ADAP2;AKAP13;ALDH1A1;ANXA4;ANXA5;APLP2;APOA1;APP;ARAP1;ARAP3;ARFGAP3;ARHGAP1;ARHGAP21;
brown	BP	GO:2001141	regulation of RNA biosynthetic process	17735	2889	1802	358	310.14	0.00086	1.154317	AATF;ABCG1;ACVR1B;AHI1;ANP32A;APP;ARHGEF11;ARID1A;ARID3A;ARID3B;ARNT;ASCC2;ASH2L;ATF5;ATF
brown	BP	GO:0002285	lymphocyte activation involved in immune...	17735	88	1802	20	9.45	0.00087	2.116402	BCL6;CD1C;CD86;EXOSC3;FOXP3;GPR183;IL23A;IL4R;JAK3;LCP1;LILRB1;MYB;PLCG2;PSEN1;PTK2B;SEMA4A;
brown	BP	GO:0030218	erythrocyte differentiation	17735	88	1802	20	9.45	0.00087	2.116402	ACIN1;ACVR1B;ARNT;ATPIF1;BCL6;CITED2;G6PD;HCLS1;HIPK2;HSPA1B;KLF13;MAEA;MAFB;MAPK14;NCOR1
brown	MF	GO:0031072	heat shock protein binding	17735	91	1802	20	9.44	0.00088	2.118644	ATF5;BCOR;C11orf73;CSNK2A1;DNAJB12;DNAJB5;DNAJB6;DNAJC13;DNAJC14;DNAJC18;DNAJC19;DNAJC5;
brown	BP	GO:0051173	positive regulation of nitrogen compound...	17735	1214	1802	164	130.32	0.00089	1.258441	AATF;ACVR1B;AHI1;APP;ARHGEF10;ARHGEF11;ARID1A;ARID3A;ARNT;ASH2L;BCL9;BRCA1;BRD4;BRE;BTG2;
brown	BP	GO:0010467	gene expression	17735	4403	1802	527	472.67	0.00092	1.114943	AATF;ABCG1;ACO1;ACVR1B;ADAR;ADK;AFF2;AGA;AHI1;ALKBH2;ALKBH3;ANP32A;APITD1;APOA1;APOBEC3
brown	BP	GO:0043412	macromolecule modification	17735	2584	1802	323	277.4	0.00093	1.164384	AATK;ACVR1B;ADAR;ADCY4;ADH5;ADPRH;AGA;ALG14;ALG5;ALKBH2;ALKBH3;ANAPC16;ANAPC4;APITD1;AP
brown	BP	GO:0034101	erythrocyte homeostasis	17735	95	1802	21	10.2	0.00096	2.058824	ACIN1;ACVR1B;ARNT;ATPIF1;BCL6;CITED2;G6PD;HCLS1;HIPK2;HMOX1;HSPA1B;KLF13;MAEA;MAFB;MAPK1
brown	BP	GO:0007167	enzyme linked receptor protein signaling...	17735	884	1802	124	94.9	0.00096	1.306639	AATF;ACVR1B;ADCY4;AHI1;AKAP13;ANKS1A;AP2A1;AP2M1;APLP2;APP;ARHGEF11;ARHGEF12;ARNT;ATP6V
brown	BP	GO:0006110	regulation of glycolysis	17735	21	1802	8	2.25	0.00098	3.555556	ARNT;FBP1;GAPDHS;GCK;HDAC4;INSR;NCOR1;PGAM1
brown	BP	GO:0042113	B cell activation	17735	169	1802	32	18.14	0.00099	1.764057	BCL6;BTK;CD86;CHRNB2;EXOSC3;FLT3;FOXP3;GPR183;HDAC4;IKZF1;IRS2;JAK3;LAT2;MYB;NFAM1;NHEJ1;NC
brown	BP	GO:0048872	homeostasis of number of cells	17735	176	1802	33	18.89	0.00099	1.746956	ACIN1;ACVR1B;ARNT;ATPIF1;ATXN2;BCL6;CITED2;CSF1;FLT3;FOXP3;G6PD;GIGYF2;HCLS1;HIPK2;HMOX1;HS
brown	BP	GO:0009889	regulation of biosynthetic process	17735	3356	1802	410	360.27	0.00099	1.138035	AATF;ABCG1;ACO1;ACVR1B;AHI1;AKR1C3;ANP32A;APP;ARHGEF11;ARID1A;ARID3A;ARID3B;ARNT;ASCC2;AS
brown	BP	GO:0002286	T cell activation involved in immune res...	17735	47	1802	13	5.05	0.00102	2.574257	BCL6;CD1C;CD86;IL23A;IL4R;JAK3;LCP1;LILRB1;MYB;PSEN1;SEMA4A;SLC11A1;STAT6
brown	CC	GO:0000123	histone acetyltransferase complex	17735	74	1802	17	7.54	0.00102	2.254642	ACTB;BRD8;BRPF3;CREBBP;EP300;ING4;KAT5;KAT6A;KAT7;POLE4;SAP130;SUPT3H;TADA2B;TAF4;UBAP2L;U
brown	BP	GO:0001818	negative regulation of cytokine producti...	17735	135	1802	27	14.49	0.00102	1.863354	APOA1;BCL6;C1QTNF3;FOXP3;FURIN;GBA;HMOX1;IL23A;IL6R;JAK3;KAT5;KLF4;LILRB1;MEFV;NFKB1;NLRP12
brown	MF	GO:0005099	Ras GTPase activator activity	17735	112	1802	23	11.62	0.00103	1.979346	ALDH1A1;ARAP1;ARAP3;ARHGAP1;ARHGAP26;ARHGAP27;C6orf170;DOCK2;GDI2;GMIP;PREX1;RALBP1;RAL
brown	BP	GO:0070727	cellular macromolecule localization	17735	960	1802	133	103.06	0.00104	1.29051	ADAR;AHI1;AKR1C3;AP1M1;AP2A1;AP2M1;AP3B1;APC2;ARCN1;ARF6;ARFGAP3;ARL1;ARL6;BBS4;BCL6;BICD
brown	BP	GO:0045912	negative regulation of carbohydrate meta...	17735	26	1802	9	2.79	0.00106	3.225806	C1QTNF3;FBP1;GCK;GRB10;GSK3A;HDAC4;PLEK;TGFB1;TINF2
brown	BP	GO:0030593	neutrophil chemotaxis	17735	53	1802	14	5.69	0.00108	2.460457	AMICA1;C5AR1;CAMK1D;CCL3;CSF3R;CXCR2;IL23A;ITGAM;ITGB2;MPP1;NOD2;PIK3CD;SYK;TIRAP
brown	BP	GO:0001816	cytokine production	17735	430	1802	67	46.16	0.00109	1.451473	AGPAT1;APOA1;ARNT;BCL6;BTK;BTN3A1;C1QTNF3;C3;CCL3;CD14;CD86;CRTAM;CSF1R;FOXP3;FURIN;G6PD;
brown	BP	GO:0006952	defense response	17735	1119	1802	152	120.13	0.00109	1.265296	ADAR;AOAH;AP1M1;AP2A1;AP2M1;APOA1;APOBEC3C;APP;ARF1;B4GALT1;BCL6;BECN1;BNIP3;BTK;C1QTNF
brown	MF	GO:0031683	G-protein beta/gamma-subunit complex bin...	17735	22	1802	8	2.28	0.00111	3.508772	GNA11;GNA12;GNA15;GNAI2;GNAL;GNAS;GNAT2;PIK3R5
brown	BP	GO:0010970	microtubule-based transport	17735	65	1802	16	6.98	0.00113	2.292264	AP3B1;APP;ARHGAP21;BICD2;BLOC1S3;CDC42;IFT43;IFT46;KIF13A;KIF1B;MAP1S;MAP2K1;NDEL1;PAFAH1B
brown	MF	GO:0016702	oxidoreductase activity, acting on singl...	17735	80	1802	18	8.3	0.00118	2.168675	ADO;ALKBH2;ALKBH3;ALOX15B;BCO2;FTO;HAAO;HGD;KDM2A;KDM3B;KDM4B;KDM5B;KDM6B;PHF2;PHF8;
brown	BP	GO:0045935	positive regulation of nucleobase-contai...	17735	1189	1802	160	127.64	0.00122	1.253526	AATF;ACVR1B;AHI1;APP;ARHGEF10;ARHGEF11;ARID1A;ARID3A;ARNT;ASH2L;BCL9;BRCA1;BRD4;BRE;BTG2;
brown	BP	GO:0009893	positive regulation of metabolic process	17735	2010	1802	256	215.78	0.00124	1.186394	AATF;ABCG1;ACVR1B;ADCY4;AGPAT1;AHI1;AKR1C3;ANAPC4;APITD1;APOA1;APP;ARHGEF10;ARHGEF11;AR
brown	CC	GO:0015629	actin cytoskeleton	17735	366	1802	56	37.27	0.00127	1.502549	ACTA1;ACTG1;AFAP1;APC2;BIN2;BRCA1;CAP1;CAPZB;CDC42EP4;CDH1;CFL1;CNN2;COPB2;CORO1C;CTDP1;
brown	BP	GO:0016482	cytoplasmic transport	17735	719	1802	103	77.19	0.00128	1.33437	ADAR;AKR1C3;AP1M1;AP2A1;AP3B1;APC2;BCL6;BLOC1S3;C11orf73;C1QTNF3;CCL3;CDH1;DAB2;DAG1;DEN
brown	BP	GO:0031062	positive regulation of histone methylati...	17735	13	1802	6	1.4	0.00133	4.285714	BRCA1;DNMT3B;JARID2;MYB;RTF1;WDR61
brown	MF	GO:0043167	ion binding	17735	5761	1802	653	597.63	0.00135	1.092649	AATK;ABAT;ABCC5;ABCC6;ABCG1;ACO1;ACOX1;ACSM1;ACSM3;ACSS2;ACTA1;ACTB;ACTG1;ACTN1;ACVR1B;
brown	MF	GO:0016701	oxidoreductase activity, acting on singl...	17735	81	1802	18	8.4	0.00137	2.142857	ADO;ALKBH2;ALKBH3;ALOX15B;BCO2;FTO;HAAO;HGD;KDM2A;KDM3B;KDM4B;KDM5B;KDM6B;PHF2;PHF8;
brown	MF	GO:0005070	SH3/SH2 adaptor activity	17735	50	1802	13	5.19	0.00138	2.504817	ARHGAP1;CRKL;GAB2;GRB10;GRB2;LASP1;SH2D1A;SH2D3C;SH3BGR;SLA;SOCS2;SRC;TP53BP2
brown	BP	GO:0080134	regulation of response to stress	17735	737	1802	105	79.12	0.00139	1.327098	AOAH;AP1M1;AP2A1;AP2M1;APOA1;ARF1;ATG7;BCL6;BECN1;BRCA1;BRE;BTK;C1QTNF3;C3;C4BPB;CASP9;C
brown	BP	GO:0031329	regulation of cellular catabolic process	17735	450	1802	69	48.31	0.00143	1.428276	ADAP2;ALDH1A1;ARAP1;ARAP3;ARFGAP3;ARHGAP26;ARHGAP27;ARHGEF10;ARNT;ATG7;BCL6;BECN1;BTG2
brown	BP	GO:0031326	regulation of cellular biosynthetic proc...	17735	3328	1802	405	357.27	0.00145	1.133596	AATF;ABCG1;ACO1;ACVR1B;AHI1;AKR1C3;ANP32A;APP;ARHGEF11;ARID1A;ARID3A;ARID3B;ARNT;ASCC2;AS
brown	MF	GO:0017124	SH3 domain binding	17735	115	1802	23	11.93	0.00149	1.927913	ACP1;ADAM19;ARHGAP1;ARHGAP26;ARHGAP27;ARHGAP31;CASP9;CBL;DENND1A;ELMO1;ELMO2;GRB2;H
brown	BP	GO:0060627	regulation of vesicle-mediated transport	17735	216	1802	38	23.19	0.00149	1.638637	AHI1;ARF6;ATXN2;B4GALT1;C3;CAMK1D;CBL;CD14;CNN2;DAB2;DOCK2;GAB2;HCK;HIP1;HMOX1;HNRNPK;L
brown	BP	GO:0006898	receptor-mediated endocytosis	17735	132	1802	26	14.17	0.00158	1.834862	ADRBK2;AHI1;ARF6;ARHGAP27;ATXN2;CAP1;CBL;CLTC;CLTCL1;CXCR2;DAB2;DNM2;GRB2;HFE;HIP1;HNRNP
brown	MF	GO:0051213	dioxygenase activity	17735	82	1802	18	8.51	0.00159	2.115159	ADO;ALKBH2;ALKBH3;ALOX15B;BCO2;FTO;HAAO;HGD;KDM2A;KDM3B;KDM4B;KDM5B;KDM6B;PHF2;PHF8;
brown	BP	GO:0006464	cellular protein modification process	17735	2482	1802	309	266.45	0.00159	1.159692	AATK;ACVR1B;ADAR;ADCY4;ADH5;ADPRH;AGA;ALG14;ALG5;ANAPC16;ANAPC4;APITD1;APLP2;APOA1;APP;
brown	BP	GO:0036211	protein modification process	17735	2482	1802	309	266.45	0.00159	1.159692	AATK;ACVR1B;ADAR;ADCY4;ADH5;ADPRH;AGA;ALG14;ALG5;ANAPC16;ANAPC4;APITD1;APLP2;APOA1;APP;

brown	BP	GO:0051650	establishment of vesicle localization	17735	55	1802	14	5.9	0.0016	2.372881	ARCN1;ARF1;ARL6;BBS4;BLOC1S3;COPA;COPB2;MAP2K1;MYO7A;PSEN1;SEC16A;SEC31A;SNAP29;WIPI1
brown	CC	GO:0030132	clathrin coat of coated pit	17735	14	1802	6	1.43	0.00161	4.195804	AP2A1;AP2M1;CLTA;CLTC;CLTCL1;DAB2
brown	BP	GO:0002697	regulation of immune effector process	17735	217	1802	38	23.3	0.00163	1.630901	AP1M1;AP2A1;AP2M1;APOA1;ARF1;BCL6;BTK;C3;C4BPB;CD86;CRTAM;DOCK2;ELMO1;FOXP3;GAB2;HCK;HN
brown	BP	GO:0045089	positive regulation of innate immune res...	17735	167	1802	31	17.93	0.00164	1.728946	BTK;CD14;CD1D;CD86;COLEC12;CRTAM;ELK1;HCK;IRAK2;KLRC4-KLRK1;MAP2K1;MAP2K3;MAPK14;MYD88;N
brown	BP	GO:0051056	regulation of small GTPase mediated sign...	17735	367	1802	58	39.4	0.00164	1.472081	ADAP2;AKAP13;ALDH1A1;APOA1;ARAP1;ARAP3;ARF6;ARFGAP3;ARHGAP1;ARHGAP24;ARHGAP25;ARHGAP2
brown	MF	GO:0016740	transferase activity	17735	1705	1802	213	176.87	0.00164	1.204274	AATK;ABAT;ACVR1B;ADK;ADRBK2;AGPAT1;AKAP13;ALAS1;ALG1L2;ALG5;ALPK1;ALPK3;ASH2L;B3GNT9;B4GA
brown	BP	GO:0030217	T cell differentiation	17735	160	1802	30	17.18	0.00165	1.746217	AP3B1;BCL6;CD1D;CD3D;CD86;CTNNB1;DOCK2;EGR1;FLT3;FOXP3;IKZF1;IL23A;IL4R;JAK3;LILRB2;MYB;NCOR
brown	MF	GO:0003924	GTPase activity	17735	232	1802	39	24.07	0.00165	1.620274	ARF1;ARF3;ARF6;ARL1;CDC42;DNM2;EHD1;EHD3;GNA11;GNA12;GNA15;GNAI2;GNAL;GNAS;GNAT2;GNB1;(
brown	BP	GO:0046039	GTP metabolic process	17735	352	1802	56	37.79	0.00167	1.481874	ADAP2;ALDH1A1;ARAP1;ARAP3;ARF1;ARF3;ARF6;ARFGAP3;ARHGAP26;ARHGAP27;ARHGEF10;ARL1;BCL6;C
brown	BP	GO:1901068	guanosine-containing compound metabolic ...	17735	360	1802	57	38.65	0.00171	1.474774	ADAP2;ALDH1A1;ARAP1;ARAP3;ARF1;ARF3;ARF6;ARFGAP3;ARHGAP26;ARHGAP27;ARHGEF10;ARL1;BCL6;C
brown	BP	GO:1901136	carbohydrate derivative catabolic proces...	17735	564	1802	83	60.55	0.00171	1.370768	ACIN1;ADAL;ADAP2;ALDH1A1;AMPD3;APOBEC3C;ARAP1;ARAP3;ARF1;ARF3;ARF6;ARFGAP3;ARHGAP26;ARI
brown	BP	GO:0008152	metabolic process	17735	9452	1802	1067	1014.69	0.00171	1.051553	AATF;AATK;ABAT;ABCC5;ABCG1;ABHD4;ACIN1;ACO1;ACOX1;ACSM1;ACSM3;ACSS2;ACTB;ACVR1B;ACYP1;A(
brown	BP	GO:0034613	cellular protein localization	17735	956	1802	131	102.63	0.00173	1.27643	ADAR;AHI1;AKR1C3;AP1M1;AP2A1;AP2M1;AP3B1;APC2;ARCN1;ARF6;ARFGAP3;ARL1;ARL6;BBS4;BCL6;BICD
brown	BP	GO:0042269	regulation of natural killer cell mediat...	17735	18	1802	7	1.93	0.00177	3.626943	CRTAM;KLRC4-KLRK1;LILRB1;PVR;SH2D1A;STAT5A;STAT5B
brown	BP	GO:0002573	myeloid leukocyte differentiation	17735	133	1802	26	14.28	0.00177	1.820728	ACIN1;CBFA2T3;CCL3;CDC42;CITED2;CSF1;CSF1R;CTNNB1;GAB2;GNAS;HAX1;HCLS1;IKZF1;IL23A;LILRB1;MA
brown	BP	GO:0038127	ERBB signaling pathway	17735	161	1802	30	17.28	0.00182	1.736111	ADCY4;AP2A1;AP2M1;APLP2;APP;CASP9;CBL;CDC42;CLTA;CLTC;EPS15L1;FOXO4;GRB2;GSK3A;MAP2K1;PHL
brown	BP	GO:0030098	lymphocyte differentiation	17735	226	1802	39	24.26	0.00188	1.607585	AP3B1;BCL6;BTK;CD1D;CD3D;CD86;CTNNB1;DOCK2;EGR1;FLT3;FOXP3;GPR183;HDAC4;IKZF1;IL23A;IL4R;JAK
brown	CC	GO:0005794	Golgi apparatus	17735	1158	1802	148	117.92	0.00188	1.255088	AATF;ABCG1;ACO1;AP1M1;AP2A1;AP3B1;APC2;APP;ARAP1;ARCN1;ARF1;ARF3;ARF6;ARFGAP3;ARHGAP21;/
brown	BP	GO:0002699	positive regulation of immune effector p...	17735	100	1802	21	10.74	0.0019	1.955307	C3;CD86;CRTAM;FOXP3;GAB2;HMOX1;IL23A;IL4R;KLRC4-KLRK1;LILRB1;MYB;NOD2;PVR;SH2D1A;STAT5A;ST
brown	BP	GO:0006739	NADP metabolic process	17735	28	1802	9	3.01	0.00192	2.990033	G6PD;GCK;GLYR1;HIBADH;NADK;PGAM1;PGD;TALDO1;TKT
brown	BP	GO:0050690	regulation of defense response to virus ...	17735	28	1802	9	3.01	0.00192	2.990033	AP1M1;AP2A1;AP2M1;ARF1;DOCK2;ELMO1;HCK;PACS1;RAC1
brown	BP	GO:0042058	regulation of epidermal growth factor re...	17735	62	1802	15	6.66	0.00192	2.252252	AP2A1;AP2M1;APLP2;APP;CBL;CDC42;CLTA;CLTC;EPS15L1;GRB2;PSEN1;PTPRJ;RAB7A;SH3KBP1;SPRY2
brown	BP	GO:0050663	cytokine secretion	17735	94	1802	20	10.09	0.00204	1.982161	APOA1;BTN3A1;C1QTNF3;CCL3;CD14;CRTAM;CSF1R;FOXP3;GLMN;IL4R;LCP2;LILRB1;NLRC4;NLRP12;NLRP3;
brown	BP	GO:0002706	regulation of lymphocyte mediated immuni...	17735	75	1802	17	8.05	0.00212	2.111801	BCL6;BTK;C3;CRTAM;FOXP3;IL23A;KLRC4-KLRK1;LILRB1;NOD2;PVR;SH2D1A;STAT5A;STAT5B;STAT6;TAP2;TC
brown	BP	GO:0048522	positive regulation of cellular process	17735	3140	1802	382	337.08	0.00213	1.133262	AATF;ACIN1;ACVR1B;ADAMTSL4;ADAR;ADCY4;ADK;AGPAT1;AHI1;AKR1C3;ALOX15B;ANAPC4;AP3B1;APITD1
brown	BP	GO:0006954	inflammatory response	17735	489	1802	73	52.49	0.00218	1.390741	AOAH;APOA1;B4GALT1;BCL6;C1QTNF3;C3;C4BPB;C5AR1;CAMK1D;CCL3;CD14;CD163;CD44;CD97;CELA1;CSI
brown	BP	GO:0007243	intracellular protein kinase cascade	17735	879	1802	121	94.36	0.00218	1.282323	AKR1C3;BRAP;BTK;C1QTNF3;C5AR1;CAMKK2;CANT1;CCL3;CD14;CD44;CDC42;CFLAR;CRKL;CSF1R;CTNNB1;D
brown	BP	GO:0009306	protein secretion	17735	170	1802	31	18.25	0.0022	1.69863	APOA1;ARFGAP3;BLOC1S3;BTN3A1;C1QTNF3;CCL3;CD14;CRTAM;CSF1R;FOXP3;GLMN;GNAS;IL4R;LCP2;LILR
brown	BP	GO:0002768	immune response-regulating cell surface ...	17735	142	1802	27	15.24	0.00222	1.771654	BTK;BTN3A1;C5AR1;CD3D;FOXP3;HLA-DPA1;KLRC4-KLRK1;LAT2;LCP2;LILRB1;LILRB2;NFAM1;NFKB1;NFKBIA;
brown	BP	GO:0006184	GTP catabolic process	17735	341	1802	54	36.61	0.00222	1.475007	ADAP2;ALDH1A1;ARAP1;ARAP3;ARF1;ARF3;ARF6;ARFGAP3;ARHGAP26;ARHGAP27;ARHGEF10;ARL1;BCL6;C
brown	BP	GO:0051254	positive regulation of RNA metabolic pro...	17735	1063	1802	143	114.11	0.00223	1.253177	AATF;ACVR1B;AHI1;APP;ARHGEF11;ARID1A;ARID3A;ARNT;ASH2L;BCL9;BRCA1;BRD4;BTG2;CALCOCO1;CAM
brown	BP	GO:0002328	pro-B cell differentiation	17735	10	1802	5	1.07	0.00225	4.672897	FLCN;FLT3;NOTCH1;PRKDC;SOX4
brown	BP	GO:0051571	positive regulation of histone H3-K4 met...	17735	10	1802	5	1.07	0.00225	4.672897	BRCA1;DNMT3B;MYB;RTF1;WDR61
brown	BP	GO:0019538	protein metabolic process	17735	3895	1802	466	418.13	0.00225	1.114486	AATF;AATK;ABCG1;ACO1;ACTB;ACVR1B;ADAM19;ADAR;ADCY4;ADH5;ADPRH;AGA;AGBL2;ALB;ALG14;ALG5;
brown	BP	GO:0043112	receptor metabolic process	17735	108	1802	22	11.59	0.00226	1.898188	ADRBK2;AHI1;ATXN2;CAPN1;CLTC;CNPY2;CXCR2;DAB2;DNM2;ECE1;FURIN;GRB2;HNRNPK;LILRB1;LMTK2;M
brown	CC	GO:0043228	non-membrane-bounded organelle	17735	3122	1802	362	317.92	0.00238	1.138651	AATF;ACTA1;ACTB;ACTG1;ACTN1;ADAR;AFAP1;AHI1;ALDOC;ANKRA2;ANXA11;APBB1IP;APC2;APITD1;APP;A
brown	CC	GO:0043232	intracellular non-membrane-bounded organ...	17735	3122	1802	362	317.92	0.00238	1.138651	AATF;ACTA1;ACTB;ACTG1;ACTN1;ADAR;AFAP1;AHI1;ALDOC;ANKRA2;ANXA11;APBB1IP;APC2;APITD1;APP;A
brown	BP	GO:0010604	positive regulation of macromolecule met...	17735	1869	1802	237	200.64	0.00241	1.18122	AATF;ACVR1B;ADCY4;AHI1;ANAPC4;APITD1;APP;ARHGEF11;ARID1A;ARID3A;ARNT;ASH2L;ATG7;ATP6V0D1;
brown	BP	GO:0031668	cellular response to extracellular stimu...	17735	136	1802	26	14.6	0.00246	1.780822	AKR1C3;ALB;ATG7;BECN1;CTSD;DAP;FBXO22;GCK;HFE;HMOX1;HSPA5;KIAA1324;LZTS1;MAX;NBR1;NUAK2;I
brown	BP	GO:0043470	regulation of carbohydrate catabolic pro...	17735	29	1802	9	3.11	0.00252	2.893891	ARNT;FBP1;GAPDHS;GCK;GSK3A;HDAC4;INSR;NCOR1;PGAM1
brown	BP	GO:0043471	regulation of cellular carbohydrate cata...	17735	29	1802	9	3.11	0.00252	2.893891	ARNT;FBP1;GAPDHS;GCK;GSK3A;HDAC4;INSR;NCOR1;PGAM1
brown	CC	GO:0044433	cytoplasmic vesicle part	17735	418	1802	61	42.57	0.00252	1.432934	ACTN1;ALB;ANXA7;AP1M1;AP2A1;AP2M1;AP3B1;APP;ARCN1;ARHGAP21;ATP6V0A1;ATP6V0C;ATP6V0D1;B
brown	BP	GO:1901069	guanosine-containing compound catabolic ...	17735	343	1802	54	36.82	0.00253	1.466594	ADAP2;ALDH1A1;ARAP1;ARAP3;ARF1;ARF3;ARF6;ARFGAP3;ARHGAP26;ARHGAP27;ARHGEF10;ARL1;BCL6;C
brown	BP	GO:0002715	regulation of natural killer cell mediat...	17735	19	1802	7	2.04	0.00255	3.431373	CRTAM;KLRC4-KLRK1;LILRB1;PVR;SH2D1A;STAT5A;STAT5B
brown	BP	GO:0030705	cytoskeleton-dependent intracellular tra...	17735	70	1802	16	7.51	0.00258	2.130493	AP3B1;APP;ARHGAP21;BICD2;BLOC1S3;CDC42;IFT43;IFT46;KIF13A;KIF1B;MAP1S;MAP2K1;NDEL1;PAFAH1B:
brown	BP	GO:0043170	macromolecule metabolic process	17735	7334	1802	840	787.32	0.00258	1.066911	AATF;AATK;ABCC5;ABCG1;ACIN1;ACO1;ACTB;ACVR1B;ADAM19;ADAR;ADCY4;ADH5;ADK;ADPRH;ADRBK2;AI
brown	BP	GO:0060397	JAK-STAT cascade involved in growth horm...	17735	24	1802	8	2.58	0.00263	3.100775	IRS2;JAK3;PTPN1;PTPN6;SOCS2;STAT3;STAT5A;STAT5B
brown	BP	GO:0007165	signal transduction	17735	4165	1802	495	447.12	0.00267	1.107085	AATF;ACVR1B;ADAP2;ADAR;ADCY4;ADRBK2;AGPAT1;AHI1;AKAP13;AKR1C3;ALDH1A1;ANKS1A;ANP32A;AN)
brown	CC	GO:0012506	vesicle membrane	17735	371	1802	55	37.78	0.00284	1.455797	ANXA7;AP1M1;AP2A1;AP2M1;AP3B1;ARCN1;ARHGAP21;ATP6V0A1;ATP6V0C;ATP6V0D1;BSG;CACNG8;CAM
brown	BP	GO:0045087	innate immune response	17735	550	1802	80	59.04	0.00284	1.355014	ADAR;APP;BTK;C1RL;C3;C4BPB;CALCOCO2;CAMK2G;CCL3;CD14;CD1D;CD209;CD44;CD86;CLEC4C;COLEC12;
brown	MF	GO:0005100	Rho GTPase activator activity	17735	36	1802	10	3.73	0.00285	2.680965	ARAP1;ARAP3;ARHGAP1;ARHGAP26;ARHGAP27;DOCK2;GMIP;PREX1;RALBP1;SRGAP2
brown	BP	GO:0030183	B cell differentiation	17735	77	1802	17	8.27	0.00285	2.055623	BCL6;BTK;FLT3;GPR183;HDAC4;IKZF1;JAK3;MYB;NFAM1;NHEJ1;PLCG2;POU2F2;PRKDC;PTK2B;STAT5A;STAT!
brown	BP	GO:0051648	vesicle localization	17735	77	1802	17	8.27	0.00285	2.055623	ARCN1;ARF1;ARL6;BBS4;BLOC1S3;COPA;COPB2;KIF13A;MAP2K1;MYO7A;NDEL1;PAFAH1B1;PSEN1;SEC16A;:
brown	MF	GO:0019900	kinase binding	17735	397	1802	59	41.18	0.00289	1.432734	ACTB;ATF7;CASP9;CCNK;CDC42;CTNNB1;DUSP12;FAM58A;FOXO4;GRB2;GSK3A;GTF2I;HCLS1;HDAC4;IFNAR:
brown	BP	GO:0042981	regulation of apoptotic process	17735	1197	1802	158	128.5	0.0029	1.229572	AATF;ACTN1;ACVR1B;ADAMTSL4;ADAR;AHI1;AKR1C3;ALB;ALOX15B;AMIGO2;ANXA4;ANXA5;APP;ATF5;ATG
brown	BP	GO:0009056	catabolic process	17735	1989	1802	250	213.52	0.00291	1.170851	ABAT;ABHD4;ACIN1;ACOX1;ADAL;ADAP2;ADH5;AKR1C3;ALDH1A1;ALDH2;ALDH3B1;ALDOC;AMPD3;ANAPC
brown	BP	GO:0002244	hematopoietic progenitor cell differenti...	17735	35	1802	10	3.76	0.00292	2.659574	CITED2;FLCN;FLT3;MLF1;NOTCH1;PLEK;PRKDC;PSEN1;SOX4;TGFB1
brown	MF	GO:0016409	palmitoyltransferase activity	17735	20	1802	7	2.07	0.00293	3.381643	CPT1A;CPT2;SPTLC2;ZDHHC18;ZDHHC3;ZDHHC5;ZDHHC7
brown	BP	GO:0050867	positive regulation of cell activation	17735	239	1802	40	25.66	0.00293	1.558846	ADK;AP3B1;BCL6;BLOC1S3;BTK;CD1D;CD3D;CD86;CDC42;CHRNB2;FOXP3;GAB2;GPR183;GRB2;HLA-DPA1;IC
brown	BP	GO:0007173	epidermal growth factor receptor signali...	17735	159	1802	29	17.07	0.00298	1.698887	ADCY4;AP2A1;AP2M1;APLP2;APP;CASP9;CBL;CDC42;CLTA;CLTC;EPS15L1;FOXO4;GRB2;GSK3A;MAP2K1;PHL
brown	CC	GO:0030666	endocytic vesicle membrane	17735	109	1802	21	11.1	0.00302	1.891892	AP2A1;AP2M1;ATP6V0A1;ATP6V0C;ATP6V0D1;CACNG8;CAMK2G;CLTA;CLTC;HLA-B;HLA-C;HLA-DPA1;HLA-E
brown	BP	GO:0002429	immune response-activating cell surface ...	17735	131	1802	25	14.06	0.00302	1.778094	BTK;BTN3A1;C5AR1;CD3D;FOXP3;HLA-DPA1;KLRC4-KLRK1;LAT2;LCP2;NFAM1;NFKB1;NFKBIA;PAK1;PIK3CD;I
brown	CC	GO:0005769	early endosome	17735	190	1802	32	19.35	0.00302	1.653747	ARF6;ATP6V0D1;CXCR7;ECE1;EHD1;EPHB1;FAM21A;FAM21C;FIG4;FKBP15;HFE;HLA-B;HLA-C;HLA-E;HSD17B
brown	MF	GO:0005083	small GTPase regulator activity	17735	295	1802	46	30.6	0.0032	1.503268	ADAP2;AKAP13;ALDH1A1;ARAP1;ARAP3;ARFGAP3;ARHGAP1;ARHGAP26;ARHGAP27;ARHGDIB;ARHGEF10;A
brown	MF	GO:0017166	vinculin binding	17735	11	1802	5	1.14	0.00323	4.385965	ACTN1;CTNNA1;DAG1;PXN;TLN1
brown	CC	GO:0019897	extrinsic to plasma membrane	17735	82	1802	17	8.35	0.0033	2.035928	APC2;CDH1;CTNNA1;CTNNB1;GNA11;GNA12;GNA15;GNAI2;GNAL;GNAS;GNAT2;GNB1;GNB5;HCK;NUMB;R/
brown	MF	GO:0042802	identical protein binding	17735	852	1802	113	88.38	0.00339	1.27857	ABAT;ABCG1;ACTG1;ADH5;ANXA9;APLP2;APOA1;APP;ARID3A;ATG7;ATPIF1;B4GALT1;BHLHE40;BNIP3;BTK;(
brown	CC	GO:0032991	macromolecular complex	17735	3893	1802	442	396.43	0.00347	1.114951	ABAT;ACTA1;ACTB;ACTG1;ADAR;AHI1;ALB;ANAPC16;ANAPC4;ANXA11;AP1M1;AP2A1;AP2M1;AP3B1;APC2;
brown	CC	GO:0005884	actin filament	17735	50	1802	12	5.09	0.00368	2.357564	ACTA1;ACTG1;APC2;BRCA1;FKBP15;GAS7;HCK;LCP1;MYO1F;PAK1;RCSD1;TPM4

brown	CC	GO:0005776	autophagic vacuole	17735	38	1802	10	3.87	0.00383	2.583979	DAPK2;NBR1;RAB23;SQSTM1;TBC1D14;TM9SF1;TP53INP2;ULK1;WIPI1;WIPI2
brown	CC	GO:0030659	cytoplasmic vesicle membrane	17735	360	1802	53	36.66	0.00387	1.445717	ANXA7;AP1M1;AP2A1;AP2M1;AP3B1;ARCN1;ARHGAP21;ATP6V0A1;ATP6V0C;ATP6V0D1;BSG;CACNG8;CAM
brown	CC	GO:0030670	phagocytic vesicle membrane	17735	51	1802	12	5.19	0.00438	2.312139	ATP6V0A1;ATP6V0C;ATP6V0D1;HLA-B;HLA-C;HLA-E;RAB11FIP1;RAB23;RAB43;RAB7A;RAB8A;SLC11A1
brown	MF	GO:0035258	steroid hormone receptor binding	17735	63	1802	14	6.54	0.00449	2.140673	BRCA1;CTNNB1;EP300;KAT5;NCOA6;NSD1;PHB2;PPID;PRKCB;RNF14;STAT3;STAT5B;WIPI1;YWHAH
brown	CC	GO:0043234	protein complex	17735	3294	1802	377	335.44	0.00452	1.123897	ABAT;ACTA1;ACTB;ACTG1;AHI1;ALB;ANAPC16;ANAPC4;ANXA11;AP1M1;AP2A1;AP2M1;AP3B1;APC2;APITD
brown	CC	GO:0000139	Golgi membrane	17735	540	1802	74	54.99	0.00492	1.345699	ABCG1;AP1M1;AP2A1;APC2;ARAP1;ARCN1;ARF1;ARF3;ARFGAP3;ARHGAP21;ARL1;B3GNT9;B4GALT1;B4GAL
brown	MF	GO:0005525	GTP binding	17735	366	1802	54	37.97	0.00494	1.422175	ACSM1;ARF1;ARF3;ARF6;ARL1;ARL6;CDC42;DNM2;EHD1;EHD3;GIMAP4;GIMAP8;GNA11;GNA12;GNA15;GN
brown	CC	GO:0000313	organellar ribosome	17735	52	1802	12	5.3	0.00518	2.264151	DAP3;MRP63;MRPL11;MRPL27;MRPL34;MRPL35;MRPL36;MRPL43;MRPL51;MRPS14;MRPS21;MRPS3
brown	CC	GO:0005761	mitochondrial ribosome	17735	52	1802	12	5.3	0.00518	2.264151	DAP3;MRP63;MRPL11;MRPL27;MRPL34;MRPL35;MRPL36;MRPL43;MRPL51;MRPS14;MRPS21;MRPS3
brown	MF	GO:0003779	actin binding	17735	351	1802	52	36.41	0.00528	1.428179	ACTN1;AFAP1;CAP1;CAPZB;CFL1;CFL2;CNN2;CORO1C;COTL1;CTNNA1;CYFIP1;DAG1;DBNL;EPB41;EPB41L3;E
brown	MF	GO:0032403	protein complex binding	17735	327	1802	49	33.92	0.00533	1.444575	ACTN1;ACVR1B;ATP6V0D1;CTNNB1;DAB2;DOCK2;DOK3;FCAR;FKBP1A;FLCN;FRS3;GNA11;GNA12;GNA15;G
brown	MF	GO:0005048	signal sequence binding	17735	22	1802	7	2.28	0.00535	3.070175	BRAP;BRE;KDELR1;KPNB1;NFKBIA;PEX7;SRP54
brown	MF	GO:0032451	demethylase activity	17735	22	1802	7	2.28	0.00535	3.070175	ALKBH2;ALKBH3;FTO;KDM2A;KDM5B;PHF2;PHF8
brown	CC	GO:0044429	mitochondrial part	17735	740	1802	97	75.36	0.00538	1.287155	ABAT;ACSM1;ACSM3;ADAP2;ALAS1;ALDH2;ATP5S;ATPIF1;BCKDHA;BCL2L13;BNIP3;C19orf12;CHCHD6;CHC
brown	MF	GO:0017016	Ras GTPase binding	17735	120	1802	22	12.45	0.00567	1.767068	BICD2;CDC42EP4;CYFIP1;DENND5A;ERC1;GOLGA5;MAP2K1;NCF2;NDRG1;NXT1;RAB11FIP4;RAB7A;RAB8A;R
brown	CC	GO:0008180	signalosome	17735	34	1802	9	3.46	0.00573	2.601156	BASP1;COPS7A;COPS8;DYNLL1;GRB2;HSPA1B;HSPA5;MYH9;NOD2
brown	MF	GO:0008092	cytoskeletal protein binding	17735	627	1802	85	65.04	0.00586	1.306888	AATF;ACTA1;ACTB;ACTN1;AFAP1;ALDOC;APC2;ARHGAP26;ARHGEF10;B4GALT1;BBS4;BRCA1;CAP1;CAPZB;C
brown	CC	GO:0005774	vacuolar membrane	17735	183	1802	30	18.64	0.00594	1.609442	AP1M1;AP2M1;ATP6V0A1;ATP6V0C;ATP6V0D1;CD1D;CD68;GBA;HGSNAT;HLA-DPA1;IGF2R;LAMP1;LAPTM
brown	CC	GO:0030117	membrane coat	17735	73	1802	15	7.43	0.00609	2.018843	AP1M1;AP2A1;AP2M1;AP3B1;ARCN1;ARL6;CLTA;CLTC;CLTCL1;COPA;COPB2;COPZ2;DAB2;SEC23B;SEC31A
brown	CC	GO:0048475	coated membrane	17735	73	1802	15	7.43	0.00609	2.018843	AP1M1;AP2A1;AP2M1;AP3B1;ARCN1;ARL6;CLTA;CLTC;CLTCL1;COPA;COPB2;COPZ2;DAB2;SEC23B;SEC31A
brown	MF	GO:0017137	Rab GTPase binding	17735	46	1802	11	4.77	0.0063	2.30608	BICD2;DENND5A;ERC1;GOLGA5;NDRG1;RAB11FIP4;RAB8A;RAC1;RIN3;RPH3A;ULK1
brown	CC	GO:0030120	vesicle coat	17735	41	1802	10	4.18	0.00686	2.392344	AP2A1;ARCN1;CLTA;CLTC;CLTCL1;COPA;COPB2;COPZ2;SEC23B;SEC31A
brown	CC	GO:0005765	lysosomal membrane	17735	139	1802	24	14.15	0.00687	1.696113	AP1M1;AP2M1;CD1D;CD68;GBA;HGSNAT;HLA-DPA1;IGF2R;LAMP1;LAPTM5;MARCH8;MYO7A;NCSTN;NEU1
brown	CC	GO:0030176	integral to endoplasmic reticulum membra...	17735	95	1802	18	9.67	0.00689	1.861427	BSCL2;DPAGT1;ELOVL1;HLA-B;HLA-C;HLA-DPA1;HLA-E;HM13;HSPA5;RRBP1;RTN1;RTN4;SGPL1;SLC27A5;SP
brown	MF	GO:0035591	signaling adaptor activity	17735	66	1802	14	6.85	0.00694	2.043796	ARHGAP1;CRKL;GAB2;GRB10;GRB2;LASP1;SH2D1A;SH2D3C;SH3BGR;SLA;SOCS2;SRC;TP53BP2;TRAT1
brown	CC	GO:0045121	membrane raft	17735	178	1802	29	18.13	0.00736	1.599559	ARID3A;BSG;BTK;CBL;CD14;DAG1;EPHB1;FLOT2;FURIN;GNAI2;HCK;HK1;HMOX1;IGF1R;INSR;KCNA5;LAT2;M
brown	MF	GO:0008565	protein transporter activity	17735	87	1802	17	9.03	0.00742	1.882614	ABCG1;AP2A1;ARFGAP3;C11orf73;COX18;KPNB1;LRP1;MRPL45;NUPL2;RAMP1;RUFY1;SEC61G;SNX1;TOMM
brown	MF	GO:0051020	GTPase binding	17735	145	1802	25	15.04	0.00752	1.662234	BICD2;BNIP3;CDC42EP4;CYFIP1;DENND5A;ERC1;GNB1;GOLGA5;MAP2K1;NCF2;NDRG1;NXT1;RAB11FIP4;RA
brown	MF	GO:0030675	Rac GTPase activator activity	17735	13	1802	5	1.35	0.00753	3.703704	ARHGAP1;ARHGAP27;DOCK2;RALBP1;SRGAP2
brown	MF	GO:0043325	phosphatidylinositol-3,4-bisphosphate bi...	17735	13	1802	5	1.35	0.00753	3.703704	ADAP2;ARAP3;GAB2;PLEK;ZFYVE1
brown	MF	GO:0043560	insulin receptor substrate binding	17735	13	1802	5	1.35	0.00753	3.703704	GRB2;IGF1R;IL4R;INSR;PRKCD
brown	MF	GO:0046965	retinoid X receptor binding	17735	13	1802	5	1.35	0.00753	3.703704	MED25;NCOA6;NCOR1;NSD1;VDR
brown	MF	GO:0048029	monosaccharide binding	17735	60	1802	13	6.22	0.00758	2.090032	ALKBH3;BSG;CD209;COLEC12;FBP1;G6PD;GCK;HK2;IGF2R;MANBA;PHYH;PLOD1;PYGL
brown	MF	GO:0005544	calcium-dependent phospholipid binding	17735	29	1802	8	3.01	0.00772	2.657807	ANXA11;ANXA4;ANXA5;ANXA7;ANXA9;DYSF;RPH3A;SYT2
brown	CC	GO:0042995	cell projection	17735	1194	1802	147	121.59	0.00774	1.208981	ABAT;ACP1;ACTB;ACTN1;ADCY4;AHI1;ALDOC;ANKS1A;APBB1IP;APC2;APP;ARAP3;ARF6;ARHGAP1;ARHGAP2
brown	CC	GO:0031975	envelope	17735	839	1802	107	85.44	0.00813	1.252341	ADAP2;ANXA11;ANXA7;ATP5S;ATPIF1;BCL2L13;BNIP3;C19orf12;CERS2;CHCHD6;CHCHD7;CLPX;COX11;COX1
brown	MF	GO:0003713	transcription coactivator activity	17735	270	1802	41	28.01	0.00815	1.463763	ARID1A;ARNT;BRCA1;CALCOCO1;CARM1;CD3D;CITED2;CREBBP;CTNNB1;EP300;GMEB1;GTF2F1;HIPK2;ING4
brown	CC	GO:0030140	trans-Golgi network transport vesicle	17735	24	1802	7	2.44	0.00818	2.868852	AP2A1;CLTA;CLTC;CLTCL1;FURIN;IGF2R;SPG21
brown	CC	GO:0005905	coated pit	17735	55	1802	12	5.6	0.00828	2.142857	AP2A1;AP2M1;APP;CLTA;CLTC;CLTCL1;DAB2;EPS15L1;ITSN1;LRP1;LRP10;RAB35
brown	CC	GO:0030863	cortical cytoskeleton	17735	55	1802	12	5.6	0.00828	2.142857	ACTB;CAP1;CAPZB;CFL1;EPB41;LASP1;MAPRE1;MPP1;MYH9;MYO1F;SPTB;TPM4
brown	MF	GO:0003682	chromatin binding	17735	327	1802	48	33.92	0.00847	1.415094	APITD1;ARID3A;BCL6;BRD3;CALCOCO1;CBFA2T3;CBX1;CDC5L;CITED2;CREBBP;CTNNB1;CUX1;EP300;ESR1;E
brown	CC	GO:0005834	heterotrimeric G-protein complex	17735	36	1802	9	3.67	0.00855	2.452316	GNA11;GNA12;GNA15;GNAI2;GNAL;GNAS;GNAT2;GNB1;GNB5
brown	CC	GO:0044451	nucleoplasm part	17735	823	1802	105	83.81	0.00859	1.252834	ACIN1;ACTB;AFF2;APITD1;ARNT;ASH2L;ATF5;BASP1;BRD8;BRPF3;CASC3;CBY1;CDC5L;CHD8;CPSF7;CREBBP;
brown	CC	GO:0005798	Golgi-associated vesicle	17735	62	1802	13	6.31	0.00865	2.060222	AP2A1;ARCN1;CLTA;CLTC;CLTCL1;COPA;COPB2;COPZ2;FURIN;IGF2R;PACS1;SPG21;SPPL3
brown	MF	GO:0031490	chromatin DNA binding	17735	30	1802	8	3.11	0.00959	2.572347	BCL6;EP300;HMGN3;HMGN4;NOTCH1;RCC1;RXRA;SRF
brown	CC	GO:0005913	cell-cell adherens junction	17735	43	1802	10	4.38	0.00974	2.283105	ACTN1;CDH1;CTNNA1;CTNNB1;CTNND1;MYH9;NDRG1;PVRL1;VCL;ZYX
brown	MF	GO:0032393	MHC class I receptor activity	17735	19	1802	6	1.97	0.0102	3.045685	HLA-B;HLA-C;HLA-E;KIR3DL1;LILRB1;LILRB2
brown	CC	GO:0030660	Golgi-associated vesicle membrane	17735	37	1802	9	3.77	0.0103	2.387268	AP2A1;ARCN1;CLTA;CLTC;CLTCL1;COPA;COPB2;COPZ2;SPPL3
brown	MF	GO:0019001	guanyl nucleotide binding	17735	380	1802	54	39.42	0.01039	1.369863	ACSM1;ARF1;ARF3;ARF6;ARL1;ARL6;CDC42;DNM2;EHD1;EHD3;GIMAP4;GIMAP8;GNA11;GNA12;GNA15;GN
brown	MF	GO:0032561	guanyl ribonucleotide binding	17735	380	1802	54	39.42	0.01039	1.369863	ACSM1;ARF1;ARF3;ARF6;ARL1;ARL6;CDC42;DNM2;EHD1;EHD3;GIMAP4;GIMAP8;GNA11;GNA12;GNA15;GN
brown	MF	GO:0042288	MHC class I protein binding	17735	14	1802	5	1.45	0.01072	3.448276	KLRC4-KLRK1;LILRB1;LILRB2;TAP2;TAPBP
brown	CC	GO:0055037	recycling endosome	17735	71	1802	14	7.23	0.01139	1.936376	ABCG1;ARF6;CXCR7;EHD1;EHD3;HFE;LMTK2;NDRG1;PLEKHB2;RAB11FIP1;RAB11FIP4;RAB8A;SCAMP2;VAM
brown	MF	GO:0035091	phosphatidylinositol binding	17735	150	1802	25	15.56	0.01148	1.606684	ADAP2;ARAP1;ARAP3;BTK;DAB2;EPB41;GAB2;HIP1;PHF12;PITPNA;PLEK;RAB35;SH3PXD2B;SH3YL1;SNX1;SN
brown	CC	GO:0030662	coated vesicle membrane	17735	145	1802	24	14.77	0.0115	1.624915	AP1M1;AP2A1;AP2M1;AP3B1;ARCN1;CLTA;CLTC;CLTCL1;COPA;COPB2;COPZ2;DAB2;DENND1A;DNAJC5;HIP
brown	MF	GO:0042169	SH2 domain binding	17735	31	1802	8	3.22	0.01177	2.484472	DAG1;INSR;KHDRBS2;LAT2;LILRB1;SH3PXD2B;SQSTM1;SRC
brown	MF	GO:0042277	peptide binding	17735	143	1802	24	14.83	0.01204	1.618341	APOA1;BRAP;BRE;CD1C;CD209;CLTA;ECE1;FURIN;IGF1R;INSR;KDELR1;KPNB1;LDLRAD3;MAML1;NFKBIA;NO
brown	MF	GO:0035257	nuclear hormone receptor binding	17735	128	1802	22	13.28	0.01208	1.656627	BRCA1;CTNNB1;EP300;HMGN3;KAT5;MED12;MED25;NCOA6;NCOR1;NSD1;PHB2;PPID;PRKCB;RNF14;RXRA;
brown	CC	GO:0031253	cell projection membrane	17735	177	1802	28	18.02	0.0123	1.553829	APC2;ARF6;ARL6;B4GALT1;BBS4;CFL1;CTNNB1;EMR2;EPB41L3;EVC;FZD1;GNA12;ITGA5;KCNC3;LCP1;MAPR
brown	MF	GO:0042803	protein homodimerization activity	17735	545	1802	73	56.54	0.01325	1.291121	ABAT;ABCG1;ADH5;ANXA9;ARID3A;ATG7;ATPIF1;B4GALT1;BHLHE40;BNIP3;CALCOCO2;CARM1;CARS;CCBL1
brown	MF	GO:0005547	phosphatidylinositol-3,4,5-trisphosphate...	17735	20	1802	6	2.07	0.01331	2.898551	ADAP2;ARAP1;ARAP3;BTK;GAB2;ZFYVE1
brown	CC	GO:0000407	pre-autophagosomal structure	17735	15	1802	5	1.53	0.01366	3.267974	NBR1;SQSTM1;ULK1;WIPI1;WIPI2
brown	CC	GO:0031306	intrinsic to mitochondrial outer membran...	17735	15	1802	5	1.53	0.01366	3.267974	BNIP3;CPT1A;FIS1;FUNDC1;MFN2
brown	MF	GO:0004713	protein tyrosine kinase activity	17735	145	1802	24	15.04	0.01417	1.595745	AATK;BTK;CAMKK2;CSF1R;DYRK4;EPHB1;EPHB2;FLT3;HCK;IGF1R;IGF2R;INSR;JAK1;JAK3;LMTK2;MAP2K1;M
brown	MF	GO:0030674	protein binding, bridging	17735	130	1802	22	13.49	0.01436	1.630838	ADAP2;ARHGAP1;ARHGAP26;COL8A2;CRKL;CUX1;DAB2;FSCN1;GAB2;GRB10;GRB2;HSPA5;LASP1;SH2D1A;S
brown	CC	GO:0032587	ruffle membrane	17735	59	1802	12	6.01	0.01448	1.996672	CFL1;EMR2;ITGA5;LCP1;PLEK;PTPRJ;RAC1;RHOA;SH3YL1;SPRY2;TIRAP;TLN1
brown	MF	GO:0004029	aldehyde dehydrogenase (NAD) activity	17735	10	1802	4	1.04	0.0145	3.846154	ADH5;ALDH1A1;ALDH2;ALDH9A1
brown	CC	GO:0044431	Golgi apparatus part	17735	654	1802	84	66.6	0.01492	1.261261	ABCG1;AP1M1;AP2A1;APC2;ARAP1;ARCN1;ARF1;ARF3;ARFGAP3;ARHGAP21;ARL1;B3GNT9;B4GALT1;B4GAL
brown	MF	GO:0008168	methyltransferase activity	17735	177	1802	28	18.36	0.01542	1.525054	ASH2L;BCDIN3D;CARM1;COQ3;COQ5;DNMT3B;DPH5;EMG1;HENMT1;LCMT2;METTL10;METTL23;METTL5;
brown	MF	GO:0000989	transcription factor binding transcripti...	17735	498	1802	67	51.66	0.01562	1.296942	ARID1A;ARNT;ATF5;BASP1;BCOR;BHLHE40;BRCA1;CALCOCO1;CARM1;CD3D;CITED2;CREBBP;CTNNB1;DNM

brown	CC	GO:0031967	organelle envelope	17735	825	1802	103	84.01	0.01639	1.226045	ADAP2;ANXA11;ANXA7;ATP5S;ATPIF1;BCL2L13;BNIP3;C19orf12;CERS2;CHCHD6;CHCHD7;CLPX;COX11;COX1
brown	MF	GO:0033218	amide binding	17735	147	1802	24	15.25	0.01659	1.57377	APOA1;BRAP;BRE;CD1C;CD209;CLTA;ECE1;FURIN;IGF1R;INSR;KDELR1;KPNB1;LDLRAD3;MAML1;NFKBIA;NOI
brown	MF	GO:0001085	RNA polymerase II transcription factor b...	17735	73	1802	14	7.57	0.01675	1.849406	BHLHE40;CITED2;CREBBP;CTNNB1;EP300;HCLS1;HIPK2;KLF4;NCOR1;RBPJ;SIN3A;SPEN;STAT3;WWP2
brown	CC	GO:0030133	transport vesicle	17735	142	1802	23	14.46	0.0168	1.590595	AP2A1;BLOC1S3;CHGA;CLTA;CLTC;CLTCL1;CRISPLD2;FURIN;GNAS;HCK;HLA-B;HLA-C;HLA-DPA1;HLA-E;IGF2R
brown	CC	GO:0019898	extrinsic to membrane	17735	127	1802	21	12.93	0.01746	1.62413	APC2;CDH1;CTNNA1;CTNNB1;EPB41;EPB41L3;GNA11;GNA12;GNA15;GNAI2;GNAL;GNAS;GNAT2;GNB1;GNE
brown	MF	GO:0046966	thyroid hormone receptor binding	17735	27	1802	7	2.8	0.0176	2.5	HMGN3;MED12;NCOA6;NCOR1;NSD1;TAF11;THRAP3
brown	CC	GO:0031227	intrinsic to endoplasmic reticulum membr...	17735	112	1802	19	11.41	0.0179	1.665206	BSCL2;DPAGT1;ELOVL1;EXTL3;HLA-B;HLA-C;HLA-DPA1;HLA-E;HM13;HSPA5;RRBP1;RTN1;RTN4;SGPL1;SLC27
brown	MF	GO:0051427	hormone receptor binding	17735	148	1802	24	15.35	0.01792	1.563518	BRCA1;CTNNB1;EP300;HMGN3;JAK1;KAT5;MED12;MED25;NCOA6;NCOR1;NSD1;PHB2;PPID;PRKCB;RNF14;F
brown	CC	GO:0035869	ciliary transition zone	17735	16	1802	5	1.63	0.01824	3.067485	AHI1;CC2D2A;TCTN1;TMEM237;TMEM67
brown	MF	GO:0031267	small GTPase binding	17735	133	1802	22	13.8	0.0184	1.594203	BICD2;CDC42EP4;CYFIP1;DENND5A;ERC1;GOLGA5;MAP2K1;NCF2;NDRG1;NXT1;RAB11FIP4;RAB7A;RAB8A;R
brown	CC	GO:0005740	mitochondrial envelope	17735	520	1802	68	52.95	0.01854	1.28423	ADAP2;ATP5S;ATPIF1;BCL2L13;BNIP3;C19orf12;CHCHD6;CHCHD7;CLPX;COX11;COX17;COX18;COX5A;COX6A
brown	CC	GO:0048471	perinuclear region of cytoplasm	17735	469	1802	62	47.76	0.01932	1.298157	AATK;ANP32A;APC2;APP;ARF1;ARF3;ATXN2;CALCOCO2;CASC3;CDH1;CKAP4;CSF1;CSNK1D;CTIF;CTNNB1;CTS
brown	CC	GO:0071556	integral to lumenal side of endoplasmic ...	17735	28	1802	7	2.85	0.01953	2.45614	HLA-B;HLA-C;HLA-DPA1;HLA-E;HM13;SPPL3;TAPBP
brown	CC	GO:0030130	clathrin coat of trans-Golgi network ves...	17735	11	1802	4	1.12	0.01968	3.571429	AP2A1;CLTA;CLTC;CLTCL1
brown	CC	GO:0031256	leading edge membrane	17735	98	1802	17	9.98	0.01979	1.703407	APC2;CFL1;EMR2;EPB41L3;FZD1;ITGA5;KCNC3;LCP1;PLEK;PTPRJ;RAC1;RHOA;SH3YL1;SPRY2;TIRAP;TLN1;VAS
brown	MF	GO:0004553	hydrolase activity, hydrolyzing O-glycos...	17735	89	1802	16	9.23	0.01991	1.733478	AMY2B;CD44;FUCA2;GBA;GLB1;GLB1L;GM2A;HEXB;LYG1;MAN2A2;MAN2B2;MANBA;MGAM;NAGA;NEU1;P
brown	MF	GO:0060090	binding, bridging	17735	142	1802	23	14.73	0.02042	1.561439	ADAP2;ARHGAP1;ARHGAP26;COL8A2;CRKL;CUX1;DAB2;FSCN1;GAB2;GRB10;GRB2;HSPA5;LASP1;PDCD6;SH
brown	MF	GO:0001102	RNA polymerase II activating transcripti...	17735	34	1802	8	3.53	0.02052	2.266289	BHLHE40;CITED2;CREBBP;CTNNB1;EP300;HIPK2;NCOR1;SIN3A
brown	MF	GO:0070008	serine-type exopeptidase activity	17735	11	1802	4	1.14	0.02095	3.508772	CPVL;CTSA;PRCP;SCPEP1
brown	MF	GO:0005097	Rab GTPase activator activity	17735	54	1802	11	5.6	0.02104	1.964286	C6orf170;GDI2;TBC1D1;TBC1D14;TBC1D19;TBC1D2;TBC1D20;TBC1D2B;TBC1D5;TBC1D8;TBC1D9
brown	CC	GO:0030135	coated vesicle	17735	242	1802	35	24.64	0.02115	1.420455	AP1M1;AP2A1;AP2M1;AP3B1;ARCN1;ATP6V0D1;CLTA;CLTC;CLTCL1;COPA;COPB2;COPZ2;DAB2;DENND1A;D
brown	MF	GO:0001076	RNA polymerase II transcription factor b...	17735	97	1802	17	10.06	0.02118	1.689861	BHLHE40;CITED2;CREBBP;HDGF;HIPK2;KLF4;MED12;NACC2;NOTCH1;PHF12;SIN3A;SMARCA2;SOX4;SPEN;SF
brown	MF	GO:0001191	RNA polymerase II transcription factor b...	17735	28	1802	7	2.9	0.02143	2.413793	BHLHE40;CITED2;CREBBP;HDGF;PHF12;SIN3A;SPEN
brown	MF	GO:0005158	insulin receptor binding	17735	28	1802	7	2.9	0.02143	2.413793	DOK3;FRS3;GRB10;IGF1R;IRS2;PTPN1;TLN1
brown	MF	GO:0016903	oxidoreductase activity, acting on the a...	17735	41	1802	9	4.25	0.02237	2.117647	ADH5;AKR1C3;ALDH1A1;ALDH2;ALDH3B1;ALDH9A1;BCKDHA;DHTKD1;GAPDHS
brown	CC	GO:0005802	trans-Golgi network	17735	115	1802	19	11.71	0.02314	1.622545	AP1M1;ARL1;ATXN2;BECN1;CBY1;CDH1;CLTA;CLTC;CLTCL1;FURIN;GNAS;HLA-DPA1;KIAA1324;KIF13A;OPTN
brown	MF	GO:0030246	carbohydrate binding	17735	215	1802	32	22.3	0.02315	1.434978	ALKBH3;BSG;CD209;CD93;CLC;CLEC4C;COLEC12;DGCR2;ENDOU;FBP1;FCN1;G6PD;GCK;HK2;IGF2R;KLRC4-KL
brown	MF	GO:0016741	transferase activity, transferring one-c...	17735	183	1802	28	18.98	0.0232	1.475237	ASH2L;BCDIN3D;CARM1;COQ3;COQ5;DNMT3B;DPH5;EMG1;HENMT1;LCMT2;METTL10;METTL23;METTL5;N
brown	MF	GO:0008270	zinc ion binding	17735	1977	1802	231	205.09	0.02339	1.126335	ADAM19;ADAP2;ADH5;AGBL2;ALB;AMZ2;ANPEP;APOBEC3C;ARAP1;ARAP3;ARFGAP3;ATF7;BAZ2A;BCL6;BRA
brown	CC	GO:0008305	integrin complex	17735	29	1802	7	2.95	0.02355	2.372881	ITGA5;ITGAE;ITGAM;ITGAX;ITGB2;ITGB4;MYH9
brown	CC	GO:0017053	transcriptional repressor complex	17735	63	1802	12	6.42	0.02369	1.869159	CSNK2A1;HDAC4;HDGF;NACC2;NCOR1;PHF12;RCOR1;SIN3A;SKI;SPEN;TBL1X;YWHAB
brown	CC	GO:0030312	external encapsulating structure	17735	17	1802	5	1.73	0.02372	2.890173	B4GALT1;DAG1;GRIN2D;P2RX1;SLC11A1
brown	MF	GO:0008484	sulfuric ester hydrolase activity	17735	17	1802	5	1.76	0.0255	2.840909	ARSD;ARSG;GALNS;GNS;SULF2
brown	MF	GO:0042054	histone methyltransferase activity	17735	49	1802	10	5.08	0.02679	1.968504	ASH2L;CARM1;MLL2;NSD1;PRMT5;PRMT6;SETD1B;SETDB1;SETMAR;WDR82
brown	CC	GO:0005901	caveola	17735	57	1802	11	5.8	0.02707	1.896552	CBL;FLOT2;HCK;HMOX1;IGF1R;INSR;KCNA5;MYOF;PTRF;SPRED1;SRC
brown	CC	GO:0012510	trans-Golgi network transport vesicle me...	17735	12	1802	4	1.22	0.02718	3.278689	AP2A1;CLTA;CLTC;CLTCL1
brown	CC	GO:0016580	Sin3 complex	17735	12	1802	4	1.22	0.02718	3.278689	CSNK2A1;NCOR1;PHF12;SIN3A
brown	CC	GO:0036038	TCTN-B9D complex	17735	12	1802	4	1.22	0.02718	3.278689	AHI1;CC2D2A;TCTN1;TMEM67
brown	CC	GO:0070822	Sin3-type complex	17735	12	1802	4	1.22	0.02718	3.278689	CSNK2A1;NCOR1;PHF12;SIN3A
brown	CC	GO:0071203	WASH complex	17735	12	1802	4	1.22	0.02718	3.278689	CAPZB;FAM21A;FAM21C;KIAA0196
brown	MF	GO:0019902	phosphatase binding	17735	115	1802	19	11.93	0.02738	1.592624	ANAPC4;AP3B1;CDH1;CTNNB1;CTNND1;ELL;ENSA;GCK;GRB2;LILRB1;LILRB2;MAGI2;MAP3K5;SH3YL1;SMG7;
brown	CC	GO:0031966	mitochondrial membrane	17735	496	1802	64	50.51	0.02801	1.267076	ATP5S;ATPIF1;BCL2L13;BNIP3;C19orf12;CHCHD6;CLPX;COX11;COX18;COX5A;COX6A1;CPOX;CPT1A;CPT2;CY
brown	CC	GO:0005801	cis-Golgi network	17735	30	1802	7	3.05	0.02809	2.295082	COPZ2;GOLGA2;GOLGA5;GOLIM4;GOSR1;KDELR1;LIMK2
brown	MF	GO:0048306	calcium-dependent protein binding	17735	36	1802	8	3.73	0.02844	2.144772	ANXA11;ANXA7;PDCD6;RBM22;SEC31A;SLC9A1;TNNC1;TSG101
brown	MF	GO:0008198	ferrous iron binding	17735	12	1802	4	1.24	0.02889	3.225806	ALKBH2;ALKBH3;FTO;HAAO
brown	MF	GO:0000988	protein binding transcription factor act...	17735	514	1802	67	53.32	0.02903	1.256564	ARID1A;ARNT;ATF5;BASP1;BCOR;BHLHE40;BRCA1;CALCOCO1;CARM1;CD3D;CITED2;CREBBP;CTNNB1;DNMT
brown	MF	GO:0016620	oxidoreductase activity, acting on the a...	17735	30	1802	7	3.11	0.03072	2.250804	ADH5;AKR1C3;ALDH1A1;ALDH2;ALDH3B1;ALDH9A1;GAPDHS
brown	CC	GO:0031234	extrinsic to internal side of plasma mem...	17735	51	1802	10	5.19	0.0309	1.926782	GNA11;GNA12;GNA15;GNAI2;GNAL;GNAS;GNAT2;GNB1;GNB5;HCK
brown	CC	GO:0030496	midbody	17735	88	1802	15	8.96	0.03163	1.674107	ANXA11;CDC42;CTDP1;CTNND1;DNM2;FLCN;GNAI2;HSPA5;KIF13A;MAPRE3;RAB11FIP4;RALB;RHOA;SSH1;T
brown	CC	GO:0016605	PML body	17735	73	1802	13	7.43	0.03171	1.749664	ELF4;HIPK2;MAX;MKNK2;N4BP1;NR2C1;RPAIN;SKI;TOP3A;TP53INP2;TRIM27;TRIM8;UBN1
brown	MF	GO:0042287	MHC protein binding	17735	24	1802	6	2.49	0.03233	2.409639	KLRC4-KLRK1;LILRB1;LILRB2;MARCH8;TAP2;TAPBP
brown	MF	GO:0042605	peptide antigen binding	17735	18	1802	5	1.87	0.03237	2.673797	CD209;MAML1;SLC7A5;TAP2;TAPBP
brown	MF	GO:0003712	transcription cofactor activity	17735	475	1802	62	49.28	0.03383	1.258117	ARID1A;ARNT;ATF5;BASP1;BCOR;BRCA1;CALCOCO1;CARM1;CD3D;CITED2;CREBBP;CTNNB1;DNMT3B;ENO1
brown	CC	GO:0000790	nuclear chromatin	17735	152	1802	23	15.48	0.0345	1.485788	ARID1A;BRD4;CALCOCO1;CBX1;CITED2;CREBBP;CSNK2A1;DNMT3B;H1F0;HNRNPK;KDM2A;KLF4;NACC2;NCC
brown	CC	GO:0031300	intrinsic to organelle membrane	17735	218	1802	31	22.2	0.03514	1.396396	BNIP3;BSCL2;COX18;CPT1A;CUX1;DPAGT1;ECE1;ELOVL1;EXTL3;FIS1;FUNDC1;GALNT2;HLA-B;HLA-C;HLA-DPA
brown	MF	GO:0004725	protein tyrosine phosphatase activity	17735	103	1802	17	10.69	0.03583	1.590271	ACP1;DUSP12;DUSP18;EYA3;MAP2K1;MDP1;MTMR14;MTMR3;PTPLA;PTPMT1;PTPN1;PTPN6;PTPN9;PTPRJ;
brown	CC	GO:0002102	podosome	17735	13	1802	4	1.32	0.03617	3.030303	BIN2;SH3PXD2B;SVIL;TPM4
brown	CC	GO:0005938	cell cortex	17735	186	1802	27	18.94	0.03752	1.425554	ACTB;ARF6;CAP1;CAPZB;CFL1;CTNNB1;DBNL;DVL3;EPB41;GCK;LASP1;MAEA;MAP2K1;MAPRE1;MARK2;MPP
brown	CC	GO:0030137	COPI-coated vesicle	17735	19	1802	5	1.93	0.0376	2.590674	ARCN1;COPA;COPB2;COPZ2;PACS1
brown	CC	GO:0044448	cell cortex part	17735	90	1802	15	9.16	0.03776	1.637555	ACTB;CAP1;CAPZB;CFL1;EPB41;GCK;LASP1;MAEA;MAPRE1;MARK2;MPP1;MYH9;MYO1F;SPTB;TPM4
brown	CC	GO:0005743	mitochondrial inner membrane	17735	347	1802	46	35.34	0.03777	1.301641	ATP5S;ATPIF1;CHCHD6;CLPX;COX11;COX18;COX5A;COX6A1;CPOX;CPT1A;CPT2;CYB5R3;CYP27A1;DNAJC19;
brown	MF	GO:0050681	androgen receptor binding	17735	38	1802	8	3.94	0.03824	2.030457	BRCA1;CTNNB1;EP300;KAT5;NSD1;PRKCB;RNF14;WIPI1
brown	CC	GO:0032588	trans-Golgi network membrane	17735	32	1802	7	3.26	0.03889	2.147239	AP1M1;CLTA;CLTC;GNAS;HLA-DPA1;KIF13A;SCAMP2
brown	MF	GO:0030331	estrogen receptor binding	17735	25	1802	6	2.59	0.03893	2.316602	CTNNB1;NCOA6;NSD1;PHB2;PPID;WIPI1
brown	MF	GO:0046914	transition metal ion binding	17735	2217	1802	254	229.99	0.03932	1.104396	ADAM19;ADAP2;ADH5;AGBL2;ALB;ALKBH2;ALKBH3;ALOX15B;AMZ2;ANPEP;AOC2;APLP2;APOBEC3C;APP;AI
brown	CC	GO:0030054	cell junction	17735	756	1802	92	76.99	0.03948	1.19496	AATF;ACTN1;AFAP1;AHI1;AMICA1;ANO7;APBB1IP;ARHGAP21;ARHGAP24;ARHGAP26;ARHGAP31;B4GALT1;
brown	MF	GO:0016798	hydrolase activity, acting on glycosyl b...	17735	112	1802	18	11.62	0.03968	1.549053	ADPRH;AMY2B;CD44;FUCA2;GBA;GLB1;GLB1L;GM2A;HEXB;LYG1;MAN2A2;MAN2B2;MANBA;MGAM;NAGA
brown	MF	GO:0019903	protein phosphatase binding	17735	74	1802	13	7.68	0.03978	1.692708	ANAPC4;AP3B1;CDH1;CTNNB1;CTNND1;ENSA;GCK;GRB2;LILRB1;LILRB2;MAP3K5;SMG7;SOD1
brown	MF	GO:0019200	carbohydrate kinase activity	17735	19	1802	5	1.97	0.04029	2.538071	GCK;HK1;HK2;NAGK;PFKFB3

brown	CC	GO:0031301	integral to organelle membrane	17735	196	1802	28	19.96	0.04138	1.402806	BNIP3;BSCL2;COX18;CPT1A;CUX1;DPAGT1;ELOVL1;FIS1;FUNDC1;GALNT2;HLA-B;HLA-C;HLA-DPA1;HLA-E;HM
brown	CC	GO:0034708	methyltransferase complex	17735	61	1802	11	6.21	0.04232	1.771337	ACTB;ASH2L;CHD8;JARID2;MAX;MLL2;NCOA6;SETD1B;TAF4;TRMT61B;WDR82
brown	MF	GO:0019213	deacetylase activity	17735	32	1802	7	3.32	0.04238	2.108434	CES1;ESD;HDAC4;NACC2;NDST1;NDST2;SIN3A
brown	CC	GO:0031901	early endosome membrane	17735	84	1802	14	8.55	0.04372	1.637427	EHD1;EPHB1;FAM21A;FAM21C;FIG4;HLA-B;HLA-C;HLA-E;HSD17B6;MARCH8;RAB5B;RAB5C;RUFY1;SNX1
brown	MF	GO:0016706	oxidoreductase activity, acting on paire...	17735	46	1802	9	4.77	0.04418	1.886792	ALKBH2;ALKBH3;FTO;KDM2A;KDM5B;PHF8;PHYH;PLOD1;TET3
brown	CC	GO:0044455	mitochondrial membrane part	17735	148	1802	22	15.07	0.04504	1.459854	ATPIF1;BNIP3;COX18;COX6A1;CPT1A;FIS1;FUNDC1;IMMP2L;MFN2;MICU1;MRPL45;NDUFA12;NDUFA7;NDU
brown	CC	GO:0005791	rough endoplasmic reticulum	17735	47	1802	9	4.79	0.04513	1.878914	GLUL;HM13;PLOD1;PSEN1;RP9;RPN1;SPPL3;SRPR;STAU1
brown	CC	GO:0031941	filamentous actin	17735	20	1802	5	2.04	0.04606	2.45098	ACTG1;BRCA1;MYO1F;PAK1;TPM4
brown	CC	GO:0000785	chromatin	17735	300	1802	40	30.55	0.04626	1.309329	ARID1A;BAZ2A;BRD4;CALCOCO1;CBX1;CBX6;CHEK1;CITED2;CREBBP;CSNK2A1;DNMT3B;DSCC1;EXOSC3;H1F
brown	CC	GO:0030126	COPI vesicle coat	17735	14	1802	4	1.43	0.04666	2.797203	ARCN1;COPA;COPB2;COPZ2
brown	CC	GO:0030313	cell envelope	17735	14	1802	4	1.43	0.04666	2.797203	DAG1;GRIN2D;P2RX1;SLC11A1
brown	CC	GO:0031307	integral to mitochondrial outer membrane	17735	14	1802	4	1.43	0.04666	2.797203	BNIP3;CPT1A;FIS1;FUNDC1
brown	CC	GO:0044462	external encapsulating structure part	17735	14	1802	4	1.43	0.04666	2.797203	DAG1;GRIN2D;P2RX1;SLC11A1
brown	CC	GO:0043005	neuron projection	17735	627	1802	77	63.85	0.04702	1.205951	ABAT;ACP1;ACTB;ACTN1;ADCY4;ALDOC;ANKS1A;APP;ATP6V0D1;BASP1;BECN1;BNIP3;CACNG8;CDC42;CDH
brown	MF	GO:0015078	hydrogen ion transmembrane transporter a...	17735	99	1802	16	10.27	0.04821	1.557936	ATP5S;ATP6V0A1;ATP6V0C;ATP6V0D1;ATP6V1B2;COX11;COX5A;COX6A1;CYB5A;SLC11A1;SLC36A1;SLC9A1;
brown	MF	GO:0001190	RNA polymerase II transcription factor b...	17735	33	1802	7	3.42	0.04916	2.046784	CITED2;CREBBP;HIPK2;KLF4;NOTCH1;SMARCA2;SOX4
brown	MF	GO:0004683	calmodulin-dependent protein kinase acti...	17735	20	1802	5	2.07	0.04929	2.415459	CAMK1D;CAMK2G;CAMKK2;DAPK2;PHKA2
brown	MF	GO:0048365	Rac GTPase binding	17735	20	1802	5	2.07	0.04929	2.415459	CYFIP1;NCF2;RAB7A;RALBP1;SRGAP2
brown	MF	GO:0004089	carbonate dehydratase activity	17735	14	1802	4	1.45	0.04945	2.758621	CA13;CA14;CA3;CA6
brown	MF	GO:0005159	insulin-like growth factor receptor bind...	17735	14	1802	4	1.45	0.04945	2.758621	INSR;SOCS2;YWHAG;YWHAH
brown	MF	GO:0015929	hexosaminidase activity	17735	14	1802	4	1.45	0.04945	2.758621	CD44;GM2A;HEXB;NAGA
blue	CC	GO:0005622	intracellular	17735	12344	2487	1968	1738.17	1.00E-30	1.132225	AAK1;AARS;AARS2;ABCA2;ABCB1;ABCC1;ABCF1;ABCF2;ABHD6;ABL1;ABL2;ABLIM1;ACAA2;ACACA;ACACB;A
blue	CC	GO:0044424	intracellular part	17735	12103	2487	1926	1704.23	3.00E-30	1.130129	AAK1;AARS;AARS2;ABCA2;ABCB1;ABCC1;ABCF1;ABCF2;ABHD6;ABL1;ABL2;ABLIM1;ACAA2;ACACA;ACACB;A
blue	CC	GO:0005634	nucleus	17735	5873	2487	1073	826.98	1.00E-29	1.297492	ABCC1;ABCF1;ABL1;ACLY;ACO2;ACTR8;ADD1;AGBL5;AGPAT3;AHNAK;AIF1;AIFM1;AKAP17A;AKAP8;AKNA;A
blue	CC	GO:0044428	nuclear part	17735	2482	2487	538	349.49	2.60E-29	1.539386	ABCF1;ABL1;ACTR8;AGPAT3;AKAP17A;AKAP8;AMOTL1;ANAPC1;ANAPC13;ANAPC5;ANAPC7;ANKLE2;ANKS1
blue	CC	GO:0043231	intracellular membrane-bounded organelle	17735	9560	2487	1585	1346.15	1.40E-27	1.177432	AAK1;AARS2;ABCA2;ABCB1;ABCC1;ABCF1;ABCF2;ABHD6;ABL1;ACAA2;ACACA;ACACB;ACAD8;ACE;ACER2;AC
blue	CC	GO:0043227	membrane-bounded organelle	17735	9590	2487	1588	1350.37	2.40E-27	1.175974	AAK1;AARS2;ABCA2;ABCB1;ABCC1;ABCF1;ABCF2;ABHD6;ABL1;ACAA2;ACACA;ACACB;ACAD8;ACE;ACER2;AC
blue	CC	GO:0070013	intracellular organelle lumen	17735	2677	2487	560	376.95	1.90E-26	1.485608	ABCF1;ABL1;ACAD8;ACO2;ACOX2;ACOX3;ACTR8;AKAP17A;AKAP8;ALDH1B1;ALDH5A1;ALDH6A1;ALDH7A1;
blue	CC	GO:0031974	membrane-enclosed lumen	17735	2770	2487	571	390.05	2.40E-25	1.463915	ABCF1;ABL1;ACAD8;ACN9;ACO2;ACOX2;ACOX3;ACTR8;AIFM1;AK2;AKAP17A;AKAP8;ALDH1B1;ALDH5A1;AL
blue	CC	GO:0043233	organelle lumen	17735	2722	2487	563	383.29	2.50E-25	1.468862	ABCF1;ABL1;ACAD8;ACO2;ACOX2;ACOX3;ACTR8;AKAP17A;AKAP8;ALDH1B1;ALDH5A1;ALDH6A1;ALDH7A1;
blue	CC	GO:0031981	nuclear lumen	17735	2101	2487	457	295.84	8.10E-25	1.544754	ABCF1;ABL1;ACTR8;AKAP17A;AKAP8;ANAPC1;ANAPC5;ANAPC7;ANKS1B;APBB1;ARNTL2;ASCC1;ASXL1;ATF6
blue	CC	GO:0043229	intracellular organelle	17735	10522	2487	1699	1481.61	1.80E-24	1.146726	AAK1;AARS2;ABCA2;ABCB1;ABCC1;ABCF1;ABCF2;ABHD6;ABL1;ABL2;ABLIM1;ACAA2;ACACA;ACACB;ACAD8;
blue	CC	GO:0043226	organelle	17735	10552	2487	1702	1485.83	2.90E-24	1.145488	AAK1;AARS2;ABCA2;ABCB1;ABCC1;ABCF1;ABCF2;ABHD6;ABL1;ABL2;ABLIM1;ACAA2;ACACA;ACACB;ACAD8;
blue	CC	GO:0044446	intracellular organelle part	17735	6219	2487	1092	875.7	5.00E-23	1.247002	ABCA2;ABCB1;ABCF1;ABCF2;ABL1;ACAA2;ACAD8;ACER2;ACN9;ACO2;ACOX2;ACOX3;ACSL5;ACTR10;ACTR8;
blue	CC	GO:0044422	organelle part	17735	6321	2487	1104	890.06	1.80E-22	1.240366	ABCA2;ABCB1;ABCF1;ABCF2;ABL1;ACAA2;ACAD8;ACER2;ACN9;ACO2;ACOX2;ACOX3;ACSL5;ACTR10;ACTR8;
blue	MF	GO:0000166	nucleotide binding	17735	2385	2487	500	346.98	1.00E-20	1.441005	AAK1;AARS;AARS2;ABCA2;ABCB1;ABCC1;ABCC10;ABCF1;ABCF2;ABL1;ABL2;ACACA;ACACB;ACAD8;ACLY;AC
blue	MF	GO:1901265	nucleoside phosphate binding	17735	2386	2487	500	347.13	1.10E-20	1.440383	AAK1;AARS;AARS2;ABCA2;ABCB1;ABCC1;ABCC10;ABCF1;ABCF2;ABL1;ABL2;ACACA;ACACB;ACAD8;ACLY;AC
blue	MF	GO:0005524	ATP binding	17735	1462	2487	338	212.7	2.00E-20	1.589093	AAK1;AARS;AARS2;ABCA2;ABCB1;ABCC1;ABCC10;ABCF1;ABCF2;ABL1;ABL2;ACACA;ACACB;ACLY;ACSL5;ACT
blue	MF	GO:0030554	adenyl nucleotide binding	17735	1495	2487	343	217.5	3.70E-20	1.577011	AAK1;AARS;AARS2;ABCA2;ABCB1;ABCC1;ABCC10;ABCF1;ABCF2;ABL1;ABL2;ACACA;ACACB;ACLY;ACSL5;ACT
blue	MF	GO:0032559	adenyl ribonucleotide binding	17735	1491	2487	341	216.92	8.40E-20	1.572008	AAK1;AARS;AARS2;ABCA2;ABCB1;ABCC1;ABCC10;ABCF1;ABCF2;ABL1;ABL2;ACACA;ACACB;ACLY;ACSL5;ACT
blue	MF	GO:0036094	small molecule binding	17735	2525	2487	519	367.35	1.20E-19	1.412822	AAK1;AARS;AARS2;ABCA2;ABCB1;ABCC1;ABCC10;ABCF1;ABCF2;ABL1;ABL2;ACACA;ACACB;ACAD8;ACLY;AC
blue	CC	GO:0005654	nucleoplasm	17735	1481	2487	323	208.54	1.70E-17	1.548864	ABCF1;AKAP17A;ANAPC1;ANAPC5;ANAPC7;ANKS1B;APBB1;ARNTL2;ASCC1;ATM;ATXN2L;BEX1;BLM;BRD1;B
blue	MF	GO:0032549	ribonucleoside binding	17735	1809	2487	383	263.18	2.60E-16	1.455278	AAK1;AARS;AARS2;ABCA2;ABCB1;ABCC1;ABCC10;ABCF1;ABCF2;ABL1;ABL2;ACACA;ACACB;ACLY;ACSL5;ACT
blue	MF	GO:0001882	nucleoside binding	17735	1818	2487	384	264.49	3.50E-16	1.451851	AAK1;AARS;AARS2;ABCA2;ABCB1;ABCC1;ABCC10;ABCF1;ABCF2;ABL1;ABL2;ACACA;ACACB;ACLY;ACSL5;ACT
blue	MF	GO:1901363	heterocyclic compound binding	17735	5302	2487	939	771.36	6.90E-16	1.21733	AAK1;AARS;AARS2;ABCA2;ABCB1;ABCC1;ABCC10;ABCF1;ABCF2;ABL1;ABL2;ACACA;ACACB;ACAD8;ACLY;AC
blue	BP	GO:0046483	heterocycle metabolic process	17735	5361	2487	961	795.62	1.40E-15	1.207863	AARS;AARS2;ABCA2;ABCC1;ABL1;ABLIM1;ACACA;ACACB;ACAD8;ACLY;ACTR8;ACVR1;ADAT1;ADCY6;ADCY7;
blue	MF	GO:0032550	purine ribonucleoside binding	17735	1805	2487	379	262.6	1.50E-15	1.44326	AAK1;AARS;AARS2;ABCA2;ABCB1;ABCC1;ABCC10;ABCF1;ABCF2;ABL1;ABL2;ACACA;ACACB;ACLY;ACSL5;ACT
blue	CC	GO:0032991	macromolecular complex	17735	3893	2487	702	548.18	1.70E-15	1.280601	ABCA2;ABCF1;ABCF2;ACLY;ACTR10;ACTR8;ACVR1;ADD1;AIF1;AKAP17A;ALMS1;AMOTL1;ANAPC1;ANAPC13;
blue	MF	GO:0001883	purine nucleoside binding	17735	1807	2487	379	262.89	1.80E-15	1.441668	AAK1;AARS;AARS2;ABCA2;ABCB1;ABCC1;ABCC10;ABCF1;ABCF2;ABL1;ABL2;ACACA;ACACB;ACLY;ACSL5;ACT
blue	MF	GO:0035639	purine ribonucleoside triphosphate bindi...	17735	1796	2487	377	261.29	1.90E-15	1.442841	AAK1;AARS;AARS2;ABCA2;ABCB1;ABCC1;ABCC10;ABCF1;ABCF2;ABL1;ABL2;ACACA;ACACB;ACLY;ACSL5;ACT
blue	MF	GO:0032553	ribonucleotide binding	17735	1846	2487	385	268.56	2.60E-15	1.433572	AAK1;AARS;AARS2;ABCA2;ABCB1;ABCC1;ABCC10;ABCF1;ABCF2;ABL1;ABL2;ACACA;ACACB;ACLY;ACSL5;ACT
blue	MF	GO:0017076	purine nucleotide binding	17735	1836	2487	383	267.11	3.00E-15	1.433866	AAK1;AARS;AARS2;ABCA2;ABCB1;ABCC1;ABCC10;ABCF1;ABCF2;ABL1;ABL2;ACACA;ACACB;ACLY;ACSL5;ACT
blue	MF	GO:0032555	purine ribonucleotide binding	17735	1831	2487	381	266.38	5.40E-15	1.430288	AAK1;AARS;AARS2;ABCA2;ABCB1;ABCC1;ABCC10;ABCF1;ABCF2;ABL1;ABL2;ACACA;ACACB;ACLY;ACSL5;ACT
blue	BP	GO:0006139	nucleobase-containing compound metabolic...	17735	5209	2487	934	773.06	5.60E-15	1.208186	AARS;AARS2;ABCA2;ABCC1;ABL1;ABLIM1;ACACA;ACACB;ACAD8;ACLY;ACTR8;ACVR1;ADAT1;ADCY6;ADCY7;
blue	BP	GO:0006725	cellular aromatic compound metabolic pro...	17735	5371	2487	957	797.1	1.10E-14	1.200602	AARS;AARS2;ABCA2;ABCC1;ABL1;ABLIM1;ACACA;ACACB;ACAD8;ACLY;ACTR8;ACVR1;ADAT1;ADCY6;ADCY7;
blue	MF	GO:0097159	organic cyclic compound binding	17735	5372	2487	942	781.54	1.20E-14	1.205313	AAK1;AARS;AARS2;ABCA2;ABCB1;ABCC1;ABCC10;ABCF1;ABCF2;ABL1;ABL2;ACACA;ACACB;ACAD8;ACLY;AC
blue	CC	GO:0044464	cell part	17735	14451	2487	2148	2034.85	1.30E-14	1.055606	AAK1;AARS;AARS2;ABCA2;ABCB1;ABCC1;ABCC10;ABCF1;ABCF2;ABHD6;ABL1;ABL2;ABLIM1;ACAA2;ACACA;
blue	CC	GO:0005623	cell	17735	14452	2487	2148	2034.99	1.40E-14	1.055533	AAK1;AARS;AARS2;ABCA2;ABCB1;ABCC1;ABCC10;ABCF1;ABCF2;ABHD6;ABL1;ABL2;ABLIM1;ACAA2;ACACA;
blue	BP	GO:0034641	cellular nitrogen compound metabolic pro...	17735	5595	2487	989	830.34	2.70E-14	1.191078	AARS;AARS2;ABCA2;ABCC1;ABL1;ABLIM1;ACACA;ACACB;ACAD8;ACE;ACER2;ACLY;ACTR8;ACVR1;ADAT1;AD
blue	BP	GO:0044237	cellular metabolic process	17735	8597	2487	1432	1275.86	4.60E-14	1.12238	AAK1;AARS;AARS2;ABCA2;ABCC1;ABCF1;ABL1;ABL2;ABLIM1;ACAA2;ACACA;ACACB;ACAD8;ACE;ACER2;ACLY
blue	BP	GO:1901360	organic cyclic compound metabolic proces...	17735	5578	2487	983	827.82	9.00E-14	1.187456	AARS;AARS2;ABCA2;ABCC1;ABL1;ABLIM1;ACAA2;ACACA;ACACB;ACAD8;ACLY;ACOX2;ACTR8;ACVR1;ADAT1;
blue	CC	GO:0044451	nucleoplasm part	17735	823	2487	193	115.89	1.00E-13	1.665372	AKAP17A;ANKS1B;APBB1;ARNTL2;ASCC1;ATXN2L;BEX1;BLM;BRD1;BRPF1;C1D;CBX5;CCNT1;CD3EAP;CDKN2
blue	MF	GO:0005488	binding	17735	11799	2487	1845	1716.57	1.00E-13	1.074818	AAK1;AARS;AARS2;ABCA2;ABCB1;ABCC1;ABCC10;ABCF1;ABCF2;ABL1;ABL2;ABLIM1;ACAA2;ACACA;ACACB;
blue	BP	GO:0044260	cellular macromolecule metabolic process	17735	6621	2487	1140	982.61	1.10E-13	1.160175	AAK1;AARS;AARS2;ABCA2;ABCF1;ABL1;ABL2;ABLIM1;ACAD8;ACER2;ACTR8;ACVR1;ADAT1;ADCY3;ADCY6;AD
blue	CC	GO:0005737	cytoplasm	17735	9016	2487	1433	1269.55	1.50E-13	1.128746	AAK1;AARS;AARS2;ABCA2;ABCB1;ABCC1;ABCF1;ABCF2;ABHD6;ABL1;ABL2;ABLIM1;ACAA2;ACACA;ACACB;A
blue	MF	GO:0043168	anion binding	17735	2346	2487	460	341.31	1.60E-13	1.347748	AAK1;AARS;AARS2;ABCA2;ABCB1;ABCC1;ABCC10;ABCF1;ABCF2;ABL1;ABL2;ACACA;ACACB;ACAD8;ACE;ACLY
blue	BP	GO:0044238	primary metabolic process	17735	8755	2487	1450	1299.31	2.50E-13	1.115977	AAK1;AARS;AARS2;ABCA2;ABCC1;ABCF1;ABL1;ABL2;ABLIM1;ACAA2;ACACA;ACACB;ACAD8;ACE;ACER2;ACLY
blue	BP	GO:0090304	nucleic acid metabolic process	17735	4431	2487	802	657.6	3.30E-13	1.219586	AARS;AARS2;ABCA2;ABL1;ABLIM1;ACAD8;ACTR8;ACVR1;ADAT1;AIFM1;AIFM2;AKAP17A;AKAP8;AKNA;ALX3
blue	CC	GO:0043234	protein complex	17735	3294	2487	596	463.83	3.80E-13	1.284954	ABCA2;ABCF2;ACLY;ACTR10;ACTR8;ACVR1;ADD1;AIF1;ALMS1;AMOTL1;ANAPC1;ANAPC13;ANAPC5;ANAPC7

blue	BP	GO:0071704	organic substance metabolic process	17735	8996	2487	1481	1335.08	8.30E-13	1.109297 AAK1;AARS;AARS2;ABCA2;ABCC1;ABCF1;ABL1;ABL2;ABLIM1;ACAA2;ACACA;ACACB;ACAD8;ACE;ACER2;ACL
blue	BP	GO:0006807	nitrogen compound metabolic process	17735	5942	2487	1026	881.84	6.00E-12	1.163476 AARS;AARS2;ABCA2;ABCC1;ABL1;ABLIM1;ACACA;ACACB;ACAD8;ACE;ACER2;ACLY;ACTR8;ACVR1;ADAT1;AD
blue	MF	GO:0003676	nucleic acid binding	17735	3299	2487	602	479.95	1.60E-11	1.254297 AARS;AARS2;ABCF1;ABL1;ADAT1;AHDC1;AIFM1;AIFM2;AKAP1;AKAP17A;AKAP8;AKNA;ALX3;APOBEC3F;AQ
blue	BP	GO:0008152	metabolic process	17735	9452	2487	1537	1402.75	1.70E-11	1.095705 AAK1;AARS;AARS2;ABCA2;ABCB1;ABCC1;ABCF1;ABL1;ABL2;ABLIM1;ACAA2;ACACA;ACACB;ACAD8;ACE;ACE
blue	MF	GO:0004386	helicase activity	17735	143	2487	53	20.8	2.00E-11	2.548077 BLM;CHD1L;CHD2;CHD3;CHD4;CHD6;DDX10;DDX17;DDX18;DDX19A;DDX19B;DDX20;DDX23;DDX24;DDX27;
blue	MF	GO:0005515	protein binding	17735	7306	2487	1206	1062.91	2.30E-11	1.134621 AAK1;ABCB1;ABCF1;ABL1;ABL2;ABLIM1;ACAA2;ACACA;ACACB;ACE;ACOX2;ACOX3;ACTR8;ACVR1;ADAM11;
blue	BP	GO:0010467	gene expression	17735	4403	2487	785	653.44	2.60E-11	1.201334 AAK1;AARS;AARS2;ABCA2;ABCF1;ABL1;ABLIM1;ACAD8;ACTR8;ACVR1;ADAT1;AFG3L2;AKAP17A;AKNA;AKT2
blue	BP	GO:0006396	RNA processing	17735	646	2487	158	95.87	3.30E-11	1.648065 AARS;ADAT1;AKAP17A;AQR;BICD1;C1D;C22orf28;CCAR1;CDKAL1;CDKN2A;CELF1;CLK2;CNOT10;CPSF3;CSTF
blue	MF	GO:0008026	ATP-dependent helicase activity	17735	106	2487	43	15.42	5.20E-11	2.788586 BLM;CHD1L;CHD2;CHD3;CHD4;CHD6;DDX10;DDX17;DDX18;DDX19A;DDX19B;DDX20;DDX23;DDX24;DDX27;
blue	MF	GO:0070035	purine NTP-dependent helicase activity	17735	106	2487	43	15.42	5.20E-11	2.788586 BLM;CHD1L;CHD2;CHD3;CHD4;CHD6;DDX10;DDX17;DDX18;DDX19A;DDX19B;DDX20;DDX23;DDX24;DDX27;
blue	MF	GO:0016887	ATPase activity	17735	353	2487	98	51.36	5.80E-11	1.9081 ABCA2;ABCB1;ABCC1;ABCC10;ABCF1;ABCF2;ATP10A;ATP13A1;ATP1A1;ATP1A3;ATP1B3;ATP2A2;ATP2A3;A
blue	BP	GO:0043170	macromolecule metabolic process	17735	7334	2487	1226	1088.42	7.50E-11	1.126403 AAK1;AARS;AARS2;ABCA2;ABCF1;ABL1;ABL2;ABLIM1;ACACA;ACAD8;ACE;ACER2;ACTR8;ACVR1;ADAM11;AD
blue	CC	GO:0005730	nucleolus	17735	622	2487	146	87.58	1.10E-10	1.667047 ABL1;ATF6B;BLM;BMS1;C14orf169;C16orf88;C1D;CBX5;CCND2;CCT5;CD3EAP;CDKN2A;CEP85;CHD3;CIRH1A
blue	CC	GO:0043228	non-membrane-bounded organelle	17735	3122	2487	553	439.61	1.60E-10	1.257933 ABCA2;ABCF1;ABL1;ABL2;ABLIM1;ACTR10;ACTR8;ADD1;AIF1;AK5;AKAP8;AKAP9;ALMS1;ANK3;ANKS1B;APO
blue	CC	GO:0043232	intracellular non-membrane-bounded organ...	17735	3122	2487	553	439.61	1.60E-10	1.257933 ABCA2;ABCF1;ABL1;ABL2;ABLIM1;ACTR10;ACTR8;ADD1;AIF1;AK5;AKAP8;AKAP9;ALMS1;ANK3;ANKS1B;APO
blue	CC	GO:0016604	nuclear body	17735	276	2487	78	38.86	5.00E-10	2.007205 AKAP17A;ANKS1B;APBB1;ATXN2L;BLM;CBX5;CDKN2A;CHEK2;CLK2;CSTF2;CTR9;DDX20;DDX42;DDX46;DGKC
blue	MF	GO:0042623	ATPase activity, coupled	17735	278	2487	80	40.44	5.40E-10	1.978239 ABCA2;ABCB1;ABCC1;ABCC10;ATP10A;ATP13A1;ATP1A1;ATP1A3;ATP1B3;ATP2A2;ATP2A3;ATP5A1;ATP5B;
blue	BP	GO:0016070	RNA metabolic process	17735	3911	2487	697	580.42	9.00E-10	1.200855 AARS;AARS2;ABCA2;ABL1;ABLIM1;ACAD8;ACTR8;ACVR1;ADAT1;AKAP17A;AKNA;ALX3;APBB1;APOBEC3F;AC
blue	BP	GO:0009987	cellular process	17735	12861	2487	1986	1908.67	2.60E-09	1.040515 AAK1;AARS;AARS2;ABCA2;ABCB1;ABCC1;ABCC10;ABCF1;ABHD6;ABL1;ABL2;ABLIM1;ACAA2;ACACA;ACACB;
blue	CC	GO:0005829	cytosol	17735	2362	2487	426	332.59	4.10E-09	1.280856 AARS;ABL1;ABL2;ACACA;ACACB;ACLY;ADD1;ADSS;AGBL5;AHSA1;AIF1;AIFM1;AIFM2;AK2;AK5;AKAP1;AKAP9
blue	MF	GO:0003723	RNA binding	17735	851	2487	184	123.81	7.00E-09	1.486148 AARS;AARS2;ABCF1;ADAT1;AKAP1;AKAP17A;APOBEC3F;AQR;ASCC1;ATXN1L;C1D;CALR;CBX7;CCNT1;CELF1;
blue	BP	GO:0006397	mRNA processing	17735	405	2487	102	60.11	2.30E-08	1.696889 AKAP17A;AQR;CCAR1;CELF1;CNOT10;CPSF3;CSTF1;CSTF2;CSTF3;CTNNBL1;DBR1;DDX20;DDX23;DDX46;DDX
blue	BP	GO:0008380	RNA splicing	17735	328	2487	86	48.68	4.30E-08	1.766639 AKAP17A;AQR;C22orf28;CCAR1;CELF1;CLK2;CPSF3;CSTF1;CSTF2;CSTF3;CTNNBL1;DBR1;DDX20;DDX23;DDX4
blue	CC	GO:0005739	mitochondrion	17735	1488	2487	281	209.53	4.60E-08	1.341097 AARS2;ABCF2;ABHD6;ABL1;ACAA2;ACACA;ACACB;ACAD8;ACLY;ACN9;ACO2;ACOX3;ACSL5;AFG3L2;AGK;AG
blue	BP	GO:0006996	organelle organization	17735	2139	2487	401	317.44	4.90E-08	1.263231 AARS2;ABL1;ABL2;ABLIM1;ACTR8;ADD1;AFG3L2;AIF1;AIFM1;AIFM2;AKAP8;AKT2;AKT3;ALMS1;ALS2CL;ANA
blue	CC	GO:0005681	spliceosomal complex	17735	147	2487	46	20.7	6.60E-08	2.222222 AKAP17A;AQR;CTNNBL1;DDX20;DDX23;DHX15;DHX35;DHX38;EFTUD2;EIF4A3;GEMIN4;GEMIN5;GPATCH1;
blue	BP	GO:0006259	DNA metabolic process	17735	866	2487	184	128.52	1.10E-07	1.431684 ABL1;ACTR8;AIFM1;AIFM2;AKAP8;APBB1;APOBEC3F;ATM;AXIN2;BAHD1;BATF;BAZ1B;BCL11B;BLM;BRD2;C
blue	CC	GO:0005694	chromosome	17735	631	2487	136	88.85	1.40E-07	1.53067 ACTR8;AKAP8;ASXL1;ATM;BAHD1;BAZ1B;BLM;CAPN2;CBX2;CBX5;CBX7;CCND2;CD3EAP;CDKN2A;CENPN;CE
blue	BP	GO:0051276	chromosome organization	17735	700	2487	154	103.89	1.40E-07	1.482337 AIFM2;AKAP8;APBB1;ARID1B;ASXL1;ATM;AXIN2;BAHD1;BAZ1B;BLM;BPTF;BRD1;BRD2;BRD7;BRPF1;C14orf1
blue	CC	GO:0070603	SWI/SNF-type complex	17735	60	2487	25	8.45	1.60E-07	2.95858 ACTR8;ARID1B;BAZ1B;BPTF;CHD3;CHD4;HDAC1;INO80;MTA1;MTA2;MTA3;NCR1;NFRKB;PHF10;RBBP4;RBB
blue	CC	GO:0044427	chromosomal part	17735	538	2487	118	75.76	3.60E-07	1.55755 ACTR8;ASXL1;ATM;BAZ1B;BLM;CAPN2;CBX2;CBX5;CBX7;CCND2;CDKN2A;CENPN;CENPO;CHEK2;CTC1;CTCF;
blue	CC	GO:0000792	heterochromatin	17735	60	2487	24	8.45	6.90E-07	2.840237 BAZ1B;CBX2;CBX5;CBX7;CDKN2A;DNMT1;DNMT3A;FOXC1;HMGA1;INCENP;MBD1;MBD2;MECP2;MPHOSP
blue	CC	GO:0044454	nuclear chromosome part	17735	255	2487	65	35.91	8.70E-07	1.810081 ACTR8;ASXL1;BAZ1B;BLM;CBX5;CBX7;CTC1;DFFA;DFFB;DNMT3A;FOXC1;GATA3;HDAC1;HELB;INCENP;ING2;
blue	BP	GO:0000377	RNA splicing, via transesterification re...	17735	210	2487	58	31.17	1.10E-06	1.860764 AQR;CCAR1;CELF1;CPSF3;CSTF1;CSTF2;CSTF3;DBR1;DDX20;DDX23;DHX35;DHX38;DHX9;EFTUD2;EIF4A3;GE
blue	BP	GO:0000398	mRNA splicing, via spliceosome	17735	210	2487	58	31.17	1.10E-06	1.860764 AQR;CCAR1;CELF1;CPSF3;CSTF1;CSTF2;CSTF3;DBR1;DDX20;DDX23;DHX35;DHX38;DHX9;EFTUD2;EIF4A3;GE
blue	BP	GO:0000375	RNA splicing, via transesterification re...	17735	215	2487	59	31.91	1.10E-06	1.84895 AQR;CCAR1;CELF1;CPSF3;CSTF1;CSTF2;CSTF3;DBR1;DDX20;DDX23;DHX35;DHX38;DHX9;EFTUD2;EIF4A3;GE
blue	CC	GO:0015630	microtubule cytoskeleton	17735	853	2487	169	120.11	1.40E-06	1.407044 ABCA2;ACTR10;AK5;AKAP9;ALMS1;ARL3;ATM;AXIN1;AXIN2;BBS2;BBS9;BICD1;BIRC6;BRSK2;BSN;C2CD3;CAL
blue	CC	GO:0071013	catalytic step 2 spliceosome	17735	79	2487	28	11.12	1.60E-06	2.517986 AQR;DDX23;DHX35;DHX38;EFTUD2;EIF4A3;GPATCH1;HNRNPA2B1;HNRNPA3;HNRNPH1;HNRNPM;HNRNPR
blue	CC	GO:0016607	nuclear speck	17735	149	2487	43	20.98	2.00E-06	2.049571 AKAP17A;APBB1;ATXN2L;CLK2;CTR9;DDX42;DDX46;DGKQ;EFTUD2;EIF4A3;EIF4ENIF1;EP400;LUC7L2;MAGO
blue	MF	GO:0016874	ligase activity	17735	483	2487	108	70.27	2.00E-06	1.536929 AARS;AARS2;ACACA;ACACB;ACLY;ACSL5;ADSS;AMFR;ANAPC5;ARIH2;ATG10;ATG3;BIRC6;BTRC;C22orf28;CA
blue	MF	GO:0017111	nucleoside-triphosphatase activity	17735	756	2487	156	109.99	2.10E-06	1.418311 ABCA2;ABCB1;ABCC1;ABCC10;ABCF1;ABCF2;AFG3L2;ARL3;ATAD3C;ATP10A;ATP13A1;ATP1A1;ATP1A3;ATP1
blue	MF	GO:0008134	transcription factor binding	17735	451	2487	102	65.61	2.20E-06	1.554641 ADD1;APBB1;ASXL1;BAZ1B;BCL10;BCL2;BEX1;BPTF;BRD7;CAMTA2;CAND2;CBX5;CDKN2A;CHD4;CIITA;CREG
blue	MF	GO:0019899	enzyme binding	17735	1084	2487	211	157.71	2.60E-06	1.337899 ABL1;ADAMTS4;ADCY6;ALS2CL;ANAPC5;ANAPC7;ANKLE2;ANXA2;ARHGEF16;ARHGEF7;ATG13;ATG3;AXIN1;
blue	CC	GO:0044444	cytoplasmic part	17735	6562	2487	1025	924	2.70E-06	1.109307 AAK1;AARS;AARS2;ABCA2;ABCB1;ABCC1;ABCF1;ABCF2;ABHD6;ABL1;ABL2;ACAA2;ACACA;ACACB;ACAD8;AC
blue	MF	GO:0016462	pyrophosphatase activity	17735	791	2487	161	115.08	3.30E-06	1.399027 ABCA2;ABCB1;ABCC1;ABCC10;ABCF1;ABCF2;AFG3L2;ARL3;ATAD3C;ATP10A;ATP13A1;ATP1A1;ATP1A3;ATP1
blue	MF	GO:0016817	hydrolase activity, acting on acid anhyd...	17735	797	2487	162	115.95	3.30E-06	1.397154 ABCA2;ABCB1;ABCC1;ABCC10;ABCF1;ABCF2;AFG3L2;ARL3;ATAD3C;ATP10A;ATP13A1;ATP1A1;ATP1A3;ATP1
blue	MF	GO:0016818	hydrolase activity, acting on acid anhyd...	17735	793	2487	161	115.37	3.80E-06	1.39551 ABCA2;ABCB1;ABCC1;ABCC10;ABCF1;ABCF2;AFG3L2;ARL3;ATAD3C;ATP10A;ATP13A1;ATP1A1;ATP1A3;ATP1
blue	BP	GO:0007049	cell cycle	17735	1388	2487	264	205.99	4.60E-06	1.281616 ABCB1;ABL1;ACTR8;ACVR1;AIF1;AKAP8;AKAP9;AKT2;ALMS1;ANAPC1;ANAPC13;ANAPC5;ANAPC7;ANK3;AN
blue	BP	GO:0034660	ncRNA metabolic process	17735	285	2487	71	42.3	4.70E-06	1.678487 AARS;AARS2;ADAT1;C1D;C22orf28;CDKAL1;CDKN2A;DARS2;DDX20;DDX47;DDX51;DDX56;DIS3L;DKC1;DRO
blue	BP	GO:0034645	cellular macromolecule biosynthetic proc...	17735	4170	2487	706	618.86	4.80E-06	1.140807 AARS;AARS2;ABCA2;ABCF1;ABL1;ABLIM1;ACAD8;ACER2;ACTR8;ACVR1;AKAP17A;AKNA;AKT2;ALG8;ALG9;AL
blue	BP	GO:0016568	chromatin modification	17735	434	2487	99	64.41	5.00E-06	1.537028 APBB1;ARID1B;ASXL1;BAHD1;BAZ1B;BPTF;BRD1;BRD2;BRD7;BRPF1;C14orf169;CBX2;CBX7;CDKN2A;CENPN;
blue	CC	GO:0000785	chromatin	17735	300	2487	71	42.24	5.10E-06	1.680871 ACTR8;ASXL1;BAZ1B;CAPN2;CBX2;CBX5;CBX7;CCND2;CDKN2A;CTR9;DFFA;DFFB;DNMT1;DNMT3A;EXOSC10
blue	CC	GO:0031967	organelle envelope	17735	825	2487	161	116.17	6.40E-06	1.3859 ABCF1;ABCF2;ABL1;ACAA2;ACN9;ACSL5;AFG3L2;AGK;AGPAT3;AIFM1;AIFM2;AK2;AKAP1;ALDH18A1;ALDH3
blue	BP	GO:1901576	organic substance biosynthetic process	17735	5154	2487	855	764.89	7.00E-06	1.117808 AARS;AARS2;ABCA2;ABCF1;ABL1;ABLIM1;ACAA2;ACACA;ACACB;ACAD8;ACER2;ACLY;ACN9;ACOX2;ACSL5;A
blue	BP	GO:0006310	DNA recombination	17735	212	2487	56	31.46	7.40E-06	1.780038 ACTR8;ATM;BATF;BCL11B;BLM;CD40LG;DCLRE1C;EME2;FAN1;FEN1;FOXP1;HSPD1;IL10;IL4;IL7R;INO80;LEF1
blue	CC	GO:0000228	nuclear chromosome	17735	294	2487	69	41.4	9.30E-06	1.666667 ACTR8;ASXL1;BAZ1B;BLM;CBX5;CBX7;CTC1;DFFA;DFFB;DNMT3A;FOXC1;GATA3;HDAC1;HELB;INCENP;ING2;
blue	BP	GO:0000723	telomere maintenance	17735	65	2487	24	9.65	9.40E-06	2.487047 ATM;BLM;CTC1;DCLRE1C;DKC1;FEN1;LIG1;MYC;PARP1;PINX1;POLA1;POLA2;POLE;RFC1;RPA1;RPA2;SMG6;
blue	MF	GO:0003724	RNA helicase activity	17735	28	2487	14	4.07	9.80E-06	3.439803 DDX10;DDX17;DDX18;DDX20;DDX23;DDX24;DDX56;DDX6;DHX15;DHX16;DHX9;EIF4A3;G3BP1;SUPV3L
blue	BP	GO:0009059	macromolecule biosynthetic process	17735	4294	2487	722	637.26	9.90E-06	1.132976 AARS;AARS2;ABCA2;ABCF1;ABL1;ABLIM1;ACAD8;ACER2;ACTR8;ACVR1;AGMAT;AKAP17A;AKNA;AKT2;ALAD
blue	CC	GO:0031975	envelope	17735	839	2487	162	118.14	1.10E-05	1.371254 ABCF1;ABCF2;ABL1;ACAA2;ACN9;ACSL5;AFG3L2;AGK;AGPAT3;AIFM1;AIFM2;AK2;AKAP1;ALDH18A1;ALDH3
blue	BP	GO:0032200	telomere organization	17735	66	2487	24	9.79	1.30E-05	2.451481 ATM;BLM;CTC1;DCLRE1C;DKC1;FEN1;LIG1;MYC;PARP1;PINX1;POLA1;POLA2;POLE;RFC1;RPA1;RPA2;SMG6;
blue	BP	GO:0000278	mitotic cell cycle	17735	777	2487	158	115.31	1.30E-05	1.370219 ABCB1;ABL1;ACTR8;ACVR1;AIF1;AKAP8;AKAP9;ALMS1;ANAPC1;ANAPC13;ANAPC5;ANAPC7;ANKLE2;APBB1;
blue	BP	GO:0044249	cellular biosynthetic process	17735	5073	2487	840	752.87	1.30E-05	1.11573 AARS;AARS2;ABCA2;ABCF1;ABL1;ABLIM1;ACACA;ACACB;ACAD8;ACER2;ACLY;ACN9;ACOX2;ACSL5;ACTR8;A
blue	BP	GO:0006354	DNA-dependent transcription, elongation	17735	106	2487	33	15.73	1.60E-05	2.097902 AXIN1;CCNT1;CTR9;ELP3;ERCC3;GTF2A2;HTATSF1;IKBKAP;LDB1;LEO1;POLR1A;POLR1B;POLR2B;POLR2C;PO
blue	MF	GO:0003682	chromatin binding	17735	327	2487	76	47.57	1.60E-05	1.597646 APBB1;BAHD1;BAZ1B;BRD2;CAMTA2;CBX2;CBX5;CCNT1;CHD6;CRAMP1L;CTCF;DNAJC1;DNMT3A;EP400;EZ
blue	BP	GO:0022613	ribonucleoprotein complex biogenesis	17735	233	2487	59	34.58	1.70E-05	1.706189 ATXN2L;BICD1;BMS1;C1D;CDKN2A;CELF1;CIRBP;DDX20;DDX23;DDX47;DDX51;DDX56;DDX6;DKC1;DROSHA;
blue	MF	GO:0004812	aminoacyl-tRNA ligase activity	17735	44	2487	18	6.4	1.80E-05	2.8125 AARS;AARS2;DARS2;EARS2;EPRS;FARSB;GARS;HARS;IARS;IARS2;KARS;LARS;LARS2;MARS;NARS;SARS;TARS;
blue	MF	GO:0016875	ligase activity, forming carbon-oxygen b...	17735	44	2487	18	6.4	1.80E-05	2.8125 AARS;AARS2;DARS2;EARS2;EPRS;FARSB;GARS;HARS;IARS;IARS2;KARS;LARS;LARS2;MARS;NARS;SARS;TARS;
blue	MF	GO:0016876	ligase activity, forming aminoacyl-tRNA ...	17735	44	2487	18	6.4	1.80E-05	2.8125 AARS;AARS2;DARS2;EARS2;EPRS;FARSB;GARS;HARS;IARS;IARS2;KARS;LARS;LARS2;MARS;NARS;SARS;TARS;

blue	MF	GO:0042393	histone binding	17735	104	2487	32	15.13	1.80E-05	2.115003	APBB1;BAZ1B;BRD1;BRD2;BRD7;CBX2;CBX5;CBX7;ING1;ING2;ING5;IPO9;KDM4A;L3MBTL1;L3MBTL2;LEF1;M
blue	BP	GO:0022402	cell cycle process	17735	1059	2487	205	157.16	1.90E-05	1.304403	ABCB1;ABL1;ACTR8;ACVR1;AIF1;AKAP8;AKAP9;AKT2;ALMS1;ANAPC1;ANAPC13;ANAPC5;ANAPC7;ANK3;ANI
blue	BP	GO:0009058	biosynthetic process	17735	5219	2487	860	774.54	2.00E-05	1.110336	AARS;AARS2;ABCA2;ABCF1;ABL1;ABLIM1;ACAA2;ACACA;ACACB;ACAD8;ACER2;ACLY;ACN9;ACOX2;ACSL5;A(
blue	BP	GO:0007163	establishment or maintenance of cell pol...	17735	113	2487	34	16.77	2.60E-05	2.02743	AMOTL1;ATN1;BRSK2;CCR7;CDC42BPB;CDK5RAP2;CKAP5;CYTH1;CYTH3;DLG3;DOCK7;DST;DYNLT1;EZR;FBF
blue	MF	GO:0008094	DNA-dependent ATPase activity	17735	70	2487	24	10.18	2.80E-05	2.357564	BLM;BPTF;CHD1L;CHD2;CHD3;CHD4;DHX9;ERCC3;FBXO18;G3BP1;HELB;MCM4;MCM6;MSH6;MYO18A;RAD
blue	BP	GO:0006200	ATP catabolic process	17735	73	2487	25	10.83	2.80E-05	2.308403	ABCA2;ABCC1;ACLY;ATP5A1;ATP5B;ATP5J;ATP5J2;ATP6V1H;ATP8B1;ATP8B2;BLM;CARNS1;CHD1L;HSPD1;IN
blue	MF	GO:0043167	ion binding	17735	5761	2487	923	838.14	3.30E-05	1.101248	AAK1;AARS;AARS2;ABCA2;ABCB1;ABCC1;ABCC10;ABCF1;ABCF2;ABL1;ABL2;ABLIM1;ACACA;ACACB;ACAD8;A
blue	BP	GO:0032862	activation of Rho GTPase activity	17735	20	2487	11	2.97	3.40E-05	3.703704	ARHGEF16;CCR7;DOCK7;EPHA1;EPHA2;FOXJ1;SCRIB;SYDE1;TAX1BP3;TBC1D7;TSC1
blue	CC	GO:0005657	replication fork	17735	47	2487	18	6.62	3.40E-05	2.719033	BAZ1B;BLM;DNMT1;HELB;MCM3;POLA1;POLA2;POLE;POLE3;RAD18;RFC1;RPA1;RPA2;TP53;TP53BP1;UBE2E
blue	MF	GO:0003678	DNA helicase activity	17735	42	2487	17	6.11	3.70E-05	2.782324	BLM;CHD1L;CHD2;CHD3;CHD4;DHX9;ERCC3;FBXO18;G3BP1;HELB;INO80;MCM4;MCM6;RUVBL1;SUPV3L1;X
blue	CC	GO:0005874	microtubule	17735	329	2487	73	46.33	4.00E-05	1.575653	ARL3;AXIN1;AXIN2;BICD1;CALM3;CAMSAP1;CCT3;CCT5;CCT6A;CCT7;CCT8;CDC16;CDK5RAP2;CKAP5;DCTN1
blue	BP	GO:0006403	RNA localization	17735	139	2487	39	20.63	4.10E-05	1.890451	BICD1;CKAP5;CPSF3;DDX19A;DDX19B;DHX38;EIF4A3;EIF5A;EXOSC10;GLE1;HHEX;HNRNPA2B1;KHDRBS1;KH
blue	BP	GO:0006338	chromatin remodeling	17735	97	2487	30	14.4	4.30E-05	2.083333	ARID1B;BAHD1;BAZ1B;BPTF;CDKN2A;CENPN;CENPO;CHD1L;CHD6;HDAC1;HMGA1;INO80;MLL4;MYC;NASP;
blue	CC	GO:0000790	nuclear chromatin	17735	152	2487	40	21.4	4.90E-05	1.869159	ACTR8;ASXL1;CBX5;CBX7;DFFA;DFFB;DNMT3A;FOXC1;GATA3;HDAC1;ING2;INO80;KDM1A;KDM4C;LDB1;MF
blue	BP	GO:0006325	chromatin organization	17735	531	2487	112	78.8	5.00E-05	1.42132	APBB1;ARID1B;ASXL1;BAHD1;BAZ1B;BPTF;BRD1;BRD2;BRD7;BRPF1;C14orf169;CBX2;CBX7;CDAN1;CDKN2A;
blue	CC	GO:0044429	mitochondrial part	17735	740	2487	142	104.2	5.30E-05	1.362764	ABCF2;ACAA2;ACAD8;ACN9;ACO2;ACSL5;AFG3L2;AGK;AIFM1;AIFM2;AK2;AKAP1;ALDH18A1;ALDH1B1;ALDH
blue	BP	GO:0060255	regulation of macromolecule metabolic pr...	17735	4178	2487	696	620.05	5.80E-05	1.12249	AAK1;ABCA2;ABCF1;ABL1;ABLIM1;ACAD8;ACE;ACER2;ACTR8;ACVR1;ADCY3;ADCY6;ADCY7;ADCY9;ADD1;AK
blue	BP	GO:0006281	DNA repair	17735	388	2487	86	57.58	6.20E-05	1.493574	ABL1;ACTR8;APBB1;ATM;AXIN2;BAZ1B;BLM;CEP164;CHD1L;CHEK2;CINP;CSNK1E;CUL4A;DCLRE1C;DDB1;DT
blue	MF	GO:0003824	catalytic activity	17735	5264	2487	846	765.83	6.30E-05	1.104684	AAK1;AARS;AARS2;ABCA2;ABCB1;ABCC1;ABCC10;ABCF1;ABCF2;ABHD15;ABHD6;ABL1;ABL2;ACAA2;ACACA;
blue	MF	GO:0004003	ATP-dependent DNA helicase activity	17735	32	2487	14	4.66	6.50E-05	3.004292	BLM;CHD1L;CHD2;CHD3;CHD4;DHX9;ERCC3;FBXO18;G3BP1;HELB;MCM4;MCM6;XRCC5;XRCC6
blue	BP	GO:0050657	nucleic acid transport	17735	132	2487	37	19.59	6.50E-05	1.888719	CKAP5;CPSF3;DDX19A;DDX19B;DHX38;EIF4A3;EIF5A;GLE1;HHEX;HNRNPA2B1;KHDRBS1;KHSRP;KIF5C;MAG(
blue	BP	GO:0050658	RNA transport	17735	132	2487	37	19.59	6.50E-05	1.888719	CKAP5;CPSF3;DDX19A;DDX19B;DHX38;EIF4A3;EIF5A;GLE1;HHEX;HNRNPA2B1;KHDRBS1;KHSRP;KIF5C;MAG(
blue	BP	GO:0051236	establishment of RNA localization	17735	132	2487	37	19.59	6.50E-05	1.888719	CKAP5;CPSF3;DDX19A;DDX19B;DHX38;EIF4A3;EIF5A;GLE1;HHEX;HNRNPA2B1;KHDRBS1;KHSRP;KIF5C;MAG(
blue	BP	GO:0043038	amino acid activation	17735	51	2487	19	7.57	6.80E-05	2.509908	AARS;AARS2;DARS2;EARS2;EPRS;FARSB;GARS;HARS;IARS;IARS2;KARS;LARS;LARS2;MARS;NARS;PET112;SAR
blue	BP	GO:0043039	tRNA aminoacylation	17735	51	2487	19	7.57	6.80E-05	2.509908	AARS;AARS2;DARS2;EARS2;EPRS;FARSB;GARS;HARS;IARS;IARS2;KARS;LARS;LARS2;MARS;NARS;PET112;SAR
blue	BP	GO:0071840	cellular component organization or bioge...	17735	4237	2487	704	628.8	7.20E-05	1.119593	AAK1;AARS2;ABL1;ABL2;ABLIM1;ACACA;ACACB;ACTR8;ADD1;AFG3L2;AIF1;AIFM1;AIFM2;AKAP8;AKT2;AKT3
blue	MF	GO:0003677	DNA binding	17735	2332	2487	400	339.27	7.80E-05	1.179002	ABL1;AHDC1;AIFM1;AIFM2;AKAP8;AKNA;ALX3;ARHGAP35;ARID1B;ARNTL2;ASXL1;ATF6B;ATM;ATXN1L;BAC
blue	BP	GO:0034470	ncRNA processing	17735	198	2487	50	29.38	7.90E-05	1.701838	AARS;ADAT1;C1D;C22orf28;CDKAL1;CDKN2A;DDX47;DDX51;DDX56;DKC1;DROSHA;EIF4A3;EXOSC10;FTSJ2;F
blue	BP	GO:0006418	tRNA aminoacylation for protein translat...	17735	48	2487	18	7.12	9.50E-05	2.52809	AARS;AARS2;DARS2;EARS2;EPRS;FARSB;GARS;HARS;IARS;IARS2;KARS;LARS;LARS2;MARS;NARS;SARS;TARS;Y
blue	CC	GO:0000428	DNA-directed RNA polymerase complex	17735	101	2487	29	14.22	9.80E-05	2.039381	CD3EAP;CTR9;ELP3;ERCC3;ERCC5;GTF2A2;IKBKAP;INTS1;INTS4;INTS7;INTS9;LEO1;MMS19;POLR1A;POLR1B;
blue	CC	GO:0055029	nuclear DNA-directed RNA polymerase comp...	17735	101	2487	29	14.22	9.80E-05	2.039381	CD3EAP;CTR9;ELP3;ERCC3;ERCC5;GTF2A2;IKBKAP;INTS1;INTS4;INTS7;INTS9;LEO1;MMS19;POLR1A;POLR1B;
blue	MF	GO:0005083	small GTPase regulator activity	17735	295	2487	67	42.92	1.00E-04	1.561044	AGAP5;AGAP6;ALS2CL;ARHGAP15;ARHGAP29;ARHGAP33;ARHGAP35;ARHGEF1;ARHGEF16;ARHGEF18;ARH(
blue	BP	GO:0002200	somatic diversification of immune recept...	17735	53	2487	19	7.87	0.00012	2.414231	BATF;BCL11B;CD40LG;CTNNBL1;FOXP1;HSPD1;IL10;IL4;LEF1;MLH1;MSH6;PAXIP1;PMS2;PMS2P5;RNF8;TCF3
blue	CC	GO:0030880	RNA polymerase complex	17735	102	2487	29	14.36	0.00012	2.019499	CD3EAP;CTR9;ELP3;ERCC3;ERCC5;GTF2A2;IKBKAP;INTS1;INTS4;INTS7;INTS9;LEO1;MMS19;POLR1A;POLR1B;
blue	BP	GO:0018130	heterocycle biosynthetic process	17735	3650	2487	611	541.69	0.00012	1.127951	ABCA2;ABL1;ABLIM1;ACACA;ACACB;ACAD8;ACTR8;ACVR1;ADCY6;ADCY7;ADSS;AK2;AK5;AKAP17A;AKNA;AL
blue	CC	GO:0015030	Cajal body	17735	47	2487	17	6.62	0.00013	2.567976	ANKS1B;DDX20;DDX42;DDX46;DKC1;EFTUD2;GAR1;GEMIN4;GEMIN5;LSG1;LSM10;NOLC1;NOP10;PRPF3;PR
blue	BP	GO:0042254	ribosome biogenesis	17735	151	2487	40	22.41	0.00013	1.784917	BMS1;C1D;CDKN2A;DDX47;DDX51;DDX56;DKC1;DROSHA;EFTUD1;EIF4A3;EXOSC10;FTSJ2;FTSJ3;GAR1;GEMI
blue	BP	GO:0022403	cell cycle phase	17735	852	2487	165	126.44	0.00013	1.304967	ABCB1;ABL1;ACTR8;ACVR1;AIF1;AKAP8;AKAP9;ALMS1;ANAPC1;ANAPC13;ANAPC5;ANAPC7;ANKLE2;APBB1;
blue	BP	GO:0019219	regulation of nucleobase-containing comp...	17735	3414	2487	574	506.66	0.00014	1.13291	ABCA2;ABL1;ABLIM1;ACAD8;ACTR8;ACVR1;ADCY7;ADD1;AGAP5;AGAP6;AHSA1;AKAP17A;AKNA;AKT2;APBB
blue	BP	GO:0046112	nucleobase biosynthetic process	17735	13	2487	8	1.93	0.00015	4.145078	CAD;CECR1;DHODH;GART;GMPS;PAICS;PRPS1;SHMT1
blue	BP	GO:0006337	nucleosome disassembly	17735	16	2487	9	2.37	0.00015	3.797468	BAZ1B;HMGA1;SMARCA4;SMARCC1;SMARCC2;SMARCD1;SMARCD3;SMARCE1;SUPT16H
blue	BP	GO:0031498	chromatin disassembly	17735	16	2487	9	2.37	0.00015	3.797468	BAZ1B;HMGA1;SMARCA4;SMARCC1;SMARCC2;SMARCD1;SMARCD3;SMARCE1;SUPT16H
blue	BP	GO:0032986	protein-DNA complex disassembly	17735	16	2487	9	2.37	0.00015	3.797468	BAZ1B;HMGA1;SMARCA4;SMARCC1;SMARCC2;SMARCD1;SMARCD3;SMARCE1;SUPT16H
blue	MF	GO:0035064	methylated histone residue binding	17735	38	2487	15	5.53	0.00015	2.712477	CBX2;CBX5;CBX7;ING1;ING2;ING5;KDM4A;L3MBTL1;L3MBTL2;MPHOSPH8;NCAPD3;NCAPG2;PHF13;TP53BP
blue	BP	GO:0051325	interphase	17735	414	2487	89	61.44	0.00015	1.448568	ABCB1;ABL1;ACVR1;AIF1;AKAP9;ALMS1;ANAPC5;APBB1;ATM;BCL2;BID;BLM;BRD7;BRSK2;CASP2;CCND1;CD
blue	BP	GO:0006984	ER-nucleus signaling pathway	17735	99	2487	29	14.69	0.00017	1.974132	AARS;ADD1;AMFR;ATG10;ATP2A2;CALR;CCND1;CUL7;DCTN1;DDIT3;DERL2;EDEM1;EIF2AK3;EIF2AK4;GOSR2
blue	MF	GO:0060589	nucleoside-triphosphatase regulator acti...	17735	447	2487	93	65.03	0.00017	1.430109	AGAP5;AGAP6;AHSA1;ALS2CL;ANKRD27;ARHGAP15;ARHGAP17;ARHGAP18;ARHGAP29;ARHGAP32;ARHGAF
blue	CC	GO:0016581	NuRD complex	17735	17	2487	9	2.39	0.00018	3.76569	CHD3;CHD4;HDAC1;MTA1;MTA2;MTA3;RBBP4;RBBP7;SALL2
blue	CC	GO:0090545	CHD-type complex	17735	17	2487	9	2.39	0.00018	3.76569	CHD3;CHD4;HDAC1;MTA1;MTA2;MTA3;RBBP4;RBBP7;SALL2
blue	BP	GO:0044271	cellular nitrogen compound biosynthetic ...	17735	3726	2487	621	552.97	0.00018	1.123027	ABCA2;ABL1;ABLIM1;ACACA;ACACB;ACAD8;ACER2;ACTR8;ACVR1;ADCY6;ADCY7;ADSS;AGMAT;AK2;AK5;AK
blue	BP	GO:0034976	response to endoplasmic reticulum stress	17735	114	2487	32	16.92	0.00019	1.891253	AARS;ADD1;AIFM1;AMFR;ATG10;BCL2;BRSK2;CALR;CCND1;CREB3L2;CUL7;DCTN1;DDIT3;DERL2;EDEM1;EIF
blue	BP	GO:0009056	catabolic process	17735	1989	2487	349	295.18	0.00019	1.182329	ABCA2;ABCC1;ABL1;ABL2;ACAD8;ACE;ACLY;ACOX2;ACOX3;ADPGK;ADSS;AFG3L2;AGAP5;AGAP6;AHSA1;AIF
blue	MF	GO:0070491	repressing transcription factor binding	17735	43	2487	16	6.26	2.00E-04	2.555911	CBX5;CHD4;GTF2A2;HDAC1;HHEX;MTA1;MTA2;MYC;PPARA;RELA;RUNX1;RUNX2;TBP;TCF3;TCF7L2;ZMYND
blue	BP	GO:0046034	ATP metabolic process	17735	124	2487	34	18.4	2.00E-04	1.847826	ABCA2;ABCC1;ACLY;AK2;AK5;ATP1A1;ATP1A3;ATP2A2;ATP2A3;ATP5A1;ATP5B;ATP5J;ATP5J2;ATP6V1H;ATP
blue	BP	GO:0080090	regulation of primary metabolic process	17735	4337	2487	714	643.65	2.00E-04	1.109299	ABCA2;ABCF1;ABL1;ABLIM1;ACACB;ACAD8;ACE;ACER2;ACN9;ACTR8;ACVR1;ADCY3;ADCY6;ADCY7;ADCY9;A
blue	CC	GO:0071564	npBAF complex	17735	11	2487	7	1.55	0.00021	4.516129	PHF10;SMARCA4;SMARCC1;SMARCC2;SMARCD1;SMARCD3;SMARCE1
blue	BP	GO:0015931	nucleobase-containing compound transport	17735	154	2487	40	22.85	0.00021	1.750547	CKAP5;CPSF3;DDX19A;DDX19B;DHX38;EIF4A3;EIF5A;GLE1;HHEX;HNRNPA2B1;KHDRBS1;KHSRP;KIF5C;MAG(
blue	BP	GO:0016071	mRNA metabolic process	17735	607	2487	122	90.08	0.00021	1.354352	AKAP17A;AQR;ATM;CCAR1;CELF1;CNOT10;CPSF3;CSTF1;CSTF2;CSTF3;CTNNBL1;DBR1;DCP1B;DDX20;DDX23
blue	BP	GO:0006351	transcription, DNA-dependent	17735	3208	2487	540	476.09	0.00021	1.134239	ABCA2;ABL1;ABLIM1;ACAD8;ACTR8;ACVR1;AKAP17A;AKNA;ALX3;APBB1;ARHGAP35;ARID1B;ARNTL2;ASCC1
blue	BP	GO:0051329	interphase of mitotic cell cycle	17735	408	2487	87	60.55	0.00023	1.436829	ABCB1;ABL1;ACVR1;AIF1;AKAP9;ALMS1;ANAPC5;APBB1;ATM;BCL2;BID;BLM;BRD7;BRSK2;CASP2;CCND1;CD
blue	BP	GO:0045786	negative regulation of cell cycle	17735	453	2487	95	67.23	0.00023	1.41306	AKT2;ANAPC1;ANAPC5;ANAPC7;APBB1;ATM;BCL2;BLM;BMP2;C11orf82;CALR;CAPN3;CASP2;CCND1;CDC16,
blue	CC	GO:0030529	ribonucleoprotein complex	17735	573	2487	111	80.68	0.00023	1.375806	ABCF1;AKAP17A;APOBEC3F;AQR;ATXN2L;BTBD2;CALR;CELF1;CIRBP;CPSF3;CSNK1E;CTNNBL1;DDX20;DDX23
blue	BP	GO:0034620	cellular response to unfolded protein	17735	87	2487	26	12.91	0.00025	2.013943	AARS;ADD1;AMFR;CALR;CCND1;CUL7;DCTN1;DDIT3;DERL2;EDEM1;EIF2AK3;EIF2AK4;GOSR2;HSP90B1;HYOU
blue	BP	GO:1901362	organic cyclic compound biosynthetic pro...	17735	3752	2487	623	556.83	0.00026	1.118833	ABCA2;ABL1;ABLIM1;ACAA2;ACACA;ACACB;ACAD8;ACOX2;ACTR8;ACVR1;ADCY6;ADCY7;ADSS;AK2;AK5;AKA
blue	BP	GO:0035023	regulation of Rho protein signal transdu...	17735	166	2487	42	24.64	0.00027	1.704545	ALS2CL;ARHGAP15;ARHGAP29;ARHGAP35;ARHGEF1;ARHGEF16;ARHGEF18;ARHGEF3;ARHGEF4;ARHGEF5;A
blue	BP	GO:0051345	positive regulation of hydrolase activit...	17735	450	2487	94	66.78	0.00028	1.407607	ACER2;ADCY3;ADCY6;ADCY7;ADCY9;ADD1;AHSA1;AIFM1;AKT2;ARHGAP15;ARHGAP29;ARHGAP35;ARHGEF1
blue	BP	GO:0019438	aromatic compound biosynthetic process	17735	3638	2487	605	539.91	0.00028	1.120557	ABCA2;ABL1;ABLIM1;ACACA;ACACB;ACAD8;ACTR8;ACVR1;ADCY6;ADCY7;ADSS;AK2;AK5;AKAP17A;AKNA;AL
blue	BP	GO:0031060	regulation of histone methylation	17735	24	2487	11	3.56	0.00029	3.089888	CTCF;CTR9;DNMT1;GATA3;KDM1A;MECP2;MLL4;PAXIP1;PYGO2;RNF20;SNW1
blue	CC	GO:0005759	mitochondrial matrix	17735	286	2487	62	40.27	0.00029	1.539608	ACAD8;ACO2;ALDH1B1;ALDH5A1;ALDH6A1;ALDH7A1;ATP5A1;ATP5B;BDH1;BLOC1S1;D2HGDH;DARS2;DLD;

blue	MF	GO:0030695	GTPase regulator activity	17735	436	2487	90	63.43	0.00029	1.418887	AGAP5;AGAP6;ALS2CL;ANKRD27;ARHGAP15;ARHGAP17;ARHGAP18;ARHGAP29;ARHGAP32;ARHGAP33;ARH
blue	BP	GO:0010468	regulation of gene expression	17735	3347	2487	560	496.72	0.00029	1.127396	AAK1;ABCA2;ABCF1;ABL1;ABLIM1;ACAD8;ACTR8;ACVR1;AKAP17A;AKNA;AKT2;ANK3;APBA1;APBA2;APBB1;
blue	BP	GO:0032856	activation of Ras GTPase activity	17735	28	2487	12	4.16	0.00033	2.884615	AKT2;ARHGEF16;CCR7;DOCK7;EPHA1;EPHA2;FOXJ1;SCRIB;SYDE1;TAX1BP3;TBC1D7;TSC1
blue	BP	GO:0002562	somatic diversification of immune recept...	17735	48	2487	17	7.12	0.00033	2.38764	BATF;BCL11B;CD40LG;FOXP1;HSPD1;IL10;IL4;LEF1;MLH1;MSH6;PAXIP1;PMS2;RNF8;TCF3;TCF7;UNG;XRCC6
blue	BP	GO:0016444	somatic cell DNA recombination	17735	48	2487	17	7.12	0.00033	2.38764	BATF;BCL11B;CD40LG;FOXP1;HSPD1;IL10;IL4;LEF1;MLH1;MSH6;PAXIP1;PMS2;RNF8;TCF3;TCF7;UNG;XRCC6
blue	BP	GO:0019222	regulation of metabolic process	17735	4834	2487	787	717.4	0.00033	1.097017	AAK1;ABCA2;ABCF1;ABL1;ABL2;ABLIM1;ACACA;ACACB;ACAD8;ACE;ACER2;ACLY;ACN9;ACTR8;ACVR1;ADCY
blue	BP	GO:0051171	regulation of nitrogen compound metaboli...	17735	3496	2487	582	518.83	0.00035	1.121755	ABCA2;ABL1;ABLIM1;ACAD8;ACTR8;ACVR1;ADCY7;ADD1;AGAP5;AGAP6;AHSA1;AKAP17A;AKNA;AKT2;APBB
blue	MF	GO:0016879	ligase activity, forming carbon-nitrogen...	17735	330	2487	71	48.01	0.00036	1.478859	ACACA;ACACB;ADSS;AMFR;ANAPC5;ARIH2;ATG10;ATG3;BIRC6;BTRC;CAD;CARNS1;CBLB;CTPS2;DTL;FBXO21
blue	BP	GO:0044248	cellular catabolic process	17735	1692	2487	299	251.11	0.00036	1.190713	ABCA2;ABCC1;ABL1;ABL2;ACAD8;ACE;ACLY;ACOX2;ACOX3;ADSS;AGAP5;AGAP6;AHSA1;AIFM1;AKT2;ALDH5
blue	CC	GO:0005819	spindle	17735	232	2487	52	32.67	0.00037	1.591674	ALMS1;ARL3;ATM;BIRC6;CALM3;CDC16;CDC25B;CDK5RAP2;CEP85;CEP89;CIAO1;CKAP5;DCTN1;DIAPH1;DY
blue	CC	GO:0016514	SWI/SNF complex	17735	15	2487	8	2.11	0.00039	3.791469	ARID1B;NCR1;SMARCA4;SMARCC1;SMARCC2;SMARCD1;SMARCD3;SMARCE1
blue	BP	GO:0006986	response to unfolded protein	17735	133	2487	35	19.74	0.00039	1.77305	AARS;ADD1;AMFR;ATF6B;CALR;CCND1;CREB3L2;CUL7;DCTN1;DDIT3;DERL2;DNAJB1;EDEM1;EIF2AK3;EIF2A
blue	CC	GO:0019867	outer membrane	17735	145	2487	36	20.42	0.00039	1.762977	ACSL5;AIFM2;AKAP1;ANKH;BAD;BCL2;BID;BRI3BP;CYB5B;DHCR7;EMD;GUCY2D;HADHB;ITPR3;MAVS;MGST1
blue	MF	GO:0003712	transcription cofactor activity	17735	475	2487	96	69.11	4.00E-04	1.38909	ARHGAP35;ARID1B;ASXL1;ATN1;BATF3;BCL10;BCL9L;BRD7;C1D;CBFA2T2;CCAR1;CIITA;CREG1;CREM;CRTC1
blue	CC	GO:0016363	nuclear matrix	17735	84	2487	24	11.83	0.00041	2.02874	AKAP8;ATN1;BLM;CAD;DNMT3A;HLCS;HNRNPM;KIF4A;MBD1;NONO;NUMA1;PAXIP1;POLA1;PSPC1;RUVBL1
blue	CC	GO:0031461	cullin-RING ubiquitin ligase complex	17735	89	2487	25	12.53	0.00042	1.995211	ANAPC1;ANAPC13;ANAPC5;ANAPC7;BTRC;CCNF;CDC16;CUL1;CUL4A;CUL7;CUL9;DCAF16;DCAF4;DCAF5;DC
blue	BP	GO:0045058	T cell selection	17735	25	2487	11	3.71	0.00044	2.96496	BCL11B;BCL2;CARD11;CCR7;CD3E;CD4;CD74;GATA3;ITPKB;MINK1;SPN
blue	BP	GO:0051028	mRNA transport	17735	114	2487	31	16.92	0.00044	1.832151	CPSF3;DDX19A;DDX19B;DHX38;EIF4A3;EIF5A;GLE1;HHEX;KHSRP;KIF5C;MAGOH;MYO1C;NUP133;NUP155;N
blue	CC	GO:0005635	nuclear envelope	17735	314	2487	66	44.21	0.00046	1.492875	ABCF1;ABL1;AGPAT3;ANKLE2;ATP2A3;BCL2;CBX5;CCND2;CLIC1;DDX19A;DDX19B;DGKH;DHCR7;DNAJC1;DN
blue	BP	GO:0034654	nucleobase-containing compound biosynthe...	17735	3583	2487	594	531.75	0.00046	1.117066	ABCA2;ABL1;ABLIM1;ACACA;ACACB;ACAD8;ACTR8;ACVR1;ADCY6;ADCY7;ADSS;AK2;AK5;AKAP17A;AKNA;AL
blue	BP	GO:0048011	nerve growth factor receptor signaling p...	17735	223	2487	52	33.09	0.00048	1.571472	ADCY3;ADCY6;ADCY7;ADCY9;AP2B1;ARHGEF1;ARHGEF16;ARHGEF18;ARHGEF3;ARHGEF4;ARHGEF6;ARHGE
blue	MF	GO:0005089	Rho guanyl-nucleotide exchange factor ac...	17735	73	2487	22	10.62	0.00049	2.071563	ALS2CL;ARHGEF1;ARHGEF16;ARHGEF18;ARHGEF3;ARHGEF4;ARHGEF5;ARHGEF6;ARHGEF7;ARHGEF9;DOCK
blue	MF	GO:0008186	RNA-dependent ATPase activity	17735	22	2487	10	3.2	5.00E-04	3.125	DDX17;DDX18;DDX20;DDX23;DDX24;DDX56;DHX9;EIF4A3;G3BP1;SUPV3L1
blue	CC	GO:0034399	nuclear periphery	17735	100	2487	27	14.08	5.00E-04	1.917614	AKAP8;ATN1;BLM;CAD;DNMT3A;HLCS;HNRNPM;KIF4A;MAPT;MBD1;NONO;NUMA1;NUP205;NUP93;PAXIP
blue	BP	GO:0008088	axon cargo transport	17735	33	2487	13	4.9	0.00051	2.653061	AP3D1;AP3S1;APBA1;BLOC1S1;CNTNAP1;DST;HTT;KIF3B;KIF4A;KLC2;MAPT;SNAPIN;SPG7
blue	BP	GO:0035966	response to topologically incorrect prot...	17735	140	2487	36	20.78	0.00052	1.732435	AARS;ADD1;AMFR;ATF6B;CALR;CCND1;CREB3L2;CUL7;DCTN1;DDIT3;DERL2;DNAJB1;EDEM1;EIF2AK3;EIF2A
blue	BP	GO:0030968	endoplasmic reticulum unfolded protein r...	17735	86	2487	25	12.76	0.00053	1.959248	AARS;ADD1;AMFR;CALR;CCND1;CUL7;DCTN1;DDIT3;DERL2;EDEM1;EIF2AK3;EIF2AK4;GOSR2;HSP90B1;HYO
blue	MF	GO:0019901	protein kinase binding	17735	357	2487	75	51.94	0.00053	1.443974	ABL1;ADCY6;ARHGEF7;ATG13;AXIN1;AXIN2;BAD;BCL10;BRSK2;CCND1;CCND2;CCNT1;CD3E;CD4;CD8A;CDC
blue	BP	GO:0045934	negative regulation of nucleobase-contai...	17735	932	2487	174	138.32	0.00055	1.257953	ARHGAP35;ASXL1;ATN1;ATP8B1;AXIN1;BAHD1;BLM;BMP2;BPTF;BRD7;BTRC;C14orf169;C1D;CALR;CAPN3;C
blue	BP	GO:0016570	histone modification	17735	268	2487	60	39.77	0.00058	1.508675	APBB1;ASXL1;BAZ1B;BRD1;BRD7;BRPF1;C14orf169;CSRP2BP;CTCF;CTR9;DNMT1;EHMT1;EP400;GATA3;GTF
blue	BP	GO:0044085	cellular component biogenesis	17735	1686	2487	296	250.22	6.00E-04	1.182959	AARS2;ABL2;ACACA;ACACB;ADD1;AIF1;AIFM1;ALAD;ALDH5A1;ALMS1;AMFR;ANK3;AP2B1;APBA1;ARG1;AR
blue	BP	GO:0016569	covalent chromatin modification	17735	274	2487	61	40.66	0.00061	1.500246	APBB1;ASXL1;BAZ1B;BRD1;BRD7;BRPF1;C14orf169;CSRP2BP;CTCF;CTR9;DNMT1;DNMT3A;EHMT1;EP400;G
blue	BP	GO:0051726	regulation of cell cycle	17735	736	2487	141	109.23	0.00062	1.290854	ABL1;AIF1;AKT2;ANAPC1;ANAPC5;ANAPC7;APBB1;ATM;AXIN2;BAD;BCL2;BID;BLM;BMP2;BRD7;BTRC;C11or
blue	BP	GO:0031323	regulation of cellular metabolic process	17735	4397	2487	717	652.55	0.00063	1.098766	ABCA2;ABCF1;ABL1;ABL2;ABLIM1;ACACA;ACACB;ACAD8;ACER2;ACLY;ACN9;ACTR8;ACVR1;ADCY3;ADCY6;A
blue	CC	GO:0031968	organelle outer membrane	17735	138	2487	34	19.43	0.00065	1.749871	ACSL5;AIFM2;AKAP1;BAD;BCL2;BID;BRI3BP;CYB5B;DHCR7;EMD;GUCY2D;HADHB;ITPR3;MAVS;MGST1;MLXI
blue	BP	GO:0035967	cellular response to topologically incor...	17735	92	2487	26	13.65	0.00066	1.904762	AARS;ADD1;AMFR;CALR;CCND1;CUL7;DCTN1;DDIT3;DERL2;EDEM1;EIF2AK3;EIF2AK4;GOSR2;HSP90B1;HYO
blue	MF	GO:0005085	guanyl-nucleotide exchange factor activi...	17735	176	2487	42	25.61	0.00066	1.639984	ALS2CL;ANKRD27;ARHGEF1;ARHGEF16;ARHGEF18;ARHGEF3;ARHGEF4;ARHGEF5;ARHGEF6;ARHGEF7;ARHG
blue	BP	GO:0006260	DNA replication	17735	275	2487	61	40.81	0.00067	1.494732	BLM;CALR;CDAN1;CHTF8;CINP;CIZ1;CTC1;DKC1;DNAJA3;DTL;FEN1;GINS4;GTPBP4;HELB;HMGA1;ING5;INO8
blue	BP	GO:2000112	regulation of cellular macromolecule bio...	17735	3117	2487	520	462.59	0.00068	1.124106	ABCA2;ABCF1;ABL1;ABLIM1;ACAD8;ACER2;ACTR8;ACVR1;AKAP17A;AKNA;AKT2;APBB1;ARHGAP35;ARID1B;
blue	BP	GO:0006271	DNA strand elongation involved in DNA re...	17735	34	2487	13	5.05	0.00071	2.574257	FEN1;GINS4;LIG1;LIG3;MCM2;MCM3;MCM4;MCM6;POLA1;POLA2;RFC1;RPA1;RPA2
blue	BP	GO:0006302	double-strand break repair	17735	112	2487	30	16.62	0.00071	1.805054	APBB1;ATM;BAZ1B;BLM;CHEK2;DCLRE1C;FAN1;FEN1;FOXM1;INO80;LIG1;LIG3;MAD2L2;MDC1;MLH1;PALB
blue	BP	GO:0006974	response to DNA damage stimulus	17735	608	2487	119	90.23	0.00072	1.318852	ABL1;ACTR8;APBB1;ATM;AXIN2;BATF;BAZ1B;BCL2;BLM;CASP2;CCND1;CD74;CDKN2A;CEP164;CHD1L;CHEK
blue	BP	GO:1901575	organic substance catabolic process	17735	1842	2487	320	273.37	0.00073	1.170575	ABCA2;ABCC1;ACAD8;ACE;ACLY;ACOX2;ACOX3;ADPGK;ADSS;AFG3L2;AGAP5;AGAP6;AHSA1;AIFM1;AKT2;AL
blue	BP	GO:0030010	establishment of cell polarity	17735	69	2487	21	10.24	0.00074	2.050781	AMOTL1;BRSK2;CCR7;CDK5RAP2;CYTH1;CYTH3;DOCK7;DYNLT1;FBF1;FOXJ1;FRMD4A;GATA3;HTT;JAM3;KA
blue	BP	GO:0010556	regulation of macromolecule biosynthetic...	17735	3197	2487	532	474.46	0.00074	1.121275	ABCA2;ABCF1;ABL1;ABLIM1;ACAD8;ACER2;ACTR8;ACVR1;AKAP17A;AKNA;AKT2;APBB1;ARHGAP35;ARID1B;
blue	BP	GO:0006541	glutamine metabolic process	17735	19	2487	9	2.82	0.00077	3.191489	ALDH5A1;CAD;CTPS2;GGH;GLS2;GMPS;LGSN;MECP2;PFAS
blue	CC	GO:0000151	ubiquitin ligase complex	17735	150	2487	36	21.12	0.00077	1.704545	ANAPC1;ANAPC13;ANAPC5;ANAPC7;ATG3;BTRC;CCNF;CDC16;CUL1;CUL4A;CUL7;CUL9;DCAF16;DCAF4;DCA
blue	BP	GO:1901605	alpha-amino acid metabolic process	17735	174	2487	42	25.82	0.00078	1.626646	ACAD8;ADSS;AGMAT;ALDH18A1;ALDH5A1;ALDH6A1;ALDH7A1;ARG1;CAD;CTPS2;DLD;DLST;DNMT3A;EIF5A;
blue	BP	GO:0051172	negative regulation of nitrogen compound...	17735	945	2487	175	140.25	0.00079	1.247772	ARHGAP35;ASXL1;ATN1;ATP8B1;AXIN1;BAHD1;BLM;BMP2;BPTF;BRD7;BTRC;C14orf169;C1D;CALR;CAPN3;C
blue	MF	GO:0000988	protein binding transcription factor act...	17735	514	2487	101	74.78	8.00E-04	1.350629	ARHGAP35;ARID1B;ASXL1;ATN1;BATF3;BCL10;BCL9L;BRD7;C1D;CBFA2T2;CCAR1;CIITA;CREG1;CREM;CRTC1
blue	BP	GO:0006915	apoptotic process	17735	1562	2487	275	231.81	8.00E-04	1.186316	AARS;ABL1;ACAA2;ACER2;ACSL5;ACVR1;ADD1;AIFM1;AIFM2;AKT2;ALMS1;ALX3;AMBRA1;APBB1;ARHGEF16
blue	MF	GO:0000989	transcription factor binding transcripti...	17735	498	2487	98	72.45	9.00E-04	1.352657	ARHGAP35;ARID1B;ASXL1;ATN1;BATF3;BCL10;BCL9L;BRD7;C1D;CBFA2T2;CCAR1;CIITA;CREG1;CREM;CRTC1
blue	BP	GO:0016043	cellular component organization	17735	4124	2487	673	612.03	0.00094	1.099619	AAK1;AARS2;ABL1;ABL2;ABLIM1;ACACA;ACACB;ACTR8;ADD1;AFG3L2;AIF1;AIFM1;AIFM2;AKAP8;AKT2;AKT3
blue	MF	GO:0047485	protein N-terminus binding	17735	86	2487	24	12.51	0.00096	1.918465	ATM;CALM3;CSNK2A2;DCN;EIF5A;ERCC3;ERCC5;EXOC2;EXOC4;GRIN2C;MECP2;NCOR2;PARP1;PEX5;RELA;R
blue	BP	GO:0006261	DNA-dependent DNA replication	17735	99	2487	27	14.69	0.00096	1.837985	BLM;CIZ1;DNAJA3;FEN1;GINS4;HELB;HMGA1;INO80;LIG1;LIG3;MCM2;MCM3;MCM4;MCM6;POLA1;POLA2;
blue	BP	GO:0051569	regulation of histone H3-K4 methylation	17735	16	2487	8	2.37	0.00098	3.375527	CTR9;DNMT1;GATA3;KDM1A;MLL4;PAXIP1;PYGO2;SNW1
blue	BP	GO:0006520	cellular amino acid metabolic process	17735	461	2487	93	68.42	0.001	1.359252	AARS;AARS2;ACAD8;ADSS;AGMAT;ALDH18A1;ALDH5A1;ALDH6A1;ALDH7A1;ARG1;ATIC;CAD;CARNS1;CKM;
blue	CC	GO:0016591	DNA-directed RNA polymerase II, holoenzy...	17735	89	2487	24	12.53	0.00102	1.915403	CTR9;ELP3;ERCC3;ERCC5;GTF2A2;IKBKAP;INTS1;INTS4;INTS7;INTS9;LEO1;MMS19;POLR2B;POLR2C;POLR2J;
blue	BP	GO:0012501	programmed cell death	17735	1575	2487	276	233.74	0.00104	1.180799	AARS;ABL1;ACAA2;ACER2;ACSL5;ACVR1;ADD1;AIFM1;AIFM2;AKT2;ALMS1;ALX3;AMBRA1;APBB1;ARHGEF16
blue	BP	GO:0016054	organic acid catabolic process	17735	198	2487	46	29.38	0.00107	1.565691	ACAD8;ACOX2;ACOX3;AKT2;ALDH5A1;ALDH6A1;ALDH7A1;AMACR;ARG1;CAD;DLD;DLST;ECI2;ENOSF1;ETFD
blue	BP	GO:0046395	carboxylic acid catabolic process	17735	198	2487	46	29.38	0.00107	1.565691	ACAD8;ACOX2;ACOX3;AKT2;ALDH5A1;ALDH6A1;ALDH7A1;AMACR;ARG1;CAD;DLD;DLST;ECI2;ENOSF1;ETFD
blue	MF	GO:0002039	p53 binding	17735	49	2487	16	7.13	0.00108	2.244039	AXIN1;BLM;BRD7;CDKN2A;EHMT1;HSPD1;HTT;KDM1A;RFWD3;RNF20;SMARCA4;TAF1;TAF3;TP53;TP53BP1;
blue	BP	GO:0051168	nuclear export	17735	120	2487	31	17.81	0.00113	1.740595	CALR;CDKN2A;CPSF3;DDX19B;DHX38;DUSP16;EIF5A;EMD;GLE1;HHEX;HSPA9;HTT;KHDRBS1;LSG1;MAGOH;
blue	BP	GO:0019048	virus-host interaction	17735	371	2487	77	55.06	0.00116	1.398474	ATP6V1H;BAD;BICD1;BTRC;CBX5;CCNT1;CD247;CD4;CRTC1;CRTC3;CUL1;CUL4A;CUL7;DDB1;DYNC1I2;DYNL
blue	BP	GO:2000113	negative regulation of cellular macromol...	17735	923	2487	170	136.98	0.0012	1.241057	ACER2;ARHGAP35;ASXL1;ATN1;ATP8B1;BAHD1;BLM;BMP2;BPTF;BRD7;BTRC;C14orf169;C1D;CALR;CAPN3;C
blue	CC	GO:0005643	nuclear pore	17735	75	2487	21	10.56	0.00122	1.988636	DDX19A;DDX19B;EIF5A;GLE1;IPO5;IPO9;MYO1C;NUP133;NUP155;NUP188;NUP205;NUP210;NUP214;NUP6
blue	BP	GO:0046578	regulation of Ras protein signal transdu...	17735	276	2487	60	40.96	0.00124	1.464844	AGAP5;AGAP6;AKT2;ALS2CL;ARHGAP15;ARHGAP29;ARHGAP35;ARHGEF1;ARHGEF16;ARHGEF18;ARHGEF3;
blue	CC	GO:0005640	nuclear outer membrane	17735	21	2487	9	2.96	0.00127	3.040541	DHCR7;EMD;GUCY2D;ITPR3;RETSAT;SIGMAR1;SYNE1;SYNE2;TMEM10
blue	BP	GO:0050821	protein stabilization	17735	67	2487	20	9.94	0.00128	2.012072	AAK1;CALR;CDKN2A;COG7;DNAJA3;FLNA;GTPBP4;HCFC1;HPS4;HSPD1;MDM4;MUL1;SMAD3;STXBP1;TCF3;

blue	BP	GO:0009113	purine nucleobase biosynthetic process	17735	10	2487	6	1.48	0.0013	4.054054	CECR1;GART;GMPS;PAICS;PRPS1;SHMT1
blue	BP	GO:0016032	viral reproduction	17735	689	2487	131	102.25	0.0013	1.281174	AP2B1;APOBEC3F;ATP6V1H;BAD;BCL2;BICD1;BTRC;CBX5;CCNT1;CD247;CD4;CRTC1;CRTC3;CUL1;CUL4A;CUL
blue	BP	GO:0006399	tRNA metabolic process	17735	121	2487	31	17.96	0.00131	1.726058	AARS;AARS2;ADAT1;C22orf28;CDKAL1;DARS2;EARS2;EPRS;FARSB;GARS;HARS;IARS;IARS2;KARS;LARS;LARS2
blue	BP	GO:0016072	rRNA metabolic process	17735	111	2487	29	16.47	0.00133	1.760777	C1D;CDKN2A;DDX47;DDX51;DDX56;DIS3L;DKC1;DROSHA;EIF4A3;EXOSC10;FTSJ2;FTSJ3;GAR1;GEMIN4;HEAT
blue	CC	GO:0005813	centrosome	17735	374	2487	74	52.66	0.00133	1.405241	AK5;AKAP9;ALMS1;ARL3;AXIN2;BRSK2;C2CD3;CALM3;CCDC64;CCNF;CCT5;CCT8;CDC16;CDC25B;CDC42BPG;
blue	BP	GO:0051056	regulation of small GTPase mediated sign...	17735	367	2487	76	54.47	0.00133	1.395263	AGAP5;AGAP6;AKT2;ALS2CL;ARHGAP15;ARHGAP17;ARHGAP18;ARHGAP29;ARHGAP32;ARHGAP35;ARHGAP
blue	BP	GO:0007266	Rho protein signal transduction	17735	200	2487	46	29.68	0.00134	1.549865	ALS2CL;ARHGAP15;ARHGAP29;ARHGAP35;ARHGEF1;ARHGEF16;ARHGEF18;ARHGEF3;ARHGEF4;ARHGEF5;A
blue	CC	GO:0046930	pore complex	17735	91	2487	24	12.81	0.00142	1.873536	BCL2;DDX19A;DDX19B;EIF5A;GLE1;IPO5;IPO9;MYO1C;NUP133;NUP155;NUP188;NUP205;NUP210;NUP214;
blue	BP	GO:0044764	multi-organism cellular process	17735	691	2487	131	102.55	0.00145	1.277426	AP2B1;APOBEC3F;ATP6V1H;BAD;BCL2;BICD1;BTRC;CBX5;CCNT1;CD247;CD4;CRTC1;CRTC3;CUL1;CUL4A;CUL
blue	BP	GO:0008219	cell death	17735	1741	2487	301	258.38	0.00145	1.164951	AARS;ABL1;ACAA2;ACER2;ACO2;ACSL5;ACVR1;ADD1;AFG3L2;AIFM1;AIFM2;AKT2;ALMS1;ALX3;AMBRA1;AP
blue	BP	GO:0030163	protein catabolic process	17735	531	2487	104	78.8	0.00148	1.319797	ACE;AFG3L2;AMFR;ANAPC1;ANAPC5;ANAPC7;ARIH2;ATM;AXIN1;BTRC;CBLB;CCNF;CDC16;CHEK2;CLN8;CSN
blue	MF	GO:0016740	transferase activity	17735	1705	2487	290	248.05	0.00148	1.169119	AAK1;ABL1;ABL2;ACAA2;ACLY;ACVR1;ADPGK;AGK;AGPAT3;AGPAT6;AGXT2L2;AK2;AK5;AKT2;AKT3;ALDH18A
blue	CC	GO:0090544	BAF-type complex	17735	14	2487	7	1.97	0.0015	3.553299	PHF10;SMARCA4;SMARCC1;SMARCC2;SMARCD1;SMARCD3;SMARCE1
blue	BP	GO:0016265	death	17735	1743	2487	301	258.68	0.00156	1.1636	AARS;ABL1;ACAA2;ACER2;ACO2;ACSL5;ACVR1;ADD1;AFG3L2;AIFM1;AIFM2;AKT2;ALMS1;ALX3;AMBRA1;AP
blue	MF	GO:0004004	ATP-dependent RNA helicase activity	17735	21	2487	9	3.06	0.00161	2.941176	DDX18;DDX20;DDX23;DDX24;DDX56;DHX9;EIF4A3;G3BP1;SUPV3L1
blue	BP	GO:0010558	negative regulation of macromolecule bio...	17735	961	2487	175	142.62	0.00172	1.227037	ACER2;APBB1;ARHGAP35;ASB1;ASXL1;ATN1;ATP8B1;AXIN1;BAHD1;BLM;BMP2;BPTF;BRD7;BTRC;C14orf169
blue	BP	GO:0022616	DNA strand elongation	17735	37	2487	13	5.49	0.00177	2.367942	FEN1;GINS4;LIG1;LIG3;MCM2;MCM3;MCM4;MCM6;POLA1;POLA2;RFC1;RPA1;RPA2
blue	BP	GO:0032774	RNA biosynthetic process	17735	3293	2487	542	488.71	0.00177	1.109042	ABCA2;ABL1;ABLIM1;ACAD8;ACTR8;ACVR1;AKAP17A;AKNA;ALX3;APBB1;ARHGAP35;ARID1B;ARNTL2;ASCC1
blue	BP	GO:0006987	activation of signaling protein activity...	17735	64	2487	19	9.5	0.00179	2	ADD1;CALR;CUL7;DCTN1;DDIT3;EDEM1;EIF2AK3;GOSR2;HSP90B1;HYOU1;KDELR3;MBTPS1;PDIA5;PDIA6;SH
blue	BP	GO:0032259	methylation	17735	139	2487	34	20.63	0.00185	1.648085	CTCF;CTR9;DNMT1;DNMT3A;EHMT1;FAM103A1;FTSJ2;FTSJ3;GATA3;HEMK1;ICMT;KDM1A;MECP2;MLL;MLL
blue	BP	GO:0016445	somatic diversification of immunoglobuli...	17735	46	2487	15	6.83	0.00191	2.196193	BATF;CD40LG;CTNNBL1;FOXP1;HSPD1;IL10;IL4;MLH1;MSH6;PAXIP1;PMS2;PMS2P5;RNF8;TCF3;UNG
blue	BP	GO:0043632	modification-dependent macromolecule cat...	17735	395	2487	80	58.62	0.00194	1.364722	AMFR;ANAPC1;ANAPC5;ANAPC7;ARIH2;AXIN1;BTRC;CCNF;CDC16;CSNK1E;CUL1;CUL4A;CUL7;CUL9;DDB1;D
blue	BP	GO:0006099	tricarboxylic acid cycle	17735	29	2487	11	4.3	0.00195	2.55814	ACO2;DLD;DLST;FH;IDH3A;NNT;OGDH;PDHA1;PDHB;SDHA;SDHB
blue	MF	GO:0016772	transferase activity, transferring phosp...	17735	889	2487	160	129.34	0.00195	1.23705	AAK1;ABL1;ABL2;ACVR1;ADPGK;AGK;AK2;AK5;AKT2;AKT3;ALDH18A1;ATM;BAZ1B;BRSK2;C9orf96;CAD;CARD
blue	BP	GO:0044093	positive regulation of molecular functio...	17735	1099	2487	197	163.1	0.00195	1.207848	ABL1;ABL2;ACER2;ADCY3;ADCY6;ADCY7;ADCY9;ADD1;AHSA1;AIFM1;AKT2;ANAPC1;ANAPC5;ANAPC7;ANK3;
blue	MF	GO:0003697	single-stranded DNA binding	17735	61	2487	18	8.87	0.00202	2.029312	BLM;CRY2;CTC1;ERCC5;FUBP1;HNRNPA2B1;HSPD1;MCM4;MCM6;MLH1;PMS2;PMS2P5;RPA1;RPA2;SSBP2;
blue	BP	GO:0000086	G2/M transition of mitotic cell cycle	17735	145	2487	35	21.52	0.00204	1.626394	ABCB1;AKAP9;ALMS1;ANAPC5;BRSK2;CDC25B;CDK5RAP2;CDKN2A;CDKN2B;CEP164;CEP72;CHEK2;CKAP5;C
blue	CC	GO:0031966	mitochondrial membrane	17735	496	2487	93	69.84	0.00206	1.331615	ACAA2;ACSL5;AFG3L2;AGK;AIFM1;AIFM2;AK2;AKAP1;ALDH18A1;ALDH3A2;APOOL;ATP5A1;ATP5B;ATP5J;AT
blue	CC	GO:0017053	transcriptional repressor complex	17735	63	2487	18	8.87	0.00207	2.029312	C1D;CBX5;CHD3;CHD4;CORO2A;DDX20;HDAC1;JAZF1;MTA1;MTA2;MTA3;NCOR2;RBBP4;RBBP7;SALL2;SMA
blue	BP	GO:0032321	positive regulation of Rho GTPase activi...	17735	65	2487	19	9.65	0.00219	1.968912	ARHGAP15;ARHGAP29;ARHGAP35;ARHGEF16;ARHGEF5;CCR7;DOCK7;EPHA1;EPHA2;FOXJ1;MYO9B;NCKAP1
blue	CC	GO:0044450	microtubule organizing center part	17735	94	2487	24	13.24	0.00229	1.812689	AKAP9;ALMS1;CCNF;CDK5RAP2;CEP104;CEP164;CKAP5;CROCC;EXOC7;FBF1;HERC2;LCK;NEURL4;ODF2;PCIF
blue	BP	GO:0006511	ubiquitin-dependent protein catabolic pr...	17735	386	2487	78	57.29	0.00234	1.361494	AMFR;ANAPC1;ANAPC5;ANAPC7;ARIH2;AXIN1;BTRC;CCNF;CDC16;CSNK1E;CUL1;CUL4A;CUL7;CUL9;DDB1;D
blue	BP	GO:0051252	regulation of RNA metabolic process	17735	2953	2487	488	438.25	0.00234	1.11352	ABCA2;ABL1;ABLIM1;ACAD8;ACTR8;ACVR1;AKAP17A;AKNA;APBB1;ARHGAP35;ARID1B;ARNTL2;ASCC1;ASXL
blue	CC	GO:0032993	protein-DNA complex	17735	110	2487	27	15.49	0.00235	1.74306	CTC1;H3F3C;HELB;HHEX;HIST1H2AC;HIST1H2AK;HIST1H2BN;HIST2H2AC;HIST2H2BE;HIST2H3D;HP1BP3;KAT
blue	BP	GO:0033554	cellular response to stress	17735	1209	2487	214	179.43	0.00237	1.192666	AARS;ABL1;ACTR8;ADD1;AIFM1;AKT2;AMFR;APBB1;ARG1;ARHGEF6;ATG10;ATG13;ATG14;ATG3;ATG9B;ATM
blue	BP	GO:0009889	regulation of biosynthetic process	17735	3356	2487	550	498.06	0.00237	1.104285	ABCA2;ABCF1;ABL1;ABLIM1;ACAD8;ACER2;ACN9;ACTR8;ACVR1;ADCY7;AKAP17A;AKNA;AKT2;APBB1;ARHGA
blue	BP	GO:0019941	modification-dependent protein catabolic...	17735	392	2487	79	58.18	0.00238	1.357855	AMFR;ANAPC1;ANAPC5;ANAPC7;ARIH2;AXIN1;BTRC;CCNF;CDC16;CSNK1E;CUL1;CUL4A;CUL7;CUL9;DDB1;D
blue	BP	GO:0010605	negative regulation of macromolecule met...	17735	1359	2487	238	201.69	0.00241	1.180029	ABL1;ACER2;ANAPC1;ANAPC5;ANAPC7;APBB1;ARHGAP35;ASB1;ASXL1;ATN1;ATP8B1;AXIN1;BAHD1;BBS2;B
blue	BP	GO:0051253	negative regulation of RNA metabolic pro...	17735	852	2487	156	126.44	0.00242	1.233787	ARHGAP35;ASXL1;ATN1;ATP8B1;AXIN1;BAHD1;BMP2;BPTF;BRD7;BTRC;C14orf169;C1D;CALR;CAPN3;CBFA2
blue	BP	GO:0006364	rRNA processing	17735	105	2487	27	15.58	0.00245	1.732991	C1D;CDKN2A;DDX47;DDX51;DDX56;DKC1;EIF4A3;EXOSC10;FTSJ2;FTSJ3;GAR1;GEMIN4;HEATR1;LAS1L;NOL9
blue	BP	GO:0044403	symbiosis, encompassing mutualism throug...	17735	445	2487	88	66.04	0.00248	1.332526	ATP6V0E1;ATP6V1D;ATP6V1F;ATP6V1H;BAD;BICD1;BTRC;CAMP;CBX5;CCNT1;CD247;CD4;CRTC1;CRTC3;CUL
blue	BP	GO:0044419	interspecies interaction between organis...	17735	445	2487	88	66.04	0.00248	1.332526	ATP6V0E1;ATP6V1D;ATP6V1F;ATP6V1H;BAD;BICD1;BTRC;CAMP;CBX5;CCNT1;CD247;CD4;CRTC1;CRTC3;CUL
blue	MF	GO:0048037	cofactor binding	17735	255	2487	54	37.1	0.00249	1.455526	ACAD8;ACLY;ACOX2;ACOX3;AGXT2L2;AIFM1;AIFM2;ALDH6A1;ASPDH;CREG1;D2HGDH;DHCR24;DHODH;DLD
blue	BP	GO:0045061	thymic T cell selection	17735	18	2487	8	2.67	0.00252	2.996255	CARD11;CCR7;CD3E;CD74;GATA3;ITPKB;MINK1;SPN
blue	BP	GO:0031327	negative regulation of cellular biosynth...	17735	1000	2487	180	148.41	0.00252	1.212856	ACER2;ARHGAP35;ASB1;ASXL1;ATN1;ATP8B1;AXIN1;BAHD1;BLM;BMP2;BPTF;BRD7;BTRC;C14orf169;C1D;CA
blue	CC	GO:0043596	nuclear replication fork	17735	27	2487	10	3.8	0.00257	2.631579	BAZ1B;HELB;MCM3;POLA1;POLA2;POLE;POLE3;RPA1;RPA2;ZMIZ2
blue	BP	GO:0006767	water-soluble vitamin metabolic process	17735	66	2487	19	9.79	0.00265	1.940756	FASN;GPHN;GSTO1;MCCC1;MMACHC;MMADHC;MTHFD1;MTHFR;NFS1;PNPO;PPCS;PSAT1;SHMT1;SLC23A2
blue	BP	GO:0032075	positive regulation of nuclease activity	17735	66	2487	19	9.79	0.00265	1.940756	ADD1;CALR;CUL7;DCTN1;DDIT3;EDEM1;EIF2AK3;GOSR2;HSP90B1;HYOU1;KDELR3;MBTPS1;PDIA5;PDIA6;SH
blue	BP	GO:0010629	negative regulation of gene expression	17735	909	2487	165	134.9	0.00265	1.223128	ARHGAP35;ASXL1;ATN1;ATP8B1;BAHD1;BBS2;BMP2;BPTF;BRD7;BTRC;C14orf169;C1D;CALR;CAPN3;CBFA2T
blue	BP	GO:0031326	regulation of cellular biosynthetic proc...	17735	3328	2487	545	493.9	0.00266	1.103462	ABCA2;ABCF1;ABL1;ABLIM1;ACAD8;ACER2;ACN9;ACTR8;ACVR1;ADCY7;AKAP17A;AKNA;AKT2;APBB1;ARHGA
blue	CC	GO:0043601	nuclear replisome	17735	19	2487	8	2.68	0.00271	2.985075	HELB;MCM3;POLA1;POLA2;POLE;POLE3;RPA1;RPA2
blue	MF	GO:0004518	nuclease activity	17735	161	2487	37	23.42	0.00271	1.579846	CPSF3;DBR1;DCLRE1C;DFFA;DFFB;DIS3L;DNASE1;DROSHA;EME2;ERCC5;EXD2;EXOG;EXOSC10;FAN1;FEN1;G
blue	MF	GO:0005088	Ras guanyl-nucleotide exchange factor ac...	17735	108	2487	27	15.71	0.00283	1.718651	ALS2CL;ARHGEF1;ARHGEF16;ARHGEF18;ARHGEF3;ARHGEF4;ARHGEF5;ARHGEF6;ARHGEF7;ARHGEF9;DENN
blue	CC	GO:0005740	mitochondrial envelope	17735	520	2487	96	73.22	0.00286	1.311117	ABCF2;ACAA2;ACN9;ACSL5;AFG3L2;AGK;AIFM1;AIFM2;AK2;AKAP1;ALDH18A1;ALDH3A2;APOOL;ATP5A1;AT
blue	BP	GO:0009199	ribonucleoside triphosphate metabolic pr...	17735	477	2487	93	70.79	0.00294	1.313745	ABCA2;ABCC1;ACLY;ADSS;AGAP5;AGAP6;AK2;AK5;AKT2;ARHGAP15;ARHGAP29;ARHGAP35;ARHGEF16;ARH
blue	BP	GO:0009116	nucleoside metabolic process	17735	602	2487	114	89.34	0.00297	1.276024	ABCA2;ABCC1;ACACA;ACACB;ACLY;ADSS;AGAP5;AGAP6;AHSA1;AK2;AK5;AKT2;APOBEC3F;ARHGAP15;ARHG
blue	MF	GO:0019900	kinase binding	17735	397	2487	78	57.76	0.00299	1.350416	ABL1;ADCY6;ARHGEF7;ATG13;AXIN1;AXIN2;BAD;BCL10;BRSK2;CCND1;CCND2;CCNT1;CD3E;CD4;CD8A;CDC2
blue	MF	GO:0042162	telomeric DNA binding	17735	15	2487	7	2.18	0.003	3.211009	HNRNPA2B1;NCL;SMG6;TERF2;TP53BP1;XRCC5;XRCC6
blue	BP	GO:0010833	telomere maintenance via telomere length...	17735	39	2487	13	5.79	0.00302	2.24525	DKC1;FEN1;LIG1;PINX1;POLA1;POLA2;POLE;RFC1;RPA1;RPA2;TERF2;TERF2IP;TNKS1BP1
blue	BP	GO:0007005	mitochondrion organization	17735	230	2487	50	34.13	0.00302	1.464987	AARS2;AFG3L2;AIFM1;AIFM2;AKT3;ATG3;BAD;BCL2;BID;BIK;CDKN2A;CHCHD3;CLN8;COX10;DARS2;DHODH;
blue	MF	GO:0019904	protein domain specific binding	17735	535	2487	101	77.83	0.00302	1.2977	ABL1;APBA1;ARHGAP17;ARHGEF16;ARHGEF4;ATN1;AXIN1;AXIN2;BAIAP2L1;BCL2;BIK;C9orf89;CADM1;CALM
blue	BP	GO:0007265	Ras protein signal transduction	17735	378	2487	76	56.1	0.00303	1.354724	AGAP5;AGAP6;AIF1;AKT2;ALS2CL;ARHGAP15;ARHGAP29;ARHGAP35;ARHGEF1;ARHGEF16;ARHGEF18;ARHG
blue	BP	GO:0051603	proteolysis involved in cellular protein...	17735	413	2487	82	61.29	0.00305	1.337902	AMFR;ANAPC1;ANAPC5;ANAPC7;ARIH2;AXIN1;BTRC;CCNF;CDC16;CSNK1E;CUL1;CUL4A;CUL7;CUL9;DDB1;D
blue	BP	GO:0042454	ribonucleoside catabolic process	17735	431	2487	85	63.96	0.00314	1.328956	ABCA2;ABCC1;ACLY;ADSS;AGAP5;AGAP6;AKT2;APOBEC3F;ARHGAP15;ARHGAP29;ARHGAP35;ARHGEF16;AR
blue	CC	GO:0042101	T cell receptor complex	17735	12	2487	6	1.69	0.00334	3.550296	BCL10;CARD11;CD247;CD3E;CD4;CD8A
blue	CC	GO:0071565	nBAF complex	17735	12	2487	6	1.69	0.00334	3.550296	SMARCA4;SMARCC1;SMARCC2;SMARCD1;SMARCD3;SMARCE1
blue	MF	GO:0001103	RNA polymerase II repressing transcripti...	17735	19	2487	8	2.76	0.00334	2.898551	CHD4;GTF2A2;HDAC1;MTA1;MTA2;PPARA;TBP;TCF7L2
blue	BP	GO:0043414	macromolecule methylation	17735	128	2487	31	19	0.00336	1.631579	CTCF;CTR9;DNMT1;DNMT3A;EHMT1;FTSJ2;FTSJ3;GATA3;HEMK1;ICMT;KDM1A;MECP2;MLL;MLL4;MPHOSPH
blue	BP	GO:0051294	establishment of spindle orientation	17735	15	2487	7	2.23	0.00337	3.139013	CDK5RAP2;DYNLT1;HTT;MAP4;NDC80;NUMA1;ZW10

blue	BP	GO:0009892	negative regulation of metabolic process	17735	1456	2487	252	216.08	0.00338	1.166235	ABL1;ACER2;ANAPC1;ANAPC5;ANAPC7;ANKLE2;APBB1;ARHGAP35;ASB1;ASXL1;ATN1;ATP8B1;AXIN1;BAHD1
blue	BP	GO:2001141	regulation of RNA biosynthetic process	17735	2889	2487	476	428.75	0.00339	1.110204	ABCA2;ABL1;ABLIM1;ACAD8;ACTR8;ACVR1;AKAP17A;AKNA;APBB1;ARHGAP35;ARID1B;ARNTL2;ASCC1;ASXL
blue	BP	GO:0045892	negative regulation of transcription, DN...	17735	823	2487	150	122.14	0.00348	1.228099	ARHGAP35;ASXL1;ATN1;ATP8B1;BAHD1;BMP2;BPTF;BRD7;BTRC;C14orf169;C1D;CALR;CAPN3;CBFA2T2;CBX
blue	BP	GO:0009890	negative regulation of biosynthetic proc...	17735	1014	2487	181	150.49	0.00353	1.202738	ACER2;APBB1;ARHGAP35;ASB1;ASXL1;ATN1;ATP8B1;AXIN1;BAHD1;BLM;BMP2;BPTF;BRD7;BTRC;C14orf169
blue	BP	GO:0043547	positive regulation of GTPase activity	17735	150	2487	35	22.26	0.0037	1.572327	AKT2;ARHGAP15;ARHGAP29;ARHGAP35;ARHGEF16;ARHGEF5;ARHGEF6;ARHGEF7;AXIN1;AXIN2;CCR7;DOCK
blue	BP	GO:0006406	mRNA export from nucleus	17735	63	2487	18	9.35	0.00371	1.925134	CPSF3;DDX19B;DHX38;EIF5A;GLE1;HHEX;MAGOH;NUP133;NUP155;NUP214;NXF1;RNPS1;SMG6;SRRM1;SRS
blue	BP	GO:0006152	purine nucleoside catabolic process	17735	422	2487	83	62.63	0.00376	1.325243	ABCA2;ABCC1;ACLY;ADSS;AGAP5;AGAP6;AKT2;ARHGAP15;ARHGAP29;ARHGAP35;ARHGEF16;ARHGEF5;ARH
blue	BP	GO:0046130	purine ribonucleoside catabolic process	17735	422	2487	83	62.63	0.00376	1.325243	ABCA2;ABCC1;ACLY;ADSS;AGAP5;AGAP6;AKT2;ARHGAP15;ARHGAP29;ARHGAP35;ARHGEF16;ARHGEF5;ARH
blue	BP	GO:0006482	protein demethylation	17735	19	2487	8	2.82	0.00379	2.836879	C14orf169;KDM1A;KDM2B;KDM4A;KDM4C;KDM5C;PPME1;UBE2E
blue	BP	GO:0008214	protein dealkylation	17735	19	2487	8	2.82	0.00379	2.836879	C14orf169;KDM1A;KDM2B;KDM4A;KDM4C;KDM5C;PPME1;UBE2E
blue	BP	GO:0044257	cellular protein catabolic process	17735	428	2487	84	63.52	0.00379	1.322418	AMFR;ANAPC1;ANAPC5;ANAPC7;ARIH2;AXIN1;BTRC;CCNF;CDC16;CHEK2;CLN8;CSNK1E;CUL1;CUL4A;CUL7;C
blue	CC	GO:0005856	cytoskeleton	17735	1762	2487	286	248.11	0.00383	1.152715	ABCA2;ABL1;ABL2;ABLIM1;ACTR10;ADD1;AIF1;AK5;AKAP9;ALMS1;ANK3;ANKS1B;ARHGAP32;ARHGAP35;AR
blue	BP	GO:0016447	somatic recombination of immunoglobulin ...	17735	40	2487	13	5.94	0.00388	2.188552	BATF;CD40LG;FOXP1;HSPD1;IL10;IL4;MLH1;MSH6;PAXIP1;PMS2;RNF8;TCF3;UNG
blue	BP	GO:0032320	positive regulation of Ras GTPase activi...	17735	98	2487	25	14.54	0.00389	1.719395	AKT2;ARHGAP15;ARHGAP29;ARHGAP35;ARHGEF16;ARHGEF5;CCR7;DOCK7;EPHA1;EPHA2;FOXJ1;MYO9B;N
blue	BP	GO:0006405	RNA export from nucleus	17735	73	2487	20	10.83	0.00392	1.846722	CPSF3;DDX19B;DHX38;EIF5A;GLE1;HHEX;KHDRBS1;MAGOH;NUP133;NUP155;NUP214;NXF1;RNPS1;SMG6;S
blue	CC	GO:0044430	cytoskeletal part	17735	1266	2487	211	178.27	0.00392	1.183598	ABCA2;ACTR10;ADD1;AIF1;AK5;AKAP9;ALMS1;ANKS1B;ARHGAP32;ARL3;ARPC2;ARPC3;ATM;AXIN1;AXIN2;E
blue	BP	GO:0031123	RNA 3'-end processing	17735	93	2487	24	13.8	0.00394	1.73913	CNOT10;CPSF3;CSTF1;CSTF2;CSTF3;DHX38;EIF4A3;EIF4B;EIF4G1;INTS1;LEO1;MAGOH;MLH1;MTPAP;PABPC
blue	BP	GO:0051701	interaction with host	17735	411	2487	81	61	0.00394	1.327869	ATP6V0E1;ATP6V1D;ATP6V1F;ATP6V1H;BAD;BICD1;BTRC;CAMP;CBX5;CCNT1;CD247;CD4;CRTC1;CRTC3;CUI
blue	CC	GO:0005680	anaphase-promoting complex	17735	20	2487	8	2.82	0.00396	2.836879	ANAPC1;ANAPC13;ANAPC5;ANAPC7;CDC16;CUL7;FZR1;MAD2L2
blue	MF	GO:0031625	ubiquitin protein ligase binding	17735	148	2487	34	21.53	0.00396	1.579192	AXIN1;AXIN2;BCL10;BCL2;BID;CALR;CHEK2;CUL1;CUL4A;CUL7;CUL9;DLG3;FAF1;HERC2;KDM4A;MUL1;NEDD
blue	MF	GO:0044389	small conjugating protein ligase binding	17735	148	2487	34	21.53	0.00396	1.579192	AXIN1;AXIN2;BCL10;BCL2;BID;CALR;CHEK2;CUL1;CUL4A;CUL7;CUL9;DLG3;FAF1;HERC2;KDM4A;MUL1;NEDD
blue	CC	GO:0005815	microtubule organizing center	17735	482	2487	89	67.87	0.00396	1.31133	ABCA2;AK5;AKAP9;ALMS1;ARL3;AXIN2;BBS2;BBS9;BIRC6;BRSK2;C2CD3;CALM3;CCDC64;CCNF;CCT5;CCT8;CI
blue	BP	GO:0006355	regulation of transcription, DNA-depende...	17735	2869	2487	472	425.78	0.00396	1.108554	ABCA2;ABL1;ABLIM1;ACAD8;ACTR8;ACVR1;AKAP17A;AKNA;APBB1;ARHGAP35;ARID1B;ARNTL2;ASCC1;ASXL
blue	BP	GO:0006913	nucleocytoplasmic transport	17735	342	2487	69	50.76	0.00419	1.359338	AXIN1;AXIN2;CALR;CBLB;CDKN2A;CPSF3;DDX19B;DHX38;DUSP16;EDAR;EIF5A;EMD;FAF1;FGF2;FLNA;GLE1;H
blue	CC	GO:0000781	chromosome, telomeric region	17735	52	2487	15	7.32	0.00428	2.04918	ATM;BLM;CHEK2;CTC1;PINX1;RNF8;SLX4;SMG6;TEP1;TERF2;TERF2IP;TNKS1BP1;TP53BP1;XRCC5;XRCC6
blue	BP	GO:0009063	cellular amino acid catabolic process	17735	109	2487	27	16.18	0.00429	1.668727	ACAD8;ALDH5A1;ALDH6A1;ALDH7A1;ARG1;CAD;DLD;DLST;ENOSF1;ETHE1;GLUD1;GOT1;GOT2;GPT;GPT2;H
blue	BP	GO:0006312	mitotic recombination	17735	36	2487	12	5.34	0.00432	2.247191	BLM;FEN1;LIG1;MLH1;POLA1;POLA2;POLE;RFC1;RPA1;RPA2;TEP1;TOP2B
blue	BP	GO:0006366	transcription from RNA polymerase II pro...	17735	1444	2487	249	214.3	0.00432	1.161923	ABCA2;ABL1;ABLIM1;ACVR1;ARNTL2;ASXL1;ATN1;AXIN1;BATF3;BCL11B;BCL9L;BEX1;BMP2;BPTF;BRD2;BRD
blue	BP	GO:0043085	positive regulation of catalytic activit...	17735	908	2487	163	134.75	0.0044	1.209647	ABL1;ABL2;ACER2;ADCY3;ADCY6;ADCY7;ADCY9;ADD1;AHSA1;AIFM1;AKT2;ANAPC1;ANAPC5;ANAPC7;ARHG
blue	BP	GO:0071824	protein-DNA complex subunit organization	17735	157	2487	36	23.3	0.00444	1.545064	BAZ1B;BRD2;CENPN;CENPO;CTCF;GTF2A2;H3F3C;HIST1H2AC;HIST1H2AK;HIST1H2BN;HIST2H2AC;HIST2H2B
blue	BP	GO:0032507	maintenance of protein location in cell	17735	94	2487	24	13.95	0.00456	1.72043	ANK3;BICD1;CD4;EZR;FAF1;FLNA;FLNB;IL10;KDELR2;KDELR3;NDC80;PCM1;PDIA3;PKD1;RACGAP1;SEH1L;SH.
blue	CC	GO:0005776	autophagic vacuole	17735	38	2487	12	5.35	0.00459	2.242991	AMBRA1;ATG14;ATG9B;ENTPD4;FYCO1;GABARAPL2;HTT;IRGM;MAP1LC3B2;RAB24;RPN2;TECPR1
blue	MF	GO:0031267	small GTPase binding	17735	133	2487	31	19.35	0.00459	1.602067	ALS2CL;ANXA2;ARHGEF16;BICD1;CCDC64;CDC42BPB;DIAPH1;DOCK10;DOCK7;DOCK9;EXOC2;FGD1;FLNA;FN
blue	BP	GO:0009119	ribonucleoside metabolic process	17735	556	2487	105	82.51	0.00463	1.272573	ABCA2;ABCC1;ACACA;ACACB;ACLY;ADSS;AGAP5;AGAP6;AK2;AK5;AKT2;APOBEC3F;ARHGAP15;ARHGAP29;A
blue	MF	GO:0004519	endonuclease activity	17735	102	2487	25	14.84	0.00523	1.684636	CPSF3;DBR1;DCLRE1C;DNASE1;DROSHA;EME2;ERCC5;EXOG;FAN1;FEN1;G3BP1;MRPL44;PMS2;POP1;RNASE
blue	MF	GO:0003713	transcription coactivator activity	17735	270	2487	55	39.28	0.00539	1.400204	ARID1B;ASXL1;BCL10;BCL9L;BRD7;CCAR1;CIITA;CREM;CRTC1;CRTC3;DDX17;ECD;FGF2;GATA3;GMEB2;GTF2
blue	CC	GO:0005667	transcription factor complex	17735	297	2487	58	41.82	0.00553	1.386896	ARNTL2;ASCC1;BEX1;CD3EAP;CREG1;CREM;ERCC3;ERCC5;ETS1;FOXC1;FOXI1;FOXJ1;FOXJ3;FOXK1;FOXK2;FC
blue	CC	GO:0030894	replisome	17735	21	2487	8	2.96	0.00562	2.702703	HELB;MCM3;POLA1;POLA2;POLE;POLE3;RPA1;RPA2
blue	CC	GO:0080008	Cul4-RING ubiquitin ligase complex	17735	21	2487	8	2.96	0.00562	2.702703	CUL4A;DCAF16;DCAF4;DCAF5;DCAF7;DCAF8;DDB1;DTL
blue	CC	GO:0035097	histone methyltransferase complex	17735	59	2487	16	8.31	0.00627	1.925391	CBX5;EZH1;HCFC1;LAS1L;MGA;MLL;MLL4;PAXIP1;RBBP4;RBBP7;RUVBL1;SETD1A;STK38;TAF1;TAF6;TEX1(
blue	MF	GO:0017016	Ras GTPase binding	17735	120	2487	28	17.46	0.00672	1.603666	ALS2CL;ANXA2;ARHGEF16;BICD1;CCDC64;CDC42BPB;DIAPH1;DOCK10;DOCK7;DOCK9;EXOC2;FLNA;FMNL3;I
blue	CC	GO:0005720	nuclear heterochromatin	17735	26	2487	9	3.66	0.00697	2.459016	CBX5;DNMT3A;FOXC1;MPHOSPH8;NCAPD3;PHC1;SATB1;TCP1;TNKS1BP1
blue	MF	GO:0016301	kinase activity	17735	762	2487	135	110.86	0.00735	1.217752	AAK1;ABL1;ABL2;ACVR1;ADPGK;AGK;AK2;AK5;AKT2;AKT3;ALDH18A1;ATM;BAZ1B;BRSK2;C9orf96;CAD;CARI
blue	CC	GO:0017119	Golgi transport complex	17735	10	2487	5	1.41	0.00749	3.546099	COG1;COG2;COG5;COG7;GOLGA3
blue	MF	GO:0016893	endonuclease activity, active with eithe...	17735	39	2487	12	5.67	0.00752	2.116402	DBR1;DNASE1;DROSHA;FAN1;FEN1;MRPL44;POP1;RNASEH1;RPP14;RPP21;SLX4;TATDN2
blue	CC	GO:0031965	nuclear membrane	17735	194	2487	40	27.32	0.00774	1.464129	ABL1;ATP2A3;BCL2;CCND2;CLIC1;DDX19A;DDX19B;DGKH;DHCR7;DNAJC1;DTL;EMD;FZR1;GTPBP4;GUCY2D;I
blue	MF	GO:0051087	chaperone binding	17735	44	2487	13	6.4	0.00801	2.03125	AHSA1;ATP1A1;BAG3;CALR;DNAJC1;GRPEL1;HSPD1;OGDH;ST13;STIP1;TBCC;TP53;TSC1
blue	CC	GO:0000421	autophagic vacuole membrane	17735	18	2487	7	2.53	0.00834	2.766798	ATG9B;ENTPD4;GABARAPL2;IRGM;MAP1LC3B2;RPN2;TECPR1
blue	CC	GO:0034708	methyltransferase complex	17735	61	2487	16	8.59	0.00881	1.862631	CBX5;EZH1;HCFC1;LAS1L;MGA;MLL;MLL4;PAXIP1;RBBP4;RBBP7;RUVBL1;SETD1A;STK38;TAF1;TAF6;TEX1(
blue	CC	GO:0000922	spindle pole	17735	94	2487	22	13.24	0.01016	1.661631	ALMS1;BIRC6;CALM3;CDC25B;CDK5RAP2;CEP85;CEP89;CKAP5;DCTN1;FBF1;KNTC1;NUMA1;NUP62;ODF2;P(
blue	MF	GO:0034061	DNA polymerase activity	17735	36	2487	11	5.24	0.0109	2.099237	DKC1;MYBBP1A;PAPD7;POLA1;POLA2;POLB;POLE;POLE3;POLG;POLH;TEP1
blue	MF	GO:0003684	damaged DNA binding	17735	46	2487	13	6.69	0.01188	1.943199	CRY2;DDB1;ERCC3;FEN1;LOC100133315;MSH6;NEIL2;POLB;POLH;RAD18;TP53;TP53BP1;XP(
blue	MF	GO:0017048	Rho GTPase binding	17735	51	2487	14	7.42	0.01204	1.886792	ARHGEF16;CDC42BPB;DIAPH1;DOCK10;DOCK7;DOCK9;FLNA;FMNL3;KCTD13;KIF3B;OCRL;PARD6A;SRGAP1;1
blue	CC	GO:0005721	centromeric heterochromatin	17735	11	2487	5	1.55	0.01216	3.225806	BAZ1B;CBX5;DNMT1;INCENP;NCAPD3
blue	CC	GO:0031252	cell leading edge	17735	260	2487	50	36.61	0.01259	1.365747	AAK1;ABL1;AIF1;AKT2;AMOTL1;APBB1;ARHGEF4;ARHGEF6;ARHGEF7;ARPC2;ARPC3;CDC42BPA;CDC42BPB;(
blue	MF	GO:0016773	phosphotransferase activity, alcohol gro...	17735	706	2487	124	102.71	0.01293	1.207283	AAK1;ABL1;ABL2;ACVR1;ADPGK;AGK;AKT2;AKT3;ATM;BAZ1B;BRSK2;C9orf96;CAD;CCL8;CCND1;CDC42BPA;(
blue	MF	GO:0016741	transferase activity, transferring one-c...	17735	183	2487	38	26.62	0.01363	1.427498	ASMT;ATIC;CAD;DNMT1;DNMT3A;EHMT1;EZH1;FTSJ2;FTSJ3;GART;HEMK1;IBA57;ICMT;METTL13;METTL16;
blue	CC	GO:0000152	nuclear ubiquitin ligase complex	17735	24	2487	8	3.38	0.01378	2.366864	ANAPC1;ANAPC13;ANAPC5;ANAPC7;CDC16;CUL7;FZR1;MAD2L2
blue	MF	GO:0017110	nucleoside-diphosphatase activity	17735	11	2487	5	1.6	0.01395	3.125	ENTPD4;ENTPD5;ENTPD8;NUDT9;SLC25A42
blue	MF	GO:0003899	DNA-directed RNA polymerase activity	17735	42	2487	12	6.11	0.01404	1.963993	CD3EAP;POLA1;POLR1A;POLR1B;POLR2B;POLR2C;POLR2J;POLR3A;POLR3B;POLR3C;POLR3E;RPAP1
blue	MF	GO:0034062	RNA polymerase activity	17735	42	2487	12	6.11	0.01404	1.963993	CD3EAP;POLA1;POLR1A;POLR1B;POLR2B;POLR2C;POLR2J;POLR3A;POLR3B;POLR3C;POLR3E;RPAP1
blue	MF	GO:0004521	endoribonuclease activity	17735	47	2487	13	6.84	0.01429	1.900585	CPSF3;DBR1;DROSHA;FEN1;MRPL44;POP1;RNASE2;RNASE3;RNASE4;RNASEH1;RPP14;RPP21;SMG6
blue	MF	GO:0004143	diacylglycerol kinase activity	17735	15	2487	6	2.18	0.0145	2.752294	AGK;CERK;DGKA;DGKD;DGKH;DGKQ
blue	MF	GO:0015278	calcium-release channel activity	17735	15	2487	6	2.18	0.0145	2.752294	CYTH3;ITPR3;JPH3;RASA3;RYR2;TPCN1
blue	MF	GO:0030983	mismatched DNA binding	17735	15	2487	6	2.18	0.0145	2.752294	MLH1;MSH6;PMS2;PMS2CL;PMS2P5;XPC
blue	MF	GO:0032452	histone demethylase activity	17735	15	2487	6	2.18	0.0145	2.752294	C14orf169;KDM1A;KDM2B;KDM4A;KDM4C;KDM5C
blue	MF	GO:0070577	histone acetyl-lysine binding	17735	15	2487	6	2.18	0.0145	2.752294	BAZ1B;BRD2;BRD7;MLL;SMARCA4;TAF1
blue	MF	GO:0043566	structure-specific DNA binding	17735	219	2487	44	31.86	0.01497	1.381042	AKAP8;BLM;CRY2;CTC1;CTCF;ERCC5;FEN1;FOXC1;FOXI1;FOXJ1;FOXJ3;FOXK1;FOXK2;FOXM1;FOXP1;FOXP4;I
blue	CC	GO:0000795	synaptonemal complex	17735	29	2487	9	4.08	0.0151	2.205882	BLM;INCENP;LIG3;MLH1;RPA1;SYCE2;TEX12;TOP2B;UBE2I

blue	MF	GO:0001085	RNA polymerase II transcription factor b...	17735	73	2487	18	10.62	0.01525	1.694915	BEX1;CHD4;DHX9;GTF2A2;HDAC1;KDM1A;LDB1;LMO2;LPIN1;MAD2L2;MTA1;MTA2;PPARA;SMAD3;TBP;TCF
blue	CC	GO:0000118	histone deacetylase complex	17735	49	2487	13	6.9	0.0157	1.884058	CBX5;CHD3;CHD4;HDAC1;ING2;MBD2;MTA1;MTA2;MTA3;RBBP4;RBBP7;SALL2;SAP30
blue	CC	GO:0005669	transcription factor TFIID complex	17735	20	2487	7	2.82	0.01565	2.48227	TAF1;TAF10;TAF3;TAF6;TAF8;TBP;TP53
blue	MF	GO:0004540	ribonuclease activity	17735	68	2487	17	9.89	0.01578	1.718908	CPSF3;DBR1;DIS3L;DROSHA;EXOSC10;FEN1;MRPL44;PAN2;PARN;POP1;RNASE2;RNASE3;RNASE4;RNASEH1;
blue	CC	GO:0008180	signalosome	17735	34	2487	10	4.79	0.0158	2.087683	AMOTL1;ATP5A1;COPS7B;DOCK7;EPB41L2;HSP90AB1;HSPA1L;PLCG1;VPRBP;WDR6
blue	CC	GO:0043209	myelin sheath	17735	34	2487	10	4.79	0.0158	2.087683	ANXA2;ATP1A3;BCL2;EXOC4;ITPR3;JAM3;MPZ;PLLP;SCRIB;SERINC5
blue	MF	GO:0030971	receptor tyrosine kinase binding	17735	33	2487	10	4.8	0.01583	2.083333	ARHGEF16;DNAJA3;HYAL2;IRS1;NRG1;PIK3R2;PITPNM3;PLCG1;SHC1;TP53
blue	MF	GO:0050662	coenzyme binding	17735	179	2487	37	26.04	0.01583	1.420891	ACAD8;ACOX2;ACOX3;AIFM1;AIFM2;ALDH6A1;ASPDH;CREG1;D2HGDH;DHCR24;DHODH;DLD;DUOX1;ECI2;E
blue	MF	GO:0051020	GTPase binding	17735	145	2487	31	21.1	0.0163	1.469194	ALS2CL;ANXA2;ARHGEF16;BICD1;CCDC64;CDC42BPB;DIAPH1;DOCK10;DOCK7;DOCK9;EXOC2;FGD1;FLNA;FN
blue	MF	GO:0005543	phospholipid binding	17735	487	2487	88	70.85	0.01672	1.242061	AGAP5;AGAP6;AKT2;AKT3;ANXA2;ANXA6;APBA1;ARHGAP15;ARHGAP32;ARHGAP33;ARHGEF1;ARHGEF16;A
blue	MF	GO:0032947	protein complex scaffold	17735	48	2487	13	6.98	0.01705	1.862464	AXIN1;BAZ1B;CAPN3;CD3E;CHCHD3;EIF3B;LAMTOR1;LDLRAP1;MAPK8IP3;MMS19;NUP62;SHANK1;SMARCD
blue	CC	GO:0000242	pericentriolar material	17735	12	2487	5	1.69	0.01846	2.95858	AKAP9;CDK5RAP2;LCK;PCM1;TCP1
blue	CC	GO:0000782	telomere cap complex	17735	12	2487	5	1.69	0.01846	2.95858	CTC1;TERF2;TERF2IP;XRCC5;XRCC6
blue	CC	GO:0000783	nuclear telomere cap complex	17735	12	2487	5	1.69	0.01846	2.95858	CTC1;TERF2;TERF2IP;XRCC5;XRCC6
blue	MF	GO:0003887	DNA-directed DNA polymerase activity	17735	29	2487	9	4.22	0.01846	2.132701	MYBBP1A;PAPD7;POLA1;POLA2;POLB;POLE;POLE3;POLG;POLH
blue	CC	GO:0005743	mitochondrial inner membrane	17735	347	2487	63	48.86	0.01914	1.289398	ACAA2;ACSL5;AFG3L2;AIFM1;AK2;ALDH18A1;ALDH3A2;APOOL;ATP5A1;ATP5B;ATP5J;ATP5J2;BDH1;CHCHD
blue	MF	GO:0016667	oxidoreductase activity, acting on a sul...	17735	54	2487	14	7.86	0.01988	1.78117	DLD;ENOX2;GSTO1;MSRB2;MSRB3;NXN;PCYOX1;PCYOX1L;PDIA3;PDIA4;PDIA5;PDIA6;TXN;TXNRD3
blue	MF	GO:0017025	TBP-class protein binding	17735	16	2487	6	2.33	0.0204	2.575107	CAND2;GTF2A2;HHEX;TAF1;THRA;YEATS2
blue	MF	GO:0030331	estrogen receptor binding	17735	25	2487	8	3.64	0.02141	2.197802	DDX17;DYX1C1;LEF1;MMS19;PPARGC1B;PRMT2;RNF4;TAF1C
blue	CC	GO:0005741	mitochondrial outer membrane	17735	118	2487	25	16.62	0.0223	1.504212	ACSL5;AIFM2;AKAP1;BAD;BCL2;BID;BRI3BP;CYB5B;HADHB;MAVS;MGST1;MLXIP;MTOR;MUL1;MYO19;PI4KE
blue	MF	GO:0016779	nucleotidyltransferase activity	17735	120	2487	26	17.46	0.02234	1.489118	ABL1;CD3EAP;DKC1;GALT;MTPAP;MYBBP1A;PAPD7;POLA1;POLA2;POLB;POLE;POLE3;POLG;POLH;POLR1A;F
blue	CC	GO:0016605	PML body	17735	73	2487	17	10.28	0.02311	1.653696	BLM;CBX5;CHEK2;EIF4ENIF1;IKBKE;MTOR;RFWD3;RNF4;RPA1;RPA2;SATB1;TCF7L2;TP53;TRIM16;UBE2I;USP
blue	CC	GO:0005876	spindle microtubule	17735	41	2487	11	5.77	0.02324	1.906412	ARL3;CALM3;CDC16;KIF3B;KIF4A;KNTC1;NEIL2;NUMA1;POLB;PRC1;ZW10
blue	MF	GO:0004715	non-membrane spanning protein tyrosine k...	17735	45	2487	12	6.55	0.02415	1.832061	ABL1;ABL2;BAZ1B;FYN;LCK;PEAK1;PTK2;SGK223;STK16;TNK2;TXK;TYK2
blue	CC	GO:0019866	organelle inner membrane	17735	377	2487	67	53.09	0.02482	1.262008	ACAA2;ACSL5;AFG3L2;AIFM1;AK2;ALDH18A1;ALDH3A2;APOOL;ATP5A1;ATP5B;ATP5J;ATP5J2;BDH1;CHCHD
blue	MF	GO:0051082	unfolded protein binding	17735	127	2487	27	18.48	0.02541	1.461039	AFG3L2;CALR;CCT3;CCT5;CCT6A;CCT7;CCT8;DNAJA3;DNAJA4;DNAJB1;DNAJB13;DNAJC1;DNAJC11;DNAJC16
blue	CC	GO:0005811	lipid particle	17735	42	2487	11	5.91	0.0276	1.861252	AIFM2;ANXA2;G0S2;IRAK1;LSS;METTL7A;NSDHL;PLIN2;PRPF19;SCCPDH;SIGMAR1
blue	CC	GO:0044665	MLL1/2 complex	17735	27	2487	8	3.8	0.02831	2.105263	HCFC1;LAS1L;MGA;MLL;RUVBL1;TAF1;TAF6;TEX10
blue	CC	GO:0071339	MLL1 complex	17735	27	2487	8	3.8	0.02831	2.105263	HCFC1;LAS1L;MGA;MLL;RUVBL1;TAF1;TAF6;TEX10
blue	CC	GO:0031519	PcG protein complex	17735	37	2487	10	5.21	0.02834	1.919386	ASXL1;CBX2;CBX7;CSNK2A2;EZH1;KDM2B;PCGF1;PHC1;RBBP4;RBBP7
blue	MF	GO:0042625	ATPase activity, coupled to transmembran...	17735	67	2487	16	9.75	0.02844	1.641026	ATP10A;ATP13A1;ATP1A1;ATP1A3;ATP1B3;ATP2A2;ATP2A3;ATP5A1;ATP5B;ATP6V0E1;ATP6V1F;ATP6V1H;A
blue	MF	GO:0000217	DNA secondary structure binding	17735	13	2487	5	1.89	0.03025	2.645503	BLM;ERCC5;MSH6;RAD18;XPC
blue	MF	GO:0016888	endodeoxyribonuclease activity, producin...	17735	13	2487	5	1.89	0.03025	2.645503	DNASE1;FAN1;FEN1;SLX4;TATDN2
blue	MF	GO:0034593	phosphatidylinositol bisphosphate phosph...	17735	13	2487	5	1.89	0.03025	2.645503	INPP4A;INPP5B;INPP5E;OCRL;SYNJ2
blue	MF	GO:0070742	C2H2 zinc finger domain binding	17735	13	2487	5	1.89	0.03025	2.645503	LEF1;MBD2;SRRM2;U2AF2;ZXDC
blue	MF	GO:0008022	protein C-terminus binding	17735	158	2487	32	22.99	0.03067	1.39191	ABL1;ATP2A2;AXIN1;BCL10;CADM1;CIITA;DLG3;DNM1;DST;ERCC3;HIC2;HSPG2;LCK;MAP2K7;MDC1;MYO1C
blue	CC	GO:0032587	ruffle membrane	17735	59	2487	14	8.31	0.03195	1.684717	AIF1;AKT2;ARHGEF4;DIAPH1;EPHA2;EZR;KANK1;MACF1;NF2;PDE9A;PIP5K1C;PPP1R9B;RASGRP2;THEM4
blue	MF	GO:0008135	translation factor activity, nucleic aci...	17735	79	2487	18	11.49	0.03269	1.56658	ABCF1;EEF2;EEF2K;EFTUD1;EIF1AD;EIF2B1;EIF2S3;EIF3A;EIF3B;EIF4B;EIF4G1;EIF4G2;EIF5A;EIF5B;GCN1L1;GI
blue	MF	GO:0003774	motor activity	17735	130	2487	27	18.91	0.03338	1.427816	DCTN1;DNAH1;DNHD1;DYNC1H1;DYNC1I2;DYNC1LI2;DYNLT1;KIF13B;KIF16B;KIF21B;KIF3B;KIF4A;KIF5C;KLC
blue	MF	GO:0016891	endoribonuclease activity, producing 5'-...	17735	27	2487	8	3.93	0.03372	2.035623	DBR1;DROSHA;FEN1;MRPL44;POP1;RNASEH1;RPP14;RPP21
blue	CC	GO:0009295	nucleoid	17735	38	2487	10	5.35	0.03377	1.869159	ATP5B;DNAJA3;HADHA;HADHB;HSPA9;LONP2;POLG;SUPV3L1;VDAC1;VDAC2
blue	MF	GO:0008168	methyltransferase activity	17735	177	2487	35	25.75	0.03413	1.359223	ASMT;DNMT1;DNMT3A;EHMT1;EZH1;FTSJ2;FTSJ3;GART;HEMK1;IBA57;ICMT;METTL13;METTL16;METTL2A;
blue	MF	GO:0019903	protein phosphatase binding	17735	74	2487	17	10.77	0.03461	1.578459	ANAPC5;ANAPC7;ANKLE2;BAD;BCL2;DLG3;EIF2AK3;FLT4;HSP90B1;MAP2K7;PHACTR4;PPME1;PPP1R16B;PPI
blue	CC	GO:0030532	small nuclear ribonucleoprotein complex	17735	33	2487	9	4.65	0.03464	1.935484	DDX23;GEMIN4;LSM10;PRPF4;PRPF8;SF3B3;SNRNP200;SNRNP40;TGS1
blue	CC	GO:0031090	organelle membrane	17735	2366	2487	362	333.16	0.03626	1.086565	ABCA2;ABCB1;ABL1;ACAA2;ACER2;ACSL5;AFG3L2;AGK;AGPAT3;AGPAT6;AIFM1;AIFM2;AK2;AKAP1;ALDH18A
blue	MF	GO:0030506	ankyrin binding	17735	18	2487	6	2.62	0.03661	2.290076	FLNC;NRCAM;OBSCN;PLEC;SPTBN1;TNKS1BP1
blue	MF	GO:0016881	acid-amino acid ligase activity	17735	299	2487	55	43.5	0.03722	1.264368	AMFR;ANAPC5;ARIH2;ATG10;ATG3;BIRC6;BTRC;CARNS1;CBLB;DTL;FBXO21;FBXW2;HECTD1;HERC1;HERC2;H
blue	MF	GO:0051059	NF-kappaB binding	17735	23	2487	7	3.35	0.03994	2.089552	BCL10;CDKN2A;DNAJA3;FAF1;HIF1AN;RELA;SETD6
blue	CC	GO:0042470	melanosome	17735	95	2487	20	13.38	0.0403	1.494768	ANXA2;ANXA6;ATP1A1;ATP1B3;FASN;GANAB;GGH;HPS4;HSP90AB1;HSP90B1;HSPA8;MLANA;PDIA3;PDIA4;I
blue	CC	GO:0048770	pigment granule	17735	95	2487	20	13.38	0.0403	1.494768	ANXA2;ANXA6;ATP1A1;ATP1B3;FASN;GANAB;GGH;HPS4;HSP90AB1;HSP90B1;HSPA8;MLANA;PDIA3;PDIA4;I
blue	CC	GO:0001741	XY body	17735	10	2487	4	1.41	0.04068	2.836879	RAD18;SIN3B;SMARCC1;UBE2B
blue	CC	GO:0031082	BLOC complex	17735	10	2487	4	1.41	0.04068	2.836879	BLOC1S1;PI4K2A;SNAP47;SNAPIN
blue	CC	GO:0031083	BLOC-1 complex	17735	10	2487	4	1.41	0.04068	2.836879	BLOC1S1;PI4K2A;SNAP47;SNAPIN
blue	CC	GO:0032300	mismatch repair complex	17735	10	2487	4	1.41	0.04068	2.836879	MLH1;MSH6;PMS2;PMS2P5
blue	MF	GO:0019902	phosphatase binding	17735	115	2487	24	16.73	0.04087	1.434549	ANAPC5;ANAPC7;ANKLE2;BAD;BCL2;CHCHD3;CRY2;CSRNP2;DLG3;EIF2AK3;ELFN2;FLT4;HSP90B1;MAP2K7;P
blue	MF	GO:0052742	phosphatidylinositol kinase activity	17735	14	2487	5	2.04	0.04156	2.45098	ATM;PI4K2A;PI4KA;PI4KB;PIK3R2
blue	MF	GO:0000049	tRNA binding	17735	33	2487	9	4.8	0.04163	1.875	AARS;AARS2;DARS2;EIF2AK4;KARS;MARS;NSUN2;XPO5;YARS
blue	MF	GO:0000287	magnesium ion binding	17735	180	2487	35	26.19	0.04241	1.336388	ABL1;ABL2;ADSS;ATP10A;ATP8B1;ATP8B2;ATP9B;BRSK2;CDC42BPA;CDC42BPB;CDC42BPG;CERK;ENO2;EPHX
blue	CC	GO:0000784	nuclear chromosome, telomeric region	17735	24	2487	7	3.38	0.04251	2.071006	CTC1;SLX4;TERF2;TERF2IP;TNKS1BP1;XRCC5;XRCC6
blue	MF	GO:0016829	lyase activity	17735	151	2487	30	21.97	0.04449	1.365498	ACO2;ADCY3;ADCY6;ADCY7;ADCY9;AGXT2L2;ALAD;DERA;ECHDC2;ENO2;FASN;FH;GGCX;GOT1;GUCY2D;HAC
blue	MF	GO:0034595	phosphatidylinositol phosphate 5-phospha...	17735	10	2487	4	1.45	0.04523	2.758621	INPP5B;INPP5E;OCRL;SYNJ2
blue	MF	GO:0036002	pre-mRNA binding	17735	10	2487	4	1.45	0.04523	2.758621	AQR;HNRNPA2B1;PTBP1;ZRSR2
blue	MF	GO:0070016	armadillo repeat domain binding	17735	10	2487	4	1.45	0.04523	2.758621	AXIN1;AXIN2;LEF1;TCF7L2
blue	CC	GO:0072686	mitotic spindle	17735	15	2487	5	2.11	0.04881	2.369668	DIAPH1;MAP4;RACGAP1;TADA2A;YEATS2
blue	CC	GO:0016592	mediator complex	17735	35	2487	9	4.93	0.04904	1.825558	MED11;MED24;MED27;MED29;MED30;MED31;MED6;PPARGC1B;RBM14
black	BP	GO:0043170	macromolecule metabolic process	17735	7334	228	120	95.33	0.00019	1.258785	ACY1;ADAT3;AKT1S1;AMH;AMN;APBA3;ASCL2;BOP1;BRSK1;C1orf85;C5;CBX4;CCDC85B;CDK16;CEBPB;CEBP
black	MF	GO:0003677	DNA binding	17735	2332	228	48	31.17	0.0012	1.539942	ASCL2;C1orf85;CBX4;CEBPB;CEBPD;CENPB;CWC22;GLI4;H1FX;H2AFX;HES4;HIC1;HMG20B;JUNB;JUND;KLF2;
black	MF	GO:0046914	transition metal ion binding	17735	2217	228	46	29.63	0.0013	1.552481	ADAT3;CYLD;CYP51A1;EGLN2;GLI4;HIC1;KLF2;LOXL1;MEX3D;MIB2;MMP23B;NEURL;NPEPL1;NR2F6;OTUD7A
black	BP	GO:0010467	gene expression	17735	4403	228	77	57.23	0.00135	1.345448	ADAT3;AMH;APBA3;ASCL2;BOP1;C1orf85;CBX4;CCDC85B;CEBPB;CEBPD;CENPB;CLASRP;CTU1;CWC22;CYLD;

black	BP	GO:0044260	cellular macromolecule metabolic process	17735	6621	228	106	86.06	0.00217	1.231699	ADAT3;AKT1S1;AMH;ASCL2;BOP1;BRSK1;C1orf85;C5;CBX4;CCDC85B;CDK16;CEBPB;CEBPD;CENPB;CLASRP;C
black	MF	GO:0008270	zinc ion binding	17735	1977	228	41	26.42	0.0025	1.551855	ADAT3;CYLD;GLI4;HIC1;KLF2;MEX3D;MIB2;MMP23B;NEURL;NR2F6;OTUD7A;PEX10;PGLYRP2;PRDM8;REPIN
black	BP	GO:0008630	intrinsic apoptotic signaling pathway in...	17735	45	228	4	0.58	0.00272	6.896552	BBC3;HIC1;MBD4;UACA
black	MF	GO:0019783	small conjugating protein-specific prote...	17735	52	228	4	0.69	0.0051	5.797101	CYLD;UFSP1;UFSP2;USP33
black	BP	GO:0032526	response to retinoic acid	17735	92	228	5	1.2	0.00695	4.166667	GDAP1;OVCA2;OXT;WNT6;ZNF35
black	BP	GO:0009296	flagellum assembly	17735	10	228	2	0.13	0.00706	15.38462	BBS5;NEURL
black	BP	GO:0014061	regulation of norepinephrine secretion	17735	12	228	2	0.16	0.01018	12.5	C5;OXT
black	BP	GO:0043064	flagellum organization	17735	12	228	2	0.16	0.01018	12.5	BBS5;NEURL
black	MF	GO:0016790	thiolester hydrolase activity	17735	102	228	5	1.36	0.0119	3.676471	ACOT2;CYLD;UFSP1;UFSP2;USP33
black	BP	GO:0048243	norepinephrine secretion	17735	13	228	2	0.17	0.01193	11.76471	C5;OXT
black	BP	GO:1901362	organic cyclic compound biosynthetic pro...	17735	3752	228	63	48.77	0.01224	1.291778	AMH;ASCL2;C1orf85;CBX4;CCDC85B;CEBPB;CEBPD;CENPB;CHCHD10;CYLD;CYP51A1;EGLN2;EID2;GMPPB;GI
black	MF	GO:0016594	glycine binding	17735	13	228	2	0.17	0.0126	11.76471	GRIN1;GRIN3B
black	BP	GO:0046717	acid secretion	17735	38	228	3	0.49	0.01306	6.122449	KISS1R;OXT;UCN
black	BP	GO:0016070	RNA metabolic process	17735	3911	228	65	50.83	0.01345	1.278772	ADAT3;AMH;ASCL2;BOP1;C1orf85;CBX4;CCDC85B;CEBPB;CEBPD;CENPB;CLASRP;CTU1;CWC22;CYLD;EGLN2;
black	BP	GO:0006957	complement activation, alternative pathw...	17735	14	228	2	0.18	0.0138	11.11111	C5;CFD
black	BP	GO:0051205	protein insertion into membrane	17735	14	228	2	0.18	0.0138	11.11111	BBC3;GRIN3B
black	CC	GO:0030313	cell envelope	17735	14	228	2	0.18	0.014	11.11111	GRIN1;GRIN3B
black	CC	GO:0044462	external encapsulating structure part	17735	14	228	2	0.18	0.014	11.11111	GRIN1;GRIN3B
black	MF	GO:0008234	cysteine-type peptidase activity	17735	147	228	6	1.96	0.0141	3.061224	CYLD;OTUD7A;RCE1;UFSP1;UFSP2;USP33
black	MF	GO:0017091	AU-rich element binding	17735	14	228	2	0.19	0.0146	10.52632	EXOSC9;MEX3D
black	BP	GO:0018130	heterocycle biosynthetic process	17735	3650	228	61	47.44	0.0154	1.285835	AMH;ASCL2;C1orf85;CBX4;CCDC85B;CEBPB;CEBPD;CENPB;CHCHD10;CYLD;EGLN2;EID2;GMPPB;GRIN1;HES4
black	BP	GO:0034654	nucleobase-containing compound biosynthe...	17735	3583	228	60	46.57	0.01575	1.288383	AMH;ASCL2;C1orf85;CBX4;CCDC85B;CEBPB;CEBPD;CENPB;CHCHD10;CYLD;EGLN2;EID2;GMPPB;GRIN1;HES4
black	BP	GO:0015874	norepinephrine transport	17735	15	228	2	0.19	0.01579	10.52632	C5;OXT
black	CC	GO:0000315	organellar large ribosomal subunit	17735	15	228	2	0.2	0.016	10	MRPL10;MRPL41
black	CC	GO:0005762	mitochondrial large ribosomal subunit	17735	15	228	2	0.2	0.016	10	MRPL10;MRPL41
black	MF	GO:0001664	G-protein coupled receptor binding	17735	195	228	7	2.61	0.016	2.681992	C5;CALY;NPB;OXT;UCN;USP33;WNT6
black	BP	GO:0097190	apoptotic signaling pathway	17735	202	228	7	2.63	0.01653	2.661597	BBC3;CYLD;HIC1;MBD4;STK11;TRADD;UACA
black	BP	GO:1900117	regulation of execution phase of apoptos...	17735	202	228	7	2.63	0.01653	2.661597	BBC3;CYLD;HIC1;MBD4;STK11;TRADD;UACA
black	BP	GO:0034645	cellular macromolecule biosynthetic proc...	17735	4170	228	68	54.2	0.01709	1.254613	AMH;ASCL2;C1orf85;CBX4;CCDC85B;CEBPB;CEBPD;CENPB;CYLD;EGLN2;EID2;GMPPB;GPAA1;GRIN1;HES4;H
black	BP	GO:0097193	intrinsic apoptotic signaling pathway	17735	116	228	5	1.51	0.01771	3.311258	BBC3;HIC1;MBD4;STK11;UACA
black	BP	GO:0009226	nucleotide-sugar biosynthetic process	17735	16	228	2	0.21	0.01789	9.52381	GMPPB;UGP2
black	BP	GO:0042347	negative regulation of NF-kappaB import ...	17735	16	228	2	0.21	0.01789	9.52381	CYLD;UACA
black	BP	GO:0009314	response to radiation	17735	305	228	9	3.96	0.01838	2.272727	BRSK1;GRIN1;H2AFX;JUNB;JUND;MBD4;NR2F6;STK11;UACA
black	MF	GO:0003676	nucleic acid binding	17735	3299	228	57	44.09	0.0187	1.29281	ASCL2;C1orf85;CBX4;CEBPB;CEBPD;CENPB;CTU1;CWC22;EXOSC6;EXOSC9;GLI4;H1FX;H2AFX;HES4;HIC1;HM
black	MF	GO:0005488	binding	17735	11799	228	170	157.68	0.0188	1.078133	ACOT2;ACY1;ADAT3;AKT1S1;ALKBH6;AMH;ANGPTL6;APBA3;ASCL2;ATP6V1E1;BBC3;BBS5;BOP1;BRSK1;C1o
black	BP	GO:0006351	transcription, DNA-dependent	17735	3208	228	54	41.7	0.02079	1.294964	AMH;ASCL2;C1orf85;CBX4;CCDC85B;CEBPB;CEBPD;CENPB;CYLD;EGLN2;EID2;GRIN1;HES4;HIC1;HMG20B;IN
black	CC	GO:0030312	external encapsulating structure	17735	17	228	2	0.22	0.021	9.090909	GRIN1;GRIN3B
black	MF	GO:0042165	neurotransmitter binding	17735	17	228	2	0.23	0.0212	8.695652	GRIN1;GRIN3B
black	BP	GO:0019438	aromatic compound biosynthetic process	17735	3638	228	60	47.29	0.02139	1.268767	AMH;ASCL2;C1orf85;CBX4;CCDC85B;CEBPB;CEBPD;CENPB;CHCHD10;CYLD;EGLN2;EID2;GMPPB;GRIN1;HES4
black	MF	GO:0016278	lysine N-methyltransferase activity	17735	45	228	3	0.6	0.0221	5	METTL21A;PRDM8;SUV420H2
black	MF	GO:0016279	protein-lysine N-methyltransferase activ...	17735	45	228	3	0.6	0.0221	5	METTL21A;PRDM8;SUV420H2
black	BP	GO:0009059	macromolecule biosynthetic process	17735	4294	228	69	55.81	0.02236	1.236338	AMH;ASCL2;C1orf85;CBX4;CCDC85B;CEBPB;CEBPD;CENPB;CYLD;EGLN2;EID2;GMPPB;GPAA1;GRIN1;HES4;H
black	BP	GO:0070536	protein K63-linked deubiquitination	17735	18	228	2	0.23	0.02243	8.695652	CYLD;USP33
black	BP	GO:0044271	cellular nitrogen compound biosynthetic ...	17735	3726	228	61	48.43	0.02337	1.25955	AMH;ASCL2;C1orf85;CBX4;CCDC85B;CEBPB;CEBPD;CENPB;CHCHD10;CYLD;EGLN2;EID2;GMPPB;GRIN1;HES4
black	MF	GO:0004970	ionotropic glutamate receptor activity	17735	18	228	2	0.24	0.0236	8.333333	GRIN1;GRIN3B
black	BP	GO:0018022	peptidyl-lysine methylation	17735	19	228	2	0.25	0.02485	8	METTL21A;SUV420H2
black	MF	GO:0003700	sequence-specific DNA binding transcript...	17735	996	228	21	13.31	0.0256	1.577761	ASCL2;C1orf85;CEBPB;CEBPD;HIC1;HMG20B;JUNB;JUND;KLF2;MYPOP;NPAS1;NR2F6;SNAPC2;TBX6;USF2;ZF
black	BP	GO:0042308	negative regulation of protein import in...	17735	49	228	3	0.64	0.02572	4.6875	CYLD;FAM89B;UACA
black	MF	GO:0001071	nucleic acid binding transcription facto...	17735	998	228	21	13.34	0.0261	1.574213	ASCL2;C1orf85;CEBPB;CEBPD;HIC1;HMG20B;JUNB;JUND;KLF2;MYPOP;NPAS1;NR2F6;SNAPC2;TBX6;USF2;ZF
black	MF	GO:0005234	extracellular-glutamate-gated ion channe...	17735	19	228	2	0.25	0.0262	8	GRIN1;GRIN3B
black	BP	GO:0071704	organic substance metabolic process	17735	8996	228	130	116.93	0.02787	1.111776	ACOT2;ACY1;ADAT3;AKT1S1;AMH;AMN;APBA3;ASCL2;BOP1;BRSK1;C1orf85;C5;CBX4;CCDC85B;CDK16;CEBI
black	CC	GO:0000178	exosome (RNase complex)	17735	20	228	2	0.26	0.028	7.692308	EXOSC6;EXOSC9
black	BP	GO:0007218	neuropeptide signaling pathway	17735	89	228	4	1.16	0.02871	3.448276	KISS1R;NPB;PCSK1N;UCN
black	MF	GO:0008093	cytoskeletal adaptor activity	17735	20	228	2	0.27	0.0288	7.407407	GAS2L1;NCK2
black	BP	GO:0010468	regulation of gene expression	17735	3347	228	55	43.5	0.03024	1.264368	AMH;APBA3;ASCL2;C1orf85;CBX4;CCDC85B;CEBPB;CENPB;CWC22;CYLD;EGLN2;EID2;GAS2L1;GRIN1;HES4;H
black	BP	GO:0090304	nucleic acid metabolic process	17735	4431	228	70	57.59	0.03066	1.215489	ADAT3;AMH;ASCL2;BOP1;C1orf85;CBX4;CCDC85B;CEBPB;CEBPD;CENPB;CLASRP;CTU1;CWC22;CYLD;EGLN2;
black	BP	GO:0042035	regulation of cytokine biosynthetic proc...	17735	91	228	4	1.18	0.03082	3.389831	CEBPB;HSPB1;LTB;ZFPM1
black	BP	GO:0017015	regulation of transforming growth factor...	17735	92	228	4	1.2	0.0319	3.333333	EID2;FAM89B;SKOR1;STK11
black	BP	GO:0051668	localization within membrane	17735	22	228	2	0.29	0.03274	6.896552	BBC3;GRIN3B
black	BP	GO:0006355	regulation of transcription, DNA-depende...	17735	2869	228	48	37.29	0.03298	1.287208	AMH;ASCL2;C1orf85;CBX4;CCDC85B;CEBPB;CENPB;CYLD;EGLN2;EID2;GRIN1;HES4;HIC1;HMG20B;INO80B;IF
black	BP	GO:0032774	RNA biosynthetic process	17735	3293	228	54	42.8	0.03306	1.261682	AMH;ASCL2;C1orf85;CBX4;CCDC85B;CEBPB;CEBPD;CENPB;CYLD;EGLN2;EID2;GRIN1;HES4;HIC1;HMG20B;IN
black	MF	GO:0005184	neuropeptide hormone activity	17735	22	228	2	0.29	0.0345	6.896552	OXT;UCN
black	MF	GO:0051861	glycolipid binding	17735	22	228	2	0.29	0.0345	6.896552	GLTPD2;GPAA1
black	BP	GO:0008152	metabolic process	17735	9452	228	135	122.85	0.03541	1.098901	ACOT2;ACY1;ADAT3;AKT1S1;AMH;AMN;APBA3;ASCL2;BBC3;BOP1;BRSK1;C1orf85;C5;CACNA1A;CBX4;CCDC
black	BP	GO:0051252	regulation of RNA metabolic process	17735	2953	228	49	38.38	0.03555	1.276707	AMH;ASCL2;C1orf85;CBX4;CCDC85B;CEBPB;CENPB;CWC22;CYLD;EGLN2;EID2;GRIN1;HES4;HIC1;HMG20B;IN
black	BP	GO:0046823	negative regulation of nucleocytoplasmic...	17735	56	228	3	0.73	0.03624	4.109589	CYLD;FAM89B;UACA
black	BP	GO:2001141	regulation of RNA biosynthetic process	17735	2889	228	48	37.55	0.03679	1.278296	AMH;ASCL2;C1orf85;CBX4;CCDC85B;CEBPB;CENPB;CYLD;EGLN2;EID2;GRIN1;HES4;HIC1;HMG20B;INO80B;IF
black	BP	GO:0060255	regulation of macromolecule metabolic pr...	17735	4178	228	66	54.3	0.0368	1.21547	AKT1S1;AMH;APBA3;ASCL2;C1orf85;C5;CBX4;CCDC85B;CEBPB;CENPB;CWC22;CYLD;DBNDD2;EGLN2;EID2;G
black	BP	GO:0031326	regulation of cellular biosynthetic proc...	17735	3328	228	54	43.26	0.03956	1.248266	AMH;ASCL2;C1orf85;CBX4;CCDC85B;CEBPB;CENPB;CYLD;EGLN2;EID2;GRIN1;HES4;HIC1;HMG20B;HSPB1;IN

black	BP	GO:2000112	regulation of cellular macromolecule bio...	17735	3117	228	51	40.51	0.04004	1.258948	AMH;ASCL2;C1orf85;CBX4;CCDC85B;CEBPB;CENPB;CYLD;EGLN2;EID2;GRIN1;HES4;HIC1;HMG20B;HSPB1;IN
black	BP	GO:1901576	organic substance biosynthetic process	17735	5154	228	79	66.99	0.04018	1.17928	AMH;ASCL2;C1orf85;CBX4;CCDC85B;CEBPB;CEBPD;CENPB;CHCHD10;CSNK1G2;CYLD;CYP51A1;EGLN2;EID2;
black	MF	GO:0001540	beta-amyloid binding	17735	24	228	2	0.32	0.0405	6.25	APBA3;DLGAP3
black	MF	GO:0016811	hydrolase activity, acting on carbon-nit...	17735	57	228	3	0.76	0.0406	3.947368	ACY1;PDF;PGLYRP2
black	BP	GO:0042108	positive regulation of cytokine biosynth...	17735	59	228	3	0.77	0.04133	3.896104	HSPB1;LTB;ZFPM1
black	BP	GO:0044058	regulation of digestive system process	17735	25	228	2	0.32	0.04145	6.25	OXT;UCN
black	BP	GO:0010556	regulation of macromolecule biosynthetic...	17735	3197	228	52	41.55	0.04191	1.251504	AMH;ASCL2;C1orf85;CBX4;CCDC85B;CEBPB;CENPB;CYLD;EGLN2;EID2;GRIN1;HES4;HIC1;HMG20B;HSPB1;IN
black	BP	GO:0090287	regulation of cellular response to growt...	17735	146	228	5	1.9	0.04193	2.631579	EID2;FAM89B;FGFBP3;SKOR1;STK11
black	BP	GO:0042089	cytokine biosynthetic process	17735	101	228	4	1.31	0.04267	3.053435	CEBPB;HSPB1;LTB;ZFPM1
black	CC	GO:0043679	axon terminus	17735	59	228	3	0.78	0.043	3.846154	GRIN1;OXT;UCN
black	BP	GO:0000291	nuclear-transcribed mRNA catabolic proce...	17735	26	228	2	0.34	0.04453	5.882353	EXOSC6;EXOSC9
black	BP	GO:0032570	response to progesterone stimulus	17735	26	228	2	0.34	0.04453	5.882353	JUNB;OXT
black	BP	GO:0043114	regulation of vascular permeability	17735	26	228	2	0.34	0.04453	5.882353	FGFBP3;UCN
black	BP	GO:0043928	exonucleolytic nuclear-transcribed mRNA ...	17735	26	228	2	0.34	0.04453	5.882353	EXOSC6;EXOSC9
black	BP	GO:0045933	positive regulation of muscle contractio...	17735	26	228	2	0.34	0.04453	5.882353	OXT;UCN
black	BP	GO:0034470	ncRNA processing	17735	198	228	6	2.57	0.0448	2.33463	ADAT3;BOP1;CTU1;EXOSC6;EXOSC9;RPP25
black	BP	GO:0009889	regulation of biosynthetic process	17735	3356	228	54	43.62	0.04544	1.237964	AMH;ASCL2;C1orf85;CBX4;CCDC85B;CEBPB;CENPB;CYLD;EGLN2;EID2;GRIN1;HES4;HIC1;HMG20B;HSPB1;IN
black	CC	GO:0000785	chromatin	17735	300	228	8	3.96	0.046	2.020202	CEBPB;H1FX;H2AFX;HIC1;INO80B;JUNB;JUND;MBD4
black	BP	GO:0042107	cytokine metabolic process	17735	104	228	4	1.35	0.04666	2.962963	CEBPB;HSPB1;LTB;ZFPM1
black	BP	GO:0032941	secretion by tissue	17735	62	228	3	0.81	0.04676	3.703704	NEURL;UCN;USF2
black	BP	GO:0090317	negative regulation of intracellular pro...	17735	62	228	3	0.81	0.04676	3.703704	CYLD;FAM89B;UACA
black	MF	GO:0008066	glutamate receptor activity	17735	26	228	2	0.35	0.0468	5.714286	GRIN1;GRIN3B

ADHFE1;ADNP;ADORA2A;ADPRHL2;AES;AFF1;AFMID;AFTPH;AHCY;AHCYL1;AIP;AK1;AK4;AKAP10;AKR1A1;ALG12;ALKBH7;ALS2;AMT;ANAPC11;ANAPC2;ANKRD17;ANKRD23;ANKRD34A;ANKRD54;ANO6;ANO9;AP1G1;AP1G2;AP1S1;APBB3;APOE;APPL2;APRT;ARC;ARFGEF1;ARFIP2;ARFRP1;ARHGAP4;ARHG

;AFTPH;AHCY;AHCYL1;AIP;AK1;AK4;AKAP10;AKR1A1;ALG12;ALKBH7;ALS2;AMT;ANAPC11;ANAPC2;ANKRD23;AP1G1;AP1G2;AP1S1;APOE;APPL2;APRT;ARC;ARFGEF1;ARFIP2;ARFRP1;ARHGAP4;ARHGEF25;ARIH1;ASH1L;ASPSCR1;ATAD3B;ATL3;ATP5D;ATP5G2;ATP5G3;ATP5H;ATP6V0E2;ATP6V1C1;AUP1;B

ADHFE1;ADNP;ADORA2A;ADPRHL2;AES;AFF1;AFMID;AFTPH;AHCY;AHCYL1;AIP;AK1;AK4;AKAP10;AKR1A1;ALG12;ALKBH7;ALS2;AMT;ANAPC11;ANAPC2;ANKRD17;ANKRD23;ANKRD34A;ANKRD54;AP1G1;AP1G2;AP1S1;APBB3;APOE;APPL2;APRT;ARC;ARFGEF1;ARFIP2;ARFRP1;ARHGAP4;ARHGEF25;ARIH1;

E1;ADPRHL2;AFMID;AFTPH;AHCY;AHCYL1;AIP;AK1;AK4;AKAP10;AKR1A1;ALG12;ALKBH7;ALS2;AMT;ANAPC11;ANAPC2;ANKRD17;ANKRD23;ANKRD34A;ANKRD54;AP1G1;AP1G2;AP1S1;APBB3;APOE;APPL2;APRT;ARC;ARFGEF1;ARFIP2;ARFRP1;ARHGAP4;ARHGEF25;ARIH1;ARNTL;ASB6;ASH1L;ASPSCR1;ATA

13;ADAMTSL2;ADCK5;ADHFE1;ADPRHL2;AFMID;AHCY;AHCYL1;AK1;AK4;AKR1A1;ALG12;ALKBH7;AMDHD2;AMT;ANAPC11;ANAPC2;APRT;ARFRP1;ARIH1;ASH1L;ATAD3B;ATF7IP;ATG4D;ATHL1;ATL3;ATP5D;ATP5H;ATP6V0E2;ATP6V1C1;B3GALT6;B3GAT3;B4GALNT4;B4GALT2;B4GALT7;BCS1L;BMP2K;BRAF;

;ATP5G2;ATP5G3;ATP5H;BCS1L;BOK;BOLA1;C12orf10;C19orf70;C1QBP;CCDC51;CCS;CDS2;CECR5;CHCHD5;CISD3;CLPP;CNTLN;COMTD1;COQ7;COQ9;COX4I1;CPSF4;CPT1C;CTU2;CYC1;CYP2E1;DDX28;DGUOK;DHRS4;DIAPH2;DNAJC30;DNLZ;DRG2;DTYMK;DUS2L;ECI1;EHHADH;ELK3;ETFB;FANCG;FASTK;FD

S;AFF1;AFMID;AFTPH;AHCY;AHCYL1;AIP;AK1;AK4;AKAP10;ALG12;ALKBH7;ALS2;AMT;ANAPC11;ANAPC2;ANKRD17;ANKRD23;ANKRD54;AP1G1;AP1G2;AP1S1;APOE;APPL2;APRT;ARC;ARFGEF1;ARFRP1;ARHGAP4;ARL6IP4;ARNTL;ASH1L;ASPSCR1;ASXL2;ATAD3B;ATF4;ATF7IP;ATL3;ATP5D;ATP5G2;ATP5G3;

ORA2A;ADPRHL2;AES;AFF1;AFMID;AFTPH;AHCY;AHCYL1;AIP;AK1;AK4;AKAP10;ALG12;ALKBH7;ALS2;AMT;ANAPC11;ANAPC2;ANKRD17;ANKRD23;ANKRD54;AP1G1;AP1G2;AP1S1;APBB3;APOE;APPL2;APRT;ARC;ARFGEF1;ARFRP1;ARHGAP4;ARHGEF25;ARL6IP4;ARNTL;ASH1L;ASPSCR1;ASXL2;ATAD3B;ATF4;

S;AFF1;AFMID;AFTPH;AHCY;AHCYL1;AIP;AK1;AK4;AKAP10;ALG12;ALKBH7;ALS2;AMT;ANAPC11;ANAPC2;ANKRD17;ANKRD23;ANKRD54;AP1G1;AP1G2;AP1S1;APOE;APPL2;APRT;ARC;ARFGEF1;ARFRP1;ARHGAP4;ARL6IP4;ARNTL;ASH1L;ASXL2;ATAD3B;ATF4;ATF7IP;ATL3;ATP5D;ATP5G2;ATP5G3;ATP5H;AT

ORA2A;ADPRHL2;AES;AFF1;AFMID;AFTPH;AHCY;AHCYL1;AIP;AK1;AK4;AKAP10;ALG12;ALKBH7;ALS2;AMT;ANAPC11;ANAPC2;ANKRD17;ANKRD23;ANKRD54;AP1G1;AP1G2;AP1S1;APBB3;APOE;APPL2;APRT;ARC;ARFGEF1;ARFRP1;ARHGAP4;ARHGEF25;ARL6IP4;ARNTL;ASH1L;ASXL2;ATAD3B;ATF4;ATF7IP;AT

S;CDS2;CHCHD5;CLPP;COQ7;COX4I1;CPT1C;CYC1;DDX28;DGUOK;DTYMK;ECI1;ETFB;FDX1L;FDXR;FPGS;GCDH;GFER;GIMAP5;GOLPH3;HCCS;HSD17B8;MRPL12;MRPL20;MRPL23;MRPL52;MRPL55;MRPS11;MRPS12;MRPS15;MRPS18A;MRPS26;MTFP1;MUT;NAGS;NDUFA11;NDUFA13;NDUFA3;NDUFA8;ND

K4;ALG12;ALS2;ANAPC11;ANAPC2;ANKRD23;AP1G1;AP1G2;AP1S1;APPL2;ARC;ARFGEF1;ARHGEF25;ARNTL;ASPSCR1;ATAD3B;ATF4;ATF7IP;ATL3;ATP5D;ATP5G2;ATP5G3;ATP5H;ATP6V0E2;ATRIP;AUP1;B3GALT6;B3GAT3;B4GALNT4;B4GALT2;B4GALT7;BABAM1;BAIAP2L2;BCS1L;BOK;BRF1;BRF2;BUB1;BU

LG12;ALS2;ANAPC11;ANAPC2;AP1G1;AP1G2;AP1S1;APPL2;ARC;ARFGEF1;ARNTL;ATAD3B;ATF4;ATF7IP;ATL3;ATP5D;ATP5G2;ATP5G3;ATP5H;ATP6V0E2;ATRIP;AUP1;B3GALT6;B3GAT3;B4GALNT4;B4GALT2;B4GALT7;BABAM1;BAIAP2L2;BCS1L;BOK;BRF1;BRF2;BUB1;BUB1B;C17orf49;C17orf70;C19orf10;C

;ATP5D;ATRIP;BRF1;BRF2;BUB1;BUB1B;C17orf49;C17orf70;C19orf10;C1orf86;C1QBP;CANX;CCDC101;CCNL2;CDC25A;CDC6;CDCA8;CDK12;CDK13;CENPF;CHCHD5;CHRAC1;CIB1;CLPP;CLSPN;COL11A2;COL6A1;COL6A2;COL9A2;CPSF2;CPSF4;CREB3;CTSF;DDX21;DDX28;DEAF1;DGUOK;DIAPH2;DTD1;DTYMK

;CPT1C;CYC1;DTYMK;ECI1;FDXR;FPGS;GCDH;GFER;GIMAP5;GOLPH3;HCCS;HSD17B8;MTFP1;NDUFA11;NDUFA13;NDUFA3;NDUFA8;NDUFB10;NDUFB11;NDUFB7;NDUFB8;NDUFB9;NDUFS6;NDUFS7;NDUFS8;NDUFV1;NME4;PDK4;PEMT;PPOX;RHOT2;SLC25A19;SLC25A25;SLC25A29;SLC25A35;SLC25A5;SL

;HCCS;LEMD2;MTFP1;NDUFA11;NDUFA13;NDUFA3;NDUFA8;NDUFB10;NDUFB11;NDUFB7;NDUFB8;NDUFB9;NDUFS6;NDUFS7;NDUFS8;NDUFV1;NME4;P2RX7;PDK4;PPOX;SLC25A19;SLC25A25;SLC25A29;SLC25A35;SLC25A5;SLC25A6;STOML2;SURF1;TAZ;TIMM10;TIMM13;TIMM50;TOR1AIP1;YME1L

;HCCS;MTFP1;NDUFA11;NDUFA13;NDUFA3;NDUFA8;NDUFB10;NDUFB11;NDUFB7;NDUFB8;NDUFB9;NDUFS6;NDUFS7;NDUFS8;NDUFV1;NME4;PDK4;PPOX;SLC25A19;SLC25A25;SLC25A29;SLC25A35;SLC25A5;SLC25A6;STOML2;SURF1;TAZ;TIMM10;TIMM13;TIMM50;YME1L

A8;NDUFB10;NDUFB11;NDUFB7;NDUFB8;NDUFB9;NDUFS6;NDUFS7;NDUFS8;NDUFV1;SDHAF2;TAZ;TXN2;TXNRD1

;ATP5D;ATRIP;BRF1;BRF2;BUB1;BUB1B;C17orf49;C17orf70;C19orf10;C1orf86;C1QBP;CANX;CCDC101;CCNL2;CDC25A;CDC6;CDCA8;CDK12;CDK13;CENPF;CHRAC1;CIB1;CLPP;CLSPN;COL11A2;COL6A1;COL6A2;COL9A2;CPSF2;CPSF4;CREB3;CTSF;DDX21;DDX28;DEAF1;DGUOK;DIAPH2;DTD1;DTYMK;DUSP2;

GALT6;B3GAT3;B4GALT2;B4GALT7;BABAM1;BRAF;BUB1B;C19orf10;CANX;CCDC101;CCNL2;CD36;CDC25A;CDC6;CDK12;CDK13;CEL;CLCF1;CLSPN;CSNK1G1;CTU2;DERL3;DGUOK;DOK7;DOLK;DPM3;DTX3;DUSP10;DUSP2;DUSP3;DVL1;E4F1;EHHADH;EIF4G3;ELP4;EPC1;ETF1;EXOSC5;FASTK;FBXL15;FBXL6;FE

UFS8;NDUFV1

UFS8;NDUFV1

UFS8;NDUFV1

LT6;B3GAT3;B4GALT2;B4GALT7;BABAM1;BRAF;BUB1B;C19orf10;C1QBP;CANX;CAPRIN1;CCDC101;CCNL2;CD36;CDC25A;CDC6;CDK12;CDK13;CEL;CLCF1;CLSPN;CSNK1G1;DALRD3;DERL3;DGUOK;DNAJC17;DNAJC30;DOK7;DOLK;DPM3;DTX3;DUSP10;DUSP2;DUSP3;DVL1;E4F1;EEF1D;EEF1G;EHHADH;EIF1;E

US1;PUSL1;RPUSD1;RPUSD3;SPATA20;TOP1;TOP2A;TOP3B;TRUB

DH;HSD17B8;MRPL12;MRPL20;MRPL23;MRPL52;MRPL55;MRPS11;MRPS12;MRPS15;MRPS18A;MRPS26;MUT;NAGS;NUDT1;PARS2;PDK4;POLRMT;QARS;SARDH;SARS2;SDHAF1;SIRT3;SLC25A5;TUF

;ATP5D;ATRIP;BRF1;BRF2;BUB1;BUB1B;C17orf49;C17orf70;C19orf10;C1orf86;C1QBP;CANX;CCDC101;CCNL2;CDC25A;CDC6;CDCA8;CDK12;CDK13;CENPF;CHRAC1;CIB1;CLPP;CLSPN;COL11A2;COL6A1;COL6A2;COL9A2;CPSF2;CPSF4;CREB3;CTSF;DDX21;DDX28;DEAF1;DGUOK;DIAPH2;DTD1;DTYMK;DUSP2;

M;APPL2;ARFGEF1;ARIH1;ARNTL;ATF4;ATF7IP;ATP5D;ATP5G2;ATP5G3;ATP5H;ATP6V0E2;ATP6V1C1;BABAM1;BAIAP2L2;BCS1L;BEST4;BRF1;BTBD6;BUB1;BUB1B;C17orf49;C1orf86;C8G;CANX;CAPRIN1;CCDC101;CD8B;CDC6;CDCA8;CDK12;CDK13;CENPF;CENPM;CENPT;CHRAC1;CLASP1;CLCF1;CLIP1;COL6

YC1;ECI1;FDXR;FPGS;GCDH;GIMAP5;HCCS;MTFP1;NDUFA11;NDUFA13;NDUFA3;NDUFA8;NDUFB10;NDUFB11;NDUFB7;NDUFB8;NDUFB9;NDUFS6;NDUFS7;NDUFS8;NDUFV1;NME4;PDK4;PEMT;PPOX;RHOT2;SLC25A19;SLC25A25;SLC25A29;SLC25A35;SLC25A5;SLC25A6;SLC44A1;SMCR7;SPG20;STOML2;S

PS12;MRPS15;MRPS18A;MRPS26;MRPS34;QTRT1;RPL13;RPL18;RPL18A;RPL28;RPL29;RPL38;RPL8;RPLP1;RPLP2;RPS15;RPS2;RPS28;RPS6KL

7;NDUFB8;NDUFB9;NDUFS6;NDUFS7;NDUFS8;NDUFV1;SDHAF2;TAZ

;HIPK1;INO80E;IRF3;MAPK1;MC1R;MCM7;MCRS1;MGMT;MPG;MRPS11;MRPS26;MSH3;MUS81;MUTYH;NEIL1;NIPBL;NSMCE1;NTHL1;NUDT1;OGG1;PARP3;PHLDA3;PIDD;PNKP;POLD1;POLD4;POLM;POLR2F;POLR2G;POLR2I;POLR2L;PSMA7;PSMB4;PSMB5;PSMB7;PSMD9;RAD21;RAD9A;RBBP5;RECQL4;R

UFS8;NDUFV1

JB1;BUB1B;CDK10;CDK12;CDK13;CDS2;CHRAC1;CNTLN;COMTD1;CPT1C;CSNK1G1;DGUOK;DOLK;DPM3;DTYMK;ELP4;EPC1;ETNK2;FASTK;FBL;FICD;FLAD1;FUK;GAMT;GLYCTK;GSTT1;GYG1;HIPK1;HIPK3;ILVBL;IRAK4;KAT2A;KCNH4;KHK;LATS1;LATS2;LCMT1;LTBP4;LTK;MAGI3;MAP3K10;MAP4K2;MAPK1;MA

IMT;PRMT7;RBBP5;RNMTL1;SARDH;SETD2;SETD7;SUV420H1;TRMT1;TRMT112;TRMT2A;TRMT61A;TRMU;WHSC1L

RS1;MGMT;MPG;MSH3;MUS81;MUTYH;NEIL1;NSMCE1;NTHL1;NUDT1;OGG1;PARP3;PNKP;POLD1;POLD4;POLM;POLR2F;POLR2G;POLR2I;POLR2L;RAD21;RAD9A;RECQL4;RNF169;RTEL1;SETX;STRA13;STUB1;SWI5;TAOK3;TMEM161A;TOP2A;TOPBP1;TP73;TREX2;TRIP

FS7;NDUFS8;NDUFV1;SURF1

NDUFB8;NDUFB9;NDUFS6;NDUFS7;NDUFS8;NDUFV1;PPOX;RHOT2;SLC25A6;SURF1;TIMM10;TIMM5

TAD3B;ATL3;ATP5D;ATP5G2;ATP5G3;ATP5H;ATP6V0E2;AUP1;B3GALT6;B3GAT3;B4GALNT4;B4GALT2;B4GALT7;BAIAP2L2;BCS1L;BOK;CANX;CCS;CD36;CDS2;CHMP4A;CHMP6;CLCN4;CLIP1;COQ7;COX4I1;CPT1C;CREB3;CREB3L4;CYC1;CYP1B1;CYP2E1;DECR2;DERL3;DHRS4;DOLK;DPM3;DSE;ECI1;ENTPD7;E

;NDUFS7;NDUFS8;NDUFV1;SURF1

IMT;PRMT7;RBBP5;RNMTL1;SARDH;SETD2;SETD7;SUV420H1;TRMT1;TRMT112;TRMT2A;TRMT61A;TRMU;WHSC1L

S13;ADCK5;ADHFE1;ADNP;ADORA2A;ADPRHL2;AES;AFF1;AFMID;AFTPH;AHCY;AHCYL1;AIP;AK1;AK4;AKAP10;AKR1A1;ALG12;ALKBH7;ALS2;AMT;ANAPC11;ANAPC2;ANKRD17;ANKRD23;ANKRD34A;ANKRD54;ANO6;ANO9;AP1G1;AP1G2;AP1S1;APBB3;APOE;APOM;APPL2;APRT;ARC;ARFGEF1;ARFIP2;ARFR

S13;ADCK5;ADHFE1;ADNP;ADORA2A;ADPRHL2;AES;AFF1;AFMID;AFTPH;AHCY;AHCYL1;AIP;AK1;AK4;AKAP10;AKR1A1;ALG12;ALKBH7;ALS2;AMT;ANAPC11;ANAPC2;ANKRD17;ANKRD23;ANKRD34A;ANKRD54;ANO6;ANO9;AP1G1;AP1G2;AP1S1;APBB3;APOE;APOM;APPL2;APRT;ARC;ARFGEF1;ARFIP2;ARFR

L18A;RPL28;RPL29;RPL38;RPL8;RPLP1;RPLP2;RPS15;RPS2;RPS2

EF1;ARIH1;ARNTL;ASB3;ASB6;ATF4;ATG4D;ATP6AP2;AVPI1;B3GALT6;B3GAT3;B4GALT2;B4GALT7;BABAM1;BRAF;BUB1B;C19orf10;C1QBP;C8G;CANX;CAPN10;CAPRIN1;CCDC101;CCNL2;CD36;CDC25A;CDC6;CDK12;CDK13;CEL;CLCF1;CLPP;CLSPN;CSNK1G1;CTSC;CTSF;DALRD3;DERL3;DGUOK;DNAJC17;DN

B3GAT3;B4GALT2;B4GALT7;BABAM1;BRAF;BUB1B;C19orf10;CANX;CCDC101;CCNL2;CD36;CDC25A;CDC6;CDK12;CDK13;CEL;CLCF1;CLSPN;CSNK1G1;DERL3;DGUOK;DOK7;DOLK;DPM3;DTX3;DUSP10;DUSP2;DUSP3;DVL1;E4F1;EHHADH;EIF4G3;ELP4;EPC1;ETF1;FASTK;FBXL15;FBXL6;FBXO2;FBXO24;FBXW11

B3GAT3;B4GALT2;B4GALT7;BABAM1;BRAF;BUB1B;C19orf10;CANX;CCDC101;CCNL2;CD36;CDC25A;CDC6;CDK12;CDK13;CEL;CLCF1;CLSPN;CSNK1G1;DERL3;DGUOK;DOK7;DOLK;DPM3;DTX3;DUSP10;DUSP2;DUSP3;DVL1;E4F1;EHHADH;EIF4G3;ELP4;EPC1;ETF1;FASTK;FBXL15;FBXL6;FBXO2;FBXO24;FBXW11

DHAF2;SURF1;TAZ

TAZ

TAZ

OLK;DTYMK;ETNK2;FASTK;FICD;FLAD1;FUK;GLYCTK;HIPK1;HIPK3;IRAK4;KCNH4;KHK;LATS1;LATS2;LTBP4;LTK;MAGI3;MAP3K10;MAP4K2;MAPK1;MAPK11;MATK;MVK;MYLK3;NME3;NME4;NPRL2;NRP1;OXSR1;PANK4;PAPSS1;PCYT2;PDK4;PFKFB2;PHKG1;PIK3C3;PIK3CB;PIK3CG;PIP5K1A;PIP5K1B;PIP5KL1;

f49;C17orf70;C1orf86;C1QBP;CCDC101;CCNL2;CDC25A;CDC6;CDCA8;CDK12;CDK13;CENPF;CHRAC1;CIB1;CLSPN;CPSF2;CPSF4;CREB3;DDX21;DDX28;DEAF1;DIAPH2;DTD1;DUSP2;DUSP3;E2F1;E4F1;EAF1;EDF1;ELP4;EPC1;ERCC1;ETV3;EXOSC5;FANCG;FBL;GEMIN7;GLTSCR2;GTF2A1;GTF3C5;H2AFY2;HDAC1

MID;AHCY;AHCYL1;AIP;AK1;AK4;AKR1A1;ALG12;ALS2;AMDHD2;AMT;ANAPC11;ANAPC2;ANKRD23;APOE;APOM;APRT;ARFGEF1;ARFRP1;ARHGAP4;ARIH1;ARL6IP4;ARNTL;ASB3;ASB6;ASH1L;ASXL2;ATF4;ATF7IP;ATG4D;ATL3;ATP5D;ATP5G2;ATP5G3;ATP5H;ATRIP;AVPI1;B3GALT6;B3GAT3;B4GALT2;B4GALT

DORA2A;AES;AFF1;AFMID;AHCY;AHCYL1;AIP;AK1;AK4;AKR1A1;ALG12;ALS2;AMDHD2;AMT;ANAPC11;ANAPC2;ANKRD23;APOE;APOM;APRT;ARFGEF1;ARFRP1;ARHGAP4;ARIH1;ARL6IP4;ARNTL;ASB3;ASB6;ASH1L;ASXL2;ATF4;ATF7IP;ATG4D;ATHL1;ATL3;ATP5D;ATP5G2;ATP5G3;ATP5H;ATP6AP2;ATRIP;AV

RFGEF1;ARHGAP4;ARHGEF25;ARIH1;ASPSCR1;ATP6V1C1;BRAF;BUB1;BUB1B;C1QBP;CAPN10;CAPRIN1;CCS;CDC25A;CDC6;CDCA8;CEL;CENPF;CENPM;CENPT;CHMP4A;CHMP6;CLASP1;CLIP1;CREB3;CRYL1;DGUOK;DIAPH2;DPP7;DTYMK;DUSP23;DUSP3;DVL1;EEF1D;EEF1G;EHHADH;EIF2B2;EIF2B4;EIF3F;EIF

;CCNL2;CDC25A;CDC6;CDK12;CDK13;CIB1;CLSPN;CPSF2;CPSF4;CREB3;DEAF1;DUSP2;DUSP3;E2F1;E4F1;EAF1;EDF1;ELP4;EPC1;ERCC1;ETV3;FANCG;FBL;GEMIN7;GTF2A1;GTF3C5;HDAC10;HDAC11;HES6;HINFP;HIPK1;HIPK3;IRF3;KAT2A;KDM6A;KIF20A;LSM2;MAML2;MAPK1;MAPK11;MBD3;MCM7;MCRS1

12;CDK13;CLCF1;CLSPN;COQ7;CSNK1G1;DGUOK;DOK7;DUSP10;DUSP2;DUSP3;DVL1;EIF4G3;FASTK;FNIP2;GLYCTK;GNB2L1;HIPK3;HPX;HRAS;KIDINS220;LAMTOR2;LATS1;LATS2;LIME1;LPAR1;LTK;MAGI3;MAP3K10;MAP4K2;MAPK1;MAPK11;MATK;MCM7;MLST8;MYLK3;NDUFA3;NDUFA8;NDUFB10;NDUF
7;NDUFB8;NDUFB9;NDUFS6;NDUFS7;NDUFS8;NDUFV1;SDHAF2;SIRT3;SURF1;TAZ;TBRG4
6;NDUFS7;NDUFS8;NDUFV1;TXNRD1

1;ATG4D;ATP6V0E2;ATP6V1C1;CANX;CCDC101;CCHCR1;CD36;CENPF;CHMP4A;CHMP6;CIB1;CREB3;ERP29;EXOC6B;FBXW11;GAPVD1;GFER;GGA1;GNB2L1;HINFP;HOOK3;IPO8;KCNN4;KIF20A;KRT18;LATS1;LIME1;LMF1;LRRC32;MAMDC4;MAPK1;MED1;MIA3;MTM1;NDUFA13;NECAB3;NF1;NFKBIL1;NSF;N
IG4;PLEKHG5;PSD;RABGEF1;RGL1;RINL;SERGEF;SH2D3A;SOS1;SPATA13;TAGAP;TBC1D10A;VAV
RP4B;LSM14A;MAPK1;MIF4GD;MRPL12;MRPL20;MRPL23;MRPL24;MRPL52;MRPL55;MRPS11;MRPS12;MRPS15;MRPS18A;NDUFA13;PARS2;QARS;RPL13;RPL18;RPL18A;RPL28;RPL29;RPL38;RPL8;RPLP1;RPLP2;RPS15;RPS2;RPS28;RPS6KB2;SARS2;SSR1;SSR4;TPR;TUFM;VARS2;WI
ARNTL;ASPSCR1;ATG4D;ATP6V0E2;ATP6V1C1;BUB1B;CANX;CCDC101;CCHCR1;CD36;CENPF;CHMP4A;CHMP6;CIB1;CLASP1;CRB3;CREB3;CUTA;DOK7;DVL1;ERP29;EXOC6B;FBXW11;GAPVD1;GET4;GFER;GGA1;GNB2L1;GRASP;HINFP;HOOK3;IPO8;KCNN4;KIF20A;KRT18;LAMTOR2;LATS1;LATS2;LIME1;LMF1;
TNK2;FASTK;FUK;GLYCTK;HIPK1;HIPK3;IRAK4;KCNH4;KHK;LATS1;LATS2;LTBP4;LTK;MAGI3;MAP3K10;MAP4K2;MAPK1;MAPK11;MATK;MVK;MYLK3;NME3;NME4;NPRL2;NRP1;OXSR1;PANK4;PAPSS1;PDK4;PFKFB2;PHKG1;PIK3C3;PIK3CB;PIK3CG;PIP5K1A;PIP5K1B;PIP5KL1;PMVK;PNKP;POLR2F;POLR2G;POL
COX4I1;CPT1C;CYC1;DTYMK;ECI1;EPC1;FDXR;FPGS;GCDH;GCHFR;GFER;GHDC;GIMAP5;GOLPH3;HCCS;HSD17B8;IPO8;LEMD2;MAD1L1;MRPS15;MTFP1;NDUFA11;NDUFA13;NDUFA3;NDUFA8;NDUFB10;NDUFB11;NDUFB7;NDUFB8;NDUFB9;NDUFS6;NDUFS7;NDUFS8;NDUFV1;NME4;NUP153;P2RX7;PDK4;

COX4I1;CPT1C;CYC1;DTYMK;ECI1;EPC1;FDXR;FPGS;GCDH;GCHFR;GFER;GHDC;GIMAP5;GOLPH3;GRIN3A;HCCS;HSD17B8;IPO8;LEMD2;MAD1L1;MRPS15;MTFP1;NDUFA11;NDUFA13;NDUFA3;NDUFA8;NDUFB10;NDUFB11;NDUFB7;NDUFB8;NDUFB9;NDUFS6;NDUFS7;NDUFS8;NDUFV1;NME4;NUP153;P2R

1;ATG4D;ATP6V0E2;ATP6V1C1;CANX;CCHCR1;CD36;CENPF;CHMP4A;CHMP6;CREB3;ERP29;EXOC6B;FBXW11;GAPVD1;GFER;GGA1;HOOK3;IPO8;KCNN4;KIF20A;KRT18;LATS1;LIME1;LMF1;LRRC32;MAMDC4;MAPK1;MED1;MIA3;MTM1;NDUFA13;NECAB3;NF1;NFKBIL1;NSF;NUP153;OGG1;P2RX7;PANX1;P
PLP2;RPS15;RPS2;RPS28;RPS6KB2;TPI
;DNASE1L2;E4F1;ERCC1;EXOSC5;FANCG;GTF2H2D;H2AFJ;H2AFY2;HINFP;HRAS;ID3;INO80E;MC1R;MCM7;MCRS1;MED1;MGMT;MKI67;MLL5;MPG;MSH3;MUS81;MUTYH;NAA10;NCAPH;NCAPH2;NEIL1;NSMCE1;NTHL1;NUDT1;NUSAP1;OBFC1;OGG1;PARP10;PARP3;PICK1;PNKP;POLD1;POLD4;POLM;POL

S4;ECI1;EHHADH;ETFB;FADS3;FDX1L;FDXR;GCDH;GNG8;GPX4;GYG1;HCCS;MUT;NDOR1;NDUFA11;NDUFA13;NDUFA3;NDUFA8;NDUFB10;NDUFB11;NDUFB7;NDUFB8;NDUFB9;NDUFS6;NDUFS7;NDUFS8;NDUFV1;PDK4;PGM2;PHKB;PHKG1;PPOX;PPP1R3F;PRKAR2A;SDHAF2;SIRT3;SLC25A5;SLC25A6;STX1
ND1;DUS2L;EEF1A2;EEF1D;EEF1G;EIF1;EIF2B2;EIF2B4;EIF3F;EIF3G;EIF3I;EIF3K;EIF4G3;ETF1;EXOSC5;FBL;HBP1;HNRNPH3;LARP4B;LSM2;LSM4;MAZ;MIF4GD;MRPL12;MRPL20;MRPL23;NHP2;NOL12;NSRP1;NUDT1;NUDT16L1;PARK7;PATL1;POLR2G;PUS1;PUSL1;RBMXL1;RBPMS;RC3H1;RNMTL1;RPL13;RP
GLYCTK;HIPK1;HIPK3;IRAK4;KCNH4;KHK;LATS1;LATS2;LTBP4;LTK;MAP3K10;MAP4K2;MAPK1;MAPK11;MATK;MVK;MYLK3;NPRL2;NRP1;OXSR1;PANK4;PAPSS1;PDK4;PFKFB2;PHKG1;PIK3C3;PIK3CB;PIK3CG;PIP5K1A;PIP5K1B;PIP5KL1;PNKP;POLR2F;POLR2G;POLR2I;POLR2L;RABEP2;ROCK2;RPS6KB2;RPS6KC

COQ7;CREB3;DERL3;DUSP10;DUSP3;E2F1;EIF2B4;ERCC1;FANCG;FEM1B;FNIP2;GTF2H2D;HINFP;HIPK1;HIPK3;HRAS;INO80E;IRAK4;IRF3;MAGI3;MAP3K10;MAP4K2;MAPK1;MAPK11;MC1R;MCM7;MCRS1;MGMT;MKI67;MPG;MRPS11;MRPS26;MSH3;MUS81;MUTYH;NEIL1;NIPBL;NSMCE1;NTHL1;NUDT1;O
L18A;RPL28;RPL29;RPL38;RPL8;RPLP1;RPLP2;RPS15;RPS2;RPS2
2;MRPL20;MRPL23;MRPL24;MRPL38;MRPL52;MRPL54;MRPL55;MRPS11;MRPS12;MRPS15;MRPS18A;MRPS26;MRPS34;NHP2;NSRP1;PATL1;PIH1D1;POP7;QTRT1;RBMXL1;RC3H1;RPL13;RPL18;RPL18A;RPL28;RPL29;RPL38;RPL8;RPLP1;RPLP2;RPS15;RPS2;RPS28;RPS6KL1;RQCD1;RRP9;SF3B5;SNRNP25;SN

RK7;PLA2G6;POLRMT;SDHAF1;SLC25A5;SLC25A6;SMCR7;STOML2;TAZ;TIMM10;TIMM13;TIMM50;TP73;YME1L1;YWHA

DORA2A;AES;AFF1;AFMID;AHCY;AHCYL1;AIP;AK1;AK4;AKR1A1;ALG12;ALS2;AMDHD2;AMT;ANAPC11;ANAPC2;ANKRD23;APOE;APOM;APRT;ARFGEF1;ARFRP1;ARHGAP4;ARIH1;ARL6IP4;ARNTL;ASB3;ASB6;ASH1L;ASXL2;ATF4;ATF7IP;ATG4D;ATHL1;ATL3;ATP5D;ATP5G2;ATP5G3;ATP5H;ATP6AP2;ATRIP;AV

H1;ARNTL;ATF4;ATF7IP;ATP5D;ATP5G2;ATP5G3;ATP5H;ATP6V0E2;ATP6V1C1;BABAM1;BAIAP2L2;BCS1L;BEST4;BRF1;BUB1;BUB1B;C17orf49;C1orf86;C8G;CCDC101;CD8B;CDC6;CDCA8;CDK12;CDK13;CENPF;CENPM;CENPT;CHRAC1;CLASP1;CLCF1;CLIP1;COL6A1;COL6A2;CPSF2;CPSF4;CTU2;DEAF1;DLGAP5
1B;C17orf49;C17orf70;C1orf86;C1QBP;CCDC101;CCNL2;CDC25A;CDC6;CDCA8;CDK12;CDK13;CENPF;CHRAC1;CIB1;CLSPN;CPSF2;CPSF4;CREB3;DDX21;DDX28;DEAF1;DIAPH2;DTD1;DUSP2;DUSP3;E2F1;E4F1;EAF1;EDF1;ELP4;EPC1;ERCC1;ETV3;EXOSC5;FANCG;FBL;GCHFR;GEMIN7;GHDC;GLTSCR2;GOLPH3;
THL1;OGG1;PHKE
RMU;WHSC1L

PS28;SCARB2;SLC25A6;SSR1;SSR4;TIMM10;TIMM13;TIMM50;WDR24;YWHA
1;BABAM1;BCS1L;BUB1;BUB1B;C11orf30;C12orf44;C17orf49;C1QBP;C21orf2;CAPN10;CCDC101;CDC25A;CDC6;CDCA8;CDK13;CENPF;CENPM;CENPT;CEP55;CHD7;CHRAC1;CLASP1;CLIP1;COQ7;CORO6;DAAM1;DIAPH2;DLGAP5;DVL1;DYM;DYNC1LI1;E4F1;ELP4;EPC1;ERCC1;FANCG;FBXW5;FGD6;FSD1;GAS
B;HINFP;HRAS;MAD1L1;MCM7;PIDD;PSMA7;PSMB4;PSMB5;PSMB7;PSMD9;RAD9A;TAOK3;TOP2A;TP73;TPF

1;ARHGAP4;ATF4;ATL3;ATP5D;ATP5G2;ATP5G3;ATP5H;AVPI1;BRAF;C19orf10;CCNL2;CD36;CDC25A;CDC6;CDK12;CDK13;CDS2;CLCF1;CLSPN;COQ7;CSNK1G1;DCXR;DGUOK;DOK7;DOLK;DPM3;DTYMK;DUSP10;DUSP2;DUSP3;DVL1;EIF4G3;ETNK2;FASTK;FBXW11;FDXR;FGD6;FICD;FLAD1;FNIP2;GCDH;GLYCT
FF1;AFMID;AHCY;AHCYL1;AIP;AK1;AK4;AKR1A1;ALG12;ALS2;AMDHD2;AMT;ANAPC11;ANAPC2;ANKRD23;APOE;APOM;APRT;ARFGEF1;ARFRP1;ARHGAP4;ARIH1;ARL6IP4;ARNTL;ASB3;ASB6;ASH1L;ASXL2;ATF4;ATF7IP;ATG4D;ATHL1;ATL3;ATP5D;ATP5G2;ATP5G3;ATP5H;ATP6AP2;ATRIP;AVPI1;B3GALT6;B
AP4;ATF4;ATL3;ATP5D;ATP5G2;ATP5G3;ATP5H;AVPI1;BRAF;C19orf10;CCNL2;CD36;CDC25A;CDC6;CDK12;CDK13;CDS2;CLCF1;CLSPN;COQ7;CSNK1G1;DCXR;DGUOK;DOK7;DOLK;DPM3;DTYMK;DUSP10;DUSP2;DUSP3;DVL1;EIF4G3;ETNK2;FASTK;FBXW11;FDXR;FGD6;FICD;FLAD1;FNIP2;GLYCTK;GNAI3;GNB

8;STRA13;SUV420H1;SYCE1L;TOP2A;TOP3B;TOPBP1;TTN
ADPRHL2;AFMID;AHCY;AHCYL1;AMDHD2;ARFRP1;ATAD3B;ATF7IP;ATG4D;ATHL1;ATL3;ATP5D;ATP5H;ATP6V0E2;ATP6V1C1;BCS1L;C12orf5;CAPN10;CD38;CDC25A;CDC6;CECR5;CEL;CHD7;CHTF18;CLPP;CPSF2;CPSF3L;CTSC;CTSF;DDX21;DDX28;DDX39A;DDX43;DDX49;DLGAP5;DNAL4;DNASE1L2;DPP7;DPF
11;FSD1;GNAI3;HAUS8;HOOK3;KIF15;KIF24;KRT18;LATS1;LATS2;LRRC45;MAD1L1;MAPK1;MZT2B;NEIL1;NUBP2;PARP3;PHF1;PLA2G6;PRKAR2A;PROCR;PXK;RASSF7;ROCK2;SAC3D1;SEPT1;SSNA1;TACC1;TCEA2;THAP11;TNFAIP3;TOP2A;TOPBP1;TUBGCP3;ZNF27
TOP2A;TOP3B;TTN;USP9X
D3B;ATL3;ATP5D;BCS1L;BMP2K;BRAF;BUB1;BUB1B;C8G;CBR1;CD320;CD38;CDC6;CDK10;CDK12;CDK13;CELF2;CELF6;CHD7;CHTF18;CLCN4;CLPP;CRYL1;CSNK1G1;DALRD3;DCXR;DDX21;DDX28;DDX39A;DDX43;DDX49;DECR2;DGUOK;DHDH;DHRS3;DHRS4;DHRS4L2;DNAJC17;DND1;DOM3Z;DRG2;DTYMK;D
K7;DUSP10;DUSP2;DUSP3;DVL1;GNB2L1;HIPK3;HRAS;KIDINS220;LAMTOR2;LATS1;LATS2;LIME1;LPAR1;MAGI3;MAP3K10;MAP4K2;MAPK1;MAPK11;NF1;NPRL2;P2RX7;PDE6G;PFKFB2;PHPT1;PIK3CB;PIK3CG;PPP1R3F;PREB;PRKAR2A;PTPRC;SPRED2;SSR1;TAOK3;TNFAIP3;TNFRSF4;TNFSF15;TP73;TRAF2;TR

3;DEAF1;E2F1;EAF1;EDF1;ELP4;EPC1;ETV3;FANCG;FBL;GEMIN7;GTF2A1;GTF3C5;HDAC10;HDAC11;HES6;HINFP;HIPK1;HIPK3;KAT2A;KDM6A;MAML2;MBD3;MCRS1;MED1;MED13L;MED18;MLL3;MLL5;NHP2;NSRP1;OGG1;PATL1;PHF16;PHF20;PIN1;PIP5K1A;POLR2F;POLR2G;POLR2I;POLR2L;POLR3K;PUS1
D3B;ATL3;ATP5D;BCS1L;BMP2K;BRAF;BUB1;BUB1B;CBR1;CD38;CDC6;CDK10;CDK12;CDK13;CELF2;CELF6;CHD7;CHTF18;CLCN4;CLPP;CRYL1;CSNK1G1;DALRD3;DCXR;DDX21;DDX28;DDX39A;DDX43;DDX49;DECR2;DGUOK;DHDH;DHRS3;DHRS4;DHRS4L2;DNAJC17;DND1;DOM3Z;DRG2;DTYMK;DUS1L;DUS2

D3B;ATL3;ATP5D;BCS1L;BMP2K;BRAF;BUB1;BUB1B;CBR1;CD38;CDC6;CDK10;CDK12;CDK13;CELF2;CELF6;CHD7;CHTF18;CLCN4;CLPP;CRYL1;CSNK1G1;DALRD3;DCXR;DDX21;DDX28;DDX39A;DDX43;DDX49;DECR2;DGUOK;DHDH;DHRS3;DHRS4;DHRS4L2;DNAJC17;DND1;DOM3Z;DRG2;DTYMK;DUS1L;DUS2L

5;RPS2;RPS28;RQCD1;UPF2;WIBG;ZC3HAV1

5;RPS2;RPS28;RQCD1;UPF2;WIBG

RPS28;RSF1;SP1;TCEB2

RPS28;RSF1;SP1;TCEB2

7;PSMD9;TOP2A;TP73;TPR

D1L1;MAPK1;MYH10;MZT2B;NUSAP1;ROCK2;SAC3D1;SLC25A5;TADA3;THAP11;TOPBP1;TUBGCP3

AMDHD2;AMT;ANKRD23;APOE;APRT;ARFGEF1;ARFRP1;ARHGAP4;ATF4;ATL3;ATP5D;ATP5G2;ATP5G3;ATP5H;B3GALT6;B3GAT3;B4GALT2;B4GALT7;CBR1;CD36;CDS2;CEL;COQ7;COQ9;COX4I1;CPT1C;CRYL1;CYC1;CYP1B1;CYP2E1;DALRD3;DCXR;DECR2;DGUOK;DHRS4;DSE;DTD1;DTYMK;ECI1;EHHADH;ETFB

P8

K7;DUSP10;DUSP2;DUSP3;DVL1;GNB2L1;HIPK3;HRAS;KIDINS220;LAMTOR2;LATS1;LATS2;LIME1;LPAR1;MAGI3;MAP3K10;MAP4K2;MAPK1;MAPK11;NF1;NPRL2;P2RX7;PDE6G;PFKFB2;PIK3CB;PIK3CG;PREB;PRKAR2A;PTPRC;SPRED2;SSR1;TAOK3;TNFAIP3;TNFRSF4;TNFSF15;TP73;TRAF2;TRAF4;TRAF6;TRIB3

3CG;PIP5K1A;PIP5K1B;PLA2G6;PMVK;PTDSS2;SLC44A1;SPHK2;TAZ;ZP3

NB2L1;HPX;IRF3;KAT2A;KRT18;MAGI3;MAPK1;MICB;NUP153;POLR2F;POLR2G;POLR2I;POLR2L;PSMA7;PSMB4;PSMB5;PSMB7;RPL13;RPL18;RPL18A;RPL28;RPL29;RPL38;RPL8;RPLP1;RPLP2;RPS15;RPS2;RPS28;RSF1;SIVA1;SLC25A5;SLC25A6;SP1;TCEB2;THOC6;TOP1;TOP2A;UCKL1;ZC3HAV

4G3;GADD45GIP1;GNB2L1;GTF2A1;HPX;IRF3;KAT2A;KRT18;MAGI3;MAPK1;MICB;MZF1;NUP153;POLR2F;POLR2G;POLR2I;POLR2L;PSMA7;PSMB4;PSMB5;PSMB7;PSMD9;RPL13;RPL18;RPL18A;RPL28;RPL29;RPL38;RPL8;RPLP1;RPLP2;RPS15;RPS2;RPS28;RSF1;SIVA1;SLC25A5;SLC25A6;SP1;TCEB2;THOC6;TO

OLRMT;TUT1

4G3;GADD45GIP1;GNB2L1;GTF2A1;HPX;IRF3;KAT2A;KRT18;MAGI3;MAPK1;MICB;MZF1;NUP153;POLR2F;POLR2G;POLR2I;POLR2L;PSMA7;PSMB4;PSMB5;PSMB7;PSMD9;RPL13;RPL18;RPL18A;RPL28;RPL29;RPL38;RPL8;RPLP1;RPLP2;RPS15;RPS2;RPS28;RSF1;SIVA1;SLC25A5;SLC25A6;SP1;TCEB2;THOC6;TO

RPLP1;RPLP2;RPS15;RPS2;RPS28;RQCD1;UPF2;WIBG;ZC3HAV1

EF1;ARFRP1;ARNTL;ASPSCR1;ATG4D;ATP6V0E2;ATP6V1C1;BUB1B;CANX;CCDC101;CCHCR1;CD36;CEL;CENPF;CHMP4A;CHMP6;CIB1;CLASP1;CPSF2;CRB3;CREB3;CUTA;DDX39A;DOK7;DVL1;ERP29;EXOC6B;FBXW11;GAPVD1;GET4;GFER;GGA1;GNB2L1;GRASP;HINFP;HOOK3;IPO8;KCNN4;KIF20A;KRT18;LAM

3;DHRS4;DHRS4L2;DUS1L;DUS2L;DUS3L;EHHADH;FADS3;FDXR;GCDH;GFER;GPX4;GSTT1;HSD11B1L;HSD17B1;HSD17B12;HSD17B8;KDM3A;KDM5A;KDM6A;LEPRE1;LEPREL2;MECR;NDOR1;NDUFA13;NDUFA3;NDUFA8;NDUFB10;NDUFB7;NDUFB8;NDUFB9;NDUFS6;NDUFS7;NDUFS8;NDUFV1;OGFOD2;P4H

K7;DUSP10;DUSP2;DUSP3;DVL1;GNB2L1;HIPK3;HRAS;KIDINS220;LAMTOR2;LATS1;LATS2;LIME1;LPAR1;MAGI3;MAP3K10;MAP4K2;MAPK1;MAPK11;NF1;P2RX7;PDE6G;PIK3CB;PIK3CG;PREB;PRKAR2A;PTPRC;SPRED2;SSR1;TAOK3;TNFAIP3;TNFRSF4;TNFSF15;TP73;TRAF2;TRAF4;TRAF6;TRIB3;TTN;WNT7A;Z

IME1;LPAR1;MAGI3;MAP3K10;MAP4K2;MAPK1;MAPK11;NF1;P2RX7;PDE6G;PIK3CB;PIK3CG;SPRED2;TAOK3;TNFAIP3;TNFSF15;TP73;TRAF2;TRAF6;TRIB3;WNT7A;ZEB2;ZFP91

APK11;NF1;P2RX7;PDE6G;PIK3CB;PIK3CG;SPRED2;TAOK3;TP73;TRAF2;TRAF6;TRIB3;WNT7A;ZEB2

B;HINFP;HRAS;MAD1L1;MCM7;PIDD;PSMA7;PSMB4;PSMB5;PSMB7;PSMD9;RAD9A;TAOK3;TOP2A;TP73;TPR

PLEKHG4;PLEKHG5;SOS1;SPATA13;STMN3;VAV3

ORA2A;AES;AFF1;AFTPH;AHCYL1;AHSA2;AIP;ALS2;ANAPC2;ANKRD23;ANKRD54;AP1G1;AP1G2;APOE;APPL2;ARFGEF1;ARFIP2;ARFRP1;ARHGAP4;ARIH1;ARL6IP4;ARNTL;ASPSCR1;ASXL2;ATF4;ATF7IP;ATL3;ATP6AP2;ATP6V1C1;ATRIP;AVEN;BABAM1;BAIAP2L2;BCL7B;BCS1L;BHLHE41;BOK;BRAF;BRAT1;BRF1

SP1;CRB3;CREB3;DOK7;DVL1;ERP29;FBXW11;GET4;GFER;GGA1;GNB2L1;HOOK3;IPO8;KRT18;LAMTOR2;LATS1;LATS2;LIME1;MAPK1;MED1;NDUFA13;NF1;NFKBIL1;NIPBL;OGG1;PEX16;PEX6;PICK1;PIP5K1A;PLEKHF1;RAD21;RALGAPB;RBPMS;RPL13;RPL18;RPL18A;RPL28;RPL29;RPL38;RPL8;RPLP1;RPLP2;RP

F1;CLSPN;DOK7;DUSP10;DUSP2;DUSP3;DVL1;EIF4G3;FNIP2;GNB2L1;HIPK3;HPX;HRAS;KIDINS220;LAMTOR2;LATS1;LATS2;LIME1;LPAR1;MAGI3;MAP3K10;MAP4K2;MAPK1;MAPK11;MCM7;MLST8;NF1;NOG;NPRL2;NRP1;P2RX7;PDE6G;PFKFB2;PIK3CB;PIK3CG;PIN1;PLA2G6;PPP1R14A;PPP1R14B;PREB;PRKA

SP1;CRB3;CREB3;DOK7;DVL1;ERP29;FBXW11;GET4;GFER;GGA1;GNB2L1;HOOK3;IPO8;KRT18;LAMTOR2;LATS1;LATS2;LIME1;MAPK1;MED1;NDUFA13;NF1;NFKBIL1;NIPBL;OGG1;PEX16;PEX6;PICK1;PIP5K1A;PLEKHF1;RAD21;RALGAPB;RBPMS;RPL13;RPL18;RPL18A;RPL28;RPL29;RPL38;RPL8;RPLP1;RPLP2;R

NAI3;GNB2L1;GNG8;GNGT2;HRAS;ITPA;LSM2;LSM4;MLST8;MPG;MTG1;MUTYH;NF1;NT5C;NTHL1;NUDT1;NUDT18;OGG1;PATL1;PEX6;PICK1;PIN1;PIP5K1A;PNKP;POLD1;PXK;RAB21;RAB33A;RAC3;RALGAPB;RASA2;RC3H1;RNASEH2C;RPL13;RPL18;RPL18A;RPL28;RPL29;RPL38;RPL8;RPLP1;RPLP2;RPS15;RPS

NDUFA13;NF1;NFKBIL1;OGG1;PEX16;PEX6;RAD21;RBPMS;RPL13;RPL18;RPL18A;RPL28;RPL29;RPL38;RPL8;RPLP1;RPLP2;RPS15;RPS2;RPS28;SCARB2;SLC25A6;SSR1;SSR4;TIMM10;TIMM13;TIMM50;TLR7;TOR1AIP1;TPR;TRPS1;UBL4A;WDR24;YWHA

LI1;E4F1;FSD1;GNAI3;GNB2L1;HAUS8;KIF20A;LATS1;LATS2;MAD1L1;MYH10;NCAPH;NUSAP1;PARD6G;PIK3C3;PIK3CB;PIN1;RABEP2;RAD21;RHOC;ROCK2;SAC3D1;SEPT1;SPG20;STAMBP;STRA13;TACC1;TOP1;TOP2A;TXNL4A;USP8;USP9X;WNT7A

GEF1;ARIH1;ARL6IP4;ARNTL;ASB3;ASB6;ASH1L;ASXL2;ATF4;ATF7IP;ATRIP;AVPI1;B3GALT6;B3GAT3;B4GALT2;B4GALT7;BABAM1;BHLHE41;BRAF;BRF1;BRF2;BUB1B;C11orf30;C17orf70;C19orf10;C1orf86;C1QBP;CANX;CAPRIN1;CCDC101;CCNL2;CD36;CD38;CDC25A;CDC6;CDK12;CDK13;CEL;CELF2;CELF6;C

FB11;NDUFB7;NDUFB8;NDUFB9;NDUFS6;NDUFS7;NDUFS8;NDUFV1;PGM2;PHKB;PHKG1;PPP1R3F;PRKAR2A;SDHAF2;SIRT3;SLC25A5;SLC25A6;STX1A;SURF1;TAZ;TBRG4
2RX6;P2RX7;PMM1;RAC3;SLC1A3;SPTBN4;STRN;TMPRSS5;TOP1;TXN2;TXNRD1;UBXN1;VPS1
MT112;WHSC1L

I1;TRMT112;WHSC1L

13;VAV3
I3;GNB2L1;KCNN4;KLHL12;MAL;MYH10;NSF;P2RX7;PICK1;PIP5K1A;PLEKHF1;PREB;SEC24B;SLC25A5;SNF8;SPTBN4;SSNA1;STAM;TAZ;TIMM10;TIMM13;TIMM50;TMED9;TOR1AIP1;UBL4A;VAV3;ZFYVE2
M1B;HERC3;KLHL12;KLHL17;MARCH9;MYLIP;NEURL2;NSMCE1;PIN1;PSMA7;PSMB4;PSMB5;PSMB7;PSMD9;RLIM;RNF115;RNF169;RNF181;RNF25;RUSC1;SH3RF1;SPSB2;SPSB3;STRA13;STUB1;TCEB2;TNFAIP3;TRAF2;TRAF6;TRIB3;TRIM24;TRIP12;TSPAN17;UBA7;UBQLN1;UBXN1;ZFP9

2;ALS2;AMDHD2;AMT;ANKRD23;APOE;APOM;APRT;ARFGEF1;ARFRP1;ARHGAP4;ATF4;ATL3;ATP5D;ATP5G2;ATP5G3;ATP5H;B3GALT6;B3GAT3;B4GALT2;B4GALT7;BTN2A1;CBR1;CD36;CDS2;CEL;COQ7;COQ9;COX4I1;CPT1C;CRYL1;CYB561D2;CYC1;CYP1B1;CYP2E1;DALRD3;DCXR;DDT;DECR2;DGUOK;DHRS3

3XW5;FEM1B;HERC3;JOSD2;KAT2A;KLHL12;KLHL17;MARCH9;MYLIP;NEURL2;NSMCE1;PIN1;PSMA7;PSMB4;PSMB5;PSMB7;PSMD9;RLIM;RNF115;RNF169;RNF181;RNF25;RUSC1;SENP2;SH3RF1;SPSB2;SPSB3;STAMBP;STRA13;STUB1;TCEB2;TNFAIP3;TRAF2;TRAF6;TRIB3;TRIM24;TRIP12;TSPAN17;UBA7;UBC

N1;PIP5K1A;PLEKHG4;PLEKHG5;PSD;RALGAPB;RASA2;SGSM3;SMAP1;SOS1;SPATA13;STMN3;TNK1;USP6NL;VAV3

ARNTL;ASPSCR1;ATP5D;ATP5H;BUB1B;CANX;CAPN10;CCDC101;CCHCR1;CCS;CD36;CD38;CENPF;CHD7;CHMP4A;CHMP6;CIB1;CLASP1;CPSF2;CRB3;CREB3;DDX39A;DLGAP5;DOK7;DVL1;ERP29;EXOC6B;FAM21B;FBXW11;GET4;GFER;GGA1;GIMAP5;GNB2L1;HOOK3;IPO8;KCNN4;KLHL12;KRT18;LAMTOR2;LA
13;CLCF1;CLSPN;CSNK1G1;DGUOK;DOK7;DUSP10;DUSP2;DUSP3;DVL1;EIF4G3;FASTK;FNIP2;GLYCTK;GNB2L1;HIPK3;HPX;HRAS;KIDINS220;LAMTOR2;LATS1;LATS2;LIME1;LPAR1;LTK;MAGI3;MAP3K10;MAP4K2;MAPK1;MAPK11;MATK;MLST8;MYLK3;NF1;NOG;NPRL2;NRP1;OXSR1;P2RX7;PDE6G;PDK4;PICK1
ERLIN2;ERP44;FADS3;FAF2;FAM69B;FKBP2;HSD17B12;JAGN1;JSRP1;KRTCAP2;LMF1;LMF2;MAN1B1;MIA3;MOGS;MPDU1;NECAB3;P4HTM;PANX1;PCYT2;PEMT;PEX16;PIGQ;POMT1;PPM1L;PREB;PTDSS2;PTPLB;RAB21;SEC22B;SEC24B;SLC37A4;SOAT1;SOAT2;SPPL2A;SPPL2B;SSR1;SSR4;STS;TLR7;TM7SF2;
I3;GNB2L1;KCNN4;KLHL12;MAL;MYH10;NSF;P2RX7;PICK1;PIP5K1A;PLEKHF1;PREB;SEC24B;SLC25A5;SNF8;SPTBN4;SSNA1;STAM;TAZ;TIMM10;TIMM13;TIMM50;TMED9;TOR1AIP1;UBL4A;VAV3;ZFYVE2

HL12;KRT18;LIME1;LMF1;MAPK1;MED1;MTM1;NDUFA13;NF1;NFKBIL1;NUP153;OGG1;P2RX7;PEX16;PEX6;PIK3C3;PREB;PTPRC;RBPMS;RPL13;RPL18;RPL18A;RPL28;RPL29;RPL38;RPL8;RPLP1;RPLP2;RPS15;RPS2;RPS28;SCARB2;SEC22B;SEC24B;SLC25A6;SSR1;SSR4;THOC6;TIMM10;TIMM13;TIMM50;TLR7;
G1;PATL1;PIN1;PIP5K1A;RSRC1;SENP2;SNRPC;SPTBN4;THAP7;TIMM50;TOPBP1;TUT1;WRAP53
;CDC25A;CDC6;CDK12;CLCF1;CLSPN;DOK7;DUSP10;DUSP2;DUSP3;DVL1;EIF4G3;FBXO2;FEM1B;FNIP2;GNB2L1;HIPK3;HPX;HRAS;KDM3A;KIDINS220;LAMTOR2;LATS1;LATS2;LIME1;LPAR1;MAGI3;MAP3K10;MAP4K2;MAPK1;MAPK11;MLST8;NF1;NIPBL;NOG;NRP1;P2RX7;PDE6G;PIK3CB;PIK3CG;PIN1;PLA2G
10;CDK12;CDK13;CHD7;CHTF18;CLCN4;CLPP;CSNK1G1;DALRD3;DDX21;DDX28;DDX39A;DDX43;DDX49;DGUOK;DTYMK;DYNC1LI1;EIF2B2;ETNK2;FASTK;FICD;FIGNL2;FLAD1;FPGS;FUK;GLYCTK;HELZ;HIPK1;HIPK3;IRAK4;KHK;KIF15;KIF20A;KIF24;KIF7;LATS1;LATS2;LTK;MAGI3;MAP3K10;MAP4K2;MAPK1;MAP

SM4;MAPK11;NSRP1;PATL1;POLR2F;POLR2G;POLR2I;POLR2L;PRMT7;PSMA7;PSMB4;PSMB5;PSMB7;PSMD9;RBMXL1;RC3H1;RPL13;RPL18;RPL18A;RPL28;RPL29;RPL38;RPL8;RPLP1;RPLP2;RPS15;RPS2;RPS28;RQCD1;RSRC1;SF3B5;SNRNP25;SNRPC;THOC6;TUT1;TXNL4A;UPF2;WDR83;WIBG;YTHDC1;YWHA

;BUB1;BUB1B;C17orf70;C1orf86;C1QBP;CANX;CAPRIN1;CCDC28B;CCDC61;CDC6;CDCA8;CDK12;CENPF;CENPM;CENPT;CEP55;CHRAC1;CLASP1;CLIP1;CNTLN;CORO6;CTTNBP2NL;DAAM1;DDX21;DDX28;DIAPH2;DLGAP5;DNAL4;DTD1;DVL1;DYNC1LI1;E4F1;EDF1;ELMO3;ERCC1;EXOSC5;FBL;FBXW11;FGD6;F
;BUB1;BUB1B;C17orf70;C1orf86;C1QBP;CANX;CAPRIN1;CCDC28B;CCDC61;CDC6;CDCA8;CDK12;CENPF;CENPM;CENPT;CEP55;CHRAC1;CLASP1;CLIP1;CNTLN;CORO6;CTTNBP2NL;DAAM1;DDX21;DDX28;DIAPH2;DLGAP5;DNAL4;DTD1;DVL1;DYNC1LI1;E4F1;EDF1;ELMO3;ERCC1;EXOSC5;FBL;FBXW11;FGD6;F
;CDK10;CDK12;CDK13;CHD7;CHTF18;CLCN4;CLPP;CSNK1G1;DALRD3;DDX21;DDX28;DDX39A;DDX43;DDX49;DGUOK;DTYMK;DYNC1LI1;EIF2B2;ETNK2;FASTK;FICD;FIGNL2;FLAD1;FPGS;FUK;GLYCTK;HELZ;HIPK1;HIPK3;IRAK4;KHK;KIF15;KIF20A;KIF24;KIF7;LATS1;LATS2;LTK;MAGI3;MAP3K10;MAP4K2;MAPK

11;ANAPC2;APOE;APOM;ARFGEF1;ARIH1;ARL6IP4;ARNTL;ASB3;ASB6;ASH1L;ASXL2;ATF4;ATF7IP;ATG4D;ATP6AP2;ATRIP;AVPI1;B3GALT6;B3GAT3;B4GALT2;B4GALT7;BABAM1;BHLHE41;BRAF;BRF1;BRF2;BUB1B;C11orf30;C17orf70;C19orf10;C1orf86;C1QBP;C8G;CANX;CAPN10;CAPRIN1;CCDC101;CCNL2;
FBXW11;FBXW5;GNB2L1;HERC3;LSM2;LSM4;MAN1B1;MPG;MUTYH;MYLIP;NTHL1;OGG1;PATL1;PNKP;POLD1;PSMA7;PSMB4;PSMB5;PSMB7;PSMD9;RC3H1;RLIM;RNASEH2C;RPL13;RPL18;RPL18A;RPL28;RPL29;RPL38;RPL8;RPLP1;RPLP2;RPS15;RPS2;RPS28;RQCD1;STUB1;TLK2;TRIP12;UBA7;UBXN1;UPF2
;WNT7A;ZEB2
F1;CLSPN;DOK7;DUSP10;DUSP2;DUSP3;DVL1;EIF4G3;FNIP2;GNB2L1;HIPK3;HPX;HRAS;KIDINS220;LAMTOR2;LATS1;LATS2;LIME1;LPAR1;MAGI3;MAP3K10;MAP4K2;MAPK1;MAPK11;MLST8;NF1;NOG;NRP1;P2RX7;PDE6G;PIK3CB;PIK3CG;PIN1;PLA2G6;PREB;PRKAR2A;PTPRC;RBPMS;SENP2;SPRED2;SPTBN4;
;CDK10;CDK12;CDK13;CHD7;CHTF18;CLCN4;CLPP;CSNK1G1;DALRD3;DDX21;DDX28;DDX39A;DDX43;DDX49;DGUOK;DTYMK;DYNC1LI1;EIF2B2;ETNK2;FASTK;FICD;FIGNL2;FLAD1;FPGS;FUK;GLYCTK;HELZ;HIPK1;HIPK3;IRAK4;KHK;KIF15;KIF20A;KIF24;KIF7;LATS1;LATS2;LTK;MAGI3;MAP3K10;MAP4K2;MAPK

G4;PLEKHG5;PSD;RABGEF1;RALGAPB;RASA2;RGL1;RHPN1;SERGEF;SGSM3;SMAP1;SOS1;SPATA13;TBC1D10A;TBC1D10C;TBC1D17;USP6NL;VAV3

M1N;PPP2R3B;PPTC7;PTPRC;TIMM50;UBASH3E
ER;GGA1;IPO8;KRT18;LIME1;MAPK1;MED1;NDUFA13;NF1;NFKBIL1;OGG1;PEX16;PEX6;PICK1;PIP5K1A;RBPMS;RPL13;RPL18;RPL18A;RPL28;RPL29;RPL38;RPL8;RPLP1;RPLP2;RPS15;RPS2;RPS28;SCARB2;SEC24B;SLC25A6;SSR1;SSR4;STAM;STX1A;SYNGR1;SYTL1;TIMM10;TIMM13;TIMM50;TLR7;TPR;TRPS1;V
AR1;MLST8;NF1;PIN1;PIP5K1A;PLEKHG4;PLEKHG5;PSD;RAC3;RALGAPB;RASA2;RGL1;RHOC;RHOT2;SGSM3;SMAP1;SOS1;SPATA13;STMN3;TAGAP;TNK1;USP6NL;VAV3
SH3RF1;SLC25A5;TIMM50;TNFRSF25;TNFSF12;TP73;TRAF2;TRIB3
SH3RF1;SLC25A5;TIMM50;TNFRSF25;TNFSF12;TP73;TRAF2;TRIB3
;FICD;GNAI3;GNB2L1;GNG8;GNGT2;HRAS;MACROD1;MLST8;MRI1;MTG1;NF1;NME3;NME4;NT5C;NUDT1;NUDT18;PANK4;PAPSS1;PEX6;PICK1;PIN1;PIP5K1A;PXK;QTRT1;RAB21;RAB33A;RAC3;RALGAPB;RASA2;SMAP1;SOS1;STMN3;STOML2;SURF1;TNNT3;TPM2;UCK1;UCKL1;USP6NL;VAV

LI1;E2F1;E4F1;FANCG;FEM1B;GAS2L3;HBP1;HINFP;HRAS;LATS1;LATS2;MAD1L1;MCM7;MLL5;PIDD;PPP2R3B;PSMA7;PSMB4;PSMB5;PSMB7;PSMD9;RAD9A;SGSM3;TAOK3;TBRG4;TNFAIP3;TOP2A;TP73;TPR;ZBTB1

;FBXL15;FBXO2;FBXO44;FBXW11;FBXW5;GNB2L1;GYG1;HERC3;LSM2;LSM4;MAN1B1;MPG;MUTYH;MYLIP;NAGLU;NTHL1;OGG1;P2RX7;PATL1;PGM2;PHKB;PHKG1;PNKP;POLD1;PSMA7;PSMB4;PSMB5;PSMB7;PSMD9;RC3H1;RLIM;RNASEH2C;RPL13;RPL18;RPL18A;RPL28;RPL29;RPL38;RPL8;RPLP1;RPLP2;F

LGAP5;DYNC1LI1;E4F1;FSD1;HAUS8;KIF15;KIF20A;LATS1;LATS2;MAD1L1;NCAPH;NIPBL;NUSAP1;PIN1;RAD21;SAC3D1;STRA13;TADA3;TPR;TTN;TXNL4A;USP9X
2;TAOK3;TNFAIP3;TP73;TRIB3

RIN1;CCNL2;CD36;CDC25A;CDC6;CDK12;CLCF1;CLSPN;DOK7;DUSP10;DUSP2;DUSP3;DVL1;EIF1;EIF2B2;EIF2B4;EIF3K;EIF4G3;ETF1;FBXO2;FBXW11;FEM1B;FNIP2;GNB2L1;HIPK3;HPX;HRAS;KDM3A;KIDINS220;LAMTOR2;LARP4B;LATS1;LATS2;LIME1;LPAR1;LSM14A;MAGI3;MAP3K10;MAP4K2;MAPK1;MAPK

CLIP1;DLGAP5;DYNC1LI1;E4F1;FSD1;HAUS8;KIF15;LATS1;LATS2;MAD1L1;MTFP1;NCAPH;NIPBL;NUSAP1;PIN1;RAD21;SAC3D1;STRA13;TADA3;TPR;TTN;TXNL4A;USP9X
3;TNFAIP3;TP73;TRIB3
IP3;TP73;TRIB3
LGAP5;DYNC1LI1;E4F1;FSD1;HAUS8;KIF15;LATS1;LATS2;MAD1L1;NCAPH;NIPBL;NUSAP1;PIN1;RAD21;SAC3D1;STRA13;TADA3;TPR;TTN;TXNL4A;USP9X
LGAP5;DYNC1LI1;E4F1;FSD1;HAUS8;KIF15;LATS1;LATS2;MAD1L1;NCAPH;NIPBL;NUSAP1;PIN1;RAD21;SAC3D1;STRA13;TADA3;TPR;TTN;TXNL4A;USP9X

ERLIN2;ERP44;FADS3;FAF2;FAM69B;FKBP2;HSD17B12;JAGN1;JSRP1;KRTCAP2;LMF1;LMF2;MAN1B1;MIA3;MOGS;MPDU1;NECAB3;P4HTM;PANX1;PCYT2;PEMT;PEX16;PIGQ;POMT1;PPM1L;PREB;PTDSS2;PTPLB;RAB21;SEC22B;SEC24B;SLC37A4;SOAT1;SOAT2;SPPL2A;SPPL2B;SSR1;SSR4;STS;TLR7;TM7SF2;
SC5;FGD6;FICD;GNAI3;GNB2L1;GNG8;GNGT2;HRAS;ITPA;LSM2;LSM4;MLST8;MPG;MTG1;MUTYH;NF1;NT5C;NTHL1;NUDT1;NUDT18;OGG1;PATL1;PEX6;PICK1;PIN1;PIP5K1A;PNKP;POLD1;PXK;RAB21;RAB33A;RAC3;RALGAPB;RASA2;RC3H1;RNASEH2C;RPL13;RPL18;RPL18A;RPL28;RPL29;RPL38;RPL8;RPLP1
F1;SIGIRR;SP1;TCEB2;TNFAIP3;TOP2A;TRAF6;ZC3HAV1
NCG;FEM1B;GAS2L3;HBP1;HINFP;HRAS;MAD1L1;MCM7;MLL5;PIDD;PPP2R3B;PSMA7;PSMB4;PSMB5;PSMB7;PSMD9;RAD9A;SGSM3;TAOK3;TBRG4;TOP2A;TP73;TPI
D6;FICD;GNAI3;GNB2L1;GNG8;GNGT2;HRAS;ITPA;MLST8;MTG1;NF1;NME3;NME4;NUDT1;PEX6;PICK1;PIN1;PIP5K1A;RAB21;RAB33A;RAC3;RALGAPB;RASA2;SMAP1;SOS1;STMN3;STOML2;SURF1;UCK1;UCKL1;USP6NL;VAV
5;SEC24B;SEMA6A;STK25;TACC1;TMED9
N10;CCHCR1;CCS;CD36;CHMP4A;CHMP6;CPSF2;CREB3;DDX39A;ERP29;FAM21B;FBXW11;GFER;GGA1;GIMAP5;HOOK3;IPO8;KLHL12;KRT18;LIME1;LMF1;MAPK1;MED1;MTM1;NDUFA13;NF1;NFKBIL1;NSRP1;NUP153;OGG1;P2RX7;PEX16;PEX6;PICK1;PIK3C3;PIP5K1A;PREB;PTPRC;RBPMS;RHOT2;RPL13;RPL
MDEC1;ADAMTS13;ADAMTSL2;ADHFE1;ADNP;ADORA2A;ADPRHL2;AES;AFF1;AFTPH;AHCY;AHCYL1;AHSA2;AIP;AK1;AK4;ALKBH7;ALS2;AMDHD2;ANAPC11;ANAPC2;ANKMY1;ANKRD17;ANKRD23;ANKRD54;AP1G1;AP1G2;APOE;APOM;APPL2;APRT;ARFGEF1;ARFIP2;ARFRP1;ARHGAP4;ARHGEF25;ARHGEF2
7;NRP1;P2RX6;P2RX7;PMM1;RAC3;SLC1A3;SPTBN4;STRN;TMPRSS5;TOP1;TXN2;TXNRD1;UBXN1;VPS1

P2K;BRAF;BUB1;BUB1B;CDC6;CDK10;CDK12;CDK13;CHD7;CHTF18;CLCN4;CLPP;CSNK1G1;DALRD3;DDX21;DDX28;DDX39A;DDX43;DDX49;DGUOK;DRG2;DTYMK;DYNC1LI1;EEF1A2;EIF2B2;ETNK2;FASTK;FICD;FIGNL2;FLAD1;FPGS;FUK;GIMAP5;GLYCTK;GNAI3;GTPBP3;GTPBP6;HELZ;HIPK1;HIPK3;HRAS;IFT27
FICD;GNAI3;GNB2L1;GNG8;GNGT2;HRAS;ITPA;LSM2;LSM4;MLST8;MPG;MTG1;MUTYH;NF1;NT5C;NTHL1;NUDT1;NUDT18;OGG1;PATL1;PEX6;PICK1;PIN1;PIP5K1A;PNKP;POLD1;PXK;RAB21;RAB33A;RAC3;RALGAPB;RASA2;RC3H1;RNASEH2C;RPL13;RPL18;RPL18A;RPL28;RPL29;RPL38;RPL8;RPLP1;RPLP2;RP
FICD;GNAI3;GNB2L1;GNG8;GNGT2;HRAS;ITPA;LSM2;LSM4;MLST8;MPG;MTG1;MUTYH;NF1;NT5C;NTHL1;NUDT1;NUDT18;OGG1;PATL1;PEX6;PICK1;PIN1;PIP5K1A;PNKP;POLD1;PXK;RAB21;RAB33A;RAC3;RALGAPB;RASA2;RC3H1;RNASEH2C;RPL13;RPL18;RPL18A;RPL28;RPL29;RPL38;RPL8;RPLP1;RPLP2;RP
D;FBXW11;GNB2L1;HIPK3;HPX;HRAS;KIDINS220;LAMTOR2;LEMD2;LGALS1;LIME1;LPAR1;MAGI3;MAP3K10;MAP4K2;MAPK1;MAPK11;MC1R;NENF;NF1;P2RX7;PDE6G;PHLDA3;PIK3CB;PIK3CG;PIN1;PLA2G6;PLEKHG5;RHOC;S100A13;SERPINF2;SH3RF1;SLC35B2;SPRED2;TAOK3;TLR7;TMEM101;TNFAIP3;TNI
;H2AFJ;H2AFY2;INO80E;MAD1L1;MBD3;MBD5;MCM7;MCRS1;MKI67;MSH3;NCAPH;NCAPH2;NEIL1;NSMCE1;NUSAP1;OBFC1;PBX4;POLD1;RAD21;RAD9A;REC8;RFX3;RNF169;SETD2;SETD7;STRA13;SUDS3;SUV420H1;SWI5;SYCE1L;TELO2;THAP7;TOP1;TOP2A;TOP3B;TOPBP1;TP73;TPR;TRIM24;TTN;WHSC1
A2;COL6A1;COL6A2;COL9A2;CREB3;CREB3L4;CRELD2;CTSC;CYP1B1;CYP2E1;DERL3;DOLK;DPM3;DSE;DUS2L;ERGIC3;ERLIN2;ERP29;ERP44;FADS3;FAF2;FAM69B;FBXO2;FCRLB;FKBP2;GHDC;HSD17B12;JAGN1;JSRP1;KRTCAP2;LEPRE1;LEPREL2;LMF1;LMF2;MAL;MAN1B1;MIA3;MOGS;MPDU1;MR1;NCEH1;NI

ATP5G2;ATP5G3;ATP5H;CDS2;DCXR;DGUOK;DOLK;DPM3;DTYMK;ETNK2;FDXR;FGD6;FICD;FLAD1;GNAI3;GNB2L1;GNG8;GNGT2;GPX4;HRAS;ISYNA1;ITPA;MC1R;MLST8;MPG;MTG1;MTM1;MTMR1;MUTYH;MVK;NF1;NME3;NME4;NT5C;NTHL1;NUDT1;NUDT18;OGG1;PANK4;PAPSS1;PCYT2;PEMT;PEX6;PFK

PHPT1;PNKP;PPAP2C;PPM1D;PPM1J;PPM1L;PPM1N;PPP2R3B;PPTC7;PTPRC;TIMM50;UBASH3E
FICD;GNAI3;GNB2L1;GNG8;GNGT2;HRAS;ITPA;LSM2;LSM4;MLST8;MPG;MTG1;MUTYH;NF1;NT5C;NTHL1;NUDT1;NUDT18;OGG1;PATL1;PEX6;PICK1;PIN1;PIP5K1A;PNKP;POLD1;PXK;RAB21;RAB33A;RAC3;RALGAPB;RASA2;RC3H1;RNASEH2C;RPL13;RPL18;RPL18A;RPL28;RPL29;RPL38;RPL8;RPLP1;RPLP2;RP

F4;TRAF6;WNT7A;ZEB2

3;TP73

PVD1;GGA1;GOLPH3;IRAK4;IRF3;LAMTOR2;MAL;MAPK1;MTM1;NIPA2;PIK3C3;PLEKHF1;RAB21;RABEP2;RABEPK;RABGEF1;RPS6KC1;RUSC1;SNF8;SNX20;SPPL2A;SPPL2B;STAM;STAMBP;STS;TLR7;TMEM9;TRAF6;USP8;VPS16;VPS41;WASH3P;ZFYVE27;ZP
UV420H1;TPR
P2K;BRAF;BUB1;BUB1B;CDC6;CDK10;CDK12;CDK13;CHD7;CHTF18;CLCN4;CLPP;CSNK1G1;DALRD3;DDX21;DDX28;DDX39A;DDX43;DDX49;DGUOK;DRG2;DTYMK;DYNC1LI1;EEF1A2;EIF2B2;ETNK2;FASTK;FICD;FIGNL2;FLAD1;FPGS;FUK;GIMAP5;GLYCTK;GNAI3;GTPBP3;GTPBP6;HELZ;HIPK1;HIPK3;HRAS;IFT27

D3B;ATL3;ATP5D;BCS1L;BMP2K;BRAF;BUB1;BUB1B;CDC6;CDK10;CDK12;CDK13;CEL;CHD7;CHTF18;CLCN4;CLPP;CRYL1;CSNK1G1;CTSC;DALRD3;DDX21;DDX28;DDX39A;DDX43;DDX49;DGUOK;DRG2;DTYMK;DUS1L;DUS2L;DUS3L;DYNC1LI1;EEF1A2;EIF2B2;ETNK2;FASTK;FBLN7;FDXR;FICD;FIGNL2;FLAD1;FPG

P2K;BRAF;BUB1;BUB1B;CDC6;CDK10;CDK12;CDK13;CHD7;CHTF18;CLCN4;CLPP;CSNK1G1;DALRD3;DDX21;DDX28;DDX39A;DDX43;DDX49;DGUOK;DRG2;DTYMK;DYNC1LI1;EEF1A2;EIF2B2;ETNK2;FASTK;FICD;FIGNL2;FLAD1;FPGS;FUK;GIMAP5;GLYCTK;GNAI3;GTPBP3;GTPBP6;HELZ;HIPK1;HIPK3;HRAS;IFT27
LC22A17;SLC37A4;SLC39A13;SPPL2A;SPPL2B;ST3GAL3;ST6GALNAC
P2K;BRAF;BUB1;BUB1B;CDC6;CDK10;CDK12;CDK13;CHD7;CHTF18;CLCN4;CLPP;CSNK1G1;DALRD3;DDX21;DDX28;DDX39A;DDX43;DDX49;DGUOK;DRG2;DTYMK;DYNC1LI1;EEF1A2;EIF2B2;ETNK2;FASTK;FICD;FIGNL2;FLAD1;FPGS;FUK;GIMAP5;GLYCTK;GNAI3;GTPBP3;GTPBP6;HELZ;HIPK1;HIPK3;HRAS;IFT27
P2K;BRAF;BUB1;BUB1B;CDC6;CDK10;CDK12;CDK13;CHD7;CHTF18;CLCN4;CLPP;CSNK1G1;DALRD3;DDX21;DDX28;DDX39A;DDX43;DDX49;DGUOK;DRG2;DTYMK;DYNC1LI1;EEF1A2;EIF2B2;ETNK2;FASTK;FICD;FIGNL2;FLAD1;FPGS;FUK;GIMAP5;GLYCTK;GNAI3;GTPBP3;GTPBP6;HELZ;HIPK1;HIPK3;HRAS;IFT27
P2K;BRAF;BUB1;BUB1B;CDC6;CDK10;CDK12;CDK13;CHD7;CHTF18;CLCN4;CLPP;CSNK1G1;DALRD3;DDX21;DDX28;DDX39A;DDX43;DDX49;DGUOK;DRG2;DTYMK;DYNC1LI1;EEF1A2;EIF2B2;ETNK2;FASTK;FICD;FIGNL2;FLAD1;FPGS;FUK;GIMAP5;GLYCTK;GNAI3;GTPBP3;GTPBP6;HELZ;HIPK1;HIPK3;HRAS;IFT27

RAF;BUB1;BUB1B;CDC6;CDK10;CDK12;CDK13;CHD7;CHTF18;CLCN4;CLPP;CSNK1G1;DALRD3;DDX21;DDX28;DDX39A;DDX43;DDX49;DGUOK;DRG2;DTYMK;DYNC1LI1;EEF1A2;EIF2B2;ETNK2;FASTK;FICD;FIGNL2;FLAD1;FPGS;FUK;GIMAP5;GLYCTK;GNAI3;GTPBP3;GTPBP6;HELZ;HIPK1;HIPK3;HRAS;IFT27;IRAK

A4;SLC39A13;SPPL2A;SPPL2B;ST3GAL3;ST6GALNAC
TUT1

PVD1;IQGAP2;ITSN2;LAMTOR2;MAP4K2;NF1;PCP2;PLEKHG4;PLEKHG5;PSD;RABEP2;RABGEF1;RALGAPB;RASA2;RGL1;RHPN1;RINL;SERGEF;SGSM3;SH2D3A;SMAP1;SOS1;SPATA13;TAGAP;TBC1D10A;TBC1D10C;TBC1D17;USP6NL;VAV

FRP1;ARL16;ARNTL;ASH1L;ASXL2;ATAD3B;ATF4;ATL3;ATP5D;BCS1L;BHLHE41;BMP2K;BRAF;BUB1;BUB1B;C17orf49;C17orf70;C1QBP;CAPRIN1;CBR1;CD320;CD38;CDC6;CDK10;CDK12;CDK13;CELF2;CELF6;CENPT;CHD7;CHRAC1;CHTF18;CLCN4;CLIP1;CLPP;CLSPN;CPSF2;CPSF4;CREB3;CREB3L4;CRIP1;CRYL1;
NKRD23;ANKRD54;APPL2;APRT;ARFGEF1;ARHGAP4;ARL6IP4;ARNTL;ASH1L;ASXL2;ATF4;ATF7IP;ATRIP;AUP1;BABAM1;BHLHE41;BMP2K;BOK;BRAF;BRAT1;BRF1;BRF2;BUB1;BUB1B;C11orf30;C12orf10;C17orf49;C17orf70;C1orf86;C1QBP;CAPS;CCDC101;CCDC106;CCDC51;CCHCR1;CCNL2;CCS;CD38;CDC25
QGAP2;ITSN2;LAMTOR2;MAP4K2;NF1;PCP2;PLEKHG4;PLEKHG5;PSD;RABEP2;RABGEF1;RALGAPB;RASA2;RGL1;RHPN1;RINL;SERGEF;SGSM3;SH2D3A;SMAP1;SOS1;SPATA13;TAGAP;TBC1D10A;TBC1D10C;TBC1D17;USP6NL;VAV
P2K;BRAF;BUB1;BUB1B;CDC6;CDK10;CDK12;CDK13;CHD7;CHTF18;CLCN4;CLPP;CSNK1G1;DALRD3;DDX21;DDX28;DDX39A;DDX43;DDX49;DGUOK;DRG2;DTYMK;DYNC1LI1;EEF1A2;EIF2B2;ETNK2;FASTK;FICD;FIGNL2;FLAD1;FPGS;FUK;GIMAP5;GLYCTK;GNAI3;GTPBP3;GTPBP6;HELZ;HIPK1;HIPK3;HRAS;IFT27

TA;CYP2E1;DAAM1;DIAPH2;DOK7;DUSP2;DVL1;DYM;EHHADH;FAF2;FBXW5;FGD6;GCHFR;GNB2L1;GRASP;GRIN3A;HDAC10;HINFP;IPO8;KAT2A;KCNN4;KIF20A;LAMB1;LAT;LATS1;LIME1;LSM2;MAP4K2;MAPK1;MC1R;MLL5;MSH3;NDUFS7;NIPBL;NOXA1;NOXO1;PANX1;PFKFB2;PHKG1;PICK1;PIN1;PIP5K1A;
;ADD3;ADI1;ADRM1;AEBP1;AEBP2;AFF4;AGAP2;AGAP3;AGL;AGPAT5;AHR;AIM2;AIMP1;AIMP2;AKAP11;AKAP7;AKAP8L;AKIP1;AKIRIN1;AKR1B1;AKT1;AKTIP;ALG2;ALKBH4;ALKBH5;ALKBH8;ALS2CR8;ANAPC10;ANG;ANKLE1;ANKRD12;ANKRD28;ANKRD32;ANKRD34B;ANKRD49;ANLN;ANP32E;ANXA1;AP1A
BLIM2;ACAD10;ACAD9;ACADM;ACADSB;ACADVL;ACAT1;ACAT2;ACBD3;ACBD5;ACBD6;ACD;ACOT13;ACOT7;ACP2;ACSF2;ACSF3;ACSL4;ACSL6;ACSS1;ACTL6A;ACTN4;ADA;ADAM10;ADAM15;ADAMTS5;ADAP1;ADC;ADCK1;ADCK2;ADCK3;ADD3;ADI1;ADORA1;ADRM1;AEBP1;AEBP2;AFF4;AGAP2;AGAP3;AGL
BLIM2;ACAD10;ACAD9;ACADM;ACADSB;ACADVL;ACAT1;ACAT2;ACBD3;ACBD5;ACBD6;ACD;ACOT13;ACOT7;ACP2;ACSF2;ACSF3;ACSL4;ACSL6;ACSS1;ACTL6A;ACTN4;ADA;ADAM10;ADAM15;ADAMTS5;ADAP1;ADC;ADCK1;ADCK2;ADCK3;ADD3;ADI1;ADORA1;ADRM1;AEBP1;AEBP2;AFF4;AGAP2;AGAP3;AGL
BI2;ABLIM2;ACAD10;ACAD9;ACADM;ACADSB;ACADVL;ACAT1;ACAT2;ACBD3;ACBD5;ACBD6;ACD;ACOT13;ACOT7;ACP2;ACSF2;ACSF3;ACSL4;ACSL6;ACSS1;ACTL6A;ACTL9;ACTN4;ACTR1A;ACTR6;ADA;ADAM10;ADAM15;ADAMTS5;ADAP1;ADC;ADCK1;ADCK2;ADCK3;ADD3;ADI1;ADORA1;ADRM1;AEBP1;AEB
BI2;ABLIM2;ACAD10;ACAD9;ACADM;ACADSB;ACADVL;ACAT1;ACAT2;ACBD3;ACBD5;ACBD6;ACD;ACOT13;ACOT7;ACP2;ACSF2;ACSF3;ACSL4;ACSL6;ACSS1;ACTL6A;ACTL9;ACTN4;ACTR1A;ACTR6;ADA;ADAM10;ADAM15;ADAMTS5;ADAP1;ADC;ADCK1;ADCK2;ADCK3;ADD3;ADI1;ADORA1;ADRM1;AEBP1;AEB
BCE1;ABHD10;ABHD14B;ABI1;ABI2;ABI3;ABLIM2;ABR;ACAD10;ACAD9;ACADM;ACADSB;ACADVL;ACAT1;ACAT2;ACBD3;ACBD5;ACBD6;ACD;ACOT13;ACOT7;ACP2;ACSF2;ACSF3;ACSL4;ACSL6;ACSS1;ACTL6A;ACTL9;ACTN4;ACTR1A;ACTR6;ACVR2A;ACY3;ADA;ADAM10;ADAM15;ADAMTS5;ADAP1;ADC;ADCK
BCE1;ABHD10;ABHD14B;ABI1;ABI2;ABI3;ABLIM2;ABR;ACAD10;ACAD9;ACADM;ACADSB;ACADVL;ACAT1;ACAT2;ACBD3;ACBD5;ACBD6;ACOT13;ACOT7;ACP2;ACSF2;ACSF3;ACSL4;ACSL6;ACSS1;ACTL9;ACTN4;ACTR1A;ACTR6;ACVR2A;ACY3;ADA;ADAM10;ADAM15;ADAMTS5;ADAP1;ADC;ADCK1;ADCK2;ADC
BCE1;ABHD10;ABHD14B;ABI1;ABI2;ABI3;ABLIM2;ABR;ACAD10;ACAD9;ACADM;ACADSB;ACADVL;ACAT1;ACAT2;ACBD3;ACBD5;ACBD6;ACD;ACOT13;ACOT7;ACP2;ACSF2;ACSF3;ACSL4;ACSL6;ACSS1;ACTL6A;ACTL9;ACTN4;ACTR1A;ACTR6;ACVR2A;ACY3;ADA;ADAM10;ADAM15;ADAMTS5;ADAP1;ADC;ADCK
BCD3;ABCE1;ABHD10;ABHD14B;ABI1;ABI2;ABI3;ABLIM2;ABR;ACAD10;ACAD9;ACADM;ACADSB;ACADVL;ACAT1;ACAT2;ACBD3;ACBD5;ACBD6;ACD;ACOT13;ACOT7;ACP2;ACSF2;ACSF3;ACSL4;ACSL6;ACSS1;ACTL6A;ACTL9;ACTN4;ACTR1A;ACTR6;ACVR1C;ACVR2A;ACVRL1;ACY3;ADA;ADAM10;ADAM15;ADA
BCD3;ABCE1;ABHD10;ABHD14B;ABI1;ABI2;ABI3;ABLIM2;ABR;ACAD10;ACAD9;ACADM;ACADSB;ACADVL;ACAT1;ACAT2;ACBD3;ACBD5;ACBD6;ACD;ACOT13;ACOT7;ACP2;ACSF2;ACSF3;ACSL4;ACSL6;ACSS1;ACTL6A;ACTL9;ACTN4;ACTR1A;ACTR6;ACVR1C;ACVR2A;ACVRL1;ACY3;ADA;ADAM10;ADAM15;ADA
0;ABI1;ABI2;ABR;ACAD10;ACAD9;ACADM;ACADSB;ACADVL;ACAT1;ACAT2;ACBD3;ACBD5;ACOT13;ACOT7;ACP2;ACSF2;ACSF3;ACSL4;ACSL6;ACSS1;ACTN4;ACTR1A;ADA;ADAM10;ADAM15;ADC;ADCK1;ADCK2;ADCK3;ADD3;ADI1;ADORA1;ADRBK1;ADSSL1;AFF4;AGAP2;AGBL3;AGGF1;AGL;AGPAT5;AGPS;AH
T1;ACAT2;ACBD3;ACBD5;ACD;ACP2;ACSL4;ACSL6;ACSS1;ACTL6A;ACTN4;ACTR1A;ADA;ADAM10;ADD3;ADORA1;AEBP2;AFF4;AGAP2;AGPAT5;AGPS;AHR;AIFM3;AK3;AKAP11;AKAP8L;AKIRIN1;AKT1;AKTIP;ALDH4A1;ALDOA;ALG1;ALG10;ALG11;ALG13;ALG2;ALG3;ALG6;ALKBH5;ALS2CR8;ANAPC10;ANG;ANK
DORA1;ADRBK1;ADRM1;AEBP1;AEBP2;AFF4;AGAP3;AGER;AGGF1;AGL;AHR;AIDA;AIM2;AIMP1;AIMP2;AKAP7;AKT1;AKTIP;ALG1;ALG10;ALG10B;ALG11;ALG13;ALG2;ALG3;ALG6;ALKBH5;ALKBH8;ANAPC10;ANG;ANGPT1;ANKRD13C;ANKRD49;ANXA1;APC;APEX1;APEX2;APH1A;APLF;APOBEC3D;APTX;ARAF;
T1;ACAT2;ACBD3;ACBD5;ACD;ACP2;ACSL4;ACSL6;ACSS1;ACTL6A;ACTN4;ACTR1A;ADA;ADAM10;ADD3;ADORA1;AEBP2;AFF4;AGAP2;AGPAT5;AGPS;AHR;AIFM3;AK3;AKAP11;AKAP8L;AKIRIN1;AKT1;AKTIP;ALDH4A1;ALDOA;ALG1;ALG10;ALG11;ALG13;ALG2;ALG3;ALG6;ALKBH5;ALS2CR8;ANAPC10;ANG;ANK
CADVL;ACAP1;ACAP2;ACAT1;ACD;ACOT7;ACSF2;ACSF3;ACSL4;ACSL6;ACSS1;ACTL6A;ACVR1C;ACVR2A;ACVRL1;ADA;ADAM10;ADAP1;ADC;ADI1;ADM2;ADORA1;ADRBK1;ADRM1;ADSSL1;AEBP1;AEBP2;AFF4;AGAP2;AGAP3;AGER;AGFG2;AGGF1;AGL;AGPAT5;AGPS;AHR;AIDA;AIFM3;AIM2;AIMP1;AIMP2;AK
P3;AIDA;AIMP1;AIMP2;AKT1;AKTIP;ALG1;ALG10;ALG10B;ALG11;ALG13;ALG2;ALG3;ALG6;ANAPC10;ANG;ANGPT1;ANXA1;APC;APH1A;ARAF;ARFGAP1;ARID5A;ARRB1;ARSA;ARSK;ASB13;ASB7;ASPH;ATG5;ATP7A;ATPBD4;ATXN7L3;AURKC;B2M;B3GALNT1;B3GALT2;B3GALTL;B3GNT1;B3GNT2;B3GNT5;B4
CADVL;ACAP1;ACAP2;ACAT1;ACAT2;ACBD3;ACD;ACOT7;ACSF2;ACSF3;ACSL4;ACSL6;ACSS1;ACTL6A;ACVR1C;ACVR2A;ACVRL1;ADA;ADAM10;ADAM15;ADAM22;ADAMTS5;ADAMTS6;ADAP1;ADC;ADI1;ADM2;ADORA1;ADRBK1;ADRM1;ADSSL1;AEBP1;AEBP2;AFF4;AGAP2;AGAP3;AGBL3;AGER;AGFG2;AGGF1
10;ADAM15;ADAM22;ADAMTS5;ADAMTS6;ADC;ADI1;ADM2;ADORA1;ADRBK1;ADRM1;AEBP1;AEBP2;AFF4;AGAP2;AGAP3;AGBL3;AGER;AGGF1;AGL;AHR;AIDA;AIM2;AIMP1;AIMP2;AKAP7;AKT1;AKTIP;ALG1;ALG10;ALG10B;ALG11;ALG13;ALG2;ALG3;ALG6;ALKBH5;ALKBH8;AMD1;ANAPC10;ANG;ANGPT1;
DSB;ACADVL;ACAP1;ACAP2;ACAT1;ACAT2;ACBD3;ACD;ACOT7;ACSF2;ACSF3;ACSL4;ACSL6;ACSS1;ACTL6A;ACVR1C;ACVR2A;ACVRL1;ADA;ADAM10;ADAM15;ADAM22;ADAMTS5;ADAMTS6;ADAP1;ADC;ADI1;ADM2;ADORA1;ADRBK1;ADRM1;ADSSL1;AEBP1;AEBP2;AFF4;AGAP2;AGAP3;AGBL3;AGER;AGFG2
;ABCE1;ABCF3;ABI1;ABI2;ABI3;ABLIM2;ABR;ACAD10;ACAD9;ACADM;ACADSB;ACADVL;ACAP1;ACAP2;ACAT1;ACBD3;ACBD5;ACBD6;ACD;ACOT7;ACSF2;ACSF3;ACSL4;ACSL6;ACSS1;ACTL6A;ACTN4;ACTR1A;ACVR1C;ACVR2A;ACVRL1;ACY3;ADA;ADAM10;ADAM15;ADAM22;ADAMTS5;ADAMTS6;ADAP1;ADC
1;AKTIP;ALKBH5;ALS2CR8;ANAPC10;ANG;ANKLE1;ANKRD28;AP1AR;APEH;APEX1;API5;APPL1;APTX;ARFGAP1;ARFGAP2;ARID4A;ARID5A;ARL4A;ASNA1;ATF1;ATF2;ATP11B;ATPAF2;ATRX;ATXN7L3;BANF1;BAP1;BAZ1A;BCAS2;BCLAF1;BDP1;BMI1;BNIP1;BNIP2;BNIP3L;BRCA2;BRCC3;BRIP1;BRWD1;BTD;BUD
DSB;ACADVL;ACAP1;ACAP2;ACAT1;ACAT2;ACBD3;ACD;ACOT7;ACSF2;ACSF3;ACSL4;ACSL6;ACSS1;ACTL6A;ACVR1C;ACVR2A;ACVRL1;ADA;ADAM10;ADAM15;ADAM22;ADAMTS5;ADAMTS6;ADAP1;ADC;ADI1;ADM2;ADORA1;ADRBK1;ADRM1;ADSSL1;AEBP1;AEBP2;AFF4;AGAP2;AGAP3;AGBL3;AGER;AGFG2

;AGAP3;AGBL3;AGFG2;AKAP8L;ANKIB1;ANKMY2;APEX2;APOBEC3D;APTX;ARAP2;ARFGAP1;ARFGAP2;ARHGEF2;ARID2;ASAP3;ASTL;ATF2;ATRX;BAZ1A;BAZ2B;BIRC2;BIRC3;BMI1;BMP1;C11orf54;C1orf27;C4orf21;CA12;CA5BP1;CBLL1;CDADC1;CGRRF1;CHD5;CHFR;CHMP1A;CHORDC1;CHURC1;CNBP;CPD;C

L4;ACSL6;AGPAT5;AGPS;AIFM3;AKIRIN1;AKTIP;ALG1;ALG10;ALG11;ALG13;ALG2;ALG3;ALG6;ANKRD13C;ANO5;ANXA1;AP1B1;AP1S2;AP2A2;AP3S2;APEH;APEX2;APH1A;APPBP2;APPL1;ARFGAP1;ARFGAP2;ARFGEF2;ARFIP1;ARL6IP1;ARL6IP5;ARL8B;ARMC10;ARRB1;ASPH;ATAD1;ATAD3A;ATG9A;ATL2;ATP

DERL1;DNAJB2;DNAJB9;DNAJC10;EDEM3;ERCC8;ERLEC1;ERLIN1;FBXL3;FBXL4;FBXL5;FBXO11;FBXO31;FBXO4;FBXO45;FBXO5;FBXO8;FBXW4;FBXW7;GIPC1;HACE1;HDAC6;HECTD2;HECTD3;HECW2;HERC4;HSPBP1;ITCH;JKAMP;KLHL20;LNX1;LONP1;LRRK2;MAD2L1;MDM2;NDFIP1;NEDD4;NPLOC4;PARK2;P

;CUL5;DERL1;DNAJB2;DNAJB9;DNAJC10;EDEM3;ERCC8;ERLEC1;ERLIN1;FBXL3;FBXL4;FBXL5;FBXO11;FBXO31;FBXO4;FBXO45;FBXO5;FBXO8;FBXW4;FBXW7;GIPC1;HACE1;HDAC6;HECTD2;HECTD3;HECW2;HERC4;HSPBP1;IDE;ITCH;JKAMP;KEAP1;KLHL20;LNX1;LONP1;LRRK2;MAD2L1;MDM2;NDFIP1;NEDD4

AP2;AGPS;AHR;AK3;AKAP11;AKAP8L;AKT1;AKTIP;ALDH4A1;ALKBH5;ALS2CR8;ANAPC10;ANG;ANKRD28;AP1AR;APEX1;APPL1;APTX;ARFGAP2;ARG2;ARID4A;ARID5A;ARL4A;ARSA;ARSK;ASNA1;ATAD3A;ATF1;ATF2;ATP5C1;ATP5E;ATP5F1;ATPAF2;ATRX;ATXN7L3;AUH;B2M;BANF1;BAP1;BAZ1A;BCAS2;BCAT2;

DERL1;DNAJB2;DNAJB9;DNAJC10;EDEM3;ERCC8;ERLEC1;ERLIN1;FBXL3;FBXL4;FBXL5;FBXO11;FBXO31;FBXO4;FBXO45;FBXO5;FBXO8;FBXW4;FBXW7;GIPC1;HACE1;HDAC6;HECTD2;HECTD3;HECW2;HERC4;HSPBP1;ITCH;JKAMP;KLHL20;LNX1;LRRK2;MAD2L1;MDM2;NDFIP1;NEDD4;NPLOC4;PARK2;PCNP;PP

K1;CDK2;CDK7;CHFR;CLN6;CNOT2;CNOT3;CNOT6;CNOT6L;CNOT7;CNOT8;CRBN;CST3;CUL2;CUL4B;CUL5;CYCS;DCP1A;DCP2;DCPS;DDB2;DERL1;DIS3;DNA2;DNAJB2;DNAJB9;DNAJC10;DNASE1L1;DNASE2;EDEM3;EIF3E;EIF4E;ENDOG;ERCC2;ERCC8;ERLEC1;ERLIN1;FBXL3;FBXL4;FBXL5;FBXO11;FBXO31;FBXC

DERL1;DNAJB2;DNAJB9;DNAJC10;EDEM3;ERCC8;ERLEC1;ERLIN1;FBXL3;FBXL4;FBXL5;FBXO11;FBXO31;FBXO4;FBXO45;FBXO5;FBXO8;FBXW4;FBXW7;GIPC1;HACE1;HDAC6;HECTD2;HECTD3;HECW2;HERC4;HSPBP1;ITCH;JKAMP;KLHL20;LNX1;LONP1;LRRK2;MAD2L1;MDM2;NDFIP1;NEDD4;NPLOC4;PARK2;P

P2;AFF4;AGAP2;AGPS;AHR;AK3;AKAP11;AKAP8L;AKT1;AKTIP;ALDH4A1;ALDOA;ALKBH5;ALS2CR8;ANAPC10;ANG;ANKRD28;AP1AR;APEX1;APPL1;APTX;ARFGAP2;ARG2;ARID4A;ARID5A;ARL4A;ARSA;ARSK;ASNA1;ATAD3A;ATF1;ATF2;ATP5C1;ATP5E;ATP5F1;ATPAF2;ATRX;ATXN7L3;AUH;B2M;BANF1;BAP1;B

;CDC26;CDC27;CDC73;CDK1;CDK2;CHFR;CISH;COPS2;COPS4;COPS5;COPS6;CRBN;CUL2;CUL4B;CUL5;DAXX;DCAF10;DCAF11;DCAF13;DDB2;DNAJB2;DTX2;DZIP3;ENY2;ERCC8;FANCL;FBXL3;FBXL5;FBXO11;FBXO3;FBXO31;FBXO33;FBXO4;FBXO45;FBXO5;FBXW7;FEM1A;FEM1C;G2E3;GPS1;HACE1;HDAC6;HE

2;CUL4B;CUL5;DERL1;DNAJB2;DNAJB9;DNAJC10;EDEM3;ERCC8;ERLEC1;ERLIN1;FBXL3;FBXL4;FBXL5;FBXO11;FBXO31;FBXO4;FBXO45;FBXO5;FBXO8;FBXW4;FBXW7;GIPC1;HACE1;HDAC6;HECTD2;HECTD3;HECW2;HERC4;HSPBP1;IDE;ITCH;JKAMP;KEAP1;KLHL20;LNX1;LONP1;LRRK2;MAD2L1;MDM2;NDFIP1

P2;AFF4;AGAP2;AGPS;AHR;AK3;AKAP11;AKAP8L;AKT1;AKTIP;ALDH4A1;ALDOA;ALKBH5;ALS2CR8;ANAPC10;ANG;ANKRD28;AP1AR;APEX1;APPL1;APTX;ARFGAP2;ARG2;ARID4A;ARID5A;ARL2;ARL4A;ARSA;ARSK;ASNA1;ATAD3A;ATF1;ATF2;ATP5C1;ATP5E;ATP5F1;ATPAF2;ATRX;ATXN7L3;AUH;B2M;BANF1;B

AURKC;AZI1;BACH1;BAG6;BAP1;BCAT1;BCR;BIRC2;BORA;BRCA2;BRCC3;BRIP1;CAB39;CAB39L;CAMK1;CAMK2D;CASC5;CASP3;CASP8AP2;CCNA2;CCNC;CCND3;CCNE2;CCNG1;CCNG2;CCNH;CCNL1;CCNT2;CCNY;CCNYL1;CCP110;CCPG1;CD28;CD2AP;CDC14A;CDC25C;CDC26;CDC27;CDC37;CDC7;CDC73;CDK

A;AIMP2;AKT1;AKTIP;ALG1;ALG10;ALG10B;ALG11;ALG13;ALG2;ALG3;ALG6;ANAPC10;ANG;ANGPT1;ANXA1;APC;ARAF;ARFGAP1;ARID5A;ARRB1;ARSA;ARSK;ASB13;ASB7;ASPH;ATG5;ATP7A;ATPBD4;ATXN7L3;AURKC;B3GALNT1;B3GALT2;B3GALTL;B3GNT1;B3GNT2;B3GNT5;B4GALT3;B4GALT6;BAG4;BAG6

A;AIMP2;AKT1;AKTIP;ALG1;ALG10;ALG10B;ALG11;ALG13;ALG2;ALG3;ALG6;ANAPC10;ANG;ANGPT1;ANXA1;APC;ARAF;ARFGAP1;ARID5A;ARRB1;ARSA;ARSK;ASB13;ASB7;ASPH;ATG5;ATP7A;ATPBD4;ATXN7L3;AURKC;B3GALNT1;B3GALT2;B3GALTL;B3GNT1;B3GNT2;B3GNT5;B4GALT3;B4GALT6;BAG4;BAG6

;ABCE1;ABCF3;ABLIM2;ACAD10;ACAD9;ACADM;ACADSB;ACADVL;ACAP1;ACAP2;ACAT1;ACBD3;ACBD5;ACBD6;ACOT7;ACSF2;ACSF3;ACSL4;ACSL6;ACSS1;ACTL6A;ACTN4;ACTR1A;ACVR1C;ACVR2A;ACVRL1;ACY3;ADA;ADAM10;ADAM15;ADAM22;ADAMTS5;ADAMTS6;ADAP1;ADCK1;ADCK2;ADCK3;ADD3;A

8;ANAPC10;ANG;ANKRD28;AP1AR;APEX1;APPL1;APTX;ARFGAP2;ARID4A;ARID5A;ARL4A;ASNA1;ATF1;ATF2;ATPAF2;ATRX;ATXN7L3;BANF1;BAP1;BAZ1A;BCAS2;BCLAF1;BDP1;BMI1;BRCA2;BRWD1;BTD;BYSL;C8orf40;CAMK2D;CAMK4;CAPG;CASC5;CASP3;CBX3;CCDC86;CCNA2;CCNC;CCNE2;CCNH;CCNL1;C

A;AIMP2;AKT1;AKTIP;ALG1;ALG10;ALG10B;ALG11;ALG13;ALG2;ALG3;ALG6;ALKBH5;ALKBH8;ANAPC10;ANG;ANGPT1;ANXA1;APC;APEX1;APOBEC3D;ARAF;ARFGAP1;ARID5A;ARRB1;ARSA;ARSK;ASB13;ASB7;ASPH;ATG5;ATP7A;ATPBD4;ATRX;ATXN7L3;AURKC;B3GALNT1;B3GALT2;B3GALTL;B3GNT1;B3GNT

BK1;ADSSL1;AGBL3;AGL;AHR;AIM2;AIMP1;AIMP2;AKAP5;AKAP7;AKR1B1;AKT1;ALDOA;ALKBH8;AMD1;ANAPC10;AP1B1;AP1S2;AP2A2;APC;APIP;APLF;APPL1;AQP11;ARAP2;ARFGAP1;ARFGEF2;ARFIP1;ARHGAP10;ARHGAP11A;ARHGAP12;ARHGAP22;ARHGAP5;ARHGDIA;ARHGEF2;ARL6IP1;ARRB1;ATG4C;

3;ABCE1;ABCF3;ABHD10;ABHD12;ABHD13;ABHD14B;ABHD3;ACAD10;ACAD9;ACADM;ACADSB;ACADVL;ACAT1;ACAT2;ACOT13;ACOT7;ACP2;ACPL2;ACSF2;ACSF3;ACSL4;ACSL6;ACSS1;ACVR1C;ACVR2A;ACVRL1;ACY3;ADA;ADAM10;ADAM15;ADAM22;ADAMTS5;ADAMTS6;ADC;ADCK1;ADCK2;ADCK3;ADI1;

;AKT1;ALKBH5;ALKBH8;ANG;ANKRD49;APEX1;ARGLU1;ARHGAP22;ARHGEF2;ARID2;ARID4A;ARID4B;ARID5A;ARID5B;ARRB1;ASF1A;ASPH;ATAD2;ATF1;ATF2;ATRX;ATXN7L3;AUH;BACH1;BAZ1A;BAZ2B;BBX;BCAS2;BCLAF1;BDP1;BIRC2;BLOC1S2;BLZF1;BMI1;BMPR1A;BMPR2;BRCA2;BRD9;BRIP1;BRMS1;BRN

I2;ACAD10;ACAD9;ACADM;ACADSB;ACADVL;ACD;ACSF2;ACSF3;ACSL4;ACSL6;ACSS1;ACTL6A;ACTN4;ACTR1A;ACVR1C;ACVR2A;ACVRL1;ADA;ADCK1;ADCK2;ADCK3;ADORA1;ADRBK1;ADSSL1;AEBP1;AEBP2;AGAP2;AGAP3;AGGF1;AGPS;AHR;AIFM3;AIM2;AIMP1;AK3;AKAP7;AKAP8L;AKD1;AKT1;ALKBH8;ANG

AP7;AKT1;ALKBH5;ALKBH8;ANG;ANKRD49;APEX1;APEX2;APLF;APOBEC3D;APTX;ARGLU1;ARHGAP22;ARHGEF2;ARID2;ARID4A;ARID4B;ARID5A;ARID5B;ARRB1;ASF1A;ASPH;ASTE1;ATAD2;ATF1;ATF2;ATRX;ATXN7L3;AUH;BACH1;BANF1;BAZ1A;BAZ2B;BBX;BCAS2;BCLAF1;BDP1;BIRC2;BLOC1S2;BLZF1;BMI1;

10;EDEM3;ERCC8;ERLEC1;ERLIN1;FBXL3;FBXL5;FBXO31;FBXO4;FBXO45;FBXO5;FBXW7;GIPC1;HECTD3;HSPBP1;JKAMP;KEAP1;KLHL20;LRRK2;MAD2L1;MDM2;NPLOC4;PCNP;PMAIP1;PPP2CB;PSMA1;PSMA2;PSMA3;PSMA4;PSMA6;PSMA8;PSMB1;PSMB3;PSMC1;PSMC2;PSMC3;PSMC5;PSMC6;PSMD10;PS

;AGAP3;AGBL3;AGFG2;AKAP8L;ANG;ANKIB1;ANKMY2;APEX2;APOBEC3D;APTX;ARAP2;ARFGAP1;ARFGAP2;ARHGEF2;ARID2;ASAP3;ASTL;ATF2;ATP2C1;ATP7A;ATRX;BAZ1A;BAZ2B;BIRC2;BIRC3;BMI1;BMP1;C11orf54;C1orf27;C4orf21;CA12;CA5BP1;CBLL1;CDADC1;CDIPT;CGRRF1;CHD5;CHFR;CHMP1A;CHO

I2;ACAD10;ACAD9;ACADM;ACADSB;ACADVL;ACD;ACSF2;ACSF3;ACSL4;ACSL6;ACSS1;ACTL6A;ACTN4;ACTR1A;ACVR1C;ACVR2A;ACVRL1;ADA;ADCK1;ADCK2;ADCK3;ADORA1;ADRBK1;ADSSL1;AEBP1;AEBP2;AGAP2;AGAP3;AGGF1;AGPS;AHR;AIFM3;AIM2;AIMP1;AK3;AKAP7;AKAP8L;AKD1;AKT1;ALKBH8;ANG

CDK1;CDK2;CHFR;CISH;CRBN;CUL2;CUL4B;CUL5;DAXX;DCAF10;DCAF11;DCAF13;DDB2;DNAJB2;DTX2;DZIP3;ERCC8;FANCL;FBXL3;FBXL5;FBXO11;FBXO3;FBXO31;FBXO33;FBXO4;FBXO45;FBXO5;FBXW7;FEM1A;FEM1C;G2E3;HACE1;HDAC6;HECTD2;HECTD3;HECW2;HERC4;HLTF;HSPBP1;ITCH;KCTD11;KEAP1

D;ACTL6A;ACVR2A;ACVRL1;ADA;ADAP1;ADC;ADI1;ADRM1;ADSSL1;AEBP1;AEBP2;AFF4;AGAP2;AGAP3;AGER;AGFG2;AGGF1;AHR;AIM2;AIMP1;AIMP2;AK3;AKAP5;AKAP7;AKD1;AKT1;ALDH4A1;ALDOA;ALKBH5;ALKBH8;AMD1;ANG;ANKRD49;APEX1;APEX2;APIP;APLF;APOBEC3D;APTX;ARAP2;ARF4;ARFGAP1

DAM22;ADAMTS5;ADAMTS6;ADAP1;ADD3;ADI1;ADSSL1;AEBP1;AEBP2;AGAP2;AGAP3;AGBL3;AGFG2;AIFM3;AKAP8L;ALKBH4;ALKBH5;ALKBH8;ANG;ANKIB1;ANKMY2;ANXA1;APEX1;APEX2;APIP;APLF;APOBEC3D;APTX;ARAF;ARAP2;ARFGAP1;ARFGAP2;ARG2;ARHGEF2;ARID2;ARMC1;ARSA;ARSK;ASAP3;AS

A;ADC;ADI1;ADRM1;ADSSL1;AEBP1;AEBP2;AFF4;AGER;AGL;AGPAT5;AHR;AIM2;AIMP1;AIMP2;AKAP5;AKR1B1;AKT1;ALDH4A1;ALDOA;ALG1;ALG10;ALG10B;ALG11;ALG13;ALG2;ALG3;ALG6;AMD1;ANG;ANGPT1;ANKRD49;ANXA1;APEX1;APIP;ARG2;ARGLU1;ARHGAP22;ARHGEF2;ARID2;ARID4A;ARID4B;AR

10;EDEM3;ERCC8;ERLEC1;ERLIN1;FBXL3;FBXL5;FBXO31;FBXO4;FBXO45;FBXO5;FBXW7;GIPC1;HECTD3;HSPBP1;JKAMP;KLHL20;LRRK2;MAD2L1;MDM2;NPLOC4;PCNP;PPP2CB;PSMA1;PSMA2;PSMA3;PSMA4;PSMA6;PSMA8;PSMB1;PSMB3;PSMC1;PSMC2;PSMC3;PSMC5;PSMC6;PSMD10;PSMD13;PSMD14

LKBH5;ALKBH8;ANG;ANKRD49;APEX1;APH1A;APTX;ARGLU1;ARHGAP22;ARHGEF2;ARID2;ARID4A;ARID4B;ARID5A;ARID5B;ARRB1;ASF1A;ASGR2;ASPH;ASTL;ATAD2;ATF1;ATF2;ATP7A;ATRX;ATXN7L3;BACH1;BAG6;BAZ1A;BAZ2B;BBX;BCAS2;BCLAF1;BDP1;BIRC2;BLOC1S2;BLZF1;BMI1;BMPR1A;BMPR2;BRC

CDK1;CDK2;CHFR;CISH;CRBN;CUL2;CUL4B;CUL5;DAXX;DCAF10;DCAF11;DCAF13;DDB2;DNAJB2;DTX2;DZIP3;ERCC8;FANCL;FBXL3;FBXL5;FBXO11;FBXO3;FBXO31;FBXO33;FBXO4;FBXO45;FBXO5;FBXW7;FEM1A;FEM1C;G2E3;HACE1;HDAC6;HECTD2;HECTD3;HECW2;HERC4;HLTF;HSPBP1;ITCH;KCTD11;KEAP1

IP2;AKT1;ALG1;ALG10;ALG10B;ALG11;ALG13;ALG2;ALG3;ALG6;ANG;ANKRD49;APEX1;ARGLU1;ARHGAP22;ARHGEF2;ARID2;ARID4A;ARID4B;ARID5A;ARID5B;ARRB1;ASF1A;ASPH;ATAD2;ATF1;ATF2;ATP7A;ATRX;ATXN7L3;B3GALNT1;B3GALT2;B3GALTL;B3GNT1;B3GNT2;B3GNT5;B4GALT3;B4GALT6;BACH1

N1B;CHFR;CLN3;CLN5;CLN6;CRBN;CST3;CUL2;CUL4B;CUL5;DERL1;DNAJB2;DNAJB9;DNAJC10;EDEM3;ERCC8;ERLEC1;ERLIN1;FBXL3;FBXL4;FBXL5;FBXO11;FBXO31;FBXO4;FBXO45;FBXO5;FBXO8;FBXW4;FBXW7;GIPC1;GPC1;HACE1;HDAC6;HECTD2;HECTD3;HECW2;HERC4;HGS;HSPBP1;IDE;ITCH;JKAMP;KEA

AIM2;AIMP1;AIMP2;AKT1;ALG1;ALG10;ALG10B;ALG11;ALG13;ALG2;ALG3;ALG6;AMD1;ANG;ANGPT1;ANKRD13C;ANKRD49;APEX1;ARGLU1;ARHGAP22;ARHGEF2;ARID2;ARID4A;ARID4B;ARID5A;ARID5B;ARRB1;ASF1A;ASPH;ATAD2;ATF1;ATF2;ATP7A;ATRX;ATXN7L3;AZIN1;B3GALNT1;B3GALT2;B3GALTL;B3

SSL1;AEBP1;AEBP2;AFF4;AGAP2;AGAP3;AGER;AGFG2;AGGF1;AHR;AIM2;AIMP1;AIMP2;AK3;AKAP5;AKAP7;AKD1;AKT1;ALDOA;ALKBH5;ALKBH8;AMD1;ANG;ANKRD49;APEX1;APEX2;APLF;APOBEC3D;APTX;ARAP2;ARF4;ARFGAP1;ARFGAP2;ARGLU1;ARHGAP22;ARHGAP5;ARHGEF2;ARID2;ARID4A;ARID4B;A

AT1;ACD;ACSL6;ACSS1;ACTL6A;ACTN4;ACVR1C;ACVR2A;ACVRL1;ACY3;ADA;ADAM10;ADAM15;ADAM22;ADAMTS5;ADAP1;ADD3;ADI1;ADM2;ADORA1;ADRBK1;ADRM1;AEBP1;AFF4;AGAP3;AGER;AGGF1;AGL;AHR;AIDA;AIM2;AIMP1;AIMP2;AKAP11;AKAP5;AKAP7;AKAP8L;AKIP1;AKT1;AKTIP;ALCAM;ALDH

2;C14orf166;CASP8;CC2D1A;CCDC14;CCDC92;CCP110;CDC14A;CDC27;CDK1;CDK2;CDKL2;CEP120;CEP135;CEP152;CEP170;CEP19;CEP250;CEP290;CEP350;CEP44;CEP57;CEP57L1;CEP70;CEP95;CEP97;CETN3;CHMP1A;CKAP2;CLIC4;CNTROB;CSK;CSNK1A1;DAPK3;DCTN2;DCTN4;DCTN6;DYNC2LI1;DYNLRB1;

;ACVRL1;ADA;ADC;ADI1;ADRM1;ADSSL1;AEBP1;AEBP2;AFF4;AGER;AGL;AGPAT5;AGPS;AHR;AIM2;AIMP1;AIMP2;AKAP5;AKR1B1;AKT1;ALDH4A1;ALDOA;ALG1;ALG10;ALG10B;ALG11;ALG13;ALG2;ALG3;ALG6;AMD1;ANG;ANGPT1;ANKRD13C;ANKRD49;ANXA1;APEX1;APIP;ARG2;ARGLU1;ARHGAP22;ARHG

ACVR2A;ACVRL1;ADA;ADC;ADI1;ADRM1;ADSSL1;AEBP1;AEBP2;AFF4;AGER;AGL;AGPAT5;AGPS;AHR;AIM2;AIMP1;AIMP2;AKAP5;AKR1B1;AKT1;ALDH4A1;ALDOA;ALG1;ALG10;ALG10B;ALG11;ALG13;ALG2;ALG3;ALG6;AMD1;ANG;ANGPT1;ANKRD13C;ANKRD49;ANXA1;APEX1;APIP;ARG2;ARGLU1;ARHGAP22

TF1;ATF2;ATPAF2;ATXN7L3;BANF1;BDP1;BMI1;BRCA2;CAMK2D;CAMK4;CASC5;CASP3;CCNA2;CCNC;CCNE2;CCNH;CCNL1;CCNT2;CD2BP2;CDC25C;CDC26;CDC27;CDC40;CDC7;CDC73;CDK1;CDK2;CDK7;CDK8;CDK9;CDKN1B;CDKN2AIP;CDT1;CEBPA;CENPK;CENPQ;CHAF1B;CHD9;CHFR;CKS1B;CLK3;CLP1;COI

DAM22;ADAMTS5;ADAMTS6;ADAP1;ADD3;ADI1;ADSSL1;AEBP1;AEBP2;AGAP2;AGAP3;AGBL3;AGFG2;AGL;AIFM3;AKAP8L;ALKBH4;ALKBH5;ALKBH8;ANG;ANKIB1;ANKMY2;ANXA1;APEX1;APEX2;APIP;APLF;APOBEC3D;APTX;ARAF;ARAP2;ARFGAP1;ARFGAP2;ARG2;ARHGEF2;ARID2;ARMC1;ARSA;ARSK;ASAP

;ALG11;ALG13;ALG2;ALG3;ALG6;ANKLE1;ANKRD13C;ANO5;AP1B1;AP1S2;AP2A2;AP3S2;AP4S1;APEH;APH1A;APPBP2;APPL1;ARFGAP1;ARFGAP2;ARFGEF2;ARFIP1;ARL6IP1;ARL6IP5;ARMC10;ARRB1;ASPH;ATL2;ATP10D;ATP11B;ATP11C;ATP2C1;ATP8A1;B2M;B3GALNT1;B3GALT2;B3GALTL;B3GNT1;B3GNT

CLK1;CLK3;CLK4;CLNS1A;CLP1;CNOT2;CNOT3;CNOT6;CNOT6L;CNOT7;CNOT8;CPSF1;CPSF6;CSTF2T;DCAF13;DCPS;DDX41;DDX54;DGCR14;DGCR8;DICER1;DIS3;DUSP11;EIF4E;ELAC2;ERCC2;ERI1;ERN1;FARS2;FCF1;FDXACB1;FRG1;FTSJ1;GEMIN2;GEMIN6;GTF2F2;GTF2H1;GTF2H4;HNRNPH2;HNRNPL;HNRPL

A1L;DCAF13;DCP1A;DCP2;DDX3X;DDX41;DGCR14;DICER1;DNAJC21;DYRK1A;DYRK2;EIF4E;ERI1;FMR1;FRG1;FXR1;FXR2;GAPDH;GEMIN2;GEMIN6;HNRNPH2;HNRNPL;HNRPLL;HSPA14;IGHMBP2;IVNS1ABP;KIAA1967;KRR1;LIN28A;LSM1;LSM11;LSM3;LSM6;MBNL1;MKRN3;MOV10;MRPL1;MRPL13;MRPL15

D;ACTL6A;ACVR2A;ACVRL1;ADA;ADAP1;ADC;ADI1;ADRM1;ADSSL1;AEBP1;AEBP2;AFF4;AGAP2;AGAP3;AGER;AGFG2;AGGF1;AHR;AIM2;AIMP1;AIMP2;AK3;AKAP5;AKAP7;AKD1;AKT1;ALDH4A1;ALDOA;ALKBH5;ALKBH8;AMD1;ANG;ANGPT1;ANKRD49;APC;APEX1;APEX2;APIP;APLF;APOBEC3D;APTX;ARAP2;A

P1;ADRM1;ADSSL1;AEBP1;AEBP2;AFF4;AGAP2;AGAP3;AGER;AGFG2;AGGF1;AHR;AIM2;AIMP1;AIMP2;AK3;AKAP5;AKAP7;AKD1;AKT1;ALDH4A1;ALDOA;ALKBH5;ALKBH8;AMD1;ANG;ANKRD49;APEX1;APEX2;APLF;APOBEC3D;APTX;ARAP2;ARF4;ARFGAP1;ARFGAP2;ARGLU1;ARHGAP22;ARHGAP5;ARHGEF2

FBXO30;FBXO4;FEM1A;G2E3;GCLM;GSS;HACE1;HECTD2;HECTD3;HECW2;HERC4;ITCH;KLHL20;KLHL9;LNX1;LRSAM1;MALT1;MARCH1;MARCH5;MARCH6;MDM2;MIB1;NEDD4;NSMCE2;PARK2;PAX6;PELI1;PIAS4;PJA2;RBBP6;RBCK1;RBX1;RCHY1;RIMKLA;RIMKLB;RING1;RNF103;RNF13;RNF135;RNF139;RNF

4;PPP2R5A;PPP2R5B;PPP2R5D;PPP2R5E;PPP3CA;PPP3CB;PPP4C;PPP4R2;PPP4R4;SHOC2;SMEK1;SMEK2;STRN3;STRN4

BCD3;ABCE1;ABHD10;ABI1;ABI2;ABI3;ABLIM2;ABR;ACADM;ACADSB;ACADVL;ACAP1;ACAP2;ACAT1;ACD;ACOT7;ACP2;ACSF2;ACSF3;ACSL4;ACSL6;ACSS1;ACTL6A;ACTN4;ACTR1A;ACVR1C;ACVR2A;ACVRL1;ACY3;ADA;ADAM10;ADAM15;ADAM22;ADAP1;ADC;ADCK3;ADI1;ADM2;ADORA1;ADRBK1;ADRM1;A

2;CAMK2D;CASC5;CCNA2;CCNE2;CCNG1;CCNG2;CCNH;CCNY;CCP110;CD28;CD2AP;CDC25C;CDC26;CDC27;CDC7;CDC73;CDK1;CDK11A;CDK11B;CDK2;CDK7;CDKN1B;CDKN1C;CDKN2C;CDKN3;CDT1;CENPE;CENPK;CENPL;CENPQ;CENPW;CEP135;CEP152;CEP250;CEP290;CEP57;CEP70;CETN3;CHFR;CHMP1A

CDC26;CDC27;CDK1;CDK2;CDK7;CDKN1B;CHFR;CHID1;CLN3;CLN5;CLN6;CNOT2;CNOT3;CNOT6;CNOT6L;CNOT7;CNOT8;CRBN;CST3;CTBS;CUL2;CUL4B;CUL5;CYCS;DCP1A;DCP2;DCPS;DDB2;DERL1;DIS3;DNA2;DNAJB2;DNAJB9;DNAJC10;DNASE1L1;DNASE2;EDEM3;EIF3E;EIF4E;ENDOG;ERCC2;ERCC8;ERLEC1

FBXO4;FEM1A;G2E3;HACE1;HECTD2;HECTD3;HECW2;HERC4;ITCH;KLHL20;KLHL9;LNX1;LRSAM1;MALT1;MARCH1;MARCH5;MARCH6;MDM2;MIB1;NEDD4;NSMCE2;PARK2;PAX6;PELI1;PIAS4;PJA2;RBBP6;RBCK1;RBX1;RCHY1;RING1;RNF103;RNF13;RNF135;RNF139;RNF144B;RNF146;RNF187;RNF2;RNF217;

L;CNOT2;CNOT3;CNOT6;CNOT6L;CNOT7;CNOT8;CPSF1;CPSF6;CSTF2T;DCP1A;DCP2;DCPS;DDX41;DGCR14;DIS3;EIF3E;EIF4E;ERCC2;ERN1;FRG1;GEMIN2;GEMIN6;GTF2F2;GTF2H1;GTF2H4;HNRNPH2;HNRNPL;HNRPLL;JMJD6;KIAA1967;KIN;LSM1;LSM11;LSM3;LSM6;LUC7L3;MAPKAPK2;MBNL1;MBNL2;MNA

P1;ADRM1;ADSSL1;AEBP1;AEBP2;AFF4;AGAP2;AGAP3;AGER;AGFG2;AGGF1;AHR;AIM2;AIMP1;AIMP2;AK3;AKAP5;AKAP7;AKD1;AKT1;ALDOA;ALKBH5;ALKBH8;AMD1;ANG;ANKRD49;APEX1;APEX2;APLF;APOBEC3D;APTX;ARAP2;ARF4;ARFGAP1;ARFGAP2;ARGLU1;ARHGAP22;ARHGAP5;ARHGEF2;ARID2;AR

FBXO4;FEM1A;G2E3;HACE1;HECTD2;HECTD3;HECW2;HERC4;ITCH;KLHL20;KLHL9;LNX1;LRSAM1;MALT1;MARCH1;MARCH5;MARCH6;MDM2;MIB1;NEDD4;PARK2;PAX6;PELI1;PJA2;RBBP6;RBCK1;RBX1;RCHY1;RING1;RNF103;RNF13;RNF135;RNF139;RNF144B;RNF146;RNF187;RNF2;RNF217;RNF220;RNF31;

CHFR;DDB2;DTX2;DZIP3;ERCC8;FANCL;FARS2;FARSA;FBXL3;FBXL5;FBXO11;FBXO3;FBXO30;FBXO4;FDXACB1;FEM1A;G2E3;GCLM;GSS;HACE1;HARS2;HECTD2;HECTD3;HECW2;HERC4;HLTF;ITCH;KLHL20;KLHL9;LIG4;LNX1;LRRC47;LRSAM1;LTN1;MALT1;MARCH1;MARCH5;MARCH6;MARCH7;MARS2;MDM2;N

;ADAMTS6;ADM2;ADORA1;ADRBK1;AEBP1;AGAP2;AGAP3;AGBL3;AIDA;AIMP1;AIMP2;AKT1;AKTIP;ALG1;ALG10;ALG10B;ALG11;ALG13;ALG2;ALG3;ALG6;ANAPC10;ANG;ANGPT1;ANXA1;APC;APEH;APH1A;ARAF;ARFGAP1;ARID5A;ARRB1;ARSA;ARSK;ASB13;ASB7;ASGR2;ASPH;ASTL;ATG4A;ATG4B;ATG4C;A

CDC92;CCP110;CDC14A;CDC27;CDK2;CDKL2;CEP120;CEP135;CEP152;CEP170;CEP19;CEP250;CEP290;CEP350;CEP44;CEP57;CEP70;CEP95;CEP97;CETN3;CKAP2;CLIC4;CNTROB;CSK;CSNK1A1;DCTN2;DCTN4;DCTN6;DYNLRB1;FAM161A;FGFR1OP;FLII;FOPNL;GNAI1;HAUS1;HAUS3;HAUS6;HAUS7;HOOK2;IFT2

AD10;ACAD9;ACADM;ACADSB;ACADVL;ACSF2;ACSF3;ACSL4;ACSL6;ACSS1;ACTL6A;ACTR1A;ACVR1C;ACVR2A;ACVRL1;ADCK1;ADCK2;ADCK3;ADRBK1;ADSSL1;AGAP2;AGAP3;AGPS;AIFM3;AK3;AKAP7;AKD1;AKT1;ANKK1;APLF;ARAF;ARF4;ARHGAP5;ARL13B;ARL15;ARL2;ARL4A;ARL5A;ARL5B;ARL8B;ASNA1;A

BXL5;FBXO11;FBXO3;FBXO30;FBXO4;FEM1A;G2E3;GCLM;GSS;HACE1;HECTD2;HECTD3;HECW2;HERC4;ITCH;KLHL20;KLHL9;LNX1;LRSAM1;MALT1;MARCH1;MARCH5;MARCH6;MDM2;MIB1;MTHFSD;NADSYN1;NEDD4;NSMCE2;PARK2;PAX6;PC;PELI1;PIAS4;PJA2;RBBP6;RBCK1;RBX1;RCHY1;RIMKLA;RIMKLB

;BRCA2;BRCC3;BRIP1;CAB39;CAB39L;CAMK2D;CASC5;CCNA2;CCNE2;CCNG1;CCNG2;CCNH;CCNY;CCP110;CD28;CD2AP;CDC25C;CDC26;CDC27;CDC7;CDC73;CDK1;CDK11A;CDK11B;CDK2;CDK7;CDK9;CDKN1B;CDKN1C;CDKN2C;CDKN3;CDT1;CENPE;CENPK;CENPL;CENPQ;CENPW;CEP120;CEP135;CEP152;CE

AD10;ACAD9;ACADM;ACADSB;ACADVL;ACSF2;ACSF3;ACSL4;ACSL6;ACSS1;ACTL6A;ACTR1A;ACVR1C;ACVR2A;ACVRL1;ADCK1;ADCK2;ADCK3;ADRBK1;ADSSL1;AGAP2;AGAP3;AGPS;AIFM3;AK3;AKAP7;AKD1;AKT1;ANKK1;APLF;ARAF;ARF4;ARHGAP5;ARL13B;ARL15;ARL2;ARL4A;ARL5A;ARL5B;ARL8B;ASNA1;A

8B;ASPM;AURKC;AZI1;BBS7;BLOC1S2;BRCA2;C12orf52;C14orf166;CASP8;CBX3;CC2D1A;CCDC14;CCDC92;CCP110;CCT2;CDC14A;CDC27;CDC42EP2;CDK1;CDK2;CDKL2;CENPE;CEP120;CEP135;CEP152;CEP170;CEP19;CEP250;CEP290;CEP350;CEP44;CEP57;CEP57L1;CEP70;CEP95;CEP97;CETN3;CHMP1A;CKA

KAP7;AKT1;AKTIP;ANAPC10;ANG;ANXA1;AP1B1;AP1S2;AP2A2;AP3M1;AP3S2;APC;APEX1;API5;APOBEC3D;APOO;APPBP2;APPL1;ARFGEF2;ARHGEF2;ARID4A;ARID5A;ARL6IP1;ARL8B;ARPC4;ARRB1;ASPM;ATF1;ATG12;ATP5C1;ATP5E;ATP5F1;ATP5I;ATP5L;ATP5O;ATP6AP1;ATP6V1G1;ATXN7L3;AURKC;B2M

G3;ALG6;ANKRD13C;ANO5;APEX1;APH1A;AQP11;ARFGAP1;ARL6IP1;ARL6IP5;ARMC10;ARSA;ARSK;ASNA1;ASPH;ATL2;ATP10D;ATP11B;ATP11C;ATP7A;ATP8A1;B2M;B3GALTL;BCAP29;BCAP31;BET1;BGLAP;BNIP1;BNIP3L;CALCRL;CALU;CAMK2D;CAMLG;CCDC88A;CDIPT;CDS1;CEPT1;CERCAM;CERS5;CHERP

;AGAP2;AGAP3;AGER;AGFG2;AHR;AIDA;AIM2;AIMP2;AKAP5;AKT1;AKTIP;ANAPC10;ANG;ANGPT1;ANKRD13C;ANKRD49;ANXA1;APC;APEX1;ARAF;ARAP2;ARFGAP1;ARFGAP2;ARGLU1;ARHGAP22;ARHGAP5;ARHGEF2;ARID2;ARID4A;ARID4B;ARID5A;ARID5B;ARL2;ARPP19;ARRB1;ASAP3;ASF1A;ASPH;ATAD1

ALNT1;B3GALT2;B3GNT1;B3GNT2;B3GNT5;B4GALT3;B4GALT6;BCAP31;BET1;BLZF1;C1GALT1;C3orf58;CDIPT;CHPF;CHPF2;CHST13;CHST14;CHST2;CHSY1;CLN3;CLSTN3;CNIH;COG3;COG4;COG6;COPE;COPZ1;CORO7;CSGALNACT1;CSGALNACT2;EBAG9;ENTPD6;FAM198B;FNDC3A;FUT4;GALNT1;GALNT3;GA

;AGAP2;AGAP3;AGER;AGFG2;AHR;AIDA;AIM2;AIMP2;AKAP5;AKT1;AKTIP;ANAPC10;ANG;ANGPT1;ANKRD49;ANXA1;APC;APEX1;ARAF;ARAP2;ARFGAP1;ARFGAP2;ARGLU1;ARHGAP22;ARHGAP5;ARHGEF2;ARID2;ARID4A;ARID4B;ARID5A;ARID5B;ARL2;ARPP19;ARRB1;ARV1;ASAP3;ASF1A;ASPH;ASTL;ATAD2

AB39;CAMK1;CAMK2D;CAMK4;CCND3;CCNH;CDC7;CDK1;CDK11A;CDK11B;CDK17;CDK18;CDK2;CDK5R1;CDK7;CDK8;CDK9;CDKL1;CDKL2;CHUK;CLK1;CLK3;CLK4;CPNE3;CSNK1A1;CSNK1A1L;CSNK1G3;CSNK2B;DAPK3;DMPK;DYRK1A;DYRK1B;DYRK2;ENG;ERCC2;ERN1;FAM20C;GAK;GRK6;GTF2H1;GTF2H4;IC

;BRCA2;CAMK2D;CASC5;CCNA2;CCNE2;CCNG1;CCNG2;CCNH;CCNY;CCP110;CD28;CD2AP;CDC25C;CDC26;CDC27;CDC7;CDC73;CDK1;CDK11A;CDK11B;CDK2;CDK7;CDKN1B;CDKN1C;CDKN2C;CDKN3;CDT1;CENPE;CENPK;CENPL;CENPQ;CENPW;CEP120;CEP135;CEP152;CEP250;CEP290;CEP57;CEP70;CETN3;C

.DA;ADAP1;ADRM1;ADSSL1;AEBP1;AEBP2;AFF4;AGAP2;AGAP3;AGER;AGFG2;AGGF1;AHR;AIM2;AIMP1;AIMP2;AK3;AKAP5;AKAP7;AKD1;AKR1B1;AKT1;ALDH4A1;ALDOA;ALKBH5;ALKBH8;AMD1;ANG;ANKRD49;APEX1;APEX2;APLF;APOBEC3D;APTX;ARAP2;ARF4;ARFGAP1;ARFGAP2;ARGLU1;ARHGAP22;ARH

PAT5;AGPS;AK3;AKD1;AKT1;ALG1;ALG10;ALG10B;ALG11;ALG13;ALG2;ALG3;ALG6;ALKBH8;ANKK1;ARAF;ASMTL;AURKC;B3GALNT1;B3GALT2;B3GALTL;B3GNT1;B3GNT2;B3GNT5;B4GALT3;B4GALT6;BCAT1;BCAT2;BCR;BMPR1A;BMPR2;BRCA2;C1GALT1;CAB39;CAMK1;CAMK2D;CAMK4;CCBL2;CCND3;CCNH

AD10;ACAD9;ACADM;ACADSB;ACADVL;ACSF2;ACSF3;ACSL4;ACSL6;ACSS1;ACTL6A;ACTN4;ACTR1A;ACVR1C;ACVR2A;ACVRL1;ADA;ADAP1;ADCK1;ADCK2;ADCK3;ADORA1;ADRBK1;ADSSL1;AGAP2;AGAP3;AGPS;AIFM3;AK3;AKAP7;AKD1;AKT1;ANKK1;APLF;ARAF;ARF4;ARHGAP5;ARL13B;ARL15;ARL2;ARL4A;A

SF2;ACSF3;ACSL4;ACSL6;ACSS1;ACTL6A;ACTR1A;ACVR1C;ACVR2A;ACVRL1;ADCK1;ADCK2;ADCK3;ADRBK1;ADSSL1;AGAP2;AGAP3;AK3;AKD1;AKT1;ANKK1;ARAF;ARF4;ARHGAP5;ARL13B;ARL15;ARL2;ARL4A;ARL5A;ARL5B;ARL8B;ASNA1;ATAD1;ATAD2;ATAD2B;ATAD3A;ATL2;ATP10D;ATP11B;ATP11C;ATP13

1;MARCH5;MARCH6;NEDD4;PARK2;PELI1;PSMA1;PSMA2;PSMA3;PSMA4;PSMA6;PSMA8;PSMB1;PSMB3;PSMC1;PSMC2;PSMC3;PSMC5;PSMC6;PSMD10;PSMD13;PSMD14;PSMD4;PSMD8;PSME1;RBCK1;RNF146;RNF187;RNF31;RNF6;SKP2;TNKS2;TOPORS;UBC;UBE2A;UBE2D1;UBE2D3;UBE2E1;UBE2G1;U

ADORA1;ADRBK1;ADRM1;AEBP2;AGAP2;AGAP3;AGER;AGFG2;AHR;AIDA;AIFM3;AIM2;AIMP2;AKAP5;AKT1;AKTIP;ANAPC10;ANG;ANGPT1;ANGPTL4;ANKRD13C;ANKRD49;ANXA1;APC;APEX1;APH1A;APTX;ARAF;ARAP2;ARFGAP1;ARFGAP2;ARGLU1;ARHGAP22;ARHGAP5;ARHGEF2;ARID2;ARID4A;ARID4B;A

SF2;ACSF3;ACSL4;ACSL6;ACSS1;ACTL6A;ACTR1A;ACVR1C;ACVR2A;ACVRL1;ADA;ADCK1;ADCK2;ADCK3;ADORA1;ADRBK1;ADSSL1;AGAP2;AGAP3;AK3;AKAP7;AKD1;AKT1;ANKK1;ARAF;ARF4;ARHGAP5;ARL13B;ARL15;ARL2;ARL4A;ARL5A;ARL5B;ARL8B;ASNA1;ATAD1;ATAD2;ATAD2B;ATAD3A;ATL2;ATP10D;A

SF2;ACSF3;ACSL4;ACSL6;ACSS1;ACTL6A;ACTR1A;ACVR1C;ACVR2A;ACVRL1;ADCK1;ADCK2;ADCK3;ADRBK1;ADSSL1;AGAP2;AGAP3;AK3;AKAP7;AKD1;AKT1;ANKK1;ARAF;ARF4;ARHGAP5;ARL13B;ARL15;ARL2;ARL4A;ARL5A;ARL5B;ARL8B;ASNA1;ATAD1;ATAD2;ATAD2B;ATAD3A;ATL2;ATP10D;ATP11B;ATP11C

SF2;ACSF3;ACSL4;ACSL6;ACSS1;ACTL6A;ACTN4;ACTR1A;ACVR1C;ACVR2A;ACVRL1;ADA;ADCK1;ADCK2;ADCK3;ADORA1;ADRBK1;ADSSL1;AGAP2;AGAP3;AK3;AKAP7;AKD1;AKT1;ANKK1;ARAF;ARF4;ARHGAP5;ARL13B;ARL15;ARL2;ARL4A;ARL5A;ARL5B;ARL8B;ASNA1;ATAD1;ATAD2;ATAD2B;ATAD3A;ATL2;AT

10;ANG;ANLN;ANXA1;AP1AR;APC;AQP11;ARFGAP1;ARFGEF2;ARHGAP10;ARHGEF2;ARID2;ARID5A;ARL2;ARPC4;ARPP19;ARRB1;ASF1A;ASPM;ATG12;ATG4B;ATG4C;ATG5;ATG9A;ATL2;ATP2C1;ATP6V1G1;ATP7A;ATXN7L3;AURKC;BAG4;BAG6;BAIAP2;BAP1;BAZ1A;BCR;BET1;BLOC1S2;BLZF1;BMF;BMI1;BNIF

SF2;ACSF3;ACSL4;ACSL6;ACSS1;ACTL6A;ACTR1A;ACVR1C;ACVR2A;ACVRL1;ADCK1;ADCK2;ADCK3;ADRBK1;ADSSL1;AGAP2;AGAP3;AK3;AKAP7;AKD1;AKT1;ANKK1;ARAF;ARF4;ARHGAP5;ARL13B;ARL15;ARL2;ARL4A;ARL5A;ARL5B;ARL8B;ASNA1;ATAD1;ATAD2;ATAD2B;ATAD3A;ATL2;ATP10D;ATP11B;ATP11C

SF2;ACSF3;ACSL4;ACSL6;ACSS1;ACTL6A;ACTR1A;ACVR1C;ACVR2A;ACVRL1;ADCK1;ADCK2;ADCK3;ADRBK1;ADSSL1;AGAP2;AGAP3;AK3;AKAP7;AKD1;AKT1;ANKK1;ARAF;ARF4;ARHGAP5;ARL13B;ARL15;ARL2;ARL4A;ARL5A;ARL5B;ARL8B;ASNA1;ATAD1;ATAD2;ATAD2B;ATAD3A;ATL2;ATP10D;ATP11B;ATP11C

.KTIP;ANAPC10;ANG;ANGPT1;ANKRD13C;ANKRD49;APC;APEX1;APTX;ARAF;ARFGAP1;ARGLU1;ARHGAP22;ARHGEF2;ARID2;ARID4A;ARID4B;ARID5A;ARID5B;ARRB1;ASF1A;ASGR2;ASPH;ASTL;ATAD1;ATAD2;ATF2;ATG5;ATP7A;ATRX;ATXN7L3;BACH1;BAG4;BAG6;BAZ1A;BAZ2B;BBX;BCLAF1;BDP1;BIRC2;BLC

APPBP2;ARFGAP1;ARFGEF2;ARFIP1;ARHGEF2;ARL6IP1;ARRB1;ASPH;ATG4C;ATP2C1;ATP5C1;ATP5E;ATP5F1;ATP5I;ATP5L;ATP5O;BCAP29;BCAP31;BET1;BLOC1S2;BLZF1;BMPR1A;BUD13;C12orf52;CAMK1;CAMK2D;CAMK4;CCDC90A;CD27;CDC37;CDC40;CDK1;CDK5R1;CEP57;CHCHD4;CHERP;CHM;CHML;C

SF2;ACSF3;ACSL4;ACSL6;ACSS1;ACTL6A;ACTR1A;ACVR1C;ACVR2A;ACVRL1;ADCK1;ADCK2;ADCK3;ADRBK1;ADSSL1;AGAP2;AGAP3;AK3;AKAP7;AKD1;AKT1;ANKK1;ARAF;ARF4;ARHGAP5;ARL13B;ARL15;ARL2;ARL4A;ARL5A;ARL5B;ARL8B;ASNA1;ATAD1;ATAD2;ATAD2B;ATAD3A;ATL2;ATP10D;ATP11B;ATP11C

NOT6;CNOT6L;CNOT7;CNOT8;CPSF1;CPSF6;CSTF2T;DCPS;DDX41;DGCR14;EIF4E;ERCC2;ERN1;FRG1;GEMIN2;GEMIN6;GTF2F2;GTF2H1;GTF2H4;HNRNPH2;HNRNPL;HNRPLL;JMJD6;KIAA1967;KIN;LSM1;LSM11;LSM3;LSM6;LUC7L3;MBNL1;MBNL2;MNAT1;MOV10;NAA38;NUDT21;PAIP1;PAN3;PAPD4;PAPOL

SF2;ACSF3;ACSL4;ACSL6;ACSS1;ACTL6A;ACTR1A;ACVR1C;ACVR2A;ACVRL1;ADCK1;ADCK2;ADCK3;ADRBK1;ADSSL1;AGAP2;AGAP3;AK3;AKAP7;AKD1;AKT1;ANKK1;ARAF;ARF4;ARHGAP5;ARL13B;ARL15;ARL2;ARL4A;ARL5A;ARL5B;ARL8B;ASNA1;ATAD1;ATAD2;ATAD2B;ATAD3A;ATL2;ATP10D;ATP11B;ATP11C

HGEF2;ARID2;ARID4A;ARID4B;ARID5A;ARID5B;ARRB1;ASF1A;ASPH;ATAD2;ATF2;ATRX;ATXN7L3;BACH1;BAZ1A;BAZ2B;BBX;BCLAF1;BDP1;BIRC2;BLOC1S2;BLZF1;BMI1;BMPR1A;BMPR2;BRCA2;BRD9;BRIP1;BRMS1;BRMS1L;BRWD1;BTAF1;BZW1;C12orf52;C14orf166;CAMK4;CARHSP1;CASP8AP2;CBFB;CBX3

AXX;DDX3X;DYRK1A;EAF2;EIF3E;FAM76B;FRG1;FYTTD1;GATAD2A;GEMIN2;GEMIN6;HDAC5;HIRA;KLHL20;LRCH4;LUC7L3;MDM2;MEF2C;MORC3;NOC3L;NOP58;NSMCE2;NUDT21;OIP5;PHAX;PHF5A;PIAS4;PLCB1;PLRG1;PNISR;PPIG;PPP1CC;PRPF18;PRPF31;PRPF40A;PRPF6;PSKH1;PTEN;RB1;RBM11;RBM2

ARID4B;ARID5A;ARID5B;ARRB1;ATF1;ATF2;ATRX;AUH;BACH1;BANF1;BAZ2B;BBX;BCLAF1;BDP1;BLZF1;BMI1;BRCA2;BRD9;BRIP1;BTAF1;C10orf2;CARHSP1;CASP8AP2;CBFB;CC2D1A;CDK9;CDKN2AIP;CDT1;CEBPA;CEBPG;CEBPZ;CENPW;CERS5;CGGBP1;CHD1;CHD5;CHD9;CHERP;CNBP;CNOT6;CNOT7;CNOT8

CASP3;CCNA2;CCNG1;CCNH;CDK1;CDK2;CDK7;CDK9;CDKN1B;CEBPG;CHAF1A;CHAF1B;CIDEB;CRY1;CUL4B;DCLRE1A;DDB2;DEK;DGKZ;DNA2;DYRK1A;DYRK2;E2F7;EEF1E1;ENDOV;EPC2;ERCC2;ERCC8;FAM175A;FANCB;FANCL;FANCM;FBXO31;FBXO45;FBXW7;GEN1;GNL1;GTF2H1;GTF2H4;HMGB1;HMGB2;I

HGAP22;ARHGEF2;ARID2;ARID4A;ARID4B;ARID5A;ARID5B;ARRB1;ASF1A;ASPH;ATAD2;ATF2;ATRX;ATXN7L3;BACH1;BAZ1A;BAZ2B;BBX;BCLAF1;BDP1;BIRC2;BLOC1S2;BLZF1;BMI1;BMPR1A;BMPR2;BRCA2;BRD9;BRIP1;BRMS1;BRMS1L;BRWD1;BTAF1;BZW1;C12orf52;C14orf166;CAMK4;CARHSP1;CASP8AP2

CH1;MCOLN1;MITD1;NDFIP2;PIKFYVE;RAB27B;RNF13;SLC29A3;SNX16;STARD3;STARD3NL;TMEM106B;TMEM55A;VAC14;VAMP7;VPS18;VPS28;VPS33B;VPS37A;VPS37B;VPS4A;VPS4I

KRD49;ANXA1;APEX1;ARGLU1;ARHGAP22;ARHGEF2;ARID2;ARID4A;ARID4B;ARID5A;ARID5B;ARPP19;ARRB1;ASF1A;ASPH;ATAD2;ATF2;ATRX;ATXN7L3;BACH1;BAZ1A;BAZ2B;BBX;BCLAF1;BDP1;BIRC2;BLOC1S2;BLZF1;BMI1;BMPR1A;BMPR2;BRCA2;BRD9;BRIP1;BRMS1;BRMS1L;BRWD1;BTAF1;BZW1;C12or

1;CD2BP2;CDC73;CDK2;CDK7;CDK8;CDK9;CEBPA;CHFR;CLK3;COIL;CPSF1;CPSF6;CREB1;CSNK1A1;CTBP1;CXXC1;DACH1;DAPK3;DAXX;DDX3X;DEPDC1;DMAP1;DPY30;DR1;DYRK1A;E2F4;E2F6;E2F7;EAF2;EED;EIF3E;ELL2;ENY2;EPC2;ERCC2;FAM60A;FAM76B;FANCB;FANCL;FANCM;FOXH1;FOXN2;FRG1;FYTTD

1S2;AP2A2;AP3M1;AP3S2;APPBP2;APPL1;ARF4;ARFGAP1;ARFGAP2;ARFGEF2;ARFIP1;ARHGEF2;ARL6IP1;ARRB1;ASPH;ATG16L2;ATG4A;ATG4B;ATG4C;ATG9A;ATP6V1G1;BCAP29;BCAP31;BET1;BLZF1;BMPR1A;C5orf30;CALCRL;CAMK1;CAMK4;CARD8;CCDC91;CCT6B;CD27;CD274;CDC37;CDK1;CDK5R1;CEP

C25C;CDC26;CDC27;CDK1;CDK11A;CDK11B;CDK2;CENPE;CENPK;CENPL;CENPQ;CENPW;CETN3;CHFR;CHMP1A;CSNK1A1;DAPK3;DCLRE1A;DCTN2;DDX11;DIS3L2;DYNLT3;EREG;FBXO5;GORASP1;HAUS1;HAUS3;HAUS6;HAUS7;HELLS;ITGB3BP;KATNB1;KIAA1009;KIF11;KIF18A;KIF20B;KIF22;KIF23;KIF2A;KIFC1

C25C;CDC26;CDC27;CDK1;CDK11A;CDK11B;CDK2;CENPE;CENPK;CENPL;CENPQ;CENPW;CETN3;CHFR;CHMP1A;CSNK1A1;DAPK3;DCLRE1A;DCTN2;DDX11;DIS3L2;DYNLT3;EREG;FBXO5;GORASP1;HAUS1;HAUS3;HAUS6;HAUS7;HELLS;ITGB3BP;KATNB1;KIAA1009;KIF11;KIF18A;KIF20B;KIF22;KIF23;KIF2A;KIFC1

3S2;AP4S1;APH1A;ARF4;ARFGAP1;ARFGAP2;ARFGEF2;ARFIP1;ARHGEF2;ARRB1;ATG9A;ATP2C1;ATP7A;B2M;B3GALNT1;B3GALT2;B3GNT1;B3GNT2;B3GNT5;B4GALT3;B4GALT6;BCAP31;BET1;BGLAP;BLZF1;BMP1;C1GALT1;C3orf58;CALU;CCDC88A;CCDC91;CD46;CDC42EP1;CDIPT;CEP57;CHPF;CHPF2;CHST1

D2;ARID4A;ARID4B;ARID5A;ARID5B;ARRB1;ASF1A;ASPH;ATAD2;ATF2;ATRX;ATXN7L3;BACH1;BAZ1A;BAZ2B;BBX;BCLAF1;BDP1;BIRC2;BLOC1S2;BLZF1;BMI1;BMPR1A;BMPR2;BRCA2;BRD9;BRIP1;BRMS1;BRMS1L;BRWD1;BTAF1;BZW1;C12orf52;C14orf166;CAMK4;CARHSP1;CASP8AP2;CBFB;CBX3;CCDC59;C

C25C;CDC26;CDC27;CDK1;CDK11A;CDK11B;CDK2;CENPE;CENPK;CENPL;CENPQ;CENPW;CETN3;CHFR;CHMP1A;CSNK1A1;DAPK3;DCLRE1A;DCTN2;DDX11;DIS3L2;DYNLT3;EREG;FBXO5;GORASP1;HAUS1;HAUS3;HAUS6;HAUS7;HELLS;ITGB3BP;KATNB1;KIAA1009;KIF11;KIF18A;KIF20B;KIF22;KIF23;KIF2A;KIFC1

27;CDC7;CDK1;CDK2;CDK7;CDK9;CDKN1B;CDKN1C;CDKN2C;CDKN3;CDT1;CGRRF1;CHFR;CRLF3;CUL2;CUL5;DBF4;DGKZ;DLG1;DNA2;ERCC2;ERN1;FAM175A;FBXO31;GAS2;GAS2L2;GAS6;HORMAD1;HSF1;IL12A;IL8;ILK;JMY;KAT2B;KIF20B;MAD2L1;MAPKAPK2;MCM5;MDM2;MEN1;MSH2;MTBP;MUC1;NBN;

KRD13C;ANKRD49;ANXA1;APEX1;ARGLU1;ARHGAP22;ARHGEF2;ARID2;ARID4A;ARID4B;ARID5A;ARID5B;ARPP19;ARRB1;ASF1A;ASPH;ATAD2;ATF2;ATRX;ATXN7L3;BACH1;BAZ1A;BAZ2B;BBX;BCLAF1;BDP1;BIRC2;BLOC1S2;BLZF1;BMI1;BMPR1A;BMPR2;BRCA2;BRD9;BRIP1;BRMS1;BRMS1L;BRWD1;BTAF1;B

CKS1B;CUL2;CUL5;DBF4;DDX3X;DGKZ;DLG1;E2F4;E2F6;E2F7;EIF4E;FBXO31;FBXO5;GPR132;MCM5;MDM2;MNAT1;MUC1;NBN;NFATC1;ORC2;ORC3;ORC4;PKD2;PPAT;PPP3CA;PPP6C;PSMA1;PSMA2;PSMA3;PSMA4;PSMA6;PSMA8;PSMB1;PSMB3;PSMC1;PSMC2;PSMC3;PSMC5;PSMC6;PSMD10;PSMD13;P

D2;ARID4A;ARID4B;ARID5A;ARID5B;ARRB1;ASF1A;ASPH;ATAD2;ATF2;ATRX;ATXN7L3;BACH1;BAZ1A;BAZ2B;BBX;BCLAF1;BDP1;BIRC2;BLOC1S2;BLZF1;BMI1;BMPR1A;BMPR2;BRCA2;BRD9;BRIP1;BRMS1;BRMS1L;BRWD1;BTAF1;BZW1;C12orf52;C14orf166;CAMK4;CARHSP1;CASP8AP2;CBFB;CBX3;CCDC59;C

P1R3D;PPP2CB;PPP2R1A;PPP2R2A;PPP3CA;PPP3CB;PPP4C;PPP5C;PPP6C;PTEN;RPAP2;SSU72

C25C;CDC26;CDC27;CDK1;CDK11A;CDK11B;CDK2;CENPE;CENPK;CENPL;CENPQ;CENPW;CETN3;CHFR;CHMP1A;CSNK1A1;DAPK3;DCLRE1A;DCTN2;DDX11;DIS3L2;DYNLT3;EREG;FBXO5;GORASP1;HAUS1;HAUS3;HAUS6;HAUS7;HELLS;ITGB3BP;KATNB1;KIAA1009;KIF11;KIF18A;KIF20B;KIF22;KIF23;KIF2A;KIFC1

AT2;ACBD3;ACOT13;ACOT7;ACSF2;ACSF3;ACSL4;ACSL6;ACSS1;ADC;ADCK1;ADCK2;ADCK3;AFF4;AGAP2;AGPAT5;AGPS;AIFM3;AIM2;AK3;AKR7A2;ALDH4A1;ALKBH4;ANXA1;APEX1;APEX2;ARAF;ARG2;ARL2;ARMC1;ARMC10;ATAD1;ATAD3A;ATP5C1;ATP5E;ATP5F1;ATP5I;ATP5L;ATP5O;ATPAF2;AUH;AZI2;BC

S;ITGB3BP;KAT2B;KAT8;KIF22;LRWD1;MAD2L1;MIS18BP1;MLF1IP;NDE1;NSL1;NUDCD2;NUF2;NUP37;NUP85;OIP5;ORC2;PMF1;PPP1CC;PPP1R12A;PPP2CB;PPP2R1A;PPP2R5A;PSEN2;RANGAP1;SEC13;SEPT7;SGOL2;SKA2;SPAG5;SS18L1;STAG1;STAG2;SUMO3;SUV39H1;SUV39H2;WDHD1;XPO1;ZWILCH

ATP7A;B2M;B3GALNT1;B3GALT2;B3GNT1;B3GNT2;B3GNT5;B4GALT3;B4GALT6;BCAP31;BET1;BLZF1;C1GALT1;C3orf58;CDIPT;CHPF;CHPF2;CHST13;CHST14;CHST2;CHSY1;CLIP3;CLN3;CLSTN3;CNIH;COG3;COG4;COG6;COPE;COPZ1;CORO7;CSGALNACT1;CSGALNACT2;EBAG9;ENTPD6;FAM198B;FNDC3A;FUT

1S2;AP2A2;AP3M1;AP3S2;APPBP2;ARF4;ARFGAP1;ARFGAP2;ARFGEF2;ARFIP1;ARHGEF2;ARL6IP1;ARRB1;ASPH;ATG16L2;ATG4A;ATG4B;ATG4C;ATG9A;ATP6V1G1;BCAP29;BCAP31;BET1;BLZF1;BMPR1A;C5orf30;CALCRL;CAMK1;CAMK4;CARD8;CCDC91;CCT6B;CD27;CD274;CDC37;CDK1;CDK5R1;CEP290;CE

PSMC5;PSMC6;PSMD10;PSMD13;PSMD14;PSMD4;PSMD8;PSME1;RAD23A;SHFM1;TXNL1;UBE3A;UBR1;UCHL5;USP14

IP5;ARMC10;ASPH;ATL2;ATP10D;ATP11C;B3GALTL;BCAP29;BCAP31;BET1;BNIP1;CAMK2D;CDIPT;CDS1;CEPT1;CERS5;CHERP;CISD2;CLN3;CLN6;CLSTN3;CNIH;COL4A3BP;CTDNEP1;CYP2S1;CYP2U1;CYP2W1;CYP4F22;DDOST;DEGS1;DEGS2;DERL1;DHRS9;DLG1;DMPK;DNAJB9;DOLPP1;DPM1;DTNBP1;EDEM3

D2;ARID4A;ARID4B;ARID5A;ARID5B;ARRB1;ASF1A;ASPH;ATAD2;ATF2;ATRX;ATXN7L3;BACH1;BAZ1A;BAZ2B;BBX;BCLAF1;BDP1;BIRC2;BLOC1S2;BLZF1;BMI1;BMPR1A;BMPR2;BRCA2;BRD9;BRIP1;BRMS1;BRMS1L;BRWD1;BTAF1;BZW1;C12orf52;C14orf166;CAMK4;CARHSP1;CASP8AP2;CBFB;CBX3;CCDC59;C

ASF1A;ASTE1;ATF1;ATF2;ATG12;ATG4B;ATG4C;ATG5;ATG9A;ATP7A;ATRX;BACH1;BAG6;BCLAF1;BMPR2;BRCA2;BRCC3;BRIP1;CASP3;CCM2;CCNA2;CCNG1;CCNH;CD27;CDK1;CDK2;CDK7;CDK9;CDKN1B;CEBPG;CHAF1A;CHAF1B;CIDEB;CLN3;COL4A3BP;COPS5;CREB1;CRY1;CST3;CUL2;CUL4B;CXCL10;CXXC1;

R;AIM2;AKAP5;AKT1;ANKRD49;APC;APEX1;ARAP2;ARFGAP1;ARFGAP2;ARGLU1;ARHGAP22;ARHGAP5;ARHGEF2;ARID2;ARID4A;ARID4B;ARID5A;ARID5B;ARL2;ARRB1;ASAP3;ASF1A;ASPH;ATAD2;ATF2;ATRX;ATXN7L3;AZIN1;BACH1;BAZ1A;BAZ2B;BBX;BCLAF1;BDP1;BIN1;BIRC2;BLOC1S2;BLZF1;BMI1;BMPR

AURKC;BCR;BMPR1A;BMPR2;CAB39;CAMK1;CAMK2D;CAMK4;CCND3;CCNH;CDC7;CDIPT;CDK1;CDK11A;CDK11B;CDK17;CDK18;CDK2;CDK5R1;CDK7;CDK8;CDK9;CDKL1;CDKL2;CDS1;CEPT1;CHUK;CKB;CLK1;CLK3;CLK4;CLP1;CMPK1;COASY;COL4A3BP;CPNE3;CRCP;CRIM1;CRLS1;CSK;CSNK1A1;CSNK1A1L;CSI

D23A;RBCK1;RNF31;SIRT2;TAB2;TDG;TOLLIP;UBE2N;UBR5;UCHL3;USP16;USP25;USPL1;VPS36;ZFAND6;ZRANB1;ZRANB3

;ARHGAP22;ARHGEF2;ARID2;ARID4A;ARID4B;ARID5A;ARID5B;ARRB1;ASF1A;ASPH;ATAD2;ATF1;ATF2;ATRX;ATXN7L3;BACH1;BAZ1A;BAZ2B;BBX;BCLAF1;BDP1;BIRC2;BLOC1S2;BLZF1;BMI1;BMPR1A;BMPR2;BRCA2;BRD9;BRIP1;BRMS1;BRMS1L;BRWD1;BTAF1;BZW1;C10orf2;C12orf52;C14orf166;CAMK4;C

IP5;ARMC10;ASPH;ATL2;ATP10D;ATP11C;B3GALTL;BCAP29;BCAP31;BET1;BNIP1;CAMK2D;CDIPT;CDS1;CEPT1;CERS5;CHERP;CISD2;CLN3;CLN6;CLSTN3;CNIH;COL4A3BP;CTDNEP1;CYP2S1;CYP2U1;CYP2W1;CYP4F22;DDOST;DEGS1;DEGS2;DERL1;DHRS9;DLG1;DMPK;DNAJB9;DOLPP1;DPM1;DTNBP1;EDEM3

CPEB4;CPSF1;CPSF6;CSTF2T;DAZAP1;DCP2;DCPS;DDX11;DDX3X;DDX41;DDX50;DDX54;DDX59;DENR;DGCR8;DHFRL1;DHX30;DHX36;DICER1;DIS3;DIS3L2;DUSP11;DZIP3;EEF1A1;EEF1B2;EEFSEC;EIF1AX;EIF2A;EIF2B5;EIF2S2;EIF3D;EIF3E;EIF3J;EIF4E;EIF4E2;EIF4E3;EIF5;EIF5A2;EIF6;ERAL1;ERI1;EWSR1;FARS2

';AKT1;AKTIP;ANAPC10;ANG;ANXA1;AP1B1;AP1S2;AP2A2;AP3M1;AP3S2;APC;APEX1;APPBP2;APPL1;ARFGEF2;ARHGEF2;ARID4A;ARID5A;ARL6IP1;ARL8B;ARPC4;ARRB1;ASPM;ATF1;ATG12;ATP5C1;ATP5E;ATP5F1;ATP5I;ATP5L;ATP5O;ATP6AP1;ATP6V1G1;ATXN7L3;AURKC;B2M;BAG6;BAP1;BAZ1A;BBIP1;B

IP5;ARMC10;ARSA;ARSK;ASPH;ATL2;ATP10D;ATP11C;B2M;B3GALTL;BCAP29;BCAP31;BET1;BNIP1;CALU;CAMK2D;CDIPT;CDS1;CEPT1;CERCAM;CERS5;CHERP;CISD2;CLN3;CLN6;CLSTN3;CNIH;CNPY3;COL24A1;COL4A3BP;COL7A1;CTDNEP1;CYP2S1;CYP2U1;CYP2W1;CYP4F22;DDOST;DEGS1;DEGS2;DERL1;DH

.DAP1;AGAP2;AGAP3;AGFG2;AKT1;ALDH4A1;ANAPC10;ARAP2;ARF4;ARFGAP1;ARFGAP2;ARHGAP5;ARL2;ARL8B;ARRB1;ASAP3;ATG12;ATG16L2;ATG4A;ATG4B;ATG4C;ATG5;ATG9A;ATL2;ATP5C1;ATP5E;ATP5F1;ATP5I;ATP5L;ATP5O;AUH;BAG6;BAP1;BCAT1;BCAT2;BCKDHB;BDH2;BIN1;BIRC2;BNIP1;BNIP2;

;ARHGAP22;ARHGEF2;ARID2;ARID4A;ARID4B;ARID5A;ARID5B;ARRB1;ASF1A;ASPH;ATAD2;ATF1;ATF2;ATRX;ATXN7L3;BACH1;BAZ1A;BAZ2B;BBX;BCLAF1;BDP1;BIRC2;BLOC1S2;BLZF1;BMI1;BMPR1A;BMPR2;BRCA2;BRD9;BRIP1;BRMS1;BRMS1L;BRWD1;BTAF1;BZW1;C10orf2;C12orf52;C14orf166;CAMK4;C

BPG;CHAF1A;CHAF1B;CRY1;CUL4B;DCLRE1A;DDB2;DEK;DNA2;ENDOV;EPC2;ERCC2;ERCC8;FAM175A;FANCB;FANCL;FANCM;GEN1;GTF2H1;GTF2H4;HMGB1;HMGB2;IGHMBP2;INO80C;INO80D;JMY;KIAA0101;KIF22;KIN;LIG4;MCM9;MEN1;MMS22L;MNAT1;MORF4L2;MSH2;MUM1;NBN;NSMCE2;NUDT16

;ANXA1;APEX2;ARG2;ARL2;ATAD3A;ATP5C1;ATP5E;ATP5F1;ATP5I;ATP5L;ATP5O;AUH;BCAT2;BCKDHB;BMF;BNIP3L;C10orf2;C14orf2;CASP8;CBR4;CCDC90A;CHCHD4;CISD1;CISD2;CNP;COASY;COQ10B;COQ2;COX16;COX6C;COX7A2;COX7A2L;COX7B;COX7C;CRAT;CRLS1;CYCS;DDX3X;DECR1;DHFRL1;DHRS1

EMIN6;GTF2F2;HNRNPH2;HNRNPL;HNRPLL;IVNS1ABP;JMJD6;KIAA1967;LSM1;LSM3;LSM6;LUC7L3;MBNL1;MBNL2;NAA38;NUDT21;PAPOLA;PCF11;PHAX;PHF5A;PLRG1;POLR2E;POLR2K;PPIG;PPIL3;PPP2R1A;PPP4R2;PPWD1;PRPF18;PRPF31;PRPF38B;PRPF39;PRPF40A;PRPF4B;PRPF6;PSIP1;PTBP2;PUF60;C

P22;ARHGEF2;ARID2;ARID4A;ARID4B;ARID5A;ARID5B;ARRB1;ASF1A;ASGR2;ASPH;ASTL;ATAD2;ATF2;ATP7A;ATRX;ATXN7L3;BACH1;BAG6;BAZ1A;BAZ2B;BBX;BCLAF1;BDP1;BIRC2;BLOC1S2;BLZF1;BMI1;BMPR1A;BMPR2;BRCA2;BRD9;BRIP1;BRMS1;BRMS1L;BRWD1;BTAF1;BZW1;C12orf52;C14orf166;CAMK

SF2;ACSF3;ACSL4;ACSL6;ACSS1;ACTL6A;ACTR1A;ACVR1C;ACVR2A;ACVRL1;ADCK1;ADCK2;ADCK3;ADRBK1;AK3;AKD1;AKT1;ANKK1;ARAF;ASNA1;ATAD1;ATAD2;ATAD2B;ATAD3A;ATP10D;ATP11B;ATP11C;ATP13A2;ATP13A3;ATP2B1;ATP2C1;ATP6AP1;ATP7A;ATP8A1;ATP8A2;ATP8B4;ATPBD4;ATRX;AURKC;

;ADAM10;ADAP1;ADM2;ADORA1;ADRBK1;ADSSL1;AGAP2;AGAP3;AGFG2;AGPAT5;AIDA;AK3;AKAP5;AKT1;AKTIP;ALDOA;ANG;ANGPT1;APC;ARAF;ARAP2;ARF4;ARFGAP1;ARFGAP2;ARHGAP5;ARL2;ARL8B;ARRB1;ARSA;ARSK;ASAP3;ATL2;ATP2B1;ATP2C1;ATP5C1;ATP5E;ATP5F1;ATP5I;ATP5L;ATP5O;ATP7A;

5;CASP3;CCNA2;CCNC;CCND3;CCNE2;CCNG1;CCNG2;CCNH;CCNL1;CCNT2;CCNY;CCNYL1;CD28;CDC25C;CDC26;CDC27;CDC37;CDC7;CDC73;CDK1;CDK2;CDK5R1;CDK5RAP3;CDK7;CDK9;CDKN1B;CDKN1C;CDKN2C;CDKN3;CDT1;CGRRF1;CHFR;CHMP1A;CHORDC1;CKS1B;CKS2;CNPPD1;COPS5;CRLF3;CTBP1;CL

3C;ANXA1;AP1AR;AP1B1;AP1S2;AP2A2;AP3M1;AP3S2;APC;APPBP2;APPL1;ARF4;ARFGAP1;ARFGAP2;ARFGEF2;ARFIP1;ARHGDIA;ARHGEF2;ARL2;ARL6IP1;ARRB1;ASPH;ATG16L2;ATG4A;ATG4B;ATG4C;ATG9A;ATP6V1G1;AURKC;BAG4;BAG6;BCAP29;BCAP31;BET1;BLZF1;BMPR1A;BORA;C5orf30;CALCRL;CAI

;AGER;AHR;AIM2;AKAP5;AKT1;ALDOA;AMD1;ANG;ANKRD49;APEX1;APIP;ARG2;ARGLU1;ARHGAP22;ARHGEF2;ARID2;ARID4A;ARID4B;ARID5A;ARID5B;ARRB1;ASF1A;ASNSD1;ASPH;ATAD2;ATF1;ATF2;ATP2B1;ATP2C1;ATP5C1;ATP5E;ATP5F1;ATP5I;ATP5L;ATP5O;ATP7A;ATRX;ATXN7L3;AZIN1;BACH1;BAZ1

D3;ADORA1;AFF4;AGAP2;AKAP11;AKT1;AKTIP;ALDOA;ALKBH8;ALS2CR8;ANG;ANKRD26;ANKRD32;ANLN;ANXA1;AP1AR;APC;APEX1;APOBEC3D;APPBP2;APTX;ARFGAP2;ARFGEF2;ARHGDIA;ARHGEF2;ARID5A;ARL2;ARL4A;ARL8B;ARPC4;ARRB1;ASNA1;ASPM;ATAD3A;ATRX;AURKC;AZI1;BAIAP2;BANF1;BAP1

D3;ADORA1;AFF4;AGAP2;AKAP11;AKT1;AKTIP;ALDOA;ALKBH8;ALS2CR8;ANG;ANKRD26;ANKRD32;ANLN;ANXA1;AP1AR;APC;APEX1;APOBEC3D;APPBP2;APTX;ARFGAP2;ARFGEF2;ARHGDIA;ARHGEF2;ARID5A;ARL2;ARL4A;ARL8B;ARPC4;ARRB1;ASNA1;ASPM;ATAD3A;ATRX;AURKC;AZI1;BAIAP2;BANF1;BAP1

SF2;ACSF3;ACSL4;ACSL6;ACSS1;ACTL6A;ACTR1A;ACVR1C;ACVR2A;ACVRL1;ADCK1;ADCK2;ADCK3;ADRBK1;AK3;AKAP7;AKD1;AKT1;ANKK1;ARAF;ASNA1;ATAD1;ATAD2;ATAD2B;ATAD3A;ATP10D;ATP11B;ATP11C;ATP13A2;ATP13A3;ATP2B1;ATP2C1;ATP6AP1;ATP7A;ATP8A1;ATP8A2;ATP8B4;ATPBD4;ATRX;
CDKN1B;CEP120;CEP135;CEP152;CEP250;CEP57;CEP57L1;CETN3;CHORDC1;CKAP2;CKS2;CLIP3;CNP;CNTROB;CRIPT;DCTN2;DICER1;ECT2;FBXO5;FER;FGFR1OP;FOPNL;GAPDH;GAS2L2;GCC2;HAUS1;HAUS3;HAUS6;HAUS7;HDAC3;HDAC6;HOOK1;HOOK2;KATNB1;KIAA0101;KIF11;KIF18A;KIF23;KIF2A;LMNA;
AKAP5;AKT1;ALDH4A1;ALDOA;AMD1;ANG;ANKRD49;APEX1;ARGLU1;ARHGAP22;ARHGEF2;ARID2;ARID4A;ARID4B;ARID5A;ARID5B;ARRB1;ASF1A;ASPH;ATAD2;ATF1;ATF2;ATP2B1;ATP2C1;ATP5C1;ATP5E;ATP5F1;ATP5I;ATP5L;ATP5O;ATP7A;ATRX;ATXN7L3;BACH1;BAZ1A;BAZ2B;BBX;BCLAF1;BDP1;BIRC2;B
CYBASC3;DTNBP1;EEA1;EPS15;FGD2;HGS;HLA-A;HLA-DMA;HLA-DPB1;HLA-DRA;HPS6;LAMTOR3;LDLR;LEPROT;LLGL1;MAP3K7;MARCH1;MCOLN1;MITD1;MTMR2;NDFIP1;NDFIP2;PIKFYVE;RAB10;RAB11FIP2;RAB12;RAB14;RAB22A;RAB27B;RAB5A;RAP2A;RAP2C;RET;RNF13;SCAMP1;SCYL2;SH3GL1;SLC29A
1;UBE2K;UBE2Q2;UBE2T;UBE3A;ZNRF1
P1;SENP6;UCHL5;USP1;USP12;USP15;USP16;USP19;USP21;USP25;USP37;USP44;USP46;USP5;USP6;USPL1;YOD1;ZRANB1
;C1GALT1;DDOST;DHPS;DOHH;DOLPP1;DPM1;EDEM3;EIF5A2;ESCO1;ESCO2;GALNT1;GALNT3;GALNT4;GALNT7;GAS6;GCNT1;GCNT4;GFPT1;GMPPA;LMAN1;MAN1A1;MAN1A2;MAN2A1;MANEA;MGAT1;MGAT2;MGAT4A;MPI;MUC1;MUC20;MUC5B;MUC6;PGAP1;PGM3;PIGA;PIGB;PIGF;PIGK;PIGM;PIGS
CTSS;CYBASC3;DTNBP1;EEA1;EPS15;FGD2;HGS;HLA-A;HLA-DMA;HLA-DPB1;HLA-DRA;HPS6;JAK2;LAMTOR3;LDLR;LEPROT;LLGL1;MAP3K7;MARCH1;MCOLN1;MITD1;MTMR2;NDFIP1;NDFIP2;PIKFYVE;RAB10;RAB11FIP2;RAB12;RAB14;RAB22A;RAB27B;RAB5A;RAP2A;RAP2C;RET;RNF13;SCAMP1;SCYL2;SH3
1;RNF31;SIRT2;TAB2;UBE2N;UBR5;UCHL3;USP16;VPS36;ZFAND6;ZRANB1;ZRANB3
SF2;ACSF3;ACSL4;ACSL6;ACSS1;ACTL6A;ACTR1A;ACVR1C;ACVR2A;ACVRL1;ADCK1;ADCK2;ADCK3;ADRBK1;AK3;AKAP7;AKD1;AKT1;ANKK1;ARAF;ASNA1;ATAD1;ATAD2;ATAD2B;ATAD3A;ATP10D;ATP11B;ATP11C;ATP13A2;ATP13A3;ATP2B1;ATP2C1;ATP6AP1;ATP7A;ATP8A1;ATP8A2;ATP8B4;ATPBD4;ATRX;
CC2;GOLGA4;GOPC;INSIG1;LLGL1;LMAN1;MON2;NAPG;OSBPL5;PRKCI;RAB10;RAB14;RAB2A;RAB33B;RAB3IP;RAB6A;RAB9B;RER1;RNF139;SAR1A;SAR1B;SCAMP1;SCAMP3;SCFD1;SCYL1;SEC13;SEC23A;SEC24A;SLC30A6;SNX8;SPAST;STEAP2;STX16;STX4;STX5;STX7;TMED10;TMED2;TMX1;TRAPPC1;TRAPP
R;AIM2;AKAP5;AKT1;ANKRD49;APEX1;ARAP2;ARFGAP1;ARFGAP2;ARGLU1;ARHGAP22;ARHGAP5;ARHGEF2;ARID2;ARID4A;ARID4B;ARID5A;ARID5B;ARL2;ARRB1;ASAP3;ASF1A;ASPH;ATAD2;ATF2;ATRX;ATXN7L3;BACH1;BAZ1A;BAZ2B;BBX;BCLAF1;BDP1;BIN1;BIRC2;BLOC1S2;BLZF1;BMI1;BMPR1A;BMPR2;
AKAP5;AKT1;ALDOA;AMD1;ANG;ANKRD49;APEX1;ARGLU1;ARHGAP22;ARHGEF2;ARID2;ARID4A;ARID4B;ARID5A;ARID5B;ARRB1;ASF1A;ASPH;ATAD2;ATF1;ATF2;ATP2B1;ATP2C1;ATP5C1;ATP5E;ATP5F1;ATP5I;ATP5L;ATP5O;ATP7A;ATRX;ATXN7L3;BACH1;BAZ1A;BAZ2B;BBX;BCLAF1;BDP1;BIRC2;BLOC1S2;B
YRK2;ERCC8;FBXL3;FBXL4;FBXL5;FBXO11;FBXO31;FBXO4;FBXO8;FBXW4;FBXW7;KEAP1;KLHL20;KLHL22;KLHL7;KLHL9;NEDD4;RBCK1;RBX1;RCHY1;RING1;RNF11;RNF144B;RNF2;RNF31;RNF40;RNF7;SKP2;SMURF2;TRAF7;UBE2A;UBE2E1;UBE2N;UBE4A;UBOX5;UBR1;WWP
EMIN2;GEMIN6;GNL1;GNL3;GTPBP10;KRR1;LSM6;MBNL1;MINA;MPHOSPH6;NOC4L;NOL6;NOP2;NOP56;NOP58;NSA2;NUDT16;PAN3;PES1;PHAX;PIN4;PRPF31;PRPF6;PSIP1;PTBP2;PTEN;PUM2;RBM5;RPF1;RPL11;RPL26;RPL7;RPS24;RPS7;RRP1;RRP36;RRP8;RRS1;RSL24D1;SART1;SCAF11;SF3A1;SF3A2;SF
CNY;CCP110;CDC25C;CDC7;CDC73;CDK1;CDK2;CDK7;CDKN1B;CDKN1C;CDKN2C;CDKN3;CDT1;CEP135;CEP152;CEP250;CEP290;CEP57;CEP70;CHMP1A;CKS1B;CRLF3;CUL2;CUL4B;CUL5;DBF4;DCTN2;DDX11;DDX3X;DGKZ;DLG1;DNA2;E2F4;E2F6;E2F7;EIF4E;FBXO31;FBXO5;FGFR1OP;GMNN;GPR132;KPNA2;
5;AKAP7;AKT1;AKTIP;ALKBH5;ANG;ANGPT1;ANKRD13C;ANXA1;AP1AR;AP1B1;AP1S2;AP2A2;AP3M1;AP3S2;APC;APOO;APPBP2;APPL1;ARF4;ARFGAP1;ARFGAP2;ARFGEF2;ARFIP1;ARHGDIA;ARHGEF2;ARL2;ARL6IP1;ARRB1;ARV1;ASPH;ATG16L2;ATG4A;ATG4B;ATG4C;ATG9A;ATP10D;ATP11B;ATP11C;ATP6
P1;ADM2;ADORA1;ADRBK1;ADSSL1;AGAP2;AGAP3;AGFG2;AGPAT5;AIDA;AK3;AKAP5;AKT1;AKTIP;ALDOA;ANG;ANGPT1;APC;ARAF;ARAP2;ARF4;ARFGAP1;ARFGAP2;ARHGAP5;ARL2;ARL8B;ARRB1;ARSA;ARSK;ASAP3;ATL2;ATP2B1;ATP2C1;ATP5C1;ATP5E;ATP5F1;ATP5I;ATP5L;ATP5O;ATP7A;AURKC;BAG4;
DOA;AMD1;ANG;ANKRD49;APEX1;ARGLU1;ARHGAP22;ARHGEF2;ARID2;ARID4A;ARID4B;ARID5A;ARID5B;ARRB1;ASF1A;ASPH;ATAD2;ATF1;ATF2;ATP2B1;ATP2C1;ATP5C1;ATP5E;ATP5F1;ATP5I;ATP5L;ATP5O;ATP7A;ATRX;ATXN7L3;BACH1;BAZ1A;BAZ2B;BBX;BCLAF1;BDP1;BIRC2;BLOC1S2;BLZF1;BMI1;BMF
PPWD1;PRPF18;PRPF31;PRPF38B;PRPF4B;PRPF6;PTBP2;RALY;RBM5;RHEB;RNPC3;SART1;SF3A1;SF3A2;SF3B1;SF3B14;SKIV2L2;SLU7;SMNDC1;SNRNP35;SNRNP48;SNRPB;SNRPB2;SNRPD1;SNRPE;SNRPG;SREK1;SUGP1;TXNL4B;U2AF1;UPF1;WBP4;XAB2;ZCCHC8;ZCRB1;ZNF32
CNY;CCP110;CDC25C;CDC7;CDC73;CDK1;CDK2;CDK7;CDKN1B;CDKN1C;CDKN2C;CDKN3;CDT1;CEP135;CEP152;CEP250;CEP290;CEP57;CEP70;CHMP1A;CKS1B;CRLF3;CUL2;CUL4B;CUL5;DBF4;DCTN2;DDX11;DDX3X;DGKZ;DLG1;DNA2;E2F4;E2F6;E2F7;EIF4E;FBXO31;FBXO5;FGFR1OP;GMNN;GPR132;KPNA2;
26;CDC27;CDC7;CDC73;CDK1;CDK2;CDK7;CDK9;CDKN1B;CDKN1C;CDKN2C;CDKN3;CDT1;CGRRF1;CHFR;CHMP1A;CRLF3;CUL2;CUL5;DBF4;DGKZ;DLG1;DNA2;ERCC2;ERN1;FAM175A;FBXO31;FBXO5;GAS2;GAS2L2;GAS6;GMNN;HDAC3;HORMAD1;HPGD;HSF1;IL12A;IL8;ILK;JMY;KAT2B;KIF20B;MAD2L1;MAPK
NDFIP1;NDFIP2;PELI1;PSMA1;PSMA2;PSMA3;PSMA4;PSMA6;PSMA8;PSMB1;PSMB3;PSMC1;PSMC2;PSMC3;PSMC5;PSMC6;PSMD10;PSMD13;PSMD14;PSMD4;PSMD8;PSME1;PTEN;RASSF1;RCHY1;RIPK2;SUMO3;TICAM1;TOPORS;UBC;UBE2D1;UBE2E1;UBE2N;WFS
3;LYST;MAP2K2;MON2;MTMR2;PIKFYVE;RAB10;RAB12;RAB14;RAB9B;RHOBTB3;SCYL2;SLC18A2;SLC30A6;SNX16;SNX17;SNX4;SNX8;SPAG9;STAM2;STX16;STX5;TOM1;UBC;VAMP7;VPS13A;VPS26A;VPS28;VPS36;VPS37A;VPS37B;VPS4A;VPS4B;VPS54;VTA1;YKT6;ZFYVE1
4;CD63;CDK2;CDKN1B;CHMP1A;CHMP1B;CHMP2A;CHMP2B;CLIP3;CLN3;CRK;CST3;CTNS;CTSS;CYBASC3;DERL1;DTNBP1;EEA1;EPS15;EXPH5;FAM109B;FGD2;FGFR4;FRS2;GIF;GPC1;HGS;HLA-A;HLA-DMA;HLA-DPB1;HLA-DRA;HPS6;IL15;JAK2;KCNQ1;KIAA1033;KIFC1;LAMTOR3;LDLR;LEPROT;LLGL1;LRP6;MA

B1;PSMB3;PSMC1;PSMC2;PSMC3;PSMC5;PSMC6;PSMD10;PSMD13;PSMD14;PSMD4;PSMD8;PSME1;RB1;RFWD2;RPS27L;TP63;TRIAP1;UB
USP37;USP44;USP46;USP6;USPL1;VCPIP1;YOD1;ZRANB1
CDK2;CDKN1B;CDT1;CHFR;DBF4;DGKZ;DLG1;DNA2;ERCC2;FAM175A;FBXO31;HORMAD1;HSF1;MAD2L1;MAPKAPK2;MCM5;MDM2;MSH2;MUC1;NBN;ORC2;ORC3;ORC4;PRCC;PSMA1;PSMA2;PSMA3;PSMA4;PSMA6;PSMA8;PSMB1;PSMB3;PSMC1;PSMC2;PSMC3;PSMC5;PSMC6;PSMD10;PSMD13;PSMD14
PSMB1;PSMB3;PSMC1;PSMC2;PSMC3;PSMC5;PSMC6;PSMD10;PSMD13;PSMD14;PSMD4;PSMD8;PSME1;PTEN;TOPORS;UBC;UBE2D1;UBE2E1;UBE2N;XRCC
;PSMB1;PSMB3;PSMC1;PSMC2;PSMC3;PSMC5;PSMC6;PSMD10;PSMD13;PSMD14;PSMD4;PSMD8;PSME1;RB1;RFWD2;RPS27L;TP63;TRIAP1;UB
CDK2;CDK9;CDKN1B;CDT1;CHFR;CRLF3;DBF4;DGKZ;DLG1;DNA2;ERCC2;FAM175A;FBXO31;HORMAD1;HSF1;MAD2L1;MAPKAPK2;MCM5;MDM2;MSH2;MUC1;NBN;ORC2;ORC3;ORC4;PKD2;PRCC;PSMA1;PSMA2;PSMA3;PSMA4;PSMA6;PSMA8;PSMB1;PSMB3;PSMC1;PSMC2;PSMC3;PSMC5;PSMC6;PSMD1
DXACB1;FRG1;FTSJ1;GEMIN2;GEMIN6;HARS2;HSD17B10;INTS12;INTS2;INTS5;INTS6;INTS8;KIAA0391;KRR1;LIN28A;LSM6;MARS2;MEPCE;METTL1;MKI67IP;MOCS3;MPHOSPH6;MTO1;NARS2;NOC4L;NOL6;NOP2;NOP56;NOP58;NSA2;NUDT16;OSGEPL1;PES1;PHAX;PIN4;PIWIL4;POLG2;PPA1;PUS10;PUS7L;I
PSMC1;PSMC2;PSMC3;PSMC5;PSMC6;PSMD10;PSMD13;PSMD14;PSMD4;PSMD8;PSME1;RFWD2;RPS27L;TP63;TRIAP1;UB
28;CD2AP;CDC25C;CDC26;CDC27;CDK1;CDK11A;CDK11B;CDK2;CENPE;CENPK;CENPL;CENPQ;CENPW;CEP120;CETN3;CHFR;CHMP1A;CKS2;CSNK1A1;DAPK3;DCLRE1A;DCTN2;DDX11;DICER1;DIS3L2;DYNLT3;EHMT2;EREG;FANCM;FBXO5;GORASP1;HAUS1;HAUS3;HAUS6;HAUS7;HDAC3;HELLS;HORMAD1;HSF
2;MUC1;NBN;PRCC;PSMA1;PSMA2;PSMA3;PSMA4;PSMA6;PSMA8;PSMB1;PSMB3;PSMC1;PSMC2;PSMC3;PSMC5;PSMC6;PSMD10;PSMD13;PSMD14;PSMD4;PSMD8;PSME1;RAD17;RB1;RFWD2;RPS27L;TP63;TRIAP1;TTK;UBC;UBE2D1;UBE2E1;USP44;ZWILC
PSMA4;PSMA6;PSMA8;PSMB1;PSMB3;PSMC1;PSMC2;PSMC3;PSMC5;PSMC6;PSMD10;PSMD13;PSMD14;PSMD4;PSMD8;PSME1;PTEN;TOPORS;UBC;UBE2D1;UBE2E1;UBE2N;USP44;XRCC
A;BMPR2;CAB39;CAMK1;CAMK2D;CAMK4;CCND3;CCNH;CDC7;CDK1;CDK11A;CDK11B;CDK17;CDK18;CDK2;CDK5R1;CDK7;CDK8;CDK9;CDKL1;CDKL2;CHUK;CKB;CLK1;CLK3;CLK4;CLP1;CMPK1;COASY;COL4A3BP;CPNE3;CRIM1;CSK;CSNK1A1;CSNK1A1L;CSNK1G3;CSNK2B;DAK;DAPK3;DCAKD;DCK;DGKE;DGKZ
E2;CCNG1;CCNG2;CCNT2;CCNY;CCP110;CD2AP;CDC14A;CDC25C;CDC26;CDC7;CDK1;CDK2;CDK7;CENPE;CENPW;CETN3;CHFR;CHMP1A;CHMP1B;CKAP2;CKS1B;CKS2;CNTROB;CSNK1A1;DAPK3;DAXX;DCLRE1A;DICER1;DIS3L2;DYNLT3;E2F7;ECT2;ERCC2;EREG;FBXO5;FGF7;GNAI1;GTPBP8;HAUS1;HAUS3;HAU
MA4;PSMA6;PSMA8;PSMB1;PSMB3;PSMC1;PSMC2;PSMC3;PSMC5;PSMC6;PSMD10;PSMD13;PSMD14;PSMD4;PSMD8;PSME1;PTEN;RB1;RFWD2;RPS27L;TJP3;TP63;TRIAP1;UB
DAP1;ADORA1;AGAP2;AGAP3;AGFG2;AGL;AKT1;ALDH4A1;ALDOA;ANAPC10;APC;ARAP2;ARF4;ARFGAP1;ARFGAP2;ARHGAP5;ARL2;ARL8B;ARRB1;ASAP3;ATL2;ATP5C1;ATP5E;ATP5F1;ATP5I;ATP5L;ATP5O;AUH;BAG6;BAP1;BCAT1;BCAT2;BCKDHB;BDH2;BIN1;BIRC2;BNIP2;BNIP3L;CASP3;CASP8;CCBL2;CCNI
PT1;GMPPA;LMAN1;MAGT1;MAN1A1;MAN1A2;MAN2A1;MANEA;MGAT1;MGAT2;MGAT4A;MPI;OSTC;PGM3;PRKCSH;SAR1B;SEC13;SEC23A;SEC24A;ST8SIA4;STT3B;UBE2J1;UGGT
K3;MEF2A;MEF2C;NFKB2;NFKBIB;PELI1;RIPK2;RPS6KA3;RPS6KA5;TAB1;TAB2;TAB3;TLR6;UBC;UBE2N
;PSMB3;PSMC1;PSMC2;PSMC3;PSMC5;PSMC6;PSMD10;PSMD13;PSMD14;PSMD4;PSMD8;PSME1;PTEN;UBC;UBE2D1;UBE2E1;USP4
RL1;DHRS9;DMPK;DOLPP1;ELOVL4;ERN1;EXTL2;FITM1;FMO5;HLA-A;HLA-DPB1;HLA-DRA;INSIG1;INSIG2;L2HGDH;LEMD3;LFNG;MARCH6;MFF;PEX11A;PEX11G;PEX12;PEX13;PEX2;PEX3;PGAP1;PGAP3;PIGB;PIGK;PIGS;PIGZ;PKD2;PORCN;PQLC2;RER1;RFNG;RHOT1;SACM1L;SAMD8;SEC61A1;SERAC1;SGMS1
AB39;CAMK1;CAMK2D;CAMK4;CCND3;CCNH;CDC7;CDK1;CDK11A;CDK11B;CDK17;CDK18;CDK2;CDK5R1;CDK7;CDK8;CDK9;CDKL1;CDKL2;CHUK;CLK1;CLK3;CLK4;COL4A3BP;CPNE3;CRIM1;CSK;CSNK1A1;CSNK1A1L;CSNK1G3;CSNK2B;DAPK3;DMPK;DYRK1A;DYRK1B;DYRK2;EFNA3;ENG;EPHB3;EPHB6;ERCC2;
CKDHB;C10orf2;CBR4;DECR1;DHFRL1;DHX30;DLAT;DNA2;ECHS1;ERAL1;ETFA;FARS2;GLS;GRPEL2;GUF1;HARS2;HMGCL;HSD17B10;HSPE1;IDH2;IDH3B;IDH3G;KIAA1967;LONP1;MARS2;MCEE;MCL1;MDH2;MRPL13;MRPL18;MRPL28;MRPL3;MRPL32;MRPL37;MRPL39;MRPL40;MRPL42;MRPL47;MRPL48;MI
;AIM2;AKAP5;AKR1B1;AKT1;ALDH4A1;ALDOA;AMD1;ANG;ANKRD49;APEX1;ARGLU1;ARHGAP22;ARHGEF2;ARID2;ARID4A;ARID4B;ARID5A;ARID5B;ARRB1;ASF1A;ASPH;ATAD2;ATF1;ATF2;ATP2B1;ATP2C1;ATP5C1;ATP5E;ATP5F1;ATP5I;ATP5L;ATP5O;ATP7A;ATRX;ATXN7L3;BACH1;BAZ1A;BAZ2B;BBX;BCLAF
PT1;GMPPA;LMAN1;MAN1A1;MAN1A2;MAN2A1;MANEA;MGAT1;MGAT2;MGAT4A;MPI;OSTC;PGM3;PRKCSH;SAR1B;SEC13;SEC23A;SEC24A;STT3B;UBE2J1;UGGT
;INSIG1;INSIG2;MID1IP1;MLYCD;NQO1;NR1H2;NR3C1;PDHX;PDK1;PDK2;PDP1;PRKAA1;PRKAB2;PRKAG2;PSMA1;PSMA2;PSMA3;PSMA4;PSMA6;PSMA8;PSMB1;PSMB3;PSMC1;PSMC2;PSMC3;PSMC5;PSMC6;PSMD10;PSMD13;PSMD14;PSMD4;PSMD8;PSME1;PTGS2;REST;SCAP;SIRT1;SMEK1;SMEK2;SREB
APKAPK3;MEF2A;MEF2C;NFKB2;NFKBIB;PELI1;RIPK2;RPS6KA3;RPS6KA5;TAB1;TAB2;TAB3;TLR6;UBC;UBE2N
NRNPH2;HNRNPL;JMJD6;LSM1;LSM3;MBNL1;MBNL2;NAA38;NUDT21;PAPOLA;PCF11;PHAX;PHF5A;PLRG1;POLR2E;POLR2K;PPIL3;PPWD1;PRPF31;PRPF4B;PRPF6;PSIP1;PTBP2;RALY;RBFOX3;RBM25;RBM4;RBM5;SART1;SCAF11;SF3A1;SF3A2;SF3B1;SF3B14;SFSWAP;SKIV2L2;SLU7;SMNDC1;SNRPB;SNRPB2;
5A;RAB8B;RAB9B;RAP1B;RHEB;TRIM23
DK5R1;CDK5RAP3;CDK7;CDKN1B;CDKN1C;CDKN2C;CDKN3;CKS1B;CKS2;CNPPD1;GTF2H1;KAT2B;MEN1;MNAT1;PKD2;PSMD10;PTEN;TFAP4
S;HLA-DMA;HLA-DRA;KCNQ1;LAMTOR3;LDLR;MAP2K2;MARCH1;MCOLN1;MITD1;NDFIP2;PIKFYVE;RAB14;RAB27B;RNF13;SDF4;SLC29A3;SLC40A1;SNX16;STARD3;STARD3NL;TMEM106B;TMEM55A;TPT1;UNC13D;VAC14;VAMP7;VPS18;VPS28;VPS33B;VPS36;VPS37A;VPS37B;VPS4A;VPS4
PT1;GMPPA;LMAN1;MAN1A1;MAN1A2;MAN2A1;MANEA;MGAT1;MGAT2;MGAT4A;MPI;OSTC;PGM3;PRKCSH;SAR1B;SEC13;SEC23A;SEC24A;STT3B;UBE2J1;UGGT
;PSMB3;PSMC1;PSMC2;PSMC3;PSMC5;PSMC6;PSMD10;PSMD13;PSMD14;PSMD4;PSMD8;PSME1;PTEN;SKP2;UBC;UBE2D1;UBE2E1;USP4
Y1;RNF11;RNF144B;RNF146;RNF217;SIAH1;SMURF2;SUMO3;UBE3A;UBR5;WWP1
CNE2;CCNH;CCNL1;CCNT2;CCNY;CCNYL1;CDC25C;CDC37;CDK5R1;CDK5RAP3;CDKN2C;CEP152;CEP250;CNPPD1;CRY1;DAB2IP;DIXDC1;DLG1;DUSP19;EEF1A1;FAS;FBXO5;FER;FGFR1OP;FIZ1;GLRX3;GSTP1;HDAC5;HDAC7;HDAC9;HINT1;IBTK;IGBP1;ILK;ITGAV;JAK2;JUP;KAT2B;KIF11;LIPE;LLGL1;MAP3K1;MAF
AB39;CAMK1;CAMK2D;CAMK4;CCND3;CCNH;CDC7;CDK1;CDK11A;CDK11B;CDK17;CDK18;CDK2;CDK5R1;CDK7;CDK8;CDK9;CDKL1;CDKL2;CHUK;CLK1;CLK3;CLK4;CLP1;COASY;COL4A3BP;CPNE3;CRIM1;CSK;CSNK1A1;CSNK1A1L;CSNK1G3;CSNK2B;DAK;DAPK3;DCAKD;DCK;DGKE;DGKZ;DMPK;DYRK1A;DYRK1E
ASPH;ATG4C;BCAP29;BCAP31;BLZF1;BMPR1A;CAMK1;CAMK4;CD27;CDC37;CDK1;CDK5R1;CEP57;CHCHD4;CHERP;CHM;CHML;COG3;COPZ1;CSK;DAB2IP;DDOST;DERL1;DMAP1;DYRK2;ECT2;ERBB2IP;EXPH5;GAS6;GCC2;GIPC1;GOLGA4;GOPC;GRPEL2;HDAC6;HGS;INPP5K;IPO11;IPO13;IPO4;JAK2;JUN;JUP;KA
7;FEM1A;HSPBP1;LRRK2;MAD2L1;MALT1;NDFIP1;NDFIP2;PELI1;PSMA1;PSMA2;PSMA3;PSMA4;PSMA6;PSMA8;PSMB1;PSMB3;PSMC1;PSMC2;PSMC3;PSMC5;PSMC6;PSMD10;PSMD13;PSMD14;PSMD4;PSMD8;PSME1;PTEN;RASSF1;RCHY1;RIPK2;RNF139;SUMO3;TGFBR1;TICAM1;TOPORS;UBC;UBE2D1;U
PEX2;ARFGAP1;ARL2;ATAD3A;ATP11B;ATP5C1;ATP5E;ATP5F1;ATP5I;ATP5L;ATP5O;BMF;BNIP1;BNIP2;BNIP3L;BRIP1;C14orf2;CAPG;CASP8;CBX3;CCDC90A;CEP70;CEPT1;CERS5;CHCHD4;CISD1;CISD2;CNP;COASY;COQ10B;COQ2;COX16;COX6C;COX7A2;COX7A2L;COX7B;COX7C;CRAT;CRLS1;CST3;CTDNEP1;
;CAMK4;CD27;CDC40;CDK1;CEP57;CHCHD4;CHERP;CLIC2;COG3;CPSF1;CSK;DAB2IP;DDOST;DMAP1;DYRK2;ECT2;EEA1;EIF4E;EIF6;ERO1L;FYTTD1;GAS6;GCC2;GOLGA4;GOPC;GRPEL2;GSTM2;HGS;HMGXB4;HOOK1;HOOK2;IBTK;INSIG1;IPO11;IPO13;JAK2;JUN;JUP;KPNA2;KPNA3;KPNA5;LLGL1;LMAN1;LMN
USP46;USP6;VCPIP1;YOD1;ZRANB1
MC2;PSMC3;PSMC5;PSMC6;PSMD10;PSMD13;PSMD14;PSMD4;PSMD8;PSME1;UBC;UBE2D1;UBE2E1;USP4
MCM5;MNAT1;ORC2;ORC3;ORC4;POLD2;PSMA1;PSMA2;PSMA3;PSMA4;PSMA6;PSMA8;PSMB1;PSMB3;PSMC1;PSMC2;PSMC3;PSMC5;PSMC6;PSMD10;PSMD13;PSMD14;PSMD4;PSMD8;PSME1;RB1;RFC2;RFC3;RFC5;SKP2;TFAP4;UBC;WEE
RPL28;MRPL3;MRPL32;MRPL37;MRPL39;MRPL40;MRPL42;MRPL47;MRPL48;MRPL50;MRPS10;MRPS17;MRPS18B;MRPS18C;MRPS2;MRPS22;MRPS28;MRPS31;MRPS33;NUFIP2;PRMT3;RBM3;RPL11;RPL15;RPL17;RPL22L1;RPL23;RPL26;RPL26L1;RPL27;RPL30;RPL31;RPL34;RPL35;RPL36A;RPL36AL;RPL39;
SNA1;BAP1;BCAS2;BCLAF1;BMI1;BRWD1;BTD;BYSL;C8orf40;CAMK4;CAPG;CCDC86;CD2AP;CDKN2AIP;CENPW;COIL;CRBN;CUTC;DACH1;DAXX;DCAF13;DCLRE1A;DDX11;DDX50;DDX54;DNAJB9;DNTTIP2;ERI1;ESF1;FAM32A;FBXO11;FBXW7;FCF1;FMR1;FRG1;FXR1;G2E3;GNL3;GORAB;GRWD1;GTPBP10;GZF

EFSEC;EIF1AX;EIF2A;EIF2B5;EIF2S2;EIF3D;EIF3E;EIF3J;EIF4E;EIF4E2;EIF4E3;EIF5;EIF5A2;FARS2;FARSA;FDXACB1;FMR1;FXR1;GAPDH;GFM2;GUF1;HARS2;HBS1L;HRSP12;IGF2BP3;IGHMBP2;IMPACT;IREB2;LIN28A;MARS2;MRPL1;MRPL13;MRPL15;MRPL18;MRPL19;MRPL2;MRPL22;MRPL28;MRPL3;MRPL32;

RPF31;PRPF6;RBM3;RICTOR;RNF135;SEC61A1;SRP9;UHMK1
RID5A;ARRB1;ATG5;BAG4;BAG6;BMI1;BMPR1A;BMPR2;BRMS1;CAB39;CAMK1;CASP3;CCDC88A;CCNA2;CCNC;CCND3;CCNE2;CCNG1;CCNH;CCNL1;CCNT2;CCNY;CCNYL1;CD28;CD80;CD81;CDC25C;CDC26;CDC27;CDC37;CDK1;CDK2;CDK5R1;CDK5RAP3;CDK7;CDK9;CDKN1B;CDKN1C;CDKN2C;CDKN3;CENPE;
MED8;MED
SMC1;PSMC2;PSMC3;PSMC5;PSMC6;PSMD10;PSMD13;PSMD14;PSMD4;PSMD8;PSME1;PTEN;TOPORS;UBC;UBE2D1;UBE2E1;UBE2N
DNA2;E2F7;FBXO31;MDM2;MUC1;NBN;PKD2;PKIA;PSMA1;PSMA2;PSMA3;PSMA4;PSMA6;PSMA8;PSMB1;PSMB3;PSMC1;PSMC2;PSMC3;PSMC5;PSMC6;PSMD10;PSMD13;PSMD14;PSMD4;PSMD8;PSME1;PTEN;RAD17;RB1;RFWD2;RPS27L;TFAP4;TJP3;TP63;TRIAP1;UB
;INTS2;INTS5;INTS6;INTS8;KIAA0391;KRR1;LIN28A;LSM6;METTL1;MOCS3;MPHOSPH6;MTO1;NOC4L;NOL6;NOP2;NOP56;NOP58;NSA2;NUDT16;OSGEPL1;PES1;PIN4;PUS10;PUS7L;RPF1;RPL11;RPL26;RPL7;RPS24;RPS7;RRP1;RRP36;RRP8;SIRT1;SKIV2L2;SMAD2;SUV39H1;TRDMT1;TRMT12;TRNT1;TRUB1;T
SMA8;PSMB1;PSMB3;PSMC1;PSMC2;PSMC3;PSMC5;PSMC6;PSMD10;PSMD13;PSMD14;PSMD4;PSMD8;PSME1;PTEN;TOPORS;UBC;UBE2D1;UBE2E1;UBE2N;USP4
;PSMC2;PSMC3;PSMC5;PSMC6;PSMD10;PSMD13;PSMD14;PSMD4;PSMD8;PSME1;PTEN;UBC;UBE2D1;UBE2E1
;C14orf2;CBX3;CCDC90A;CNP;COQ10B;COX6C;COX7A2;COX7A2L;COX7B;COX7C;CRAT;CRLS1;CYCS;DHFRL1;DHRS1;ERAL1;ERN1;FAM169A;GPAM;GPD2;GRPEL2;GUF1;HIGD1A;HMGCL;HSD17B10;IDH2;IMMP1L;KMO;L2HGDH;LEMD3;LMNB2;MATR3;MCU;MDH2;MPV17;MTHFD2L;NDUFA1;NDUFA4;NDUF
G;PEX12;PEX13;PEX14;PEX2;PEX3;PNPLA8;RAB8E
G;PEX12;PEX13;PEX14;PEX2;PEX3;PNPLA8;RAB8E
2;HNRNPL;JMJD6;LSM3;MBNL1;MBNL2;NAA38;NUDT21;PAPOLA;PCF11;PHAX;PHF5A;PLRG1;POLR2E;POLR2K;PPIL3;PPWD1;PRPF31;PRPF4B;PRPF6;PSIP1;PTBP2;RALY;RBFOX3;RBM25;RBM4;RBM5;SART1;SCAF11;SF3A1;SF3A2;SF3B1;SF3B14;SFSWAP;SKIV2L2;SLU7;SNRPB;SNRPB2;SNRPD1;SNRPE;SNRPG
2;HNRNPL;JMJD6;LSM3;MBNL1;MBNL2;NAA38;NUDT21;PAPOLA;PCF11;PHAX;PHF5A;PLRG1;POLR2E;POLR2K;PPIL3;PPWD1;PRPF31;PRPF4B;PRPF6;PSIP1;PTBP2;RALY;RBFOX3;RBM25;RBM4;RBM5;SART1;SCAF11;SF3A1;SF3A2;SF3B1;SF3B14;SFSWAP;SKIV2L2;SLU7;SNRPB;SNRPB2;SNRPD1;SNRPE;SNRPG
3;PEX14;PEX2;PEX3;PMAIP1;PMPCA;RAB6A;RAB8B;RPL11;RPL15;RPL17;RPL23;RPL26;RPL26L1;RPL27;RPL30;RPL31;RPL34;RPL35;RPL36A;RPL39;RPL41;RPL7;RPL9;RPS15A;RPS24;RPS27;RPS29;RPS3A;RPS7;SEC11A;SEC11C;SEC61A1;SNX16;SPCS2;SPCS3;SRP14;SRP19;SRP9;SSR3;TIMM17A;TIMM22;TIMM2
2;TGFA;TTK
K3;MEF2A;MEF2C;NFKB2;NFKBIB;PELI1;RIPK2;RPS6KA3;RPS6KA5;TAB1;TAB2;TAB3;TLR10;TLR6;UBC;UBE2N
PEX2;ARFGAP1;ARL2;ATAD3A;ATP11B;ATP5C1;ATP5E;ATP5F1;ATP5I;ATP5L;ATP5O;BMF;BNIP1;BNIP2;BNIP3L;BRIP1;C14orf2;CAPG;CASP8;CBX3;CCDC90A;CEP70;CEPT1;CERS5;CHCHD4;CISD1;CISD2;CNP;COASY;COQ10B;COQ2;COX16;COX6C;COX7A2;COX7A2L;COX7B;COX7C;CRAT;CRLS1;CST3;CTDNEP1;
TMED10;TMED2;TMX1;TRAPPC1;TRAPPC2P1;TRAPPC8;VAMP7;YKT6
IRPG;SRSF10;SRSF6
PSMC3;PSMC5;PSMC6;PSMD10;PSMD13;PSMD14;PSMD4;PSMD8;PSME1;RFWD2;TRIAP1;UBC
PSMC3;PSMC5;PSMC6;PSMD10;PSMD13;PSMD14;PSMD4;PSMD8;PSME1;RFWD2;TRIAP1;UBC
PSMC3;PSMC5;PSMC6;PSMD10;PSMD13;PSMD14;PSMD4;PSMD8;PSME1;RFWD2;TRIAP1;UBC
PSMC3;PSMC5;PSMC6;PSMD10;PSMD13;PSMD14;PSMD4;PSMD8;PSME1;RFWD2;TRIAP1;UBC
CF3;ACAD10;ACAD9;ACADM;ACADSB;ACADVL;ACBD3;ACBD5;ACBD6;ACOT7;ACSF2;ACSF3;ACSL4;ACSL6;ACSS1;ACTL6A;ACTR1A;ACVR1C;ACVR2A;ACVRL1;ADAP1;ADCK1;ADCK2;ADCK3;ADRBK1;ADSSL1;AGAP2;AGAP3;AGPS;AIFM3;AK3;AKAP7;AKD1;AKT1;ANG;ANKK1;ARAF;ARAP2;ARF4;ARHGAP5;ARL13
;MEF2A;MEF2C;NFKB2;NFKBIB;PELI1;RIPK2;RPS6KA3;RPS6KA5;TAB1;TAB2;TAB3;TBK1;TICAM1;UBC
88B;CCP110;CDK1;CDK2;CDK5R1;CDKN1B;CENPE;CEP120;CEP135;CEP152;CEP250;CEP57;CEP57L1;CETN3;CHORDC1;CKAP2;CKS2;CLIP3;CLN3;CNP;CNTROB;CRIPT;DCTN2;DICER1;DNAH6;DTNBP1;DYNLRB1;ECT2;FBXO5;FER;FGFR1OP;FOPNL;GAPDH;GAS2L2;GCC2;HAUS1;HAUS3;HAUS6;HAUS7;HDAC3;HD
XACB1;FIGNL1;GSS;GTPBP10;HMGCL;HPRT1;ICK;IDH2;IDH3B;IDH3G;IDI1;LHPP;MAP3K13;MAP3K7;MAP3K8;MAST2;MAST4;MSH2;MST4;MTHFD2;NLK;NT5C3;NUDT16;OPA1;PAPOLA;PDXK;PGM3;PPA1;PPM1B;PRKACB;PRPS2;PRPSAP1;PRTFDC1;PTEN;REV1;RPS6KA3;RPS6KA4;RPS6KA5;S100P;SNRK;STK3
M11;MCM5;MNAT1;ORC2;ORC3;ORC4;POLD2;PSMA1;PSMA2;PSMA3;PSMA4;PSMA6;PSMA8;PSMB1;PSMB3;PSMC1;PSMC2;PSMC3;PSMC5;PSMC6;PSMD10;PSMD13;PSMD14;PSMD4;PSMD8;PSME1;RB1;RFC2;RFC3;RFC5;SKP2;TFAP4;UBC;WEE
IDE1;NSL1;NUDCD2;NUF2;NUP37;NUP85;ORC2;PMF1;PPP1CC;PPP1R12A;PSEN2;RANGAP1;SEC13;SEPT7;SGOL2;SKA2;SPAG5;SS18L1;SUMO3;XPO1;ZWILCH
RS9;DMPK;DOLPP1;ELOVL4;ERN1;FITM1;HLA-A;HLA-DPB1;HLA-DRA;INSIG1;INSIG2;L2HGDH;LEMD3;LFNG;MARCH6;MFF;PEX11A;PEX11G;PEX12;PEX13;PEX2;PEX3;PIGK;PIGS;PKD2;PORCN;PQLC2;RER1;RFNG;RHOT1;SACM1L;SAMD8;SEC61A1;SGMS1;SGMS2;SLC24A6;SSR3;ST8SIA4;STEAP2;SYP;TECR;TIM
BP2;APPL1;ARFIP1;ARHGEF2;ARL2;ARL6IP1;ASPH;ATG4C;ATG9A;AURKC;BAG4;BAG6;BCAP29;BCAP31;BLZF1;BMPR1A;CAMK1;CAMK4;CASC5;CD27;CD63;CDC37;CDK1;CDK5R1;CEP57;CEP57L1;CHCHD4;CHERP;CHM;CHML;CLIP3;COG3;COPZ1;CSK;CTDNEP1;DAB2IP;DBN1;DDOST;DENND4C;DERL1;DLG1;D

;ATP5F1;ATP5I;ATP5L;ATP5O;BMF;BNIP3L;C14orf2;CASP8;CCDC90A;CISD1;CISD2;CNP;COASY;COQ10B;COQ2;COX16;COX6C;COX7A2;COX7A2L;COX7B;COX7C;CRAT;CRLS1;CYCS;DDX3X;DHFRL1;DHRS1;DMPK;ERAL1;GK;GPAM;GPAT2;GPD2;GRPEL2;GUF1;HIGD1A;HMGCL;HSD17B10;IDH2;IMMP1L;KMO;L2
A;MEF2C;NFKB2;NFKBIB;RIPK2;RPS6KA3;RPS6KA5;TAB1;TAB2;TAB3;TBK1;TICAM1;UBC
PSMC3;PSMC5;PSMC6;PSMD10;PSMD13;PSMD14;PSMD4;PSMD8;PSME1;RFWD2;TRIAP1;UBC
PSMC3;PSMC5;PSMC6;PSMD10;PSMD13;PSMD14;PSMD4;PSMD8;PSME1;RFWD2;TRIAP1;UBC
PSMC3;PSMC5;PSMC6;PSMD10;PSMD13;PSMD14;PSMD4;PSMD8;PSME1;RFWD2;TRIAP1;UBC
K3;MEF2A;MEF2C;NFKB2;NFKBIB;PELI1;RIPK2;RPS6KA3;RPS6KA5;TAB1;TAB2;TAB3;TBK1;TICAM1;TLR6;UBC;UBE2N
;CABIN1;CASC5;CBX3;CDC73;CDK2;CDK9;CENPK;CENPQ;CENPW;CHD1;CHD5;CHD9;CTBP1;CXXC1;DAPK3;DEK;DMAP1;DOT1L;DPY30;DR1;EHMT2;EID1;ELK4;ENY2;EPC2;FAM175A;HDAC3;HDAC5;HDAC6;HDAC8;HDAC9;HELLS;HIRA;HLTF;HMGN5;HNF1A;HSF4;ING3;ITGB3BP;JAK2;JMJD6;KAT2B;KAT8;LRWD

ORA1;ADRBK1;AGAP2;AGAP3;AIM2;AKAP5;AKT1;AKTIP;ALKBH5;ANG;ANXA1;AP1AR;AP1B1;AP1S2;AP2A2;AP3M1;AP3S2;APOO;APPBP2;APPL1;ARF4;ARFGAP1;ARFGAP2;ARFGEF2;ARFIP1;ARHGEF2;ARL6IP1;ARL6IP5;ARPP19;ARRB1;ARV1;ASPH;ATG16L2;ATG4A;ATG4B;ATG4C;ATG9A;ATP10D;ATP11B;ATP
DAP1;ADORA1;AGAP2;AGAP3;AGFG2;AGL;AKT1;ALDH4A1;ALDOA;ANAPC10;APC;ARAP2;ARF4;ARFGAP1;ARFGAP2;ARHGAP5;ARL2;ARL8B;ARRB1;ASAP3;ATG12;ATG16L2;ATG4A;ATG4B;ATG4C;ATG5;ATG9A;ATL2;ATP5C1;ATP5E;ATP5F1;ATP5I;ATP5L;ATP5O;AUH;BAG6;BAP1;BCAT1;BCAT2;BCKDHB;BDH2

;LDLR;LPCAT1;LPCAT2;LPCAT4;MBOAT1;NQO1;PDE1B;PLA2G15;PLA2G4A;PNPLA6;PNPLA8;PSMA1;PSMA2;PSMA3;PSMA4;PSMA6;PSMA8;PSMB1;PSMB3;PSMC1;PSMC2;PSMC3;PSMC5;PSMC6;PSMD10;PSMD13;PSMD14;PSMD4;PSMD8;PSME1;SAMD8;SAT1;SCARB1;SGMS1;SGMS2;SLC27A1;SLC44A3;S
KD1;APPBP2;ARF4;ARHGAP5;ARL2;ARL8B;ASNA1;ATAD1;ATAD2;ATAD2B;ATAD3A;ATL2;ATP10D;ATP11B;ATP11C;ATP13A2;ATP13A3;ATP2B1;ATP2C1;ATP5C1;ATP5E;ATP5F1;ATP5I;ATP5L;ATP5O;ATP6AP1;ATP7A;ATP8A1;ATP8A2;ATP8B4;ATRX;BRIP1;BTAF1;C10orf2;CCNH;CDK7;CENPE;CGN;CHD1;CHD5;
KD1;APPBP2;ARF4;ARHGAP5;ARL2;ARL8B;ASNA1;ATAD1;ATAD2;ATAD2B;ATAD3A;ATL2;ATP10D;ATP11B;ATP11C;ATP13A2;ATP13A3;ATP2B1;ATP2C1;ATP5C1;ATP5E;ATP5F1;ATP5I;ATP5L;ATP5O;ATP6AP1;ATP7A;ATP8A1;ATP8A2;ATP8B4;ATRX;BRIP1;BTAF1;C10orf2;CCNH;CDK7;CENPE;CGN;CHD1;CHD5;
BP2;APPL1;ARFIP1;ARHGEF2;ARL2;ARL6IP1;ASPH;ATG4C;ATG9A;AURKC;BAG4;BAG6;BCAP29;BCAP31;BLZF1;BMPR1A;CAMK1;CAMK4;CASC5;CD27;CD63;CDC37;CDK1;CDK5R1;CEP57;CEP57L1;CHCHD4;CHERP;CHM;CHML;CLIP3;COG3;COPZ1;CSK;CTDNEP1;DAB2IP;DBN1;DDOST;DENND4C;DERL1;DLG1;D
PSMC3;PSMC5;PSMC6;PSMD10;PSMD13;PSMD14;PSMD4;PSMD8;PSME1;RFWD2;TRIAP1;UBC
2;MTHFD2L;MTHFSD;NADKD1;NADSYN1;NAMPT;PANK1;PANK3;PDHX;PDK1;PDK2;PDP1;PDSS1;PDXK;PTS;QDPR;QPRT;RFK;SLC25A1;SPR;TECR;TPK
O;LCAT;LDLR;LPCAT1;LPCAT2;LPCAT4;MBOAT1;MTHFD2L;MTRR;NQO1;PDE1B;PHGDH;PLA2G15;PLA2G4A;PNPLA6;PNPLA8;PSMA1;PSMA2;PSMA3;PSMA4;PSMA6;PSMA8;PSMB1;PSMB3;PSMC1;PSMC2;PSMC3;PSMC5;PSMC6;PSMD10;PSMD13;PSMD14;PSMD4;PSMD8;PSME1;SAMD8;SAT1;SCARB1;SG
;GPAM;GPAT2;GPCPD1;IDI1;IMPA1;INPP5D;INPP5K;LCAT;LCLAT1;LPCAT1;LPCAT2;LPCAT4;LPGAT1;MBOAT1;MTMR2;MTMR6;MTMR7;PGAP1;PGAP3;PI4K2B;PIGA;PIGB;PIGF;PIGK;PIGM;PIGS;PIGW;PIGY;PIGZ;PIK3C2A;PIK3CA;PIKFYVE;PLA2G4A;PLD2;PLD3;PLD4;PNPLA2;PNPLA8;PTEN;SACM1L;SAMD8;SE
KD1;APPBP2;ARF4;ARHGAP5;ARL2;ARL8B;ASNA1;ATAD1;ATAD2;ATAD2B;ATAD3A;ATL2;ATP10D;ATP11B;ATP11C;ATP13A2;ATP13A3;ATP2B1;ATP2C1;ATP5C1;ATP5E;ATP5F1;ATP5I;ATP5L;ATP5O;ATP6AP1;ATP6V1G1;ATP7A;ATP8A1;ATP8A2;ATP8B4;ATRX;BRIP1;BTAF1;C10orf2;CCNH;CDK7;CENPE;CGN;C
KD1;APPBP2;ARF4;ARHGAP5;ARL2;ARL8B;ASNA1;ATAD1;ATAD2;ATAD2B;ATAD3A;ATL2;ATP10D;ATP11B;ATP11C;ATP13A2;ATP13A3;ATP2B1;ATP2C1;ATP5C1;ATP5E;ATP5F1;ATP5I;ATP5L;ATP5O;ATP6AP1;ATP7A;ATP8A1;ATP8A2;ATP8B4;ATRX;BRIP1;BTAF1;C10orf2;CCNH;CDK7;CENPE;CGN;CHD1;CHD5;
HRS9;DKK3;DLAT;FGFR4;GNMT;INSIG1;INSIG2;KMO;MID1IP1;MLYCD;NQO1;NR1H2;NR3C1;OXCT1;PDHX;PDK1;PDK2;PDP1;PDSS1;PRKAA1;PRKAB2;PRKAG2;PSMA1;PSMA2;PSMA3;PSMA4;PSMA6;PSMA8;PSMB1;PSMB3;PSMC1;PSMC2;PSMC3;PSMC5;PSMC6;PSMD10;PSMD13;PSMD14;PSMD4;PSMD8;P
CCNL1;CCNT2;CCNY;CCNYL1;CDC25C;CDC37;CDK5R1;CDK5RAP3;CDKN2C;CEP152;CEP250;CNPPD1;CRY1;DAB2IP;DIXDC1;DLG1;DUSP19;EEF1A1;FBXO5;FER;FGFR1OP;FIZ1;GLRX3;GSTP1;HDAC5;HDAC7;HDAC9;HINT1;IBTK;IGBP1;ILK;ITGAV;JAK2;JUP;KAT2B;KIF11;LIPE;LLGL1;MAP3K1;MAP3K11;MAP3K13;
SENP1;SOCS5;SUMO1;SUMO2;TAF9;TRIB2;UBE2V2;UCHL5;USP14;USP44;USP5
MSH2;MUC1;NBN;PSMA1;PSMA2;PSMA3;PSMA4;PSMA6;PSMA8;PSMB1;PSMB3;PSMC1;PSMC2;PSMC3;PSMC5;PSMC6;PSMD10;PSMD13;PSMD14;PSMD4;PSMD8;PSME1;RAD17;RBBP8;RFWD2;RINT1;RPS27L;TAOK1;TIPRL;TP63;TRIAP1;UB
MC2;PSMC3;PSMC5;PSMC6;PSMD10;PSMD13;PSMD14;PSMD4;PSMD8;PSME1;UBC;UBE2D1;UBE2E1;USP4
MC2;PSMC3;PSMC5;PSMC6;PSMD10;PSMD13;PSMD14;PSMD4;PSMD8;PSME1;UBC;UBE2D1;UBE2E1;USP4
D28;CDC25C;CDC26;CDC27;CDC7;CDC73;CDK1;CDK2;CDK9;CDKN1B;CDT1;CHFR;CHMP1A;CHORDC1;CRLF3;CUL4B;DAPK3;DBF4;DDX3X;DGKZ;DLG1;DNA2;E2F7;ECT2;ERCC2;EREG;FAM175A;FBXO31;FBXO5;FBXW7;HDAC8;HORMAD1;HSF1;KIF20B;KIF23;MAD2L1;MAPKAPK2;MCM5;MDM2;MSH2;MUC1;

A;MEF2C;NFKB2;NFKBIB;RIPK2;RPS6KA3;RPS6KA5;TAB1;TAB2;TAB3;TBK1;TICAM1;UBC

;GPS1;GPS2;GSTP1;HDAC3;IGBP1;IKBKG;ITCH;JUN;KIAA1804;MAP2K2;MAP2K4;MAP3K11;MAP3K13;MAP3K2;MAP3K7;MAP4K1;MAP4K3;MAP4K5;MAPK8;MAPK8IP1;MAPK8IP2;MAPKAPK2;MAPKAPK3;MAPKBP1;MDFIC;MEF2A;MEF2C;MEN1;PKN1;PLCB1;PTGER4;PTPLAD1;RAP2A;RB1CC1;RBM4;RIPK2;I
GATAD2A;LUC7L3;MEF2C;NOC3L;PHF5A;PLCB1;PLRG1;PNISR;PPIG;PPP1CC;PRPF18;PRPF31;PRPF40A;PRPF6;PSKH1;RBM11;RBM25;RBM27;RBM39;RBM4;RCHY1;RFWD2;RING1;SF3B1;SLU7;SMNDC1;SRSF10;SRSF3;SUMO1;TOPORS;U2AF1;WBP
C5;SMC6;SP3;SUMO1;TDG;TDP2;THAP1;TOPORS;TP53INP1;ZMYM2
.;NBN;PSMA1;PSMA2;PSMA3;PSMA4;PSMA6;PSMA8;PSMB1;PSMB3;PSMC1;PSMC2;PSMC3;PSMC5;PSMC6;PSMD10;PSMD13;PSMD14;PSMD4;PSMD8;PSME1;RAD17;RBBP8;RFWD2;RINT1;RPS27L;TAOK1;TIPRL;TP63;TRIAP1;UB

MB1;PSMB3;PSMC1;PSMC2;PSMC3;PSMC5;PSMC6;PSMD10;PSMD13;PSMD14;PSMD4;PSMD8;PSME1;UB(
MC1;PSMC2;PSMC3;PSMC5;PSMC6;PSMD10;PSMD13;PSMD14;PSMD4;PSMD8;PSME1;RFWD2;RRP8;TRIAP1;UB(
DCS5;SUMO1;SUMO2;TAF9;TRIB2;UBE2V2;USP44;USP5
S2;MRPS22;MRPS28;MRPS31;MRPS33;RBM3;RPL11;RPL15;RPL17;RPL23;RPL26;RPL26L1;RPL27;RPL30;RPL31;RPL34;RPL35;RPL36A;RPL39;RPL39L;RPL41;RPL7;RPL9;RPS15A;RPS24;RPS27;RPS29;RPS3A;RPS7;ZCCHC1
HAUS7;KIAA0101;KIF11;NDE1;PKD2;PLK4;PPP1R12A;SASS6;TUBE1;XPO1
B4A;SEC62;SNX2;TIMM17A;TIMM22;TIMM23;TNPO1;TOMM20;TOMM40;TOMM5;TSNAX;USO1;VPS26A;XPO1;ZFYVE1(
IAUS6;HAUS7;KIAA0101;KIF11;NDE1;PKD2;PLK4;PPP1R12A;SASS6;TUBE1;XPO1
3P;LEPREL4;LIG4;MAD2L1;MKI67IP;MLF1IP;NDE1;NOL6;NSL1;NSMCE2;NUDCD2;NUF2;NUP37;NUP85;ORC2;PES1;PMF1;PPP1CC;RANGAP1;RGS12;RRS1;SEPT7;SGOL2;SKA2;SMARCA5;SMC2;SMC4;SMC5;SMC6;SPAG5;SS18L1;SUV39H1;SYCP2;SYN1;TUBG1;WAPAL;ZWILCI
ATP5E;ATP5F1;ATP5I;ATP5L;ATP5O;BMF;BNIP3L;C14orf2;CASP8;CCDC90A;CHCHD4;CISD1;CISD2;CNP;COASY;COQ10B;COQ2;COX16;COX6C;COX7A2;COX7A2L;COX7B;COX7C;CRAT;CRLS1;CYCS;DDX3X;DHFRL1;DHRS1;DMPK;ERAL1;GK;GPAM;GPAT2;GPD2;GRPEL2;GUF1;HIGD1A;HMGCL;HSD17B10;IDH2;IN
PSMC1;PSMC2;PSMC3;PSMC5;PSMC6;PSMD10;PSMD13;PSMD14;PSMD4;PSMD8;PSME1;SAR1B;SEC13;SEC23A;SEC24A;SEC61A1;TAPBPL;UBC;UBE2/

;GPS1;GPS2;GSTP1;HDAC3;IGBP1;IKBKG;ITCH;JUN;KIAA1804;MAP2K2;MAP2K4;MAP3K11;MAP3K13;MAP3K2;MAP3K7;MAP4K1;MAP4K3;MAP4K5;MAPK8;MAPK8IP1;MAPK8IP2;MAPKAPK2;MAPKAPK3;MAPKBP1;MDFIC;MEF2A;MEF2C;MEN1;PKN1;PLCB1;PTGER4;PTPLAD1;RAP2A;RB1CC1;RBM4;RIPK2;I

ARPC4;ATL2;ATPAF2;AURKC;BAG4;BBS10;BBS12;BCLAF1;BMF;C10orf2;CAPG;CAPZA1;CAPZA2;CASP8;CBR4;CCDC103;CCDC39;CCT2;CCT6B;CD2AP;CDC42EP2;CDK1;CDKN1B;CENPE;CENPW;CEP57;CHAF1A;CHAF1B;CLIP3;COG4;CORO1A;CPSF6;CRBN;CRTC2;CSNK2B;CUTC;CYBA;DDX3X;DECR1;DHPS;DLG1;I

P1L;GRIK5;INSIG1;LRMP;LYST;PIK3C2A;PRKCI;RSG1;SAR1B;SEC13;SEC23A;SEC24A;STX5;SYP;TMED10;TMED2;USO1;VAMP7;VPS33B;WASL;ZFYVE1
L9;USP21;USP25;USP37;USP44;USP46;USP5;USP6;YOD1;ZRANB1
A5;MID1IP1;MTRF1;PLEKHH2;RDX;SPTBN2;SPTBN5;TRPV4;TWF1;TWF2;UPF1
PDP1;PPEF1;PPM1B;PPM1G;PPP1CA;PPP1CB;PPP1CC;PPP1R12A;PPP2CB;PPP2R1A;PPP2R2A;PPP2R3A;PPP2R4;PPP2R5A;PPP3CA;PPP3CB;PPP5C;PPP6C;PTEN;PTP4A1;PTPN12;PTPN2;PTPN22;PTPN4;PTPN7;PTPRO;RPAP2;RPRD1A;SMEK1;SMEK2;SMPD1;SSU72;SYMPI
RPL27;RPL30;RPL31;RPL34;RPL35;RPL36A;RPL39;RPL39L;RPL41;RPL7;RPL9;ZCCHC1
PL47;MRPL48;MRPS10;MRPS17;MRPS18B;MRPS18C;MRPS2;MRPS22;MRPS31;MRPS33;RPL11;RPL15;RPL17;RPL22L1;RPL23;RPL26;RPL26L1;RPL27;RPL30;RPL31;RPL34;RPL35;RPL36A;RPL36AL;RPL39;RPL39L;RPL41;RPL7;RPL9;RPS15A;RPS24;RPS27;RPS27L;RPS29;RPS3A;RP
N4;ACVRL1;ADAM10;ADAM15;ADRM1;AEBP2;AGAP2;AGER;AHR;AIM2;AKIP1;AKT1;AKTIP;ALCAM;ALDOA;ANAPC10;ANG;ANGPT1;ANGPTL4;ANLN;ANXA1;AP1AR;AP1B1;AP1S2;AP2A2;APC;APPL1;AQP11;ARFGAP1;ARFGEF2;ARHGAP10;ARHGAP42;ARHGAP5;ARHGDIA;ARHGEF2;ARID2;ARID5A;ARL2;ARPC4
IAPKAPK3;MEF2A;MEF2C;NFKB2;NFKBIB;PELI1;RIPK2;RPS6KA3;RPS6KA5;TAB1;TAB2;TAB3;TBK1;TICAM1;TLR6;UBC;UBE2N
ARPC4;ATL2;ATPAF2;AURKC;BAG4;BBS10;BBS12;BCLAF1;BMF;C10orf2;CAPG;CAPZA1;CAPZA2;CASP8;CBR4;CCDC103;CCDC39;CCT2;CCT6B;CD2AP;CDC42EP2;CDK1;CDKN1B;CENPE;CENPW;CEP57;CHAF1A;CHAF1B;CLIP3;COG4;CORO1A;CPSF6;CRBN;CRTC2;CSNK2B;CUTC;CYBA;DDX3X;DECR1;DHPS;DLG1;I
K2;PEX1;PEX11A;PEX11G;PEX12;PEX13;PEX14;PEX2;PEX3;PNPLA8;RAB8B;SCP2
K2;PEX1;PEX11A;PEX11G;PEX12;PEX13;PEX14;PEX2;PEX3;PNPLA8;RAB8B;SCP2

14;RAB3IP;RAB9B;SCAMP1;SCAMP3;SCFD1;SLC30A6;STEAP2;STX4;STX7;VAMP4;VAMP7;VPS13/
IB3;PSMC1;PSMC2;PSMC3;PSMC5;PSMC6;PSMD10;PSMD13;PSMD14;PSMD4;PSMD8;PSME1;SCARB

HMT2;TK1;TP63;USP16
.;PAPD4;PEX2;POLR2E;POLR3D;POLR3G;POLR3H;RECQL5;RPAP2;RPRD1A;SHFM1;TAF12;TAF13;TAF2;TAF5;TAF7;TAF9;TAF9B;TBPL
PSMA2;PSMA3;PSMA4;PSMA6;PSMA8;PSMB1;PSMB3;PSMC1;PSMC2;PSMC3;PSMC5;PSMC6;PSMD10;PSMD13;PSMD14;PSMD4;PSMD8;PSME1;RFWD2;RPS27L;RRP8;SIRT1;SMYD2;TFAP4;TP63;TRIAP1;UB
SMC5;PSMC6;PSMD10;PSMD13;PSMD14;PSMD4;PSMD8;PSME1
7;EEF1A1;EEFSEC;EIF5;ERAL1;FPGT;GBP3;GBP6;GCH1;GFM2;GIMAP2;GIMAP7;GMPPA;GNA13;GNAI1;GNAO1;GNL1;GNL3;GTPBP10;GTPBP2;GTPBP8;GUCY2C;GUF1;HBS1L;KRAS;LRRK2;MFN1;MMAA;NIN;NKIRAS1;NRAS;NUDT16;OLA1;OPA1;PCK2;RAB10;RAB11B;RAB12;RAB14;RAB18;RAB1B;RAB22A;RAI
4;PSMA6;PSMC2;PSMC3;UPF1;XRN1
44;CEP95;CKAP2;DDX11;ECT2;ERCC2;FAM110A;FBXO5;HAUS1;HAUS3;HAUS6;HAUS7;HDAC3;KATNB1;KIAA1009;KIF11;KIF18A;KIF20B;KIF22;KIF23;KIF2A;KIF3A;KIFC1;MAD2L1;MAP2K5;MAP9;MZT1;NDE1;NEK7;NUDCD2;NUP85;PIN4;PKD2;PPP2CB;PTP4A1;RANGAP1;RASSF1;RB1;RIF1;SEPT7;SKA2;SPAG5

;PIWIL4;PNRC2;PQBP1;PSMA2;PSMA4;PSMA6;PSMC2;PSMC3;PUM2;RBM4;TDRD6;TIA1;UHMK1;UPF1;XRN1
L2;IDE;IDI1;IMPDH2;MAP2K2;MFF;MLYCD;MPV17;NUDT12;PEX1;PEX11A;PEX11G;PEX12;PEX13;PEX14;PEX2;PEX3;PNPLA8;PXT1;RAB8B;SCP2;TMEM135;ZADH
L2;IDE;IDI1;IMPDH2;MAP2K2;MFF;MLYCD;MPV17;NUDT12;PEX1;PEX11A;PEX11G;PEX12;PEX13;PEX14;PEX2;PEX3;PNPLA8;PXT1;RAB8B;SCP2;TMEM135;ZADH

(1;TMX3;TXNDC12;TXNDC17;TXNL1;TXNRD2
APPL1;ARHGEF2;ARPP19;ARRB1;ATF2;ATP6V1G1;BCR;BIN1;BLZF1;BORA;BRCA2;C10orf2;C14orf166;CAB39;CARHSP1;CASP8;CBX3;CCDC88A;CCNA2;CCNC;CCND3;CCNE2;CCNH;CCNL1;CCNT2;CCNY;CCNYL1;CD28;CDC25C;CDC27;CDC37;CDC42EP2;CDC73;CDK5R1;CDK5RAP3;CDKN1B;CDKN2C;CEP152;CEP
3;ARRB1;ATF1;ATF2;ATRX;BACH1;BANF1;BAZ2B;BBX;BCLAF1;BDP1;BLZF1;BMI1;BRCA2;BRIP1;BTAF1;C10orf2;CARHSP1;CASP8AP2;CBFB;CC2D1A;CDK9;CDT1;CEBPA;CEBPG;CEBPZ;CENPW;CERS5;CGGBP1;CHD1;CHD5;CHD9;CNBP;CREB1;CREB5;CREBL2;CREBZF;CRY1;CXXC1;DACH1;DBP;DDB2;DDX11;DDX3
UVBL2;SIRT1;SUZ12;TAF7;TAF9;WDR5
.;PEX2;POLR2E;RECQL5;RPAP2;RPRD1A;SHFM1;TAF12;TAF13;TAF2;TAF5;TAF7;TAF9;TAF9B;TBPL

ENPQ;CENPW;CHMP1A;CREB1;CSNK1A1;DAPK3;DAXX;DCTN2;DDX11;DMAP1;DYNLT3;EED;EHMT2;EIF3E;ESCO1;ESCO2;FAM60A;FER;GABPA;GTPBP10;H2AFV;H2AFZ;HDAC8;HELLS;HIRA;HMGB1;HMGB2;HMGN5;HORMAD1;INO80C;ITGB3BP;JUN;KAT2B;KAT8;KIF22;KLHDC3;LEPREL4;LIG4;LRWD1;MAD2L
.;PEX2;POLR2E;POLR3D;POLR3G;POLR3H;RECQL5;RPAP2;RPRD1A;SHFM1;TAF12;TAF13;TAF2;TAF5;TAF7;TAF9;TAF9B;TBPL
.;PEX2;POLR2E;POLR3D;POLR3G;POLR3H;RECQL5;RPAP2;RPRD1A;SHFM1;TAF12;TAF13;TAF2;TAF5;TAF7;TAF9;TAF9B;TBPL
L;USP37;USP38;USP44;USP45;USP46;USP5;USP51;USP6;USPL1;ZRANB1
7;EEF1A1;EEFSEC;EIF5;ERAL1;FPGT;GBP3;GBP6;GCH1;GFM2;GIMAP2;GIMAP7;GMPPA;GNA13;GNAI1;GNAO1;GNL1;GNL3;GTPBP10;GTPBP2;GTPBP8;GUCY2C;GUF1;HBS1L;KRAS;LRRK2;MFN1;MMAA;NIN;NKIRAS1;NRAS;NUDT16;OLA1;OPA1;PCK2;PRPS2;RAB10;RAB11B;RAB12;RAB14;RAB18;RAB1B;RAB2
7;EEF1A1;EEFSEC;EIF5;ERAL1;FPGT;GBP3;GBP6;GCH1;GFM2;GIMAP2;GIMAP7;GMPPA;GNA13;GNAI1;GNAO1;GNL1;GNL3;GTPBP10;GTPBP2;GTPBP8;GUCY2C;GUF1;HBS1L;KRAS;LRRK2;MFN1;MMAA;NIN;NKIRAS1;NRAS;NUDT16;OLA1;OPA1;PCK2;PRPS2;RAB10;RAB11B;RAB12;RAB14;RAB18;RAB1B;RAB2
MYSM1;OTUD5;PIGK;SENP1;SENP6;SENP7;UCHL3;UCHL5;USP1;USP12;USP14;USP15;USP16;USP19;USP21;USP25;USP31;USP37;USP38;USP44;USP45;USP46;USP5;USP51;USP6;USPL1;VCPIP1;YOD1;ZRANB1

;CCDC90A;CNP;COQ10B;COX6C;COX7A2;COX7A2L;COX7B;COX7C;CRAT;CRLS1;CYCS;DHFRL1;DHRS1;ERAL1;GPAM;GPD2;GRPEL2;GUF1;HIGD1A;HMGCL;HSD17B10;IDH2;IMMP1L;KMO;L2HGDH;MCU;MDH2;MPV17;MTHFD2L;NDUFA1;NDUFA4;NDUFA5;NDUFA6;NDUFAF1;NDUFB1;NDUFB3;NDUFB5;NDU

APK6;MAPK8;NLK;PPP4C;RIPK3;STK10;TGFBR2

X5;TMED10;TMED2;TMED5;TMED7;UGGT2;VMA21;VMP1
UVBL2;SIRT1;SUZ12;TAF7;TAF9;WDR5
ABCD2;ABCD3;ABCE1;ABHD10;ABHD12;ABHD13;ABHD14B;ABHD3;ABI1;ABI2;ABI3;ABLIM2;ABR;ACAD10;ACAD9;ACADM;ACADSB;ACADVL;ACAT1;ACAT2;ACBD3;ACBD5;ACBD6;ACD;ACOT13;ACOT7;ACP2;ACPL2;ACSF2;ACSF3;ACSL4;ACSL6;ACSS1;ACTL6A;ACTL9;ACTN4;ACTR1A;ACTR6;ACVR1C;ACVR2A;A
GL1;SLC9A6;SNX13;SNX16;SNX2;SNX4;SNX8;STAM2;STX7;TMEM184A;VAC14;ZFYVE1

MPK;L2HGDH;LRRK2;MFF;NDUFA1;NDUFA4;NDUFA5;NDUFA6;NDUFAF1;NDUFB1;NDUFB3;NDUFB5;NDUFS2;NDUFS4;NDUFS5;OPA1;PPIF;RHOT1;SDHD;SLC24A6;TIMM17A;TIMM23;TMEM70;TOMM20;TOMM40;TOMM5;UQCRB;UQCRC1;UQCRC2;UQCRI
RCC1
DRA;INSIG1;INSIG2;MARCH6;PGAP1;PGAP3;PIGB;PIGK;PIGS;PIGZ;PKD2;PORCN;SACM1L;SAMD8;SEC61A1;SERAC1;SSR3;TECR;TMEM66;WFS1

Q1;KIFC1;LDLR;LLGL1;LRP6;MAP2K2;MARCH1;MTMR2;NIPA1;PIKFYVE;PRDX3;PTP4A1;RAB14;RAB22A;RAB5A;RABEP1;RNF11;SH3GL1;SIAH1;SLC9A6;SNX13;SNX16;SNX17;SNX2;SNX3;SNX4;SNX8;STAM2;STEAP2;STX7;TMEM184A;TOM1;TRIM3;VAC14;VPS18;VPS4B;WDFY1;ZFYVE1
TAD1;ATAD2;ATP10D;ATP11B;ATP11C;ATP13A2;ATP13A3;ATP2B1;ATP2C1;ATP5C1;ATP5E;ATP5F1;ATP5I;ATP5L;ATP5O;ATP6AP1;ATP7A;ATP8A1;ATP8A2;ATP8B4;BRIP1;CCNH;CDK7;CHD1;CHD5;CRBN;DDX11;DDX3X;DDX41;DDX50;DDX54;DDX59;DHX30;DHX36;DHX40;DICER1;DNA2;DNAH6;ERCC2;FANC
DA1;TAF12;TAF2;TAF5;TAF7;TAF9;TAF9B;WDR5;ZZZ3
DNAJC24;DNAJC25;DNAJC27;ERO1LB;GRPEL2;HSPE1;LMAN1;LRPAP1;MKKS;NDUFAF1;PFDN4;PFDN5;PPIB;RP2;RUVBL2;SCAP;SERPINH1;SIL1;SLMAP;SRSF10;TBCA;TOMM20;TOR1A;TRAP1;VBP1
ABCD2;ABCD3;ABCE1;ABCF3;ABHD10;ABHD12;ABHD13;ABHD14B;ABHD3;ABI1;ABI2;ABI3;ABLIM2;ABR;ACAD10;ACAD9;ACADM;ACADSB;ACADVL;ACAP1;ACAP2;ACAT1;ACAT2;ACBD3;ACBD5;ACBD6;ACD;ACOT13;ACOT7;ACP2;ACPL2;ACSF2;ACSF3;ACSL4;ACSL6;ACSS1;ACTL6A;ACTN4;ACTR1A;ACVR1C;AC
B1;LRRCC1;MZT1;NEK1;NIN;PAX2;PLK4;POC1B;ROCK1;SASS6;TNKS2;TOPORS;TUBD1;TUBE1;TUBG1;TUBG
LG1;DUSP11;DUSP19;G6PC3;GDPD3;GDPD5;GPCPD1;IMPA1;IMPAD1;INPP5D;INPP5K;LHPP;MINPP1;MTMR2;MTMR6;MTMR7;MTMR9;NANP;NAPEPLD;NT5C3;NUDT16;PDE1B;PDE3B;PDE4A;PDE4D;PDE7A;PDP1;PGAM5;PGAP1;PIKFYVE;PLCB1;PLCB2;PLCB3;PLCD3;PLCL1;PLCXD2;PLD2;PLD3;PLD4;PPEF1;

RPS18B;MRPS18C;MRPS22;MRPS28;MRPS31;MRPS3
RPS18B;MRPS18C;MRPS22;MRPS28;MRPS31;MRPS3
7;RPL9;ZCCHC1

IV2L2;SLU7;SNRPB;SNRPB2;SNRPD1;SNRPE;SNRPG;U2AF1;XAB2;ZCCHC
2;GAA;HLA-DMA;HLA-DPB1;HLA-DRA;LMBRD1;MARCH1;MCOLN1;MFSD8;OSTM1;PNPLA7;PQLC2;PSEN2;RAB12;RNF13;SLC17A5;SLC29A3;TLR8;TMEM106B;TMEM55A;TRIM23;VAMP7;VPS18;VPS33B;ZNRF
R1

19;FGFR1OP;GAS6;GMFB;HMGB1;IBTK;KAT2B;MAP2K2;MAPK8IP1;MAPK8IP2;MBIP;PIK3CA;PKIA;PKIB;PRKAG2;PRKAR1A;PRKAR1B;SPA17;STRADA;TRIB2
19;FGFR1OP;GAS6;GMFB;GSTP1;HMGB1;IBTK;KAT2B;LRP6;MALT1;MAP2K2;MAPK8IP1;MAPK8IP2;MBIP;PIK3CA;PKIA;PKIB;PRKAG2;PRKAR1A;PRKAR1B;SPA17;STRADA;TAB1;TRIB2;VAC14

ERBB2IP;ERN1;FAM169A;FAM188A;GCH1;IL4I1;INTS2;LEMD3;LMNB2;MATR3;MIB1;MRPL19;NEK1;NOC4L;NRM;NUCB2;NUP54;NUP85;PARP16;PLCB1;PNPLA7;PSEN2;PTCHD2;PTGDS;RAE1;RANGAP1;RECQL5;RNF13;SIRT1;SUMO1;TMEM201;TMEM38B;TMEM48;TMPO;TOR1A;TSGA10;UNC50;WTA
T1;CERS5;CST3;CTDNEP1;CYHR1;DMPK;DTX2;EIF5A2;ERBB2IP;ERN1;FAM169A;FAM188A;FANCL;GCH1;GMCL1;IL4I1;INTS2;IPO11;IPO13;IPO4;KPNA3;LEMD3;LMNA;LMNB2;MAD2L1;MATR3;MIB1;MRPL19;MTMR6;NEK1;NOC4L;NRM;NUCB2;NUP37;NUP54;NUP62CL;NUP85;PARP16;PLCB1;PNPLA7;PRKCZ

PITPNM1;PKN1;PKN2;PPP1CC;RALA;SEPT7;SPAST;TOPORS;TXNDC9;VPS37B;VPS4A
4GALT3;B4GALT6;C1GALT1;CHPF;CHPF2;CHSY1;CSGALNACT1;CSGALNACT2;DDOST;DPM1;EXTL2;FUT4;GALNT1;GALNT3;GALNT4;GALNT7;GCNT1;GCNT2;GCNT4;LFNG;MGAT1;MGAT2;MGAT4A;OSTC;PIGA;PIGB;PIGM;PIGZ;PLOD3;POGLUT1;POMT2;RFNG;STT3B;TYMP;UGCG;UGGT2;UGT2B1
E1;BAP1;BRCC3;C11orf54;CDC14A;CDC25C;CDKN3;CES4A;CNOT6;CNOT6L;CNOT7;CNP;CTDNEP1;CTDSP1;CTDSPL2;CYCS;DAGLB;DAPP1;DCP2;DICER1;DIS3;DIS3L2;DLG1;DNA2;DNASE1L1;DNASE2;DUSP11;DUSP19;ELAC2;ENDOG;ENDOV;ERI1;ERI2;ERN1;FANCM;G6PC3;GDPD3;GDPD5;GEN1;GPCPD1;HRSP
1B;PTPN23;RAB3IP;RPGR;RSG1;TOPORS;WDR3
N7;CLN3;CLN5;CTNS;CYBASC3;DIRC2;DRAM2;GAA;HLA-DMA;HLA-DPB1;HLA-DRA;HOOK1;HOOK2;LMBRD1;MARCH1;MCOLN1;MFSD8;MTMR2;OSTM1;PNPLA7;PQLC2;PSEN2;RAB12;RNF13;SLC17A5;SLC29A3;TLR8;TMEM106B;TMEM55A;TRIM23;VAMP7;VMP1;VPS18;VPS33B;VPS4B;ZNRF

37;NUP85;ORC2;PMF1;PPP1CC;RANGAP1;SEPT7;SGOL2;SKA2;SPAG5;SS18L1;ZWILCH
;NUP37;NUP85;ORC2;PMF1;PPP1CC;RANGAP1;SEPT7;SGOL2;SKA2;SPAG5;SS18L1;ZWILCH

MTMR7;PDP1;PGAM5;PPEF1;PPM1B;PPM1G;PPP1CA;PPP1CB;PPP1CC;PPP1R15B;PPP1R3D;PPP2CB;PPP2R1A;PPP2R2A;PPP3CA;PPP3CB;PPP4C;PPP5C;PPP6C;PTEN;PTP4A1;PTPDC1;PTPN12;PTPN2;PTPN22;PTPN23;PTPN4;PTPN7;PTPRO;RNGTT;RPAP2;SSU72;TENC1;UBLCP1

KMO;MARCH5;MCL1;MFF;MFN1;MSTO1;MTX2;MTX3;OPA1;PGAM5;PMAIP1;PPP1CC;RHOT1;RPS6KB1;TMEM173;TOMM20;TOMM40;TOMM
LNT1;GALNT3;GOLPH3L;HACE1;LLGL1;LPCAT2;MGAT2;NAGPA;NCS1;PITPNM1;RAB14;RAB27B;SAR1B;SCFD1;SGMS1;TMBIM4;TMED2;VCPIP1;VRK

;EML2;ERCC2;GOPC;GRIP1;IFT52;ITGB3BP;JAK2;KPNA3;LIG4;MID1IP1;MLLT4;MSH2;PEX1;PEX12;PFKM;PLEKHB1;PPP2CB;PRDX3;PRKAA1;SCLT1;SIAH1;SIRT1;SLC9A3R2;SNX17;TAF13;TRIM3;VPS4A;VPS4B;XRCC

RAD51B;RAD51C;RECQL;RFC3;RUVBL2;SMARCA1;TOP1MT

EB1;CRTC2;CTBP1;DDX54;DMAP1;DR1;DRAP1;DYRK1B;E2F6;E2F7;EID1;ELK4;ENY2;ESR2;FOXH1;GABPA;GMNN;GPS2;GRIP1;HCFC2;HDAC3;HDAC5;HDAC7;HDAC9;HIRA;HSF2;HSF4;JMY;JUN;JUP;KAT2B;MED12L;MED13;MED14;MED15;MED16;MED21;MED22;MED23;MED26;MED4;MED7;MED8;MED9;MI

/39H2;SUZ12;TRIM28;UBE2A

KAG2;TRIB2

AP3K14;MAP3K2;MAP3K7;MAP3K8;MAP4K1;MAPK6;MAPK8;NLK;NSMAF;PPP4C;RGS12;RIPK3;SMAD2;SMAD4;SMAD5;STK10;TGFBR2
SRM
GAP10;ARHGAP11A;ARHGAP12;ARHGAP22;ARHGAP42;ARHGAP5;ARHGDIA;ARRB1;ASAP3;AZIN1;BCR;BNIP2;CAB39;CARD8;CASP8AP2;CDC42EP2;CDK5R1;CDKN1B;CHM;CHML;CHN2;CTSH;DAB2IP;DBF4;DEPDC1;DEPDC1B;DNAJC10;DUSP19;EBAG9;ECT2;ELMOD2;FAM13B;FRS2;GAS6;GDI1;GIT1;GMFB;GU
;CSNK1A1;DAPK3;DAXX;DCTN2;DDX11;DMAP1;DYNLT3;EED;EIF3E;ESCO1;ESCO2;FAM60A;FER;GABPA;H2AFV;H2AFZ;HELLS;HIRA;HMGN5;INO80C;ITGB3BP;JUN;KAT2B;KAT8;KIF22;KLHDC3;LEPREL4;LRWD1;MAD2L1;MAF;MEF2A;MEN1;MIS18BP1;MLF1IP;MMS22L;MUC1;NBN;NDE1;NEDD4;NFATC1;NSL1

11A;ARHGAP12;ARHGAP22;ARHGAP42;ARHGAP5;ARHGDIA;ARHGEF10L;ARHGEF2;ARL2;ARRB1;ASAP3;BCR;BNIP2;CDC42EP2;CHM;CHML;CHN2;CYTH2;DAB2IP;DENND1B;DENND1C;DENND4A;DENND4C;DEPDC1;DEPDC1B;DIS3;DOCK11;ECT2;ECT2L;EIF2B5;ELMOD2;FAM13B;FBXO8;FGD2;FGD3;FGD4;FN
;TRIB2

;G6PC3;IMPA1;IMPAD1;INPP5D;INPP5K;LHPP;MINPP1;MTMR2;MTMR6;MTMR7;MTMR9;NANP;NT5C3;NUDT16;PDP1;PGAM5;PIKFYVE;PPEF1;PPM1B;PPM1G;PPP1CA;PPP1CB;PPP1CC;PPP1R15B;PPP1R3D;PPP2CB;PPP2R1A;PPP2R2A;PPP3CA;PPP3CB;PPP4C;PPP5C;PPP6C;PTEN;PTP4A1;PTPDC1;PTPN12;P
;CBR4;CTBP1;DBI;DECR1;DHFRL1;ERO1L;ERO1LB;ETFA;FMO5;GALE;GAPDH;GCH1;GRHPR;HMGCL;HPGD;IDH2;IDH3B;IDH3G;KMO;MICAL1;MTO1;MTRR;NDUFS2;NOS3;PHGDH;QDPR;SCP2;SIRT1;SIRT2;SPR;SUOX;TGDS;TXNRD2;UGDH
GAP12;ARHGAP22;ARHGAP42;ARHGAP5;ARHGDIA;ARRB1;ASAP3;BCR;BNIP2;CDC42EP2;CHM;CHML;CHN2;DAB2IP;DEPDC1;DEPDC1B;ECT2;ELMOD2;FAM13B;GDI1;GIT1;JUN;LLGL1;LRRK2;PLCB1;PLXNB2;PTPLAD1;RAB3GAP2;RABEP1;RANGAP1;RASA1;RGS1;RGS12;RGS18;RGS5;RIN1;RP2;SH3BP1;SIPA1;SY
4A;SEPT7;SPAG16;UHMK1;WDPCP;WDR35
ABHD12;ABHD13;ABHD14B;ABHD3;ACAD10;ACOT13;ACOT7;ACP2;ACPL2;ACY3;ADA;ADAM10;ADAM15;ADAM22;ADAMTS5;ADAMTS6;ADORA1;AEBP1;AGAP3;AGBL3;AGL;AKD1;AKR7A2;ANG;APEH;APEX1;APEX2;APH1A;APLF;APOBEC3D;APPBP2;APTX;ARF4;ARG2;ARHGAP5;ARL2;ARL8B;ARSA;ARSK;ASN

11A;ARHGAP12;ARHGAP22;ARHGAP42;ARHGAP5;ARHGDIA;ARHGEF10L;ARHGEF2;ARL2;ARRB1;ASAP3;BCR;BNIP2;CDC42EP2;CHM;CHML;CHN2;CYTH2;DAB2IP;DENND1B;DENND1C;DENND4A;DENND4C;DEPDC1;DEPDC1B;DIS3;DNAJC10;DOCK11;ECT2;ECT2L;EIF2B5;ELMOD2;FAM13B;FBXO8;FGD2;FGD3
JBP1;NUBPL;PPAT;REV3L
JBP1;NUBPL;PPAT;REV3L
NSIG2;MARCH6;PIGK;PIGS;PKD2;PORCN;SACM1L;SAMD8;SEC61A1;SSR3;TECR;TMEM66;WFS1
MGCS1;ING3;KAT2B;KAT8;LCAT;LCLAT1;LPCAT1;LPCAT2;LPCAT4;LPGAT1;MBOAT1;MCAT;MGEA5;NAA16;NAA20;NAA30;NAA50;NAA60;NAT1;OXSM;PDHX;PIGW;PLA2G15;PORCN;SAT1;SCP2;SLC27A3;SPTLC1;SPTSSA;TADA1;TAF12;TAF5;TAF9;TGM4;WDR5;YKT6;ZDHHC13;ZDHHC14;ZDHHC16;ZDHHC17;ZD
T3;GOLPH3L;HACE1;LLGL1;NAGPA;NCS1;PITPNM1;RYR1;SAR1B;SCFD1;SGMS1;TMED2

B3GNT5;B4GALT3;B4GALT6;C1GALT1;CHPF;CHPF2;CHSY1;CSGALNACT1;CSGALNACT2;DDOST;DPM1;EXTL2;FUT4;GALNT1;GALNT3;GALNT4;GALNT7;GCNT1;GCNT2;GCNT4;GLT8D1;GLT8D2;GTDC1;GXYLT1;HPRT1;LFNG;MGAT1;MGAT2;MGAT4A;NAMPT;OSTC;PARP11;PARP16;PARP8;PIGA;PIGB;PIGM;PIGZ
GTF2IRD2B;HIST1H2BC;HIST1H2BD;HIST2H2BF;HKR1;LDHB;LIAS;MEAF6;METTL3;NAP1L4;NPR2;PCID2;PDE6B;PMS2P1;POLR1C;PPIE;PPIH;PRPSAP2;RBFA;RPP38;RSAD1;SIRT5;SUPT7L;TCEA3;TGFB3;WDR77;ZNF133;ZNF202;ZNF211;ZNF232;ZNF266;ZNF329;ZNF343;ZNF419;ZNF44;ZNF530;ZNF542;ZNF544;
2IRD2;GTF2IRD2B;HIST1H2BC;HIST1H2BD;HIST2H2BF;HKR1;LDHB;MEAF6;METTL3;NAP1L4;NPR2;PCID2;PDE6B;PMS2P1;POLR1C;PPIE;PPIH;PRPSAP2;RBFA;RPP38;RSAD1;SIRT5;SUPT7L;TCEA3;TGFB3;WDR77;ZNF133;ZNF202;ZNF211;ZNF232;ZNF266;ZNF329;ZNF343;ZNF419;ZNF44;ZNF530;ZNF542;ZNF54
GTF2IRD2B;HIST1H2BC;HIST1H2BD;HIST2H2BF;HKR1;HSD17B7;LDHB;LIAS;MEAF6;METTL3;NAP1L4;NPR2;PCID2;PDE6B;PMS2P1;POLR1C;PPIE;PPIH;PRPSAP2;RBFA;RPP38;RSAD1;SIRT5;SUPT7L;TCEA3;TGFB3;WDR77;ZNF133;ZNF202;ZNF211;ZNF232;ZNF266;ZNF329;ZNF343;ZNF419;ZNF44;ZNF530;ZNF54
D2B;HIST1H2BC;HIST1H2BD;HIST2H2BF;HKR1;LDHB;MEAF6;METTL3;NAP1L4;NPR2;PCID2;PDE6B;PMS2P1;POLR1C;PPIE;PPIH;PRPSAP2;RBFA;RPP38;SIRT5;SUPT7L;TCEA3;TGFB3;WDR77;ZNF133;ZNF202;ZNF211;ZNF232;ZNF266;ZNF329;ZNF343;ZNF419;ZNF44;ZNF530;ZNF542;ZNF544;ZNF563;ZNF671;ZN
IRD2;GTF2IRD2B;HIST1H2BC;HIST1H2BD;HIST2H2BF;HKR1;LDHB;MEAF6;METTL3;NAP1L4;NPR2;OVGP1;PCID2;PDE6B;PMS2P1;POLR1C;PPIE;PPIH;PRPSAP2;RBFA;RPP38;RSAD1;SIRT5;SUPT7L;TCEA3;TGFB3;WDR77;ZNF133;ZNF202;ZNF211;ZNF232;ZNF266;ZNF329;ZNF343;ZNF419;ZNF44;ZNF530;ZNF542;
T1H2BD;HIST2H2BF;HKR1;MEAF6;METTL3;NAP1L4;PCID2;PMS2P1;POLR1C;PPIE;PPIH;RBFA;RPP38;SIRT5;SUPT7L;TCEA3;TGFB3;WDR77;ZNF133;ZNF202;ZNF211;ZNF232;ZNF266;ZNF329;ZNF343;ZNF419;ZNF44;ZNF530;ZNF542;ZNF544;ZNF563;ZNF671;ZNF69;ZNF773;ZNF78
D2B;HIST1H2BC;HIST1H2BD;HIST2H2BF;HKR1;LDHB;MEAF6;METTL3;NAP1L4;NPR2;PCID2;PDE6B;PMS2P1;POLR1C;PPIE;PPIH;PRPSAP2;RBFA;RPP38;RSAD1;SIRT5;SUPT7L;TCEA3;TGFB3;WDR77;ZNF133;ZNF202;ZNF211;ZNF232;ZNF266;ZNF329;ZNF343;ZNF419;ZNF44;ZNF530;ZNF542;ZNF544;ZNF563;ZNF

F329;ZNF343;ZNF419;ZNF44;ZNF530;ZNF542;ZNF544;ZNF563;ZNF671;ZNF69;ZNF773;ZNF789
;COQ6;DNAH3;DSN1;DUSP6;EXOSC2;EXOSC7;FANCE;FGR;GALNT12;GEMIN8;GPD1;GTF2IRD2;GTF2IRD2B;HIST1H2BC;HIST1H2BD;HIST2H2BF;HKR1;HSD17B7;LDHB;LETMD1;LIAS;LUC7L;MEAF6;METTL17;METTL3;NAP1L4;NUP43;NVL;OVGP1;PEX11B;PMS2P1;POLR1C;PPIE;PPIH;RBFA;RDH16;RPP38;RSAD1
;COQ6;DNAH3;DSN1;DUSP6;EXOSC2;EXOSC7;FANCE;FGR;GALNT12;GEMIN8;GPD1;GTF2IRD2;GTF2IRD2B;HIST1H2BC;HIST1H2BD;HIST2H2BF;HKR1;HSD17B7;LDHB;LETMD1;LIAS;LUC7L;MEAF6;METTL17;METTL3;NAP1L4;NUP43;NVL;OVGP1;PEX11B;PMS2P1;POLR1C;PPIE;PPIH;RBFA;RDH16;RPP38;RSAD1
H2;DUSP6;EXOSC2;EXOSC7;FANCE;FGR;GALNT12;GEMIN8;GPD1;GTF2IRD2;GTF2IRD2B;HIST1H2BC;HIST1H2BD;HIST2H2BF;HKR1;HSD17B7;IL24;ILKAP;LDHB;LIAS;MEAF6;METTL17;METTL3;NAP1L4;NPR2;PCID2;PDE6B;PMS2P1;POLR1C;PPIE;PPIH;PRPSAP2;RBFA;RPP38;RSAD1;SIRT5;SLC25A17;SLC25A4;SU
PCID2;POLR1C;PPIE;PPIH;RBFA;RPP38;SIRT5;SUPT7L;TCEA3;TGFB3;WDR77;ZNF133;ZNF202;ZNF211;ZNF232;ZNF266;ZNF329;ZNF343;ZNF419;ZNF44;ZNF530;ZNF542;ZNF544;ZNF563;ZNF671;ZNF69;ZNF773;ZNF78
NF266;ZNF329;ZNF343;ZNF419;ZNF44;ZNF530;ZNF542;ZNF544;ZNF563;ZNF671;ZNF69;ZNF773;ZNF789
RD2;GTF2IRD2B;HIST1H2BC;HIST1H2BD;HIST2H2BF;HKR1;HSD17B7;LDHB;METTL3;NPR2;NVL;PMS2P1;POLR1C;PPIE;PRR3;RDH16;SIRT5;TCEA3;ZNF133;ZNF202;ZNF211;ZNF232;ZNF266;ZNF329;ZNF343;ZNF419;ZNF44;ZNF530;ZNF542;ZNF544;ZNF563;ZNF671;ZNF69;ZNF773;ZNF78

RD2;GTF2IRD2B;HIST1H2BC;HIST1H2BD;HIST2H2BF;HKR1;HSD17B7;LDHB;METTL3;NPR2;NVL;PMS2P1;POLR1C;PPIE;PRR3;RDH16;SIRT5;TCEA3;ZNF133;ZNF202;ZNF211;ZNF232;ZNF266;ZNF329;ZNF343;ZNF419;ZNF44;ZNF530;ZNF542;ZNF544;ZNF563;ZNF671;ZNF69;ZNF773;ZNF78
TTL3;PMS2P1;POLR1C;PPIE;PRR3;TCEA3;ZNF133;ZNF202;ZNF211;ZNF232;ZNF266;ZNF329;ZNF343;ZNF419;ZNF44;ZNF530;ZNF542;ZNF544;ZNF563;ZNF671;ZNF69;ZNF773;ZNF78
;SIRT5;SLC25A17;SLC25A4;UQCC
;CDK20;CDK5RAP1;CIAPIN1;CLYBL;COQ10A;COQ6;CRIP3;DEPDC7;DNAH3;DPH2;DSN1;DUSP6;EXOSC2;EXOSC7;FANCE;FGR;GALNT12;GEMIN8;GPD1;GTF2IRD2;GTF2IRD2B;HIST1H2BC;HIST1H2BD;HIST2H2BF;HKR1;HSD17B7;ILKAP;LDHB;LETMD1;LIAS;LUC7L;MEAF6;METTL17;METTL3;NAP1L4;NUP43;NVL;

H2;DUSP6;EXOSC2;EXOSC7;FANCE;FGR;GALNT12;GEMIN8;GPD1;GTF2IRD2;GTF2IRD2B;HIST1H2BC;HIST1H2BD;HIST2H2BF;HKR1;HSD17B7;IL24;ILKAP;KLKB1;LDHB;LIAS;MEAF6;METTL17;METTL3;NAP1L4;NPR2;NUP43;OVGP1;PCID2;PDE6B;PMS2P1;POLR1C;PPIE;PPIH;PRPSAP2;RBFA;RDH16;RPP38;RSAD1
VGP1;PDE6B;PRPSAP2;PRR3;RSAD1;SIRT5;SMYD5;TCEA3;ZNF133;ZNF185;ZNF202;ZNF211;ZNF232;ZNF266;ZNF329;ZNF343;ZNF419;ZNF44;ZNF530;ZNF542;ZNF544;ZNF563;ZNF671;ZNF69;ZNF773;ZNF78
IF211;ZNF232;ZNF266;ZNF329;ZNF343;ZNF419;ZNF44;ZNF530;ZNF542;ZNF544;ZNF563;ZNF671;ZNF69;ZNF773;ZNF789

2;CDK20;CDK5RAP1;CIAPIN1;CLYBL;COQ10A;COQ6;CRIP3;DEPDC7;DNAH3;DPH2;DSN1;DUSP6;EXOSC2;EXOSC7;FANCE;FGR;GALNT12;GEMIN8;GPD1;GTF2IRD2;GTF2IRD2B;HIST1H2BC;HIST1H2BD;HIST2H2BF;HKR1;HPCAL4;HSD17B7;ILKAP;LDHB;LETMD1;LIAS;LUC7L;MEAF6;METTL17;METTL3;NAP1L4;NU
C2;EXOSC7;FANCE;FGR;GALNT12;GEMIN8;GPD1;GTF2IRD2;GTF2IRD2B;HIST1H2BC;HIST1H2BD;HIST2H2BF;HKR1;HSD17B7;IL24;ILKAP;KLKB1;LDHB;LIAS;MEAF6;METTL17;METTL3;NAP1L4;NPR2;NUP43;OVGP1;PCID2;PDE6B;PMS2P1;POLR1C;PPIE;PPIH;PRPSAP2;RBFA;RDH16;RPP38;RSAD1;SIRT5;SLC25A1

DE6B;PRPSAP2;PRR3;RSAD1;SIRT5;SMYD5;TCEA3;ZNF133;ZNF185;ZNF202;ZNF211;ZNF232;ZNF266;ZNF329;ZNF343;ZNF419;ZNF44;ZNF530;ZNF542;ZNF544;ZNF563;ZNF671;ZNF69;ZNF773;ZNF78

ETTL17;METTL3;PCID2;POLR1C;PPIE;PPIH;RBFA;RPP38;SIRT5;SUPT7L;TCEA3;TGFB3;WDR77;ZNF133;ZNF202;ZNF211;ZNF232;ZNF266;ZNF329;ZNF343;ZNF419;ZNF44;ZNF530;ZNF542;ZNF544;ZNF563;ZNF671;ZNF69;ZNF773;ZNF78
CRIP3;CXCL16;DNAH3;DNAJC9;DSN1;EXOSC2;EXOSC7;FANCE;FGR;GEMIN8;GPD1;GTF2IRD2;GTF2IRD2B;HIST1H2BC;HIST1H2BD;HIST2H2BF;HKR1;HPCAL4;HSD17B7;IL24;ILKAP;ITGA10;KLKB1;LDHB;LETMD1;LIAS;LUC7L;MEAF6;METTL17;METTL3;NAP1L4;NPR2;NUP43;NVL;OCM;OVGP1;PDE6B;PEX11B;PM

2B;HKR1;HPCAL4;ILKAP;ITGA10;LIAS;METTL17;NPR2;NVL;OCM;OVGP1;PDE6B;PMS2P1;PRPSAP2;PRR3;RSAD1;SIRT5;SMYD5;TCEA3;ZNF133;ZNF185;ZNF202;ZNF211;ZNF232;ZNF266;ZNF329;ZNF343;ZNF419;ZNF44;ZNF530;ZNF542;ZNF544;ZNF563;ZNF671;ZNF69;ZNF773;ZNF78

D1;SIRT5;SUPT7L;TCEA3;TGFB3;WDR77;ZNF133;ZNF202;ZNF211;ZNF232;ZNF266;ZNF329;ZNF343;ZNF419;ZNF44;ZNF530;ZNF542;ZNF544;ZNF563;ZNF671;ZNF69;ZNF773;ZNF78

NCE;FGR;GALNT12;GEMIN8;GPD1;GTF2IRD2;GTF2IRD2B;HIST1H2BC;HIST1H2BD;HIST2H2BF;HKR1;HSD17B7;IL24;ILKAP;KLKB1;LDHB;LIAS;MEAF6;METTL17;METTL3;NAP1L4;NPR2;NUP43;OVGP1;PCID2;PDE6B;PMS2P1;POLR1C;PPIE;PPIH;PRPSAP2;RBFA;RDH16;RPP38;SIRT5;SLC25A17;SUPT7L;TCEA3;TGF

IIN8;GTF2IRD2;GTF2IRD2B;HIST1H2BC;HIST1H2BD;HIST2H2BF;HKR1;IL24;ILKAP;LIAS;MEAF6;METTL17;METTL3;NAP1L4;PCID2;PMS2P1;POLR1C;PPIE;PPIH;RBFA;RPP38;SIRT5;SUPT7L;TCEA3;TGFB3;WDR77;ZNF133;ZNF202;ZNF211;ZNF232;ZNF266;ZNF329;ZNF343;ZNF419;ZNF44;ZNF530;ZNF542;ZNF544;
SUPT7L;TCEA3;TGFB3;WDR77;ZNF133;ZNF202;ZNF211;ZNF232;ZNF266;ZNF329;ZNF343;ZNF419;ZNF44;ZNF530;ZNF542;ZNF544;ZNF563;ZNF671;ZNF69;ZNF773;ZNF78
;COQ6;DNAH3;DSN1;DUSP6;EXOSC2;EXOSC7;FANCE;FGR;GALNT12;GEMIN8;GPD1;GTF2IRD2;GTF2IRD2B;HIST1H2BC;HIST1H2BD;HIST2H2BF;HKR1;HSD17B7;LDHB;LETMD1;LIAS;LUC7L;MEAF6;METTL17;METTL3;NAP1L4;NUP43;NVL;OVGP1;PEX11B;PMS2P1;POLR1C;PPIE;PPIH;RBFA;RDH16;RPP38;RSAD1

;COQ6;DNAH3;DSN1;DUSP6;EXOSC2;EXOSC7;FANCE;FGR;GALNT12;GEMIN8;GPD1;GTF2IRD2;GTF2IRD2B;HIST1H2BC;HIST1H2BD;HIST2H2BF;HKR1;HSD17B7;LDHB;LETMD1;LIAS;LUC7L;MEAF6;METTL17;METTL3;NAP1L4;NUP43;NVL;OVGP1;PEX11B;PMS2P1;POLR1C;PPIE;PPIH;RBFA;RDH16;RPP38;RSAD1

IST1H2BC;HIST1H2BD;HIST2H2BF;HKR1;LUC7L;MEAF6;METTL3;NAP1L4;NUP43;NVL;PMS2P1;POLR1C;PPIE;PPIH;RPP38;SUPT7L;TCEA3;TGFB3;WDR77;ZNF133;ZNF202;ZNF211;ZNF232;ZNF266;ZNF329;ZNF343;ZNF419;ZNF44;ZNF530;ZNF542;ZNF544;ZNF563;ZNF671;ZNF69;ZNF773;ZNF78

SUPT7L;TCEA3;TGFB3;WDR77;ZNF133;ZNF202;ZNF211;ZNF232;ZNF266;ZNF329;ZNF343;ZNF419;ZNF44;ZNF530;ZNF542;ZNF544;ZNF563;ZNF671;ZNF69;ZNF773;ZNF78

7;ZNF133;ZNF202;ZNF211;ZNF232;ZNF266;ZNF329;ZNF343;ZNF419;ZNF44;ZNF530;ZNF542;ZNF544;ZNF563;ZNF671;ZNF69;ZNF773;ZNF78
7L;TCEA3;TGFB3;WDR77;ZNF133;ZNF202;ZNF211;ZNF232;ZNF266;ZNF329;ZNF343;ZNF419;ZNF44;ZNF530;ZNF542;ZNF544;ZNF563;ZNF671;ZNF69;ZNF773;ZNF78

2;ZNF211;ZNF266;ZNF329;ZNF343;ZNF419;ZNF44;ZNF530;ZNF542;ZNF544;ZNF563;ZNF671;ZNF69;ZNF773;ZNF78

;LIAS;MEAF6;METTL17;NPR2;PCID2;POLR1C;PPIE;PRPSAP2;RSAD1;SIRT5;SUPT7L;TCEA3;TGFB3;WDR77;ZNF133;ZNF202;ZNF211;ZNF232;ZNF266;ZNF329;ZNF343;ZNF419;ZNF44;ZNF530;ZNF542;ZNF544;ZNF563;ZNF671;ZNF69;ZNF773;ZNF78

2;ZNF211;ZNF266;ZNF329;ZNF343;ZNF419;ZNF44;ZNF530;ZNF542;ZNF544;ZNF563;ZNF671;ZNF69;ZNF773;ZNF78
7;ZNF133;ZNF202;ZNF211;ZNF232;ZNF266;ZNF329;ZNF343;ZNF419;ZNF44;ZNF530;ZNF542;ZNF544;ZNF563;ZNF671;ZNF69;ZNF773;ZNF78

3;TGFB3;WDR77;ZNF133;ZNF202;ZNF211;ZNF232;ZNF266;ZNF329;ZNF343;ZNF419;ZNF44;ZNF530;ZNF542;ZNF544;ZNF563;ZNF671;ZNF69;ZNF773;ZNF78

2;ZNF211;ZNF266;ZNF329;ZNF343;ZNF419;ZNF44;ZNF530;ZNF542;ZNF544;ZNF563;ZNF671;ZNF69;ZNF773;ZNF78

;LIAS;MEAF6;METTL17;NPR2;PCID2;POLR1C;PPIE;PRPSAP2;RSAD1;SIRT5;SUPT7L;TCEA3;TGFB3;WDR77;ZNF133;ZNF202;ZNF211;ZNF232;ZNF266;ZNF329;ZNF343;ZNF419;ZNF44;ZNF530;ZNF542;ZNF544;ZNF563;ZNF671;ZNF69;ZNF773;ZNF78

32
IN8;GTF2IRD2;GTF2IRD2B;HIST1H2BC;HIST1H2BD;HIST2H2BF;HKR1;IL24;ILKAP;KLKB1;LIAS;MEAF6;METTL17;METTL3;NAP1L4;OVGP1;PCID2;PMS2P1;POLR1C;PPIE;PPIH;RBFA;RPP38;SIRT5;SUPT7L;TCEA3;TGFB3;WDR77;ZNF133;ZNF202;ZNF211;ZNF232;ZNF266;ZNF329;ZNF343;ZNF419;ZNF44;ZNF530;ZI

MEAF6;METTL17;NPR2;PCID2;POLR1C;PPIE;PRPSAP2;RSAD1;SIRT5;SUPT7L;TCEA3;TGFB3;WDR77;ZNF133;ZNF202;ZNF211;ZNF232;ZNF266;ZNF329;ZNF343;ZNF419;ZNF44;ZNF530;ZNF542;ZNF544;ZNF563;ZNF671;ZNF69;ZNF773;ZNF78

X;RPS5;RPS6;RPS8;RPSA
RPL36;RPL37;RPL37A;RPL4;RPL5;RPL7A;RPS11;RPS12;RPS13;RPS14;RPS16;RPS18;RPS19;RPS20;RPS21;RPS23;RPS25;RPS27A;RPS3;RPS4X;RPS5;RPS6;RPS8;RPS
L36;RPL37;RPL37A;RPL4;RPL5;RPL7A;RPS11;RPS12;RPS13;RPS14;RPS16;RPS18;RPS19;RPS20;RPS21;RPS23;RPS25;RPS27A;RPS3;RPS4X;RPS5;RPS6;RPS8;RPS
PS3;RPS4X;RPS5;RPS6;RPS8;RPSA
PL37;RPL37A;RPL4;RPL5;RPL7A;RPS11;RPS12;RPS13;RPS14;RPS16;RPS18;RPS19;RPS20;RPS21;RPS23;RPS25;RPS27A;RPS3;RPS4X;RPS5;RPS6;RPS8;RPSA;SRPR
PL37;RPL37A;RPL4;RPL5;RPL7A;RPS11;RPS12;RPS13;RPS14;RPS16;RPS18;RPS19;RPS20;RPS21;RPS23;RPS25;RPS27A;RPS3;RPS4X;RPS5;RPS6;RPS8;RPSA;SRPR
PL37;RPL37A;RPL4;RPL5;RPL7A;RPS11;RPS12;RPS13;RPS14;RPS16;RPS18;RPS19;RPS20;RPS21;RPS23;RPS25;RPS27A;RPS3;RPS4X;RPS5;RPS6;RPS8;RPSA;SRPR
PL37;RPL37A;RPL4;RPL5;RPL7A;RPS11;RPS12;RPS13;RPS14;RPS16;RPS18;RPS19;RPS20;RPS21;RPS23;RPS25;RPS27A;RPS3;RPS4X;RPS5;RPS6;RPS8;RPSA;TRNAU1A
PL37;RPL37A;RPL4;RPL5;RPL7A;RPS11;RPS12;RPS13;RPS14;RPS16;RPS18;RPS19;RPS20;RPS21;RPS23;RPS25;RPS27A;RPS3;RPS4X;RPS5;RPS6;RPS8;RPSA;SRPR
PL32;RPL35A;RPL36;RPL37;RPL37A;RPL4;RPL5;RPL7A;RPS11;RPS12;RPS13;RPS14;RPS16;RPS18;RPS19;RPS20;RPS21;RPS23;RPS25;RPS27A;RPS3;RPS4X;RPS5;RPS6;RPS8;RPS
PL37;RPL37A;RPL4;RPL5;RPL7A;RPS11;RPS12;RPS13;RPS14;RPS16;RPS18;RPS19;RPS20;RPS21;RPS23;RPS25;RPS27A;RPS3;RPS4X;RPS5;RPS6;RPS8;RPSA;SRPR
PL37;RPL37A;RPL4;RPL5;RPL7A;RPS11;RPS12;RPS13;RPS14;RPS16;RPS18;RPS19;RPS20;RPS21;RPS23;RPS25;RPS27A;RPS3;RPS4X;RPS5;RPS6;RPS8;RPS
;RPL35A;RPL36;RPL37;RPL37A;RPL4;RPL5;RPL7A;RPS11;RPS12;RPS13;RPS14;RPS16;RPS18;RPS19;RPS20;RPS21;RPS23;RPS25;RPS27A;RPS3;RPS4X;RPS5;RPS6;RPS8;RPS
;RPL36;RPL37;RPL37A;RPL4;RPL5;RPL7A;RPS11;RPS12;RPS13;RPS14;RPS16;RPS18;RPS19;RPS20;RPS21;RPS23;RPS25;RPS27A;RPS3;RPS4X;RPS5;RPS6;RPS8;RPSA;SRPR
;RPL36;RPL37;RPL37A;RPL4;RPL5;RPL7A;RPS11;RPS12;RPS13;RPS14;RPS16;RPS18;RPS19;RPS20;RPS21;RPS23;RPS25;RPS27A;RPS3;RPS4X;RPS5;RPS6;RPS8;RPS
;RPL36;RPL37;RPL37A;RPL4;RPL5;RPL7A;RPS11;RPS12;RPS13;RPS14;RPS16;RPS18;RPS19;RPS20;RPS21;RPS23;RPS25;RPS27A;RPS3;RPS4X;RPS5;RPS6;RPS8;RPS
L35A;RPL36;RPL37;RPL37A;RPL4;RPL5;RPL7A;RPS11;RPS12;RPS13;RPS14;RPS16;RPS18;RPS19;RPS20;RPS21;RPS23;RPS25;RPS27A;RPS3;RPS4X;RPS5;RPS6;RPS8;RPS
L36;RPL37;RPL37A;RPL4;RPL5;RPL7A;RPS11;RPS12;RPS13;RPS14;RPS16;RPS18;RPS19;RPS20;RPS21;RPS23;RPS25;RPS27A;RPS3;RPS4X;RPS5;RPS6;RPS8;RPS
RPL36;RPL37;RPL37A;RPL4;RPL5;RPL7A;RPS11;RPS12;RPS13;RPS14;RPS16;RPS18;RPS19;RPS20;RPS21;RPS23;RPS25;RPS27A;RPS3;RPS4X;RPS5;RPS6;RPS8;RPSA;UX
L36;RPL37;RPL37A;RPL4;RPL5;RPL7A;RPS11;RPS12;RPS13;RPS14;RPS16;RPS18;RPS19;RPS20;RPS21;RPS23;RPS25;RPS27A;RPS3;RPS4X;RPS5;RPS6;RPS8;RPS
PL37;RPL37A;RPL4;RPL5;RPL7A;RPS11;RPS12;RPS13;RPS14;RPS16;RPS18;RPS19;RPS20;RPS21;RPS23;RPS25;RPS27A;RPS3;RPS4X;RPS5;RPS6;RPS8;RPS
PL32;RPL35A;RPL36;RPL37;RPL37A;RPL4;RPL5;RPL7A;RPS11;RPS12;RPS13;RPS14;RPS16;RPS18;RPS19;RPS20;RPS21;RPS23;RPS25;RPS27A;RPS3;RPS4X;RPS5;RPS6;RPS8;RPS
L36;RPL37;RPL37A;RPL4;RPL5;RPL7A;RPS11;RPS12;RPS13;RPS14;RPS16;RPS18;RPS19;RPS20;RPS21;RPS23;RPS25;RPS27A;RPS3;RPS4X;RPS5;RPS6;RPS8;RPS
PL37;RPL37A;RPL4;RPL5;RPL7A;RPS11;RPS12;RPS13;RPS14;RPS16;RPS18;RPS19;RPS20;RPS21;RPS23;RPS25;RPS27A;RPS3;RPS4X;RPS5;RPS6;RPS8;RPS
PL37;RPL37A;RPL4;RPL5;RPL7A;RPS11;RPS12;RPS13;RPS14;RPS16;RPS18;RPS19;RPS20;RPS21;RPS23;RPS25;RPS27A;RPS3;RPS4X;RPS5;RPS6;RPS8;RPSA;SRPRB;TOMM
32;RPL35A;RPL36;RPL37;RPL37A;RPL4;RPL5;RPL7A;RPS11;RPS12;RPS13;RPS14;RPS16;RPS18;RPS19;RPS20;RPS21;RPS23;RPS25;RPS27A;RPS3;RPS4X;RPS5;RPS6;RPS8;RPS
PL37;RPL37A;RPL4;RPL5;RPL7A;RPS11;RPS12;RPS13;RPS14;RPS16;RPS18;RPS19;RPS20;RPS21;RPS23;RPS25;RPS27A;RPS3;RPS4X;RPS5;RPS6;RPS8;RPS
L36;RPL37;RPL37A;RPL4;RPL5;RPL7A;RPS11;RPS12;RPS13;RPS14;RPS16;RPS18;RPS19;RPS20;RPS21;RPS23;RPS25;RPS27A;RPS3;RPS4X;RPS5;RPS6;RPS8;RPS
PL24;RPL27A;RPL3;RPL32;RPL35A;RPL36;RPL37;RPL37A;RPL4;RPL5;RPL7A;RPS11;RPS12;RPS13;RPS14;RPS16;RPS18;RPS19;RPS20;RPS21;RPS23;RPS25;RPS27A;RPS3;RPS4X;RPS5;RPS6;RPS8;RPSA;SRPRB;TRNAU1A
PL23A;RPL24;RPL27A;RPL3;RPL32;RPL35A;RPL36;RPL37;RPL37A;RPL4;RPL5;RPL7A;RPS11;RPS12;RPS13;RPS14;RPS16;RPS18;RPS19;RPS20;RPS21;RPS23;RPS25;RPS27A;RPS3;RPS4X;RPS5;RPS6;RPS8;RPSA;SNRPD2;SNRPD
RPL35A;RPL36;RPL37;RPL37A;RPL4;RPL5;RPL7A;RPS11;RPS12;RPS13;RPS14;RPS16;RPS18;RPS19;RPS20;RPS21;RPS23;RPS25;RPS27A;RPS3;RPS4X;RPS5;RPS6;RPS8;RPSA;SNUPN;SRPRB;TOMM
4;RPL19;RPL22;RPL23A;RPL24;RPL27A;RPL3;RPL32;RPL35A;RPL36;RPL37;RPL37A;RPL4;RPL5;RPL7A;RPS11;RPS12;RPS13;RPS14;RPS16;RPS18;RPS19;RPS20;RPS21;RPS23;RPS25;RPS27A;RPS3;RPS4X;RPS5;RPS6;RPS8;RPS
RPL36;RPL37;RPL37A;RPL4;RPL5;RPL7A;RPS11;RPS12;RPS13;RPS14;RPS16;RPS18;RPS19;RPS20;RPS21;RPS23;RPS25;RPS27A;RPS3;RPS4X;RPS5;RPS6;RPS8;RPSA;SNUPN;SRPRB;TOMM
A;RPL24;RPL27A;RPL3;RPL32;RPL35A;RPL36;RPL37;RPL37A;RPL4;RPL5;RPL7A;RPS11;RPS12;RPS13;RPS14;RPS16;RPS18;RPS19;RPS20;RPS21;RPS23;RPS25;RPS27A;RPS3;RPS4X;RPS5;RPS6;RPS8;RPSA;SNRPD2;SNRPD3;SNUP
3A;RPL14;RPL19;RPL22;RPL23A;RPL24;RPL27A;RPL3;RPL32;RPL35A;RPL36;RPL37;RPL37A;RPL4;RPL5;RPL7A;RPS11;RPS12;RPS13;RPS14;RPS16;RPS18;RPS19;RPS20;RPS21;RPS23;RPS25;RPS27A;RPS3;RPS4X;RPS5;RPS6;RPS8;RPSA;SKP
3A;RPL14;RPL19;RPL22;RPL23A;RPL24;RPL27A;RPL3;RPL32;RPL35A;RPL36;RPL37;RPL37A;RPL4;RPL5;RPL7A;RPS11;RPS12;RPS13;RPS14;RPS16;RPS18;RPS19;RPS20;RPS21;RPS23;RPS25;RPS27A;RPS3;RPS4X;RPS5;RPS6;RPS8;RPSA;SKP
4;RPL19;RPL22;RPL23A;RPL24;RPL27A;RPL3;RPL32;RPL35A;RPL36;RPL37;RPL37A;RPL4;RPL5;RPL7A;RPS11;RPS12;RPS13;RPS14;RPS16;RPS18;RPS19;RPS20;RPS21;RPS23;RPS25;RPS27A;RPS3;RPS4X;RPS5;RPS6;RPS8;RPS
RPL3;RPL32;RPL35A;RPL36;RPL37;RPL37A;RPL4;RPL5;RPL7A;RPS11;RPS12;RPS13;RPS14;RPS16;RPS18;RPS19;RPS20;RPS21;RPS23;RPS25;RPS27A;RPS3;RPS4X;RPS5;RPS6;RPS8;RPSA;SNUPN;SRPRB;TOMM
RPL32;RPL35A;RPL36;RPL37;RPL37A;RPL4;RPL5;RPL7A;RPS11;RPS12;RPS13;RPS14;RPS16;RPS18;RPS19;RPS20;RPS21;RPS23;RPS25;RPS27A;RPS3;RPS4X;RPS5;RPS6;RPS8;RPSA;SNUPN;SRPRB;STX8;TOMM6;TRAPPC
RPL32;RPL35A;RPL36;RPL37;RPL37A;RPL4;RPL5;RPL7A;RPS11;RPS12;RPS13;RPS14;RPS16;RPS18;RPS19;RPS20;RPS21;RPS23;RPS25;RPS27A;RPS3;RPS4X;RPS5;RPS6;RPS8;RPS
;RPL36;RPL37;RPL37A;RPL4;RPL5;RPL7A;RPS11;RPS12;RPS13;RPS14;RPS16;RPS18;RPS19;RPS20;RPS21;RPS23;RPS25;RPS27A;RPS3;RPS4X;RPS5;RPS6;RPS8;RPSA;ZGLP
L3;RPL32;RPL35A;RPL36;RPL37;RPL37A;RPL4;RPL5;RPL7A;RPS11;RPS12;RPS13;RPS14;RPS16;RPS18;RPS19;RPS20;RPS21;RPS23;RPS25;RPS27A;RPS3;RPS4X;RPS5;RPS6;RPS8;RPSA;SKP1;USP4
;RPL37A;RPL4;RPL5;RPL7A
PL37;RPL37A;RPL4;RPL5;RPL7A
RPL27A;RPL3;RPL32;RPL35A;RPL36;RPL37;RPL37A;RPL4;RPL5;RPL7A;RPS11;RPS12;RPS13;RPS14;RPS16;RPS18;RPS19;RPS20;RPS21;RPS23;RPS25;RPS27A;RPS3;RPS4X;RPS5;RPS6;RPS8;RPSA;SNUPN;SRPRB;TOMM
RPL27A;RPL3;RPL32;RPL35A;RPL36;RPL37;RPL37A;RPL4;RPL5;RPL7A;RPS11;RPS12;RPS13;RPS14;RPS16;RPS18;RPS19;RPS20;RPS21;RPS23;RPS25;RPS27A;RPS3;RPS4X;RPS5;RPS6;RPS8;RPSA;SNUPN;SRPRB;TOMM
4;RPL27A;RPL3;RPL32;RPL35A;RPL36;RPL37;RPL37A;RPL4;RPL5;RPL7A;RPS11;RPS12;RPS13;RPS14;RPS16;RPS18;RPS19;RPS20;RPS21;RPS23;RPS25;RPS27A;RPS3;RPS4X;RPS5;RPS6;RPS8;RPSA;SNUPN;SRPRB;STX8;TOMM6;TRAPPC3;UX
L3;RPL32;RPL35A;RPL36;RPL37;RPL37A;RPL4;RPL5;RPL7A;RPS11;RPS12;RPS13;RPS14;RPS16;RPS18;RPS19;RPS20;RPS21;RPS23;RPS25;RPS27A;RPS3;RPS4X;RPS5;RPS6;RPS8;RPSA;SKP1;USP4
;RPL22;RPL23A;RPL24;RPL27A;RPL3;RPL32;RPL35A;RPL36;RPL37;RPL37A;RPL4;RPL5;RPL7A;RPS11;RPS12;RPS13;RPS14;RPS16;RPS18;RPS19;RPS20;RPS21;RPS23;RPS25;RPS27A;RPS3;RPS4X;RPS5;RPS6;RPS8;RPSA;SNUPN;SRPRB;TOMM
;RPL22;RPL23A;RPL24;RPL27A;RPL3;RPL32;RPL35A;RPL36;RPL37;RPL37A;RPL4;RPL5;RPL7A;RPS11;RPS12;RPS13;RPS14;RPS16;RPS18;RPS19;RPS20;RPS21;RPS23;RPS25;RPS27A;RPS3;RPS4X;RPS5;RPS6;RPS8;RPSA;SNUPN;SRPRB;TOMM

MB6;RAN;RPIA;RPL10;RPL10A;RPL12;RPL13A;RPL14;RPL19;RPL22;RPL23A;RPL24;RPL27A;RPL3;RPL32;RPL35A;RPL36;RPL37;RPL37A;RPL4;RPL5;RPL7A;RPS11;RPS12;RPS13;RPS14;RPS16;RPS18;RPS19;RPS20;RPS21;RPS23;RPS25;RPS27A;RPS3;RPS4X;RPS5;RPS6;RPS8;RPSA;SKP1;SNRPD2;SNRPD3;SNUPN;SU
PL12;RPL13A;RPL14;RPL19;RPL22;RPL23A;RPL24;RPL27A;RPL3;RPL32;RPL35A;RPL36;RPL37;RPL37A;RPL4;RPL5;RPL7A;RPS11;RPS12;RPS13;RPS14;RPS16;RPS18;RPS19;RPS20;RPS21;RPS23;RPS25;RPS27A;RPS3;RPS4X;RPS5;RPS6;RPS8;RPSA;SKP
6;MRPS7;MYL12A;MYL5;NACA;NDUFC2;NPM1;PCNA;POLR2H;POMP;POP4;PSMA5;PSMB6;RAN;RPL10;RPL10A;RPL12;RPL13A;RPL14;RPL19;RPL22;RPL23A;RPL24;RPL27A;RPL3;RPL32;RPL35A;RPL36;RPL37;RPL37A;RPL4;RPL5;RPL7A;RPS11;RPS12;RPS13;RPS14;RPS16;RPS18;RPS19;RPS20;RPS21;RPS23;RP
PL36;RPL37;RPL37A;RPL4;RPL5;RPL7A;RPS11;RPS12;RPS13;RPS14;RPS16;RPS18;RPS19;RPS20;RPS21;RPS23;RPS25;RPS27A;RPS3;RPS4X;RPS5;RPS6;RPS8;RPS
PL36;RPL37;RPL37A;RPL4;RPL5;RPL7A;RPS11;RPS12;RPS13;RPS14;RPS16;RPS18;RPS19;RPS20;RPS21;RPS23;RPS25;RPS27A;RPS3;RPS4X;RPS5;RPS6;RPS8;RPS
PL36;RPL37;RPL37A;RPL4;RPL5;RPL7A;RPS11;RPS12;RPS13;RPS14;RPS16;RPS18;RPS19;RPS20;RPS21;RPS23;RPS25;RPS27A;RPS3;RPS4X;RPS5;RPS6;RPS8;RPS
PL37;RPL37A;RPL4;RPL5;RPL7A;RPS11;RPS12;RPS13;RPS14;RPS16;RPS18;RPS19;RPS20;RPS21;RPS23;RPS25;RPS27A;RPS3;RPS4X;RPS5;RPS6;RPS8;RPS
A;RPL14;RPL19;RPL22;RPL23A;RPL24;RPL27A;RPL3;RPL32;RPL35A;RPL36;RPL37;RPL37A;RPL4;RPL5;RPL7A;RPS11;RPS12;RPS13;RPS14;RPS16;RPS18;RPS19;RPS20;RPS21;RPS23;RPS25;RPS27A;RPS3;RPS4X;RPS5;RPS6;RPS8;RPSA;USP42;ZGLP
PL36;RPL37;RPL37A;RPL4;RPL5;RPL7A;RPS11;RPS12;RPS13;RPS14;RPS16;RPS18;RPS19;RPS20;RPS21;RPS23;RPS25;RPS27A;RPS3;RPS4X;RPS5;RPS6;RPS8;RPS
PL3;RPL32;RPL35A;RPL36;RPL37;RPL37A;RPL4;RPL5;RPL7A;RPS11;RPS12;RPS13;RPS14;RPS16;RPS18;RPS19;RPS20;RPS21;RPS23;RPS25;RPS27A;RPS3;RPS4X;RPS5;RPS6;RPS8;RPS
RPL27A;RPL3;RPL32;RPL35A;RPL36;RPL37;RPL37A;RPL4;RPL5;RPL7A;RPS11;RPS12;RPS13;RPS14;RPS16;RPS18;RPS19;RPS20;RPS21;RPS23;RPS25;RPS27A;RPS3;RPS4X;RPS5;RPS6;RPS8;RPSA;SNRPD2;SNRPD3;SNUP
A;RPL14;RPL19;RPL22;RPL23A;RPL24;RPL27A;RPL3;RPL32;RPL35A;RPL36;RPL37;RPL37A;RPL4;RPL5;RPL7A;RPS11;RPS12;RPS13;RPS14;RPS16;RPS18;RPS19;RPS20;RPS21;RPS23;RPS25;RPS27A;RPS3;RPS4X;RPS5;RPS6;RPS8;RPSA;USP42;ZGLP
;RPL22;RPL23A;RPL24;RPL27A;RPL3;RPL32;RPL35A;RPL36;RPL37;RPL37A;RPL4;RPL5;RPL7A;RPS11;RPS12;RPS13;RPS14;RPS16;RPS18;RPS19;RPS20;RPS21;RPS23;RPS25;RPS27A;RPS3;RPS4X;RPS5;RPS6;RPS8;RPSA;SNUPN;SRPRB;TOMM
L27A;RPL3;RPL35A;RPL37;RPL4;RPL5;RPL7A;RPS11;RPS13;RPS14;RPS16;RPS18;RPS20;RPS25;RPS3;RPS4X;RPS5;SNRPD3;SNUPN;TRNAU1AI
RPL24;RPL27A;RPL3;RPL32;RPL35A;RPL36;RPL37;RPL37A;RPL4;RPL5;RPL7A;RPS11;RPS12;RPS13;RPS14;RPS16;RPS18;RPS19;RPS20;RPS21;RPS23;RPS25;RPS27A;RPS3;RPS4X;RPS5;RPS6;RPS8;RPSA;SNUPN;SRPRB;STX8;TOMM6;TRAPPC3;UX
;MYL5;NOP16;NPM1;PCNA;POLR2H;POP4;PRDX1;RAN;RPL10;RPL10A;RPL12;RPL13A;RPL14;RPL19;RPL22;RPL23A;RPL24;RPL27A;RPL3;RPL32;RPL35A;RPL36;RPL37;RPL37A;RPL4;RPL5;RPL7A;RPS11;RPS12;RPS13;RPS14;RPS16;RPS18;RPS19;RPS20;RPS21;RPS23;RPS25;RPS27A;RPS3;RPS4X;RPS5;RPS6;RPS
;MYL5;NOP16;NPM1;PCNA;POLR2H;POP4;PRDX1;RAN;RPL10;RPL10A;RPL12;RPL13A;RPL14;RPL19;RPL22;RPL23A;RPL24;RPL27A;RPL3;RPL32;RPL35A;RPL36;RPL37;RPL37A;RPL4;RPL5;RPL7A;RPS11;RPS12;RPS13;RPS14;RPS16;RPS18;RPS19;RPS20;RPS21;RPS23;RPS25;RPS27A;RPS3;RPS4X;RPS5;RPS6;RPS
L14;RPL19;RPL22;RPL23A;RPL24;RPL27A;RPL3;RPL32;RPL35A;RPL36;RPL37;RPL37A;RPL4;RPL5;RPL7A;RPS11;RPS12;RPS13;RPS14;RPS16;RPS18;RPS19;RPS20;RPS21;RPS23;RPS25;RPS27A;RPS3;RPS4X;RPS5;RPS6;RPS8;RPSA;SNUPN;SRPRB;TOMM
L14;RPL19;RPL22;RPL23A;RPL24;RPL27A;RPL3;RPL32;RPL35A;RPL36;RPL37;RPL37A;RPL4;RPL5;RPL7A;RPS11;RPS12;RPS13;RPS14;RPS16;RPS18;RPS19;RPS20;RPS21;RPS23;RPS25;RPS27A;RPS3;RPS4X;RPS5;RPS6;RPS8;RPSA;SNUPN;SRPRB;TOMM6;VDAC
;RPL23A;RPL24;RPL27A;RPL3;RPL32;RPL35A;RPL36;RPL37;RPL37A;RPL4;RPL5;RPL7A;RPS11;RPS12;RPS13;RPS14;RPS16;RPS18;RPS19;RPS20;RPS21;RPS23;RPS25;RPS27A;RPS3;RPS4X;RPS5;RPS6;RPS8;RPSA;SNUPN;SRPRB;STX8;TOMM6;TRAPPC3;UX
23A;RPL24;RPL27A;RPL3;RPL32;RPL35A;RPL36;RPL37;RPL37A;RPL4;RPL5;RPL7A;RPS11;RPS12;RPS13;RPS14;RPS16;RPS18;RPS19;RPS20;RPS21;RPS23;RPS25;RPS27A;RPS3;RPS4X;RPS5;RPS6;RPS8;RPSA;SKP1;USP4
RPS8;RPSA;SNRPD2;SNRPD3;SNUPN
MDH1;MED10;MRPS16;MRPS7;MYL12A;MYL5;NDUFC2;NOP16;NPM1;PARL;PCNA;POLR2H;POMP;POP4;PRDX1;PSMA5;PSMB6;RAN;RPL10;RPL10A;RPL12;RPL13A;RPL14;RPL19;RPL22;RPL23A;RPL24;RPL27A;RPL3;RPL32;RPL35A;RPL36;RPL37;RPL37A;RPL4;RPL5;RPL7A;RPS11;RPS12;RPS13;RPS14;RPS16;
MDH1;MED10;MRPS16;MRPS7;MYL12A;MYL5;NDUFC2;NOP16;NPM1;PARL;PCNA;POLR2H;POP4;PRDX1;PSMA5;PSMB6;RAN;RPL10;RPL10A;RPL12;RPL13A;RPL14;RPL19;RPL22;RPL23A;RPL24;RPL27A;RPL3;RPL32;RPL35A;RPL36;RPL37;RPL37A;RPL4;RPL5;RPL7A;RPS11;RPS12;RPS13;RPS14;RPS16;RPS18;
L19;RPL22;RPL23A;RPL24;RPL27A;RPL3;RPL32;RPL35A;RPL36;RPL37;RPL37A;RPL4;RPL5;RPL7A;RPS11;RPS12;RPS13;RPS14;RPS16;RPS18;RPS19;RPS20;RPS21;RPS23;RPS25;RPS27A;RPS3;RPS4X;RPS5;RPS6;RPS8;RPSA;SKP1;USP4
23A;RPL24;RPL27A;RPL3;RPL32;RPL35A;RPL36;RPL37;RPL37A;RPL4;RPL5;RPL7A;RPS11;RPS12;RPS13;RPS14;RPS16;RPS18;RPS19;RPS20;RPS21;RPS23;RPS25;RPS27A;RPS3;RPS4X;RPS5;RPS6;RPS8;RPSA;SKP1;USP4
SA

;NACA;NAP1L1;NPM1;PCNA;PER2;POLR2H;PPIA;RAN;RPL10;RPL10A;RPL12;RPL13A;RPL14;RPL19;RPL22;RPL23A;RPL24;RPL27A;RPL3;RPL32;RPL35A;RPL36;RPL37;RPL37A;RPL4;RPL5;RPL7A;RPS11;RPS12;RPS13;RPS14;RPS16;RPS18;RPS19;RPS20;RPS21;RPS23;RPS25;RPS27A;RPS3;RPS4X;RPS5;RPS6;RPS8;
CA;NPM1;PCNA;PER2;POLR2H;POP4;PSMA5;PSMB6;RAN;RPL10;RPL10A;RPL12;RPL13A;RPL14;RPL19;RPL22;RPL23A;RPL24;RPL27A;RPL3;RPL32;RPL35A;RPL36;RPL37;RPL37A;RPL4;RPL5;RPL7A;RPS11;RPS12;RPS13;RPS14;RPS16;RPS18;RPS19;RPS20;RPS21;RPS23;RPS25;RPS27A;RPS3;RPS4X;RPS5;RPS6;R
L22;RPL23A;RPL24;RPL27A;RPL3;RPL32;RPL35A;RPL36;RPL37;RPL37A;RPL4;RPL5;RPL7A;RPS11;RPS12;RPS13;RPS14;RPS16;RPS18;RPS19;RPS20;RPS21;RPS23;RPS25;RPS27A;RPS3;RPS4X;RPS5;RPS6;RPS8;RPSA;SNUPN;SRPRB;STX8;TOMM6;TRAPPC3;UXT;VDAC
;MRPS7;MYL12A;MYL5;NACA;NAP1L1;NDUFC2;PARL;POMP;PPIA;PRDX1;PSMA5;PSMB6;RAN;RPIA;RPL10;RPL10A;RPL12;RPL13A;RPL14;RPL19;RPL22;RPL23A;RPL24;RPL27A;RPL3;RPL32;RPL35A;RPL36;RPL37;RPL37A;RPL4;RPL5;RPL7A;RPS11;RPS12;RPS13;RPS14;RPS16;RPS18;RPS19;RPS20;RPS21;RPS2
;NACA;NAP1L1;NPM1;PCNA;PER2;POLR2H;PPIA;RAN;RPL10;RPL10A;RPL12;RPL13A;RPL14;RPL19;RPL22;RPL23A;RPL24;RPL27A;RPL3;RPL32;RPL35A;RPL36;RPL37;RPL37A;RPL4;RPL5;RPL7A;RPS11;RPS12;RPS13;RPS14;RPS16;RPS18;RPS19;RPS20;RPS21;RPS23;RPS25;RPS27A;RPS3;RPS4X;RPS5;RPS6;RPS8;
;RPL13A;RPL14;RPL19;RPL22;RPL23A;RPL24;RPL27A;RPL3;RPL32;RPL35A;RPL36;RPL37;RPL37A;RPL4;RPL5;RPL7A;RPS11;RPS12;RPS13;RPS14;RPS16;RPS18;RPS19;RPS20;RPS21;RPS23;RPS25;RPS27A;RPS3;RPS4X;RPS5;RPS6;RPS8;RPSA;SKP1;SRPRB;TOMM6;TRNAU1AP;USP42;UXT;ZGLF
CNA;PER2;POLR2H;POP4;PPIA;PSMA5;PSMB6;RAN;RPL10;RPL10A;RPL12;RPL13A;RPL14;RPL19;RPL22;RPL23A;RPL24;RPL27A;RPL3;RPL32;RPL35A;RPL36;RPL37;RPL37A;RPL4;RPL5;RPL7A;RPS11;RPS12;RPS13;RPS14;RPS16;RPS18;RPS19;RPS20;RPS21;RPS23;RPS25;RPS27A;RPS3;RPS4X;RPS5;RPS6;RPS8;R
4;PSMA5;PSMB6;RAN;RPL10;RPL10A;RPL12;RPL13A;RPL14;RPL19;RPL22;RPL23A;RPL24;RPL27A;RPL3;RPL32;RPL35A;RPL36;RPL37;RPL37A;RPL4;RPL5;RPL7A;RPS11;RPS12;RPS13;RPS14;RPS16;RPS18;RPS19;RPS20;RPS21;RPS23;RPS25;RPS27A;RPS3;RPS4X;RPS5;RPS6;RPS8;RPSA;SNRPD2;SNRPD3;SNUPN;
NPA1;HS3ST3B1;ICT1;ITGB1BP1;MDH1;MRPS16;MRPS7;MYL12A;MYL5;NACA;NAP1L1;NBPF10;NDUFC2;NPM1;PARL;PCNA;PDCL3;PER2;POLR2H;POMP;PPIA;PRDX1;PSMA5;PSMB6;RAN;RANGRF;RPIA;RPL10;RPL10A;RPL12;RPL13A;RPL14;RPL19;RPL22;RPL23A;RPL24;RPL27A;RPL3;RPL32;RPL35A;RPL36;RF
;EIF3H;EIF3M;FAU;GSTM3;GTF3C6;HMGN1;HNRNPA1;HS3ST3B1;ICT1;ISY1;ITGB1BP1;MDH1;MED10;MRPS16;MRPS7;MYL12A;MYL5;NACA;NAP1L1;NBPF10;NDUFC2;NOP16;NPM1;PARL;PCNA;PDCL3;PER2;POLR2H;POMP;POP4;PPIA;PRDX1;PSMA5;PSMB6;RAN;RANGRF;RPIA;RPL10;RPL10A;RPL12;RPL1
RPL12;RPL13A;RPL14;RPL19;RPL22;RPL23A;RPL24;RPL27A;RPL3;RPL32;RPL35A;RPL36;RPL37;RPL37A;RPL4;RPL5;RPL7A;RPS11;RPS12;RPS13;RPS14;RPS16;RPS18;RPS19;RPS20;RPS21;RPS23;RPS25;RPS27A;RPS3;RPS4X;RPS5;RPS6;RPS8;RPSA;SNRPD3;UXT;ZGLP1;ZNRD

PIA;RAN;RPL10;RPL10A;RPL12;RPL13A;RPL14;RPL19;RPL22;RPL23A;RPL24;RPL27A;RPL3;RPL32;RPL35A;RPL36;RPL37;RPL37A;RPL4;RPL5;RPL7A;RPS11;RPS12;RPS13;RPS14;RPS16;RPS18;RPS19;RPS20;RPS21;RPS23;RPS25;RPS27A;RPS3;RPS4X;RPS5;RPS6;RPS8;RPSA;SKP1;SNRPD2;SNRPD3;SNUPN;SSBP1;S

L14;RPL19;RPL22;RPL23A;RPL24;RPL27A;RPL3;RPL32;RPL35A;RPL36;RPL37;RPL37A;RPL4;RPL5;RPL7A;RPS11;RPS12;RPS13;RPS14;RPS16;RPS18;RPS19;RPS20;RPS21;RPS23;RPS25;RPS27A;RPS3;RPS4X;RPS5;RPS6;RPS8;RPSA;SNUPN;SRPRB;STX8;TOMM6;TRAPPC3;UXT;VDAC
;NACA;NAP1L1;NPM1;PCNA;PER2;POLR2H;POP4;PPIA;PSMA5;PSMB6;RAN;RPIA;RPL10;RPL10A;RPL12;RPL13A;RPL14;RPL19;RPL22;RPL23A;RPL24;RPL27A;RPL3;RPL32;RPL35A;RPL36;RPL37;RPL37A;RPL4;RPL5;RPL7A;RPS11;RPS12;RPS13;RPS14;RPS16;RPS18;RPS19;RPS20;RPS21;RPS23;RPS25;RPS27A;RP
L14;RPL19;RPL22;RPL23A;RPL24;RPL27A;RPL3;RPL32;RPL35A;RPL36;RPL37;RPL37A;RPL4;RPL5;RPL7A;RPS11;RPS12;RPS13;RPS14;RPS16;RPS18;RPS19;RPS20;RPS21;RPS23;RPS25;RPS27A;RPS3;RPS4X;RPS5;RPS6;RPS8;RPSA;SNUPN;SRPRB;STX8;TOMM6;TRAPPC3;UXT;VDAC
6;HMGN1;HNRNPA1;HS3ST3B1;ICT1;ISY1;MDH1;MED10;MRPS16;MRPS7;MYL12A;MYL5;NACA;NAP1L1;NDUFC2;NOP16;NPM1;PARL;PCNA;PER2;POLR2H;POMP;POP4;PPIA;PRDX1;PSMA5;PSMB6;RAN;RANGRF;RPIA;RPL10;RPL10A;RPL12;RPL13A;RPL14;RPL19;RPL22;RPL23A;RPL24;RPL27A;RPL3;RPL32;R
;EIF3H;EIF3M;FAU;GSTM3;GTF3C6;HMGN1;HNRNPA1;HS3ST3B1;ICT1;ISY1;ITGB1BP1;MDH1;MED10;MRPS16;MRPS7;MYL12A;MYL5;NACA;NAP1L1;NBPF10;NDUFC2;NOP16;NPM1;PARL;PCNA;PDCL3;PER2;POLR2H;POMP;POP4;PPIA;PRDX1;PSMA5;PSMB6;RAN;RANGRF;RPIA;RPL10;RPL10A;RPL12;RPL1
MED10;NACA;NAP1L1;NPM1;PCNA;PER2;POLR2H;POP4;PPIA;PSMA5;PSMB6;RAN;RPIA;RPL10;RPL10A;RPL12;RPL13A;RPL14;RPL19;RPL22;RPL23A;RPL24;RPL27A;RPL3;RPL32;RPL35A;RPL36;RPL37;RPL37A;RPL4;RPL5;RPL7A;RPS11;RPS12;RPS13;RPS14;RPS16;RPS18;RPS19;RPS20;RPS21;RPS23;RPS25;RPS2
6;HMGN1;HNRNPA1;HS3ST3B1;ICT1;ISY1;MDH1;MED10;MRPS16;MRPS7;MYL12A;MYL5;NACA;NAP1L1;NDUFC2;NOP16;NPM1;PARL;PCNA;PER2;POLR2H;POMP;POP4;PPIA;PRDX1;PSMA5;PSMB6;RAN;RANGRF;RPIA;RPL10;RPL10A;RPL12;RPL13A;RPL14;RPL19;RPL22;RPL23A;RPL24;RPL27A;RPL3;RPL32;R
;NPM1;PCNA;PER2;POLR2H;POP4;PPIA;PSMA5;PSMB6;RAN;RPIA;RPL10;RPL10A;RPL12;RPL13A;RPL14;RPL19;RPL22;RPL23A;RPL24;RPL27A;RPL3;RPL32;RPL35A;RPL36;RPL37;RPL37A;RPL4;RPL5;RPL7A;RPS11;RPS12;RPS13;RPS14;RPS16;RPS18;RPS19;RPS20;RPS21;RPS23;RPS25;RPS27A;RPS3;RPS4X;RPS5
0;RPL10A;RPL12;RPL13A;RPL14;RPL19;RPL22;RPL23A;RPL24;RPL27A;RPL3;RPL32;RPL35A;RPL36;RPL37;RPL37A;RPL4;RPL5;RPL7A;RPS11;RPS12;RPS13;RPS14;RPS16;RPS18;RPS19;RPS20;RPS21;RPS23;RPS25;RPS27A;RPS3;RPS4X;RPS5;RPS6;RPS8;RPSA;SKP1;SRPRB;TOMM6;TRNAU1AP;USP42;UXT;ZGLF
L10A;RPL12;RPL13A;RPL14;RPL19;RPL22;RPL23A;RPL24;RPL27A;RPL3;RPL32;RPL35A;RPL36;RPL37;RPL37A;RPL4;RPL5;RPL7A;RPS11;RPS12;RPS13;RPS14;RPS16;RPS18;RPS19;RPS20;RPS21;RPS23;RPS25;RPS27A;RPS3;RPS4X;RPS5;RPS6;RPS8;RPSA;SKP1;SNRPD2;SNRPD3;SNUPN;SSBP1;STX8;SUGT1;TOMM
AP1L1;NPM1;PCNA;PER2;POLR2H;POP4;PPIA;PSMA5;PSMB6;RAN;RPIA;RPL10;RPL10A;RPL12;RPL13A;RPL14;RPL19;RPL22;RPL23A;RPL24;RPL27A;RPL3;RPL32;RPL35A;RPL36;RPL37;RPL37A;RPL4;RPL5;RPL7A;RPS11;RPS12;RPS13;RPS14;RPS16;RPS18;RPS19;RPS20;RPS21;RPS23;RPS25;RPS27A;RPS3;RPS4
RPS16;MRPS7;NACA;NAP1L1;NPM1;PCNA;PER2;POLR2H;PPIA;RAN;RPL10;RPL10A;RPL12;RPL13A;RPL14;RPL19;RPL22;RPL23A;RPL24;RPL27A;RPL3;RPL32;RPL35A;RPL36;RPL37;RPL37A;RPL4;RPL5;RPL7A;RPS11;RPS12;RPS13;RPS14;RPS16;RPS18;RPS19;RPS20;RPS21;RPS23;RPS25;RPS27A;RPS3;RPS4X;RPS

;NACA;NAP1L1;NPM1;PCNA;PER2;POLR2H;POP4;PPIA;PSMA5;PSMB6;RAN;RPIA;RPL10;RPL10A;RPL12;RPL13A;RPL14;RPL19;RPL22;RPL23A;RPL24;RPL27A;RPL3;RPL32;RPL35A;RPL36;RPL37;RPL37A;RPL4;RPL5;RPL7A;RPS11;RPS12;RPS13;RPS14;RPS16;RPS18;RPS19;RPS20;RPS21;RPS23;RPS25;RPS27A;RP
MRPS7;NACA;NAP1L1;NPM1;PCNA;PER2;POLR2H;PPIA;RAN;RPL10;RPL10A;RPL12;RPL13A;RPL14;RPL19;RPL22;RPL23A;RPL24;RPL27A;RPL3;RPL32;RPL35A;RPL36;RPL37;RPL37A;RPL4;RPL5;RPL7A;RPS11;RPS12;RPS13;RPS14;RPS16;RPS18;RPS19;RPS20;RPS21;RPS23;RPS25;RPS27A;RPS3;RPS4X;RPS5;RPS6

RPS16;MRPS7;NACA;NAP1L1;NPM1;PCNA;PER2;POLR2H;PPIA;RAN;RPL10;RPL10A;RPL12;RPL13A;RPL14;RPL19;RPL22;RPL23A;RPL24;RPL27A;RPL3;RPL32;RPL35A;RPL36;RPL37;RPL37A;RPL4;RPL5;RPL7A;RPS11;RPS12;RPS13;RPS14;RPS16;RPS18;RPS19;RPS20;RPS21;RPS23;RPS25;RPS27A;RPS3;RPS4X;RPS
RPL12;RPL13A;RPL14;RPL19;RPL22;RPL23A;RPL24;RPL27A;RPL3;RPL32;RPL35A;RPL36;RPL37;RPL37A;RPL4;RPL5;RPL7A;RPS11;RPS12;RPS13;RPS14;RPS16;RPS18;RPS19;RPS20;RPS21;RPS23;RPS25;RPS27A;RPS3;RPS4X;RPS5;RPS6;RPS8;RPSA;SNRPD3;UXT;ZGLP1;ZNRD

3B1;ISY1;MED10;NACA;NAP1L1;NPM1;PCNA;PER2;POLR2H;POP4;PPIA;PSMA5;PSMB6;RAN;RPIA;RPL10;RPL10A;RPL12;RPL13A;RPL14;RPL19;RPL22;RPL23A;RPL24;RPL27A;RPL3;RPL32;RPL35A;RPL36;RPL37;RPL37A;RPL4;RPL5;RPL7A;RPS11;RPS12;RPS13;RPS14;RPS16;RPS18;RPS19;RPS20;RPS21;RPS23;R
RPL12;RPL13A;RPL14;RPL19;RPL22;RPL23A;RPL24;RPL27A;RPL3;RPL32;RPL35A;RPL36;RPL37;RPL37A;RPL4;RPL5;RPL7A;RPS11;RPS12;RPS13;RPS14;RPS16;RPS18;RPS19;RPS20;RPS21;RPS23;RPS25;RPS27A;RPS3;RPS4X;RPS5;RPS6;RPS8;RPSA;SNRPD3;UXT;ZGLP1;ZNRD
RPL12;RPL13A;RPL14;RPL19;RPL22;RPL23A;RPL24;RPL27A;RPL3;RPL32;RPL35A;RPL36;RPL37;RPL37A;RPL4;RPL5;RPL7A;RPS11;RPS12;RPS13;RPS14;RPS16;RPS18;RPS19;RPS20;RPS21;RPS23;RPS25;RPS27A;RPS3;RPS4X;RPS5;RPS6;RPS8;RPSA;SNRPD3;UXT;ZGLP1;ZNRD

MED10;MRPS16;MRPS7;NACA;NAP1L1;NPM1;PCNA;PER2;POLR2H;POP4;PPIA;PSMA5;PSMB6;RAN;RPL10;RPL10A;RPL12;RPL13A;RPL14;RPL19;RPL22;RPL23A;RPL24;RPL27A;RPL3;RPL32;RPL35A;RPL36;RPL37;RPL37A;RPL4;RPL5;RPL7A;RPS11;RPS12;RPS13;RPS14;RPS16;RPS18;RPS19;RPS20;RPS21;RPS23;I
RPL12;RPL13A;RPL14;RPL19;RPL22;RPL23A;RPL24;RPL27A;RPL3;RPL32;RPL35A;RPL36;RPL37;RPL37A;RPL4;RPL5;RPL7A;RPS11;RPS12;RPS13;RPS14;RPS16;RPS18;RPS19;RPS20;RPS21;RPS23;RPS25;RPS27A;RPS3;RPS4X;RPS5;RPS6;RPS8;RPSA;SNRPD3;UXT;ZGLP1;ZNRD
RPL12;RPL13A;RPL14;RPL19;RPL22;RPL23A;RPL24;RPL27A;RPL3;RPL32;RPL35A;RPL36;RPL37;RPL37A;RPL4;RPL5;RPL7A;RPS11;RPS12;RPS13;RPS14;RPS16;RPS18;RPS19;RPS20;RPS21;RPS23;RPS25;RPS27A;RPS3;RPS4X;RPS5;RPS6;RPS8;RPSA;SNRPD3;UXT;ZGLP1;ZNRD

RPS19;RPS25;RPS6;ZNRD1

RNPA1;HS3ST3B1;ICT1;ISY1;MDH1;MED10;MRPS16;MRPS7;NACA;NAP1L1;NDUFC2;NPM1;PCNA;PER2;POLR2H;POP4;PPIA;PRDX1;PSMA5;PSMB6;RAN;RARRES3;RPIA;RPL10;RPL10A;RPL12;RPL13A;RPL14;RPL19;RPL22;RPL23A;RPL24;RPL27A;RPL3;RPL32;RPL35A;RPL36;RPL37;RPL37A;RPL4;RPL5;RPL7A;RI

A;RPL12;RPL14;RPL19;RPL22;RPL23A;RPL24;RPL27A;RPL3;RPL35A;RPL37;RPL4;RPL5;RPL7A;RPS11;RPS13;RPS14;RPS16;RPS18;RPS20;RPS25;RPS3;RPS4X;RPS5;SNRPD3;SNUPN;SPAG7;SSBP1;TRNAU1AP;ZGLP1;ZNRD

RPL13A;RPL14;RPL19;RPL22;RPL23A;RPL24;RPL27A;RPL3;RPL32;RPL35A;RPL36;RPL37;RPL37A;RPL4;RPL5;RPL7A;RPS11;RPS12;RPS13;RPS14;RPS16;RPS18;RPS19;RPS20;RPS21;RPS23;RPS25;RPS27A;RPS3;RPS4X;RPS5;RPS6;RPS8;RPSA;SNUPN;SRPRB;STX8;TOMM6;TRAPPC3;UXT;VDAC

PD2;SNRPD3;SNUPN

MED10;MRPS16;MRPS7;NACA;NAP1L1;NPM1;PARL;PCNA;PER2;POLR2H;POP4;PPIA;PSMA5;PSMB6;RAN;RPL10;RPL10A;RPL12;RPL13A;RPL14;RPL19;RPL22;RPL23A;RPL24;RPL27A;RPL3;RPL32;RPL35A;RPL36;RPL37;RPL37A;RPL4;RPL5;RPL7A;RPS11;RPS12;RPS13;RPS14;RPS16;RPS18;RPS19;RPS20;RPS21;R

GN1;HNRNPA1;HS3ST3B1;ICT1;ISY1;MDH1;MED10;MRPS16;MRPS7;NACA;NAP1L1;NDUFC2;NPM1;PARL;PCNA;PER2;POLR2H;POP4;PPIA;PRDX1;PSMA5;PSMB6;RAN;RARRES3;RPIA;RPL10;RPL10A;RPL12;RPL13A;RPL14;RPL19;RPL22;RPL23A;RPL24;RPL27A;RPL3;RPL32;RPL35A;RPL36;RPL37;RPL37A;RPL4;I

HS3ST3B1;ICT1;ISY1;MDH1;MED10;MRPS16;MRPS7;NACA;NAP1L1;NPM1;PARL;PCNA;PER2;POLR2H;POP4;PPIA;PSMA5;PSMB6;RAN;RARRES3;RPIA;RPL10;RPL10A;RPL12;RPL13A;RPL14;RPL19;RPL22;RPL23A;RPL24;RPL27A;RPL3;RPL32;RPL35A;RPL36;RPL37;RPL37A;RPL4;RPL5;RPL7A;RPS11;RPS12;RPS13

RNPA1;HS3ST3B1;ICT1;ISY1;MDH1;MED10;MRPS16;MRPS7;NACA;NAP1L1;NPM1;PARL;PCNA;PER2;POLR2H;POP4;PPIA;PSMA5;PSMB6;RAN;RARRES3;RPIA;RPL10;RPL10A;RPL12;RPL13A;RPL14;RPL19;RPL22;RPL23A;RPL24;RPL27A;RPL3;RPL32;RPL35A;RPL36;RPL37;RPL37A;RPL4;RPL5;RPL7A;RPS11;RPS12

RDX1;PSMA5;PSMB6;RAN;RPL3;RPL36;RPL4;RPL5;RPS13;RPS14;RPS19;RPS25;RPS27A;RPS6;SKP1;SNRPD2;SNRPD3;SNUPN;ZNRD1

H;POP4;PRDX1;PSMA5;PSMB6;RAN;RPL3;RPL36;RPL4;RPL5;RPS13;RPS14;RPS19;RPS25;RPS27A;RPS6;SKP1;SNRPD2;SNRPD3;SSBP1;SUCLG1;ZNRD1

RAN;RPL3;RPL36;RPL4;RPL5;RPS13;RPS14;RPS19;RPS25;RPS27A;RPS6;SKP1;SNRPD2;SNRPD3;ZNRD1

OLR2H;POP4;PRDX1;RAN;RPL10A;RPL12;RPL14;RPL19;RPL22;RPL23A;RPL24;RPL27A;RPL3;RPL35A;RPL37;RPL4;RPL5;RPL7A;RPS11;RPS13;RPS14;RPS16;RPS18;RPS20;RPS25;RPS3;RPS4X;RPS5;SNRPD3;SNUPN;SPAG7;SRPRB;SSBP1;SUCLG1;TRNAU1AP;VDAC3;ZGLP1;ZNRD

OLR2H;POP4;PRDX1;RAN;RPL10A;RPL12;RPL14;RPL19;RPL22;RPL23A;RPL24;RPL27A;RPL3;RPL35A;RPL37;RPL4;RPL5;RPL7A;RPS11;RPS13;RPS14;RPS16;RPS18;RPS20;RPS25;RPS3;RPS4X;RPS5;SNRPD3;SNUPN;SPAG7;SRPRB;SSBP1;SUCLG1;TRNAU1AP;VDAC3;ZGLP1;ZNRD

SMA5;PSMB6;RAN;RPL3;RPL36;RPL4;RPL5;RPS13;RPS14;RPS19;RPS25;RPS27A;RPS6;SKP1;SNRPD2;SNRPD3;SSBP1;SUCLG1;ZNRD1

SMA5;PSMB6;RAN;RPL3;RPL36;RPL4;RPL5;RPS13;RPS14;RPS19;RPS25;RPS27A;RPS6;SKP1;SNRPD2;SNRPD3;SSBP1;SUCLG1;ZNRD1

2;EIF3H;EIF3M;FAU;GSTM3;GTF3C6;HMGN1;HNRNPA1;HS3ST3B1;ICT1;ISY1;ITGB1BP1;MDH1;MED10;MRPS16;MRPS7;MYL12A;MYL5;NACA;NAP1L1;NBPF10;NDUFC2;NOP16;NPM1;PARL;PCNA;PDCL3;PER2;POLR2H;POMP;POP4;PPIA;PRDX1;PSMA5;PSMB6;RAN;RANGRF;RPIA;RPL10;RPL10A;RPL12;RPL1

2;EIF3H;EIF3M;FAU;GSTM3;GTF3C6;HMGN1;HNRNPA1;HS3ST3B1;ICT1;ISY1;ITGB1BP1;MDH1;MED10;MRPS16;MRPS7;MYL12A;MYL5;NACA;NAP1L1;NBPF10;NDUFC2;NOP16;NPM1;PARL;PCNA;PDCL3;PER2;POLR2H;POMP;POP4;PPIA;PRDX1;PSMA5;PSMB6;RAN;RANGRF;RPIA;RPL10;RPL10A;RPL12;RPL1

S19;RPS21;RPS25;RPS6;RPS8;RPSA;SNRPD2;SNRPD3;SNUPN

NAP1L1;NOP16;NPM1;PARL;PCNA;PER2;POLR2H;POMP;POP4;PPIA;PRDX1;PSMA5;PSMB6;RAN;RANGRF;RPL23A;RPL3;RPL36;RPL4;RPL5;RPS13;RPS14;RPS19;RPS25;RPS27A;RPS3;RPS6;RPSA;SKP1;SNRPD2;SNRPD3;SNUPN;SPAG7;SUGT1;THYN1;TRNAU1AP;UXT;ZGLP1;ZNRD

J;GSTM3;GTF3C6;HDHD1;HMGN1;HNRNPA1;HS3ST3B1;ICT1;IL20RB;ISY1;ITGB1BP1;MDH1;MED10;MRPS16;MRPS7;MYL12A;NACA;NAP1L1;NDUFC2;NPM1;PARL;PCNA;PDCL3;PER2;POLR2H;POP4;PPIA;PRDX1;PSMA5;PSMB6;RAN;RANGRF;RARRES3;RPIA;RPL10;RPL10A;RPL12;RPL13A;RPL14;RPL19;RPL22

PM1;PCNA;POLR2H;POMP;PSMA5;PSMB6;RAN;RPL23A;SKP1;SNUPN;STX8;SUCLG1;SUGT1;TRAPPC3;UQCRFS1;UXT;VDAC3

);ARHGEF19;ARL11;ARL4D;ASAP1;ATF6;ATP6V1A;ATXN1;ATXN7;BCL2L11;BMX;C3AR1;CACNA1E;CBS;CCR1;CCR2;CD59;CDA;CDC42EP3;CDH2;CDK14;CDK4;CDKL5;CEP63;CHRNA10;CLEC1A;CNTNAP3;COMMD7;CSRNP1;CUL3;CXCR1;CYTH4;DNAJB11;DNAJC3;DOCK1;DOCK4;DOK4;EMR3;EXT1;FCGR1A;FCGF

);ARHGEF19;ARL11;ARL4D;ASAP1;ATF6;ATP6V1A;ATXN1;ATXN7;BCL2L11;BMX;C3AR1;CACNA1E;CBS;CCR1;CCR2;CD59;CDA;CDC42EP3;CDH2;CDK14;CDK4;CDKL5;CEP63;CHRNA10;CLEC1A;CNTNAP3;COMMD7;CSRNP1;CUL3;CXCR1;CYTH4;DNAJB11;DNAJC3;DOCK1;DOCK4;DOK4;EMR3;EXT1;FCGR1A;FCGF

.1;ARL4D;ASAP1;ATF6;ATP6V1A;ATXN1;ATXN7;BCL2L11;BMX;C3AR1;CBS;CCR1;CCR2;CD59;CDA;CDC42EP3;CDH2;CDK14;CDK4;CDKL5;CEP63;CLEC1A;CNTNAP3;COMMD7;CSRNP1;CUL3;CXCR1;CYTH4;DNAJB11;DNAJC3;DOCK1;DOCK4;DOK4;EMR3;EXT1;FCGR1A;FCGR1B;FFAR2;FFAR3;FGF13;FLOT1;FOXO3

);ARHGEF19;ARL11;ARL4D;ASAP1;ATF6;ATP6V1A;ATXN1;ATXN7;BCL2L11;BMX;C3AR1;CACNA1E;CBS;CCR1;CCR2;CD59;CDA;CDC42EP3;CDH2;CDK14;CDK4;CDKL5;CEP63;CHRNA10;CLEC1A;CNTNAP3;COMMD7;CSRNP1;CUL3;CXCR1;CYTH4;DNAJB11;DNAJC3;DOCK1;DOCK4;DOK4;EMR3;EXT1;FCGR1A;FCGF

APH1B;APOBEC3A;AQP9;ARHGAP19;ARHGEF19;ARL11;ARL4D;ARSB;ASAP1;ATF6;ATP6V1A;ATP7B;ATXN1;ATXN7;AVIL;BCL2L11;BEST1;BMX;BST1;C3AR1;CA4;CACNA1E;CASP5;CBS;CCR1;CCR2;CD177;CD55;CD59;CDA;CDC42EP3;CDH2;CDK14;CDK4;CDKL5;CEP63;CHRNA10;CLEC1A;CNGA4;CNTNAP3;COMN

ENTPD1;F5;FCGR1A;FPR2;GNAQ;H3F3B;HIF1A;HMGCR;HP;HPSE;IL10RB;IL18RAP;IL1B;IL1RN;JMJD1C;LAMP2;LYN;LYVE1;MRVI1;NAIP;NFE2;NFE2L2;ORM1;ORM2;OSM;PAPSS2;PLAU;PPAP2B;PROK2;RAB27A;SBNO2;SCARF1;SLC8A1;SOD2;TLR2;TLR4;TLR5;TNFRSF1A;TREM1;WNT10B;ZFP3

'BB;DNAJC3;EBP;FCGR1A;FCGR1B;FCGR3B;FOXO3;FYB;GIMAP1;HCAR2;HIF1A;IFNAR1;IFNGR2;IGSF6;IL10RB;IL18RAP;IL1B;IL1R1;IL1R2;IL1RN;IRAK3;ITGAD;KLF6;LILRB3;LMO4;LYN;NAIP;NCF4;NFIL3;NPEPPS;ORM1;ORM2;OSBPL1A;OSM;PAK2;PDE4B;PELI2;PGLYRP1;PIAS1;PICALM;PNP;PTPRE;PVRL2;RAB27
ARHGAP19;ARHGEF19;ARL11;ARL4D;ASAP1;ATF6;ATP6V1A;ATXN1;ATXN7;BCL2L11;BMX;C3AR1;CACNA1E;CASP5;CBS;CCR1;CCR2;CD59;CDA;CDC42EP3;CDH2;CDK14;CDK4;CDKL5;CEP63;CLEC1A;CNTNAP3;COMMD7;CSRNP1;CUL3;CXCR1;CYTH4;DGAT2;DNAJB11;DNAJC3;DOCK1;DOCK4;DOK4;EMR3;EXT1
BB;DNAJC3;FCGR1A;FCGR1B;FPR2;HIF1A;HP;IFNAR1;IFNGR2;IL10RB;IL18RAP;IL1B;IL1R1;IL1RN;INHBB;IRAK3;LILRB3;LYN;NAIP;ORM1;ORM2;OSM;PAK2;PELI2;PGLYRP1;PIAS1;PLD1;PROK2;PVRL2;RAB27A;SBNO2;SOCS3;SRPK1;SRPK2;TARBP2;TLR2;TLR4;TLR5;TNFRSF1A;ZFP3
9;ARL4D;ARMCX6;ASAP1;ASTN2;ATF6;ATP11A;ATP2C2;ATP5G1;ATP6V1A;ATP7B;ATP9A;AVL9;BCL2L11;BEST1;BMX;BST1;C19orf59;C3AR1;C7orf53;CA4;CACNA1E;CCR1;CCR2;CD177;CD300LD;CD55;CD59;CDC42EP3;CDH2;CDK14;CDK4;CDKL5;CEACAM3;CEACAM4;CHRNA10;CLDN6;CLEC1A;CLMN;CNGA4;
L1B;IL1RN;LYN;NAIP;ORM1;ORM2;OSM;PROK2;SBNO2;TLR2;TLR4;TLR5;TNFRSF1A;ZFP3
OBEC3A;ARL11;ARL4D;ARPC5;ARPC5L;ASAP1;ATF6;ATP11A;ATP2C2;ATP7B;ATXN1;ATXN7;AVIL;BCL2L11;BEND7;BMX;CA4;CBS;CCDC64B;CCR1;CCR2;CCT4;CD300LD;CD59;CDA;CDC42EP3;CDH2;CDK14;CDK4;CDKL5;CEP63;CHRNA10;CIR1;CLDN6;CLMN;CNTNAP3;COMMD7;CR1;CTBP2;CUL3;CXCR1;CYBB;D
;KLF6;KPNA1;KPNA4;NUP50;NUPL1;PIAS1;SOCS3;TNFRSF1A

H;SOCS3;TARBP2;TRIB1
H;SOCS3;TARBP2;TRIB1

;CR1;CXCR1;DOCK1;DOCK4;EXT1;FCGR1A;FPR1;FPR2;GSK3B;HMGCR;HPSE;IL1B;INHBB;KAL1;LYN;MYO10;NCOA1;NFE2L2;OSM;PAK2;PDE4B;PGLYRP1;PLAU;PLD1;PLXNC1;PROK2;RASGRP4;RS1;SBNO2;SCARF1;SDCBP;SLC8A1;SOD2;SOS2;TLR2;TLR4;TLR5;TNFRSF1A;TNFSF14;WAC;ZFP3
L2;OSM;PAK2;PELI2;PIAS1;PLAU;PPAP2B;PVRL2;SBNO2;SCARF1;SDCBP;SOCS3;STK3;TARBP2;TLR2;TLR4;TLR5;TNFRSF1A;UIMC1;WAC;ZAK;ZFP3
;KLF6;KPNA1;KPNA4;MME;NUP50;NUPL1;PHB;PIAS1;SOCS3;TNFRSF1A

ACAM4;CLEC1A;CR1;CYBB;EBP;ENTPD1;FCGR1A;FFAR2;FFAR3;FLOT1;GNAQ;GPR17;HAS1;HCAR3;IFNAR1;IFNGR2;IGSF6;IL10RB;IL13RA1;IL1R1;IQGAP1;ITGAD;KCNE1;KCNJ15;KCNS1;LILRB3;LYN;LYVE1;MME;MYO10;NAIP;NCF4;OSM;PDZD3;PHB;PICALM;PLB1;RAB27A;RAPGEF2;RHOH;RS1;SDCBP;SHROOM
S;CCR1;CCR2;CDH2;CDKL5;CEP63;CUL3;CYTH4;DOCK1;DOCK4;DOK4;FGF13;FLOT1;FOXO3;FPR1;FYB;GPR89A;GSK3B;GTSE1;HIF1A;HMGCR;HPSE;IFNAR1;IL1B;IL1RN;IQGAP1;IRAK3;LIMD1;LITAF;LYN;MCTP2;MLLT11;OSM;PDZD3;PDZD8;PELI2;PIAS1;PLD1;PROK2;RAB1A;RAB27A;RAB31;RAPGEF2;RASGRP4;
3ST1;C3AR1;CA4;CACNA1E;CCR1;CCR2;CD177;CD300LD;CD55;CD59;CDH2;CDK14;CDKL5;CEACAM4;CHRNA10;CLDN6;CLEC1A;CNTNAP3;CR1;CXCR1;CYBB;CYTH4;EBP;EMR3;ENTPD1;F5;FAM120A;FCGR1A;FCGR1B;FCGR1C;FCGR2A;FCGR3B;FFAR2;FFAR3;FGF13;FLOT1;FPR1;FPR2;GNAQ;GPR141;GPR17;GPI

YB;IFNAR1;IFNGR2;IGSF6;IL10RB;IL18RAP;IL1B;IL1R1;IL1R2;IL1RN;IRAK3;ITGAD;LYN;NAIP;NCF4;NFIL3;OSM;PAK2;PELI2;PGLYRP1;PIAS1;PNP;PVRL2;RAB27A;SBNO2;SECTM1;SOCS3;SRPK1;SRPK2;TLR2;TLR4;TLR5;TNFSF14;TREM
3ST1;C3AR1;CA4;CACNA1E;CCR1;CCR2;CD177;CD300LD;CD55;CD59;CDH2;CDK14;CDKL5;CEACAM4;CHRNA10;CLDN6;CLEC1A;CNTNAP3;CR1;CXCR1;CYBB;CYTH4;EBP;EMR3;ENTPD1;F5;FAM120A;FCGR1A;FCGR1B;FCGR1C;FCGR2A;FCGR3B;FFAR2;FFAR3;FGF13;FLOT1;FPR1;FPR2;GNAQ;GPR141;GPR17;GPI
SBNO2;SCARF1;TLR2;TLR4;TNFRSF1A;WAC;ZFP3
1;CACNA1E;CBS;CCR1;CCR2;CD177;CD55;CD59;CDK4;CEP63;CLEC1A;CR1;CXCR1;CYBB;DNAJB11;DNAJC3;DOCK1;EDEM2;ENTPD1;F5;FCGR1A;FCGR1B;FOXO3;FPR2;GNAQ;GSK3B;GTSE1;H3F3B;HIF1A;HMGCR;HP;HPSE;HSPA6;IDH1;IFNAR1;IFNGR2;IL10RB;IL18RAP;IL1B;IL1R1;IL1RN;INHBB;IRAK3;JMJD1C;L

AR1;CACNA1E;CBS;CCR1;CCR2;CD55;CD59;CDH2;CDK14;CDKL5;CR1;CUL3;CYTH4;DOCK4;FCGR1A;FCGR1B;FLOT1;FPR1;FYB;GPR89A;GPSM2;GSK3B;HEY1;HIF1A;HMGCR;HP;HPSE;IFNAR1;IFNGR2;IL1B;IL1RN;IRAK3;LIMD1;LITAF;LYN;MLLT11;MYO5A;NCOA1;NFE2L2;NPTN;OSM;PAK2;PELI2;PGLYRP1;PHB;P
;APAF1;APBB2;APH1B;APOBEC3A;AQP9;ARHGAP19;ARHGEF19;ARL11;ARL4D;ARPC5;ARPC5L;ARSB;ASAP1;ATF6;ATP11A;ATP2C2;ATP5G1;ATP6V1A;ATP7B;ATP9A;ATXN1;ATXN7;AVIL;AVL9;BCL2L11;BMX;BRWD3;C3AR1;CACNA1E;CASP4;CASP5;CBS;CCR1;CCR2;CD177;CD55;CD59;CDA;CDC123;CDC42EP3;
NAJC3;FCGR1A;FCGR1B;FOXO3;GPR17;GSK3B;HAS1;HIF1A;HMGCR;IFNAR1;IFNGR2;IL1B;IL1R1;IL1RN;INHBB;IRAK3;KLF6;KPNA1;KPNA4;LITAF;LYN;MME;MYO5A;NFE2L2;NPTN;NUP50;NUPL1;PDE4B;PDK3;PHB;PIAS1;PTPRE;RAB31;RNF111;SLC8A1;SOCS3;SOD2;TARBP2;TLR2;TLR4;TNFRSF1A;TRIB1;UBR2;V
6;ATP11A;ATP2C2;ATP5G1;ATP6V1A;ATP7B;ATP9A;AVL9;BCL2L11;BEST1;BMX;BST1;C19orf59;C3AR1;C7orf53;CA4;CACNA1E;CCR1;CCR2;CD177;CD300LD;CD55;CD59;CDH2;CDKL5;CEACAM3;CEACAM4;CHRNA10;CLDN6;CLEC1A;CLMN;CNGA4;CNTNAP3;CR1;CXCR1;CYBB;DGAT2;DNAJC3;EBP;EDEM2;ELO
MMD7;CXCR1;DGAT2;DNAJB11;DNAJC3;DOCK4;FCGR1A;FCGR1B;FOXO3;GPR17;GSK3B;HAS1;HIF1A;HMGCR;HP;IFNAR1;IFNGR2;IL1B;IL1R1;IL1RN;INHBB;IQGAP1;IRAK3;KLF6;KPNA1;KPNA4;LITAF;LYN;MME;MYO5A;NCOA1;NFE2L2;NPEPPS;NPTN;NUP50;NUPL1;OPLAH;PAPSS2;PDE4B;PDK3;PHB;PIAS1;PTF
GPR89A;IL1B;ITM2B;LAMP2;MME;MYO5A;NCF4;PICALM;RAB27A;RAB31;SDCBP;SEC24D;SMAGP;SNAP23;STX12;STX3;STX6;SYN2;SYNGR3;TRIM9;VAMP3;WIPF1;WLS;YIPF

NO2;SOCS3;TARBP2;TLR2;TLR4;TLR5;TNFRSF1A;ZFP3

.RN;IRAK3;KLF6;KPNA1;KPNA4;MME;NUP50;NUPL1;PHB;PIAS1;SOCS3;TNFRSF1A

PM6;TRPV5;VAMP3
D1;FFAR2;FFAR3;GPR17;HAS1;HCAR3;IFNAR1;IFNGR2;IGSF6;IL10RB;IL13RA1;IL1R1;ITGAD;KCNE1;KCNJ15;KCNS1;LILRB3;LYN;LYVE1;MME;NCF4;OSM;PHB;RAPGEF2;SDCBP;SIT1;SLC22A1;SLC22A14;SLC22A4;SLC8A1;ST3GAL5;TLR2;TLR4;TM9SF2;TNFRSF1A;TRPV
S;CCR1;CDH2;CDK14;CDKL5;CUL3;CYTH4;DOCK4;FLOT1;FPR1;GPR89A;GPSM2;GSK3B;HCAR2;HEY1;HIF1A;HMGCR;HPSE;IFNAR1;IFNGR2;IL1B;IL1RN;INHBB;IQGAP1;IRAK3;LIMD1;LITAF;LYN;MARCKS;MLLT11;MYO5A;NPTN;OSM;PAK2;PELI2;PHB;PIAS1;PLAU;PPAP2B;PROK2;PTPRE;RAPGEF2;RASGRP4;RHO

2;CDK14;CLEC1A;COMMD7;CSRNP1;CUL3;CXCR1;DOCK1;DOK4;EMR3;FCGR1A;FCGR1B;FFAR2;FFAR3;FOXO3;FPR1;FPR2;FYB;GNAQ;GPR141;GPR17;GPR97;GPSM2;GSK3B;HCAR3;HEY1;HIF1A;IFNAR1;IFNGR2;IGSF6;IL13RA1;IL18RAP;IL1B;IL1R1;IL1RN;INHBB;IRAK3;ITGAD;KLF6;KPNA1;KPNA4;LILRB3;LIMD1

S;CCR1;CDH2;CDK14;CDKL5;CUL3;CYTH4;DOCK4;FLOT1;FPR1;GPR89A;GPSM2;GSK3B;HCAR2;HEY1;HIF1A;HMGCR;HPSE;IFNAR1;IFNGR2;IL1B;IL1RN;INHBB;IQGAP1;IRAK3;LIMD1;LITAF;LYN;MARCKS;MLLT11;MYO5A;NPTN;OSM;PAK2;PELI2;PHB;PIAS1;PLAU;PPAP2B;PROK2;PTPRE;RAPGEF2;RASGRP4;RHO
FFAR3;GPR17;HAS1;HCAR3;IFNAR1;IFNGR2;IGSF6;IL10RB;IL13RA1;IL1R1;ITGAD;KCNE1;KCNJ15;KCNS1;LILRB3;LYN;LYVE1;MME;NCF4;OSM;PHB;RAPGEF2;SDCBP;SIT1;SLC22A1;SLC22A14;SLC22A4;SLC8A1;ST3GAL5;TLR2;TLR4;TM9SF2;TNFRSF1A;TRPV
APOBEC3A;AQP9;ARHGAP19;ARHGEF19;ARL11;ARL4D;ARPC5;ARPC5L;ASAP1;ATF6;ATP11A;ATP6V1A;ATP7B;ATP9A;ATXN1;ATXN7;AVIL;BCL2L11;BEST1;BMX;BRWD3;C3AR1;CACNA1E;CASP4;CASP5;CBS;CCR1;CCR2;CD177;CD55;CD59;CDA;CDC123;CDC42EP3;CDH2;CDK14;CDK4;CDKL5;CENPV;CEP63;CHF
GPR89A;IL1B;ITM2B;LAMP2;MME;MYO5A;NCF4;PICALM;RAB27A;RAB31;SDCBP;SEC24D;SMAGP;SNAP23;STX12;STX3;STX6;SYN2;SYNGR3;TRIM9;VAMP3;WIPF1;WLS;YIPF
2;ANXA3;APAF1;APBB2;APH1B;APOA1BP;APOBEC3A;ARHGEF19;ARL11;ARL4D;ARPC5;ARPC5L;ARSB;ASAP1;ATF6;ATP11A;ATP2C2;ATP5G1;ATP6V1A;ATP7B;ATP9A;ATXN1;ATXN7;AVIL;BCL2L11;BEND7;BMX;BST1;CA4;CACNA1E;CAPN12;CAPN13;CAPN8;CBS;CCDC64B;CCR1;CCR2;CCT4;CD300LD;CD59;CDA
AK3;LITAF;LYN;OSM;PELI2;PIAS1;PROK2;RAPGEF2;RHOH;SDCBP;SECTM1;SOCS3;SRPK1;SRPK2;STK3;TLR2;TLR4;TNFRSF1A;TNFSF14;TRIB1;WLS;ZAK;ZFP3
DH2;CDK14;CDKL5;CUL3;CYTH4;DOCK4;FLOT1;FPR1;GPR89A;GPSM2;GSK3B;HEY1;HIF1A;HMGCR;HPSE;IFNAR1;IFNGR2;IL1B;IL1RN;IRAK3;LIMD1;LITAF;LYN;MLLT11;MYO5A;NPTN;OSM;PAK2;PELI2;PHB;PIAS1;PLAU;PPAP2B;PROK2;PTPRE;RASGRP4;RHOH;RNF111;RS1;SDCBP;SECTM1;SH3GLB1;SOCS3;SOS

1A;ATP2C2;ATP5G1;ATP6V1A;ATP7B;ATP9A;AVL9;BEST1;BST1;C19orf59;C3AR1;C7orf53;CA4;CACNA1E;CCR1;CCR2;CD177;CD300LD;CD55;CD59;CDH2;CEACAM3;CEACAM4;CHRNA10;CLDN6;CLEC1A;CLMN;CNGA4;CNTNAP3;CR1;CXCR1;CYBB;DGAT2;EBP;ELOVL5;EMR3;ENTPD1;EXT1;FCGR1A;FCGR1B;FC
L11;BRWD3;C3AR1;CACNA1E;CBS;CCR1;CCR2;CD177;CD55;CD59;CDC42EP3;CDK4;CDKL5;CHRNA10;DGAT2;DOCK1;ENTPD1;F5;FGF13;FLI1;FOXO3;GNAQ;GPR89A;GSK3B;H3F3B;HCAR2;HIF1A;HMGCR;HP;HPSE;IL1B;IL1RN;INHBB;IQGAP1;JMJD1C;KCNE1;LAMP2;LIMD1;LYN;MARCKS;MLLT11;MRVI1;MYO1

iALNT14;GIMAP1;GLIPR2;GOLGA7B;GPR89A;ITM2B;LITAF;LYN;MYO5A;NEDD9;NMNAT2;PICALM;PLD1;PPAP2B;PRRG4;RAB1A;RAB27A;RAB31;RTN3;SEC24D;SECTM1;SH3GLB1;SNAP23;ST3GAL4;ST3GAL5;ST6GALNAC2;STX11;STX12;STX6;TNFRSF1A;USP32;WL
GPR89A;IL1B;ITM2B;LAMP2;MME;MYO5A;NCF4;PICALM;RAB27A;RAB31;SDCBP;SEC24D;SMAGP;SNAP23;STX12;STX3;STX6;SYN2;SYNGR3;TRIM9;VAMP3;WIPF1;WLS;YIPF
2;TLR4;TNFRSF1A;TRIB1;UBR2;WNT10E

CDC42EP3;CDK4;CYBB;DGAT2;DMXL2;DNAJC3;DOCK4;EBP;EDEM2;ELOVL5;EXT1;FCGR1A;FCGR1B;GABARAPL1;GALNT14;GIMAP1;GLIPR2;GNAQ;GOLGA7B;GPR89A;HMGCR;ITM2B;KAZN;KPNA1;LAMP2;LBR;LMNB1;MBOAT2;MRVI1;NMNAT2;NUP50;NUPL1;PICALM;PLD1;RAB27A;RAB31;RANBP1;RTN3;SI

.NKRD6;ANTXR2;ANXA3;AP3B2;APAF1;APH1B;APOA1BP;APOBEC3A;ARHGAP19;ARL4D;ARPC5;ARPC5L;ARSB;ASAP1;ATF6;ATP11A;ATP2C2;ATP5G1;ATP6V1A;ATP7B;ATP9A;ATXN1;ATXN7;AVIL;AVL9;BCL2L11;BEND5;BEST1;BMX;C21orf59;CA4;CAPN8;CBS;CCDC125;CCR2;CCT4;CDA;CDC123;CDC42EP3;CDK

ASAP1;ATP2C2;ATP5G1;ATP7B;CA4;CACNA1E;CBS;CCR2;CD55;CDA;CDKL5;COQ4;CYBB;DCTPP1;DGAT2;DOCK1;DOCK4;DPYD;EBP;ELOVL5;ENO3;EXT1;GBE1;GDE1;GMPR2;GNAQ;GSK3B;HAL;HAS1;HCAR2;HIF1A;HMGCR;HPSE;IDH1;IDS;IL1B;IL1RN;INPP5A;IQGAP1;JHDM1D;KDM1B;LBR;LYN;LYVE1;MARCKS;
1A;ATP2C2;ATP5G1;ATP6V1A;ATP7B;ATP9A;AVL9;BEST1;C19orf59;C3AR1;C7orf53;CACNA1E;CCR1;CCR2;CD300LD;CD55;CDH2;CEACAM3;CEACAM4;CHRNA10;CLDN6;CLEC1A;CLMN;CNGA4;CNTNAP3;CR1;CXCR1;CYBB;DGAT2;EBP;ELOVL5;EMR3;ENTPD1;EXT1;FCGR1A;FCGR1B;FCGR1C;FCGR2A;FFAR2;FF/
)SBPL11;OSBPL1A;OXER1;PICALM;PLD1;RAPGEF2;RASGRP4;RS1;SBF2;SH3GLB1;SNTA1;SNTG2;SOS2;STX3;TIAM2;TLR2;TLR4;WDFY

TXN1;ATXN7;BMX;CBS;CCR2;CDA;CDK4;CDKL5;DCTPP1;DGAT2;DNAJB11;DNAJC3;DOCK1;DOCK4;DPYD;DUSP13;FGF13;FPR1;FYB;GMPR2;GNAQ;GSK3B;HMGCR;IDH1;IL1B;IL1RN;INPP5A;IQGAP1;IRAK3;LIMD1;LYN;MAK;MBOAT2;NMNAT2;NPTN;NT5C2;NUDT5;OSM;OXER1;PAK2;PANK2;PAPSS2;PDE4B;PD
.1E;CBS;CCR1;CCR2;CD55;CDK4;COMMD7;CXCR1;DGAT2;DNAJB11;DNAJC3;DOCK1;DOCK4;EBP;EDEM2;EXT1;FCGR1A;FCGR1B;FOXO3;FPR1;FPR2;GPR17;GSK3B;HAS1;HIF1A;HMGCR;HP;HSPA6;IDH1;IFNAR1;IFNGR2;IL1B;IL1R1;IL1RN;INHBB;IQGAP1;IRAK3;KAL1;KLF6;KPNA1;KPNA4;LITAF;LYN;MME;MYO1(

TXN1;ATXN7;BMX;CBS;CCR2;CDA;CDK4;CDKL5;DCTPP1;DGAT2;DNAJB11;DNAJC3;DOCK1;DOCK4;DPYD;DUSP13;ELOVL5;FGF13;FPR1;FYB;GMPR2;GNAQ;GSK3B;HMGCR;IDH1;IL1B;IL1RN;INPP5A;IQGAP1;IRAK3;LIMD1;LYN;MAK;MBOAT2;NMNAT2;NPTN;NT5C2;NUDT5;OSM;OXER1;PAK2;PANK2;PAPSS2;P

;AP3B2;APAF1;APBB2;APH1B;APOBEC3A;AQP9;ARHGAP19;ARHGEF19;ARL11;ARL4D;ARPC5;ARPC5L;ARSB;ASAP1;ATF6;ATP11A;ATP2C2;ATP5G1;ATP6V1A;ATP7B;ATP9A;ATXN1;ATXN7;AVIL;AVL9;BCL2L11;BEST1;BMX;BRWD3;BST1;C3AR1;CA4;CACNA1E;CASP4;CASP5;CBS;CCNI;CCR1;CCR2;CD177;CD55;(

GPR89A;IL1B;IL1RN;ITM2B;LAMP2;MME;MYO5A;NCF4;PICALM;RAB27A;RAB31;SDCBP;SEC24D;SMAGP;SNAP23;STX12;STX3;STX6;SYN2;SYNGR3;TRIM9;VAMP3;WIPF1;WLS;YIPF

TP2C2;ATP5G1;ATP7B;CA4;CACNA1E;CBS;CCR2;CDA;CDKL5;COQ4;DCTPP1;DGAT2;DOCK1;DOCK4;DPYD;EBP;ELOVL5;ENO3;EXT1;GBE1;GDE1;GMPR2;GNAQ;GSK3B;HAL;HAS1;HIF1A;HMGCR;HPSE;IDH1;IDS;IQGAP1;LBR;LYVE1;MARCKS;MBOAT2;ME2;MME;MYO5A;NCOA1;NCOA2;NMNAT2;NPL;NT5C2;NU

DOCK4;DPYD;GMPR2;GNAQ;GSK3B;HMGCR;IDH1;IQGAP1;NMNAT2;NT5C2;NUDT5;OXER1;PANK2;PAPSS2;PDE4B;PDZD3;PNP;RAB1A;RAB31;TGM3

331;SEC24D;SMAGP;VAMP3;WLS
CTPP1;DGAT2;DOCK1;DOCK4;DPYD;GMPR2;GNAQ;GSK3B;HMGCR;IDH1;INPP5A;IQGAP1;MBOAT2;NMNAT2;NT5C2;NUDT5;OXER1;PANK2;PAPSS2;PDE4B;PDZD3;PFKFB4;PGS1;PLB1;PLBD1;PLD1;PNP;PPAP2B;RAB1A;RAB31;SH3GLB1;TGM3

PP1;DOCK1;DOCK4;DPYD;GMPR2;GNAQ;GSK3B;HMGCR;IDH1;IQGAP1;NMNAT2;NT5C2;NUDT5;OXER1;PANK2;PAPSS2;PDE4B;PDZD3;PNP;RAB1A;RAB31;TGM3

LYRP1;PIAS1;PVRL2;RAB27A;SOCS3;SRPK1;SRPK2;TLR2;TLR4;TLR5

A;ARHGAP19;ARHGEF19;ARL11;ARL4D;ARPC5;ARPC5L;ASAP1;ATF6;ATP6V1A;ATXN1;ATXN7;AVIL;BCL2L11;BEST1;BMX;BRWD3;C3AR1;CACNA1E;CASP4;CASP5;CBS;CCR1;CCR2;CD55;CD59;CDA;CDC123;CDC42EP3;CDH2;CDK14;CDK4;CDKL5;CENPV;CEP63;CHRNA10;CIR1;CLEC1A;CLMN;CNTNAP3;COMMD7

AIP2;WDFY3

331;SEC24D;SMAGP;VAMP3;WLS

DPYD;GMPR2;GNAQ;GSK3B;HMGCR;IDH1;IQGAP1;NMNAT2;NT5C2;NUDT5;OXER1;PANK2;PAPSS2;PDE4B;PDZD3;PNP;RAB1A;RAB31;TGM3

HIF1A;HMGCR;HPSE;IL1B;IL1RN;IRAK3;LITAF;LYN;NPTN;OSM;PAK2;PELI2;PPAP2B;PROK2;PVRL2;RASGRP4;RNF111;SCARF1;SDCBP;SECTM1;STK3;TLR2;TLR4;TLR5;TNFRSF1A;UIMC1;WAC;WLS;WNT10B;ZAK

MD7;CXCR1;DGAT2;DNAJB11;DNAJC3;EDEM2;FCGR1A;FCGR1B;FOXO3;GPR17;GSK3B;HAS1;HIF1A;HMGCR;HSPA6;IDH1;IFNAR1;IFNGR2;IL1B;IL1R1;IL1RN;INHBB;IRAK3;KLF6;KPNA1;KPNA4;LITAF;LYN;MME;MYO5A;NFE2L2;NPTN;NUP50;NUPL1;PDCD7;PDE4B;PDK3;PHB;PIAS1;PTPRE;RAB31;RNF111;SLC8

I5;FGF13;GNAQ;GPSM2;IQGAP1;KAL1;NAIP;PAK2;PDZD3;PPP1R3B;PPP4R1;RANBP1;RAPGEF2;RASGRP4;RHOH;ROPN1L;SBF2;SERPINB1;SOCS3;SOS2;STK3;TBC1D30;TIAM2;TNFSF14;TRIB1

;CCR1;CCR2;CD55;CD59;CDC123;CDH2;CDK4;CDKL5;CENPV;CR1;CSRNP1;CUL3;DNAJB11;DNAJC3;E2F3;ETS2;FCGR1A;FGF13;FOXO3;FPR1;FYB;GPR89A;GSK3B;GTSE1;HCAR2;HEY1;HIF1A;HMGCR;HP;HPSE;IFNAR1;IL1B;IL1RN;INHBB;IQGAP1;IRAK3;ITM2B;JHDM1D;KCNE1;KLF6;KPNA1;LIMD1;LITAF;LMO4;LY
;TRIB1

D30;TIAM2

P4R1;PTPRE;SBF2
3;DOCK1;DOCK4;FGF13;FPR1;GNAQ;GSK3B;HMGCR;IL1B;IL1RN;IQGAP1;IRAK3;LYN;NPTN;OSM;OXER1;PAK2;PDK3;PDZD3;PELI2;PPAP2B;PROK2;RHOH;SDCBP;SOCS3;STK3;TARBP2;TLR4;TMEM132D;TNFRSF1A;TPX2;TRIB1;ZA

IRAK3;LIMD1;LYN;PHB;PTPRE;RHOH;SOCS3;STK3;TARBP2;TLR4;TNFRSF1A;TNFSF14;TRIB1;UBR2;ZFP36
LYN;MME;NPEPPS;OSM;PDK3;PLAU;RS1;SLC12A6;SLC8A1;SOD2;TLR4;TLR5;TNFRSF1A;TNFSF14;UIMC1;ZAK
MARCKS;MYO5A;OSM;PNP;RAB27A;RAB31;SNAP23;STX12;STX3;SYN2;TLR2;TRIM9;VAMP3
IFRSF1A;WAC
;GMPR2;GNAQ;GSK3B;HMGCR;IQGAP1;NT5C2;OXER1;PANK2;PAPSS2;PDE4B;PDZD3;PNP;RAB1A;RAB31;TGM3
N;PAK2;PDE4B;PGLYRP1;PLD1;PVRL2;SOD2;TARBP2;TLR2;TLR4;TNFRSF1A;TRIB1
I;EXT1;FGF13;FPR1;FPR2;GSK3B;HAS1;HIF1A;HIST1H1T;IL1B;KAL1;KIAA0319;LIMD1;LYN;MYO10;MYO5A;NRD1;PAK2;PDE4B;PLAU;PLD1;PLXNC1;PPAP2B;PROK2;PVRL2;ROPN1L;SDCBP;SLC8A1;SOS2;TLR2;TREM1;TRIB1;ZSWIM

RAB31;SEC24D;SMAGP;VAMP3;WLS
2

N;MRVI1;NFE2;NFE2L2;PAPSS2;PLAU;PPAP2B;RAB27A;SLC8A1;TLR4;TREM1;WNT10B

B;IRAK3;LILRB3;LYN;ORM1;ORM2;PAK2;PELI2;PGLYRP1;PIAS1;PNP;PTPRE;PVRL2;SIT1;SOCS3;TARBP2;TLR2;TLR4;TLR5;ZFP3

5;DOCK1;DOCK4;DPYD;EDEM2;EXT1;GALNT14;GNAQ;GSK3B;HAS1;HIF1A;HMGCR;HPSE;IDS;IL1B;IQGAP1;ITM2B;KCNE1;LYVE1;NPL;OXER1;PANK2;PAPSS2;PDE4B;PDZD3;PFKFB4;PGLYRP1;PNP;RAB1A;RAB31;SEC24D;ST3GAL4;ST3GAL5;ST6GALNAC2;TGM
TNFRSF1A;ZFP36
2;HEY1;HIVEP1;HMGCR;HP;IL1B;INHBB;IQGAP1;IRAK3;ITM2B;LIMD1;LRRFIP1;LYN;NCOA2;NFIL3;NR6A1;PAK2;PDZD3;PHB;PIAS1;PPAP2B;RHOH;S100A11;SBNO2;SOCS3;TARBP2;TDRD9;TLE4;TNFRSF1A;TRIB1;TSHZ3;UBR2;UIMC1;WAC;WNT10B;ZFP36;ZNF217;ZNF43

3;DOCK1;DOCK4;FGF13;FPR1;GNAQ;GSK3B;HMGCR;IL1B;IL1RN;IQGAP1;IRAK3;LYN;NPTN;OSM;OXER1;PAK2;PDZD3;PELI2;PPAP2B;PROK2;RHOH;SDCBP;SOCS3;STK3;TARBP2;TLR4;TMEM132D;TNFRSF1A;TPX2;TRIB1;ZA
;GNAQ;GSK3B;HMGCR;IQGAP1;PANK2;PAPSS2;PNP;RAB1A;RAB31;TGM3

;PVRL2;RAB27A;SBNO2;TARBP2;TLR2;TLR4

SE;IDH1;INHBB;IRAK3;MXD1;NCF4;NCOA1;NCOA2;NR6A1;PTPRE;PVRL2;S100A11;SBF2;SDCBP;SH3GLB1;SOS2;STK3;TARBP2;TLR2;TRIM

R2;CD59;CDC123;CDH2;CDK4;CDKL5;CENPV;CSRNP1;CUL3;DNAJB11;DNAJC3;E2F3;ETS2;FCGR1A;FGF13;FOXO3;FPR1;GPR89A;GSK3B;GTSE1;HCAR2;HEY1;HIF1A;HMGCR;HP;HPSE;IL1B;IL1RN;INHBB;IQGAP1;ITM2B;JHDM1D;KCNE1;KLF6;LIMD1;LITAF;LMO4;LYN;MLLT11;MYO10;NCOA1;NCOA2;NFE2;NFE2L
;CXCR1;DNAH14;DOCK1;DOCK4;FGF13;FPR1;FPR2;GSK3B;HAS1;HIF1A;HIST1H1T;IL1B;KAL1;KIAA0319;LIMD1;LYN;LYVE1;MYO5A;NRD1;PDE4B;PLAU;PPAP2B;ROPN1L;SDCBP;SLC8A1;TLR2;TREM1;TRIB1;WIPF1;ZSWIM

2;STK3;TLR2;TNFSF14;WNT10B;ZA

NKRD6;ANTXR2;ANXA3;AP3B2;APAF1;APBB2;APH1B;APOA1BP;APOBEC3A;AQP9;ARHGAP19;ARHGEF19;ARL11;ARL4D;ARPC5;ARPC5L;ARSB;ASAP1;ATF6;ATP11A;ATP2C2;ATP5G1;ATP6V1A;ATP7B;ATP9A;ATXN1;ATXN7;AVIL;AVL9;BCL2L11;BEND5;BEST1;BMX;BST1;C21orf59;C3AR1;CA4;CACNA1E;CAPN1
NKRD6;ANTXR2;ANXA3;AP3B2;APAF1;APBB2;APH1B;APOA1BP;APOBEC3A;AQP9;ARHGAP19;ARHGEF19;ARL11;ARL4D;ARPC5;ARPC5L;ARSB;ASAP1;ATF6;ATP11A;ATP2C2;ATP5G1;ATP6V1A;ATP7B;ATP9A;ATXN1;ATXN7;AVIL;AVL9;BCL2L11;BEND5;BEST1;BMX;BST1;C21orf59;C3AR1;CA4;CACNA1E;CAPN1

K3;SOD2;TLR2;TLR4;TNFRSF1A;TRIB1;WNT10
;BCL2L11;CA4;CDK4;COQ4;CYBB;DGAT2;DMXL2;DNAJC3;EBP;EDEM2;ELOVL5;EXT1;FCGR1A;FCGR1B;GABARAPL1;GALNT14;GIMAP1;GLIPR2;GNAQ;GOLGA7B;GPR89A;HMGCR;HPSE;ITM2B;KAZN;KIAA0319;LAMP2;LBR;LITAF;LMNB1;LYN;MBOAT2;MRVI1;NMNAT2;NUP50;NUPL1;OSBPL1A;PGS1;PHB;PICA
;IQGAP1;LYVE1;NPL;PDE4B;PGLYRP1;PNP;RAB1A;RAB31;TGM

QGAP1;IRAK3;LYN;NPTN;OSM;PAK2;PELI2;PPAP2B;PROK2;RHOH;SDCBP;SOCS3;STK3;TARBP2;TLR4;TNFRSF1A;TPX2;TRIB1;ZA
B;HMGCR;IL1B;IL1RN;INPP5A;IQGAP1;IRAK3;LIMD1;LYN;MAK;NPTN;OSM;PAK2;PDK3;PELI2;PPAP2B;PPP4R1;PROK2;PTPRE;RHOH;SDCBP;SOCS3;SRPK1;SRPK2;STK3;TARBP2;TLR2;TLR4;TNFRSF1A;TPX2;TRIB1;ZA

TK3;TLR2;TNFSF14;WNT10B;ZA

BP1;RAPGEF2;STK3;TBC1D30;TIAM2

RASGRP4;RHOH;SBF2;SOS2;TBC1D30;TIAM2

D;SH3GLB1;ST3GAL4;ST3GAL5;ST6GALNAC2;STX12;STX6;TNFRSF1A;WL

RASGRP4;RHOH;SBF2;SOS2;TBC1D30;TIAM2
A;PLD1;RAB27A;RAB31;STX12;STX6;TLR4;TM9SF2;VAMP

APAF1;APH1B;APOA1BP;ARHGAP19;ARSB;ATF6;ATP11A;ATP2C2;ATP5G1;ATP6V1A;ATP7B;ATP9A;AVL9;BCL2L11;BEND5;BEST1;BMX;C21orf59;CA4;CAPN8;CBS;CCR2;CCT4;CDA;CDK14;CDK4;CDKL5;CEP63;COQ4;CUL3;CYBB;DCTPP1;DGAT2;DMXL2;DNAH14;DNAJB11;DNAJC3;DOCK1;DOCK4;DPYD;EBP;ECH

144;TMCO3
L2;S100A11;SBF2;SDCBP;SH3GLB1;TARBP2;TRIM9

4D;STX6;TARBP2;TLR4

4;FAM65B;FGF13;FRMD4B;GSK3B;IQGAP1;KPNA1;LRRC6;LYN;MAK;MME;MYO10;MYO5A;NEDD9;PDZD3;RAB27A;ROPN1L;S100A11;SLC8A1;SNAP23;STX3;TIAM2;TNFRSF1A;TRIM9;TRPM6;TSHZ3;VAMP

LK;PARD3;PDE5A;PDGFA;PDGFRA;PF4;PPBP;PRKAR2B;PROS1;SELP;SERPINE1;SLC8A3;SPARC;TFPI;TPM1;TREML1;VEGFC;VIL1;VW
5A;PDGFA;PDGFRA;PF4;PPBP;PRKAR2B;PROS1;SELP;SERPINE1;SLC8A3;SPARC;TFPI;TREML1;VEGFC;VWI
5A;PDGFA;PDGFRA;PF4;PPBP;PRKAR2B;PROS1;SELP;SERPINE1;SLC8A3;SPARC;TFPI;TREML1;VEGFC;VWI
5A;PDGFA;PDGFRA;PF4;PPBP;PRKAR2B;PROS1;SELP;SERPINE1;SLC8A3;SPARC;TFPI;TREML1;VEGFC;VWI
P;SERPINE1;SPARC;TREML1;VEGFC;VWF

5A;PDGFA;PDGFRA;PF4;PPBP;PRKAR2B;PROS1;SELP;SERPINE1;SLC8A3;SPARC;TFPI;TREML1;VEGFC;VWI
N1;MYLK;PARD3;PDE5A;PDGFA;PDGFRA;PF4;PPBP;PRKAR2B;PROS1;PTGES;SELP;SERPINE1;SLC8A3;SPARC;TFPI;TNFSF4;TPM1;TREML1;VEGFC;VIL1;VW

REML1;VEGFC;VWF

;VWF
PBP;PROS1;SELP;SERPINE1;SPARC;TNFSF4;TREML1;VEGFC;VWI
;GUCY1B3;HOMER2;ITGA2B;ITGB3;LTBP1;MAP1B;MGLL;MMRN1;PCSK6;PDE5A;PDGFA;PDGFRA;PF4;PPBP;PRKAR2B;PROS1;PTGS1;SELP;SERPINE1;SH3TC2;SLC6A4;SLC8A3;SPARC;SYTL4;TFPI;TPM1;TREML1;VEGFC;VIL1;VWF;WASF
OS1;SELP;SERPINE1;SPARC;SYTL4;TNFSF4;VEGFC;VWF
;PROS1;SELP;SERPINE1;SPARC;SYTL4;TNFSF4;VEGFC;VWF
VF
B3;HIST1H2BJ;ITGA2B;ITGB3;MGLL;MMRN1;MYLK;PARD3;PDE5A;PDGFA;PDGFRA;PDLIM1;PF4;PLOD2;PPBP;PRKAR2B;PROS1;PTGES;PTGS1;SELP;SERPINE1;SLC6A4;SLC8A3;SPARC;TFPI;TGFB1I1;TNFSF4;TPM1;TREML1;VEGFC;VIL1;VW

ELP;SERPINE1;SPARC;STON2;SYTL4;VEGFC;VWF
LP;SERPINE1;SPARC;SYTL4;TREML1;VEGFC;VWF
AM;F2RL3;GFI1B;GP6;GP9;GRB14;GUCY1A3;GUCY1B3;HOMER2;ITGA2B;ITGB3;ITGB5;KCND3;MAP1B;MEIS1;MGLL;MMRN1;MYL9;MYLK;NAT8B;NRGN;PARD3;PCSK6;PCYT1B;PDE5A;PDGFA;PDGFRA;PEAR1;PF4;PGRMC1;PLXNB3;PPBP;PRKAR2B;PROS1;PTGS1;RGS6;SDC4;SELP;SERPINE1;SH3TC2;SLC6A4;S
SELP;SERPINE1;SPARC;SYTL4;TREML1;VEGFC;VWF
CLEC1B;CLU;CMTM5;CXCL5;EGF;ESAM;F2RL3;FSTL1;GNAZ;GNG11;GP6;GP9;GRB14;GRK5;GUCY1A3;GUCY1B3;HIST1H2BJ;HOMER2;ITGA2B;ITGB3;ITGB5;LTBP1;MAOB;MGLL;MMRN1;MYL9;MYLK;NRGN;PARD3;PCSK6;PDE5A;PDGFA;PDGFRA;PDLIM1;PF4;PF4V1;PGRMC1;PKHD1L1;PLOD2;PLXNB3;PPBP;PR
PINE1;SPARC;SYTL4;TREML1;VEGFC;VWF
RAB6B;SELP;SERPINE1;SPARC;SYTL4;TREML1;VEGFC;VWF
;CD9;CDK2AP1;CDKN1A;CLDN5;CLEC1B;CLU;CXCL5;DNM3;EGF;ESAM;F2RL3;FSTL1;GFI1B;GNAZ;GNG11;GP6;GP9;GRB14;GRK5;GUCY1A3;GUCY1B3;HIST1H2AG;HIST1H2BH;HIST1H2BJ;HOMER2;HRASLS;ITGA2B;ITGB3;ITGB5;KCND3;LTBP1;MAOB;MAP1B;MEIS1;MGLL;MMD;MMRN1;MYL9;MYLK;NRGN;PA
N;EGF;ESAM;F2RL3;GNAZ;GNG11;GP6;GP9;GRB14;GRK5;HOMER2;ITGA2B;ITGB3;ITGB5;KCND3;LY6G6F;MAP1B;MFAP3L;MGLL;MMD;MYLK;PARD3;PDGFRA;PEAR1;PLXNB3;PRKAR2B;RGS6;RHOBTB1;SDC4;SDPR;SELP;SERPINE1;SH3TC2;SLC24A3;SLC6A4;SLC8A3;SYTL4;TFPI;TNFSF4;TPM1;TREML1;TSPAN9
;ESAM;F2RL3;GNAZ;GNG11;GP6;GP9;GRB14;GRK5;HOMER2;ITGA2B;ITGB3;ITGB5;KCND3;LY6G6F;MAP1B;MFAP3L;MGLL;MMD;MYLK;PARD3;PDGFRA;PEAR1;PLXNB3;PRKAR2B;RGS6;RHOBTB1;SDC4;SDPR;SELP;SERPINE1;SH3TC2;SLC24A3;SLC6A4;SLC8A3;SYTL4;TFPI;TNFSF4;TPM1;TREML1;TSPAN9;TUBB
AM;F2RL3;GFI1B;GP6;GP9;GRB14;GUCY1A3;GUCY1B3;HOMER2;ITGA2B;ITGB3;ITGB5;KCND3;MAP1B;MEIS1;MGLL;MMRN1;MYL9;MYLK;NAT8B;NRGN;PARD3;PCSK6;PCYT1B;PDE5A;PDGFA;PDGFRA;PEAR1;PF4;PGRMC1;PLXNB3;PPBP;PRKAR2B;PROS1;PTGS1;RGS6;SDC4;SELP;SERPINE1;SH3TC2;SLC6A4;S
L1;HOMER2;ITGA2B;ITGB3;LTBP1;MAOB;MYL9;PARD3;PDGFA;PDGFRA;PF4;PGRMC1;PLXNB3;PPBP;PRKAR2B;PTGES;PTGS1;SELP;SERPINE1;SLC6A4;SLC8A3;SMOX;SPARC;TGFB1I1;TNFSF4;TPM1;VEGFC;VIL
C2L;CLU;CMTM5;EGF;ELOVL7;ESAM;F2RL3;FAM26E;GNAZ;GNG11;GP6;GP9;GRB14;GRK5;HOMER2;HRASLS;ITGA2B;ITGB3;ITGB5;KCND3;LY6G6F;MAOB;MAP1B;MFAP3L;MGLL;MMD;MYLK;NAT8B;PARD3;PCSK6;PCYT1B;PDGFA;PDGFRA;PEAR1;PGRMC1;PKHD1L1;PLOD2;PLXNB3;PRKAR2B;PROS1;PTCRA;P
FRA;PF4;PGRMC1;PLXNB3;PPBP;PROS1;SELP;SERPINE1;TPM1;VEGFC;VIL1

;CD9;CDK2AP1;CDKN1A;CLDN5;CLEC1B;CLU;CXCL5;DNM3;EGF;ESAM;F2RL3;FSTL1;GFI1B;GNAZ;GNG11;GP6;GP9;GRB14;GRK5;GUCY1A3;GUCY1B3;HIST1H2AG;HIST1H2BH;HIST1H2BJ;HOMER2;HRASLS;ITGA2B;ITGB3;ITGB5;KCND3;LTBP1;MAOB;MAP1B;MEIS1;MGLL;MMD;MMRN1;MYL9;MYLK;NAT8B;NI
PDGFA;PF4;PPBP;PRKAR2B;PROS1;RAB6B;SELP;SERPINE1;SLC8A3;SPARC;STON2;SYTL4;TNFSF4;TOM1L1;VEGFC;VWI

;PRKAR2B;SERPINE1;SLC6A4;SLC8A3;SPARC;TGFB1I1;TNFSF4;VIL1
F;F2RL3;FSTL1;GNAZ;GNG11;GP6;GRB14;GRK5;GUCY1A3;GUCY1B3;HOMER2;ITGA2B;ITGB3;ITGB5;KCND3;LTBP1;MAOB;MGLL;NRGN;PARD3;PCSK6;PDE5A;PDGFA;PDGFRA;PF4;PLXNB3;PRKAR2B;PTGES;RAB6B;RGS6;RHOBTB1;SELP;SERPINE1;SH3TC2;SLC6A4;SLC8A3;SPARC;SYTL4;TGFB1I1;TNFSF4;TREM

F;F2RL3;FSTL1;GNAZ;GNG11;GP6;GRB14;GRK5;GUCY1A3;GUCY1B3;HOMER2;ITGA2B;ITGB3;ITGB5;KCND3;LTBP1;MAOB;MGLL;NRGN;PARD3;PCSK6;PDE5A;PDGFA;PDGFRA;PF4;PLXNB3;PRKAR2B;PTGES;RAB6B;RGS6;RHOBTB1;SELP;SERPINE1;SH3TC2;SLC6A4;SPARC;SYTL4;TGFB1I1;TNFSF4;TREML1;VEGF
F;F2RL3;FSTL1;GNAZ;GNG11;GP6;GRB14;GRK5;GUCY1A3;GUCY1B3;HOMER2;ITGA2B;ITGB3;ITGB5;KCND3;LTBP1;MAOB;MGLL;NRGN;PARD3;PCSK6;PDE5A;PDGFA;PDGFRA;PF4;PLXNB3;PRKAR2B;PTGES;RAB6B;RGS6;RHOBTB1;SELP;SERPINE1;SH3TC2;SLC6A4;SPARC;SYTL4;TGFB1I1;TNFSF4;TREML1;VEGF

A3;SPARC;TNFSF4;TPM1
L;SERPINE1;SLC6A4;SPARC;TGFB1I1;TNFSF4;TSC22D1;VEGFC

P1B;MMRN1;MYLK;PARD3;PCSK6;PDGFA;PEAR1;PF4;PPBP;PRKAR2B;PROS1;RAB6B;SELP;SERPINE1;SLC24A3;SLC35D3;SLC6A4;SLC8A3;SPARC;SYTL4;TNFSF4;VEGFC;VWI
3;GP6;GRB14;ITGA2B;ITGB3;KCND3;LTBP1;MAOB;MAP1B;MMRN1;MYLK;PARD3;PCSK6;PDGFA;PDGFRA;PEAR1;PF4;PPBP;PRKAR2B;PROS1;RAB6B;SELP;SERPINE1;SLC24A3;SLC35D3;SLC6A4;SLC8A3;SPARC;STON2;SYTL4;TNFSF4;TOM1L1;TPM1;VEGFC;VIL1;VW
FSF4;VEGFC
LP;SERPINE1;TPM1;TUBA8;TUBB1;VEGFC;VIL1
F4V1;PPBP;SERPINE1;TGFB1I1;TNFSF4;VEGFC;VWF
PDGFA;PF4;PPBP;PRKAR2B;PROS1;RAB6B;SELP;SERPINE1;SLC8A3;SPARC;STON2;SYTL4;TNFSF4;TOM1L1;VEGFC;VWI

RD3;PDGFA;PDGFRA;PF4;PRKAR2B;PTGES;SELP;SERPINE1;SLC6A4;SLC8A3;SPARC;TGFB1I1;TNFSF4;VEGFC;VIL1

;PPBP;PROS1;SELP;SERPINE1;SLC6A4;SPARC;TNFSF4;VEGFC

;KCND3;MAOB;MAP1B;MMRN1;MYLK;PARD3;PCSK6;PDGFA;PEAR1;PF4;PPBP;PRKAR2B;PROS1;RAB6B;SELP;SERPINE1;SLC24A3;SLC35D3;SLC6A4;SLC8A3;SPARC;STON2;SYTL4;TNFSF4;TOM1L1;VEGFC;VW

RKAR2B;SELP;SERPINE1;STON2;SYTL4;TNFSF4;TPM1;VEGFC;VIL1

VEGFC;VIL1
;KCND3;MAOB;MAP1B;MMRN1;MYLK;PARD3;PCSK6;PDGFA;PEAR1;PF4;PPBP;PRKAR2B;PROS1;RAB6B;SELP;SERPINE1;SLC24A3;SLC35D3;SLC6A4;SLC8A3;SPARC;STON2;SYTL4;TNFSF4;TOM1L1;VEGFC;VW

;MMD;PDGFRA;PEAR1;RGS6;SDC4;SDPR;SELP;SLC6A4;TNFSF4;TPM1;TSPAN9;VSIG2;VWF

1I1;VEGFC;VIL1

RA;PF4;PROS1;RGS6;SELP;SERPINE1;SLC6A4;TGFB1I1;TNFSF4;TPM1;VEGFC;VIL1;WASF1

C4;SELP;SLC6A4;TNFSF4;TSPAN9;VSIG2

FPI;TNFSF4;VEGFC;VWF

L1;GNAZ;GNG11;GP6;GRB14;GRK5;GUCY1A3;GUCY1B3;HOMER2;ITGA2B;ITGB3;ITGB5;LTBP1;MAOB;MGLL;MYLK;NRGN;PARD3;PCSK6;PDE5A;PDGFA;PDGFRA;PF4;PLXNB3;PRKAR2B;PTGES;PTGS1;RAB6B;RGS6;RHOBTB1;SELP;SERPINE1;SLC6A4;SLC8A3;SMOX;SPARC;TGFB1I1;TNFSF4;TPM1;TREML1;VEGF

/EGFC;VIL1
/EGFC;VIL1

;GNG11;GP6;GRB14;GRK5;GUCY1A3;GUCY1B3;HOMER2;ITGA2B;ITGB3;ITGB5;LTBP1;MGLL;NRGN;PARD3;PCSK6;PDE5A;PDGFA;PDGFRA;PF4;PLXNB3;PRKAR2B;PTGES;RAB6B;RGS6;RHOBTB1;SELP;SERPINE1;SPARC;TGFB1I1;TNFSF4;TREML1;VEGFC;VIL
C4;SELP;SLC6A4;TNFSF4;TSPAN9;VSIG2

;F2RL3;FAM26E;GNAZ;GNG11;GP6;GP9;HRASLS;ITGA2B;ITGB3;ITGB5;KCND3;LY6G6F;MAOB;MFAP3L;MGLL;MMD;NAT8B;PCYT1B;PDGFRA;PEAR1;PGRMC1;PKHD1L1;PLOD2;PLXNB3;PRKAR2B;PROS1;PTCRA;PTGES;PTGS1;RGS6;SDC4;SDPR;SEC14L5;SELP;SLC24A3;SLC35D3;SLC6A4;SLC8A3;ST7;STON2;SYT
31I1;TPM1;VWF
31I1;TPM1;VWF

K6;PDGFA;PF4;PF4V1;PKHD1L1;PPBP;PROS1;SDC4;SELP;SERPINE1;SPARC;TFPI;TNFSF4;VEGFC;VW

VIL1

PDE5A;PDGFA;PDGFRA;PRKAR2B;RGS6;RHOBTB1;SELP;SERPINE1;SLC6A4;SYTL4;TGFB1I1;VEGFC;WASF3

PDE5A;PDGFA;PDGFRA;PRKAR2B;RGS6;RHOBTB1;SELP;SERPINE1;SLC6A4;SYTL4;TGFB1I1;VEGFC;WASF3
;PTGS1;SERPINE1;SLC6A4;SPARC;TGFB1I1;TNFSF4;TSC22D1;VEGFC

DGFRA;SLC6A4;VIL1;VWF

CLEC1B;CLU;CXCL5;EGF;ESAM;F2RL3;FSTL1;GFI1B;GNAZ;GNG11;GP6;GP9;GRB14;GRK5;GUCY1A3;GUCY1B3;HOMER2;ITGA2B;ITGB3;ITGB5;LTBP1;MAOB;MAP1B;MEIS1;MGLL;MMRN1;MYL9;MYLK;NRGN;PARD3;PCSK6;PDE5A;PDGFA;PDGFRA;PDLIM1;PF4;PLXNB3;PPBP;PRKAR2B;PROS1;PTCRA;PTGES;PTG

MTM5;CTTN;CXCL5;DNM3;EGF;ELOVL7;ENKUR;GFI1B;GNAZ;GP6;GRB14;GRK5;GUCY1B3;HIST1H2AG;HIST1H2BH;HIST1H2BJ;HOMER2;ITGA2B;ITGB3;ITGB5;LTBP1;MAOB;MAP1B;MEIS1;MGLL;MYLK;NRGN;PARD3;PCSK6;PDGFA;PDGFRA;PF4;PF4V1;PGRMC1;PLXNB3;PPBP;PRKAR2B;PTCRA;RGS6;SDC4;SD

N1A;CLU;CTTN;DNM3;EGF;ELOVL7;GNAZ;GRB14;GUCY1A3;GUCY1B3;HRASLS;ITGA2B;ITGB3;ITGB5;MAOB;MAP1B;MGLL;MMD;MMRN1;MYL9;MYLK;PARD3;PCSK6;PCYT1B;PDE5A;PDGFA;PF4;PGRMC1;PKHD1L1;PLOD2;PPBP;PRKAR2B;PROS1;PTGES;PTGS1;RAB6B;RHOBTB1;SDC4;SDPR;SELP;SERPINE1;SH

ERPINE1;SLC6A4;SLC8A3;SMOX;SPARC;TGFB1I1;TNFSF4;TPM1;VEGFC;VIL1

;SELP;SLC6A4;TPM1;TUBA8;TUBB1;VIL1;VWF;WASF3

TNFSF4;VEGFC
6;SH3TC2;SLC6A4;TGFB1I1;TPM1;VEGFC;WASF3
MYLK;NAT8B;PARD3;PCSK6;PCYT1B;PDE5A;PDGFA;PDGFRA;PF4;PGRMC1;PLXNB3;RGS6;SERPINE1;SH3TC2;SLC6A4;SLC8A3;SPARC;TGFB1I1;TNFSF4;TPM1;VEGFC;VIL1;VWF;WASF

MEIS1;MYL9;MYLK;NAT8B;NRGN;PARD3;PCSK6;PCYT1B;PDE5A;PDGFA;PDGFRA;PF4;PGRMC1;PLXNB3;RGS6;SDC4;SERPINE1;SH3TC2;SLC6A4;SLC8A3;SPARC;TGFB1I1;TNFSF4;TPM1;VEGFC;VIL1;VWF;WASF

1;VWF;WASF3
GB5;LTBP1;MGLL;PARD3;PCSK6;PDGFA;PDGFRA;PF4;PRKAR2B;RGS6;SERPINE1;TGFB1I1;VEGFC;VIL

MYLK;PDE5A;PRKAR2B;PTGS1;SH3TC2;SLC6A4;TPM1;VEGFC;WASF3

GRMC1;PLXNB3;RGS6;SH3TC2;SLC6A4;SLC8A3;VEGFC;WASF3

GB5;MAP1B;MEIS1;MYL9;MYLK;NAT8B;NRGN;PARD3;PCSK6;PCYT1B;PDGFA;PDGFRA;PF4;PGRMC1;PLXNB3;RGS6;SDC4;SERPINE1;SH3TC2;SLC6A4;SLC8A3;SPARC;TGFB1I1;TNFSF4;TPM1;VEGFC;VWF;WASF

;MAP1B;MYLK;PDE5A;PDGFA;PDGFRA;PF4;PPBP;PRKAR2B;RGS6;SDC4;SELP;SERPINE1;SLC6A4;SYTL4;TGFB1I1;TNFSF4;TPM1;TSC22D1;VEGFC;VIL1;WASF

3;TPM1;VIL1;WASF3
L;MAOB;MAP1B;MEIS1;MGLL;PCSK6;PDE5A;PDGFA;PDGFRA;PF4;PROS1;PTCRA;PTGES;RGS6;SERPINE1;SLC6A4;SYTL4;TGFB1I1;TNFSF4;TPM1;TSC22D1;VIL

3;SPARC;TGFB1I1;TNFSF4;VEGFC;VIL1
YL9;MYLK;NRGN;PARD3;PCYT1B;PDGFA;PDGFRA;PF4;PGRMC1;PLXNB3;RGS6;SDC4;SERPINE1;SH3TC2;SLC6A4;SLC8A3;SPARC;TGFB1I1;TNFSF4;TPM1;VEGFC;VWF;WASF

FRA;PF4;PGRMC1;PLXNB3;SERPINE1;SLC6A4;TGFB1I1;TPM1;VEGFC;VIL1;WASF3
GFB1I1;TPM1;VIL1;WASF3

3P5;CALD1;CAV2;CCDC3;CD9;CDK2AP1;CDKN1A;CLU;CTTN;DNM3;EGF;ELOVL7;GNAZ;GRB14;GRK5;GUCY1A3;GUCY1B3;HOMER2;HRASLS;ITGA2B;ITGB3;ITGB5;MAOB;MAP1B;MGLL;MMD;MMRN1;MYL9;MYLK;PARD3;PCSK6;PCYT1B;PDE5A;PDGFA;PDGFRA;PDLIM1;PF4;PGRMC1;PKHD1L1;PLOD2;PPBP;PR

2;CD9;CDK2AP1;CDKN1A;CLDN5;CLEC1B;CLEC2L;CLU;CMTM5;CTDSPL;CTTN;CXCL5;DNM3;EGF;ELOVL7;ENDOD1;ENKUR;FSTL1;FSTL4;GFI1B;GNAZ;GP6;GRB14;GRK5;GUCY1A3;GUCY1B3;HIST1H2AG;HIST1H2BH;HIST1H2BJ;HOMER2;ITGA2B;ITGB3;ITGB5;KCND3;LTBP1;MAOB;MAP1B;MEIS1;MGLL;MYL9;M
1;PLOD2;PROS1;PTGES;PTGS1;RAB6B;SELP;SLC6A4;SYTL4
S1;PTGS1;TFPI;VWF

DGFRA;PF4;PF4V1;PLOD2;PRKAR2B;PTGES;RAB6B;RHOBTB1;SDPR;SELP;TUBA8;TUBB1;VIL1

);FBXO7;FECH;FKBP1B;FTL;GATA1;GFAP;GLRX5;GPX1;GYPA;HBB;HBD;HBG1;HBG2;HBZ;HPS1;KCNH2;KEL;KLF1;KRT1;MXI1;MYL4;NEDD4L;PDE3A;PRDX5;RBM38;RHAG;RNF10;SGIP1;SLC25A37;SLC2A4;SLC4A1;SNCA;SOX6;SPTA1;TAL1;TESC;TGM2;TRIM10;UTS2R;YBX

ESC;TRIM1(

KLF1;NEDD4L;PRDX5;RHAG;RNF10;SGIP1;SLC25A37;SLC2A4;SLC4A1;SNCA;SOX6;SPTA1;TAL1;TGM2;TRIM10;UTS2I
AL1;TESC;TRIM1(
ESC;TRIM1(

CE;SEC14L4;SLC14A1;SLC25A37;SLC2A4;SLC38A5;SLC4A1;SLC6A19;SLC6A8;SLC6A9;XK

TA1;VIPR2
SLC2A4;SLC38A5;SLC4A1;SLC6A19;SLC6A8;SLC6A9

12;SLC4A1;SOX6;SPTA1;TAL1;TESC

A;TGM2;UTS2R

Q1;HBZ;HMBS;IGF2BP2;MYL4;NEDD4L;OAZ1;ODC1;PDE3A;PPM1A;PRDX5;PRDX6;RAP1GAP;RBM38;RHOV;RPL3L;SELENBP1;SHARPIN;SIAH2;SNCA;SPTA1;STRADB;TMOD1;UBA52;UBB

9A

1;KRT1;LGALS3;LYL1;PBX1;POLL;RAG1;RILP;SFRP2;SNCA;SOX6;SPTA1;TAL1;TESC;TRIM10;UBA52;UBB

4;SLC4A1;SNCA;TGM2;UTS2R
GM2;UTS2R

;KCNH2;MARCH2;MFSD2B;MRC2;MXI1;NEDD4L;NUDT4;OSBP2;RAB3IL1;RHAG;RILP;RPL3L;SELENBP1;SFRP2;SGIP1;SLC14A1;SLC25A37;SLC25A39;SLC2A4;SLC38A5;SLC4A1;SLC6A19;SLC6A8;SLC6A9;SNCA;STRADB;STX2;TESC;TGM2;TSPO2;UBA52;UBB;UTS2R;VTI1B;X

;KCNH2;MARCH2;MFSD2B;MRC2;MXI1;NEDD4L;NUDT4;OSBP2;RAB3IL1;RHAG;RILP;RPL3L;SELENBP1;SFRP2;SGIP1;SLC14A1;SLC25A37;SLC25A39;SLC2A4;SLC38A5;SLC4A1;SLC6A19;SLC6A8;SLC6A9;SNCA;STRADB;STX2;TESC;TGM2;TSPO2;UBA52;UBB;UTS2R;VTI1B;X

BZ;HPS1;KLF1;KRT1;LYL1;MBNL3;MEF2B;MYL4;ODC1;PBX1;RAG1;RBM38;SFRP2;SHARPIN;SIM2;SOX6;SPTA1;STX2;TAL1;TESC;TGM2;TRIM10;WNT9A
ILP;RPL3L;SELENBP1;SFRP2;SLC14A1;SLC2A4;SLC38A5;SLC4A1;SLC6A19;SLC6A8;SNCA;STRADB;STX2;TESC;UBA52;VTI1B;XK

YL4;PBX1;PDE3A;PRDX5;RBM38;RNF10;SFRP2;SGIP1;SNCA;SOX6;TAL1;TESC;UBA52;UBB;UTS2R;WNT9A

);TESC

;KCNH2;MXI1;NEDD4L;OSBP2;RHAG;RILP;RPL3L;SFRP2;SLC14A1;SLC25A37;SLC2A4;SLC38A5;SLC4A1;SLC6A19;SLC6A8;SLC6A9;SNCA;STRADB;STX2;TESC;TGM2;UBA52;UBB;UTS2R;VTI1B;XI

H;HMBS;MYL4;NT5M;OAZ1;ODC1;PDE3A;PRDX5;RAP1GAP;SLC25A39;SLC6A8;SNCA;SPTA1;VIPR2

JBB

;CTSE;DARC;DPM2;E2F2;ECSIT;EHD2;EPB42;EPB49;ESPN;FBXO17;FBXO7;FBXO9;FECH;FHL2;FKBP1B;FKBP8;FTL;GATA1;GFAP;GPX1;GSPT1;GYPA;GYPC;HBA2;HBB;HEMGN;HEPACAM2;HPS1;IGF2BP2;ITLN1;KANK2;KCNH2;KEL;KLC3;KRT1;LGALS3;LYL1;MEF2B;MXI1;MYL4;NEDD4L;PBX1;PLVAP;PPM1A;PRDX

orf78;CA1;CDC34;CHPT1;CR1L;CSDA;CTSE;DCAF12;DPM2;ECSIT;EHD2;EMID1;EPB42;EPB49;ESPN;FBXO7;FBXO9;FECH;FHL2;FKBP1B;FKBP8;FTL;GALNT5;GCAT;GFAP;GLRX5;GMPR;GPX1;GUK1;GYPC;HAGH;HBA2;HBB;HBD;HBG1;HBG2;HBM;HBQ1;HBZ;HEPACAM2;HMBS;HPS1;IFI27;IGF2BP2;KANK2;KCNH2

CSIT;EHD2;EPB42;ESPN;FECH;FHL2;FKBP1B;GATA1;GFAP;GLRX5;GPX1;HBB;HBD;HBG1;HBG2;HBZ;HEMGN;HPS1;IGF2BP2;KCNH2;KEL;KLF1;KRT1;LGALS3;LYL1;MBNL3;MEF2B;MYL4;NEDD4L;ODC1;PBX1;PDE3A;POLL;PRDX5;RAG1;RBM38;RNF10;SFRP2;SGIP1;SHARPIN;SIAH2;SIM2;SLC6A8;SNCA;SOX6;SPTA

CA;STRADB;TGM2;UBA52;UBB;WNT9A

LC2A4;SNCA;SOX6;STRADB;TESC;TFDP1;UBA52;UBB;WNT9A
A1;SLC6A19;SLC6A8;SLC6A9
RDX5;PRDX6;PTPRN;SHARPIN;SLC2A4;SNCA;SOX6;STRADB;TESC;TFDP1;UBA52;UBB;WNT9A

KANK2;MBNL3;MXI1;NEDD4L;NFIX;PBX1;PDE3A;PPM1A;PRDX5;RAG1;RBM38;RNF10;SESN3;SFRP2;SIAH2;SIM2;SNCA;SOX6;SPTA1;STRADB;TAL1;TESC;UBA52;UBB;VIPR2;WNT9A;YBX

ST2;BPGM;CA1;CDC34;CHPT1;CNN1;CSDA;CTSE;DARC;DPCD;DYRK3;E2F2;ECSIT;EHD2;EPB42;EPB49;ESPN;FAM107A;FBXO7;FBXO9;FECH;FHL2;FKBP1B;FKBP8;FTL;GATA1;GFAP;GLRX5;GPX1;GSPT1;GYPA;HBA2;HBB;HBD;HBG1;HBG2;HBQ1;HBZ;HEMGN;HEPACAM2;HPS1;IFI27;IGF2BP2;ITLN1;KANK2;KCNH

KANK2;KRT1;MBNL3;MXI1;NEDD4L;NFIX;PBX1;PDE3A;PPM1A;PRDX5;RAG1;RBM38;RNF10;SESN3;SFRP2;SHARPIN;SIAH2;SIM2;SNCA;SOX6;SPTA1;STRADB;TAL1;TESC;UBA52;UBB;UTS2R;VIPR2;WNT9A;YBX
X1

;SNCA;TESC;XK

;RAG1;SFRP2;SHARPIN;SIAH2;SNCA;STRADB;TGM2;TSTA3;UBA52;UBB;WNT9A

;RAG1;SFRP2;SHARPIN;SIAH2;SNCA;STRADB;TGM2;TSTA3;UBA52;UBB;WNT9A

TESC;TFDP1;UBA52;UBB;VIPR2

RX5;GPX1;HBZ;HPS1;KLF1;KRT1;LYL1;MBNL3;MEF2B;MYL4;ODC1;PBX1;PDE3A;RAG1;RBM38;RNF10;SFRP2;SHARPIN;SIAH2;SIM2;SNCA;SOX6;SPTA1;STRADB;STX2;TAL1;TESC;TGM2;TMOD1;TRIM10;UTS2R;WNT9A;YBX

VRB;C5orf4;C9orf3;CA1;CDC34;CHPT1;CNN1;CSDA;CTSE;DARC;DPM2;DYRK3;E2F2;ECSIT;EHD2;EPB42;EPB49;ESPN;FBXO17;FBXO7;FBXO9;FECH;FHL2;FKBP1B;FKBP8;FTL;GATA1;GCAT;GFAP;GLRX5;GMPR;GPX1;GSPT1;GUK1;GYPA;GYPC;HAGH;HBA2;HBB;HBD;HBG1;HBG2;HBM;HBQ1;HBZ;HEMGN;HEPACA

LC6A8;SLC6A9;SPTA1;TMPRSS9;TSPAN7;VIPR2

orf77;C1orf116;C5orf4;CHPT1;CR1L;DARC;DPM2;EHD2;EPB42;EPB49;FECH;FKBP1B;FKBP8;GALNT5;GATA1;GFAP;GPR146;GYPA;GYPB;GYPC;HEPACAM2;HPS1;IFI27;ITLN1;KCNH2;KEL;KRT1;LGALS3;MARCH2;MFSD2B;MRC2;OR2W3;OSBP2;PAQR9;PDE3A;PDZK1IP1;PLEK2;PLVAP;PTPRF;PTPRN;RAP1GAP;RH
PB42;EPB49;FECH;FHL2;FKBP1B;FKBP8;FTL;GALNT5;GCAT;GFAP;GLRX5;GMPR;GPX1;GUK1;GYPC;HAGH;HBA2;HBB;HBD;HBG1;HBG2;HBM;HBQ1;HBZ;HEPACAM2;HMBS;HPS1;IFI27;IGF2BP2;KANK2;LGALS3;MARCH2;MBNL3;MYL4;NEDD4L;NT5M;OAZ1;ODC1;PDE3A;PLVAP;PPM1A;PRDX5;PRDX6;RAP1GAI
TCD3;S1PR1;SCML4;SERTAD3;SRSF1;STAT4;TARBP1;TBRG1;TRAF5;UPF3A;ZBTB24;ZFP28;ZMYND15;ZNF234;ZNF251;ZNF331;ZNF514;ZNF528;ZNF548;ZNF585B;ZNF841;ZNF850;ZNF88
TCD3;S1PR1;SCML4;SERTAD3;SRSF1;STAT4;TARBP1;TBRG1;TRAF5;UPF3A;ZBTB24;ZFP28;ZMYND15;ZNF234;ZNF251;ZNF331;ZNF514;ZNF528;ZNF548;ZNF585B;ZNF841;ZNF850;ZNF88
PIGL;PTCD3;S1PR1;SCML4;SERTAD3;SLC11A2;SRSF1;STAT4;TARBP1;TBRG1;TRAF5;UPF3A;ZBTB24;ZFP28;ZMYND15;ZNF234;ZNF251;ZNF331;ZNF514;ZNF528;ZNF548;ZNF585B;ZNF841;ZNF850;ZNF88
L;SCML4;SERTAD3;SRSF1;STAT4;TARBP1;TBRG1;TRAF5;TSEN2;UPF3A;ZBTB24;ZFP28;ZMYND15;ZNF234;ZNF251;ZNF331;ZNF514;ZNF528;ZNF548;ZNF585B;ZNF841;ZNF850;ZNF88
PIGL;PTCD3;S1PR1;SCML4;SERTAD3;SLC11A2;SRSF1;STAT4;TARBP1;TBRG1;TRAF5;UPF3A;ZBTB24;ZFP28;ZMYND15;ZNF234;ZNF251;ZNF331;ZNF514;ZNF528;ZNF548;ZNF585B;ZNF841;ZNF850;ZNF88
PIGL;PTCD3;S1PR1;SCML4;SERTAD3;SLC11A2;SRSF1;STAT4;TARBP1;TBRG1;TRAF5;UPF3A;ZBTB24;ZFP28;ZMYND15;ZNF234;ZNF251;ZNF331;ZNF514;ZNF528;ZNF548;ZNF585B;ZNF841;ZNF850;ZNF88
L;STAT4;TARBP1;TRAF5;TSEN2;UPF3A;ZBTB24;ZFP28;ZMYND15;ZNF234;ZNF251;ZNF331;ZNF514;ZNF528;ZNF548;ZNF585B;ZNF841;ZNF850;ZNF88
UPF3A;ZBTB24;ZFP28;ZMYND15;ZNF234;ZNF251;ZNF331;ZNF514;ZNF528;ZNF548;ZNF585B;ZNF841;ZNF850;ZNF88
TRAF5;UPF3A;ZBTB24;ZFP28;ZMYND15;ZNF234;ZNF251;ZNF331;ZNF514;ZNF528;ZNF548;ZNF585B;ZNF841;ZNF850;ZNF88
UPF3A;ZBTB24;ZFP28;ZMYND15;ZNF234;ZNF251;ZNF331;ZNF514;ZNF528;ZNF548;ZNF585B;ZNF841;ZNF850;ZNF88
UPF3A;ZBTB24;ZFP28;ZMYND15;ZNF234;ZNF251;ZNF331;ZNF514;ZNF528;ZNF548;ZNF585B;ZNF841;ZNF850;ZNF88
UPF3A;ZBTB24;ZFP28;ZMYND15;ZNF234;ZNF251;ZNF331;ZNF514;ZNF528;ZNF548;ZNF585B;ZNF841;ZNF850;ZNF88
L;STAT4;TARBP1;TBRG1;TRAF5;TSEN2;UPF3A;ZBTB24;ZFP28;ZMYND15;ZNF234;ZNF251;ZNF331;ZNF514;ZNF528;ZNF548;ZNF585B;ZNF841;ZNF850;ZNF88
F5;ZBTB24;ZFP28;ZMYND15;ZNF234;ZNF251;ZNF331;ZNF514;ZNF528;ZNF548;ZNF585B;ZNF841;ZNF850;ZNF88
4;ZFP28;ZNF234;ZNF251;ZNF331;ZNF514;ZNF528;ZNF548;ZNF585B;ZNF831;ZNF841;ZNF850;ZNF88
F5;ZBTB24;ZFP28;ZMYND15;ZNF234;ZNF251;ZNF331;ZNF514;ZNF528;ZNF548;ZNF585B;ZNF841;ZNF850;ZNF88
T4;TARBP1;TRAF5;ZBTB24;ZFP28;ZMYND15;ZNF234;ZNF251;ZNF331;ZNF514;ZNF528;ZNF548;ZNF585B;ZNF841;ZNF850;ZNF88
2B;PER3;PIGG;PIGL;PTCD3;S1PR1;SCML4;SERTAD3;SRSF1;STAT4;TARBP1;TBRG1;TRAF5;TSEN2;UPF3A;ZBTB24;ZFP28;ZMYND15;ZNF234;ZNF251;ZNF331;ZNF514;ZNF528;ZNF548;ZNF585B;ZNF841;ZNF850;ZNF88
RBP1;TRAF5;ZBTB24;ZFP28;ZMYND15;ZNF234;ZNF251;ZNF331;ZNF514;ZNF528;ZNF548;ZNF585B;ZNF841;ZNF850;ZNF88
RBP1;TRAF5;ZBTB24;ZFP28;ZMYND15;ZNF234;ZNF251;ZNF331;ZNF514;ZNF528;ZNF548;ZNF585B;ZNF841;ZNF850;ZNF88
D15;ZNF234;ZNF251;ZNF331;ZNF514;ZNF528;ZNF548;ZNF585B;ZNF841;ZNF850;ZNF88
D15;ZNF234;ZNF251;ZNF331;ZNF514;ZNF528;ZNF548;ZNF585B;ZNF841;ZNF850;ZNF88
585B;ZNF831;ZNF841;ZNF850;ZNF88
3;SLC11A2;STAT4;TARBP1;TBRG1;TRAF5;UPF3A;ZBTB24;ZFP28;ZMYND15;ZNF234;ZNF251;ZNF331;ZNF514;ZNF528;ZNF548;ZNF585B;ZNF841;ZNF850;ZNF88
RBP1;TRAF5;ZBTB24;ZFP28;ZMYND15;ZNF234;ZNF251;ZNF331;ZNF514;ZNF528;ZNF548;ZNF585B;ZNF841;ZNF850;ZNF88
P1;TBRG1;TRAF5;UPF3A;ZBTB24;ZFP28;ZMYND15;ZNF234;ZNF251;ZNF331;ZNF514;ZNF528;ZNF548;ZNF585B;ZNF841;ZNF850;ZNF88
D15;ZNF234;ZNF251;ZNF331;ZNF514;ZNF528;ZNF548;ZNF585B;ZNF841;ZNF850;ZNF88
F5;ZBTB24;ZFP28;ZMYND15;ZNF234;ZNF251;ZNF331;ZNF514;ZNF528;ZNF548;ZNF585B;ZNF841;ZNF850;ZNF88
TERFD2;NARF;NR3C2;OFD1;OSBPL3;PER3;PIGG;PIGL;PTCD3;RIC3;S100PBP;SCML4;SEC22C;SERTAD3;SLC11A2;SRSF1;STAT4;SUN1;TARBP1;TBRG1;TSEN2;UPF3A;WDR75;ZBTB24;ZFP28;ZMYND15;ZNF234;ZNF251;ZNF331;ZNF514;ZNF528;ZNF548;ZNF585B;ZNF841;ZNF85
PS25;MTERFD2;NARF;NR3C2;OFD1;OSBPL3;PER3;PIGG;PIGL;PTCD3;RIC3;S100PBP;SCML4;SEC22C;SEPT8;SERTAD3;SLC11A2;SRSF1;STAT4;SUN1;TARBP1;TBRG1;TRAF5;TSEN2;UPF3A;WDR75;ZBTB24;ZFP28;ZMYND15;ZNF234;ZNF251;ZNF331;ZNF514;ZNF528;ZNF548;ZNF585B;ZNF841;ZNF85
TERFD2;NARF;NR3C2;OFD1;OSBPL3;PER3;PIGG;PIGL;PTCD3;RIC3;S100PBP;SCML4;SEC22C;SERTAD3;SLC11A2;SRSF1;STAT4;SUN1;TARBP1;TBRG1;TSEN2;UPF3A;WDR75;ZBTB24;ZFP28;ZMYND15;ZNF234;ZNF251;ZNF331;ZNF514;ZNF528;ZNF548;ZNF585B;ZNF841;ZNF85
PS25;MTERFD2;NARF;NR3C2;OFD1;OSBPL3;PER3;PIGG;PIGL;PTCD3;RIC3;S100PBP;SCML4;SEC22C;SEPT8;SERTAD3;SLC11A2;SRSF1;STAT4;SUN1;TARBP1;TBRG1;TRAF5;TSEN2;UPF3A;WDR75;ZBTB24;ZFP28;ZMYND15;ZNF234;ZNF251;ZNF331;ZNF514;ZNF528;ZNF548;ZNF585B;ZNF841;ZNF85
D7;MOAP1;MRPS25;MTERFD2;NARF;NR3C2;OFD1;OSBPL3;PAIP2B;PER3;PIGG;PIGL;PTCD3;RIC3;S100PBP;SCML4;SEC22C;SEPT8;SERTAD3;SLC11A2;SRSF1;STAT4;SUN1;TARBP1;TBRG1;TRAF5;TSEN2;UPF3A;WDR75;ZBTB24;ZFP28;ZMYND15;ZNF234;ZNF251;ZNF331;ZNF514;ZNF528;ZNF548;ZNF585B;ZNF8
;MRPS25;MTERFD2;NR3C2;PAIP2B;PER3;PIGG;PIGL;PTCD3;S1PR1;SCML4;SERTAD3;SLC11A2;SRSF1;STAT4;TARBP1;TBRG1;TRAF5;TSEN2;UPF3A;ZBTB24;ZFP28;ZMYND15;ZNF234;ZNF251;ZNF331;ZNF514;ZNF528;ZNF548;ZNF585B;ZNF841;ZNF850;ZNF88
;TARBP1;TRAF5;UPF3A;ZBTB24;ZFP28;ZMYND15;ZNF234;ZNF251;ZNF331;ZNF514;ZNF528;ZNF548;ZNF585B;ZNF841;ZNF850;ZNF88
1A2;SRSF1;STAT4;TARBP1;TBRG1;TRAF5;TSEN2;UPF3A;ZBTB24;ZFP28;ZMYND15;ZNF234;ZNF251;ZNF331;ZNF514;ZNF528;ZNF548;ZNF585B;ZNF841;ZNF850;ZNF88
;SLC11A2;SRSF1;STAT4;TARBP1;TBRG1;TRAF5;TSEN2;UPF3A;ZBTB24;ZFP28;ZMYND15;ZNF234;ZNF251;ZNF331;ZNF514;ZNF528;ZNF548;ZNF585B;ZNF841;ZNF850;ZNF88
1A2;SRSF1;STAT4;TARBP1;TBRG1;TRAF5;TSEN2;UPF3A;ZBTB24;ZFP28;ZMYND15;ZNF234;ZNF251;ZNF331;ZNF514;ZNF528;ZNF548;ZNF585B;ZNF841;ZNF850;ZNF88
585B;ZNF831;ZNF841;ZNF850;ZNF88
1;STAT4;TARBP1;TSEN2;UPF3A;ZBTB24;ZFP28;ZNF234;ZNF251;ZNF331;ZNF514;ZNF528;ZNF548;ZNF585B;ZNF831;ZNF841;ZNF850;ZNF88
L;STAT4;TARBP1;TBRG1;TRAF5;TSEN2;UPF3A;ZBTB24;ZFP28;ZMYND15;ZNF234;ZNF251;ZNF331;ZNF514;ZNF528;ZNF548;ZNF585B;ZNF841;ZNF850;ZNF88
P1;TRAF5;UPF3A;ZBTB24;ZFP28;ZMYND15;ZNF234;ZNF251;ZNF331;ZNF514;ZNF528;ZNF548;ZNF585B;ZNF841;ZNF850;ZNF88

2B;PER3;PIGG;PIGL;PTCD3;S1PR1;SCML4;SERTAD3;SRSF1;STAT4;TARBP1;TBRG1;TRAF5;TSEN2;UPF3A;ZBTB24;ZFP28;ZMYND15;ZNF234;ZNF251;ZNF331;ZNF514;ZNF528;ZNF548;ZNF585B;ZNF841;ZNF850;ZNF88

1A2;SRSF1;STAT4;TARBP1;TBRG1;TRAF5;TSEN2;UPF3A;ZBTB24;ZFP28;ZMYND15;ZNF234;ZNF251;ZNF331;ZNF514;ZNF528;ZNF548;ZNF585B;ZNF841;ZNF850;ZNF88
TARBP1;TSEN2;UPF3A;ZBTB24;ZFP28;ZNF234;ZNF251;ZNF331;ZNF514;ZNF528;ZNF548;ZNF585B;ZNF831;ZNF841;ZNF850;ZNF88
D7;MOAP1;MRPS25;MTERFD2;NARF;NR3C2;OFD1;OSBPL3;PAIP2B;PER3;PIGG;PIGL;PTCD3;RIC3;S100PBP;SCML4;SEC22C;SEPT8;SERTAD3;SLC11A2;SRSF1;STAT4;SUN1;TARBP1;TBRG1;TRAF5;TSEN2;UPF3A;WDR75;ZBTB24;ZFP28;ZMYND15;ZNF234;ZNF251;ZNF331;ZNF514;ZNF528;ZNF548;ZNF585B;ZNF8

D15;ZNF234;ZNF251;ZNF331;ZNF514;ZNF528;ZNF548;ZNF585B;ZNF841;ZNF850;ZNF88
;SLC11A2;SRSF1;STAT4;TARBP1;TBRG1;TRAF5;TSEN2;UPF3A;ZBTB24;ZFP28;ZMYND15;ZNF234;ZNF251;ZNF331;ZNF514;ZNF528;ZNF548;ZNF585B;ZNF841;ZNF850;ZNF88
;MOAP1;MRPS25;MTERFD2;NR3C2;PAIP2B;PER3;PIGG;PIGL;PTCD3;S1PR1;SCML4;SERTAD3;SLC11A2;SRSF1;STAT4;TARBP1;TBRG1;TRAF5;TSEN2;UPF3A;ZBTB24;ZFP28;ZMYND15;ZNF234;ZNF251;ZNF331;ZNF514;ZNF528;ZNF548;ZNF585B;ZNF841;ZNF850;ZNF88

F1;STAT4;SUN1;TARBP1;TBRG1;TSEN2;UPF3A;WDR75;ZBTB24;ZFP28;ZMYND15;ZNF234;ZNF251;ZNF331;ZNF514;ZNF528;ZNF548;ZNF585B;ZNF841;ZNF85
D15;ZNF234;ZNF251;ZNF331;ZNF514;ZNF528;ZNF548;ZNF585B;ZNF841;ZNF850;ZNF88

DL;KCTD7;MOAP1;MRPS25;MTERFD2;NARF;NR3C2;OFD1;OSBPL3;PAIP2B;PER3;PIGG;PIGL;PTCD3;RIC3;S100PBP;S1PR1;SCML4;SEC22C;SEMA6B;SEPT8;SERTAD3;SLC11A2;SRSF1;STAT4;SUN1;TARBP1;TBRG1;TRAF5;TSEN2;UPF3A;WDR75;ZBTB24;ZFP28;ZMYND15;ZNF234;ZNF251;ZNF331;ZNF514;ZNF528;
DL;KCTD7;MOAP1;MRPS25;MTERFD2;NARF;NR3C2;OFD1;OSBPL3;PAIP2B;PER3;PIGG;PIGL;PTCD3;RIC3;S100PBP;S1PR1;SCML4;SEC22C;SEMA6B;SEPT8;SERTAD3;SLC11A2;SRSF1;STAT4;SUN1;TARBP1;TBRG1;TRAF5;TSEN2;UPF3A;WDR75;ZBTB24;ZFP28;ZMYND15;ZNF234;ZNF251;ZNF331;ZNF514;ZNF528;

NR3C2;PAIP2B;PER3;PIGG;PIGL;PTCD3;S1PR1;SCML4;SERTAD3;SLC11A2;SRSF1;STAT4;TARBP1;TBRG1;TRAF5;TSEN2;UPF3A;ZBTB24;ZFP28;ZMYND15;ZNF234;ZNF251;ZNF331;ZNF514;ZNF528;ZNF548;ZNF585B;ZNF841;ZNF850;ZNF88

NR3C2;PAIP2B;PER3;PIGG;PIGL;PTCD3;S1PR1;SCML4;SERTAD3;SRSF1;STAT4;TARBP1;TBRG1;TRAF5;TSEN2;UPF3A;ZBTB24;ZFP28;ZMYND15;ZNF234;ZNF251;ZNF331;ZNF514;ZNF528;ZNF548;ZNF585B;ZNF841;ZNF850;ZNF88

ARF;NR3C2;OFD1;OSBPL3;PAIP2B;PTCD3;RIC3;S100PBP;S1PR1;SCML4;SEPT8;SERTAD3;SLC11A2;SRSF1;STAT4;SUN1;TARBP1;TBRG1;TRAF5;TSEN2;UPF3A;ZBTB24;ZFP28;ZMYND15;ZNF234;ZNF251;ZNF331;ZNF514;ZNF528;ZNF548;ZNF585B;ZNF831;ZNF841;ZNF850;ZNF88

HNRPDL;KCTD7;MFSD7;MOAP1;MRPS25;MTERFD2;NR3C2;OFD1;PAIP2B;PER3;PIGG;PIGL;PTCD3;S1PR1;SCML4;SEC22C;SEMA6B;SEPT8;SERTAD3;SLC11A2;SRSF1;STAT4;SUN1;TARBP1;TBRG1;TRAF5;TSEN2;UPF3A;ZBTB24;ZFP28;ZMYND15;ZNF234;ZNF251;ZNF331;ZNF514;ZNF528;ZNF548;ZNF585B;ZNF84

;ZNF528;ZNF548;ZNF585B;ZNF831;ZNF841;ZNF850;ZNF88(

850

;ZNF528;ZNF548;ZNF585B;ZNF831;ZNF841;ZNF850;ZNF88(

P70;TNFRSF13C
HR3;CELSR1;CNR2;CNTNAP2;CR2;CTGF;CXCR5;DIRAS1;ENAH;EVC2;FCER2;FCRL1;FCRL2;FCRL3;FCRL5;GNG7;HLA-DOA;HLA-DOB;HTR3A;ICOSLG;KCNG1;KCNH8;KCNIP2;L1CAM;MS4A1;NETO1;P2RX5;PARM1;PAWR;PCDH9;PIK3C2B;PMEPA1;PPAPDC1B;PTPRK;QSOX2;RASGRP3;SCN4A;SIGLEC6;SLC15A2;SLC
HR3;CELSR1;CNR2;CNTNAP2;CR2;CTGF;CXCR5;DIRAS1;ENAH;EVC2;FCER2;FCRL1;FCRL2;FCRL3;FCRL5;GNG7;HLA-DOA;HLA-DOB;HTR3A;ICOSLG;KCNG1;KCNH8;KCNIP2;L1CAM;MS4A1;NETO1;P2RX5;PARM1;PAWR;PCDH9;PIK3C2B;PMEPA1;PPAPDC1B;PTPRK;QSOX2;RASGRP3;SCN4A;SIGLEC6;SLC15A2;SLC
R;SWAP70;TNFRSF13C
TNFRSF13C
DOA;HLA-DOB;IRF4;MS4A1;PAWR;PAX5;POU2AF1;SWAP70;TNFRSF13C
SLG;KCNG1;KCNIP2;MS4A1;P2RX5;PTPRK;RASGRP3;SCN4A;SIGLEC6;SLC15A2;TSPAN13
FCER2;GNG7;HLA-DOA;HLA-DOB;HTR3A;ICOSLG;KCNG1;KCNIP2;L1CAM;MS4A1;P2RX5;PTPRK;RASGRP3;SCN4A;SIGLEC6;SLC15A2;TNFRSF13C;TSPAN13
SLG;KCNG1;KCNIP2;MS4A1;P2RX5;PTPRK;RASGRP3;SCN4A;SIGLEC6;SLC15A2;TSPAN13
PAWR;SWAP70;TNFRSF13C

CR2;CXCR5;DTX1;ENAH;FCER2;HLA-DOA;HLA-DOB;ICOSLG;IRF4;L1CAM;MS4A1;PAWR;PAX5;POU2AF1;SWAP70;TNFRSF13(

EC17A;CNR2;CNTNAP2;CR2;CXCR5;DENND5B;EVC2;FCER2;FCRL1;FCRL2;FCRL3;FCRL5;GYLTL1B;HLA-DOA;HLA-DOB;HS3ST1;HTR3A;ICOSLG;KCNG1;KCNH8;KCNIP2;L1CAM;LARGE;MARCH3;MOXD1;MS4A1;NETO1;NIPAL4;P2RX5;PARM1;PCDH9;PMEPA1;PPAPDC1B;PTPRK;QSOX2;RASGRP3;SCN4A;SEL1L3;S

R1;CLEC17A;CNR2;CNTNAP2;CR2;CXCR5;DENND5B;EVC2;FCER2;FCRL1;FCRL2;FCRL3;FCRL5;GYLTL1B;HLA-DOA;HLA-DOB;HS3ST1;HTR3A;ICOSLG;KCNG1;KCNH8;KCNIP2;L1CAM;LARGE;MARCH3;MOXD1;MS4A1;NETO1;NIPAL4;P2RX5;PARM1;PCDH9;PMEPA1;PPAPDC1B;PTPRK;QSOX2;RASGRP3;SCN4A;SEI

/R;TNFRSF13(

D83;CDHR3;CELSR1;CLEC17A;CNR2;CNTNAP2;CORO2B;CR2;CTGF;CXCR5;DENND5B;DIRAS1;ENAH;EVC2;FARP2;FCER2;FCRL1;FCRL2;FCRL3;FCRL5;GGA2;GNG7;GYLTL1B;HLA-DOA;HLA-DOB;HS3ST1;HTR3A;ICOSLG;KCNG1;KCNH8;KCNIP2;KLHL14;L1CAM;LARGE;MARCH3;MOXD1;MS4A1;NETO1;NIPAL4;P2R

;GHRL;GNG7;HTR3A;IRF4;L1CAM;LAMA5;NXPH4;P2RX5;PAWR;PEG10;PMEPA1;PNOC;PRICKLE1;PTPRK;SPRY1;STAP1

R1;CLEC17A;CNR2;CNTNAP2;CR2;CXCR5;DENND5B;EVC2;FARP2;FCER2;FCRL1;FCRL2;FCRL3;FCRL5;GGA2;GNG7;GYLTL1B;HLA-DOA;HLA-DOB;HS3ST1;HTR3A;ICOSLG;KCNG1;KCNH8;KCNIP2;KLHL14;L1CAM;LARGE;MARCH3;MOXD1;MS4A1;NETO1;NIPAL4;P2RX5;PARM1;PCDH9;PMEPA1;PPAPDC1B;PTPRK;

);CD72;CD79A;CD79B;CD83;CDCA7L;CDHR3;CELSR1;CNR2;CNTNAP2;COCH;COL19A1;COL4A3;COL4A4;CORO2B;CR2;CTGF;CXCR5;DIRAS1;DPF3;DTX1;E2F5;EBF1;EGR3;ENAH;EVC2;FARP2;FCRL2;FCRLA;GGA2;GHRL;GNG7;GYLTL1B;HLA-DOA;HTR3A;ICOSLG;IRF4;KCNG1;KCNH8;KCNIP2;L1CAM;LAMA5;LAMC

;PTPRK;SIGLEC6

;PTPRK;SIGLEC6

CD79B;CD83;CDHR3;CELSR1;CNR2;CNTNAP2;COCH;COL19A1;COL4A3;COL4A4;CORO2B;CR2;CTGF;CXCR5;DIRAS1;DPF3;DTX1;E2F5;EGR3;ENAH;EVC2;FARP2;FCRL2;FCRLA;GGA2;GHRL;GNG7;GYLTL1B;HLA-DOA;HTR3A;ICOSLG;IRF4;KCNG1;KCNH8;KCNIP2;L1CAM;LAMA5;LAMC1;LARGE;MAP2;MICAL3;MMF

OL19A1;COL4A3;COL4A4;CTGF;CXCR5;DPF3;DTX1;E2F5;EBF1;EGR3;ENAH;FARP2;GHRL;GNG7;HLA-DOA;HTR3A;ICOSLG;IRF4;KCNG1;KCNH8;KCNIP2;L1CAM;LAMA5;LAMC1;MACROD2;MAP2;MMP11;MYBPC2;NETO1;P2RX5;PAWR;PAX5;PEG10;PNOC;PRICKLE1;RAPGEF5;SCN4A;SEMA3G;SOBP;SPRY1;STRBI

F;CXCR5;DPF3;DTX1;E2F5;EBF1;EGR3;ENAH;FARP2;GHRL;HLA-DOA;IRF4;KCNIP2;L1CAM;LAMA5;LAMC1;MACROD2;MAP2;MMP11;P2RX5;PAX5;PEG10;PRICKLE1;RAPGEF5;SEMA3G;SOBP;SPRY1;STRBP;SWAP70;TCF4;TCL1

COL4A4;CTGF;CXCR5;DPF3;DTX1;E2F5;EBF1;EGR3;ENAH;FARP2;GHRL;GNG7;HLA-DOA;HTR3A;ICOSLG;IRF4;KCNG1;KCNH8;KCNIP2;L1CAM;LAMA5;LAMC1;MACROD2;MAP2;MMP11;MYBPC2;NETO1;P2RX5;PAWR;PAX5;PEG10;PNOC;PRICKLE1;RAPGEF5;SCN4A;SEMA3G;SOBP;SPRY1;STRBP;SWAP70;TCF4;T

LSR1;CNR2;CNTNAP2;COL4A3;COL4A4;CR2;CTGF;CXCR5;DIRAS1;DTX1;E2F5;EGR3;ENAH;EVC2;FARP2;FCER2;GHRL;GNG7;HLA-DOA;HLA-DOB;HTR3A;ICOSLG;IRF4;KCNIP2;L1CAM;LAMA5;LAMC1;MAP2;MS4A1;NETO1;NXPH4;P2RX5;PAWR;PAX5;PEG10;PIK3C2B;PKIG;PLEKHG1;PMEPA1;PNOC;POU2AF1;PR

M;LAMC1;MACROD2;MAP2;P2RX5;PAX5;PRICKLE1;RAPGEF5;TCF4

AP2;TCF4

;DIRAS1;DTX1;E2F5;EGR3;ENAH;EVC2;FARP2;FCRL2;GHRL;GNG7;HTR3A;ICOSLG;IRF4;KCNG1;KCNH8;KCNIP2;L1CAM;LAMA5;NETO1;NXPH4;P2RX5;PAWR;PEG10;PIK3C2B;PKIG;PLEKHG1;PMEPA1;PNOC;PRICKLE1;PTPRK;RAPGEF5;RASGRP3;SIGLEC6;SNX22;SPRY1;STAP
;DIRAS1;DTX1;E2F5;EGR3;ENAH;EVC2;FARP2;FCRL2;GHRL;GNG7;HTR3A;ICOSLG;IRF4;KCNG1;KCNH8;KCNIP2;L1CAM;LAMA5;NETO1;NXPH4;P2RX5;PAWR;PEG10;PIK3C2B;PKIG;PLEKHG1;PMEPA1;PNOC;PRICKLE1;PTPRK;RAPGEF5;RASGRP3;SIGLEC6;SPRY1;STAP
;DIRAS1;DTX1;E2F5;EGR3;ENAH;EVC2;FARP2;FCRL2;GHRL;GNG7;HTR3A;ICOSLG;IRF4;KCNG1;KCNH8;KCNIP2;L1CAM;LAMA5;NETO1;NXPH4;P2RX5;PAWR;PEG10;PIK3C2B;PKIG;PLEKHG1;PMEPA1;PNOC;PRICKLE1;PTPRK;RAPGEF5;RASGRP3;SIGLEC6;SPRY1;STAP
4
;COL4A4;CTGF;CXCR5;DPF3;DTX1;E2F5;EGR3;ENAH;FARP2;GHRL;HLA-DOA;IRF4;KCNIP2;L1CAM;LAMA5;LAMC1;MACROD2;MAP2;P2RX5;PAX5;PEG10;PRICKLE1;RAPGEF5;SOBP;SPRY1;STRBP;SWAP70;TCF4;TCL1

R5;DPF3;DTX1;E2F5;EGR3;ENAH;FARP2;GHRL;HLA-DOA;IRF4;KCNIP2;L1CAM;LAMA5;LAMC1;MACROD2;MAP2;P2RX5;PAX5;PEG10;PRICKLE1;RAPGEF5;SOBP;SPRY1;SWAP70;TCF

AH;FARP2;FCRLA;GHRL;HLA-DOA;IRF4;KCNIP2;L1CAM;LAMA5;LAMC1;MAP2;PAX5;PEG10;PRICKLE1;SPRY1;STRBP;TCF4;TCL1

A4;CTGF;CXCR5;DTX1;E2F5;EGR3;ENAH;FARP2;GHRL;HLA-DOA;IRF4;KCNIP2;L1CAM;LAMA5;LAMC1;MACROD2;MAP2;PAX5;PEG10;PRICKLE1;SOBP;SPRY1;STRBP;TCF4;TCL1

;COL4A4;CTGF;CXCR5;DPF3;DTX1;E2F5;EBF1;EGR3;ENAH;FARP2;FCRLA;GHRL;HLA-DOA;IRF4;KCNIP2;L1CAM;LAMA5;LAMC1;MACROD2;MAP2;MMP11;P2RX5;PAX5;PEG10;PRICKLE1;RAPGEF5;SEMA3G;SOBP;SPRY1;STRBP;SWAP70;TCF4;TCL1

AP2;TCF4

X1;ENAH;FARP2;FCRLA;GHRL;HLA-DOA;IRF4;KCNIP2;L1CAM;LAMA5;LAMC1;MAP2;PAX5;PEG10;PRICKLE1;SPRY1;STRBP;TCF4;TCL1A

;DIRAS1;DTX1;E2F5;ENAH;EVC2;FARP2;GHRL;GNG7;HTR3A;ICOSLG;IRF4;KCNIP2;L1CAM;LAMA5;NXPH4;P2RX5;PAWR;PEG10;PIK3C2B;PKIG;PLEKHG1;PMEPA1;PNOC;PRICKLE1;PTPRK;RAPGEF5;RASGRP3;SPRY1;STAP1
CAM;P2RX5;PAWR;PEG10;PLEKHG1;PMEPA1;PRICKLE1;RAPGEF5;RASGRP3;SPRY1;TNFRSF13C

AMC1;PTPRK

MC1;MAP2;STRBP;TCF4;TCL1A

A3;TEX101;THSD7A

STYK1;TGFBR3;TLR3

STYK1;TGFBR3;TLR3

'O6;SLC4A4;STYK1;TGFBR3;TLR3

'O6;PDGFD;PHLDA1;PHLDB2;SLC4A4;STYK1;TGFBR3;TLR[illegible]

TGFBR3;TLR[illegible]

TGFBR3;TLR3

1;OAS2;OAS3;OASL;PLSCR1;RSAD2;TRIM22
;OAS1;OAS2;OAS3;OASL;PLSCR1;RSAD2;TRIM22
X1;OAS1;OAS2;OAS3;OASL;PLSCR1;RSAD2;USP18;XAF1;ZBP1
ISG15;LILRB5;MT2A;MX1;OAS1;OAS2;OAS3;OASL;PLSCR1;RSAD2;SIGLEC1;TRIM22;USP18;XAF1;ZBP

F7;ISG15;MX1;OAS1;OAS2;OAS3;OASL;PLSCR1;RSAD2;TRIM22

F7;ISG15;MX1;OAS1;OAS2;OAS3;OASL;PLSCR1;RSAD2;TRIM22
;OAS1;OAS2;OAS3;OASL;PLSCR1;RSAD2;TRIM22
T2A;MX1;OAS1;OAS2;OAS3;OASL;PLSCR1;RSAD2;TRIM22;USP18;XAF1;ZBP1
1;USP18;XAF1;ZBP1

1;ZBP1

ISG15;MT2A;MX1;OAS1;OAS2;OAS3;OASL;PLSCR1;RSAD2;TRIM22;USP18;XAF1;ZBP1

F7;ISG15;MX1;OAS1;OAS2;OAS3;OASL;PLSCR1;RSAD2;TRIM22

ISG15;LILRB5;MT2A;MX1;OAS1;OAS2;OAS3;OASL;PLSCR1;PNPT1;RSAD2;SIGLEC1;TOR1B;TRIM22;USP18;XAF1;ZBP
M22

OAS2;OAS3;OASL;PLSCR1;PNPT1;TOR1B;USP18;XAF1;ZBP1
M22
M22
OASL;PNPT1;USP18;XAF1;ZBP1

M22

ZBP1

OAS1;OAS2;OAS3;OASL;PLSCR1;PNPT1;RTP4;TOR1B;USP18;XAF1;ZBP1
OASL;PNPT1;USP18;XAF1;ZBP1

I6;IFIH1;IFIT1;IFIT2;IFIT3;IFITM3;IRF7;ISG15;LAMP3;MT2A;MX1;OAS1;OAS2;OAS3;OASL;OTOF;PLSCR1;PNPT1;RSAD2;RSPH9;RTP4;SAMD4A;SPATS2L;TDRD7;TOR1B;TRIM22;TRIM69;USP18;XAF1;ZBP1;ZCCHC

RD7;TRIM22

3;IFIT5;IFITM3;IRF7;ISG15;LILRB5;LY6E;MT2A;MX1;OAS1;OAS2;OAS3;OASL;PLSCR1;PNPT1;RSAD2;RTP4;SIGLEC1;TOR1B;TRIM22;USP18;XAF1;ZBP
RD7;TRIM22
;PLSCR1;USP18;XAF1;ZBP1

PLSCR1;USP18;XAF1;ZBP1

G15;LILRB5;LY6E;MT2A;MX1;OAS1;OAS2;OAS3;OASL;OTOF;PLSCR1;RSAD2;USP18;XAF1;ZBP1;ZCCHC[illegible]

IRF7;ISG15;LAMP3;MT2A;MX1;OAS1;OAS2;OAS3;OASL;OTOF;PLSCR1;PNPT1;RSAD2;RSPH9;TDRD7;TOR1B;TRIM22;USP18;XAF1;ZBP

5;LY6E;MT2A;MX1;OAS1;OAS2;OAS3;OASL;PLSCR1;RSAD2;USP18;XAF1;ZBP[illegible]
G15;LILRB5;LY6E;MT2A;MX1;OAS1;OAS2;OAS3;OASL;OTOF;PLSCR1;RSAD2;USP18;XAF1;ZBP
G15;LILRB5;LY6E;MT2A;MX1;OAS1;OAS2;OAS3;OASL;OTOF;PLSCR1;RSAD2;USP18;XAF1;ZBP

F7;ISG15;LILRB5;LY6E;MT2A;MX1;OAS1;OAS2;OAS3;OASL;PLSCR1;PNPT1;RSAD2;USP18;XAF1;ZBP[illegible]

I6;IFIH1;IFIT1;IFIT2;IFIT3;IFIT5;IFITM3;IRF7;ISG15;LAMP3;MT2A;MX1;OAS1;OAS2;OAS3;OASL;OTOF;PARP12;PHF11;PLSCR1;PNPT1;RSAD2;RSPH9;RTP4;SAMD4A;SPATS2L;TDRD7;TOR1B;TRIM22;TRIM69;USP18;XAF1;ZBP1;ZCCHC

I6;IFIH1;IFIT1;IFIT2;IFIT3;IFIT5;IFITM3;IRF7;ISG15;LAMP3;MT2A;MX1;OAS1;OAS2;OAS3;OASL;OTOF;PARP12;PHF11;PLSCR1;PNPT1;RSAD2;RSPH9;RTP4;SAMD4A;SPATS2L;TDRD7;TOR1B;TRIM22;TRIM69;USP18;XAF1;ZBP1;ZCCHC

OASL;PLSCR1;PNPT1;RSAD2;SAMD4[illegible]

LSCR1;PNPT1;TDRD7;ZBP1;ZCCHC[illegible]

IFIT3;IFITM3;IRF7;ISG15;LILRB5;LY6E;MT2A;MX1;OAS1;OAS2;OAS3;OASL;OTOF;PLSCR1;PNPT1;RSAD2;RSPH9;RTP4;SIGLEC1;TDRD7;TOR1B;TRIM69;USP18;XAF1;ZBP1;ZCCHC

I6;IFIH1;IFIT1;IFIT2;IFIT3;IFIT5;IFITM3;IRF7;ISG15;LAMP3;LY6E;MT2A;MX1;OAS1;OAS2;OAS3;OASL;OTOF;PARP12;PHF11;PLSCR1;PNPT1;RSAD2;RSPH9;RTP4;SAMD4A;SIGLEC1;SPATS2L;TDRD7;TOR1B;TRIM22;TRIM69;USP18;XAF1;ZBP1;ZCCHC
I6;IFIH1;IFIT1;IFIT2;IFIT3;IFIT5;IFITM3;IRF7;ISG15;LAMP3;LY6E;MT2A;MX1;OAS1;OAS2;OAS3;OASL;OTOF;PARP12;PHF11;PLSCR1;PNPT1;RSAD2;RSPH9;RTP4;SAMD4A;SIGLEC1;SPATS2L;TDRD7;TOR1B;TRIM22;TRIM69;USP18;XAF1;ZBP1;ZCCHC

;PARP12;PHF11;PLSCR1;RSAD2;RUFY4;TOR1B;TRIM22;TRIM69;XAF1;ZCCHC2

4A

SL;PARP12;PLSCR1;PNPT1;TDRD7;TOR1B;ZBP1;ZCCHC2

SL;PARP12;PLSCR1;PNPT1;TDRD7;TOR1B;ZBP1;ZCCHC2

IFIT3;IFITM3;IRF7;ISG15;LAMP3;LILRB5;LY6E;MT2A;MX1;OAS1;OAS2;OAS3;OASL;OTOF;PLSCR1;PNPT1;RSAD2;RSPH9;RTP4;SIGLEC1;TDRD7;TOR1B;TRIM69;USP18;XAF1;ZBP1;ZCCHC

RP12;PNPT1;RSAD2;TOR1B;TRIM22;TRIM69;USP18;ZBP1

5;LY6E;MT2A;MX1;OAS1;OAS2;OAS3;OASL;PHF11;PLSCR1;PNPT1;RSAD2;SAMD4A;TRIM22;USP18;XAF1;ZBP

G15;LILRB5;LY6E;MT2A;MX1;OAS1;OAS2;OAS3;OASL;OTOF;PHF11;PLSCR1;PNPT1;RSAD2;SAMD4A;TDRD7;TRIM22;USP18;XAF1;ZBP

IM69;XAF1;ZCCHC2

X1;OAS1;OAS2;OAS3;OASL;OTOF;PARP12;PHF11;PLSCR1;PNPT1;RSAD2;RSPH9;SPATS2L;TDRD7;TOR1B;TRIM22;TRIM69;USP18;XAF1;ZBP

:

A7A;SIGLEC8;SLC29A1;SMPD3;SRGAP3;TE(
A7A;SIGLEC8;SLC29A1;SMPD3;SRGAP3;TE(

A7A;SIGLEC8;SLC29A1;SMPD3;SRGAP3;TE(

MA7A;SIGLEC8;SLC29A1;SRGAP3;TEC;THBS4

11;SLAMF7;SMAD7;SPON2;VIT;WNT1
11;SLAMF7;SMAD7;SPON2;VIT;WNT1
G1;LAG3;LGR6;NMUR1;NPC1;PDGFRB;PTCH1;PTGDR;S1PR5;SLAMF6;SLAMF7
R2DL1;KLRD1;LAG3;NCAM1;NKG7;NMUR1;NPC1;PCDH1;PDGFRB;PTCH1;RGS9;SGCD;SMAD7;SYT11;SYTL2
ASLG;FCRL6;FEZ1;FZD4;GPR114;GPR153;GPR56;GPR68;GZMB;KIR2DL1;KLRD1;KLRG1;LAG3;LGR6;LIM2;NCAM1;NKG7;NMUR1;NPC1;PCDH1;PDGFRB;PDZD4;PTCH1;PTGDR;RAB7L1;RGS9;S1PR5;SGCD;SLAMF6;SLAMF7;SLC1A7;SMAD7;SYT11;SYTL2;TIGIT;WNT1;XPNPEP

ASLG;FCRL6;FEZ1;FZD4;GPR114;GPR153;GPR56;GPR68;GZMB;KIR2DL1;KLRD1;KLRG1;LAG3;LGR6;LIM2;NCAM1;NKG7;NMUR1;NPC1;PCDH1;PDGFRB;PTCH1;PTGDR;RAB7L1;RGS9;S1PR5;SGCD;SLAMF6;SLAMF7;SLC1A7;SMAD7;SYT11;SYTL2;TIGIT;WNT1;XPNPEP

RADD;EFNA5;ERBB2;F2R;FASLG;FZD4;GFI1;GPR114;GPR56;GPR68;KIR2DL1;KLRD1;KLRG1;LAG3;MTSS1;NCAM1;NMUR1;PDGFRB;PIK3R3;PPP2R2B;PTCH1;PTGDR;RAB7L1;RASGEF1A;RCAN2;RGS9;SH2D2A;SMAD7;USP28;WNT1;YPEL1;YWHAQ;ZFYVE2
GPR68;KIR2DL1;KLRD1;KLRG1;LAG3;MTSS1;NCAM1;NMUR1;PDGFRB;PIK3R3;PTCH1;PTGDR;RGS9;SMAD7;WNT1;ZFYVE28
R1;NPC1;PDGFRB;PTCH1;PTGDR;S1PR5
DUSP8;EDARADD;EFNA5;ERBB2;F2R;FASLG;FZD4;GFI1;GPR114;GPR56;GPR68;KIR2DL1;KLRD1;KLRG1;LAG3;MTSS1;NCALD;NCAM1;NMUR1;NPC1;PCDH1;PDGFRB;PIK3R3;PPP2R2B;PTCH1;PTGDR;RAB7L1;RASGEF1A;RCAN2;RGS9;SH2D2A;SMAD7;USP28;WNT1;YPEL1;YWHAQ;ZFYVE2
DUSP8;EDARADD;EFNA5;ERBB2;F2R;FASLG;FZD4;GFI1;GPR114;GPR56;GPR68;KIR2DL1;KLRD1;KLRG1;LAG3;MTSS1;NCALD;NCAM1;NMUR1;PCDH1;PDGFRB;PIK3R3;PPP2R2B;PTCH1;PTGDR;RAB7L1;RASGEF1A;RCAN2;RGS9;SH2D2A;SMAD7;USP28;WNT1;YPEL1;YWHAQ;ZFYVE2
DUSP8;EDARADD;EFNA5;ERBB2;F2R;FASLG;FZD4;GFI1;GPR114;GPR56;GPR68;KIR2DL1;KLRD1;KLRG1;LAG3;MTSS1;NCALD;NCAM1;NMUR1;PCDH1;PDGFRB;PIK3R3;PPP2R2B;PTCH1;PTGDR;RAB7L1;RASGEF1A;RCAN2;RGS9;SH2D2A;SMAD7;USP28;WNT1;YPEL1;YWHAQ;ZFYVE2
CX3CR1;CXCR6;DLG5;DTHD1;DUSP8;EDARADD;EFNA5;EOMES;ERBB2;F2R;FASLG;FEZ1;FZD4;GFI1;GPR114;GPR56;GPR68;HOXC4;HOXC5;KIR2DL1;KLRD1;KLRG1;LAG3;LDB2;LLGL2;MTSS1;MYO3B;NCAM1;NEB;NMUR1;NPC1;NUAK1;PAK6;PATL2;PDGFRB;PIK3R3;PODN;PPP2R2B;PTCH1;PTGDR;RAB7L1;RASG

R1;NPC1;PDGFRB;PTCH1;PTGDR;S1PR5
;TIGIT

;SH2D1B;SLAMF7;SPON2

R1;NPC1;PDGFRB;PTCH1;PTGDR;RGS9;S1PR5;SMAD7
R1;NPC1;PDGFRB;PTCH1;PTGDR;RGS9;S1PR5;SMAD7

CX3CR1;CXCR6;DLG5;DTHD1;DUSP8;EDARADD;EFNA5;EOMES;ERBB2;F2R;FASLG;FEZ1;FZD4;GFI1;GPR114;GPR56;GPR68;HOXC4;HOXC5;KIR2DL1;KLRD1;KLRG1;LAG3;LDB2;LLGL2;MTSS1;MYO3B;NCAM1;NEB;NMUR1;NPC1;NUAK1;PAK6;PATL2;PDGFRB;PIK3R3;PODN;PPP2R2B;PTCH1;PTGDR;RAB7L1;RASG

IF7;SPON2;TBX21

G3;NCAM1;NPC1;NUAK1;PDGFRB;PTCH1;RASGEF1A;RGS9;SH2D1B;SMAD7;TBX21;WNT1;YPEL1;ZFYVE28

3;CHST10;CLIC3;CX3CR1;CXCR6;DLG5;DTHD1;DUSP8;EDARADD;EFNA5;EOMES;EPDR1;ERBB2;F2R;FASLG;FEZ1;FZD4;GFI1;GPR114;GPR56;GPR68;GZMB;GZMH;KIF19;KIR2DL1;KLRD1;KLRG1;LAG3;LDB2;LIM2;LLGL2;MTSS1;MYO3B;NCALD;NCAM1;NEB;NMUR1;NPC1;NUAK1;PAK6;PATL2;PCDH1;PDGFRB;PIK

RADD;EFNA5;ERBB2;F2R;FASLG;FEZ1;FZD4;GFI1;GPR114;GPR56;GPR68;KIR2DL1;KLRD1;KLRG1;LAG3;MTSS1;NCAM1;NMUR1;NPC1;NUAK1;PDGFRB;PIK3R3;PPP2R2B;PTCH1;PTGDR;RAB7L1;RASGEF1A;RCAN2;RGS9;SH2D2A;SMAD7;USP28;WNT1;YPEL1;YWHAQ;ZFYVE2

HOXC4;HOXC5;LDB2;LIM2;MSC;MTSS1;NCAM1;NEB;PCDH1;PDGFRB;PPP2R2B;PTCH1;RCAN2;RGS9;S1PR5;SGCD;SH2D2A;SMAD7;SPON2;TBX21;WNT1;YPEL

EPB41L4A;EPHX4;ERBB2;F2R;FASLG;FCRL6;FZD4;GPR114;GPR153;GPR56;GPR68;GZMB;ITPRIPL1;JAKMIP1;KIR2DL1;KLRD1;KLRG1;LAG3;LGR6;LIM2;MANEAL;NCALD;NCAM1;NKG7;NMUR1;NPC1;PCDH1;PDGFRB;PTCH1;PTGDR;RGS9;S1PR5;SCD5;SGCD;SLAMF6;SLAMF7;SLC1A7;SMAD7;SYT11;SYTL2;TIGIT;

H1;PDGFRB;SYT11

3;NCAM1;PIK3R3;SH2D1B;SLAMF7;SPON2;TBX21;TIGIT;WNT1

L1;RASGEF1A;RCAN2;RGS9;SMAD7;USP28;WNT1;YPEL1;YWHAC

XCR6;DLG5;DTHD1;DUSP8;EDARADD;EFNA5;EOMES;ERBB2;F2R;FASLG;FEZ1;FZD4;GFI1;GNLY;GPR114;GPR56;GPR68;KIR2DL1;KLRD1;KLRG1;LAG3;MTSS1;MYO3B;NCAM1;NMUR1;NPC1;NUAK1;PDGFRB;PIK3R3;PPP2R2B;PTCH1;PTGDR;RAB7L1;RASGEF1A;RCAN2;RGS9;SH2D1B;SH2D2A;SLAMF7;SMAD7;S

CX3CR1;CXCR6;DLG5;DTHD1;DUSP8;EDARADD;EFNA5;EOMES;ERBB2;F2R;FASLG;FEZ1;FZD4;GFI1;GNLY;GPR114;GPR56;GPR68;HOXC4;HOXC5;KIR2DL1;KLRD1;KLRG1;LAG3;LDB2;LLGL2;MTSS1;MYO3B;NCAM1;NEB;NMUR1;NPC1;NUAK1;PAK6;PATL2;PDGFRB;PIK3R3;PODN;PPP2R2B;PTCH1;PTGDR;RAB7L1

N2;TBX21

'NT1
G1;LAG3;MTSS1;NCAM1;NPC1;NUAK1;PDGFRB;PIK3R3;PPP2R2B;PTGDR;SH2D1B;SLAMF7;SMAD7;SPON2;USP28;WNT1

;CHST10;CLIC3;CX3CR1;CXCR6;DLG5;DTHD1;DUSP8;EDARADD;EFNA5;EOMES;EPDR1;ERBB2;F2R;FASLG;FEZ1;FZD4;GFI1;GPR114;GPR56;GPR68;GZMB;GZMH;HOXC4;HOXC5;KIF19;KIR2DL1;KLRD1;KLRG1;LAG3;LDB2;LIM2;LLGL2;MSC;MTSS1;MYO3B;NCALD;NCAM1;NEB;NMUR1;NPC1;NUAK1;PAK6;PATL2
EPHX4;ERBB2;F2R;FASLG;FCRL6;FZD4;GPR114;GPR153;GPR56;GPR68;ITPRIPL1;KIR2DL1;KLRD1;KLRG1;LAG3;LGR6;LIM2;MANEAL;NCAM1;NKG7;NMUR1;NPC1;PCDH1;PDGFRB;PTCH1;PTGDR;S1PR5;SCD5;SGCD;SLAMF6;SLAMF7;SLC1A7;SYT11;TIGIT;TM4SF19;VANGL1;XPNPEP
2;CLIC3;CX3CR1;CXCR6;DLG5;EFNA5;EPB41L4A;EPHX4;ERBB2;F2R;FASLG;FCRL6;FEZ1;FZD4;GPR114;GPR153;GPR56;GPR68;GZMB;ITPRIPL1;JAKMIP1;KIR2DL1;KLRD1;KLRG1;LAG3;LGR6;LIM2;MANEAL;NCALD;NCAM1;NKG7;NMUR1;NPC1;PCDH1;PDGFRB;PPP2R2B;PTCH1;PTGDR;RAB11FIP5;RAB7L1;RGS9;

HOXC4;HOXC5;LDB2;LIM2;MSC;MTSS1;NCAM1;NEB;PCDH1;PDGFRB;PPP2R2B;PTCH1;PTGDR;RCAN2;RGS9;S1PR5;SGCD;SH2D2A;SMAD7;SPON2;TBX21;VANGL1;WNT1;YPEL
'11

HOXC4;HOXC5;LDB2;LIM2;MSC;MTSS1;NCAM1;NEB;PCDH1;PDGFRB;PPP2R2B;PTCH1;RCAN2;RGS9;S1PR5;SGCD;SH2D2A;SMAD7;SOX13;SPON2;TBX21;WNT1;YPEL

HOXC4;HOXC5;LDB2;LIM2;MSC;MTSS1;NCAM1;NEB;NUAK1;PDGFRB;PPP2R2B;PTCH1;S1PR5;SGCD;SH2D2A;SMAD7;SPON2;TBX21;WNT

';SYTL2;ZFYVE28

28

28

';MSC;MTSS1;NEB;PDGFRB;PPP2R2B;PTCH1;SGCD;SMAD7;TBX21;WNT

F2R;FASLG;FCRL6;FZD4;GPR114;GPR153;GPR56;GPR68;ITPRIPL1;KIR2DL1;KLRD1;KLRG1;LAG3;LGR6;LIM2;MANEAL;NCAM1;NKG7;NMUR1;NPC1;PCDH1;PDGFRB;PTCH1;PTGDR;S1PR5;SCD5;SGCD;SLAMF6;SLAMF7;SLC1A7;SYT11;TIGIT;TM4SF19;VANGL
;SMAD7;SPON2

HOXC4;HOXC5;LDB2;LIM2;MSC;MTSS1;NCAM1;NEB;NUAK1;PCDH1;PDGFRB;PPP2R2B;PTCH1;PTGDR;RCAN2;RGS9;S1PR5;SGCD;SH2D2A;SMAD7;SOX13;SPON2;TBX21;VANGL1;WNT1;YPEL

D2L1;RHOB;SNCG;TSPO;TSPYL5;USP47;YAP1
WRN

FOS;HMGA2;HUS1;HYAL3;ITGB1;PIK3R1;PLK3;RGS14;RHOB;RNF168;TIPIN;TRIM32;TSPYL5;UNC119;USF1;USP47;WRN;YAP

3;PIK3R1;RGS14;TIPIN;TRIM32;UNC119;USF1;USP47;WRN

ERCC6;FANCC;FANCD2;FOXO1;GADD45G;GTF2H3;HMGA2;HUS1;HUS1B;IER3;MAPK3;MASTL;MCM8;MORF4L1;MRE11A;OTUB1;PARG;PARP2;PGAP2;PLK3;POLR2D;PSMB10;PSMB8;PSMB9;PSMD5;PSME2;PTPN11;PYCARD;RAD50;RAD52;RNF168;SFN;SHPRH;SIRT6;SMC1B;SMC3;TIPIN;USP10;USP47;WRN;

ST1H2AD;HIST1H2BK;HIST1H2BO;HIST2H2AB;HMGA2;HUS1;HUS1B;KIFAP3;LRPPRC;MORF4L1;MPHOSPH10;MRE11A;NPM2;NR1H3;NUFIP1;NUP107;NUP160;ORC5;PBRM1;PCGF2;PDS5A;PPP2CA;PRDM7;PRIM2;PTGES3;RAD50;RARA;RBMX;RNF168;SEPT2;SGOL1;SHPRH;SIRT6;SIRT7;SMC1B;SMC3;SPI1;TE

HIST1H2BO;HIST2H2AB;HMGA2;HUS1;HUS1B;MORF4L1;MRE11A;NPM2;NR1H3;NUFIP1;NUP107;NUP160;ORC5;PBRM1;PCGF2;PDS5A;PPP2CA;PRIM2;PTGES3;RAD50;RARA;RBMX;RNF168;SEPT2;SGOL1;SHPRH;SIRT6;SIRT7;SMC1B;SMC3;SPI1;TERF1;TIPIN;TNKS;TTC37;UHRF

;HOMER3;HTR3B;KCNB1;MAGEE1;NRXN1;PDE2A;RGS14;SRCIN1;ZACN

GADD45G;GCLC;GPD1L;GRB7;HEXIM2;HHATL;INPP5J;NR1H3;OTUB1;PDCL;PPP2CA;PRNP;PSMB10;PSMB8;PSMB9;PSMD5;PSME2;PYCARD;RARA;RGS14;RGS2;RNF168;SET;SFN;SMAD6;SPI1;SRCIN1;SYNCRIP;THBS1;UBE2

NAJA2;DRD3;DUSP1;ELANE;EPO;ERCC4;ERCC6;FADD;FANCD2;FKBPL;FOS;FOXO1;GCLC;GPLD1;HMGA2;HPN;HUS1;HYAL3;IGFBP2;INSRR;ITGB1;ITPR1;LTBR;MAP7;MAPK3;MMP2;MSTN;PDE2A;PDIA2;PIK3R1;PKD2L1;PLK3;PRKCE;RGS14;RHOB;RNF168;SDC2;SNCG;ST8SIA1;THBS1;TIPIN;TRIM32;TSC22D3;TSI

GPD1L;HPN;HTR3B;NRXN1;PDE2A;PKD2L1;PLP2;SCN1B;SLC30A4;SLC39A1;SLC39A6;STIM2;TCIRG1;THBS1;WNK2;WNK3

N;ITGB1;KIAA1462;KRIT1;MTDH;OBSL1;PANX2;PDZD2;RAB13;RPGRIP1L;SCN1B;SDCCAG8;SHROOM2;WNK3

37;HEXIM2;HHATL;INPP5J;NR1H3;OTUB1;PDCL;PPP2CA;PRNP;PSMB10;PSMB8;PSMB9;PSMD5;PSME2;PYCARD;RARA;RGS14;RGS2;RNF168;SET;SFN;SMAD6;SPI1;SRCIN1;SYNCRIP;UBE2

C5;PBRM1;PCGF2;RAD50;RARA;RBMX;SGOL1;SIRT6;SIRT7;SMC1B;SMC3;SPI1;TERF1;TIPIN;TNKS;UHRF2

N3;DNAJC2;DPEP3;DRD3;EIF4EBP1;EML4;ERCC4;FANCD2;FBXO43;HAUS2;HMGA2;HSP90AA1;HUS1;ITGB1;MASTL;MCM8;MPHOSPH9;MRE11A;NEK4;NPAT;NPM2;NUP107;NUP160;ORC5;PBRM1;PDS5A;PIWIL2;PLK3;PPP2CA;PRIM2;PSMB10;PSMB8;PSMB9;PSMD5;PSME2;PSRC1;RAD50;RAD52;RANBP2;R

BMX;SGOL1;SIRT6;SIRT7;SMC1B;SMC3;SPI1;TERF1;TIPIN;TNKS;UHRF2

R3B;KCNB1;MAGEE1;RGS14;SRCIN1;ZACN

KN2D;CENPC1;CENPJ;CEP192;CEP76;CLASP2;CLOCK;CSPP1;CYP1A1;DCTN3;DNAJC2;DPEP3;DRD3;DUSP1;EIF4EBP1;EML4;ERCC4;FANCD2;FBXO43;GADD45G;GSPT2;HAUS2;HDAC2;HES1;HEXIM2;HMGA2;HSP90AA1;HUS1;HUS1B;ITGB1;LIN37;MAPK3;MASTL;MCM8;MPHOSPH9;MRE11A;NANOS3;NEK4;N

PF3B;XPOT

PF3B;XPOT

PF3B;XPOT

EP76;CLASP2;CLOCK;CSPP1;CYP1A1;DCTN3;DNAJC2;DPEP3;DRD3;EIF4EBP1;EML4;ERCC4;FANCD2;FBXO43;HAUS2;HMGA2;HSP90AA1;HUS1;HUS1B;ITGB1;MASTL;MCM8;MPHOSPH9;MRE11A;NEK4;NPAT;NPM2;NUP107;NUP160;ORC5;PBRM1;PDS5A;PIWIL2;PLK3;PPP2CA;PRIM2;PRKCE;PRNP;PSMB10;P

PPP2CA;PRNP;PSMB10;PSMB8;PSMB9;PSMD5;PSME2;PYCARD;RGS14;RGS2;SET;SFN;SMAD6;SPI1;SRCIN1;UBE2

1A1;DCTN3;DNAJC2;DRD3;EIF4EBP1;EML4;HAUS2;HES1;HMGA2;HSP90AA1;HUS1;ITGB1;LIN37;MASTL;MCM8;MPHOSPH9;NEK4;NPAT;NPM2;NUP107;NUP160;ORC5;PBRM1;PDS5A;PLK3;PRIM2;PSMB10;PSMB8;PSMB9;PSMD5;PSME2;PSRC1;RANBP2;RBL1;RGS14;SDCCAG8;SEPT2;SGOL1;SIRT7;SMAD6;

ASF1B;ATL1;ATP2A1;ATP2B2;ATP6V0A2;ATP6V0B;ATP6V1G2;ATPAF1;ATXN10;ATXN3;AURKAIP1;AZU1;B9D2;BAG5;BAK1;BANP;BAX;BCL3;BIN3;BMPR1B;CACNB2;CAMK2B;CAND1;CASP7;CBX8;CD151;CD3G;CDC23;CDH5;CDK5;CDKN2D;CENPC1;CENPJ;CEP192;CEP68;CEP76;CHRNA3;CIB2;CLASP2;CLIC5;CI

3

PF3B;XPOT

2;FBXO43;HAUS2;HMGA2;MASTL;MPHOSPH9;MRE11A;NEK4;NPM2;NUP107;NUP160;PBRM1;PDS5A;PIWIL2;PPP2CA;PSRC1;RAD50;RAD52;RANBP2;RGS14;RSPH1;SEPT2;SGOL1;SIRT7;SMC1B;SMC3;SPICE1;TERF1;TIPIN;TNKS;UBD;UBE2C;UBE2

CK;CSPP1;CYP1A1;DRD3;EIF4EBP1;FBXO43;GADD45G;HDAC2;HES1;HEXIM2;HMGA2;HUS1;HUS1B;ITGB1;MASTL;MCM8;MRE11A;NANOS3;NPM2;ORC5;PLK3;PRKCE;PRNP;PSMB10;PSMB8;PSMB9;PSMD5;PSME2;PSRC1;PTPN11;RARA;RBL1;RHOB;SERTAD1;SFN;SMAD6;SMC3;SPHK1;SPICE1;TERF1;THBS1;

;GRM4;HOMER3;HTR3B;KCNB1;MAGEE1;NRXN1;NUFIP1;PDE2A;RABAC1;RGS14;SEPT5;SRCIN1;SYPL1;SYPL2;SYT6;SYT9;ZACN

;MORF4L1;NPM2;NR1H3;NUFIP1;PCGF2;PDS5A;RARA;RBMX;SHPRH;SIRT6;SIRT7;SMC3;SPI1;TIPIN;TTC37;UHRF2

.6;STIM2;TRPM4
ADNP2;ADRA1A;AFAP1L2;AGPAT2;AGTRAP;AIPL1;AMOT;AMOTL2;AMPD2;AMPH;ANK2;ANKRD2;ANKRD42;ANO2;ANXA2P2;AP1M2;AP1S3;AP2S1;AP4E1;AP4M1;APLP1;AQP8;AR;ARF5;ARHGAP9;ARHGEF40;ARL2BP;ARL8A;ARMCX3;ARPC1B;ARRB2;ARSJ;ART1;AS3MT;ASCC3;ASF1B;ASL;ASPHD1;ASS1;ATA
CASR;CDK5;CP;DRD3;EFCAB4A;EPO;FTH1;GJA4;GPD1L;HPN;ITPR1;KCNA3;KCNH3;KCNJ12;PDE2A;PKD2L1;PRKCE;SCN1B;SLC24A2;SLC30A4;SLC31A2;SLC39A1;SLC39A6;SLC4A8;SLC9B2;STIM2;TCIRG1;TCN1;TSPO;WNK2;WNK3
FAT1;FZD9;GABARAP;GNB2;HHATL;INSL3;KPTN;LRPPRC;MAGEE1;MAP6;MAP7;MT1G;MTDH;OBSL1;PDE2A;PRKCE;RAB40C;SEPT2;SERBP1;SET;SNCG;SRFBP1;SYT6;TPD52;TRIM3

ASF1B;ATL1;ATP2A1;ATP2B2;ATP6V0A2;ATP6V0B;ATP6V1G2;ATPAF1;ATXN10;ATXN3;AURKAIP1;AZU1;B9D2;BAG5;BAK1;BANP;BAX;BCL3;BIN3;BMPR1B;CACNB2;CAMK2B;CAND1;CASP7;CBX8;CD151;CD3G;CDC23;CDH5;CDK5;CDKN2D;CENPC1;CENPJ;CEP192;CEP68;CEP76;CHRNA3;CIB2;CLASP2;CLIC5;CI

HUS1;HUS1B;MCM8;MRE11A;NPM2;ORC5;PLK3;PRKCE;PSMB10;PSMB8;PSMB9;PSMD5;PSME2;PSRC1;PTPN11;SMAD6;SMC3;SPICE1;TERF1;TIPIN;UBD;UBE2C;UHRF2;USP47

3;LUM;MFAP2;SMC3;TNC

K4;NPM2;NUP107;NUP160;PBRM1;PDS5A;PSRC1;RANBP2;RGS14;SEPT2;SGOL1;SIRT7;SMC3;SPICE1;TERF1;TIPIN;TNKS;UBD;UBE2C;UBE2S

HK1;ST8SIA1

2;AGPAT2;AGTRAP;AIPL1;AMOT;AMOTL2;AMPH;ANK2;ANKRD2;ANKRD42;ANO2;ANXA2P2;AP1M2;AP1S3;AP2S1;AP4E1;AP4M1;APLP1;AQP8;AR;ARF5;ARL2BP;ARL8A;ARMCX3;ARPC1B;ARRB2;ARSJ;ART1;AS3MT;ASCC3;ASF1B;ASPHD1;ATAD5;ATE1;ATL1;ATMIN;ATP2A1;ATP2B2;ATP6V0A2;ATP6V0B;ATP

MR11;MTMR8;PDE2A;PDE4C;PDE8A;PDP2;PPAP2A;PPM1M;PPP2CA;PTP4A2;PTP4A3;PTPN11;PTPN14;PTPN21;SMPDL3B;SSH3

PF3B;XPOT

2;AGPAT2;AGTRAP;AIPL1;AMOT;AMOTL2;AMPH;ANK2;ANKRD2;ANKRD42;ANO2;ANXA2P2;AP1M2;AP1S3;AP2S1;AP4E1;AP4M1;APLP1;AQP8;AR;ARF5;ARL2BP;ARL8A;ARMCX3;ARPC1B;ARRB2;ARSJ;ART1;AS3MT;ASCC3;ASF1B;ASPHD1;ATAD5;ATE1;ATL1;ATMIN;ATP2A1;ATP2B2;ATP6V0A2;ATP6V0B;ATP
3;HPN;HTR3B;ITPR1;KCNA3;KCNB1;KCNH3;KCNJ12;KCNQ5;PANX2;PDE2A;PKD2L1;SCN1B;SHROOM2;SLC24A2;STIM2;TRPM4;ZACN
3;HPN;HTR3B;ITPR1;KCNA3;KCNB1;KCNH3;KCNJ12;KCNQ5;PANX2;PDE2A;PKD2L1;SCN1B;SHROOM2;SLC24A2;STIM2;TRPM4;ZACN

HBS1;WNT11

P90AA1;HUS1;ITGB1;MASTL;MCM8;NPAT;ORC5;PLK3;PRIM2;PSMB10;PSMB8;PSMB9;PSMD5;PSME2;RBL1;SDCCAG8;SMAD6;TERF1;TIPIN;TUBA1A;TUBA4A;USP47

TAD5;ATE1;ATMIN;ATR;ATXN3;AURKAIP1;B9D2;BANP;BARD1;BAX;BCL3;BEX4;C10orf137;C19orf40;CABP1;CAMK2B;CAMKK1;CAND1;CASK;CASP7;CBX8;CCDC19;CCNJ;CDC14B;CDC23;CDK5;CDK6;CDKN2D;CDYL;CENPC1;CERS4;CERS6;CITED4;CLOCK;CMAS;CPE;CSE1L;CTNNBIP1;CTNND2;CWC27;DCAF17;D

;CENPC1;CENPJ;CEP192;CHRNA3;CTNNBIP1;DARS;DDX1;DGAT1;DISP1;DNER;DNM1L;DYNC2H1;EIF4EBP1;FADD;FANCC;FHOD3;FUZ;GJA4;GJC1;GNPAT;GPM6B;GPX3;GRB7;HAS3;HAUS2;HES1;HIST1H1C;HIST1H2AD;HIST1H2BK;HIST1H2BO;HIST2H2AB;HMGA2;HSP90AA1;IHH;INPP5J;INTU;ITGB1;KCNA3;K

;ADCK4;ADNP2;ADRA1A;AFAP1L2;AGPAT2;AGTRAP;AIPL1;AMOT;AMOTL2;AMPD2;AMPH;ANK2;ANKRD2;ANKRD42;ANO2;ANXA2P2;AP1M2;AP1S3;AP2S1;AP4E1;AP4M1;APLP1;AQP8;AR;ARF5;ARHGAP9;ARHGEF40;ARL2BP;ARL8A;ARMCX3;ARPC1B;ARRB2;ARSJ;ART1;AS3MT;ASCC3;ASF1B;ASL;ASPHD1;A

4A;USP47
3;SLC30A4;SPI1;TERF1;TNKS;TSPO;UBE2C;UBE2S;WNK2;WNK3

T2;AGTRAP;AIPL1;AMOT;AMOTL2;AMPH;ANKRD2;ANKRD42;ANO2;ANXA2P2;AP1M2;AP1S3;AP2S1;AP4E1;AP4M1;APLP1;AQP8;AR;ARF5;ARL2BP;ARL8A;ARMCX3;ARRB2;ARSJ;ART1;AS3MT;ASCC3;ASF1B;ASPHD1;ATAD5;ATE1;ATL1;ATMIN;ATP2A1;ATP2B2;ATP6V0A2;ATP6V0B;ATP6V1G2;ATPAF1;ATR;A

DKN2D;COL18A1;CTLA4;DDAH2;DEDD2;DNM1L;DUSP1;EDNRB;EI24;EPO;ERCC6;F3;FADD;FOXO1;GCLC;GPLD1;GRM4;HDAC2;HMGA2;HPN;IER3;INSL3;ITGB1;ITPR1;KRIT1;LTBR;MEIS3;MET;MTCH1;MTDH;NANOS3;NFKBID;NOL3;NOS1AP;OGT;PCGF2;PGAP2;PIK3R1;PLK3;POR;PPP2CA;PRKCE;PRNP;PSMB10
T2;AGTRAP;AIPL1;AMOT;AMOTL2;AMPH;ANKRD2;ANKRD42;ANO2;ANXA2P2;AP1M2;AP1S3;AP2S1;AP4E1;AP4M1;APLP1;AQP8;AR;ARF5;ARL2BP;ARL8A;ARMCX3;ARRB2;ARSJ;ART1;AS3MT;ASCC3;ASF1B;ASPHD1;ATAD5;ATE1;ATL1;ATMIN;ATP2A1;ATP2B2;ATP6V0A2;ATP6V0B;ATP6V1G2;ATPAF1;ATR;A
P90AA1;HUS1;ITGB1;MASTL;MCM8;NPAT;ORC5;PLK3;PRIM2;PSMB10;PSMB8;PSMB9;PSMD5;PSME2;RBL1;SDCCAG8;SMAD6;TERF1;TIPIN;TUBA1A;TUBA4A;USP47

DKN2D;COL18A1;CTLA4;DDAH2;DEDD2;DNM1L;DUSP1;EDNRB;EI24;EPO;ERCC6;F3;FADD;FOXO1;GCLC;GPLD1;GRM4;HDAC2;HMGA2;HPN;IER3;INSL3;ITGB1;ITPR1;KRIT1;LTBR;MEIS3;MTCH1;MTDH;NANOS3;NFKBID;NOL3;NOS1AP;OGT;PCGF2;PGAP2;PIK3R1;PLK3;POR;PPP2CA;PRKCE;PRNP;PSMB10;PSM
CDK5;CDKN2D;COL18A1;CTLA4;DDAH2;DEDD2;DNM1L;DUSP1;EDNRB;EI24;EPO;ERCC6;F3;FADD;FOXO1;GCLC;GPLD1;GRM4;HDAC2;HMGA2;HPN;IER3;INSL3;ITGB1;ITPR1;KRIT1;LTBR;MEIS3;MET;MTCH1;MTDH;NANOS3;NFKBID;NOL3;NOS1AP;OGT;PCGF2;PGAP2;PIK3R1;PLK3;POR;PPP2CA;PRKCE;PRNP;
H3;HMGA2;HUS1;HUS1B;MCM8;MORF4L1;MRE11A;OTUB1;PARP2;POLR2D;RAD50;RAD52;RNF168;SHPRH;SIRT6;SMC1B;SMC3;USP10;USP47;WRN

IK3;HPN;KCNB1;NOV;PLK3;RARA;SDC2;SHROOM2;SNCG;SRCIN1;SRR

MR8;MVP;NUP107;NUP160;NUP88;OIT3;RANBP2;ROGDI;SLC16A3;SUN3;TMEM97;TNKS;XPOT

PRHL1;ADRA1A;AFAP1L2;AGAP7;AGPAT2;AIPL1;AMOT;AMOTL2;AMPD2;AMPH;ANGPTL1;ANK2;ANKRD42;AOC3;AP1M2;AP1S3;AP2S1;AP4E1;AP4M1;APLP1;APOBEC2;AQP8;AR;ARF5;ARHGAP9;ARHGEF40;ARL2BP;ARL8A;ARMCX3;ARPC1B;ARRB2;ARSJ;ART1;ARTN;AS3MT;ASB12;ASB14;ASCC3;ASF1B;ASL;

L1;SMC1B;SMC3
E;RGS14;SLC24A2;SNCG
2;SGOL1;SMC3;SNCG;SPICE1;SPIN1;TERF1;TNKS
68;SYNCRIP;THBS1;TRMT6;UPF3B

D;ERRFI1;GADD45G;GLIS2;HDAC2;HEXIM2;HMGA2;KIAA1244;MAPK3;MASTL;MPHOSPH10;NFIB;NFKBID;PEX19;POR;PPP2CA;PRNP;PSMB10;PSMB8;PSMB9;PSMD5;PSME2;PYCARD;RECK;RGS14;RGS2;SFN;SMAD6;SPOCK3;SRCIN1;TERF1;THBS1;TNKS;TRIM37;UBE2C;USP47;WFDC2;ZC3H12A;ZFP9

3;CDK5;CNIH2;CP;CPT1B;DRD3;EPO;FTH1;GPD1L;HPN;HSP90AA1;HTR3B;MFSD6;NRXN1;NUP107;NUP160;NUP88;PDE2A;PEX19;PKD2L1;PLP2;RANBP2;SCN1B;SLC15A3;SLC16A3;SLC24A2;SLC30A4;SLC38A1;SLC39A1;SLC39A4;SLC39A6;SLC44A4;SLC4A8;SLC7A10;SLCO2B1;SPNS2;STIM2;TCIRG1;THBS1;WNK

;TSPYL1;TSPYL5

2;AGTRAP;AMOT;AMOTL2;AMPD2;AMPH;ANK2;ANKRD2;ANXA2P2;AP1M2;AP1S3;AP2S1;AP4E1;AP4M1;APLP1;ARF5;ARHGAP9;ARL2BP;ARL8A;ARMCX3;ARRB2;ARSJ;ART1;AS3MT;ASL;ASPHD1;ASS1;ATL1;ATP2A1;ATP2B2;ATP6V0A2;ATP6V0B;ATP6V1G2;ATPAF1;ATXN10;ATXN3;AURKAIP1;AZU1;B3GNT8

H1;NFKBID;OGT;PIK3R1;PPP2CA;PRKCE;PYCARD;RHOB;SAV1;SHQ1;SPI1;TERF1;THBS1;TNFRSF12A;TNFSF8;TSPO;UBD;UNC5C;WNT11;ZC3H8

.;UBE2C;UHRF2
.3;BMPR1B;C10orf137;CAND1;CASK;CBX8;CDK5;CDYL;CITED4;CLOCK;CTNNBIP1;CTNND2;DDX1;DEDD2;DNAJC2;DPPA4;DRD3;EDNRB;EIF1B;EIF2S1;EIF4EBP1;EIF4EBP3;ELF3;ELP2;ENPP7;EPO;ERCC6;ESRRA;ESRRB;FADD;FKTN;FOS;FOXD4L1;FOXO1;FRYL;FSTL3;FUBP3;GCLC;GLIS2;GRB7;GTF2H3;HAS3;HDAC

NP;BCL3;BMPR1B;C10orf137;CAND1;CARD9;CASK;CBX8;CDK5;CDYL;CITED4;CLOCK;CTNNBIP1;CTNND2;DDX1;DEDD2;DNAJC2;DPPA4;DRD3;EDNRB;EIF1B;EIF2S1;EIF4EBP1;EIF4EBP3;ELANE;ELF3;ELP2;ENPP7;EPO;ERCC6;ESRRA;ESRRB;FADD;FKTN;FOS;FOXD4L1;FOXO1;FRYL;FSTL3;FUBP3;GCLC;GLIS2;GRB7;
AT1;STAT2;TRAFD1;TRIM5;UBE2L6
NG1;SP100;STAT1;STAT2;TAP1;TRAFD1;TRIM5;UBE2L6

AT1;STAT2;TAP1;TRAFD1;TRIM5;UBE2L6

;RNASEL;SERPING1;SP100;STAT1;STAT2;TAP1;TRAFD1;TRIM5;UBE2L6

TAT1;STAT2;TAP1;TRIM5
100;STAT1;STAT2;TGIF1;TRAFD1;TRIM5;UBE2L6

321D1;PARP9;PML;RNASEL;SERPING1;SP100;STAT1;STAT2;TAP1;TRAFD1;TRIM5;UBE2L6

E2L6

;HCAR1;ICAM1;IFI16;IFI35;IRF1;IRF9;MB21D1;NRARP;PARP9;PML;RAB20;RNASEL;SEPT4;SERPING1;SHISA5;SORT1;SP100;SP110;STAT1;STAT2;TAP1;TGIF1;TRAFD1;TRIM38;TRIM5;UBE2L

E2L6

100;STAT1;STAT2;TGIF1;TRAFD1;TRIM5;UBE2L6

STAT2;TGIF1;TRIM5;UBE2L6

TRAFD1;TRIM38;TRIM5

21D1;NRARP;PML;RAB20;RNASEL;SEPT4;SHISA5;SORT1;SP100;SP110;STAT1;STAT2;TGIF1;TRIM38;TRIM5;UBE2L

PT4;SHISA5;SORT1;SP100;SP110;STAT1;STAT2;TGIF1;TRIM38;TRIM5;UBE2L

100;STAT1;TGIF1;TRAFD1;TRIM21;UBE2L6;WAR

GBP2;GBP4;GBP5;IFI16;IFI35;IRF1;IRF9;LACTB;LAP3;LRRC18;MB21D1;MEX3B;NUDT11;PARP14;PARP9;PML;PSTPIP2;RAB20;RMI2;RNASEL;SDC3;SEPT4;SERPING1;SHISA5;SMTNL1;SORT1;SP100;STAT1;STAT2;TAP1;TRIM21;TRIM5;UBE2L6;WAR

;ICAM1;IFI16;IFI35;IRF1;IRF9;MB21D1;NRARP;PARP14;PARP9;PML;RAB20;RNASEL;SEPT4;SERPING1;SHISA5;SMTNL1;SORT1;SP100;SP110;STAT1;STAT2;TAP1;TGIF1;TRAFD1;TRIM21;TRIM38;TRIM5;UBE2L6;WARS;ZNF88

[illegible]FI35;IRF1;IRF9;MB21D1;NRARP;PARP14;PARP9;PML;RAB20;RNASEL;SEPT4;SERPING1;SHISA5;SMTNL1;SORT1;SP100;SP110;STAT1;STAT2;TAP1;TGIF1;TRAFD1;TRIM21;TRIM38;TRIM5;UBE2L6;WARS;ZNF88

[illegible];IFI16;IRF1;IRF9;LAP3;MEX3B;NRARP;PARP14;PML;RMI2;RNASEL;SDC3;SEPT4;SERPING1;SORT1;SP100;SP110;STAT1;STAT2;TGIF1;TRIM21;TRIM5;UBE2L6;WARS;ZNF88

[illegible]L;SDC3;SEPT4;SERPING1;TRIM21;TRIM5;UBE2L6;WAR[illegible]

PT4;SHISA5;SORT1;SP100;SP110;STAT1;STAT2;TGIF1;TRIM38;TRIM5;UBE2L
PT4;SHISA5;SORT1;SP100;SP110;STAT1;STAT2;TGIF1;TRIM38;TRIM5;UBE2L

.D1;MEX3B;PARP9;PML;PSTPIP2;RAB20;RNASEL;SDC3;SEPT4;SERPING1;SHISA5;SMTNL1;SORT1;STAT1;STAT2;TAP1;TRIM21;TRIM5;UBE2L6;WAR

F1;TRIM21;TRIM5;ZNF88

1;ACYP2;ADAL;ADAP2;ADAR;ADCY4;ADH5;ADK;ADO;ADSL;AFAP1;AFF2;AGA;AGBL2;AGPAT1;AHI1;AKAP13;AKR1C3;ALAS1;ALB;ALDH1A1;ALDH2;ALDH3B1;ALDH9A1;ALDOC;ALG14;ALG5;ALKBH2;ALKBH3;ALOX15B;ALPK3;AMIGO2;AMPD3;ANAPC16;ANAPC4;ANKFY1;ANKRA2;ANKRD37;ANKS1A;ANO7;AN
1;ACYP2;ADAL;ADAP2;ADAR;ADCY4;ADH5;ADK;ADO;ADSL;AFAP1;AFF2;AGA;AGBL2;AGPAT1;AHI1;AKAP13;AKR1C3;ALAS1;ALB;ALDH1A1;ALDH2;ALDH3B1;ALDH9A1;ALDOC;ALG14;ALG5;ALKBH2;ALKBH3;ALOX15B;ALPK3;AMIGO2;AMPD3;ANAPC16;ANAPC4;ANKFY1;ANKRA2;ANKRD37;ANKS1A;ANO10;AN
2;ADAL;ADAP2;ADAR;ADCY4;ADH5;ADK;ADO;ADSL;AFAP1;AGA;AGBL2;AGPAT1;AHI1;AKAP13;AKR1C3;ALAS1;ALB;ALDH1A1;ALDH2;ALDH3B1;ALDH9A1;ALDOC;ALG14;ALG5;ALKBH3;ALOX15B;AMPD3;ANAPC16;ANAPC4;ANKFY1;ANKRA2;ANKRD37;ANKS1A;ANO7;ANP32A;ANPEP;ANXA11;ANXA4;ANXA5
1;ACYP2;ADAP2;ADAR;ADH5;ADK;ADO;ADSL;AFF2;AGA;AGPAT1;AHI1;AKAP13;AKR1C3;ALAS1;ALB;ALDH2;ALDH9A1;ALDOC;ALG14;ALG5;ALKBH2;ALKBH3;ALPK3;AMIGO2;ANAPC16;ANAPC4;ANKFY1;ANKRA2;ANKRD37;ANKS1A;ANO7;ANP32A;ANPEP;ANXA11;ANXA5;ANXA7;AP1M1;AP2A1;AP2M1;AP3B1
2;ADAR;ADH5;ADK;ADO;ADSL;AFF2;AGA;AGPAT1;AHI1;AKAP13;AKR1C3;ALAS1;ALB;ALDH2;ALDH9A1;ALDOC;ALG14;ALG5;ALKBH2;ALKBH3;ALPK3;AMIGO2;ANAPC16;ANAPC4;ANKFY1;ANKRA2;ANKRD37;ANKS1A;ANO7;ANP32A;ANPEP;ANXA11;ANXA7;AP1M1;AP2A1;AP2M1;AP3B1;APC2;APITD1;APLP2;
1;ACYP2;ADAP2;ADAR;ADH5;ADK;ADO;ADSL;AFAP1;AFF2;AGA;AGPAT1;AHI1;AKAP13;AKR1C3;ALAS1;ALB;ALDH2;ALDH9A1;ALDOC;ALG14;ALG5;ALKBH2;ALKBH3;ALPK3;AMIGO2;ANAPC16;ANAPC4;ANKFY1;ANKRA2;ANKRD37;ANKS1A;ANO7;ANP32A;ANPEP;ANXA11;ANXA5;ANXA7;AP1M1;AP2A1;AP2M1
1;ACYP2;ADAP2;ADAR;ADH5;ADK;ADO;ADSL;AFAP1;AFF2;AGA;AGPAT1;AHI1;AKAP13;AKR1C3;ALAS1;ALB;ALDH2;ALDH9A1;ALDOC;ALG14;ALG5;ALKBH2;ALKBH3;ALPK3;AMIGO2;ANAPC16;ANAPC4;ANKFY1;ANKRA2;ANKRD37;ANKS1A;ANO7;ANP32A;ANPEP;ANXA11;ANXA7;AP1M1;AP2A1;AP2M1;AP3B1
AL;ADAP2;ADH5;ADK;ADO;ADSL;AGA;AGBL2;AGPAT1;AHI1;AKAP13;ALAS1;ALB;ALDH1A1;ALDH2;ALDH9A1;ALDOC;ALG14;ALG5;ALKBH3;AMPD3;ANAPC4;ANKFY1;ANKRA2;ANO7;ANP32A;ANPEP;ANXA11;ANXA7;ANXA9;AP1M1;AP2A1;AP2M1;AP3B1;APBB1IP;APC2;APITD1;APOA1;APP;ARAP1;ARAP3;AR
L4;ADAP2;ADAR;ADCY4;ADH5;AFAP1;AGA;AHI1;AKAP13;ALB;ALDOC;ALKBH3;AMICA1;AMIGO2;ANAPC4;ANKFY1;ANKRA2;ANKS1A;ANP32A;ANXA11;ANXA7;ANXA9;AP1M1;AP2A1;AP2M1;AP3B1;APC2;APITD1;APLP2;APOA1;APOBEC3C;APP;ARAP1;ARF1;ARF6;ARHGAP1;ARHGAP21;ARHGAP24;ARHGAP2
HI1;ALAS1;ALB;ALDH2;ALG14;ALG5;ALKBH2;ALKBH3;ANAPC16;ANAPC4;ANKFY1;ANP32A;ANXA11;ANXA7;AP1M1;AP2A1;AP2M1;AP3B1;APC2;APITD1;APOA1;APP;ARAP1;ARCN1;ARF1;ARF3;ARF6;ARFGAP3;ARHGAP21;ARHGEF10;ARID1A;ARL1;ARL6;ARNT;ARSD;ARSG;ASAH1;ASH2L;ATF5;ATF7;ATP5S;AT
HI1;ALAS1;ALB;ALDH2;ALG14;ALG5;ALKBH2;ALKBH3;ANAPC16;ANAPC4;ANKFY1;ANP32A;ANXA11;ANXA7;AP1M1;AP2A1;AP2M1;AP3B1;APC2;APITD1;APOA1;APP;ARAP1;ARCN1;ARF1;ARF3;ARF6;ARFGAP3;ARHGAP21;ARHGEF10;ARID1A;ARL1;ARL6;ARNT;ARSD;ARSG;ASAH1;ASH2L;ATF5;ATF7;ATP5S;AT
3;ACTG1;ACTN1;ACVR1B;ACYP2;ADAL;ADAP2;ADAR;ADCY4;ADH5;ADK;ADO;ADSL;AFAP1;AFF2;AGA;AGBL2;AGPAT1;AHI1;AKAP13;AKR1C3;ALAS1;ALB;ALDH1A1;ALDH2;ALDH3B1;ALDH9A1;ALDOC;ALG14;ALG5;ALKBH2;ALKBH3;ALOX15B;ALPK3;ALPL;AMICA1;AMIGO2;AMPD3;ANAPC16;ANAPC4;ANKFY1;
3;ACTG1;ACTN1;ACVR1B;ACYP2;ADAL;ADAP2;ADAR;ADCY4;ADH5;ADK;ADO;ADSL;AFAP1;AFF2;AGA;AGBL2;AGPAT1;AHI1;AKAP13;AKR1C3;ALAS1;ALB;ALDH1A1;ALDH2;ALDH3B1;ALDH9A1;ALDOC;ALG14;ALG5;ALKBH2;ALKBH3;ALOX15B;ALPK3;ALPL;AMICA1;AMIGO2;AMPD3;ANAPC16;ANAPC4;ANKFY1;
F6;ARFGAP3;ARHGAP27;ARL1;ATXN2;B4GALT1;BIN2;BLOC1S3;BRPF3;C3;CAMK1D;CAP1;CBL;CCL3;CD14;CD209;CD93;CDC42SE1;CFL1;CLCN5;CLTA;CLTC;CLTCL1;CNN2;COLEC12;COPA;COPB2;COPZ2;CORO1C;CTNNB1;CUX1;CXCR2;DAB2;DBNL;DENND1A;DNM2;DOCK2;DYSF;EHD1;EHD3;ELMO1;ELMO2;E
1;ACTN1;ACVR1B;ADAL;ADAM19;ADAMTSL4;ADAP2;ADAR;ADCY4;ADH5;ADK;ADO;ADPRH;ADRBK2;AFAP1;AFF2;AGA;AGBL2;AHI1;AKAP13;ALAS1;ALB;ALDH1A1;ALDOC;ALKBH2;ALKBH3;ALOX15B;ALPK1;ALPK3;ALPL;AMICA1;AMIGO2;AMPD3;AMY2B;AMZ2;ANAPC4;ANKFY1;ANKRA2;ANKS1A;ANP32A;A
;AMPD3;ANAPC4;ANKRA2;ANO7;ANPEP;ANXA7;ANXA9;AP1M1;AP2A1;AP2M1;APBB1IP;APITD1;APP;ARAP1;ARAP3;ARCN1;ARF1;ARFGAP3;ARHGAP1;ARHGAP24;ARHGAP25;ARHGAP26;ARHGAP30;ARHGAP31;ARHGDIB;ARHGEF10;ARHGEF11;ARHGEF12;ATP6V1B2;BLOC1S3;BPNT1;BRPF3;BTK;BUB3;C11
25;ARHGAP26;ARHGAP27;ARHGAP30;ARHGAP31;ARHGDIB;ARHGEF10;ARHGEF11;ARHGEF12;ARL1;ARL6;BCL6;BRAP;CDC42;CDC42EP4;CFL1;CRKL;CSF1;CTNNAL1;DBNL;DNMBP;DOCK2;DOCK5;DOCK6;DOCK8;DOK3;ELMO1;FBP1;FLCN;GDI2;GIT2;GMIP;GNA12;GNB1;GRB2;HMOX1;IQSEC1;ITSN1;KSR1;LIM
9;CD93;CDC42SE1;CLCN5;CLTA;CLTC;CLTCL1;CNN2;COLEC12;CORO1C;CXCR2;DAB2;DBNL;DENND1A;DNM2;DOCK2;EHD1;ELMO1;ELMO2;EPS15L1;FCN1;FKBP15;GRB2;HCK;HFE;HIP1;HNRNPK;IGF2R;ITSN1;LDLRAD3;LILRB1;LRP1;MAGI2;MERTK;MYO7A;NME1;NOD2;PACSIN2;PECAM1;PPT1;PTPN1;PTX3;R
RNT;ASH2L;ATPIF1;B4GALT1;BCL6;BECN1;BLOC1S3;BNIP3;BSG;BTK;BTN3A1;C1RL;C3;C4BPB;C5AR1;CALCOCO2;CAMK1D;CAMK2G;CBFA2T3;CCL3;CD14;CD1C;CD1D;CD209;CD3D;CD44;CD86;CD93;CD97;CDC42;CDH1;CHRNB2;CITED2;CLEC4C;CLTA;CLTC;CNN2;COLEC12;CREBBP;CRKL;CRTAM;CSF1;CSF1R;
SD;CTSZ;CXCR2;DAPK2;GALNS;GBA;GLB1;GM2A;GNS;HCK;HEXB;HGSNAT;HLA-DPA1;IGF2R;KIAA1324;LAMP1;LAPTM5;LIPA;MAN2B2;MANBA;MARCH8;MYO7A;NAAA;NAGA;NBR1;NCF2;NCSTN;NEU1;PLBD2;PPT1;PRCP;PSAP;PSEN1;RAB23;RAB7A;RNASET2;RRAGB;RRAGC;SFTPB;SIAE;SLC11A1;SLC36A1;S
ARHGAP21;ARHGAP30;ARHGDIB;ATP6V0A1;ATP6V0C;ATP6V0D1;ATP6V1B2;BECN1;BICD2;BLOC1S3;BSG;BTK;C3;CACNG8;CAMK2G;CD93;CDC42;CHGA;CLTA;CLTC;CLTCL1;COPA;COPB2;COPZ2;CRISPLD2;CTNNA1;CTSB;CTSD;DAB2;DAPK2;DENND1A;DNAJC5;DYSF;ECE1;EHD1;EHD3;ENO1;ERMAP;ESD;F13A
LDOC;ALKBH3;AP2M1;ATP5S;ATPIF1;BCKDHA;BCL2L13;BCO2;BNIP3;BRD8;BSG;C10orf10;C14orf159;C17orf89;C19orf12;C22orf32;C6orf57;C7orf55;CDC42SE1;CHCHD1;CHCHD2;CHCHD6;CHCHD7;CLPX;CLTC;COA5;COQ3;COQ5;COX11;COX17;COX18;COX5A;COX6A1;CPOX;CPT1A;CPT2;CS;CTSA;CTSB;CTSD
F1;ARF3;ARF6;ARFGAP3;ARHGAP21;ARL1;ARL6;ATP5S;ATP6V0A1;ATP6V0C;ATP6V0D1;ATPIF1;B3GNT9;B4GALT1;B4GALT4;B4GALT5;BBS4;BCL2L13;BNIP3;BSCL2;BSG;C19orf12;C3orf52;CACNG8;CAMK2G;CANT1;CBFA2T3;CD14;CD1C;CD1D;CD68;CDC42;CERS2;CHCHD6;CHGA;CHMP4B;CHST11;CHST15;C
1;GM2A;GNS;HCK;HEXB;HGSNAT;HLA-DPA1;IGF2R;KIAA1324;LAMP1;LAPTM5;LIPA;MAN2B2;MANBA;MARCH8;MYO7A;NAAA;NAGA;NBR1;NCF2;NCSTN;NEU1;PLBD2;PPT1;PRCP;PSAP;PSEN1;RAB7A;RNASET2;RRAGB;RRAGC;SFTPB;SIAE;SLC11A1;SLC36A1;SLC48A1;SQSTM1;SRC;TIAL1;TLR9;TM9SF1;TMB
1;GM2A;GNS;HCK;HEXB;HGSNAT;HLA-DPA1;IGF2R;KIAA1324;LAMP1;LAPTM5;LIPA;MAN2B2;MANBA;MARCH8;MYO7A;NAAA;NAGA;NBR1;NCF2;NCSTN;NEU1;PLBD2;PPT1;PRCP;PSAP;PSEN1;RAB7A;RNASET2;RRAGB;RRAGC;SFTPB;SIAE;SLC11A1;SLC36A1;SLC48A1;SQSTM1;SRC;TIAL1;TLR9;TM9SF1;TMB
2;CSF1;CSF1R;CTNNB1;DAB2;DOCK2;EGR1;FLCN;FLT3;FOXP3;G6PD;GAB2;GNAS;GPR183;HAX1;HCLS1;HDAC4;HIPK2;HLA-B;HSPA1B;IKZF1;IL23A;IL4R;JAK3;KAT6A;KLF13;KLF4;LILRB1;LILRB2;MAEA;MAFB;MAPK14;MERTK;MKNK2;MLF1;MMP9;MYB;MYH9;NBEAL2;NCOA6;NCOR1;NFAM1;NFKBIA;NHEJ1;N
C;ATPIF1;BCOR;BICD2;BNIP3;BRCA1;CASC3;CASP9;CBX1;CCNK;CDC42;CDC42EP4;CDH1;CFLAR;CITED2;COTL1;CTNNB1;CTNND1;CYB5A;CYFIP1;DENND5A;DNM2;DUSP12;DVL3;DYNLL1;EGR1;ELL;ENSA;ERC1;ESR1;FAM58A;FOXO4;FOXP3;FURIN;GCK;GLMN;GNB1;GOLGA5;GRB2;GSK3A;GTF2I;HCLS1;HDAC4
ARL1;ARL6;BBS4;BCL6;BICD2;BLOC1S3;BNIP3;C11orf73;C1QTNF3;CCL3;CD209;CDC42;CDH1;CHMP4B;CLTA;CLTC;CLTCL1;COPA;COPB2;COPZ2;CPT1A;CPT2;CTSA;CUX1;DAB2;DAG1;DENND3;DNAJC19;DNM2;DSCR3;EHD1;EHD3;EIF2D;ERC1;ERGIC1;FAM160A2;FAM21A;FAM21C;FBXO22;FGF9;FIS1;FZD1;G
3;ALPK3;AMIGO2;ANAPC16;ANAPC4;ANKRA2;ANKRD37;ANKS1A;ANO7;ANP32A;ANXA11;ANXA7;APITD1;APLP2;APOA1;APOBEC3C;ARID1A;ARID3A;ARID3B;ARNT;ASB8;ASH2L;ATF5;ATF7;ATP6V0A1;ATXN2;BASP1;BAZ2A;BCAS3;BCCIP;BCL2L13;BCL6;BCL9;BCOR;BCORL1;BECN1;BEX2;BHLHE40;BIVM;BNIP
;BCL9;BLOC1S3;BRPF3;BSG;C1QTNF3;C3;C4BPB;C5AR1;C6orf89;CAMK1D;CAP1;CAPZB;CCL3;CD14;CD163;CD44;CD97;CDC42;CELA1;CFL1;CFLAR;CNN2;CSF1;CSF1R;CTNNA1;CXCR2;DGKG;DOCK6;DOCK8;DYSF;EHD1;EHD3;EMR2;F11R;F13A1;F2RL2;FOXP3;GBA;GNA11;GNA12;GNA15;GNAI2;GNAS;GNB1;G

LOT2;GOLIM4;HLA-B;HLA-C;HLA-DPA1;HLA-E;IGF2R;ITSN1;NCF2;RAB11FIP1;RAB11FIP4;RAB23;RAB35;RAB43;RAB5B;RAB5C;RAB7A;RAB8A;SLC11A1;SRGAP2;SYK;SYT2;TIRAP;TLR9;VAMP2
AMICA1;ANXA5;ANXA7;AOAH;AP1M1;AP2A1;AP2M1;AP3B1;APBB1IP;APITD1;APOA1;APOBEC3C;APP;ARF1;ARNT;ASH2L;ATG7;ATP2B4;ATP6V0D1;B4GALT1;BCCIP;BCL6;BCL9;BECN1;BLOC1S3;BNIP3;BRCA1;BRE;BRPF3;BSG;BTG2;BTK;C11orf73;C1QTNF3;C1RL;C3;C4BPB;C5AR1;C6orf89;CA3;CALCOCO2;CA
2;CRKL;CSF1;CSF1R;CTNNB1;DAB2;DOCK2;EGR1;FLCN;FLT3;FOXP3;G6PD;GAB2;GNAS;GPR183;HAX1;HCLS1;HDAC4;HIPK2;HLA-B;HSPA1B;IKZF1;IL23A;IL4R;JAK3;JARID2;KAT6A;KLF13;KLF4;LILRB1;LILRB2;MAEA;MAFB;MAPK14;MERTK;MKNK2;MLF1;MMP9;MYB;MYH9;NBEAL2;NCOA6;NCOR1;NFAM1;NFKI
CAP1;CAPZB;CDC42;CFL1;DGKG;DOCK6;DOCK8;EHD1;EHD3;F11R;F13A1;F2RL2;GNA11;GNA12;GNA15;GNAI2;GNAS;GNB1;GRB2;HSPA5;IRF2;ITGA5;ITGAM;ITGAX;ITGB2;ITPK1;KCNMB3;KCNMB4;KIF3C;LCP2;LEFTY2;MAFG;MAPK14;MERTK;MFN2;MYB;P2RX1;PECAM1;PHF21A;PIK3R5;PLAUR;PLCG2;PLEK;
C4BPB;CAP1;CAPZB;CDC42;CFL1;DGKG;DOCK6;DOCK8;EHD1;EHD3;F11R;F13A1;F2RL2;GNA11;GNA12;GNA15;GNAI2;GNAS;GNB1;GRB2;HSPA5;IRF2;ITGA5;ITGAM;ITGAX;ITGB2;ITPK1;KCNMB3;KCNMB4;KIF3C;LCP2;LEFTY2;MAFG;MAPK14;MERTK;MFN2;MYB;P2RX1;PECAM1;PHF21A;PIK3R5;PLAUR;PLCG
209;CD3D;CD86;CD93;CDC42;CFL1;CHRNB2;CSF1;CTNNB1;CXCR2;DGKG;DOCK2;EGR1;ELF4;EXOSC3;F13A1;F2RL2;FKBP1A;FLT3;FOXP3;GAB2;GLMN;GNA11;GNA12;GNA15;GNAI2;GNB1;GPR183;GRB2;HDAC4;HLA-DPA1;HMOX1;HSPA5;ICOS;IKZF1;IL17RA;IL23A;IL4R;IRS2;ITGAM;JAK3;KLRC4-KLRK1;KRT2;I
CAP1;CAPZB;CDC42;CFL1;DGKG;DOCK6;DOCK8;EHD1;EHD3;F11R;F13A1;F2RL2;GNA11;GNA12;GNA15;GNAI2;GNAS;GNB1;GRB2;HSPA5;IRF2;ITGA5;ITGAM;ITGAX;ITGB2;ITPK1;KCNMB3;KCNMB4;KIF3C;LCP2;LEFTY2;MAFG;MAPK14;MERTK;MFN2;MYB;P2RX1;PECAM1;PHF21A;PIK3R5;PLAUR;PLCG2;PLEK;
ATG7;ATP6V0A1;ATP6V0C;ATP6V0D1;ATP6V1B2;BCL6;BLOC1S3;BTN3A1;C11orf73;C1QTNF3;CCL3;CD14;CDC42;CDH1;CHMP4B;CLTA;CLTC;CLTCL1;COG8;COPA;COPB2;COPZ2;COX18;CRTAM;CSF1R;CTSA;DAB2;DAG1;DENND1A;DNAJC14;DNAJC19;DNM2;EHD1;EHD3;EIF2D;ERC1;FAM160A2;FGF9;FIS1;FO
2;CRKL;CSF1;CSF1R;CTNNB1;DAB2;DOCK2;EGR1;EXOSC3;FLCN;FLT3;FOXP3;G6PD;GAB2;GNAS;GPR183;HAX1;HCLS1;HDAC4;HIPK2;HLA-B;HSPA1B;ICOS;IKZF1;IL23A;IL4R;JAK3;JARID2;KAT6A;KLF13;KLF4;LILRB1;LILRB2;MAEA;MAFB;MAPK14;MERTK;MKNK2;MLF1;MMP9;MYB;MYH9;NBEAL2;NCOA6;NCOR1
ARHGAP21;ARHGAP30;ARHGDIB;ATP6V0A1;ATP6V0C;ATP6V0D1;ATP6V1B2;BECN1;BLOC1S3;BSG;C3;CACNG8;CAMK2G;CD93;CDC42;CHGA;CLTA;CLTC;CLTCL1;COPA;COPB2;COPZ2;CRISPLD2;CTNNA1;CTSB;CTSD;DAB2;DENND1A;DNAJC5;DYSF;ECE1;EHD1;EHD3;ENO1;ESD;F13A1;FLOT2;FURIN;GCK;GNAS
;C1RL;C3;C4BPB;C5AR1;CAMK1D;CCL3;CD14;CD1D;CD209;CD3D;CD86;CDC42;CDH1;CHRNB2;COLEC12;CRTAM;CSF1;CTNNB1;DOCK2;ELK1;ELMO1;FKBP1A;FLCN;FLT3;FOXP3;GAB2;GLMN;GNAS;GPR183;GRB2;HAX1;HCK;HCLS1;HLA-B;HLA-C;HLA-DPA1;HLA-E;HMOX1;HSPA1B;ICOS;IFNAR2;IKZF1;IL23A;IL4
RF1;ARFGAP3;ARHGAP21;ARL1;ARL6;B4GALT1;BBS4;BCL6;BICD2;BLOC1S3;BNIP3;BRPF3;BTN3A1;C11orf73;C1QTNF3;CAMK2G;CAP1;CCL3;CD14;CD209;CDC42;CDH1;CFL1;CHMP4B;CHRNB2;CLTA;CLTC;CLTCL1;COPA;COPB2;COPZ2;COX18;CPT1A;CPT2;CRTAM;CSF1R;CTSA;CUX1;DAB2;DAG1;DENND3;DNA
FGAP3;ARL1;ARL6;ATG7;ATP6V0A1;ATP6V0C;ATP6V0D1;ATP6V1B2;ATXN2;BBS4;BCL6;BICD2;BLOC1S3;BSCL2;BTN3A1;BUB3;C11orf73;C1QTNF3;C3;CASC3;CBY1;CCL3;CD14;CDC42;CDH1;CHKA;CHMP4B;CHST11;CLTA;CLTC;CLTCL1;COG8;COPA;COPB2;COPZ2;COX18;CPT1A;CPT2;CRTAM;CSF1R;CTNNB1;C
P25;ARHGAP26;ARHGAP27;ARHGAP30;ARHGAP31;ARHGDIB;ARHGEF11;ARHGEF12;ARL1;BCL2L13;C6orf170;CASP9;CCL3;CFLAR;CLPX;CTSA;DBNL;DNAJB6;DOCK2;GDI2;GIT2;GM2A;GMIP;GTF2F1;ITSN1;MAP2K1;MAP3K5;MMP14;NLRP12;PLEKHG6;PPP1R12B;PREX1;PRKCD;PRKRA;PSAP;PSME3;RALBP1;R
NAS;HAX1;HCLS1;HIPK2;HSPA1B;IKZF1;IL23A;KAT6A;KLF13;LILRB1;MAEA;MAFB;MAPK14;MMP9;MYH9;NBEAL2;NCOA6;NCOR1;NFKBIA;NME1;PAF1;PAFAH1B1;PIK3CD;PKNOX1;PSEN1;PTK2B;RCOR1;RNF41;SRF;STAT5A;STAT5B;TFE3;TGFB1;THOC5;TIRAP;WDR6
FIP1;DAG1;DBNL;DYSF;EMR2;EPB41L3;FZD1;GAS7;GNAS;GSN;HAX1;ITGA5;ITGB4;ITSN1;KCNC3;LCP1;MTMR14;MYH9;NDEL1;NME2;NRBP1;PAFAH1B1;PAK1;PLEK;PTK2B;PTPRJ;PXN;RAC1;RHOA;SH3YL1;SPRY2;SRGAP2;SSH1;TIRAP;TLN1;VASP;VIM;WASF2
;ARHGEF10;ARHGEF11;ARHGEF12;BCL6;BRAP;CDC42EP4;CFL1;CRKL;CSF1;CTNNAL1;DBNL;DNMBP;DOK3;ELMO1;FBP1;FLCN;GIT2;GMIP;GNA12;GNB1;GRB2;IQSEC1;ITSN1;KSR1;LIMK1;MAP2K1;MAPK13;MAPK14;MAPKAPK5;MFN2;NDEL1;NOTCH2;PAFAH1B1;PLEKHG6;PREX1;PTK2B;RAC1;RAF1;RALB;RA
PC4;ANP32A;ANXA11;APITD1;APOA1;APP;ARID1A;ARNT;ARSD;ARSG;ASAH1;ASH2L;ATF5;ATF7;BASP1;BAZ2A;BCKDHA;BHLHE40;BNIP3;BRCA1;BRD4;BRD8;BRIX1;BRPF3;CALCOCO1;CAMK2G;CARM1;CASC3;CASP6;CBFA2T3;CBX1;CBY1;CCRN4L;CDC5L;CDV3;CENPH;CERKL;CES1;CFL1;CFL2;CHCHD1;CHCHD
GDIB;ATP6V0A1;ATP6V0C;ATP6V0D1;ATP6V1B2;BECN1;BICD2;BLOC1S3;BSG;BTK;CACNG8;CAMK2G;CD93;CDC42;CHGA;CLTA;CLTC;CLTCL1;COPA;COPB2;COPZ2;CRISPLD2;CTNNA1;CTSB;CTSD;DAB2;DAPK2;DENND1A;DNAJC5;DYSF;ECE1;EHD1;EHD3;ERMAP;ESD;F13A1;FLOT2;FURIN;GCK;GNAS;GOLIM4;HA
;PTPRJ;PXN;TBL1X;VCL
AI2;GNB1;GRB2;HSPA5;LCP2;LEFTY2;MAPK14;MERTK;P2RX1;PECAM1;PIK3R5;PLCG2;PLEK;PRKCB;PRKCD;PSAP;PTPN1;PTPRJ;RAC1;RAC2;RAF1;RHOA;SERPINA1;SOD1;SRC;SRF;SYK;TGFB1;TLN1;VAV1;VCL
K;LRP1;MERTK;MYO7A;NOD2;PECAM1;PTX3;RAB7A;RAC1;SLC11A1;SYK;TGFB1
ANO10;ANXA4;ANXA5;APP;ARF6;ARHGEF11;ARHGEF12;ASAH1;ATF5;ATG7;ATPIF1;ATXN2;B4GALT1;BCL2L13;BCL6;BEAN1;BECN1;BEX2;BNIP3;BRCA1;BRE;BSCL2;BTG2;BTK;C19orf12;C5AR1;CAMK1D;CASP6;CASP9;CBL;CCL3;CCNB1IP1;CD14;CD44;CDC42;CDH1;CERKL;CFL1;CFLAR;CHD8;CHST11;CITED2;CS
ANO10;ANXA4;ANXA5;APP;ARF6;ARHGEF11;ARHGEF12;ASAH1;ATF5;ATG7;ATPIF1;ATXN2;B4GALT1;BCL2L13;BCL6;BEAN1;BECN1;BEX2;BNIP3;BRCA1;BRE;BSCL2;BTG2;BTK;C19orf12;C5AR1;CAMK1D;CASP6;CASP9;CBL;CCL3;CCNB1IP1;CD14;CD44;CDC42;CDH1;CERKL;CFL1;CFLAR;CHD8;CHST11;CITED2;CS
PC4;ANP32A;ANXA11;APITD1;APOA1;APP;ARID1A;ARNT;ARSD;ARSG;ASAH1;ASH2L;ATF5;ATF7;BASP1;BAZ2A;BCKDHA;BHLHE40;BNIP3;BRCA1;BRD4;BRD8;BRIX1;BRPF3;CALCOCO1;CAMK2G;CARM1;CASC3;CASP6;CBFA2T3;CBX1;CBY1;CCRN4L;CDC5L;CDV3;CENPH;CERKL;CES1;CFL1;CFL2;CHCHD1;CHD8;C
3;PGAM1;PGD;PGK1;PGM1;TALDO1;TKT
N1;ARF1;ARF6;ARFGAP3;ARHGAP21;ARL1;ARL6;B4GALT1;BBS4;BCL6;BICD2;BLOC1S3;BNIP3;BRPF3;BTN3A1;BUB3;C11orf73;C1QTNF3;CAMK2G;CAP1;CASC3;CCL3;CD14;CD209;CDC42;CDH1;CFL1;CHMP4B;CHRNB2;CLTA;CLTC;CLTCL1;COPA;COPB2;COPZ2;COX18;CPT1A;CPT2;CRTAM;CSF1R;CTNNB1;CTSA
I1;APC2;APLP2;APOA1;APP;ARAP1;ARAP3;ARF1;ARF6;ARFGAP3;ARHGAP1;ARHGAP24;ARHGAP25;ARHGAP26;ARHGAP27;ARHGAP30;ARHGAP31;ARHGDIB;ARHGEF10;ARHGEF11;ARHGEF12;ARNT;ATG7;BBS4;BCDIN3D;BCL6;BECN1;BNIP3;BRAP;BRCA1;BRE;BTK;BTN3A1;C1QTNF3;C1RL;C3;C4BPB;C5AR1;C
F3;BSG;C4BPB;C6orf89;CAP1;CAPZB;CD44;CDC42;CFL1;CFLAR;CNN2;DGKG;DOCK6;DOCK8;DYSF;EHD1;EHD3;F11R;F13A1;F2RL2;GNA11;GNA12;GNA15;GNAI2;GNAS;GNB1;GRB2;GSN;HMOX1;HSPA5;IRF2;ITGA5;ITGAM;ITGAX;ITGB2;ITPK1;KCNMB3;KCNMB4;KIF3C;LCP2;LEFTY2;MAFG;MAPK14;MERTK;M
ATG7;ATP6V0A1;ATP6V0C;ATP6V0D1;ATP6V1B2;BCL6;BLOC1S3;BTN3A1;C11orf73;C1QTNF3;CCL3;CD14;CDC42;CDH1;CHMP4B;CLTA;CLTC;CLTCL1;COG8;COPA;COPB2;COPZ2;COX18;CRTAM;CSF1R;CTSA;DAB2;DAG1;DENND1A;DNAJC14;DNAJC19;DNM2;EHD1;EHD3;EIF2D;ERC1;FAM160A2;FGF9;FIS1;FO
A;ANXA11;APITD1;APOA1;ARID1A;ARNT;ARSD;ARSG;ASAH1;ASH2L;ATF5;ATF7;BASP1;BAZ2A;BCKDHA;BHLHE40;BNIP3;BRCA1;BRD4;BRD8;BRIX1;BRPF3;CALCOCO1;CAMK2G;CARM1;CASC3;CASP6;CBFA2T3;CBX1;CBY1;CCRN4L;CDC5L;CDV3;CENPH;CERKL;CES1;CFL1;CFL2;CHCHD1;CHD8;CHEK1;CITED2;CL
SEN1;RAB23;SQSTM1;TBC1D14;TP53INP2;ULK1;WIPI1;WIPI2
LL;TBC1D2;VCL
C4BPB;CAP1;CAPZB;CDC42;CDH1;CFL1;COPA;DGKG;DOCK6;DOCK8;EHD1;EHD3;EPHB2;F11R;F13A1;F2RL2;GNA11;GNA12;GNA15;GNAI2;GNAS;GNB1;GRB2;HK2;HSPA5;IRF2;ITGA5;ITGAM;ITGAX;ITGB2;ITPK1;KCNMB3;KCNMB4;KIF3C;LCP2;LEFTY2;MAFG;MAPK14;MERTK;MFN2;MYB;P2RX1;PECAM1;PHF
F11;ARID1A;ARID3A;ARID3B;ARNT;ASCC2;ASH2L;ATF5;ATF7;ATXN2;BASP1;BAZ2A;BBS4;BCDIN3D;BCL6;BCL9;BCOR;BCORL1;BHLHE40;BRCA1;BRD3;BRD4;BRD8;BTG2;BTK;C1QTNF3;C3;C4BPB;CALCOCO1;CAMK1D;CAMKK2;CAMTA1;CARM1;CASC3;CASZ1;CBFA2T3;CBL;CBX1;CBX6;CBY1;CCL3;CCNK;CCRN4
F2;ITGA5;ITGB4;LASP1;LIMK1;MAP2K1;MSN;PAK1;PNMA1;PTK2B;PXN;SH3KBP1;STARD8;TLN1;TNS3;VASP;VCL;ZYX
AT1;AHI1;AKAP13;AKR1C3;ALB;ALDH1A1;ALOX15B;AMIGO2;ANAPC4;ANKS1A;ANP32A;ANXA4;ANXA5;ANXA7;AP2A1;AP2M1;AP3B1;APBB1IP;APC2;APITD1;APLP2;APOA1;APOBEC3C;APP;ARAP1;ARAP3;ARF1;ARF3;ARF6;ARFGAP3;ARHGAP1;ARHGAP21;ARHGAP23;ARHGAP24;ARHGAP25;ARHGAP26;ARH
F2;AGPAT1;AHI1;AKAP13;AKR1C3;ALB;ALDH1A1;ALKBH2;ALKBH3;ALOX15B;AMICA1;AMIGO2;ANAPC4;ANKS1A;ANP32A;ANXA4;ANXA5;ANXA7;AOAH;AP1M1;AP2A1;AP2M1;AP3B1;APBB1IP;APC2;APITD1;APLP2;APOA1;APOBEC3C;APP;ARAP1;ARAP3;ARF1;ARF3;ARF6;ARFGAP3;ARHGAP1;ARHGAP21;AR
F2;ITGA5;LASP1;LIMK1;MAP2K1;MSN;PAK1;PNMA1;PTK2B;PXN;SH3KBP1;STARD8;TLN1;TNS3;VASP;VCL;ZYX
ARL1;ARL6;ATG7;ATP6V0A1;ATP6V0C;ATP6V0D1;ATP6V1B2;BBS4;BCL6;BICD2;BLOC1S3;BTN3A1;BUB3;C11orf73;C1QTNF3;CBY1;CCL3;CD14;CDC42;CDH1;CHMP4B;CLTA;CLTC;CLTCL1;COG8;COPA;COPB2;COPZ2;COX18;CRTAM;CSF1R;CTNNB1;CTSA;DAB2;DAG1;DENND1A;DNAJC14;DNAJC19;DNM2;DNM
;LASP1;LIMK1;MAP2K1;MSN;PAK1;PNMA1;PTK2B;PXN;SH3KBP1;STARD8;TLN1;TNS3;VASP;VCL;ZYX
;FLT3;FOXP3;GAB2;GNAS;GPR183;HAX1;HCLS1;HDAC4;HLA-B;IKZF1;IL23A;IL4R;JAK3;LILRB1;LILRB2;MAPK14;MERTK;MMP9;MYB;MYH9;NCOR1;NFAM1;NHEJ1;NME1;PAFAH1B1;PIK3CD;PKNOX1;PLCG2;POU2F2;PRKDC;PSEN1;PTK2B;RNF41;SEMA4A;SOD1;SOX4;SRF;STAT5A;STAT5B;STAT6;SYK;TFE3;TGFB
;BNIP3;BRCA1;BRD8;BRPF3;CAMK2G;CARM1;CASC3;CASP6;CBFA2T3;CBX1;CBY1;CCRN4L;CDC5L;CENPH;CHD8;CHEK1;CPSF7;CREBBP;CRNKL1;CSNK2A1;CTDP1;CTDSP2;CTNNB1;DAG1;DSCC1;DUT;EGR1;ELF4;ELL;ELL3;EP300;ESR1;EYA3;FANCF;FOXJ2;FOXN3;FOXO4;FOXP3;FUS;GATAD2B;GCK;GIT2;GTF2F1
ARF6;ARHGAP21;ARHGAP26;ARHGDIB;ARHGEF10;ARHGEF11;ARID1A;ARL1;ASH2L;ATG7;ATP6V0A1;ATP6V0C;ATP6V0D1;ATP6V1B2;BAZ2A;BBS4;BCL6;BCOR;BCORL1;BECN1;BICD2;BLOC1S3;BNIP3;BOD1;BRCA1;BRD3;BRD4;BRD8;BRE;BRPF3;BSCL2;BUB3;C11orf73;CAP1;CAPZB;CARM1;CBX6;CCL3;CCNB1I
D2;PACSIN2;PPT1;PTPN1;PTX3;RAB5B;RAB5C;RAC1;RUFY1;SLC11A1;SYK;TGFB1;VAMP8
;ARNT;ASH2L;ATF5;ATF7;BASP1;BAZ2A;BCCIP;BCOR;BHLHE40;BNIP3;BRCA1;BRD4;BRD8;BRE;BRIX1;BRPF3;CALCOCO1;CAMK2G;CARM1;CASC3;CASP6;CBFA2T3;CBX1;CBX6;CBY1;CCRN4L;CDC5L;CDV3;CENPH;CERKL;CERS2;CFL1;CFL2;CHCHD1;CHD8;CHEK1;CITED2;COPS7A;COPS8;CPSF7;CPT2;CREBBP;CRI
PFKFB3;PGAM1;PGD;PGK1;PGM1;TALDO1;TKT
ANXA4;ANXA5;APP;ARF6;ARHGEF11;ARHGEF12;ATF5;ATG7;ATPIF1;B4GALT1;BCL2L13;BCL6;BECN1;BEX2;BNIP3;BRCA1;BRE;BTG2;BTK;C5AR1;CAMK1D;CASP6;CASP9;CBL;CCL3;CCNB1IP1;CD14;CD44;CDC42;CDH1;CERKL;CFL1;CFLAR;CHD8;CHST11;CITED2;CSNK2A1;CTNNA1;CTNNB1;CTSB;CXCR2;CXCR7;D
M1;AP2A1;AP2M1;AP3B1;APC2;APOA1;APOBR;APP;ARAP3;ARCN1;ARF1;ARF3;ARF6;ARFGAP3;ARHGAP21;ARHGAP27;ARL1;ARL6;ATG7;ATP2B4;ATP5S;ATP6V0A1;ATP6V0C;ATP6V0D1;ATP6V1B2;ATXN2;B4GALT1;BBS4;BCL6;BICD2;BIN2;BLOC1S3;BNIP3;BRPF3;BTN3A1;C11orf73;C1QTNF3;C3;CA13;CA14;
H3;ANAPC4;ANP32A;APITD1;APLP2;APOA1;APOBEC3C;APP;ARAP1;ARAP3;ARFGAP3;ARHGAP26;ARHGAP27;ARHGEF10;ARHGEF11;ARID1A;ARID3A;ARID3B;ARL1;ARNT;ASCC2;ASH2L;ATF5;ATF7;ATG7;ATP6V0D1;ATPIF1;ATXN2;BASP1;BAZ2A;BBS4;BCCIP;BCDIN3D;BCL2L13;BCL6;BCL9;BCO2;BCOR;BCORL1
;APOA1;APOBEC3C;APP;ARHGEF11;ARID1A;ARID3A;ARID3B;ARNT;ASCC2;ASH2L;ATF5;ATF7;ATG7;ATP6V0D1;ATXN2;BASP1;BAZ2A;BBS4;BCCIP;BCDIN3D;BCL6;BCL9;BCOR;BCORL1;BHLHE40;BRCA1;BRD3;BRD4;BRD8;BRE;BTG2;BTK;BUB3;C1QTNF3;C3;C4BPB;C5AR1;CALCOCO1;CAMK1D;CAMKK2;CAMTA
YB;MYO1F;NOD2;PIK3CD;PLCG2;PRAM1;PSEN1;PTK2B;SEMA4A;SLC11A1;STAT6;SYK;TGFB1;TNFSF1
YB;MYO1F;NOD2;PIK3CD;PLCG2;PRAM1;PSEN1;PTK2B;SEMA4A;SLC11A1;STAT6;SYK;TGFB1;TNFSF1
;CD1D;CD209;CD86;CD93;CFLAR;CREBBP;CXCR7;DAG1;DYNLL1;EIF4A2;EIF4H;EP300;F11R;GRB2;HCK;HIPK2;HLA-B;HLA-C;HNRNPK;INSR;ITGA5;KAT5;KPNB1;MAP3K5;NCF2;NFKBIA;NLRX1;NOD2;NUP98;PACS1;PILRA;PRMT6;PSMB2;PVR;PVRL1;RAB11FIP4;RCOR1;RHOA;RPLP0;RXRA;SLC11A1;SPEN;SRC;ST
P26;ARHGAP27;ARHGAP30;ARHGAP31;ARHGDIB;ARHGEF11;ARHGEF12;C6orf170;DOCK2;GDI2;GIT2;GMIP;PLEKHG6;PREX1;RALBP1;RALGAPA2;RAP1GAP2;RGS10;RGS16;RGS3;RIN2;RIN3;SIPA1L1;SIPA1L2;SMAP2;SRGAP2;STARD8;TBC1D1;TBC1D14;TBC1D19;TBC1D2;TBC1D20;TBC1D2B;TBC1D5;TBC1D8;
ANXA4;ANXA5;APP;ARF6;ARHGEF11;ARHGEF12;ATF5;ATG7;ATPIF1;B4GALT1;BCL2L13;BCL6;BECN1;BEX2;BNIP3;BRCA1;BRE;BTG2;BTK;C5AR1;CAMK1D;CASP6;CASP9;CBL;CCL3;CCNB1IP1;CD14;CD44;CDC42;CDH1;CERKL;CFL1;CFLAR;CHD8;CHST11;CITED2;CSNK2A1;CTNNA1;CTNNB1;CTSB;CXCR2;CXCR7;D
4;RAB35;RAB5B;RAB5C;RAB7A;RAC1;RPN1;SLC1A5;TPP1;YWHAE
4;RAB35;RAB5B;RAB5C;RAB7A;RAC1;RPN1;SLC1A5;TPP1;YWHAE
P3;ARHGAP21;ARHGAP23;ARHGAP24;ARHGAP25;ARHGAP26;ARHGAP27;ARHGEF11;ARHGEF12;ARL6;BIN2;BTK;CMIP;DAB2;DNM2;DOK3;DYSF;ELMO1;ELMO2;EPB41;FAM129B;GAB2;GAB3;GRB10;HIP1;IQSEC1;IRS2;ITSN1;KIF1B;MYOF;OSBP;PHF12;PHLPP1;PITPNA;PLCG2;PLCL2;PLEK;PLEKHB2;PLEKHG6;
V0C;ATP6V0D1;ATP6V1B2;CASP9;CBL;CDC42;CLTA;CLTC;CSF1R;CTNNB1;EIF4EBP2;ELK1;EPHB1;EPHB2;EPS15L1;FGF9;FLT3;FOXO4;FRS3;FURIN;GAB2;GIGYF2;GRB10;GRB2;GSK3A;IGF1R;IGF2R;INSR;IRS2;ITGA5;ITSN1;JAK3;LCP2;LRP1;MAP2K1;MAPK13;MAPK14;MKNK1;MYD88;MYOF;NCSTN;NDST1;NFKE
GDIB;ATP6V0A1;ATP6V0C;ATP6V0D1;ATP6V1B2;BECN1;BLOC1S3;BSG;CACNG8;CAMK2G;CD93;CDC42;CHGA;CLTA;CLTC;CLTCL1;COPA;COPB2;COPZ2;CRISPLD2;CTNNA1;CTSB;CTSD;DAB2;DENND1A;DNAJC5;DYSF;ECE1;EHD1;EHD3;ESD;F13A1;FLOT2;FURIN;GCK;GNAS;GOLIM4;HAX1;HCK;HEXB;HIP1;HLA-B
;CTNND1;DAG1;FAM129B;HCK;IRF2;ITGA5;LASP1;LIMK1;MAP2K1;MSN;MYH9;NDRG1;PAK1;PNMA1;PTK2B;PVRL1;PXN;SH3KBP1;STARD8;TJP2;TLN1;TNS3;VASP;VCL;ZYX
CXCR7;ECE1;EHD1;EHD3;EPHB1;FAM21A;FAM21C;FIG4;FKBP15;FLOT2;GOLIM4;GRB2;HFE;HLA-B;HLA-C;HLA-DPA1;HLA-E;HSD17B6;IGF2R;INSR;IRAK2;KIAA0196;KIAA1324;KIF13A;LAMP1;LMTK2;MAP2K1;MARCH8;MGRN1;MYD88;NBR1;NDRG1;NUMB;OCIAD2;PLEKHB2;PLIN3;PRAF2;PTPN1;RAB11FIP1;R
PITD1;APLP2;APOA1;APP;ARAP1;ARAP3;ARFGAP3;ARHGAP26;ARHGAP27;ARHGEF10;ARHGEF11;ARID1A;ARID3A;ARID3B;ARNT;ASCC2;ASH2L;ATF5;ATF7;ATG7;ATP6V0D1;ATPIF1;ATXN2;BASP1;BAZ2A;BCCIP;BCDIN3D;BCL6;BCL9;BCO2;BCOR;BCORL1;BECN1;BHLHE40;BRCA1;BRD3;BRD4;BRD8;BRE;BTG2;
;HK2;INSR;MGAM;NAGA;NCOR1;PFKFB3;PGAM1;PGD;PGK1;PGM1;PHKA2;PYGL;TALDO1;TKT
DAP2;ADAR;ADCY4;ADH5;ADK;ADRBK2;AFF2;AGPAT1;AHI1;AKAP13;AKR1C3;ALB;ALDH1A1;ALDH2;ALDH9A1;ALKBH2;ALKBH3;ALOX15B;AMICA1;AMIGO2;ANAPC4;ANKS1A;ANP32A;ANXA4;ANXA5;ANXA7;AOAH;AP1M1;AP2A1;AP2M1;AP3B1;APBB1IP;APC2;APITD1;APLP2;APOA1;APOBEC3C;APP;ARAP1;A
CA1;ANKS1A;ANP32A;ANXA11;AP1M1;AP2A1;AP2M1;AP3B1;APC2;APOA1;APOBR;APP;ARAP1;ARAP3;ARCN1;ARF1;ARF3;ARF6;ARFGAP3;ARHGAP21;ARHGAP27;ARL1;ARL6;ATG7;ATP2B4;ATP5S;ATP6V0A1;ATP6V0C;ATP6V0D1;ATP6V1B2;ATXN2;B4GALT1;BBS4;BCL6;BICD2;BIN2;BLOC1S3;BNIP3;BRCA1;E
AKAP13;ALB;ALKBH2;ALKBH3;ALOX15B;ALPL;AMPD3;AMY2B;AMZ2;ANKFY1;ANPEP;ANXA11;ANXA4;ANXA5;ANXA7;ANXA9;AOC2;APLP2;APOBEC3C;APP;ARAP1;ARAP3;ARFGAP3;ARL1;ARL6;ARSD;ARSG;ASH2L;ATF7;ATP2B4;ATP5S;B4GALT1;B4GALT4;B4GALT5;BAZ2A;BCKDHA;BCL6;BCO2;BPNT1;BRAP;BR
AP1;ARHGAP24;ARHGAP25;ARHGAP26;ARHGAP27;ARHGAP30;ARHGAP31;ARHGDIB;ARHGEF10;ARHGEF11;ARHGEF12;ARL1;ARL6;ASB8;BCL6;BNIP3;BRAP;BRCA1;BTK;C1QTNF3;C5AR1;CAMKK2;CANT1;CASP9;CBL;CCL3;CD14;CD209;CD44;CDC42;CDC42EP4;CFL1;CFLAR;CRKL;CSF1;CSF1R;CTNNAL1;CTNNB

1;HK2;INSR;MGAM;NCOR1;PFKFB3;PGAM1;PGD;PGK1;PGM1;PHKA2;PYGL;TALDO1;TKT
5;ATF7;BASP1;BAZ2A;BHLHE40;BNIP3;BRCA1;BRD4;BRD8;BRIX1;BRPF3;CALCOCO1;CAMK2G;CARM1;CASC3;CASP6;CBFA2T3;CBX1;CBY1;CCRN4L;CDC5L;CDV3;CENPH;CERKL;CFL1;CFL2;CHCHD1;CHD8;CHEK1;CITED2;CPSF7;CPT2;CREBBP;CRNKL1;CSNK2A1;CTDP1;CTDSP2;CTNNB1;CTSB;DAG1;DAP3;DEDD;

PFKFB3;PGAM1;PGD;PGK1;PGM1;TALDO1;TKT
H9;NME2;PAK1;PLEK;PTPRJ;RAC1;RHOA;SH3YL1;SPRY2;TIRAP;TLN1;WASF2
A1;CTNNB1;CTNND1;DAG1;FAM129B;HCK;IRF2;ITGA5;LASP1;LIMK1;MAP2K1;MSN;MYH9;NDRG1;PAK1;PNMA1;PPL;PTK2B;PVRL1;PXN;SH3KBP1;STARD8;TJP2;TLN1;TNS3;VASP;VCL;ZYX
PHB1;FAM21A;FAM21C;FIG4;GOLIM4;HLA-B;HLA-C;HLA-DPA1;HLA-E;HSD17B6;INSR;IRAK2;KIF13A;LAMP1;MARCH8;MYD88;NDRG1;PLEKHB2;PLIN3;PRAF2;RAB11FIP4;RAB23;RAB5B;RAB5C;RIPK1;RUFY1;SCAMP2;SLC11A1;SLC48A1;SNX1;SPG21;STEAP3;TLR9;TMBIM1;TPCN2;TSG101;VAMP8;VPS25;WIPI
L3;CERKL;CHEK1;CHKA;CSF1R;CSNK1D;CSNK2A1;DAPK1;DAPK2;DGKG;DYRK4;EIF2AK1;EPHB1;EPHB2;FKBP1A;FLT3;GCK;GSK3A;HCK;HIPK2;HK1;HK2;IGF1R;IGF2R;INSR;IP6K1;IRAK2;ITPK1;JAK1;JAK3;KSR1;LIMK1;LIMK2;LMTK2;LRRK1;MAP2K1;MAP2K3;MAP3K3;MAP3K5;MAP4K4;MAPK13;MAPK14;MAPKAP

PRKCD;PSEN1;RAB23;SQSTM1;TBC1D14;TP53INP2;ULK1;VDR;WIPI1;WIPI2
B1;GM2A;GNS;HEXB;HGSNAT;HLA-DPA1;IGF2R;LAMP1;LAPTM5;MARCH8;MYO7A;NCSTN;NEU1;PLBD2;PSAP;PSEN1;RAB7A;RNASET2;SLC36A1;SLC48A1;TLR9;TM9SF1;TMBIM1;TPCN2;TPP1;TXNDC5;VAMP8;VCAN;WIPI1;WIPI2;ZER
L3;CERKL;CHEK1;CHKA;CSF1R;CSNK1D;CSNK2A1;DAPK1;DAPK2;DGKG;DYRK4;EIF2AK1;EPHB1;EPHB2;FKBP1A;FLT3;GCK;GSK3A;HCK;HIPK2;HK1;HK2;IGF1R;IGF2R;INSR;IP6K1;IRAK2;ITPK1;JAK1;JAK3;KSR1;LIMK1;LIMK2;LMTK2;LRRK1;MAP2K1;MAP2K3;MAP3K3;MAP3K5;MAP4K4;MAPK13;MAPK14;MAPKAP
SVIL;TNNC1;TRIOBP
D3;EPHB1;FAM21A;FAM21C;FIG4;GOLIM4;HLA-B;HLA-C;HLA-DPA1;HLA-E;HSD17B6;INSR;IRAK2;KIF13A;LAMP1;MARCH8;MYD88;NDRG1;PLEKHB2;PLIN3;PRAF2;RAB11FIP4;RAB23;RAB5B;RAB5C;RIPK1;RUFY1;SCAMP2;SLC11A1;SLC48A1;SNX1;SPG21;STEAP3;TLR9;TMBIM1;TPCN2;TSG101;VAMP8;VPS25;
.;CCL3;CD1D;CD209;CD86;CD93;CFLAR;CREBBP;CXCR7;DAG1;DYNLL1;EIF4A2;EIF4H;EP300;F11R;GRB2;HCK;HIPK2;HLA-B;HLA-C;HNRNPK;INSR;ITGA5;KAT5;KPNB1;MAP3K5;NCF2;NFKBIA;NLRX1;NOD2;NUP98;PACS1;PILRA;PRMT6;PSMB2;PVR;PVRL1;RAB11FIP4;RCOR1;RHOA;RPLP0;RXRA;SLC11A1;SPEN;S
.;CCL3;CD1D;CD209;CD86;CD93;CFLAR;CREBBP;CXCR7;DAG1;DYNLL1;EIF4A2;EIF4H;EP300;F11R;GRB2;HCK;HIPK2;HLA-B;HLA-C;HNRNPK;INSR;ITGA5;KAT5;KPNB1;MAP3K5;NCF2;NFKBIA;NLRX1;NOD2;NUP98;PACS1;PILRA;PRMT6;PSMB2;PVR;PVRL1;RAB11FIP4;RCOR1;RHOA;RPLP0;RXRA;SLC11A1;SPEN;S
TNNB1;CXCR2;DOCK2;EGR1;ELF4;EXOSC3;FKBP1A;FLT3;FOXP3;GAB2;GLMN;GPR183;GRB2;HDAC4;HLA-DPA1;HMOX1;ICOS;IKZF1;IL23A;IL4R;IRS2;ITGAM;JAK3;KLRC4-KLRK1;LAT2;LCP1;LCP2;LILRB1;LILRB2;MERTK;MYB;MYH9;MYO1F;NCOR1;NCSTN;NDRG1;NFAM1;NHEJ1;NOD2;PAK1;PFDN1;PIK3CD;PKN

EK1;CSF1R;CSNK1D;CSNK2A1;DAPK1;DAPK2;DYRK4;EIF2AK1;EPHB1;EPHB2;FKBP1A;FLT3;GSK3A;HCK;HIPK2;IGF1R;IGF2R;INSR;IRAK2;JAK1;JAK3;KSR1;LIMK1;LIMK2;LMTK2;LRRK1;MAP2K1;MAP2K3;MAP3K3;MAP3K5;MAP4K4;MAPK13;MAPK14;MAPKAPK5;MARK2;MARK3;MAST3;MERTK;MKNK1;MKNK2
TB;ACTG1;ACTN1;ACVR1B;ACYP1;ACYP2;ADAL;ADAMTSL4;ADAP2;ADAR;ADCY4;ADH5;ADK;ADPRH;ADRBK2;ADSL;AFF2;AGA;AGPAT1;AHI1;AKAP13;AKR1C3;ALAS1;ALB;ALDH1A1;ALDH2;ALDH3B1;ALDH9A1;ALDOC;ALG14;ALG5;ALKBH2;ALKBH3;ALOX15B;ALPL;AMICA1;AMIGO2;AMPD3;ANAPC16;ANAPC
SR1;KIAA0196;KIF13A;LMTK2;MAP2K1;MGRN1;RAB35;RAB7A;SNX1;SNX27;SQSTM1;TRAK1;TSG101;VPS25;VPS35;WAS;WIPI
DSR1;KIF13A;KIF1C;NAPA;NRBP1;OPTN;PACS1;RAB8A;SCAMP2;SEC16A;SEC22A;SEC23B;SEC31A;SNX1;STX18;TAPBP;TRAPPC2;TXNDC5;VAMP2;VAMP8;WIPI1
8;CHEK1;CREBBP;DNMT3B;EP300;EYA3;FLCN;FOXP3;H2AFY;HDAC4;HMGN3;IKZF1;ING4;JARID2;KAT5;KAT6A;KAT7;KDM2A;KDM3B;KDM4B;KDM5B;KDM6B;MIS18A;MLL2;MSL3P1;MYB;NACC2;NCOR1;NSD1;PADI4;PAF1;PHF21A;PHF8;POLE4;POU5F1;PRKCB;PRKD2;PRMT5;PRMT6;RCOR1;RERE;RTF1;SAP1
AKAP13;ALB;ALKBH2;ALKBH3;ALOX15B;ALPL;AMPD3;AMY2B;AMZ2;ANKFY1;ANPEP;ANXA11;ANXA4;ANXA5;ANXA7;ANXA9;AOC2;APLP2;APOBEC3C;APP;ARAP1;ARAP3;ARFGAP3;ARL1;ARL6;ARSD;ARSG;ASH2L;ATF7;ATP2B4;ATP5S;B4GALT1;B4GALT4;B4GALT5;BAZ2A;BCKDHA;BCL6;BCO2;BPNT1;BRAP;BR

LK1;WIPI1;WIPI2
X1;ITSN1;NME2;NRBP1;PTK2B;PXN;RAC1;RHOA;SRGAP2;SSH1;VASP;WASF2
3A;IRS2;ITSN1;MAP2K1;MAPK13;MAPK14;MYD88;NCSTN;NFKB1;NFKBIA;PHLPP1;PREX1;PRKACA;PRKCD;PSEN1;RAC1;RAF1;RALB;RHOA;RPS6KA1;RTN4;SPRY2;SQSTM1;SRC;STAT3;ULK1;VAV1;YWHAE
M1;AP2A1;AP2M1;AP3B1;APC2;APOA1;APOBR;APP;ARAP3;ARCN1;ARF1;ARF3;ARF6;ARFGAP3;ARHGAP21;ARHGAP27;ARL1;ARL6;ATG7;ATP2B4;ATP5S;ATP6V0A1;ATP6V0C;ATP6V0D1;ATP6V1B2;ATXN2;B4GALT1;BBS4;BCL6;BICD2;BIN2;BLOC1S3;BNIP3;BRPF3;BTN3A1;C11orf73;C1QTNF3;C3;CA13;CA14;

KCD;PSEN1;RAB23;SQSTM1;TBC1D14;TP53INP2;ULK1;WIPI1;WIPI2
SR1;ELF4;EXOSC3;FKBP1A;FLT3;FOXP3;GLMN;GPR183;GRB2;HDAC4;HLA-DPA1;ICOS;IKZF1;IL23A;IL4R;IRS2;ITGAM;JAK3;KLRC4-KLRK1;LAT2;LCP1;LILRB1;LILRB2;MERTK;MYB;MYH9;NCOR1;NCSTN;NFAM1;NHEJ1;NOD2;PAK1;PFDN1;PIK3CD;PKNOX1;PLCG2;POU2F2;PRKCB;PRKCD;PRKDC;PSEN1;PTK2B;PTP
ELK1;FKBP1A;FOXP3;GAB2;HCK;HLA-B;HLA-C;HLA-DPA1;HLA-E;HMOX1;IFNAR2;IL23A;IL4R;IRAK2;ITGB2;JAK1;JAK3;KIR3DL1;KLRC4-KLRK1;LAT2;LCP2;LILRA1;LILRB1;LILRB2;MAP2K1;MAP2K3;MAPK14;MYB;MYD88;MYO1F;NFAM1;NFKB1;NFKBIA;NLRC4;NOD2;PAK1;PIK3CD;PLCG2;POLR3F;PRAM1;PRKCB;P
APOA1;APP;ARNT;ARSD;ARSG;ASB8;ASH2L;ATG7;ATP6V0D1;ATXN2;B3GNT9;B4GALT1;B4GALT4;B4GALT5;BAZ2A;BCCIP;BCOR;BNIP3;BRCA1;BRD4;BRD8;BRPF3;BTG2;BTK;BUB3;C3;C5AR1;CAMK2G;CAMKK2;CARM1;CARS;CASC3;CBL;CCL3;CCNB1IP1;CCNK;CD44;CDC42;CDKL3;CFL1;CHEK1;CLPX;CNOT4;CC
AP27;ARHGEF10;ARHGEF11;ARID1A;ARID3A;ARID3B;ARNT;ASCC2;ASH2L;ATF5;ATF7;ATP6V0D1;ATPIF1;BASP1;BAZ2A;BCDIN3D;BCL6;BCL9;BCOR;BCORL1;BHLHE40;BRCA1;BRD3;BRD4;BRD8;BRE;BTG2;BTK;CALCOCO1;CAMK1D;CAMKK2;CAMTA1;CAP1;CARM1;CASZ1;CBFA2T3;CBL;CBX1;CBX6;CBY1;CCL3;
FL1;CFLAR;CNN2;CORO1C;CSF1R;DOCK2;DYNLL1;ELMO1;EPB41;EPB41L3;ERMN;FAM101B;FHDC1;FSCN1;GAS7;GRB2;GSN;HAX1;HCK;HCLS1;IQSEC1;KCNA5;LCP1;LIMK1;LRP1;MICAL2;MYH11;MYH9;MYL6;MYL6B;MYO1F;MYO7A;NUAK2;PACSIN2;PAFAH1B1;PAK1;PFDN1;PLEK;PPM1F;PREX1;PRKCD;PTK2B
AP27;ARHGEF10;ARHGEF11;ARID1A;ARID3A;ARID3B;ARNT;ASCC2;ASH2L;ATF5;ATF7;ATP6V0D1;ATPIF1;BASP1;BAZ2A;BCDIN3D;BCL6;BCL9;BCOR;BCORL1;BHLHE40;BRCA1;BRD3;BRD4;BRD8;BRE;BTG2;BTK;CALCOCO1;CAMK1D;CAMKK2;CAMTA1;CAP1;CARM1;CASZ1;CBFA2T3;CBL;CBX1;CBX6;CBY1;CCL3;
PLP2;APOA1;APP;ARAP1;ARAP3;ARFGAP3;ARHGAP26;ARHGAP27;ARHGEF10;ARHGEF11;ARID1A;ARID3A;ARID3B;ARNT;ASCC2;ASH2L;ATF5;ATF7;ATG7;ATP6V0D1;ATPIF1;ATXN2;BASP1;BAZ2A;BBS4;BCCIP;BCDIN3D;BCL6;BCL9;BCOR;BCORL1;BHLHE40;BRCA1;BRD3;BRD4;BRD8;BRE;BSCL2;BTG2;BTK;BUB3
BBP;CXCR7;DAG1;DYNLL1;EIF4A2;EIF4H;EP300;F11R;GRB2;HCK;HIPK2;HLA-B;HLA-C;HNRNPK;INSR;ITGA5;KAT5;KPNB1;MAP3K5;NFKBIA;NLRX1;NUP98;PACS1;PILRA;PRMT6;PSMB2;PVR;PVRL1;RAB11FIP4;RCOR1;RHOA;RPLP0;RXRA;SPEN;SRC;STAT3;SYK;TAF4;TAP2;TBC1D20;TGFB1;THOC5;TRIM25;TSG10
RF6;ARFGAP3;ARL6;ATG7;ATP6V0A1;ATP6V0C;ATP6V0D1;ATP6V1B2;ATXN2;BCL6;BLOC1S3;BTN3A1;C11orf73;C1QTNF3;C3;CA13;CA14;CA3;CA6;CAMK2G;CASC3;CCL3;CD14;CD209;CDC42;CDH1;CHKA;CHMP4B;CHRNB2;CLTA;CLTC;CLTCL1;COG8;COPA;COPB2;COPZ2;COX18;CPT1A;CPT2;CRTAM;CSF1R;CT
N1;RAB23;RNF185;SQSTM1;TBC1D14;TM9SF1;TP53INP2;ULK1;UVRAG;WIPI1;WIPI2

;ARHGAP26;ARHGAP27;ARHGAP30;ARHGAP31;ARHGDIB;ARHGEF10;ARHGEF11;ARHGEF12;ATPIF1;C6orf170;CDC42SE1;DENND1A;DENND3;DENND5A;DNAJB6;DNMBP;DOCK2;DOCK5;DOCK6;DOCK8;EIF2B3;GDI2;GIT2;GMIP;IQSEC1;ITSN1;MAP4K4;MAPRE3;PLEKHG6;PREX1;RALBP1;RALGAPA2;RAP1GAP
ARF3;ARFGAP3;ARHGAP21;ARL1;ATP6V0A1;ATP6V0C;ATP6V0D1;ATP6V1B2;B3GNT9;B4GALT1;B4GALT4;B4GALT5;BNIP3;BSCL2;BSG;C3orf52;CACNG8;CAMK2G;CANT1;CBFA2T3;CDC42;CDC42EP4;CERS2;CHGA;CHST11;CHST15;CHST7;CKAP4;CLCN5;CLTA;CLTC;CLTCL1;COG8;COPA;COPB2;COPZ2;CUX1;C
AP2;ADAR;ADCY4;ADH5;ADK;ADPRH;ADRBK2;ADSL;AFF2;AGA;AGPAT1;AHI1;AKR1C3;ALAS1;ALB;ALDH1A1;ALDH2;ALDH3B1;ALDH9A1;ALDOC;ALG14;ALG5;ALKBH2;ALKBH3;ALOX15B;AMPD3;ANAPC16;ANAPC4;ANP32A;AOAH;AOC2;APITD1;APLP2;APOA1;APOBEC3C;APP;ARAP1;ARAP3;ARF1;ARF3;ARF6;
;SLC11A1;SRGAP2;SYK;TLR9

CDH1;CLTA;CLTC;CLTCL1;COPA;COPB2;COPZ2;CTSA;DAB2;DAG1;DNAJC19;EHD1;EHD3;EIF2D;FGF9;FIS1;FZD1;GCC1;HCLS1;IL23A;IMMP2L;JAK3;KDELR1;KIF13A;KPNB1;LCP1;LTBP2;MAPK14;MFN2;MRPL45;MYO7A;NAPA;NFKBIA;NLRP12;NLRP3;NUP98;NUPL2;NXT1;OPTN;OS9;PACS1;PDCD6;PEX7;PMPCB
5;ATF7;BASP1;BAZ2A;BCDIN3D;BCL6;BCL9;BCOR;BCORL1;BHLHE40;BRCA1;BRD3;BRD4;BRD8;BTG2;BTK;CALCOCO1;CAMK1D;CAMKK2;CAMTA1;CARM1;CASZ1;CBFA2T3;CBL;CBX1;CBX6;CBY1;CCNK;CCRN4L;CD3D;CD86;CDC5L;CDH1;CELA1;CELF3;CFLAR;CHD8;CHEK1;CITED2;CNOT1;CNOT4;CREBBP;C
ALG5;ALKBH2;ALKBH3;ANAPC16;ANAPC4;ANP32A;AOAH;APITD1;APLP2;APOA1;APOBEC3C;APP;ARHGEF11;ARID1A;ARID3A;ARID3B;ARNT;ARSD;ARSG;ASB8;ASCC2;ASH2L;ATF5;ATF7;ATG7;ATP6V0D1;ATXN2;B3GNT9;B4GALT1;B4GALT4;B4GALT5;BASP1;BAZ2A;BCCIP;BCDIN3D;BCL6;BCL9;BCOR;BCORL1;B
L;SYK;TGFB1;TLN1;VCL
;ARHGAP26;ARHGAP27;ARHGAP30;ARHGAP31;ARHGDIB;ARHGEF10;ARHGEF11;ARHGEF12;C6orf170;CDC42SE1;DENND1A;DENND3;DENND5A;DNMBP;DOCK2;DOCK5;DOCK6;DOCK8;EIF2B3;GDI2;GIT2;GMIP;IQSEC1;ITSN1;MAP4K4;MAPRE3;PLEKHG6;PREX1;RALBP1;RALGAPA2;RAP1GAP2;RAPGEFL1;RC
ACYP1;ACYP2;ADAL;ADAM19;ADAMTSL4;ADAR;ADCY4;ADH5;ADK;ADO;ADPRH;ADRBK2;ADSL;AGA;AGBL2;AGPAT1;AKAP13;AKR1C3;ALAS1;ALDH1A1;ALDH2;ALDH3B1;ALDH9A1;ALDOC;ALG1L2;ALG5;ALKBH2;ALKBH3;ALOX15B;ALPK1;ALPK3;ALPL;AMPD3;AMY2B;AMZ2;ANAPC4;ANPEP;AOAH;AOC2;APO
DC5;VCAN
42;CHRNB2;COLEC12;CRTAM;ELK1;FOXP3;GAB2;GPR183;GRB2;HCK;HLA-DPA1;HMOX1;ICOS;IKZF1;IL23A;IL4R;IL6R;IRAK2;IRS2;KLRC4-KLRK1;LAT2;LCP2;LILRB1;LILRB2;MAP2K1;MAP2K3;MAPK14;MYB;MYD88;NFAM1;NFKB1;NFKBIA;NLRC4;NOD2;PAK1;PIK3CD;PLCG2;POLR3F;PRKCB;PRKD2;PSEN1;PTK2B
5;ATF7;ATXN2;BASP1;BAZ2A;BCL6;BCL9;BCOR;BCORL1;BHLHE40;BRCA1;BRD3;BRD4;BRD8;BTK;CALCOCO1;CAMK1D;CAMKK2;CAMTA1;CARM1;CASC3;CASZ1;CBFA2T3;CBL;CBX1;CBX6;CBY1;CCNK;CCRN4L;CD3D;CD86;CDC42;CDC5L;CDH1;CELA1;CFLAR;CHD8;CHEK1;CITED2;CNOT1;CNOT4;CREBBP;CSNK2
5;ATF7;ATXN2;BASP1;BAZ2A;BCL6;BCL9;BCOR;BCORL1;BHLHE40;BRCA1;BRD3;BRD4;BRD8;BTK;CALCOCO1;CAMK1D;CAMKK2;CAMTA1;CARM1;CASC3;CASZ1;CBFA2T3;CBL;CBX1;CBX6;CBY1;CCL3;CCNK;CCRN4L;CD3D;CD86;CDC42;CDC5L;CDH1;CELA1;CFLAR;CHD8;CHEK1;CITED2;CNOT1;CNOT4;CNPY2;CF
IDAC4;ING4;JARID2;KAT5;KAT6A;KAT7;KDM2A;KDM5B;KDM6B;MLL2;MYB;NACC2;NCOR1;NSD1;PAF1;PHF8;POLE4;POU5F1;PRKCB;PRKD2;PRMT5;PRMT6;RCOR1;RTF1;SAP130;SETD1B;SIN3A;SUPT3H;TGFB1;USP22;WDR61;WDR82;YEATS
L;WDR82
AP2M1;APBB1IP;APOA1;ARAP1;ARAP3;ARHGAP21;ARHGAP23;ARHGAP24;ARHGAP25;ARHGAP26;ARHGAP27;ARHGEF11;ARHGEF12;ARL6;BIN2;BTK;CD14;CD1C;CD1D;CMIP;DAB2;DNM2;DOK3;DYSF;ELMO1;ELMO2;EPB41;ESR1;FABP6;FAM129B;GAB2;GAB3;GPR183;GRB10;HDLBP;HIP1;HSD17B4;IQSEC1
NUB1;PIM2;PRKCD;PSEN1;TNFRSF1B;TP53INP2;ULK1;UVRAG
IDAC4;ING4;JARID2;KAT5;KAT6A;KAT7;KDM2A;KDM5B;KDM6B;MLL2;MYB;NACC2;NCOR1;NSD1;PAF1;PHF8;POLE4;PRKCB;PRKD2;PRMT5;PRMT6;RCOR1;RTF1;SAP130;SETD1B;SIN3A;SUPT3H;TGFB1;USP22;WDR61;WDR82;YEATS

FL1;CFLAR;CNN2;CORO1C;CSF1R;DOCK2;DYNLL1;ELMO1;EPB41;EPB41L3;ERMN;FAM101B;FHDC1;FSCN1;GAS7;GRB2;GSN;HAX1;HCK;HCLS1;IQSEC1;LCP1;LIMK1;LRP1;MICAL2;MYH11;MYH9;MYO1F;NUAK2;PACSIN2;PAFAH1B1;PAK1;PFDN1;PLEK;PPM1F;PREX1;PRKCD;PTK2B;PTPN1;RAC1;RAC2;RHOA;SIP

D;RTF1;SETD1B;WDR61;WDR82
D;RTF1;SETD1B;WDR61;WDR82
D;CSNK2A1;DAPK1;DAPK2;DYRK4;EIF2AK1;FKBP1A;GSK3A;HIPK2;KSR1;LIMK1;LIMK2;LMTK2;LRRK1;MAP2K1;MAP2K3;MAP3K3;MAP3K5;MAP4K4;MAPK13;MAPK14;MAPKAPK5;MARK2;MARK3;MAST3;MKNK1;MKNK2;NEK3;NEK6;NUAK2;PAK1;PHKA2;PIM1;PIM2;PINK1;PRKACA;PRKCB;PRKCD;PRKD2;PRK
LDH2;ALDH3B1;ALKBH2;ALKBH3;ALPL;AMICA1;ANKS1A;ANP32A;ANXA4;ANXA5;AP1M1;AP2A1;AP2M1;APBB1IP;APC2;APITD1;APLP2;APOA1;APP;ARAP1;ARAP3;ARF1;ARF3;ARF6;ARFGAP3;ARHGAP1;ARHGAP21;ARHGAP23;ARHGAP24;ARHGAP25;ARHGAP26;ARHGAP27;ARHGAP30;ARHGAP31;ARHGDIB;

CRTAM;DOCK2;ELK1;ELMO1;FOXP3;GBA;HCK;IFNAR2;IL17RA;IL23A;IRAK2;JAK1;KLF4;KLRC4-KLRK1;LILRB1;MAP2K1;MAP2K3;MAPK13;MAPK14;MEFV;MYD88;MYO1F;NFKB1;NFKBIA;NLRC4;NLRP12;NLRP3;NLRP6;NOD2;OSMR;PACS1;PCBP2;POLR3F;PRKCD;PTPN1;PTPN6;PVR;RAC1;RIPK1;RPS6KA1;SAMH
AN
1;AP3B1;APC2;APOA1;APOBR;APP;ARCN1;ARF1;ARFGAP3;ARHGAP21;ARL1;ARL6;ATP2B4;ATP5S;ATP6V0A1;ATP6V0C;ATP6V0D1;ATP6V1B2;ATXN2;B4GALT1;BBS4;BCL6;BICD2;BIN2;BLOC1S3;BNIP3;BRPF3;BTN3A1;C11orf73;C1QTNF3;C3;CA13;CA14;CA3;CA6;CACNG8;CAMK1D;CAMK2G;CAP1;CCL3;CD14

GB2;KCNA5;MAML1;MAP2K1;MAP2K3;MAP3K5;MAPKAPK5;MICALCL;MSN;NBR1;NEK6;NME1;NOD2;PAK1;PGAM1;PLEK;PRKAB1;PRKACA;PRKCB;PRKCD;PTK2B;PTPN1;PTPN6;PTPRJ;PXN;RAB8A;RAC1;RAF1;SKI;SPDYA;SPRED1;SPRY2;SQSTM1;STAT3;SYK;TBC1D14;TIRAP;TNIP1;TPCN2;ULK1;YWHAG;ZC3H
;LILRB1;LILRB2;MAP2K1;MAP2K3;MAPK14;MYD88;NFAM1;NFKB1;NFKBIA;NOD2;PAK1;PIK3CD;PLCG2;PRKCB;PRKD2;PSEN1;PTPRJ;RIPK1;RPS6KA1;SPG21;SYK;TIRAP;TLR9;TNIP1;TRAT1;UBE2V1;VASP;WAS;XIAP
D14;CD1C;CD1D;CD209;CD3D;CD44;CD86;CD97;CDH1;CLEC4C;COLEC12;CREBBP;CRTAM;CSF1;CSF1R;CTSB;DAPK1;DOCK2;EGR1;ELK1;ENDOU;EP300;EXOSC3;FCAR;FCN1;FKBP1A;FOXP3;GAB2;GPR183;GTPBP1;HCK;HFE;HLA-B;HLA-C;HLA-DPA1;HLA-E;HMOX1;HRH2;ICOS;IFNAR2;IGF1R;IL23A;IL4R;IL6R;IR
ID3A;ARNT;ASH2L;ATG7;ATP6V0D1;BCL9;BECN1;BRCA1;BRD4;BRE;BTG2;C3;C5AR1;CALCOCO1;CAMKK2;CAP1;CBFA2T3;CD3D;CD44;CD86;CDC42;CDH1;CELA1;CELF3;CHD8;CHEK1;CITED2;CNPY2;CPT1A;CREBBP;CSF1;CSF1R;CSNK1D;CTDP1;CTDSP2;CTNNB1;DAB2;DBNL;DISC1;DNM2;DNMT3B;DVL3;ECE1
FOXP3;GLMN;GRB2;HLA-DPA1;ICOS;IKZF1;IL23A;IL4R;ITGAM;JAK3;KLRC4-KLRK1;LCP1;LILRB1;LILRB2;MYB;MYH9;NCOR1;NCSTN;NHEJ1;NOD2;PAK1;PIK3CD;PKNOX1;PRKDC;PSEN1;PTPN6;RAC1;SEMA4A;SLC11A1;SOD1;SOX4;SRC;SRF;STAT5A;STAT5B;STAT6;SYK;TGFB1;VAV1;WA
CNK;CDKL3;CERKL;CHEK1;CHKA;CSF1R;CSNK1D;CSNK2A1;CTDP1;DAPK1;DAPK2;DGKG;DPAGT1;DYRK4;EIF2AK1;EIF2B3;EPHB1;EPHB2;FKBP1A;FLT3;GCK;GSK3A;HCK;HIPK2;HK1;HK2;IGF1R;IGF2R;INSR;IP6K1;IRAK2;ITPK1;JAK1;JAK3;KSR1;LIMK1;LIMK2;LMTK2;LRRK1;MAP2K1;MAP2K3;MAP3K3;MAP3K5;M
K14;NFKBIA;NME1;PAF1;PTK2B;RCOR1;RNF41;STAT5A;STAT5B;TFE3;THOC5;WDR61
CN1;BNIP3;BRCA1;BRE;BTG2;C11orf73;CASP9;CCNK;CD44;CDC42;CHCHD6;CHEK1;CITED2;CREBBP;CRKL;CSNK1D;CTDSP2;CTNNA1;CTSD;DAB2;DAP;DBNL;DCLRE1B;DVL3;EEPD1;EGR1;EID3;EIF2AK1;EIF2B3;ELK1;EP300;EPHB1;EXTL3;EYA3;FAM129A;FAM162A;FANCF;FBXO22;FOXN3;FOXO4;FTO;G6PD;GCK
;MAP2K1;MAP2K3;MAPK14;MYD88;NFAM1;NFKB1;NFKBIA;NOD2;PAK1;PIK3CD;PLCG2;PRKCB;PRKD2;PSEN1;PTPRJ;RIPK1;RPS6KA1;SPG21;SYK;TIRAP;TLR9;TNIP1;TRAT1;UBE2V1;VASP;WAS;XIAP

CALCOCO1;CASP9;CBL;CDC5L;CDH1;CHD8;CITED2;CTNND1;DAG1;DAP;DAZAP2;DBNL;DENND1A;DNMBP;DYNLL1;ELMO1;ELMO2;ERC1;ETV6;FOXJ2;FZD1;GABRR2;GRB2;HCLS1;HSPA5;INSR;KHDRBS2;KPNB1;LAT2;LILRB1;LIN7B;LRRFIP2;MED12;MLF1;MRPL17;MYD88;MYO7A;NOD2;OSTF1;PINK1;POU2F2;

2;CHEK1;CREBBP;CSF1R;DPAGT1;DPH5;EP300;EPHB2;FKBP1A;FKBP5;FKBP9;FLCN;FLT3;FOXP3;FURIN;GALNT2;HAX1;HCK;HCLS1;HDAC4;HIPK2;IL23A;IL6R;ING4;INSR;ITGA5;ITGB2;JAK1;JAK3;KAT5;KAT6A;KAT7;LMTK2;MAN2A2;MAPK13;MAPK14;MKNK1;NEK6;NOD2;PADI2;PADI4;PAK1;PINK1;POLE4;PPID

7;BASP1;BAZ2A;BCL6;BCL9;BCOR;BCORL1;BHLHE40;BRCA1;BRD3;BRD4;BRD8;BTK;CALCOCO1;CAMK1D;CAMKK2;CAMTA1;CARM1;CASZ1;CBFA2T3;CBL;CBX1;CBX6;CBY1;CCNK;CCRN4L;CD3D;CD86;CDC5L;CDH1;CELA1;CFLAR;CHD8;CHEK1;CITED2;CNOT1;CNOT4;CREBBP;CSNK2A1;CTNNB1;CTNND1;CUX1;
STAT5B;STAT6;SYK;TAP2;TGFB1;TNFSF13
ARHGAP23;ARHGAP24;ARHGAP25;ARHGAP26;ARHGAP27;ARHGAP30;ARHGAP31;ARHGDIB;ARHGEF10;ARHGEF11;ARHGEF12;ARL1;ATPIF1;BCCIP;BCL2L13;C3;C6orf170;CABLES2;CASP9;CAST;CCL3;CDC42SE1;CFLAR;CLPX;CSNK2A1;CTSA;DBNL;DENND1A;DENND3;DENND5A;DNAJB6;DNMBP;DOCK2;DOC
7;BASP1;BAZ2A;BCL6;BCL9;BCOR;BCORL1;BHLHE40;BRCA1;BRD3;BRD4;BRD8;BTK;CALCOCO1;CAMK1D;CAMKK2;CAMTA1;CARM1;CASZ1;CBFA2T3;CBL;CBX1;CBX6;CBY1;CCL3;CCNK;CCRN4L;CD3D;CD86;CDC5L;CDH1;CELA1;CFLAR;CHD8;CHEK1;CITED2;CNOT1;CNOT4;CREBBP;CSNK2A1;CTDP1;CTNNB1;CT
SLC11A1;STAT6;TGFB1;TNFSF13
;PKNOX1;RCOR1;SRF;STAT5A;STAT5B
DNAJC5B;FKBP5;HSCB;LIMK1;NFKB1;NFKBIA;PPID;UNC45B
CALCOCO1;CAMKK2;CAP1;CBFA2T3;CD3D;CD86;CDC42;CDH1;CELA1;CELF3;CHD8;CHEK1;CITED2;CREBBP;CTDP1;CTNNB1;DAB2;DNM2;DVL3;EGR1;ELF4;ELK1;ELL;ELL3;EP300;ESR1;EYA3;FLCN;FOXJ2;FOXO4;FOXP3;FZD1;GLI1;GNAL;GNAS;GSK3A;GTF2F1;GTPBP1;HAX1;HCLS1;HDAC4;HEXB;HIPK2;HIVEP3;H
C;APP;ARHGEF11;ARID1A;ARID3A;ARID3B;ARNT;ASCC2;ASH2L;ASPRV1;ATF5;ATF7;ATXN2;BASP1;BAZ2A;BBS4;BCDIN3D;BCL6;BCL9;BCOR;BCORL1;BHLHE40;BRCA1;BRD3;BRD4;BRD8;BTG2;BTK;C1QTNF3;C3;C4BPB;CALCOCO1;CAMK1D;CAMKK2;CAMTA1;CARM1;CARS;CASC3;CASZ1;CBFA2T3;CBL;CBX1;CE
LP2;APOA1;APOBEC3C;APP;ARNT;ARSD;ARSG;ASB8;ASH2L;ATG7;ATP6V0D1;B3GNT9;B4GALT1;B4GALT4;B4GALT5;BAZ2A;BCCIP;BCDIN3D;BCOR;BRCA1;BRD4;BRD8;BRPF3;BTG2;BTK;BUB3;C3;C5AR1;CAMK2G;CAMKK2;CARM1;CBL;CCL3;CCNB1IP1;CCNK;CD44;CDC42;CDKL3;CFL1;CHEK1;CNOT4;COPS7A;
4;NCOR1;PKNOX1;RCOR1;SRF;STAT5A;STAT5B
0A1;ATP6V0C;ATP6V0D1;ATP6V1B2;CASP9;CBL;CDC42;CHST11;CITED2;CLTA;CLTC;CSF1R;CTNNB1;DAB2;EGR1;EIF4EBP2;ELK1;EPHB1;EPHB2;EPS15L1;F11R;FBN2;FGF9;FKBP1A;FLCN;FLT3;FOXO4;FRS3;FURIN;FZD1;GAB2;GDF15;GIGYF2;GRB10;GRB2;GSK3A;HIPK2;HSPA5;IGF1R;IGF2R;INSR;IRS2;ITGA5;ITS

D2;PFDN1;PIK3CD;PLCG2;POU2F2;PRKCB;PRKCD;PRKDC;PTK2B;STAT5A;STAT5B;STAT6;SYK;TGFB1;TIRAP;TNFSF13
PA1B;JAK3;KLF13;LIPA;MAEA;MAFB;MAPK14;MYB;NCOR1;NCSTN;PIK3CD;PKNOX1;RCOR1;SOD1;SRF;STAT5A;STAT5B;TGFB1
H2L;ATF5;ATF7;ATXN2;BASP1;BAZ2A;BCL6;BCL9;BCOR;BCORL1;BHLHE40;BRCA1;BRD3;BRD4;BRD8;BTK;C1QTNF3;C3;CALCOCO1;CAMK1D;CAMKK2;CAMTA1;CAP1;CARM1;CASC3;CASZ1;CBFA2T3;CBL;CBX1;CBX6;CBY1;CCL3;CCNK;CCRN4L;CD3D;CD86;CDC42;CDC5L;CDH1;CELA1;CFLAR;CHD8;CHEK1;CITE

SP22;YEATS4
;NLRP3;NLRX1;NOD2;PCBP2;POMC;RAC1;SLC11A1;TGFB1;TLR9;TRIM25;TRIM27
GAPA2;SRGAP2;TBC1D1;TBC1D14;TBC1D19;TBC1D2;TBC1D20;TBC1D2B;TBC1D5;TBC1D8;TBC1D9
2;BUB3;C11orf73;C1QTNF3;CASC3;CDC42;CDH1;CLTA;CLTC;CLTCL1;COPA;COPB2;COPZ2;COX18;CTNNB1;CTSA;DAB2;DAG1;DNAJC19;EHD1;EHD3;EIF2D;EPB41L3;FGF9;FIS1;FLCN;FZD1;GCC1;HCLS1;IL23A;IMMP2L;JAK3;KDELR1;KIF13A;KPNB1;LCP1;LTBP2;MAGI2;MAPK14;MAPRE1;MFN2;MRPL45;MYO7A

GBA;GLMN;HLA-B;HMOX1;IL17RA;IL23A;IL4R;IL6R;JAK3;KAT5;KLF4;KLRC4-KLRK1;LCP2;LILRB1;LILRB2;LIPA;MAP2K3;MAPK13;MEFV;MYD88;NFAM1;NFKB1;NFKBIA;NLRC4;NLRP12;NLRP3;NLRX1;NOD2;NOTCH1;NOTCH2;PCBP2;PIK3CD;POLR3F;POMC;PRKCD;PRKD2;PTAFR;RAC1;RIPK1;SLC11A1;SOD1;STA
3;C1RL;C3;C4BPB;C5AR1;CALCOCO2;CAMK1D;CAMK2G;CCL3;CD14;CD163;CD1D;CD209;CD300C;CD44;CD86;CD97;CELA1;CLEC4C;COLEC12;COTL1;CREBBP;CRTAM;CSF1;CSF1R;CSF3R;CTSB;CXCR2;DAPK1;DOCK2;EGR1;ELK1;ELMO1;EMR2;EP300;F11R;FCN1;FOXP3;GBA;HCK;HDAC4;HLA-B;HLA-C;HLA-DPA

1;SOD1;UGT8
PHYH;PLOD1;TET3
CALCOCO1;CAMKK2;CAP1;CBFA2T3;CD3D;CD86;CDC42;CDH1;CELA1;CELF3;CHD8;CHEK1;CITED2;CREBBP;CTDP1;CTNNB1;DAB2;DNM2;DVL3;EGR1;ELF4;ELK1;ELL;ELL3;EP300;ESR1;EYA3;FLCN;FOXJ2;FOXO4;FOXP3;FZD1;GLI1;GNAL;GNAS;GSK3A;GTF2F1;GTPBP1;HAX1;HCLS1;HDAC4;HEXB;HIPK2;HIVEP3;H
D1A;ARID3A;ARNT;ASH2L;ATG7;ATP6V0D1;BCL9;BECN1;BRCA1;BRD4;BRE;BTG2;C3;C5AR1;CALCOCO1;CAMK1D;CAMKK2;CAP1;CBFA2T3;CCL3;CD3D;CD44;CD86;CDC42;CDH1;CELA1;CELF3;CHD8;CHEK1;CITED2;CNN2;CNPY2;CPT1A;CREBBP;CSF1;CSF1R;CSNK1D;CTDP1;CTDSP2;CTNNB1;DAB2;DBNL;DISC1
CTNNA1;CTNNAL1;DAPK1;DYNLL2;EPB41;FAM101B;FKBP15;FSCN1;GAS7;GSN;HAX1;HCK;HDAC4;LASP1;LCP1;LSP1;MAEA;MYH11;MYH9;MYL6;MYL6B;MYO1F;MYO7A;PAK1;PGM1;PPP1R12B;RCSD1;SH3PXD2B;SPTB;SVIL;TLN1;TNNC1;TPM3;TPM4;TRIOBP;VASP;VCL;WAS;WASF2;ZNF174;ZY
ND3;DNAJC19;ERGIC1;FAM160A2;FGF9;FIS1;FZD1;GCC1;GOSR1;HCLS1;IL23A;IMMP2L;JAK3;JPH4;KIF13A;KPNB1;LMTK2;MAP2K1;MAPK14;MFN2;MGRN1;NFKBIA;NLRP12;NLRP3;NRBP1;NUP98;NUPL2;NXT1;OPTN;PACS1;PEX7;PLCG2;PMPCB;POU5F1;PPP1R10;PRKACA;PTK2B;PTTG1IP;RAB23;RAB7A;RBM

ADAL;ADAM19;ADAP2;ADAR;ADCY4;ADH5;ADK;ADO;ADPRH;ADRBK2;AGBL2;AKAP13;ALAS1;ALB;ALKBH2;ALKBH3;ALOX15B;ALPK1;ALPK3;ALPL;AMPD3;AMY2B;AMZ2;ANKFY1;ANPEP;ANXA11;ANXA4;ANXA5;ANXA7;ANXA9;AOC2;APLP2;APOBEC3C;APP;ARAP1;ARAP3;ARF1;ARF3;ARF6;ARFGAP3;ARL1;AR
PHYH;PLOD1;TET3

CL3;CD14;CD1D;CD44;CD86;CDC42;CHEK1;COLEC12;CRTAM;DAB2;DBNL;DOCK2;DVL3;EIF2AK1;ELK1;ELMO1;EPHB1;EYA3;FOXP3;GBA;HCK;HIPK2;IFNAR2;IGF1R;IL17RA;IL23A;IRAK2;JAK1;KIAA1324;KLF4;KLRC4-KLRK1;LILRB1;LZTS1;MAP2K1;MAP2K3;MAP3K5;MAP4K4;MAPK13;MAPK14;MEFV;MYD88;M
;CPT1A;CSNK1D;DAB2;DAP;DAPK1;DAPK2;DISC1;DNAJB6;FBP1;FBXO22;FURIN;GAPDHS;GCK;GIT2;GLMN;GMIP;GSK3A;GTPBP1;HDAC4;INSR;IRS2;KIAA1324;LZTS1;MAP2K1;N4BP1;NCOR1;NDEL1;NUB1;PAFAH1B1;PGAM1;PIM2;PREX1;PRKACA;PRKCD;PSEN1;PSME3;PTK2B;RAD23B;RALBP1;RALGAPA2;RG
H2L;ATF5;ATF7;ATXN2;BASP1;BAZ2A;BCL6;BCL9;BCOR;BCORL1;BHLHE40;BRCA1;BRD3;BRD4;BRD8;BTK;C1QTNF3;C3;CALCOCO1;CAMK1D;CAMKK2;CAMTA1;CAP1;CARM1;CASC3;CASZ1;CBFA2T3;CBL;CBX1;CBX6;CBY1;CCL3;CCNK;CCRN4L;CD3D;CD86;CDC42;CDC5L;CDH1;CELA1;CFLAR;CHD8;CHEK1;CITE
CLS1;KHDRBS2;OSTF1;PTTG1;SH3BGR;SH3KBP1;SIRPA;TP53BP2;UVRAG;VASP;ZFP106
LRB1;MAGI2;MAP2K1;MERTK;NAPA;NOD2;NOTCH1;PACSIN2;PPT1;PRAM1;PTPN1;PTX3;RAB3D;RAB5B;RAB5C;RAC1;RALB;RUFY1;SLC11A1;SYK;TGFB1;VAMP8
;IGF2R;LDLRAD3;LILRB1;LRP1;MAGI2;PPT1;RAC1;RAMP1;SORL1;ULK1
PHYH;PLOD1;TET3
ARNT;ARSD;ARSG;ASB8;ASH2L;ATG7;ATP6V0D1;B3GNT9;B4GALT1;B4GALT4;B4GALT5;BAZ2A;BCCIP;BCOR;BRCA1;BRD4;BRD8;BRPF3;BTG2;BTK;BUB3;C3;C5AR1;CAMK2G;CAMKK2;CARM1;CBL;CCL3;CCNB1IP1;CCNK;CD44;CDC42;CDKL3;CFL1;CHEK1;CNOT4;COPS7A;COPS8;CREBBP;CSF1;CSF1R;CSNK1D;C
ARNT;ARSD;ARSG;ASB8;ASH2L;ATG7;ATP6V0D1;B3GNT9;B4GALT1;B4GALT4;B4GALT5;BAZ2A;BCCIP;BCOR;BRCA1;BRD4;BRD8;BRPF3;BTG2;BTK;BUB3;C3;C5AR1;CAMK2G;CAMKK2;CARM1;CBL;CCL3;CCNB1IP1;CCNK;CD44;CDC42;CDKL3;CFL1;CHEK1;CNOT4;COPS7A;COPS8;CREBBP;CSF1;CSF1R;CSNK1D;C

MOX1;IL23A;IL4R;JAK3;KLRC4-KLRK1;LILRB1;MYB;NOD2;PACS1;PCBP2;PRAM1;PVR;RAC1;SH2D1A;SIN3A;STAT5A;STAT5B;STAT6;SYK;TAP2;TGFB1;TNFSF1

NFKB1;NFKBIA;NLRC4;NOD2;POLR3F;PVR;RIPK1;RPS6KA1;SH2D1A;SIN3A;STAT5A;STAT5B;TIRAP;TLR9;TNIP1;UBE2V1;XIA

26;ARHGAP27;ARHGAP30;ARHGAP31;ARHGDIB;ARHGEF10;ARHGEF11;ARHGEF12;BCL6;CDC42;CSF1;DNMBP;FBP1;FLCN;GDI2;GIT2;GMIP;IQSEC1;ITSN1;MAP2K1;MFN2;NDEL1;NOTCH2;PAFAH1B1;PLEKHG6;PREX1;PTK2B;RAC1;RAC2;RAF1;RALBP1;RALGAPA2;RAP1GAP2;RGL3;RHOA;SEMA4D;SIPA1L1;SIP

ALT1;B4GALT4;B4GALT5;BCDIN3D;BTK;CAMK1D;CAMK2G;CAMKK2;CARM1;CASS4;CCBL1;CCDC111;CCL3;CCNK;CDKL3;CERKL;CERS2;CHEK1;CHKA;CHST11;CHST15;CHST7;COQ3;COQ5;CPT1A;CPT2;CREBBP;CS;CSF1R;CSNK1D;CSNK2A1;CTDP1;DAPK1;DAPK2;DGKG;DNMT3B;DPAGT1;DPH5;DYRK4;EIF2AK1;E

1;NHEJ1;PIK3CD;PKNOX1;PRKDC;SEMA4A;SOD1;SOX4;SRF;STAT5A;STAT5B;STAT6;SYK;TGFB

GNB5;GTPBP1;GTPBP5;MFN2;MX2;NKIRAS2;RAB23;RAB35;RAB3D;RAB43;RAB5B;RAB5C;RAB7A;RAC1;RAC2;RALB;RHEBL1;RHOA;RRAGC;RRAS2;SRP54;SRPR;ZFHX3

DC42;DNM2;GIT2;GMIP;GNA11;GNA12;GNA15;GNAI2;GNAL;GNAS;GNAT2;GNB1;GNB5;GTPBP1;MAP2K1;MX2;NDEL1;NME1;NME2;NUDT15;PAFAH1B1;PREX1;PTK2B;RAB23;RAB35;RAB3D;RAB43;RAB5B;RAB5C;RAB7A;RALBP1;RALGAPA2;RGS10;RGS16;RGS3;RRAGC;SEMA4D;SIPA1L1;SMAP2;SPRY2;SRG

DC42;DNM2;GIT2;GMIP;GNA11;GNA12;GNA15;GNAI2;GNAL;GNAS;GNAT2;GNB1;GNB5;GTPBP1;IMPDH1;MAP2K1;MX2;NDEL1;NME1;NME2;NUDT15;PAFAH1B1;PREX1;PTK2B;RAB23;RAB35;RAB3D;RAB43;RAB5B;RAB5C;RAB7A;RALBP1;RALGAPA2;RGS10;RGS16;RGS3;RRAGC;SEMA4D;SIPA1L1;SMAP2;SI

HGAP27;ARHGEF10;ARL1;BCL6;CD44;CDC42;CHI3L2;DNM2;GALNS;GBA;GIT2;GLB1;GM2A;GMIP;GNA11;GNA12;GNA15;GNAI2;GNAL;GNAS;GNAT2;GNB1;GNB5;GNS;GSK3A;GTPBP1;HEXB;HGSNAT;HSPA5;LYG1;MANBA;MAP2K1;MLH3;MX2;MYH9;NAGA;NAGK;NDEL1;NUDT15;NUDT7;PAFAH1B1;PDE7B;P

CYP2;ADAL;ADAM19;ADAP2;ADAR;ADCY4;ADH5;ADK;ADPRH;ADRBK2;ADSL;AFF2;AGA;AGBL2;AGPAT1;AHI1;AKR1C3;ALAS1;ALB;ALDH1A1;ALDH2;ALDH3B1;ALDH9A1;ALDOC;ALG14;ALG5;ALKBH2;ALKBH3;ALOX15B;AMPD3;AMY2B;AMZ2;ANAPC16;ANAPC4;ANP32A;ANPEP;AOAH;AOC2;APITD1;APLP2;AP

2;BUB3;C11orf73;C1QTNF3;CDC42;CDH1;CLTA;CLTC;CLTCL1;COPA;COPB2;COPZ2;COX18;CTNNB1;CTSA;DAB2;DAG1;DNAJC19;EHD1;EHD3;EIF2D;EPB41L3;FGF9;FIS1;FLCN;FZD1;GCC1;HCLS1;IL23A;IMMP2L;JAK3;KDELR1;KIF13A;KPNB1;LCP1;LTBP2;MAGI2;MAPK14;MAPRE1;MFN2;MRPL45;MYO7A;NACC

PK14;MMP9;MYH9;NME1;PAFAH1B1;PIK3CD;PSEN1;STAT5A;TFE3;TGFB1;THOC5

PP1;PRKACA;PRKCD;PSEN1;PTK2B;PTPRJ;PXN;RAB7A;RAF1;RBPJ;SH3KBP1;SPRY2;SRC;TGFB1;YWHAB

3;LILRB2;MERTK;MYB;NCOR1;NFAM1;NHEJ1;PIK3CD;PKNOX1;PLCG2;POU2F2;PRKDC;PTK2B;RNF41;SEMA4A;SOD1;SOX4;SRF;STAT5A;STAT5B;STAT6;SYK;TGFB

ARL1;ATP6V0A1;ATP6V1B2;ATXN2;B3GNT9;B4GALT1;B4GALT4;B4GALT5;BECN1;BHLHE40;BICD2;BSG;CALCOCO2;CANT1;CBFA2T3;CBY1;CDC42;CDH1;CERKL;CHST11;CHST15;CHST7;CLCN5;CLTA;CLTC;CLTCL1;COG8;COPA;COPB2;COPZ2;CRYZ;CSNK1D;CUX1;DENND5A;DNM2;DNMBP;DYRK4;EPB41;ERC1;E

AT5B;STAT6;SYK;TAP2;TGFB1;TNFSF1

NOD2;NOTCH1;NOTCH2;SYK;TRIM27

FB1;TNFSF1

L;APOA1;APP;ARAP1;ARF6;ARHGEF10;ARHGEF11;ARID1A;ARID3A;ARNT;ASH2L;ATG7;ATP6V0D1;B4GALT1;BBS4;BCL2L13;BCL6;BCL9;BECN1;BLOC1S3;BNIP3;BRCA1;BRD4;BRE;BTG2;BTK;C1QTNF3;C3;C5AR1;C6orf89;CALCOCO1;CAMK1D;CAMKK2;CANT1;CAP1;CAPN1;CAPNS1;CARM1;CASP6;CASP9;CBFA2

F1;CSF1R;CXCR2;EMR2;F11R;FOXP3;GBA;HCK;HDAC4;HMOX1;IL17RA;IL23A;IL4R;IL6R;IRAK2;ITGB2;KDM6B;KLF4;LIPA;LTA4H;LTB4R;MAP2K3;MAPK13;MEFV;MMP25;MYD88;NDST1;NFAM1;NFKB1;NLRC4;NLRP12;NLRP3;NLRP6;NOD2;NOTCH1;NOTCH2;OSMR;P2RX1;PARP4;PIK3CD;PNMA1;PRKCD;PTAFR

AB2;DAG1;DAPK1;DAPK2;DBNL;DVL3;ELK1;EPHB1;ERC1;ESR1;FGF9;FKBP1A;FLCN;FLT3;GBA;GNA12;GNAI2;GPR183;GRB2;HAX1;HCLS1;HIPK2;HMOX1;IFNAR2;IGF1R;IL23A;IL6R;INSR;IRAK2;IRS2;ITSN1;JAK1;JAK3;KLF4;MAGI2;MAP2K1;MAP2K3;MAP3K3;MAP3K5;MAP4K4;MAPK13;MAPK14;MAPKAPK5;M

B1;LTBP2;NLRC4;NLRP12;NLRP3;NOD2;NOTCH1;NOTCH2;PAFAH1B1;PLEK;POU2F2;PPID;RHBDF2;STEAP3;SYK;TGFB1;TRIM2

;PAK1;PIK3CD;PLCG2;PRKCB;PRKD2;PSEN1;PTPRJ;SPG21;SYK;TRAT1;UBE2V1;VASP;WAS

DC42;DNM2;GIT2;GMIP;GNA11;GNA12;GNA15;GNAI2;GNAL;GNAS;GNAT2;GNB1;GNB5;GTPBP1;MAP2K1;MX2;NDEL1;NUDT15;PAFAH1B1;PREX1;PTK2B;RAB23;RAB35;RAB3D;RAB43;RAB5B;RAB5C;RAB7A;RALBP1;RALGAPA2;RGS10;RGS16;RGS3;RRAGC;SEMA4D;SIPA1L1;SMAP2;SPRY2;SRGAP2;SRP54;T

KK2;CBFA2T3;CD3D;CD86;CDH1;CELA1;CELF3;CHD8;CHEK1;CITED2;CREBBP;CTDP1;CTNNB1;DAB2;DNM2;DVL3;EGR1;ELF4;ELK1;ELL;ELL3;EP300;ESR1;FLCN;FOXJ2;FOXO4;FOXP3;FZD1;GLI1;GTF2F1;GTPBP1;HAX1;HCLS1;HDAC4;HEXB;HIPK2;HIVEP3;HMGN3;HNRNPD;HNRNPK;IKZF1;IL23A;JAK3;KAT5;KAT6

;AMZ2;ANAPC16;ANAPC4;ANPEP;APITD1;APLP2;APOA1;APP;ARNT;ARSD;ARSG;ASB8;ASH2L;ASPRV1;ATG7;ATP6V0D1;ATXN2;B3GNT9;B4GALT1;B4GALT4;B4GALT5;BAZ2A;BCCIP;BCOR;BNIP3;BRCA1;BRD4;BRD8;BRPF3;BTG2;BTK;BUB3;C1RL;C3;C4BPB;C5AR1;CAMK2G;CAMKK2;CANT1;CAPN1;CARM1;CA

AGI2;PSEN1;RAMP1;SMURF1;TGFB1;TRAT1;ULK1

RAP3;ARF1;ARHGAP21;ARHGAP24;ARHGAP26;ARHGDIB;ARHGEF10;ARID1A;ARL6;ATXN2;BASP1;BAZ2A;BBS4;BCL6;BHLHE40;BICD2;BIN2;BOD1;BRCA1;BRD4;BRIX1;BUB3;CACNG8;CALCOCO1;CALCOCO2;CAP1;CAPZB;CASS4;CBFA2T3;CBX1;CBX6;CBY1;CC2D2A;CCNB1IP1;CCRN4L;CDC42;CDC42EP4;CDC42

RAP3;ARF1;ARHGAP21;ARHGAP24;ARHGAP26;ARHGDIB;ARHGEF10;ARID1A;ARL6;ATXN2;BASP1;BAZ2A;BBS4;BCL6;BHLHE40;BICD2;BIN2;BOD1;BRCA1;BRD4;BRIX1;BUB3;CACNG8;CALCOCO1;CALCOCO2;CAP1;CAPZB;CASS4;CBFA2T3;CBX1;CBX6;CBY1;CC2D2A;CCNB1IP1;CCRN4L;CDC42;CDC42EP4;CDC42

BCL9;BRCA1;BRD4;BRE;BTG2;C3;C5AR1;CALCOCO1;CAMKK2;CBFA2T3;CCL3;CD3D;CD44;CD86;CDC42;CDH1;CELA1;CELF3;CHD8;CHEK1;CITED2;CNN2;CNPY2;CREBBP;CSF1;CSF1R;CSNK1D;CTDP1;CTDSP2;CTNNB1;DAB2;DBNL;DISC1;DNM2;DNMT3B;DVL3;ECE1;EGR1;ELF4;ELK1;ELL;ELL3;EP300;ESR1;EXTL3

PRKCD;PSEN1;RAB23;SQSTM1;TBC1D14;TP53INP2;ULK1;VDR;WIPI1;WIPI2

SG;CACNG8;CAMK2G;CHGA;CLTA;CLTC;CLTCL1;COPA;COPB2;COPZ2;DAB2;DENND1A;DNAJC5;DYSF;F13A1;FLOT2;HIP1;HLA-B;HLA-C;HLA-DPA1;HLA-E;KIAA0319L;KIF1B;LEFTY2;MARCH8;MYOF;PECAM1;RAB11FIP1;RAB23;RAB43;RAB7A;RAB8A;SEC23B;SEC31A;SERPINA1;SH3KBP1;SLC11A1;SNX19;SNX24;

DC42;DNM2;GIT2;GMIP;GNA11;GNA12;GNA15;GNAI2;GNAL;GNAS;GNAT2;GNB1;GNB5;GTPBP1;MAP2K1;MX2;NDEL1;NUDT15;PAFAH1B1;PREX1;PTK2B;RAB23;RAB35;RAB3D;RAB43;RAB5B;RAB5C;RAB7A;RALBP1;RALGAPA2;RGS10;RGS16;RGS3;RRAGC;SEMA4D;SIPA1L1;SMAP2;SPRY2;SRGAP2;SRP54;T

1;SOD1;UGT8

FF2;AGA;AGBL2;AHI1;AKR1C3;ALAS1;ALB;ALG14;ALG5;ALKBH2;ALKBH3;AMZ2;ANAPC16;ANAPC4;ANP32A;ANPEP;AOAH;APITD1;APLP2;APOA1;APOBEC3C;APP;ARHGEF11;ARID1A;ARID3A;ARID3B;ARNT;ARSD;ARSG;ASB8;ASCC2;ASH2L;ASPRV1;ATF5;ATF7;ATG7;ATP6V0D1;ATXN2;B3GNT9;B4GALT1;B4GA

A4;ANXA5;AP2A1;AP2M1;APBB1IP;APC2;APLP2;APOA1;APP;ARAP1;ARAP3;ARF1;ARF3;ARF6;ARFGAP3;ARHGAP1;ARHGAP21;ARHGAP23;ARHGAP24;ARHGAP25;ARHGAP26;ARHGAP27;ARHGAP30;ARHGAP31;ARHGDIB;ARHGEF10;ARHGEF11;ARHGEF12;ARID1A;ARL1;ARL6;ARNT;ASB8;ATP6V0A1;ATP6V

K2G;CHGA;CLTA;CLTC;CLTCL1;COPA;COPB2;COPZ2;DAB2;DENND1A;DNAJC5;DYSF;FLOT2;GRB2;HIP1;HLA-B;HLA-C;HLA-DPA1;HLA-E;KIF1B;MARCH8;MYOF;PECAM1;RAB11FIP1;RAB23;RAB43;RAB7A;RAB8A;SEC23B;SEC31A;SH3KBP1;SLC11A1;SNX19;SNX24;SPPL3;SRI;SV2B;SYT2;TMEM67;ULK1;VAMP2;V

CREBBP;CRTAM;CSF1;CSF1R;CTSB;DAPK1;EGR1;ELK1;EP300;FCN1;HCK;HLA-B;HLA-C;HLA-DPA1;HLA-E;IFNAR2;IL23A;IRAK2;IRF2;JAK1;JAK3;KLRC4-KLRK1;LILRB1;MAP2K1;MAP2K3;MAP3K5;MAPK14;MEFV;MX2;MYD88;MYO1F;NCF2;NFKB1;NFKBIA;NLRC4;NLRP3;NLRX1;NOD2;NUB1;PCBP2;PIK3CD;POLR3

5B;SYK

SEC31A;SNAP29;WIPI1

2;IRS2;ITGB2;KCNA5;KIAA1598;MAML1;MAP2K1;MAP2K3;MAP3K5;MAPKAPK5;MICALCL;MSN;NBR1;NEK6;NME1;NOD2;PAK1;PGAM1;PLEK;PRKAB1;PRKACA;PRKCB;PRKCD;PTK2B;PTPN1;PTPN6;PTPRJ;PXN;RAB8A;RAC1;RAF1;SKI;SPDYA;SPRED1;SPRY2;SQSTM1;STAT3;SYK;TBC1D14;TIRAP;TNIP1;TPCN2;U

7;ATPIF1;B4GALT1;BCL2L13;BCL6;BECN1;BEX2;BNIP3;BRCA1;BTG2;BTK;C5AR1;CAMK1D;CASP6;CASP9;CBL;CCL3;CD44;CDC42;CDH1;CERKL;CFL1;CFLAR;CHD8;CHST11;CITED2;CSNK2A1;CTNNA1;CTNNB1;CTSB;CXCR7;DAB2;DAP;DAP3;DAPK1;DAPK2;DEDD;DNAJB6;DNAJC5;DNM2;DPF2;EGR1;EP300;ESR1;

4;APOBEC3C;ARAP1;ARAP3;ARF1;ARF3;ARF6;ARFGAP3;ARHGAP26;ARHGAP27;ARHGEF10;ARL1;ARNT;ATG2A;ATG7;BCKDHA;BCL6;BCO2;BECN1;BNIP3;BSCL2;BTG2;BUB3;CAPN1;CASC3;CBR3;CCBL1;CCRN4L;CD44;CDC42;CHI3L2;CLC;CLPX;CNOT1;CNOT4;CPT1A;CPT2;CRYZ;CSAD;CSNK1D;CTIF;CTSD;DAB2;

OS;IKZF1;IL23A;IL4R;IRS2;KLRC4-KLRK1;LILRB1;LILRB2;MYB;NOD2;PAK1;PLEK;PTPN6;PTPRJ;RAC1;RPS6KA1;SRC;STAT5A;STAT5B;STAT6;SYK;TGFB1;TIRAP;TNFSF13;VAV

PP1;PRKACA;PRKCD;PSEN1;PTK2B;PTPRJ;PXN;RAB7A;RAF1;SH3KBP1;SPRY2;SRC;TGFB1;YWHAB

;RAB11FIP1;RAB23;RAB43;RAB7A;RAB8A;SLC11A1;SYT2;VAMP2

PLCG2;PRKCB;PRKD2;PSEN1;PTPRJ;SPG21;SYK;TRAT1;UBE2V1;VASP;WAS

6;LMTK2;MAP2K1;MARCH8;MGRN1;NUMB;PTPN1;RAB5B;RAB5C;RIN3;RUFY1;SNX1;SNX27;TRAK1;TSG101;UVRAG;VAMP8;WASF2

RHGEF11;ARHGEF12;C6orf170;DENND1A;DENND3;DENND5A;DNMBP;DOCK2;GDI2;GIT2;GMIP;IQSEC1;ITSN1;MAP4K4;MAPRE3;PLEKHG6;PREX1;RALBP1;RALGAPA2;RCC1;RIN2;RIN3;SMAP2;SRGAP2;TBC1D1;TBC1D14;TBC1D19;TBC1D2;TBC1D20;TBC1D2B;TBC1D5;TBC1D8;TBC1D9;VAV1;WA

AC1;ST14

C10orf88;C1QTNF3;CALCOCO2;CARM1;CARS;CASC3;CBX1;CBY1;CCBL1;CDC42;CHKA;CLDN16;CLDN9;CPOX;CPT1A;CSF1;CSF1R;DAPK1;DAPK2;DYNLL1;ECE1;EID3;EIF2AK1;ESR1;FLT3;FOXP3;G6PD;GLUL;GOLGA5;GRB2;GSTA4;HEXB;HM13;HMOX1;HSD17B4;IGF1R;IL6R;IRAK2;KCNRG;KLRC4-KLRK1;LCP1;LILR

APITD1;APOA1;APOBR;APP;ARCN1;ARID1A;ARL6;ARNT;ASH2L;ATF5;ATP5S;ATP6V0A1;ATP6V0C;ATP6V0D1;ATP6V1B2;ATPIF1;ATXN2;BASP1;BAZ2A;BBS4;BCCIP;BCKDHA;BCOR;BECN1;BIN2;BLOC1S3;BOD1;BRAP;BRCA1;BRD8;BRE;BRPF3;BUB3;CACNG8;CAMK1D;CAMK2G;CAPZB;CASC3;CASP9;CBL;CBX1;

IK2G;CHGA;CLTA;CLTC;CLTCL1;COPA;COPB2;COPZ2;DAB2;DENND1A;DNAJC5;DYSF;FLOT2;HIP1;HLA-B;HLA-C;HLA-DPA1;HLA-E;KIF1B;MARCH8;MYOF;PECAM1;RAB11FIP1;RAB23;RAB43;RAB7A;RAB8A;SEC23B;SEC31A;SH3KBP1;SLC11A1;SNX19;SNX24;SPPL3;SRI;SV2B;SYT2;TMEM67;ULK1;VAMP2;VAMP

1;APP;ARCN1;ARID1A;ARL6;ARNT;ASH2L;ATF5;ATP5S;ATP6V0A1;ATP6V0C;ATP6V0D1;ATP6V1B2;ATPIF1;BASP1;BAZ2A;BBS4;BCCIP;BCKDHA;BCOR;BECN1;BIN2;BLOC1S3;BOD1;BRAP;BRCA1;BRD8;BRE;BRPF3;BUB3;CACNG8;CAMK1D;CAMK2G;CAPZB;CASC3;CASP9;CBL;CBX1;CBX6;CC2D2A;CCNK;CD3D;CD
LT4;B4GALT5;BSG;CANT1;CBFA2T3;CDC42;CHST11;CHST15;CHST7;CLCN5;CLTA;CLTC;CLTCL1;COG8;COPA;COPB2;COPZ2;CUX1;DNM2;ERC1;ERGIC1;FIG4;FURIN;GALNT11;GALNT2;GCC1;GCNT7;GOLGA2;GOLGA5;GOLGB1;GOLIM4;GOSR1;HLA-B;HLA-C;HLA-DPA1;HLA-E;KIF13A;MAN2A2;NDST1;NDST2;NOT
AI2;GNAL;GNAS;GNAT2;GTPBP1;GTPBP5;INSR;LRRK1;MFN2;MX2;NKIRAS2;NME1;NUDT2;RAB19;RAB23;RAB35;RAB36;RAB3D;RAB40B;RAB42;RAB43;RAB5B;RAB5C;RAB7A;RAB8A;RABL5;RAC1;RAC2;RALB;RHEBL1;RHOA;RRAGB;RRAGC;RRAS2;SEPHS1;SRP54;SRPR;THG1L;ZFHX

RMN;FBXO25;FHDC1;FKBP15;FSCN1;GAS7;GSN;HIP1;LASP1;LCP1;LSP1;MAEA;MAP1S;MEFV;MICAL2;MYH11;MYH9;MYO1F;MYO7A;RCSD1;SPTB;SSH1;SSH2;SVIL;TLN1;TMSB10;TMSB15B;TNNC1;TPM3;TPM4;TRIOBP;VASP;VCL;WAS;WASF2;WIPF2;YWHA
NAI2;GNAL;GNAS;GNAT2;GRB10;HCLS1;IGF1R;IRS2;ITGA5;KAT5;LRP1;MAX;MLH3;MMP14;NDEL1;NFKBIA;NME1;PAF1;PAFAH1B1;PIK3R5;PTK2B;PTPN1;PXN;RPAIN;SH2B3;SIN3A;SLC27A5;SMURF1;SRC;SYK;TGFBI;TIMP2;TLN1;ULK1;YWHA

HD7;CLPX;COQ3;COX11;COX17;COX18;COX5A;COX6A1;CPOX;CPT1A;CPT2;CS;CYB5A;CYB5R3;CYP27A1;DAP3;DNAJC19;FDX1;FIS1;FUNDC1;GHITM;HAAO;HIBADH;HK1;HK2;IMMP2L;LRRC59;ME3;MFN2;MICU1;MRP63;MRPL11;MRPL17;MRPL27;MRPL34;MRPL35;MRPL36;MRPL43;MRPL45;MRPL51;MRPS1
AC1;RAF1;RALBP1;RIN3;RPH3A;SRGAP2;TRIOBP;ULK1;VCL

DH1;CFL1;CFL2;CNN2;CORO1C;COTL1;CTNNA1;CYFIP1;DAG1;DBNL;DNM2;DYNLL2;EPB41;EPB41L3;ERMN;FAM101B;FBXO25;FHDC1;FKBP15;FSCN1;GAS7;GLI1;GSN;HIP1;KATNA1;KCNA5;KIF1B;LASP1;LCP1;LMTK2;LSP1;MAEA;MAP1S;MAPRE1;MAPRE3;MEFV;MICAL2;MSN;MYH11;MYH9;MYO1F;MYO7A;
;MARCH8;MYO7A;NCSTN;NEU1;PSAP;PSEN1;RAB7A;SLC36A1;SLC48A1;TLR9;TM9SF1;TMBIM1;TPCN2;VAMP8;WIPI1;WIPI2;ZER

;PSAP;PSEN1;RAB7A;SLC36A1;SLC48A1;TLR9;TM9SF1;TMBIM1;TPCN2;VAMP
CS1;SPPL3;TAP2;TAPB

YOF;NFAM1;P2RX1;PPT1;PSEN1;PTK2B;PTRF;RIPK1;SLC9A1;SPRED1;SRC;TNFRSF1
122;TOMM7;XPO6;XPO
B3D;RAB7A;RAB8A;RAC1;RAF1;RALBP1;RIN3;RPH3A;SRGAP2;TRIOBP;ULK1;VCL

4;ARHGAP31;ARL6;ATP6V0D1;ATP6V1B2;B4GALT1;BASP1;BBS4;BECN1;BNIP3;BRCA1;C6orf170;CACNG8;CAPZB;CBY1;CC2D2A;CCDC135;CDC42;CDH1;CFL1;CTNNA1;CTNNB1;CTNND1;CYFIP1;DAG1;DBNL;DENND1A;DISC1;DNAH17;DNM2;DYSF;EFHC1;EMR2;EPB41L3;EPHB1;EPHB2;ERMN;EVC;FKBP15;FK
17;COX18;COX5A;COX6A1;CPOX;CPT1A;CPT2;CYB5A;CYB5R3;CYP27A1;DAG1;DNAJC19;FIS1;FUNDC1;GHITM;GRIN2D;HAAO;HAX1;HIPK2;HK1;HK2;IGF2R;IMMP2L;KPNB1;LRRC59;MFN2;MGST3;MICU1;MRPL17;MRPL45;MRPS14;MRPS23;MRTO4;MX2;MYOF;NDEL1;NDUFA12;NDUFA7;NDUFAB1;NDUFAF4
;KAT5;KAT6A;KLF7;LPIN2;MAML1;MAML3;MAX;MED12;MTF1;NCOA6;NFYC;PHF2;PRKCB;RNF14;RXRA;SAP130;SMARCA2;SMARCD2;SOX4;SUPT3H;TAF11;TAF4;TCF20;THRAP3;TRERF1;USP22;YY

YA3;FOXP3;GLI1;H2AFY;HESX1;HMGN3;HMGN4;JARID2;MBD6;MED12;MLH3;MYB;NCOA6;NCOR1;NOTCH1;NSD1;PAF1;PBX2;PHF21A;PHF8;PLAC8;PRKCB;PRMT5;RCC1;RCOR1;RERE;RXRA;SIN3A;SKI;SRF;TADA2B;TRERF1;TTC5;WDR8

CRNKL1;CSNK2A1;CTNNB1;ELF4;ELL;ELL3;EP300;EYA3;FANCF;FOXJ2;FOXN3;FOXO4;FOXP3;GATAD2B;GTF2F1;HAX1;HCLS1;HDAC4;HDGF;HIPK2;HSPA1B;ING4;INTS3;JARID2;KAT5;KAT6A;KAT7;KLF4;MAFB;MAML1;MAML3;MAX;MED12;MKNK2;MLL2;N4BP1;NACC2;NCOA6;NCOR1;NEK6;NFYC;NR2C1;PAF1

AI2;GNAL;GNAS;GNAT2;GTPBP1;GTPBP5;INSR;LRRK1;MFN2;MX2;NKIRAS2;NME1;NUDT2;RAB19;RAB23;RAB35;RAB36;RAB3D;RAB40B;RAB42;RAB43;RAB5B;RAB5C;RAB7A;RAB8A;RABL5;RAC1;RAC2;RALB;RHEBL1;RHOA;RRAGB;RRAGC;RRAS2;SEPHS1;SRP54;SRPR;THG1L;ZFHX
AI2;GNAL;GNAS;GNAT2;GTPBP1;GTPBP5;INSR;LRRK1;MFN2;MX2;NKIRAS2;NME1;NUDT2;RAB19;RAB23;RAB35;RAB36;RAB3D;RAB40B;RAB42;RAB43;RAB5B;RAB5C;RAB7A;RAB8A;RABL5;RAC1;RAC2;RALB;RHEBL1;RHOA;RRAGB;RRAGC;RRAS2;SEPHS1;SRP54;SRPR;THG1L;ZFHX

P8
X12;SNX19;SNX24;SNX27;SNX30;SNX33;TIRAP;WIPI1;WIPI2;ZFYVE1
1;HLA-B;HLA-C;HLA-DPA1;HLA-E;SEC23B;SEC31A;SV2B;SYT2;VAMP2

D2;NUP98;PEX7;PPID;SLC7A5;SRP54;TAP2;TAPBP;TPP1
STAT3;STAT5B;TAF11;THRAP3;VDR;WIPI1;YWHA
E1;MSN;PLEK;PTPRJ;RAB35;RAC1;RHOA;SH3YL1;SPRY2;TIRAP;TLN1;TMEM67;VAS
;CHKA;CPOX;CSF1;CSF1R;DYNLL1;ECE1;EIF2AK1;FLT3;FOXP3;G6PD;GOLGA5;GSTA4;HEXB;HM13;HMOX1;HSD17B4;IL6R;IRAK2;KLRC4-KLRK1;LILRB1;MAP3K5;MAX;MTERFD3;MYH9;NACC2;NAGA;NFKB1;NLRC4;NR2C1;NRBP1;PAFAH1B1;PCYT1A;PDCD6;PEX7;PLEK;PLOD1;PRKRA;PVRL1;PYGL;RAB11FIP4;SE

AP2K3;MERTK;PRKCD;PTK2B;SRC;SYK;TP53RK;TRIM2
H2D3C;SH3BGR;SLA;SOCS2;SRC;TIRAP;TP53BP2;TRAT1

LT4;B4GALT5;BSG;CANT1;CBFA2T3;CDC42;CHST11;CHST15;CHST7;CLCN5;CLTA;CLTC;CLTCL1;COG8;COPA;COPB2;COPZ2;CUX1;DNM2;DNMBP;ERC1;ERGIC1;FIG4;FURIN;GALNT11;GALNT2;GCC1;GCNT7;GOLGA2;GOLGA5;GOLGB1;GOLIM4;GOSR1;HLA-B;HLA-C;HLA-DPA1;HLA-E;IGF2R;KIF13A;MAN2A2;MM
METTL6;MLL2;N6AMT1;N6AMT2;NSD1;PRMT5;PRMT6;SETD1B;SETDB1;SETMAR;TFB2M;THUMPD2;TRMT11;TRMT61B;WDR
T3B;ENO1;EP300;ERF;FOXP3;GMEB1;GTF2F1;HDAC4;HDGF;HIPK2;ING4;KAT5;KAT6A;KDM5B;KLF4;KLF7;LPIN2;MAML1;MAML3;MAX;MED12;MTF1;NACC2;NCOA6;NCOR1;NFYC;NOTCH1;NSD1;PHF12;PHF2;PRKCB;RNF14;RXRA;SAP130;SIN3A;SKI;SMARCA2;SMARCD2;SOX4;SPEN;SRF;SRSF2;SUPT3H;TAF1

17;COX18;COX5A;COX6A1;CPOX;CPT1A;CPT2;CYB5A;CYB5R3;CYP27A1;DNAJC19;FIS1;FUNDC1;GHITM;HAAO;HAX1;HIPK2;HK1;HK2;IGF2R;IMMP2L;KPNB1;LRRC59;MFN2;MGST3;MICU1;MRPL17;MRPL45;MRPS14;MRPS23;MRTO4;MX2;MYOF;NDEL1;NDUFA12;NDUFA7;NDUFAB1;NDUFAF4;NDUFB2;NDU
D2;NUP98;PEX7;PPID;SLC7A5;SRP54;TAP2;TAPBP;TPP1

;PRG2;RAB3D;SEC23B;SEC31A;SPG21;TGOLN2;VAMP2;YIPF3
35;HCK;MSN;NUMB;PLAUR;RAC1;ST14

A5;SPCS1;SPPL3;TAP2;TAPBP
XRA;SOCS2;STAT3;STAT5B;TAF11;THRAP3;VDR;WIPI1;YWHAH

AC1;RAF1;RALBP1;RIN3;RPH3A;SRGAP2;TRIOBP;ULK1;VCL
A1;CPOX;CPT1A;CPT2;CYB5A;CYB5R3;CYP27A1;DNAJC19;FIS1;FUNDC1;GHITM;HAAO;HK1;HK2;IMMP2L;MFN2;MICU1;MRPL17;MRPL45;NDUFA12;NDUFA7;NDUFAB1;NDUFAF4;NDUFB2;NDUFC1;NDUFS3;NDUFV2;NLRX1;OCIAD2;PHB2;PINK1;PMPCB;PSEN1;PTPMT1;RAF1;RNF185;ROMO1;SDHC;SLC25A1
SB;CXCR7;CYFIP1;DNAJB6;DOCK6;ECE1;EHD1;EHD3;EIF4H;GALNT2;GLB1;GSN;HFE;HSPA1B;KAT5;LMTK2;MAGI2;MANF;MAP1S;MAP2K1;MAPRE3;MT1E;MTMR14;NANOS1;NDRG1;NME1;NRBP1;OPTN;PAFAH1B1;PLA2G16;PPM1F;PREX1;PRKCD;PRKRA;PSEN1;PTK2B;RAB8A;RAP1GAP2;SEC23B;SEC31A;TN

SF
HKA2
2D1A;SH2D3C;SH3BGR;SLA;SOCS2;SRC;TIRAP;TP53BP2;TRAT1

NAJC5;ECE1;FURIN;HIP1;HLA-B;HLA-C;HLA-DPA1;HLA-E;IGF2R;PACS1;PPT1;RAB35;RPH3A;SEC23B;SEC31A;SPG21;SV2B;SYT2;TPRG1L;VAMP2;WIPI1
F;THRAP3;TRERF1

;RAC1;SCAMP2;TGOLN2;WIPI1
RK1;MAN2A2;MAN2B2;MANBA;MGAM;NOMO2;NOMO3;PHYH;PLOD1;PRG2;PTX3;PYGL;SELL;SIGLEC7;SIGLEC9;UAP1;VCAN
METTL6;MLL2;N6AMT1;N6AMT2;NSD1;PRMT5;PRMT6;SETD1B;SETDB1;SETMAR;TFB2M;THUMPD2;TRMT11;TRMT61B;WDR
AP;BRCA1;BRPF3;CA13;CA3;CA6;CASZ1;CBFA2T3;CBL;CNOT4;CPM;CREBBP;CRYZ;DHX34;DPF2;DUSP12;EGR1;EP300;ESR1;FBXO40;FOXP3;FUS;GATAD2B;GIT2;GLI1;GTF3A;HDAC4;HIVEP3;IKZF1;ING4;KAT6A;KAT7;KDM2A;KDM4B;KDM5B;KLF13;KLF4;KLF7;KLF9;KPNB1;LASP1;LIMK1;LIMK2;LOC440434;LTA4

SOD1;SPRED1;TRPC4AP;WNK1
B5A;CYB5R3;CYP27A1;DNAJC19;FIS1;FUNDC1;GHITM;HAAO;HK1;HK2;IMMP2L;MFN2;MICU1;MRPL17;MRPL45;NDUFA12;NDUFA7;NDUFAB1;NDUFAF4;NDUFB2;NDUFC1;NDUFS3;NDUFV2;NLRX1;OCIAD2;PHB2;PINK1;PMPCB;PSEN1;PTPMT1;RAF1;RNF185;ROMO1;SDHC;SLC25A14;SLC25A26;SLC25A33;S

T3B;ENO1;EP300;ERF;FOXP3;GMEB1;GTF2F1;HDAC4;HDGF;HIPK2;ING4;KAT5;KAT6A;KDM5B;KLF4;KLF7;LPIN2;MAML1;MAML3;MAX;MED12;MTF1;NACC2;NCOA6;NCOR1;NFYC;NOTCH1;NSD1;PHF12;PHF2;PRKCB;RNF14;RXRA;SAP130;SIN3A;SKI;SMARCA2;SMARCD2;SOX4;SPEN;SRF;SRSF2;SUPT3H;TAF1

TC19

;EP300;ERF;FOXP3;GMEB1;GTF2F1;HDAC4;HDGF;HIPK2;ING4;KAT5;KAT6A;KDM5B;KLF7;LPIN2;MAML1;MAML3;MAX;MED12;MTF1;NCOA6;NCOR1;NFYC;NSD1;PHF12;PHF2;PRKCB;RNF14;RXRA;SAP130;SIN3A;SKI;SMARCA2;SMARCD2;SOX4;SPEN;SRSF2;SUPT3H;TAF11;TAF4;TBL1X;TCF20;TFB2M;THRAP3
OR1;PHF12;RCC1;RXRA;SCRT2;SIN3A;SMARCA2;SRF;STAT6;YY1
A1;HLA-E;HM13;HSPA5;MFN2;MICU1;QSOX1;RRBP1;RTN1;RTN4;SGPL1;SLC27A5;SPCS1;SPPL3;ST3GAL2;TAP2;TAPBP
;PTRHD1;SSH1;SSH2

1;MYH9;MYO1F;MYO7A;NRBP1;PAFAH1B1;PSEN1;PTK2B;PXN;RHOA;SPTB;TPM4

GHITM;IMMP2L;MICU1;MRPL17;MRPL45;NDUFA12;NDUFA7;NDUFAB1;NDUFB2;NDUFC1;NDUFS3;NDUFV2;OCIAD2;PHB2;PMPCB;PSEN1;PTPMT1;ROMO1;SDHC;SLC25A14;SLC25A26;SLC25A33;SLC25A44;SRC;TIMM8A;TIMM9;TK2;TOMM22;TTC19;UQCR10;UQCR11;USMG

RAP1;ARAP3;ARFGAP3;ATF7;BAZ2A;BCL6;BRAP;BRCA1;BRPF3;CA13;CA3;CA6;CASZ1;CBFA2T3;CBL;CNOT4;COX11;COX17;CPM;CREBBP;CRYZ;CYP27A1;CYP2R1;CYP4F3;DHX34;DPF2;DUSP12;EGR1;ENDOU;EP300;ESR1;FBXO40;FDX1;FOXP3;FTO;FUS;GALNT2;GATAD2B;GIT2;GLI1;GLUL;GTF3A;HAAO;HDAC4
BRCA1;CACNG8;CASS4;CDH1;CHRNB1;CHRNB2;CLDN16;CLDN9;CNN2;CTNNA1;CTNNB1;CTNND1;CYFIP1;DAG1;DENND1A;DISC1;DNM2;DNMBP;EPB41L3;F11R;FAM129B;FLCN;FSCN1;GABRR2;GRIN2D;GTF2F1;HCK;IQSEC1;IRF2;ITGA5;ITGB4;ITSN1;KCNA5;LASP1;LCP1;LIMK1;LIN7B;LRFN1;LZTS1;MAGI2;M
;NEU1;PHKA2;SMUG1

M13;HSPA5;MICU1;QSOX1;RRBP1;RTN1;RTN4;SGPL1;SLC27A5;SPCS1;SPPL3;ST3GAL2;TAP2;TAPB

JFAB1;NDUFB2;NDUFC1;NDUFS3;NDUFV2;SDHC;TOMM22;TOMM7;USMG5

0;H2AFY;HIST3H2A;HMGN3;HMGN4;HNRNPK;IKZF1;KAT5;KAT6A;KDM2A;KLF4;MAU2;NACC2;NCOR1;PHF12;PPP1R10;RCC1;RXRA;SCRT2;SIN3A;SMARCA2;SRF;STAT6;TOX4;WDR61;WDR82;YY1

1;CTNNB1;CYFIP1;DAG1;DBNL;DENND1A;DISC1;DNM2;EPB41L3;EPHB1;EPHB2;ERMN;FKBP15;FKBP1A;FZD1;GLUL;GNAS;GNB1;GPRIN1;IGF1R;ITSN1;KATNA1;KCNC3;KCND1;KIAA1598;KIF1B;LAMP1;LIMK1;LIN7B;LRFN1;LZTS1;MAGI2;MAP1S;MAP2K1;MAX;MBP;NDEL1;NEK3;NGDN;NMB;P2RX1;PAFAH1B1
;SLC9A8;UQCR10;UQCR11;ZER1

CAD8;ACE;ACER2;ACLY;ACN9;ACO2;ACOX2;ACOX3;ACSL5;ACTR10;ACTR8;ADCY3;ADCY6;ADD1;ADSS;AFG3L2;AGBL5;AGK;AGMAT;AGPAT3;AGPAT6;AGXT2L2;AHNAK;AHSA1;AIF1;AIFM1;AIFM2;AK2;AK5;AKAP1;AKAP17A;AKAP3;AKAP8;AKAP9;AKNA;AKT2;AKT3;ALAD;ALDH18A1;ALDH1B1;ALDH3A2;ALDH5
CAD8;ACE;ACER2;ACLY;ACN9;ACO2;ACOX2;ACOX3;ACSL5;ACTR10;ACTR8;ADCY3;ADCY6;ADD1;ADSS;AFG3L2;AGBL5;AGK;AGMAT;AGPAT3;AGPAT6;AGXT2L2;AHNAK;AHSA1;AIF1;AIFM1;AIFM2;AK2;AK5;AKAP1;AKAP17A;AKAP3;AKAP8;AKAP9;AKNA;AKT2;AKT3;ALAD;ALDH18A1;ALDH1B1;ALDH3A2;ALDH5
KT2;AKT3;ALDH1B1;ALDH6A1;ALDH7A1;ALX3;AMOTL1;ANAPC1;ANAPC13;ANAPC5;ANAPC7;ANKLE2;ANKRD11;ANKS1B;ANP32B;APBA1;APBB1;APOBEC3F;AQR;ARHGAP35;ARHGAP39;ARID1B;ARIH2;ARL3;ARNTL2;ASCC1;ASXL1;ATF6B;ATM;ATN1;ATP1A3;ATP2A3;ATP5A1;ATXN1L;ATXN2L;AXIN1;AXIN2;B
B;APBB1;APOBEC3F;AQR;ARID1B;ARNTL2;ASCC1;ASXL1;ATF6B;ATM;ATN1;ATP2A3;ATP5A1;ATXN2L;BAHD1;BAZ1B;BCL2;BEX1;BLM;BMS1;BPTF;BRD1;BRPF1;C14orf169;C16orf88;C1D;CAD;CALM3;CBX2;CBX5;CBX7;CCAR1;CCND1;CCND2;CCNT1;CCT5;CD3EAP;CDC16;CDC25B;CDKN2A;CENPN;CENPO;CEP8
LY;ACN9;ACO2;ACOX2;ACOX3;ACSL5;ACTR8;ADCY6;ADD1;AFG3L2;AGBL5;AGK;AGMAT;AGPAT3;AGPAT6;AGXT2L2;AHNAK;AHSA1;AIF1;AIFM1;AIFM2;AK2;AKAP1;AKAP17A;AKAP3;AKAP8;AKAP9;AKNA;AKT2;AKT3;ALDH18A1;ALDH1B1;ALDH3A2;ALDH5A1;ALDH6A1;ALDH7A1;ALG8;ALG9;ALMS1;ALS2CL;A
LY;ACN9;ACO2;ACOX2;ACOX3;ACSL5;ACTR8;ADCY6;ADD1;AFG3L2;AGBL5;AGK;AGMAT;AGPAT3;AGPAT6;AGXT2L2;AHNAK;AHSA1;AIF1;AIFM1;AIFM2;AK2;AKAP1;AKAP17A;AKAP3;AKAP8;AKAP9;AKNA;AKT2;AKT3;ALDH18A1;ALDH1B1;ALDH3A2;ALDH5A1;ALDH6A1;ALDH7A1;ALG8;ALG9;ALMS1;ALS2CL;A
AMACR;ANAPC1;ANAPC5;ANAPC7;ANKS1B;APBB1;ARNTL2;ASCC1;ASXL1;ATF6B;ATM;ATN1;ATP5A1;ATP5B;ATXN2L;BAZ1B;BDH1;BEX1;BLM;BLOC1S1;BMS1;BRD1;BRPF1;C14orf169;C16orf88;C1D;CAD;CALM3;CALR;CBX5;CBX7;CCAR1;CCND1;CCND2;CCNT1;CCT5;CD3EAP;CD4;CD74;CDC16;CDC25B;CDKN
DH6A1;ALDH7A1;AMACR;ANAPC1;ANAPC5;ANAPC7;ANKS1B;APBB1;ARNTL2;ASCC1;ASXL1;ATF6B;ATM;ATN1;ATP5A1;ATP5B;ATXN2L;BAZ1B;BDH1;BEX1;BLM;BLOC1S1;BMS1;BRD1;BRPF1;C14orf169;C16orf88;C1D;CAD;CALM3;CALR;CBX5;CBX7;CCAR1;CCND1;CCND2;CCNT1;CCT5;CD3EAP;CD4;CD74;CDC
AMACR;ANAPC1;ANAPC5;ANAPC7;ANKS1B;APBB1;ARNTL2;ASCC1;ASXL1;ATF6B;ATM;ATN1;ATP5A1;ATP5B;ATXN2L;BAZ1B;BDH1;BEX1;BLM;BLOC1S1;BMS1;BRD1;BRPF1;C14orf169;C16orf88;C1D;CAD;CALM3;CALR;CBX5;CBX7;CCAR1;CCND1;CCND2;CCNT1;CCT5;CD3EAP;CD4;CD74;CDC16;CDC25B;CDKN
B;ATM;ATN1;ATXN2L;BAZ1B;BEX1;BLM;BMS1;BRD1;BRPF1;C14orf169;C16orf88;C1D;CAD;CALM3;CBX5;CBX7;CCAR1;CCND1;CCND2;CCNT1;CCT5;CD3EAP;CDC16;CDC25B;CDKN2A;CENPN;CENPO;CEP85;CHD3;CHD4;CHEK2;CIITA;CIRBP;CIRH1A;CLEC3B;CLK2;CORO2A;CPSF3;CREG1;CREM;CSRP2BP;CSTB;C
ACE;ACER2;ACLY;ACN9;ACO2;ACOX2;ACOX3;ACSL5;ACTR10;ACTR8;ADCY6;ADD1;AFG3L2;AGBL5;AGK;AGMAT;AGPAT3;AGPAT6;AGXT2L2;AHNAK;AHSA1;AIF1;AIFM1;AIFM2;AK2;AK5;AKAP1;AKAP17A;AKAP3;AKAP8;AKAP9;AKNA;AKT2;AKT3;ALDH18A1;ALDH1B1;ALDH3A2;ALDH5A1;ALDH6A1;ALDH7A1;A
ACE;ACER2;ACLY;ACN9;ACO2;ACOX2;ACOX3;ACSL5;ACTR10;ACTR8;ADCY6;ADD1;AFG3L2;AGBL5;AGK;AGMAT;AGPAT3;AGPAT6;AGXT2L2;AHNAK;AHSA1;AIF1;AIFM1;AIFM2;AK2;AK5;AKAP1;AKAP17A;AKAP3;AKAP8;AKAP9;AKNA;AKT2;AKT3;ALDH18A1;ALDH1B1;ALDH3A2;ALDH5A1;ALDH6A1;ALDH7A1;A
ADD1;AFG3L2;AGK;AGPAT3;AGPAT6;AIF1;AIFM1;AIFM2;AK2;AK5;AKAP1;AKAP17A;AKAP8;AKAP9;ALDH18A1;ALDH1B1;ALDH3A2;ALDH5A1;ALDH6A1;ALDH7A1;ALG8;ALG9;ALMS1;AMACR;AMFR;AMOTL1;ANAPC1;ANAPC13;ANAPC5;ANAPC7;ANKLE2;ANKS1B;AP2B1;AP3D1;AP3S1;APBB1;APOBEC3F;APO
ADD1;AFG3L2;AGK;AGPAT3;AGPAT6;AIF1;AIFM1;AIFM2;AK2;AK5;AKAP1;AKAP17A;AKAP8;AKAP9;ALDH18A1;ALDH1B1;ALDH3A2;ALDH5A1;ALDH6A1;ALDH7A1;ALG8;ALG9;ALMS1;AMACR;AMFR;AMOTL1;ANAPC1;ANAPC13;ANAPC5;ANAPC7;ANK3;ANKLE2;ANKS1B;AP2B1;AP3D1;AP3S1;APBB1;APOBEC3
OX2;ACOX3;ACSL5;ACTR8;ACVR1;ADCY3;ADCY6;ADCY7;ADCY9;ADSS;AFG3L2;AGK;AHCYL2;AIFM1;AIFM2;AK2;AK5;AKAP17A;AKT2;AKT3;ALDH18A1;ARHGAP35;ARHGEF5;ARL10;ARL13A;ARL3;ASPDH;ATAD3C;ATM;ATP10A;ATP13A1;ATP1A1;ATP1A3;ATP2A2;ATP2A3;ATP5A1;ATP5B;ATP8B1;ATP8B2;ATP9
OX2;ACOX3;ACSL5;ACTR8;ACVR1;ADCY3;ADCY6;ADCY7;ADCY9;ADSS;AFG3L2;AGK;AHCYL2;AIFM1;AIFM2;AK2;AK5;AKAP17A;AKT2;AKT3;ALDH18A1;ARHGAP35;ARHGEF5;ARL10;ARL13A;ARL3;ASPDH;ATAD3C;ATM;ATP10A;ATP13A1;ATP1A1;ATP1A3;ATP2A2;ATP2A3;ATP5A1;ATP5B;ATP8B1;ATP8B2;ATP9
R8;ACVR1;ADCY3;ADCY6;ADCY7;ADCY9;AFG3L2;AGK;AK2;AK5;AKT2;AKT3;ALDH18A1;ATAD3C;ATM;ATP10A;ATP13A1;ATP1A1;ATP1A3;ATP2A2;ATP2A3;ATP5A1;ATP5B;ATP8B1;ATP8B2;ATP9B;BAZ1B;BLM;BMS1;BRSK2;C22orf28;C9orf96;CAD;CARNS1;CBWD3;CBWD6;CCT3;CCT5;CCT6A;CCT7;CCT8;CDC4
R8;ACVR1;ADCY3;ADCY6;ADCY7;ADCY9;AFG3L2;AGK;AK2;AK5;AKT2;AKT3;ALDH18A1;ATAD3C;ATM;ATP10A;ATP13A1;ATP1A1;ATP1A3;ATP2A2;ATP2A3;ATP5A1;ATP5B;ATP8B1;ATP8B2;ATP9B;BAZ1B;BLM;BMS1;BRSK2;C22orf28;C9orf96;CAD;CARNS1;CBWD3;CBWD6;CCT3;CCT5;CCT6A;CCT7;CCT8;CDC4
R8;ACVR1;ADCY3;ADCY6;ADCY7;ADCY9;AFG3L2;AGK;AK2;AK5;AKT2;AKT3;ALDH18A1;ATAD3C;ATM;ATP10A;ATP13A1;ATP1A1;ATP1A3;ATP2A2;ATP2A3;ATP5A1;ATP5B;ATP8B1;ATP8B2;ATP9B;BAZ1B;BLM;BMS1;BRSK2;C22orf28;C9orf96;CAD;CARNS1;CBWD3;CBWD6;CCT3;CCT5;CCT6A;CCT7;CCT8;CDC4
OX2;ACOX3;ACSL5;ACTR8;ACVR1;ADCY3;ADCY6;ADCY7;ADCY9;ADSS;AFG3L2;AGK;AHCYL2;AIFM1;AIFM2;AK2;AK5;AKAP17A;AKT2;AKT3;ALDH18A1;ARHGAP35;ARHGEF5;ARL10;ARL13A;ARL3;ASPDH;ATAD3C;ATM;ATP10A;ATP13A1;ATP1A1;ATP1A3;ATP2A2;ATP2A3;ATP5A1;ATP5B;ATP8B1;ATP8B2;ATP9
RPF1;C14orf169;C1D;CALM3;CBX5;CCAR1;CCND1;CCNT1;CD3EAP;CDC16;CDC25B;CDKN2A;CENPN;CENPO;CHD3;CHD4;CHEK2;CIITA;CIRBP;CLK2;CORO2A;CPSF3;CREG1;CREM;CSRP2BP;CSTF1;CSTF2;CSTF3;CTCF;CTR9;CUL1;DDB1;DDIT3;DDX20;DDX23;DDX42;DDX46;DFFA;DFFB;DGKQ;DHX33;DHX38;DHX
R8;ACVR1;ADCY3;ADCY6;ADCY7;ADCY9;ADSS;AFG3L2;AGK;AK2;AK5;AKT2;AKT3;ALDH18A1;ARHGAP35;ARHGEF5;ARL10;ARL13A;ARL3;ATAD3C;ATM;ATP10A;ATP13A1;ATP1A1;ATP1A3;ATP2A2;ATP2A3;ATP5A1;ATP5B;ATP8B1;ATP8B2;ATP9B;BAZ1B;BLM;BMS1;BRSK2;C22orf28;C9orf96;CAD;CARNS1;CBW
R8;ACVR1;ADCY3;ADCY6;ADCY7;ADCY9;ADSS;AFG3L2;AGK;AK2;AK5;AKT2;AKT3;ALDH18A1;ARHGAP35;ARHGEF5;ARL10;ARL13A;ARL3;ATAD3C;ATM;ATP10A;ATP13A1;ATP1A1;ATP1A3;ATP2A2;ATP2A3;ATP5A1;ATP5B;ATP8B1;ATP8B2;ATP9B;BAZ1B;BLM;BMS1;BRSK2;C22orf28;C9orf96;CAD;CARNS1;CBW
OX2;ACOX3;ACSL5;ACTR8;ACVR1;ADAT1;ADCY3;ADCY6;ADCY7;ADCY9;ADSS;AFG3L2;AGK;AHCYL2;AHDC1;AIFM1;AIFM2;AK2;AK5;AKAP1;AKAP17A;AKAP8;AKNA;AKT2;AKT3;ALDH18A1;ALX3;APOBEC3F;AQR;ARHGAP35;ARHGEF5;ARID1B;ARIH2;ARL10;ARL13A;ARL3;ARNTL2;ASCC1;ASPDH;ASXL1;ATAD3C;
ADD1;ADSS;AGAP5;AGAP6;AHSA1;AIFM1;AIFM2;AK2;AK5;AKAP17A;AKAP8;AKNA;AKT2;ALAD;ALDH18A1;ALDH6A1;ALX3;APBB1;APOBEC3F;AQR;ARHGAP15;ARHGAP29;ARHGAP35;ARHGEF16;ARHGEF5;ARHGEF6;ARHGEF7;ARID1B;ARNTL2;ASCC1;ASMT;ASPDH;ASXL1;ATF6B;ATIC;ATM;ATN1;ATP1A1;ATP
R8;ACVR1;ADCY3;ADCY6;ADCY7;ADCY9;ADSS;AFG3L2;AGK;AK2;AK5;AKT2;AKT3;ALDH18A1;ARHGAP35;ARHGEF5;ARL10;ARL13A;ARL3;ATAD3C;ATM;ATP10A;ATP13A1;ATP1A1;ATP1A3;ATP2A2;ATP2A3;ATP5A1;ATP5B;ATP8B1;ATP8B2;ATP9B;BAZ1B;BLM;BMS1;BRSK2;C22orf28;C9orf96;CAD;CARNS1;CBW
;ANAPC5;ANAPC7;ANXA2;AP2B1;AP3D1;AP3S1;APBA1;APOBEC3F;AQR;ARHGEF7;ARID1B;ARL3;ARNTL2;ARPC2;ARPC3;ASCC1;ASXL1;ATG13;ATG3;ATM;ATP1A1;ATP1A3;ATP1B3;ATP5A1;ATP5B;ATP5J;ATP5J2;ATP6V0E1;ATP6V1D;ATP6V1F;ATP6V1H;ATXN2L;AXIN1;AXIN2;BAHD1;BAZ1B;BBS1;BBS2;BBS9;B
R8;ACVR1;ADCY3;ADCY6;ADCY7;ADCY9;ADSS;AFG3L2;AGK;AK2;AK5;AKT2;AKT3;ALDH18A1;ARHGAP35;ARHGEF5;ARL10;ARL13A;ARL3;ATAD3C;ATM;ATP10A;ATP13A1;ATP1A1;ATP1A3;ATP2A2;ATP2A3;ATP5A1;ATP5B;ATP8B1;ATP8B2;ATP9B;BAZ1B;BLM;BMS1;BRSK2;C22orf28;C9orf96;CAD;CARNS1;CBW
R8;ACVR1;ADCY3;ADCY6;ADCY7;ADCY9;ADSS;AFG3L2;AGK;AK2;AK5;AKT2;AKT3;ALDH18A1;ARHGAP35;ARHGEF5;ARL10;ARL13A;ARL3;ATAD3C;ATM;ATP10A;ATP13A1;ATP1A1;ATP1A3;ATP2A2;ATP2A3;ATP5A1;ATP5B;ATP8B1;ATP8B2;ATP9B;BAZ1B;BLM;BMS1;BRSK2;C22orf28;C9orf96;CAD;CARNS1;CBW
R8;ACVR1;ADCY3;ADCY6;ADCY7;ADCY9;ADSS;AFG3L2;AGK;AK2;AK5;AKT2;AKT3;ALDH18A1;ARHGAP35;ARHGEF5;ARL10;ARL13A;ARL3;ATAD3C;ATM;ATP10A;ATP13A1;ATP1A1;ATP1A3;ATP2A2;ATP2A3;ATP5A1;ATP5B;ATP8B1;ATP8B2;ATP9B;BAZ1B;BLM;BMS1;BRSK2;C22orf28;C9orf96;CAD;CARNS1;CBW
R8;ACVR1;ADCY3;ADCY6;ADCY7;ADCY9;ADSS;AFG3L2;AGK;AK2;AK5;AKT2;AKT3;ALDH18A1;ARHGAP35;ARHGEF5;ARL10;ARL13A;ARL3;ATAD3C;ATM;ATP10A;ATP13A1;ATP1A1;ATP1A3;ATP2A2;ATP2A3;ATP5A1;ATP5B;ATP8B1;ATP8B2;ATP9B;BAZ1B;BLM;BMS1;BRSK2;C22orf28;C9orf96;CAD;CARNS1;CBW
R8;ACVR1;ADCY3;ADCY6;ADCY7;ADCY9;ADSS;AFG3L2;AGK;AK2;AK5;AKT2;AKT3;ALDH18A1;ARHGAP35;ARHGEF5;ARL10;ARL13A;ARL3;ATAD3C;ATM;ATP10A;ATP13A1;ATP1A1;ATP1A3;ATP2A2;ATP2A3;ATP5A1;ATP5B;ATP8B1;ATP8B2;ATP9B;BAZ1B;BLM;BMS1;BRSK2;C22orf28;C9orf96;CAD;CARNS1;CBW
ADD1;ADSS;AGAP5;AGAP6;AHSA1;AIFM1;AIFM2;AK2;AK5;AKAP17A;AKAP8;AKNA;AKT2;ALDH6A1;ALX3;APBB1;APOBEC3F;AQR;ARHGAP15;ARHGAP29;ARHGAP35;ARHGEF16;ARHGEF5;ARHGEF6;ARHGEF7;ARID1B;ARNTL2;ASCC1;ASPDH;ASXL1;ATF6B;ATIC;ATM;ATN1;ATP1A1;ATP1A3;ATP2A2;ATP2A3;AT
ADD1;ADSS;AGAP5;AGAP6;AHSA1;AIFM1;AIFM2;AK2;AK5;AKAP17A;AKAP8;AKNA;AKT2;ALAD;ALDH6A1;ALX3;APBB1;APOBEC3F;AQR;ARHGAP15;ARHGAP29;ARHGAP35;ARHGEF16;ARHGEF5;ARHGEF6;ARHGEF7;ARID1B;ARNTL2;ASCC1;ASMT;ASPDH;ASXL1;ATF6B;ATIC;ATM;ATN1;ATP1A1;ATP1A3;ATP2A
OX2;ACOX3;ACSL5;ACTR8;ACVR1;ADAT1;ADCY3;ADCY6;ADCY7;ADCY9;ADSS;AFG3L2;AGK;AHCYL2;AHDC1;AIFM1;AIFM2;AK2;AK5;AKAP1;AKAP17A;AKAP8;AKNA;AKT2;AKT3;ALDH18A1;ALX3;APOBEC3F;AQR;ARHGAP35;ARHGEF5;ARID1B;ARIH2;ARL10;ARL13A;ARL3;ARNTL2;ASCC1;ASPDH;ASXL1;ATAD3C;
ACACB;ACAD8;ACE;ACER2;ACLY;ACN9;ACO2;ACOX2;ACOX3;ACSL5;ACTR10;ACTR8;ACVR1;ADAM11;ADAM23;ADCY3;ADCY6;ADCY7;ADCY9;ADD1;ADSS;AFG3L2;AGBL5;AGK;AGMAT;AGPAT3;AGPAT6;AGXT2L2;AHNAK;AHSA1;AIF1;AIFM1;AIFM2;AK2;AK5;AKAP1;AKAP17A;AKAP3;AKAP8;AKAP9;AKNA;AKT2;
ACACB;ACAD8;ACE;ACER2;ACLY;ACN9;ACO2;ACOX2;ACOX3;ACSL5;ACTR10;ACTR8;ACVR1;ADAM11;ADAM23;ADCY3;ADCY6;ADCY7;ADCY9;ADD1;ADSS;AFG3L2;AGBL5;AGK;AGMAT;AGPAT3;AGPAT6;AGXT2L2;AHNAK;AHSA1;AIF1;AIFM1;AIFM2;AK2;AK5;AKAP1;AKAP17A;AKAP3;AKAP8;AKAP9;AKNA;AKT2;
CY6;ADCY7;ADD1;ADSS;AGAP5;AGAP6;AGMAT;AGPAT6;AHSA1;AIFM1;AIFM2;AK2;AK5;AKAP17A;AKAP8;AKNA;AKT2;ALAD;ALDH18A1;ALDH5A1;ALDH6A1;ALDH7A1;ALX3;APBB1;APOBEC3F;AQR;ARG1;ARHGAP15;ARHGAP29;ARHGAP35;ARHGEF16;ARHGEF5;ARHGEF6;ARHGEF7;ARID1B;ARNTL2;ASCC1;A
;ACN9;ACO2;ACOX2;ACOX3;ACSL5;ACTR8;ACVR1;ADAT1;ADCY3;ADCY6;ADCY7;ADCY9;ADD1;ADIPOR2;ADPGK;ADSS;AFG3L2;AGAP5;AGAP6;AGBL5;AGK;AGMAT;AGPAT3;AGPAT6;AHCYL2;AHSA1;AIFM1;AIFM2;AK2;AK5;AKAP17A;AKAP8;AKNA;AKT2;AKT3;ALAD;ALDH18A1;ALDH3A2;ALDH5A1;ALDH6A1;
ADCY6;ADCY7;ADD1;ADSS;AGAP5;AGAP6;AHSA1;AIFM1;AIFM2;AK2;AK5;AKAP17A;AKAP8;AKNA;AKT2;ALAD;ALDH18A1;ALDH6A1;ALX3;AMACR;APBB1;APOBEC3F;AQR;ARHGAP15;ARHGAP29;ARHGAP35;ARHGEF16;ARHGEF5;ARHGEF6;ARHGEF7;ARID1B;ARNTL2;ASCC1;ASMT;ASPDH;ASXL1;ATF6B;ATIC;
A;CHD3;CHD4;CHEK2;CLK2;CORO2A;CPSF3;CREG1;CREM;CSRP2BP;CSTF2;CTR9;DDX20;DDX42;DDX46;DGKQ;DKC1;EFTUD2;EIF4A3;EIF4ENIF1;ELP3;EP400;ERCC3;ERCC5;ETS1;EZH1;FIP1L1;FOXC1;FOXI1;FOXJ1;FOXJ3;FOXK1;FOXK2;FOXM1;FOXP1;FOXP4;GAR1;GEMIN4;GEMIN5;GTF2A2;GTF3C1;GTF3C2;G
ACAD8;ACE;ACLY;ACO2;ACOX2;ACOX3;ACSL5;ACTR8;ACVR1;ADAM11;ADAM23;ADAMTS10;ADAMTS17;ADAMTS4;ADAT1;ADCY3;ADCY6;ADCY7;ADCY9;ADD1;ADIPOR2;ADPGK;ADSS;AFG3L2;AGAP5;AGAP6;AGBL5;AGK;AGMAT;AGXT2L2;AHCYL2;AHDC1;AHNAK;AHSA1;AIF1;AIFM1;AIFM2;AIG1;AK2;AK5;A
DCY7;ADCY9;ADD1;AFG3L2;AGBL5;AHSA1;AIFM1;AIFM2;AKAP17A;AKAP8;AKNA;AKT2;AKT3;ALG8;ALG9;ALX3;AMFR;ANAPC1;ANAPC13;ANAPC5;ANAPC7;ANKLE2;APBB1;APOBEC3F;AQR;ARHGAP35;ARID1B;ARIH2;ARNTL2;ASB1;ASB16;ASCC1;ASMT;ASXL1;ATF6B;ATG10;ATG3;ATM;ATN1;ATP8B1;ATXN1L
CAD8;ACE;ACER2;ACLY;ACN9;ACO2;ACOX2;ACOX3;ACSL5;ACTR10;ADCY3;ADD1;ADSS;AFG3L2;AGBL5;AGK;AGMAT;AGPAT3;AGPAT6;AGXT2L2;AHSA1;AIF1;AIFM1;AIFM2;AK2;AK5;AKAP1;AKAP3;AKAP8;AKAP9;AKT2;AKT3;ALAD;ALDH18A1;ALDH1B1;ALDH3A2;ALDH5A1;ALDH6A1;ALDH7A1;ALG8;ALG9;AL
;ACOX2;ACOX3;ACSL5;ACTR8;ACVR1;ADCY3;ADCY6;ADCY7;ADCY9;ADSS;AFG3L2;AGK;AGXT2L2;AIFM1;AIFM2;AK2;AK5;AKT2;AKT3;ALDH18A1;ALDH6A1;ANXA2;APBA1;ARHGAP35;ARHGEF5;ARL10;ARL13A;ARL3;ATAD3C;ATM;ATP10A;ATP13A1;ATP1A1;ATP1A3;ATP2A2;ATP2A3;ATP5A1;ATP5B;ATP8B1;A
;ACN9;ACO2;ACOX2;ACOX3;ACSL5;ACTR8;ACVR1;ADAM11;ADAM23;ADAMTS10;ADAMTS17;ADAMTS4;ADAT1;ADCY3;ADCY6;ADCY7;ADCY9;ADD1;ADIPOR2;ADPGK;ADSS;AFG3L2;AGAP5;AGAP6;AGBL5;AGK;AGMAT;AGPAT3;AGPAT6;AHSA1;AIFM1;AIFM2;AK2;AK5;AKAP17A;AKAP8;AKNA;AKT2;AKT3;AL
;APBB1;APOBEC3F;AQR;ARHGAP35;ARID1B;ARNTL2;ASCC1;ASXL1;ATF6B;ATM;ATN1;ATP8B1;ATXN1L;AXIN1;AXIN2;BACH2;BAHD1;BATF;BATF3;BAZ1B;BCL10;BCL11B;BCL9L;BEX1;BICD1;BLM;BMP2;BPTF;BRD2;BRD7;BRPF1;BTRC;C14orf169;C1D;C22orf28;CALR;CAMTA2;CAND2;CAPN3;CARD11;CBFA2T2;
;ANXA2;AP2B1;AP3D1;AP3S1;APBA1;APOBEC3F;ARHGEF7;ARID1B;ARL3;ARNTL2;ARPC2;ARPC3;ASCC1;ASXL1;ATG13;ATG3;ATM;ATP1A1;ATP1A3;ATP1B3;ATP5A1;ATP5B;ATP5J;ATP5J2;ATP6V0E1;ATP6V1D;ATP6V1F;ATP6V1H;AXIN1;AXIN2;BAHD1;BAZ1B;BBS1;BBS2;BBS9;BCL10;BCL2;BET1L;BEX1;BICD1;

;ACN9;ACO2;ACOX2;ACOX3;ACSL5;ACTR8;ACVR1;ADAM11;ADAM23;ADAMTS10;ADAMTS17;ADAMTS4;ADAT1;ADCY3;ADCY6;ADCY7;ADCY9;ADD1;ADIPOR2;ADPGK;ADSS;AFG3L2;AGAP5;AGAP6;AGBL5;AGK;AGMAT;AGPAT3;AGPAT6;AHSA1;AIFM1;AIFM2;AK2;AK5;AKAP17A;AKAP8;AKNA;AKT2;AKT3;AL

CY6;ADCY7;ADD1;ADSS;AGAP5;AGAP6;AGK;AGMAT;AGPAT6;AHSA1;AIFM1;AIFM2;AK2;AK5;AKAP17A;AKAP8;AKNA;AKT2;ALAD;ALDH18A1;ALDH5A1;ALDH6A1;ALDH7A1;ALX3;APBB1;APOBEC3F;AQR;ARG1;ARHGAP15;ARHGAP29;ARHGAP35;ARHGEF16;ARHGEF5;ARHGEF6;ARHGEF7;ARID1B;ARNTL2;ASC

;ARHGAP35;ARID1B;ARIH2;ARNTL2;ASCC1;ASXL1;ATF6B;ATM;ATXN1L;BACH2;BAHCC1;BAHD1;BATF;BATF3;BCL11B;BCL2;BLM;BPTF;BRD7;BRPF1;C1D;CALR;CAMTA2;CBX2;CBX7;CC2D1B;CCAR1;CCNT1;CDK5RAP2;CDKN2A;CELF1;CHD1L;CHD2;CHD3;CHD4;CHD6;CHTF8;CIC;CIITA;CIRBP;CIZ1;CPSF3;CRAMI

R2;ACLY;ACN9;ACO2;ACOX2;ACOX3;ACSL5;ACTR8;ACVR1;ADAM11;ADAM23;ADAMTS10;ADAMTS17;ADAMTS4;ADAT1;ADCY3;ADCY6;ADCY7;ADCY9;ADD1;ADIPOR2;ADPGK;ADSS;AFG3L2;AGAP5;AGAP6;AGBL5;AGK;AGMAT;AGPAT3;AGPAT6;AHCYL2;AHSA1;AIFM1;AIFM2;AK2;AK5;AKAP17A;AKAP8;AKNA

DDX31;DDX42;DDX46;DDX47;DDX51;DDX52;DDX55;DDX56;DDX6;DHX15;DHX16;DHX32;DHX33;DHX35;DHX37;DHX38;DHX57;DHX9;EIF4A3;EP400;ERCC3;FBXO18;G3BP1;HELB;INO80;MCM2;MCM3;MCM4;MCM6;RAD54L2;RUVBL1;SMARCA4;SMARCAL1;SNRNP200;SRCAP;SUPV3L1;XRCC5;XRC

ADAM23;ADAMTS10;ADAMTS4;ADCY3;ADCY6;ADD1;ADIPOR2;AFG3L2;AGBL5;AHNAK;AHSA1;AIF1;AIFM1;AIG1;AKAP1;AKAP17A;AKAP3;AKAP8;AKAP9;AKT2;AKT3;ALAD;ALDH5A1;ALDH7A1;ALS2CL;AMACR;AMBRA1;AMFR;AMOTL1;ANAPC5;ANAPC7;ANK3;ANKLE2;ANKRD27;ANKS1B;ANKS6;ANKZF1;ANX

;ALX3;ANK3;APBA1;APBA2;APBB1;APOBEC3F;AQR;ARHGAP35;ARID1B;ARNTL2;ASCC1;ASMT;ASXL1;ATF6B;ATN1;ATP8B1;ATXN1L;AVPR2;AXIN1;AXIN2;BACH2;BAHD1;BATF;BATF3;BAZ1B;BBS2;BCL10;BCL11B;BCL2;BCL9L;BEX1;BICD1;BLM;BMP2;BPTF;BRD2;BRD7;BRPF1;BTRC;C14orf169;C1D;C22orf28;C2

1;CSTF2;CSTF3;CTNNBL1;DBR1;DDX17;DDX20;DDX23;DDX46;DDX47;DDX51;DDX56;DHX15;DHX16;DHX35;DHX38;DHX9;DKC1;DROSHA;EFTUD2;EIF4A3;EIF4B;EIF4G1;ERCC3;EXOSC10;FAM103A1;FIP1L1;FTSJ2;FTSJ3;GAR1;GEMIN4;GEMIN5;GPATCH1;HEATR1;HNRNPA0;HNRNPA2B1;HNRNPA3;HNRNPH1;

DDX31;DDX42;DDX46;DDX47;DDX51;DDX52;DDX55;DDX56;DDX6;DHX15;DHX16;DHX33;DHX35;DHX37;DHX38;DHX57;DHX9;EIF4A3;ERCC3;FBXO18;G3BP1;HELB;MCM4;MCM6;SNRNP200;SUPV3L1;XRCC5;XRC

DDX31;DDX42;DDX46;DDX47;DDX51;DDX52;DDX55;DDX56;DDX6;DHX15;DHX16;DHX33;DHX35;DHX37;DHX38;DHX57;DHX9;EIF4A3;ERCC3;FBXO18;G3BP1;HELB;MCM4;MCM6;SNRNP200;SUPV3L1;XRCC5;XRC

TP5A1;ATP5B;ATP5J;ATP5J2;ATP6V0E1;ATP6V1D;ATP6V1F;ATP6V1H;ATP8B1;ATP8B2;ATP9B;BLM;BPTF;CARNS1;CCT8;CHD1L;CHD2;CHD3;CHD4;CHD6;DDX10;DDX17;DDX18;DDX19A;DDX19B;DDX20;DDX23;DDX24;DDX27;DDX31;DDX42;DDX46;DDX47;DDX51;DDX52;DDX55;DDX56;DDX6;DHX15;DHX16;

AM23;ADAMTS10;ADAMTS17;ADAMTS4;ADAT1;ADCY3;ADCY6;ADCY7;ADCY9;ADD1;AFG3L2;AGBL5;AGMAT;AHSA1;AIFM1;AIFM2;AKAP17A;AKAP8;AKNA;AKT2;AKT3;ALAD;ALG8;ALG9;ALX3;AMFR;AMZ1;ANAPC1;ANAPC13;ANAPC5;ANAPC7;ANK3;ANKLE2;APBA1;APBA2;APBB1;APOBEC3F;APOL3;APOLD

;CLEC3B;CSTB;CTCF;DDX17;DDX18;DDX24;DDX31;DDX46;DDX47;DDX51;DDX52;DDX56;DHX15;DHX33;DHX9;DIEXF;DKC1;DNAJB1;DROSHA;EFCAB4B;EIF3A;ELP3;EPHX2;EXOSC10;EZR;FEN1;FOXK1;FTSJ2;FTSJ3;GAR1;GATA3;GEMIN4;GGN;GNL2;GNL3L;GTF3C1;GTPBP4;HADHA;HEATR1;HNRNPA2B1;HNRN

BEC3F;ARHGAP32;ARHGAP35;ARHGAP39;ARL3;ARPC2;ARPC3;ASXL1;ATF6B;ATM;ATP5B;ATXN2L;AXIN1;AXIN2;BAHD1;BAIAP2L1;BAZ1B;BBS2;BBS9;BICD1;BIRC6;BLM;BMS1;BRK1;BRSK2;BSN;BTBD2;C14orf169;C16orf88;C1D;C2CD3;CALM3;CAMSAP1;CAPN2;CAPN3;CBX2;CBX5;CBX7;CCDC64;CCND2;CCN

BEC3F;ARHGAP32;ARHGAP35;ARHGAP39;ARL3;ARPC2;ARPC3;ASXL1;ATF6B;ATM;ATP5B;ATXN2L;AXIN1;AXIN2;BAHD1;BAIAP2L1;BAZ1B;BBS2;BBS9;BICD1;BIRC6;BLM;BMS1;BRK1;BRSK2;BSN;BTBD2;C14orf169;C16orf88;C1D;C2CD3;CALM3;CAMSAP1;CAPN2;CAPN3;CBX2;CBX5;CBX7;CCDC64;CCND2;CCN

;DKC1;EFTUD2;EIF4A3;EIF4ENIF1;EP400;GAR1;GEMIN4;GEMIN5;HNRNPM;IKBKE;LSG1;LSM10;LUC7L2;MAGOH;MBD1;MTOR;NACC1;NCOR2;NOLC1;NONO;NOP10;NXF1;PHC1;PNN;POLDIP3;PRPF19;PRPF3;PRPF4;PRPF40B;PRPF8;PSME4;PSPC1;PTK2;RFWD3;RNF4;RNPS1;RPA1;RPA2;RREB1;SART3;SATB1

TP6V0E1;ATP6V1D;ATP6V1F;ATP6V1H;ATP8B1;ATP8B2;ATP9B;BLM;BPTF;CCT8;CHD1L;CHD2;CHD3;CHD4;CHD6;DDX10;DDX17;DDX18;DDX19A;DDX19B;DDX20;DDX23;DDX24;DDX27;DDX31;DDX42;DDX46;DDX47;DDX51;DDX52;DDX55;DDX56;DDX6;DHX15;DHX16;DHX33;DHX35;DHX37;DHX38;DHX57;

R;ARHGAP35;ARID1B;ARNTL2;ASCC1;ASXL1;ATF6B;ATM;ATN1;ATP8B1;ATXN1L;AXIN1;BACH2;BAHD1;BATF;BATF3;BAZ1B;BCL10;BCL11B;BCL9L;BEX1;BICD1;BLM;BMP2;BPTF;BRD2;BRD7;BRPF1;BTRC;C14orf169;C1D;C22orf28;CALR;CAMTA2;CAND2;CAPN3;CARD11;CBFA2T2;CBX2;CBX5;CBX7;CCAR1;CCN

ACAD8;ACE;ACER2;ACLY;ACN9;ACO2;ACOX2;ACOX3;ACSL5;ACTR10;ACTR8;ACVR1;ADAM11;ADAM23;ADAT1;ADCY3;ADCY6;ADCY7;ADCY9;ADD1;ADIPOR2;ADPGK;ADSS;AFG3L2;AGAP5;AGAP6;AGBL5;AGK;AGMAT;AGPAT3;AGPAT6;AHCYL2;AHSA1;AIF1;AIFM1;AIFM2;AK2;AK5;AKAP17A;AKAP3;AKAP8;AK

;AKT2;ALAD;ALDH7A1;ALMS1;AMOTL1;ANAPC1;ANAPC5;ANAPC7;AP2B1;ARG1;ARHGAP15;ARHGAP17;ARHGAP18;ARHGAP29;ARHGAP32;ARHGAP35;ARHGAP39;ARHGEF1;ARHGEF16;ARHGEF18;ARHGEF3;ARHGEF4;ARHGEF6;ARHGEF7;ARHGEF9;ASMT;ATG13;ATG3;ATIC;ATOX1;ATP6V1D;ATP6V1F;ATP

CIRBP;CPSF3;CSTF1;CSTF2;CSTF3;DARS2;DDX10;DDX17;DDX18;DDX19A;DDX19B;DDX24;DDX31;DDX42;DDX46;DDX47;DDX51;DDX52;DDX55;DDX56;DDX6;DHX9;DIS3L;DKC1;DNMT1;DROSHA;EARS2;EDC3;EEF2;EEF2K;EFTUD1;EIF1AD;EIF2AK4;EIF2B1;EIF2S3;EIF3A;EIF3B;EIF4A3;EIF4B;EIF4G1;EIF4G2;EIF5

47;DHX15;DHX16;DHX35;DHX38;DHX9;EFTUD2;EIF4A3;EIF4B;EIF4G1;ERCC3;FAM103A1;FIP1L1;GEMIN4;GEMIN5;GPATCH1;HNRNPA0;HNRNPA2B1;HNRNPA3;HNRNPH1;HNRNPM;HNRNPR;HNRNPU;HSPA8;KHDRBS1;KHSRP;KIAA1429;LEO1;LOC100130932;LSM10;MAGOH;MLH1;MTPAP;NONO;PABPC1;

6;DDX47;DHX15;DHX16;DHX35;DHX38;DHX9;EFTUD2;EIF4A3;GEMIN4;GEMIN5;GPATCH1;HNRNPA0;HNRNPA2B1;HNRNPA3;HNRNPH1;HNRNPM;HNRNPR;HNRNPU;HSPA8;KHSRP;KIAA1429;LOC100130932;LSM10;MAGOH;NONO;PABPC1;PNN;POLR2B;POLR2C;POLR2J;PPP1R9B;PRPF19;PRPF3;PRPF4;PR

MAT;AGXT2L2;AIFM1;AIFM2;AK2;AKAP1;AKAP8;ALDH18A1;ALDH1B1;ALDH3A2;ALDH5A1;ALDH6A1;ALDH7A1;AMACR;APOOL;ATIC;ATP5A1;ATP5B;ATP5J;ATP5J2;BAD;BCL2;BDH1;BID;BIK;BLOC1S1;BRI3BP;C15orf48;C15orf62;C1D;C22orf29;CASP2;CCT7;CD3EAP;CDKN2A;CERK;CHCHD3;CLIC1;CLN8;CLPB;

PC1;ANAPC13;ANAPC5;ANAPC7;ANKLE2;ANXA2;AP2B1;APBB1;ARHGEF18;ARHGEF5;ARID1B;ARPC2;ARPC3;ASXL1;ATG13;ATG14;ATG3;ATG9B;ATM;ATP6V0E1;ATP6V1D;ATP6V1F;ATP6V1H;AXIN1;AXIN2;BAD;BAHD1;BAIAP2L1;BAZ1B;BBS2;BCL2;BICD1;BID;BIK;BIRC6;BLM;BLOC1S1;BMS1;BPTF;BRD1;BRD

HNRNPA2B1;HNRNPA3;HNRNPH1;HNRNPM;HNRNPR;HNRNPU;HSPA8;LOC100130932;MAGOH;PABPC1;PNN;PRPF19;PRPF3;PRPF4;PRPF8;RBM17;RBM28;SCAF8;SF1;SF3A3;SF3B2;SF3B3;SNRNP200;SNRNP40;SNRNP70;SNW1;SRRM1;SRRM2;SYF2;TFIP11;U2AF1L4;U2AF2;ZRSR

LR;CD40LG;CDAN1;CDKN2A;CENPN;CENPO;CEP164;CHD1L;CHEK2;CHTF8;CINP;CIZ1;CSNK1E;CTC1;CTCF;CUL4A;DCLRE1C;DDB1;DFFA;DFFB;DKC1;DNAJA3;DNASE1;DNMT1;DNMT3A;DTL;EHMT1;EME2;ERCC3;ERCC5;FAN1;FANCI;FEN1;FOXM1;FOXP1;FZR1;GADD45A;GINS4;GTPBP4;H3F3C;HELB;HEMK1;H

NPO;CHEK2;CTC1;CTCF;CTR9;DCTN1;DFFA;DFFB;DNMT1;DNMT3A;EHMT1;EXOSC10;FLNA;FOXC1;GATA3;H3F3C;HDAC1;HELB;HIST1H2AC;HIST1H2AK;HIST1H2BN;HIST2H2AC;HIST2H2BE;HIST2H3D;HLCS;HMGA1;HP1BP3;INCENP;ING2;INO80;INTS7;KAT6B;KDM1A;KDM4C;KIF4A;KNTC1;L3MBTL1;LDB1;LIG

69;CBX2;CBX7;CDAN1;CDKN2A;CENPN;CENPO;CHD1L;CHD3;CHD4;CHD6;CIT;CSRP2BP;CTC1;CTCF;CTR9;DCLRE1C;DFFB;DKC1;DNMT1;DNMT3A;EHMT1;EP400;FEN1;GATA3;GTF3C4;H3F3C;HCFC1;HDAC1;HIST1H2AC;HIST1H2AK;HIST1H2BN;HIST2H2AC;HIST2H2BE;HIST2H3D;HLCS;HMG20A;HMGA1;HP1B

P7;RUVBL1;SALL2;SMARCA4;SMARCC1;SMARCC2;SMARCD1;SMARCD3;SMARCE1;TOP2E

CTR9;DCTN1;DFFA;DFFB;DNMT1;DNMT3A;EXOSC10;FLNA;FOXC1;GATA3;H3F3C;HDAC1;HELB;HIST1H2AC;HIST1H2AK;HIST1H2BN;HIST2H2AC;HIST2H2BE;HIST2H3D;HLCS;HMGA1;HP1BP3;INCENP;ING2;INO80;KAT6B;KDM1A;KDM4C;KNTC1;L3MBTL1;LDB1;LIG3;MBD1;MBD2;MCM2;MCM3;MECP2;MLH1

8;NCAPD3;PHC1;RNF20;RRP1B;SATB1;TCP1;TNKS1BP1;TOP2B;UBE2B;UHRF1

INO80;KDM1A;KDM4C;LDB1;LIG3;MCM2;MCM3;MLH1;MPHOSPH8;MSH6;NCAPD3;NDC80;NFRKB;NR1D1;PHC1;POGZ;POLA1;POLA2;POLE;POLE3;RBBP4;RPA1;RPA2;RUNX2;RUNX3;RUVBL1;SATB1;SLX4;SMARCA4;SMARCD3;SOX18;SYCE2;TBP;TCF3;TCF7L2;TCP1;TERF2;TERF2IP;TEX12;TIMELESS;TNKS1BP

MIN4;GEMIN5;GPATCH1;HNRNPA0;HNRNPA2B1;HNRNPA3;HNRNPH1;HNRNPM;HNRNPR;HNRNPU;MAGOH;PABPC1;PNN;POLR2B;POLR2C;POLR2J;PRPF19;PRPF3;PRPF4;PRPF8;PTBP1;RBFOX2;RBM15B;RNPS1;SF1;SF3A3;SF3B2;SF3B3;SFPQ;SMC1A;SNRNP200;SNRNP40;SNRNP70;SNW1;SRRM1;SRRM2;S

MIN4;GEMIN5;GPATCH1;HNRNPA0;HNRNPA2B1;HNRNPA3;HNRNPH1;HNRNPM;HNRNPR;HNRNPU;MAGOH;PABPC1;PNN;POLR2B;POLR2C;POLR2J;PRPF19;PRPF3;PRPF4;PRPF8;PTBP1;RBFOX2;RBM15B;RNPS1;SF1;SF3A3;SF3B2;SF3B3;SFPQ;SMC1A;SNRNP200;SNRNP40;SNRNP70;SNW1;SRRM1;SRRM2;S

MIN4;GEMIN5;GPATCH1;HNRNPA0;HNRNPA2B1;HNRNPA3;HNRNPH1;HNRNPM;HNRNPR;HNRNPU;KHSRP;MAGOH;PABPC1;PNN;POLR2B;POLR2C;POLR2J;PRPF19;PRPF3;PRPF4;PRPF8;PTBP1;RBFOX2;RBM15B;RNPS1;SF1;SF3A3;SF3B2;SF3B3;SFPQ;SMC1A;SNRNP200;SNRNP40;SNRNP70;SNW1;SRRM1;SF

M3;CAMSAP1;CCDC64;CCNF;CCT3;CCT5;CCT6A;CCT7;CCT8;CDC16;CDC25B;CDC42BPG;CDK5RAP2;CEP104;CEP164;CEP72;CEP85;CEP89;CHD3;CHD4;CIAO1;CKAP5;CNTRL;CROCC;DCTN1;DCTN5;DDHD2;DHX9;DIAPH1;DIS3L;DNAH1;DNHD1;DNM1;DST;DTL;DYNC1H1;DYNC1I2;DYNC1LI2;DYNLT1;EMD;EML

;HNRNPU;MAGOH;PABPC1;PNN;PRPF19;PRPF8;SF3A3;SF3B2;SF3B3;SNRNP200;SNRNP40;SNW1;SRRM1;SRRM2;SYF2;TFIP11

H;MBD1;NONO;NXF1;PNN;POLDIP3;PRPF19;PRPF3;PRPF4;PRPF40B;PRPF8;PSME4;PSPC1;RNPS1;RREB1;SART3;SETD1A;SF3A3;SNRNP70;SON;SRRM1;SRRM2;SRSF5;TFIP11;THOC1;U2AF1L4;WBP11;YLPM1;ZBTB16;ZC3H1

D;CARNS1;CBLB;CTPS2;DARS2;DTL;DTX4;EARS2;EPRS;FARSB;FBXO21;FBXW2;GARS;GART;GMPS;HARS;HECTD1;HERC1;HERC2;HLCS;HUWE1;IARS;IARS2;IRAK1;KARS;KCMF1;KCTD10;KCTD13;LARS;LARS2;LGSN;LIG1;LIG3;LIPT2;LMO7;MARS;MCCC1;MCCC2;MID2;MTHFD1;MTHFD1L;MTHFS;MUL1;MYCBP2;

LB3;ATP2A2;ATP2A3;ATP5A1;ATP5B;ATP5J;ATP5J2;ATP6V0E1;ATP6V1D;ATP6V1F;ATP6V1H;ATP8B1;ATP8B2;ATP9B;BLM;BMS1;BPTF;CARNS1;CCT8;CHD1L;CHD2;CHD3;CHD4;CHD6;CLPB;DCTN1;DDX10;DDX17;DDX18;DDX19A;DDX19B;DDX20;DDX23;DDX24;DDX27;DDX31;DDX42;DDX46;DDX47;DDX51;D

1;DDIT3;DGKQ;DHX33;DHX9;DIP2A;DNAJA3;DNMT1;DRG1;ERCC3;ETS1;FAF1;FLNA;FOSB;FOXC1;FOXI1;FOXJ1;FOXJ3;FOXK1;FOXK2;FOXM1;FOXP1;FOXP4;GATA3;GTF2A2;HDAC1;HHEX;HIF1AN;HMGA1;HTT;HYAL1;KAT6B;KCTD1;KDM1A;LDB1;LEF1;LMO2;LPIN1;MAD2L2;MAPK9;MECP2;MED24;MED30;MI

AXIN2;BACE1;BAD;BCL10;BCL2;BICD1;BID;BRSK2;CAD;CALM3;CALR;CAMTA2;CASP2;CBX5;CCDC64;CCND1;CCND2;CCNT1;CD3E;CD4;CD74;CD8A;CDC25B;CDC42BPB;CDK5RAP2;CDKN2A;CDKN2B;CHCHD3;CHEK2;CRADD;CRY2;CSRNP2;CSTB;CTNNBL1;CUL1;CUL4A;CUL7;CUL9;DFFB;DGKQ;DHCR24;DIAPH1;

;ACER2;ACLY;ACN9;ACO2;ACOX2;ACOX3;ACSL5;ADD1;ADSS;AFG3L2;AGBL5;AGK;AGMAT;AGPAT3;AGPAT6;AGXT2L2;AHSA1;AIF1;AIFM1;AIFM2;AK2;AK5;AKAP1;AKAP3;AKAP8;AKAP9;AKT2;AKT3;ALAD;ALDH18A1;ALDH1B1;ALDH3A2;ALDH5A1;ALDH6A1;ALDH7A1;ALG8;ALG9;ALMS1;ALS2CL;AMACR;AM

LB3;ATP2A2;ATP2A3;ATP5A1;ATP5B;ATP5J;ATP5J2;ATP6V0E1;ATP6V1D;ATP6V1F;ATP6V1H;ATP8B1;ATP8B2;ATP9B;BLM;BMS1;BPTF;CARNS1;CCT8;CHD1L;CHD2;CHD3;CHD4;CHD6;CLPB;DCTN1;DDX10;DDX17;DDX18;DDX19A;DDX19B;DDX20;DDX23;DDX24;DDX27;DDX31;DDX42;DDX46;DDX47;DDX51;D

LB3;ATP2A2;ATP2A3;ATP5A1;ATP5B;ATP5J;ATP5J2;ATP6V0E1;ATP6V1D;ATP6V1F;ATP6V1H;ATP8B1;ATP8B2;ATP9B;BLM;BMS1;BPTF;CARNS1;CCT8;CHD1L;CHD2;CHD3;CHD4;CHD6;CLPB;DCTN1;DDX10;DDX17;DDX18;DDX19A;DDX19B;DDX20;DDX23;DDX24;DDX27;DDX31;DDX42;DDX46;DDX47;DDX51;D

LB3;ATP2A2;ATP2A3;ATP5A1;ATP5B;ATP5J;ATP5J2;ATP6V0E1;ATP6V1D;ATP6V1F;ATP6V1H;ATP8B1;ATP8B2;ATP9B;BLM;BMS1;BPTF;CARNS1;CCT8;CHD1L;CHD2;CHD3;CHD4;CHD6;CLPB;DCTN1;DDX10;DDX17;DDX18;DDX19A;DDX19B;DDX20;DDX23;DDX24;DDX27;DDX31;DDX42;DDX46;DDX47;DDX51;D

KLE2;APBB1;ARL3;ATM;AXIN2;BAD;BCL2;BID;BIRC6;BLCAP;BLM;BMP2;BRD7;BRSK2;BTRC;C11orf82;CALR;CAPN3;CASP2;CCAR1;CCND1;CCND2;CCNDBP1;CCNF;CCNT1;CDC16;CDC25B;CDK5RAP2;CDKN2A;CDKN2B;CENPN;CENPO;CEP164;CEP72;CHD3;CHEK2;CHTF8;CINP;CIT;CKAP5;CNTRL;CRADD;CROCC;

HA;EARS2;EIF4A3;EPRS;EXOSC10;FARSB;FTSJ2;FTSJ3;GAR1;GARS;GEMIN4;GEMIN5;HARS;HEATR1;IARS;IARS2;INTS1;INTS4;INTS7;INTS9;KARS;LARS;LARS2;LAS1L;MARS;NARS;NOL9;NOLC1;NOP10;NOP14;NSUN2;NSUN4;PA2G4;PDCD11;PET112;PLD6;POP1;PUS3;QTRTD1;RPP14;RPP21;RRP1B;SARS;SMAD

X3;APBB1;ARHGAP35;ARID1B;ARNTL2;ASCC1;ASMT;ASXL1;ATF6B;ATG10;ATN1;ATP8B1;ATXN1L;AXIN1;B3GALT4;BACH2;BAHD1;BATF;BATF3;BAZ1B;BCL10;BCL11B;BCL2;BCL9L;BEX1;BLM;BMP2;BPTF;BRD2;BRD7;BRPF1;BTRC;C14orf169;C1D;C1GALT1C1;CALR;CAMTA2;CAND2;CAPN3;CARD11;CBFA2T2;

CENPO;CHD1L;CHD3;CHD4;CHD6;CSRP2BP;CTCF;CTR9;DNMT1;DNMT3A;EHMT1;EP400;GATA3;GTF3C4;HCFC1;HDAC1;HLCS;HMG20A;HMGA1;HUWE1;ING2;ING5;INO80;KAT6B;KDM1A;KDM2B;KDM4A;KDM4C;KDM5C;L3MBTL1;L3MBTL2;LDB1;LEF1;LEO1;LPIN1;MAP3K12;MECP2;MLL;MLL4;MYC;NASP;

;FOXC1;GATA3;H3F3C;HDAC1;HIST1H2AC;HIST1H2AK;HIST1H2BN;HIST2H2AC;HIST2H2BE;HIST2H3D;HLCS;HMGA1;HP1BP3;INCENP;ING2;INO80;KAT6B;KDM1A;KDM4C;L3MBTL1;LDB1;MBD1;MBD2;MCM2;MECP2;MPHOSPH8;MSH6;NCAPD3;NFRKB;NR1D1;PHC1;POGZ;POLA1;RAD18;RBBP4;RNF20;RRP1

A2;ANKLE2;APOOL;ATP2A3;ATP5A1;ATP5B;ATP5J;ATP5J2;BAD;BCL2;BDH1;BID;BIK;BLOC1S1;BRI3BP;CBX5;CCND2;CHCHD3;CLIC1;COX10;COX15;COX5B;COX8A;CYB5B;DDX19A;DDX19B;DGKH;DHCR7;DHODH;DNAJC1;DNAJC11;DNASE1;DST;DTL;EFHD1;EIF5A;EMD;ETFDH;EXOG;FAF1;FZR1;GLE1;GOT2;GRA

CTR8;ACVR1;ADCY6;ADCY7;ADSS;AGK;AGMAT;AGPAT3;AGPAT6;AK2;AK5;AKAP17A;AKNA;AKT2;ALAD;ALDH18A1;ALDH7A1;ALG8;ALG9;ALX3;AMACR;APBB1;ARG1;ARHGAP35;ARID1B;ARNTL2;ASB1;ASCC1;ASMT;ASPDH;ASXL1;ATF6B;ATG10;ATIC;ATM;ATN1;ATP1A1;ATP1A3;ATP2A2;ATP2A3;ATP5A1;ATP

;LIG1;LIG3;MDC1;MLH1;MND1;MSH6;NFRKB;NONO;PALB2;PAXIP1;PMS2;PMS2P5;POLA1;POLA2;POLE;RAD18;RAD51D;RBM14;RFC1;RNF8;RPA1;RPA2;RUVBL1;SFPQ;SLX4;SMC1A;SUPV3L1;TCF3;TCF7;TEP1;TERF2IP;TOP2B;TP53BP1;UBE2B;UNG;XRCC3;XRCC5;XRCC6;ZFYVE2

INO80;KDM1A;KDM4C;LDB1;LIG3;MCM2;MCM3;MLH1;MPHOSPH8;MSH6;NCAPD2;NCAPD3;NDC80;NFRKB;NR1D1;PHC1;PINX1;POGZ;POLA1;POLA2;POLE;POLE3;RBBP4;RPA1;RPA2;RUNX2;RUNX3;RUVBL1;SATB1;SLX4;SMARCA4;SMARCD3;SMARCE1;SMC1A;SOX18;SYCE2;TBP;TCF3;TCF7L2;TCP1;TERF2;

EP1;TERF2;TERF2IP;TNKS1BP1;XRCC5;XRCC6;YLPM1

;ALG8;ALG9;ALX3;APBB1;ARG1;ARHGAP35;ARID1B;ARNTL2;ASB1;ASCC1;ASMT;ASXL1;ATF6B;ATG10;ATN1;ATP8B1;ATXN1L;AXIN1;B3GALT4;BACH2;BAHD1;BATF;BATF3;BAZ1B;BCL10;BCL11B;BCL2;BCL9L;BEX1;BLM;BMP2;BPTF;BRD2;BRD7;BRPF1;BTRC;C14orf169;C1D;C1GALT1C1;CALR;CAMTA2;CAND2;

A2;ANKLE2;APOOL;ATP2A3;ATP5A1;ATP5B;ATP5J;ATP5J2;BAD;BCL2;BDH1;BID;BIK;BLOC1S1;BRI3BP;CBX5;CCND2;CHCHD3;CLIC1;COX10;COX15;COX5B;COX8A;CYB5B;DDX19A;DDX19B;DGKH;DHCR7;DHODH;DNAJC1;DNAJC11;DNASE1;DST;DTL;EFHD1;EIF5A;EMD;ETFDH;EXOG;FAF1;FZR1;GLE1;GOT2;GRA

EP1;TERF2;TERF2IP;TNKS1BP1;XRCC5;XRCC6;YLPM1

ATM;BCL2;BID;BIRC6;BLM;BRD7;BRSK2;BTRC;CASP2;CCND1;CCNF;CDC16;CDC25B;CDK5RAP2;CDKN2A;CDKN2B;CENPN;CENPO;CEP164;CEP72;CHEK2;CIT;CKAP5;CNTRL;CRADD;CSNK1E;CSNK2A2;CUL1;CUL4A;DCTN1;DYNC1H1;DYNC1I2;DYNLT1;FEN1;FHL1;FLNA;FOXC1;FOXM1;FZR1;GINS4;HAUS5;HDAC

VR1;ADCY6;ADCY7;ADSS;AGK;AGMAT;AGPAT3;AGPAT6;AK2;AK5;AKAP17A;AKNA;AKT2;ALAD;ALDH18A1;ALDH7A1;ALG8;ALG9;ALX3;AMACR;APBB1;ARG1;ARHGAP35;ARID1B;ARNTL2;ASB1;ASCC1;ASMT;ASPDH;ASXL1;ATF6B;ATG10;ATIC;ATM;ATN1;ATP1A1;ATP1A3;ATP2A2;ATP2A3;ATP5A1;ATP5B;ATP

R2J;POLR3A;POLR3B;POLR3C;POLR3E;SSRP1;SUPT16H;SUPT4H1;SUPT6H;TAF1;TAF10;TAF1C;TAF6;TBP;TCEB1;TCEB3;THOC1;TSFM;UBT

1;FOXC1;FOXP1;FOXP4;GON4L;HCFC1;HHEX;ING2;KDM1A;L3MBTL1;LDB1;LEF1;LMO2;MBD2;MECP2;MNT;MSH6;MTA1;MTA2;MTA3;NCOR2;NFATC3;NKAP;NUP62;PATZ1;PHC1;PHF13;POLA1;POLE;POLG;POLR2B;POLR3A;RELA;RNF20;RNF4;RNF8;RPA1;RUNX2;SATB1;SIN3B;SMAD3;SMARCC1;SMARCC2;

DYNC1H1;EFTUD1;EIF3A;EIF4A3;EXOSC10;FAM103A1;FTSJ2;FTSJ3;GAR1;GEMIN4;GEMIN5;GNL2;GNL3L;GTPBP4;HEATR1;LAS1L;LSG1;NOL9;NOLC1;NOP10;NOP14;NSUN4;PA2G4;PDCD11;PRPF19;RCL1;RRN3;RRP1B;SDAD1;SF1;SF3A3;SNRNP200;SRSF5;TGS1;TSC1;TSR1;TSR2;USP39;UTP14A;UTP20;WBP1

ARS

ARS

ARS

MCM2;MLL;MPHOSPH8;NASP;NCAPD2;NCAPD3;NCAPG2;PHF13;PYGO2;RBBP4;RNF20;RNF8;SMARCA4;TAF1;TP53BP1;UHRF1
KLE2;APBB1;ATM;AXIN2;BCL2;BID;BIRC6;BLM;BRD7;BRSK2;BTRC;C11orf82;CALR;CAPN3;CASP2;CCND1;CCNF;CDC16;CDC25B;CDK5RAP2;CDKN2A;CDKN2B;CENPN;CENPO;CEP164;CEP72;CHD3;CHEK2;CIT;CKAP5;CNTRL;CRADD;CSNK1E;CSNK2A2;CSRP2BP;CTCF;CUL1;CUL4A;DCTN1;DDB1;DDIT3;DHCR24;D
CTR8;ACVR1;ADCY6;ADCY7;ADSS;AGK;AGMAT;AGPAT3;AGPAT6;AK2;AK5;AKAP17A;AKNA;AKT2;ALAD;ALDH18A1;ALDH7A1;ALG8;ALG9;ALX3;AMACR;AP3D1;APBB1;ARG1;ARHGAP35;ARID1B;ARNTL2;ASB1;ASCC1;ASMT;ASPDH;ASXL1;ATF6B;ATG10;ATIC;ATM;ATN1;ATP1A1;ATP1A3;ATP2A2;ATP2A3;ATP5
1;FOXJ1;FRMD4A;GATA3;HTT;JAM3;KANK1;MACF1;MAP4;NCKAP1L;NDC80;NUMA1;PARVA;PTK2;SCRIB;SFRP5;SPN;SYNE2;VANGL2;ZW10
51D;RBBP4;RFC1;SMARCA4;SMARCAL1;TOP2B;XRCC3;XRCC5;XRCC6
NO80;MACF1;MDN1;MLH1;MSH6;MYO9B;PMS2;PMS2P5;PSMC4;PSMD6;WRNIP1
ACE;ACLY;ACO2;ACOX2;ACOX3;ACSL5;ACTR8;ACVR1;ADAM11;ADAM23;ADAMTS10;ADAMTS17;ADAMTS4;ADAT1;ADCY3;ADCY6;ADCY7;ADCY9;ADD1;ADPGK;ADSS;AFG3L2;AGAP5;AGAP6;AGBL5;AGK;AGMAT;AGXT2L2;AIF1;AIFM1;AIFM2;AK2;AK5;AKAP8;AKT2;AKT3;ALAD;ALDH18A1;ALDH6A1;AMFR;AM

3;UHRF1;ZMIZ2
RCC5;XRCC6
;DNAH1;DNM1;DST;DYNC1H1;DYNC1I2;DYNC1LI2;DYNLT1;EMD;EML3;FKBP4;HAUS5;HSPH1;INCENP;INO80;INVS;KIF13B;KIF16B;KIF21B;KIF3B;KIF4A;KIF5C;KLC1;KLC2;KNTC1;LZTS2;MACF1;MAP1A;MAP1LC3B2;MAP4;MAPRE2;MAPT;MID2;NAV1;NEIL2;NICN1;NINL;NUMA1;ODF2;PCNT;POLB;PRC1;RACGA
SRP;KIF5C;MAGOH;MYO1C;NUP133;NUP155;NUP188;NUP205;NUP210;NUP214;NUP62;NUP93;NXF1;POM121;POM121C;RNPS1;SEH1L;SIDT1;SIDT2;SMG6;SRRM1;SRSF5;THOC1;THOC2;TSC1;U2AF
RBBP4;RBBP7;RNF8;RUVBL1;SATB1;SCMH1;SMARCA4;SMARCC1;SMARCC2;SMARCD1;SMARCD3;SMARCE1;SUPT4H1;SUPT6H;TTF1
PHOSPH8;MSH6;NCAPD3;NFRKB;NR1D1;PHC1;POGZ;RBBP4;RUNX2;RUNX3;RUVBL1;SATB1;SLX4;SMARCA4;SMARCD3;SOX18;TBP;TCF3;TCF7L2;TCP1;TIMELESS;TNKS1BP1;TP53;UBE2B;UHRF
CENPN;CENPO;CHD1L;CHD3;CHD4;CHD6;CSRP2BP;CTCF;CTR9;DNMT1;DNMT3A;EHMT1;EP400;GATA3;GTF3C4;H3F3C;HCFC1;HDAC1;HIST1H2AC;HIST1H2AK;HIST1H2BN;HIST2H2AC;HIST2H2BE;HIST2H3D;HLCS;HMG20A;HMGA1;HP1BP3;HUWE1;ING2;ING5;INO80;KAT6B;KDM1A;KDM2B;KDM4A;KDM4C
3A2;ALDH5A1;ALDH6A1;ALDH7A1;APOOL;ATP5A1;ATP5B;ATP5J;ATP5J2;BAD;BCL2;BDH1;BID;BIK;BLOC1S1;BRI3BP;CHCHD3;COX10;COX15;COX5B;COX8A;CYB5B;D2HGDH;DARS2;DHODH;DLD;DLST;DNAJA3;DNAJC11;EARS2;EFHD1;ETFDH;ETHE1;EXOG;FH;FXN;GARS;GLS2;GLUD1;GOT2;GPT2;GRAMD4;G
AP17A;AKNA;AKT2;ANAPC1;ANAPC5;ANAPC7;ANK3;ANKLE2;APBA1;APBA2;APBB1;APOBEC3F;ARHGAP35;ARID1B;ARNTL2;ASB1;ASCC1;ASXL1;ATG10;ATN1;ATP8B1;ATXN1L;AVPR2;AXIN1;AXIN2;BAHD1;BAZ1B;BBS2;BCL10;BCL11B;BCL2;BCL9L;BEX1;BLM;BMP2;BPTF;BRD2;BRD7;BRPF1;BTRC;C14orf169;C
L;EME2;ERCC3;ERCC5;FAN1;FANCI;FEN1;FOXM1;FZR1;GADD45A;HERC2;HMGA1;HUWE1;INO80;LIG1;LIG3;LOC100133315;MAD2L2;MDC1;MLH1;MMS19;MSH6;NEIL2;NFRKB;NONO;PALB2;PAPD7;PARP1;PAXIP1;PMS2;PMS2CL;PMS2P5;POLA1;POLB;POLE;POLG;POLH;POLR2B;POLR2C;POLR2J;PRPF19;RA
ACACB;ACAD8;ACE;ACER2;ACLY;ACO2;ACOX2;ACOX3;ACSL5;ACVR1;ADAM11;ADAM23;ADAMTS10;ADAMTS17;ADAMTS4;ADAT1;ADCY3;ADCY6;ADCY7;ADCY9;ADPGK;ADSS;AFG3L2;AGBL5;AGK;AGMAT;AGPAT3;AGPAT6;AGXT2L2;AHCYL2;AIFM1;AIFM2;AK2;AK5;AKT2;AKT3;ALAD;ALDH18A1;ALDH1B1;AL

OH;MYO1C;NUP133;NUP155;NUP188;NUP205;NUP210;NUP214;NUP62;NUP93;NXF1;POM121;POM121C;RNPS1;SEH1L;SIDT1;SIDT2;SMG6;SRRM1;SRSF5;THOC1;THOC2;TSC1;U2AF1
OH;MYO1C;NUP133;NUP155;NUP188;NUP205;NUP210;NUP214;NUP62;NUP93;NXF1;POM121;POM121C;RNPS1;SEH1L;SIDT1;SIDT2;SMG6;SRRM1;SRSF5;THOC1;THOC2;TSC1;U2AF1
OH;MYO1C;NUP133;NUP155;NUP188;NUP205;NUP210;NUP214;NUP62;NUP93;NXF1;POM121;POM121C;RNPS1;SEH1L;SIDT1;SIDT2;SMG6;SRRM1;SRSF5;THOC1;THOC2;TSC1;U2AF1
S;TARS;YARS
S;TARS;YARS
3;ALAD;ALDH5A1;ALMS1;ALS2CL;AMFR;AMIGO1;ANAPC1;ANAPC13;ANAPC5;ANAPC7;ANK3;ANKLE2;ANXA2;AP2B1;AP3D1;APBA1;APBB1;ARG1;ARHGAP15;ARHGAP35;ARHGAP39;ARHGEF1;ARHGEF16;ARHGEF18;ARHGEF4;ARHGEF5;ARHGEF6;ARHGEF7;ARID1B;ARL3;ARPC2;ARPC3;ASXL1;ATG13;ATG14;
H2;BAHCC1;BAHD1;BATF;BATF3;BCL11B;BCL2;BLM;BPTF;BRD7;BRPF1;C1D;CALR;CAMTA2;CBX2;CC2D1B;CCNT1;CDK5RAP2;CDKN2A;CHD1L;CHD2;CHD3;CHD4;CHD6;CHTF8;CIC;CIITA;CRAMP1L;CREB3L2;CREM;CRY2;CSRNP2;CTC1;CTCF;CXXC5;DDB1;DDIT3;DDX20;DFFB;DHX33;DHX9;DNAJC1;DNMT1;DNM
FTSJ3;GAR1;GEMIN4;HEATR1;INTS1;INTS4;INTS7;INTS9;KARS;LAS1L;NOL9;NOLC1;NOP10;NOP14;NSUN2;NSUN4;PA2G4;PDCD11;POP1;PUS3;QTRTD1;RPP14;RPP21;RRP1B;SARS;SMAD3;SRRT;TRIT1;TSR2;TYW1;UTP14A;UTP20;WBP11;WDR4;WDR55;ZCCHC1
YARS
POLR2B;POLR2C;POLR2J;POLR3A;POLR3C;RPRD1B;TAF1;TAF10;TAF3;TAF6;TAF8;TBP;TP53;TRRA
POLR2B;POLR2C;POLR2J;POLR3A;POLR3C;RPRD1B;TAF1;TAF10;TAF3;TAF6;TAF8;TBP;TP53;TRRA
GEF3;ARHGEF4;ARHGEF5;ARHGEF6;ARHGEF7;ARHGEF9;CDC42BPA;CDC42BPB;CDC42BPG;CIT;CYTH1;CYTH3;DENND2D;DENND4B;DNAJA3;DOCK10;DOCK9;EVI5L;FGD1;GBF1;HERC1;KALRN;MADD;MCF2L;MINK1;MYO9B;NCKAP1L;NET1;OBSCN;OCRL;PLEKHG2;PSD4;RAB3GAP1;RABGAP1;RALGDS;RANBP1
3;TCF7;UNG;XRCC6
POLR2B;POLR2C;POLR2J;POLR3A;POLR3C;RPRD1B;TAF1;TAF10;TAF3;TAF6;TAF8;TBP;TP53;TRRA
AD;ALDH18A1;ALX3;APBB1;ARHGAP35;ARID1B;ARNTL2;ASCC1;ASMT;ASPDH;ASXL1;ATF6B;ATIC;ATN1;ATP1A1;ATP1A3;ATP2A2;ATP2A3;ATP5A1;ATP5B;ATP5J;ATP5J2;ATP8B1;ATXN1L;AVPR2;AXIN1;BACH2;BAHD1;BATF;BATF3;BAZ1B;BCL10;BCL11B;BCL9L;BEX1;BLM;BMP2;BPTF;BRD2;BRD7;BRPF1;BTRC
PF4;SRRM2;TGS1
IN4;GNL2;GNL3L;GTPBP4;HEATR1;LAS1L;LSG1;NOL9;NOLC1;NOP10;NOP14;NSUN4;PA2G4;PDCD11;RCL1;RRN3;RRP1B;SDAD1;TSC1;TSR1;TSR2;UTP14A;UTP20;WBP11;WDR55;ZNHIT
ATM;BCL2;BID;BIRC6;BLM;BRD7;BRSK2;CALR;CASP2;CCND1;CCNF;CDC16;CDC25B;CDK5RAP2;CDKN2A;CDKN2B;CENPN;CENPO;CEP164;CEP72;CHD3;CHEK2;CIT;CKAP5;CNTRL;CRADD;CSNK1E;CUL1;CUL4A;DCTN1;DMWD;DYNC1H1;DYNC1I2;DYNLT1;FEN1;FHL1;FLNA;FOXM1;FZR1;GINS4;HAUS5;HGF;HYA
1;ARHGAP15;ARHGAP29;ARHGAP35;ARHGEF16;ARHGEF5;ARHGEF6;ARHGEF7;ARID1B;ARNTL2;ASCC1;ASXL1;ATN1;ATP8B1;ATXN1L;AVPR2;AXIN1;AXIN2;BAHD1;BAZ1B;BCL10;BCL11B;BCL9L;BEX1;BLM;BMP2;BPTF;BRD2;BRD7;BRPF1;BTRC;C14orf169;C1D;CALM3;CALR;CAMTA2;CAND2;CAPN3;CARD11;C

1;UHRF1
C25B;CDK5RAP2;CDKN2A;CDKN2B;CEP164;CEP72;CHEK2;CKAP5;CNTRL;CRADD;CSNK1E;CUL1;CUL4A;DCTN1;DYNC1H1;DYNC1I2;FEN1;FHL1;FOXM1;FZR1;GINS4;HYAL1;ING5;INO80;KHDRBS1;KLF11;KRT7;LIG1;LSM10;MCM2;MCM3;MCM4;MCM6;MTA3;MYC;NINL;NUMA1;ODF2;PCM1;PCNT;PKD1;POLA1
2;HSP90B1;HYOU1;KDELR3;MBTPS1;PDIA5;PDIA6;SHC1;SREBF2;SYVN1;TATDN2;TP53;TSPYL2;VCP;XBP
33;ARHGAP35;ARHGEF1;ARHGEF16;ARHGEF18;ARHGEF3;ARHGEF4;ARHGEF5;ARHGEF6;ARHGEF7;ARHGEF9;AXIN1;AXIN2;CDC42BPA;CDC42BPB;CDC42BPG;CIT;CYTH1;CYTH3;DENND2D;DENND4B;DNAJA3;DNAJC1;DOCK10;DOCK7;DOCK9;EIF2B1;EVI5L;FGD1;GBF1;GRPEL1;HERC1;HERC2;HMHA1;IPO5;K

AP17A;AKNA;ALAD;ALDH7A1;ALX3;APBB1;ARG1;ARHGAP35;ARID1B;ARNTL2;ASCC1;ASMT;ASPDH;ASXL1;ATF6B;ATIC;ATN1;ATP1A1;ATP1A3;ATP2A2;ATP2A3;ATP5A1;ATP5B;ATP5J;ATP5J2;ATP8B1;ATXN1L;AVPR2;AXIN1;BACH2;BAHD1;BATF;BATF3;BAZ1B;BCL10;BCL11B;BCL9L;BEX1;BLM;BMP2;BPTF;BRD
2AK3;EIF2AK4;GOSR2;HSP90B1;HYOU1;KDELR3;MBTPS1;PDIA5;PDIA6;SCAMP5;SHC1;SYVN1;TATDN2;TP53;TSPYL2;VCP;XBP
M1;AKT2;ALDH1B1;ALDH5A1;ALDH6A1;ALDH7A1;AMACR;AMBRA1;AMFR;ANAPC1;ANAPC5;ANAPC7;APOBEC3F;ARG1;ARHGAP15;ARHGAP29;ARHGAP35;ARHGEF16;ARHGEF5;ARHGEF6;ARHGEF7;ARIH2;ASPDH;ATG10;ATG13;ATG14;ATG3;ATG9B;ATM;ATP5A1;ATP5B;ATP5J;ATP5J2;ATP6V1H;ATP8B1;AT
3
8B1;ATP8B2;BAD;BLM;CARNS1;CHD1L;ENTPD5;HSPD1;INO80;MACF1;MDN1;MLH1;MSH6;MYO9B;NDUFS1;PMS2;PMS2P5;PSMC4;PSMD6;WRNIP1
DD1;AGAP5;AGAP6;AHSA1;AKAP17A;AKNA;AKT2;ANAPC1;ANAPC5;ANAPC7;ANKLE2;APBB1;ARHGAP15;ARHGAP29;ARHGAP35;ARHGEF16;ARHGEF5;ARHGEF6;ARHGEF7;ARID1B;ARNTL2;ASB1;ASCC1;ASXL1;ATG10;ATN1;ATP8B1;ATXN1L;AVPR2;AXIN1;AXIN2;BAD;BAHD1;BAZ1B;BCL10;BCL11B;BCL2;BCL9

OH;MYO1C;NUP133;NUP155;NUP188;NUP205;NUP210;NUP214;NUP62;NUP93;NXF1;POM121;POM121C;RNPS1;SEH1L;SIDT1;SIDT2;SLC25A42;SLC35B1;SLC35D1;SMG6;SRRM1;SRSF5;THOC1;THOC2;TSC1;U2AF
3;DDX46;DDX47;DDX6;DHX15;DHX16;DHX35;DHX38;DHX9;EDC3;EDC4;EFTUD2;EIF4A3;EIF4B;EIF4G1;ELAVL1;ERCC3;EXOSC10;FAM103A1;FIP1L1;GEMIN4;GEMIN5;GPATCH1;HNRNPA0;HNRNPA2B1;HNRNPA3;HNRNPH1;HNRNPM;HNRNPR;HNRNPU;HSPA8;KHDRBS1;KHSRP;KIAA1429;LEO1;LOC10013093
;ASXL1;ATF6B;ATN1;ATP8B1;ATXN1L;AXIN1;BACH2;BAHD1;BATF;BATF3;BAZ1B;BCL10;BCL11B;BCL9L;BEX1;BLM;BMP2;BPTF;BRD2;BRD7;BRPF1;BTRC;C14orf169;C1D;CALR;CAMTA2;CAND2;CAPN3;CARD11;CBFA2T2;CBX2;CBX5;CBX7;CCAR1;CCNT1;CD3EAP;CDH13;CDK5RAP2;CDKN2A;CDKN2B;CDON;CE
C25B;CDK5RAP2;CDKN2A;CDKN2B;CEP164;CEP72;CHEK2;CKAP5;CNTRL;CRADD;CSNK1E;CUL1;CUL4A;DCTN1;DYNC1H1;DYNC1I2;FEN1;FHL1;FOXM1;FZR1;GINS4;HYAL1;ING5;INO80;KHDRBS1;KLF11;LIG1;LSM10;MCM2;MCM3;MCM4;MCM6;MTA3;MYC;NINL;NUMA1;ODF2;PCM1;PCNT;PKD1;POLA1;POL
;CDK5RAP2;CDKN2A;CDKN2B;CHEK2;CRADD;CSNK2A2;CUL1;CUL4A;DDB1;DDIT3;DHCR24;DST;DTL;EIF4G2;ERCC3;ETS1;FHL1;FOXC1;FOXM1;FZR1;GADD45A;GATA3;HDAC1;HEXIM1;HTRA2;INCA1;ING5;INTS7;KHDRBS1;KNTC1;MACF1;MAD2L1BP;MAD2L2;MAP2K6;MCM2;MCM3;MCM4;MCM6;MDC1;MD
;DDX6;DHX15;DHX35;DHX38;DHX9;DKC1;DRG1;EDC3;EDC4;EEF2;EFTUD2;EIF2AK4;EIF4A3;EPRS;G3BP1;GAR1;GCN1L1;GEMIN4;GEMIN5;GPATCH1;GTF3C1;HEATR1;HNRNPA0;HNRNPA2B1;HNRNPA3;HNRNPAB;HNRNPH1;HNRNPM;HNRNPR;HNRNPU;HSPA8;ILF2;ILF3;KHSRP;LARP7;LOC100130932;LSM10
J1;KDELR3;MBTPS1;PDIA5;PDIA6;RHBDD1;SHC1;SYVN1;TATDN2;TSPYL2;VCP;XBP1
P17A;AKNA;ALAD;ALDH18A1;ALX3;AMACR;APBB1;ARHGAP35;ARID1B;ARNTL2;ASCC1;ASMT;ASPDH;ASXL1;ATF6B;ATIC;ATN1;ATP1A1;ATP1A3;ATP2A2;ATP2A3;ATP5A1;ATP5B;ATP5J;ATP5J2;ATP8B1;ATXN1L;AVPR2;AXIN1;BACH2;BAHD1;BATF;BATF3;BAZ1B;BCL10;BCL11B;BCL9L;BEX1;BLM;BMP2;BPTF;
RHGEF6;ARHGEF7;ARHGEF9;CCR7;DOCK7;EPHA1;EPHA2;FGD1;FOXJ1;GPR55;KALRN;KANK1;KCTD13;MCF2L;MTOR;MYO9B;NCKAP1L;NET1;OBSCN;OCRL;PLEKHG2;PTK2;RASGRF2;SCRIB;SYDE1;TAX1BP3;TBC1D7;TIAM1;TNFAIP1;TRIO;TSC1;VAV
16;ARHGEF5;ARHGEF6;ARHGEF7;AXIN1;AXIN2;BAD;BMP2;CALM3;CALR;CASP1;CCR7;CDKN2A;CUL7;DCTN1;DDIT3;DNAJA3;DNAJC1;DOCK7;EDEM1;EIF2AK3;EPHA1;EPHA2;FGF2;FGFR1;FOXJ1;GABARAPL2;GOSR2;GPR55;HSP90B1;HSPD1;HTRA2;HYOU1;ITPR3;KDELR3;LCK;MBTPS1;MSH6;MUL1;MYC;MYO
AD;ALX3;APBB1;ARHGAP35;ARID1B;ARNTL2;ASCC1;ASMT;ASPDH;ASXL1;ATF6B;ATIC;ATN1;ATP1A1;ATP1A3;ATP2A2;ATP2A3;ATP5A1;ATP5B;ATP5J;ATP5J2;ATP8B1;ATXN1L;AVPR2;AXIN1;BACH2;BAHD1;BATF;BATF3;BAZ1B;BCL10;BCL11B;BCL9L;BEX1;BLM;BMP2;BPTF;BRD2;BRD7;BRPF1;BTRC;C14orf169

DLST;DNAJA3;EARS2;ETFDH;ETHE1;FH;FXN;GARS;GLS2;GLUD1;GOT2;GPT2;GRPEL1;HADH;HADHA;HADHB;HSPA1L;HSPA9;HSPD1;IARS2;IDH3A;IVD;KARS;LARS2;MCCC1;MCCC2;MIPEP;MRPL49;MRPS24;MRPS36;MRPS9;NDUFA10;NDUFA9;NFS1;NSUN4;OGDH;PCCA;PCCB;PDHA1;PDHB;PDPR;PITRM1;POL

HGAP35;ARHGEF1;ARHGEF16;ARHGEF18;ARHGEF3;ARHGEF4;ARHGEF5;ARHGEF6;ARHGEF7;ARHGEF9;AXIN1;AXIN2;CDC42BPA;CDC42BPB;CDC42BPG;CIT;CYTH1;CYTH3;DENND2D;DENND4B;DNAJA3;DOCK10;DOCK7;DOCK9;EIF2B1;EVI5L;FGD1;GBF1;HERC1;HERC2;HMHA1;IPO5;KALRN;LAMTOR1;MADD;
APOBEC3F;ARHGAP35;ARID1B;ARNTL2;ASCC1;ASXL1;ATN1;ATP8B1;ATXN1L;AVPR2;AXIN1;AXIN2;BAHD1;BAZ1B;BBS2;BCL10;BCL11B;BCL2;BCL9L;BEX1;BLM;BMP2;BPTF;BRD2;BRD7;BRPF1;BTRC;C14orf169;C1D;CALR;CAMTA2;CAND2;CAPN3;CARD11;CBFA2T2;CBX2;CBX5;CBX7;CCAR1;CCDC88C;CCNT1;C

3;ADCY6;ADCY7;ADCY9;ADD1;AGAP5;AGAP6;AHSA1;AIFM1;AKAP17A;AKNA;AKT2;ANAPC1;ANAPC5;ANAPC7;ANK3;ANKLE2;APBA1;APBA2;APBB1;APOBEC3F;ARHGAP15;ARHGAP29;ARHGAP35;ARHGEF16;ARHGEF5;ARHGEF6;ARHGEF7;ARID1B;ARNTL2;ASB1;ASCC1;ASXL1;ATG10;ATG14;ATN1;ATP6V1H;A
1;ARHGAP15;ARHGAP29;ARHGAP35;ARHGEF16;ARHGEF5;ARHGEF6;ARHGEF7;ARID1B;ARNTL2;ASCC1;ASXL1;ATN1;ATP8B1;ATXN1L;AVPR2;AXIN1;AXIN2;BAHD1;BAZ1B;BCL10;BCL11B;BCL9L;BEX1;BLM;BMP2;BPTF;BRD2;BRD7;BRPF1;BTRC;C14orf169;C1D;CALM3;CALR;CAMTA2;CAND2;CAPN3;CARD11;
;FBXW2;GART;GMPS;HECTD1;HERC1;HERC2;HLCS;HUWE1;IRAK1;KCTD10;KCTD13;LGSN;LMO7;MCCC1;MTHFD1;MTHFD1L;MTHFS;MUL1;PAICS;PCCA;PET112;PFAS;PJA1;PPCS;PPIL2;PRPF19;RFWD3;RNF125;RNF20;RNF4;RNF43;RNF8;SYVN1;TNFAIP1;TRAF3;TRIM17;TRIM56;TRIM68;TTC3;TTL;TTLL3;TTLL
A1;ALDH6A1;ALDH7A1;AMACR;AMBRA1;AMFR;ANAPC1;ANAPC5;ANAPC7;APOBEC3F;ARG1;ARHGAP15;ARHGAP29;ARHGAP35;ARHGEF16;ARHGEF5;ARHGEF6;ARHGEF7;ARIH2;ASPDH;ATG10;ATG13;ATG14;ATG3;ATG9B;ATM;ATP5A1;ATP5B;ATP5J;ATP5J2;ATP6V1H;ATP8B1;ATP8B2;AXIN1;AXIN2;BAD;B
NLT1;FBF1;HAUS5;INCENP;INVS;JTB;KIF3B;KIF4A;KNTC1;MAD2L1BP;MAD2L2;MAP4;MAP7D3;MMS19;MYC;NEIL2;NSUN2;NUMA1;NUP62;ODF2;PCIF1;PINX1;POLB;PRC1;PRPF19;RAB11FIP3;RACGAP1;RCC2;RPS6KA2;SEPT6;SPECC1L;TADA2A;TBCCD1;TUBGCP4;TUBGCP5;TUBGCP6;YEATS2;ZW1

K4;GOSR2;HSP90AB1;HSP90B1;HSPA1L;HSPA4;HSPA8;HSPD1;HSPH1;HYOU1;KDELR3;MBTPS1;PDIA5;PDIA6;RHBDD1;SHC1;SYVN1;TATDN2;TSPYL2;VCP;XBP
L;MLXIP;MTOR;MUL1;MYO19;PI4KB;PLD6;RETSAT;SAMM50;SIGMAR1;SLC24A1;SMCR7L;SOX10;SYNE1;SYNE2;TMEM109;TOMM34;VAMP1;VAT1;VDAC1;VDAC
;CRTC3;CTCF;DDIT3;DDX17;ECD;FGF2;GATA3;GMEB2;GTF2A2;HCFC1;HDAC1;HMGA1;HSBP1;HTATIP2;ICMT;IL31RA;JAZF1;KCTD1;KDM1A;L3MBTL2;LDB1;LPIN1;LPXN;MAGED1;MBD1;MECP2;MED11;MED24;MED27;MED30;MED31;MED6;MEIS2;MKL1;MMS19;MNT;MYCBP;NAB2;NCOR2;NFE2L1;NMI;NR
L;SATB1;SCAF8;SFPQ;SNW1;SRRM1;TEP1;THOC1;TP53;UHRF1
AF7;DCAF8;DDB1;DTL;FBXW8;FZR1;KCTD10;KCTD13;KLHL3;MAD2L2;TNFAIP1

UP188;NUP205;NUP210;NUP214;NUP62;NUP93;NXF1;POM121;POM121C;RNPS1;SEH1L;SMG6;SRRM1;SRSF5;THOC1;THOC2;U2AF
ASE1;DST;DTL;EIF5A;EMD;FAF1;FZR1;GLE1;GTPBP4;GUCY2D;HTATIP2;INTS1;IPO5;IPO9;ITPR3;LPIN1;MNS1;MYO1C;NUP133;NUP155;NUP188;NUP205;NUP210;NUP214;NUP62;NUP93;NXF1;PARP1;PCM1;POLA1;POM121;POM121C;POU6F1;RETSAT;RNF43;S100A6;SCRN1;SEH1L;SIGMAR1;SMAD3;SUN2;
X3;APBB1;ARHGAP35;ARID1B;ARNTL2;ASCC1;ASPDH;ASXL1;ATF6B;ATIC;ATN1;ATP1A1;ATP1A3;ATP2A2;ATP2A3;ATP5A1;ATP5B;ATP5J;ATP5J2;ATP8B1;ATXN1L;AVPR2;AXIN1;BACH2;BAHD1;BATF;BATF3;BAZ1B;BCL10;BCL11B;BCL9L;BEX1;BLM;BMP2;BPTF;BRD2;BRD7;BRPF1;BTRC;C14orf169;C1D;CAD;C
F7;ARHGEF9;BAD;CALM3;CASP2;DDIT4;DUSP7;FGD1;HDAC1;IKBKB;IRAK1;IRS1;ITPR3;KALRN;MAGED1;MAPK7;MAPKAP1;MCF2L;MTOR;NET1;OBSCN;OMG;PCSK5;PDPK1;PIK3R2;PLCG1;PLEKHG2;PRDM4;PRKCA;PSENEN;RALGDS;RAPGEF1;RASGRF2;RELA;RPS6KA2;SHC1;THEM4;TIAM1;TRIO;VAV2;YWHA
10;DOCK9;FGD1;KALRN;MCF2L;NET1;OBSCN;PLEKHG2;RASGRF2;TIAM1;TRIO;VAV

1;POLA1;PSPC1;RUVBL1;SATB1;SCAF8;SFPQ;SNW1;SRRM1;TEP1;THOC1;TP53;UHRF1

K4;F12;GOSR2;HSP90AB1;HSP90B1;HSPA1L;HSPA4;HSPA8;HSPD1;HSPH1;HYOU1;KDELR3;MBTPS1;PDIA5;PDIA6;RHBDD1;SHC1;SYVN1;TATDN2;TSPYL2;VCP;XBP
J1;KDELR3;MBTPS1;PDIA5;PDIA6;SHC1;SYVN1;TATDN2;TSPYL2;VCP;XBP
25B;CDK5RAP2;CDKN2A;CDKN2B;CHEK2;DNAJA3;DPYSL2;ELAVL1;EPHA1;FAF1;FLNA;FOXM1;GYS1;HNRNPA0;HSP90AB1;IKBKB;IL12RB2;IL31RA;IRS1;JTB;KIF13B;LAX1;LCK;MAD2L2;MAP2K6;MAP2K7;MAP3K12;MAPK7;MAPK8IP3;MAPK9;MAPT;MAVS;NEK9;PAM;PARD6A;PARN;PDPK1;PKD1;PPME1;PRC1;
BFA2T2;CBX2;CBX5;CBX7;CDAN1;CDKN2A;CHCHD3;CIITA;CRY2;CTCF;CTR9;DDIT3;DDX20;DNAJA3;DNMT1;DNMT3A;DRD4;EHMT1;EZH1;FGF2;FGFR1;FOSB;FOXJ1;FOXK1;FOXM1;FOXP1;GABBR1;GATA3;GRM2;GTPBP4;HCFC1;HDAC1;HEXIM1;HHEX;HIC2;HIF1AN;HMG20A;HMGA1;HNRNPA2B1;HSBP1;HSP
3C4;HCFC1;HDAC1;HLCS;HUWE1;ING5;KAT6B;KDM1A;KDM2B;KDM4A;KDM4C;KDM5C;LDB1;LEF1;LEO1;LPIN1;MAP3K12;MECP2;MLL;MLL4;PAXIP1;PCGF1;PHF15;PIM3;POLE3;PRKCA;PRMT2;PYGO2;RBM14;RNF20;RNF8;RUVBL1;SATB1;SETD1A;SNW1;SRCAP;TADA2A;TAF1;TAF10;TCF3;TP53;TRIM16;TRR
HGEF4;ARHGEF5;ARHGEF6;ARHGEF7;ARPC2;ARPC3;ATG13;ATG14;ATG3;ATG9B;ATXN2L;AXIN1;BAD;BAHD1;BAIAP2L1;BBS1;BBS2;BCL10;BCL2;BICD1;BID;BIK;BLM;BMS1;BRD2;C1D;C2CD3;CADM1;CADM2;CALR;CAPN3;CCDC88C;CCR7;CD3E;CD74;CDAN1;CDH13;CDH24;CDKN2A;CELF1;CENPN;CENPO;CEP
ATA3;GTF3C4;HCFC1;HDAC1;HLCS;HUWE1;ING5;KAT6B;KDM1A;KDM2B;KDM4A;KDM4C;KDM5C;LDB1;LEF1;LEO1;LPIN1;MAP3K12;MECP2;MLL;MLL4;PAXIP1;PCGF1;PHF15;PIM3;POLE3;PRKCA;PRMT2;PYGO2;RBM14;RNF20;RNF8;RUVBL1;SATB1;SETD1A;SNW1;SRCAP;TADA2A;TAF1;TAF10;TCF3;TP53;TRI
f82;CALR;CAPN3;CASP2;CCND1;CCND2;CCNDBP1;CCNF;CCNT1;CDC16;CDC25B;CDK5RAP2;CDKN2A;CDKN2B;CHEK2;CRADD;CSNK2A2;CTCF;CUL1;CUL4A;DDB1;DDIT3;DHCR24;DST;DTL;EIF4G2;ERCC3;ETS1;FGF2;FGFR1;FHL1;FOXC1;FOXM1;FZR1;GADD45A;GATA3;GTPBP4;HCFC1;HDAC1;HEXIM1;HSPA8;H
DCY7;ADCY9;ADD1;AGAP5;AGAP6;AHSA1;AKAP17A;AKNA;AKT2;ANAPC1;ANAPC5;ANAPC7;ANKLE2;APBB1;ARHGAP15;ARHGAP29;ARHGAP35;ARHGEF16;ARHGEF5;ARHGEF6;ARHGEF7;ARID1B;ARNTL2;ASB1;ASCC1;ASXL1;ATG10;ATG14;ATN1;ATP8B1;ATXN1L;AVPR2;AXIN1;AXIN2;BAD;BAHD1;BAZ1B;BCL
P;MTOR;MUL1;MYO19;PI4KB;PLD6;RETSAT;SAMM50;SIGMAR1;SMCR7L;SOX10;SYNE1;SYNE2;TMEM109;TOMM34;VAMP1;VAT1;VDAC1;VDAC
J1;KDELR3;MBTPS1;PDIA5;PDIA6;RHBDD1;SHC1;SYVN1;TATDN2;TSPYL2;VCP;XBP
EF9;CYTH1;CYTH3;DENND2D;DENND4B;DOCK10;DOCK7;DOCK9;EIF2B1;FGD1;GBF1;HERC1;HERC2;KALRN;LAMTOR1;MADD;MCF2L;NET1;OBSCN;PLEKHG2;PSD4;RABIF;RALGDS;RALGPS1;RANBP10;RAPGEF1;RASGRF2;RASGRP2;SBF1;TIAM1;TRIO;VAV
0;INPPL1;KCTD13;KITLG;KRT7;LIG1;LIG3;MCM2;MCM3;MCM3AP;MCM4;MCM6;NASP;NF2;PAPD7;PDGFC;PINX1;POLA1;POLA2;POLB;POLE;POLE3;POLG;POLH;RBBP4;RBBP7;RBM14;RFC1;RNASEH1;RPA1;RPA2;RRM1;SHC1;SMC1A;SSRP1;SUPT16H;TERF2;TERF2IP;TNFAIP1;TNKS1BP1;TOP2B;TP53;TSPYL2;
ARNTL2;ASCC1;ASXL1;ATN1;ATP8B1;ATXN1L;AXIN1;BAHD1;BAZ1B;BCL10;BCL11B;BCL2;BCL9L;BEX1;BLM;BMP2;BPTF;BRD2;BRD7;BRPF1;BTRC;C14orf169;C1D;CALR;CAMTA2;CAND2;CAPN3;CARD11;CBFA2T2;CBX2;CBX5;CBX7;CCAR1;CCNT1;CCR7;CDAN1;CDH13;CDK5RAP2;CDKN2A;CDKN2B;CDON;CELS

2;PAPD7;POLA1;RNF8;RPA1;RPA2;SLX4;TDP1;TERF2IP;TP53;TP53BP1;VCP;XRCC5;XRCC6;ZFYVE2
2;CINP;CRADD;CSNK1E;CUL4A;DCLRE1C;DDB1;DDIT3;DDIT4;DTL;EME2;ERCC3;ERCC5;FAN1;FANCI;FEN1;FOXM1;FZR1;GADD45A;HERC2;HMGA1;HUWE1;IKBKE;ING2;INO80;INTS7;KDM1A;LIG1;LIG3;LOC100133315;MAD2L2;MAP2K6;MDC1;MLH1;MMS19;MRPS9;MSH6;MYC;NEIL2;NFATC2;NFRKB;NONO;
LDH1B1;ALDH5A1;ALDH6A1;ALDH7A1;AMACR;AMFR;ANAPC1;ANAPC5;ANAPC7;APOBEC3F;ARG1;ARHGAP15;ARHGAP29;ARHGAP35;ARHGEF16;ARHGEF5;ARHGEF6;ARHGEF7;ARIH2;ASPDH;ATM;ATP5A1;ATP5B;ATP5J;ATP5J2;ATP6V1H;ATP8B1;ATP8B2;AXIN1;AXIN2;BAD;BLM;BTRC;CAD;CALM3;CARNS1;
NK1;MAP4;NDC80;NUMA1;PTK2;SCRIB;ZW1
ARNTL2;ASB1;ASCC1;ASXL1;ATN1;ATP8B1;ATXN1L;AXIN1;BAHD1;BAZ1B;BCL10;BCL11B;BCL2;BCL9L;BEX1;BLM;BMP2;BPTF;BRD2;BRD7;BRPF1;BTRC;C14orf169;C1D;CALR;CAMTA2;CAND2;CAPN3;CARD11;CBFA2T2;CBX2;CBX5;CBX7;CCAR1;CCNT1;CCR7;CD3E;CD4;CDAN1;CDH13;CDK5RAP2;CDKN2A;CDK

F5;DCAF7;DCAF8;DDB1;DTL;FBXL12;FBXO21;FBXW8;FZR1;KCTD10;KCTD13;KLHL3;LMO7;MAD2L2;PPIL2;PRPF19;RNF20;RNF8;TCEB1;TNFAIP1;UBE2B;UBE2L
;GGH;GLS2;GLUD1;GMPS;GOT1;GOT2;GPT;GPT2;HPD;HTT;IBA57;IVD;KYNU;LGSN;MCCC1;MCCC2;MECP2;MTHFD1;MTHFR;NFS1;OGDH;PCBD1;PDPR;PFAS;PSAT1;SEPHS2;SHMT1;SM
BFA2T2;CBX2;CBX5;CBX7;CDAN1;CDKN2A;CHCHD3;CIITA;CRY2;CTCF;CTR9;DDIT3;DDX20;DNAJA3;DNMT1;DNMT3A;DRD4;EHMT1;EZH1;FGF2;FGFR1;FOSB;FOXJ1;FOXK1;FOXM1;FOXP1;GABBR1;GATA3;GRM2;GTPBP4;HCFC1;HDAC1;HEXIM1;HHEX;HIC2;HIF1AN;HMG20A;HMGA1;HNRNPA2B1;HSBP1;HSP
;CRTC3;CRY2;CTCF;DDIT3;DDX17;ECD;FGF2;GATA3;GMEB2;GTF2A2;HCFC1;HDAC1;HMGA1;HSBP1;HTATIP2;ICMT;IL31RA;JAZF1;KCTD1;KDM1A;L3MBTL2;LDB1;LMO2;LPIN1;LPXN;MAGED1;MBD1;MECP2;MED11;MED24;MED27;MED30;MED31;MED6;MEIS2;MKL1;MMS19;MNT;MTA2;MYCBP;NAB2;NCO
5;ARHGEF18;ARHGEF3;ARHGEF4;ARHGEF6;ARHGEF7;ARHGEF9;ATG3;ATM;ATN1;AXIN1;BAD;BAG3;BCL10;BCL11B;BCL2;BID;BIK;BIRC6;BLCAP;BMP2;BRSK2;C11orf82;C1D;C22orf29;CADM1;CALR;CAPN3;CARD11;CARD14;CARD16;CASP1;CASP10;CASP2;CCAR1;CCR7;CD3E;CD40LG;CD74;CDKN2A;CHEK2;CL
;CRTC3;CTCF;DDIT3;DDX17;ECD;FGF2;GATA3;GMEB2;GTF2A2;HCFC1;HDAC1;HMGA1;HSBP1;HTATIP2;ICMT;IL31RA;JAZF1;KCTD1;KDM1A;L3MBTL2;LDB1;LMO2;LPIN1;LPXN;MAGED1;MBD1;MECP2;MED11;MED24;MED27;MED30;MED31;MED6;MEIS2;MKL1;MMS19;MNT;MTA2;MYCBP;NAB2;NCOR2;NF
3;ALAD;ALDH5A1;ALMS1;ALS2CL;AMFR;AMIGO1;ANAPC1;ANAPC13;ANAPC5;ANAPC7;ANK3;ANKLE2;ANXA2;AP2B1;AP3D1;APBA1;APBB1;ARG1;ARHGAP15;ARHGAP35;ARHGAP39;ARHGEF1;ARHGEF16;ARHGEF18;ARHGEF4;ARHGEF5;ARHGEF6;ARHGEF7;ARID1B;ARL3;ARPC2;ARPC3;ASXL1;ATG13;ATG14;
PA2;SLA2;SMARCA4;SMARCC1;SMARCE1;SRRM2;STXBP1;TP53;TSC1
POLB;POLE;POLG;RFC1;RNASEH1;RPA1;RPA2;SMC1A;TERF2;TOP2B;WRNIP

CNDP2;CTPS2;DARS2;DLD;DLST;DNMT3A;DUOX1;EARS2;EIF5A;ENOSF1;EPRS;ETHE1;FARSB;FASN;GARS;GATA3;GGH;GGT7;GGTA1P;GLS2;GLUD1;GMPS;GOT1;GOT2;GPT;GPT2;GSTM4;HARS;HPD;HTT;IARS;IARS2;IBA57;IVD;KARS;KYNU;LARS;LARS2;LGSN;MARS;MCCC1;MCCC2;MECP2;MGST1;MTHFD1;MT
RPRD1B;TAF1;TAF10;TAF3;TAF6;TAF8;TBP;TP53;TRRAI
5;ARHGEF18;ARHGEF3;ARHGEF4;ARHGEF6;ARHGEF7;ARHGEF9;ATG3;ATM;ATN1;AXIN1;BAD;BAG3;BCL10;BCL11B;BCL2;BID;BIK;BIRC6;BLCAP;BMP2;BRSK2;C11orf82;C1D;C22orf29;CADM1;CALR;CAPN3;CARD11;CARD14;CARD16;CASP1;CASP10;CASP2;CCAR1;CCR7;CD3E;CD40LG;CD74;CDKN2A;CHEK2;CL
H;ETHE1;GLUD1;GOT1;GOT2;GPT;GPT2;HACL1;HADH;HADHA;HADHB;HPD;IBA57;IRS1;IVD;KYNU;LONP2;LPIN1;MCCC1;MCCC2;MTOR;OGDH;PCBD1;PCCA;PCCB;PCYOX1;PCYOX1L;PDPR;PEX5;PPARA;PPARD;SHMT
H;ETHE1;GLUD1;GOT1;GOT2;GPT;GPT2;HACL1;HADH;HADHA;HADHB;HPD;IBA57;IRS1;IVD;KYNU;LONP2;LPIN1;MCCC1;MCCC2;MTOR;OGDH;PCBD1;PCCA;PCCB;PCYOX1;PCYOX1L;PDPR;PEX5;PPARA;PPARD;SHMT
;USP7
NUP133;NUP155;NUP214;NXF1;PKD1;RNPS1;SMG6;SRRM1;SRSF5;TCF7L2;THOC1;THOC2;TSC1;TXN;U2AF2;XPO
T1;EIF4G1;ERCC3;FYN;GTF2A2;HCFC1;HDAC1;HMGA1;HSPA8;HSPD1;HTATIP2;HYAL2;IL16;IL2RB;IL2RG;IPO5;KARS;KCTD5;KDM4A;KLC1;KRT7;LCK;MAVS;MFGE8;MMP1;NFX1;NMT2;NXF1;PAAF1;PLCG1;POLA1;RBM15B;RELA;RNF216;RRAGA;SATB1;SCRIB;SEPT6;SF3B2;SLAMF1;SLC20A2;SMAD3;SNAPIN;SN
CBFA2T2;CBX2;CBX5;CBX7;CDAN1;CDKN2A;CHCHD3;CIITA;CIRBP;CRY2;CTCF;CTR9;DDIT3;DDX20;DNAJA3;DNMT1;DNMT3A;EHMT1;EIF2AK3;EIF2AK4;EIF2B1;EIF4A3;EPRS;EZH1;FGF2;FGFR1;FOSB;FOXJ1;FOXK1;FOXM1;FOXP1;GATA3;GTPBP4;HCFC1;HDAC1;HEXIM1;HHEX;HIC2;HIF1AN;HMG20A;HMGA1;H
2;NUP93;NXF1;POM121;POM121C;SEH1L;TNPO2;XPO
ARHGEF4;ARHGEF5;ARHGEF6;ARHGEF7;ARHGEF9;CCR7;CYTH1;CYTH3;DOCK7;EPHA1;EPHA2;FGD1;FOXJ1;FOXM1;GBF1;GPR55;ICMT;ITPKB;KALRN;KANK1;KCTD13;KITLG;MCF2L;MTOR;MYO9B;NCKAP1L;NET1;NRG1;NUP62;OBSCN;OCRL;PLEKHG2;PSD4;PTK2;RAB3GAP1;RASA3;RASA4;RASAL3;RASGRF2;S

TSC1;UBE2B;USP13;VHL;ZBED

L7;DDB1;DYNC1I2;DYNLT1;EIF4G1;ERCC3;FYN;GTF2A2;HCFC1;HDAC1;HMGA1;HSPA8;HSPD1;HTATIP2;HTATSF1;HYAL2;IL16;IL2RB;IL2RG;IPO5;KARS;KCTD5;KDM4A;KLC1;KRT7;LCK;LEF1;MAVS;MFGE8;MMP1;NFX1;NMT1;NMT2;NUP133;NUP155;NUP188;NUP205;NUP210;NUP214;NUP62;NUP93;NXF1;PA

;MARS;NARS;NSUN2;PET112;POP1;PUS3;QTRTD1;RPP14;RPP21;SARS;TARS;TRIT1;TYW1;WDR4;YARS

TR1;LAS1L;NOL9;NOLC1;NOP10;NOP14;NSUN4;PA2G4;PDCD11;RRP1B;TSR2;UTP14A;UTP20;WBP11;WDR5

;CDK5RAP2;CEP104;CEP164;CEP72;CEP85;CEP89;CHD3;CHD4;CKAP5;CNTRL;CROCC;DCTN1;DCTN5;DDHD2;DHX9;DIS3L;DTL;DYNC1H1;DYNC1I2;DYNC1LI2;EXOC7;FBF1;HAUS5;HERC2;JTB;KDM4A;KIAA0368;KIAA0586;KIAA0753;KIF3B;LCK;MAP7D3;MCM3;MID2;MYCBP;NCAPG;NEURL4;NPHP4;ODF2;PCM1

P39;ARHGEF1;ARHGEF16;ARHGEF18;ARHGEF3;ARHGEF4;ARHGEF5;ARHGEF6;ARHGEF7;ARHGEF9;CCR7;CDON;CYTH1;CYTH3;DOCK7;EPHA1;EPHA2;FGD1;FOXJ1;FOXM1;GBF1;GPR55;HMHA1;ICMT;INPP5B;ITPKB;KALRN;KANK1;KCTD13;KITLG;MCF2L;MTOR;MYO9B;NCKAP1L;NET1;NRG1;NUP62;OBSCN;OC

RHGEF6;ARHGEF7;ARHGEF9;CCR7;CDH13;DOCK7;EPHA1;EPHA2;FGD1;FOXJ1;GPR55;KALRN;KANK1;KCTD13;MCF2L;MTOR;MYO9B;NCKAP1L;NET1;OBSCN;OCRL;PHACTR4;PLEKHG2;PTK2;RASGRF2;SCRIB;SRGAP1;SYDE1;TAX1BP3;TBC1D7;TIAM1;TNFAIP1;TRIO;TSC1;VANGL2;VAV

NUP62;NUP93;NXF1;POM121;POM121C;SEH1L;TNPO2;VDAC1;VDAC2;XPO5

L7;DDB1;DYNC1I2;DYNLT1;EIF4G1;ERCC3;FYN;GTF2A2;HCFC1;HDAC1;HMGA1;HSPA8;HSPD1;HTATIP2;HTATSF1;HYAL2;IL16;IL2RB;IL2RG;IPO5;KARS;KCTD5;KDM4A;KLC1;KRT7;LCK;LEF1;MAVS;MFGE8;MMP1;NFX1;NMT1;NMT2;NUP133;NUP155;NUP188;NUP205;NUP210;NUP214;NUP62;NUP93;NXF1;PA

BB1;ARHGEF16;ARHGEF18;ARHGEF3;ARHGEF4;ARHGEF6;ARHGEF7;ARHGEF9;ATG3;ATM;ATN1;AXIN1;AXIN2;BAD;BAG3;BCL10;BCL11B;BCL2;BID;BIK;BIRC6;BLCAP;BMP2;BRSK2;C11orf82;C1D;C22orf29;CADM1;CALR;CAPN3;CARD11;CARD14;CARD16;CASP1;CASP10;CASP2;CCAR1;CCR7;CD3E;CD40LG;CD7

K1E;CUL1;CUL4A;CUL7;CUL9;DDB1;DDIT3;DERL2;DTL;DYX1C1;EDEM1;FAF1;FBXL12;FBXO21;FLNA;FOXRED2;FYN;FZR1;GABARAPL2;GRIN2C;HECTD1;HERC2;HSP90AB1;HSP90B1;HTRA2;HUWE1;IL10;KCTD10;KCTD13;KIAA0368;LNPEP;LONP2;MAD2L2;MDM4;MTOR;NEDD8;NKD1;NRG1;PAN2;PCYOX1;PJA1

A1;ALG8;ALG9;ASMT;ATAT1;ATIC;ATM;B3GALT4;B3GNTL1;BAZ1B;BRSK2;C1GALT1C1;C9orf96;CAD;CARD11;CCL8;CCND1;CD3EAP;CDC42BPA;CDC42BPB;CDC42BPG;CDKAL1;CERK;CHEK2;CHST12;CIT;CKM;CLK2;COX10;CSNK1E;CSNK2A2;CSRP2BP;DAD1;DCLK2;DCTN5;DDR1;DFFB;DGKA;DGKD;DGKH;DGKQ

BB1;ARHGEF16;ARHGEF18;ARHGEF3;ARHGEF4;ARHGEF6;ARHGEF7;ARHGEF9;ATG3;ATM;ATN1;AXIN1;AXIN2;BAD;BAG3;BCL10;BCL11B;BCL2;BID;BIK;BIRC6;BLCAP;BMP2;BRSK2;C11orf82;C1D;C22orf29;CADM1;CALR;CAPN3;CARD11;CARD14;CARD16;CASP1;CASP10;CASP2;CCAR1;CCR7;CD3E;CD40LG;CD7

);C1D;CALR;CAPN3;CBFA2T2;CBX2;CBX5;CBX7;CDAN1;CDKN2A;CHCHD3;CIITA;CIRBP;CRY2;CTCF;CTR9;DDIT3;DDX20;DNAJA3;DNMT1;DNMT3A;EHMT1;EIF2AK3;EIF2AK4;EIF2B1;EIF4A3;EPRS;EZH1;FGF2;FGFR1;FOSB;FOXJ1;FOXK1;FOXM1;FOXP1;GATA3;GTPBP4;HCFC1;HDAC1;HEXIM1;HHEX;HIC2;HIF1AN;

L;ASXL1;ATF6B;ATN1;ATP8B1;ATXN1L;AXIN1;BACH2;BAHD1;BATF;BATF3;BAZ1B;BCL10;BCL11B;BCL9L;BEX1;BLM;BMP2;BPTF;BRD2;BRD7;BRPF1;BTRC;C14orf169;C1D;CALR;CAMTA2;CAND2;CAPN3;CARD11;CBFA2T2;CBX2;CBX5;CBX7;CCAR1;CCNT1;CD3EAP;CDH13;CDK5RAP2;CDKN2A;CDKN2B;CDON;CEI

C1;SYVN1;TATDN2;TSPYL2;XBP1

L4;MPHOSPH8;MTA2;MTR;NSUN2;PAXIP1;PLD6;PRMT2;PYGO2;RNF20;RNMT;SATB1;SETD1A;SETD6;SNW1;TGS1;TPMT;UHRF1;USP7;WDR

DIT3;DERL2;DTL;EDEM1;EXOSC10;FAF1;FBXL12;FBXO21;FOXRED2;FZR1;HECTD1;HERC2;HSP90AB1;HSP90B1;HUWE1;KCTD10;KCTD13;KIAA0368;MAD2L2;NEDD8;PAN2;PCYOX1;PPP2R5C;PSMC4;PSMD1;PSMD11;PSMD2;PSMD6;PSMD7;PSME4;PTK2;RHBDD1;RNF20;RNF216;RNF4;RNF43;RNF8;SEC61B;SY

D11;CCL8;CCND1;CD3EAP;CDC42BPA;CDC42BPB;CDC42BPG;CERK;CHEK2;CIT;CKM;CLK2;CSNK1E;CSNK2A2;DCLK2;DDR1;DGKA;DGKD;DGKH;DGKQ;DKC1;DLG3;DSTYK;EEF2K;EFNA4;EIF2AK3;EIF2AK4;EPHA1;EPHA2;ERBB3;ERCC3;FAM20B;FASTKD5;FGFR1;FGFRL1;FGGY;FLT4;FN3KRP;FYN;GALK2;GALT;GK5;

;ANXA2;ARHGAP15;ARHGAP29;ARHGAP35;ARHGEF16;ARHGEF5;ARHGEF6;ARHGEF7;AVPR2;AXIN1;AXIN2;BAD;BCL10;BCL2;BEX1;BMP2;BTRC;CALM3;CALR;CAPN3;CARD11;CARD14;CASP1;CCND1;CCND2;CCR7;CD4;CD74;CDC16;CDC25B;CDKN2A;CRTC1;CRTC3;CUL1;CUL7;CXCL1;CYTL1;DCTN1;DDIT3;DNA

TDP1;WBP11;XPC

NTRL;CSNK1E;DCTN1;DYNC1H1;DYNC1I2;FHL1;FOXM1;KHDRBS1;MTA3;NINL;NUMA1;ODF2;PCM1;PCNT;SFI1;TAF1;TOP2B;TUBB;TUBGCP4;TUBGCP5;TUBGCP6;YWHAE

TP5J2;BAD;BCL2;BDH1;BID;BIK;BRI3BP;CHCHD3;COX10;COX15;COX5B;COX8A;CYB5B;DHODH;DNAJC11;EFHD1;ETFDH;EXOG;GOT2;GRAMD4;HADHA;HADHB;HERC2;HSPD1;HTRA2;IMMT;LETM1;LOC494141;MAVS;MCCC1;MCCC2;MGST1;MLXIP;MTCH2;MTOR;MUL1;MYO19;NAT8L;NDUFA10;NDUFA2;NDU

RCC2;SMARCE1;ZBTB16

L;NET1;OCRL;SCRIB;SYDE1;TAX1BP3;TBC1D7;TSC1

1;PCM1;PCNT;RAB11FIP3;SFI1;STARD9;TCP1;TUBGCP4;TUBGCP5;TUBGCP6

DIT3;DERL2;DTL;EDEM1;FAF1;FBXL12;FBXO21;FOXRED2;FZR1;HECTD1;HERC2;HSP90AB1;HSP90B1;HUWE1;KCTD10;KCTD13;KIAA0368;MAD2L2;NEDD8;PAN2;PPP2R5C;PSMC4;PSMD1;PSMD11;PSMD2;PSMD6;PSMD7;PSME4;PTK2;RHBDD1;RNF20;RNF216;RNF4;RNF43;RNF8;SEC61B;SYVN1;TAF1;TCEB1;

L1;ATN1;ATP8B1;ATXN1L;AXIN1;BAHD1;BAZ1B;BCL10;BCL11B;BCL9L;BEX1;BLM;BMP2;BPTF;BRD2;BRD7;BRPF1;BTRC;C14orf169;C1D;CALR;CAMTA2;CAND2;CAPN3;CARD11;CBFA2T2;CBX2;CBX5;CBX7;CCAR1;CCNT1;CDH13;CDK5RAP2;CDKN2A;CDKN2B;CDON;CELF1;CELSR2;CHCHD3;CHD2;CHD3;CHD4;CH

76B;LEF1;MCM3;MPHOSPH8;POLA1;POLA2;POLE;POLE3;RPA1;RPA2;TCF3;TCF7L2;TERF2;TERF2IP;XRCC5;XRCC6

M;AXIN1;AXIN2;BAD;BATF;BAZ1B;BCL2;BLM;BRSK2;C11orf82;CALR;CAPN3;CASP2;CCND1;CCR7;CD74;CDKN2A;CEP164;CHD1L;CHEK2;CINP;CLPB;CRADD;CREB3L2;CSNK1E;CUL4A;CUL7;DCLRE1C;DCTN1;DDB1;DDIT3;DDIT4;DERL2;DHX9;DTL;DUOX1;DUSP7;DVL2;EDEM1;EIF2AK3;EIF2AK4;EIF2B1;EME2;ERC

AP35;ARID1B;ARNTL2;ASB1;ASCC1;ASXL1;ATN1;ATP8B1;ATXN1L;AVPR2;AXIN1;BAHD1;BAZ1B;BCL10;BCL11B;BCL2;BCL9L;BEX1;BLM;BMP2;BPTF;BRD2;BRD7;BRPF1;BTRC;C14orf169;C1D;CALR;CAMTA2;CAND2;CAPN3;CARD11;CBFA2T2;CBX2;CBX5;CBX7;CCAR1;CCNT1;CCR7;CD3E;CD4;CDAN1;CDH13;CDK

DIT3;DERL2;DTL;EDEM1;FAF1;FBXL12;FBXO21;FOXRED2;FZR1;HECTD1;HERC2;HSP90AB1;HSP90B1;HUWE1;KCTD10;KCTD13;KIAA0368;MAD2L2;NEDD8;PAN2;PCYOX1;PPP2R5C;PSMC4;PSMD1;PSMD11;PSMD2;PSMD6;PSMD7;PSME4;PTK2;RHBDD1;RNF20;RNF216;RNF4;RNF43;RNF8;SEC61B;SYVN1;TAF1

LM;BMP2;BPTF;BRD7;BTRC;C14orf169;C1D;CALR;CAPN3;CBFA2T2;CBX2;CBX5;CBX7;CCDC23;CDAN1;CDC16;CDKN2A;CELF1;CHCHD3;CIITA;CIRBP;CLN8;CRY2;CTCF;CTR9;DDIT3;DDIT4;DDX20;DNAJA3;DNAJC1;DNMT1;DNMT3A;DROSHA;DUSP16;DUSP7;EHMT1;EIF2AK3;EIF2AK4;EIF2B1;EIF4A3;EPHA1;EPR

T2;CBX2;CBX5;CBX7;CDKN2A;CHCHD3;CIITA;CRY2;CTCF;CTR9;DDIT3;DDX20;DNAJA3;DNMT1;DNMT3A;EHMT1;EZH1;FGF2;FGFR1;FOSB;FOXJ1;FOXK1;FOXM1;FOXP1;GATA3;HCFC1;HDAC1;HEXIM1;HHEX;HIC2;HIF1AN;HMG20A;HMGA1;HNRNPA2B1;HSBP1;HSPA8;ID1;IL4;ILF3;IRF8;JAZF1;KAT6B;KCTD1;KD

);NOLC1;NOP10;NOP14;NSUN4;PA2G4;PDCD11;RRP1B;TSR2;UTP14A;UTP20;WBP11;WDR5

L1;CUL4A;CUL7;DDB1;DYNC1I2;DYNLT1;EIF4G1;ERCC3;FYN;GPX2;GTF2A2;HCFC1;HDAC1;HMGA1;HSPA8;HSPD1;HTATIP2;HYAL2;IL10;IL16;IL2RB;IL2RG;IPO5;IRF8;KARS;KCTD5;KDM4A;KLC1;KRT7;LCK;LEF1;MAVS;MFGE8;MMP1;NFX1;NMT2;NXF1;PAAF1;PLCG1;POLA1;RBM15B;RELA;RNF216;RRAGA;SATB1

L1;CUL4A;CUL7;DDB1;DYNC1I2;DYNLT1;EIF4G1;ERCC3;FYN;GPX2;GTF2A2;HCFC1;HDAC1;HMGA1;HSPA8;HSPD1;HTATIP2;HYAL2;IL10;IL16;IL2RB;IL2RG;IPO5;IRF8;KARS;KCTD5;KDM4A;KLC1;KRT7;LCK;LEF1;MAVS;MFGE8;MMP1;NFX1;NMT2;NXF1;PAAF1;PLCG1;POLA1;RBM15B;RELA;RNF216;RRAGA;SATB1

D;DUOX1;ECI2;ETFDH;FASN;FOXRED2;GLUD1;GOT1;GOT2;GPT;GPT2;H6PD;HACL1;HADH;HADHA;HADHB;HIF1AN;IDH3A;IVD;KDM1A;KYNU;LMO2;MIOX;NDUFA9;NFS1;NNT;OGDH;PARP1;PDXDC1;PDXDC2P;PGAM2;PNPO;PSAT1;PYGB;SDHA;SDHB;SHMT1;TP53I3;TXNRD3;TYW1;UXS

ALR;CAPN3;CBFA2T2;CBX2;CBX5;CBX7;CDAN1;CDKN2A;CHCHD3;CIITA;CIRBP;CLK2;CRY2;CTCF;CTR9;DDIT3;DDX20;DNAJA3;DNMT1;DNMT3A;DRD4;EHMT1;EIF2AK3;EIF2AK4;EIF2B1;EIF4A3;EPRS;EZH1;FAM132A;FGF2;FGFR1;FOSB;FOXJ1;FOXK1;FOXM1;FOXP1;GABBR1;GATA3;GRM2;GTPBP4;HCFC1;HDAC

2;SLC2A1;SLC46A1;THTPA;TKTL1;VNN3

C1;SYVN1;TATDN2;TSPYL2;XBP1

2;CBX2;CBX5;CBX7;CDKN2A;CELF1;CHCHD3;CIITA;CRY2;CTCF;CTR9;DDIT3;DDX20;DNAJA3;DNMT1;DNMT3A;DROSHA;EHMT1;EZH1;FGF2;FGFR1;FOSB;FOXJ1;FOXK1;FOXM1;FOXP1;FYN;GATA3;HCFC1;HDAC1;HEXIM1;HHEX;HIC2;HIF1AN;HMG20A;HMGA1;HSBP1;HSPA8;ID1;IL4;ILF3;INPPL1;IRF8;JAZF1;KA

AP35;ARID1B;ARNTL2;ASB1;ASCC1;ASXL1;ATN1;ATP8B1;ATXN1L;AVPR2;AXIN1;BAHD1;BAZ1B;BCL10;BCL11B;BCL2;BCL9L;BEX1;BLM;BMP2;BPTF;BRD2;BRD7;BRPF1;BTRC;C14orf169;C1D;CALR;CAMTA2;CAND2;CAPN3;CARD11;CBFA2T2;CBX2;CBX5;CBX7;CCAR1;CCNT1;CCR7;CD3E;CD4;CDAN1;CDH13;CDK

3BP1;MRPL44;PAN2;PARN;PMS2;POLA1;POLG;POP1;REXO1;RNASE2;RNASE3;RNASE4;RNASEH1;RPP14;RPP21;SLX4;SMG6;SND1;TATDN2;TDP1;ZC3H12B;ZC3H12

D2D;DENND4B;DOCK10;DOCK9;FGD1;KALRN;MADD;MCF2L;NET1;OBSCN;PLEKHG2;RANBP10;RASGRF2;SBF1;TIAM1;TRIO;VAV2

P5B;ATP5J;ATP5J2;BAD;BCL2;BDH1;BID;BIK;BLOC1S1;BRI3BP;CHCHD3;COX10;COX15;COX5B;COX8A;CYB5B;DHODH;DNAJC11;EFHD1;ETFDH;EXOG;GOT2;GRAMD4;HADHA;HADHB;HERC2;HSPD1;HTRA2;IMMT;LETM1;LOC494141;MAVS;MCCC1;MCCC2;MGST1;MLXIP;MTCH2;MTOR;MUL1;MYO19;NAT8L;

GEF5;ARHGEF6;ARHGEF7;ATP1A1;ATP1A3;ATP2A2;ATP2A3;ATP5A1;ATP5B;ATP5J;ATP5J2;ATP6V1H;ATP8B1;ATP8B2;AXIN1;AXIN2;BAD;BLM;CAD;CARNS1;CCR7;CHD1L;CTPS2;DNM1;DOCK7;EFTUD1;EFTUD2;EIF2S3;ENTPD5;EPHA1;EPHA2;FGD1;FOXJ1;GFM1;GNG10;GNL2;GNL3L;GTPBP4;HSPD1;INO80;LS

AP29;ARHGAP35;ARHGEF16;ARHGEF5;ARHGEF6;ARHGEF7;ATIC;ATP1A1;ATP1A3;ATP2A2;ATP2A3;ATP5A1;ATP5B;ATP5J;ATP5J2;ATP6V1H;ATP8B1;ATP8B2;AXIN1;AXIN2;BAD;BLM;CAD;CARNS1;CCR7;CECR1;CHD1L;CTPS2;DCTD;DERA;DHODH;DNAJC1;DNM1;DNMT3A;DOCK7;EFTUD1;EFTUD2;EIF2S3;ENT

25B;CDK5RAP2;CDKN2A;CDKN2B;CHEK2;DGKQ;DNAJA3;DPYSL2;ELAVL1;EPHA1;FAF1;FLNA;FOXM1;GYS1;HNRNPA0;HSP90AB1;IKBKB;IL12RB2;IL31RA;IRS1;JTB;KIF13B;LAX1;LCK;MAD2L2;MAP2K6;MAP2K7;MAP3K12;MAPK7;MAPK8IP3;MAPK9;MAPT;MAVS;NEK9;PAM;PARD6A;PARN;PDPK1;PIK3IP1;PKD1

DNAJA3;EARS2;FXN;GFM1;GRPEL1;HGF;HSPA4;HSPA9;HTRA2;HTT;LETM1;LIG3;LPIN1;MAPK9;MIPEP;MUL1;NRF1;PAM16;PET112;PEX5;PLD6;POLG;PPARGC1B;RNASEH1;SAMM50;SMAD3;SMCR7L;SPG7;SUPV3L1;TAMM41;TIMM17B;TOMM34;TP53;VAT

M3;CARD11;CARD14;CASP2;CCDC88C;CD3E;CIT;CLCN3;CLEC3B;CNTNAP1;CRADD;CSRP2BP;CTR9;DLG3;DRD4;DVL2;EPB41L2;EVL;FAF1;FOXJ1;FOXJ3;FOXM1;GATA3;GRIN2C;HHEX;HIF1AN;HNRNPM;HSP90AB1;INPPL1;IRS1;KCTD13;KHDRBS1;L3MBTL1;LAX1;LCK;LDB1;LEF1;MAPT;MAVS;MBD2;MDC1;MECP

GEF3;ARHGEF4;ARHGEF5;ARHGEF6;ARHGEF7;ARHGEF9;CCR7;CDH13;CDKN2A;CYTH1;CYTH3;DHCR24;DOCK7;EPHA1;EPHA2;FGD1;FGF2;FOXJ1;FOXM1;G3BP1;GBF1;GPR55;GRAP2;ICMT;ITPKB;KALRN;KANK1;KCTD13;KITLG;MCF2L;MTOR;MYO9B;NCKAP1L;NET1;NISCH;NRG1;NUP62;OBSCN;OCRL;PHACTR4

DIT3;DERL2;DTL;DYX1C1;EDEM1;FAF1;FBXL12;FBXO21;FOXRED2;FZR1;HECTD1;HERC2;HSP90AB1;HSP90B1;HTRA2;HUWE1;KCTD10;KCTD13;KIAA0368;LONP2;MAD2L2;NEDD8;PAN2;PCYOX1;PPP2R5C;PSMC4;PSMD1;PSMD11;PSMD2;PSMD6;PSMD7;PSME4;PTK2;RHBDD1;RNF20;RNF216;RNF4;RNF43;RN

RHGEF5;ARHGEF6;ARHGEF7;ATP5A1;ATP5B;ATP5J;ATP5J2;ATP6V1H;ATP8B1;ATP8B2;AXIN1;AXIN2;BLM;CARNS1;CCR7;CECR1;CHD1L;DNM1;DOCK7;EFTUD1;EFTUD2;EIF2S3;ENTPD4;EPHA1;EPHA2;FGD1;FOXJ1;GFM1;GNG10;GNL2;GNL3L;GTPBP4;HSPD1;INO80;LSG1;MACF1;MDN1;MLH1;MSH6;MTOR;M

H8;MTA2;NSUN2;PAXIP1;PLD6;PRMT2;PYGO2;RNF20;RNMT;SATB1;SETD1A;SETD6;SNW1;TGS1;UHRF1;USP7;WDR

1;BBS2;BCL2;BLM;BMP2;BPTF;BRD7;BTRC;C14orf169;C1D;CALR;CAPN3;CBFA2T2;CBX2;CBX5;CBX7;CCDC23;CDAN1;CDC16;CDKN2A;CDKN2B;CELF1;CHCHD3;CIITA;CIRBP;CLK2;CLN8;CRY2;CTCF;CTR9;CYP2D6;DDIT3;DDIT4;DDX20;DNAJA3;DNAJC1;DNMT1;DNMT3A;DRD4;DROSHA;DUSP16;DUSP7;EHMT1;E

.1;ATN1;ATP8B1;ATXN1L;AXIN1;BAHD1;BAZ1B;BCL10;BCL11B;BCL9L;BEX1;BLM;BMP2;BPTF;BRD2;BRD7;BRPF1;BTRC;C14orf169;C1D;CALR;CAMTA2;CAND2;CAPN3;CARD11;CBFA2T2;CBX2;CBX5;CBX7;CCAR1;CCNT1;CDH13;CDK5RAP2;CDKN2A;CDKN2B;CDON;CELSR2;CHCHD3;CHD2;CHD3;CHD4;CHD6;CH

2;CBX5;CBX7;CDKN2A;CHCHD3;CIITA;CRY2;CTCF;CTR9;DDIT3;DDX20;DNAJA3;DNMT1;DNMT3A;EHMT1;EZH1;FGF2;FGFR1;FOSB;FOXJ1;FOXK1;FOXM1;FOXP1;GATA3;HCFC1;HDAC1;HEXIM1;HHEX;HIC2;HIF1AN;HMG20A;HMGA1;HSBP1;HSPA8;ID1;IL4;ILF3;IRF8;JAZF1;KAT6B;KCTD1;KDM1A;KDM2B;KDM4

;C1D;CALR;CAPN3;CBFA2T2;CBX2;CBX5;CBX7;CDAN1;CDKN2A;CHCHD3;CIITA;CIRBP;CLK2;CRY2;CTCF;CTR9;DDIT3;DDX20;DNAJA3;DNMT1;DNMT3A;DRD4;EHMT1;EIF2AK3;EIF2AK4;EIF2B1;EIF4A3;EPRS;EZH1;FAM132A;FGF2;FGFR1;FOSB;FOXJ1;FOXK1;FOXM1;FOXP1;GABBR1;GATA3;GRM2;GTPBP4;HCFC

7;EPHA1;EPHA2;FOXJ1;MYO9B;NCKAP1L;NET1;OCRL;RAB3GAP1;RABGAP1;RACGAP1;RAP1GDS1;RASA3;RASA4;RASAL3;RGS13;RGS17;SCRIB;SNX9;SYDE1;SYNGAP1;TAX1BP3;TBC1D7;TSC1

SF5;THOC1;THOC2;U2AF2

HGEF6;ARHGEF7;ATP5A1;ATP5B;ATP5J;ATP5J2;ATP6V1H;ATP8B1;ATP8B2;AXIN1;AXIN2;BLM;CARNS1;CCR7;CECR1;CHD1L;DNM1;DOCK7;EFTUD1;EFTUD2;EIF2S3;EPHA1;EPHA2;FGD1;FOXJ1;GFM1;GNG10;GNL2;GNL3L;GTPBP4;HSPD1;INO80;LSG1;MACF1;MDN1;MLH1;MSH6;MTOR;MYO9B;NCKAP1L;NET

HGEF6;ARHGEF7;ATP5A1;ATP5B;ATP5J;ATP5J2;ATP6V1H;ATP8B1;ATP8B2;AXIN1;AXIN2;BLM;CARNS1;CCR7;CECR1;CHD1L;DNM1;DOCK7;EFTUD1;EFTUD2;EIF2S3;EPHA1;EPHA2;FGD1;FOXJ1;GFM1;GNG10;GNL2;GNL3L;GTPBP4;HSPD1;INO80;LSG1;MACF1;MDN1;MLH1;MSH6;MTOR;MYO9B;NCKAP1L;NET

CUL9;DDB1;DDIT3;DERL2;DTL;DYX1C1;EDEM1;FAF1;FBXL12;FBXO21;FOXRED2;FZR1;HECTD1;HERC2;HSP90AB1;HSP90B1;HTRA2;HUWE1;KCTD10;KCTD13;KIAA0368;LONP2;MAD2L2;NEDD8;PAN2;PCYOX1;PPP2R5C;PSMC4;PSMD1;PSMD11;PSMD2;PSMD6;PSMD7;PSME4;PTK2;RHBDD1;RNF20;RNF216;RN

RHGAP39;ARL3;ARPC2;ARPC3;ATM;AXIN1;AXIN2;BAIAP2L1;BBS2;BBS9;BICD1;BIRC6;BRK1;BRSK2;BSN;C2CD3;CALM3;CAMSAP1;CCDC64;CCNF;CCT3;CCT5;CCT6A;CCT7;CCT8;CDC16;CDC25B;CDC42BPA;CDC42BPB;CDC42BPG;CDK5RAP2;CEP104;CEP164;CEP72;CEP85;CEP89;CHD3;CHD4;CIAO1;CIT;CKAP5;C

CKAP1L;NET1;OCRL;RAB3GAP1;RASA3;RASA4;RASAL3;SCRIB;SYDE1;SYNGAP1;TAX1BP3;TBC1D7;TSC1

SRRM1;SRSF5;THOC1;THOC2;TSC1;U2AF2

BS2;BBS9;BICD1;BIRC6;BRSK2;C2CD3;CALM3;CAMSAP1;CCDC64;CCNF;CCT3;CCT5;CCT6A;CCT7;CCT8;CDC16;CDC25B;CDC42BPA;CDC42BPB;CDC42BPG;CDK5RAP2;CEP104;CEP164;CEP72;CEP85;CEP89;CHD3;CHD4;CIAO1;CKAP5;CNTRL;CROCC;DCTN1;DCTN5;DDHD2;DFNB31;DHX9;DIAPH1;DIS3L;DNAH1;

1;PAN2;PARN;RNPS1;SRRM1;SRSF5;SUPV3L1;TNKS1BP1;U2AF2;ZCCHC11

.1;CUL4A;CUL7;DDB1;DYNC1I2;DYNLT1;EIF4G1;ERCC3;FYN;GTF2A2;HCFC1;HDAC1;HMGA1;HSPA8;HSPD1;HTATIP2;HYAL2;IL16;IL2RB;IL2RG;IPO5;KARS;KCTD5;KDM4A;KLC1;KRT7;LCK;MAVS;MFGE8;MMP1;NFX1;NMT2;NXF1;PAAF1;PLCG1;POLA1;RBM15B;RELA;RNF216;RRAGA;SATB1;SCRIB;SEPT6;SF3B2;

8;PA2G4;RAD18;RELA;RNF20;RNF8;SMAD3;SNX9;TNFRSF14;TP53;TRAF1;TRAF3;UBE2B;UBE2L3;UBXN7;USP13;USP7;XRCC5

8;PA2G4;RAD18;RELA;RNF20;RNF8;SMAD3;SNX9;TNFRSF14;TP53;TRAF1;TRAF3;UBE2B;UBE2L3;UBXN7;USP13;USP7;XRCC5

DC16;CDC25B;CDC42BPG;CDK5RAP2;CEP104;CEP164;CEP72;CEP85;CEP89;CHD3;CHD4;CKAP5;CNTRL;CROCC;DCTN1;DCTN5;DDHD2;DHX9;DIS3L;DTL;DYNC1H1;DYNC1I2;DYNC1LI2;EXOC7;EZR;FAM110B;FBF1;HAUS5;HERC2;JTB;KDM4A;KIAA0368;KIAA0586;KIAA0753;KIF3B;LCK;LZTS2;MAP7D3;MCM3;MI

.1;ATN1;ATP8B1;ATXN1L;AXIN1;BAHD1;BAZ1B;BCL10;BCL11B;BCL9L;BEX1;BLM;BMP2;BPTF;BRD2;BRD7;BRPF1;BTRC;C14orf169;C1D;CALR;CAMTA2;CAND2;CAPN3;CARD11;CBFA2T2;CBX2;CBX5;CBX7;CCAR1;CCNT1;CDH13;CDK5RAP2;CDKN2A;CDKN2B;CDON;CELSR2;CHCHD3;CHD2;CHD3;CHD4;CHD6;CH

HHEX;HSP90AB1;HSPA9;HTATIP2;HTT;HYAL2;IL10;ING1;IPO5;IPO9;KANK1;KHDRBS1;KPNA6;LSG1;MAGOH;MAPK7;MAVS;MBTPS1;MCM3AP;MLXIP;MTOR;MYBBP1A;NODAL;NUP133;NUP155;NUP205;NUP214;NUP62;NXF1;PDIA3;PKD1;POLA2;RNPS1;SEC61B;SFRP5;SMAD3;SMG6;SPTBN1;SRRM1;SRSF5;S

PD;IBA57;IVD;KYNU;MCCC1;MCCC2;OGDH;PCBD1;PCYOX1;PCYOX1L;PDPR;SHMT1

7;CALR;CAMTA2;CBFA2T2;CBX2;CBX7;CCNT1;CDH13;CDKN2A;CDKN2B;CDON;CHCHD3;CHD2;CHD3;CHD4;CIAO1;CIITA;CPSF3;CREG1;CREM;CRTC1;CRY2;CSRNP2;CSTF1;CSTF2;CSTF3;CTR9;CYTL1;DDIT3;DDX17;DDX20;DHX38;DNAJA3;DNMT1;DNMT3A;DVL2;ECD;EHMT1;EIF2AK3;EIF4A3;ELP3;ERCC3;ETS1;

AP15;ARHGAP29;ARHGAP35;ARHGEF16;ARHGEF5;ARHGEF6;ARHGEF7;AVPR2;AXIN1;AXIN2;BAD;BCL2;BMP2;BTRC;CALM3;CALR;CARD14;CASP1;CCND1;CCND2;CCR7;CD4;CD74;CDC16;CDC25B;CDKN2A;CUL1;CUL7;CXCL1;DCTN1;DDIT3;DNAJA3;DNAJC1;DOCK7;DRD4;DVL2;EDEM1;EIF2AK3;EPHA1;EPHA2

E;HIST2H3D;HMGA1;HP1BP3;KAT6B;MCM2;NASP;POGZ;RBBP4;RBBP7;RNF4;RNF8;RPA1;RPA2;RUVBL1;SMARCA4;SMARCC1;SMARCC2;SMARCD1;SMARCD3;SMARCE1;SUPT16H;TAF1;THRA;TSPYL2

ANK1;SUFU;SUN2;SYNE1;SYNE2;TAF3;TAF8;THRA

MNL3;GGA3;IPO5;KCTD13;KIF16B;KIF3B;MAPKAP1;MYO5B;OCRL;PARD6A;PEX5;PRKCH;RAB11FIP3;RAB3GAP1;RANBP10;SRGAP1;TNFAIP1;ZFYVE20

RHGAP35;ARHGEF16;ARHGEF5;ARHGEF6;ARHGEF7;ATP1A1;ATP1A3;ATP2A2;ATP2A3;ATP5A1;ATP5B;ATP5J;ATP5J2;ATP6V1H;ATP8B1;ATP8B2;AXIN1;AXIN2;BAD;BLM;CAD;CARNS1;CCR7;CECR1;CHD1L;CTPS2;DHODH;DNM1;DNMT3A;DOCK7;EFTUD1;EFTUD2;EIF2S3;ENTPD4;ENTPD5;EPHA1;EPHA2;FGD1

2;RNASE3;RNASE4;RNASEH1;RPP14;RPP21;SLX4;SMG6;TATDN2;ZC3H12B;ZC3H12D

A2;HCFC1;HMGA1;HTATIP2;ICMT;IL31RA;KDM1A;LPIN1;MAGED1;MED24;MED27;MED30;MED6;MKL1;MMS19;MNT;MYCBP;PCBD1;PPARGC1B;PRMT2;RBM14;RNF20;RNF4;SIK1;SMARCA4;SMARCC1;SMARCC2;SMARCD1;SMARCD3;SMARCE1;SNW1;SOX10;SRCAP;TAF1;TAF10;TCF3;TRIP4;TRRAP;UBE2L3

OXM1;FOXP1;FOXP4;GTF2A2;GTF3C1;GTF3C2;GTF3C4;HMGA1;HOXA9;ING2;KDM1A;LDB1;LEF1;LMO2;LPIN1;MED27;MMS19;MTA2;NFATC2;NPAS2;PARP1;PRMT2;RBL2;RBM14;RELA;RUNX2;SMAD3;SNAPC4;TAF1;TAF10;TAF3;TAF6;TAF8;TBP;TCF3;TCF7;TCF7L2;TEAD2;TFDP2;TLE1;TP53;TRRAP;XRCC

PO5;KCTD13;KIF16B;KIF3B;MAPKAP1;MYO5B;OCRL;PARD6A;PRKCH;RAB11FIP3;RAB3GAP1;RANBP10;SRGAP1;TNFAIP1;ZFYVE20

D11;CCL8;CCND1;CDC42BPA;CDC42BPB;CDC42BPG;CERK;CHEK2;CIT;CKM;CLK2;CSNK1E;CSNK2A2;DCLK2;DDR1;DGKA;DGKD;DGKH;DGKQ;DLG3;DSTYK;EEF2K;EFNA4;EIF2AK3;EIF2AK4;EPHA1;EPHA2;ERBB3;ERCC3;FAM20B;FASTKD5;FGFR1;FGFRL1;FGGY;FLT4;FN3KRP;FYN;GALK2;GK5;GNE;GUCY2D;HKDC1

INTS1;ITPR3;LPIN1;NUP155;NUP205;NUP210;NUP62;NUP93;PCM1;POM121;POM121C;POU6F1;RETSAT;SCRN1;SIGMAR1;SMAD3;SUN2;SYNE1;SYNE2;TEX10;TMEM109;TMEM57;TXLNG;WDR3;WHSC

CIF1;PRC1;RAB11FIP3;TBCCD1;TUBGCP4;TUBGCP5;TUBGCP6;ZW10

TNFAIP1

CDC42BPG;CIT;CYTH3;DCTN1;DIAPH1;DPP4;DST;EPHA2;EVL;EZR;FGD1;INPPL1;KANK1;LRRC16A;MACF1;MAPK8IP3;MCF2L;MYO1D;NF2;PARD6A;PDE9A;PHACTR4;PIP5K1C;PLCG1;PPP1R9B;PTK2;PTPN13;RASGRP2;S100A6;SCRIB;SLC39A14;SNX9;SPTBN1;SYNE2;THEM4;TRPV1;TSC

CDC42BPB;CDC42BPG;CERK;CHEK2;CIT;CLK2;CSNK1E;CSNK2A2;DCLK2;DDR1;DGKA;DGKD;DGKH;DGKQ;DSTYK;EEF2K;EFNA4;EIF2AK3;EIF2AK4;EPHA1;EPHA2;ERBB3;ERCC3;FAM20B;FASTKD5;FGFR1;FGFRL1;FGGY;FLT4;FYN;GALK2;GK5;GNE;GUCY2D;HKDC1;IKBKB;IKBKE;IP6K2;IRAK1;ITPKB;KALRN;LCK;LMT

METTL2A;METTL2B;METTL7A;METTL7B;MLL;MLL4;MTR;NSUN2;NSUN4;PDPR;PRDM2;PRMT2;RNMT;SETD1A;SETD6;SHMT1;SMYD3;TGS1;THUMPD3;TPMT;TRMT2B;WDR4;WHSC

FUBP1;HNRNPA2B1;HSPD1;MCM4;MCM6;MECP2;MLH1;MSH6;NKAP;PMS2;PMS2CL;PMS2P5;RAD18;RPA1;RPA2;SATB1;SMAD3;SSBP2;TARDBP;TDP1;TEF;TERF2;THRA;UHRF1;WBP11;XPC;XRCC5;XRCC

:7L2;TP53;TP53BP1

RPP14;RPP21;SMG6

ETFDH;FASN;FOXRED2;GLUD1;H6PD;HACL1;HADH;HADHA;HADHB;IDH3A;IVD;KDM1A;MIOX;NDUFA9;NNT;OGDH;PARP1;PNPO;SDHA;TP53I3;TXNRD3;TYW1;UXS1
MNL3;GGA3;IPO5;KCTD13;KIF16B;KIF3B;MAPKAP1;MYO5B;OCRL;PARD6A;PEX5;PRKCH;RAB11FIP3;RAB3GAP1;RANBP10;SRGAP1;TNFAIP1;ZFYVE2(
RHGEF18;ARHGEF3;ARHGEF4;ARHGEF5;ARHGEF6;ARHGEF7;ARHGEF9;BAD;BDH1;CDC42BPA;CDC42BPB;CDC42BPG;CERK;CIT;CYTH1;CYTH3;DGKA;DGKD;DGKH;DNM1;DOCK10;DOCK9;FGD1;ING2;INPPL1;IPCEF1;IRS1;ITPR3;KALRN;KIF16B;LANCL2;LDLRAP1;MCF2L;MFGE8;MPRIP;MYO1E;NET1;NISCH;OBS
)1

3;COX15;COX5B;COX8A;DHODH;DNAJC11;EFHD1;ETFDH;EXOG;GOT2;HADHA;HADHB;HERC2;HSPD1;IMMT;LETM1;LOC494141;MCCC1;MCCC2;MGST1;MTCH2;NDUFA10;NDUFA2;NDUFA9;NDUFB4;NDUFB6;NDUFS1;NDUFV3;NNT;PAM16;POLG;SAMM50;SCO1;SCO2;SDHA;SDHB;SFXN1;SFXN2;SLC25A12;

3;PLD6;SAMM50;SMCR7L;SOX10;TOMM34;VAMP1;VAT1;VDAC1;VDAC2
OLR1B;POLR2B;POLR2C;POLR2J;POLR3A;POLR3B;POLR3C;POLR3E;RPAP1;TEP1;ZCCHC1
7;ZBTB16

3;COX15;COX5B;COX8A;DHODH;DNAJC11;EFHD1;EMD;ETFDH;EXOG;GOT2;HADHA;HADHB;HERC2;HSPD1;IMMT;LETM1;LOC494141;MCCC1;MCCC2;MGST1;MTCH2;NDUFA10;NDUFA2;NDUFA9;NDUFB4;NDUFB6;NDUFS1;NDUFV3;NNT;PAM16;POLG;SAMM50;SCO1;SCO2;SDHA;SDHB;SFXN1;SFXN2;SIGM
;DNAJC7;GRPEL1;HSP90AB1;HSP90B1;HSPA8;HSPA9;HSPD1;HTRA2;MDN1;SPG7;ST13;TCP1;TTC1

ATP8B1;ATP8B2;ATP9B;PCYOX1

;NCL;PABPC1;PCGF1;PEX26;PEX5;SAE1;SHANK1;SREBF2;STIP1;TAX1BP3;TERF2;TOP2B;UBE2I;USP7;XRCC5;XRCC6

FM1;PET112;TSFM
1;KLC2;MYH3;MYH7B;MYO15A;MYO18A;MYO19;MYO1C;MYO1D;MYO1E;MYO5B;MYO9B;SMC1A;STARD

METTL2B;METTL7A;METTL7B;MLL;MLL4;MTR;NSUN2;NSUN4;PDPR;PRDM2;PRMT2;RNMT;SETD1A;SETD6;SMYD3;TGS1;THUMPD3;TPMT;TRMT2B;WDR4;WHS(
P1R9B;RPA2;TP53;VCP

A1;ALDH3A2;ALG8;ALG9;AMFR;ANKLE2;AP2B1;AP3D1;AP3S1;APOOL;ARHGAP32;ARL3;ATF6B;ATG9B;ATP10A;ATP2A2;ATP2A3;ATP5A1;ATP5B;ATP5J;ATP5J2;ATP6V0E1;ATP6V1D;ATP6V1F;ATP6V1H;B3GALT4;BACE1;BAD;BBS1;BBS2;BBS9;BCL2;BDH1;BET1L;BID;BIK;BLOC1S1;BRI3BP;C1GALT1C1;CALR;CCN

HUWE1;IRAK1;KCTD10;KCTD13;LMO7;MUL1;PAICS;PJA1;PPCS;PPIL2;PRPF19;RFWD3;RNF125;RNF20;RNF4;RNF43;RNF8;SYVN1;TNFAIP1;TRAF3;TRIM17;TRIM56;TRIM68;TTC3;TTL;TTLL3;TTLL5;UBE2B;UBE2D2;UBE2F;UBE2G2;UBE2I;UBE2L3;UBE2Q1;UBE2Z;UBE3C;UBR4;UBR7;UHRF1;VH

PDIA6;PMEL;SLC1A4;SLC2A1;SND1;YWHAE
PDIA6;PMEL;SLC1A4;SLC2A1;SND1;YWHAE

HACTR4;PPARA;PPME1;PPP1R16B;PPP1R9B;RPA2;SFI1;SMAD3;TP53;VCP

K2;FAN1;FARSB;FEN1;FOXK1;FOXK2;HACL1;IDH3A;MAP2K7;MAP3K12;MSH6;MTPAP;PI4K2A;PRPS1;RNASEH1;RPS6KA2;SIK1;SIK2;STK36;STK38;TSSK3;TSSK4

CL1;HADHA;HADHB;HMGA1;NEIL2;PAICS;PAM;PCBD1;PDXDC1;PDXDC2P;POLB;SHMT1;UXS1;XRCC5;XRCC6

D;CENPB;CFD;CLASRP;CSNK1G2;CTU1;CWC22;CYLD;DBNDD2;DCAF15;DNAJC4;EGLN2;EID2;EXOSC6;EXOSC9;FAM109A;FBXL14;GAS2L1;GMPPB;GPAA1;GRIN1;H1FX;H2AFX;HES4;HIC1;HMG20B;HSPB1;IDUA;INO80B;IRF2BP1;JUNB;JUND;KISS1R;KLF2;LOXL1;LTB;MAST1;MAT2B;MBD4;METTL21A;MEX3D;M
MBD4;MYPOP;NPAS1;NR2F6;OTUD7A;PRDM8;REPIN1;SSBP4;TAF6L;TBX6;TIGD5;USF2;ZDHHC1;ZFPM1;ZNF219;ZNF35;ZNF358;ZNF414;ZNF444;ZNF564;ZNF575;ZNF579;ZNF628;ZNF638;ZNF668;ZNF764;ZNF771;ZNF784;ZNF787;ZNF837;ZNF865;ZSCAN1
A;PDF;PEX10;PGLYRP2;PRDM8;REPIN1;RNF126;USP33;ZDHHC1;ZDHHC24;ZFPM1;ZNF219;ZNF35;ZNF358;ZNF414;ZNF428;ZNF444;ZNF564;ZNF575;ZNF579;ZNF628;ZNF638;ZNF668;ZNF764;ZNF771;ZNF784;ZNF787;ZNF804A;ZNF837;ZNF865;ZSCAN10;ZSWIM
EGLN2;EID2;EXOSC6;EXOSC9;GAS2L1;GRIN1;HES4;HIC1;HMG20B;HSPB1;INO80B;IRF2BP1;JUNB;JUND;KLF2;LTB;MEX3D;MRPL10;MRPL4;MRPL41;MTIF2;MYPOP;NCK2;NPAS1;NR2F6;PABPN1;PCSK1N;PDF;PRDM8;PSMD12;RCE1;RPP25;SKOR1;SNAPC2;SNRPA;SUV420H2;TAF6L;TBX6;UCN;USF2;UTF1;WNT

SNK1G2;CTU1;CWC22;CYLD;DBNDD2;DCAF15;DNAJC4;EGLN2;EID2;EXOSC6;EXOSC9;FAM109A;FBXL14;GMPPB;GPAA1;GRIN1;H1FX;H2AFX;HES4;HIC1;HMG20B;HSPB1;IDUA;INO80B;IRF2BP1;JUNB;JUND;KISS1R;KLF2;LOXL1;MAST1;MAT2B;MBD4;METTL21A;MGAT4B;MRPL10;MRPL4;MRPL41;MTIF2;MY
1;RNF126;USP33;ZDHHC1;ZDHHC24;ZFPM1;ZNF219;ZNF35;ZNF358;ZNF414;ZNF428;ZNF444;ZNF564;ZNF575;ZNF579;ZNF628;ZNF638;ZNF668;ZNF764;ZNF771;ZNF784;ZNF787;ZNF804A;ZNF837;ZNF865;ZSCAN10;ZSWIM

RIN1;HES4;HIC1;HMG20B;INO80B;IRF2BP1;JUNB;JUND;KLF2;LTB4R2;MAT2B;MYPOP;NCK2;NPAS1;NR2F6;OAT;PABPN1;PRDM8;SKOR1;SNAPC2;SQLE;SUV420H2;TAF6L;TBX6;UCN;UGP2;USF2;UTF1;WNT6;ZFPM1;ZNF219;ZNF35;ZNF358;ZNF414;ZNF444;ZNF564;ZNF575;ZNF579;ZNF628;ZNF638;ZNF668;Z

EID2;EXOSC6;EXOSC9;GRIN1;HES4;HIC1;HMG20B;HSPB1;INO80B;IRF2BP1;JUNB;JUND;KLF2;MYPOP;NCK2;NPAS1;NR2F6;PABPN1;PRDM8;PSMD12;RPP25;SKOR1;SNAPC2;SNRPA;SUV420H2;TAF6L;TBX6;USF2;UTF1;WNT6;ZFPM1;ZNF219;ZNF35;ZNF358;ZNF414;ZNF444;ZNF564;ZNF575;ZNF579;ZNF628;Z

;HIC1;HMG20B;INO80B;IRF2BP1;JUNB;JUND;KLF2;LTB4R2;MAT2B;MYPOP;NCK2;NPAS1;NR2F6;OAT;PABPN1;PRDM8;SKOR1;SNAPC2;SUV420H2;TAF6L;TBX6;UCN;UGP2;USF2;UTF1;WNT6;ZFPM1;ZNF219;ZNF35;ZNF358;ZNF414;ZNF444;ZNF564;ZNF575;ZNF579;ZNF628;ZNF638;ZNF668;ZNF747;ZNF764;
;HIC1;HMG20B;INO80B;IRF2BP1;JUNB;JUND;KLF2;LTB4R2;MAT2B;MYPOP;NCK2;NPAS1;NR2F6;PABPN1;PRDM8;SKOR1;SNAPC2;SUV420H2;TAF6L;TBX6;UCN;UGP2;USF2;UTF1;WNT6;ZFPM1;ZNF219;ZNF35;ZNF358;ZNF414;ZNF444;ZNF564;ZNF575;ZNF579;ZNF628;ZNF638;ZNF668;ZNF747;ZNF764;ZNF7

IC1;HMG20B;HSPB1;INO80B;IRF2BP1;JUNB;JUND;KLF2;MAT2B;MGAT4B;MRPL10;MRPL4;MRPL41;MTIF2;MYPOP;NCK2;NPAS1;NR2F6;PABPN1;PDF;PRDM8;REPIN1;SKOR1;SNAPC2;SUV420H2;TAF6L;TBX6;UCN;UGP2;USF2;UTF1;WNT6;ZDHHC1;ZFPM1;ZNF219;ZNF35;ZNF358;ZNF414;ZNF444;ZNF564;ZNF

G20B;JUNB;JUND;KLF2;MBD4;MEX3D;MTIF2;MYPOP;NPAS1;NR2F6;OTUD7A;PABPN1;PRDM8;REPIN1;RPP25;SNRPA;SSBP4;TAF6L;TBX6;TIGD5;USF2;ZDHHC1;ZFPM1;ZNF219;ZNF35;ZNF358;ZNF414;ZNF444;ZNF564;ZNF575;ZNF579;ZNF628;ZNF638;ZNF668;ZNF747;ZNF764;ZNF771;ZNF784;ZNF787;ZNF8
rf85;C1QTNF4;C5;C9orf69;CACNA1A;CALY;CBX4;CCDC85B;CDK16;CEBPB;CEBPD;CENPB;CLEC11A;COPB1;CORO1B;CPLX1;CSNK1G2;CTU1;CWC22;CYLD;CYP51A1;CYTIP;DBNDD2;DLGAP3;DNAJC4;DOK2;EGLN2;EID2;EXOSC6;EXOSC9;FAM108A1;FAM109A;FAM89B;FBXL14;FBXL8;FGFBP3;GAS2L1;GFOD1;GJ
O80B;IRF2BP1;JUNB;JUND;KLF2;MYPOP;NCK2;NPAS1;NR2F6;PABPN1;PRDM8;SKOR1;SNAPC2;SUV420H2;TAF6L;TBX6;USF2;UTF1;WNT6;ZFPM1;ZNF219;ZNF35;ZNF358;ZNF414;ZNF444;ZNF564;ZNF575;ZNF579;ZNF628;ZNF638;ZNF668;ZNF747;ZNF764;ZNF771;ZNF784;ZNF787;ZNF837;ZNF865;ZSCAN1

;HIC1;HMG20B;INO80B;IRF2BP1;JUNB;JUND;KLF2;LTB4R2;MAT2B;MYPOP;NCK2;NPAS1;NR2F6;PABPN1;PRDM8;SKOR1;SNAPC2;SUV420H2;TAF6L;TBX6;UCN;UGP2;USF2;UTF1;WNT6;ZFPM1;ZNF219;ZNF35;ZNF358;ZNF414;ZNF444;ZNF564;ZNF575;ZNF579;ZNF628;ZNF638;ZNF668;ZNF747;ZNF764;ZNF7

IC1;HMG20B;HSPB1;INO80B;IRF2BP1;JUNB;JUND;KLF2;LTB;MAT2B;MGAT4B;MRPL10;MRPL4;MRPL41;MTIF2;MYPOP;NCK2;NPAS1;NR2F6;PABPN1;PDF;PRDM8;REPIN1;SKOR1;SNAPC2;SUV420H2;TAF6L;TBX6;UCN;UGP2;USF2;UTF1;WNT6;ZDHHC1;ZFPM1;ZNF219;ZNF35;ZNF358;ZNF414;ZNF444;ZNF564

;HIC1;HMG20B;INO80B;IRF2BP1;JUNB;JUND;KLF2;LTB4R2;LTC4S;MAT2B;MYPOP;NCK2;NPAS1;NR2F6;PABPN1;PRDM8;SKOR1;SNAPC2;SUV420H2;TAF6L;TBX6;UCN;UGP2;USF2;UTF1;WNT6;ZFPM1;ZNF219;ZNF35;ZNF358;ZNF414;ZNF444;ZNF564;ZNF575;ZNF579;ZNF628;ZNF638;ZNF668;ZNF747;ZNF76

PM1;ZNF219;ZNF35;ZNF444;ZNF628;ZSCAN10

PM1;ZNF219;ZNF35;ZNF444;ZNF628;ZSCAN10

B;CEBPD;CENPB;CFD;CHCHD10;CLASRP;CSNK1G2;CTU1;CWC22;CYLD;CYP51A1;DBNDD2;DCAF15;DNAJC4;EGLN2;EID2;EXOSC6;EXOSC9;FAM109A;FBXL14;GAS2L1;GMPPB;GPAA1;GRIN1;H1FX;H2AFX;HES4;HIC1;HMG20B;HSPB1;IDUA;INO80B;IRF2BP1;JUNB;JUND;KISS1R;KLF2;LOXL1;LTB;LTB4R2;LTC4S;

IC1;HMG20B;HSPB1;INO80B;IRF2BP1;JUNB;JUND;KLF2;MEX3D;MTIF2;MYPOP;NCK2;NR2F6;PRDM8;SKOR1;SUV420H2;TAF6L;TBX6;UCN;USF2;UTF1;WNT6;ZFPM1;ZNF219;ZNF35;ZNF358;ZNF414;ZNF564;ZNF575;ZNF579;ZNF638;ZNF668;ZNF747;ZNF764;ZNF771;ZNF784;ZNF787;ZNF837;ZNF865;ZSCAN1
EID2;EXOSC6;EXOSC9;GRIN1;H1FX;H2AFX;HES4;HIC1;HMG20B;HSPB1;INO80B;IRF2BP1;JUNB;JUND;KLF2;MBD4;MYPOP;NCK2;NPAS1;NR2F6;PABPN1;PRDM8;PSMD12;REPIN1;RPP25;SKOR1;SNAPC2;SNRPA;SUV420H2;TAF6L;TBX6;UCN;USF2;UTF1;WNT6;ZFPM1;ZNF219;ZNF35;ZNF358;ZNF414;ZNF444;Z

RF2BP1;JUNB;JUND;KLF2;MYPOP;NCK2;NR2F6;PRDM8;SKOR1;SUV420H2;TAF6L;TBX6;USF2;UTF1;WNT6;ZFPM1;ZNF219;ZNF35;ZNF358;ZNF414;ZNF564;ZNF575;ZNF579;ZNF638;ZNF668;ZNF747;ZNF764;ZNF771;ZNF784;ZNF787;ZNF837;ZNF865;ZSCAN1
O80B;IRF2BP1;JUNB;JUND;KLF2;MYPOP;NCK2;NPAS1;NR2F6;PABPN1;PRDM8;SKOR1;SNAPC2;SUV420H2;TAF6L;TBX6;USF2;UTF1;WNT6;ZFPM1;ZNF219;ZNF35;ZNF358;ZNF414;ZNF444;ZNF564;ZNF575;ZNF579;ZNF628;ZNF638;ZNF668;ZNF747;ZNF764;ZNF771;ZNF784;ZNF787;ZNF837;ZNF865;ZSCAN1

85B;CDK16;CEBPB;CEBPD;CENPB;CFD;CHCHD10;CLASRP;CSNK1G2;CTU1;CWC22;CYLD;CYP51A1;DBNDD2;DCAF15;DNAJC4;EGLN2;EID2;EXOSC6;EXOSC9;FAM109A;FBXL14;GAS2L1;GMPPB;GPAA1;GRIN1;H1FX;H2AFX;HES4;HIC1;HIGD2A;HMG20B;HSPB1;IDUA;INO80B;IRF2BP1;JUNB;JUND;KISS1R;KLF2;LO
IO80B;IRF2BP1;JUNB;JUND;KLF2;MYPOP;NCK2;NR2F6;PRDM8;SKOR1;SUV420H2;TAF6L;TBX6;USF2;UTF1;WNT6;ZFPM1;ZNF219;ZNF35;ZNF358;ZNF414;ZNF564;ZNF575;ZNF579;ZNF638;ZNF668;ZNF747;ZNF764;ZNF771;ZNF784;ZNF787;ZNF837;ZNF865;ZSCAN1

RF2BP1;JUNB;JUND;KLF2;MYPOP;NCK2;NR2F6;PRDM8;SKOR1;SUV420H2;TAF6L;TBX6;USF2;UTF1;WNT6;ZFPM1;ZNF219;ZNF35;ZNF358;ZNF414;ZNF564;ZNF575;ZNF579;ZNF638;ZNF668;ZNF747;ZNF764;ZNF771;ZNF784;ZNF787;ZNF837;ZNF865;ZSCAN1
AS2L1;GRIN1;H2AFX;HES4;HIC1;HMG20B;HSPB1;INO80B;IRF2BP1;JUNB;JUND;KISS1R;KLF2;LTB;MEX3D;MTIF2;MYPOP;NCK2;NR2F6;PRDM8;PSMD12;SKOR1;SOCS1;STK11;SUV420H2;TAF6L;TBX6;UCN;USF2;UTF1;VAPB;WNT6;YIF1A;ZFPM1;ZNF219;ZNF35;ZNF358;ZNF414;ZNF564;ZNF575;ZNF579;ZNF63
O80B;IRF2BP1;JUNB;JUND;KLF2;LTB;LTB4R2;MTIF2;MYPOP;NCK2;NR2F6;PRDM8;SKOR1;STK11;SUV420H2;TAF6L;TBX6;UCN;USF2;UTF1;WNT6;ZFPM1;ZNF219;ZNF35;ZNF358;ZNF414;ZNF564;ZNF575;ZNF579;ZNF638;ZNF668;ZNF747;ZNF764;ZNF771;ZNF784;ZNF787;ZNF837;ZNF865;ZSCAN1

O80B;IRF2BP1;JUNB;JUND;KLF2;MTIF2;MYPOP;NCK2;NR2F6;PRDM8;SKOR1;SUV420H2;TAF6L;TBX6;UCN;USF2;UTF1;WNT6;ZFPM1;ZNF219;ZNF35;ZNF358;ZNF414;ZNF564;ZNF575;ZNF579;ZNF638;ZNF668;ZNF747;ZNF764;ZNF771;ZNF784;ZNF787;ZNF837;ZNF865;ZSCAN1
GMPPB;GPAA1;GRIN1;HES4;HIC1;HMG20B;HSPB1;INO80B;IRF2BP1;JUNB;JUND;KLF2;LTB;LTB4R2;LTC4S;MAT2B;MGAT4B;MRPL10;MRPL4;MRPL41;MTIF2;MYPOP;NCK2;NPAS1;NR2F6;OAT;PABPN1;PDF;PHOSPHO1;PRDM8;REPIN1;SKOR1;SNAPC2;SQLE;STK11;SUV420H2;TAF6L;TBX6;UCN;UGP2;USF2;UT

O80B;IRF2BP1;JUNB;JUND;KLF2;LTB;MTIF2;MYPOP;NCK2;NR2F6;PRDM8;SKOR1;SUV420H2;TAF6L;TBX6;UCN;USF2;UTF1;WNT6;ZFPM1;ZNF219;ZNF35;ZNF358;ZNF414;ZNF564;ZNF575;ZNF579;ZNF638;ZNF668;ZNF747;ZNF764;ZNF771;ZNF784;ZNF787;ZNF837;ZNF865;ZSCAN1

O80B;IRF2BP1;JUNB;JUND;KLF2;LTB;LTB4R2;MTIF2;MYPOP;NCK2;NR2F6;PRDM8;SKOR1;STK11;SUV420H2;TAF6L;TBX6;UCN;USF2;UTF1;WNT6;ZFPM1;ZNF219;ZNF35;ZNF358;ZNF414;ZNF564;ZNF575;ZNF579;ZNF638;ZNF668;ZNF747;ZNF764;ZNF771;ZNF784;ZNF787;ZNF837;ZNF865;ZSCAN1

EF25;ARHGEF26;ARIH1;ARL16;ARL6IP4;ARNTL;ASB6;ASH1L;ASPSCR1;ASXL2;ATAD3B;ATF4;ATF7IP;ATG4D;ATL3;ATP5D;ATP5G2;ATP5G3;ATP5H;ATP6V0E2;ATP6V1C1;ATRIP;AUP1;AVEN;B3GALT6;B3GAT3;B4GALNT4;B4GALT2;B4GALT7;BABAM1;BAIAP2L2;BCS1L;BHLHE41;BMP2K;BOK;BOLA1;BRAF;BRAT1
3GALT6;B3GAT3;B4GALNT4;B4GALT2;B4GALT7;BAIAP2L2;BCS1L;BOK;BOLA1;BRAF;BTBD6;BUB1;BUB1B;C12orf10;C12orf44;C19orf10;C19orf70;C1QBP;CANX;CAPN10;CAPRIN1;CCDC47;CCDC51;CCDC78;CCS;CD320;CD36;CD8B;CDC25A;CDC6;CDCA8;CDS2;CECR5;CEL;CENPF;CENPM;CENPT;CEP55;CHCHD
ARL6IP4;ARNTL;ASB6;ASH1L;ASPSCR1;ASXL2;ATAD3B;ATF4;ATF7IP;ATG4D;ATL3;ATP5D;ATP5G2;ATP5G3;ATP5H;ATP6V0E2;ATP6V1C1;ATRIP;AUP1;B3GALT6;B3GAT3;B4GALNT4;B4GALT2;B4GALT7;BABAM1;BAIAP2L2;BCS1L;BHLHE41;BMP2K;BOK;BOLA1;BRAF;BRAT1;BRF1;BRF2;BTBD6;BUB1;BUB1B;C11
D3B;ATF4;ATG4D;ATL3;ATP5D;ATP5G2;ATP5G3;ATP5H;ATP6V0E2;ATP6V1C1;AUP1;B3GALT6;B3GAT3;B4GALNT4;B4GALT2;B4GALT7;BABAM1;BAIAP2L2;BCS1L;BOK;BOLA1;BRAF;BTBD6;BUB1;BUB1B;C12orf10;C12orf44;C17orf70;C19orf10;C19orf24;C19orf70;C1orf198;C1QBP;C21orf2;CANX;CAPN10;CA
;BUB1;BUB1B;C12orf5;CAPN10;CBR1;CCS;CD38;CDC25A;CDC6;CDK10;CDK12;CDK13;CDS2;CDYL2;CECR5;CEL;CHD7;CHRAC1;CHTF18;CLPP;CNTLN;COMTD1;COQ7;COX4I1;CPSF2;CPSF3L;CPT1C;CRYL1;CSNK1G1;CTSC;CTSF;CYP1B1;CYP2E1;DALRD3;DCXR;DDT;DDX21;DDX28;DDX39A;DDX43;DDX49;DECR2;D
X1L;FDXR;FIBP;FPGS;GADD45GIP1;GCDH;GFER;GIMAP5;GLYCTK;GOLPH3;GPX4;GTPBP3;HCCS;HDHD3;HINT2;HSD17B8;ISOC2;MACROD1;MAD1L1;MAPK1;MECR;METAP1D;METTL12;MMAB;MPDU1;MPV17L2;MRPL12;MRPL20;MRPL23;MRPL24;MRPL38;MRPL52;MRPL54;MRPL55;MRPS11;MRPS12;MRP
ATP5H;ATP6V0E2;ATRIP;AUP1;B3GALT6;B3GAT3;B4GALNT4;B4GALT2;B4GALT7;BABAM1;BAIAP2L2;BCS1L;BHLHE41;BMP2K;BOK;BOLA1;BRAF;BRAT1;BRF1;BRF2;BUB1;BUB1B;C11orf30;C12orf10;C17orf49;C17orf70;C19orf10;C19orf70;C1orf86;C1QBP;CANX;CAPS;CCDC101;CCDC106;CCDC47;CCDC51;CC
ATF7IP;ATL3;ATP5D;ATP5G2;ATP5G3;ATP5H;ATP6V0E2;ATP6V1C1;ATRIP;AUP1;B3GALT6;B3GAT3;B4GALNT4;B4GALT2;B4GALT7;BABAM1;BAIAP2L2;BCS1L;BHLHE41;BMP2K;BOK;BOLA1;BRAF;BRAT1;BRF1;BRF2;BTBD6;BUB1;BUB1B;C11orf30;C12orf10;C17orf49;C17orf70;C19orf10;C19orf70;C1orf86;C1
P6V0E2;ATRIP;AUP1;B3GALT6;B3GAT3;B4GALNT4;B4GALT2;B4GALT7;BABAM1;BAIAP2L2;BCS1L;BHLHE41;BMP2K;BOK;BOLA1;BRAF;BRAT1;BRF1;BRF2;BUB1;BUB1B;C11orf30;C12orf10;C17orf49;C17orf70;C19orf10;C19orf70;C1orf86;C1QBP;CANX;CAPS;CCDC101;CCDC106;CCDC47;CCDC51;CCDC78;CCH
L3;ATP5D;ATP5G2;ATP5G3;ATP5H;ATP6V0E2;ATP6V1C1;ATRIP;AUP1;B3GALT6;B3GAT3;B4GALNT4;B4GALT2;B4GALT7;BABAM1;BAIAP2L2;BCS1L;BHLHE41;BMP2K;BOK;BOLA1;BRAF;BRAT1;BRF1;BRF2;BTBD6;BUB1;BUB1B;C11orf30;C12orf10;C17orf49;C17orf70;C19orf10;C19orf70;C1orf86;C1QBP;CANX
UFB10;NDUFB11;NDUFB7;NDUFB8;NDUFB9;NDUFS6;NDUFS7;NDUFS8;NDUFV1;NME4;NUDT1;PARS2;PDK4;PEMT;POLRMT;PPOX;QARS;RHOT2;SARDH;SARS2;SDHAF1;SIRT3;SLC25A19;SLC25A25;SLC25A29;SLC25A35;SLC25A5;SLC25A6;SLC44A1;SMCR7;SPG20;STOML2;SURF1;TAZ;TIMM10;TIMM13;TIMI
B1B;C17orf49;C17orf70;C19orf10;C1orf86;C1QBP;CANX;CCDC101;CCDC28B;CCDC61;CCNL2;CCS;CD36;CDC25A;CDC6;CDCA8;CDK12;CDK13;CDS2;CENPF;CENPM;CENPT;CEP55;CHCHD5;CHMP4A;CHMP6;CHRAC1;CIB1;CLASP1;CLCN4;CLIP1;CLPP;CLSPN;CNTLN;COL11A2;COL6A1;COL6A2;COL9A2;COQ7;CO
1orf86;C1QBP;CANX;CCDC101;CCDC28B;CCDC61;CCNL2;CCS;CD36;CDC25A;CDC6;CDCA8;CDK12;CDK13;CDS2;CENPF;CENPM;CENPT;CEP55;CHCHD5;CHMP4A;CHMP6;CHRAC1;CIB1;CLASP1;CLCN4;CLIP1;CLPP;CLSPN;CNTLN;COL11A2;COL6A1;COL6A2;COL9A2;COQ7;COX4I1;CPSF2;CPSF4;CPT1C;CREB3;CR
;DUSP2;DUSP3;E2F1;E4F1;EAF1;ECI1;EDF1;ELP4;EPC1;ERCC1;ERP29;ERP44;ETFB;ETV3;EXOSC5;FANCG;FBL;FDX1L;FDXR;FPGS;GCDH;GEMIN7;GFER;GLTSCR2;GOLPH3;GTF2A1;GTF3C5;H2AFY2;HDAC10;HDAC11;HES6;HINFP;HIPK1;HIPK3;HSD17B8;IMP4;INO80E;IRF3;KAT2A;KDM5A;KDM6A;KIF20A;KRT18;
C25A6;SLC44A1;SMCR7;SPG20;STOML2;SURF1;TAZ;TIMM10;TIMM13;TIMM50;TOMM40L;USP30;YME1

DUSP3;E2F1;E4F1;EAF1;ECI1;EDF1;ELP4;EPC1;ERCC1;ERP29;ERP44;ETFB;ETV3;EXOSC5;FANCG;FBL;FDX1L;FDXR;FPGS;GCDH;GEMIN7;GLTSCR2;GTF2A1;GTF3C5;H2AFY2;HDAC10;HDAC11;HES6;HINFP;HIPK1;HIPK3;HSD17B8;IMP4;INO80E;IRF3;KAT2A;KDM5A;KDM6A;KIF20A;KRT18;LEPRE1;LEPREL2;LSM2;N
3XO2;FBXO24;FBXW11;FBXW5;FEM1B;FICD;FKBP11;FKBP2;FNIP2;GLYCTK;GNB2L1;GTPBP3;HDAC10;HDAC11;HERC3;HIPK3;HPX;HRAS;JOSD2;KAT2A;KDM3A;KDM6A;KIDINS220;KLHL12;KLHL17;LAMTOR2;LATS1;LATS2;LCMT1;LIME1;LMF1;LPAR1;LTK;MAGI3;MAN1B1;MAP3K10;MAP4K2;MAPK1;MAPK11;

IF2B2;EIF2B4;EIF3F;EIF3G;EIF3I;EIF3K;EIF4G3;ELP4;EPC1;ERLIN2;ERP29;ERP44;ETF1;FASTK;FBXL15;FBXL6;FBXO2;FBXO24;FBXW11;FBXW5;FEM1B;FICD;FKBP11;FKBP2;FLAD1;FNIP2;GFER;GLYCTK;GNB2L1;HDAC10;HDAC11;HERC3;HIPK3;HPX;HRAS;JAG2;JOSD2;KAT2A;KDM3A;KDM6A;KIDINS220;KLHL12;K

DUSP3;E2F1;E4F1;EAF1;ECI1;EDF1;ELP4;EPC1;ERCC1;ERP29;ERP44;ETFB;ETV3;EXOSC5;FANCG;FBL;FDX1L;FDXR;FPGS;GCDH;GEMIN7;GLTSCR2;GTF2A1;GTF3C5;H2AFY2;HDAC10;HDAC11;HES6;HINFP;HIPK1;HIPK3;HSD17B8;IMP4;INO80E;IRF3;KAT2A;KDM5A;KDM6A;KIF20A;KRT18;LEPRE1;LEPREL2;LSM2;N
A1;COL6A2;CPSF2;CPSF4;CTU2;DEAF1;DLGAP5;DNAL4;DPM3;DVL1;DYNC1LI1;E2F1;E4F1;EDF1;EEF1A2;EEF1D;EEF1G;EIF2B2;EIF2B4;EIF3F;EIF3G;EIF3I;EIF3K;EIF4G3;ELP4;EPC1;ERCC1;ERLIN2;ETV3;EXOC6B;EXOSC5;FAF2;FAM21B;FAM98B;FANCG;FBL;FBXL15;FBXO2;FBXO24;FBXO44;FBXW11;FBXW5;FSD1
JRF1;TAZ;TIMM10;TIMM13;TIMM50;TOMM40L;USP30;YME1I

NF169;RTEL1;SETX;SPRED2;STRA13;STUB1;SWI5;TAOK3;TLK2;TMEM161A;TOP2A;TOPBP1;TP73;TREX2;TRIP12;VA

APK11;MATK;MCRS1;METTL12;METTL22;MFNG;MGMT;MLL3;MLL5;MMAB;MVK;MYLK3;NAA10;NAGS;NAT14;NAT6;NAT9;NCOA3;NME3;NME4;NPRL2;NRP1;NSUN5;NSUN5P2;OXSR1;PANK4;PAPSS1;PARP10;PARP3;PCYT2;PDK4;PEMT;PFKFB2;PHF20;PHKG1;PIGQ;PIK3C3;PIK3CB;PIK3CG;PIP5K1A;PIP5K1B

PC1;ERGIC3;ERLIN2;ERP44;FADS3;FAF2;FAM21B;FAM69B;FBXO2;FDXR;FKBP2;FPGS;GCDH;GCHFR;GGA1;GIMAP5;GOLPH3;HCCS;HIP1R;HOOK3;HRAS;HSD17B12;IRAK4;IRF3;JAGN1;JSRP1;KRTCAP2;LAMTOR2;LEMD2;LMF1;LMF2;MAN1B1;MAP4K2;MARCH9;MFNG;MIA3;MOGS;MPDU1;MRPS15;MTFP1;ND

P1;ARHGAP4;ARHGEF25;ARHGEF26;ARIH1;ARL16;ARL6IP4;ARNTL;ASB6;ASH1L;ASPSCR1;ASXL2;ATAD3B;ATF4;ATF7IP;ATG4D;ATL3;ATP5D;ATP5G2;ATP5G3;ATP5H;ATP6AP2;ATP6V0E2;ATP6V1C1;ATRIP;AUP1;AVEN;B3GALT6;B3GAT3;B4GALNT4;B4GALT2;B4GALT7;BABAM1;BAI1;BAIAP2L2;BCS1L;BEST4;B
P1;ARHGAP4;ARHGEF25;ARHGEF26;ARIH1;ARL16;ARL6IP4;ARNTL;ASB6;ASH1L;ASPSCR1;ASXL2;ATAD3B;ATF4;ATF7IP;ATG4D;ATL3;ATP5D;ATP5G2;ATP5G3;ATP5H;ATP6AP2;ATP6V0E2;ATP6V1C1;ATRIP;AUP1;AVEN;B3GALT6;B3GAT3;B4GALNT4;B4GALT2;B4GALT7;BABAM1;BAI1;BAIAP2L2;BCS1L;BEST4;B

AJC30;DOK7;DOLK;DPM3;DPP7;DPP8;DSE;DTX3;DUSP10;DUSP2;DUSP3;DVL1;E4F1;EEF1D;EEF1G;EHHADH;EIF1;EIF2B2;EIF2B4;EIF3F;EIF3G;EIF3I;EIF3K;EIF4G3;ELP4;EPC1;ERLIN2;ERP29;ERP44;ETF1;FASTK;FBXL15;FBXL6;FBXO2;FBXO24;FBXO44;FBXW11;FBXW5;FEM1B;FICD;FKBP11;FKBP2;FLAD1;FNIP2;G
.;FBXW5;FEM1B;FICD;FKBP11;FKBP2;FNIP2;GLYCTK;GNB2L1;HDAC10;HDAC11;HERC3;HIPK3;HPX;HRAS;JOSD2;KAT2A;KDM3A;KDM6A;KIDINS220;KLHL12;KLHL17;LAMTOR2;LATS1;LATS2;LCMT1;LIME1;LMF1;LPAR1;LTK;MAGI3;MAN1B1;MAP3K10;MAP4K2;MAPK1;MAPK11;MARCH9;MATK;MBD3;MCRS1
.;FBXW5;FEM1B;FICD;FKBP11;FKBP2;FNIP2;GLYCTK;GNB2L1;HDAC10;HDAC11;HERC3;HIPK3;HPX;HRAS;JOSD2;KAT2A;KDM3A;KDM6A;KIDINS220;KLHL12;KLHL17;LAMTOR2;LATS1;LATS2;LCMT1;LIME1;LMF1;LPAR1;LTK;MAGI3;MAN1B1;MAP3K10;MAP4K2;MAPK1;MAPK11;MARCH9;MATK;MBD3;MCRS1

PMVK;PNKP;POLD1;POLD4;POLM;POLR2F;POLR2G;POLR2I;POLR2L;POLR3K;POLRMT;PTDSS2;RABEP2;ROCK2;RPS6KB2;RPS6KC1;RPS6KL1;SPEG;SPHK2;STK25;TAOK3;TBRG4;TLK2;TNK1;TRIM24;TRPT1;TSSK6;TTN;TUT1;UCK1;UCKL1;ULK3;ZAP
);HDAC11;HES6;HINFP;HIPK1;HIPK3;IMP4;INO80E;IRF3;KAT2A;KDM5A;KDM6A;KIF20A;KRT18;LSM2;MAML2;MAPK1;MAPK11;MBD3;MCM7;MCRS1;MED1;MED13L;MED18;MGMT;MKI67;MLL3;MLL5;MOB2;MPG;MSH3;MUS81;MUTYH;NCOA3;NDUFA13;NHP2;NLE1;NMRAL1;NOL12;NR2C2AP;NSRP1;NTI
7;BABAM1;BHLHE41;BRAF;BRF1;BRF2;BUB1B;C11orf30;C12orf44;C17orf70;C19orf10;C1orf86;C1QBP;CANX;CAPRIN1;CBR1;CCDC101;CCNL2;CCS;CD36;CD38;CDC25A;CDC6;CDK12;CDK13;CDS2;CEL;CELF2;CELF6;CENPF;CHD7;CHTF18;CIB1;CLCF1;CLSPN;COQ7;COQ9;COX4I1;CPSF2;CPSF4;CPT1C;CREB3;CR
PI1;B3GALT6;B3GAT3;B4GALT2;B4GALT7;BABAM1;BHLHE41;BOK;BRAF;BRF1;BRF2;BTN2A1;BUB1B;C11orf30;C12orf44;C17orf70;C19orf10;C1orf86;C1QBP;C8G;CANX;CAPN10;CAPRIN1;CBR1;CCDC101;CCNL2;CCS;CD36;CD38;CDC25A;CDC6;CDK12;CDK13;CDS2;CEL;CELF2;CELF6;CENPF;CHD7;CHTF18;CIB1
3G;EIF3I;EIF3K;EIF4G3;ENGASE;ETF1;ETNK2;EXOSC5;FAM13A;FBXO2;FBXW11;FLAD1;FPGS;GAMT;GAPVD1;GCHFR;GEMIN7;GET4;GOLPH3;GSTT1;GYG1;HRAS;HSD17B1;IRAK4;IRF3;KHK;KIDINS220;KIF15;LARP4B;LATS1;LATS2;LGALS4;LIMS2;LSM2;LSM4;MAD1L1;MAPK1;MAPK11;MATK;MCM7;MLST8;MR
.;MED1;MED13L;MED18;MGMT;MLL3;MLL5;MPG;MUTYH;NCOA3;NDUFA13;NHP2;NR2C2AP;NSRP1;NTHL1;NUP153;OGG1;PATL1;PHF16;PHF20;PIN1;PIP5K1A;POLD1;POLD4;POLR2F;POLR2G;POLR2I;POLR2L;POLR3K;POU2F1;PPP2R3B;PSMA7;PSMB4;PSMB5;PSMB7;PSMD9;PUS1;RAD21;RAD9A;RBBP5;R

37;NDUFB8;NDUFB9;NDUFS6;NDUFS7;NDUFS8;NDUFV1;NF1;NOG;NPRL2;NRP1;OXSR1;P2RX7;PDE6G;PDK4;PFKFB2;PICK1;PIK3CB;PIK3CG;PIN1;PIP5K1A;PIP5K1B;PLA2G6;PNKP;POLR2F;POLR2G;POLR2I;POLR2L;PPP1R14A;PPP1R14B;PREB;PRKAR2A;PTPRC;RBPMS;ROCK2;RSRC1;SDHAF2;SENP2;SPRED2;S

NUP153;OGG1;P2RX7;PANX1;PEX16;PEX6;PICK1;PIK3C3;PIP5K1A;PREB;RAB21;RAB25;RAB33A;RABEP2;RABGEF1;RBPMS;RINL;RPL13;RPL18;RPL18A;RPL28;RPL29;RPL38;RPL8;RPLP1;RPLP2;RPS15;RPS2;RPS28;S100A13;SCARB2;SEC22B;SEC24B;SENP2;SERGEF;SLC25A6;SNF8;SNX20;SPTBN4;SSR1;SSR4;STA

LRRC32;MAL;MAMDC4;MAPK1;MED1;MIA3;MTM1;NDUFA13;NECAB3;NF1;NFKBIL1;NIPBL;NSF;NUP153;OGG1;P2RX7;PANX1;PEX16;PEX6;PICK1;PIK3C3;PIP5K1A;PLEKHF1;PREB;RAB21;RAB25;RAB33A;RABEP2;RABGEF1;RAD21;RALGAPB;RBPMS;RINL;RPL13;RPL18;RPL18A;RPL28;RPL29;RPL38;RPL8;RPLP
R2I;POLR2L;RABEP2;ROCK2;RPS6KB2;RPS6KC1;RPS6KL1;SPEG;SPHK2;STK25;TAOK3;TBRG4;TLK2;TNK1;TRIM24;TSSK6;TTN;UCK1;UCKL1;ULK3;ZAP7
PEMT;PHF20;PPOX;RHOT2;SENP2;SLC25A19;SLC25A25;SLC25A29;SLC25A35;SLC25A5;SLC25A6;SLC29A2;SLC44A1;SMCR7;SPG20;STOML2;SURF1;TAZ;TIMM10;TIMM13;TIMM50;TOMM40L;TOR1AIP1;TPR;TRAF6;USP30;YME1

K7;PDK4;PEMT;PHF20;PPOX;RHOT2;SENP2;SLC25A19;SLC25A25;SLC25A29;SLC25A35;SLC25A5;SLC25A6;SLC29A2;SLC44A1;SMCR7;SPG20;STOML2;SURF1;TAZ;TIMM10;TIMM13;TIMM50;TOMM40L;TOR1AIP1;TPR;TRAF6;USP30;YME1

EX16;PEX6;PICK1;PIK3C3;PIP5K1A;PREB;RAB21;RAB25;RAB33A;RABEP2;RABGEF1;RBPMS;RINL;RPL13;RPL18;RPL18A;RPL28;RPL29;RPL38;RPL8;RPLP1;RPLP2;RPS15;RPS2;RPS28;S100A13;SCARB2;SEC22B;SEC24B;SENP2;SERGEF;SLC25A6;SNF8;SNX20;SSR1;SSR4;STAM;STX1A;SYNGR1;SYTL1;TIMM10;TIMN

R2F;POLR2G;POLR2I;POLR2L;PRMT7;RAD21;RAD9A;REC8;RECQL4;RNF169;RSF1;RTEL1;SETX;SLC50A1;STOML2;STRA13;STUB1;SWI5;TAOK3;TMEM161A;TOP1;TOP2A;TOP3B;TOPBP1;TP73;TPR;TREX2;TRIP12;TSSK6;TTN;WRAF

A;SURF1;TAZ;TBRG4;TXN2;TXNRD1;TYSND
L18;RPL18A;RPL28;RPL29;RPL38;RPL8;RPLP1;RPLP2;RPS15;RPS2;RPUSD1;RPUSD3;RRP9;SNRPC;THOC6;TIMM50;TLR7;TPR;TRMT1;TRMT2A;TRMU;TRUB2;TUFM;TUT1;UPF2;WIBG;WRAP53;ZC3HA
1;RPS6KL1;SPEG;SPHK2;STK25;TAOK3;TBRG4;TLK2;TNK1;TRIM24;TRPT1;TSSK6;TTN;UCK1;UCKL1;ULK3;ZAP7

GG1;PAFAH1B2;PARK7;PARP3;PDK4;PHLDA3;PIDD;PIK3C3;PLA2G6;PNKP;POLD1;POLD4;POLM;POLR2F;POLR2G;POLR2I;POLR2L;PREB;PSMA7;PSMB4;PSMB5;PSMB7;PSMD9;RAD21;RAD9A;RBBP5;RECQL4;RNF169;RTEL1;SERPINF2;SETX;SH2D3A;SH3RF1;SPRED2;SSR1;STRA13;STUB1;SWI5;TAOK3;TCEB2;T

RPC;TBL3;TOP1;TXNL4A;WDR83;WRAP

PI1;B3GALT6;B3GAT3;B4GALT2;B4GALT7;BABAM1;BHLHE41;BRAF;BRF1;BRF2;BTN2A1;BUB1B;C11orf30;C17orf70;C19orf10;C1orf86;C1QBP;C8G;CANX;CAPN10;CAPRIN1;CBR1;CCDC101;CCNL2;CD36;CD38;CDC25A;CDC6;CDK12;CDK13;CDS2;CEL;CELF2;CELF6;CENPF;CHD7;CHTF18;CIB1;CLCF1;CLPP;CLSPN

;DNAL4;DPM3;DVL1;DYNC1LI1;E2F1;E4F1;EDF1;EEF1A2;EEF1D;EEF1G;EIF2B2;EIF2B4;EIF3F;EIF3G;EIF3I;EIF3K;EIF4G3;ELP4;EPC1;ERCC1;ERLIN2;ETV3;EXOC6B;EXOSC5;FAF2;FAM21B;FAM98B;FANCG;FBXL15;FBXO2;FBXO24;FBXO44;FBXW11;FBXW5;FSD1;FXYD2;GAS2L3;GCHFR;GEMIN7;GET4;GGA1;GNAI
GTF2A1;GTF3C5;H2AFY2;HDAC10;HDAC11;HES6;HINFP;HIPK1;HIPK3;IMP4;INO80E;IPO8;IRF3;KAT2A;KDM5A;KDM6A;KIF20A;KRT18;LAT;LEMD2;LSM2;LSM4;MAD1L1;MAML2;MAPK1;MAPK11;MBD3;MCM7;MCRS1;MED1;MED13L;MED18;MGMT;MKI67;MLL3;MLL5;MOB2;MPG;MRPS15;MSH3;MUS81;M

2L3;GET4;GFER;GIMAP5;GNAI3;GOLPH3;H2AFJ;H2AFY2;HAUS8;HDAC10;HDAC11;HOOK3;HPS3;HRAS;ITPA;KAT2A;KDM3A;KDM5A;KDM6A;KIF15;KIF20A;KIF24;KLHL12;KLHL17;LATS1;LATS2;MAD1L1;MAPK1;MBD3;MCRS1;MLL3;MLL5;MLST8;MRPL12;MSH3;MTFP1;MTM1;MYH10;MYLK3;NAGLU;NCAPH;

K;GNAI3;GNB2L1;GNG8;GNGT2;GPX4;HIPK3;HPX;HRAS;HSD17B12;ISYNA1;ITPA;KIDINS220;LAMTOR2;LATS1;LATS2;LIME1;LPAR1;LTK;MAGI3;MAP3K10;MAP4K2;MAPK1;MAPK11;MATK;MC1R;MCM7;MLST8;MPG;MTG1;MTM1;MTMR1;MUTYH;MVK;MYLK3;NDUFA3;NDUFA8;NDUFB10;NDUFB7;NDUFB8
3GAT3;B4GALT2;B4GALT7;BABAM1;BHLHE41;BRAF;BRF1;BRF2;BTN2A1;BUB1B;C11orf30;C17orf70;C19orf10;C1orf86;C1QBP;C8G;CANX;CAPN10;CAPRIN1;CCDC101;CCNL2;CD36;CD38;CDC25A;CDC6;CDK12;CDK13;CDS2;CEL;CELF2;CELF6;CENPF;CHD7;CHTF18;CIB1;CLCF1;CLPP;CLSPN;CPSF2;CPSF4;CPT1C
2L1;GNG8;GNGT2;GPX4;HIPK3;HPX;HRAS;ISYNA1;ITPA;KIDINS220;LAMTOR2;LATS1;LATS2;LIME1;LPAR1;LTK;MAGI3;MAP3K10;MAP4K2;MAPK1;MAPK11;MATK;MC1R;MCM7;MLST8;MPG;MTG1;MTM1;MTMR1;MUTYH;MVK;MYLK3;NDUFA3;NDUFA8;NDUFB10;NDUFB7;NDUFB8;NDUFB9;NDUFS6;NDUFS

P8;DTD1;DUSP10;DUSP2;DUSP23;DUSP3;DYNC1LI1;EEF1A2;EIF3F;ENGASE;ENTPD7;ERCC1;ERI3;EXD3;EXOSC5;FAHD2B;FIGNL2;FXYD2;GNAI3;GNG8;GNGT2;GTPBP3;GZMM;HAGHL;HDAC10;HDAC11;HDHD3;HELZ;HEXDC;HINT2;HRAS;ITPA;JOSD2;KIAA2018;KIF15;KIF20A;KIF24;KIF7;LONRF3;LYZ;MACROD1;

US1L;DUS2L;DUS3L;DYNC1LI1;EEF1A2;EHHADH;EIF2B2;EIF3G;ETNK2;FASTK;FDXR;FICD;FIGNL2;FLAD1;FPGS;FUK;GCDH;GFER;GIMAP5;GLYCTK;GNAI3;GTPBP3;GTPBP6;HELZ;HIPK1;HIPK3;HNRNPH3;HRAS;HSD11B1L;HSD17B1;HSD17B12;HSD17B8;IFT27;ILVBL;IRAK4;ISYNA1;ITPA;KHK;KIF15;KIF20A;KIF24;K
AF4;TRAF6;TRIB3;TTN;VAV3;WNT7A;WRAP53;ZBTB17;ZEB2;ZFP91;ZF

;RBBP5;RFX3;RLIM;RSRC1;SENP2;SNF8;SNRPC;SPTBN4;STRA13;SUDS3;TADA3;TCEA2;THAP7;TIMM50;TOPBP1;TP73;TUT1;WRAP
;DUS3L;DYNC1LI1;EEF1A2;EHHADH;EIF2B2;EIF3G;ETNK2;FASTK;FDXR;FICD;FIGNL2;FLAD1;FPGS;FUK;GCDH;GFER;GIMAP5;GLYCTK;GNAI3;GTPBP3;GTPBP6;HELZ;HIPK1;HIPK3;HNRNPH3;HRAS;HSD11B1L;HSD17B1;HSD17B12;HSD17B8;IFT27;IRAK4;ISYNA1;ITPA;KHK;KIF15;KIF20A;KIF24;KIF7;LATS1;LATS2;L

.;DUS3L;DYNC1LI1;EEF1A2;EHHADH;EIF2B2;EIF3G;ETNK2;FASTK;FDXR;FICD;FIGNL2;FLAD1;FPGS;FUK;GCDH;GFER;GIMAP5;GLYCTK;GNAI3;GTPBP3;GTPBP6;HELZ;HIPK1;HIPK3;HNRNPH3;HRAS;HSD11B1L;HSD17B1;HSD17B12;HSD17B8;IFT27;IRAK4;ISYNA1;ITPA;KHK;KIF15;KIF20A;KIF24;KIF7;LATS1;LATS2;L

;ETNK2;FADS3;FDXR;FGD6;FICD;FLAD1;FPGS;GAMT;GCDH;GCHFR;GNAI3;GNB2L1;GNG8;GNGT2;GSTT1;GYG1;HAGHL;HRAS;HSD17B1;HSD17B12;HSD17B8;ISYNA1;ITPA;KDM3A;KHK;LHB;LMF1;MACROD1;MAPK1;MC1R;MECR;MED1;MLST8;MMAB;MPG;MRI1;MTG1;MTM1;MTMR1;MUT;MUTYH;MVD;M

;TTN;VAV3;WNT7A;ZBTB17;ZEB2;ZFP91;ZP

P1;TOP2A;TPR;UCKL1;ZC3HAV1;ZNF274;ZNF394;ZNF446;ZNF500;ZSCAN

P1;TOP2A;TPR;UCKL1;ZC3HAV1;ZNF274;ZNF394;ZNF446;ZNF500;ZSCAN

ITOR2;LATS1;LATS2;LIME1;LMF1;LRRC32;MAL;MAMDC4;MAPK1;MED1;MIA3;MTM1;NDUFA13;NECAB3;NF1;NFKBIL1;NIPBL;NSF;NUP153;OGG1;OSBPL9;P2RX7;PANX1;PEX16;PEX6;PICK1;PIK3C3;PIP5K1A;PLA2G6;PLEKHF1;PREB;RAB21;RAB25;RAB33A;RABEP2;RABGEF1;RAD21;RALGAPB;RBPMS;RINL;RP

ITM;PAOX;PARK7;PPOX;PTGES2;PYCRL;PYROXD2;SARDH;SDR39U1;SETD2;SURF1;TM7SF2;TXN2;TXNRD1;UEVLD;VKOR

BTB17;ZEB2;ZFP91;ZP

1;BUB1;BUB1B;C12orf44;C19orf25;C19orf57;C19orf60;C1orf86;C1QBP;C1QTNF6;C8G;CANX;CAPN10;CCDC101;CCDC130;CCDC28B;CCDC47;CCNL2;CCS;CD320;CD36;CD8B;CDC25A;CDC6;CDCA8;CDK10;CDK12;CDK13;CDYL2;CENPF;CENPT;CHD7;CHMP6;CHRAC1;CIB1;CLASP1;CLCF1;CLDN7;CLIP1;CLPP;CLS

PS15;RPS2;RPS28;SCARB2;SEC24B;SLC25A6;SPTBN4;SSNA1;SSR1;SSR4;STAM;STX1A;SYNGR1;SYTL1;TIMM10;TIMM13;TIMM50;TLR7;TOR1AIP1;TPR;TRPS1;UBL4A;VPS16;VPS41;VTI1A;WDR24;YWHAZ;ZFYVE27;Z

AR2A;PTPRC;RBPMS;SENP2;SPRED2;SPTBN4;SSR1;TADA3;TAOK3;TNFAIP3;TNFRSF4;TNFSF15;TP73;TRAF2;TRAF4;TRAF6;TRIB3;TTN;VAV3;WNT7A;ZBTB17;ZEB2;ZFP91;Z

PS15;RPS2;RPS28;SCARB2;SEC24B;SLC25A6;SPTBN4;SSNA1;SSR1;SSR4;STAM;STX1A;SYNGR1;SYTL1;TIMM10;TIMM13;TIMM50;TLR7;TOR1AIP1;TPR;TRPS1;UBL4A;VPS16;VPS41;VTI1A;WDR24;YWHAZ;ZFYVE27;Z

S2;RPS28;RQCD1;SMAP1;SOS1;STMN3;TNNT3;TPM2;UPF2;USP6NL;VAV3;WIBG;ZC3HA

ENPF;CHD7;CHTF18;CIB1;CLCF1;CLSPN;CPSF2;CPSF4;CREB3;CREB3L4;CSDE1;CSNK1G1;CTU2;DALRD3;DDX39A;DEAF1;DERL3;DGUOK;DNAJC17;DNAJC30;DNASE1L2;DOK7;DOLK;DPM3;DSE;DTX3;DUSP10;DUSP2;DUSP3;DVL1;E2F1;E4F1;EAF1;EDF1;EEF1D;EEF1G;EHHADH;EIF1;EIF2B2;EIF2B4;EIF3F;EIF3G;E

;DHRS4;DOLK;DPM3;DSE;DTD1;DTYMK;DUSP10;ECI1;EDF1;EHHADH;ETFB;ETNK2;FADS3;FDX1L;FDXR;FGD6;FICD;FLAD1;FPGS;GAMT;GCDH;GCHFR;GIMAP5;GNAI3;GNB2L1;GNG8;GNGT2;GPX4;GSTT1;GYG1;HAGHL;HCCS;HINT2;HPX;HRAS;HSD17B1;HSD17B12;HSD17B8;ISYNA1;ITPA;KDM3A;KHK;LHB;LM

QLN1;UBXN1;USP34;USP8;USP9X;ZFP

AT;LATS1;LATS2;LIME1;LMF1;LRRC32;LTBP4;MAL;MAP4K2;MAPK1;MED1;MIA3;MTM1;MYH10;NDUFA13;NECAB3;NF1;NFKBIL1;NIPBL;NSF;NSRP1;NUP153;NUSAP1;OGG1;P2RX7;PANX1;PCSK4;PEX16;PEX6;PFKFB2;PHPT1;PICK1;PIK3C3;PIK3CG;PIP5K1A;PLA2G6;PLEKHF1;PREB;PRKAR2A;PSMD9;PTPRC;RA
L;PIK3CB;PIK3CG;PIN1;PLA2G6;POLR2F;POLR2G;POLR2I;POLR2L;PREB;PRKAR2A;PTPRC;RBPMS;ROCK2;RSRC1;SENP2;SPRED2;SPTBN4;SSR1;TADA3;TAOK3;TLK2;TLR7;TNFAIP3;TNFRSF4;TNFSF15;TNK1;TP73;TRAF2;TRAF4;TRAF6;TRIB3;TRIM24;TSSK6;TTN;ULK3;WNT7A;ZAP70;ZBTB17;ZEB2;ZFP91;Z
TMED1;TMED3;TMED9;TMEM147;TMEM189;UBXN1;USE1;VKORC1;YIPF5;ZFYVE

TPR;TRAPPC2L;TRAPPC4;TRPS1;UPF2;USE1;WDR24;YIPF5;YWH

6;PREB;PRKAR2A;PSMA7;PSMB4;PSMB5;PSMB7;PSMD9;PTPRC;RBPMS;SENP2;SPRED2;SPTBN4;SSR1;STRA13;STUB1;TADA3;TAOK3;TNFAIP3;TNFRSF4;TNFSF15;TP73;TRAF2;TRAF4;TRAF6;TRIB3;TRIP12;TTN;UBQLN1;UBXN1;WNT7A;ZBTB17;ZEB2;ZFP91;Z
K11;MATK;MCM7;MKI67;MMAB;MSH3;MVD;MVK;MYH10;MYLK3;NDUFA13;NME3;NME4;NSF;NUBP2;OXSR1;P2RX6;P2RX7;PANK4;PAPSS1;PARS2;PDK4;PEX6;PFKFB2;PHKG1;PIK3C3;PIK3CB;PIK3CG;PIP5K1A;PIP5K1B;PIP5KL1;PMVK;PNKP;QARS;RABEP2;RECQL4;ROCK2;RPS6KB2;RPS6KC1;RPS6KL1;RTEL

Z;ZC3HA

RMD3;FSD1;GAS2L3;GLTSCR2;GNAI3;GNB2L1;GRIN3A;H2AFJ;H2AFY2;HAUS8;HIP1R;HOOK3;IFFO1;IMP4;INO80E;IQGAP2;ITGB1BP2;KAT2A;KDM5A;KIF15;KIF20A;KIF24;KIF7;KLHL17;KRT18;LARP4B;LATS1;LATS2;LRRC45;LSM14A;MAD1L1;MAPK1;MBD3;MBD5;MC1R;MCM7;MCRS1;MED1;MKI67;MOB2;M
RMD3;FSD1;GAS2L3;GLTSCR2;GNAI3;GNB2L1;GRIN3A;H2AFJ;H2AFY2;HAUS8;HIP1R;HOOK3;IFFO1;IMP4;INO80E;IQGAP2;ITGB1BP2;KAT2A;KDM5A;KIF15;KIF20A;KIF24;KIF7;KLHL17;KRT18;LARP4B;LATS1;LATS2;LRRC45;LSM14A;MAD1L1;MAPK1;MBD3;MBD5;MC1R;MCM7;MCRS1;MED1;MKI67;MOB2;M
1;MAPK11;MATK;MCM7;MKI67;MMAB;MSH3;MVD;MVK;MYH10;MYLK3;NDUFA13;NME3;NME4;NSF;NUBP2;OXSR1;P2RX6;P2RX7;PANK4;PAPSS1;PARS2;PDK4;PEX6;PFKFB2;PHKG1;PIK3C3;PIK3CB;PIK3CG;PIP5K1A;PIP5K1B;PIP5KL1;PMVK;PNKP;PRKAR2A;QARS;RABEP2;RECQL4;ROCK2;RPS6KB2;RPS6KC

CD36;CD38;CDC25A;CDC6;CDK12;CDK13;CEL;CELF2;CELF6;CENPF;CHD7;CHTF18;CIB1;CLCF1;CLPP;CLSPN;CPSF2;CPSF4;CREB3;CREB3L4;CRIP1;CSDE1;CSNK1G1;CTSC;CTSF;CTU2;DALRD3;DDX39A;DEAF1;DERL3;DGUOK;DNAJC17;DNAJC30;DNASE1L2;DND1;DOK7;DOLK;DPM3;DPP7;DPP8;DSE;DTX3;DUSP1
;USP30;USP34;USP8;USP9X;WIBG;YME1L1;ZC3HA

SSR1;TADA3;TAOK3;TNFAIP3;TNFRSF4;TNFSF15;TP73;TRAF2;TRAF4;TRAF6;TRIB3;TTN;WNT7A;ZBTB17;ZEB2;ZFP91;Z
1;MAPK11;MATK;MCM7;MKI67;MMAB;MSH3;MVD;MVK;MYH10;MYLK3;NDUFA13;NME3;NME4;NSF;NUBP2;OXSR1;P2RX6;P2RX7;PANK4;PAPSS1;PARS2;PDK4;PEX6;PFKFB2;PHKG1;PIK3C3;PIK3CB;PIK3CG;PIP5K1A;PIP5K1B;PIP5KL1;PMVK;PNKP;PRKAR2A;QARS;RABEP2;RECQL4;ROCK2;RPS6KB2;RPS6KC

'PS16;VPS41;VTI1A;WDR24;YWHAZ;ZI

RPS15;RPS2;RPS28;RQCD1;SGSM3;STUB1;TLK2;TNFAIP3;TRAF2;TRIM24;TRIP12;UBA7;UBXN1;UPF2;USE1;USP30;USP34;USP8;USP9X;WIBG;YME1L1;ZC3HA'

11;MIF4GD;MLST8;MYLIP;NDUFA13;NF1;NIPBL;NOG;NRP1;P2RX7;PDE6G;PIK3CB;PIK3CG;PIN1;PLA2G6;PREB;PRKAR2A;PSMA7;PSMB4;PSMB5;PSMB7;PSMD9;PTPRC;RBPMS;RPS6KB2;SENP2;SPRED2;SPTBN4;SSR1;STRA13;STUB1;TADA3;TAOK3;TLK2;TNFAIP3;TNFRSF4;TNFSF15;TP73;TPR;TRAF2;TRAF4;TF

TMED1;TMED3;TMED9;TMEM147;TMEM189;UBXN1;USE1;VKORC1;YIPF5;ZFYVE

L;RPLP2;RPS15;RPS2;RPS28;RQCD1;SMAP1;SOS1;STMN3;STS;TNNT3;TPM2;UPF2;USP6NL;VAV3;WIBG;ZC3HA\

L18;RPL18A;RPL28;RPL29;RPL38;RPL8;RPLP1;RPLP2;RPS15;RPS2;RPS28;RSRC1;SCARB2;SEC22B;SEC24B;SLC25A5;SLC25A6;SNF8;SSNA1;SSR1;SSR4;STAM;STOML2;STX1A;SYNGR1;SYTL1;THOC6;TIMM10;TIMM13;TIMM50;TLR7;TMED9;TPR;TRAPPC2L;TRAPPC4;TRPS1;UPF2;USE1;USP6NL;VPS16;VPS41;VTI1

6;ARIH1;ARL16;ARL6IP4;ARNTL;ASH1L;ASPSCR1;ASXL2;ATAD3B;ATF4;ATF7IP;ATL3;ATP5D;ATP5G2;ATP5G3;ATP6AP2;ATP6V1C1;ATRIP;AVEN;B3GAT3;B4GALT2;B4GALT7;BABAM1;BAIAP2L2;BCL7B;BCS1L;BHLHE41;BMP2K;BOK;BRAF;BRAT1;BRF1;BRF2;BUB1;BUB1B;C12orf44;C17orf49;C17orf70;C19orf25

;IRAK4;KHK;KIF15;KIF20A;KIF24;KIF7;LATS1;LATS2;LTK;MAGI3;MAP3K10;MAP4K2;MAPK1;MAPK11;MATK;MCM7;MKI67;MMAB;MSH3;MTG1;MVD;MVK;MYH10;MYLK3;NDOR1;NDUFA13;NDUFV1;NME3;NME4;NSF;NUBP2;OXSR1;P2RX6;P2RX7;PANK4;PAPSS1;PARS2;PDE6G;PDK4;PEX6;PFKFB2;PHKG1;P

'S15;RPS2;RPS28;RQCD1;SMAP1;SOS1;STMN3;TNNT3;TPM2;UPF2;USP6NL;VAV3;WIBG;ZC3HA\

'S15;RPS2;RPS28;RQCD1;SMAP1;SOS1;STMN3;TNNT3;TPM2;UPF2;USP6NL;VAV3;WIBG;ZC3HA\

P2;TP73;TRAF2;TRAF4;TRAF6;TRIB3;WNT7A;ZC3HAV1;ZEB2;ZF

LI

DUFB8;NECAB3;P4HTM;PANX1;PCYT2;PEMT;PEX16;PHEX;PIGQ;POMT1;PPM1L;PREB;PTDSS2;PTPLB;RAB21;RTN4R;SEC22B;SEC24B;SELM;SLC26A11;SLC37A4;SOAT1;SOAT2;SPPL2A;SPPL2B;SSR1;SSR4;STS;TLR7;TM7SF2;TMED1;TMED3;TMED9;TMEM147;TMEM189;TMTC2;TRAPPC2L;TRAPPC4;TRAPPC6A;

KFB2;PGM2;PHEX;PICK1;PIGQ;PIK3C3;PIK3CB;PIK3CG;PIN1;PIP5K1A;PIP5K1B;PLA2G6;PLCD1;PMM1;PMVK;PNKP;PPAP2C;PPM1L;PTDSS2;PXK;RAB21;RAB33A;RAC3;RALGAPB;RASA2;SLC44A1;SMAP1;SMPD2;SOS1;SPHK2;STMN3;STOML2;STS;SURF1;TAZ;TNNT3;TPM2;TXNRD1;UCK1;UCKL1;USP6NL;VAV3;

'S15;RPS2;RPS28;RQCD1;SMAP1;SOS1;STMN3;TNNT3;TPM2;UPF2;USP6NL;VAV3;WIBG;ZC3HA\

;IRAK4;KHK;KIF15;KIF20A;KIF24;KIF7;LATS1;LATS2;LTK;MAGI3;MAP3K10;MAP4K2;MAPK1;MAPK11;MATK;MCM7;MKI67;MMAB;MSH3;MTG1;MVD;MVK;MYH10;MYLK3;NDUFA13;NME3;NME4;NSF;NUBP2;OXSR1;P2RX6;P2RX7;PANK4;PAPSS1;PARS2;PDE6G;PDK4;PEX6;PFKFB2;PHKG1;PIK3C3;PIK3CB;PIK

S;FUK;GCDH;GCHFR;GFER;GIMAP5;GLYCTK;GNAI3;GRIN3A;GTPBP3;GTPBP6;HELZ;HIPK1;HIPK3;HRAS;HSD17B12;IFT27;ILVBL;IRAK4;KHK;KIF15;KIF20A;KIF24;KIF7;LATS1;LATS2;LEPRE1;LEPREL2;LTK;MAGI3;MAP3K10;MAP4K2;MAPK1;MAPK11;MATK;MCM7;MKI67;MMAB;MSH3;MTG1;MUT;MVD;MVK;MY

;IRAK4;KHK;KIF15;KIF20A;KIF24;KIF7;LATS1;LATS2;LTK;MAGI3;MAP3K10;MAP4K2;MAPK1;MAPK11;MATK;MCM7;MKI67;MMAB;MSH3;MTG1;MVD;MVK;MYH10;MYLK3;NDUFA13;NME3;NME4;NSF;NUBP2;OXSR1;P2RX6;P2RX7;PANK4;PAPSS1;PARS2;PDK4;PEX6;PFKFB2;PHKG1;PIK3C3;PIK3CB;PIK3CG;PIF

;IRAK4;KHK;KIF15;KIF20A;KIF24;KIF7;LATS1;LATS2;LTK;MAGI3;MAP3K10;MAP4K2;MAPK1;MAPK11;MATK;MCM7;MKI67;MMAB;MSH3;MTG1;MVD;MVK;MYH10;MYLK3;NDUFA13;NME3;NME4;NSF;NUBP2;OXSR1;P2RX6;P2RX7;PANK4;PAPSS1;PARS2;PDK4;PEX6;PFKFB2;PHKG1;PIK3C3;PIK3CB;PIK3CG;PIF

;IRAK4;KHK;KIF15;KIF20A;KIF24;KIF7;LATS1;LATS2;LTK;MAGI3;MAP3K10;MAP4K2;MAPK1;MAPK11;MATK;MCM7;MKI67;MMAB;MSH3;MTG1;MVD;MVK;MYH10;MYLK3;NDUFA13;NME3;NME4;NSF;NUBP2;OXSR1;P2RX6;P2RX7;PANK4;PAPSS1;PARS2;PDK4;PEX6;PFKFB2;PHKG1;PIK3C3;PIK3CB;PIK3CG;PIF

;IRAK4;KHK;KIF15;KIF20A;KIF24;KIF7;LATS1;LATS2;LTK;MAGI3;MAP3K10;MAP4K2;MAPK1;MAPK11;MATK;MCM7;MKI67;MMAB;MSH3;MTG1;MVD;MVK;MYH10;MYLK3;NDUFA13;NME3;NME4;NSF;NUBP2;OXSR1;P2RX6;P2RX7;PANK4;PAPSS1;PARS2;PDE6G;PDK4;PEX6;PFKFB2;PHKG1;PIK3C3;PIK3CB;PIK

4;KHK;KIF15;KIF20A;KIF24;KIF7;LATS1;LATS2;LTK;MAGI3;MAP3K10;MAP4K2;MAPK1;MAPK11;MATK;MCM7;MKI67;MMAB;MSH3;MTG1;MVD;MVK;MYH10;MYLK3;NDUFA13;NME3;NME4;NSF;NUBP2;OXSR1;P2RX6;P2RX7;PANK4;PAPSS1;PARS2;PDK4;PEX6;PFKFB2;PHKG1;PIK3C3;PIK3CB;PIK3CG;PIP5K1A

;CSDE1;CSNK1G1;CYB5D2;CYC1;CYP1B1;CYP2E1;DALRD3;DCXR;DDX21;DDX28;DDX39A;DDX43;DDX49;DEAF1;DECR2;DGUOK;DHDH;DHRS3;DHRS4;DHRS4L2;DNAJC17;DNASE1L2;DND1;DOM3Z;DRG2;DTYMK;DUS1L;DUS2L;DUS3L;DYNC1LI1;E2F1;E4F1;EDF1;EEF1A2;EEF1D;EEF1G;EHHADH;EIF1;EIF2B2;EIF

A;CDC6;CDCA8;CDK12;CDK13;CDYL2;CELF2;CELF6;CENPF;CENPM;CENPT;CHD7;CHRAC1;CHTF18;CIB1;CLSPN;COMMD5;COQ7;COX4I1;CPSF2;CPSF3L;CPSF4;CREB3;CREB3L4;CUEDC2;DDX21;DDX28;DDX39A;DEAF1;DGCR6;DGCR6L;DHRS4;DIAPH2;DLGAP5;DND1;DPP8;DTD1;DUSP10;DUSP2;DUSP23;DUSP3

;IRAK4;KHK;KIF15;KIF20A;KIF24;KIF7;LATS1;LATS2;LTK;MAGI3;MAP3K10;MAP4K2;MAPK1;MAPK11;MATK;MCM7;MKI67;MMAB;MSH3;MTG1;MVD;MVK;MYH10;MYLK3;NDUFA13;NME3;NME4;NSF;NUBP2;OXSR1;P2RX6;P2RX7;PANK4;PAPSS1;PARS2;PDK4;PEX6;PFKFB2;PHKG1;PIK3C3;PIK3CB;PIK3CG;PIF

PPP1R3F;PRDM1;PRKAR2A;PTPRC;RAD9A;RANBP9;SCARB2;SERPINB9;SERPINF2;SGSM3;SP1;SPG20;SPHK2;SPRED2;SPTBN4;STRN;STUB1;SUDS3;SYTL1;TCEB2;TNFAIP3;TNIP2;TOP2A;TP73;TPR;TRAF2;TRAF4;TRAF6;TRIB3;TTN;TUT1;TYSND1;UBQLN1;UBXN1;ZF

R;APC;APEH;APEX1;APEX2;API5;APLF;APPBP2;APPL1;APTX;ARFGAP1;ARFGAP2;ARGLU1;ARHGAP22;ARID2;ARID4A;ARID4B;ARID5A;ARID5B;ARL2;ARL4A;ARRB1;ARVCF;ASF1A;ASNA1;ASPM;ATAD2;ATF1;ATF2;ATP11B;ATPAF2;ATRX;ATXN7L3;AURKC;BACH1;BAG4;BAG6;BAIAP2;BANF1;BAP1;BAZ1A;BAZ2B;

L;AGPAT5;AGPS;AHR;AIFM3;AIM2;AIMP1;AIMP2;AK3;AKAP11;AKAP7;AKAP8L;AKIP1;AKIRIN1;AKR1B1;AKR7A2;AKT1;AKTIP;ALDH4A1;ALDOA;ALG1;ALG10;ALG11;ALG13;ALG2;ALG3;ALG6;ALKBH4;ALKBH5;ALKBH8;ALS2CR8;ANAPC10;ANG;ANKLE1;ANKMY2;ANKRD12;ANKRD13C;ANKRD28;ANKRD32;ANKF

L;AGPAT5;AGPS;AHR;AIFM3;AIM2;AIMP1;AIMP2;AK3;AKAP11;AKAP7;AKAP8L;AKIP1;AKIRIN1;AKR1B1;AKR7A2;AKT1;AKTIP;ALDH4A1;ALDOA;ALG1;ALG10;ALG11;ALG13;ALG2;ALG3;ALG6;ALKBH4;ALKBH5;ALKBH8;ALS2CR8;ANAPC10;ANG;ANKLE1;ANKMY2;ANKRD12;ANKRD13C;ANKRD28;ANKRD32;ANKF

BP2;AFF4;AGAP2;AGAP3;AGL;AGPAT5;AGPS;AHR;AIFM3;AIM2;AIMP1;AIMP2;AK3;AKAP11;AKAP7;AKAP8L;AKIP1;AKIRIN1;AKR1B1;AKR7A2;AKT1;AKTIP;ALDH4A1;ALDOA;ALG1;ALG10;ALG11;ALG13;ALG2;ALG3;ALG6;ALKBH4;ALKBH5;ALKBH8;ALS2CR8;ANAPC10;ANG;ANKLE1;ANKMY2;ANKRD12;ANKRD13

BP2;AFF4;AGAP2;AGAP3;AGL;AGPAT5;AGPS;AHR;AIFM3;AIM2;AIMP1;AIMP2;AK3;AKAP11;AKAP7;AKAP8L;AKIP1;AKIRIN1;AKR1B1;AKR7A2;AKT1;AKTIP;ALDH4A1;ALDOA;ALG1;ALG10;ALG11;ALG13;ALG2;ALG3;ALG6;ALKBH4;ALKBH5;ALKBH8;ALS2CR8;ANAPC10;ANG;ANKLE1;ANKMY2;ANKRD12;ANKRD13

1;ADCK2;ADCK3;ADD3;ADI1;ADORA1;ADRBK1;ADRM1;ADSSL1;AEBP1;AEBP2;AFF4;AGAP2;AGAP3;AGBL3;AGGF1;AGL;AGPAT5;AGPS;AHR;AIDA;AIFM3;AIM2;AIMP1;AIMP2;AK3;AKAP11;AKAP5;AKAP7;AKAP8L;AKIP1;AKIRIN1;AKR1B1;AKR7A2;AKT1;AKTIP;ALDH4A1;ALDOA;ALG1;ALG10;ALG11;ALG13;ALG

K3;ADD3;ADI1;ADORA1;ADRBK1;ADRM1;ADSSL1;AEBP1;AFF4;AGAP2;AGAP3;AGBL3;AGGF1;AGL;AGPAT5;AGPS;AHR;AIDA;AIFM3;AIM2;AIMP1;AIMP2;AK3;AKAP11;AKAP5;AKAP7;AKAP8L;AKR1B1;AKR7A2;AKT1;AKTIP;ALDH4A1;ALDOA;ALG1;ALG10;ALG11;ALG13;ALG2;ALG3;ALG6;ALKBH4;ALKBH8;AMD

1;ADCK2;ADCK3;ADD3;ADI1;ADORA1;ADRBK1;ADRM1;ADSSL1;AEBP1;AEBP2;AFF4;AGAP2;AGAP3;AGBL3;AGGF1;AGL;AGPAT5;AGPS;AHR;AIDA;AIFM3;AIM2;AIMP1;AIMP2;AK3;AKAP11;AKAP5;AKAP7;AKAP8L;AKIP1;AKIRIN1;AKR1B1;AKR7A2;AKT1;AKTIP;ALDH4A1;ALDOA;ALG1;ALG10;ALG11;ALG13;ALG

MTS5;ADAP1;ADC;ADCK1;ADCK2;ADCK3;ADD3;ADI1;ADORA1;ADRBK1;ADRM1;ADSSL1;AEBP1;AEBP2;AFF4;AGAP2;AGAP3;AGBL3;AGER;AGGF1;AGL;AGPAT5;AGPS;AHR;AIDA;AIFM3;AIM2;AIMP1;AIMP2;AK3;AKAP11;AKAP5;AKAP7;AKAP8L;AKIP1;AKIRIN1;AKR1B1;AKR7A2;AKT1;AKTIP;ALCAM;ALDH4A1;A

MTS5;ADAP1;ADC;ADCK1;ADCK2;ADCK3;ADD3;ADI1;ADORA1;ADRBK1;ADRM1;ADSSL1;AEBP1;AEBP2;AFF4;AGAP2;AGAP3;AGBL3;AGER;AGGF1;AGL;AGPAT5;AGPS;AHR;AIDA;AIFM3;AIM2;AIMP1;AIMP2;AK3;AKAP11;AKAP5;AKAP7;AKAP8L;AKIP1;AKIRIN1;AKR1B1;AKR7A2;AKT1;AKTIP;ALCAM;ALDH4A1;A

HR;AIDA;AIFM3;AIM2;AIMP1;AIMP2;AK3;AKAP5;AKAP7;AKR1B1;AKR7A2;AKT1;AKTIP;ALDH4A1;ALDOA;ALG1;ALG10;ALG11;ALG13;ALG2;ALG3;ALG6;ALKBH4;ALKBH8;AMD1;ANAPC10;ANKRD13C;ANLN;ANO5;ANP32E;ANXA1;AP1AR;AP1B1;AP1S2;AP2A2;AP3M1;AP3S2;AP4S1;APC;APEX1;APEX2;APH1A;A

KLE1;ANKRD13C;ANKRD26;ANKRD28;ANKRD32;ANLN;ANO5;ANXA1;AP1AR;AP1B1;AP1S2;AP2A2;AP3S2;APC;APEH;APEX1;APEX2;APH1A;API5;APPBP2;APPL1;APTX;ARFGAP1;ARFGAP2;ARFGEF2;ARFIP1;ARG2;ARHGEF2;ARID4A;ARID5A;ARL2;ARL4A;ARL6IP1;ARL6IP5;ARL8B;ARMC10;ARPC4;ARRB1;ARSA;A

;ARFGAP1;ARFGEF2;ARGLU1;ARHGAP22;ARHGEF2;ARID2;ARID4A;ARID4B;ARID5A;ARID5B;ARRB1;ARSA;ARSK;ASB13;ASB7;ASF1A;ASGR2;ASPH;ASTE1;ATAD1;ATAD2;ATF1;ATF2;ATG5;ATP7A;ATPBD4;ATRX;ATXN7L3;AUH;AURKC;B2M;B3GALNT1;B3GALT2;B3GALTL;B3GNT1;B3GNT2;B3GNT5;B4GALT3;B4(

KLE1;ANKRD13C;ANKRD26;ANKRD28;ANKRD32;ANLN;ANO5;ANXA1;AP1AR;AP1B1;AP1S2;AP2A2;AP3S2;APC;APEH;APEX1;APEX2;APH1A;API5;APPBP2;APPL1;APTX;ARFGAP1;ARFGAP2;ARFGEF2;ARFIP1;ARG2;ARHGEF2;ARID4A;ARID5A;ARL2;ARL4A;ARL6IP1;ARL6IP5;ARL8B;ARMC10;ARPC4;ARRB1;ARSA;A

K3;AKAP5;AKAP7;AKD1;AKR1B1;AKR7A2;AKT1;AKTIP;ALDH4A1;ALDOA;ALG1;ALG10;ALG10B;ALG11;ALG13;ALG2;ALG3;ALG6;ALKBH5;ALKBH8;AMD1;ANAPC10;ANG;ANGPT1;ANGPTL4;ANKRD13C;ANKRD49;ANXA1;APC;APEX1;APEX2;APH1A;APIP;APLF;APOBEC3D;APTX;ARAF;ARAP2;ARF4;ARFGAP1;ARFG

GALT3;B4GALT6;BAG2;BAG4;BAG6;BAP1;BBS10;BBS12;BCR;BIRC2;BMI1;BMPR1A;BMPR2;BNIP3L;BRCA2;BRCC3;BRMS1;BTBD1;C1GALT1;CAB39;CAMK1;CAMK2D;CAMK4;CASP3;CASP8;CBLL1;CCDC88A;CCNA2;CCNC;CCND3;CCNE2;CCNG1;CCNH;CCNL1;CCNT2;CCNY;CCNYL1;CCT2;CCT6B;CD28;CD80;CD81

L;AGL;AGPAT5;AGPS;AHR;AIDA;AIM2;AIMP1;AIMP2;AK3;AKAP5;AKAP7;AKD1;AKR1B1;AKR7A2;AKT1;AKTIP;ALDH4A1;ALDOA;ALG1;ALG10;ALG10B;ALG11;ALG13;ALG2;ALG3;ALG6;ALKBH5;ALKBH8;AMD1;ANAPC10;ANG;ANGPT1;ANGPTL4;ANKRD49;ANXA1;APC;APEH;APEX1;APEX2;APH1A;APIP;APLF;APC

ANKRD13C;ANKRD49;ANXA1;APC;APEH;APEX1;APEX2;APH1A;APIP;APLF;APOBEC3D;APTX;ARAF;ARFGAP1;ARFGEF2;ARGLU1;ARHGAP22;ARHGEF2;ARID2;ARID4A;ARID4B;ARID5A;ARID5B;ARRB1;ARSA;ARSK;ASB13;ASB7;ASF1A;ASGR2;ASPH;ASTE1;ASTL;ATAD1;ATAD2;ATF1;ATF2;ATG4A;ATG4B;ATG4C;AT

2;AGGF1;AGL;AGPAT5;AGPS;AHR;AIDA;AIM2;AIMP1;AIMP2;AK3;AKAP5;AKAP7;AKD1;AKR1B1;AKR7A2;AKT1;AKTIP;ALDH4A1;ALDOA;ALG1;ALG10;ALG10B;ALG11;ALG13;ALG2;ALG3;ALG6;ALKBH5;ALKBH8;AMD1;ANAPC10;ANG;ANGPT1;ANGPTL4;ANKRD13C;ANKRD49;ANXA1;APC;APEH;APEX1;APEX2;AP

K1;ADCK2;ADCK3;ADD3;ADI1;ADM2;ADORA1;ADRBK1;ADRM1;ADSSL1;AEBP1;AEBP2;AFF4;AGAP2;AGAP3;AGBL3;AGER;AGFG2;AGGF1;AGL;AGPS;AHR;AIDA;AIFM3;AIM2;AIMP1;AIMP2;AK3;AKAP11;AKAP5;AKAP7;AKAP8L;AKD1;AKIP1;AKT1;AKTIP;ALCAM;ALDH4A1;ALDOA;ALG13;ALG2;ALKBH4;ALKBH5;

13;BYSL;C8orf40;CAMK2D;CAMK4;CAPG;CASC5;CASP3;CASP8;CBX3;CCDC86;CCNA2;CCNC;CCNE2;CCNH;CCNL1;CCNT2;CD2AP;CD2BP2;CDC25C;CDC26;CDC27;CDC40;CDC7;CDC73;CDK1;CDK2;CDK5R1;CDK7;CDK8;CDK9;CDKN1B;CDKN2AIP;CDT1;CEBPA;CENPE;CENPK;CENPQ;CENPW;CEP70;CEPT1;CERS5;(

2;AGGF1;AGL;AGPAT5;AGPS;AHR;AIDA;AIFM3;AIM2;AIMP1;AIMP2;AK3;AKAP5;AKAP7;AKD1;AKR1B1;AKR7A2;AKT1;AKTIP;ALDH4A1;ALDOA;ALG1;ALG10;ALG10B;ALG11;ALG13;ALG2;ALG3;ALG6;ALKBH5;ALKBH8;AMD1;ANAPC10;ANG;ANGPT1;ANGPTL4;ANKRD13C;ANKRD49;ANXA1;APC;APEH;APEX1;AP

REB5;CSRP1;CXXC1;CYHR1;DBF4;DDX41;DEF8;DNAJC21;DNPEP;DPP3;DTNB;DTX2;DZIP3;EEA1;EGLN1;EHMT2;ERI2;ESR2;EWSR1;FANCL;FBXO11;FBXO30;FHL3;FIZ1;G2E3;GATAD2A;GCH1;GIT1;GTF2B;GTF2E1;GZF1;HDAC6;HLTF;IDE;IGHMBP2;IKZF2;IKZF5;ING3;INTS12;KIN;KLF16;KLF3;KLF5;LANCL1;LIMS1;
10D;ATP11B;ATP11C;ATP2C1;ATP5C1;ATP5E;ATP5F1;ATP5I;ATP5L;ATP5O;ATP6AP1;ATP6V1G1;ATP8A1;B2M;B3GALNT1;B3GALT2;B3GALTL;B3GNT1;B3GNT2;B3GNT5;B4GALT3;B4GALT6;BBS7;BCAP29;BCAP31;BET1;BLZF1;BMF;BNIP1;BNIP3L;BRIP1;C14orf2;C1GALT1;C3orf58;CAMK2D;CAPG;CASP8;CBX3;
CNP;PPP2CB;PSMA1;PSMA2;PSMA3;PSMA4;PSMA6;PSMA8;PSMB1;PSMB3;PSMC1;PSMC2;PSMC3;PSMC5;PSMC6;PSMD10;PSMD13;PSMD14;PSMD4;PSMD8;PSME1;PTEN;RAD23A;RBBP6;RBCK1;RBX1;RCHY1;RNF103;RNF11;RNF144B;RNF146;RNF187;RNF217;RNF40;RNF6;SENP1;SIAH1;SIRT1;SIRT2;SKP
;NPLOC4;OMA1;PARK2;PCNP;PMAIP1;PPP2CB;PSMA1;PSMA2;PSMA3;PSMA4;PSMA6;PSMA8;PSMB1;PSMB3;PSMC1;PSMC2;PSMC3;PSMC5;PSMC6;PSMD10;PSMD13;PSMD14;PSMD4;PSMD8;PSME1;PTEN;RAD23A;RBBP6;RBCK1;RBX1;RCHY1;RNF103;RNF11;RNF144B;RNF146;RNF187;RNF217;RNF40;R
;BCKDHB;BCLAF1;BDP1;BLZF1;BMI1;BRCA2;BRWD1;BTD;BYSL;C10orf2;C8orf40;CALU;CAMK2D;CAMK4;CAPG;CASC5;CASP3;CBR4;CBX3;CCDC86;CCNA2;CCNC;CCNE2;CCNH;CCNL1;CCNT2;CD2AP;CD2BP2;CDC25C;CDC26;CDC27;CDC40;CDC7;CDC73;CDK1;CDK2;CDK7;CDK8;CDK9;CDKN1B;CDKN2AIP;CDT1;
P2CB;PSMA1;PSMA2;PSMA3;PSMA4;PSMA6;PSMA8;PSMB1;PSMB3;PSMC1;PSMC2;PSMC3;PSMC5;PSMC6;PSMD10;PSMD13;PSMD14;PSMD4;PSMD8;PSME1;PTEN;RAD23A;RBBP6;RBCK1;RBX1;RCHY1;RNF103;RNF11;RNF144B;RNF146;RNF187;RNF217;RNF40;RNF6;SENP1;SIAH1;SIRT1;SIRT2;SKP2;SMU
04;FBXO45;FBXO5;FBXO8;FBXW4;FBXW7;GIPC1;GPC1;GTF2H1;GTF2H4;HACE1;HDAC6;HECTD2;HECTD3;HECW2;HERC4;HMGB1;HMGB2;HSPBP1;IDE;ITCH;JKAMP;KEAP1;KLHL20;LIN28A;LNX1;LONP1;LRRK2;LSM1;LSM3;LSM6;MAD2L1;MDM2;MGEA5;MNAT1;NDFIP1;NEDD4;NGLY1;NPLOC4;NUDT16;OMA
CNP;PPP2CB;PSMA1;PSMA2;PSMA3;PSMA4;PSMA6;PSMA8;PSMB1;PSMB3;PSMC1;PSMC2;PSMC3;PSMC5;PSMC6;PSMD10;PSMD13;PSMD14;PSMD4;PSMD8;PSME1;PTEN;RAD23A;RBBP6;RBCK1;RBX1;RCHY1;RNF103;RNF11;RNF144B;RNF146;RNF187;RNF217;RNF40;RNF6;SENP1;SIAH1;SIRT1;SIRT2;SKP
AZ1A;BCAS2;BCAT2;BCKDHB;BCLAF1;BDP1;BLZF1;BMI1;BRCA2;BRWD1;BTD;BYSL;C10orf2;C8orf40;CALU;CAMK2D;CAMK4;CAPG;CASC5;CASP3;CBR4;CBX3;CCDC86;CCNA2;CCNC;CCNE2;CCNH;CCNL1;CCNT2;CD2AP;CD2BP2;CDC25C;CDC26;CDC27;CDC40;CDC7;CDC73;CDK1;CDK2;CDK7;CDK8;CDK9;CDKN
CTD2;HECTD3;HECW2;HERC4;HLTF;HSPBP1;ITCH;KCTD11;KEAP1;KLHL15;KLHL20;KLHL22;KLHL7;KLHL9;LNX1;LRR1;LRRK2;LRSAM1;LTN1;MAD2L1;MALT1;MARCH1;MARCH5;MARCH6;MARCH7;MDM2;MED8;MKRN2;MKRN3;MYSM1;NDFIP1;NDFIP2;NEDD4;NSMCE2;OTUD5;PARK2;PAX6;PCNP;PELI1;PELI3
;NEDD4;NPLOC4;OMA1;PARK2;PCNP;PMAIP1;PPP2CB;PSMA1;PSMA2;PSMA3;PSMA4;PSMA6;PSMA8;PSMB1;PSMB3;PSMC1;PSMC2;PSMC3;PSMC5;PSMC6;PSMD10;PSMD13;PSMD14;PSMD4;PSMD8;PSME1;PTEN;RAB12;RAD23A;RBBP6;RBCK1;RBX1;RCHY1;RNF103;RNF11;RNF144B;RNF146;RNF187;RN
AP1;BAZ1A;BCAS2;BCAT2;BCKDHB;BCLAF1;BDP1;BLZF1;BMI1;BRCA2;BRWD1;BTD;BYSL;C10orf2;C8orf40;CALU;CAMK2D;CAMK4;CAPG;CASC5;CASP3;CBR4;CBX3;CCDC86;CCNA2;CCNC;CCNE2;CCNH;CCNL1;CCNT2;CD2AP;CD2BP2;CDC25C;CDC26;CDC27;CDC40;CDC7;CDC73;CDK1;CDK2;CDK7;CDK8;CDK9;
1;CDK11A;CDK11B;CDK2;CDK5R1;CDK5RAP3;CDK7;CDK9;CDKL1;CDKN1B;CDKN1C;CDKN2C;CDKN3;CDT1;CENPE;CENPK;CENPL;CENPQ;CENPW;CEP120;CEP135;CEP152;CEP250;CEP290;CEP57;CEP70;CETN3;CGRRF1;CHAF1A;CHAF1B;CHFR;CHMP1A;CHMP1B;CHORDC1;CKAP2;CKS1B;CKS2;CNPPD1;CNTRO
;BAP1;BCR;BIRC2;BMI1;BMPR1A;BMPR2;BRCA2;BRCC3;BRMS1;BTBD1;C1GALT1;CAB39;CAMK1;CAMK2D;CAMK4;CASP3;CBLL1;CCDC88A;CCNA2;CCNC;CCND3;CCNE2;CCNG1;CCNH;CCNL1;CCNT2;CCNY;CCNYL1;CD80;CD81;CDC25C;CDC26;CDC27;CDC37;CDC73;CDK1;CDK11A;CDK11B;CDK17;CDK18;CDK2
;BAP1;BCR;BIRC2;BMI1;BMPR1A;BMPR2;BRCA2;BRCC3;BRMS1;BTBD1;C1GALT1;CAB39;CAMK1;CAMK2D;CAMK4;CASP3;CBLL1;CCDC88A;CCNA2;CCNC;CCND3;CCNE2;CCNG1;CCNH;CCNL1;CCNT2;CCNY;CCNYL1;CD80;CD81;CDC25C;CDC26;CDC27;CDC37;CDC73;CDK1;CDK11A;CDK11B;CDK17;CDK18;CDK2
DI1;ADRBK1;ADSSL1;AEBP1;AEBP2;AGAP2;AGAP3;AGBL3;AGFG2;AGL;AGPS;AIFM3;AK3;AKAP7;AKAP8L;AKD1;AKT1;ALKBH4;ALKBH5;ALKBH8;ANG;ANKIB1;ANKK1;ANKMY2;ANXA1;APEX1;APEX2;APIP;APLF;APOBEC3D;APTX;ARAF;ARAP2;ARF4;ARFGAP1;ARFGAP2;ARG2;ARHGAP5;ARHGEF2;ARID2;ARL13
CNT2;CD2AP;CD2BP2;CDC25C;CDC26;CDC27;CDC40;CDC7;CDC73;CDK1;CDK2;CDK7;CDK8;CDK9;CDKN1B;CDKN2AIP;CDT1;CEBPA;CENPE;CENPK;CENPQ;CENPW;CHAF1B;CHD9;CHFR;CHMP1A;CKS1B;CLIC4;CLK3;CLP1;COIL;CPSF1;CPSF6;CRBN;CREB1;CSNK1A1;CTBP1;CUL2;CUTC;CXXC1;DACH1;DAPK3;DA
2;B3GNT5;B4GALT3;B4GALT6;BAG4;BAG6;BAP1;BCR;BIRC2;BMI1;BMPR1A;BMPR2;BRCA2;BRCC3;BRMS1;BTBD1;C1GALT1;CAB39;CAMK1;CAMK2D;CAMK4;CASP3;CBLL1;CCDC88A;CCNA2;CCNC;CCND3;CCNE2;CCNG1;CCNH;CCNL1;CCNT2;CCNY;CCNYL1;CD80;CD81;CDC25C;CDC26;CDC27;CDC37;CDC73;C
ATP6V1G1;ATP7A;AZI1;AZIN1;BACH1;BAG4;BAG6;BAIAP2;BANF1;BCAP31;BCAT1;BCL2L15;BCR;BIRC2;BLOC1S2;BMF;BNIP2;BNIPL;CAB39;CAB39L;CACNB3;CACNB4;CALB1;CAMK2D;CAMK4;CAPZA1;CAPZA2;CARHSP1;CASC5;CASP3;CASP8;CCDC88A;CCNE2;CCP110;CCT2;CD28;CDC25C;CDC26;CDC27;CDC
ADORA1;ADRBK1;ADSSL1;AEBP1;AGAP3;AGBL3;AGL;AGPAT5;AGPS;AIFM3;AK3;AKAP7;AKD1;AKR1B1;AKR7A2;AKT1;ALDH16A1;ALDH4A1;ALDOA;ALG1;ALG10;ALG10B;ALG11;ALG13;ALG2;ALG3;ALG6;ALKBH4;ALKBH5;ALKBH8;AMD1;ANAPC10;ANG;ANKK1;APEH;APEX1;APEX2;APH1A;APIP;APLF;APOBEC
MS1L;BRWD1;BTAF1;BUD13;BZW1;C10orf2;C12orf52;C14orf166;CAMK4;CARHSP1;CASP8AP2;CBFB;CBX3;CCDC59;CCNA2;CCNC;CCNH;CCNL1;CCNT2;CD28;CD2BP2;CD80;CDC40;CDC73;CDCA7;CDK1;CDK11A;CDK11B;CDK2;CDK5R1;CDK5RAP3;CDK7;CDK8;CDK9;CDKN1B;CDKN1C;CDT1;CEBPA;CEBPG;CEBP
;ANKK1;APEX1;APEX2;APLF;APTX;ARAF;ARF4;ARHGAP5;ARID2;ARID4A;ARID4B;ARID5A;ARID5B;ARL13B;ARL15;ARL2;ARL4A;ARL5A;ARL5B;ARL8B;ARRB1;ASNA1;ATAD1;ATAD2;ATAD2B;ATAD3A;ATF1;ATF2;ATL2;ATP10D;ATP11B;ATP11C;ATP13A2;ATP13A3;ATP2B1;ATP2C1;ATP6AP1;ATP7A;ATP8A1;ATP8
BMPR1A;BMPR2;BRCA2;BRCC3;BRD9;BRIP1;BRMS1;BRMS1L;BRWD1;BTAF1;BUD13;BZW1;C10orf2;C12orf52;C14orf166;CAMK4;CARHSP1;CASP5;CASP3;CASP8AP2;CBFB;CBX3;CCDC59;CCDC88A;CCNA2;CCNC;CCNE2;CCNH;CCNL1;CCNT2;CD28;CD2BP2;CD80;CDC25C;CDC40;CDC7;CDC73;CDCA7;CDK1;CD
MD13;PSMD14;PSMD4;PSMD8;PSME1;PTEN;RAD23A;RBCK1;RBX1;RCHY1;RNF103;RNF187;SENP1;SIAH1;SIRT1;SIRT2;SKP2;SOCS5;SOCS6;STT3B;SUMO1;SUMO2;TAF9;TBL1XR1;TOPORS;TRIB2;TRIM13;UBC;UBE2D1;UBE2D3;UBE2E1;UBE2J1;UBE2V2;UBE2W;UCHL5;USP14;USP19;USP44;USP5;WFS1;WW
RDC1;CHURC1;CNBP;CPD;CREB5;CSRP1;CUTC;CXXC1;CYCS;CYHR1;CYP2S1;CYP2U1;CYP2W1;CYP4F22;DBF4;DCP2;DDX41;DEF8;DNAJC21;DNPEP;DPP3;DTNB;DTX2;DYRK2;DZIP3;EEA1;EGLN1;EHMT2;ERI2;ESR2;EWSR1;F8;FANCL;FBXL5;FBXO11;FBXO30;FHL3;FIZ1;G2E3;GALNT1;GALNT3;GATAD2A;GCH1;G
;ANKK1;APEX1;APEX2;APLF;APTX;ARAF;ARF4;ARHGAP5;ARID2;ARID4A;ARID4B;ARID5A;ARID5B;ARL13B;ARL15;ARL2;ARL4A;ARL5A;ARL5B;ARL8B;ARRB1;ASNA1;ATAD1;ATAD2;ATAD2B;ATAD3A;ATF1;ATF2;ATL2;ATP10D;ATP11B;ATP11C;ATP13A2;ATP13A3;ATP2B1;ATP2C1;ATP5O;ATP6AP1;ATP7A;ATP8
;KLHL15;KLHL20;KLHL22;KLHL7;KLHL9;LNX1;LRR1;LRRK2;LRSAM1;LTN1;MAD2L1;MALT1;MARCH1;MARCH5;MARCH6;MARCH7;MDM2;MED8;MKRN2;MKRN3;NDFIP1;NDFIP2;NEDD4;NSMCE2;PARK2;PAX6;PCNP;PELI1;PELI3;PIAS4;PJA2;PSMA1;PSMA2;PSMA3;PSMA4;PSMA6;PSMA8;PSMB1;PSMB3;PSM
;ARFGAP2;ARG2;ARGLU1;ARHGAP22;ARHGAP5;ARHGEF2;ARID2;ARID4A;ARID4B;ARID5A;ARID5B;ARL2;ARL8B;ARRB1;ASAP3;ASF1A;ASNSD1;ASPH;ASTE1;ATAD2;ATF1;ATF2;ATL2;ATP2B1;ATP2C1;ATP5C1;ATP5E;ATP5F1;ATP5I;ATP5L;ATP5O;ATP7A;ATRX;ATXN7L3;AUH;AZIN1;BACH1;BANF1;BAZ1A;BAZ
GR1;ASNA1;ASPH;ASTL;ATF2;ATP10D;ATP11B;ATP11C;ATP13A2;ATP13A3;ATP2B1;ATP2C1;ATP7A;ATP8A1;ATP8A2;ATP8B4;ATRX;ATXN7L3;B4GALT3;B4GALT6;BAZ1A;BAZ2B;BGLAP;BIRC2;BIRC3;BMI1;BMP1;BMPR1A;BMPR2;BNIP2;BRCC3;BRIP1;C11orf54;C1GALT1;C1orf27;C1R;C4orf21;CA12;CA5BP1;C
RID5A;ARID5B;ARPP19;ARRB1;ASF1A;ASNSD1;ASPH;ATAD2;ATF1;ATF2;ATP2B1;ATP2C1;ATP5C1;ATP5E;ATP5F1;ATP5I;ATP5L;ATP5O;ATP7A;ATPBD4;ATRX;ATXN7L3;AZIN1;B3GALNT1;B3GALT2;B3GALTL;B3GNT1;B3GNT2;B3GNT5;B4GALT3;B4GALT6;BACH1;BAZ1A;BAZ2B;BBX;BCAT1;BCAT2;BCLAF1;BDH2;
;PSMD4;PSMD8;PSME1;PTEN;RAD23A;RBCK1;RBX1;RCHY1;RNF103;RNF187;SENP1;SIAH1;SIRT1;SIRT2;SKP2;SOCS5;STT3B;SUMO1;SUMO2;TAF9;TBL1XR1;TOPORS;TRIB2;TRIM13;UBC;UBE2D1;UBE2D3;UBE2E1;UBE2J1;UBE2V2;UBE2W;USP19;USP44;USP5;WFS1;WWP1;YOD1;ZNF
A2;BRD9;BRIP1;BRMS1;BRMS1L;BRWD1;BTAF1;BUD13;BZW1;C10orf2;C12orf52;C14orf166;CAMK4;CARHSP1;CASP3;CASP8AP2;CBFB;CBX3;CCDC59;CCNA2;CCNC;CCNH;CCNL1;CCNT2;CD28;CD2BP2;CD46;CD80;CDC40;CDC73;CDCA7;CDK1;CDK11A;CDK11B;CDK2;CDK5R1;CDK5RAP3;CDK7;CDK8;CDK9;CDK
;KLHL15;KLHL20;KLHL22;KLHL7;KLHL9;LNX1;LRR1;LRRK2;LRSAM1;LTN1;MAD2L1;MALT1;MARCH1;MARCH5;MARCH6;MARCH7;MDM2;MED8;MKRN2;MKRN3;NDFIP1;NDFIP2;NEDD4;PARK2;PAX6;PCNP;PELI1;PELI3;PJA2;PSMA1;PSMA2;PSMA3;PSMA4;PSMA6;PSMA8;PSMB1;PSMB3;PSMC1;PSMC2;PSM
;BAZ1A;BAZ2B;BBX;BCLAF1;BDP1;BIRC2;BLOC1S2;BLZF1;BMI1;BMPR1A;BMPR2;BRCA2;BRD9;BRIP1;BRMS1;BRMS1L;BRWD1;BTAF1;BZW1;C10orf2;C12orf52;C14orf166;C1GALT1;CAMK4;CARHSP1;CASP8AP2;CBFB;CBX3;CCDC59;CCDC88A;CCNA2;CCNC;CCNE2;CCNH;CCNL1;CCNT2;CD28;CD80;CDC25C;C
P1;KLHL20;LDLR;LNX1;LONP1;LPCAT1;LRRK2;LRSAM1;MAD2L1;MDM2;MGEA5;NDFIP1;NEDD4;NGLY1;NPLOC4;OMA1;OSBPL7;PARK2;PCNP;PMAIP1;PPP2CB;PPP2R3A;PSMA1;PSMA2;PSMA3;PSMA4;PSMA6;PSMA8;PSMB1;PSMB3;PSMC1;PSMC2;PSMC3;PSMC5;PSMC6;PSMD10;PSMD13;PSMD14;PSMD
GNT1;B3GNT2;B3GNT5;B4GALT3;B4GALT6;BACH1;BAZ1A;BAZ2B;BBX;BCLAF1;BDP1;BIRC2;BLOC1S2;BLZF1;BMI1;BMPR1A;BMPR2;BRCA2;BRD9;BRIP1;BRMS1;BRMS1L;BRWD1;BTAF1;BZW1;C10orf2;C12orf52;C14orf166;C1GALT1;CAMK4;CARHSP1;CASP8AP2;CBFB;CBX3;CCDC59;CCDC88A;CCNA2;CCNC;
RID5A;ARID5B;ARL2;ARL8B;ARRB1;ASAP3;ASF1A;ASPH;ASTE1;ATAD2;ATF1;ATF2;ATL2;ATP2B1;ATP2C1;ATP5C1;ATP5E;ATP5F1;ATP5I;ATP5L;ATP5O;ATP7A;ATRX;ATXN7L3;AUH;BACH1;BANF1;BAZ1A;BAZ2B;BBX;BCAS2;BCLAF1;BDP1;BIN1;BIRC2;BLOC1S2;BLZF1;BMI1;BMPR1A;BMPR2;BNIP2;BRCA2;BRC
4A1;ALDOA;ALG2;ALKBH8;ANG;ANGPT1;ANGPTL4;ANGPTL7;ANKIB1;ANKRD13C;ANKRD28;ANKRD44;ANLN;ANXA1;AP1B1;AP2A2;AP3M1;APC;APEX1;APH1A;API5;APIP;APLF;APPBP2;APPL1;APTX;ARAF;ARF4;ARFGAP1;ARFGEF2;ARFIP1;ARHGAP10;ARHGAP22;ARHGAP42;ARHGAP5;ARHGDIA;ARHGEF2;A
FAM110A;FAM161A;FGFR1OP;FLII;FOPNL;GNAI1;HAUS1;HAUS3;HAUS6;HAUS7;HOOK2;IFT122;IFT140;IFT20;IFT57;IFT74;IFT80;IFT81;IFT88;KATNB1;KEAP1;KIAA1009;KIAA1731;KIF18A;KIF20B;KIF23;KIF2A;KIF3A;KIFC1;KIFC3;LRRCC1;MAP3K11;MARK4;MIB1;MKKS;MKS1;MLF1IP;MZT1;NDE1;NDN;NDRG2;
EF2;ARID2;ARID4A;ARID4B;ARID5A;ARID5B;ARPP19;ARRB1;ASF1A;ASNSD1;ASPH;ATAD2;ATF1;ATF2;ATP2B1;ATP2C1;ATP5C1;ATP5E;ATP5F1;ATP5I;ATP5L;ATP5O;ATP7A;ATRX;ATXN7L3;AZIN1;B3GALNT1;B3GALT2;B3GALTL;B3GNT1;B3GNT2;B3GNT5;B4GALT3;B4GALT6;BACH1;BAZ1A;BAZ2B;BBX;BCAT1;
;ARHGEF2;ARID2;ARID4A;ARID4B;ARID5A;ARID5B;ARPP19;ARRB1;ASF1A;ASNSD1;ASPH;ATAD2;ATF1;ATF2;ATP2B1;ATP2C1;ATP5C1;ATP5E;ATP5F1;ATP5I;ATP5L;ATP5O;ATP7A;ATPBD4;ATRX;ATXN7L3;AZIN1;B3GALNT1;B3GALT2;B3GALTL;B3GNT1;B3GNT2;B3GNT5;B4GALT3;B4GALT6;BACH1;BAZ1A;BAZ
L;CPSF1;CPSF6;CREB1;CSNK1A1;CTBP1;CUL2;CXXC1;DACH1;DAPK3;DAXX;DBF4;DDB2;DDX11;DDX3X;DEPDC1;DGCR8;DIS3;DMAP1;DNA2;DPY30;DR1;DYRK1A;E2F4;E2F6;E2F7;EAF2;EED;EIF3E;ELL2;ENY2;EPC2;ERCC2;ERCC8;ESF1;ESR2;FAM60A;FAM76B;FANCB;FANCL;FANCM;FBXO5;FBXW7;FMR1;FOXH1
3;ASGR1;ASNA1;ASPH;ASTL;ATF2;ATP10D;ATP11B;ATP11C;ATP13A2;ATP13A3;ATP2B1;ATP2C1;ATP7A;ATP8A1;ATP8A2;ATP8B4;ATRX;ATXN7L3;B4GALT3;B4GALT6;BAZ1A;BAZ2B;BGLAP;BIRC2;BIRC3;BMI1;BMP1;BMPR1A;BMPR2;BNIP2;BRCC3;BRIP1;C11orf54;C1GALT1;C1orf27;C1R;C4orf21;CA12;CA5B
2;B3GNT5;B4GALT3;B4GALT6;BCAP29;BCAP31;BET1;BLZF1;BNIP1;BNIP2;BNIP3L;BRIP1;C1GALT1;C3orf58;CAMK2D;CAPG;CASC3;CD274;CD46;CD59;CDC42EP1;CDC42EP2;CDIPT;CDS1;CEP70;CEPT1;CERS5;CHERP;CHMP1A;CHPF;CHPF2;CHST13;CHST14;CHST2;CHSY1;CISD2;CLIC4;CLN3;CLN6;CLSTN3;CNIH;
L;HSD17B10;INTS12;INTS2;INTS5;INTS6;INTS8;IVNS1ABP;JMJD6;KIAA0391;KIAA1967;KIN;KRR1;LIN28A;LSM1;LSM11;LSM3;LSM6;LUC7L3;MBNL1;MBNL2;METTL1;MNAT1;MOCS3;MOV10;MPHOSPH6;MRM1;MTO1;NAA38;NOC4L;NOL6;NOP2;NOP56;NOP58;NSA2;NUDT16;NUDT21;OSGEPL1;PAIP1;PAN3
;MRPL18;MRPL19;MRPL2;MRPL22;MRPL28;MRPL3;MRPL32;MRPL37;MRPL39;MRPL40;MRPL42;MRPL47;MRPL48;MRPL50;MRPS10;MRPS17;MRPS18B;MRPS18C;MRPS2;MRPS22;MRPS28;MRPS31;MRPS33;NAA38;NOP56;NOP58;NSA2;NUFIP2;PAN3;PES1;PHF5A;PIN4;PIWIL4;PLRG1;PNRC2;PPIL3;PPWD
RF4;ARFGAP1;ARFGAP2;ARG2;ARGLU1;ARHGAP22;ARHGAP5;ARHGEF2;ARID2;ARID4A;ARID4B;ARID5A;ARID5B;ARL2;ARL8B;ARRB1;ARSA;ARSK;ARV1;ASAP3;ASF1A;ASNSD1;ASPH;ASTE1;ATAD2;ATF1;ATF2;ATL2;ATP2B1;ATP2C1;ATP5C1;ATP5E;ATP5F1;ATP5I;ATP5L;ATP5O;ATP7A;ATRX;ATXN7L3;AUH;AZ
;ARID2;ARID4A;ARID4B;ARID5A;ARID5B;ARL2;ARL8B;ARRB1;ASAP3;ASF1A;ASPH;ASTE1;ATAD2;ATF1;ATF2;ATL2;ATP2B1;ATP2C1;ATP5C1;ATP5E;ATP5F1;ATP5I;ATP5L;ATP5O;ATP7A;ATRX;ATXN7L3;AUH;BACH1;BANF1;BAZ1A;BAZ2B;BBX;BCAS2;BCLAF1;BDH2;BDP1;BIN1;BIRC2;BLOC1S2;BLZF1;BMI1;BMP
144B;RNF146;RNF187;RNF2;RNF217;RNF220;RNF31;RNF40;RNF6;RNF7;SIAH1;SKP2;SMURF2;SUMO1;SUMO3;TOPORS;TRAF7;TRIM11;TRIM13;TRIM2;TRIM23;TRIM28;TRIM33;TRIM59;TTLL1;TTLL12;UBA3;UBE2A;UBE2D1;UBE2D3;UBE2E1;UBE2G1;UBE2J1;UBE2J2;UBE2K;UBE2M;UBE2N;UBE2O;UBE2Q2;

ADSSL1;AEBP1;AEBP2;AFF4;AGAP2;AGAP3;AGER;AGFG2;AGGF1;AGL;AGPAT5;AGPS;AHR;AIDA;AIFM3;AIM2;AIMP1;AIMP2;AK3;AKAP11;AKAP5;AKAP7;AKD1;AKIP1;AKR1B1;AKR7A2;AKT1;AKTIP;ALCAM;ALDH4A1;ALDOA;ALG1;ALG10;ALG10B;ALG11;ALG13;ALG2;ALG3;ALG6;ALKBH5;ALKBH8;AMD1;ANAP
;CKS1B;CRLF3;CSNK1A1;CUL2;CUL4B;CUL5;DAPK3;DBF4;DCLRE1A;DCTN2;DDX11;DDX3X;DGKZ;DIS3L2;DLG1;DNA2;DYNLT3;DYRK1A;E2F4;E2F6;E2F7;EIF4E;EREG;FBXO31;FBXO5;FER;FGFR1OP;GMNN;GORASP1;GPR132;HAUS1;HAUS3;HAUS6;HAUS7;HDAC3;HELLS;ITGB3BP;KATNB1;KIAA1009;KIF11;KIF18
;ERLIN1;FBXL3;FBXL4;FBXL5;FBXO11;FBXO31;FBXO4;FBXO45;FBXO5;FBXO8;FBXW4;FBXW7;FUCA1;GAA;GIPC1;GPC1;GTF2H1;GTF2H4;GUSB;HACE1;HDAC6;HECTD2;HECTD3;HECW2;HERC4;HEXA;HGS;HMGB1;HMGB2;HMMR;HSPBP1;IDE;ITCH;JKAMP;KEAP1;KLHL20;LDLR;LIN28A;LNX1;LONP1;LPCAT1;LR
RNF220;RNF31;RNF40;RNF6;RNF7;SIAH1;SKP2;SMURF2;SUMO1;SUMO3;TOPORS;TRAF7;TRIM11;TRIM13;TRIM2;TRIM23;TRIM28;TRIM33;TRIM59;UBE2A;UBE2D1;UBE2D3;UBE2E1;UBE2G1;UBE2J1;UBE2J2;UBE2K;UBE2M;UBE2N;UBE2O;UBE2Q2;UBE2T;UBE2W;UBE3A;UBE4A;UBOX5;UBR1;UBR3;UBR5;
T1;MOV10;NAA38;NUDT16;NUDT21;PAIP1;PAN3;PAPD4;PAPD5;PAPOLA;PAPOLG;PCF11;PHAX;PHF5A;PLRG1;PNRC2;POLR2E;POLR2K;PPIL3;PPP2R1A;PPP2R2A;PPP4R2;PPWD1;PRPF18;PRPF31;PRPF38B;PRPF39;PRPF4B;PRPF6;PSIP1;PSMA1;PSMA2;PSMA3;PSMA4;PSMA6;PSMA8;PSMB1;PSME
ID4A;ARID4B;ARID5A;ARID5B;ARL2;ARL8B;ARRB1;ASAP3;ASF1A;ASPH;ASTE1;ATAD2;ATF1;ATF2;ATL2;ATP2B1;ATP2C1;ATP5C1;ATP5E;ATP5F1;ATP5I;ATP5L;ATP5O;ATP7A;ATRX;ATXN7L3;AUH;BACH1;BANF1;BAZ1A;BAZ2B;BBX;BCAS2;BCLAF1;BDH2;BDP1;BIN1;BIRC2;BLOC1S2;BLZF1;BMI1;BMPR1A;BMP
;RNF40;RNF6;SIAH1;SKP2;SMURF2;TOPORS;TRAF7;TRIM11;TRIM13;TRIM2;TRIM23;TRIM28;TRIM33;TRIM59;UBE2A;UBE2D1;UBE2D3;UBE2E1;UBE2G1;UBE2J1;UBE2J2;UBE2K;UBE2M;UBE2N;UBE2O;UBE2Q2;UBE2T;UBE2W;UBE3A;UBE4A;UBOX5;UBR1;UBR3;UBR5;WWP1;ZNF738;ZNF
MEX3C;MIB1;MKRN2;MKRN3;MSL2;MTHFSD;NADSYN1;NARS2;NEDD4;NSMCE2;PARK2;PAX6;PCNP;PELI1;PELI3;PIAS4;PJA2;POLG2;RARS2;RBBP6;RBCK1;RBX1;RCHY1;RFWD2;RIMKLA;RIMKLB;RING1;RNF103;RNF123;RNF13;RNF135;RNF138;RNF139;RNF144B;RNF146;RNF187;RNF19A;RNF2;RNF217;RNF220;
TG5;ATP7A;ATPBD4;ATXN7L3;AURKC;B2M;B3GALNT1;B3GALT2;B3GALTL;B3GNT1;B3GNT2;B3GNT5;B4GALT3;B4GALT6;BAG2;BAG4;BAG6;BAP1;BBS10;BBS12;BCR;BIRC2;BMI1;BMP1;BMPR1A;BMPR2;BNIP3L;BRCA2;BRCC3;BRMS1;BTBD1;C1GALT1;C1QA;C1QB;C1QC;C1R;CAB39;CAMK1;CAMK2D;CAMK
;IFT74;IFT81;IFT88;KATNB1;KEAP1;KIAA1009;KIAA1731;KIF20B;KIF23;KIF2A;KIF3A;KIFC3;LRRCC1;MAP3K11;MARK4;MIB1;MKKS;MKS1;MLF1IP;MZT1;NDE1;NDN;NDRG2;NEDD1;NEK1;NFU1;NIN;NME7;NUDT21;ODF2L;PAN3;PAX2;PCGF5;PIBF1;PLK1S1;PLK4;POC1B;POLR3H;PPP1R12A;PPP2R3C;PPP4C;P
TAD1;ATAD2;ATAD2B;ATAD3A;ATL2;ATP10D;ATP11B;ATP11C;ATP13A2;ATP13A3;ATP2B1;ATP2C1;ATP6AP1;ATP7A;ATP8A1;ATP8A2;ATP8B4;ATPBD4;ATRX;AURKC;BBS10;BBS12;BCR;BDH2;BMPR1A;BMPR2;BRIP1;BTAF1;C10orf2;CAMK1;CAMK2D;CAMK4;CBR4;CBWD1;CBWD2;CCT2;CCT6B;CDC7;CDK1;C
;RING1;RNF103;RNF13;RNF135;RNF139;RNF144B;RNF146;RNF187;RNF2;RNF217;RNF220;RNF31;RNF40;RNF6;RNF7;SIAH1;SKP2;SMURF2;SUMO1;SUMO3;TOPORS;TRAF7;TRIM11;TRIM13;TRIM2;TRIM23;TRIM28;TRIM33;TRIM59;TTLL1;TTLL12;UBA3;UBE2A;UBE2D1;UBE2D3;UBE2E1;UBE2G1;UBE2J1;UB
P250;CEP290;CEP57;CEP70;CETN3;CGRRF1;CHFR;CHMP1A;CHORDC1;CKAP2;CKS1B;CKS2;CNTROB;CRLF3;CSNK1A1;CUL2;CUL4B;CUL5;DAPK3;DAXX;DBF4;DCLRE1A;DCTN2;DDX11;DDX3X;DGKZ;DICER1;DIS3L2;DLG1;DNA2;DYNLT3;E2F4;E2F6;E2F7;ECT2;EHMT2;EIF4E;ERCC2;EREG;ERN1;FAM175A;FANCM;
TAD1;ATAD2;ATAD2B;ATAD3A;ATL2;ATP10D;ATP11B;ATP11C;ATP13A2;ATP13A3;ATP2B1;ATP2C1;ATP6AP1;ATP7A;ATP8A1;ATP8A2;ATP8B4;ATPBD4;ATRX;AURKC;BBS10;BBS12;BCR;BDH2;BMPR1A;BMPR2;BRIP1;BTAF1;C10orf2;CAMK1;CAMK2D;CAMK4;CBR4;CBWD1;CBWD2;CCT2;CCT6B;CDC7;CDK1;C
P2;CLIC4;CNP;CNTROB;CSK;CSNK1A1;DAPK3;DCTN2;DCTN4;DCTN6;DDX11;DGCR8;DLG1;DNAH6;DNAI2;DYNC2LI1;DYNLRB1;DYNLT3;ECT2;EML2;EML5;ERCC2;FAM110A;FAM161A;FBXO5;FER;FGFR1OP;FLII;FNTA;FOPNL;GAPDH;GAS2L2;GNAI1;GTF2F2;HAUS1;HAUS3;HAUS6;HAUS7;HDAC3;HDAC6;HOOK
;BAG6;BAP1;BAZ1A;BBIP1;BBS7;BCAS2;BCKDHB;BIRC2;BLOC1S2;BMF;BMI1;BNIP1;BRCA2;BRCC3;BTBD1;BUD13;C14orf166;C14orf2;C1QA;C1QB;C2orf49;C3orf58;CACNA1H;CACNB3;CACNB4;CAMK2D;CAPG;CAPZA1;CAPZA2;CARD8;CARHSP1;CASC5;CASP8;CATSPERB;CATSPERG;CBLL1;CBX3;CCDC53;CCN
;CISD2;CLEC2D;CLN3;CLN5;CLN6;CLSTN3;CNBP;CNIH;CNPY3;COL24A1;COL4A3BP;COL7A1;CRABP2;CRAT;CRIPAK;CST3;CTDNEP1;CYB5R4;CYP2S1;CYP2U1;CYP2W1;CYP4F22;DBI;DDOST;DDRGK1;DDX41;DEGS1;DEGS2;DERL1;DHRS1;DHRS9;DLG1;DMPK;DNAJB9;DNAJC10;DNASE1L1;DOLPP1;DPM1;DTNBP1
;ATAD2;ATF2;ATG5;ATP7A;ATRX;ATXN7L3;AZIN1;BACH1;BAG4;BAG6;BAZ1A;BAZ2B;BBX;BCLAF1;BDP1;BIN1;BIRC2;BIRC3;BLOC1S2;BLZF1;BMI1;BMPR1A;BMPR2;BNIP2;BRCA2;BRCC3;BRD9;BRIP1;BRMS1;BRMS1L;BRWD1;BTAF1;BZW1;C12orf52;C14orf166;CAB39;CAB39L;CALCRL;CAMK1;CAMK4;CARHSP
LNT4;GALNT7;GCNT1;GCNT2;GCNT4;GLCE;GOLGA4;GOLPH3L;GOLT1B;GOPC;GORASP1;GPSM1;GXYLT1;HACE1;HLA-A;HLA-DPB1;HLA-DRA;HS2ST1;HS6ST1;LEPROT;LFNG;LLGL1;LMAN1;LMAN2;LPCAT1;LPCAT2;MAN1A1;MAN1A2;MAN2A1;MANEA;MBTPS2;MGAT1;MGAT2;MGAT4A;MST4;NAA60;NAGPA

;ATF2;ATG5;ATRX;ATXN7L3;AZIN1;BACH1;BAG4;BAG6;BAZ1A;BAZ2B;BBX;BCLAF1;BDP1;BIN1;BIRC2;BLOC1S2;BLZF1;BMI1;BMPR1A;BMPR2;BNIP2;BRCA2;BRCC3;BRD9;BRIP1;BRMS1;BRMS1L;BRWD1;BTAF1;BZW1;C12orf52;C14orf166;CAB39;CAB39L;CALCRL;CAMK1;CAMK4;CARHSP1;CASP3;CASP8AP2;C
K;ILK;KIAA1804;KNDC1;LRRK2;MAP2K2;MAP2K4;MAP2K5;MAP3K1;MAP3K11;MAP3K13;MAP3K14;MAP3K2;MAP3K7;MAP3K8;MAP4K1;MAP4K3;MAP4K5;MAPK6;MAPK8;MAPKAPK2;MAPKAPK3;MARK4;MAST2;MAST4;MNAT1;MST4;MYLK4;NEK1;NEK7;NLK;PAK4;PBK;PDIK1L;PDK1;PDK2;PHKG2;PIK3CA
CHFR;CHMP1A;CKS1B;CKS2;CRLF3;CSNK1A1;CUL2;CUL4B;CUL5;DAPK3;DBF4;DCLRE1A;DCTN2;DDX11;DDX3X;DGKZ;DICER1;DIS3L2;DLG1;DNA2;DYNLT3;E2F4;E2F6;E2F7;EHMT2;EIF4E;EREG;FANCM;FBXO31;FBXO5;FGFR1OP;GMNN;GORASP1;GPR132;HAUS1;HAUS3;HAUS6;HAUS7;HDAC3;HELLS;HORMAD
HGAP5;ARHGEF2;ARID2;ARID4A;ARID4B;ARID5A;ARID5B;ARL2;ARL8B;ARRB1;ARV1;ASAP3;ASF1A;ASPH;ASTE1;ATAD2;ATF1;ATF2;ATL2;ATP2B1;ATP2C1;ATP5C1;ATP5E;ATP5F1;ATP5I;ATP5L;ATP5O;ATP7A;ATRX;ATXN7L3;AUH;BACH1;BANF1;BAZ1A;BAZ2B;BBX;BCAS2;BCLAF1;BDH2;BDP1;BIN1;BIRC2;BLOC
H;CDC7;CDIPT;CDK1;CDK11A;CDK11B;CDK17;CDK18;CDK2;CDK5R1;CDK7;CDK8;CDK9;CDKL1;CDKL2;CDS1;CEPT1;CERS5;CHM;CHML;CHPF;CHPF2;CHST13;CHST14;CHST2;CHSY1;CHUK;CKB;CLK1;CLK3;CLK4;CLP1;CMPK1;COASY;COL4A3BP;COMT;COQ2;CPNE3;CRAT;CRCP;CRIM1;CRLS1;CROT;CSGALNACT1;C
ARL5A;ARL5B;ARL8B;ASNA1;ATAD1;ATAD2;ATAD2B;ATAD3A;ATL2;ATP10D;ATP11B;ATP11C;ATP13A2;ATP13A3;ATP2B1;ATP2C1;ATP6AP1;ATP7A;ATP8A1;ATP8A2;ATP8B4;ATPBD4;ATRX;AURKC;BBS10;BBS12;BCR;BDH2;BMPR1A;BMPR2;BRIP1;BTAF1;C10orf2;CALB1;CAMK1;CAMK2D;CAMK4;CBR4;CBWD
A2;ATP13A3;ATP2B1;ATP2C1;ATP6AP1;ATP7A;ATP8A1;ATP8A2;ATP8B4;ATPBD4;ATRX;AURKC;BBS10;BBS12;BCR;BMPR1A;BMPR2;BRIP1;BTAF1;C10orf2;CAMK1;CAMK2D;CAMK4;CBWD1;CBWD2;CCT2;CCT6B;CDC7;CDK1;CDK11A;CDK11B;CDK17;CDK18;CDK2;CDK7;CDK8;CDK9;CDKL1;CDKL2;CENPE;CHD
UBE2K;UBE2N;UBE2Q2;UBE2T;UBE2V2;UBE2W;UBE3A;UBE4A;UBOX5;UBR5;ZNF738;ZNRF
RID5A;ARID5B;ARL2;ARPP19;ARRB1;ARRDC3;ARV1;ASAP3;ASF1A;ASGR2;ASPH;ASTL;ATAD1;ATAD2;ATF2;ATG5;ATP7A;ATRX;ATXN7L3;AZIN1;BACH1;BAG4;BAG6;BAZ1A;BAZ2B;BBX;BCAP31;BCLAF1;BDP1;BIN1;BIRC2;BIRC3;BLOC1S2;BLZF1;BMI1;BMPR1A;BMPR2;BNIP2;BRCA2;BRCC3;BRD9;BRIP1;BRMS1
TP11B;ATP11C;ATP13A2;ATP13A3;ATP2B1;ATP2C1;ATP6AP1;ATP7A;ATP8A1;ATP8A2;ATP8B4;ATPBD4;ATRX;AURKC;BBS10;BBS12;BCR;BMPR1A;BMPR2;BRIP1;BTAF1;C10orf2;CAMK1;CAMK2D;CAMK4;CBWD1;CBWD2;CCT2;CCT6B;CDC7;CDK1;CDK11A;CDK11B;CDK17;CDK18;CDK2;CDK7;CDK8;CDK9;CDK
C;ATP13A2;ATP13A3;ATP2B1;ATP2C1;ATP6AP1;ATP7A;ATP8A1;ATP8A2;ATP8B4;ATPBD4;ATRX;AURKC;BBS10;BBS12;BCR;BMPR1A;BMPR2;BRIP1;BTAF1;C10orf2;CAMK1;CAMK2D;CAMK4;CBWD1;CBWD2;CCT2;CCT6B;CDC7;CDK1;CDK11A;CDK11B;CDK17;CDK18;CDK2;CDK7;CDK8;CDK9;CDKL1;CDKL2;CENI
TP10D;ATP11B;ATP11C;ATP13A2;ATP13A3;ATP2B1;ATP2C1;ATP6AP1;ATP7A;ATP8A1;ATP8A2;ATP8B4;ATPBD4;ATRX;AURKC;BBS10;BBS12;BCR;BMPR1A;BMPR2;BRIP1;BTAF1;C10orf2;CAMK1;CAMK2D;CAMK4;CBWD1;CBWD2;CCT2;CCT6B;CDC7;CDK1;CDK11A;CDK11B;CDK17;CDK18;CDK2;CDK7;CDK8;CD
P1;BNIP3L;BORA;BRCA2;BRCC3;BRD9;BRWD1;C10orf2;CABIN1;CAPG;CAPZA1;CAPZA2;CASC5;CASP3;CBX3;CCDC88A;CCDC88B;CCNA2;CCNG1;CCNG2;CCP110;CD28;CD2AP;CDC25C;CDC26;CDC27;CDC42EP2;CDC73;CDK1;CDK11A;CDK11B;CDK2;CDK5R1;CDK9;CDKN1B;CEBPA;CENPE;CENPK;CENPL;CENPQ;
C;ATP13A2;ATP13A3;ATP2B1;ATP2C1;ATP6AP1;ATP7A;ATP8A1;ATP8A2;ATP8B4;ATPBD4;ATRX;AURKC;BBS10;BBS12;BCR;BMPR1A;BMPR2;BRIP1;BTAF1;C10orf2;CAMK1;CAMK2D;CAMK4;CBWD1;CBWD2;CCT2;CCT6B;CDC7;CDK1;CDK11A;CDK11B;CDK17;CDK18;CDK2;CDK7;CDK8;CDK9;CDKL1;CDKL2;CENI
C;ATP13A2;ATP13A3;ATP2B1;ATP2C1;ATP6AP1;ATP7A;ATP8A1;ATP8A2;ATP8B4;ATPBD4;ATRX;AURKC;BBS10;BBS12;BCR;BMPR1A;BMPR2;BRIP1;BTAF1;C10orf2;CAMK1;CAMK2D;CAMK4;CBWD1;CBWD2;CCT2;CCT6B;CDC7;CDK1;CDK11A;CDK11B;CDK17;CDK18;CDK2;CDK7;CDK8;CDK9;CDKL1;CDKL2;CENI
OC1S2;BLZF1;BMI1;BMPR1A;BMPR2;BRCA2;BRCC3;BRD9;BRIP1;BRMS1;BRMS1L;BRWD1;BTAF1;BZW1;C12orf52;C14orf166;CAB39;CAMK1;CAMK4;CARHSP1;CASP3;CASP8AP2;CBFB;CBX3;CCDC59;CCDC88A;CCNA2;CCNC;CCND3;CCNE2;CCNG1;CCNH;CCNL1;CCNT2;CCNY;CCNYL1;CD28;CD46;CD80;CD81;CI
CHMP2A;CHMP2B;CLIC2;CLN3;CNP;COG3;COG4;COG6;COL4A3BP;COPE;COPZ1;CPSF1;CSK;DAB2IP;DDOST;DERL1;DMAP1;DPY30;DTNBP1;DYRK2;ECT2;EEA1;EIF4E;EIF6;EPS15;ERBB2IP;ERO1L;EXOC5;EXPH5;FAM109B;FYTTD1;GAS6;GCC2;GIPC1;GOLGA4;GOPC;GRPEL2;GSTM2;HDAC6;HGS;HMGXB4;HOOK
C;ATP13A2;ATP13A3;ATP2B1;ATP2C1;ATP6AP1;ATP7A;ATP8A1;ATP8A2;ATP8B4;ATPBD4;ATRX;AURKC;BBS10;BBS12;BCR;BMPR1A;BMPR2;BRIP1;BTAF1;C10orf2;CAMK1;CAMK2D;CAMK4;CBWD1;CBWD2;CCT2;CCT6B;CDC7;CDK1;CDK11A;CDK11B;CDK17;CDK18;CDK2;CDK7;CDK8;CDK9;CDKL1;CDKL2;CENI
A;PAPOLG;PCF11;PHAX;PHF5A;PLRG1;POLR2E;POLR2K;PPIL3;PPP4R2;PPWD1;PRPF18;PRPF31;PRPF38B;PRPF39;PRPF40A;PRPF4B;PRPF6;PSIP1;PTBP2;PUF60;QKI;RALY;RBFOX3;RBM10;RBM11;RBM25;RBM27;RBM39;RBM4;RBM5;RNGTT;RNPC3;SART1;SCAF11;SF3A1;SF3A2;SF3B1;SF3B14;SFSWAP;SKIV2
C;ATP13A2;ATP13A3;ATP2B1;ATP2C1;ATP6AP1;ATP7A;ATP8A1;ATP8A2;ATP8B4;ATPBD4;ATRX;AURKC;BBS10;BBS12;BCR;BMPR1A;BMPR2;BRIP1;BTAF1;C10orf2;CAMK1;CAMK2D;CAMK4;CBWD1;CBWD2;CCT2;CCT6B;CDC7;CDK1;CDK11A;CDK11B;CDK17;CDK18;CDK2;CDK7;CDK8;CDK9;CDKL1;CDKL2;CENI
;CCDC59;CCDC88A;CCNA2;CCNC;CCNH;CCNL1;CCNT2;CD28;CD80;CDC73;CDCA7;CDK1;CDK11A;CDK11B;CDK2;CDK5R1;CDK5RAP3;CDK7;CDK8;CDK9;CDKN1B;CDKN1C;CDT1;CEBPA;CEBPG;CEBPZ;CENPK;CEP290;CGGBP1;CHAF1A;CHAF1B;CHD1;CHD5;CHD9;CHMP1A;CHUK;CHURC1;CKAP2;CNBP;CNOT2;C
5;RBM27;RBM39;RBM4;RCHY1;RFWD2;RING1;RNF2;RNF6;SART1;SF3B1;SIRT1;SKIL;SLU7;SMC5;SMC6;SMNDC1;SP3;SRSF10;SRSF3;SS18L1;SUMO1;SUMO3;TAF9;TDG;TDP2;THAP1;TOLLIP;TOPORS;TP53INP1;U2AF1;USPL1;WBP4;XPO1;ZMYM
3;CNP;COPS5;CPEB2;CPEB3;CPEB4;CPSF1;CPSF6;CREB1;CREB5;CREBL2;CREBZF;CRY1;CSTF2T;CXXC1;DACH1;DAZAP1;DBF4;DBP;DCP2;DCPS;DDB2;DDX11;DDX3X;DDX41;DDX50;DDX54;DDX59;DEK;DENND4A;DENR;DGCR8;DHFRL1;DHX30;DHX36;DHX40;DICER1;DIS3;DIS3L2;DMTF1;DNA2;DNAJC21;DNASE1
GHMBP2;IKBKG;INO80C;INO80D;JMY;KIAA0101;KIF22;KIN;LIG4;MAPKAPK2;MCL1;MCM9;MCTS1;MDM2;MEN1;MMS22L;MNAT1;MNDA;MORF4L2;MSH2;MUC1;MUM1;NBN;NEK1;NSMCE2;NUDT16;PMAIP1;PMS1;POLD2;POLG2;POLI;POLK;POLQ;POLR2E;POLR2K;PPP1R15A;PPP4C;PPP4R2;PSMA1;PSMA
;CBFB;CBX3;CCDC59;CCDC88A;CCNA2;CCNC;CCNH;CCNL1;CCNT2;CD28;CD80;CDC73;CDCA7;CDK1;CDK11A;CDK11B;CDK2;CDK5R1;CDK5RAP3;CDK7;CDK8;CDK9;CDKN1B;CDKN1C;CDT1;CEBPA;CEBPG;CEBPZ;CENPK;CEP290;CGGBP1;CHAF1A;CHAF1B;CHD1;CHD5;CHD9;CHMP1A;CHUK;CHURC1;CKAP2;CNE

rf52;C14orf166;CALCRL;CAMK4;CARHSP1;CASP8AP2;CBFB;CBX3;CCDC59;CCDC88A;CCNA2;CCNC;CCNH;CCNL1;CCNT2;CD28;CD80;CDC73;CDCA7;CDK1;CDK11A;CDK11B;CDK2;CDK5R1;CDK5RAP3;CDK7;CDK8;CDK9;CDKN1B;CDKN1C;CDT1;CEBPA;CEBPG;CEBPZ;CENPK;CEP290;CGGBP1;CHAF1A;CHAF1B;CHI
1;GATAD2A;GEMIN2;GEMIN6;GPS2;GTF2E2;GTF2F2;GTF2H1;GTF2H4;HDAC3;HDAC5;HDAC6;HDAC7;HDAC8;HDAC9;HIRA;HMGB1;HNF1A;ING3;INO80C;INTS12;INTS2;INTS5;INTS6;INTS8;IVNS1ABP;JUN;KAT2B;KAT8;KLHL20;LRCH4;LUC7L3;MAP3K7;MBIP;MDM2;MED12L;MED13;MED14;MED15;MED16;M
P290;CEP57;CHCHD4;CHERP;CHM;CHML;CHMP1A;CHMP1B;CHMP2A;CHMP2B;CLEC5A;CLEC6A;CLIP3;COG3;COG4;COG6;COPE;COPZ1;CRIPT;CSK;CXCL10;DAB2IP;DDOST;DENND1B;DENND4C;DERL1;DLG1;DLG4;DMAP1;DPH3;DYRK2;ECT2;EIF5A2;EPB41L5;EPS15;ERBB2IP;EXOC1;EXOC3;EXOC5;EXOC6;EXC
;KLHL9;LRRCC1;MAD2L1;MAP9;MIS18BP1;MLF1IP;NDE1;NEDD1;NEK1;NSL1;NSMCE2;NUDC;NUF2;NUP37;NUP85;OIP5;PAPD5;PBK;PDS5B;PES1;PHIP;PMF1;PPP1CC;PPP1R12A;PPP5C;PTEN;RANGAP1;RB1;RBBP8;RPS27;RRS1;SEC13;SEPT7;SETDB2;SGOL2;SIRT2;SKA2;SMC2;SMC4;SMC5;SPAG5;SSSCA1;STA
;KLHL9;LRRCC1;MAD2L1;MAP9;MIS18BP1;MLF1IP;NDE1;NEDD1;NEK1;NSL1;NSMCE2;NUDC;NUF2;NUP37;NUP85;OIP5;PAPD5;PBK;PDS5B;PES1;PHIP;PMF1;PPP1CC;PPP1R12A;PPP5C;PTEN;RANGAP1;RB1;RBBP8;RPS27;RRS1;SEC13;SEPT7;SETDB2;SGOL2;SIRT2;SKA2;SMC2;SMC4;SMC5;SPAG5;SSSCA1;STA
13;CHST14;CHST2;CHSY1;CLIP3;CLN3;CLN5;CLSTN3;CNIH;COG3;COG4;COG6;COL4A3BP;COPE;COPZ1;CORO7;CSGALNACT1;CSGALNACT2;CSK;CYBA;DBI;DLG1;DPY30;DRAM2;EAPP;EBAG9;ENTPD6;ERGIC2;F2RL1;FAM109B;FAM134B;FAM198B;FAM3C;FBXW7;FGD2;FGD3;FGD4;FGF7;FNDC3A;FUT4;G2E3;G
CNA2;CCNC;CCNH;CCNL1;CCNT2;CD28;CD80;CDC73;CDCA7;CDK1;CDK11A;CDK11B;CDK2;CDK5R1;CDK5RAP3;CDK7;CDK8;CDK9;CDKN1B;CDKN1C;CDT1;CEBPA;CEBPG;CEBPZ;CENPK;CEP290;CGGBP1;CHAF1A;CHAF1B;CHD1;CHD5;CHD9;CHMP1A;CHUK;CHURC1;CKAP2;CNBP;CNOT2;CNOT3;CNOT6;CNOT7
;KLHL9;LRRCC1;MAD2L1;MAP9;MARCH5;MFF;MIS18BP1;MLF1IP;MTFR1;NDE1;NEDD1;NEK1;NSL1;NSMCE2;NUDC;NUF2;NUP37;NUP85;OIP5;OPA1;PAPD5;PBK;PDS5B;PES1;PEX11A;PEX11G;PHIP;PMF1;PPP1CC;PPP1R12A;PPP5C;PTEN;RANGAP1;RB1;RBBP8;RPS27;RRS1;SEC13;SEPT7;SETDB2;SGOL2;SIRT
;ORC2;ORC3;ORC4;PKD2;PLAGL1;PPM1G;PPP1R15A;PRCC;PRKAA1;PRKAB2;PRKAG2;PSMA1;PSMA2;PSMA3;PSMA4;PSMA6;PSMA8;PSMB1;PSMB3;PSMC1;PSMC2;PSMC3;PSMC5;PSMC6;PSMD10;PSMD13;PSMD14;PSMD4;PSMD8;PSME1;RAD17;RASSF1;RB1;RBBP8;RFC2;RFC3;RFC5;RFWD2;RHEB;RINT1;
ZW1;C12orf52;C14orf166;CALCRL;CAMK4;CARHSP1;CASP8AP2;CBFB;CBX3;CCDC59;CCDC88A;CCNA2;CCNC;CCNH;CCNL1;CCNT2;CD28;CD80;CDC73;CDCA7;CDK1;CDK11A;CDK11B;CDK2;CDK5R1;CDK5RAP3;CDK7;CDK8;CDK9;CDKN1B;CDKN1C;CDT1;CEBPA;CEBPG;CEBPZ;CENPK;CEP290;CGGBP1;CHAF1A;C
SMD14;PSMD4;PSMD8;PSME1;PTEN;RB1;RBBP8;RFWD2;RHOU;RPS27L;RPS6KB1;SKP2;TJP3;TP63;TRIAP1;UBC;USP37;WEI
CNA2;CCNC;CCNH;CCNL1;CCNT2;CD28;CD80;CDC73;CDCA7;CDK1;CDK11A;CDK11B;CDK2;CDK5R1;CDK5RAP3;CDK7;CDK8;CDK9;CDKN1B;CDKN1C;CDT1;CEBPA;CEBPG;CEBPZ;CENPK;CEP290;CGGBP1;CHAF1A;CHAF1B;CHD1;CHD5;CHD9;CHMP1A;CHUK;CHURC1;CKAP2;CLK1;CLK3;CLK4;CNBP;CNOT2;CNOT

;KLHL9;LRRCC1;MAD2L1;MAP9;MDK;MIS18BP1;MLF1IP;MPHOSPH6;NDE1;NEDD1;NEK1;NSL1;NSMCE2;NUDC;NUF2;NUP37;NUP85;OIP5;PAPD5;PBK;PDS5B;PES1;PHIP;PMF1;PPP1CC;PPP1R12A;PPP5C;PTEN;RANGAP1;RB1;RBBP8;RPS27;RRS1;SEC13;SEPT7;SETDB2;SGOL2;SIRT2;SKA2;SMC2;SMC4;SMC5;
AT2;BCKDHB;BMF;BNIP1;BNIP3L;C10orf2;C14orf2;C21orf33;C2orf47;C5orf54;CA5BP1;CASP3;CASP8;CBR4;CCBL2;CCDC136;CCDC58;CCDC90A;CCDC90B;CDK1;CDK7;CHCHD4;CHMP2B;CISD1;CISD2;CKB;CLIC4;CLN3;CMC1;CNP;COASY;COL4A3BP;COMT;COQ10B;COQ2;COX16;COX6C;COX7A2;COX7A2L;CO

T4;GALNT1;GALNT3;GALNT4;GALNT7;GAS6;GCNT1;GCNT2;GCNT4;GLCE;GOLGA4;GOLPH3L;GOLT1B;GOPC;GORASP1;GPC1;GPSM1;GXYLT1;HACE1;HLA-A;HLA-DPB1;HLA-DRA;HS2ST1;HS6ST1;LEPROT;LFNG;LLGL1;LMAN1;LMAN2;LPCAT1;LPCAT2;MAN1A1;MAN1A2;MAN2A1;MANEA;MAP6D1;MBTPS2;M
EP57;CHCHD4;CHERP;CHM;CHML;CHMP1A;CHMP1B;CHMP2A;CHMP2B;CLEC5A;CLEC6A;CLIP3;COG3;COG4;COG6;COPE;COPZ1;CSK;CXCL10;DAB2IP;DDOST;DENND1B;DENND4C;DERL1;DMAP1;DPH3;DYRK2;ECT2;EIF5A2;EPS15;ERBB2IP;EXOC1;EXOC3;EXOC5;EXOC6;EXOC8;EXPH5;F2RL1;GAS6;GCC2;GDI1

;EIF5A2;ELOVL4;EPT1;ERGIC2;ERLIN1;ERN1;ERO1L;ERO1LB;EXTL2;FAM69A;FAR2;FDFT1;FITM1;FMO5;G6PC3;GPSM1;HLA-A;HLA-DPB1;HLA-DRA;HMOX2;IER3IP1;INSIG1;INSIG2;ITPR2;JKAMP;KTN1;LCLAT1;LMAN1;LMAN2;LPCAT1;LPCAT2;LPCAT4;LPGAT1;LRMP;LRRC8C;MAGT1;MAPK8IP1;MARCH1;MAR
CNA2;CCNC;CCNH;CCNL1;CCNT2;CD28;CD80;CDC73;CDCA7;CDK1;CDK11A;CDK11B;CDK2;CDK5R1;CDK5RAP3;CDK7;CDK8;CDK9;CDKN1B;CDKN1C;CDT1;CEBPA;CEBPG;CEBPZ;CENPK;CEP290;CGGBP1;CHAF1A;CHAF1B;CHD1;CHD5;CHD9;CHMP1A;CHUK;CHURC1;CKAP2;CNBP;CNOT2;CNOT3;CNOT6;CNOT7
DAB2IP;DAXX;DCLRE1A;DDB2;DDX11;DDX3X;DEK;DERL1;DGKZ;DIXDC1;DNA2;DNAJB9;DNAJC10;DUSP19;DYRK1A;DYRK2;E2F7;ECT2;EEF1E1;EGLN1;EIF2A;EIF2B5;ENDOV;EPC2;ERCC2;ERCC8;ERN1;ERO1L;F2RL1;FAM175A;FANCB;FANCL;FANCM;FAS;FBXO31;FBXO45;FBXW7;FCER1A;FER;FNIP1;GAB1;GAS
1A;BMPR2;BNIP2;BRCA2;BRCC3;BRD9;BRIP1;BRMS1;BRMS1L;BRWD1;BTAF1;BZW1;C12orf52;C14orf166;CALCRL;CAMK4;CARHSP1;CASP8AP2;CBFB;CBX3;CCDC59;CCDC88A;CCNA2;CCNC;CCNH;CCNL1;CCNT2;CD28;CD80;CDC42EP2;CDC73;CDCA7;CDK1;CDK11A;CDK11B;CDK2;CDK5R1;CDK5RAP3;CDK7;C
NK1G3;CSNK2B;DAK;DAPK3;DCAKD;DCK;DGKE;DGKZ;DLG1;DMPK;DYRK1A;DYRK1B;DYRK2;EFNA3;ENG;EPHB3;EPHB6;EPT1;ERCC2;ERN1;ETNK1;FAM20C;FASTKD1;FASTKD3;FER;FGFR1OP;FGFR4;FN3K;FPGT;GAK;GK;GMPPA;GRK6;GTF2H1;GTF2H4;GUCY2C;ICK;ILK;IPMK;ISPD;ITPKC;JAK2;KIAA1804;KNDC1;

ARHSP1;CASP8AP2;CBFB;CBX3;CCDC59;CCNA2;CCNC;CCNH;CCNL1;CCNT2;CD28;CD80;CDC40;CDC73;CDCA7;CDK1;CDK11A;CDK11B;CDK2;CDK5R1;CDK5RAP3;CDK7;CDK8;CDK9;CDKN1B;CDKN1C;CDT1;CEBPA;CEBPG;CEBPZ;CENPK;CEP290;CGGBP1;CHAF1A;CHAF1B;CHD1;CHD5;CHD9;CHMP1A;CHUK;CHU
;EIF5A2;ELOVL4;EPT1;ERGIC2;ERLIN1;ERN1;ERO1L;ERO1LB;EXTL2;FAM69A;FAR2;FDFT1;FITM1;FMO5;G6PC3;GPSM1;HLA-A;HLA-DPB1;HLA-DRA;HMOX2;IER3IP1;INSIG1;INSIG2;ITPR2;JKAMP;KTN1;LCLAT1;LMAN1;LMAN2;LPCAT1;LPCAT2;LPCAT4;LPGAT1;LRMP;LRRC8C;MAGT1;MAPK8IP1;MARCH1;MAR
;FARSA;FDXACB1;FMR1;FXR1;FXR2;FYTTD1;HBS1L;HNRNPH2;HNRNPL;HNRPLL;IGF2BP3;IGHMBP2;IMPDH2;IREB2;JMJD6;KIAA0020;KIN;KRR1;LARP1B;LARP4;LIN28A;LONP1;LRRC47;LSM1;LSM11;LSM3;LSM6;LUC7L3;MATR3;MBNL1;MBNL2;MCTS1;MEF2C;METTL1;MEX3C;MKI67IP;MOV10;MPHOSPH6;M
BS7;BCKDHB;BIRC2;BLOC1S2;BMF;BMI1;BNIP1;BRCA2;BRCC3;BTBD1;BUD13;C14orf166;C14orf2;C1QA;C1QB;C2orf49;C3orf58;CACNA1H;CACNB3;CACNB4;CAMK2D;CAPG;CAPZA1;CAPZA2;CARD8;CARHSP1;CASC5;CASP8;CATSPERB;CATSPERG;CBLL1;CBX3;CCDC53;CCNC;CCND3;CCNH;CCNY;CCT2;CD2AP;
HRS9;DLG1;DMPK;DNAJB9;DNAJC10;DOLPP1;DPM1;DTNBP1;EDEM3;EIF5A2;ELOVL4;EPT1;ERGIC2;ERLEC1;ERLIN1;ERN1;ERO1L;ERO1LB;EXTL2;FAM69A;FAR2;FDFT1;FITM1;FKBP10;FKBP7;FMO5;G6PC3;GAS6;GPSM1;HLA-A;HLA-DPB1;HLA-DRA;HMOX2;IER3IP1;INSIG1;INSIG2;ITPR2;JKAMP;KDELC2;KTN1;I
BNIP3L;CASP3;CASP8;CCBL2;CCNH;CDC26;CDC27;CDC42EP2;CDK1;CDK2;CDK7;CHFR;CHN2;CISD2;CLN3;CLN6;CNN3;CNOT2;CNOT3;CNOT6;CNOT6L;CNOT7;CNOT8;CNP;CNR1;COLQ;COMT;CRAT;CRBN;CRK;CROT;CST3;CTSH;CUL2;CUL4B;CUL5;CYCS;DAB2IP;DAPK3;DCP1A;DCP2;DCPS;DDB2;DDX3X;DECR1;
ARHSP1;CASP8AP2;CBFB;CBX3;CCDC59;CCNA2;CCNC;CCNH;CCNL1;CCNT2;CD28;CD80;CDC40;CDC73;CDCA7;CDK1;CDK11A;CDK11B;CDK2;CDK5R1;CDK5RAP3;CDK7;CDK8;CDK9;CDKN1B;CDKN1C;CDT1;CEBPA;CEBPG;CEBPZ;CENPK;CEP290;CGGBP1;CHAF1A;CHAF1B;CHD1;CHD5;CHD9;CHMP1A;CHUK;CHU
;PMS1;POLD2;POLG2;POLI;POLK;POLQ;POLR2E;POLR2K;PPP4C;PPP4R2;PSMD14;RAD17;RAD23A;RAD51AP1;RAD51B;RAD51C;RBBP8;RBX1;RECQL;RECQL5;REV1;REV3L;RFC2;RFC3;RFC5;RPS27L;RRM2B;RUVBL2;SFR1;SHFM1;SIRT1;SLC30A9;SMARCA5;SMARCAD1;SMC2;SMC4;SMC5;SMC6;SMG1;SUMO1;
;DHX30;DLAT;DMPK;DNA2;ECHS1;ERAL1;ETFA;FARS2;GK;GLS;GPAM;GPAT2;GPD2;GRPEL2;GUF1;HARS2;HIGD1A;HMGCL;HSD17B10;HSPE1;IDH2;IDH3B;IDH3G;IMMP1L;KIAA1967;KMO;L2HGDH;LONP1;LRRK2;MAPK8IP1;MARCH5;MARS2;MCEE;MCL1;MCU;MDH2;MFF;MFN1;MPV17;MRPL13;MRPL18;M
QKI;RALY;RBFOX3;RBM10;RBM11;RBM25;RBM39;RBM4;RBM5;RNPC3;SART1;SCAF11;SF3A1;SF3A2;SF3B1;SF3B14;SFSWAP;SKIV2L2;SLU7;SNRNP27;SNRNP35;SNRNP48;SNRPB;SNRPB2;SNRPD1;SNRPE;SNRPG;SREK1;SREK1IP1;SRSF10;SRSF11;SRSF3;SRSF6;SRSF8;SUGP1;THOC3;THOC7;TIA1;TRA2
4;CARHSP1;CASP8AP2;CBFB;CBX3;CCDC59;CCNA2;CCNC;CCNH;CCNL1;CCNT2;CD28;CD46;CD80;CDC73;CDCA7;CDK1;CDK11A;CDK11B;CDK2;CDK5R1;CDK5RAP3;CDK7;CDK8;CDK9;CDKN1B;CDKN1C;CDKN2AIP;CDT1;CEBPA;CEBPG;CEBPZ;CENPK;CEP290;CFH;CFP;CGGBP1;CHAF1A;CHAF1B;CHD1;CHD5;CHD
BBS10;BBS12;BCR;BMPR1A;BMPR2;BRIP1;BTAF1;C10orf2;CAMK1;CAMK2D;CAMK4;CBWD1;CBWD2;CCT2;CCT6B;CDC7;CDK1;CDK11A;CDK11B;CDK17;CDK18;CDK2;CDK7;CDK8;CDK9;CDKL1;CDKL2;CENPE;CHD1;CHD5;CHD9;CHUK;CKB;CLCN7;CLK1;CLK3;CLK4;CLP1;CMPK1;COASY;CSK;CSNK1A1;CSNK1A1L
AURKC;BAG4;BCR;BIN1;BIRC3;BMPR1A;BMPR2;BNIP2;CAB39;CACNB4;CALCRL;CAMK1;CAMK2D;CAMK4;CASC5;CASP3;CCDC88A;CCNA2;CCNC;CCND3;CCNE2;CCNG1;CCNH;CCNL1;CCNT2;CCNY;CCNYL1;CD2BP2;CD80;CD81;CDC25C;CDC37;CDC42EP2;CDIPT;CDK1;CDK11A;CDK11B;CDK17;CDK18;CDK2;CDI
UL2;CUL4B;CUL5;DAPK3;DBF4;DDX3X;DGKZ;DICER1;DLG1;DNA2;DYNLT3;E2F7;ECT2;EIF4E;ERCC2;EREG;ERN1;FAM175A;FBXO31;FBXO5;FBXW7;FIGNL1;GAS2;GAS2L2;GAS6;GMNN;GTF2H1;HDAC3;HDAC8;HORMAD1;HPGD;HSF1;IL12A;IL8;ILK;JMY;JUN;KAT2B;KIAA0101;KIF20B;KIF23;LEMD3;LRP6;MAD2L
MK1;CAMK4;CARD8;CASC5;CCDC91;CCT6B;CD27;CD274;CD63;CD81;CDC37;CDK1;CDK5R1;CEP120;CEP250;CEP290;CEP57;CEP57L1;CHCHD4;CHERP;CHM;CHML;CHMP1A;CHMP1B;CHMP2A;CHMP2B;CLEC5A;CLEC6A;CLIP3;COG3;COG4;COG6;COLQ;COPE;COPZ1;CRIPT;CSK;CTDNEP1;CXCL10;DAB2IP;DBN1
A;BAZ2B;BBX;BCLAF1;BDH2;BDP1;BIRC2;BLOC1S2;BLZF1;BMI1;BMPR1A;BMPR2;BRCA2;BRD9;BRIP1;BRMS1;BRMS1L;BRWD1;BTAF1;BZW1;C10orf2;C12orf52;C14orf166;CALCRL;CAMK4;CARHSP1;CASP8AP2;CBFB;CBX3;CCDC59;CCNA2;CCNC;CCNH;CCNL1;CCNT2;CD28;CD80;CDC40;CDC73;CDCA7;CDK1;C
L;BAZ1A;BBS7;BCAS2;BCLAF1;BIN1;BLOC1S2;BMF;BMI1;BRCA2;BRWD1;BTBD1;BTD;BYSL;C10orf2;C12orf52;C14orf166;C8orf40;CAMK4;CAPG;CAPZA1;CAPZA2;CARHSP1;CASC5;CASP8;CBX3;CC2D1A;CCDC14;CCDC39;CCDC6;CCDC86;CCDC92;CCP110;CCT2;CD2AP;CDC14A;CDC27;CDC42EP1;CDC42EP2;CD
L;BAZ1A;BBS7;BCAS2;BCLAF1;BIN1;BLOC1S2;BMF;BMI1;BRCA2;BRWD1;BTBD1;BTD;BYSL;C10orf2;C12orf52;C14orf166;C8orf40;CAMK4;CAPG;CAPZA1;CAPZA2;CARHSP1;CASC5;CASP8;CBX3;CC2D1A;CCDC14;CCDC39;CCDC6;CCDC86;CCDC92;CCP110;CCT2;CD2AP;CDC14A;CDC27;CDC42EP1;CDC42EP2;CD

AURKC;BBS10;BBS12;BCR;BMPR1A;BMPR2;BRIP1;BTAF1;C10orf2;CAMK1;CAMK2D;CAMK4;CBWD1;CBWD2;CCT2;CCT6B;CDC7;CDK1;CDK11A;CDK11B;CDK17;CDK18;CDK2;CDK7;CDK8;CDK9;CDKL1;CDKL2;CENPE;CHD1;CHD5;CHD9;CHUK;CKB;CLCN7;CLK1;CLK3;CLK4;CLP1;CMPK1;COASY;CSK;CSNK1A1;CSN
MAP6D1;MARK4;MID1IP1;NDE1;NEK7;NIN;PEX14;PKD2;PLK1S1;PLK4;PPP1R12A;PPP4C;PRKCZ;RB1;RNF19A;RSPH4A;SASS6;SENP6;SKA2;SLAIN2;SPAG5;SPAST;SPIRE2;SS18;TBCD;TPPP3;TRPV4;TTK;TTLL1;TUBA1B;TUBE1;TUBG1;TUBGCP2;WEE1;XPC
LOC1S2;BLZF1;BMI1;BMPR1A;BMPR2;BRCA2;BRD9;BRIP1;BRMS1;BRMS1L;BRWD1;BTAF1;BZW1;C10orf2;C12orf52;C14orf166;CALCRL;CAMK4;CARHSP1;CASP8AP2;CBFB;CBX3;CCDC59;CCNA2;CCNC;CCNH;CCNL1;CCNT2;CD28;CD80;CDC40;CDC73;CDCA7;CDK1;CDK11A;CDK11B;CDK2;CDK5R1;CDK5RAP3;
A3;SLC9A6;SNX10;SNX13;SNX16;SNX18;SNX2;SNX25;SNX3;SNX4;SNX8;STAM2;STARD3;STARD3NL;STEAP2;STX7;TAB1;TAB2;TAB3;TBK1;TICAM1;TLR8;TMEM106B;TMEM184A;TMEM55A;UBC;UBE2D3;VAC14;VAMP7;VPS18;VPS26A;VPS28;VPS29;VPS33B;VPS37A;VPS37B;VPS4A;VPS4B;VTA1;WDR44;ZFYVI

;PIGW;PRKCSH;PROC;PROZ;SAR1B;SEC13;SEC23A;SEC24A;STT3B;SUMF2;UGGT
GL1;SLC29A3;SLC9A6;SNX10;SNX13;SNX16;SNX18;SNX2;SNX25;SNX3;SNX4;SNX8;STAM2;STARD3;STARD3NL;STEAP2;STX7;TAB1;TAB2;TAB3;TBK1;TICAM1;TLR8;TMEM106B;TMEM184A;TMEM55A;UBC;UBE2D3;VAC14;VAMP7;VPS18;VPS26A;VPS28;VPS29;VPS33B;VPS37A;VPS37B;VPS4A;VPS4B;VTA1;WI

AURKC;BBS10;BBS12;BCR;BMPR1A;BMPR2;BRIP1;BTAF1;C10orf2;CAMK1;CAMK2D;CAMK4;CBWD1;CBWD2;CCT2;CCT6B;CDC7;CDK1;CDK11A;CDK11B;CDK17;CDK18;CDK2;CDK7;CDK8;CDK9;CDKL1;CDKL2;CENPE;CHD1;CHD5;CHD9;CHUK;CKB;CLCN7;CLK1;CLK3;CLK4;CLP1;CMPK1;COASY;CSK;CSNK1A1;CSN
PC2P1;TRAPPC8;USO1;VAMP4;VAMP7;VPS13A;YKT
BNIP2;BRCA2;BRCC3;BRD9;BRIP1;BRMS1;BRMS1L;BRWD1;BTAF1;BZW1;C12orf52;C14orf166;CALCRL;CAMK4;CARHSP1;CASP8AP2;CBFB;CBX3;CCDC59;CCDC88A;CCNA2;CCNC;CCNH;CCNL1;CCNT2;CD28;CD80;CDC42EP2;CDC73;CDCA7;CDK1;CDK11A;CDK11B;CDK2;CDK5R1;CDK5RAP3;CDK7;CDK8;CDK9;C
LZF1;BMI1;BMPR1A;BMPR2;BRCA2;BRD9;BRIP1;BRMS1;BRMS1L;BRWD1;BTAF1;BZW1;C10orf2;C12orf52;C14orf166;CALCRL;CAMK4;CARHSP1;CASP8AP2;CBFB;CBX3;CCDC59;CCNA2;CCNC;CCNH;CCNL1;CCNT2;CD28;CD80;CDC40;CDC73;CDCA7;CDK1;CDK11A;CDK11B;CDK2;CDK5R1;CDK5RAP3;CDK7;CDK

SWAP;SIRT1;SKIV2L2;SLU7;SNRPB;SNRPD1;SNRPE;SNRPG;SRSF10;SRSF6;SURF6;SUV39H1;UTP11L;UTP14C;UTP15;UTP23;WDR
LRWD1;LSM11;MAP3K11;MCM5;MDM2;MNAT1;MTBP;MUC1;NBN;NDE1;NEDD1;NFATC1;ORC2;ORC3;ORC4;PKD2;PKIA;PLCB1;PLK4;POLD2;PPAT;PPP2R1A;PPP3CA;PPP6C;PSMA1;PSMA2;PSMA3;PSMA4;PSMA6;PSMA8;PSMB1;PSMB3;PSMC1;PSMC2;PSMC3;PSMC5;PSMC6;PSMD10;PSMD13;PSMD14;PS
V1G1;ATP8A1;AURKC;BAG4;BAG6;BCAP29;BCAP31;BET1;BLZF1;BMPR1A;BORA;BUD13;C5orf30;CALCRL;CAMK1;CAMK4;CARD8;CASC5;CCDC91;CCT6B;CD27;CD274;CD63;CD81;CDC37;CDC40;CDK1;CDK5R1;CEP120;CEP250;CEP290;CEP57;CEP57L1;CHCHD4;CHERP;CHM;CHML;CHMP1A;CHMP1B;CHMP2
BCR;BIN1;BIRC3;BMPR1A;BMPR2;BNIP2;CAB39;CACNB4;CALCRL;CAMK1;CAMK2D;CAMK4;CASC5;CASP3;CCDC88A;CCNA2;CCNC;CCND3;CCNE2;CCNG1;CCNH;CCNL1;CCNT2;CCNY;CCNYL1;CD2BP2;CD80;CD81;CDC25C;CDC37;CDC42EP2;CDIPT;CDK1;CDK11A;CDK11B;CDK17;CDK18;CDK2;CDK5R1;CDK5RA
PR1A;BMPR2;BRCA2;BRD9;BRIP1;BRMS1;BRMS1L;BRWD1;BTAF1;BZW1;C10orf2;C12orf52;C14orf166;CALCRL;CAMK4;CARHSP1;CASP8AP2;CBFB;CBX3;CCDC59;CCNA2;CCNC;CCNH;CCNL1;CCNT2;CD28;CD80;CDC40;CDC73;CDCA7;CDK1;CDK11A;CDK11B;CDK2;CDK5R1;CDK5RAP3;CDK7;CDK8;CDK9;CDKN1

LRWD1;LSM11;MAP3K11;MCM5;MDM2;MNAT1;MTBP;MUC1;NBN;NDE1;NEDD1;NFATC1;ORC2;ORC3;ORC4;PKD2;PKIA;PLCB1;PLK4;POLD2;PPAT;PPP2R1A;PPP3CA;PPP6C;PSMA1;PSMA2;PSMA3;PSMA4;PSMA6;PSMA8;PSMB1;PSMB3;PSMC1;PSMC2;PSMC3;PSMC5;PSMC6;PSMD10;PSMD13;PSMD14;PS
APK2;MCM5;MDM2;MEN1;MSH2;MTBP;MUC1;NBN;ORC2;ORC3;ORC4;PKD2;PLAGL1;PPM1G;PPP1R15A;PRCC;PRKAA1;PRKAB2;PRKACB;PRKAG2;PSMA1;PSMA2;PSMA3;PSMA4;PSMA6;PSMA8;PSMB1;PSMB3;PSMC1;PSMC2;PSMC3;PSMC5;PSMC6;PSMD10;PSMD13;PSMD14;PSMD4;PSMD8;PSME1;PTE

AP2K2;MAP3K7;MARCH1;MCOLN1;MITD1;MTMR2;NDFIP1;NDFIP2;NIPA1;OCIAD1;PIKFYVE;PKN1;PRDX3;PRKCI;PRKCZ;PTP4A1;PTPN23;RAB10;RAB11B;RAB11FIP2;RAB12;RAB14;RAB22A;RAB27B;RAB4A;RAB5A;RABEP1;RAP2A;RAP2C;RET;RNF11;RNF13;SCAMP1;SCOC;SCYL2;SDF4;SH3GL1;SIAH1;SLC29A3

4;PSMD4;PSMD8;PSME1;RAD17;RB1;RBBP8;RFC2;RFC3;RFC5;RFWD2;RINT1;RPS27L;TAOK1;TIPRL;TP63;TRIAP1;TTK;UBC;UBE2D1;UBE2E1;USP44;WEE1;ZWILC

0;PSMD13;PSMD14;PSMD4;PSMD8;PSME1;RAD17;RASSF1;RB1;RBBP8;RFC2;RFC3;RFC5;RFWD2;RINT1;RPS27L;RRP8;TAOK1;TFAP4;TIPRL;TP63;TRIAP1;TTK;UBC;UBE2D1;UBE2E1;USP44;WEE1;ZWILC
RARS2;RPF1;RPL11;RPL26;RPL7;RPS24;RPS7;RRP1;RRP36;RRP8;SEPSECS;SIRT1;SKIV2L2;SMAD2;SNRPB;SNRPD1;SNRPE;SNRPG;SUV39H1;TARS2;TRDMT1;TRMT12;TRNT1;TRUB1;TYW3;TYW5;UTP11L;UTP14C;UTP15;UTP23;VARS;WARS2;WDR36;XR

F1;ITGB3BP;KATNB1;KIAA1009;KIF11;KIF18A;KIF20B;KIF22;KIF23;KIF2A;KIFC1;KLHDC3;KLHL9;KPNA2;LEPREL4;LFNG;LRRCC1;MAD2L1;MAP9;MDK;MIS18BP1;MLF1IP;MPHOSPH6;MSH2;NBN;NDE1;NEDD1;NEK1;NEK7;NSL1;NSMCE2;NUDC;NUF2;NUP37;NUP85;OIP5;OSGIN2;PAPD5;PBK;PDS5B;PES1;PHIP;P

;DLG1;DMPK;DYRK1A;DYRK1B;DYRK2;EFNA3;ENG;EPHB3;EPHB6;ERCC2;ERN1;ETNK1;FAM20C;FASTKD1;FASTKD3;FER;FGFR1OP;FGFR4;FN3K;GAK;GK;GRK6;GTF2H1;GTF2H4;GUCY2C;ICK;ILK;IPMK;ITPKC;JAK2;KIAA1804;KNDC1;LRRK2;MAP2K2;MAP2K4;MAP2K5;MAP3K1;MAP3K11;MAP3K13;MAP3K14;M
JS6;HAUS7;HELLS;ITGB3BP;KATNB1;KIAA1009;KIF11;KIF20B;KIF23;KIF2A;KIFC1;KLHL22;KLHL9;LIG4;LRP6;LRRCC1;MAD2L1;MAP9;MDK;MEN1;MIS18BP1;MITD1;NDE1;NEDD1;NEK1;NEK7;NSL1;NSMCE2;NUDC;NUF2;NUP37;OIP5;PAPD5;PARD6B;PDCD6IP;PDS5B;PKN2;PMF1;PPP1CA;PPP1CB;PPP1CC;PRPF

H;CDC26;CDC27;CDC42EP2;CDK1;CDK2;CDK7;CDKN1B;CHFR;CHID1;CHN2;CLN3;CLN5;CLN6;CNN3;CNOT2;CNOT3;CNOT6;CNOT6L;CNOT7;CNOT8;CNP;CNR1;COLQ;COMT;CRAT;CRBN;CRK;CROT;CST3;CTBS;CTSH;CUL2;CUL4B;CUL5;CYCS;DAB2IP;DAGLB;DCP1A;DCP2;DCPS;DDB2;DDX3X;DECR1;DERL1;DHPS

L;SGMS2;SLC24A6;SSR3;ST8SIA4;STEAP2;SYP;TECR;TIMM17A;TIMM23;TMEM66;TMEM70;TOMM40;WFS
ERN1;FAM20C;FASTKD1;FASTKD3;FER;FGFR1OP;FGFR4;GAK;GRK6;GTF2H1;GTF2H4;GUCY2C;ICK;ILK;JAK2;KIAA1804;KNDC1;LRRK2;MAP2K2;MAP2K4;MAP2K5;MAP3K1;MAP3K11;MAP3K13;MAP3K14;MAP3K2;MAP3K7;MAP3K8;MAP4K1;MAP4K3;MAP4K5;MAPK6;MAPK8;MAPKAPK2;MAPKAPK3;MARK4
RPS17;MRPS18B;MRPS18C;MRPS22;MRPS28;MRPS31;MRPS33;MTERF;NARS2;NR3C1;OXCT1;PC;PCK2;PDHX;PDK1;PDK2;PDP1;PIN4;PMPCA;POLDIP2;POLG2;PPIF;RARS2;SHMT2;SUCLA2;SUCLG2;SUOX;TARS2;TFAM;TOP1MT;WAR
F1;BDP1;BIRC2;BLOC1S2;BLZF1;BMI1;BMPR1A;BMPR2;BRCA2;BRD9;BRIP1;BRMS1;BRMS1L;BRWD1;BTAF1;BZW1;C10orf2;C12orf52;C14orf166;CACNA1H;CALCRL;CAMK4;CARHSP1;CASP8AP2;CBFB;CBX3;CCDC59;CCNA2;CCNC;CCNH;CCNL1;CCNT2;CD28;CD80;CDC40;CDC73;CDCA7;CDK1;CDK11A;CDK11B

F1;STRADA;WDTC

;SNRPD1;SNRPE;SNRPG;SRSF10;SRSF11;SRSF3;SRSF6;SUGP1;TIA1;TRA2A;TRA2B;U2AF1;WBP4;XAB2;ZCCHC

P3K11;MAP3K13;MAP3K2;MAPK6;MAPK8IP1;MAPK8IP2;MEF2A;MSH2;NBEA;NFATC1;PARK2;PAX6;PDE3B;PDLIM5;PFKL;PFKM;PJA2;PKN1;PLK1S1;PPP1CB;PPP1CC;PPP1R12A;PPP2R5A;PRDX3;PRKAA1;PRKAG2;PRKCSH;PRKCZ;PRPSAP1;PTEN;PTPN22;RAB11FIP2;RB1;RB1CC1;RHEB;RNF138;RPS6KA3;RPS6K
3;DYRK2;EFNA3;ENG;EPHB3;EPHB6;ERCC2;ERN1;ETNK1;FAM20C;FASTKD1;FASTKD3;FER;FGFR1OP;FGFR4;GAK;GK;GRK6;GTF2H1;GTF2H4;GUCY2C;ICK;ILK;IPMK;ITPKC;JAK2;KIAA1804;KNDC1;LRRK2;MAP2K2;MAP2K4;MAP2K5;MAP3K1;MAP3K11;MAP3K13;MAP3K14;MAP3K2;MAP3K7;MAP3K8;MAP4K1;
ATNB1;KLHL2;KPNA2;KPNA3;KPNA5;LMNA;MDFIC;MDM2;MFF;MGEA5;MTX2;MTX3;NAGPA;NAPB;NAPG;NCKIPSD;NEDD4;NFASC;NFKBIE;NOP58;NUP54;PACS2;PAN3;PEX1;PEX12;PEX13;PEX14;PEX2;PEX3;PGAP1;PHIP;PKD2;PKIA;PMPCA;PPP3CA;PRKCI;PSMD10;PTGS2;RAB10;RAB3GAP2;RAB6A;RAB8B;R
UBE2E1;UBE2N;UBR5;USP44;VPS28;WFS
CYCS;CYHR1;DDX3X;DHFRL1;DHRS1;DMPK;DTX2;EIF5A2;ERAL1;ERBB2IP;ERN1;FAM169A;FAM188A;FANCL;GCH1;GK;GMCL1;GPAM;GPAT2;GPD2;GRPEL2;GUF1;HIGD1A;HMGCL;HSD17B10;IDH2;IL4I1;IMMP1L;INTS2;IPO11;IPO13;IPO4;KMO;KPNA3;L2HGDH;LEMD3;LMNA;LMNB2;LRRK2;MAD2L1;MAPK8I
A;LYST;MALT1;MAP2K2;MDFIC;MDM2;MFF;MGEA5;MON2;MTMR2;MTX2;MTX3;NAGPA;NCKIPSD;NEDD4;NEMF;NFKBIE;NOP58;OSBPL5;P2RX4;PEX1;PEX12;PEX13;PEX14;PEX2;PEX3;PHAX;PHIP;PKD2;PKIA;PMPCA;PPP3CA;PSMD10;PTGS2;RAB10;RAB12;RAB14;RAB2A;RAB3IP;RAB6A;RAB8B;RAB9B;RAE1

RPL39L;RPL41;RPL7;RPL9;RPS15A;RPS24;RPS27;RPS27L;RPS29;RPS3A;RPS7;SERP1;ZCCHC
F1;HAUS7;HINT3;HMGN5;IDH3G;INO80C;JMJD6;KIAA0020;KIF20B;KLHL7;KRR1;LEPREL4;LIN28A;LYAR;MAK16;MAN1A2;MBIP;MDFIC;MDM2;MINA;MKI67IP;MORF4L2;MPHOSPH6;NBN;NEK1;NKRF;NMD3;NOC2L;NOC3L;NOC4L;NOL6;NOP2;NOP56;NOP58;NSA2;NUDT16;OLA1;OXR1;PES1;PHF6;PIN4;PLK4;

MRPL37;MRPL42;MRPL47;MRPL48;MRPS10;MRPS17;MRPS18B;MRPS18C;MRPS2;MRPS33;MTIF3;MTRF1;NARS2;NCK1;PAIP1;PAIP2;PIWIL4;POLG2;PPA1;PPP1R15A;PPP1R15B;PUM2;QKI;RARS2;RBM3;RBM4;RMND1;RNF139;RPL11;RPL15;RPL17;RPL22L1;RPL23;RPL26;RPL26L1;RPL27;RPL30;RPL31;RPL34

CHORDC1;CKS1B;CKS2;CLIP3;CLN3;CLN6;CNPPD1;CPEB2;CPEB3;CREBL2;CRIPAK;CRK;CSK;CSNK2B;CTBP1;CXXC1;DAB2IP;DAPK3;DAXX;DDX11;DDX3X;DLG1;DNAJB2;DNAJB9;DNAJC10;DUSP19;DYRK1A;ECT2;EEFSEC;EID1;EIF2A;EIF2B5;EIF3E;EIF4E;EIF4E2;EIF4E3;EIF5;EIF5A2;ENG;EREG;ERN1;FBXO4;FBXO5

YW3;TYW5;UTP11L;UTP14C;UTP15;UTP23;WDR

A5;NDUFA6;NDUFAF1;NDUFB1;NDUFB3;NDUFB5;NDUFS2;NDUFS4;NDUFS5;NIPSNAP1;NRM;OMA1;OPA1;PC;PLA2G4A;PMPCA;PPIF;PSEN2;RNF13;SDHD;SHMT2;SIRT1;SLC24A6;SLC25A1;SLC25A10;SLC25A11;SLC25A22;SLC25A24;SLC25A3;SLC25A32;SLC25A34;SLC25A36;SLC25A38;SLC25A40;SLC25A43;S

;SRSF10;SRSF11;SRSF3;SRSF6;SUGP1;TIA1;TRA2A;TRA2B;U2AF1;WBP4;XAB2;ZCCHC
;SRSF10;SRSF11;SRSF3;SRSF6;SUGP1;TIA1;TRA2A;TRA2B;U2AF1;WBP4;XAB2;ZCCHC
23;TIMM8B;TOMM20;TOMM40;TOMM5;TRAM1;TRNT1;VAMP7;ZFAND6;ZFYVE

CYCS;CYHR1;DDX3X;DHFRL1;DHRS1;DMPK;DTX2;EIF5A2;ERAL1;ERBB2IP;ERN1;FAM169A;FAM188A;FANCL;GCH1;GK;GMCL1;GPAM;GPAT2;GPD2;GRPEL2;GUF1;HIGD1A;HMGCL;HSD17B10;IDH2;IL4I1;IMMP1L;INTS2;IPO11;IPO13;IPO4;KMO;KPNA3;L2HGDH;LEMD3;LMNA;LMNB2;LRRK2;MAD2L1;MAPK8I

B;ARL15;ARL2;ARL4A;ARL5A;ARL5B;ARL8B;ASNA1;ATAD1;ATAD2;ATAD2B;ATAD3A;ATL2;ATP10D;ATP11B;ATP11C;ATP13A2;ATP13A3;ATP2B1;ATP2C1;ATP6AP1;ATP7A;ATP8A1;ATP8A2;ATP8B4;ATPBD4;ATRX;AURKC;BBS10;BBS12;BCR;BMPR1A;BMPR2;BRIP1;BTAF1;C10orf2;CAMK1;CAMK2D;CAMK4;CBI

AC6;HOOK1;HOOK2;IGBP1;KATNB1;KIAA0101;KIF11;KIF18A;KIF20B;KIF22;KIF23;KIF27;KIF2A;KIF3A;KIF5B;KIFC1;KIFC3;KPNA2;KTN1;LMNA;LYST;MAP3K11;MAP6D1;MARK4;MID1IP1;MKKS;NDE1;NEK7;NIN;NME7;OPA1;PEX1;PEX13;PEX14;PKD2;PLK1S1;PLK4;PPP1R12A;PPP4C;PRKCZ;RAB6A;RB1;RHOT1;R
8L;TDP2;WEE

M17A;TIMM23;TMEM66;TMEM70;TOMM40;WF
LG4;DMAP1;DYRK2;ECT2;ERBB2IP;EXOC8;EXPH5;FCHO2;FGF7;FGFR1OP;FOPNL;GAS6;GCC2;GIPC1;GOLGA4;GOPC;GRIK5;GRPEL2;HDAC6;HDAC8;HGS;INPP5K;IPO11;IPO13;IPO4;JAK2;JUN;JUP;KATNB1;KIF5B;KLHL2;KPNA2;KPNA3;KPNA5;LAMTOR3;LEPROT;LMNA;LRWD1;MARCH5;MDFIC;MDM2;MFF;MG

2HGDH;LRRK2;MAPK8IP1;MARCH5;MCL1;MCU;MDH2;MFF;MFN1;MPV17;MSTO1;MTHFD2L;MTX2;MTX3;NDUFA1;NDUFA4;NDUFA5;NDUFA6;NDUFAF1;NDUFB1;NDUFB3;NDUFB5;NDUFS2;NDUFS4;NDUFS5;NIPSNAP1;OMA1;OPA1;PC;PGAM5;PLA2G4A;PMAIP1;PMPCA;PNPLA7;PPIF;PPP1CC;PSEN2;RHO

D1;MAP3K7;MAPK8;MBIP;MBTD1;MEN1;MIS18BP1;MLF1IP;MORF4L2;MSL2;MSL3;MUC1;MYSM1;NAA60;NOC2L;NR3C1;OIP5;PHF17;PKN1;PRDM5;PRMT1;RB1;RCBTB1;REST;RING1;RNF2;RNF40;RPS6KA4;RPS6KA5;RRP8;RUVBL2;RYBP;SETDB2;SIRT1;SIRT2;SMARCA1;SMARCA5;SMARCAD1;SMARCB1;SRE

11C;ATP6V1G1;ATP8A1;BCAP29;BCAP31;BET1;BLZF1;BMPR1A;BUD13;C5orf30;CA12;CACNB4;CALCRL;CAMK1;CAMK4;CARD8;CCDC91;CCT6B;CD27;CD274;CDC37;CDC40;CDK1;CDK5R1;CEP290;CEP57;CHCHD4;CHERP;CHM;CHML;CHMP1A;CHMP1B;CHMP2A;CHMP2B;CLEC5A;CLEC6A;CLIP3;CLN3;CNR1;C
;BIN1;BIRC2;BNIP1;BNIP2;BNIP3L;CASP3;CASP8;CCBL2;CCNH;CDC26;CDC27;CDC42EP2;CDK1;CDK2;CDK7;CDKN1B;CHFR;CHID1;CHN2;CISD2;CLN3;CLN5;CLN6;CNN3;CNOT2;CNOT3;CNOT6;CNOT6L;CNOT7;CNOT8;CNP;CNR1;COLQ;COMT;CRAT;CRBN;CRK;CROT;CST3;CTBS;CTSH;CUL2;CUL4B;CUL5;CYCS;DA

MPD1;SMPD4;SMPDL3A;SPTLC1;SRM;SULT1E
CHD9;CRBN;DCTN2;DDX11;DDX3X;DDX41;DDX50;DDX54;DDX59;DHX30;DHX36;DHX40;DICER1;DNA2;DNAH6;DNAI2;DYNC2LI1;DYNLRB1;DYNLT3;EEF1A1;EEFSEC;EIF5;ENTPD6;ERCC2;FANCM;FIGNL1;GBP3;GBP6;GFM2;GNA13;GNAI1;GNAO1;GNL1;GNL3;GTF2F2;GTF2H1;GTF2H4;GTPBP10;GTPBP2;GUF1
CHD9;CRBN;DCP2;DCPS;DCTN2;DDX11;DDX3X;DDX41;DDX50;DDX54;DDX59;DHX30;DHX36;DHX40;DICER1;DNA2;DNAH6;DNAI2;DOLPP1;DYNC2LI1;DYNLRB1;DYNLT3;EEF1A1;EEFSEC;EIF5;ENTPD6;ERCC2;FANCM;FIGNL1;GBP3;GBP6;GFM2;GNA13;GNAI1;GNAO1;GNL1;GNL3;GTF2F2;GTF2H1;GTF2H4;GTF
LG4;DMAP1;DYRK2;ECT2;ERBB2IP;EXOC8;EXPH5;FCHO2;FGF7;FGFR1OP;FOPNL;GAS6;GCC2;GIPC1;GOLGA4;GOPC;GRIK5;GRPEL2;HDAC6;HDAC8;HGS;INPP5K;IPO11;IPO13;IPO4;JAK2;JUN;JUP;KATNB1;KIF5B;KLHL2;KPNA2;KPNA3;KPNA5;LAMTOR3;LEPROT;LMNA;LRWD1;MARCH5;MDFIC;MDM2;MFF;MG

MS1;SGMS2;SLC27A1;SLC44A3;SMPD1;SMPD4;SMPDL3A;SPTLC1;SRM;SULT1A1;SULT1
RAC1;SERINC1;SGMS1;SGMS2;SLC27A1;SLC44A3;SPTLC1;SYNJ1;VAC1
HD1;CHD5;CHD9;CRBN;DCP2;DCPS;DCTN2;DDX11;DDX3X;DDX41;DDX50;DDX54;DDX59;DHX30;DHX36;DHX40;DICER1;DNA2;DNAH6;DNAI2;DOLPP1;DYNC2LI1;DYNLRB1;DYNLT3;EEF1A1;EEFSEC;EIF5;ENTPD6;ERCC2;FANCM;FIGNL1;GBP3;GBP6;GFM2;GNA13;GNAI1;GNAO1;GNL1;GNL3;GTF2F2;GTF2H1;G
CHD9;CRBN;DCP2;DCPS;DCTN2;DDX11;DDX3X;DDX41;DDX50;DDX54;DDX59;DHX30;DHX36;DHX40;DICER1;DNA2;DNAH6;DNAI2;DOLPP1;DYNC2LI1;DYNLRB1;DYNLT3;EEF1A1;EEFSEC;EIF5;ENTPD6;ERCC2;FANCM;FIGNL1;GBP3;GBP6;GFM2;GNA13;GNAI1;GNAO1;GNL1;GNL3;GTF2F2;GTF2H1;GTF2H4;GTF
SME1;PTGS2;REST;SCAP;SCP2;SIRT1;SMEK1;SMEK2;SREBF1;STARD3;STRADA;SULT1A1;SULT1B1;WDT
MAP3K2;MAPK6;MAPK8IP1;MAPK8IP2;MEF2A;MSH2;NBEA;NFATC1;PARK2;PAX6;PDE3B;PDLIM5;PJA2;PKN1;PLK1S1;PPP1CB;PPP1CC;PPP1R12A;PRDX3;PRKAG2;PRKCSH;PRKCZ;PTEN;RAB11FIP2;RB1CC1;RHEB;RNF138;RPS6KA3;RPS6KA4;RPTOR;SIRT1;SPAG16;SPAG9;SREBF1;STK11IP;TFPT;TGFBR2;TICAM

NBN;NSMCE2;NUDT16;ORC2;ORC3;ORC4;PHIP;PKD2;PKIA;PKN2;PLCB1;PLK4;PRCC;PSMA1;PSMA2;PSMA3;PSMA4;PSMA6;PSMA8;PSMB1;PSMB3;PSMC1;PSMC2;PSMC3;PSMC5;PSMC6;PSMD10;PSMD13;PSMD14;PSMD4;PSMD8;PSME1;PTEN;RAD17;RASSF1;RB1;RBBP8;RFC2;RFC3;RFC5;RFWD2;RINT1;RI

RPS6KA3;RPS6KA5;SPAG9;TAB1;TAB2;TAB3;TAOK1;TLR6;UBC;VEGFA;ZMYND11;ZNF622;ZNF67

MMP1L;KMO;L2HGDH;LRRK2;MAPK8IP1;MARCH5;MCL1;MCU;MDH2;MFF;MFN1;MPV17;MSTO1;MTHFD2L;MTX2;MTX3;NDUFA1;NDUFA4;NDUFA5;NDUFA6;NDUFAF1;NDUFB1;NDUFB3;NDUFB5;NDUFS2;NDUFS4;NDUFS5;NIPSNAP1;OMA1;OPA1;PC;PGAM5;PLA2G4A;PMAIP1;PMPCA;PNPLA7;PPIF;PPP1

RPS6KA3;RPS6KA5;SPAG9;TAB1;TAB2;TAB3;TAOK1;TLR6;UBC;VEGFA;ZMYND11;ZNF622;ZNF67

DLG4;ECT2;EPS15;FAS;FBXO5;FCHO1;FCHO2;FER;FERMT3;GCH1;GFPT1;GLS;GMNN;GNMT;GOPC;GRHPR;HDAC6;HLA-DMA;HMGB1;HMGCL;HNF1A;HPRT1;HSF4;IDE;IGHMBP2;IL1RAP;ILK;IMPDH2;INSIG1;JAK2;JMY;JUP;KCNA2;KCNAB2;KCTD11;KCTD12;KCTD18;KCTD2;KCTD3;KCTD6;KCTD9;KIF23;KPNA3;L

4;ARPP19;ARRB1;ASF1A;ASPM;ATAD1;ATG12;ATG4B;ATG4C;ATG5;ATG9A;ATL2;ATP10D;ATP11B;ATP11C;ATP2C1;ATP6V1G1;ATP7A;ATP8A1;ATP8A2;ATPAF2;ATXN7L3;AURKC;B3GNT2;BAG4;BAG6;BAIAP2;BAP1;BAZ1A;BBIP1;BBS10;BBS12;BBS7;BCAP31;BCLAF1;BCR;BET1;BIN1;BIRC2;BLOC1S2;BLZF1;BM

DLG4;ECT2;EPS15;FAS;FBXO5;FCHO1;FCHO2;FER;FERMT3;GCH1;GFPT1;GLS;GMNN;GNMT;GOPC;GRHPR;HDAC6;HLA-DMA;HMGB1;HMGCL;HNF1A;HPRT1;HSF4;IDE;IGHMBP2;IL1RAP;ILK;IMPDH2;INSIG1;JAK2;JMY;JUP;KCNA2;KCNAB2;KCTD11;KCTD12;KCTD18;KCTD2;KCTD3;KCTD6;KCTD9;KIF23;KPNA3;L

327B;RAB28;RAB2A;RAB2B;RAB33B;RAB37;RAB39B;RAB4A;RAB5A;RAB6A;RAB8B;RAB9B;RABL3;RALA;RAP1A;RAP1B;RAP2A;RAP2C;RASL11A;RHEB;RHOBTB3;RHOQ;RHOT1;RHOU;RIT1;RNGTT;RP2;RSG1;RTKN;SAR1A;SAR1B;SEPT10;SEPT14;SEPT7;SEPT9;SUCLG2;TRIM23;TUBA1B;TUBD1;TUBE1;TUBG1;TU

;SPAST;TBL1XR1;TOPORS;TTK;TUBG1;TUBG2;TUBGCP2;VRK1;WDR6

250;CNPPD1;CRY1;CSK;CST3;CSTA;CUL2;CUL4B;CUL5;DAB2IP;DAXX;DIAPH3;DIXDC1;DLG1;DLG4;DNAJC10;DOCK11;DUSP19;DZIP3;ECT2;EEF1A1;EID1;EIF4E2;ESR2;EXPH5;FANCL;FAS;FBXO5;FER;FGD2;FGD3;FGD4;FGFR1OP;FIZ1;FMNL1;FMNL2;FXYD7;FZD6;GCLM;GCSH;GDI1;GLRX3;GMNN;GOLGA4;GSTM

3X;DDX41;DEK;DENND4A;DMTF1;DNA2;DNASE1L1;DNASE2;DOT1L;DR1;DRAP1;E2F4;E2F6;E2F7;EBF4;ELF2;ELK4;ERCC2;ESR2;FANCM;FOXH1;FOXN2;GABPA;GABPB1;GATAD2A;GEN1;GPBP1;GRHL1;GTF2B;GTF2E1;GTF2E2;GTF2F2;GZF1;H2AFV;H2AFZ;HDAC3;HDAC5;HDGFRP2;HDX;HELLS;HLF;HLTF;HMGB

1;MAF;MEF2A;MEN1;MIS18BP1;MKI67IP;MLF1IP;MMS22L;MSH2;MUC1;NBN;NDE1;NEDD4;NFATC1;NOL6;NSL1;NSMCE2;NUDCD2;NUF2;NUP37;NUP85;OIP5;ORC2;ORC3;ORC4;PAX6;PDS5B;PES1;PHOX2A;PLCB1;PMF1;POLG2;POT1;PPP1CC;PPP1R12A;PPP2CB;PPP2R1A;PPP2R5A;PRPF4B;PSEN2;PSIP1;PU

2A;RAB27B;RAB28;RAB2A;RAB2B;RAB33B;RAB37;RAB39B;RAB4A;RAB5A;RAB6A;RAB8B;RAB9B;RABL3;RALA;RAP1A;RAP1B;RAP2A;RAP2C;RASL11A;RHEB;RHOBTB3;RHOQ;RHOT1;RHOU;RIT1;RNGTT;RP2;RSG1;RTKN;SAR1A;SAR1B;SEPT10;SEPT14;SEPT7;SEPT9;SUCLG2;TMEM173;TRIM23;TUBA1B;TUBD1

2A;RAB27B;RAB28;RAB2A;RAB2B;RAB33B;RAB37;RAB39B;RAB4A;RAB5A;RAB6A;RAB8B;RAB9B;RABL3;RALA;RAP1A;RAP1B;RAP2A;RAP2C;RASL11A;RHEB;RHOBTB3;RHOQ;RHOT1;RHOU;RIT1;RNGTT;RP2;RSG1;RTKN;SAR1A;SAR1B;SEPT10;SEPT14;SEPT7;SEPT9;SUCLG2;TMEM173;TRIM23;TUBA1B;TUBD1

JFS2;NDUFS4;NDUFS5;NIPSNAP1;OMA1;OPA1;PC;PLA2G4A;PMPCA;PPIF;PSEN2;SDHD;SHMT2;SLC24A6;SLC25A1;SLC25A10;SLC25A11;SLC25A22;SLC25A24;SLC25A3;SLC25A32;SLC25A34;SLC25A36;SLC25A38;SLC25A40;SLC25A43;SLC25A46;SPNS1;TIMM17A;TIMM22;TIMM23;TIMM8B;TMEM70;UCP2;UC

CVRL1;ACY3;ADA;ADAM10;ADAM15;ADAM22;ADAMTS5;ADAMTS6;ADAP1;ADC;ADCK1;ADCK2;ADCK3;ADD3;ADI1;ADM2;ADORA1;ADRBK1;ADRM1;ADSSL1;AEBP1;AEBP2;AFF4;AGAP2;AGAP3;AGBL3;AGER;AGFG2;AGGF1;AGL;AGPAT5;AGPS;AHR;AIDA;AIFM3;AIM2;AIMP1;AIMP2;AK3;AKAP11;AKAP5;AK[illegible]

M;GTF2F2;GTF2H1;GTF2H4;HLTF;IDE;IGHMBP2;KATNB1;KIF18A;KIF20B;LONP1;LONRF1;MNAT1;MSH2;NBN;PEX1;PMS1;POLQ;PPP2R4;PSMC1;PSMC2;PSMC5;PSMC6;RAD51B;RAD51C;RECQL;RECQL5;RFC3;RHOBTB3;RUVBL2;SKIV2L;SKIV2L2;SMARCA1;SMARCA5;SPAST;TOP1MT;UPF1;VPS4A;VPS4B;YTH[illegible]

VR2A;ACVRL1;ACY3;ADA;ADAM10;ADAM15;ADAM22;ADAMTS5;ADAMTS6;ADAP1;ADC;ADCK1;ADCK2;ADCK3;ADD3;ADI1;ADM2;ADORA1;ADRBK1;ADRM1;ADSSL1;AEBP1;AEBP2;AFF4;AGAP2;AGAP3;AGBL3;AGER;AGFG2;AGGF1;AGL;AGPAT5;AGPS;AHR;AIDA;AIFM3;AIM2;AIMP1;AIMP2;AK3;AKAP11;AK[illegible]

PPM1B;PPM1G;PPP1CA;PPP1CB;PPP1CC;PPP1R15B;PPP1R3D;PPP2CB;PPP2R1A;PPP2R2A;PPP3CA;PPP3CB;PPP4C;PPP5C;PPP6C;PTEN;PTP4A1;PTPDC1;PTPN12;PTPN2;PTPN22;PTPN23;PTPN4;PTPN7;PTPRO;RNGTT;RPAP2;SACM1L;SGPP1;SMPD1;SMPD4;SMPDL3A;SSU72;SYNJ1;TDP2;TENC1;UBLCP1;VPS[illegible]

[illegible];PSEN2;PTCHD2;PTGDS;RAE1;RANGAP1;RECQL5;RNF13;SEC13;SIRT1;SLC22A18;SLC2A9;SREBF1;SUMO1;TMEM201;TMEM38B;TMEM48;TMPO;TNKS2;TNPO1;TOR1A;TREX1;TSGA10;UBXN4;UNC50;WTAP;XP[illegible]

[illegible]12;IAH1;IMPA1;IMPAD1;INPP5D;INPP5K;LHPP;LIPE;LIPN;LYPLA1;LYPLAL1;MINPP1;MTMR2;MTMR6;MTMR7;MTMR9;MYSM1;N4BP2;NANP;NAPEPLD;NT5C3;NUDT16;PDE1B;PDE3B;PDE4A;PDE4D;PDE7A;PDP1;PGAM5;PGAP1;PGAP3;PIKFYVE;PLA2G15;PLA2G4A;PLCB1;PLCB2;PLCB3;PLCD3;PLCL1;PLCXD2

EF2A;MUC1;MYSM1;NCOA4;NCOA7;NFKB2;NFKBIB;NOC2L;NRIP1;PBXIP1;PEX14;PFDN5;PKN1;PLRG1;PMF1;PPP1R13L;PQBP1;PRPF6;PSIP1;PSMC3;PSMC5;RB1;RBBP8;RELB;RFXAP;RIPK3;RXRB;RYBP;SCAI;SIRT1;SKIL;SLC30A9;SMARCB1;SP4;SS18;SUB1;TADA1;TAF12;TAF7;TAF9;TAF9B;TBL1XR1;TBPL1;TDG

UCA1B;HMGB1;JUN;LLGL1;LRRK2;MALT1;MAP2K2;MAPK8IP2;MMP15;MMP17;MMP24;PIK3CA;PLCB1;PLXNB2;PPP2R4;PRKAG2;PSMD14;PTPLAD1;RAB3GAP2;RABEP1;RANGAP1;RASA1;RCVRN;RGS1;RGS12;RGS18;RGS5;RIN1;RP2;RPS27L;SH3BP1;SIPA1;STRADA;SYDE2;TAB1;TBC1D10B;TBC1D12;TBC1D1

;NSMCE2;NUDCD2;NUF2;NUP37;NUP85;OIP5;ORC2;ORC3;ORC4;PAX6;PDS5B;PHOX2A;PLCB1;PMF1;POT1;PPP1CC;PPP1R12A;PPP2CB;PPP2R1A;PPP2R5A;PSEN2;PSIP1;PURA;PURB;RAD17;RANGAP1;RB1;RFC2;RFC3;RFC5;RIF1;RING1;RNF2;RNF40;RRP8;RUVBL2;SEC13;SEPT7;SFR1;SGOL2;SIRT1;SKA2;SMA

IP1;GDI1;GIT1;GOPC;GPS1;GPS2;GPSM1;IQSEC2;JUN;KNDC1;LAMTOR3;LLGL1;LRRK2;MAP4K1;MAP4K3;MAP4K5;MCF2;MON2;P2RY12;PLCB1;PLEKHG3;PLXNB2;PTGIR;PTPLAD1;RAB3GAP2;RAB3IP;RABEP1;RALGPS2;RANGAP1;RAPGEF6;RASA1;RCBTB2;RGS1;RGS12;RGS18;RGS5;RIC8A;RIC8B;RIN1;RP2;RP

PTPN2;PTPN22;PTPN23;PTPN4;PTPN7;PTPRO;RNGTT;RPAP2;SACM1L;SGPP1;SSU72;SYNJ1;TENC1;UBLCP1;VPS2

YDE2;TBC1D10B;TBC1D12;TBC1D13;TBC1D15;TBC1D22A;TBC1D23;TBC1D25;TBC1D4;TBC1D8B;TBCD;TSC2;USP

A1;ASTE1;ASTL;ATAD1;ATAD2;ATAD2B;ATAD3A;ATG4A;ATG4B;ATG4C;ATL2;ATP10D;ATP11B;ATP11C;ATP13A2;ATP13A3;ATP2B1;ATP2C1;ATP5C1;ATP5E;ATP5F1;ATP5I;ATP5L;ATP5O;ATP6AP1;ATP6V1G1;ATP7A;ATP8A1;ATP8A2;ATP8B4;ATRX;BAP1;BMP1;BRCC3;BRIP1;BTAF1;BTD;C10orf2;C11orf54;C1F

;FGD4;FNIP1;GDI1;GIT1;GOPC;GPS1;GPS2;GPSM1;GRPEL2;IQSEC2;JUN;KNDC1;LAMTOR3;LLGL1;LRRK2;MAP4K1;MAP4K3;MAP4K5;MCF2;MON2;P2RY12;PLCB1;PLEKHG3;PLXNB2;PTGIR;PTPLAD1;RAB3GAP2;RAB3IP;RABEP1;RALGPS2;RANGAP1;RAPGEF6;RASA1;RCBTB2;RGS1;RGS12;RGS18;RGS5;RIC8A;R

DHHC2;ZDHHC20;ZDHHC21;ZDHHC

;PLOD3;POGLUT1;POMT2;PPAT;QPRT;RFNG;ST8SIA4;STT3B;TNKS2;TYMP;UGCG;UGGT2;UGT2B

ZNF563;ZNF671;ZNF69;ZNF773;ZNF78

4;ZNF563;ZNF671;ZNF69;ZNF773;ZNF78

2;ZNF544;ZNF563;ZNF671;ZNF69;ZNF773;ZNF78

F69;ZNF773;ZNF78

;ZNF544;ZNF563;ZNF671;ZNF69;ZNF773;ZNF78

671;ZNF69;ZNF773;ZNF78

;SIRT5;SLC25A17;SLC25A4;SUPT7L;TCEA3;TGFB3;UQCC;WDR77;ZNF133;ZNF202;ZNF211;ZNF232;ZNF266;ZNF329;ZNF343;ZNF419;ZNF44;ZNF530;ZNF542;ZNF544;ZNF563;ZNF671;ZNF69;ZNF773;ZNF7

;SIRT5;SLC25A17;SLC25A4;SUPT7L;TCEA3;TGFB3;UQCC;WDR77;ZNF133;ZNF202;ZNF211;ZNF232;ZNF266;ZNF329;ZNF343;ZNF419;ZNF44;ZNF530;ZNF542;ZNF544;ZNF563;ZNF671;ZNF69;ZNF773;ZNF7

IPT7L;TCEA3;TGFB3;WDR77;ZNF133;ZNF202;ZNF211;ZNF232;ZNF266;ZNF329;ZNF343;ZNF419;ZNF44;ZNF530;ZNF542;ZNF544;ZNF563;ZNF671;ZNF69;ZNF773;ZNF7

;OVGP1;PDE6B;PEX11B;PMS2P1;POLR1C;PPIE;PPIH;RBFA;RDH16;RPP38;RSAD1;SDCCAG3;SIRT5;SLC25A17;SLC25A4;STMN1;SUPT7L;TCEA3;TGFB3;UQCC;WDR77;ZNF133;ZNF185;ZNF202;ZNF211;ZNF232;ZNF266;ZNF329;ZNF343;ZNF419;ZNF44;ZNF530;ZNF542;ZNF544;ZNF563;ZNF671;ZNF69;ZNF773;ZN

L;SIRT5;SLC25A17;SLC25A4;SUPT7L;TCEA3;TGFB3;WDR77;ZNF133;ZNF202;ZNF211;ZNF232;ZNF266;ZNF329;ZNF343;ZNF419;ZNF44;ZNF530;ZNF542;ZNF544;ZNF563;ZNF671;ZNF69;ZNF773;ZNF7

P43;NVL;OVGP1;PDE6B;PEX11B;PMS2P1;POLR1C;PPIE;PPIH;RBFA;RDH16;RPP38;RSAD1;SDCCAG3;SIRT5;SLC25A17;SLC25A4;STMN1;SUPT7L;TCEA3;TGFB3;UQCC;WDR77;ZNF133;ZNF185;ZNF202;ZNF211;ZNF232;ZNF266;ZNF329;ZNF343;ZNF419;ZNF44;ZNF530;ZNF542;ZNF544;ZNF563;ZNF671;ZNF69;ZN
7;SUPT7L;TCEA3;TGFB3;WDR77;ZNF133;ZNF202;ZNF211;ZNF232;ZNF266;ZNF329;ZNF343;ZNF419;ZNF44;ZNF530;ZNF542;ZNF544;ZNF563;ZNF671;ZNF69;ZNF773;ZNF7[illegible]

MS2P1;POLR1C;PPIE;PPIH;PRPSAP2;PRR3;RDH16;RPP38;RSAD1;SIRT5;SLC25A17;SLC25A4;SMYD5;STMN1;TCEA3;TGFB3;WDR77;ZNF133;ZNF185;ZNF202;ZNF211;ZNF232;ZNF266;ZNF329;ZNF343;ZNF419;ZNF44;ZNF530;ZNF542;ZNF544;ZNF563;ZNF671;ZNF69;ZNF773;ZNF7

B3;WDR77;ZNF133;ZNF202;ZNF211;ZNF232;ZNF266;ZNF329;ZNF343;ZNF419;ZNF44;ZNF530;ZNF542;ZNF544;ZNF563;ZNF671;ZNF69;ZNF773;ZNF7[illegible]

ZNF563;ZNF671;ZNF69;ZNF773;ZNF78

;SIRT5;SLC25A17;SLC25A4;STMN1;SUPT7L;TCEA3;TGFB3;UQCC;WDR77;ZNF133;ZNF185;ZNF202;ZNF211;ZNF232;ZNF266;ZNF329;ZNF343;ZNF419;ZNF44;ZNF530;ZNF542;ZNF544;ZNF563;ZNF671;ZNF69;ZNF773;ZNF7

;SIRT5;SLC25A17;SLC25A4;STMN1;SUPT7L;TCEA3;TGFB3;UQCC;WDR77;ZNF133;ZNF185;ZNF202;ZNF211;ZNF232;ZNF266;ZNF329;ZNF343;ZNF419;ZNF44;ZNF530;ZNF542;ZNF544;ZNF563;ZNF671;ZNF69;ZNF773;ZNF7

NF542;ZNF544;ZNF563;ZNF671;ZNF69;ZNF773;ZNF78

GT1;TRAPPC3;UX

S25;RPS27A;RPS3;RPS4X;RPS5;RPS6;RPS8;RPSA;SKP1;SNRPD2;SNRPD3;SNUPN;STX8;SUCLG1;SUGT1;TRAPPC3;UQCRFS1;UXT;VDA

8;RPSA;SSBP1;SUGT1;UXT;ZNRD
8;RPSA;SSBP1;SUGT1;UXT;ZNRD

RPS18;RPS19;RPS20;RPS21;RPS23;RPS25;RPS27A;RPS3;RPS4X;RPS5;RPS6;RPS8;RPSA;SKP1;SNRPD2;SNRPD3;SNUPN;SRPRB;SSBP1;STX8;SUCLG1;SUGT1;TICAM2;TOMM6;TRAPPC3;UQCRFS1;UXT;VDAC3;ZNR
RPS19;RPS20;RPS21;RPS23;RPS25;RPS27A;RPS3;RPS4X;RPS5;RPS6;RPS8;RPSA;SKP1;SNRPD2;SNRPD3;SNUPN;SRPRB;SSBP1;STX8;SUCLG1;SUGT1;TICAM2;TOMM6;TRAPPC3;UQCRFS1;UXT;VDAC3;ZNR

RPSA;SNRPD3;SRPRB;SSBP1;TRNAU1AP;UXT;ZGLP1;ZNRD
PS8;RPSA;SNRPD2;SNRPD3;SNUPN;SRPRB;TRNAU1AP;UXT;ZGLP1;ZNRD

;RPS25;RPS27A;RPS3;RPS4X;RPS5;RPS6;RPS8;RPSA;SKP1;SNRPD2;SNRPD3;SNUPN;SRPRB;SSBP1;STX8;SUCLG1;SUGT1;TICAM2;TOMM6;TRAPPC3;UQCRFS1;UXT;VDA
RPSA;SNRPD3;SRPRB;SSBP1;TRNAU1AP;UXT;ZGLP1;ZNRD

PSA;SNRPD2;SNRPD3;SNUPN;SSBP1;UXT;ZGLP1;ZNRD
UXT;ZGLP1;ZNRD
L37;RPL37A;RPL4;RPL5;RPL7A;RPS11;RPS12;RPS13;RPS14;RPS16;RPS18;RPS19;RPS20;RPS21;RPS23;RPS25;RPS27A;RPS3;RPS4X;RPS5;RPS6;RPS8;RPSA;SKP1;SNRPD2;SNRPD3;SNUPN;SRPRB;SSBP1;STX8;SUCLG1;SUGT1;TICAM2;TOMM6;TRAPPC3;TRNAU1AP;UQCRFS1;UXT;VDA
3A;RPL14;RPL19;RPL22;RPL23A;RPL24;RPL27A;RPL3;RPL32;RPL35A;RPL36;RPL37;RPL37A;RPL4;RPL5;RPL7A;RPS11;RPS12;RPS13;RPS14;RPS16;RPS18;RPS19;RPS20;RPS21;RPS23;RPS25;RPS27A;RPS3;RPS4X;RPS5;RPS6;RPS8;RPSA;SKP1;SNRPD2;SNRPD3;SNUPN;SPAG7;SRPRB;SSBP1;STX8;SUCLG1;SUGT1;T

TX8;SUGT1;TOMM6;UX

S3;RPS4X;RPS5;RPS6;RPS8;RPSA;SNRPD2;SNRPD3;SNUPN;SRPRB;SSBP1;UXT;ZGLP1;ZNRD

PL35A;RPL36;RPL37;RPL37A;RPL4;RPL5;RPL7A;RPS11;RPS12;RPS13;RPS14;RPS16;RPS18;RPS19;RPS20;RPS21;RPS23;RPS25;RPS27A;RPS3;RPS4X;RPS5;RPS6;RPS8;RPSA;SKP1;SNRPD2;SNRPD3;SNUPN;SPAG7;SRPRB;SSBP1;STX8;SUCLG1;SUGT1;THYN1;TICAM2;TOMM6;TRAPPC3;TRNAU1AP;UQCRFS1;UXT;
3A;RPL14;RPL19;RPL22;RPL23A;RPL24;RPL27A;RPL3;RPL32;RPL35A;RPL36;RPL37;RPL37A;RPL4;RPL5;RPL7A;RPS11;RPS12;RPS13;RPS14;RPS16;RPS18;RPS19;RPS20;RPS21;RPS23;RPS25;RPS27A;RPS3;RPS4X;RPS5;RPS6;RPS8;RPSA;SKP1;SNRPD2;SNRPD3;SNUPN;SPAG7;SRPRB;SSBP1;STX8;SUCLG1;SUGT1;T
7A;RPS3;RPS4X;RPS5;RPS6;RPS8;RPSA;SNRPD2;SNRPD3;SNUPN;SRPRB;SSBP1;UXT;ZGLP1;ZNRD
PL35A;RPL36;RPL37;RPL37A;RPL4;RPL5;RPL7A;RPS11;RPS12;RPS13;RPS14;RPS16;RPS18;RPS19;RPS20;RPS21;RPS23;RPS25;RPS27A;RPS3;RPS4X;RPS5;RPS6;RPS8;RPSA;SKP1;SNRPD2;SNRPD3;SNUPN;SPAG7;SRPRB;SSBP1;STX8;SUCLG1;SUGT1;THYN1;TICAM2;TOMM6;TRAPPC3;TRNAU1AP;UQCRFS1;UXT;
;RPS6;RPS8;RPSA;SNRPD2;SNRPD3;SNUPN;SRPRB;SSBP1;UXT;ZGLP1;ZNRD

M6;UX
X;RPS5;RPS6;RPS8;RPSA;SNRPD2;SNRPD3;SNUPN;SRPRB;SSBP1;UXT;ZGLP1;ZNRD
5;RPS6;RPS8;RPSA;SNRPD3;SRPRB;SSBP1;TRNAU1AP;UXT;ZGLP1;ZNRD

S3;RPS4X;RPS5;RPS6;RPS8;RPSA;SNRPD2;SNRPD3;SNUPN;SRPRB;SSBP1;UXT;ZGLP1;ZNRD
;RPS8;RPSA;SNRPD3;SRPRB;SSBP1;TRNAU1AP;UXT;ZGLP1;ZNRD

5;RPS6;RPS8;RPSA;SNRPD3;SRPRB;SSBP1;TRNAU1AP;UXT;ZGLP1;ZNRD

PS25;RPS27A;RPS3;RPS4X;RPS5;RPS6;RPS8;RPSA;SNRPD2;SNRPD3;SNUPN;SRPRB;SSBP1;UXT;ZGLP1;ZNRD

RPS25;RPS27A;RPS3;RPS4X;RPS5;RPS6;RPS8;RPSA;SKP1;SNRPD2;SNRPD3;SNUPN;SRPRB;SSBP1;TOMM6;TRNAU1AP;USP42;UXT;ZGLP1;ZNRD

PS11;RPS12;RPS13;RPS14;RPS16;RPS18;RPS19;RPS20;RPS21;RPS23;RPS25;RPS27A;RPS3;RPS4X;RPS5;RPS6;RPS8;RPSA;SKP1;SNRPD2;SNRPD3;SNUPN;SRPRB;SSBP1;SUCLG1;TOMM6;TRNAU1AP;UQCRFS1;USP42;UXT;ZGLP1;ZNRI

PS23;RPS25;RPS27A;RPS3;RPS4X;RPS5;RPS6;RPS8;RPSA;SKP1;SNRPD2;SNRPD3;SNUPN;SRPRB;SSBP1;TOMM6;TRNAU1AP;USP42;UXT;ZGLP1;ZNRI
RPL5;RPL7A;RPS11;RPS12;RPS13;RPS14;RPS16;RPS18;RPS19;RPS20;RPS21;RPS23;RPS25;RPS27A;RPS3;RPS4X;RPS5;RPS6;RPS8;RPSA;SKP1;SNRPD2;SNRPD3;SNUPN;SRPRB;SSBP1;SUCLG1;TOMM6;TRNAU1AP;UQCRFS1;USP42;UXT;ZGLP1;ZNRI
3;RPS14;RPS16;RPS18;RPS19;RPS20;RPS21;RPS23;RPS25;RPS27A;RPS3;RPS4X;RPS5;RPS6;RPS8;RPSA;SKP1;SNRPD2;SNRPD3;SNUPN;SRPRB;SSBP1;SUCLG1;TOMM6;TRNAU1AP;USP42;UXT;ZGLP1;ZNRI
2;RPS13;RPS14;RPS16;RPS18;RPS19;RPS20;RPS21;RPS23;RPS25;RPS27A;RPS3;RPS4X;RPS5;RPS6;RPS8;RPSA;SKP1;SNRPD2;SNRPD3;SNUPN;SRPRB;SSBP1;SUCLG1;TOMM6;TRNAU1AP;USP42;UXT;ZGLP1;ZNRI

3A;RPL14;RPL19;RPL22;RPL23A;RPL24;RPL27A;RPL3;RPL32;RPL35A;RPL36;RPL37;RPL37A;RPL4;RPL5;RPL7A;RPS11;RPS12;RPS13;RPS14;RPS16;RPS18;RPS19;RPS20;RPS21;RPS23;RPS25;RPS27A;RPS3;RPS4X;RPS5;RPS6;RPS8;RPSA;SKP1;SNRPD2;SNRPD3;SNUPN;SPAG7;SRPRB;SSBP1;STX8;SUCLG1;SUGT1;T
3A;RPL14;RPL19;RPL22;RPL23A;RPL24;RPL27A;RPL3;RPL32;RPL35A;RPL36;RPL37;RPL37A;RPL4;RPL5;RPL7A;RPS11;RPS12;RPS13;RPS14;RPS16;RPS18;RPS19;RPS20;RPS21;RPS23;RPS25;RPS27A;RPS3;RPS4X;RPS5;RPS6;RPS8;RPSA;SKP1;SNRPD2;SNRPD3;SNUPN;SPAG7;SRPRB;SSBP1;STX8;SUCLG1;SUGT1;T

2;RPL23A;RPL24;RPL27A;RPL3;RPL32;RPL35A;RPL36;RPL37;RPL37A;RPL4;RPL5;RPL7A;RPS11;RPS12;RPS13;RPS14;RPS16;RPS18;RPS19;RPS20;RPS21;RPS23;RPS25;RPS27A;RPS3;RPS4X;RPS5;RPS6;RPS8;RPSA;SKP1;SNRPD2;SNRPD3;SNUPN;SRPRB;SSBP1;STX8;SUCLG1;SUGT1;TICAM2;TOMM6;TRAPPC3;TRN

R1B;FFAR2;FFAR3;FGF13;FLOT1;FOXO3;FPR1;FPR2;FYB;GNAQ;GPR141;GPR17;GPR89A;GPR97;GPSM2;GRIP2;GSK3B;GTSE1;HCAR2;HCAR3;HEY1;HIF1A;HMGCR;HPSE;IFNAR1;IFNGR2;IGSF6;IL10RB;IL13RA1;IL18RAP;IL1B;IL1R1;IL1RN;INHBB;IQGAP1;IRAK3;ITGAD;KCNE1;KCNH7;KCNJ15;KCNS1;KLF6;KPNA1;

R1B;FFAR2;FFAR3;FGF13;FLOT1;FOXO3;FPR1;FPR2;FYB;GNAQ;GPR141;GPR17;GPR89A;GPR97;GPSM2;GRIP2;GSK3B;GTSE1;HCAR2;HCAR3;HEY1;HIF1A;HMGCR;HPSE;IFNAR1;IFNGR2;IGSF6;IL10RB;IL13RA1;IL18RAP;IL1B;IL1R1;IL1RN;INHBB;IQGAP1;IRAK3;ITGAD;KCNE1;KCNH7;KCNJ15;KCNS1;KLF6;KPNA1;

3;FPR1;FPR2;FYB;GNAQ;GPR141;GPR17;GPR89A;GPR97;GPSM2;GSK3B;GTSE1;HCAR3;HEY1;HIF1A;HMGCR;HPSE;IFNAR1;IFNGR2;IGSF6;IL10RB;IL13RA1;IL18RAP;IL1B;IL1R1;IL1RN;INHBB;IQGAP1;IRAK3;ITGAD;KLF6;KPNA1;KPNA4;LILRB3;LIMD1;LITAF;LYN;MCTP2;MLLT11;MYO5A;NCOA1;NCOA2;NEDD9;N

R1B;FFAR2;FFAR3;FGF13;FLOT1;FOXO3;FPR1;FPR2;FYB;GNAQ;GPR141;GPR17;GPR89A;GPR97;GPSM2;GRIP2;GSK3B;GTSE1;HCAR2;HCAR3;HEY1;HIF1A;HMGCR;HPSE;IFNAR1;IFNGR2;IGSF6;IL10RB;IL13RA1;IL18RAP;IL1B;IL1R1;IL1RN;INHBB;INPP5A;IQGAP1;IRAK3;ITGAD;KCNH7;KCNJ15;KCNS1;KLF6;KPNA1

MD7;CR1;CSRNP1;CST7;CUL3;CXCR1;CYBB;CYTH4;DGAT2;DNAJB11;DNAJC3;DOCK1;DOCK4;DOK4;EBP;EDEM2;EMR3;ENTPD1;EXT1;F5;FCGR1A;FCGR1B;FCGR3B;FFAR2;FFAR3;FGF13;FLOT1;FOXO3;FPR1;FPR2;FYB;GNAQ;GPR141;GPR17;GPR89A;GPR97;GPSM2;GSK3B;GTSE1;H3F3B;HAS1;HCAR3;HEY1;HIF1

'A;RASGRP4;RHOH;SBNO2;SEC24D;SECTM1;SIT1;SOCS3;SOD2;SRPK1;SRPK2;STK3;TARBP2;TLR2;TLR4;TLR5;TNFSF14;TREM1;TREML2;WNT10B;ZFP36;ZFP36
1;FCGR1A;FCGR1B;FFAR2;FFAR3;FGF13;FLOT1;FOXO3;FPR1;FPR2;FYB;GNAQ;GPR141;GPR17;GPR89A;GPR97;GPSM2;GSK3B;GTSE1;HAS1;HCAR3;HEY1;HIF1A;HMGCR;HP;HPSE;IFNAR1;IFNGR2;IGSF6;IL10RB;IL13RA1;IL18RAP;IL1B;IL1R1;IL1RN;INHBB;IQGAP1;IRAK3;ITGAD;KLF6;KPNA1;KPNA4;LILRB3;LIMD

CNTNAP3;COQ4;CR1;CXCR1;CYBB;CYTH4;DGAT2;DMXL2;DNAJC3;DOCK1;DOCK4;EBP;EDEM2;ELOVL5;EMR3;ENTPD1;EXT1;F5;FAM120A;FCGR1A;FCGR1B;FCGR1C;FCGR2A;FCGR3B;FFAR2;FFAR3;FGF13;FLOT1;FOXO3;FPR1;FPR2;GABARAPL1;GALNT14;GDE1;GIMAP1;GLIPR2;GNAQ;GOLGA7B;GPR141;GPR

GAT2;DMXL2;DNAJB11;DNAJC3;DOCK1;DOCK4;DOK4;DPYD;E2F3;ELOVL5;ETS2;EVI5;EXT1;F5;FCGR1A;FCGR1B;FCGR1C;FCGR2A;FCGR3B;FGF13;FLOT1;FOXO3;FPR1;FYB;GABARAPL1;GCM1;GNAQ;GPSM2;GSK3B;H3F3B;HEY1;HIF1A;HIVEP1;HMGCR;HP;HPSE;IDH1;IL10RB;IL13RA1;IL1B;IL1R1;IL1R2;IL1RN;IN

4;SIT1;SLC12A6;SLC22A1;SLC22A14;SLC22A4;SLC8A1;SNTG2;ST3GAL5;STX3;TGM3;TLR2;TLR4;TM9SF2;TNFRSF1A;TRPM6;TRPV5;VAMI
RHOH;SDCBP;SECTM1;SLC8A1;SOCS3;SOS2;SRPK1;SRPK2;STK3;TEAD3;TIAM2;TLR2;TLR4;TNFRSF1A;TNFSF14;TREM1;TRIB1;UBR2;WLS;ZAK;ZFP
R84;GPR97;GPSM2;GRIP2;GSK3B;HAS1;HCAR2;HCAR3;IFNAR1;IFNGR2;IGSF6;IL10RB;IL13RA1;IL1R1;IL1R2;IL1RN;IQGAP1;ITGAD;ITM2B;ITPRIP;KAL1;KCNE1;KCNH7;KCNJ15;KCNS1;KIAA0319;LAMP2;LILRB3;LITAF;LRRC4;LYN;LYVE1;MARCKS;MCMBP;MME;MYO10;NAIP;NCF4;NEDD9;NFE2L2;NPTN;OSM;OX

R84;GPR97;GRIP2;GSK3B;HAS1;HCAR2;HCAR3;IFNAR1;IFNGR2;IGSF6;IL10RB;IL13RA1;IL1R1;IL1R2;IL1RN;IQGAP1;ITGAD;ITM2B;ITPRIP;KAL1;KCNE1;KCNH7;KCNJ15;KCNS1;KIAA0319;LAMP2;LILRB3;LITAF;LRRC4;LYN;LYVE1;MARCKS;MCMBP;MME;MYO10;NAIP;NCF4;NFE2L2;NPTN;OSM;OXER1;PAK2;PDZD

AMP2;LILRB3;LIMD1;LYN;LYVE1;MRVI1;NAIP;NFE2;NFE2L2;NPEPPS;ORM1;ORM2;OSM;PAK2;PAPSS2;PDK3;PELI2;PGLYRP1;PIAS1;PLAU;PLD1;POLD3;PPAP2B;PROK2;PVRL2;RAB27A;RTN3;SBNO2;SCARF1;SDCBP;SLC12A6;SLC8A1;SOCS3;SOD2;SRPK1;SRPK2;STK3;STX3;TARBP2;TLR2;TLR4;TLR5;TNFRSF1A;TI

IAS1;PLAU;PPAP2B;PROK2;PTPRE;PVRL2;RASGRP4;RHOH;RNF111;RS1;SBNO2;SCARF1;SDCBP;SECTM1;SH3GLB1;SOCS3;SOS2;STK3;TARBP2;TIAM2;TLR2;TLR4;TLR5;TNFRSF1A;TNFSF14;TRIB1;UBR2;UIMC1;WAC;WLS;WNT10B;ZAK;ZFP3
CDH2;CDK14;CDK4;CDKL5;CENPV;CEP63;CHRNA10;CLDN6;CLEC1A;CLMN;CNTNAP3;COMMD7;CSRNP1;CTBP2;CUL3;CXCR1;CYBB;CYTH4;DGAT2;DMRTC2;DNAH14;DNAJB11;DNAJC3;DOCK1;DOCK4;DOK4;DUSP13;E2F3;EBP;EMR3;ENTPD1;EVI5;EXT1;F5;FAM65B;FCGR1A;FCGR1B;FFAR2;FFAR3;FGF13;FLO
VNT10
VL5;EMR3;ENTPD1;EXT1;FCGR1A;FCGR1B;FCGR1C;FCGR2A;FCGR3B;FFAR2;FFAR3;FLOT1;FPR1;FPR2;GALNT14;GDE1;GIMAP1;GNAQ;GPR141;GPR17;GPR84;GPR89A;GPR97;GSK3B;HAS1;HCAR2;HCAR3;HMGCR;HPSE;IFNAR1;IFNGR2;IGSF6;IL10RB;IL13RA1;IL18RAP;IL1R1;IL1R2;IQGAP1;ITGAD;ITM2B;KCNE
PRE;RAB31;RNF111;SLC8A1;SOCS3;SOD2;STX3;TARBP2;TLR2;TLR4;TNFRSF1A;TRIB1;UBR2;WNT10

H;RNF111;RS1;SDCBP;SECTM1;SH3GLB1;SOCS3;SOS2;STK3;TG;TIAM2;TLR2;TLR4;TLR5;TNFRSF1A;TNFSF14;TRIB1;UBR2;VAMP3;WLS;WNT10B;ZA

;LYN;MLLT11;NEDD9;NPTN;NUP50;NUPL1;OXER1;PAK2;PDZD3;PELI2;PIAS1;PPAP2B;PROK2;PTPRE;RASGRP4;REPS2;RNF111;RS1;SOCS3;SOS2;STK3;TIAM2;TLE4;TLR2;TLR4;TNFRSF1A;TRIB1;WLS;WNT1

H;RNF111;RS1;SDCBP;SECTM1;SH3GLB1;SOCS3;SOS2;STK3;TG;TIAM2;TLR2;TLR4;TLR5;TNFRSF1A;TNFSF14;TRIB1;UBR2;WAC;WLS;WNT10B;ZA

RNA10;CIR1;CLEC1A;CLMN;CNTNAP3;COMMD7;CR1;CSRNP1;CST7;CTBP2;CUL3;CXCR1;CYTH4;DGAT2;DMRTC2;DNAJB11;DNAJC3;DOCK1;DOCK4;DOK4;E2F3;EMR3;ENTPD1;ETS2;EXT1;F5;FCGR1A;FCGR1B;FFAR2;FFAR3;FGF13;FLI1;FLOT1;FOXO3;FPR1;FPR2;FYB;GCM1;GNAQ;GPR141;GPR17;GPR89A;GPR

A;CDC42EP3;CDH2;CDK14;CDK19;CDK4;CDKL5;CEP63;CHRNA10;CIR1;CLDN6;CLEC1A;CLMN;CNGA4;CNTNAP3;COMMD7;CR1;CSRNP1;CTBP2;CUL3;CXCR1;CYBB;CYTH4;DCTPP1;DGAT2;DHRS12;DHRS13;DHRS7;DHRSX;DMRTC2;DMXL2;DNAH14;DNAJB11;DNAJC3;DOCK1;DOCK4;DOK4;DPYD;E2F3;EDEM2;E

2;STK3;TIAM2;TLR2;TLR4;TLR5;TNFRSF1A;TNFSF14;TRIB1;UBR2;WLS;WNT10B;ZA

GR1C;FCGR2A;FCGR3B;FFAR2;FFAR3;FLOT1;FPR1;FPR2;GALNT14;GDE1;GIMAP1;GPR141;GPR17;GPR84;GPR89A;GPR97;HAS1;HCAR2;HCAR3;HMGCR;IFNAR1;IFNGR2;IGSF6;IL10RB;IL13RA1;IL18RAP;IL1R1;IL1R2;ITGAD;ITM2B;KCNE1;KCNH7;KCNJ15;KCNS1;KIAA0319;KPNA1;LAMP2;LBR;LILRA6;LILRB3;LRR
0;MYO5A;NFE2;NFE2L2;NPTN;OSBPL11;OSM;PAPSS2;PDE4B;PDK3;PIF1;PLAU;POLD3;PPAP2B;PROK2;RAB27A;RAPGEF2;SBF2;SH3GLB1;SLC22A1;SLC22A4;SLC8A1;SNTA1;SOD2;STX12;SYN2;TG;TLR2;TLR4;TMX4;TNFSF14;TREM1;TRIM9;USP3;WNT10B;ZFP36;ZFP3

DCBP;SEC24D;SH3GLB1;SMAGP;ST3GAL4;ST3GAL5;ST6GALNAC2;STX12;STX6;TNFRSF1A;TOR1AIP2;VAMP3;WDFY3;WI

(14;CDK4;CDKL5;CENPV;CEP63;CIR1;CLMN;COQ4;CST7;CUL3;CYBB;DCTPP1;DGAT2;DMXL2;DNAH14;DNAJB11;DNAJC3;DOCK1;DOCK4;DPYD;DUSP13;EBP;ECHDC3;EDEM2;ELOVL5;ENO3;EVI5;EXT1;F5;FAM120A;FAM65B;FCGR1A;FCGR1B;FGF13;FLOT1;FOXO3;FPR1;FRMD4B;FRY;FYB;GABARAPL1;GALNT14

;MBOAT2;ME2;MME;MYO5A;NCF4;NCOA1;NCOA2;NMNAT2;NPL;NT5C2;NUDT5;NUP50;NUPL1;OLAH;OPLAH;OSBPL1A;OXER1;PANK2;PAPSS2;PDE4B;PDK3;PDZD3;PFKFB4;PGS1;PLB1;PLBD1;PLD1;PNP;PNPLA1;PPAP2B;PPP1R3B;RAB1A;RAB31;SCARF1;SH3GLB1;SLC22A1;SLC22A4;SOD2;SQRDL;ST3GAL4;S
AR3;FLOT1;FPR1;FPR2;GALNT14;GDE1;GIMAP1;GPR141;GPR17;GPR84;GPR89A;GPR97;HAS1;HCAR2;HCAR3;HMGCR;IFNAR1;IFNGR2;IGSF6;IL10RB;IL13RA1;IL18RAP;IL1R1;IL1R2;ITGAD;ITM2B;KCNE1;KCNH7;KCNJ15;KCNS1;KIAA0319;KPNA1;LAMP2;LBR;LILRA6;LILRB3;LRRC4;LYN;LYVE1;MANSC1;MBOAT2

)K3;PDZD3;PELI2;PFKFB4;PGS1;PLB1;PLBD1;PLD1;PNP;PPAP2B;PPP4R1;PROK2;PTPRE;RAB1A;RAB31;RHOH;SDCBP;SH3GLB1;SOCS3;SRPK1;SRPK2;STK3;TARBP2;TGM3;TLR2;TLR4;TMEM132D;TNFRSF1A;TPX2;TRIB1;ZA
0;MYO5A;NCOA1;NFE2L2;NPEPPS;NPTN;NUP50;NUPL1;OPLAH;PAK2;PAPSS2;PDCD7;PDE4B;PDK3;PDZD3;PHB;PIAS1;PLAU;PLD1;PLXNC1;PNP;PROK2;PTPRE;RAB31;RNF111;SDCBP;SLC22A1;SLC8A1;SOCS3;SOD2;SOS2;STX3;TARBP2;TLR2;TLR4;TNFRSF1A;TRIB1;TRPM6;UBR2;WNT1(

DE4B;PDK3;PDZD3;PELI2;PFKFB4;PGS1;PLB1;PLBD1;PLD1;PNP;PPAP2B;PPP4R1;PROK2;PTPRE;RAB1A;RAB31;RHOH;SDCBP;SH3GLB1;SOCS3;SRPK1;SRPK2;STK3;TARBP2;TGM3;TLR2;TLR4;TMEM132D;TNFRSF1A;TPX2;TRIB1;ZA

CD59;CDA;CDC123;CDC42EP3;CDH2;CDK14;CDK4;CDKL5;CENPV;CEP63;CHRNA10;CLDN6;CLEC1A;CLMN;CNGA4;CNTNAP3;COMMD7;CR1;CSRNP1;CTBP2;CUL3;CXCR1;CYBB;CYTH4;DGAT2;DMRTC2;DNAH14;DNAJB11;DNAJC3;DOCK1;DOCK4;DOK4;DUSP13;E2F3;EBP;ELMOD3;EMR3;ENTPD1;ETS2;EVI5;EXT

JDT5;NUP50;NUPL1;OLAH;OPLAH;OSBPL1A;OXER1;PANK2;PAPSS2;PDE4B;PDK3;PDZD3;PFKFB4;PGS1;PLB1;PLBD1;PLD1;PNP;PPAP2B;RAB1A;RAB31;SCARF1;SLC22A1;SLC22A4;SOD2;SQRDL;ST3GAL4;TG;TGM3;TIAM2;TMLHE;TNFRSF

7;CR1;CSRNP1;CST7;CTBP2;CUL3;CXCR1;CYTH4;DGAT2;DMRTC2;DNAJB11;DNAJC3;DOCK1;DOCK4;DOK4;E2F3;EMR3;ETS2;EXT1;FCGR1A;FCGR1B;FFAR2;FFAR3;FGF13;FLOT1;FOXO3;FPR1;FPR2;FYB;GCM1;GNAQ;GPR141;GPR17;GPR89A;GPR97;GPSM2;GSK3B;GTSE1;HAS1;HCAR2;HCAR3;HEY1;HIF1A;HIVE

8A1;SOCS3;SOD2;TARBP2;TLR2;TLR4;TNFRSF1A;TRIB1;UBR2;WNT10

YN;MLLT11;MYO10;NCOA1;NCOA2;NFE2;NFE2L2;NFIL3;NFYA;NPTN;NRD1;OSBPL11;OSM;PAK2;PDCD7;PELI2;PHB;PIAS1;PICALM;PNP;PPAP2B;PROK2;PVRL2;RAB27A;RANBP1;RASGRP4;RNF111;SCARF1;SDCBP;SECTM1;SH3GLB1;SLC8A1;SOCS3;SOD2;SRPK1;SRPK2;STK3;SYNGR3;TARBP2;TEAD3;TLR2;TLR4

L2;NFYA;NPTN;NRD1;OSM;PAK2;PDCD7;PELI2;PHB;PIAS1;PICALM;PNP;PPAP2B;PROK2;PVRL2;RAB27A;RANBP1;RASGRP4;RNF111;SCARF1;SDCBP;SECTM1;SH3GLB1;SLC8A1;SOCS3;SOD2;SRPK1;SRPK2;STK3;TARBP2;TEAD3;TLR2;TLR4;TLR5;TNFRSF1A;TNFSF14;TPX2;TRIB1;TSHZ3;UIMC1;VAMP3;WAC;WLS;

2;CAPN13;CAPN8;CASP4;CASP5;CBS;CCDC125;CCNJL;CCR1;CCR2;CCT4;CD177;CD300LD;CD55;CD59;CDA;CDC123;CDC42EP3;CDH2;CDK14;CDK4;CDKL5;CEACAM4;CENPV;CEP63;CHRNA10;CIR1;CLDN6;CLEC1A;CLMN;CNTNAP3;COQ4;CR1;CSRNP1;CST7;CTBP2;CUL3;CXCR1;CYBB;CYTH4;DCTPP1;DGAT2;DM

2;CAPN13;CAPN8;CASP4;CASP5;CBS;CCDC125;CCNJL;CCR1;CCR2;CCT4;CD177;CD300LD;CD55;CD59;CDA;CDC123;CDC42EP3;CDH2;CDK14;CDK4;CDKL5;CEACAM4;CENPV;CEP63;CHRNA10;CIR1;CLDN6;CLEC1A;CLMN;CNTNAP3;COQ4;CR1;CSRNP1;CST7;CTBP2;CUL3;CXCR1;CYBB;CYTH4;DCTPP1;DGAT2;DM

LM;PLD1;RAB27A;RAB31;RTN3;SBF2;SDCBP;SEC24D;SH3GLB1;SLC15A4;SMAGP;SOD2;SQRDL;ST3GAL4;ST3GAL5;ST6GALNAC2;STX12;STX6;TIMM44;TLR4;TM9SF2;TNFRSF1A;TOR1AIP2;VAMP3;W

HDC3;EDEM2;ELOVL5;ENO3;EXT1;F5;FAM65B;FCGR1A;FCGR1B;FLOT1;FOXO3;FPR1;FYB;GABARAPL1;GALNT14;GBE1;GIMAP1;GLIPR2;GMPR2;GOLGA7B;GPR89A;GPSM2;GRIP2;GSDMC;GSK3B;GTSE1;HAL;HIF1A;HMGCR;HPSE;IDH1;IDS;IFNGR2;IL1B;INHBB;ITM2B;KCNE1;KIAA0319;KPNA1;KPNA4;LAMP2;L

LC8A3;SPARC;TFPI;TGFB1I1;TNFSF4;TPM1;TREML1;VEGFC;VIL1;VWF;WASF

KAR2B;PROS1;PTGES;PTGS1;RAB6B;RGS6;RHOBTB1;SELP;SERPINE1;SLC6A4;SLC8A3;SMOX;SPARC;TFPI;TGFB1I1;TNFSF4;TPM1;TREML1;VEGFC;VIL1;VW

RD3;PCSK6;PDE5A;PDGFA;PDGFRA;PEAR1;PF4;PGRMC1;PLOD2;PLXNB3;PPBP;PRKAR2B;PROS1;PTCRA;PTGES;PTGS1;RAB6B;RGS6;RHOBTB1;SDC4;SELP;SERPINE1;SH3TC2;SLC24A3;SLC6A4;SLC8A3;SMOX;SPARC;STON2;SYTL4;TGFB1I1;TNFSF4;TOM1L1;TPM1;TREML1;TSC22D1;TUBA8;TUBB1;VEGFC;VIL1;
;TUBB1;VEPH1;VSIG2;VW
1;VEPH1;VSIG2;VW
LC8A3;SPARC;TFPI;TGFB1I1;TNFSF4;TPM1;TREML1;VEGFC;VIL1;VWF;WASF

TGES;PTGS1;RAB6B;RGS6;RHOBTB1;SDC4;SDPR;SEC14L5;SELP;SERPINE1;SH3TC2;SLC24A3;SLC35D3;SLC6A4;SLC8A3;ST7;STON2;SYTL4;TFPI;TMEM40;TNFSF4;TOM1L1;TPM1;TREML1;TSPAN33;TSPAN9;TUBB1;VEGFC;VEPH1;VSIG2;V

RGN;PARD3;PCSK6;PCYT1B;PDE5A;PDGFA;PDGFRA;PEAR1;PF4;PGRMC1;PLOD2;PLXNB3;PPBP;PRKAR2B;PROS1;PTCRA;PTGES;PTGS1;RAB6B;RGS6;RHOBTB1;SDC4;SELP;SERPINE1;SH3TC2;SLC24A3;SLC35D3;SLC6A4;SLC8A3;SMOX;SPARC;STON2;SYTL4;TFPI;TGFB1I1;TNFSF4;TOM1L1;TPM1;TREML1;TSC22

L1;VEGFC;VIL1;WASF

C;VIL1;WASF
C;VIL1;WASF

C;VIL

L4;TFPI;TMEM40;TNFSF4;TPM1;TREML1;TSPAN33;TSPAN9;VSIG2;VW

GS1;RAB6B;RGS6;RHOBTB1;SDC4;SELP;SERPINE1;SH3TC2;SLC6A4;SLC8A3;SPARC;SPOCD1;STON2;SYTL4;TFPI;TGFB1I1;TNFSF4;TPM1;TREML1;TSC22D1;VEGFC;VIL1;VWF;WASF3;ZNF4

PR;SELP;SERPINE1;SH3BGRL2;SLC6A4;SLC8A3;SPARC;STON2;SYTL4;TGFB1I1;TNFSF4;TOM1L1;TPM1;TREML1;VEGFC;VIL1;VWF;WASI

H3TC2;SLC6A4;SLC8A3;SMOX;SPARC;STON2;SYTL4;TFPI;TOM1L1;TPM1;TREML1;VEGFC;VW

KAR2B;PROS1;PTGES;PTGS1;PVALB;RAB6B;RGS6;RHOBTB1;SDC4;SDPR;SELP;SERPINE1;SH3TC2;SLC6A4;SLC8A3;SMOX;SPARC;STON2;SYTL4;TFPI;TGFB1I1;TOM1L1;TPM1;TREML1;TSC22D1;TUBA8;TUBB1;VEGFC;VIL1;VWF;WAS

YLK;NRGN;PARD3;PCSK6;PDE5A;PDGFA;PDGFRA;PDLIM1;PF4;PF4V1;PGRMC1;PLOD2;PLXNB3;PPBP;PRKAR2B;PROS1;PTCRA;PTGES;PTGS1;PVALB;RAB6B;RBPMS2;RGS6;RHOBTB1;SDC4;SDPR;SELP;SERPINE1;SH3BGRL2;SLC6A4;SLC8A3;SPARC;STON2;SYTL4;TGFB1I1;TNFSF4;TOM1L1;TPM1;TREML1;TRIM4

5;PRDX6;PTPRN;RAB3IL1;RAG1;RAP1GAP;RHAG;RILP;RIOK3;RNF10;SELENBP1;SFRP2;SGIP1;SHARPIN;SIAH2;SIM2;SLC2A4;SLC4A1;SNCA;SOX6;SPDYC;SPTA1;STRADB;STX2;TAL1;TESC;TFDP1;TGM2;TMCC2;TMOD1;TNS1;TUBB2B;UBA52;UBB;UBXN6;WNT9A;XK;YB

;KLC3;LGALS3;MARCH2;MBNL3;MYL4;NEDD4L;NT5M;NUDT4;OAZ1;ODC1;PBX1;PDE3A;PLEK2;PLVAP;PPM1A;PRDX5;PRDX6;RAP1GAP;RBM38;RHOV;RILP;RNF10;RPL3L;SELENBP1;SFRP2;SGIP1;SHARPIN;SIAH2;SLC25A37;SLC25A39;SLC2A4;SLC4A1;SNCA;SPDYC;SPTA1;ST6GALNAC4;STRADB;STX2;TESC;TG

1;STX2;TAL1;TESC;TGM2;TMOD1;TRIM10;TUBB2B;UBA52;UBB;UTS2R;WNT9A;YB

I2;KEL;KLF1;KRT1;LGALS3;LYL1;MBNL3;MEF2B;MXI1;MYL4;NEDD4L;NUDT4;ODC1;OSBP2;PBX1;PDE3A;PLEK2;POLL;PPM1A;PRDX5;PRDX6;PTPRF;PTPRN;RAG1;RAP1GAP;RBM38;RHAG;RHOV;RILP;RIOK3;RNF10;RPL3L;RUNDC3A;SESN3;SFRP2;SGIP1;SHARPIN;SIAH2;SIM2;SLC14A1;SLC25A37;SLC2A4;SLC38A

AM2;HPS1;IGF2BP2;ITLN1;KANK2;KCNH2;KEL;KLC3;KLF1;KRT1;LGALS3;LYL1;MARCH2;MBNL3;MEF2B;MKRN1;MRC2;MXI1;MYL4;NEDD4L;NFIX;NT5M;NUDT4;OSBP2;PBX1;PDE3A;PLEK2;PLVAP;POLL;POLR1D;PPM1A;PRDX5;PRDX6;PTPRF;PTPRN;RAB3IL1;RAG1;RAP1GAP;RBM38;RHAG;RHOV;RILP;RIOK3;RI

IAG;RHCE;RHOV;RILP;SEC14L4;SELENBP1;SFRP2;SGIP1;SHISA4;SHISA7;SLC14A1;SLC25A37;SLC25A39;SLC2A4;SLC38A5;SLC4A1;SLC6A19;SLC6A8;SLC6A9;SNCA;SPTA1;ST6GALNAC4;STX2;TESC;TGM2;TMC5;TMCC2;TMEM158;TMEM56;TMEM98;TMOD1;TMPRSS9;TSPAN5;TSPAN7;TSPO2;TUBB2A;TUBB2B;
P;RBM38;RHOV;RILP;RPL3L;SELENBP1;SGIP1;SHARPIN;SIAH2;SLC25A37;SLC25A39;SLC2A4;SLC4A1;SNCA;SPTA1;ST6GALNAC4;STRADB;STX2;TGM2;TMEM98;TMOD1;TSPO2;UBA52;UBB;UTS2R;VTI1B;YB

331;ZNF841;ZNF850;ZNF88

341;ZNF85

ZNF548;ZNF585B;ZNF831;ZNF841;ZNF850;ZNF8[illegible]
ZNF548;ZNF585B;ZNF831;ZNF841;ZNF850;ZNF8[illegible]

1;ZNF850;ZNF8[illegible]

2A5;SLC5A9;SPRY1;SWAP70;SYNPO;TCL1A;TNFRSF13C;TSPAN1
2A5;SLC5A9;SPRY1;SWAP70;SYNPO;TNFRSF13C;TSPAN1

IGLEC6;SLC15A2;SLC2A5;SLC5A9;SLC9A7;SYBU;TNFRSF13C;TSPAN13;TSPAN
L1L3;SIGLEC6;SLC15A2;SLC2A5;SLC5A9;SLC9A7;SYBU;TNFRSF13C;TSPAN13;TSPAN

X5;PARM1;PAWR;PCDH9;PIK3C2B;PMEPA1;PPAPDC1B;PRICKLE1;PTPRK;QSOX2;RASGRP3;SCN4A;SEL1L3;SEMA3G;SIGLEC6;SLC15A2;SLC2A5;SLC5A9;SLC9A7;SNX22;SPRY1;SWAP70;SYBU;SYNPO;TNFRSF13C;TSPAN13;TSPAN

;QSOX2;RASGRP3;SCN4A;SEL1L3;SIGLEC6;SLC15A2;SLC2A5;SLC5A9;SLC9A7;SYBU;TNFRSF13C;TSPAN13;TSPAN

1;LARGE;MACROD2;MAP2;MICAL3;MMP11;MS4A1;MYBPC2;NETO1;NXPH4;OLFML2A;OSBPL10;P2RX5;PAWR;PAX5;PCDH9;PEG10;PIK3C2B;PKIG;PLEKHG1;PMEPA1;PNOC;PRICKLE1;PTPRK;QRSL1;QSOX2;RAPGEF5;RASGRP3;SCN4A;SEMA3G;SIGLEC6;SLC15A2;SLC2A5;SLC5A9;SLC9A7;SNX22;SOBP;SPRY1;S

P11;MS4A1;MYBPC2;NETO1;NXPH4;OLFML2A;P2RX5;PAWR;PAX5;PCDH9;PEG10;PIK3C2B;PKIG;PLEKHG1;PMEPA1;PNOC;PRICKLE1;PTPRK;QRSL1;QSOX2;RAPGEF5;RASGRP3;SIGLEC6;SLC15A2;SLC2A5;SLC5A9;SLC9A7;SNX22;SPRY1;STAG3;STAP1;STRBP;SWAP70;SYNPO;TCF4;TCL1A;TNFRSF1

P;SWAP70;TCF4;TCL1A;TNFRSF13

TCL1A;TNFRSF13

ICKLE1;PTPRK;RAPGEF5;RASGRP3;SLC15A2;SOBP;SPRY1;STAP1;STRBP;SWAP70;TNFRSF13

GEF1A;RCAN2;RGS9;S1PR5;SH2D2A;SH3RF2;SLAMF7;SMAD7;SYTL2;TBX21;TIGIT;USP28;VIT;WNT1;YPEL1;YWHAQ;ZFYVE28;ZNF6[illegible]

GEF1A;RCAN2;RGS9;S1PR5;SH2D1B;SH2D2A;SH3RF2;SLAMF7;SMAD7;SYTL2;TBX21;TIGIT;USP28;VIT;WNT1;YPEL1;YWHAQ;ZFYVE28;ZNF6[illegible]

3R3;PODN;PPP2R2B;PTCH1;PTGDR;PYHIN1;RAB7L1;RASGEF1A;RCAN2;RGS9;S1PR5;SGCD;SH2D1B;SH2D2A;SLAMF7;SLC1A7;SMAD7;SPON2;SYTL2;TBX21;TIGIT;USP28;VIT;WNT1;YPEL1;YWHAQ;ZFYVE

TM4SF19;VANGL1;WNT1;XPNPEF

PON2;TBX21;USP28;WNT1;YPEL1;YWHAQ;ZFYVE2

L;RASGEF1A;RCAN2;RGS9;S1PR5;SH2D1B;SH2D2A;SH3RF2;SLAMF7;SMAD7;SYTL2;TBX21;TIGIT;USP28;VIT;WNT1;YPEL1;YWHAQ;ZFYVE28;ZNF6

;PCDH1;PDGFRB;PIK3R3;PODN;PPP2R2B;PTCH1;PTGDR;PYHIN1;RAB7L1;RASGEF1A;RCAN2;RGS9;S1PR5;SGCD;SH2D1B;SH2D2A;SLAMF7;SLC1A7;SMAD7;SPON2;SYTL2;TBX21;TIGIT;USP28;VANGL1;VIT;WNT1;YPEL1;YWHAQ;ZFYVE

S1PR5;SCD5;SGCD;SLAMF6;SLAMF7;SLC1A7;SMAD7;SYT11;SYTL2;TIGIT;TM4SF19;VANGL1;WNT1;XPNPEP2;ZFYVE2

;YAP

RF1;TIPIN;TNKS;TTC37;UHRF

PO;TSPYL5;UNC119;USF1;USP47;VGF;WRN;YAP

BL1;RGS14;RSPH1;SDCCAG8;SEPT2;SGOL1;SIRT7;SMAD6;SMC1B;SMC3;SPICE1;TERF1;TIPIN;TNKS;TUBA1A;TUBA4A;UBD;UBE2C;UBE2S;USP4

PAT;NPM2;NR2C2;NUP107;NUP160;ORC5;PBRM1;PDS5A;PIWIL2;PLK3;PPP2CA;PRIM2;PRKCE;PRNP;PSMB10;PSMB8;PSMB9;PSMD5;PSME2;PSRC1;PTPN11;RAD50;RAD52;RANBP2;RARA;RBL1;RGS14;RGS2;RHOB;RSPH1;SDCCAG8;SEPT2;SEPT5;SERTAD1;SFN;SGOL1;SIRT7;SMAD6;SMC1B;SMC3;SPHK1;SPI

SMB8;PSMB9;PSMD5;PSME2;PSRC1;PTPN11;RAD50;RAD52;RANBP2;RBL1;RGS14;RHOB;RSPH1;SDCCAG8;SEPT2;SGOL1;SIRT7;SMAD6;SMC1B;SMC3;SPICE1;TERF1;THBS1;TIPIN;TNKS;TUBA1A;TUBA4A;UBD;UBE2C;UBE2S;UHRF2;USP4

SMC3;SPHK1;SPICE1;TERF1;TIPIN;TNKS;TUBA1A;TUBA4A;UBD;UBE2C;UBE2S;USP4

LINT1;CNTN2;COL16A1;COL18A1;COL23A1;COL4A2;COL9A1;CPE;CRTAP;CSPG5;CTNNBIP1;DAAM2;DARS;DCTN3;DDX1;DEDD2;DGAT1;DISP1;DNAH9;DNAJC2;DNER;DNM1L;DRD3;DSTN;DYNC2H1;EI24;EIF4EBP1;ELANE;ELF3;ELFN1;EML4;EPHA4;ERCC4;ERCC6;ESRRB;EXOSC4;FADD;FANCC;FANCD2;FAT1;FB

TIPIN;TRIM32;UBD;UBE2C;UHRF2;USP4

D5;ATE1;ATL1;ATMIN;ATP2A1;ATP2B2;ATP6V0A2;ATP6V0B;ATP6V1G2;ATPAF1;ATR;ATXN10;ATXN3;AURKAIP1;AZU1;B3GNT8;B9D2;BAG5;BAK1;BANP;BARD1;BAX;BCAN;BCAS1;BCKDK;BCL3;BEX4;BFAR;BIN3;C10orf137;C19orf40;CA7;CABP1;CAMK2B;CAMKK1;CAND1;CAPN11;CARD9;CASK;CASP7;CBX8;C

LINT1;CNTN2;COL16A1;COL18A1;COL23A1;COL4A2;COL9A1;CPE;CRTAP;CSPG5;CTNNBIP1;DAAM2;DARS;DCTN3;DDX1;DEDD2;DGAT1;DISP1;DNAH9;DNAJC2;DNER;DNM1L;DRD3;DSTN;DYNC2H1;EI24;EIF4EBP1;ELANE;ELF3;ELFN1;EML4;EPHA4;ERCC4;ERCC6;ESRRB;EXOSC4;FADD;FANCC;FANCD2;FAT1;FB

6V1G2;ATPAF1;ATR;ATXN3;AURKAIP1;AZU1;B3GNT8;B9D2;BAK1;BANP;BARD1;BAX;BCAN;BCKDK;BCL3;BEX4;BFAR;BIN3;C10orf137;C19orf40;CABP1;CAMK2B;CAMKK1;CAND1;CAPN11;CASK;CASP7;CBX8;CCDC15;CCDC19;CCIN;CCNJ;CDC14B;CDC23;CDK5;CDK6;CDKN2D;CDYL;CENPC1;CENPJ;CEP128;CEP

6V1G2;ATPAF1;ATR;ATXN3;AURKAIP1;AZU1;B3GNT8;B9D2;BAK1;BANP;BARD1;BAX;BCAN;BCKDK;BCL3;BEX4;BFAR;BIN3;C10orf137;C19orf40;CABP1;CAMK2B;CAMKK1;CAND1;CAPN11;CASK;CASP7;CBX8;CCDC15;CCDC19;CCIN;CCNJ;CDC14B;CDC23;CDK5;CDK6;CDKN2D;CDYL;CENPC1;CENPJ;CEP128;CEP

DN;DDX1;DEDD2;DIP2C;DNAJC2;DNAJC8;DNTTIP1;DPPA4;DUSP1;EDNRB;EI24;EIF2S1;EIF4EBP1;ELF3;ELP2;EPM2A;ERCC4;ERCC6;ERRFI1;ESRRA;ESRRB;ETV3L;EXOSC4;FANCC;FANCD2;FAT1;FBXO32;FBXO43;FGF18;FHOD3;FKBPL;FKTN;FOS;FOXD4L1;FOXO1;FSTL3;FUBP3;GADD45G;GATAD1;GLIS2;GNPTAB;

CNB1;KCNQ5;KCTD4;KIFAP3;LAMA3;LIMA1;MAP1LC3A;MTDH;MYO1B;NCBP1;NCBP2;NIP7;NPM2;NRXN1;NUFIP1;NUP107;PANX2;PCBD2;PDSS2;PPP2CA;PRKCE;PRNP;PSMD5;PSRC1;PVRL4;PYCARD;RAB34;RAD52;RBMX;RPGRIP1L;SEPT2;SET;SHPRH;SHQ1;SHROOM1;SMAD6;SPICE1;SRR;SRSF9;SSH3;TAF1

SS1;ATAD5;ATE1;ATL1;ATMIN;ATP2A1;ATP2B2;ATP6V0A2;ATP6V0B;ATP6V1G2;ATPAF1;ATR;ATXN10;ATXN3;AURKAIP1;AZU1;B3GNT8;B9D2;BAG5;BAK1;BANP;BARD1;BAX;BCAN;BCAS1;BCKDK;BCL3;BEX4;BFAR;BIN3;C10orf137;C19orf40;CA7;CABP1;CAMK2B;CAMKK1;CAND1;CAPN11;CARD9;CASK;CASP7

TXN3;AURKAIP1;AZU1;B3GNT8;B9D2;BAK1;BANP;BARD1;BAX;BCAN;BCKDK;BCL3;BEX4;BFAR;C10orf137;C19orf40;CABP1;CAMK2B;CAMKK1;CAND1;CAPN11;CASK;CASP7;CBX8;CCDC19;CCNJ;CDC14B;CDC23;CDK5;CDK6;CDKN2D;CDYL;CENPC1;CERS1;CERS4;CERS6;CHIC2;CHST1;CITED4;CLASP2;CLIC5;CLIN

);PSMB8;PSMB9;PSMD5;PSME2;PYCARD;RASSF6;RHOB;SAV1;SET;SFN;SHQ1;SMAD6;SPHK1;SPI1;TERF1;THBS1;TNFRSF12A;TNFSF8;TRIM32;TSC22D3;TSPO;UBD;UNC5C;USP47;WNK3;WNT11;WRN;YAP1;ZC3H8;ZNF2

TXN3;AURKAIP1;AZU1;B3GNT8;B9D2;BAK1;BANP;BARD1;BAX;BCAN;BCKDK;BCL3;BEX4;BFAR;C10orf137;C19orf40;CABP1;CAMK2B;CAMKK1;CAND1;CAPN11;CASK;CASP7;CBX8;CCDC19;CCNJ;CDC14B;CDC23;CDK5;CDK6;CDKN2D;CDYL;CENPC1;CERS1;CERS4;CERS6;CHIC2;CHST1;CITED4;CLASP2;CLIC5;CLIN

IB8;PSMB9;PSMD5;PSME2;PYCARD;RASSF6;RHOB;SAV1;SET;SFN;SHQ1;SMAD6;SPHK1;SPI1;TERF1;THBS1;TNFRSF12A;TNFSF8;TRIM32;TSC22D3;TSPO;UBD;UNC5C;USP47;WNK3;WNT11;WRN;YAP1;ZC3H8;ZNF2

PSMB10;PSMB8;PSMB9;PSMD5;PSME2;PYCARD;RASSF6;RHOB;SAV1;SET;SFN;SHQ1;SMAD6;SPHK1;SPI1;TERF1;THBS1;TNFRSF12A;TNFSF8;TRIM32;TSC22D3;TSPO;UBD;UNC5C;USP47;WNK3;WNT11;WRN;YAP1;ZC3H8;ZNF205;ZNF7

;ASPHD1;ASS1;ATAD5;ATE1;ATG2B;ATL1;ATMIN;ATP2A1;ATP2B2;ATP6V0A2;ATP6V0B;ATP6V1G2;ATR;ATXN10;ATXN3;AURKAIP1;AZU1;B3GNT8;B9D2;BAG5;BAI3;BAIAP3;BAK1;BANP;BARD1;BAX;BCAN;BCKDK;BCL3;BFAR;BIN3;BMPR1B;C10orf137;C19orf40;CA7;CABP1;CACNB2;CAMK2B;CAMKK1;CAND1

2;WNK

;B9D2;BAG5;BAK1;BAX;BCAN;BCKDK;BCL3;BFAR;CA7;CABP1;CAMK2B;CAMKK1;CAPN11;CASK;CASP7;CCDC19;CCIN;CD151;CDC23;CDK5;CDK6;CDKN2D;CENPC1;CENPJ;CEP128;CEP192;CEP76;CERS1;CERS4;CERS6;CHIC2;CHST1;CLASP2;CLIC5;CLINT1;CLOCK;CLTB;CNIH2;COL16A1;COL18A1;COL23A1;COL4

2;HES1;HEXIM2;HHATL;HMBOX1;HMGA2;HOXB6;HTRA3;HUS1;IHH;KCNH3;KLF12;L3MBTL3;LEFTY1;LRPPRC;LUM;MAPK10;MAPK3;MED17;MESP2;MET;MKL2;MLLT10;MLX;MORF4L1;MRE11A;MSTN;MTDH;NAA15;NANOG;NANOS3;NCBP1;NCBP2;NFIB;NFKBID;NPAT;NPM2;NR1H3;NR2C2;NUFIP1;PAX9;P

GTF2H3;HAS3;HDAC2;HES1;HEXIM2;HHATL;HMBOX1;HMGA2;HOXB6;HPN;HTRA3;HUS1;IHH;KCNH3;KLF12;L3MBTL3;LEFTY1;LRPPRC;LUM;MAPK10;MAPK3;MED17;MESP2;MET;MKL2;MLLT10;MLX;MORF4L1;MRE11A;MSTN;MTDH;NAA15;NANOG;NANOS3;NCBP1;NCBP2;NFIB;NFKBID;NPAT;NPM2;NR1

P32A;ANPEP;ANXA11;ANXA4;ANXA5;ANXA7;ANXA9;AOC2;AP1M1;AP2A1;AP2M1;AP3B1;APBB1IP;APC2;APITD1;APLP2;APOA1;APOBEC3C;APP;ARAP1;ARAP3;ARCN1;ARF1;ARF3;ARF6;ARFGAP3;ARHGAP1;ARHGAP21;ARHGAP24;ARHGAP25;ARHGAP26;ARHGAP27;ARHGAP30;ARHGAP31;ARHGDIB;ARHGE

NO7;ANP32A;ANPEP;ANXA11;ANXA4;ANXA5;ANXA7;ANXA9;AOC2;AP1M1;AP2A1;AP2M1;AP3B1;APBB1IP;APC2;APITD1;APLP2;APOA1;APOBEC3C;APP;ARAP1;ARAP3;ARCN1;ARF1;ARF3;ARF6;ARFGAP3;ARHGAP1;ARHGAP21;ARHGAP23;ARHGAP24;ARHGAP25;ARHGAP26;ARHGAP27;ARHGAP30;ARHGAP

;ANXA7;ANXA9;AOC2;AP1M1;AP2A1;AP2M1;AP3B1;APBB1IP;APC2;APITD1;APOA1;APOBEC3C;APP;ARAP1;ARAP3;ARCN1;ARF1;ARF3;ARF6;ARFGAP3;ARHGAP1;ARHGAP21;ARHGAP24;ARHGAP25;ARHGAP26;ARHGAP27;ARHGAP30;ARHGAP31;ARHGDIB;ARHGEF10;ARHGEF11;ARHGEF12;ARID3A;ARL1;AR

L;APC2;APITD1;APLP2;APOA1;APOBEC3C;APP;ARAP1;ARCN1;ARF1;ARF3;ARF6;ARFGAP3;ARHGAP1;ARHGAP21;ARHGAP30;ARHGDIB;ARID1A;ARID3A;ARID3B;ARL1;ARL6;ARNT;ARSD;ARSG;ASAH1;ASB8;ASH2L;ATF5;ATF7;ATP5S;ATP6V0A1;ATP6V0C;ATP6V0D1;ATP6V1B2;ATPIF1;ATXN2;B3GNT9;B4GALT1;

APOA1;APOBEC3C;APP;ARAP1;ARCN1;ARF1;ARF3;ARF6;ARFGAP3;ARHGAP1;ARHGAP21;ARHGAP30;ARHGDIB;ARID1A;ARID3A;ARID3B;ARL1;ARL6;ARNT;ARSD;ARSG;ASAH1;ASB8;ASH2L;ATF5;ATF7;ATP5S;ATP6V0A1;ATP6V0C;ATP6V0D1;ATP6V1B2;ATPIF1;ATXN2;B3GNT9;B4GALT1;B4GALT4;B4GALT5;BA

L;AP3B1;APBB1IP;APC2;APITD1;APLP2;APOA1;APOBEC3C;APP;ARAP1;ARAP3;ARCN1;ARF1;ARF3;ARF6;ARFGAP3;ARHGAP1;ARHGAP21;ARHGAP24;ARHGAP26;ARHGAP30;ARHGDIB;ARHGEF10;ARID1A;ARID3A;ARID3B;ARL1;ARL6;ARNT;ARSD;ARSG;ASAH1;ASB8;ASH2L;ATF5;ATF7;ATP5S;ATP6V0A1;ATP6V

;APBB1IP;APC2;APITD1;APLP2;APOA1;APOBEC3C;APP;ARAP1;ARAP3;ARCN1;ARF1;ARF3;ARF6;ARFGAP3;ARHGAP1;ARHGAP21;ARHGAP24;ARHGAP26;ARHGAP30;ARHGDIB;ARHGEF10;ARID1A;ARID3A;ARID3B;ARL1;ARL6;ARNT;ARSD;ARSG;ASAH1;ASB8;ASH2L;ATF5;ATF7;ATP5S;ATP6V0A1;ATP6V0C;ATP6

CN1;ARF1;ARF3;ARF6;ARFGAP3;ARHGAP1;ARHGAP21;ARHGAP24;ARHGAP25;ARHGAP26;ARHGAP30;ARHGAP31;ARHGDIB;ARHGEF10;ARHGEF11;ARHGEF12;ARL1;ARL6;ARSD;ARSG;ASAH1;ATP5S;ATP6V0A1;ATP6V0C;ATP6V0D1;ATP6V1B2;ATPIF1;ATXN2;B3GNT9;B4GALT1;B4GALT4;B4GALT5;BBS4;BCKD

6;ARHGAP27;ARHGAP31;ARHGEF10;ARHGEF11;ARHGEF12;ARID1A;ARID3A;ARL1;ARL6;ARNT;ASH2L;ATF5;ATF7;ATG2A;ATG7;ATP2B4;ATP6V0A1;ATP6V0C;ATP6V0D1;ATPIF1;ATXN2;B4GALT1;BASP1;BAZ2A;BBS4;BCAS3;BCCIP;BCKDHA;BCL6;BCL9;BCOR;BECN1;BHLHE40;BICD2;BIN2;BLOC1S3;BNIP3;BRAP

P6V0A1;ATP6V0C;ATP6V0D1;ATPIF1;B3GNT9;B4GALT1;B4GALT4;B4GALT5;BASP1;BAZ2A;BBS4;BCCIP;BCKDHA;BCL2L13;BCL6;BCOR;BHLHE40;BIN2;BNIP3;BOD1;BRCA1;BRD4;BRD8;BRE;BRIX1;BRPF3;BSCL2;BSG;BUB3;C19orf12;C3orf52;CACNG8;CALCOCO1;CAMK2G;CANT1;CAP1;CAPZB;CARM1;CASC3;C

P6V0A1;ATP6V0C;ATP6V0D1;ATPIF1;B3GNT9;B4GALT1;B4GALT4;B4GALT5;BASP1;BAZ2A;BBS4;BCCIP;BCKDHA;BCL2L13;BCL6;BCOR;BHLHE40;BIN2;BNIP3;BOD1;BRCA1;BRD4;BRD8;BRE;BRIX1;BRPF3;BSCL2;BSG;BUB3;C19orf12;C3orf52;CACNG8;CALCOCO1;CAMK2G;CANT1;CAP1;CAPZB;CARM1;CASC3;C

ANKRA2;ANKRD37;ANKS1A;ANO10;ANO7;ANP32A;ANPEP;ANXA11;ANXA4;ANXA5;ANXA7;ANXA9;AOC2;AP1M1;AP2A1;AP2M1;AP3B1;APBB1IP;APC2;APITD1;APLP2;APOA1;APOBEC3C;APOBR;APP;ARAP1;ARAP3;ARCN1;ARF1;ARF3;ARF6;ARFGAP3;ARHGAP1;ARHGAP21;ARHGAP23;ARHGAP24;ARHGAP25

ANKRA2;ANKRD37;ANKS1A;ANO10;ANO7;ANP32A;ANPEP;ANXA11;ANXA4;ANXA5;ANXA7;ANXA9;AOC2;AP1M1;AP2A1;AP2M1;AP3B1;APBB1IP;APC2;APITD1;APLP2;APOA1;APOBEC3C;APOBR;APP;ARAP1;ARAP3;ARCN1;ARF1;ARF3;ARF6;ARFGAP3;ARHGAP1;ARHGAP21;ARHGAP23;ARHGAP24;ARHGAP25

PS15L1;ERC1;ERGIC1;F13A1;FAM160A2;FAM21A;FAM21C;FCN1;FKBP15;GAB2;GCC1;GOLGA5;GOSR1;GRB2;GSN;HCK;HFE;HIP1;HMOX1;HNRNPK;HSPA5;IGF2R;ITSN1;KDELR1;KIF13A;KIF1C;LAT2;LDLRAD3;LEFTY2;LILRB1;LIN7B;LMTK2;LRP1;MAGI2;MAP2K1;MERTK;MYO1F;MYO7A;NAPA;NME1;NOD2;NOT

NPEP;ANXA11;ANXA4;ANXA5;ANXA7;ANXA9;AOC2;AP1M1;AP2A1;AP2M1;AP3B1;APBB1IP;APC2;APITD1;APLP2;APOA1;APOBEC3C;APP;ARAP1;ARAP3;ARF1;ARF3;ARF6;ARFGAP3;ARHGAP1;ARHGAP21;ARHGAP23;ARHGAP24;ARHGAP25;ARHGAP26;ARHGAP27;ARHGAP31;ARHGEF10;ARHGEF11;ARHGEF

1orf73;CA13;CA3;CAMK2G;CAPZB;CARM1;CARS;CASC3;CASP6;CASP9;CBL;CBR3;CCBL1;CCL3;CDC42;CENPH;CHEK1;CHKA;CHMP4B;CLTA;CLTC;CNOT1;CNOT4;COPA;COPB2;CRKL;CRYZ;CSNK1D;CSNK2A1;CTNNA1;CTNNAL1;CTNNB1;CTNND1;CUX1;CYB5R3;DBNL;DNM2;DOCK2;DOCK6;DOCK8;DYNLL1;DYNL

IK1;LRRK1;MAP2K1;MAPK13;MAPK14;MAPKAPK5;MFN2;NDEL1;NKIRAS2;NOTCH2;PAFAH1B1;PLEKHG6;PREX1;PTK2B;RAB19;RAB23;RAB35;RAB36;RAB3D;RAB40B;RAB42;RAB43;RAB5B;RAB5C;RAB7A;RAB8A;RABL5;RAC1;RAC2;RAF1;RALB;RALBP1;RALGAPA2;RAP1GAP2;RAPGEFL1;RGL3;RGL4;RHEBL1;RH

AB5B;RAB5C;RAB7A;RAC1;RAMP1;RIN2;RIN3;RUFY1;SH3KBP1;SLC11A1;SNX1;SORL1;SYK;TGFB1;ULK1;VAMP8;WAS

CSF3R;CTNNB1;CTSB;CTSD;CXCR2;DAB2;DAPK1;DNM2;DOCK2;DYNLL1;DYNLL2;EGR1;ELF4;ELK1;ELMO1;EMR2;ENDOU;EP300;EXOSC3;F11R;FCAR;FCN1;FKBP1A;FLCN;FLT3;FOXP3;G6PD;GAB2;GALNT2;GLMN;GNAS;GPR183;GRB2;GTPBP1;HAX1;HCK;HCLS1;HDAC4;HFE;HIPK2;HLA-B;HLA-C;HLA-DPA1;HLA-

LC48A1;SQSTM1;SRC;TBC1D14;TIAL1;TLR9;TM9SF1;TMBIM1;TP53INP2;TPCN2;TPP1;TXNDC5;ULK1;USP4;UVRAG;VAMP8;VCAN;WIPI1;WIPI2;ZER

1;FLOT2;FURIN;GCK;GNAS;GOLIM4;GRB2;GSN;HAX1;HCK;HEXB;HFE;HIP1;HLA-B;HLA-C;HLA-DPA1;HLA-E;HSPA5;IGF2R;ITSN1;KIAA0319L;KIF1B;LAMP1;LEFTY2;LTBP2;MARCH8;MMP14;MSN;MYH11;MYH9;MYO7A;MYOF;NBR1;NCF2;NCSTN;NEU1;NOD2;PACS1;PAFAH1B1;PDCD6;PECAM1;POMC;PPT1;PR

;CYB5A;CYB5R1;CYB5R3;CYP27A1;DAP3;DHTKD1;DISC1;DNAJC19;DUT;DYNLL1;ESRP2;FAHD1;FAM136A;FAM162A;FDX1;FIS1;FUNDC1;GCK;GHITM;GIMAP8;GLUL;GM2A;GRN;GSR;GTPBP5;HAAO;HAX1;HCLS1;HDDC2;HIBADH;HK1;HK2;HRK;HSCB;HSPA1B;IMMP2L;IQCE;ISCU;KIF1B;LIPT1;LRRC59;LRRK1;LY

HST7;CKAP4;CLCN5;CLPX;CLTA;CLTC;CLTCL1;CNOT1;COG8;COPA;COPB2;COPZ2;COX11;COX18;COX5A;COX6A1;CPOX;CPT1A;CPT2;CUX1;CYB5A;CYB5R3;CYP27A1;CYP2R1;CYP4F3;DAB2;DENND1A;DNAJC14;DNAJC19;DNAJC5;DNM2;DPAGT1;DYSF;ECE1;EHD1;EHD3;ELOVL1;ELOVL3;EPHB1;ERC1;ERGIC1;EV

IM1;TPCN2;TPP1;TXNDC5;USP4;UVRAG;VAMP8;VCAN

IM1;TPCN2;TPP1;TXNDC5;USP4;UVRAG;VAMP8;VCAN

ME1;NOTCH1;NOTCH2;PAF1;PAFAH1B1;PIK3CD;PKNOX1;PLCG2;PLEK;POU2F2;PRKDC;PSEN1;PTK2B;RCOR1;RNF41;SEMA4A;SGPL1;SH2B3;SOD1;SOX4;SPTB;SRF;STAT5A;STAT5B;STAT6;STK4;SYK;TACC3;TFE3;TGFB1;THOC5;TIRAP;WDR

;HMOX1;HNRNPUL1;HSPA1B;HSPA5;IFNAR2;IL6R;IRS2;ITGB2;KCNA5;KIAA1598;LILRB1;LILRB2;MAGI2;MAML1;MAP2K1;MAP2K3;MAP3K5;MAPKAPK5;MBP;MFN2;MICALCL;MSN;MTF1;NACC2;NAE1;NBR1;NCF2;NCOA6;NCOR1;NDRG1;NEK6;NFKBIA;NME1;NOD2;NOTCH1;NR2C1;NXT1;OS9;PAF1;PAFAH1

CC1;GNAS;GOLGA5;GOSR1;HCLS1;HGSNAT;IFT43;IFT46;IL23A;IMMP2L;JAK3;JPH4;KDELR1;KIAA0196;KIF13A;KIF1B;KIF1C;KPNB1;LCP1;LMTK2;LTBP2;MAP1S;MAP2K1;MAPK14;MFN2;MGRN1;MICU1;MRPL45;MX2;MYO7A;NAPA;NDEL1;NFKBIA;NLRP12;NLRP3;NRBP1;NUP98;NUPL2;NXT1;OPTN;OS9;PACS

3;BRCA1;BRD3;BRD4;BRD8;BRE;BRIX1;BRPF3;BTK;BUB3;C11orf73;CALCOCO1;CALCOCO2;CAMK1D;CAMK2G;CAMTA1;CARM1;CASC3;CASP6;CASP9;CASZ1;CBFA2T3;CBL;CBR3;CBX1;CBX6;CBY1;CCBL1;CCNB1IP1;CCRN4L;CDC5L;CDCA4;CDV3;CELF3;CENPH;CERKL;CERS2;CFL1;CFL2;CHCHD1;CHD8;CHEK1;C

RB2;GSN;HCK;HDAC4;HMOX1;HSPA5;IL17RA;IL23A;IL4R;IL6R;IRAK2;IRF2;ITGA5;ITGAM;ITGAX;ITGB2;ITGB4;ITPK1;KCNMB3;KCNMB4;KDM6B;KIF3C;KLF4;LCP2;LEFTY2;LIPA;LTA4H;LTB4R;MAFG;MAP2K1;MAP2K3;MAPK13;MAPK14;MAX;MEFV;MERTK;MFN2;MMP25;MYB;MYD88;MYOF;NDEL1;NDST1;NF

AMK1D;CAMK2G;CAP1;CAPZB;CASC3;CASP9;CCL3;CCNK;CD14;CD163;CD1D;CD209;CD300C;CD44;CD86;CD97;CDC42;CELA1;CFL1;CFLAR;CHCHD6;CHEK1;CHRNB2;CITED2;CLEC4C;CNN2;COLEC12;COTL1;CREBBP;CRKL;CRTAM;CSF1;CSF1R;CSF3R;CSNK1D;CTDSP2;CTNNA1;CTSB;CTSD;CXCR2;DAB2;DAP;DAPI
BIA;NHEJ1;NME1;NOTCH1;NOTCH2;PAF1;PAFAH1B1;PIK3CD;PKNOX1;PLCG2;PLEK;POU2F2;PRKDC;PSEN1;PTK2B;RCOR1;RNF41;SBDS;SEMA4A;SGPL1;SH2B3;SOD1;SOX4;SPTB;SRF;STAT5A;STAT5B;STAT6;STK4;SYK;TACC3;TFE3;TGFB1;THOC5;TIRAP;WDR
PRCP;PRKACA;PRKCB;PRKCD;PSAP;PSEN1;PTPN1;PTPN6;PTPRJ;RAC1;RAC2;RAF1;RCOR1;RHOA;SELL;SELPLG;SERPINA1;SH2B3;SIN3A;SIRPA;SLC7A5;SLC7A7;SOD1;SRC;SRF;SYK;TGFB1;TLN1;VAV1;VCL;WA
2;PLEK;PRCP;PRKACA;PRKCB;PRKCD;PSAP;PTPN1;PTPN6;PTPRJ;RAC1;RAC2;RAF1;RCOR1;RHOA;SELL;SELPLG;SERPINA1;SH2B3;SIN3A;SIRPA;SLC7A5;SLC7A7;SOD1;SRC;SRF;SYK;TGFB1;TLN1;VAV1;VCL;WA
LAT2;LCP1;LCP2;LEFTY2;LILRB1;LILRB2;MAPK14;MERTK;MYB;MYH9;MYO1F;NCOR1;NCSTN;NDRG1;NFAM1;NHEJ1;NOD2;P2RX1;PAK1;PECAM1;PFDN1;PIK3CD;PIK3R5;PKNOX1;PLCG2;PLEK;POU2F2;PRAM1;PREX1;PRKCB;PRKCD;PRKDC;PRMT5;PSAP;PSEN1;PTK2B;PTPN1;PTPN6;PTPRJ;RAC1;RAC2;RAF1;RH
PRCP;PRKACA;PRKCB;PRKCD;PSAP;PTPN1;PTPN6;PTPRJ;RAC1;RAC2;RAF1;RCOR1;RHOA;SELL;SELPLG;SERPINA1;SH2B3;SIN3A;SIRPA;SLC7A5;SLC7A7;SOD1;SRC;SRF;SYK;TGFB1;TLN1;VAV1;VCL;WA
XP3;FZD1;GCC1;GDI2;GLMN;GNAS;GOSR1;HCLS1;IL23A;IL4R;IMMP2L;JAK3;KDELR1;KIF13A;KPNB1;LCP1;LCP2;LILRB1;LIN7B;LMTK2;LRP1;LTBP2;MAPK14;MFN2;MRPL45;MX2;MYH9;MYO7A;NAPA;NFKBIA;NLRC4;NLRP12;NLRP3;NOD2;NOTCH1;NOTCH2;NUP35;NUP98;NUPL2;NXT1;OPTN;OS9;PACS1;PAF
L;NFAM1;NFKBIA;NHEJ1;NME1;NOTCH1;NOTCH2;PAF1;PAFAH1B1;PIK3CD;PKNOX1;PLCG2;PLEK;POU2F2;PRKDC;PSEN1;PTK2B;RCOR1;RNF41;SBDS;SEMA4A;SGPL1;SH2B3;SOD1;SOX4;SPTB;SRF;STAT5A;STAT5B;STAT6;STK4;SYK;TACC3;TFE3;TGFB1;THOC5;TIRAP;TNFSF13;WDR
;GOLIM4;GRB2;GSN;HAX1;HCK;HEXB;HIP1;HLA-B;HLA-C;HLA-DPA1;HLA-E;HSPA5;IGF2R;ITSN1;KIF1B;LAMP1;LEFTY2;LTBP2;MARCH8;MMP14;MSN;MYH11;MYH9;MYO7A;MYOF;NCF2;NCSTN;NEU1;PACS1;PECAM1;POMC;PPT1;PRG2;RAB11FIP1;RAB11FIP4;RAB23;RAB35;RAB3D;RAB43;RAB5B;RAB5C;RAB
R;IL6R;IRAK2;IRS2;ITGB2;JAK1;JAK3;KIR3DL1;KLF13;KLRC4-KLRK1;LAT2;LCP2;LILRA1;LILRB1;LILRB2;MAFB;MAP2K1;MAP2K3;MAPK14;MERTK;MPP1;MSN;MYB;MYD88;MYO1F;NFAM1;NFKB1;NFKBIA;NLRC4;NME1;NOD2;NOTCH1;PACS1;PAF1;PAK1;PCBP2;PIK3CD;PLCG2;POLR3F;PRAM1;PRKCB;PRKD2;PS
JC19;DNAJC5;DNM2;DSCR3;EHD1;EHD3;EIF2D;ERC1;ERGIC1;F13A1;FAM160A2;FAM21A;FAM21C;FBXO22;FGF9;FIS1;FOXP3;FURIN;FZD1;GAB2;GCC1;GCK;GLMN;GLUL;GNAS;GOLGA5;GOSR1;HCK;HCLS1;HGSNAT;HMGN3;HMOX1;HSPA5;IFT43;IFT46;IL23A;IL4R;IMMP2L;IRS2;JAK3;JPH4;KCNA5;KCNC3;KC
TSA;DAB2;DAG1;DENND1A;DNAJC14;DNAJC19;DNM2;DNMT3B;EHD1;EHD3;EIF2D;EPB41L3;ERC1;FAM160A2;FBN2;FGF9;FIS1;FLCN;FOXP3;FTO;FZD1;GCC1;GDI2;GLMN;GM2A;GNAS;GOSR1;HCLS1;HDLBP;HEXB;HNRNPK;IL23A;IL4R;IMMP2L;IRS2;JAK3;KATNA1;KDELR1;KIF13A;KPNB1;LCP1;LCP2;LILRB1;LI
ALGAPA2;RAP1GAP2;RGS10;RGS16;RGS3;RIN2;RIN3;SIPA1L1;SIPA1L2;SMAP2;SPRY2;SRGAP2;STARD8;STK4;TBC1D1;TBC1D14;TBC1D19;TBC1D2;TBC1D20;TBC1D2B;TBC1D5;TBC1D8;TBC1D9;TIMP

LBP1;RALGAPA2;RHOA;RRAS2;RSU1;SEMA4D;SIPA1L1;SMAP2;SPRY2;SQSTM1;SRC;SRGAP2;TBC1D2;TIMP2;TRIM67;ULK1;VAV1;WASF2;YWHA
7;CHD8;CHEK1;CITED2;CLPX;COL15A1;COL5A2;COL8A2;COQ3;COX17;CPOX;CPSF7;CPT2;CREBBP;CRNKL1;CS;CSNK2A1;CTDP1;CTDSP2;CTNNB1;CTSA;CTSB;CTSD;CYP27A1;DAG1;DAP3;DAPK2;DEDD;DNAJC14;DNMT3B;DSCC1;DUT;EGR1;ELF4;ELL;ELL3;EMG1;EP300;ESR1;EXOSC1;EXOSC3;EXOSC8;EYA3;F13
AX1;HCK;HEXB;HFE;HIP1;HLA-B;HLA-C;HLA-DPA1;HLA-E;HSPA5;IGF2R;ITSN1;KIAA0319L;KIF1B;LAMP1;LEFTY2;MARCH8;MMP14;MYH11;MYO7A;MYOF;NBR1;NCF2;NCSTN;NEU1;PACS1;PDCD6;PECAM1;POMC;PPT1;PRG2;PSEN1;PTPN1;RAB11FIP1;RAB11FIP4;RAB23;RAB35;RAB3D;RAB43;RAB5B;RAB5C;R

NK2A1;CTNNA1;CTNNB1;CTSB;CTSD;CXCR2;CXCR7;DAB2;DAP;DAP3;DAPK1;DAPK2;DBNL;DEDD;DNAJB6;DNAJC5;DNM2;DPF2;DYNLL1;DYNLL2;EGR1;ELMO1;ELMO2;EP300;ESR1;FAIM;FAM129B;FAM162A;FIG4;FIS1;FLCN;FLT3;FOSL2;FURIN;FUS;GBA;GHITM;GIGYF2;GLO1;GNB1;GNRH1;GRN;GSN;H1F0;H
NK2A1;CTNNA1;CTNNB1;CTSB;CTSD;CXCR2;CXCR7;DAB2;DAP;DAP3;DAPK1;DAPK2;DBNL;DEDD;DNAJB6;DNAJC5;DNM2;DPF2;DYNLL1;DYNLL2;EGR1;ELMO1;ELMO2;EP300;ESR1;FAIM;FAM129B;FAM162A;FIG4;FIS1;FLCN;FLT3;FOSL2;FURIN;FUS;GBA;GHITM;GIGYF2;GLO1;GNB1;GNRH1;GRN;GSN;H1F0;H
HEK1;CITED2;CLPX;COL15A1;COL5A2;COL8A2;COQ3;CPSF7;CPT2;CREBBP;CRNKL1;CS;CSNK2A1;CTDP1;CTDSP2;CTNNB1;CTSA;CTSB;CTSD;CYP27A1;DAG1;DAP3;DAPK2;DEDD;DNAJC14;DNMT3B;DSCC1;DUT;EGR1;ELF4;ELL;ELL3;EMG1;EP300;ESR1;EXOSC1;EXOSC3;EXOSC8;EYA3;F13A1;FANCF;FDX1;FLT3;F

A;CUX1;DAB2;DAG1;DENND3;DNAJC19;DNAJC5;DNM2;DOCK2;DSCR3;EHD1;EHD3;EIF2D;EPB41L3;ERC1;ERGIC1;F13A1;FAM160A2;FAM21A;FAM21C;FBXO22;FGF9;FIS1;FLCN;FOXP3;FURIN;FZD1;GAB2;GCC1;GCK;GLMN;GLUL;GNAS;GOLGA5;GOSR1;HCK;HCLS1;HGSNAT;HMGN3;HMOX1;HSPA5;IFT43;IFT4
ACNG8;CAMK1D;CANT1;CARM1;CASP9;CBL;CBY1;CCL3;CD14;CD1D;CD3D;CD44;CD86;CDC42;CDH1;CFLAR;CHD8;CHEK1;CHRNB2;CHST11;CITED2;CLTA;CLTC;COLEC12;CREBBP;CRTAM;CSF1;CSF1R;CSNK1D;CSNK2A1;CTNNA1;CTNNB1;CTNND1;DAB2;DAG1;DAPK1;DBNL;DISC1;DNMBP;DOCK2;DVL3;EGR1;
FN2;MYB;MYOF;P2RX1;PAK1;PECAM1;PHF21A;PIK3R5;PLAUR;PLCG2;PLEK;PRCP;PRKACA;PRKCB;PRKCD;PSAP;PTPN1;PTPN6;PTPRJ;RAC1;RAC2;RAF1;RCOR1;RHOA;SELL;SELPLG;SERPINA1;SH2B3;SIN3A;SIRPA;SLC11A1;SLC7A5;SLC7A7;SOD1;SRC;SRF;SYK;TGFB1;TLN1;VAV1;VCL;WA
XP3;FZD1;GCC1;GDI2;GLMN;GNAS;GOSR1;HCLS1;IL23A;IL4R;IMMP2L;JAK3;KDELR1;KIF13A;KPNB1;LCP1;LCP2;LILRB1;LIN7B;LMTK2;LRP1;LTBP2;MAPK14;MFN2;MRPL45;MX2;MYH9;MYO7A;NAPA;NFKBIA;NLRC4;NLRP12;NLRP3;NOD2;NOTCH1;NOTCH2;NUP35;NUP98;NUPL2;NXT1;OPTN;OS9;PACS1;PAF
PX;COL15A1;COL5A2;COL8A2;COQ3;CPSF7;CPT2;CREBBP;CRNKL1;CS;CSNK2A1;CTDP1;CTDSP2;CTNNB1;CTSA;CTSB;CTSD;CYP27A1;DAG1;DAP3;DAPK2;DEDD;DNAJC14;DNMT3B;DSCC1;DUT;EGR1;ELF4;ELL;ELL3;EMG1;EP300;ESR1;EXOSC1;EXOSC3;EXOSC8;EYA3;FANCF;FDX1;FLT3;FOXJ2;FOXN3;FOXO4;FC

21A;PIK3R5;PLAUR;PLCG2;PLEK;PRCP;PRKACA;PRKCB;PRKCD;PSAP;PTPN1;PTPN6;PTPRJ;RAC1;RAC2;RAF1;RCOR1;RHOA;SELL;SELPLG;SERPINA1;SH2B3;SIN3A;SIRPA;SLC7A5;SLC7A7;SOD1;SRC;SRF;STAT5A;STAT5B;SYK;TGFB1;TLN1;UTS2;VAV1;VCL;WAS;WNK
4L;CD3D;CD44;CD86;CDC42;CDC5L;CDH1;CELA1;CELF3;CFLAR;CHD8;CHEK1;CITED2;CNN2;CNOT1;CNOT4;CNPY2;CREBBP;CSF1;CSNK1D;CSNK2A1;CTIF;CTNNB1;CTNND1;CUX1;CUX2;DAB2;DAP;DAPK1;DEDD;DNM2;DNMT3B;DPF2;DVL3;DYNLL1;EGR1;EID2B;EID3;EIF2AK1;EIF2B3;EIF4A2;EIF4EBP2;EIF4H;EL

GAP27;ARHGAP30;ARHGAP31;ARHGDIB;ARHGEF10;ARHGEF11;ARHGEF12;ARID1A;ARID3A;ARID3B;ARL1;ARL6;ARNT;ASB8;ASCC2;ASH2L;ATF5;ATF7;ATG7;ATP6V0A1;ATP6V0C;ATP6V0D1;ATP6V1B2;ATPIF1;ATXN2;B4GALT1;BASP1;BAZ2A;BBS4;BCCIP;BCDIN3D;BCL2L13;BCL6;BCL9;BCO2;BCOR;BCORL1;B
HGAP23;ARHGAP24;ARHGAP25;ARHGAP26;ARHGAP27;ARHGAP30;ARHGAP31;ARHGDIB;ARHGEF10;ARHGEF11;ARHGEF12;ARID1A;ARID3A;ARID3B;ARL1;ARL6;ARNT;ASB8;ASCC2;ASH2L;ATF5;ATF7;ATG7;ATP6V0A1;ATP6V0C;ATP6V0D1;ATP6V1B2;ATPIF1;ATXN2;B4GALT1;BASP1;BAZ2A;BBS4;BCCIP;BCDI

T3B;EHD1;EHD3;EIF2D;EPB41L3;ERC1;FAM160A2;FBN2;FGF9;FIS1;FLCN;FOXP3;FZD1;GCC1;GDI2;GLMN;GNAS;GOSR1;HCLS1;IL23A;IL4R;IMMP2L;JAK3;KATNA1;KDELR1;KIF13A;KPNB1;LCP1;LCP2;LILRB1;LIN7B;LMTK2;LRP1;LTBP2;MAGI2;MAPK14;MAPRE1;MFN2;MLH3;MRPL45;MX2;MYH9;MYO7A;NACC

1;THOC
;GTF3A;H1F0;HAX1;HCLS1;HDAC4;HDGF;HIPK2;HNRNPD;HNRNPF;HNRNPK;HNRNPUL1;HSPA1B;ING4;INTS3;IRF2;JARID2;KAT5;KAT6A;KAT7;KDM2A;KLF4;KPNB1;LZTS1;MAFB;MAFG;MAML1;MAML3;MAP2K3;MAPK14;MAU2;MAX;MED12;MED25;MIS18A;MKNK2;MLL2;N4BP1;NACC2;NCOA6;NCOR1;NEK
IP1;CCNK;CDC42;CENPH;CFL1;CFLAR;CHCHD6;CHD8;CHEK1;CLTC;CLTCL1;CNN2;COPA;COPB2;CORO1C;COX18;CREBBP;CSF1R;CSNK1D;CTDP1;CTNNB1;CTSD;DAB2;DAG1;DAP3;DCLRE1B;DISC1;DNAJB6;DNAJC19;DNMT3B;DOCK2;DSCC1;DYNLL1;DYSF;ELMO1;ENSA;EP300;EPB41;EPB41L3;ERMN;EYA3;FAM

NKL1;CSNK2A1;CTDP1;CTDSP2;CTNNB1;CTSB;CWC15;DAG1;DAP3;DEDD;DHX8;DNAJC14;DNMT3B;DSCC1;DUT;DYNLL1;EGR1;ELF4;ELL;ELL3;EMG1;EP300;ESR1;EXOSC1;EXOSC3;EXOSC8;EYA3;FANCF;FOXJ2;FOXN3;FOXO4;FOXP3;FUS;GATAD2B;GCK;GIT2;GM2A;GRB2;GTF2F1;GTF3A;H1F0;H2AFY;HAX1;HCL

AB2;DAP;DAP3;DAPK1;DAPK2;DBNL;DEDD;DNAJB6;DNAJC5;DNM2;DPF2;DYNLL1;DYNLL2;EGR1;ELMO1;ELMO2;EP300;ESR1;FAIM;FAM129B;FAM162A;FIS1;FLCN;FLT3;FURIN;GHITM;GLO1;GNB1;GNRH1;GSN;H1F0;HCK;HCLS1;HERPUD1;HIP1;HIPK2;HK2;HMOX1;HRK;HSPA1B;HSPA5;IGF1R;IL6R;ING4;IRS2
CA3;CA6;CACNG8;CAMK1D;CAMK2G;CAP1;CASC3;CBL;CCL3;CD14;CD209;CD93;CDC42;CDC42SE1;CDH1;CFL1;CHKA;CHMP4B;CHRNB1;CHRNB2;CLCN5;CLDN16;CLTA;CLTC;CLTCL1;CNN2;CNNM4;COG8;COLEC12;COPA;COPB2;COPZ2;CORO1C;COX17;COX18;CPNE7;CPT1A;CPT2;CRTAM;CSF1R;CTNNB1;CTS
;BECN1;BHLHE40;BRCA1;BRD3;BRD4;BRD8;BRE;BSCL2;BTG2;BTK;BUB3;C1QTNF3;C3;C4BPB;C5AR1;CALCOCO1;CAMK1D;CAMKK2;CAMTA1;CAP1;CARM1;CASC3;CASP9;CAST;CASZ1;CBFA2T3;CBL;CBX1;CBX6;CBY1;CCL3;CCNK;CCRN4L;CD3D;CD44;CD86;CDC42;CDC5L;CDH1;CELA1;CELF3;CFLAR;CHD8;CHEK
1;CARM1;CASC3;CASZ1;CBFA2T3;CBL;CBX1;CBX6;CBY1;CCL3;CCNK;CCRN4L;CD3D;CD44;CD86;CDC42;CDC5L;CDH1;CELA1;CELF3;CFLAR;CHD8;CHEK1;CITED2;CNN2;CNOT1;CNOT4;CNPY2;CREBBP;CSF1;CSF1R;CSNK1D;CSNK2A1;CTDP1;CTDSP2;CTIF;CTNNB1;CTNND1;CUX1;CUX2;DAB2;DAP;DAPK1;DBNL;DI

AT3;SYK;TAF4;TAP2;TBC1D20;TGFB1;THOC5;TNIP1;TRIM25;TSG101;UBN1;VIM;WWP2;ZY
TBC1D
AB2;DAP;DAP3;DAPK1;DAPK2;DBNL;DEDD;DNAJB6;DNAJC5;DNM2;DPF2;DYNLL1;DYNLL2;EGR1;ELMO1;ELMO2;EP300;ESR1;FAIM;FAM129B;FAM162A;FIS1;FLCN;FLT3;FURIN;GHITM;GLO1;GNB1;GNRH1;GSN;H1F0;HCK;HCLS1;HERPUD1;HIP1;HIPK2;HK2;HMOX1;HRK;HSPA1B;HSPA5;IGF1R;IL6R;ING4;IRS2

PLEKHM1P;PLEKHO2;PREX1;PRKD2;PTAFR;RAB35;RPH3A;SH2B3;SH3PXD2B;SH3YL1;SNX1;SNX12;SNX19;SNX24;SNX27;SNX30;SNX33;SYT2;TBC1D2;TBC1D2B;TIRAP;TRIOBP;VAV1;WIPI1;WIPI2;ZFYVE
1;NFKBIA;PHLPP1;PID1;PREX1;PRKAB1;PRKACA;PRKCB;PRKCD;PRKD2;PSEN1;PTK2B;PTPN1;PTPN6;PTPRJ;PXN;RAB7A;RAC1;RAF1;RALB;RBPJ;RHOA;RPS6KA1;RTN4;SGPL1;SH3KBP1;SIPA1L1;SOCS2;SPRY2;SQSTM1;SRC;STAT3;STAT5A;STAT5B;SULF2;TGFB1;TLR9;TRAT1;TXNIP;ULK1;VAV1;YWHAB;ZFP1
;HLA-C;HLA-DPA1;HLA-E;HSPA5;IGF2R;ITSN1;KIF1B;LAMP1;LEFTY2;MARCH8;MMP14;MYH11;MYO7A;MYOF;NCF2;NCSTN;NEU1;PACS1;PECAM1;POMC;PPT1;PRG2;RAB11FIP1;RAB11FIP4;RAB23;RAB35;RAB3D;RAB43;RAB5B;RAB5C;RAB7A;RAB8A;RAC1;RGL4;RIN3;RPH3A;RPN1;SEC23B;SEC31A;SERPINA1

AB11FIP4;RAB23;RAB35;RAB5B;RAB5C;RAB7A;RAB8A;RIN3;RIPK1;RUFY1;SCAMP2;SLA;SLC11A1;SLC48A1;SNX1;SNX27;SPG21;SQSTM1;SRC;STEAP3;TLR9;TMBIM1;TPCN2;TRAK1;TSG101;UVRAG;VAMP8;VPS25;VPS35;WASF2;WIP
BTK;BUB3;C1QTNF3;C3;C5AR1;CALCOCO1;CAMK1D;CAMKK2;CAMTA1;CAP1;CARM1;CASC3;CASZ1;CBFA2T3;CBL;CBX1;CBX6;CBY1;CCL3;CCNK;CCRN4L;CD3D;CD44;CD86;CDC42;CDC5L;CDH1;CELA1;CELF3;CFLAR;CHD8;CHEK1;CHRNB2;CITED2;CNOT1;CNOT4;CNPY2;CPT1A;CPT2;CREBBP;CSF1;CSF1R;CSNK

ARAP3;ARF1;ARF3;ARF6;ARFGAP3;ARHGAP1;ARHGAP21;ARHGAP23;ARHGAP24;ARHGAP25;ARHGAP26;ARHGAP27;ARHGAP30;ARHGAP31;ARHGDIB;ARHGEF10;ARHGEF11;ARHGEF12;ARID1A;ARID3A;ARID3B;ARL1;ARL6;ARNT;ASB8;ASCC2;ASH2L;ATF5;ATF7;ATG7;ATP2B4;ATP6V0A1;ATP6V0C;ATP6V0D1
BRPF3;BSCL2;BSG;BTN3A1;BUB3;C11orf73;C1QTNF3;C3;C5AR1;CA13;CA14;CA3;CA6;CACNG8;CAMK1D;CAMK2G;CAP1;CASC3;CBL;CBY1;CCDC135;CCL3;CD14;CD209;CD93;CDC42;CDC42SE1;CDH1;CELF3;CFL1;CHKA;CHMP4B;CHRNB1;CHRNB2;CHST11;CITED2;CLCN5;CLDN16;CLTA;CLTC;CLTCL1;CNN2;CNN
CA1;BRPF3;BTK;CA13;CA14;CA3;CA6;CACNA2D4;CAMKK2;CANT1;CAPN1;CAPNS1;CARS;CASZ1;CBFA2T3;CBL;CCNB1IP1;CD209;CD93;CD97;CDH1;CELA1;CHI3L2;CHKA;CHRNB1;CHRNB2;CLPX;CNOT4;COL5A2;COLEC12;COX11;COX17;COX5A;CPM;CPPED1;CREBBP;CRYZ;CTDSP2;CYB5A;CYB5D1;CYP27A1;CY
1;DAB2;DAG1;DAPK1;DAPK2;DBNL;DGKG;DISC1;DNMBP;DOCK2;DOCK5;DOCK6;DOCK8;DOK3;DVL3;EIF4EBP2;ELK1;ELMO1;EP300;EPHB1;ERC1;ESR1;FBP1;FGF9;FIS1;FKBP1A;FLCN;FLT3;FOXO4;GAB2;GBA;GCK;GDI2;GIT2;GMIP;GNA12;GNAI2;GNB1;GPR183;GRB2;GSK3A;GSN;HAX1;HCLS1;HDAC4;HIPK2;H

;DNAJC14;DNMT3B;DSCC1;DUT;EGR1;ELF4;ELL;ELL3;EMG1;EP300;ESR1;EXOSC1;EXOSC3;EXOSC8;EYA3;FANCF;FOXJ2;FOXN3;FOXO4;FOXP3;FUS;GATAD2B;GCK;GIT2;GM2A;GTF2F1;GTF3A;H1F0;H2AFY;HAX1;HCLS1;HDAC4;HDGF;HIPK2;HMOX1;HNRNPD;HNRNPF;HNRNPK;HNRNPUL1;HSPA1B;ING4;INTS3

PK5;MARK2;MARK3;MAST3;MERTK;MKNK1;MKNK2;MPP1;NADK;NAGK;NEK3;NEK6;NME1;NME2;NRBP1;NUAK2;PAK1;PFKFB3;PGK1;PHKA2;PIK3CD;PIM1;PIM2;PINK1;PIP4K2C;POLR2A;PRKAB1;PRKACA;PRKCB;PRKCD;PRKD2;PRKDC;PSTK;PTK2B;RAF1;RIPK1;RPS6KA1;SEPHS1;SIK3;SQSTM1;SRC;STK24;STK

PK5;MARK2;MARK3;MAST3;MERTK;MKNK1;MKNK2;NADK;NEK3;NEK6;NME2;NRBP1;NUAK2;PAK1;PFKFB3;PHKA2;PIK3CD;PIM1;PIM2;PINK1;PIP4K2C;POLR2A;PRKAB1;PRKACA;PRKCB;PRKCD;PRKD2;PRKDC;PTK2B;RAF1;RIPK1;RPS6KA1;SIK3;SQSTM1;SRC;STK24;STK32B;STK35;STK4;STK40;SYK;TK2;TP53RK

WIPI

RC;STAT3;SYK;TAF11;TAF4;TAP2;TBC1D20;TGFB1;THOC5;TIRAP;TNIP1;TRIM25;TSG101;UBN1;VIM;WWP2;ZY

RC;STAT3;SYK;TAF11;TAF4;TAP2;TBC1D20;TGFB1;THOC5;TIRAP;TNIP1;TRIM25;TSG101;UBN1;VIM;WWP2;ZY

OX1;PLCG2;POU2F2;PRAM1;PREX1;PRKCB;PRKCD;PRKDC;PSEN1;PTK2B;PTPN6;RAC1;RNF41;SELPLG;SEMA4A;SLC11A1;SOD1;SOX4;SRC;SRF;STAT5A;STAT5B;STAT6;SYK;TGFB1;TIRAP;TNFSF13;VAV1;W

;NEK3;NEK6;NME2;NRBP1;NUAK2;PAK1;PHKA2;PIM1;PIM2;PINK1;POLR2A;PRKAB1;PRKACA;PRKCB;PRKCD;PRKD2;PRKDC;PTK2B;RAF1;RIPK1;RPS6KA1;SIK3;SQSTM1;SRC;STK24;STK32B;STK35;STK4;STK40;SYK;TP53RK;TRIM27;ULK1;VRK3;WNK1;XRCC6BP

4;ANKS1A;ANO10;ANP32A;ANPEP;ANXA11;ANXA4;ANXA5;ANXA7;ANXA9;AOAH;AOC2;AP1M1;AP2A1;AP2M1;AP3B1;APBB1IP;APC2;APITD1;APLP2;APOA1;APOBEC3C;APP;ARAP1;ARAP3;ARCN1;ARF1;ARF3;ARF6;ARFGAP3;ARHGAP1;ARHGAP21;ARHGAP23;ARHGAP24;ARHGAP25;ARHGAP26;ARHGAP27;

L30;SETD1B;SIN3A;SMARCA2;SMARCD2;SUPT3H;TET3;TGFB1;UBN1;USP22;UTP3;WDR61;WDR82;YEATS

CA1;BRPF3;BTK;CA13;CA14;CA3;CA6;CACNA2D4;CAMKK2;CANT1;CAPN1;CAPNS1;CARS;CASZ1;CBFA2T3;CBL;CCNB1IP1;CD209;CD93;CD97;CDH1;CELA1;CLPX;CNOT4;COL5A2;COLEC12;COX11;COX17;COX5A;CPM;CPPED1;CREBBP;CRYZ;CTDSP2;CYB5A;CYB5D1;CYP27A1;CYP2R1;CYP4F3;DAG1;DGKG;DHX3

CA3;CA6;CACNG8;CAMK1D;CAMK2G;CAP1;CASC3;CBL;CCL3;CD14;CD209;CD93;CDC42;CDC42SE1;CDH1;CFL1;CHKA;CHMP4B;CHRNB1;CHRNB2;CLCN5;CLDN16;CLTA;CLTC;CLTCL1;CNN2;CNNM4;COG8;COLEC12;COPA;COPB2;COPZ2;CORO1C;COX17;COX18;CPNE7;CPT1A;CPT2;CRTAM;CSF1R;CTNNB1;CTS

N6;RAC1;RNF41;SEMA4A;SLC11A1;SOD1;SOX4;SRC;SRF;STAT5A;STAT5B;STAT6;SYK;TGFB1;TIRAP;TNFSF13;VAV1;WA

RKD2;PSEN1;PTPN1;PTPN6;PTPRJ;PVR;RIPK1;RPS6KA1;SAMHD1;SELL;SH2D1A;SIN3A;SLC11A1;SPG21;STAT5A;STAT5B;STAT6;SYK;TAP2;TGFB1;TIRAP;TLR9;TNFSF13;TNIP1;TRAT1;TRIM27;UBE2V1;VASP;WAS;XI

OPS7A;COPS8;CREBBP;CSF1;CSF1R;CSNK1D;CSNK2A1;CTDP1;CTDSP2;CTIF;DAB2;DAPK1;DAPK2;DBNL;DENND3;DISC1;DNAJB12;DNAJB5;DNAJB6;DNAJC14;DNAJC18;DNAJC19;DNAJC5;DNAJC5B;DNMT3B;DPAGT1;DPH5;DSCC1;DUSP12;DUSP18;DVL3;DYRK4;EGR1;EIF2AK1;EIF2B3;EIF4A2;EIF4EBP2;EIF4H;EI

CCNK;CCRN4L;CD3D;CD86;CDC42;CDC5L;CDH1;CELA1;CELF3;CFLAR;CHD8;CHEK1;CITED2;CNOT1;CNOT4;CREBBP;CSNK2A1;CTDP1;CTDSP2;CTNNB1;CTNND1;CUX1;CUX2;DAB2;DAP;DEDD;DNAJB6;DNM2;DNMT3B;DPF2;DSCC1;DVL3;DYNLL1;EGR1;EID2B;EID3;ELF4;ELK1;ELL;ELL3;ENO1;EP300;ERC1;ERF;ES

;PTPN1;RAC1;RAC2;RHOA;SIPA1L1;SPTB;SRF;SRGAP2;SRI;SSH1;SSH2;TLN1;TMSB10;TMSB15B;TNNC1;TPM3;TPM4;TRIOBP;VASP;VIM;WAS;WASF

CCNK;CCRN4L;CD3D;CD86;CDC42;CDC5L;CDH1;CELA1;CELF3;CFLAR;CHD8;CHEK1;CHRNB2;CITED2;CNOT1;CNOT4;CREBBP;CSNK2A1;CTDP1;CTDSP2;CTNNB1;CTNND1;CUX1;CUX2;DAB2;DAP;DEDD;DNAJB6;DNM2;DNMT3B;DPF2;DSCC1;DVL3;DYNLL1;EGR1;EID2B;EID3;ELF4;ELK1;ELL;ELL3;ENO1;EP300;ER

;C1QTNF3;C3;C4BPB;C5AR1;CALCOCO1;CAMK1D;CAMKK2;CAMTA1;CAP1;CARM1;CASC3;CASZ1;CBFA2T3;CBL;CBX1;CBX6;CBY1;CCL3;CCNK;CCRN4L;CD3D;CD44;CD86;CDC42;CDC5L;CDH1;CELA1;CELF3;CFLAR;CHD8;CHEK1;CHRNB2;CITED2;CNOT1;CNOT4;CPT1A;CPT2;CREBBP;CSF1;CSF1R;CSNK1D;CSNK2

1;UBN1;VIM;WWP2;ZY

SA;DAB2;DAG1;DENND1A;DNAJC14;DNAJC19;DNM2;EHD1;EHD3;EIF2D;ERC1;FABP6;FAM160A2;FGF9;FIS1;FLVCR2;FOXP3;FZD1;GCC1;GCK;GDI2;GLMN;GLUL;GNAS;GOSR1;GRB10;GSK3A;HCLS1;HDLBP;HK1;HK2;HMGN3;HNRNPK;IL23A;IL4R;IMMP2L;INSR;IRS2;JAK3;KCNA5;KCNG2;KDELR1;KIF13A;KPNB1

2;RAPGEFL1;RCC1;RGL3;RGL4;RGS10;RGS16;RGS3;RIN2;RIN3;SH2D3C;SIPA1L1;SIPA1L2;SMAP2;SRGAP2;STARD8;TBC1D1;TBC1D14;TBC1D19;TBC1D2;TBC1D20;TBC1D2B;TBC1D5;TBC1D8;TBC1D9;VAV1;WA

YB5A;CYB5R3;CYP2R1;CYP4F3;DAB2;DENND1A;DNAJC14;DNAJC5;DNM2;DOCK2;DPAGT1;DSCR3;DYSF;ELOVL1;ELOVL3;EPS15L1;ERC1;ERGIC1;EXTL3;FIG4;FKBP1A;FLOT2;FURIN;GALNT11;GALNT2;GCC1;GCNT7;GOLGA2;GOLGA5;GOLGB1;GOLIM4;GOSR1;GRB2;HAX1;HERPUD1;HIP1;HIPK2;HLA-B;HLA-C;HL

ARFGAP3;ARHGAP26;ARHGAP27;ARHGEF10;ARHGEF11;ARID1A;ARID3A;ARID3B;ARL1;ARNT;ARSD;ARSG;ASAH1;ASB8;ASCC2;ASH2L;ATF5;ATF7;ATG2A;ATG7;ATP2B4;ATP5S;ATP6V0D1;ATPIF1;ATXN2;B3GNT9;B4GALT1;B4GALT4;B4GALT5;BASP1;BAZ2A;BCCIP;BCDIN3D;BCKDHA;BCL6;BCL9;BCO2;BCOR;B

;POU5F1;PPP1R10;PRKACA;PTTG1IP;RAB23;RAB7A;RAMP1;RBM22;RERE;RHOA;RPAIN;RPH3A;RPL6;RPLP0;RPN1;RPS10;RPS9;SEC23B;SEC61G;SLC11A1;SMURF1;SNX1;SOX4;SPCS1;SRP54;SRPR;SSR2;STAT3;STX18;TACC3;TAP2;TGFB1;TIMM8A;TIMM9;TLR9;TNPO3;TOMM22;TOMM7;TRAK1;TXNIP;XPO6;X

SNK2A1;CTDP1;CTNNB1;CTNND1;CUX1;CUX2;DAB2;DAP;DEDD;DNM2;DNMT3B;DPF2;DVL3;DYNLL1;EGR1;EID2B;EID3;ELF4;ELK1;ELL;ELL3;ENO1;EP300;ERC1;ERF;ESR1;ESRP2;EYA3;FAM120B;FAM58A;FLCN;FOSL2;FOXJ2;FOXN3;FOXO4;FOXP3;FZD1;GAS7;GLI1;GLO1;GTF2F1;GTF2H5;GTF3A;GTPBP1;HAX1

BHLHE40;BNIP3;BRCA1;BRD3;BRD4;BRD8;BRE;BRPF3;BTG2;BTK;BUB3;BZW2;C3;C5AR1;CALCOCO1;CAMK1D;CAMK2G;CAMKK2;CAMTA1;CANT1;CAPN1;CARM1;CARS;CASC3;CASZ1;CBFA2T3;CBL;CBX1;CBX6;CBY1;CCL3;CCNB1IP1;CCNK;CCRN4L;CD3D;CD44;CD86;CDC42;CDC5L;CDH1;CDKL3;CELA1;CELF3;C

C1;RGL3;RGL4;RGS10;RGS16;RGS3;RIN2;RIN3;SH2D3C;SIPA1L1;SIPA1L2;SMAP2;SRGAP2;STARD8;TBC1D1;TBC1D14;TBC1D19;TBC1D2;TBC1D20;TBC1D2B;TBC1D5;TBC1D8;TBC1D9;VAV1;WA

BEC3C;ARF1;ARF3;ARF6;ARL1;ARSD;ARSG;ASAH1;ASH2L;ASPRV1;ATG7;ATP2B4;ATP6V0C;ATP6V0D1;ATP6V1B2;B3GNT9;B4GALT1;B4GALT4;B4GALT5;BBS4;BCDIN3D;BCKDHA;BCO2;BCOR;BPNT1;BRAP;BRCA1;BTK;C1RL;C3orf33;CA13;CA14;CA3;CA6;CAMK1D;CAMK2G;CAMKK2;CANT1;CAPN1;CAPNS1;CA

;PTPN6;PTPRJ;PVR;RAC1;RIPK1;RPS6KA1;SH2D1A;SIN3A;SLC11A1;SPG21;SRC;STAT5A;STAT5B;STAT6;SYK;TAP2;TGFB1;TIRAP;TLR9;TNFSF13;TNIP1;TRAT1;UBE2V1;VASP;VAV1;WAS;XI

A1;CTIF;CTNNB1;CTNND1;CUX1;CUX2;DAB2;DAP;DAPK1;DEDD;DNM2;DNMT3B;DPF2;DSCC1;DVL3;DYNLL1;EGR1;EID2B;EID3;EIF2AK1;EIF2B3;EIF4A2;EIF4EBP2;EIF4H;ELF4;ELK1;ELL;ELL3;ENO1;EP300;ERC1;ERF;ESR1;EYA3;FAM120B;FAM129A;FAM58A;FLCN;FOSL2;FOXJ2;FOXN3;FOXO4;FOXP3;FZD1;GAS

REBBP;CSNK2A1;CTDP1;CTIF;CTNNB1;CTNND1;CUX1;CUX2;DAB2;DAP;DAPK1;DEDD;DNM2;DNMT3B;DPF2;DSCC1;DVL3;DYNLL1;EGR1;EID2B;EID3;EIF2AK1;EIF2B3;EIF4A2;EIF4EBP2;EIF4H;ELF4;ELK1;ELL;ELL3;ENO1;EP300;ERC1;ERF;ESR1;EYA3;FAM120B;FAM129A;FAM58A;FLCN;FOSL2;FOXJ2;FOXN3;FOX

;IRS2;ITSN1;KIF1B;MARK2;MYOF;NDUFAB1;NSFL1C;OSBP;OSBPL2;PCYT1A;PHF12;PHLPP1;PITPNA;PLCG2;PLCL2;PLEK;PLEKHB2;PLEKHG6;PLEKHM1P;PLEKHO2;PLIN1;PRAM1;PREX1;PRKD2;PSAP;PTAFR;PYGL;RAB35;RBP5;RPH3A;RUFY1;SELL;SH2B3;SH3PXD2B;SH3YL1;SNX1;SNX12;SNX19;SNX24;SNX27;SN

A1L1;SPTB;SRF;SSH1;SSH2;TLN1;TMSB10;TMSB15B;TRIOBP;VASP;WAS;WASF

:DC;RAF1;RIPK1;RPS6KA1;SIK3;SQSTM1;STK24;STK32B;STK35;STK4;STK40;TP53RK;ULK1;WNK1;XRCC6BP
ARHGEF10;ARHGEF11;ARHGEF12;ARID1A;ARL1;ARL6;ARNT;ASB8;ASH2L;ATG7;ATP6V0A1;ATP6V0C;ATP6V0D1;ATP6V1B2;B4GALT1;BBS4;BCCIP;BCDIN3D;BCL6;BCL9;BECN1;BIN2;BLOC1S3;BNIP3;BPNT1;BRAP;BRCA1;BRD8;BRE;BSG;BTG2;BTK;BTN3A1;C11orf73;C1QTNF3;C3;C5AR1;CACNG8;CALCOCO1;C

D1;SH2D1A;SIN3A;STAT5A;STAT5B;TGFB1;TIRAP;TLR9;TNFRSF1B;TNIP1;UBE2V1;XIA

4;CD209;CD93;CDC42;CDC42SE1;CDH1;CFL1;CHKA;CHMP4B;CHRNB1;CHRNB2;CLDN16;CLTA;CLTC;CLTCL1;CNN2;CNNM4;COLEC12;COPA;COPB2;CORO1C;COX17;CPT1A;CPT2;CRTAM;CSF1R;CTNNB1;CUX1;CXCR2;DAB2;DAG1;DAPK1;DENND1A;DENND3;DNAJC19;DNAJC5;DNM2;DOCK2;DSCR3;EHD1;EHD

C:

AK2;IRF2;ITGB2;JAK1;JAK3;KIR3DL1;KLRC4-KLRK1;LAT2;LCP1;LCP2;LILRA1;LILRB1;LILRB2;LTB4R;MAP2K1;MAP2K3;MAP3K5;MAPK14;MBP;MEFV;MX2;MYB;MYD88;MYO1F;NCF2;NFAM1;NFKB1;NFKBIA;NLRC4;NLRP3;NLRX1;NOD2;NOTCH1;NOTCH2;NUB1;PAK1;PCBP2;PIK3CD;PLCG2;PNMA1;POLR3F;POU
;EGR1;ELF4;ELK1;ELL;ELL3;EP300;ESR1;EXTL3;EYA3;FAM129A;FBXO22;FKBP1A;FLCN;FLT3;FOXJ2;FOXO4;FOXP3;FURIN;FZD1;GAPDHS;GBA;GCK;GLI1;GLMN;GNAI2;GNAL;GNAS;GRB10;GRB2;GSK3A;GTF2F1;GTPBP1;HAX1;HCLS1;HDAC4;HDGF;HERPUD1;HEXB;HIPK2;HIVEP3;HMGN3;HMOX1;HNRNPD;HNR

AP4K4;MAPK13;MAPK14;MAPKAPK5;MARK2;MARK3;MAST3;MERTK;MKNK1;MKNK2;MPP1;NADK;NAGK;NEK3;NEK6;NME1;NME2;NMNAT1;NRBP1;NUAK2;PAK1;PCYT1A;PFKFB3;PGK1;PHKA2;PIK3CD;PIM1;PIM2;PINK1;PIP4K2C;POLE4;POLR2A;POLR3F;PRIM1;PRKAB1;PRKACA;PRKCB;PRKCD;PRKD2;PRKI

;GNB1;GRB2;GSK3A;GTF2H5;HDGF;HERPUD1;HFE;HIPK2;HMOX1;HSPA5;IGF1R;ING4;INTS3;IRAK2;KAT5;KIAA1324;LZTS1;MAP2K1;MAP2K3;MAP3K5;MAP4K4;MAPK14;MAPKAPK5;MAX;MLH3;MRPS35;MYOF;NACC2;NBR1;NCOA6;NCOR1;NDEL1;NDNL2;NDRG1;NEK6;NFKBIA;NHEJ1;NOD2;NOTCH1;NSMC

PRKCD;PSEN1;PTTG1;PXN;RHOA;RIPK1;SH3BGR;SH3KBP1;SH3PXD2B;SIRPA;SKI;SQSTM1;SRC;TACC3;TBL1X;TLN1;TP53BP2;UVRAG;VASP;WBP5;XPA;YWHAB;YWHAG;YWHAH;ZFP1(

;PPM1F;PRKACA;PRKCB;PRKCD;PRKD2;PRKDC;PRMT5;PRMT6;PSEN1;PTK2B;PTPN6;PXN;RAB3D;RAF1;RPN1;RTF1;SAP130;SEC31A;SEMA4D;SIN3A;SOX4;SPRED1;SPRY2;SRC;STAT5A;STK4;STT3A;SUPT3H;SYK;TGFB1;TTLL4;UAP1;UGGT1;USP22;WDR61;YEATS4;ZDHH(

CUX2;DAB2;DAP;DEDD;DNM2;DNMT3B;DPF2;DVL3;DYNLL1;EGR1;EID2B;EID3;ELF4;ELK1;ELL;ELL3;ENO1;EP300;ERC1;ERF;ESR1;EYA3;FAM120B;FAM58A;FLCN;FOSL2;FOXJ2;FOXN3;FOXO4;FOXP3;FZD1;GAS7;GLI1;GLO1;GTF2F1;GTF2H5;GTF3A;HAX1;HCK;HCLS1;HDAC4;HDGF;HESX1;HEXB;HIP1;HIPK2;HIVE

K5;DOCK6;DOCK8;DYNLL1;EIF2B3;ENSA;ESR1;FURIN;GDI2;GIT2;GLMN;GM2A;GMIP;GTF2F1;IQSEC1;ITSN1;KLF4;LMTK2;MAP2K1;MAP3K5;MAP4K4;MAPRE3;MMP14;NCOR1;NLRP12;NOTCH1;OAZ2;PAPLN;PIK3R5;PLEKHG6;PPP1R10;PPP1R11;PPP1R12B;PPP2R2D;PREX1;PRKCD;PRKRA;PSAP;PSMD3;PSME
NND1;CUX1;CUX2;DAB2;DAP;DEDD;DNM2;DNMT3B;DPF2;DVL3;DYNLL1;EGR1;EID2B;EID3;ELF4;ELK1;ELL;ELL3;ENO1;EP300;ERC1;ERF;ESR1;EYA3;FAM120B;FAM58A;FLCN;FOSL2;FOXJ2;FOXN3;FOXO4;FOXP3;FZD1;GAS7;GLI1;GLO1;GTF2F1;GTF2H5;GTF3A;HAX1;HCK;HCLS1;HDAC4;HDGF;HESX1;HEXB;HIP

IMGN3;HNRNPD;HNRNPK;IGF1R;IKZF1;IL23A;INSR;JAK3;KAT5;KAT6A;KLF13;KLF4;KLF7;KLRC4-KLRK1;LILRB1;LPIN2;MAFB;MAML1;MAML3;MAP2K1;MAP2K3;MAPK14;MAPRE3;MED12;MED25;MLL2;MTF1;MYB;NCOA6;NFIC;NFKB1;NFKBIA;NFYC;NOD2;NOTCH1;NSD1;PAF1;PBX2;PHF2;PHF8;PID1;PIM2;PK
3X6;CBY1;CCL3;CCNK;CCRN4L;CD3D;CD44;CD86;CDC42;CDC5L;CDH1;CELA1;CELF3;CFLAR;CHD8;CHEK1;CITED2;CNN2;CNOT1;CNOT4;CNPY2;CPSF7;CREBBP;CRNKL1;CSF1;CSNK1D;CSNK2A1;CTDP1;CTIF;CTNNB1;CTNND1;CUX1;CUX2;CWC15;DAB2;DAP;DAPK1;DEDD;DHX8;DNM2;DNMT3B;DPF2;DVL3;DYN
COPS8;CREBBP;CSF1;CSF1R;CSNK1D;CSNK2A1;CTDP1;CTDSP2;DAB2;DAPK1;DAPK2;DBNL;DISC1;DNMT3B;DPAGT1;DPH5;DSCC1;DUSP12;DUSP18;DVL3;DYRK4;EGR1;EIF2AK1;EP300;EPHB1;EPHB2;ERC1;EXOSC3;EXTL3;EYA3;F13A1;FAM129A;FAM58A;FBXO22;FBXO25;FKBP1A;FKBP5;FKBP9;FLCN;FLT3;FNT

N1;JAK1;JAK3;LCP2;LEFTY2;LRP1;LTBP2;MAGI2;MAP2K1;MAPK13;MAPK14;MKNK1;MYD88;MYOF;NCOR1;NCSTN;NDST1;NFKB1;NFKBIA;NOTCH1;PHLPP1;PID1;POU5F1;PREX1;PRKAB1;PRKACA;PRKCB;PRKCD;PRKD2;PSEN1;PTK2B;PTPN1;PTPN6;PTPRJ;PXN;RAB7A;RAC1;RAF1;RALB;RBPJ;RHOA;RPS6KA1;R

D2;CNOT1;CNOT4;CNPY2;CREBBP;CSNK2A1;CTDP1;CTIF;CTNNB1;CTNND1;CUX1;CUX2;DAB2;DAP;DAPK1;DEDD;DNM2;DNMT3B;DPF2;DSCC1;DVL3;DYNLL1;EGR1;EID2B;EID3;EIF2AK1;EIF2B3;EIF4A2;EIF4EBP2;EIF4H;ELF4;ELK1;ELL;ELL3;ENO1;EP300;ERC1;ERF;ESR1;EYA3;FAM120B;FAM129A;FAM58A;FBP

;NACC2;NAPA;NFKBIA;NLRP12;NLRP3;NUP98;NUPL2;NXT1;OPTN;OS9;PACS1;PAK1;PDCD6;PEX7;PID1;PMPCB;POU5F1;PPP1R10;PRKACA;PTTG1IP;RAB23;RAB7A;RALB;RALGAPA2;RAMP1;RBM22;RERE;RHOA;RPAIN;RPH3A;RPL6;RPLP0;RPN1;RPS10;RPS9;RRAGB;RRAGC;SEC23B;SEC61G;SH3PXD2B;SIN3A;

T5A;STAT5B;SULF2;SYK;TGFB1;TIRAP;TLR9;TRIM25;TRIM27;ZNF28
.1;HLA-E;HMOX1;IFNAR2;IL17RA;IL23A;IL4R;IL6R;IRAK2;IRF2;ITGAX;ITGB2;JAK1;JAK3;KDM6B;KLF4;KLRC4-KLRK1;LILRA1;LILRB1;LILRB2;LIPA;LSP1;LTA4H;LTB4R;MAP2K1;MAP2K3;MAP3K5;MAPK13;MAPK14;MEFV;MICU1;MLF2;MMP25;MX2;MYD88;MYO1F;NCF2;NDST1;NFAM1;NFKB1;NFKBIA;NLRC4;NLI

IMGN3;HNRNPD;HNRNPK;IGF1R;IKZF1;IL23A;INSR;JAK3;KAT5;KAT6A;KLF13;KLF4;KLF7;LILRB1;LPIN2;MAFB;MAML1;MAML3;MAP2K1;MAP2K3;MAPK14;MAPRE3;MED12;MED25;MLL2;MTF1;MYB;NCOA6;NFIC;NFKB1;NFKBIA;NFYC;NOTCH1;NSD1;PAF1;PBX2;PHF2;PHF8;PID1;PIM2;PKNOX1;PLAC8;POLR2/
1;DNM2;DNMT3B;DVL3;ECE1;EGR1;ELF4;ELK1;ELL;ELL3;EP300;ESR1;EXTL3;EYA3;FAM129A;FBXO22;FGF9;FKBP1A;FLCN;FLT3;FOXJ2;FOXO4;FOXP3;FURIN;FZD1;GAPDHS;GBA;GCK;GLI1;GLMN;GNAI2;GNAL;GNAS;GRB10;GRB2;GSK3A;GTF2F1;GTPBP1;HAX1;HCLS1;HDAC4;HDGF;HERPUD1;HEXB;HIPK2;HIVE

M22;RBM8A;RERE;RHOA;RPAIN;RPL6;RPLP0;RPN1;RPS10;RPS9;SEC16A;SEC22A;SEC23B;SEC31A;SEC61G;SLC11A1;SMG7;SMURF1;SNX1;SNX27;SOX4;SPCS1;SRI;SRP54;SRPR;SRSF2;SRSF4;SRSF7;SSR2;STAT3;STX18;TACC3;TAP2;TGFB1;THOC5;TIMM8A;TIMM9;TLR9;TNPO3;TOMM22;TOMM7;TPCN2;TRAK1;

L6;ARSD;ARSG;ASH2L;ATF7;ATP2B4;ATP5S;ATP6V1B2;B4GALT1;B4GALT4;B4GALT5;BAZ2A;BCKDHA;BCL6;BCO2;BPNT1;BRAP;BRCA1;BRPF3;BTK;CA13;CA14;CA3;CA6;CACNA2D4;CAMK1D;CAMK2G;CAMKK2;CANT1;CAPN1;CAPNS1;CARS;CASZ1;CBFA2T3;CBL;CBR3;CCBL1;CCNB1IP1;CD209;CD93;CD97;CD(

YO1F;NBR1;NCOR1;NDEL1;NEK6;NFKB1;NFKBIA;NLRC4;NLRP12;NLRP3;NLRP6;NOD2;NSMCE4A;OSMR;PACS1;PAFAH1B1;PAK1;PCBP2;PLAUR;PLEK;POLR3F;PRKCD;PTK2B;PTPN1;PTPN6;PTPRJ;PVR;RAC1;RIPK1;RPS6KA1;SAMHD1;SETMAR;SH2D1A;SIN3A;SPRED1;STAT5A;STAT5B;STK24;SYK;TBC1D14;TGFB
S10;RGS16;RGS3;SEMA4D;SIPA1L1;SMAP2;SPRY2;SRGAP2;TBC1D14;TBC1D2;TNFRSF1B;TNNC1;TP53INP2;TRIM67;ULK1;UVRA(
D2;CNOT1;CNOT4;CREBBP;CSNK2A1;CTDP1;CTIF;CTNNB1;CTNND1;CUX1;CUX2;DAB2;DAP;DAPK1;DEDD;DNM2;DNMT3B;DPF2;DSCC1;DVL3;DYNLL1;EGR1;EID2B;EID3;EIF2AK1;EIF2B3;EIF4A2;EIF4EBP2;EIF4H;ELF4;ELK1;ELL;ELL3;ENO1;EP300;ERC1;ERF;ESR1;EYA3;FAM120B;FAM129A;FAM58A;FBP1;FLCN;

SNK2A1;CTDP1;CTDSP2;DAB2;DAPK1;DAPK2;DBNL;DISC1;DNMT3B;DPAGT1;DPH5;DSCC1;DUSP12;DUSP18;DVL3;DYRK4;EGR1;EIF2AK1;EP300;EPHB1;EPHB2;ERC1;EXTL3;EYA3;F13A1;FAM129A;FAM58A;FBXO22;FBXO25;FKBP1A;FKBP5;FKBP9;FLCN;FLT3;FNTB;FOXP3;FURIN;FZD1;G6PD;GALNT11;GALNT2;
SNK2A1;CTDP1;CTDSP2;DAB2;DAPK1;DAPK2;DBNL;DISC1;DNMT3B;DPAGT1;DPH5;DSCC1;DUSP12;DUSP18;DVL3;DYRK4;EGR1;EIF2AK1;EP300;EPHB1;EPHB2;ERC1;EXTL3;EYA3;F13A1;FAM129A;FAM58A;FBXO22;FBXO25;FKBP1A;FKBP5;FKBP9;FLCN;FLT3;FNTB;FOXP3;FURIN;FZD1;G6PD;GALNT11;GALNT2;

A1L2;SMAP2;SPRY2;SQSTM1;SRGAP2;STARD8;TBC1D2;TIMP2;TRIM67;VAV
EIF2B3;ELOVL1;ELOVL3;EMG1;EP300;EPHB1;EPHB2;EXTL3;F13A1;FKBP1A;FLT3;FNTB;GALNT11;GALNT2;GCK;GCNT7;GSK3A;GSTA4;HCK;HENMT1;HGSNAT;HIPK2;HK1;HK2;IGF1R;IGF2R;INSR;IP6K1;IRAK2;ITPK1;JAK1;JAK3;KAT5;KAT6A;KAT7;KSR1;LCMT2;LIMK1;LIMK2;LIPT1;LMTK2;LPCAT3;LRRK1;MAP2K1

AP2;SRP54;TBC1D
PRY2;SRGAP2;SRP54;TBC1D
REX1;PRKCD;PTK2B;RAB23;RAB35;RAB3D;RAB43;RAB5B;RAB5C;RAB7A;RALBP1;RALGAPA2;RGS10;RGS16;RGS3;RNF213;RRAGC;SEMA4D;SIPA1L1;SLC9A1;SMAP2;SMUG1;SPRY2;SRGAP2;SRP54;TBC1D2;TGFB1;VCA
POA1;APOBEC3C;APOBR;APP;ARAP1;ARAP3;ARF1;ARF3;ARF6;ARFGAP3;ARHGAP26;ARHGAP27;ARHGEF10;ARHGEF11;ARID1A;ARID3A;ARID3B;ARL1;ARNT;ARSD;ARSG;ASAH1;ASB8;ASCC2;ASH2L;ASPRV1;ATF5;ATF7;ATG2A;ATG7;ATP2B4;ATP5S;ATP6V0D1;ATPIF1;ATXN2;B3GNT9;B4GALT1;B4GALT4;B4G
2;NAPA;NFKBIA;NLRP12;NLRP3;NUP98;NUPL2;NXT1;OPTN;OS9;PACS1;PAK1;PDCD6;PEX7;PID1;PMPCB;POU5F1;PPP1R10;PRKACA;PTTG1IP;RAB23;RAB7A;RALB;RALGAPA2;RAMP1;RBM22;RERE;RHOA;RPAIN;RPH3A;RPL6;RPLP0;RPN1;RPS10;RPS9;RRAGB;RRAGC;SEC23B;SEC61G;SH3PXD2B;SIN3A;SLC11A

RGIC1;ESD;ESR1;FABP6;FIG4;FURIN;GALNT11;GALNT2;GCC1;GCNT7;GDI2;GIMAP8;GOLGA2;GOLGA5;GOLGB1;GOLIM4;GOLM1;GOSR1;GRB2;H1F0;HCK;HIP1;HLA-B;HLA-C;HLA-DPA1;HLA-E;IGF2R;KCNA5;KDELR1;KIF13A;KIF1C;KRT2;LMTK2;LOC388152;LZTS1;MAN2A2;MAP2K1;MAPRE1;MMP14;NCSTN;N

2T3;CBL;CCL3;CD14;CD1D;CD3D;CD44;CD86;CDC42;CDC42EP4;CDH1;CELA1;CELF3;CFL1;CFLAR;CHD8;CHEK1;CHRNB2;CITED2;CNPY2;CPT1A;CREBBP;CRTAM;CSF1;CSF1R;CSNK1D;CSNK2A1;CTDP1;CTDSP2;CTNNA1;CTNNB1;CXCR2;DAB2;DAPK1;DAPK2;DBNL;DEDD;DISC1;DNM2;DNMT3B;DOCK2;DPF2;DVL
;PTX3;RAC1;SAAL1;SERPINA1;SLC11A1;STAT3;STAT5A;STAT5B;SYK;TGFB1;TIRAP;TLR9;TNFRSF1B;TNIP1;XIA
ARK2;MERTK;MKNK1;MKNK2;MYD88;NBR1;NCOR1;NDST1;NEK6;NFKBIA;NKIRAS2;NLRP12;NLRP3;NOD2;NOTCH1;PAFAH1B1;PAK1;PIK3R5;PIM2;PINK1;PLEK;PRDX4;PRKACA;PRKCB;PRKCD;PRKD2;PSAP;PSEN1;PTK2B;PTPN1;PTPN6;PTPRJ;PXN;RAF1;RGS3;RHOA;RIPK1;RNF41;RPS6KA1;SH2D3C;SLC44A2;S

3C1D
A;KLF13;KLF4;KLF7;LILRB1;LPIN2;MAFB;MAML1;MAML3;MAP2K1;MAP2K3;MAPK14;MAPRE3;MED12;MED25;MLL2;MTF1;MYB;NCOA6;NFIC;NFKB1;NFKBIA;NFYC;NOTCH1;NSD1;PAF1;PBX2;PHF2;PHF8;PID1;PIM2;PKNOX1;PLAC8;POLR2A;POMC;POU2F2;POU5F1;PRKD2;PRKDC;RBM20;RBM22;RBPJ;RIPK

RS;CASC3;CASP6;CASP9;CBL;CCL3;CCNB1IP1;CCNK;CD44;CD86;CDC42;CDH1;CDKL3;CELA1;CFL1;CFLAR;CHEK1;CHST11;CHST15;CHST7;CLPX;CNOT4;COPS7A;COPS8;CPM;CPVL;CREBBP;CSF1;CSF1R;CSNK1D;CSNK2A1;CTDP1;CTDSP2;CTIF;CTNNB1;CTSA;CTSB;CTSD;CTSZ;DAB2;DAG1;DAPK1;DAPK2;DBNL;DE

SE1;CDC5L;CDH1;CDV3;CENPH;CERKL;CFL1;CFL2;CHCHD1;CHEK1;CITED2;CKAP4;CLIP2;CLPX;CLTC;CLTCL1;CNFN;CNN2;CNOT1;COPB2;CORO1C;COTL1;CPT2;CREBBP;CSNK1D;CSNK2A1;CTDP1;CTNNA1;CTNNAL1;CTNNB1;CTSB;DAG1;DAP3;DAPK1;DBNL;DCLRE1B;DEDD;DISC1;DNAH17;DNAJB6;DNAJC14;D
SE1;CDC5L;CDH1;CDV3;CENPH;CERKL;CFL1;CFL2;CHCHD1;CHEK1;CITED2;CKAP4;CLIP2;CLPX;CLTC;CLTCL1;CNFN;CNN2;CNOT1;COPB2;CORO1C;COTL1;CPT2;CREBBP;CSNK1D;CSNK2A1;CTDP1;CTNNA1;CTNNAL1;CTNNB1;CTSB;DAG1;DAP3;DAPK1;DBNL;DCLRE1B;DEDD;DISC1;DNAH17;DNAJB6;DNAJC14;D
;EYA3;FAM129A;FBXO22;FGF9;FKBP1A;FLCN;FLT3;FOXJ2;FOXO4;FOXP3;FURIN;FZD1;GAPDHS;GBA;GCK;GLI1;GLMN;GNAI2;GSK3A;GTF2F1;GTPBP1;HAX1;HCLS1;HDAC4;HDGF;HERPUD1;HEXB;HIPK2;HIVEP3;HMGN3;HMOX1;HNRNPD;HNRNPK;HSPA5;IGF1R;IKZF1;IL23A;IL6R;INSR;IRAK2;IRS2;ITGA5;JAK3;J

;SPPL3;SRI;SV2B;SYT2;TGFB1;TMEM67;ULK1;VAMP2;VAMP
3C1D

LT4;B4GALT5;BASP1;BAZ2A;BBS4;BCCIP;BCDIN3D;BCL6;BCL9;BCOR;BCORL1;BECN1;BHLHE40;BNIP3;BRCA1;BRD3;BRD4;BRD8;BRE;BRPF3;BTG2;BTK;BUB3;BZW2;C1QTNF3;C1RL;C3;C4BPB;C5AR1;CALCOCO1;CAMK1D;CAMK2G;CAMKK2;CAMTA1;CANT1;CAPN1;CARM1;CARS;CASC3;CASP6;CASP9;CASZ1;C

C;ATP6V0D1;ATP6V1B2;B4GALT1;BBS4;BCL6;BCL9;BNIP3;BRAP;BRCA1;BRD8;BRE;BSG;BTK;BTN3A1;C1QTNF3;C3;C5AR1;CACNG8;CALCOCO1;CAMK2G;CAMKK2;CANT1;CAP1;CARM1;CASP9;CBL;CBY1;CC2D2A;CCL3;CD101;CD14;CD209;CD3D;CD44;CD53;CD86;CD97;CDC42;CDC42EP4;CDC42SE1;CELA1;C
AMP8;WA
F;PRKCD;PTAFR;PTPN1;PTPN6;PVR;RIPK1;RPS6KA1;SAMHD1;SH2D1A;SIN3A;SLC11A1;STAT5A;STAT5B;SYK;TGFB1;TIRAP;TLR9;TNIP1;TRIM25;TXNIP;UBE2V1;XIA

LK1;YWHAG;ZC3HC
FAIM;FAM129B;FAM162A;FIS1;FLCN;FLT3;FURIN;GLO1;GNRH1;HCK;HCLS1;HERPUD1;HIP1;HIPK2;HMOX1;HRK;HSPA1B;HSPA5;IGF1R;IL6R;ING4;IRS2;ITGA5;ITSN1;JAK3;KIAA1324;KLF4;LILRB1;MAEA;MAP3K5;MMP9;NACC2;NAE1;NCF2;NDUFS3;NFKB1;NLRC4;NLRP12;NLRP3;NME1;NME2;NOD2;NOTCH1;
DAP;DAPK1;DAPK2;DEDD;DENND3;DHTKD1;DISC1;DNAJB6;DNM2;ECE1;EIF4A2;ENO1;ESD;EXOSC1;EXOSC3;EXOSC8;FBP1;FBXO22;FIS1;FUNDC1;FURIN;G6PD;GALNS;GAPDHS;GBA;GCK;GIT2;GLB1;GLMN;GLUL;GLYR1;GM2A;GMIP;GNA11;GNA12;GNA15;GNAI2;GNAL;GNAS;GNAT2;GNB1;GNB5;GNS;GSK3

B1;LRRK1;MAP1S;MAP3K5;MAX;MTERFD3;MYD88;MYH9;NACC2;NAGA;NFKB1;NFKBIA;NLRC4;NME1;NR2C1;NRBP1;PAFAH1B1;PAK1;PCYT1A;PDCD6;PEX7;PIP4K2C;PLEK;PLOD1;PRKRA;PVRL1;PYGL;RAB11FIP4;RAF1;RIPK1;SETMAR;SLC11A1;SOD1;SPATA24;SPPL3;SQSTM1;SRC;SRF;SRGAP2;STAT6;STK4;S
CBX6;CC2D2A;CCNK;CCRN4L;CD3D;CDC42;CDC5L;CDH1;CENPH;CFLAR;CHD8;CHRNB1;CHRNB2;CLIP2;CLPX;CLTA;CLTC;CLTCL1;CNOT1;COG8;COPA;COPB2;COPS7A;COPS8;COPZ2;COX6A1;CPSF7;CREBBP;CRNKL1;CSF1;CSF1R;CSF2RB;CSNK1D;CSNK2A1;CTDP1;CTNNA1;CTNNB1;CWC15;CYB5R3;CYFIP1;DAB

C42;CDC5L;CDH1;CENPH;CFLAR;CHD8;CHRNB1;CHRNB2;CLIP2;CLPX;CLTA;CLTC;CLTCL1;COG8;COPA;COPB2;COPS7A;COPS8;COPZ2;COX6A1;CPSF7;CREBBP;CSF1;CSF1R;CSF2RB;CSNK1D;CSNK2A1;CTDP1;CTNNA1;CTNNB1;CYB5R3;CYFIP1;DAB2;DAG1;DISC1;DNAH17;DNM2;DSCR3;DYNLL1;DYNLL2;EID3;EI
TCH1;NOTCH2;OSBP;PSEN1;QSOX1;RAB36;RAC1;RNF24;SEC16A;SEC23B;SLC35A2;SLC9A8;SPPL3;ST3GAL2;STX18;TAPBP;TLR9;TMEM132A;WIPI1;XYLT1;ZDHH

4;MRPS21;MRPS35;MTERFD3;NDUFA12;NDUFA7;NDUFAB1;NDUFAF4;NDUFB2;NDUFC1;NDUFS3;NDUFV2;NLRX1;OCIAD2;PHB2;PINK1;PMPCB;PPA2;PSEN1;PTPMT1;RAF1;RNF185;ROMO1;SDHC;SIRT4;SLC25A14;SLC25A26;SLC25A33;SLC25A44;SNN;SOD1;SRC;TFB2M;TIMM8A;TIMM9;TK2;TMEM14C;TO

NDEL1;NDRG1;NEK6;NME1;PACSIN2;PAFAH1B1;PXN;RCSD1;RHOA;SBDS;SPTB;SSH1;SSH2;SVIL;TLN1;TMEM67;TMSB10;TMSB15B;TNNC1;TPM3;TPM4;TRIOBP;TTLL4;VASP;VCL;WAS;WASF2;WIPF2;YWH

BP1A;FSCN1;FZD1;GAS7;GLI1;GLUL;GNA12;GNAS;GNAT2;GNB1;GPRIN1;GSN;GTF2I;HAX1;HCK;IFT46;IGF1R;IQCB1;ITGA5;ITSN1;KATNA1;KCNC3;KCND1;KIAA1598;KIF1B;LAMP1;LCP1;LIMK1;LIN7B;LOXHD1;LRFN1;LZTS1;MAGI2;MAP1S;MAP2K1;MAPRE1;MAX;MBP;MERTK;MPP1;MSN;MTMR14;MYH9;MY
;NDUFB2;NDUFC1;NDUFS3;NDUFV2;NLRP6;NLRX1;NUP35;NUP98;NUPL2;NXT1;OCIAD2;P2RX1;PAFAH1B1;PAK1;PDCD6;PHB2;PINK1;PMPCB;PSEN1;PTPMT1;RAC2;RAF1;RCC1;RNF185;ROMO1;RRP12;RTN4;SDHC;SLC11A1;SLC25A14;SLC25A26;SLC25A33;SLC25A44;SNN;SP140;SRC;TIMM8A;TIMM9;TK2;T

;PBX2;PHF12;PHF21A;PKNOX1;POLE4;POLR2A;POLR3F;POU5F1;PRKDC;RBM8A;RCOR1;RERE;RNF34;RPAIN;RPRD2;RTF1;SAP130;SETD1B;SIN3A;SKI;SMAD9;SPEN;SRP54;SRSF2;SRSF4;SUPT3H;TADA2B;TAF11;TAF4;TBL1X;THRAP3;TOE1;TOP3A;TP53INP2;TRIM27;TRIM8;UBAP2L;UBN1;USP22;WDR61;WDR

ETMAR;SLC11A1;SOD1;SPATA24;SPPL3;SQSTM1;SRF;SRGAP2;STK4;SUMF1;TGFB1;TIMM9;TIRAP;TKT;TNNC1;TPD52L2;TRIM8;XPA;ZNF1

MP14;NDST1;NDST2;NOTCH1;NOTCH2;NSFL1C;OSBP;PACS1;PSEN1;QSOX1;RAB36;RAC1;RNF24;SEC16A;SEC23B;SLC35A2;SLC9A8;SPG21;SPPL3;ST3GAL2;STX18;SULF2;TAPBP;TGFB1;TLR9;TMEM132A;VCAN;WIPI1;XYLT1;ZDHHC3;ZFYV

1;TAF4;TBL1X;TCF20;TFB2M;THRAP3;TRERF1;TSG101;USP22;YWHAB;Y

FC1;NDUFS3;NDUFV2;NLRP6;NLRX1;NUP35;NUP98;NUPL2;NXT1;OCIAD2;PAFAH1B1;PAK1;PDCD6;PHB2;PINK1;PMPCB;PSEN1;PTPMT1;RAC2;RAF1;RCC1;RNF185;ROMO1;RRP12;RTN4;SDHC;SLC25A14;SLC25A26;SLC25A33;SLC25A44;SNN;SP140;SRC;TIMM8A;TIMM9;TK2;TMEM14C;TMEM18;TMEM38A;

.4;SLC25A26;SLC25A33;SLC25A44;SNN;SRC;TIMM8A;TIMM9;TK2;TMEM14C;TOMM22;TOMM7;TTC19;TXNIP;UQCR10;UQCR11;USMG
FRSF1B;TP53BP2;TPD52L2;TRAIP;TRAPPC2;YWHAB;ZFYVE

H;MAN2A2;MAN2B2;MARCH8;MEFV;MGRN1;MICAL2;MLL2;MMP14;MMP25;MMP9;MT1E;MTF1;NANOS1;NBR1;NR2C1;NSD1;NUPL2;P2RX1;PAPLN;PHF12;PHF2;PHF21A;PHF23;PHF8;PIAS3;PLAGL2;PMPCB;PPP1R10;PRKCB;PTPN1;PXN;RBM20;RBM22;RERE;RFFL;RNF114;RNF122;RNF14;RNF167;RNF17(

SLC25A44;SNN;SRC;TIMM8A;TIMM9;TK2;TMEM14C;TOMM22;TOMM7;TTC19;UQCR10;UQCR11;USMG

1;TAF4;TBL1X;TCF20;TFB2M;THRAP3;TRERF1;TSG101;USP22;YWHAB;Y

;TRERF1;TSG101;USP22;YWHAB;Y

;HIVEP3;IKZF1;ING4;ISCU;KAT6A;KAT7;KDM2A;KDM4B;KDM5B;KLF13;KLF4;KLF7;KLF9;KPNB1;LASP1;LIMK1;LIMK2;LOC440434;LOXL3;LTA4H;MAN2A2;MAN2B2;MARCH8;MEFV;MGRN1;MICAL2;MLL2;MMP14;MMP25;MMP9;MT1E;MTF1;NANOS1;NBR1;NR2C1;NSD1;NUDT7;NUPL2;P2RX1;PAPLN;PHF12;
AP2K1;MSN;MYH9;NAF1;NDRG1;NEU1;PAK1;PECAM1;PNMA1;PPL;PRKCD;PTK2B;PTPRJ;PVR;PVRL1;PXN;RHOA;RPH3A;SH3KBP1;SH3PXD2B;SIPA1L1;SNAP29;SRGAP2;STARD8;STRAP;SV2B;SVIL;SYT2;TBC1D2;TJP2;TLN1;TNS3;TPRG1L;TRIM25;UBN1;VAMP2;VASP;VCL;YWHAH;ZY

;PAK1;PIAS3;PPT1;PREX1;PSEN1;PTK2B;RAB8A;RGS10;RHOA;SH3KBP1;SIPA1L1;SLC38A7;SMURF1;SNAP29;SOD1;SRGAP2;TGFB1;TIMP2;TNFRSF1B;ULK1;VAMP

5A1;ALDH6A1;ALDH7A1;ALG8;ALG9;ALMS1;ALS2CL;ALX3;AMACR;AMBRA1;AMFR;AMOTL1;ANAPC1;ANAPC13;ANAPC5;ANAPC7;ANK3;ANKLE2;ANKRD11;ANKRD27;ANKS1B;ANKS6;ANKZF1;ANO8;ANP32B;ANXA2;ANXA6;AP2B1;AP3D1;AP3S1;APBA1;APBB1;APOBEC3F;APOL3;APOOL;AQP3;AQR;ARG1;ARI
5A1;ALDH6A1;ALDH7A1;ALG8;ALG9;ALMS1;ALS2CL;ALX3;AMACR;AMBRA1;AMFR;AMOTL1;ANAPC1;ANAPC13;ANAPC5;ANAPC7;ANK3;ANKLE2;ANKRD11;ANKRD27;ANKS1B;ANKS6;ANP32B;ANXA2;ANXA6;AP2B1;AP3D1;AP3S1;APBA1;APBB1;APOBEC3F;APOL3;APOOL;AQP3;AQR;ARG1;ARHGAP15;ARHGA
ACH2;BAHD1;BAIAP2L1;BATF;BATF3;BAZ1B;BBS9;BCL10;BCL11B;BCL2;BCL9L;BDH1;BET1L;BEX1;BLM;BLMH;BMS1;BPTF;BRD1;BRD2;BRD7;BRPF1;BSN;BTRC;C11orf82;C12orf39;C14orf169;C15orf48;C16orf88;C1D;C1orf114;C1orf61;C2CD2;C5orf20;C9orf89;CAD;CALM3;CALR;CAMTA2;CAND2;CAPN2;CAPI
35;CHD3;CHD4;CHEK2;CIITA;CIRBP;CIRH1A;CLEC3B;CLIC1;CLK2;COPS7B;CORO2A;CPSF3;CREG1;CREM;CSNK2A2;CSRP2BP;CSTB;CSTF1;CSTF2;CSTF3;CTC1;CTCF;CTNNBL1;CTR9;CUL1;CUL7;DDB1;DDIT3;DDX17;DDX18;DDX19A;DDX19B;DDX20;DDX23;DDX24;DDX31;DDX42;DDX46;DDX47;DDX51;DDX52;DD
LX3;AMACR;AMBRA1;AMFR;AMOTL1;ANAPC1;ANAPC13;ANAPC5;ANAPC7;ANK3;ANKLE2;ANKRD11;ANKRD27;ANKS1B;ANP32B;ANXA2;ANXA6;AP2B1;AP3D1;AP3S1;APBA1;APBB1;APOBEC3F;APOOL;AQR;ARHGAP32;ARHGAP35;ARHGAP39;ARID1B;ARIH2;ARL3;ARNTL2;ARPC2;ASCC1;ASXL1;ATF6B;ATG1
LX3;AMACR;AMBRA1;AMFR;AMOTL1;ANAPC1;ANAPC13;ANAPC5;ANAPC7;ANK3;ANKLE2;ANKRD11;ANKRD27;ANKS1B;ANP32B;ANXA2;ANXA6;AP2B1;AP3D1;AP3S1;APBA1;APBB1;APOBEC3F;APOOL;AQR;ARHGAP32;ARHGAP35;ARHGAP39;ARID1B;ARIH2;ARL3;ARNTL2;ARPC2;ASCC1;ASXL1;ATF6B;ATG1
2A;CENPN;CENPO;CEP85;CES2;CHD3;CHD4;CHEK2;CIITA;CIRBP;CIRH1A;CLEC3B;CLK2;COL17A1;COL27A1;COL5A1;COL6A3;CORO2A;CPSF3;CREG1;CREM;CSRP2BP;CSTB;CSTF1;CSTF2;CSTF3;CTC1;CTCF;CTR9;CUBN;CUL1;D2HGDH;DARS2;DCN;DDB1;DDIT3;DDX17;DDX18;DDX20;DDX23;DDX24;DDX31;DDX4
16;CDC25B;CDKN2A;CENPN;CENPO;CEP85;CES2;CHD3;CHD4;CHEK2;CIITA;CIRBP;CIRH1A;CLEC3B;CLK2;COL17A1;COL27A1;COL5A1;COL6A3;CORO2A;CPSF3;CREG1;CREM;CSRP2BP;CSTB;CSTF1;CSTF2;CSTF3;CTC1;CTCF;CTR9;CUBN;CUL1;D2HGDH;DARS2;DBH;DCN;DDB1;DDIT3;DDX17;DDX18;DDX20;DDX
2A;CENPN;CENPO;CEP85;CES2;CHD3;CHD4;CHEK2;CIITA;CIRBP;CIRH1A;CLEC3B;CLK2;COL17A1;COL27A1;COL5A1;COL6A3;CORO2A;CPSF3;CREG1;CREM;CSRP2BP;CSTB;CSTF1;CSTF2;CSTF3;CTC1;CTCF;CTR9;CUBN;CUL1;D2HGDH;DARS2;DBH;DCN;DDB1;DDIT3;DDX17;DDX18;DDX20;DDX23;DDX24;DDX31;
STF1;CSTF2;CSTF3;CTC1;CTCF;CTR9;CUL1;DDB1;DDIT3;DDX17;DDX18;DDX20;DDX23;DDX24;DDX31;DDX42;DDX46;DDX47;DDX51;DDX52;DDX56;DFFA;DFFB;DGKQ;DHX15;DHX33;DHX38;DHX9;DIEXF;DKC1;DNAJB1;DNMT3A;DROSHA;DUSP7;EFCAB4B;EFTUD2;EIF3A;EIF4A3;EIF4ENIF1;ELAVL1;ELP3;EP400
LG8;ALG9;ALMS1;ALS2CL;ALX3;AMACR;AMBRA1;AMFR;AMOTL1;ANAPC1;ANAPC13;ANAPC5;ANAPC7;ANK3;ANKLE2;ANKRD11;ANKRD27;ANKS1B;ANP32B;ANXA2;ANXA6;AP2B1;AP3D1;AP3S1;APBA1;APBB1;APOBEC3F;APOOL;AQR;ARHGAP32;ARHGAP35;ARHGAP39;ARID1B;ARIH2;ARL3;ARNTL2;ARPC2
LG8;ALG9;ALMS1;ALS2CL;ALX3;AMACR;AMBRA1;AMFR;AMOTL1;ANAPC1;ANAPC13;ANAPC5;ANAPC7;ANK3;ANKLE2;ANKRD11;ANKRD27;ANKS1B;ANP32B;ANXA2;ANXA6;AP2B1;AP3D1;AP3S1;APBA1;APBB1;APOBEC3F;APOOL;AQR;ARHGAP32;ARHGAP35;ARHGAP39;ARID1B;ARIH2;ARL3;ARNTL2;ARPC2
OL;AQR;ARHGAP32;ARID1B;ARL3;ARNTL2;ARPC2;ARPC3;ASCC1;ASXL1;ATF6B;ATG9B;ATM;ATN1;ATP10A;ATP2A2;ATP2A3;ATP5A1;ATP5B;ATP5J;ATP5J2;ATP6V0E1;ATP6V1D;ATP6V1F;ATP6V1H;ATXN2L;AXIN1;AXIN2;B3GALT4;BACE1;BAD;BAHD1;BAZ1B;BBS1;BBS2;BBS9;BCL2;BDH1;BET1L;BEX1;BICD1;BI
F;APOOL;AQR;ARHGAP32;ARID1B;ARL3;ARNTL2;ARPC2;ARPC3;ASCC1;ASXL1;ATF6B;ATG9B;ATM;ATN1;ATP10A;ATP2A2;ATP2A3;ATP5A1;ATP5B;ATP5J;ATP5J2;ATP6V0E1;ATP6V1D;ATP6V1F;ATP6V1H;ATXN2L;AXIN1;AXIN2;B3GALT4;BACE1;BAD;BAHD1;BAZ1B;BBS1;BBS2;BBS9;BCL2;BDH1;BET1L;BEX1;BI
3;BAZ1B;BDH1;BLM;BMS1;BRSK2;C22orf28;C9orf96;CAD;CARNS1;CBWD3;CBWD6;CCT3;CCT5;CCT6A;CCT7;CCT8;CDC42BPA;CDC42BPB;CDC42BPG;CELF1;CERK;CHD1L;CHD2;CHD3;CHD4;CHD6;CHEK2;CHST12;CIITA;CIRBP;CIT;CKM;CLCN3;CLCN6;CLK2;CLPB;CREG1;CRY2;CSNK1E;CSNK2A2;CSTF2;CTPS2;CU
3;BAZ1B;BDH1;BLM;BMS1;BRSK2;C22orf28;C9orf96;CAD;CARNS1;CBWD3;CBWD6;CCT3;CCT5;CCT6A;CCT7;CCT8;CDC42BPA;CDC42BPB;CDC42BPG;CELF1;CERK;CHD1L;CHD2;CHD3;CHD4;CHD6;CHEK2;CHST12;CIITA;CIRBP;CIT;CKM;CLCN3;CLCN6;CLK2;CLPB;CREG1;CRY2;CSNK1E;CSNK2A2;CSTF2;CTPS2;CU
2BPA;CDC42BPB;CDC42BPG;CERK;CHD1L;CHD2;CHD3;CHD4;CHD6;CHEK2;CIITA;CIT;CKM;CLCN3;CLCN6;CLK2;CLPB;CSNK1E;CSNK2A2;CTPS2;CUL9;DARS2;DCLK2;DDR1;DDX10;DDX17;DDX18;DDX19A;DDX19B;DDX20;DDX23;DDX24;DDX27;DDX31;DDX42;DDX46;DDX47;DDX51;DDX52;DDX55;DDX56;DDX6;
2BPA;CDC42BPB;CDC42BPG;CERK;CHD1L;CHD2;CHD3;CHD4;CHD6;CHEK2;CHST12;CIITA;CIT;CKM;CLCN3;CLCN6;CLK2;CLPB;CSNK1E;CSNK2A2;CTPS2;CUL9;DARS2;DCLK2;DDR1;DDX10;DDX17;DDX18;DDX19A;DDX19B;DDX20;DDX23;DDX24;DDX27;DDX31;DDX42;DDX46;DDX47;DDX51;DDX52;DDX55;DDX5
2BPA;CDC42BPB;CDC42BPG;CERK;CHD1L;CHD2;CHD3;CHD4;CHD6;CHEK2;CHST12;CIITA;CIT;CKM;CLCN3;CLCN6;CLK2;CLPB;CSNK1E;CSNK2A2;CTPS2;CUL9;DARS2;DCLK2;DDR1;DDX10;DDX17;DDX18;DDX19A;DDX19B;DDX20;DDX23;DDX24;DDX27;DDX31;DDX42;DDX46;DDX47;DDX51;DDX52;DDX55;DDX5
3;BAZ1B;BDH1;BLM;BMS1;BRSK2;C22orf28;C9orf96;CAD;CARNS1;CBWD3;CBWD6;CCT3;CCT5;CCT6A;CCT7;CCT8;CDC42BPA;CDC42BPB;CDC42BPG;CELF1;CERK;CHD1L;CHD2;CHD3;CHD4;CHD6;CHEK2;CHST12;CIITA;CIRBP;CIT;CKM;CLCN3;CLCN6;CLK2;CLPB;CREG1;CRY2;CSNK1E;CSNK2A2;CSTF2;CTPS2;CU
9;DKC1;DROSHA;DUSP7;EFTUD2;EIF4A3;EIF4ENIF1;ELAVL1;ELP3;EP400;ERCC3;ERCC5;ETS1;EZH1;FANCI;FEN1;FIP1L1;FOXC1;FOXI1;FOXJ1;FOXJ3;FOXK1;FOXK2;FOXM1;FOXP1;FOXP4;FZR1;GAR1;GATA3;GEMIN4;GEMIN5;GINS4;GTF2A2;GTF3C1;GTF3C2;GTF3C4;HCFC1;HDAC1;HIST2H3D;HMGA1;HNRNPA
VD3;CBWD6;CCT3;CCT5;CCT6A;CCT7;CCT8;CDC42BPA;CDC42BPB;CDC42BPG;CERK;CHD1L;CHD2;CHD3;CHD4;CHD6;CHEK2;CHST12;CIITA;CIT;CKM;CLCN3;CLCN6;CLK2;CLPB;CSNK1E;CSNK2A2;CTPS2;CUL9;DARS2;DCLK2;DDR1;DDX10;DDX17;DDX18;DDX19A;DDX19B;DDX20;DDX23;DDX24;DDX27;DDX31;D
VD3;CBWD6;CCT3;CCT5;CCT6A;CCT7;CCT8;CDC42BPA;CDC42BPB;CDC42BPG;CERK;CHD1L;CHD2;CHD3;CHD4;CHD6;CHEK2;CHST12;CIITA;CIT;CKM;CLCN3;CLCN6;CLK2;CLPB;CSNK1E;CSNK2A2;CTPS2;CUL9;DARS2;DCLK2;DDR1;DDX10;DDX17;DDX18;DDX19A;DDX19B;DDX20;DDX23;DDX24;DDX27;DDX31;D
ATF6B;ATM;ATP10A;ATP13A1;ATP1A1;ATP1A3;ATP2A2;ATP2A3;ATP5A1;ATP5B;ATP8B1;ATP8B2;ATP9B;ATXN1L;BACH2;BAHCC1;BAHD1;BATF;BATF3;BAZ1B;BCL11B;BCL2;BDH1;BLM;BMS1;BPTF;BRD7;BRPF1;BRSK2;C1D;C22orf28;C9orf96;CAD;CALR;CAMTA2;CARNS1;CBWD3;CBWD6;CBX2;CBX7;CC2D1B
P1A3;ATP2A2;ATP2A3;ATP5A1;ATP5B;ATP5J;ATP5J2;ATP6V1H;ATP8B1;ATP8B2;ATXN1L;AVPR2;AXIN1;AXIN2;BACH2;BAD;BAHD1;BATF;BATF3;BAZ1B;BCL10;BCL11B;BCL9L;BEX1;BICD1;BLM;BMP2;BPTF;BRD2;BRD7;BRPF1;BTRC;C14orf169;C1D;C22orf28;CAD;CALM3;CALR;CAMTA2;CAND2;CAPN3;CARD11
VD3;CBWD6;CCT3;CCT5;CCT6A;CCT7;CCT8;CDC42BPA;CDC42BPB;CDC42BPG;CERK;CHD1L;CHD2;CHD3;CHD4;CHD6;CHEK2;CHST12;CIITA;CIT;CKM;CLCN3;CLCN6;CLK2;CLPB;CSNK1E;CSNK2A2;CTPS2;CUL9;DARS2;DCLK2;DDR1;DDX10;DDX17;DDX18;DDX19A;DDX19B;DDX20;DDX23;DDX24;DDX27;DDX31;D
3CL10;BCL2;BET1L;BEX1;BICD1;BIRC6;BLOC1S1;BPTF;BRD1;BRPF1;BTBD2;BTRC;C1D;C22orf28;CACNA1F;CACNA1I;CACNB1;CAD;CALM3;CALR;CAMSAP1;CAPN3;CARD11;CASP1;CATSPER1;CATSPER2;CBX2;CBX5;CBX7;CCND1;CCND2;CCNF;CCNT1;CCT3;CCT5;CCT6A;CCT7;CCT8;CD1A;CD247;CD3E;CD3EAP;C
VD3;CBWD6;CCT3;CCT5;CCT6A;CCT7;CCT8;CDC42BPA;CDC42BPB;CDC42BPG;CERK;CHD1L;CHD2;CHD3;CHD4;CHD6;CHEK2;CHST12;CIITA;CIT;CKM;CLCN3;CLCN6;CLK2;CLPB;CSNK1E;CSNK2A2;CTPS2;CUL9;DARS2;DCLK2;DDR1;DDX10;DDX17;DDX18;DDX19A;DDX19B;DDX20;DDX23;DDX24;DDX27;DDX31;D
VD3;CBWD6;CCT3;CCT5;CCT6A;CCT7;CCT8;CDC42BPA;CDC42BPB;CDC42BPG;CERK;CHD1L;CHD2;CHD3;CHD4;CHD6;CHEK2;CIITA;CIT;CKM;CLCN3;CLCN6;CLK2;CLPB;CSNK1E;CSNK2A2;CTPS2;CUL9;DARS2;DCLK2;DDR1;DDX10;DDX17;DDX18;DDX19A;DDX19B;DDX20;DDX23;DDX24;DDX27;DDX31;DDX42;DI
VD3;CBWD6;CCT3;CCT5;CCT6A;CCT7;CCT8;CDC42BPA;CDC42BPB;CDC42BPG;CERK;CHD1L;CHD2;CHD3;CHD4;CHD6;CHEK2;CHST12;CIITA;CIT;CKM;CLCN3;CLCN6;CLK2;CLPB;CREG1;CSNK1E;CSNK2A2;CTPS2;CUL9;DARS2;DCLK2;DDR1;DDX10;DDX17;DDX18;DDX19A;DDX19B;DDX20;DDX23;DDX24;DDX27;D
VD3;CBWD6;CCT3;CCT5;CCT6A;CCT7;CCT8;CDC42BPA;CDC42BPB;CDC42BPG;CERK;CHD1L;CHD2;CHD3;CHD4;CHD6;CHEK2;CHST12;CIITA;CIT;CKM;CLCN3;CLCN6;CLK2;CLPB;CSNK1E;CSNK2A2;CTPS2;CUL9;DARS2;DCLK2;DDR1;DDX10;DDX17;DDX18;DDX19A;DDX19B;DDX20;DDX23;DDX24;DDX27;DDX31;D
VD3;CBWD6;CCT3;CCT5;CCT6A;CCT7;CCT8;CDC42BPA;CDC42BPB;CDC42BPG;CERK;CHD1L;CHD2;CHD3;CHD4;CHD6;CHEK2;CHST12;CIITA;CIT;CKM;CLCN3;CLCN6;CLK2;CLPB;CSNK1E;CSNK2A2;CTPS2;CUL9;DARS2;DCLK2;DDR1;DDX10;DDX17;DDX18;DDX19A;DDX19B;DDX20;DDX23;DDX24;DDX27;DDX31;D
TP5A1;ATP5B;ATP5J;ATP5J2;ATP6V1H;ATP8B1;ATP8B2;ATXN1L;AVPR2;AXIN1;AXIN2;BACH2;BAD;BAHD1;BATF;BATF3;BAZ1B;BCL10;BCL11B;BCL9L;BEX1;BICD1;BLM;BMP2;BPTF;BRD2;BRD7;BRPF1;BTRC;C14orf169;C1D;C22orf28;CAD;CALM3;CALR;CAMTA2;CAND2;CAPN3;CARD11;CARNS1;CBFA2T2;CBX2
2;ATP2A3;ATP5A1;ATP5B;ATP5J;ATP5J2;ATP6V1H;ATP8B1;ATP8B2;ATXN1L;AVPR2;AXIN1;AXIN2;BACH2;BAD;BAHD1;BATF;BATF3;BAZ1B;BCL10;BCL11B;BCL9L;BEX1;BICD1;BLM;BMP2;BPTF;BRD2;BRD7;BRPF1;BTRC;C14orf169;C1D;C22orf28;CAD;CALM3;CALR;CAMTA2;CAND2;CAPN3;CARD11;CARNS1;CI
ATF6B;ATM;ATP10A;ATP13A1;ATP1A1;ATP1A3;ATP2A2;ATP2A3;ATP5A1;ATP5B;ATP8B1;ATP8B2;ATP9B;ATXN1L;BACH2;BAHCC1;BAHD1;BATF;BATF3;BAZ1B;BCL11B;BCL2;BDH1;BLM;BMS1;BPTF;BRD7;BRPF1;BRSK2;C1D;C22orf28;C9orf96;CAD;CALR;CAMTA2;CARNS1;CBWD3;CBWD6;CBX2;CBX7;CC2D1B
;AKT3;ALAD;ALDH18A1;ALDH1B1;ALDH3A2;ALDH5A1;ALDH6A1;ALDH7A1;ALG8;ALG9;ALMS1;ALS2CL;ALX3;AMACR;AMBRA1;AMFR;AMIGO1;AMOTL1;ANAPC1;ANAPC13;ANAPC5;ANAPC7;ANK3;ANKH;ANKLE2;ANKRD11;ANKRD27;ANKS1B;ANKS6;ANKZF1;ANO8;ANP32B;ANXA2;ANXA6;AP2B1;AP3D1;AP3
;AKT3;ALAD;ALDH18A1;ALDH1B1;ALDH3A2;ALDH5A1;ALDH6A1;ALDH7A1;ALG8;ALG9;ALMS1;ALS2CL;ALX3;AMACR;AMBRA1;AMFR;AMIGO1;AMOTL1;ANAPC1;ANAPC13;ANAPC5;ANAPC7;ANK3;ANKH;ANKLE2;ANKRD11;ANKRD27;ANKS1B;ANKS6;ANKZF1;ANO8;ANP32B;ANXA2;ANXA6;AP2B1;AP3D1;AP3
SMT;ASPDH;ASXL1;ATF6B;ATIC;ATM;ATN1;ATP1A1;ATP1A3;ATP2A2;ATP2A3;ATP5A1;ATP5B;ATP5J;ATP5J2;ATP6V1H;ATP8B1;ATP8B2;ATXN1L;AVPR2;AXIN1;AXIN2;BACE1;BACH2;BAD;BAHD1;BATF;BATF3;BAZ1B;BCL10;BCL11B;BCL9L;BEX1;BICD1;BLM;BMP2;BPTF;BRD2;BRD7;BRPF1;BTRC;C14orf169;C1I
LDH7A1;ALG8;ALG9;ALMS1;ALX3;AMACR;AMBRA1;AMFR;ANAPC1;ANAPC13;ANAPC5;ANAPC7;ANKLE2;APBB1;APOBEC3F;AQR;ARG1;ARHGAP15;ARHGAP29;ARHGAP35;ARHGEF16;ARHGEF5;ARHGEF6;ARHGEF7;ARID1B;ARIH2;ARNTL2;ASB1;ASB16;ASCC1;ASMT;ASPDH;ASXL1;ATF6B;ATG10;ATG13;ATG
ATM;ATN1;ATP1A1;ATP1A3;ATP2A2;ATP2A3;ATP5A1;ATP5B;ATP5J;ATP5J2;ATP6V1H;ATP8B1;ATP8B2;ATXN1L;AVPR2;AXIN1;AXIN2;BACH2;BAD;BAHD1;BATF;BATF3;BAZ1B;BCL10;BCL11B;BCL9L;BEX1;BICD1;BLM;BMP2;BPTF;BRD2;BRD7;BRPF1;BTRC;C14orf169;C1D;C22orf28;CAD;CALM3;CALR;CAMTA2;
TF3C4;HCFC1;HDAC1;HMGA1;HNRNPM;HOXA9;IKBKAP;IKBKE;ING2;ING5;INTS1;INTS4;INTS7;INTS9;JAZF1;KAT6B;KDM1A;LAS1L;LDB1;LEF1;LEO1;LMO2;LPIN1;LSG1;LSM10;LUC7L2;MAGOH;MBD1;MBD2;MED11;MED24;MED27;MED29;MED30;MED31;MED6;MGA;MLL;MLL4;MMS19;MTA1;MTA2;MTA3;
KAP1;AKAP17A;AKAP3;AKAP8;AKAP9;AKNA;AKT2;AKT3;ALAD;ALDH18A1;ALDH5A1;ALDH6A1;ALDH7A1;ALS2CL;ALX3;AMACR;AMBRA1;AMFR;AMOTL1;AMZ1;ANAPC5;ANAPC7;ANK3;ANKLE2;ANKRD27;ANKS1B;ANKS6;ANKZF1;ANXA2;ANXA6;AP2B1;AP3S1;APBA1;APBA2;APBB1;APOBEC3F;APOL3;APOLD
;AVPR2;AXIN1;AXIN2;B3GALT4;BACH2;BAG3;BAHD1;BATF;BATF3;BAZ1B;BCL10;BCL11B;BCL2;BCL9L;BEX1;BICD1;BIRC6;BLM;BLMH;BLOC1S1;BMP2;BPTF;BRD1;BRD2;BRD7;BRPF1;BRSK2;BTRC;C14orf169;C1D;C1GALT1C1;C22orf28;CAD;CALM3;CALR;CAMTA2;CAND2;CAPN3;CARD11;CARD14;CBFA2T2;CB
MS1;ALS2CL;AMACR;AMBRA1;AMFR;AMOTL1;ANAPC1;ANAPC5;ANAPC7;ANK3;ANKLE2;ANKRD27;ANKS1B;ANKS6;ANP32B;ANXA2;ANXA6;AP2B1;AP3D1;AP3S1;APBA1;APBB1;APOBEC3F;APOL3;APOOL;AQP3;ARG1;ARHGAP15;ARHGAP17;ARHGAP18;ARHGAP29;ARHGAP32;ARHGAP33;ARHGAP35;ARHGA
ATP8B2;ATP9B;BAZ1B;BLM;BMS1;BRSK2;C22orf28;C9orf96;CAD;CARNS1;CBWD3;CBWD6;CCL8;CCT3;CCT5;CCT6A;CCT7;CCT8;CDC42BPA;CDC42BPB;CDC42BPG;CECR1;CERK;CHD1L;CHD2;CHD3;CHD4;CHD6;CHEK2;CHST12;CIITA;CIT;CKM;CLCN3;CLCN6;CLEC3B;CLK2;CLPB;COL5A1;CREG1;CSNK1E;CSNK2A2
DH18A1;ALDH1B1;ALDH3A2;ALDH5A1;ALDH6A1;ALDH7A1;ALG8;ALG9;ALMS1;ALX3;AMACR;AMFR;AMZ1;ANAPC1;ANAPC13;ANAPC5;ANAPC7;ANKLE2;APBB1;APOBEC3F;APOL3;APOLD1;AQR;ARG1;ARHGAP15;ARHGAP29;ARHGAP35;ARHGEF16;ARHGEF5;ARHGEF6;ARHGEF7;ARID1B;ARIH2;ARNTL2;ASB
;CBX2;CBX5;CBX7;CCAR1;CCNT1;CD3EAP;CD40LG;CDAN1;CDH13;CDK5RAP2;CDKAL1;CDKN2A;CDKN2B;CDON;CELF1;CELSR2;CENPN;CENPO;CEP164;CHCHD3;CHD1L;CHD2;CHD3;CHD4;CHD6;CHEK2;CHTF8;CIAO1;CIC;CIITA;CINP;CIRH1A;CIZ1;CLK2;CMKLR1;CNOT10;CPSF3;CREB3L2;CREG1;CREM;CRTC1;CR
;BIRC6;BLOC1S1;BPTF;BRD1;BRPF1;BTRC;C1D;C22orf28;CACNA1F;CACNA1I;CACNB1;CAD;CALM3;CALR;CAMSAP1;CAPN3;CARD11;CASP1;CATSPER1;CATSPER2;CBX2;CBX5;CBX7;CCND1;CCND2;CCNF;CCNT1;CCT3;CCT5;CCT6A;CCT7;CCT8;CD1A;CD247;CD3E;CD3EAP;CD4;CD74;CD8A;CDC16;CDC25B;CDK5F

AD;ALDH18A1;ALDH1B1;ALDH3A2;ALDH5A1;ALDH6A1;ALDH7A1;ALG8;ALG9;ALMS1;ALX3;AMACR;AMFR;AMZ1;ANAPC1;ANAPC13;ANAPC5;ANAPC7;ANK3;ANKLE2;APBA1;APBA2;APBB1;APOBEC3F;APOL3;APOLD1;AQR;ARG1;ARHGAP15;ARHGAP29;ARHGAP35;ARHGEF16;ARHGEF5;ARHGEF6;ARHGEF7;A

CC1;ASMT;ASPDH;ASXL1;ATF6B;ATIC;ATM;ATN1;ATP1A1;ATP1A3;ATP2A2;ATP2A3;ATP5A1;ATP5B;ATP5J;ATP5J2;ATP6V1H;ATP8B1;ATP8B2;ATXN1L;AVPR2;AXIN1;AXIN2;BACE1;BACH2;BAD;BAHD1;BATF;BATF3;BAZ1B;BCL10;BCL11B;BCL9L;BEX1;BICD1;BLM;BMP2;BPTF;BRD2;BRD7;BRPF1;BTRC;C14orf16

P1L;CREB3L2;CREM;CRY2;CSRNP2;CSTF1;CSTF2;CSTF3;CTC1;CTCF;CXXC5;DARS2;DBF4B;DDB1;DDIT3;DDX10;DDX17;DDX18;DDX19A;DDX19B;DDX20;DDX23;DDX24;DDX27;DDX31;DDX42;DDX46;DDX47;DDX51;DDX52;DDX55;DDX56;DDX6;DFFB;DHX15;DHX16;DHX33;DHX35;DHX37;DHX38;DHX57;DHX9;D

A;AKT2;AKT3;ALAD;ALDH18A1;ALDH1B1;ALDH3A2;ALDH5A1;ALDH6A1;ALDH7A1;ALG8;ALG9;ALMS1;ALX3;AMACR;AMBRA1;AMFR;AMZ1;ANAPC1;ANAPC13;ANAPC5;ANAPC7;ANK3;ANKLE2;AP3D1;APBA1;APBA2;APBB1;APOBEC3F;APOL3;APOLD1;AQR;ARG1;ARHGAP15;ARHGAP29;ARHGAP35;ARHGEF1

A2;ANXA6;AP2B1;AP3S1;APBA1;APBA2;APBB1;APOBEC3F;ARHGAP17;ARHGAP32;ARHGAP33;ARHGEF1;ARHGEF16;ARHGEF4;ARHGEF6;ARHGEF7;ARID1B;ARIH2;ARL3;ARNTL2;ARPC2;ARPC3;ASB16;ASMT;ASXL1;ATG13;ATG14;ATG3;ATIC;ATM;ATN1;ATOX1;ATP10A;ATP1A1;ATP2A2;ATP5A1;ATP5B;ATP6V

CD3;CALR;CAMTA2;CAND2;CAPN2;CAPN3;CARD11;CASP1;CASP2;CBFA2T2;CBX2;CBX5;CBX7;CCAR1;CCDC88C;CCNT1;CD3EAP;CDH13;CDK5RAP2;CDKAL1;CDKN2A;CDKN2B;CDON;CELF1;CELSR2;CHCHD3;CHD2;CHD3;CHD4;CHD6;CHEK2;CIAO1;CIC;CIITA;CIRBP;CIRH1A;CLEC3B;CLK2;CMKLR1;CNOT10;COG

HNRNPM;HNRNPR;HNRNPU;HSPA8;INTS1;INTS4;INTS7;INTS9;KARS;KHDRBS1;KHSRP;KIAA1429;LARP7;LAS1L;LEO1;LOC100130932;LSM10;MAGOH;MLH1;MRPL44;MTPAP;NOL9;NOLC1;NONO;NOP10;NOP14;NSUN2;NSUN4;PA2G4;PABPC1;PABPC4;PAN2;PARN;PDCD11;PHRF1;PNN;POLR2B;POLR2C;POL

DHX33;DHX35;DHX37;DHX38;DHX57;DHX9;DNAH1;DYNC1H1;EIF4A3;ERCC3;FBXO18;G3BP1;HELB;HSPA8;HSPD1;INO80;LONP2;MACF1;MCM4;MCM6;MDN1;MLH1;MSH6;MYO18A;MYO1D;MYO1E;MYO9B;PCYOX1;PMS2;PMS2P5;PSMC4;PSMD6;RAD51D;RBBP4;RFC1;SMARCA4;SMARCAL1;SNRNP200;SU

D1;AQR;ARG1;ARHGAP35;ARID1B;ARIH2;ARNTL2;ASB1;ASB16;ASCC1;ASMT;ASXL1;ATF6B;ATG10;ATG3;ATM;ATN1;ATP8B1;ATXN1L;AVPR2;AXIN1;AXIN2;B3GALT4;BACE1;BACH2;BAG3;BAHD1;BATF;BATF3;BAZ1B;BBS2;BCL10;BCL11B;BCL2;BCL9L;BEX1;BICD1;BIRC6;BLM;BLMH;BLOC1S1;BMP2;BPTF;BRD1;

PA3;HSPA8;HSPA9;IKBKAP;ILF2;ILF3;IPO5;ITPR3;KDM2B;KDM4A;LBH;LEO1;MDN1;MED27;MPHOSPH8;MYBBP1A;MYC;MYO1C;NAT10;NCL;NF2;NFATC3;NOL10;NOL11;NOL9;NOLC1;NOM1;NONO;NOP10;NOP14;NSUN2;PA2G4;PARN;PARP1;PDCD11;PINX1;PNO1;POLA1;POLR1A;POLR1B;POP1;PSPC1;PTB

F;CCT3;CCT5;CCT6A;CCT7;CCT8;CD3EAP;CDC16;CDC25B;CDC42BPA;CDC42BPB;CDC42BPG;CDH23;CDK5RAP2;CDKN2A;CENPN;CENPO;CEP104;CEP164;CEP72;CEP85;CEP89;CHD3;CHD4;CHEK2;CIAO1;CIRBP;CIRH1A;CIT;CKAP5;CLEC3B;CNTRL;CORO2A;CROCC;CSTB;CTC1;CTCF;CTR9;DCLK2;DCTN1;DCTN5;D

F;CCT3;CCT5;CCT6A;CCT7;CCT8;CD3EAP;CDC16;CDC25B;CDC42BPA;CDC42BPB;CDC42BPG;CDH23;CDK5RAP2;CDKN2A;CENPN;CENPO;CEP104;CEP164;CEP72;CEP85;CEP89;CHD3;CHD4;CHEK2;CIAO1;CIRBP;CIRH1A;CIT;CKAP5;CLEC3B;CNTRL;CORO2A;CROCC;CSTB;CTC1;CTCF;CTR9;DCLK2;DCTN1;DCTN5;D

L;SETD1A;SF3A3;SFPQ;SNRNP70;SON;SRRM1;SRRM2;SRSF5;TCF7L2;TFIP11;TGS1;THOC1;TP53;TRIM16;U2AF1L4;UBE2I;USP7;WBP11;YLPM1;ZBTB16;ZC3H14;ZNF4

DHX9;DYNC1H1;EIF4A3;ERCC3;FBXO18;G3BP1;HELB;HSPA8;LONP2;MCM4;MCM6;MSH6;MYO18A;MYO1D;MYO1E;PCYOX1;RAD51D;RBBP4;RFC1;SMARCA4;SMARCAL1;SNRNP200;SUPV3L1;TOP2B;XRCC3;XRCC5;XRC

NT1;CD3EAP;CDH13;CDK5RAP2;CDKAL1;CDKN2A;CDKN2B;CDON;CELF1;CELSR2;CHCHD3;CHD2;CHD3;CHD4;CHD6;CHEK2;CIAO1;CIC;CIITA;CIRH1A;CLK2;CMKLR1;CNOT10;CPSF3;CREB3L2;CREG1;CREM;CRTC1;CRTC3;CRY2;CSNK2A2;CSRNP2;CSTF1;CSTF2;CSTF3;CTCF;CTNNBL1;CTR9;CYTL1;DARS2;DBR1;DC

AP9;AKNA;AKT2;AKT3;ALAD;ALDH18A1;ALDH3A2;ALDH5A1;ALDH6A1;ALDH7A1;ALG8;ALG9;ALMS1;ALS2CL;ALX3;AMACR;AMBRA1;AMFR;AMIGO1;AMIGO3;AMOTL1;ANAPC1;ANAPC13;ANAPC5;ANAPC7;ANK3;ANKLE2;ANKRD27;ANXA2;AP2B1;AP3D1;AP3S1;APBA1;APBA2;APBB1;APOBEC3F;APOL3;APO

6V1H;AXIN1;BAD;BAG3;BAIAP2L1;BCL10;BCL2;BICD1;BID;BLMH;BLOC1S1;BTRC;C2CD2;C9orf89;CABP4;CAD;CALM3;CALR;CAMP;CAPN3;CARD11;CASP1;CASP10;CASP2;CBLB;CCND1;CCND2;CCT3;CCT5;CCT6A;CCT7;CCT8;CDC16;CDC25B;CDK5RAP2;CDKN2A;CDKN2B;CENPN;CENPO;CEP164;CEP72;CHMP4

A;EIF5B;ELAVL1;EPRS;EXOSC10;FAM103A1;FARSB;FIP1L1;FUBP1;G3BP1;GAR1;GCN1L1;GEMIN5;GFM1;HEXIM1;HNRNPA0;HNRNPA2B1;HNRNPA3;HNRNPAB;HNRNPH1;HNRNPM;HNRNPR;HNRNPU;HSP90B1;HTATSF1;ILF2;ILF3;KARS;KDM2B;KHDRBS1;KHSRP;LARP1;LARP7;LOC100130932;LSM10;MAGOH;

PAN2;PARN;PDCD11;PHRF1;PNN;POLR2B;POLR2C;POLR2J;PRPF19;PRPF3;PRPF4;PRPF40B;PRPF8;PTBP1;RBFOX2;RBM15B;RBM17;RBM26;RBM28;RBM4B;RNASE4;RNMT;RNPS1;SCAF1;SCAF8;SF1;SF3A3;SF3B2;SF3B3;SFPQ;SMC1A;SNRNP200;SNRNP40;SNRNP70;SNW1;SON;SRRM1;SRRM2;SRSF5;SUGP2;

PF40B;PRPF8;PTBP1;RBFOX2;RBM15B;RBM17;RBM28;RBM4B;RNPS1;SCAF1;SCAF8;SF1;SF3A3;SF3B2;SF3B3;SFPQ;SMC1A;SNRNP200;SNRNP40;SNRNP70;SNW1;SON;SRRM1;SRRM2;SRSF5;SUGP2;SYF2;TARDBP;TFIP11;TGS1;THOC1;THOC2;U2AF1L4;U2AF2;USP39;WBP11;ZRS

COX10;COX15;COX5B;COX8A;CTPS2;CYB5B;CYP2D6;D2HGDH;DARS2;DCAF5;DDIT4;DDX23;DHODH;DHX32;DLD;DLST;DNAJA3;DNAJC11;DPYSL2;EARS2;ECHDC2;ECI2;EFHD1;ENOSF1;ETFDH;ETHE1;EXOG;FAM110B;FASN;FASTKD5;FEN1;FH;FITM2;FKBP4;FTSJ2;FXN;FYN;G0S2;GARS;GBF1;GFM1;GLOD4;GLS2

2;BRD7;BRK1;BRPF1;BRSK2;C14orf169;CALR;CAMP;CAMSAP1;CAPN3;CBX2;CBX7;CCDC88C;CCNF;CCR7;CDAN1;CDC16;CDC25B;CDC42BPA;CDC42BPB;CDC42BPG;CDK5RAP2;CDKN2A;CENPN;CENPO;CEP164;CEP72;CHCHD3;CHD1L;CHD3;CHD4;CHD6;CHEK2;CHMP5;CIT;CKAP5;CLCN3;CLN8;CNTNAP1;COG

ERC2;HGF;HHEX;HIST1H2AC;HIST1H2AK;HIST1H2BN;HIST2H2AC;HIST2H2BE;HIST2H3D;HMGA1;HP1BP3;HSPD1;HUWE1;IL10;IL4;IL7R;ING5;INO80;INPPL1;KAT6B;KCTD13;KITLG;KRBA2;KRT7;LEF1;LIG1;LIG3;LOC100133315;MAD2L2;MCM2;MCM3;MCM3AP;MCM4;MCM6;MDC1;MLH1;MMS19;MND1;MP

1;LIG3;MBD1;MBD2;MCM2;MCM3;MDC1;MECP2;MLH1;MPHOSPH8;MSH6;MTA3;NCAPD2;NCAPD3;NCAPG;NDC80;NFRKB;NR1D1;NUMA1;NUP133;PHC1;PINX1;POGZ;POLA1;POLA2;POLE;POLE3;PPP2R5C;RAD18;RBBP4;RBM19;RCC2;RFC1;RNF20;RNF8;RPA1;RPA2;RRP1B;RUNX2;RUNX3;RUVBL1;SATB1

P3;HUWE1;ING2;ING5;INO80;KAT6B;KDM1A;KDM2B;KDM4A;KDM4C;KDM5C;L3MBTL1;L3MBTL2;LDB1;LEF1;LEO1;LIG1;LPIN1;MAP3K12;MCM2;MECP2;MLL;MLL4;MTA2;MYC;NASP;NCAPD2;NCAPD3;NCAPG;NCAPG2;NDC80;PAPD7;PARP1;PAXIP1;PCGF1;PHF13;PHF15;PIM3;PINX1;POGZ;POLA1;POLA2;P

;MPHOSPH8;MSH6;MTA3;NCAPD2;NCAPD3;NCAPG;NDC80;NFRKB;NR1D1;NUP133;PHC1;PINX1;POGZ;POLA1;POLA2;POLE;POLE3;PPP2R5C;RAD18;RBBP4;RCC2;RFC1;RNF20;RNF8;RPA1;RPA2;RRP1B;RUNX2;RUNX3;RUVBL1;SATB1;SCMH1;SEH1L;SEPT6;SLX4;SMARCA4;SMARCD3;SMC1A;SMG6;SNW1;SC

P1;TOP2B;TP53;UBE2B;UBE2I;UHRF1;XRCC5;XRCC6;ZMIZ

RSF5;SYF2;TFIP11;TGS1;U2AF2;USP39;ZRSR

RSF5;SYF2;TFIP11;TGS1;U2AF2;USP39;ZRSR

RM2;SRSF5;SYF2;TFIP11;TGS1;U2AF2;USP39;ZRSR

3;EXOC7;EZR;FAM110B;FBF1;FKBP4;HAUS5;HERC2;HSPH1;INCENP;INO80;INPP5B;INVS;JTB;KARS;KDM4A;KIAA0368;KIAA0586;KIAA0753;KIF13B;KIF16B;KIF21B;KIF3B;KIF4A;KIF5C;KLC1;KLC2;KNTC1;LCK;LZTS2;MACF1;MAD2L1BP;MAD2L2;MAP1A;MAP1LC3B2;MAP4;MAP7D3;MAPRE2;MAPT;MCM3;MID2

NARS;NFX1;PAICS;PCCA;PCCB;PET112;PFAS;PJA1;PPCS;PPIL2;PRPF19;RAD18;RCL1;RFWD3;RNF125;RNF144A;RNF20;RNF216;RNF4;RNF43;RNF8;SAE1;SARS;SYVN1;TARS;TNFAIP1;TRAF3;TRIM17;TRIM56;TRIM68;TTC3;TTL;TTLL3;TTLL5;UBE2B;UBE2D2;UBE2F;UBE2G2;UBE2I;UBE2L3;UBE2Q1;UBE2Z;UBE3

DX52;DDX55;DDX56;DDX6;DHX15;DHX16;DHX32;DHX33;DHX35;DHX37;DHX38;DHX57;DHX9;DNAH1;DNHD1;DNM1;DYNC1H1;DYNC1I2;DYNC1LI2;DYNLT1;EEF2;EFTUD1;EFTUD2;EHD4;EIF2S3;EIF4A3;EIF5B;ENTPD8;EP400;ERCC3;FBXO18;G3BP1;GFM1;GNG10;GNL2;GNL3L;GTPBP4;HELB;HSPA8;HSPD1;IN

ED6;MEIS2;MTA1;MTA2;MTOR;MYBBP1A;MYC;NAB2;NUP62;PARD6A;PARP1;PDCD11;PPARA;PRMT2;RBFOX2;RELA;RNF4;RUNX1;RUNX2;SETD6;SMAD3;SMARCA4;SMARCD3;SNW1;SOX10;STK36;SUFU;TAF1;TBP;TCF3;TCF7L2;TFDP2;THRA;TLE1;TNFRSF10A;TOB2;TP53;TP53BP1;UBE2I;UBXN7;USP7;VHL;Y

DIS3L;DLG3;DNAJA3;DOCK10;DOCK7;DOCK9;DPP4;DPYSL2;EIF2AK3;ELAVL1;ELFN2;EPHA1;EXOC2;FAF1;FGD1;FLNA;FLT4;FMNL3;FOXM1;FXN;GABARAPL2;GGA3;GSTM4;GYS1;HDAC1;HERC2;HLCS;HMGA1;HNRNPA0;HOXA9;HSP90AB1;HSP90B1;HSPA9;HYAL2;IKBKB;IL12RB2;IL31RA;IPO5;IRS1;JTB;KCTD13

BRA1;AMFR;AMOTL1;ANAPC1;ANAPC5;ANAPC7;ANK3;ANKLE2;ANKRD27;ANXA2;ANXA6;AP2B1;AP3D1;AP3S1;APBA1;APOBEC3F;APOOL;ARG1;ARHGAP15;ARHGAP17;ARHGAP18;ARHGAP29;ARHGAP32;ARHGAP35;ARHGAP39;ARHGEF1;ARHGEF16;ARHGEF18;ARHGEF3;ARHGEF4;ARHGEF6;ARHGEF7;ARI

DX52;DDX55;DDX56;DDX6;DHX15;DHX16;DHX32;DHX33;DHX35;DHX37;DHX38;DHX57;DHX9;DNAH1;DNHD1;DNM1;DYNC1H1;DYNC1I2;DYNC1LI2;DYNLT1;EEF2;EFTUD1;EFTUD2;EHD4;EIF2S3;EIF4A3;EIF5B;ENTPD4;ENTPD5;ENTPD8;EP400;ERCC3;FBXO18;G3BP1;GFM1;GMPS;GNG10;GNL2;GNL3L;GTPBP

DX52;DDX55;DDX56;DDX6;DHX15;DHX16;DHX32;DHX33;DHX35;DHX37;DHX38;DHX57;DHX9;DNAH1;DNHD1;DNM1;DYNC1H1;DYNC1I2;DYNC1LI2;DYNLT1;EEF2;EFTUD1;EFTUD2;EHD4;EIF2S3;EIF4A3;EIF5B;ENTPD4;ENTPD5;ENTPD8;EP400;ERCC3;FBXO18;G3BP1;GFM1;GMPS;GNG10;GNL2;GNL3L;GTPBP

DX52;DDX55;DDX56;DDX6;DHX15;DHX16;DHX32;DHX33;DHX35;DHX37;DHX38;DHX57;DHX9;DNAH1;DNHD1;DNM1;DYNC1H1;DYNC1I2;DYNC1LI2;DYNLT1;EEF2;EFTUD1;EFTUD2;EHD4;EIF2S3;EIF4A3;EIF5B;ENTPD4;ENTPD5;ENTPD8;EP400;ERCC3;FBXO18;G3BP1;GFM1;GMPS;GNG10;GNL2;GNL3L;GTPBP

CSNK1E;CSNK2A2;CSRP2BP;CTCF;CUL1;CUL4A;DBF4B;DCTN1;DDB1;DDIT3;DHCR24;DMWD;DST;DTL;DYNC1H1;DYNC1I2;DYNLT1;EIF4G2;ERCC3;ETS1;FANCI;FEN1;FGF2;FGFR1;FHL1;FLNA;FOXC1;FOXM1;FZR1;GADD45A;GATA3;GINS4;GTPBP4;HAUS5;HCFC1;HDAC1;HEXIM1;HGF;HHEX;HSPA8;HTRA2;HTT;H

3;SRRT;TARS;TGS1;TRIT1;TSR2;TYW1;UTP14A;UTP20;WBP11;WDR4;WDR55;YARS;ZCCHC

CBX2;CBX5;CBX7;CCAR1;CCNT1;CCR7;CD3EAP;CD4;CDAN1;CDH13;CDK5RAP2;CDKN2A;CDKN2B;CDON;CELSR2;CHCHD3;CHD2;CHD3;CHD4;CHD6;CHEK2;CHST12;CHTF8;CIAO1;CIC;CIITA;CINP;CIRBP;CIRH1A;CIZ1;CMKLR1;COG2;COG7;CPSF3;CREB3L2;CREG1;CREM;CRTC1;CRTC3;CRY2;CSNK2A2;CSRNP2;CS

PAXIP1;PCGF1;PHF13;PHF15;PIM3;POLE3;PRKCA;PRMT2;PYGO2;RBBP4;RBBP7;RBL2;RBM14;RNF20;RNF8;RUVBL1;SATB1;SCMH1;SETD1A;SMARCA4;SMARCAL1;SMARCC1;SMARCC2;SMARCD1;SMARCD3;SMARCE1;SNW1;SRCAP;SUPT4H1;SUPT6H;TADA2A;TAF1;TAF10;TCF3;TERF2;TP53;TRIM16;TRRAP;T

LB;RUNX2;RUNX3;RUVBL1;SATB1;SLX4;SMARCA4;SMARCD3;SNW1;SOX18;TBP;TCF3;TCF7L2;TCP1;TIMELESS;TNKS1BP1;TOP2B;TP53;UBE2B;UHRF

AMD4;GTPBP4;GUCY2D;HADHA;HADHB;HERC2;HSPD1;HTATIP2;HTRA2;IMMT;INTS1;IPO5;IPO9;ITPR3;LETM1;LOC494141;LPIN1;MAVS;MCCC1;MCCC2;MGST1;MLXIP;MNS1;MTCH2;MTOR;MUL1;MYO19;MYO1C;NAT8L;NDUFA10;NDUFA2;NDUFA9;NDUFB4;NDUFB6;NDUFS1;NDUFV3;NNT;NUP133;NUP15

P5B;ATP5J;ATP5J2;ATP8B1;ATXN1L;AVPR2;AXIN1;B3GALT4;BACH2;BAHD1;BATF;BATF3;BAZ1B;BCL10;BCL11B;BCL2;BCL9L;BEX1;BLM;BMP2;BPTF;BRD2;BRD7;BRPF1;BTRC;C14orf169;C1D;C1GALT1C1;CAD;CALR;CAMTA2;CAND2;CAPN3;CARD11;CARNS1;CBFA2T2;CBX2;CBX5;CBX7;CCAR1;CCNT1;CCR7;CD3

TERF2IP;TEX12;TIMELESS;TNKS1BP1;TOP2B;TP53;UBE2B;UBE2I;UHRF1;XRCC5;XRCC6;ZMIZ

CAPN3;CARD11;CBFA2T2;CBX2;CBX5;CBX7;CCAR1;CCNT1;CCR7;CD3E;CD3EAP;CD4;CDAN1;CDH13;CDK5RAP2;CDKN2A;CDKN2B;CDON;CELSR2;CHCHD3;CHD2;CHD3;CHD4;CHD6;CHEK2;CHST12;CHTF8;CIAO1;CIC;CIITA;CINP;CIRBP;CIRH1A;CIZ1;CMKLR1;COG2;COG7;COL5A1;CPSF3;CREB3L2;CREG1;CREM;

AMD4;GRIN2C;GTPBP4;GUCY2D;HADHA;HADHB;HERC2;HSPD1;HTATIP2;HTRA2;IMMT;INTS1;IPO5;IPO9;ITPR3;LETM1;LOC494141;LPIN1;MAVS;MCCC1;MCCC2;MGST1;MLXIP;MNS1;MTCH2;MTOR;MUL1;MYO19;MYO1C;NAT8L;NDUFA10;NDUFA2;NDUFA9;NDUFB4;NDUFB6;NDUFS1;NDUFV3;NNT;NUP13

1;HGF;HTT;HYAL1;INCENP;ING5;INO80;JTB;KHDRBS1;KIF3B;KLF11;KNTC1;L3MBTL1;LIG1;LMLN;LRP5;LSM10;LZTS2;MAD2L1BP;MAD2L2;MAP4;MAPRE2;MCM2;MCM3;MCM4;MCM6;MTA3;MYC;NCAPD2;NCAPD3;NCAPG;NCAPG2;NDC80;NEK9;NINL;NME6;NOLC1;NSUN2;NUMA1;NUP133;ODF2;PAPD7;P

5J;ATP5J2;ATP8B1;ATXN1L;AVPR2;AXIN1;B3GALT4;BACH2;BAHD1;BATF;BATF3;BAZ1B;BCL10;BCL11B;BCL2;BCL9L;BDH1;BEX1;BLM;BMP2;BPTF;BRD2;BRD7;BRPF1;BTRC;C14orf169;C1D;C1GALT1C1;CAD;CALR;CAMTA2;CAND2;CAPN3;CARD11;CARNS1;CBFA2T2;CBX2;CBX5;CBX7;CCAR1;CCNT1;CCR7;CD3E

SMARCE1;SMC1A;SNAPC4;SOX10;SSRP1;TADA2A;TCF3;TCF7;TCF7L2;TERF2;THRA;TLE1;TOP2B;TP53;TTF1;UHRF1;WHSC1;ZKSCAN2;ZNF5

1;WDR55;ZNHIT6;ZRS

MWD;DST;DTL;DYNC1H1;DYNC1I2;DYNLT1;EIF4G2;ERCC3;FEN1;FHL1;FLNA;FOXM1;FZR1;GADD45A;GINS4;HAUS5;HGF;HTRA2;HTT;HYAL1;ILF3;INCENP;ING2;ING5;INO80;INTS7;JTB;KHDRBS1;KIF3B;KLF11;KNTC1;KRT7;L3MBTL1;LEF1;LIG1;LIG3;LMLN;LRP5;LSM10;LZTS2;MACF1;MAD2L1BP;MAD2L2;MAP
A1;ATP5B;ATP5J;ATP5J2;ATP8B1;ATXN1L;AVPR2;AXIN1;B3GALT4;BACH2;BAHD1;BATF;BATF3;BAZ1B;BCL10;BCL11B;BCL2;BCL9L;BDH1;BEX1;BLM;BMP2;BPTF;BRD2;BRD7;BRPF1;BTRC;C14orf169;C1D;C1GALT1C1;CAD;CALR;CAMTA2;CAND2;CAPN3;CARD11;CARNS1;CBFA2T2;CBX2;CBX5;CBX7;CCAR1;CC

Z1;ANKZF1;ANXA2;ANXA6;APBA1;APOBEC3F;ARG1;ARHGAP29;ARHGAP35;ARHGEF5;ARIH2;ARL10;ARL13A;ARL3;ASXL1;ATAD3C;ATM;ATOX1;ATP10A;ATP13A1;ATP1A1;ATP1A3;ATP2A2;ATP2A3;ATP5A1;ATP5B;ATP8B1;ATP8B2;ATP9B;BAZ1B;BCL11B;BLM;BMS1;BPTF;BRD1;BRPF1;BRSK2;BSN;C11orf95;

P1;RANBP10;RCC2;RP1L1;SARM1;STIM1;TBCC;TCP1;TCP11L1;TPPP;TTLL3;TUBA3D;TUBB;TUBGCP4;TUBGCP5;TUBGCP6;ZW1

;KDM5C;L3MBTL1;L3MBTL2;LDB1;LEF1;LEO1;LPIN1;MAP3K12;MCM2;MECP2;MLL;MLL4;MTA2;MYC;NASP;PAXIP1;PCGF1;PHF13;PHF15;PIM3;POLE3;PRKCA;PRMT2;PYGO2;RBBP4;RBBP7;RBL2;RBM14;RNF20;RNF8;RUVBL1;SATB1;SCMH1;SETD1A;SMARCA4;SMARCAL1;SMARCC1;SMARCC2;SMARCD1;S
RPEL1;HADH;HADHA;HADHB;HERC2;HSPA1L;HSPA9;HSPD1;HTRA2;IARS2;IDH3A;IMMT;IVD;KARS;LARS2;LETM1;LOC494141;MAVS;MCCC1;MCCC2;MGST1;MIPEP;MLXIP;MRPL49;MRPS24;MRPS36;MRPS9;MTCH2;MTOR;MUL1;MYO19;NAT8L;NDUFA10;NDUFA2;NDUFA9;NDUFB4;NDUFB6;NDUFS1;NDUF
1D;C2CD3;CALM3;CALR;CAMTA2;CAND2;CAPN3;CARD11;CARD14;CBFA2T2;CBLB;CBX2;CBX5;CBX7;CCAR1;CCDC23;CCDC88C;CCND1;CCND2;CCNT1;CCR7;CD3E;CD4;CD74;CDAN1;CDC16;CDC25B;CDH13;CDK5RAP2;CDKN2A;CDKN2B;CDON;CELF1;CELSR2;CHCHD3;CHD2;CHD3;CHD4;CHD6;CHEK2;CHMP5;
D18;RAD51D;RBM14;RFC1;RFWD3;RNF8;RPA1;RPA2;RUVBL1;SFPQ;SLX4;SMC1A;SSRP1;SUPT16H;TDP1;TERF2IP;TP53;TP53BP1;UBE2B;UHRF1;UNG;USP7;VCP;WDR33;WRNIP1;XPC;XRCC3;XRCC5;XRCC6;ZFYVE
LDH3A2;ALDH5A1;ALDH6A1;ALDH7A1;ALG8;ALG9;AMACR;AMFR;AMZ1;ANAPC5;APOBEC3F;ARG1;ARIH2;ARL3;ASMT;ASPDH;ATAD3C;ATAT1;ATG10;ATG3;ATIC;ATM;ATP10A;ATP13A1;ATP1A1;ATP1A3;ATP1B3;ATP2A2;ATP2A3;ATP5A1;ATP5B;ATP5J;ATP5J2;ATP6V0E1;ATP6V1D;ATP6V1F;ATP6V1H;ATP8

ATG3;ATG9B;ATM;ATP10A;ATP6V0E1;ATP6V1D;ATP6V1F;ATP6V1H;ATP8B1;ATP9B;ATXN2L;AXIN1;AXIN2;BAD;BAHD1;BAIAP2L1;BAZ1B;BBS1;BBS2;BCL10;BCL11B;BCL2;BCL9L;BICD1;BID;BIK;BIRC6;BLM;BLOC1S1;BMP2;BMS1;BPTF;BRD1;BRD2;BRD7;BRK1;BRPF1;BRSK2;C14orf169;C1D;C22orf28;C2CD3;C
MT3A;EIF4G1;EIF4G2;ELP3;EME2;EP400;ERCC3;ERCC5;ETS1;FAN1;FANCI;FBXL19;FBXO18;FBXO21;FEN1;FLYWCH1;FOSB;FOXC1;FOXI1;FOXJ1;FOXJ3;FOXK1;FOXK2;FOXM1;FOXP1;FOXP4;FUBP1;G3BP1;GABPB2;GATA3;GMEB2;GON4L;GTF3C1;GTF3C2;GTF3C4;H3F3C;HDAC1;HEMK1;HHEX;HIC2;HIST1H2AC;

0;RASA3;RASA4;RASAL3;RASGRF2;SBF1;SGSM2;SRGAP1;SYDE1;SYNGAP1;TBC1D16;TBC1D22B;TBC1D24;TBC1D7;TBC1D9B;TGFBRAP1;TIAM1;TNIK;TRIO;VAV2;VPS39;WDR

;C14orf169;C1D;CAD;CALR;CAMTA2;CAND2;CAPN3;CARD11;CBFA2T2;CBX2;CBX5;CBX7;CCAR1;CCNT1;CD3EAP;CDH13;CDK5RAP2;CDKN2A;CDKN2B;CDON;CECR1;CELSR2;CHCHD3;CHD2;CHD3;CHD4;CHD6;CHEK2;CIAO1;CIC;CIITA;CIRH1A;CMKLR1;COX10;COX15;CPSF3;CREB3L2;CREG1;CREM;CRTC1;CRT

L1;ILF3;INCENP;ING2;ING5;INO80;JTB;KHDRBS1;KIF3B;KLF11;KNTC1;KRT7;L3MBTL1;LIG1;LIG3;LMLN;LRP5;LSM10;LZTS2;MAD2L1BP;MAD2L2;MAP4;MAPRE2;MCM2;MCM3;MCM4;MCM6;MLH1;MND1;MNS1;MSH6;MTA3;MYC;NCAPD2;NCAPD3;NCAPG;NCAPG2;NDC80;NEK9;NINL;NME6;NOLC1;NSUN2;
BFA2T2;CBX2;CBX5;CBX7;CCAR1;CCNT1;CCR7;CDAN1;CDH13;CDK5RAP2;CDKN2A;CDKN2B;CDON;CELF1;CELSR2;CHCHD3;CHD2;CHD3;CHD4;CHD6;CHEK2;CIAO1;CIC;CIITA;CIRH1A;CIZ1;CLK2;CMKLR1;CREB3L2;CREG1;CREM;CRTC1;CRTC3;CRY2;CSNK2A2;CSRNP2;CTC1;CTCF;CTR9;CUL7;CYTL1;DCTN1;DDI

1;POLA2;POLE;PPP2R5C;PRMT2;PSMC4;PSMD1;PSMD11;PSMD2;PSMD6;PSMD7;PSME4;RBL2;RFC1;RFWD3;RPA1;RPA2;SFI1;SMARCA4;TAF1;TCF3;TFDP2;TIMELESS;TOP2B;TP53;TUBB;TUBGCP4;TUBGCP5;TUBGCP6;UBE2L3;YWHAE;ZC3H1

ALRN;LAMTOR1;MADD;MCF2L;MINK1;MYO9B;NCKAP1L;NET1;OBSCN;OCRL;PLEKHG2;PSD4;RAB3GAP1;RABGAP1;RABIF;RACGAP1;RALGDS;RALGPS1;RANBP10;RAP1GDS1;RAPGEF1;RASA3;RASA4;RASAL3;RASGRF2;RASGRP2;RGS13;RGS17;SBF1;SGSM2;SIPA1L3;SRGAP1;SYDE1;SYNGAP1;TBC1D16;TBC1D

2;BRD7;BRPF1;BTRC;C14orf169;C1D;CAD;CALR;CAMTA2;CAND2;CAPN3;CARD11;CARNS1;CBFA2T2;CBX2;CBX5;CBX7;CCAR1;CCNT1;CD3EAP;CDH13;CDK5RAP2;CDKN2A;CDKN2B;CDON;CECR1;CELSR2;CHCHD3;CHD2;CHD3;CHD4;CHD6;CHEK2;CIAO1;CIC;CIITA;CIRH1A;CMKLR1;CNDP2;COX10;COX15;CPSF

P8B2;AXIN1;AXIN2;BAD;BCL2;BDH1;BLM;BTRC;CAD;CALM3;CARNS1;CBLB;CCNF;CCR7;CDC16;CDKN2A;CECR1;CES2;CHD1L;CHEK2;CLN8;CNOT10;CSNK1E;CUL1;CUL4A;CUL7;CUL9;CYP2D6;DAGLA;DBH;DCN;DCP1B;DDB1;DDHD2;DDIT3;DDIT4;DDX6;DERA;DERL2;DFFA;DFFB;DIS3L;DLD;DLST;DNAJC1;DNASE

L;BEX1;BLM;BMP2;BPTF;BRD2;BRD7;BRPF1;BTRC;C14orf169;C1D;C2CD3;CALM3;CALR;CAMTA2;CAND2;CAPN3;CARD11;CARD14;CBFA2T2;CBLB;CBX2;CBX5;CBX7;CCAR1;CCDC23;CCDC88C;CCND1;CCND2;CCNT1;CCR7;CD3E;CD4;CD74;CDAN1;CDC16;CDC25B;CDH13;CDK5RAP2;CDKN2A;CDKN2B;CDON;C

2;LSM10;MAGOH;MLH1;MTPAP;NONO;NUP214;PABPC1;PAN2;PARN;PDCD11;PHRF1;PNN;POLR2B;POLR2C;POLR2J;PRKCA;PRPF19;PRPF3;PRPF4;PRPF40B;PRPF8;PSMC4;PSMD1;PSMD11;PSMD2;PSMD6;PSMD7;PSME4;PTBP1;RBFOX2;RBM15B;RBM17;RBM26;RBM28;RBM4B;RNASE4;RNMT;RNPS1;RPL
LSR2;CHCHD3;CHD2;CHD3;CHD4;CHD6;CHEK2;CIAO1;CIC;CIITA;CIRH1A;CMKLR1;CPSF3;CREB3L2;CREG1;CREM;CRTC1;CRTC3;CRY2;CSNK2A2;CSRNP2;CSTF1;CSTF2;CSTF3;CTCF;CTR9;CYTL1;DDIT3;DDX17;DDX20;DHX33;DHX38;DIDO1;DNAJA3;DNMT1;DNMT3A;DRG1;DVL2;ECD;EHMT1;EIF2AK3;EIF4A3;EL
A2;POLE;PPP2R5C;PRMT2;PSMC4;PSMD1;PSMD11;PSMD2;PSMD6;PSMD7;PSME4;RBL2;RFC1;RFWD3;RPA1;RPA2;SFI1;SMARCA4;TAF1;TCF3;TFDP2;TOP2B;TP53;TUBB;TUBGCP4;TUBGCP5;TUBGCP6;UBE2L3;YWHAE;ZC3H1
M4;MYBBP1A;MYC;NME6;PA2G4;PDCD4;PKD1;PPP1R13B;PPP1R9B;PPP2R5C;PRDM4;PRKAG1;PSMC4;PSMD1;PSMD11;PSMD2;PSMD6;PSMD7;PSME4;RFWD3;RPA1;RPA2;RPS6KA2;RUNX3;SCRIB;SMAD3;SMARCA4;SMC1A;TAF6;TAOK2;TCF7L2;TOP2B;TP53;TRIM35;TRRAP;TSC1;TSPYL2;VASH1;WDR6;XP
;LSM14B;MAGOH;MRPL16;MRPL33;MRPL44;MRPL49;MRPS24;MRPS27;MRPS36;MRPS5;MRPS9;MSI2;NCL;NFATC2;NOP10;NOP14;NSUN4;NUP62;PA2G4;PABPC1;PABPC4;PAN2;PNN;POP1;PRPF19;PRPF3;PRPF4;PRPF8;RBM14;RBM17;RBM28;RPL21;RRP1B;RSL1D1;SCAF8;SF1;SF3A3;SF3B2;SF3B3;SMG6;

RD2;BRD7;BRPF1;BTRC;C14orf169;C1D;CAD;CALR;CAMTA2;CAND2;CAPN3;CARD11;CBFA2T2;CBX2;CBX5;CBX7;CCAR1;CCNT1;CD3EAP;CDH13;CDK5RAP2;CDKN2A;CDKN2B;CDON;CECR1;CELSR2;CHCHD3;CHD2;CHD3;CHD4;CHD6;CHEK2;CIAO1;CIC;CIITA;CIRH1A;CMKLR1;COX10;COX15;CPSF3;CREB3L2;C

B;NCKAP1L;NET1;NLRP1;NOD1;NODAL;OCRL;PDIA5;PDIA6;PLCG1;PPARGC1B;PRKCA;RAB3GAP1;RABGAP1;RACGAP1;RAP1GDS1;RASA3;RASA4;RASAL3;RGS13;RGS17;RYR2;S100A8;S100A9;SCRIB;SHC1;SMAD3;SNX9;SYDE1;SYNGAP1;SYVN1;TATDN2;TAX1BP3;TBC1D7;TNF;TNFRSF10A;TSC1;TSPYL2;TXK;U
;C1D;CAD;CALR;CAMTA2;CAND2;CAPN3;CARD11;CBFA2T2;CBX2;CBX5;CBX7;CCAR1;CCNT1;CD3EAP;CDH13;CDK5RAP2;CDKN2A;CDKN2B;CDON;CECR1;CELSR2;CHCHD3;CHD2;CHD3;CHD4;CHD6;CHEK2;CIAO1;CIC;CIITA;CIRH1A;CMKLR1;COX10;COX15;CPSF3;CREB3L2;CREG1;CREM;CRTC1;CRTC3;CRY2;CS

G;PPM1K;SHC1;SUPV3L1;TP53;VDAC1;VDAC

MCF2L;MINK1;MYO9B;NCKAP1L;NET1;OBSCN;OCRL;PLEKHG2;PSD4;RAB3GAP1;RABGAP1;RABIF;RACGAP1;RALGDS;RALGPS1;RANBP10;RAP1GDS1;RAPGEF1;RASA3;RASA4;RASAL3;RASGRF2;RASGRP2;RGS13;RGS17;SBF1;SGSM2;SIPA1L3;SRGAP1;SYDE1;SYNGAP1;TBC1D16;TBC1D22B;TBC1D24;TBC1D7;TE
DH13;CDK5RAP2;CDKN2A;CDKN2B;CDON;CELF1;CELSR2;CHCHD3;CHD2;CHD3;CHD4;CHD6;CHEK2;CIAO1;CIC;CIITA;CIRBP;CIRH1A;CLEC3B;CLK2;CMKLR1;COG7;CREB3L2;CREG1;CREM;CRTC1;CRTC3;CRY2;CSNK2A2;CSRNP2;CTCF;CTR9;CYTL1;DDIT3;DDX17;DDX20;DHX33;DHX9;DNAJA3;DNAJC1;DNMT1;DN

ATP8B1;ATXN1L;AVPR2;AXIN1;AXIN2;BAD;BAHD1;BAZ1B;BBS2;BCL10;BCL11B;BCL2;BCL9L;BEX1;BICD1;BLM;BMP2;BPTF;BRD2;BRD7;BRPF1;BTRC;C14orf169;C1D;C2CD3;CALM3;CALR;CAMTA2;CAND2;CAPN3;CARD11;CARD14;CASP1;CBFA2T2;CBLB;CBX2;CBX5;CBX7;CCAR1;CCDC23;CCDC88C;CCND1;CCND
CBFA2T2;CBX2;CBX5;CBX7;CCAR1;CCNT1;CCR7;CDAN1;CDH13;CDK5RAP2;CDKN2A;CDKN2B;CDON;CELF1;CELSR2;CHCHD3;CHD2;CHD3;CHD4;CHD6;CHEK2;CIAO1;CIC;CIITA;CIRH1A;CIZ1;CLK2;CMKLR1;CREB3L2;CREG1;CREM;CRTC1;CRTC3;CRY2;CSNK2A2;CSRNP2;CTC1;CTCF;CTR9;CUL7;CYTL1;DCTN1;DDIT
5;UBE2B;UBE2D2;UBE2F;UBE2G2;UBE2I;UBE2L3;UBE2Q1;UBE2Z;UBE3C;UBR4;UBR7;UHRF1;VH
CL2;BDH1;BLM;BTRC;CAD;CARNS1;CCNF;CCR7;CDC16;CDKN2A;CECR1;CHD1L;CHEK2;CLN8;CNOT10;CSNK1E;CUL1;CUL4A;CUL7;CUL9;CYP2D6;DBH;DCN;DCP1B;DDB1;DDIT3;DDIT4;DDX6;DERA;DERL2;DFFA;DFFB;DIS3L;DLD;DLST;DNAJC1;DNASE1;DNM1;DOCK7;DPYSL2;DRAM1;DROSHA;DTL;DUOX1;DYX1

1D1;NRG1;PCBD1;PPARGC1B;PRMT2;RAD54L2;RBFOX2;RBM14;RNF20;RNF4;SAP30;SF1;SIK1;SIN3B;SMARCA4;SMARCC1;SMARCC2;SMARCD1;SMARCD3;SMARCE1;SND1;SNW1;SOX10;SRCAP;SUFU;TADA2A;TAF1;TAF10;TCF3;TFCP2L1;TFDP2;TLE1;TP53BP1;TRIP4;TRRAP;UBE2L3;ZHX2;ZHX3;ZMIZ2;ZMYN

SYNE1;SYNE2;TEX10;TMEM109;TMEM57;TNPO2;TUBB;TXLNG;WDR3;WHSC1;XPC
ALR;CAMTA2;CAND2;CAPN3;CARD11;CBFA2T2;CBX2;CBX5;CBX7;CCAR1;CCNT1;CD3EAP;CDH13;CDK5RAP2;CDKN2A;CDKN2B;CDON;CECR1;CELSR2;CHCHD3;CHD2;CHD3;CHD4;CHD6;CHEK2;CIAO1;CIC;CIITA;CIRH1A;CMKLR1;CPSF3;CREB3L2;CREG1;CREM;CRTC1;CRTC3;CRY2;CSNK2A2;CSRNP2;CSTF1;CSTF

PRKAG1;PTK2;RACGAP1;RAD54L2;RELA;RYR2;SIK1;SKAP1;SMAD3;STK38;STK39;STXBP1;SUFU;TAOK2;TCF3;TCF7L2;TOP2B;TP53;TRAF3;UTR
A8;ID1;IL4;ILF3;INPPL1;IRF8;JAZF1;KAT6B;KCTD1;KDM1A;KDM2B;KDM4A;KHDRBS1;KLF11;L3MBTL1;LANCL2;LDB1;LEF1;LIG3;LPIN1;LRP8;MAD2L2;MAGED1;MBD1;MBD2;MDM4;MECP2;MEIS2;MLH1;MPHOSPH8;MSH6;MTA2;MXD3;MYBBP1A;MYC;NAB2;NACC1;NCOR2;NF2;NFX1;NKAP;NODAL;NR1D1;N
AP;UBE2B;UHRF1;YEATS
164;CHEK2;CIAO1;CIRBP;CLDN15;CLDN20;CNTNAP1;COL17A1;COX10;COX15;CRTC1;CRTC3;CTCF;DDX20;DDX23;DDX47;DDX51;DDX56;DDX6;DGKD;DGKH;DIAPH1;DKC1;DROSHA;DST;DYNC1H1;EFTUD1;EHD4;EIF2AK3;EIF3A;EIF4A3;EPHA1;EPHA2;EPRS;EPS8;EVL;EXOSC10;EZR;FAF1;FAM103A1;FBF1;FGD1
IM16;TRRAP;UBE2B;UHRF1;YEATS
TRA2;HYAL1;INCA1;ING5;INO80;INTS7;KHDRBS1;KNTC1;L3MBTL1;LEF1;LRP5;MACF1;MAD2L1BP;MAD2L2;MADD;MAP2K6;MCM2;MCM3;MCM4;MCM6;MDC1;MDM4;MNT;MTA3;MYBBP1A;MYC;NME6;NUP214;PA2G4;PDCD4;PHACTR4;PIM3;PKD1;PPP1R13B;PPP1R9B;PPP2R5C;PRDM4;PRKAG1;PRKCA;
.10;BCL11B;BCL2;BCL9L;BEX1;BLM;BMP2;BPTF;BRD2;BRD7;BRPF1;BTRC;C14orf169;C1D;CALM3;CALR;CAMTA2;CAND2;CAPN3;CARD11;CARD14;CBFA2T2;CBX2;CBX5;CBX7;CCAR1;CCDC23;CCDC88C;CCND1;CCND2;CCNT1;CCR7;CD3E;CD4;CD74;CDAN1;CDC16;CDC25B;CDH13;CDK5RAP2;CDKN2A;CDKN2B

TTF1;WRNIP
R2;CHCHD3;CHD2;CHD3;CHD4;CHD6;CHEK2;CIAO1;CIC;CIITA;CIRBP;CIRH1A;CIZ1;CMKLR1;CREB3L2;CREG1;CREM;CRTC1;CRTC3;CRY2;CSNK2A2;CSRNP2;CTC1;CTCF;CTR9;CYTL1;DDIT3;DDX17;DDX20;DHX33;DNAJA3;DNAJC1;DNMT1;DNMT3A;DVL2;ECD;EHMT1;EIF2AK3;EIF2AK4;EIF2B1;EIF3B;EIF4A3;EIF4

PALB2;PAPD7;PARP1;PAXIP1;PMS2;PMS2CL;PMS2P5;POLA1;POLB;POLE;POLG;POLH;POLR2B;POLR2C;POLR2J;PPP2R5C;PRPF19;PSMC4;PSMD1;PSMD11;PSMD2;PSMD6;PSMD7;PSME4;RAD18;RAD51D;RBM14;RFC1;RFWD3;RNF8;RPA1;RPA2;RUVBL1;SFPQ;SGK1;SLX4;SMC1A;SNW1;SSRP1;SUPT16H;TAF1
CBLB;CCNF;CCR7;CDC16;CDKN2A;CECR1;CHD1L;CHEK2;CLN8;CNOT10;CSNK1E;CUL1;CUL4A;CUL7;CUL9;CYP2D6;DAGLA;DBH;DCN;DCP1B;DDB1;DDHD2;DDIT3;DDIT4;DDX6;DERA;DERL2;DFFA;DFFB;DIS3L;DLD;DLST;DNAJC1;DNASE1;DNM1;DOCK7;DPYSL2;DROSHA;DTL;DYX1C1;ECD;ECI2;EDC3;EDC4;EDEN

N2B;CDON;CELSR2;CHCHD3;CHD2;CHD3;CHD4;CHD6;CHEK2;CIAO1;CIC;CIITA;CIRBP;CIRH1A;CIZ1;CMKLR1;CREB3L2;CREG1;CREM;CRTC1;CRTC3;CRY2;CSNK2A2;CSRNP2;CTC1;CTCF;CTR9;CYTL1;DDIT3;DDX17;DDX20;DHX33;DNAJA3;DNAJC1;DNMT1;DNMT3A;DVL2;ECD;EHMT1;EIF2AK3;EIF2AK4;EIF2B1;EI

A8;ID1;IL10;IL4;ILF3;INPPL1;IRF8;JAZF1;KAT6B;KCTD1;KDM1A;KDM2B;KDM4A;KHDRBS1;KLF11;L3MBTL1;LANCL2;LDB1;LEF1;LIG3;LPIN1;LRP8;MAD2L2;MAGED1;MBD1;MBD2;MDM4;MECP2;MEIS2;MLH1;MPHOSPH8;MSH6;MTA2;MXD3;MYBBP1A;MYC;NAB2;NACC1;NCOR2;NF2;NFX1;NKAP;NODAL;NR1
R2;NFE2L1;NMI;NR1D1;NRG1;PCBD1;PER1;PPARGC1B;PRMT2;RAD54L2;RBFOX2;RBM14;RNF20;RNF4;SAP30;SF1;SIK1;SIN3B;SMAD3;SMARCA4;SMARCC1;SMARCC2;SMARCD1;SMARCD3;SMARCE1;SND1;SNW1;SOX10;SRCAP;SUFU;TADA2A;TAF1;TAF10;TCF3;TFCP2L1;TFDP2;TLE1;TP53BP1;TRIP4;TRRAP;
N8;CRADD;CSNK2A2;CSRNP2;CSTB;CTNNBL1;CUL1;CUL4A;CXCR3;CYFIP2;DAD1;DBH;DDIT3;DDIT4;DDX19A;DDX20;DDX47;DFFA;DFFB;DHCR24;DHODH;DIDO1;DNAJA3;DNAJB13;DNASE1;DRAM1;EDAR;EIF2AK3;EIF5A;EPHA2;ERBB3;ERCC3;ERCC5;ETS1;FAF1;FAIM3;FCER1G;FGD1;FGF2;FGFR1;FLT4;FOXC1;
E2L1;NMI;NR1D1;NRG1;PCBD1;PPARGC1B;PRMT2;RAD54L2;RBFOX2;RBM14;RNF20;RNF4;SAP30;SF1;SIK1;SIN3B;SMARCA4;SMARCC1;SMARCC2;SMARCD1;SMARCD3;SMARCE1;SND1;SNW1;SOX10;SRCAP;SUFU;TADA2A;TAF1;TAF10;TCF3;TFCP2L1;TFDP2;TLE1;TP53BP1;TRIP4;TRRAP;UBE2L3;ZHX2;ZHX3;
ATG3;ATG9B;ATM;ATP10A;ATP6V0E1;ATP6V1D;ATP6V1F;ATP6V1H;ATP8B1;ATP9B;ATXN2L;AXIN1;AXIN2;BAD;BAHD1;BAIAP2L1;BAZ1B;BBS1;BBS2;BCL10;BCL11B;BCL2;BCL9L;BICD1;BID;BIK;BIRC6;BLM;BLOC1S1;BMP2;BMS1;BPTF;BRD1;BRD2;BRD7;BRK1;BRPF1;BRSK2;C14orf169;C22orf28;C2CD3;CABP4

HFD1L;MTHFR;MTHFS;MTR;NARS;NFS1;OGDH;PCBD1;PCYOX1;PCYOX1L;PDPR;PET112;PFAS;PPCS;PSAT1;PSMC4;PSMD1;PSMD11;PSMD2;PSMD6;PSMD7;PSME4;PTDSS1;SARS;SEPHS2;SERINC5;SHMT1;SLC25A15;SLC46A1;SLC7A6;SMS;TARS;VNN3;YA

N8;CRADD;CSNK2A2;CSRNP2;CSTB;CTNNBL1;CUL1;CUL4A;CXCR3;CYFIP2;DAD1;DBH;DDIT3;DDIT4;DDX19A;DDX20;DDX47;DFFA;DFFB;DHCR24;DHODH;DIDO1;DNAJA3;DNAJB13;DNASE1;DRAM1;EDAR;EIF2AK3;EIF5A;EPHA2;ERBB3;ERCC3;ERCC5;ETS1;FAF1;FAIM3;FCER1G;FGD1;FGF2;FGFR1;FLT4;FOXC1;

ID1;SNW1;SRCAP;SUPT6H;TAF1;TBP;TCEB1;THOC1;THOC2;TNFRSF14;TP53;UBE2I;UBR4;UNG;USP7;VDAC1;VPRBP;YWHAE;ZC3H7
ISBP1;HSPA8;ID1;IL4;ILF3;INPPL1;IRF8;JAZF1;KAT6B;KCTD1;KDM1A;KDM2B;KDM4A;KHDRBS1;KLF11;L3MBTL1;LANCL2;LDB1;LEF1;LPIN1;LRP8;MAD2L2;MAGED1;MBD1;MBD2;MDM4;MECP2;MEIS2;MPHOSPH8;MTA2;MXD3;MYBBP1A;MYC;NAB2;NACC1;NCOR2;NF2;NFX1;NKAP;NODAL;NR1D1;NRG1;NR

CRIB;SYDE1;SYNGAP1;TAX1BP3;TBC1D7;TIAM1;TNFAIP1;TRIO;TSC1;VAV

AF1;PARD6A;PCSK5;PLCG1;POLA1;POLR2B;POLR2C;POLR2J;POM121;PSMC4;PSMD1;PSMD11;PSMD2;PSMD6;PSMD7;PSME4;RBM15B;RELA;RNF216;RNMT;RPL21;RRAGA;SATB1;SCRIB;SEH1L;SEPT6;SF3B2;SLAMF1;SLC20A2;SLPI;SMAD3;SMARCA4;SNAPIN;SND1;SNW1;SRCAP;SSRP1;SUPT16H;SUPT4H1;S

1;PCNT;PDE4DIP;PLEKHA7;RAB11FIP3;RAB3GAP1;RABGAP1;SERPINB6;SFI1;STARD9;TBCCD1;TCHP;TCP1;TSPYL2;TTLL5;TUBGCP4;TUBGCP5;TUBGCP6;WDR67;ZFYVE2
RL;PIK3R2;PLEKHG2;PSD4;PTK2;RAB3GAP1;RACGAP1;RALGDS;RAPGEF1;RASA3;RASA4;RASAL3;RASGRF2;RASGRP2;RHOBTB2;RHOF;SCRIB;SIPA1L3;SRGAP1;SYDE1;SYNGAP1;TAX1BP3;TBC1D7;TIAM1;TNFAIP1;TRIO;TSC1;VAV

AF1;PARD6A;PCSK5;PLCG1;POLA1;POLR2B;POLR2C;POLR2J;POM121;PSMC4;PSMD1;PSMD11;PSMD2;PSMD6;PSMD7;PSME4;RBM15B;RELA;RNF216;RNMT;RPL21;RRAGA;SATB1;SCRIB;SEH1L;SEPT6;SF3B2;SLAMF1;SLC20A2;SLPI;SMAD3;SMARCA4;SNAPIN;SND1;SNW1;SRCAP;SSRP1;SUPT16H;SUPT4H1;S
74;CDKN2A;CHEK2;CLN8;CRADD;CSNK2A2;CSRNP2;CSTB;CTNNBL1;CUL1;CUL4A;CXCR3;CYFIP2;DAD1;DAGLA;DBH;DCTN1;DDIT3;DDIT4;DDX19A;DDX20;DDX47;DFFA;DFFB;DHCR24;DHODH;DIDO1;DNAJA3;DNAJB13;DNASE1;DRAM1;DYNC1H1;EDAR;EIF2AK3;EIF4G1;EIF4G2;EIF5A;EPHA2;ERBB3;ERCC3;ER
1;PPP2R5C;PSMC4;PSMD1;PSMD11;PSMD2;PSMD6;PSMD7;PSME4;PTK2;RELA;RHBDD1;RNF20;RNF216;RNF4;RNF43;RNF8;SEC61B;SERPINE2;SMAD3;SPG7;SYVN1;TAF1;TCEB1;TNF;TNFAIP1;TTC3;UBE2B;UBE2D2;UBE2G2;UBE2I;UBE2L3;UBE3C;UBQLN4;UFD1L;UHRF1;USP11;USP13;USP20;USP24;USP27X
;DHDDS;DKC1;DLG3;DLST;DNMT1;DNMT3A;DSTYK;EEF2K;EFNA4;EHMT1;EIF2AK3;EIF2AK4;ELP3;EPHA1;EPHA2;ERBB3;ERCC3;EXT2;EZH1;FAM20B;FASN;FASTKD5;FGFR1;FGFRL1;FGGY;FKRP;FLT4;FN3KRP;FTSJ2;FTSJ3;FUT11;FUT2;FYN;GAL3ST4;GALK2;GALNT10;GALNT6;GALNT9;GALT;GART;GGT7;GGTA1

74;CDKN2A;CHEK2;CLN8;CRADD;CSNK2A2;CSRNP2;CSTB;CTNNBL1;CUL1;CUL4A;CXCR3;CYFIP2;DAD1;DAGLA;DBH;DCTN1;DDIT3;DDIT4;DDX19A;DDX20;DDX47;DFFA;DFFB;DHCR24;DHODH;DIDO1;DNAJA3;DNAJB13;DNASE1;DRAM1;DYNC1H1;EDAR;EIF2AK3;EIF4G1;EIF4G2;EIF5A;EPHA2;ERBB3;ERCC3;ER

;HMG20A;HMGA1;HSBP1;HSPA8;ID1;IL10;IL4;ILF3;INPPL1;IRF8;JAZF1;KAT6B;KCTD1;KDM1A;KDM2B;KDM4A;KHDRBS1;KLF11;L3MBTL1;LANCL2;LDB1;LEF1;LPIN1;LRP8;MAD2L2;MAGED1;MBD1;MBD2;MDM4;MECP2;MEIS2;MPHOSPH8;MTA2;MXD3;MYBBP1A;MYC;NAB2;NACC1;NCOR2;NF2;NFX1;NKAP;

LSR2;CHCHD3;CHD2;CHD3;CHD4;CHD6;CHEK2;CIAO1;CIC;CIITA;CIRH1A;CMKLR1;CPSF3;CREB3L2;CREG1;CREM;CRTC1;CRTC3;CRY2;CSNK2A2;CSRNP2;CSTF1;CSTF2;CSTF3;CTCF;CTR9;CYTL1;DDIT3;DDX17;DDX20;DHX33;DHX38;DIDO1;DNAJA3;DNMT1;DNMT3A;DRG1;DVL2;ECD;EHMT1;EIF2AK3;EIF4A3;EL

YVN1;TAF1;TCEB1;TNFAIP1;TTC3;UBE2B;UBE2D2;UBE2G2;UBE2I;UBE2L3;UBE3C;UBQLN4;UFD1L;UHRF1;USP11;USP13;USP20;USP24;USP27X;USP35;USP36;USP39;USP40;USP48;USP54;USP7;V

GNE;GUCY2D;HKDC1;HMGXB3;IKBKB;IKBKE;IP6K2;IRAK1;ITPKB;KALRN;LCK;LMTK3;MAP2K6;MAP2K7;MAP3K12;MAP3K4;MAP3K9;MAPK7;MAPK9;MINK1;MPP2;MTOR;MTPAP;MYBBP1A;NDUFA10;NEK9;NME6;NOL9;OBSCN;PACSIN1;PAPD7;PASK;PDPK1;PEAK1;PFKP;PI4K2A;PI4KA;PI4KAP2;PI4KB;PIK3R2
AJA3;DNAJC1;DOCK7;DRD4;DVL2;EDEM1;EFCAB4B;EIF2AK3;EPHA1;EPHA2;ERBB3;FGF2;FGFR1;FOXJ1;FXN;FZR1;GABARAPL2;GATA3;GIPR;GMFG;GOSR2;GPR55;GSTO1;GTF2A2;GTF3C4;HGF;HSP90AB1;HSP90B1;HSPD1;HTRA2;HYOU1;IKBKB;IL10;IL4;IRAK1;IRS1;ITPR3;JTB;KDELR3;KDM1A;KITLG;LCK;LRP8;

UFA9;NDUFB4;NDUFB6;NDUFS1;NDUFV3;NNT;OGDH;PAM16;PI4KB;PLD6;POLG;PRKCA;SAMM50;SCO1;SCO2;SDHA;SDHB;SFXN1;SFXN2;SFXN3;SLC25A12;SLC25A15;SLC25A23;SLC25A27;SLC25A42;SLC25A45;SMCR7L;SNPH;SOX10;SPG7;STAR;TAMM41;THEM4;TIMM17B;TOMM34;VAMP1;VAT1;VDAC1;V

TNFAIP1;TTC3;UBE2B;UBE2D2;UBE2G2;UBE2I;UBE2L3;UBE3C;UBQLN4;UFD1L;UHRF1;USP11;USP13;USP20;USP24;USP27X;USP35;USP36;USP39;USP40;USP48;USP54;USP7;V
HD6;CHEK2;CIAO1;CIC;CIITA;CIRH1A;CLK2;CMKLR1;CREB3L2;CREG1;CREM;CRTC1;CRTC3;CRY2;CSNK2A2;CSRNP2;CTCF;CTR9;CYTL1;DDIT3;DDX17;DDX20;DHX33;DNAJA3;DNMT1;DNMT3A;DVL2;ECD;EHMT1;EIF2AK3;EIF4A3;ELP3;ERCC3;ETS1;EZH1;FADS1;FGF2;FGFR1;FLNA;FOSB;FOXC1;FOXI1;FOXJ1;FOX

C3;ERCC5;ETS1;FADS1;FAN1;FANCI;FEN1;FLT4;FOXM1;FXN;FZR1;GADD45A;GOSR2;HERC2;HGF;HIF1AN;HMGA1;HSP90B1;HUWE1;HYOU1;IKBKE;IL18;IL18BP;IL26;ING2;INO80;INTS7;IRAK1;KDELR3;KDM1A;LARP1;LIG1;LIG3;LOC100133315;MAD2L2;MAP2K6;MAP2K7;MAP3K12;MAP3K4;MAP3K9;MAPK7;
5RAP2;CDKN2A;CDKN2B;CDON;CELSR2;CHCHD3;CHD2;CHD3;CHD4;CHD6;CHEK2;CIAO1;CIC;CIITA;CIRBP;CIRH1A;CIZ1;CLK2;CMKLR1;CREB3L2;CREG1;CREM;CRTC1;CRTC3;CRY2;CSNK2A2;CSRNP2;CTC1;CTCF;CTR9;CYTL1;DDIT3;DDX17;DDX20;DHCR7;DHX33;DLD;DNAJA3;DNAJC1;DNMT1;DNMT3A;DRD4;
1;TCEB1;TNFAIP1;TTC3;UBE2B;UBE2D2;UBE2G2;UBE2I;UBE2L3;UBE3C;UBQLN4;UFD1L;UHRF1;USP11;USP13;USP20;USP24;USP27X;USP35;USP36;USP39;USP40;USP48;USP54;USP7;V
S;EZH1;F2;FGF2;FGFR1;FLNA;FOSB;FOXJ1;FOXK1;FOXM1;FOXP1;FYN;FZR1;GABARAPL2;GADD45A;GATA3;GLG1;GMFG;GRIN2C;GTPBP4;HCFC1;HDAC1;HEXIM1;HHEX;HIC2;HIF1AN;HMG20A;HMGA1;HNRNPA2B1;HSBP1;HSP90AB1;HSPA8;HYAL2;ID1;IL10;IL4;ILF3;INCA1;INPPL1;IRF8;JAZF1;KAT6B;KCTD1;K
M1A;KDM2B;KDM4A;KHDRBS1;KLF11;L3MBTL1;LANCL2;LDB1;LEF1;LPIN1;LRP8;MAD2L2;MAGED1;MBD1;MBD2;MDM4;MECP2;MEIS2;MPHOSPH8;MTA2;MXD3;MYBBP1A;MYC;NAB2;NACC1;NCOR2;NFX1;NKAP;NODAL;NR1D1;NRG1;NRIP2;PA2G4;PABPC1;PARP1;PATZ1;PDCD4;PER1;PPARA;PPARD;PPA

1;SCRIB;SEPT6;SF3B2;SLAMF1;SLC20A2;SMAD3;SMARCA4;SNAPIN;SND1;SNW1;SRCAP;SUPT6H;TAF1;TARDBP;TBP;TCEB1;THOC1;THOC2;TNF;TNFRSF14;TP53;UBE2I;UBR4;UNG;USP7;VDAC1;VPRBP;YWHAE;ZC3H
1;SCRIB;SEPT6;SF3B2;SLAMF1;SLC20A2;SMAD3;SMARCA4;SNAPIN;SND1;SNW1;SRCAP;SUPT6H;TAF1;TARDBP;TBP;TCEB1;THOC1;THOC2;TNF;TNFRSF14;TP53;UBE2I;UBR4;UNG;USP7;VDAC1;VPRBP;YWHAE;ZC3H

C1;HEXIM1;HHEX;HIC2;HIF1AN;HMG20A;HMGA1;HSBP1;HSPA8;ID1;IL10;IL4;ILF3;INPPL1;IRF8;JAZF1;KAT6B;KCTD1;KDM1A;KDM2B;KDM4A;KHDRBS1;KLF11;L3MBTL1;LANCL2;LDB1;LEF1;LPIN1;LRP8;MAD2L2;MAGED1;MBD1;MBD2;MDM4;MECP2;MEIS2;MPHOSPH8;MTA2;MXD3;MYBBP1A;MYC;NAB2;N

T6B;KCTD1;KDM1A;KDM2B;KDM4A;KHDRBS1;KLF11;L3MBTL1;LANCL2;LDB1;LEF1;LPIN1;LRP8;MAD2L2;MAGED1;MBD1;MBD2;MDM4;MECP2;MEIS2;MLL4;MPHOSPH8;MTA2;MXD3;MYBBP1A;MYC;NAB2;NACC1;NCOR2;NFX1;NKAP;NODAL;NR1D1;NRG1;NRIP2;PA2G4;PARP1;PATZ1;PDCD4;PER1;PPARA;
5RAP2;CDKN2A;CDKN2B;CDON;CELSR2;CHCHD3;CHD2;CHD3;CHD4;CHD6;CHEK2;CIAO1;CIC;CIITA;CIRBP;CIRH1A;CIZ1;CLK2;CMKLR1;CREB3L2;CREG1;CREM;CRTC1;CRTC3;CRY2;CSNK2A2;CSRNP2;CTC1;CTCF;CTR9;CYTL1;DDIT3;DDX17;DDX20;DHX33;DLD;DNAJA3;DNAJC1;DNMT1;DNMT3A;DRD4;DVL2;EC

NDUFA10;NDUFA2;NDUFA9;NDUFB4;NDUFB6;NDUFS1;NDUFV3;NNT;OGDH;PAM16;PI4KB;PLD6;POLG;PRKCA;SAMM50;SCO1;SCO2;SDHA;SDHB;SFXN1;SFXN2;SFXN3;SLC25A12;SLC25A15;SLC25A23;SLC25A27;SLC25A42;SLC25A45;SMCR7L;SNPH;SOX10;SPG7;STAR;TAMM41;THEM4;TIMM17B;TOMM34;
G1;MACF1;MDN1;MLH1;MSH6;MTOR;MYO9B;NCKAP1L;NDUFS1;NET1;NME6;OCRL;PMS2;PMS2P5;PSMC4;PSMD6;PTK2;PTPRN2;RAB3GAP1;RABGAP1;RABL2A;RABL2B;RACGAP1;RAP1GDS1;RASA3;RASA4;RASAL3;RGS13;RGS17;RHOF;SCRIB;SNX9;SYDE1;SYNGAP1;TAX1BP3;TBC1D7;TBCC;TSC1;UCK2;W
TPD4;ENTPD5;EPHA1;EPHA2;FGD1;FOXJ1;GABARAPL2;GFM1;GNG10;GNL2;GNL3L;GTPBP4;HSPD1;INO80;LSG1;MACF1;MCCC2;MDN1;MLH1;MSH6;MTOR;MYO9B;NCKAP1L;NDUFS1;NET1;NME6;NT5E;NUDT9;OCRL;PMS2;PMS2P5;PPCS;PRPS1;PSMC4;PSMD6;PTK2;PTPRN2;QTRTD1;RAB3GAP1;RABGAP1;
;PPME1;PRC1;PRKAG1;PTK2;RACGAP1;RAD54L2;RELA;RYR2;SIK1;SKAP1;SLC2A1;SMAD3;STK38;STK39;STXBP1;SUFU;TAOK2;TCF3;TCF7L2;TOP2B;TP53;TRAF3;UTR

2;MKL1;MTOR;MTSS1L;NKD1;NLRP1;NOD1;NUP62;PHRF1;PKD1;PLEKHA2;PLSCR4;POLR2J;PPARA;PPARGC1B;PTK2;PTTG2;RAPGEF1;RELA;REPS1;RUNX2;SCAF1;SFRP5;SH3BP5;SHANK1;SKAP1;SNX6;SOCS7;SON;SRRM2;ST13;STX1B;STXBP1;SYNGAP1;TCF7L2;TFDP2;THRA;TNFAIP1;TNK2;TRIM16;U2AF2;UB
;PLEKHG2;PSD4;PTK2;RAB3GAP1;RALGDS;RASA3;RASA4;RASAL3;RASGRF2;RASGRP2;RREB1;SCRIB;SHC1;SRGAP1;SYDE1;SYNGAP1;TAX1BP3;TBC1D7;TIAM1;TNFAIP1;TP53;TRIO;TSC1;VANGL2;VAV
NF8;SEC61B;SYVN1;TAF1;TCEB1;TNFAIP1;TTC3;UBE2B;UBE2D2;UBE2G2;UBE2I;UBE2L3;UBE3C;UBQLN4;UFD1L;UHRF1;USP11;USP13;USP20;USP24;USP27X;USP35;USP36;USP39;USP40;USP48;USP54;USP7;V
YO9B;NCKAP1L;NET1;NT5E;NUDT9;OCRL;PMS2;PMS2P5;PSMC4;PSMD6;PTK2;PTPRN2;RAB3GAP1;RABGAP1;RABL2A;RABL2B;RACGAP1;RAP1GDS1;RASA3;RASA4;RASAL3;RGS13;RGS17;RHOF;SCRIB;SNX9;SYDE1;SYNGAP1;TAX1BP3;TBC1D7;TBCC;TSC1;WRNI

EIF2AK3;EIF2AK4;EIF2B1;EIF4A3;EPHA1;EPRS;EZH1;F2;FAM132A;FGF2;FGFR1;FLNA;FOSB;FOXJ1;FOXK1;FOXM1;FOXP1;FYN;FZR1;GABARAPL2;GABBR1;GADD45A;GATA3;GLG1;GMFG;GRIN2C;GRM2;GTPBP4;HCFC1;HDAC1;HEXIM1;HHEX;HIC2;HIF1AN;HMG20A;HMGA1;HNRNPA2B1;HSBP1;HSP90AB1;HSF
HEK2;CIAO1;CIC;CIITA;CIRH1A;CMKLR1;CREB3L2;CREG1;CREM;CRTC1;CRTC3;CRY2;CSNK2A2;CSRNP2;CTCF;CTR9;CYTL1;DDIT3;DDX17;DDX20;DHX33;DNAJA3;DNMT1;DNMT3A;DVL2;ECD;EHMT1;EIF2AK3;EIF4A3;ELP3;ERCC3;ETS1;EZH1;FADS1;FGF2;FGFR1;FLNA;FOSB;FOXC1;FOXI1;FOXJ1;FOXJ3;FOXK1;FC
4A;KHDRBS1;KLF11;L3MBTL1;LANCL2;LDB1;LEF1;LPIN1;LRP8;MAD2L2;MAGED1;MBD1;MBD2;MDM4;MECP2;MEIS2;MPHOSPH8;MTA2;MXD3;MYBBP1A;MYC;NAB2;NACC1;NCOR2;NFX1;NKAP;NODAL;NR1D1;NRG1;NRIP2;PA2G4;PARP1;PATZ1;PDCD4;PER1;PPARA;PPARD;PPARGC1B;PRMT2;RAMP3;RBBF
C1;HDAC1;HEXIM1;HHEX;HIC2;HIF1AN;HMG20A;HMGA1;HSBP1;HSPA8;ID1;IL10;IL4;ILF3;INPPL1;IRF8;JAZF1;KAT6B;KCTD1;KDM1A;KDM2B;KDM4A;KHDRBS1;KLF11;L3MBTL1;LANCL2;LDB1;LEF1;LPIN1;LRP8;MAD2L2;MAGED1;MBD1;MBD2;MDM4;MECP2;MEIS2;MPHOSPH8;MTA2;MXD3;MYBBP1A;MYC;I

1;NT5E;NUDT9;OCRL;PMS2;PMS2P5;PSMC4;PSMD6;PTK2;PTPRN2;RAB3GAP1;RABGAP1;RABL2A;RABL2B;RACGAP1;RAP1GDS1;RASA3;RASA4;RASAL3;RGS13;RGS17;RHOF;SCRIB;SNX9;SYDE1;SYNGAP1;TAX1BP3;TBC1D7;TBCC;TSC1;WRNII
1;NT5E;NUDT9;OCRL;PMS2;PMS2P5;PSMC4;PSMD6;PTK2;PTPRN2;RAB3GAP1;RABGAP1;RABL2A;RABL2B;RACGAP1;RAP1GDS1;RASA3;RASA4;RASAL3;RGS13;RGS17;RHOF;SCRIB;SNX9;SYDE1;SYNGAP1;TAX1BP3;TBC1D7;TBCC;TSC1;WRNII

NF4;RNF43;RNF8;SEC61B;SYVN1;TAF1;TCEB1;TNFAIP1;TTC3;UBE2B;UBE2D2;UBE2G2;UBE2I;UBE2L3;UBE3C;UBQLN4;UFD1L;UHRF1;USP11;USP13;USP20;USP24;USP27X;USP35;USP36;USP39;USP40;USP48;USP54;USP7;V
CNTRL;CORO2A;CROCC;DCLK2;DCTN1;DCTN5;DDHD2;DDX20;DFNB31;DGKQ;DHCR24;DHX9;DIAPH1;DIS3L;DNAH1;DNAJA3;DNAJC7;DNHD1;DNM1;DRG1;DST;DSTYK;DTL;DYNC1H1;DYNC1I2;DYNC1LI2;DYNLT1;EMD;EML3;EPB41L2;EPPK1;EPS8;EVL;EXOC7;EZR;FAM110B;FBF1;FGD1;FKBP4;FLNA;FLNB;FLNC

DNAJA3;DNHD1;DNM1;DST;DTL;DYNC1H1;DYNC1I2;DYNC1LI2;DYNLT1;EMD;EML3;EPB41L2;EPS8;EXOC7;EZR;FAM110B;FBF1;FKBP4;FLNB;FYN;GRIN2C;GYS2;HAUS5;HERC2;HSPH1;INCENP;INO80;INPPL1;INVS;JTB;KDM4A;KIAA0368;KIAA0586;KIAA0753;KIF13B;KIF16B;KIF21B;KIF3B;KIF4A;KIF5C;KLC1;KLC

;SLAMF1;SLC20A2;SMAD3;SNAPIN;SND1;SNW1;SRCAP;SUPT6H;TAF1;TBP;TCEB1;THOC1;THOC2;TNFRSF14;TP53;UBE2I;UBR4;UNG;USP7;VDAC1;VPRBP;YWHAE;ZC3H7

D2;MYCBP;NCAPG;NEURL4;NINL;NPHP4;ODF2;PCIF1;PCM1;PCNT;PDE4DIP;PLEKHA7;PTK2;RAB11FIP3;RAB3GAP1;RABGAP1;RTTN;RUVBL1;SERPINB6;SFI1;STARD9;TBCCD1;TCHP;TCP1;TCTN2;TMEM216;TRIP4;TSPYL2;TTLL5;TUBGCP4;TUBGCP5;TUBGCP6;WDR67;ZFYVE2
HEK2;CIAO1;CIC;CIITA;CIRH1A;CMKLR1;CREB3L2;CREG1;CREM;CRTC1;CRTC3;CRY2;CSNK2A2;CSRNP2;CTCF;CTR9;CYTL1;DDIT3;DDX17;DDX20;DHX33;DNAJA3;DNMT1;DNMT3A;DVL2;ECD;EHMT1;EIF2AK3;EIF4A3;ELP3;ERCC3;ETS1;EZH1;FADS1;FGF2;FGFR1;FLNA;FOSB;FOXC1;FOXI1;FOXJ1;FOXJ3;FOXK1;FC
SUFU;TCF7L2;TGS1;THOC1;THOC2;THRA;TNF;TP53;TSC1;TXN;U2AF2;XPO5;ZFYVE

EZH1;FGF2;FGFR1;FOSB;FOXC1;FOXI1;FOXJ1;FOXK2;FOXM1;FOXP1;FUBP1;GABPB2;GATA3;GMEB2;GTF2A2;HCFC1;HDAC1;HEXIM1;HGF;HHEX;HIF1AN;HMG20A;HSBP1;HTATIP2;HTATSF1;HYAL2;ID1;IKBKAP;IKZF3;IL10;IL26;IL4;INO80;IRAK1;IRF8;ITGA6;JAZF1;KDM1A;KDM2B;KDM4C;KLF11;LDB1;LEF1;LE(
2;ERBB3;FGF2;FGFR1;FOXJ1;FXN;FZR1;GABARAPL2;GIPR;GMFG;GOSR2;GPR55;GTF3C4;HGF;HSP90AB1;HSP90B1;HSPD1;HTRA2;HYOU1;IRAK1;IRS1;ITPR3;JTB;KDELR3;KITLG;LCK;LRP8;MADD;MAGED1;MAP2K6;MAP2K7;MAP3K4;MAP3K9;MAPK8IP3;MBTPS1;MSH6;MUL1;MYC;MYO9B;NCKAP1L;NET1;NLR

L;FOXJ1;GFM1;GNG10;GNL2;GNL3L;GTPBP4;HSPD1;INO80;LSG1;MACF1;MCCC2;MDN1;MLH1;MSH6;MTOR;MYO9B;NCKAP1L;NDUFS1;NET1;NME6;NT5E;NUDT9;OCRL;PMS2;PMS2P5;PPCS;PSMC4;PSMD6;PTK2;PTPRN2;QTRTD1;RAB3GAP1;RABGAP1;RABL2A;RABL2B;RACGAP1;RAP1GDS1;RASA3;RASA4;

;ZMIZ

.;HMGXB3;IKBKB;IKBKE;IP6K2;IRAK1;ITPKB;KALRN;LCK;LMTK3;MAP2K6;MAP2K7;MAP3K12;MAP3K4;MAP3K9;MAPK7;MAPK9;MINK1;MPP2;MTOR;NEK9;NME6;NOL9;OBSCN;PACSIN1;PASK;PDPK1;PEAK1;PFKP;PI4K2A;PI4KA;PI4KAP2;PI4KB;PIK3R2;PIK3R4;PIM3;PIP4K2A;PIP4K2B;PIP5K1C;PKN3;POLR2B;F

K3;MAP2K6;MAP2K7;MAP3K12;MAP3K4;MAP3K9;MAPK7;MAPK9;MINK1;MTOR;NDUFA10;NEK9;NOL9;OBSCN;PACSIN1;PASK;PDPK1;PEAK1;PFKP;PI4K2A;PI4KA;PI4KAP2;PI4KB;PIK3R2;PIK3R4;PIM3;PIP4K2A;PIP4K2B;PIP5K1C;PKN3;POLR2B;POLR2J;PRKAG1;PRKCA;PRKCH;PRKCQ;PRKX;PTK2;RIOK1;RPS6I

CN;PAFAH2;PASK;PEBP1;PIGU;PLA2G7;PLCD4;PLCG1;PLCH2;PLEKHA2;PLEKHA7;PLEKHG2;PSD4;RALGPS1;RASA3;RASA4;RASAL3;RASGRF2;SBF1;SEC14L2;SH2B1;SH3PXD2A;SHC1;SKAP1;SNX29;SNX6;SNX9;SPTBN1;SYNGAP1;TECPR1;TEX2;TIAM1;TRIO;TULP3;VAV2;ZCCHC14;ZFYVE26;ZFYVE

SLC25A15;SLC25A23;SLC25A27;SLC25A42;SLC25A45;STAR;TAMM41;THEM4;TIMM17B;VDAC1;VDAC

AR1;SLC25A12;SLC25A15;SLC25A23;SLC25A27;SLC25A42;SLC25A45;SMAD3;STAR;SUN2;TAMM41;THEM4;TIMM17B;VDAC1;VDAC

ND2;CD1A;CD4;CD74;CDKAL1;CHCHD3;CHMP4C;CHMP5;CHMP7;CHST12;CLCN3;CLCN6;CLIC1;CLN8;CLSTN1;COG1;COG2;COG5;COG7;COPG2;COX10;COX15;COX5B;COX8A;CREB3L2;CUBN;CYB5B;CYP2D6;DAD1;DBH;DDX19A;DDX19B;DERL2;DGKH;DHCR24;DHCR7;DHDDS;DHODH;DNAJC1;DNAJC11;DOPE

GAT4B;MMP23B;MRPL10;MRPL4;MRPL41;MTIF2;MYPOP;NCK2;NEURL;NPAS1;NPEPL1;NR2F6;OTUD7A;PABPN1;PCSK1N;PDF;PDXP;PGLYRP2;PRDM8;PSMD12;RCE1;REPIN1;RPP25;SKOR1;SNAPC2;SNRPA;SOCS1;STK11;SUV420H2;TAF6L;TBX6;TFR2;TOR2A;TPST2;UCN;UFSP2;UGP2;USF2;USP33;UTF1;VAP

T6;ZFPM1;ZNF219;ZNF35;ZNF358;ZNF414;ZNF444;ZNF564;ZNF575;ZNF579;ZNF628;ZNF638;ZNF668;ZNF747;ZNF764;ZNF771;ZNF784;ZNF787;ZNF837;ZNF865;ZSCAN

POP;NCK2;NEURL;NPAS1;NR2F6;PABPN1;PDF;PDXP;PRDM8;PSMD12;RCE1;REPIN1;RPP25;SKOR1;SNAPC2;SNRPA;SOCS1;STK11;SUV420H2;TAF6L;TBX6;TOR2A;TPST2;UCN;UGP2;USF2;USP33;UTF1;VAPB;WNT6;YIF1A;ZDHHC1;ZFPM1;ZNF219;ZNF35;ZNF358;ZNF414;ZNF444;ZNF564;ZNF575;ZNF579;ZNF6

NF747;ZNF764;ZNF771;ZNF784;ZNF787;ZNF837;ZNF865;ZSCAN1

NF638;ZNF668;ZNF747;ZNF764;ZNF771;ZNF784;ZNF787;ZNF837;ZNF865;ZSCAN1

ZNF771;ZNF784;ZNF787;ZNF837;ZNF865;ZSCAN1
71;ZNF784;ZNF787;ZNF837;ZNF865;ZSCAN1

575;ZNF579;ZNF628;ZNF638;ZNF668;ZNF747;ZNF764;ZNF771;ZNF784;ZNF787;ZNF837;ZNF865;ZSCAN1

37;ZNF865;ZSCAN1
D3;GLI4;GLTPD2;GMPPB;GPAA1;GPN1;GRIN1;GRIN3B;H1FX;H2AFX;HCN2;HES4;HIC1;HMG20B;HSPB1;IDUA;INO80B;IRF2BP1;JUNB;JUND;KIFC2;KISS1R;KLF2;LOXL1;LRRC26;LTB;LTC4S;MAST1;MAT2B;MBD4;MEX3D;MGAT4B;MIB2;MMP23B;MTIF2;MYPOP;NCK2;NDUFAF3;NEURL;NPAS1;NPB;NPEPL1;NR2

71;ZNF784;ZNF787;ZNF837;ZNF865;ZSCAN1

;ZNF575;ZNF579;ZNF628;ZNF638;ZNF668;ZNF747;ZNF764;ZNF771;ZNF784;ZNF787;ZNF837;ZNF865;ZSCAN1

4;ZNF771;ZNF784;ZNF787;ZNF837;ZNF865;ZSCAN1

MAST1;MAT2B;MBD4;METTL21A;MEX3D;MGAT4B;MMP23B;MRPL10;MRPL4;MRPL41;MTIF2;MYPOP;NCK2;NEURL;NPAS1;NPEPL1;NR2F6;NUDT3;OAT;OTUD7A;PABPN1;PCSK1N;PDF;PDXP;PGLYRP2;PHOSPHO1;PRDM8;PSMD12;RCE1;REPIN1;RPP25;SKOR1;SNAPC2;SNRPA;SOCS1;SQLE;STK11;SUV420H2;T

NF564;ZNF575;ZNF579;ZNF628;ZNF638;ZNF668;ZNF747;ZNF764;ZNF771;ZNF784;ZNF787;ZNF837;ZNF865;ZSCAN1

XL1;LTB;LTB4R2;LTC4S;MAST1;MAT2B;MBD4;METTL21A;MEX3D;MGAT4B;MMP23B;MRPL10;MRPL4;MRPL41;MTIF2;MYPOP;NCK2;NEURL;NPAS1;NPEPL1;NR2F6;NUDT3;OAT;OTUD7A;PABPN1;PCSK1N;PDF;PDXP;PGLYRP2;PHOSPHO1;PRDM8;PSMD12;RCE1;REPIN1;RPP25;SKOR1;SNAPC2;SNRPA;SOCS1;

3;ZNF668;ZNF747;ZNF764;ZNF771;ZNF784;ZNF787;ZNF837;ZNF865;ZSCAN1

F1;VAPB;WNT6;ZDHHC1;ZFPM1;ZNF219;ZNF35;ZNF358;ZNF414;ZNF444;ZNF564;ZNF575;ZNF579;ZNF628;ZNF638;ZNF668;ZNF747;ZNF764;ZNF771;ZNF784;ZNF787;ZNF837;ZNF865;ZSCAN1

l;BRF1;BRF2;BTBD6;BUB1;BUB1B;C11orf30;C12orf10;C12orf44;C12orf5;C17orf49;C17orf70;C19orf10;C19orf24;C19orf70;C1orf198;C1orf86;C1QBP;C21orf2;CANX;CAPN10;CAPRIN1;CAPS;CBR1;CCDC101;CCDC106;CCDC28B;CCDC47;CCDC51;CCDC61;CCDC78;CCHCR1;CCNL2;CCS;CD320;CD36;CD38;CD8B

5;CHMP4A;CHMP6;CIB1;CISD3;CLASP1;CLCN4;CLIP1;CLPP;CNTLN;COL11A2;COL6A1;COL6A2;COL9A2;COMTD1;COQ7;COQ9;COX4I1;CPSF4;CPT1C;CREB3;CREB3L4;CRELD2;CRIP2;CRYL1;CTSC;CTSF;CTU2;CYC1;CYP1B1;CYP2E1;DCUN1D3;DDX28;DECR2;DERL3;DGUOK;DHRS4;DIAPH2;DNAJC30;DNAL4;DNLZ

orf30;C12orf10;C12orf44;C17orf49;C17orf70;C19orf10;C19orf24;C19orf70;C1orf198;C1orf86;C1QBP;C21orf2;CANX;CAPN10;CAPRIN1;CAPS;CBR1;CCDC101;CCDC106;CCDC28B;CCDC47;CCDC51;CCDC61;CCDC78;CCHCR1;CCNL2;CCS;CD320;CD36;CD38;CD8B;CD99;CDC25A;CDC6;CDCA8;CDK12;CDK13;CDS

PRIN1;CAPS;CBR1;CCDC28B;CCDC47;CCDC51;CCDC78;CCHCR1;CCS;CD320;CD36;CD8B;CD99;CDC25A;CDC6;CDCA8;CDS2;CECR5;CEL;CELF2;CELF6;CEMP1;CENPF;CENPM;CENPT;CEP55;CHCHD5;CHMP4A;CHMP6;CHTF18;CIB1;CISD3;CLASP1;CLCN4;CLIP1;CLPP;CNTLN;COL11A2;COL6A1;COL6A2;COL9A2;CC

DGUOK;DHDH;DHRS3;DHRS4;DHRS4L2;DLGAP5;DNAL4;DNASE1L2;DOLK;DPM3;DPP7;DPP8;DSE;DTD1;DTX3;DTYMK;DUS1L;DUS2L;DUS3L;DUSP10;DUSP2;DUSP23;DUSP3;DYNC1LI1;E4F1;ECI1;EEF1A2;EHHADH;EIF3F;ELP4;ENGASE;ENTPD7;EPC1;ERCC1;ERI3;ERP29;ERP44;ETNK2;EXD3;EXOSC5;FADS3;FAHI

S15;MRPS18A;MRPS26;MRPS34;MTFP1;MTG1;MUT;MUTYH;NAGS;NAT6;NDUFA11;NDUFA13;NDUFA3;NDUFA8;NDUFB10;NDUFB11;NDUFB7;NDUFB8;NDUFB9;NDUFS6;NDUFS7;NDUFS8;NDUFV1;NME3;NME4;NT5C;NTHL1;NUDT1;NUDT8;OGG1;PARK7;PARS2;PDK4;PEMT;PICK1;PLA2G6;POLRMT;PPOX

DC78;CCHCR1;CCNL2;CCS;CD320;CD36;CD38;CD8B;CDC25A;CDC6;CDCA8;CDK12;CDK13;CDS2;CDYL2;CECR5;CELF2;CELF6;CENPF;CENPM;CENPT;CHCHD5;CHD7;CHMP4A;CHMP6;CHRAC1;CHTF18;CIB1;CISD3;CLASP1;CLCN4;CLIP1;CLPP;CLSPN;CNTLN;COL11A2;COL6A1;COL6A2;COL9A2;COMMD5;COMTD1

QBP;CANX;CAPRIN1;CAPS;CCDC101;CCDC106;CCDC28B;CCDC47;CCDC51;CCDC61;CCDC78;CCHCR1;CCNL2;CCS;CD320;CD36;CD38;CD8B;CDC25A;CDC6;CDCA8;CDK12;CDK13;CDS2;CDYL2;CECR5;CELF2;CELF6;CENPF;CENPM;CENPT;CEP55;CHCHD5;CHD7;CHMP4A;CHMP6;CHRAC1;CHTF18;CIB1;CISD3;CLAS

HCR1;CCNL2;CCS;CD320;CD36;CD38;CD8B;CDC25A;CDC6;CDCA8;CDK12;CDK13;CDS2;CDYL2;CECR5;CELF2;CELF6;CENPF;CENPM;CENPT;CHCHD5;CHD7;CHMP4A;CHMP6;CHRAC1;CHTF18;CIB1;CISD3;CLASP1;CLCN4;CLIP1;CLPP;CLSPN;CNTLN;COL11A2;COL6A1;COL6A2;COL9A2;COMMD5;COMTD1;COQ7;C

;CAPRIN1;CAPS;CCDC101;CCDC106;CCDC28B;CCDC47;CCDC51;CCDC61;CCDC78;CCHCR1;CCNL2;CCS;CD320;CD36;CD38;CD8B;CDC25A;CDC6;CDCA8;CDK12;CDK13;CDS2;CDYL2;CECR5;CELF2;CELF6;CENPF;CENPM;CENPT;CEP55;CHCHD5;CHD7;CHMP4A;CHMP6;CHRAC1;CHTF18;CIB1;CISD3;CLASP1;CLCN4

M50;TOMM40L;TUFM;USP30;YME

X4I1;CPSF2;CPSF4;CPT1C;CREB3;CREB3L4;CTSF;CYC1;CYP1B1;CYP2E1;DAAM1;DDX21;DDX28;DEAF1;DECR2;DERL3;DGUOK;DHRS4;DIAPH2;DLGAP5;DNAL4;DOLK;DPM3;DSE;DTD1;DTYMK;DUSP2;DUSP3;DVL1;DYNC1LI1;E2F1;E4F1;EAF1;ECI1;EDF1;ELP4;ENTPD7;EPC1;ERCC1;ERGIC3;ERLIN2;ERP29;ERP44;

EB3L4;CTSF;CYC1;CYP1B1;CYP2E1;DAAM1;DDX21;DDX28;DEAF1;DECR2;DERL3;DGUOK;DHRS4;DIAPH2;DLGAP5;DNAL4;DOLK;DPM3;DSE;DTD1;DTYMK;DUSP2;DUSP3;DVL1;DYNC1LI1;E2F1;E4F1;EAF1;ECI1;EDF1;ELP4;ENTPD7;EPC1;ERCC1;ERGIC3;ERLIN2;ERP29;ERP44;ETFB;ETV3;EXOSC5;FADS3;FAF2;FAN

LEPRE1;LEPREL2;LSM2;MAML2;MAPK1;MAPK11;MBD3;MCM7;MCRS1;MED1;MED13L;MED18;MGMT;MKI67;MLL3;MLL5;MOB2;MPG;MRPL12;MRPL20;MRPL23;MRPL52;MRPL55;MRPS11;MRPS12;MRPS15;MRPS18A;MRPS26;MSH3;MUS81;MUT;MUTYH;NAGLU;NAGS;NCOA3;NDUFA13;NDUFA8;NDUF

MAML2;MAPK1;MAPK11;MBD3;MCM7;MCRS1;MED1;MED13L;MED18;MGMT;MKI67;MLL3;MLL5;MOB2;MPG;MRPL12;MRPL20;MRPL23;MRPL52;MRPL55;MRPS11;MRPS12;MRPS15;MRPS18A;MRPS26;MSH3;MUS81;MUT;MUTYH;NAGLU;NAGS;NCOA3;NDUFA13;NHP2;NLE1;NMRAL1;NOL12;NR2C2AP;

MARCH9;MATK;MBD3;MCRS1;METAP1D;MGMT;MLL3;MLL5;MLST8;MOGS;MPG;MTM1;MUTYH;MYLIP;MYLK3;NAA10;NCOA3;NEURL2;NF1;NHP2;NIPBL;NOG;NPRL2;NRP1;NSMCE1;NTHL1;OGG1;OXSR1;P2RX7;PARP10;PARP3;PDE6G;PDK4;PHEX;PHF16;PHF20;PHPT1;PICK1;PIGQ;PIK3CB;PIK3CG;PIN1;PL

LHL17;LAMTOR2;LARP4B;LATS1;LATS2;LCMT1;LIME1;LMF1;LPAR1;LSM14A;LTBP4;LTK;MAGI3;MAN1B1;MAP3K10;MAP4K2;MAPK1;MAPK11;MARCH9;MATK;MBD3;MCRS1;METAP1D;MIF4GD;MLL3;MLL5;MLST8;MOGS;MPDU1;MRPL12;MRPL20;MRPL23;MRPL24;MRPL52;MRPL55;MRPS11;MRPS12;MRI

MAML2;MAPK1;MAPK11;MBD3;MCM7;MCRS1;MED1;MED13L;MED18;MGMT;MKI67;MLL3;MLL5;MOB2;MPG;MRPL12;MRPL20;MRPL23;MRPL52;MRPL55;MRPS11;MRPS12;MRPS15;MRPS18A;MRPS26;MSH3;MUS81;MUT;MUTYH;NAGLU;NAGS;NCOA3;NDUFA13;NHP2;NLE1;NMRAL1;NOL12;NR2C2AP;

;FXYD2;GAS2L3;GCHFR;GEMIN7;GET4;GGA1;GNAI3;GNB2L1;GNG8;GNGT2;GRIN3A;GTF2A1;GTF3C5;H2AFJ;H2AFY2;HAUS8;HDAC10;HDAC11;HES6;HNRNPH3;HOOK3;IFFO1;IFT27;IMP4;INO80E;IPO8;IQGAP2;KAT2A;KCNH4;KCNN4;KCNQ4;KDM5A;KDM6A;KIF15;KIF20A;KIF24;KIF7;KLHL12;KRT18;KRTCAP2

;PIP5KL1;PMVK;PNKP;PNMT;POLD1;POLD4;POLM;POLR2F;POLR2G;POLR2I;POLR2L;POLR3K;POLRMT;POMT1;PRMT7;PTDSS2;QPCTL;QTRT1;RABEP2;RABGGTA;RBBP5;RNMTL1;ROCK2;RPS6KB2;RPS6KC1;RPS6KL1;SARDH;SAT2;SETD2;SETD7;SOAT1;SOAT2;SPEG;SPHK2;ST3GAL3;ST6GALNAC6;STK25;SUV42

DUFA11;NDUFA13;NDUFA3;NDUFA8;NDUFB10;NDUFB11;NDUFB7;NDUFB8;NDUFB9;NDUFS6;NDUFS7;NDUFS8;NDUFV1;NECAB3;NME4;NOSIP;NUP153;P2RX7;P4HTM;PANX1;PCSK4;PCYT2;PDK4;PEMT;PEX16;PEX6;PHF20;PICK1;PIGQ;PLEKHF1;POMT1;PPM1L;PPOX;PREB;PTDSS2;PTGES2;PTPLB;QPCTL;RA

BHLHE41;BMP2K;BOK;BOLA1;BRAF;BRAT1;BRF1;BRF2;BTBD6;BTN1A1;BTN2A1;BUB1;BUB1B;C11orf30;C12orf10;C12orf44;C12orf5;C17orf49;C17orf70;C19orf10;C19orf24;C19orf70;C1orf198;C1orf86;C1QBP;C21orf2;C8G;CANX;CAPN10;CAPRIN1;CAPS;CBR1;CCDC101;CCDC106;CCDC28B;CCDC47;CCDC51

BHLHE41;BMP2K;BOK;BOLA1;BRAF;BRAT1;BRF1;BRF2;BTBD6;BTN1A1;BTN2A1;BUB1;BUB1B;C11orf30;C12orf10;C12orf44;C12orf5;C17orf49;C17orf70;C19orf10;C19orf24;C19orf70;C1orf198;C1orf86;C1QBP;C21orf2;C8G;CANX;CAPN10;CAPRIN1;CAPS;CBR1;CCDC101;CCDC106;CCDC28B;CCDC47;CCDC51

FER;GLYCTK;GNB2L1;GZMM;HDAC10;HDAC11;HERC3;HIPK3;HPX;HRAS;JAG2;JOSD2;KAT2A;KDM3A;KDM6A;KIDINS220;KLHL12;KLHL17;LAMTOR2;LARP4B;LATS1;LATS2;LCMT1;LHB;LIME1;LMF1;LONRF3;LPAR1;LSM14A;LTBP4;LTK;MAGI3;MAN1B1;MAP3K10;MAP4K2;MAPK1;MAPK11;MARCH9;MATK;ME

;METAP1D;MLL3;MLL5;MLST8;MOGS;MTM1;MYLIP;MYLK3;NAA10;NCOA3;NEURL2;NF1;NIPBL;NOG;NPRL2;NRP1;NSMCE1;OXSR1;P2RX7;PARP10;PARP3;PDE6G;PDK4;PHEX;PHF16;PHF20;PHPT1;PICK1;PIGQ;PIK3CB;PIK3CG;PIN1;PLA2G6;PMM1;POLR2F;POLR2G;POLR2I;POLR2L;POMT1;PPM1D;PPM1J;PP

;METAP1D;MLL3;MLL5;MLST8;MOGS;MTM1;MYLIP;MYLK3;NAA10;NCOA3;NEURL2;NF1;NIPBL;NOG;NPRL2;NRP1;NSMCE1;OXSR1;P2RX7;PARP10;PARP3;PDE6G;PDK4;PHEX;PHF16;PHF20;PHPT1;PICK1;PIGQ;PIK3CB;PIK3CG;PIN1;PLA2G6;PMM1;POLR2F;POLR2G;POLR2I;POLR2L;POMT1;PPM1D;PPM1J;PP

HL1;NUP153;NUSAP1;OBFC1;OGG1;PAFAH1B2;PATL1;PHF16;PHF20;PIN1;PIP5K1A;PNKP;POLD1;POLD4;POLR2F;POLR2G;POLR2I;POLR2L;POLR3K;POP7;POU2F1;PPP2R3B;PSMA7;PSMB4;PSMB5;PSMB7;PSMD9;PUS1;RAD21;RAD9A;RBBP5;REC8;RFX3;RLIM;RNF169;RPS15;RPS19BP1;RPS2;RPS6KB2;RSF1;F

EB3L4;CRYL1;CSDE1;CSNK1G1;CTU2;CYB561D2;CYC1;CYP1B1;CYP2E1;DALRD3;DCXR;DDT;DDX39A;DEAF1;DECR2;DERL3;DGUOK;DHRS3;DHRS4;DNAJC17;DNAJC30;DNASE1L2;DOK7;DOLK;DPM3;DSE;DTD1;DTX3;DTYMK;DUSP10;DUSP2;DUSP3;DVL1;E2F1;E4F1;EAF1;ECI1;EDF1;EEF1D;EEF1G;EHHADH;EIF1

L;CLCF1;CLPP;CLSPN;COQ7;COQ9;COX4I1;CPSF2;CPSF4;CPT1C;CREB3;CREB3L4;CRIP1;CRYL1;CSDE1;CSNK1G1;CTAGE5;CTSC;CTSF;CTU2;CYB561D2;CYC1;CYP1B1;CYP2E1;DALRD3;DCXR;DDT;DDX39A;DEAF1;DECR2;DERL3;DGUOK;DHDH;DHRS3;DHRS4;DNAJC17;DNAJC30;DNASE1L2;DND1;DOK7;DOLK;DPM

RI1;MTM1;MTMR1;MVD;MVK;MYLK3;NARFL;NDOR1;NFKBIL1;NGFRAP1;NOSIP;NRP1;NSF;NT5C;PAFAH1B2;PAFAH1B3;PAPSS1;PARD6G;PATL1;PDE6G;PEX6;PFKFB2;PGM2;PHKB;PHKG1;PHPT1;PIK3C3;PIK3CB;PIK3CG;PIP5K1A;PIP5K1B;PLA2G6;PLCD1;PLEKHG5;PMM1;PMVK;PNMT;PRKAR2A;PRMT7;PSMA

RFX3;RLIM;RNF169;RPS15;RPS19BP1;RPS2;RPS6KB2;RSF1;RSRC1;SENP2;SETX;SF3B5;SIVA1;SNF8;SNRPC;SP1;SPTBN4;STRA13;SUDS3;TADA3;TCEA2;TCEB2;THAP7;TIMM50;TLE2;TOP1;TOP2A;TOPBP1;TP73;TPR;TRIP12;TUT1;TXNL4A;WDR18;WIBG;WRAP53;YWHAZ;ZBT

PTBN4;SSR1;SURF1;TADA3;TAOK3;TAZ;TLK2;TLR7;TNFAIP3;TNFRSF4;TNFSF15;TNK1;TOP1;TP73;TRAF2;TRAF4;TRAF6;TRIB3;TRIM24;TSSK6;TTN;ULK3;VAV3;WNT7A;ZAP70;ZBTB17;ZEB2;ZFP91;Z

M;STX1A;SYNGR1;SYTL1;TIMM10;TIMM13;TIMM50;TLR7;TMED1;TMED3;TNFRSF4;TNFSF15;TOMM40L;TPR;TRAF2;TRAF6;TRPS1;UEVLD;USE1;VPS13B;VPS16;VPS41;VTI1A;WDR24;YIPF5;YWHAZ;

1;RPLP2;RPS15;RPS2;RPS28;S100A13;SCARB2;SEC22B;SEC24B;SENP2;SERGEF;SLC25A6;SNF8;SNX20;SPTBN4;SSNA1;SSR1;SSR4;STAM;STX1A;SYNGR1;SYTL1;TIMM10;TIMM13;TIMM50;TLR7;TMED1;TMED3;TMSB4X;TNFRSF4;TNFSF15;TOMM40L;TOR1AIP1;TPR;TRAF2;TRAF6;TRPS1;UBL4A;UEVLD;USE1;VP

M13;TIMM50;TLR7;TMED1;TMED3;TNFRSF4;TNFSF15;TOMM40L;TPR;TRAF2;TRAF6;TRPS1;UEVLD;USE1;VPS13B;VPS16;VPS41;VTI1A;WDR24;YIPF5;YWHAZ;

LK2;TMEM161A;TOP2A;TOPBP1;TP73;TPR;TRAF2;TRAF4;TRAF6;TREX2;TRIB3;TRIP12;TXNRD1;ULK3;VAV3;WNT7A;ZBTB17;Z

N;COQ7;COQ9;CPSF2;CPSF4;CPT1C;CREB3;CREB3L4;CRIP1;CRYL1;CSDE1;CSNK1G1;CTSC;CTSF;CTU2;CYP2E1;DALRD3;DCXR;DDT;DDX39A;DEAF1;DECR2;DERL3;DGUOK;DHDH;DHRS3;DHRS4;DNAJC17;DNAJC30;DNASE1L2;DND1;DOK7;DOLK;DPM3;DPP7;DPP8;DSE;DTD1;DTX3;DTYMK;DUSP10;DUSP2;DUSP

3;GNG8;GNGT2;GRIN3A;GTF2A1;GTF3C5;HAUS8;HDAC10;HDAC11;HES6;HOOK3;IFFO1;IFT27;INO80E;IPO8;IQGAP2;KAT2A;KCNH4;KCNN4;KCNQ4;KDM5A;KDM6A;KIF15;KIF20A;KIF24;KIF7;KLHL12;KRT18;KRTCAP2;LAMB1;LAMTOR2;LAT;LATS1;LATS2;LIME1;MAD1L1;MAPK1;MBD3;MCM7;MCRS1;MED1;M
MUTYH;NCOA3;NDUFA13;NHP2;NIPBL;NLE1;NMRAL1;NOL12;NR2C2AP;NSRP1;NTHL1;NUP153;NUSAP1;OBFC1;OGG1;P2RX7;PAFAH1B2;PATL1;PHF16;PHF20;PIN1;PIP5K1A;PNKP;POLD1;POLD4;POLR2F;POLR2G;POLR2I;POLR2L;POLR3K;POP7;POU2F1;PPP2R3B;PSMA7;PSMB4;PSMB5;PSMB7;PSMD9;PUS1

NCAPH2;NCOA3;NDUFS7;NDUFS8;NEURL2;NF1;NIPBL;NUP153;NUSAP1;OBFC1;P2RX7;PARK7;PARP10;PEX16;PEX6;PHF1;PHF16;PHF20;PHPT1;PIK3C3;PIN1;PIP5K1A;PLA2G6;PLEKHF1;POLD1;POLD4;POLRMT;PREB;PRMT7;RAC3;RAD21;RANBP9;RBBP5;REC8;ROCK2;RSF1;RTEL1;SAC3D1;SDHAF1;SEC24B;SE

;NDUFB9;NDUFS6;NDUFS7;NDUFS8;NDUFV1;NF1;NME3;NME4;NOG;NPRL2;NRP1;NT5C;NTHL1;NUDT1;NUDT18;OGG1;OXSR1;P2RX7;PANK4;PAPSS1;PCYT2;PDE6G;PDK4;PEMT;PEX6;PFKFB2;PGM2;PHEX;PHPT1;PICK1;PIGQ;PIK3C3;PIK3CB;PIK3CG;PIN1;PIP5K1A;PIP5K1B;PLA2G6;PLCD1;PMM1;PMVK;PNK
C;CREB3;CREB3L4;CRYL1;CSDE1;CSNK1G1;CTSC;CTSF;CTU2;CYP2E1;DALRD3;DCXR;DDX39A;DEAF1;DECR2;DERL3;DGUOK;DHDH;DHRS3;DHRS4;DNAJC17;DNAJC30;DNASE1L2;DOK7;DOLK;DPM3;DPP7;DPP8;DSE;DTD1;DTX3;DTYMK;DUSP10;DUSP2;DUSP3;DVL1;E2F1;E4F1;EAF1;ECI1;EDF1;EEF1D;EEF1G;EH
S7;NDUFS8;NDUFV1;NF1;NME3;NME4;NOG;NPRL2;NRP1;NT5C;NTHL1;NUDT1;NUDT18;OGG1;OXSR1;P2RX7;PANK4;PAPSS1;PCYT2;PDE6G;PDK4;PEMT;PEX6;PFKFB2;PGM2;PHPT1;PICK1;PIGQ;PIK3C3;PIK3CB;PIK3CG;PIN1;PIP5K1A;PIP5K1B;PLA2G6;PLCD1;PMM1;PMVK;PNKP;POLR2F;POLR2G;POLR2I;POL

MAN1B1;MBLAC1;MCM7;METAP1D;MMP28;MOGS;MPG;MSH3;MST1;MTG1;MTM1;MTMR1;MUS81;MUTYH;MYH10;NAALADL1;NAGLU;NAPSA;NCEH1;NEIL1;NSF;NT5C;NTHL1;NUDT1;NUDT14;NUDT17;NUDT18;NUDT22;NUDT8;OGG1;PAFAH1B2;PAFAH1B3;PARK7;PCSK4;PDE6G;PEX6;PFKFB2;PGP;PHE

KIF7;LATS1;LATS2;LEPRE1;LEPREL2;LTK;MAGI3;MAP3K10;MAP4K2;MAPK1;MAPK11;MATK;MCM7;MECR;MKI67;MMAB;MRPL23;MSH3;MTG1;MUT;MVD;MVK;MYH10;MYLK3;NDOR1;NDUFA13;NDUFV1;NME3;NME4;NMRAL1;NSF;NT5C;NUBP2;OGFOD2;OXSR1;P2RX6;P2RX7;P4HTM;PANK4;PAPSS1;PARS

LTK;MAGI3;MAP3K10;MAP4K2;MAPK1;MAPK11;MATK;MCM7;MECR;MKI67;MMAB;MRPL23;MSH3;MTG1;MVD;MVK;MYH10;MYLK3;NDOR1;NDUFA13;NDUFV1;NME3;NME4;NMRAL1;NSF;NT5C;NUBP2;OXSR1;P2RX6;P2RX7;PANK4;PAPSS1;PARS2;PDE6G;PDK4;PEX6;PFKFB2;PHKG1;PIK3C3;PIK3CB;PIK3C

.TK;MAGI3;MAP3K10;MAP4K2;MAPK1;MAPK11;MATK;MCM7;MECR;MKI67;MMAB;MRPL23;MSH3;MTG1;MVD;MVK;MYH10;MYLK3;NDOR1;NDUFA13;NDUFV1;NME3;NME4;NMRAL1;NSF;NT5C;NUBP2;OXSR1;P2RX6;P2RX7;PANK4;PAPSS1;PARS2;PDE6G;PDK4;PEX6;PFKFB2;PHKG1;PIK3C3;PIK3CB;PIK3C

VK;NAGLU;NAGS;NCOA3;NDUFA11;NDUFA13;NDUFA3;NDUFA8;NDUFB10;NDUFB11;NDUFB7;NDUFB8;NDUFB9;NDUFS6;NDUFS7;NDUFS8;NDUFV1;NF1;NME3;NME4;NOSIP;NT5C;NTHL1;NUDT1;NUDT18;NUP153;OGG1;PANK4;PAOX;PAPSS1;PARS2;PCYT2;PDK4;PEMT;PEX6;PFKFB2;PGM2;PHKB;PHKG1;

L13;RPL18;RPL18A;RPL28;RPL29;RPL38;RPL8;RPLP1;RPLP2;RPS15;RPS2;RPS28;S100A13;SCARB2;SEC22B;SEC24B;SENP2;SERGEF;SLC25A6;SNF8;SNX20;SOAT1;SOAT2;SPTBN4;SSNA1;SSR1;SSR4;STAM;STX1A;SYNGR1;SYTL1;THOC6;TIMM10;TIMM13;TIMM50;TLR7;TMED1;TMED3;TMSB4X;TNFRSF4;TNFSF1

PN;COL11A2;COL6A1;COL6A2;COMMD4;COMMD5;CORO6;CPSF2;CRB3;CREB3;CRELD2;CRYL1;CSNK1G1;CTSC;CTTNBP2NL;CTU2;CUEDC2;CUTA;CXorf40A;CYP2E1;DAAM1;DDX39A;DEAF1;DECR2;DERL3;DHRS4;DIAPH2;DLGAP5;DNAJC17;DNAJC30;DOK7;DPM3;DUSP2;DVL1;DYM;E2F1;E4F1;EAF1;EDF1;EEF

IF3I;EIF3K;EIF4G3;ELF1;ELK3;ELP4;EPC1;ERCC1;ERLIN2;ERP29;ERP44;ETF1;ETV2;ETV3;EXOSC5;FANCG;FASTK;FBL;FBXL15;FBXL6;FBXO2;FBXO24;FBXW11;FBXW5;FEM1B;FICD;FKBP11;FKBP2;FLAD1;FNIP2;GEMIN7;GFER;GLYCTK;GNB2L1;GPBP1L1;GTF2A1;GTF2H2D;GTF3C5;GTPBP3;GYG1;H2AFJ;H2AFY2;H

F1;MACROD1;MAPK1;MC1R;MECR;MED1;MLST8;MMAB;MPG;MRI1;MTG1;MTM1;MTMR1;MUT;MUTYH;MVD;MVK;NAGLU;NAGS;NCEH1;NCOA3;NDOR1;NDUFA11;NDUFA13;NDUFA3;NDUFA8;NDUFB10;NDUFB11;NDUFB7;NDUFB8;NDUFB9;NDUFS6;NDUFS7;NDUFS8;NDUFV1;NF1;NME3;NME4;NOSIP;

BEPK;RAD21;RALGAPB;RBPMS;RFX3;RHOT2;RPL13;RPL18;RPL18A;RPL28;RPL29;RPL38;RPL8;RPLP1;RPLP2;RPS15;RPS2;RPS28;RSRC1;S100A13;SCARB2;SEC22B;SEC24B;SEMA6A;SERGEF;SERPINF2;SLC17A9;SLC25A5;SLC25A6;SNF8;SPTBN4;SSNA1;SSR1;SSR4;STAM;STK25;STOML2;STX1A;SYNGR1;SYTL1;TA

l;SARS2;SETX;SPEG;SPHK2;STK25;TAOK3;TLK2;TNK1;TOP1;TOP2A;TOP3B;TPR;TRIB3;TRMU;TSSK6;TTN;TUT1;UBA7;UCK1;UCKL1;ULK3;VARS2;YME1L1;ZAI

RPL12;MRPL20;MRPL23;MRPL24;MRPL38;MRPL52;MRPL54;MRPL55;MRPS11;MRPS12;MRPS15;MRPS18A;MRPS26;MRPS34;MSH3;MTM1;MUS81;MYH10;MYLIP;MYLK3;MZT2B;NCAPH;NCAPH2;NDOR1;NEIL1;NHP2;NLE1;NMRAL1;NOL12;NRP1;NSMCE1;NUBP2;NUP153;NUSAP1;OBFC1;P2RX6;PAFAH1B
RPL12;MRPL20;MRPL23;MRPL24;MRPL38;MRPL52;MRPL54;MRPL55;MRPS11;MRPS12;MRPS15;MRPS18A;MRPS26;MRPS34;MSH3;MTM1;MUS81;MYH10;MYLIP;MYLK3;MZT2B;NCAPH;NCAPH2;NDOR1;NEIL1;NHP2;NLE1;NMRAL1;NOL12;NRP1;NSMCE1;NUBP2;NUP153;NUSAP1;OBFC1;P2RX6;PAFAH1B
l;RPS6KL1;RTEL1;SARS2;SETX;SPEG;SPHK2;STK25;TAOK3;TLK2;TNK1;TOP1;TOP2A;TOP3B;TPR;TRIB3;TRMU;TSSK6;TTN;TUT1;UBA7;UCK1;UCKL1;ULK3;VARS2;YME1L1;ZAI

);DUSP2;DUSP3;DVL1;E2F1;E4F1;EAF1;EDF1;EEF1D;EEF1G;EHHADH;EIF1;EIF2B2;EIF2B4;EIF3F;EIF3G;EIF3I;EIF3K;EIF4G3;ELF1;ELK3;ELP4;EPC1;ERCC1;ERLIN2;ERP29;ERP44;ETF1;ETV2;ETV3;EXOSC5;FANCG;FASTK;FBL;FBXL15;FBXL6;FBXO2;FBXO24;FBXO44;FBXW11;FBXW5;FEM1B;FICD;FKBP11;FKBP2;FLA

l;RPS6KL1;RTEL1;SARS2;SETX;SPEG;SPHK2;STK25;TAOK3;TLK2;TNK1;TOP1;TOP2A;TOP3B;TPR;TRIB3;TRMU;TSSK6;TTN;TUT1;UBA7;UCK1;UCKL1;ULK3;VARS2;YME1L1;ZAI

RAF6;TRIB3;TRIP12;TTN;UBQLN1;UBXN1;WIBG;WNT7A;ZBTB17;ZEB2;ZFP91;Z

1A;WASH3P;WDR24;YIPF5;YWHAZ;Z
5;C19orf57;C19orf60;C1orf86;C1QBP;C1QTNF6;C8G;CANX;CAPN10;CAPRIN1;CAPS;CBR1;CCDC101;CCDC130;CCDC28B;CCDC47;CCNL2;CCS;CD320;CD36;CD38;CD8B;CDC25A;CDC6;CDCA8;CDK10;CDK12;CDK13;CDYL2;CEL;CELF2;CELF6;CENPF;CENPT;CHD7;CHMP4A;CHMP6;CHRAC1;CHTF18;CIB1;CISD3;CL

IK3C3;PIK3CB;PIK3CG;PIP5K1A;PIP5K1B;PIP5KL1;PMVK;PNKP;PRKAR2A;QARS;RAB21;RAB25;RAB33A;RABEP2;RAC3;RECQL4;RHOC;RHOT2;ROCK2;RPS6KB2;RPS6KC1;RPS6KL1;RTEL1;SARS2;SEPT1;SETX;SPEG;SPHK2;STK25;TAOK3;TLK2;TNK1;TOP1;TOP2A;TOP3B;TPR;TRIB3;TRMU;TSSK6;TTN;TUBB6;TUFM;

;UBQLN1;UBXN1;UCKL1;USE1;VKORC1;YIPF5;ZFAND2B;ZFYVE27;

Z

3CG;PIP5K1A;PIP5K1B;PIP5KL1;PMVK;PNKP;PRKAR2A;QARS;RAB21;RAB25;RAB33A;RABEP2;RAC3;RECQL4;RHOC;RHOT2;ROCK2;RPS6KB2;RPS6KC1;RPS6KL1;RTEL1;SARS2;SEPT1;SETX;SPEG;SPHK2;STK25;TAOK3;TLK2;TNK1;TOP1;TOP2A;TOP3B;TPR;TRIB3;TRMU;TSSK6;TTN;TUBB6;TUFM;TUT1;UBA7;UCK

YH10;MYLK3;NDOR1;NDUFA13;NDUFV1;NME3;NME4;NRP1;NSF;NUBP2;OGFOD2;OXSR1;P2RX6;P2RX7;P4HTM;PANK4;PAPSS1;PARS2;PCOLCE;PDE6G;PDK4;PEX6;PFKFB2;PHKG1;PHLDA3;PIK3C3;PIK3CB;PIK3CG;PIN1;PIP5K1A;PIP5K1B;PIP5KL1;PLEKHA4;PMVK;PNKP;PPOX;PRKAR2A;QARS;RAB21;RAB25;R

P5K1A;PIP5K1B;PIP5KL1;PMVK;PNKP;QARS;RAB21;RAB25;RAB33A;RABEP2;RAC3;RECQL4;RHOC;RHOT2;ROCK2;RPS6KB2;RPS6KC1;RPS6KL1;RTEL1;SARS2;SEPT1;SETX;SPEG;SPHK2;STK25;TAOK3;TLK2;TNK1;TOP1;TOP2A;TOP3B;TPR;TRIB3;TRMU;TSSK6;TTN;TUBB6;TUFM;TUT1;UBA7;UCK1;UCKL1;ULK3;VA

P5K1A;PIP5K1B;PIP5KL1;PMVK;PNKP;QARS;RAB21;RAB25;RAB33A;RABEP2;RAC3;RECQL4;RHOC;RHOT2;ROCK2;RPS6KB2;RPS6KC1;RPS6KL1;RTEL1;SARS2;SEPT1;SETX;SPEG;SPHK2;STK25;TAOK3;TLK2;TNK1;TOP1;TOP2A;TOP3B;TPR;TRIB3;TRMU;TSSK6;TTN;TUBB6;TUFM;TUT1;UBA7;UCK1;UCKL1;ULK3;VA
P5K1A;PIP5K1B;PIP5KL1;PMVK;PNKP;QARS;RAB21;RAB25;RAB33A;RABEP2;RAC3;RECQL4;RHOC;RHOT2;ROCK2;RPS6KB2;RPS6KC1;RPS6KL1;RTEL1;SARS2;SEPT1;SETX;SPEG;SPHK2;STK25;TAOK3;TLK2;TNK1;TOP1;TOP2A;TOP3B;TPR;TRIB3;TRMU;TSSK6;TTN;TUBB6;TUFM;TUT1;UBA7;UCK1;UCKL1;ULK3;VA
3CG;PIP5K1A;PIP5K1B;PIP5KL1;PMVK;PNKP;PRKAR2A;QARS;RAB21;RAB25;RAB33A;RABEP2;RAC3;RECQL4;RHOC;RHOT2;ROCK2;RPS6KB2;RPS6KC1;RPS6KL1;RTEL1;SARS2;SEPT1;SETX;SPEG;SPHK2;STK25;TAOK3;TLK2;TNK1;TOP1;TOP2A;TOP3B;TPR;TRIB3;TRMU;TSSK6;TTN;TUBB6;TUFM;TUT1;UBA7;UCK

;PIP5K1B;PIP5KL1;PMVK;PNKP;QARS;RAB21;RAB25;RAB33A;RABEP2;RAC3;RECQL4;RHOC;RHOT2;ROCK2;RPS6KB2;RPS6KC1;RPS6KL1;RTEL1;SARS2;SEPT1;SETX;SPEG;SPHK2;STK25;TAOK3;TLK2;TNK1;TOP1;TOP2A;TOP3B;TPR;TRIB3;TRMU;TSSK6;TTN;TUBB6;TUFM;TUT1;UBA7;UCK1;UCKL1;ULK3;VARS2;YI

2B4;EIF3F;EIF3G;EIF3I;EIF3K;EIF4G3;ELF1;ELK3;ERCC1;ERI3;ETF1;ETNK2;ETV2;ETV3;EXD3;EXOSC5;FADS3;FANCG;FASTK;FBL;FDXR;FICD;FIGNL2;FKBP11;FKBP2;FLAD1;FPGS;FUK;GCDH;GFER;GIMAP5;GLYCTK;GNAI3;GPBP1L1;GTF2A1;GTF3C5;GTPBP3;GTPBP6;H2AFJ;H2AFY2;HBP1;HELZ;HES6;HES7;HINFP;H
;DYM;E2F1;E4F1;EAF1;EDF1;EEF1A2;EEF1D;EHHADH;EIF3G;EIF3K;ELF1;ELK3;ELP4;EPC1;ERCC1;ETV2;ETV3;EXOSC5;FAM53A;FANCG;FBL;FBXO38;FBXW11;FEM1B;FIBP;FNIP2;FSD1;GADD45GIP1;GCHFR;GEMIN7;GHDC;GLTSCR2;GNB2L1;GOLPH3;GPBP1L1;GTF2A1;GTF2H2D;GTF3C5;GTPBP3;H2AFJ;H2AFY2

P5K1A;PIP5K1B;PIP5KL1;PMVK;PNKP;QARS;RAB21;RAB25;RAB33A;RABEP2;RAC3;RECQL4;RHOC;RHOT2;ROCK2;RPS6KB2;RPS6KC1;RPS6KL1;RTEL1;SARS2;SEPT1;SETX;SPEG;SPHK2;STK25;TAOK3;TLK2;TNK1;TOP1;TOP2A;TOP3B;TPR;TRIB3;TRMU;TSSK6;TTN;TUBB6;TUFM;TUT1;UBA7;UCK1;UCKL1;ULK3;VA

BBX;BCAS2;BCL2L15;BCLAF1;BDP1;BHLHB9;BIN1;BIRC2;BIRC3;BLOC1S2;BLZF1;BMI1;BNIP1;BNIP2;BNIP3L;BNIPL;BRCA2;BRCC3;BRIP1;BRMS1;BRMS1L;BRWD1;BTAF1;BTBD10;BTD;BUD13;BYSL;C11orf1;C11orf49;C11orf54;C12orf52;C14orf166;C8orf40;C8orf58;C9orf72;CABIN1;CALB1;CALML6;CAMK1;CA
RD34B;ANKRD49;ANLN;ANO5;ANP32E;ANXA1;AP1AR;AP1B1;AP1S2;AP2A2;AP3M1;AP3S2;AP4S1;APC;APEH;APEX1;APEX2;APH1A;API5;APLF;APPBP2;APPL1;APTX;AQP11;ARAF;ARF4;ARFGAP1;ARFGAP2;ARFGEF2;ARFIP1;ARG2;ARGLU1;ARHGAP22;ARHGEF2;ARID2;ARID4A;ARID4B;ARID5A;ARID5B;ARL13
RD34B;ANKRD49;ANLN;ANO5;ANP32E;ANXA1;AP1AR;AP1B1;AP1S2;AP2A2;AP3M1;AP3S2;AP4S1;APC;APEH;APEX1;APEX2;APH1A;API5;APLF;APPBP2;APPL1;APTX;AQP11;ARAF;ARF4;ARFGAP1;ARFGAP2;ARFGEF2;ARFIP1;ARG2;ARGLU1;ARHGAP22;ARHGEF2;ARID2;ARID4A;ARID4B;ARID5A;ARID5B;ARL13
C;ANKRD26;ANKRD28;ANKRD32;ANKRD34B;ANKRD49;ANLN;ANO5;ANP32E;ANXA1;AP1AR;AP1B1;AP1S2;AP2A2;AP3M1;AP3S2;AP4S1;APC;APEH;APEX1;APEX2;APH1A;API5;APLF;APOBEC3D;APPBP2;APPL1;APTX;AQP11;ARAF;ARF4;ARFGAP1;ARFGAP2;ARFGEF2;ARFIP1;ARG2;ARGLU1;ARHGAP22;ARHGD
C;ANKRD26;ANKRD28;ANKRD32;ANKRD34B;ANKRD49;ANLN;ANO5;ANP32E;ANXA1;AP1AR;AP1B1;AP1S2;AP2A2;AP3M1;AP3S2;AP4S1;APC;APEH;APEX1;APEX2;APH1A;API5;APLF;APOBEC3D;APPBP2;APPL1;APTX;AQP11;ARAF;ARF4;ARFGAP1;ARFGAP2;ARFGEF2;ARFIP1;ARG2;ARGLU1;ARHGAP22;ARHGD
2;ALG3;ALG6;ALKBH4;ALKBH5;ALKBH8;ALS2CR8;AMD1;ANAPC10;ANG;ANKLE1;ANKMY2;ANKRD12;ANKRD13C;ANKRD26;ANKRD28;ANKRD32;ANKRD34B;ANKRD49;ANLN;ANO5;ANP32E;ANXA1;AP1AR;AP1B1;AP1S2;AP2A2;AP3M1;AP3S2;AP4S1;APC;APEH;APEX1;APEX2;APH1A;API5;APIP;APLF;APOBEC3
1;ANAPC10;ANKRD12;ANKRD13C;ANKRD34B;ANLN;ANO5;ANP32E;ANXA1;AP1AR;AP1B1;AP1S2;AP2A2;AP3M1;AP3S2;AP4S1;APC;APEH;APEX1;APEX2;APH1A;API5;APIP;APLF;APOBEC3D;APPBP2;APPL1;APTX;AQP11;ARAF;ARAP2;ARF4;ARFGAP1;ARFGAP2;ARFGEF2;ARFIP1;ARG2;ARHGAP10;ARHGAP11A
2;ALG3;ALG6;ALKBH4;ALKBH5;ALKBH8;ALS2CR8;AMD1;ANAPC10;ANG;ANKLE1;ANKMY2;ANKRD12;ANKRD13C;ANKRD26;ANKRD28;ANKRD32;ANKRD34B;ANKRD49;ANLN;ANO5;ANP32E;ANXA1;AP1AR;AP1B1;AP1S2;AP2A2;AP3M1;AP3S2;AP4S1;APC;APEH;APEX1;APEX2;APH1A;API5;APIP;APLF;APOBEC3
ALDOA;ALG1;ALG10;ALG10B;ALG11;ALG13;ALG2;ALG3;ALG6;ALKBH4;ALKBH5;ALKBH8;ALS2CR8;AMD1;ANAPC10;ANG;ANGPT1;ANKLE1;ANKMY2;ANKRD12;ANKRD13C;ANKRD26;ANKRD28;ANKRD32;ANKRD34B;ANKRD49;ANLN;ANO5;ANP32E;ANXA1;AP1AR;AP1B1;AP1S2;AP2A2;AP3M1;AP3S2;AP4S1;AI
ALDOA;ALG1;ALG10;ALG10B;ALG11;ALG13;ALG2;ALG3;ALG6;ALKBH4;ALKBH5;ALKBH8;ALS2CR8;AMD1;ANAPC10;ANG;ANGPT1;ANKLE1;ANKMY2;ANKRD12;ANKRD13C;ANKRD26;ANKRD28;ANKRD32;ANKRD34B;ANKRD49;ANLN;ANO5;ANP32E;ANXA1;AP1AR;AP1B1;AP1S2;AP2A2;AP3M1;AP3S2;AP4S1;AI
PIP;APLF;APOBEC3D;APPBP2;APPL1;AQP11;ARAF;ARAP2;ARF4;ARFGAP1;ARFGAP2;ARFGEF2;ARFIP1;ARG2;ARHGAP10;ARHGAP11A;ARHGAP12;ARHGAP22;ARHGAP5;ARHGDIA;ARHGEF2;ARL2;ARL6IP1;ARL6IP5;ARL8B;ARMC1;ARMC10;ARRB1;ARSA;ARSK;ASNA1;ASPH;ASTL;ATAD1;ATAD3A;ATG12;ATG4
RSK;ASNA1;ASPH;ASPM;ATAD1;ATAD3A;ATF1;ATF2;ATG9A;ATL2;ATP10D;ATP11B;ATP11C;ATP2C1;ATP5C1;ATP5E;ATP5F1;ATP5I;ATP5L;ATP5O;ATP6AP1;ATP6V1G1;ATP7A;ATP8A1;ATPAF2;ATRX;ATXN7L3;AUH;AURKC;AZI1;B2M;B3GALNT1;B3GALT2;B3GALTL;B3GNT1;B3GNT2;B3GNT5;B4GALT3;B4GALT
GALT6;BACH1;BAG2;BAG4;BAG6;BANF1;BAP1;BAZ1A;BAZ2B;BBS10;BBS12;BBX;BCAS2;BCLAF1;BCR;BDP1;BIRC2;BLOC1S2;BLZF1;BMI1;BMPR1A;BMPR2;BNIP3L;BRCA2;BRCC3;BRD9;BRIP1;BRMS1;BRMS1L;BRWD1;BTAF1;BTBD1;BUD13;BZW1;C10orf2;C12orf52;C14orf166;C1GALT1;CAB39;CALCRL;CAMK1
RSK;ASNA1;ASPH;ASPM;ATAD1;ATAD3A;ATF1;ATF2;ATG9A;ATL2;ATP10D;ATP11B;ATP11C;ATP2C1;ATP5C1;ATP5E;ATP5F1;ATP5I;ATP5L;ATP5O;ATP6AP1;ATP6V1G1;ATP7A;ATP8A1;ATPAF2;ATRX;ATXN7L3;AUH;AURKC;AZI1;B2M;B3GALNT1;B3GALT2;B3GALTL;B3GNT1;B3GNT2;B3GNT5;B4GALT3;B4GALT
AP2;ARFGEF2;ARG2;ARGLU1;ARHGAP22;ARHGAP5;ARHGEF2;ARID2;ARID4A;ARID4B;ARID5A;ARID5B;ARL2;ARL8B;ARPP19;ARRB1;ARSA;ARSK;ARV1;ASAP3;ASB13;ASB7;ASF1A;ASGR2;ASNSD1;ASPH;ASTE1;ATAD1;ATAD2;ATF1;ATF2;ATG12;ATG16L2;ATG4A;ATG4B;ATG4C;ATG5;ATG9A;ATL2;ATP2B1;ATP2
;CDC25C;CDC26;CDC27;CDC37;CDC73;CDK1;CDK11A;CDK11B;CDK17;CDK18;CDK2;CDK5R1;CDK5RAP3;CDK7;CDK9;CDKL1;CDKN1B;CDKN1C;CDKN2C;CDKN3;CENPE;CHCHD4;CHFR;CHM;CHORDC1;CHUK;CISH;CKS1B;CKS2;CLIP3;CLK1;CLK3;CLK4;CLN3;CLN5;CLN6;CNPPD1;COG3;COL4A3BP;COPS2;COPS4;C
OBEC3D;APTX;ARAF;ARAP2;ARF4;ARFGAP1;ARFGAP2;ARG2;ARGLU1;ARHGAP22;ARHGAP5;ARHGEF2;ARID2;ARID4A;ARID4B;ARID5A;ARID5B;ARL2;ARL8B;ARPP19;ARRB1;ARSA;ARSK;ARV1;ASAP3;ASB13;ASB7;ASF1A;ASGR2;ASNSD1;ASPH;ASTE1;ASTL;ATAD2;ATF1;ATF2;ATG4A;ATG4B;ATG4C;ATG5;ATL2;A
TG5;ATP7A;ATPBD4;ATRX;ATXN7L3;AUH;AURKC;AZIN1;B2M;B3GALNT1;B3GALT2;B3GALTL;B3GNT1;B3GNT2;B3GNT5;B4GALT3;B4GALT6;BACH1;BAG2;BAG4;BAG6;BANF1;BAP1;BAZ1A;BAZ2B;BBS10;BBS12;BBX;BCAS2;BCLAF1;BCR;BDP1;BIRC2;BLOC1S2;BLZF1;BMI1;BMP1;BMPR1A;BMPR2;BNIP3L;BRCA
H1A;APIP;APLF;APOBEC3D;APTX;ARAF;ARAP2;ARF4;ARFGAP1;ARFGAP2;ARFGEF2;ARG2;ARGLU1;ARHGAP22;ARHGAP5;ARHGEF2;ARID2;ARID4A;ARID4B;ARID5A;ARID5B;ARL2;ARL8B;ARPP19;ARRB1;ARSA;ARSK;ARV1;ASAP3;ASB13;ASB7;ASF1A;ASGR2;ASNSD1;ASPH;ASTE1;ASTL;ATAD1;ATAD2;ATF1;ATF
ALKBH8;ANG;ANGPT1;ANGPTL4;ANGPTL7;ANKIB1;ANKK1;ANKMY2;ANKRD13C;ANKRD28;ANKRD44;ANLN;ANXA1;AP1B1;AP2A2;AP3M1;APC;APEX1;APEX2;APH1A;API5;APIP;APLF;APOBEC3D;APPBP2;APPL1;APTX;ARAF;ARAP2;ARF4;ARFGAP1;ARFGAP2;ARFGEF2;ARFIP1;ARG2;ARHGAP10;ARHGAP12;ARH
CHAF1A;CHAF1B;CHD9;CHFR;CHMP1A;CKS1B;CLIC4;CLK3;CLP1;COIL;COPS2;COPS4;COPS5;COPS6;CPSF1;CPSF6;CRBN;CREB1;CSNK1A1;CSNK2B;CST3;CTBP1;CTDNEP1;CUL2;CUTC;CXXC1;CYHR1;DACH1;DAPK3;DAXX;DBF4;DCAF13;DCLRE1A;DDB2;DDX11;DDX3X;DDX41;DDX50;DDX54;DEPDC1;DGCR14;DG
EX2;APH1A;APIP;APLF;APOBEC3D;APTX;ARAF;ARAP2;ARF4;ARFGAP1;ARFGAP2;ARFGEF2;ARG2;ARGLU1;ARHGAP22;ARHGAP5;ARHGEF2;ARID2;ARID4A;ARID4B;ARID5A;ARID5B;ARL2;ARL8B;ARPP19;ARRB1;ARRDC3;ARSA;ARSK;ARV1;ASAP3;ASB13;ASB7;ASF1A;ASGR2;ASNSD1;ASPH;ASTE1;ASTL;ATAD1;A

LIN28A;LNX1;LNX2;LONRF1;LRSAM1;LTN1;MAN2A1;MAN2B1;MAN2C1;MAP3K1;MARCH1;MARCH5;MARCH6;MARCH7;MATR3;MBNL1;MBNL2;MBTD1;MDM2;MEX3C;MIB1;MICAL1;MICALL1;MICALL2;MKRN2;MKRN3;MMP15;MMP17;MMP24;MNAT1;MORC2;MORC3;MPI;MSL2;MTF2;MYNN;NAPEPLD;

;CCDC90A;CCZ1;CD164;CD46;CD63;CDIPT;CDS1;CEP70;CEPT1;CERS5;CHERP;CHMP1A;CHMP1B;CHMP2A;CHMP2B;CHPF;CHPF2;CHST13;CHST14;CHST2;CHSY1;CISD1;CISD2;CLCN7;CLIC4;CLIP3;CLN3;CLN5;CLN6;CLSTN3;CNIH;CNP;COASY;COG3;COG4;COG6;COL4A3BP;COPE;COPZ1;COQ10B;COQ2;CORO1A

P2;SMURF2;SOCS5;STT3B;SUMO1;SUMO2;SUMO3;TAF9;TBL1XR1;TOPORS;TRIB2;TRIM13;UBA6;UBC;UBE2A;UBE2D1;UBE2D3;UBE2E1;UBE2G1;UBE2J1;UBE2K;UBE2N;UBE2V2;UBE2W;UBE3A;UBE4A;UBR1;UBR3;UBR5;UCHL3;UCHL5;USP1;USP12;USP14;USP15;USP16;USP19;USP21;USP25;USP31;USP37;

NF6;SENP1;SIAH1;SIRT1;SIRT2;SKP2;SMURF2;SOCS5;SOCS6;STT3B;SUMO1;SUMO2;SUMO3;TAF9;TBL1XR1;TOPORS;TRIB2;TRIM13;UBA6;UBC;UBE2A;UBE2D1;UBE2D3;UBE2E1;UBE2G1;UBE2J1;UBE2K;UBE2N;UBE2V2;UBE2W;UBE3A;UBE4A;UBR1;UBR3;UBR5;UCHL3;UCHL5;USP1;USP12;USP14;USP15;US

CEBPA;CENPE;CENPK;CENPQ;CENPW;CERCAM;CHAF1B;CHD9;CHFR;CHMP1A;CKS1B;CLIC4;CLK3;CLN5;CLN6;CLP1;CNPY3;COIL;COL24A1;COL7A1;CPSF1;CPSF6;CRAT;CRBN;CREB1;CROT;CSNK1A1;CTBP1;CTSS;CUL2;CUTC;CXXC1;DACH1;DAPK3;DAXX;DBF4;DCAF13;DCLRE1A;DDB2;DDX11;DDX3X;DDX50;DD

RF2;SOCS5;STT3B;SUMO1;SUMO2;SUMO3;TAF9;TBL1XR1;TOPORS;TRIB2;TRIM13;UBA6;UBC;UBE2A;UBE2D1;UBE2D3;UBE2E1;UBE2G1;UBE2J1;UBE2K;UBE2N;UBE2V2;UBE2W;UBE3A;UBE4A;UBR1;UBR3;UBR5;UCHL3;UCHL5;USP1;USP12;USP14;USP15;USP16;USP19;USP21;USP25;USP31;USP37;USP38;U

A1;PAIP1;PAN3;PAPD4;PAPD5;PARK2;PCNP;PMAIP1;PNRC2;PPP2CB;PPP2R1A;PPP2R2A;PSMA1;PSMA2;PSMA3;PSMA4;PSMA6;PSMA8;PSMB1;PSMB3;PSMC1;PSMC2;PSMC3;PSMC5;PSMC6;PSMD10;PSMD13;PSMD14;PSMD4;PSMD8;PSME1;PTEN;RAB12;RAD23A;RBBP6;RBBP8;RBCK1;RBX1;RCHY1;RNAS

P2;SMURF2;SOCS5;STT3B;SUMO1;SUMO2;SUMO3;TAF9;TBL1XR1;TOPORS;TRIB2;TRIM13;UBA6;UBC;UBE2A;UBE2D1;UBE2D3;UBE2E1;UBE2G1;UBE2J1;UBE2K;UBE2N;UBE2V2;UBE2W;UBE3A;UBE4A;UBR1;UBR3;UBR5;UCHL3;UCHL5;USP1;USP12;USP14;USP15;USP16;USP19;USP21;USP25;USP31;USP37;

1B;CDKN2AIP;CDT1;CEBPA;CENPE;CENPK;CENPQ;CENPW;CERCAM;CHAF1B;CHD9;CHFR;CHMP1A;CKS1B;CLIC4;CLK3;CLN5;CLN6;CLP1;CNPY3;COIL;COL24A1;COL7A1;CPSF1;CPSF6;CRAT;CRBN;CREB1;CROT;CSNK1A1;CTBP1;CTSS;CUL2;CUTC;CXXC1;DACH1;DAPK3;DAXX;DBF4;DCAF13;DCLRE1A;DDB2;DDX1

;PIAS4;PJA2;PSMA1;PSMA2;PSMA3;PSMA4;PSMA6;PSMA8;PSMB1;PSMB3;PSMC1;PSMC2;PSMC3;PSMC5;PSMC6;PSMD10;PSMD13;PSMD14;PSMD4;PSMD8;PSME1;PTEN;RASSF1;RBBP6;RBCK1;RBX1;RCHY1;RFWD2;RING1;RIPK2;RNF103;RNF11;RNF123;RNF13;RNF135;RNF138;RNF139;RNF144B;RNF14

NF217;RNF40;RNF6;SENP1;SIAH1;SIRT1;SIRT2;SKP2;SMURF2;SOCS5;SOCS6;STT3B;SUMO1;SUMO2;SUMO3;TAF9;TBL1XR1;TOPORS;TRIB2;TRIM13;UBA6;UBC;UBE2A;UBE2D1;UBE2D3;UBE2E1;UBE2G1;UBE2J1;UBE2K;UBE2N;UBE2V2;UBE2W;UBE3A;UBE4A;UBR1;UBR3;UBR5;UCHL3;UCHL5;USP1;USP12;U

CDKN1B;CDKN2AIP;CDT1;CEBPA;CENPE;CENPK;CENPQ;CENPW;CERCAM;CHAF1B;CHCHD4;CHD9;CHFR;CHMP1A;CKS1B;CLIC4;CLK3;CLN5;CLN6;CLP1;CNPY3;COIL;COL24A1;COL7A1;CPSF1;CPSF6;CRAT;CRBN;CREB1;CROT;CSNK1A1;CTBP1;CTSS;CUL2;CUTC;CXXC1;CYCS;DACH1;DAPK3;DAXX;DBF4;DCAF13;

B;COPS5;CREBL2;CRLF3;CSNK1A1;CTBP1;CUL2;CUL4B;CUL5;DAPK3;DAXX;DBF4;DCLRE1A;DCTN2;DDX11;DDX3X;DGKZ;DICER1;DIS3L2;DLG1;DMTF1;DNA2;DYNLT3;DYRK1A;E2F4;E2F6;E2F7;ECT2;EHMT2;EID1;EIF4E;ERBB2IP;ERCC2;EREG;ERH;ERN1;ESCO1;ESCO2;FAM175A;FAM32A;FANCM;FBXO31;FBXO

;CDK5R1;CDK5RAP3;CDK7;CDK9;CDKL1;CDKN1B;CDKN1C;CDKN2C;CDKN3;CENPE;CHFR;CHM;CHORDC1;CHUK;CISH;CKS1B;CKS2;CLIP3;CLK1;CLK3;CLK4;CNPPD1;COG3;COL4A3BP;COPS2;COPS4;COPS5;COPS6;CRBN;CREB1;CREBL2;CRIPAK;CRK;CRY1;CSK;CSNK1A1;CSNK1G3;CSNK2B;CSTA;CTBP1;CTDNEP1;

;CDK5R1;CDK5RAP3;CDK7;CDK9;CDKL1;CDKN1B;CDKN1C;CDKN2C;CDKN3;CENPE;CHFR;CHM;CHORDC1;CHUK;CISH;CKS1B;CKS2;CLIP3;CLK1;CLK3;CLK4;CNPPD1;COG3;COL4A3BP;COPS2;COPS4;COPS5;COPS6;CRBN;CREB1;CREBL2;CRIPAK;CRK;CRY1;CSK;CSNK1A1;CSNK1G3;CSNK2B;CSTA;CTBP1;CTDNEP1;

;ARL15;ARL2;ARL4A;ARL5A;ARL5B;ARL8B;ARMC1;ARSA;ARSK;ASAP3;ASGR1;ASNA1;ASPH;ASTL;ATAD1;ATAD2;ATAD2B;ATAD3A;ATF2;ATL2;ATP10D;ATP11B;ATP11C;ATP13A2;ATP13A3;ATP2B1;ATP2C1;ATP6AP1;ATP7A;ATP8A1;ATP8A2;ATP8B4;ATPBD4;ATRX;ATXN7L3;AURKC;B4GALT3;B4GALT6;BAZ1A

XX;DBF4;DCAF13;DCLRE1A;DDB2;DDX11;DDX3X;DDX50;DDX54;DEPDC1;DGCR8;DIS3;DMAP1;DNA2;DNAJB9;DNTTIP2;DPY30;DR1;DYRK1A;E2F4;E2F6;E2F7;EAF2;EED;EIF3E;EIF6;ELL2;ENY2;EPC2;ERCC2;ERCC8;ERI1;ESF1;ESR2;FAM32A;FAM60A;FAM76B;FANCB;FANCL;FANCM;FBXO11;FBXO5;FBXW7;FCF1

DK1;CDK11A;CDK11B;CDK17;CDK18;CDK2;CDK5R1;CDK5RAP3;CDK7;CDK9;CDKL1;CDKN1B;CDKN1C;CDKN2C;CDKN3;CENPE;CHFR;CHM;CHORDC1;CHUK;CISH;CKS1B;CKS2;CLIP3;CLK1;CLK3;CLK4;CNPPD1;COG3;COL4A3BP;COPS2;COPS4;COPS5;COPS6;CRBN;CREB1;CREBL2;CRIPAK;CRK;CRY1;CSK;CSNK1A1

37;CDK1;CDK2;CDK5R1;CDKN1B;CDKN2C;CDT1;CENPE;CENPK;CENPL;CENPQ;CEP135;CEP152;CEP250;CEP290;CEP57;CEP70;CHAF1B;CHMP1B;CHMP2A;CHMP2B;CHN2;CHUK;CIDEB;CISH;CKB;CLIC4;CLNS1A;CMPK1;CNBP;CNOT2;CNOT3;CNOT6;CNOT6L;CNOT7;CNOT8;COL4A3BP;COMT;COPE;COPZ1;CORC

3D;APPBP2;APTX;ARAF;ARF4;ARG2;ARHGAP5;ARL2;ARL8B;ARSA;ARSK;ASMTL;ASNA1;ASNSD1;ASPH;ASTE1;ASTL;ATAD1;ATAD2;ATAD2B;ATAD3A;ATG4A;ATG4B;ATG4C;ATL2;ATP10D;ATP11B;ATP11C;ATP13A2;ATP13A3;ATP2B1;ATP2C1;ATP5C1;ATP5E;ATP5F1;ATP5I;ATP5L;ATP5O;ATP6AP1;ATP6V1G1;A

Z;CENPK;CEP290;CGGBP1;CHAF1A;CHAF1B;CHD1;CHD5;CHD9;CHERP;CHMP1A;CHUK;CHURC1;CKAP2;CLK1;CLK3;CLK4;CLNS1A;CLP1;CNBP;CNOT2;CNOT3;CNOT6;CNOT6L;CNOT7;CNOT8;CNP;COMMD6;COPS2;COPS5;CPSF1;CPSF6;CRABP2;CRCP;CREB1;CREB5;CREBL2;CREBZF;CRK;CRLF3;CRTC2;CRY1;CST

A2;ATP8B4;ATPBD4;ATRX;AUH;AURKC;BACH1;BANF1;BAZ2B;BBS10;BBS12;BBX;BCLAF1;BCR;BDH2;BDP1;BLZF1;BMI1;BMPR1A;BMPR2;BRCA2;BRD9;BRIP1;BTAF1;C10orf2;CAMK1;CAMK2D;CAMK4;CARHSP1;CASP8AP2;CBFB;CBR4;CBWD1;CBWD2;CC2D1A;CCT2;CCT6B;CDC7;CDK1;CDK11A;CDK11B;CDK1

K11A;CDK11B;CDK2;CDK5R1;CDK5RAP3;CDK7;CDK8;CDK9;CDKN1B;CDKN1C;CDT1;CEBPA;CEBPG;CEBPZ;CENPK;CENPQ;CENPW;CEP290;CGGBP1;CHAF1A;CHAF1B;CHD1;CHD5;CHD9;CHERP;CHMP1A;CHUK;CHURC1;CKAP2;CLK1;CLK3;CLK4;CLNS1A;CLP1;CNBP;CNOT2;CNOT3;CNOT6;CNOT6L;CNOT7;CNO

P1;YOD1;ZNF

T1;GPC1;GTF2B;GTF2E1;GZF1;HDAC6;HEPH;HLTF;HMGCL;IDE;IDI1;IGHMBP2;IKZF2;IKZF5;ING3;INTS12;JMJD6;KIN;KLF16;KLF3;KLF5;LANCL1;LIMS1;LIN28A;LNX1;LNX2;LONRF1;LOX;LRSAM1;LTN1;MAN2A1;MAN2B1;MAN2C1;MAP3K1;MARCH1;MARCH5;MARCH6;MARCH7;MATR3;MBNL1;MBNL2;MBTD1

A1;ATP8A2;ATP8B4;ATPBD4;ATRX;AUH;AURKC;BACH1;BANF1;BAZ2B;BBS10;BBS12;BBX;BCLAF1;BCR;BDH2;BDP1;BLZF1;BMI1;BMPR1A;BMPR2;BRCA2;BRD9;BRIP1;BTAF1;C10orf2;CALB1;CAMK1;CAMK2D;CAMK4;CARHSP1;CASP8AP2;CBFB;CBR4;CBWD1;CBWD2;CC2D1A;CCT2;CCT6B;CDC7;CDK1;CDK11A

C1;PSMC2;PSMC3;PSMC5;PSMC6;PSMD10;PSMD13;PSMD14;PSMD4;PSMD8;PSME1;PTEN;RASSF1;RBBP6;RBCK1;RBX1;RCHY1;RFWD2;RING1;RIPK2;RNF103;RNF11;RNF123;RNF13;RNF135;RNF138;RNF139;RNF144B;RNF146;RNF187;RNF19A;RNF2;RNF217;RNF220;RNF31;RNF40;RNF6;RNF7;RYBP;SENP6

B;BBX;BCAS2;BCAT1;BCAT2;BCKDHB;BCLAF1;BDH2;BDP1;BIN1;BIRC2;BLOC1S2;BLZF1;BMI1;BMPR1A;BMPR2;BNIP2;BRCA2;BRCC3;BRD9;BRIP1;BRMS1;BRMS1L;BRWD1;BTAF1;BUD13;BZW1;C10orf2;C12orf52;C14orf166;CACNB4;CALCRL;CAMK4;CARHSP1;CASC5;CASP3;CASP8AP2;CBFB;CBX3;CCBL2;CCD

CNA1H;CALB1;CALML4;CALML6;CALU;CAPN14;CAPS2;CBLL1;CD248;CDADC1;CDC7;CDH26;CDHR1;CDHR5;CDIPT;CDK2;CDK5R1;CEPT1;CETN3;CGRRF1;CHD5;CHFR;CHMP1A;CHN2;CHORDC1;CHPF;CHSY1;CHURC1;CISD1;CISD2;CLEC7A;CLSTN3;CMC1;CNBP;CNOT6;CNOT6L;COMT;COPS5;CPD;CREB5;CSGAI

BDP1;BIRC2;BLOC1S2;BLZF1;BMI1;BMPR1A;BMPR2;BRCA2;BRD9;BRIP1;BRMS1;BRMS1L;BRWD1;BTAF1;BZW1;C10orf2;C12orf52;C14orf166;C1GALT1;CACNA1H;CALCRL;CAMK4;CARHSP1;CASP8AP2;CBFB;CBR4;CBX3;CCDC59;CCDC88A;CCNA2;CCNC;CCNE2;CCNH;CCNL1;CCNT2;CD28;CD80;CD81;CDC25C

N1B;CDKN1C;CDKN2AIP;CDT1;CEBPA;CEBPG;CEBPZ;CENPK;CEP290;CFH;CFP;CGGBP1;CHAF1A;CHAF1B;CHCHD4;CHD1;CHD5;CHD9;CHERP;CHMP1A;CHUK;CHURC1;CKAP2;CLK1;CLK3;CLK4;CLN3;CLN5;CLNS1A;CLP1;CNBP;CNOT2;CNOT3;CNOT6;CNOT6L;CNOT7;CNOT8;COG3;COMMD6;COPS2;COPS5;CPE

C3;PSMC5;PSMC6;PSMD10;PSMD13;PSMD14;PSMD4;PSMD8;PSME1;PTEN;RASSF1;RBBP6;RBCK1;RBX1;RCHY1;RFWD2;RING1;RIPK2;RNF103;RNF11;RNF123;RNF13;RNF135;RNF138;RNF139;RNF144B;RNF146;RNF187;RNF19A;RNF2;RNF217;RNF220;RNF31;RNF40;RNF6;RNF7;RYBP;SIAH1;SIRT1;SKP2;SM

C40;CDC7;CDC73;CDCA7;CDK1;CDK11A;CDK11B;CDK2;CDK5R1;CDK5RAP3;CDK7;CDK8;CDK9;CDKN1B;CDKN1C;CDT1;CEBPA;CEBPG;CEBPZ;CENPK;CEP290;CERCAM;CGGBP1;CHAF1A;CHAF1B;CHD1;CHD5;CHD9;CHMP1A;CHPF;CHPF2;CHST13;CHST14;CHSY1;CHUK;CHURC1;CKAP2;CLIP3;CLP1;CNBP;CNOT

4;PSMD8;PSME1;PTEN;RAB12;RAD23A;RBBP6;RBCK1;RBX1;RCHY1;RNF103;RNF11;RNF144B;RNF146;RNF187;RNF217;RNF40;RNF6;SENP1;SIAH1;SIRT1;SIRT2;SKP2;SMAD4;SMURF2;SOCS5;SOCS6;STT3B;SUMO1;SUMO2;SUMO3;TAF9;TBL1XR1;TIMP1;TOPORS;TPRKB;TRIB2;TRIM13;UBA6;UBC;UBE2A;UBE

CCNE2;CCNH;CCNL1;CCNT2;CD28;CD80;CDC25C;CDC40;CDC7;CDC73;CDCA7;CDK1;CDK11A;CDK11B;CDK2;CDK5R1;CDK5RAP3;CDK7;CDK8;CDK9;CDKN1B;CDKN1C;CDT1;CEBPA;CEBPG;CEBPZ;CENPK;CEP290;CERCAM;CGGBP1;CHAF1A;CHAF1B;CHD1;CHD5;CHD9;CHMP1A;CHPF;CHPF2;CHST13;CHST14;CH

C3;BRD9;BRIP1;BRMS1;BRMS1L;BRWD1;BTAF1;BUD13;BZW1;C10orf2;C12orf52;C14orf166;CACNB4;CALCRL;CAMK4;CARHSP1;CASC5;CASP3;CASP8AP2;CBFB;CBX3;CCDC59;CCDC88A;CCNA2;CCNC;CCNE2;CCNH;CCNL1;CCNT2;CD28;CD2BP2;CD80;CDC25C;CDC40;CDC42EP2;CDC7;CDC73;CDCA7;CDK1;CDK

RID2;ARID4B;ARID5A;ARID5B;ARL13B;ARL2;ARL4A;ARL6IP1;ARL6IP5;ARL8B;ARPC4;ARPP19;ARRB1;ARRDC3;ARVCF;ASAP3;ASB13;ASF1A;ASGR1;ASGR2;ASPH;ASPM;ATAD2B;ATF2;ATG12;ATG4B;ATG5;ATG9A;ATL2;ATP10D;ATP11B;ATP11C;ATP13A2;ATP2B1;ATP5F1;ATP5O;ATP6V1G1;ATP7A;ATP8A1;ATP

NEDD1;NEK1;NEK7;NFU1;NIN;NME7;NUDCD2;NUDT21;ODF2L;PAN3;PAX2;PCGF5;PDCD6IP;PDE4D;PIBF1;PKD2;PLK1S1;PLK4;POC1B;POLR3H;PPP1R12A;PPP2R3C;PPP4C;PPP4R2;PRKACB;PSEN2;PSKH1;PSMA1;PTPN23;RAB3IP;RASSF1;RBBP6;RBM39;RELB;RFWD2;RIC8B;RNF19A;ROCK1;RPGR;RPS7;RSG1;

CAT2;BCLAF1;BDH2;BDP1;BIRC2;BLOC1S2;BLZF1;BMI1;BMPR1A;BMPR2;BRCA2;BRD9;BRIP1;BRMS1;BRMS1L;BRWD1;BTAF1;BZW1;C10orf2;C12orf52;C14orf166;C1GALT1;CACNA1H;CALCRL;CAMK4;CARHSP1;CASP8AP2;CBFB;CBR4;CBX3;CCDC59;CCDC88A;CCNA2;CCNC;CCNE2;CCNH;CCNL1;CCNT2;CD28

2B;BBX;BCAT1;BCAT2;BCLAF1;BDH2;BDP1;BIRC2;BLOC1S2;BLZF1;BMI1;BMPR1A;BMPR2;BRCA2;BRD9;BRIP1;BRMS1;BRMS1L;BRWD1;BTAF1;BZW1;C10orf2;C12orf52;C14orf166;C1GALT1;CACNA1H;CALCRL;CAMK4;CARHSP1;CASP8AP2;CBFB;CBR4;CBX3;CCBL2;CCDC59;CCDC88A;CCNA2;CCNC;CCNE2;CC

;FOXN2;FRG1;FYTTD1;GATAD2A;GEMIN2;GEMIN6;GMNN;GNL3;GPS2;GRHL1;GTF2B;GTF2E1;GTF2E2;GTF2F2;GTF2H1;GTF2H4;GZF1;HAT1;HDAC3;HDAC5;HDAC6;HDAC7;HDAC8;HDAC9;HIRA;HMGB1;HMGB2;HMGN5;HNF1A;HNRNPH2;HNRNPL;ING3;INO80C;INTS12;INTS2;INTS5;INTS6;INTS8;ITGB3BP;I

1;CACNA1H;CALB1;CALML4;CALML6;CALU;CAPN14;CAPS2;CBLL1;CD248;CDADC1;CDC7;CDH26;CDHR1;CDHR5;CDIPT;CDK2;CDK5R1;CEPT1;CETN3;CGRRF1;CHD5;CHFR;CHID1;CHMP1A;CHN2;CHORDC1;CHPF;CHSY1;CHURC1;CISD1;CISD2;CLEC7A;CLSTN3;CMC1;CNBP;CNOT6;CNOT6L;COMT;COPS5;CPD;C

COG3;COG4;COG6;COL4A3BP;COPE;COPZ1;CORO1A;CORO7;CSGALNACT1;CSGALNACT2;CST3;CTDNEP1;CYBA;CYHR1;CYP2S1;CYP2U1;CYP2W1;CYP4F22;DDOST;DEGS1;DEGS2;DENND4C;DERL1;DHRS9;DLG1;DLG4;DMPK;DNAJB9;DOLPP1;DPM1;DTNBP1;DTX2;EBAG9;EDEM3;EIF5A2;ELOVL4;ENTPD6;EPN1

;PAPD4;PAPOLA;PAPOLG;PCF11;PES1;PHAX;PHF5A;PIN4;PLRG1;POLR2E;POLR2K;PPIG;PPIL3;PPP2R1A;PPP4R2;PPWD1;PRPF18;PRPF31;PRPF38B;PRPF39;PRPF40A;PRPF4B;PRPF6;PSIP1;PTBP2;PUF60;PUS10;PUS7L;QKI;RALY;RBFOX3;RBM10;RBM11;RBM25;RBM27;RBM3;RBM39;RBM4;RBM5;RBMS1;RN

1;PQBP1;PRMT3;PRPF18;PRPF31;PRPF38B;PRPF4B;PRPF6;PSMA1;PSMA2;PSMA4;PSMA6;PSMC2;PSMC3;PTBP2;PUF60;PUM2;RALY;RBM3;RBM4;RBM5;RHEB;RNPC3;RPL11;RPL15;RPL17;RPL22L1;RPL23;RPL26;RPL26L1;RPL27;RPL30;RPL31;RPL34;RPL35;RPL36A;RPL36AL;RPL39;RPL39L;RPL41;RPL7;RPL9;

IN1;B3GNT1;B3GNT2;B4GALT3;B4GALT6;BACH1;BANF1;BAZ1A;BAZ2B;BBX;BCAS2;BCAT1;BCAT2;BCKDHB;BCLAF1;BDH2;BDP1;BIN1;BIRC2;BLOC1S2;BLZF1;BMI1;BMPR1A;BMPR2;BNIP2;BRCA2;BRCC3;BRD9;BRIP1;BRMS1;BRMS1L;BRWD1;BTAF1;BTD;BUD13;BZW1;C10orf2;C12orf52;C14orf166;CACNB4;

R1A;BMPR2;BNIP2;BRCA2;BRCC3;BRD9;BRIP1;BRMS1;BRMS1L;BRWD1;BTAF1;BUD13;BZW1;C10orf2;C12orf52;C14orf166;CACNB4;CALCRL;CAMK4;CARHSP1;CASC5;CASP3;CASP8AP2;CBFB;CBX3;CCBL2;CCDC59;CCDC88A;CCNA2;CCNC;CCNE2;CCNH;CCNL1;CCNT2;CD28;CD2BP2;CD80;CDC25C;CDC40;CD

;UBE2T;UBE2V2;UBE2W;UBE3A;UBE4A;UBOX5;UBR1;UBR3;UBR5;WWP1;ZNF738;ZN

C10;ANG;ANGPT1;ANGPTL4;ANGPTL7;ANKRD13C;ANKRD49;ANLN;ANXA1;AP1AR;AP1B1;AP1S2;AP2A2;AP3M1;AP3S2;APC;APEX1;APEX2;APH1A;API5;APIP;APLF;APOBEC3D;APPBP2;APPL1;APTX;AQP11;ARAF;ARAP2;ARF4;ARFGAP1;ARFGAP2;ARFGEF2;ARFIP1;ARG2;ARGLU1;ARHGAP10;ARHGAP11A;ARH

A;KIF20B;KIF22;KIF23;KIF2A;KIFC1;KLHL9;KPNA2;LIN52;LIN54;LIN9;LRRCC1;LRWD1;LSM11;MAD2L1;MAP3K11;MAP9;MCM5;MDK;MDM2;MIS18BP1;MLF1IP;MNAT1;MPHOSPH6;MTBP;MUC1;NBN;NDE1;NEDD1;NEK1;NEK7;NFATC1;NSL1;NSMCE2;NUDC;NUF2;NUP37;NUP85;OIP5;ORC2;ORC3;ORC4;PAP

RK2;LRSAM1;LSM1;LSM3;LSM6;MAD2L1;MDM2;MGEA5;MNAT1;MTMR2;NDFIP1;NEDD4;NGLY1;NPLOC4;NUDT16;OMA1;OSBPL7;PAIP1;PAN3;PAPD4;PAPD5;PARK2;PCNP;PHKG2;PMAIP1;PNRC2;PPP1CA;PPP1CB;PPP1R3D;PPP2CB;PPP2R1A;PPP2R2A;PPP2R3A;PSMA1;PSMA2;PSMA3;PSMA4;PSMA6;PSM

WWP1;ZNF738;ZNF

3;PSMC1;PSMC2;PSMC3;PSMC5;PSMC6;PSMD10;PSMD13;PSMD14;PSMD4;PSMD8;PSME1;PTBP2;PUF60;QKI;RALY;RBFOX3;RBM10;RBM11;RBM25;RBM27;RBM39;RBM4;RBM5;RNGTT;RNH1;RNPC3;RPL11;RPL15;RPL17;RPL23;RPL26;RPL26L1;RPL27;RPL30;RPL31;RPL34;RPL35;RPL36A;RPL39;RPL41;RPL

2;BNIP2;BRCA2;BRCC3;BRD9;BRIP1;BRMS1;BRMS1L;BRWD1;BTAF1;BUD13;BZW1;C10orf2;C12orf52;C14orf166;CACNB4;CALCRL;CAMK4;CARHSP1;CASC5;CASP3;CASP8AP2;CBFB;CBX3;CCBL2;CCDC59;CCDC88A;CCNA2;CCNC;CCNE2;CCNH;CCNL1;CCNT2;CD28;CD2BP2;CD80;CDC25C;CDC40;CDC42EP2;CD

RNF31;RNF40;RNF6;RNF7;SIAH1;SKP2;SLC27A1;SLC27A3;SLC27A4;SMURF2;SUCLA2;SUCLG2;SUMO1;SUMO3;TARS2;TOPORS;TRAF7;TRIM11;TRIM13;TRIM2;TRIM23;TRIM28;TRIM33;TRIM39;TRIM41;TRIM59;TTLL1;TTLL12;UBA2;UBA3;UBA6;UBE2A;UBE2D1;UBE2D3;UBE2E1;UBE2G1;UBE2J1;UBE2J2;UB

4;CAPN14;CAPN7;CARD17;CASP3;CASP8;CBLL1;CCDC88A;CCNA2;CCNC;CCND3;CCNE2;CCNG1;CCNH;CCNL1;CCNT2;CCNY;CCNYL1;CCT2;CCT6B;CD28;CD46;CD80;CD81;CDC25C;CDC26;CDC27;CDC37;CDC73;CDK1;CDK11A;CDK11B;CDK17;CDK18;CDK2;CDK5R1;CDK5RAP3;CDK7;CDK9;CDKL1;CDKN1B;CDKN

P4R2;PRKACB;PSEN2;PSMA1;RAB3IP;RBM39;RFWD2;RIC8B;RNF19A;ROCK1;RPGR;SASS6;SCLT1;SLAIN2;SLC9A3R1;SMAD4;TNKS2;TOPORS;TRNT1;TUBD1;TUBE1;TUBG1;TUBG2;TUBGCP2;TXNDC9;VPS37A;WDR35;WRAF

DK11A;CDK11B;CDK17;CDK18;CDK2;CDK7;CDK8;CDK9;CDKL1;CDKL2;CENPE;CHD1;CHD5;CHD9;CHFR;CHUK;CKB;CLCN7;CLK1;CLK3;CLK4;CLP1;CMPK1;CNP;COASY;CPEB2;CPEB3;CPEB4;CPSF6;CRCP;CRY1;CSK;CSNK1A1;CSNK1A1L;CSNK1G3;CSTF2T;CTBP1;DACH1;DAK;DAPK3;DAZAP1;DCAKD;DCK;DDX11;D

E2J2;UBE2K;UBE2M;UBE2N;UBE2O;UBE2Q2;UBE2T;UBE2V2;UBE2W;UBE3A;UBE4A;UBOX5;UBR1;UBR3;UBR5;WWP1;ZNF738;ZN

FBXO31;FBXO5;FBXW7;FGFR1OP;GAS2;GAS2L2;GAS6;GMNN;GORASP1;GPR132;HAUS1;HAUS3;HAUS6;HAUS7;HDAC3;HDAC8;HELLS;HORMAD1;HSF1;IL12A;IL8;ILK;ITGB3BP;JMY;KAT2B;KATNB1;KIAA1009;KIF11;KIF18A;KIF20B;KIF22;KIF23;KIF2A;KIFC1;KLHDC3;KLHL9;KPNA2;LEPREL4;LFNG;LRRCC1;LRWD

DK11A;CDK11B;CDK17;CDK18;CDK2;CDK7;CDK8;CDK9;CDKL1;CDKL2;CENPE;CHD1;CHD5;CHD9;CHFR;CHUK;CKB;CLCN7;CLK1;CLK3;CLK4;CLP1;CMPK1;CNP;COASY;CPEB2;CPEB3;CPEB4;CPSF6;CRCP;CRY1;CSK;CSNK1A1;CSNK1A1L;CSNK1G3;CSTF2T;CTBP1;DACH1;DAK;DAPK3;DAZAP1;DCAKD;DCK;DDX11;D

1;HOOK2;IFT122;IFT140;IFT20;IFT57;IFT74;IFT80;IFT81;IFT88;KATNB1;KEAP1;KIAA1009;KIAA1731;KIF11;KIF18A;KIF20B;KIF22;KIF23;KIF27;KIF2A;KIF3A;KIF5B;KIFC1;KIFC3;KLC4;LANCL1;LRRCC1;LYST;MAD2L1;MAP2K2;MAP2K5;MAP3K11;MAP9;MARK4;MAST2;MIB1;MID1IP1;MKKS;MKS1;MLF1IP;MZT1;N

C;CCND3;CCNH;CCNY;CCT2;CD2AP;CD2BP2;CD48;CDC14A;CDC26;CDC27;CDC37;CDC40;CDC73;CDK1;CDK2;CDK5R1;CDK7;CDK8;CDK9;CEBPA;CENPE;CENPK;CENPW;CEP170;CEP19;CEP250;CEP290;CEP350;CEP44;CEP57;CEP57L1;CEP95;CGN;CHAF1A;CHAF1B;CHM;CHML;CHUK;CISD2;CKAP2;CLCN2;CLIC2

L;EDEM3;EIF5A2;ELOVL4;EPM2AIP1;EPT1;ERGIC2;ERLEC1;ERLIN1;ERN1;ERO1L;ERO1LB;EXTL2;FAM134A;FAM134B;FAM172A;FAM69A;FAR2;FBXW7;FDFT1;FGFR4;FITM1;FKBP10;FKBP7;FMO5;FNDC3B;G6PC3;GAS6;GBA2;GOLT1B;GPSM1;GRIP1;GSTM2;HACE1;HLA-A;HLA-DPB1;HLA-DRA;HMOX2;HPS6;HS

P1;CASC5;CASP3;CASP8AP2;CBFB;CBX3;CCDC59;CCDC88A;CCNA2;CCNC;CCND3;CCNE2;CCNG1;CCNH;CCNL1;CCNT2;CCNY;CCNYL1;CD28;CD2BP2;CD80;CD81;CDC25C;CDC26;CDC27;CDC37;CDC42EP2;CDC73;CDCA7;CDK1;CDK11A;CDK11B;CDK2;CDK5R1;CDK5RAP3;CDK7;CDK8;CDK9;CDKN1B;CDKN1C;CDK

;NCS1;NDFIP1;NDFIP2;NOS3;NOTCH3;NRAS;PAQR3;PDCD10;PGAP3;PIKFYVE;PITPNM1;PJA2;PNPLA8;PSEN2;RAB12;RAB14;RAB2A;RAB2B;RAB33B;RAB6A;RER1;RFNG;RHOU;RNF13;ROCK1;SACM1L;SAR1B;SCAMP3;SCAP;SCFD1;SCOC;SCYL1;SEC23A;SEC24A;SGMS1;SGMS2;SLC30A5;SLC30A6;SLC30A7;SLC

CBFB;CBX3;CCDC59;CCDC88A;CCNA2;CCNC;CCND3;CCNE2;CCNG1;CCNH;CCNL1;CCNT2;CCNY;CCNYL1;CD28;CD46;CD80;CD81;CDC25C;CDC26;CDC27;CDC37;CDC42EP2;CDC73;CDCA7;CDK1;CDK11A;CDK11B;CDK2;CDK5R1;CDK5RAP3;CDK7;CDK8;CDK9;CDKN1B;CDKN1C;CDKN2C;CDKN3;CDT1;CEBPA;CEBP
;PKN1;PKN2;PLK4;PPP4C;PRKAA1;PRKAB2;PRKACB;PRKCI;PRKCZ;PRKD3;PRPF4B;PSKH1;PTK6;RABEP1;RIOK2;RIPK2;RIPK3;ROCK1;RPS6KA3;RPS6KA4;RPS6KA5;RPS6KB1;SLK;SMG1;SNRK;STK10;STK17A;STK17B;STK32C;STK38L;STRADA;TAOK1;TBK1;TESK1;TESK2;TGFBR1;TGFBR2;TLK1;TRPM7;TTK;UHMK1;
)1;HSF1;ITGB3BP;KATNB1;KIAA1009;KIF11;KIF18A;KIF20B;KIF22;KIF23;KIF2A;KIFC1;KLHDC3;KLHL9;KPNA2;LEPREL4;LFNG;LRRCC1;LRWD1;LSM11;MAD2L1;MAP3K11;MAP9;MCM5;MDK;MDM2;MIS18BP1;MLF1IP;MNAT1;MPHOSPH6;MSH2;MTBP;MUC1;NBN;NDE1;NEDD1;NEK1;NEK7;NFATC1;NSL1;NSM
C1S2;BLZF1;BMI1;BMPR1A;BMPR2;BNIP2;BRCA2;BRCC3;BRD9;BRIP1;BRMS1;BRMS1L;BRWD1;BTAF1;BUD13;BZW1;C10orf2;C12orf52;C14orf166;CACNA1H;CACNB4;CALCRL;CAMK4;CARHSP1;CASC5;CASP3;CASP8AP2;CBFB;CBX3;CCBL2;CCDC59;CCDC88A;CCNA2;CCNC;CCNE2;CCNH;CCNL1;CCNT2;CD28;C
CSGALNACT2;CSK;CSNK1A1;CSNK1A1L;CSNK1G3;CSNK2B;CXXC1;DAK;DAPK3;DCAKD;DCK;DCTN6;DDOST;DGKE;DGKZ;DHPS;DLAT;DLG1;DMPK;DOT1L;DPM1;DSEL;DYRK1A;DYRK1B;DYRK2;EED;EFNA3;EHMT2;EIF2B5;ELOVL4;ENG;EPHB3;EPHB6;EPT1;ERCC2;ERN1;ESCO1;ESCO2;ETNK1;EXTL2;FAM20C;FASTI
1;CBWD2;CCT2;CCT6B;CDC7;CDIPT;CDK1;CDK11A;CDK11B;CDK17;CDK18;CDK2;CDK7;CDK8;CDK9;CDKL1;CDKL2;CENPE;CHD1;CHD5;CHD9;CHFR;CHUK;CKB;CLCN7;CLK1;CLK3;CLK4;CLP1;CMPK1;CNP;COASY;CPEB2;CPEB3;CPEB4;CPSF6;CRABP2;CRCP;CRY1;CSK;CSNK1A1;CSNK1A1L;CSNK1G3;CSTF2T;CTBP1
1;CHD5;CHD9;CHUK;CKB;CLCN7;CLK1;CLK3;CLK4;CLP1;CMPK1;COASY;CSK;CSNK1A1;CSNK1A1L;CSNK1G3;DAK;DAPK3;DCAKD;DCK;DDX11;DDX3X;DDX41;DDX50;DDX54;DDX59;DGKE;DGKZ;DHX30;DHX36;DHX40;DICER1;DMPK;DNA2;DNAH6;DNAJA1;DNAJC27;DYRK1A;DYRK1B;DYRK2;EEF1A1;EEFSEC;EIF5

;BRMS1L;BRWD1;BTAF1;BZW1;C12orf52;C14orf166;CAB39;CAB39L;CALCRL;CAMK1;CAMK4;CARD8;CARHSP1;CASC5;CASP3;CASP8;CASP8AP2;CBFB;CBX3;CCDC59;CCDC88A;CCNA2;CCNC;CCND3;CCNE2;CCNG1;CCNH;CCNL1;CCNT2;CCNY;CCNYL1;CD27;CD28;CD2BP2;CD46;CD80;CD81;CDC25C;CDC26;CD(
L1;CDKL2;CENPE;CHD1;CHD5;CHD9;CHUK;CKB;CLCN7;CLK1;CLK3;CLK4;CLP1;CMPK1;COASY;CSK;CSNK1A1;CSNK1A1L;CSNK1G3;DAK;DAPK3;DCAKD;DCK;DDX11;DDX3X;DDX41;DDX50;DDX54;DDX59;DGKE;DGKZ;DHX30;DHX36;DHX40;DICER1;DMPK;DNA2;DNAH6;DNAJA1;DNAJC27;DYRK1A;DYRK1B;DYRK
PE;CHD1;CHD5;CHD9;CHUK;CKB;CLCN7;CLK1;CLK3;CLK4;CLP1;CMPK1;COASY;CSK;CSNK1A1;CSNK1A1L;CSNK1G3;DAK;DAPK3;DCAKD;DCK;DDX11;DDX3X;DDX41;DDX50;DDX54;DDX59;DGKE;DGKZ;DHX30;DHX36;DHX40;DICER1;DMPK;DNA2;DNAH6;DNAJA1;DNAJC27;DYRK1A;DYRK1B;DYRK2;EEF1A1;EEFS
K9;CDKL1;CDKL2;CENPE;CHD1;CHD5;CHD9;CHUK;CKB;CLCN7;CLK1;CLK3;CLK4;CLP1;CMPK1;COASY;CSK;CSNK1A1;CSNK1A1L;CSNK1G3;DAK;DAPK3;DCAKD;DCK;DDX11;DDX3X;DDX41;DDX50;DDX54;DDX59;DGKE;DGKZ;DHX30;DHX36;DHX40;DICER1;DMPK;DNA2;DNAH6;DNAJA1;DNAJC27;DYRK1A;DYRK1
CENPW;CEP120;CEP135;CEP152;CEP250;CEP57;CEP57L1;CETN3;CHAF1A;CHAF1B;CHCHD4;CHD1;CHD5;CHD9;CHFR;CHMP1A;CHORDC1;CKAP2;CKS2;CLIC4;CLIP3;CLN3;CLN5;CLN6;CNN3;CNP;CNTROB;COG4;COL4A3BP;COPE;COPZ1;CORO1A;CRIPAK;CRIPT;CRK;CSNK1A1;CTBP1;CTDNEP1;CXADR;CXXC1;CY
PE;CHD1;CHD5;CHD9;CHUK;CKB;CLCN7;CLK1;CLK3;CLK4;CLP1;CMPK1;COASY;CSK;CSNK1A1;CSNK1A1L;CSNK1G3;DAK;DAPK3;DCAKD;DCK;DDX11;DDX3X;DDX41;DDX50;DDX54;DDX59;DGKE;DGKZ;DHX30;DHX36;DHX40;DICER1;DMPK;DNA2;DNAH6;DNAJA1;DNAJC27;DYRK1A;DYRK1B;DYRK2;EEF1A1;EEFS
PE;CHD1;CHD5;CHD9;CHUK;CKB;CLCN7;CLK1;CLK3;CLK4;CLP1;CMPK1;COASY;CSK;CSNK1A1;CSNK1A1L;CSNK1G3;DAK;DAPK3;DCAKD;DCK;DDX11;DDX3X;DDX41;DDX50;DDX54;DDX59;DGKE;DGKZ;DHX30;DHX36;DHX40;DICER1;DMPK;DNA2;DNAH6;DNAJA1;DNAJC27;DYRK1A;DYRK1B;DYRK2;EEF1A1;EEFS
DC25C;CDC26;CDC27;CDC37;CDC73;CDCA7;CDK1;CDK11A;CDK11B;CDK2;CDK5R1;CDK5RAP3;CDK7;CDK8;CDK9;CDKN1B;CDKN1C;CDKN2AIP;CDKN2C;CDKN3;CDT1;CEBPA;CEBPG;CEBPZ;CENPE;CENPK;CEP290;CFH;CFP;CGGBP1;CHAF1A;CHAF1B;CHD1;CHD5;CHD9;CHMP1A;CHORDC1;CHUK;CHURC1;CKAP
1;HOOK2;IBTK;INPP5K;INSIG1;IPO11;IPO13;IPO4;JAK2;JUN;JUP;KATNB1;KIAA1033;KIF3A;KIF5B;KLHL2;KPNA2;KPNA3;KPNA5;LLGL1;LMAN1;LMNA;LRP6;LYST;MALT1;MAP2K2;MCU;MDFIC;MDM2;MFF;MGEA5;MKKS;MON2;MRPL18;MTMR2;MTX2;MTX3;NAGPA;NAPB;NAPG;NCKIPSD;NDE1;NEDD4;NEMF
PE;CHD1;CHD5;CHD9;CHUK;CKB;CLCN7;CLK1;CLK3;CLK4;CLP1;CMPK1;COASY;CSK;CSNK1A1;CSNK1A1L;CSNK1G3;DAK;DAPK3;DCAKD;DCK;DDX11;DDX3X;DDX41;DDX50;DDX54;DDX59;DGKE;DGKZ;DHX30;DHX36;DHX40;DICER1;DMPK;DNA2;DNAH6;DNAJA1;DNAJC27;DYRK1A;DYRK1B;DYRK2;EEF1A1;EEFS
2L2;SLU7;SMNDC1;SNRNP27;SNRNP35;SNRNP48;SNRPB;SNRPB2;SNRPD1;SNRPE;SNRPG;SREK1;SREK1IP1;SRSF10;SRSF11;SRSF3;SRSF6;SRSF8;SSU72;SUGP1;SUPT5H;SYMPK;THOC3;THOC7;TIA1;TRA2A;TRA2B;TXNL4B;U2AF1;WBP4;WTAP;XAB2;ZCCHC8;ZCRB1;ZNF326;ZRAN
PE;CHD1;CHD5;CHD9;CHUK;CKB;CLCN7;CLK1;CLK3;CLK4;CLP1;CMPK1;COASY;CSK;CSNK1A1;CSNK1A1L;CSNK1G3;DAK;DAPK3;DCAKD;DCK;DDX11;DDX3X;DDX41;DDX50;DDX54;DDX59;DGKE;DGKZ;DHX30;DHX36;DHX40;DICER1;DMPK;DNA2;DNAH6;DNAJA1;DNAJC27;DYRK1A;DYRK1B;DYRK2;EEF1A1;EEFS
NOT3;CNOT6;CNOT7;CNOT8;COMMD6;COPS2;COPS5;CPEB2;CPEB3;CRABP2;CREB1;CREB5;CREBL2;CREBZF;CRK;CRLF3;CRTC2;CRY1;CST3;CTBP1;CTDSP1;CUL2;CXXC1;DAB2IP;DACH1;DAPK3;DAXX;DBP;DDX3X;DDX41;DDX54;DEK;DENND4A;DEPDC1;DICER1;DKK3;DLG4;DMAP1;DMTF1;DNA2;DNTTIP2;DPY

1L1;DNASE2;DOT1L;DR1;DRAP1;DUSP11;DZIP3;E2F4;E2F6;E2F7;EBF4;EEF1A1;EEF1B2;EEFSEC;EIF1AX;EIF2A;EIF2B5;EIF2S2;EIF3D;EIF3E;EIF3J;EIF4E;EIF4E2;EIF4E3;EIF5;EIF5A2;EIF6;ELF2;ELK4;ENDOG;ERAL1;ERCC2;ERI1;ERI2;ESR2;EWSR1;FAM200A;FAM200B;FANCM;FARS2;FARSA;FDXACB1;FIZ1;FMR1;FO
A2;PSMA3;PSMA4;PSMA6;PSMA8;PSMB1;PSMB3;PSMC1;PSMC2;PSMC3;PSMC5;PSMC6;PSMD10;PSMD13;PSMD14;PSMD4;PSMD8;PSME1;RAD17;RAD23A;RAD51AP1;RAD51B;RAD51C;RASSF1;RBBP8;RBX1;RECQL;RECQL5;REV1;REV3L;RFC2;RFC3;RFC5;RFWD2;RIF1;RINT1;RPS27L;RRM2B;RUVBL2;SESN1
3P;CNOT2;CNOT3;CNOT6;CNOT7;CNOT8;COMMD6;COPS2;COPS5;CPEB2;CPEB3;CRABP2;CREB1;CREB5;CREBL2;CREBZF;CRK;CRLF3;CRTC2;CRY1;CST3;CTBP1;CTDSP1;CUL2;CXXC1;DAB2IP;DACH1;DAPK3;DAXX;DBP;DDX3X;DDX41;DDX54;DEK;DENND4A;DEPDC1;DICER1;DKK3;DLG4;DMAP1;DMTF1;DNA2;D

D1;CHD5;CHD9;CHMP1A;CHUK;CHURC1;CKAP2;CNBP;CNOT2;CNOT3;CNOT6;CNOT7;CNOT8;COMMD6;COPS2;COPS5;CPEB2;CPEB3;CRABP2;CREB1;CREB5;CREBL2;CREBZF;CRK;CRLF3;CRTC2;CRY1;CST3;CTBP1;CTDNEP1;CTDSP1;CUL2;CXXC1;DAB2IP;DACH1;DAPK3;DAXX;DBP;DDX3X;DDX41;DDX54;DEK;DI
MED21;MED22;MED23;MED26;MED28;MED4;MED7;MED8;MED9;MEF2C;MEN1;MIER1;MINA;MMS22L;MNAT1;MORC3;MSL2;MSL3;NAA16;NFATC1;NFYB;NOC3L;NOP58;NR4A1;NR4A3;NRIP1;NSMCE2;NUDT21;OIP5;PBX3;PCF11;PELP1;PEX2;PHAX;PHF17;PHF5A;PIAS4;PLCB1;PLRG1;PMF1;PNISR;POLR2E
OC8;EXPH5;F2RL1;GAS6;GCC2;GDI1;GIPC1;GOLGA4;GOLT1B;GOPC;GORASP1;GPAM;GRPEL2;HDAC6;HGS;HLA-DMA;HOOK1;HOOK2;INPP5K;IPO11;IPO13;IPO4;JAK2;JUN;JUP;KATNB1;KIF18A;KIF5B;KLHL2;KPNA2;KPNA3;KPNA5;LIN7A;LIN7C;LMAN2;LMNA;LRSAM1;LRWD1;LYST;MCOLN1;MDFIC;MDM2;MF
AG1;STAG2;TAOK1;TGFA;TTK;TTYH1;TXNL4B;UBE2D1;UBE2E1;USP16;USP37;USP44;VCPIP1;VRK1;WAPAL;WEE1;XPO1;ZNF830;ZWIL(
AG1;STAG2;TAOK1;TGFA;TTK;TTYH1;TXNL4B;UBE2D1;UBE2E1;USP16;USP37;USP44;VCPIP1;VRK1;WAPAL;WEE1;XPO1;ZNF830;ZWIL(
GAK;GALNT1;GALNT3;GALNT4;GALNT7;GAS6;GCC2;GCNT1;GCNT2;GCNT4;GKAP1;GLA;GLCE;GOLGA4;GOLPH3L;GOLT1B;GOPC;GORAB;GORASP1;GPC1;GPSM1;GRHL1;GXYLT1;HACE1;HDAC5;HLA-A;HLA-DPB1;HLA-DRA;HS2ST1;HS6ST1;IER3IP1;IFT20;IFT57;IL15;IMPAD1;KIFC3;KLF5;KLHL20;LAPTM4A;LDLR;
7;CNOT8;COMMD6;COPS2;COPS5;CRABP2;CREB1;CREB5;CREBL2;CREBZF;CRK;CRLF3;CRTC2;CRY1;CTBP1;CTDSP1;CUL2;CXXC1;DAB2IP;DACH1;DAPK3;DAXX;DBP;DDX3X;DDX41;DDX54;DEK;DENND4A;DEPDC1;DICER1;DKK3;DLG4;DMAP1;DMTF1;DNTTIP2;DPY30;DR1;DRAP1;DYRK1B;E2F4;E2F6;E2F7;EAF2;
2;SKA2;SMC2;SMC4;SMC5;SPAG5;SSSCA1;STAG1;STAG2;TAOK1;TGFA;TTK;TTYH1;TXNL4B;UBE2D1;UBE2E1;USP16;USP37;USP44;VCPIP1;VRK1;WAPAL;WEE1;XPO1;ZNF830;ZWIL(
RPS27L;RPTOR;RRP8;SART1;SESN1;SKIL;STRADA;TAOK1;TFAP4;TGFBR1;TIPRL;TP53INP1;TP63;TRIAP1;TSC2;TTK;UBC;UBE2D1;UBE2E1;UHMK1;USP44;WEE1;ZWIL
CHAF1B;CHD1;CHD5;CHD9;CHMP1A;CHUK;CHURC1;CKAP2;CNBP;CNOT2;CNOT3;CNOT6;CNOT7;CNOT8;COMMD6;COPS2;COPS5;CPEB2;CPEB3;CRABP2;CREB1;CREB5;CREBL2;CREBZF;CRK;CRLF3;CRTC2;CRY1;CST3;CTBP1;CTDNEP1;CTDSP1;CUL2;CXXC1;DAB2IP;DACH1;DAPK3;DAXX;DBP;DDX3X;DDX41;DD

T3;CNOT6;CNOT7;CNOT8;COMMD6;COPS2;COPS5;CRABP2;CREB1;CREB5;CREBL2;CREBZF;CRK;CRLF3;CRTC2;CRY1;CTBP1;CTDSP1;CUL2;CXXC1;DAB2IP;DACH1;DAPK3;DAXX;DBP;DDX3X;DDX41;DDX54;DEK;DENND4A;DEPDC1;DICER1;DKK3;DLG4;DMAP1;DMTF1;DNTTIP2;DPY30;DR1;DRAP1;DYRK1B;E2F4;

SPAG5;SSSCA1;STAG1;STAG2;TAOK1;TGFA;TTK;TTYH1;TXNL4B;UBE2D1;UBE2E1;USP16;USP37;USP44;VCPIP1;VRK1;WAPAL;WEE1;XPO1;ZNF830;ZWIL(
X7B;COX7C;CRAT;CRLS1;CRY1;CXorf23;CYBA;CYCS;DBI;DCAKD;DCPS;DDX3X;DECR1;DEGS1;DHFRL1;DHRS1;DHX30;DLAT;DMPK;DNA2;DNAJC15;DNAJC27;ECH1;ECHS1;EEFSEC;EFHA1;ELAC2;ENDOG;ERAL1;ESR2;ETFA;FARS2;FASTKD1;FASTKD3;FDPS;FOXRED1;FUNDC2;GBAS;GCSH;GFM2;GK;GLRX;GLRX2;G

GAT1;MGAT2;MGAT4A;MST4;MUC1;MUC20;MUC5B;MUC6;NAA60;NAGPA;NCS1;NDFIP1;NDFIP2;NOS3;NOTCH3;NRAS;PAQR3;PDCD10;PGAP3;PIKFYVE;PITPNM1;PJA2;PNPLA8;PROC;PROZ;PSEN2;RAB12;RAB14;RAB27B;RAB2A;RAB2B;RAB33B;RAB6A;RER1;RFNG;RHOU;RNF13;ROCK1;SACM1L;SAR1B;SC/
;GIPC1;GOLGA4;GOLT1B;GOPC;GORASP1;GPAM;GRPEL2;HDAC6;HGS;HLA-DMA;HOOK1;HOOK2;INPP5K;IPO11;IPO13;IPO4;JAK2;JUN;JUP;KATNB1;KIF18A;KLHL2;KPNA2;KPNA3;KPNA5;LIN7A;LIN7C;LMAN2;LMNA;LRSAM1;LYST;MCOLN1;MDFIC;MDM2;MFF;MGEA5;MON1A;MON2;MSR1;MTX2;MTX3;NA(

CH5;MARCH6;MBOAT1;NCLN;NOTCH3;NPLOC4;NUCB2;NUS1;OSBPL5;OSTC;PARP16;PGAP1;PGAP3;PIGA;PIGB;PIGF;PIGK;PIGM;PIGS;PIGW;PIGY;PIGZ;PITPNM1;PJA2;PKD2;PLA2G4A;PLD2;PLD3;PLOD3;PNPLA2;PNPLA6;PNPLA8;POMT2;PORCN;PSEN2;PSKH1;PTGS2;PTPLAD1;PTPLAD2;RAB10;RAB14;RAB2/
7;CNOT8;COMMD6;COPS2;COPS5;CRABP2;CREB1;CREB5;CREBL2;CREBZF;CRK;CRLF3;CRTC2;CRY1;CTBP1;CTDSP1;CUL2;CXXC1;DAB2IP;DACH1;DAPK3;DAXX;DBP;DDX3X;DDX41;DDX54;DEK;DENND4A;DEPDC1;DICER1;DKK3;DLG4;DMAP1;DMTF1;DNTTIP2;DPY30;DR1;DRAP1;DYRK1B;E2F4;E2F6;E2F7;EAF2;
5;GEN1;GFPT1;GLRX2;GNL1;GPS1;GPS2;GSTM2;GSTP1;GTF2H1;GTF2H4;HDAC3;HDAC6;HMGB1;HMGB2;HSF1;IGBP1;IGHMBP2;IKBKG;IL18;INO80C;INO80D;INSIG1;INSIG2;IREB2;ITCH;JAK2;JMY;JUN;KIAA0101;KIAA1804;KIF22;KIN;KLHDC3;LAMB2;LIG4;LMNA;LONP1;MAP2K2;MAP2K4;MAP2K5;MAP3K11;N
DK8;CDK9;CDKN1B;CDKN1C;CDT1;CEBPA;CEBPG;CEBPZ;CENPK;CEP290;CGGBP1;CHAF1A;CHAF1B;CHD1;CHD5;CHD9;CHMP1A;CHN2;CHUK;CHURC1;CKAP2;CLK1;CLK3;CLK4;CNBP;CNN3;CNOT2;CNOT3;CNOT6;CNOT7;CNOT8;COMMD6;COMT;COPS2;COPS5;CRABP2;CREB1;CREB5;CREBL2;CREBZF;CRK;CRL
LRRK2;MAP2K2;MAP2K4;MAP2K5;MAP3K1;MAP3K11;MAP3K13;MAP3K14;MAP3K2;MAP3K7;MAP3K8;MAP4K1;MAP4K3;MAP4K5;MAPK6;MAPK8;MAPKAPK2;MAPKAPK3;MARK4;MAST2;MAST4;MED21;MNAT1;MOCS3;MST4;MYLK4;N4BP2;NADKD1;NANS;NEK1;NEK7;NLK;NME7;PAK4;PAN3;PANK1;PAI

URC1;CKAP2;CLP1;CNBP;CNOT2;CNOT3;CNOT6;CNOT7;CNOT8;COMMD6;COPS2;COPS5;CPSF1;CRABP2;CRCP;CREB1;CREB5;CREBL2;CREBZF;CRK;CRLF3;CRTC2;CRY1;CTBP1;CTDSP1;CUL2;CXXC1;DAB2IP;DACH1;DAPK3;DAXX;DBP;DDX3X;DDX41;DDX54;DEK;DENND4A;DEPDC1;DICER1;DKK3;DLG4;DMAP1;D
CH5;MARCH6;MBOAT1;NCLN;NOTCH3;NUS1;OSBPL5;OSTC;PARP16;PGAP1;PGAP3;PIGA;PIGB;PIGF;PIGK;PIGM;PIGS;PIGW;PIGY;PIGZ;PITPNM1;PJA2;PKD2;PLA2G4A;PLD2;PLD3;PLOD3;PNPLA2;PNPLA6;PNPLA8;POMT2;PORCN;PSEN2;PSKH1;PTGS2;PTPLAD1;PTPLAD2;RAB10;RAB2A;RAB2B;RDH10;RINT1;I
RM1;MRPL1;MRPL18;MRPL3;MRPS17;MTHFSD;MTIF3;MTRF1;MTRF1L;NAA38;NKRF;NOL6;NOP2;NOP56;NOP58;NUDT16;NUDT21;NUFIP2;PAIP1;PAPOLA;PAPOLG;PHAX;PIWIL4;PPIL4;PRKCSH;PRPF31;PRPF6;PSMA1;PSMA6;PTBP2;PUF60;PUM2;PURB;PUS10;PUS7L;QKI;RAD51AP1;RAE1;RALY;RBFOX3;RE
CD48;CDC14A;CDC26;CDC27;CDC37;CDC73;CDK1;CDK2;CDK5R1;CDK7;CDK8;CDK9;CEBPA;CENPE;CENPK;CENPW;CEP170;CEP19;CEP250;CEP290;CEP350;CEP44;CEP57;CEP57L1;CEP95;CGN;CHAF1A;CHAF1B;CHM;CHML;CHUK;CISD2;CKAP2;CLCN2;CLIC2;CLIC4;CLP1;CNOT7;CNP;COG3;COG4;COG6;COPE;C
LCLAT1;LMAN1;LMAN2;LPCAT1;LPCAT2;LPCAT4;LPGAT1;LRMP;LRPAP1;LRRC8C;MAGT1;MAPK8IP1;MARCH1;MARCH5;MARCH6;MBOAT1;MINPP1;NCLN;NOTCH3;NUS1;OSBPL5;OSBPL7;OSTC;P4HA1;P4HB;PACS2;PARP16;PGAP1;PGAP3;PIGA;PIGB;PIGF;PIGK;PIGM;PIGS;PIGW;PIGY;PIGZ;PITPNM1;PJA2;PK
DERL1;DIS3;DNA2;DNAJB2;DNAJB9;DNAJC10;DNASE1L1;DNASE2;ECH1;ECHS1;ECT2;EDEM3;EEFSEC;EIF3E;EIF4E;EIF5;ELMOD2;ENDOG;EPHB3;ERCC2;ERCC8;ERLEC1;ERLIN1;FBXL3;FBXL4;FBXL5;FBXO11;FBXO31;FBXO4;FBXO45;FBXO5;FBXO8;FBXW4;FBXW7;FGD2;FGD3;FGD4;FNBP1L;GALC;GBA2;GCH1;G
URC1;CKAP2;CLP1;CNBP;CNOT2;CNOT3;CNOT6;CNOT7;CNOT8;COMMD6;COPS2;COPS5;CPSF1;CRABP2;CRCP;CREB1;CREB5;CREBL2;CREBZF;CRK;CRLF3;CRTC2;CRY1;CTBP1;CTDSP1;CUL2;CXXC1;DAB2IP;DACH1;DAPK3;DAXX;DBP;DDX3X;DDX41;DDX54;DEK;DENND4A;DEPDC1;DICER1;DKK3;DLG4;DMAP1;D
TAOK1;TCEA1;TDG;TDP2;TFPT;TONSL;TREX1;TRIM28;UBC;UBE2A;UBE2D3;UBE2N;UBE2T;UBE2V2;UBE2W;UBR5;UCHL5;UPF1;USP1;XAB2;XRCC1;XRCC4;ZRAN
RPL28;MRPL3;MRPL32;MRPL37;MRPL39;MRPL40;MRPL42;MRPL47;MRPL48;MRPS17;MRPS18B;MRPS18C;MRPS22;MRPS28;MRPS31;MRPS33;MSTO1;MTERF;MTHFD2L;MTX2;MTX3;NARS2;NDUFA1;NDUFA4;NDUFA5;NDUFA6;NDUFAF1;NDUFB1;NDUFB3;NDUFB5;NDUFS2;NDUFS4;NDUFS5;NIPSNAP1;N
A;TRA2B;TXNL4B;U2AF1;WBP4;WTAP;XAB2;ZCCHC8;ZCRB1;ZNF326;ZRAN
D9;CHMP1A;CHUK;CHURC1;CKAP2;CLK1;CLK3;CLK4;CLP1;CNBP;CNOT2;CNOT3;CNOT6;CNOT6L;CNOT7;CNOT8;COG3;COMMD6;COPS2;COPS5;CPEB2;CPEB3;CRABP2;CREB1;CREB5;CREBL2;CREBZF;CRK;CRLF3;CRTC2;CRY1;CTBP1;CTDSP1;CTSH;CUL2;CXXC1;DAB2IP;DACH1;DAPK3;DAXX;DBP;DDX3X;DDX41
;CSNK1G3;DAK;DAPK3;DCAKD;DCK;DDX11;DDX3X;DDX41;DDX50;DDX54;DDX59;DGKE;DGKZ;DHX30;DHX36;DHX40;DICER1;DMPK;DNA2;DNAH6;DNAJA1;DYRK1A;DYRK1B;DYRK2;EPHB3;EPHB6;ERCC2;ERN1;ETNK1;FANCM;FARS2;FARSA;FDXACB1;FER;FGFR4;FIGNL1;GAK;GK;GRK6;GSS;GTF2F2;GUCY2C;H/
K5R1;CDK5RAP3;CDK7;CDK9;CDKL1;CDKN1B;CDKN1C;CDKN2C;CDKN3;CDS1;CENPE;CEPT1;CERS5;CHN2;CHORDC1;CHUK;CKS1B;CKS2;CLIP3;CLK1;CLK3;CLK4;CLN3;CMAHP;CMPK1;CNN3;CNP;CNPPD1;COASY;COL4A3BP;CREB1;CREBL2;CRIPAK;CRK;CRLS1;CSGALNACT1;CSK;CSNK1A1;CSNK2B;CTBP1;CTDNE
1;MAPKAPK2;MCM5;MDM2;MEN1;MNAT1;MSH2;MTBP;MUC1;NBN;NEK7;NR4A3;NSMCE2;NUDT16;ORC2;ORC3;ORC4;PES1;PGGT1B;PHIP;PKD2;PKIA;PKN2;PLAGL1;PLCB1;PLK4;PPM1G;PPP1R15A;PRCC;PRKAA1;PRKAB2;PRKACB;PRKAG2;PSMA1;PSMA2;PSMA3;PSMA4;PSMA6;PSMA8;PSMB1;PSMB3;PS
L;DDOST;DENND1B;DENND4C;DERL1;DLG1;DLG4;DMAP1;DPH3;DYRK2;ECT2;EIF5A2;EPB41L5;EPS15;ERBB2IP;EXOC1;EXOC3;EXOC5;EXOC6;EXOC8;EXPH5;F2RL1;FBXW7;FCHO2;FGF7;FGFR1OP;FOPNL;GAS6;GCC2;GDI1;GIPC1;GOLGA4;GOLT1B;GOPC;GORASP1;GPAM;GRIK5;GRIP1;GRPEL2;HDAC6;HDAC8;I
DK11A;CDK11B;CDK2;CDK5R1;CDK5RAP3;CDK7;CDK8;CDK9;CDKN1B;CDKN1C;CDT1;CEBPA;CEBPG;CEBPZ;CENPK;CEP290;CGGBP1;CHAF1A;CHAF1B;CHD1;CHD5;CHD9;CHMP1A;CHUK;CHURC1;CKAP2;CLP1;CMPK1;CNBP;CNOT2;CNOT3;CNOT6;CNOT7;CNOT8;COASY;COMMD6;COPS2;COPS5;CPSF1;CRABI
C42SE2;CDK1;CDK2;CDK5R1;CDKL2;CDKN2AIP;CENPE;CENPK;CENPL;CENPQ;CENPW;CEP120;CEP135;CEP152;CEP170;CEP19;CEP250;CEP290;CEP350;CEP44;CEP57;CEP57L1;CEP70;CEP95;CEP97;CETN3;CGN;CHMP1A;CKAP2;CLIC4;CLK3;CLNS1A;CNN3;CNOT3;CNOT7;CNP;CNTROB;COIL;CORO1A;CRBN;CRE
C42SE2;CDK1;CDK2;CDK5R1;CDKL2;CDKN2AIP;CENPE;CENPK;CENPL;CENPQ;CENPW;CEP120;CEP135;CEP152;CEP170;CEP19;CEP250;CEP290;CEP350;CEP44;CEP57;CEP57L1;CEP70;CEP95;CEP97;CETN3;CGN;CHMP1A;CKAP2;CLIC4;CLK3;CLNS1A;CNN3;CNOT3;CNOT7;CNP;CNTROB;COIL;CORO1A;CRBN;CRE

NK1A1L;CSNK1G3;DAK;DAPK3;DCAKD;DCK;DDX11;DDX3X;DDX41;DDX50;DDX54;DDX59;DGKE;DGKZ;DHX30;DHX36;DHX40;DICER1;DMPK;DNA2;DNAH6;DNAJA1;DYRK1A;DYRK1B;DYRK2;EPHB3;EPHB6;ERCC2;ERN1;ETNK1;FANCM;FARS2;FARSA;FDXACB1;FER;FGFR4;FIGNL1;GAK;GK;GRK6;GRPEL2;GSS;GTF

CDK7;CDK8;CDK9;CDKN1B;CDKN1C;CDT1;CEBPA;CEBPG;CEBPZ;CENPK;CEP290;CGGBP1;CHAF1A;CHAF1B;CHD1;CHD5;CHD9;CHMP1A;CHUK;CHURC1;CKAP2;CLP1;CMPK1;CNBP;CNOT2;CNOT3;CNOT6;CNOT7;CNOT8;COASY;COMMD6;COPS2;COPS5;CPSF1;CRABP2;CRCP;CREB1;CREB5;CREBL2;CREBZF;CRI
E16;ZNF

DR44;ZFYVE16;ZNF

NK1A1L;CSNK1G3;DAK;DAPK3;DCAKD;DCK;DDX11;DDX3X;DDX41;DDX50;DDX54;DDX59;DGKE;DGKZ;DHX30;DHX36;DHX40;DICER1;DMPK;DNA2;DNAH6;DNAJA1;DYRK1A;DYRK1B;DYRK2;EPHB3;EPHB6;ERCC2;ERN1;ETNK1;FANCM;FARS2;FARSA;FDXACB1;FER;FGFR4;FIGNL1;GAK;GK;GRK6;GSS;GTF2F2;GU

CDKN1B;CDKN1C;CDT1;CEBPA;CEBPG;CEBPZ;CENPK;CEP290;CGGBP1;CHAF1A;CHAF1B;CHD1;CHD5;CHD9;CHMP1A;CHN2;CHUK;CHURC1;CKAP2;CLK1;CLK3;CLK4;CNBP;CNN3;CNOT2;CNOT3;CNOT6;CNOT7;CNOT8;COMMD6;COPS2;COPS5;CRABP2;CREB1;CREB5;CREBL2;CREBZF;CRK;CRLF3;CRTC2;CRY1;CS
8;CDK9;CDKN1B;CDKN1C;CDT1;CEBPA;CEBPG;CEBPZ;CENPK;CEP290;CGGBP1;CHAF1A;CHAF1B;CHD1;CHD5;CHD9;CHMP1A;CHUK;CHURC1;CKAP2;CLP1;CMPK1;CNBP;CNOT2;CNOT3;CNOT6;CNOT7;CNOT8;COASY;COMMD6;COPS2;COPS5;CPSF1;CRABP2;CRCP;CREB1;CREB5;CREBL2;CREBZF;CRK;CRLF3;CF

MD4;PSMD8;PSME1;PTEN;RAD17;RB1;RBBP8;RFC2;RFC3;RFC5;RFWD2;RHOU;RPS27L;RPS6KB1;SKP2;TAF2;TFAP4;TJP3;TP63;TRIAP1;TUBG1;TUBG2;TUBGCP2;UBC;USP37;WE
A;CHMP2B;CLEC5A;CLEC6A;CLIP3;COG3;COG4;COG6;COL4A3BP;COLQ;COPE;COPZ1;CPSF1;CRIPT;CSK;CTDNEP1;CXCL10;DAB2IP;DBN1;DDOST;DENND1B;DENND4C;DERL1;DLG1;DLG4;DMAP1;DPH3;DYRK2;ECT2;EIF4E;EIF5A2;ENY2;EPB41L5;EPS15;ERBB2IP;EXOC1;EXOC3;EXOC5;EXOC6;EXOC8;EXPH5;F2R
P3;CDK7;CDK9;CDKL1;CDKN1B;CDKN1C;CDKN2C;CDKN3;CDS1;CENPE;CEPT1;CERS5;CHN2;CHORDC1;CHUK;CKS1B;CKS2;CLIP3;CLK1;CLK3;CLK4;CLN3;CMAHP;CMPK1;CNN3;CNP;CNPPD1;COASY;COL4A3BP;CREB1;CREBL2;CRIPAK;CRK;CRLS1;CSGALNACT1;CSK;CSNK1A1;CSNK2B;CTBP1;CTDNEP1;CTDSP1;CT
B;CDKN1C;CDT1;CEBPA;CEBPG;CEBPZ;CENPK;CEP290;CGGBP1;CHAF1A;CHAF1B;CHD1;CHD5;CHD9;CHMP1A;CHUK;CHURC1;CKAP2;CLP1;CMPK1;CNBP;CNOT2;CNOT3;CNOT6;CNOT7;CNOT8;COASY;COMMD6;COPS2;COPS5;CPSF1;CRABP2;CRCP;CREB1;CREB5;CREBL2;CREBZF;CRK;CRLF3;CRTC2;CRY1;CTB

MD4;PSMD8;PSME1;PTEN;RAD17;RB1;RBBP8;RFC2;RFC3;RFC5;RFWD2;RHOU;RPS27L;RPS6KB1;SKP2;TAF2;TFAP4;TJP3;TP63;TRIAP1;TUBG1;TUBG2;TUBGCP2;UBC;USP37;WEE1;ZNF6
EN;PTGS2;RAD17;RASSF1;RB1;RBBP8;RFC2;RFC3;RFC5;RFWD2;RHEB;RINT1;RPS27L;RPTOR;RRP8;SART1;SESN1;SIPA1;SKIL;STRADA;TAOK1;TFAP4;TGFBR1;THAP5;TIPRL;TP53INP1;TP63;TRIAP1;TSC2;TTK;UBC;UBE2D1;UBE2E1;UHMK1;USP44;WEE1;ZWIL

;SLC40A1;SLC9A6;SNX10;SNX13;SNX16;SNX17;SNX18;SNX2;SNX25;SNX3;SNX4;SNX8;SPAST;STAM2;STARD3;STARD3NL;STEAP2;STX7;TAB1;TAB2;TAB3;TBK1;TFRC;TICAM1;TLR8;TMEM106B;TMEM184A;TMEM55A;TOM1;TPT1;TRIM3;UBC;UBE2D3;UNC13D;USP6;VAC14;VAMP4;VAMP7;VPS18;VPS26A;VP

PIWIL4;PLCB1;PLK1S1;PMF1;PMS1;PPP1CC;PPP1R12A;PPP5C;PSMD13;PTEN;RAD51B;RAD51C;RANGAP1;RB1;RBBP8;RBM7;RPS27;RRS1;SEC13;SENP6;SEPT7;SETDB2;SGOL2;SIRT2;SKA2;SMC2;SMC4;SMC5;SPAG5;SPIRE2;SSSCA1;STAG1;STAG2;SUV39H2;SYCP2;SYCP3;TAOK1;TGFA;TTK;TTYH1;TUBG1;TXNL

AP3K2;MAP3K7;MAP3K8;MAP4K1;MAP4K3;MAP4K5;MAPK6;MAPK8;MAPKAPK2;MAPKAPK3;MARK4;MAST2;MAST4;MNAT1;MST4;MYLK4;N4BP2;NADKD1;NEK1;NEK7;NLK;NME7;PAK4;PAN3;PANK1;PANK3;PBK;PDIK1L;PDK1;PDK2;PDXK;PFKL;PFKM;PGM2L1;PHKG2;PI4K2B;PIK3C2A;PIK3CA;PIKFYVE;PKN
40A;RAB10;RABEP1;RALA;RASA1;RB1;RBBP8;ROCK1;SEPT10;SEPT14;SEPT7;SEPT9;SETDB2;SGOL2;SIRT2;SKA2;SMC2;SMC4;SMC5;SPAG5;SPAST;SPIRE2;SSSCA1;STAG1;STAG2;SYCP2;SYCP3;TGFA;TUBA1B;USP16;USP37;USP44;VEGFA;VEGFB;VPS4A;VPS4B;VRK1;WAPAL;WEE1;ZNF830;ZWIL

;DIS3;DNA2;DNAJB2;DNAJB9;DNAJC10;DNASE1L1;DNASE2;ECH1;ECHS1;ECT2;EDEM3;EEFSEC;EIF3E;EIF4E;EIF5;ELMOD2;ENDOG;EPHB3;ERCC2;ERCC8;ERLEC1;ERLIN1;FBXL3;FBXL4;FBXL5;FBXO11;FBXO31;FBXO4;FBXO45;FBXO5;FBXO8;FBXW4;FBXW7;FGD2;FGD3;FGD4;FUCA1;FUT4;GAA;GALC;GALE;GAP

;MAST2;MAST4;MNAT1;MST4;MYLK4;NEK1;NEK7;NLK;PAK4;PAN3;PBK;PDIK1L;PDK1;PDK2;PHKG2;PIK3CA;PKN1;PKN2;PLK4;POLR2E;PPP4C;PRKAA1;PRKAB2;PRKACB;PRKCI;PRKCZ;PRKD3;PRPF4B;PSKH1;PTK6;PTK7;RABEP1;RET;RIOK2;RIPK2;RIPK3;ROCK1;RPS6KA3;RPS6KA4;RPS6KA5;RPS6KB1;RYK;SCYL1

;CDK2;CDK5R1;CDK5RAP3;CDK7;CDK8;CDK9;CDKN1B;CDKN1C;CDT1;CEBPA;CEBPG;CEBPZ;CENPK;CEP290;CGGBP1;CHAF1A;CHAF1B;CHD1;CHD5;CHD9;CHMP1A;CHUK;CHURC1;CKAP2;CLP1;CMPK1;CNBP;CNOT2;CNOT3;CNOT6;CNOT7;CNOT8;COASY;COMMD6;COPS2;COPS5;CPSF1;CRABP2;CRCP;CREB1;

KA4;RPTOR;SIRT1;SPAG16;SPAG9;SREBF1;STK11IP;STRADA;TAX1BP1;TFPT;TGFBR2;TICAM1;TMEM173;TOLLIP;TPRKB;TRPV4;TWF2;USP3
MAP4K3;MAP4K5;MAPK6;MAPK8;MAPKAPK2;MAPKAPK3;MARK4;MAST2;MAST4;MNAT1;MST4;MYLK4;N4BP2;NADKD1;NEK1;NEK7;NLK;PAK4;PAN3;PANK1;PANK3;PBK;PDIK1L;PDK1;PDK2;PDXK;PFKL;PFKM;PGM2L1;PHKG2;PI4K2B;PIK3C2A;PIK3CA;PIKFYVE;PKN1;PKN2;PLK4;POLR2E;PPP4C;PRKAA1;PRK
ANGAP1;RBCK1;RPGR;RPH3AL;RPL11;RPL15;RPL17;RPL23;RPL26;RPL26L1;RPL27;RPL30;RPL31;RPL34;RPL35;RPL36A;RPL39;RPL41;RPL7;RPL9;RPS15A;RPS24;RPS27;RPS29;RPS3A;RPS7;SAR1A;SAR1B;SEC11A;SEC11C;SEC13;SEC23A;SEC24A;SEC61A1;SEC62;SERAC1;SIL1;SIRT1;SLU7;SMAD4;SNX13;SNX16;S

P1;MARCH5;MATR3;MCL1;MCU;MDH2;MFF;MFN1;MIB1;MPV17;MRPL19;MSTO1;MTHFD2L;MTMR6;MTX2;MTX3;NDUFA1;NDUFA4;NDUFA5;NDUFA6;NDUFAF1;NDUFB1;NDUFB3;NDUFB5;NDUFS2;NDUFS4;NDUFS5;NEK1;NIPSNAP1;NOC4L;NRM;NUCB2;NUP37;NUP54;NUP62CL;NUP85;OMA1;OPA1;PA
;RANGAP1;RBCK1;RNF139;RPL11;RPL15;RPL17;RPL23;RPL26;RPL26L1;RPL27;RPL30;RPL31;RPL34;RPL35;RPL36A;RPL39;RPL41;RPL7;RPL9;RPS15A;RPS24;RPS27;RPS29;RPS3A;RPS7;RYR1;SAR1A;SAR1B;SCFD1;SCYL2;SEC11A;SEC11C;SEC13;SEC23A;SEC24A;SEC61A1;SIRT1;SLC30A6;SMAD4;SMG1;SMG5;SN

;PNMA3;POLR1E;PPP1CA;PPP1CB;PPP1CC;PWP2;RASL11A;RBBP6;RBM34;RBM4;RECQL5;REXO4;RPF1;RPF2;RPL11;RPL23;RPL35;RPL9;RPS24;RPS3A;RPS7;RRP1;RRP36;RRP8;RRS1;RSL24D1;RSPH4A;SEPT7;SIRT1;SKIV2L2;SLC30A5;SMARCA5;SMARCB1;SRP19;STAU2;STX16;SUB1;SURF6;TAF13;TAF1A;TBCA;

4;RPL35;RPL36A;RPL36AL;RPL39;RPL39L;RPL41;RPL7;RPL9;RPS15A;RPS24;RPS27;RPS27L;RPS29;RPS3A;RPS6KA3;RPS6KB1;RPS7;SARNP;SEC11A;SEC11C;SELT;SEPSECS;SERP1;SPCS2;SPCS3;SRP14;SRP19;SRP9;SSR3;TACO1;TARS2;TIA1;TRAM1;UHMK1;UPF1;USP16;VARS;W

;FBXW7;FCER1A;FEM1A;FER;FGF7;FGFR1OP;FGFR4;FMR1;FNIP1;FNTA;FRS2;FXR1;GAB1;GAPDH;GAS6;GFPT1;GIPC1;GMFB;GPS1;GPS2;GSTP1;GTF2H1;GUF1;HDAC6;HRSP12;HSPBP1;IBTK;IDE;IFT52;IFT88;IGBP1;IGF2BP3;IKBKG;IL12A;IL15;IL6ST;IL8;ILK;IMPACT;INPP5K;IREB2;ITCH;ITGA1;JAK2;JUN;KAT2B;

SLC25A46;SPNS1;TIMM17A;TIMM22;TIMM23;TIMM8B;TMEM201;TMEM70;TMPO;UCP2;UCP3;UNC50;UQCRB;UQCRC1;UQCRC2;UQCRH;UQC

P1;MARCH5;MATR3;MCL1;MCU;MDH2;MFF;MFN1;MIB1;MPV17;MRPL19;MSTO1;MTHFD2L;MTMR6;MTX2;MTX3;NDUFA1;NDUFA4;NDUFA5;NDUFA6;NDUFAF1;NDUFB1;NDUFB3;NDUFB5;NDUFS2;NDUFS4;NDUFS5;NEK1;NIPSNAP1;NOC4L;NRM;NUCB2;NUP37;NUP54;NUP62CL;NUP85;OMA1;OPA1;PA

R4;CBWD1;CBWD2;CCBL2;CCT2;CCT6B;CDC7;CDK1;CDK11A;CDK11B;CDK17;CDK18;CDK2;CDK7;CDK8;CDK9;CDKL1;CDKL2;CENPE;CFH;CHD1;CHD5;CHD9;CHST14;CHUK;CKB;CLCN7;CLK1;CLK3;CLK4;CLP1;CMPK1;COASY;CPNE1;CRABP2;CSK;CSNK1A1;CSNK1A1L;CSNK1G3;CXCL6;DAK;DAPK3;DAPP1;DBI;DCA

RNF19A;RPGR;RSPH4A;SASS6;SENP6;SKA2;SLAIN2;SPAG5;SPAST;SPIRE2;SS18;TBCD;TPPP3;TRPV4;TTK;TTLL1;TUBA1B;TUBD1;TUBE1;TUBG1;TUBG2;TUBGCP2;WEE1;XP

SEA5;MORC3;MPP5;MTX2;MTX3;MZT1;NAGPA;NAPB;NAPG;NCKIPSD;NEDD4;NFASC;NFKBIE;NIN;NME7;NOP58;NUP54;PACS2;PAN3;PAX6;PES1;PEX1;PEX12;PEX13;PEX14;PEX2;PEX3;PGAP1;PHIP;PIKFYVE;PKD2;PKIA;PMAIP1;PMPCA;PPP2R5A;PPP3CA;PRKCI;PRKCZ;PSMD10;PTGS2;RAB10;RAB3GAP2;RAB

T1;RNF144B;RPS6KB1;SDHD;SFXN4;SHMT2;SLC24A6;SLC25A1;SLC25A10;SLC25A11;SLC25A22;SLC25A24;SLC25A3;SLC25A32;SLC25A34;SLC25A36;SLC25A38;SLC25A40;SLC25A43;SLC25A46;SLC27A3;SPNS1;TIMM17A;TIMM22;TIMM23;TIMM8B;TMEM173;TMEM70;TOMM20;TOMM40;TOMM5;UCP2;UC

BF1;SS18L1;SUPT5H;SUV39H2;SUZ12;SYCP3;TADA1;TAF12;TAF5;TAF7;TAF9;TBL1XR1;TET1;TLK1;TP63;UBE2A;UBE2E1;UBE2N;UBR5;USP16;USP21;VEGFA;VPS72;WDR5;ZMYN

OG3;COG4;COG6;COL4A3BP;COPE;COPZ1;CPSF1;CREBL2;CSK;CTNS;CXCL10;CYB5R4;DAB2IP;DDOST;DENND1B;DENND4C;DERL1;DMAP1;DPH3;DTNBP1;DYRK2;ECT2;EIF4E;EIF5A2;ENY2;EPS15;ERBB2IP;EXOC1;EXOC3;EXOC5;EXOC6;EXOC8;EXPH5;F2RL1;FABP5;FCER1A;FMR1;FOLR2;FYTTD1;G6PC3;GAS6;
AB2IP;DAGLB;DAPK3;DCP1A;DCP2;DCPS;DDB2;DDX3X;DECR1;DERL1;DHPS;DIS3;DNA2;DNAJB2;DNAJB9;DNAJC10;DNASE1L1;DNASE2;ECH1;ECHS1;ECT2;EDEM3;EEFSEC;EIF3E;EIF4E;EIF5;ELMOD2;ENDOG;EPHB3;ERCC2;ERCC8;ERLEC1;ERLIN1;FBXL3;FBXL4;FBXL5;FBXO11;FBXO31;FBXO4;FBXO45;FBXO5;FB

;HBS1L;HELLS;HLTF;IDE;IGHMBP2;KATNB1;KIF11;KIF18A;KIF20B;KIF22;KIF23;KIF27;KIF2A;KIF3A;KIF5B;KIFC1;KIFC3;KLC4;KRAS;LONP1;LONRF1;LRRK2;MCM5;MCM9;MFN1;MMAA;MNAT1;MOV10;MSH2;MYO1G;MYO5C;NBN;NKIRAS1;NRAS;NUBP1;OPA1;ORC4;PEX1;PMS1;POLQ;PPP2R4;PSMC1;PSMC2;
PBP10;GTPBP2;GUF1;HBS1L;HELLS;HLTF;IDE;IGHMBP2;KATNB1;KIF11;KIF18A;KIF20B;KIF22;KIF23;KIF27;KIF2A;KIF3A;KIF5B;KIFC1;KIFC3;KLC4;KRAS;LHPP;LONP1;LONRF1;LRRK2;MCM5;MCM9;MFN1;MMAA;MNAT1;MOV10;MSH2;MYO1G;MYO5C;NBN;NKIRAS1;NRAS;NUBP1;NUDT12;NUDT16;OPA1;ORC
SEA5;MORC3;MPP5;MTX2;MTX3;MZT1;NAGPA;NAPB;NAPG;NCKIPSD;NEDD4;NFASC;NFKBIE;NIN;NME7;NOP58;NUP54;PACS2;PAN3;PAX6;PES1;PEX1;PEX12;PEX13;PEX14;PEX2;PEX3;PGAP1;PHIP;PIKFYVE;PKD2;PKIA;PMAIP1;PMPCA;PPP2R5A;PPP3CA;PRKCI;PRKCZ;PSMD10;PTGS2;RAB10;RAB3GAP2;RAB

GTF2H4;GTPBP10;GTPBP2;GUF1;HBS1L;HELLS;HLTF;IDE;IGHMBP2;KATNB1;KIF11;KIF18A;KIF20B;KIF22;KIF23;KIF27;KIF2A;KIF3A;KIF5B;KIFC1;KIFC3;KLC4;KRAS;LHPP;LONP1;LONRF1;LRRK2;MCM5;MCM9;MFN1;MMAA;MNAT1;MOV10;MSH2;MYO1G;MYO5C;NBN;NKIRAS1;NRAS;NUBP1;NUDT12;NUDT16;
PBP10;GTPBP2;GUF1;HBS1L;HELLS;HLTF;IDE;IGHMBP2;KATNB1;KIF11;KIF18A;KIF20B;KIF22;KIF23;KIF27;KIF2A;KIF3A;KIF5B;KIFC1;KIFC3;KLC4;KRAS;LHPP;LONP1;LONRF1;LRRK2;MCM5;MCM9;MFN1;MMAA;MNAT1;MOV10;MSH2;MYO1G;MYO5C;NBN;NKIRAS1;NRAS;NUBP1;NUDT12;NUDT16;OPA1;ORC

M1;TMEM173;TPRKB;TRPV4;TWF2;USP3

PS27L;RRP8;SENP6;SIRT2;SMC5;STAG2;TAOK1;TFAP4;TGFA;TIPRL;TJP3;TP63;TRIAP1;TTK;UBC;UBE2D1;UBE2E1;USP19;USP44;WAPAL;WEE1;XPO1;ZWI

CC;PSEN2;RHOT1;RNF144B;RPS6KB1;SDHD;SFXN4;SHMT2;SLC24A6;SLC25A1;SLC25A10;SLC25A11;SLC25A22;SLC25A24;SLC25A3;SLC25A32;SLC25A34;SLC25A36;SLC25A38;SLC25A40;SLC25A43;SLC25A46;SLC27A3;SPNS1;SUOX;TIMM17A;TIMM22;TIMM23;TIMM8B;TMEM173;TMEM70;TOMM20;TOMM

IN7A;LLGL1;LNX1;LNX2;LONP1;MALT1;MAP3K1;MAPK8IP2;MCU;MDM2;MFF;MID1IP1;MITF;MKI67IP;MKKS;MNAT1;MPP6;MPP7;MSTO1;MTMR2;MUC20;NAPB;NAPG;NCK1;NDUFAF1;NDUFS4;NDUFS5;NUBPL;NUDT21;P2RX4;PARD6B;PDSS1;PEX13;PEX14;PFKL;PFKM;PFN2;PIK3C2A;PMAIP1;PPAT;PPP2R

IF;BMI1;BMP1;BMPR2;BNIP1;BNIP3L;BORA;BRCA2;BRCC3;BRD9;BRWD1;BYSL;C10orf2;C1QL3;C5orf30;CABIN1;CACNA1H;CACNB3;CACNB4;CAMK1;CAMK2D;CAPG;CAPZA1;CAPZA2;CASC5;CASP3;CASP8;CBLL1;CBR4;CBX3;CCDC103;CCDC39;CCDC88A;CCDC88B;CCNA2;CCNG1;CCNG2;CCP110;CCT2;CCT6B;

IN7A;LLGL1;LNX1;LNX2;LONP1;MALT1;MAP3K1;MAPK8IP2;MCU;MDM2;MFF;MID1IP1;MITF;MKI67IP;MKKS;MNAT1;MPP6;MPP7;MSTO1;MTMR2;MUC20;NAPB;NAPG;NCK1;NDUFAF1;NDUFS4;NDUFS5;NUBPL;NUDT21;P2RX4;PARD6B;PDSS1;PEX13;PEX14;PFKL;PFKM;PFN2;PIK3C2A;PMAIP1;PPAT;PPP2R

IBG2;URG

12;GSTP1;HACE1;HAUS7;HDAC3;HDAC5;HDAC6;HDAC7;HDAC9;HINT1;IBTK;IGBP1;ILK;INF2;ITGAV;JAK2;JKAMP;JUP;KAT2B;KAT8;KIAA1967;KIF11;KPNA2;LIPE;LLGL1;LONP1;LRPAP1;LRRK2;MALT1;MAP3K1;MAP3K11;MAP3K13;MAP3K2;MAPK6;MAPK8;MAPK8IP1;MAPK8IP2;MARCH5;MARCH6;MAST2;MD
1;HMGB2;HMGN5;HMGXB4;HNF1A;HNRNPL;HSF1;HSF2;HSF4;HSF5;IFT57;IGHMBP2;IKZF2;IKZF5;IMPDH2;IRF5;JRKL;JUN;KIF22;KIN;KLF16;KLF3;KLF5;KLLN;LCOR;LCORL;LEMD3;LIG4;LIN28A;LIN54;LONP1;LRWD1;LUC7L3;MAF;MAF1;MCM5;MCM9;MED26;MEF2A;MEF2C;MEF2D;MEN1;MIER1;MIER2;MIER

JRA;PURB;RAD17;RANGAP1;RB1;RBBP6;REV3L;RFC2;RFC3;RFC5;RGS12;RIF1;RING1;RNF2;RNF40;RRP8;RRS1;RUVBL2;SEC13;SEPT7;SETDB2;SFR1;SGOL2;SIRT1;SKA2;SMARCA5;SMARCAD1;SMARCB1;SMC2;SMC4;SMC5;SMC6;SMCHD1;SPAG5;SS18L1;STAG1;STAG2;SUMO3;SUV39H1;SUV39H2;SUZ12;SYCF

;TUBE1;TUBG1;TUBG2;URG
;TUBE1;TUBG1;TUBG2;URG

CP3;UQCRB;UQCRC1;UQCRC2;UQCRH;UQCF

AP7;AKAP8L;AKIP1;AKIRIN1;AKR1B1;AKR7A2;AKT1;AKTIP;ALCAM;ALDH4A1;ALDOA;ALG1;ALG10;ALG10B;ALG11;ALG13;ALG2;ALG3;ALG6;ALKBH4;ALKBH5;ALKBH8;ALS2CR8;AMD1;AMMECR1;ANAPC10;ANG;ANGPT1;ANGPTL4;ANGPTL7;ANKAR;ANKLE1;ANKMY2;ANKRD12;ANKRD13C;ANKRD26;ANKRD28

[illegible]

AP5;AKAP7;AKAP8L;AKD1;AKIP1;AKR1B1;AKR7A2;AKT1;AKTIP;ALCAM;ALDH16A1;ALDH4A1;ALDOA;ALG1;ALG10;ALG10B;ALG11;ALG13;ALG2;ALG3;ALG6;ALKBH4;ALKBH5;ALKBH8;ALS2CR8;AMD1;AMMECR1;ANAPC10;ANG;ANGPT1;ANGPTL4;ANGPTL7;ANKIB1;ANKK1;ANKMY2;ANKRD13C;ANKRD28;ANK

2;PLD2;PLD3;PLD4;PNKD;PNPLA2;PNPLA6;PNPLA8;PPEF1;PPM1B;PPM1G;PPP1CA;PPP1CB;PPP1CC;PPP1R15B;PPP1R3D;PPP2CB;PPP2R1A;PPP2R2A;PPP3CA;PPP3CB;PPP4C;PPP5C;PPP6C;PROCA1;PSMD14;PTEN;PTER;PTP4A1;PTPDC1;PTPN12;PTPN2;PTPN22;PTPN23;PTPN4;PTPN7;PTPRO;PTRH2;RAD51C;

;TDP2;TFEC;TMF1;TOB1;TONSL;TRIM28;UBE3A;USP16;USP21;YAF2;ZEB1;ZFX;ZHX1;ZNF136;ZNF281;ZN

3;TBC1D15;TBC1D22A;TBC1D23;TBC1D25;TBC1D4;TBC1D8B;TBCD;TRIM23;TSC2;UBA2;USP6;VAC1

RCA5;SMARCAD1;SMC2;SMC4;SMC5;SMC6;SPAG5;SS18L1;STAG1;STAG2;SUMO3;SUV39H1;SUV39H2;SUZ12;SYCP2;SYN1;TFPT;TMPO;TNKS2;TONSL;TP63;TRIM28;UBE2A;UCHL5;UPF1;WAPAL;WDHD1;XPO1;ZRANB3;ZWIL

GR;RTKN;SH3BP1;SIPA1;ST5;SYDE2;TBC1D10B;TBC1D12;TBC1D13;TBC1D15;TBC1D22A;TBC1D23;TBC1D25;TBC1D4;TBC1D8B;TBCD;TBXA2R;TSC2;USP6;WA

;CAPN14;CAPN7;CARD17;CASP3;CASP8;CCNH;CDADC1;CDC14A;CDC25C;CDK7;CDKN3;CENPE;CES4A;CGN;CHD1;CHD5;CHD9;CHID1;CHMP1A;CNOT6;CNOT6L;CNOT7;CNP;COPS5;CPD;CRBN;CTBS;CTDNEP1;CTDSP1;CTDSPL2;CTSH;CTSO;CTSS;CTSW;CYCS;DAGLB;DAPP1;DCLRE1A;DCP1A;DCP2;DCPS;DCTN2

IC8B;RIN1;RP2;RPGR;RTKN;SH3BP1;SIPA1;ST5;SYDE2;TBC1D10B;TBC1D12;TBC1D13;TBC1D15;TBC1D22A;TBC1D23;TBC1D25;TBC1D4;TBC1D8B;TBCD;TBXA2R;TSC2;USP6;WA

F7

IF773;ZNF7

'HYN1;TICAM2;TOMM6;TRAPPC3;TRNAU1AP;UQCRFS1;UXT;VDAC3;ZGLP1;ZNR

VDAC3;ZGLP1;ZNR
'HYN1;TICAM2;TOMM6;TRAPPC3;TRNAU1AP;UQCRFS1;UXT;VDAC3;ZGLP1;ZNR

VDAC3;ZGLP1;ZNR

HYN1;TICAM2;TOMM6;TRAPPC3;TRNAU1AP;UQCRFS1;UXT;VDAC3;ZGLP1;ZNR
HYN1;TICAM2;TOMM6;TRAPPC3;TRNAU1AP;UQCRFS1;UXT;VDAC3;ZGLP1;ZNR

IAU1AP;UQCRFS1;USP42;UXT;ZGLP1;ZNR

;KPNA4;LILRB3;LIMD1;LITAF;LYN;MARCKS;MCTP2;MLLT11;MYO5A;NCOA1;NCOA2;NEDD9;NFE2;NFE2L2;NPTN;NR6A1;NUP50;NUPL1;OSM;OXER1;PAK2;PDE4B;PDK3;PDZD3;PDZD8;PELI2;PHB;PIAS1;PLAU;PLD1;PLXNC1;PPAP2B;PPP4R1;PROK2;PTPRE;RAB1A;RAB27A;RAB31;RANBP1;RAPGEF2;RASGRP4;R

;KPNA4;LILRB3;LIMD1;LITAF;LYN;MARCKS;MCTP2;MLLT11;MYO5A;NCOA1;NCOA2;NEDD9;NFE2;NFE2L2;NPTN;NR6A1;NUP50;NUPL1;OSM;OXER1;PAK2;PDE4B;PDK3;PDZD3;PDZD8;PELI2;PHB;PIAS1;PLAU;PLD1;PLXNC1;PPAP2B;PPP4R1;PROK2;PTPRE;RAB1A;RAB27A;RAB31;RANBP1;RAPGEF2;RASGRP4;R

IFE2L2;NPTN;NR6A1;NUP50;NUPL1;OSM;OXER1;PAK2;PDE4B;PDK3;PDZD3;PDZD8;PELI2;PHB;PIAS1;PLAU;PLD1;PLXNC1;PPAP2B;PPP4R1;PROK2;PTPRE;RAB1A;RAB27A;RAB31;RANBP1;RAPGEF2;RASGRP4;RASSF3;REPS2;RHOH;RNF111;ROPN1L;RS1;S100A11;SDCBP;SECTM1;SH3GLB1;SIT1;SLC8A1;SOCS3;

L;KPNA4;LILRB3;LIMD1;LITAF;LYN;MARCKS;MCTP2;MLLT11;MYO5A;NCF4;NCOA1;NCOA2;NEDD9;NFE2;NFE2L2;NPTN;NR6A1;NUP50;NUPL1;OSM;OXER1;PAK2;PDE4B;PDK3;PDZD3;PDZD8;PELI2;PHB;PIAS1;PLAU;PLD1;PLXNC1;PPAP2B;PPP4R1;PROK2;PTPRE;RAB1A;RAB27A;RAB31;RANBP1;RAPGEF2;RAS(

LA;HMGCR;HP;HPSE;HSPA6;IDH1;IFNAR1;IFNGR2;IGSF6;IL10RB;IL13RA1;IL18RAP;IL1B;IL1R1;IL1R2;IL1RN;INHBB;IQGAP1;IRAK3;ITGAD;JMJD1C;KAL1;KLF6;KPNA1;KPNA4;LAMP2;LILRB3;LIMD1;LITAF;LYN;LYVE1;MCTP2;MLLT11;MME;MRVI1;MYO10;MYO5A;NAIP;NCF4;NCOA1;NCOA2;NEDD9;NFE2;NFE2L2

)1;LITAF;LYN;MCTP2;MLLT11;MME;MYO5A;NCOA1;NCOA2;NEDD9;NFE2L2;NPEPPS;NPTN;NR6A1;NUP50;NUPL1;OPLAH;OSM;OXER1;PAK2;PAPSS2;PDE4B;PDK3;PDZD3;PDZD8;PELI2;PHB;PIAS1;PLAU;PLD1;PLXNC1;POLD3;PPAP2B;PPP4R1;PROK2;PTPRE;RAB1A;RAB27A;RAB31;RANBP1;RAPGEF2;RASGRP4

17;GPR84;GPR89A;GPR97;GRIP2;GSK3B;HAS1;HCAR2;HCAR3;HMGCR;HPSE;IFNAR1;IFNGR2;IGSF6;IL10RB;IL13RA1;IL18RAP;IL1R1;IL1R2;IL1RN;INPP5A;IQGAP1;ITGAD;ITM2B;ITPRIP;KAL1;KAZN;KCNE1;KCNH7;KCNJ15;KCNS1;KIAA0319;KPNA1;LAMP2;LBR;LILRA6;LILRB3;LITAF;LMNB1;LRG1;LRRC4;LYN;LYV

MP3;INHBB;INPP5A;IQGAP1;IRAK3;ITM2B;JHDM1D;JMJD1C;KAL1;KCNE1;KCNS1;KIAA0319;KPNA1;LBR;LILRB3;LIMD1;LITAF;LMNB1;LMO4;LRRFIP1;LYN;MAK;MARCKS;MME;MPZL3;MRVI1;MXD1;MYO10;MYO5A;NAIP;NCF4;NCOA1;NCOA2;NECAB2;NEDD9;NFE2;NFE2L2;NFYA;NHP2L1;NPTN;NR6A1;NRD1;

ER1;PAK2;PDE4B;PDZD3;PHB;PICALM;PLAU;PLB1;PLXNC1;PPAP2B;PTPRE;PVRL2;RAB27A;RAPGEF2;RASGRP4;RASSF3;RHOH;RS1;SDCBP;SECTM1;SHROOM4;SIT1;SLC12A6;SLC22A1;SLC22A14;SLC22A4;SLC26A8;SLC2A11;SLC8A1;SMAGP;SNAP23;SNTA1;SNTG2;ST3GAL5;STX3;STX6;TBC1D30;TGM3;TLR2;TL

3;PHB;PICALM;PLAU;PLB1;PLXNC1;PPAP2B;PTPRE;PVRL2;RAB27A;RAPGEF2;RASGRP4;RASSF3;RHOH;RS1;SDCBP;SECTM1;SHROOM4;SIT1;SLC12A6;SLC22A1;SLC22A14;SLC22A4;SLC26A8;SLC2A11;SLC8A1;SMAGP;SNAP23;SNTA1;SNTG2;ST3GAL5;STX3;STX6;TBC1D30;TGM3;TLR2;TLR4;TLR5;TM9SF2;TNFR

REM1;TRIB1;UIMC1;USP3;WAC;WNT10B;ZAK;ZFP

T1;FOXO3;FPR1;FPR2;FRMD4B;FYB;GCM1;GIMAP1;GNAQ;GPR141;GPR17;GPR89A;GPR97;GPSM2;GRIP2;GSK3B;GTSE1;H3F3B;HAS1;HCAR2;HCAR3;HEY1;HIF1A;HIST1H1T;HMGCR;HP;HPSE;IFNAR1;IFNGR2;IGSF6;IL10RB;IL13RA1;IL18RAP;IL1B;IL1R1;IL1RN;INHBB;INPP5A;INSC;IQGAP1;IRAK3;ITGAD;ITM2E

1;KCNH7;KCNJ15;KCNS1;KIAA0319;KPNA1;LAMP2;LBR;LILRA6;LILRB3;LRRC4;LYN;LYVE1;MANSC1;MBOAT2;MCTP2;MEGF9;MME;MPZL3;MRVI1;MYO10;NAIP;NCF4;NPTN;NUP50;NUPL1;OSM;OXER1;PCNX;PDZD3;PHB;PICALM;PLB1;PLD1;PLXDC2;PLXNC1;PPAP2B;PRRG4;PTPRE;PVRL2;RAB27A;RAPGEF2;R

)7;GPSM2;GSK3B;GTSE1;H3F3B;HAS1;HCAR2;HCAR3;HEY1;HIF1A;HIVEP1;HMGCR;HP;HPSE;IFNAR1;IFNGR2;IGSF6;IL10RB;IL13RA1;IL18RAP;IL1B;IL1R1;IL1RN;INHBB;IQGAP1;IRAK3;ITGAD;ITM2B;JHDM1D;JMJD1C;KCNE1;KCNH7;KDM1B;KIAA0319;KLF6;KPNA1;KPNA4;LAMP2;LILRB3;LIMD1;LITAF;LMO4;LR

FCAB2;ELOVL5;EMR3;ENO3;ENTPD1;ETS2;EVI5;EXT1;F5;FAM120A;FCGR1A;FCGR1B;FCGR1C;FCGR2A;FCGR3B;FFAR2;FFAR3;FGF13;FLI1;FLOT1;FOXO3;FPR1;FYB;GABARAPL1;GBE1;GCM1;GDE1;GIMAP1;GMPR2;GNAQ;GPSM2;GSK3B;H3F3B;HEY1;HIF1A;HIST1H1T;HIVEP1;HMGCR;HP;HPSE;HSPA6;IDH1;ID

C4;LYN;LYVE1;MANSC1;MBOAT2;MCTP2;MEGF9;MME;MPZL3;MRVI1;NCF4;NPTN;NUP50;NUPL1;OSM;OXER1;PCNX;PHB;PLB1;PLXDC2;PLXNC1;PPAP2B;PRRG4;PTPRE;PVRL2;RAPGEF2;RNF130;RNF145;RNF149;RNF19B;RTN3;SCARF1;SDCBP;SECTM1;SIT1;SLC12A6;SLC15A4;SLC22A1;SLC22A14;SLC22A4;SI

4;GBE1;GDE1;GIMAP1;GLIPR2;GLT1D1;GMPR2;GNAQ;GOLGA7B;GPR89A;GPSM2;GRIP2;GSDMC;GSK3B;GTSE1;HAL;HEY1;HIF1A;HIVEP1;HMGCR;HPSE;IDH1;IDS;IFNGR2;IL1B;IL1RN;INHBB;INSC;IQGAP1;IRAK3;ITM2B;KAZN;KCNE1;KIAA0319;KLF6;KPNA1;KPNA4;LAMP2;LBR;LIMD1;LITAF;LRRC6;LRRFIP1;LYN

ST3GAL5;TG;TGM3;TIAM2;TMLHE;TMX4;TNFRSF1A;WNT1

2;MCTP2;MEGF9;MME;MPZL3;MRVI1;NCF4;NPTN;NUP50;NUPL1;OSM;OXER1;PCNX;PHB;PLB1;PLXDC2;PLXNC1;PPAP2B;PRRG4;PTPRE;PVRL2;RAPGEF2;RNF130;RNF145;RNF149;RNF19B;RTN3;SCARF1;SDCBP;SECTM1;SIT1;SLC12A6;SLC15A4;SLC22A1;SLC22A14;SLC22A4;SLC26A8;SLC2A11;SLC45A4;SLC8A

T1;F5;FAM65B;FCGR1A;FCGR1B;FFAR2;FFAR3;FGF13;FLI1;FLOT1;FOXO3;FPR1;FPR2;FRMD4B;FYB;GCM1;GIMAP1;GNAQ;GPR141;GPR17;GPR89A;GPR97;GPSM2;GRIP2;GSK3B;GTSE1;H3F3B;HAS1;HCAR2;HCAR3;HEY1;HIF1A;HIST1H1T;HMGCR;HP;HPSE;IDH1;IFNAR1;IFNGR2;IGSF6;IL10RB;IL13RA1;IL18RAP

P1;HMGCR;HP;HPSE;IFNAR1;IFNGR2;IGSF6;IL10RB;IL13RA1;IL18RAP;IL1B;IL1R1;IL1RN;INHBB;IQGAP1;IRAK3;ITGAD;ITM2B;JHDM1D;JMJD1C;KCNE1;KCNH7;KDM1B;KIAA0319;KLF6;KPNA1;KPNA4;LILRB3;LIMD1;LITAF;LMO4;LRRFIP1;LYN;MAK;MARCKS;MCTP2;MLLT11;MYO10;MYO5A;NAIP;NCOA1;NCOA

;TLR5;TNFRSF1A;TNFSF14;TPX2;TRIB1;TSHZ3;UIMC1;VAMP3;WAC;WIPF1;WLS;WNT10B;ZAK;ZFF

;WNT10B;ZAK;ZFP

RTC2;DMXL2;DNAH14;DNAJB11;DNAJC3;DOCK1;DOCK4;DPYD;DUSP13;E2F3;EBP;ECHDC3;EDEM2;ELMOD3;ELOVL5;EMR3;ENO3;ENTPD1;ETS2;EVI5;EXT1;F5;FAM120A;FAM49A;FAM65B;FCGR1A;FCGR1B;FCGR1C;FCGR2A;FCGR3B;FFAR2;FFAR3;FGF13;FLI1;FLOT1;FOXO3;FPR1;FPR2;FRMD4B;FRY;FYB;GA
RTC2;DMXL2;DNAH14;DNAJB11;DNAJC3;DOCK1;DOCK4;DPYD;DUSP13;E2F3;EBP;ECHDC3;EDEM2;ELMOD3;ELOVL5;EMR3;ENO3;ENTPD1;ETS2;EVI5;EXT1;F5;FAM120A;FAM49A;FAM65B;FCGR1A;FCGR1B;FCGR1C;FCGR2A;FCGR3B;FFAR2;FFAR3;FGF13;FLI1;FLOT1;FOXO3;FPR1;FPR2;FRMD4B;FRY;FYB;GA

BR;LIMD1;LITAF;LYN;MARCKS;MBOAT2;ME2;MME;MRPL9;MRVI1;MXD1;MYO10;MYO5A;MZT2A;NCF4;NEDD9;NFE2L2;NLN;NMNAT2;NPEPPS;NRD1;NT5C2;OPLAH;OSBPL1A;PAK2;PANK2;PAPSS2;PDE4B;PDK3;PDRG1;PDZD3;PELI2;PFKFB4;PGS1;PHB;PICALM;PLBD1;PLD1;PLIN5;PNP;POLD3;PPAP2B;PPP1F

VWF;WAS

D1;TUBA8;TUBB1;VEGFC;VIL1;VWF;WAS

40;TUBA8;TUBB1;VEGFC;VEPH1;VIL1;VWF;WASF3;ZNF4

M2;TMEM98;TMOD1;TNS1;TRIM10;TRIM50;TSPO2;TSTA3;TTC25;TUBB2A;TUBB2B;UBA52;UBB;UBXN6;UTS2R;VTI1B;WDR45;YE

A5;SLC4A1;SLC6A19;SLC6A8;SLC6A9;SNCA;SOX6;SPDYC;SPTA1;STRADB;STX2;TAL1;TESC;TFDP1;TGM2;TMOD1;TNS1;TRIM10;TSTA3;TUBB2A;TUBB2B;UBA52;UBB;UTS2R;VIPR2;VTI1B;WDR45;WNT9A;XK;Y

NF10;RPL3L;SEC14L4;SELENBP1;SFRP2;SGIP1;SHARPIN;SIAH2;SIM2;SLC2A4;SLC4A1;SNCA;SOX6;SPDYC;SPTA1;STRADB;STX2;TAL1;TESC;TFDP1;TGM2;TMCC2;TMEM158;TMOD1;TNS1;TRIM10;TRIM50;TRIM58;TSPO2;TSTA3;TUBB2A;TUBB2B;UBA52;UBB;UBXN6;VWCE;WDR45;WNT9A;XK;Y

UBA52;UBB;UTS2R;VIPR2;VTI1B;WNT9A;

STAG3;STAP1;STRBP;SWAP70;SYNPO;TCF4;TCL1A;TNFRSF1

CE1;SPIN1;TERF1;THBS1;TIPIN;TNKS;TRIM32;TUBA1A;TUBA4A;UBD;UBE2C;UBE2S;UHRF2;USP[illegible]

LN1;FHOD3;FOXD4L1;FTH1;FUZ;GABARAP;GALR2;GJA4;GJC1;GNG4;GNPAT;GPM6B;GPX3;GRB7;GSPT2;HAS3;HAUS2;HDAC2;HES1;HIST1H1C;HIST1H2AD;HIST1H2BK;HIST1H2BO;HIST2H2AB;HMGA2;HPN;HSP90AA1;HTRA3;IGFBP2;IHH;INPP5J;INSRR;INTU;ISL2;ITGA7;ITGB1;KCNA3;KCNB1;KCNQ5;KCTD4;[illegible]

CCDC15;CCDC19;CCDC50;CCIN;CCNJ;CD151;CDC14B;CDC23;CDK5;CDK6;CDKN2D;CDYL;CENPC1;CENPJ;CEP128;CEP192;CEP68;CEP76;CERS1;CERS4;CERS6;CHIC2;CHRNA3;CHST1;CITED4;CLASP2;CLIC5;CLINT1;CLLU1;CLOCK;CLTB;CMAS;CNIH2;COL16A1;COL18A1;COL23A1;COL4A2;COL9A1;COX6B1;CPE;CP

LN1;FHOD3;FOXD4L1;FTH1;FUZ;GABARAP;GALR2;GJA4;GJC1;GNG4;GNPAT;GPM6B;GPX3;GRB7;GSPT2;HAS3;HAUS2;HDAC2;HES1;HIST1H1C;HIST1H2AD;HIST1H2BK;HIST1H2BO;HIST2H2AB;HMGA2;HPN;HSP90AA1;HTRA3;IGFBP2;IHH;INPP5J;INSRR;INTU;ISG20;ISL2;ITGA7;ITGB1;KCNA3;KCNB1;KCNQ5;K

192;CEP68;CEP76;CERS1;CERS4;CERS6;CHIC2;CHRNA3;CHST1;CITED4;CLASP2;CLIC5;CLINT1;CLOCK;CLTB;CMAS;CNIH2;COL16A1;COL18A1;COL23A1;COL4A2;COL9A1;COX6B1;CPE;CPT1B;CRTAP;CSE1L;CSPG5;CSPP1;CTLA4;CTNNBIP1;CTNND2;CWC27;CYP11B1;CYP1A1;CYP2A7;CYP4V2;DBT;DCAF17;DCTN3

192;CEP68;CEP76;CERS1;CERS4;CERS6;CHIC2;CHRNA3;CHST1;CITED4;CLASP2;CLIC5;CLINT1;CLOCK;CLTB;CMAS;CNIH2;COL16A1;COL18A1;COL23A1;COL4A2;COL9A1;COX6B1;CPE;CPT1B;CRTAP;CSE1L;CSPG5;CSPP1;CTLA4;CTNNBIP1;CTNND2;CWC27;CYP11B1;CYP1A1;CYP2A7;CYP4V2;DBT;DCAF17;DCTN3

GTF2H3;GTF3C3;GVINP1;HAUS2;HDAC2;HELQ;HES1;HEXIM2;HIST1H1C;HIST1H2AD;HIST1H2BK;HIST1H2BO;HIST2H2AB;HLX;HMBOX1;HMGA2;HN1;HNRNPA1L2;HOXB6;HPN;HUS1;HUS1B;IER3;IL15RA;INTS10;IPO7;IPPK;ISG20;ISL2;JDP2;KIAA1161;KIFAP3;KLF12;KPTN;L3MBTL3;LIN37;LRPPRC;LTV1;MAFF;

B;TERF1;THBS1;TNKS;TRIM37;TRIP6;TSPAN4;TSPYL1;TSPYL5;TTBK2;TUBA1A;TUBA1C;TUBA4A;UBD;UBE2C;UBE2S;WDR19;WNT

7;CBX8;CCDC15;CCDC19;CCDC50;CCIN;CCNJ;CD151;CDC14B;CDC23;CDK5;CDK6;CDKN2D;CDYL;CENPC1;CENPJ;CEP128;CEP192;CEP68;CEP76;CERS1;CERS4;CERS6;CHIC2;CHRNA3;CHST1;CITED4;CLASP2;CLIC5;CLINT1;CLLU1;CLOCK;CLTB;CMAS;CNIH2;COL16A1;COL18A1;COL23A1;COL4A2;COL9A1;COX6B1;

IT1;CLOCK;CLTB;CMAS;CNIH2;COL16A1;COL18A1;COL23A1;COL4A2;COL9A1;COX6B1;CPE;CPT1B;CRTAP;CSE1L;CSPG5;CTLA4;CTNNBIP1;CTNND2;CWC27;CYP11B1;CYP1A1;CYP2A7;CYP4V2;DBT;DCAF17;DDAH2;DDN;DDX1;DEDD2;DGAT1;DHRS7B;DHX29;DIP2C;DNAH2;DNAH9;DNAJC2;DNAJC8;DNER;DNM

IT1;CLOCK;CLTB;CMAS;CNIH2;COL16A1;COL18A1;COL23A1;COL4A2;COL9A1;COX6B1;CPE;CPT1B;CRTAP;CSE1L;CSPG5;CTLA4;CTNNBIP1;CTNND2;CWC27;CYP11B1;CYP1A1;CYP2A7;CYP4V2;DBT;DCAF17;DDAH2;DDN;DDX1;DEDD2;DGAT1;DHRS7B;DHX29;DIP2C;DNAH2;DNAH9;DNAJC2;DNAJC8;DNER;DNM

;CARD9;CASK;CASP7;CASR;CBX8;CCIN;CCL25;CCL27;CCL28;CD151;CD300A;CD3G;CDC14B;CDC23;CDH5;CDK5;CDK6;CDKN2D;CDYL;CENPC1;CENPJ;CEP192;CEP68;CEP76;CERS1;CERS4;CERS6;CHRM4;CHRM5;CHRNA3;CHST1;CIB2;CITED4;CLASP2;CLCN1;CLDN10;CLDN11;CLIC5;CLINT1;CLOCK;CLTB;CMAS;CI

A2;COL9A1;COX6B1;CPE;CPT1B;CRTAP;CRYZL1;CSPG5;CTLA4;CTNNBIP1;CYP11B1;CYP1A1;CYP2A7;CYP4V2;DARS;DBT;DCTN3;DDAH2;DDN;DDX1;DGAT1;DHRS7B;DHX29;DNAH2;DNAH9;DNAJC2;DNER;DNM1L;DOK1;DRD3;DSTN;DYNC1I1;DYNC2H1;EBPL;ECE2;EI24;EIF2S1;EIF4EBP1;EIF4EBP3;ELANE;ELF3;E

BRM1;PCBD2;PCGF2;PDE2A;PDE8A;PDS5A;PIAS2;PIK3R1;PIWIL2;PLK3;PPP1R8;PPP2CA;PRDM7;PRNP;PRR13;PSRC1;PTPN14;PYCARD;RALGAPA1;RARA;RBL1;RBMX;RFXANK;RGS14;RGS2;RHOG;RNF168;SBNO1;SCRT1;SERTAD1;SET;SHOX2;SIRT7;SMAD6;SMC3;SNAPC3;SPHK1;SPI1;SRA1;SRFBP1;SYNCRIP;TA

H3;NR2C2;NUFIP1;PAX9;PBRM1;PCBD2;PCGF2;PDE2A;PDE8A;PDS5A;PIAS2;PIK3R1;PIWIL2;PLK3;POLR2D;PPP1R8;PPP2CA;PRDM7;PRNP;PRR13;PSRC1;PTPN14;PYCARD;RALGAPA1;RARA;RBL1;RBMX;RFXANK;RGS14;RGS2;RHOG;RNF168;SBNO1;SCRT1;SERTAD1;SET;SHOX2;SIRT7;SMAD6;SMC3;SNAPC3;SP

F10;ARHGEF11;ARHGEF12;ARID1A;ARID3A;ARID3B;ARL1;ARL6;ARNT;ARSD;ARSG;ASAH1;ASB8;ASH2L;ATF5;ATF7;ATG7;ATP5S;ATP6V0A1;ATP6V0C;ATP6V0D1;ATP6V1B2;ATPIF1;ATXN2;B3GNT9;B4GALT1;B4GALT4;B4GALT5;BASP1;BAZ2A;BBS4;BCAS3;BCCIP;BCDIN3D;BCKDHA;BCL2L13;BCL6;BCL9;BCO2;
31;ARHGDIB;ARHGEF10;ARHGEF11;ARHGEF12;ARID1A;ARID3A;ARID3B;ARL1;ARL6;ARNT;ARSD;ARSG;ASAH1;ASB8;ASH2L;ATF5;ATF7;ATG7;ATP5S;ATP6V0A1;ATP6V0C;ATP6V0D1;ATP6V1B2;ATPIF1;ATXN2;B3GNT9;B4GALT1;B4GALT4;B4GALT5;BASP1;BAZ2A;BBS4;BCAS3;BCCIP;BCDIN3D;BCKDHA;BCL2L1
RL6;ARNT;ARSD;ARSG;ASAH1;ASB8;ATF5;ATF7;ATG7;ATP5S;ATP6V0A1;ATP6V0C;ATP6V0D1;ATP6V1B2;ATPIF1;ATXN2;B3GNT9;B4GALT1;B4GALT4;B4GALT5;BASP1;BBS4;BCAS3;BCDIN3D;BCKDHA;BCL2L13;BCL9;BCO2;BCORL1;BECN1;BEX2;BEX5;BHLHE40;BICD2;BIN2;BIVM;BLOC1S3;BNIP3;BOD1;BPNT1;
B4GALT4;B4GALT5;BASP1;BAZ2A;BBS4;BCAS3;BCCIP;BCKDHA;BCL2L13;BCL6;BCL9;BCO2;BCOR;BCORL1;BECN1;BEX2;BHLHE40;BICD2;BIVM;BLOC1S3;BNIP3;BRCA1;BRD3;BRD4;BRD8;BRE;BRIX1;BRPF3;BSCL2;BSG;BTK;BUB3;C10orf10;C11orf73;C14orf159;C17orf89;C19orf12;C22orf32;C3;C3orf52;C6orf17
SP1;BAZ2A;BBS4;BCAS3;BCCIP;BCKDHA;BCL2L13;BCL6;BCL9;BCO2;BCOR;BCORL1;BECN1;BEX2;BHLHE40;BICD2;BIVM;BLOC1S3;BNIP3;BRCA1;BRD3;BRD4;BRD8;BRE;BRIX1;BRPF3;BSCL2;BSG;BTK;BUB3;C10orf10;C11orf73;C14orf159;C17orf89;C19orf12;C22orf32;C3orf52;C6orf170;C6orf57;C7orf55;CACN
C;ATP6V0D1;ATP6V1B2;ATPIF1;ATXN2;B3GNT9;B4GALT1;B4GALT4;B4GALT5;BASP1;BAZ2A;BBS4;BCAS3;BCCIP;BCKDHA;BCL2L13;BCL6;BCL9;BCO2;BCOR;BCORL1;BECN1;BEX2;BHLHE40;BICD2;BIN2;BIVM;BLOC1S3;BNIP3;BOD1;BRCA1;BRD3;BRD4;BRD8;BRE;BRIX1;BRPF3;BSCL2;BSG;BTK;BUB3;C10orf10;
V0D1;ATP6V1B2;ATPIF1;ATXN2;B3GNT9;B4GALT1;B4GALT4;B4GALT5;BASP1;BAZ2A;BBS4;BCAS3;BCCIP;BCKDHA;BCL2L13;BCL6;BCL9;BCO2;BCOR;BCORL1;BECN1;BEX2;BHLHE40;BICD2;BIN2;BIVM;BLOC1S3;BNIP3;BOD1;BRCA1;BRD3;BRD4;BRD8;BRE;BRIX1;BRPF3;BSCL2;BSG;BTK;BUB3;C10orf10;C11orf
HA;BCL2L13;BCO2;BECN1;BHLHE40;BICD2;BLOC1S3;BNIP3;BPNT1;BRCA1;BRD8;BRPF3;BSCL2;BSG;BTK;BUB3;C10orf10;C11orf73;C14orf159;C17orf89;C19orf12;C22orf32;C3orf52;C6orf57;C7orf55;CA13;CA3;CACNG8;CALCOCO2;CAMK2G;CANT1;CAP1;CAPZB;CARM1;CARS;CASC3;CASP6;CASP9;CBFA2T3;
;BRCA1;BRD3;BRD4;BRE;BRPF3;BSG;BTG2;BTK;BTN3A1;BUB3;C10orf88;C11orf73;C16orf70;C1QTNF3;C3;C5AR1;CALCOCO1;CALCOCO2;CAMK1D;CAMK2G;CAMKK2;CAP1;CAPN1;CAPNS1;CAPZB;CARM1;CARS;CASC3;CASP6;CASP9;CAST;CBFA2T3;CBL;CBX1;CBX6;CBY1;CCBL1;CCL3;CCNB1IP1;CCNK;CD163;
ASP6;CBFA2T3;CBX1;CBX6;CBY1;CC2D2A;CCRN4L;CD14;CD1C;CD1D;CD68;CDC42;CDC5L;CDV3;CENPH;CERKL;CERS2;CES1;CFL1;CFL2;CHCHD1;CHCHD6;CHCHD7;CHD8;CHEK1;CHGA;CHMP4B;CHST11;CHST15;CHST7;CITED2;CKAP4;CLCN5;CLIP2;CLPX;CLTA;CLTC;CLTCL1;CNN2;CNOT1;COG8;COL15A1;COL5
ASP6;CBFA2T3;CBX1;CBX6;CBY1;CC2D2A;CCRN4L;CD14;CD1C;CD1D;CD68;CDC42;CDC5L;CDV3;CENPH;CERKL;CERS2;CES1;CFL1;CFL2;CHCHD1;CHCHD6;CHCHD7;CHD8;CHEK1;CHGA;CHMP4B;CHST11;CHST15;CHST7;CITED2;CKAP4;CLCN5;CLIP2;CLPX;CLTA;CLTC;CLTCL1;CNN2;CNOT1;COG8;COL15A1;COL5
;ARHGAP26;ARHGAP27;ARHGAP30;ARHGAP31;ARHGDIB;ARHGEF10;ARHGEF11;ARHGEF12;ARID1A;ARID3A;ARID3B;ARL1;ARL6;ARNT;ARSD;ARSG;ASAH1;ASB8;ASH2L;ATF5;ATF7;ATG7;ATP2B4;ATP5S;ATP6V0A1;ATP6V0C;ATP6V0D1;ATP6V1B2;ATPIF1;ATXN2;B3GNT9;B4GALT1;B4GALT4;B4GALT5;BASP1
;ARHGAP26;ARHGAP27;ARHGAP30;ARHGAP31;ARHGDIB;ARHGEF10;ARHGEF11;ARHGEF12;ARID1A;ARID3A;ARID3B;ARL1;ARL6;ARNT;ARSD;ARSG;ASAH1;ASB8;ASH2L;ATF5;ATF7;ATG7;ATP2B4;ATP5S;ATP6V0A1;ATP6V0C;ATP6V0D1;ATP6V1B2;ATPIF1;ATXN2;B3GNT9;B4GALT1;B4GALT4;B4GALT5;BASP1
TCH1;NRBP1;OPTN;P2RX1;PACS1;PACSIN2;PAK1;PECAM1;PIK3CD;PLEK;PLIN3;PPT1;PRAM1;PSAP;PSEN1;PTPN1;PTX3;RAB3D;RAB43;RAB5B;RAB5C;RAB7A;RAB8A;RAC1;RALB;RAMP1;RIN2;RIN3;RUFY1;SCAMP2;SEC16A;SEC22A;SEC23B;SEC31A;SERPINA1;SFT2D3;SH3KBP1;SLC11A1;SNAP29;SNX1;SOD1;SC
12;ARID1A;ARID3A;ARID3B;ARL1;ARL6;ARNT;ARSD;ARSG;ASH2L;ATF5;ATF7;ATG2A;ATG7;ATP2B4;ATP5S;ATP6V0A1;ATP6V0C;ATP6V0D1;ATP6V1B2;ATPIF1;ATXN2;B4GALT1;B4GALT4;B4GALT5;BASP1;BAZ2A;BBS4;BCAS3;BCCIP;BCKDHA;BCL6;BCL9;BCO2;BCOR;BECN1;BHLHE40;BICD2;BIN2;BLOC1S3;BNIP
L2;EIF2B3;EIF4A2;EIF4H;ELMO1;ELMO2;ENO1;ENOPH1;ERC1;EXOSC1;EXOSC3;EXOSC8;FABP6;FAHD1;FAM129B;FAM96B;FBP1;FKBP1A;FOXO4;FSCN1;G6PD;GAB2;GAPDHS;GCK;GDI2;GIMAP4;GLI1;GLUL;GMIP;GNAI2;GNAS;GRB10;GRB2;GSK3A;GSN;GSR;GSTA4;GTPBP1;HAAO;HCK;HDAC4;HGD;HK1;HK2;
HOA;RIN2;RRAGC;RRAS2;RSU1;SEMA4D;SH2D3C;SIPA1L1;SIPA1L2;SMAP2;SPRY2;SQSTM1;SRC;SRGAP2;STARD8;TBC1D2;TIMP2;TRIM67;ULK1;VAV1;WASF2;YWH

E;HMOX1;HRH2;HSPA1B;ICOS;IFNAR2;IGF1R;IKZF1;IL23A;IL4R;IL6R;IRAK2;IRF2;IRS2;ITGA5;ITGAM;ITGAX;ITGB2;JAK1;JAK3;JARID2;KAT6A;KIF3C;KIR3DL1;KLF13;KLF4;KLRC4-KLRK1;LAT2;LCP1;LCP2;LILRA1;LILRB1;LILRB2;LTB4R;MAEA;MAFB;MAP2K1;MAP2K3;MAP3K5;MAPK14;MARCH8;MBP;MEFV;MERT

G2;PSEN1;PTPN1;RAB11FIP1;RAB11FIP4;RAB23;RAB35;RAB3D;RAB43;RAB5B;RAB5C;RAB7A;RAB8A;RAC1;RGL4;RIN3;RPH3A;RPN1;SEC23B;SEC31A;SERPINA1;SH3KBP1;SLC11A1;SLC1A5;SNX19;SNX24;SOD1;SPG21;SPPL3;SQSTM1;SRGAP2;SRI;SV2B;SYK;SYT2;TBC1D2;TGFB1;TGFBI;TGOLN2;TIRAP;TLN1;TL
RM4;MAP2K1;MAPK14;ME3;MFN2;MICU1;MRP63;MRPL11;MRPL14;MRPL17;MRPL21;MRPL27;MRPL30;MRPL34;MRPL35;MRPL36;MRPL43;MRPL45;MRPL46;MRPL51;MRPL53;MRPS14;MRPS21;MRPS23;MRPS30;MRPS35;MRPS6;MSRA;MTCP1;MTERFD3;NDUFA12;NDUFA7;NDUFAB1;NDUFAF4;NDUFB2
C;EXTL3;FAM21A;FAM21C;FIG4;FIS1;FKBP1A;FLOT2;FUNDC1;FURIN;GALNT11;GALNT2;GBA;GCC1;GCNT7;GHITM;GNAS;GOLGA2;GOLGA5;GOLGB1;GOLIM4;GOSR1;GRB2;HAAO;HAX1;HERPUD1;HGSNAT;HIP1;HIPK2;HK1;HK2;HLA-B;HLA-C;HLA-DPA1;HLA-E;HM13;HMOX1;HSD17B3;HSD17B4;HSD17B6;HS

31;PAK1;PARP4;PCBP2;PEX7;PGAM1;PIAS3;PINK1;PLAUR;PLEK;POLR2A;POU5F1;PREX1;PRKAB1;PRKACA;PRKCB;PRKCD;PRKDC;PTK2B;PTPN1;PTPN6;PTPRJ;PXN;RAB11FIP4;RAB3D;RAB7A;RAB8A;RAC1;RAF1;RALBP1;RIN3;RPH3A;RXRA;SELL;SERPINA1;SH3YL1;SKI;SMG7;SOD1;SP2;SPDYA;SPOP;SPRED1;SPF
1;PAFAH1B1;PDCD6;PEX7;PLCG2;PMPCB;POU5F1;PPP1R10;PRKACA;PSEN1;PTK2B;PTTG1IP;RAB23;RAB35;RAB43;RAB7A;RAB8A;RAMP1;RBM22;RBM8A;RERE;RHOA;RPAIN;RPH3A;RPL6;RPLP0;RPN1;RPS10;RPS9;SCAMP2;SEC16A;SEC22A;SEC23B;SEC31A;SEC61G;SLC11A1;SLC25A14;SLC9A1;SMG7;SMUR
TED2;CLPX;CMIP;CNOT4;COPS7A;COPS8;CPM;CPSF7;CPT2;CREBBP;CRNKL1;CSNK1D;CSNK2A1;CTDP1;CTDSP2;CTNNB1;CTNND1;CTSA;CTSB;CUX1;CUX2;CWC15;DAB2;DAG1;DAP3;DAZAP2;DCLRE1B;DEDD;DHX8;DNAJB6;DNAJC14;DNMT3B;DPF2;DSCC1;DSCR3;DUSP12;DUSP18;DUT;DYNLL1;EGR1;EHD3;E
AM1;NFKB1;NLRC4;NLRP12;NLRP3;NLRP6;NMNAT1;NOD2;NOTCH1;NOTCH2;OSMR;P2RX1;PAK1;PARP4;PECAM1;PHF21A;PIK3CD;PIK3R5;PLAUR;PLCG2;PLEK;PNMA1;POU5F1;PRCP;PRKACA;PRKCB;PRKCD;PSAP;PTAFR;PTPN1;PTPN6;PTPRJ;PTX3;RAC1;RAC2;RAF1;RCOR1;RHOA;SAAL1;SELL;SELPLG;SERPIN

K1;DBNL;DCLRE1B;DGKG;DNAJB5;DNAJB6;DOCK2;DOCK6;DOCK8;DVL3;DYSF;EEPD1;EGR1;EHD1;EHD3;EID3;EIF2AK1;EIF2B3;ELK1;ELMO1;EMR2;EP300;EPHB1;EXTL3;EYA3;F11R;F13A1;F2RL2;FAM129A;FAM162A;FANCF;FBXO22;FCN1;FLCN;FOXN3;FOXO4;FOXP3;FTO;FUNDC1;G6PD;GBA;GCK;GNA11;GNA

HOA;RNF41;RPS6KA1;SELPLG;SEMA4A;SERPINA1;SLC11A1;SOD1;SOX4;SRC;SRF;STAT5A;STAT5B;STAT6;SYK;TGFB1;TIRAP;TLN1;TNFSF13;VAV1;VCL;W

AH1B1;PDCD6;PEX7;PLEK;PMPCB;POU2F2;POU5F1;PPID;PPP1R10;PPT1;PRAF2;PRKACA;PRKCB;PSEN1;PTTG1IP;RAB11FIP1;RAB19;RAB23;RAB35;RAB36;RAB3D;RAB40B;RAB43;RAB5B;RAB5C;RAB7A;RAB8A;RAMP1;RBM22;RERE;RHBDF2;RHOA;RPAIN;RPH3A;RPL6;RPLP0;RPN1;RPS10;RPS9;RRBP1;SCAMP

37A;RAB8A;RAC1;RGL4;RIN3;RPH3A;RPN1;SEC23B;SEC31A;SERPINA1;SH3KBP1;SLC11A1;SLC1A5;SNX19;SNX24;SPG21;SPPL3;SRGAP2;SRI;SV2B;SYK;SYT2;TBC1D2;TGFB1;TGFBI;TGOLN2;TIRAP;TLN1;TLR9;TMEM67;TPP1;TPRG1L;ULK1;VAMP2;VAMP8;VIM;WAS;WIPI1;YIPF3;YWH
EN1;PTK2B;PTPN1;PTPN6;PTPRJ;PVR;RAC1;RCOR1;RIPK1;RNF41;RPS6KA1;SAMHD1;SELL;SH2D1A;SIN3A;SLC11A1;SOD1;SPG21;SRC;STAT5A;STAT5B;STAT6;SYK;TAP2;TFE3;TGFB1;THOC5;TIRAP;TLR9;TNFSF13;TNIP1;TRAT1;TRIM27;UBE2V1;VASP;VAV1;WAS;WDR61;X
NG2;KCNMB4;KDELR1;KIAA0196;KIF13A;KIF1B;KIF1C;KPNB1;LAT2;LCP1;LCP2;LEFTY2;LILRB1;LIN7B;LMTK2;LTBP2;MAP1S;MAP2K1;MAPK14;MERTK;MFN2;MGRN1;MICU1;MRPL45;MX2;MYH9;MYO1F;MYO7A;NAPA;NDEL1;NFKBIA;NLRC4;NLRP12;NLRP3;NMB;NOD2;NOTCH1;NOTCH2;NRBP1;NUP98;NUP
N7B;LMTK2;LRP1;LRP10;LTBP2;MAGI2;MAGOHB;MAPK14;MAPRE1;MEST;MFN2;MLH3;MRPL45;MX2;MYH9;MYO7A;NACC2;NAPA;NCOR1;NFKB1;NFKBIA;NLRC4;NLRP12;NLRP3;NMB;NOD2;NOTCH1;NOTCH2;NUP35;NUP98;NUPL2;NXT1;OPTN;OS9;OSBP;OSBPL2;PACS1;PAFAH1B1;PAK1;PDCD6;PEX7;PID

3A1;FANCF;FDX1;FLT3;FOXJ2;FOXN3;FOXO4;FOXP3;FURIN;FUS;GALNS;GATAD2B;GBA;GCK;GIT2;GLB1;GM2A;GNS;GOLIM4;GTF2F1;GTF3A;H1F0;H2AFY;HAX1;HCLS1;HDAC4;HDGF;HEXB;HIBADH;HIPK2;HMOX1;HNRNPD;HNRNPF;HNRNPK;HNRNPUL1;HSD17B4;HSPA1B;HSPA5;IGF2R;ING4;INTS3;IP6K1;IQS
RAB7A;RAB8A;RAC1;RGL4;RIN3;RPH3A;RPN1;SEC23B;SEC31A;SERPINA1;SH3KBP1;SLC11A1;SLC1A5;SNX19;SNX24;SOD1;SPG21;SPPL3;SQSTM1;SRGAP2;SRI;SV2B;SYK;SYT2;TBC1D2;TGFB1;TGOLN2;TIRAP;TLR9;TM9SF1;TMEM67;TP53INP2;TPP1;TPRG1L;ULK1;VAMP2;VAMP8;WIPI1;YIPF3;YWH

CK;HCLS1;HERPUD1;HEXB;HIP1;HIPK2;HK2;HMOX1;HRK;HSPA1B;HSPA5;IGF1R;IL6R;ING4;IRS2;ITGA5;ITGB2;ITSN1;JAK3;KCNC3;KIAA0196;KIAA1324;KIF1B;KLF4;KPNB1;LAMP1;LILRB1;MAEA;MAGEH1;MAP1S;MAP3K5;MAPK14;MAX;MKNK2;MMP9;MRPS30;NACC2;NAE1;NCF2;NCSTN;NDUFS3;NEK6;NFKB
CK;HCLS1;HERPUD1;HEXB;HIP1;HIPK2;HK2;HMOX1;HRK;HSPA1B;HSPA5;IGF1R;IL6R;ING4;IRS2;ITGA5;ITGB2;ITSN1;JAK3;KCNC3;KIAA0196;KIAA1324;KIF1B;KLF4;KPNB1;LAMP1;LILRB1;MAEA;MAGEH1;MAP1S;MAP3K5;MAPK14;MAX;MKNK2;MMP9;MRPS30;NACC2;NAE1;NCF2;NCSTN;NDUFS3;NEK6;NFKB
FOXJ2;FOXN3;FOXO4;FOXP3;FURIN;FUS;GALNS;GATAD2B;GBA;GCK;GIT2;GLB1;GM2A;GNS;GOLIM4;GTF2F1;GTF3A;H1F0;H2AFY;HAX1;HCLS1;HDAC4;HDGF;HEXB;HIBADH;HIPK2;HMOX1;HNRNPD;HNRNPF;HNRNPK;HNRNPUL1;HSD17B4;HSPA1B;HSPA5;ING4;INTS3;IP6K1;IQSEC1;IRF2;ISG20L2;JARID2;KAT

6;IL23A;IL4R;IMMP2L;IRS2;JAK3;JPH4;KCNA5;KCNC3;KCNG2;KCNMB4;KDELR1;KIAA0196;KIF13A;KIF1B;KIF1C;KPNB1;LAT2;LCP1;LCP2;LEFTY2;LILRB1;LIN7B;LMTK2;LTBP2;MAGI2;MAP1S;MAP2K1;MAPK14;MAPRE1;MERTK;MFN2;MGRN1;MICU1;MRPL45;MX2;MYH9;MYO1F;MYO7A;NACC2;NAPA;NDEL1;
EIF2AK1;ELK1;ELMO1;EP300;EPHB1;EPS15L1;ESR1;EVC;EYA3;FAM162A;FBN2;FBP1;FGF9;FIS1;FKBP1A;FLCN;FLT3;FOXP3;FURIN;FZD1;GAB2;GBA;GDI2;GIT2;GLI1;GMIP;GNAI2;GPR183;GRB10;GRB2;GSK3A;HAX1;HCK;HCLS1;HIPK2;HLA-B;HLA-C;HLA-DPA1;HLA-E;HMGN3;HMOX1;HRK;HSPA5;IFNAR2;IGF1R

AH1B1;PDCD6;PEX7;PID1;PLEK;PMPCB;POU2F2;POU5F1;PPID;PPP1R10;PPT1;PRAF2;PRKACA;PRKCB;PSEN1;PTTG1IP;RAB11FIP1;RAB19;RAB23;RAB35;RAB36;RAB3D;RAB40B;RAB43;RAB5B;RAB5C;RAB7A;RAB8A;RAMP1;RBM22;RERE;RHBDF2;RHOA;RPAIN;RPH3A;RPL6;RPLP0;RPN1;RPS10;RPS9;RRBP1;SC
OXP3;FURIN;FUS;GALNS;GATAD2B;GBA;GCK;GIT2;GLB1;GM2A;GNS;GOLIM4;GTF2F1;GTF3A;H1F0;H2AFY;HAX1;HCLS1;HDAC4;HDGF;HEXB;HIBADH;HIPK2;HMOX1;HNRNPD;HNRNPF;HNRNPK;HNRNPUL1;HSD17B4;HSPA1B;HSPA5;ING4;INTS3;IP6K1;IQSEC1;IRF2;ISG20L2;JARID2;KAT5;KAT6A;KAT7;KDM2A;

F4;ELK1;ELL;ELL3;ENO1;EP300;ERC1;ERF;ESR1;ESRP2;EYA3;FAM120B;FAM129A;FAM58A;FGF9;FLCN;FOSL2;FOXJ2;FOXN3;FOXO4;FOXP3;FTO;FZD1;GAS7;GIGYF2;GLI1;GLMN;GLO1;GNAS;GNRH1;GTF2F1;GTF2H5;GTF3A;H2AFY;HAX1;HCK;HCLS1;HDAC4;HDGF;HENMT1;HESX1;HEXB;HIP1;HIPK2;HIVEP3;HN

ECN1;BEX2;BHLHE40;BLOC1S3;BNIP3;BRAP;BRCA1;BRD3;BRD4;BRD8;BRE;BSG;BTG2;BTG3;BTK;BTN3A1;BUB3;C11orf31;C1QTNF3;C3;C5AR1;C6orf89;CABLES2;CACNG8;CALCOCO1;CAMK1D;CAMK2G;CAMKK2;CAMTA1;CANT1;CAP1;CAPN1;CAPNS1;CAPZB;CARM1;CASC3;CASP6;CASP9;CASZ1;CBFA2T3;CE
IN3D;BCL2L13;BCL6;BCL9;BCO2;BCOR;BCORL1;BECN1;BEX2;BHLHE40;BLOC1S3;BNIP3;BRAP;BRCA1;BRD3;BRD4;BRD8;BRE;BSCL2;BSG;BTG2;BTG3;BTK;BTN3A1;BUB3;C11orf31;C1QTNF3;C1RL;C3;C4BPB;C5AR1;C6orf89;CABLES2;CACNG8;CALCOCO1;CAMK1D;CAMK2G;CAMKK2;CAMTA1;CANT1;CAP1;CAP

2;NAPA;NFKBIA;NLRC4;NLRP12;NLRP3;NOD2;NOTCH1;NOTCH2;NUP35;NUP98;NUPL2;NXT1;OPTN;OS9;PACS1;PAFAH1B1;PAK1;PDCD6;PEX7;PID1;PLEK;PMPCB;POU2F2;POU5F1;PPID;PPP1R10;PPT1;PRAF2;PRKACA;PRKCB;PSEN1;PTTG1IP;RAB11FIP1;RAB19;RAB23;RAB35;RAB36;RAB3D;RAB40B;RAB43;

K6;NFKB1;NFYC;NMNAT1;NOTCH1;NOTCH2;NR2C1;NRBP1;NUP98;PAF1;PBX2;PCBP1;PCBP2;PHF12;PHF21A;PKNOX1;POLE4;POLR2A;POLR3F;POLR3GL;POU5F1;PPID;PRIM1;PRKCD;PRKDC;PSMB2;PSMD3;PSME3;PSMF1;PTRF;RAD1;RAD23B;RBM8A;RBPJ;RCC1;RCOR1;RERE;RFC4;RNF34;RPA3;RPAIN;RPRD
101B;FAM160A2;FAM162A;FHDC1;FIG4;FIS1;FLCN;FOXP3;FSCN1;GAS7;GBA;GOLGA5;GOLGB1;GRB2;GSN;H1F0;H2AFY;HAX1;HCK;HCLS1;HDAC4;HEXB;HIP1;HIPK2;HIST3H2A;HK2;HMGN3;HRK;IKZF1;IMMP2L;ING4;INSR;IQSEC1;JARID2;KAT5;KAT6A;KAT7;KATNA1;KDM2A;KDM3B;KDM4B;KDM5B;KDM6B;

LS1;HDAC4;HDGF;HIPK2;HMOX1;HNRNPD;HNRNPF;HNRNPK;HNRNPUL1;HSPA1B;HSPA5;IGF2R;ING4;INTS3;IP6K1;IQSEC1;IRF2;ISG20L2;JARID2;KAT5;KAT6A;KAT7;KDM2A;KDM5B;KLF4;KPNB1;LRRC59;LZTS1;MAEA;MAFB;MAFG;MAML1;MAML3;MAP2K3;MAPK14;MAU2;MAX;MED12;MED25;MGST3;MID

;ITGA5;ITGB2;ITSN1;JAK3;KIAA1324;KIF1B;KLF4;KPNB1;LAMP1;LILRB1;MAEA;MAGEH1;MAP1S;MAP3K5;MAPK14;MAX;MKNK2;MMP9;MRPS30;NACC2;NAE1;NCF2;NCSTN;NDUFS3;NEK6;NFKB1;NFKBIA;NLRC4;NLRP12;NLRP3;NME1;NME2;NOD2;NOTCH1;NOTCH2;NUAK2;NUDT2;P2RX1;PAK1;PDCD5;PDC
A;CUX1;CXCR2;DAB2;DAG1;DAPK1;DBNL;DENND1A;DENND3;DNAJC14;DNAJC19;DNAJC5;DNM2;DOCK2;DSCR3;DYNLL1;DYNLL2;DYSF;EHD1;EHD3;EIF2D;ELMO1;ELMO2;ENSA;EPS15L1;ERC1;ERGIC1;F13A1;FABP6;FAM160A2;FAM21A;FAM21C;FBXO22;FCN1;FGF9;FIS1;FKBP15;FKBP1A;FLVCR2;FOXP3;FUR
K1;CHRNB2;CITED2;CLPX;CNN2;CNOT1;CNOT4;CNPY2;CPT1A;CPT2;CREBBP;CSF1;CSF1R;CSNK1D;CSNK2A1;CTDP1;CTDSP2;CTIF;CTNNB1;CTNND1;CTSA;CTSB;CUX1;CUX2;DAB2;DAP;DAPK1;DAPK2;DBNL;DEDD;DISC1;DNAJB6;DNM2;DNMT3B;DPF2;DSCC1;DUSP12;DVL3;DYNLL1;ECE1;EGR1;EID2B;EID3;EIF2
EDD;DISC1;DNM2;DNMT3B;DPF2;DSCC1;DVL3;DYNLL1;ECE1;EGR1;EID2B;EID3;EIF2AK1;EIF2B3;EIF4A2;EIF4EBP2;EIF4H;ELF4;ELK1;ELL;ELL3;ENO1;EP300;ERC1;ERF;ESR1;ESRP2;EXTL3;EYA3;FAM120B;FAM129A;FAM58A;FBP1;FBXO22;FGF9;FKBP1A;FLCN;FLT3;FOSL2;FOXJ2;FOXN3;FOXO4;FOXP3;FTO;FURI

;ITGA5;ITGB2;ITSN1;JAK3;KIAA1324;KIF1B;KLF4;KPNB1;LILRB1;MAEA;MAGEH1;MAP1S;MAP3K5;MAPK14;MAX;MKNK2;MMP9;MRPS30;NACC2;NAE1;NCF2;NCSTN;NDUFS3;NEK6;NFKB1;NFKBIA;NLRC4;NLRP12;NLRP3;NME1;NME2;NOD2;NOTCH1;NOTCH2;NUAK2;NUDT2;P2RX1;PAK1;PDCD5;PDCD6;PHL

;SH3KBP1;SLC11A1;SLC1A5;SNX19;SNX24;SPG21;SPPL3;SRGAP2;SRI;SV2B;SYK;SYT2;TBC1D2;TGFB1;TGOLN2;TIRAP;TLR9;TMEM67;TPP1;TPRG1L;ULK1;VAMP2;VAMP8;WIPI1;YIPF3;YWH

1D;CSNK2A1;CTDP1;CTDSP2;CTIF;CTNNB1;CTNND1;CUX1;CUX2;DAB2;DAP;DAPK1;DAPK2;DBNL;DEDD;DISC1;DNAJB6;DNM2;DNMT3B;DPF2;DSCC1;DUSP12;DVL3;DYNLL1;ECE1;EGR1;EID2B;EID3;EIF2AK1;EIF2B3;EIF4A2;EIF4EBP2;EIF4H;ELF4;ELK1;ELL;ELL3;ENO1;EP300;ERC1;ERF;ESR1;ESRP2;EXTL3;EYA3;

;ATP6V1B2;ATPIF1;ATXN2;B4GALT1;BASP1;BAZ2A;BBS4;BCCIP;BCDIN3D;BCL2L13;BCL6;BCL9;BCO2;BCOR;BCORL1;BECN1;BEX2;BHLHE40;BICD2;BLOC1S3;BNIP3;BRAP;BRCA1;BRD3;BRD4;BRD8;BRE;BRPF3;BSCL2;BSG;BTG2;BTG3;BTK;BTN3A1;BUB3;C11orf31;C1QTNF3;C1RL;C3;C4BPB;C5AR1;C6orf89;CAB
IM4;COG8;COLEC12;COPA;COPB2;COPZ2;CORO1C;COX17;COX18;CPNE7;CPT1A;CPT2;CRTAM;CSF1;CSF1R;CSF3R;CTNNB1;CTSA;CUX1;CXCR2;DAB2;DAG1;DAPK1;DBNL;DENND1A;DENND3;DISC1;DNAAF2;DNAH17;DNAJC14;DNAJC19;DNAJC5;DNM2;DNMT3B;DOCK2;DSCR3;DYNLL1;DYNLL2;DYSF;EHD1;EH
P2R1;CYP4F3;DAG1;DGKG;DHTKD1;DHX34;DNMT3B;DPF2;DUSP12;DYRK4;ECE1;EFHA2;EFHC1;EGR1;EHD1;EHD3;ELAC1;EMR2;ENDOU;ENO1;ENOPH1;EP300;EPS15L1;ESR1;EXTL3;EYA3;F13A1;FAHD1;FAHD2A;FBN2;FBP1;FBXO40;FCN1;FDX1;FKBP9;FNTB;FOXP3;FTO;FUCA2;FURIN;FUS;GALNS;GALNT2;GA
IMOX1;IFNAR2;IGF1R;IL23A;IL6R;ING4;INSR;IQSEC1;IRAK2;IRS2;ITSN1;JAK1;JAK3;KAT5;KLF4;KSR1;LAT2;LIMK1;LRRK1;MAGI2;MAP2K1;MAP2K3;MAP3K3;MAP3K5;MAP4K4;MAPK13;MAPK14;MAPKAPK5;MARK2;MERTK;MFN2;MGRN1;MKNK1;MKNK2;MYD88;NACC2;NBR1;NCOR1;NDEL1;NDRG1;NDST1;

;IP6K1;IQSEC1;IRF2;ISG20L2;JARID2;KAT5;KAT6A;KAT7;KDM2A;KDM5B;KLF4;KPNB1;LZTS1;MAEA;MAFB;MAFG;MAML1;MAML3;MAP2K3;MAPK14;MAU2;MAX;MED12;MED25;MIDN;MIS18A;MKNK2;MLH3;MLL2;MLLT1;MRTO4;MSN;MYB;N4BP1;NACC2;NCF2;NCOA6;NCOR1;NEK6;NFKB1;NFYC;NMNAT1

32B;STK35;STK4;STK40;SYK;TJP2;TK2;TP53RK;TRIM27;ULK1;VRK3;WNK1;XRCC6BP

;TRIM27;ULK1;VRK3;WNK1;XRCC6BP

ARHGAP30;ARHGAP31;ARHGDIB;ARHGEF10;ARHGEF11;ARHGEF12;ARID1A;ARID3A;ARID3B;ARL1;ARL6;ARNT;ARSD;ARSG;ASAH1;ASB8;ASCC2;ASH2L;ATF5;ATF7;ATG2A;ATG7;ATP2B4;ATP5S;ATP6V0A1;ATP6V0C;ATP6V0D1;ATP6V1B2;ATPIF1;ATXN2;B3GNT9;B4GALT1;B4GALT4;B4GALT5;BASP1;BAZ2A;B

34;DNMT3B;DPF2;DUSP12;DYRK4;ECE1;EFHA2;EFHC1;EGR1;EHD1;EHD3;ELAC1;EMR2;ENDOU;ENO1;ENOPH1;EP300;EPS15L1;ESR1;EXTL3;EYA3;F13A1;FAHD1;FAHD2A;FBN2;FBP1;FBXO40;FCN1;FDX1;FKBP9;FNTB;FOXP3;FTO;FURIN;FUS;GALNS;GALNT2;GATAD2B;GCK;GDPD1;GIT2;GLI1;GLO1;GLUL;GME

A;CUX1;CXCR2;DAB2;DAG1;DAPK1;DBNL;DENND1A;DENND3;DNAJC14;DNAJC19;DNAJC5;DNM2;DOCK2;DSCR3;DYNLL1;DYNLL2;DYSF;EHD1;EHD3;EIF2D;ELMO1;ELMO2;ENSA;EPS15L1;ERC1;ERGIC1;F13A1;FABP6;FAM160A2;FAM21A;FAM21C;FBXO22;FCN1;FGF9;FIS1;FKBP15;FKBP1A;FLVCR2;FOXP3;FU

P300;EPHB1;EPHB2;ERC1;EXTL3;EYA3;F13A1;FAM129A;FAM58A;FBXO22;FBXO25;FKBP15;FKBP1A;FKBP5;FKBP9;FLCN;FLT3;FNTB;FOXP3;FURIN;FZD1;G6PD;GALNT11;GALNT2;GBA;GCNT7;GIGYF2;GLMN;GNA11;GNA15;GNAI2;GSK3A;HAX1;HCK;HCLS1;HDAC4;HDGF;HERPUD1;HEXB;HIPK2;HSCB;HSPA1B;H
R1;ESRP2;EXTL3;EYA3;FAM120B;FAM58A;FLCN;FOSL2;FOXJ2;FOXN3;FOXO4;FOXP3;FZD1;GAS7;GIT2;GLI1;GLO1;GMIP;GNAI2;GNAL;GNAS;GSK3A;GTF2F1;GTF2H5;GTF3A;GTPBP1;HAX1;HCK;HCLS1;HDAC4;HDGF;HERPUD1;HESX1;HEXB;HIP1;HIPK2;HIVEP3;HMGN3;HMOX1;HNRNPD;HNRNPF;HNRNPK;HN

C1;ERF;ESR1;ESRP2;EXTL3;EYA3;FAM120B;FAM58A;FLCN;FOSL2;FOXJ2;FOXN3;FOXO4;FOXP3;FZD1;GAS7;GIT2;GLI1;GLO1;GMIP;GNAI2;GNAL;GNAS;GSK3A;GTF2F1;GTF2H5;GTF3A;GTPBP1;HAX1;HCK;HCLS1;HDAC4;HDGF;HERPUD1;HESX1;HEXB;HIP1;HIPK2;HIVEP3;HMGN3;HMOX1;HNRNPD;HNRNPF;HN
A1;CTDP1;CTDSP2;CTIF;CTNNB1;CTNND1;CUX1;CUX2;DAB2;DAP;DAPK1;DBNL;DEDD;DISC1;DNAJB6;DNM2;DNMT3B;DPF2;DSCC1;DUSP12;DVL3;DYNLL1;ECE1;EGR1;EID2B;EID3;EIF2AK1;EIF2B3;EIF4A2;EIF4EBP2;EIF4H;ELF4;ELK1;ELL;ELL3;ENO1;EP300;ERC1;ERF;ESR1;ESRP2;EXTL3;EYA3;FAM120B;FAM1

;LCP1;LCP2;LILRB1;LIN7B;LMTK2;LRP1;LRP10;LTBP2;MAGOHB;MAPK14;MFN2;MRPL45;MX2;MYH9;MYO7A;NAPA;NCOR1;NFKB1;NFKBIA;NLRC4;NLRP12;NLRP3;NMB;NOD2;NOTCH1;NOTCH2;NUP35;NUP98;NUPL2;NXT1;OPTN;OS9;OSBP;OSBPL2;P2RX1;PACS1;PAFAH1B1;PDCD6;PEX7;PID1;PINK1;PLEK;P

A-DPA1;HLA-E;HM13;HMOX1;HSD17B3;HSPA5;IGF2R;ITSN1;JAK1;JAK3;JPH4;KDELR1;KIF13A;KIF1B;KPNB1;KSR1;LPCAT3;LPIN2;LRP1;LRP10;LRRC59;MAN2A2;MARCH8;MBOAT7;MEST;MFSD2A;MGST3;MRPS14;MRPS23;MRTO4;MX2;MYOF;NDEL1;NDST1;NDST2;NLRP6;NOMO2;NOTCH1;NOTCH2;NRBP1;
CORL1;BECN1;BHLHE40;BNIP3;BPNT1;BRCA1;BRD3;BRD4;BRD8;BRE;BRPF3;BSG;BTG2;BTK;BUB3;BZW2;C1QTNF3;C3;C5AR1;CA13;CA3;CA6;CALCOCO1;CAMK1D;CAMK2G;CAMKK2;CAMTA1;CANT1;CAP1;CAPN1;CARM1;CARS;CASC3;CASZ1;CBFA2T3;CBL;CBR3;CBX1;CBX6;CBY1;CCBL1;CCL3;CCNB1IP1;CCN

PO7;YWHAB;YWHAG;YWHAH;ZDHH
;HCK;HCLS1;HDAC4;HDGF;HESX1;HEXB;HIP1;HIPK2;HIVEP3;HMGN3;HMOX1;HNRNPD;HNRNPF;HNRNPK;HNRNPUL1;IFNAR2;IKZF1;IL23A;ING4;INSR;IRAK2;IRF2;JAK3;JARID2;KAT5;KAT6A;KAT7;KDM2A;KDM3B;KDM4B;KDM5B;KDM6B;KHDRBS2;KLF13;KLF4;KLF7;KLF9;LILRB1;LOXL3;LPIN2;LZTS1;MAFB;M
CENPH;CFL1;CFLAR;CHD8;CHEK1;CHST11;CHST15;CHST7;CITED2;CLPX;CLTC;CNOT1;CNOT4;CNPY2;COPS7A;COPS8;CPSF7;CREBBP;CRNKL1;CSF1;CSF1R;CSNK1D;CSNK2A1;CTDP1;CTDSP2;CTIF;CTNNB1;CTNND1;CUX1;CUX2;CWC15;CXCR2;DAB2;DAP;DAPK1;DAPK2;DBNL;DCLRE1B;DEDD;DENND3;DHX8;DIS

ARM1;CARS;CASP6;CASP9;CASS4;CBL;CBR3;CCBL1;CCDC111;CCL3;CCNB1IP1;CCNK;CD101;CD44;CDC42;CDKL3;CELA1;CERKL;CERS2;CES1;CFLAR;CHD8;CHEK1;CHKA;CHST11;CHST15;CHST7;CLC;CLPX;CNOT4;COQ3;COQ5;COX11;COX5A;COX6A1;CPM;CPOX;CPPED1;CPT1A;CPT2;CPVL;CREBBP;CRYZ;CS;CSAD

7;GCK;GIGYF2;GLI1;GLO1;GRB10;GSK3A;GTF2F1;GTF2H5;GTF3A;HAX1;HCK;HCLS1;HDAC4;HDGF;HESX1;HEXB;HIP1;HIPK2;HIVEP3;HMGN3;HMOX1;HNRNPD;HNRNPK;HNRNPUL1;IFNAR2;IGF1R;IKZF1;IL23A;ING4;INSR;IRAK2;IRF2;IRS2;JAK3;JARID2;KAT5;KAT6A;KAT7;KDM2A;KDM3B;KDM4B;KDM5B;KDM
O4;FOXP3;FZD1;GAS7;GCK;GIGYF2;GLI1;GLMN;GLO1;GRB10;GSK3A;GTF2F1;GTF2H5;GTF3A;HAX1;HCK;HCLS1;HDAC4;HDGF;HESX1;HEXB;HIP1;HIPK2;HIVEP3;HMGN3;HMOX1;HNRNPD;HNRNPK;HNRNPUL1;IFNAR2;IGF1R;IKZF1;IL23A;IL6R;ING4;INSR;IRAK2;IRF2;IRS2;JAK3;JARID2;KAT5;KAT6A;KAT7;KDM2

NX30;SNX33;SYT2;TBC1D2;TBC1D2B;TIRAP;TRIOBP;VAV1;WIPI1;WIPI2;ZFYV

AMK1D;CAMK2G;CAMKK2;CANT1;CAP1;CARM1;CASP9;CBL;CBY1;CC2D2A;CCL3;CCNK;CD101;CD14;CD209;CD3D;CD44;CD53;CD68;CD86;CD97;CDC42;CDC42EP4;CDC42SE1;CDH1;CELA1;CERKL;CFL1;CFLAR;CHCHD6;CHD8;CHEK1;CHKA;CHRNB1;CHRNB2;CHST11;CITED2;CLTA;CLTC;CLTCL1;CNN2;COL15A1

3;ELMO1;ELMO2;ERC1;ERGIC1;F13A1;FABP6;FAM160A2;FAM21A;FAM21C;FCN1;FGF9;FIS1;FKBP1A;FLVCR2;FOXP3;FURIN;FZD1;GAB2;GABRR2;GCC1;GCK;GLMN;GLUL;GNAI2;GNAS;GOLGA5;GOSR1;GRB10;GRB2;GSK3A;HCK;HCLS1;HDLBP;HFE;HGSNAT;HIP1;HK1;HK2;HMGN3;HMOX1;HNRNPK;HSPA5;IF

2F2;PRAM1;PRG2;PRKCB;PRKCD;PRKD2;PRKRA;PSEN1;PTAFR;PTK2B;PTPN1;PTPN6;PTPRJ;PVR;PVRL1;RFX1;RIPK1;RPS6KA1;SAMHD1;SELL;SEMA4A;SEMA4D;SH2D1A;SIN3A;SLC11A1;SP2;SPG21;STAT5A;STAT5B;STAT6;SYK;TAP2;TAPBP;TFE3;TGFB1;TIRAP;TLR9;TNFRSF1B;TNFSF13;TNIP1;TRAT1;TRIM25;T
NPK;HSPA5;IGF1R;IKZF1;IL23A;IL6R;INSR;IRAK2;IRS2;ITGA5;JAK3;JARID2;KAT5;KAT6A;KLF13;KLF4;KLF7;KLRC4-KLRK1;LILRB1;LPIN2;MAFB;MAGI2;MAML1;MAML3;MAP2K1;MAP2K3;MAP3K3;MAP3K5;MAPK14;MAPKAPK5;MAPRE3;MED12;MED25;MLL2;MTF1;MYB;NCOA6;NCOR1;NDNL2;NFIC;NFKB1;NF

C;PSTK;PTK2B;RAF1;RIPK1;RPS6KA1;SEPHS1;SIK3;SQSTM1;SRC;STK24;STK32B;STK35;STK4;STK40;SYK;THG1L;TJP2;TK2;TP53RK;TRIM27;UAP1;ULK1;VRK3;WNK1;XRCC6B

E4A;NUAK2;OS9;PAFAH1B1;PAK1;PARP4;PDCD6;POLR2A;PRKCD;PRKDC;PRMT6;PSEN1;PSMB2;PSMD3;PSME3;PSMF1;PTK2B;PTPN1;PTTG1;PXN;RAB23;RAD1;RAD23B;RAD54B;RBM22;RCSD1;RFC4;RHOA;RIPK1;ROMO1;RPA3;RPAIN;RPS6KA1;SEC31A;SETMAR;SH2D3C;SIN3A;SLC6A12;SMUG1;SOD1;SOX

EP3;HMGN3;HMOX1;HNRNPD;HNRNPK;HNRNPUL1;IFNAR2;IKZF1;IL23A;ING4;INSR;IRAK2;IRF2;JAK3;JARID2;KAT5;KAT6A;KAT7;KDM2A;KDM3B;KDM4B;KDM5B;KDM6B;KHDRBS2;KLF13;KLF4;KLF7;KLF9;LILRB1;LOXL3;LPIN2;LZTS1;MAFB;MAML1;MAML3;MAP2K1;MAP2K3;MAPK13;MAPK14;MAPRE3;MED

3;PSMF1;PTTG1;RALBP1;RALGAPA2;RAP1GAP2;RAPGEFL1;RCC1;RGL3;RGL4;RGS10;RGS16;RGS3;RIN2;RIN3;RPS6KA1;SERPINA1;SERPINB8;SH2D3C;SIPA1L1;SIPA1L2;SKI;SMAP2;SPINK2;SPINT1;SPRED1;SPRY2;SRGAP2;STARD8;STK4;TBC1D1;TBC1D14;TBC1D19;TBC1D2;TBC1D20;TBC1D2B;TBC1D5;TBC1D8
1;HIPK2;HIVEP3;HMGN3;HMOX1;HNRNPD;HNRNPK;HNRNPUL1;IFNAR2;IKZF1;IL23A;ING4;INSR;IRAK2;IRF2;JAK3;JARID2;KAT5;KAT6A;KAT7;KDM2A;KDM3B;KDM4B;KDM5B;KDM6B;KHDRBS2;KLF13;KLF4;KLF7;KLF9;LILRB1;LOXL3;LPIN2;LZTS1;MAFB;MAML1;MAML3;MAP2K1;MAP2K3;MAPK13;MAPK14;

NOX1;PLAC8;POLR2A;POMC;POU2F2;POU5F1;PRKCD;PRKD2;PRKDC;PTX3;RAC1;RAF1;RAMP1;RBM20;RBM22;RBPJ;RIPK1;RNF14;RPS6KA1;RTF1;RXRA;SETMAR;SIN3A;SKI;SLC11A1;SMAD9;SMARCA2;SOX4;SPEN;SQSTM1;SRF;SSBP3;STAT3;STAT5A;STAT5B;STAT6;SUPT3H;TAF11;TAF15;TAF4;TASP1;TBL1X
LL1;ECE1;EGR1;EID2B;EID3;EIF2AK1;EIF2B3;EIF4A2;EIF4EBP2;EIF4H;ELAC1;ELF4;ELK1;ELL;ELL3;EMG1;ENO1;EP300;ERC1;ERF;ESR1;ESRP2;ETV6;EXOSC1;EXOSC3;EXOSC8;EYA3;FAM120B;FAM129A;FAM58A;FGF9;FKBP1A;FLCN;FOSL2;FOXJ2;FOXN3;FOXO4;FOXP3;FTO;FURIN;FUS;FZD1;GAS7;GATAD2B;GIG
B;FOXP3;FTO;FURIN;FZD1;G6PD;GALNT11;GALNT2;GBA;GCNT7;GLMN;GNA11;GNA15;GNAI2;GNAS;GSK3A;HAX1;HCK;HCLS1;HDAC4;HDGF;HENMT1;HERPUD1;HIPK2;HSPA5;IGF1R;IL23A;IL6R;ING4;INSR;IRAK2;ITGA5;ITGB2;JAK1;JAK3;JARID2;KAT5;KAT6A;KAT7;KDM2A;KDM5B;KDM6B;KIAA0317;KLHL21

TN4;SGPL1;SH3KBP1;SIPA1L1;SKI;SMAD9;SMURF1;SOCS2;SPRY2;SQSTM1;SRC;STAT3;STAT5A;STAT5B;STRAP;SULF2;SYK;TGFB1;TLR9;TRAT1;TXNIP;ULK1;VAV1;XIAP;YWHAB;ZFP1

1;FLCN;FOSL2;FOXJ2;FOXN3;FOXO4;FOXP3;FZD1;GAS7;GCK;GIGYF2;GLI1;GLMN;GLO1;GNAI2;GNAL;GNAS;GRB10;GSK3A;GTF2F1;GTF2H5;GTF3A;HAX1;HCK;HCLS1;HDAC4;HDGF;HESX1;HEXB;HIP1;HIPK2;HIVEP3;HMGN3;HMOX1;HNRNPD;HNRNPK;HNRNPUL1;IFNAR2;IGF1R;IKZF1;IL23A;IL6R;ING4;INSR;I

SLC11A1;SMURF1;SNX1;SOX4;SPCS1;SRP54;SRPR;SSR2;STAT3;STAU1;STX18;TACC3;TAP2;TGFB1;TIMM8A;TIMM9;TINF2;TLN1;TLR9;TNPO3;TOMM22;TOMM7;TRAK1;TXNIP;UGT8;USP4;VCL;XPO6;XPO7;YWHAB;YWHAG;YWHAH;ZDHHC18;ZDHH

RP12;NLRP3;NLRP6;NLRX1;NOD2;NOTCH1;NOTCH2;NUB1;OSMR;P2RX1;PACS1;PARP4;PCBP2;PIK3CD;PLAC8;PNMA1;POLR3F;PRG2;PRKCD;PTAFR;PTPN1;PTPN6;PTX3;PVR;RAB23;RAC1;RIPK1;ROMO1;RPS6KA1;SAAL1;SAMHD1;SERPINA1;SH2D1A;SIN3A;SLC11A1;SP140;STAT3;STAT5A;STAT5B;SYK;TAPBP;

A;POMC;POU2F2;POU5F1;PRKD2;PRKDC;RAC1;RAF1;RAMP1;RBM20;RBM22;RBPJ;RIPK1;RNF14;RPS6KA1;RTF1;RXRA;SETMAR;SIN3A;SKI;SLC11A1;SMAD9;SMARCA2;SOX4;SPEN;SQSTM1;SRF;SSBP3;STAT3;STAT5A;STAT5B;STAT6;SUPT3H;TAF11;TAF15;TAF4;TASP1;TBL1X;TFB2M;TFE3;TGFB1;THRAP3;TIM
P3;HMGN3;HMOX1;HNRNPD;HNRNPK;HSPA5;IGF1R;IKZF1;IL23A;IL6R;INSR;IRAK2;IRS2;ITGA5;JAK3;JARID2;KAT5;KAT6A;KLF13;KLF4;KLF7;KLRC4-KLRK1;LILRB1;LPIN2;MAFB;MAGI2;MAML1;MAML3;MAP2K1;MAP2K3;MAP3K3;MAP3K5;MAPK14;MAPKAPK5;MAPRE3;MED12;MED25;MLL2;MTF1;MYB;NC

TRAPPC2;TXNIP;WIPI1;XPO6;XPO7;ZC3

42;CDH1;CDKL3;CELA1;CHD8;CHEK1;CHI3L2;CHKA;CHRNB1;CHRNB2;CHST15;CLCN5;CLPX;CNOT4;COL5A2;COLEC12;COX11;COX17;COX5A;CPM;CPPED1;CREBBP;CRISPLD2;CRYZ;CSAD;CSF1R;CSNK1D;CSNK2A1;CTDSP2;CYB5A;CYB5D1;CYB5R3;CYP27A1;CYP2R1;CYP4F3;DAB2;DAG1;DAPK1;DAPK2;DGKG;

1;TIRAP;TLR9;TNFRSF1B;TNIP1;UBE2V1;ULK1;XI

;FOSL2;FOXJ2;FOXN3;FOXO4;FOXP3;FZD1;GAS7;GCK;GIGYF2;GLI1;GLMN;GLO1;GNAI2;GNAL;GNAS;GRB10;GSK3A;GTF2F1;GTF2H5;GTF3A;HAX1;HCK;HCLS1;HDAC4;HDGF;HESX1;HEXB;HIP1;HIPK2;HIVEP3;HMGN3;HMOX1;HNRNPD;HNRNPK;HNRNPUL1;IFNAR2;IGF1R;IKZF1;IL23A;ING4;INSR;IRAK2;IRF2;IR

GBA;GCNT7;GLMN;GNA11;GNA15;GNAI2;GSK3A;HAX1;HCK;HCLS1;HDAC4;HDGF;HERPUD1;HIPK2;HSPA5;IGF1R;IL23A;IL6R;ING4;INSR;IRAK2;ITGA5;ITGB2;JAK1;JAK3;JARID2;KAT5;KAT6A;KAT7;KDM2A;KDM5B;KDM6B;KIAA0317;KLHL21;KSR1;LIMK1;LIPT1;LMTK2;MAML1;MAN2A2;MANBA;MAP2K1;MAP
GBA;GCNT7;GLMN;GNA11;GNA15;GNAI2;GSK3A;HAX1;HCK;HCLS1;HDAC4;HDGF;HERPUD1;HIPK2;HSPA5;IGF1R;IL23A;IL6R;ING4;INSR;IRAK2;ITGA5;ITGB2;JAK1;JAK3;JARID2;KAT5;KAT6A;KAT7;KDM2A;KDM5B;KDM6B;KIAA0317;KLHL21;KSR1;LIMK1;LIPT1;LMTK2;MAML1;MAN2A2;MANBA;MAP2K1;MAP

;MAP2K3;MAP3K3;MAP3K5;MAP4K4;MAPK13;MAPK14;MAPKAPK5;MARK2;MARK3;MAST3;MBOAT7;MERTK;METTL10;METTL23;METTL5;METTL6;MGST3;MKNK1;MKNK2;MLL2;MPP1;N6AMT1;N6AMT2;NADK;NAGK;NDST1;NDST2;NEK3;NEK6;NME1;NME2;NMNAT1;NRBP1;NSD1;NTPCR;NUAK2;PAK1;P

ALT5;BASP1;BAZ2A;BBS4;BCCIP;BCDIN3D;BCKDHA;BCL2L13;BCL6;BCL9;BCO2;BCOR;BCORL1;BECN1;BHLHE40;BNIP3;BPNT1;BRCA1;BRD3;BRD4;BRD8;BRE;BRPF3;BSCL2;BSG;BTG2;BTK;BUB3;BZW2;C1QTNF3;C1RL;C3;C4BPB;C5AR1;CA13;CA14;CA3;CA6;CALCOCO1;CAMK1D;CAMK2G;CAMKK2;CAMTA1;CA
1;SMURF1;SNX1;SOX4;SPCS1;SRP54;SRPR;SSR2;STAT3;STX18;TACC3;TAP2;TGFB1;TIMM8A;TIMM9;TINF2;TLN1;TLR9;TNPO3;TOMM22;TOMM7;TRAK1;TXNIP;UGT8;USP4;VCL;XPO6;XPO7;YWHAB;YWHAG;YWHAH;ZDHHC18;ZDHH

DST1;NDST2;NOTCH1;NOTCH2;NSFL1C;NUCB1;OPTN;OSBP;PACS1;PAK1;PLA2G12A;PLIN3;PPT1;PRKD2;PSAP;PSEN1;QSOX1;RAB36;RAB43;RAB7A;RAB8A;RAC1;RNF122;RNF24;RRAGB;SCAMP2;SEC14L1;SEC16A;SEC23B;SETDB1;SLC1A5;SLC35A2;SLC9A8;SNX1;SPG21;SPPL3;ST3GAL2;STX18;SULF2;TAPBP;T

3;ECE1;EGR1;ELF4;ELK1;ELL;ELL3;EP300;EPHB1;EPHB2;ESR1;EVC;EXTL3;EYA3;FAM129A;FAM162A;FBN2;FBXO22;FGF9;FIG4;FIS1;FKBP1A;FLCN;FLT3;FOSL2;FOXJ2;FOXO4;FOXP3;FURIN;FZD1;GAB2;GAPDHS;GBA;GCK;GLI1;GLMN;GLUL;GNAI2;GNAL;GNAS;GPR183;GRB10;GRB2;GRN;GSK3A;GTF2F1;GTPBP

LC9A1;SNIP1;SOCS2;SOD1;SPRED1;SPRY2;SQSTM1;SRC;STAT3;STAT5A;STAT5B;STK4;SYK;TFG;TGFB1;TIMP2;TIRAP;TLR9;TNIP1;UBE2V1;WNK1;YWH

1;RNF14;RPS6KA1;RTF1;RXRA;SIN3A;SKI;SLC11A1;SMAD9;SMARCA2;SOX4;SPEN;SQSTM1;SRF;SSBP3;STAT3;STAT5A;STAT5B;STAT6;SUPT3H;TAF11;TAF15;TAF4;TASP1;TBL1X;TFB2M;TFE3;TGFB1;THRAP3;TLR9;TNIP1;TP53INP2;TRERF1;TTC5;UBE2V1;USP22;VDR;WDR61;YEATS4;YWHAH;ZMIZ1;ZNF287;ZN

DD;DENND3;DISC1;DNAJB12;DNAJB5;DNAJB6;DNAJC14;DNAJC18;DNAJC19;DNAJC5;DNAJC5B;DNMT3B;DPAGT1;DPH5;DSCC1;DUSP12;DUSP18;DVL3;DYRK4;ECE1;EGR1;EIF2AK1;EIF2B3;EIF4A2;EIF4EBP2;EIF4H;ENDOU;EP300;EPHB1;EPHB2;ERC1;EXTL3;EYA3;F13A1;FAM129A;FAM58A;FBXO22;FBXO25;FK

NM2;DNMBP;DNMT3B;DOCK2;DPF2;DSCC1;DYNLL1;DYNLL2;EID3;ELL3;ELMO1;ELMO2;EMG1;ENO1;EPB41;EPB41L3;ERMN;ESRP2;EVC;EXOSC1;EXOSC3;EXOSC8;FAM101B;FAM110C;FAM96B;FBXO40;FKBP15;FNTB;FOXJ2;FSCN1;G6PD;GAS7;GM2A;GNAI2;GSN;H1F0;H2AFY;HAX1;HCK;HDAC4;HENMT1;HI
NM2;DNMBP;DNMT3B;DOCK2;DPF2;DSCC1;DYNLL1;DYNLL2;EID3;ELL3;ELMO1;ELMO2;EMG1;ENO1;EPB41;EPB41L3;ERMN;ESRP2;EVC;EXOSC1;EXOSC3;EXOSC8;FAM101B;FAM110C;FAM96B;FBXO40;FKBP15;FNTB;FOXJ2;FSCN1;G6PD;GAS7;GM2A;GNAI2;GSN;H1F0;H2AFY;HAX1;HCK;HDAC4;HENMT1;HI
ARID2;KAT5;KAT6A;KLF13;KLF4;KLF7;LILRB1;LPIN2;MAFB;MAGI2;MAML1;MAML3;MAP2K1;MAP2K3;MAP3K3;MAP3K5;MAPK14;MAPKAPK5;MAPRE3;MED12;MED25;MLL2;MTF1;MYB;NCOA6;NCOR1;NDNL2;NFIC;NFKB1;NFKBIA;NFYC;NOD2;NOTCH1;NSD1;NUB1;PAF1;PAK1;PBX2;PHF2;PHF8;PIAS3;PID

CBFA2T3;CBL;CBX1;CBX6;CBY1;CCL3;CCNB1IP1;CCNK;CCRN4L;CD3D;CD44;CD86;CDC42;CDC5L;CDH1;CDKL3;CELA1;CELF3;CENPH;CFL1;CFLAR;CHD8;CHEK1;CHI3L2;CHST11;CHST15;CHST7;CITED2;CLPX;CLTC;CNN2;CNOT1;CNOT4;CNPY2;COPS7A;COPS8;CPM;CPOX;CPSF7;CPVL;CREBBP;CRNKL1;CSF1;CSF1

ERKL;CFL1;CFLAR;CHD8;CHKA;CHRNB1;CHRNB2;CHST11;CITED2;CLTA;CLTC;CLTCL1;COL15A1;COLEC12;CORO1C;CREBBP;CRKL;CSF1;CSF1R;CSF2RB;CSF3R;CSNK1D;CSNK2A1;CTDSP2;CTNNA1;CTNNAL1;CTNNB1;CTNND1;CXCR2;CXCR7;DAB2;DAG1;DAP;DAP3;DAPK1;DAPK2;DBNL;DEDD;DGKG;DISC1;DNM

NOTCH2;NUAK2;NUDT2;P2RX1;PDCD5;PDCD6;PIM1;PIM2;PINK1;PLAC8;PLAGL2;PPID;PPM1F;PPT1;PRKCD;PRKDC;PRKRA;PSEN1;PSMB2;PSMD3;PSME3;PSMF1;PTK2B;RAF1;RHOA;RIPK1;RNF41;RPS6KA1;RTN4;SCRT2;SEMA4D;SIN3A;SKI;SOCS2;SOD1;SOX4;SPRY2;SRC;SSBP3;STAT5A;STAT5B;STEAP3;STK2
A;GTPBP1;H1F0;HAAO;HDAC4;HERPUD1;HEXB;HGD;HGSNAT;HIBADH;HK1;HK2;HMOX1;HNRNPD;HSD17B14;HSD17B4;HSD17B6;HSPA1B;HSPA5;INSR;IRS2;KIAA0317;KIAA1324;KPNB1;LAMP1;LIPA;LSM5;LSM7;LTA4H;LYG1;LYSMD1;LZTS1;MANBA;MAP2K1;MGAM;MLH3;MX2;MYH9;N4BP1;NAGA;NAGK;

UMF1;TFG;TGFB1;THG1L;TIMM9;TIRAP;TKT;TNNC1;TPCN2;TPD52L2;TPRG1L;TRIM8;VIM;WAS;XPA;ZNF1
2;DAG1;DAP3;DHX8;DISC1;DNAH17;DNM2;DSCR3;DYNLL1;DYNLL2;EID3;EIF2B3;EIF4A2;EIF4H;ELL;ELL3;ENO1;EP300;EPB41;ERC1;EXOSC1;EXOSC3;EXOSC8;EYA3;FAM110C;FAM160A2;FAM21A;FAM21C;FAM96B;FANCF;FBXO25;FIS1;FKBP15;FLOT2;FNTB;FOXJ2;FOXN3;FOXO4;FOXP3;GABRR2;GAS7;GLMN

F2B3;EIF4A2;EIF4H;ELL;ELL3;ENO1;EP300;EPB41;ERC1;EXOSC1;EXOSC3;EXOSC8;EYA3;FAM110C;FAM160A2;FAM21A;FAM21C;FAM96B;FANCF;FBXO25;FIS1;FKBP15;FLOT2;FNTB;FOXJ2;FOXN3;FOXO4;FOXP3;GABRR2;GAS7;GLMN;GLUL;GNA11;GNA12;GNA15;GNAI2;GNAL;GNAS;GNAT2;GNB1;GNB5;GOS

MM22;TOMM7;TTC19;TXNIP;UQCR10;UQCR11;USM

O7A;NDEL1;NEK3;NGDN;NMB;NME2;NRBP1;P2RX1;PAFAH1B1;PAK1;PIAS3;PLEK;PPT1;PREX1;PSEN1;PTK2B;PTPRJ;PXN;RAB35;RAB8A;RAC1;RAF1;RGS10;RHOA;RTN4;SH3KBP1;SH3PXD2B;SH3YL1;SIPA1L1;SLC38A7;SMURF1;SNAP29;SOD1;SPRY2;SRGAP2;SSH1;SVIL;TCTN1;TGFB1;TIMP2;TIRAP;TLN1;TMEM
MEM14C;TMEM18;TMEM38A;TMEM43;TOMM22;TOMM7;TRIM27;TTC19;TXNIP;UQCR10;UQCR11;USMG5;XPO6;XPO7;ZC3H

32;YEATS4;YWHAB;YY1;ZFHX3;ZM

TMEM43;TOMM22;TOMM7;TRIM27;TTC19;TXNIP;UQCR10;UQCR11;USMG5;XPO6;XPO7;ZC3H

);RNF185;RNF213;RNF24;RNF34;RNF39;RNF41;RNPEP;RP9;RPH3A;RUFY1;RXRA;SCRT2;SEC23B;SETDB1;SETMAR;SH3RF3;SIRT4;SKI;SMAP2;SOD1;SP140;SP2;SQSTM1;SRSF7;TADA2B;TAF15;TCF20;TIMM9;TOE1;TOP3A;TRAIP;TRERF1;TRIM25;TRIM27;TRIM67;TRIM8;USP22;VDR;XIAP;XRN2;YY1;ZBTB37;ZBT

PHF2;PHF21A;PHF23;PHF8;PIAS3;PIM1;PLAGL2;PLOD1;PMPCB;PPP1R10;PRKCB;PTPN1;PXN;RBM20;RBM22;RERE;RFFL;RNF114;RNF122;RNF14;RNF167;RNF170;RNF185;RNF213;RNF24;RNF34;RNF39;RNF41;RNPEP;RP9;RPH3A;RUFY1;RXRA;SCRT2;SEC23B;SETDB1;SETMAR;SH3RF3;SIRT4;SKI;SMAP2;SOD1

HGAP15;ARHGAP17;ARHGAP18;ARHGAP29;ARHGAP32;ARHGAP33;ARHGAP35;ARHGAP39;ARHGEF1;ARHGEF16;ARHGEF18;ARHGEF3;ARHGEF4;ARHGEF5;ARHGEF6;ARHGEF7;ARHGEF9;ARID1B;ARIH2;ARL10;ARL13A;ARL3;ARNTL2;ARPC2;ARPC3;ASB1;ASCC1;ASMT;ASXL1;ATF6B;ATG10;ATG13;ATG14;ATG
AP17;ARHGAP18;ARHGAP29;ARHGAP32;ARHGAP33;ARHGAP35;ARHGAP39;ARHGEF1;ARHGEF16;ARHGEF18;ARHGEF3;ARHGEF4;ARHGEF5;ARHGEF6;ARHGEF7;ARHGEF9;ARID1B;ARIH2;ARL3;ARNTL2;ARPC2;ARPC3;ASCC1;ASMT;ASXL1;ATF6B;ATG10;ATG13;ATG14;ATG3;ATG9B;ATIC;ATM;ATN1;ATOX1;AT
N3;CASP2;CBFA2T2;CBLB;CBX2;CBX5;CBX7;CC2D1B;CCAR1;CCND1;CCND2;CCNDBP1;CCNF;CCNT1;CCT5;CD3EAP;CDAN1;CDC16;CDC25B;CDKN2A;CDKN2B;CDR2;CELF1;CENPN;CENPO;CEP164;CEP85;CERK;CHCHD3;CHD1L;CHD2;CHD3;CHD4;CHD6;CHEK2;CHMP5;CHMP7;CHTF8;CIC;CIITA;CINP;CIRBP;CIRH
X56;DFFA;DFFB;DGKH;DGKQ;DHCR7;DHX15;DHX33;DHX35;DHX38;DHX9;DIEXF;DKC1;DNAJB1;DNAJC1;DNASE1;DNMT3A;DOCK7;DROSHA;DST;DTL;DUSP7;EFCAB4B;EFTUD2;EIF3A;EIF4A3;EIF4ENIF1;EIF5A;ELAVL1;ELP3;EMD;EP400;EPB41L2;EPHX2;ERCC3;ERCC5;ETS1;EXOSC10;EZH1;EZR;FAF1;FAM175B;F
4;ATG9B;ATIC;ATM;ATN1;ATP10A;ATP1A1;ATP1A3;ATP1B3;ATP2A2;ATP2A3;ATP5A1;ATP5B;ATP5J;ATP5J2;ATP6V0E1;ATP6V1D;ATP6V1F;ATP6V1H;ATP8B1;ATP9B;ATXN1L;ATXN2L;AVPR2;AXIN1;AXIN2;B3GALT4;BACE1;BACH2;BAD;BAHD1;BAIAP2L1;BATF;BATF3;BAZ1B;BBS1;BBS2;BBS9;BCL10;BCL11B;B
4;ATG9B;ATIC;ATM;ATN1;ATP10A;ATP1A1;ATP1A3;ATP1B3;ATP2A2;ATP2A3;ATP5A1;ATP5B;ATP5J;ATP5J2;ATP6V0E1;ATP6V1D;ATP6V1F;ATP6V1H;ATP8B1;ATP9B;ATXN1L;ATXN2L;AVPR2;AXIN1;AXIN2;B3GALT4;BACE1;BACH2;BAD;BAHD1;BAIAP2L1;BATF;BATF3;BAZ1B;BBS1;BBS2;BBS9;BCL10;BCL11B;B
2;DDX46;DDX47;DDX51;DDX52;DDX56;DFFA;DFFB;DGKQ;DHX15;DHX33;DHX38;DHX9;DIEXF;DKC1;DLD;DLST;DNAJA3;DNAJB1;DNMT3A;DROSHA;DUSP7;EARS2;ECI2;EFCAB4B;EFTUD2;EIF3A;EIF4A3;EIF4ENIF1;ELAVL1;ELP3;EP400;EPHX2;ERCC3;ERCC5;ETFDH;ETHE1;ETS1;EXOSC10;EZH1;EZR;F2;FANCI;FEI
23;DDX24;DDX31;DDX42;DDX46;DDX47;DDX51;DDX52;DDX56;DFFA;DFFB;DGKQ;DHX15;DHX33;DHX38;DHX9;DIEXF;DKC1;DLD;DLST;DNAJA3;DNAJB1;DNMT3A;DROSHA;DUSP7;EARS2;ECI2;EFCAB4B;EFTUD2;EIF3A;EIF4A3;EIF4ENIF1;ELAVL1;ELP3;EP400;EPHX2;ERCC3;ERCC5;ETFDH;ETHE1;ETS1;EXOSC10;
DDX42;DDX46;DDX47;DDX51;DDX52;DDX56;DFFA;DFFB;DGKQ;DHX15;DHX33;DHX38;DHX9;DIEXF;DKC1;DLD;DLST;DNAJA3;DNAJB1;DNMT3A;DROSHA;DUSP7;EARS2;ECI2;EFCAB4B;EFTUD2;EIF3A;EIF4A3;EIF4ENIF1;ELAVL1;ELP3;EP400;EPHX2;ERCC3;ERCC5;ETFDH;ETHE1;ETS1;EXOSC10;EZH1;EZR;F2;FANC
;EPHX2;ERCC3;ERCC5;ETS1;EXOSC10;EZH1;EZR;FANCI;FEN1;FIP1L1;FOXC1;FOXI1;FOXJ1;FOXJ3;FOXK1;FOXK2;FOXM1;FOXP1;FOXP4;FTSJ2;FTSJ3;FZR1;GAR1;GATA3;GEMIN4;GEMIN5;GGN;GINS4;GNL2;GNL3L;GTF2A2;GTF3C1;GTF3C2;GTF3C4;GTPBP4;HADHA;HCFC1;HDAC1;HEATR1;HELB;HIST2H3D;HLCS
;ARPC3;ASCC1;ASXL1;ATF6B;ATG14;ATG9B;ATIC;ATM;ATN1;ATP10A;ATP1A1;ATP1A3;ATP1B3;ATP2A2;ATP2A3;ATP5A1;ATP5B;ATP5J;ATP5J2;ATP6V0E1;ATP6V1D;ATP6V1F;ATP6V1H;ATP8B1;ATP9B;ATXN1L;ATXN2L;AVPR2;AXIN1;AXIN2;B3GALT4;BACE1;BACH2;BAD;BAHD1;BAIAP2L1;BATF;BATF3;BAZ1B
;ARPC3;ASCC1;ASXL1;ATF6B;ATG14;ATG9B;ATIC;ATM;ATN1;ATP10A;ATP1A1;ATP1A3;ATP1B3;ATP2A2;ATP2A3;ATP5A1;ATP5B;ATP5J;ATP5J2;ATP6V0E1;ATP6V1D;ATP6V1F;ATP6V1H;ATP8B1;ATP9B;ATXN1L;ATXN2L;AVPR2;AXIN1;AXIN2;B3GALT4;BACE1;BACH2;BAD;BAHD1;BAIAP2L1;BATF;BATF3;BAZ1B
D;BIK;BIRC6;BLM;BLOC1S1;BMS1;BPTF;BRD1;BRI3BP;BRPF1;BRSK2;C14orf169;C16orf88;C1D;C1GALT1C1;C2CD3;CAD;CALM3;CALR;CAMSAP1;CAPN2;CBX2;CBX5;CBX7;CCAR1;CCDC64;CCND1;CCND2;CCNF;CCNT1;CCT3;CCT5;CCT6A;CCT7;CCT8;CD1A;CD3EAP;CD4;CD74;CDC16;CDC25B;CDC42BPA;CDC42I
CD1;BID;BIK;BIRC6;BLM;BLOC1S1;BMS1;BPTF;BRD1;BRI3BP;BRPF1;BRSK2;C14orf169;C16orf88;C1D;C1GALT1C1;C2CD3;CAD;CALM3;CALR;CAMSAP1;CAPN2;CAPN3;CBX2;CBX5;CBX7;CCAR1;CCDC64;CCND1;CCND2;CCNF;CCNT1;CCT3;CCT5;CCT6A;CCT7;CCT8;CD1A;CD3EAP;CD4;CD74;CDC16;CDC25B;CDC4
L9;D2HGDH;DARS2;DCLK2;DDR1;DDX10;DDX17;DDX18;DDX19A;DDX19B;DDX20;DDX23;DDX24;DDX27;DDX31;DDX42;DDX46;DDX47;DDX51;DDX52;DDX55;DDX56;DDX6;DGKA;DGKD;DGKH;DGKQ;DHCR24;DHODH;DHX15;DHX16;DHX32;DHX33;DHX35;DHX37;DHX38;DHX57;DHX9;DLD;DNAH1;DNAJA3;D
L9;D2HGDH;DARS2;DCLK2;DDR1;DDX10;DDX17;DDX18;DDX19A;DDX19B;DDX20;DDX23;DDX24;DDX27;DDX31;DDX42;DDX46;DDX47;DDX51;DDX52;DDX55;DDX56;DDX6;DGKA;DGKD;DGKH;DGKQ;DHCR24;DHODH;DHX15;DHX16;DHX32;DHX33;DHX35;DHX37;DHX38;DHX57;DHX9;DLD;DNAH1;DNAJA3;D
DGKA;DGKD;DGKH;DGKQ;DHX15;DHX16;DHX32;DHX33;DHX35;DHX37;DHX38;DHX57;DHX9;DNAH1;DNAJA3;DNAJA4;DSTYK;DYNC1H1;DYNC1LI2;EARS2;EEF2K;EHD4;EIF2AK3;EIF2AK4;EIF4A3;ENTPD8;EP400;EPHA1;EPHA2;EPRS;ERBB3;ERCC3;FAM20B;FARSB;FBXO18;FGFR1;FKBP4;FLT4;FYN;G3BP1;GALK
6;DDX6;DGKA;DGKD;DGKH;DGKQ;DHX15;DHX16;DHX32;DHX33;DHX35;DHX37;DHX38;DHX57;DHX9;DNAH1;DNAJA3;DNAJA4;DSTYK;DYNC1H1;DYNC1LI2;EARS2;EEF2K;EHD4;EIF2AK3;EIF2AK4;EIF4A3;ENTPD8;EP400;EPHA1;EPHA2;EPRS;ERBB3;ERCC3;FAM20B;FARSB;FBXO18;FGFR1;FKBP4;FLT4;FYN;G3B
6;DDX6;DGKA;DGKD;DGKH;DGKQ;DHX15;DHX16;DHX32;DHX33;DHX35;DHX37;DHX38;DHX57;DHX9;DNAH1;DNAJA3;DNAJA4;DSTYK;DYNC1H1;DYNC1LI2;EARS2;EEF2K;EHD4;EIF2AK3;EIF2AK4;EIF4A3;ENTPD8;EP400;EPHA1;EPHA2;EPRS;ERBB3;ERCC3;FAM20B;FARSB;FBXO18;FGFR1;FKBP4;FLT4;FYN;G3B
BN;CUL9;D2HGDH;DARS2;DBH;DCLK2;DDR1;DDX10;DDX17;DDX18;DDX19A;DDX19B;DDX20;DDX23;DDX24;DDX27;DDX31;DDX42;DDX46;DDX47;DDX51;DDX52;DDX55;DDX56;DDX6;DGKA;DGKD;DGKH;DGKQ;DHCR24;DHODH;DHX15;DHX16;DHX32;DHX33;DHX35;DHX37;DHX38;DHX57;DHX9;DLD;DNAH
A0;HNRNPA2B1;HNRNPA3;HNRNPH1;HNRNPM;HNRNPR;HNRNPU;HOXA9;IKBKAP;IKBKE;ING2;ING5;INTS1;INTS4;INTS7;INTS9;IP6K2;ITPR3;JAZF1;KAT6B;KDM1A;KHSRP;L3MBTL1;LARP7;LAS1L;LDB1;LEF1;LEO1;LIG1;LIG3;LMO2;LPIN1;LSG1;LSM10;LUC7L2;MAD2L2;MAFK;MAGOH;MAP2K6;MAPK7;MAPK9;
DX42;DDX46;DDX47;DDX51;DDX52;DDX55;DDX56;DDX6;DGKA;DGKD;DGKH;DGKQ;DHX15;DHX16;DHX32;DHX33;DHX35;DHX37;DHX38;DHX57;DHX9;DNAH1;DNAJA3;DNAJA4;DNM1;DRG1;DSTYK;DYNC1H1;DYNC1LI2;EARS2;EEF2;EEF2K;EFTUD1;EFTUD2;EHD4;EIF2AK3;EIF2AK4;EIF2B1;EIF2S3;EIF4A3;EIF
DDX42;DDX46;DDX47;DDX51;DDX52;DDX55;DDX56;DDX6;DGKA;DGKD;DGKH;DGKQ;DHX15;DHX16;DHX32;DHX33;DHX35;DHX37;DHX38;DHX57;DHX9;DNAH1;DNAJA3;DNAJA4;DNM1;DRG1;DSTYK;DYNC1H1;DYNC1LI2;EARS2;EEF2;EEF2K;EFTUD1;EFTUD2;EHD4;EIF2AK3;EIF2AK4;EIF2B1;EIF2S3;EIF4A3;EIF
;CCAR1;CCNT1;CCT3;CCT5;CCT6A;CCT7;CCT8;CDC42BPA;CDC42BPB;CDC42BPG;CDK5RAP2;CDKN2A;CELF1;CERK;CHD1L;CHD2;CHD3;CHD4;CHD6;CHEK2;CHST12;CHTF8;CIC;CIITA;CIRBP;CIT;CIZ1;CKM;CLCN3;CLCN6;CLK2;CLPB;CPSF3;CRAMP1L;CREB3L2;CREG1;CREM;CRY2;CSNK1E;CSNK2A2;CSRNP2;CSTF
L;CARNS1;CBFA2T2;CBX2;CBX5;CBX7;CCAR1;CCNT1;CCR7;CD3EAP;CD40LG;CDAN1;CDH13;CDK5RAP2;CDKAL1;CDKN2A;CDKN2B;CDON;CECR1;CELF1;CELSR2;CENPN;CENPO;CEP164;CHCHD3;CHD1L;CHD2;CHD3;CHD4;CHD6;CHEK2;CHTF8;CIAO1;CIC;CIITA;CINP;CIRH1A;CIZ1;CLK2;CMKLR1;CNOT10;COX10;
DX42;DDX46;DDX47;DDX51;DDX52;DDX55;DDX56;DDX6;DGKA;DGKD;DGKH;DGKQ;DHX15;DHX16;DHX32;DHX33;DHX35;DHX37;DHX38;DHX57;DHX9;DNAH1;DNAJA3;DNAJA4;DNM1;DRG1;DSTYK;DYNC1H1;DYNC1LI2;EARS2;EEF2;EEF2K;EFTUD1;EFTUD2;EHD4;EIF2AK3;EIF2AK4;EIF2B1;EIF2S3;EIF4A3;EIF
D4;CD74;CD8A;CDC16;CDC25B;CDK5RAP2;CDKN2A;CELF1;CENPN;CENPO;CEP85;CEP89;CHD3;CHD4;CHMP7;CHRNA2;CHRNA5;CIAO1;CIRBP;CKAP5;CLIC1;CNIH3;CNTNAP1;COG1;COG2;COG5;COG7;COPG2;COPS7B;CORO2A;CPSF3;CREG1;CREM;CSNK1E;CSNK2A2;CSRP2BP;CSTF2;CTC1;CTNNBL1;CTR9;CU
DX42;DDX46;DDX47;DDX51;DDX52;DDX55;DDX56;DDX6;DGKA;DGKD;DGKH;DGKQ;DHX15;DHX16;DHX32;DHX33;DHX35;DHX37;DHX38;DHX57;DHX9;DNAH1;DNAJA3;DNAJA4;DNM1;DRG1;DSTYK;DYNC1H1;DYNC1LI2;EARS2;EEF2;EEF2K;EFTUD1;EFTUD2;EHD4;EIF2AK3;EIF2AK4;EIF2B1;EIF2S3;EIF4A3;EIF
X46;DDX47;DDX51;DDX52;DDX55;DDX56;DDX6;DGKA;DGKD;DGKH;DGKQ;DHX15;DHX16;DHX32;DHX33;DHX35;DHX37;DHX38;DHX57;DHX9;DNAH1;DNAJA3;DNAJA4;DNM1;DRG1;DSTYK;DYNC1H1;DYNC1LI2;EARS2;EEF2;EEF2K;EFTUD1;EFTUD2;EHD4;EIF2AK3;EIF2AK4;EIF2B1;EIF2S3;EIF4A3;EIF5B;ENTP
DX31;DDX42;DDX46;DDX47;DDX51;DDX52;DDX55;DDX56;DDX6;DGKA;DGKD;DGKH;DGKQ;DHODH;DHX15;DHX16;DHX32;DHX33;DHX35;DHX37;DHX38;DHX57;DHX9;DNAH1;DNAJA3;DNAJA4;DNM1;DRG1;DSTYK;DYNC1H1;DYNC1LI2;EARS2;EEF2;EEF2K;EFTUD1;EFTUD2;EHD4;EIF2AK3;EIF2AK4;EIF2B1;EI
DX42;DDX46;DDX47;DDX51;DDX52;DDX55;DDX56;DDX6;DGKA;DGKD;DGKH;DGKQ;DHX15;DHX16;DHX32;DHX33;DHX35;DHX37;DHX38;DHX57;DHX9;DNAH1;DNAJA3;DNAJA4;DNM1;DRG1;DSTYK;DYNC1H1;DYNC1LI2;EARS2;EEF2;EEF2K;EFTUD1;EFTUD2;EHD4;EIF2AK3;EIF2AK4;EIF2B1;EIF2S3;EIF4A3;EIF
DX42;DDX46;DDX47;DDX51;DDX52;DDX55;DDX56;DDX6;DGKA;DGKD;DGKH;DGKQ;DHX15;DHX16;DHX32;DHX33;DHX35;DHX37;DHX38;DHX57;DHX9;DNAH1;DNAJA3;DNAJA4;DNM1;DRG1;DSTYK;DYNC1H1;DYNC1LI2;EARS2;EEF2;EEF2K;EFTUD1;EFTUD2;EHD4;EIF2AK3;EIF2AK4;EIF2B1;EIF2S3;EIF4A3;EIF
2;CBX5;CBX7;CCAR1;CCNT1;CCR7;CD3EAP;CD40LG;CDAN1;CDH13;CDK5RAP2;CDKAL1;CDKN2A;CDKN2B;CDON;CECR1;CELF1;CELSR2;CENPN;CENPO;CEP164;CHCHD3;CHD1L;CHD2;CHD3;CHD4;CHD6;CHEK2;CHTF8;CIAO1;CIC;CIITA;CINP;CIRH1A;CIZ1;CLK2;CMKLR1;CNOT10;CPSF3;CREB3L2;CREG1;CREM;C
BFA2T2;CBX2;CBX5;CBX7;CCAR1;CCNT1;CCR7;CD3EAP;CD40LG;CDAN1;CDH13;CDK5RAP2;CDKAL1;CDKN2A;CDKN2B;CDON;CECR1;CELF1;CELSR2;CENPN;CENPO;CEP164;CHCHD3;CHD1L;CHD2;CHD3;CHD4;CHD6;CHEK2;CHTF8;CIAO1;CIC;CIITA;CINP;CIRH1A;CIZ1;CLK2;CMKLR1;CNOT10;COX10;COX15;CPSI
;CCAR1;CCNT1;CCT3;CCT5;CCT6A;CCT7;CCT8;CDC42BPA;CDC42BPB;CDC42BPG;CDK5RAP2;CDKN2A;CELF1;CERK;CHD1L;CHD2;CHD3;CHD4;CHD6;CHEK2;CHST12;CHTF8;CIC;CIITA;CIRBP;CIT;CIZ1;CKM;CLCN3;CLCN6;CLK2;CLPB;CPSF3;CRAMP1L;CREB3L2;CREG1;CREM;CRY2;CSNK1E;CSNK2A2;CSRNP2;CSTF
3S1;APBA1;APBA2;APBB1;APOBEC3F;APOL3;APOLD1;APOOL;AQP3;AQR;ARG1;ARHGAP15;ARHGAP17;ARHGAP18;ARHGAP29;ARHGAP32;ARHGAP33;ARHGAP35;ARHGAP39;ARHGEF1;ARHGEF16;ARHGEF18;ARHGEF3;ARHGEF4;ARHGEF5;ARHGEF6;ARHGEF7;ARHGEF9;ARID1B;ARIH2;ARL10;ARL13A;ARL3;
3S1;APBA1;APBA2;APBB1;APOBEC3F;APOL3;APOLD1;APOOL;AQP3;AQR;ARG1;ARHGAP15;ARHGAP17;ARHGAP18;ARHGAP29;ARHGAP32;ARHGAP33;ARHGAP35;ARHGAP39;ARHGEF1;ARHGEF16;ARHGEF18;ARHGEF3;ARHGEF4;ARHGEF5;ARHGEF6;ARHGEF7;ARHGEF9;ARID1B;ARIH2;ARL10;ARL13A;ARL3;
D;C22orf28;CAD;CALM3;CALR;CAMTA2;CAND2;CAPN3;CARD11;CARNS1;CBFA2T2;CBX2;CBX5;CBX7;CCAR1;CCNT1;CCR7;CD3EAP;CD40LG;CDAN1;CDH13;CDK5RAP2;CDKAL1;CDKN2A;CDKN2B;CDON;CECR1;CELF1;CELSR2;CENPN;CENPO;CEP164;CHCHD3;CHD1L;CHD2;CHD3;CHD4;CHD6;CHEK2;CHTF8;CIA
14;ATG3;ATG9B;ATIC;ATM;ATN1;ATP1A1;ATP1A3;ATP2A2;ATP2A3;ATP5A1;ATP5B;ATP5J;ATP5J2;ATP6V1H;ATP8B1;ATP8B2;ATXN1L;AVPR2;AXIN1;AXIN2;B3GALT4;BACE1;BACH2;BAD;BAG3;BAHD1;BATF;BATF3;BAZ1B;BCL10;BCL11B;BCL2;BCL9L;BDH1;BEX1;BICD1;BIRC6;BLM;BLMH;BLOC1S1;BMP2;BPTI
;CAND2;CAPN3;CARD11;CARNS1;CBFA2T2;CBX2;CBX5;CBX7;CCAR1;CCNT1;CCR7;CD3EAP;CD40LG;CDAN1;CDH13;CDK5RAP2;CDKAL1;CDKN2A;CDKN2B;CDON;CECR1;CELF1;CELSR2;CENPN;CENPO;CEP164;CHCHD3;CHD1L;CHD2;CHD3;CHD4;CHD6;CHEK2;CHTF8;CIAO1;CIC;CIITA;CINP;CIRH1A;CIZ1;CLK2;CL
MTOR;NACC1;NCOR2;NFATC2;NOLC1;NONO;NOP10;NPAS2;NXF1;PARP1;PAXIP1;PHC1;PHF15;PNN;POLDIP3;POLE3;POLR2B;POLR2C;POLR2J;POLR3A;POLR3C;PPARGC1B;PRMT2;PRPF19;PRPF3;PRPF4;PRPF40B;PRPF8;PSME4;PSPC1;PTK2;RBBP4;RBBP7;RBL2;RBM14;RELA;RFWD3;RNF4;RNPS1;RPA1;RPA2
1;AQR;ARG1;ARHGAP15;ARHGAP17;ARHGAP29;ARHGAP32;ARHGAP33;ARHGAP35;ARHGEF1;ARHGEF16;ARHGEF18;ARHGEF3;ARHGEF4;ARHGEF5;ARHGEF6;ARHGEF7;ARHGEF9;ARID1B;ARIH2;ARL10;ARL13A;ARL3;ARNTL2;ARPC2;ARPC3;ASB16;ASCC1;ASMT;ASPDH;ASXL1;ATAD3C;ATF6B;ATG13;ATG14;A
X2;CBX5;CBX7;CCAR1;CCDC23;CCDC88C;CCL8;CCND1;CCND2;CCNF;CCNT1;CCR7;CCT3;CCT5;CCT6A;CCT7;CCT8;CD3E;CD3EAP;CD4;CD40LG;CD74;CDAN1;CDC16;CDC25B;CDC42BPA;CDC42BPB;CDC42BPG;CDH13;CDK5RAP2;CDKAL1;CDKN2A;CDKN2B;CDON;CELF1;CELSR2;CENPN;CENPO;CEP164;CHCHD3;
AP39;ARHGEF1;ARHGEF16;ARHGEF18;ARHGEF3;ARHGEF4;ARHGEF5;ARHGEF6;ARHGEF7;ARHGEF9;ARIH2;ARL3;ARNTL2;ARPC2;ARPC3;ASCC1;ASMT;ATF6B;ATG10;ATG13;ATG14;ATG3;ATG9B;ATIC;ATM;ATN1;ATOX1;ATP10A;ATP1A1;ATP1A3;ATP1B3;ATP2A2;ATP2A3;ATP5A1;ATP5B;ATP5J;ATP5J2;ATP6V
;CTPS2;CUL9;CYTH3;D2HGDH;DARS2;DBH;DCLK2;DDR1;DDX10;DDX17;DDX18;DDX19A;DDX19B;DDX20;DDX23;DDX24;DDX27;DDX31;DDX42;DDX46;DDX47;DDX51;DDX52;DDX55;DDX56;DDX6;DGKA;DGKD;DGKH;DGKQ;DHCR24;DHODH;DHX15;DHX16;DHX32;DHX33;DHX35;DHX37;DHX38;DHX57;DHX9;
1;ASB16;ASCC1;ASMT;ASPDH;ASXL1;ATF6B;ATG10;ATG3;ATIC;ATM;ATN1;ATP1A1;ATP1A3;ATP2A2;ATP2A3;ATP5A1;ATP5B;ATP5J;ATP5J2;ATP6V1H;ATP8B1;ATP8B2;ATXN1L;AVPR2;AXIN1;AXIN2;B3GALT4;BACE1;BACH2;BAD;BAG3;BAHD1;BATF;BATF3;BAZ1B;BCL10;BCL11B;BCL2;BCL9L;BDH1;BEX1;BICD
TC3;CRY2;CSNK1E;CSNK2A2;CSRNP2;CSTF1;CSTF2;CSTF3;CTC1;CTCF;CTNNBL1;CTR9;CUL4A;CYTL1;DARS2;DBR1;DCLRE1C;DCP1B;DDB1;DDIT3;DDX17;DDX20;DDX23;DDX24;DDX46;DDX47;DDX51;DDX56;DDX6;DFFA;DFFB;DHX15;DHX16;DHX33;DHX35;DHX38;DHX9;DIDO1;DIS3L;DKC1;DNAJA3;DNASE1;D
AP2;CDKN2A;CENPN;CENPO;CEP85;CEP89;CHD3;CHD4;CHMP7;CHRNA2;CHRNA5;CIAO1;CKAP5;CLIC1;CNIH3;CNTNAP1;COG1;COG2;COG5;COG7;COPG2;COPS7B;CORO2A;CPSF3;CREG1;CREM;CSNK2A2;CSRP2BP;CSTF2;CTNNBL1;CTR9;CUL1;CUL4A;CUL7;CUL9;DAD1;DCAF16;DCAF4;DCAF5;DCAF7;DCAF8

ARID1B;ARIH2;ARNTL2;ASB1;ASB16;ASCC1;ASMT;ASPDH;ASXL1;ATF6B;ATG10;ATG3;ATIC;ATM;ATN1;ATP1A1;ATP1A3;ATP2A2;ATP2A3;ATP5A1;ATP5B;ATP5J;ATP5J2;ATP6V1H;ATP8B1;ATP8B2;ATXN1L;AVPR2;AXIN1;AXIN2;B3GALT4;BACE1;BACH2;BAD;BAG3;BAHD1;BATF;BATF3;BAZ1B;BBS2;BCL10;BCL1
9;C1D;C22orf28;CAD;CALM3;CALR;CAMTA2;CAND2;CAPN3;CARD11;CARNS1;CBFA2T2;CBX2;CBX5;CBX7;CCAR1;CCNT1;CCR7;CD3EAP;CD40LG;CDAN1;CDH13;CDK5RAP2;CDKAL1;CDKN2A;CDKN2B;CDON;CECR1;CELF1;CELSR2;CENPN;CENPO;CEP164;CERK;CHCHD3;CHD1L;CHD2;CHD3;CHD4;CHD6;CHEK2;
DIS3L;DKC1;DNAJC1;DNMT1;DNMT3A;DROSHA;EARS2;EDC3;EEF2;EEF2K;EFTUD1;EHD4;EIF1AD;EIF2AK4;EIF2B1;EIF2S3;EIF3A;EIF3B;EIF4A3;EIF4B;EIF4G1;EIF4G2;EIF5A;EIF5B;ELAVL1;ELP3;EME2;ENOX2;EP400;EPRS;ERCC3;ERCC5;ETS1;EXD2;EXOG;EXOSC10;FAM103A1;FAN1;FANCI;FARSB;FBXL19;FBXO18
5;ARHGEF5;ARHGEF6;ARHGEF7;ARID1B;ARIH2;ARNTL2;ASB1;ASB16;ASCC1;ASMT;ASPDH;ASXL1;ATF6B;ATG10;ATG13;ATG14;ATG3;ATG9B;ATIC;ATM;ATN1;ATP1A1;ATP1A3;ATP2A2;ATP2A3;ATP5A1;ATP5B;ATP5J;ATP5J2;ATP6V1H;ATP8B1;ATP8B2;ATXN1L;AVPR2;AXIN1;AXIN2;B3GALT4;BACE1;BACH2;B

V1D;ATP6V1F;ATP6V1H;ATP8B1;AXIN1;AXIN2;BACE1;BAD;BAG3;BAHD1;BAIAP2L1;BATF;BAZ1B;BBS1;BBS2;BBS9;BCL10;BCL2;BCL9L;BET1L;BEX1;BICD1;BID;BIK;BIRC6;BLCAP;BLM;BLMH;BLOC1S1;BMP2;BMP8A;BMP8B;BPTF;BRD1;BRD2;BRD7;BRSK2;BTBD11;BTBD2;BTRC;C1D;C22orf28;C22orf29;C9orf89;
7;CPSF3;CREB3L2;CREG1;CREM;CRTC1;CRTC3;CRY2;CSNK2A2;CSRNP2;CSTF1;CSTF2;CSTF3;CTCF;CTNNBL1;CTR9;CYTL1;DARS2;DBR1;DCP1B;DDIT3;DDX17;DDX20;DDX23;DDX46;DDX47;DDX51;DDX56;DDX6;DHCR24;DHX15;DHX16;DHX33;DHX35;DHX38;DHX9;DIDO1;DKC1;DNAJA3;DNAJC1;DNMT1;DNM
R2J;POP1;PPP1R9B;PRPF19;PRPF3;PRPF4;PRPF40B;PRPF8;PTBP1;PUS3;QTRTD1;RBFOX2;RBM15B;RBM17;RBM26;RBM28;RBM4B;RBM6;RCL1;RNASE4;RNMT;RNPS1;RPP14;RPP21;RRP1B;SARS;SART3;SCAF1;SCAF8;SF1;SF3A3;SF3B2;SF3B3;SFPQ;SMAD3;SMC1A;SNRNP200;SNRNP40;SNRNP70;SNW1;SON;

JPV3L1;TOP2B;VCP;WRNIP1;XRCC3;XRCC5;XR
;BRD2;BRD7;BRPF1;BRSK2;BTRC;C14orf169;C1D;C1GALT1C1;C22orf28;C2CD3;CAD;CALM3;CALR;CAMTA2;CAND2;CAPN2;CAPN3;CAPN5;CARD11;CARD14;CASP1;CASP10;CASP2;CBFA2T2;CBLB;CBX2;CBX5;CBX7;CCAR1;CCDC23;CCDC88C;CCL8;CCND1;CCND2;CCNF;CCNT1;CCR7;CCT3;CCT5;CCT6A;CCT7;CC
P1;PTK2;RBL2;RBM19;RBM28;RBM4B;RCC2;RCL1;RNF20;RNMT;RPP21;RRN3;RRP1B;RSL1D1;SDAD1;SENP5;SMG6;SNAPIN;SPG11;SPTBN1;SRP68;SRSF5;SSRP1;TAOK2;TCOF1;TEX10;TFCP2L1;THTPA;TP53;TRIM68;TSPYL2;TSR1;TTF1;UBE2I;UBTF;URB1;URB2;USP36;UTP14A;UTP20;WDR3;WDR55;WHSC1;Z
DHD2;DDX17;DDX18;DDX20;DDX24;DDX31;DDX46;DDX47;DDX51;DDX52;DDX56;DDX6;DFFA;DFFB;DFNB31;DGKQ;DHCR24;DHX15;DHX33;DHX9;DIAPH1;DIEXF;DIS3L;DKC1;DNAH1;DNAJA3;DNAJB1;DNAJC7;DNHD1;DNM1;DNMT1;DNMT3A;DRG1;DROSHA;DST;DSTYK;DTL;DYNC1H1;DYNC1I2;DYNC1LI2;D
DHD2;DDX17;DDX18;DDX20;DDX24;DDX31;DDX46;DDX47;DDX51;DDX52;DDX56;DDX6;DFFA;DFFB;DFNB31;DGKQ;DHCR24;DHX15;DHX33;DHX9;DIAPH1;DIEXF;DIS3L;DKC1;DNAH1;DNAJA3;DNAJB1;DNAJC7;DNHD1;DNM1;DNMT1;DNMT3A;DRG1;DROSHA;DST;DSTYK;DTL;DYNC1H1;DYNC1I2;DYNC1LI2;D

P1B;DDIT3;DDX17;DDX20;DDX23;DDX24;DDX46;DDX47;DDX51;DDX56;DDX6;DHX15;DHX16;DHX33;DHX35;DHX38;DHX9;DIDO1;DIS3L;DKC1;DNAJA3;DNMT1;DNMT3A;DRG1;DROSHA;DVL2;EARS2;ECD;EDC3;EDC4;EFTUD2;EHMT1;EIF2AK3;EIF4A3;EIF4B;EIF4G1;EIF4G2;ELAVL1;ELP3;EPRS;ERCC3;ETS1;EXC
LD1;AQP3;AQR;ARG1;ARHGAP15;ARHGAP17;ARHGAP18;ARHGAP29;ARHGAP32;ARHGAP33;ARHGAP35;ARHGAP39;ARHGEF1;ARHGEF16;ARHGEF18;ARHGEF3;ARHGEF4;ARHGEF5;ARHGEF6;ARHGEF7;ARHGEF9;ARID1B;ARIH2;ARL10;ARL13A;ARL3;ARNTL2;ARPC2;ARPC3;ASB1;ASB16;ASCC1;ASMT;ASPDH
C;CHMP5;CHMP7;CIAO1;CKAP5;CKM;CMBL;CNDP2;CNOT10;CNTRL;COG5;CSNK1E;CSNK2A2;CTPS2;CUBN;CUL1;CUL7;DCP1B;DCTD;DCTN1;DCTN5;DDIT4;DDX20;DDX6;DFFA;DFFB;DGKA;DGKQ;DHCR24;DNAJA3;DOCK9;DPP9;DPYSL2;DUSP7;DVL2;DYNC1H1;DYNC1I2;DYNC1LI2;EDC3;EDC4;EEF2;EEF2K;EIF2
;MARS;MBD2;MECP2;MRPL16;MRPL44;MRPS5;MSI2;MTPAP;NCL;NOL9;NOM1;NONO;NOP14;NSUN2;NXF1;PA2G4;PABPC1;PABPC1L;PABPC4;PARN;PDCD11;PDCD4;PET112;PINX1;PNO1;POLDIP3;PPARGC1B;PPRC1;PRPF8;PSPC1;PTBP1;PUM1;PUS3;RAVER1;RBFOX2;RBM14;RBM15B;RBM17;RBM19;RBM
SYF2;TARDBP;TFIP11;TGS1;THOC1;THOC2;TNKS1BP1;U2AF1L4;U2AF2;USP39;WBP11;WDR33;ZRS

;GLUD1;GNL3L;GOT2;GPRC5C;GPT2;GRAMD4;GRPEL1;H6PD;HADH;HADHA;HADHB;HCFC1;HEATR1;HEBP2;HEMK1;HERC2;HKDC1;HLCS;HSP90AB1;HSPA1L;HSPA9;HSPD1;HTRA2;IARS2;IBA57;IDH3A;ILF3;IMMT;ISCA2;IVD;JTB;KARS;KIAA0141;KRT5;KYNU;LARS2;LDHA;LETM1;LIG1;LIPT2;LOC494141;LYRM1
1;COG2;CORO2A;COX10;CROCC;CSRP2BP;CTC1;CTCF;CTR9;CXCL1;DARS2;DCLRE1C;DCTN1;DFFB;DHODH;DIAPH1;DKC1;DNAJA3;DNM1;DNMT1;DNMT3A;DOCK7;DOPEY2;DPYSL2;DST;DYNC1H1;DYNC1LI2;DYNLT1;EARS2;EFTUD1;EHMT1;EIF2AK3;EP400;EPB41L2;EPHA1;EPS8;ETS1;EVL;EZR;FEN1;FGD1;FIT

PHOSPH8;MSH6;MTA2;MYC;MYO18A;NASP;NCAPD2;NCAPD3;NCAPG;NCAPG2;NEIL2;NF2;NFRKB;NONO;NT5E;NYNRIN;PALB2;PAPD7;PARP1;PAXIP1;PDGFC;PHF13;PINX1;PLD6;PMS2;PMS2CL;PMS2P5;POLA1;POLA2;POLB;POLE;POLE3;POLG;POLH;POLR2B;POLR2C;POLR2J;PRPF19;PTTG2;RAD18;RAD51D;
;SCMH1;SEH1L;SEPT6;SETD1A;SIN3B;SLX4;SMARCA4;SMARCC1;SMARCD3;SMARCE1;SMC1A;SMG6;SNW1;SOX18;SSRP1;SUPT16H;SYCE2;TADA2A;TBP;TCF3;TCF7L2;TCP1;TEP1;TERF2;TERF2IP;TEX12;TIMELESS;TNKS1BP1;TOP2B;TP53;TP53BP1;UBE2B;UBE2I;UHRF1;VCP;WHSC1;XRCC5;XRCC6;ZMIZ2;ZNF3
POLE;POLE3;PRKCA;PRMT2;PTTG2;PYGO2;RBBP4;RBBP7;RBL2;RBM14;RFC1;RNF20;RNF4;RNF8;RPA1;RPA2;RUVBL1;SATB1;SCMH1;SEH1L;SETD1A;SMARCA4;SMARCAL1;SMARCC1;SMARCC2;SMARCD1;SMARCD3;SMARCE1;SMC1A;SMG6;SNW1;SRCAP;SUPT16H;SUPT4H1;SUPT6H;SUPV3L1;TADA2A;TAF1;

X18;SYCE2;TBP;TCF3;TCF7L2;TCP1;TEP1;TERF2;TERF2IP;TEX12;TIMELESS;TNKS1BP1;TOP2B;TP53;TP53BP1;UBE2B;UBE2I;UHRF1;VCP;XRCC5;XRCC6;ZMIZ2;ZNF330;ZW

2;MMS19;MYC;MYCBP;MYCBP2;NAV1;NCAPG;NEIL2;NEURL4;NICN1;NINL;NPHP4;NSUN2;NUMA1;NUP62;ODF2;OTUD7B;PCIF1;PCM1;PCNT;PDE4DIP;PEA15;PINX1;PLEKHA7;POLB;POLR2C;PRC1;PRPF19;PTK2;RAB11FIP3;RAB3GAP1;RABGAP1;RACGAP1;RANBP10;RCC2;RNF4;RP1L1;RPS6KA2;RTTN;RUVBL1

C;UBR4;UBR7;UHRF1;UNKL;VHL;YA
IO80;KIF13B;KIF16B;KIF21B;KIF3B;KIF4A;KIF5C;KLC1;KLC2;LONP2;LSG1;MACF1;MCM2;MCM3;MCM4;MCM6;MDN1;MLH1;MSH6;MYH3;MYH7B;MYO15A;MYO18A;MYO19;MYO1C;MYO1D;MYO1E;MYO5B;MYO9B;NAV1;PCYOX1;PMS2;PMS2P5;PSMC4;PSMD6;RAB11A;RABL2A;RABL2B;RAD51D;RAD54L2
EATS2;ZMYN
;KDM1A;KDM4A;KDM4C;KIF13B;KIF16B;KIF3B;LAX1;LCK;LDB1;LONP2;LPIN1;LUC7L2;MAD2L2;MAP2K6;MAP2K7;MAP3K12;MAPK7;MAPK8IP3;MAPK9;MAPKAP1;MAPT;MAVS;MDM4;MTA1;MUL1;MYO5B;NCOR2;NEDD8;NEK9;NFRKB;NLRP1;OCRL;PA2G4;PAM;PARD6A;PARN;PCCA;PDE4DIP;PDPK1;PEX26
HGEF9;ARL3;ARPC2;ASMT;ATF6B;ATG13;ATG14;ATG3;ATG9B;ATIC;ATM;ATN1;ATOX1;ATP10A;ATP1A1;ATP1A3;ATP1B3;ATP2A2;ATP2A3;ATP5A1;ATP5B;ATP5J;ATP5J2;ATP6V0E1;ATP6V1D;ATP6V1F;ATP6V1H;ATP8B1;ATP9B;ATXN2L;AVPR2;AXIN1;AXIN2;B3GALT4;BACE1;BAD;BAG3;BAIAP2L1;BCL10;BCL
4;HELB;HSPA8;HSPD1;INO80;KIF13B;KIF16B;KIF21B;KIF3B;KIF4A;KIF5C;KLC1;KLC2;LONP2;LSG1;MACF1;MCM2;MCM3;MCM4;MCM6;MDN1;MLH1;MSH6;MYH3;MYH7B;MYO15A;MYO18A;MYO19;MYO1C;MYO1D;MYO1E;MYO5B;MYO9B;NAV1;NUDT9;PCYOX1;PMS2;PMS2P5;PSMC4;PSMD6;RAB11A;RA
4;HELB;HSPA8;HSPD1;INO80;IRGM;KIF13B;KIF16B;KIF21B;KIF3B;KIF4A;KIF5C;KLC1;KLC2;LONP2;LSG1;MACF1;MCM2;MCM3;MCM4;MCM6;MDN1;MLH1;MSH6;MYH3;MYH7B;MYO15A;MYO18A;MYO19;MYO1C;MYO1D;MYO1E;MYO5B;MYO9B;NAV1;NUDT9;PCYOX1;PMS2;PMS2P5;PSMC4;PSMD6;RAB1
4;HELB;HSPA8;HSPD1;INO80;KIF13B;KIF16B;KIF21B;KIF3B;KIF4A;KIF5C;KLC1;KLC2;LONP2;LSG1;MACF1;MCM2;MCM3;MCM4;MCM6;MDN1;MLH1;MSH6;MYH3;MYH7B;MYO15A;MYO18A;MYO19;MYO1C;MYO1D;MYO1E;MYO5B;MYO9B;NAV1;NUDT9;PCYOX1;PMS2;PMS2P5;PSMC4;PSMD6;RAB11A;RA
HYAL1;ILF3;INCA1;INCENP;ING1;ING2;ING5;INO80;INTS7;JTB;KHDRBS1;KIF3B;KLF11;KNTC1;KRT7;L3MBTL1;LEF1;LIG1;LIG3;LMLN;LRP5;LSM10;LZTS2;MACF1;MAD2L1BP;MAD2L2;MADD;MAP2K6;MAP4;MAPK7;MAPRE2;MCM2;MCM3;MCM4;MCM6;MDC1;MDM4;MLH1;MND1;MNS1;MNT;MPHOSPH8;M

TF1;CSTF2;CSTF3;CTC1;CTCF;CTR9;CYTL1;DAD1;DARS2;DCN;DDIT3;DDX17;DDX20;DHDDS;DHX33;DHX38;DIDO1;DKC1;DNAJA3;DNAJC1;DNMT1;DNMT3A;DPH1;DRG1;DTL;DVL2;EARS2;ECD;EDEM1;EEF2;EEF2K;EHMT1;EIF2AK3;EIF2AK4;EIF2B1;EIF2S3;EIF3A;EIF3B;EIF4A3;EIF4B;EIF4G1;EIF4G2;EIF5A;EIF5B
SPYL2;TTF1;UBE2B;UHRF1;YEA

55;NUP188;NUP205;NUP210;NUP214;NUP62;NUP93;NXF1;OGDH;PAM16;PARP1;PCM1;PI4KB;PLD6;POLA1;POLG;POM121;POM121C;POU6F1;PRKCA;RETSAT;RNF43;S100A6;SAMM50;SCO1;SCO2;SCRN1;SDHA;SDHB;SEH1L;SFXN1;SFXN2;SFXN3;SIGMAR1;SLC25A12;SLC25A15;SLC25A23;SLC25A27;SLC25
E;CD3EAP;CD4;CD74;CDAN1;CDH13;CDK5RAP2;CDKN2A;CDKN2B;CDON;CECR1;CELSR2;CHCHD3;CHD2;CHD3;CHD4;CHD6;CHEK2;CHST12;CHTF8;CIAO1;CIC;CIITA;CINP;CIRBP;CIRH1A;CIZ1;CKM;CLK2;CLN8;CMKLR1;CNDP2;COG2;COG7;COL5A1;COX10;COX15;CPSF3;CREB3L2;CREG1;CREM;CRTC1;CRTC3;C

CRTC1;CRTC3;CRY2;CSNK2A2;CSRNP2;CSTF1;CSTF2;CSTF3;CTC1;CTCF;CTR9;CYTL1;DAD1;DARS2;DCN;DDIT3;DDX17;DDX20;DHDDS;DHX33;DHX38;DIDO1;DKC1;DNAJA3;DNAJC1;DNMT1;DNMT3A;DPH1;DRG1;DTL;DVL2;EARS2;ECD;EDEM1;EEF2;EEF2K;EHMT1;EIF2AK3;EIF2AK4;EIF2B1;EIF2S3;EIF3A;EIF3B;
3;NUP155;NUP188;NUP205;NUP210;NUP214;NUP62;NUP93;NXF1;OGDH;PAM16;PARP1;PCM1;PI4KB;PLD6;POLA1;POLG;POM121;POM121C;POU6F1;PRKCA;RETSAT;RNF43;S100A6;SAMM50;SCO1;SCO2;SCRN1;SDHA;SDHB;SEH1L;SFXN1;SFXN2;SFXN3;SIGMAR1;SLC25A12;SLC25A15;SLC25A23;SLC25A2

CM1;PCNT;PHF13;PIM3;PINX1;PKD1;POGZ;POLA1;POLA2;POLE;PPP1R9B;PPP2R5C;PRC1;PRKCA;PRMT2;PSMC4;PSMD1;PSMD11;PSMD2;PSMD6;PSMD7;PSME4;RACGAP1;RBBP4;RBL2;RCC2;RFC1;RFWD3;RNF8;RPA1;RPA2;RPS6KA2;RUVBL1;SCRIB;SEH1L;SFI1;SH2B1;SIK1;SMAD3;SMARCA4;SMC1A;SUN2;
E;CD3EAP;CD4;CD74;CDAN1;CDH13;CDK5RAP2;CDKN2A;CDKN2B;CDON;CECR1;CELSR2;CHCHD3;CHD2;CHD3;CHD4;CHD6;CHEK2;CHST12;CHTF8;CIAO1;CIC;CIITA;CINP;CIRBP;CIRH1A;CIZ1;CKM;CLK2;CLN8;CMKLR1;CNDP2;COG2;COG7;COX10;COX15;CPSF3;CREB3L2;CREG1;CREM;CRTC1;CRTC3;CRY2;CSNK

2K6;MAP4;MAPRE2;MCM2;MCM3;MCM4;MCM6;MDC1;MDM4;MLH1;MND1;MNS1;MSH6;MTA3;MYBBP1A;MYC;NCAPD2;NCAPD3;NCAPG;NCAPG2;NDC80;NEK9;NINL;NME6;NOLC1;NSUN2;NUMA1;NUP133;ODF2;PA2G4;PAPD7;PCM1;PCNT;PHF13;PINX1;PKD1;PLD6;PMS2;PMS2P5;POGZ;POLA1;POLA2
NT1;CCR7;CD3E;CD3EAP;CD4;CD74;CDAN1;CDH13;CDK5RAP2;CDKN2A;CDKN2B;CDON;CECR1;CELSR2;CHCHD3;CHD2;CHD3;CHD4;CHD6;CHEK2;CHST12;CHTF8;CIAO1;CIC;CIITA;CINP;CIRBP;CIRH1A;CIZ1;CKM;CLK2;CLN8;CMKLR1;CNDP2;COG2;COG7;COL5A1;COX10;COX15;CPSF3;CREB3L2;CREG1;CREM;C

C14orf169;C22orf28;C9orf96;CABP4;CACNA1F;CACNA2D3;CAD;CALM3;CALR;CAPN2;CAPN3;CAPNS2;CARNS1;CBFA2T2;CBLB;CBWD3;CBWD6;CCL8;CCT3;CCT5;CCT6A;CCT7;CCT8;CD4;CDC42BPA;CDC42BPB;CDC42BPG;CDH13;CDH20;CDH23;CDH24;CDHR2;CDKAL1;CECR1;CELSR2;CELSR3;CERK;CHD1L;CHD

MARCD3;SMARCE1;SNW1;SRCAP;SUPT16H;SUPT4H1;SUPT6H;SUPV3L1;TADA2A;TAF1;TAF10;TCF3;TERF2;TP53;TRIM16;TRRAP;TSPYL2;TTF1;UBE2B;UHRF1;YEA
V3;NFS1;NNT;NSUN4;OGDH;PAM16;PCCA;PCCB;PDHA1;PDHB;PDPR;PI4KB;PITRM1;PLD6;POLG;PPM1K;PRKCA;SAMM50;SCO1;SCO2;SDHA;SDHB;SFXN1;SFXN2;SFXN3;SHC1;SLC25A12;SLC25A15;SLC25A23;SLC25A27;SLC25A42;SLC25A45;SMCR7L;SNPH;SOX10;SPG7;STAR;SUPV3L1;SYNE2;TAMM41;THEM
;CIAO1;CIC;CIITA;CIRBP;CIRH1A;CIZ1;CLEC3B;CLK2;CLN8;CMKLR1;COG7;CREB3L2;CREG1;CREM;CRTC1;CRTC3;CRY2;CSNK1E;CSNK2A2;CSRNP2;CTC1;CTCF;CTR9;CUL1;CUL4A;CUL7;CYTL1;DCTN1;DDIT3;DDIT4;DDX17;DDX20;DHX33;DHX9;DNAJA3;DNAJC1;DNMT1;DNMT3A;DOCK7;DRD4;DROSHA;DUSP16;

31;ATP8B2;ATP9B;B3GALT4;B3GNTL1;BACE1;BAZ1B;BDH1;BIRC6;BLM;BLMH;BMP2;BMS1;BPTF;BRSK2;BTRC;C14orf169;C1GALT1C1;C22orf28;C9orf96;CAD;CAPN2;CAPN3;CAPN5;CARD11;CARNS1;CASP1;CASP10;CASP2;CBLB;CCL8;CCND1;CCT8;CD3EAP;CDC25B;CDC42BPA;CDC42BPB;CDC42BPG;CDKAL1

ABP4;CACNA1F;CACNA1I;CACNB1;CADM1;CADM2;CALR;CAMP;CAMSAP1;CAPN3;CATSPER1;CBFA2T2;CBX2;CBX7;CCDC64;CCDC88C;CCNF;CCR7;CD3E;CD74;CDAN1;CDC16;CDC25B;CDC42BPA;CDC42BPB;CDC42BPG;CDH13;CDH23;CDH24;CDHR2;CDK5RAP2;CDKN2A;CELF1;CELSR2;CELSR3;CENPN;CENPO
;HIST1H2AK;HIST1H2BN;HIST2H2AC;HIST2H2BE;HIST2H3D;HMG20A;HMGA1;HMGXB3;HNRNPA2B1;HNRNPAB;HNRNPU;HOMEZ;HOXA9;HOXB2;HP1BP3;HSPD1;HUWE1;IKBKAP;IKZF3;ILF2;ILF3;ING2;INO80;IRF8;IWS1;JRK;KAT6B;KDM1A;KDM2B;KDM5C;KHDRBS1;KHSRP;KIF4A;KLF11;LDB1;LEF1;LHX4;LIG

C3;CRY2;CSNK2A2;CSRNP2;CSTF1;CSTF2;CSTF3;CTCF;CTPS2;CTR9;CYTL1;DCTD;DDIT3;DDX17;DDX20;DHODH;DHX33;DHX38;DIDO1;DNAJA3;DNMT1;DNMT3A;DRD4;DRG1;DTL;DVL2;ECD;EHMT1;EIF2AK3;EIF4A3;ELP3;ENTPD8;ERCC3;ETS1;EZH1;FADS1;FGF2;FGFR1;FLNA;FOSB;FOXC1;FOXI1;FOXJ1;FOXJ3;F

;NUMA1;NUP133;ODF2;PAPD7;PCM1;PCNT;PHF13;PINX1;PKD1;PLD6;PMS2;PMS2P5;POGZ;POLA1;POLA2;POLE;PPP1R9B;PPP2R5C;PRC1;PRMT2;PSMC4;PSMD1;PSMD11;PSMD2;PSMD6;PSMD7;PSME4;RACGAP1;RAD51D;RBL2;RCC2;RFC1;RFWD3;RNF4;RNF8;RPA1;RPA2;RPS6KA2;RUVBL1;SEH1L;SFI1;SH
T3;DDX17;DDX20;DHX33;DNAJA3;DNAJC1;DNMT1;DNMT3A;DOCK7;DRD4;DVL2;ECD;EDEM1;EHMT1;EIF2AK3;EIF4A3;ELP3;EPHA1;EPHA2;ERCC3;ETS1;EZH1;FADS1;FGD1;FGF2;FGFR1;FLNA;FOSB;FOXC1;FOXI1;FOXJ1;FOXJ3;FOXK1;FOXK2;FOXM1;FOXP1;FOXP4;GABARAPL2;GABBR1;GABPB2;GATA3;GIPR;

22B;TBC1D24;TBC1D7;TBC1D9B;TGFBRAP1;TIAM1;TNIK;TNK2;TRIO;TSC1;VAV2;VPS39;WDR

3;CREB3L2;CREG1;CREM;CRTC1;CRTC3;CRY2;CSNK2A2;CSRNP2;CSTF1;CSTF2;CSTF3;CTCF;CTPS2;CTR9;CYTL1;DCTD;DDIT3;DDX17;DDX20;DHODH;DHX33;DHX38;DIDO1;DNAJA3;DNMT1;DNMT3A;DRD4;DRG1;DTL;DVL2;ECD;EHMT1;EIF2AK3;EIF4A3;ELP3;ENTPD8;ERCC3;ETS1;EZH1;FADS1;FGF2;FGFR1;FLN

1;DNM1;DOCK7;DPYSL2;DRAM1;DROSHA;DTL;DUOX1;DYX1C1;ECD;ECI2;EDC3;EDC4;EDEM1;EFTUD1;EFTUD2;EIF2S3;EIF4A3;EIF4B;EIF4G1;ENO2;ENOSF1;ENTPD4;ENTPD5;EPHA1;EPHA2;EPHX1;EPHX2;ERCC3;ERCC5;ETFDH;ETHE1;EXOSC10;FAF1;FBXL12;FBXO21;FEN1;FGD1;FGF2;FLNA;FOXJ1;FOXRED2;F

ELF1;CELSR2;CHCHD3;CHD2;CHD3;CHD4;CHD6;CHEK2;CIAO1;CIC;CIITA;CIRBP;CIRH1A;CIZ1;CLEC3B;CLK2;CLN8;CMKLR1;CREB3L2;CREG1;CREM;CRTC1;CRTC3;CRY2;CSNK1E;CSNK2A2;CSRNP2;CTC1;CTCF;CTR9;CUL1;CUL4A;CUL7;CYTL1;DCTN1;DDIT3;DDIT4;DDX17;DDX20;DHCR7;DHX33;DLD;DNAJA3;DNA

21;SCAF1;SCAF8;SF1;SF3A3;SF3B2;SF3B3;SFPQ;SMC1A;SMG6;SNRNP200;SNRNP40;SNRNP70;SNW1;SON;SRRM1;SRRM2;SRSF5;SUGP2;SUPV3L1;SYF2;TARDBP;TFIP11;TGS1;THOC1;THOC2;TNKS1BP1;U2AF1L4;U2AF2;USP39;WBP11;WDR33;ZHX2;ZR
P3;ERCC3;ETS1;EZH1;FADS1;FGF2;FGFR1;FLNA;FOSB;FOXC1;FOXI1;FOXJ1;FOXJ3;FOXK1;FOXK2;FOXM1;FOXP1;FOXP4;FUBP1;GABPB2;GATA3;GMEB2;GON4L;GTF2A2;GTF3C1;GTF3C2;GTF3C4;HABP4;HCFC1;HDAC1;HEXIM1;HGF;HHEX;HIC2;HIF1AN;HMG20A;HMGA1;HNRNPAB;HOMEZ;HOXA9;HOXB2;HS

C;ZW
SND1;SNRNP200;SNRNP40;SNRNP70;SNW1;SRP68;SRRM1;SRRM2;SYF2;TEP1;TFIP11;TGS1;TNRC6A;TNRC6B;U2AF1L4;U2AF2;UTP14A;UTP20;ZNHIT6;ZR

REG1;CREM;CRTC1;CRTC3;CRY2;CSNK2A2;CSRNP2;CSTF1;CSTF2;CSTF3;CTCF;CTPS2;CTR9;CYTL1;DBH;DCTD;DDIT3;DDX17;DDX20;DHCR24;DHCR7;DHODH;DHX33;DHX38;DIDO1;DNAJA3;DNMT1;DNMT3A;DRD4;DRG1;DTL;DVL2;ECD;EHMT1;EIF2AK3;EIF4A3;ELP3;ENTPD8;ERCC3;ETS1;EZH1;FADS1;FGF2;FG

HRF1;VCP;XBI
NK2A2;CSRNP2;CSTF1;CSTF2;CSTF3;CTCF;CTPS2;CTR9;CYTL1;DBH;DCTD;DDIT3;DDX17;DDX20;DHODH;DHX33;DHX38;DIDO1;DNAJA3;DNMT1;DNMT3A;DRD4;DRG1;DTL;DVL2;ECD;EHMT1;EIF2AK3;EIF4A3;ELP3;ENTPD8;ERCC3;ETS1;EZH1;FADS1;FGF2;FGFR1;FLNA;FOSB;FOXC1;FOXI1;FOXJ1;FOXJ3;FOXK1;

3C1D9B;TGFBRAP1;TIAM1;TNIK;TNK2;TRIO;TSC1;VAV2;VPS39;WDR
NMT3A;DROSHA;DVL2;ECD;EHMT1;EIF2AK3;EIF2AK4;EIF2B1;EIF3B;EIF4A3;EIF4B;EIF4G1;EIF4G2;EIF5A;EIF5B;ELAVL1;ELP3;EPHX2;EPRS;ERCC3;ETS1;EXOSC10;EZH1;F12;FADS1;FGF2;FGFR1;FLNA;FOSB;FOXC1;FOXI1;FOXJ1;FOXJ3;FOXK1;FOXK2;FOXM1;FOXP1;FOXP4;FUBP1;FYN;GABPB2;GATA3;GCN1L1;GL

D2;CCNT1;CCR7;CD3E;CD4;CD74;CDAN1;CDC16;CDC25B;CDH13;CDK5RAP2;CDKN2A;CDKN2B;CDON;CELF1;CELSR2;CHCHD3;CHD2;CHD3;CHD4;CHD6;CHEK2;CHMP5;CIAO1;CIC;CIITA;CIRBP;CIRH1A;CIZ1;CLEC3B;CLK2;CLN8;CMKLR1;COG7;CREB3L2;CREG1;CREM;CRTC1;CRTC3;CRY2;CSNK1E;CSNK2A2;CSRN
T3;DDX17;DDX20;DHX33;DNAJA3;DNAJC1;DNMT1;DNMT3A;DOCK7;DRD4;DVL2;ECD;EDEM1;EHMT1;EIF2AK3;EIF4A3;ELP3;EPHA1;EPHA2;ERCC3;ETS1;EZH1;FADS1;FGD1;FGF2;FGFR1;FLNA;FOSB;FOXC1;FOXI1;FOXJ1;FOXJ3;FOXK1;FOXK2;FOXM1;FOXP1;FOXP4;GABARAPL2;GABBR1;GABPB2;GATA3;GIPR;

C1;ECD;ECI2;EDC3;EDC4;EDEM1;EFTUD1;EFTUD2;EIF2S3;EIF4A3;EIF4B;EIF4G1;ENOSF1;ENTPD4;EPHA1;EPHA2;EPHX1;EPHX2;ERCC3;ERCC5;ETFDH;ETHE1;EXOSC10;FAF1;FBXL12;FBXO21;FEN1;FGD1;FOXJ1;FOXRED2;FZR1;GABARAPL2;GALT;GFM1;GLUD1;GNG10;GNL2;GNL3L;GOT1;GOT2;GPT;GPT2;GSTM

2;CSTF3;CTCF;CTPS2;CTR9;CYTL1;DCTD;DDIT3;DDX17;DDX20;DHODH;DHX33;DHX38;DIDO1;DNAJA3;DNMT1;DNMT3A;DRD4;DRG1;DTL;DVL2;ECD;EHMT1;EIF2AK3;EIF4A3;ELP3;ENTPD8;ERCC3;ETS1;EZH1;FADS1;FGF2;FGFR1;FLNA;FOSB;FOXC1;FOXI1;FOXJ1;FOXJ3;FOXK1;FOXK2;FOXM1;FOXP1;FOXP4;FU

NRG1;NRIP2;PA2G4;PABPC1;PARP1;PATZ1;PDCD4;PER1;PPARA;PPARD;PPARGC1B;PRKCA;PRMT2;PTBP1;RAD18;RAMP3;RBBP7;RBFOX2;RELA;RFX5;RREB1;RUNX2;RUNX3;SALL2;SAP30;SATB1;SCMH1;SFPQ;SIK1;SIN3B;SLA2;SMAD3;SMARCA4;SMARCC2;SMARCE1;SMC1A;SNW1;SNX6;SOX10;SOX18;SREBF

;FKBP4;FLNA;FLNC;FOXJ1;FSCN2;FTSJ2;FTSJ3;FXN;GAR1;GEMIN4;GEMIN5;GNL2;GNL3L;GTF2A2;GTPBP4;H3F3C;HACL1;HAUS5;HCFC1;HEATR1;HIST1H2AC;HIST1H2AK;HIST1H2BN;HIST2H2AC;HIST2H2BE;HIST2H3D;HLA-DRB1;HMGA1;HP1BP3;HSP90B1;HSPA4;HSPD1;HYAL1;IFT172;IKBKAP;IL2RB;INADL;I

PRMT2;PSMC4;PSMD1;PSMD11;PSMD2;PSMD6;PSMD7;PSME4;RACGAP1;RBL2;RFWD3;RNF4;RPA1;RPA2;RPRD1B;RPS6KA2;RUNX3;SCRIB;SH2B1;SIK1;SMAD3;SMARCA4;SMC1A;SON;SRSF5;TAF6;TAOK2;TCF3;TCF7L2;TIMELESS;TNF;TOP2B;TP53;TRIM35;TRRAP;TSC1;TSPYL2;TXLNG;UBE2B;UBE2L3;VASH1
;CDON;CELF1;CELSR2;CHCHD3;CHD2;CHD3;CHD4;CHD6;CHEK2;CHMP5;CIAO1;CIC;CIITA;CIRBP;CIRH1A;CIZ1;CLK2;CLN8;CMKLR1;CREB3L2;CREG1;CREM;CRTC1;CRTC3;CRY2;CSNK1E;CSNK2A2;CSRNP2;CTC1;CTCF;CTR9;CUL1;CUL7;CYP2D6;CYTL1;DCTN1;DDIT3;DDIT4;DDX17;DDX20;DHX33;DLD;DLG3;DNAJ

B;EIF4G1;EIF4G2;EIF5A;EIF5B;ELAVL1;ELP3;EPRS;ERCC3;ETS1;EZH1;FADS1;FGF2;FGFR1;FLNA;FOSB;FOXC1;FOXI1;FOXJ1;FOXJ3;FOXK1;FOXK2;FOXM1;FOXP1;FOXP4;GABPB2;GATA3;GCN1L1;GMEB2;GON4L;GTF2A2;GTPBP4;HABP4;HCFC1;HDAC1;HEXIM1;HGF;HHEX;HIC2;HIF1AN;HMG20A;HMGA1;HNRNF

;TAOK2;TDP1;TERF2;TERF2IP;TIMELESS;TMEM109;TP53;TP53BP1;UBE2B;UHRF1;UNG;USP7;VCP;WDR33;WRNIP1;XPC;XRCC3;XRCC5;XRCC6;ZBTB40;ZFYV
M1;EFTUD1;EFTUD2;EIF2S3;EIF4A3;EIF4B;EIF4G1;ENO2;ENOSF1;ENTPD4;ENTPD5;EPHA1;EPHA2;EPHX2;ERCC3;ERCC5;ETFDH;ETHE1;EXOSC10;FAF1;FBXL12;FBXO21;FEN1;FGD1;FGF2;FLNA;FOXJ1;FOXRED2;FUT2;FYN;FZR1;GABARAPL2;GALT;GFM1;GK5;GLUD1;GNG10;GNL2;GNL3L;GNPDA1;GOT1;GOT2;G

F3B;EIF4A3;EIF4B;EIF4G1;EIF4G2;EIF5A;EIF5B;ELAVL1;ELP3;EPRS;ERCC3;ETS1;EZH1;F2;FADS1;FGF2;FGFR1;FLNA;FOSB;FOXC1;FOXI1;FOXJ1;FOXJ3;FOXK1;FOXK2;FOXM1;FOXP1;FOXP4;GABPB2;GATA3;GCN1L1;GMEB2;GON4L;GTF2A2;GTPBP4;HABP4;HCFC1;HDAC1;HEXIM1;HGF;HHEX;HIC2;HIF1AN;HMG

LD1;NRG1;NRIP2;PA2G4;PABPC1;PARP1;PATZ1;PDCD4;PER1;PPARA;PPARD;PPARGC1B;PRKCA;PRMT2;PTBP1;RAD18;RAMP3;RBBP7;RBFOX2;RELA;RFX5;RREB1;RUNX2;RUNX3;SALL2;SAP30;SATB1;SCMH1;SFPQ;SIK1;SIN3B;SLA2;SMAD3;SMARCA4;SMARCC2;SMARCE1;SMC1A;SNW1;SNX6;SOX10;SOX18;S
UBE2L3;ZHX2;ZHX3;ZMIZ2;ZMYN
FXN;FYN;G0S2;GADD45A;GATA3;GLS2;GRAMD4;HDAC1;HGF;HSP90AB1;HSP90B1;HSPA9;HSPD1;HTATIP2;HTRA2;HTT;HYAL2;ID1;IKBIP;IKBKE;IKZF3;IL10;IL18;IL2RA;IL2RB;IL31RA;IL4;ING2;ING5;INTS1;IP6K2;ITGA6;JTB;KALRN;KDM1A;KDM2B;KIAA0141;KITLG;KLF11;LCK;LEF1;LGMN;LHX4;MADD;MAGED1;
ZMIZ2;ZMYN
4;CACNA1F;CACNA1I;CACNB1;CADM1;CADM2;CALR;CAMP;CAMSAP1;CAPN3;CATSPER1;CBFA2T2;CBX2;CBX7;CCDC64;CCDC88C;CCNF;CCR7;CD3E;CD74;CDAN1;CDC16;CDC25B;CDC42BPA;CDC42BPB;CDC42BPG;CDH13;CDH23;CDH24;CDHR2;CDK5RAP2;CDKN2A;CELF1;CELSR2;CELSR3;CENPN;CENPO;CEP

FXN;FYN;G0S2;GADD45A;GATA3;GLS2;GRAMD4;HDAC1;HGF;HSP90AB1;HSP90B1;HSPA9;HSPD1;HTATIP2;HTRA2;HTT;HYAL2;ID1;IKBIP;IKBKE;IKZF3;IL10;IL18;IL2RA;IL2RB;IL31RA;IL4;ING2;ING5;INTS1;IP6K2;ITGA6;ITM2C;JTB;KALRN;KDM1A;KDM2B;KIAA0141;KITLG;KLF11;LCK;LEF1;LGMN;LHX4;MADD;M

IP2;PA2G4;PARP1;PASK;PATZ1;PDCD4;PER1;PPARA;PPARD;PPARGC1B;PRMT2;RAMP3;RBBP7;RBFOX2;RELA;RFX5;RNF8;RREB1;RUNX2;RUNX3;SALL2;SAP30;SATB1;SCMH1;SFPQ;SIK1;SIN3B;SLA2;SMAD3;SMARCA4;SMARCC2;SMARCE1;SMC1A;SNW1;SNX6;SOX10;SOX18;SREBF2;SUFU;SUPT4H1;TAF3;TCF

;UPT6H;TAF1;TAF10;TAF6;TARDBP;TBP;TCEB1;TCEB3;THOC1;THOC2;TNF;TNFRSF14;TP53;UBE2I;UBR4;UNG;USP7;VDAC1;VPRBP;XRCC5;XRCC6;YWHAE;ZC3H7B;ZKSCAN1;ZKSCAN2;ZKSCAN5;ZNF263;ZNF397;ZNF445;ZNF496;ZSCAN16;ZSCAN2;ZSCAN21;ZSCAN22;ZSCAN

;UPT6H;TAF1;TAF10;TAF6;TARDBP;TBP;TCEB1;TCEB3;THOC1;THOC2;TNF;TNFRSF14;TP53;UBE2I;UBR4;UNG;USP7;VDAC1;VPRBP;XRCC5;XRCC6;YWHAE;ZC3H7B;ZKSCAN1;ZKSCAN2;ZKSCAN5;ZNF263;ZNF397;ZNF445;ZNF496;ZSCAN16;ZSCAN2;ZSCAN21;ZSCAN22;ZSCAN

CC5;ETS1;FAF1;FAIM3;FCER1G;FGD1;FGF2;FGFR1;FGGY;FLT4;FLVCR1;FOXC1;FXN;FYN;G0S2;GADD45A;GARS;GATA3;GLS2;GRAMD4;HDAC1;HGF;HSP90AB1;HSP90B1;HSPA9;HSPD1;HTATIP2;HTRA2;HTT;HYAL2;ID1;IKBIP;IKBKE;IKZF3;IL10;IL18;IL2RA;IL2RB;IL31RA;IL4;ING1;ING2;ING5;INTS1;IP6K2;ITGA6;IT

;USP35;USP36;USP39;USP40;USP48;USP54;USP7;V

P;GK5;GNE;GOT1;GOT2;GPHN;GPT;GPT2;GSTM4;GSTO1;GTF3C4;GUCY2D;GYS1;GYS2;HADHA;HADHB;HCFC1;HEMK1;HHAT;HKDC1;HMGXB3;HRASLS2;HYAL1;IBA57;ICMT;IKBKB;IKBKE;ILF2;IP6K2;IRAK1;ITPKB;KALRN;KAT6B;LCK;LIPT2;LMTK3;MAP2K6;MAP2K7;MAP3K12;MAP3K4;MAP3K9;MAPK7;MAPK9

CC5;ETS1;FAF1;FAIM3;FCER1G;FGD1;FGF2;FGFR1;FGGY;FLT4;FLVCR1;FOXC1;FXN;FYN;G0S2;GADD45A;GARS;GATA3;GLS2;GRAMD4;HDAC1;HGF;HSP90AB1;HSP90B1;HSPA9;HSPD1;HTATIP2;HTRA2;HTT;HYAL2;ID1;IKBIP;IKBKE;IKZF3;IL10;IL18;IL2RA;IL2RB;IL31RA;IL4;ING1;ING2;ING5;INTS1;IP6K2;ITGA6;IT

NODAL;NR1D1;NRG1;NRIP2;PA2G4;PARP1;PASK;PATZ1;PDCD4;PER1;PPARA;PPARD;PPARGC1B;PRMT2;RAMP3;RBBP7;RBFOX2;RELA;RFX5;RNF8;RREB1;RUNX2;RUNX3;SALL2;SAP30;SATB1;SCMH1;SFPQ;SIK1;SIN3B;SLA2;SMAD3;SMARCA4;SMARCC2;SMARCE1;SMC1A;SNW1;SNX6;SOX10;SOX18;SREBF2;S

P3;ERCC3;ETS1;EZH1;FADS1;FGF2;FGFR1;FLNA;FOSB;FOXC1;FOXI1;FOXJ1;FOXJ3;FOXK1;FOXK2;FOXM1;FOXP1;FOXP4;FUBP1;GABPB2;GATA3;GMEB2;GON4L;GTF2A2;GTF3C1;GTF3C2;GTF3C4;HABP4;HCFC1;HDAC1;HELB;HEXIM1;HGF;HHEX;HIC2;HIF1AN;HMG20A;HMGA1;HNRNPAB;HOMEZ;HOXA9;HOXE

;PIK3R4;PIM3;PIP4K2A;PIP4K2B;PIP5K1C;PKN3;POLA1;POLA2;POLB;POLE;POLE3;POLG;POLH;POLR1A;POLR1B;POLR2B;POLR2C;POLR2J;POLR3A;POLR3B;POLR3C;POLR3E;PPIP5K1;PRKAG1;PRKCA;PRKCH;PRKCQ;PRKX;PRPS1;PTK2;RIOK1;RPAP1;RPS6KA2;SBK1;SEPHS2;SGK1;SGK196;SGK223;SGK494;SHC1;

MADD;MAGED1;MAP2K6;MAP2K7;MAP3K4;MAP3K9;MAPK8IP3;MAVS;MBTPS1;MLL;MSH6;MUL1;MYC;MYO9B;NCKAP1L;NET1;NLRP1;NOD1;NODAL;NRG1;OCRL;PDE6H;PDGFC;PDIA5;PDIA6;PDPK1;PHACTR4;PILRB;PITRM1;PKD1;PLCG1;PPARGC1B;PRKAG1;PRKCA;PRKCH;PRKCQ;PRLR;PSENEN;PSMC4;PS

'DA

J3;FOXK1;FOXK2;FOXM1;FOXP1;FOXP4;GABPB2;GATA3;GMEB2;GON4L;GTF2A2;HABP4;HCFC1;HDAC1;HEXIM1;HGF;HHEX;HIC2;HIF1AN;HMG20A;HMGA1;HNRNPA2B1;HNRNPAB;HNRNPH1;HSBP1;HSPA8;HTATIP2;HTATSF1;HYAL2;ID1;IKBKAP;IKBKB;IKZF3;IL10;IL16;IL26;IL31RA;IL4;ILF2;ILF3;ING1;ING2;IN

;MAPK8IP3;MAPK9;MATN2;MBTPS1;MDC1;MDM4;MGST1;MINK1;MLH1;MMS19;MRPS9;MSH6;MTOR;MUL1;MYBBP1A;MYC;NEIL2;NET1;NFATC2;NFRKB;NOD1;NONO;NRBP2;OMG;PALB2;PAPD7;PARP1;PAXIP1;PDCD4;PDIA5;PDIA6;PMS2;PMS2CL;PMS2P5;POLA1;POLB;POLE;POLG;POLH;POLR2B;POLR2

DVL2;ECD;EHMT1;EIF2AK3;EIF2AK4;EIF2B1;EIF3B;EIF4A3;EIF4B;EIF4G1;EIF4G2;EIF5A;EIF5B;ELAVL1;ELP3;EPRS;ERCC3;ETS1;EZH1;F2;FADS1;FAM132A;FGF2;FGFR1;FITM2;FLNA;FOSB;FOXC1;FOXI1;FOXJ1;FOXJ3;FOXK1;FOXK2;FOXM1;FOXP1;FOXP4;GABBR1;GABPB2;GATA3;GCN1L1;GIPR;GMEB2;GON4L;G

DM1A;KDM2B;KDM4A;KHDRBS1;KLF11;L3MBTL1;LANCL2;LAX1;LDB1;LEF1;LIG3;LPIN1;LRP5;LRP8;MAD2L2;MAGED1;MBD1;MBD2;MDM4;MECP2;MEIS2;MLH1;MLL4;MPHOSPH8;MSH6;MTA2;MXD3;MYBBP1A;MYC;NAB2;NACC1;NCOR2;NF2;NFX1;NKAP;NODAL;NR1D1;NRG1;NRIP2;NXN;PA2G4;PABPC1;

RGC1B;PRMT2;PTBP1;RAMP3;RBBP7;RBFOX2;RELA;RFX5;RREB1;RUNX2;RUNX3;SALL2;SAP30;SATB1;SCMH1;SFPQ;SIK1;SIN3B;SLA2;SMAD3;SMARCA4;SMARCC2;SMARCE1;SNW1;SNX6;SOX10;SOX18;SREBF2;SUFU;SUPT4H1;TAF3;TARDBP;TCF7L2;TFCP2L1;TGIF2;THRA;TIMELESS;TLE1;TNF;TP53;TXN;U2A

ACC1;NCOR2;NF2;NFX1;NKAP;NODAL;NR1D1;NRG1;NRIP2;PA2G4;PARP1;PASK;PATZ1;PDCD4;PER1;PPARA;PPARD;PPARGC1B;PRKCA;PRMT2;RAMP3;RBBP7;RBFOX2;RELA;RFX5;RNF8;RREB1;RUNX2;RUNX3;SALL2;SAP30;SATB1;SCMH1;SFPQ;SIK1;SIN3B;SLA2;SMAD3;SMARCA4;SMARCC2;SMARCE1;SMC1

PPARD;PPARGC1B;PRMT2;RAMP3;RBBP7;RBFOX2;RELA;RFX5;RREB1;RUNX2;RUNX3;SALL2;SAP30;SATB1;SCMH1;SFPQ;SIDT1;SIDT2;SIK1;SIN3B;SLA2;SMAD3;SMARCA4;SMARCC2;SMARCE1;SND1;SNW1;SNX6;SOX10;SOX18;SREBF2;SRRT;SUFU;SUPT4H1;TAF3;TCF7L2;TFCP2L1;TGIF2;THRA;TIMELESS;TLE1

CD;EHMT1;EIF2AK3;EIF2AK4;EIF2B1;EIF3B;EIF4A3;EIF4B;EIF4G1;EIF4G2;EIF5A;EIF5B;ELAVL1;ELP3;EPRS;ERCC3;ETS1;EZH1;FADS1;FAM132A;FGF2;FGFR1;FITM2;FLNA;FOSB;FOXC1;FOXI1;FOXJ1;FOXJ3;FOXK1;FOXK2;FOXM1;FOXP1;FOXP4;GABBR1;GABPB2;GATA3;GCN1L1;GIPR;GMEB2;GON4L;GRM2;GTF2

VAMP1;VAT1;VDAC1;VDA

RNII

RABL2A;RABL2B;RACGAP1;RAP1GDS1;RASA3;RASA4;RASAL3;RGS13;RGS17;RHOF;RYR2;SCRIB;SNX9;SYDE1;SYNGAP1;TAX1BP3;TBC1D7;TBCC;TSC1;UCK2;UHRF1;WRNI

E2I;VCP;WBP1;WBP11;YWHAE;ZXI

PA8;HYAL2;ID1;IL10;IL4;ILF3;INCA1;INPPL1;IRF8;JAZF1;KAT6B;KCTD1;KDM1A;KDM2B;KDM4A;KHDRBS1;KIAA0226;KLF11;L3MBTL1;LANCL2;LAX1;LDB1;LEF1;LIG3;LPIN1;LRP5;LRP8;MAD2L2;MAGED1;MBD1;MBD2;MDM4;MECP2;MEIS2;MLH1;MLL4;MPHOSPH8;MSH6;MTA2;MTOR;MXD3;MYBBP1A;MYC;N
OXK2;FOXM1;FOXP1;FOXP4;GABPB2;GATA3;GMEB2;GON4L;GTF2A2;HABP4;HCFC1;HDAC1;HEXIM1;HGF;HHEX;HIC2;HIF1AN;HMG20A;HMGA1;HNRNPAB;HSBP1;HSPA8;HTATIP2;HTATSF1;HYAL2;ID1;IKBKAP;IKBKB;IKZF3;IL10;IL16;IL26;IL31RA;IL4;ILF2;ILF3;ING1;ING2;ING5;INO80;IRAK1;IRF8;ITGA6;JAZF1;
P7;RBFOX2;RELA;RFX5;RREB1;RUNX2;RUNX3;SALL2;SAP30;SATB1;SCMH1;SFPQ;SIK1;SIN3B;SLA2;SMAD3;SMARCA4;SMARCC2;SMARCE1;SNW1;SNX6;SOX10;SOX18;SREBF2;SUFU;SUPT4H1;TAF3;TCF7L2;TFCP2L1;TGIF2;THRA;TIMELESS;TLE1;TNF;TP53;TXN;UBE2B;UBE2I;UHRF1;VHL;WHSC1;XRCC5;XRCC6;
NAB2;NACC1;NCOR2;NF2;NFX1;NKAP;NODAL;NR1D1;NRG1;NRIP2;PA2G4;PARP1;PASK;PATZ1;PDCD4;PER1;PPARA;PPARD;PPARGC1B;PRKCA;PRMT2;RAMP3;RBBP7;RBFOX2;RELA;RFX5;RNF8;RREB1;RUNX2;RUNX3;SALL2;SAP30;SATB1;SCMH1;SFPQ;SIK1;SIN3B;SLA2;SMAD3;SMARCA4;SMARCC2;SMARCE

C;FNBP1;FRMD4A;FSCN2;FYN;GPHN;GRIN2C;GYS2;HAUS5;HERC2;HNRNPH1;HSBP1;HSPH1;INCENP;INO80;INPP5B;INPP5E;INPPL1;INVS;IP6K2;IPP;JTB;KALRN;KARS;KDM4A;KIAA0368;KIAA0586;KIAA0753;KIF13B;KIF16B;KIF21B;KIF3B;KIF4A;KIF5C;KITLG;KLC1;KLC2;KLHL3;KNTC1;KRT10;KRT5;KRT7;KRTAP5-

C2;KNTC1;KRT10;KRT5;KRT7;KRTAP5-1;LANCL2;LCK;LDLRAP1;LPXN;LZTS2;MACF1;MAD2L1BP;MAD2L2;MAP1A;MAP1LC3B2;MAP4;MAP7D3;MAPRE2;MAPT;MCM3;MID2;MINK1;MMS19;MNS1;MYC;MYCBP;MYH3;MYH7B;MYO15A;MYO18A;MYO19;MYO1C;MYO1D;MYO1E;MYO5B;MYO9B;MYOM1;NAV1

OXK2;FOXM1;FOXP1;FOXP4;GABPB2;GATA3;GMEB2;GON4L;GTF2A2;HABP4;HCFC1;HDAC1;HEXIM1;HGF;HHEX;HIC2;HIF1AN;HMG20A;HMGA1;HNRNPAB;HSBP1;HSPA8;HTATIP2;HTATSF1;HYAL2;ID1;IKBKAP;IKBKB;IKZF3;IL10;IL16;IL26;IL31RA;IL4;ILF2;ILF3;ING1;ING2;ING5;INO80;IRAK1;IRF8;ITGA6;JAZF1;

D1;LMO2;LPIN1;LRP5;LRP8;LSM10;MAD2L2;MAGED1;MAGOH;MAPK7;MAVS;MBD1;MBD2;MCF2L;MDM4;MECP2;MED24;MED27;MED30;MED31;MED6;MEIS2;MKL1;MLL;MNT;MTA2;MYC;NCOR2;NEDD8;NFATC2;NFATC3;NFE2L1;NFRKB;NFX1;NKAP;NLRC5;NMI;NODAL;NPAS2;NR1D1;NRF1;NRIP2;PAM;
P1;NOD1;NODAL;NRG1;OCRL;PDE6H;PDGFC;PDIA5;PDIA6;PDPK1;PHACTR4;PILRB;PITRM1;PKD1;PLCG1;PPARGC1B;PRKAG1;PRKCA;PRLR;PSENEN;PSMC4;PSMD1;PSMD11;PSMD2;PSMD6;PSMD7;PSME4;PTK2;RAB3GAP1;RABGAP1;RACGAP1;RAP1GDS1;RAPGEF1;RASA3;RASA4;RASAL3;RFC1;RGS13;RGS1

RASAL3;RGS13;RGS17;RHOF;SCRIB;SNX9;SYDE1;SYNGAP1;TAX1BP3;TBC1D7;TBCC;TSC1;UCK2;WRNI

POLR2J;PPIP5K1;PRKAG1;PRKCA;PRKCH;PRKCQ;PRKX;PRPS1;PTK2;RIOK1;RPS6KA2;SBK1;SEPHS2;SGK1;SGK196;SGK223;SGK494;SHC1;SHPK;SIK1;SIK2;SRPK3;STK16;STK36;STK38;STK39;TAF1;TAOK2;TNIK;TNK2;TRIO;TSSK3;TSSK4;TXK;TYK2;UCK2;UL

KA2;SBK1;SGK1;SGK196;SGK223;SGK494;SHC1;SHPK;SIK1;SIK2;SRPK3;STK16;STK36;STK38;STK39;TAF1;TAOK2;TNIK;TNK2;TRIO;TRRAP;TSSK3;TSSK4;TXK;TYK2;UCK2;UL

Y2;DRAM1;DRD4;DST;DTL;EDEM1;EFHD1;EHD4;EIF2AK3;EIF5A;EMD;ENTPD4;ENTPD5;EPHX1;ETFDH;EXOG;EXT2;FADS1;FADS2;FAF1;FAM20B;FITM2;FKRP;FNDC5;FUT11;FUT2;FZR1;GABARAPL2;GAL3ST4;GALNT10;GALNT6;GALNT9;GBF1;GGA3;GGCX;GGTA1P;GLG1;GOLGA1;GOLGA3;GOLGA7;GORASP2;G

B;WNT6;YIF1A;ZDHHC1;ZFPM1;ZNF219;ZNF35;ZNF358;ZNF414;ZNF444;ZNF564;ZNF575;ZNF579;ZNF628;ZNF638;ZNF668;ZNF747;ZNF764;ZNF771;ZNF784;ZNF787;ZNF837;ZNF865;ZSCAN

28;ZNF638;ZNF668;ZNF747;ZNF764;ZNF771;ZNF784;ZNF787;ZNF837;ZNF865;ZSCAN

F6;NUDT3;NUTF2;OAT;OTUD7A;OXT;PABPN1;PCSK1N;PDF;PDXP;PDZD7;PEX10;PGLYRP2;PHOSPHO1;PLSCR3;PODXL2;PRDM8;PSD3;RASL10A;RDH14;REPIN1;RNF126;RPP25;RRAS;SCARF2;SCGB3A1;SKOR1;SNRPA;SOCS1;SQLE;SSBP4;STBD1;STK11;SUV420H2;TAF6L;TBX6;TCTEX1D4;TIGD5;TMEM102;TOR2

'AF6L;TBX6;TFR2;TOR2A;TPRA1;TPST2;UCN;UFSP2;UGP2;USF2;USP33;UTF1;VAPB;WNT6;YIF1A;ZDHHC1;ZFPM1;ZNF219;ZNF35;ZNF358;ZNF414;ZNF444;ZNF564;ZNF575;ZNF579;ZNF628;ZNF638;ZNF668;ZNF747;ZNF764;ZNF771;ZNF784;ZNF787;ZNF837;ZNF865;ZSCAI

:SQLE;STK11;SUV420H2;TAF6L;TBX6;TFR2;TOR2A;TPRA1;TPST2;TRADD;UACA;UCN;UFSP2;UGP2;USF2;USP33;UTF1;VAPB;WNT6;YIF1A;ZDHHC1;ZFPM1;ZNF219;ZNF35;ZNF358;ZNF414;ZNF444;ZNF564;ZNF575;ZNF579;ZNF628;ZNF638;ZNF668;ZNF747;ZNF764;ZNF771;ZNF784;ZNF787;ZNF837;ZNF865;ZSC

;CD99;CDC25A;CDC6;CDCA8;CDK12;CDK13;CDS2;CDYL2;CECR5;CEL;CELF2;CELF6;CEMP1;CENPF;CENPM;CENPT;CEP55;CHCHD5;CHD7;CHMP4A;CHMP6;CHRAC1;CHTF18;CIB1;CISD3;CLASP1;CLCN4;CLIP1;CLPP;CLSPN;CNT
;DOLK;DPM3;DPP7;DRG2;DSE;DTYMK;DUS2L;DUSP10;DUSP23;DUSP3;DVL1;DYM;DYNC1LI1;ECI1;EEF1A2;EEF1D;EEF1G;EHHADH;EIF2B2;EIF2B4;EIF3F;EIF3G;EIF3I;EIF3K;EIF4G3;ELK3;ENGASE;ENTPD7;ERGIC3;ERLIN2
S2;CDYL2;CECR5;CEL;CELF2;CELF6;CEMP1;CENPF;CENPM;CENPT;CEP55;CHCHD5;CHD7;CHMP4A;CHMP6;CHRAC1;CHTF18;CIB1;CISD3;CLASP1;CLCN4;CLIP1;CLPP;CLSPN;CNTLN;COL11A2;COL6A1;COL6A2;COL9A2;COMMD4
OMMD4;COMTD1;COQ7;COQ9;COX4I1;CPSF3L;CPSF4;CPT1C;CREB3;CREB3L4;CRELD2;CRIP1;CRIP2;CRYL1;CSDE1;CSNK1G1;CTSC;CTSF;CTU2;CUEDC2;CYC1;CYP1B1;CYP2E1;DAAM1;DALRD3;DCUN1D3;DDT;DDX28;DDX39A;DEA
D2B;FASTK;FBL;FBXL15;FBXL6;FBXO2;FBXO24;FBXW11;FBXW5;FDXR;FEM1B;FICD;FIGNL2;FKBP11;FKBP2;FLAD1;FPGS;FUK;FXYD2;GAMT;GCDH;GFER;GLYCTK;GNAI3;GNG8;GNGT2;GPX4;GSTT1;GTPBP3;GYG1;GZMM;HAGHL;HCCS;HD
;PPTC7;PTGES2;PTRH1;PUS1;QARS;QTRT1;RHOT2;RNMTL1;SARDH;SARS2;SDHAF1;SDHAF2;SIRT3;SIVA1;SLC25A19;SLC25A25;SLC25A29;SLC25A35;SLC25A5;SLC25A6;SLC44A1;SLMO1;SMCR7;SPG20;STOML2;SURF1;TAOK3;TAZ;TBRG4;TIMM10;TIMM13;TI
1;COQ7;COQ9;COX4I1;CPSF2;CPSF3L;CPSF4;CPT1C;CREB3;CREB3L4;CRELD2;CTSC;CTSF;CTU2;CUEDC2;CYC1;CYP1B1;CYP2E1;DDX21;DDX28;DDX39A;DEAF1;DECR2;DERL3;DGCR6;DGCR6L;DGUOK;DHRS4;DIAPH2;DLGAP5;DI
SP1;CLCN4;CLIP1;CLPP;CLSPN;CNTLN;COL11A2;COL6A1;COL6A2;COL9A2;COMMD5;COMTD1;COQ7;COQ9;CORO6;COX4I1;CPSF2;CPSF3L;CPSF4;CPT1C;CREB3;CREB3L4;CRELD2;CTSC;CTSF;CTTNBP2NL;CTU2;CUEDC2;CYC1;CY
OQ9;COX4I1;CPSF2;CPSF3L;CPSF4;CPT1C;CREB3;CREB3L4;CRELD2;CTSC;CTSF;CTU2;CUEDC2;CYC1;CYP1B1;CYP2E1;DDX21;DDX28;DDX39A;DEAF1;DECR2;DERL3;DGCR6;DGCR6L;DGUOK;DHRS4;DIAPH2;DLGAP5;DNAJC30;DI
4;CLIP1;CLPP;CLSPN;CNTLN;COL11A2;COL6A1;COL6A2;COL9A2;COMMD5;COMTD1;COQ7;COQ9;CORO6;COX4I1;CPSF2;CPSF3L;CPSF4;CPT1C;CREB3;CREB3L4;CRELD2;CTSC;CTSF;CTTNBP2NL;CTU2;CUEDC2;CYC1;CYP1B1;CYF

ETFB;ETV3;EXOSC5;FADS3;FAF2;FAM21B;FAM69B;FANCG;FBL;FBXO2;FBXW11;FDX1L;FDXR;FKBP2;FPGS;FSD1;GAS2L3;GCDH;GCHFR;GEMIN7;GFER;GGA1;GHDC;GIMAP5;GLTSCR2;GNAI3;GNB2L1;GOLPH3;GRIN3A;GTF2A
M21B;FAM69B;FANCG;FBL;FBXW11;FDX1L;FDXR;FKBP2;FPGS;FSD1;GAS2L3;GCDH;GCHFR;GEMIN7;GFER;GGA1;GHDC;GIMAP5;GLTSCR2;GNAI3;GNB2L1;GOLPH3;GRIN3A;GTF2A1;GTF3C5;H2AFJ;H2AFY2;HAUS8;HCCS;HD
B7;NHP2;NLE1;NME4;NMRAL1;NOL12;NR2C2AP;NSRP1;NTHL1;NUDT1;NUP153;NUSAP1;OBFC1;OGG1;PAFAH1B2;PAOX;PARS2;PATL1;PDK4;PHF16;PHF20;PIN1;PIP5K1A;PNKP;POLD1;POLD4;POLR2F;POLR2G;POLR2I;POLR2L;POLR3K;POL

NSRP1;NTHL1;NUDT1;NUP153;NUSAP1;OBFC1;OGG1;PAFAH1B2;PAOX;PARS2;PATL1;PDK4;PHF16;PHF20;PIN1;PIP5K1A;PNKP;POLD1;POLD4;POLR2F;POLR2G;POLR2I;POLR2L;POLR3K;POLRMT;POP7;POU2F1;PPP2R3B;PSMA7;PSMB4;PS
A2G6;PMM1;POLR2F;POLR2G;POLR2I;POLR2L;POMT1;PPM1D;PPM1J;PPM1L;PPP2R3B;PREB;PRKAR2A;PRMT7;PSMA7;PSMB4;PSMB5;PSMB7;PSMD9;PTPRC;PUS1;PUSL1;QPCTL;QTRT1;RABGGTA;RBBP5;RBPMS;RLIM;RNF115;RNF169;RNF18

PS15;MRPS18A;MTM1;MYLIP;MYLK3;NAA10;NCOA3;NDUFA13;NEURL2;NF1;NIPBL;NOG;NPRL2;NRP1;NSMCE1;OXSR1;P2RX7;PARP10;PARP3;PARS2;PDE6G;PDK4;PHEX;PHF16;PHF20;PHPT1;PICK1;PIGQ;PIK3CB;PIK3CG;PIN1;PL

NSRP1;NTHL1;NUDT1;NUP153;NUSAP1;OBFC1;OGG1;PAFAH1B2;PAOX;PARS2;PATL1;PDK4;PHF16;PHF20;PIN1;PIP5K1A;PNKP;POLD1;POLD4;POLR2F;POLR2G;POLR2I;POLR2L;POLR3K;POLRMT;POP7;POU2F1;PPP2R3B;PSMA7;PSMB4;PS
;LAMB1;LAMTOR2;LARP4B;LAT;LATS1;LATS2;LIME1;LSM14A;LSM2;LSM4;MAD1L1;MAPK1;MBD3;MCM7;MCRS1;MED1;MED13L;MED18;MICB;MLL3;MLL5;MR1;MRPL12;MRPL20;MRPL23;MRPL24;MRPL38;MRPL52;MR

20H1;TADA3;TAOK3;TAZ;TBRG4;TGM1;TIPARP;TLK2;TNK1;TRIM24;TRMT1;TRMT112;TRMT2A;TRMT61A;TRMU;TRPT1;TSSK6;TTN;TUT1;UCK1;UCKL1;ULK3;WHSC1L1;ZAP70;ZC3HAV1;ZD

AB21;RABEPK;RHOT2;SCARB2;SEC22B;SEC24B;SENP2;SLC22A17;SLC25A19;SLC25A25;SLC25A29;SLC25A35;SLC25A5;SLC25A6;SLC26A11;SLC29A2;SLC2A8;SLC35B2;SLC35D2;SLC37A4;SLC39A13;SLC44A1;SLC50A1;SMCR7;SNF8;SNTB

;CCDC61;CCDC78;CCHCR1;CCNL2;CCS;CD320;CD36;CD38;CD7;CD8B;CD99;CDC25A;CDC6;CDCA8;CDK12;CDK13;CDS2;CDYL2;CECR5;CEL;CELF2;CELF6;CEMP1;CENPF;CENPM;CENPT;CEP55;CHCHD5;CHD7;CHMP4A;CHMP6;CHR
;CCDC61;CCDC78;CCHCR1;CCNL2;CCS;CD320;CD36;CD38;CD7;CD8B;CD99;CDC25A;CDC6;CDCA8;CDK12;CDK13;CDS2;CDYL2;CECR5;CEL;CELF2;CELF6;CEMP1;CENPF;CENPM;CENPT;CEP55;CHCHD5;CHD7;CHMP4A;CHMP6;CHR

3D3;MCRS1;METAP1D;MIF4GD;MLL3;MLL5;MLST8;MMP28;MOGS;MPDU1;MRPL12;MRPL20;MRPL23;MRPL24;MRPL52;MRPL55;MRPS11;MRPS12;MRPS15;MRPS18A;MST1;MTM1;MYLIP;MYLK3;NAA10;NAALADL1;NAPSA;NCOA3;NDUF
PM1L;PPP2R3B;PREB;PRKAR2A;PRMT7;PSMA7;PSMB4;PSMB5;PSMB7;PSMD9;PTPRC;QPCTL;RABGGTA;RBBP5;RBPMS;RLIM;RNF115;RNF169;RNF181;RNF25;ROCK2;RSRC1;RUSC1;SDHAF2;SEC24B;SENP2;SETD2;SETD7;SH3RF1;SIRT3;SPI
PM1L;PPP2R3B;PREB;PRKAR2A;PRMT7;PSMA7;PSMB4;PSMB5;PSMB7;PSMD9;PTPRC;QPCTL;RABGGTA;RBBP5;RBPMS;RLIM;RNF115;RNF169;RNF181;RNF25;ROCK2;RSRC1;RUSC1;SDHAF2;SEC24B;SENP2;SETD2;SETD7;SH3RF1;SIRT3;SPI

RSRC1;SAP30L;SDHAF2;SENP2;SERGEF;SETX;SF3B5;SIVA1;SLC29A2;SNF8;SNRPC;SP1;SPTBN4;STRA13;SUDS3;SUV420H1;SWI5;SYCE1L;TADA3;TBL3;TCEA2;TCEB2;THAP7;TIMM50;TLE2;TOP1;TOP2A;TOPBP1;TP73;TPR;TRIM24
L;EIF2B2;EIF2B4;EIF3F;EIF3G;EIF3I;EIF3K;EIF4G3;ELF1;ELK3;ELP4;EPC1;EPG5;ERCC1;ERLIN2;ERP29;ERP44;ETF1;ETFB;ETNK2;ETV2;ETV3;EXOSC5;FADS3;FANCG;FASTK;FBL;FBXL15;FBXL6;FBXO2;FBXO24;F
M3;DPP7;DPP8;DSE;DTD1;DTX3;DTYMK;DUSP10;DUSP2;DUSP3;DVL1;E2F1;E4F1;EAF1;ECI1;EDF1;EEF1D;EEF1G;EHHADH;EIF1;EIF2B2;EIF2B4;EIF3F;EIF3G;EIF3I;EIF3K;EIF4G3;ELF1;ELK3;ELP4;EPC1;EPG5;ERCC1
A7;PSMB4;PSMB5;PSMB7;PSMD9;PTGES2;QARS;RAC3;RANBP9;RGL1;RHOC;RHOT2;RNF115;RNF25;ROCK2;RPL13;RPL18;RPL18A;RPL28;RPL29;RPL38;RPL8;RPLP1;RPLP2;RPS15;RPS2;RPS28;RQCD1;S100A13;SEC24B;SERPINB9;S

PS13B;VPS16;VPS41;VTI1A;WDR24;WNT7A;YIPF5;YWHAZ;ZFYVE27

3;DVL1;E2F1;E4F1;EAF1;ECI1;EDF1;EEF1D;EEF1G;EHHADH;EIF1;EIF2B2;EIF2B4;EIF3F;EIF3G;EIF3I;EIF3K;EIF4G3;ELF1;ELK3;ELP4;EPC1;ERCC1;ERLIN2;ERP29;ERP44;ETF1;ETNK2;ETV2;ETV3;EXOSC5;FADS3;FANC

MED13L;MED18;MICB;MLL3;MLL5;MR1;MSH3;MYH10;MZT2B;NARFL;NAT9;NCAPH;NDUFA11;NDUFA13;NDUFA3;NDUFA8;NDUFB10;NDUFB11;NDUFB7;NDUFB8;NDUFB9;NDUFS6;NDUFS7;NDUFS8;NDUFV1;NEURL2;NIPBL;N

L;RAD21;RAD9A;RBBP5;REC8;RFX3;RLIM;RNF169;RPS15;RPS19BP1;RPS2;RPS6KB2;RRP9;RSF1;RSRC1;SAP30L;SDHAF2;SENP2;SERGEF;SETX;SF3B5;SIVA1;SLC29A2;SNF8;SNRNP25;SNRPC;SP1;SPTBN4;STOML2;STRA13;STUB1;SUDS3

EMA6A;SERPINF2;SETD2;SETD7;SLC25A5;SLC25A6;SMCR7;SPG20;SPTBN4;STMN3;STOML2;STRA13;SUDS3;SUV420H1;SYCE1L;TACC1;TADA3;TAZ;TIMM10;TIMM13;TIMM50;TLK2;TMED9;TMSB4X;TOP1;TOP2A;TOR1AIP1;TP73;TP

KP;POLR2F;POLR2G;POLR2I;POLR2L;PPAP2C;PPM1D;PPM1J;PPM1L;PPP1R14A;PPP1R14B;PPP2R3B;PREB;PRKAR2A;PTDSS2;PTPRC;PXK;RAB21;RAB33A;RAC3;RALGAPB;RASA2;RBPMS;ROCK2;RSRC1;SDHAF2;SENP2;SLC44A1;SMAP1;SMPD2;SO

IHADH;EIF1;EIF2B2;EIF2B4;EIF3F;EIF3G;EIF3I;EIF3K;EIF4G3;ELF1;ELK3;ELP4;EPC1;ERCC1;ERLIN2;ERP29;ERP44;ETF1;ETNK2;ETV2;ETV3;EXOSC5;FADS3;FANCG;FASTK;FBL;FBXL15;FBXL6;FBXO2;FBXO24;FBX

.R2L;PPAP2C;PPM1D;PPM1J;PPM1L;PPP1R14A;PPP1R14B;PPP2R3B;PREB;PRKAR2A;PTDSS2;PTPRC;PXK;RAB21;RAB33A;RAC3;RALGAPB;RASA2;RBPMS;ROCK2;RSRC1;SDHAF2;SENP2;SLC44A1;SMAP1;SMPD2;SOS1;SPHK2;SPRED2;SPTBN4;SS

X;PHKB;PHPT1;PICK1;PLA2G6;PLCD1;PNKP;POLD1;POP7;PPAP2C;PPM1D;PPM1J;PPM1L;PPM1N;PPP2R3B;PPTC7;PRSS22;PRSS53;PSMA7;PSMB4;PSMB5;PSMB7;PTPRC;PTRH1;QPCTL;RAB21;RAB33A;RAC3;RAD9A;RECQL4;REXO1L1;RHBDD3;RHOT2;

2;PDE6G;PDK4;PEX6;PFKFB2;PHKG1;PIK3C3;PIK3CB;PIK3CG;PIP5K1A;PIP5K1B;PIP5KL1;PMVK;PNKP;POLD1;PPOX;PRKAR2A;PYCRL;QARS;R3HCC1;RAB21;RAB25;RAB33A;RABEP2;RAC3;RBMXL1;RBPMS;RECQL4;RHOC;RHOT2;ROC

G;PIP5K1A;PIP5K1B;PIP5KL1;PMVK;PNKP;POLD1;PPOX;PRKAR2A;PYCRL;QARS;R3HCC1;RAB21;RAB25;RAB33A;RABEP2;RAC3;RBMXL1;RBPMS;RECQL4;RHOC;RHOT2;ROCK2;RPS6KB2;RPS6KC1;RPS6KL1;RTEL1;SARS2;SDR39U1;SI

G;PIP5K1A;PIP5K1B;PIP5KL1;PMVK;PNKP;POLD1;PPOX;PRKAR2A;PYCRL;QARS;R3HCC1;RAB21;RAB25;RAB33A;RABEP2;RAC3;RBMXL1;RBPMS;RECQL4;RHOC;RHOT2;ROCK2;RPS6KB2;RPS6KC1;RPS6KL1;RTEL1;SARS2;SDR39U1;SI

PICK1;PIK3C3;PIK3CB;PIK3CG;PIN1;PIP5K1A;PIP5K1B;PLA2G6;PMM1;PMVK;PNKP;PNMT;PPAP2C;PPM1L;PPOX;PRKAR2A;PSMA7;PSMB4;PSMB5;PSMB7;PSMD9;PTDSS2;PTGES2;PTPLB;PXK;PYCRL;QARS;QTRT1;RAB21;RAB33A;RAC3;RALGAPB;RASA2;RGL1;S

15;TOMM40L;TOR1AIP1;TPR;TRAF2;TRAF6;TRPS1;UBL4A;UEVLD;UPF2;USE1;VPS13B;VPS16;VPS41;VTI1A;WDR24;WNT7A;YIPF5;YWHAZ;ZFYVE2[illegible]

[illegible]1D;EEF1G;EHHADH;EIF2B2;EIF2B4;EIF3F;EIF3G;EIF3I;EIF3K;ELF1;ELK3;ELMO3;ELP4;ERCC1;ERLIN2;ETF1;EXD3;EXOSC5;FAF2;FANCG;FASTK;FBL;FBXL15;FBXO2;FBXO44;FBXW11;FBXW5;FBXW9;FCGR3A;FCHSD2;FDXR;

BP1;HDAC10;HDAC11;HERC3;HES6;HES7;HINFP;HIPK1;HIPK3;HIVEP2;HNRNPH3;HPX;HRAS;ID3;IKZF4;IMP4;INO80E;IRAK4;IRF3;JAG2;JOSD2;KAT2A;KCNH4;KDM3A;KDM5A;KDM6A;KIDINS220;KLI

;NOXA1;NOXO1;NT5C;NTHL1;NUDT1;NUDT18;NUP153;OGG1;P2RX7;PAFAH1B2;PAFAH1B3;PANK4;PAOX;PAPSS1;PARS2;PCYT2;PDK4;PEMT;PEX6;PFKFB2;PGM2;PHKB;PHKG1;PICK1;PIGQ;PIK3C3;PIK3CB;PIK3CG;PIN1;PIP5K1A;PIP5K1B;PLA2G6;PLCD1;PMM1

CC1;THOC6;TIMM10;TIMM13;TIMM50;TLR7;TMED9;TMSB4X;TNFRSF4;TNFSF15;TOR1AIP1;TPR;TRAF2;TRAF6;TRAPPC2L;TRAPPC4;TRPS1;TTN;UBL4A;UPF2;USE1;USP6NL;VPS16;VPS41;VTI1A;WASH3P;WDR24;WNT7A;YII

2;PARP3;PATL1;PBX4;PHF1;PLA2G6;PNKP;POLD1;POLR2F;POLRMT;POP7;PRKAR2A;PROCR;PUS1;PXK;QTRT1;RAC3;RAD21;RAD9A;RANBP9;RASSF7;RC3H1;REC8;RFX3;RINL;RNF169;ROCK2;RPL13;RPL18;RPL18A;RPL28;RPL29;RPL38;RPL8;F
2;PARP3;PATL1;PBX4;PHF1;PLA2G6;PNKP;POLD1;POLR2F;POLRMT;POP7;PRKAR2A;PROCR;PUS1;PXK;QTRT1;RAC3;RAD21;RAD9A;RANBP9;RASSF7;RC3H1;REC8;RFX3;RINL;RNF169;ROCK2;RPL13;RPL18;RPL18A;RPL28;RPL29;RPL38;RPL8;F

.D1;FNIP2;GEMIN7;GFER;GLYCTK;GNB2L1;GPBP1L1;GTF2A1;GTF2H2D;GTF3C5;GTPBP3;GYG1;GZMM;H2AFJ;H2AFY2;HBP1;HDAC10;HDAC11;HERC3;HES6;HES7;HINFP;HIPK1;HIPK3;HIVEP2;HNRNPH3;HP

ASP1;CLCF1;CLCN4;CLDN7;CLIP1;CLPP;CLSPN;COL11A2;COL6A1;COL6A2;COMMD4;COMMD5;COQ7;CORO6;CPSF2;CPSF4;CRB3;CREB3;CREB3L4;CRELD1;CRELD2;CRIP1;CRIP2;CRYL1;CSDE1;CSNK1G1;CTSC;CTTNBP2NL;CTU2;CUE

;TUT1;UBA7;UCK1;UCKL1;ULK3;VARS2;YME1L1;ZA

1;UCKL1;ULK3;VARS2;YME1L1;ZA

AB33A;RABEP2;RAC3;RECQL4;RHOC;RHOT2;ROCK2;RPL29;RPS6KB2;RPS6KC1;RPS6KL1;RTEL1;SARS2;SEPT1;SETX;SIRT3;SLC1A3;SOAT1;SOAT2;SPEG;SPHK2;STK25;STOML2;TAOK3;TLK2;TNK1;TOP1;TOP2A;TOP3B;TPR;

RS2;YME1L1;ZA

RS2;YME1L1;ZA
RS2;YME1L1;ZA
1;UCKL1;ULK3;VARS2;YME1L1;ZA

ME1L1;ZA

IPK1;HIPK3;HIVEP2;HNRNPH3;HRAS;HSD11B1L;HSD17B1;HSD17B12;HSD17B8;IFT27;IKZF4;ILVBL;IRAK4;IRF3;ISYNA1;ITPA;KDM3A;KDM5A;KHK;KIAA2018;KIF15;KIF20A;KIF24;KIF7;KLF10;LAF
2;HBP1;HDAC10;HDAC11;HELZ;HERC3;HES6;HES7;HEXDC;HINFP;HIPK1;HIPK3;HIVEP2;HNRNPH3;HRAS;ID3;IKZF4;IMP4;INO80E;IPO8;IRF3;ISOC2;KAT2A;KDM3A;KDM5A;KDM6A;KIAA2018;KIF20A;KLF10;KRT18;LAT;L

RS2;YME1L1;ZA

MK2D;CAMK4;CAPG;CAPN7;CARD8;CASC5;CASP3;CASP8;CASP8AP2;CBFB;CBX3;CC2D1A;CCDC59;CCDC86;CCDC91;CCNA2;CCNC;CCND3;CCNE2;CCNG1;CCNH;CCNL1;CCNT2;CCNY;CCT2;CD2AP;CD2BP2;CDC14A;CDC25C;CDC
3;ARL2;ARL4A;ARL6IP1;ARL6IP5;ARL8B;ARMC1;ARMC10;ARRB1;ARSA;ARSK;ARVCF;ASF1A;ASNA1;ASPH;ASPM;ASTL;ATAD1;ATAD2;ATAD3A;ATF1;ATF2;ATG5;ATG9A;ATL2;ATP10D;ATP11B;ATP11C;ATP13A2;ATP2C1;ATP5C1;ATP5E;ATP5F1;
3;ARL2;ARL4A;ARL6IP1;ARL6IP5;ARL8B;ARMC1;ARMC10;ARRB1;ARSA;ARSK;ARVCF;ASF1A;ASNA1;ASPH;ASPM;ASTL;ATAD1;ATAD2;ATAD3A;ATF1;ATF2;ATG5;ATG9A;ATL2;ATP10D;ATP11B;ATP11C;ATP13A2;ATP2C1;ATP5C1;ATP5E;ATP5F1;
DIA;ARHGEF2;ARID2;ARID4A;ARID4B;ARID5A;ARID5B;ARL13B;ARL2;ARL4A;ARL6IP1;ARL6IP5;ARL8B;ARMC1;ARMC10;ARPC4;ARRB1;ARSA;ARSK;ARVCF;ASF1A;ASNA1;ASPH;ASPM;ASTL;ATAD1;ATAD2;ATAD3A;ATF1;ATF2;ATG5;ATG9A;ATL2;A
DIA;ARHGEF2;ARID2;ARID4A;ARID4B;ARID5A;ARID5B;ARL13B;ARL2;ARL4A;ARL6IP1;ARL6IP5;ARL8B;ARMC1;ARMC10;ARPC4;ARRB1;ARSA;ARSK;ARVCF;ASF1A;ASNA1;ASPH;ASPM;ASTL;ATAD1;ATAD2;ATAD3A;ATF1;ATF2;ATG5;ATG9A;ATL2;A
D;APPBP2;APPL1;APTX;AQP11;ARAF;ARAP2;ARF4;ARFGAP1;ARFGAP2;ARFGEF2;ARFIP1;ARG2;ARGLU1;ARHGAP10;ARHGAP11A;ARHGAP12;ARHGAP22;ARHGAP5;ARHGDIA;ARHGEF10L;ARHGEF2;ARID2;ARID4A;ARID4B;ARID5A;ARID5B;ARL13B;ARL
;ARHGAP12;ARHGAP22;ARHGAP5;ARHGDIA;ARHGEF10L;ARHGEF2;ARID4B;ARL2;ARL4A;ARL6IP1;ARL6IP5;ARL8B;ARMC1;ARMC10;ARMCX5-GPRASP2;ARPC4;ARPP19;ARRB1;ARRDC3;ARSA;ARSK;ARVCF;ASAP3;ASMTL;ASNA1;ASPH;ASPM;ASTL;ATAD
D;APPBP2;APPL1;APTX;AQP11;ARAF;ARAP2;ARF4;ARFGAP1;ARFGAP2;ARFGEF2;ARFIP1;ARG2;ARGLU1;ARHGAP10;ARHGAP11A;ARHGAP12;ARHGAP22;ARHGAP42;ARHGAP5;ARHGDIA;ARHGEF10L;ARHGEF2;ARID2;ARID4A;ARID4B;ARID5A;ARID5B;A
PC;APEH;APEX1;APEX2;APH1A;API5;APIP;APLF;APOBEC3D;APPBP2;APPL1;APTX;AQP11;ARAF;ARAP2;ARF4;ARFGAP1;ARFGAP2;ARFGEF2;ARFIP1;ARG2;ARGLU1;ARHGAP10;ARHGAP11A;ARHGAP12;ARHGAP22;ARHGAP42;ARHGAP5;ARHGDIA;ARHGEF10
PC;APEH;APEX1;APEX2;APH1A;API5;APIP;APLF;APOBEC3D;APPBP2;APPL1;APTX;AQP11;ARAF;ARAP2;ARF4;ARFGAP1;ARFGAP2;ARFGEF2;ARFIP1;ARG2;ARGLU1;ARHGAP10;ARHGAP11A;ARHGAP12;ARHGAP22;ARHGAP42;ARHGAP5;ARHGDIA;ARHGEF10
C;ATG5;ATG9A;ATL2;ATP10D;ATP11B;ATP11C;ATP13A2;ATP2C1;ATP5C1;ATP5E;ATP5F1;ATP5I;ATP5L;ATP5O;ATP6AP1;ATP6V1G1;ATP7A;ATP8A1;ATPAF2;AUH;AZI1;AZI2;AZIN1;B2M;B3GALNT1;B3GALT2;B3GALTL;B3GNT1;B3GNT2;B3GNT5;
T6;BANF1;BAP1;BAZ1A;BBS7;BCAP29;BCAP31;BCAS2;BCAT2;BCKDHB;BCLAF1;BDP1;BET1;BLOC1S2;BLZF1;BMF;BMI1;BNIP1;BNIP2;BNIP3L;BRCA2;BRCC3;BRIP1;BRWD1;BTD;BUD13;BYSL;C10orf2;C12orf52;C14orf166;C14orf2;C
;CAMK2D;CAMK4;CAMLG;CARHSP1;CASC5;CASP3;CASP8;CASP8AP2;CBFB;CBLL1;CBX3;CCDC59;CCDC88A;CCNA2;CCNC;CCND3;CCNE2;CCNG1;CCNH;CCNL1;CCNT2;CCNY;CCNYL1;CCT2;CCT6B;CD28;CD2BP2;CD80;CD81;CDC25C;CDC26;CD
T6;BANF1;BAP1;BAZ1A;BBS7;BCAP29;BCAP31;BCAS2;BCAT2;BCKDHB;BCLAF1;BDP1;BET1;BLOC1S2;BLZF1;BMF;BMI1;BNIP1;BNIP2;BNIP3L;BRCA2;BRCC3;BRIP1;BRWD1;BTD;BUD13;BYSL;C10orf2;C12orf52;C14orf166;C14orf2;C
2C1;ATP5C1;ATP5E;ATP5F1;ATP5I;ATP5L;ATP5O;ATP7A;ATPBD4;ATRX;ATXN7L3;AUH;AURKC;AZIN1;B2M;B3GALNT1;B3GALT2;B3GALTL;B3GNT1;B3GNT2;B3GNT5;B4GALT3;B4GALT6;BACH1;BAG2;BAG4;BAG6;BANF1;BAP1;BAZ1A;BAZ2B;B
OPS5;COPS6;CPEB2;CPEB3;CRBN;CREB1;CREBL2;CRIPAK;CRK;CRY1;CSK;CSNK1A1;CSNK1G3;CSNK2B;CSTA;CTBP1;CTDNEP1;CTDSP1;CUL2;CUL4B;CUL5;CXXC1;DAB2IP;DAPK3;DAPP1;DAXX;DCAF10;DCAF11;DCAF13;DDB2;DDOST;DDX11;D
ATP2B1;ATP2C1;ATP5C1;ATP5E;ATP5F1;ATP5I;ATP5L;ATP5O;ATP7A;ATPBD4;ATRX;ATXN7L3;AUH;AURKC;AZIN1;B2M;B3GALNT1;B3GALT2;B3GALTL;B3GNT1;B3GNT2;B3GNT5;B4GALT3;B4GALT6;BACH1;BAG2;BAG4;BAG6;BANF1;BAP1;BAZ1
2;BRCC3;BRD9;BRIP1;BRMS1;BRMS1L;BRWD1;BTAF1;BTBD1;BUD13;BZW1;C10orf2;C12orf52;C14orf166;C1GALT1;C1QA;C1QB;C1QC;C1R;CAB39;CALCRL;CAMK1;CAMK2D;CAMK4;CAMLG;CAPN14;CAPN7;CARD17;CARHSP1;CASC5;CASP3;CAS
2;ATG4A;ATG4B;ATG4C;ATG5;ATL2;ATP2B1;ATP2C1;ATP5C1;ATP5E;ATP5F1;ATP5I;ATP5L;ATP5O;ATP7A;ATPBD4;ATRX;ATXN7L3;AUH;AURKC;AZIN1;B2M;B3GALNT1;B3GALT2;B3GALTL;B3GNT1;B3GNT2;B3GNT5;B4GALT3;B4GALT6;BACH1;BA
GAP22;ARHGAP42;ARHGAP5;ARHGDIA;ARHGEF2;ARID2;ARID4A;ARID4B;ARID5A;ARID5B;ARL13B;ARL15;ARL2;ARL4A;ARL5A;ARL5B;ARL6IP1;ARL6IP5;ARL8B;ARMC1;ARMCX5-GPRASP2;ARPC4;ARPP19;ARRB1;ARRDC3;ARSA;ARSK;ARVCF;ASAP3;AS
CR8;DIS3;DMAP1;DMPK;DNA2;DNAJB9;DNTTIP2;DPY30;DR1;DTX2;DYRK1A;E2F4;E2F6;E2F7;EAF2;EED;EIF3E;EIF5A2;EIF6;ELL2;ENY2;EPC2;ERBB2IP;ERCC2;ERCC8;ERI1;ERN1;ESF1;ESR2;FAM169A;FAM175A;FAM188A;FAM
TAD2;ATF1;ATF2;ATG12;ATG16L2;ATG4A;ATG4B;ATG4C;ATG5;ATG9A;ATL2;ATP2B1;ATP2C1;ATP5C1;ATP5E;ATP5F1;ATP5I;ATP5L;ATP5O;ATP7A;ATPBD4;ATRX;ATXN7L3;AUH;AURKC;AZIN1;B2M;B3GALNT1;B3GALT2;B3GALTL;B3GNT1;B3GN

NFXL1;NPLOC4;NR1D2;NR1H2;NR3C1;NR4A1;NR4A3;NSMCE2;P2RX4;PAN3;PARK2;PCGF5;PCGF6;PDCD2;PDLIM2;PDLIM5;PDXK;PEX12;PEX2;PHC3;PHF17;PHF20L1;PHF6;PHF7;PIAS4;PJA2;PLAG1;PLAGL1;PNKD;PNMA3;POLR2K;PRDM10;PRDM5;P
A;CORO7;COX16;COX6C;COX7A2;COX7A2L;COX7B;COX7C;CRAT;CRLS1;CSGALNACT1;CSGALNACT2;CST3;CTDNEP1;CTNS;CYBA;CYBASC3;CYCS;CYP2S1;CYP2U1;CYP2W1;CYP4F22;DDOST;DDX3X;DEGS1;DEGS2;DENND4C;DERL1;I
USP38;USP44;USP45;USP46;USP5;USP51;USP53;USP6;USPL1;WFS1;WWP1;YOD1;ZMPSTE24;ZNRF1;ZRA
SP16;USP19;USP21;USP25;USP31;USP37;USP38;USP44;USP45;USP46;USP5;USP51;USP53;USP6;USPL1;WFS1;WWP1;YOD1;ZMPSTE24;ZNRF1;ZRA
DX54;DECR1;DEPDC1;DGCR8;DHFRL1;DHX30;DIS3;DLAT;DMAP1;DNA2;DNAJB9;DNAJC10;DNTTIP2;DPY30;DR1;DYRK1A;E2F4;E2F6;E2F7;EAF2;ECHS1;EDEM3;EED;EIF3E;EIF6;ELL2;ENY2;EPC2;ERAL1;ERCC2;ERCC8;ERI1;ERLEC1
USP44;USP45;USP46;USP5;USP51;USP53;USP6;USPL1;WFS1;WWP1;YOD1;ZNRF1;ZRA
SEH2A;RNF103;RNF11;RNF144B;RNF146;RNF187;RNF217;RNF40;RNF6;RNH1;RPL11;RPL15;RPL17;RPL23;RPL26;RPL26L1;RPL27;RPL30;RPL31;RPL34;RPL35;RPL36A;RPL39;RPL41;RPL7;RPL9;RPS15A;RPS24;RPS27;RPS29;RPS3A;RPS7;SI
USP38;USP44;USP45;USP46;USP5;USP51;USP53;USP6;USPL1;WFS1;WWP1;YOD1;ZMPSTE24;ZNRF1;ZRA
L1;DDX3X;DDX50;DDX54;DECR1;DEPDC1;DGCR8;DHFRL1;DHX30;DIS3;DLAT;DMAP1;DNA2;DNAJB9;DNAJC10;DNTTIP2;DPY30;DR1;DYRK1A;E2F4;E2F6;E2F7;EAF2;ECHS1;EDEM3;EED;EIF3E;EIF6;ELL2;ENY2;EPC2;ERAL1;ERCC2;EI
6;RNF187;RNF19A;RNF2;RNF217;RNF220;RNF31;RNF40;RNF6;RNF7;RYBP;SENP1;SENP6;SIAH1;SIRT1;SKP2;SMURF2;SOCS4;SOCS5;SOCS6;SPOPL;SUMO1;SUMO2;SUMO3;SUZ12;TGFBR1;TICAM1;TNKS2;TOLLIP;TOPORS;TRAF7;TRIM13;TR
SP14;USP15;USP16;USP19;USP21;USP25;USP31;USP37;USP38;USP44;USP45;USP46;USP5;USP51;USP53;USP6;USPL1;WFS1;WWP1;YOD1;ZMPSTE24;ZNRF1;ZRA
DCLRE1A;DDB2;DDX11;DDX3X;DDX50;DDX54;DECR1;DEPDC1;DGCR8;DHFRL1;DHX30;DIS3;DLAT;DMAP1;DNA2;DNAJB9;DNAJC10;DNTTIP2;DPY30;DR1;DYRK1A;E2F4;E2F6;E2F7;EAF2;ECHS1;EDEM3;EED;EIF3E;EIF6;ELL2;ENY2;EI
5;FBXW7;FER;FGFR1OP;FIGNL1;GAK;GAS2;GAS2L2;GAS6;GMNN;GNAI1;GORASP1;GPR132;GPS1;GPS2;GTF2H1;HACE1;HAUS1;HAUS3;HAUS6;HAUS7;HDAC3;HDAC8;HELLS;HORMAD1;HPGD;HSF1;IL12A;IL8;ILK;ITGB3BP;JMY;JUN;KAT2
;CTDSP1;CUL2;CUL4B;CUL5;CXXC1;DAB2IP;DAPK3;DAPP1;DAXX;DCAF10;DCAF11;DCAF13;DDB2;DDOST;DDX11;DHPS;DLG1;DMAP1;DMPK;DNAJB2;DNAJB9;DNAJC10;DOHH;DOLPP1;DOT1L;DPH3;DPM1;DPY30;DR1;DTX2;DUSP11;DUSP19;DYI
;CTDSP1;CUL2;CUL4B;CUL5;CXXC1;DAB2IP;DAPK3;DAPP1;DAXX;DCAF10;DCAF11;DCAF13;DDB2;DDOST;DDX11;DHPS;DLG1;DMAP1;DMPK;DNAJB2;DNAJB9;DNAJC10;DOHH;DOLPP1;DOT1L;DPH3;DPM1;DPY30;DR1;DTX2;DUSP11;DUSP19;DYI
;BAZ2B;BBS10;BBS12;BCR;BGLAP;BIRC2;BIRC3;BMI1;BMP1;BMPR1A;BMPR2;BNIP2;BRCC3;BRIP1;BTAF1;C10orf2;C11orf54;C1GALT1;C1orf27;C1R;C4orf21;CA12;CA5BP1;CACNA1H;CALB1;CALML4;CALML6;CALU;CAMK1;CAMK2D;CAMK4;C
;FER;FMR1;FOXH1;FOXN2;FRG1;FXR1;FYTTD1;G2E3;GABPA;GATAD2A;GEMIN2;GEMIN6;GMCL1;GMNN;GNL3;GORAB;GPS2;GRHL1;GRWD1;GTF2B;GTF2E1;GTF2E2;GTF2F2;GTF2H1;GTF2H4;GTPBP10;GZF1;HAT1;HAUS7;HDAC3;HD
;CSNK1G3;CSNK2B;CSTA;CTBP1;CTDNEP1;CTDSP1;CUL2;CUL4B;CUL5;CXXC1;DAB2IP;DAPK3;DAPP1;DAXX;DCAF10;DCAF11;DCAF13;DDB2;DDOST;DDX11;DHPS;DLG1;DMAP1;DMPK;DNAJB2;DNAJB9;DNAJC10;DOHH;DOLPP1;DOT1L;DPH3;DPM
D7;COX19;CPNE3;CRK;CSK;CSNK1A1;CSNK2B;CTBP1;CTSH;CUL2;CUL5;CYCS;DAK;DAPP1;DAXX;DCK;DCP1A;DCP2;DCPS;DCTN2;DCTN4;DCTN6;DDX3X;DENND1B;DENND1C;DENND4C;DEPDC1B;DHPS;DIAPH3;DICER1;DIS3;DIXDC1;DLG1;DMPK
TP7A;ATP8A1;ATP8A2;ATP8B4;ATPBD4;ATRX;AUH;AURKC;AZIN1;B3GALNT1;B3GALT2;B3GALTL;B3GNT1;B3GNT2;B3GNT5;B4GALT3;B4GALT6;BAP1;BCAT1;BCAT2;BCKDHB;BCR;BDH2;BIRC2;BIRC3;BMI1;BMP1;BMPR1A;BMPR2;BRCA2;BRCC3;BR
TF2T;CTBP1;CTDSP1;CUL2;CXXC1;DAB2IP;DACH1;DAPK3;DAXX;DBP;DCAF13;DCP1A;DCP2;DCPS;DDX3X;DDX41;DDX54;DEK;DENND4A;DEPDC1;DGCR14;DGCR8;DICER1;DIS3;DKK3;DLG4;DMAP1;DMTF1;DNA2;DNTTIP2;DPY30;DR1;DRAP
7;CDK18;CDK2;CDK7;CDK8;CDK9;CDKL1;CDKL2;CDKN2AIP;CDT1;CEBPA;CEBPG;CEBPZ;CENPE;CENPW;CERS5;CGGBP1;CHD1;CHD5;CHD9;CHERP;CHFR;CHUK;CKB;CLCN7;CLK1;CLK3;CLK4;CLP1;CMPK1;CNBP;CNOT6;CNOT7;CNOT8;CNP;
T8;CNP;COMMD6;COPS2;COPS5;CPSF1;CPSF6;CRABP2;CRCP;CREB1;CREB5;CREBL2;CREBZF;CRK;CRLF3;CRTC2;CRY1;CST3;CSTF2T;CTBP1;CTDSP1;CUL2;CUL4B;CXXC1;CYCS;DAB2IP;DACH1;DAPK3;DAXX;DBF4;DBP;DCAF13;DCLRE1A;DC

L;MDM2;MEX3C;MIB1;MICAL1;MICALL1;MICALL2;MKRN2;MKRN3;MMP15;MMP17;MMP24;MNAT1;MORC2;MORC3;MPI;MSL2;MTF2;MTRR;MYNN;NAPEPLD;NFU1;NFXL1;NOS3;NPLOC4;NR1D2;NR1H2;NR3C1;NR4A1;NR4A3;NSMCE2;NUDT16;P
;CDK11B;CDK17;CDK18;CDK2;CDK7;CDK8;CDK9;CDKL1;CDKL2;CDKN2AIP;CDT1;CEBPA;CEBPG;CEBPZ;CENPE;CENPW;CERS5;CGGBP1;CHD1;CHD5;CHD9;CHERP;CHFR;CHUK;CKB;CLCN7;CLK1;CLK3;CLK4;CLP1;CMPK1;CNBP;CNOT6;CNOT
S;SIAH1;SIRT1;SKP2;SMURF2;SOCS4;SOCS5;SOCS6;SPOPL;SUMO1;SUMO2;SUMO3;SUZ12;TGFBR1;TICAM1;TNKS2;TOLLIP;TOPORS;TRAF7;TRIM13;TRIM23;TRIM28;TRIM33;TRIM39;TRIM41;UBA2;UBA3;UBA5;UBA6;UBC;UBE2A;UBE2D1;U
C59;CCDC88A;CCNA2;CCNC;CCNE2;CCNH;CCNL1;CCNT2;CD28;CD2BP2;CD80;CDC25C;CDC40;CDC42EP2;CDC7;CDC73;CDCA7;CDK1;CDK11A;CDK11B;CDK2;CDK5R1;CDK5RAP3;CDK7;CDK8;CDK9;CDKN1B;CDKN1C;CDS1;CDT1;CEBPA;CE
LNACT1;CSGALNACT2;CSNK2B;CSRP1;CTDSP1;CUTC;CXXC1;CYB561;CYB5R4;CYBA;CYBASC3;CYBRD1;CYCS;CYHR1;CYP2S1;CYP2U1;CYP2W1;CYP4F22;DAGLB;DAK;DBF4;DCP2;DDX11;DDX41;DDX59;DEF8;DGCR8;DGKE;DGKZ;DICER1;DMPK
;CDC40;CDC7;CDC73;CDCA7;CDIPT;CDK1;CDK11A;CDK11B;CDK2;CDK5R1;CDK5RAP3;CDK7;CDK8;CDK9;CDKN1B;CDKN1C;CDS1;CDT1;CEBPA;CEBPG;CEBPZ;CENPK;CEP290;CEPT1;CERCAM;CERS5;CGGBP1;CHAF1A;CHAF1B;CHD1;CHD

B2;CPEB3;CPSF1;CPSF6;CRABP2;CRCP;CREB1;CREB5;CREBL2;CREBZF;CRK;CRLF3;CRTC2;CRY1;CSTF2T;CTBP1;CTDSP1;CTSH;CUL2;CXXC1;DAB2IP;DACH1;DAPK3;DAXX;DBP;DCAF13;DCP1A;DCP2;DCPS;DDOST;DDX3X;DDX41;DDX54;DE
URF2;SOCS4;SOCS5;SOCS6;SPOPL;SUMO3;SUZ12;TGFBR1;TICAM1;TNKS2;TOPORS;TRAF7;TRIM13;TRIM23;TRIM28;TRIM33;TRIM39;TRIM41;UBA6;UBC;UBE2A;UBE2D1;UBE2D3;UBE2E1;UBE2G1;UBE2K;UBE2M;UBE2N;UBE2Q2;UBE2T;UE
2;CNOT3;CNOT6;CNOT7;CNOT8;COG3;COMMD6;COPS2;COPS5;CPEB2;CPEB3;CPSF1;CRABP2;CRCP;CREB1;CREB5;CREBL2;CREBZF;CRK;CRLF3;CRTC2;CRY1;CSGALNACT1;CSGALNACT2;CST3;CTBP1;CTDSP1;CUL2;CXXC1;DAB2IP;DACH1;DA
E2D1;UBE2D3;UBE2E1;UBE2G1;UBE2J1;UBE2K;UBE2N;UBE2V2;UBE2W;UBE3A;UBE4A;UBR1;UBR3;UBR5;UCHL3;UCHL5;USP1;USP12;USP14;USP15;USP16;USP19;USP21;USP25;USP31;USP37;USP38;USP44;USP45;USP46;USP5;USP51;USP
ST2;CHSY1;CHUK;CHURC1;CKAP2;CLIP3;CLP1;CNBP;CNOT2;CNOT3;CNOT6;CNOT7;CNOT8;COG3;COMMD6;COPS2;COPS5;CPEB2;CPEB3;CPSF1;CRABP2;CRCP;CREB1;CREB5;CREBL2;CREBZF;CRK;CRLF3;CRTC2;CRY1;CSGALNACT1;CSGALNACT
11A;CDK11B;CDK2;CDK5R1;CDK5RAP3;CDK7;CDK8;CDK9;CDKN1B;CDKN1C;CDT1;CEBPA;CEBPG;CEBPZ;CENPK;CENPQ;CENPW;CEP290;CGGBP1;CHAF1A;CHAF1B;CHD1;CHD5;CHD9;CHERP;CHMP1A;CHN2;CHUK;CHURC1;CKAP2;CLK1;CLK
AF2;ATRX;ATXN7L3;B2M;BAG2;BAG4;BAG6;BAIAP2;BANF1;BAP1;BAZ1A;BAZ2B;BBIP1;BBS10;BBS12;BBS7;BCAP31;BCAS2;BCKDHB;BCL2A1;BCLAF1;BCR;BET1;BIN1;BIRC2;BIRC3;BLOC1S2;BLZF1;BMF;BMI1;BMP1;BMPR1A;BMPR2;BNIP
SASS6;SCLT1;SCYL1;SLAIN2;SLC9A3R1;SLMAP;SMAD4;SMEK1;SMEK2;SPAST;TNKS2;TOPORS;TRNT1;TUBD1;TUBE1;TUBG1;TUBG2;TUBGCP2;TXNDC9;VPS37A;WDR35;WRAP73;V
3;CD80;CD81;CDC25C;CDC40;CDC7;CDC73;CDCA7;CDIPT;CDK1;CDK11A;CDK11B;CDK2;CDK5R1;CDK5RAP3;CDK7;CDK8;CDK9;CDKN1B;CDKN1C;CDS1;CDT1;CEBPA;CEBPG;CEBPZ;CENPK;CEP290;CEPT1;CERCAM;CERS5;CGGBP1;CHAF1A
NH;CCNL1;CCNT2;CD28;CD80;CD81;CDC25C;CDC40;CDC7;CDC73;CDCA7;CDIPT;CDK1;CDK11A;CDK11B;CDK2;CDK5R1;CDK5RAP3;CDK7;CDK8;CDK9;CDKN1B;CDKN1C;CDS1;CDT1;CEBPA;CEBPG;CEBPZ;CENPK;CEP290;CEPT1;CERCAM;C
YNS1ABP;JMJD6;JUN;KAT2B;KAT8;KIF20B;KIF23;KLHL20;KPNA2;KPNA3;KPNA5;LIG4;LIN52;LIN54;LIN9;LMNA;LRCH4;LSM11;LUC7L3;MAP3K7;MAPK8;MAPKAPK2;MAPKAPK3;MBIP;MCL1;MCM5;MDM2;MED12L;MED13;MED14;MED15;
REB5;CSGALNACT1;CSGALNACT2;CSNK2B;CSRP1;CTBS;CTDSP1;CUTC;CXXC1;CYB561;CYB5R4;CYBA;CYBASC3;CYBRD1;CYCS;CYHR1;CYP2S1;CYP2U1;CYP2W1;CYP4F22;DAGLB;DAK;DBF4;DCP2;DDX11;DDX41;DDX59;DEF8;DGCR8;DGKE;DC
L;EPS15;EPT1;ERBB2IP;ERGIC2;ERLIN1;ERN1;ERO1L;ERO1LB;EXOC3;EXTL2;FAM169A;FAM188A;FAM69A;FANCL;FAR2;FCHO1;FCHO2;FDFT1;FITM1;FMO5;FNDC3A;FRS2;FUT4;G6PC3;GALNT1;GALNT3;GALNT4;GALNT7;
GTT;RNPC3;RPF1;RPL11;RPL26;RPL7;RPS24;RPS7;RRP1;RRP36;RRP8;SART1;SCAF11;SF3A1;SF3A2;SF3B1;SF3B14;SFSWAP;SIRT1;SKIV2L2;SLU7;SMAD2;SMNDC1;SNRNP27;SNRNP35;SNRNP48;SNRPB;SNRPB2;SNRPD1;SNRPE;S
RPS15A;RPS24;RPS27;RPS27L;RPS29;RPS3A;RPS7;RRP1;SART1;SERP1;SF3A1;SF3A2;SF3B1;SF3B14;SKIV2L2;SLU7;SMNDC1;SNRNP35;SNRNP48;SNRPB;SNRPB2;SNRPD1;SNRPE;SNRPG;SREK1;SRP14;SRP19;SRP9;SUGP1;TAF9;TDRD6
CALCRL;CAMK4;CARHSP1;CASC5;CASP3;CASP8AP2;CBFB;CBX3;CCBL2;CCDC59;CCDC88A;CCNA2;CCNC;CCNE2;CCNH;CCNL1;CCNT2;CD28;CD2BP2;CD80;CDC25C;CDC40;CDC42EP2;CDC7;CDC73;CDCA7;CDK1;CDK11A;CDK11B;CDK2;CDK
C42EP2;CDC7;CDC73;CDCA7;CDK1;CDK11A;CDK11B;CDK2;CDK5R1;CDK5RAP3;CDK7;CDK8;CDK9;CDKN1B;CDKN1C;CDT1;CEBPA;CEBPG;CEBPZ;CENPK;CENPQ;CENPW;CEP290;CGGBP1;CHAF1A;CHAF1B;CHD1;CHD5;CHD9;CHERP;CHMP1A;

GAP12;ARHGAP22;ARHGAP42;ARHGAP5;ARHGDIA;ARHGEF10L;ARHGEF2;ARID2;ARID4A;ARID4B;ARID5A;ARID5B;ARL13B;ARL15;ARL2;ARL4A;ARL5A;ARL5B;ARL6IP1;ARL8B;ARPC4;ARPP19;ARRB1;ARRDC3;ARSA;ARSK;ARV1;ARVCF;ASAP3;ASB13
D5;PBK;PDS5B;PES1;PHIP;PKD2;PKIA;PKN2;PLCB1;PLK4;PMF1;POLD2;PPAT;PPP1CC;PPP1R12A;PPP2R1A;PPP3CA;PPP5C;PPP6C;PRCC;PRDM5;PSMA1;PSMA2;PSMA3;PSMA4;PSMA6;PSMA8;PSMB1;PSMB3;PSMC1;PSMC2;PSMC3;PSMC5;PSI
MA8;PSMB1;PSMB3;PSMC1;PSMC2;PSMC3;PSMC5;PSMC6;PSMD10;PSMD13;PSMD14;PSMD4;PSMD8;PSME1;PTEN;RAB12;RAD23A;RBBP6;RBBP8;RBCK1;RBX1;RCHY1;RNASEH2A;RNF103;RNF11;RNF144B;RNF146;RNF187;RNF217;RNF40;RNF6;RI

L7;RPL9;RPS15A;RPS24;RPS27;RPS29;RPS3A;RPS7;SART1;SCAF11;SF3A1;SF3A2;SF3B1;SF3B14;SFSWAP;SKIV2L2;SLU7;SMG1;SMG5;SMG9;SMNDC1;SNRNP27;SNRNP35;SNRNP48;SNRPB;SNRPB2;SNRPD1;SNRPE;SNRPG;SREK1;SREK1IP
C7;CDC73;CDCA7;CDK1;CDK11A;CDK11B;CDK2;CDK5R1;CDK5RAP3;CDK7;CDK8;CDK9;CDKN1B;CDKN1C;CDT1;CEBPA;CEBPG;CEBPZ;CENPK;CENPQ;CENPW;CEP290;CGGBP1;CHAF1A;CHAF1B;CHD1;CHD5;CHD9;CHERP;CHMP1A;CHN2;CHU

E2K;UBE2M;UBE2N;UBE2O;UBE2Q2;UBE2T;UBE2V2;UBE2W;UBE3A;UBE4A;UBOX5;UBR1;UBR3;UBR5;VARS;WARS2;WWP1;ZNF738;ZNRF1;ZI
1C;CDKN2C;CDKN3;CEBPG;CENPE;CFH;CFP;CHCHD4;CHFR;CHM;CHORDC1;CHPF;CHPF2;CHST13;CHST14;CHSY1;CHUK;CISH;CKS1B;CKS2;CLIP3;CLK1;CLK3;CLK4;CLN3;CLN5;CLN6;CNPPD1;COG3;COL4A3BP;COPS2;COPS4;COPS5;COPS6;CPD;CPEB

DX3X;DDX41;DDX50;DDX54;DDX59;DECR1;DGKE;DGKZ;DHFRL1;DHRS1;DHRS11;DHRS9;DHX30;DHX36;DHX40;DICER1;DMPK;DNA2;DNAH6;DNAJA1;DNAJC27;DYRK1A;DYRK1B;DYRK2;EEF1A1;EEFSEC;EIF5;EPHB3;EPHB6;ERAL1;ERCC2;

D1;LSM11;MAD2L1;MAP3K11;MAP9;MAPKAPK2;MCM5;MDK;MDM2;MEN1;MIS18BP1;MITD1;MLF1IP;MNAT1;MPHOSPH6;MSH2;MTBP;MUC1;NBN;NDE1;NEDD1;NEK1;NEK7;NFATC1;NSL1;NSMCE2;NUDC;NUDT16;NUF2;NUP37;NUP85;OI
DX3X;DDX41;DDX50;DDX54;DDX59;DECR1;DGKE;DGKZ;DHFRL1;DHRS1;DHRS11;DHRS9;DHX30;DHX36;DHX40;DICER1;DMPK;DNA2;DNAH6;DNAJA1;DNAJC27;DYRK1A;DYRK1B;DYRK2;EEF1A1;EEFSEC;EIF5;EPHB3;EPHB6;ERAL1;ERCC2;
IDE1;NDN;NDRG2;NEDD1;NEK1;NEK7;NFU1;NIN;NME7;NUDC;NUDCD2;NUDT21;NUP85;ODF2L;PAN3;PAX2;PBXIP1;PCGF5;PDCD6IP;PDE4D;PIBF1;PIN4;PKD2;PLK1S1;PLK4;POC1B;POLR3H;PPP1R12A;PPP2CB;P
;CLIC4;CLP1;CNOT3;CNOT7;CNP;COG3;COG4;COG6;COPE;COPS2;COPS4;COPS5;COPS6;COPZ1;CORO1A;CPSF1;CPSF6;CRBN;CRCP;CREB1;CSNK1A1;CSNK1A1L;CSNK2B;CTBP1;CTDNEP1;CUL2;CUL4B;CUL5;CXADR;CXXC1;CYBA;CY
SD17B10;HSPA13;IER3IP1;INF2;INPP5K;INSIG1;INSIG2;ITPR2;JKAMP;KDELC2;KEAP1;KIAA0020;KTN1;LCLAT1;LMAN1;LMAN2;LPCAT1;LPCAT2;LPCAT4;LPGAT1;LRMP;LRP6;LRPAP1;LRRC8C;MAGT1;MAN1A1;MAP2K2;MAPK8IP1
N2C;CDKN3;CDT1;CEBPA;CEBPG;CEBPZ;CENPE;CENPK;CEP290;CGGBP1;CHAF1A;CHAF1B;CHD1;CHD5;CHD9;CHMP1A;CHN2;CHORDC1;CHUK;CHURC1;CISD1;CISD2;CKAP2;CKS1B;CKS2;CLIP3;CLK1;CLK3;CLK4;CLN3;CLN6;CNBP;CNN3;CNOT2
33A1;SLC35A1;SLC35A3;SLC35A4;SLC35A5;SLC35B3;SLC35B4;SLC35C1;SMPD4;SREBF1;ST8SIA4;STEAP2;STEAP4;STX16;STX5;SVIP;TLR8;TMBIM4;TMCO1;TMED2;TMED7;TMEM167A;TMEM167B;TMF1;TNKS2;TRIM23;UGCG;UNC50;USO1;VAC

G;CEBPZ;CENPE;CENPK;CEP290;CFH;CFP;CGGBP1;CHAF1A;CHAF1B;CHD1;CHD5;CHD9;CHMP1A;CHN2;CHORDC1;CHUK;CHURC1;CKAP2;CKS1B;CKS2;CLIP3;CLK1;CLK3;CLK4;CLN3;CLN6;CNBP;CNN3;CNOT2;CNOT3;CNOT6;CNOT7;CNOT8;CNPP
VRK1;VRK2;WE
CE2;NUDC;NUF2;NUP37;NUP85;OIP5;ORC2;ORC3;ORC4;OSGIN2;PAPD5;PBK;PDS5B;PES1;PHIP;PIWIL4;PKD2;PKIA;PLCB1;PLK1S1;PLK4;PMF1;PMS1;POLD2;PPAT;PPP1CC;PPP1R12A;PPP2R1A;PPP3CA;PPP5C;PPP6C;PSMA1;PSMA2;PSMA3
D2BP2;CD80;CDC25C;CDC40;CDC42EP2;CDC7;CDC73;CDCA7;CDK1;CDK11A;CDK11B;CDK2;CDK5R1;CDK5RAP3;CDK7;CDK8;CDK9;CDKN1B;CDKN1C;CDT1;CEBPA;CEBPG;CEBPZ;CENPK;CENPQ;CENPW;CEP290;CGGBP1;CHAF1A;CHAF1B;CHI
KD1;FASTKD3;FBXO11;FDFT1;FDPS;FER;FGFR1OP;FGFR4;FN3K;FNTA;FPGT;FTSJ1;FTSJD1;FUT4;GAK;GALNT1;GALNT3;GALNT4;GALNT7;GAPDH;GCNT1;GCNT2;GCNT4;GCSH;GFPT1;GGCT;GGPS1;GK;GLT8D1;GLT8D2;GMPPA;GNMT;GPAM;G
;DACH1;DAK;DAPK3;DAZAP1;DCAKD;DCK;DDX11;DDX3X;DDX41;DDX50;DDX54;DDX59;DECR1;DGKE;DGKZ;DHFRL1;DHRS1;DHRS11;DHRS9;DHX30;DHX36;DHX40;DICER1;DMPK;DNA2;DNAH6;DNAJA1;DNAJC27;DPM1;DYRK1A;DYRK1B;DYI
;EPHB3;EPHB6;ERAL1;ERCC2;ERN1;ETNK1;FANCM;FARS2;FARSA;FDXACB1;FER;FGFR4;FIGNL1;FPGT;GAK;GBP3;GBP6;GCH1;GFM2;GIMAP2;GIMAP7;GK;GMPPA;GNA13;GNAI1;GNAO1;GNL1;GNL3;GRK6;GSS;GTF2F2;GTPBP10;GTPBP2;G

C27;CDC37;CDC42EP2;CDC73;CDCA7;CDK1;CDK11A;CDK11B;CDK2;CDK5R1;CDK5RAP3;CDK7;CDK8;CDK9;CDKN1B;CDKN1C;CDKN2AIP;CDKN2C;CDKN3;CDT1;CEBPA;CEBPG;CEBPZ;CENPE;CENPK;CEP290;CFH;CFP;CGGBP1;CHAF1A;CHAF1B;CHD
2;EEF1A1;EEFSEC;EIF5;EPHB3;EPHB6;ERAL1;ERCC2;ERN1;ETNK1;FANCM;FARS2;FARSA;FDXACB1;FER;FGFR4;FIGNL1;FPGT;GAK;GBP3;GBP6;GCH1;GFM2;GIMAP2;GIMAP7;GK;GMPPA;GNA13;GNAI1;GNAO1;GNL1;GNL3;GRK6;GSS;GTF2F
EC;EIF5;EPHB3;EPHB6;ERAL1;ERCC2;ERN1;ETNK1;FANCM;FARS2;FARSA;FDXACB1;FER;FGFR4;FIGNL1;FPGT;GAK;GBP3;GBP6;GCH1;GFM2;GIMAP2;GIMAP7;GK;GMPPA;GNA13;GNAI1;GNAO1;GNL1;GNL3;GRK6;GSS;GTF2F2;GTPBP10;GT
B;DYRK2;EEF1A1;EEFSEC;EIF5;EPHB3;EPHB6;ERAL1;ERCC2;ERN1;ETNK1;FANCM;FARS2;FARSA;FDXACB1;FER;FGFR4;FIGNL1;FPGT;GAK;GBP3;GBP6;GCH1;GFM2;GIMAP2;GIMAP7;GK;GMPPA;GNA13;GNAI1;GNAO1;GNL1;GNL3;GRK6;GSS;
YBA;CYTH2;DAPK3;DBN1;DCLRE1A;DCTN2;DDX11;DDX3X;DEK;DIAPH3;DICER1;DIS3L2;DLG1;DLG4;DMAP1;DMPK;DNA2;DOT1L;DPY30;DR1;DTNBP1;DYNLT3;ECT2;EEA1;EHMT2;EID1;EIF2A;EIF6;ELK4;ENY2;EPB41L5;EPC2;EPS15;I
EC;EIF5;EPHB3;EPHB6;ERAL1;ERCC2;ERN1;ETNK1;FANCM;FARS2;FARSA;FDXACB1;FER;FGFR4;FIGNL1;FPGT;GAK;GBP3;GBP6;GCH1;GFM2;GIMAP2;GIMAP7;GK;GMPPA;GNA13;GNAI1;GNAO1;GNL1;GNL3;GRK6;GSS;GTF2F2;GTPBP10;GT
EC;EIF5;EPHB3;EPHB6;ERAL1;ERCC2;ERN1;ETNK1;FANCM;FARS2;FARSA;FDXACB1;FER;FGFR4;FIGNL1;FPGT;GAK;GBP3;GBP6;GCH1;GFM2;GIMAP2;GIMAP7;GK;GMPPA;GNA13;GNAI1;GNAO1;GNL1;GNL3;GRK6;GRPEL2;GSS;GTF2F2;GTP
2;CKS1B;CKS2;CLIP3;CLK1;CLK3;CLK4;CLN3;CLN6;CLP1;CNBP;CNOT2;CNOT3;CNOT6;CNOT6L;CNOT7;CNOT8;CNPPD1;COG3;COMMD6;COPS2;COPS5;CPEB2;CPEB3;CRABP2;CREB1;CREB5;CREBL2;CREBZF;CRIPAK;CRK;CRLF3;CRTC2;CRY1;CS
;NFASC;NFKBIE;NME7;NOC2L;NOP58;NUP54;NUS1;OPA1;OSBPL5;P2RX4;PACS2;PAN3;PEX1;PEX12;PEX13;PEX14;PEX2;PEX3;PGAP1;PHAX;PHIP;PIKFYVE;PKD2;PKIA;PLEKHA8;PMAIP1;PMPCA;PPIF;PPP1CC;PPP1R12A;PPP3CA;PRKAB
EC;EIF5;EPHB3;EPHB6;ERAL1;ERCC2;ERN1;ETNK1;FANCM;FARS2;FARSA;FDXACB1;FER;FGFR4;FIGNL1;FPGT;GAK;GBP3;GBP6;GCH1;GFM2;GIMAP2;GIMAP7;GK;GMPPA;GNA13;GNAI1;GNAO1;GNL1;GNL3;GRK6;GSS;GTF2F2;GTPBP10;GT

EC;EIF5;EPHB3;EPHB6;ERAL1;ERCC2;ERN1;ETNK1;FANCM;FARS2;FARSA;FDXACB1;FER;FGFR4;FIGNL1;FPGT;GAK;GBP3;GBP6;GCH1;GFM2;GIMAP2;GIMAP7;GK;GMPPA;GNA13;GNAI1;GNAO1;GNL1;GNL3;GRK6;GSS;GTF2F2;GTPBP10;GT
'30;DR1;DRAP1;DYRK1B;DYRK2;E2F4;E2F6;E2F7;EAF2;EAPP;EBF4;EED;EEF1A1;EEFSEC;EGLN1;EHMT2;EID1;EIF2A;EIF2B5;EIF3E;EIF4E;EIF4E2;EIF4E3;EIF5;EIF5A2;ELF2;ELK4;ELL2;ELOF1;ENG;ENY2;EPC2;ERCC2;EREG;ERN1;

XH1;FOXN2;FXR1;FXR2;FYTTD1;GABPA;GABPB1;GATAD2A;GEN1;GIN1;GPATCH2;GPATCH3;GPBP1;GPKOW;GRHL1;GTF2B;GTF2E1;GTF2E2;GTF2F2;GZF1;H2AFV;H2AFZ;HBS1L;HDAC3;HDAC5;HDGFRP2;HDX
;SFR1;SHFM1;SIRT1;SLC30A9;SMARCA5;SMARCAD1;SMC2;SMC4;SMC5;SMC6;SMG1;SMYD2;SUMO1;TAF9;TAOK1;TCEA1;TDG;TDP2;TFAP4;TFPT;TIPRL;TLK1;TONSL;TOPORS;TP63;TREX1;TRIAP1;TRIM28;UBC;UBE2A;UBE2D3;UBE2N;UBE2T;UBE2V2;UE
NTTIP2;DPY30;DR1;DRAP1;DYRK1B;DYRK2;E2F4;E2F6;E2F7;EAF2;EAPP;EBF4;EED;EEF1A1;EEFSEC;EGLN1;EHMT2;EID1;EIF2A;EIF2B5;EIF3E;EIF4E;EIF4E2;EIF4E3;EIF5;EIF5A2;ELF2;ELK4;ELL2;ELOF1;ENG;ENY2;EPC2;ERCC2;EI

ENND4A;DEPDC1;DICER1;DKK3;DLAT;DLG4;DMAP1;DMTF1;DNA2;DNTTIP2;DPY30;DR1;DRAP1;DYRK1B;DYRK2;E2F4;E2F6;E2F7;EAF2;EAPP;EBF4;EED;EEF1A1;EEFSEC;EGLN1;EHMT2;EID1;EIF2A;EIF2B5;EIF3E;EIF4E;EIF4E2;EIF4E
;POLR3D;POLR3G;POLR3H;PPIG;PPP1CA;PPP1CB;PPP1CC;PRPF18;PRPF31;PRPF40A;PRPF6;PSKH1;PTEN;RARG;RB1;RBBP8;RBM11;RBM25;RBM27;RBM39;RBM4;RCHY1;RECQL5;REST;RFWD2;RING1;RNF2;RNF6;RPAP2;RPRD1A;RUVBL2;S
F;MGEA5;MON1A;MON2;MPP5;MSR1;MTX2;MTX3;NACAD;NAGPA;NAPB;NAPG;NCKIPSD;NDFIP1;NDFIP2;NECAP1;NECAP2;NEDD4;NFASC;NFKBIE;NMD3;NOP58;NR1H2;NUP37;NUP54;NUP62CL;NUP85;NXT2;PACS2;PAN3;PDCD6IP;PEX1;PEX1

;LEPROT;LFNG;LLGL1;LMAN1;LMAN2;LPCAT1;LPCAT2;MAN1A1;MAN1A2;MAN2A1;MANEA;MAP2K2;MAP6D1;MAP9;MARCH1;MBTPS2;MDFIC;MED15;MGAT1;MGAT2;MGAT4A;MST4;MUC1;MUC20;MUC5B;MUC6;NAA60;NAGPA;NCS1;NDFIP1;
EAPP;EBF4;EED;EEF1A1;EGLN1;EHMT2;EID1;EIF2A;ELF2;ELK4;ELL2;ELOF1;ENG;ENY2;EPC2;ERCC2;EREG;ERN1;ESF1;ESR2;EWSR1;F2RL1;FBXO5;FBXW7;FER;FHOD1;FIZ1;FLII;FNIP1;FOXH1;FOXN2;FZD2;FZD6;GABPA;GABPB1;G

X54;DEK;DENND4A;DEPDC1;DICER1;DKK3;DLAT;DLG4;DMAP1;DMTF1;DNA2;DNTTIP2;DPY30;DR1;DRAP1;DYRK1B;DYRK2;E2F4;E2F6;E2F7;EAF2;EAPP;EBF4;EED;EEF1A1;EEFSEC;EGLN1;EHMT2;EID1;EIF2A;EIF2B5;EIF3E;EIF4E;EIF4

E2F6;E2F7;EAF2;EAPP;EBF4;EED;EEF1A1;EGLN1;EHMT2;EID1;EIF2A;ELF2;ELK4;ELL2;ELOF1;ENG;ENY2;EPC2;ERCC2;EREG;ERN1;ESF1;ESR2;EWSR1;F2RL1;FBXO5;FBXW7;FER;FHOD1;FIZ1;FLII;FNIP1;FOXH1;FOXN2;FZD2;FZD6

LS;GLT8D1;GPAM;GPAT2;GPD2;GRPEL2;GSTP1;GTPBP10;GTPBP8;GUF1;HARS2;HEBP1;HIGD1A;HINT3;HMGCL;HRSP12;HSD17B10;HSDL1;HSDL2;HSPE1;IDE;IDH2;IDH3B;IDH3G;IDI1;IMMP1L;IMPA1;IREB2;ISCA1;KIAA0391;I

AMP3;SCAP;SCFD1;SCOC;SCYL1;SDF4;SEC23A;SEC24A;SGMS1;SGMS2;SLC30A5;SLC30A6;SLC30A7;SLC33A1;SLC35A1;SLC35A3;SLC35A4;SLC35A5;SLC35B3;SLC35B4;SLC35C1;SMPD4;SREBF1;ST8SIA4;STEAP2;STEAP4;STX16;STX5;SVIP;TLR8;T
CAD;NAGPA;NAPB;NAPG;NCKIPSD;NDFIP1;NDFIP2;NECAP1;NECAP2;NEDD4;NFASC;NFKBIE;NMD3;NOP58;NR1H2;NUP37;NUP54;NUP62CL;NUP85;NXT2;PACS2;PAN3;PDCD6IP;PEX1;PEX12;PEX13;PEX14;PEX2;PEX3;PGAP1;PHAX;PHIP;PITPNM1;

A;RAB2B;RAE1;RDH10;RINT1;RNF103;RNF13;RNF139;RYR1;RYR3;SACM1L;SAMD8;SAR1B;SC5DL;SCAP;SCFD1;SEC11A;SEC11C;SEC13;SEC23A;SEC24A;SEC61A1;SEC62;SEL1L;SELK;SERAC1;SERINC1;SERP1;SGPP1;SLC27
EAPP;EBF4;EED;EEF1A1;EGLN1;EHMT2;EID1;EIF2A;ELF2;ELK4;ELL2;ELOF1;ENG;ENY2;EPC2;ERCC2;EREG;ERN1;ESF1;ESR2;EWSR1;F2RL1;FBXO5;FBXW7;FER;FHOD1;FIZ1;FLII;FNIP1;FOXH1;FOXN2;FZD2;FZD6;GABPA;GABPB1;G
MAP3K13;MAP3K2;MAP3K7;MAP4K1;MAP4K3;MAP4K5;MAPK8;MAPK8IP1;MAPK8IP2;MAPKAPK2;MAPKAPK3;MAPKBP1;MBIP;MBTPS2;MCL1;MCM9;MCTS1;MDFIC;MDM2;MEF2A;MEF2C;MEN1;MMS22L;MNAT1;MNDA;MORF4L2;MPV17;N
LF3;CRTC2;CRY1;CST3;CTBP1;CTDSP1;CUL2;CXXC1;DAB2IP;DACH1;DAPK3;DAXX;DBP;DDX11;DDX3X;DDX41;DDX54;DEK;DENND4A;DEPDC1;DICER1;DKK3;DLG4;DMAP1;DMTF1;DNA2;DNAJB9;DNAJC10;DNTTIP2;DPY30;DR1;DRAP1;DYRK1B;E
NK3;PAPD4;PAPD5;PAPOLA;PAPOLG;PBK;PDIK1L;PDK1;PDK2;PDXK;PFKL;PFKM;PGM2L1;PHKG2;PI4K2B;PIGF;PIK3C2A;PIK3CA;PIKFYVE;PKN1;PKN2;PLK4;POLD2;POLG2;POLI;POLK;POLQ;POLR1E;POLR2E;POLR2K;POLR3D;POLR3G;POLR3H;PPIP5K

MTF1;DNTTIP2;DPY30;DR1;DRAP1;DYRK1B;E2F4;E2F6;E2F7;EAF2;EAPP;EBF4;EED;EEF1A1;EGLN1;EHMT2;EID1;EIF2A;ELF2;ELK4;ELL2;ELOF1;ENG;ENY2;EPC2;ERCC2;EREG;ERN1;ESF1;ESR2;EWSR1;F2RL1;FBXO5;FBXW7;FER;FH
RNF103;RNF13;RNF139;RYR1;RYR3;SACM1L;SAMD8;SAR1B;SC5DL;SCAP;SCFD1;SEC11A;SEC11C;SEC13;SEC23A;SEC24A;SEC61A1;SEC62;SEL1L;SELK;SERAC1;SERINC1;SERP1;SGPP1;SLC27A4;SLC33A1;SLC39A7;SLC9A
M10;RBM11;RBM12B;RBM15;RBM18;RBM25;RBM27;RBM3;RBM34;RBM39;RBM4;RBM41;RBM42;RBM43;RBM44;RBM47;RBM5;RBM7;RBMS1;RNASEH2A;RNPC3;RPF1;RPL11;RPL15;RPL26;RPL30;RPL31;RPL34;RPL35;RPL
OPS2;COPS4;COPS5;COPS6;COPZ1;CORO1A;CPSF1;CPSF6;CRBN;CRCP;CREB1;CSNK1A1;CSNK2B;CTBP1;CTDNEP1;CUL2;CUL4B;CUL5;CXADR;CXXC1;CYBA;CYCS;DACH1;DCAF10;DCAF11;DCAF13;DCTN2;DCTN6;DCUN1D1;DDB2;DD
D2;PLA2G4A;PLD2;PLD3;PLOD3;PNPLA2;PNPLA6;PNPLA8;POGLUT1;POMT2;PORCN;PPIB;PRKCSH;PROC;PROZ;PSEN2;PSKH1;PTGS2;PTPLAD1;PTPLAD2;RAB10;RAB2A;RAB2B;RCN2;RCN3;RDH10;RINT1;RNF103;RNF13;RNF
CSH;GFM2;GIPC1;GIT1;GLA;GLS;GNA13;GNAI1;GNAO1;GNL1;GNL3;GPC1;GPI;GPR65;GSTM2;GTF2H1;GTF2H4;HACE1;HDAC6;HECTD2;HECTD3;HECW2;HERC4;HEXA;HMGB1;HMGB2;HMGCL;HMOX2;HPRT1;HSD17B10;HSPBP1;IDE;I
MTF1;DNTTIP2;DPY30;DR1;DRAP1;DYRK1B;E2F4;E2F6;E2F7;EAF2;EAPP;EBF4;EED;EEF1A1;EGLN1;EHMT2;EID1;EIF2A;ELF2;ELK4;ELL2;ELOF1;ENG;ENY2;EPC2;ERCC2;EREG;ERN1;ESF1;ESR2;EWSR1;F2RL1;FBXO5;FBXW7;FER;FH

IR3C1;OMA1;OPA1;OXCT1;PC;PCK2;PDHX;PDK1;PDK2;PDP1;PGAM5;PIN4;PLA2G4A;PMAIP1;PMPCA;PNPLA7;POLDIP2;POLG2;PPIF;PPP1CC;PSEN2;RARS2;RHOT1;RNF144B;RPS6KB1;SDHD;SFXN4;SHMT2;SLC24A6;SLC25A1;SLC25A10;SLC25A11;SLC25A

;DDX54;DEK;DENND4A;DEPDC1;DGCR8;DICER1;DKK3;DLG4;DMAP1;DMTF1;DNTTIP2;DPY30;DR1;DRAP1;DTNBP1;DYRK1B;E2F4;E2F6;E2F7;EAF2;EAPP;EBF4;EED;EEF1A1;EEFSEC;EGLN1;EHMT2;EID1;EIF2A;EIF2B5;EIF3E;EIF4E;E
ARS2;HELLS;HLTF;HSPA13;HSPA14;HSPE1;ICK;IDE;IDH3G;IGHMBP2;ILK;IPMK;ITPKC;JAK2;KIAA1804;KIF11;KIF18A;KIF20B;KIF22;KIF23;KIF27;KIF2A;KIF3A;KIF5B;KIFC1;KIFC3;LACE1;LIG4;LONP1;LRRK2;MAP2K2;MAP2K4;M
P1;CTDSP1;CTNS;CXXC1;DAB2IP;DAPK3;DAPP1;DAXX;DCAKD;DCK;DDX11;DDX3X;DEGS1;DEGS2;DGKE;DGKZ;DHFRL1;DLAT;DLG1;DMPK;DNA2;DNAJB9;DNAJC10;DOLPP1;DPM1;DUSP11;DUSP19;DYRK1A;DYRK1B;DYRK2;ECT2;EEFSEC;EIF
MC1;PSMC2;PSMC3;PSMC5;PSMC6;PSMD10;PSMD13;PSMD14;PSMD4;PSMD8;PSME1;PTEN;PTGS2;RAD17;RASSF1;RB1;RBBP8;RFC2;RFC3;RFC5;RFWD2;RHEB;RINT1;RPS15A;RPS27L;RPS6KB1;RPTOR;RRP8;SART1;SENP6;SESN1;SIPA1;SIRT1;
HGS;HLA-DMA;HNF1A;HOMER1;HOOK1;HOOK2;INPP5K;IPO11;IPO13;IPO4;JAK2;JUN;JUP;KATNB1;KIF18A;KIF5B;KLHL2;KPNA2;KPNA3;KPNA5;LAMTOR3;LEPROT;LIN7A;LIN7C;LMAN2;LMNA;LRSAM1;LRWD1;LYST;MAPK8;MARCH5;MCOLN1;I
2;CRCP;CREB1;CREB5;CREBL2;CREBZF;CRK;CRLF3;CRTC2;CRY1;CTBP1;CTDSP1;CUL2;CXXC1;DAB2IP;DACH1;DAPK3;DAXX;DBP;DCAKD;DCK;DDX3X;DDX41;DDX54;DEK;DENND4A;DEPDC1;DHFRL1;DICER1;DKK3;DLG4;DMAP1;DMTF1;DNTT
B1;CRIPT;CSK;CSNK1A1;CST3;CSTA;CUTC;DACH1;DAPK3;DAXX;DBN1;DCAF13;DCLRE1A;DCP1A;DCP2;DCTN2;DCTN4;DCTN6;DDX11;DDX3X;DDX50;DDX54;DGCR8;DHX30;DLG1;DLG4;DMAP1;DNA2;DNAH6;DNAI2;DNAJB9;DNAJC21
B1;CRIPT;CSK;CSNK1A1;CST3;CSTA;CUTC;DACH1;DAPK3;DAXX;DBN1;DCAF13;DCLRE1A;DCP1A;DCP2;DCTN2;DCTN4;DCTN6;DDX11;DDX3X;DDX50;DDX54;DGCR8;DHX30;DLG1;DLG4;DMAP1;DNA2;DNAH6;DNAI2;DNAJB9;DNAJC21

F2F2;GUCY2C;HARS2;HELLS;HLTF;HSPA13;HSPA14;HSPE1;ICK;IDE;IDH3G;IGHMBP2;ILK;IPMK;ITPKC;JAK2;KIAA1804;KIF11;KIF18A;KIF20B;KIF22;KIF23;KIF27;KIF2A;KIF3A;KIF5B;KIFC1;KIFC3;LACE1;LIG4;LONP1;LRRK2;MA

K;CRLF3;CRTC2;CRY1;CTBP1;CTDSP1;CUL2;CXXC1;DAB2IP;DACH1;DAPK3;DAXX;DBP;DCAKD;DCK;DDX3X;DDX41;DDX54;DEK;DENND4A;DEPDC1;DHFRL1;DICER1;DKK3;DLG4;DMAP1;DMTF1;DNTTIP2;DPY30;DR1;DRAP1;DYRK1B;E2F4;E2I

CY2C;HARS2;HELLS;HLTF;HSPA13;HSPA14;HSPE1;ICK;IDE;IDH3G;IGHMBP2;ILK;IPMK;ITPKC;JAK2;KIAA1804;KIF11;KIF18A;KIF20B;KIF22;KIF23;KIF27;KIF2A;KIF3A;KIF5B;KIFC1;KIFC3;LACE1;LIG4;LONP1;LRRK2;MAP2K2;MA

ST3;CTBP1;CTDSP1;CUL2;CXXC1;DAB2IP;DACH1;DAPK3;DAXX;DBP;DDX11;DDX3X;DDX41;DDX54;DEK;DENND4A;DEPDC1;DICER1;DKK3;DLG4;DMAP1;DMTF1;DNA2;DNAJB9;DNAJC10;DNTTIP2;DPY30;DR1;DRAP1;DYRK1B;E2F4;E2F6;E2F7;E

RTC2;CRY1;CTBP1;CTDSP1;CUL2;CXXC1;DAB2IP;DACH1;DAPK3;DAXX;DBP;DCAKD;DCK;DDX3X;DDX41;DDX54;DEK;DENND4A;DEPDC1;DHFRL1;DICER1;DKK3;DLG4;DMAP1;DMTF1;DNTTIP2;DPY30;DR1;DRAP1;DYRK1B;E2F4;E2F6;E2F7;E

L1;FBXW7;FCHO2;FGF7;FGFR1OP;FITM1;FMR1;FOPNL;FYTTD1;GAS6;GCC2;GDF9;GDI1;GIPC1;GLTPD1;GOLGA4;GOLT1B;GOPC;GORASP1;GPAM;GRIK5;GRIP1;GRPEL2;HDAC6;HDAC8;HEXA;HGS;HLA-DMA;HNF1A;HOMER1;HOOK1;HOOK2;IN

TNS;CXXC1;DAB2IP;DAPK3;DAPP1;DAXX;DCAKD;DCK;DDX11;DDX3X;DEGS1;DEGS2;DGKE;DGKZ;DHFRL1;DLG1;DMPK;DNA2;DNAJB9;DNAJC10;DOLPP1;DPM1;DUSP11;DUSP19;DYRK1A;DYRK1B;DYRK2;ECT2;EEFSEC;EIF2A;EIF5;ELMOD2;EN

P1;CTDSP1;CUL2;CXXC1;DAB2IP;DACH1;DAPK3;DAXX;DBP;DCAKD;DCK;DDX3X;DDX41;DDX54;DEK;DENND4A;DEPDC1;DHFRL1;DICER1;DKK3;DLG4;DMAP1;DMTF1;DNTTIP2;DPY30;DR1;DRAP1;DYRK1B;E2F4;E2F6;E2F7;EAF2;EAPP;EB

S28;VPS29;VPS33B;VPS36;VPS37A;VPS37B;VPS4A;VPS4B;VTA1;WDFY1;WDR44;YKT6;ZFYVE16;ZNRF1;ZN

4B;UBC;UBE2D1;UBE2E1;USP16;USP37;USP44;UTP14C;VCPIP1;VRK1;WAPAL;WEE1;XPO1;ZNF830;ZWIL

N1;PKN2;PLK4;POLR2E;PPIP5K2;PPP4C;PRKAA1;PRKAB2;PRKACB;PRKCI;PRKCZ;PRKD3;PRPF4B;PRPS2;PSKH1;PTK6;PTK7;RABEP1;RBKS;RET;RFK;RIOK2;RIPK2;RIPK3;ROCK1;RP2;RPS6KA3;RPS6KA4;RPS6KA5;RPS6KB1;RYK;SCYL1;SCYL2;SGM

DH;GBA2;GCH1;GCSH;GFM2;GIPC1;GIT1;GK;GLA;GLS;GNA13;GNAI1;GNAO1;GNL1;GNL3;GPC1;GPD2;GPI;GPR65;GTF2H1;GTF2H4;GUSB;HACE1;HDAC6;HECTD2;HECTD3;HECW2;HERC4;HEXA;HGS;HMGB1;HMGB2;HMGCL;HMMR;HMOX

;SCYL2;SLK;SMG1;SNRK;STK10;STK17A;STK17B;STK32C;STK38L;STRADA;TAOK1;TBK1;TESK1;TESK2;TGFA;TGFBR1;TGFBR2;TIE1;TLK1;TRIM28;TRPM7;TTK;UHMK1;VRK1;VRK2;WEE1;Y

CREB5;CREBL2;CREBZF;CRK;CRLF3;CRTC2;CRY1;CTBP1;CTDSP1;CUL2;CXXC1;DAB2IP;DACH1;DAPK3;DAXX;DBP;DCAKD;DCK;DDX3X;DDX41;DDX54;DEK;DENND4A;DEPDC1;DHFRL1;DICER1;DKK3;DLG4;DMAP1;DMTF1;DNTTIP2;DPY30;DR1;

AB2;PRKACB;PRKCI;PRKCZ;PRKD3;PRPF4B;PSKH1;PTK6;PTK7;RABEP1;RBKS;RET;RFK;RIOK2;RIPK2;RIPK3;ROCK1;RPS6KA3;RPS6KA4;RPS6KA5;RPS6KB1;RYK;SCYL1;SCYL2;SLK;SMG1;SNRK;STK10;STK17A;STK17B;STK32C;STK38L;STRADA;T

NX17;SNX2;SNX8;SPCS2;SPCS3;SRP14;SRP19;SRP9;SSR3;STAM2;STON1;STRADA;STX16;STX4;STX5;STX7;STXBP4;SYNGR2;SYTL3;TGFBR1;TIMM17A;TIMM22;TIMM23;TIMM8B;TLK1;TMED10;TMED2;TMEM173;TMEM30A

RP16;PC;PGAM5;PLA2G4A;PLCB1;PMAIP1;PMPCA;PNPLA7;PPIF;PPP1CC;PRKCZ;PSEN2;PTCHD2;PTGDS;RAE1;RANGAP1;RECQL5;RHOT1;RNF13;RNF144B;RPS6KB1;SDHD;SEC13;SFXN4;SHMT2;SIRT1;SLC22A18;SLC24A6;SLC25A1;SLC25A10;SLC2

K16;SNX8;SPAST;SPCS2;SPCS3;SRP14;SRP19;SRP9;SRSF10;SRSF11;SRSF3;SRSF6;SSR3;STEAP2;STRADA;STX5;TGFBR1;THOC3;THOC7;TIMM17A;TIMM22;TIMM23;TIMM8B;TMED10;TMED2;TMEM173;TMEM30A;TMEM30B;

TCEA1;TCF25;TDP2;THAP2;TWISTNB;UBLCP1;UTP11L;UTP14C;UTP15;UTP23;VRK1;WDR36;WDR43;WRB;XPO1;ZBTB33;ZC3H15;ZCCHC17;ZCCHC7;ZMAT3;ZNF146;ZNF22;ZNF415;ZNF622;ZNF

IAA1804;KLHDC3;KNDC1;KRAS;LIN28A;LMNA;LPAR2;LRP6;LRRK2;LRRN3;MAD2L1;MALT1;MAP2K2;MAP2K5;MAP3K1;MAP3K11;MAP3K13;MAP3K2;MAP3K7;MAP4K1;MAP4K5;MAPK8;MAPK8IP1;MAPK8IP2;MAPKAP

RP16;PC;PGAM5;PLA2G4A;PLCB1;PMAIP1;PMPCA;PNPLA7;PPIF;PPP1CC;PRKCZ;PSEN2;PTCHD2;PTGDS;RAE1;RANGAP1;RECQL5;RHOT1;RNF13;RNF144B;RPS6KB1;SDHD;SEC13;SFXN4;SGMS2;SHMT2;SIRT1;SLC22A18;SLC24A6;SLC25A1;SLC25A1

KD;DCK;DDX11;DDX3X;DDX41;DDX50;DDX54;DDX59;DECR1;DGKE;DGKZ;DHX30;DHX36;DHX40;DICER1;DMPK;DNA2;DNAH6;DNAJA1;DNAJC27;DYRK1A;DYRK1B;DYRK2;EEA1;EEF1A1;EEFSEC;EGLN1;EIF5;EPHB3;EPHB6;ERAL1;ERCC2;ER

3IP;RAB6A;RAB8B;RANGAP1;RAP2A;RB1;RBCK1;RHOQ;RPGR;RPH3AL;RPL11;RPL15;RPL17;RPL23;RPL26;RPL26L1;RPL27;RPL30;RPL31;RPL34;RPL35;RPL36A;RPL39;RPL41;RPL7;RPL9;RPS15A;RPS24;RPS27;RPS29;RPS3A;RPS7;RS

P3;UQCRB;UQCRC1;UQCRC2;UQCRH;UQCRQ;V

CC2;GDF9;GDI1;GIF;GIPC1;GLS;GLTPD1;GOLGA4;GOLT1B;GOPC;GORASP1;GPAM;GRPEL2;HDAC6;HGS;HLA-DMA;HNF1A;HOOK1;HOOK2;INPP5K;IPO11;IPO13;IPO4;ITGAV;ITPR2;JAK2;JUN;JUP;KATNB1;KCNQ1;KIF18A;KLHL2;KPNA
XO8;FBXW4;FBXW7;FGD2;FGD3;FGD4;FNBP1L;FUCA1;FUT4;GAA;GALC;GALE;GAPDH;GBA2;GCH1;GCSH;GFM2;GIPC1;GIT1;GK;GLA;GLS;GNA13;GNAI1;GNAO1;GNL1;GNL3;GPC1;GPD2;GPI;GPR65;GSTM2;GTF2H1;GTF2H4;GUSB;

SMC3;PSMC5;PSMC6;RAB11B;RAB14;RAB18;RAB22A;RAB27B;RAB28;RAB2A;RAB4A;RAB5A;RAB6A;RAB8B;RAD17;RAD51B;RAD51C;RALA;RAP1A;RAP1B;RAP2A;RAP2C;RASL11A;RECQL;RECQL5;RFC2;RFC3;RFC5;RHEB;RHOBTB3;RH
;PEX1;PMS1;POLQ;PPA1;PPP2R4;PSMC1;PSMC2;PSMC3;PSMC5;PSMC6;RAB11B;RAB14;RAB18;RAB22A;RAB27B;RAB28;RAB2A;RAB4A;RAB5A;RAB6A;RAB8B;RAD17;RAD51B;RAD51C;RALA;RAP1A;RAP1B;RAP2A;RAP2C;RASL11A;RE
3IP;RAB6A;RAB8B;RANGAP1;RAP2A;RB1;RBCK1;RHOQ;RPGR;RPH3AL;RPL11;RPL15;RPL17;RPL23;RPL26;RPL26L1;RPL27;RPL30;RPL31;RPL34;RPL35;RPL36A;RPL39;RPL41;RPL7;RPL9;RPS15A;RPS24;RPS27;RPS29;RPS3A;RPS7;RS

OPA1;ORC4;PEX1;PMS1;POLQ;PPA1;PPP2R4;PSMC1;PSMC2;PSMC3;PSMC5;PSMC6;RAB11B;RAB14;RAB18;RAB22A;RAB27B;RAB28;RAB2A;RAB4A;RAB5A;RAB6A;RAB8B;RAD17;RAD51B;RAD51C;RALA;RAP1A;RAP1B;RAP2A;RAP2C;R
;PEX1;PMS1;POLQ;PPA1;PPP2R4;PSMC1;PSMC2;PSMC3;PSMC5;PSMC6;RAB11B;RAB14;RAB18;RAB22A;RAB27B;RAB28;RAB2A;RAB4A;RAB5A;RAB6A;RAB8B;RAD17;RAD51B;RAD51C;RALA;RAP1A;RAP1B;RAP2A;RAP2C;RASL11A;RE

I40;TOMM5;UCP2;UCP3;UQCRB;UQCRC1;UQCRC2;UQCRH;UQCRQ;VI

1A;PPP5C;PRKAA1;PRKCSH;PRKCZ;PSMD10;PSMG1;PTGER4;QPRT;RASA1;RDX;RICTOR;RIPK3;RYR3;SCUBE1;SENP6;SEPT7;SEPT9;SHMT2;SKAP2;SKIL;SLAIN2;SLC9A3R1;SLC9A3R2;SMAD2;SMAD4;SMARCAD1;SPAG9;SPAST;SPTBN2;SPTBN

CD28;CD2AP;CD2BP2;CD47;CDC25C;CDC26;CDC27;CDC42EP1;CDC42EP2;CDC42SE2;CDC73;CDHR1;CDK1;CDK11A;CDK11B;CDK2;CDK5R1;CDK9;CDKN1B;CDKN2AIP;CDKN2C;CEBPA;CENPE;CENPK;CENPL;CENPQ;CENPW;CEP120;CEP135;C

1A;PPP5C;PRKAA1;PRKCSH;PRKCZ;PSMD10;PSMG1;PTGER4;QPRT;RASA1;RDX;RICTOR;RIPK3;RYR3;SCUBE1;SENP6;SEPT7;SEPT9;SHMT2;SKAP2;SKIL;SLAIN2;SLC9A3R1;SLC9A3R2;SMAD2;SMAD4;SMARCAD1;SPAG9;SPAST;SPTBN2;SPTBN

M2;MEF2A;MEF2D;MSH2;NBEA;NEDD4;NFATC1;NLK;NOTCH3;NRIP1;NUDT21;PARK2;PAX6;PDE3B;PDE4D;PDLIM5;PFKL;PFKM;PFN1;PIAS4;PJA2;PKD2;PKN1;PKN2;PLCB1;PLK1S1;POT1;PPP1CB;PPP1CC;PPP1R12A;PPP1R3D;PPP

3;MIS18BP1;MITF;MNDA;MSH2;MSL3;MTERF;MTERFD1;MTF2;MUC1;MYNN;MYSM1;NBN;NDN;NFATC1;NFIA;NFKB2;NFYB;NKRF;NR1D2;NR1H2;NR3C1;NR4A1;NR4A3;NUCB2;ORC2;ORC3;ORC4;PAIP1;PAPD5;PAX2;PAX6;PBX3;P

2;SYCP3;SYN1;TFPT;TMPO;TNKS2;TONSL;TP63;TRIM28;TUBG1;UBE2A;UCHL5;UPF1;WAPAL;WDHD1;XPO1;ZFR;ZMYND11;ZRANB3;ZWI

3;ANKRD32;ANKRD34B;ANKRD46;ANKRD49;ANLN;ANO5;ANP32E;ANXA1;AP1AR;AP1B1;AP1S2;AP2A2;AP3M1;AP3S2;AP4S1;APC;APEH;APEX1;APEX2;APH1A;API5;APIP;APLF;APOBEC3D;APOO;APPBP2;APPL1;APTX;AQP11;ARAF;ARAP2;ARF4;ARFGAP1;AR

KRD44;ANLN;ANP32E;ANXA1;AP1B1;AP1S2;AP2A2;AP3M1;AP3S2;AP4S1;APC;APEH;APEX1;APEX2;APH1A;API5;APIP;APLF;APOBEC3D;APPBP2;APPL1;APTX;AQP11;ARAF;ARAP2;ARF4;ARFGAP1;ARFGAP2;ARFGEF2;ARFIP1;ARG2;ARHGAP10;ARHGAP11A;A

;RBBP8;REXO4;RNASEH2A;RNGTT;RPAP2;SACM1L;SERAC1;SGPP1;SMPD1;SMPD4;SMPDL3A;SSU72;SYNJ1;TATDN1;TATDN3;TDP2;TENC1;TREX1;UBLCP1;UCHL3;UCHL5;USP1;USP12;USP14;USP15;USP16;USP19;USP21;USP25;USP31;U

2;DDX11;DDX3X;DDX41;DDX50;DDX54;DDX59;DHX30;DHX36;DHX40;DICER1;DIS3;DIS3L2;DLG1;DNA2;DNAH6;DNAI2;DNASE1L1;DNASE2;DNPEP;DOLPP1;DPEP2;DPP3;DUSP11;DUSP19;DYNC2LI1;DYNLRB1;DYNLT3;EDEM3;EEF1A1;EEFSEC;

ASSF3;REPS2;RHOH;RNF111;ROPN1L;RS1;S100A11;SBF2;SDCBP;SECTM1;SH3GLB1;SIT1;SLC8A1;SOCS3;SOD2;SOS2;SRPK1;SRPK2;STK3;STX12;SYN2;TEAD3;TG;TIAM2;TLE4;TLR2;TLR4;TLR5;TNFRSF1A;TNFSF14;TREM1;T
ASSF3;REPS2;RHOH;RNF111;ROPN1L;RS1;S100A11;SBF2;SDCBP;SECTM1;SH3GLB1;SIT1;SLC8A1;SOCS3;SOD2;SOS2;SRPK1;SRPK2;STK3;STX12;SYN2;TEAD3;TG;TIAM2;TLE4;TLR2;TLR4;TLR5;TNFRSF1A;TNFSF14;TREM1;T
SOD2;SOS2;SRPK1;SRPK2;STK3;TEAD3;TG;TIAM2;TLE4;TLR2;TLR4;TLR5;TNFRSF1A;TNFSF14;TREM1;TRIB1;UBR2;WLS;WNT10B;ZAK;ZFP36;ZNI
GRP4;RASSF3;REPS2;RHOH;RNF111;ROPN1L;RS1;S100A11;SBF2;SDCBP;SECTM1;SH3GLB1;SIT1;SLC8A1;SOCS3;SOD2;SOS2;SRPK1;SRPK2;STK3;STX12;SYN2;TEAD3;TG;TIAM2;TLE4;TLR2;TLR4;TLR5;TNFRSF1A;TNFSF14;TRI
2;NFIL3;NPEPPS;NPTN;NQO2;NR6A1;NUP50;NUPL1;OPLAH;ORM1;ORM2;OSM;OXER1;PAK2;PAPSS2;PDCD7;PDE4B;PDK3;PDZD3;PDZD8;PELI2;PGLYRP1;PHB;PIAS1;PLAU;PLD1;PLXNC1;PNP;POLD3;PPAP2B;PPP4R1;PROK2;PTI

4;RASSF3;REPS2;RHOH;RNF111;ROPN1L;RS1;S100A11;SCARF1;SDCBP;SECTM1;SH3GLB1;SIT1;SLC12A6;SLC8A1;SOCS3;SOD2;SOS2;SRPK1;SRPK2;STK3;STX3;TARBP2;TEAD3;TG;TIAM2;TLE4;TLR2;TLR4;TLR5;TNFRSF1A;TNFS

E1;MANSC1;MARCKS;MBOAT2;MCMBP;MCTP2;MEGF9;MME;MPZL3;MRVI1;MYO10;NAIP;NCF4;NFE2L2;NMNAT2;NPTN;NUP50;NUPL1;OSBPL1A;OSM;OXER1;PAK2;PCNX;PDZD3;PGS1;PHB;PICALM;PLAU;PLB1;PLD1;PLXDC2;PLXN

NT5C2;NUP50;ORM1;OSBPL1A;OSM;PAK2;PDK3;PDRG1;PDZD3;PELI2;PHB;PHC2;PIAS1;PICALM;PLAU;PLXDC2;PLXNC1;POLD3;PPAP2B;PPP1R3B;PPP3CC;PPP3R1;PPP4R1;PROK2;PRUNE;PTPRE;PVRL2;RAB27A;RANBP1;RAPGEF2;I

R4;TLR5;TM9SF2;TNFRSF1A;TNFSF14;TREM1;TREML2;TRPM6;TRPV5;UBR2;VAMP3;WNT

SF1A;TNFSF14;TREM1;TREML2;TRPM6;TRPV5;UBR2;VAMP3;WNT

3;JHDM1D;JMJD1C;KAL1;KAZN;KCNE1;KCNH7;KCNJ15;KCNS1;KDM1B;KIAA0319;KLF6;KPNA1;KPNA4;LAMP2;LILRB3;LIMD1;LITAF;LMNB1;LMO4;LRG1;LRRC6;LYN;LYVE1;MAK;MARCKS;MCMBP;MCTP2;MLLT11;MME;MPZL3;MSL1;MY(

RHOH;RNF130;RNF145;RNF149;RNF19B;RS1;RTN3;SCARF1;SDCBP;SEC24D;SECTM1;SHROOM4;SIT1;SLC12A6;SLC15A4;SLC22A1;SLC22A14;SLC22A4;SLC26A8;SLC2A11;SLC45A4;SLC8A1;SMAGP;SNTG2;ST3GAL4;ST3GAL5;ST6GA

RFIP1;LYN;MAK;MARCKS;MCTP2;MLLT11;MRVI1;MYO10;MYO5A;NAIP;NCOA1;NCOA2;NEDD9;NFE2;NFE2L2;NFIL3;NFYA;NPTN;NR6A1;NRBF2;NRD1;NUP50;NUPL1;ORM1;ORM2;OSBPL11;OSM;OTX1;OXER1;PAK2;PAPSS2;PDCD7;

S;IL10RB;IL13RA1;IL1B;IL1R1;IL1R2;IL1RN;IMP3;INHBB;INPP5A;IQGAP1;IRAK3;ITGAD;ITM2B;JHDM1D;JMJD1C;KAL1;KCNE1;KCNS1;KDM1B;KIAA0232;KIAA0319;KLF6;KPNA1;LBR;LILRB3;LIMD1;LITAF;LMNB1;LMO4;LRRFIP1;LYI

LC26A8;SLC2A11;SLC45A4;SLC8A1;SMAGP;ST3GAL4;ST3GAL5;ST6GALNAC2;STX12;STX3;STX6;SUSD3;SYNGR3;TLR2;TLR4;TLR5;TM9SF2;TMCC1;TMCC3;TMCO3;TMEM132D;TMEM154;TMX4;TNFRSF1A;TNFSF14;TOR1AIP2;TRI

;MAK;MARCKS;MBOAT2;MCMBP;ME2;MME;MRPL9;MRVI1;MXD1;MYO10;MYO5A;MZT2A;NAIP;NCF4;NCOA2;NECAB2;NEDD9;NFE2;NFE2L2;NLN;NMNAT2;NPEPPS;NPL;NQO2;NRBF2;NRD1;NT5C2;OPLAH;OSBPL1A;PAK2;PANK2;PAPSS2;

1;SMAGP;ST3GAL4;ST3GAL5;ST6GALNAC2;STX12;STX3;STX6;SUSD3;SYNGR3;TLR2;TLR4;TLR5;TM9SF2;TMCC1;TMCC3;TMCO3;TMEM132D;TMEM154;TMX4;TNFRSF1A;TNFSF14;TOR1AIP2;TREM1;TREML2;TRPM6;TRPV5;TSPA

;IL1B;IL1R1;IL1RN;INHBB;INPP5A;INSC;IQGAP1;IRAK3;ITGAD;ITM2B;JHDM1D;JMJD1C;KAL1;KAZN;KCNE1;KCNH7;KCNJ15;KCNS1;KDM1B;KIAA0319;KLF6;KPNA1;KPNA4;LAMP2;LILRB3;LIMD1;LITAF;LMNB1;LMO4;LRG1;LRRC

2;NEDD9;NFE2;NFE2L2;NFIL3;NFYA;NPTN;NR6A1;NRBF2;NRD1;NUP50;NUPL1;ORM1;ORM2;OSBPL11;OSM;OTX1;OXER1;PAK2;PDCD7;PDE4B;PDK3;PDZD3;PDZD8;PELI2;PGLYRP1;PHB;PIAS1;PICALM;PIF1;PLAU;PLD1;PLXNC1;PN

BARAPL1;GALNT14;GBE1;GCM1;GDE1;GIMAP1;GLIPR2;GLT1D1;GMPR2;GNAQ;GOLGA7B;GPR141;GPR17;GPR84;GPR89A;GPR97;GPSM2;GRIP2;GSDMC;GSK3B;GTSE1;H3F3B;HAL;HAS1;HCAR2;HCAR3;HEY1;HIF1A;HIST1H1T;HIVEP1;HMGC
BARAPL1;GALNT14;GBE1;GCM1;GDE1;GIMAP1;GLIPR2;GLT1D1;GMPR2;GNAQ;GOLGA7B;GPR141;GPR17;GPR84;GPR89A;GPR97;GPSM2;GRIP2;GSDMC;GSK3B;GTSE1;H3F3B;HAL;HAS1;HCAR2;HCAR3;HEY1;HIF1A;HIST1H1T;HIVEP1;HMGC

R3B;PPP3CC;PPP3R1;PRRG4;RAB1A;RAB27A;RAB31;RHOH;RTN3;SBF2;SDCBP;SEC24D;SECTM1;SH3GLB1;SHROOM4;SLC15A4;SLC22A4;SLC8A1;SMAGP;SNAP23;SOCS3;SOD2;SOS2;SQRDL;SRPK1;ST3GAL4;ST3GAL5;ST6GALNAC2;STK3;S

KIFAP3;KPTN;L3MBTL3;LAMA3;LIMA1;LUM;MAP1LC3A;MAP6;MAP7;MAPK3;MASTL;MET;MFAP2;MMP2;MORF4L1;MRE11A;MTCH1;MTDH;MTX1;MYO1B;NCBP1;NCBP2;NCF1;NEK4;NFIB;NIP7;NOL8;NOV;NPM2;NR1H3;NRTN;NRXN1;NUFIP

T1B;CRTAP;CRYZL1;CSE1L;CSPG5;CSPP1;CTLA4;CTNNBIP1;CTNND2;CWC27;CYP11B1;CYP1A1;CYP2A7;CYP4V2;DARS;DBT;DCAF17;DCTN3;DDAH2;DDHD1;DDN;DDX1;DEDD2;DGAT1;DHRS7B;DHX29;DIP2C;DNAH2;DNAH9;DNAJC2;DNAJC

CTD4;KIFAP3;KPTN;L3MBTL3;LAMA3;LIMA1;LUM;MAP1LC3A;MAP6;MAP7;MAPK3;MASTL;MET;MFAP2;MMP2;MORF4L1;MPHOSPH10;MRE11A;MTCH1;MTDH;MTX1;MYO1B;NCBP1;NCBP2;NCF1;NEK4;NFIB;NIP7;NOL8;NOV;NPM2;NR1H3;N

3;DDAH2;DDN;DDX1;DEDD2;DGAT1;DHRS7B;DHX29;DIP2C;DNAH2;DNAH9;DNAJC2;DNAJC8;DNER;DNM1L;DNTTIP1;DPPA4;DRD3;DSTN;DUSP1;DYNC1I1;DYNC2H1;EBPL;ECE2;EDNRB;EI24;EIF2S1;EIF4EBP1;ELANE;ELF3;ELP2;EML

3;DDAH2;DDN;DDX1;DEDD2;DGAT1;DHRS7B;DHX29;DIP2C;DNAH2;DNAH9;DNAJC2;DNAJC8;DNER;DNM1L;DNTTIP1;DPPA4;DRD3;DSTN;DUSP1;DYNC1I1;DYNC2H1;EBPL;ECE2;EDNRB;EI24;EIF2S1;EIF4EBP1;ELANE;ELF3;ELP2;EML

;MAGEE1;MAPK10;MAPK3;MASTL;MCM8;MED17;MEIS3;MESP2;METTL8;MKL2;MLLT10;MLX;MMP2;MORF4L1;MPHOSPH10;MPHOSPH9;MRE11A;MT1G;MTAP;MTDH;MTL5;MTMR8;MVP;MYEF2;NAA15;NAA35;NANOG;NANOS3;NAP

;CPE;CPT1B;CRTAP;CRYZL1;CSE1L;CSPG5;CSPP1;CTLA4;CTNNBIP1;CTNND2;CWC27;CYP11B1;CYP1A1;CYP2A7;CYP4V2;DARS;DBT;DCAF17;DCTN3;DDAH2;DDHD1;DDN;DDX1;DEDD2;DGAT1;DHRS7B;DHX29;DIP2C;DNAH2;DNAH9;DNAJC2

M1L;DNTTIP1;DPPA4;DRD3;DUSP1;DYNC2H1;EBPL;ECE2;EDNRB;EI24;EIF2S1;EIF4EBP1;ELANE;ELF3;ELP2;ENPP7;EPHA4;EPM2A;ERAP1;ERCC4;ERCC6;ERMP1;ERRFI1;ESRRA;ESRRB;ETV3L;EXOSC4;FAAH;FANCC;FANCD2;FASTKD2;FA

M1L;DNTTIP1;DPPA4;DRD3;DUSP1;DYNC2H1;EBPL;ECE2;EDNRB;EI24;EIF2S1;EIF4EBP1;ELANE;ELF3;ELP2;ENPP7;EPHA4;EPM2A;ERAP1;ERCC4;ERCC6;ERMP1;ERRFI1;ESRRA;ESRRB;ETV3L;EXOSC4;FANCC;FANCD2;FASTKD2;FAT1;FBX

NIH2;CNTN2;COL16A1;COL18A1;COL23A1;COL4A2;COL9A1;COX6B1;CP;CPE;CPT1B;CPXM2;CRTAP;CRYZL1;CSE1L;CSPG5;CSPP1;CTLA4;CTNNBIP1;CTNND2;CWC27;CXCL3;CYP11B1;CYP1A1;CYP4V2;DAAM2;DARS;DBT;DCAF17;DCTN3;DDA

ENPP7;EPHA4;EPM2A;ERAP1;ERMP1;EXOSC4;FADD;FAH;FANCC;FASTKD2;FAT1;FHOD3;FKBP14;FKTN;FMO4;FOXO1;FSTL3;FTH1;FUT1;FUT3;FUT7;FUT8;FZD9;G3BP2;GABARAP;GALK1;GATM;GCLC;GJC1;GNB2;GNPAT;GNPNAT1;GNPTA

AF4B;TCERG1;TCF12;TERF1;TFB1M;TFEB;THBS1;THRSP;TIPIN;TNFSF8;TNKS;TRAK2;TRIM32;TRIM37;TRIP6;TRMT6;TSC22D3;TTF2;UBP1;UPF3B;USF1;USP47;WNT11;YAP1;ZBTB44;ZC3H12A;ZC3H8;ZFP90;ZFPL1;ZIK1;ZKSCAN

HK1;SPI1;SRA1;SRFBP1;SYNCRIP;TAF4B;TCERG1;TCF12;TERF1;TFB1M;TFEB;THBS1;THRSP;TIPIN;TNFSF8;TNKS;TRAK2;TRIM32;TRIM37;TRIP6;TRMT6;TSC22D3;TTF2;UBP1;UPF3B;USF1;USP47;WNT11;YAP1;ZBTB44;ZC3H12A;ZC

BCOR;BCORL1;BECN1;BEX2;BEX5;BHLHE40;BICD2;BIN2;BIVM;BLOC1S3;BNIP3;BOD1;BPNT1;BRAP;BRCA1;BRD3;BRD4;BRD8;BRE;BRIX1;BRPF3;BSCL2;BSG;BTG3;BTK;BUB3;C10orf10;C11orf73;C14orf159;C17orf89;C19orf12;C22orf32;C

.3;BCL6;BCL9;BCO2;BCOR;BCORL1;BECN1;BEX2;BEX5;BHLHE40;BICD2;BIN2;BIVM;BLOC1S3;BNIP3;BOD1;BPNT1;BRAP;BRCA1;BRD3;BRD4;BRD8;BRE;BRIX1;BRPF3;BSCL2;BSG;BTG3;BTK;BUB3;C10orf10;C11orf73;C14orf159;C17orf89;C19

BRAP;BRCA1;BRD4;BRD8;BRE;BRPF3;BSCL2;BSG;BTG3;BTK;BUB3;C10orf10;C11orf73;C14orf159;C17orf89;C19orf12;C22orf32;C3orf52;C6orf170;C6orf57;C6orf89;C7orf55;CA13;CA3;CA6;CACNG8;CALCOCO1;CALCOCO2;CAMK1D;CAM

70;C6orf57;C7orf55;CACNG8;CALCOCO1;CALCOCO2;CAMK1D;CAMK2G;CAMTA1;CANT1;CARM1;CASC3;CASP6;CASP9;CASZ1;CBFA2T3;CBL;CBR3;CBX1;CBX6;CBY1;CC2D2A;CCBL1;CCDC135;CCNB1IP1;CCRN4L;CD14;CD1C;CD1

G8;CALCOCO1;CALCOCO2;CAMK1D;CAMK2G;CAMTA1;CANT1;CARM1;CASC3;CASP6;CASP9;CASZ1;CBFA2T3;CBL;CBR3;CBX1;CBX6;CBY1;CC2D2A;CCBL1;CCDC135;CCNB1IP1;CCRN4L;CD14;CD1C;CD1D;CD68;CD86;CD93;CDC4

C11orf73;C14orf159;C17orf89;C19orf12;C22orf32;C3;C3orf52;C6orf170;C6orf57;C7orf55;CACNG8;CALCOCO1;CALCOCO2;CAMK1D;CAMK2G;CAMTA1;CANT1;CAP1;CAPZB;CARM1;CASC3;CASP6;CASP9;CASS4;CASZ1;CBFA2T3;C

73;C14orf159;C17orf89;C19orf12;C22orf32;C3orf52;C6orf170;C6orf57;C7orf55;CACNG8;CALCOCO1;CALCOCO2;CAMK1D;CAMK2G;CAMTA1;CANT1;CAP1;CAPZB;CARM1;CASC3;CASP6;CASP9;CASS4;CASZ1;CBFA2T3;CBL;CBR3;C

CBL;CBR3;CBY1;CCBL1;CCL3;CCRN4L;CD14;CD1C;CD1D;CD68;CD93;CDC42;CDC42SE1;CDH1;CENPH;CERKL;CERS2;CES1;CFL1;CHCHD1;CHCHD2;CHCHD6;CHCHD7;CHEK1;CHGA;CHKA;CHMP4B;CHST11;CHST15;CHST7;CKAP4;CLCN5;CL

CD1D;CD3D;CD44;CD53;CD82;CD86;CD93;CDC42;CDC42EP4;CDC5L;CDH1;CDNF;CENPH;CERS2;CFL1;CFL2;CFLAR;CHCHD1;CHCHD6;CHD8;CHEK1;CHKA;CHMP4B;CITED2;CLDN16;CLDN9;CLPX;CLTC;CLTCL1;CMTM1;CNN2;CNOT1;CNOT4;CN

A2;COL8A2;COPA;COPB2;COPS7A;COPS8;COPZ2;COQ3;COX11;COX17;COX18;COX5A;COX6A1;CPOX;CPSF7;CPT1A;CPT2;CREBBP;CRNKL1;CS;CSNK1D;CSNK2A1;CTDP1;CTDSP2;CTNNB1;CTSA;CTSB;CTSD;CUX1;CWC15;CYB5A;CYB5

A2;COL8A2;COPA;COPB2;COPS7A;COPS8;COPZ2;COQ3;COX11;COX17;COX18;COX5A;COX6A1;CPOX;CPSF7;CPT1A;CPT2;CREBBP;CRNKL1;CS;CSNK1D;CSNK2A1;CTDP1;CTDSP2;CTNNB1;CTSA;CTSB;CTSD;CUX1;CWC15;CYB5A;CYB5

L;BAZ2A;BBS4;BCAS3;BCCIP;BCDIN3D;BCKDHA;BCL2L13;BCL6;BCL9;BCO2;BCOR;BCORL1;BECN1;BEX2;BEX5;BHLHE40;BICD2;BIN2;BIVM;BLOC1S3;BNIP3;BOD1;BPNT1;BRAP;BRCA1;BRD3;BRD4;BRD8;BRE;BRIX1;BRPF3;BSCL2;BSG;BTG3;BTK;BT

L;BAZ2A;BBS4;BCAS3;BCCIP;BCDIN3D;BCKDHA;BCL2L13;BCL6;BCL9;BCO2;BCOR;BCORL1;BECN1;BEX2;BEX5;BHLHE40;BICD2;BIN2;BIVM;BLOC1S3;BNIP3;BOD1;BPNT1;BRAP;BRCA1;BRD3;BRD4;BRD8;BRE;BRIX1;BRPF3;BSCL2;BSG;BTG3;BTK;BT

ORL1;STX18;SYK;TAPBP;TGFB1;TLN1;TRAPPC2;TXNDC5;ULK1;VAMP2;VAMP8;VCL;VPS35;WASF2;W

P3;BPNT1;BRAP;BRCA1;BRD3;BRD4;BRE;BRPF3;BSG;BTG2;BTK;BTN3A1;BUB3;C10orf88;C11orf31;C11orf73;C16orf70;C17orf85;C1QTNF3;C3;C3orf33;C5AR1;CA13;CA14;CA3;CA6;CACNA2D4;CALCOCO1;CALCOCO2;CAMK1D;CAMK2G;CAMKK

HMOX1;HNRNPD;HSD17B14;HSPA1B;IMPDH1;IRAK2;IRF2;IRS2;ISCU;ITPK1;ITSN1;JAK1;JAK3;KAT5;KIF3C;KPNB1;LCP1;LCP2;LIMK1;LPIN2;LSM5;LSM7;LTA4H;MAP1S;MAP2K1;MAP2K3;MAP3K3;MAPK13;MAPK14;MAPRE1;MEFV;MFN2;MGRN1;MI

K;MKNK2;MLF1;MMP9;MPP1;MSN;MX2;MYB;MYD88;MYH9;MYO1F;NBEAL2;NCF2;NCOA6;NCOR1;NCSTN;NDRG1;NFAM1;NFKB1;NFKBIA;NHEJ1;NLRC4;NLRP3;NLRX1;NME1;NOD2;NOTCH1;NOTCH2;NUB1;PACS1;PAF1;PAF

R9;TM9SF1;TMEM67;TP53INP2;TPP1;TPRG1L;ULK1;VAMP2;VAMP8;VIM;WAS;WIPI1;YIPF3;YWH

2;NDUFC1;NDUFS3;NDUFV2;NFKB1;NIT2;NLRX1;NOL7;NUDT2;OCIAD2;OXNAD1;PAF1;PET117;PFDN2;PHB2;PHYH;PID1;PIGH;PINK1;PMPCB;PPA2;PPL;PRDX4;PRKACA;PRKCD;PROSC;PSAP;PSEN1;PSTK;PTPMT1;PTRF;PXMP2;RAB35;RAB3D;RA

SPA5;IGF2R;IMMP2L;INSR;IRAK2;JPH4;KDELR1;KIF13A;KIF1B;KPNB1;KSR1;LAMP1;LAPTM5;LPCAT3;LPIN2;LRRC59;MAN2A2;MARCH8;MBOAT7;MEST;MFN2;MFSD2A;MGST3;MICU1;MRPL17;MRPL45;MRPS14;MRPS23;MRTO4;MYD88;MY

RY2;SQSTM1;SRGAP2;SRI;STAT3;SYK;TBC1D14;TGFB1;TIRAP;TNFRSF1B;TNIP1;TPCN2;TRIOBP;TRPC4AP;TSG101;TXNIP;UBE4B;ULK1;VCL;WNK1;YWHAB;YWHAG;YWHAH;ZC3HC1;ZFH

F1;SNX1;SNX27;SOD1;SOX4;SPCS1;SQSTM1;SRI;SRP54;SRPR;SRSF2;SRSF4;SRSF7;SSR2;STAT3;STX18;TACC3;TAP2;TAPBP;TGFB1;THOC5;TIMM8A;TIMM9;TLR9;TNNC1;TNPO3;TOMM22;TOMM7;TPCN2;TRAK1;TRAPPC2;TSG101;T

ID2B;EID3;ELAC1;ELF4;ELK1;ELL;ELL3;EMG1;ENO1;ENOPH1;EP300;EPB41;EPS15L1;ERF;ESD;ESR1;ESRP2;ETV6;EXOSC1;EXOSC3;EXOSC8;EYA3;FABP6;FAM120B;FAM129A;FAM192A;FAM96B;FANCF;FANK1;FBXO22;FBXO25;FE

A1;SH2B3;SIN3A;SIRPA;SLC11A1;SLC7A5;SLC7A7;SOD1;SRC;SRF;STAT3;STAT5A;STAT5B;STK24;SYK;TGFB1;TIRAP;TLN1;TLR9;TNFRSF1B;TNIP1;VAV1;VCL;WAS;X

A12;GNA15;GNAI2;GNAS;GNB1;GPX7;GRB2;GSK3A;GSN;GTF2H5;HCK;HDAC4;HDGF;HERPUD1;HFE;HIPK2;HLA-B;HLA-C;HLA-DPA1;HLA-E;HMOX1;HSPA1B;HSPA5;IFNAR2;IGF1R;IL17RA;IL23A;IL4R;IL6R;ING4;INTS3;IRAK

2;SEC16A;SEC22A;SEC23B;SEC31A;SEC61G;SFT2D3;SLC11A1;SLC7A6OS;SMURF1;SNAP29;SNX1;SNX12;SNX19;SNX24;SNX27;SNX30;SOX4;SPCS1;SRP54;SRPR;SSR2;STAT3;STEAP3;STX18;SYK;TACC3;TAP2;TGFB1;TIMM8A;TIMN

L2;NXT1;OPTN;OS9;P2RX1;PACS1;PAFAH1B1;PAK1;PDCD6;PECAM1;PEX7;PIK3CD;PINK1;PLCG2;PLEK;PMPCB;POU2F2;POU5F1;PPID;PPP1R10;PPT1;PRAM1;PRKACA;PSAP;PSEN1;PTK2B;PTTG1IP;RAB23;RAB35;RAB3D;RAB43;RAB7A
1;PLEK;PMPCB;POU2F2;POU5F1;PPID;PPP1R10;PPT1;PRAF2;PRKACA;PRKCB;PRKCD;PSAP;PSEN1;PTTG1IP;RAB11FIP1;RAB19;RAB23;RAB35;RAB36;RAB3D;RAB40B;RAB43;RAB5B;RAB5C;RAB7A;RAB8A;RALB;RALGAPA2;RAMP1;RBM2

EC1;IRF2;ISG20L2;JARID2;KAT5;KAT6A;KAT7;KDM2A;KDM5B;KLF4;KPNB1;LEFTY2;LRRC59;LZTS1;MAEA;MAFB;MAFG;MAML1;MAML3;MAP2K3;MAPK14;MAU2;MAX;ME3;MED12;MED25;MIDN;MIS18A;MKNK2;MLH3;MLL2;MLLT1;MN

1;NFKBIA;NLRC4;NLRP12;NLRP3;NME1;NME2;NOD2;NOTCH1;NOTCH2;NUAK2;NUDT2;OPTN;P2RX1;PAK1;PARP4;PDCD5;PDCD6;PHLPP1;PIM1;PIM2;PINK1;PLAC8;PLAGL2;PLCG2;PNMA1;PPID;PPM1F;PPT1;PREX1;PRKCB;PRK
1;NFKBIA;NLRC4;NLRP12;NLRP3;NME1;NME2;NOD2;NOTCH1;NOTCH2;NUAK2;NUDT2;OPTN;P2RX1;PAK1;PARP4;PDCD5;PDCD6;PHLPP1;PIM1;PIM2;PINK1;PLAC8;PLAGL2;PLCG2;PNMA1;PPID;PPM1F;PPT1;PREX1;PRKCB;PRK
T5;KAT6A;KAT7;KDM2A;KDM5B;KLF4;KPNB1;LEFTY2;LRRC59;LZTS1;MAEA;MAFB;MAFG;MAML1;MAML3;MAP2K3;MAPK14;MAU2;MAX;ME3;MED12;MED25;MIDN;MIS18A;MKNK2;MLH3;MLL2;MLLT1;MMP14;MRP63;MRPL11;MRPL27

NFKBIA;NLRC4;NLRP12;NLRP3;NMB;NOD2;NOTCH1;NOTCH2;NRBP1;NUP98;NUPL2;NXT1;OPTN;OS9;P2RX1;PACS1;PAFAH1B1;PAK1;PDCD6;PECAM1;PEX7;PID1;PIK3CD;PINK1;PLCG2;PLEK;PLXNA2;PMPCB;POU2F2;POU5F1;PPID;PP
;IL17RA;IL23A;IL4R;IL6R;INSR;IQSEC1;IRAK2;ITGA5;ITGB2;ITSN1;JAK1;JAK3;KIAA1324;KIR3DL1;KLF4;KLRC4-KLRK1;KREMEN1;LAT2;LCP2;LILRA1;LILRB1;LILRB2;LRP1;LZTS1;MAGI2;MAP2K1;MAP2K3;MAP3K3;MAP3K5;M

CAMP2;SEC16A;SEC22A;SEC23B;SEC31A;SEC61G;SFT2D3;SLC11A1;SLC7A6OS;SMURF1;SNAP29;SNX1;SNX12;SNX19;SNX24;SNX27;SNX30;SOX4;SPCS1;SRP54;SRPR;SSR2;STAT3;STEAP3;STX18;SYK;TACC3;TAP2;TGFB1;TIMM8
KDM5B;KLF4;KPNB1;LRRC59;LZTS1;MAEA;MAFB;MAFG;MAML1;MAML3;MAP2K3;MAPK14;MAU2;MAX;ME3;MED12;MED25;MIDN;MIS18A;MKNK2;MLH3;MLL2;MLLT1;MMP14;MRP63;MRPL11;MRPL27;MRPL34;MRPL35;MRPL36;MRPL4

MGN3;HMOX1;HNRNPD;HNRNPF;HNRNPK;HNRNPUL1;IFNAR2;IFT46;IKZF1;IL23A;ING4;INSR;IRAK2;IRF2;JAK3;JARID2;KAT5;KAT6A;KAT7;KDM2A;KDM3B;KDM4B;KDM5B;KDM6B;KHDRBS2;KIAA0430;KLF13;KLF4;KLF7;K

L;CBX1;CBX6;CBY1;CC2D2A;CCL3;CCNK;CCRN4L;CD101;CD14;CD1D;CD209;CD3D;CD44;CD53;CD86;CD97;CDC42;CDC42EP4;CDC42SE1;CDC5L;CDH1;CELA1;CELF3;CERKL;CFL1;CFLAR;CHD8;CHEK1;CHKA;CHRNB1;CHRNB2;CHST11;CITED2;
N1;CAPNS1;CAPZB;CARM1;CASC3;CASP6;CASP9;CAST;CASZ1;CBFA2T3;CBL;CBX1;CBX6;CBY1;CC2D2A;CCL3;CCNK;CCRN4L;CD101;CD14;CD1D;CD209;CD3D;CD44;CD53;CD86;CD97;CDC42;CDC42EP4;CDC42SE1;CDC5L;CDH1;CELA1;CELF3;C

RAB5B;RAB5C;RAB7A;RAB8A;RALB;RALGAPA2;RAMP1;RBM22;RERE;RHBDF2;RHOA;RPAIN;RPH3A;RPL6;RPLP0;RPN1;RPS10;RPS9;RRAGB;RRAGC;RRBP1;SCAMP2;SEC16A;SEC22A;SEC23B;SEC31A;SEC61G;SFT2D3;SH3PXD2B;SIN3A

2;RPS6KA1;RTF1;RXRA;SAP130;SBDS;SETD1B;SF3B4;SIN3A;SKI;SMAD9;SMARCA2;SMUG1;SMURF1;SNAPC5;SNRPA1;SNRPF;SP140;SPATA24;SPEN;SQSTM1;SRP54;SRSF2;SRSF4;SRSF7;STAT3;STAT5A;STAT5B;STK24;SUPT3H;TADA2B;T
KIAA1324;KIF13A;KLHL21;LCP1;LIMK1;LRP1;MAEA;MAP1S;MAP2K1;MAPRE1;MAPRE3;MARK2;MAU2;MFN2;MICAL2;MIS12;MIS18A;MLH3;MLL2;MSL3P1;MYB;MYH11;MYH9;MYO1F;MYO7A;NACC2;NAP1L3;NAP1L5;NCF2;NCOR1;NDEL1;NI

N;MIS18A;MKNK2;MLH3;MLL2;MLLT1;MRPS14;MRPS23;MRTO4;MSN;MX2;MYB;MYH9;MYOF;N4BP1;NACC2;NCF2;NCOA6;NCOR1;NDEL1;NEK6;NFKB1;NFYC;NLRP6;NMNAT1;NOD2;NOL7;NOTCH1;NOTCH2;NPM3;NR2C1;NRBP1;NUP35;NUP98

CD6;PHLPP1;PIM1;PIM2;PINK1;PLAC8;PLAGL2;PLCG2;PNMA1;PPID;PPM1F;PPT1;PREX1;PRKCB;PRKCD;PRKDC;PRKRA;PSEN1;PSMB2;PSMD3;PSME3;PSMF1;PTK2B;PTPN6;RAC1;RAF1;RALB;RASSF5;RFFL;RHOA;RIPK1;RNF34;RNF4
RIN;FZD1;GAB2;GABRR2;GCC1;GCK;GDI2;GLMN;GLUL;GNAI2;GNAS;GOLGA5;GOLIM4;GOSR1;GRB10;GRB2;GSK3A;GSN;HCK;HCLS1;HDLBP;HFE;HGSNAT;HIP1;HK1;HK2;HMGN3;HMOX1;HNRNPK;HSPA5;IFT43;IFT46;IGF2R;IL23A;IL4R;IMI
AK1;EIF2B3;EIF4A2;EIF4EBP2;EIF4H;ELF4;ELK1;ELL;ELL3;ENO1;EP300;ERC1;ERF;ESR1;ESRP2;EXTL3;EYA3;FAM120B;FAM129A;FAM162A;FAM58A;FBP1;FBXO22;FGF9;FIS1;FKBP15;FKBP1A;FLCN;FLT3;FOSL2;FOXJ2;I
N;FZD1;G6PD;GAPDHS;GAS7;GBA;GCK;GIGYF2;GLI1;GLMN;GLO1;GNAI2;GNAS;GNRH1;GRB10;GSK3A;GTF2F1;GTF2H5;GTF3A;GTPBP1;H2AFY;HAX1;HCK;HCLS1;HDAC4;HDGF;HENMT1;HERPUD1;HESX1;HEXB;HIP1;HI

PP1;PIM1;PIM2;PINK1;PLAC8;PLAGL2;PNMA1;PPID;PPM1F;PPT1;PREX1;PRKCB;PRKCD;PRKDC;PRKRA;PSEN1;PSMB2;PSMD3;PSME3;PSMF1;PTK2B;PTPN6;RAC1;RAF1;RALB;RASSF5;RFFL;RHOA;RIPK1;RNF34;RNF41;RPS6KA1;RRA

;FAM120B;FAM129A;FAM58A;FBP1;FBXO22;FKBP15;FKBP1A;FLCN;FLT3;FOSL2;FOXJ2;FOXN3;FOXO4;FOXP3;FURIN;FZD1;G6PD;GAPDHS;GAS7;GBA;GCK;GIGYF2;GIT2;GLI1;GLMN;GLO1;GMIP;GNAI2;GNAL;GNAS;GRB1

LES2;CACNG8;CALCOCO1;CAMK1D;CAMK2G;CAMKK2;CAMTA1;CANT1;CAP1;CAPN1;CAPNS1;CAPZB;CARM1;CASC3;CASP6;CASP9;CAST;CASZ1;CBFA2T3;CBL;CBX1;CBX6;CBY1;CC2D2A;CCL3;CCNK;CCRN4L;CD101;CD14;CD1D;CD209;CD3D;CD
HD3;EIF2D;ELMO1;ELMO2;EMR2;ENSA;EPB41L3;EPHB1;EPS15L1;ERC1;ERGIC1;F11R;F13A1;FABP6;FAM160A2;FAM21A;FAM21C;FBN2;FBXO22;FCN1;FGF9;FIS1;FKBP15;FKBP1A;FLCN;FLVCR2;FOXP3;FSCN1;FTO;FURIN;FZD1;GAB2;GAE
ATAD2B;GBA;GCK;GDPD1;GIT2;GLB1;GLB1L;GLI1;GLO1;GLUL;GMEB1;GMIP;GNA11;GNA12;GNA15;GNAI2;GNAL;GNAS;GNAT2;GNS;GSN;GTF3A;GTPBP5;HAAO;HDAC4;HDDC2;HDDC3;HENMT1;HEXB;HGD;HIVEP3;HMOX1;HSCB;HSPA5;IKZF1;I
NEK6;NFAM1;NFKBIA;NKIRAS2;NLRP12;NLRP3;NOD2;NOTCH1;NOTCH2;PAFAH1B1;PAK1;PHLPP1;PIK3CD;PIK3R5;PIM2;PINK1;PLCG2;PLCL2;PLEK;PLEKHG6;PLEKHM1P;PPM1F;PRDX4;PREX1;PRKACA;PRKCB;PRKCD;PRKD2;PSAP;PSEN1;P

l;NOL7;NOTCH1;NOTCH2;NPM3;NR2C1;NRBP1;NUP98;PAF1;PBX2;PCBP1;PCBP2;PHF12;PHF2;PHF21A;PHF8;PIGH;PKNOX1;PNMA1;POLE4;POLR2A;POLR3F;POLR3GL;POP5;POU5F1;PPID;PRIM1;PRKCD;PRKDC;PSMB2;PSMD3;PSME3;PSMF1;P

3S4;BCCIP;BCDIN3D;BCKDHA;BCL2L13;BCL6;BCL9;BCO2;BCOR;BCORL1;BEAN1;BECN1;BEX2;BHLHE40;BICD2;BIN2;BLOC1S3;BNIP3;BOD1;BPNT1;BRAP;BRCA1;BRD3;BRD4;BRD8;BRE;BRPF3;BSCL2;BSG;BTBD9;BTG2;BTG3;BTK;BTN3A1;BUB3;BZW

B1;GMIP;GNA11;GNA12;GNA15;GNAI2;GNAL;GNAS;GNAT2;GNS;GSN;GTF3A;GTPBP5;HAAO;HDAC4;HDDC2;HDDC3;HENMT1;HGD;HIVEP3;HMOX1;HSCB;HSPA5;IKZF1;IMPDH1;ING4;IRF2BPL;ISCU;ITGA5;ITGAE;ITGAM;ITGAX;ITGB2;ITPI

RIN;FZD1;GAB2;GABRR2;GCC1;GCK;GDI2;GLMN;GLUL;GNAI2;GNAS;GOLGA5;GOLIM4;GOSR1;GRB10;GRB2;GSK3A;GSN;HCK;HCLS1;HDLBP;HFE;HGSNAT;HIP1;HK1;HK2;HMGN3;HMOX1;HNRNPK;HSPA5;IFT43;IFT46;IGF2R;IL23A;IL4R;IMI

HSPA5;IGF1R;IL23A;IL6R;IMMP2L;ING4;INSR;IRAK2;ITGA5;ITGB2;JAK1;JAK3;JARID2;KAT5;KAT6A;KAT7;KDM2A;KDM5B;KDM6B;KIAA0317;KLHL21;KSR1;LIMK1;LIPT1;LMTK2;MAML1;MAN2A2;MANBA;MAP2K1;MAP2K3;MAP3
RNPUL1;HSPA5;IFNAR2;IGF1R;IKZF1;IL23A;ING4;INSR;IRAK2;IRF2;JAK3;JARID2;KAT5;KAT6A;KAT7;KDM2A;KDM3B;KDM4B;KDM5B;KDM6B;KHDRBS2;KLF13;KLF4;KLF7;KLF9;LILRB1;LOXL3;LPIN2;LZTS1;MAFB;MAML1;MAML

RNPK;HNRNPUL1;HSPA5;IFNAR2;IGF1R;IKZF1;IL23A;ING4;INSR;IRAK2;IRF2;JAK3;JARID2;KAT5;KAT6A;KAT7;KDM2A;KDM3B;KDM4B;KDM5B;KDM6B;KHDRBS2;KLF13;KLF4;KLF7;KLF9;KLRC4-KLRK1;LILRB1;LOXL3;LPIN2;LZ
29A;FAM58A;FBP1;FBXO22;FKBP1A;FLCN;FLT3;FOSL2;FOXJ2;FOXN3;FOXO4;FOXP3;FURIN;FZD1;G6PD;GAPDHS;GAS7;GBA;GCK;GIGYF2;GIT2;GLI1;GLMN;GLO1;GMIP;GNAI2;GNAL;GNAS;GRB10;GSK3A;GTF2F1;GTF2H

PMPCB;POU2F2;POU5F1;PPID;PPP1R10;PPT1;PRAF2;PRKACA;PRKCB;PRKCD;PSAP;PSEN1;PTTG1IP;RAB11FIP1;RAB19;RAB23;RAB35;RAB36;RAB3D;RAB40B;RAB43;RAB5B;RAB5C;RAB7A;RAB8A;RAMP1;RBM22;RBM8A;RERE;RHBDF2;R

NUP35;NUP98;NUPL2;NXT1;ORMDL1;OS9;OSBP;OST4;PAFAH1B1;PAK1;PCYT1A;PDCD6;PECAM1;PIGC;PIGH;PLAUR;PLOD1;PSEN1;PTPLA;PTPN1;QSOX1;RAB11FIP1;RAB23;RAB35;RAB36;RAB43;RAB7A;RAB8A;RAC1;RAC2;RCC1;RHBDF2
IK;CCRN4L;CD3D;CD44;CD86;CDC42;CDC5L;CDH1;CDKL3;CELA1;CELF3;CENPH;CERS2;CFL1;CFLAR;CHD8;CHEK1;CHKA;CHRNB2;CHST11;CHST15;CHST7;CITED2;CLPX;CLTC;CNOT1;CNOT4;CNPY2;COPS7A;COPS8;COQ3;COQ5;COX17;COX5

AML1;MAML3;MAP2K1;MAP2K3;MAPK13;MAPK14;MAPRE3;MED12;MED25;MLL2;MLLT1;MSL3P1;MTERFD3;MTF1;MYB;MYCL1;NACC2;NCOA6;NCOR1;NDNL2;NFAM1;NFIC;NFKB1;NFKBIA;NFYC;NHLH1;NLRC4;NLRP12;NLRP3;NI
C1;DNAJB12;DNAJB5;DNAJB6;DNAJC14;DNAJC18;DNAJC19;DNAJC5;DNAJC5B;DNM2;DNMT3B;DPAGT1;DPF2;DPH5;DSCC1;DUSP12;DUSP18;DUT;DVL3;DYNLL1;DYRK4;ECE1;EEPD1;EGR1;EID2B;EID3;EIF2AK1;EIF2B3;EIF4A2;EIF

);CSF1R;CSNK1D;CSNK2A1;CTDP1;CTDSP2;CTSA;CTSB;CTSD;CTSZ;CYB5A;CYB5R1;CYB5R3;CYP27A1;CYP2R1;CYP4F3;DAPK1;DAPK2;DCLRE1B;DDTL;DGKG;DHTKD1;DHX34;DHX8;DNAH17;DNM2;DNMT3B;DPAGT1;DPH5;DUS4L;DUSP12;DUSP

6B;KHDRBS2;KLF13;KLF4;KLF7;KLF9;LILRB1;LOXL3;LPIN2;LZTS1;MAFB;MAML1;MAML3;MAP2K1;MAP2K3;MAPK13;MAPK14;MAPKAPK5;MAPRE3;MED12;MED25;MKNK1;MKNK2;MLL2;MLLT1;MSL3P1;MTERFD3;MTF1;MYB;N
2A;KDM3B;KDM4B;KDM5B;KDM6B;KHDRBS2;KLF13;KLF4;KLF7;KLF9;LILRB1;LOXL3;LPIN2;LZTS1;MAFB;MAML1;MAML3;MAP2K1;MAP2K3;MAPK13;MAPK14;MAPKAPK5;MAPRE3;MARCH8;MED12;MED25;MKNK1;MKNK2;N

L;COL5A2;COLEC12;CORO1C;CREBBP;CRKL;CRYZ;CSF1;CSF1R;CSF2RB;CSF3R;CSNK1D;CSNK2A1;CTDSP2;CTNNA1;CTNNAL1;CTNNB1;CTNND1;CTSB;CTSD;CUX2;CXCR2;CXCR7;CYP27A1;CYP2R1;CYP4F3;DAB2;DAG1;DAP;DAP3;DAPK1;DAPK2;

T43;IFT46;IL23A;IL4R;IMMP2L;INSR;IRS2;ITSN1;JAK3;JPH4;KCNA5;KCNC3;KCND1;KCNG2;KCNK6;KCNMB3;KCNMB4;KIAA0196;KIF13A;KIF1B;KIF1C;KPNB1;LASP1;LAT2;LCP2;LEFTY2;LILRB1;LILRB2;LIN7B;LMTK2;LRP1;LRP10;LTBP2

RIM27;TXNIP;UBE2V1;VASP;WAS;XIAP;YTH

KBIA;NFYC;NOD2;NOTCH1;NSD1;NUB1;PAF1;PAK1;PBX2;PHF2;PHF8;PIAS3;PID1;PIK3R5;PIM1;PIM2;PKNOX1;PLAC8;POLR2A;POMC;POU2F2;POU5F1;PRKACA;PRKCD;PRKD2;PRKDC;PSEN1;PSMB2;PSMD3;PSME3;PSMF1;PTK2

4;SPDYA;SPRED1;SQSTM1;SRF;SRPR;STK24;SYK;TBC1D14;TIRAP;TLN1;TLR9;TP53INP2;TP53TG1;TPP1;TTC5;UBE2V1;UBE4B;ULK1;UVRAG;WIPI1;WIPI2;XIAP;XPA;XRCC6BP1;XYLT1;YY1;ZSW

D12;MED25;MLL2;MLLT1;MSL3P1;MTERFD3;MTF1;MYB;MYCL1;NACC2;NCOA6;NCOR1;NDNL2;NFAM1;NFIC;NFKB1;NFKBIA;NFYC;NHLH1;NLRC4;NLRP12;NLRP3;NME2;NOD2;NOTCH1;NOTCH2;NR2C1;NSD1;PADI4;PAF1;PBX2;PFDN1

;TBC1D9;TIMP2;TXNIP;VAV1;WAS;WNK1;XIAP;YWH

MAPRE3;MED12;MED25;MLL2;MLLT1;MSL3P1;MTERFD3;MTF1;MYB;MYCL1;NACC2;NCOA6;NCOR1;NDNL2;NFAM1;NFIC;NFKB1;NFKBIA;NFYC;NHLH1;NLRC4;NLRP12;NLRP3;NME2;NOD2;NOTCH1;NOTCH2;NR2C1;NSD1;PADI4;PAF1

;TFB2M;TFE3;TGFB1;THRAP3;TIMP2;TINF2;TLR9;TNFSF13;TNIP1;TP53INP2;TRERF1;TTC5;UBE2V1;USP22;VDR;WDR61;YEATS4;YWHAH;ZMIZ1;ZNF287;ZN

YF2;GLI1;GLMN;GLO1;GMEB1;GNAS;GNRH1;GTF2F1;GTF2H5;GTF2I;GTF3A;H2AFY;HAX1;HCK;HCLS1;HDAC4;HDGF;HENMT1;HESX1;HEXB;HIP1;HIPK2;HIVEP3;HMGN3;HMOX1;HNRNPD;HNRNPF;HNRNPK;HNRNPUL1;HSPA1

;KSR1;LIMK1;LIPT1;LMTK2;MAML1;MAN2A2;MANBA;MAP2K1;MAP2K3;MAP3K3;MAP3K5;MAP4K4;MAPK13;MAPK14;MAPKAPK5;MARCH8;MARK2;MERTK;MGRN1;MKNK1;MKNK2;MLL2;MLLT1;MSRA;MTMR3;MYB;N4BP1;N6AMT1;NACC2;N

RAK2;IRF2;IRS2;JAK3;JARID2;KAT5;KAT6A;KAT7;KDM2A;KDM3B;KDM4B;KDM5B;KDM6B;KHDRBS2;KLF13;KLF4;KLF7;KLF9;KLRC4-KLRK1;LILRB1;LOXL3;LPIN2;LZTS1;MAFB;MAML1;MAML3;MAP2K1;MAP2K3;MAPK13;M

TGFB1;TIAL1;TIRAP;TLR9;TNFRSF1B;TNIP1;TRAT1;TRIM25;TXNIP;UBE2V1;WAS;X

P2;TINF2;TLR9;TNFSF13;TNIP1;TP53INP2;TRERF1;TTC5;UBE2V1;USP22;VDR;WDR61;YEATS4;YWHAH;ZMIZ1;ZNF287;ZN

DA6;NCOR1;NDNL2;NFIC;NFKB1;NFKBIA;NFYC;NOD2;NOTCH1;NSD1;NUB1;PAF1;PAK1;PBX2;PHF2;PHF8;PIAS3;PID1;PIK3R5;PIM1;PIM2;PKNOX1;PLAC8;POLR2A;POMC;POU2F2;POU5F1;PPM1F;PRKAB1;PRKACA;PRKCD;PRKD2;

DHTKD1;DHX34;DHX8;DNAH17;DNM2;DNMT3B;DPF2;DUS4L;DUSP12;DYRK4;ECE1;EFHA2;EFHC1;EGR1;EHD1;EHD3;EIF2AK1;EIF4A2;ELAC1;EMR2;ENDOU;ENO1;ENOPH1;EP300;EPB41;EPHB1;EPHB2;EPS15L1;ESR1;EXTL3;EYA3;F13A1;FAH

S2;JAK3;JARID2;KAT5;KAT6A;KAT7;KDM2A;KDM3B;KDM4B;KDM5B;KDM6B;KHDRBS2;KLF13;KLF4;KLF7;KLF9;KLRC4-KLRK1;LILRB1;LOXL3;LPIN2;LZTS1;MAFB;MAML1;MAML3;MAP2K1;MAP2K3;MAPK13;MAPK14;MAPK

2K3;MAP3K3;MAP3K5;MAP4K4;MAPK13;MAPK14;MAPKAPK5;MARCH8;MARK2;MERTK;MGRN1;MKNK1;MKNK2;MLL2;MLLT1;MSRA;MTMR3;MYB;N4BP1;N6AMT1;NACC2;NAE1;NCOR1;NDNL2;NDUFAB1;NEK3;NEK6;NLRP12;NOD2;NSD1;NU

2K3;MAP3K3;MAP3K5;MAP4K4;MAPK13;MAPK14;MAPKAPK5;MARCH8;MARK2;MERTK;MGRN1;MKNK1;MKNK2;MLL2;MLLT1;MSRA;MTMR3;MYB;N4BP1;N6AMT1;NACC2;NAE1;NCOR1;NDNL2;NDUFAB1;NEK3;NEK6;NLRP12;NOD2;NSD1;NU

ARP4;PCYT1A;PFKFB3;PGK1;PHKA2;PIGC;PIGH;PIK3CD;PIM1;PIM2;PINK1;PIP4K2C;POLE4;POLR2A;POLR3F;PRIM1;PRKAB1;PRKACA;PRKCB;PRKCD;PRKD2;PRKDC;PRMT5;PRMT6;PSTK;PTK2B;PYGL;RABGGTB;RAF1;RIPK1;RPN1;RPS6KA1;S

NT1;CAP1;CAPN1;CARM1;CARS;CASC3;CASP6;CASP9;CAST;CASZ1;CBFA2T3;CBL;CBR3;CBX1;CBX6;CBY1;CCBL1;CCL3;CCNB1IP1;CCNK;CCRN4L;CD3D;CD44;CD86;CDC42;CDC5L;CDH1;CDKL3;CELA1;CELF3;CENPH;CERS2;CES1;CFL1;CFLA

TBC1D14;TGFB1;TGOLN2;TLR9;TMEM132A;TMEM43;TRAPPC2;VCAN;WIPI1;XYLT1;YIPF3;ZDHHC3;ZDHHC7;ZFY

1;HAX1;HCK;HCLS1;HDAC4;HDGF;HERPUD1;HEXB;HIP1;HIPK2;HIVEP3;HLA-DPA1;HMGN3;HMOX1;HNRNPD;HNRNPK;HRK;HSPA1B;HSPA5;ICOS;IGF1R;IKZF1;IL23A;IL4R;IL6R;ING4;INSR;IRAK2;IRS2;ITGA5;ITSN1;JAK3

F

KBP15;FKBP1A;FKBP5;FKBP9;FLCN;FLT3;FNTB;FOXP3;FURIN;FZD1;G6PD;GALNT11;GALNT2;GBA;GCNT7;GIGYF2;GLMN;GNA11;GNA15;GNAI2;GSK3A;HAX1;HCK;HCLS1;HDAC4;HDGF;HERPUD1;HEXB;HIPK2;HM13;HMOX1;HSCB;H

P1;HIPK2;HIST3H2A;HMGN3;HMGN4;HMOX1;HNRNPK;HSD17B14;HYLS1;IFT43;IFT46;IKZF1;IP6K1;IQCB1;IQSEC1;ISG20L2;JAK1;JAK3;KAT5;KAT6A;KATNA1;KDM2A;KDM5B;KIF13A;KIF1B;KIF1C;KIF3C;KLF4;KLHL21;KRT2;LASP1;L
P1;HIPK2;HIST3H2A;HMGN3;HMGN4;HMOX1;HNRNPK;HSD17B14;HYLS1;IFT43;IFT46;IKZF1;IP6K1;IQCB1;IQSEC1;ISG20L2;JAK1;JAK3;KAT5;KAT6A;KATNA1;KDM2A;KDM5B;KIF13A;KIF1B;KIF1C;KIF3C;KLF4;KLHL21;KRT2;LASP1;L
1;PIK3R5;PIM1;PIM2;PKNOX1;PLAC8;POLR2A;POMC;POU2F2;POU5F1;PPM1F;PRKAB1;PRKACA;PRKCD;PRKD2;PRKDC;PSEN1;PSMB2;PSMD3;PSME3;PSMF1;PTK2B;PXN;RAC1;RAF1;RAMP1;RBM20;RBM22;RBPJ;RHOA;RIPK1;RN

R;CSNK1D;CSNK2A1;CTDP1;CTDSP2;CTIF;CTNNB1;CTNND1;CTSA;CTSB;CTSD;CTSZ;CUX1;CUX2;CWC15;CXCR2;DAB2;DAG1;DAP;DAPK1;DAPK2;DBNL;DCLRE1B;DEDD;DENND3;DHX8;DISC1;DNAJB12;DNAJB5;DNAJB6;DNAJC14;DNAJ

2;DNMBP;DOCK2;DOCK5;DOCK6;DOCK8;DOK3;DPF2;DVL3;EGR1;EIF4A2;EIF4EBP2;ELK1;ELMO1;EMR2;EP300;EPHB1;EPHB2;EPS15L1;ERC1;ESR1;EVC;EXTL3;F11R;FAM162A;FBN2;FBP1;FCN1;FGF9;FIS1;FKBP1A;FLCN;FLT3;FOXO4;FOXP3

4;STK4;TEX261;TGFB1;TIAL1;TMBIM6;TNFRSF9;TP53BP2;TRAIP;TXNDC5;TXNIP;UNC5B;XIAP;XPA;ZC3H
NBR1;NCOR1;NDEL1;NUB1;NUDT15;NUDT7;OS9;PAFAH1B1;PCBP2;PDE7B;PEX7;PFKFB3;PGAM1;PGD;PGK1;PGM1;PHKA2;PHYH;PIM2;PINK1;PLA2G12A;PLA2G16;PLBD2;PLCG2;PLIN1;PPT1;PREX1;PRKACA;PRKCD;PSEN1;PSMB2;PSMD3;PSME3;PS

;GLUL;GNA11;GNA12;GNA15;GNAI2;GNAL;GNAS;GNAT2;GNB1;GNB5;GOSR1;GRB2;GSK3A;GSN;GTF2F1;GTPBP1;H1F0;H2AFY;HAX1;HCK;HCLS1;HDAC4;HDGF;HDLBP;HENMT1;HFE;HIST3H2A;HLA-B;HLA-C;HLA-DPA1;HLA-E;HNRNP

R1;GRB2;GSK3A;GSN;GTF2F1;GTPBP1;HAX1;HCK;HCLS1;HDAC4;HDGF;HFE;HLA-B;HLA-C;HLA-DPA1;HLA-E;HSPA1B;HSPA5;IFT46;IL23A;IL6R;IMMP2L;ING4;INSR;INTS3;ITGA5;ITGAE;ITGAM;ITGAX;ITGB2;ITGB4;JARID2;K

M237;TMEM67;TNFRSF1B;TTLL4;ULK1;VAMP2;VASP;VIM;WA

B47;ZBTB5;ZC3H13;ZC3H3;ZC3H7A;ZC3HC1;ZCCHC24;ZCCHC3;ZDHHC18;ZDHHC3;ZDHHC5;ZDHHC7;ZFAND2A;ZFAND3;ZFHX3;ZFP106;ZFP30;ZFP36L2;ZFP37;ZFYVE1;ZMIZ1;ZNF10;ZNF14;ZNF140;ZNF16;ZNF165;ZNF174;ZNF

1;SP140;SP2;SQSTM1;SRSF7;TADA2B;TAF15;TBXAS1;TCF20;TIMM9;TOE1;TOP3A;TRAIP;TRERF1;TRIM25;TRIM27;TRIM67;TRIM8;USP22;VDR;XIAP;XRN2;YY1;ZBTB37;ZBTB47;ZBTB5;ZC3H13;ZC3H3;ZC3H7A;ZC3HC1;ZCCHC24;Z[illegible]

3;ATG9B;ATIC;ATM;ATN1;ATOX1;ATP10A;ATP1A1;ATP1A3;ATP1B3;ATP2A2;ATP2A3;ATP5A1;ATP5B;ATP5J;ATP5J2;ATP6V0E1;ATP6V1D;ATP6V1F;ATP6V1H;ATP8B1;ATP9B;ATRN;ATXN1L;ATXN2L;AVPR2;AXIN1;AXIN2;B3GALT4;BACE1;BACH2;BAD;BA
TP10A;ATP1A1;ATP1A3;ATP1B3;ATP2A2;ATP2A3;ATP5A1;ATP5B;ATP5J;ATP5J2;ATP6V0E1;ATP6V1D;ATP6V1F;ATP6V1H;ATP8B1;ATP9B;ATRN;ATXN1L;ATXN2L;AVPR2;AXIN1;AXIN2;B3GALT4;BACE1;BACH2;BAD;BAG3;BAHD1;BAIAP2L1;BATF;BATF3;B
1A;CIZ1;CLCC1;CLEC3B;CLIC1;CLK2;CLSTN1;CLUAP1;CMTM2;COG5;COPS7B;CORO2A;CPSF3;CRADD;CRAMP1L;CREB3L2;CREG1;CREM;CRTC1;CRTC3;CRY2;CSNK1E;CSNK2A2;CSRNP2;CSRP2BP;CSTB;CSTF1;CSTF2;CSTF3;CTC
FANCI;FEN1;FIP1L1;FOXC1;FOXI1;FOXJ1;FOXJ3;FOXK1;FOXK2;FOXM1;FOXP1;FOXP4;FTSJ2;FTSJ3;FZR1;GAR1;GATA3;GEMIN4;GEMIN5;GGN;GINS4;GLE1;GNL2;GNL3L;GPATCH1;GTF2A2;GTF3C1;GTF3C2;GTF3C4;GTPBP
CL2;BCL9L;BDH1;BET1L;BEX1;BICD1;BID;BIK;BIRC6;BLM;BLMH;BLOC1S1;BMS1;BPTF;BRD1;BRD2;BRD7;BRI3BP;BRPF1;BRSK2;BSN;BTRC;C11orf82;C12orf39;C14orf169;C15orf48;C15orf62;C16orf80;C16orf88;C1D;C1GALT1C1;
CL2;BCL9L;BDH1;BET1L;BEX1;BICD1;BID;BIK;BIRC6;BLM;BLMH;BLOC1S1;BMS1;BPTF;BRD1;BRD2;BRD7;BRI3BP;BRPF1;BRSK2;BSN;BTRC;C11orf82;C12orf39;C14orf169;C15orf48;C15orf62;C16orf80;C16orf88;C1D;C1GALT1C1;
N1;FH;FIP1L1;FOXC1;FOXI1;FOXJ1;FOXJ3;FOXK1;FOXK2;FOXM1;FOXP1;FOXP4;FOXRED2;FTSJ2;FTSJ3;FXN;FZR1;GANAB;GAR1;GARS;GATA3;GBF1;GEMIN4;GEMIN5;GGN;GINS4;GLS2;GLUD1;GNL2;GNL3L;GOT2;GPC2;GP
;EZH1;EZR;F2;FANCI;FEN1;FH;FIP1L1;FOXC1;FOXI1;FOXJ1;FOXJ3;FOXK1;FOXK2;FOXM1;FOXP1;FOXP4;FOXRED2;FTSJ2;FTSJ3;FXN;FZR1;GANAB;GAR1;GARS;GATA3;GBF1;GEMIN4;GEMIN5;GGN;GINS4;GLS2;GLUD1;GNL2;
CI;FEN1;FH;FIP1L1;FOXC1;FOXI1;FOXJ1;FOXJ3;FOXK1;FOXK2;FOXM1;FOXP1;FOXP4;FOXRED2;FTSJ2;FTSJ3;FXN;FZR1;GANAB;GAR1;GARS;GATA3;GBF1;GEMIN4;GEMIN5;GGN;GINS4;GLS2;GLUD1;GNL2;GNL3L;GOT2;GPC
;HMGA1;HNRNPA0;HNRNPA2B1;HNRNPA3;HNRNPH1;HNRNPM;HNRNPR;HNRNPU;HOXA9;HSPA8;HSPA9;IKBKAP;IKBKE;ILF2;ILF3;INCENP;ING2;ING5;INO80;INTS1;INTS4;INTS7;INTS9;IP6K2;IPO5;ITPR3;JAZF1;KAT6
;BBS1;BBS2;BBS9;BCL10;BCL11B;BCL2;BCL9L;BDH1;BET1L;BEX1;BICD1;BID;BIK;BIRC6;BLM;BLMH;BLOC1S1;BMS1;BPTF;BRD1;BRD2;BRD7;BRI3BP;BRK1;BRPF1;BRSK2;BSN;BTBD2;BTRC;C11orf82;C12orf39;C14orf169;C15orf48;C
;BBS1;BBS2;BBS9;BCL10;BCL11B;BCL2;BCL9L;BDH1;BET1L;BEX1;BICD1;BID;BIK;BIRC6;BLM;BLMH;BLOC1S1;BMS1;BPTF;BRD1;BRD2;BRD7;BRI3BP;BRK1;BRPF1;BRSK2;BSN;BTBD2;BTRC;C11orf82;C12orf39;C14orf169;C15orf48;C
BPB;CDC42BPG;CDK5RAP2;CDKAL1;CDKN2A;CENPN;CENPO;CEP104;CEP164;CEP72;CEP85;CEP89;CES2;CHCHD3;CHD3;CHD4;CHEK2;CHMP4C;CHMP5;CHMP7;CHST12;CIAO1;CIITA;CIRBP;CIRH1A;CKAP5;CLCN3;CLCN6;CLEC3B;CLIC
42BPA;CDC42BPB;CDC42BPG;CDH23;CDK5RAP2;CDKAL1;CDKN2A;CENPN;CENPO;CEP104;CEP164;CEP72;CEP85;CEP89;CES2;CHCHD3;CHD3;CHD4;CHEK2;CHMP4C;CHMP5;CHMP7;CHST12;CIAO1;CIITA;CIRBP;CIRH1A;CKAP5;CLCN3;
NAJA4;DNM1;DNMT1;DRG1;DSTYK;DUOX1;DYNC1H1;DYNC1LI2;EARS2;EEF2;EEF2K;EFTUD1;EFTUD2;EHD4;EIF2AK3;EIF2AK4;EIF2B1;EIF2S3;EIF3B;EIF4A3;EIF4B;EIF5B;ELAVL1;ENOX2;ENTPD8;EP400;EPHA1;EPHA2;EPRS;ERBB3;E
NAJA4;DNM1;DNMT1;DRG1;DSTYK;DUOX1;DYNC1H1;DYNC1LI2;EARS2;EEF2;EEF2K;EFTUD1;EFTUD2;EHD4;EIF2AK3;EIF2AK4;EIF2B1;EIF2S3;EIF3B;EIF4A3;EIF4B;EIF5B;ELAVL1;ENOX2;ENTPD8;EP400;EPHA1;EPHA2;EPRS;ERBB3;E
2;GARS;GART;GK5;GLUD1;GMPS;GNE;GPHN;GUCY2D;HARS;HELB;HKDC1;HLCS;HNRNPU;HSP90AB1;HSP90B1;HSPA1L;HSPA4;HSPA8;HSPA9;HSPD1;HSPH1;HYOU1;IARS;IARS2;IKBKB;IKBKE;ILF2;INO80;IP6K2;IRAK1;ITM2C;ITF
P1;GAL3ST4;GALK2;GARS;GART;GK5;GLUD1;GMPS;GNE;GPHN;GRPEL1;GUCY2D;HARS;HCN3;HELB;HKDC1;HLCS;HNRNPU;HSP90AB1;HSP90B1;HSPA1L;HSPA4;HSPA8;HSPA9;HSPD1;HSPH1;HYOU1;IARS;IARS2;IKBKB;IKBKE;ILF2;
P1;GAL3ST4;GALK2;GARS;GART;GK5;GLUD1;GMPS;GNE;GPHN;GUCY2D;HARS;HCN3;HELB;HKDC1;HLCS;HNRNPU;HSP90AB1;HSP90B1;HSPA1L;HSPA4;HSPA8;HSPA9;HSPD1;HSPH1;HYOU1;IARS;IARS2;IKBKB;IKBKE;ILF2;INO80;I
1;DNAJA3;DNAJA4;DNM1;DNMT1;DRG1;DSTYK;DUOX1;DYNC1H1;DYNC1LI2;EARS2;EEF2;EEF2K;EFTUD1;EFTUD2;EHD4;EIF2AK3;EIF2AK4;EIF2B1;EIF2S3;EIF3B;EIF4A3;EIF4B;EIF5B;ELAVL1;ENOX2;ENTPD8;EP400;EPHA1;EPHA2;EP
MBD1;MBD2;MCM2;MCM3;MCM4;MCM6;MDC1;MED11;MED24;MED27;MED29;MED30;MED31;MED6;MGA;MLL;MLL4;MMS19;MTA1;MTA2;MTA3;MTOR;MYC;MYO1C;NACC1;NCL;NCOR2;NFATC2;NOLC1;NONO;NOP10;NPAS2;NR1D1;NUMA
5B;ENTPD8;EP400;EPHA1;EPHA2;EPRS;ERBB3;ERCC3;FAM20B;FARSB;FBXO18;FGFR1;FKBP4;FLT4;FYN;G3BP1;GAL3ST4;GALK2;GARS;GART;GFM1;GIMAP6;GK5;GLUD1;GMPS;GNE;GNL2;GNL3L;GPHN;GPN2;GPN3;GTPBP4;GUCY2I
5B;ENTPD8;EP400;EPHA1;EPHA2;EPRS;ERBB3;ERCC3;FAM20B;FARSB;FBXO18;FGFR1;FKBP4;FLT4;FYN;G3BP1;GAL3ST4;GALK2;GARS;GART;GFM1;GIMAP6;GK5;GLUD1;GMPS;GNE;GNL2;GNL3L;GPHN;GPN2;GPN3;GTPBP4;GUCY2I
1;CSTF2;CSTF3;CTC1;CTCF;CTPS2;CUBN;CUL9;CXXC5;CYB5B;CYP20A1;CYP2D6;D2HGDH;DARS2;DBF4B;DCLK2;DDB1;DDIT3;DDR1;DDX10;DDX17;DDX18;DDX19A;DDX19B;DDX20;DDX23;DDX24;DDX27;DDX31;DDX42;DDX46;D
COX15;CPSF3;CREB3L2;CREG1;CREM;CRTC1;CRTC3;CRY2;CSNK1E;CSNK2A2;CSRNP2;CSTF1;CSTF2;CSTF3;CTC1;CTCF;CTNNBL1;CTPS2;CTR9;CUL4A;CUL7;CYP2D6;CYTL1;DARS2;DBR1;DCLRE1C;DCP1B;DCTD;DCTN1;DDB1;DDIT3
5B;ENTPD8;EP400;EPHA1;EPHA2;EPRS;ERBB3;ERCC3;FAM20B;FARSB;FBXO18;FGFR1;FKBP4;FLT4;FYN;G3BP1;GAL3ST4;GALK2;GARS;GART;GFM1;GIMAP6;GK5;GLUD1;GMPS;GNE;GNL2;GNL3L;GPHN;GPN2;GPN3;GTPBP4;GUCY2I
L1;CUL4A;CUL7;CUL9;DAD1;DCAF16;DCAF4;DCAF5;DCAF7;DCAF8;DCTN1;DDB1;DDX19A;DDX19B;DDX20;DDX23;DDX6;DFNB31;DHX15;DHX35;DHX38;DHX9;DIAPH1;DIS3L;DKC1;DLST;DNAH1;DNAJA3;DNHD1;DNM1;DOCK7;DPYSL2
5B;ENTPD8;EP400;EPHA1;EPHA2;EPRS;ERBB3;ERCC3;FAM20B;FARSB;FBXO18;FGFR1;FKBP4;FLT4;FYN;G3BP1;GAL3ST4;GALK2;GARS;GART;GFM1;GIMAP6;GK5;GLUD1;GMPS;GNE;GNL2;GNL3L;GPHN;GPN2;GPN3;GTPBP4;GUCY2I
D8;EP400;EPHA1;EPHA2;EPRS;ERBB3;ERCC3;FAM20B;FARSB;FBXO18;FGFR1;FKBP4;FLT4;FYN;G3BP1;GALK2;GARS;GART;GFM1;GIMAP6;GK5;GLUD1;GMPS;GNE;GNL2;GNL3L;GPHN;GPN2;GPN3;GTPBP4;GUCY2D;HARS;HELB;HHA
F2S3;EIF4A3;EIF5B;ENTPD8;EP400;EPHA1;EPHA2;EPRS;ERBB3;ERCC3;FAM20B;FARSB;FBXO18;FGFR1;FKBP4;FLT4;FYN;G3BP1;GAL3ST4;GALK2;GARS;GART;GFM1;GIMAP6;GK5;GLUD1;GMPS;GNE;GNL2;GNL3L;GPHN;GPN2;GPN3;
5B;ENTPD8;EP400;EPHA1;EPHA2;EPRS;ERBB3;ERCC3;FAM20B;FARSB;FBXO18;FGFR1;FKBP4;FLT4;FYN;G3BP1;GAL3ST4;GALK2;GARS;GART;GFM1;GIMAP6;GK5;GLUD1;GMPS;GNE;GNL2;GNL3L;GPHN;GPN2;GPN3;GRPEL1;GTPBP
5B;ENTPD8;EP400;EPHA1;EPHA2;EPRS;ERBB3;ERCC3;FAM20B;FARSB;FBXO18;FGFR1;FKBP4;FLT4;FYN;G3BP1;GAL3ST4;GALK2;GARS;GART;GFM1;GIMAP6;GK5;GLUD1;GMPS;GNE;GNL2;GNL3L;GPHN;GPN2;GPN3;GTPBP4;GUCY2I
RTC1;CRTC3;CRY2;CSNK1E;CSNK2A2;CSRNP2;CSTF1;CSTF2;CSTF3;CTC1;CTCF;CTNNBL1;CTPS2;CTR9;CUL4A;CUL7;CYTL1;DARS2;DBR1;DCLRE1C;DCP1B;DCTD;DCTN1;DDB1;DDIT3;DDX17;DDX20;DDX23;DDX24;DDX46;DDX47;DI
F3;CREB3L2;CREG1;CREM;CRTC1;CRTC3;CRY2;CSNK1E;CSNK2A2;CSRNP2;CSTF1;CSTF2;CSTF3;CTC1;CTCF;CTNNBL1;CTPS2;CTR9;CUL4A;CUL7;CYP2D6;CYTL1;DARS2;DBH;DBR1;DCLRE1C;DCP1B;DCTD;DCTN1;DDB1;DDIT3;DDX1
1;CSTF2;CSTF3;CTC1;CTCF;CTPS2;CUBN;CUL9;CXXC5;CYB5B;CYP20A1;CYP2D6;D2HGDH;DARS2;DBF4B;DCLK2;DDB1;DDIT3;DDR1;DDX10;DDX17;DDX18;DDX19A;DDX19B;DDX20;DDX23;DDX24;DDX27;DDX31;DDX42;DDX46;D
ARNTL2;ARPC2;ARPC3;ASB1;ASCC1;ASMT;ASXL1;ATF6B;ATG10;ATG13;ATG14;ATG3;ATG9B;ATIC;ATM;ATN1;ATOX1;ATP10A;ATP1A1;ATP1A3;ATP1B3;ATP2A2;ATP2A3;ATP5A1;ATP5B;ATP5J;ATP5J2;ATP6V0E1;ATP6V1D;ATP6V1F;ATP6V1H;ATP8B1;ATP8B
ARNTL2;ARPC2;ARPC3;ASB1;ASCC1;ASMT;ASXL1;ATF6B;ATG10;ATG13;ATG14;ATG3;ATG9B;ATIC;ATM;ATN1;ATOX1;ATP10A;ATP1A1;ATP1A3;ATP1B3;ATP2A2;ATP2A3;ATP5A1;ATP5B;ATP5J;ATP5J2;ATP6V0E1;ATP6V1D;ATP6V1F;ATP6V1H;ATP8B1;ATP8B
O1;CIC;CIITA;CINP;CIRH1A;CIZ1;CKM;CLK2;CMKLR1;CNDP2;CNOT10;COX10;COX15;CPSF3;CREB3L2;CREG1;CREM;CRTC1;CRTC3;CRY2;CSNK1E;CSNK2A2;CSRNP2;CSTF1;CSTF2;CSTF3;CTC1;CTCF;CTNNBL1;CTPS2;CTR9;CUL4A;CUL7;
F;BRD1;BRD2;BRD7;BRPF1;BRSK2;BTRC;C14orf169;C1D;C1GALT1C1;C22orf28;CAD;CALM3;CALR;CAMTA2;CAND2;CAPN3;CARD11;CARD14;CARNS1;CBFA2T2;CBX2;CBX5;CBX7;CCAR1;CCDC23;CCDC88C;CCL8;CCND1;CCND2;CCNF;CCNT1;CC
N8;CMKLR1;CNOT10;COX10;COX15;CPSF3;CREB3L2;CREG1;CREM;CRTC1;CRTC3;CRY2;CSNK1E;CSNK2A2;CSRNP2;CSTF1;CSTF2;CSTF3;CTC1;CTCF;CTNNBL1;CTPS2;CTR9;CUBN;CUL4A;CUL7;CYP2D6;CYTL1;DARS2;DBH;DBR1;DCLF
;RPRD1B;RREB1;RUNX2;RUVBL1;SALL2;SAP30;SART3;SATB1;SETD1A;SF3A3;SFPQ;SMAD3;SMARCC2;SMARCE1;SNAPC4;SNRNP70;SNW1;SON;SRRM1;SRRM2;SRSF5;STK38;SUPT4H1;TADA2A;TAF1;TAF10;TAF3;TAF6;TAF8;TBP;TCF
ATG3;ATIC;ATM;ATN1;ATOX1;ATP10A;ATP13A1;ATP1A1;ATP1A3;ATP2A2;ATP2A3;ATP5A1;ATP5B;ATP6V1D;ATP6V1F;ATP6V1H;ATP8B1;ATP8B2;ATP9B;ATRN;ATXN1L;AXIN1;AXIN2;BACE1;BACH2;BAD;BAG3;BAHCC1;BAHD1;BAIAP2L1;BATF;BATF3;BAZ1
;CHD1L;CHD2;CHD3;CHD4;CHD6;CHEK2;CHMP5;CHST12;CHTF8;CIAO1;CIC;CIITA;CINP;CIRBP;CIRH1A;CIZ1;CLK2;CLN8;CMKLR1;CNOT10;COG2;COG7;COPS7B;CPSF3;CREB3L2;CREG1;CREM;CRTC1;CRTC3;CRY2;CSNK1E;CSNK2A2;
V0E1;ATP6V1D;ATP6V1F;ATP6V1H;ATP8B1;ATP9B;ATRN;ATXN2L;AVPR2;AXIN1;AXIN2;B3GALT4;BACE1;BAD;BAG3;BAIAP2L1;BATF;BBS1;BBS2;BBS9;BCL10;BCL2;BDH1;BET1L;BEX1;BICD1;BID;BIK;BIRC6;BLM;BLMH;BLOC1S1;BPTF;BRD2;BRD7;
DLD;DNAH1;DNAJA3;DNAJA4;DNM1;DRG1;DSTYK;DYNC1H1;DYNC1LI2;EARS2;ECI2;EEF2;EEF2K;EFTUD1;EFTUD2;EHD4;EIF2AK3;EIF2AK4;EIF2B1;EIF2S3;EIF4A3;EIF5B;ENTPD8;EP400;EPHA1;EPHA2;EPRS;ERBB3;ERCC3;ETFDH;FAM
1;BIRC6;BLM;BLMH;BLOC1S1;BMP2;BPTF;BRD1;BRD2;BRD7;BRPF1;BRSK2;BTRC;C14orf169;C1D;C1GALT1C1;C22orf28;C2CD3;CAD;CALM3;CALR;CAMTA2;CAND2;CAPN2;CAPN3;CAPN5;CARD11;CARD14;CARNS1;CASP1;CASP10;CASP2;CBFA2T2
NMT1;DNMT3A;DRG1;DROSHA;DTL;DVL2;EARS2;ECD;EDC3;EDC4;EFTUD2;EHMT1;EIF2AK3;EIF4A3;EIF4B;EIF4G1;EIF4G2;ELAVL1;ELP3;EME2;EPRS;ERCC3;ERCC5;ETS1;EXOSC10;EZH1;FADS1;FAM103A1;FAN1;FANCI;FA
;DCTN1;DDB1;DDX19A;DDX19B;DDX20;DFNB31;DHX9;DIAPH1;DIS3L;DLST;DNAH1;DNAJA3;DNHD1;DNM1;DOCK7;DPYSL2;DST;DTL;DYNC1H1;DYNC1I2;DYNC1LI2;DYNLT1;EIF2B1;EIF3A;EIF3B;EIF4A3;EIF4B;EIF4G1;EIF4G2;EI

1B;BCL2;BCL9L;BDH1;BEX1;BICD1;BIRC6;BLM;BLMH;BLOC1S1;BMP2;BPTF;BRD1;BRD2;BRD7;BRPF1;BRSK2;BTRC;C14orf169;C1D;C1GALT1C1;C22orf28;C2CD3;CAD;CALM3;CALR;CAMTA2;CAND2;CAPN2;CAPN3;CAPN5;CARD11;CARD14;CARNS1
CHST12;CHTF8;CIAO1;CIC;CIITA;CINP;CIRH1A;CIZ1;CKM;CLK2;CLN8;CMKLR1;CNDP2;CNOT10;COX10;COX15;CPSF3;CREB3L2;CREG1;CREM;CRTC1;CRTC3;CRY2;CSNK1E;CSNK2A2;CSRNP2;CSTF1;CSTF2;CSTF3;CTC1;CTCF;CTNNBL1;C
;FBXO21;FEN1;FIP1L1;FLYWCH1;FOSB;FOXC1;FOXI1;FOXJ1;FOXJ3;FOXK1;FOXK2;FOXM1;FOXP1;FOXP4;FUBP1;G3BP1;GABPB2;GAR1;GATA3;GCN1L1;GEMIN5;GFM1;GMEB2;GON4L;GPANK1;GPATCH1;GPATCH4;GPA
AD;BAG3;BAHD1;BATF;BATF3;BAZ1B;BBS2;BCL10;BCL11B;BCL2;BCL9L;BDH1;BEX1;BICD1;BIRC6;BLM;BLMH;BLOC1S1;BMP2;BPTF;BRD1;BRD2;BRD7;BRPF1;BRSK2;BTRC;C14orf169;C1D;C1GALT1C1;C22orf28;C2CD3;CAD;CALM3;CALR;CAMTA2;C

;CACNA1I;CAD;CADM1;CALM3;CALR;CAMP;CAMSAP1;CAMTA2;CAND2;CAPN2;CAPN3;CARD11;CARD14;CASP1;CASP10;CASP2;CATSPER1;CATSPER2;CBFA2T2;CBLB;CBX2;CBX5;CBX7;CCAR1;CCDC141;CCDC64;CCDC88C;CCL8;CCND1;CCND2
T3A;DPH1;DRG1;DROSHA;DVL2;EARS2;ECD;EDC3;EDC4;EEF2;EEF2K;EFTUD2;EHMT1;EIF2AK3;EIF2AK4;EIF2B1;EIF2S3;EIF3A;EIF3B;EIF4A3;EIF4B;EIF4G1;EIF4G2;EIF5A;EIF5B;ELAVL1;ELP3;EPHX2;EPRS;ERCC3;ETS1
;SRRM1;SRRM2;SRRT;SRSF5;SUGP2;SUPV3L1;SYF2;TARDBP;TFIP11;TGS1;THOC1;THOC2;TNKS1BP1;TRIT1;TSR2;TYW1;U2AF1L4;U2AF2;USP39;UTP14A;UTP20;WBP11;WDR33;WDR4;WDR55;ZCCHC11;Z

T8;CD3E;CD3EAP;CD4;CD40LG;CD74;CDAN1;CDC16;CDC25B;CDC42BPA;CDC42BPB;CDC42BPG;CDH13;CDK5RAP2;CDKAL1;CDKN2A;CDKN2B;CDON;CELF1;CELSR2;CENPN;CENPO;CEP164;CHCHD3;CHD1L;CHD2;CHD3;CHD4;CHD6;CHEK2;CHM
3ED6;ZBTB9;ZCCHC11;ZHX3;ZNF330;ZN
YNLT1;EDC3;EDC4;EFCAB4B;EHMT1;EIF2AK4;EIF3A;ELP3;EMD;EML3;EPB41L2;EPHX2;EPPK1;EPS8;EVL;EXOC7;EXOSC10;EZR;FAM110B;FBF1;FEN1;FGD1;FKBP4;FLNA;FLNB;FLNC;FNBP1;FOXC1;FOXK1;FRMD4A;FSCN2;FTSJ2;F
YNLT1;EDC3;EDC4;EFCAB4B;EHMT1;EIF2AK4;EIF3A;ELP3;EMD;EML3;EPB41L2;EPHX2;EPPK1;EPS8;EVL;EXOC7;EXOSC10;EZR;FAM110B;FBF1;FEN1;FGD1;FKBP4;FLNA;FLNB;FLNC;FNBP1;FOXC1;FOXK1;FRMD4A;FSCN2;FTSJ2;F

SC10;EZH1;FADS1;FAM103A1;FARSB;FEN1;FGF2;FGFR1;FIP1L1;FLNA;FOSB;FOXC1;FOXI1;FOXJ1;FOXJ3;FOXK1;FOXK2;FOXM1;FOXP1;FOXP4;FTSJ2;FTSJ3;FUBP1;GABPB2;GAR1;GARS;GATA3;GEMIN4;GEMIN5;GMEB2;
;ASXL1;ATF6B;ATG10;ATG13;ATG14;ATG3;ATG9B;ATIC;ATM;ATN1;ATOX1;ATP10A;ATP1A1;ATP1A3;ATP1B3;ATP2A2;ATP2A3;ATP5A1;ATP5B;ATP5J;ATP5J2;ATP6V0E1;ATP6V1D;ATP6V1F;ATP6V1H;ATP8B1;ATP8B2;ATP9B;ATXN1L;ATXN2L;AVPR2;AXIN
AK4;EIF2B1;EIF2S3;EIF3A;EIF3B;EIF4A3;EIF4B;EIF4ENIF1;EIF4G1;EIF4G2;EIF5A;EIF5B;ELAVL1;ENO2;ENOX2;EPHX2;EPRS;EVL;EXOC7;EZR;FAF1;FARSB;FASN;FGD1;FGF2;FGFR1;FHL1;FKBP4;FLNA;FLNB;FLNC;FXN;FYN;FZR1;G3E
26;RBM28;RBM33;RBM4B;RBM6;RCAN3;RNASEH1;RNMT;RNPS1;RPL21;RPP14;RSL1D1;SARS;SART3;SCAF1;SCAF4;SCAF8;SECISBP2;SETD1A;SF1;SFPQ;SLC4A1AP;SLTM;SNRNP70;SON;SRBD1;SRP68;SRRM1;SRRM2;SRSF5;SUGP2;SUPT

;MAPK9;MARS;MAVS;MCCC1;MCCC2;MCCD1;MGST1;MIPEP;MLXIP;MMACHC;MMADHC;MRPL16;MRPL33;MRPL44;MRPL49;MRPS24;MRPS27;MRPS36;MRPS5;MRPS9;MSRB2;MSRB3;MTCH2;MTHFD1;MTHFD1L;MTOR;MTPAP;MUL1;MY
I2;FKBP4;FLNA;FLNB;FMNL3;FOXJ1;FOXP1;FSCN2;FXN;FZR1;GADD45A;GATA3;GBF1;GFM1;GORASP2;GRID2IP;GRPEL1;GTF3C4;H3F3C;HAUS5;HCFC1;HDAC1;HGF;HIST1H2AC;HIST1H2AK;HIST1H2BN;HIST2H2AC;HIST2H2BE;HIST

RBBP4;RBBP7;RBM14;RFC1;RFWD3;RNASEH1;RNF8;RPA1;RPA2;RRM1;RUVBL1;SFPQ;SH2B1;SHC1;SLX4;SMARCAL1;SMC1A;SMG6;SSRP1;SUPT16H;SUPV3L1;TCF3;TCF7;TCF7L2;TDP1;TEP1;TERF2;TERF2IP;TNFAIP1;TNKS1BP1;TOP2B;TP5
30;ZW
;TAF10;TCF3;TCF7L2;TEP1;TERF2;TERF2IP;TNKS1BP1;TOP2B;TP53;TRIM16;TRRAP;TSPYL2;TTF1;UBE2B;UHRF1;XPC;XRCC5;XRCC6;YEATS2;YLPM1;Z

L;SARM1;SEPT6;SERPINB6;SFI1;SPECC1L;SPTAN1;STARD9;STIM1;TADA2A;TBCC;TBCCD1;TCHP;TCP1;TCP11L1;TCTN2;TIMM17B;TMEM216;TPPP;TRIP4;TSPYL2;TTLL3;TTLL5;TUBA3D;TUBB;TUBGCP4;TUBGCP5;TUBGCP6;WDR67;

;RBBP4;RFC1;RHOF;RUVBL1;SMARCA4;SMARCAL1;SMC1A;SNRNP200;SPATA5;SPG7;SRCAP;STARD9;SUPV3L1;TBCC;THTPA;TOP2B;TUBA3D;TUBB;VCP;WRNIP1;XRCC3;XRCC5;

;PEX5;PHACTR4;PHRF1;PIK3IP1;PKD1;PLSCR4;POLB;POLG;PPARA;PPME1;PPP1R16B;PPP1R9B;PRC1;PRKAG1;PRKCA;PRKCH;PTK2;RAB11FIP3;RAB3GAP1;RACGAP1;RAD18;RAD54L2;RANBP10;RBBP4;RELA;RNF20;RNF8;RPA2;RPRD1B;RRN
2;BDH1;BET1L;BICD1;BID;BIK;BIRC6;BLMH;BLOC1S1;BRI3BP;BRSK2;BTBD2;BTRC;C12orf39;C15orf48;C15orf62;C1D;C1GALT1C1;C22orf29;C2CD2;C9orf89;CABP4;CACNB1;CAD;CADM1;CALM3;CALR;CAMP;CAPN3;CARD11;CASP1;CASP10;C
BL2A;RABL2B;RAD51D;RAD54L2;RBBP4;RFC1;RHOF;RUVBL1;SLC25A42;SMARCA4;SMARCAL1;SMC1A;SNRNP200;SPATA5;SPG7;SRCAP;STARD9;SUPV3L1;TBCC;THTPA;TOP2B;TUBA3D;TUBB;VCP;WRNIP1;XRCC3;XRCC5;
1A;RABL2A;RABL2B;RAD51D;RAD54L2;RBBP4;RFC1;RHOF;RUVBL1;SLC25A42;SMARCA4;SMARCAL1;SMC1A;SNRNP200;SPATA5;SPG7;SRCAP;STARD9;SUPV3L1;TBCC;THTPA;TOP2B;TUBA3D;TUBB;VCP;WRNIP1;XRCC3;XRCC5;
BL2A;RABL2B;RAD51D;RAD54L2;RBBP4;RFC1;RHOF;RUVBL1;SLC25A42;SMARCA4;SMARCAL1;SMC1A;SNRNP200;SPATA5;SPG7;SRCAP;STARD9;SUPV3L1;TBCC;THTPA;TOP2B;TUBA3D;TUBB;VCP;WRNIP1;XRCC3;XRCC5;
SH6;MTA3;MYBBP1A;MYC;NASP;NCAPD2;NCAPD3;NCAPG;NCAPG2;NDC80;NEK9;NINL;NME6;NOLC1;NSUN2;NUMA1;NUP133;NUP214;ODF2;PA2G4;PAPD7;PARD6A;PCM1;PCNT;PDCD4;PHACTR4;PHF13;PIM3;PINX1;PKD1;PLD6;PMS2;PMS2P5;P

;ELAVL1;ELP3;ENTPD5;EPRS;ERCC3;ETS1;EXT2;EZH1;FADS1;FARSB;FEN1;FGF2;FGFR1;FKRP;FLNA;FOSB;FOXC1;FOXI1;FOXJ1;FOXJ3;FOXK1;FOXK2;FOXM1;FOXP1;FOXP4;FUBP1;FUT11;FUT2;GABPB2;GAL3S

A42;SLC25A45;SMAD3;SMCR7L;SNPH;SOX10;SPG7;STAR;SUN2;SYNE1;SYNE2;TAMM41;TEX10;THEM4;TIMM17B;TMEM109;TMEM57;TNPO2;TOMM34;TUBB;TXLNG;VAMP1;VAT1;VDAC1;VDAC2;WDR3;WHSC1;
RY2;CSNK2A2;CSRNP2;CSTF1;CSTF2;CSTF3;CTC1;CTCF;CTPS2;CTR9;CYTL1;DAD1;DARS2;DBH;DCN;DCTD;DDIT3;DDX17;DDX20;DHCR24;DHCR7;DHDDS;DHODH;DHX33;DHX38;DIDO1;DKC1;DLD;DNAJA3;DNAJC1;DNMT1;DNMT3A;

EIF4A3;EIF4B;EIF4G1;EIF4G2;EIF5A;EIF5B;ELAVL1;ELP3;ENTPD5;EPRS;ERCC3;ETS1;EXT2;EZH1;F2;FADS1;FARSB;FEN1;FGF2;FGFR1;FKRP;FLNA;FOSB;FOXC1;FOXI1;FOXJ1;FOXJ3;FOXK1;FOXK2;FOXM1;FOXP1;
7;SLC25A42;SLC25A45;SMAD3;SMCR7L;SNPH;SOX10;SPG7;STAR;SUN2;SYNE1;SYNE2;TAMM41;TEX10;THEM4;TIMM17B;TMEM109;TMEM57;TNPO2;TOMM34;TUBB;TXLNG;VAMP1;VAT1;VDAC1;VDAC2;WDR3;WHSC1;

TADA2A;TAF1;TCF3;TFDP2;TIMELESS;TNF;TOP2B;TP53;TRIM35;TRRAP;TUBB;TUBGCP4;TUBGCP5;TUBGCP6;UBE2I;UBE2L3;YWHAE;ZC3H12D;ZW
2A2;CSRNP2;CSTF1;CSTF2;CSTF3;CTC1;CTCF;CTPS2;CTR9;CYTL1;DAD1;DARS2;DBH;DCN;DCTD;DDIT3;DDX17;DDX20;DHDDS;DHODH;DHX33;DHX38;DIDO1;DKC1;DLD;DNAJA3;DNAJC1;DNMT1;DNMT3A;DPH1;DRD4;DRG1;DTL;DU

;POLE;PPP1R9B;PPP2R5C;PRC1;PRKAG1;PRMT2;PSMC4;PSMD1;PSMD11;PSMD2;PSMD6;PSMD7;PSME4;RACGAP1;RAD51D;RBL2;RCC2;RFC1;RFWD3;RNF4;RNF8;RPA1;RPA2;RPRD1B;RPS6KA2;RUVBL1;SEH1L;SF1;SFI1;SH2B1;SMAD3;SMARCA4;SM
RTC1;CRTC3;CRY2;CSNK2A2;CSRNP2;CSTF1;CSTF2;CSTF3;CTC1;CTCF;CTPS2;CTR9;CYTL1;DAD1;DARS2;DBH;DCN;DCTD;DDIT3;DDX17;DDX20;DHCR24;DHCR7;DHDDS;DHODH;DHX33;DHX38;DIDO1;DKC1;DLD;DNAJA3;DNAJC1;DNM

D2;CHD3;CHD4;CHD6;CHEK2;CHST12;CIITA;CIT;CIZ1;CKM;CLCN3;CLCN6;CLEC3B;CLK2;CLPB;CLSTN1;CNDP2;COL27A1;COL5A1;COX5B;CPSF3;CRB2;CREG1;CSNK1E;CSNK2A2;CTCF;CTPS2;CUBN;CUL9;CXXC5;CYB561D1;CYB5B;CYP20A1;C

I4;TIMM17B;TOMM34;TP53;VAMP1;VAT1;VDAC1;VD
DUSP7;DVL2;DYX1C1;ECD;EDEM1;EEF2K;EHD4;EHMT1;EIF2AK3;EIF2AK4;EIF2B1;EIF3B;EIF4A3;EIF4B;EIF4G1;EIF4G2;EIF5A;EIF5B;ELAVL1;ELP3;EPHA1;EPHX2;EPRS;ERBB3;ERCC3;ETS1;EXOSC10;EZH1;F12;F2;FADS1;FA

;CECR1;CERK;CES2;CHD1L;CHD2;CHD3;CHD4;CHD6;CHEK2;CHST12;CIT;CKM;CLK2;CLPB;CMBL;CNDP2;COX10;COX15;COX5B;COX8A;CPSF3;CREG1;CSNK1E;CSNK2A2;CSRP2BP;CTPS2;CYB5RL;CYP20A1;CYP2D6;D2HGDH;DAD1;DAGLA;DARS2;

;CEP164;CEP72;CHAD;CHCHD3;CHD1L;CHD3;CHD4;CHD6;CHEK2;CHMP4C;CHMP5;CHMP7;CIAO1;CIRBP;CIT;CKAP5;CLCN3;CLDN15;CLDN20;CLN8;CNTNAP1;COG1;COG2;COL17A1;COL27A1;COL5A1;COL6A3;CORO2A;COX10;COX15;CROCC;C
1;LIG3;LMO2;LOC100133315;LONP2;MAFK;MBD1;MBD2;MCM2;MCM3;MCM3AP;MCM4;MCM6;MECP2;MED6;MEIS2;MGA;MKL1;MLH1;MLL;MLL4;MLXIP;MND1;MNT;MSH6;MTA1;MTA2;MTA3;MTOR;MXD3;MYBBP1A;MYC;MYO18A;NCL;NCOR

OXK1;FOXK2;FOXM1;FOXP1;FOXP4;FUBP1;FXN;GABBR1;GABPB2;GARS;GART;GATA3;GIPR;GMEB2;GMPS;GON4L;GPHN;GRM2;GTF2A2;GTF3C1;GTF3C2;GTF3C4;HABP4;HCFC1;HDAC1;HELB;HEXIM1;HGF;HHEX;HIC2;HIF1AN;

2B1;SMARCA4;SMC1A;STARD9;SUN2;SYCE2;TADA2A;TAF1;TCF3;TFDP2;TIMELESS;TNF;TOP2B;TP53;TUBB;TUBGCP4;TUBGCP5;TUBGCP6;UBE2B;UBE2I;UBE2L3;YWHAE;ZC3H12D;ZNF318;ZV
GMEB2;GON4L;GOSR2;GRM2;GTF2A2;GTPBP4;HABP4;HCFC1;HDAC1;HEXIM1;HGF;HHEX;HIC2;HIF1AN;HMG20A;HMGA1;HNRNPA2B1;HNRNPAB;HNRNPH1;HSBP1;HSP90B1;HSPA8;HTATIP2;HTATSF1;HYAL2;HYOU1;ID1;IKBKAP;IKBKB

IA;FOSB;FOXC1;FOXI1;FOXJ1;FOXJ3;FOXK1;FOXK2;FOXM1;FOXP1;FOXP4;FUBP1;FXN;GABBR1;GABPB2;GARS;GART;GATA3;GGT7;GIPR;GMEB2;GMPS;GON4L;GOT1;GRM2;GTF2A2;GTF3C1;GTF3C2;GTF3C4;HABP4;HCFC1;HDAC

UT2;FYN;FZR1;GABARAPL2;GALT;GFM1;GK5;GLUD1;GNG10;GNL2;GNL3L;GNPDA1;GOT1;GOT2;GPC2;GPT;GPT2;GRIN2C;GSTM4;GSTO1;GTPBP4;H6PD;HACL1;HADH;HADHA;HADHB;HECTD1;HERC2;HKDC1;HPD;HRASLS2;HSP90AB1;HSP

JC1;DNMT1;DNMT3A;DOCK7;DRD4;DUSP16;DUSP7;DVL2;DYX1C1;ECD;EDEM1;EEF2K;EHD4;EHMT1;EIF2AK3;EIF2AK4;EIF2B1;EIF3B;EIF4A3;EIF4B;EIF4G1;EIF4G2;EIF5A;EIF5B;ELAVL1;ELP3;EPHA1;EPHA2;EPHX2;EPRS;ERBB

3P1;HSPA8;HTATIP2;HTATSF1;HYAL2;ID1;IKBKAP;IKBKB;IKZF3;IL10;IL16;IL26;IL31RA;IL4;ILF2;ILF3;ING1;ING2;ING5;INO80;IRAK1;IRF8;ITGA6;IWS1;JAZF1;KANK1;KAT6B;KCTD1;KDM1A;KDM2B;KDM4A;KDM4C;KDM5

FR1;FLNA;FOSB;FOXC1;FOXI1;FOXJ1;FOXJ3;FOXK1;FOXK2;FOXM1;FOXP1;FOXP4;FUBP1;FXN;GABBR1;GABPB2;GARS;GART;GATA3;GIPR;GMEB2;GMPS;GON4L;GPHN;GRM2;GTF2A2;GTF3C1;GTF3C2;GTF3C4;HABP4;HCFC1;HDA

FOXK2;FOXM1;FOXP1;FOXP4;FUBP1;FXN;GABBR1;GABPB2;GARS;GART;GATA3;GIPR;GMEB2;GMPS;GON4L;GRM2;GTF2A2;GTF3C1;GTF3C2;GTF3C4;HABP4;HCFC1;HDAC1;HELB;HEXIM1;HGF;HHEX;HIC2;HIF1AN;HMG20A;HMG

LG1;GMEB2;GON4L;GTF2A2;GTPBP4;HABP4;HCFC1;HDAC1;HEMK1;HEXIM1;HGF;HHEX;HIC2;HIF1AN;HMG20A;HMGA1;HNRNPA0;HNRNPA2B1;HNRNPAB;HNRNPH1;HNRNPU;HPS4;HSBP1;HSPA8;HSPD1;HTATIP2;HTATSF1;

NP2;CSTB;CTC1;CTCF;CTR9;CUL1;CUL4A;CUL7;CXCL1;CYP2D6;CYTL1;DCTN1;DDIT3;DDIT4;DDX17;DDX20;DHCR24;DHCR7;DHX33;DHX9;DLD;DLG3;DNAJA3;DNAJC1;DNMT1;DNMT3A;DOCK7;DRD4;DROSHA;DUSP16;DUSP7;DVL2;DYX1C

GMEB2;GON4L;GOSR2;GRM2;GTF2A2;GTPBP4;HABP4;HCFC1;HDAC1;HEXIM1;HGF;HHEX;HIC2;HIF1AN;HMG20A;HMGA1;HNRNPA2B1;HNRNPAB;HNRNPH1;HSBP1;HSP90AB1;HSP90B1;HSPA8;HTATIP2;HTATSF1;HYAL2;HYOU1;ID1;IKBK

I4;GSTO1;GTPBP4;HACL1;HADH;HADHA;HADHB;HECTD1;HERC2;HPD;HSP90AB1;HSP90B1;HSPD1;HTRA2;HUWE1;HYAL1;IBA57;IL10;INO80;IRGM;IRS1;IVD;KCTD10;KCTD13;KIAA0226;KIAA0368;KIF16B;KYNU;LARP1;LONP2;LPIN1;L

BP1;GABBR1;GABPB2;GARS;GART;GATA3;GIPR;GMEB2;GMPS;GON4L;GRM2;GTF2A2;GTF3C1;GTF3C2;GTF3C4;HABP4;HCFC1;HDAC1;HELB;HEXIM1;HGF;HHEX;HIC2;HIF1AN;HMG20A;HMGA1;HNRNPAB;HOMEZ;HOXA9;HOXB2;H

2;SUFU;SUPT4H1;TAF3;TARDBP;TCF7L2;TERF2;TERF2IP;TFCP2L1;TGIF2;THRA;TIMELESS;TLE1;TNF;TP53;TSPYL2;TTF1;TXN;U2AF2;UBE2B;UBE2I;UHRF1;VHL;WHSC1;XRCC5;XRCC6;YEATS2;ZBED6;ZBTB16;ZBTB7A;ZHX2;ZHX3;ZMYND8;Z

NO80;INPPL1;IRAK1;ISCA2;ITGA6;ITPR3;KANK1;KAT6B;KCNA6;KCNC4;KCTD1;KCTD10;KCTD13;KCTD5;KIF3B;KNTC1;KRT5;LAS1L;LDB1;LPXN;LRRC16A;LRRC4B;LSG1;MACF1;MAPT;MBD2;MCM2;MDM4;MDN1;MECP2;MED24;

;WDR6;XPC;ZC3H12D;ZV

A3;DNAJC1;DNMT1;DNMT3A;DOCK7;DRD4;DUSP16;DUSP7;DVL2;DYX1C1;ECD;EDEM1;EEF2K;EHD4;EHMT1;EIF2AK3;EIF2AK4;EIF2B1;EIF3B;EIF4A3;EIF4B;EIF4G1;EIF4G2;EIF5A;EIF5B;ELAVL1;ELFN2;ELP3;EPHA1;EPHA2;EPR

AB;HSBP1;HSPA8;HTATIP2;HTATSF1;HYAL2;ID1;IKBKAP;IKBKB;IKZF3;IL10;IL16;IL26;IL31RA;IL4;ILF2;ILF3;ING1;ING2;ING5;INO80;INPPL1;IRAK1;IRF8;IRS1;ITGA6;JAZF1;KANK1;KAT6B;KCTD1;KCTD13;KI

PC2;GPT;GPT2;GRIN2C;GTPBP4;H6PD;HACL1;HADH;HADHA;HADHB;HECTD1;HERC2;HKDC1;HPD;HRASLS2;HSP90AB1;HSP90B1;HSPD1;HSPG2;HTRA2;HUWE1;HYAL1;HYAL2;IBA57;IL10;INO80;INPP5E;IRS1;IVD;KCTD10;KCTD13;KIAA

20A;HMGA1;HNRNPAB;HSBP1;HSPA8;HSPH1;HTATIP2;HTATSF1;HYAL2;ID1;IKBKAP;IKBKB;IKZF3;IL10;IL16;IL18;IL26;IL27;IL31RA;IL4;ILF2;ILF3;ING1;ING2;ING5;INO80;INPPL1;IRAK1;IRF8;IRS1;ITGA

REBF2;SUFU;SUPT4H1;TAF3;TARDBP;TCF7L2;TERF2;TERF2IP;TFCP2L1;TGIF2;THRA;TIMELESS;TLE1;TNF;TP53;TSPYL2;TTF1;TXN;U2AF2;UBE2B;UBE2I;UHRF1;VHL;WHSC1;XRCC5;XRCC6;YEATS2;ZBED6;ZBTB16;ZBTB7A;ZHX2;ZHX3;ZMY

;MAP2K6;MAP2K7;MAP3K9;MAPK7;MAPK9;MAPT;MCF2L;MDM4;MECP2;MKL1;MLH1;MLL;MNT;MSH6;MUL1;MYBBP1A;MYC;MYO18A;NACC1;NCKAP1L;NDUFS1;NET1;NISCH;NLRP1;NME6;NMT1;NOD1;NODAL;NRBP2;NRG1;N

164;CEP72;CHAD;CHCHD3;CHD1L;CHD3;CHD4;CHD6;CHEK2;CHMP4C;CHMP5;CHMP7;CIAO1;CIRBP;CIT;CKAP5;CLCN3;CLDN15;CLDN20;CLN8;CNTNAP1;COG1;COG2;COL17A1;COL27A1;COL5A1;COL6A3;CORO2A;COX10;COX15;CROCC;CRTC

AGED1;MAP2K6;MAP2K7;MAP3K9;MAPK7;MAPK9;MAPT;MCF2L;MDM4;MECP2;MKL1;MLH1;MLL;MNT;MSH6;MUL1;MYBBP1A;MYC;MYO18A;NACC1;NCKAP1L;NDUFS1;NET1;NISCH;NLRP1;NME6;NMT1;NOD1;NODAL;NRBP2;

7L2;TERF2;TFCP2L1;TGIF2;THRA;TIMELESS;TLE1;TNF;TNRC6A;TP53;TSC1;TSPYL2;TTF1;TXN;UBE2B;UBE2I;UHRF1;VHL;WHSC1;XRCC5;XRCC6;YEATS2;ZBED6;ZBTB16;ZBTB7A;ZHX2;ZHX3;ZMYND8;ZNF263;ZNF496;Z

M2C;JTB;KALRN;KDM1A;KDM2B;KIAA0141;KITLG;KLF11;LCK;LEF1;LGMN;LHX4;MADD;MAGED1;MAP2K6;MAP2K7;MAP3K9;MAPK7;MAPK9;MAPT;MCF2L;MDM4;MECP2;MKL1;MLH1;MLL;MNT;MSH6;MTPAP;MUL1;MYBB

;MBOAT4;MED24;METTL13;METTL16;METTL2A;METTL2B;METTL7A;METTL7B;MGAT5;MGST1;MINK1;MLL;MLL4;MPP2;MTOR;MTPAP;MTR;MYBBP1A;NAA40;NAT10;NAT8L;NDUFA10;NEK9;NFS1;NME6;NMT1;NMT2;NOL9;NSUN2;NSUN4;

M2C;JTB;KALRN;KDM1A;KDM2B;KIAA0141;KITLG;KLF11;LCK;LEF1;LGMN;LHX4;MADD;MAGED1;MAP2K6;MAP2K7;MAP3K9;MAPK7;MAPK9;MAPT;MCF2L;MDM4;MECP2;MKL1;MLH1;MLL;MNT;MSH6;MTPAP;MUL1;MYBB

SUFU;SUPT4H1;TAF3;TARDBP;TCF7L2;TERF2;TFCP2L1;TGIF2;THRA;TIMELESS;TLE1;TNF;TNRC6A;TP53;TSC1;TSPYL2;TTF1;TXN;UBE2B;UBE2I;UHRF1;VHL;WHSC1;XRCC5;XRCC6;YEATS2;ZBED6;ZBTB16;ZBTB7A;ZHX2;ZHX3;ZMYI

32;HSBP1;HSPA8;HTATIP2;HTATSF1;HYAL2;ID1;IKBKAP;IKBKB;IKZF3;IL10;IL16;IL26;IL31RA;IL4;ILF2;ILF3;ING1;ING2;ING5;INO80;IRAK1;IRF8;ITGA6;IWS1;JAZF1;KANK1;KAT6B;KCTD1;KDM1A;KDM2B;KDM4A;KDM4C

;SHPK;SIK1;SIK2;SRPK3;STK16;STK36;STK38;STK39;TAF1;TAOK2;TEP1;TNIK;TNK2;TRIO;TRRAP;TSSK3;TSSK4;TXK;TYK2;UCK2;ULK2;ZCCH
MD1;PSMD11;PSMD2;PSMD6;PSMD7;PSME4;PTK2;RAB3GAP1;RABGAP1;RACGAP1;RAP1GDS1;RAPGEF1;RASA3;RASA4;RASAL3;RELA;RFC1;RGS13;RGS17;RYR2;S100A12;S100A8;S100A9;SAE1;SCRIB;SERINC5;SGK1;SHC1;SLX4;SMAD3;SMA

NG5;INO80;IRAK1;IRF8;ITGA6;JAZF1;KANK1;KAT6B;KCTD1;KDM1A;KDM2B;KDM4A;KDM4C;KDM5C;KHDRBS1;KHSRP;KLF11;KLHL31;KRBA2;L3MBTL1;L3MBTL2;LANCL2;LBH;LDB1;LEF1;LEO1;LMO2;LPIN1;LPXN;LRP5

C;POLR2J;PPARGC1B;PPP2R5C;PRPF19;PSMC4;PSMD1;PSMD11;PSMD2;PSMD6;PSMD7;PSME4;PTK2;RAD18;RAD51D;RBBP7;RBM14;RFC1;RFWD3;RHBDD1;RNF8;RPA1;RPA2;RPS6KA2;RUVBL1;SCAMP5;SEMA4C;SFPQ;SFRP5;SGK1;SHC1;SL
RM2;GTF2A2;GTPBP4;HABP4;HCFC1;HDAC1;HEXIM1;HGF;HHEX;HIC2;HIF1AN;HMG20A;HMGA1;HNRNPAB;HSBP1;HSP90AB1;HSPA8;HSPH1;HTATIP2;HTATSF1;HYAL2;ID1;IKBKAP;IKBKB;IKZF3;IL10;IL16;IL18;IL

;PARP1;PASK;PATZ1;PDCD4;PDPK1;PER1;PLAT;PPARA;PPARD;PPARGC1B;PRKCA;PRMT2;PSMC4;PSMD1;PSMD11;PSMD2;PSMD6;PSMD7;PTBP1;RAD18;RAMP3;RBBP7;RBFOX2;RELA;RFX5;RNF8;RREB1;RUNX2;RUNX3;SALL2;SAP30;SAT
F2;UBE2B;UBE2I;UHRF1;VHL;WHSC1;XRCC5;XRCC6;YEATS2;ZBED6;ZBTB16;ZBTB7A;ZHX2;ZHX3;ZMYND8;ZNF263;ZNF496;Z

LA;SNW1;SNX6;SOX10;SOX18;SREBF2;SUFU;SUPT4H1;TAF3;TARDBP;TCF7L2;TERF2;TFCP2L1;TGIF2;THRA;TIMELESS;TLE1;TNF;TNRC6A;TP53;TSC1;TSPYL2;TTF1;TXN;UBE2B;UBE2I;UHRF1;VHL;WHSC1;XRCC5;XRCC6;YEATS2;ZBE

;TNF;TNRC6A;TNRC6B;TNRC6C;TP53;TXN;UBE2B;UBE2I;UHRF1;VHL;WHSC1;XPO5;XRCC5;XRCC6;YEATS2;ZBED6;ZBTB16;ZBTB7A;ZCCHC11;ZHX2;ZHX3;ZMYND8;ZNF263;ZNF496;Z
A2;GTPBP4;HABP4;HCFC1;HDAC1;HEXIM1;HGF;HHEX;HIC2;HIF1AN;HMG20A;HMGA1;HNRNPAB;HSBP1;HSP90AB1;HSPA8;HTATIP2;HTATSF1;HYAL2;ID1;IKBKAP;IKBKB;IKZF3;IL10;IL16;IL26;IL27;IL31RA;IL4;I

NAB2;NACC1;NCOR2;NF2;NFX1;NKAP;NODAL;NR1D1;NRBP2;NRG1;NRIP2;NXN;PA2G4;PABPC1;PARP1;PASK;PATZ1;PDCD4;PDPK1;PER1;PIK3IP1;PLAT;PPARA;PPARD;PPARGC1B;PRKCA;PRMT2;PSMC4;PSMD1;PSMD11;PSMD2;PSMD6;PS
KANK1;KAT6B;KCTD1;KDM1A;KDM2B;KDM4A;KDM4C;KDM5C;KHDRBS1;KHSRP;KLF11;KLHL31;KRBA2;L3MBTL1;L3MBTL2;LANCL2;LBH;LDB1;LEF1;LEO1;LMO2;LPIN1;LPXN;LRP5;LRP8;LZTR1;MAD2L2;MAGED1;MAM
YEATS2;ZBED6;ZBTB16;ZBTB7A;ZHX2;ZHX3;ZMYND8;ZNF263;ZNF496;Z
1;SMC1A;SNW1;SNX6;SOX10;SOX18;SREBF2;SUFU;SUPT4H1;TAF3;TARDBP;TCF7L2;TERF2;TFCP2L1;TGIF2;THRA;TIMELESS;TLE1;TNF;TNRC6A;TP53;TSC1;TSPYL2;TTF1;TXN;UBE2B;UBE2I;UHRF1;VHL;WHSC1;XRCC5;XRCC6;YEAT

1;KY;LANCL2;LCK;LDLRAP1;LMOD3;LPXN;LZTS2;MACF1;MAD2L1BP;MAD2L2;MAP1A;MAP1LC3B2;MAP2K6;MAP4;MAP7D3;MAPRE2;MAPT;MCM3;MID2;MINK1;MMS19;MNS1;MPRIP;MYC;MYCBP;MYCBP2;MYH3;MYH7B;MYO

;NCAPG;NEFH;NEIL2;NEURL4;NF2;NICN1;NINL;NPHP4;NSUN2;NUMA1;NUP62;ODF2;PCIF1;PCM1;PCNT;PDE4DIP;PEA15;PINX1;PLEKHA7;PNN;POLB;PRC1;PRPF19;PTK2;RAB11FIP3;RAB3GAP1;RABGAP1;RACGAP1;RANBP10;RCC2;RP1L1;

KANK1;KAT6B;KCTD1;KDM1A;KDM2B;KDM4A;KDM4C;KDM5C;KHDRBS1;KHSRP;KLF11;KLHL31;KRBA2;L3MBTL1;L3MBTL2;LANCL2;LBH;LDB1;LEF1;LEO1;LMO2;LPIN1;LPXN;LRP5;LRP8;LZTR1;MAD2L2;MAGED1;MAM

PARP1;PAXIP1;PER1;PHRF1;PKD1;POLA1;POLR2B;POLR2C;POLR2J;PPARA;PPARD;PPARGC1B;PRDM4;RAI1;RBBP7;RBM14;RELA;RFX5;RGMB;RNF4;RNMT;RNPS1;RPRD1B;RREB1;RUNX1;RUNX2;RUNX3;RUVBL1;RYR2;SALL2;SAP30;S
7;RYR2;S100A12;S100A8;S100A9;SAE1;SCRIB;SERINC5;SHC1;SLX4;SMAD3;SNX9;SYDE1;SYNGAP1;SYVN1;TAOK2;TATDN2;TAX1BP3;TBC1D7;TNF;TNFRSF10A;TNFRSF11A;TNIK;TSC1;TSPYL2;TXK;UHRF1;VANGL2;VAV2;VCP;X

GOSR2;GOT2;GPR89B;GPRC5C;GRAMD4;GSTM4;GTPBP4;GUCY2D;HADHA;HADHB;HERC2;HHAT;HLA-DRB1;HSP90B1;HSPA8;HSPD1;HTRA2;HTT;HYAL2;ICMT;IKBIP;IKBKE;IMMT;INPP5B;INPP5E;INTS1;IRAK1;IRGM;ITM2C;ITPR3;JPH3;KD[illegible]

2A;TPRN;TRADD;UACA;UCN;UGP2;USF2;USP33;UTF1;VAPB;WNT6;YIF1A;ZDHHC1;ZDHHC24;ZFPM1;ZNF219;ZNF35;ZNF358;ZNF414;ZNF428;ZNF444;ZNF564;ZNF575;ZNF579;ZNF628;ZNF638;ZNF668;ZNF747;ZNF764;ZNF771;ZN

CA
