# Supplementary material for: Meditation and vacation effects have an impact on disease-associated molecular phenotypes
Source: Transl Psychiatry. 2016 Aug 30;6(8):e880–. doi: 10.1038/tp.2016.164 (PMC5022094; doi:10.1038/tp.2016.164)
Supplement: Supplementary Table 6 [file tp2016164x5.pdf]

Supplementary Table 6. Gene sets from the MsigDB database enriched for the coexpression network modules identified in Supplementary Table 4.

module	set_idx	set_description	overlap	red balls	black balls	sample siz	fold_enricl	pv
blue	8430	GSE10325_LUPUS_CD4_TCELL_VS_LUPUS_MYELOID_U	88	195	16058	2978	2.462957	0
blue	9362	GSE22886_NAIVE_TCELL_VS_MONOCYTE_UP	88	196	16057	2978	2.450391	0
brown	8431	GSE10325_LUPUS_CD4_TCELL_VS_LUPUS_MYELOID_DI	88	200	16053	2190	3.265443	0
brown	8433	GSE10325_LUPUS_BCELL_VS_LUPUS_MYELOID_DN	91	200	16053	2190	3.376765	0
brown	9375	GSE22886_NAIVE_CD8_TCELL_VS_MONOCYTE_DN	81	199	16054	2190	3.020796	0
brown	9655	GSE29618_BCELL_VS_MONOCYTE_DN	79	199	16054	2190	2.946208	0
brown	9667	GSE29618_BCELL_VS_MONOCYTE_DAY7_FLU_VACCINE	79	198	16055	2190	2.961088	0
brown	9672	GSE29618_MONOCYTE_VS_PDC_DAY7_FLU_VACCINE_	76	200	16053	2190	2.820155	0
brown	9674	GSE29618_MONOCYTE_VS_MDC_DAY7_FLU_VACCINE_	75	200	16053	2190	2.783048	0
cyan	6178	SANA_RESPONSE_TO_IFNG_UP	18	70	16183	77	54.27718	0
cyan	8573	GSE13484_UNSTIM_VS_YF17D_VACCINE_STIM_PBMC_	23	190	16063	77	25.55154	0
cyan	8579	GSE13485_CTRL_VS_DAY7_YF17D_VACCINE_PBMC_DN	24	198	16055	77	25.5852	0
cyan	8585	GSE13485_DAY1_VS_DAY7_YF17D_VACCINE_PBMC_DI	17	194	16059	77	18.49652	0
cyan	8595	GSE13485_PRE_VS_POST_YF17D_VACCINATION_PBMC	17	195	16058	77	18.40167	0
cyan	8619	GSE14000_UNSTIM_VS_4H_LPS_DC_DN	20	193	16060	77	21.87336	0
cyan	8661	GSE1432_CTRL_VS_IFNG_6H_MICROGLIA_DN	18	197	16056	77	19.28631	0
cyan	8663	GSE1432_CTRL_VS_IFNG_24H_MICROGLIA_DN	20	192	16061	77	21.98728	0
cyan	9225	GSE18791_CTRL_VS_NEWCASTLE_VIRUS_DC_6H_DN	18	197	16056	77	19.28631	0
cyan	9227	GSE18791_CTRL_VS_NEWCASTLE_VIRUS_DC_8H_DN	21	180	16073	77	24.62576	0
cyan	9229	GSE18791_CTRL_VS_NEWCASTLE_VIRUS_DC_10H_DN	17	183	16070	77	19.60833	0
cyan	9243	GSE18791_UNSTIM_VS_NEWCATSLE_VIRUS_DC_6H_DI	25	186	16067	77	28.37069	0
cyan	9245	GSE18791_UNSTIM_VS_NEWCATSLE_VIRUS_DC_10H_I	20	180	16073	77	23.4531	0
cyan	9523	GSE2706_2H_VS_8H_R848_STIM_DC_DN	18	171	16082	77	22.21873	0
greenyello	2653	GNF2_IL2RB	16	44	16209	150	39.40121	0
greenyello	2684	GNF2_PTPN4	14	49	16204	150	30.9581	0
greenyello	5151	HAHTOLA_SEZARY_SYNDROM_DN	13	38	16215	150	37.06825	0
greenyello	9473	GSE26495_NAIVE_VS_PD1HIGH_CD8_TCELL_DN	40	197	16056	150	22.00068	0
greenyello	9475	GSE26495_NAIVE_VS_PD1LOW_CD8_TCELL_DN	42	198	16055	150	22.98404	0
greenyello	9786	GSE34205_HEALTHY_VS_RSV_INF_INFANT_PBMC_UP	22	193	16060	150	12.35116	0
lightcyan	3654	MODULE_171	14	122	16131	62	30.08223	0
lightcyan	4650	REACTOME_INTERFERON_ALPHA_BETA_SIGNALING	14	49	16204	62	74.89862	0
lightcyan	4656	REACTOME_INTERFERON_SIGNALING	18	137	16116	62	34.44243	0
lightcyan	4747	REACTOME_CYTOKINE_SIGNALING_IN_IMMUNE_SYSTE	18	238	16015	62	19.8261	0
lightcyan	4891	FULCHER_INFLAMMATORY_RESPONSE_LECTIN_VS_LPS	28	437	15816	62	16.79649	0
lightcyan	5010	TAKEDA_TARGETS_OF_NUP98_HOXA9_FUSION_3D_UF	31	162	16091	62	50.16358	0
lightcyan	5012	TAKEDA_TARGETS_OF_NUP98_HOXA9_FUSION_8D_UF	24	138	16115	62	45.59046	0
lightcyan	5014	TAKEDA_TARGETS_OF_NUP98_HOXA9_FUSION_10D_U	27	178	16075	62	39.76359	0
lightcyan	5016	TAKEDA_TARGETS_OF_NUP98_HOXA9_FUSION_16D_U	18	162	16091	62	29.12724	0
lightcyan	5221	GRAESSMANN_APOPTOSIS_BY_SERUM_DEPRIVATION_	25	475	15778	62	13.79711	0
lightcyan	5235	GRAESSMANN_RESPONSE_TO_MC_AND_SERUM_DEPR	19	177	16076	62	28.13988	0
lightcyan	5510	FARMER_BREAST_CANCER_CLUSTER_1	15	41	16212	62	95.90677	0
lightcyan	5531	BOWIE_RESPONSE_TO_TAMOXIFEN	9	18	16235	62	131.0726	0
lightcyan	5552	XU_AKT1_TARGETS_6HR	10	25	16228	62	104.8581	0
lightcyan	5561	EINAV_INTERFERON_SIGNATURE_IN_CANCER	12	27	16226	62	116.509	0
lightcyan	5569	DAUER_STAT3_TARGETS_DN	22	50	16203	62	115.3439	0
lightcyan	5586	SEITZ_NEOPLASTIC_TRANSFORMATION_BY_8P_DELETI	15	59	16194	62	66.64707	0
lightcyan	5782	NUYTTEN_NIPP1_TARGETS_UP	29	683	15570	62	11.13061	0
lightcyan	5784	NUYTTEN_EZH2_TARGETS_UP	29	946	15307	62	8.036162	0
lightcyan	6099	DER_IFN_ALPHA_RESPONSE_UP	12	72	16181	62	43.69086	0
lightcyan	6128	RADAEVA_RESPONSE_TO_IFNA1_UP	14	51	16202	62	71.96142	0
lightcyan	6147	DER_IFN_BETA_RESPONSE_UP	15	101	16152	62	38.93245	0
lightcyan	6179	SANA_TNF_SIGNALING_UP	12	73	16180	62	43.09236	0
lightcyan	6322	ROETH_TERT_TARGETS_UP	9	14	16239	62	168.5219	0
lightcyan	6341	BENNETT_SYSTEMIC_LUPUS_ERYTHEMATOSUS	15	29	16224	62	135.5923	0
lightcyan	6393	LIANG_SILENCED_BY_METHYLATION_2	11	44	16209	62	65.53629	0

lightcyan	6428	DEBIASI_APOPTOSIS_BY_REOVIRUS_INFECTION_UP	19	293	15960	62	16.99917	0
lightcyan	6508	BROWNE_INTERFERON_RESPONSIVE_GENES	19	68	16185	62	73.24644	0
lightcyan	7082	MOSERLE_IFNA_RESPONSE	22	31	16222	62	186.0385	0
lightcyan	7326	ZHANG_INTERFERON_RESPONSE	10	22	16231	62	119.1569	0
lightcyan	7843	STAMBOLSKY_TARGETS_OF_MUTATED_TP53_DN	12	45	16208	62	69.90538	0
lightcyan	8130	BOSCO_INTERFERON_INDUCED_ANTIVIRAL_MODULE	28	76	16177	62	96.5798	0
lightcyan	8257	LTE2_UP.V1_DN	25	176	16077	62	37.23653	0
lightcyan	8259	MEK_UP.V1_DN	18	173	16080	62	27.27522	0
lightcyan	8385	HECKER_IFNB1_TARGETS	34	93	16160	62	95.83802	0
lightcyan	8435	GSE10325_CD4_TCELL_VS_LUPUS_CD4_TCELL_DN	31	184	16069	62	44.16576	0
lightcyan	8437	GSE10325_BCELL_VS_LUPUS_BCELL_DN	21	199	16054	62	27.66356	0
lightcyan	8439	GSE10325_MYELOID_VS_LUPUS_MYELOID_DN	29	198	16055	62	38.395	0
lightcyan	8573	GSE13484_UNSTIM_VS_YF17D_VACCINE_STIM_PBMC_	40	190	16063	62	55.18846	0
lightcyan	8577	GSE13485_CTRL_VS_DAY3_YF17D_VACCINE_PBMC_DN	43	193	16060	62	58.4054	0
lightcyan	8579	GSE13485_CTRL_VS_DAY7_YF17D_VACCINE_PBMC_DN	50	198	16055	62	66.19827	0
lightcyan	8583	GSE13485_DAY1_VS_DAY3_YF17D_VACCINE_PBMC_DI	24	194	16059	62	32.43033	0
lightcyan	8585	GSE13485_DAY1_VS_DAY7_YF17D_VACCINE_PBMC_DI	45	194	16059	62	60.80687	0
lightcyan	8589	GSE13485_DAY3_VS_DAY7_YF17D_VACCINE_PBMC_DI	49	180	16073	62	71.36174	0
lightcyan	8592	GSE13485_DAY7_VS_DAY21_YF17D_VACCINE_PBMC_U	38	196	16057	62	50.82406	0
lightcyan	8595	GSE13485_PRE_VS_POST_YF17D_VACCINATION_PBMC	42	195	16058	62	56.46203	0
lightcyan	8615	GSE14000_UNSTIM_VS_4H_LPS_DC_TRANSLATED_RNA	26	178	16075	62	38.29087	0
lightcyan	8617	GSE14000_UNSTIM_VS_16H_LPS_DC_TRANSLATED_RN	21	189	16064	62	29.12724	0
lightcyan	8619	GSE14000_UNSTIM_VS_4H_LPS_DC_DN	28	193	16060	62	38.03142	0
lightcyan	8663	GSE1432_CTRL_VS_IFNG_24H_MICROGLIA_DN	18	192	16061	62	24.57611	0
lightcyan	9199	GSE17974_IL4_AND_ANTI_IL12_VS_UNTREATED_24H_	24	161	16092	62	39.07754	0
lightcyan	9223	GSE18791_CTRL_VS_NEWCASTLE_VIRUS_DC_4H_DN	32	169	16084	62	49.63695	0
lightcyan	9225	GSE18791_CTRL_VS_NEWCASTLE_VIRUS_DC_6H_DN	36	197	16056	62	47.9047	0
lightcyan	9227	GSE18791_CTRL_VS_NEWCASTLE_VIRUS_DC_8H_DN	36	180	16073	62	52.42903	0
lightcyan	9229	GSE18791_CTRL_VS_NEWCASTLE_VIRUS_DC_10H_DN	26	183	16070	62	37.24467	0
lightcyan	9231	GSE18791_CTRL_VS_NEWCASTLE_VIRUS_DC_12H_DN	24	178	16075	62	35.34542	0
lightcyan	9233	GSE18791_CTRL_VS_NEWCASTLE_VIRUS_DC_14H_DN	18	159	16094	62	29.67681	0
lightcyan	9235	GSE18791_CTRL_VS_NEWCASTLE_VIRUS_DC_16H_DN	25	178	16075	62	36.81814	0
lightcyan	9243	GSE18791_UNSTIM_VS_NEWCATSLE_VIRUS_DC_6H_DI	37	186	16067	62	52.14716	0
lightcyan	9245	GSE18791_UNSTIM_VS_NEWCATSLE_VIRUS_DC_10H_I	33	180	16073	62	48.05995	0
lightcyan	9247	GSE18791_UNSTIM_VS_NEWCATSLE_VIRUS_DC_18H_I	27	159	16094	62	44.51522	0
lightcyan	9389	GSE22886_CTRL_VS_LPS_24H_DC_DN	28	195	16058	62	37.64136	0
lightcyan	9459	GSE24634_IL4_VS_CTRL_TREATED_NAIVE_CD4_TCELL_	19	187	16066	62	26.63507	0
lightcyan	9501	GSE2706_UNSTIM_VS_8H_R848_DC_DN	20	183	16070	62	28.64974	0
lightcyan	9515	GSE2706_R848_VS_R848_AND_LPS_2H_STIM_DC_DN	15	160	16093	62	24.57611	0
lightcyan	9523	GSE2706_2H_VS_8H_R848_STIM_DC_DN	15	171	16082	62	22.99519	0
lightcyan	9789	GSE34205_HEALTHY_VS_FLU_INF_INFANT_PBMC_DN	34	193	16060	62	46.18101	0
lightcyan	9791	GSE34205_RSV_VS_FLU_INF_INFANT_PBMC_DN	18	192	16061	62	24.57611	0
lightcyan	9957	GSE3982_CTRL_VS_LPS_48H_DC_DN	17	184	16069	62	24.21993	0
lightcyan	10127	GSE6269_HEALTHY_VS_FLU_INF_PBMC_DN	22	157	16096	62	36.73372	0
lightcyan	10134	GSE6269_FLU_VS_E_COLI_INF_PBMC_UP	23	162	16091	62	37.21814	0
lightcyan	10136	GSE6269_FLU_VS_STREP_AUREUS_INF_PBMC_UP	25	171	16082	62	38.32532	0
lightcyan	10138	GSE6269_FLU_VS_STREP_PNEUMO_INF_PBMC_UP	30	167	16086	62	47.09195	0
magenta	6110	KLEIN_PRIMARY_EFFUSION_LYMPHOMA_DN	16	57	16196	167	27.31884	0
magenta	6306	HADDAD_B_LYMPHOCYTE_PROGENITOR	27	267	15986	167	9.841687	0
magenta	8423	GSE10325_CD4_TCELL_VS_BCELL_DN	55	184	16069	167	29.09122	0
magenta	8426	GSE10325_BCELL_VS_MYELOID_UP	44	187	16066	167	22.89961	0
magenta	8429	GSE10325_LUPUS_CD4_TCELL_VS_LUPUS_BCELL_DN	48	186	16067	167	25.1157	0
magenta	8432	GSE10325_LUPUS_BCELL_VS_LUPUS_MYELOID_UP	32	188	16065	167	16.56568	0
magenta	9323	GSE22886_TCELL_VS_BCELL_NAIVE_DN	42	172	16081	167	23.765	0
magenta	9325	GSE22886_CD8_TCELL_VS_BCELL_NAIVE_DN	33	172	16081	167	18.6725	0
magenta	9327	GSE22886_CD4_TCELL_VS_BCELL_NAIVE_DN	30	173	16080	167	16.87688	0
magenta	9364	GSE22886_NAIVE_BCELL_VS_NEUTROPHIL_UP	28	193	16060	167	14.11945	0
magenta	9366	GSE22886_NAIVE_BCELL_VS_DC_UP	24	180	16073	167	12.97645	0
magenta	9368	GSE22886_NAIVE_BCELL_VS_MONOCYTE_UP	43	168	16085	167	24.91014	0
magenta	9654	GSE29618_BCELL_VS_MONOCYTE_UP	46	182	16071	167	24.59821	0

magenta	9656	GSE29618_BCELL_VS_PDC_UP	28	187	16066	167	14.57248	0
magenta	9658	GSE29618_BCELL_VS_MDC_UP	37	184	16069	167	19.57046	0
magenta	9666	GSE29618_BCELL_VS_MONOCYTE_DAY7_FLU_VACCINE	45	184	16069	167	23.80191	0
magenta	9668	GSE29618_BCELL_VS_PDC_DAY7_FLU_VACCINE_UP	26	183	16070	167	13.82736	0
magenta	9670	GSE29618_BCELL_VS_MDC_DAY7_FLU_VACCINE_UP	41	182	16071	167	21.92449	0
magenta	9963	GSE3982_MEMORY_CD4_TCELL_VS_BCELL_DN	33	191	16062	167	16.81503	0
magenta	10064	GSE3982_BCELL_VS_CENT_MEMORY_CD4_TCELL_UP	22	191	16062	167	11.21002	0
magenta	10066	GSE3982_BCELL_VS_NKCELL_UP	26	192	16061	167	13.1792	0
magenta	10145	GSE6269_STREP_AUREUS_VS_STREP_PNEUMO_INF_PE	22	161	16092	167	13.29884	0
pink	2574	GNF2_BNIP3L	29	67	16186	228	30.85474	0
pink	2596	GNF2_PRDX2	16	31	16222	228	36.7923	0
pink	2598	GNF2_RAD23A	30	68	16185	228	31.4493	0
pink	2610	GNF2_ANK1	35	73	16180	228	34.17778	0
pink	2625	GNF2_CDC27	26	59	16194	228	31.41377	0
pink	2662	GNF2_MAP2K3	33	74	16179	228	31.7893	0
pink	2681	GNF2_PCAF	18	35	16218	228	36.6609	0
pink	2702	GNF2_SPTA1	39	80	16173	228	34.75148	0
pink	2703	GNF2_SPTB	35	73	16180	228	34.17778	0
pink	2706	GNF2_TAL1	34	72	16181	228	33.6624	0
pink	5115	JAATINEN_HEMATOPOIETIC_STEM_CELL_DN	29	223	16030	228	9.270258	0
pink	7484	VALK_AML_CLUSTER_7	17	27	16226	228	44.8832	0
pink	7485	VALK_AML_CLUSTER_8	18	26	16227	228	49.35121	0
pink	9787	GSE34205_HEALTHY_VS_RSV_INF_INFANT_PBMC_DN	46	194	16059	228	16.90265	0
pink	9790	GSE34205_RSV_VS_FLU_INF_INFANT_PBMC_UP	60	155	16098	228	27.59423	0
purple	4693	REACTOME_HEMOSTASIS	29	394	15859	157	7.619661	0
purple	7825	RAGHAVACHARI_PLATELET_SPECIFIC_GENES	23	70	16183	157	34.01447	0
purple	7913	WIERENGA_STAT5A_TARGETS_DN	32	205	16048	157	16.15958	0
purple	10224	GSE9006_HEALTHY_VS_TYPE_1_DIABETES_PBMC_AT_[	27	194	16059	157	14.40774	0
red	9359	GSE22886_NAIVE_TCELL_VS_NEUTROPHIL_DN	41	163	16090	524	7.801866	0
red	9365	GSE22886_NAIVE_BCELL_VS_NEUTROPHIL_DN	46	198	16055	524	7.20601	0
red	9660	GSE29618_MONOCYTE_VS_PDC_UP	39	198	16055	524	6.109444	0
red	9662	GSE29618_MONOCYTE_VS_MDC_UP	39	198	16055	524	6.109444	0
red	10050	GSE3982_NEUTROPHIL_VS_EFF_MEMORY_CD4_TCELL_	36	192	16061	524	5.81572	0
red	10052	GSE3982_NEUTROPHIL_VS_CENT_MEMORY_CD4_TCEL	38	195	16058	524	6.044373	0
red	10054	GSE3982_NEUTROPHIL_VS_NKCELL_UP	37	190	16063	524	6.040187	0
salmon	955	TRANSLATION	20	168	16085	127	15.23528	0
salmon	1197	STRUCTURAL_CONSTITUENT_OF_RIBOSOME	30	78	16175	127	49.22168	0
salmon	1239	RNA_BINDING	25	244	16009	127	13.11233	0
salmon	1310	STRUCTURAL_MOLECULE_ACTIVITY	31	191	16062	127	20.77104	0
salmon	2348	MORF_ACTG1	39	136	16117	127	36.69911	0
salmon	2349	MORF_ANP32B	21	195	16058	127	13.78207	0
salmon	2382	MORF_EIF3S6	21	119	16134	127	22.58407	0
salmon	2383	MORF_EIF4A2	19	137	16116	127	17.74855	0
salmon	2410	MORF_JUND	15	64	16189	127	29.99446	0
salmon	2425	MORF_NME2	30	152	16101	127	25.2585	0
salmon	2427	MORF_NPM1	42	160	16093	127	33.5938	0
salmon	2460	MORF_RAN	29	265	15988	127	14.00496	0
salmon	2476	MORF_TPT1	35	101	16152	127	44.34825	0
salmon	2479	MORF_UBE2I	26	235	16018	127	14.15909	0
salmon	2492	GCM_PSME1	16	85	16168	127	24.08967	0
salmon	2496	GCM_ACTG1	21	123	16130	127	21.84963	0
salmon	2510	GCM_CSNK2B	16	99	16154	127	20.68305	0
salmon	2538	GCM_NPM1	31	116	16137	127	34.20058	0
salmon	2561	GCM_TPT1	28	69	16184	127	51.93244	0
salmon	2633	GNF2_DAP3	28	118	16135	127	30.36728	0
salmon	2639	GNF2_EIF3S6	41	119	16134	127	44.0927	0
salmon	2641	GNF2_FBL	39	144	16109	127	34.66027	0
salmon	2677	GNF2_NPM1	19	70	16183	127	34.73645	0
salmon	2704	GNF2_ST13	29	63	16190	127	58.90976	0
salmon	2711	GNF2_TPT1	18	37	16216	127	62.25878	0

salmon	2789	KEGG_RIBOSOME	37	84	16169	127	56.37055	0
salmon	3521	MODULE_29	15	26	16227	127	73.83253	0
salmon	3522	MODULE_32	30	232	16021	127	16.54867	0
salmon	3571	MODULE_83	47	309	15944	127	19.46566	0
salmon	3602	MODULE_114	40	330	15923	127	15.51229	0
salmon	3636	MODULE_151	39	311	15942	127	16.04848	0
salmon	4122	REACTOME_TRANSLATION	40	144	16109	127	35.54899	0
salmon	4131	REACTOME_FORMATION_OF_THE_TERNARY_COMPLEX	20	47	16206	127	54.45803	0
salmon	4200	REACTOME_SRP_DEPENDENT_COTRANSLATIONAL_PRC	39	108	16145	127	46.21369	0
salmon	4316	REACTOME_ACTIVATION_OF_THE_MRNA_UPON_BIND	20	54	16199	127	47.39866	0
salmon	4361	REACTOME_PEPTIDE_CHAIN_ELONGATION	38	84	16169	127	57.89408	0
salmon	4429	REACTOME_METABOLISM_OF_PROTEINS	40	394	15859	127	12.99253	0
salmon	4437	REACTOME_3_UTR_MEDIATED_TRANSLATIONAL_REGU	39	103	16150	127	48.45708	0
salmon	4535	REACTOME_METABOLISM_OF_MRNA	40	208	16045	127	24.61084	0
salmon	4549	REACTOME_METABOLISM_OF_RNA	43	251	16002	127	21.92424	0
salmon	4694	REACTOME_INFLUENZA_LIFE_CYCLE	40	134	16119	127	38.2019	0
salmon	4695	REACTOME_INFLUENZA_VIRAL_RNA_TRANSCRIPTION_	39	100	16153	127	49.91079	0
salmon	4752	REACTOME_NONSENSE_MEDIATED_DECAY_ENHANCED	38	105	16148	127	46.31526	0
salmon	4982	OSMAN_BLADDER_CANCER_DN	30	391	15862	127	9.819159	0
salmon	7207	CHNG_MULTIPLE_MYELOMA_HYPERPLOID_UP	19	50	16203	127	48.63102	0
salmon	7433	HSIAO_HOUSEKEEPING_GENES	39	385	15868	127	12.96384	0
salmon	7690	DANG_MYC_TARGETS_UP	19	135	16118	127	18.01149	0
salmon	7840	MARTENS_TRETINOIN_RESPONSE_DN	42	786	15467	127	6.838432	0
salmon	7991	BILANGES_SERUM_AND_RAPAMYCIN_SENSITIVE_GENE	24	67	16186	127	45.84228	0
salmon	8133	PECE_MAMMARY_STEM_CELL_DN	22	131	16122	127	21.49222	0
salmon	8609	GSE14000_TRANSLATED_RNA_VS_MRNA_DC_DN	20	181	16072	127	14.14104	0
salmon	9364	GSE22886_NAIVE_BCELL_VS_NEUTROPHIL_UP	21	193	16060	127	13.92489	0
turquoise	4914	DIAZ_CHRONIC_MEYLOGENOUS_LEUKEMIA_UP	495	1378	14875	4169	1.400418	0
turquoise	5088	KINSEY_TARGETS_OF_EWSR1_FLII_FUSION_UP	437	1200	15053	4169	1.419717	0
turquoise	5142	RODRIGUES_THYROID_CARCINOMA_POORLY_DIFFEREN	282	616	15637	4169	1.78472	0
turquoise	5144	RODRIGUES_THYROID_CARCINOMA_ANAPLASTIC_UP	305	675	15578	4169	1.761561	0
turquoise	5306	HAMAI_APOPTOSIS_VIA_TRAIL_UP	281	552	15701	4169	1.984581	0
turquoise	5507	SCHLOSSER_SERUM_RESPONSE_DN	275	667	15586	4169	1.607343	0
turquoise	5946	GEORGES_TARGETS_OF_MIR192_AND_MIR215	342	843	15410	4169	1.581613	0
turquoise	5947	SHEN_SMARCA2_TARGETS_UP	212	420	15833	4169	1.967833	0
turquoise	7095	ZHANG_BREAST_CANCER_PROGENITORS_UP	184	413	15840	4169	1.736878	0
turquoise	7455	MILI_PSEUDOPODIA_HAPTOTAXIS_UP	292	505	15748	4169	2.254203	0
turquoise	7922	PILON_KLF1_TARGETS_DN	714	1952	14301	4169	1.426002	0
turquoise	7932	JOHNSTONE_PARVB_TARGETS_3_DN	422	882	15371	4169	1.865286	0
turquoise	8224	PIGF_UP.V1_UP	102	171	16082	4169	2.325443	0
brown	8427	GSE10325_BCELL_VS_MYELOID_DN	73	199	16054	2190	2.722446	1.11E-16
salmon	7499	JISON_SICKLE_CELL_DISEASE_DN	19	175	16078	127	13.89458	1.11E-16
yellow	6005	NIKOLSKY_BREAST_CANCER_16P13_AMPLICON	41	108	16145	1426	4.326873	1.11E-16
yellow	7869	LU_EZH2_TARGETS_UP	68	262	15991	1426	2.95816	1.11E-16
brown	10275	GSE9988_ANTI_TREM1_VS_CTRL_TREATED_MONOCYT	72	197	16056	2190	2.712412	2.22E-16
cyan	8615	GSE14000_UNSTIM_VS_4H_LPS_DC_TRANSLATED_RNA	16	178	16075	77	18.9733	2.22E-16
magenta	7066	SMID_BREAST_CANCER_NORMAL_LIKE_UP	29	407	15846	167	6.934588	2.22E-16
purple	4324	REACTOME_RESPONSE_TO_ELEVATED_PLATELET_CYTC	15	73	16180	157	21.2717	2.22E-16
red	9672	GSE29618_MONOCYTE_VS_PDC_DAY7_FLU_VACCINE_	35	200	16053	524	5.428006	2.22E-16
red	9977	GSE3982_EOSINOPHIL_VS_NEUTROPHIL_DN	33	178	16075	524	5.750375	2.22E-16
red	10131	GSE6269_HEALTHY_VS_STREP_AUREUS_INF_PBMC_DN	32	165	16088	524	6.015452	2.22E-16
salmon	2303	MORF_CCNI	15	87	16166	127	22.06489	2.22E-16
brown	7838	MARTENS_BOUND_BY_PML_RARA_FUSION	123	439	15814	2190	2.079362	3.33E-16
lightcyan	8823	GSE16755_CTRL_VS_IFNA_TREATED_MAC_DN	15	189	16064	62	20.80517	3.33E-16
salmon	2645	GNF2_GLTSCR2	11	31	16222	127	45.41097	3.33E-16
brown	9363	GSE22886_NAIVE_TCELL_VS_MONOCYTE_DN	72	200	16053	2190	2.671726	4.44E-16
salmon	2499	GCM_APEX1	16	113	16140	127	18.12055	4.44E-16
lightcyan	5530	BOWIE_RESPONSE_TO_EXTRACELLULAR_MATRIX	8	17	16236	62	123.3624	6.66E-16
lightcyan	8661	GSE1432_CTRL_VS_IFNG_6H_MICROGLIA_DN	15	197	16056	62	19.96029	6.66E-16
purple	4763	REACTOME_PLATELET_ACTIVATION_SIGNALING_AND_	20	180	16073	157	11.50248	6.66E-16

salmon	9360	GSE22886_NAIVE_TCELL_VS_DC_UP	19	193	16060	127	12.59871	6.66E-16
brown	10277	GSE9988_ANTI_TREM1_VS_VEHICLE_TREATED_MONO(	71	197	16056	2190	2.67474	7.77E-16
turquoise	8574	GSE13485_CTRL_VS_DAY1_YF17D_VACCINE_PBMC_UP	102	194	16059	4169	2.049746	7.77E-16
cyan	8667	GSE1432_1H_VS_24H_IFNG_MICROGLIA_DN	16	197	16056	77	17.14338	8.88E-16
brown	8425	GSE10325_CD4_TCELL_VS_MYELOID_DN	71	198	16055	2190	2.661231	1.11E-15
brown	9662	GSE29618_MONOCYTE_VS_MDC_UP	71	198	16055	2190	2.661231	1.11E-15
turquoise	4981	OSMAN_BLADDER_CANCER_UP	173	394	15859	4169	1.711794	1.33E-15
turquoise	4872	SENGUPTA_NASOPHARYNGEAL_CARCINOMA_WITH_LM	155	342	15911	4169	1.766881	1.55E-15
turquoise	6227	FLECHNER_BIOPSY_KIDNEY_TRANSPLANT_OK_VS_DON	217	526	15727	4169	1.608332	1.55E-15
brown	9381	GSE22886_NAIVE_CD4_TCELL_VS_MONOCYTE_DN	71	200	16053	2190	2.634619	1.89E-15
cyan	4656	REACTOME_INTERFERON_SIGNALING	14	137	16116	77	21.57001	2.44E-15
lightcyan	6518	KRASNOSELSKAYA_ILF3_TARGETS_UP	9	32	16221	62	73.72833	2.44E-15
lightcyan	6968	WALLACE_PROSTATE_CANCER_RACE_UP	16	267	15986	62	15.70907	2.44E-15
pink	6416	IVANOVA_HEMATOPOIESIS_MATURE_CELL	27	279	15974	228	6.898557	2.44E-15
lightcyan	8199	ERB2_UP.V1_DN	14	181	16072	62	20.27642	4.88E-15
cyan	8589	GSE13485_DAY3_VS_DAY7_YF17D_VACCINE_PBMC_DN	15	180	16073	77	17.58983	5.00E-15
lightcyan	9509	GSE2706_UNSTIM_VS_8H_LPS_AND_R848_DC_DN	14	182	16071	62	20.16501	5.33E-15
red	10225	GSE9006_HEALTHY_VS_TYPE_1_DIABETES_PBMC_AT_[	32	185	16068	524	5.365133	5.77E-15
lightcyan	4731	REACTOME_IMMUNE_SYSTEM	23	801	15452	62	7.527264	6.00E-15
red	10143	GSE6269_E_COLI_VS_STREP_PNEUMO_INF_PBMC_DN	30	162	16091	524	5.743921	6.22E-15
turquoise	7407	ZHANG_TLX_TARGETS_36HR_DN	96	183	16070	4169	2.045134	6.66E-15
cyan	8435	GSE10325_CD4_TCELL_VS_LUPUS_CD4_TCELL_DN	15	184	16069	77	17.20744	6.88E-15
red	9375	GSE22886_NAIVE_CD8_TCELL_VS_MONOCYTE_DN	33	199	16054	524	5.143552	7.77E-15
salmon	521	CELLULAR_BIOSYNTHETIC_PROCESS	21	289	15964	127	9.299322	8.22E-15
salmon	707	MACROMOLECULE_BIOSYNTHETIC_PROCESS	21	289	15964	127	9.299322	8.22E-15
turquoise	7903	GABRIELY_MIR21_TARGETS	130	276	15977	4169	1.836267	8.22E-15
cyan	5784	NUYTTEN_EZH2_TARGETS_UP	27	946	15307	77	6.024423	8.44E-15
turquoise	6428	DEBIASI_APOPTOSIS_BY_REOVIRUS_INFECTION_UP	136	293	15960	4169	1.80956	8.44E-15
red	9363	GSE22886_NAIVE_TCELL_VS_MONOCYTE_DN	33	200	16053	524	5.117834	9.10E-15
turquoise	7930	JOHNSTONE_PARVB_TARGETS_2_DN	146	322	15931	4169	1.76766	9.77E-15
lightcyan	7605	UROSEVIC_RESPONSE_TO_IMIQUIMOD	8	23	16230	62	91.18093	1.31E-14
lightcyan	6932	HELLER_SILENCED_BY_METHYLATION_UP	15	243	16010	62	16.1818	1.35E-14
lightcyan	8590	GSE13485_DAY3_VS_DAY21_YF17D_VACCINE_PBMC_U	14	195	16058	62	18.82068	1.40E-14
turquoise	7185	CHEN_HOXA5_TARGETS_9HR_UP	107	214	16039	4169	1.949268	1.40E-14
cyan	8577	GSE13485_CTRL_VS_DAY3_YF17D_VACCINE_PBMC_DN	15	193	16060	77	16.40502	1.41E-14
cyan	6233	DER_IFN_GAMMA_RESPONSE_UP	11	68	16185	77	34.14496	1.59E-14
cyan	6508	BROWNE_INTERFERON_RESPONSIVE_GENES	11	68	16185	77	34.14496	1.59E-14
salmon	7692	DANG_BOUND_BY_MYC	36	1049	15204	127	4.391944	1.60E-14
lightcyan	6167	GRANDVAUX_IRF3_TARGETS_UP	7	14	16239	62	131.0726	2.79E-14
magenta	10130	GSE6269_HEALTHY_VS_STREP_AUREUS_INF_PBMC_UP	19	179	16074	167	10.33041	2.82E-14
turquoise	5141	DODD_NASOPHARYNGEAL_CARCINOMA_DN	454	1311	14942	4169	1.350065	2.93E-14
cyan	7305	ICHIBA_GRAFT_VERSUS_HOST_DISEASE_D7_UP	12	98	16155	77	25.84628	3.06E-14
salmon	7996	BILANGES_SERUM_RESPONSE_TRANSLATION	10	32	16221	127	39.99262	3.31E-14
lightcyan	1639	V$ISRE_01	14	208	16045	62	17.64439	3.44E-14
salmon	5742	PUJANA_BRCA1_PCC_NETWORK	43	1553	14700	127	3.543454	4.12E-14
red	8427	GSE10325_BCELL_VS_MYELOID_DN	32	199	16054	524	4.987687	4.91E-14
red	9381	GSE22886_NAIVE_CD4_TCELL_VS_MONOCYTE_DN	32	200	16053	524	4.962748	5.66E-14
red	9674	GSE29618_MONOCYTE_VS_MDC_DAY7_FLU_VACCINE_	32	200	16053	524	4.962748	5.66E-14
black	3169	chr19p13	36	467	15786	283	4.427236	6.00E-14
salmon	7236	WANG_TUMOR_INVASIVENESS_UP	22	363	15890	127	7.756144	7.39E-14
red	3534	MODULE_45	53	520	15733	524	3.161366	9.68E-14
cyan	4645	REACTOME_INTERFERON_GAMMA_SIGNALING	10	57	16196	77	37.03121	1.12E-13
lightcyan	9507	GSE2706_UNSTIM_VS_2H_LPS_AND_R848_DC_DN	13	181	16072	62	18.82811	1.36E-13
lightcyan	9857	GSE360_HIGH_DOSE_B_MALAYI_VS_M_TUBERCULOSIS	13	181	16072	62	18.82811	1.36E-13
magenta	8502	GSE12366_NAIVE_VS_MEMORY_BCELL_UP	18	170	16083	167	10.30483	1.45E-13
lightcyan	9405	GSE24081_CONTROLLER_VS_PROGRESSOR_HIV_SPECIF	13	182	16071	62	18.72465	1.46E-13
lightcyan	8805	GSE15930_STIM_VS_STIM_AND_IFNAB_48H_CD8_T_C	13	186	16067	62	18.32197	1.93E-13
brown	9369	GSE22886_NAIVE_BCELL_VS_MONOCYTE_DN	67	199	16054	2190	2.498683	2.16E-13
magenta	9328	GSE22886_NAIVE_VS_IGG_IGA_MEMORY_BCELL_UP	18	174	16079	167	10.06793	2.18E-13
cyan	8823	GSE16755_CTRL_VS_IFNA_TREATED_MAC_DN	14	189	16064	77	15.6354	2.25E-13

blue	8424	GSE10325_CD4_TCELL_VS_MYELOID_UP	79	194	16059	2978	2.222461	2.35E-13
lightcyan	5545	XU_HGF_TARGETS_INDUCED_BY_AKT1_6HR	7	18	16235	62	101.9453	2.55E-13
red	9655	GSE29618_BCELL_VS_MONOCYTE_DN	31	199	16054	524	4.831821	2.97E-13
salmon	2396	MORF_G22P1	16	170	16083	127	12.04484	3.01E-13
cyan	9457	GSE24634_IL4_VS_CTRL_TREATED_NAIVE_CD4_TCELL_	14	193	16060	77	15.31135	3.01E-13
salmon	5081	TIEN_INTESTINE_PROBIOTICS_6HR_UP	11	54	16199	127	26.06926	3.08E-13
blue	8454	GSE11057_CD4_CENT_MEM_VS_PBMC_UP	79	195	16058	2978	2.211064	3.25E-13
turquoise	8587	GSE13485_DAY1_VS_DAY21_YF17D_VACCINE_PBMC_[	93	184	16069	4169	1.970456	3.25E-13
lightcyan	3793	MODULE_345	11	111	16142	62	25.97835	3.45E-13
cyan	5561	EINAV_INTERFERON_SIGNATURE_IN_CANCER	8	27	16226	77	62.54161	3.61E-13
cyan	8665	GSE1432_1H_VS_6H_IFNG_MICROGLIA_DN	14	196	16057	77	15.07699	3.72E-13
salmon	5084	TIEN_INTESTINE_PROBIOTICS_24HR_DN	17	205	16048	127	10.61268	4.19E-13
blue	2312	MORF_HDAC2	101	277	15976	2978	1.989988	4.89E-13
red	10133	GSE6269_HEALTHY_VS_STREP_PNEUMO_INF_PBMC_D	28	165	16088	524	5.263521	4.91E-13
salmon	7137	GRADE_COLON_CANCER_UP	30	812	15441	127	4.728191	6.23E-13
salmon	7848	KIM_BIPOLAR_DISORDER_OLIGODENDROCYTE_DENSIT	27	646	15607	127	5.348858	6.24E-13
brown	10141	GSE6269_E_COLI_VS_STREP_AUREUS_INF_PBMC_DN	60	172	16081	2190	2.588882	6.81E-13
lightcyan	8158	ALTEMEIER_RESPONSE_TO_LPS_WITH_MECHANICAL_V	11	118	16135	62	24.43726	6.84E-13
brown	9671	GSE29618_BCELL_VS_MDC_DAY7_FLU_VACCINE_DN	66	199	16054	2190	2.461389	7.20E-13
lightcyan	5087	ZHOU_INFLAMMATORY_RESPONSE_LPS_UP	15	319	15934	62	12.32657	7.21E-13
turquoise	2244	SCGGAAGY_V$ELK1_02	403	1162	15091	4169	1.352074	7.76E-13
turquoise	10118	GSE39820_TGFBETA1_VS_TGFBETA3_IN_IL6_TREATED_	97	197	16056	4169	1.919584	7.78E-13
lightcyan	8064	YANG_BCL3_TARGETS_UP	15	322	15931	62	12.21173	8.25E-13
turquoise	7651	PYEON_CANCER_HEAD_AND_NECK_VS_CERVICAL_UP	92	184	16069	4169	1.949268	9.78E-13
lightcyan	7683	ZHAN_MULTIPLE_MYELOMA_LB_DN	8	38	16215	62	55.18846	1.25E-12
grey60	6239	NAKAJIMA_EOSINOPHIL	7	30	16223	44	86.19015	1.26E-12
cyan	6099	DER_IFN_ALPHA_RESPONSE_UP	10	72	16181	77	29.31638	1.32E-12
brown	10293	GSE9988_ANTI_TREM1_AND_LPS_VS_CTRL_TREATED_I	65	197	16056	2190	2.448705	1.40E-12
yellow	3331	chr9q34	51	199	16054	1426	2.920997	1.40E-12
magenta	3750	MODULE_292	15	120	16133	167	12.16542	1.48E-12
pink	2985	BIOCARTA_AHSP_PATHWAY	8	13	16240	228	43.86775	1.61E-12
yellow	8609	GSE14000_TRANSLATED_RNA_VS_MRNA_DC_DN	48	181	16072	1426	3.022572	1.66E-12
lightgreen	9473	GSE26495_NAIVE_VS_PD1HIGH_CD8_TCELL_DN	11	197	16056	42	21.60781	1.93E-12
lightcyan	9237	GSE18791_CTRL_VS_NEWCASTLE_VIRUS_DC_18H_DN	12	173	16080	62	18.18348	1.96E-12
lightcyan	9839	GSE360_L_DONOVANI_VS_M_TUBERCULOSIS_DC_DN	12	173	16080	62	18.18348	1.96E-12
lightgreen	9475	GSE26495_NAIVE_VS_PD1LOW_CD8_TCELL_DN	11	198	16055	42	21.49868	2.04E-12
cyan	9231	GSE18791_CTRL_VS_NEWCASTLE_VIRUS_DC_12H_DN	13	178	16075	77	15.4158	2.09E-12
blue	9380	GSE22886_NAIVE_CD4_TCELL_VS_MONOCYTE_UP	77	194	16059	2978	2.166196	2.24E-12
cyan	8130	BOSCO_INTERFERON_INDUCED_ANTIVIRAL_MODULE	10	76	16177	77	27.77341	2.31E-12
pink	7909	CHYLA_CBFA2T3_TARGETS_DN	21	213	16040	228	7.028107	2.52E-12
lightcyan	9511	GSE2706_R848_VS_LPS_2H_STIM_DC_DN	11	134	16119	62	21.51938	2.81E-12
magenta	9637	GSE29614_DAY3_VS_DAY7_TIV_FLU_VACCINE_PBMC_	17	175	16078	167	9.454269	2.93E-12
magenta	3669	MODULE_188	15	127	16126	167	11.49488	3.44E-12
salmon	5910	BENPORATH_MYC_MAX_TARGETS	28	754	15499	127	4.752438	3.77E-12
magenta	9338	GSE22886_IGG_IGA_MEMORY_BCELL_VS_BLOOD_PLAS	17	179	16074	167	9.243	4.23E-12
lightcyan	9953	GSE3982_CTRL_VS_LPS_4H_MAC_DN	12	187	16066	62	16.82215	4.94E-12
lightcyan	6178	SANA_RESPONSE_TO_IFNG_UP	9	70	16183	62	33.70438	5.07E-12
cyan	4747	REACTOME_CYTOKINE_SIGNALING_IN_IMMUNE_SYSTE	14	238	16015	77	12.41635	5.27E-12
salmon	7280	FLOTHO_PEDIATRIC_ALL_THERAPY_RESPONSE_UP	10	52	16201	127	24.61084	7.10E-12
salmon	7846	KIM_ALL_DISORDERS_OLIGODENDROCYTE_NUMBER_C	27	717	15536	127	4.819194	7.11E-12
magenta	8396	KAECH_DAY15_EFF_VS_MEMORY_CD8_TCELL_UP	17	185	16068	167	8.943227	7.22E-12
cyan	5217	MARKEY_RB1_ACUTE_LOF_UP	13	197	16056	77	13.929	7.63E-12
blue	4986	GINESTIER_BREAST_CANCER_20Q13_AMPLIFICATION_I	70	173	16080	2978	2.208314	7.69E-12
cyan	4829	BERTUCCI_MEDULLARY_VS_DUCTAL_BREAST_CANCER_	13	198	16055	77	13.85865	8.14E-12
lightcyan	8665	GSE1432_1H_VS_6H_IFNG_MICROGLIA_DN	12	196	16057	62	16.0497	8.60E-12
lightcyan	3688	MODULE_208	10	108	16145	62	24.2727	8.85E-12
salmon	7703	WONG_EMBRYONIC_STEM_CELL_CORE	19	327	15926	127	7.435936	9.12E-12
red	9371	GSE22886_NAIVE_CD8_TCELL_VS_NEUTROPHIL_DN	26	160	16093	524	5.040291	9.42E-12
salmon	367	BIOSYNTHETIC_PROCESS	21	418	15835	127	6.429435	1.05E-11
red	8433	GSE10325_LUPUS_BCELL_VS_LUPUS_MYELOID_DN	29	200	16053	524	4.49749	1.10E-11

magenta	9663	GSE29618_MONOCYTE_VS_MDC_DN	17	190	16063	167	8.707879	1.11E-11
blue	9374	GSE22886_NAIVE_CD8_TCELL_VS_MONOCYTE_UP	76	196	16057	2978	2.116247	1.21E-11
turquoise	4903	PUIFFE_INVASION_INHIBITED_BY_ASCITES_DN	72	137	16116	4169	2.048866	1.40E-11
turquoise	6482	CUI_TCF21_TARGETS_2_DN	276	759	15494	4169	1.41765	1.53E-11
pink	3908	MODULE_539	8	16	16237	228	35.64254	1.55E-11
magenta	9334	GSE22886_NAIVE_BCELL_VS_BLOOD_PLASMA_CELL_UI	17	194	16059	167	8.528335	1.55E-11
magenta	9673	GSE29618_MONOCYTE_VS_PDC_DAY7_FLU_VACCINE_	17	195	16058	167	8.4846	1.68E-11
lightcyan	9201	GSE17974_IL4_AND_ANTI_IL12_VS_UNTREATED_48H_	11	159	16094	62	18.13583	1.83E-11
blue	7044	MITSIADES_RESPONSE_TO_APLIDIN_DN	89	246	16007	2978	1.97453	1.93E-11
lightcyan	3535	MODULE_46	14	332	15921	62	11.05431	2.01E-11
turquoise	5432	SEIDEN_ONCOGENESIS_BY_MET	51	85	16168	4169	2.339122	2.12E-11
cyan	9223	GSE18791_CTRL_VS_NEWCASTLE_VIRUS_DC_4H_DN	12	169	16084	77	14.98778	2.21E-11
lightcyan	5237	GRAESSMANN_APOPTOSIS_BY_DOXORUBICIN_UP	22	1072	15181	62	5.379845	2.31E-11
salmon	6640	MODY_HIPPOCAMPUS_PRENATAL	9	41	16212	127	28.09238	2.31E-11
lightcyan	2257	STTTCRNTTT_V$IRF_Q6	11	163	16090	62	17.69078	2.40E-11
lightcyan	9932	GSE37416_CTRL_VS_48H_F_TULARENSIS_LVS_NEUTRO	11	163	16090	62	17.69078	2.40E-11
magenta	5140	DODD_NASOPHARYNGEAL_CARCINOMA_UP	44	1439	14814	167	2.975836	2.41E-11
lightcyan	3750	MODULE_292	10	120	16133	62	21.84543	2.56E-11
salmon	5454	LI_AMPLIFIED_IN_LUNG_CANCER	14	160	16093	127	11.19793	2.68E-11
lightcyan	3563	MODULE_75	14	340	15913	62	10.79421	2.77E-11
salmon	9788	GSE34205_HEALTHY_VS_FLU_INF_INFANT_PBMC_UP	15	193	16060	127	9.946351	2.78E-11
yellow	7898	LI_DCP2_BOUND_MRNA	30	88	16165	1426	3.885551	2.89E-11
salmon	6194	POMEROY_MEDULLOBLASTOMA_PROGNOSIS_DN	9	42	16211	127	27.42351	2.92E-11
lightcyan	3853	MODULE_436	10	122	16131	62	21.48731	3.02E-11
blue	4984	GINESTIER_BREAST_CANCER_ZNF217_AMPLIFIED_DN	106	314	15939	2978	1.842405	3.15E-11
cyan	6143	WIELAND_UP_BY_HBV_INFECTION	10	99	16154	77	21.321	3.46E-11
magenta	3630	MODULE_145	13	102	16151	167	12.40396	3.77E-11
blue	2323	MORF_PRKDC	73	189	16064	2978	2.107997	3.81E-11
red	3572	MODULE_84	46	480	15773	524	2.972479	3.84E-11
turquoise	6864	MARSON_BOUND_BY_FOXP3_UNSTIMULATED	394	1161	15092	4169	1.323018	4.16E-11
brown	5038	MULLIGHAN_MLL_SIGNATURE_2_UP	104	409	15844	2190	1.88712	4.38E-11
salmon	2466	MORF_SART1	10	62	16191	127	20.64135	4.52E-11
brown	10131	GSE6269_HEALTHY_VS_STREP_AUREUS_INF_PBMC_DN	55	165	16088	2190	2.47382	4.70E-11
greenyello	2623	GNF2_CD7	9	38	16215	150	25.66263	4.95E-11
turquoise	7034	ACEVEDO_LIVER_CANCER_UP	325	929	15324	4169	1.363858	4.95E-11
blue	8448	GSE11057_NAIVE_CD4_VS_PBMC_CD4_TCELL_UP	74	194	16059	2978	2.081799	5.62E-11
blue	9370	GSE22886_NAIVE_CD8_TCELL_VS_NEUTROPHIL_UP	75	198	16055	2978	2.067307	6.15E-11
salmon	5744	PUJANA_ATM_PCC_NETWORK	35	1317	14936	127	3.401043	6.28E-11
magenta	5965	SHIN_B_CELL_LYMPHOMA_CLUSTER_9	7	15	16238	167	45.41756	6.40E-11
pink	7921	PILON_KLF1_TARGETS_UP	27	428	15825	228	4.496956	6.61E-11
brown	7931	JOHNSTONE_PARVB_TARGETS_3_UP	104	412	15841	2190	1.873379	6.93E-11
lightcyan	7498	JISON_SICKLE_CELL_DISEASE_UP	11	180	16073	62	16.01998	7.04E-11
brown	9659	GSE29618_BCELL_VS_MDC_DN	62	199	16054	2190	2.312214	7.09E-11
purple	525	BLOOD_COAGULATION	9	38	16215	157	24.51844	7.46E-11
purple	835	COAGULATION	9	38	16215	157	24.51844	7.46E-11
brown	2674	GNF2_MYD88	29	60	16193	2190	3.58704	7.80E-11
yellow	10238	GSE9006_TYPE_1_VS_TYPE_2_DIABETES_PBMC_AT_DX	48	200	16053	1426	2.735428	7.82E-11
salmon	2347	MORF_ACP1	15	208	16045	127	9.229066	8.07E-11
turquoise	5599	DACOSTA_UV_RESPONSE_VIA_ERCC3_DN	294	829	15424	4169	1.382593	8.19E-11
brown	8590	GSE13485_DAY3_VS_DAY21_YF17D_VACCINE_PBMC_U	61	195	16058	2190	2.321585	8.37E-11
brown	10052	GSE3982_NEUTROPHIL_VS_CENT_MEMORY_CD4_TCEL	61	195	16058	2190	2.321585	8.37E-11
blue	2436	MORF_PPP1CC	65	163	16090	2978	2.176379	9.00E-11
brown	6328	THEILGAARD_NEUTROPHIL_AT_SKIN_WOUND_DN	67	224	16029	2190	2.219812	9.11E-11
turquoise	7989	LEE_BMP2_TARGETS_DN	300	850	15403	4169	1.375954	9.58E-11
brown	2614	GNF2_CASP1	42	111	16142	2190	2.80812	1.02E-10
magenta	7416	SHEDDEN_LUNG_CANCER_GOOD_SURVIVAL_A12	18	251	16002	167	6.979364	1.08E-10
cyan	9389	GSE22886_CTRL_VS_LPS_24H_DC_DN	12	195	16058	77	12.98941	1.18E-10
blue	5742	PUJANA_BRCA1_PCC_NETWORK	380	1553	14700	2978	1.33543	1.18E-10
cyan	8592	GSE13485_DAY7_VS_DAY21_YF17D_VACCINE_PBMC_U	12	196	16057	77	12.92314	1.25E-10
salmon	7540	YAMASHITA_LIVER_CANCER_WITH_EPCAM_UP	9	49	16204	127	23.50587	1.29E-10

magenta	9661	GSE29618_MONOCYTE_VS_PDC_DN	16	192	16061	167	8.110279	1.35E-10
yellow	3413	chr16p13	52	230	16023	1426	2.576852	1.36E-10
pink	3516	MODULE_24	24	350	15903	228	4.88812	1.50E-10
blue	348	NUCLEOBASENUCLEOSIDENUCLEOTIDE_AND_NUCLEIC_	291	1136	15117	2978	1.398053	1.55E-10
magenta	3758	MODULE_301	14	139	16114	167	9.802352	1.63E-10
salmon	8699	GSE1460_DP_THYMOCYTE_VS_NAIVE_CD4_TCELL_ADU	14	183	16070	127	9.790543	1.63E-10
blue	2325	MORF_RAD23A	111	342	15911	2978	1.771355	1.64E-10
lightcyan	8621	GSE14000_UNSTIM_VS_16H_LPS_DC_DN	11	195	16058	62	14.78768	1.67E-10
brown	9660	GSE29618_MONOCYTE_VS_PDC_UP	61	198	16055	2190	2.28641	1.68E-10
turquoise	8406	GSE10239_NAIVE_VS_MEMORY_CD8_TCELL_UP	92	198	16055	4169	1.811441	1.69E-10
lightcyan	5048	TONKS_TARGETS_OF_RUNX1_RUNX1T1_FUSION_MON	11	196	16057	62	14.71223	1.76E-10
salmon	7993	BILANGES_RAPAMYCIN_SENSITIVE_VIA_TSC1_AND_TS(	10	71	16182	127	18.02484	1.83E-10
lightcyan	5217	MARKEY_RB1_ACUTE_LOF_UP	11	197	16056	62	14.63755	1.86E-10
brown	9997	GSE3982_MAST_CELL_VS_NEUTROPHIL_DN	56	175	16078	2190	2.374868	1.88E-10
lightcyan	6125	BECKER_TAMOXIFEN_RESISTANCE_UP	7	42	16211	62	43.69086	2.01E-10
yellow	6877	SANSOM_APC_TARGETS_REQUIRE_MYC	47	199	16054	1426	2.691899	2.20E-10
yellow	10230	GSE9006_HEALTHY_VS_TYPE_2_DIABETES_PBMC_AT_I	47	199	16054	1426	2.691899	2.20E-10
pink	6102	WELCH_GATA1_TARGETS	8	21	16232	228	27.15622	2.31E-10
greenyello	9477	GSE26495_PD1HIGH_VS_PD1LOW_CD8_TCELL_DN	14	159	16094	150	9.540545	2.35E-10
red	8425	GSE10325_CD4_TCELL_VS_MYELOID_DN	27	198	16055	524	4.229615	2.38E-10
red	9357	GSE22886_DC_VS_MONOCYTE_DN	27	198	16055	524	4.229615	2.38E-10
purple	274	HEMOSTASIS	9	43	16210	157	21.66746	2.48E-10
greenyello	5115	JAATINEN_HEMATOPOIETIC_STEM_CELL_DN	16	223	16030	150	7.77423	2.49E-10
cyan	5010	TAKEDA_TARGETS_OF_NUP98_HOXA9_FUSION_3D_UF	11	162	16091	77	14.33245	2.60E-10
yellow	5646	DAIRKEE_TERT_TARGETS_UP	68	350	15903	1426	2.214394	2.76E-10
magenta	3710	MODULE_238	14	145	16108	167	9.396738	2.87E-10
red	8594	GSE13485_PRE_VS_POST_YF17D_VACCINATION_PBMC	24	159	16094	524	4.681838	2.90E-10
red	8431	GSE10325_LUPUS_CD4_TCELL_VS_LUPUS_MYELOID_DI	27	200	16053	524	4.187319	3.00E-10
turquoise	5742	PUJANA_BRCA1_PCC_NETWORK	502	1553	14700	4169	1.260184	3.49E-10
salmon	7212	MALONEY_RESPONSE_TO_17AAG_DN	10	76	16177	127	16.839	3.66E-10
red	10031	GSE3982_MAC_VS_NEUTROPHIL_DN	24	161	16092	524	4.623678	3.78E-10
salmon	8418	GSE10239_NAIVE_VS_DAY4.5_EFF_CD8_TCELL_UP	14	196	16057	127	9.14117	4.06E-10
magenta	5436	KLEIN_TARGETS_OF_BCR_ABL1_FUSION	9	43	16210	167	20.37	4.30E-10
red	9377	GSE22886_NAIVE_CD4_TCELL_VS_NEUTROPHIL_DN	24	162	16091	524	4.595137	4.30E-10
yellow	7840	MARTENS_TRETINOIN_RESPONSE_DN	121	786	15467	1426	1.754595	4.42E-10
blue	2300	MORF_BUB3	93	275	15978	2978	1.845691	4.57E-10
brown	5036	MULLIGHAN_MLL_SIGNATURE_1_UP	94	371	15882	2190	1.88037	4.66E-10
purple	831	WOUND_HEALING	9	46	16207	157	20.25436	4.72E-10
brown	10133	GSE6269_HEALTHY_VS_STREP_PNEUMO_INF_PBMC_D	53	165	16088	2190	2.383863	4.90E-10
lightgreen	2623	GNF2_CD7	6	38	16215	42	61.1015	5.33E-10
lightgreen	5151	HAHTOLA_SEZARY_SYNDROM_DN	6	38	16215	42	61.1015	5.33E-10
purple	8458	GSE11057_PBMC_VS_MEM_CD4_TCELL_UP	15	192	16061	157	8.087679	5.40E-10
blue	2345	MORF_XRCC5	82	233	16020	2978	1.920732	5.63E-10
purple	4807	PICCALUGA_ANGIOIMMUNOBLASTIC_LYMPHOMA_UP	14	163	16090	157	8.891485	6.01E-10
salmon	2665	GNF2_MBD4	7	25	16228	127	35.83339	6.44E-10
salmon	8146	HOLLEMAN_ASPARAGINASE_RESISTANCE_B_ALL_UP	7	25	16228	127	35.83339	6.44E-10
salmon	7253	LEE_LIVER_CANCER_SURVIVAL_DN	13	168	16085	127	9.902934	6.61E-10
red	9940	GSE37416_0H_VS_24H_F_TULARENSIS_LVS_NEUTROPI	26	193	16060	524	4.17848	6.74E-10
purple	502	REGULATION_OF_BODY_FLUID_LEVELS	9	48	16205	157	19.41043	7.07E-10
brown	8453	GSE11057_CD4_EFF_MEM_VS_PBMC_DN	59	195	16058	2190	2.245468	7.31E-10
turquoise	2272	RCGCANGCGY_V$NRF1_Q6	305	881	15372	4169	1.349664	7.39E-10
yellow	3169	chr19p13	82	467	15786	1426	2.001294	7.43E-10
red	3498	MODULE_5	38	383	15870	524	3.077422	7.77E-10
salmon	2391	MORF_FBL	12	138	16115	127	11.12838	8.10E-10
cyan	9203	GSE17974_IL4_AND_ANTI_IL12_VS_UNTREATED_72H_	11	181	16072	77	12.82794	8.51E-10
brown	7348	RUTELLA_RESPONSE_TO_CSF2RB_AND_IL4_DN	82	312	15941	2190	1.950512	9.41E-10
red	9942	GSE37416_0H_VS_48H_F_TULARENSIS_LVS_NEUTROPI	26	196	16057	524	4.114523	9.46E-10
red	2646	GNF2_HCK	18	94	16159	524	5.939459	9.85E-10
blue	10132	GSE6269_HEALTHY_VS_STREP_PNEUMO_INF_PBMC_U	69	186	16067	2978	2.024627	1.01E-09
red	10058	GSE3982_NEUTROPHIL_VS_TH2_UP	24	169	16084	524	4.404806	1.04E-09

turquoise	5997	NIKOLSKY_BREAST_CANCER_8Q12_Q22_AMPLICON	53	97	16156	4169	2.130128	1.08E-09
lightgreen	8445	GSE11057_NAIVE_VS_EFF_MEMORY_CD4_TCELL_DN	9	190	16063	42	18.33045	1.08E-09
brown	8458	GSE11057_PBMC_VS_MEM_CD4_TCELL_UP	58	192	16061	2190	2.2419	1.09E-09
brown	9982	GSE3982_EOSINOPHIL_VS_EFF_MEMORY_CD4_TCELL_	58	192	16061	2190	2.2419	1.09E-09
magenta	3804	MODULE_361	13	133	16120	167	9.512809	1.09E-09
cyan	6147	DER_IFN_BETA_RESPONSE_UP	9	101	16152	77	18.80892	1.09E-09
magenta	5898	PASQUALUCCI_LYMPHOMA_BY_GC_STAGE_DN	14	161	16092	167	8.4629	1.15E-09
blue	8428	GSE10325_LUPUS_CD4_TCELL_VS_LUPUS_BCELL_UP	71	194	16059	2978	1.997402	1.16E-09
magenta	5979	MORI_PLASMA_CELL_DN	8	33	16220	167	23.59354	1.17E-09
red	9667	GSE29618_BCELL_VS_MONOCYTE_DAY7_FLU_VACCINE	26	198	16055	524	4.072962	1.18E-09
lightcyan	9525	GSE2706_2H_VS_8H_LPS_STIM_DC_DN	10	177	16076	62	14.81046	1.20E-09
cyan	9859	GSE360_LOW_DOSE_B_MALAYI_VS_M_TUBERCULOSIS	11	187	16066	77	12.41635	1.20E-09
blue	9376	GSE22886_NAIVE_CD4_TCELL_VS_NEUTROPHIL_UP	72	198	16055	2978	1.984614	1.23E-09
brown	10015	GSE3982_DC_VS_NEUTROPHIL_DN	56	183	16070	2190	2.271048	1.23E-09
magenta	10062	GSE3982_BCELL_VS_EFF_MEMORY_CD4_TCELL_UP	15	193	16060	167	7.563991	1.38E-09
cyan	8807	GSE15930_STIM_VS_STIM_AND_TRICHOSTATINA_48H_	11	190	16063	77	12.2203	1.42E-09
red	10141	GSE6269_E_COLI_VS_STREP_AUREUS_INF_PBMC_DN	24	172	16081	524	4.327978	1.50E-09
greenyello	9308	GSE22886_NAIVE_CD8_TCELL_VS_MEMORY_TCELL_UP	14	183	16070	150	8.289326	1.51E-09
blue	10021	GSE3982_DC_VS_EFF_MEMORY_CD4_TCELL_DN	64	169	16084	2978	2.066817	1.55E-09
lightcyan	6704	BROWNE_HCMV_INFECTION_6HR_UP	7	56	16197	62	32.76815	1.66E-09
turquoise	4844	SENGUPTA_NASOPHARYNGEAL_CARCINOMA_UP	116	276	15977	4169	1.638515	1.69E-09
lightcyan	9527	GSE2706_2H_VS_8H_R848_AND_LPS_STIM_DC_DN	10	184	16069	62	14.24702	1.75E-09
cyan	9463	GSE24634_IL4_VS_CTRL_TREATED_NAIVE_CD4_TCELL_	11	194	16059	77	11.96834	1.77E-09
blue	2304	MORF_DNMT1	49	116	16137	2978	2.305403	1.85E-09
magenta	6388	KUMAR_TARGETS_OF_MLL_AF9_FUSION	20	376	15877	167	5.176774	1.87E-09
lightcyan	9859	GSE360_LOW_DOSE_B_MALAYI_VS_M_TUBERCULOSIS	10	187	16066	62	14.01846	2.05E-09
brown	9449	GSE24634_TEFF_VS_TCONV_DAY3_IN_CULTURE_DN	58	195	16058	2190	2.207409	2.08E-09
turquoise	5238	GRAESSMANN_APOPTOSIS_BY_DOXORUBICIN_DN	540	1705	14548	4169	1.234727	2.15E-09
magenta	6365	ZHAN_MULTIPLE_MYELOMA_CD1_VS_CD2_DN	9	51	16202	167	17.17471	2.16E-09
cyan	8439	GSE10325_MYELOID_VS_LUPUS_MYELOID_DN	11	198	16055	77	11.72655	2.20E-09
purple	7498	JISON_SICKLE_CELL_DISEASE_UP	14	180	16073	157	8.051734	2.21E-09
red	6796	KRIGE_RESPONSE_TO_TOSEDOSTAT_24HR_UP	57	759	15494	524	2.329353	2.28E-09
turquoise	4893	GARY_CD5_TARGETS_DN	164	426	15827	4169	1.500845	2.43E-09
brown	8455	GSE11057_CD4_CENT_MEM_VS_PBMC_DN	57	191	16062	2190	2.214782	2.50E-09
magenta	9342	GSE22886_IGM_MEMORY_BCELL_VS_BLOOD_PLASMA	14	171	16082	167	7.967994	2.54E-09
brown	8449	GSE11057_NAIVE_CD4_VS_PBMC_CD4_TCELL_DN	58	196	16057	2190	2.196147	2.58E-09
brown	10295	GSE9988_ANTI_TREM1_AND_LPS_VS_VEHICLE_TREATE	58	196	16057	2190	2.196147	2.58E-09
lightgreen	2684	GNF2_PTPN4	6	49	16204	42	47.38484	2.64E-09
magenta	7061	SMID_BREAST_CANCER_LUMINAL_B_DN	21	425	15828	167	4.808919	2.68E-09
blue	2346	MORF_AATF	73	205	16048	2978	1.94347	2.73E-09
greenyello	9791	GSE34205_RSV_VS_FLU_INF_INFANT_PBMC_DN	14	192	16061	150	7.900764	2.82E-09
turquoise	8199	ERB2_UP.V1_DN	83	181	16072	4169	1.787727	2.85E-09
purple	5070	SENESE_HDAC1_AND_HDAC2_TARGETS_DN	14	184	16069	157	7.876696	2.95E-09
cyan	4650	REACTOME_INTERFERON_ALPHA_BETA_SIGNALING	7	49	16204	77	30.15399	2.97E-09
purple	3536	MODULE_47	14	186	16067	157	7.792001	3.39E-09
red	6778	MCLACHLAN_DENTAL_CARIES_UP	27	223	16030	524	3.755443	3.46E-09
turquoise	8354	TBK1.DF_DN	115	276	15977	4169	1.62439	3.62E-09
brown	10283	GSE9988_LPS_VS_CTRL_TREATED_MONOCYTE_DN	58	198	16055	2190	2.173963	3.91E-09
brown	10291	GSE9988_LOW_LPS_VS_VEHICLE_TREATED_MONOCYTI	58	198	16055	2190	2.173963	3.91E-09
salmon	5745	PUJANA_CHEK2_PCC_NETWORK	24	757	15496	127	4.057375	3.92E-09
cyan	4731	REACTOME_IMMUNE_SYSTEM	19	801	15452	77	5.006842	3.99E-09
cyan	6068	GRANDVAUX_IFN_RESPONSE_NOT_VIA_IRF3	5	14	16239	77	75.38497	4.05E-09
brown	3534	MODULE_45	118	520	15733	2190	1.684101	4.17E-09
lightcyan	1720	V$IRF7_01	10	202	16051	62	12.97748	4.31E-09
lightcyan	4189	REACTOME_ANTIVIRAL_MECHANISM_BY_IFN_STIMULA	7	64	16189	62	28.67213	4.35E-09
lightgreen	4919	DEURIG_T_CELL_PROLYMPHOCYTIC_LEUKEMIA_DN	10	308	15945	42	12.56416	4.44E-09
red	50	MEMBRANE_PART	82	1316	14937	524	1.932681	4.50E-09
salmon	2540	GCM_PFN1	8	50	16203	127	20.47622	4.54E-09
magenta	3853	MODULE_436	12	122	16131	167	9.572789	4.55E-09
pink	7918	ACOSTA_PROLIFERATION_INDEPENDENT_MYC_TARGET	13	110	16143	228	8.424601	4.62E-09

brown	10289	GSE9988_LOW_LPS_VS_CTRL_TREATED_MONOCYTE_D	58	199	16054	2190	2.163039	4.81E-09
blue	2328	MORF_RAD54L	45	105	16148	2978	2.33901	4.85E-09
red	2577	GNF2_CARD15	15	70	16183	524	6.646538	4.88E-09
turquoise	6442	BLALOCK_ALZHEIMERS_DISEASE_DN	373	1128	15125	4169	1.289144	4.89E-09
turquoise	2467	MORF_SKP1A	89	200	16053	4169	1.734849	4.90E-09
magenta	10017	GSE3982_DC_VS_BCELL_DN	14	180	16073	167	7.569594	4.95E-09
red	9352	GSE22886_NEUTROPHIL_VS_DC_UP	21	142	16111	524	4.587047	5.53E-09
turquoise	4983	GINESTIER_BREAST_CANCER_ZNF217_AMPLIFIED_UP	43	75	16178	4169	2.235161	5.62E-09
purple	7921	PILON_KLF1_TARGETS_UP	20	428	15825	157	4.83749	5.77E-09
salmon	7655	CAIRO_HEPATOBLASTOMA_CLASSES_UP	21	591	15662	127	4.547384	5.84E-09
cyan	3572	MODULE_84	15	480	15773	77	6.596185	5.99E-09
cyan	10138	GSE6269_FLU_VS_STREP_PNEUMO_INF_PBMC_UP	10	167	16086	77	12.6394	6.07E-09
blue	8459	GSE11057_PBMC_VS_MEM_CD4_TCELL_DN	69	193	16060	2978	1.951195	6.11E-09
turquoise	2001	CATTTCA,MIR-203	106	251	16002	4169	1.646394	6.15E-09
blue	9358	GSE22886_NAIVE_TCELL_VS_NEUTROPHIL_UP	70	197	16056	2978	1.939281	6.37E-09
brown	10127	GSE6269_HEALTHY_VS_FLU_INF_PBMC_DN	49	157	16096	2190	2.316252	6.41E-09
red	8158	ALTEMEIER_RESPONSE_TO_LPS_WITH_MECHANICAL_V	19	118	16135	524	4.994291	7.05E-09
brown	2705	GNF2_STAT6	31	78	16175	2190	2.949555	7.09E-09
magenta	4913	HUTTMANN_B_CLL_POOR_SURVIVAL_DN	9	58	16195	167	15.1019	7.11E-09
magenta	5971	MORI_LARGE_PRE_BII_LYMPHOCYTE_DN	9	58	16195	167	15.1019	7.11E-09
blue	2363	MORF_CDC16	35	73	16180	2978	2.616701	7.18E-09
cyan	9853	GSE360_T_GONDII_VS_M_TUBERCULOSIS_DC_DN	10	171	16082	77	12.34374	7.63E-09
turquoise	182	INTRACELLULAR_ORGANELLE_PART	371	1125	15128	4169	1.285651	7.64E-09
lightcyan	5409	MAHADEVAN_RESPONSE_TO_MP470_UP	5	19	16234	62	68.98557	7.65E-09
blue	10053	GSE3982_NEUTROPHIL_VS_CENT_MEMORY_CD4_TCEL	69	194	16059	2978	1.941137	7.80E-09
turquoise	9313	GSE22886_NAIVE_CD4_TCELL_VS_MEMORY_TCELL_DN	87	196	16057	4169	1.730473	8.42E-09
cyan	9237	GSE18791_CTRL_VS_NEWCASTLE_VIRUS_DC_18H_DN	10	173	16080	77	12.20104	8.53E-09
brown	10050	GSE3982_NEUTROPHIL_VS_EFF_MEMORY_CD4_TCELL_	56	192	16061	2190	2.164593	8.57E-09
turquoise	8001	FEVR_CTNNB1_TARGETS_DN	193	526	15727	4169	1.430452	8.62E-09
turquoise	8225	VEGF_A_UP.V1_DN	80	176	16077	4169	1.772062	8.99E-09
blue	2398	MORF_GNB1	96	302	15951	2978	1.734895	9.22E-09
blue	6224	PENG_GLUTAMINE_DEPRIVATION_DN	101	323	15930	2978	1.706584	9.99E-09
blue	2330	MORF_RFC4	57	150	16103	2978	2.073922	1.03E-08
lightcyan	5277	MISSIAGLIA_REGULATED_BY_METHYLATION_UP	8	113	16140	62	18.55895	1.05E-08
red	10048	GSE3982_NEUTROPHIL_VS_BASOPHIL_UP	23	175	16078	524	4.076543	1.06E-08
cyan	5235	GRAESSMANN_RESPONSE_TO_MC_AND_SERUM_DEPR	10	177	16076	77	11.92531	1.06E-08
cyan	9525	GSE2706_2H_VS_8H_LPS_STIM_DC_DN	10	177	16076	77	11.92531	1.06E-08
turquoise	6366	MOREAUX_MULTIPLE_MYELOMA_BY_TACI_DN	78	171	16082	4169	1.77828	1.13E-08
salmon	6840	HILLION_HMGA1B_TARGETS	9	80	16173	127	14.39734	1.19E-08
salmon	2350	MORF_AP2M1	13	213	16040	127	7.810765	1.19E-08
cyan	6428	DEBIASI_APOPTOSIS_BY_REOVIRUS_INFECTION_UP	12	293	15960	77	8.64483	1.22E-08
lightcyan	6638	JACKSON_DNMT1_TARGETS_UP	7	74	16179	62	24.79752	1.22E-08
greenyello	10091	GSE3982_CENT_MEMORY_CD4_TCELL_VS_NKCELL_DN	13	181	16072	150	7.782284	1.28E-08
lightcyan	6760	ZHU_CMV_8_HR_UP	6	43	16210	62	36.57839	1.31E-08
turquoise	3	ORGANELLE_PART	371	1130	15123	4169	1.279962	1.34E-08
brown	10056	GSE3982_NEUTROPHIL_VS_TH1_UP	51	170	16083	2190	2.226438	1.40E-08
turquoise	8648	GSE14308_TH17_VS_INDUCED_TREG_UP	84	189	16064	4169	1.732683	1.41E-08
cyan	9402	GSE24026_PD1_LIGATION_VS_CTRL_IN_ACT_TCELL_LIN	10	183	16070	77	11.53431	1.46E-08
blue	6670	DEBIASI_APOPTOSIS_BY_REOVIRUS_INFECTION_DN	89	276	15977	2978	1.759907	1.49E-08
lightcyan	6792	DOUGLAS_BMI1_TARGETS_DN	11	299	15954	62	9.644136	1.50E-08
cyan	5782	NUYTTEN_NIPP1_TARGETS_UP	17	683	15570	77	5.25377	1.51E-08
lightcyan	7818	BROWNE_HCMV_INFECTION_4HR_UP	6	44	16209	62	35.74707	1.52E-08
blue	80	NUCLEUS	322	1333	14920	2978	1.318362	1.60E-08
cyan	5531	BOWIE_RESPONSE_TO_TAMOXIFEN	5	18	16235	77	58.63276	1.71E-08
lightgreen	8450	GSE11057_EFF_MEM_VS_CENT_MEM_CD4_TCELL_UP	8	181	16072	42	17.10392	1.73E-08
greenyello	2689	GNF2_RAB7L1	7	33	16220	150	22.98404	1.73E-08
turquoise	5785	NUYTTEN_EZH2_TARGETS_DN	328	984	15269	4169	1.299512	1.76E-08
cyan	9459	GSE24634_IL4_VS_CTRL_TREATED_NAIVE_CD4_TCELL_	10	187	16066	77	11.28759	1.80E-08
turquoise	5100	KIM_WT1_TARGETS_DN	166	443	15810	4169	1.460851	1.82E-08
lightgreen	7333	CHAN_INTERFERON_PRODUCING_DENDRITIC_CELL	4	12	16241	42	128.9921	1.88E-08

lightyellow	6349	NAKAJIMA_MAST_CELL	5	42	16211	33	58.63276	2.03E-08
turquoise	5910	BENPORATH_MYC_MAX_TARGETS	260	754	15499	4169	1.344323	2.11E-08
turquoise	8349	STK33_UP	109	265	15988	4169	1.603549	2.12E-08
lightcyan	9503	GSE2706_UNSTIM_VS_2H_LPS_DC_DN	9	177	16076	62	13.32941	2.17E-08
yellow	7137	GRADE_COLON_CANCER_UP	118	812	15441	1426	1.656304	2.25E-08
lightcyan	5812	HASEGAWA_TUMORIGENESIS_BY_RET_C634R	4	9	16244	62	116.509	2.38E-08
turquoise	4323	REACTOME_GENERIC_TRANSCRIPTION_PATHWAY	127	321	15932	4169	1.542412	2.42E-08
lightcyan	7417	COLINA_TARGETS_OF_4EBP1_AND_4EBP2	11	315	15938	62	9.154275	2.57E-08
greenyello	4919	DEURIG_T_CELL_PROLYMPHOCYTIC_LEUKEMIA_DN	16	308	15945	150	5.628745	2.61E-08
red	4976	SMIRNOV_CIRCULATING_ENDOTHELIOCYTES_IN_CANC	21	155	16098	524	4.202327	2.70E-08
turquoise	5783	NUYTTEN_NIPP1_TARGETS_DN	270	790	15463	4169	1.332411	2.84E-08
pink	6174	ROSS_AML_OF_FAB_M7_TYPE	10	67	16186	228	10.63957	2.99E-08
salmon	9971	GSE3982_EOSINOPHIL_VS_MAST_CELL_DN	12	190	16063	127	8.082719	3.07E-08
pink	8074	STEINER_ERYTHROCYTE_MEMBRANE_GENES	6	15	16238	228	28.51404	3.21E-08
lightgreen	9374	GSE22886_NAIVE_CD8_TCELL_VS_MONOCYTE_UP	8	196	16057	42	15.79495	3.22E-08
salmon	7320	NUTT_GBM_VS_AO_GLIOMA_DN	7	42	16211	127	21.3294	3.24E-08
lightcyan	9825	GSE360_DC_VS_MAC_B_MALAYI_HIGH_DOSE_DN	9	186	16067	62	12.68444	3.34E-08
red	10271	GSE9988_ANTI_TREM1_VS_LOW_LPS_MONOCYTE_DN	23	186	16067	524	3.835457	3.40E-08
lightcyan	6604	ZHANG_ANTIVIRAL_RESPONSE_TO_RIBAVIRIN_UP	5	25	16228	62	52.42903	3.43E-08
lightcyan	8567	GSE13484_3H_UNSTIM_VS_YF17D_VACCINE_STIM_PB	9	187	16066	62	12.61661	3.50E-08
red	6626	MCLACHLAN_DENTAL_CARIES_DN	25	218	16035	524	3.557016	3.96E-08
lightcyan	8807	GSE15930_STIM_VS_STIM_AND_TRICHOSTATINA_48H_	9	190	16063	62	12.4174	4.01E-08
lightcyan	9715	GSE30083_SP1_VS_SP4_THYMOCYTE_DN	9	190	16063	62	12.4174	4.01E-08
magenta	10068	GSE3982_BCELL_VS_TH1_UP	13	179	16074	167	7.068176	4.06E-08
brown	10054	GSE3982_NEUTROPHIL_VS_NKCELL_UP	54	190	16063	2190	2.109257	4.16E-08
blue	2334	MORF_RRM1	42	101	16152	2978	2.269534	4.49E-08
pink	2250	GATAAGR_V$GATA_C	16	210	16043	228	5.431245	4.56E-08
lightcyan	9457	GSE24634_IL4_VS_CTRL_TREATED_NAIVE_CD4_TCELL_	9	193	16060	62	12.22439	4.59E-08
greenyello	7333	CHAN_INTERFERON_PRODUCING_DENDRITIC_CELL	5	12	16241	150	45.14722	4.71E-08
lightcyan	9451	GSE24634_TEFF_VS_TCONV_DAY5_IN_CULTURE_DN	9	194	16059	62	12.16137	4.80E-08
turquoise	5782	NUYTTEN_NIPP1_TARGETS_UP	237	683	15570	4169	1.352787	5.02E-08
cyan	7326	ZHANG_INTERFERON_RESPONSE	5	22	16231	77	47.97226	5.17E-08
red	118	INTEGRAL_TO_MEMBRANE	66	1025	15228	524	1.997204	5.25E-08
salmon	10126	GSE6269_HEALTHY_VS_FLU_INF_PBMC_UP	11	161	16092	127	8.743728	5.29E-08
blue	10130	GSE6269_HEALTHY_VS_STREP_AUREUS_INF_PBMC_UF	63	179	16074	2978	1.920863	5.40E-08
pink	8041	JUBAN_TARGETS_OF_SPI1_AND_FLI1_UP	12	112	16141	228	7.637688	5.59E-08
cyan	6582	ZHU_CMV_ALL_UP	8	112	16141	77	15.07699	5.60E-08
red	5013	TAKEDA_TARGETS_OF_NUP98_HOXA9_FUSION_8D_DN	23	191	16062	524	3.735053	5.61E-08
magenta	8504	GSE12845_IGD_POS_VS_NEG_BLOOD_BCELL_UP	13	184	16069	167	6.876106	5.62E-08
purple	8160	ACEVEDO_FGFR1_TARGETS_IN_PROSTATE_CANCER_M	14	233	16020	157	6.220224	5.97E-08
cyan	9201	GSE17974_IL4_AND_ANTI_IL12_VS_UNTREATED_48H_	9	159	16094	77	11.94781	5.98E-08
cyan	9247	GSE18791_UNSTIM_VS_NEWCATSLE_VIRUS_DC_18H_[	9	159	16094	77	11.94781	5.98E-08
lightcyan	6490	CHANG_IMMORTALIZED_BY_HPV31_DN	6	55	16198	62	28.59765	6.03E-08
brown	9944	GSE37416_12H_VS_24H_F_TULARENSIS_LVS_NEUTROI	55	197	16056	2190	2.071982	6.08E-08
brown	8580	GSE13485_CTRL_VS_DAY21_YF17D_VACCINE_PBMC_U	54	192	16061	2190	2.087286	6.11E-08
red	9455	GSE24634_TEFF_VS_TCONV_DAY10_IN_CULTURE_DN	23	192	16061	524	3.715599	6.18E-08
turquoise	2159	ATTCTTT,MIR-186	95	227	16026	4169	1.631546	6.27E-08
turquoise	9638	GSE29615_CTRL_VS_DAY3_LAIV_IFLU_VACCINE_PBMC_	74	165	16088	4169	1.748435	6.35E-08
cyan	7843	STAMBOLSKY_TARGETS_OF_MUTATED_TP53_DN	6	45	16208	77	28.14372	6.52E-08
greenyello	2664	GNF2_MATK	6	24	16229	150	27.08833	6.56E-08
pink	1874	V$GATA_Q6	13	137	16116	228	6.764278	6.67E-08
red	9891	GSE36392_TYPE_2_MYELOID_VS_EOSINOPHIL_IL25_TR	22	178	16075	524	3.833583	6.83E-08
turquoise	2140	TGCTTTG,MIR-330	109	270	15983	4169	1.573854	6.87E-08
lightcyan	4645	REACTOME_INTERFERON_GAMMA_SIGNALING	6	57	16196	62	27.59423	7.50E-08
lightcyan	7242	TSAI_RESPONSE_TO_RADIATION_THERAPY	5	29	16224	62	45.19744	7.58E-08
turquoise	2048	TAGCTTT,MIR-9	84	195	16058	4169	1.67937	7.86E-08
turquoise	8415	GSE10239_MEMORY_VS_KLRG1HIGH_EFF_CD8_TCELL_	85	198	16055	4169	1.673614	7.94E-08
brown	7922	PILON_KLF1_TARGETS_DN	340	1952	14301	2190	1.292673	7.94E-08
red	8453	GSE11057_CD4_EFF_MEM_VS_PBMC_DN	23	195	16058	524	3.658436	8.25E-08
lightcyan	4664	REACTOME_RIG_I_MDA5_MEDIATED_INDUCTION_OF_	6	58	16195	62	27.11846	8.34E-08

magenta	7571	MIKKELSEN_MEF_ICP_WITH_H3K27ME3	11	128	16125	167	8.363726	8.36E-08
red	6240	BROWN_MYELOID_CELL_DEVELOPMENT_UP	20	151	16102	524	4.108235	8.51E-08
magenta	8828	GSE17580_UNINFECTED_VS_S_MANSONI_INF_TEFF_UI	13	191	16062	167	6.624103	8.72E-08
magenta	9675	GSE29618_MONOCYTE_VS_MDC_DAY7_FLU_VACCINE_	13	191	16062	167	6.624103	8.72E-08
lightcyan	1866	V$IRF_Q6	9	208	16045	62	11.34282	8.74E-08
turquoise	1947	TGTTTAC,MIR-30A-5P,MIR-30C,MIR-30D,MIR-30B,MIR-	185	514	15739	4169	1.40317	8.74E-08
lightgreen	5115	JAATINEN_HEMATOPOIETIC_STEM_CELL_DN	8	223	16030	42	13.88255	8.75E-08
brown	9938	GSE37416_0H_VS_12H_F_TULARENSIS_LVS_NEUTROPI	54	194	16059	2190	2.065768	8.92E-08
red	41	INTRINSIC_TO_MEMBRANE	66	1041	15212	524	1.966507	9.38E-08
turquoise	9493	GSE26928_EFF_MEM_VS_CENTR_MEM_CD4_TCELL_DI	68	149	16104	4169	1.779198	9.40E-08
red	59	MEMBRANE	90	1601	14652	524	1.743626	9.75E-08
red	5183	DELYS_THYROID_CANCER_UP	34	383	15870	524	2.753483	9.78E-08
magenta	9664	GSE29618_PDC_VS_MDC_UP	13	193	16060	167	6.555459	9.86E-08
magenta	9676	GSE29618_PDC_VS_MDC_DAY7_FLU_VACCINE_UP	13	193	16060	167	6.555459	9.86E-08
blue	6761	WELCSH_BRCA1_TARGETS_DN	51	136	16117	2978	2.046634	9.95E-08
turquoise	5733	SPIELMAN_LYMPHOBLAST_EUROPEAN_VS_ASIAN_DN	205	581	15672	4169	1.375559	1.00E-07
red	225	PLASMA_MEMBRANE_PART	58	867	15386	524	2.074967	1.02E-07
turquoise	9931	GSE37416_CTRL_VS_24H_F_TULARENSIS_LVS_NEUTRO	85	199	16054	4169	1.665204	1.04E-07
brown	7855	VERHAAK_GLIOBLASTOMA_NEURAL	55	200	16053	2190	2.040902	1.06E-07
lightcyan	8129	BOSCO_TH1_CYTOTOXIC_MODULE	7	101	16152	62	18.16848	1.08E-07
red	9677	GSE29618_PDC_VS_MDC_DAY7_FLU_VACCINE_DN	23	198	16055	524	3.603005	1.09E-07
red	10234	GSE9006_TYPE_1_DIABETES_AT_DX_VS_4MONTH_POS	23	198	16055	524	3.603005	1.09E-07
blue	9976	GSE3982_EOSINOPHIL_VS_NEUTROPHIL_UP	64	186	16067	2978	1.877915	1.11E-07
purple	4521	REACTOME_FORMATION_OF_FIBRIN_CLOT_CLOTTING_	6	25	16228	157	24.84535	1.12E-07
red	10015	GSE3982_DC_VS_NEUTROPHIL_DN	22	183	16070	524	3.728841	1.12E-07
lightcyan	6098	JECHLINGER_EPITHELIAL_TO_MESENCHYMAL_TRANSIT	6	61	16192	62	25.78477	1.13E-07
salmon	2378	MORF_DEK	13	259	15994	127	6.423525	1.20E-07
red	9665	GSE29618_PDC_VS_MDC_DN	23	199	16054	524	3.5849	1.20E-07
greenyello	7831	LI_INDUCED_T_TO_NATURAL_KILLER_UP	15	300	15953	150	5.417667	1.20E-07
turquoise	5809	WEI_MYCN_TARGETS_WITH_E_BOX	256	754	15499	4169	1.323641	1.25E-07
blue	9653	GSE29617_DAY3_VS_DAY7_TIV_FLU_VACCINE_PBMC_	60	171	16082	2978	1.914979	1.26E-07
lightcyan	7601	TSAI_DNAJB4_TARGETS_UP	4	13	16240	62	80.66005	1.34E-07
magenta	3607	MODULE_119	11	134	16119	167	7.98923	1.34E-07
red	10056	GSE3982_NEUTROPHIL_VS_TH1_UP	21	170	16083	524	3.831533	1.37E-07
turquoise	62	CYTOPLASM	595	1950	14303	4169	1.189554	1.43E-07
red	6287	HESS_TARGETS_OF_HOXA9_AND_MEIS1_DN	14	77	16176	524	5.639486	1.43E-07
salmon	29	RIBONUCLEOPROTEIN_COMPLEX	10	140	16113	127	9.14117	1.44E-07
purple	3131	chr4q12	6	26	16227	157	23.88976	1.45E-07
turquoise	1958	TGCACTT,MIR-519C,MIR-519B,MIR-519A	145	387	15866	4169	1.460692	1.45E-07
red	6820	CREIGHTON_ENDOCRINE_THERAPY_RESISTANCE_3	47	644	15609	524	2.263676	1.50E-07
turquoise	2112	ACTTTAT,MIR-142-5P	102	252	16001	4169	1.577979	1.54E-07
brown	10285	GSE9988_LPS_VS_VEHICLE_TREATED_MONOCYTE_DN	54	197	16056	2190	2.034309	1.55E-07
cyan	9235	GSE18791_CTRL_VS_NEWCASTLE_VIRUS_DC_16H_DN	9	178	16075	77	10.67248	1.58E-07
cyan	9977	GSE3982_EOSINOPHIL_VS_NEUTROPHIL_DN	9	178	16075	77	10.67248	1.58E-07
brown	9984	GSE3982_EOSINOPHIL_VS_CENT_MEMORY_CD4_TCELL	52	187	16066	2190	2.063722	1.59E-07
turquoise	2442	MORF_PPP6C	51	103	16150	4169	1.930343	1.61E-07
red	9354	GSE22886_NEUTROPHIL_VS_MONOCYTE_UP	18	129	16124	524	4.327978	1.70E-07
turquoise	6792	DOUGLAS_BMI1_TARGETS_DN	117	299	15954	4169	1.525514	1.71E-07
salmon	5732	SPIELMAN_LYMPHOBLAST_EUROPEAN_VS_ASIAN_UP	17	475	15778	127	4.580207	1.72E-07
red	826	RESPONSE_TO_EXTERNAL_STIMULUS	25	235	16018	524	3.2997	1.73E-07
cyan	7498	JISON_SICKLE_CELL_DISEASE_UP	9	180	16073	77	10.5539	1.73E-07
blue	7655	CAIRO_HEPATOBLASTOMA_CLASSES_UP	158	591	15662	2978	1.459078	1.78E-07
lightgreen	3052	BIOCARTA_NKCELLS_PATHWAY	4	20	16233	42	77.39524	1.81E-07
turquoise	5745	PUJANA_CHEK2_PCC_NETWORK	256	757	15496	4169	1.318396	1.82E-07
turquoise	8348	STK33_SKM_UP	104	259	15994	4169	1.565436	1.86E-07
lightcyan	6068	GRANDVAUX_IFN_RESPONSE_NOT_VIA_IRF3	4	14	16239	62	74.89862	1.87E-07
lightgreen	3419	chr12p13	7	164	16089	42	16.51728	1.88E-07
brown	9447	GSE24634_TREG_VS_TCONV_POST_DAY10_IL4_CONVE	53	193	16060	2190	2.038018	1.89E-07
brown	9977	GSE3982_EOSINOPHIL_VS_NEUTROPHIL_DN	50	178	16075	2190	2.08468	1.94E-07
purple	6162	TENEDINI_MEGAKARYOCYTE_MARKERS	8	65	16188	157	12.74121	2.01E-07

greenyello	10186	GSE7764_NKCELL_VS_SPLENOCYTE_UP	12	191	16062	150	6.807539	2.06E-07
cyan	9761	GSE3337_CTRL_VS_4H_IFNG_IN_CD8POS_DC_DN	9	184	16069	77	10.32446	2.09E-07
magenta	3793	MODULE_345	10	111	16142	167	8.76787	2.11E-07
turquoise	1948	GTGCAAT,MIR-25,MIR-32,MIR-92,MIR-363,MIR-367	108	272	15981	4169	1.547948	2.14E-07
purple	5051	TONKS_TARGETS_OF_RUNX1_RUNX1T1_FUSION_ERYTI	5	15	16238	157	34.50743	2.19E-07
lightcyan	6582	ZHU_CMV_ALL_UP	7	112	16141	62	16.38407	2.20E-07
lightcyan	8299	MEL18_DN.V1_DN	7	112	16141	62	16.38407	2.20E-07
blue	4801	PARENT_MTOR_SIGNALING_UP	144	530	15723	2978	1.482844	2.25E-07
cyan	6341	BENNETT_SYSTEMIC_LUPUS_ERYTHEMATOSUS	5	29	16224	77	36.39275	2.27E-07
turquoise	8969	GSE17721_CPG_VS_GARDIQUIMOD_1H_BMDM_DN	82	193	16060	4169	1.656373	2.28E-07
turquoise	80	NUCLEUS	421	1333	14920	4169	1.231271	2.29E-07
turquoise	9738	GSE31082_DP_VS_CD8_SP_THYMOCYTE_UP	83	196	16057	4169	1.650911	2.29E-07
purple	6088	GNATENKO_PLATELET_SIGNATURE	7	45	16208	157	16.10347	2.29E-07
lightcyan	8000	FEVR_CTNNB1_TARGETS_UP	13	582	15671	62	5.855476	2.31E-07
salmon	4828	ONKEN_UVEAL_MELANOMA_DN	17	486	15767	127	4.47654	2.38E-07
cyan	8567	GSE13484_3H_UNSTIM_VS_YF17D_VACCINE_STIM_PB	9	187	16066	77	10.15883	2.40E-07
greenyello	7668	HAHTOLA_CTCL_PATHOGENESIS	5	16	16237	150	33.86042	2.52E-07
magenta	4973	VECCHI_GASTRIC_CANCER_EARLY_DN	14	246	16007	167	5.538727	2.52E-07
salmon	2542	GCM_PPP1CC	7	56	16197	127	15.99705	2.54E-07
magenta	9330	GSE22886_NAIVE_VS_IGM_MEMORY_BCELL_UP	12	175	16078	167	6.673601	2.57E-07
brown	7418	CHEN_METABOLIC_SYNDROM_NETWORK	205	1094	15159	2190	1.390676	2.63E-07
lightcyan	9713	GSE30083_SP1_VS_SP3_THYMOCYTE_DN	8	171	16082	62	12.2641	2.66E-07
magenta	5976	MORI_MATURE_B_LYMPHOCYTE_UP	9	87	16166	167	10.06793	2.66E-07
blue	2336	MORF_SMC1L1	29	62	16191	2978	2.55279	2.70E-07
greenyello	9374	GSE22886_NAIVE_CD8_TCELL_VS_MONOCYTE_UP	12	196	16057	150	6.633878	2.73E-07
magenta	9635	GSE29614_CTRL_VS_DAY7_TIV_FLU_VACCINE_PBMC_I	12	176	16077	167	6.635683	2.73E-07
greenyello	4565	REACTOME_GPCR_LIGAND_BINDING	13	235	16018	150	5.994014	2.76E-07
cyan	8385	HECKER_IFNB1_TARGETS	7	93	16160	77	15.88759	2.77E-07
purple	8385	HECKER_IFNB1_TARGETS	9	93	16160	157	10.01829	2.80E-07
turquoise	7395	LEE_RECENT_THYMIC_EMIGRANT	90	218	16035	4169	1.609488	2.88E-07
turquoise	2320	MORF_PSMC1	80	188	16065	4169	1.658952	2.95E-07
turquoise	6637	WELCSH_BRCA1_TARGETS_UP	80	188	16065	4169	1.658952	2.95E-07
turquoise	1998	ATGTTAA,MIR-302C	86	206	16047	4169	1.627544	2.95E-07
turquoise	10202	GSE7852_THYMUS_VS_FAT_TREG_UP	83	197	16056	4169	1.642531	2.97E-07
purple	7371	SWEET_LUNG_CANCER_KRAS_DN	16	351	15902	157	4.718965	2.98E-07
red	10149	GSE7400_CTRL_VS_CSF3_IN_VIVO_TREATED_PBMC_DI	21	178	16075	524	3.65933	3.00E-07
salmon	9675	GSE29618_MONOCYTE_VS_MDC_DAY7_FLU_VACCINE_	11	191	16062	127	7.370367	3.01E-07
pink	5045	TONKS_TARGETS_OF_RUNX1_RUNX1T1_FUSION_HSC_	14	183	16070	228	5.453504	3.08E-07
red	9787	GSE34205_HEALTHY_VS_RSV_INF_INFANT_PBMC_DN	22	194	16059	524	3.517412	3.15E-07
turquoise	4952	HORIUCHI_WTAP_TARGETS_DN	117	302	15951	4169	1.51036	3.15E-07
cyan	8064	YANG_BCL3_TARGETS_UP	11	322	15931	77	7.210736	3.24E-07
pink	7973	VANDESLUIS_NORMAL_EMBRYOS_DN	6	21	16232	228	20.36717	3.25E-07
blue	239	RNA_METABOLIC_PROCESS	193	757	15496	2978	1.391459	3.26E-07
salmon	5915	STARK_PREFRONTAL_CORTEX_22Q11_DELETION_DN	17	498	15755	127	4.368672	3.35E-07
turquoise	1960	CAGTATT,MIR-200B,MIR-200C,MIR-429	150	408	15845	4169	1.433286	3.41E-07
cyan	8621	GSE14000_UNSTIM_VS_16H_LPS_DC_DN	9	195	16058	77	9.742058	3.42E-07
brown	7351	RUTELLA_RESPONSE_TO_HGF_VS_CSF2RB_AND_IL4_U	90	398	15855	2190	1.67822	3.43E-07
magenta	7380	HOFFMANN_SMALL_PRE_BII_TO_IMMATURE_B_LYMP	8	66	16187	167	11.79677	3.64E-07
blue	1220	HELICASE_ACTIVITY	25	50	16203	2978	2.728845	3.64E-07
red	10074	GSE3982_BASOPHIL_VS_CENT_MEMORY_CD4_TCELL_U	22	196	16057	524	3.48152	3.77E-07
lightcyan	5149	GOZGIT_ESR1_TARGETS_DN	13	608	15645	62	5.605077	3.80E-07
turquoise	6463	RAMALHO_STEMNESS_UP	84	201	16052	4169	1.629239	3.83E-07
red	3535	MODULE_46	30	332	15921	524	2.802757	3.84E-07
purple	3553	MODULE_64	17	404	15849	157	4.356136	3.84E-07
blue	10039	GSE3982_MAC_VS_CENT_MEMORY_CD4_TCELL_DN	60	176	16077	2978	1.860576	3.94E-07
salmon	8578	GSE13485_CTRL_VS_DAY7_YF17D_VACCINE_PBMC_UP	10	156	16097	127	8.203614	3.97E-07
magenta	3605	MODULE_117	19	472	15781	167	3.917677	4.00E-07
turquoise	2180	TAATGTG,MIR-323	65	145	16108	4169	1.74762	4.01E-07
turquoise	1944	GCACTTT,MIR-17-5P,MIR-20A,MIR-106A,MIR-106B,MIR	188	534	15719	4169	1.372519	4.05E-07
red	10046	GSE3982_NEUTROPHIL_VS_BCELL_UP	20	166	16087	524	3.737009	4.09E-07

blue	8444	GSE11057_NAIVE_VS_EFF_MEMORY_CD4_TCELL_UP	64	192	16061	2978	1.81923	4.11E-07
salmon	10135	GSE6269_FLU_VS_E_COLI_INF_PBMC_DN	9	120	16133	127	9.598228	4.11E-07
brown	2709	GNF2_TNFRSF1B	25	65	16188	2190	2.854408	4.21E-07
magenta	6195	LU_IL4_SIGNALING	9	92	16161	167	9.520763	4.31E-07
brown	2656	GNF2_ITGB2	23	57	16196	2190	2.994625	4.43E-07
turquoise	2104	TTTGTAG,MIR-520D	112	288	15965	4169	1.516098	4.48E-07
salmon	143	RIBOSOME	6	38	16215	127	20.2068	4.55E-07
red	6794	KRIGE_RESPONSE_TO_TOSEDOSTAT_6HR_UP	59	929	15324	524	1.969874	4.56E-07
brown	9677	GSE29618_PDC_VS_MDC_DAY7_FLU_VACCINE_DN	53	198	16055	2190	1.986553	4.58E-07
red	7858	HIRSCH_CELLULAR_TRANSFORMATION_SIGNATURE_UI	24	231	16022	524	3.222564	4.72E-07
red	9369	GSE22886_NAIVE_BCELL_VS_MONOCYTE_DN	22	199	16054	524	3.429034	4.91E-07
turquoise	9933	GSE37416_CTRL_VS_48H_F_TULARENSIS_LVS_NEUTRO	82	196	16057	4169	1.631021	4.96E-07
blue	10023	GSE3982_DC_VS_CENT_MEMORY_CD4_TCELL_DN	61	181	16072	2978	1.839332	4.97E-07
yellow	5175	ENK_UV_RESPONSE_KERATINOCYTE_UP	74	470	15783	1426	1.794518	4.98E-07
magenta	7487	VALK_AML_CLUSTER_10	6	30	16223	167	19.46467	5.21E-07
purple	3495	MODULE_2	15	321	15932	157	4.83749	5.21E-07
lightgreen	8389	KAECH_NAIVE_VS_DAY15_EFF_CD8_TCELL_DN	7	191	16062	42	14.18237	5.28E-07
lightgreen	2717	GNF2_ZAP70	4	26	16227	42	59.5348	5.53E-07
magenta	8393	KAECH_DAY8_EFF_VS_DAY15_EFF_CD8_TCELL_DN	12	188	16065	167	6.212129	5.56E-07
brown	10022	GSE3982_DC_VS_CENT_MEMORY_CD4_TCELL_UP	52	194	16059	2190	1.989258	5.59E-07
greenyello	5908	BENPORATH_PRC2_TARGETS	15	339	15914	150	4.794395	5.76E-07
red	227	PLASMA_MEMBRANE	66	1095	15158	524	1.869528	5.89E-07
turquoise	2103	ACCAAAG,MIR-9	160	444	15809	4169	1.404878	5.93E-07
lightcyan	8235	WNT_UP.V1_DN	7	130	16123	62	14.11551	6.07E-07
lightcyan	4872	SENGUPTA_NASOPHARYNGEAL_CARCINOMA_WITH_LM	10	342	15911	62	7.665063	6.08E-07
lightgreen	10255	GSE9650_NAIVE_VS_EFF_CD8_TCELL_DN	7	195	16058	42	13.89145	6.08E-07
salmon	2678	GNF2_NS	6	40	16213	127	19.19646	6.24E-07
brown	2622	GNF2_CD53	23	58	16195	2190	2.942993	6.42E-07
turquoise	8986	GSE17721_LPS_VS_PAM3CSK4_1H_BMDM_UP	79	188	16065	4169	1.638215	6.44E-07
green	4787	REACTOME_GAP_JUNCTION_ASSEMBLY	7	9	16244	1305	9.68676	6.60E-07
red	4954	GAL_LEUKEMIC_STEM_CELL_DN	23	219	16034	524	3.257512	6.76E-07
blue	307	BIOPOLYMER_METABOLIC_PROCESS	352	1529	14724	2978	1.256447	6.81E-07
red	9984	GSE3982_EOSINOPHIL_VS_CENT_MEMORY_CD4_TCELL	21	187	16066	524	3.483212	6.88E-07
salmon	2311	MORF_HDAC1	12	253	16000	127	6.070026	6.88E-07
lightgreen	8387	KAECH_NAIVE_VS_DAY8_EFF_CD8_TCELL_DN	7	199	16054	42	13.61223	6.97E-07
purple	7704	WONG_ADULT_TISSUE_STEM_MODULE	21	629	15624	157	3.456229	7.12E-07
red	6889	FOSTER_TOLERANT_MACROPHAGE_UP	18	142	16111	524	3.931755	7.30E-07
grey60	5013	TAKEDA_TARGETS_OF_NUP98_HOXA9_FUSION_8D_DN	7	191	16062	44	13.53772	7.36E-07
brown	2650	GNF2_ICAM3	18	39	16214	2190	3.42529	7.39E-07
magenta	4845	SENGUPTA_NASOPHARYNGEAL_CARCINOMA_DN	14	269	15984	167	5.065156	7.43E-07
lightcyan	7929	JOHNSTONE_PARVB_TARGETS_2_UP	7	134	16119	62	13.69415	7.46E-07
lightcyan	10146	GSE6566_STRONG_VS_WEAK_DC_STIMULATED_CD4_T	7	134	16119	62	13.69415	7.46E-07
lightcyan	8667	GSE1432_1H_VS_24H_IFNG_MICROGLIA_DN	8	197	16056	62	10.64549	7.80E-07
purple	3576	MODULE_88	21	633	15620	157	3.434389	7.89E-07
red	3564	MODULE_76	12	64	16189	524	5.81572	7.93E-07
pink	6353	RADMACHER_AML_PROGNOSIS	9	73	16180	228	8.788572	8.00E-07
turquoise	5600	DACOSTA_UV_RESPONSE_VIA_ERCC3_COMMON_DN	167	469	15784	4169	1.388178	8.03E-07
lightcyan	6881	RIGGINS_TAMOXIFEN_RESISTANCE_DN	8	198	16055	62	10.59172	8.11E-07
magenta	9340	GSE22886_IGG_IGA_MEMORY_BCELL_VS_BM_PLASMA	12	195	16058	167	5.989129	8.21E-07
brown	4084	PID_VEGFR1_2_PATHWAY	25	67	16186	2190	2.769202	8.24E-07
turquoise	6869	ZHENG_FOXP3_TARGETS_IN_THYMUS_UP	80	192	16061	4169	1.62439	8.28E-07
turquoise	10106	GSE39820_CTRL_VS_TGFBETA1_IL6_IL23A_CD4_TCELL_	80	192	16061	4169	1.62439	8.28E-07
salmon	10235	GSE9006_TYPE_1_DIABETES_AT_DX_VS_4MONTH_POS	10	169	16084	127	7.572567	8.31E-07
lightcyan	6169	LEE_LIVER_CANCER_MYC_E2F1_UP	5	46	16207	62	28.49404	8.33E-07
turquoise	2324	MORF_RAD21	76	180	16073	4169	1.646049	8.35E-07
cyan	9233	GSE18791_CTRL_VS_NEWCASTLE_VIRUS_DC_14H_DN	8	159	16094	77	10.62027	8.35E-07
magenta	5974	MORI_IMMATURE_B_LYMPHOCYTE_UP	7	51	16202	167	13.35811	8.44E-07
turquoise	1469	V$ELK1_02	95	238	16015	4169	1.556139	8.53E-07
lightcyan	4719	REACTOME_INNATE_IMMUNE_SYSTEM	8	200	16053	62	10.48581	8.75E-07
magenta	2847	KEGG_B_CELL_RECEPTOR_SIGNALING_PATHWAY	8	74	16179	167	10.52144	8.90E-07

purple	2830	KEGG_FOCAL_ADHESION	11	172	16081	157	6.620612	8.91E-07
purple	235	REGULATION_OF_BIOLOGICAL_QUALITY	15	335	15918	157	4.635327	8.92E-07
cyan	3793	MODULE_345	7	111	16142	77	13.31122	9.29E-07
red	5115	JAATINEN_HEMATOPOIETIC_STEM_CELL_DN	23	223	16030	524	3.199081	9.30E-07
turquoise	3711	MODULE_239	55	119	16134	4169	1.801845	9.36E-07
greenyello	8450	GSE11057_EFF_MEM_VS_CENT_MEM_CD4_TCELL_UP	11	181	16072	150	6.585009	9.39E-07
purple	3498	MODULE_5	16	383	15870	157	4.324691	9.50E-07
red	8455	GSE11057_CD4_CENT_MEM_VS_PBMC_DN	21	191	16062	524	3.410265	9.76E-07
lightcyan	5966	ZHANG_RESPONSE_TO_IKK_INHIBITOR_AND_TNF_UP	8	203	16050	62	10.33084	9.79E-07
turquoise	2141	TTTTGAG,MIR-373	83	202	16051	4169	1.601874	1.04E-06
greenyello	8829	GSE17580_UNINFECTED_VS_S_MANSONI_INF_TEFF_DI	11	183	16070	150	6.513042	1.05E-06
purple	4824	LIU_PROSTATE_CANCER_DN	16	386	15867	157	4.29108	1.05E-06
cyan	5530	BOWIE_RESPONSE_TO_EXTRACELLULAR_MATRIX	4	17	16236	77	49.66539	1.06E-06
magenta	9336	GSE22886_NAIVE_BCELL_VS_BM_PLASMA_CELL_UP	12	200	16053	167	5.839401	1.07E-06
turquoise	4943	BORCZUK_MALIGNANT_MESOTHELIOMA_UP	115	302	15951	4169	1.484542	1.09E-06
brown	9357	GSE22886_DC_VS_MONOCYTE_DN	52	198	16055	2190	1.949071	1.10E-06
brown	9365	GSE22886_NAIVE_BCELL_VS_NEUTROPHIL_DN	52	198	16055	2190	1.949071	1.10E-06
brown	2850	KEGG_LEUKOCYTE_TRANSENDOTHELIAL_MIGRATION	32	99	16154	2190	2.398856	1.10E-06
turquoise	5911	BENPORATH_CYCLING_GENES	212	622	15631	4169	1.328762	1.13E-06
brown	2833	KEGG_ADHERENS_JUNCTION	25	68	16185	2190	2.728478	1.14E-06
turquoise	5915	STARK_PREFRONTAL_CORTEX_22Q11_DELETION_DN	175	498	15755	4169	1.369968	1.16E-06
lightcyan	6686	ZHU_CMV_24_HR_UP	6	90	16163	62	17.47634	1.17E-06
turquoise	4957	BASAKI_YBX1_TARGETS_DN	132	357	15896	4169	1.441476	1.19E-06
turquoise	3305	chr8q21	24	38	16215	4169	2.462234	1.20E-06
turquoise	6796	KRIGE_RESPONSE_TO_TOSEDOSTAT_24HR_UP	252	759	15494	4169	1.294376	1.21E-06
magenta	5969	MORI_PRE_BI_LYMPHOCYTE_DN	8	77	16176	167	10.11152	1.21E-06
blue	3524	MODULE_35	12	16	16237	2978	4.093267	1.22E-06
lightcyan	3533	MODULE_44	9	285	15968	62	8.278268	1.24E-06
turquoise	6962	FINETTI_BREAST_CANCERS_KINOME_GRAY	13	15	16238	4169	3.378732	1.25E-06
red	9986	GSE3982_EOSINOPHIL_VS_NKCELL_UP	20	178	16075	524	3.485076	1.25E-06
red	9938	GSE37416_0H_VS_12H_F_TULARENSIS_LVS_NEUTROPI	21	194	16059	524	3.357529	1.26E-06
turquoise	6794	KRIGE_RESPONSE_TO_TOSEDOSTAT_6HR_UP	301	929	15324	4169	1.263143	1.27E-06
blue	2444	MORF_PRKAG1	69	218	16035	2978	1.727434	1.29E-06
red	6416	IVANOVA_HEMATOPOIESIS_MATURE_CELL	26	279	15974	524	2.890489	1.29E-06
brown	9665	GSE29618_PDC_VS_MDC_DN	52	199	16054	2190	1.939276	1.30E-06
brown	10272	GSE9988_ANTI_TREM1_VS_ANTI_TREM1_AND_LPS_M	52	199	16054	2190	1.939276	1.30E-06
lightcyan	1820	V$ICSBP_Q6	8	211	16042	62	9.939153	1.31E-06
purple	6032	ONDER_CDH1_TARGETS_2_UP	12	217	16036	157	5.724735	1.32E-06
brown	7482	VALK_AML_CLUSTER_5	16	33	16220	2190	3.598284	1.34E-06
turquoise	2075	CATGTAA,MIR-496	67	155	16098	4169	1.685174	1.35E-06
turquoise	7531	CHIANG_LIVER_CANCER_SUBCLASS_UNANNOTATED_D	78	188	16065	4169	1.617478	1.38E-06
lightcyan	9221	GSE18791_CTRL_VS_NEWCASTLE_VIRUS_DC_2H_DN	7	147	16106	62	12.4831	1.39E-06
blue	8422	GSE10325_CD4_TCELL_VS_BCELL_UP	63	194	16059	2978	1.772343	1.39E-06
turquoise	6994	DE_YY1_TARGETS_DN	42	84	16169	4169	1.949268	1.40E-06
lightcyan	6229	LEE_LIVER_CANCER_E2F1_UP	5	51	16202	62	25.70051	1.41E-06
cyan	9828	GSE360_DC_VS_MAC_M_TUBERCULOSIS_UP	8	171	16082	77	9.874991	1.45E-06
cyan	10136	GSE6269_FLU_VS_STREP_AUREUS_INF_PBMC_UP	8	171	16082	77	9.874991	1.45E-06
magenta	10128	GSE6269_HEALTHY_VS_E_COLI_INF_PBMC_UP	11	170	16083	167	6.297393	1.46E-06
salmon	7556	YAGI_AML_WITH_INV_16_TRANSLOCATION	14	377	15876	127	4.752438	1.48E-06
red	8449	GSE11057_NAIVE_CD4_VS_PBMC_CD4_TCELL_DN	21	196	16057	524	3.323269	1.49E-06
turquoise	183	MACROMOLECULAR_COMPLEX	283	868	15385	4169	1.271067	1.50E-06
yellow	5732	SPIELMAN_LYMPHOBLAST_EUROPEAN_VS_ASIAN_UP	73	475	15778	1426	1.751634	1.50E-06
turquoise	4857	GAZDA_DIAMOND_BLACKFAN_ANEMIA_PROGENITOR_	34	63	16190	4169	2.103972	1.50E-06
red	7316	MARTINELLI_IMMATURE_NEUTROPHIL_DN	6	13	16240	524	14.31562	1.54E-06
magenta	5093	SABATES_COLORECTAL_ADENOMA_DN	11	171	16082	167	6.260567	1.55E-06
pink	7914	WIERENGA_STAT5A_TARGETS_GROUP1	11	126	16127	228	6.223301	1.61E-06
turquoise	7400	SHEDDEN_LUNG_CANCER_POOR_SURVIVAL_A6	155	434	15819	4169	1.392335	1.62E-06
salmon	9983	GSE3982_EOSINOPHIL_VS_EFF_MEMORY_CD4_TCELL_	10	182	16071	127	7.031669	1.63E-06
blue	2375	MORF_DDB1	74	240	16013	2978	1.682788	1.64E-06
green	8591	GSE13485_DAY3_VS_DAY21_YF17D_VACCINE_PBMC_I	21	83	16170	1305	3.151115	1.65E-06

lightgreen	8008	WINZEN_DEGRADED_VIA_KHSRP	5	78	16175	42	24.80617	1.66E-06
salmon	6159	POMEROY_MEDULLOBLASTOMA_DESMOPLASIC_VS_CI	6	47	16206	127	16.33741	1.67E-06
salmon	7468	IRITANI_MAD1_TARGETS_DN	6	47	16206	127	16.33741	1.67E-06
salmon	6677	WHITESIDE_CISPLATIN_RESISTANCE_DN	4	12	16241	127	42.65879	1.68E-06
lightcyan	2841	KEGG_RIG_I_LIKE_RECEPTOR_SIGNALING_PATHWAY	5	53	16200	62	24.73068	1.71E-06
blue	5744	PUJANA_ATM_PCC_NETWORK	306	1317	14936	2978	1.268074	1.72E-06
blue	2371	MORF_CUL1	30	70	16183	2978	2.33901	1.72E-06
turquoise	10110	GSE39820_CTRL_VS_TGFBETA3_IL6_IL23A_CD4_TCELL_	80	195	16058	4169	1.5994	1.73E-06
blue	7499	JISON_SICKLE_CELL_DISEASE_DN	58	175	16078	2978	1.808834	1.73E-06
cyan	6760	ZHU_CMV_8_HR_UP	5	43	16210	77	24.54394	1.75E-06
turquoise	5245	GAUSSMANN_MLL_AF4_FUSION_TARGETS_C_UP	66	153	16100	4169	1.681722	1.76E-06
cyan	3853	MODULE_436	7	122	16131	77	12.11103	1.76E-06
red	8774	GSE15767_MED_VS_SCS_MAC_LN_UP	20	182	16071	524	3.408481	1.78E-06
turquoise	1966	ACTGAAA,MIR-30A-3P,MIR-30E-3P	74	177	16076	4169	1.629897	1.81E-06
brown	6403	REN_ALVEOLAR_RHABDOMYOSARCOMA_DN	85	384	15869	2190	1.642771	1.82E-06
greenyello	7588	ONO_AML1_TARGETS_UP	5	23	16230	150	23.55507	1.84E-06
greenyello	9315	GSE22886_NAIVE_TCELL_VS_NKCELL_DN	11	194	16059	150	6.143746	1.86E-06
cyan	7307	ICHIBA_GRAFT_VERSUS_HOST_DISEASE_35D_UP	7	123	16130	77	12.01256	1.86E-06
brown	8592	GSE13485_DAY7_VS_DAY21_YF17D_VACCINE_PBMC_U	51	196	16057	2190	1.931094	1.87E-06
lightcyan	9711	GSE30083_SP1_VS_SP2_THYMOCYTE_DN	7	154	16099	62	11.91569	1.90E-06
lightcyan	7305	ICHIBA_GRAFT_VERSUS_HOST_DISEASE_D7_UP	6	98	16155	62	16.0497	1.93E-06
red	3563	MODULE_75	29	340	15913	524	2.645583	1.95E-06
brown	4976	SMIRNOV_CIRCULATING_ENDOTHELIOCYTES_IN_CANC	43	155	16098	2190	2.058857	1.95E-06
cyan	9148	GSE17721_12H_VS_24H_GARDIQUIMOD_BMDM_UP	8	178	16075	77	9.486648	1.95E-06
magenta	4944	BORCZUK_MALIGNANT_MESOTHELIOMA_DN	8	82	16171	167	9.494961	1.96E-06
turquoise	4808	PICCALUGA_ANGIOIMMUNOBLASTIC_LYMPHOMA_DN	59	133	16120	4169	1.729426	2.05E-06
pink	10137	GSE6269_FLU_VS_STREP_AUREUS_INF_PBMC_DN	12	156	16097	228	5.483468	2.07E-06
brown	2577	GNF2_CARD15	25	70	16183	2190	2.650522	2.12E-06
salmon	7531	CHIANG_LIVER_CANCER_SUBCLASS_UNANNOTATED_D	10	188	16065	127	6.807254	2.19E-06
turquoise	9936	GSE37416_0H_VS_6H_F_TULARENSIS_LVS_NEUTROPH	80	196	16057	4169	1.59124	2.20E-06
cyan	9803	GSE360_CTRL_VS_M_TUBERCULOSIS_DC_DN	8	181	16072	77	9.329411	2.21E-06
cyan	9857	GSE360_HIGH_DOSE_B_MALAYI_VS_M_TUBERCULOSIS	8	181	16072	77	9.329411	2.21E-06
red	7912	WIERENGA_STAT5A_TARGETS_UP	21	201	16052	524	3.2406	2.24E-06
turquoise	3232	chr5q14	21	32	16221	4169	2.558415	2.26E-06
brown	10046	GSE3982_NEUTROPHIL_VS_BCELL_UP	45	166	16087	2190	2.011842	2.27E-06
salmon	9981	GSE3982_EOSINOPHIL_VS_BASOPHIL_DN	10	189	16064	127	6.771237	2.30E-06
purple	4530	REACTOME_SMOOTH_MUSCLE_CONTRACTION	5	23	16230	157	22.50485	2.31E-06
turquoise	2020	ATACTGT,MIR-144	74	178	16075	4169	1.62074	2.33E-06
purple	931	RESPONSE_TO_WOUNDING	10	153	16100	157	6.766163	2.33E-06
pink	6274	GUO_HEX_TARGETS_DN	8	62	16191	228	9.198076	2.34E-06
turquoise	2023	GTATTAT,MIR-369-3P	69	163	16090	4169	1.650301	2.35E-06
purple	10239	GSE9006_TYPE_1_VS_TYPE_2_DIABETES_PBMC_AT_DX	11	190	16063	157	5.993396	2.37E-06
blue	2537	GCM_NF2	86	293	15960	2978	1.601916	2.39E-06
salmon	8410	GSE10239_NAIVE_VS_KLRG1HIGH_EFF_CD8_TCELL_UP	10	190	16063	127	6.735599	2.41E-06
brown	4890	FULCHER_INFLAMMATORY_RESPONSE_LECTIN_VS_LPS	114	558	15695	2190	1.516213	2.41E-06
red	6358	TAVOR_CEBPA_TARGETS_UP	10	48	16205	524	6.461912	2.42E-06
salmon	71	NON_MEMBRANE_BOUND_ORGANELLE	17	575	15678	127	3.783649	2.42E-06
salmon	177	INTRACELLULAR_NON_MEMBRANE_BOUND_ORGANEL	17	575	15678	127	3.783649	2.42E-06
blue	8688	GSE1460_INTRATHYMIC_T_PROGENITOR_VS_NAIVE_CI	63	197	16056	2978	1.745353	2.49E-06
greenyello	5907	BENPORATH_ES_WITH_H3K27ME3	20	654	15599	150	3.313558	2.49E-06
red	10269	GSE9988_ANTI_TREM1_VS_LPS_MONOCYTE_DN	20	186	16067	524	3.33518	2.50E-06
greenyello	6268	MATSUDA_NATURAL_KILLER_DIFFERENTIATION	16	433	15820	150	4.003818	2.55E-06
blue	6286	LI_WILMS_TUMOR_VS_FETAL_KIDNEY_1_DN	53	157	16096	2978	1.842405	2.57E-06
red	5966	ZHANG_RESPONSE_TO_IKK_INHIBITOR_AND_TNF_UP	21	203	16050	524	3.208673	2.63E-06
turquoise	7937	BRUINS_UVC_RESPONSE_LATE	332	1045	15208	4169	1.238578	2.65E-06
brown	2816	KEGG_LYSOSOME	35	117	16136	2190	2.220095	2.69E-06
brown	9940	GSE37416_0H_VS_24H_F_TULARENSIS_LVS_NEUTROPH	50	193	16060	2190	1.922658	2.69E-06
cyan	8805	GSE15930_STIM_VS_STIM_AND_IFNAB_48H_CD8_T_C	8	186	16067	77	9.07862	2.71E-06
red	6316	VERHAAK_AML_WITH_NPM1_MUTATED_UP	19	171	16082	524	3.446353	2.73E-06
red	6403	REN_ALVEOLAR_RHABDOMYOSARCOMA_DN	31	384	15869	524	2.503991	2.75E-06

brown	4021	PID_AJDISS_2PATHWAY	18	42	16211	2190	3.180626	2.80E-06
cyan	9953	GSE3982_CTRL_VS_LPS_4H_MAC_DN	8	187	16066	77	9.030072	2.82E-06
salmon	8408	GSE10239_NAIVE_VS_KLRG1INT_EFF_CD8_TCELL_UP	10	194	16059	127	6.596721	2.90E-06
salmon	10047	GSE3982_NEUTROPHIL_VS_BCELL_DN	10	194	16059	127	6.596721	2.90E-06
red	10273	GSE9988_ANTI_TREM1_VS_ANTI_TREM1_AND_LPS_M	19	172	16081	524	3.426316	2.98E-06
brown	9346	GSE22886_DAY0_VS_DAY1_MONOCYTE_IN_CULTURE_	51	199	16054	2190	1.901983	3.02E-06
brown	8442	GSE10856_CTRL_VS_TNFRSF6B_IN_MACROPHAGE_UP	47	178	16075	2190	1.959599	3.02E-06
purple	826	RESPONSE_TO_EXTERNAL_STIMULUS	12	235	16018	157	5.286245	3.03E-06
cyan	9461	GSE24634_IL4_VS_CTRL_TREATED_NAIVE_CD4_TCELL_	8	189	16064	77	8.934515	3.06E-06
turquoise	6031	DING_LUNG_CANCER_EXPRESSION_BY_COPY_NUMBEF	47	100	16153	4169	1.832312	3.18E-06
blue	10128	GSE6269_HEALTHY_VS_E_COLI_INF_PBMC_UP	56	170	16083	2978	1.797827	3.21E-06
black	5307	HAMAI_APOPTOSIS_VIA_TRAIL_DN	13	155	16098	283	4.816802	3.23E-06
pink	5120	GRAHAM_CML_QUIESCENT_VS_NORMAL_QUIESCENT_	9	86	16167	228	7.460067	3.24E-06
turquoise	679	CELLULAR_PROTEIN_METABOLIC_PROCESS	320	1005	15248	4169	1.241325	3.28E-06
brown	9319	GSE22886_NAIVE_CD8_TCELL_VS_NKCELL_DN	49	189	16064	2190	1.924083	3.30E-06
red	5599	DACOSTA_UV_RESPONSE_VIA_ERCC3_DN	52	829	15424	524	1.945589	3.31E-06
purple	2831	KEGG_ECM_RECEPTOR_INTERACTION	7	66	16187	157	10.97964	3.31E-06
turquoise	779	PROTEIN_METABOLIC_PROCESS	350	1111	15142	4169	1.228162	3.34E-06
salmon	3848	MODULE_429	4	14	16239	127	36.56468	3.35E-06
purple	3541	MODULE_52	15	373	15880	157	4.163095	3.37E-06
lightgreen	345	SIGNAL_TRANSDUCTION	14	1331	14922	42	4.070373	3.38E-06
salmon	6439	ZHOU_TNF_SIGNALING_4HR	6	53	16200	127	14.48789	3.44E-06
cyan	6285	NEMETH_INFLAMMATORY_RESPONSE_LPS_UP	6	87	16166	77	14.5571	3.46E-06
red	2710	GNF2_TNFSF10	8	30	16223	524	8.271247	3.46E-06
salmon	8392	KAECH_DAY8_EFF_VS_DAY15_EFF_CD8_TCELL_UP	10	198	16055	127	6.463453	3.49E-06
lightcyan	8366	KRAS.600_UP.V1_DN	7	169	16084	62	10.85808	3.53E-06
cyan	3477	ST_TYPE_I_INTERFERON_PATHWAY	3	7	16246	77	90.46197	3.53E-06
brown	9379	GSE22886_NAIVE_CD4_TCELL_VS_DC_DN	51	200	16053	2190	1.892473	3.53E-06
purple	6347	DORSEY_GAB2_TARGETS	5	25	16228	157	20.70446	3.59E-06
greenyello	10082	GSE3982_EFF_MEMORY_VS_CENT_MEMORY_CD4_TCE	10	168	16085	150	6.449603	3.59E-06
red	136	INTEGRAL_TO_PLASMA_MEMBRANE	47	721	15532	524	2.021924	3.63E-06
lightcyan	8159	ACEVEDO_FGFR1_TARGETS_IN_PROSTATE_CANCER_M	8	242	16011	62	8.665956	3.64E-06
salmon	9355	GSE22886_NEUTROPHIL_VS_MONOCYTE_DN	10	199	16054	127	6.430974	3.65E-06
turquoise	6863	MARSON_BOUND_BY_FOXP3_STIMULATED	312	978	15275	4169	1.243705	3.65E-06
purple	6174	ROSS_AML_OF_FAB_M7_TYPE	7	67	16186	157	10.81576	3.67E-06
blue	7397	LEE_DIFFERENTIATING_T_LYMPHOCYTE	62	195	16058	2978	1.735265	3.69E-06
turquoise	10116	GSE39820_TGFBETA3_IL6_VS_TGFBETA3_IL6_IL23A_TF	76	186	16067	4169	1.592951	3.72E-06
lightcyan	6735	SATO_SILENCED_BY_METHYLATION_IN_PANCREATIC_C	9	326	15927	62	7.237136	3.75E-06
cyan	5569	DAUER_STAT3_TARGETS_DN	5	50	16203	77	21.10779	3.75E-06
lightcyan	9828	GSE360_DC_VS_MAC_M_TUBERCULOSIS_UP	7	171	16082	62	10.73109	3.81E-06
brown	10058	GSE3982_NEUTROPHIL_VS_TH2_UP	45	169	16084	2190	1.976129	3.82E-06
turquoise	4969	WANG_CLIM2_TARGETS_DN	73	177	16076	4169	1.607871	3.84E-06
red	9997	GSE3982_MAST_CELL_VS_NEUTROPHIL_DN	19	175	16078	524	3.367579	3.85E-06
brown	5854	RICKMAN_METASTASIS_DN	59	244	16009	2190	1.794534	3.99E-06
yellow	2244	SCGGAAGY_V$ELK1_02	146	1162	15091	1426	1.432058	4.06E-06
red	8458	GSE11057_PBMC_VS_MEM_CD4_TCELL_UP	20	192	16061	524	3.230956	4.08E-06
turquoise	2249	MGGAAGTG_V$GABP_B	239	725	15528	4169	1.285173	4.10E-06
yellow	186	MITOCHONDRION	54	327	15926	1426	1.882175	4.16E-06
greenyello	4368	REACTOME_CLASS_A1_RHODOPSIN_LIKE_RECEPTORS	10	171	16082	150	6.336452	4.21E-06
lightcyan	6804	KIM_LRRC3B_TARGETS	4	29	16224	62	36.15795	4.24E-06
brown	2685	GNF2_PTPN6	19	47	16206	2190	3.000165	4.27E-06
lightgreen	10082	GSE3982_EFF_MEMORY_VS_CENT_MEMORY_CD4_TCE	6	168	16085	42	13.82058	4.30E-06
turquoise	1942	AGCACTT,MIR-93,MIR-302A,MIR-302B,MIR-302C,MIR-3	113	303	15950	4169	1.45391	4.30E-06
cyan	8474	GSE11864_CSF1_IFNG_VS_CSF1_PAM3CYS_IN_MAC_U	8	198	16055	77	8.528401	4.32E-06
red	8576	GSE13485_CTRL_VS_DAY3_YF17D_VACCINE_PBMC_UP	17	145	16108	524	3.636496	4.39E-06
brown	6287	HESS_TARGETS_OF_HOXA9_AND_MEIS1_DN	26	77	16176	2190	2.505948	4.44E-06
turquoise	9683	GSE29618_PRE_VS_DAY7_FLU_VACCINE_PDC_DN	79	196	16057	4169	1.571349	4.48E-06
cyan	8437	GSE10325_BCELL_VS_LUPUS_BCELL_DN	8	199	16054	77	8.485545	4.48E-06
blue	5745	PUJANA_CHEK2_PCC_NETWORK	187	757	15496	2978	1.348201	4.51E-06
blue	9403	GSE24026_PD1_LIGATION_VS_CTRL_IN_ACT_TCELL_LIN	61	192	16061	2978	1.733954	4.53E-06

red	10061	GSE3982_BCELL_VS_BASOPHIL_DN	19	177	16076	524	3.329527	4.56E-06
salmon	5031	RHEIN_ALL_GLUCOCORTICOID_THERAPY_DN	13	358	15895	127	4.647187	4.58E-06
lightcyan	9834	GSE360_L_DONOVANI_VS_B_MALAYI_HIGH_DOSE_DC_	7	176	16077	62	10.42623	4.61E-06
cyan	6968	WALLACE_PROSTATE_CANCER_RACE_UP	9	267	15986	77	7.114986	4.63E-06
blue	9992	GSE3982_MAST_CELL_VS_DC_UP	59	184	16069	2978	1.75002	4.66E-06
turquoise	9003	GSE17721_POLYIC_VS_CPG_0.5H_BMDM_DN	76	187	16066	4169	1.584432	4.70E-06
brown	8622	GSE14000_4H_VS_16H_LPS_DC_TRANSLATED_RNA_UF	48	186	16067	2190	1.915216	4.75E-06
blue	8712	GSE1460_NAIVE_CD4_TCELL_ADULT_BLOOD_VS_THYM	57	176	16077	2978	1.767547	4.77E-06
turquoise	1969	TACTTGA,MIR-26A,MIR-26B	105	278	15975	4169	1.472469	4.84E-06
salmon	2690	GNF2_RAN	7	86	16167	127	10.41668	4.85E-06
lightgreen	2653	GNF2_IL2RB	4	44	16209	42	35.17965	4.85E-06
lightcyan	4657	REACTOME_NEGATIVE_REGULATORS_OF_RIG_I_MDA5	4	30	16223	62	34.95269	4.88E-06
purple	2835	KEGG_GAP_JUNCTION	7	70	16183	157	10.35223	4.94E-06
red	10231	GSE9006_HEALTHY_VS_TYPE_2_DIABETES_PBMC_AT_I	19	178	16075	524	3.310822	4.95E-06
greenyello	1012	CELLULAR_DEFENSE_RESPONSE	6	48	16205	150	13.54417	4.98E-06
blue	3645	MODULE_160	11	15	16238	2978	4.002306	5.06E-06
turquoise	2124	ACCATTT,MIR-522	62	145	16108	4169	1.666961	5.06E-06
brown	9443	GSE24634_TREG_VS_TCONV_POST_DAY5_IL4_CONVER	50	197	16056	2190	1.88362	5.06E-06
greenyello	356	CELL_SURFACE_RECEPTOR_LINKED_SIGNAL_TRANSDUC	16	457	15796	150	3.793552	5.08E-06
lightyellow	5047	TONKS_TARGETS_OF_RUNX1_RUNX1T1_FUSION_GRAN	3	17	16236	33	86.91444	5.09E-06
red	2620	GNF2_CD33	10	52	16201	524	5.964841	5.21E-06
turquoise	935	CELLULAR_MACROMOLECULE_METABOLIC_PROCESS	322	1017	15236	4169	1.234345	5.24E-06
lightcyan	5141	DODD_NASOPHARYNGEAL_CARCINOMA_DN	17	1311	14942	62	3.399289	5.24E-06
red	6314	THEILGAARD_NEUTROPHIL_AT_SKIN_WOUND_UP	12	76	16177	524	4.897449	5.27E-06
black	7719	MIKKELSEN_NPC_HCP_WITH_H3K27ME3	13	162	16091	283	4.608668	5.27E-06
blue	9786	GSE34205_HEALTHY_VS_RSV_INF_INFANT_PBMC_UP	61	193	16060	2978	1.724969	5.45E-06
salmon	5797	LOPEZ_MBD_TARGETS	21	893	15360	127	3.009523	5.48E-06
magenta	6310	ZHAN_MULTIPLE_MYELOMA_CD2_UP	6	44	16209	167	13.27137	5.49E-06
magenta	8561	GSE13411_PLASMA_CELL_VS_MEMORY_BCELL_DN	11	195	16058	167	5.490035	5.54E-06
lightcyan	8569	GSE13484_12H_UNSTIM_VS_YF17D_VACCINE_STIM_PI	7	181	16072	62	10.13821	5.55E-06
lightcyan	8909	GSE17721_CTRL_VS_GARDIQUIMOD_12H_BMDM_DN	7	181	16072	62	10.13821	5.55E-06
lightcyan	9203	GSE17974_IL4_AND_ANTI_IL12_VS_UNTREATED_72H_	7	181	16072	62	10.13821	5.55E-06
lightcyan	9803	GSE360_CTRL_VS_M_TUBERCULOSIS_DC_DN	7	181	16072	62	10.13821	5.55E-06
red	9936	GSE37416_0H_VS_6H_F_TULARENSIS_LVS_NEUTROPH	20	196	16057	524	3.165018	5.59E-06
red	10038	GSE3982_MAC_VS_CENT_MEMORY_CD4_TCELL_UP	20	196	16057	524	3.165018	5.59E-06
cyan	3534	MODULE_45	12	520	15733	77	4.871029	5.61E-06
red	14	INTRINSIC_TO_PLASMA_MEMBRANE	47	733	15520	524	1.988823	5.64E-06
brown	2716	GNF2_VAV1	16	36	16217	2190	3.298427	5.64E-06
turquoise	42	CYTOPLASMIC_PART	395	1277	14976	4169	1.20589	5.64E-06
turquoise	8583	GSE13485_DAY1_VS_DAY3_YF17D_VACCINE_PBMC_DI	78	194	16059	4169	1.567453	5.73E-06
lightcyan	10247	GSE9037_CTRL_VS_LPS_4H_STIM_IRAK4_KO_BMDM_I	7	182	16071	62	10.08251	5.75E-06
red	10288	GSE9988_LOW_LPS_VS_CTRL_TREATED_MONOCYTE_U	19	180	16073	524	3.274035	5.84E-06
black	7559	MEISSNER_NPC_HCP_WITH_H3K4ME2_AND_H3K27ME	14	189	16064	283	4.254155	5.84E-06
brown	9367	GSE22886_NAIVE_BCELL_VS_DC_DN	50	198	16055	2190	1.874106	5.90E-06
lightcyan	6233	DER_IFN_GAMMA_RESPONSE_UP	5	68	16185	62	19.27538	5.94E-06
turquoise	5240	GRAESSMANN_RESPONSE_TO_MC_AND_DOXORUBICII	244	746	15507	4169	1.275125	6.18E-06
cyan	4891	FULCHER_INFLAMMATORY_RESPONSE_LECTIN_VS_LPS	11	437	15816	77	5.313174	6.33E-06
blue	1375	ATPASE_ACTIVITY	38	103	16150	2978	2.013517	6.40E-06
turquoise	6383	NOUZOVA_TRETINOIN_AND_H4_ACETYLATION	59	137	16116	4169	1.678932	6.47E-06
purple	6303	TAVOR_CEBPA_TARGETS_DN	5	28	16225	157	18.48612	6.48E-06
magenta	6361	VERHAAK_AML_WITH_NPM1_MUTATED_DN	12	238	16015	167	4.90706	6.53E-06
lightcyan	9027	GSE17721_PAM3CSK4_VS_GADIQUIMOD_4H_BMDM_	7	186	16067	62	9.865678	6.64E-06
blue	9474	GSE26495_NAIVE_VS_PD1LOW_CD8_TCELL_UP	60	190	16063	2978	1.723481	6.68E-06
purple	3527	MODULE_38	15	395	15858	157	3.931226	6.75E-06
pink	6246	SCHURINGA_STAT5A_TARGETS_UP	5	20	16233	228	17.82127	6.79E-06
blue	8612	GSE14000_TRANSLATED_RNA_VS_MRNA_16H_LPS_DC	46	134	16119	2978	1.873535	6.95E-06
lightgreen	9308	GSE22886_NAIVE_CD8_TCELL_VS_MEMORY_TCELL_UP	6	183	16070	42	12.68774	7.03E-06
brown	7433	HSIAO_HOUSEKEEPING_GENES	83	385	15868	2190	1.599951	7.07E-06
lightcyan	8522	GSE12845_PRE_GC_VS_DARKZONE_GC_TONSIL_BCELL_	7	188	16065	62	9.760724	7.12E-06
turquoise	2054	GTACTGT,MIR-101	87	223	16030	4169	1.520954	7.14E-06

magenta	8546	GSE13411_NAIVE_VS_IGM_MEMORY_BCELL_UP	10	163	16090	167	5.970758	7.15E-06
turquoise	9736	GSE31082_DP_VS_CD4_SP_THYMOCYTE_UP	78	195	16058	4169	1.559415	7.17E-06
lightcyan	6623	HAN_JNK_SINGALING_UP	4	33	16220	62	31.77517	7.23E-06
magenta	946	HUMORAL_IMMUNE_RESPONSE	5	27	16226	167	18.02284	7.25E-06
lightcyan	8923	GSE17721_LPS_VS_POLYIC_8H_BMDM_DN	7	189	16064	62	9.70908	7.37E-06
turquoise	7692	DANG_BOUND_BY_MYC	330	1049	15204	4169	1.226422	7.39E-06
lightgreen	10160	GSE7460_CD8_TCELL_VS_CD4_TCELL_ACT_UP	6	185	16068	42	12.55058	7.48E-06
blue	61	NUCLEAR_PART	143	557	15696	2978	1.401166	7.49E-06
lightgreen	3519	MODULE_27	7	285	15968	42	9.504678	7.55E-06
turquoise	2064	ATATGCA,MIR-448	75	186	16067	4169	1.571991	7.59E-06
turquoise	10248	GSE9037_WT_VS_IRAK4_KO_BMDM_UP	75	186	16067	4169	1.571991	7.59E-06
brown	9921	GSE36476_YOUNG_VS_OLD_DONOR_MEMORY_CD4_T	48	189	16064	2190	1.884816	7.61E-06
brown	10280	GSE9988_LPS_VS_LPS_AND_ANTI_TREM1_MONOCYTE_	48	189	16064	2190	1.884816	7.61E-06
turquoise	5176	ENK_UV_RESPONSE_KERATINOCYTE_DN	162	468	15785	4169	1.349494	7.70E-06
turquoise	2133	AAGCACT,MIR-520F	84	214	16039	4169	1.530267	7.74E-06
blue	2381	MORF_EIF3S2	72	241	16012	2978	1.630513	7.82E-06
pink	5150	HAHTOLA_SEZARY_SYNDROM_UP	9	96	16157	228	6.682977	8.08E-06
turquoise	1979	TTTGCAC,MIR-19A,MIR-19B	161	465	15788	4169	1.349816	8.08E-06
magenta	635	IMMUNE_RESPONSE	11	203	16050	167	5.273679	8.13E-06
blue	8692	GSE1460_INTRATHYMIC_T_PROGENITOR_VS_THYMIC_	59	187	16066	2978	1.721945	8.19E-06
greenyello	4365	REACTOME_SIGNALING_BY_GPCR	15	421	15832	150	3.86057	8.28E-06
salmon	2498	GCM_ANP32B	5	36	16217	127	17.7745	8.36E-06
brown	5298	MCBRYAN_PUBERTAL_BREAST_6_7WK_UP	46	179	16074	2190	1.907191	8.37E-06
cyan	6124	IIZUKA_LIVER_CANCER_PROGRESSION_G1_G2_UP	3	9	16244	77	70.35931	8.41E-06
greenyello	10160	GSE7460_CD8_TCELL_VS_CD4_TCELL_ACT_UP	10	185	16068	150	5.856937	8.45E-06
turquoise	205	PROTEIN_COMPLEX	242	742	15511	4169	1.27149	8.47E-06
cyan	5195	CASTELLANO_NRAS_TARGETS_UP	5	59	16194	77	17.88796	8.58E-06
turquoise	8421	GSE10239_MEMORY_VS_DAY4.5_EFF_CD8_TCELL_DN	79	199	16054	4169	1.54766	8.75E-06
magenta	6377	BASSO_CD40_SIGNALING_UP	8	100	16153	167	7.785868	8.76E-06
pink	75	CYTOSOLIC_PART	5	21	16232	228	16.97264	8.81E-06
magenta	10264	GSE9650_EXHAUSTED_VS_MEMORY_CD8_TCELL_UP	10	167	16086	167	5.827746	8.85E-06
purple	8007	TORCHIA_TARGETS_OF_EWSR1_FLI1_FUSION_DN	13	307	15946	157	4.38368	9.00E-06
lightgreen	2845	KEGG_NATURAL_KILLER_CELL_MEDIATED_CYTOTOXICI	5	110	16143	42	17.58983	9.03E-06
red	931	RESPONSE_TO_WOUNDING	17	153	16100	524	3.446353	9.09E-06
purple	7879	FIGUEROA_AML_METHYLATION_CLUSTER_1_UP	8	107	16146	157	7.739985	9.20E-06
magenta	807	IMMUNE_SYSTEM_PROCESS	13	289	15964	167	4.377867	9.22E-06
purple	4673	REACTOME_PLATELET_AGGREGATION_PLUG_FORMAT	5	30	16223	157	17.25372	9.25E-06
lightgreen	8391	KAECH_NAIVE_VS_MEMORY_CD8_TCELL_DN	6	192	16061	42	12.09301	9.26E-06
turquoise	9430	GSE24142_ADULT_VS_FETAL_DN3_THYMOCYTE_UP	76	190	16063	4169	1.559415	9.34E-06
brown	7370	SWEET_LUNG_CANCER_KRAS_UP	92	441	15812	2190	1.548241	9.43E-06
purple	3544	MODULE_55	19	624	15629	157	3.152121	9.48E-06
lightgreen	9786	GSE34205_HEALTHY_VS_RSV_INF_INFANT_PBMC_UP	6	193	16060	42	12.03035	9.53E-06
lightgreen	10259	GSE9650_NAIVE_VS_MEMORY_CD8_TCELL_DN	6	193	16060	42	12.03035	9.53E-06
turquoise	4947	GRABARCZYK_BCL11B_TARGETS_UP	38	78	16175	4169	1.899287	9.60E-06
blue	9979	GSE3982_EOSINOPHIL_VS_BCELL_DN	60	192	16061	2978	1.705528	9.61E-06
turquoise	9752	GSE32423_CTRL_VS_IL7_IL4_MEMORY_CD8_TCELL_UP	74	184	16069	4169	1.56789	9.71E-06
brown	2646	GNF2_HCK	29	94	16159	2190	2.2896	9.72E-06
purple	7248	VART_KSHV_INFECTION_ANGIOGENIC_MARKERS_UP	9	142	16111	157	6.561272	9.73E-06
lightgreen	8422	GSE10325_CD4_TCELL_VS_BCELL_UP	6	194	16059	42	11.96834	9.82E-06
blue	821	TRANSCRIPTION	166	667	15586	2978	1.358286	9.92E-06
salmon	679	CELLULAR_PROTEIN_METABOLIC_PROCESS	22	1005	15248	127	2.801473	9.94E-06
turquoise	2565	GCM_ZNF198	51	115	16138	4169	1.728916	9.96E-06
blue	10055	GSE3982_NEUTROPHIL_VS_NKCELL_DN	58	184	16069	2978	1.720359	1.00E-05
turquoise	2326	MORF_RAD23B	72	178	16075	4169	1.576936	1.01E-05
turquoise	6407	BROWNE_HCMV_INFECTION_16HR_UP	82	209	16044	4169	1.529569	1.01E-05
lightgreen	8401	GOLDRATH_NAIVE_VS_MEMORY_CD8_TCELL_DN	6	195	16058	42	11.90696	1.01E-05
red	9414	GSE24142_EARLY_THYMIC_PROGENITOR_VS_DN2_THY	19	187	16066	524	3.151478	1.02E-05
turquoise	307	BIOPOLYMER_METABOLIC_PROCESS	463	1529	14724	4169	1.180525	1.02E-05
lightyellow	8576	GSE13485_CTRL_VS_DAY3_YF17D_VACCINE_PBMC_UP	5	145	16108	33	16.98328	1.02E-05
magenta	7204	QI_PLASMACYTOMA_UP	12	249	16004	167	4.690282	1.03E-05

salmon	9387	GSE22886_IL2_VS_IL15_STIM_NKCELL_DN	9	177	16076	127	6.507273	1.04E-05
turquoise	2455	MORF_RAB5A	44	95	16158	4169	1.805638	1.04E-05
turquoise	2156	GTTATAT,MIR-410	39	81	16172	4169	1.877073	1.05E-05
magenta	4219	REACTOME_ANTIGEN_ACTIVATES_B_CELL_RECEPTOR_	5	29	16224	167	16.77989	1.05E-05
purple	3523	MODULE_33	13	312	15941	157	4.313429	1.07E-05
blue	4307	REACTOME_PROCESSING_OF_CAPPED_INTRON_CONT/	46	136	16117	2978	1.845983	1.08E-05
lightcyan	4951	HORIUCHI_WTAP_TARGETS_UP	8	281	15972	62	7.463207	1.09E-05
purple	6320	PETROVA_ENDOTHELIUM_LYMPHATIC_VS_BLOOD_DN	9	144	16109	157	6.470143	1.09E-05
magenta	9653	GSE29617_DAY3_VS_DAY7_TIV_FLU_VACCINE_PBMC_	10	171	16082	167	5.691424	1.09E-05
turquoise	3212	chr3q26	25	44	16209	4169	2.215078	1.10E-05
turquoise	3784	MODULE_332	25	44	16209	4169	2.215078	1.10E-05
red	5848	RICKMAN_TUMOR_DIFFERENTIATED_WELL_VS_POORL	27	333	15920	524	2.514906	1.12E-05
greenyello	8389	KAECH_NAIVE_VS_DAY15_EFF_CD8_TCELL_DN	10	191	16062	150	5.672949	1.12E-05
lightcyan	8078	PLASARI_TGFB1_TARGETS_10HR_DN	7	202	16051	62	9.084238	1.14E-05
turquoise	9926	GSE37416_CTRL_VS_6H_F_TULARENSIS_LVS_NEUTROP	77	194	16059	4169	1.547357	1.14E-05
blue	9360	GSE22886_NAIVE_TCELL_VS_DC_UP	60	193	16060	2978	1.696691	1.15E-05
pink	3703	MODULE_227	4	11	16242	228	25.92185	1.15E-05
greenyello	8391	KAECH_NAIVE_VS_MEMORY_CD8_TCELL_DN	10	192	16061	150	5.643403	1.17E-05
blue	9472	GSE26495_NAIVE_VS_PD1HIGH_CD8_TCELL_UP	59	189	16064	2978	1.703723	1.18E-05
blue	182	INTRACELLULAR_ORGANELLE_PART	261	1125	15128	2978	1.266184	1.18E-05
salmon	9366	GSE22886_NAIVE_BCELL_VS_DC_UP	9	180	16073	127	6.398819	1.19E-05
blue	3	ORGANELLE_PART	262	1130	15123	2978	1.265411	1.19E-05
blue	8011	YUAN_ZNF143_PARTNERS	13	21	16232	2978	3.37857	1.19E-05
salmon	935	CELLULAR_MACROMOLECULE_METABOLIC_PROCESS	22	1017	15236	127	2.768417	1.20E-05
turquoise	10162	GSE7460_TREG_VS_TCONV_ACT_UP	74	185	16068	4169	1.559415	1.22E-05
greenyello	10259	GSE9650_NAIVE_VS_MEMORY_CD8_TCELL_DN	10	193	16060	150	5.614162	1.23E-05
blue	3716	MODULE_244	57	181	16072	2978	1.71872	1.23E-05
lightcyan	3572	MODULE_84	10	480	15773	62	5.461358	1.25E-05
red	9277	GSE20366_CD103_POS_VS_CD103_KLRG1_DP_TREG_D	18	173	16080	524	3.227221	1.25E-05
salmon	8153	HOLLEMAN_VINCRISTINE_RESISTANCE_ALL_DN	4	19	16234	127	26.9424	1.26E-05
pink	7188	LIN_NPAS4_TARGETS_DN	7	56	16197	228	8.910636	1.26E-05
salmon	8127	BOUDOUKHA_BOUND_BY_IGF2BP2	7	100	16153	127	8.958346	1.32E-05
greenyello	9372	GSE22886_NAIVE_CD8_TCELL_VS_DC_UP	10	195	16058	150	5.556581	1.34E-05
blue	3598	MODULE_110	11	16	16237	2978	3.752162	1.35E-05
purple	4928	CHARAFE_BREAST_CANCER_LUMINAL_VS_MESENCHYM	15	419	15834	157	3.706049	1.36E-05
turquoise	5901	BENPORATH_NANOG_TARGETS	291	918	15335	4169	1.235811	1.36E-05
lightgreen	227	PLASMA_MEMBRANE	12	1095	15158	42	4.240835	1.37E-05
cyan	5221	GRAESSMANN_APOPTOSIS_BY_SERUM_DEPRIVATION_	11	475	15778	77	4.88812	1.39E-05
greenyello	5905	BENPORATH_SUZ12_TARGETS	18	612	15641	150	3.186863	1.40E-05
turquoise	5744	PUJANA_ATM_PCC_NETWORK	403	1317	14936	4169	1.192946	1.41E-05
turquoise	3422	chr6q22	26	47	16206	4169	2.156637	1.41E-05
turquoise	9340	GSE22886_IGG_IGA_MEMORY_BCELL_VS_BM_PLASMA	77	195	16058	4169	1.539422	1.42E-05
purple	4237	REACTOME_CELL_SURFACE_INTERACTIONS_AT_THE_V.	7	82	16171	157	8.837269	1.42E-05
turquoise	7187	LIN_NPAS4_TARGETS_UP	62	149	16104	4169	1.62221	1.43E-05
blue	9964	GSE3982_DC_VS_MAC_LPS_STIM_UP	58	186	16067	2978	1.70186	1.44E-05
purple	3494	MODULE_1	13	321	15932	157	4.192492	1.45E-05
red	4894	ZHOU_INFLAMMATORY_RESPONSE_LIVE_UP	29	377	15876	524	2.385937	1.45E-05
red	10282	GSE9988_LPS_VS_CTRL_TREATED_MONOCYTE_UP	18	175	16078	524	3.190338	1.46E-05
lightcyan	4638	REACTOME_TRAF3_DEPENDENT_IRF_ACTIVATION_PAT	3	13	16240	62	60.49504	1.47E-05
lightcyan	7394	LEE_SP4_THYMOCYTE	3	13	16240	62	60.49504	1.47E-05
magenta	9296	GSE20715_0H_VS_24H_OZONE_TLR4_KO_LUNG_UP	10	177	16076	167	5.498495	1.47E-05
salmon	10124	GSE5960_TH1_VS_ANERGIC_TH1_UP	9	185	16068	127	6.225878	1.48E-05
blue	8706	GSE1460_CD4_THYMOCYTE_VS_THYMIC_STROMAL_CE	57	182	16071	2978	1.709276	1.48E-05
red	7042	ACEVEDO_METHYLATED_IN_LIVER_CANCER_DN	37	542	15711	524	2.117409	1.50E-05
turquoise	6163	TARTE_PLASMA_CELL_VS_PLASMABLAST_DN	112	307	15946	4169	1.422268	1.50E-05
cyan	6518	KRASNOSELSKAYA_ILF3_TARGETS_UP	4	32	16221	77	26.38474	1.51E-05
brown	8603	GSE13738_RESTING_VS_TCR_ACTIVATED_CD4_TCELL_[	49	199	16054	2190	1.827395	1.52E-05
brown	9348	GSE22886_DAY0_VS_DAY7_MONOCYTE_IN_CULTURE_	49	199	16054	2190	1.827395	1.52E-05
brown	9361	GSE22886_NAIVE_TCELL_VS_DC_DN	49	199	16054	2190	1.827395	1.52E-05
turquoise	1949	TGAATGT,MIR-181A,MIR-181B,MIR-181C,MIR-181D	145	416	15837	4169	1.358865	1.52E-05

midnightb	9958	GSE3982_MEMORY_CD4_TCELL_VS_TH1_UP	7	184	16069	71	8.708742	1.53E-05
salmon	6480	HEDENFALK_BREAST_CANCER_BRACX_UP	4	20	16233	127	25.59528	1.56E-05
cyan	5277	MISSIAGLIA_REGULATED_BY_METHYLATION_UP	6	113	16140	77	11.20768	1.57E-05
turquoise	2139	GTGCAAA,MIR-507	46	102	16151	4169	1.758164	1.57E-05
lightcyan	8222	MTOR_UP.V1_UP	6	141	16112	62	11.15511	1.58E-05
red	9998	GSE3982_MAST_CELL_VS_BCELL_UP	18	176	16077	524	3.172211	1.58E-05
magenta	9333	GSE22886_IGA_VS_IGM_MEMORY_BCELL_DN	10	179	16074	167	5.437059	1.63E-05
cyan	5044	TONKS_TARGETS_OF_RUNX1_RUNX1T1_FUSION_HSC_	7	171	16082	77	8.640617	1.63E-05
turquoise	4966	WANG_LMO4_TARGETS_UP	121	337	15916	4169	1.399771	1.64E-05
magenta	4873	SENGUPTA_NASOPHARYNGEAL_CARCINOMA_WITH_LM	8	109	16144	167	7.142998	1.65E-05
turquoise	5083	TIEN_INTESTINE_PROBIOTICS_24HR_UP	183	545	15708	4169	1.30905	1.66E-05
purple	8564	GSE13484_UNSTIM_VS_12H_YF17D_VACCINE_STIM_PI	10	191	16062	157	5.420015	1.67E-05
cyan	8198	EGFR_UP.V1_UP	7	172	16081	77	8.590381	1.70E-05
red	10290	GSE9988_LOW_LPS_VS_VEHICLE_TREATED_MONOCYTI	18	177	16076	524	3.154289	1.71E-05
red	9463	GSE24634_IL4_VS_CTRL_TREATED_NAIVE_CD4_TCELL_	19	194	16059	524	3.037765	1.72E-05
brown	6363	APPEL_IMATINIB_RESPONSE	14	31	16222	2190	3.351628	1.74E-05
lightcyan	5590	KANG_GIST_WITH_PDGFRA_UP	4	41	16212	62	25.57514	1.75E-05
turquoise	9230	GSE18791_CTRL_VS_NEWCASTLE_VIRUS_DC_12H_UP	77	196	16057	4169	1.531568	1.75E-05
salmon	2692	GNF2_RBBP6	6	70	16183	127	10.9694	1.76E-05
cyan	8259	MEK_UP.V1_DN	7	173	16080	77	8.540725	1.76E-05
lightcyan	6703	BAELDE_DIABETIC_NEPHROPATHY_DN	9	396	15857	62	5.957845	1.79E-05
turquoise	8740	GSE15324_ELF4_KO_VS_WT_ACTIVATED_CD8_TCELL_U	76	193	16060	4169	1.535175	1.80E-05
pink	3549	MODULE_60	17	368	15885	228	3.293061	1.80E-05
brown	9879	GSE360_T_GONDII_VS_B_MALAYI_HIGH_DOSE_MAC_D	46	184	16069	2190	1.855365	1.81E-05
magenta	7053	SMID_BREAST_CANCER_RELAPSE_IN_BONE_DN	11	221	16032	167	4.844149	1.81E-05
blue	2377	MORF_DEAF1	24	56	16197	2978	2.33901	1.84E-05
cyan	7863	CHICAS_RB1_TARGETS_CONFLUENT	11	490	15763	77	4.738484	1.85E-05
brown	2647	GNF2_HLA-C	18	47	16206	2190	2.842262	1.85E-05
brown	2694	GNF2_S100A4	18	47	16206	2190	2.842262	1.85E-05
turquoise	2452	MORF_RAB11A	30	58	16195	4169	2.016485	1.88E-05
magenta	9999	GSE3982_MAST_CELL_VS_BCELL_DN	10	182	16071	167	5.347437	1.88E-05
lightcyan	8362	KRAS.300_UP.V1_DN	5	86	16167	62	15.241	1.88E-05
magenta	544	MULTICELLULAR_ORGANISMAL_DEVELOPMENT	21	729	15524	167	2.803553	1.90E-05
lightcyan	246	RESPONSE_TO_VIRUS	4	42	16211	62	24.96621	1.93E-05
lightcyan	6356	ZHAN_MULTIPLE_MYELOMA_HP_UP	4	42	16211	62	24.96621	1.93E-05
greenyello	7719	MIKKELSEN_NPC_HCP_WITH_H3K27ME3	9	162	16091	150	6.01963	1.94E-05
brown	2969	BIOCARTA_ERK_PATHWAY	12	24	16229	2190	3.710731	1.95E-05
brown	4494	REACTOME_SEMA4D_INDUCED_CELL_MIGRATION_ANI	12	24	16229	2190	3.710731	1.95E-05
purple	4976	SMIRNOV_CIRCULATING_ENDOTHELIOCYTES_IN_CANC	9	155	16098	157	6.010972	1.97E-05
turquoise	5792	BUYTAERT_PHOTODYNAMIC_THERAPY_STRESS_UP	250	778	15475	4169	1.252743	1.97E-05
cyan	8257	LTE2_UP.V1_DN	7	176	16077	77	8.395145	1.97E-05
red	2674	GNF2_MYD88	10	60	16193	524	5.169529	1.97E-05
purple	7443	MEISSNER_BRAIN_HCP_WITH_H3K4ME3_AND_H3K27M	21	779	15474	157	2.790716	1.97E-05
salmon	9979	GSE3982_EOSINOPHIL_VS_BCELL_DN	9	192	16061	127	5.998893	1.98E-05
cyan	7294	SEKI_INFLAMMATORY_RESPONSE_LPS_UP	5	70	16183	77	15.07699	1.99E-05
cyan	7457	LIU_VAV3_PROSTATE_CARCINOGENESIS_UP	5	70	16183	77	15.07699	1.99E-05
red	8933	GSE17721_POLYIC_VS_PAM3CSK4_1H_BMDM_DN	18	179	16074	524	3.119046	1.99E-05
purple	8453	GSE11057_CD4_EFF_MEM_VS_PBMC_DN	10	195	16058	157	5.308836	1.99E-05
blue	8687	GSE1460_INTRATHYMIC_T_PROGENITOR_VS_CD4_THY	59	192	16061	2978	1.677103	1.99E-05
blue	9394	GSE22886_NAIVE_CD4_TCELL_VS_12H_ACT_TH2_UP	59	192	16061	2978	1.677103	1.99E-05
lightcyan	9197	GSE17974_IL4_AND_ANTI_IL12_VS_UNTREATED_12H_	6	147	16106	62	10.6998	2.00E-05
turquoise	1957	TTGCACT,MIR-130A,MIR-301,MIR-130B	130	368	15885	4169	1.377201	2.00E-05
brown	7350	RUTELLA_RESPONSE_TO_HGF_DN	55	234	16019	2190	1.744361	2.00E-05
cyan	8158	ALTEMEIER_RESPONSE_TO_LPS_WITH_MECHANICAL_V	6	118	16135	77	10.73278	2.01E-05
cyan	9503	GSE2706_UNSTIM_VS_2H_LPS_DC_DN	7	177	16076	77	8.347714	2.04E-05
salmon	2715	GNF2_UBE2I	5	43	16210	127	14.88097	2.04E-05
salmon	5305	LUI_THYROID_CANCER_PAX8_PPARG_DN	5	43	16210	127	14.88097	2.04E-05
greenyello	3961	PID_IL12_2PATHWAY	6	61	16192	150	10.6577	2.04E-05
magenta	14	INTRINSIC_TO_PLASMA_MEMBRANE	21	733	15520	167	2.788254	2.06E-05
greenyello	5906	BENPORATH_EED_TARGETS	18	630	15623	150	3.09581	2.07E-05

turquoise	5067	SENESE_HDAC1_TARGETS_UP	144	415	15838	4169	1.352745	2.11E-05
turquoise	6688	BURTON_ADIPOGENESIS_11	28	53	16200	4169	2.059604	2.12E-05
black	7725	MIKKELSEN_MEF_HCP_WITH_H3K27ME3	17	300	15953	283	3.254429	2.13E-05
red	9944	GSE37416_12H_VS_24H_F_TULARENSIS_LVS_NEUTROI	19	197	16056	524	2.991504	2.13E-05
pink	1599	V$GATA_C	12	196	16057	228	4.364393	2.14E-05
red	10284	GSE9988_LPS_VS_VEHICLE_TREATED_MONOCYTE_UP	18	180	16073	524	3.101718	2.15E-05
turquoise	8781	GSE15930_NAIVE_VS_24H_IN_VITRO_STIM_INFAB_CD	77	197	16056	4169	1.523794	2.16E-05
purple	7863	CHICAS_RB1_TARGETS_CONFLUENT	16	490	15763	157	3.38032	2.16E-05
blue	10017	GSE3982_DC_VS_BCELL_DN	56	180	16073	2978	1.697948	2.16E-05
purple	6403	REN_ALVEOLAR_RHABDOMYOSARCOMA_DN	14	384	15869	157	3.77425	2.18E-05
magenta	41	INTRINSIC_TO_MEMBRANE	26	1041	15212	167	2.430747	2.21E-05
turquoise	9747	GSE32423_MEMORY_VS_NAIVE_CD8_TCELL_IL7_IL4_D	76	194	16059	4169	1.527262	2.22E-05
salmon	2608	GNF2_XRCC5	6	73	16180	127	10.51861	2.24E-05
salmon	6839	HILLION_HMGA1_TARGETS	6	73	16180	127	10.51861	2.24E-05
salmon	9372	GSE22886_NAIVE_CD8_TCELL_VS_DC_UP	9	195	16058	127	5.906602	2.24E-05
salmon	9895	GSE36392_TYPE_2_MYELOID_VS_NEUTROPHIL_IL25_TI	9	195	16058	127	5.906602	2.24E-05
lightcyan	5050	TONKS_TARGETS_OF_RUNX1_RUNX1T1_FUSION_ERYTI	6	150	16103	62	10.48581	2.24E-05
turquoise	2009	GTGCCAT,MIR-183	65	160	16093	4169	1.583781	2.26E-05
purple	10148	GSE7400_CTRL_VS_CSF3_IN_VIVO_TREATED_PBMC_UI	10	198	16055	157	5.228399	2.28E-05
turquoise	8530	GSE13306_LAMINA_PROPRIA_VS_SPLEEN_TREG_UP	75	191	16062	4169	1.530839	2.28E-05
brown	9458	GSE24634_IL4_VS_CTRL_TREATED_NAIVE_CD4_TCELL_	47	191	16062	2190	1.826223	2.28E-05
blue	10014	GSE3982_DC_VS_NEUTROPHIL_UP	60	197	16056	2978	1.662241	2.29E-05
blue	605	RNA_PROCESSING	53	168	16085	2978	1.721771	2.31E-05
salmon	2591	GNF2_HDAC1	7	109	16144	127	8.218666	2.32E-05
purple	4431	REACTOME_MUSCLE_CONTRACTION	5	36	16217	157	14.3781	2.34E-05
purple	5657	GRUETZMANN_PANCREATIC_CANCER_UP	13	336	15917	157	4.005327	2.34E-05
turquoise	8642	GSE14308_TH1_VS_INDUCED_TREG_UP	74	188	16065	4169	1.53453	2.34E-05
turquoise	8652	GSE14308_NAIVE_CD4_TCELL_VS_INDUCED_TREG_UP	74	188	16065	4169	1.53453	2.34E-05
yellow	10126	GSE6269_HEALTHY_VS_FLU_INF_PBMC_UP	31	161	16092	1426	2.194572	2.35E-05
magenta	2899	KEGG_PRIMARY_IMMUNODEFICIENCY	5	34	16219	167	14.31226	2.36E-05
cyan	8681	GSE1448_CTRL_VS_ANTI_VBETA5_DP_THYMOCYTE_DN	7	181	16072	77	8.163235	2.36E-05
turquoise	2148	ATGTACA,MIR-493	101	274	15979	4169	1.437052	2.37E-05
turquoise	6862	ZHENG_BOUND_BY_FOXP3	158	463	15790	4169	1.330386	2.38E-05
turquoise	8189	ZWANG_TRANSIENTLY_UP_BY_1ST_EGF_PULSE_ONLY	444	1472	14781	4169	1.175917	2.40E-05
turquoise	9257	GSE20151_CTRL_VS_FUSOBACT_NUCLEATUM_NEUTRC	73	185	16068	4169	1.538342	2.40E-05
cyan	7068	SMID_BREAST_CANCER_BASAL_UP	11	504	15749	77	4.606859	2.40E-05
turquoise	1976	ACTGTGA,MIR-27A,MIR-27B	144	416	15837	4169	1.349494	2.42E-05
red	8346	STK33_NOMO_UP	23	271	15982	524	2.632454	2.42E-05
pink	4781	REACTOME_METABOLISM_OF_PORPHYRINS	4	13	16240	228	21.93387	2.44E-05
cyan	6657	GEISS_RESPONSE_TO_DSRNA_UP	4	36	16217	77	23.4531	2.44E-05
turquoise	5623	MARTORIATI_MDM4_TARGETS_FETAL_LIVER_DN	160	470	15783	4169	1.327161	2.45E-05
red	7908	CHYLA_CBFA2T3_TARGETS_UP	27	348	15905	524	2.406505	2.46E-05
red	9348	GSE22886_DAY0_VS_DAY7_MONOCYTE_IN_CULTURE_	19	199	16054	524	2.961439	2.46E-05
magenta	8374	KRAS.LUNG_UP.V1_DN	7	84	16169	167	8.110279	2.48E-05
pink	5012	TAKEDA_TARGETS_OF_NUP98_HOXA9_FUSION_8D_UI	10	138	16115	228	5.165586	2.50E-05
turquoise	911	PROTEIN_MODIFICATION_PROCESS	189	569	15684	4169	1.294945	2.51E-05
turquoise	8417	GSE10239_KLRG1INT_VS_KLRG1HIGH_EFF_CD8_TCELL_	71	179	16074	4169	1.546347	2.52E-05
cyan	8721	GSE14769_UNSTIM_VS_80MIN_LPS_BMDM_DN	7	183	16070	77	8.074019	2.53E-05
turquoise	5784	NUYTTEN_EZH2_TARGETS_UP	297	946	15307	4169	1.223959	2.54E-05
grey60	5015	TAKEDA_TARGETS_OF_NUP98_HOXA9_FUSION_10D_D	5	130	16123	44	14.20717	2.56E-05
turquoise	8961	GSE17721_PAM3CSK4_VS_CPG_12H_BMDM_DN	70	176	16077	4169	1.550554	2.58E-05
yellow	218	MITOCHONDRIAL_MEMBRANE_PART	15	52	16201	1426	3.287774	2.59E-05
greenyello	9725	GSE30962_PRIMARY_VS_SECONDARY_CHRONIC_LCMV	9	168	16085	150	5.804643	2.60E-05
magenta	8550	GSE13411_NAIVE_BCELL_VS_PLASMA_CELL_UP	10	189	16064	167	5.149384	2.60E-05
lightgreen	2837	KEGG_ANTIGEN_PROCESSING_AND_PRESENTATION	4	67	16186	42	23.10306	2.62E-05
brown	9455	GSE24634_TEFF_VS_TCONV_DAY10_IN_CULTURE_DN	47	192	16061	2190	1.816712	2.64E-05
grey60	5017	TAKEDA_TARGETS_OF_NUP98_HOXA9_FUSION_16D_D	5	131	16122	44	14.09872	2.66E-05
salmon	2467	MORF_SKP1A	9	200	16053	127	5.758937	2.74E-05
pink	7912	WIERENGA_STAT5A_TARGETS_UP	12	201	16052	228	4.255826	2.75E-05
lightcyan	8293	ALK_DN.V1_DN	5	93	16160	62	14.09383	2.75E-05

blue	8696	GSE1460_DP_THYMOCYTE_VS_NAIVE_CD4_TCELL_COR	59	194	16059	2978	1.659813	2.80E-05
red	3596	MODULE_108	8	39	16214	524	6.362498	2.82E-05
lightgreen	59	MEMBRANE	14	1601	14652	42	3.383927	2.84E-05
yellow	6876	SANSOM_APC_MYC_TARGETS	37	209	16044	1426	2.01776	2.85E-05
salmon	6095	LEE_LIVER_CANCER_MYC_UP	5	46	16207	127	13.91048	2.85E-05
red	7348	RUTELLA_RESPONSE_TO_CSF2RB_AND_IL4_DN	25	312	15941	524	2.485351	2.87E-05
red	3622	MODULE_134	6	20	16233	524	9.305153	2.88E-05
turquoise	9127	GSE17721_0.5H_VS_12H_CPG_BMDM_DN	74	189	16064	4169	1.526411	2.89E-05
cyan	8571	GSE13484_12H_VS_3H_YF17D_VACCINE_STIM_PBMC_	7	187	16066	77	7.901313	2.91E-05
cyan	9300	GSE2197_IMMUNOSUPPRESSIVE_DNA_VS_UNTREATED	7	187	16066	77	7.901313	2.91E-05
brown	6931	STEARMAN_LUNG_CANCER_EARLY_VS_LATE_DN	20	57	16196	2190	2.604021	2.92E-05
salmon	2387	MORF_ERH	7	113	16140	127	7.92774	2.93E-05
magenta	8458	GSE11057_PBMC_VS_MEM_CD4_TCELL_UP	10	192	16061	167	5.068925	2.97E-05
turquoise	4919	DEURIG_T_CELL_PROLYMPHOCYTIC_LEUKEMIA_DN	111	308	15945	4169	1.404992	3.00E-05
lightyellow	6954	MARTINEZ_RB1_TARGETS_DN	7	457	15796	33	7.543996	3.01E-05
pink	10139	GSE6269_FLU_VS_STREP_PNEUMO_INF_PBMC_DN	10	141	16112	228	5.05568	3.01E-05
cyan	7683	ZHAN_MULTIPLE_MYELOMA_LB_DN	4	38	16215	77	22.21873	3.04E-05
turquoise	9141	GSE17721_0.5H_VS_4H_GARDIQUIMOD_BMDM_DN	72	183	16070	4169	1.533851	3.05E-05
turquoise	10243	GSE9037_CTRL_VS_LPS_4H_STIM_BMDM_DN	72	183	16070	4169	1.533851	3.05E-05
salmon	2244	SCGGAAGY_V$ELK1_02	23	1162	15091	127	2.533095	3.05E-05
purple	6703	BAELDE_DIABETIC_NEPHROPATHY_DN	14	396	15857	157	3.659879	3.06E-05
blue	5158	PROVENZANI_METASTASIS_UP	56	182	16071	2978	1.679289	3.07E-05
turquoise	724	BIOPOLYMER_MODIFICATION	194	588	15665	4169	1.286252	3.09E-05
magenta	8495	GSE12366_GC_VS_NAIVE_BCELL_DN	10	193	16060	167	5.042661	3.11E-05
magenta	8496	GSE12366_GC_VS_MEMORY_BCELL_UP	10	193	16060	167	5.042661	3.11E-05
red	9967	GSE3982_DC_VS_NEUTROPHIL_LPS_STIM_DN	17	168	16085	524	3.138643	3.11E-05
lightgreen	8288	ESC_J1_UP_LATE.V1_UP	5	142	16111	42	13.62592	3.11E-05
lightgreen	9702	GSE29618_LAIV_VS_TIV_FLU_VACCINE_DAY7_BCELL_U	5	142	16111	42	13.62592	3.11E-05
cyan	9131	GSE17721_0.5H_VS_8H_CPG_BMDM_DN	7	189	16064	77	7.817701	3.11E-05
cyan	9547	GSE27786_LIN_NEG_VS_BCELL_DN	7	189	16064	77	7.817701	3.11E-05
magenta	5127	GRAHAM_CML_DIVIDING_VS_NORMAL_QUIESCENT_D	7	87	16166	167	7.830615	3.12E-05
lightgreen	2664	GNF2_MATK	3	24	16229	42	48.37202	3.13E-05
turquoise	2090	AGCATTA,MIR-155	53	125	16128	4169	1.65298	3.15E-05
yellow	7250	MOOTHA_HUMAN_MITODB_6_2002	61	411	15842	1426	1.691617	3.17E-05
greenyello	7591	ONO_FOXP3_TARGETS_DN	5	40	16213	150	13.54417	3.18E-05
magenta	270	SYSTEM_DEVELOPMENT	18	583	15670	167	3.004838	3.19E-05
blue	9909	GSE36476_CTRL_VS_TSST_ACT_16H_MEMORY_CD4_T(	60	199	16054	2978	1.645535	3.20E-05
brown	7204	QI_PLASMACYTOMA_UP	57	249	16004	2190	1.698889	3.22E-05
pink	3267	chr1q32	9	114	16139	228	5.62777	3.25E-05
magenta	6033	ONDER_CDH1_TARGETS_2_DN	14	374	15879	167	3.64312	3.26E-05
yellow	2283	GGGCGGR_V$SP1_Q6	288	2655	13598	1426	1.236352	3.26E-05
brown	7406	ZHANG_TLX_TARGETS_36HR_UP	50	210	16043	2190	1.767015	3.27E-05
turquoise	2012	GTTTGTT,MIR-495	83	218	16035	4169	1.484305	3.27E-05
purple	8110	PEDRIOLI_MIR31_TARGETS_DN	13	347	15906	157	3.878357	3.27E-05
red	5073	SENESE_HDAC3_TARGETS_UP	32	456	15797	524	2.176644	3.33E-05
cyan	8959	GSE17721_PAM3CSK4_VS_CPG_8H_BMDM_DN	7	191	16062	77	7.73584	3.33E-05
lightcyan	691	RESPONSE_TO_BIOTIC_STIMULUS	5	97	16156	62	13.51264	3.37E-05
blue	9476	GSE26495_PD1HIGH_VS_PD1LOW_CD8_TCELL_UP	52	166	16087	2978	1.709638	3.41E-05
brown	5045	TONKS_TARGETS_OF_RUNX1_RUNX1T1_FUSION_HSC_	45	183	16070	2190	1.824949	3.44E-05
brown	9948	GSE3982_CTRL_VS_PMA_STIM_EOSINOPHIL_UP	45	183	16070	2190	1.824949	3.44E-05
cyan	10050	GSE3982_NEUTROPHIL_VS_EFF_MEMORY_CD4_TCELL_	7	192	16061	77	7.695549	3.44E-05
lightcyan	4628	REACTOME_TRAF6_MEDIATED_IRF7_ACTIVATION	3	17	16236	62	46.26091	3.46E-05
blue	4909	CASORELLI_ACUTE_PROMYELOCYTIC_LEUKEMIA_DN	160	653	15600	2978	1.337259	3.49E-05
brown	6240	BROWN_MYELOID_CELL_DEVELOPMENT_UP	39	151	16102	2190	1.916801	3.49E-05
brown	9928	GSE37416_CTRL_VS_12H_F_TULARENSIS_LVS_NEUTRO	47	194	16059	2190	1.797983	3.49E-05
greenyello	3222	chr1q23	6	67	16186	150	9.703284	3.51E-05
turquoise	1538	ACTAYRNNNCCCR_UNKNOWN	150	439	15814	4169	1.332074	3.52E-05
turquoise	3334	chr2q33	36	76	16177	4169	1.846675	3.54E-05
turquoise	4991	GARGALOVIC_RESPONSE_TO_OXIDIZED_PHOSPHOLIPID	36	76	16177	4169	1.846675	3.54E-05
cyan	8495	GSE12366_GC_VS_NAIVE_BCELL_DN	7	193	16060	77	7.655676	3.56E-05

green	4779	REACTOME_GAP_JUNCTION_TRAFFICKING	8	18	16235	1305	5.535292	3.56E-05
turquoise	4754	REACTOME_ANTIGEN_PROCESSING_UBIQUITINATION_	74	190	16063	4169	1.518377	3.56E-05
turquoise	8610	GSE14000_TRANSLATED_RNA_VS_MRNA_4H_LPS_DC_	74	190	16063	4169	1.518377	3.56E-05
turquoise	9121	GSE17721_0.5H_VS_24H_PAM3CSK4_BMDM_DN	74	190	16063	4169	1.518377	3.56E-05
turquoise	5073	SENESE_HDAC3_TARGETS_UP	155	456	15797	4169	1.325161	3.57E-05
cyan	5139	WATANABE_ULCERATIVE_COLITIS_WITH_CANCER_DN	3	14	16239	77	45.23098	3.58E-05
red	10286	GSE9988_LOW_LPS_VS_ANTI_TREM1_AND_LPS_MON(	18	187	16066	524	2.98561	3.59E-05
blue	4416	REACTOME_MRNA_PROCESSING	49	154	16099	2978	1.736538	3.61E-05
blue	2308	MORF_FEN1	26	65	16188	2978	2.183076	3.62E-05
cyan	8583	GSE13485_DAY1_VS_DAY3_YF17D_VACCINE_PBMC_DI	7	194	16059	77	7.616214	3.68E-05
cyan	9763	GSE3337_CTRL_VS_16H_IFNG_IN_CD8POS_DC_DN	7	194	16059	77	7.616214	3.68E-05
yellow	2645	GNF2_GLTSCR2	11	31	16222	1426	4.044315	3.71E-05
brown	10143	GSE6269_E_COLI_VS_STREP_PNEUMO_INF_PBMC_DN	41	162	16091	2190	1.878271	3.71E-05
brown	9837	GSE360_L_DONOVANI_VS_B_MALAYI_LOW_DOSE_DC_	46	189	16064	2190	1.806282	3.73E-05
magenta	5879	RICKMAN_HEAD_AND_NECK_CANCER_A	6	61	16192	167	9.572789	3.74E-05
blue	2362	MORF_CDC10	46	142	16111	2978	1.767984	3.76E-05
turquoise	2014	CTTGTAT,MIR-381	72	184	16069	4169	1.525514	3.77E-05
turquoise	8461	GSE11864_UNTREATED_VS_CSF1_IN_MAC_DN	72	184	16069	4169	1.525514	3.77E-05
salmon	4917	CASORELLI_APL_SECONDARY_VS_DE_NOVO_DN	3	9	16244	127	42.65879	3.78E-05
greenyello	1322	RECEPTOR_ACTIVITY	14	423	15830	150	3.586162	3.79E-05
greenyello	4366	REACTOME_PEPTIDE_LIGAND_BINDING_RECEPTORS	7	100	16153	150	7.584733	3.87E-05
brown	2619	GNF2_CD1D	17	45	16208	2190	2.803663	3.88E-05
pink	1502	V$GATA3_01	11	176	16077	228	4.455318	3.92E-05
red	8012	KOINUMA_TARGETS_OF_SMAD2_OR_SMAD3	46	768	15485	524	1.8578	3.95E-05
magenta	3535	MODULE_46	13	332	15921	167	3.810854	3.96E-05
red	7654	CAIRO_HEPATOBLASTOMA_DN	19	206	16047	524	2.860807	3.97E-05
blue	10041	GSE3982_MAC_VS_NKCELL_DN	53	171	16082	2978	1.691565	3.98E-05
brown	8722	GSE14769_UNSTIM_VS_120MIN_LPS_BMDM_UP	47	195	16058	2190	1.788762	4.01E-05
turquoise	2121	AGTCTTA,MIR-499	33	68	16185	4169	1.891937	4.01E-05
lightcyan	6689	BROWNE_HCMV_INFECTION_12HR_UP	5	101	16152	62	12.97748	4.10E-05
turquoise	9695	GSE29618_PRE_VS_DAY7_POST_LAIV_FLU_VACCINE_P	76	197	16056	4169	1.504004	4.11E-05
cyan	9511	GSE2706_R848_VS_LPS_2H_STIM_DC_DN	6	134	16119	77	9.45125	4.12E-05
greenyello	8129	BOSCO_TH1_CYTOTOXIC_MODULE	7	101	16152	150	7.509637	4.13E-05
brown	6304	LENAOUR_DENDRITIC_CELL_MATURATION_DN	34	126	16127	2190	2.002617	4.16E-05
cyan	6815	MONNIER_POSTRADIATION_TUMOR_ESCAPE_DN	9	352	15901	77	5.396879	4.18E-05
blue	8432	GSE10325_LUPUS_BCELL_VS_LUPUS_MYELOID_UP	57	188	16065	2978	1.654725	4.18E-05
brown	3010	BIOCARTA_INTEGRIN_PATHWAY	15	37	16216	2190	3.0087	4.19E-05
brown	4671	REACTOME_IL_2_SIGNALING	15	37	16216	2190	3.0087	4.19E-05
turquoise	2420	MORF_MBD4	39	85	16168	4169	1.78874	4.21E-05
yellow	8773	GSE15750_DAY6_VS_DAY10_TRAF6KO_EFF_CD8_TCELI	34	189	16064	1426	2.050365	4.23E-05
purple	8162	LIM_MAMMARY_STEM_CELL_UP	14	408	15845	157	3.552236	4.24E-05
turquoise	9706	GSE29618_LAIV_VS_TIV_FLU_VACCINE_DAY7_PDC_UP	75	194	16059	4169	1.507166	4.24E-05
lightcyan	9797	GSE360_CTRL_VS_T_GONDII_DC_DN	6	168	16085	62	9.362327	4.25E-05
lightcyan	9850	GSE360_T_GONDII_VS_B_MALAYI_LOW_DOSE_DC_UP	6	168	16085	62	9.362327	4.25E-05
greenyello	8525	GSE13229_IMM_VS_MATURE_NKCELL_DN	9	179	16074	150	5.447933	4.27E-05
turquoise	2435	MORF_PPP1CA	67	169	16084	4169	1.545574	4.28E-05
brown	9420	GSE24142_EARLY_THYMIC_PROGENITOR_VS_DN2_THY	46	190	16063	2190	1.796775	4.28E-05
turquoise	8346	STK33_NOMO_UP	99	271	15982	4169	1.424189	4.29E-05
salmon	4702	REACTOME_HOST_INTERACTIONS_OF_HIV_FACTORS	7	120	16133	127	7.465289	4.31E-05
salmon	183	MACROMOLECULAR_COMPLEX	19	868	15385	127	2.801326	4.34E-05
yellow	3343	chr11q13	38	221	16032	1426	1.959771	4.35E-05
salmon	6399	TAKAO_RESPONSE_TO_UVB_RADIATION_UP	6	82	16171	127	9.364125	4.36E-05
lightcyan	10245	GSE9037_CTRL_VS_LPS_1H_STIM_IRAK4_KO_BMDM_I	6	169	16084	62	9.306929	4.39E-05
brown	7454	MILI_PSEUDOPODIA_CHEMOTAXIS_DN	90	446	15807	2190	1.497604	4.42E-05
blue	1004	REGULATION_OF_TRANSCRIPTION	127	501	15752	2978	1.383486	4.47E-05
turquoise	4371	REACTOME_CELL_CYCLE_MITOTIC	109	304	15949	4169	1.397831	4.50E-05
magenta	5016	TAKEDA_TARGETS_OF_NUP98_HOXA9_FUSION_16D_U	9	162	16091	167	5.406853	4.54E-05
pink	4544	REACTOME_AMINE_COMPOUND_SLC_TRANSPORTERS	4	15	16238	228	19.00936	4.56E-05
lightgreen	10077	GSE3982_BASOPHIL_VS_NKCELL_DN	5	154	16099	42	12.56416	4.59E-05
blue	1093	RNA_HELICASE_ACTIVITY	13	23	16230	2978	3.084781	4.63E-05

turquoise	9107	GSE17721_0.5H_VS_8H_POLYIC_BMDM_DN	72	185	16068	4169	1.517268	4.64E-05
salmon	779	PROTEIN_METABOLIC_PROCESS	22	1111	15142	127	2.534186	4.64E-05
salmon	5909	BENPORATH_MYC_TARGETS_WITH_EBOX	9	214	16039	127	5.382184	4.65E-05
turquoise	5794	FERREIRA_EWINGS_SARCOMA_UNSTABLE_VS_STABLE_	63	157	16096	4169	1.564381	4.66E-05
lightcyan	8197	EGFR_UP.V1_DN	6	171	16082	62	9.198076	4.69E-05
lightcyan	9847	GSE360_L_MAJOR_VS_M_TUBERCULOSIS_DC_DN	6	171	16082	62	9.198076	4.69E-05
lightcyan	7978	AZARE_NEOPLASTIC_TRANSFORMATION_BY_STAT3_DN	5	104	16149	62	12.60313	4.72E-05
black	7840	MARTENS_TRETINOIN_RESPONSE_DN	30	786	15467	283	2.192027	4.73E-05
red	9408	GSE24142_EARLY_THYMIC_PROGENITOR_VS_DN2_THY	18	191	16062	524	2.923085	4.75E-05
purple	6219	COLLER_MYC_TARGETS_DN	3	8	16245	157	38.82086	4.78E-05
salmon	2301	MORF_CTBP1	8	166	16087	127	6.167536	4.78E-05
magenta	118	INTEGRAL_TO_MEMBRANE	25	1025	15228	167	2.37374	4.79E-05
yellow	3602	MODULE_114	51	330	15923	1426	1.76145	4.80E-05
magenta	3652	MODULE_169	7	93	16160	167	7.325414	4.81E-05
turquoise	2243	GTGACGY_V$E4F1_Q6	194	592	15661	4169	1.277561	4.82E-05
turquoise	2489	GCM_ERBB2IP	31	63	16190	4169	1.918328	4.86E-05
blue	1116	ATP_DEPENDENT_HELICASE_ACTIVITY	14	26	16227	2978	2.938756	4.88E-05
cyan	5966	ZHANG_RESPONSE_TO_IKK_INHIBITOR_AND_TNF_UP	7	203	16050	77	7.278549	4.91E-05
purple	8055	PEDERSEN_METASTASIS_BY_ERBB2_ISOFORM_7	13	361	15892	157	3.72795	4.92E-05
purple	227	PLASMA_MEMBRANE	25	1095	15158	157	2.363523	4.93E-05
blue	7608	FONTAINE_FOLLICULAR_THYROID_ADENOMA_UP	26	66	16187	2978	2.149999	4.93E-05
purple	9640	GSE29615_CTRL_VS_DAY7_LAIV_FLU_VACCINE_PBMC_	8	135	16118	157	6.134654	4.98E-05
magenta	3563	MODULE_75	13	340	15913	167	3.721187	5.06E-05
turquoise	6207	FLECHNER_BIOPSY_KIDNEY_TRANSPLANT_REJECTED_V	163	486	15767	4169	1.307534	5.06E-05
red	9982	GSE3982_EOSINOPHIL_VS_EFF_MEMORY_CD4_TCELL_	18	192	16061	524	2.90786	5.09E-05
blue	3621	MODULE_133	10	15	16238	2978	3.63846	5.12E-05
turquoise	2177	TAATAAT,MIR-126	68	173	16080	4169	1.532373	5.18E-05
magenta	136	INTEGRAL_TO_PLASMA_MEMBRANE	20	721	15532	167	2.699677	5.18E-05
turquoise	9562	GSE27786_BCELL_VS_CD4_TCELL_UP	75	195	16058	4169	1.499437	5.18E-05
lightcyan	6104	LEE_LIVER_CANCER_MYC_TGFA_UP	4	54	16199	62	19.41816	5.26E-05
lightcyan	6136	LEE_LIVER_CANCER_ACOX1_UP	4	54	16199	62	19.41816	5.26E-05
turquoise	2321	MORF_PSMC2	49	115	16138	4169	1.661116	5.30E-05
lightcyan	8988	GSE17721_LPS_VS_PAM3CSK4_2H_BMDM_UP	6	175	16078	62	8.987834	5.33E-05
lightgreen	9477	GSE26495_PD1HIGH_VS_PD1LOW_CD8_TCELL_DN	5	159	16094	42	12.16906	5.34E-05
turquoise	9155	GSE17974_0H_VS_2H_IN_VITRO_ACT_CD4_TCELL_DN	74	192	16061	4169	1.502561	5.35E-05
turquoise	9317	GSE22886_NAIVE_VS_MEMORY_TCELL_DN	74	192	16061	4169	1.502561	5.35E-05
magenta	3929	PID_BCR_5PATHWAY	6	65	16188	167	8.983694	5.38E-05
blue	9391	GSE22886_NAIVE_CD4_TCELL_VS_12H_ACT_TH1_DN	59	198	16055	2978	1.626281	5.39E-05
lightgreen	225	PLASMA_MEMBRANE_PART	10	867	15386	42	4.463393	5.47E-05
purple	5072	SENESE_HDAC2_TARGETS_DN	7	101	16152	157	7.174812	5.52E-05
brown	8460	GSE11864_UNTREATED_VS_CSF1_IN_MAC_UP	44	181	16072	2190	1.804112	5.61E-05
brown	8666	GSE1432_1H_VS_24H_IFNG_MICROGLIA_UP	46	192	16061	2190	1.778058	5.64E-05
lightgreen	10037	GSE3982_MAC_VS_EFF_MEMORY_CD4_TCELL_DN	5	161	16092	42	12.01789	5.67E-05
greenyello	4383	REACTOME_CHEMOKINE_RECEPTORS_BIND_CHEMOKI	5	45	16208	150	12.03926	5.68E-05
lightcyan	8695	GSE1460_DP_VS_CD4_THYMOCYTE_DN	6	177	16076	62	8.886277	5.68E-05
lightcyan	9499	GSE2706_UNSTIM_VS_2H_R848_DC_DN	6	177	16076	62	8.886277	5.68E-05
turquoise	8634	GSE14308_TH2_VS_INDUCED_TREG_UP	72	186	16067	4169	1.509111	5.69E-05
cyan	1866	V$IRF_Q6	7	208	16045	77	7.103584	5.73E-05
purple	7988	LEE_BMP2_TARGETS_UP	18	650	15603	157	2.866771	5.74E-05
greenyello	10132	GSE6269_HEALTHY_VS_STREP_PNEUMO_INF_PBMC_U	9	186	16067	150	5.242903	5.76E-05
brown	6785	MARCHINI_TRABECTEDIN_RESISTANCE_DN	16	42	16211	2190	2.827223	5.79E-05
lightcyan	8855	GSE17721_CTRL_VS_POLYIC_4H_BMDM_DN	6	178	16075	62	8.836354	5.86E-05
lightcyan	9090	GSE17721_LPS_VS_GARDIQUIMOD_24H_BMDM_UP	6	178	16075	62	8.836354	5.86E-05
lightcyan	9795	GSE360_CTRL_VS_L_MAJOR_DC_DN	6	178	16075	62	8.836354	5.86E-05
red	2614	GNF2_CASP1	13	111	16142	524	3.632642	5.87E-05
purple	2206	TTTNNANAGCYR_UNKNOWN	7	102	16151	157	7.104471	5.88E-05
red	5150	HAHTOLA_SEZARY_SYNDROM_UP	12	96	16157	524	3.877147	5.92E-05
cyan	3533	MODULE_44	8	285	15968	77	5.924994	5.98E-05
purple	3496	MODULE_3	13	368	15885	157	3.657038	5.98E-05
yellow	3193	chr22q13	30	161	16092	1426	2.123779	5.99E-05

brown	10286	GSE9988_LOW_LPS_VS_ANTI_TREM1_AND_LPS_MONO	45	187	16066	2190	1.785913	6.03E-05
purple	8586	GSE13485_DAY1_VS_DAY21_YF17D_VACCINE_PBMC_U	9	179	16074	157	5.205031	6.10E-05
blue	7092	TOYOTA_TARGETS_OF_MIR34B_AND_MIR34C	114	444	15809	2978	1.401299	6.10E-05
red	6482	CUI_TCF21_TARGETS_2_DN	45	759	15494	524	1.838963	6.11E-05
lightgreen	3341	chr5q11	3	30	16223	42	38.69762	6.21E-05
lightgreen	3835	MODULE_410	3	30	16223	42	38.69762	6.21E-05
turquoise	3585	MODULE_97	41	92	16161	4169	1.737391	6.22E-05
blue	9488	GSE26928_NAIVE_VS_CENT_MEMORY_CD4_TCELL_UP	55	182	16071	2978	1.649302	6.24E-05
purple	7885	FIGUEROA_AML_METHYLATION_CLUSTER_4_UP	7	103	16150	157	7.035496	6.26E-05
cyan	1820	V$ICSBP_Q6	7	211	16042	77	7.002585	6.27E-05
blue	5143	RODRIGUES_THYROID_CARCINOMA_POORLY_DIFFEREN	178	746	15507	2978	1.302237	6.27E-05
brown	4914	DIAZ_CHRONIC_MEYLOGENOUS_LEUKEMIA_UP	234	1378	14875	2190	1.260248	6.27E-05
salmon	2576	GNF2_BUB3	4	28	16225	127	18.28234	6.29E-05
blue	9442	GSE24634_TREG_VS_TCONV_POST_DAY5_IL4_CONVER	59	199	16054	2978	1.618109	6.31E-05
turquoise	9934	GSE37416_0H_VS_3H_F_TULARENSIS_LVS_NEUTROPH	75	196	16057	4169	1.491787	6.31E-05
turquoise	6865	MARSON_BOUND_BY_E2F4_UNSTIMULATED	224	699	15554	4169	1.249317	6.39E-05
salmon	8949	GSE17721_PAM3CSK4_VS_CPG_0.5H_BMDM_DN	8	173	16080	127	5.917983	6.41E-05
brown	2849	KEGG_FC_GAMMA_R_MEDIATED_PHAGOCYTOSIS	27	93	16160	2190	2.154618	6.42E-05
brown	7301	CHAUHAN_RESPONSE_TO_METHOXYESTRADIOL_DN	28	98	16155	2190	2.120417	6.48E-05
lightgreen	3160	chr6p11	2	5	16248	42	154.7905	6.49E-05
greenyello	9319	GSE22886_NAIVE_CD8_TCELL_VS_NKCELL_DN	9	189	16064	150	5.159683	6.52E-05
lightcyan	9478	GSE26669_CTRL_VS_COSTIM_BLOCK_MLR_CD4_TCELL_	6	182	16071	62	8.642148	6.63E-05
turquoise	61	NUCLEAR_PART	183	557	15696	4169	1.280848	6.68E-05
cyan	6686	ZHU_CMV_24_HR_UP	5	90	16163	77	11.72655	6.72E-05
lightgreen	10025	GSE3982_DC_VS_NKCELL_DN	5	167	16086	42	11.58611	6.75E-05
lightcyan	9402	GSE24026_PD1_LIGATION_VS_CTRL_IN_ACT_TCELL_LIN	6	183	16070	62	8.594923	6.84E-05
brown	8702	GSE1460_CD4_THYMOCYTE_VS_NAIVE_CD4_TCELL_CO	47	199	16054	2190	1.752807	6.86E-05
red	2682	GNF2_PECAM1	9	56	16197	524	4.984903	6.90E-05
brown	8664	GSE1432_1H_VS_6H_IFNG_MICROGLIA_UP	45	188	16065	2190	1.776414	6.91E-05
lightgreen	10176	GSE7460_FOXP3_MUT_VS_HET_ACT_TCONV_UP	5	168	16085	42	11.51715	6.94E-05
yellow	7740	YAO_TEMPORAL_RESPONSE_TO_PROGESTERONE_CLUS	31	170	16083	1426	2.078389	6.96E-05
purple	4868	DAVICIONI_TARGETS_OF_PAX_FOXO1_FUSIONS_UP	10	226	16027	157	4.580632	6.98E-05
pink	3544	MODULE_55	22	624	15629	228	2.513256	6.99E-05
greenyello	7017	WORSCHECH_TUMOR_REJECTION_UP	5	47	16206	150	11.52695	7.03E-05
lightcyan	8093	KRIEG_KDM3A_TARGETS_NOT_HYPOXIA	6	184	16069	62	8.548212	7.05E-05
blue	2437	MORF_PPP2CA	41	125	16128	2978	1.790122	7.07E-05
purple	7891	FIGUEROA_AML_METHYLATION_CLUSTER_7_UP	7	105	16148	157	6.901486	7.08E-05
lightgreen	3553	MODULE_64	7	404	15849	42	6.705033	7.08E-05
purple	3915	MODULE_557	3	9	16244	157	34.50743	7.12E-05
salmon	10090	GSE3982_CENT_MEMORY_CD4_TCELL_VS_NKCELL_UP	8	176	16077	127	5.817108	7.23E-05
purple	8369	KRAS.600.LUNG.BREAST_UP.V1_UP	9	183	16070	157	5.09126	7.23E-05
turquoise	7098	BERNARD_PPAPDC1B_TARGETS_UP	22	40	16213	4169	2.144195	7.24E-05
lightcyan	9505	GSE2706_UNSTIM_VS_8H_LPS_DC_DN	6	185	16068	62	8.502005	7.26E-05
turquoise	3317	chr13q21	7	7	16246	4169	3.898537	7.28E-05
brown	3958	PID_MET_PATHWAY	24	79	16174	2190	2.254621	7.29E-05
brown	2695	GNF2_SELL	17	47	16206	2190	2.684358	7.38E-05
lightgreen	7066	SMID_BREAST_CANCER_NORMAL_LIKE_UP	7	407	15846	42	6.65561	7.42E-05
lightcyan	5195	CASTELLANO_NRAS_TARGETS_UP	4	59	16194	62	17.77255	7.47E-05
lightcyan	8629	GSE14308_TH2_VS_TH1_DN	6	186	16067	62	8.456296	7.48E-05
lightcyan	9930	GSE37416_CTRL_VS_24H_F_TULARENSIS_LVS_NEUTRO	6	186	16067	62	8.456296	7.48E-05
magenta	3553	MODULE_64	14	404	15849	167	3.372591	7.50E-05
lightgreen	136	INTEGRAL_TO_PLASMA_MEMBRANE	9	721	15532	42	4.830493	7.56E-05
magenta	2851	KEGG_INTESTINAL_IMMUNE_NETWORK_FOR_IGA_PRO	5	43	16210	167	11.31667	7.57E-05
blue	10060	GSE3982_BCELL_VS_BASOPHIL_UP	54	179	16074	2978	1.646454	7.62E-05
midnightb	4323	REACTOME_GENERIC_TRANSCRIPTION_PATHWAY	8	321	15932	71	5.705059	7.66E-05
green	2627	GNF2_CDH3	7	15	16238	1305	5.812056	7.66E-05
turquoise	8777	GSE15930_NAIVE_VS_24H_IN_VITRO_STIM_CD8_TCELL	75	197	16056	4169	1.484215	7.66E-05
blue	8452	GSE11057_CD4_EFF_MEM_VS_PBMC_UP	58	196	16057	2978	1.615031	7.69E-05
magenta	7265	BOYLAN_MULTIPLE_MYELOMA_C_D_UP	8	135	16118	167	5.76731	7.70E-05
lightcyan	4959	NOJIMA_SFRP2_TARGETS_DN	3	22	16231	62	35.74707	7.73E-05

lightcyan	5629	YAN_ESCAPE_FROM_ANOIKIS	3	22	16231	62	35.74707	7.73E-05
lightcyan	7693	DORN_ADENOVIRUS_INFECTION_12HR_UP	3	22	16231	62	35.74707	7.73E-05
turquoise	1946	TGCTGCT,MIR-15A,MIR-16,MIR-15B,MIR-195,MIR-424,I	171	517	15736	4169	1.289458	7.73E-05
lightgreen	10257	GSE9650_NAIVE_VS_EXHAUSTED_CD8_TCELL_DN	5	172	16081	42	11.24931	7.76E-05
greenyello	7282	VILIMAS_NOTCH1_TARGETS_UP	5	48	16205	150	11.28681	7.79E-05
brown	6241	NADLER_OBESITY_UP	19	56	16197	2190	2.517996	7.79E-05
salmon	2612	GNF2_APEX1	6	91	16162	127	8.438003	7.83E-05
purple	4830	BERTUCCI_MEDULLARY_VS_DUCTAL_BREAST_CANCER_	8	144	16109	157	5.751238	7.86E-05
turquoise	2548	GCM_RAB10	68	175	16078	4169	1.51486	7.86E-05
yellow	5493	LUI_THYROID_CANCER_CLUSTER_3	10	28	16225	1426	4.070577	7.87E-05
brown	9410	GSE24142_EARLY_THYMIC_PROGENITOR_VS_DN3_THY	45	189	16064	2190	1.767015	7.91E-05
blue	7285	SPIRA_SMOKERS_LUNG_CANCER_DN	12	21	16232	2978	3.11868	7.92E-05
cyan	7791	NIELSEN_SYNOVIAL_SARCOMA_DN	3	18	16235	77	35.17965	7.93E-05
turquoise	8814	GSE16522_MEMORY_VS_NAIVE_CD8_TCELL_UP	74	194	16059	4169	1.487071	7.93E-05
turquoise	9206	GSE17974_1H_VS_72H_UNTREATED_IN_VITRO_CD4_T	74	194	16059	4169	1.487071	7.93E-05
turquoise	9928	GSE37416_CTRL_VS_12H_F_TULARENSIS_LVS_NEUTRO	74	194	16059	4169	1.487071	7.93E-05
salmon	5175	ENK_UV_RESPONSE_KERATINOCYTE_UP	13	470	15783	127	3.539772	7.95E-05
red	2666	GNF2_MCL1	9	57	16196	524	4.897449	7.96E-05
blue	9661	GSE29618_MONOCYTE_VS_PDC_DN	57	192	16061	2978	1.620252	8.00E-05
cyan	3498	MODULE_5	9	383	15870	77	4.960056	8.00E-05
magenta	225	PLASMA_MEMBRANE_PART	22	867	15386	167	2.469566	8.04E-05
turquoise	1803	V$NRF1_Q6	88	239	16014	4169	1.435445	8.05E-05
red	9361	GSE22886_NAIVE_TCELL_VS_DC_DN	18	199	16054	524	2.805574	8.12E-05
lightcyan	9131	GSE17721_0.5H_VS_8H_CPG_BMDM_DN	6	189	16064	62	8.322069	8.18E-05
blue	2380	MORF_EI24	45	142	16111	2978	1.72955	8.21E-05
greenyello	8401	GOLDRATH_NAIVE_VS_MEMORY_CD8_TCELL_DN	9	195	16058	150	5.000923	8.29E-05
lightgreen	2689	GNF2_RAB7L1	3	33	16220	42	35.17965	8.29E-05
turquoise	2692	GNF2_RBBP6	33	70	16183	4169	1.837882	8.31E-05
salmon	5602	DIRMEIER_LMP1_RESPONSE_LATE_DN	4	30	16223	127	17.06352	8.32E-05
red	2619	GNF2_CD1D	8	45	16208	524	5.514165	8.33E-05
blue	4377	REACTOME_CYTOSOLIC_TRNA_AMINOACYLATION	13	24	16229	2978	2.956249	8.41E-05
brown	2661	GNF2_LYN	12	27	16226	2190	3.298427	8.46E-05
salmon	2324	MORF_RAD21	8	180	16073	127	5.687839	8.46E-05
salmon	10117	GSE39820_TGFBETA3_IL6_VS_TGFBETA3_IL6_IL23A_TF	8	180	16073	127	5.687839	8.46E-05
turquoise	4888	WILCOX_PRESPONSE_TO_ROGESTERONE_UP	55	135	16118	4169	1.588293	8.49E-05
purple	6936	HELLER_HDAC_TARGETS_SILENCED_BY_METHYLATION_	13	381	15872	157	3.532257	8.50E-05
lightgreen	1324	TRANSMEMBRANE_RECEPTOR_ACTIVITY	6	285	15968	42	8.146867	8.51E-05
magenta	7662	GUTIERREZ_WALDENSTROEMS_MACROGLOBULINEMIA	3	9	16244	167	32.44112	8.55E-05
red	10032	GSE3982_MAC_VS_BCELL_UP	17	182	16071	524	2.897209	8.56E-05
red	5394	PEREZ_TP63_TARGETS	23	294	15959	524	2.426514	8.57E-05
lightgreen	14	INTRINSIC_TO_PLASMA_MEMBRANE	9	733	15520	42	4.751413	8.58E-05
brown	4610	REACTOME_IL_3_5_AND_GM_CSF_SIGNALING	15	39	16214	2190	2.854408	8.63E-05
lightcyan	6388	KUMAR_TARGETS_OF_MLL_AF9_FUSION	8	376	15877	62	5.577557	8.65E-05
red	7855	VERHAAK_GLIOBLASTOMA_NEURAL	18	200	16053	524	2.791546	8.66E-05
lightcyan	8342	HOXA9_DN.V1_UP	6	191	16062	62	8.234927	8.67E-05
red	2699	GNF2_SPI1	7	34	16219	524	6.385889	8.78E-05
salmon	8460	GSE11864_UNTREATED_VS_CSF1_IN_MAC_UP	8	181	16072	127	5.656414	8.80E-05
blue	8482	GSE11924_TFH_VS_TH2_CD4_TCELL_UP	58	197	16056	2978	1.606833	8.98E-05
lightcyan	5709	FURUKAWA_DUSP6_TARGETS_PCI35_UP	4	62	16191	62	16.91259	9.07E-05
brown	6584	BURTON_ADIPOGENESIS_8	24	80	16173	2190	2.226438	9.10E-05
turquoise	3178	chr5q21	14	21	16232	4169	2.599025	9.13E-05
lightcyan	8495	GSE12366_GC_VS_NAIVE_BCELL_DN	6	193	16060	62	8.149591	9.18E-05
red	8186	ZWANG_CLASS_3_TRANSIENTLY_INDUCED_BY_EGF	18	201	16052	524	2.777658	9.23E-05
turquoise	2201	TMTCGCGANR_UNKNOWN	62	157	16096	4169	1.53955	9.26E-05
brown	4693	REACTOME_HEMOSTASIS	80	394	15859	2190	1.506896	9.26E-05
turquoise	8779	GSE15930_NAIVE_VS_24H_IN_VITRO_STIM_IL12_CD8_	75	198	16055	4169	1.476718	9.28E-05
purple	225	PLASMA_MEMBRANE_PART	21	867	15386	157	2.50746	9.29E-05
greenyello	9321	GSE22886_NAIVE_CD4_TCELL_VS_NKCELL_DN	9	198	16055	150	4.925152	9.32E-05
brown	6135	FLECHNER_BIOPSY_KIDNEY_TRANSPLANT_REJECTED_V	25	85	16168	2190	2.182783	9.37E-05
turquoise	2197	TGCGCANK_UNKNOWN	167	505	15748	4169	1.289219	9.43E-05

lightgreen	1322	RECEPTOR_ACTIVITY	7	423	15830	42	6.403861	9.45E-05
blue	5920	BENPORATH_ES_CORE_NINE_CORRELATED	31	87	16166	2978	1.944694	9.47E-05
turquoise	2283	GGGCGGR_V$SP1_Q6	759	2655	13598	4169	1.114497	9.52E-05
lightgreen	3652	MODULE_169	4	93	16160	42	16.64414	9.52E-05
turquoise	5269	BERENJENO_TRANSFORMED_BY_RHOA_UP	169	512	15741	4169	1.286822	9.57E-05
blue	2315	MORF_MSH2	24	61	16192	2978	2.147288	9.74E-05
blue	2668	GNF2_MCM5	24	61	16192	2978	2.147288	9.74E-05
red	9005	GSE17721_POLYIC_VS_CPG_1H_BMDM_DN	17	184	16069	524	2.865717	9.80E-05
magenta	10060	GSE3982_BCELL_VS_BASOPHIL_UP	9	179	16074	167	4.893353	9.82E-05
cyan	6128	RADAEVA_RESPONSE_TO_IFNA1_UP	4	51	16202	77	16.55513	9.83E-05
red	7351	RUTELLA_RESPONSE_TO_HGF_VS_CSF2RB_AND_IL4_U	28	398	15855	524	2.182113	9.83E-05
blue	3678	MODULE_198	78	285	15968	2978	1.493684	9.86E-05
blue	6148	MANALO_HYPOXIA_DN	78	285	15968	2978	1.493684	9.86E-05
lightgreen	10091	GSE3982_CENT_MEMORY_CD4_TCELL_VS_NKCELL_DN	5	181	16072	42	10.68995	9.87E-05
red	5850	RICKMAN_TUMOR_DIFFERENTIATED_WELL_VS_MODEI	10	72	16181	524	4.307941	9.91E-05
yellow	8514	GSE12845_IGD_NEG_BLOOD_VS_PRE_GC_TONSIL_BCE	33	189	16064	1426	1.99006	9.96E-05
grey60	9988	GSE3982_EOSINOPHIL_VS_TH1_UP	5	173	16080	44	10.67591	0.0001
turquoise	1964	AATGTGA,MIR-23A,MIR-23B	123	356	15897	4169	1.346966	0.000101
magenta	5897	PASQUALUCCI_LYMPHOMA_BY_GC_STAGE_UP	11	267	15986	167	4.009576	0.000101
purple	1291	COLLAGEN_BINDING	3	10	16243	157	31.05669	0.000101
purple	4362	REACTOME_COMMON_PATHWAY	3	10	16243	157	31.05669	0.000101
turquoise	2168	ATTACAT,MIR-380-3P	37	82	16171	4169	1.759096	0.000101
blue	886	REGULATION_OF_NUCLEOBASENUCLEOSIDENUCLEOTIL	136	552	15701	2978	1.344648	0.000101
magenta	6116	TARTE_PLASMA_CELL_VS_PLASMABLAST_UP	12	315	15938	167	3.707556	0.000103
red	7922	PILON_KLF1_TARGETS_DN	92	1952	14301	524	1.461875	0.000103
blue	3187	chr11q12	34	99	16154	2978	1.874358	0.000104
yellow	4984	GINESTIER_BREAST_CANCER_ZNF217_AMPLIFIED_DN	48	314	15939	1426	1.742311	0.000104
brown	4763	REACTOME_PLATELET_ACTIVATION_SIGNALING_AND_/	43	180	16073	2190	1.772905	0.000104
blue	2342	MORF_UNG	28	76	16177	2978	2.010728	0.000104
red	7266	BOYLAN_MULTIPLE_MYELOMA_C_D_DN	20	240	16013	524	2.584765	0.000105
red	8347	STK33_SKM_DN	20	240	16013	524	2.584765	0.000105
greenyello	8162	LIM_MAMMARY_STEM_CELL_UP	13	408	15845	150	3.452435	0.000106
green	9365	GSE22886_NAIVE_BCELL_VS_NEUTROPHIL_DN	32	198	16055	1305	2.012833	0.000108
brown	5217	MARKEY_RB1_ACUTE_LOF_UP	46	197	16056	2190	1.73293	0.000109
brown	9453	GSE24634_TEFF_VS_TCONV_DAY7_IN_CULTURE_DN	46	197	16056	2190	1.73293	0.000109
lightcyan	709	RESPONSE_TO_OTHER_ORGANISM	4	65	16188	62	16.13201	0.000109
magenta	9250	GSE19825_NAIVE_VS_IL2RAHIGH_DAY3_EFF_CD8_TCEI	8	142	16111	167	5.483006	0.00011
yellow	8314	RB_DN.V1_UP	25	128	16125	1426	2.226097	0.00011
turquoise	2106	AACTGAC,MIR-223	40	91	16162	4169	1.713643	0.00011
salmon	9829	GSE360_DC_VS_MAC_M_TUBERCULOSIS_DN	8	187	16066	127	5.474925	0.00011
brown	9778	GSE339_CD8POS_VS_CD4CD8DN_DC_IN_CULTURE_UP	44	186	16067	2190	1.755614	0.000111
cyan	6377	BASSO_CD40_SIGNALING_UP	5	100	16153	77	10.5539	0.000111
brown	7134	CHUNG_BLISTER_CYTOTOXICITY_DN	16	44	16209	2190	2.698713	0.000111
magenta	5588	YORDY_RECIPROCAL_REGULATION_BY_ETS1_AND_SP1	6	74	16179	167	7.891083	0.000112
turquoise	3665	MODULE_182	41	94	16159	4169	1.700426	0.000112
purple	2806	KEGG_CYTOKINE_CYTOKINE_RECEPTOR_INTERACTION	9	194	16059	157	4.802581	0.000113
purple	10022	GSE3982_DC_VS_CENT_MEMORY_CD4_TCELL_UP	9	194	16059	157	4.802581	0.000113
cyan	9199	GSE17974_IL4_AND_ANTI_IL12_VS_UNTREATED_24H_	6	161	16092	77	7.866258	0.000114
salmon	8432	GSE10325_LUPUS_BCELL_VS_LUPUS_MYELOID_UP	8	188	16065	127	5.445803	0.000115
magenta	6242	SANSOM_APC_TARGETS_DN	12	319	15934	167	3.661067	0.000115
salmon	2381	MORF_EIF3S2	9	241	16012	127	4.779201	0.000116
magenta	8368	KRAS.600.LUNG.BREAST_UP.V1_DN	9	183	16070	167	4.786394	0.000116
yellow	8310	CRX_NRL_DN.V1_UP	24	121	16132	1426	2.260684	0.000117
purple	6934	HELLER_HDAC_TARGETS_UP	11	289	15964	157	3.940295	0.000117
lightcyan	7415	HAN_SATB1_TARGETS_DN	8	393	15860	62	5.336288	0.000118
turquoise	5743	PUJANA_BRCA2_PCC_NETWORK	141	418	15835	4169	1.315057	0.000118
greenyello	4831	SCHUETZ_BREAST_CANCER_DUCTAL_INVASIVE_UP	11	303	15950	150	3.933619	0.000118
turquoise	3412	chr6q14	16	26	16227	4169	2.3991	0.000118
salmon	9314	GSE22886_NAIVE_TCELL_VS_NKCELL_UP	8	189	16064	127	5.41699	0.000119
brown	2615	GNF2_CASP4	11	24	16229	2190	3.401503	0.000119

brown	2673	GNF2_MSN	11	24	16229	2190	3.401503	0.000119
greenyello	9492	GSE26928_EFF_MEM_VS_CENTR_MEM_CD4_TCELL_UI	8	160	16093	150	5.417667	0.000119
red	8567	GSE13484_3H_UNSTIM_VS_YF17D_VACCINE_STIM_PB	17	187	16066	524	2.819743	0.00012
turquoise	86	ORGANELLE_MEMBRANE	101	284	15969	4169	1.386451	0.00012
turquoise	8657	GSE14308_INDUCED_VS_NATURAL_TREG_DN	73	193	16060	4169	1.474576	0.00012
brown	3988	PID_TCPTP_PATHWAY	15	40	16213	2190	2.783048	0.000121
lightcyan	635	IMMUNE_RESPONSE	6	203	16050	62	7.748133	0.000121
magenta	10070	GSE3982_BCELL_VS_TH2_UP	9	184	16069	167	4.760381	0.000121
purple	8449	GSE11057_NAIVE_CD4_VS_PBMC_CD4_TCELL_DN	9	196	16057	157	4.753575	0.000122
red	6328	THEILGAARD_NEUTROPHIL_AT_SKIN_WOUND_DN	19	224	16029	524	2.630921	0.000123
purple	3619	MODULE_131	4	27	16226	157	15.33664	0.000124
magenta	3530	MODULE_41	14	424	15829	167	3.213507	0.000125
salmon	6073	BASSO_B_LYMPHOCYTE_NETWORK	7	142	16111	127	6.308695	0.000125
turquoise	9502	GSE2706_UNSTIM_VS_2H_LPS_DC_UP	66	171	16082	4169	1.504698	0.000126
brown	3116	BIOCARTA_UCALPAIN_PATHWAY	9	17	16236	2190	3.929009	0.000126
turquoise	64	ORGANELLE_LUMEN	147	439	15814	4169	1.305433	0.000126
turquoise	146	MEMBRANE_ENCLOSED_LUMEN	147	439	15814	4169	1.305433	0.000126
brown	9865	GSE360_L_DONOVANI_VS_B_MALAYI_HIGH_DOSE_MA	44	187	16066	2190	1.746226	0.000126
green	2696	GNF2_SERPINB5	7	16	16237	1305	5.448803	0.000127
lightgreen	9726	GSE30962_ACUTE_VS_CHRONIC_LCMV_PRIMARY_INF_	5	191	16062	42	10.13027	0.000127
blue	10057	GSE3982_NEUTROPHIL_VS_TH1_DN	57	195	16058	2978	1.595325	0.000127
red	10129	GSE6269_HEALTHY_VS_E_COLI_INF_PBMC_DN	14	136	16117	524	3.192945	0.000128
cyan	1616	V$STAT3_01	3	21	16232	77	30.15399	0.000128
red	9644	GSE29615_CTRL_VS_LAIV_FLU_VACCINE_PBMC_UP	15	153	16100	524	3.0409	0.000129
blue	2347	MORF_ACP1	60	208	16045	2978	1.574334	0.000129
turquoise	4680	REACTOME_DNA_REPLICATION	71	187	16066	4169	1.480193	0.000129
turquoise	7253	LEE_LIVER_CANCER_SURVIVAL_DN	65	168	16085	4169	1.508362	0.000129
turquoise	167	NUCLEOPLASM_PART	76	203	16050	4169	1.459551	0.00013
cyan	5237	GRAESSMANN_APOPTOSIS_BY_DOXORUBICIN_UP	15	1072	15181	77	2.953516	0.00013
brown	2680	GNF2_PAK2	12	28	16225	2190	3.180626	0.00013
magenta	6223	PENG_RAPAMYCIN_RESPONSE_UP	9	186	16067	167	4.709195	0.000131
brown	2621	GNF2_CD48	13	32	16221	2190	3.014969	0.000132
brown	8003	FARDIN_HYPOXIA_11	13	32	16221	2190	3.014969	0.000132
salmon	8943	GSE17721_POLYIC_VS_PAM3CSK4_12H_BMDM_DN	8	192	16061	127	5.332349	0.000133
blue	8771	GSE15750_DAY6_VS_DAY10_EFF_CD8_TCELL_DN	56	191	16062	2978	1.60016	0.000133
turquoise	8106	FORTSCHEGGER_PHF8_TARGETS_UP	88	242	16011	4169	1.41765	0.000134
red	4908	CASORELLI_ACUTE_PROMYELOCYTIC_LEUKEMIA_UP	16	171	16082	524	2.902192	0.000135
purple	6482	CUI_TCF21_TARGETS_2_DN	19	759	15494	157	2.591467	0.000135
black	3265	chr19q13	25	637	15616	283	2.253968	0.000135
yellow	3635	MODULE_150	7	15	16238	1426	5.318887	0.000135
brown	2457	MORF_RAC1	48	210	16043	2190	1.696334	0.000136
red	10280	GSE9988_LPS_VS_LPS_AND_ANTI_TREM1_MONOCYTE_	17	189	16064	524	2.789905	0.000136
salmon	7476	ROME_INSULIN_TARGETS_IN_MUSCLE_UP	12	429	15824	127	3.579759	0.000136
lightgreen	8424	GSE10325_CD4_TCELL_VS_MYELOID_UP	5	194	16059	42	9.973613	0.000137
magenta	227	PLASMA_MEMBRANE	25	1095	15158	167	2.221994	0.000137
salmon	4697	REACTOME_HIV_INFECTION	8	193	16060	127	5.30472	0.000137
salmon	9786	GSE34205_HEALTHY_VS_RSV_INF_INFANT_PBMC_UP	8	193	16060	127	5.30472	0.000137
salmon	5500	LUI_TARGETS_OF_PAX8_PPARG_FUSION	4	34	16219	127	15.05604	0.000138
blue	2783	KEGG_AMINOACYL_TRNA_BIOSYNTHESIS	18	41	16212	2978	2.396059	0.000138
blue	2532	GCM_MLL	51	170	16083	2978	1.637307	0.000139
turquoise	3141	chr8q22	26	52	16201	4169	1.949268	0.000139
magenta	9384	GSE22886_TH1_VS_TH2_48H_ACT_UP	8	147	16106	167	5.296509	0.00014
brown	6249	GALINDO_IMMUNE_RESPONSE_TO_ENTEROTOXIN	24	82	16171	2190	2.172135	0.00014
turquoise	8724	GSE14769_UNSTIM_VS_240MIN_LPS_BMDM_UP	74	197	16056	4169	1.464425	0.00014
turquoise	9604	GSE27786_NKCELL_VS_MONO_MAC_UP	74	197	16056	4169	1.464425	0.00014
pink	6005	NIKOLSKY_BREAST_CANCER_16P13_AMPLICON	8	108	16145	228	5.280377	0.00014
blue	2517	GCM_DPF2	14	28	16225	2978	2.728845	0.00014
greenyello	1675	V$PAX4_02	8	164	16089	150	5.285528	0.000142
purple	4910	CORRE_MULTIPLE_MYELOMA_UP	5	52	16201	157	9.954067	0.000142
salmon	8997	GSE17721_LPS_VS_PAM3CSK4_12H_BMDM_DN	8	194	16059	127	5.277376	0.000142

yellow	7468	IRITANI_MAD1_TARGETS_DN	13	47	16206	1426	3.152532	0.000142
magenta	8522	GSE12845_PRE_GC_VS_DARKZONE_GC_TONSIL_BCELL_	9	188	16065	167	4.659097	0.000143
red	8497	GSE12366_GC_VS_MEMORY_BCELL_DN	17	190	16063	524	2.775221	0.000145
brown	8695	GSE1460_DP_VS_CD4_THYMOCYTE_DN	42	177	16076	2190	1.761025	0.000146
green	4877	TURASHVILI_BREAST_DUCTAL_CARCINOMA_VS_DUCTA	26	150	16103	1305	2.158764	0.000147
turquoise	5265	BARRIER_CANCER_RELAPSE_TUMOR_SAMPLE_UP	11	15	16238	4169	2.858927	0.000147
salmon	9008	GSE17721_POLYIC_VS_CPG_4H_BMDM_UP	8	195	16058	127	5.250313	0.000147
cyan	5629	YAN_ESCAPE_FROM_ANOIKIS	3	22	16231	77	28.78335	0.000148
greenyello	10112	GSE39820_IL1B_IL6_VS_IL1B_IL6_IL23A_TREATED_CD4	8	165	16088	150	5.253495	0.000148
magenta	1493	V$TAL1ALPHAE47_01	9	189	16064	167	4.634445	0.000148
grey60	6012	NIKOLSKY_BREAST_CANCER_19Q13.4_AMPLICON	2	7	16246	44	105.539	0.000149
lightgreen	8399	GOLDRATH_NAIVE_VS_EFF_CD8_TCELL_DN	5	198	16055	42	9.772126	0.00015
turquoise	8896	GSE17721_CTRL_VS_GARDIQUIMOD_0.5H_BMDM_UP	72	191	16062	4169	1.469606	0.000151
brown	2749	KEGG_OTHER_GLYCAN_DEGRADATION	8	14	16239	2190	4.240835	0.000151
lightcyan	7569	MIKKELSEN_MCV6_LCP_WITH_H3K4ME3	5	133	16120	62	9.855081	0.000151
salmon	8395	KAECH_DAY8_EFF_VS_MEMORY_CD8_TCELL_DN	8	196	16057	127	5.223526	0.000153
salmon	10263	GSE9650_EFFECTOR_VS_MEMORY_CD8_TCELL_DN	8	196	16057	127	5.223526	0.000153
blue	2311	MORF_HDAC1	70	253	16000	2978	1.510033	0.000153
lightgreen	9324	GSE22886_CD8_TCELL_VS_BCELL_NAIVE_UP	5	199	16054	42	9.72302	0.000154
brown	9898	GSE36392_EOSINOPHIL_VS_NEUTROPHIL_IL25_TREATE	43	183	16070	2190	1.743841	0.000155
cyan	5854	RICKMAN_METASTASIS_DN	7	244	16009	77	6.055514	0.000155
red	6741	GENTILE_UV_LOW_DOSE_DN	9	62	16191	524	4.502493	0.000156
magenta	7478	VALK_AML_CLUSTER_1	4	27	16226	167	14.41827	0.000157
lightcyan	3607	MODULE_119	5	134	16119	62	9.781536	0.000157
brown	6634	RODWELL_AGING_KIDNEY_UP	87	443	15810	2190	1.457488	0.000158
salmon	8386	KAECH_NAIVE_VS_DAY8_EFF_CD8_TCELL_UP	8	197	16056	127	5.19701	0.000158
yellow	5915	STARK_PREFRONTAL_CORTEX_22Q11_DELETION_DN	68	498	15755	1426	1.556301	0.000159
blue	229	NUCLEOLUS	39	121	16132	2978	1.75909	0.000159
brown	4891	FULCHER_INFLAMMATORY_RESPONSE_LECTIN_VS_LPS	86	437	15816	2190	1.460516	0.00016
red	8764	GSE15733_BM_VS_SPLEEN_MEMORY_CD4_TCELL_UP	15	156	16097	524	2.982421	0.00016
magenta	5242	GAUSSMANN_MLL_AF4_FUSION_TARGETS_A_DN	6	79	16174	167	7.391647	0.000161
greenyello	1315	RECEPTOR_BINDING	10	262	15991	150	4.135623	0.000162
lightcyan	6303	TAVOR_CEBPA_TARGETS_DN	3	28	16225	62	28.08698	0.000162
lightcyan	7359	MUELLER_COMMON_TARGETS_OF_AML_FUSIONS_DN	3	28	16225	62	28.08698	0.000162
turquoise	9103	GSE17721_0.5H_VS_12H_POLYIC_BMDM_DN	70	185	16068	4169	1.475122	0.000162
turquoise	9924	GSE37416_CTRL_VS_3H_F_TULARENSIS_LVS_NEUTROP	70	185	16068	4169	1.475122	0.000162
turquoise	2247	TTGTTT_V$FOXO4_01	480	1632	14621	4169	1.146628	0.000162
blue	2458	MORF_RAF1	36	109	16144	2978	1.80254	0.000163
brown	9623	GSE28237_EARLY_VS_LATE_GC_BCELL_DN	44	189	16064	2190	1.727748	0.000163
purple	7342	POTTI_TOPOTECAN_SENSITIVITY	7	120	16133	157	6.0388	0.000164
brown	6796	KRIGE_RESPONSE_TO_TOSEDOSTAT_24HR_UP	137	759	15494	2190	1.339579	0.000164
brown	2995	BIOCARTA_IL2_PATHWAY	10	21	16232	2190	3.534029	0.000165
purple	2671	GNF2_MMP1	4	29	16224	157	14.27894	0.000165
purple	3898	MODULE_521	4	29	16224	157	14.27894	0.000165
magenta	2832	KEGG_CELL_ADHESION_MOLECULES_CAMS	7	113	16140	167	6.02888	0.000165
turquoise	4751	REACTOME_CLASS_I_MHC_MEDIATED_ANTIGEN_PROC	83	227	16026	4169	1.425456	0.000165
brown	2643	GNF2_FOS	15	41	16212	2190	2.715169	0.000167
yellow	9951	GSE3982_CTRL_VS_IGE_STIM_MAST_CELL_DN	31	178	16075	1426	1.984978	0.000167
turquoise	1694	V$TGIF_01	74	198	16055	4169	1.457029	0.000168
turquoise	8793	GSE15930_NAIVE_VS_72H_IN_VITRO_STIM_IFNAB_CD	74	198	16055	4169	1.457029	0.000168
turquoise	9311	GSE22886_UNSTIM_VS_STIM_MEMORY_TCELL_DN	74	198	16055	4169	1.457029	0.000168
turquoise	2531	GCM_MAX	17	29	16224	4169	2.285349	0.000169
red	7307	ICHIBA_GRAFT_VERSUS_HOST_DISEASE_35D_UP	13	123	16130	524	3.278238	0.000169
cyan	7605	UROSEVIC_RESPONSE_TO_IMIQUIMOD	3	23	16230	77	27.5319	0.000169
turquoise	3176	chr5q12	19	34	16219	4169	2.178594	0.000169
yellow	26	MITOCHONDRIAL_PART	26	139	16114	1426	2.131928	0.00017
turquoise	4967	WANG_LMO4_TARGETS_DN	115	333	15920	4169	1.346342	0.00017
blue	9232	GSE18791_CTRL_VS_NEWCASTLE_VIRUS_DC_14H_UP	57	197	16056	2978	1.579128	0.000172
blue	9326	GSE22886_CD4_TCELL_VS_BCELL_NAIVE_UP	57	197	16056	2978	1.579128	0.000172
blue	9392	GSE22886_NAIVE_CD4_TCELL_VS_48H_ACT_TH1_UP	57	197	16056	2978	1.579128	0.000172

brown	9147	GSE17721_4_VS_24H_GARDIQUIMOD_BMDM_DN	45	195	16058	2190	1.712645	0.000172
red	10127	GSE6269_HEALTHY_VS_FLU_INF_PBMC_DN	15	157	16096	524	2.963424	0.000172
brown	3498	MODULE_5	77	383	15870	2190	1.492043	0.000173
greenyello	3616	MODULE_128	6	89	16164	150	7.304719	0.000173
turquoise	8184	ZWANG_CLASS_1_TRANSIENTLY_INDUCED_BY_EGF	143	428	15825	4169	1.302549	0.000173
magenta	9788	GSE34205_HEALTHY_VS_FLU_INF_INFANT_PBMC_UP	9	193	16060	167	4.538395	0.000174
turquoise	7378	HOFFMANN_LARGE_TO_SMALL_PRE_BII_LYMPHOCYTE	62	160	16093	4169	1.510683	0.000174
greenyello	459	DEFENSE_RESPONSE	9	215	16038	150	4.535721	0.000174
magenta	8173	SMIRNOV_RESPONSE_TO_IR_6HR_DN	7	114	16139	167	5.975995	0.000175
turquoise	9224	GSE18791_CTRL_VS_NEWCASTLE_VIRUS_DC_6H_UP	73	195	16058	4169	1.459452	0.000175
turquoise	9608	GSE27786_NKTCELL_VS_NEUTROPHIL_UP	73	195	16058	4169	1.459452	0.000175
turquoise	9897	GSE36392_EOSINOPHIL_VS_MAC_IL25_TREATED_LUNG	73	195	16058	4169	1.459452	0.000175
cyan	9869	GSE360_L_DONOVANI_VS_M_TUBERCULOSIS_MAC_DN	6	174	16079	77	7.278549	0.000175
turquoise	6430	IVANOVA_HEMATOPOIESIS_LATE_PROGENITOR	168	514	15739	4169	1.27423	0.000175
salmon	9353	GSE22886_NEUTROPHIL_VS_DC_DN	8	200	16053	127	5.119055	0.000175
salmon	9388	GSE22886_CTRL_VS_LPS_24H_DC_UP	8	200	16053	127	5.119055	0.000175
cyan	7061	SMID_BREAST_CANCER_LUMINAL_B_DN	9	425	15828	77	4.469885	0.000176
blue	9816	GSE360_DC_VS_MAC_UP	53	180	16073	2978	1.606986	0.000176
red	9836	GSE360_L_DONOVANI_VS_B_MALAYI_LOW_DOSE_DC_	16	175	16078	524	2.835856	0.000176
lightyellow	5049	TONKS_TARGETS_OF_RUNX1_RUNX1T1_FUSION_MON	3	54	16199	33	27.36195	0.000176
purple	4831	SCHUETZ_BREAST_CANCER_DUCTAL_INVASIVE_UP	11	303	15950	157	3.758235	0.000177
brown	6588	VERRECCHIA_EARLY_RESPONSE_TO_TGFB1	17	50	16203	2190	2.523297	0.000178
magenta	3519	MODULE_27	11	285	15968	167	3.75634	0.000179
lightcyan	7348	RUTELLA_RESPONSE_TO_CSF2RB_AND_IL4_DN	7	312	15941	62	5.881462	0.000179
red	7836	KAMIKUBO_MYELOID_CEBPA_NETWORK	6	27	16226	524	6.892706	0.000181
turquoise	8711	GSE1460_NAIVE_CD4_TCELL_CORD_BLOOD_VS_THYMI	72	192	16061	4169	1.461951	0.000181
turquoise	8932	GSE17721_POLYIC_VS_PAM3CSK4_1H_BMDM_UP	72	192	16061	4169	1.461951	0.000181
purple	2282	CATTGTYY_V$SOX9_B1	11	304	15949	157	3.745872	0.000182
purple	4388	REACTOME_P130CAS_LINKAGE_TO_MAPK_SIGNALING_	3	12	16241	157	25.88057	0.000182
purple	4393	REACTOME_GRB2_SOS_PROVIDES_LINKAGE_TO_MAPK	3	12	16241	157	25.88057	0.000182
purple	5124	GRAHAM_CML_DIVIDING_VS_NORMAL_DIVIDING_UP	3	12	16241	157	25.88057	0.000182
purple	6762	DASU_IL6_SIGNALING_SCAR_DN	3	12	16241	157	25.88057	0.000182
salmon	6116	TARTE_PLASMA_CELL_VS_PLASMABLAST_UP	10	315	15938	127	4.062742	0.000183
purple	5120	GRAHAM_CML_QUIESCENT_VS_NORMAL_QUIESCENT_	6	86	16167	157	7.222486	0.000184
cyan	8961	GSE17721_PAM3CSK4_VS_CPG_12H_BMDM_DN	6	176	16077	77	7.195838	0.000186
turquoise	5386	LASTOWSKA_NEUROBLASTOMA_COPY_NUMBER_DN	244	781	15472	4169	1.217981	0.000186
magenta	1595	V$OCT1_Q6	9	195	16058	167	4.491847	0.000188
salmon	6615	ZHANG_RESPONSE_TO_CANTHARIDIN_DN	5	68	16185	127	9.410028	0.000189
brown	2858	KEGG_REGULATION_OF_ACTIN_CYTOSKELETON	42	179	16074	2190	1.741348	0.00019
brown	9967	GSE3982_DC_VS_NEUTROPHIL_LPS_STIM_DN	40	168	16085	2190	1.767015	0.000192
red	6963	MASSARWEH_TAMOXIFEN_RESISTANCE_UP	33	523	15730	524	1.957107	0.000193
brown	9111	GSE17721_4H_VS_24H_POLYIC_BMDM_DN	45	196	16057	2190	1.703907	0.000194
brown	9942	GSE37416_0H_VS_48H_F_TULARENSIS_LVS_NEUTROPI	45	196	16057	2190	1.703907	0.000194
brown	4492	REACTOME_SEMA4D_IN_SEMAPHORIN_SIGNALING	12	29	16224	2190	3.070949	0.000195
greenyello	3567	MODULE_79	6	91	16162	150	7.144176	0.000195
blue	9882	GSE360_T_GONDII_VS_M_TUBERCULOSIS_MAC_UP	54	185	16068	2978	1.593055	0.000196
turquoise	2051	AAACCAC,MIR-140	41	96	16157	4169	1.665	0.000196
greenyello	10257	GSE9650_NAIVE_VS_EXHAUSTED_CD8_TCELL_DN	8	172	16081	150	5.03969	0.000196
blue	2480	MORF_UBE2N	32	94	16159	2978	1.857937	0.000197
cyan	8889	GSE17721_CTRL_VS_CPG_6H_BMDM_DN	6	178	16075	77	7.114986	0.000198
salmon	8145	HOLLEMAN_VINCRISTINE_RESISTANCE_B_ALL_DN	3	15	16238	127	25.59528	0.000198
purple	6256	ALCALAY_AML_BY_NPM1_LOCALIZATION_UP	7	124	16129	157	5.844	0.000201
blue	2593	GNF2_MLH1	18	42	16211	2978	2.33901	0.000201
blue	4398	REACTOME_TRNA_AMINOACYLATION	18	42	16211	2978	2.33901	0.000201
turquoise	9343	GSE22886_IGM_MEMORY_BCELL_VS_BLOOD_PLASMA	74	199	16054	4169	1.449707	0.000201
turquoise	9556	GSE27786_LIN_NEG_VS_ERYTHROBLAST_UP	74	199	16054	4169	1.449707	0.000201
greenyello	345	SIGNAL_TRANSDUCTION	26	1331	14922	150	2.116594	0.000202
red	2233	TGANTCA_V$AP1_C	49	895	15358	524	1.698147	0.000202
turquoise	2013	TATTATA,MIR-374	86	238	16015	4169	1.408715	0.000202
yellow	9596	GSE27786_CD8_TCELL_VS_MONO_MAC_UP	33	196	16057	1426	1.918986	0.000203

blue	2366	MORF_CDK2	26	71	16182	2978	1.998591	0.000204
tan	10068	GSE3982_BCELL_VS_TH1_UP	8	179	16074	145	5.009594	0.000205
lightcyan	6889	FOSTER_TOLERANT_MACROPHAGE_UP	5	142	16111	62	9.230463	0.000206
red	5934	AMIT_EGF_RESPONSE_40_MCF10A	5	18	16235	524	8.615882	0.000207
greenyello	3653	MODULE_170	6	92	16161	150	7.066522	0.000208
magenta	6299	YU_MYC_TARGETS_DN	5	53	16200	167	9.181448	0.000208
purple	2822	KEGG_VASCULAR_SMOOTH_MUSCLE_CONTRACTION	6	88	16165	157	7.058338	0.000208
red	4906	THUM_SYSTOLIC_HEART_FAILURE_UP	26	374	15879	524	2.156274	0.000209
cyan	9033	GSE17721_PAM3CSK4_VS_GADIQUIMOD_12H_BMDM_	6	180	16073	77	7.035931	0.00021
brown	8910	GSE17721_CTRL_VS_GARDIQUIMOD_24H_BMDM_UP	44	191	16062	2190	1.709656	0.00021
purple	6324	LI_WILMS_TUMOR_VS_FETAL_KIDNEY_1_UP	8	166	16087	157	4.989026	0.000211
cyan	3201	chr1p22	4	62	16191	77	13.61793	0.000211
purple	136	INTEGRAL_TO_PLASMA_MEMBRANE	18	721	15532	157	2.584468	0.000211
magenta	1461	AACTTT_UNKNOWN	30	1473	14780	167	1.982146	0.000212
salmon	7002	DAIRKEE_CANCER_PRONE_RESPONSE_BPA_E2	6	109	16144	127	7.044571	0.000212
red	5792	BUYTAERT_PHOTODYNAMIC_THERAPY_STRESS_UP	44	778	15475	524	1.754185	0.000213
red	8324	RPS14_DN.V1_UP	16	178	16075	524	2.788061	0.000214
red	8901	GSE17721_CTRL_VS_GARDIQUIMOD_2H_BMDM_DN	16	178	16075	524	2.788061	0.000214
turquoise	7860	CHICAS_RB1_TARGETS_SENESCENT	166	509	15744	4169	1.271429	0.000215
purple	2262	YTATTTTNR_V$MEF2_02	15	535	15718	157	2.902494	0.000215
purple	2687	GNF2_PTX3	4	31	16222	157	13.35772	0.000215
purple	5857	FRIDMAN_IMMORTALIZATION_DN	4	31	16222	157	13.35772	0.000215
red	7068	SMID_BREAST_CANCER_BASAL_UP	32	504	15749	524	1.969344	0.000216
yellow	6797	KRIGE_RESPONSE_TO_TOSEDOSTAT_24HR_DN	118	980	15273	1426	1.372366	0.000216
cyan	8479	GSE11864_CSF1_PAM3CYS_VS_CSF1_IFNG_PAM3CYS_	6	181	16072	77	6.997058	0.000216
cyan	9507	GSE2706_UNSTIM_VS_2H_LPS_AND_R848_DC_DN	6	181	16072	77	6.997058	0.000216
red	6578	LU_AGING_BRAIN_UP	19	234	16019	524	2.518489	0.000216
salmon	2504	GCM_CBFB	5	70	16183	127	9.14117	0.000217
turquoise	8256	CAMP_UP.V1_UP	72	193	16060	4169	1.454376	0.000218
turquoise	9550	GSE27786_LIN_NEG_VS_CD8_TCELL_UP	72	193	16060	4169	1.454376	0.000218
turquoise	9598	GSE27786_NKCELL_VS_NKTCELL_UP	72	193	16060	4169	1.454376	0.000218
turquoise	9724	GSE30962_PRIMARY_VS_SECONDARY_CHRONIC_LCMV	72	193	16060	4169	1.454376	0.000218
turquoise	10108	GSE39820_CTRL_VS_TGFBETA3_IL6_CD4_TCELL_UP	72	193	16060	4169	1.454376	0.000218
turquoise	10168	GSE7460_CTRL_VS_TGFB_TREATED_ACT_FOXP3_HET_T	72	193	16060	4169	1.454376	0.000218
blue	7456	MILI_PSEUDOPODIA_HAPTOTAXIS_DN	154	647	15606	2978	1.299048	0.000218
turquoise	2095	CTTTGTA,MIR-524	126	372	15881	4169	1.320472	0.000218
lightgreen	118	INTEGRAL_TO_MEMBRANE	10	1025	15228	42	3.775377	0.000219
lightcyan	5771	GROSS_ELK3_TARGETS_DN	3	31	16222	62	25.36889	0.00022
purple	3616	MODULE_128	6	89	16164	157	6.979031	0.000222
brown	6955	MARTINEZ_TP53_TARGETS_UP	99	522	15731	2190	1.407519	0.000222
cyan	9405	GSE24081_CONTROLLER_VS_PROGRESSOR_HIV_SPECIF	6	182	16071	77	6.958613	0.000223
red	9443	GSE24634_TREG_VS_TCONV_POST_DAY5_IL4_CONVER	17	197	16056	524	2.676609	0.000224
turquoise	5061	UDAYAKUMAR_MED1_TARGETS_UP	52	130	16123	4169	1.559415	0.000226
turquoise	4637	REACTOME_TRAF6_MEDIATED_INDUCTION_OF_NFKB_	33	73	16180	4169	1.762352	0.000226
turquoise	9105	GSE17721_0.5H_VS_4H_POLYIC_BMDM_DN	71	190	16063	4169	1.456822	0.000226
turquoise	6815	MONNIER_POSTRADIATION_TUMOR_ESCAPE_DN	120	352	15901	4169	1.329047	0.000227
turquoise	5858	SCHAEFFER_PROSTATE_DEVELOPMENT_6HR_UP	60	155	16098	4169	1.509111	0.000227
brown	9825	GSE360_DC_VS_MAC_B_MALAYI_HIGH_DOSE_DN	43	186	16067	2190	1.715714	0.000227
brown	4085	PID_THROMBIN_PAR1_PATHWAY	15	42	16211	2190	2.650522	0.000228
brown	5450	MARTIN_INTERACT_WITH_HDAC	15	42	16211	2190	2.650522	0.000228
cyan	9501	GSE2706_UNSTIM_VS_8H_R848_DC_DN	6	183	16070	77	6.920588	0.000229
blue	613	REGULATION_OF_GENE_EXPRESSION	143	595	15658	2978	1.31168	0.00023
cyan	5440	RASHI_RESPONSE_TO_IONIZING_RADIATION_2	5	117	16136	77	9.020424	0.000232
purple	4489	REACTOME_G_ALPHA_I_SIGNALLING_EVENTS	7	127	16126	157	5.705953	0.000233
yellow	2715	GNF2_UBE2I	12	43	16210	1426	3.18073	0.000233
lightgreen	7304	GOLDRATH_ANTIGEN_RESPONSE	6	343	15910	42	6.769263	0.000234
brown	7312	LINDSTEDT_DENDRITIC_CELL_MATURATION_D	20	65	16188	2190	2.283527	0.000234
turquoise	8472	GSE11864_CSF1_VS_CSF1_IFNG_PAM3CYS_IN_MAC_U	70	187	16066	4169	1.459345	0.000235
turquoise	8931	GSE17721_POLYIC_VS_PAM3CSK4_0.5H_BMDM_DN	70	187	16066	4169	1.459345	0.000235
turquoise	9093	GSE17721_0.5H_VS_12H_LPS_BMDM_DN	70	187	16066	4169	1.459345	0.000235

cyan	9302	GSE2197_CPG_DNA_VS_UNTREATED_IN_DC_UP	6	184	16069	77	6.882976	0.000236
cyan	9527	GSE2706_2H_VS_8H_R848_AND_LPS_STIM_DC_DN	6	184	16069	77	6.882976	0.000236
pink	3576	MODULE_88	21	633	15620	228	2.364908	0.000237
blue	9983	GSE3982_EOSINOPHIL_VS_EFF_MEMORY_CD4_TCELL_	53	182	16071	2978	1.589327	0.000239
greenyello	9487	GSE26928_NAIVE_VS_EFF_MEMORY_CD4_TCELL_DN	8	177	16076	150	4.897326	0.000239
cyan	6634	RODWELL_AGING_KIDNEY_UP	9	443	15810	77	4.288265	0.000239
red	6862	ZHENG_BOUND_BY_FOXP3	30	463	15790	524	2.009752	0.000239
turquoise	9495	GSE26928_EFF_MEMORY_VS_CXCR5_POS_CD4_TCELL_	64	168	16085	4169	1.485157	0.000243
turquoise	5903	BENPORATH_SOX2_TARGETS	216	685	15568	4169	1.22932	0.000243
cyan	9505	GSE2706_UNSTIM_VS_8H_LPS_DC_DN	6	185	16068	77	6.84577	0.000243
cyan	10162	GSE7460_TREG_VS_TCONV_ACT_UP	6	185	16068	77	6.84577	0.000243
purple	5058	PAPASPYRIDONOS_UNSTABLE_ATEROSCLEROTIC_PLAQ	4	32	16221	157	12.94029	0.000244
purple	6843	LEIN_ASTROCYTE_MARKERS	4	32	16221	157	12.94029	0.000244
turquoise	9995	GSE3982_MAST_CELL_VS_MAC_DN	69	184	16069	4169	1.461951	0.000244
lightgreen	8189	ZWANG_TRANSIENTLY_UP_BY_1ST_EGF_PULSE_ONLY	12	1472	14781	42	3.154697	0.000246
blue	4435	REACTOME_MRNA_SPLICING	35	107	16146	2978	1.785226	0.000246
yellow	9255	GSE19825_NAIVE_VS_DAY3_EFF_CD8_TCELL_DN	33	198	16055	1426	1.899603	0.000246
tan	9465	GSE25087_FETAL_VS_ADULT_TREG_DN	8	184	16069	145	4.873463	0.000247
brown	8474	GSE11864_CSF1_IFNG_VS_CSF1_PAM3CYS_IN_MAC_U	45	198	16055	2190	1.686696	0.000248
purple	9513	GSE2706_R848_VS_LPS_8H_STIM_DC_DN	8	170	16083	157	4.871637	0.000248
lightgreen	41	INTRINSIC_TO_MEMBRANE	10	1041	15212	42	3.717351	0.000249
purple	7061	SMID_BREAST_CANCER_LUMINAL_B_DN	13	425	15828	157	3.166564	0.00025
purple	3567	MODULE_79	6	91	16162	157	6.825646	0.00025
cyan	9133	GSE17721_0.5H_VS_24H_CPG_BMDM_DN	6	186	16067	77	6.808965	0.000251
brown	4028	PID_ECADHERIN_NASCENTAJ_PATHWAY	14	38	16215	2190	2.734223	0.000252
magenta	976	REGULATION_OF_MULTICELLULAR_ORGANISMAL_PRO	7	121	16132	167	5.630277	0.000252
yellow	8416	GSE10239_KLRG1INT_VS_KLRG1HIGH_EFF_CD8_TCELL_	31	182	16071	1426	1.941352	0.000252
turquoise	7411	ZHANG_TLX_TARGETS_UP	38	88	16165	4169	1.683459	0.000253
red	3649	MODULE_165	9	66	16187	524	4.229615	0.000253
lightgreen	1012	CELLULAR_DEFENSE_RESPONSE	3	48	16205	42	24.18601	0.000256
magenta	7500	POOLA_INVASIVE_BREAST_CANCER_UP	10	249	16004	167	3.908568	0.000257
red	6312	ABBUD_LIF_SIGNALING_1_UP	7	40	16213	524	5.428006	0.000257
purple	5044	TONKS_TARGETS_OF_RUNX1_RUNX1T1_FUSION_HSC_	8	171	16082	157	4.843148	0.000258
cyan	8723	GSE14769_UNSTIM_VS_120MIN_LPS_BMDM_DN	6	187	16066	77	6.772554	0.000258
purple	14	INTRINSIC_TO_PLASMA_MEMBRANE	18	733	15520	157	2.542157	0.000258
turquoise	1996	GTGCCTT,MIR-506	207	654	15599	4169	1.233941	0.000259
red	9803	GSE360_CTRL_VS_M_TUBERCULOSIS_DC_DN	16	181	16072	524	2.74185	0.000259
red	10076	GSE3982_BASOPHIL_VS_NKCELL_UP	16	181	16072	524	2.74185	0.000259
brown	2682	GNF2_PECAM1	18	56	16197	2190	2.38547	0.000259
turquoise	69	NUCLEAR_LUMEN	125	370	15883	4169	1.317073	0.00026
turquoise	3201	chr1p22	29	62	16191	4169	1.823509	0.00026
turquoise	4408	REACTOME_CYCLIN_E_ASSOCIATED_EVENTS_DURING_	29	62	16191	4169	1.823509	0.00026
greenyello	3340	chr11q25	3	14	16239	150	23.21857	0.00026
red	8305	EIF4E_DN	11	96	16157	524	3.554051	0.000261
turquoise	10120	GSE39820_TGFBETA1_VS_TGFBETA3_IN_IL6_IL23A_TR	67	178	16075	4169	1.467427	0.000263
salmon	6805	ZHONG_SECRETOME_OF_LUNG_CANCER_AND_MACRO	5	73	16180	127	8.765505	0.000264
blue	2333	MORF_RPA2	54	187	16066	2978	1.576017	0.000265
pink	1541	V$GATA1_03	10	183	16070	228	3.89536	0.000265
cyan	9037	GSE17721_PAM3CSK4_VS_GADIQUIMOD_24H_BMDM_	6	188	16065	77	6.736529	0.000265
purple	3653	MODULE_170	6	92	16161	157	6.751454	0.000266
brown	2999	BIOCARTA_IL6_PATHWAY	10	22	16231	2190	3.373391	0.000266
brown	4306	REACTOME_EGFR_DOWNREGULATION	10	22	16231	2190	3.373391	0.000266
brown	4496	REACTOME_LYSOSOME_VESICLE_BIOGENESIS	10	22	16231	2190	3.373391	0.000266
yellow	3571	MODULE_83	46	309	15944	1426	1.696732	0.000266
purple	5645	MOHANKUMAR_TLX1_TARGETS_DN	7	130	16123	157	5.574277	0.000269
lightyellow	10137	GSE6269_FLU_VS_STREP_AUREUS_INF_PBMC_DN	4	156	16097	33	12.62859	0.000269
turquoise	9030	GSE17721_PAM3CSK4_VS_GADIQUIMOD_8H_BMDM_	71	191	16062	4169	1.449194	0.000271
turquoise	9060	GSE17721_POLYIC_VS_GARDIQUIMOD_2H_BMDM_UP	71	191	16062	4169	1.449194	0.000271
salmon	10084	GSE3982_EFF_MEMORY_CD4_TCELL_VS_NKCELL_UP	7	161	16092	127	5.56419	0.000271
magenta	3960	PID_INTEGRIN3_PATHWAY	4	31	16222	167	12.55785	0.000272

cyan	8617	GSE14000_UNSTIM_VS_16H_LPS_DC_TRANSLATED_RN	6	189	16064	77	6.700886	0.000273
cyan	7511	BOYAULT_LIVER_CANCER_SUBCLASS_G5_DN	3	27	16226	77	23.4531	0.000276
blue	9873	GSE360_L_MAJOR_VS_B_MALAYI_HIGH_DOSE_MAC_D	53	183	16070	2978	1.580642	0.000277
turquoise	8202	GCNP_SHH_UP_EARLY.V1_UP	60	156	16097	4169	1.499437	0.000277
brown	9351	GSE22886_DAY1_VS_DAY7_MONOCYTE_IN_CULTURE_	45	199	16054	2190	1.67822	0.000279
brown	9373	GSE22886_NAIVE_CD8_TCELL_VS_DC_DN	45	199	16054	2190	1.67822	0.000279
brown	6957	MARTINEZ_RB1_AND_TP53_TARGETS_UP	98	519	15734	2190	1.401355	0.00028
cyan	8638	GSE14308_TH1_VS_TH17_UP	6	190	16063	77	6.665619	0.000281
cyan	9520	GSE2706_LPS_VS_R848_AND_LPS_8H_STIM_DC_UP	6	190	16063	77	6.665619	0.000281
cyan	3654	MODULE_171	5	122	16131	77	8.650735	0.000282
turquoise	1986	ATGCTGC,MIR-103,MIR-107	70	188	16065	4169	1.451583	0.000282
turquoise	9548	GSE27786_LIN_NEG_VS_CD4_TCELL_UP	70	188	16065	4169	1.451583	0.000282
turquoise	9943	GSE37416_0H_VS_48H_F_TULARENSIS_LVS_NEUTROPH	70	188	16065	4169	1.451583	0.000282
lightcyan	8215	CSR_LATE_UP.V1_DN	5	152	16101	62	8.623196	0.000282
turquoise	9807	GSE360_CTRL_VS_L_MAJOR_MAC_DN	65	172	16081	4169	1.473284	0.000283
blue	2306	MORF_ESPL1	23	61	16192	2978	2.057817	0.000284
blue	2332	MORF_RPA1	23	61	16192	2978	2.057817	0.000284
brown	6316	VERHAAK_AML_WITH_NPM1_MUTATED_UP	40	171	16082	2190	1.736014	0.000285
brown	2963	BIOCARTA_EGF_PATHWAY	12	30	16223	2190	2.968584	0.000285
brown	975	CYTOKINE_SECRETION	8	15	16238	2190	3.958113	0.000285
red	345	SIGNAL_TRANSDUCTION	66	1331	14922	524	1.538042	0.000286
midnightb	4808	PICCALUGA_ANGIOIMMUNOBLASTIC_LYMPHOMA_DN	5	133	16120	71	8.605846	0.000287
cyan	8719	GSE14769_UNSTIM_VS_60MIN_LPS_BMDM_DN	6	191	16062	77	6.63072	0.000289
purple	8100	ALFANO_MYC_TARGETS	9	220	16033	157	4.235003	0.00029
brown	9120	GSE17721_0.5H_VS_24H_PAM3CSK4_BMDM_UP	43	188	16065	2190	1.697462	0.00029
yellow	8318	RB_P130_DN.V1_UP	22	113	16140	1426	2.219005	0.000291
greenyello	539	VIRAL_REPRODUCTION	4	35	16218	150	12.38324	0.000292
greenyello	2898	KEGG_GRAFT_VERSUS_HOST_DISEASE	4	35	16218	150	12.38324	0.000292
blue	1277	ATPASE_ACTIVITY_COUPLED	29	84	16169	2978	1.884202	0.000293
turquoise	8880	GSE17721_CTRL_VS_CPG_0.5H_BMDM_UP	69	185	16068	4169	1.454049	0.000293
red	8721	GSE14769_UNSTIM_VS_80MIN_LPS_BMDM_DN	16	183	16070	524	2.711884	0.000293
purple	8325	IL15_UP.V1_DN	7	132	16121	157	5.489819	0.000295
pink	7053	SMID_BREAST_CANCER_RELAPSE_IN_BONE_DN	11	221	16032	228	3.548127	0.000296
tan	9472	GSE26495_NAIVE_VS_PD1HIGH_CD8_TCELL_UP	8	189	16064	145	4.744536	0.000296
magenta	809	ANATOMICAL_STRUCTURE_DEVELOPMENT	18	696	15557	167	2.516983	0.000297
red	7632	SAKAI_CHRONIC_HEPATITIS_VS_LIVER_CANCER_UP	10	82	16171	524	3.782582	0.000297
cyan	9737	GSE31082_DP_VS_CD4_SP_THYMOCYTE_DN	6	192	16061	77	6.596185	0.000297
turquoise	7446	WENDT_COHESIN_TARGETS_UP	17	30	16223	4169	2.209171	0.000297
yellow	8121	FOSTER_KDM1A_TARGETS_DN	33	200	16053	1426	1.880607	0.000298
lightgreen	7813	BROWNE_HCMV_INFECTION_30MIN_DN	4	125	16128	42	12.38324	0.000298
purple	7977	AZARE_NEOPLASTIC_TRANSFORMATION_BY_STAT3_UI	3	14	16239	157	22.18335	0.000298
turquoise	3191	chr14q13	12	18	16235	4169	2.599025	0.000298
turquoise	8404	GSE10094_LCMV_VS_LISTERIA_IND_EFF_CD4_TCELL_U	73	198	16055	4169	1.437339	0.000298
turquoise	3501	MODULE_8	132	395	15858	4169	1.302802	0.000299
purple	5062	UDAYAKUMAR_MED1_TARGETS_DN	9	221	16032	157	4.21584	0.0003
red	2643	GNF2_FOS	7	41	16212	524	5.295615	0.000302
red	4987	OSWALD_HEMATOPOIETIC_STEM_CELL_IN_COLLAGEN_	18	221	16032	524	2.526286	0.000303
brown	7349	RUTELLA_RESPONSE_TO_HGF_UP	79	402	15851	2190	1.458446	0.000303
turquoise	1	NUCLEOPLASM	94	267	15986	4169	1.372519	0.000303
brown	2620	GNF2_CD33	17	52	16201	2190	2.426247	0.000304
blue	2339	MORF_TERF1	24	65	16188	2978	2.015147	0.000306
green	6133	YAGI_AML_RELAPSE_PROGNOSIS	10	35	16218	1305	3.558402	0.000307
brown	2654	GNF2_INPP5D	15	43	16210	2190	2.588882	0.000307
greenyello	1278	G_PROTEIN_COUPLED_RECEPTOR_ACTIVITY	6	99	16154	150	6.566869	0.000309
yellow	7255	MOOTHA_MITOCHONDRIA	59	427	15826	1426	1.574846	0.00031
red	8220	AKT_UP.V1_UP	14	148	16105	524	2.934057	0.000311
lightyellow	5016	TAKEDA_TARGETS_OF_NUP98_HOXA9_FUSION_16D_U	4	162	16091	33	12.16087	0.000311
lightgreen	5134	WAMUNYOKOLI_OVARIAN_CANCER_GRADES_1_2_UP	4	127	16126	42	12.18823	0.000316
red	5017	TAKEDA_TARGETS_OF_NUP98_HOXA9_FUSION_16D_D	13	131	16122	524	3.07804	0.000317
green	2697	GNF2_SERPINI2	5	9	16244	1305	6.919115	0.000317

turquoise	8031	IKEDA_MIR30_TARGETS_UP	46	113	16140	4169	1.587015	0.000317
red	3332	chr15q	3	5	16248	524	18.61031	0.000317
black	8188	ZWANG_TRANSIENTLY_UP_BY_2ND_EGF_PULSE_ONLY	34	1044	15209	283	1.870361	0.000318
turquoise	2046	TTGCCAA,MIR-182	102	294	15959	4169	1.352554	0.000318
red	2878	KEGG_LEISHMANIA_INFECTION	9	68	16185	524	4.105214	0.000319
pink	2778	KEGG_PORPHYRIN_AND_CHLOROPHYLL_METABOLISM	4	24	16229	228	11.88085	0.000321
turquoise	2337	MORF_SP3	35	80	16173	4169	1.70561	0.000323
turquoise	2453	MORF_RAB1A	71	192	16061	4169	1.441646	0.000323
turquoise	8640	GSE14308_TH1_VS_NAIVE_CD4_TCELL_UP	71	192	16061	4169	1.441646	0.000323
turquoise	10104	GSE39820_CTRL_VS_TGFBETA1_IL6_CD4_TCELL_UP	71	192	16061	4169	1.441646	0.000323
pink	1366	STRUCTURAL_CONSTITUENT_OF_CYTOSKELETON	5	43	16210	228	8.288964	0.000324
lightgreen	5313	MCBRYAN_PUBERTAL_TGFB1_TARGETS_DN	3	52	16201	42	22.32555	0.000325
salmon	9438	GSE24634_NAIVE_CD4_TCELL_VS_DAY10_IL4_CONV_T	7	166	16087	127	5.396594	0.000327
brown	9114	GSE17721_0.5H_VS_12H_PAM3CSK4_BMDM_UP	43	189	16064	2190	1.688481	0.000328
brown	9461	GSE24634_IL4_VS_CTRL_TREATED_NAIVE_CD4_TCELL_	43	189	16064	2190	1.688481	0.000328
turquoise	3636	MODULE_151	107	311	15942	4169	1.341297	0.000329
magenta	7878	DUTERTRE_ESTRADIOL_RESPONSE_24HR_DN	14	466	15787	167	2.923878	0.000329
purple	6735	SATO_SILENCED_BY_METHYLATION_IN_PANCREATIC_C	11	326	15927	157	3.493084	0.000332
brown	381	PROGRAMMED_CELL_DEATH	77	391	15862	2190	1.461515	0.000336
blue	5506	SCHLOSSER_MYC_TARGETS_AND_SERUM_RESPONSE_U	19	47	16206	2978	2.2063	0.000337
turquoise	10179	GSE7460_WT_VS_FOXP3_HET_ACT_TCONV_DN	70	189	16064	4169	1.443903	0.000337
magenta	4446	REACTOME_AXON_GUIDANCE	9	211	16042	167	4.151233	0.000337
lightcyan	8372	KRAS.KIDNEY_UP.V1_DN	4	87	16166	62	12.05265	0.000337
purple	8324	RPS14_DN.V1_UP	8	178	16075	157	4.652687	0.000338
yellow	148	MITOCHONDRIAL_INNER_MEMBRANE	15	64	16189	1426	2.671316	0.000338
yellow	4597	REACTOME_RESPIRATORY_ELECTRON_TRANSPORT	15	64	16189	1426	2.671316	0.000338
yellow	5797	LOPEZ_MBD_TARGETS	108	893	15360	1426	1.378435	0.000339
cyan	7868	GREGORY_SYNTHETIC_LETHAL_WITH_IMATINIB	5	127	16126	77	8.310154	0.000339
salmon	5950	SAKAI_TUMOR_INFILTRATING_MONOCYTES_DN	5	77	16176	127	8.310154	0.000339
salmon	6189	SANA_RESPONSE_TO_IFNG_DN	5	77	16176	127	8.310154	0.000339
brown	2940	BIOCARTA_CELL2CELL_PATHWAY	7	12	16241	2190	4.329186	0.00034
turquoise	2535	GCM_MYST2	65	173	16080	4169	1.464768	0.000342
brown	4134	REACTOME_GAP_JUNCTION_DEGRADATION	6	9	16244	2190	4.947641	0.000347
brown	6137	IIZUKA_LIVER_CANCER_PROGRESSION_G2_G3_DN	6	9	16244	2190	4.947641	0.000347
greenyello	7248	VART_KSHV_INFECTION_ANGIOGENIC_MARKERS_UP	7	142	16111	150	5.341362	0.000349
yellow	6175	MOOTHA_VOXPHOS	18	85	16168	1426	2.413613	0.000349
salmon	902	RIBOSOME_BIOGENESIS_AND_ASSEMBLY	3	18	16235	127	21.3294	0.000349
blue	1686	V$SOX9_B1	57	202	16051	2978	1.540041	0.00035
cyan	1490	V$IRF1_01	6	198	16055	77	6.396301	0.00035
purple	2858	KEGG_REGULATION_OF_ACTIN_CYTOSKELETON	8	179	16074	157	4.626695	0.000351
blue	64	ORGANELLE_LUMEN	109	439	15814	2978	1.355098	0.000351
blue	146	MEMBRANE_ENCLOSED_LUMEN	109	439	15814	2978	1.355098	0.000351
yellow	1197	STRUCTURAL_CONSTITUENT_OF_RIBOSOME	17	78	16175	1426	2.484096	0.000353
purple	4612	REACTOME_PLATELET_HOMEOSTASIS	5	63	16190	157	8.216055	0.000353
purple	5231	CONCANNON_APOPTOSIS_BY_EPOXOMICIN_UP	9	226	16027	157	4.122569	0.000354
brown	3475	SIG_INSULIN_RECEPTOR_PATHWAY_IN_CARDIAC_MYO	16	48	16205	2190	2.47382	0.000354
turquoise	8608	GSE14000_TRANSLATED_RNA_VS_MRNA_DC_UP	73	199	16054	4169	1.430117	0.000354
blue	6610	KAAB_HEART_ATRIUM_VS_VENTRICLE_DN	64	233	16020	2978	1.499108	0.000357
magenta	8034	LIU_TOPBP1_TARGETS	3	14	16239	167	20.855	0.000357
brown	7036	ACEVEDO_LIVER_TUMOR_VS_NORMAL_ADJACENT_TIS	146	831	15422	2190	1.303891	0.000357
lightcyan	8380	LEF1_UP.V1_DN	5	160	16093	62	8.192036	0.000357
greenyello	1783	V$CRX_Q4	8	188	16065	150	4.61078	0.000359
pink	7304	GOLDRATH_ANTIGEN_RESPONSE	14	343	15910	228	2.909595	0.000359
yellow	8	ORGANELLAR_RIBOSOME	8	22	16231	1426	4.144588	0.000359
yellow	194	MITOCHONDRIAL_RIBOSOME	8	22	16231	1426	4.144588	0.000359
turquoise	2105	TGCCTTA,MIR-124A	162	500	15753	4169	1.263126	0.000359
turquoise	5570	PATIL_LIVER_CANCER	219	700	15553	4169	1.219685	0.00036
magenta	8366	KRAS.600_UP.V1_DN	8	169	16084	167	4.607023	0.000361
turquoise	1471	V$CETS1P54_01	87	245	16008	4169	1.384378	0.000362
brown	807	IMMUNE_SYSTEM_PROCESS	60	289	15964	2190	1.540788	0.000363

salmon	2435	MORF_PPP1CA	7	169	16084	127	5.300797	0.000364
salmon	8966	GSE17721_CPG_VS_GARDIQUIMOD_0.5H_BMDM_UP	7	169	16084	127	5.300797	0.000364
turquoise	151	MICROTUBULE_ORGANIZING_CENTER	29	63	16190	4169	1.794565	0.000365
blue	9322	GSE22886_TCELL_VS_BCELL_NAIVE_UP	56	198	16055	2978	1.543589	0.000368
turquoise	4556	REACTOME_MITOTIC_M_M_G1_PHASES	63	167	16086	4169	1.470706	0.000368
turquoise	8440	GSE10463_CD40L_AND_VA347_VS_CD40L_IN_DC_UP	63	167	16086	4169	1.470706	0.000368
turquoise	10156	GSE7460_CTRL_VS_TGFB_TREATED_ACT_TREG_UP	72	196	16057	4169	1.432116	0.000369
turquoise	10159	GSE7460_CD8_TCELL_VS_TREG_ACT_DN	72	196	16057	4169	1.432116	0.000369
brown	9416	GSE24142_EARLY_THYMIC_PROGENITOR_VS_DN3_THY	43	190	16063	2190	1.679594	0.00037
yellow	3514	MODULE_22	12	45	16208	1426	3.039364	0.00037
blue	9987	GSE3982_EOSINOPHIL_VS_NKCELL_DN	53	185	16068	2978	1.563554	0.000371
blue	10051	GSE3982_NEUTROPHIL_VS_EFF_MEMORY_CD4_TCELL_	53	185	16068	2978	1.563554	0.000371
brown	2935	BIOCARTA_CCR3_PATHWAY	9	19	16234	2190	3.515429	0.000371
greenyello	7559	MEISSNER_NPC_HCP_WITH_H3K4ME2_AND_H3K27ME	8	189	16064	150	4.586384	0.000372
magenta	2026	GTGGTGA,MIR-197	5	60	16193	167	8.110279	0.000373
brown	6285	NEMETH_INFLAMMATORY_RESPONSE_LPS_UP	24	87	16166	2190	2.0473	0.000374
turquoise	2154	CAGCACT,MIR-512-3P	49	123	16130	4169	1.553076	0.000377
pink	5279	TANG_SENESCENCE_TP53_TARGETS_UP	4	25	16228	228	11.40561	0.000378
salmon	7771	KYNG_RESPONSE_TO_H2O2_VIA_ERCC6_DN	4	44	16209	127	11.63422	0.000379
brown	2618	GNF2_CD14	13	35	16218	2190	2.756543	0.00038
turquoise	8604	GSE13738_RESTING_VS_BYSTANDER_ACTIVATED_CD4_	62	164	16089	4169	1.473837	0.000382
black	8252	SRC_UP.V1_UP	9	127	16126	283	4.06992	0.000383
brown	5270	BERENJENO_TRANSFORMED_BY_RHOA_DN	72	362	15891	2190	1.476092	0.000383
brown	8605	GSE13738_RESTING_VS_BYSTANDER_ACTIVATED_CD4_	44	196	16057	2190	1.666042	0.000383
green	8593	GSE13485_DAY7_VS_DAY21_YF17D_VACCINE_PBMC_D	19	101	16152	1305	2.342908	0.000385
lightgreen	50	MEMBRANE_PART	11	1316	14937	42	3.234603	0.000385
greenyello	10220	GSE8678_IL7R_LOW_VS_HIGH_EFF_CD8_TCELL_UP	8	190	16063	150	4.562246	0.000385
lightyellow	3523	MODULE_33	5	312	15941	33	7.892871	0.000385
turquoise	3637	MODULE_152	48	120	16133	4169	1.559415	0.000386
blue	9276	GSE20366_CD103_POS_VS_CD103_KLRG1_DP_TREG_U	49	168	16085	2978	1.591826	0.000386
grey60	1503	V$EVI1_01	2	11	16242	44	67.16116	0.000388
cyan	1720	V$IRF7_01	6	202	16051	77	6.269641	0.00039
salmon	9947	GSE37416_12H_VS_48H_F_TULARENSIS_LVS_NEUTROI	7	171	16082	127	5.238799	0.000391
turquoise	3571	MODULE_83	106	309	15944	4169	1.337362	0.000395
cyan	6179	SANA_TNF_SIGNALING_UP	4	73	16180	77	11.56591	0.000396
greenyello	1657	V$FREAC7_01	7	145	16108	150	5.230851	0.000396
red	8929	GSE17721_LPS_VS_POLYIC_24H_BMDM_DN	16	188	16065	524	2.63976	0.000397
red	10042	GSE3982_MAC_VS_TH1_UP	16	188	16065	524	2.63976	0.000397
green	5905	BENPORATH_SUZ12_TARGETS	73	612	15641	1305	1.485575	0.000398
magenta	59	MEMBRANE	31	1601	14652	167	1.884462	0.000398
lightyellow	8200	ERB2_UP.V1_UP	4	173	16080	33	11.38763	0.000399
brown	10225	GSE9006_HEALTHY_VS_TYPE_1_DIABETES_PBMC_AT_D	42	185	16068	2190	1.684872	0.000401
turquoise	9568	GSE27786_BCELL_VS_NKTCELL_UP	70	190	16063	4169	1.436303	0.000401
grey60	9354	GSE22886_NEUTROPHIL_VS_MONOCYTE_UP	4	129	16124	44	11.45384	0.000402
greenyello	7589	ONO_AML1_TARGETS_DN	4	38	16215	150	11.40561	0.000403
blue	2522	GCM_GSPT1	48	164	16089	2978	1.597373	0.000403
brown	9969	GSE3982_MAC_VS_NEUTROPHIL_LPS_STIM_DN	37	157	16096	2190	1.749007	0.000405
salmon	6807	ZHONG_SECRETOME_OF_LUNG_CANCER_AND_FIBROB	6	123	16130	127	6.24275	0.000408
blue	8602	GSE13738_RESTING_VS_TCR_ACTIVATED_CD4_TCELL_U	51	177	16076	2978	1.572555	0.000408
greenyello	5063	ODONNELL_TFRC_TARGETS_UP	11	350	15903	150	3.40539	0.000409
magenta	50	MEMBRANE_PART	27	1316	14937	167	1.996756	0.000409
salmon	2369	MORF_CSNK2B	9	286	15967	127	4.027229	0.000413
yellow	4704	REACTOME_RESPIRATORY_ELECTRON_TRANSPORT_AT	17	79	16174	1426	2.452651	0.000413
greenyello	50	MEMBRANE_PART	25	1316	14937	150	2.058384	0.000414
green	5907	BENPORATH_ES_WITH_H3K27ME3	77	654	15599	1305	1.466344	0.000414
brown	3051	BIOCARTA_RAS_PATHWAY	10	23	16230	2190	3.226722	0.000414
purple	6746	WANG_SMARCE1_TARGETS_UP	9	231	16022	157	4.033336	0.000415
brown	8888	GSE17721_CTRL_VS_CPG_6H_BMDM_UP	43	191	16062	2190	1.6708	0.000416
cyan	7082	MOSERLE_IFNA_RESPONSE	3	31	16222	77	20.4269	0.000418
turquoise	9023	GSE17721_PAM3CSK4_VS_GADIQUIMOD_1H_BMDM_	69	187	16066	4169	1.438498	0.000418

turquoise	8785	GSE15930_NAIVE_VS_48H_IN_VITRO_STIM_IL12_CD8_	73	200	16053	4169	1.422966	0.00042
red	9031	GSE17721_PAM3CSK4_VS_GADIQUIMOD_8H_BMDM_	16	189	16064	524	2.625793	0.000421
red	10153	GSE7460_TCONV_VS_TREG_THYMUS_DN	16	189	16064	524	2.625793	0.000421
blue	2539	GCM_NUMA1	21	55	16198	2978	2.083845	0.000423
pink	4914	DIAZ_CHRONIC_MEYLOGENOUS_LEUKEMIA_UP	35	1378	14875	228	1.810579	0.000424
salmon	5646	DAIRKEE_TERT_TARGETS_UP	10	350	15903	127	3.656468	0.000424
midnightb	3265	chr19q13	10	637	15616	71	3.59365	0.000425
magenta	7395	LEE_RECENT_THYMIC_EMIGRANT	9	218	16035	167	4.017937	0.000428
purple	4482	REACTOME_GPCR_DOWNSTREAM_SIGNALING	11	336	15917	157	3.389123	0.000428
turquoise	2097	ATGCAGT,MIR-217	41	99	16154	4169	1.614546	0.00043
purple	5723	TOMLINS_PROSTATE_CANCER_DN	4	37	16216	157	11.1916	0.000432
purple	6376	ZHAN_MULTIPLE_MYELOMA_DN	4	37	16216	157	11.1916	0.000432
purple	7004	YAMASHITA_METHYLATED_IN_PROSTATE_CANCER	4	37	16216	157	11.1916	0.000432
brown	5848	RICKMAN_TUMOR_DIFFERENTIATED_WELL_VS_POORL	67	333	15920	2190	1.493207	0.000432
turquoise	179	CENTROSOME	26	55	16198	4169	1.842945	0.000434
brown	9760	GSE3337_CTRL_VS_4H_IFNG_IN_CD8POS_DC_UP	41	180	16073	2190	1.690444	0.000434
turquoise	2043	ATAGGAA,MIR-202	39	93	16160	4169	1.63487	0.000435
lightcyan	9793	GSE360_CTRL_VS_L_DONOVANI_DC_DN	5	167	16086	62	7.848658	0.000435
magenta	6318	BASSO_CD40_SIGNALING_DN	5	62	16191	167	7.848658	0.000435
turquoise	8907	GSE17721_CTRL_VS_GARDIQUIMOD_8H_BMDM_DN	68	184	16069	4169	1.440764	0.000436
blue	601	REGULATION_OF_CELLULAR_METABOLIC_PROCESS	163	700	15553	2978	1.270862	0.000437
turquoise	8789	GSE15930_NAIVE_VS_72H_IN_VITRO_STIM_CD8_TCELI	72	197	16056	4169	1.424846	0.000438
turquoise	8795	GSE15930_NAIVE_VS_72H_IN_VITRO_STIM_TRICHOST/	72	197	16056	4169	1.424846	0.000438
turquoise	8934	GSE17721_POLYIC_VS_PAM3CSK4_2H_BMDM_UP	72	197	16056	4169	1.424846	0.000438
lightcyan	2842	KEGG_CYTOSOLIC_DNA_SENSING_PATHWAY	3	39	16214	62	20.16501	0.000438
purple	6038	ONDER_CDH1_SIGNALING_VIA_CTNNB1	5	66	16187	157	7.842598	0.000439
purple	9892	GSE36392_TYPE_2_MYELOID_VS_MAC_IL25_TREATED_	7	141	16112	157	5.139405	0.00044
brown	529	RAS_PROTEIN_SIGNAL_TRANSDUCTION	19	63	16190	2190	2.238218	0.000442
brown	7980	DELPUECH_FOXO3_TARGETS_UP	19	63	16190	2190	2.238218	0.000442
greenyello	8422	GSE10325_CD4_TCELL_VS_BCELL_UP	8	194	16059	150	4.468179	0.000442
pink	7825	RAGHAVACHARI_PLATELET_SPECIFIC_GENES	6	70	16183	228	6.11015	0.000443
red	593	INFLAMMATORY_RESPONSE	11	102	16151	524	3.34499	0.000444
lightyellow	8353	KRAS.DF.V1_UP	4	178	16075	33	11.06776	0.000444
magenta	6937	HELLER_HDAC_TARGETS_SILENCED_BY_METHYLATION_	10	267	15986	167	3.645069	0.000446
red	8445	GSE11057_NAIVE_VS_EFF_MEMORY_CD4_TCELL_DN	16	190	16063	524	2.611973	0.000447
greenyello	10142	GSE6269_E_COLI_VS_STREP_PNEUMO_INF_PBMC_UP	7	148	16105	150	5.12482	0.000448
purple	8188	ZWANG_TRANSIENTLY_UP_BY_2ND_EGF_PULSE_ONLY	22	1044	15209	157	2.181504	0.000448
purple	6578	LU_AGING_BRAIN_UP	9	234	16019	157	3.981627	0.000456
greenyello	10255	GSE9650_NAIVE_VS_EFF_CD8_TCELL_DN	8	195	16058	150	4.445265	0.000458
cyan	7235	ISHIKAWA_STING_SIGNALING	2	7	16246	77	60.30798	0.000458
purple	5103	KIM_WT1_TARGETS_12HR_UP	7	142	16111	157	5.103212	0.000459
yellow	5998	NIKOLSKY_BREAST_CANCER_8Q23_Q24_AMPLICON	24	132	16121	1426	2.072294	0.000459
purple	3502	MODULE_9	6	102	16151	157	6.089547	0.000463
grey60	1073	CARBOHYDRATE_BINDING	3	56	16197	44	19.78856	0.000464
greenyello	1455	AAANWWTGC_UNKNOWN	7	149	16104	150	5.090425	0.000466
turquoise	1817	V$E4F1_Q6	88	250	16003	4169	1.372285	0.000466
red	807	IMMUNE_SYSTEM_PROCESS	21	289	15964	524	2.253843	0.000467
brown	2453	MORF_RAB1A	43	192	16061	2190	1.662098	0.000467
brown	8662	GSE1432_CTRL_VS_IFNG_24H_MICROGLIA_UP	43	192	16061	2190	1.662098	0.000467
blue	1510	V$MZF1_02	54	191	16062	2978	1.543012	0.000468
lightgreen	4173	REACTOME_IMMUNOREGULATORY_INTERACTIONS_BE	3	59	16194	42	19.67676	0.000472
blue	9316	GSE22886_NAIVE_VS_MEMORY_TCELL_UP	51	178	16075	2978	1.56372	0.000472
purple	3563	MODULE_75	11	340	15913	157	3.349251	0.000473
turquoise	9295	GSE20715_0H_VS_6H_OZONE_TLR4_KO_LUNG_DN	66	178	16075	4169	1.445525	0.000473
turquoise	9771	GSE339_CD4POS_VS_CD4CD8DN_DC_DN	66	178	16075	4169	1.445525	0.000473
red	6298	HALMOS_CEBPA_TARGETS_UP	7	44	16209	524	4.934551	0.000473
red	8719	GSE14769_UNSTIM_VS_60MIN_LPS_BMDM_DN	16	191	16062	524	2.598297	0.000473
turquoise	9759	GSE32423_IL7_VS_IL7_IL4_NAIVE_CD8_TCELL_DN	70	191	16062	4169	1.428783	0.000477
turquoise	10206	GSE7852_LN_VS_FAT_TCONV_UP	70	191	16062	4169	1.428783	0.000477
pink	6243	ADDYA_ERYTHROID_DIFFERENTIATION_BY_HEMIN	6	71	16182	228	6.024092	0.000478

lightcyan	7869	LU_EZH2_TARGETS_UP	6	262	15991	62	6.003324	0.000478
cyan	3611	MODULE_123	6	210	16043	77	6.030798	0.000479
yellow	9326	GSE22886_CD4_TCELL_VS_BCELL_NAIVE_UP	32	197	16056	1426	1.851389	0.000484
yellow	6697	ABE_INNER_EAR	11	40	16213	1426	3.134344	0.000484
purple	6203	MANALO_HYPOXIA_UP	8	188	16065	157	4.405204	0.000487
purple	9894	GSE36392_TYPE_2_MYELOID_VS_NEUTROPHIL_IL25_TI	8	188	16065	157	4.405204	0.000487
turquoise	2338	MORF_SOD1	96	277	15976	4169	1.351117	0.000489
turquoise	6940	WONG_MITOCHONDRIA_GENE_MODULE	77	214	16039	4169	1.402745	0.000489
red	6788	BILD_HRAS_ONCOGENIC_SIGNATURE	18	230	16023	524	2.427431	0.00049
magenta	6130	TARTE_PLASMA_CELL_VS_B_LYMPHOCYTE_DN	4	36	16217	167	10.81371	0.00049
red	7844	STAMBOLSKY_RESPONSE_TO_VITAMIN_D3_UP	9	72	16181	524	3.877147	0.000491
lightyellow	5045	TONKS_TARGETS_OF_RUNX1_RUNX1T1_FUSION_HSC_	4	183	16070	33	10.76536	0.000493
lightyellow	8369	KRAS.600.LUNG.BREAST_UP.V1_UP	4	183	16070	33	10.76536	0.000493
blue	2310	MORF_HAT1	50	174	16079	2978	1.568302	0.000496
blue	1885	V$AP2_Q3	58	209	16044	2978	1.514574	0.000496
cyan	6158	BROCKE_APOPTOSIS_REVERSED_BY_IL6	5	138	16115	77	7.647751	0.000497
turquoise	8864	GSE17721_CTRL_VS_PAM3CSK4_0.5H_BMDM_UP	69	188	16065	4169	1.430846	0.000497
salmon	9890	GSE36392_TYPE_2_MYELOID_VS_EOSINOPHIL_IL25_TR	7	178	16075	127	5.032779	0.000498
blue	944	TRANSCRIPTION_FROM_RNA_POLYMERASE_II_PROMC	99	396	15857	2978	1.364422	0.000498
purple	356	CELL_SURFACE_RECEPTOR_LINKED_SIGNAL_TRANSDUC	13	457	15796	157	2.944835	0.000499
turquoise	896	CENTROSOME_CYCLE	8	10	16243	4169	3.118829	0.000501
red	8927	GSE17721_LPS_VS_POLYIC_16H_BMDM_DN	16	192	16061	524	2.584765	0.000501
red	10103	GSE39820_CTRL_VS_IL1B_IL6_IL23A_CD4_TCELL_DN	16	192	16061	524	2.584765	0.000501
turquoise	2032	GTGACTT,MIR-224	54	140	16113	4169	1.503721	0.000503
cyan	6623	HAN_JNK_SINGALING_UP	3	33	16220	77	19.1889	0.000504
cyan	7816	BROWNE_HCMV_INFECTION_2HR_UP	3	33	16220	77	19.1889	0.000504
purple	6621	GEORGANTAS_HSC_MARKERS	5	68	16185	157	7.611933	0.000504
brown	1025	APOPTOSIS_GO	76	390	15863	2190	1.446233	0.000506
salmon	599	RIBONUCLEOPROTEIN_COMPLEX_BIOGENESIS_AND_AS	5	84	16169	127	7.617642	0.000507
brown	9806	GSE360_CTRL_VS_L_MAJOR_MAC_UP	42	187	16066	2190	1.666852	0.000508
cyan	7373	HINATA_NFKB_TARGETS_KERATINOCYTE_UP	4	78	16175	77	10.82451	0.000509
blue	5048	TONKS_TARGETS_OF_RUNX1_RUNX1T1_FUSION_MON	55	196	16057	2978	1.531495	0.00051
blue	5987	KAUFFMANN_DNA_REPAIR_GENES	62	227	16026	2978	1.490647	0.000513
cyan	6890	FOSTER_TOLERANT_MACROPHAGE_DN	8	391	15862	77	4.31873	0.000516
pink	4631	REACTOME_FACTORS_INVOLVED_IN_MEGAKARYOCYTI	7	100	16153	228	4.989956	0.000517
turquoise	8614	GSE14000_UNSTIM_VS_4H_LPS_DC_TRANSLATED_RNA	72	198	16055	4169	1.41765	0.000518
turquoise	9393	GSE22886_NAIVE_CD4_TCELL_VS_48H_ACT_TH1_DN	72	198	16055	4169	1.41765	0.000518
turquoise	10114	GSE39820_TGFBETA1_IL6_VS_TGFBETA1_IL6_IL23A_TF	72	198	16055	4169	1.41765	0.000518
turquoise	10148	GSE7400_CTRL_VS_CSF3_IN_VIVO_TREATED_PBMC_UI	72	198	16055	4169	1.41765	0.000518
magenta	4152	REACTOME_DEVELOPMENTAL_BIOLOGY	11	323	15930	167	3.314418	0.000519
blue	2396	MORF_G22P1	49	170	16083	2978	1.573099	0.00052
brown	6537	BLALOCK_ALZHEIMERS_DISEASE_UP	250	1535	14718	2190	1.208707	0.00052
purple	9420	GSE24142_EARLY_THYMIC_PROGENITOR_VS_DN2_THY	8	190	16063	157	4.358833	0.000522
lightyellow	6115	SANA_TNF_SIGNALING_DN	3	78	16175	33	18.94289	0.000523
greenyello	8387	KAECH_NAIVE_VS_DAY8_EFF_CD8_TCELL_DN	8	199	16054	150	4.355913	0.000524
brown	8457	GSE11057_NAIVE_VS_MEMORY_CD4_TCELL_DN	43	193	16060	2190	1.653486	0.000525
red	7177	PODAR_RESPONSE_TO_ADAPHOSTIN_UP	13	138	16115	524	2.921908	0.000525
brown	7408	ZHANG_TLX_TARGETS_60HR_UP	57	275	15978	2190	1.538267	0.000525
turquoise	2015	AAAGGAT,MIR-501	44	109	16144	4169	1.573721	0.000525
brown	4116	PID_FAK_PATHWAY	18	59	16194	2190	2.264175	0.000531
red	9447	GSE24634_TREG_VS_TCONV_POST_DAY10_IL4_CONVE	16	193	16060	524	2.571372	0.000531
cyan	6792	DOUGLAS_BMI1_TARGETS_DN	7	299	15954	77	4.941624	0.000532
lightyellow	8139	GENTLES_LEUKEMIC_STEM_CELL_DN	2	17	16236	33	57.94296	0.000533
blue	2165	CAGCAGG,MIR-370	41	136	16117	2978	1.645333	0.000535
lightcyan	6033	ONDER_CDH1_TARGETS_2_DN	7	374	15879	62	4.90646	0.000535
blue	9378	GSE22886_NAIVE_CD4_TCELL_VS_DC_UP	54	192	16061	2978	1.534975	0.000537
lightcyan	3617	MODULE_129	5	175	16078	62	7.489862	0.000539
lightcyan	9195	GSE17974_IL4_AND_ANTI_IL12_VS_UNTREATED_6H_A	5	175	16078	62	7.489862	0.000539
purple	8455	GSE11057_CD4_CENT_MEM_VS_PBMC_DN	8	191	16062	157	4.336012	0.000541
turquoise	9686	GSE29618_PRE_VS_DAY7_POST_LAIV_FLU_VACCINE_B	67	182	16071	4169	1.435176	0.000541

turquoise	9805	GSE360_CTRL_VS_L_DONOVANI_MAC_DN	67	182	16071	4169	1.435176	0.000541
blue	854	REGULATION_OF_METABOLIC_PROCESS	164	708	15545	2978	1.264211	0.000542
pink	9056	GSE17721_POLYIC_VS_GARDIQUIMOD_0.5H_BMDM_U	9	165	16088	228	3.888278	0.000542
greenyello	2845	KEGG_NATURAL_KILLER_CELL_MEDIATED_CYTOTOXICI	6	110	16143	150	5.910182	0.000544
blue	4442	REACTOME_TRANSCRIPTION	51	179	16074	2978	1.554984	0.000545
lightgreen	5709	FURUKAWA_DUSP6_TARGETS_PCI35_UP	3	62	16191	42	18.72465	0.000546
greenyello	931	RESPONSE_TO_WOUNDING	7	153	16100	150	4.957342	0.000546
purple	2268	TGGAAA_V$NFAT_Q4_01	28	1506	14747	157	1.924717	0.000546
lightcyan	3660	MODULE_177	4	99	16154	62	10.59172	0.000551
lightcyan	8961	GSE17721_PAM3CSK4_VS_CPG_12H_BMDM_DN	5	176	16077	62	7.447306	0.000553
lightcyan	8974	GSE17721_CPG_VS_GARDIQUIMOD_6H_BMDM_UP	5	176	16077	62	7.447306	0.000553
lightcyan	9042	GSE17721_LPS_VS_CPG_2H_BMDM_UP	5	176	16077	62	7.447306	0.000553
red	8477	GSE11864_CSF1_IFNG_VS_CSF1_IFNG_PAM3CYS_IN_M	15	175	16078	524	2.658615	0.000554
brown	5657	GRUETZMANN_PANCREATIC_CANCER_UP	67	336	15917	2190	1.479875	0.000558
salmon	8156	HOLLEMAN_ASPARAGINASE_RESISTANCE_ALL_UP	3	21	16232	127	18.28234	0.000559
blue	2679	GNF2_PA2G4	27	79	16174	2978	1.865286	0.00056
purple	3606	MODULE_118	11	347	15906	157	3.281687	0.00056
red	7144	BOQUEST_STEM_CELL_DN	16	194	16059	524	2.558118	0.000562
turquoise	4718	REACTOME_MYD88_MAL_CASCADE_INITIATED_ON_PL	34	79	16174	4169	1.677851	0.000563
lightyellow	5908	BENPORATH_PRC2_TARGETS	5	339	15914	33	7.264235	0.000563
brown	4151	REACTOME_SIGNALING_BY_SCF_KIT	21	74	16179	2190	2.10609	0.000564
turquoise	9262	GSE20366_EX_VIVO_VS_DEC205_CONVERSION_UP	70	192	16061	4169	1.421342	0.000565
magenta	3503	MODULE_11	13	435	15818	167	2.908514	0.000566
turquoise	2440	MORF_PPP2R5E	35	82	16171	4169	1.66401	0.000566
turquoise	1463	V$ELK1_01	87	248	16005	4169	1.367632	0.000566
blue	2384	MORF_EIF4E	28	83	16170	2978	1.841148	0.000567
lightcyan	9296	GSE20715_0H_VS_24H_OZONE_TLR4_KO_LUNG_UP	5	177	16076	62	7.405231	0.000567
turquoise	2114	AACATTC,MIR-409-3P	50	128	16125	4169	1.522866	0.000567
greenyello	5736	THEODOROU_MAMMARY_TUMORIGENESIS	3	18	16235	150	18.05889	0.000568
salmon	9654	GSE29618_BCELL_VS_MONOCYTE_UP	7	182	16071	127	4.922168	0.000568
brown	9858	GSE360_LOW_DOSE_B_MALAYI_VS_M_TUBERCULOSIS	42	188	16065	2190	1.657986	0.00057
cyan	5149	GOZGIT_ESR1_TARGETS_DN	10	608	15645	77	3.471676	0.000572
blue	1369	TRANSCRIPTION_FACTOR_BINDING	74	282	15971	2978	1.43216	0.000574
salmon	4723	REACTOME_SCF_BETA_TRCP_MEDIATED_DEGRADATIO	4	49	16204	127	10.44705	0.000574
brown	2642	GNF2_FGR	12	32	16221	2190	2.783048	0.000574
brown	3037	BIOCARTA_PDGF_PATHWAY	12	32	16221	2190	2.783048	0.000574
brown	2817	KEGG_ENDOCYTOSIS	39	171	16082	2190	1.692614	0.000576
purple	3626	MODULE_138	5	70	16183	157	7.394449	0.000576
purple	8056	PEDERSEN_TARGETS_OF_611CTF_ISOFORM_OF_ERBB2	5	70	16183	157	7.394449	0.000576
lightyellow	5013	TAKEDA_TARGETS_OF_NUP98_HOXA9_FUSION_8D_DN	4	191	16062	33	10.31445	0.000579
lightcyan	5173	ENK_UV_RESPONSE_EPIDERMIS_UP	6	272	15981	62	5.782614	0.000583
turquoise	2158	ACACTAC,MIR-142-3P	49	125	16128	4169	1.528226	0.000583
greenyello	41	INTRINSIC_TO_MEMBRANE	21	1041	15212	150	2.185802	0.000584
lightyellow	3748	MODULE_289	3	81	16172	33	18.2413	0.000584
purple	6532	KUNINGER_IGF1_VS_PDGFB_TARGETS_DN	4	40	16213	157	10.35223	0.000584
salmon	9950	GSE3982_CTRL_VS_IGE_STIM_MAST_CELL_UP	7	183	16070	127	4.895271	0.000587
purple	4909	CASORELLI_ACUTE_PROMYELOCYTIC_LEUKEMIA_DN	16	653	15600	157	2.536534	0.000589
turquoise	8529	GSE13229_MATURE_VS_INTMATURE_NKCELL_DN	69	189	16064	4169	1.423275	0.00059
turquoise	9131	GSE17721_0.5H_VS_8H_CPG_BMDM_DN	69	189	16064	4169	1.423275	0.00059
turquoise	9468	GSE25087_TREG_VS_TCONV_FETUS_UP	69	189	16064	4169	1.423275	0.00059
turquoise	1525	V$NRF2_01	92	265	15988	4169	1.353454	0.000593
brown	4048	PID_ECADHERIN_KERATINOCYTE_PATHWAY	9	20	16233	2190	3.339658	0.000594
red	8401	GOLDRATH_NAIVE_VS_MEMORY_CD8_TCELL_DN	16	195	16058	524	2.544999	0.000594
brown	4583	REACTOME_SIGNALING_BY_ILS	26	100	16153	2190	1.92958	0.000595
blue	8504	GSE12845_IGD_POS_VS_NEG_BLOOD_BCELL_UP	52	184	16069	2978	1.542391	0.000596
blue	10029	GSE3982_DC_VS_TH2_DN	52	184	16069	2978	1.542391	0.000596
lightcyan	9842	GSE360_L_MAJOR_VS_B_MALAYI_HIGH_DOSE_DC_UP	5	179	16074	62	7.322491	0.000597
turquoise	2174	GTAAACC,MIR-299-5P	21	42	16211	4169	1.949268	0.000599
blue	69	NUCLEAR_LUMEN	93	370	15883	2978	1.371798	0.0006
red	7350	RUTELLA_RESPONSE_TO_HGF_DN	18	234	16019	524	2.385937	0.000601

purple	3516	MODULE_24	11	350	15903	157	3.253558	0.000601
purple	5063	ODONNELL_TFRC_TARGETS_UP	11	350	15903	157	3.253558	0.000601
red	5697	HASINA_NOL7_TARGETS_UP	4	13	16240	524	9.543746	0.000606
salmon	9666	GSE29618_BCELL_VS_MONOCYTE_DAY7_FLU_VACCINE	7	184	16069	127	4.868667	0.000606
salmon	9792	GSE360_CTRL_VS_L_DONOVANI_DC_UP	7	184	16069	127	4.868667	0.000606
blue	3771	MODULE_318	13	28	16225	2978	2.533927	0.000607
turquoise	9351	GSE22886_DAY1_VS_DAY7_MONOCYTE_IN_CULTURE_	72	199	16054	4169	1.410526	0.000611
brown	3015	BIOCARTA_PYK2_PATHWAY	11	28	16225	2190	2.915574	0.000612
red	4890	FULCHER_INFLAMMATORY_RESPONSE_LECTIN_VS_LPS	33	558	15695	524	1.834349	0.000613
magenta	1492	V$TAL1BETAE47_01	8	183	16070	167	4.254573	0.000613
magenta	9651	GSE29617_CTRL_VS_DAY7_TIV_FLU_VACCINE_PBMC_2	8	183	16070	167	4.254573	0.000613
greenyello	8764	GSE15733_BM_VS_SPLEEN_MEMORY_CD4_TCELL_UP	7	156	16097	150	4.862009	0.000613
blue	9390	GSE22886_NAIVE_CD4_TCELL_VS_12H_ACT_TH1_UP	54	193	16060	2978	1.527022	0.000615
blue	7643	FOURNIER_ACINAR_DEVELOPMENT_LATE_2	71	269	15984	2978	1.440505	0.000615
brown	3572	MODULE_84	90	480	15773	2190	1.391524	0.000616
red	459	DEFENSE_RESPONSE	17	215	16038	524	2.452521	0.000619
purple	1608	V$TATA_C	8	195	16058	157	4.247068	0.00062
brown	9978	GSE3982_EOSINOPHIL_VS_BCELL_UP	38	166	16087	2190	1.698889	0.000624
red	6730	SESTO_RESPONSE_TO_UV_C5	7	46	16207	524	4.720005	0.000625
blue	2024	TCCCCAC,MIR-491	19	49	16204	2978	2.116247	0.000627
blue	2399	MORF_GSPT1	19	49	16204	2978	2.116247	0.000627
red	9934	GSE37416_0H_VS_3H_F_TULARENSIS_LVS_NEUTROPH	16	196	16057	524	2.532014	0.000628
red	10192	GSE7852_TREG_VS_TCONV_LN_UP	16	196	16057	524	2.532014	0.000628
blue	960	CATABOLIC_PROCESS	56	202	16051	2978	1.513023	0.000631
brown	8000	FEVR_CTNNB1_TARGETS_UP	106	582	15671	2190	1.351675	0.000631
blue	10025	GSE3982_DC_VS_NKCELL_DN	48	167	16086	2978	1.568677	0.000631
green	3846	MODULE_426	14	66	16187	1305	2.641844	0.000634
turquoise	8132	PECE_MAMMARY_STEM_CELL_UP	53	138	16115	4169	1.497264	0.000635
brown	3992	PID_TXA2PATHWAY	17	55	16198	2190	2.293906	0.000636
yellow	2871	KEGG_PARKINSONS_DISEASE	20	104	16149	1426	2.191849	0.000636
turquoise	7740	YAO_TEMPORAL_RESPONSE_TO_PROGESTERONE_CLUS	63	170	16083	4169	1.444752	0.000637
lightyellow	10074	GSE3982_BASOPHIL_VS_CENT_MEMORY_CD4_TCELL_U	4	196	16057	33	10.05133	0.000638
brown	10016	GSE3982_DC_VS_BCELL_UP	42	189	16064	2190	1.649214	0.00064
purple	1723	V$STAT5B_01	8	196	16057	157	4.2254	0.000641
purple	3852	MODULE_433	4	41	16212	157	10.09974	0.000643
purple	6465	MCDOWELL_ACUTE_LUNG_INJURY_UP	4	41	16212	157	10.09974	0.000643
salmon	3442	chr17p12	3	22	16231	127	17.45132	0.000644
pink	1542	V$GATA1_04	9	169	16084	228	3.796247	0.000644
turquoise	2128	GTTAAAG,MIR-302B	27	59	16194	4169	1.784076	0.000645
turquoise	2085	ATACCTC,MIR-202	58	154	16099	4169	1.46828	0.000645
purple	6978	IWANAGA_CARCINOGENESIS_BY_KRAS_PTEN_DN	10	298	15955	157	3.473902	0.000646
salmon	5677	KIM_MYC_AMPLIFICATION_TARGETS_UP	7	186	16067	127	4.816315	0.000647
salmon	7220	ALONSO_METASTASIS_UP	7	186	16067	127	4.816315	0.000647
greenyello	2224	YNTTTNNNANGCARM_UNKNOWN	4	43	16210	150	10.07938	0.00065
brown	320	REGULATION_OF_CYTOKINE_SECRETION	7	13	16240	2190	3.996171	0.000651
magenta	607	EXTRACELLULAR_STRUCTURE_ORGANIZATION_AND_BI	3	17	16236	167	17.17471	0.000652
pink	9240	GSE18791_UNSTIM_VS_NEWCATSLE_VIRUS_DC_2H_UI	7	104	16149	228	4.798035	0.000654
red	694	PROTEIN_KINASE_CASCADE	20	276	15977	524	2.247621	0.000654
purple	7945	KIM_GLIS2_TARGETS_UP	5	72	16181	157	7.189048	0.000656
greenyello	2227	YTAAYNGCT_UNKNOWN	6	114	16139	150	5.702807	0.000658
brown	7456	MILI_PSEUDOPODIA_HAPTOTAXIS_DN	116	647	15606	2190	1.330587	0.000659
purple	6642	ZHU_CMV_ALL_DN	6	109	16144	157	5.698475	0.000659
red	8344	STK33_DN	19	256	15997	524	2.302056	0.000664
magenta	13	PROTEINACEOUS_EXTRACELLULAR_MATRIX	5	68	16185	167	7.156129	0.000667
blue	5743	PUJANA_BRCA2_PCC_NETWORK	103	418	15835	2978	1.344837	0.000667
salmon	9032	GSE17721_PAM3CSK4_VS_GADIQUIMOD_12H_BMDM_	7	187	16066	127	4.79056	0.000667
turquoise	9117	GSE17721_0.5H_VS_4H_PAM3CSK4_BMDM_DN	66	180	16073	4169	1.429464	0.00067
magenta	7885	FIGUEROA_AML_METHYLATION_CLUSTER_4_UP	6	103	16150	167	5.669322	0.000676
blue	1087	DNA_HELICASE_ACTIVITY	12	25	16228	2978	2.619691	0.000677
blue	706	MRNA_PROCESSING_GO_0006397	25	72	16181	2978	1.895031	0.000677

pink	6773	DAZARD_UV_RESPONSE_CLUSTER_G2	4	29	16224	228	9.832426	0.00068
brown	7311	LINDSTEDT_DENDRITIC_CELL_MATURATION_C	19	65	16188	2190	2.16935	0.000681
tan	1127	OXIDOREDUCTASE_ACTIVITY_GO_0016616	4	45	16208	145	9.963525	0.000682
magenta	8516	GSE12845_IGD_NEG_BLOOD_VS_DARKZONE_GC_TONS	8	186	16067	167	4.185951	0.000682
blue	6118	PENG_LEUCINE_DEPRIVATION_DN	52	185	16068	2978	1.534053	0.000684
brown	7864	WANG_RESPONSE_TO_GSK3_INHIBITOR_SB216763_UI	71	363	15890	2190	1.451581	0.000684
red	8218	AKT_UP_MTOR_DN.V1_UP	14	160	16093	524	2.714003	0.000684
magenta	998	ORGAN_DEVELOPMENT	12	388	15865	167	3.010001	0.000684
cyan	8115	PHONG_TNF_RESPONSE_NOT_VIA_P38	7	312	15941	77	4.735723	0.000684
brown	6629	MCCLUNG_CREB1_TARGETS_UP	21	75	16178	2190	2.078009	0.000685
lightgreen	1443	CYTOKINE_ACTIVITY	3	67	16186	42	17.32729	0.000685
salmon	9649	GSE29617_CTRL_VS_DAY3_TIV_FLU_VACCINE_PBMC_2	7	188	16065	127	4.765078	0.000689
blue	4355	REACTOME_P75_NTR_RECEPTOR_MEDIATED_SIGNALLI	26	76	16177	2978	1.867104	0.000691
turquoise	8124	RAO_BOUND_BY_SALL4_ISOFORM_B	138	423	15830	4169	1.271863	0.000692
lightcyan	8734	GSE15324_NAIVE_VS_ACTIVATED_CD8_TCELL_UP	5	185	16068	62	7.085004	0.000693
lightcyan	8891	GSE17721_CTRL_VS_CPG_8H_BMDM_DN	5	185	16068	62	7.085004	0.000693
lightcyan	6890	FOSTER_TOLERANT_MACROPHAGE_DN	7	391	15862	62	4.693136	0.000696
turquoise	6681	DAZARD_RESPONSE_TO_UV_NHEK_DN	105	310	15943	4169	1.320472	0.000698
purple	6247	HADDAD_T_LYMPHOCYTE_AND_NK_PROGENITOR_UP	5	73	16180	157	7.090568	0.000698
brown	2297	MORF_BECN1	26	101	16152	2190	1.910475	0.000701
yellow	6006	NIKOLSKY_BREAST_CANCER_16Q24_AMPLICON	12	48	16205	1426	2.849404	0.000701
blue	9451	GSE24634_TEFF_VS_TCONV_DAY5_IN_CULTURE_DN	54	194	16059	2978	1.519151	0.000703
purple	3727	MODULE_259	4	42	16211	157	9.859266	0.000705
cyan	6135	FLECHNER_BIOPSY_KIDNEY_TRANSPLANT_REJECTED_V	4	85	16168	77	9.933079	0.000705
cyan	6988	FUJII_YBX1_TARGETS_UP	3	37	16216	77	17.11443	0.000707
turquoise	3579	MODULE_91	19	37	16216	4169	2.001951	0.000709
magenta	3865	MODULE_456	6	104	16149	167	5.614809	0.000711
purple	7204	QI_PLASMACYTOMA_UP	9	249	16004	157	3.74177	0.000712
greenyello	9716	GSE30083_SP2_VS_SP3_THYMOCYTE_UP	7	160	16093	150	4.740458	0.000713
magenta	191	EXTRACELLULAR_MATRIX	5	69	16184	167	7.052417	0.000713
red	3605	MODULE_117	29	472	15781	524	1.905716	0.000714
red	2695	GNF2_SELL	7	47	16206	524	4.619579	0.000715
purple	3504	MODULE_12	10	302	15951	157	3.42789	0.000715
brown	9808	GSE360_CTRL_VS_T_GONDII_MAC_UP	42	190	16063	2190	1.640534	0.000716
salmon	5507	SCHLOSSER_SERUM_RESPONSE_DN	14	667	15586	127	2.686161	0.000718
turquoise	9643	GSE29615_DAY3_VS_DAY7_LAIV_FLU_VACCINE_PBMC_	60	161	16092	4169	1.452871	0.000719
turquoise	5034	MULLIGHAN_NPM1_MUTATED_SIGNATURE_2_UP	49	126	16127	4169	1.516098	0.00072
turquoise	4161	REACTOME_TRIF_MEDIATED_TLR3_SIGNALING	31	71	16182	4169	1.702178	0.000722
greenyello	1920	V$NFAT_Q4_01	8	209	16044	150	4.147496	0.000723
cyan	7417	COLINA_TARGETS_OF_4EBP1_AND_4EBP2	7	315	15938	77	4.69062	0.000724
lightcyan	8563	GSE13484_UNSTIM_VS_3H_YF17D_VACCINE_STIM_PB	5	187	16066	62	7.009229	0.000727
lightcyan	9865	GSE360_L_DONOVANI_VS_B_MALAYI_HIGH_DOSE_MA	5	187	16066	62	7.009229	0.000727
turquoise	9566	GSE27786_BCELL_VS_NKCELL_UP	68	187	16066	4169	1.41765	0.000728
blue	5386	LASTOWSKA_NEUROBLASTOMA_COPY_NUMBER_DN	178	781	15472	2978	1.243878	0.000729
turquoise	8755	GSE15659_CD45RA_NEG_CD4_TCELL_VS_NONSUPPRES	64	174	16079	4169	1.433945	0.000729
lightgreen	7061	SMID_BREAST_CANCER_LUMINAL_B_DN	6	425	15828	42	5.463193	0.000729
pink	8323	RPS14_DN.V1_DN	9	172	16081	228	3.730034	0.000731
brown	2284	TCANNTGAY_V$SREBP1_01	77	401	15852	2190	1.425069	0.000731
red	4747	REACTOME_CYTOKINE_SIGNALING_IN_IMMUNE_SYSTE	18	238	16015	524	2.345837	0.000733
pink	4842	KIM_RESPONSE_TO_TSA_AND_DECITABINE_UP	7	106	16147	228	4.707506	0.000733
purple	2289	WGGAATGY_V$TEF1_Q6	10	303	15950	157	3.416577	0.000734
black	9170	GSE17974_CTRL_VS_ACT_IL4_AND_ANTI_IL12_1H_CD4	10	168	16085	283	3.418518	0.000734
brown	9657	GSE29618_BCELL_VS_PDC_DN	43	196	16057	2190	1.628178	0.000735
brown	9946	GSE37416_12H_VS_48H_F_TULARENSIS_LVS_NEUTROI	43	196	16057	2190	1.628178	0.000735
grey60	4458	REACTOME_NUCLEOTIDE_LIKE_PURINERGIC_RECEPTOI	2	15	16238	44	49.25152	0.000735
grey60	4461	REACTOME_EICOSANOID_LIGAND_BINDING_RECEPTOI	2	15	16238	44	49.25152	0.000735
magenta	7687	ZHAN_MULTIPLE_MYELOMA_PR_DN	4	40	16213	167	9.732335	0.000736
lightcyan	1491	V$IRF2_01	4	107	16146	62	9.799819	0.000739
lightcyan	7573	MIKKELSEN_MEF_LCP_WITH_H3K4ME3	4	107	16146	62	9.799819	0.000739
greenyello	9718	GSE30083_SP2_VS_SP4_THYMOCYTE_UP	7	161	16092	150	4.711014	0.000739

turquoise	2164	RRCCGTTA_UNKNOWN	34	80	16173	4169	1.656878	0.00074
red	9373	GSE22886_NAIVE_CD8_TCELL_VS_DC_DN	16	199	16054	524	2.493843	0.000741
blue	4982	OSMAN_BLADDER_CANCER_DN	97	391	15862	2978	1.353954	0.000743
purple	8526	GSE13229_IMM_VS_INTMATURE_NKCELL_UP	7	154	16099	157	4.705559	0.000745
lightcyan	9428	GSE24142_ADULT_VS_FETAL_DN2_THYMOCYTE_UP	5	188	16065	62	6.971946	0.000745
black	10230	GSE9006_HEALTHY_VS_TYPE_2_DIABETES_PBMC_AT_[	11	199	16054	283	3.174583	0.000749
salmon	7036	ACEVEDO_LIVER_TUMOR_VS_NORMAL_ADJACENT_TIS	16	831	15422	127	2.464046	0.000755
salmon	8892	GSE17721_CTRL_VS_CPG_12H_BMDM_UP	7	191	16062	127	4.690234	0.000756
salmon	9703	GSE29618_LAIV_VS_TIV_FLU_VACCINE_DAY7_BCELL_D	7	191	16062	127	4.690234	0.000756
lightgreen	9492	GSE26928_EFF_MEM_VS_CENTR_MEM_CD4_TCELL_UI	4	160	16093	42	9.674405	0.000757
brown	1896	V$SRF_Q4	40	179	16074	2190	1.658427	0.000757
brown	9823	GSE360_DC_VS_MAC_T_GONDII_DN	40	179	16074	2190	1.658427	0.000757
brown	9851	GSE360_T_GONDII_VS_B_MALAYI_LOW_DOSE_DC_DN	40	179	16074	2190	1.658427	0.000757
blue	8451	GSE11057_EFF_MEM_VS_CENT_MEM_CD4_TCELL_DN	50	177	16076	2978	1.54172	0.000759
red	7831	LI_INDUCED_T_TO_NATURAL_KILLER_UP	21	300	15953	524	2.171202	0.000759
turquoise	8503	GSE12366_NAIVE_VS_MEMORY_BCELL_DN	67	184	16069	4169	1.419576	0.000761
blue	2640	GNF2_ELAC2	18	46	16207	2978	2.135618	0.000761
turquoise	4922	LIU_VMYB_TARGETS_UP	47	120	16133	4169	1.526927	0.000763
lightcyan	9127	GSE17721_0.5H_VS_12H_CPG_BMDM_DN	5	189	16064	62	6.935057	0.000763
lightgreen	7374	HINATA_NFKB_TARGETS_KERATINOCYTE_DN	2	16	16237	42	48.37202	0.000765
purple	4611	REACTOME_NITRIC_OXIDE_STIMULATES_GUANYLATE_(	3	19	16234	157	16.34563	0.000765
yellow	7033	ACEVEDO_NORMAL_TISSUE_ADJACENT_TO_LIVER_TUI	46	324	15929	1426	1.61818	0.000765
brown	3472	ST_STAT3_PATHWAY	6	10	16243	2190	4.452877	0.000767
greenyello	2419	MORF_MAP3K14	5	78	16175	150	6.945726	0.000769
lightcyan	3729	MODULE_262	2	11	16242	62	47.66276	0.00077
lightcyan	5694	SEMBA_FHIT_TARGETS_UP	2	11	16242	62	47.66276	0.00077
purple	5751	KHETCHOUMIAN_TRIM24_TARGETS_UP	4	43	16210	157	9.629981	0.000771
purple	7222	CROMER_TUMORIGENESIS_UP	4	43	16210	157	9.629981	0.000771
red	6304	LENAOUR_DENDRITIC_CELL_MATURATION_DN	12	126	16127	524	2.954017	0.000772
red	7914	WIERENGA_STAT5A_TARGETS_GROUP1	12	126	16127	524	2.954017	0.000772
cyan	8168	DURAND_STROMA_MAX_UP	6	230	16023	77	5.506381	0.000772
lightcyan	6819	CREIGHTON_ENDOCRINE_THERAPY_RESISTANCE_2	7	398	15855	62	4.610593	0.000772
lightcyan	7351	RUTELLA_RESPONSE_TO_HGF_VS_CSF2RB_AND_IL4_U	7	398	15855	62	4.610593	0.000772
red	8541	GSE13306_RA_VS_UNTREATED_TREG_DN	14	162	16091	524	2.680497	0.000774
salmon	4772	REACTOME_SCFSKP2_MEDIATED_DEGRADATION_OF_F	4	53	16200	127	9.658595	0.000774
salmon	8705	GSE1460_CD4_THYMOCYTE_VS_NAIVE_CD4_TCELL_AD	7	192	16061	127	4.665805	0.00078
salmon	8941	GSE17721_POLYIC_VS_PAM3CSK4_8H_BMDM_DN	7	192	16061	127	4.665805	0.00078
black	10238	GSE9006_TYPE_1_VS_TYPE_2_DIABETES_PBMC_AT_DX	11	200	16053	283	3.15871	0.000781
lightcyan	9062	GSE17721_POLYIC_VS_GARDIQUIMOD_4H_BMDM_UP	5	190	16063	62	6.898557	0.000781
lightcyan	9105	GSE17721_0.5H_VS_4H_POLYIC_BMDM_DN	5	190	16063	62	6.898557	0.000781
lightcyan	9520	GSE2706_LPS_VS_R848_AND_LPS_8H_STIM_DC_UP	5	190	16063	62	6.898557	0.000781
greenyello	299	VIRAL_GENOME_REPLICATION	3	20	16233	150	16.253	0.000783
greenyello	3622	MODULE_134	3	20	16233	150	16.253	0.000783
magenta	9270	GSE20366_TREG_VS_NAIVE_CD4_TCELL_DEC205_CON\	8	190	16063	167	4.097825	0.000784
red	9887	GSE360_HIGH_DOSE_B_MALAYI_VS_M_TUBERCULOSIS	15	181	16072	524	2.570484	0.000787
greenyello	7441	MEISSNER_BRAIN_HCP_WITH_H3K27ME3	6	118	16135	150	5.509492	0.000788
cyan	7858	HIRSCH_CELLULAR_TRANSFORMATION_SIGNATURE_UI	6	231	16022	77	5.482543	0.00079
pink	7576	MIKKELSEN_IPS_ICP_WITH_H3K4ME3_AND_H327ME3	6	78	16175	228	5.483468	0.00079
yellow	3123	chr8q24	23	129	16124	1426	2.032133	0.000792
brown	2978	BIOCARTA_FMLP_PATHWAY	12	33	16220	2190	2.698713	0.000792
lightyellow	5680	KIM_MYCN_AMPLIFICATION_TARGETS_DN	3	90	16163	33	16.41717	0.000794
greenyello	8537	GSE13306_TREG_RA_VS_TCONV_RA_DN	7	163	16090	150	4.653211	0.000795
lightcyan	807	IMMUNE_SYSTEM_PROCESS	6	289	15964	62	5.44246	0.0008
lightcyan	6934	HELLER_HDAC_TARGETS_UP	6	289	15964	62	5.44246	0.0008
blue	3174	chr16q22	39	130	16123	2978	1.637307	0.0008
brown	9504	GSE2706_UNSTIM_VS_8H_LPS_DC_UP	42	191	16062	2190	1.631944	0.000801
brown	10207	GSE7852_LN_VS_FAT_TCONV_DN	42	191	16062	2190	1.631944	0.000801
black	3418	chr16p11	7	87	16166	283	4.620893	0.000803
purple	9272	GSE20366_CD103_POS_VS_NEG_TREG_KLRG1NEG_UP	7	156	16097	157	4.645231	0.000804
lightgreen	6268	MATSUDA_NATURAL_KILLER_DIFFERENTIATION	6	433	15820	42	5.362257	0.000804

salmon	8397	KAECH_DAY15_EFF_VS_MEMORY_CD8_TCELL_DN	7	193	16060	127	4.64163	0.000804
salmon	8878	GSE17721_CTRL_VS_PAM3CSK4_24H_BMDM_UP	7	193	16060	127	4.64163	0.000804
salmon	9013	GSE17721_POLYIC_VS_CPG_8H_BMDM_DN	7	193	16060	127	4.64163	0.000804
salmon	9067	GSE17721_POLYIC_VS_GARDIQUIMOD_8H_BMDM_DN	7	193	16060	127	4.64163	0.000804
salmon	9802	GSE360_CTRL_VS_M_TUBERCULOSIS_DC_UP	7	193	16060	127	4.64163	0.000804
purple	4365	REACTOME_SIGNALING_BY_GPCR	12	421	15832	157	2.950754	0.000808
turquoise	7722	MIKKELSEN_NPC_ICP_WITH_H3K4ME3	126	383	15870	4169	1.282547	0.000808
red	5154	HAHTOLA_MYCOSIS_FUNGOIDES_CD4_UP	8	62	16191	524	4.002216	0.000809
magenta	8499	GSE12366_PLASMA_CELL_VS_NAIVE_BCELL_DN	8	191	16062	167	4.076371	0.000811
magenta	8675	GSE14350_IL2RB_KO_VS_WT_TREG_DN	8	191	16062	167	4.076371	0.000811
lightgreen	5540	HERNANDEZ_ABERRANT_MITOSIS_BY_DOCETACEL_2NI	3	71	16182	42	16.35111	0.000812
red	502	REGULATION_OF_BODY_FLUID_LEVELS	7	48	16205	524	4.523338	0.000814
turquoise	3520	MODULE_28	17	32	16221	4169	2.071098	0.000815
cyan	9711	GSE30083_SP1_VS_SP2_THYMOCYTE_DN	5	154	16099	77	6.853179	0.000817
red	2849	KEGG_FC_GAMMA_R_MEDIATED_PHAGOCYTOSIS	10	93	16160	524	3.33518	0.000818
turquoise	2525	GCM_IL6ST	25	54	16199	4169	1.804878	0.000818
lightcyan	8687	GSE1460_INTRATHYMIC_T_PROGENITOR_VS_CD4_THY	5	192	16061	62	6.826697	0.000819
lightcyan	8927	GSE17721_LPS_VS_POLYIC_16H_BMDM_DN	5	192	16061	62	6.826697	0.000819
lightcyan	9537	GSE27786_LSK_VS_NKCELL_DN	5	192	16061	62	6.826697	0.000819
brown	1752	V$MAZR_01	43	197	16056	2190	1.619913	0.00082
lightyellow	8052	PEDERSEN_METASTASIS_BY_ERBB2_ISOFORM_4	3	91	16162	33	16.23676	0.00082
turquoise	9145	GSE17721_0.5H_VS_24H_GARDIQUIMOD_BMDM_DN	69	191	16062	4169	1.408372	0.000822
turquoise	9555	GSE27786_LIN_NEG_VS_NKTCELL_DN	69	191	16062	4169	1.408372	0.000822
turquoise	9560	GSE27786_LIN_NEG_VS_MONO_MAC_UP	69	191	16062	4169	1.408372	0.000822
red	7628	WORSCHECH_TUMOR_EVASION_AND_TOLEROGENICIT	4	14	16239	524	8.86205	0.000826
cyan	4032	PID_IFNGPATHWAY	3	39	16214	77	16.23676	0.000826
blue	5484	MATTIOLI_MGUS_VS_PCL	32	101	16152	2978	1.729169	0.000828
salmon	8995	GSE17721_LPS_VS_PAM3CSK4_8H_BMDM_DN	7	194	16059	127	4.617704	0.000829
lightgreen	3552	MODULE_63	4	164	16089	42	9.438444	0.00083
lightgreen	10098	GSE3982_NKCELL_VS_TH2_UP	4	164	16089	42	9.438444	0.00083
purple	2235	CAGGTA_V$AREB6_01	15	609	15644	157	2.54981	0.000832
red	9910	GSE36476_CTRL_VS_TSST_ACT_40H_MEMORY_CD4_T(	15	182	16071	524	2.556361	0.000833
midnightb	9368	GSE22886_NAIVE_BCELL_VS_MONOCYTE_UP	5	168	16085	71	6.812961	0.000835
brown	2848	KEGG_FC_EPSILON_RI_SIGNALING_PATHWAY	19	66	16187	2190	2.136481	0.000837
red	8345	STK33_NOMO_DN	19	261	15992	524	2.257955	0.000838
red	5773	GROSS_HYPOXIA_VIA_ELK3_DN	13	145	16108	524	2.78085	0.000838
grey60	5818	GUENTHER_GROWTH_SPHERICAL_VS_ADHERENT_UP	2	16	16237	44	46.1733	0.000839
purple	6591	ZHU_CMV_24_HR_DN	5	76	16177	157	6.810677	0.000839
purple	2211	WTTGKCTG_UNKNOWN	12	423	15830	157	2.936803	0.000841
greenyello	2279	CTAWWWATA_V$RSRFC4_Q2	9	267	15986	150	3.65236	0.000843
greenyello	6968	WALLACE_PROSTATE_CANCER_RACE_UP	9	267	15986	150	3.65236	0.000843
blue	6394	IVANOVA_HEMATOPOIESIS_EARLY_PROGENITOR	118	492	15761	2978	1.308958	0.000845
lightgreen	10112	GSE39820_IL1B_IL6_VS_IL1B_IL6_IL23A_TREATED_CD4	4	165	16088	42	9.381241	0.000849
yellow	9893	GSE36392_TYPE_2_MYELOID_VS_MAC_IL25_TREATED_	31	195	16058	1426	1.811929	0.000851
turquoise	2813	KEGG_UBIQUITIN_MEDIATED_PROTEOLYSIS	50	130	16123	4169	1.499437	0.000856
cyan	6796	KRIGE_RESPONSE_TO_TOSEDOSTAT_24HR_UP	11	759	15494	77	3.0591	0.000857
turquoise	7509	BOYAULT_LIVER_CANCER_SUBCLASS_G3_UP	68	188	16065	4169	1.410109	0.000859
lightcyan	7334	WUNDER_INFLAMMATORY_RESPONSE_AND_CHOLESTI	3	49	16204	62	16.0497	0.000859
blue	9336	GSE22886_NAIVE_BCELL_VS_BM_PLASMA_CELL_UP	55	200	16053	2978	1.500865	0.000864
blue	9353	GSE22886_NEUTROPHIL_VS_DC_DN	55	200	16053	2978	1.500865	0.000864
yellow	3345	chr17q25	27	162	16091	1426	1.899603	0.000865
purple	8381	LEF1_UP.V1_UP	7	158	16095	157	4.586431	0.000866
greenyello	1460	V$AP4_01	8	215	16038	150	4.031752	0.000869
turquoise	1669	V$GABP_B	87	251	16002	4169	1.351286	0.000869
blue	9888	GSE360_LOW_DOSE_B_MALAYI_VS_M_TUBERCULOSIS	50	178	16075	2978	1.533059	0.00087
tan	5638	OXFORD_RALA_OR_RALB_TARGETS_UP	4	48	16205	145	9.340805	0.000872
brown	9772	GSE339_CD8POS_VS_CD4CD8DN_DC_UP	41	186	16067	2190	1.635913	0.000873
red	3747	MODULE_288	5	24	16229	524	6.461912	0.000874
lightcyan	286	MULTI_ORGANISM_PROCESS	4	112	16141	62	9.362327	0.000876
salmon	8403	GOLDRATH_EFF_VS_MEMORY_CD8_TCELL_DN	7	196	16057	127	4.570585	0.00088

red	8829	GSE17580_UNINFECTED_VS_S_MANSONI_INF_TEFF_DI	15	183	16070	524	2.542391	0.000881
turquoise	8720	GSE14769_UNSTIM_VS_80MIN_LPS_BMDM_UP	71	198	16055	4169	1.39796	0.000882
turquoise	9173	GSE17974_CTRL_VS_ACT_IL4_AND_ANTI_IL12_2H_CD4	71	198	16055	4169	1.39796	0.000882
turquoise	3509	MODULE_17	115	346	15907	4169	1.295756	0.000883
purple	7654	CAIRO_HEPATOBLASTOMA_DN	8	206	16047	157	4.020283	0.000886
red	2705	GNF2_STAT6	9	78	16175	524	3.578905	0.000888
magenta	7451	ZHAN_EARLY_DIFFERENTIATION_GENES_DN	4	42	16211	167	9.268891	0.000888
turquoise	3312	chr13q12	27	60	16193	4169	1.754342	0.000888
yellow	7853	KIM_ALL_DISORDERS_DURATION_CORR_DN	24	138	16115	1426	1.982194	0.000889
cyan	5568	DAUER_STAT3_TARGETS_UP	3	40	16213	77	15.83084	0.00089
cyan	10127	GSE6269_HEALTHY_VS_FLU_INF_PBMC_DN	5	157	16096	77	6.722227	0.000891
yellow	1207	HISTONE_DEACETYLASE_BINDING	5	10	16243	1426	5.698808	0.000893
purple	7392	LEE_INTRATHYMIC_T_PROGENITOR	3	20	16233	157	15.52834	0.000893
brown	10260	GSE9650_EFFECTOR_VS_EXHAUSTED_CD8_TCELL_UP	42	192	16061	2190	1.623445	0.000895
magenta	8390	KAECH_NAIVE_VS_MEMORY_CD8_TCELL_UP	8	194	16059	167	4.013334	0.000897
turquoise	10242	GSE9037_CTRL_VS_LPS_4H_STIM_BMDM_UP	67	185	16068	4169	1.411903	0.000898
lightyellow	6388	KUMAR_TARGETS_OF_MLL_AF9_FUSION	5	376	15877	33	6.549404	0.000898
lightcyan	8400	GOLDRATH_NAIVE_VS_MEMORY_CD8_TCELL_UP	5	196	16057	62	6.687377	0.000898
lightcyan	8704	GSE1460_CD4_THYMOCYTE_VS_NAIVE_CD4_TCELL_AD	5	196	16057	62	6.687377	0.000898
salmon	6215	NING_CHRONIC_OBSTRUCTIVE_PULMONARY_DISEASE_	6	143	16110	127	5.369638	0.000902
brown	6885	STEIN_ESRRA_TARGETS_UP	73	379	15874	2190	1.429464	0.000907
green	5906	BENPORATH_EED_TARGETS	73	630	15623	1305	1.44313	0.000907
salmon	9358	GSE22886_NAIVE_TCELL_VS_NEUTROPHIL_UP	7	197	16056	127	4.547384	0.000907
brown	1669	V$GABP_B	52	251	16002	2190	1.537514	0.000909
purple	5325	BUSA_SAM68_TARGETS_DN	2	5	16248	157	41.40892	0.00091
purple	7795	DASU_IL6_SIGNALING_DN	2	5	16248	157	41.40892	0.00091
purple	7899	WAGSCHAL_EHMT2_TARGETS_UP	2	5	16248	157	41.40892	0.00091
blue	8690	GSE1460_INTRATHYMIC_T_PROGENITOR_VS_NAIVE_CI	54	196	16057	2978	1.503649	0.000913
blue	9399	GSE22886_UNSTIM_VS_IL2_STIM_NKCELL_DN	54	196	16057	2978	1.503649	0.000913
blue	9540	GSE27786_LSK_VS_ERYTHROBLAST_UP	54	196	16057	2978	1.503649	0.000913
brown	10072	GSE3982_BASOPHIL_VS_EFF_MEMORY_CD4_TCELL_UF	43	198	16055	2190	1.611731	0.000914
purple	7249	VART_KSHV_INFECTION_ANGIOGENIC_MARKERS_DN	6	116	16137	157	5.354601	0.000914
brown	4595	REACTOME_RECYCLING_PATHWAY_OF_L1	10	25	16228	2190	2.968584	0.000916
greenyello	10025	GSE3982_DC_VS_NKCELL_DN	7	167	16086	150	4.541756	0.000917
red	635	IMMUNE_RESPONSE	16	203	16050	524	2.444703	0.000917
purple	6493	CHIBA_RESPONSE_TO_TSA_UP	4	45	16208	157	9.201982	0.000917
purple	6549	CHIBA_RESPONSE_TO_TSA	4	45	16208	157	9.201982	0.000917
blue	4699	REACTOME_HIV_LIFE_CYCLE	35	114	16139	2978	1.675606	0.000919
lightcyan	9453	GSE24634_TEFF_VS_TCONV_DAY7_IN_CULTURE_DN	5	197	16056	62	6.65343	0.000919
lightcyan	9944	GSE37416_12H_VS_24H_F_TULARENSIS_LVS_NEUTROI	5	197	16056	62	6.65343	0.000919
yellow	5598	DACOSTA_UV_RESPONSE_VIA_ERCC3_UP	43	300	15953	1426	1.633658	0.00092
lightcyan	3672	MODULE_191	2	12	16241	62	43.69086	0.000922
lightcyan	4640	REACTOME_NFKB_ACTIVATION_THROUGH_FADD_RIP1	2	12	16241	62	43.69086	0.000922
lightcyan	6677	WHITESIDE_CISPLATIN_RESISTANCE_DN	2	12	16241	62	43.69086	0.000922
magenta	5030	RHEIN_ALL_GLUCOCORTICOID_THERAPY_UP	5	73	16180	167	6.665983	0.000922
turquoise	8854	GSE17721_CTRL_VS_POLYIC_4H_BMDM_UP	70	195	16058	4169	1.399475	0.000923
turquoise	9344	GSE22886_IGM_MEMORY_BCELL_VS_BM_PLASMA_CE	70	195	16058	4169	1.399475	0.000923
blue	5853	RICKMAN_METASTASIS_UP	83	328	15925	2978	1.381062	0.000925
magenta	9344	GSE22886_IGM_MEMORY_BCELL_VS_BM_PLASMA_CE	8	195	16058	167	3.992753	0.000928
lightyellow	7069	SMID_BREAST_CANCER_BASAL_DN	6	574	15679	33	5.148242	0.000928
red	9616	GSE27786_NEUTROPHIL_VS_MONO_MAC_UP	15	184	16069	524	2.528574	0.000932
red	9781	GSE339_EX_VIVO_VS_IN_CULTURE_CD4POS_DC_DN	15	184	16069	524	2.528574	0.000932
pink	5184	DELYS_THYROID_CANCER_DN	9	178	16075	228	3.604302	0.000932
salmon	9393	GSE22886_NAIVE_CD4_TCELL_VS_48H_ACT_TH1_DN	7	198	16055	127	4.524417	0.000934
salmon	9395	GSE22886_NAIVE_CD4_TCELL_VS_12H_ACT_TH2_DN	7	198	16055	127	4.524417	0.000934
lightcyan	8297	BMI1_DN.V1_DN	4	114	16139	62	9.198076	0.000936
pink	3774	MODULE_322	5	54	16199	228	6.600471	0.00094
lightcyan	1490	V$IRF1_01	5	198	16055	62	6.619827	0.00094
lightcyan	8793	GSE15930_NAIVE_VS_72H_IN_VITRO_STIM_IFNAB_CD	5	198	16055	62	6.619827	0.00094
lightcyan	9480	GSE26669_CTRL_VS_COSTIM_BLOCK_MLR_CD8_TCELL_	5	198	16055	62	6.619827	0.00094

purple	8348	STK33_SKM_UP	9	259	15994	157	3.5973	0.000941
turquoise	2461	MORF_RAP1A	47	121	16132	4169	1.514308	0.000941
tan	1396	OXIDOREDUCTASE_ACTIVITY_ACTING_ON_CH_OH_GRO	4	49	16204	145	9.150176	0.000943
brown	2624	GNF2_CD97	13	38	16215	2190	2.538921	0.000944
brown	2927	BIOCARTA_BIOPEPTIDES_PATHWAY	13	38	16215	2190	2.538921	0.000944
brown	4856	GAZDA_DIAMOND_BLACKFAN_ANEMIA_PROGENITOR_	13	38	16215	2190	2.538921	0.000944
purple	6115	SANA_TNF_SIGNALING_DN	5	78	16175	157	6.636044	0.000944
purple	6182	SWEET_KRAS_TARGETS_UP	5	78	16175	157	6.636044	0.000944
cyan	3336	chr22q12	4	92	16161	77	9.177301	0.000949
turquoise	5008	TAKEDA_TARGETS_OF_NUP98_HOXA9_FUSION_6HR_U	30	69	16184	4169	1.695016	0.000949
greenyello	10007	GSE3982_MAST_CELL_VS_NKCELL_DN	7	168	16085	150	4.514722	0.00095
brown	10091	GSE3982_CENT_MEMORY_CD4_TCELL_VS_NKCELL_DN	40	181	16072	2190	1.640102	0.000951
lightgreen	6918	KONDO_PROSTATE_CANCER_WITH_H3K27ME3	3	75	16178	42	15.47905	0.000952
turquoise	3172	chr11q22	18	35	16218	4169	2.004962	0.000953
red	5507	SCHLOSSER_SERUM_RESPONSE_DN	37	667	15586	524	1.720593	0.000959
blue	4925	CHARAFE_BREAST_CANCER_LUMINAL_VS_BASAL_UP	87	347	15906	2978	1.368354	0.00096
brown	492	REGULATION_OF_DEVELOPMENTAL_PROCESS	74	386	15867	2190	1.422767	0.000962
salmon	9346	GSE22886_DAY0_VS_DAY1_MONOCYTE_IN_CULTURE_	7	199	16054	127	4.501682	0.000962
blue	6786	BILD_MYC_ONCOGENIC_SIGNATURE	53	192	16061	2978	1.50655	0.000965
blue	10102	GSE39820_CTRL_VS_IL1B_IL6_IL23A_CD4_TCELL_UP	53	192	16061	2978	1.50655	0.000965
turquoise	8943	GSE17721_POLYIC_VS_PAM3CSK4_12H_BMDM_DN	69	192	16061	4169	1.401037	0.000966
red	5777	GROSS_HYPOXIA_VIA_HIF1A_DN	10	95	16158	524	3.264966	0.000966
blue	7454	MILI_PSEUDOPODIA_CHEMOTAXIS_DN	108	446	15807	2978	1.321593	0.000966
turquoise	5097	NAGASHIMA_NRG1_SIGNALING_DN	22	46	16207	4169	1.864518	0.000966
purple	2807	KEGG_CHEMOKINE_SIGNALING_PATHWAY	7	161	16092	157	4.500969	0.000967
lightcyan	8295	BMI1_DN_MEL18_DN.V1_DN	4	115	16138	62	9.118093	0.000967
red	5085	ZHOU_INFLAMMATORY_RESPONSE_FIMA_UP	26	414	15839	524	1.947939	0.000968
pink	7216	WINTER_HYPOXIA_METAGENE	10	216	16037	228	3.300236	0.000968
grey60	9359	GSE22886_NAIVE_TCELL_VS_NEUTROPHIL_DN	4	163	16090	44	9.064696	0.000968
cyan	9515	GSE2706_R848_VS_R848_AND_LPS_2H_STIM_DC_DN	5	160	16093	77	6.596185	0.00097
brown	10098	GSE3982_NKCELL_VS_TH2_UP	37	164	16089	2190	1.674354	0.000972
lightgreen	3079	BIOCARTA_TOB1_PATHWAY	2	18	16235	42	42.99735	0.000972
greenyello	10021	GSE3982_DC_VS_EFF_MEMORY_CD4_TCELL_DN	7	169	16084	150	4.488008	0.000983
cyan	3535	MODULE_46	7	332	15921	77	4.450438	0.000985
red	8255	CAMP_UP.V1_DN	15	185	16068	524	2.514906	0.000985
red	9275	GSE20366_CD103_KLRG1_DP_VS_DN_TREG_DN	15	185	16068	524	2.514906	0.000985
yellow	8133	PECE_MAMMARY_STEM_CELL_DN	23	131	16122	1426	2.001108	0.000985
lightgreen	7656	CAIRO_HEPATOBLASTOMA_CLASSES_DN	4	172	16081	42	8.999446	0.000991
lightgreen	10073	GSE3982_BASOPHIL_VS_EFF_MEMORY_CD4_TCELL_DN	4	172	16081	42	8.999446	0.000991
magenta	10140	GSE6269_E_COLI_VS_STREP_AUREUS_INF_PBMC_UP	7	152	16101	167	4.481997	0.000991
blue	8586	GSE13485_DAY1_VS_DAY21_YF17D_VACCINE_PBMC_U	50	179	16074	2978	1.524494	0.000997
brown	3928	PID_ENDOTHELINPATHWAY	17	57	16196	2190	2.213418	0.000999
purple	288	POSITIVE_REGULATION_OF_CELL_PROLIFERATION	6	118	16135	157	5.263845	0.000999
blue	2490	GCM_GSTA4	22	62	16191	2978	1.9366	0.001001
red	5067	SENESE_HDAC1_TARGETS_UP	26	415	15838	524	1.943245	0.001002
red	8349	STK33_UP	19	265	15988	524	2.223873	0.001004
green	2055	KRCTCNNNNMANAGC_UNKNOWN	11	47	16206	1305	2.914861	0.001005
pink	6936	HELLER_HDAC_TARGETS_SILENCED_BY_METHYLATION_	14	381	15872	228	2.6194	0.001006
turquoise	8827	GSE17580_TREG_VS_TEFF_S_MANSONI_INF_DN	68	189	16064	4169	1.402648	0.00101
blue	2433	MORF_PHB	37	123	16130	2978	1.641744	0.001017
tan	8700	GSE1460_DP_THYMOCYTE_VS_THYMIC_STROMAL_CEL	7	176	16077	145	4.458111	0.001022
salmon	6853	GAVIN_FOXP3_TARGETS_CLUSTER_T7	5	98	16155	127	6.529407	0.001023
salmon	7301	CHAUHAN_RESPONSE_TO_METHOXYESTRADIOL_DN	5	98	16155	127	6.529407	0.001023
cyan	10134	GSE6269_FLU_VS_E_COLI_INF_PBMC_UP	5	162	16091	77	6.514751	0.001025
turquoise	1943	CTACCTC,LET-7A,LET-7B,LET-7C,LET-7D,LET-7E,LET-7F,M	112	337	15916	4169	1.295656	0.001025
cyan	6932	HELLER_SILENCED_BY_METHYLATION_UP	6	243	16010	77	5.211801	0.001027
magenta	2917	BIOCARTA_ACE2_PATHWAY	2	5	16248	167	38.92934	0.001028
magenta	5611	FUNG_IL2_TARGETS_WITH_STAT5_BINDING_SITES_T1	2	5	16248	167	38.92934	0.001028
magenta	7090	BASSO_HAIRY_CELL_LEUKEMIA_UP	2	5	16248	167	38.92934	0.001028
turquoise	4558	REACTOME_MAP_KINASE_ACTIVATION_IN_TLR_CASCA	23	49	16204	4169	1.829925	0.001033

lightgreen	7854	VERHAAK_GLIOBLASTOMA_PRONEURAL	4	174	16079	42	8.896004	0.001034
purple	3922	MODULE_573	3	21	16232	157	14.7889	0.001035
brown	273	ESTABLISHMENT_OF_PROTEIN_LOCALIZATION	39	176	16077	2190	1.644528	0.001036
yellow	7207	CHNG_MULTIPLE_MYELOMA_HYPERPLOID_UP	12	50	16203	1426	2.735428	0.001037
blue	8618	GSE14000_UNSTIM_VS_4H_LPS_DC_UP	54	197	16056	2978	1.496016	0.001038
red	9824	GSE360_DC_VS_MAC_B_MALAYI_HIGH_DOSE_UP	15	186	16067	524	2.501385	0.00104
blue	560	REGULATION_OF_TRANSCRIPTION_FROM_RNA_POLYM	66	251	16002	2978	1.43509	0.001041
lightgreen	7704	WONG_ADULT_TISSUE_STEM_MODULE	7	629	15624	42	4.306571	0.001042
greenyello	6859	GAVIN_PDE3B_TARGETS	3	22	16231	150	14.77545	0.001043
red	6396	MARTINEZ_RESPONSE_TO_TRABECTEDIN	7	50	16203	524	4.342405	0.001044
green	1135	AMINE_RECEPTOR_ACTIVITY	6	16	16237	1305	4.670402	0.001049
cyan	4807	PICCALUGA_ANGIOIMMUNOBLASTIC_LYMPHOMA_UP	5	163	16090	77	6.474783	0.001054
turquoise	2163	ATCATGA,MIR-433	43	109	16144	4169	1.537955	0.001054
brown	5059	DITTMER_PTHLH_TARGETS_UP	27	109	16144	2190	1.838344	0.001055
lightgreen	3617	MODULE_129	4	175	16078	42	8.84517	0.001056
turquoise	9143	GSE17721_0.5H_VS_8H_GARDIQUIMOD_BMDM_DN	67	186	16067	4169	1.404312	0.001057
red	360	CYCLIC_NUCLEOTIDE_METABOLIC_PROCESS	3	7	16246	524	13.29308	0.001058
purple	3609	MODULE_121	5	80	16173	157	6.470143	0.001059
lightgreen	356	CELL_SURFACE_RECEPTOR_LINKED_SIGNAL_TRANSDUC	6	457	15796	42	5.08065	0.001063
brown	9478	GSE26669_CTRL_VS_COSTIM_BLOCK_MLR_CD4_TCELL_	40	182	16071	2190	1.63109	0.001064
grey60	3548	MODULE_59	2	18	16235	44	41.04293	0.001066
lightgreen	6385	MOREAUX_B_LYMPHOCYTE_MATURATION_BY_TACI_U	3	78	16175	42	14.8837	0.001067
greenyello	225	PLASMA_MEMBRANE_PART	18	867	15386	150	2.24955	0.001067
turquoise	10216	GSE8515_CTRL_VS_IL6_4H_STIM_MAC_UP	63	173	16080	4169	1.419698	0.00107
turquoise	6764	MARTINEZ_RESPONSE_TO_TRABECTEDIN_DN	91	266	15987	4169	1.33371	0.001075
brown	4066	PID_IL8CXCR2_PATHWAY	12	34	16219	2190	2.619339	0.001076
brown	4080	PID_EPOPATHWAY	12	34	16219	2190	2.619339	0.001076
blue	9318	GSE22886_NAIVE_CD8_TCELL_VS_NKCELL_UP	51	184	16069	2978	1.512729	0.001078
lightyellow	8294	ALK_DN.V1_UP	3	100	16153	33	14.77545	0.001078
tan	1186	RIBONUCLEASE_ACTIVITY	3	23	16230	145	14.62039	0.001081
tan	4462	REACTOME_ACTIVATION_OF_GENES_BY_ATF4	3	23	16230	145	14.62039	0.001081
turquoise	9071	GSE17721_POLYIC_VS_GARDIQUIMOD_16H_BMDM_D	70	196	16057	4169	1.392335	0.001081
red	3544	MODULE_55	35	624	15629	524	1.739745	0.001081
purple	2055	KRCTCNNNNMANAGC_UNKNOWN	4	47	16206	157	8.810408	0.001082
yellow	15	ORGANELLE_INNER_MEMBRANE	15	71	16182	1426	2.407947	0.001083
red	2814	KEGG_SNARE_INTERACTIONS_IN_VESICULAR_TRANSPC	6	37	16216	524	5.029812	0.001084
red	4601	REACTOME_IL1_SIGNALING	6	37	16216	524	5.029812	0.001084
yellow	9590	GSE27786_CD8_TCELL_VS_NKTCELL_UP	29	181	16072	1426	1.826137	0.001085
turquoise	3738	MODULE_277	24	52	16201	4169	1.799325	0.00109
pink	1645	V$LMO2COM_02	9	182	16071	228	3.525087	0.00109
turquoise	3341	chr5q11	16	30	16223	4169	2.07922	0.001095
turquoise	1851	V$IRF1_Q6	73	206	16047	4169	1.38152	0.001097
blue	9364	GSE22886_NAIVE_BCELL_VS_NEUTROPHIL_UP	53	193	16060	2978	1.498744	0.001098
blue	9530	GSE27786_LSK_VS_BCELL_UP	53	193	16060	2978	1.498744	0.001098
blue	9536	GSE27786_LSK_VS_NKCELL_UP	53	193	16060	2978	1.498744	0.001098
red	4179	REACTOME_PROTEOLYTIC_CLEAVAGE_OF_SNARE_CON	4	15	16238	524	8.271247	0.001098
yellow	214	RIBOSOMAL_SUBUNIT	7	20	16233	1426	3.989165	0.001098
lightcyan	5332	DARWICHE_SKIN_TUMOR_PROMOTER_UP	4	119	16134	62	8.811602	0.001098
yellow	9322	GSE22886_TCELL_VS_BCELL_NAIVE_UP	31	198	16055	1426	1.784475	0.001101
yellow	9448	GSE24634_TEFF_VS_TCONV_DAY3_IN_CULTURE_UP	31	198	16055	1426	1.784475	0.001101
cyan	7049	SAGIV_CD24_TARGETS_DN	3	43	16210	77	14.72637	0.001101
blue	368	TRANSCRIPTION_DNA_DEPENDENT	131	558	15695	2978	1.281286	0.001103
magenta	7443	MEISSNER_BRAIN_HCP_WITH_H3K4ME3_AND_H3K27N	18	779	15474	167	2.248807	0.001105
turquoise	8801	GSE15930_STIM_VS_STIM_AND_TRICHOSTATINA_24H_	66	183	16070	4169	1.40603	0.001106
brown	8570	GSE13484_12H_VS_3H_YF17D_VACCINE_STIM_PBMC_	42	194	16059	2190	1.606708	0.001111
brown	9315	GSE22886_NAIVE_TCELL_VS_NKCELL_DN	42	194	16059	2190	1.606708	0.001111
brown	9463	GSE24634_IL4_VS_CTRL_TREATED_NAIVE_CD4_TCELL_	42	194	16059	2190	1.606708	0.001111
magenta	8267	PRC2_EZH2_UP.V1_DN	7	155	16098	167	4.395248	0.001111
red	7312	LINDSTEDT_DENDRITIC_CELL_MATURATION_D	8	65	16188	524	3.817499	0.001111
cyan	10112	GSE39820_IL1B_IL6_VS_IL1B_IL6_IL23A_TREATED_CD4	5	165	16088	77	6.396301	0.001113

cyan	3563	MODULE_75	7	340	15913	77	4.345722	0.00113
turquoise	10208	GSE7852_THYMUS_VS_FAT_TCONV_UP	69	193	16060	4169	1.393777	0.001132
pink	8368	KRAS.600.LUNG.BREAST_UP.V1_DN	9	183	16070	228	3.505824	0.001133
greenyello	4482	REACTOME_GPCR_DOWNSTREAM_SIGNALING	10	336	15917	150	3.224802	0.001134
red	8173	SMIRNOV_RESPONSE_TO_IR_6HR_DN	11	114	16139	524	2.992885	0.001135
blue	1334	ATP_DEPENDENT_DNA_HELICASE_ACTIVITY	7	11	16242	2978	3.473075	0.001136
turquoise	8023	GOBERT_OLIGODENDROCYTE_DIFFERENTIATION_UP	174	554	15699	4169	1.22445	0.001144
brown	4171	REACTOME_MEMBRANE_TRAFFICKING	28	115	16138	2190	1.806964	0.001146
tan	6797	KRIGE_RESPONSE_TO_TOSEDOSTAT_24HR_DN	19	980	15273	145	2.173167	0.001146
red	5050	TONKS_TARGETS_OF_RUNX1_RUNX1T1_FUSION_ERYTI	13	150	16103	524	2.688155	0.001148
brown	2996	BIOCARTA_IL3_PATHWAY	7	14	16239	2190	3.710731	0.00115
magenta	3480	SIG_BCR_SIGNALING_PATHWAY	4	45	16208	167	8.650965	0.001154
brown	4892	GARY_CD5_TARGETS_UP	86	464	15789	2190	1.375529	0.001155
greenyello	4489	REACTOME_G_ALPHA_I_SIGNALLING_EVENTS	6	127	16126	150	5.119055	0.001156
grey60	4368	REACTOME_CLASS_A1_RHODOPSIN_LIKE_RECEPTORS	4	171	16082	44	8.640617	0.001157
salmon	182	INTRACELLULAR_ORGANELLE_PART	19	1125	15128	127	2.161379	0.001159
red	8994	GSE17721_LPS_VS_PAM3CSK4_8H_BMDM_UP	15	188	16065	524	2.474775	0.001159
red	10180	GSE7460_WT_VS_FOXP3_HET_ACT_WITH_TGFB_TCON	15	188	16065	524	2.474775	0.001159
brown	8942	GSE17721_POLYIC_VS_PAM3CSK4_12H_BMDM_UP	39	177	16076	2190	1.635237	0.001159
red	5780	GROSS_HYPOXIA_VIA_ELK3_AND_HIF1A_UP	12	132	16121	524	2.819743	0.001163
lightyellow	6347	DORSEY_GAB2_TARGETS	2	25	16228	33	39.40121	0.001165
midnightb	9486	GSE26928_NAIVE_VS_EFF_MEMORY_CD4_TCELL_UP	5	181	16072	71	6.323632	0.001165
turquoise	8763	GSE15659_NONSUPPRESSIVE_TCELL_VS_ACTIVATED_TI	61	167	16086	4169	1.424016	0.001167
red	10236	GSE9006_1MONTH_VS_4MONTH_POST_TYPE_1_DIABI	14	169	16084	524	2.56947	0.001168
blue	2529	GCM_MAP4K4	47	167	16086	2978	1.535997	0.001169
green	3258	chr15q15	14	70	16183	1305	2.490881	0.00117
brown	3507	MODULE_15	66	339	15914	2190	1.444886	0.001171
purple	2836	KEGG_COMPLEMENT_AND_COAGULATION_CASCADES	4	48	16205	157	8.626858	0.001172
purple	5351	BEGUM_TARGETS_OF_PAX3_FOXO1_FUSION_UP	4	48	16205	157	8.626858	0.001172
salmon	5484	MATTIOLI_MGUS_VS_PCL	5	101	16152	127	6.335464	0.001172
cyan	8659	GSE1432_CTRL_VS_IFNG_1H_MICROGLIA_DN	5	167	16086	77	6.319698	0.001174
magenta	3684	MODULE_204	5	77	16176	167	6.319698	0.001174
red	6078	ROSS_AML_WITH_CBFB_MYH11_FUSION	7	51	16202	524	4.257259	0.001177
cyan	6393	LIANG_SILENCED_BY_METHYLATION_2	3	44	16209	77	14.39168	0.001178
turquoise	2053	ATGTTTC,MIR-494	52	138	16115	4169	1.469014	0.001178
turquoise	2096	CTGTTAC,MIR-194	38	94	16159	4169	1.576004	0.00118
green	2206	TTTNNANAGCYR_UNKNOWN	18	102	16151	1305	2.197836	0.001183
turquoise	8620	GSE14000_UNSTIM_VS_16H_LPS_DC_UP	68	190	16063	4169	1.395266	0.001185
turquoise	8680	GSE1448_CTRL_VS_ANTI_VBETA5_DP_THYMOCYTE_UF	68	190	16063	4169	1.395266	0.001185
turquoise	9264	GSE20366_EX_VIVO_VS_HOMEOSTATIC_CONVERSION_	68	190	16063	4169	1.395266	0.001185
cyan	4655	REACTOME_REGULATION_OF_IFNA_SIGNALING	2	11	16242	77	38.3778	0.001185
cyan	6432	RUAN_RESPONSE_TO_TNF_UP	2	11	16242	77	38.3778	0.001185
salmon	5079	TIEN_INTESTINE_PROBIOTICS_2HR_UP	3	27	16226	127	14.2196	0.001188
purple	3018	BIOCARTA_MCALPAIN_PATHWAY	3	22	16231	157	14.11668	0.00119
purple	5639	OXFORD_RALA_OR_RALB_TARGETS_DN	3	22	16231	157	14.11668	0.00119
lightgreen	10203	GSE7852_THYMUS_VS_FAT_TREG_DN	4	181	16072	42	8.55196	0.001197
yellow	9572	GSE27786_BCELL_VS_NEUTROPHIL_UP	31	199	16054	1426	1.775508	0.001197
tan	8446	GSE11057_NAIVE_VS_CENT_MEMORY_CD4_TCELL_UP	7	181	16072	145	4.334959	0.001202
turquoise	18	PEROXISOMAL_MEMBRANE	9	13	16240	4169	2.698987	0.001204
turquoise	112	MICROBODY_MEMBRANE	9	13	16240	4169	2.698987	0.001204
turquoise	3297	chr6q16	9	13	16240	4169	2.698987	0.001204
lightcyan	7794	DASU_IL6_SIGNALING_UP	3	55	16198	62	14.29883	0.001204
greenyello	9279	GSE20366_TREG_VS_NAIVE_CD4_TCELL_DN	7	175	16078	150	4.334133	0.001204
cyan	9797	GSE360_CTRL_VS_T_GONDII_DC_DN	5	168	16085	77	6.282081	0.001205
cyan	9815	GSE360_CTRL_VS_M_TUBERCULOSIS_MAC_DN	5	168	16085	77	6.282081	0.001205
salmon	3	ORGANELLE_PART	19	1130	15123	127	2.151815	0.001221
lightgreen	4980	JAEGER_METASTASIS_DN	4	182	16071	42	8.504971	0.001221
red	9410	GSE24142_EARLY_THYMIC_PROGENITOR_VS_DN3_THY	15	189	16064	524	2.461681	0.001223
brown	394	IMMUNE_SYSTEM_DEVELOPMENT	20	73	16180	2190	2.033277	0.001224
purple	2286	TTCYNRGAA_V$STAT5B_01	9	269	15984	157	3.463571	0.001226

blue	10027	GSE3982_DC_VS_TH1_DN	51	185	16068	2978	1.504552	0.001228
magenta	7710	CHANDRAN_METASTASIS_DN	9	253	16000	167	3.462096	0.001233
tan	8187	ZWANG_DOWN_BY_2ND_EGF_PULSE	8	235	16018	145	3.815818	0.001236
red	9280	GSE20715_WT_VS_TLR4_KO_LUNG_UP	14	170	16083	524	2.554356	0.001237
magenta	7295	SEKI_INFLAMMATORY_RESPONSE_LPS_DN	3	21	16232	167	13.90334	0.001237
cyan	10245	GSE9037_CTRL_VS_LPS_1H_STIM_IRAK4_KO_BMDM_D	5	169	16084	77	6.244909	0.001238
purple	3701	MODULE_223	6	123	16130	157	5.049868	0.001239
purple	5159	PROVENZANI_METASTASIS_DN	6	123	16130	157	5.049868	0.001239
turquoise	8737	GSE15324_NAIVE_VS_ACTIVATED_ELF4_KO_CD8_TCEL	67	187	16066	4169	1.396802	0.00124
turquoise	9171	GSE17974_CTRL_VS_ACT_IL4_AND_ANTI_IL12_1H_CD4	67	187	16066	4169	1.396802	0.00124
magenta	8810	GSE15930_STIM_VS_STIM_AND_IFNAB_72H_CD8_T_C	7	158	16095	167	4.311794	0.001241
lightcyan	3701	MODULE_223	4	123	16130	62	8.525046	0.001242
lightcyan	5338	DARWICHE_SQUAMOUS_CELL_CARCINOMA_UP	4	123	16130	62	8.525046	0.001242
blue	7745	SENGUPTA_EBNA1_ANTICORRELATED	43	150	16103	2978	1.564538	0.001244
magenta	2844	KEGG_HEMATOPOIETIC_CELL_LINEAGE	5	78	16175	167	6.238676	0.001244
greenyello	1735	V$TBP_01	7	176	16077	150	4.309508	0.001245
greenyello	9742	GSE32423_MEMORY_VS_NAIVE_CD8_TCELL_UP	7	176	16077	150	4.309508	0.001245
greenyello	10085	GSE3982_EFF_MEMORY_CD4_TCELL_VS_NKCELL_DN	7	176	16077	150	4.309508	0.001245
yellow	10169	GSE7460_CTRL_VS_TGFB_TREATED_ACT_FOXP3_HET_T	30	191	16062	1426	1.790201	0.001246
lightgreen	10174	GSE7460_FOXP3_MUT_VS_WT_ACT_WITH_TGFB_TCOI	4	183	16070	42	8.458496	0.001246
blue	8562	GSE13484_UNSTIM_VS_3H_YF17D_VACCINE_STIM_PB	53	194	16059	2978	1.491018	0.001247
blue	10047	GSE3982_NEUTROPHIL_VS_BCELL_DN	53	194	16059	2978	1.491018	0.001247
turquoise	4863	IGARASHI_ATF4_TARGETS_DN	34	82	16171	4169	1.616466	0.001248
lightcyan	6116	TARTE_PLASMA_CELL_VS_PLASMABLAST_UP	6	315	15938	62	4.993241	0.001248
brown	4162	REACTOME_CELL_CELL_COMMUNICATION	24	94	16159	2190	1.894841	0.001249
purple	7563	MEISSNER_NPC_HCP_WITH_H3K4ME2	11	383	15870	157	2.973225	0.001253
midnightb	9774	GSE339_CD4POS_VS_CD8POS_DC_IN_CULTURE_UP	5	184	16069	71	6.22053	0.001253
blue	6602	TAKAO_RESPONSE_TO_UVB_RADIATION_DN	30	95	16158	2978	1.723481	0.001256
turquoise	7810	BAE_BRCA1_TARGETS_UP	30	70	16183	4169	1.670801	0.001259
yellow	4927	CHARAFE_BREAST_CANCER_LUMINAL_VS_MESENCHYM	54	405	15848	1426	1.519682	0.001262
turquoise	2302	MORF_CCNF	32	76	16177	4169	1.641489	0.001262
purple	3679	MODULE_199	4	49	16204	157	8.450799	0.001266
purple	6459	HENDRICKS_SMARCA4_TARGETS_UP	4	49	16204	157	8.450799	0.001266
brown	295	CELL_DEVELOPMENT	93	510	15743	2190	1.353325	0.001268
cyan	9054	GSE17721_LPS_VS_CPG_24H_BMDM_UP	5	170	16083	77	6.208174	0.001271
blue	510	RNA_BIOSYNTHETIC_PROCESS	131	560	15693	2978	1.27671	0.001274
yellow	8163	LIM_MAMMARY_STEM_CELL_DN	53	396	15857	1426	1.525438	0.001279
tan	10033	GSE3982_MAC_VS_BCELL_DN	7	183	16070	145	4.287582	0.001281
red	2184	GTAGGCA,MIR-189	5	26	16227	524	5.964841	0.001282
turquoise	199	CONDENSED_CHROMOSOME	17	33	16220	4169	2.008337	0.001283
turquoise	7284	SPIRA_SMOKERS_LUNG_CANCER_UP	17	33	16220	4169	2.008337	0.001283
green	7201	HUANG_DASATINIB_RESISTANCE_DN	13	63	16190	1305	2.569957	0.001283
brown	6745	BROWNE_HCMV_INFECTION_24HR_UP	32	138	16115	2190	1.720919	0.001284
blue	4575	REACTOME_EFFECTS_OF_PIP2_HYDROLYSIS	10	20	16233	2978	2.728845	0.001285
grey60	9965	GSE3982_DC_VS_MAC_LPS_STIM_DN	4	176	16077	44	8.395145	0.001287
red	9062	GSE17721_POLYIC_VS_GARDIQUIMOD_4H_BMDM_UP	15	190	16063	524	2.448724	0.001289
lightyellow	5905	BENPORATH_SUZ12_TARGETS	6	612	15641	33	4.82858	0.001292
turquoise	3369	chr3q25	21	44	16209	4169	1.860665	0.001295
brown	9986	GSE3982_EOSINOPHIL_VS_NKCELL_UP	39	178	16075	2190	1.62605	0.001296
turquoise	8093	KRIEG_KDM3A_TARGETS_NOT_HYPOXIA	66	184	16069	4169	1.398388	0.001298
turquoise	8983	GSE17721_CPG_VS_GARDIQUIMOD_24H_BMDM_DN	66	184	16069	4169	1.398388	0.001298
blue	9486	GSE26928_NAIVE_VS_EFF_MEMORY_CD4_TCELL_UP	50	181	16072	2978	1.507649	0.001299
blue	8042	JUBAN_TARGETS_OF_SPI1_AND_FLI1_DN	28	87	16166	2978	1.756498	0.001301
greenyello	2268	TGGAAA_V$NFAT_Q4_01	26	1506	14747	150	1.870642	0.001305
turquoise	4183	REACTOME_CELL_CYCLE	123	377	15876	4169	1.271936	0.001305
brown	2984	BIOCARTA_GH_PATHWAY	10	26	16227	2190	2.854408	0.00131
blue	7989	LEE_BMP2_TARGETS_DN	190	850	15403	2978	1.219954	0.001312
grey60	9990	GSE3982_EOSINOPHIL_VS_TH2_UP	4	177	16076	44	8.347714	0.001314
salmon	4217	REACTOME_ACTIVATION_OF_NF_KAPPAB_IN_B_CELLS	4	61	16192	127	8.391894	0.001315
salmon	6806	ZHONG_SECRETOME_OF_LUNG_CANCER_AND_ENDOT	4	61	16192	127	8.391894	0.001315

brown	7500	POOLA_INVASIVE_BREAST_CANCER_UP	51	249	16004	2190	1.520058	0.001317
grey60	3819	MODULE_385	2	20	16233	44	36.93864	0.001319
red	235	REGULATION_OF_BIOLOGICAL_QUALITY	22	335	15918	524	2.036949	0.001323
salmon	5493	LUI_THYROID_CANCER_CLUSTER_3	3	28	16225	127	13.71175	0.001323
brown	1909	V$SRF_Q5_01	40	184	16069	2190	1.613361	0.001324
brown	9465	GSE25087_FETAL_VS_ADULT_TREG_DN	40	184	16069	2190	1.613361	0.001324
brown	9849	GSE360_T_GONDII_VS_B_MALAYI_HIGH_DOSE_DC_DN	40	184	16069	2190	1.613361	0.001324
purple	6327	AFFAR_YY1_TARGETS_UP	7	170	16083	157	4.262683	0.001326
greenyello	3580	MODULE_92	5	88	16165	150	6.156439	0.001326
greenyello	5184	DELYS_THYROID_CANCER_DN	7	178	16075	150	4.261086	0.001328
greenyello	1324	TRANSMEMBRANE_RECEPTOR_ACTIVITY	9	285	15968	150	3.421684	0.001331
brown	10031	GSE3982_MAC_VS_NEUTROPHIL_DN	36	161	16092	2190	1.659457	0.001336
turquoise	7958	LINSLEY_MIR16_TARGETS	72	204	16049	4169	1.375954	0.001338
turquoise	7332	YOSHIMURA_MAPK8_TARGETS_DN	109	329	15924	4169	1.291613	0.001338
blue	5741	PUJANA_XPRSS_INT_NETWORK	47	168	16085	2978	1.526854	0.00134
turquoise	4941	LAIHO_COLORECTAL_CANCER_SERRATED_UP	42	107	16146	4169	1.530267	0.001345
blue	1304	RNA_DEPENDENT_ATPASE_ACTIVITY	9	17	16236	2978	2.889365	0.001346
blue	6797	KRIGE_RESPONSE_TO_TOSEDOSTAT_24HR_DN	216	980	15273	2978	1.202919	0.001346
purple	8038	KATSANOU_ELAVL1_TARGETS_DN	6	125	16128	157	4.96907	0.001346
brown	8476	GSE11864_CSF1_IFNG_VS_CSF1_IFNG_PAM3CYS_IN_M	41	190	16063	2190	1.601473	0.001349
greenyello	3747	MODULE_288	3	24	16229	150	13.54417	0.001353
purple	3554	MODULE_65	2	6	16247	157	34.50743	0.001356
purple	3957	PID_INTEGRIN_CS_PATHWAY	3	23	16230	157	13.50291	0.001359
purple	4950	NEWMAN_ERCC6_TARGETS_DN	3	23	16230	157	13.50291	0.001359
purple	6255	WANG_IMMORTALIZED_BY_HOXA9_AND_MEIS1_DN	3	23	16230	157	13.50291	0.001359
red	9458	GSE24634_IL4_VS_CTRL_TREATED_NAIVE_CD4_TCELL_	15	191	16062	524	2.435904	0.001359
turquoise	8450	GSE11057_EFF_MEM_VS_CENT_MEM_CD4_TCELL_UP	65	181	16072	4169	1.400027	0.001359
turquoise	8636	GSE14308_TH2_VS_NATURAL_TREG_UP	65	181	16072	4169	1.400027	0.001359
brown	6011	NIKOLSKY_BREAST_CANCER_19Q13.1_AMPLICON	9	22	16231	2190	3.036052	0.001367
brown	7684	ZHAN_MULTIPLE_MYELOMA_HP_DN	14	44	16209	2190	2.361374	0.001367
turquoise	1965	CACTGTG,MIR-128A,MIR-128B	99	295	15958	4169	1.308323	0.001368
brown	8400	GOLDRATH_NAIVE_VS_MEMORY_CD8_TCELL_UP	42	196	16057	2190	1.590313	0.001371
brown	9934	GSE37416_0H_VS_3H_F_TULARENSIS_LVS_NEUTROPH	42	196	16057	2190	1.590313	0.001371
brown	10074	GSE3982_BASOPHIL_VS_CENT_MEMORY_CD4_TCELL_U	42	196	16057	2190	1.590313	0.001371
red	6746	WANG_SMARCE1_TARGETS_UP	17	231	16022	524	2.282649	0.001372
purple	6316	VERHAAK_AML_WITH_NPM1_MUTATED_UP	7	171	16082	157	4.237755	0.001372
lightgreen	10196	GSE7852_TREG_VS_TCONV_FAT_UP	4	188	16065	42	8.233536	0.001377
lightgreen	10201	GSE7852_LN_VS_FAT_TREG_DN	4	188	16065	42	8.233536	0.001377
red	6525	WANG_CISPLATIN_RESPONSE_AND_XPC_DN	16	211	16042	524	2.352013	0.001378
red	4926	CHARAFE_BREAST_CANCER_LUMINAL_VS_BASAL_DN	25	402	15851	524	1.928929	0.001379
turquoise	5071	SENESE_HDAC2_TARGETS_UP	41	104	16149	4169	1.536923	0.001382
blue	4826	LIU_SOX4_TARGETS_DN	75	295	15958	2978	1.387548	0.001382
red	8198	EGFR_UP.V1_UP	14	172	16081	524	2.524654	0.001383
blue	2448	MORF_PTPN11	33	108	16145	2978	1.667627	0.001385
lightcyan	3669	MODULE_188	4	127	16126	62	8.256541	0.001398
salmon	4408	REACTOME_CYCLIN_E_ASSOCIATED_EVENTS_DURING_	4	62	16191	127	8.256541	0.001398
green	7560	MEISSNER_BRAIN_HCP_WITH_H3K4ME2_AND_H3K27M	9	35	16218	1305	3.202562	0.001404
lightgreen	1517	V$PBX1_01	4	189	16064	42	8.189972	0.001404
yellow	9626	GSE2826_WT_VS_BTK_KO_BCELL_UP	29	184	16069	1426	1.796363	0.00141
greenyello	7133	CHUNG_BLISTER_CYTOTOXICITY_UP	6	132	16121	150	4.925152	0.00141
turquoise	8126	VANOEVELEN_MYOGENESIS_SIN3A_TARGETS	74	211	16042	4169	1.367259	0.001411
midnightb	9921	GSE36476_YOUNG_VS_OLD_DONOR_MEMORY_CD4_T	5	189	16064	71	6.055965	0.001411
brown	1335	TRANSCRIPTION_FACTOR_ACTIVITY	56	280	15973	2190	1.484292	0.001412
blue	9372	GSE22886_NAIVE_CD8_TCELL_VS_DC_UP	53	195	16058	2978	1.483372	0.001412
blue	9673	GSE29618_MONOCYTE_VS_PDC_DAY7_FLU_VACCINE_	53	195	16058	2978	1.483372	0.001412
blue	9996	GSE3982_MAST_CELL_VS_NEUTROPHIL_UP	53	195	16058	2978	1.483372	0.001412
blue	10088	GSE3982_EFF_MEMORY_CD4_TCELL_VS_TH2_UP	46	164	16089	2978	1.530815	0.001413
greenyello	1543	V$HFH1_01	7	180	16073	150	4.213741	0.001417
cyan	948	DNA_DAMAGE_RESPONSESIGNAL_TRANSDUCTION_BY_	2	12	16241	77	35.17965	0.001418
turquoise	4997	GARGALOVIC_RESPONSE_TO_OXIDIZED_PHOSPHOLIPID	8	11	16242	4169	2.8353	0.001422

blue	7875	DUTERTRE_ESTRADIOL_RESPONSE_6HR_UP	57	213	16040	2978	1.460509	0.001423
grey60	10076	GSE3982_BASOPHIL_VS_NKCELL_UP	4	181	16072	44	8.163235	0.001427
lightgreen	9266	GSE20366_EX_VIVO_VS_DEC205_CONVERSION_NAIVE_	4	190	16063	42	8.146867	0.001431
magenta	1495	V$HEN1_01	7	162	16091	167	4.20533	0.001434
magenta	8278	PKCA_DN.V1_UP	6	119	16134	167	4.90706	0.001434
lightcyan	6634	RODWELL_AGING_KIDNEY_UP	7	443	15810	62	4.142249	0.001438
brown	1037	RHO_PROTEIN_SIGNAL_TRANSDUCTION	12	35	16218	2190	2.544501	0.001439
brown	3940	PID_GMCSF_PATHWAY	12	35	16218	2190	2.544501	0.001439
brown	6133	YAGI_AML_RELAPSE_PROGNOSIS	12	35	16218	2190	2.544501	0.001439
turquoise	2017	CTACTGT,MIR-199A	60	165	16088	4169	1.41765	0.001443
cyan	9195	GSE17974_IL4_AND_ANTI_IL12_VS_UNTREATED_6H_A	5	175	16078	77	6.030798	0.001445
cyan	9753	GSE32423_CTRL_VS_IL7_IL4_MEMORY_CD8_TCELL_DN	5	175	16078	77	6.030798	0.001445
salmon	7250	MOOTHA_HUMAN_MITODB_6_2002	10	411	15842	127	3.11378	0.001447
turquoise	9649	GSE29617_CTRL_VS_DAY3_TIV_FLU_VACCINE_PBMC_2	67	188	16065	4169	1.389372	0.001452
turquoise	10100	GSE39820_CTRL_VS_IL1B_IL6_CD4_TCELL_UP	67	188	16065	4169	1.389372	0.001452
salmon	4440	REACTOME_G1_S_TRANSITION	5	106	16147	127	6.036622	0.001453
salmon	4771	REACTOME_S_PHASE	5	106	16147	127	6.036622	0.001453
turquoise	2508	GCM_CSNK1A1	18	36	16217	4169	1.949268	0.001453
turquoise	2438	MORF_PPP2R4	23	50	16203	4169	1.793327	0.001454
lightgreen	8803	GSE15930_STIM_VS_STIM_AND_IL-12_48H_CD8_T_CE	4	191	16062	42	8.104213	0.001459
red	5251	GAUSSMANN_MLL_AF4_FUSION_TARGETS_F_UP	13	154	16099	524	2.618333	0.001459
salmon	2201	TMTCGCGANR_UNKNOWN	6	157	16096	127	4.890817	0.001461
turquoise	1835	GCGNNANTTCC_UNKNOWN	46	120	16133	4169	1.494439	0.001461
red	8262	RAF_UP.V1_UP	14	173	16080	524	2.51006	0.001462
red	9650	GSE29617_CTRL_VS_DAY7_TIV_FLU_VACCINE_PBMC_2	14	173	16080	524	2.51006	0.001462
black	7439	MEISSNER_NPC_HCP_WITH_H3_UNMETHYLATED	16	392	15861	283	2.344126	0.001463
brown	991	REGULATION_OF_PROGRAMMED_CELL_DEATH	61	311	15942	2190	1.455656	0.001463
purple	8371	KRAS.BREAST_UP.V1_UP	5	86	16167	157	6.018738	0.001465
salmon	2325	MORF_RAD23A	9	342	15911	127	3.367799	0.001465
magenta	3523	MODULE_33	10	312	15941	167	3.119338	0.001465
turquoise	2460	MORF_RAN	90	265	15988	4169	1.324031	0.001468
turquoise	10072	GSE3982_BASOPHIL_VS_EFF_MEMORY_CD4_TCELL_UF	70	198	16055	4169	1.378271	0.001471
brown	9087	GSE17721_LPS_VS_GARDIQUIMOD_12H_BMDM_DN	40	185	16068	2190	1.60464	0.001474
brown	9424	GSE24142_DN2_VS_DN3_THYMOCYTE_FETAL_UP	40	185	16068	2190	1.60464	0.001474
turquoise	2021	TGTATGA,MIR-485-3P	51	136	16117	4169	1.461951	0.001476
red	3553	MODULE_64	25	404	15849	524	1.91938	0.001476
blue	9875	GSE360_L_MAJOR_VS_B_MALAYI_LOW_DOSE_MAC_D	50	182	16071	2978	1.499365	0.001478
salmon	4140	REACTOME_SIGNALING_BY_WNT	4	63	16190	127	8.125484	0.001483
pink	7641	KOBAYASHI_EGFR_SIGNALING_24HR_UP	6	88	16165	228	4.860347	0.001485
turquoise	9169	GSE17974_CTRL_VS_ACT_IL4_AND_ANTI_IL12_0.5H_CI	63	175	16078	4169	1.403473	0.001488
green	9221	GSE18791_CTRL_VS_NEWCASTLE_VIRUS_DC_2H_DN	23	147	16106	1305	1.948649	0.001491
black	5132	WAMUNYOKOLI_OVARIAN_CANCER_LMP_UP	12	250	16003	283	2.756693	0.001492
brown	4491	REACTOME_CD28_DEPENDENT_VAV1_PATHWAY	6	11	16242	2190	4.04807	0.001496
brown	9579	GSE27786_CD4_TCELL_VS_NKCELL_DN	41	191	16062	2190	1.593089	0.001499
brown	10064	GSE3982_BCELL_VS_CENT_MEMORY_CD4_TCELL_UP	41	191	16062	2190	1.593089	0.001499
salmon	2338	MORF_SOD1	8	277	15976	127	3.696069	0.001502
turquoise	3602	MODULE_114	109	330	15923	4169	1.287699	0.001502
brown	636	HEMOPOIETIC_OR_LYMPHOID_ORGAN_DEVELOPMENT	19	69	16184	2190	2.043591	0.001503
brown	4007	PID_IL2_1PATHWAY	16	54	16199	2190	2.198951	0.001503
turquoise	7255	MOOTHA_MITOCHONDRIA	137	427	15826	4169	1.250819	0.001504
red	9132	GSE17721_0.5H_VS_24H_CPG_BMDM_UP	15	193	16060	524	2.410661	0.001507
red	10109	GSE39820_CTRL_VS_TGFBETA3_IL6_CD4_TCELL_DN	15	193	16060	524	2.410661	0.001507
greenyello	9405	GSE24081_CONTROLLER_VS_PROGRESSOR_HIV_SPECIF	7	182	16071	150	4.167436	0.001509
blue	2378	MORF_DEK	67	259	15994	2978	1.411835	0.001511
turquoise	4412	REACTOME_P53_DEPENDENT_G1_DNA_DAMAGE_RESI	24	53	16200	4169	1.765375	0.001514
turquoise	4772	REACTOME_SCFSKP2_MEDIATED_DEGRADATION_OF_F	24	53	16200	4169	1.765375	0.001514
turquoise	5137	MAYBURD_RESPONSE_TO_L663536_DN	24	53	16200	4169	1.765375	0.001514
lightcyan	8107	FORTSCHEGGER_PHF8_TARGETS_DN	9	718	15535	62	3.285942	0.001515
purple	10233	GSE9006_TYPE_1_DIABETES_AT_DX_VS_1MONTH_POS	7	174	16079	157	4.16469	0.001516
lightgreen	8457	GSE11057_NAIVE_VS_MEMORY_CD4_TCELL_DN	4	193	16060	42	8.020232	0.001516

cyan	8695	GSE1460_DP_VS_CD4_THYMOCYTE_DN	5	177	16076	77	5.962653	0.001519
purple	7571	MIKKELSEN_MEF_ICP_WITH_H3K27ME3	6	128	16125	157	4.852607	0.00152
brown	9349	GSE22886_DAY0_VS_DAY7_MONOCYTE_IN_CULTURE_	42	197	16056	2190	1.58224	0.001521
turquoise	8280	MTOR_UP.N4.V1_UP	66	185	16068	4169	1.390829	0.001521
turquoise	8891	GSE17721_CTRL_VS_CPG_8H_BMDM_DN	66	185	16068	4169	1.390829	0.001521
blue	4343	REACTOME_NRAGE_SIGNALS_DEATH_THROUGH_JNK	16	41	16212	2978	2.12983	0.001524
black	6691	IVANOVA_HEMATOPOIESIS_STEM_CELL_AND_PROGEN	21	588	15665	283	2.051111	0.001532
magenta	9841	GSE360_L_MAJOR_VS_T_GONDII_DC_DN	7	164	16089	167	4.154046	0.001539
purple	598	RESPONSE_TO_STRESS	12	454	15799	157	2.736272	0.001539
greenyello	8379	KRAS.PROSTATE_UP.V1_UP	5	91	16162	150	5.95348	0.00154
purple	4405	REACTOME_INTEGRIN_ALPHAIIB_BETA3_SIGNALING	3	24	16229	157	12.94029	0.001542
purple	5720	LIEN_BREAST_CARCINOMA_METAPLASTIC	3	24	16229	157	12.94029	0.001542
lightgreen	7144	BOQUEST_STEM_CELL_DN	4	194	16059	42	7.978891	0.001545
turquoise	4730	REACTOME_ACTIVATED_TLR4_SIGNALLING	36	89	16164	4169	1.576936	0.001548
pink	3413	chr16p13	10	230	16023	228	3.099352	0.001552
turquoise	2073	CAGCTTT,MIR-320	75	215	16038	4169	1.359955	0.001555
tan	4449	REACTOME_PERK_REGULATED_GENE_EXPRESSION	3	26	16227	145	12.93342	0.001556
cyan	9431	GSE24142_ADULT_VS_FETAL_DN3_THYMOCYTE_DN	5	178	16075	77	5.929155	0.001557
greenyello	8369	KRAS.600.LUNG.BREAST_UP.V1_UP	7	183	16070	150	4.144663	0.001558
pink	3523	MODULE_33	12	312	15941	228	2.741734	0.001559
turquoise	4827	ONKEN_UVEAL_MELANOMA_UP	231	761	15492	4169	1.183393	0.00156
magenta	4224	REACTOME_SIGNALING_BY_THE_B_CELL_RECEPTOR_B	6	121	16132	167	4.825951	0.001562
blue	8478	GSE11864_CSF1_PAM3CYS_VS_CSF1_IFNG_PAM3CYS_	49	178	16075	2978	1.502398	0.001563
purple	2196	TGACATY_UNKNOWN	13	518	15735	157	2.59805	0.001569
salmon	6091	BYSTRYKH_HEMATOPOIESIS_STEM_CELL_AND_BRAIN_	4	64	16189	127	7.998524	0.001573
turquoise	2190	SNACANNNYSYAGA_UNKNOWN	35	86	16167	4169	1.586614	0.001575
blue	2616	GNF2_CASP8	12	27	16226	2978	2.42564	0.001576
turquoise	7703	WONG_EMBRYONIC_STEM_CELL_CORE	108	327	15926	4169	1.28759	0.001579
lightgreen	3535	MODULE_46	5	332	15921	42	5.827955	0.001579
turquoise	7878	DUTERTRE_ESTRADIOL_RESPONSE_24HR_DN	148	466	15787	4169	1.238162	0.001581
purple	5626	BORLAK_LIVER_CANCER_EGF_UP	4	52	16201	157	7.963253	0.001582
purple	8359	CORDENONSI_YAP_CONSERVED_SIGNATURE	4	52	16201	157	7.963253	0.001582
purple	9354	GSE22886_NEUTROPHIL_VS_MONOCYTE_UP	6	129	16124	157	4.81499	0.001582
brown	6801	DURCHDEWALD_SKIN_CARCINOGENESIS_DN	48	233	16020	2190	1.528885	0.001583
midnightb	8448	GSE11057_NAIVE_CD4_VS_PBMC_CD4_TCELL_UP	5	194	16059	71	5.899884	0.001584
brown	1627	V$EGR1_01	49	239	16014	2190	1.521555	0.001584
blue	9806	GSE360_CTRL_VS_L_MAJOR_MAC_UP	51	187	16066	2978	1.488461	0.001585
red	2806	KEGG_CYTOKINE_CYTOKINE_RECEPTOR_INTERACTION	15	194	16059	524	2.398235	0.001587
red	10105	GSE39820_CTRL_VS_TGFBETA1_IL6_CD4_TCELL_DN	15	194	16059	524	2.398235	0.001587
magenta	1973	AGTTCTC,MIR-146A,MIR-146B	4	49	16204	167	7.944764	0.001589
magenta	7567	MIKKELSEN_MCV6_ICP_WITH_H3K27ME3	4	49	16204	167	7.944764	0.001589
blue	7819	BROWNE_HCMV_INFECTION_4HR_DN	59	223	16030	2978	1.443963	0.001591
turquoise	8279	MTOR_UP.N4.V1_DN	65	182	16071	4169	1.392335	0.001593
magenta	1488	V$HEN1_02	7	165	16088	167	4.12887	0.001593
turquoise	4550	REACTOME_MITOTIC_G1_G1_S_PHASES	49	130	16123	4169	1.469448	0.001593
lightgreen	7588	ONO_AML1_TARGETS_UP	2	23	16230	42	33.6501	0.001594
turquoise	1416	PHOSPHATASE_REGULATOR_ACTIVITY	13	23	16230	4169	2.203521	0.001595
yellow	8436	GSE10325_BCELL_VS_LUPUS_BCELL_UP	25	152	16101	1426	1.874608	0.001597
blue	10254	GSE9650_NAIVE_VS_EFF_CD8_TCELL_UP	53	196	16057	2978	1.475804	0.001597
grey60	556	AMINO_ACID_CATABOLIC_PROCESS	2	22	16231	44	33.58058	0.001599
grey60	7419	MCMURRAY_TP53_HRAS_COOPERATION_RESPONSE_U	2	22	16231	44	33.58058	0.001599
yellow	9929	GSE37416_CTRL_VS_12H_F_TULARENSIS_LVS_NEUTRO	30	194	16059	1426	1.762518	0.001601
lightgreen	8791	GSE15930_NAIVE_VS_72H_IN_VITRO_STIM_IL12_CD8_	4	196	16057	42	7.897473	0.001604
turquoise	2683	GNF2_PPP6C	19	39	16214	4169	1.899287	0.001605
cyan	7334	WUNDER_INFLAMMATORY_RESPONSE_AND_CHOLESTI	3	49	16204	77	12.92314	0.001611
turquoise	4386	REACTOME_CELL_CYCLE_CHECKPOINTS	43	111	16142	4169	1.510244	0.001612
brown	6556	BURTON_ADIPOGENESIS_5	29	123	16130	2190	1.749775	0.001614
turquoise	6484	PAL_PRMT5_TARGETS_UP	68	192	16061	4169	1.380732	0.001616
black	2188	RYTGCNWTGGNR_UNKNOWN	7	98	16155	283	4.102221	0.001617
turquoise	2292	MORF_ATOX1	33	80	16173	4169	1.608146	0.00162

midnightb	8430	GSE10325_LUPUS_CD4_TCELL_VS_LUPUS_MYELOID_U	5	195	16058	71	5.869628	0.00162
magenta	2894	KEGG_ASTHMA	3	23	16230	167	12.69435	0.001622
magenta	7495	VALK_AML_WITH_EVI1	3	23	16230	167	12.69435	0.001622
purple	6557	WESTON_VEGFA_TARGETS	5	88	16165	157	5.881948	0.001623
purple	8378	KRAS.PROSTATE_UP.V1_DN	5	88	16165	157	5.881948	0.001623
lightcyan	5006	GARGALOVIC_RESPONSE_TO_OXIDIZED_PHOSPHOLIPID	3	61	16192	62	12.89238	0.001625
purple	6954	MARTINEZ_RB1_TARGETS_DN	12	457	15796	157	2.71831	0.001627
green	8824	GSE17580_TREG_VS_TEFF_UP	27	184	16069	1305	1.827549	0.001631
turquoise	5950	SAKAI_TUMOR_INFILTRATING_MONOCYTES_DN	32	77	16176	4169	1.620171	0.001635
turquoise	37	MICROTUBULE_CYTOSKELETON	53	143	16110	4169	1.444912	0.001636
red	5087	ZHOU_INFLAMMATORY_RESPONSE_LPS_UP	21	319	15934	524	2.041883	0.001638
brown	6223	PENG_RAPAMYCIN_RESPONSE_UP	40	186	16067	2190	1.596013	0.001639
red	9516	GSE2706_R848_VS_R848_AND_LPS_8H_STIM_DC_UP	13	156	16097	524	2.584765	0.00164
turquoise	4185	REACTOME_ORC1_REMOVAL_FROM_CHROMATIN	28	65	16188	4169	1.67937	0.001641
red	3396	chr6p22	8	69	16184	524	3.596194	0.001645
purple	2280	TAAWWATAG_V$RSRFC4_Q2	6	130	16123	157	4.777952	0.001646
purple	5262	LANDIS_ERBB2_BREAST_TUMORS_324_DN	6	130	16123	157	4.777952	0.001646
turquoise	3715	MODULE_243	31	74	16179	4169	1.633171	0.001646
green	1247	NEUROTRANSMITTER_RECEPTOR_ACTIVITY	7	23	16230	1305	3.790471	0.001648
red	5568	DAUER_STAT3_TARGETS_UP	6	40	16213	524	4.652576	0.001649
midnightb	9362	GSE22886_NAIVE_TCELL_VS_MONOCYTE_UP	5	196	16057	71	5.839681	0.001657
purple	4951	HORIUCHI_WTAP_TARGETS_UP	9	281	15972	157	3.315661	0.001657
greenyello	10027	GSE3982_DC_VS_TH1_DN	7	185	16068	150	4.099856	0.001657
lightcyan	1312	DOUBLE_STRANDED_RNA_BINDING	2	16	16237	62	32.76815	0.00166
lightcyan	5839	WOTTON_RUNX_TARGETS_UP	2	16	16237	62	32.76815	0.00166
salmon	10037	GSE3982_MAC_VS_EFF_MEMORY_CD4_TCELL_DN	6	161	16092	127	4.769306	0.00166
cyan	7573	MIKKELSEN_MEF_LCP_WITH_H3K4ME3	4	107	16146	77	7.890763	0.001662
green	7839	MARTENS_TRETINOIN_RESPONSE_UP	57	478	15775	1305	1.485149	0.001663
brown	9589	GSE27786_CD8_TCELL_VS_NKCELL_DN	41	192	16061	2190	1.584791	0.001664
brown	10004	GSE3982_MAST_CELL_VS_CENT_MEMORY_CD4_TCELL_	41	192	16061	2190	1.584791	0.001664
brown	10020	GSE3982_DC_VS_EFF_MEMORY_CD4_TCELL_UP	41	192	16061	2190	1.584791	0.001664
lightgreen	1853	V$HMGIY_Q6	4	198	16055	42	7.817701	0.001665
salmon	618	PROTEIN_RNA_COMPLEX_ASSEMBLY	4	65	16188	127	7.875469	0.001666
red	6921	KONDO_EZH2_TARGETS	15	195	16058	524	2.385937	0.001669
purple	5455	LINDGREN_BLADDER_CANCER_CLUSTER_2B	10	338	15915	157	3.06279	0.00167
cyan	3003	BIOCARTA_IL22BP_PATHWAY	2	13	16240	77	32.47353	0.001671
cyan	7601	TSAI_DNAJB4_TARGETS_UP	2	13	16240	77	32.47353	0.001671
blue	8134	ABRAMSON_INTERACT_WITH_AIRE	17	45	16208	2978	2.061794	0.001671
yellow	4180	REACTOME_RESOLUTION_OF_AP_SITES_VIA_THE_MUL	6	16	16237	1426	4.274106	0.001671
yellow	6017	NIKOLSKY_BREAST_CANCER_22Q13_AMPLICON	6	16	16237	1426	4.274106	0.001671
purple	9525	GSE2706_2H_VS_8H_LPS_STIM_DC_DN	7	177	16076	157	4.094102	0.001672
cyan	8569	GSE13484_12H_UNSTIM_VS_YF17D_VACCINE_STIM_PI	5	181	16072	77	5.830882	0.001676
cyan	9046	GSE17721_LPS_VS_CPG_6H_BMDM_UP	5	181	16072	77	5.830882	0.001676
blue	9960	GSE3982_MEMORY_CD4_TCELL_VS_TH2_UP	50	183	16070	2978	1.491172	0.001678
cyan	8000	FEVR_CTNNB1_TARGETS_UP	9	582	15671	77	3.264092	0.001679
red	1952	TGCACTG,MIR-148A,MIR-152,MIR-148B	19	277	15976	524	2.127532	0.001682
brown	967	PROTEIN_LOCALIZATION	42	198	16055	2190	1.574249	0.001684
brown	8447	GSE11057_NAIVE_VS_CENT_MEMORY_CD4_TCELL_DN	42	198	16055	2190	1.574249	0.001684
blue	515	CELLULAR_CATABOLIC_PROCESS	52	192	16061	2978	1.478124	0.001693
greenyello	3652	MODULE_169	5	93	16160	150	5.825448	0.001696
lightcyan	7309	LINDSTEDT_DENDRITIC_CELL_MATURATION_A	3	62	16191	62	12.68444	0.001703
yellow	2873	KEGG_HUNTINGTONS_DISEASE	26	161	16092	1426	1.840609	0.001705
turquoise	9249	GSE19825_NAIVE_VS_IL2RALOW_DAY3_EFF_CD8_TCEL	70	199	16054	4169	1.371345	0.001709
turquoise	9592	GSE27786_CD8_TCELL_VS_ERYTHROBLAST_UP	70	199	16054	4169	1.371345	0.001709
cyan	9509	GSE2706_UNSTIM_VS_8H_LPS_AND_R848_DC_DN	5	182	16071	77	5.798844	0.001717
red	9834	GSE360_L_DONOVANI_VS_B_MALAYI_HIGH_DOSE_DC_	14	176	16077	524	2.467275	0.00172
red	10085	GSE3982_EFF_MEMORY_CD4_TCELL_VS_NKCELL_DN	14	176	16077	524	2.467275	0.00172
turquoise	5446	IWANAGA_E2F1_TARGETS_INDUCED_BY_SERUM	16	31	16222	4169	2.012148	0.001728
turquoise	2290	GCCATNTTG_V$YY1_Q6	130	404	15849	4169	1.25448	0.001733
brown	932	DETECTION_OF_BIOTIC_STIMULUS	5	8	16245	2190	4.638413	0.001737

brown	5201	BERENJENO_TRANSFORMED_BY_RHOA_REVERSIBLY_U	5	8	16245	2190	4.638413	0.001737
purple	3041	BIOCARTA_EDG1_PATHWAY	3	25	16228	157	12.42268	0.00174
purple	6042	ROZANOV_MMP14_TARGETS_SUBSET	3	25	16228	157	12.42268	0.00174
brown	2718	KEGG_GLYCOLYSIS_GLUCONEOGENESIS	14	45	16208	2190	2.308899	0.001742
brown	6088	GNATENKO_PLATELET_SIGNATURE	14	45	16208	2190	2.308899	0.001742
blue	6795	KRIGE_RESPONSE_TO_TOSEDOSTAT_6HR_DN	195	880	15373	2978	1.209374	0.001752
greenyello	7266	BOYLAN_MULTIPLE_MYELOMA_C_D_DN	8	240	16013	150	3.611778	0.001752
blue	5240	GRAESSMANN_RESPONSE_TO_MC_AND_DOXORUBICIN	168	746	15507	2978	1.229078	0.001754
red	9946	GSE37416_12H_VS_48H_F_TULARENSIS_LVS_NEUTROI	15	196	16057	524	2.373763	0.001756
pink	6506	LEE_AGING_CEREBELLUM_DN	5	62	16191	228	5.748797	0.001757
blue	6008	NIKOLSKY_BREAST_CANCER_17Q11_Q21_AMPLICON	31	101	16152	2978	1.675132	0.001758
lightgreen	5164	COLDREN_GEFITINIB_RESISTANCE_DN	4	201	16052	42	7.701019	0.001759
greenyello	1786	V$FOXM1_01	7	187	16066	150	4.056007	0.001762
greenyello	9728	GSE30962_ACUTE_VS_CHRONIC_LCMV_SECONDARY_II	7	187	16066	150	4.056007	0.001762
pink	8978	GSE17721_CPG_VS_GARDIQUIMOD_12H_BMDM_UP	8	158	16095	228	3.609372	0.001763
midnightb	7557	YAGI_AML_WITH_T_9_11_TRANSLOCATION	4	118	16135	71	7.759847	0.001764
salmon	8865	GSE17721_CTRL_VS_PAM3CSK4_0.5H_BMDM_DN	6	163	16090	127	4.710787	0.001768
blue	9305	GSE22045_TREG_VS_TCONV_DN	49	179	16074	2978	1.494004	0.001777
purple	4970	VECCHI_GASTRIC_CANCER_ADVANCED_VS_EARLY_UP	6	132	16121	157	4.705559	0.001779
lightyellow	8278	PKCA_DN.V1_UP	3	119	16134	33	12.41635	0.001779
purple	6044	ROZANOV_MMP14_TARGETS_UP	8	230	16023	157	3.600775	0.001789
lightyellow	3224	chr11q14	2	31	16222	33	31.77517	0.001792
brown	9843	GSE360_L_MAJOR_VS_B_MALAYI_HIGH_DOSE_DC_DN	39	181	16072	2190	1.599099	0.001792
brown	10076	GSE3982_BASOPHIL_VS_NKCELL_UP	39	181	16072	2190	1.599099	0.001792
turquoise	9902	GSE36476_CTRL_VS_TSST_ACT_16H_MEMORY_CD4_T(	69	196	16057	4169	1.372444	0.001792
magenta	5811	LIAO_METASTASIS	13	494	15759	167	2.561141	0.001795
lightyellow	4909	CASORELLI_ACUTE_PROMYELOCYTIC_LEUKEMIA_DN	6	653	15600	33	4.525407	0.0018
cyan	9993	GSE3982_MAST_CELL_VS_DC_DN	5	184	16069	77	5.735813	0.001801
blue	9226	GSE18791_CTRL_VS_NEWCASTLE_VIRUS_DC_8H_UP	53	197	16056	2978	1.468312	0.001803
blue	9234	GSE18791_CTRL_VS_NEWCASTLE_VIRUS_DC_16H_UP	53	197	16056	2978	1.468312	0.001803
cyan	5288	ROYLANCE_BREAST_CANCER_16Q_COPY_NUMBER_UP	3	51	16202	77	12.41635	0.001808
turquoise	4429	REACTOME_METABOLISM_OF_PROTEINS	127	394	15859	4169	1.256635	0.001814
lightyellow	5907	BENPORATH_ES_WITH_H3K27ME3	6	654	15599	33	4.518488	0.001814
red	9007	GSE17721_POLYIC_VS_CPG_2H_BMDM_DN	14	177	16076	524	2.453336	0.001814
blue	7028	MCCABE_HOXC6_TARGETS_CANCER_UP	11	24	16229	2978	2.501441	0.001816
red	4995	GARGALOVIC_RESPONSE_TO_OXIDIZED_PHOSPHOLIPID	5	28	16225	524	5.538781	0.001816
red	4176	REACTOME_BOTULINUM_NEUROTOXICITY	4	17	16236	524	7.298159	0.001819
red	6487	ZHANG_RESPONSE_TO_CANTHARIDIN_UP	4	17	16236	524	7.298159	0.001819
brown	3406	chr12q13	40	187	16066	2190	1.587478	0.00182
brown	10213	GSE8384_CTRL_VS_B_ABORTUS_4H_MAC_CELL_LINE_I	40	187	16066	2190	1.587478	0.00182
black	4912	HUTTMANN_B_CLL_POOR_SURVIVAL_UP	12	256	15997	283	2.692083	0.001821
salmon	6172	FERNANDEZ_BOUND_BY_MYC	6	164	16089	127	4.682063	0.001823
yellow	2729	KEGG_OXIDATIVE_PHOSPHORYLATION	19	105	16148	1426	2.062426	0.001824
magenta	7872	HOELZEL_NF1_TARGETS_DN	5	85	16168	167	5.724903	0.001825
magenta	10113	GSE39820_IL1B_IL6_VS_IL1B_IL6_IL23A_TREATED_CD4	7	169	16084	167	4.031145	0.001827
brown	693	PROTEIN_SECRETION	10	27	16226	2190	2.748689	0.001832
brown	7258	DAVIES_MULTIPLE_MYELOMA_VS_MGUS_DN	10	27	16226	2190	2.748689	0.001832
purple	5126	GRAHAM_CML_DIVIDING_VS_NORMAL_QUIESCENT_U	7	180	16073	157	4.025867	0.00184
brown	9422	GSE24142_EARLY_THYMIC_PROGENITOR_VS_DN3_THY	41	193	16060	2190	1.57658	0.001843
brown	9804	GSE360_CTRL_VS_L_DONOVANI_MAC_UP	41	193	16060	2190	1.57658	0.001843
brown	10109	GSE39820_CTRL_VS_TGFBETA3_IL6_CD4_TCELL_DN	41	193	16060	2190	1.57658	0.001843
brown	10122	GSE5463_CTRL_VS_DEXAMETHASONE_TREATED_THYM	41	193	16060	2190	1.57658	0.001843
cyan	9044	GSE17721_LPS_VS_CPG_4H_BMDM_UP	5	185	16068	77	5.704809	0.001844
cyan	10160	GSE7460_CD8_TCELL_VS_CD4_TCELL_ACT_UP	5	185	16068	77	5.704809	0.001844
magenta	3937	PID_INTEGRIN1_PATHWAY	4	51	16202	167	7.633204	0.001845
red	5217	MARKEY_RB1_ACUTE_LOF_UP	15	197	16056	524	2.361714	0.001845
salmon	7164	BHATI_G2M_ARREST_BY_2METHOXYESTRADIOL_DN	5	112	16141	127	5.713231	0.001854
lightcyan	7858	HIRSCH_CELLULAR_TRANSFORMATION_SIGNATURE_UI	5	231	16022	62	5.674138	0.00186
black	9345	GSE22886_IGM_MEMORY_BCELL_VS_BM_PLASMA_CE	10	190	16063	283	3.022689	0.00186
turquoise	9017	GSE17721_POLYIC_VS_CPG_16H_BMDM_DN	65	183	16070	4169	1.384726	0.001861

turquoise	5912	BENPORATH_PROLIFERATION	54	147	16106	4169	1.432116	0.001871
turquoise	8214	CSR_EARLY_UP.V1_UP	54	147	16106	4169	1.432116	0.001871
greenyello	8617	GSE14000_UNSTIM_VS_16H_LPS_DC_TRANSLATED_RN	7	189	16064	150	4.013086	0.001872
greenyello	10153	GSE7460_TCONV_VS_TREG_THYMUS_DN	7	189	16064	150	4.013086	0.001872
lightcyan	7459	CROONQUIST_IL6_DEPRIVATION_UP	2	17	16236	62	30.84061	0.001877
turquoise	4745	REACTOME_ADAPTIVE_IMMUNE_SYSTEM	156	496	15757	4169	1.226153	0.001879
turquoise	8501	GSE12366_PLASMA_CELL_VS_MEMORY_BCELL_DN	68	193	16060	4169	1.373578	0.00188
turquoise	10049	GSE3982_NEUTROPHIL_VS_BASOPHIL_DN	68	193	16060	4169	1.373578	0.00188
blue	4325	REACTOME_TRANSPORT_OF_MATURE_TRANSCRIPT_TO	19	53	16200	2978	1.95653	0.001882
turquoise	5086	ZHOU_INFLAMMATORY_RESPONSE_FIMA_DN	85	250	16003	4169	1.325503	0.001882
lightgreen	5326	KAN_RESPONSE_TO_ARSENIC_TRIOXIDE	3	95	16158	42	12.2203	0.001883
turquoise	2350	MORF_AP2M1	74	213	16040	4169	1.354421	0.001883
greenyello	4095	PID_MYC_REPRESSPATHWAY	4	57	16196	150	7.603743	0.001883
magenta	6626	MCLACHLAN_DENTAL_CARIES_DN	8	218	16035	167	3.571499	0.001886
purple	1106	PHOSPHORUS_OXYGEN_LYASE_ACTIVITY	2	7	16246	157	29.5778	0.001886
purple	5583	TERAMOTO_OPN_TARGETS_CLUSTER_8	2	7	16246	157	29.5778	0.001886
purple	5635	OXFORD_RALB_TARGETS_UP	2	7	16246	157	29.5778	0.001886
yellow	3636	MODULE_151	43	311	15942	1426	1.575876	0.001886
pink	3692	MODULE_212	11	277	15976	228	2.830816	0.001889
turquoise	68	ENDOPLASMIC_RETICULUM	90	267	15986	4169	1.314114	0.001894
lightcyan	6158	BROCKE_APOPTOSIS_REVERSED_BY_IL6	4	138	16115	62	7.59841	0.001896
purple	6364	YAGI_AML_FAB_MARKERS	7	181	16072	157	4.003625	0.001899
salmon	5847	RICKMAN_TUMOR_DIFFERENTIATED_WELL_VS_POORL	7	224	16029	127	3.999262	0.001899
brown	5792	BUYTAERT_PHOTODYNAMIC_THERAPY_STRESS_UP	133	778	15475	2190	1.268707	0.0019
brown	7648	HOSHIDA_LIVER_CANCER_SUBCLASS_S1	46	223	16030	2190	1.530884	0.001902
grey60	631	AMINE_CATABOLIC_PROCESS	2	24	16229	44	30.7822	0.001903
brown	6342	XU_RESPONSE_TO_TRETINOIN_UP	7	15	16238	2190	3.463349	0.001907
blue	7709	CHANDRAN_METASTASIS_UP	54	202	16051	2978	1.458986	0.001914
greenyello	424	VIRAL_INFECTIOUS_CYCLE	3	27	16226	150	12.03926	0.001916
greenyello	2688	GNF2_RAB3A	3	27	16226	150	12.03926	0.001916
pink	6550	RODWELL_AGING_KIDNEY_DN	7	125	16128	228	3.991965	0.001918
yellow	6652	CUI_TCF21_TARGETS_2_UP	48	357	15896	1426	1.532453	0.001921
salmon	6109	NING_CHRONIC_OBSTRUCTIVE_PULMONARY_DISEASE_	5	113	16140	127	5.662672	0.001928
cyan	9865	GSE360_L_DONOVANI_VS_B_MALAYI_HIGH_DOSE_MA	5	187	16066	77	5.643795	0.001933
red	6285	NEMETH_INFLAMMATORY_RESPONSE_LPS_UP	9	87	16166	524	3.208673	0.001935
red	8447	GSE11057_NAIVE_VS_CENT_MEMORY_CD4_TCELL_DN	15	198	16055	524	2.349786	0.001939
red	9367	GSE22886_NAIVE_BCELL_VS_DC_DN	15	198	16055	524	2.349786	0.001939
cyan	213	PML_BODY	2	14	16239	77	30.15399	0.001943
cyan	759	DNA_DAMAGE_RESPONSESIGNAL_TRANSDUCTION_RES	2	14	16239	77	30.15399	0.001943
cyan	3547	MODULE_58	2	14	16239	77	30.15399	0.001943
cyan	6586	RUAN_RESPONSE_TO_TNF_TROGLITAZONE_UP	2	14	16239	77	30.15399	0.001943
lightcyan	3758	MODULE_301	4	139	16114	62	7.543746	0.001946
lightcyan	7705	NAKAYAMA_SOFT_TISSUE_TUMORS_PCA1_UP	3	65	16188	62	12.09901	0.001951
blue	10134	GSE6269_FLU_VS_E_COLI_INF_PBMC_UP	45	162	16091	2978	1.516025	0.001953
lightyellow	6283	BROWN_MYELOID_CELL_DEVELOPMENT_DN	3	123	16130	33	12.01256	0.001955
turquoise	1168	UDP_GLYCOSYLTRANSFERASE_ACTIVITY	17	34	16219	4169	1.949268	0.001958
brown	8786	GSE15930_NAIVE_VS_48H_IN_VITRO_STIM_IFNAB_CD	38	176	16077	2190	1.602361	0.001959
brown	10085	GSE3982_EFF_MEMORY_CD4_TCELL_VS_NKCELL_DN	38	176	16077	2190	1.602361	0.001959
cyan	8298	BMI1_DN.V1_UP	4	112	16141	77	7.538497	0.001964
magenta	6936	HELLER_HDAC_TARGETS_SILENCED_BY_METHYLATION_	11	381	15872	167	2.809861	0.001969
turquoise	9691	GSE29618_PRE_VS_DAY7_POST_LAIV_FLU_VACCINE_M	67	190	16063	4169	1.374747	0.001971
turquoise	5203	LINDGREN_BLADDER_CANCER_CLUSTER_1_UP	43	112	16141	4169	1.49676	0.001977
cyan	9733	GSE31082_DN_VS_CD4_SP_THYMOCYTE_DN	5	188	16065	77	5.613775	0.001979
turquoise	9734	GSE31082_DN_VS_CD8_SP_THYMOCYTE_UP	70	200	16053	4169	1.364488	0.00198
pink	731	HOMEOSTATIC_PROCESS	8	161	16092	228	3.542116	0.001984
greenyello	8675	GSE14350_IL2RB_KO_VS_WT_TREG_DN	7	191	16062	150	3.971065	0.001987
greenyello	9721	GSE30083_SP3_VS_SP4_THYMOCYTE_DN	7	191	16062	150	3.971065	0.001987
greenyello	9726	GSE30962_ACUTE_VS_CHRONIC_LCMV_PRIMARY_INF_	7	191	16062	150	3.971065	0.001987
greenyello	10207	GSE7852_LN_VS_FAT_TCONV_DN	7	191	16062	150	3.971065	0.001987
blue	9947	GSE37416_12H_VS_48H_F_TULARENSIS_LVS_NEUTROI	47	171	16082	2978	1.500067	0.001991

purple	1989	GTGGGTGK_UNKNOWN	8	234	16019	157	3.539224	0.001993
yellow	6940	WONG_MITOCHONDRIA_GENE_MODULE	32	214	16039	1426	1.704316	0.001994
blue	2468	MORF_SNRP70	21	61	16192	2978	1.878877	0.001995
brown	2860	KEGG_GNRH_SIGNALING_PATHWAY	21	81	16172	2190	1.924083	0.001995
magenta	5948	SHEN_SMARCA2_TARGETS_DN	8	220	16033	167	3.539031	0.001997
pink	6361	VERHAAK_AML_WITH_NPM1_MUTATED_DN	10	238	16015	228	2.995172	0.001997
cyan	3692	MODULE_212	6	277	15976	77	4.572085	0.002
brown	8313	RB_DN.V1_DN	26	108	16145	2190	1.786648	0.002005
greenyello	2230	YWATTWNNRGCT_UNKNOWN	4	58	16195	150	7.472644	0.002009
greenyello	2637	GNF2_DNM1	4	58	16195	150	7.472644	0.002009
brown	1996	GTGCCTT,MIR-506	114	654	15599	2190	1.293649	0.002011
yellow	2391	MORF_FBL	23	138	16115	1426	1.899603	0.002015
blue	6875	SANSOM_APC_TARGETS	49	180	16073	2978	1.485704	0.002016
brown	8940	GSE17721_POLYIC_VS_PAM3CSK4_8H_BMDM_UP	40	188	16065	2190	1.579034	0.002017
brown	9770	GSE339_CD4POS_VS_CD4CD8DN_DC_UP	40	188	16065	2190	1.579034	0.002017
magenta	8980	GSE17721_CPG_VS_GARDIQUIMOD_16H_BMDM_UP	7	172	16081	167	3.960834	0.002019
turquoise	619	POST_TRANSLATIONAL_PROTEIN_MODIFICATION	137	430	15823	4169	1.242092	0.002021
magenta	8376	KRAS.LUNG.BREAST_UP.V1_DN	5	87	16166	167	5.593296	0.002022
pink	4498	REACTOME_TRANSPORT_OF_GLUCOSE_AND_OTHER_S	5	64	16189	228	5.569147	0.002023
cyan	8717	GSE14769_UNSTIM_VS_40MIN_LPS_BMDM_DN	5	189	16064	77	5.584072	0.002025
red	4831	SCHUETZ_BREAST_CANCER_DUCTAL_INVASIVE_UP	20	303	15950	524	2.047338	0.002028
brown	5784	NUYTTEN_EZH2_TARGETS_UP	158	946	15307	2190	1.239525	0.002029
blue	8550	GSE13411_NAIVE_BCELL_VS_PLASMA_CELL_UP	51	189	16064	2978	1.47271	0.002029
brown	536	REGULATION_OF_RAS_PROTEIN_SIGNAL_TRANSDUCTI(	8	19	16234	2190	3.124826	0.002032
brown	2967	BIOCARTA_EPO_PATHWAY	8	19	16234	2190	3.124826	0.002032
brown	3071	BIOCARTA_MAL_PATHWAY	8	19	16234	2190	3.124826	0.002032
blue	8519	GSE12845_NAIVE_VS_PRE_GC_TONSIL_BCELL_DN	53	198	16055	2978	1.460897	0.002032
lightcyan	7733	YAO_TEMPORAL_RESPONSE_TO_PROGESTERONE_CLUS	3	66	16187	62	11.91569	0.002039
lightcyan	8110	PEDRIOLI_MIR31_TARGETS_DN	6	347	15906	62	4.532769	0.002039
brown	8874	GSE17721_CTRL_VS_PAM3CSK4_8H_BMDM_UP	41	194	16059	2190	1.568453	0.00204
brown	9570	GSE27786_BCELL_VS_ERYTHROBLAST_UP	41	194	16059	2190	1.568453	0.00204
yellow	9919	GSE36476_YOUNG_VS_OLD_DONOR_MEMORY_CD4_T	30	197	16056	1426	1.735678	0.00204
turquoise	8898	GSE17721_CTRL_VS_GARDIQUIMOD_1H_BMDM_UP	63	177	16076	4169	1.387615	0.002044
turquoise	8996	GSE17721_LPS_VS_PAM3CSK4_12H_BMDM_UP	63	177	16076	4169	1.387615	0.002044
salmon	6695	JIANG_HYPOXIA_NORMAL	8	291	15962	127	3.518251	0.002046
greenyello	9262	GSE20366_EX_VIVO_VS_DEC205_CONVERSION_UP	7	192	16061	150	3.950382	0.002047
lightyellow	5148	GOZGIT_ESR1_TARGETS_UP	3	125	16128	33	11.82036	0.002047
red	5863	SCHAEFFER_PROSTATE_DEVELOPMENT_48HR_DN	21	325	15928	524	2.004187	0.002053
grey60	4482	REACTOME_GPCR_DOWNSTREAM_SIGNALING	5	336	15917	44	5.496821	0.002055
turquoise	8107	FORTSCHEGGER_PHF8_TARGETS_DN	218	718	15535	4169	1.183678	0.002057
turquoise	4515	REACTOME_CDT1_ASSOCIATION_WITH_THE_CDC6_OR	24	54	16199	4169	1.732683	0.00207
purple	785	SECOND_MESSENGER_MEDIATED_SIGNALING	5	93	16160	157	5.565715	0.002071
cyan	8476	GSE11864_CSF1_IFNG_VS_CSF1_IFNG_PAM3CYS_IN_M	5	190	16063	77	5.554682	0.002072
turquoise	10036	GSE3982_MAC_VS_EFF_MEMORY_CD4_TCELL_UP	69	197	16056	4169	1.365477	0.002078
yellow	46	MITOCHONDRIAL_MEMBRANE	16	83	16170	1426	2.197131	0.002079
salmon	5155	HAHTOLA_MYCOSIS_FUNGOIDES_CD4_DN	5	115	16138	127	5.56419	0.002082
purple	1758	CTGRYYYNATT_UNKNOWN	4	56	16197	157	7.394449	0.002083
yellow	5500	LUI_TARGETS_OF_PAX8_PPARG_FUSION	9	34	16219	1426	3.017016	0.002092
lightyellow	7914	WIERENGA_STAT5A_TARGETS_GROUP1	3	126	16127	33	11.72655	0.002094
lightcyan	6440	MARCHINI_TRABECTEDIN_RESISTANCE_UP	2	18	16235	62	29.12724	0.002106
pink	7574	MIKKELSEN_IPS_WITH_HCP_H3K27ME3	4	39	16214	228	7.311291	0.002111
turquoise	2111	TACAATC,MIR-508	25	57	16196	4169	1.709885	0.002112
turquoise	4440	REACTOME_G1_S_TRANSITION	41	106	16147	4169	1.507925	0.002112
red	8100	ALFANO_MYC_TARGETS	16	220	16033	524	2.255795	0.002116
blue	1433	DNA_BINDING	119	509	15744	2978	1.275963	0.002116
cyan	9129	GSE17721_0.5H_VS_4H_CPG_BMDM_DN	5	191	16062	77	5.5256	0.00212
red	8260	MEK_UP.V1_UP	14	180	16073	524	2.412447	0.002123
turquoise	3497	MODULE_4	12	21	16232	4169	2.227735	0.002129
turquoise	7077	BONOME_OVARIAN_CANCER_POOR_SURVIVAL_DN	12	21	16232	4169	2.227735	0.002129
magenta	7704	WONG_ADULT_TISSUE_STEM_MODULE	15	629	15624	167	2.320907	0.00213

magenta	291	SYNAPTOGENESIS	2	7	16246	167	27.80667	0.00213
grey60	951	G_PROTEIN_COUPLED_RECEPTOR_PROTEIN_SIGNALIN(	4	202	16051	44	7.314581	0.002131
red	292	JAK_STAT_CASCADE	5	29	16224	524	5.347789	0.002137
brown	5395	PEREZ_TP53_AND_TP63_TARGETS	37	171	16082	2190	1.605813	0.002141
salmon	6425	JIANG_AGING_CEREBRAL_CORTEX_UP	3	33	16220	127	11.63422	0.002142
salmon	6477	HU_GENOTOXIC_DAMAGE_24HR	3	33	16220	127	11.63422	0.002142
yellow	9152	GSE17974_0H_VS_1H_IN_VITRO_ACT_CD4_TCELL_UP	27	172	16081	1426	1.789161	0.002143
purple	1874	V$GATA_Q6	6	137	16116	157	4.533823	0.002146
purple	6633	RODWELL_AGING_KIDNEY_NO_BLOOD_UP	7	185	16068	157	3.91706	0.002149
greenyello	549	CELL_CELL_SIGNALING	8	248	16005	150	3.495269	0.00215
yellow	1921	V$E2F1_Q3_01	32	215	16038	1426	1.696389	0.00215
turquoise	4140	REACTOME_SIGNALING_BY_WNT	27	63	16190	4169	1.670801	0.002157
blue	9436	GSE24634_NAIVE_CD4_TCELL_VS_DAY7_IL4_CONV_TR	52	194	16059	2978	1.462886	0.002157
turquoise	1152	TRANSFERASE_ACTIVITY_TRANSFERRING_HEXOSYL_GR	29	69	16184	4169	1.638515	0.002158
turquoise	2523	GCM_HBP1	29	69	16184	4169	1.638515	0.002158
turquoise	3440	chr15q21	29	69	16184	4169	1.638515	0.002158
turquoise	4658	REACTOME_NFKB_AND_MAP_KINASES_ACTIVATION_M	29	69	16184	4169	1.638515	0.002158
turquoise	4077	PID_P38ALPHABETADOWNSTREAMPATHWAY	18	37	16216	4169	1.896585	0.002158
turquoise	8176	GHANDHI_BYSTANDER_IRRADIATION_UP	28	66	16187	4169	1.653925	0.002162
green	1242	NEUROTRANSMITTER_BINDING	7	24	16229	1305	3.632535	0.002167
turquoise	10252	GSE9037_WT_VS_IRAK4_KO_LPS_4H_STIM_BMDM_UF	65	184	16069	4169	1.377201	0.002168
cyan	8687	GSE1460_INTRATHYMIC_T_PROGENITOR_VS_CD4_THY	5	192	16061	77	5.496821	0.002169
cyan	9791	GSE34205_RSV_VS_FLU_INF_INFANT_PBMC_DN	5	192	16061	77	5.496821	0.002169
green	1278	G_PROTEIN_COUPLED_RECEPTOR_ACTIVITY	17	99	16154	1305	2.138635	0.00217
greenyello	2806	KEGG_CYTOKINE_CYTOKINE_RECEPTOR_INTERACTION	7	194	16059	150	3.909656	0.00217
midnightb	4788	REACTOME_SYNTHESIS_OF_GLYCOSYLPHOSPHATIDYLIN	2	16	16237	71	28.61444	0.00217
brown	9291	GSE20715_0H_VS_24H_OZONE_LUNG_DN	38	177	16076	2190	1.593308	0.002177
turquoise	8945	GSE17721_POLYIC_VS_PAM3CSK4_16H_BMDM_DN	68	194	16059	4169	1.366497	0.00218
turquoise	8997	GSE17721_LPS_VS_PAM3CSK4_12H_BMDM_DN	68	194	16059	4169	1.366497	0.00218
turquoise	9470	GSE25087_TREG_VS_TCONV_ADULT_UP	68	194	16059	4169	1.366497	0.00218
turquoise	9941	GSE37416_0H_VS_24H_F_TULARENSIS_LVS_NEUTROPI	68	194	16059	4169	1.366497	0.00218
purple	282	G_PROTEIN_SIGNALING_COUPLED_TO_IP3_SECOND_M	3	27	16226	157	11.50248	0.002182
green	7439	MEISSNER_NPC_HCP_WITH_H3_UNMETHYLATED	48	392	15861	1305	1.525029	0.00219
lightgreen	1233	CHEMOKINE_ACTIVITY	2	27	16226	42	28.6649	0.002196
brown	2875	KEGG_VIBRIO_CHOLERAE_INFECTION	14	46	16207	2190	2.258706	0.002199
brown	985	REGULATION_OF_APOPTOSIS	60	310	15943	2190	1.436412	0.002199
lightcyan	6320	PETROVA_ENDOTHELIUM_LYMPHATIC_VS_BLOOD_DN	4	144	16109	62	7.28181	0.002214
lightcyan	2686	GNF2_PTPRC	3	68	16185	62	11.56523	0.002221
purple	2888	KEGG_MELANOMA	4	57	16196	157	7.264722	0.002224
purple	5787	INGRAM_SHH_TARGETS_DN	4	57	16196	157	7.264722	0.002224
purple	6678	BURTON_ADIPOGENESIS_PEAK_AT_0HR	4	57	16196	157	7.264722	0.002224
blue	2351	MORF_AP3D1	37	128	16125	2978	1.577613	0.002233
brown	8553	GSE13411_NAIVE_VS_MEMORY_BCELL_DN	40	189	16064	2190	1.57068	0.002233
grey60	1045	NITROGEN_COMPOUND_CATABOLIC_PROCESS	2	26	16227	44	28.41434	0.002234
red	8479	GSE11864_CSF1_PAM3CYS_VS_CSF1_IFNG_PAM3CYS_	14	181	16072	524	2.399119	0.002235
red	8569	GSE13484_12H_UNSTIM_VS_YF17D_VACCINE_STIM_PI	14	181	16072	524	2.399119	0.002235
cyan	3618	MODULE_130	2	15	16238	77	28.14372	0.002235
cyan	3708	MODULE_236	2	15	16238	77	28.14372	0.002235
pink	188	CORTICAL_CYTOSKELETON	3	19	16234	228	11.25554	0.002236
pink	5156	HAHTOLA_MYCOSIS_FUNGOIDES_UP	3	19	16234	228	11.25554	0.002236
lightgreen	8129	BOSCO_TH1_CYTOTOXIC_MODULE	3	101	16152	42	11.49434	0.002243
cyan	6490	CHANG_IMMORTALIZED_BY_HPV31_DN	3	55	16198	77	11.51334	0.002247
magenta	8277	PKCA_DN.V1_DN	6	130	16123	167	4.491847	0.002248
blue	703	REGULATION_OF_TRANSCRIPTIONDNA_DEPENDENT	97	404	15849	2978	1.310386	0.002248
lightgreen	459	DEFENSE_RESPONSE	4	215	16038	42	7.199557	0.002248
salmon	9342	GSE22886_IGM_MEMORY_BCELL_VS_BLOOD_PLASMA	6	171	16082	127	4.490399	0.00225
brown	7397	LEE_DIFFERENTIATING_T_LYMPHOCYTE	41	195	16058	2190	1.56041	0.002254
brown	8890	GSE17721_CTRL_VS_CPG_8H_BMDM_UP	41	195	16058	2190	1.56041	0.002254
lightcyan	4827	ONKEN_UVEAL_MELANOMA_UP	9	761	15492	62	3.100271	0.002257
yellow	2417	MORF_MAP2K2	22	131	16122	1426	1.914103	0.00226

brown	4493	REACTOME_SEMAPHORIN_INTERACTIONS	17	61	16192	2190	2.068276	0.002266
red	3616	MODULE_128	9	89	16164	524	3.136568	0.002266
cyan	9207	GSE17974_1H_VS_72H_UNTREATED_IN_VITRO_CD4_T	5	194	16059	77	5.440153	0.002269
cyan	9553	GSE27786_LIN_NEG_VS_NKCELL_DN	5	194	16059	77	5.440153	0.002269
lightcyan	3710	MODULE_238	4	145	16108	62	7.231591	0.00227
lightcyan	7580	MIKKELSEN_IPS_LCP_WITH_H3K4ME3	4	145	16108	62	7.231591	0.00227
turquoise	9119	GSE17721_0.5H_VS_8H_PAM3CSK4_BMDM_DN	64	181	16072	4169	1.378488	0.002274
purple	6856	GAVIN_FOXP3_TARGETS_CLUSTER_P4	5	95	16158	157	5.448542	0.002274
red	5895	AMIT_DELAYED_EARLY_GENES	4	18	16235	524	6.892706	0.00228
brown	5601	DIRMEIER_LMP1_RESPONSE_LATE_UP	16	56	16197	2190	2.120417	0.002282
purple	9414	GSE24142_EARLY_THYMIC_PROGENITOR_VS_DN2_THY	7	187	16066	157	3.875166	0.002283
greenyello	6284	ROETH_TERT_TARGETS_DN	2	8	16245	150	27.08833	0.002284
midnightb	5942	AMIT_SERUM_RESPONSE_120_MCF10A	3	60	16193	71	11.44577	0.002287
turquoise	2291	MORF_ATRX	67	191	16062	4169	1.36755	0.002288
turquoise	8470	GSE11864_CSF1_VS_CSF1_PAM3CYS_IN_MAC_UP	67	191	16062	4169	1.36755	0.002288
turquoise	8959	GSE17721_PAM3CSK4_VS_CPG_8H_BMDM_DN	67	191	16062	4169	1.36755	0.002288
turquoise	9899	GSE36392_EOSINOPHIL_VS_NEUTROPHIL_IL25_TREATE	67	191	16062	4169	1.36755	0.002288
lightyellow	5015	TAKEDA_TARGETS_OF_NUP98_HOXA9_FUSION_10D_D	3	130	16123	33	11.36573	0.002289
blue	8653	GSE14308_NAIVE_CD4_TCELL_VS_INDUCED_TREG_DN	51	190	16063	2978	1.464959	0.002289
blue	9971	GSE3982_EOSINOPHIL_VS_MAST_CELL_DN	51	190	16063	2978	1.464959	0.002289
turquoise	7870	LU_EZH2_TARGETS_DN	118	365	15888	4169	1.260349	0.0023
blue	2594	GNF2_MSH2	12	28	16225	2978	2.33901	0.002302
yellow	9514	GSE2706_R848_VS_R848_AND_LPS_2H_STIM_DC_UP	25	156	16097	1426	1.826541	0.002305
green	6770	MAHAJAN_RESPONSE_TO_IL1A_UP	13	67	16186	1305	2.416527	0.002305
pink	4933	DOANE_RESPONSE_TO_ANDROGEN_UP	8	165	16088	228	3.456247	0.002313
yellow	7983	KASLER_HDAC7_TARGETS_1_UP	29	190	16063	1426	1.739636	0.002317
pink	5382	RIZ_ERYTHROID_DIFFERENTIATION_CCNE1	4	40	16213	228	7.128509	0.00232
cyan	8590	GSE13485_DAY3_VS_DAY21_YF17D_VACCINE_PBMC_U	5	195	16058	77	5.412254	0.00232
cyan	9449	GSE24634_TEFF_VS_TCONV_DAY3_IN_CULTURE_DN	5	195	16058	77	5.412254	0.00232
cyan	10052	GSE3982_NEUTROPHIL_VS_CENT_MEMORY_CD4_TCEL	5	195	16058	77	5.412254	0.00232
red	5733	SPIELMAN_LYMPHOBLAST_EUROPEAN_VS_ASIAN_DN	32	581	15672	524	1.708347	0.002322
blue	2481	MORF_USP5	18	50	16203	2978	1.964768	0.002324
turquoise	6028	TCGA_GLIOBLASTOMA_COPY_NUMBER_DN	15	29	16224	4169	2.016485	0.002331
brown	9862	GSE360_L_DONOVANI_VS_T_GONDII_MAC_UP	36	166	16087	2190	1.609474	0.00234
lightcyan	6700	SARTIPY_BLUNTED_BY_INSULIN_RESISTANCE_UP	2	19	16234	62	27.59423	0.002348
lightcyan	7526	CHIANG_LIVER_CANCER_SUBCLASS_INTERFERON_UP	2	19	16234	62	27.59423	0.002348
turquoise	8498	GSE12366_PLASMA_CELL_VS_NAIVE_BCELL_UP	60	168	16085	4169	1.392335	0.002351
turquoise	10164	GSE7460_TREG_VS_TCONV_ACT_WITH_TGFB_UP	60	168	16085	4169	1.392335	0.002351
red	10078	GSE3982_BASOPHIL_VS_TH1_UP	14	182	16071	524	2.385937	0.002351
blue	2553	GCM_RING1	27	86	16167	2978	1.713461	0.002356
greenyello	3597	MODULE_109	3	29	16224	150	11.20897	0.002361
lightgreen	1268	CHEMOKINE_RECEPTOR_BINDING	2	28	16225	42	27.64116	0.002361
lightgreen	7480	VALK_AML_CLUSTER_3	2	28	16225	42	27.64116	0.002361
greenyello	9185	GSE17974_CTRL_VS_ACT_IL4_AND_ANTI_IL12_72H_CD	7	197	16056	150	3.850118	0.002366
brown	5190	CHIARADONNA_NEOPLASTIC_TRANSFORMATION_CDC2	32	143	16110	2190	1.660747	0.002366
greenyello	3503	MODULE_11	11	435	15818	150	2.739969	0.00237
tan	4323	REACTOME_GENERIC_TRANSCRIPTION_PATHWAY	9	321	15932	145	3.142701	0.002371
salmon	2345	MORF_XRCC5	7	233	16020	127	3.844784	0.002371
pink	1672	V$GATA1_05	9	204	16049	228	3.14493	0.002391
yellow	39	SPINDLE_POLE	6	17	16236	1426	4.022688	0.002392
purple	8303	NOTCH_DN.V1_DN	6	140	16113	157	4.43667	0.002393
turquoise	8940	GSE17721_POLYIC_VS_PAM3CSK4_8H_BMDM_UP	66	188	16065	4169	1.368635	0.002401
magenta	8188	ZWANG_TRANSIENTLY_UP_BY_2ND_EGF_PULSE_ONLY	21	1044	15209	167	1.957654	0.002402
turquoise	6881	RIGGINS_TAMOXIFEN_RESISTANCE_DN	69	198	16055	4169	1.358581	0.002402
turquoise	8519	GSE12845_NAIVE_VS_PRE_GC_TONSIL_BCELL_DN	69	198	16055	4169	1.358581	0.002402
turquoise	4578	REACTOME_POST_TRANSLATIONAL_PROTEIN_MODIFIC	56	155	16098	4169	1.408504	0.002404
blue	4942	LAIHO_COLORECTAL_CANCER_SERRATED_DN	26	82	16171	2978	1.730487	0.002409
turquoise	2387	MORF_ERH	43	113	16140	4169	1.483514	0.002412
turquoise	4985	GINESTIER_BREAST_CANCER_20Q13_AMPLIFICATION_U	43	113	16140	4169	1.483514	0.002412
lightyellow	6404	TRAYNOR_RETT_SYNDROM_UP	2	36	16217	33	27.36195	0.002412

lightcyan	7294	SEKI_INFLAMMATORY_RESPONSE_LPS_UP	3	70	16183	62	11.23479	0.002413
red	274	HEMOSTASIS	6	43	16210	524	4.327978	0.002414
red	1422	CYTOKINE_BINDING	6	43	16210	524	4.327978	0.002414
red	6191	LEE_LIVER_CANCER_MYC_DN	6	43	16210	524	4.327978	0.002414
red	4928	CHARAFE_BREAST_CANCER_LUMINAL_VS_MESENCHYM	25	419	15834	524	1.850667	0.002415
brown	9884	GSE360_HIGH_VS_LOW_DOSE_B_MALAYI_MAC_UP	38	178	16075	2190	1.584357	0.002417
turquoise	5207	LINDGREN_BLADDER_CANCER_CLUSTER_3_UP	104	317	15936	4169	1.279015	0.002418
purple	9410	GSE24142_EARLY_THYMIC_PROGENITOR_VS_DN3_THY	7	189	16064	157	3.834159	0.002423
red	7648	HOSHIDA_LIVER_CANCER_SUBCLASS_S1	16	223	16030	524	2.225448	0.002425
magenta	6083	PENG_LEUCINE_DEPRIVATION_UP	6	132	16121	167	4.423789	0.002427
greenyello	8447	GSE11057_NAIVE_VS_CENT_MEMORY_CD4_TCELL_DN	7	198	16055	150	3.830673	0.002434
lightgreen	3865	MODULE_456	3	104	16149	42	11.16277	0.002438
magenta	6965	MASSARWEH_RESPONSE_TO_ESTRADIOL	4	55	16198	167	7.078062	0.002439
blue	5238	GRAESSMANN_APOPTOSIS_BY_DOXORUBICIN_DN	356	1705	14548	2978	1.139553	0.00244
lightcyan	8220	AKT_UP.V1_UP	4	148	16105	62	7.085004	0.002445
brown	8948	GSE17721_PAM3CSK4_VS_CPG_0.5H_BMDM_UP	39	184	16069	2190	1.573027	0.002446
brown	9082	GSE17721_LPS_VS_GARDIQUIMOD_6H_BMDM_UP	39	184	16069	2190	1.573027	0.002446
brown	9735	GSE31082_DN_VS_CD8_SP_THYMOCYTE_DN	39	184	16069	2190	1.573027	0.002446
brown	10070	GSE3982_BCELL_VS_TH2_UP	39	184	16069	2190	1.573027	0.002446
lightyellow	3605	MODULE_117	5	472	15781	33	5.217322	0.002451
magenta	7171	RAY_TUMORIGENESIS_BY_ERBB2_CDC25A_UP	5	91	16162	167	5.347437	0.002463
green	7699	DORN_ADENOVIRUS_INFECTION_48HR_UP	5	13	16240	1305	4.790156	0.002466
brown	5723	TOMLINS_PROSTATE_CANCER_DN	12	37	16216	2190	2.40696	0.002469
turquoise	1972	CAGTGTT,MIR-141,MIR-200A	92	276	15977	4169	1.299512	0.002476
cyan	6881	RIGGINS_TAMOXIFEN_RESISTANCE_DN	5	198	16055	77	5.330251	0.002479
cyan	9357	GSE22886_DC_VS_MONOCYTE_DN	5	198	16055	77	5.330251	0.002479
brown	3161	chr12q24	41	196	16057	2190	1.552449	0.002487
brown	9606	GSE27786_NKTCELL_VS_ERYTHROBLAST_UP	41	196	16057	2190	1.552449	0.002487
blue	2562	GCM_UBE2N	43	155	16098	2978	1.514069	0.002491
blue	5504	SCHLOSSER_MYC_TARGETS_REPRESSED_BY_SERUM	43	155	16098	2978	1.514069	0.002491
magenta	3698	MODULE_220	8	228	16025	167	3.414855	0.002492
red	7878	DUTERTRE_ESTRADIOL_RESPONSE_24HR_DN	27	466	15787	524	1.797132	0.002493
red	3730	MODULE_263	5	30	16223	524	5.169529	0.002498
purple	1436	CYCLASE_ACTIVITY	2	8	16245	157	25.88057	0.002499
purple	4810	LU_TUMOR_VASCULATURE_DN	2	8	16245	157	25.88057	0.002499
brown	1852	V$SREBP1_Q6	42	202	16051	2190	1.543076	0.0025
blue	6449	RHODES_CANCER_META_SIGNATURE	21	62	16191	2978	1.848572	0.002504
brown	8015	TERAO_AOX4_TARGETS_HG_UP	10	28	16225	2190	2.650522	0.002509
blue	2808	KEGG_PHOSPHATIDYLINOSITOL_SIGNALING_SYSTEM	23	70	16183	2978	1.793241	0.00251
lightcyan	7736	YAO_TEMPORAL_RESPONSE_TO_PROGESTERONE_CLUS	3	71	16182	62	11.07656	0.002512
brown	5089	KINSEY_TARGETS_OF_EWSR1_FLII_FUSION_DN	56	287	15966	2190	1.44809	0.002514
greenyello	10063	GSE3982_BCELL_VS_EFF_MEMORY_CD4_TCELL_DN	6	148	16105	150	4.392703	0.002516
green	2296	MORF_BCL2L11	21	135	16118	1305	1.937352	0.002516
turquoise	8632	GSE14308_TH2_VS_NAIVE_CD4_TCELL_UP	65	185	16068	4169	1.369756	0.002519
lightcyan	7145	BOQUEST_STEM_CELL_CULTURED_VS_FRESH_UP	6	362	15891	62	4.344947	0.00252
red	1309	MAGNESIUM_ION_BINDING	7	58	16195	524	3.743452	0.00252
pink	3169	chr19p13	15	467	15786	228	2.289671	0.002522
purple	6339	PETROVA_PROX1_TARGETS_DN	4	59	16194	157	7.018461	0.002524
purple	6547	LINDVALL_IMMORTALIZED_BY_TERT_DN	4	59	16194	157	7.018461	0.002524
magenta	8968	GSE17721_CPG_VS_GARDIQUIMOD_1H_BMDM_UP	7	179	16074	167	3.805941	0.002527
magenta	9823	GSE360_DC_VS_MAC_T_GONDII_DN	7	179	16074	167	3.805941	0.002527
lightgreen	3584	MODULE_96	2	29	16224	42	26.68801	0.002532
lightgreen	3597	MODULE_109	2	29	16224	42	26.68801	0.002532
purple	3515	MODULE_23	11	419	15834	157	2.717769	0.002535
greenyello	593	INFLAMMATORY_RESPONSE	5	102	16151	150	5.311438	0.002544
lightcyan	7863	CHICAS_RB1_TARGETS_CONFLUENT	7	490	15763	62	3.744931	0.002546
cyan	3000	BIOCARTA_IL10_PATHWAY	2	16	16237	77	26.38474	0.002547
cyan	3062	BIOCARTA_PML_PATHWAY	2	16	16237	77	26.38474	0.002547
salmon	6262	MOREAUX_B_LYMPHOCYTE_MATURATION_BY_TACI_D	4	73	16180	127	7.012404	0.002553
red	3773	MODULE_321	10	108	16145	524	2.871961	0.002555

brown	2807	KEGG_CHEMOKINE_SIGNALING_PATHWAY	35	161	16092	2190	1.613361	0.002557
red	5174	ENK_UV_RESPONSE_EPIDERMIS_DN	27	467	15786	524	1.793284	0.002569
purple	8367	KRAS.600_UP.V1_UP	7	191	16062	157	3.794011	0.00257
salmon	2398	MORF_GNB1	8	302	15951	127	3.390103	0.002573
salmon	4943	BORCZUK_MALIGNANT_MESOTHELIOMA_UP	8	302	15951	127	3.390103	0.002573
red	3576	MODULE_88	34	633	15620	524	1.666009	0.002576
blue	2550	GCM_RAN	51	191	16062	2978	1.457289	0.002578
blue	8828	GSE17580_UNINFECTED_VS_S_MANSONI_INF_TEFF_UI	51	191	16062	2978	1.457289	0.002578
yellow	8787	GSE15930_NAIVE_VS_48H_IN_VITRO_STIM_IFNAB_CD	30	200	16053	1426	1.709642	0.002578
magenta	5024	HOEBEKE_LYMPHOID_STEM_CELL_UP	5	92	16161	167	5.289313	0.002583
magenta	7151	LABBE_TARGETS_OF_TGFB1_AND_WNT3A_UP	5	92	16161	167	5.289313	0.002583
red	6864	MARSON_BOUND_BY_FOXP3_UNSTIMULATED	55	1161	15092	524	1.469375	0.002586
grey60	1102	SUGAR_BINDING	2	28	16225	44	26.38474	0.002589
tan	8352	KRAS.DF.V1_DN	6	154	16099	145	4.367129	0.00259
salmon	8643	GSE14308_TH1_VS_INDUCED_TREG_DN	6	176	16077	127	4.362831	0.002599
salmon	10039	GSE3982_MAC_VS_CENT_MEMORY_CD4_TCELL_DN	6	176	16077	127	4.362831	0.002599
brown	6558	CHEN_LVAD_SUPPORT_OF_FAILING_HEART_UP	22	88	16165	2190	1.855365	0.002599
red	9761	GSE3337_CTRL_VS_4H_IFNG_IN_CD8POS_DC_DN	14	184	16069	524	2.360002	0.0026
greenyello	8271	JNK_DN.V1_DN	6	149	16104	150	4.363221	0.002602
lightcyan	4630	REACTOME_TRAF6_MEDIATED_NFKB_ACTIVATION	2	20	16233	62	26.21452	0.002603
yellow	4850	SOTIRIOU_BREAST_CANCER_GRADE_1_VS_3_UP	24	149	16104	1426	1.835858	0.002603
turquoise	8123	RAO_BOUND_BY_SALL4_ISOFORM_A	46	123	16130	4169	1.457989	0.002604
turquoise	94	PEROXISOMAL_PART	9	14	16239	4169	2.506202	0.002604
turquoise	162	MICROBODY_PART	9	14	16239	4169	2.506202	0.002604
turquoise	1065	PROTEIN_PHOSPHATASE_TYPE_2A_REGULATOR_ACTIV	9	14	16239	4169	2.506202	0.002604
pink	3505	MODULE_13	14	422	15831	228	2.364908	0.002604
magenta	9760	GSE3337_CTRL_VS_4H_IFNG_IN_CD8POS_DC_UP	7	180	16073	167	3.784797	0.002607
greenyello	227	PLASMA_MEMBRANE	20	1095	15158	150	1.979056	0.002608
lightcyan	7945	KIM_GLIS2_TARGETS_UP	3	72	16181	62	10.92272	0.002614
blue	7236	WANG_TUMOR_INVASIVENESS_UP	88	363	15890	2978	1.323076	0.002615
cyan	2622	GNF2_CD53	3	58	16195	77	10.91782	0.002616
cyan	8111	PHONG_TNF_TARGETS_UP	3	58	16195	77	10.91782	0.002616
lightcyan	8254	SNF5_DN.V1_UP	4	151	16102	62	6.944243	0.002629
brown	7918	ACOSTA_PROLIFERATION_INDEPENDENT_MYC_TARGET	26	110	16143	2190	1.754164	0.002636
grey60	593	INFLAMMATORY_RESPONSE	3	102	16151	44	10.8643	0.002637
red	3567	MODULE_79	9	91	16162	524	3.067633	0.002641
turquoise	777	VESICLE_MEDIATED_TRANSPORT	64	182	16071	4169	1.370914	0.002643
purple	9445	GSE24634_TREG_VS_TCONV_POST_DAY7_IL4_CONVER	7	192	16061	157	3.77425	0.002646
brown	3931	PID_RHOA_PATHWAY	13	42	16211	2190	2.297119	0.002647
turquoise	8862	GSE17721_CTRL_VS_POLYIC_24H_BMDM_UP	67	192	16061	4169	1.360427	0.002648
turquoise	9025	GSE17721_PAM3CSK4_VS_GADIQUIMOD_2H_BMDM_	67	192	16061	4169	1.360427	0.002648
brown	4008	PID_CXCR4_PATHWAY	24	99	16154	2190	1.799142	0.00265
brown	6143	WIELAND_UP_BY_HBV_INFECTION	24	99	16154	2190	1.799142	0.00265
brown	2934	BIOCARTA_CBL_PATHWAY	6	12	16241	2190	3.710731	0.002652
brown	4619	REACTOME_SIGNAL_REGULATORY_PROTEIN_SIRP_FAM	6	12	16241	2190	3.710731	0.002652
brown	5548	XU_HGF_TARGETS_INDUCED_BY_AKT1_48HR_UP	6	12	16241	2190	3.710731	0.002652
red	2803	KEGG_MAPK_SIGNALING_PATHWAY	16	225	16028	524	2.205666	0.002652
brown	1953	CACTGCC,MIR-34A,MIR-34C,MIR-449	51	257	15996	2190	1.472741	0.002658
purple	9915	GSE36476_YOUNG_VS_OLD_DONOR_MEMORY_CD4_T	6	143	16110	157	4.343593	0.002661
brown	383	HEMOPOIESIS	18	67	16186	2190	1.993825	0.002664
turquoise	2520	GCM_FANCL	13	24	16229	4169	2.111707	0.002668
turquoise	3125	chr7p21	13	24	16229	4169	2.111707	0.002668
turquoise	3570	MODULE_82	13	24	16229	4169	2.111707	0.002668
salmon	1469	V$ELK1_02	7	238	16015	127	3.764011	0.00267
salmon	8695	GSE1460_DP_VS_CD4_THYMOCYTE_DN	6	177	16076	127	4.338182	0.002673
salmon	10005	GSE3982_MAST_CELL_VS_CENT_MEMORY_CD4_TCELL_	6	177	16076	127	4.338182	0.002673
salmon	10165	GSE7460_TREG_VS_TCONV_ACT_WITH_TGFB_DN	6	177	16076	127	4.338182	0.002673
turquoise	171	INTEGRAL_TO_ORGANELLE_MEMBRANE	22	49	16204	4169	1.750363	0.002676
brown	9009	GSE17721_POLYIC_VS_CPG_4H_BMDM_DN	38	179	16074	2190	1.575506	0.002678
pink	10241	GSE9037_CTRL_VS_LPS_1H_STIM_BMDM_DN	8	169	16084	228	3.374442	0.002682

pink	10249	GSE9037_WT_VS_IRAK4_KO_BMDM_DN	8	169	16084	228	3.374442	0.002682
purple	2884	KEGG_GLIOMA	4	60	16193	157	6.901486	0.002685
purple	5128	GRAHAM_NORMAL_QUIESCENT_VS_NORMAL_DIVIDIN	4	60	16193	157	6.901486	0.002685
cyan	6703	BAELDE_DIABETIC_NEPHROPATHY_DN	7	396	15857	77	3.731175	0.002687
purple	7242	TSAI_RESPONSE_TO_RADIATION_THERAPY	3	29	16224	157	10.7092	0.002687
magenta	8569	GSE13484_12H_UNSTIM_VS_YF17D_VACCINE_STIM_PI	7	181	16072	167	3.763887	0.002689
red	9284	GSE20715_WT_VS_TLR4_KO_24H_OZONE_LUNG_UP	13	165	16088	524	2.443777	0.002696
turquoise	7111	BOCHKIS_FOXA2_TARGETS	121	377	15876	4169	1.251255	0.002698
lightyellow	2879	KEGG_PATHWAYS_IN_CANCER	4	290	15963	33	6.793312	0.002702
cyan	8078	PLASARI_TGFB1_TARGETS_10HR_DN	5	202	16051	77	5.224701	0.002703
brown	9765	GSE3337_4H_VS_16H_IFNG_IN_CD8POS_DC_DN	39	185	16068	2190	1.564524	0.002705
lightgreen	3688	MODULE_208	3	108	16145	42	10.74934	0.002713
yellow	9378	GSE22886_NAIVE_CD4_TCELL_VS_DC_UP	29	192	16061	1426	1.721515	0.002714
blue	2494	GCM_SIRT2	16	43	16210	2978	2.030768	0.002715
purple	6497	KAYO_AGING_MUSCLE_UP	7	193	16060	157	3.754695	0.002724
brown	8566	GSE13484_3H_UNSTIM_VS_YF17D_VACCINE_STIM_PB	40	191	16062	2190	1.554233	0.002726
brown	9726	GSE30962_ACUTE_VS_CHRONIC_LCMV_PRIMARY_INF_	40	191	16062	2190	1.554233	0.002726
brown	9963	GSE3982_MEMORY_CD4_TCELL_VS_BCELL_DN	40	191	16062	2190	1.554233	0.002726
blue	7653	CAIRO_HEPATOBLASTOMA_UP	52	196	16057	2978	1.447958	0.002728
brown	3726	MODULE_257	33	150	16103	2190	1.632721	0.00273
tan	5632	OXFORD_RALA_TARGETS_UP	2	9	16244	145	24.90881	0.002731
red	9061	GSE17721_POLYIC_VS_GARDIQUIMOD_2H_BMDM_DN	14	185	16068	524	2.347246	0.002732
red	9424	GSE24142_DN2_VS_DN3_THYMOCYTE_FETAL_UP	14	185	16068	524	2.347246	0.002732
red	9505	GSE2706_UNSTIM_VS_8H_LPS_DC_DN	14	185	16068	524	2.347246	0.002732
red	10010	GSE3982_MAST_CELL_VS_TH2_UP	14	185	16068	524	2.347246	0.002732
brown	4096	PID_HIF1_TFPATHWAY	17	62	16191	2190	2.034917	0.002737
red	2870	KEGG_ALZHEIMERS_DISEASE	12	146	16107	524	2.549357	0.002737
brown	9919	GSE36476_YOUNG_VS_OLD_DONOR_MEMORY_CD4_T	41	197	16056	2190	1.544568	0.002741
brown	10191	GSE7764_IL15_NK_CELL_24H_VS_SPLENOCYTE_DN	41	197	16056	2190	1.544568	0.002741
turquoise	3372	chr14q22	23	52	16201	4169	1.724353	0.002744
turquoise	6503	HU_GENOTOXIN_ACTION_DIRECT_VS_INDIRECT_24HR	23	52	16201	4169	1.724353	0.002744
yellow	10144	GSE6269_STREP_AUREUS_VS_STREP_PNEUMO_INF_PE	25	158	16095	1426	1.80342	0.002749
salmon	2326	MORF_RAD23B	6	178	16075	127	4.31381	0.002749
salmon	7744	YAO_TEMPORAL_RESPONSE_TO_PROGESTERONE_CLU!	6	178	16075	127	4.31381	0.002749
brown	1806	V$PU1_Q6	42	203	16050	2190	1.535475	0.002751
black	8407	GSE10239_NAIVE_VS_MEMORY_CD8_TCELL_DN	9	168	16085	283	3.076666	0.002756
blue	1870	V$NFY_Q6_01	59	228	16025	2978	1.412297	0.00276
pink	6537	BLALOCK_ALZHEIMERS_DISEASE_UP	35	1535	14718	228	1.625393	0.00277
turquoise	8857	GSE17721_CTRL_VS_POLYIC_6H_BMDM_DN	63	179	16074	4169	1.372111	0.002773
brown	485	NEGATIVE_REGULATION_OF_CELLULAR_PROCESS	99	562	15691	2190	1.307339	0.002774
grey60	3441	chr11p13	2	29	16224	44	25.47492	0.002776
pink	1540	V$GATA1_02	8	170	16083	228	3.354592	0.002781
brown	2666	GNF2_MCL1	16	57	16196	2190	2.083217	0.002782
lightgreen	4873	SENGUPTA_NASOPHARYNGEAL_CARCINOMA_WITH_LN	3	109	16144	42	10.65072	0.002785
grey60	6302	LENAOUR_DENDRITIC_CELL_MATURATION_UP	3	104	16149	44	10.65538	0.002786
brown	3019	BIOCARTA_PPARA_PATHWAY	15	52	16201	2190	2.140806	0.00279
turquoise	4346	REACTOME_REGULATION_OF_APOPTOSIS	24	55	16198	4169	1.70118	0.002791
brown	4104	PID_S1P_S1P2_PATHWAY	9	24	16229	2190	2.783048	0.002793
brown	6670	DEBIASI_APOPTOSIS_BY_REOVIRUS_INFECTION_DN	54	276	15977	2190	1.452025	0.002793
yellow	6000	NIKOLSKY_BREAST_CANCER_11Q12_Q14_AMPLICON	21	125	16128	1426	1.914799	0.002797
purple	7144	BOQUEST_STEM_CELL_DN	7	194	16059	157	3.73534	0.002804
pink	235	REGULATION_OF_BIOLOGICAL_QUALITY	12	335	15918	228	2.553496	0.002807
turquoise	6693	DAZARD_RESPONSE_TO_UV_SCC_UP	44	117	16136	4169	1.466116	0.002816
salmon	5776	GROSS_HYPOXIA_VIA_HIF1A_UP	4	75	16178	127	6.825407	0.002817
turquoise	147	DNA_DIRECTED_RNA_POLYMERASEII_HOLOENZYME	28	67	16186	4169	1.629239	0.002818
pink	8071	HUANG_GATA2_TARGETS_DN	5	69	16184	228	5.165586	0.002819
turquoise	2635	GNF2_DEK	25	58	16195	4169	1.680404	0.00282
magenta	364	REGULATION_OF_SYNAPSE_STRUCTURE_AND_ACTIVIT	2	8	16245	167	24.33084	0.002822
magenta	5609	FUNG_IL2_SIGNALING_2	2	8	16245	167	24.33084	0.002822
lightcyan	5251	GAUSSMANN_MLL_AF4_FUSION_TARGETS_F_UP	4	154	16099	62	6.808965	0.002823

turquoise	2149	GCATTTG,MIR-105	56	156	16097	4169	1.399475	0.002829
pink	11	BRUSH_BORDER	2	6	16247	228	23.7617	0.002832
pink	7645	BOYLAN_MULTIPLE_MYELOMA_PCA1_DN	2	6	16247	228	23.7617	0.002832
brown	1379	GTPASE_ACTIVITY	23	94	16159	2190	1.815889	0.002834
turquoise	3322	chr5q22	11	19	16234	4169	2.257048	0.002841
red	8835	GSE17721_CTRL_VS_LPS_1H_BMDM_DN	13	166	16087	524	2.429056	0.002841
red	9978	GSE3982_EOSINOPHIL_VS_BCELL_UP	13	166	16087	524	2.429056	0.002841
purple	4366	REACTOME_PEPTIDE_LIGAND_BINDING_RECEPTORS	5	100	16153	157	5.176115	0.002843
greenyello	118	INTEGRAL_TO_MEMBRANE	19	1025	15228	150	2.008501	0.002844
red	3653	MODULE_170	9	92	16161	524	3.034289	0.002846
lightgreen	5167	KOKKINAKIS_METHIONINE_DEPRIVATION_96HR_UP	3	110	16143	42	10.5539	0.002858
magenta	10033	GSE3982_MAC_VS_BCELL_DN	7	183	16070	167	3.722751	0.002859
greenyello	426	VIRAL_REPRODUCTIVE_PROCESS	3	31	16222	150	10.48581	0.002866
greenyello	3224	chr11q14	3	31	16222	150	10.48581	0.002866
black	8658	GSE1432_CTRL_VS_IFNG_1H_MICROGLIA_UP	9	169	16084	283	3.058461	0.002867
red	9762	GSE3337_CTRL_VS_16H_IFNG_IN_CD8POS_DC_UP	14	186	16067	524	2.334626	0.00287
blue	7001	DAIRKEE_CANCER_PRONE_RESPONSE_BPA	17	47	16206	2978	1.974058	0.002873
cyan	8207	HINATA_NFKB_IMMU_INF	2	17	16236	77	24.8327	0.002877
blue	4146	REACTOME_SIGNALLING_BY_NGF	53	201	16052	2978	1.439092	0.002877
turquoise	6086	GOLDRATH_HOMEOSTATIC_PROLIFERATION	59	166	16087	4169	1.385625	0.002881
lightyellow	2809	KEGG_NEUROACTIVE_LIGAND_RECEPTOR_INTERACTIO	3	141	16112	33	10.47905	0.002882
pink	5093	SABATES_COLORECTAL_ADENOMA_DN	8	171	16082	228	3.334975	0.002884
lightgreen	5666	SIMBULAN_UV_RESPONSE_NORMAL_DN	2	31	16222	42	24.96621	0.002891
lightgreen	6660	BURTON_ADIPOGENESIS_1	2	31	16222	42	24.96621	0.002891
turquoise	4806	NAKAMURA_TUMOR_ZONE_PERIPHERAL_VS_CENTRAL	176	572	15681	4169	1.19955	0.002893
blue	9860	GSE360_L_DONOVANI_VS_L_MAJOR_MAC_UP	47	174	16079	2978	1.474204	0.002901
turquoise	3162	chr2q32	17	35	16218	4169	1.893575	0.002906
turquoise	3471	ST_GA13_PATHWAY	17	35	16218	4169	1.893575	0.002906
turquoise	6539	BURTON_ADIPOGENESIS_12	17	35	16218	4169	1.893575	0.002906
blue	10033	GSE3982_MAC_VS_BCELL_DN	49	183	16070	2978	1.461349	0.002907
blue	910	MRNA_METABOLIC_PROCESS	26	83	16170	2978	1.709638	0.002907
blue	5163	COLDREN_GEFITINIB_RESISTANCE_UP	26	83	16170	2978	1.709638	0.002907
greenyello	2986	BIOCARTA_TCAPOPTOSIS_PATHWAY	2	9	16244	150	24.07852	0.002919
greenyello	3923	MODULE_575	2	9	16244	150	24.07852	0.002919
purple	3537	MODULE_48	8	249	16004	157	3.326017	0.002924
salmon	6442	BLALOCK_ALZHEIMERS_DISEASE_DN	18	1128	15125	127	2.042176	0.002928
yellow	9788	GSE34205_HEALTHY_VS_FLU_INF_INFANT_PBMC_UP	29	193	16060	1426	1.712595	0.002933
brown	8792	GSE15930_NAIVE_VS_72H_IN_VITRO_STIM_IFNAB_CD8	37	174	16079	2190	1.578127	0.002933
yellow	9922	GSE37416_CTRL_VS_0H_F_TULARENSIS_LVS_NEUTROP	23	142	16111	1426	1.846093	0.002939
turquoise	3728	MODULE_261	37	95	16158	4169	1.518377	0.00295
lightcyan	4822	HOLLMANN_APOPTOSIS_VIA_CD40_DN	5	257	15996	62	5.1001	0.002953
greenyello	6476	KAAB_HEART_ATRIUM_VS_VENTRICLE_UP	7	205	16048	150	3.69987	0.002954
lightcyan	8543	GSE13306_RA_VS_UNTREATED_TCONV_DN	4	156	16097	62	6.721671	0.002957
midnightb	5847	RICKMAN_TUMOR_DIFFERENTIATED_WELL_VS_POORL	5	224	16029	71	5.109721	0.00296
turquoise	7821	WHITFIELD_CELL_CYCLE_S	55	153	16100	4169	1.401435	0.002962
magenta	881	NEURON_DIFFERENTIATION	4	58	16195	167	6.711955	0.002963
magenta	983	REGULATION_OF_IMMUNE_SYSTEM_PROCESS	4	58	16195	167	6.711955	0.002963
brown	8547	GSE13411_NAIVE_VS_IGM_MEMORY_BCELL_DN	38	180	16073	2190	1.566753	0.002964
brown	8967	GSE17721_CPG_VS_GARDIQUIMOD_0.5H_BMDM_DN	38	180	16073	2190	1.566753	0.002964
brown	9172	GSE17974_CTRL_VS_ACT_IL4_AND_ANTI_IL12_2H_CD4	38	180	16073	2190	1.566753	0.002964
purple	625	PHOSPHOINOSITIDE_MEDIATED_SIGNALING	3	30	16223	157	10.35223	0.002965
purple	3730	MODULE_263	3	30	16223	157	10.35223	0.002965
purple	8592	GSE13485_DAY7_VS_DAY21_YF17D_VACCINE_PBMC_U	7	196	16057	157	3.697225	0.002968
lightyellow	2582	GNF2_CCNA1	2	40	16213	33	24.62576	0.002971
salmon	2493	GCM_RAD21	3	37	16216	127	10.37646	0.002982
salmon	8613	GSE14000_TRANSLATED_RNA_VS_MRNA_16H_LPS_DC	6	181	16072	127	4.242311	0.002986
brown	7220	ALONSO_METASTASIS_UP	39	186	16067	2190	1.556113	0.002988
brown	8429	GSE10325_LUPUS_CD4_TCELL_VS_LUPUS_BCELL_DN	39	186	16067	2190	1.556113	0.002988
brown	3069	BIOCARTA_HER2_PATHWAY	8	20	16233	2190	2.968584	0.002991
salmon	2437	MORF_PPP2CA	5	125	16128	127	5.119055	0.002991

brown	7596	NGO_MALIGNANT_GLIOMA_1P_LOH	7	16	16237	2190	3.246889	0.002999
brown	9737	GSE31082_DP_VS_CD4_SP_THYMOCYTE_DN	40	192	16061	2190	1.546138	0.003006
lightgreen	6801	DURCHDEWALD_SKIN_CARCINOGENESIS_DN	4	233	16020	42	6.643368	0.003006
brown	2340	MORF_TERF2IP	26	111	16142	2190	1.73836	0.00301
brown	5283	WANG_ESOPHAGUS_CANCER_VS_NORMAL_UP	26	111	16142	2190	1.73836	0.00301
red	9859	GSE360_LOW_DOSE_B_MALAYI_VS_M_TUBERCULOSIS	14	187	16066	524	2.322141	0.003014
brown	9669	GSE29618_BCELL_VS_PDC_DAY7_FLU_VACCINE_DN	41	198	16055	2190	1.536767	0.003017
brown	10234	GSE9006_TYPE_1_DIABETES_AT_DX_VS_4MONTH_POS	41	198	16055	2190	1.536767	0.003017
pink	7476	ROME_INSULIN_TARGETS_IN_MUSCLE_UP	14	429	15824	228	2.32632	0.00302
purple	2185	RYAAAKNNNNNNTTGW_UNKNOWN	4	62	16191	157	6.678858	0.003026
purple	5891	GOTZMANN_EPITHELIAL_TO_MESENCHYMAL_TRANSIT	4	62	16191	157	6.678858	0.003026
purple	6274	GUO_HEX_TARGETS_DN	4	62	16191	157	6.678858	0.003026
red	7204	QI_PLASMACYTOMA_UP	17	249	16004	524	2.117638	0.003026
magenta	8209	CYCLIN_D1_KE_.V1_DN	6	138	16115	167	4.23145	0.003029
blue	6458	KAYO_CALORIE_RESTRICTION_MUSCLE_DN	24	75	16178	2978	1.746461	0.003035
magenta	8508	GSE12845_IGD_POS_BLOOD_VS_PRE_GC_TONSIL_BCEI	7	185	16068	167	3.682505	0.003037
magenta	9292	GSE20715_0H_VS_48H_OZONE_LUNG_UP	7	185	16068	167	3.682505	0.003037
magenta	9765	GSE3337_4H_VS_16H_IFNG_IN_CD8POS_DC_DN	7	185	16068	167	3.682505	0.003037
turquoise	2138	ACTGTAG,MIR-139	42	111	16142	4169	1.475122	0.00304
brown	4050	PID_PDGFRBPATHWAY	29	128	16125	2190	1.681425	0.003044
brown	4324	REACTOME_RESPONSE_TO_ELEVATED_PLATELET_CYTC	19	73	16180	2190	1.931613	0.003045
red	3675	MODULE_195	11	129	16124	524	2.644875	0.00305
brown	2233	TGANTCA_V$AP1_C	149	895	15358	2190	1.235528	0.003051
blue	10037	GSE3982_MAC_VS_EFF_MEMORY_CD4_TCELL_DN	44	161	16092	2978	1.491543	0.003052
turquoise	8949	GSE17721_PAM3CSK4_VS_CPG_0.5H_BMDM_DN	61	173	16080	4169	1.374629	0.003053
turquoise	9650	GSE29617_CTRL_VS_DAY7_TIV_FLU_VACCINE_PBMC_2	61	173	16080	4169	1.374629	0.003053
turquoise	8816	GSE16522_MEMORY_VS_NAIVE_ANTI_CD3CD28_STIM_	67	193	16060	4169	1.353378	0.003056
blue	8386	KAECH_NAIVE_VS_DAY8_EFF_CD8_TCELL_UP	52	197	16056	2978	1.440608	0.003059
blue	9446	GSE24634_TREG_VS_TCONV_POST_DAY10_IL4_CONVE	52	197	16056	2978	1.440608	0.003059
blue	2655	GNF2_ITGAL	19	55	16198	2978	1.885384	0.003062
turquoise	9624	GSE2826_WT_VS_XID_BCELL_UP	64	183	16070	4169	1.363423	0.003064
cyan	1639	V$ISRE_01	5	208	16045	77	5.073989	0.003065
greenyello	59	MEMBRANE	26	1601	14652	150	1.759642	0.003067
blue	1234	PROTEIN_N_TERMINUS_BINDING	14	36	16217	2978	2.122435	0.003068
blue	2793	KEGG_DNA_REPLICATION	14	36	16217	2978	2.122435	0.003068
salmon	10035	GSE3982_MAC_VS_BASOPHIL_DN	6	182	16071	127	4.219001	0.003069
blue	7857	VERHAAK_GLIOBLASTOMA_MESENCHYMAL	46	170	16083	2978	1.476787	0.003078
blue	2331	MORF_RFC5	23	71	16182	2978	1.767984	0.00308
blue	3266	chr16p12	23	71	16182	2978	1.767984	0.00308
salmon	2794	KEGG_SPLICEOSOME	5	126	16127	127	5.078428	0.003095
yellow	7251	MOOTHA_PGC	52	403	15850	1426	1.47066	0.003099
salmon	4553	REACTOME_REGULATION_OF_MITOTIC_CELL_CYCLE	4	77	16176	127	6.648124	0.003099
green	5575	WANG_METHYLATED_IN_BREAST_CANCER	8	32	16221	1305	3.113602	0.003109
yellow	187	MITOCHONDRIAL_ENVELOPE	17	94	16159	1426	2.061271	0.003113
greenyello	1785	V$EFC_Q6	7	207	16046	150	3.664122	0.003117
greenyello	1871	V$SREBP_Q3	7	207	16046	150	3.664122	0.003117
blue	3693	MODULE_213	21	63	16190	2978	1.81923	0.003119
lightyellow	9840	GSE360_L_MAJOR_VS_T_GONDII_DC_UP	3	145	16108	33	10.18997	0.003119
yellow	955	TRANSLATION	26	168	16085	1426	1.763917	0.003127
magenta	3500	MODULE_7	7	186	16067	167	3.662707	0.003129
turquoise	7251	MOOTHA_PGC	128	403	15850	4169	1.238245	0.003131
blue	2794	KEGG_SPLICEOSOME	36	126	16127	2978	1.55934	0.003149
lightcyan	3238	chr4q22	2	22	16231	62	23.83138	0.003149
turquoise	5171	CHOW_RASSF1_TARGETS_UP	14	27	16226	4169	2.021464	0.00315
blue	1345	DNA_DEPENDENT_ATPASE_ACTIVITY	10	22	16231	2978	2.480768	0.003152
salmon	9088	GSE17721_LPS_VS_GARDIQUIMOD_16H_BMDM_UP	6	183	16070	127	4.195947	0.003153
salmon	10033	GSE3982_MAC_VS_BCELL_DN	6	183	16070	127	4.195947	0.003153
cyan	7309	LINDSTEDT_DENDRITIC_CELL_MATURATION_A	3	62	16191	77	10.21345	0.003164
lightcyan	3126	chr10q23	3	77	16176	62	10.21345	0.003164
lightcyan	7276	TOOKER_GEMCITABINE_RESISTANCE_UP	3	77	16176	62	10.21345	0.003164

greenyello	1685	V$RSRFC4_Q2	6	155	16098	150	4.194323	0.003165
brown	2854	KEGG_NEUROTROPHIN_SIGNALING_PATHWAY	27	117	16136	2190	1.712645	0.003167
yellow	8842	GSE17721_CTRL_VS_LPS_8H_BMDM_UP	29	194	16059	1426	1.703767	0.003167
yellow	8960	GSE17721_PAM3CSK4_VS_CPG_12H_BMDM_UP	29	194	16059	1426	1.703767	0.003167
yellow	9335	GSE22886_NAIVE_BCELL_VS_BLOOD_PLASMA_CELL_DI	29	194	16059	1426	1.703767	0.003167
greenyello	6337	MOREAUX_MULTIPLE_MYELOMA_BY_TACI_UP	9	324	15929	150	3.009815	0.003169
lightgreen	5862	SCHAEFFER_PROSTATE_DEVELOPMENT_48HR_UP	5	390	15863	42	4.961233	0.003179
red	8184	ZWANG_CLASS_1_TRANSIENTLY_INDUCED_BY_EGF	25	428	15825	524	1.811751	0.003192
purple	5557	AGARWAL_AKT_PATHWAY_TARGETS	2	9	16244	157	23.00495	0.003193
purple	8208	HINATA_NFKB_MATRIX	2	9	16244	157	23.00495	0.003193
magenta	55	BASEMENT_MEMBRANE	3	29	16224	167	10.06793	0.003199
magenta	3584	MODULE_96	3	29	16224	167	10.06793	0.003199
magenta	7577	MIKKELSEN_IPS_ICP_WITH_H3K27ME3	3	29	16224	167	10.06793	0.003199
brown	1011	SMALL_GTPASE_MEDIATED_SIGNAL_TRANSDUCTION	21	84	16169	2190	1.855365	0.003213
turquoise	91	NUCLEAR_ENVELOPE_ENDOPLASMIC_RETICULUM_NET	34	86	16167	4169	1.541282	0.003215
turquoise	8848	GSE17721_CTRL_VS_POLYIC_0.5H_BMDM_UP	63	180	16073	4169	1.364488	0.003217
greenyello	14	INTRINSIC_TO_PLASMA_MEMBRANE	15	733	15520	150	2.217326	0.003218
greenyello	2482	MORF_WNT1	4	66	16187	150	6.566869	0.003224
red	5015	TAKEDA_TARGETS_OF_NUP98_HOXA9_FUSION_10D_D	11	130	16123	524	2.62453	0.003238
salmon	8587	GSE13485_DAY1_VS_DAY21_YF17D_VACCINE_PBMC_D	6	184	16069	127	4.173143	0.003239
salmon	10115	GSE39820_TGFBETA1_IL6_VS_TGFBETA1_IL6_IL23A_TF	6	184	16069	127	4.173143	0.003239
lightyellow	5114	JAATINEN_HEMATOPOIETIC_STEM_CELL_UP	4	305	15948	33	6.459215	0.00324
yellow	8678	GSE1448_CTRL_VS_ANTI_VALPHA2_DP_THYMOCYTE_U	27	177	16076	1426	1.738619	0.00324
yellow	10165	GSE7460_TREG_VS_TCONV_ACT_WITH_TGFB_DN	27	177	16076	1426	1.738619	0.00324
lightyellow	9197	GSE17974_IL4_AND_ANTI_IL12_VS_UNTREATED_12H_	3	147	16106	33	10.05133	0.003242
lightgreen	1804	V$PEA3_Q6	4	238	16015	42	6.503802	0.003244
magenta	3278	chr14q32	5	97	16156	167	5.016668	0.00325
blue	3272	chr17p13	51	193	16060	2978	1.442187	0.003252
blue	9664	GSE29618_PDC_VS_MDC_UP	51	193	16060	2978	1.442187	0.003252
turquoise	6930	STEARMAN_LUNG_CANCER_EARLY_VS_LATE_UP	45	121	16132	4169	1.449869	0.003258
cyan	6525	WANG_CISPLATIN_RESPONSE_AND_XPC_DN	5	211	16042	77	5.001846	0.003259
purple	3960	PID_INTEGRIN3_PATHWAY	3	31	16222	157	10.01829	0.003259
purple	3984	PID_ARF6_PATHWAY	3	31	16222	157	10.01829	0.003259
purple	4761	REACTOME_PACKAGING_OF_TELOMERE_ENDS	3	31	16222	157	10.01829	0.003259
purple	7754	NAKAMURA_ADIPOGENESIS_EARLY_DN	3	31	16222	157	10.01829	0.003259
purple	8271	JNK_DN.V1_DN	6	149	16104	157	4.168683	0.003262
blue	9985	GSE3982_EOSINOPHIL_VS_CENT_MEMORY_CD4_TCELL	49	184	16069	2978	1.453407	0.003271
lightyellow	7572	MIKKELSEN_MEF_LCP_WITH_H3K27ME3	2	42	16211	33	23.4531	0.003272
brown	8681	GSE1448_CTRL_VS_ANTI_VBETA5_DP_THYMOCYTE_DN	38	181	16072	2190	1.558097	0.003276
blue	8540	GSE13306_RA_VS_UNTREATED_TREG_UP	47	175	16078	2978	1.46578	0.003276
lightcyan	8008	WINZEN_DEGRADED_VIA_KHSRP	3	78	16175	62	10.08251	0.003281
greenyello	1647	V$RFX1_01	7	209	16044	150	3.629059	0.003287
brown	9859	GSE360_LOW_DOSE_B_MALAYI_VS_M_TUBERCULOSIS	39	187	16066	2190	1.547791	0.003296
brown	9886	GSE360_HIGH_DOSE_B_MALAYI_VS_M_TUBERCULOSIS	39	187	16066	2190	1.547791	0.003296
turquoise	1961	ACATTCC,MIR-1,MIR-206	91	275	15978	4169	1.290061	0.003298
turquoise	7114	MATZUK_OVULATION	8	12	16241	4169	2.599025	0.003306
brown	8501	GSE12366_PLASMA_CELL_VS_MEMORY_BCELL_DN	40	193	16060	2190	1.538127	0.00331
brown	9101	GSE17721_12H_VS_24H_LPS_BMDM_DN	40	193	16060	2190	1.538127	0.00331
brown	9290	GSE20715_0H_VS_24H_OZONE_LUNG_UP	40	193	16060	2190	1.538127	0.00331
lightcyan	9652	GSE29617_DAY3_VS_DAY7_TIV_FLU_VACCINE_PBMC_2	4	161	16092	62	6.512923	0.003312
lightcyan	10251	GSE9037_WT_VS_IRAK4_KO_LPS_1H_STIM_BMDM_DN	4	161	16092	62	6.512923	0.003312
red	8903	GSE17721_CTRL_VS_GARDIQUIMOD_4H_BMDM_DN	13	169	16084	524	2.385937	0.003317
brown	9600	GSE27786_NKCELL_VS_ERYTHROBLAST_UP	41	199	16054	2190	1.529045	0.003317
pink	9627	GSE2826_WT_VS_BTK_KO_BCELL_DN	8	175	16078	228	3.258747	0.003322
brown	404	ESTABLISHMENT_OF_CELLULAR_LOCALIZATION	62	328	15925	2190	1.402837	0.003325
brown	7558	YAGI_AML_WITH_11Q23_REARRANGED	62	328	15925	2190	1.402837	0.003325
yellow	4135	REACTOME_BASE_EXCISION_REPAIR	6	18	16235	1426	3.799205	0.003325
salmon	8925	GSE17721_LPS_VS_POLYIC_12H_BMDM_DN	6	185	16068	127	4.150585	0.003326
brown	963	NEGATIVE_REGULATION_OF_BIOLOGICAL_PROCESS	102	585	15668	2190	1.293998	0.003338
lightgreen	7266	BOYLAN_MULTIPLE_MYELOMA_C_D_DN	4	240	16013	42	6.449603	0.003342

blue	4126	REACTOME_PYRUVATE_METABOLISM_AND_CITRIC_AC	15	40	16213	2978	2.046634	0.003343
red	2714	GNF2_TYK2	5	32	16221	524	4.846434	0.003348
red	6462	CHEN_LVAD_SUPPORT_OF_FAILING_HEART_DN	5	32	16221	524	4.846434	0.003348
turquoise	8672	GSE14350_TREG_VS_TEFF_IN_IL2RB_KO_UP	68	197	16056	4169	1.345688	0.003349
turquoise	10267	GSE9650_GP33_VS_GP276_LCMV_SPECIFIC_EXHAUSTE	68	197	16056	4169	1.345688	0.003349
salmon	1609	V$USF_C	7	248	16005	127	3.612236	0.003354
brown	4178	REACTOME_TRANS_GOLGI_NETWORK_VESICLE_BUDDI	16	58	16195	2190	2.0473	0.00337
red	3508	MODULE_16	26	453	15800	524	1.780235	0.003371
turquoise	8563	GSE13484_UNSTIM_VS_3H_YF17D_VACCINE_STIM_PB	65	187	16066	4169	1.355106	0.003372
brown	3468	ST_ADRENERGIC	10	29	16224	2190	2.559125	0.003374
brown	3976	PID_NECTIN_PATHWAY	10	29	16224	2190	2.559125	0.003374
brown	4102	PID_NCADHERINPATHWAY	10	29	16224	2190	2.559125	0.003374
brown	4205	REACTOME_GLYCOSPHINGOLIPID_METABOLISM	10	29	16224	2190	2.559125	0.003374
grey60	2742	KEGG_TRYPTOPHAN_METABOLISM	2	32	16221	44	23.08665	0.003374
grey60	3746	MODULE_287	2	32	16221	44	23.08665	0.003374
blue	928	REGULATION_OF_RNA_METABOLIC_PROCESS	98	414	15839	2978	1.291917	0.003375
red	3873	MODULE_471	3	10	16243	524	9.305153	0.003375
blue	1453	TRANSCRIPTION_COFACTOR_ACTIVITY	54	207	16046	2978	1.423745	0.003381
lightcyan	9256	GSE20151_CTRL_VS_FUSOBACT_NUCLEATUM_NEUTRC	4	162	16091	62	6.47272	0.003386
turquoise	4347	REACTOME_RNA_POL_II_TRANSCRIPTION	39	102	16151	4169	1.490617	0.003391
turquoise	2633	GNF2_DAP3	44	118	16135	4169	1.453692	0.003394
salmon	2679	GNF2_PA2G4	4	79	16174	127	6.479817	0.0034
brown	7322	PARK_APL_PATHOGENESIS_DN	14	48	16205	2190	2.164593	0.003409
magenta	8347	STK33_SKM_DN	8	240	16013	167	3.244112	0.00341
salmon	9004	GSE17721_POLYIC_VS_CPG_1H_BMDM_UP	6	186	16067	127	4.12827	0.003416
salmon	9772	GSE339_CD8POS_VS_CD4CD8DN_DC_UP	6	186	16067	127	4.12827	0.003416
yellow	8454	GSE11057_CD4_CENT_MEM_VS_PBMC_UP	29	195	16058	1426	1.69503	0.003417
yellow	8819	GSE16522_ANTI_CD3CD28_STIM_VS_UNSTIM_MEMOF	29	195	16058	1426	1.69503	0.003417
yellow	9096	GSE17721_0.5H_VS_8H_LPS_BMDM_UP	29	195	16058	1426	1.69503	0.003417
yellow	9574	GSE27786_BCELL_VS_MONO_MAC_UP	29	195	16058	1426	1.69503	0.003417
brown	6541	KAYO_AGING_MUSCLE_DN	26	112	16141	2190	1.722839	0.003429
turquoise	7250	MOOTHA_HUMAN_MITODB_6_2002	130	411	15842	4169	1.233114	0.003435
lightcyan	7760	BAUS_TFF2_TARGETS_UP	2	23	16230	62	22.79523	0.00344
pink	5639	OXFORD_RALA_OR_RALB_TARGETS_DN	3	22	16231	228	9.720694	0.003445
turquoise	2557	GCM_SUFU	31	77	16176	4169	1.569541	0.003454
turquoise	4553	REACTOME_REGULATION_OF_MITOTIC_CELL_CYCLE	31	77	16176	4169	1.569541	0.003454
salmon	80	NUCLEUS	20	1333	14920	127	1.920126	0.003465
salmon	7916	WIERENGA_PML_INTERACTOME	3	39	16214	127	9.844337	0.003468
lightgreen	1074	G_PROTEIN_COUPLED_RECEPTOR_BINDING	2	34	16219	42	22.76331	0.00347
brown	579	INTERLEUKIN_1_SECRETION	5	9	16244	2190	4.123034	0.003475
blue	9852	GSE360_T_GONDII_VS_M_TUBERCULOSIS_DC_UP	46	171	16082	2978	1.46815	0.003478
greenyello	8381	LEF1_UP.V1_UP	6	158	16095	150	4.114684	0.003479
greenyello	8188	ZWANG_TRANSIENTLY_UP_BY_2ND_EGF_PULSE_ONLY	19	1044	15209	150	1.971948	0.003479
red	9520	GSE2706_LPS_VS_R848_AND_LPS_8H_STIM_DC_UP	14	190	16063	524	2.285476	0.003479
brown	3533	MODULE_44	55	285	15968	2190	1.432212	0.003488
yellow	913	NEGATIVE_REGULATION_OF_TRANSFERASE_ACTIVITY	8	30	16223	1426	3.039364	0.003489
red	8899	GSE17721_CTRL_VS_GARDIQUIMOD_1H_BMDM_DN	13	170	16083	524	2.371902	0.003489
red	7963	PASINI_SUZ12_TARGETS_DN	18	274	15979	524	2.037625	0.003496
purple	951	G_PROTEIN_COUPLED_RECEPTOR_PROTEIN_SIGNALIN(	7	202	16051	157	3.587406	0.003506
yellow	7744	YAO_TEMPORAL_RESPONSE_TO_PROGESTERONE_CLU!	27	178	16075	1426	1.728852	0.003509
black	5291	JOHANSSON_GLIOMAGENESIS_BY_PDGFB_DN	3	18	16235	283	9.571849	0.003511
turquoise	8555	GSE13411_IGM_VS_SWITCHED_MEMORY_BCELL_DN	67	194	16059	4169	1.346402	0.003519
turquoise	8917	GSE17721_LPS_VS_POLYIC_2H_BMDM_DN	67	194	16059	4169	1.346402	0.003519
turquoise	9079	GSE17721_LPS_VS_GARDIQUIMOD_2H_BMDM_DN	67	194	16059	4169	1.346402	0.003519
turquoise	9140	GSE17721_0.5H_VS_4H_GARDIQUIMOD_BMDM_UP	67	194	16059	4169	1.346402	0.003519
turquoise	9743	GSE32423_MEMORY_VS_NAIVE_CD8_TCELL_DN	67	194	16059	4169	1.346402	0.003519
yellow	4122	REACTOME_TRANSLATION	23	144	16109	1426	1.820453	0.003521
turquoise	1692	CCGNMNNTNACG_UNKNOWN	30	74	16179	4169	1.580488	0.003522
magenta	3835	MODULE_410	3	30	16223	167	9.732335	0.003528
salmon	4550	REACTOME_MITOTIC_G1_G1_S_PHASES	5	130	16123	127	4.922168	0.003539

lightcyan	3534	MODULE_45	7	520	15733	62	3.528877	0.00354
midnightb	7294	SEKI_INFLAMMATORY_RESPONSE_LPS_UP	3	70	16183	71	9.810664	0.003547
blue	8061	WAKABAYASHI_ADIPOGENESIS_PPARG_RXRA_BOUND_	39	140	16113	2978	1.520356	0.003552
magenta	5022	BILBAN_B_CLL_LPL_UP	4	61	16192	167	6.381859	0.003559
turquoise	2058	GTAAGAT,MIR-200A	21	47	16206	4169	1.741899	0.00356
purple	6116	TARTE_PLASMA_CELL_VS_PLASMABLAST_UP	9	315	15938	157	2.95778	0.003564
turquoise	7157	WEST_ADRENOCORTICAL_TUMOR_UP	94	286	15967	4169	1.281337	0.003568
lightgreen	6196	BYSTRYKH_HEMATOPOIESIS_STEM_CELL_QTL_TRANS	7	781	15472	42	3.468417	0.003569
purple	7756	NAKAMURA_ADIPOGENESIS_LATE_DN	3	32	16221	157	9.705215	0.003571
green	1045	NITROGEN_COMPOUND_CATABOLIC_PROCESS	7	26	16227	1305	3.353109	0.003575
pink	6088	GNATENKO_PLATELET_SIGNATURE	4	45	16208	228	6.336452	0.003582
lightyellow	5618	TSUNODA_CISPLATIN_RESISTANCE_DN	2	44	16209	33	22.38705	0.003586
lightyellow	6026	CHEN_NEUROBLASTOMA_COPY_NUMBER_GAINS	2	44	16209	33	22.38705	0.003586
red	2844	KEGG_HEMATOPOIETIC_CELL_LINEAGE	8	78	16175	524	3.181249	0.003587
red	5949	LIN_SILENCED_BY_TUMOR_MICROENVIRONMENT	8	78	16175	524	3.181249	0.003587
brown	8845	GSE17721_CTRL_VS_LPS_12H_BMDM_DN	37	176	16077	2190	1.560194	0.003591
purple	2900	KEGG_HYPERTROPHIC_CARDIOMYOPATHY_HCM	4	65	16188	157	6.370603	0.003591
purple	7311	LINDSTEDT_DENDRITIC_CELL_MATURATION_C	4	65	16188	157	6.370603	0.003591
blue	4441	REACTOME_CITRIC_ACID_CYCLE_TCA_CYCLE	9	19	16234	2978	2.585221	0.003595
blue	5982	COLLIS_PRKDC_SUBSTRATES	9	19	16234	2978	2.585221	0.003595
turquoise	3705	MODULE_233	12	22	16231	4169	2.126475	0.003598
turquoise	5585	SCHRAMM_INHBA_TARGETS_DN	12	22	16231	4169	2.126475	0.003598
turquoise	7828	WANG_METASTASIS_OF_BREAST_CANCER_ESR1_UP	12	22	16231	4169	2.126475	0.003598
salmon	2320	MORF_PSMC1	6	188	16065	127	4.084352	0.0036
salmon	8947	GSE17721_POLYIC_VS_PAM3CSK4_24H_BMDM_DN	6	188	16065	127	4.084352	0.0036
salmon	9819	GSE360_DC_VS_MAC_L_DONOVANI_DN	6	188	16065	127	4.084352	0.0036
magenta	449	RESPONSE_TO_STEROID_HORMONE_STIMULUS	2	9	16244	167	21.62741	0.003603
magenta	3044	BIOCARTA_PLATELETAPP_PATHWAY	2	9	16244	167	21.62741	0.003603
magenta	3118	BIOCARTA_VITCB_PATHWAY	2	9	16244	167	21.62741	0.003603
magenta	4029	PID_INTEGRIN4_PATHWAY	2	9	16244	167	21.62741	0.003603
red	9832	GSE360_L_DONOVANI_VS_T_GONDII_DC_UP	12	151	16102	524	2.464941	0.003607
magenta	8128	BOSCO_ALLERGEN_INDUCED_TH2_ASSOCIATED_MODU	6	143	16110	167	4.083497	0.00361
lightcyan	8335	TGFB_UP.V1_DN	4	165	16088	62	6.355034	0.003616
lightcyan	10112	GSE39820_IL1B_IL6_VS_IL1B_IL6_IL23A_TREATED_CD4	4	165	16088	62	6.355034	0.003616
cyan	7443	MEISSNER_BRAIN_HCP_WITH_H3K4ME3_AND_H3K27M	10	779	15474	77	2.709601	0.003618
magenta	8826	GSE17580_TREG_VS_TEFF_S_MANSONI_INF_UP	7	191	16062	167	3.566824	0.003622
magenta	8830	GSE17580_UNINFECTED_VS_S_MANSONI_INF_TREG_U	7	191	16062	167	3.566824	0.003622
magenta	10258	GSE9650_NAIVE_VS_MEMORY_CD8_TCELL_UP	7	191	16062	167	3.566824	0.003622
greenyello	7652	PYEON_CANCER_HEAD_AND_NECK_VS_CERVICAL_DN	2	10	16243	150	21.67067	0.003627
brown	9070	GSE17721_POLYIC_VS_GARDIQUIMOD_16H_BMDM_U	39	188	16065	2190	1.539558	0.003631
brown	9894	GSE36392_TYPE_2_MYELOID_VS_NEUTROPHIL_IL25_TI	39	188	16065	2190	1.539558	0.003631
turquoise	7850	KIM_ALL_DISORDERS_CALB1_CORR_UP	155	500	15753	4169	1.208546	0.003634
blue	712	MACROMOLECULE_CATABOLIC_PROCESS	36	127	16126	2978	1.547062	0.003635
turquoise	3783	MODULE_331	28	68	16185	4169	1.60528	0.003636
brown	10270	GSE9988_ANTI_TREM1_VS_LOW_LPS_MONOCYTE_UP	40	194	16059	2190	1.530198	0.00364
red	3800	MODULE_356	11	132	16121	524	2.584765	0.003641
red	6431	LU_AGING_BRAIN_DN	11	132	16121	524	2.584765	0.003641
blue	9113	GSE17721_12H_VS_24H_POLYIC_BMDM_DN	51	194	16059	2978	1.434753	0.003642
blue	9966	GSE3982_DC_VS_NEUTROPHIL_LPS_STIM_UP	51	194	16059	2978	1.434753	0.003642
red	8342	HOXA9_DN.V1_UP	14	191	16062	524	2.27351	0.003647
red	10172	GSE7460_FOXP3_MUT_VS_WT_ACT_TCONV_UP	14	191	16062	524	2.27351	0.003647
red	10207	GSE7852_LN_VS_FAT_TCONV_DN	14	191	16062	524	2.27351	0.003647
yellow	6478	NIELSEN_GIST_VS_SYNOVIAL_SARCOMA_UP	5	13	16240	1426	4.383698	0.003647
yellow	8086	SANDERSON_PPARA_TARGETS	5	13	16240	1426	4.383698	0.003647
greenyello	1478	V$SOX5_01	7	213	16040	150	3.560908	0.003649
greenyello	1652	V$NFY_01	7	213	16040	150	3.560908	0.003649
greenyello	1877	V$SMAD_Q6	7	213	16040	150	3.560908	0.003649
salmon	2272	RCGCANGCGY_V$NRF1_Q6	15	881	15372	127	2.178939	0.00365
cyan	7569	MIKKELSEN_MCV6_LCP_WITH_H3K4ME3	4	133	16120	77	6.348208	0.003657
red	9912	GSE36476_CTRL_VS_TSST_ACT_72H_MEMORY_CD4_T(	13	171	16082	524	2.358031	0.003669

lightgreen	2898	KEGG_GRAFT_VERSUS_HOST_DISEASE	2	35	16218	42	22.11293	0.003675
lightgreen	6260	STOSSI_RESPONSE_TO_ESTRADIOL	2	35	16218	42	22.11293	0.003675
blue	8645	GSE14308_TH1_VS_NATURAL_TREG_DN	49	185	16068	2978	1.44555	0.003675
grey60	4565	REACTOME_GPCR_LIGAND_BINDING	4	235	16018	44	6.287427	0.003676
magenta	6932	HELLER_SILENCED_BY_METHYLATION_UP	8	243	16010	167	3.204061	0.003676
red	2263	TGACAGNY_V$MEIS1_01	35	672	15581	524	1.615478	0.003678
pink	1562	V$SRY_01	8	178	16075	228	3.203824	0.003683
yellow	8452	GSE11057_CD4_EFF_MEM_VS_PBMC_UP	29	196	16057	1426	1.686382	0.003683
yellow	9939	GSE37416_0H_VS_12H_F_TULARENSIS_LVS_NEUTROPI	29	196	16057	1426	1.686382	0.003683
lightyellow	6957	MARTINEZ_RB1_AND_TP53_TARGETS_UP	5	519	15734	33	4.744847	0.003689
blue	6814	MONNIER_POSTRADIATION_TUMOR_ESCAPE_UP	89	372	15881	2978	1.305738	0.003692
blue	8643	GSE14308_TH1_VS_INDUCED_TREG_DN	47	176	16077	2978	1.457451	0.003692
red	3713	MODULE_241	7	62	16191	524	3.501939	0.003692
red	7309	LINDSTEDT_DENDRITIC_CELL_MATURATION_A	7	62	16191	524	3.501939	0.003692
salmon	9434	GSE24634_NAIVE_CD4_TCELL_VS_DAY5_IL4_CONV_TR	6	189	16064	127	4.062742	0.003694
lightcyan	9848	GSE360_T_GONDII_VS_B_MALAYI_HIGH_DOSE_DC_UP	4	166	16087	62	6.316751	0.003695
turquoise	8725	GSE14769_UNSTIM_VS_240MIN_LPS_BMDM_DN	66	191	16062	4169	1.347138	0.003697
turquoise	9065	GSE17721_POLYIC_VS_GARDIQUIMOD_6H_BMDM_DN	66	191	16062	4169	1.347138	0.003697
greenyello	8261	RAF_UP.V1_DN	6	160	16093	150	4.06325	0.0037
turquoise	3360	chr2q31	24	56	16197	4169	1.670801	0.003713
salmon	6697	ABE_INNER_EAR	3	40	16213	127	9.598228	0.003728
greenyello	6990	OSADA_ASCL1_TARGETS_UP	3	34	16219	150	9.560588	0.003739
lightcyan	4949	NEWMAN_ERCC6_TARGETS_UP	2	24	16229	62	21.84543	0.003744
lightcyan	7627	WORSCHECH_TUMOR_EVASION_AND_TOLEROGENICIT	2	24	16229	62	21.84543	0.003744
midnightb	7590	ONO_FOXP3_TARGETS_UP	2	21	16232	71	21.80148	0.003745
turquoise	959	PHOSPHORYLATION	93	283	15970	4169	1.281145	0.003752
green	893	NEUROTRANSMITTER_SECRETION	4	9	16244	1305	5.535292	0.003753
green	7695	DORN_ADENOVIRUS_INFECTION_24HR_UP	4	9	16244	1305	5.535292	0.003753
tan	8753	GSE15659_CD45RA_NEG_CD4_TCELL_VS_RESTING_TRE	6	166	16087	145	4.051433	0.003753
lightyellow	5886	WU_CELL_MIGRATION	3	155	16098	33	9.532551	0.003763
red	4844	SENGUPTA_NASOPHARYNGEAL_CARCINOMA_UP	18	276	15977	524	2.022859	0.003773
lightcyan	8659	GSE1432_CTRL_VS_IFNG_1H_MICROGLIA_DN	4	167	16086	62	6.278926	0.003775
lightcyan	9134	GSE17721_4_VS_24H_CPG_BMDM_UP	4	167	16086	62	6.278926	0.003775
magenta	4225	REACTOME_EXTRACELLULAR_MATRIX_ORGANIZATION	4	62	16191	167	6.278926	0.003775
brown	6929	STEARMAN_TUMOR_FIELD_EFFECT_UP	11	34	16219	2190	2.401061	0.003778
lightyellow	6955	MARTINEZ_TP53_TARGETS_UP	5	522	15731	33	4.717578	0.003781
black	6470	SESTO_RESPONSE_TO_UV_C6	4	37	16216	283	6.208767	0.003782
turquoise	844	POSITIVE_REGULATION_OF_JNK_ACTIVITY	10	17	16236	4169	2.293257	0.003786
salmon	8620	GSE14000_UNSTIM_VS_16H_LPS_DC_UP	6	190	16063	127	4.041359	0.003791
salmon	9663	GSE29618_MONOCYTE_VS_MDC_DN	6	190	16063	127	4.041359	0.003791
yellow	9871	GSE360_L_MAJOR_VS_T_GONDII_MAC_DN	27	179	16074	1426	1.719193	0.003796
yellow	2369	MORF_CSNK2B	39	286	15967	1426	1.55422	0.003801
red	7870	LU_EZH2_TARGETS_DN	22	365	15888	524	1.869528	0.003802
purple	5239	GRAESSMANN_RESPONSE_TO_MC_AND_DOXORUBICIN	13	574	15679	157	2.344582	0.003808
pink	8818	GSE16522_ANTI_CD3CD28_STIM_VS_UNSTIM_MEMOF	8	179	16074	228	3.185926	0.00381
pink	9611	GSE27786_NKTCELL_VS_MONO_MAC_DN	8	179	16074	228	3.185926	0.00381
turquoise	7864	WANG_RESPONSE_TO_GSK3_INHIBITOR_SB216763_UF	116	363	15890	4169	1.245813	0.003813
greenyello	2807	KEGG_CHEMOKINE_SIGNALING_PATHWAY	6	161	16092	150	4.038012	0.003815
greenyello	9199	GSE17974_IL4_AND_ANTI_IL12_VS_UNTREATED_24H_	6	161	16092	150	4.038012	0.003815
turquoise	2069	ATGAAGG,MIR-205	53	148	16105	4169	1.396098	0.003817
pink	294	GLYCOPROTEIN_METABOLIC_PROCESS	5	74	16179	228	4.81656	0.003819
pink	5381	RIZ_ERYTHROID_DIFFERENTIATION	5	74	16179	228	4.81656	0.003819
red	6869	ZHENG_FOXP3_TARGETS_IN_THYMUS_UP	14	192	16061	524	2.261669	0.003821
red	8391	KAECH_NAIVE_VS_MEMORY_CD8_TCELL_DN	14	192	16061	524	2.261669	0.003821
red	8663	GSE1432_CTRL_VS_IFNG_24H_MICROGLIA_DN	14	192	16061	524	2.261669	0.003821
lightgreen	3654	MODULE_171	3	122	16131	42	9.515808	0.003827
pink	5068	SENESE_HDAC1_TARGETS_DN	9	219	16034	228	2.929524	0.003832
magenta	1497	V$TAL1BETAITF2_01	7	193	16060	167	3.529863	0.003834
magenta	1671	V$OCT1_B	7	193	16060	167	3.529863	0.003834
magenta	10187	GSE7764_NKCELL_VS_SPLENOCYTE_DN	7	193	16060	167	3.529863	0.003834

red	3999	PID_IL1PATHWAY	5	33	16220	524	4.699572	0.003843
turquoise	9321	GSE22886_NAIVE_CD4_TCELL_VS_NKCELL_DN	68	198	16055	4169	1.338891	0.003844
brown	388	INDUCTION_OF_APOPTOSIS_BY_EXTRACELLULAR_SIGN	9	25	16228	2190	2.671726	0.003849
blue	4353	REACTOME_CELL_DEATH_SIGNALLING_VIA_NRAGE_NR	19	56	16197	2978	1.851716	0.003852
blue	3996	PID_TELOMERASEPATHWAY	21	64	16189	2978	1.790804	0.003855
cyan	8124	RAO_BOUND_BY_SALL4_ISOFORM_B	7	423	15830	77	3.493015	0.003866
lightgreen	5132	WAMUNYOKOLI_OVARIAN_CANCER_LMP_UP	4	250	16003	42	6.191619	0.003867
blue	2600	GNF2_RFC4	20	60	16193	2978	1.81923	0.003869
yellow	5132	WAMUNYOKOLI_OVARIAN_CANCER_LMP_UP	35	250	16003	1426	1.595666	0.003871
blue	9246	GSE18791_UNSTIM_VS_NEWCATSLE_VIRUS_DC_18H_U	50	190	16063	2978	1.436234	0.003874
turquoise	1549	ACTWSNACTNY_UNKNOWN	35	90	16163	4169	1.516098	0.003877
magenta	6103	FRASOR_RESPONSE_TO_ESTRADIOL_UP	3	31	16222	167	9.418389	0.003877
turquoise	8628	GSE14308_TH2_VS_TH1_UP	65	188	16065	4169	1.347898	0.003885
turquoise	9077	GSE17721_LPS_VS_GARDIQUIMOD_1H_BMDM_DN	65	188	16065	4169	1.347898	0.003885
salmon	8499	GSE12366_PLASMA_CELL_VS_NAIVE_BCELL_DN	6	191	16062	127	4.0202	0.003889
salmon	9030	GSE17721_PAM3CSK4_VS_GADIQUIMOD_8H_BMDM_	6	191	16062	127	4.0202	0.003889
salmon	9994	GSE3982_MAST_CELL_VS_MAC_UP	6	191	16062	127	4.0202	0.003889
yellow	9092	GSE17721_0.5H_VS_12H_LPS_BMDM_UP	28	188	16065	1426	1.697517	0.00389
magenta	7725	MIKKELSEN_MEF_HCP_WITH_H3K27ME3	9	300	15953	167	2.919701	0.003892
brown	2843	KEGG_JAK_STAT_SIGNALING_PATHWAY	26	113	16140	2190	1.707593	0.003895
purple	4040	PID_UPA_UPAR_PATHWAY	3	33	16220	157	9.411118	0.003901
purple	5228	LANDIS_ERBB2_BREAST_TUMORS_65_DN	3	33	16220	157	9.411118	0.003901
purple	5616	WEINMANN_ADAPTATION_TO_HYPOXIA_DN	3	33	16220	157	9.411118	0.003901
turquoise	232	CHROMOSOME	45	122	16131	4169	1.437985	0.003902
magenta	7839	MARTENS_TRETINOIN_RESPONSE_UP	12	478	15775	167	2.443264	0.003914
pink	110	CELL_CORTEX_PART	3	23	16230	228	9.298055	0.003922
pink	7783	DAZARD_UV_RESPONSE_CLUSTER_G24	3	23	16230	228	9.298055	0.003922
pink	291	SYNAPTOGENESIS	2	7	16246	228	20.36717	0.003928
pink	1130	SOLUTE_SODIUM_SYMPORTER_ACTIVITY	2	7	16246	228	20.36717	0.003928
pink	4277	REACTOME_ABACAVIR_TRANSPORT_AND_METABOLISI	2	7	16246	228	20.36717	0.003928
pink	4520	REACTOME_NA_CL_DEPENDENT_NEUROTRANSMITTER	2	7	16246	228	20.36717	0.003928
pink	7256	MOOTHA_ROS	2	7	16246	228	20.36717	0.003928
greenyello	9258	GSE20366_TREG_VS_TCONV_UP	6	162	16091	150	4.013086	0.003932
blue	5074	SENESE_HDAC3_TARGETS_DN	111	479	15774	2978	1.264726	0.003934
greenyello	1979	TTTGCAC,MIR-19A,MIR-19B	11	465	15788	150	2.563197	0.003937
lightcyan	9833	GSE360_L_DONOVANI_VS_T_GONDII_DC_DN	4	169	16084	62	6.204619	0.003938
brown	860	CELLULAR_LOCALIZATION	64	343	15910	2190	1.384762	0.003942
pink	7863	CHICAS_RB1_TARGETS_CONFLUENT	15	490	15763	228	2.182197	0.003952
greenyello	413	ANATOMICAL_STRUCTURE_MORPHOGENESIS	8	274	15979	150	3.163601	0.003953
purple	9238	GSE18791_UNSTIM_VS_NEWCATSLE_VIRUS_DC_1H_UI	5	108	16145	157	4.792699	0.003959
cyan	1289	TRANSCRIPTION_REPRESSOR_ACTIVITY	4	136	16117	77	6.208174	0.00396
grey60	8347	STK33_SKM_DN	4	240	16013	44	6.156439	0.003962
brown	10061	GSE3982_BCELL_VS_BASOPHIL_DN	37	177	16076	2190	1.551379	0.003964
purple	2973	BIOCARTA_EXTRINSIC_PATHWAY	2	10	16243	157	20.70446	0.003965
purple	4296	REACTOME_PLATELET_ADHESION_TO_EXPOSED_COLL/	2	10	16243	157	20.70446	0.003965
cyan	7053	SMID_BREAST_CANCER_RELAPSE_IN_BONE_DN	5	221	16032	77	4.775519	0.00397
brown	6420	KAAB_FAILED_HEART_ATRIUM_DN	30	136	16117	2190	1.637087	0.003973
brown	10129	GSE6269_HEALTHY_VS_E_COLI_INF_PBMC_DN	30	136	16117	2190	1.637087	0.003973
turquoise	4771	REACTOME_S_PHASE	40	106	16147	4169	1.471146	0.003975
blue	1264	ATP_DEPENDENT_RNA_HELICASE_ACTIVITY	8	16	16237	2978	2.728845	0.003981
blue	2772	KEGG_ONE_CARBON_POOL_BY_FOLATE	8	16	16237	2978	2.728845	0.003981
blue	4605	REACTOME_AMINO_ACID_SYNTHESIS_AND_INTERCON	8	16	16237	2978	2.728845	0.003981
cyan	7270	FERRARI_RESPONSE_TO_FENRETINIDE_UP	2	20	16233	77	21.10779	0.003983
brown	10125	GSE5960_TH1_VS_ANERGIC_TH1_DN	38	183	16070	2190	1.541068	0.003983
salmon	8388	KAECH_NAIVE_VS_DAY15_EFF_CD8_TCELL_UP	6	192	16061	127	3.999262	0.00399
salmon	9378	GSE22886_NAIVE_CD4_TCELL_VS_DC_UP	6	192	16061	127	3.999262	0.00399
salmon	9737	GSE31082_DP_VS_CD4_SP_THYMOCYTE_DN	6	192	16061	127	3.999262	0.00399
brown	9337	GSE22886_NAIVE_BCELL_VS_BM_PLASMA_CELL_DN	39	189	16064	2190	1.531413	0.003994
brown	10024	GSE3982_DC_VS_NKCELL_UP	39	189	16064	2190	1.531413	0.003994
brown	8616	GSE14000_UNSTIM_VS_16H_LPS_DC_TRANSLATED_RN	40	195	16058	2190	1.522351	0.003997

red	10097	GSE3982_NKCELL_VS_TH1_DN	14	193	16060	524	2.249951	0.004002
red	10259	GSE9650_NAIVE_VS_MEMORY_CD8_TCELL_DN	14	193	16060	524	2.249951	0.004002
tan	6225	MAGRANGEAS_MULTIPLE_MYELOMA_IGLL_VS_IGLK_U	3	36	16217	145	9.340805	0.004003
lightcyan	4844	SENGUPTA_NASOPHARYNGEAL_CARCINOMA_UP	5	276	15977	62	4.749007	0.004004
purple	6523	LINDVALL_IMMORTALIZED_BY_TERT_UP	4	67	16186	157	6.180435	0.004006
purple	6751	JI_RESPONSE_TO_FSH_UP	4	67	16186	157	6.180435	0.004006
purple	6949	LEE_METASTASIS_AND_ALTERNATIVE_SPLICING_UP	4	67	16186	157	6.180435	0.004006
red	6863	MARSON_BOUND_BY_FOXP3_STIMULATED	47	978	15275	524	1.4906	0.004012
blue	2736	KEGG_VALINE_LEUCINE_AND_ISOLEUCINE_BIOSYNTHE	6	10	16243	2978	3.274614	0.004018
blue	5415	BAKER_HEMATOPOESIS_STAT1_TARGETS	6	10	16243	2978	3.274614	0.004018
lightcyan	9054	GSE17721_LPS_VS_CPG_24H_BMDM_UP	4	170	16083	62	6.168121	0.004022
lightcyan	9513	GSE2706_R848_VS_LPS_8H_STIM_DC_DN	4	170	16083	62	6.168121	0.004022
brown	3482	ST_B_CELL_ANTIGEN_RECEPTOR	12	39	16214	2190	2.283527	0.00403
brown	4916	CASORELLI_APL_SECONDARY_VS_DE_NOVO_UP	12	39	16214	2190	2.283527	0.00403
brown	6712	VARELA_ZMPSTE24_TARGETS_UP	12	39	16214	2190	2.283527	0.00403
yellow	3358	chr1p36	39	287	15966	1426	1.548805	0.004038
turquoise	8621	GSE14000_UNSTIM_VS_16H_LPS_DC_DN	67	195	16058	4169	1.339497	0.00404
turquoise	8770	GSE15750_DAY6_VS_DAY10_EFF_CD8_TCELL_UP	67	195	16058	4169	1.339497	0.00404
turquoise	9209	GSE17974_2H_VS_72H_UNTREATED_IN_VITRO_CD4_T	67	195	16058	4169	1.339497	0.00404
red	7647	BOYLAN_MULTIPLE_MYELOMA_PCA3_DN	7	63	16190	524	3.446353	0.004041
lightyellow	8286	ESC_J1_UP_EARLY.V1_UP	3	159	16094	33	9.292739	0.004042
lightyellow	8594	GSE13485_PRE_VS_POST_YF17D_VACCINATION_PBMC	3	159	16094	33	9.292739	0.004042
brown	8306	EIF4E_UP	22	91	16162	2190	1.794199	0.004043
pink	4902	PUIFFE_INVASION_INHIBITED_BY_ASCITES_UP	5	75	16178	228	4.752339	0.004046
lightyellow	7892	FIGUEROA_AML_METHYLATION_CLUSTER_7_DN	1	2	16251	33	246.2576	0.004057
lightcyan	3443	chr4q32	2	25	16228	62	20.97161	0.004059
salmon	2319	MORF_PCNA	4	83	16170	127	6.167536	0.00406
red	4565	REACTOME_GPCR_LIGAND_BINDING	16	235	16018	524	2.111808	0.004064
red	1526	V$CEBPB_01	15	214	16039	524	2.174101	0.004066
turquoise	2562	GCM_UBE2N	55	155	16098	4169	1.383352	0.004067
magenta	5149	GOZGIT_ESR1_TARGETS_DN	14	608	15645	167	2.240998	0.004072
pink	9018	GSE17721_POLYIC_VS_CPG_24H_BMDM_UP	8	181	16072	228	3.150722	0.004074
pink	7371	SWEET_LUNG_CANCER_KRAS_DN	12	351	15902	228	2.437097	0.004081
red	4866	DAVICIONI_MOLECULAR_ARMS_VS_ERMS_UP	19	300	15953	524	1.964421	0.004082
red	6971	SARRIO_EPITHELIAL_MESENCHYMAL_TRANSITION_DN	11	134	16119	524	2.546186	0.004084
purple	6275	HSIAO_LIVER_SPECIFIC_GENES	6	156	16097	157	3.981627	0.004086
salmon	2373	MORF_DAP3	6	193	16060	127	3.97854	0.004092
salmon	9138	GSE17721_0.5H_VS_12H_GARDIQUIMOD_BMDM_UP	6	193	16060	127	3.97854	0.004092
salmon	9466	GSE25087_FETAL_VS_ADULT_TCONV_UP	6	193	16060	127	3.97854	0.004092
salmon	10099	GSE3982_NKCELL_VS_TH2_DN	6	193	16060	127	3.97854	0.004092
turquoise	27	PROTEIN_SERINE_THREONINE_PHOSPHATASE_COMPLE	7	10	16243	4169	2.728976	0.004095
lightgreen	3401	chr1q24	2	37	16216	42	20.91763	0.0041
turquoise	9190	GSE17974_IL4_AND_ANTI_IL12_VS_UNTREATED_2H_A	58	165	16088	4169	1.370395	0.0041
lightcyan	9078	GSE17721_LPS_VS_GARDIQUIMOD_2H_BMDM_UP	4	171	16082	62	6.132051	0.004106
midnightb	6127	KIM_GERMINAL_CENTER_T_HELPER_DN	2	22	16231	71	20.8105	0.004108
lightyellow	9492	GSE26928_EFF_MEM_VS_CENTR_MEM_CD4_TCELL_UI	3	160	16093	33	9.234659	0.004113
blue	2472	MORF_THRA	14	37	16216	2978	2.065072	0.004116
black	1423	HYDROLASE_ACTIVITY_ACTING_ON_CARBON_NITROGE	3	19	16234	283	9.068068	0.004117
black	6828	LOPES_METHYLATED_IN_COLON_CANCER_UP	3	19	16234	283	9.068068	0.004117
purple	4873	SENGUPTA_NASOPHARYNGEAL_CARCINOMA_WITH_LM	5	109	16144	157	4.748729	0.004117
blue	6173	FLECHNER_PBL_KIDNEY_TRANSPLANT_OK_VS_DONOR_	41	150	16103	2978	1.491769	0.004123
tan	4536	REACTOME_MRNA_DECAY_BY_3_TO_5_EXORIBONUCL	2	11	16242	145	20.37994	0.004124
red	858	CYTOKINE_AND_CHEMOKINE_MEDIATED_SIGNALING_I	4	21	16232	524	5.908033	0.004131
red	2774	KEGG_NICOTINATE_AND_NICOTINAMIDE_METABOLISM	4	21	16232	524	5.908033	0.004131
turquoise	8060	WAKABAYASHI_ADIPOGENESIS_PPARG_BOUND_8D	187	616	15637	4169	1.183484	0.00414
turquoise	1952	TGCACTG,MIR-148A,MIR-152,MIR-148B	91	277	15976	4169	1.280747	0.004149
brown	6093	PENG_GLUCOSE_DEPRIVATION_UP	13	44	16209	2190	2.192704	0.004159
brown	6771	JIANG_AGING_HYPOTHALAMUS_UP	13	44	16209	2190	2.192704	0.004159
purple	3511	MODULE_19	8	264	15989	157	3.137039	0.004166
green	9059	GSE17721_POLYIC_VS_GARDIQUIMOD_1H_BMDM_DN	26	187	16066	1305	1.731629	0.004168

grey60	3326	chr21q22	3	120	16133	44	9.234659	0.00417
red	4891	FULCHER_INFLAMMATORY_RESPONSE_LECTIN_VS_LPS	25	437	15816	524	1.774438	0.004171
magenta	8765	GSE15733_BM_VS_SPLEEN_MEMORY_CD4_TCELL_DN	7	196	16057	167	3.475834	0.004172
magenta	10192	GSE7852_TREG_VS_TCONV_LN_UP	7	196	16057	167	3.475834	0.004172
greenyello	1520	V$S8_01	6	164	16089	150	3.964146	0.004175
greenyello	10003	GSE3982_MAST_CELL_VS_EFF_MEMORY_CD4_TCELL_[	6	164	16089	150	3.964146	0.004175
red	9441	GSE24634_TREG_VS_TCONV_POST_DAY3_IL4_CONVER	14	194	16059	524	2.238353	0.00419
red	10093	GSE3982_CENT_MEMORY_CD4_TCELL_VS_TH1_DN	14	194	16059	524	2.238353	0.00419
red	10222	GSE8868_SPLEEN_VS_INTESTINE_CD11B_POS_CD11C_I	14	194	16059	524	2.238353	0.00419
greenyello	2530	GCM_MAPK10	4	71	16182	150	6.104413	0.004195
yellow	9337	GSE22886_NAIVE_BCELL_VS_BM_PLASMA_CELL_DN	28	189	16064	1426	1.688536	0.004195
salmon	8960	GSE17721_PAM3CSK4_VS_CPG_12H_BMDM_UP	6	194	16059	127	3.958032	0.004196
cyan	6794	KRIGE_RESPONSE_TO_TOSEDOSTAT_6HR_UP	11	929	15324	77	2.499308	0.004204
pink	6332	ALCALAY_AML_BY_NPM1_LOCALIZATION_DN	8	182	16071	228	3.13341	0.004211
brown	1095	LIPID_BINDING	19	75	16178	2190	1.880104	0.004214
tan	10128	GSE6269_HEALTHY_VS_E_COLI_INF_PBMC_UP	6	170	16083	145	3.956105	0.004215
yellow	2272	RCGCANGCGY_V$NRF1_Q6	100	881	15372	1426	1.293713	0.004219
lightcyan	4926	CHARAFE_BREAST_CANCER_LUMINAL_VS_BASAL_DN	6	402	15851	62	3.912614	0.004219
brown	7236	WANG_TUMOR_INVASIVENESS_UP	67	363	15890	2190	1.369801	0.00422
brown	3773	MODULE_321	25	108	16145	2190	1.717931	0.004222
purple	4337	REACTOME_INTEGRIN_CELL_SURFACE_INTERACTIONS	4	68	16185	157	6.089547	0.004225
purple	6235	YAO_HOXA10_TARGETS_VIA_PROGESTERONE_UP	4	68	16185	157	6.089547	0.004225
purple	7176	BRUECKNER_TARGETS_OF_MIRLET7A3_DN	4	68	16185	157	6.089547	0.004225
magenta	7432	SHAFFER_IRF4_TARGETS_IN_ACTIVATED_DENDRITIC_C	4	64	16189	167	6.08271	0.004232
red	2836	KEGG_COMPLEMENT_AND_COAGULATION_CASCADES	6	48	16205	524	3.877147	0.004245
magenta	2897	KEGG_ALLOGRAFT_REJECTION	3	32	16221	167	9.124064	0.004246
greenyello	1819	V$HEB_Q6	7	219	16034	150	3.463349	0.004246
turquoise	9564	GSE27786_BCELL_VS_CD8_TCELL_UP	66	192	16061	4169	1.340122	0.004247
turquoise	9697	GSE29618_PRE_VS_DAY7_POST_TIV_FLU_VACCINE_PD	66	192	16061	4169	1.340122	0.004247
black	2022	GTTRYCATRR_UNKNOWN	8	147	16106	283	3.125502	0.004247
purple	6851	LEIN_LOCALIZED_TO_PROXIMAL_DENDRITES	3	34	16219	157	9.13432	0.004248
purple	7020	HUANG_FOXA2_TARGETS_DN	3	34	16219	157	9.13432	0.004248
blue	3586	MODULE_98	88	369	15884	2978	1.301563	0.004251
turquoise	5001	GARGALOVIC_RESPONSE_TO_OXIDIZED_PHOSPHOLIPIC	47	129	16124	4169	1.420397	0.004256
yellow	9181	GSE17974_CTRL_VS_ACT_IL4_AND_ANTI_IL12_24H_CD	29	198	16055	1426	1.669348	0.004269
blue	8509	GSE12845_IGD_POS_BLOOD_VS_PRE_GC_TONSIL_BCEI	52	200	16053	2978	1.418999	0.00427
blue	9435	GSE24634_NAIVE_CD4_TCELL_VS_DAY5_IL4_CONV_TR	52	200	16053	2978	1.418999	0.00427
blue	9913	GSE36476_CTRL_VS_TSST_ACT_72H_MEMORY_CD4_T(	52	200	16053	2978	1.418999	0.00427
lightcyan	9052	GSE17721_LPS_VS_CPG_16H_BMDM_UP	4	173	16080	62	6.06116	0.004279
cyan	4001	PID_REG_GR_PATHWAY	3	69	16184	77	9.177301	0.00428
salmon	4436	REACTOME_MRNA_SPLICING_MINOR_PATHWAY	3	42	16211	127	9.14117	0.004283
salmon	6356	ZHAN_MULTIPLE_MYELOMA_HP_UP	3	42	16211	127	9.14117	0.004283
turquoise	2027	GGGCATT,MIR-365	38	100	16153	4169	1.481444	0.004287
turquoise	9196	GSE17974_IL4_AND_ANTI_IL12_VS_UNTREATED_12H_	63	182	16071	4169	1.349494	0.00429
greenyello	1761	V$STAT5A_04	6	165	16088	150	3.940121	0.0043
salmon	8561	GSE13411_PLASMA_CELL_VS_MEMORY_BCELL_DN	6	195	16058	127	3.937735	0.004302
salmon	8804	GSE15930_STIM_VS_STIM_AND_IFNAB_48H_CD8_T_C	6	195	16058	127	3.937735	0.004302
salmon	9205	GSE17974_0.5H_VS_72H_UNTREATED_IN_VITRO_CD4_	6	195	16058	127	3.937735	0.004302
salmon	9397	GSE22886_NAIVE_CD4_TCELL_VS_48H_ACT_TH2_DN	6	195	16058	127	3.937735	0.004302
salmon	9991	GSE3982_EOSINOPHIL_VS_TH2_DN	6	195	16058	127	3.937735	0.004302
turquoise	5371	ABDULRAHMAN_KIDNEY_CANCER_VHL_UP	4	4	16249	4169	3.898537	0.004324
black	7948	MIKHAYLOVA_OXIDATIVE_STRESS_RESPONSE_VIA_VHL	2	6	16247	283	19.1437	0.004327
green	9944	GSE37416_12H_VS_24H_F_TULARENSIS_LVS_NEUTROI	27	197	16056	1305	1.706949	0.004331
brown	3017	BIOCARTA_MAPK_PATHWAY	21	86	16167	2190	1.812217	0.004331
blue	10045	GSE3982_MAC_VS_TH2_DN	50	191	16062	2978	1.428715	0.004334
tan	10229	GSE9006_HEALTHY_VS_TYPE_1_DIABETES_PBMC_4MC	6	171	16082	145	3.93297	0.004337
turquoise	2372	MORF_DAP	31	78	16175	4169	1.549418	0.004346
yellow	5503	SCHLOSSER_MYC_AND_SERUM_RESPONSE_SYNERGY	8	31	16222	1426	2.94132	0.004347
brown	3003	BIOCARTA_IL22BP_PATHWAY	6	13	16240	2190	3.42529	0.004367
brown	3884	MODULE_492	6	13	16240	2190	3.42529	0.004367

brown	7356	CHANG_CORE_SERUM_RESPONSE_DN	41	202	16051	2190	1.506336	0.004374
brown	8491	GSE11924_TH2_VS_TH17_CD4_TCELL_DN	38	184	16069	2190	1.532693	0.004383
brown	8480	GSE11924_TFH_VS_TH1_CD4_TCELL_UP	40	196	16057	2190	1.514584	0.004384
brown	8704	GSE1460_CD4_THYMOCYTE_VS_NAIVE_CD4_TCELL_AD	40	196	16057	2190	1.514584	0.004384
brown	4822	HOLLMANN_APOPTOSIS_VIA_CD40_DN	50	257	15996	2190	1.443864	0.004387
brown	8904	GSE17721_CTRL_VS_GARDIQUIMOD_6H_BMDM_UP	39	190	16063	2190	1.523353	0.004388
brown	9154	GSE17974_0H_VS_2H_IN_VITRO_ACT_CD4_TCELL_UP	39	190	16063	2190	1.523353	0.004388
blue	5644	MOHANKUMAR_TLX1_TARGETS_UP	90	379	15874	2978	1.296021	0.004388
blue	2599	GNF2_RFC3	15	41	16212	2978	1.996716	0.004393
greenyello	3605	MODULE_117	11	472	15781	150	2.525184	0.004401
greenyello	98	ANCHORED_TO_MEMBRANE	2	11	16242	150	19.70061	0.004406
greenyello	209	ANCHORED_TO_PLASMA_MEMBRANE	2	11	16242	150	19.70061	0.004406
salmon	8400	GOLDRATH_NAIVE_VS_MEMORY_CD8_TCELL_UP	6	196	16057	127	3.917644	0.00441
salmon	9683	GSE29618_PRE_VS_DAY7_FLU_VACCINE_PDC_DN	6	196	16057	127	3.917644	0.00441
salmon	10254	GSE9650_NAIVE_VS_EFF_CD8_TCELL_UP	6	196	16057	127	3.917644	0.00441
greenyello	2285	CCAWWNAAGG_V$SRF_Q4	4	72	16181	150	6.01963	0.00441
magenta	8626	GSE14026_TH1_VS_TH17_UP	6	149	16104	167	3.919061	0.004413
blue	2535	GCM_MYST2	46	173	16080	2978	1.451178	0.004413
green	1758	CTGRYYYNATT_UNKNOWN	11	56	16197	1305	2.446401	0.004419
salmon	2420	MORF_MBD4	4	85	16168	127	6.022418	0.00442
blue	8689	GSE1460_INTRATHYMIC_T_PROGENITOR_VS_NAIVE_CI	44	164	16089	2978	1.464258	0.004424
magenta	2254	TGTTTGY_V$HNF3_Q6	13	549	15704	167	2.30456	0.004432
cyan	4751	REACTOME_CLASS_I_MHC_MEDIATED_ANTIGEN_PROC	5	227	16026	77	4.649293	0.004446
purple	3610	MODULE_122	5	111	16142	157	4.663166	0.004448
purple	6525	WANG_CISPLATIN_RESPONSE_AND_XPC_DN	7	211	16042	157	3.434389	0.004449
magenta	2240	CAGGTG_V$E12_Q6	33	2020	14233	167	1.589936	0.004451
purple	8071	HUANG_GATA2_TARGETS_DN	4	69	16184	157	6.001292	0.004452
blue	238	BIOPOLYMER_CATABOLIC_PROCESS	32	111	16142	2978	1.573388	0.004455
lightcyan	9097	GSE17721_0.5H_VS_8H_LPS_BMDM_DN	4	175	16078	62	5.991889	0.004457
lightcyan	9751	GSE32423_CTRL_VS_IL4_MEMORY_CD8_TCELL_DN	4	175	16078	62	5.991889	0.004457
lightcyan	9836	GSE360_L_DONOVANI_VS_B_MALAYI_LOW_DOSE_DC_	4	175	16078	62	5.991889	0.004457
red	1949	TGAATGT,MIR-181A,MIR-181B,MIR-181C,MIR-181D	24	416	15837	524	1.789452	0.004457
brown	532	LEUKOCYTE_DIFFERENTIATION	10	30	16223	2190	2.47382	0.004459
brown	3998	PID_NETRIN_PATHWAY	10	30	16223	2190	2.47382	0.004459
brown	5817	SCIAN_INVERSED_TARGETS_OF_TP53_AND_TP73_DN	10	30	16223	2190	2.47382	0.004459
greenyello	5062	UDAYAKUMAR_MED1_TARGETS_DN	7	221	16032	150	3.432006	0.004461
magenta	6436	IVANOVA_HEMATOPOIESIS_STEM_CELL_LONG_TERM	8	251	16002	167	3.10194	0.004464
salmon	4648	REACTOME_ACTIVATION_OF_IRF3_IRF7_MEDIATED_BY	2	13	16240	127	19.68867	0.004466
salmon	4663	REACTOME_TRAF6_MEDIATED_INDUCTION_OF_TAK1_	2	13	16240	127	19.68867	0.004466
salmon	7874	DEMAGALHAES_AGING_DN	2	13	16240	127	19.68867	0.004466
turquoise	9622	GSE28237_EARLY_VS_LATE_GC_BCELL_UP	53	149	16104	4169	1.386728	0.004473
magenta	506	GENERATION_OF_NEURONS	4	65	16188	167	5.989129	0.004474
magenta	670	SYNAPSE_ORGANIZATION_AND_BIOGENESIS	2	10	16243	167	19.46467	0.004474
magenta	1295	KINASE_ACTIVATOR_ACTIVITY	2	10	16243	167	19.46467	0.004474
lightgreen	8302	PTEN_DN.V1_UP	3	129	16124	42	8.999446	0.004474
green	2341	MORF_TFDP2	26	188	16065	1305	1.722418	0.004478
purple	9201	GSE17974_IL4_AND_ANTI_IL12_VS_UNTREATED_48H_	6	159	16094	157	3.906502	0.004483
green	3996	PID_TELOMERASEPATHWAY	12	64	16189	1305	2.335201	0.004484
midnightb	7588	ONO_AML1_TARGETS_UP	2	23	16230	71	19.9057	0.004486
pink	7923	BHAT_ESR1_TARGETS_NOT_VIA_AKT1_UP	8	184	16069	228	3.099352	0.004496
cyan	8222	MTOR_UP.V1_UP	4	141	16112	77	5.988026	0.004502
brown	3004	BIOCARTA_IL7_PATHWAY	7	17	16236	2190	3.055896	0.004512
purple	345	SIGNAL_TRANSDUCTION	23	1331	14922	157	1.78889	0.004513
turquoise	9497	GSE26928_CENTR_MEMORY_VS_CXCR5_POS_CD4_TCE	56	159	16094	4169	1.37307	0.004517
yellow	9345	GSE22886_IGM_MEMORY_BCELL_VS_BM_PLASMA_CE	28	190	16063	1426	1.679649	0.00452
salmon	9401	GSE22886_UNSTIM_VS_IL15_STIM_NKCELL_DN	6	197	16056	127	3.897758	0.00452
salmon	9586	GSE27786_CD4_TCELL_VS_MONO_MAC_UP	6	197	16056	127	3.897758	0.00452
magenta	3588	MODULE_100	11	425	15828	167	2.518957	0.004527
red	3902	MODULE_528	3	11	16242	524	8.45923	0.004531
red	4904	PIEPOLI_LGI1_TARGETS_UP	3	11	16242	524	8.45923	0.004531

blue	1937	V$T3R_Q6	51	196	16057	2978	1.420113	0.004546
lightgreen	7101	EHLERS_ANEUPLOIDY_UP	2	39	16214	42	19.84493	0.004547
lightcyan	8815	GSE16522_MEMORY_VS_NAIVE_CD8_TCELL_DN	4	176	16077	62	5.957845	0.004548
lightcyan	9965	GSE3982_DC_VS_MAC_LPS_STIM_DN	4	176	16077	62	5.957845	0.004548
greenyello	1553	V$OCT1_04	6	167	16086	150	3.892934	0.004559
magenta	5050	TONKS_TARGETS_OF_RUNX1_RUNX1T1_FUSION_ERYTI	6	150	16103	167	3.892934	0.004559
lightyellow	9978	GSE3982_EOSINOPHIL_VS_BCELL_UP	3	166	16087	33	8.900876	0.004559
magenta	7891	FIGUEROA_AML_METHYLATION_CLUSTER_7_UP	5	105	16148	167	4.634445	0.004561
blue	2572	GNF2_ATM	12	30	16223	2978	2.183076	0.004567
pink	4404	REACTOME_TRANSMEMBRANE_TRANSPORT_OF_SMAI	11	311	15942	228	2.521338	0.00457
brown	464	ACTIN_FILAMENT_BASED_PROCESS	24	103	16150	2190	1.729273	0.004571
salmon	3643	MODULE_158	3	43	16210	127	8.928585	0.004578
tan	7533	COULOUARN_TEMPORAL_TGFB1_SIGNATURE_DN	5	121	16132	145	4.631804	0.004582
black	6533	MEDINA_SMARCA4_TARGETS	4	39	16214	283	5.890369	0.004584
red	1632	V$CHOP_01	14	196	16057	524	2.215513	0.004588
red	8605	GSE13738_RESTING_VS_BYSTANDER_ACTIVATED_CD4_	14	196	16057	524	2.215513	0.004588
yellow	9324	GSE22886_CD8_TCELL_VS_BCELL_NAIVE_UP	29	199	16054	1426	1.660959	0.00459
black	7441	MEISSNER_BRAIN_HCP_WITH_H3K27ME3	7	118	16135	283	3.406929	0.004598
grey60	7443	MEISSNER_BRAIN_HCP_WITH_H3K4ME3_AND_H3K27N	7	779	15474	44	3.319261	0.004601
magenta	3625	MODULE_137	11	426	15827	167	2.513044	0.004606
lightcyan	3580	MODULE_92	3	88	16165	62	8.936767	0.004607
lightyellow	2670	GNF2_MLF1	2	50	16203	33	19.70061	0.004608
turquoise	6261	YU_MYC_TARGETS_UP	19	42	16211	4169	1.763624	0.004609
blue	10081	GSE3982_BASOPHIL_VS_TH2_DN	49	187	16066	2978	1.43009	0.004611
purple	4591	REACTOME_RNA_POL_I_PROMOTER_OPENING	3	35	16218	157	8.873339	0.004614
yellow	3531	MODULE_42	7	25	16228	1426	3.191332	0.004617
purple	1508	V$EVI1_05	5	112	16141	157	4.621531	0.004621
purple	4864	ZHONG_RESPONSE_TO_AZACITIDINE_AND_TSA_UP	6	160	16093	157	3.882086	0.004622
purple	8380	LEF1_UP.V1_DN	6	160	16093	157	3.882086	0.004622
turquoise	9610	GSE27786_NKTCELL_VS_MONO_MAC_UP	67	196	16057	4169	1.332663	0.004627
turquoise	9613	GSE27786_ERYTHROBLAST_VS_NEUTROPHIL_DN	67	196	16057	4169	1.332663	0.004627
turquoise	9942	GSE37416_0H_VS_48H_F_TULARENSIS_LVS_NEUTROPI	67	196	16057	4169	1.332663	0.004627
blue	2475	MORF_TPR	39	142	16111	2978	1.498943	0.004628
blue	9702	GSE29618_LAIV_VS_TIV_FLU_VACCINE_DAY7_BCELL_U	39	142	16111	2978	1.498943	0.004628
blue	5083	TIEN_INTESTINE_PROBIOTICS_24HR_UP	124	545	15708	2978	1.24175	0.004631
salmon	8726	GSE14769_UNSTIM_VS_360MIN_LPS_BMDM_UP	6	198	16055	127	3.878072	0.004632
salmon	9159	GSE17974_0H_VS_6H_IN_VITRO_ACT_CD4_TCELL_DN	6	198	16055	127	3.878072	0.004632
salmon	9370	GSE22886_NAIVE_CD8_TCELL_VS_NEUTROPHIL_UP	6	198	16055	127	3.878072	0.004632
magenta	2895	KEGG_AUTOIMMUNE_THYROID_DISEASE	3	33	16220	167	8.847578	0.004636
lightcyan	4969	WANG_CLIM2_TARGETS_DN	4	177	16076	62	5.924184	0.00464
lightcyan	9066	GSE17721_POLYIC_VS_GARDIQUIMOD_8H_BMDM_UP	4	177	16076	62	5.924184	0.00464
lightcyan	10189	GSE7764_IL15_TREATED_VS_CTRL_NK_CELL_24H_DN	4	177	16076	62	5.924184	0.00464
lightcyan	7043	MITSIADES_RESPONSE_TO_APLIDIN_UP	6	410	15843	62	3.836271	0.004641
turquoise	3291	chr13q14	28	69	16184	4169	1.582015	0.004645
red	2246	RYTTCCTG_V$ETS2_B	46	960	15293	524	1.48624	0.004645
blue	5060	DITTMER_PTHLH_TARGETS_DN	22	69	16184	2978	1.740133	0.00465
magenta	2194	TCCATTKW_UNKNOWN	7	200	16053	167	3.406317	0.004655
blue	9826	GSE360_DC_VS_MAC_B_MALAYI_LOW_DOSE_UP	47	178	16075	2978	1.441075	0.004661
brown	3994	PID_HDAC_CLASSI_PATHWAY	17	65	16188	2190	1.940998	0.004662
blue	6311	HASLINGER_B_CLL_WITH_CHROMOSOME_12_TRISOM'	10	23	16230	2978	2.372909	0.004671
blue	2329	MORF_RFC1	31	107	16146	2978	1.5812	0.004677
purple	2902	KEGG_DILATED_CARDIOMYOPATHY	4	70	16183	157	5.91556	0.004687
turquoise	7357	CHANG_CYCLING_GENES	52	146	16107	4169	1.38852	0.00469
blue	9962	GSE3982_MEMORY_CD4_TCELL_VS_BCELL_UP	45	169	16084	2978	1.453231	0.004691
turquoise	10269	GSE9988_ANTI_TREM1_VS_LPS_MONOCYTE_DN	64	186	16067	4169	1.341432	0.004693
red	4882	TURASHVILI_BREAST_LOBULAR_CARCINOMA_VS_DUCT	6	49	16204	524	3.798021	0.004708
lightyellow	10261	GSE9650_EFFECTOR_VS_EXHAUSTED_CD8_TCELL_DN	3	168	16085	33	8.794913	0.004714
tan	9490	GSE26928_NAIVE_VS_CXCR5_POS_CD4_TCELL_UP	6	174	16079	145	3.865161	0.004718
purple	118	INTEGRAL_TO_MEMBRANE	19	1025	15228	157	1.91895	0.004726
turquoise	1971	GTATGAT,MIR-154,MIR-487	27	66	16187	4169	1.594856	0.004726

turquoise	4878	TURASHVILI_BREAST_DUCTAL_CARCINOMA_VS_LOBUL	27	66	16187	4169	1.594856	0.004726
lightcyan	946	HUMORAL_IMMUNE_RESPONSE	2	27	16226	62	19.41816	0.004726
lightcyan	7281	FLOTHO_PEDIATRIC_ALL_THERAPY_RESPONSE_DN	2	27	16226	62	19.41816	0.004726
lightcyan	8353	KRAS.DF.V1_UP	4	178	16075	62	5.890903	0.004733
lightcyan	9891	GSE36392_TYPE_2_MYELOID_VS_EOSINOPHIL_IL25_TR	4	178	16075	62	5.890903	0.004733
brown	4806	NAKAMURA_TUMOR_ZONE_PERIPHERAL_VS_CENTRAL	99	572	15681	2190	1.284484	0.004735
turquoise	9708	GSE29618_LAIV_VS_TIV_FLU_VACCINE_DAY7_MDC_UF	61	176	16077	4169	1.351197	0.004737
blue	5847	RICKMAN_TUMOR_DIFFERENTIATED_WELL_VS_POORL	57	224	16029	2978	1.388787	0.004737
salmon	8783	GSE15930_NAIVE_VS_48H_IN_VITRO_STIM_CD8_TCELI	6	199	16054	127	3.858584	0.004746
magenta	1703	CCTNTMAGA_UNKNOWN	5	106	16147	167	4.590724	0.004748
greenyello	5621	MARTORIATI_MDM4_TARGETS_NEUROEPITHELIUM_DI	5	118	16135	150	4.591243	0.004754
turquoise	6613	BROWNE_HCMV_INFECTION_18HR_UP	58	166	16087	4169	1.362139	0.004755
lightcyan	3616	MODULE_128	3	89	16164	62	8.836354	0.004755
lightgreen	5023	BILBAN_B_CLL_LPL_DN	2	40	16213	42	19.34881	0.004778
lightgreen	7591	ONO_FOXP3_TARGETS_DN	2	40	16213	42	19.34881	0.004778
yellow	9539	GSE27786_LSK_VS_NKTCELL_DN	27	182	16071	1426	1.690855	0.004779
pink	4845	SENGUPTA_NASOPHARYNGEAL_CARCINOMA_DN	10	269	15984	228	2.650003	0.004782
blue	6096	MENSSEN_MYC_TARGETS	18	53	16200	2978	1.853555	0.004784
pink	6385	MOREAUX_B_LYMPHOCYTE_MATURATION_BY_TACI_U	5	78	16175	228	4.569557	0.004785
turquoise	5029	AKL_HTLV1_INFECTION_DN	26	63	16190	4169	1.60892	0.004793
brown	9839	GSE360_L_DONOVANI_VS_M_TUBERCULOSIS_DC_DN	36	173	16080	2190	1.54435	0.004796
red	10036	GSE3982_MAC_VS_EFF_MEMORY_CD4_TCELL_UP	14	197	16056	524	2.204266	0.004797
red	10151	GSE7460_TCONV_VS_TREG_LN_DN	14	197	16056	524	2.204266	0.004797
red	10232	GSE9006_TYPE_1_DIABETES_AT_DX_VS_1MONTH_POS	14	197	16056	524	2.204266	0.004797
greenyello	1537	V$MYCMAX_02	7	224	16029	150	3.386042	0.004799
brown	8586	GSE13485_DAY1_VS_DAY21_YF17D_VACCINE_PBMC_U	37	179	16074	2190	1.534045	0.004811
cyan	1402	LIPID_TRANSPORTER_ACTIVITY	2	22	16231	77	19.1889	0.004813
red	2709	GNF2_TNFRSF1B	7	65	16188	524	3.340311	0.004813
brown	8725	GSE14769_UNSTIM_VS_240MIN_LPS_BMDM_DN	39	191	16062	2190	1.515377	0.004814
brown	9123	GSE17721_ALL_VS_24H_PAM3CSK4_BMDM_DN	39	191	16062	2190	1.515377	0.004814
brown	10175	GSE7460_FOXP3_MUT_VS_WT_ACT_WITH_TGFB_TCOI	39	191	16062	2190	1.515377	0.004814
purple	3872	MODULE_470	2	11	16242	157	18.82224	0.004816
purple	5724	TOMLINS_METASTASIS_UP	2	11	16242	157	18.82224	0.004816
purple	6433	WHITESIDE_CISPLATIN_RESISTANCE_UP	2	11	16242	157	18.82224	0.004816
purple	6507	EHRLICH_ICF_SYNDROM_UP	2	11	16242	157	18.82224	0.004816
brown	8925	GSE17721_LPS_VS_POLYIC_12H_BMDM_DN	38	185	16068	2190	1.524408	0.004817
brown	9924	GSE37416_CTRL_VS_3H_F_TULARENSIS_LVS_NEUTROP	38	185	16068	2190	1.524408	0.004817
midnightb	9514	GSE2706_R848_VS_R848_AND_LPS_2H_STIM_DC_UP	4	156	16097	71	5.869628	0.00482
turquoise	498	PROTEIN_DNA_COMPLEX_ASSEMBLY	21	48	16205	4169	1.70561	0.004821
turquoise	4338	REACTOME_REGULATION_OF_ORNITHINE_DECARBOXY	21	48	16205	4169	1.70561	0.004821
lightcyan	9629	GSE2826_XID_VS_BTK_KO_BCELL_DN	4	179	16074	62	5.857992	0.004828
lightcyan	10155	GSE7460_CTRL_VS_TGFB_TREATED_ACT_CD8_TCELL_D	4	179	16074	62	5.857992	0.004828
turquoise	3046	BIOCARTA_PROTEASOME_PATHWAY	14	28	16225	4169	1.949268	0.004829
turquoise	3353	chr12q21	14	28	16225	4169	1.949268	0.004829
blue	1713	V$SPZ1_01	50	192	16061	2978	1.421273	0.004839
blue	10059	GSE3982_NEUTROPHIL_VS_TH2_DN	50	192	16061	2978	1.421273	0.004839
brown	2487	GCM_BCL2L1	11	35	16218	2190	2.332459	0.00485
brown	4092	PID_PI3KCIAKTPATHWAY	11	35	16218	2190	2.332459	0.00485
brown	7345	RUIZ_TNC_TARGETS_UP	29	132	16121	2190	1.630473	0.004859
salmon	8785	GSE15930_NAIVE_VS_48H_IN_VITRO_STIM_IL12_CD8_	6	200	16053	127	3.839291	0.004862
greenyello	2031	AGGGCCA,MIR-328	4	74	16179	150	5.856937	0.004863
yellow	9215	GSE17974_2.5H_VS_72H_IL4_AND_ANTI_IL12_ACT_CD	28	191	16062	1426	1.670855	0.004865
turquoise	8878	GSE17721_CTRL_VS_PAM3CSK4_24H_BMDM_UP	66	193	16060	4169	1.333178	0.004866
turquoise	208	INTRINSIC_TO_ORGANELLE_MEMBRANE	22	51	16202	4169	1.681722	0.00487
turquoise	2018	CAAGGAT,MIR-362	24	57	16196	4169	1.641489	0.004877
red	2820	KEGG_APOPTOSIS	8	82	16171	524	3.026066	0.004878
lightgreen	6968	WALLACE_PROSTATE_CANCER_RACE_UP	4	267	15986	42	5.797396	0.004882
salmon	2795	KEGG_PROTEASOME	3	44	16209	127	8.725662	0.004885
salmon	3613	MODULE_125	3	44	16209	127	8.725662	0.004885
brown	2249	MGGAAGTG_V$GABP_B	122	725	15528	2190	1.248853	0.004897

blue	9308	GSE22886_NAIVE_CD8_TCELL_VS_MEMORY_TCELL_UP	48	183	16070	2978	1.431525	0.004907
red	9146	GSE17721_4_VS_24H_GARDIQUIMOD_BMDM_UP	13	177	16076	524	2.278098	0.004909
pink	9541	GSE27786_LSK_VS_ERYTHROBLAST_DN	7	148	16105	228	3.371592	0.004909
tan	3740	MODULE_279	5	123	16130	145	4.55649	0.00491
lightcyan	9033	GSE17721_PAM3CSK4_VS_GADIQUIMOD_12H_BMDM_	4	180	16073	62	5.825448	0.004924
purple	5855	FRIDMAN_SENESCENCE_UP	4	71	16182	157	5.832242	0.00493
purple	6434	LEE_CALORIE_RESTRICTION_NEOCORTEX_UP	4	71	16182	157	5.832242	0.00493
purple	7200	HUANG_DASATINIB_RESISTANCE_UP	4	71	16182	157	5.832242	0.00493
magenta	7871	HOELZEL_NF1_TARGETS_UP	5	107	16146	167	4.54782	0.00494
brown	6953	MARTINEZ_RB1_TARGETS_UP	101	586	15667	2190	1.279126	0.004944
lightyellow	8258	LTE2_UP.V1_UP	3	171	16082	33	8.640617	0.004952
yellow	7991	BILANGES_SERUM_AND_RAPAMYCIN_SENSITIVE_GENE	13	67	16186	1426	2.211478	0.004956
red	160	CYTOPLASMIC_MEMBRANE_BOUND_VESICLE	9	100	16153	524	2.791546	0.004974
lightgreen	6971	SARRIO_EPITHELIAL_MESENCHYMAL_TRANSITION_DN	3	134	16119	42	8.663646	0.004974
grey60	4610	REACTOME_IL_3_5_AND_GM_CSF_SIGNALING	2	39	16214	44	18.94289	0.004981
turquoise	4828	ONKEN_UVEAL_MELANOMA_DN	150	486	15767	4169	1.203252	0.004983
red	2618	GNF2_CD14	5	35	16218	524	4.431025	0.004987
blue	9639	GSE29615_CTRL_VS_DAY3_LAIV_IFLU_VACCINE_PBMC_	42	156	16097	2978	1.469378	0.004989
pink	308	PROTEIN_AMINO_ACID_N_LINKED_GLYCOSYLATION	3	25	16228	228	8.554211	0.004989
salmon	2445	MORF_PRKAR1A	5	141	16112	127	4.538169	0.004995
purple	2287	TATAAA_V$TATA_01	17	880	15373	157	1.999862	0.005
turquoise	2108	ATAAGCT,MIR-21	39	104	16149	4169	1.461951	0.005
brown	703	REGULATION_OF_TRANSCRIPTIONDNA_DEPENDENT	73	404	15849	2190	1.341007	0.00501
brown	7391	LEE_EARLY_T_LYMPHOCYTE_DN	15	55	16198	2190	2.024035	0.005011
purple	3535	MODULE_46	9	332	15921	157	2.806327	0.005014
lightgreen	5510	FARMER_BREAST_CANCER_CLUSTER_1	2	41	16212	42	18.87689	0.005015
magenta	9684	GSE29618_PRE_VS_DAY7_FLU_VACCINE_MDC_UP	6	153	16100	167	3.816602	0.005017
lightcyan	9068	GSE17721_POLYIC_VS_GARDIQUIMOD_12H_BMDM_U	4	181	16072	62	5.793263	0.005021
green	3067	BIOCARTA_ATRBRCA_PATHWAY	6	21	16232	1305	3.558402	0.005021
greenyello	5393	PEREZ_TP53_TARGETS	18	1001	15252	150	1.948412	0.005033
lightyellow	8198	EGFR_UP.V1_UP	3	172	16081	33	8.590381	0.005033
turquoise	363	ACTIVATION_OF_JNK_ACTIVITY	9	15	16238	4169	2.339122	0.005034
turquoise	700	MICROTUBULE_ORGANIZING_CENTER_ORGANIZATION_	9	15	16238	4169	2.339122	0.005034
turquoise	3216	chr4q24	9	15	16238	4169	2.339122	0.005034
turquoise	7987	DORMOY_ELAVL1_TARGETS	9	15	16238	4169	2.339122	0.005034
yellow	8833	GSE17721_CTRL_VS_LPS_0.5H_BMDM_DN	26	174	16079	1426	1.703092	0.005046
magenta	2926	BIOCARTA_BCR_PATHWAY	3	34	16219	167	8.587355	0.005047
magenta	7365	VANTVEER_BREAST_CANCER_BRCA1_UP	3	34	16219	167	8.587355	0.005047
greenyello	7908	CHYLA_CBFA2T3_TARGETS_UP	9	348	15905	150	2.802241	0.005047
purple	7216	WINTER_HYPOXIA_METAGENE	7	216	16037	157	3.354889	0.005049
brown	379	INTRACELLULAR_TRANSPORT	51	265	15988	2190	1.428281	0.005051
lightcyan	3567	MODULE_79	3	91	16162	62	8.642148	0.005059
brown	3175	chr11q24	12	40	16213	2190	2.226438	0.005064
brown	6608	GENTILE_UV_RESPONSE_CLUSTER_D7	12	40	16213	2190	2.226438	0.005064
blue	9253	GSE19825_CD24LOW_VS_IL2RA_HIGH_DAY3_EFF_CD8	51	197	16056	2978	1.412904	0.005065
blue	3184	chr10p15	13	34	16219	2978	2.086764	0.005065
blue	3739	MODULE_278	13	34	16219	2978	2.086764	0.005065
red	4827	ONKEN_UVEAL_MELANOMA_UP	38	761	15492	524	1.548821	0.005076
green	1649	V$RFX1_02	31	238	16015	1305	1.622213	0.005091
blue	236	DNA_METABOLIC_PROCESS	62	248	16005	2978	1.364422	0.005098
black	7403	SHEDDEN_LUNG_CANCER_GOOD_SURVIVAL_A5	5	64	16189	283	4.486804	0.005099
red	5140	DODD_NASOPHARYNGEAL_CARCINOMA_UP	64	1439	14814	524	1.379499	0.005107
turquoise	8884	GSE17721_CTRL_VS_CPG_2H_BMDM_UP	65	190	16063	4169	1.33371	0.005117
turquoise	9520	GSE2706_LPS_VS_R848_AND_LPS_8H_STIM_DC_UP	65	190	16063	4169	1.33371	0.005117
lightcyan	8565	GSE13484_UNSTIM_VS_12H_YF17D_VACCINE_STIM_PI	4	182	16071	62	5.761432	0.005119
lightcyan	8972	GSE17721_CPG_VS_GARDIQUIMOD_4H_BMDM_UP	4	182	16071	62	5.761432	0.005119
lightcyan	9100	GSE17721_12H_VS_24H_LPS_BMDM_UP	4	182	16071	62	5.761432	0.005119
tan	8602	GSE13738_RESTING_VS_TCR_ACTIVATED_CD4_TCELL_U	6	177	16076	145	3.799649	0.005124
greenyello	5938	AMIT_EGF_RESPONSE_480_MCF10A	3	38	16215	150	8.554211	0.005131
greenyello	7463	CROONQUIST_NRAS_SIGNALING_UP	3	38	16215	150	8.554211	0.005131

magenta	3688	MODULE_208	5	108	16145	167	4.505711	0.005137
red	9231	GSE18791_CTRL_VS_NEWCASTLE_VIRUS_DC_12H_DN	13	178	16075	524	2.265299	0.005145
red	9795	GSE360_CTRL_VS_L_MAJOR_DC_DN	13	178	16075	524	2.265299	0.005145
blue	8660	GSE1432_CTRL_VS_IFNG_6H_MICROGLIA_UP	49	188	16065	2978	1.422483	0.005151
greenyello	6787	BILD_E2F3_ONCOGENIC_SIGNATURE	7	227	16026	150	3.341292	0.005155
lightyellow	6915	KYNG_ENVIRONMENTAL_STRESS_RESPONSE_UP	2	53	16200	33	18.58548	0.005164
turquoise	1753	V$STAT1_02	75	224	16029	4169	1.305314	0.005168
cyan	5657	GRUETZMANN_PANCREATIC_CANCER_UP	6	336	15917	77	3.769249	0.005168
salmon	382	CENTROSOME_ORGANIZATION_AND_BIOGENESIS	2	14	16239	127	18.28234	0.005184
turquoise	8976	GSE17721_CPG_VS_GARDIQUIMOD_8H_BMDM_UP	62	180	16073	4169	1.342829	0.005186
yellow	1173	HYDROLASE_ACTIVITY_HYDROLYZING_N_GLYCOSYL_CC	4	9	16244	1426	5.065607	0.005186
yellow	4340	REACTOME_BASE_FREE_SUGAR_PHOSPHATE_REMOVA	4	9	16244	1426	5.065607	0.005186
brown	10137	GSE6269_FLU_VS_STREP_AUREUS_INF_PBMC_DN	33	156	16097	2190	1.569924	0.005189
pink	7027	BOYERINAS_ONCOFETAL_TARGETS_OF_LET7A1	2	8	16245	228	17.82127	0.005189
brown	5896	DEN_INTERACT_WITH_LCA5	9	26	16227	2190	2.568967	0.005193
magenta	3555	MODULE_66	11	433	15820	167	2.472418	0.005195
purple	7963	PASINI_SUZ12_TARGETS_DN	8	274	15979	157	3.022549	0.005196
brown	4139	REACTOME_SIGNALING_BY_RHO_GTPASES	24	104	16149	2190	1.712645	0.0052
purple	4853	GAZDA_DIAMOND_BLACKFAN_ANEMIA_ERYTHROID_D	11	461	15792	157	2.470163	0.005202
grey60	9248	GSE19825_NAIVE_VS_IL2RALOW_DAY3_EFF_CD8_TCEL	3	130	16123	44	8.524301	0.005213
lightcyan	3653	MODULE_170	3	92	16161	62	8.548212	0.005216
lightcyan	7644	BOYLAN_MULTIPLE_MYELOMA_PCA1_UP	3	92	16161	62	8.548212	0.005216
lightcyan	9088	GSE17721_LPS_VS_GARDIQUIMOD_16H_BMDM_UP	4	183	16070	62	5.729949	0.005219
yellow	4157	REACTOME_TCA_CYCLE_AND_RESPIRATORY_ELECTRON	19	115	16138	1426	1.883084	0.005221
turquoise	8030	IKEDA_MIR133_TARGETS_DN	5	6	16247	4169	3.248781	0.00523
turquoise	2532	GCM_MLL	59	170	16083	4169	1.353022	0.005231
turquoise	8024	GOBERT_OLIGODENDROCYTE_DIFFERENTIATION_DN	287	983	15270	4169	1.13823	0.005231
yellow	8928	GSE17721_LPS_VS_POLYIC_24H_BMDM_UP	28	192	16061	1426	1.662152	0.005232
red	9346	GSE22886_DAY0_VS_DAY1_MONOCYTE_IN_CULTURE_	14	199	16054	524	2.182113	0.00524
red	9659	GSE29618_BCELL_VS_MDC_DN	14	199	16054	524	2.182113	0.00524
red	9671	GSE29618_BCELL_VS_MDC_DAY7_FLU_VACCINE_DN	14	199	16054	524	2.182113	0.00524
red	7809	SASSON_RESPONSE_TO_FORSKOLIN_DN	8	83	16170	524	2.989607	0.00525
magenta	4337	REACTOME_INTEGRIN_CELL_SURFACE_INTERACTIONS	4	68	16185	167	5.724903	0.005255
cyan	1058	INDUCTION_OF_APOPTOSIS_BY_INTRACELLULAR_SIGN	2	23	16230	77	18.3546	0.005255
cyan	3949	PID_IL27PATHWAY	2	23	16230	77	18.3546	0.005255
brown	8439	GSE10325_MYELOID_VS_LUPUS_MYELOID_DN	40	198	16055	2190	1.499285	0.005255
brown	9439	GSE24634_NAIVE_CD4_TCELL_VS_DAY10_IL4_CONV_T	40	198	16055	2190	1.499285	0.005255
lightgreen	3743	MODULE_284	2	42	16211	42	18.42744	0.005257
blue	10094	GSE3982_CENT_MEMORY_CD4_TCELL_VS_TH2_UP	45	170	16083	2978	1.444683	0.005271
pink	9427	GSE24142_ADULT_VS_FETAL_EARLY_THYMIC_PROGEN	8	189	16064	228	3.017358	0.005273
brown	9153	GSE17974_0H_VS_1H_IN_VITRO_ACT_CD4_TCELL_DN	39	192	16061	2190	1.507484	0.005275
brown	9445	GSE24634_TREG_VS_TCONV_POST_DAY7_IL4_CONVER	39	192	16061	2190	1.507484	0.005275
brown	9602	GSE27786_NKCELL_VS_NEUTROPHIL_UP	39	192	16061	2190	1.507484	0.005275
brown	1607	V$SRF_C	36	174	16079	2190	1.535475	0.005282
turquoise	8558	GSE13411_SWITCHED_MEMORY_BCELL_VS_PLASMA_C	67	197	16056	4169	1.325898	0.005286
turquoise	9161	GSE17974_0H_VS_12H_IN_VITRO_ACT_CD4_TCELL_DN	67	197	16056	4169	1.325898	0.005286
brown	1585	V$SRF_Q6	38	186	16067	2190	1.516213	0.005287
brown	9072	GSE17721_POLYIC_VS_GARDIQUIMOD_24H_BMDM_U	37	180	16073	2190	1.525523	0.005289
brown	9750	GSE32423_CTRL_VS_IL4_MEMORY_CD8_TCELL_UP	37	180	16073	2190	1.525523	0.005289
midnightb	2758	KEGG_GLYCOSYLPHOSPHATIDYLINOSITOL_GPI_ANCHOI	2	25	16228	71	18.31324	0.00529
midnightb	4475	REACTOME_POST_TRANSLATIONAL_MODIFICATION_SY	2	25	16228	71	18.31324	0.00529
purple	2246	RYTTCCTG_V$ETS2_B	18	960	15293	157	1.941043	0.00529
lightgreen	4903	PUIFFE_INVASION_INHIBITED_BY_ASCITES_DN	3	137	16116	42	8.473931	0.00529
turquoise	2792	KEGG_BASAL_TRANSCRIPTION_FACTORS	15	31	16222	4169	1.886389	0.005293
turquoise	3128	chr10q21	15	31	16222	4169	1.886389	0.005293
turquoise	3307	chr12p11	15	31	16222	4169	1.886389	0.005293
blue	9718	GSE30083_SP2_VS_SP4_THYMOCYTE_UP	43	161	16092	2978	1.457644	0.005298
blue	10084	GSE3982_EFF_MEMORY_CD4_TCELL_VS_NKCELL_UP	43	161	16092	2978	1.457644	0.005298
turquoise	1985	AAGCCAT,MIR-135A,MIR-135B	97	300	15953	4169	1.260527	0.005299
purple	7408	ZHANG_TLX_TARGETS_60HR_UP	8	275	15978	157	3.011558	0.005309

red	8593	GSE13485_DAY7_VS_DAY21_YF17D_VACCINE_PBMC_[	9	101	16152	524	2.763907	0.005309
yellow	7965	BAKKER_FOXO3_TARGETS_DN	25	166	16087	1426	1.716508	0.005319
lightcyan	8965	GSE17721_PAM3CSK4_VS_CPG_24H_BMDM_DN	4	184	16069	62	5.698808	0.00532
lightcyan	9735	GSE31082_DN_VS_CD8_SP_THYMOCYTE_DN	4	184	16069	62	5.698808	0.00532
cyan	3659	MODULE_176	4	148	16105	77	5.704809	0.005341
yellow	1446	ISOMERASE_ACTIVITY	8	32	16221	1426	2.849404	0.005358
green	4867	DAVICIONI_MOLECULAR_ARMS_VS_ERMS_DN	22	153	16100	1305	1.79083	0.005366
turquoise	9059	GSE17721_POLYIC_VS_GARDIQUIMOD_1H_BMDM_DN	64	187	16066	4169	1.334259	0.00538
red	5261	LANDIS_ERBB2_BREAST_TUMORS_324_UP	11	139	16114	524	2.454597	0.005381
grey60	4365	REACTOME_SIGNALING_BY_GPCR	5	421	15832	44	4.387011	0.005388
red	9796	GSE360_CTRL_VS_T_GONDII_DC_UP	13	179	16074	524	2.252644	0.00539
red	9851	GSE360_T_GONDII_VS_B_MALAYI_LOW_DOSE_DC_DN	13	179	16074	524	2.252644	0.00539
red	10166	GSE7460_CTRL_VS_TGFB_TREATED_ACT_TCONV_UP	13	179	16074	524	2.252644	0.00539
yellow	7133	CHUNG_BLISTER_CYTOTOXICITY_UP	21	132	16121	1426	1.813257	0.005392
purple	4503	REACTOME_G_ALPHA_Z_SIGNALLING_EVENTS	3	37	16216	157	8.393699	0.005401
purple	6470	SESTO_RESPONSE_TO_UV_C6	3	37	16216	157	8.393699	0.005401
turquoise	7820	WHITFIELD_CELL_CYCLE_G1_S	49	137	16116	4169	1.394367	0.005404
tan	10060	GSE3982_BCELL_VS_BASOPHIL_UP	6	179	16074	145	3.757195	0.005408
salmon	4519	REACTOME_SYNTHESIS_OF_DNA	4	90	16163	127	5.687839	0.005414
lightcyan	8911	GSE17721_CTRL_VS_GARDIQUIMOD_24H_BMDM_DN	4	185	16068	62	5.668003	0.005422
lightcyan	9044	GSE17721_LPS_VS_CPG_4H_BMDM_UP	4	185	16068	62	5.668003	0.005422
magenta	561	POSITIVE_REGULATION_OF_T_CELL_PROLIFERATION	2	11	16242	167	17.69516	0.005431
magenta	2925	BIOCARTA_BLYMPHOCYTE_PATHWAY	2	11	16242	167	17.69516	0.005431
lightcyan	5172	CHOW_RASSF1_TARGETS_DN	2	29	16224	62	18.07898	0.00544
lightcyan	8183	ZWANG_EGF_PERSISTENTLY_UP	2	29	16224	62	18.07898	0.00544
purple	2200	TGTYNNNNNRGCARM_UNKNOWN	4	73	16180	157	5.672454	0.005441
lightyellow	10071	GSE3982_BCELL_VS_TH2_DN	3	177	16076	33	8.347714	0.00545
magenta	1851	V$IRF1_Q6	7	206	16047	167	3.307104	0.005459
green	5908	BENPORATH_PRC2_TARGETS	41	339	15914	1305	1.506285	0.005466
black	3343	chr11q13	10	221	16032	283	2.598692	0.005471
brown	5386	LASTOWSKA_NEUROBLASTOMA_COPY_NUMBER_DN	130	781	15472	2190	1.235326	0.005471
red	9379	GSE22886_NAIVE_CD4_TCELL_VS_DC_DN	14	200	16053	524	2.171202	0.005474
salmon	2346	MORF_AATF	6	205	16048	127	3.74565	0.005475
magenta	7685	ZHAN_MULTIPLE_MYELOMA_MF_DN	3	35	16218	167	8.342002	0.005479
turquoise	2038	GTCTTCC,MIR-7	52	147	16106	4169	1.379074	0.00548
blue	6739	RAMPON_ENRICHED_LEARNING_ENVIRONMENT_LATE_	9	20	16233	2978	2.45596	0.005485
black	5928	AMIT_EGF_RESPONSE_40_HELA	4	41	16212	283	5.603034	0.005493
blue	2173	CAGCCTC,MIR-485-5P	36	130	16123	2978	1.51136	0.005493
turquoise	3561	MODULE_72	92	283	15970	4169	1.267369	0.005499
purple	9883	GSE360_T_GONDII_VS_M_TUBERCULOSIS_MAC_DN	6	166	16087	157	3.74177	0.005519
greenyello	3596	MODULE_108	3	39	16214	150	8.334872	0.005522
lightcyan	8817	GSE16522_MEMORY_VS_NAIVE_ANTI_CD3CD28_STIM_	4	186	16067	62	5.63753	0.005526
lightcyan	9719	GSE30083_SP2_VS_SP4_THYMOCYTE_DN	4	186	16067	62	5.63753	0.005526
midnightb	7632	SAKAI_CHRONIC_HEPATITIS_VS_LIVER_CANCER_UP	3	82	16171	71	8.374957	0.005528
turquoise	4740	REACTOME_TOLL_RECEPTOR_CASCADES	41	111	16142	4169	1.44	0.005532
salmon	4141	REACTOME_METABOLISM_OF_NON_CODING_RNA	3	46	16207	127	8.346286	0.005536
salmon	4295	REACTOME_CDK_MEDIATED_PHOSPHORYLATION_ANC	3	46	16207	127	8.346286	0.005536
salmon	6676	BRACHAT_RESPONSE_TO_CAMPTOTHECIN_DN	3	46	16207	127	8.346286	0.005536
greenyello	7704	WONG_ADULT_TISSUE_STEM_MODULE	13	629	15624	150	2.239417	0.005537
turquoise	912	PROTEIN_AMINO_ACID_PHOSPHORYLATION	83	252	16001	4169	1.284042	0.005545
blue	2719	KEGG_CITRATE_CYCLE_TCA_CYCLE	11	27	16226	2978	2.223503	0.005549
magenta	735	CELLULAR_HOMEOSTASIS	5	110	16143	167	4.423789	0.00555
grey60	3530	MODULE_41	5	424	15829	44	4.355971	0.005551
blue	9330	GSE22886_NAIVE_VS_IGM_MEMORY_BCELL_UP	46	175	16078	2978	1.434593	0.005556
turquoise	2113	TTTGCAG,MIR-518A-2	66	194	16059	4169	1.326306	0.00556
turquoise	8866	GSE17721_CTRL_VS_PAM3CSK4_1H_BMDM_UP	66	194	16059	4169	1.326306	0.00556
turquoise	8993	GSE17721_LPS_VS_PAM3CSK4_6H_BMDM_DN	66	194	16059	4169	1.326306	0.00556
black	9591	GSE27786_CD8_TCELL_VS_NKTCELL_DN	9	187	16066	283	2.764063	0.005566
purple	41	INTRINSIC_TO_MEMBRANE	19	1041	15212	157	1.889456	0.005567
pink	7667	HAHTOLA_CTCL_CUTANEOUS	3	26	16227	228	8.225202	0.005582

turquoise	4598	REACTOME_ASPARAGINE_N_LINKED_GLYCOSYLATION	30	76	16177	4169	1.538896	0.005583
green	1447	GROWTH_FACTOR_ACTIVITY	7	28	16225	1305	3.113602	0.005587
green	4453	REACTOME_NCAM1_INTERACTIONS	7	28	16225	1305	3.113602	0.005587
turquoise	4906	THUM_SYSTOLIC_HEART_FAILURE_UP	118	374	15879	4169	1.23002	0.005592
cyan	5050	TONKS_TARGETS_OF_RUNX1_RUNX1T1_FUSION_ERYTI	4	150	16103	77	5.628745	0.005599
cyan	8559	GSE13411_SWITCHED_MEMORY_BCELL_VS_PLASMA_(	4	150	16103	77	5.628745	0.005599
red	8189	ZWANG_TRANSIENTLY_UP_BY_1ST_EGF_PULSE_ONLY	65	1472	14781	524	1.369644	0.005607
brown	697	MACROMOLECULE_LOCALIZATION	43	217	16036	2190	1.470612	0.00561
green	3271	chr18q12	8	35	16218	1305	2.846721	0.005612
green	4591	REACTOME_RNA_POL_I_PROMOTER_OPENING	8	35	16218	1305	2.846721	0.005612
yellow	2373	MORF_DAP3	28	193	16060	1426	1.65354	0.005623
yellow	9396	GSE22886_NAIVE_CD4_TCELL_VS_48H_ACT_TH2_UP	28	193	16060	1426	1.65354	0.005623
midnightb	9151	GSE17974_0H_VS_0.5H_IN_VITRO_ACT_CD4_TCELL_DI	4	163	16090	71	5.617558	0.005625
lightcyan	8723	GSE14769_UNSTIM_VS_120MIN_LPS_BMDM_DN	4	187	16066	62	5.607383	0.005631
blue	2473	MORF_TNFRSF25	51	198	16055	2978	1.405769	0.005634
brown	3660	MODULE_177	23	99	16154	2190	1.724178	0.005636
red	9783	GSE339_EX_VIVO_VS_IN_CULTURE_CD8POS_DC_DN	13	180	16073	524	2.240129	0.005644
salmon	7139	GRADE_COLON_AND_RECTAL_CANCER_UP	7	273	15980	127	3.281446	0.005646
turquoise	8187	ZWANG_DOWN_BY_2ND_EGF_PULSE	78	235	16018	4169	1.293982	0.005655
brown	4152	REACTOME_DEVELOPMENTAL_BIOLOGY	60	323	15930	2190	1.3786	0.005658
turquoise	8485	GSE11924_TFH_VS_TH17_CD4_TCELL_DN	63	184	16069	4169	1.334825	0.005658
turquoise	8600	GSE13493_CD4INTCD8POS_VS_CD8POS_THYMOCYTE_I	63	184	16069	4169	1.334825	0.005658
turquoise	9774	GSE339_CD4POS_VS_CD8POS_DC_IN_CULTURE_UP	63	184	16069	4169	1.334825	0.005658
grey60	118	INTEGRAL_TO_MEMBRANE	8	1025	15228	44	2.883016	0.005662
red	35	MEMBRANE_BOUND_VESICLE	9	102	16151	524	2.73681	0.005662
turquoise	3179	chr14q21	16	34	16219	4169	1.834606	0.005663
yellow	3956	PID_ATR_PATHWAY	9	39	16214	1426	2.630219	0.00568
greenyello	1747	CTGCAGY_UNKNOWN	13	631	15622	150	2.232319	0.005683
blue	7846	KIM_ALL_DISORDERS_OLIGODENDROCYTE_NUMBER_C	158	717	15536	2978	1.202671	0.005691
blue	2735	KEGG_VALINE_LEUCINE_AND_ISOLEUCINE_DEGRADATI	15	42	16211	2978	1.949175	0.005696
lightcyan	874	ION_HOMEOSTASIS	3	95	16158	62	8.278268	0.005703
tan	9486	GSE26928_NAIVE_VS_EFF_MEMORY_CD4_TCELL_UP	6	181	16072	145	3.715679	0.005703
brown	6476	KAAB_HEART_ATRIUM_VS_VENTRICLE_UP	41	205	16048	2190	1.484292	0.005705
purple	6800	DURCHDEWALD_SKIN_CARCINOGENESIS_UP	4	74	16179	157	5.5958	0.005709
lightyellow	5126	GRAHAM_CML_DIVIDING_VS_NORMAL_QUIESCENT_U	3	180	16073	33	8.208586	0.00571
lightyellow	8260	MEK_UP.V1_UP	3	180	16073	33	8.208586	0.00571
pink	8160	ACEVEDO_FGFR1_TARGETS_IN_PROSTATE_CANCER_M	9	233	16020	228	2.753501	0.00572
turquoise	2195	TCCCRNNRTGC_UNKNOWN	68	201	16052	4169	1.318908	0.005726
cyan	6240	BROWN_MYELOID_CELL_DEVELOPMENT_UP	4	151	16102	77	5.591468	0.005731
purple	2934	BIOCARTA_CBL_PATHWAY	2	12	16241	157	17.25372	0.005743
purple	3197	chr3q24	2	12	16241	157	17.25372	0.005743
blue	8631	GSE14308_TH2_VS_TH17_DN	49	189	16064	2978	1.414957	0.005744
purple	4450	REACTOME_G_ALPHA_Q_SIGNALLING_EVENTS	5	118	16135	157	4.386538	0.005756
purple	9193	GSE17974_IL4_AND_ANTI_IL12_VS_UNTREATED_4H_A	5	118	16135	157	4.386538	0.005756
blue	2357	MORF_BUB1B	21	66	16187	2978	1.736538	0.005762
brown	9118	GSE17721_0.5H_VS_8H_PAM3CSK4_BMDM_UP	39	193	16060	2190	1.499674	0.005773
blue	3358	chr1p36	70	287	15966	2978	1.331144	0.005773
brown	9151	GSE17974_0H_VS_0.5H_IN_VITRO_ACT_CD4_TCELL_DI	34	163	16090	2190	1.548035	0.005777
tan	1288	3_5_EXONUCLEASE_ACTIVITY	2	13	16240	145	17.24456	0.005781
turquoise	78	PROTEASOME_COMPLEX	12	23	16230	4169	2.034019	0.005784
turquoise	1258	PROTEIN_SERINE_THREONINE_PHOSPHATASE_ACTIVIT	12	23	16230	4169	2.034019	0.005784
brown	8946	GSE17721_POLYIC_VS_PAM3CSK4_24H_BMDM_UP	38	187	16066	2190	1.508104	0.005795
brown	9522	GSE2706_2H_VS_8H_R848_STIM_DC_UP	38	187	16066	2190	1.508104	0.005795
lightyellow	6364	YAGI_AML_FAB_MARKERS	3	181	16072	33	8.163235	0.005798
lightyellow	10076	GSE3982_BASOPHIL_VS_NKCELL_UP	3	181	16072	33	8.163235	0.005798
brown	10013	GSE3982_DC_VS_MAC_DN	35	169	16084	2190	1.536989	0.005799
brown	3065	BIOCARTA_RHO_PATHWAY	10	31	16222	2190	2.39402	0.005801
brown	3967	PID_AVB3_OPN_PATHWAY	10	31	16222	2190	2.39402	0.005801
brown	3977	PID_P38ALPHABETAPATHWAY	10	31	16222	2190	2.39402	0.005801
brown	9019	GSE17721_POLYIC_VS_CPG_24H_BMDM_DN	37	181	16072	2190	1.517094	0.005807

brown	9456	GSE24634_IL4_VS_CTRL_TREATED_NAIVE_CD4_TCELL_	37	181	16072	2190	1.517094	0.005807
brown	9803	GSE360_CTRL_VS_M_TUBERCULOSIS_DC_DN	37	181	16072	2190	1.517094	0.005807
cyan	1318	RECEPTOR_SIGNALING_PROTEIN_ACTIVITY	3	77	16176	77	8.223815	0.005816
cyan	7276	TOOKER_GEMCITABINE_RESISTANCE_UP	3	77	16176	77	8.223815	0.005816
purple	4615	REACTOME_AQUAPORIN_MEDIATED_TRANSPORT	3	38	16215	157	8.172813	0.005823
purple	8185	ZWANG_CLASS_2_TRANSIENTLY_INDUCED_BY_EGF	3	38	16215	157	8.172813	0.005823
black	8205	RAPA_EARLY_UP.V1_DN	8	155	16098	283	2.964186	0.005825
green	8321	CAHOY_NEURONAL	11	58	16195	1305	2.362043	0.005834
turquoise	4216	REACTOME_DOWNSTREAM_SIGNALING_EVENTS_OF_E	35	92	16161	4169	1.483139	0.005836
lightgreen	5103	KIM_WT1_TARGETS_12HR_UP	3	142	16111	42	8.175553	0.005843
lightgreen	8201	GCNP_SHH_UP_EARLY.V1_DN	3	142	16111	42	8.175553	0.005843
lightcyan	10280	GSE9988_LPS_VS_LPS_AND_ANTI_TREM1_MONOCYTE_	4	189	16064	62	5.548046	0.005844
yellow	3603	MODULE_115	7	26	16227	1426	3.068589	0.005847
turquoise	8630	GSE14308_TH2_VS_TH17_UP	65	191	16062	4169	1.326727	0.005849
turquoise	9073	GSE17721_POLYIC_VS_GARDIQUIMOD_24H_BMDM_D	65	191	16062	4169	1.326727	0.005849
salmon	4216	REACTOME_DOWNSTREAM_SIGNALING_EVENTS_OF_E	4	92	16161	127	5.56419	0.005851
tan	9488	GSE26928_NAIVE_VS_CENT_MEMORY_CD4_TCELL_UP	6	182	16071	145	3.695263	0.005855
green	317	GLUTAMATE_SIGNALING_PATHWAY	4	10	16243	1305	4.981762	0.00586
green	7697	DORN_ADENOVIRUS_INFECTION_32HR_UP	4	10	16243	1305	4.981762	0.00586
greenyello	4947	GRABARCZYK_BCL11B_TARGETS_UP	4	78	16175	150	5.556581	0.00586
salmon	4529	REACTOME_AUTODEGRADATION_OF_THE_E3_UBIQUI	3	47	16206	127	8.168705	0.005881
salmon	5505	SCHLOSSER_MYC_TARGETS_AND_SERUM_RESPONSE_[	3	47	16206	127	8.168705	0.005881
salmon	7278	BOHN_PRIMARY_IMMUNODEFICIENCY_SYNDROM_UP	3	47	16206	127	8.168705	0.005881
yellow	3145	chr11q	3	5	16248	1426	6.838569	0.005887
red	7660	GUTIERREZ_CHRONIC_LYMPHOCYTIC_LEUKEMIA_UP	3	12	16241	524	7.754294	0.005898
brown	2834	KEGG_TIGHT_JUNCTION	24	105	16148	2190	1.696334	0.0059
red	9080	GSE17721_LPS_VS_GARDIQUIMOD_4H_BMDM_UP	13	181	16072	524	2.227753	0.005908
red	9203	GSE17974_IL4_AND_ANTI_IL12_VS_UNTREATED_72H_	13	181	16072	524	2.227753	0.005908
brown	941	POSITIVE_REGULATION_OF_CELLULAR_PROCESS	100	583	15670	2190	1.272978	0.005924
brown	3050	BIOCARTA_RAC1_PATHWAY	8	22	16231	2190	2.698713	0.005925
magenta	7068	SMID_BREAST_CANCER_BASAL_UP	12	504	15749	167	2.317223	0.005926
magenta	4057	PID_SYNDECAN_1_PATHWAY	3	36	16217	167	8.110279	0.005932
brown	5297	MCBRYAN_PUBERTAL_BREAST_5_6WK_DN	28	128	16125	2190	1.623445	0.005942
purple	3499	MODULE_6	9	341	15912	157	2.73226	0.005947
turquoise	6101	PENG_GLUTAMINE_DEPRIVATION_UP	17	37	16216	4169	1.79122	0.005948
turquoise	7770	KYNG_RESPONSE_TO_H2O2_VIA_ERCC6_UP	17	37	16216	4169	1.79122	0.005948
salmon	681	RRNA_PROCESSING	2	15	16238	127	17.06352	0.005951
salmon	700	MICROTUBULE_ORGANIZING_CENTER_ORGANIZATION_	2	15	16238	127	17.06352	0.005951
salmon	1014	RRNA_METABOLIC_PROCESS	2	15	16238	127	17.06352	0.005951
lightcyan	8544	GSE13306_RA_VS_UNTREATED_MEM_CD4_TCELL_UP	4	190	16063	62	5.518846	0.005953
lightcyan	8668	GSE1432_6H_VS_24H_IFNG_MICROGLIA_UP	4	190	16063	62	5.518846	0.005953
lightcyan	9420	GSE24142_EARLY_THYMIC_PROGENITOR_VS_DN2_THY	4	190	16063	62	5.518846	0.005953
lightcyan	9426	GSE24142_ADULT_VS_FETAL_EARLY_THYMIC_PROGEN	4	190	16063	62	5.518846	0.005953
red	4199	REACTOME_BETA_DEFENSINS	2	4	16249	524	15.50859	0.005962
red	7619	WEBER_METHYLATED_ICP_IN_SPERM_DN	2	4	16249	524	15.50859	0.005962
yellow	9535	GSE27786_LSK_VS_CD8_TCELL_DN	27	185	16068	1426	1.663436	0.005967
purple	1095	LIPID_BINDING	4	75	16178	157	5.521189	0.005987
purple	7375	HINATA_NFKB_TARGETS_FIBROBLAST_UP	4	75	16178	157	5.521189	0.005987
purple	5115	JAATINEN_HEMATOPOIETIC_STEM_CELL_DN	7	223	16030	157	3.249579	0.005989
blue	4579	REACTOME_CHROMOSOME_MAINTENANCE	29	100	16153	2978	1.58273	0.005998
blue	10030	GSE3982_MAC_VS_NEUTROPHIL_UP	50	194	16059	2978	1.406621	0.006002
brown	3936	PID_NOTCH_PATHWAY	15	56	16197	2190	1.987891	0.00601
tan	8456	GSE11057_NAIVE_VS_MEMORY_CD4_TCELL_UP	6	183	16070	145	3.675071	0.006011
lightgreen	4383	REACTOME_CHEMOKINE_RECEPTORS_BIND_CHEMOKI	2	45	16208	42	17.19894	0.006015
magenta	293	NERVOUS_SYSTEM_DEVELOPMENT	8	264	15989	167	2.949193	0.006016
greenyello	3530	MODULE_41	10	424	15829	150	2.555503	0.006021
turquoise	8521	GSE12845_NAIVE_VS_DARKZONE_GC_TONSIL_BCELL_[	67	198	16055	4169	1.319202	0.006024
turquoise	9251	GSE19825_NAIVE_VS_IL2RAHIGH_DAY3_EFF_CD8_TCEI	67	198	16055	4169	1.319202	0.006024
pink	5122	GRAHAM_CML_QUIESCENT_VS_NORMAL_DIVIDING_U	4	52	16201	228	5.483468	0.006031
red	8282	PTEN_DN.V2_UP	9	103	16150	524	2.710239	0.006032

yellow	8993	GSE17721_LPS_VS_PAM3CSK4_6H_BMDM_DN	28	194	16059	1426	1.645017	0.006037
yellow	10221	GSE8678_IL7R_LOW_VS_HIGH_EFF_CD8_TCELL_DN	28	194	16059	1426	1.645017	0.006037
green	8575	GSE13485_CTRL_VS_DAY1_YF17D_VACCINE_PBMC_DN	14	83	16170	1305	2.100743	0.006043
red	5399	DACOSTA_UV_RESPONSE_VIA_ERCC3_XPCS_DN	8	85	16168	524	2.919264	0.006059
lightcyan	8830	GSE17580_UNINFECTED_VS_S_MANSONI_INF_TREG_U	4	191	16062	62	5.489951	0.006063
lightcyan	9060	GSE17721_POLYIC_VS_GARDIQUIMOD_2H_BMDM_UP	4	191	16062	62	5.489951	0.006063
lightcyan	9347	GSE22886_DAY0_VS_DAY1_MONOCYTE_IN_CULTURE_	4	191	16062	62	5.489951	0.006063
lightcyan	9350	GSE22886_DAY1_VS_DAY7_MONOCYTE_IN_CULTURE_	4	191	16062	62	5.489951	0.006063
lightcyan	9721	GSE30083_SP3_VS_SP4_THYMOCYTE_DN	4	191	16062	62	5.489951	0.006063
lightcyan	9739	GSE31082_DP_VS_CD8_SP_THYMOCYTE_DN	4	191	16062	62	5.489951	0.006063
lightyellow	9992	GSE3982_MAST_CELL_VS_DC_UP	3	184	16069	33	8.030138	0.006068
lightyellow	8039	WANG_THOC1_TARGETS_UP	1	3	16250	33	164.1717	0.006079
green	951	G_PROTEIN_COUPLED_RECEPTOR_PROTEIN_SIGNALING	27	202	16051	1305	1.664698	0.00608
red	4957	BASAKI_YBX1_TARGETS_DN	21	357	15896	524	1.82454	0.006082
blue	2319	MORF_PCNA	25	83	16170	2978	1.643882	0.006099
blue	2501	GCM_BMPR2	25	83	16170	2978	1.643882	0.006099
greenyello	3588	MODULE_100	10	425	15828	150	2.54949	0.006118
greenyello	7061	SMID_BREAST_CANCER_LUMINAL_B_DN	10	425	15828	150	2.54949	0.006118
purple	7714	MIKKELSEN_ES_ICP_WITH_H3K4ME3	13	608	15645	157	2.21347	0.006126
greenyello	1663	V$PAX8_B	4	79	16174	150	5.486245	0.006129
turquoise	1997	ACAACTT,MIR-382	26	64	16189	4169	1.583781	0.006136
brown	4030	PID_AMB2_NEUTROPHILS_PATHWAY	11	36	16217	2190	2.267669	0.00615
magenta	2216	YATTNATC_UNKNOWN	8	265	15988	167	2.938063	0.00615
lightyellow	7062	SMID_BREAST_CANCER_LUMINAL_A_UP	2	58	16195	33	16.98328	0.006155
lightyellow	10123	GSE5463_CTRL_VS_DEXAMETHASONE_TREATED_THYM	3	185	16068	33	7.986732	0.006159
black	7983	KASLER_HDAC7_TARGETS_1_UP	9	190	16063	283	2.72042	0.006161
greenyello	2950	BIOCARTA_CTL_PATHWAY	2	13	16240	150	16.66974	0.006174
lightcyan	9528	GSE27786_LSK_VS_LIN_NEG_CELL_UP	4	192	16061	62	5.461358	0.006175
lightcyan	9727	GSE30962_ACUTE_VS_CHRONIC_LCMV_PRIMARY_INF_	4	192	16061	62	5.461358	0.006175
lightcyan	9737	GSE31082_DP_VS_CD4_SP_THYMOCYTE_DN	4	192	16061	62	5.461358	0.006175
lightcyan	10104	GSE39820_CTRL_VS_TGFBETA1_IL6_CD4_TCELL_UP	4	192	16061	62	5.461358	0.006175
lightcyan	10106	GSE39820_CTRL_VS_TGFBETA1_IL6_IL23A_CD4_TCELL_	4	192	16061	62	5.461358	0.006175
purple	3605	MODULE_117	11	472	15781	157	2.412596	0.00618
red	8115	PHONG_TNF_RESPONSE_NOT_VIA_P38	19	312	15941	524	1.888866	0.00618
red	7658	CAIRO_LIVER_DEVELOPMENT_DN	13	182	16071	524	2.215513	0.006182
red	8565	GSE13484_UNSTIM_VS_12H_YF17D_VACCINE_STIM_PI	13	182	16071	524	2.215513	0.006182
brown	573	MYELOID_LEUKOCYTE_DIFFERENTIATION	5	10	16243	2190	3.710731	0.006182
brown	3021	BIOCARTA_MONOCYTE_PATHWAY	5	10	16243	2190	3.710731	0.006182
brown	4149	REACTOME_COPI_MEDIATED_TRANSPORT	5	10	16243	2190	3.710731	0.006182
brown	4799	WINTER_HYPOXIA_UP	20	83	16170	2190	1.788304	0.006188
brown	7808	SASSON_RESPONSE_TO_FORSKOLIN_UP	20	83	16170	2190	1.788304	0.006188
brown	4511	REACTOME_GOLGI_ASSOCIATED_VESICLE_BIOGENESIS	14	51	16202	2190	2.037264	0.006193
cyan	5529	PATTERSON_DOCETAXEL_RESISTANCE	2	25	16228	77	16.88623	0.006193
cyan	6565	BRACHAT_RESPONSE_TO_METHOTREXATE_UP	2	25	16228	77	16.88623	0.006193
cyan	6604	ZHANG_ANTIVIRAL_RESPONSE_TO_RIBAVIRIN_UP	2	25	16228	77	16.88623	0.006193
lightcyan	6363	APPEL_IMATINIB_RESPONSE	2	31	16222	62	16.91259	0.0062
greenyello	826	RESPONSE_TO_EXTERNAL_STIMULUS	7	235	16018	150	3.227546	0.006203
grey60	41	INTRINSIC_TO_MEMBRANE	8	1041	15212	44	2.838704	0.006207
brown	928	REGULATION_OF_RNA_METABOLIC_PROCESS	74	414	15839	2190	1.326541	0.00621
green	108	EXTRACELLULAR_REGION_PART	28	212	16041	1305	1.644922	0.006217
green	1457	V$MYOD_01	28	212	16041	1305	1.644922	0.006217
greenyello	3625	MODULE_137	10	426	15827	150	2.543505	0.006217
salmon	4250	REACTOME_P53_INDEPENDENT_G1_S_DNA_DAMAGE_	3	48	16205	127	7.998524	0.006237
salmon	6307	PARK_HSC_AND_MULTIPOTENT_PROGENITORS	3	48	16205	127	7.998524	0.006237
turquoise	4217	REACTOME_ACTIVATION_OF_NF_KAPPAB_IN_B_CELLS	25	61	16192	4169	1.597761	0.006243
pink	367	BIOSYNTHETIC_PROCESS	13	418	15835	228	2.217	0.006252
blue	9324	GSE22886_CD8_TCELL_VS_BCELL_NAIVE_UP	51	199	16054	2978	1.398704	0.006257
purple	2761	KEGG_ARACHIDONIC_ACID_METABOLISM	3	39	16214	157	7.963253	0.006264
purple	3596	MODULE_108	3	39	16214	157	7.963253	0.006264
purple	4351	REACTOME_PHASE1_FUNCTIONALIZATION_OF_COMPO	3	39	16214	157	7.963253	0.006264

turquoise	550	LIPID_BIOSYNTHETIC_PROCESS	33	86	16167	4169	1.49595	0.006275
turquoise	4820	WATANABE_RECTAL_CANCER_RADIOTHERAPY_RESPON	33	86	16167	4169	1.49595	0.006275
green	23	EXTRACELLULAR_MATRIX_PART	9	43	16210	1305	2.606736	0.006276
lightgreen	7735	YAO_TEMPORAL_RESPONSE_TO_PROGESTERONE_CLUS	2	46	16207	42	16.82505	0.006279
black	8285	ESC_J1_UP_EARLY.V1_DN	8	157	16096	283	2.926425	0.006281
turquoise	4988	OSWALD_HEMATOPOIETIC_STEM_CELL_IN_COLLAGEN_	85	260	15993	4169	1.274522	0.006283
lightcyan	8256	CAMP_UP.V1_UP	4	193	16060	62	5.43306	0.006288
blue	2591	GNF2_HDAC1	31	109	16144	2978	1.552187	0.006288
blue	9821	GSE360_DC_VS_MAC_L_MAJOR_DN	44	167	16086	2978	1.437954	0.006291
brown	3952	PID_ER_NONGENOMIC_PATHWAY	12	41	16212	2190	2.172135	0.006299
turquoise	638	PROTEIN_MODIFICATION_BY_SMALL_PROTEIN_CONJU	19	43	16210	4169	1.722609	0.006302
turquoise	1995	TCTGATC,MIR-383	19	43	16210	4169	1.722609	0.006302
lightgreen	9496	GSE26928_CENTR_MEMORY_VS_CXCR5_POS_CD4_TCE	3	146	16107	42	7.951566	0.00631
brown	8995	GSE17721_LPS_VS_PAM3CSK4_8H_BMDM_DN	39	194	16059	2190	1.491943	0.00631
brown	9331	GSE22886_NAIVE_VS_IGM_MEMORY_BCELL_DN	39	194	16059	2190	1.491943	0.00631
brown	9441	GSE24634_TREG_VS_TCONV_POST_DAY3_IL4_CONVER	39	194	16059	2190	1.491943	0.00631
brown	9567	GSE27786_BCELL_VS_NKCELL_DN	39	194	16059	2190	1.491943	0.00631
brown	9856	GSE360_HIGH_DOSE_B_MALAYI_VS_M_TUBERCULOSIS	39	194	16059	2190	1.491943	0.00631
grey60	8188	ZWANG_TRANSIENTLY_UP_BY_2ND_EGF_PULSE_ONLY	8	1044	15209	44	2.830547	0.006314
turquoise	4157	REACTOME_TCA_CYCLE_AND_RESPIRATORY_ELECTRON	42	115	16138	4169	1.423813	0.006316
turquoise	5155	HAHTOLA_MYCOSIS_FUNGOIDES_CD4_DN	42	115	16138	4169	1.423813	0.006316
red	6581	LIAN_LIPA_TARGETS_3M	6	52	16201	524	3.578905	0.006322
tan	9952	GSE3982_CTRL_VS_LPS_4H_MAC_UP	6	185	16068	145	3.63534	0.00633
turquoise	4100	PID_P53REGULATIONPATHWAY	24	58	16195	4169	1.613188	0.00633
turquoise	7853	KIM_ALL_DISORDERS_DURATION_CORR_DN	49	138	16115	4169	1.384263	0.006332
brown	3523	MODULE_33	58	312	15941	2190	1.379631	0.006333
brown	8115	PHONG_TNF_RESPONSE_NOT_VIA_P38	58	312	15941	2190	1.379631	0.006333
blue	6181	SCHUHMACHER_MYC_TARGETS_UP	24	79	16174	2978	1.658032	0.006334
turquoise	605	RNA_PROCESSING	58	168	16085	4169	1.345923	0.006336
turquoise	8886	GSE17721_CTRL_VS_CPG_4H_BMDM_UP	66	195	16058	4169	1.319505	0.006338
salmon	5141	DODD_NASOPHARYNGEAL_CARCINOMA_DN	19	1311	14942	127	1.85473	0.006355
salmon	7255	MOOTHA_MITOCHONDRIA	9	427	15826	127	2.697394	0.006356
purple	2817	KEGG_ENDOCYTOSIS	6	171	16082	157	3.632361	0.006359
purple	4368	REACTOME_CLASS_A1_RHODOPSIN_LIKE_RECEPTORS	6	171	16082	157	3.632361	0.006359
purple	5093	SABATES_COLORECTAL_ADENOMA_DN	6	171	16082	157	3.632361	0.006359
brown	9812	GSE360_CTRL_VS_B_MALAYI_LOW_DOSE_MAC_UP	37	182	16071	2190	1.508759	0.006366
brown	7214	BLUM_RESPONSE_TO_SALIRASIB_UP	46	237	16016	2190	1.440452	0.00637
brown	10215	GSE8515_CTRL_VS_IL1_4H_STIM_MAC_DN	36	176	16077	2190	1.518026	0.006379
midnightb	10092	GSE3982_CENT_MEMORY_CD4_TCELL_VS_TH1_UP	4	169	16084	71	5.418118	0.006383
turquoise	1007	STRESS_ACTIVATED_PROTEIN_KINASE_SIGNALING_PAT	20	46	16207	4169	1.695016	0.00639
turquoise	4295	REACTOME_CDK_MEDIATED_PHOSPHORYLATION_AND	20	46	16207	4169	1.695016	0.00639
turquoise	2295	MORF_BAG5	23	55	16198	4169	1.630297	0.006394
blue	8691	GSE1460_INTRATHYMIC_T_PROGENITOR_VS_NAIVE_CI	49	190	16063	2978	1.407509	0.006394
lightcyan	7144	BOQUEST_STEM_CELL_DN	4	194	16059	62	5.405055	0.006402
lightcyan	9463	GSE24634_IL4_VS_CTRL_TREATED_NAIVE_CD4_TCELL_	4	194	16059	62	5.405055	0.006402
magenta	731	HOMEOSTATIC_PROCESS	6	161	16092	167	3.626957	0.006404
greenyello	2412	MORF_LCAT	4	80	16173	150	5.417667	0.006407
magenta	6509	MCCLUNG_DELTA_FOSB_TARGETS_2WK	3	37	16216	167	7.891083	0.006408
grey60	7838	MARTENS_BOUND_BY_PML_RARA_FUSION	5	439	15814	44	4.207134	0.006415
brown	1747	CTGCAGY_UNKNOWN	107	631	15622	2190	1.258473	0.006421
red	95	CYTOPLASMIC_VESICLE	9	104	16149	524	2.684179	0.006421
magenta	6968	WALLACE_PROSTATE_CANCER_RACE_UP	8	267	15986	167	2.916056	0.006426
turquoise	1434	UBIQUITIN_PROTEIN_LIGASE_ACTIVITY	21	49	16204	4169	1.670801	0.00643
turquoise	4783	REACTOME_VIF_MEDIATED_DEGRADATION_OF_APOBI	21	49	16204	4169	1.670801	0.00643
turquoise	6941	WONG_PROTEASOME_GENE_MODULE	21	49	16204	4169	1.670801	0.00643
turquoise	7562	YOSHIOKA_LIVER_CANCER_EARLY_RECURRENCE_DN	21	49	16204	4169	1.670801	0.00643
lightgreen	9384	GSE22886_TH1_VS_TH2_48H_ACT_UP	3	147	16106	42	7.897473	0.00643
yellow	2309	MORF_GPX4	11	54	16199	1426	2.321737	0.006441
magenta	9519	GSE2706_LPS_VS_R848_AND_LPS_2H_STIM_DC_DN	5	114	16139	167	4.268568	0.006443
brown	5744	PUJANA_ATM_PCC_NETWORK	208	1317	14936	2190	1.172106	0.006456

purple	5905	BENPORATH_SUZ12_TARGETS	13	612	15641	157	2.199003	0.006461
red	5045	TONKS_TARGETS_OF_RUNX1_RUNX1T1_FUSION_HSC_	13	183	16070	524	2.203406	0.006465
red	8624	GSE14000_4H_VS_16H_LPS_DC_UP	13	183	16070	524	2.203406	0.006465
red	9229	GSE18791_CTRL_VS_NEWCASTLE_VIRUS_DC_10H_DN	13	183	16070	524	2.203406	0.006465
turquoise	9619	GSE28237_FOLLICULAR_VS_EARLY_GC_BCELL_DN	63	185	16068	4169	1.32761	0.006472
magenta	7022	MASRI_RESISTANCE_TO_TAMOXIFEN_AND_AROMATA!	2	12	16241	167	16.22056	0.006474
magenta	7637	LOPEZ_MESOTHELIOMA_SURVIVAL_DN	2	12	16241	167	16.22056	0.006474
magenta	7660	GUTIERREZ_CHRONIC_LYMPHOCYTIC_LEUKEMIA_UP	2	12	16241	167	16.22056	0.006474
yellow	9895	GSE36392_TYPE_2_MYELOID_VS_NEUTROPHIL_IL25_TI	28	195	16058	1426	1.636581	0.006476
tan	9762	GSE3337_CTRL_VS_16H_IFNG_IN_CD8POS_DC_UP	6	186	16067	145	3.615795	0.006494
brown	5501	SCHLOSSER_SERUM_RESPONSE_UP	27	123	16130	2190	1.629101	0.006497
turquoise	2034	GAGACTG,MIR-452	32	83	16170	4169	1.50305	0.006497
turquoise	6620	GENTILE_UV_HIGH_DOSE_DN	95	295	15958	4169	1.255461	0.006501
blue	6537	BLALOCK_ALZHEIMERS_DISEASE_UP	318	1535	14718	2978	1.130648	0.006507
midnightb	8533	GSE13306_TREG_VS_TCONV_LAMINA_PROPRIA_DN	4	170	16083	71	5.386247	0.006515
midnightb	10094	GSE3982_CENT_MEMORY_CD4_TCELL_VS_TH2_UP	4	170	16083	71	5.386247	0.006515
lightcyan	6921	KONDO_EZH2_TARGETS	4	195	16058	62	5.377337	0.006518
lightcyan	9551	GSE27786_LIN_NEG_VS_CD8_TCELL_DN	4	195	16058	62	5.377337	0.006518
lightcyan	10052	GSE3982_NEUTROPHIL_VS_CENT_MEMORY_CD4_TCEL	4	195	16058	62	5.377337	0.006518
grey60	2809	KEGG_NEUROACTIVE_LIGAND_RECEPTOR_INTERACTIO	3	141	16112	44	7.859284	0.006528
grey60	7523	CHIANG_LIVER_CANCER_SUBCLASS_CTNNB1_DN	3	141	16112	44	7.859284	0.006528
brown	1374	OXIDOREDUCTASE_ACTIVITY_ACTING_ON_THE_ALDEH'	7	18	16235	2190	2.886124	0.006534
brown	3330	chr10p14	7	18	16235	2190	2.886124	0.006534
brown	4604	REACTOME_REGULATION_OF_SIGNALING_BY_CBL	7	18	16235	2190	2.886124	0.006534
brown	6440	MARCHINI_TRABECTEDIN_RESISTANCE_UP	7	18	16235	2190	2.886124	0.006534
purple	6082	BYSTRYKH_HEMATOPOIESIS_STEM_CELL_AND_BRAIN_	6	172	16081	157	3.611243	0.006537
purple	8077	PLASARI_TGFB1_TARGETS_10HR_UP	6	172	16081	157	3.611243	0.006537
yellow	1252	METHYLTRANSFERASE_ACTIVITY	8	33	16220	1426	2.763058	0.006539
yellow	2798	KEGG_BASE_EXCISION_REPAIR	8	33	16220	1426	2.763058	0.006539
pink	8374	KRAS.LUNG_UP.V1_DN	5	84	16169	228	4.24316	0.006541
purple	3519	MODULE_27	8	285	15968	157	2.905889	0.006544
lightgreen	7142	GRADE_COLON_VS_RECTAL_CANCER_DN	2	47	16206	42	16.46707	0.006547
salmon	197	CHROMOSOMAL_PART	4	95	16158	127	5.388479	0.006548
lightgreen	10067	GSE3982_BCELL_VS_NKCELL_DN	3	148	16105	42	7.844112	0.006551
cyan	8285	ESC_J1_UP_EARLY.V1_DN	4	157	16096	77	5.377781	0.006567
purple	7715	MIKKELSEN_ES_ICP_WITH_H3K4ME3_AND_H3K27ME3	4	77	16176	157	5.377781	0.006567
lightyellow	1151	LYASE_ACTIVITY	2	60	16193	33	16.41717	0.006574
lightyellow	5224	VANHARANTA_UTERINE_FIBROID_DN	2	60	16193	33	16.41717	0.006574
turquoise	114	EARLY_ENDOSOME	10	18	16235	4169	2.165854	0.006574
turquoise	4298	REACTOME_ENOS_ACTIVATION_AND_REGULATION	10	18	16235	4169	2.165854	0.006574
turquoise	6624	KANG_DOXORUBICIN_RESISTANCE_DN	10	18	16235	4169	2.165854	0.006574
turquoise	9195	GSE17974_IL4_AND_ANTI_IL12_VS_UNTREATED_6H_A	60	175	16078	4169	1.336641	0.006582
black	8821	GSE16522_ANTI_CD3CD28_STIM_VS_UNSTIM_NAIVE_(	9	192	16061	283	2.692083	0.006585
turquoise	6822	CREIGHTON_ENDOCRINE_THERAPY_RESISTANCE_5	135	436	15817	4169	1.207116	0.006597
lightcyan	6462	CHEN_LVAD_SUPPORT_OF_FAILING_HEART_DN	2	32	16221	62	16.38407	0.006597
salmon	4783	REACTOME_VIF_MEDIATED_DEGRADATION_OF_APOBI	3	49	16204	127	7.835288	0.006607
pink	4917	CASORELLI_APL_SECONDARY_VS_DE_NOVO_DN	2	9	16244	228	15.84113	0.00661
pink	5696	SHANK_TAL1_TARGETS_DN	2	9	16244	228	15.84113	0.00661
pink	5816	SCIAN_INVERSED_TARGETS_OF_TP53_AND_TP73_UP	2	9	16244	228	15.84113	0.00661
blue	9104	GSE17721_0.5H_VS_4H_POLYIC_BMDM_UP	45	172	16081	2978	1.427884	0.006617
blue	2502	GCM_CALM1	30	105	16148	2978	1.55934	0.006625
lightcyan	3161	chr12q24	4	196	16057	62	5.349901	0.006635
blue	8517	GSE12845_IGD_NEG_BLOOD_VS_DARKZONE_GC_TON!	50	195	16058	2978	1.399408	0.006667
blue	8639	GSE14308_TH1_VS_TH17_DN	50	195	16058	2978	1.399408	0.006667
turquoise	9153	GSE17974_0H_VS_1H_IN_VITRO_ACT_CD4_TCELL_DN	65	192	16061	4169	1.319817	0.006668
turquoise	3886	MODULE_495	8	13	16240	4169	2.3991	0.006669
cyan	5398	DACOSTA_UV_RESPONSE_VIA_ERCC3_XPCS_UP	2	26	16227	77	16.23676	0.006689
yellow	2648	GNF2_HMMR	10	47	16206	1426	2.425025	0.006699
tan	1115	DRUG_BINDING	2	14	16239	145	16.01281	0.006705
tan	3767	MODULE_313	2	14	16239	145	16.01281	0.006705

tan	5499	GRASEMANN_RETINOBLASTOMA_WITH_6P_AMPLIFICA	2	14	16239	145	16.01281	0.006705
tan	5672	INAMURA_LUNG_CANCER_SCC_SUBTYPES_UP	2	14	16239	145	16.01281	0.006705
tan	6850	LEIN_LOCALIZED_TO_DISTAL_AND_PROXIMAL_DENDRI	2	14	16239	145	16.01281	0.006705
blue	761	ADAPTIVE_IMMUNE_RESPONSE	10	24	16229	2978	2.274037	0.00671
red	598	RESPONSE_TO_STRESS	25	454	15799	524	1.707994	0.006711
greenyello	2105	TGCCTTA,MIR-124A	11	500	15753	150	2.383773	0.006712
salmon	6940	WONG_MITOCHONDRIA_GENE_MODULE	6	214	16039	127	3.588123	0.006717
purple	8259	MEK_UP.V1_DN	6	173	16080	157	3.590369	0.006719
purple	8837	GSE17721_CTRL_VS_LPS_2H_BMDM_DN	6	173	16080	157	3.590369	0.006719
turquoise	7409	ZHANG_TLX_TARGETS_60HR_DN	88	271	15982	4169	1.265946	0.006724
purple	2889	KEGG_BLADDER_CANCER	3	40	16213	157	7.764172	0.006725
purple	3175	chr11q24	3	40	16213	157	7.764172	0.006725
purple	5652	JOHANSSON_BRAIN_CANCER_EARLY_VS_LATE_DN	3	40	16213	157	7.764172	0.006725
purple	5664	LI_WILMS_TUMOR_VS_FETAL_KIDNEY_2_DN	3	40	16213	157	7.764172	0.006725
blue	2592	GNF2_TDG	13	35	16218	2978	2.027142	0.006729
blue	7995	BILANGES_SERUM_SENSITIVE_VIA_TSC2	13	35	16218	2978	2.027142	0.006729
purple	738	ICOSANOID_METABOLIC_PROCESS	2	13	16240	157	15.92651	0.006744
purple	3837	MODULE_412	2	13	16240	157	15.92651	0.006744
purple	5331	NUNODA_RESPONSE_TO_DASATINIB_IMATINIB_DN	2	13	16240	157	15.92651	0.006744
purple	5368	WONG_ENDMETRIUM_CANCER_UP	2	13	16240	157	15.92651	0.006744
magenta	1970	TCTATGA,MIR-376A,MIR-376B	4	73	16180	167	5.332786	0.006752
red	6964	MASSARWEH_TAMOXIFEN_RESISTANCE_DN	14	205	16048	524	2.118246	0.006769
yellow	2603	GNF2_RRM2	9	40	16213	1426	2.564464	0.00677
brown	252	REGULATION_OF_RHO_PROTEIN_SIGNAL_TRANSDUCTI	6	14	16239	2190	3.180626	0.006779
brown	1408	OXIDOREDUCTASE_ACTIVITY_ACTING_ON_THE_ALDEHY	6	14	16239	2190	3.180626	0.006779
brown	3080	BIOCARTA_BARRESTIN_SRC_PATHWAY	6	14	16239	2190	3.180626	0.006779
lightyellow	2901	KEGG_ARRHYTHMOGENIC_RIGHT_VENTRICULAR_CARD	2	61	16192	33	16.14804	0.006789
magenta	9265	GSE20366_EX_VIVO_VS_HOMEOSTATIC_CONVERSION_	6	163	16090	167	3.582455	0.006791
red	6510	CUI_TCF21_TARGETS_DN	4	24	16229	524	5.169529	0.006795
greenyello	246	RESPONSE_TO_VIRUS	3	42	16211	150	7.739524	0.006798
greenyello	6356	ZHAN_MULTIPLE_MYELOMA_HP_UP	3	42	16211	150	7.739524	0.006798
yellow	9156	GSE17974_0H_VS_4H_IN_VITRO_ACT_CD4_TCELL_UP	26	178	16075	1426	1.66482	0.006806
turquoise	2102	AAGCAAT,MIR-137	62	182	16071	4169	1.328073	0.006808
lightgreen	6627	BURTON_ADIPOGENESIS_PEAK_AT_2HR	2	48	16205	42	16.12401	0.00682
lightgreen	7282	VILIMAS_NOTCH1_TARGETS_UP	2	48	16205	42	16.12401	0.00682
green	4849	KOBAYASHI_EGFR_SIGNALING_6HR_DN	5	16	16237	1305	3.892002	0.006831
green	6544	INGA_TP53_TARGETS	5	16	16237	1305	3.892002	0.006831
brown	6384	BRUNO_HEMATOPOIESIS	16	62	16191	2190	1.915216	0.006832
brown	4146	REACTOME_SIGNALLING_BY_NGF	40	201	16052	2190	1.476908	0.006833
brown	2445	MORF_PRKAR1A	30	141	16112	2190	1.579034	0.006843
magenta	459	DEFENSE_RESPONSE	7	215	16038	167	3.168667	0.006854
red	4731	REACTOME_IMMUNE_SYSTEM	39	801	15452	524	1.5102	0.006865
brown	5143	RODRIGUES_THYROID_CARCINOMA_POORLY_DIFFEREI	124	746	15507	2190	1.233594	0.006869
purple	1680	V$EN1_01	4	78	16175	157	5.308836	0.006871
purple	2101	CTCCAAG,MIR-432	4	78	16175	157	5.308836	0.006871
purple	2844	KEGG_HEMATOPOIETIC_CELL_LINEAGE	4	78	16175	157	5.308836	0.006871
purple	7576	MIKKELSEN_IPS_ICP_WITH_H3K4ME3_AND_H327ME3	4	78	16175	157	5.308836	0.006871
lightcyan	9365	GSE22886_NAIVE_BCELL_VS_NEUTROPHIL_DN	4	198	16055	62	5.295862	0.006873
brown	3463	SIG_IL4RECEPTOR_IN_B_LYPHOCYTES	9	27	16226	2190	2.47382	0.006874
brown	4726	REACTOME_THE_ROLE_OF_NEF_IN_HIV1_REPLICATION	9	27	16226	2190	2.47382	0.006874
brown	8152	HOLLEMAN_VINCRISTINE_RESISTANCE_ALL_UP	9	27	16226	2190	2.47382	0.006874
greenyello	9983	GSE3982_EOSINOPHIL_VS_EFF_MEMORY_CD4_TCELL_	6	182	16071	150	3.572088	0.006881
yellow	9032	GSE17721_PAM3CSK4_VS_GADIQUIMOD_12H_BMDM_	27	187	16066	1426	1.645645	0.006887
yellow	9617	GSE27786_NEUTROPHIL_VS_MONO_MAC_DN	27	187	16066	1426	1.645645	0.006887
brown	5099	KIM_WT1_TARGETS_UP	39	195	16058	2190	1.484292	0.006888
brown	8686	GSE1460_INTRATHYMIC_T_PROGENITOR_VS_CD4_THY	39	195	16058	2190	1.484292	0.006888
brown	9309	GSE22886_NAIVE_CD8_TCELL_VS_MEMORY_TCELL_DN	39	195	16058	2190	1.484292	0.006888
yellow	42	CYTOPLASMIC_PART	137	1277	14976	1426	1.222767	0.0069
greenyello	2260	YTAATTAA_V$LHX3_01	5	129	16124	150	4.199742	0.006901
greenyello	9923	GSE37416_CTRL_VS_0H_F_TULARENSIS_LVS_NEUTROF	5	129	16124	150	4.199742	0.006901

turquoise	2591	GNF2_HDAC1	40	109	16144	4169	1.430656	0.006902
magenta	1326	PROTEIN_KINASE_REGULATOR_ACTIVITY	3	38	16215	167	7.683423	0.006905
magenta	8185	ZWANG_CLASS_2_TRANSIENTLY_INDUCED_BY_EGF	3	38	16215	167	7.683423	0.006905
purple	1607	V$SRF_C	6	174	16079	157	3.569734	0.006905
pink	10135	GSE6269_FLU_VS_E_COLI_INF_PBMC_DN	6	120	16133	228	3.564254	0.006921
lightyellow	9786	GSE34205_HEALTHY_VS_RSV_INF_INFANT_PBMC_UP	3	193	16060	33	7.655676	0.006922
cyan	6186	SMITH_TERT_TARGETS_DN	3	82	16171	77	7.722363	0.006924
cyan	7807	SASSON_RESPONSE_TO_GONADOTROPHINS_DN	3	82	16171	77	7.722363	0.006924
magenta	1620	V$AHRARNT_01	5	116	16137	167	4.194972	0.006925
magenta	6323	PETROVA_ENDOTHELIUM_LYMPHATIC_VS_BLOOD_UP	5	116	16137	167	4.194972	0.006925
brown	10080	GSE3982_BASOPHIL_VS_TH2_UP	38	189	16064	2190	1.492146	0.006934
blue	2194	TCCATTKW_UNKNOWN	51	200	16053	2978	1.391711	0.006937
blue	8475	GSE11864_CSF1_IFNG_VS_CSF1_PAM3CYS_IN_MAC_D	46	177	16076	2978	1.418383	0.00694
blue	9053	GSE17721_LPS_VS_CPG_16H_BMDM_DN	46	177	16076	2978	1.418383	0.00694
turquoise	2516	GCM_DLG1	30	77	16176	4169	1.51891	0.006941
yellow	9126	GSE17721_0.5H_VS_12H_CPG_BMDM_UP	28	196	16057	1426	1.628231	0.006941
yellow	9782	GSE339_EX_VIVO_VS_IN_CULTURE_CD8POS_DC_UP	28	196	16057	1426	1.628231	0.006941
lightcyan	3630	MODULE_145	3	102	16151	62	7.710152	0.006942
greenyello	3555	MODULE_66	10	433	15820	150	2.502386	0.006942
brown	77	GOLGI_APPARATUS_PART	22	95	16158	2190	1.718654	0.006954
brown	2455	MORF_RAB5A	22	95	16158	2190	1.718654	0.006954
purple	5486	FARMER_BREAST_CANCER_APOCRINE_VS_LUMINAL	8	288	15965	157	2.875619	0.006954
brown	5312	MCBRYAN_PUBERTAL_TGFB1_TARGETS_UP	32	153	16100	2190	1.552201	0.006962
pink	8381	LEF1_UP.V1_UP	7	158	16095	228	3.1582	0.006968
blue	4708	REACTOME_LATE_PHASE_OF_HIV_LIFE_CYCLE	29	101	16152	2978	1.567059	0.006973
blue	5740	PUJANA_BREAST_CANCER_LIT_INT_NETWORK	29	101	16152	2978	1.567059	0.006973
black	1893	V$EGR_Q6	10	229	16024	283	2.507908	0.006974
red	886	REGULATION_OF_NUCLEOBASENUCLEOSIDENUCLEOTII	29	552	15701	524	1.629526	0.006976
salmon	138	SPLICEOSOME	3	50	16203	127	7.678583	0.006989
salmon	4662	REACTOME_DESTABILIZATION_OF_MRNA_BY_AUF1_H	3	50	16203	127	7.678583	0.006989
magenta	8226	VEGF_A_UP.V1_UP	6	164	16089	167	3.56061	0.00699
brown	9499	GSE2706_UNSTIM_VS_2H_R848_DC_DN	36	177	16076	2190	1.50945	0.006995
green	4566	REACTOME_RNA_POL_I_RNA_POL_III_AND_MITOCHOI	15	93	16160	1305	2.008775	0.006996
lightgreen	3582	MODULE_94	4	296	15957	42	5.229408	0.007003
tan	9921	GSE36476_YOUNG_VS_OLD_DONOR_MEMORY_CD4_T	6	189	16064	145	3.558402	0.007005
magenta	2274	CCCNNGGGAR_V$OLF1_01	8	271	15982	167	2.873014	0.007005
lightcyan	104	MICROSOME	2	33	16220	62	15.88759	0.007006
black	7377	HOFFMANN_PRE_BI_TO_LARGE_PRE_BII_LYMPHOCYTE	5	69	16184	283	4.161674	0.007008
pink	3530	MODULE_41	13	424	15829	228	2.185628	0.007012
cyan	8172	SMIRNOV_RESPONSE_TO_IR_6HR_UP	4	160	16093	77	5.276948	0.007014
turquoise	8464	GSE11864_UNTREATED_VS_CSF1_PAM3CYS_IN_MAC_I	64	189	16064	4169	1.320139	0.007016
turquoise	9040	GSE17721_LPS_VS_CPG_1H_BMDM_UP	64	189	16064	4169	1.320139	0.007016
turquoise	10034	GSE3982_MAC_VS_BASOPHIL_UP	64	189	16064	4169	1.320139	0.007016
blue	528	COFACTOR_CATABOLIC_PROCESS	5	8	16245	2978	3.411056	0.007041
midnightb	6430	IVANOVA_HEMATOPOIESIS_LATE_PROGENITOR	7	514	15739	71	3.117526	0.007048
turquoise	2271	GATTGGY_V$NFY_Q6_01	291	1003	15250	4169	1.131081	0.007054
purple	6788	BILD_HRAS_ONCOGENIC_SIGNATURE	7	230	16023	157	3.150678	0.007055
red	1077	NUCLEOTIDE_BINDING	14	206	16047	524	2.107963	0.007056
greenyello	8721	GSE14769_UNSTIM_VS_80MIN_LPS_BMDM_DN	6	183	16070	150	3.552568	0.007062
greenyello	10174	GSE7460_FOXP3_MUT_VS_WT_ACT_WITH_TGFB_TCOI	6	183	16070	150	3.552568	0.007062
red	9257	GSE20151_CTRL_VS_FUSOBACT_NUCLEATUM_NEUTRC	13	185	16068	524	2.179585	0.007063
blue	869	PIGMENT_BIOSYNTHETIC_PROCESS	7	14	16239	2978	2.728845	0.007068
blue	1320	LIGASE_ACTIVITY_FORMING_CARBON_OXYGEN_BONDS	7	14	16239	2978	2.728845	0.007068
blue	5310	HUMMERICH_BENIGN_SKIN_TUMOR_UP	7	14	16239	2978	2.728845	0.007068
blue	2497	GCM_AIP	14	39	16214	2978	1.959171	0.007075
purple	3617	MODULE_129	6	175	16078	157	3.549336	0.007095
lightgreen	7269	WU_SILENCED_BY_METHYLATION_IN_BLADDER_CANCI	2	49	16204	42	15.79495	0.007099
red	949	NUCLEOTIDE_METABOLIC_PROCESS	5	38	16215	524	4.081207	0.007125
greenyello	287	INORGANIC_ANION_TRANSPORT	2	14	16239	150	15.47905	0.007159
greenyello	6897	JI_CARCINOGENESIS_BY_KRAS_AND_STK11_DN	2	14	16239	150	15.47905	0.007159

turquoise	833	TRANSCRIPTION_INITIATION_FROM_RNA_POLYMERASI	14	29	16224	4169	1.882052	0.007159
turquoise	8058	WAKABAYASHI_ADIPOGENESIS_PPARG_BOUND_36HR	14	29	16224	4169	1.882052	0.007159
cyan	7809	SASSON_RESPONSE_TO_FORSKOLIN_DN	3	83	16170	77	7.629322	0.007159
magenta	7008	RIGGI_EWING_SARCOMA_PROGENITOR_UP	9	330	15923	167	2.654273	0.007161
turquoise	9009	GSE17721_POLYIC_VS_CPG_4H_BMDM_DN	61	179	16074	4169	1.328552	0.007163
green	7331	YOSHIMURA_MAPK8_TARGETS_UP	97	947	15306	1305	1.275689	0.007163
cyan	9652	GSE29617_DAY3_VS_DAY7_TIV_FLU_VACCINE_PBMC_2	4	161	16092	77	5.244172	0.007167
purple	1663	V$PAX8_B	4	79	16174	157	5.241635	0.007184
blue	5141	DODD_NASOPHARYNGEAL_CARCINOMA_DN	274	1311	14942	2978	1.140661	0.007204
purple	546	ANATOMICAL_STRUCTURE_FORMATION	3	41	16212	157	7.574802	0.007205
midnightb	9169	GSE17974_CTRL_VS_ACT_IL4_AND_ANTI_IL12_0.5H_CI	4	175	16078	71	5.232354	0.007206
turquoise	8413	GSE10239_MEMORY_VS_KLRG1INT_EFF_CD8_TCELL_D	66	196	16057	4169	1.312773	0.007206
turquoise	9236	GSE18791_CTRL_VS_NEWCASTLE_VIRUS_DC_18H_UP	66	196	16057	4169	1.312773	0.007206
turquoise	9946	GSE37416_12H_VS_48H_F_TULARENSIS_LVS_NEUTROI	66	196	16057	4169	1.312773	0.007206
turquoise	10089	GSE3982_EFF_MEMORY_CD4_TCELL_VS_TH2_DN	66	196	16057	4169	1.312773	0.007206
turquoise	7036	ACEVEDO_LIVER_TUMOR_VS_NORMAL_ADJACENT_TIS	244	831	15422	4169	1.144697	0.007217
purple	2222	YKACATTT_UNKNOWN	7	231	16022	157	3.137039	0.007218
red	2218	YGACNNYACAR_UNKNOWN	7	70	16183	524	3.101718	0.007226
red	3631	MODULE_146	7	70	16183	524	3.101718	0.007226
greenyello	2203	TTANTCA_UNKNOWN	14	725	15528	150	2.09234	0.007235
greenyello	1909	V$SRF_Q5_01	6	184	16069	150	3.533261	0.007246
greenyello	8435	GSE10325_CD4_TCELL_VS_LUPUS_CD4_TCELL_DN	6	184	16069	150	3.533261	0.007246
brown	7240	GRESHOCK_CANCER_COPY_NUMBER_UP	55	295	15958	2190	1.383662	0.007246
red	8238	ATM_DN.V1_UP	9	106	16147	524	2.633534	0.007255
turquoise	9917	GSE36476_YOUNG_VS_OLD_DONOR_MEMORY_CD4_T	58	169	16084	4169	1.337959	0.007281
greenyello	8351	KRAS.AMP.LUNG_UP.V1_UP	4	83	16170	150	5.221847	0.007288
purple	8257	LTE2_UP.V1_DN	6	176	16077	157	3.529169	0.007288
magenta	6935	HELLER_HDAC_TARGETS_DN	8	273	15980	167	2.851966	0.007309
greenyello	2287	TATAAA_V$TATA_01	16	880	15373	150	1.970061	0.007316
green	8845	GSE17721_CTRL_VS_LPS_12H_BMDM_DN	24	176	16077	1305	1.698328	0.007317
lightyellow	9446	GSE24634_TREG_VS_TCONV_POST_DAY10_IL4_CONVE	3	197	16056	33	7.500231	0.007323
lightgreen	7831	LI_INDUCED_T_TO_NATURAL_KILLER_UP	4	300	15953	42	5.159683	0.007337
yellow	649	DEVELOPMENT_OF_PRIMARY_SEXUAL_CHARACTERISTI	5	15	16238	1426	3.799205	0.007338
red	8070	HUANG_GATA2_TARGETS_UP	11	145	16108	524	2.353027	0.007344
red	8232	ATF2_S_UP.V1_UP	11	145	16108	524	2.353027	0.007344
greenyello	8264	PRC1_BMI_UP.V1_UP	5	131	16122	150	4.135623	0.007354
tan	10045	GSE3982_MAC_VS_TH2_DN	6	191	16062	145	3.521141	0.007361
tan	10206	GSE7852_LN_VS_FAT_TCONV_UP	6	191	16062	145	3.521141	0.007361
turquoise	2079	ATGTCAC,MIR-489	28	71	16182	4169	1.537451	0.007373
red	9576	GSE27786_CD4_VS_CD8_TCELL_UP	13	186	16067	524	2.167867	0.007379
turquoise	1963	AAAGGGA,MIR-204,MIR-211	68	203	16050	4169	1.305914	0.007379
lightgreen	6590	ZHU_CMV_8_HR_DN	2	50	16203	42	15.47905	0.007383
salmon	4629	REACTOME_CIRCADIAN_CLOCK	3	51	16202	127	7.528022	0.007384
purple	7689	DANG_REGULATED_BY_MYC_DN	7	232	16021	157	3.123517	0.007384
yellow	8513	GSE12845_IGD_NEG_BLOOD_VS_NAIVE_TONSIL_BCELL	27	188	16065	1426	1.636892	0.00739
blue	9584	GSE27786_CD4_TCELL_VS_NEUTROPHIL_UP	50	196	16057	2978	1.392268	0.007394
blue	9610	GSE27786_NKTCELL_VS_MONO_MAC_UP	50	196	16057	2978	1.392268	0.007394
blue	10263	GSE9650_EFFECTOR_VS_MEMORY_CD8_TCELL_DN	50	196	16057	2978	1.392268	0.007394
green	1270	RHODOPSIN_LIKE_RECEPTOR_ACTIVITY	12	68	16185	1305	2.197836	0.007397
greenyello	2246	RYTTCCTG_V$ETS2_B	17	960	15293	150	1.918757	0.007399
blue	5505	SCHLOSSER_MYC_TARGETS_AND_SERUM_RESPONSE_I	16	47	16206	2978	1.857937	0.007403
blue	6752	LEE_CALORIE_RESTRICTION_MUSCLE_DN	16	47	16206	2978	1.857937	0.007403
turquoise	6717	DAZARD_UV_RESPONSE_CLUSTER_G6	52	149	16104	4169	1.360563	0.007404
salmon	3525	MODULE_36	5	155	16098	127	4.12827	0.007406
salmon	5504	SCHLOSSER_MYC_TARGETS_REPRESSED_BY_SERUM	5	155	16098	127	4.12827	0.007406
magenta	247	MUSCLE_DEVELOPMENT	4	75	16178	167	5.190579	0.007422
magenta	847	NEUROGENESIS	4	75	16178	167	5.190579	0.007422
magenta	4581	REACTOME_L1CAM_INTERACTIONS	4	75	16178	167	5.190579	0.007422
magenta	7279	BOHN_PRIMARY_IMMUNODEFICIENCY_SYNDROM_DN	3	39	16214	167	7.486412	0.007425
black	3123	chr8q24	7	129	16124	283	3.116416	0.007425

lightyellow	9439	GSE24634_NAIVE_CD4_TCELL_VS_DAY10_IL4_CONV_T	3	198	16055	33	7.462351	0.007426
lightyellow	10072	GSE3982_BASOPHIL_VS_EFF_MEMORY_CD4_TCELL_UP	3	198	16055	33	7.462351	0.007426
lightcyan	7112	HONMA_DOCETAXEL_RESISTANCE	2	34	16219	62	15.4203	0.007426
yellow	2578	GNF2_CDC20	11	55	16198	1426	2.279523	0.007428
red	5488	FARMER_BREAST_CANCER_BASAL_VS_LULMINAL	18	295	15958	524	1.892573	0.007432
brown	5733	SPIELMAN_LYMPHOBLAST_EUROPEAN_VS_ASIAN_DN	99	581	15672	2190	1.264586	0.007432
greenyello	1837	V$CDX2_Q5	6	185	16068	150	3.514162	0.007433
brown	530	MYELOID_CELL_DIFFERENTIATION	10	32	16221	2190	2.319207	0.007438
brown	2714	GNF2_TYK2	10	32	16221	2190	2.319207	0.007438
brown	4587	REACTOME_SIGNAL_TRANSDUCTION_BY_L1	10	32	16221	2190	2.319207	0.007438
brown	6397	WEIGEL_OXIDATIVE_STRESS_BY_TBH_AND_H2O2	10	32	16221	2190	2.319207	0.007438
blue	921	CHROMOSOME_ORGANIZATION_AND_BIOGENESIS	33	119	16134	2978	1.513477	0.007446
pink	9720	GSE30083_SP3_VS_SP4_THYMOCYTE_UP	7	160	16093	228	3.118723	0.007446
grey60	9748	GSE32423_CTRL_VS_IL7_MEMORY_CD8_TCELL_UP	3	148	16105	44	7.487561	0.007458
cyan	7864	WANG_RESPONSE_TO_GSK3_INHIBITOR_SB216763_UP	6	363	15890	77	3.488891	0.007461
yellow	9150	GSE17974_0H_VS_0.5H_IN_VITRO_ACT_CD4_TCELL_UP	23	153	16100	1426	1.713367	0.007468
blue	158	U12_DEPENDENT_SPLICEOSOME	6	11	16242	2978	2.976922	0.007476
blue	1187	TRANSCRIPTION_ELONGATION_REGULATOR_ACTIVITY	6	11	16242	2978	2.976922	0.007476
blue	3013	BIOCARTA_TCRA_PATHWAY	6	11	16242	2978	2.976922	0.007476
blue	4439	REACTOME_PURINE_RIBONUCLEOSIDE_MONOPHOSPH	6	11	16242	2978	2.976922	0.007476
blue	5827	CAFFAREL_RESPONSE_TO_THC_8HR_5_DN	6	11	16242	2978	2.976922	0.007476
cyan	2257	STTTCRNTTT_V$IRF_Q6	4	163	16090	77	5.179826	0.007481
purple	5235	GRAESSMANN_RESPONSE_TO_MC_AND_SERUM_DEPR	6	177	16076	157	3.50923	0.007485
purple	10189	GSE7764_IL15_TREATED_VS_CTRL_NK_CELL_24H_DN	6	177	16076	157	3.50923	0.007485
red	332	PROTEIN_TETRAMERIZATION	3	13	16240	524	7.15781	0.007486
red	750	NLS_BEARING_SUBSTRATE_IMPORT_INTO_NUCLEUS	3	13	16240	524	7.15781	0.007486
red	4632	REACTOME_REGULATION_OF_IFNG_SIGNALING	3	13	16240	524	7.15781	0.007486
red	5420	SANCHEZ_MDM2_TARGETS	3	13	16240	524	7.15781	0.007486
red	7593	SETLUR_PROSTATE_CANCER_TMPRSS2_ERG_FUSION_D	3	13	16240	524	7.15781	0.007486
midnightb	8602	GSE13738_RESTING_VS_TCR_ACTIVATED_CD4_TCELL_U	4	177	16076	71	5.173231	0.007495
brown	8791	GSE15930_NAIVE_VS_72H_IN_VITRO_STIM_IL12_CD8_	39	196	16057	2190	1.476719	0.00751
brown	8822	GSE16755_CTRL_VS_IFNA_TREATED_MAC_UP	39	196	16057	2190	1.476719	0.00751
brown	10256	GSE9650_NAIVE_VS_EXHAUSTED_CD8_TCELL_UP	39	196	16057	2190	1.476719	0.00751
brown	10268	GSE9988_ANTI_TREM1_VS_LPS_MONOCYTE_UP	39	196	16057	2190	1.476719	0.00751
blue	7263	BOYLAN_MULTIPLE_MYELOMA_D_UP	24	80	16173	2978	1.637307	0.00751
red	6935	HELLER_HDAC_TARGETS_DN	17	273	15980	524	1.931472	0.00753
purple	3218	chr6p21	8	292	15961	157	2.836227	0.007531
midnightb	8175	GHANDHI_DIRECT_IRRADIATION_DN	2	30	16223	71	15.26103	0.007563
brown	8691	GSE1460_INTRATHYMIC_T_PROGENITOR_VS_NAIVE_CI	38	190	16063	2190	1.484292	0.00757
blue	9417	GSE24142_EARLY_THYMIC_PROGENITOR_VS_DN3_THY	48	187	16066	2978	1.400904	0.007572
red	1002	INTRACELLULAR_SIGNALING_CASCADE	30	580	15673	524	1.604337	0.007574
brown	2686	GNF2_PTPRC	17	68	16185	2190	1.855365	0.007574
brown	4337	REACTOME_INTEGRIN_CELL_SURFACE_INTERACTIONS	17	68	16185	2190	1.855365	0.007574
yellow	2639	GNF2_EIF3S6	19	119	16134	1426	1.819787	0.007576
green	4327	REACTOME_RNA_POL_I_TRANSCRIPTION	11	60	16193	1305	2.283308	0.007582
turquoise	2373	MORF_DAP3	65	193	16060	4169	1.312979	0.007584
turquoise	4697	REACTOME_HIV_INFECTION	65	193	16060	4169	1.312979	0.007584
turquoise	8568	GSE13484_12H_UNSTIM_VS_YF17D_VACCINE_STIM_PI	65	193	16060	4169	1.312979	0.007584
turquoise	8919	GSE17721_LPS_VS_POLYIC_4H_BMDM_DN	65	193	16060	4169	1.312979	0.007584
turquoise	9975	GSE3982_EOSINOPHIL_VS_MAC_DN	65	193	16060	4169	1.312979	0.007584
turquoise	10099	GSE3982_NKCELL_VS_TH2_DN	65	193	16060	4169	1.312979	0.007584
grey60	8626	GSE14026_TH1_VS_TH17_UP	3	149	16104	44	7.437309	0.007598
greenyello	7140	GRADE_COLON_AND_RECTAL_CANCER_DN	4	84	16169	150	5.159683	0.007598
magenta	20	EXTERNAL_SIDE_OF_PLASMA_MEMBRANE	2	13	16240	167	14.97282	0.007599
magenta	1010	CELL_MATURATION	2	13	16240	167	14.97282	0.007599
magenta	3629	MODULE_143	2	13	16240	167	14.97282	0.007599
magenta	3910	MODULE_543	2	13	16240	167	14.97282	0.007599
magenta	4871	DAVICIONI_RHABDOMYOSARCOMA_PAX_FOXO1_FUSI	2	13	16240	167	14.97282	0.007599
magenta	7011	VICENT_METASTASIS_UP	2	13	16240	167	14.97282	0.007599
midnightb	5024	HOEBEKE_LYMPHOID_STEM_CELL_UP	3	92	16161	71	7.464636	0.0076

pink	6292	NEMETH_INFLAMMATORY_RESPONSE_LPS_DN	3	29	16224	228	7.374319	0.007607
pink	7577	MIKKELSEN_IPS_ICP_WITH_H3K27ME3	3	29	16224	228	7.374319	0.007607
red	6324	LI_WILMS_TUMOR_VS_FETAL_KIDNEY_1_UP	12	166	16087	524	2.242205	0.007615
blue	1988	CTCTGGA,MIR-520A,MIR-525	37	137	16116	2978	1.473975	0.00762
brown	8423	GSE10325_CD4_TCELL_VS_BCELL_DN	37	184	16069	2190	1.492359	0.007621
brown	10158	GSE7460_CD8_TCELL_VS_TREG_ACT_UP	37	184	16069	2190	1.492359	0.007621
salmon	2119	AGTCTAG,MIR-151	2	17	16236	127	15.05604	0.00763
salmon	3797	MODULE_352	2	17	16236	127	15.05604	0.00763
salmon	3821	MODULE_388	2	17	16236	127	15.05604	0.00763
turquoise	3325	chr19p12	15	32	16221	4169	1.827439	0.007632
turquoise	5824	CAFFAREL_RESPONSE_TO_THC_UP	15	32	16221	4169	1.827439	0.007632
turquoise	5869	SCHAEFFER_PROSTATE_DEVELOPMENT_AND_CANCER_	15	32	16221	4169	1.827439	0.007632
cyan	8226	VEGF_A_UP.V1_UP	4	164	16089	77	5.148242	0.007641
cyan	9022	GSE17721_PAM3CSK4_VS_GADIQUIMOD_1H_BMDM_	4	164	16089	77	5.148242	0.007641
midnightb	9500	GSE2706_UNSTIM_VS_8H_R848_DC_UP	4	178	16075	71	5.144168	0.007642
black	7383	HOFFMANN_IMMATURE_TO_MATURE_B_LYMPHOCYT	4	45	16208	283	5.104986	0.007655
brown	168	RECEPTOR_COMPLEX	13	47	16206	2190	2.052745	0.007657
brown	9429	GSE24142_ADULT_VS_FETAL_DN2_THYMOCYTE_DN	36	178	16075	2190	1.50097	0.007661
brown	9779	GSE339_CD8POS_VS_CD4CD8DN_DC_IN_CULTURE_DN	36	178	16075	2190	1.50097	0.007661
lightgreen	5943	AMIT_SERUM_RESPONSE_240_MCF10A	2	51	16202	42	15.17554	0.007672
lightyellow	7312	LINDSTEDT_DENDRITIC_CELL_MATURATION_D	2	65	16188	33	15.15431	0.007677
purple	1814	V$AP3_Q6	6	178	16075	157	3.489515	0.007686
purple	8353	KRAS.DF.V1_UP	6	178	16075	157	3.489515	0.007686
purple	9854	GSE360_HIGH_VS_LOW_DOSE_B_MALAYI_DC_UP	6	178	16075	157	3.489515	0.007686
tan	312	CHROMATIN_ASSEMBLY	2	15	16238	145	14.94529	0.007691
tan	681	RRNA_PROCESSING	2	15	16238	145	14.94529	0.007691
tan	1014	RRNA_METABOLIC_PROCESS	2	15	16238	145	14.94529	0.007691
tan	3712	MODULE_240	2	15	16238	145	14.94529	0.007691
cyan	8348	STK33_SKM_UP	5	259	15994	77	4.074863	0.007693
magenta	2234	CAGCTG_V$AP4_Q5	22	1231	15022	167	1.739329	0.007694
pink	1546	V$HNF1_01	7	161	16092	228	3.099352	0.007695
brown	940	NEGATIVE_REGULATION_OF_DEVELOPMENTAL_PROCE	34	166	16087	2190	1.520058	0.007702
red	9144	GSE17721_0.5H_VS_24H_GARDIQUIMOD_BMDM_UP	13	187	16066	524	2.156274	0.007705
red	9591	GSE27786_CD8_TCELL_VS_NKTCELL_DN	13	187	16066	524	2.156274	0.007705
purple	1169	PHOSPHOLIPID_BINDING	3	42	16211	157	7.394449	0.007705
purple	5796	NAKAYAMA_FRA2_TARGETS	3	42	16211	157	7.394449	0.007705
brown	3083	BIOCARTA_MET_PATHWAY	11	37	16216	2190	2.20638	0.007707
brown	3462	ST_P38_MAPK_PATHWAY	11	37	16216	2190	2.20638	0.007707
tan	8459	GSE11057_PBMC_VS_MEM_CD4_TCELL_DN	6	193	16060	145	3.484652	0.00773
cyan	6276	GILDEA_METASTASIS	2	28	16225	77	15.07699	0.007732
cyan	6793	KRIGE_AMINO_ACID_DEPRIVATION	2	28	16225	77	15.07699	0.007732
blue	9156	GSE17974_0H_VS_4H_IN_VITRO_ACT_CD4_TCELL_UP	46	178	16075	2978	1.410414	0.007734
blue	9274	GSE20366_CD103_KLRG1_DP_VS_DN_TREG_UP	46	178	16075	2978	1.410414	0.007734
blue	9552	GSE27786_LIN_NEG_VS_NKCELL_UP	46	178	16075	2978	1.410414	0.007734
lightyellow	7912	WIERENGA_STAT5A_TARGETS_UP	3	201	16052	33	7.350972	0.007738
turquoise	4719	REACTOME_INNATE_IMMUNE_SYSTEM	67	200	16053	4169	1.30601	0.007766
turquoise	4715	REACTOME_APC_C_CDC20_MEDIATED_DEGRADATION	26	65	16188	4169	1.559415	0.007775
pink	8329	IL21_UP.V1_DN	6	123	16130	228	3.477321	0.007779
pink	6934	HELLER_HDAC_TARGETS_UP	10	289	15964	228	2.466612	0.007797
red	488	LOCOMOTORY_BEHAVIOR	7	71	16182	524	3.058031	0.0078
red	7200	HUANG_DASATINIB_RESISTANCE_UP	7	71	16182	524	3.058031	0.0078
cyan	10131	GSE6269_HEALTHY_VS_STREP_AUREUS_INF_PBMC_DN	4	165	16088	77	5.117041	0.007803
salmon	8930	GSE17721_POLYIC_VS_PAM3CSK4_0.5H_BMDM_UP	5	157	16096	127	4.075681	0.007807
brown	6954	MARTINEZ_RB1_TARGETS_DN	80	457	15796	2190	1.299162	0.007812
cyan	520	LIPID_METABOLIC_PROCESS	5	260	15993	77	4.059191	0.007815
greenyello	9459	GSE24634_IL4_VS_CTRL_TREATED_NAIVE_CD4_TCELL_	6	187	16066	150	3.476578	0.007818
purple	2908	BIOCARTA_AMI_PATHWAY	2	14	16239	157	14.7889	0.007818
purple	3765	MODULE_311	2	14	16239	157	14.7889	0.007818
purple	4676	REACTOME_INTRINSIC_PATHWAY	2	14	16239	157	14.7889	0.007818
purple	7458	LIU_VAV3_PROSTATE_CARCINOGENESIS_DN	2	14	16239	157	14.7889	0.007818

salmon	3673	MODULE_192	4	100	16153	127	5.119055	0.007829
magenta	9850	GSE360_T_GONDII_VS_B_MALAYI_LOW_DOSE_DC_UP	6	168	16085	167	3.475834	0.00783
magenta	10261	GSE9650_EFFECTOR_VS_EXHAUSTED_CD8_TCELL_DN	6	168	16085	167	3.475834	0.00783
yellow	6795	KRIGE_RESPONSE_TO_TOSEDOSTAT_6HR_DN	98	880	15373	1426	1.26928	0.007831
purple	3657	MODULE_174	4	81	16172	157	5.112212	0.007839
purple	3748	MODULE_289	4	81	16172	157	5.112212	0.007839
lightyellow	951	G_PROTEIN_COUPLED_RECEPTOR_PROTEIN_SIGNALING	3	202	16051	33	7.314581	0.007844
red	6822	CREIGHTON_ENDOCRINE_THERAPY_RESISTANCE_5	24	436	15817	524	1.707367	0.007844
lightcyan	22	VESICULAR_FRACTION	2	35	16218	62	14.97972	0.007856
lightcyan	7664	GUTIERREZ_MULTIPLE_MYELOMA_DN	2	35	16218	62	14.97972	0.007856
turquoise	3811	MODULE_372	11	21	16232	4169	2.042091	0.007864
red	915	MEMBRANE_FUSION	4	25	16228	524	4.962748	0.007887
red	1273	PEPTIDE_RECEPTOR_ACTIVITY	4	25	16228	524	4.962748	0.007887
purple	1896	V$SRF_Q4	6	179	16074	157	3.470021	0.007891
purple	7038	ACEVEDO_LIVER_CANCER_WITH_H3K27ME3_UP	6	179	16074	157	3.470021	0.007891
red	7418	CHEN_METABOLIC_SYNDROM_NETWORK	50	1094	15159	524	1.417604	0.007897
purple	4565	REACTOME_GPCR_LIGAND_BINDING	7	235	16018	157	3.083643	0.007898
brown	665	ACTIN_CYTOSKELETON_ORGANIZATION_AND_BIOGENE	22	96	16157	2190	1.700752	0.007902
yellow	1359	TRANSFERASE_ACTIVITY_TRANSFERRING_ONE_CARBON	8	34	16219	1426	2.681792	0.007907
yellow	4855	GAZDA_DIAMOND_BLACKFAN_ANEMIA_MYELOID_DN	8	34	16219	1426	2.681792	0.007907
turquoise	9880	GSE360_T_GONDII_VS_B_MALAYI_LOW_DOSE_MAC_U	59	173	16080	4169	1.329559	0.007929
turquoise	121	ENDOMEMBRANE_SYSTEM	69	207	16046	4169	1.299512	0.007931
pink	7787	NIELSEN_GIST	5	88	16165	228	4.050289	0.007936
red	2247	TTGTTT_V$FOXO4_01	70	1632	14621	524	1.330394	0.007954
yellow	9228	GSE18791_CTRL_VS_NEWCASTLE_VIRUS_DC_10H_UP	28	198	16055	1426	1.611784	0.007956
red	5177	LANDIS_BREAST_CANCER_PROGRESSION_UP	5	39	16214	524	3.976561	0.007961
red	7465	CROONQUIST_NRAS_VS_STROMAL_STIMULATION_UP	5	39	16214	524	3.976561	0.007961
cyan	9848	GSE360_T_GONDII_VS_B_MALAYI_HIGH_DOSE_DC_UP	4	166	16087	77	5.086215	0.007967
cyan	10046	GSE3982_NEUTROPHIL_VS_BCELL_UP	4	166	16087	77	5.086215	0.007967
red	1700	V$NKX61_01	12	167	16086	524	2.228779	0.007973
red	9585	GSE27786_CD4_TCELL_VS_NEUTROPHIL_DN	12	167	16086	524	2.228779	0.007973
red	9793	GSE360_CTRL_VS_L_DONOVANI_DC_DN	12	167	16086	524	2.228779	0.007973
lightgreen	5620	MARTORIATI_MDM4_TARGETS_NEUROEPITHELIUM_UI	3	159	16094	42	7.301438	0.007977
blue	10227	GSE9006_HEALTHY_VS_TYPE_1_DIABETES_PBMC_1MC	42	160	16093	2978	1.432644	0.007987
black	9251	GSE19825_NAIVE_VS_IL2RAHIGH_DAY3_EFF_CD8_TCEI	9	198	16055	283	2.610504	0.00799
brown	51	GOLGI_APPARATUS	41	209	16044	2190	1.455885	0.007999
grey60	3581	MODULE_93	3	152	16101	44	7.29052	0.008024
brown	799	REGULATION_OF_SMALL_GTPASE_MEDIATED_SIGNAL_	8	23	16230	2190	2.581378	0.008029
brown	859	HEART_DEVELOPMENT	8	23	16230	2190	2.581378	0.008029
brown	3110	BIOCARTA_TPO_PATHWAY	8	23	16230	2190	2.581378	0.008029
brown	4227	REACTOME_CIRCADIAN_REPRESSION_OF_EXPRESSION_	8	23	16230	2190	2.581378	0.008029
brown	4616	REACTOME_IL_RECEPTOR_SHC_SIGNALING	8	23	16230	2190	2.581378	0.008029
brown	7354	SCHRAETS_MLL_TARGETS_DN	8	23	16230	2190	2.581378	0.008029
red	1783	V$CRX_Q4	13	188	16065	524	2.144805	0.008042
red	9894	GSE36392_TYPE_2_MYELOID_VS_NEUTROPHIL_IL25_TI	13	188	16065	524	2.144805	0.008042
red	4489	REACTOME_G_ALPHA_I_SIGNALLING_EVENTS	10	127	16126	524	2.442297	0.008048
yellow	1306	NUCLEOBASENUCLEOSIDENUCLEOTIDE_AND_NUCLEIC_	4	10	16243	1426	4.559046	0.008048
yellow	7659	CAIRO_HEPATOBLASTOMA_POOR_SURVIVAL	4	10	16243	1426	4.559046	0.008048
turquoise	6394	IVANOVA_HEMATOPOIESIS_EARLY_PROGENITOR	150	492	15761	4169	1.188578	0.00805
magenta	8799	GSE15930_STIM_VS_STIM_AND_IFNAB_24H_CD8_T_C	6	169	16084	167	3.455267	0.008051
magenta	10249	GSE9037_WT_VS_IRAK4_KO_BMDM_DN	6	169	16084	167	3.455267	0.008051
brown	7630	STEIN_ESRRA_TARGETS	88	510	15743	2190	1.280566	0.008052
blue	4507	REACTOME_CD28_DEPENDENT_PI3K_AKT_SIGNALING	9	21	16232	2978	2.33901	0.008059
midnightb	3236	chr13q34	2	31	16222	71	14.76874	0.008062
blue	4347	REACTOME_RNA_POL_II_TRANSCRIPTION	29	102	16151	2978	1.551696	0.008075
blue	4819	WATANABE_RECTAL_CANCER_RADIOTHERAPY_RESPON	29	102	16151	2978	1.551696	0.008075
blue	924	RNA_SPLICING	26	89	16164	2978	1.594381	0.008082
midnightb	9467	GSE25087_FETAL_VS_ADULT_TCONV_DN	4	181	16072	71	5.058906	0.008096
midnightb	9693	GSE29618_PRE_VS_DAY7_POST_TIV_FLU_VACCINE_MC	4	181	16072	71	5.058906	0.008096
lightyellow	2928	BIOCARTA_NEUROTRANSMITTERS_PATHWAY	1	4	16249	33	123.1288	0.008098

lightyellow	6967	WALLACE_PROSTATE_CANCER_DN	1	4	16249	33	123.1288	0.008098
purple	1470	V$RSRFC4_01	6	180	16073	157	3.450743	0.008099
purple	9418	GSE24142_DN2_VS_DN3_THYMOCYTE_ADULT_UP	6	180	16073	157	3.450743	0.008099
brown	825	POSITIVE_REGULATION_OF_BIOLOGICAL_PROCESS	104	616	15637	2190	1.252974	0.008099
red	5912	BENPORATH_PROLIFERATION	11	147	16106	524	2.321013	0.008109
tan	8722	GSE14769_UNSTIM_VS_120MIN_LPS_BMDM_UP	6	195	16058	145	3.448912	0.008113
magenta	1969	TACTTGA,MIR-26A,MIR-26B	8	278	15975	167	2.800672	0.008113
lightgreen	8380	LEF1_UP.V1_DN	3	160	16093	42	7.255804	0.008115
lightcyan	8313	RB_DN.V1_DN	3	108	16145	62	7.28181	0.008122
magenta	5249	GAUSSMANN_MLL_AF4_FUSION_TARGETS_E_UP	4	77	16176	167	5.055759	0.008134
magenta	7715	MIKKELSEN_ES_ICP_WITH_H3K4ME3_AND_H3K27ME3	4	77	16176	167	5.055759	0.008134
yellow	2220	YGCGYRCGC_UNKNOWN	38	290	15963	1426	1.493481	0.008144
cyan	5920	BENPORATH_ES_CORE_NINE_CORRELATED	3	87	16166	77	7.278549	0.008149
purple	5906	BENPORATH_EED_TARGETS	13	630	15623	157	2.136174	0.00815
grey60	931	RESPONSE_TO_WOUNDING	3	153	16100	44	7.24287	0.008169
grey60	9684	GSE29618_PRE_VS_DAY7_FLU_VACCINE_MDC_UP	3	153	16100	44	7.24287	0.008169
turquoise	9226	GSE18791_CTRL_VS_NEWCASTLE_VIRUS_DC_8H_UP	66	197	16056	4169	1.306109	0.008174
turquoise	9253	GSE19825_CD24LOW_VS_IL2RA_HIGH_DAY3_EFF_CD8	66	197	16056	4169	1.306109	0.008174
turquoise	9306	GSE22886_CD8_VS_CD4_NAIVE_TCELL_UP	66	197	16056	4169	1.306109	0.008174
turquoise	9401	GSE22886_UNSTIM_VS_IL15_STIM_NKCELL_DN	66	197	16056	4169	1.306109	0.008174
turquoise	9586	GSE27786_CD4_TCELL_VS_MONO_MAC_UP	66	197	16056	4169	1.306109	0.008174
turquoise	6979	IWANAGA_CARCINOGENESIS_BY_KRAS_UP	50	143	16110	4169	1.363125	0.008177
brown	4684	REACTOME_METABOLISM_OF_CARBOHYDRATES	39	197	16056	2190	1.469223	0.008178
brown	8507	GSE12845_IGD_POS_BLOOD_VS_NAIVE_TONSIL_BCELL	39	197	16056	2190	1.469223	0.008178
brown	8618	GSE14000_UNSTIM_VS_4H_LPS_DC_UP	39	197	16056	2190	1.469223	0.008178
brown	9764	GSE3337_4H_VS_16H_IFNG_IN_CD8POS_DC_UP	39	197	16056	2190	1.469223	0.008178
brown	10232	GSE9006_TYPE_1_DIABETES_AT_DX_VS_1MONTH_POS	39	197	16056	2190	1.469223	0.008178
purple	8320	CAHOY_ASTROGLIAL	4	82	16171	157	5.049868	0.00818
blue	10265	GSE9650_EXHAUSTED_VS_MEMORY_CD8_TCELL_DN	50	197	16056	2978	1.3852	0.008186
pink	670	SYNAPSE_ORGANIZATION_AND_BIOGENESIS	2	10	16243	228	14.25702	0.008187
pink	684	HEME_BIOSYNTHETIC_PROCESS	2	10	16243	228	14.25702	0.008187
pink	2961	BIOCARTA_SKP2E2F_PATHWAY	2	10	16243	228	14.25702	0.008187
pink	3734	MODULE_272	2	10	16243	228	14.25702	0.008187
blue	7922	PILON_KLF1_TARGETS_DN	397	1952	14301	2978	1.109991	0.008209
black	2283	GGGCGGR_V$SP1_Q6	62	2655	13598	283	1.34114	0.008211
greenyello	6993	DE_YY1_TARGETS_UP	2	15	16238	150	14.44711	0.008211
salmon	4412	REACTOME_P53_DEPENDENT_G1_DNA_DAMAGE_RESI	3	53	16200	127	7.243946	0.008213
greenyello	8773	GSE15750_DAY6_VS_DAY10_TRAF6KO_EFF_CD8_TCELI	6	189	16064	150	3.439788	0.008217
greenyello	8827	GSE17580_TREG_VS_TEFF_S_MANSONI_INF_DN	6	189	16064	150	3.439788	0.008217
purple	5121	GRAHAM_CML_QUIESCENT_VS_NORMAL_QUIESCENT_	3	43	16210	157	7.222486	0.008225
purple	5363	OLSSON_E2F3_TARGETS_DN	3	43	16210	157	7.222486	0.008225
brown	1945	GGGNRMNNYCAT_UNKNOWN	18	74	16179	2190	1.80522	0.008234
turquoise	6703	BAELDE_DIABETIC_NEPHROPATHY_DN	123	396	15857	4169	1.210909	0.008246
midnightb	8462	GSE11864_UNTREATED_VS_CSF1_IFNG_IN_MAC_UP	4	182	16071	71	5.03111	0.008251
midnightb	9910	GSE36476_CTRL_VS_TSST_ACT_40H_MEMORY_CD4_T(	4	182	16071	71	5.03111	0.008251
lightgreen	9643	GSE29615_DAY3_VS_DAY7_LAIV_FLU_VACCINE_PBMC_	3	161	16092	42	7.210736	0.008254
brown	8499	GSE12366_PLASMA_CELL_VS_NAIVE_BCELL_DN	38	191	16062	2190	1.476521	0.008255
brown	8675	GSE14350_IL2RB_KO_VS_WT_TREG_DN	38	191	16062	2190	1.476521	0.008255
brown	9350	GSE22886_DAY1_VS_DAY7_MONOCYTE_IN_CULTURE_	38	191	16062	2190	1.476521	0.008255
salmon	6227	FLECHNER_BIOPSY_KIDNEY_TRANSPLANT_OK_VS_DON	10	526	15727	127	2.433011	0.008255
salmon	8001	FEVR_CTNNB1_TARGETS_DN	10	526	15727	127	2.433011	0.008255
turquoise	251	PROTEIN_FOLDING	23	56	16197	4169	1.601185	0.008262
turquoise	3709	MODULE_237	23	56	16197	4169	1.601185	0.008262
turquoise	4717	REACTOME_AUTODEGRADATION_OF_CDH1_BY_CDH1_	23	56	16197	4169	1.601185	0.008262
lightgreen	1023	REGULATION_OF_BINDING	2	53	16200	42	14.60288	0.008264
magenta	9625	GSE2826_WT_VS_XID_BCELL_DN	6	170	16083	167	3.434942	0.008277
magenta	9628	GSE2826_XID_VS_BTK_KO_BCELL_UP	6	170	16083	167	3.434942	0.008277
cyan	292	JAK_STAT_CASCADE	2	29	16224	77	14.5571	0.00828
cyan	6804	KIM_LRRC3B_TARGETS	2	29	16224	77	14.5571	0.00828
yellow	3637	MODULE_152	19	120	16133	1426	1.804622	0.008282

red	2245	TGACCTY_V$ERR1_Q2	41	862	15391	524	1.475295	0.008285
lightcyan	6407	BROWNE_HCMV_INFECTION_16HR_UP	4	209	16044	62	5.017132	0.008286
lightcyan	2716	GNF2_VAV1	2	36	16217	62	14.56362	0.008298
lightcyan	6486	SATO_SILENCED_BY_METHYLATION_IN_PANCREATIC_C	2	36	16217	62	14.56362	0.008298
lightcyan	6721	KAAB_FAILED_HEART_VENTRICLE_DN	2	36	16217	62	14.56362	0.008298
lightcyan	7901	OHGUCHI_LIVER_HNF4A_TARGETS_UP	2	36	16217	62	14.56362	0.008298
magenta	1908	V$GR_Q6_01	7	223	16030	167	3.054993	0.008302
magenta	6778	MCLACHLAN_DENTAL_CARIES_UP	7	223	16030	167	3.054993	0.008302
red	2238	TTGCWCAAY_V$CEBPB_02	6	55	16198	524	3.383692	0.008304
red	3723	MODULE_254	6	55	16198	524	3.383692	0.008304
red	3749	MODULE_291	6	55	16198	524	3.383692	0.008304
tan	8623	GSE14000_4H_VS_16H_LPS_DC_TRANSLATED_RNA_DN	6	196	16057	145	3.431316	0.008309
purple	2191	SYATTGTG_UNKNOWN	6	181	16072	157	3.431678	0.008312
lightyellow	5906	BENPORATH_EED_TARGETS	5	630	15623	33	3.90885	0.008313
pink	8370	KRAS.BREAST_UP.V1_DN	5	89	16164	228	4.00478	0.008315
lightcyan	6467	WANG_SMARCE1_TARGETS_DN	5	329	15924	62	3.983969	0.008316
brown	8784	GSE15930_NAIVE_VS_48H_IN_VITRO_STIM_IL12_CD8_	37	185	16068	2190	1.484292	0.008322
salmon	1537	V$MYCMAX_02	6	224	16029	127	3.427939	0.008324
blue	2560	GCM_TPR	12	32	16221	2978	2.046634	0.008333
red	9815	GSE360_CTRL_VS_M_TUBERCULOSIS_MAC_DN	12	168	16085	524	2.215513	0.008344
purple	3588	MODULE_100	10	425	15828	157	2.435819	0.008345
yellow	8323	RPS14_DN.V1_DN	25	172	16081	1426	1.65663	0.008353
red	6890	FOSTER_TOLERANT_MACROPHAGE_DN	22	391	15862	524	1.745212	0.008365
turquoise	8171	SMIRNOV_RESPONSE_TO_IR_2HR_DN	22	53	16200	4169	1.618261	0.008374
black	5998	NIKOLSKY_BREAST_CANCER_8Q23_Q24_AMPLICON	7	132	16121	283	3.045588	0.008381
turquoise	767	REGULATION_OF_CYCLIN_DEPENDENT_PROTEIN_KINAS	18	41	16212	4169	1.711553	0.008386
red	8827	GSE17580_TREG_VS_TEFF_S_MANSONI_INF_DN	13	189	16064	524	2.133457	0.008391
red	8998	GSE17721_LPS_VS_PAM3CSK4_16H_BMDM_UP	13	189	16064	524	2.133457	0.008391
red	9461	GSE24634_IL4_VS_CTRL_TREATED_NAIVE_CD4_TCELL_	13	189	16064	524	2.133457	0.008391
red	10080	GSE3982_BASOPHIL_VS_TH2_UP	13	189	16064	524	2.133457	0.008391
red	10179	GSE7460_WT_VS_FOXP3_HET_ACT_TCONV_DN	13	189	16064	524	2.133457	0.008391
lightgreen	5016	TAKEDA_TARGETS_OF_NUP98_HOXA9_FUSION_16D_U	3	162	16091	42	7.166226	0.008395
lightgreen	9421	GSE24142_EARLY_THYMIC_PROGENITOR_VS_DN2_THY	3	162	16091	42	7.166226	0.008395
lightgreen	9766	GSE33513_TCF7_KO_VS_HET_EARLY_THYMIC_PROGEN	3	162	16091	42	7.166226	0.008395
turquoise	8650	GSE14308_TH17_VS_NATURAL_TREG_UP	63	187	16066	4169	1.313411	0.0084
turquoise	9595	GSE27786_CD8_TCELL_VS_NEUTROPHIL_DN	63	187	16066	4169	1.313411	0.0084
turquoise	9617	GSE27786_NEUTROPHIL_VS_MONO_MAC_DN	63	187	16066	4169	1.313411	0.0084
blue	256	NEGATIVE_REGULATION_OF_NUCLEOBASENUCLEOSIDE	48	188	16065	2978	1.393453	0.008403
midnightb	8699	GSE1460_DP_THYMOCYTE_VS_NAIVE_CD4_TCELL_ADU	4	183	16070	71	5.003617	0.008408
brown	5859	SCHAEFFER_PROSTATE_DEVELOPMENT_6HR_DN	83	478	15775	2190	1.288664	0.00841
brown	1799	V$MAZ_Q6	35	173	16080	2190	1.501452	0.008422
greenyello	8445	GSE11057_NAIVE_VS_EFF_MEMORY_CD4_TCELL_DN	6	190	16063	150	3.421684	0.008422
greenyello	9266	GSE20366_EX_VIVO_VS_DEC205_CONVERSION_NAIVE_	6	190	16063	150	3.421684	0.008422
turquoise	2636	GNF2_DENR	21	50	16203	4169	1.637385	0.008452
turquoise	3255	chr4q31	21	50	16203	4169	1.637385	0.008452
brown	8328	IL2_UP.V1_UP	33	161	16092	2190	1.521169	0.008465
turquoise	533	JNK_CASCADE	19	44	16209	4169	1.683459	0.008468
turquoise	2795	KEGG_PROTEASOME	19	44	16209	4169	1.683459	0.008468
blue	5399	DACOSTA_UV_RESPONSE_VIA_ERCC3_XPCS_DN	25	85	16168	2978	1.605203	0.008469
cyan	8903	GSE17721_CTRL_VS_GARDIQUIMOD_4H_BMDM_DN	4	169	16084	77	4.995927	0.008474
cyan	9962	GSE3982_MEMORY_CD4_TCELL_VS_BCELL_UP	4	169	16084	77	4.995927	0.008474
purple	3625	MODULE_137	10	426	15827	157	2.430101	0.008476
pink	6172	FERNANDEZ_BOUND_BY_MYC	7	164	16089	228	3.042656	0.008476
brown	2987	BIOCARTA_HIVNEF_PATHWAY	15	58	16195	2190	1.919343	0.008485
turquoise	497	MEMBRANE_LIPID_BIOSYNTHETIC_PROCESS	20	47	16206	4169	1.658952	0.008486
turquoise	4529	REACTOME_AUTODEGRADATION_OF_THE_E3_UBIQUI	20	47	16206	4169	1.658952	0.008486
yellow	8939	GSE17721_POLYIC_VS_PAM3CSK4_6H_BMDM_DN	27	190	16063	1426	1.619661	0.008486
blue	3326	chr21q22	33	120	16133	2978	1.500865	0.008506
yellow	8783	GSE15930_NAIVE_VS_48H_IN_VITRO_STIM_CD8_TCELL	28	199	16054	1426	1.603685	0.008507
red	10067	GSE3982_BCELL_VS_NKCELL_DN	11	148	16105	524	2.305331	0.008515

magenta	3654	MODULE_171	5	122	16131	167	3.988662	0.008524
purple	1645	V$LMO2COM_02	6	182	16071	157	3.412823	0.008528
purple	7856	VERHAAK_GLIOBLASTOMA_CLASSICAL	6	182	16071	157	3.412823	0.008528
magenta	3965	PID_NFAT_TFPATHWAY	3	41	16212	167	7.121221	0.008532
magenta	6213	BECKER_TAMOXIFEN_RESISTANCE_DN	3	41	16212	167	7.121221	0.008532
lightgreen	9265	GSE20366_EX_VIVO_VS_HOMEOSTATIC_CONVERSION_	3	163	16090	42	7.122261	0.008537
lightcyan	735	CELLULAR_HOMEOSTASIS	3	110	16143	62	7.149413	0.00854
turquoise	67	ENDOPLASMIC_RETICULUM_MEMBRANE	30	78	16175	4169	1.499437	0.008564
turquoise	2019	GTGTTGA,MIR-505	35	94	16159	4169	1.451583	0.008564
midnightb	8539	GSE13306_TREG_VS_TCONV_DN	4	184	16069	71	4.976424	0.008567
midnightb	9204	GSE17974_0.5H_VS_72H_UNTREATED_IN_VITRO_CD4_	4	184	16069	71	4.976424	0.008567
green	4454	REACTOME_CLASS_C_3_METABOTROPIC_GLUTAMATE_	3	6	16247	1305	6.227203	0.008585
green	7081	GALI_TP53_TARGETS_APOPTOTIC_DN	3	6	16247	1305	6.227203	0.008585
midnightb	5703	HATADA_METHYLATED_IN_LUNG_CANCER_UP	5	289	15964	71	3.960476	0.0086
blue	9871	GSE360_L_MAJOR_VS_T_GONDII_MAC_DN	46	179	16074	2978	1.402535	0.008603
turquoise	8523	GSE12845_PRE_GC_VS_DARKZONE_GC_TONSIL_BCELL_	65	194	16059	4169	1.306211	0.008603
green	9529	GSE27786_LSK_VS_LIN_NEG_CELL_DN	25	188	16065	1305	1.656171	0.008607
grey60	8336	TGFB_UP.V1_UP	3	156	16097	44	7.103584	0.008614
lightyellow	3440	chr15q21	2	69	16184	33	14.2758	0.008615
lightyellow	5008	TAKEDA_TARGETS_OF_NUP98_HOXA9_FUSION_6HR_U	2	69	16184	33	14.2758	0.008615
lightyellow	5375	TANAKA_METHYLATED_IN_ESOPHAGEAL_CARCINOMA	2	69	16184	33	14.2758	0.008615
green	689	REGULATED_SECRETORY_PATHWAY	4	11	16242	1305	4.528875	0.008628
green	742	APOPTOTIC_MITOCHONDRIAL_CHANGES	4	11	16242	1305	4.528875	0.008628
green	7793	NIELSEN_SCHWANNOMA_DN	4	11	16242	1305	4.528875	0.008628
greenyello	8367	KRAS.600_UP.V1_UP	6	191	16062	150	3.40377	0.008631
greenyello	8831	GSE17580_UNINFECTED_VS_S_MANSONI_INF_TREG_D	6	191	16062	150	3.40377	0.008631
greenyello	10194	GSE7852_TREG_VS_TCONV_THYMUS_UP	6	191	16062	150	3.40377	0.008631
salmon	4515	REACTOME_CDT1_ASSOCIATION_WITH_THE_CDC6_OR	3	54	16199	127	7.109799	0.008647
grey60	7725	MIKKELSEN_MEF_HCP_WITH_H3K27ME3	4	300	15953	44	4.925152	0.008647
pink	6953	MARTINEZ_RB1_TARGETS_UP	16	586	15667	228	1.946351	0.008653
red	74	VESICLE	9	109	16144	524	2.561051	0.00866
lightgreen	10003	GSE3982_MAST_CELL_VS_EFF_MEMORY_CD4_TCELL_D	3	164	16089	42	7.078833	0.008681
lightgreen	10088	GSE3982_EFF_MEMORY_CD4_TCELL_VS_TH2_UP	3	164	16089	42	7.078833	0.008681
magenta	1602	V$NFKB_C	7	225	16028	167	3.027838	0.008697
brown	6936	HELLER_HDAC_TARGETS_SILENCED_BY_METHYLATION_	68	381	15872	2190	1.324565	0.008726
midnightb	9882	GSE360_T_GONDII_VS_M_TUBERCULOSIS_MAC_UP	4	185	16068	71	4.949524	0.008728
midnightb	10051	GSE3982_NEUTROPHIL_VS_EFF_MEMORY_CD4_TCELL_	4	185	16068	71	4.949524	0.008728
blue	9788	GSE34205_HEALTHY_VS_FLU_INF_INFANT_PBMC_UP	49	193	16060	2978	1.385631	0.008728
lightcyan	4967	WANG_LMO4_TARGETS_DN	5	333	15920	62	3.936114	0.008736
tan	2675	GNF2_MYL2	2	16	16237	145	14.01121	0.008739
magenta	8120	FOSTER_KDM1A_TARGETS_UP	6	172	16081	167	3.395001	0.008741
magenta	10257	GSE9650_NAIVE_VS_EXHAUSTED_CD8_TCELL_DN	6	172	16081	167	3.395001	0.008741
purple	5045	TONKS_TARGETS_OF_RUNX1_RUNX1T1_FUSION_HSC_	6	183	16070	157	3.394174	0.008749
lightcyan	6153	ZUCCHI_METASTASIS_UP	2	37	16216	62	14.17001	0.008751
greenyello	2887	KEGG_BASAL_CELL_CARCINOMA	3	46	16207	150	7.066522	0.008751
red	8410	GSE10239_NAIVE_VS_KLRG1HIGH_EFF_CD8_TCELL_UP	13	190	16063	524	2.122228	0.008752
red	8607	GSE13738_TCR_VS_BYSTANDER_ACTIVATED_CD4_TCEL	13	190	16063	524	2.122228	0.008752
purple	1563	V$SRF_01	3	44	16209	157	7.058338	0.008765
purple	3648	MODULE_164	3	44	16209	157	7.058338	0.008765
purple	4930	CHARAFE_BREAST_CANCER_BASAL_VS_MESENCHYMAL	3	44	16209	157	7.058338	0.008765
purple	5456	LINDGREN_BLADDER_CANCER_HIGH_RECURRENCE	3	44	16209	157	7.058338	0.008765
purple	5618	TSUNODA_CISPLATIN_RESISTANCE_DN	3	44	16209	157	7.058338	0.008765
purple	6664	BURTON_ADIPOGENESIS_7	3	44	16209	157	7.058338	0.008765
turquoise	6998	POS_RESPONSE_TO_HISTAMINE_DN	7	11	16242	4169	2.480887	0.008774
turquoise	7805	ONGUSAHA_BRCA1_TARGETS_DN	7	11	16242	4169	2.480887	0.008774
turquoise	8053	PEDERSEN_METASTASIS_BY_ERBB2_ISOFORM_5	7	11	16242	4169	2.480887	0.008774
brown	4678	REACTOME_ETHANOL_OXIDATION	3	4	16249	2190	5.566096	0.008788
purple	4866	DAVICIONI_MOLECULAR_ARMS_VS_ERMS_UP	8	300	15953	157	2.760594	0.008791
magenta	347	DEVELOPMENTAL_MATURATION	2	14	16239	167	13.90334	0.008806
magenta	810	REGULATION_OF_T_CELL_PROLIFERATION	2	14	16239	167	13.90334	0.008806

magenta	2908	BIOCARTA_AMI_PATHWAY	2	14	16239	167	13.90334	0.008806
blue	6935	HELLER_HDAC_TARGETS_DN	66	273	15980	2978	1.319441	0.00882
cyan	6316	VERHAAK_AML_WITH_NPM1_MUTATED_UP	4	171	16082	77	4.937495	0.008824
cyan	9847	GSE360_L_MAJOR_VS_M_TUBERCULOSIS_DC_DN	4	171	16082	77	4.937495	0.008824
lightgreen	8335	TGFB_UP.V1_DN	3	165	16088	42	7.035931	0.008826
turquoise	8682	GSE1448_ANTI_VALPHA2_VS_VBETA5_DP_THYMOCYTI	62	184	16069	4169	1.313637	0.008842
turquoise	9927	GSE37416_CTRL_VS_6H_F_TULARENSIS_LVS_NEUTROP	62	184	16069	4169	1.313637	0.008842
greenyello	1737	V$FOXO4_01	6	192	16061	150	3.386042	0.008844
cyan	2963	BIOCARTA_EGF_PATHWAY	2	30	16223	77	14.07186	0.008844
cyan	4854	GAZDA_DIAMOND_BLACKFAN_ANEMIA_MYELOID_UP	2	30	16223	77	14.07186	0.008844
cyan	7353	SCHRAETS_MLL_TARGETS_UP	2	30	16223	77	14.07186	0.008844
red	2187	RYTAAWNNNTGAY_UNKNOWN	5	40	16213	524	3.877147	0.008863
red	6105	FLECHNER_PBL_KIDNEY_TRANSPLANT_OK_VS_DONOR_	5	40	16213	524	3.877147	0.008863
blue	4375	REACTOME_RECRUITMENT_OF_MITOTIC_CENTROSOM	19	60	16193	2978	1.728268	0.008871
lightgreen	2655	GNF2_ITGAL	2	55	16198	42	14.07186	0.008877
lightgreen	5542	HERNANDEZ_MITOTIC_ARREST_BY_DOCETAXEL_2_UP	2	55	16198	42	14.07186	0.008877
purple	2885	KEGG_PROSTATE_CANCER	4	84	16169	157	4.929633	0.008891
purple	7149	LABBE_TGFB1_TARGETS_UP	4	84	16169	157	4.929633	0.008891
brown	3943	PID_IL4_2PATHWAY	14	53	16200	2190	1.960386	0.008893
brown	8670	GSE14350_TREG_VS_TEFF_UP	39	198	16055	2190	1.461803	0.008895
salmon	5142	RODRIGUES_THYROID_CARCINOMA_POORLY_DIFFEREI	11	616	15637	127	2.285292	0.008905
turquoise	4407	REACTOME_INSULIN_SYNTHESIS_AND_PROCESSING	9	16	16237	4169	2.192927	0.008908
turquoise	4570	REACTOME_ENERGY_DEPENDENT_REGULATION_OF_M	9	16	16237	4169	2.192927	0.008908
turquoise	8883	GSE17721_CTRL_VS_CPG_1H_BMDM_DN	54	157	16096	4169	1.340898	0.008915
brown	5237	GRAESSMANN_APOPTOSIS_BY_DOXORUBICIN_UP	171	1072	15181	2190	1.183834	0.00894
brown	265	PROTEIN_AUTOPROCESSING	9	28	16225	2190	2.38547	0.008941
brown	432	PROTEIN_AMINO_ACID_AUTOPHOSPHORYLATION	9	28	16225	2190	2.38547	0.008941
brown	3138	chr20p12	9	28	16225	2190	2.38547	0.008941
brown	7208	CHNG_MULTIPLE_MYELOMA_HYPERPLOID_DN	9	28	16225	2190	2.38547	0.008941
blue	9792	GSE360_CTRL_VS_L_DONOVANI_DC_UP	47	184	16069	2978	1.394084	0.008959
blue	9958	GSE3982_MEMORY_CD4_TCELL_VS_TH1_UP	47	184	16069	2978	1.394084	0.008959
brown	3508	MODULE_16	79	453	15800	2190	1.29425	0.008962
purple	169	SECRETORY_GRANULE	2	15	16238	157	13.80297	0.008964
purple	3656	MODULE_173	2	15	16238	157	13.80297	0.008964
purple	3737	MODULE_275	2	15	16238	157	13.80297	0.008964
purple	5893	HE_PTEN_TARGETS_UP	2	15	16238	157	13.80297	0.008964
purple	7999	DALESSIO_TSA_RESPONSE	2	15	16238	157	13.80297	0.008964
purple	1909	V$SRF_Q5_01	6	184	16069	157	3.375727	0.008974
purple	9005	GSE17721_POLYIC_VS_CPG_1H_BMDM_DN	6	184	16069	157	3.375727	0.008974
red	1532	V$CEBPB_02	14	212	16041	524	2.048304	0.008983
brown	8687	GSE1460_INTRATHYMIC_T_PROGENITOR_VS_CD4_THY	38	192	16061	2190	1.468831	0.00899
brown	8973	GSE17721_CPG_VS_GARDIQUIMOD_4H_BMDM_DN	38	192	16061	2190	1.468831	0.00899
black	2276	GGGTGGRR_V$PAX4_03	30	1093	15160	283	1.576334	0.009
cyan	3600	MODULE_112	4	172	16081	77	4.908789	0.009002
yellow	3565	MODULE_77	7	28	16225	1426	2.849404	0.009019
yellow	8834	GSE17721_CTRL_VS_LPS_1H_BMDM_UP	26	182	16071	1426	1.628231	0.009045
green	3797	MODULE_352	5	17	16236	1305	3.663061	0.009048
green	4635	REACTOME_INHIBITION_OF_VOLTAGE_GATED_CA2_CH	5	17	16236	1305	3.663061	0.009048
green	7564	MEISSNER_BRAIN_HCP_WITH_H3K4ME2	5	17	16236	1305	3.663061	0.009048
blue	8404	GSE10094_LCMV_VS_LISTERIA_IND_EFF_CD4_TCELL_U	50	198	16055	2978	1.378204	0.009048
blue	9130	GSE17721_0.5H_VS_8H_CPG_BMDM_UP	50	198	16055	2978	1.378204	0.009048
red	4847	WATANABE_COLON_CANCER_MSI_VS_MSS_DN	6	56	16197	524	3.323269	0.009054
red	5135	WAMUNYOKOLI_OVARIAN_CANCER_GRADES_1_2_DN	6	56	16197	524	3.323269	0.009054
turquoise	9579	GSE27786_CD4_TCELL_VS_NKCELL_DN	64	191	16062	4169	1.306316	0.009056
red	6430	IVANOVA_HEMATOPOIESIS_LATE_PROGENITOR	27	514	15739	524	1.629307	0.009059
greenyello	8606	GSE13738_TCR_VS_BYSTANDER_ACTIVATED_CD4_TCEI	6	193	16060	150	3.368497	0.00906
lightcyan	5657	GRUETZMANN_PANCREATIC_CANCER_UP	5	336	15917	62	3.90097	0.00906
brown	907	CYTOSKELETON_ORGANIZATION_AND_BIOGENESIS	37	186	16067	2190	1.476312	0.009076
brown	9762	GSE3337_CTRL_VS_16H_IFNG_IN_CD8POS_DC_UP	37	186	16067	2190	1.476312	0.009076
brown	9930	GSE37416_CTRL_VS_24H_F_TULARENSIS_LVS_NEUTRO	37	186	16067	2190	1.476312	0.009076

blue	7521	BOYAULT_LIVER_CANCER_SUBCLASS_G123_DN	14	40	16213	2978	1.910191	0.009085
red	1363	HEMATOPOIETIN_INTERFERON_CLASSD200_DOMAIN_(	4	26	16227	524	4.771873	0.009088
red	5011	TAKEDA_TARGETS_OF_NUP98_HOXA9_FUSION_3D_DN	4	26	16227	524	4.771873	0.009088
yellow	8785	GSE15930_NAIVE_VS_48H_IN_VITRO_STIM_IL12_CD8_	28	200	16053	1426	1.595666	0.009089
salmon	4346	REACTOME_REGULATION_OF_APOPTOSIS	3	55	16198	127	6.98053	0.009094
greenyello	5012	TAKEDA_TARGETS_OF_NUP98_HOXA9_FUSION_8D_UP	5	138	16115	150	3.925845	0.009102
magenta	1641	V$OLF1_01	7	227	16026	167	3.001161	0.009105
magenta	9254	GSE19825_NAIVE_VS_DAY3_EFF_CD8_TCELL_UP	5	124	16129	167	3.924329	0.009109
magenta	1228	KINASE_REGULATOR_ACTIVITY	3	42	16211	167	6.951668	0.00912
red	9350	GSE22886_DAY1_VS_DAY7_MONOCYTE_IN_CULTURE_	13	191	16062	524	2.111117	0.009125
red	9759	GSE32423_IL7_VS_IL7_IL4_NAIVE_CD8_TCELL_DN	13	191	16062	524	2.111117	0.009125
red	10161	GSE7460_CD8_TCELL_VS_CD4_TCELL_ACT_DN	13	191	16062	524	2.111117	0.009125
red	10175	GSE7460_FOXP3_MUT_VS_WT_ACT_WITH_TGFB_TCOI	13	191	16062	524	2.111117	0.009125
red	9646	GSE29617_CTRL_VS_TIV_FLU_VACCINE_PBMC_2008_U	12	170	16083	524	2.189448	0.009126
brown	6778	MCLACHLAN_DENTAL_CARIES_UP	43	223	16030	2190	1.431044	0.009127
purple	7345	RUIZ_TNC_TARGETS_UP	5	132	16121	157	3.921299	0.009143
brown	8780	GSE15930_NAIVE_VS_24H_IN_VITRO_STIM_INFAB_CD	36	180	16073	2190	1.484292	0.00915
brown	131	CONTRACTILE_FIBER_PART	7	19	16234	2190	2.734223	0.009161
brown	336	REGULATION_OF_ANATOMICAL_STRUCTURE_MORPHC	7	19	16234	2190	2.734223	0.009161
brown	964	REGULATION_OF_PROTEIN_SECRETION	7	19	16234	2190	2.734223	0.009161
brown	4090	PID_ARF_3PATHWAY	7	19	16234	2190	2.734223	0.009161
brown	4109	PID_EPHA2_FWDPATHWAY	7	19	16234	2190	2.734223	0.009161
brown	6571	VERRECCHIA_RESPONSE_TO_TGFB1_C1	7	19	16234	2190	2.734223	0.009161
brown	7005	OUYANG_PROSTATE_CANCER_PROGRESSION_UP	7	19	16234	2190	2.734223	0.009161
brown	7283	VILIMAS_NOTCH1_TARGETS_DN	7	19	16234	2190	2.734223	0.009161
brown	8151	HOLLEMAN_PREDNISOLONE_RESISTANCE_ALL_DN	7	19	16234	2190	2.734223	0.009161
tan	3786	MODULE_334	5	143	16110	145	3.919219	0.009165
turquoise	1659	CCAWNWWNNNGGC_UNKNOWN	28	72	16181	4169	1.516098	0.009168
cyan	8963	GSE17721_PAM3CSK4_VS_CPG_16H_BMDM_DN	4	173	16080	77	4.880414	0.009183
cyan	9052	GSE17721_LPS_VS_CPG_16H_BMDM_UP	4	173	16080	77	4.880414	0.009183
lightcyan	4915	DIAZ_CHRONIC_MEYLOGENOUS_LEUKEMIA_DN	3	113	16140	62	6.959606	0.00919
lightgreen	1073	CARBOHYDRATE_BINDING	2	56	16197	42	13.82058	0.009191
blue	2299	MORF_BUB1	17	52	16201	2978	1.784245	0.009192
turquoise	321	MACROMOLECULAR_COMPLEX_ASSEMBLY	86	267	15986	4169	1.255708	0.009196
purple	9086	GSE17721_LPS_VS_GARDIQUIMOD_12H_BMDM_UP	6	185	16068	157	3.35748	0.009202
midnightb	8432	GSE10325_LUPUS_BCELL_VS_LUPUS_MYELOID_UP	4	188	16065	71	4.870542	0.009223
midnightb	10180	GSE7460_WT_VS_FOXP3_HET_ACT_WITH_TGFB_TCON	4	188	16065	71	4.870542	0.009223
midnightb	10211	GSE7852_TREG_VS_TCONV_DN	4	188	16065	71	4.870542	0.009223
magenta	8693	GSE1460_INTRATHYMIC_T_PROGENITOR_VS_THYMIC_	6	174	16079	167	3.355978	0.009224
blue	6641	BURTON_ADIPOGENESIS_4	15	44	16209	2978	1.860576	0.009227
yellow	3551	MODULE_62	15	88	16165	1426	1.942775	0.009231
brown	3959	PID_PTP1BPATHWAY	13	48	16205	2190	2.009979	0.009235
brown	4112	PID_FGF_PATHWAY	13	48	16205	2190	2.009979	0.009235
brown	6253	ZHAN_MULTIPLE_MYELOMA_CD1_AND_CD2_DN	13	48	16205	2190	2.009979	0.009235
turquoise	634	CELLULAR_COMPONENT_ASSEMBLY	90	281	15972	4169	1.248642	0.009247
turquoise	9395	GSE22886_NAIVE_CD4_TCELL_VS_12H_ACT_TH2_DN	66	198	16055	4169	1.299512	0.009249
turquoise	9582	GSE27786_CD4_TCELL_VS_ERYTHTROBLAST_UP	66	198	16055	4169	1.299512	0.009249
turquoise	9989	GSE3982_EOSINOPHIL_VS_TH1_DN	66	198	16055	4169	1.299512	0.009249
purple	3417	chr3q21	4	85	16168	157	4.871637	0.009262
purple	7872	HOELZEL_NF1_TARGETS_DN	4	85	16168	157	4.871637	0.009262
lightgreen	8407	GSE10239_NAIVE_VS_MEMORY_CD8_TCELL_DN	3	168	16085	42	6.910289	0.009269
lightgreen	9332	GSE22886_IGA_VS_IGM_MEMORY_BCELL_UP	3	168	16085	42	6.910289	0.009269
lightgreen	9725	GSE30962_PRIMARY_VS_SECONDARY_CHRONIC_LCMV	3	168	16085	42	6.910289	0.009269
black	2271	GATTGGY_V$NFY_Q6_01	28	1003	15250	283	1.603261	0.009276
greenyello	10093	GSE3982_CENT_MEMORY_CD4_TCELL_VS_TH1_DN	6	194	16059	150	3.351134	0.00928
brown	9377	GSE22886_NAIVE_CD4_TCELL_VS_NEUTROPHIL_DN	33	162	16091	2190	1.511779	0.009292
brown	10134	GSE6269_FLU_VS_E_COLI_INF_PBMC_UP	33	162	16091	2190	1.511779	0.009292
green	8329	IL21_UP.V1_DN	18	123	16130	1305	1.822596	0.009296
turquoise	2162	TGTGTGA,MIR-377	61	181	16072	4169	1.313872	0.009307
turquoise	8446	GSE11057_NAIVE_VS_CENT_MEMORY_CD4_TCELL_UP	61	181	16072	4169	1.313872	0.009307

turquoise	9486	GSE26928_NAIVE_VS_EFF_MEMORY_CD4_TCELL_UP	61	181	16072	4169	1.313872	0.009307
blue	9242	GSE18791_UNSTIM_VS_NEWCATSLE_VIRUS_DC_6H_UI	48	189	16064	2978	1.38608	0.009308
magenta	715	NEUROLOGICAL_SYSTEM_PROCESS	7	228	16025	167	2.987998	0.009315
pink	9020	GSE17721_PAM3CSK4_VS_GADIQUIMOD_0.5H_BMDM	7	167	16086	228	2.987998	0.009315
purple	4383	REACTOME_CHEMOKINE_RECEPTORS_BIND_CHEMOKII	3	45	16208	157	6.901486	0.009326
purple	4580	REACTOME_DEPOSITION_OF_NEW_CENPA_CONTAININ	3	45	16208	157	6.901486	0.009326
purple	5426	MIDORIKAWA_AMPLIFIED_IN_LIVER_CANCER	3	45	16208	157	6.901486	0.009326
purple	5676	SASAI_RESISTANCE_TO_NEOPLASTIC_TRANSFROMATIC	3	45	16208	157	6.901486	0.009326
greenyello	253	AXON_GUIDANCE	2	16	16237	150	13.54417	0.009327
greenyello	3031	BIOCARTA_NO2IL12_PATHWAY	2	16	16237	150	13.54417	0.009327
greenyello	3038	BIOCARTA_CCR5_PATHWAY	2	16	16237	150	13.54417	0.009327
greenyello	5818	GUENTHER_GROWTH_SPHERICAL_VS_ADHERENT_UP	2	16	16237	150	13.54417	0.009327
salmon	8703	GSE1460_CD4_THYMOCYTE_VS_NAIVE_CD4_TCELL_CO	5	164	16089	127	3.901719	0.009328
green	10272	GSE9988_ANTI_TREM1_VS_ANTI_TREM1_AND_LPS_M	26	199	16054	1305	1.627209	0.009353
blue	730	NEGATIVE_REGULATION_OF_TRANSCRIPTION	43	166	16087	2978	1.413739	0.00936
red	1763	V$LHX3_01	11	150	16103	524	2.274593	0.009372
grey60	1693	V$CART1_01	3	161	16092	44	6.882976	0.009386
grey60	9822	GSE360_DC_VS_MAC_T_GONDII_UP	3	161	16092	44	6.882976	0.009386
midnightb	8553	GSE13411_NAIVE_VS_MEMORY_BCELL_DN	4	189	16064	71	4.844772	0.009392
yellow	8250	MYC_UP.V1_UP	23	156	16097	1426	1.680418	0.009398
brown	2932	BIOCARTA_CARM_ER_PATHWAY	10	33	16220	2190	2.248928	0.009409
red	5645	MOHANKUMAR_TLX1_TARGETS_DN	10	130	16123	524	2.385937	0.009419
lightgreen	9962	GSE3982_MEMORY_CD4_TCELL_VS_BCELL_UP	3	169	16084	42	6.8694	0.00942
turquoise	5084	TIEN_INTESTINE_PROBIOTICS_24HR_DN	68	205	16048	4169	1.293173	0.00942
turquoise	5608	AMUNDSON_RESPONSE_TO_ARSENITE	68	205	16048	4169	1.293173	0.00942
cyan	7246	BEIER_GLIOMA_STEM_CELL_UP	2	31	16222	77	13.61793	0.009425
purple	9285	GSE20715_WT_VS_TLR4_KO_24H_OZONE_LUNG_DN	6	186	16067	157	3.339429	0.009435
purple	10152	GSE7460_TCONV_VS_TREG_THYMUS_UP	6	186	16067	157	3.339429	0.009435
purple	3555	MODULE_66	10	433	15820	157	2.390815	0.009441
salmon	6117	WILLERT_WNT_SIGNALING	2	19	16234	127	13.4712	0.009496
salmon	7547	WANG_RECURRENT_LIVER_CANCER_UP	2	19	16234	127	13.4712	0.009496
brown	580	ALCOHOL_METABOLIC_PROCESS	18	75	16178	2190	1.781151	0.009503
pink	3336	chr22q12	5	92	16161	228	3.87419	0.009526
turquoise	8761	GSE15659_RESTING_VS_ACTIVATED_TREG_DN	58	171	16082	4169	1.322311	0.009532
turquoise	9037	GSE17721_PAM3CSK4_VS_GADIQUIMOD_24H_BMDM_	63	188	16065	4169	1.306425	0.009533
turquoise	9064	GSE17721_POLYIC_VS_GARDIQUIMOD_6H_BMDM_UP	63	188	16065	4169	1.306425	0.009533
turquoise	9647	GSE29617_CTRL_VS_TIV_FLU_VACCINE_PBMC_2008_D	63	188	16065	4169	1.306425	0.009533
turquoise	9756	GSE32423_IL7_VS_IL4_MEMORY_CD8_TCELL_UP	63	188	16065	4169	1.306425	0.009533
red	1650	V$TCF11MAFG_01	12	171	16082	524	2.176644	0.009538
red	4368	REACTOME_CLASS_A1_RHODOPSIN_LIKE_RECEPTORS	12	171	16082	524	2.176644	0.009538
red	5093	SABATES_COLORECTAL_ADENOMA_DN	12	171	16082	524	2.176644	0.009538
yellow	9190	GSE17974_IL4_AND_ANTI_IL12_VS_UNTREATED_2H_A	24	165	16088	1426	1.657835	0.009538
grey60	1495	V$HEN1_01	3	162	16091	44	6.840488	0.009546
grey60	9258	GSE20366_TREG_VS_TCONV_UP	3	162	16091	44	6.840488	0.009546
grey60	9377	GSE22886_NAIVE_CD4_TCELL_VS_NEUTROPHIL_DN	3	162	16091	44	6.840488	0.009546
cyan	9097	GSE17721_0.5H_VS_8H_LPS_BMDM_DN	4	175	16078	77	4.824638	0.009551
cyan	9751	GSE32423_CTRL_VS_IL4_MEMORY_CD8_TCELL_DN	4	175	16078	77	4.824638	0.009551
cyan	10282	GSE9988_LPS_VS_CTRL_TREATED_MONOCYTE_UP	4	175	16078	77	4.824638	0.009551
blue	8967	GSE17721_CPG_VS_GARDIQUIMOD_0.5H_BMDM_DN	46	180	16073	2978	1.394743	0.009552
brown	3002	BIOCARTA_IL2RB_PATHWAY	11	38	16215	2190	2.148318	0.009554
brown	3966	PID_EPHBFWDPATHWAY	11	38	16215	2190	2.148318	0.009554
brown	4079	PID_ECADHERIN_STABILIZATION_PATHWAY	11	38	16215	2190	2.148318	0.009554
brown	6863	MARSON_BOUND_BY_FOXP3_STIMULATED	157	978	15275	2190	1.19138	0.009555
salmon	4717	REACTOME_AUTODEGRADATION_OF_CDH1_BY_CDH1_	3	56	16197	127	6.855877	0.009555
midnightb	8691	GSE1460_INTRATHYMIC_T_PROGENITOR_VS_NAIVE_CI	4	190	16063	71	4.819274	0.009563
midnightb	9908	GSE36476_CTRL_VS_TSST_ACT_16H_MEMORY_CD4_T(	4	190	16063	71	4.819274	0.009563
midnightb	10019	GSE3982_DC_VS_BASOPHIL_DN	4	190	16063	71	4.819274	0.009563
midnightb	10274	GSE9988_ANTI_TREM1_VS_CTRL_TREATED_MONOCYT	4	190	16063	71	4.819274	0.009563
purple	3549	MODULE_60	9	368	15885	157	2.531795	0.009568
lightcyan	7395	LEE_RECENT_THYMIC_EMIGRANT	4	218	16035	62	4.810003	0.009574

blue	1285	DNA_POLYMERASE_ACTIVITY	8	18	16235	2978	2.42564	0.009576
blue	1454	INOSITOL_OR_PHOSPHATIDYLINOSITOL_KINASE_ACTIV	8	18	16235	2978	2.42564	0.009576
salmon	4952	HORIUCHI_WTAP_TARGETS_DN	7	302	15951	127	2.96634	0.009591
pink	9276	GSE20366_CD103_POS_VS_CD103_KLRG1_DP_TREG_U	7	168	16085	228	2.970212	0.009607
turquoise	186	MITOCHONDRION	103	327	15926	4169	1.227979	0.009617
red	2217	YCATTAA_UNKNOWN	22	396	15857	524	1.723176	0.009618
green	6889	FOSTER_TOLERANT_MACROPHAGE_UP	20	142	16111	1305	1.754142	0.00963
blue	2608	GNF2_XRCC5	22	73	16180	2978	1.644783	0.009639
lightcyan	8043	SERVITJA_ISLET_HNF1A_TARGETS_UP	3	115	16138	62	6.838569	0.00964
blue	10142	GSE6269_E_COLI_VS_STREP_PNEUMO_INF_PBMC_UP	39	148	16105	2978	1.438175	0.009642
blue	8527	GSE13229_IMM_VS_INTMATURE_NKCELL_DN	49	194	16059	2978	1.378489	0.00965
blue	9006	GSE17721_POLYIC_VS_CPG_2H_BMDM_UP	49	194	16059	2978	1.378489	0.00965
blue	9334	GSE22886_NAIVE_BCELL_VS_BLOOD_PLASMA_CELL_UI	49	194	16059	2978	1.378489	0.00965
blue	9413	GSE24142_DN2_VS_DN3_THYMOCYTE_DN	49	194	16059	2978	1.378489	0.00965
red	7644	BOYLAN_MULTIPLE_MYELOMA_PCA1_UP	8	92	16161	524	2.697146	0.009651
blue	8059	WAKABAYASHI_ADIPOGENESIS_PPARG_RXRA_BOUND_	177	823	15430	2978	1.173768	0.009656
cyan	4827	ONKEN_UVEAL_MELANOMA_UP	9	761	15492	77	2.496322	0.009659
red	5176	ENK_UV_RESPONSE_KERATINOCYTE_DN	25	468	15785	524	1.6569	0.009661
blue	2071	GGCAGCT,MIR-22	52	208	16045	2978	1.364422	0.009668
purple	8571	GSE13484_12H_VS_3H_YF17D_VACCINE_STIM_PBMC_	6	187	16066	157	3.321571	0.009673
blue	8315	RB_P107_DN.V1_DN	33	121	16132	2978	1.488461	0.009687
turquoise	4721	REACTOME_MITOTIC_PROMETAPHASE	32	85	16168	4169	1.467684	0.00969
turquoise	852	REGULATION_OF_CYTOSKELETON_ORGANIZATION_AN	13	27	16226	4169	1.877073	0.009703
turquoise	7076	BONOME_OVARIAN_CANCER_POOR_SURVIVAL_UP	13	27	16226	4169	1.877073	0.009703
grey60	9601	GSE27786_NKCELL_VS_ERYTHROBLAST_DN	3	163	16090	44	6.798522	0.009707
red	7185	CHEN_HOXA5_TARGETS_9HR_UP	14	214	16039	524	2.029161	0.00971
yellow	6786	BILD_MYC_ONCOGENIC_SIGNATURE	27	192	16061	1426	1.60279	0.009712
yellow	9526	GSE2706_2H_VS_8H_R848_AND_LPS_STIM_DC_UP	27	192	16061	1426	1.60279	0.009712
yellow	10011	GSE3982_MAST_CELL_VS_TH2_DN	27	192	16061	1426	1.60279	0.009712
blue	3686	MODULE_206	37	139	16114	2978	1.452766	0.009718
purple	6971	SARRIO_EPITHELIAL_MESENCHYMAL_TRANSITION_DN	5	134	16119	157	3.862772	0.009719
purple	9642	GSE29615_DAY3_VS_DAY7_LAIV_FLU_VACCINE_PBMC_	5	134	16119	157	3.862772	0.009719
magenta	3599	MODULE_111	5	126	16127	167	3.862038	0.009721
red	2847	KEGG_B_CELL_RECEPTOR_SIGNALING_PATHWAY	7	74	16179	524	2.934057	0.009724
red	4962	RODRIGUES_DCC_TARGETS_UP	2	5	16248	524	12.40687	0.009724
red	8098	LIU_IL13_MEMORY_MODEL_DN	2	5	16248	524	12.40687	0.009724
lightgreen	4368	REACTOME_CLASS_A1_RHODOPSIN_LIKE_RECEPTORS	3	171	16082	42	6.789056	0.009726
lightgreen	5044	TONKS_TARGETS_OF_RUNX1_RUNX1T1_FUSION_HSC_	3	171	16082	42	6.789056	0.009726
magenta	9042	GSE17721_LPS_VS_CPG_2H_BMDM_UP	6	176	16077	167	3.317842	0.009726
magenta	9742	GSE32423_MEMORY_VS_NAIVE_CD8_TCELL_UP	6	176	16077	167	3.317842	0.009726
magenta	23	EXTRACELLULAR_MATRIX_PART	3	43	16210	167	6.790001	0.009731
purple	3503	MODULE_11	10	435	15818	157	2.379823	0.009732
turquoise	1982	CACTTTG,MIR-520G,MIR-520H	65	195	16058	4169	1.299512	0.009736
turquoise	8936	GSE17721_POLYIC_VS_PAM3CSK4_4H_BMDM_UP	65	195	16058	4169	1.299512	0.009736
turquoise	9959	GSE3982_MEMORY_CD4_TCELL_VS_TH1_DN	65	195	16058	4169	1.299512	0.009736
cyan	9273	GSE20366_CD103_POS_VS_NEG_TREG_KLRG1NEG_DN	4	176	16077	77	4.797226	0.009738
cyan	9717	GSE30083_SP2_VS_SP3_THYMOCYTE_DN	4	176	16077	77	4.797226	0.009738
cyan	6665	KEEN_RESPONSE_TO_ROSIGLITAZONE_DN	3	93	16160	77	6.808965	0.009777
black	8331	PDGF_ERK_DN.V1_DN	7	136	16117	283	2.956012	0.009793
salmon	8490	GSE11924_TH2_VS_TH17_CD4_TCELL_UP	5	166	16087	127	3.85471	0.009797
cyan	7408	ZHANG_TLX_TARGETS_60HR_UP	5	275	15978	77	3.83778	0.009817
red	200	NUCLEAR_MEMBRANE_PART	5	41	16212	524	3.782582	0.009833
red	6465	MCDOWELL_ACUTE_LUNG_INJURY_UP	5	41	16212	524	3.782582	0.009833
red	7606	KUROZUMI_RESPONSE_TO_ONCOCYTIC_VIRUS	5	41	16212	524	3.782582	0.009833
greenyello	3827	MODULE_399	3	48	16205	150	6.772083	0.009839
tan	3641	MODULE_156	2	17	16236	145	13.18702	0.009847
tan	4626	REACTOME_DESTABILIZATION_OF_MRNA_BY_BRF1	2	17	16236	145	13.18702	0.009847
tan	4641	REACTOME_DESTABILIZATION_OF_MRNA_BY_KSRP	2	17	16236	145	13.18702	0.009847
tan	4644	REACTOME_DESTABILIZATION_OF_MRNA_BY_TRISTETI	2	17	16236	145	13.18702	0.009847
lightyellow	7445	TAVAZOIE_METASTASIS	2	74	16179	33	13.31122	0.009856

lightcyan	853	CHEMICAL_HOMEOSTASIS	3	116	16137	62	6.779616	0.009869
lightcyan	5187	CHIARADONNA_NEOPLASTIC_TRANSFORMATION_KRAS	3	116	16137	62	6.779616	0.009869
lightcyan	5259	OUELLET_OVARIAN_CANCER_INVASIVE_VS_LMP_UP	3	116	16137	62	6.779616	0.009869
salmon	4435	REACTOME_MRNA_SPLICING	4	107	16146	127	4.784164	0.009884
brown	2333	MORF_RPA2	37	187	16066	2190	1.468417	0.009885
brown	9414	GSE24142_EARLY_THYMIC_PROGENITOR_VS_DN2_THY	37	187	16066	2190	1.468417	0.009885
brown	9768	GSE339_CD4POS_VS_CD8POS_DC_UP	37	187	16066	2190	1.468417	0.009885
brown	5455	LINDGREN_BLADDER_CANCER_CLUSTER_2B	61	338	15915	2190	1.339376	0.009906
brown	7555	YAGI_AML_WITH_T_8_21_TRANSLOCATION	61	338	15915	2190	1.339376	0.009906
pink	5292	MCBRYAN_PUBERTAL_BREAST_3_4WK_UP	7	169	16084	228	2.952637	0.009907
purple	7982	WIEDERSCHAIN_TARGETS_OF_BMI1_AND_PCGF2	3	46	16207	157	6.751454	0.009907
blue	2103	ACCAAAG,MIR-9	101	444	15809	2978	1.241501	0.009908
midnightb	8687	GSE1460_INTRATHYMIC_T_PROGENITOR_VS_CD4_THY	4	192	16061	71	4.769073	0.009911
pink	742	APOPTOTIC_MITOCHONDRIAL_CHANGES	2	11	16242	228	12.96093	0.009914
pink	992	HEME_METABOLIC_PROCESS	2	11	16242	228	12.96093	0.009914
pink	3787	MODULE_335	2	11	16242	228	12.96093	0.009914
pink	5328	MCBRYAN_TERMINAL_END_BUD_UP	2	11	16242	228	12.96093	0.009914
pink	5921	PETRETTO_BLOOD_PRESSURE_UP	2	11	16242	228	12.96093	0.009914
purple	6637	WELCSH_BRCA1_TARGETS_UP	6	188	16065	157	3.303903	0.009914
turquoise	7356	CHANG_CORE_SERUM_RESPONSE_DN	67	202	16051	4169	1.293079	0.009917
cyan	8861	GSE17721_CTRL_VS_POLYIC_12H_BMDM_DN	4	177	16076	77	4.770123	0.009928
turquoise	399	ORGANELLE_ORGANIZATION_AND_BIOGENESIS	135	441	15812	4169	1.19343	0.009941
turquoise	2445	MORF_PRKAR1A	49	141	16112	4169	1.354811	0.009956
brown	1004	REGULATION_OF_TRANSCRIPTION	86	501	15752	2190	1.273943	0.009957
pink	737	GLYCOPROTEIN_BIOSYNTHETIC_PROCESS	4	60	16193	228	4.752339	0.009961
greenyello	9306	GSE22886_CD8_VS_CD4_NAIVE_TCELL_UP	6	197	16056	150	3.300102	0.009962
red	2269	TGASTMAGC_V$NFE2_01	12	172	16081	524	2.163989	0.009964
red	9323	GSE22886_TCELL_VS_BCELL_NAIVE_DN	12	172	16081	524	2.163989	0.009964
red	9325	GSE22886_CD8_TCELL_VS_BCELL_NAIVE_DN	12	172	16081	524	2.163989	0.009964
tan	6754	BROWNE_HCMV_INFECTION_6HR_DN	5	146	16107	145	3.838687	0.009971
brown	8613	GSE14000_TRANSLATED_RNA_VS_MRNA_16H_LPS_DC	36	181	16072	2190	1.476092	0.009981
magenta	9007	GSE17721_POLYIC_VS_CPG_2H_BMDM_DN	6	177	16076	167	3.299097	0.009984
lightgreen	5784	NUYTTEN_EZH2_TARGETS_UP	7	946	15307	42	2.86346	0.009998
green	9078	GSE17721_LPS_VS_GARDIQUIMOD_2H_BMDM_UP	23	171	16082	1305	1.675154	0.010004
pink	6072	HOUSTIS_ROS	3	32	16221	228	6.682977	0.010014
pink	6134	SHIPP_DLBCL_CURED_VS_FATAL_UP	3	32	16221	228	6.682977	0.010014
cyan	2621	GNF2_CD48	2	32	16221	77	13.19237	0.010023
cyan	3037	BIOCARTA_PDGF_PATHWAY	2	32	16221	77	13.19237	0.010023
brown	45	MYOFIBRIL	6	15	16238	2190	2.968584	0.010026
brown	1391	SIGNAL_SEQUENCE_BINDING	6	15	16238	2190	2.968584	0.010026
brown	3134	chr18q23	6	15	16238	2190	2.968584	0.010026
brown	3485	SA_MMP_CYTOKINE_CONNECTION	6	15	16238	2190	2.968584	0.010026
brown	4005	PID_ARF6DOWNSTREAMPATHWAY	6	15	16238	2190	2.968584	0.010026
brown	5078	HUMMEL_BURKITTS_LYMPHOMA_DN	6	15	16238	2190	2.968584	0.010026
brown	6335	YAO_HOXA10_TARGETS_VIA_PROGESTERONE_DN	6	15	16238	2190	2.968584	0.010026
brown	5239	GRAESSMANN_RESPONSE_TO_MC_AND_DOXORUBICIN	97	574	15679	2190	1.254149	0.010028
salmon	6705	DAZARD_UV_RESPONSE_CLUSTER_G1	3	57	16196	127	6.735599	0.010029
black	7765	GERHOLD_RESPONSE_TO_TZD_DN	2	9	16244	283	12.76247	0.010032
purple	4430	REACTOME_DOWNSTREAM_SIGNAL_TRANSDUCTION	4	87	16166	157	4.759646	0.010032
purple	5127	GRAHAM_CML_DIVIDING_VS_NORMAL_QUIESCENT_D	4	87	16166	157	4.759646	0.010032
purple	7466	CROONQUIST_NRAS_VS_STROMAL_STIMULATION_DN	4	87	16166	157	4.759646	0.010032
grey60	4933	DOANE_RESPONSE_TO_ANDROGEN_UP	3	165	16088	44	6.716116	0.010033
turquoise	8204	GCNP_SHH_UP_LATE.V1_UP	57	168	16085	4169	1.322718	0.010033
lightgreen	8259	MEK_UP.V1_DN	3	173	16080	42	6.71057	0.010037
magenta	4489	REACTOME_G_ALPHA_I_SIGNALLING_EVENTS	5	127	16126	167	3.831628	0.010038
salmon	8588	GSE13485_DAY3_VS_DAY7_YF17D_VACCINE_PBMC_UI	5	167	16086	127	3.831628	0.010038
turquoise	4599	REACTOME_ASSEMBLY_OF_THE_PRE_REPLICATIVE_CO	25	63	16190	4169	1.547038	0.010038
green	4087	PID_SYNDECAN_2_PATHWAY	7	31	16222	1305	2.812285	0.010049
brown	9794	GSE360_CTRL_VS_L_MAJOR_DC_UP	35	175	16078	2190	1.484292	0.010063
green	9386	GSE22886_IL2_VS_IL15_STIM_NKCELL_UP	18	124	16129	1305	1.807898	0.010084

brown	1261	SULFURIC_ESTER_HYDROLASE_ACTIVITY	5	11	16242	2190	3.373391	0.010087
brown	2997	BIOCARTA_IL4_PATHWAY	5	11	16242	2190	3.373391	0.010087
brown	4301	REACTOME_RETROGRADE_NEUROTROPHIN_SIGNALLIN	5	11	16242	2190	3.373391	0.010087
brown	5483	MYLLYKANGAS_AMPLIFICATION_HOT_SPOT_12	5	11	16242	2190	3.373391	0.010087
brown	6774	VERRECCHIA_RESPONSE_TO_TGFB1_C3	5	11	16242	2190	3.373391	0.010087
red	1921	V$E2F1_Q3_01	14	215	16038	524	2.019723	0.010091
red	8113	PHONG_TNF_RESPONSE_VIA_P38_COMPLETE	14	215	16038	524	2.019723	0.010091
magenta	278	REGULATION_OF_DEFENSE_RESPONSE	2	15	16238	167	12.97645	0.010093
magenta	7788	NIELSEN_LEIOMYOSARCOMA_CNN1_DN	2	15	16238	167	12.97645	0.010093
brown	345	SIGNAL_TRANSDUCTION	208	1331	14922	2190	1.159778	0.010098
blue	3510	MODULE_18	94	410	15843	2978	1.251275	0.010101
magenta	929	CATION_HOMEOSTASIS	4	82	16171	167	4.747481	0.010107
lightyellow	7120	MATZUK_STEROIDOGENESIS	1	5	16248	33	98.50303	0.010112
lightyellow	7985	KASLER_HDAC7_TARGETS_2_UP	1	5	16248	33	98.50303	0.010112
lightyellow	6059	ROSS_AML_WITH_PML_RARA_FUSION	2	75	16178	33	13.13374	0.010114
blue	6975	MARZEC_IL2_SIGNALING_UP	30	108	16145	2978	1.516025	0.010118
cyan	5014	TAKEDA_TARGETS_OF_NUP98_HOXA9_FUSION_10D_U	4	178	16075	77	4.743324	0.010121
cyan	8469	GSE11864_CSF1_VS_CSF1_IFNG_IN_MAC_DN	4	178	16075	77	4.743324	0.010121
cyan	8855	GSE17721_CTRL_VS_POLYIC_4H_BMDM_DN	4	178	16075	77	4.743324	0.010121
cyan	9795	GSE360_CTRL_VS_L_MAJOR_DC_DN	4	178	16075	77	4.743324	0.010121
yellow	8285	ESC_J1_UP_EARLY.V1_DN	23	157	16096	1426	1.669714	0.010125
yellow	8881	GSE17721_CTRL_VS_CPG_0.5H_BMDM_DN	23	157	16096	1426	1.669714	0.010125
brown	10214	GSE8515_CTRL_VS_IL1_4H_STIM_MAC_UP	34	169	16084	2190	1.493075	0.010132
brown	10236	GSE9006_1MONTH_VS_4MONTH_POST_TYPE_1_DIABI	34	169	16084	2190	1.493075	0.010132
blue	1902	V$AP2_Q6_01	57	232	16021	2978	1.340898	0.010154
purple	10016	GSE3982_DC_VS_BCELL_UP	6	189	16064	157	3.286422	0.010159
purple	10024	GSE3982_DC_VS_NKCELL_UP	6	189	16064	157	3.286422	0.010159
lightcyan	5568	DAUER_STAT3_TARGETS_UP	2	40	16213	62	13.10726	0.010174
purple	417	REGULATION_OF_MUSCLE_CONTRACTION	2	16	16237	157	12.94029	0.010181
purple	5527	LUND_SILENCED_BY_METHYLATION	2	16	16237	157	12.94029	0.010181
purple	6054	NELSON_RESPONSE_TO_ANDROGEN_DN	2	16	16237	157	12.94029	0.010181
purple	6378	HASLINGER_B_CLL_WITH_6Q21_DELETION	2	16	16237	157	12.94029	0.010181
purple	6718	NIELSEN_LEIOMYOSARCOMA_CNN1_UP	2	16	16237	157	12.94029	0.010181
purple	7786	NIELSEN_GIST_VS_SYNOVIAL_SARCOMA_DN	2	16	16237	157	12.94029	0.010181
greenyello	1853	V$HMGIY_Q6	6	198	16055	150	3.283434	0.010197
greenyello	8399	GOLDRATH_NAIVE_VS_EFF_CD8_TCELL_DN	6	198	16055	150	3.283434	0.010197
midnightb	3162	chr2q32	2	35	16218	71	13.08089	0.0102
green	6815	MONNIER_POSTRADIATION_TUMOR_ESCAPE_DN	41	352	15901	1305	1.450655	0.010212
greenyello	9352	GSE22886_NEUTROPHIL_VS_DC_UP	5	142	16111	150	3.815258	0.01022
brown	2368	MORF_CSNK1D	17	70	16183	2190	1.802355	0.010222
purple	5876	KOYAMA_SEMA3B_TARGETS_UP	7	247	16006	157	2.93383	0.010223
brown	8057	WAKABAYASHI_ADIPOGENESIS_PPARG_RXRA_BOUND_	30	145	16108	2190	1.535475	0.010225
salmon	6818	CREIGHTON_ENDOCRINE_THERAPY_RESISTANCE_1	9	461	15792	127	2.498454	0.010229
brown	954	PROTEIN_TRANSPORT	31	151	16102	2190	1.523611	0.010233
turquoise	1839	V$SMAD4_Q6	64	192	16061	4169	1.299512	0.01025
turquoise	9729	GSE30962_ACUTE_VS_CHRONIC_LCMV_SECONDARY_II	64	192	16061	4169	1.299512	0.01025
midnightb	9140	GSE17721_0.5H_VS_4H_GARDIQUIMOD_BMDM_UP	4	194	16059	71	4.719907	0.010267
midnightb	9380	GSE22886_NAIVE_CD4_TCELL_VS_MONOCYTE_UP	4	194	16059	71	4.719907	0.010267
midnightb	9436	GSE24634_NAIVE_CD4_TCELL_VS_DAY7_IL4_CONV_TR	4	194	16059	71	4.719907	0.010267
midnightb	9451	GSE24634_TEFF_VS_TCONV_DAY5_IN_CULTURE_DN	4	194	16059	71	4.719907	0.010267
midnightb	9706	GSE29618_LAIV_VS_TIV_FLU_VACCINE_DAY7_PDC_UP	4	194	16059	71	4.719907	0.010267
midnightb	10270	GSE9988_ANTI_TREM1_VS_LOW_LPS_MONOCYTE_UP	4	194	16059	71	4.719907	0.010267
red	6665	KEEN_RESPONSE_TO_ROSIGLITAZONE_DN	8	93	16160	524	2.668144	0.010271
lightyellow	3530	MODULE_41	4	424	15829	33	4.646369	0.010274
blue	5746	PUJANA_BRCA_CENTERED_NETWORK	32	117	16136	2978	1.492701	0.010281
turquoise	3612	MODULE_124	35	95	16158	4169	1.436303	0.010281
salmon	8407	GSE10239_NAIVE_VS_MEMORY_CD8_TCELL_DN	5	168	16085	127	3.808821	0.010282
blue	8610	GSE14000_TRANSLATED_RNA_VS_MRNA_4H_LPS_DC_	48	190	16063	2978	1.378785	0.010292
lightgreen	7545	BUDHU_LIVER_CANCER_METASTASIS_DN	1	4	16249	42	96.74405	0.010298
lightgreen	7758	VERRECCHIA_RESPONSE_TO_TGFB1_C6	1	4	16249	42	96.74405	0.010298

lightgreen	1946	TGCTGCT,MIR-15A,MIR-16,MIR-15B,MIR-195,MIR-424,I	5	517	15736	42	3.742516	0.010299
turquoise	5576	HEIDENBLAD_AMPLIFIED_IN_PANCREATIC_CANCER	14	30	16223	4169	1.819317	0.010302
red	8555	GSE13411_IGM_VS_SWITCHED_MEMORY_BCELL_DN	13	194	16059	524	2.078471	0.010318
red	8979	GSE17721_CPG_VS_GARDIQUIMOD_12H_BMDM_DN	13	194	16059	524	2.078471	0.010318
red	9470	GSE25087_TREG_VS_TCONV_ADULT_UP	13	194	16059	524	2.078471	0.010318
purple	8548	GSE13411_NAIVE_VS_SWITCHED_MEMORY_BCELL_UP	5	136	16117	157	3.805967	0.010319
blue	3982	PID_CDC42_REG_PATHWAY	11	29	16224	2978	2.070158	0.01032
blue	7491	VALK_AML_CLUSTER_15	11	29	16224	2978	2.070158	0.01032
cyan	7860	CHICAS_RB1_TARGETS_SENESCENT	7	509	15744	77	2.90284	0.010321
turquoise	1705	V$E2F1_Q3	73	223	16030	4169	1.276203	0.010336
lightcyan	4934	DOANE_RESPONSE_TO_ANDROGEN_DN	4	223	16030	62	4.702155	0.010344
magenta	4244	REACTOME_COLLAGEN_FORMATION	3	44	16209	167	6.635683	0.010366
magenta	7267	BOYLAN_MULTIPLE_MYELOMA_C_UP	3	44	16209	167	6.635683	0.010366
magenta	7842	STAMBOLSKY_TARGETS_OF_MUTATED_TP53_UP	3	44	16209	167	6.635683	0.010366
salmon	42	CYTOPLASMIC_PART	18	1277	14976	127	1.803896	0.010372
lightyellow	6354	CROMER_METASTASIS_DN	2	76	16177	33	12.96093	0.010374
yellow	8919	GSE17721_LPS_VS_POLYIC_4H_BMDM_DN	27	193	16060	1426	1.594485	0.010377
yellow	10095	GSE3982_CENT_MEMORY_CD4_TCELL_VS_TH2_DN	27	193	16060	1426	1.594485	0.010377
blue	2512	GCM_DDX5	20	65	16188	2978	1.679289	0.010382
blue	4167	REACTOME_MEIOSIS	24	82	16171	2978	1.597373	0.0104
blue	7917	ACOSTA_PROLIFERATION_INDEPENDENT_MYC_TARGET	24	82	16171	2978	1.597373	0.0104
greenyello	7644	BOYLAN_MULTIPLE_MYELOMA_PCA1_UP	4	92	16161	150	4.711014	0.0104
red	135	LIPID_RAFT	4	27	16226	524	4.595137	0.010402
red	3276	chr9q32	4	27	16226	524	4.595137	0.010402
red	3732	MODULE_265	4	27	16226	524	4.595137	0.010402
red	9798	GSE360_CTRL_VS_B_MALAYI_HIGH_DOSE_DC_UP	12	173	16080	524	2.15148	0.010405
red	9954	GSE3982_CTRL_VS_LPS_1H_NEUTROPHIL_UP	12	173	16080	524	2.15148	0.010405
magenta	6801	DURCHDEWALD_SKIN_CARCINOGENESIS_DN	7	233	16020	167	2.923878	0.010416
red	4970	VECCHI_GASTRIC_CANCER_ADVANCED_VS_EARLY_UP	10	132	16121	524	2.349786	0.010428
purple	6558	CHEN_LVAD_SUPPORT_OF_FAILING_HEART_UP	4	88	16165	157	4.705559	0.010433
purple	7787	NIELSEN_GIST	4	88	16165	157	4.705559	0.010433
red	6254	GUO_HEX_TARGETS_UP	7	75	16178	524	2.894936	0.010436
red	7091	BASSO_HAIRY_CELL_LEUKEMIA_DN	7	75	16178	524	2.894936	0.010436
purple	7565	MIKKELSEN_MCV6_HCP_WITH_H3K27ME3	7	248	16005	157	2.922	0.010437
turquoise	8394	KAECH_DAY8_EFF_VS_MEMORY_CD8_TCELL_UP	66	199	16054	4169	1.292982	0.01044
turquoise	8437	GSE10325_BCELL_VS_LUPUS_BCELL_DN	66	199	16054	4169	1.292982	0.01044
green	9069	GSE17721_POLYIC_VS_GARDIQUIMOD_12H_BMDM_D	25	191	16062	1305	1.630158	0.010441
midnightb	9340	GSE22886_IGG_IGA_MEMORY_BCELL_VS_BM_PLASMA	4	195	16058	71	4.695702	0.010448
midnightb	9904	GSE36476_CTRL_VS_TSST_ACT_40H_MEMORY_CD4_TC	4	195	16058	71	4.695702	0.010448
turquoise	2137	AGGTGCA,MIR-500	30	79	16174	4169	1.480457	0.010485
turquoise	4704	REACTOME_RESPIRATORY_ELECTRON_TRANSPORT_AT	30	79	16174	4169	1.480457	0.010485
brown	1718	V$ZIC3_01	39	200	16053	2190	1.447185	0.010485
brown	7827	KARLSSON_TGFB1_TARGETS_DN	39	200	16053	2190	1.447185	0.010485
salmon	214	RIBOSOMAL_SUBUNIT	2	20	16233	127	12.79764	0.010498
purple	3639	MODULE_154	3	47	16206	157	6.607806	0.010509
purple	4760	REACTOME_AMYLOIDS	3	47	16206	157	6.607806	0.010509
purple	6673	ZHANG_ANTIVIRAL_RESPONSE_TO_RIBAVIRIN_DN	3	47	16206	157	6.607806	0.010509
yellow	5517	ROSTY_CERVICAL_CANCER_PROLIFERATION_CLUSTER	21	140	16113	1426	1.709642	0.01051
magenta	2266	GCANCTGNY_V$MYOD_Q6	15	750	15503	167	1.946467	0.010511
cyan	8955	GSE17721_PAM3CSK4_VS_CPG_4H_BMDM_DN	4	180	16073	77	4.69062	0.010513
magenta	8510	GSE12845_IGD_POS_BLOOD_VS_DARKZONE_GC_TONS	6	179	16074	167	3.262235	0.010515
magenta	8933	GSE17721_POLYIC_VS_PAM3CSK4_1H_BMDM_DN	6	179	16074	167	3.262235	0.010515
salmon	4166	REACTOME_ER_PHAGOSOME_PATHWAY	3	58	16195	127	6.619468	0.010517
salmon	6789	BILD_SRC_ONCOGENIC_SIGNATURE	3	58	16195	127	6.619468	0.010517
pink	1478	V$SOX5_01	8	213	16040	228	2.677374	0.010517
pink	5294	MCBRYAN_PUBERTAL_BREAST_4_5WK_UP	8	213	16040	228	2.677374	0.010517
salmon	7649	HOSHIDA_LIVER_CANCER_SUBCLASS_S2	4	109	16144	127	4.696381	0.010529
grey60	9797	GSE360_CTRL_VS_T_GONDII_DC_DN	3	168	16085	44	6.596185	0.010534
turquoise	2265	CACGTG_V$MYC_Q2	267	922	15331	4169	1.128969	0.010542
pink	2005	GTGTGAG,MIR-342	4	61	16192	228	4.674432	0.010546

turquoise	1068	ACID_AMINO_ACID_LIGASE_ACTIVITY	23	57	16196	4169	1.573094	0.010551
brown	4000	PID_NFAT_3PATHWAY	14	54	16199	2190	1.924083	0.010553
blue	7558	YAGI_AML_WITH_11Q23_REARRANGED	77	328	15925	2978	1.281226	0.010555
black	7177	PODAR_RESPONSE_TO_ADAPHOSTIN_UP	7	138	16115	283	2.913172	0.01056
lightcyan	5336	DARWICHE_PAPILLOMA_RISK_HIGH_UP	3	119	16134	62	6.608702	0.010576
lightcyan	8278	PKCA_DN.V1_UP	3	119	16134	62	6.608702	0.010576
green	5149	GOZGIT_ESR1_TARGETS_DN	65	608	15645	1305	1.331474	0.010579
blue	8599	GSE13493_DP_VS_CD8POS_THYMOCYTE_DN	46	181	16072	2978	1.387037	0.010587
turquoise	71	NON_MEMBRANE_BOUND_ORGANELLE	172	575	15678	4169	1.166171	0.010596
turquoise	177	INTRACELLULAR_NON_MEMBRANE_BOUND_ORGANEL	172	575	15678	4169	1.166171	0.010596
brown	8339	SIRNA_EIF4GI_DN	20	87	16166	2190	1.706083	0.010605
brown	4821	HOLLMANN_APOPTOSIS_VIA_CD40_UP	38	194	16059	2190	1.453688	0.010624
brown	9926	GSE37416_CTRL_VS_6H_F_TULARENSIS_LVS_NEUTROP	38	194	16059	2190	1.453688	0.010624
purple	4262	REACTOME_GASTRIN_CREB_SIGNALLING_PATHWAY_V	5	137	16116	157	3.778186	0.010629
midnightb	8403	GOLDRATH_EFF_VS_MEMORY_CD8_TCELL_DN	4	196	16057	71	4.671745	0.010631
midnightb	9374	GSE22886_NAIVE_CD8_TCELL_VS_MONOCYTE_UP	4	196	16057	71	4.671745	0.010631
midnightb	9906	GSE36476_CTRL_VS_TSST_ACT_72H_MEMORY_CD4_T(	4	196	16057	71	4.671745	0.010631
midnightb	9939	GSE37416_0H_VS_12H_F_TULARENSIS_LVS_NEUTROPI	4	196	16057	71	4.671745	0.010631
midnightb	10268	GSE9988_ANTI_TREM1_VS_LPS_MONOCYTE_UP	4	196	16057	71	4.671745	0.010631
blue	6876	SANSOM_APC_MYC_TARGETS	52	209	16044	2978	1.357894	0.010633
cyan	128	NUCLEAR_BODY	2	33	16220	77	12.7926	0.010637
cyan	748	DNA_DAMAGE_RESPONSESIGNAL_TRANSDUCTION	2	33	16220	77	12.7926	0.010637
lightyellow	8190	ZWANG_EGF_INTERVAL_UP	2	77	16176	33	12.7926	0.010637
brown	3785	MODULE_333	8	24	16229	2190	2.47382	0.010647
brown	4218	REACTOME_RORA_ACTIVATES_CIRCADIAN_EXPRESSIOI	8	24	16229	2190	2.47382	0.010647
blue	8616	GSE14000_UNSTIM_VS_16H_LPS_DC_TRANSLATED_RN	49	195	16058	2978	1.371419	0.010651
blue	8709	GSE1460_CORD_VS_ADULT_BLOOD_NAIVE_CD4_TCELL	49	195	16058	2978	1.371419	0.010651
blue	9740	GSE31082_CD4_VS_CD8_SP_THYMOCYTE_UP	49	195	16058	2978	1.371419	0.010651
purple	1739	V$FOXO1_02	6	191	16062	157	3.252009	0.010664
purple	9347	GSE22886_DAY0_VS_DAY1_MONOCYTE_IN_CULTURE_	6	191	16062	157	3.252009	0.010664
purple	9408	GSE24142_EARLY_THYMIC_PROGENITOR_VS_DN2_THY	6	191	16062	157	3.252009	0.010664
brown	2008	AGGGCAG,MIR-18A	24	110	16143	2190	1.619228	0.010665
brown	2845	KEGG_NATURAL_KILLER_CELL_MEDIATED_CYTOTOXICI	24	110	16143	2190	1.619228	0.010665
brown	5786	INGRAM_SHH_TARGETS_UP	24	110	16143	2190	1.619228	0.010665
lightcyan	6248	MA_MYELOID_DIFFERENTIATION_DN	2	41	16212	62	12.78757	0.01067
turquoise	4731	REACTOME_IMMUNE_SYSTEM	234	801	15452	4169	1.138898	0.010674
turquoise	2099	ATGCTGG,MIR-338	38	105	16148	4169	1.410899	0.010677
red	2230	YWATTWNNRGCT_UNKNOWN	6	58	16195	524	3.208673	0.010697
red	3230	chr1q25	6	58	16195	524	3.208673	0.010697
red	6903	KYNG_DNA_DAMAGE_BY_UV	6	58	16195	524	3.208673	0.010697
cyan	4951	HORIUCHI_WTAP_TARGETS_UP	5	281	15972	77	3.755835	0.010709
cyan	9889	GSE360_LOW_DOSE_B_MALAYI_VS_M_TUBERCULOSIS	4	181	16072	77	4.664705	0.010713
blue	2485	GCM_BNIP1	19	61	16192	2978	1.699936	0.01072
black	5907	BENPORATH_ES_WITH_H3K27ME3	20	654	15599	283	1.756303	0.010722
turquoise	871	REGULATION_OF_JNK_ACTIVITY	10	19	16234	4169	2.051861	0.010723
turquoise	3293	chr14q12	10	19	16234	4169	2.051861	0.010723
turquoise	6759	CHEN_ETV5_TARGETS_TESTIS	10	19	16234	4169	2.051861	0.010723
turquoise	7412	MELLMAN_TUT1_TARGETS_UP	10	19	16234	4169	2.051861	0.010723
turquoise	6836	HOWLIN_CITED1_TARGETS_1_DN	15	33	16220	4169	1.772062	0.010726
turquoise	2537	GCM_NF2	93	293	15960	4169	1.23742	0.010735
red	8971	GSE17721_CPG_VS_GARDIQUIMOD_2H_BMDM_DN	13	195	16058	524	2.067812	0.010742
red	9740	GSE31082_CD4_VS_CD8_SP_THYMOCYTE_UP	13	195	16058	524	2.067812	0.010742
red	10040	GSE3982_MAC_VS_NKCELL_UP	13	195	16058	524	2.067812	0.010742
turquoise	2369	MORF_CSNK2B	91	286	15967	4169	1.240444	0.010748
brown	8628	GSE14308_TH2_VS_TH1_UP	37	188	16065	2190	1.460607	0.010753
brown	8806	GSE15930_STIM_VS_STIM_AND_TRICHOSTATINA_48H_	37	188	16065	2190	1.460607	0.010753
brown	9064	GSE17721_POLYIC_VS_GARDIQUIMOD_6H_BMDM_UP	37	188	16065	2190	1.460607	0.010753
midnightb	504	NEGATIVE_REGULATION_OF_GROWTH	2	36	16217	71	12.71753	0.01077
midnightb	7901	OHGUCHI_LIVER_HNF4A_TARGETS_UP	2	36	16217	71	12.71753	0.01077
red	1388	PROTEIN_DIMERIZATION_ACTIVITY	11	153	16100	524	2.229993	0.010781

red	9684	GSE29618_PRE_VS_DAY7_FLU_VACCINE_MDC_UP	11	153	16100	524	2.229993	0.010781
salmon	8533	GSE13306_TREG_VS_TCONV_LAMINA_PROPRIA_DN	5	170	16083	127	3.764011	0.010784
magenta	8260	MEK_UP.V1_UP	6	180	16073	167	3.244112	0.010787
lightgreen	2246	RYTTCCTG_V$ETS2_B	7	960	15293	42	2.821701	0.010791
turquoise	8717	GSE14769_UNSTIM_VS_40MIN_LPS_BMDM_DN	63	189	16064	4169	1.299512	0.010792
turquoise	9242	GSE18791_UNSTIM_VS_NEWCATSLE_VIRUS_DC_6H_UI	63	189	16064	4169	1.299512	0.010792
turquoise	9981	GSE3982_EOSINOPHIL_VS_BASOPHIL_DN	63	189	16064	4169	1.299512	0.010792
turquoise	10280	GSE9988_LPS_VS_LPS_AND_ANTI_TREM1_MONOCYTE_	63	189	16064	4169	1.299512	0.010792
lightcyan	7414	HAN_SATB1_TARGETS_UP	5	351	15902	62	3.734262	0.010808
brown	1819	V$HEB_Q6	42	219	16034	2190	1.423294	0.010833
lightgreen	3595	MODULE_107	2	61	16192	42	12.68774	0.010833
lightgreen	3961	PID_IL12_2PATHWAY	2	61	16192	42	12.68774	0.010833
lightgreen	9431	GSE24142_ADULT_VS_FETAL_DN3_THYMOCYTE_DN	3	178	16075	42	6.522071	0.010842
purple	6527	BROWNE_HCMV_INFECTION_20HR_DN	4	89	16164	157	4.652687	0.010844
pink	2276	GGGTGGRR_V$PAX4_03	25	1093	15160	228	1.630491	0.010847
red	9545	GSE27786_LSK_VS_MONO_MAC_DN	12	174	16079	524	2.139116	0.010861
brown	5259	OUELLET_OVARIAN_CANCER_INVASIVE_VS_LMP_UP	25	116	16137	2190	1.599453	0.010863
blue	2265	CACGTG_V$MYC_Q2	196	922	15331	2978	1.160203	0.010871
purple	59	MEMBRANE	25	1601	14652	157	1.616525	0.010871
brown	777	VESICLE_MEDIATED_TRANSPORT	36	182	16071	2190	1.467981	0.010872
brown	10032	GSE3982_MAC_VS_BCELL_UP	36	182	16071	2190	1.467981	0.010872
red	5054	TONKS_TARGETS_OF_RUNX1_RUNX1T1_FUSION_SUST/	5	42	16211	524	3.692521	0.010873
blue	7925	BHAT_ESR1_TARGETS_VIA_AKT1_UP	60	247	16006	2978	1.325755	0.010904
turquoise	804	NEGATIVE_REGULATION_OF_RNA_METABOLIC_PROCE!	41	115	16138	4169	1.389913	0.010911
cyan	8565	GSE13484_UNSTIM_VS_12H_YF17D_VACCINE_STIM_PI	4	182	16071	77	4.639075	0.010915
cyan	8863	GSE17721_CTRL_VS_POLYIC_24H_BMDM_DN	4	182	16071	77	4.639075	0.010915
red	4206	REACTOME_PPARA_ACTIVATES_GENE_EXPRESSION	8	94	16159	524	2.63976	0.01092
green	5253	GAUSSMANN_MLL_AF4_FUSION_TARGETS_G_UP	24	182	16071	1305	1.642339	0.010934
purple	5012	TAKEDA_TARGETS_OF_NUP98_HOXA9_FUSION_8D_UF	5	138	16115	157	3.750808	0.010945
purple	6158	BROCKE_APOPTOSIS_REVERSED_BY_IL6	5	138	16115	157	3.750808	0.010945
purple	8289	ESC_V6.5_UP_EARLY.V1_DN	5	138	16115	157	3.750808	0.010945
blue	1807	V$SP3_Q3	53	214	16039	2978	1.351671	0.010945
blue	3756	MODULE_299	12	33	16220	2978	1.984614	0.010953
blue	4311	REACTOME_DOWNSTREAM_TCR_SIGNALING	12	33	16220	2978	1.984614	0.010953
blue	4460	REACTOME_TRANSPORT_OF_MATURE_MRNA_DERIVEI	12	33	16220	2978	1.984614	0.010953
turquoise	1410	SMALL_CONJUGATING_PROTEIN_LIGASE_ACTIVITY	21	51	16202	4169	1.60528	0.010959
brown	2526	GCM_ING1	16	65	16188	2190	1.826821	0.010961
brown	2829	KEGG_VEGF_SIGNALING_PATHWAY	16	65	16188	2190	1.826821	0.010961
red	4808	PICCALUGA_ANGIOIMMUNOBLASTIC_LYMPHOMA_DN	10	133	16120	524	2.332118	0.010962
purple	8115	PHONG_TNF_RESPONSE_NOT_VIA_P38	8	312	15941	157	2.654418	0.010969
brown	9974	GSE3982_EOSINOPHIL_VS_MAC_UP	35	176	16077	2190	1.475859	0.010978
blue	8651	GSE14308_TH17_VS_NATURAL_TREG_DN	47	186	16067	2978	1.379094	0.010979
turquoise	1664	V$WHN_B	65	196	16057	4169	1.292882	0.010991
turquoise	8665	GSE1432_1H_VS_6H_IFNG_MICROGLIA_DN	65	196	16057	4169	1.292882	0.010991
turquoise	9961	GSE3982_MEMORY_CD4_TCELL_VS_TH2_DN	65	196	16057	4169	1.292882	0.010991
midnightb	9376	GSE22886_NAIVE_CD4_TCELL_VS_NEUTROPHIL_UP	4	198	16055	71	4.624555	0.011003
yellow	947	MALE_GONAD_DEVELOPMENT	3	6	16247	1426	5.698808	0.011007
yellow	5522	OHASHI_AURKA_TARGETS	3	6	16247	1426	5.698808	0.011007
tan	185	NUCLEOLAR_PART	2	18	16235	145	12.45441	0.011013
tan	902	RIBOSOME_BIOGENESIS_AND_ASSEMBLY	2	18	16235	145	12.45441	0.011013
tan	7055	SMID_BREAST_CANCER_RELAPSE_IN_LUNG_DN	2	18	16235	145	12.45441	0.011013
yellow	5305	LUI_THYROID_CANCER_PAX8_PPARG_DN	9	43	16210	1426	2.385547	0.011015
magenta	335	POSITIVE_REGULATION_OF_IMMUNE_SYSTEM_PROCE!	3	45	16208	167	6.488224	0.011024
magenta	728	REGULATION_OF_RESPONSE_TO_STIMULUS	3	45	16208	167	6.488224	0.011024
magenta	2150	CAGTCAC,MIR-134	3	45	16208	167	6.488224	0.011024
magenta	7520	BOYAULT_LIVER_CANCER_SUBCLASS_G123_UP	3	45	16208	167	6.488224	0.011024
turquoise	2210	WTGAAAT_UNKNOWN	143	471	15782	4169	1.183632	0.011028
salmon	6366	MOREAUX_MULTIPLE_MYELOMA_BY_TACI_DN	5	171	16082	127	3.741999	0.011041
brown	4747	REACTOME_CYTOKINE_SIGNALING_IN_IMMUNE_SYSTE	45	238	16015	2190	1.403217	0.011049
grey60	8258	LTE2_UP.V1_UP	3	171	16082	44	6.480463	0.01105

grey60	9852	GSE360_T_GONDII_VS_M_TUBERCULOSIS_DC_UP	3	171	16082	44	6.480463	0.01105
magenta	9618	GSE28237_FOLLICULAR_VS_EARLY_GC_BCELL_UP	6	181	16072	167	3.226189	0.011065
magenta	9679	GSE29618_PRE_VS_DAY7_FLU_VACCINE_BCELL_DN	6	181	16072	167	3.226189	0.011065
yellow	9900	GSE36392_MAC_VS_NEUTROPHIL_IL25_TREATED_LUN	27	194	16059	1426	1.586266	0.011079
blue	2197	TGCGCANK_UNKNOWN	113	505	15748	2978	1.221226	0.011089
yellow	9460	GSE24634_IL4_VS_CTRL_TREATED_NAIVE_CD4_TCELL_	26	185	16068	1426	1.601827	0.011093
turquoise	768	GOLGI_VESICLE_TRANSPORT	20	48	16205	4169	1.62439	0.011104
grey60	5586	SEITZ_NEOPLASTIC_TRANSFORMATION_BY_8P_DELETI(	2	59	16194	44	12.52157	0.011113
greenyello	2118	GAGCTGG,MIR-337	5	145	16108	150	3.736322	0.011118
cyan	8368	KRAS.600.LUNG.BREAST_UP.V1_DN	4	183	16070	77	4.613725	0.011119
cyan	8727	GSE14769_UNSTIM_VS_360MIN_LPS_BMDM_DN	4	183	16070	77	4.613725	0.011119
cyan	9425	GSE24142_DN2_VS_DN3_THYMOCYTE_FETAL_DN	4	183	16070	77	4.613725	0.011119
purple	3959	PID_PTP1BPATHWAY	3	48	16205	157	6.470143	0.011131
purple	7104	ZHENG_GLIOBLASTOMA_PLASTICITY_DN	3	48	16205	157	6.470143	0.011131
purple	7461	CROONQUIST_STROMAL_STIMULATION_UP	3	48	16205	157	6.470143	0.011131
purple	7670	VANTVEER_BREAST_CANCER_POOR_PROGNOSIS	3	48	16205	157	6.470143	0.011131
turquoise	255	PHOSPHOLIPID_BIOSYNTHETIC_PROCESS	17	39	16214	4169	1.699362	0.011162
turquoise	1974	TAGGTCA,MIR-192,MIR-215	17	39	16214	4169	1.699362	0.011162
cyan	944	TRANSCRIPTION_FROM_RNA_POLYMERASE_II_PROMO	6	396	15857	77	3.19815	0.011169
tan	1427	NUCLEASE_ACTIVITY	3	52	16201	145	6.466711	0.011173
lightcyan	5796	NAKAYAMA_FRA2_TARGETS	2	42	16211	62	12.4831	0.011176
lightgreen	513	CYTOKINE_PRODUCTION	2	62	16191	42	12.4831	0.011176
greenyello	951	G_PROTEIN_COUPLED_RECEPTOR_PROTEIN_SIGNALIN(	6	202	16051	150	3.218416	0.011178
greenyello	8078	PLASARI_TGFB1_TARGETS_10HR_DN	6	202	16051	150	3.218416	0.011178
red	9385	GSE22886_TH1_VS_TH2_48H_ACT_DN	13	196	16057	524	2.057262	0.011179
turquoise	1951	TGGTGCT,MIR-29A,MIR-29B,MIR-29C	141	464	15789	4169	1.184685	0.01118
turquoise	4892	GARY_CD5_TARGETS_UP	141	464	15789	4169	1.184685	0.01118
blue	870	PIGMENT_METABOLIC_PROCESS	7	15	16238	2978	2.546922	0.011181
blue	3991	PID_CIRCADIANPATHWAY	7	15	16238	2978	2.546922	0.011181
red	3316	chr10q26	7	76	16177	524	2.856845	0.011185
purple	9422	GSE24142_EARLY_THYMIC_PROGENITOR_VS_DN3_THY	6	193	16060	157	3.21831	0.011185
purple	9447	GSE24634_TREG_VS_TCONV_POST_DAY10_IL4_CONVE	6	193	16060	157	3.21831	0.011185
turquoise	6625	SESTO_RESPONSE_TO_UV_C0	37	102	16151	4169	1.414175	0.011186
midnightb	9346	GSE22886_DAY0_VS_DAY1_MONOCYTE_IN_CULTURE_	4	199	16054	71	4.601316	0.011193
turquoise	2748	KEGG_N_GLYCAN_BIOSYNTHESIS	19	45	16208	4169	1.646049	0.011195
brown	7929	JOHNSTONE_PARVB_TARGETS_2_UP	28	134	16119	2190	1.550753	0.011201
turquoise	3543	MODULE_54	80	248	16005	4169	1.257593	0.011205
black	5952	SAKAI_CHRONIC_HEPATITIS_VS_LIVER_CANCER_DN	3	27	16226	283	6.381233	0.011217
turquoise	2028	TGAGATT,MIR-216	33	89	16164	4169	1.445525	0.01122
grey60	8077	PLASARI_TGFB1_TARGETS_10HR_UP	3	172	16081	44	6.442785	0.011226
purple	7883	FIGUEROA_AML_METHYLATION_CLUSTER_3_UP	5	139	16114	157	3.723823	0.011267
lightgreen	3563	MODULE_75	4	340	15913	42	4.552661	0.011267
cyan	5964	SHIN_B_CELL_LYMPHOMA_CLUSTER_8	2	34	16219	77	12.41635	0.011268
cyan	6933	HELLER_SILENCED_BY_METHYLATION_DN	3	98	16155	77	6.461569	0.011268
blue	1343	STRUCTURE_SPECIFIC_DNA_BINDING	17	53	16200	2978	1.75058	0.011273
blue	3986	PID_CD8TCRPATHWAY	17	53	16200	2978	1.75058	0.011273
green	876	REGULATION_OF_TRANSPORT	10	55	16198	1305	2.264437	0.011287
turquoise	1216	ENZYME_REGULATOR_ACTIVITY	88	276	15977	4169	1.243012	0.0113
lightcyan	658	MEMBRANE_ORGANIZATION_AND_BIOGENESIS	3	122	16131	62	6.446192	0.011313
cyan	8093	KRIEG_KDM3A_TARGETS_NOT_HYPOXIA	4	184	16069	77	4.58865	0.011327
cyan	8965	GSE17721_PAM3CSK4_VS_CPG_24H_BMDM_DN	4	184	16069	77	4.58865	0.011327
cyan	9050	GSE17721_LPS_VS_CPG_12H_BMDM_UP	4	184	16069	77	4.58865	0.011327
cyan	9957	GSE3982_CTRL_VS_LPS_48H_DC_DN	4	184	16069	77	4.58865	0.011327
red	8988	GSE17721_LPS_VS_PAM3CSK4_2H_BMDM_UP	12	175	16078	524	2.126892	0.011332
lightgreen	8569	GSE13484_12H_UNSTIM_VS_YF17D_VACCINE_STIM_PI	3	181	16072	42	6.41397	0.011342
lightgreen	9203	GSE17974_IL4_AND_ANTI_IL12_VS_UNTREATED_72H_	3	181	16072	42	6.41397	0.011342
magenta	6332	ALCALAY_AML_BY_NPM1_LOCALIZATION_DN	6	182	16071	167	3.208462	0.011348
red	5713	KARAKAS_TGFB1_SIGNALING	3	15	16238	524	6.203435	0.011353
red	7953	KIM_PTEN_TARGETS_UP	3	15	16238	524	6.203435	0.011353
green	3925	PID_FANCONI_PATHWAY	9	47	16206	1305	2.384886	0.01137

black	5106	ELVIDGE_HYPOXIA_DN	7	140	16113	283	2.871555	0.011371
brown	6934	HELLER_HDAC_TARGETS_UP	53	289	15964	2190	1.361029	0.011384
turquoise	1000	GENE_SILENCING	6	9	16244	4169	2.599025	0.011397
turquoise	1141	SMALL_CONJUGATING_PROTEIN_SPECIFIC_PROTEASE_	6	9	16244	4169	2.599025	0.011397
blue	5973	MORI_SMALL_PRE_BII_LYMPHOCYTE_DN	22	74	16179	2978	1.622556	0.011399
turquoise	3624	MODULE_136	132	432	15821	4169	1.19122	0.011402
magenta	3642	MODULE_157	4	85	16168	167	4.579923	0.011428
brown	877	RECEPTOR_MEDIATED_ENDOCYTOSIS	9	29	16224	2190	2.303212	0.011446
brown	5327	LANG_MYB_FAMILY_TARGETS	9	29	16224	2190	2.303212	0.011446
brown	6292	NEMETH_INFLAMMATORY_RESPONSE_LPS_DN	9	29	16224	2190	2.303212	0.011446
red	7895	PANGAS_TUMOR_SUPPRESSION_BY_SMAD1_AND_SM/	9	114	16139	524	2.448724	0.011447
purple	9441	GSE24634_TREG_VS_TCONV_POST_DAY3_IL4_CONVER	6	194	16059	157	3.20172	0.011453
blue	4851	SOTIRIOU_BREAST_CANCER_GRADE_1_VS_3_DN	16	49	16204	2978	1.782103	0.011455
blue	655	SECONDARY_METABOLIC_PROCESS	9	22	16231	2978	2.232691	0.011457
blue	4526	REACTOME_DOUBLE_STRAND_BREAK_REPAIR	9	22	16231	2978	2.232691	0.011457
magenta	1450	EXTRACELLULAR_MATRIX_STRUCTURAL_CONSTITUENT	2	16	16237	167	12.16542	0.011457
magenta	3011	BIOCARTA_INTRINSIC_PATHWAY	2	16	16237	167	12.16542	0.011457
magenta	3240	chr6p23	2	16	16237	167	12.16542	0.011457
magenta	5300	VETTER_TARGETS_OF_PRKCA_AND_ETS1_UP	2	16	16237	167	12.16542	0.011457
magenta	8087	ROLEF_GLIS3_TARGETS	2	16	16237	167	12.16542	0.011457
brown	771	PROTEIN_PROCESSING	12	44	16209	2190	2.024035	0.011465
brown	3012	BIOCARTA_KERATINOCYTE_PATHWAY	12	44	16209	2190	2.024035	0.011465
purple	1120	HEPARIN_BINDING	2	17	16236	157	12.17909	0.011465
purple	6732	NIELSEN_LEIOMYOSARCOMA_UP	2	17	16236	157	12.17909	0.011465
purple	7951	ZHU_SKIL_TARGETS_UP	2	17	16236	157	12.17909	0.011465
midnightb	1931	V$PR_02	3	107	16146	71	6.418191	0.011467
turquoise	1984	GACTGTT,MIR-212,MIR-132	49	142	16111	4169	1.34527	0.011494
green	9512	GSE2706_R848_VS_LPS_8H_STIM_DC_UP	19	135	16118	1305	1.752842	0.011514
lightgreen	5029	AKL_HTLV1_INFECTION_DN	2	63	16190	42	12.28496	0.011524
lightgreen	5166	KOKKINAKIS_METHIONINE_DEPRIVATION_48HR_DN	2	63	16190	42	12.28496	0.011524
lightgreen	6414	NATSUME_RESPONSE_TO_INTERFERON_BETA_UP	2	63	16190	42	12.28496	0.011524
cyan	8891	GSE17721_CTRL_VS_CPG_8H_BMDM_DN	4	185	16068	77	4.563847	0.011536
cyan	9086	GSE17721_LPS_VS_GARDIQUIMOD_12H_BMDM_UP	4	185	16068	77	4.563847	0.011536
salmon	48	SMALL_NUCLEAR_RIBONUCLEOPROTEIN_COMPLEX	2	21	16232	127	12.18823	0.011544
salmon	3237	chr8q12	2	21	16232	127	12.18823	0.011544
salmon	6097	IIZUKA_LIVER_CANCER_PROGRESSION_L0_L1_DN	2	21	16232	127	12.18823	0.011544
blue	6878	SANSOM_WNT_PATHWAY_REQUIRE_MYC	15	45	16208	2978	1.81923	0.011546
salmon	6541	KAYO_AGING_MUSCLE_DN	4	112	16141	127	4.570585	0.011548
turquoise	6329	AFFAR_YY1_TARGETS_DN	64	193	16060	4169	1.292779	0.011573
turquoise	8876	GSE17721_CTRL_VS_PAM3CSK4_12H_BMDM_UP	64	193	16060	4169	1.292779	0.011573
turquoise	8906	GSE17721_CTRL_VS_GARDIQUIMOD_8H_BMDM_UP	64	193	16060	4169	1.292779	0.011573
turquoise	1981	TTGGAGA,MIR-515-5P,MIR-519E	46	132	16121	4169	1.358581	0.01158
grey60	9970	GSE3982_EOSINOPHIL_VS_MAST_CELL_UP	3	174	16079	44	6.36873	0.011581
cyan	3187	chr11q12	3	99	16154	77	6.396301	0.011581
turquoise	3496	MODULE_3	114	368	15885	4169	1.207699	0.011583
red	3813	MODULE_375	6	59	16194	524	3.154289	0.011593
purple	9191	GSE17974_IL4_AND_ANTI_IL12_VS_UNTREATED_2H_A	5	140	16113	157	3.697225	0.011596
greenyello	4108	PID_CD8TCRDOWNSTREAMPATHWAY	3	51	16202	150	6.373725	0.011612
red	8507	GSE12845_IGD_POS_BLOOD_VS_NAIVE_TONSIL_BCELL	13	197	16056	524	2.046819	0.01163
red	8558	GSE13411_SWITCHED_MEMORY_BCELL_VS_PLASMA_(	13	197	16056	524	2.046819	0.01163
magenta	9501	GSE2706_UNSTIM_VS_8H_R848_DC_DN	6	183	16070	167	3.19093	0.011635
cyan	7988	LEE_BMP2_TARGETS_UP	8	650	15603	77	2.597882	0.011659
brown	6442	BLALOCK_ALZHEIMERS_DISEASE_DN	178	1128	15125	2190	1.171117	0.011664
lightgreen	1778	V$AFP1_Q6	3	183	16070	42	6.343872	0.011682
lightgreen	8677	GSE14350_IL2RB_KO_VS_WT_TEFF_DN	3	183	16070	42	6.343872	0.011682
lightgreen	8829	GSE17580_UNINFECTED_VS_S_MANSONI_INF_TEFF_DI	3	183	16070	42	6.343872	0.011682
lightgreen	9960	GSE3982_MEMORY_CD4_TCELL_VS_TH2_UP	3	183	16070	42	6.343872	0.011682
turquoise	367	BIOSYNTHETIC_PROCESS	128	418	15835	4169	1.19381	0.011683
brown	8550	GSE13411_NAIVE_BCELL_VS_PLASMA_CELL_UP	37	189	16064	2190	1.452879	0.011683
brown	353	SECRETION_BY_CELL	23	105	16148	2190	1.625653	0.011683

lightcyan	3375	chr2p25	2	43	16210	62	12.1928	0.011693
lightcyan	7222	CROMER_TUMORIGENESIS_UP	2	43	16210	62	12.1928	0.011693
lightcyan	7452	MILI_PSEUDOPODIA	2	43	16210	62	12.1928	0.011693
brown	2080	CCTGCTG,MIR-214	42	220	16033	2190	1.416824	0.011693
purple	8052	PEDERSEN_METASTASIS_BY_ERBB2_ISOFORM_4	4	91	16162	157	4.55043	0.011696
brown	2876	KEGG_EPITHELIAL_CELL_SIGNALING_IN_HELICOBACTER	15	60	16193	2190	1.855365	0.011708
blue	9867	GSE360_L_DONOVANI_VS_B_MALAYI_LOW_DOSE_MA	46	182	16071	2978	1.379416	0.011713
blue	9999	GSE3982_MAST_CELL_VS_BCELL_DN	46	182	16071	2978	1.379416	0.011713
turquoise	3017	BIOCARTA_MAPK_PATHWAY	32	86	16167	4169	1.450618	0.011713
green	217	COLLAGEN	5	18	16235	1305	3.459557	0.011714
green	284	NEGATIVE_REGULATION_OF_DNA_METABOLIC_PROCE	5	18	16235	1305	3.459557	0.011714
green	1390	TRANSFERASE_ACTIVITY_TRANSFERRING_PENTOSYL_GI	5	18	16235	1305	3.459557	0.011714
green	3102	BIOCARTA_CTLA4_PATHWAY	5	18	16235	1305	3.459557	0.011714
green	3882	MODULE_489	5	18	16235	1305	3.459557	0.011714
green	4834	DAVICIONI_PAX_FOXO1_SIGNATURE_IN_ARMS_DN	5	18	16235	1305	3.459557	0.011714
green	6682	BANDRES_RESPONSE_TO_CARMUSTIN_WITHOUT_MGI	5	18	16235	1305	3.459557	0.011714
brown	3035	BIOCARTA_P38MAPK_PATHWAY	11	39	16214	2190	2.093233	0.011724
brown	7916	WIERENGA_PML_INTERACTOME	11	39	16214	2190	2.093233	0.011724
purple	5099	KIM_WT1_TARGETS_UP	6	195	16058	157	3.185301	0.011725
purple	6921	KONDO_EZH2_TARGETS	6	195	16058	157	3.185301	0.011725
purple	8686	GSE1460_INTRATHYMIC_T_PROGENITOR_VS_CD4_THY	6	195	16058	157	3.185301	0.011725
blue	3161	chr12q24	49	196	16057	2978	1.364422	0.011736
blue	9236	GSE18791_CTRL_VS_NEWCASTLE_VIRUS_DC_18H_UP	49	196	16057	2978	1.364422	0.011736
greenyello	10217	GSE8515_CTRL_VS_IL6_4H_STIM_MAC_DN	5	147	16106	150	3.685488	0.011746
cyan	8817	GSE16522_MEMORY_VS_NAIVE_ANTI_CD3CD28_STIM_	4	186	16067	77	4.53931	0.011748
greenyello	802	ESTABLISHMENT_AND_OR_MAINTENANCE_OF_CELL_P	2	18	16235	150	12.03926	0.01175
greenyello	7165	CLIMENT_BREAST_CANCER_COPY_NUMBER_UP	2	18	16235	150	12.03926	0.01175
greenyello	7774	ZEMBUTSU_SENSITIVITY_TO_CISPLATIN	2	18	16235	150	12.03926	0.01175
brown	2926	BIOCARTA_BCR_PATHWAY	10	34	16219	2190	2.182783	0.011755
purple	2877	KEGG_PATHOGENIC_ESCHERICHIA_COLI_INFECTION	3	49	16204	157	6.3381	0.011774
purple	3754	MODULE_297	3	49	16204	157	6.3381	0.011774
purple	3801	MODULE_357	3	49	16204	157	6.3381	0.011774
purple	7873	DEMAGALHAES_AGING_UP	3	49	16204	157	6.3381	0.011774
yellow	1240	N_METHYLTRANSFERASE_ACTIVITY	4	11	16242	1426	4.144588	0.011779
yellow	4775	REACTOME_RESOLUTION_OF_AP_SITES_VIA_THE_SINC	4	11	16242	1426	4.144588	0.011779
pink	6401	LEE_AGING_NEOCORTEX_DN	4	63	16190	228	4.526037	0.01178
pink	7129	MATZUK_SPERMATOCYTE	4	63	16190	228	4.526037	0.01178
pink	7980	DELPUECH_FOXO3_TARGETS_UP	4	63	16190	228	4.526037	0.01178
pink	6340	HOEGERKORP_CD44_TARGETS_DIRECT_DN	2	12	16241	228	11.88085	0.011787
brown	6947	COATES_MACROPHAGE_M1_VS_M2_DN	17	71	16182	2190	1.77697	0.011797
brown	7200	HUANG_DASATINIB_RESISTANCE_UP	17	71	16182	2190	1.77697	0.011797
red	6139	UEDA_PERIFERAL_CLOCK	11	155	16098	524	2.201219	0.011807
red	9723	GSE30962_PRIMARY_VS_SECONDARY_ACUTE_LCMV_II	12	176	16077	524	2.114807	0.011818
red	9965	GSE3982_DC_VS_MAC_LPS_STIM_DN	12	176	16077	524	2.114807	0.011818
yellow	9209	GSE17974_2H_VS_72H_UNTREATED_IN_VITRO_CD4_T	27	195	16058	1426	1.578131	0.011819
yellow	9551	GSE27786_LIN_NEG_VS_CD8_TCELL_DN	27	195	16058	1426	1.578131	0.011819
lightcyan	7689	DANG_REGULATED_BY_MYC_DN	4	232	16021	62	4.519744	0.011827
red	2680	GNF2_PAK2	4	28	16225	524	4.431025	0.011833
red	4649	REACTOME_ION_TRANSPORT_BY_P_TYPE_ATPASES	4	28	16225	524	4.431025	0.011833
red	5274	WANG_BARRETTS_ESOPHAGUS_AND_ESOPHAGUS_CA	4	28	16225	524	4.431025	0.011833
red	7113	WILENSKY_RESPONSE_TO_DARAPLADIB	4	28	16225	524	4.431025	0.011833
pink	3781	MODULE_329	3	34	16219	228	6.289861	0.011836
salmon	2310	MORF_HAT1	5	174	16079	127	3.677482	0.011837
salmon	10119	GSE39820_TGFBETA1_VS_TGFBETA3_IN_IL6_TREATED_	5	174	16079	127	3.677482	0.011837
salmon	10233	GSE9006_TYPE_1_DIABETES_AT_DX_VS_1MONTH_POS	5	174	16079	127	3.677482	0.011837
yellow	8487	GSE11924_TH1_VS_TH2_CD4_TCELL_DN	25	177	16076	1426	1.609833	0.011851
yellow	9387	GSE22886_IL2_VS_IL15_STIM_NKCELL_DN	25	177	16076	1426	1.609833	0.011851
yellow	9102	GSE17721_0.5H_VS_12H_POLYIC_BMDM_UP	26	186	16067	1426	1.593215	0.011853
lightgreen	5070	SENESE_HDAC1_AND_HDAC2_TARGETS_DN	3	184	16069	42	6.309394	0.011855
lightgreen	8434	GSE10325_CD4_TCELL_VS_LUPUS_CD4_TCELL_UP	3	184	16069	42	6.309394	0.011855

lightgreen	9958	GSE3982_MEMORY_CD4_TCELL_VS_TH1_UP	3	184	16069	42	6.309394	0.011855
lightgreen	10158	GSE7460_CD8_TCELL_VS_TREG_ACT_UP	3	184	16069	42	6.309394	0.011855
lightgreen	6338	CHIARETTI_ACUTE_LYMPHOBLASTIC_LEUKEMIA_ZAP70	2	64	16189	42	12.09301	0.011876
pink	3553	MODULE_64	12	404	15849	228	2.117379	0.011893
blue	2504	GCM_CBFB	21	70	16183	2978	1.637307	0.011899
salmon	4915	DIAZ_CHRONIC_MEYLOGENOUS_LEUKEMIA_DN	4	113	16140	127	4.530137	0.011901
brown	1063	HYDROLASE_ACTIVITY_ACTING_ON_ACID_ANHYDRIDES	41	214	16039	2190	1.421869	0.011901
magenta	8518	GSE12845_NAIVE_VS_PRE_GC_TONSIL_BCELL_UP	6	184	16069	167	3.173588	0.011928
magenta	9465	GSE25087_FETAL_VS_ADULT_TREG_DN	6	184	16069	167	3.173588	0.011928
purple	5715	SHETH_LIVER_CANCER_VS_TXNIP_LOSS_PAM2	5	141	16112	157	3.671003	0.011931
purple	7523	CHIANG_LIVER_CANCER_SUBCLASS_CTNNB1_DN	5	141	16112	157	3.671003	0.011931
grey60	1735	V$TBP_01	3	176	16077	44	6.296358	0.011942
greenyello	1513	V$IK2_01	6	205	16048	150	3.171317	0.011956
greenyello	1880	V$TTF1_Q6	6	205	16048	150	3.171317	0.011956
brown	9066	GSE17721_POLYIC_VS_GARDIQUIMOD_8H_BMDM_UP	35	177	16076	2190	1.467521	0.01196
brown	9296	GSE20715_0H_VS_24H_OZONE_TLR4_KO_LUNG_UP	35	177	16076	2190	1.467521	0.01196
brown	9956	GSE3982_CTRL_VS_LPS_48H_DC_UP	35	177	16076	2190	1.467521	0.01196
cyan	8563	GSE13484_UNSTIM_VS_3H_YF17D_VACCINE_STIM_PB	4	187	16066	77	4.515036	0.011963
cyan	4926	CHARAFE_BREAST_CANCER_LUMINAL_VS_BASAL_DN	6	402	15851	77	3.150417	0.011963
red	7015	ENGELMANN_CANCER_PROGENITORS_UP	5	43	16210	524	3.606648	0.011986
red	7109	ZHENG_IL22_SIGNALING_UP	5	43	16210	524	3.606648	0.011986
greenyello	2276	GGGTGGRR_V$PAX4_03	18	1093	15160	150	1.78441	0.011987
magenta	3505	MODULE_13	10	422	15831	167	2.306241	0.011987
blue	1122	LIGASE_ACTIVITY	27	96	16157	2978	1.534975	0.01199
green	2642	GNF2_FGR	7	32	16221	1305	2.724401	0.011993
green	7694	DORN_ADENOVIRUS_INFECTION_12HR_DN	7	32	16221	1305	2.724401	0.011993
lightyellow	4944	BORCZUK_MALIGNANT_MESOTHELIOMA_DN	2	82	16171	33	12.01256	0.011997
lightyellow	6694	KAYO_CALORIE_RESTRICTION_MUSCLE_UP	2	82	16171	33	12.01256	0.011997
lightyellow	7807	SASSON_RESPONSE_TO_GONADOTROPHINS_DN	2	82	16171	33	12.01256	0.011997
salmon	61	NUCLEAR_PART	10	557	15696	127	2.297601	0.012023
purple	1044	REGULATION_OF_CELL_PROLIFERATION	7	255	15998	157	2.841788	0.012028
lightgreen	9485	GSE26669_CD4_VS_CD8_TCELL_IN_MLR_COSTIM_BLO	3	185	16068	42	6.27529	0.012029
brown	2822	KEGG_VASCULAR_SMOOTH_MUSCLE_CONTRACTION	20	88	16165	2190	1.686696	0.012032
brown	3391	chr20q11	20	88	16165	2190	1.686696	0.012032
brown	3438	chr14q24	20	88	16165	2190	1.686696	0.012032
turquoise	382	CENTROSOME_ORGANIZATION_AND_BIOGENESIS	8	14	16239	4169	2.227735	0.012088
turquoise	1314	ACETYLGLUCOSAMINYLTRANSFERASE_ACTIVITY	8	14	16239	4169	2.227735	0.012088
turquoise	3274	chr11p14	8	14	16239	4169	2.227735	0.012088
turquoise	4052	PID_TCRJNKPATHWAY	8	14	16239	4169	2.227735	0.012088
turquoise	6498	VISALA_RESPONSE_TO_HEAT_SHOCK_AND_AGING_UP	8	14	16239	4169	2.227735	0.012088
red	9640	GSE29615_CTRL_VS_DAY7_LAIV_FLU_VACCINE_PBMC_	10	135	16118	524	2.297569	0.012093
red	6881	RIGGINS_TAMOXIFEN_RESISTANCE_DN	13	198	16055	524	2.036481	0.012095
red	10072	GSE3982_BASOPHIL_VS_EFF_MEMORY_CD4_TCELL_UF	13	198	16055	524	2.036481	0.012095
turquoise	305	REGULATION_OF_PROTEIN_KINASE_ACTIVITY	48	139	16114	4169	1.346257	0.012095
green	8160	ACEVEDO_FGFR1_TARGETS_IN_PROSTATE_CANCER_M	29	233	16020	1305	1.550119	0.012095
brown	2071	GGCAGCT,MIR-22	40	208	16045	2190	1.427204	0.012105
salmon	5152	HAHTOLA_MYCOSIS_FUNGOIDES_SKIN_UP	5	175	16078	127	3.656468	0.012111
salmon	9794	GSE360_CTRL_VS_L_MAJOR_DC_UP	5	175	16078	127	3.656468	0.012111
magenta	7266	BOYLAN_MULTIPLE_MYELOMA_C_D_DN	7	240	16013	167	2.838598	0.012112
lightyellow	4142	REACTOME_SYNTHESIS_OF_BILE_ACIDS_AND_BILE_SAI	1	6	16247	33	82.08586	0.012123
blue	8505	GSE12845_IGD_POS_VS_NEG_BLOOD_BCELL_DN	47	187	16066	2978	1.371719	0.012123
blue	9768	GSE339_CD4POS_VS_CD8POS_DC_UP	47	187	16066	2978	1.371719	0.012123
blue	9886	GSE360_HIGH_DOSE_B_MALAYI_VS_M_TUBERCULOSIS	47	187	16066	2978	1.371719	0.012123
turquoise	791	ENDOSOME_TRANSPORT	11	22	16231	4169	1.949268	0.01213
turquoise	4554	REACTOME_TAK1_ACTIVATES_NFKB_BY_PHOSPHORYL	11	22	16231	4169	1.949268	0.01213
turquoise	6483	VIETOR_IFRD1_TARGETS	11	22	16231	4169	1.949268	0.01213
purple	8377	KRAS.LUNG.BREAST_UP.V1_UP	4	92	16161	157	4.500969	0.012138
red	2248	RTAAACA_V$FREAC2_01	35	728	15525	524	1.49121	0.012148
turquoise	2202	TNCATNTCCYR_UNKNOWN	42	119	16134	4169	1.375954	0.012163
cyan	8522	GSE12845_PRE_GC_VS_DARKZONE_GC_TONSIL_BCELL_	4	188	16065	77	4.49102	0.01218

cyan	8994	GSE17721_LPS_VS_PAM3CSK4_8H_BMDM_UP	4	188	16065	77	4.49102	0.01218
cyan	9000	GSE17721_LPS_VS_PAM3CSK4_24H_BMDM_UP	4	188	16065	77	4.49102	0.01218
cyan	9770	GSE339_CD4POS_VS_CD4CD8DN_DC_UP	4	188	16065	77	4.49102	0.01218
lightgreen	8024	GOBERT_OLIGODENDROCYTE_DIFFERENTIATION_DN	7	983	15270	42	2.75568	0.012193
purple	5183	DELYS_THYROID_CANCER_UP	9	383	15870	157	2.432639	0.012193
pink	9742	GSE32423_MEMORY_VS_NAIVE_CD8_TCELL_UP	7	176	16077	228	2.835202	0.012195
green	9869	GSE360_L_DONOVANI_VS_M_TUBERCULOSIS_MAC_DN	23	174	16079	1305	1.646272	0.012204
grey60	6917	KONDO_PROSTATE_CANCER_HCP_WITH_H3K27ME3	2	62	16191	44	11.91569	0.01222
grey60	7550	WOO_LIVER_CANCER_RECURRENCE_DN	2	62	16191	44	11.91569	0.01222
lightcyan	6664	BURTON_ADIPOGENESIS_7	2	44	16209	62	11.91569	0.01222
turquoise	2319	MORF_PCNA	31	83	16170	4169	1.45608	0.012221
cyan	6689	BROWNE_HCMV_INFECTION_12HR_UP	3	101	16152	77	6.269641	0.012222
magenta	6633	RODWELL_AGING_KIDNEY_NO_BLOOD_UP	6	185	16068	167	3.156433	0.012226
magenta	8506	GSE12845_IGD_POS_BLOOD_VS_NAIVE_TONSIL_BCELL	6	185	16068	167	3.156433	0.012226
magenta	8734	GSE15324_NAIVE_VS_ACTIVATED_CD8_TCELL_UP	6	185	16068	167	3.156433	0.012226
magenta	10225	GSE9006_HEALTHY_VS_TYPE_1_DIABETES_PBMC_AT_[	6	185	16068	167	3.156433	0.012226
lightgreen	5930	AMIT_EGF_RESPONSE_120_HELA	2	65	16188	42	11.90696	0.012233
lightgreen	7705	NAKAYAMA_SOFT_TISSUE_TUMORS_PCA1_UP	2	65	16188	42	11.90696	0.012233
blue	2338	MORF_SOD1	66	277	15976	2978	1.300388	0.012235
cyan	3553	MODULE_64	6	404	15849	77	3.134821	0.012236
tan	1339	EXONUCLEASE_ACTIVITY	2	19	16234	145	11.79891	0.012237
tan	7845	STAMBOLSKY_RESPONSE_TO_VITAMIN_D3_DN	2	19	16234	145	11.79891	0.012237
greenyello	7849	KIM_BIPOLAR_DISORDER_OLIGODENDROCYTE_DENSIT	3	52	16201	150	6.251154	0.012241
salmon	4699	REACTOME_HIV_LIFE_CYCLE	4	114	16139	127	4.490399	0.012261
green	8584	GSE13485_DAY1_VS_DAY7_YF17D_VACCINE_PBMC_UI	14	90	16163	1305	1.937352	0.012262
brown	8594	GSE13485_PRE_VS_POST_YF17D_VACCINATION_PBMC	32	159	16094	2190	1.493627	0.012265
blue	2730	KEGG_PURINE_METABOLISM	37	141	16112	2978	1.43216	0.01227
blue	8222	MTOR_UP.V1_UP	37	141	16112	2978	1.43216	0.01227
lightyellow	7809	SASSON_RESPONSE_TO_FORSKOLIN_DN	2	83	16170	33	11.86783	0.012278
red	3632	MODULE_147	8	96	16157	524	2.584765	0.012309
grey60	9972	GSE3982_EOSINOPHIL_VS_DC_UP	3	178	16075	44	6.225613	0.012311
grey60	9986	GSE3982_EOSINOPHIL_VS_NKCELL_UP	3	178	16075	44	6.225613	0.012311
red	9291	GSE20715_0H_VS_24H_OZONE_LUNG_DN	12	177	16076	524	2.102859	0.012321
red	9503	GSE2706_UNSTIM_VS_2H_LPS_DC_DN	12	177	16076	524	2.102859	0.012321
red	9990	GSE3982_EOSINOPHIL_VS_TH2_UP	12	177	16076	524	2.102859	0.012321
turquoise	8259	MEK_UP.V1_DN	58	173	16080	4169	1.307024	0.012337
lightcyan	6304	LENAOUR_DENDRITIC_CELL_MATURATION_DN	3	126	16127	62	6.241551	0.012339
red	4588	REACTOME_FATTY_ACID_TRIACYLGLYCEROL_AND_KET(	11	156	16097	524	2.187109	0.012348
red	8767	GSE15750_WT_VS_TRAF6KO_DAY6_EFF_CD8_TCELL_D	11	156	16097	524	2.187109	0.012348
greenyello	809	ANATOMICAL_STRUCTURE_DEVELOPMENT	13	696	15557	150	2.023841	0.01235
tan	9194	GSE17974_IL4_AND_ANTI_IL12_VS_UNTREATED_6H_A	5	154	16099	145	3.639275	0.012353
blue	10003	GSE3982_MAST_CELL_VS_EFF_MEMORY_CD4_TCELL_[	42	164	16089	2978	1.397701	0.01236
magenta	1623	V$NKX25_01	4	87	16166	167	4.474637	0.012367
magenta	6294	ZHAN_MULTIPLE_MYELOMA_CD1_AND_CD2_UP	4	87	16166	167	4.474637	0.012367
yellow	173	MITOCHONDRIAL_RESPIRATORY_CHAIN	6	23	16230	1426	2.973291	0.012376
turquoise	9175	GSE17974_CTRL_VS_ACT_IL4_AND_ANTI_IL12_4H_CD4	65	197	16056	4169	1.286319	0.012379
lightgreen	7943	MIYAGAWA_TARGETS_OF_EWSR1_ETS_FUSIONS_DN	3	187	16066	42	6.208174	0.012382
blue	3948	PID_TCR_PATHWAY	20	66	16187	2978	1.653845	0.01239
black	5384	CHASSOT_SKIN_WOUND	2	10	16243	283	11.48622	0.012397
cyan	9211	GSE17974_0.5H_VS_72H_IL4_AND_ANTI_IL12_ACT_CD	4	189	16064	77	4.467258	0.012399
green	5307	HAMAI_APOPTOSIS_VIA_TRAIL_DN	21	155	16098	1305	1.687371	0.012422
purple	2855	KEGG_LONG_TERM_DEPRESSION	3	50	16203	157	6.211338	0.012439
purple	6588	VERRECCHIA_EARLY_RESPONSE_TO_TGFB1	3	50	16203	157	6.211338	0.012439
greenyello	6047	CERVERA_SDHB_TARGETS_1_UP	4	97	16156	150	4.468179	0.012451
yellow	5232	CONCANNON_APOPTOSIS_BY_EPOXOMICIN_DN	22	151	16102	1426	1.66058	0.012455
black	9146	GSE17721_4_VS_24H_GARDIQUIMOD_BMDM_UP	8	177	16076	283	2.595756	0.012469
brown	44	CONTRACTILE_FIBER	7	20	16233	2190	2.597511	0.012483
brown	3052	BIOCARTA_NKCELLS_PATHWAY	7	20	16233	2190	2.597511	0.012483
brown	4058	PID_P38_MK2PATHWAY	7	20	16233	2190	2.597511	0.012483
brown	6234	HASLINGER_B_CLL_WITH_17P13_DELETION	7	20	16233	2190	2.597511	0.012483

brown	7751	MOOTHA_GLYCOLYSIS	7	20	16233	2190	2.597511	0.012483
cyan	3218	chr6p21	5	292	15961	77	3.614348	0.012485
green	413	ANATOMICAL_STRUCTURE_MORPHOGENESIS	33	274	15979	1305	1.499983	0.012497
blue	9884	GSE360_HIGH_VS_LOW_DOSE_B_MALAYI_MAC_UP	45	178	16075	2978	1.379753	0.012497
greenyello	2286	TTCYNRGAA_V$STAT5B_01	7	269	15984	150	2.819603	0.012501
magenta	1699	V$FOXJ2_02	6	186	16067	167	3.139463	0.01253
turquoise	3183	chr4q21	25	64	16189	4169	1.522866	0.012539
turquoise	4712	REACTOME_APC_C_CDH1_MEDIATED_DEGRADATION_	25	64	16189	4169	1.522866	0.012539
turquoise	5225	VANHARANTA_UTERINE_FIBROID_WITH_7Q_DELETION	25	64	16189	4169	1.522866	0.012539
lightgreen	9419	GSE24142_DN2_VS_DN3_THYMOCYTE_ADULT_DN	3	188	16065	42	6.175152	0.01256
midnightb	6060	ZUCCHI_METASTASIS_DN	2	39	16214	71	11.73926	0.012561
midnightb	7101	EHLERS_ANEUPLOIDY_UP	2	39	16214	71	11.73926	0.012561
turquoise	7092	TOYOTA_TARGETS_OF_MIR34B_AND_MIR34C	135	444	15809	4169	1.185366	0.012568
purple	6955	MARTINEZ_TP53_TARGETS_UP	11	522	15731	157	2.181504	0.012573
cyan	2716	GNF2_VAV1	2	36	16217	77	11.72655	0.012577
purple	5198	CHEBOTAEV_GR_TARGETS_DN	4	93	16160	157	4.452572	0.01259
purple	6665	KEEN_RESPONSE_TO_ROSIGLITAZONE_DN	4	93	16160	157	4.452572	0.01259
lightgreen	6535	MAHAJAN_RESPONSE_TO_IL1A_DN	2	66	16187	42	11.72655	0.012595
turquoise	10292	GSE9988_ANTI_TREM1_AND_LPS_VS_CTRL_TREATED_I	60	180	16073	4169	1.299512	0.012601
turquoise	10294	GSE9988_ANTI_TREM1_AND_LPS_VS_VEHICLE_TREATE	60	180	16073	4169	1.299512	0.012601
purple	1847	V$HFH4_01	5	143	16110	157	3.619661	0.012621
purple	8304	NOTCH_DN.V1_UP	5	143	16110	157	3.619661	0.012621
cyan	4754	REACTOME_ANTIGEN_PROCESSING_UBIQUITINATION_	4	190	16063	77	4.443746	0.012621
cyan	10054	GSE3982_NEUTROPHIL_VS_NKCELL_UP	4	190	16063	77	4.443746	0.012621
magenta	7145	BOQUEST_STEM_CELL_CULTURED_VS_FRESH_UP	9	362	15891	167	2.419641	0.012629
brown	7848	KIM_BIPOLAR_DISORDER_OLIGODENDROCYTE_DENSIT	107	646	15607	2190	1.229251	0.01263
salmon	4299	REACTOME_MICRORNA_MIRNA_BIOGENESIS	2	22	16231	127	11.63422	0.012634
yellow	9500	GSE2706_UNSTIM_VS_8H_R848_DC_UP	25	178	16075	1426	1.600789	0.012675
brown	9105	GSE17721_0.5H_VS_4H_POLYIC_BMDM_DN	37	190	16063	2190	1.445232	0.012677
brown	9474	GSE26495_NAIVE_VS_PD1LOW_CD8_TCELL_UP	37	190	16063	2190	1.445232	0.012677
brown	10019	GSE3982_DC_VS_BASOPHIL_DN	37	190	16063	2190	1.445232	0.012677
black	6934	HELLER_HDAC_TARGETS_UP	11	289	15964	283	2.185959	0.012693
greenyello	2258	CTTTGA_V$LEF1_Q2	16	936	15317	150	1.852194	0.012723
greenyello	3553	MODULE_64	9	404	15849	150	2.413812	0.012743
yellow	7598	KESHELAVA_MULTIPLE_DRUG_RESISTANCE	14	83	16170	1426	1.922489	0.012747
green	3518	MODULE_26	22	165	16088	1305	1.660587	0.012755
green	4489	REACTOME_G_ALPHA_I_SIGNALLING_EVENTS	18	127	16126	1305	1.765191	0.012769
greenyello	1800	V$MTF1_Q4	6	208	16045	150	3.125577	0.012772
blue	2355	MORF_BMI1	23	79	16174	2978	1.588948	0.012772
blue	4720	REACTOME_REGULATION_OF_GLUCOKINASE_BY_GLUC	10	26	16227	2978	2.099111	0.01278
blue	6883	STEIN_ESRRA_TARGETS_RESPONSIVE_TO_ESTROGEN_U	10	26	16227	2978	2.099111	0.01278
turquoise	5278	MISSIAGLIA_REGULATED_BY_METHYLATION_DN	41	116	16137	4169	1.377931	0.012781
red	6630	LEE_AGING_CEREBELLUM_UP	7	78	16175	524	2.783593	0.012799
turquoise	1472	AACWWCAANK_UNKNOWN	44	126	16127	4169	1.361394	0.01281
pink	4410	REACTOME_G1_PHASE	3	35	16218	228	6.11015	0.012814
pink	5669	CEBALLOS_TARGETS_OF_TP53_AND_MYC_DN	3	35	16218	228	6.11015	0.012814
purple	3548	MODULE_59	2	18	16235	157	11.50248	0.012818
purple	7387	HOFMANN_MYELODYSPLASTIC_SYNDROM_HIGH_RISK_	2	18	16235	157	11.50248	0.012818
pink	8263	PRC1_BMI_UP.V1_DN	6	137	16116	228	3.121975	0.012818
magenta	2117	TCATCTC,MIR-143	5	135	16118	167	3.604569	0.012828
turquoise	6144	SHEPARD_BMYB_MORPHOLINO_UP	62	187	16066	4169	1.292563	0.012832
turquoise	10081	GSE3982_BASOPHIL_VS_TH2_DN	62	187	16066	4169	1.292563	0.012832
magenta	1881	V$OCT_Q6	6	187	16066	167	3.122674	0.012838
magenta	1015	SYSTEM_PROCESS	9	363	15890	167	2.412976	0.012839
red	2020	ATACTGT,MIR-144	12	178	16075	524	2.091046	0.012839
red	8442	GSE10856_CTRL_VS_TNFRSF6B_IN_MACROPHAGE_UP	12	178	16075	524	2.091046	0.012839
red	8855	GSE17721_CTRL_VS_POLYIC_4H_BMDM_DN	12	178	16075	524	2.091046	0.012839
red	9972	GSE3982_EOSINOPHIL_VS_DC_UP	12	178	16075	524	2.091046	0.012839
cyan	8725	GSE14769_UNSTIM_VS_240MIN_LPS_BMDM_DN	4	191	16062	77	4.42048	0.012846
lightyellow	3417	chr3q21	2	85	16168	33	11.58859	0.012848

brown	9792	GSE360_CTRL_VS_L_DONOVANI_DC_UP	36	184	16069	2190	1.452025	0.01285
brown	9957	GSE3982_CTRL_VS_LPS_48H_DC_DN	36	184	16069	2190	1.452025	0.01285
blue	10092	GSE3982_CENT_MEMORY_CD4_TCELL_VS_TH1_UP	43	169	16084	2978	1.388643	0.012854
lightgreen	3395	chr6q26	1	5	16248	42	77.39524	0.012856
lightgreen	7173	YAGUE_PRETUMOR_DRUG_RESISTANCE_UP	1	5	16248	42	77.39524	0.012856
grey60	9456	GSE24634_IL4_VS_CTRL_TREATED_NAIVE_CD4_TCELL_	3	181	16072	44	6.122426	0.012875
greenyello	2135	GTGTCAA,MIR-514	3	53	16200	150	6.133208	0.012889
greenyello	3140	chr4p16	4	98	16155	150	4.422585	0.012889
magenta	330	T_CELL_PROLIFERATION	2	17	16236	167	11.44981	0.012898
magenta	1465	V$EVI1_06	2	17	16236	167	11.44981	0.012898
magenta	5287	SILIGAN_TARGETS_OF_EWS_FLI1_FUSION_DN	2	17	16236	167	11.44981	0.012898
magenta	6251	HASLINGER_B_CLL_WITH_MUTATED_VH_GENES	2	17	16236	167	11.44981	0.012898
magenta	6277	ZHAN_MULTIPLE_MYELOMA_SPIKED	2	17	16236	167	11.44981	0.012898
turquoise	3775	MODULE_323	24	61	16192	4169	1.533851	0.012939
salmon	8611	GSE14000_TRANSLATED_RNA_VS_MRNA_4H_LPS_DC_	5	178	16075	127	3.594842	0.012959
salmon	8879	GSE17721_CTRL_VS_PAM3CSK4_24H_BMDM_DN	5	178	16075	127	3.594842	0.012959
salmon	9036	GSE17721_PAM3CSK4_VS_GADIQUIMOD_24H_BMDM_	5	178	16075	127	3.594842	0.012959
salmon	9817	GSE360_DC_VS_MAC_DN	5	178	16075	127	3.594842	0.012959
lightgreen	3911	MODULE_544	2	67	16186	42	11.55153	0.012962
grey60	3564	MODULE_76	2	64	16189	44	11.54332	0.012983
grey60	6844	LEIN_OLIGODENDROCYTE_MARKERS	2	64	16189	44	11.54332	0.012983
salmon	2304	MORF_DNMT1	4	116	16137	127	4.412979	0.013001
brown	8873	GSE17721_CTRL_VS_PAM3CSK4_6H_BMDM_DN	35	178	16075	2190	1.459276	0.013012
brown	9036	GSE17721_PAM3CSK4_VS_GADIQUIMOD_24H_BMDM_	35	178	16075	2190	1.459276	0.013012
brown	9972	GSE3982_EOSINOPHIL_VS_DC_UP	35	178	16075	2190	1.459276	0.013012
green	7461	CROONQUIST_STROMAL_STIMULATION_UP	9	48	16205	1305	2.335201	0.013024
turquoise	1235	PROTEIN_SERINE_THREONINE_KINASE_ACTIVITY	64	194	16059	4169	1.286115	0.013035
turquoise	9207	GSE17974_1H_VS_72H_UNTREATED_IN_VITRO_CD4_T	64	194	16059	4169	1.286115	0.013035
turquoise	9929	GSE37416_CTRL_VS_12H_F_TULARENSIS_LVS_NEUTRO	64	194	16059	4169	1.286115	0.013035
turquoise	9966	GSE3982_DC_VS_NEUTROPHIL_LPS_STIM_UP	64	194	16059	4169	1.286115	0.013035
purple	2896	KEGG_SYSTEMIC_LUPUS_ERYTHEMATOSUS	4	94	16159	157	4.405204	0.013053
purple	8333	PDGF_UP.V1_DN	4	94	16159	157	4.405204	0.013053
greenyello	3456	SA_CASPASE_CASCADE	2	19	16234	150	11.40561	0.013054
greenyello	5421	BREUHAHN_GROWTH_FACTOR_SIGNALING_IN_LIVER_	2	19	16234	150	11.40561	0.013054
greenyello	1774	V$RP58_01	5	151	16102	150	3.587859	0.013073
cyan	8821	GSE16522_ANTI_CD3CD28_STIM_VS_UNSTIM_NAIVE_	4	192	16061	77	4.397457	0.013073
blue	6064	ASTON_MAJOR_DEPRESSIVE_DISORDER_DN	36	137	16116	2978	1.434137	0.013076
grey60	5908	BENPORATH_PRC2_TARGETS	4	339	15914	44	4.358541	0.013099
pink	3627	MODULE_139	4	65	16188	228	4.386775	0.013104
pink	4427	REACTOME_METABOLISM_OF_NUCLEOTIDES	4	65	16188	228	4.386775	0.013104
lightgreen	8719	GSE14769_UNSTIM_VS_60MIN_LPS_BMDM_DN	3	191	16062	42	6.07816	0.013104
lightgreen	8831	GSE17580_UNINFECTED_VS_S_MANSONI_INF_TREG_D	3	191	16062	42	6.07816	0.013104
lightgreen	9721	GSE30083_SP3_VS_SP4_THYMOCYTE_DN	3	191	16062	42	6.07816	0.013104
lightgreen	10172	GSE7460_FOXP3_MUT_VS_WT_ACT_TCONV_UP	3	191	16062	42	6.07816	0.013104
lightgreen	10207	GSE7852_LN_VS_FAT_TCONV_DN	3	191	16062	42	6.07816	0.013104
purple	5185	CHIARADONNA_NEOPLASTIC_TRANSFORMATION_KRAS	3	51	16202	157	6.089547	0.013124
purple	5943	AMIT_SERUM_RESPONSE_240_MCF10A	3	51	16202	157	6.089547	0.013124
purple	6494	RAMASWAMY_METASTASIS_DN	3	51	16202	157	6.089547	0.013124
lightyellow	5120	GRAHAM_CML_QUIESCENT_VS_NORMAL_QUIESCENT_	2	86	16167	33	11.45384	0.013137
lightyellow	8371	KRAS.BREAST_UP.V1_UP	2	86	16167	33	11.45384	0.013137
magenta	6253	ZHAN_MULTIPLE_MYELOMA_CD1_AND_CD2_DN	3	48	16205	167	6.08271	0.01314
brown	5302	DUNNE_TARGETS_OF_AML1_MTG8_FUSION_UP	13	50	16203	2190	1.92958	0.013144
lightcyan	5393	PEREZ_TP53_TARGETS	9	1001	15252	62	2.35695	0.013147
magenta	10180	GSE7460_WT_VS_FOXP3_HET_ACT_WITH_TGFB_TCON	6	188	16065	167	3.106064	0.013152
salmon	4599	REACTOME_ASSEMBLY_OF_THE_PRE_REPLICATIVE_CO	3	63	16190	127	6.094113	0.01316
red	6310	ZHAN_MULTIPLE_MYELOMA_CD2_UP	5	44	16209	524	3.524679	0.013174
red	7134	CHUNG_BLISTER_CYTOTOXICITY_DN	5	44	16209	524	3.524679	0.013174
red	7960	CHANGOLKAR_H2AFY_TARGETS_UP	5	44	16209	524	3.524679	0.013174
turquoise	1009	CHROMATIN_ASSEMBLY_OR_DISASSEMBLY	12	25	16228	4169	1.871298	0.01318
midnightb	5382	RIZ_ERYTHROID_DIFFERENTIATION_CCNE1	2	40	16213	71	11.44577	0.013186

midnightb	6105	FLECHNER_PBL_KIDNEY_TRANSPLANT_OK_VS_DONOR_	2	40	16213	71	11.44577	0.013186
blue	2515	GCM_DFFA	34	128	16125	2978	1.449699	0.013203
lightcyan	5507	SCHLOSSER_SERUM_RESPONSE_DN	7	667	15586	62	2.751149	0.013207
brown	1002	INTRACELLULAR_SIGNALING_CASCADE	97	580	15673	2190	1.241175	0.013212
turquoise	7896	PANGAS_TUMOR_SUPPRESSION_BY_SMAD1_AND_SM	49	143	16110	4169	1.335862	0.013223
lightyellow	356	CELL_SURFACE_RECEPTOR_LINKED_SIGNAL_TRANSDUC	4	457	15796	33	4.310855	0.013244
cyan	3559	MODULE_70	2	37	16216	77	11.40962	0.013255
lightcyan	7266	BOYLAN_MULTIPLE_MYELOMA_C_D_DN	4	240	16013	62	4.369086	0.013256
grey60	5045	TONKS_TARGETS_OF_RUNX1_RUNX1T1_FUSION_HSC_	3	183	16070	44	6.055514	0.013259
purple	4988	OSWALD_HEMATOPOIETIC_STEM_CELL_IN_COLLAGEN_	7	260	15993	157	2.787139	0.013268
turquoise	9387	GSE22886_IL2_VS_IL15_STIM_NKCELL_DN	59	177	16076	4169	1.299512	0.013271
yellow	4695	REACTOME_INFLUENZA_VIRAL_RNA_TRANSCRIPTION_	16	100	16153	1426	1.823619	0.013278
turquoise	384	MICROTUBULE_BASED_PROCESS	29	77	16176	4169	1.46828	0.013279
lightgreen	9791	GSE34205_RSV_VS_FLU_INF_INFANT_PBMC_DN	3	192	16061	42	6.046503	0.013289
green	4167	REACTOME_MEIOSIS	13	82	16171	1305	1.974479	0.013296
cyan	9339	GSE22886_IGG_IGA_MEMORY_BCELL_VS_BLOOD_PLAS	4	193	16060	77	4.374672	0.013303
cyan	9447	GSE24634_TREG_VS_TCONV_POST_DAY10_IL4_CONVE	4	193	16060	77	4.374672	0.013303
cyan	9789	GSE34205_HEALTHY_VS_FLU_INF_INFANT_PBMC_DN	4	193	16060	77	4.374672	0.013303
lightcyan	8083	PLASARI_TGFB1_SIGNALING_VIA_NFIC_10HR_UP	2	46	16207	62	11.39762	0.013304
blue	3348	chr7q36	18	58	16195	2978	1.693766	0.013309
blue	4762	REACTOME_TELOMERE_MAINTENANCE	18	58	16195	2978	1.693766	0.013309
red	4656	REACTOME_INTERFERON_SIGNALING	10	137	16116	524	2.264027	0.013309
red	5206	LINDGREN_BLADDER_CANCER_CLUSTER_2A_DN	10	137	16116	524	2.264027	0.013309
turquoise	6789	BILD_SRC_ONCOGENIC_SIGNATURE	23	58	16195	4169	1.545971	0.013325
yellow	5238	GRAESSMANN_APOPTOSIS_BY_DOXORUBICIN_DN	175	1705	14548	1426	1.169843	0.013327
brown	300	ESTABLISHMENT_OF_LOCALIZATION	120	735	15518	2190	1.211667	0.013328
lightgreen	3689	MODULE_209	2	68	16185	42	11.38165	0.013333
lightgreen	3877	MODULE_481	2	68	16185	42	11.38165	0.013333
lightgreen	6621	GEORGANTAS_HSC_MARKERS	2	68	16185	42	11.38165	0.013333
purple	8762	GSE15659_NONSUPPRESSIVE_TCELL_VS_ACTIVATED_TI	5	145	16108	157	3.569734	0.013337
pink	7501	POOLA_INVASIVE_BREAST_CANCER_DN	5	100	16153	228	3.564254	0.013338
blue	6163	TARTE_PLASMA_CELL_VS_PLASMABLAST_DN	72	307	15946	2978	1.279979	0.013346
turquoise	2091	GCAAAAA,MIR-129	54	160	16093	4169	1.315756	0.013352
blue	9432	GSE24634_NAIVE_CD4_TCELL_VS_DAY3_IL4_CONV_TR	47	188	16065	2978	1.364422	0.013363
brown	3527	MODULE_38	69	395	15858	2190	1.296407	0.013368
red	8685	GSE1460_INTRATHYMIC_T_PROGENITOR_VS_DP_THYM	12	179	16074	524	2.079364	0.013375
red	8731	GSE14769_40MIN_VS_360MIN_LPS_BMDM_DN	12	179	16074	524	2.079364	0.013375
red	9629	GSE2826_XID_VS_BTK_KO_BCELL_DN	12	179	16074	524	2.079364	0.013375
lightgreen	5393	PEREZ_TP53_TARGETS	7	1001	15252	42	2.706127	0.01338
salmon	6693	DAZARD_RESPONSE_TO_UV_SCC_UP	4	117	16136	127	4.375261	0.013382
blue	3438	chr14q24	25	88	16165	2978	1.55048	0.013386
brown	1223	KINASE_ACTIVITY	61	343	15910	2190	1.319852	0.013394
yellow	2425	MORF_NME2	22	152	16101	1426	1.649655	0.013395
red	5440	RASHI_RESPONSE_TO_IONIZING_RADIATION_2	9	117	16136	524	2.385937	0.013413
red	356	CELL_SURFACE_RECEPTOR_LINKED_SIGNAL_TRANSDUC	24	457	15796	524	1.628911	0.013426
turquoise	421	NEGATIVE_REGULATION_OF_TRANSCRIPTION_DNA_DE	40	113	16140	4169	1.380013	0.01343
turquoise	1494	AAGWWRNYGGC_UNKNOWN	40	113	16140	4169	1.380013	0.01343
turquoise	224	ENDOPLASMIC_RETICULUM_PART	33	90	16163	4169	1.429464	0.013446
turquoise	4519	REACTOME_SYNTHESIS_OF_DNA	33	90	16163	4169	1.429464	0.013446
cyan	821	TRANSCRIPTION	8	667	15586	77	2.531669	0.01346
blue	2724	KEGG_ASCORBATE_AND_ALDARATE_METABOLISM	5	9	16244	2978	3.03205	0.013464
blue	5829	CAFFAREL_RESPONSE_TO_THC_8HR_3_DN	5	9	16244	2978	3.03205	0.013464
grey60	3895	MODULE_514	1	5	16248	44	73.87727	0.013465
grey60	5321	ZIRN_TRETINOIN_RESPONSE_DN	1	5	16248	44	73.87727	0.013465
magenta	9278	GSE20366_TREG_VS_NAIVE_CD4_TCELL_UP	6	189	16064	167	3.08963	0.013471
lightgreen	1479	V$E4BP4_01	3	193	16060	42	6.015174	0.013475
lightgreen	6329	AFFAR_YY1_TARGETS_DN	3	193	16060	42	6.015174	0.013475
lightgreen	10108	GSE39820_CTRL_VS_TGFBETA3_IL6_CD4_TCELL_UP	3	193	16060	42	6.015174	0.013475
yellow	7815	BROWNE_HCMV_INFECTION_1HR_DN	26	188	16065	1426	1.576266	0.0135
green	4565	REACTOME_GPCR_LIGAND_BINDING	29	235	16018	1305	1.536927	0.013501

turquoise	9527	GSE2706_2H_VS_8H_R848_AND_LPS_STIM_DC_DN	61	184	16069	4169	1.29245	0.013514
turquoise	9578	GSE27786_CD4_TCELL_VS_NKCELL_UP	61	184	16069	4169	1.29245	0.013514
turquoise	9626	GSE2826_WT_VS_BTK_KO_BCELL_UP	61	184	16069	4169	1.29245	0.013514
turquoise	9689	GSE29618_PRE_VS_DAY7_POST_TIV_FLU_VACCINE_BC	61	184	16069	4169	1.29245	0.013514
tan	6595	PAL_PRMT5_TARGETS_DN	2	20	16233	145	11.20897	0.013518
lightyellow	8006	TORCHIA_TARGETS_OF_EWSR1_FLI1_FUSION_UP	3	247	16006	33	5.981965	0.01352
green	1751	V$BACH2_01	28	225	16028	1305	1.549882	0.013523
purple	5326	KAN_RESPONSE_TO_ARSENIC_TRIOXIDE	4	95	16158	157	4.358833	0.013527
grey60	5140	DODD_NASOPHARYNGEAL_CARCINOMA_UP	9	1439	14814	44	2.310269	0.01353
red	6451	LIAN_LIPA_TARGETS_6M	6	61	16192	524	3.05087	0.013542
salmon	8964	GSE17721_PAM3CSK4_VS_CPG_24H_BMDM_UP	5	180	16073	127	3.554899	0.013545
yellow	9628	GSE2826_XID_VS_BTK_KO_BCELL_UP	24	170	16083	1426	1.609075	0.013547
brown	2285	CCAWWNAAGG_V$SRF_Q4	17	72	16181	2190	1.752289	0.013556
magenta	4745	REACTOME_ADAPTIVE_IMMUNE_SYSTEM	11	496	15757	167	2.158381	0.013585
purple	5862	SCHAEFFER_PROSTATE_DEVELOPMENT_48HR_UP	9	390	15863	157	2.388976	0.013588
red	5038	MULLIGHAN_MLL_SIGNATURE_2_UP	22	409	15844	524	1.668406	0.0136
brown	8201	GCNP_SHH_UP_EARLY.V1_DN	29	142	16111	2190	1.515651	0.013613
brown	988	NEGATIVE_REGULATION_OF_PROGRAMMED_CELL_DE/	28	136	16117	2190	1.527948	0.013626
brown	7096	ZHANG_BREAST_CANCER_PROGENITORS_DN	28	136	16117	2190	1.527948	0.013626
brown	3934	PID_LYSOPHOSPHOLIPID_PATHWAY	15	61	16192	2190	1.824949	0.013643
red	2651	GNF2_IGF1	3	16	16237	524	5.81572	0.013643
red	3840	MODULE_416	3	16	16237	524	5.81572	0.013643
red	7949	STEGER_ADIPOGENESIS_UP	3	16	16237	524	5.81572	0.013643
blue	2770	KEGG_PROPANOATE_METABOLISM	11	30	16223	2978	2.001153	0.013651
blue	4774	REACTOME_DNA_STRAND_ELONGATION	11	30	16223	2978	2.001153	0.013651
blue	5825	CAFFAREL_RESPONSE_TO_THC_DN	11	30	16223	2978	2.001153	0.013651
tan	5833	CAFFAREL_RESPONSE_TO_THC_24HR_5_DN	3	56	16197	145	6.004803	0.013658
blue	5208	LINDGREN_BLADDER_CANCER_CLUSTER_3_DN	51	207	16046	2978	1.344648	0.013659
lightgreen	2806	KEGG_CYTOKINE_CYTOKINE_RECEPTOR_INTERACTION	3	194	16059	42	5.984168	0.013662
lightgreen	10199	GSE7852_LN_VS_THYMUS_TREG_DN	3	194	16059	42	5.984168	0.013662
pink	5126	GRAHAM_CML_DIVIDING_VS_NORMAL_QUIESCENT_U	7	180	16073	228	2.772198	0.013662
cyan	7418	CHEN_METABOLIC_SYNDROM_NETWORK	11	1094	15159	77	2.122356	0.013671
black	9172	GSE17974_CTRL_VS_ACT_IL4_AND_ANTI_IL12_2H_CD4	8	180	16073	283	2.552493	0.013686
turquoise	2464	MORF_REV3L	22	55	16198	4169	1.559415	0.013691
purple	1544	V$FOXD3_01	5	146	16107	157	3.545284	0.013706
brown	7033	ACEVEDO_NORMAL_TISSUE_ADJACENT_TO_LIVER_TUI	58	324	15929	2190	1.328533	0.013706
turquoise	8342	HOXA9_DN.V1_UP	63	191	16062	4169	1.285905	0.013726
turquoise	9580	GSE27786_CD4_TCELL_VS_NKTCELL_UP	63	191	16062	4169	1.285905	0.013726
salmon	4712	REACTOME_APC_C_CDH1_MEDIATED_DEGRADATION_	3	64	16189	127	5.998893	0.01373
salmon	6826	PELLICCIOTTA_HDAC_IN_ANTIGEN_PRESENTATION_UP	3	64	16189	127	5.998893	0.01373
green	6228	GOLDRATH_IMMUNE_MEMORY	11	65	16188	1305	2.107669	0.013738
brown	9994	GSE3982_MAST_CELL_VS_MAC_UP	37	191	16062	2190	1.437665	0.01374
purple	8078	PLASARI_TGFB1_TARGETS_10HR_DN	6	202	16051	157	3.07492	0.013758
turquoise	6218	PENG_RAPAMYCIN_RESPONSE_DN	78	243	16010	4169	1.251382	0.013766
grey60	6535	MAHAJAN_RESPONSE_TO_IL1A_DN	2	66	16187	44	11.19353	0.013767
salmon	2800	KEGG_MISMATCH_REPAIR	2	23	16230	127	11.12838	0.013767
salmon	5435	CAVARD_LIVER_CANCER_MALIGNANT_VS_BENIGN	2	23	16230	127	11.12838	0.013767
cyan	9205	GSE17974_0.5H_VS_72H_UNTREATED_IN_VITRO_CD4_	4	195	16058	77	4.329804	0.01377
greenyello	6855	GAVIN_FOXP3_TARGETS_CLUSTER_P3	5	153	16100	150	3.540959	0.013773
blue	4697	REACTOME_HIV_INFECTION	48	193	16060	2978	1.357353	0.013778
blue	8397	KAECH_DAY15_EFF_VS_MEMORY_CD8_TCELL_DN	48	193	16060	2978	1.357353	0.013778
magenta	8492	GSE12366_GC_BCELL_VS_PLASMA_CELL_UP	6	190	16063	167	3.073369	0.013795
magenta	8494	GSE12366_GC_VS_NAIVE_BCELL_UP	6	190	16063	167	3.073369	0.013795
magenta	9481	GSE26669_CTRL_VS_COSTIM_BLOCK_MLR_CD8_TCELL_	6	190	16063	167	3.073369	0.013795
purple	6890	FOSTER_TOLERANT_MACROPHAGE_DN	9	391	15862	157	2.382866	0.013797
pink	4207	REACTOME_ASSOCIATION_OF_LICENSING_FACTORS_W	2	13	16240	228	10.96694	0.013803
pink	4555	REACTOME_REGULATION_OF_AMPK_ACTIVITY_VIA_LK	2	13	16240	228	10.96694	0.013803
pink	7768	MURAKAMI_UV_RESPONSE_1HR_UP	2	13	16240	228	10.96694	0.013803
blue	8510	GSE12845_IGD_POS_BLOOD_VS_DARKZONE_GC_TONS	45	179	16074	2978	1.372045	0.013805
red	2010	ACTGCAG,MIR-17-3P	8	98	16155	524	2.532014	0.013822

midnightb	1053	REGULATION_OF_CELL_GROWTH	2	41	16212	71	11.16661	0.013823
midnightb	2643	GNF2_FOS	2	41	16212	71	11.16661	0.013823
turquoise	2076	AAGCACA,MIR-218	103	331	15922	4169	1.21314	0.013824
turquoise	294	GLYCOPROTEIN_METABOLIC_PROCESS	28	74	16179	4169	1.475122	0.013827
purple	892	FATTY_ACID_METABOLIC_PROCESS	3	52	16201	157	5.97244	0.013831
purple	5313	MCBRYAN_PUBERTAL_TGFB1_TARGETS_DN	3	52	16201	157	5.97244	0.013831
pink	4268	REACTOME_TRANSCRIPTIONAL_ACTIVITY_OF_SMAD2_	3	36	16217	228	5.940424	0.013837
pink	7155	NAKAMURA_METASTASIS_MODEL_DN	3	36	16217	228	5.940424	0.013837
salmon	9889	GSE360_LOW_DOSE_B_MALAYI_VS_M_TUBERCULOSIS	5	181	16072	127	3.535259	0.013845
brown	648	NEGATIVE_REGULATION_OF_CELLULAR_COMPONENT_	8	25	16228	2190	2.374868	0.013845
brown	4046	PID_IL3_PATHWAY	8	25	16228	2190	2.374868	0.013845
grey60	3500	MODULE_7	3	186	16067	44	5.957845	0.013848
grey60	9714	GSE30083_SP1_VS_SP4_THYMOCYTE_UP	3	186	16067	44	5.957845	0.013848
grey60	9976	GSE3982_EOSINOPHIL_VS_NEUTROPHIL_UP	3	186	16067	44	5.957845	0.013848
lightgreen	8430	GSE10325_LUPUS_CD4_TCELL_VS_LUPUS_MYELOID_U	3	195	16058	42	5.95348	0.013851
lightgreen	9372	GSE22886_NAIVE_CD8_TCELL_VS_DC_UP	3	195	16058	42	5.95348	0.013851
lightcyan	2647	GNF2_HLA-C	2	47	16206	62	11.15511	0.013862
lightcyan	6667	LIU_SMARCA4_TARGETS	2	47	16206	62	11.15511	0.013862
pink	4971	VECCHI_GASTRIC_CANCER_ADVANCED_VS_EARLY_DN	5	101	16152	228	3.528965	0.013877
magenta	6221	LEE_LIVER_CANCER_DENA_UP	3	49	16204	167	5.958573	0.013893
turquoise	9159	GSE17974_0H_VS_6H_IN_VITRO_ACT_CD4_TCELL_DN	65	198	16055	4169	1.279823	0.013908
lightgreen	7145	BOQUEST_STEM_CELL_CULTURED_VS_FRESH_UP	4	362	15891	42	4.27598	0.013917
blue	1133	RNA_SPLICING_FACTOR_ACTIVITYTRANSESTERIFICATIO	8	19	16234	2978	2.297975	0.013926
blue	5476	MYLLYKANGAS_AMPLIFICATION_HOT_SPOT_23	8	19	16234	2978	2.297975	0.013926
red	8780	GSE15930_NAIVE_VS_24H_IN_VITRO_STIM_INFAB_CD	12	180	16073	524	2.067812	0.013927
turquoise	3565	MODULE_77	13	28	16225	4169	1.810035	0.013928
turquoise	4600	REACTOME_BIOSYNTHESIS_OF_THE_N_GLYCAN_PRECU	13	28	16225	4169	1.810035	0.013928
magenta	5877	KOYAMA_SEMA3B_TARGETS_DN	9	368	15885	167	2.380191	0.013929
brown	9184	GSE17974_CTRL_VS_ACT_IL4_AND_ANTI_IL12_72H_CD	36	185	16068	2190	1.444176	0.013945
brown	9485	GSE26669_CD4_VS_CD8_TCELL_IN_MLR_COSTIM_BLO	36	185	16068	2190	1.444176	0.013945
brown	9881	GSE360_T_GONDII_VS_B_MALAYI_LOW_DOSE_MAC_D	36	185	16068	2190	1.444176	0.013945
brown	10010	GSE3982_MAST_CELL_VS_TH2_UP	36	185	16068	2190	1.444176	0.013945
brown	10246	GSE9037_CTRL_VS_LPS_4H_STIM_IRAK4_KO_BMDM_U	36	185	16068	2190	1.444176	0.013945
cyan	2927	BIOCARTA_BIOPEPTIDES_PATHWAY	2	38	16215	77	11.10936	0.013949
cyan	6755	LEE_AGING_MUSCLE_UP	2	38	16215	77	11.10936	0.013949
red	6158	BROCKE_APOPTOSIS_REVERSED_BY_IL6	10	138	16115	524	2.247621	0.01395
turquoise	5133	WAMUNYOKOLI_OVARIAN_CANCER_LMP_DN	58	174	16079	4169	1.299512	0.013978
lightgreen	4890	FULCHER_INFLAMMATORY_RESPONSE_LECTIN_VS_LPS	5	558	15695	42	3.467529	0.01398
pink	7331	YOSHIMURA_MAPK8_TARGETS_UP	22	947	15306	228	1.656042	0.014
brown	724	BIOPOLYMER_MODIFICATION	98	588	15665	2190	1.23691	0.014007
cyan	1723	V$STAT5B_01	4	196	16057	77	4.307713	0.014008
cyan	9252	GSE19825_CD24LOW_VS_IL2RA_HIGH_DAY3_EFF_CD8	4	196	16057	77	4.307713	0.014008
brown	2367	MORF_CNTN1	21	95	16158	2190	1.640534	0.014014
lightcyan	5144	RODRIGUES_THYROID_CARCINOMA_ANAPLASTIC_UP	7	675	15578	62	2.718542	0.014026
turquoise	1386	SMALL_PROTEIN_CONJUGATING_ENZYME_ACTIVITY	21	52	16201	4169	1.574409	0.014028
lightgreen	8419	GSE10239_NAIVE_VS_DAY4.5_EFF_CD8_TCELL_DN	3	196	16057	42	5.923105	0.014041
lightgreen	9902	GSE36476_CTRL_VS_TSST_ACT_16H_MEMORY_CD4_T	3	196	16057	42	5.923105	0.014041
lightgreen	10156	GSE7460_CTRL_VS_TGFB_TREATED_ACT_TREG_UP	3	196	16057	42	5.923105	0.014041
lightgreen	10262	GSE9650_EFFECTOR_VS_MEMORY_CD8_TCELL_UP	3	196	16057	42	5.923105	0.014041
tan	4909	CASORELLI_ACUTE_PROMYELOCYTIC_LEUKEMIA_DN	12	653	15600	145	2.059841	0.014042
blue	138	SPLICEOSOME	16	50	16203	2978	1.746461	0.014047
grey60	9984	GSE3982_EOSINOPHIL_VS_CENT_MEMORY_CD4_TCELL	3	187	16066	44	5.925984	0.014048
pink	8636	GSE14308_TH2_VS_NATURAL_TREG_UP	7	181	16072	228	2.756882	0.014048
blue	1736	CTCNANGTGNY_UNKNOWN	21	71	16182	2978	1.614246	0.01405
turquoise	7823	WHITFIELD_CELL_CYCLE_G2_M	67	205	16048	4169	1.274156	0.014063
turquoise	2167	GGTGTGT,MIR-329	32	87	16166	4169	1.433945	0.014068
purple	8760	GSE15659_RESTING_VS_ACTIVATED_TREG_UP	5	147	16106	157	3.521166	0.014081
red	9477	GSE26495_PD1HIGH_VS_PD1LOW_CD8_TCELL_DN	11	159	16094	524	2.145842	0.014081
tan	6148	MANALO_HYPOXIA_DN	7	285	15968	145	2.753079	0.014081
blue	263	NEGATIVE_REGULATION_OF_CELLULAR_METABOLIC_P	56	231	16022	2978	1.323076	0.014092

brown	3611	MODULE_123	40	210	16043	2190	1.413612	0.014112
magenta	10194	GSE7852_TREG_VS_TCONV_THYMUS_UP	6	191	16062	167	3.057278	0.014125
lightyellow	1246	CARBOXYPEPTIDASE_ACTIVITY	1	7	16246	33	70.35931	0.014129
lightyellow	2700	GNF2_SPINK1	1	7	16246	33	70.35931	0.014129
lightyellow	5043	TONKS_TARGETS_OF_RUNX1_RUNX1T1_FUSION_SUST/	1	7	16246	33	70.35931	0.014129
greenyello	10077	GSE3982_BASOPHIL_VS_NKCELL_DN	5	154	16099	150	3.517965	0.014132
green	850	POSITIVE_REGULATION_OF_CELL_MIGRATION	3	7	16246	1305	5.337603	0.014133
green	1179	FUCOSYLTRANSFERASE_ACTIVITY	3	7	16246	1305	5.337603	0.014133
green	2700	GNF2_SPINK1	3	7	16246	1305	5.337603	0.014133
pink	8714	GSE14769_UNSTIM_VS_20MIN_LPS_BMDM_UP	6	140	16113	228	3.055075	0.01414
cyan	5481	MYLLYKANGAS_AMPLIFICATION_HOT_SPOT_3	1	3	16250	77	70.35931	0.014146
salmon	8462	GSE11864_UNTREATED_VS_CSF1_IFNG_IN_MAC_UP	5	182	16071	127	3.515835	0.01415
salmon	9478	GSE26669_CTRL_VS_COSTIM_BLOCK_MLR_CD4_TCELL_	5	182	16071	127	3.515835	0.01415
salmon	9539	GSE27786_LSK_VS_NKTCELL_DN	5	182	16071	127	3.515835	0.01415
salmon	9867	GSE360_L_DONOVANI_VS_B_MALAYI_LOW_DOSE_MA	5	182	16071	127	3.515835	0.01415
blue	1146	SINGLE_STRANDED_DNA_BINDING	12	34	16219	2978	1.926243	0.014164
blue	2899	KEGG_PRIMARY_IMMUNODEFICIENCY	12	34	16219	2978	1.926243	0.014164
green	418	RESPONSE_TO_LIGHT_STIMULUS	7	33	16220	1305	2.641844	0.014193
lightgreen	8789	GSE15930_NAIVE_VS_72H_IN_VITRO_STIM_CD8_TCELI	3	197	16056	42	5.893038	0.014233
lightgreen	9401	GSE22886_UNSTIM_VS_IL15_STIM_NKCELL_DN	3	197	16056	42	5.893038	0.014233
lightgreen	10265	GSE9650_EXHAUSTED_VS_MEMORY_CD8_TCELL_DN	3	197	16056	42	5.893038	0.014233
turquoise	5892	GOTZMANN_EPITHELIAL_TO_MESENCHYMAL_TRANSIT	60	181	16072	4169	1.292333	0.014234
turquoise	8460	GSE11864_UNTREATED_VS_CSF1_IN_MAC_UP	60	181	16072	4169	1.292333	0.014234
purple	131	CONTRACTILE_FIBER_PART	2	19	16234	157	10.89708	0.014235
purple	5156	HAHTOLA_MYCOSIS_FUNGOIDES_UP	2	19	16234	157	10.89708	0.014235
purple	5409	MAHADEVAN_RESPONSE_TO_MP470_UP	2	19	16234	157	10.89708	0.014235
purple	6571	VERRECCHIA_RESPONSE_TO_TGFB1_C1	2	19	16234	157	10.89708	0.014235
purple	6927	KANG_CISPLATIN_RESISTANCE_UP	2	19	16234	157	10.89708	0.014235
purple	7007	OUYANG_PROSTATE_CANCER_MARKERS	2	19	16234	157	10.89708	0.014235
purple	7283	VILIMAS_NOTCH1_TARGETS_DN	2	19	16234	157	10.89708	0.014235
purple	7399	FINAK_BREAST_CANCER_SDPP_SIGNATURE	2	19	16234	157	10.89708	0.014235
brown	221	INTEGRIN_COMPLEX	6	16	16237	2190	2.783048	0.014242
brown	2904	BIOCARTA_RELA_PATHWAY	6	16	16237	2190	2.783048	0.014242
brown	3000	BIOCARTA_IL10_PATHWAY	6	16	16237	2190	2.783048	0.014242
brown	3089	BIOCARTA_SPRY_PATHWAY	6	16	16237	2190	2.783048	0.014242
brown	6960	FINETTI_BREAST_CANCER_KINOME_GREEN	6	16	16237	2190	2.783048	0.014242
greenyello	1257	PROTEIN_TYROSINE_KINASE_ACTIVITY	3	55	16198	150	5.910182	0.014243
cyan	9586	GSE27786_CD4_TCELL_VS_MONO_MAC_UP	4	197	16056	77	4.285846	0.014248
grey60	9894	GSE36392_TYPE_2_MYELOID_VS_NEUTROPHIL_IL25_TI	3	188	16065	44	5.894463	0.014249
grey60	10042	GSE3982_MAC_VS_TH1_UP	3	188	16065	44	5.894463	0.014249
brown	3460	SIG_CHEMOTAXIS	11	40	16213	2190	2.040902	0.014251
brown	4119	PID_HES_HEYPATHWAY	11	40	16213	2190	2.040902	0.014251
lightcyan	3804	MODULE_361	3	133	16120	62	5.913049	0.01426
blue	8637	GSE14308_TH2_VS_NATURAL_TREG_DN	46	184	16069	2978	1.364422	0.014262
blue	9263	GSE20366_EX_VIVO_VS_DEC205_CONVERSION_DN	46	184	16069	2978	1.364422	0.014262
blue	9469	GSE25087_TREG_VS_TCONV_FETUS_DN	46	184	16069	2978	1.364422	0.014262
blue	9699	GSE29618_PRE_VS_DAY7_POST_LAIV_FLU_VACCINE_N	46	184	16069	2978	1.364422	0.014262
pink	1751	V$BACH2_01	8	225	16028	228	2.534581	0.014262
greenyello	5072	SENESE_HDAC2_TARGETS_DN	4	101	16152	150	4.291221	0.014265
brown	7476	ROME_INSULIN_TARGETS_IN_MUSCLE_UP	74	429	15824	2190	1.280159	0.014266
cyan	1491	V$IRF2_01	3	107	16146	77	5.918073	0.014267
brown	2372	MORF_DAP	18	78	16175	2190	1.712645	0.014271
red	903	ACUTE_INFLAMMATORY_RESPONSE	2	6	16247	524	10.33906	0.014277
red	5317	LUCAS_HNF4A_TARGETS_DN	2	6	16247	524	10.33906	0.014277
red	5360	RAMJAUN_APOPTOSIS_BY_TGFB1_VIA_MAPK1_UP	2	6	16247	524	10.33906	0.014277
red	7617	WEBER_METHYLATED_ICP_IN_FIBROBLAST	2	6	16247	524	10.33906	0.014277
red	8117	KINNEY_DNMT1_METHYLATION_TARGETS	2	6	16247	524	10.33906	0.014277
yellow	8392	KAECH_DAY8_EFF_VS_DAY15_EFF_CD8_TCELL_UP	27	198	16055	1426	1.55422	0.014281
red	7864	WANG_RESPONSE_TO_GSK3_INHIBITOR_SB216763_UF	20	363	15890	524	1.708935	0.014299
salmon	2488	GCM_BECN1	3	65	16188	127	5.906602	0.014313

salmon	2512	GCM_DDX5	3	65	16188	127	5.906602	0.014313
salmon	3666	MODULE_183	3	65	16188	127	5.906602	0.014313
salmon	4185	REACTOME_ORC1_REMOVAL_FROM_CHROMATIN	3	65	16188	127	5.906602	0.014313
salmon	4715	REACTOME_APC_C_CDC20_MEDIATED_DEGRADATION_	3	65	16188	127	5.906602	0.014313
salmon	6424	ZAMORA_NOS2_TARGETS_UP	3	65	16188	127	5.906602	0.014313
brown	9988	GSE3982_EOSINOPHIL_VS_TH1_UP	34	173	16080	2190	1.458553	0.014319
turquoise	4723	REACTOME_SCF_BETA_TRCP_MEDIATED_DEGRADATIO	20	49	16204	4169	1.59124	0.014327
tan	7368	AGUIRRE_PANCREATIC_CANCER_COPY_NUMBER_UP	7	286	15967	145	2.743453	0.01433
lightyellow	136	INTEGRAL_TO_PLASMA_MEMBRANE	5	721	15532	33	3.4155	0.014342
green	8289	ESC_V6.5_UP_EARLY.V1_DN	19	138	16115	1305	1.714737	0.014363
blue	697	MACROMOLECULE_LOCALIZATION	53	217	16036	2978	1.332984	0.014364
blue	1511	V$ZID_01	53	217	16036	2978	1.332984	0.014364
greenyello	7304	GOLDRATH_ANTIGEN_RESPONSE	8	343	15910	150	2.527191	0.01437
purple	1672	V$GATA1_05	6	204	16049	157	3.044773	0.014381
turquoise	15	ORGANELLE_INNER_MEMBRANE	27	71	16182	4169	1.482542	0.014384
blue	2038	GTCTTCC,MIR-7	38	147	16106	2978	1.410831	0.014384
blue	6996	MCCABE_BOUND_BY_HOXC6	79	342	15911	2978	1.260694	0.014387
yellow	8553	GSE13411_NAIVE_VS_MEMORY_BCELL_DN	26	189	16064	1426	1.567926	0.01439
magenta	1539	V$PBX1_02	4	91	16162	167	4.27795	0.014391
magenta	8379	KRAS.PROSTATE_UP.V1_UP	4	91	16162	167	4.27795	0.014391
blue	2491	GCM_HDAC1	13	38	16215	2978	1.867104	0.014402
magenta	189	TRANS_GOLGI_NETWORK	2	18	16235	167	10.81371	0.014414
magenta	217	COLLAGEN	2	18	16235	167	10.81371	0.014414
magenta	226	NEURON_PROJECTION	2	18	16235	167	10.81371	0.014414
magenta	714	POSITIVE_REGULATION_OF_T_CELL_ACTIVATION	2	18	16235	167	10.81371	0.014414
magenta	1104	RAS_GUANYL_NUCLEOTIDE_EXCHANGE_FACTOR_ACTI\	2	18	16235	167	10.81371	0.014414
magenta	4834	DAVICIONI_PAX_FOXO1_SIGNATURE_IN_ARMS_DN	2	18	16235	167	10.81371	0.014414
magenta	5992	NIKOLSKY_BREAST_CANCER_6P24_P22_AMPLICON	2	18	16235	167	10.81371	0.014414
lightgreen	8447	GSE11057_NAIVE_VS_CENT_MEMORY_CD4_TCELL_DN	3	198	16055	42	5.863276	0.014426
lightgreen	10205	GSE7852_LN_VS_THYMUS_TCONV_DN	3	198	16055	42	5.863276	0.014426
blue	7451	ZHAN_EARLY_DIFFERENTIATION_GENES_DN	14	42	16211	2978	1.81923	0.014429
lightcyan	6307	PARK_HSC_AND_MULTIPOTENT_PROGENITORS	2	48	16205	62	10.92272	0.01443
red	2142	RNTCANNRNNYNATTW_UNKNOWN	5	45	16208	524	3.446353	0.014439
red	3480	SIG_BCR_SIGNALING_PATHWAY	5	45	16208	524	3.446353	0.014439
red	3766	MODULE_312	5	45	16208	524	3.446353	0.014439
brown	369	STRIATED_MUSCLE_DEVELOPMENT	9	30	16223	2190	2.226438	0.01444
brown	2916	BIOCARTA_AT1R_PATHWAY	9	30	16223	2190	2.226438	0.01444
brown	4009	PID_IGF1_PATHWAY	9	30	16223	2190	2.226438	0.01444
brown	4069	PID_AR_NONGENOMIC_PATHWAY	9	30	16223	2190	2.226438	0.01444
brown	4421	REACTOME_GPVI_MEDIATED_ACTIVATION_CASCADE	9	30	16223	2190	2.226438	0.01444
brown	7324	PARK_TRETINOIN_RESPONSE_AND_PML_RARA_FUSIOI	9	30	16223	2190	2.226438	0.01444
turquoise	8920	GSE17721_LPS_VS_POLYIC_6H_BMDM_UP	62	188	16065	4169	1.285688	0.014456
turquoise	8947	GSE17721_POLYIC_VS_PAM3CSK4_24H_BMDM_DN	62	188	16065	4169	1.285688	0.014456
salmon	8624	GSE14000_4H_VS_16H_LPS_DC_UP	5	183	16070	127	3.496622	0.014459
salmon	9047	GSE17721_LPS_VS_CPG_6H_BMDM_DN	5	183	16070	127	3.496622	0.014459
salmon	9051	GSE17721_LPS_VS_CPG_12H_BMDM_DN	5	183	16070	127	3.496622	0.014459
salmon	9471	GSE25087_TREG_VS_TCONV_ADULT_DN	5	183	16070	127	3.496622	0.014459
magenta	8556	GSE13411_IGM_MEMORY_BCELL_VS_PLASMA_CELL_U	6	192	16061	167	3.041355	0.014461
lightgreen	5855	FRIDMAN_SENESCENCE_UP	2	71	16182	42	10.90074	0.014474
lightgreen	6443	BROWNE_HCMV_INFECTION_16HR_DN	2	71	16182	42	10.90074	0.014474
cyan	9667	GSE29618_BCELL_VS_MONOCYTE_DAY7_FLU_VACCINE	4	198	16055	77	4.2642	0.01449
red	9259	GSE20366_TREG_VS_TCONV_DN	12	181	16072	524	2.056387	0.014496
red	9889	GSE360_LOW_DOSE_B_MALAYI_VS_M_TUBERCULOSIS	12	181	16072	524	2.056387	0.014496
greenyello	9494	GSE26928_EFF_MEMORY_VS_CXCR5_POS_CD4_TCELL_	5	155	16098	150	3.495269	0.014498
purple	5956	SUNG_METASTASIS_STROMA_UP	4	97	16156	157	4.268961	0.014507
purple	1461	AACTTT_UNKNOWN	23	1473	14780	157	1.616438	0.014515
lightcyan	5334	DARWICHE_PAPILLOMA_RISK_LOW_UP	3	134	16119	62	5.868922	0.014548
purple	4748	REACTOME_MEIOTIC_SYNAPSIS	3	53	16200	157	5.859752	0.014559
grey60	455	AMINO_ACID_METABOLIC_PROCESS	2	68	16185	44	10.8643	0.014571
turquoise	2155	GTACAGG,MIR-486	19	46	16207	4169	1.610265	0.014576

turquoise	4012	PID_PLK1_PATHWAY	19	46	16207	4169	1.610265	0.014576
brown	5268	BARIS_THYROID_CANCER_DN	14	56	16197	2190	1.855365	0.01459
lightgreen	8394	KAECH_DAY8_EFF_VS_MEMORY_CD8_TCELL_UP	3	199	16054	42	5.833812	0.014621
cyan	3688	MODULE_208	3	108	16145	77	5.863276	0.014626
turquoise	6568	YAMAZAKI_TCEB3_TARGETS_DN	64	195	16058	4169	1.27952	0.014646
turquoise	9210	GSE17974_0.5H_VS_72H_IL4_AND_ANTI_IL12_ACT_CD	64	195	16058	4169	1.27952	0.014646
turquoise	9904	GSE36476_CTRL_VS_TSST_ACT_40H_MEMORY_CD4_T(	64	195	16058	4169	1.27952	0.014646
brown	621	POSITIVE_REGULATION_OF_CYTOKINE_SECRETION	4	8	16245	2190	3.710731	0.014652
brown	7073	ITO_PTTG1_TARGETS_DN	4	8	16245	2190	3.710731	0.014652
brown	7126	MATZUK_PREOVULATORY_FOLLICLE	4	8	16245	2190	3.710731	0.014652
brown	7766	MARIADASON_RESPONSE_TO_BUTYRATE_CURCUMIN_	4	8	16245	2190	3.710731	0.014652
brown	7796	BANDRES_RESPONSE_TO_CARMUSTIN_WITHOUT_MGI	4	8	16245	2190	3.710731	0.014652
grey60	9266	GSE20366_EX_VIVO_VS_DEC205_CONVERSION_NAIVE_	3	190	16063	44	5.832416	0.014656
grey60	10019	GSE3982_DC_VS_BASOPHIL_DN	3	190	16063	44	5.832416	0.014656
turquoise	2119	AGTCTAG,MIR-151	9	17	16236	4169	2.063931	0.014669
magenta	884	NEURON_DEVELOPMENT	3	50	16203	167	5.839401	0.014671
magenta	4800	WINTER_HYPOXIA_DN	3	50	16203	167	5.839401	0.014671
magenta	7099	BERNARD_PPAPDC1B_TARGETS_DN	3	50	16203	167	5.839401	0.014671
blue	7688	DANG_REGULATED_BY_MYC_UP	20	67	16186	2978	1.629161	0.014695
brown	911	PROTEIN_MODIFICATION_PROCESS	95	569	15684	2190	1.239084	0.014697
red	8869	GSE17721_CTRL_VS_PAM3CSK4_2H_BMDM_DN	11	160	16093	524	2.132431	0.014698
red	9916	GSE36476_YOUNG_VS_OLD_DONOR_MEMORY_CD4_T	11	160	16093	524	2.132431	0.014698
blue	8908	GSE17721_CTRL_VS_GARDIQUIMOD_12H_BMDM_UP	47	189	16064	2978	1.357203	0.014705
blue	9314	GSE22886_NAIVE_TCELL_VS_NKCELL_UP	47	189	16064	2978	1.357203	0.014705
turquoise	2606	GNF2_SMC4L1	31	84	16169	4169	1.438746	0.014713
black	8088	DELACROIX_RARG_BOUND_MEF	12	335	15918	283	2.057233	0.014716
turquoise	8465	GSE11864_UNTREATED_VS_CSF1_PAM3CYS_IN_MAC_I	57	171	16082	4169	1.299512	0.014723
turquoise	6270	STONER_ESOPHAGEAL_CARCINOGENESIS_UP	15	34	16219	4169	1.719943	0.014724
tan	8747	GSE15659_NAIVE_CD4_TCELL_VS_RESTING_TREG_DN	5	161	16092	145	3.481045	0.014731
green	8276	CTIP_DN.V1_UP	14	92	16161	1305	1.895236	0.014734
green	9633	GSE29614_CTRL_VS_DAY3_TIV_FLU_VACCINE_PBMC_I	14	92	16161	1305	1.895236	0.014734
blue	9382	GSE22886_TH1_VS_TH2_12H_ACT_UP	44	175	16078	2978	1.372219	0.014735
cyan	9671	GSE29618_BCELL_VS_MDC_DAY7_FLU_VACCINE_DN	4	199	16054	77	4.242772	0.014735
brown	8060	WAKABAYASHI_ADIPOGENESIS_PPARG_BOUND_8D	102	616	15637	2190	1.228878	0.014737
turquoise	7686	ZHAN_MULTIPLE_MYELOMA_MS_DN	18	43	16210	4169	1.631946	0.01476
brown	8212	CYCLIN_D1_UP.V1_UP	31	155	16098	2190	1.484292	0.01476
salmon	8434	GSE10325_CD4_TCELL_VS_LUPUS_CD4_TCELL_UP	5	184	16069	127	3.477619	0.014772
salmon	8644	GSE14308_TH1_VS_NATURAL_TREG_UP	5	184	16069	127	3.477619	0.014772
salmon	9985	GSE3982_EOSINOPHIL_VS_CENT_MEMORY_CD4_TCELL	5	184	16069	127	3.477619	0.014772
magenta	9724	GSE30962_PRIMARY_VS_SECONDARY_CHRONIC_LCMV	6	193	16060	167	3.025596	0.014802
magenta	10167	GSE7460_CTRL_VS_TGFB_TREATED_ACT_TCONV_DN	6	193	16060	167	3.025596	0.014802
magenta	10198	GSE7852_LN_VS_THYMUS_TREG_UP	6	193	16060	167	3.025596	0.014802
turquoise	8745	GSE15659_NAIVE_VS_PTPRC_NEG_CD4_TCELL_DN	52	154	16099	4169	1.316389	0.014814
magenta	8714	GSE14769_UNSTIM_VS_20MIN_LPS_BMDM_UP	5	140	16113	167	3.475834	0.014816
lightgreen	8402	GOLDRATH_EFF_VS_MEMORY_CD8_TCELL_UP	3	200	16053	42	5.804643	0.014818
yellow	3198	chr22q11	19	127	16126	1426	1.705155	0.014837
pink	8369	KRAS.600.LUNG.BREAST_UP.V1_UP	7	183	16070	228	2.726752	0.014843
pink	9425	GSE24142_DN2_VS_DN3_THYMOCYTE_FETAL_DN	7	183	16070	228	2.726752	0.014843
turquoise	3558	MODULE_69	138	457	15796	4169	1.177239	0.014852
red	157	ACTIN_CYTOSKELETON	9	119	16134	524	2.345837	0.014856
turquoise	2611	GNF2_ANP32B	16	37	16216	4169	1.685854	0.014858
turquoise	646	PROTEIN_UBIQUITINATION	17	40	16213	4169	1.656878	0.014861
brown	7806	SASSON_RESPONSE_TO_GONADOTROPHINS_UP	19	84	16169	2190	1.678664	0.014862
green	3769	MODULE_316	8	41	16212	1305	2.430128	0.014865
green	7784	DAZARD_UV_RESPONSE_CLUSTER_G28	5	19	16234	1305	3.277475	0.014867
brown	8862	GSE17721_CTRL_VS_POLYIC_24H_BMDM_UP	37	192	16061	2190	1.430177	0.014874
brown	9564	GSE27786_BCELL_VS_CD8_TCELL_UP	37	192	16061	2190	1.430177	0.014874
pink	117	CELL_CORTEX	3	37	16216	228	5.779872	0.014905
pink	2240	CAGGTG_V$E12_Q6	40	2020	14233	228	1.411586	0.01492
red	2046	TTGCCAA,MIR-182	17	294	15959	524	1.79351	0.014925

magenta	8276	CTIP_DN.V1_UP	4	92	16161	167	4.23145	0.014928
lightyellow	7902	OHGUCHI_LIVER_HNF4A_TARGETS_DN	2	92	16161	33	10.70685	0.014933
lightyellow	8377	KRAS.LUNG.BREAST_UP.V1_UP	2	92	16161	33	10.70685	0.014933
turquoise	7355	CHANG_CORE_SERUM_RESPONSE_UP	68	209	16044	4169	1.268423	0.014939
red	4914	DIAZ_CHRONIC_MEYLOGENOUS_LEUKEMIA_UP	59	1378	14875	524	1.328021	0.01496
lightcyan	6936	HELLER_HDAC_TARGETS_SILENCED_BY_METHYLATION_	5	381	15872	62	3.440225	0.014971
salmon	229	NUCLEOLUS	4	121	16132	127	4.230624	0.014976
salmon	4224	REACTOME_SIGNALING_BY_THE_B_CELL_RECEPTOR_B	4	121	16132	127	4.230624	0.014976
black	2945	BIOCARTA_COMP_PATHWAY	2	11	16242	283	10.44202	0.01498
black	4536	REACTOME_MRNA_DECAY_BY_3_TO_5_EXORIBONUCL	2	11	16242	283	10.44202	0.01498
cyan	9437	GSE24634_NAIVE_CD4_TCELL_VS_DAY7_IL4_CONV_TR	4	200	16053	77	4.221558	0.014983
cyan	9672	GSE29618_MONOCYTE_VS_PDC_DAY7_FLU_VACCINE_	4	200	16053	77	4.221558	0.014983
turquoise	8951	GSE17721_PAM3CSK4_VS_CPG_1H_BMDM_DN	59	178	16075	4169	1.292212	0.014993
purple	5149	GOZGIT_ESR1_TARGETS_DN	12	608	15645	157	2.043203	0.015001
pink	6957	MARTINEZ_RB1_AND_TP53_TARGETS_UP	14	519	15734	228	1.922912	0.015012
brown	1918	V$SP1_Q4_01	44	236	16017	2190	1.383662	0.01504
red	3082	BIOCARTA_IL1R_PATHWAY	4	30	16223	524	4.135623	0.01506
red	7997	KIM_TIAL1_TARGETS	4	30	16223	524	4.135623	0.01506
grey60	515	CELLULAR_CATABOLIC_PROCESS	3	192	16061	44	5.771662	0.01507
grey60	8458	GSE11057_PBMC_VS_MEM_CD4_TCELL_UP	3	192	16061	44	5.771662	0.01507
grey60	8662	GSE1432_CTRL_VS_IFNG_24H_MICROGLIA_UP	3	192	16061	44	5.771662	0.01507
red	8776	GSE15930_NAIVE_VS_24H_IN_VITRO_STIM_CD8_TCELI	12	182	16071	524	2.045088	0.015083
red	10035	GSE3982_MAC_VS_BASOPHIL_DN	12	182	16071	524	2.045088	0.015083
salmon	9868	GSE360_L_DONOVANI_VS_M_TUBERCULOSIS_MAC_UI	5	185	16068	127	3.458821	0.01509
salmon	9987	GSE3982_EOSINOPHIL_VS_NKCELL_DN	5	185	16068	127	3.458821	0.01509
purple	84	CELL_FRACTION	9	397	15856	157	2.346853	0.015098
brown	8516	GSE12845_IGD_NEG_BLOOD_VS_DARKZONE_GC_TONS	36	186	16067	2190	1.436412	0.015113
brown	8999	GSE17721_LPS_VS_PAM3CSK4_16H_BMDM_DN	36	186	16067	2190	1.436412	0.015113
blue	8775	GSE15767_MED_VS_SCS_MAC_LN_DN	48	194	16059	2978	1.350356	0.015136
magenta	1795	V$LBP1_Q6	6	194	16059	167	3.010001	0.015148
magenta	7144	BOQUEST_STEM_CELL_DN	6	194	16059	167	3.010001	0.015148
magenta	10047	GSE3982_NEUTROPHIL_VS_BCELL_DN	6	194	16059	167	3.010001	0.015148
yellow	8308	CRX_DN.V1_UP	17	110	16143	1426	1.76145	0.015171
turquoise	5846	GARCIA_TARGETS_OF_FLI1_AND_DAX1_DN	54	161	16092	4169	1.307584	0.015185
blue	4183	REACTOME_CELL_CYCLE	86	377	15876	2978	1.24499	0.015186
yellow	9907	GSE36476_CTRL_VS_TSST_ACT_72H_MEMORY_CD4_T(	27	199	16054	1426	1.54641	0.015187
blue	2301	MORF_CTBP1	42	166	16087	2978	1.380861	0.015192
blue	8538	GSE13306_TREG_VS_TCONV_UP	42	166	16087	2978	1.380861	0.015192
greenyello	2131	RNGTGGGC_UNKNOWN	12	638	15615	150	2.037994	0.015209
brown	4446	REACTOME_AXON_GUIDANCE	40	211	16042	2190	1.406912	0.015213
blue	8547	GSE13411_NAIVE_VS_IGM_MEMORY_BCELL_DN	45	180	16073	2978	1.364422	0.015223
blue	9809	GSE360_CTRL_VS_T_GONDII_MAC_DN	45	180	16073	2978	1.364422	0.015223
turquoise	8735	GSE15324_NAIVE_VS_ACTIVATED_CD8_TCELL_DN	61	185	16068	4169	1.285463	0.015226
turquoise	9106	GSE17721_0.5H_VS_8H_POLYIC_BMDM_UP	61	185	16068	4169	1.285463	0.015226
turquoise	10240	GSE9037_CTRL_VS_LPS_1H_STIM_BMDM_UP	61	185	16068	4169	1.285463	0.015226
midnightb	5336	DARWICHE_PAPILLOMA_RISK_HIGH_UP	3	119	16134	71	5.770979	0.015245
lightgreen	7362	VANASSE_BCL2_TARGETS_DN	2	73	16180	42	10.60209	0.015257
grey60	3272	chr17p13	3	193	16060	44	5.741757	0.01528
grey60	6329	AFFAR_YY1_TARGETS_DN	3	193	16060	44	5.741757	0.01528
grey60	9101	GSE17721_12H_VS_24H_LPS_BMDM_DN	3	193	16060	44	5.741757	0.01528
grey60	9447	GSE24634_TREG_VS_TCONV_POST_DAY10_IL4_CONVE	3	193	16060	44	5.741757	0.01528
yellow	6674	SCHLINGEMANN_SKIN_CARCINOGENESIS_TPA_DN	6	24	16229	1426	2.849404	0.015304
purple	4870	DAVICIONI_RHABDOMYOSARCOMA_PAX_FOXO1_FUSI(	3	54	16199	157	5.751238	0.015308
lightyellow	14	INTRINSIC_TO_PLASMA_MEMBRANE	5	733	15520	33	3.359585	0.015312
purple	6819	CREIGHTON_ENDOCRINE_THERAPY_RESISTANCE_2	9	398	15855	157	2.340956	0.015324
brown	4982	OSMAN_BLADDER_CANCER_DN	68	391	15862	2190	1.290689	0.015344
blue	841	NEGATIVE_REGULATION_OF_METABOLIC_PROCESS	56	232	16021	2978	1.317373	0.015345
purple	1785	V$EFC_Q6	6	207	16046	157	3.000646	0.015353
cyan	7340	POTTI_ETOPOSIDE_SENSITIVITY	2	40	16213	77	10.5539	0.015383
red	6620	GENTILE_UV_HIGH_DOSE_DN	17	295	15958	524	1.78743	0.015384

salmon	232	CHROMOSOME	4	122	16131	127	4.195947	0.015392
salmon	6074	BYSTRYKH_HEMATOPOIESIS_STEM_CELL_QTL_CIS	4	122	16131	127	4.195947	0.015392
brown	879	NOTCH_SIGNALING_PATHWAY	5	12	16241	2190	3.092275	0.015399
brown	2909	BIOCARTA_GRANULOCYTES_PATHWAY	5	12	16241	2190	3.092275	0.015399
brown	2946	BIOCARTA_VDR_PATHWAY	5	12	16241	2190	3.092275	0.015399
brown	3199	chr7p11	5	12	16241	2190	3.092275	0.015399
brown	3633	MODULE_148	5	12	16241	2190	3.092275	0.015399
brown	3674	MODULE_194	5	12	16241	2190	3.092275	0.015399
brown	4369	REACTOME_NOTCH_HLH_TRANSCRIPTION_PATHWAY	5	12	16241	2190	3.092275	0.015399
brown	6168	KUROKAWA_LIVER_CANCER_CHEMOTHERAPY_UP	5	12	16241	2190	3.092275	0.015399
brown	6997	POS_RESPONSE_TO_HISTAMINE_UP	5	12	16241	2190	3.092275	0.015399
brown	7323	PARK_TRETINOIN_RESPONSE	5	12	16241	2190	3.092275	0.015399
lightgreen	3863	MODULE_454	1	6	16247	42	64.49603	0.015407
lightgreen	4836	FOURNIER_ACINAR_DEVELOPMENT_EARLY_DN	1	6	16247	42	64.49603	0.015407
lightgreen	5806	FREDERICK_PRKCI_TARGETS	1	6	16247	42	64.49603	0.015407
lightgreen	7946	KIM_GLIS2_TARGETS_DN	1	6	16247	42	64.49603	0.015407
salmon	8649	GSE14308_TH17_VS_INDUCED_TREG_DN	5	186	16067	127	3.440225	0.015413
salmon	9001	GSE17721_LPS_VS_PAM3CSK4_24H_BMDM_DN	5	186	16067	127	3.440225	0.015413
turquoise	10210	GSE7852_TREG_VS_TCONV_UP	63	192	16061	4169	1.279207	0.015424
yellow	8613	GSE14000_TRANSLATED_RNA_VS_MRNA_16H_LPS_DC	25	181	16072	1426	1.574256	0.015428
lightcyan	8213	CSR_EARLY_UP.V1_DN	3	137	16116	62	5.740405	0.015431
red	4583	REACTOME_SIGNALING_BY_ILS	8	100	16153	524	2.481374	0.015468
magenta	2100	ACATATC,MIR-190	3	51	16202	167	5.724903	0.015472
magenta	8453	GSE11057_CD4_EFF_MEM_VS_PBMC_DN	6	195	16058	167	2.994565	0.0155
magenta	8890	GSE17721_CTRL_VS_CPG_8H_BMDM_UP	6	195	16058	167	2.994565	0.0155
turquoise	5741	PUJANA_XPRSS_INT_NETWORK	56	168	16085	4169	1.299512	0.01551
purple	50	MEMBRANE_PART	21	1316	14937	157	1.651951	0.015514
turquoise	6089	KIM_GERMINAL_CENTER_T_HELPER_UP	25	65	16188	4169	1.499437	0.015517
brown	4078	PID_KITPATHWAY	13	51	16202	2190	1.891745	0.015522
brown	5974	MORI_IMMATURE_B_LYMPHOCYTE_UP	13	51	16202	2190	1.891745	0.015522
salmon	4865	ZHONG_RESPONSE_TO_AZACITIDINE_AND_TSA_DN	3	67	16186	127	5.730286	0.015523
salmon	7688	DANG_REGULATED_BY_MYC_UP	3	67	16186	127	5.730286	0.015523
red	3657	MODULE_174	7	81	16172	524	2.680497	0.015525
red	3748	MODULE_289	7	81	16172	524	2.680497	0.015525
purple	6049	LE_EGR2_TARGETS_DN	4	99	16154	157	4.182719	0.015531
lightyellow	7041	ACEVEDO_LIVER_CANCER_WITH_H3K9ME3_DN	2	94	16159	33	10.47905	0.015553
brown	5295	MCBRYAN_PUBERTAL_BREAST_4_5WK_DN	34	174	16079	2190	1.450171	0.015559
brown	9970	GSE3982_EOSINOPHIL_VS_MAST_CELL_UP	34	174	16079	2190	1.450171	0.015559
salmon	3205	chr4q33	1	2	16251	127	63.98819	0.015567
greenyello	7863	CHICAS_RB1_TARGETS_CONFLUENT	10	490	15763	150	2.211293	0.015568
turquoise	8603	GSE13738_RESTING_VS_TCR_ACTIVATED_CD4_TCELL_I	65	199	16054	4169	1.273391	0.015591
turquoise	8783	GSE15930_NAIVE_VS_48H_IN_VITRO_STIM_CD8_TCELI	65	199	16054	4169	1.273391	0.015591
turquoise	9167	GSE17974_0H_VS_72H_IN_VITRO_ACT_CD4_TCELL_DN	65	199	16054	4169	1.273391	0.015591
yellow	81	SPINDLE	8	38	16215	1426	2.399498	0.015592
yellow	143	RIBOSOME	8	38	16215	1426	2.399498	0.015592
black	5025	HOEBEKE_LYMPHOID_STEM_CELL_DN	5	84	16169	283	3.418518	0.015618
greenyello	9304	GSE22045_TREG_VS_TCONV_UP	5	158	16095	150	3.428903	0.015632
salmon	62	CYTOPLASM	24	1950	14303	127	1.575094	0.015651
lightgreen	2827	KEGG_TGF_BETA_SIGNALING_PATHWAY	2	74	16179	42	10.45882	0.015655
lightgreen	3655	MODULE_172	2	74	16179	42	10.45882	0.015655
magenta	1991	ACACTGG,MIR-199A,MIR-199B	5	142	16111	167	3.426879	0.015665
magenta	8288	ESC_J1_UP_LATE.V1_UP	5	142	16111	167	3.426879	0.015665
pink	1822	V$TEF1_Q6	7	185	16068	228	2.697274	0.015669
pink	10010	GSE3982_MAST_CELL_VS_TH2_UP	7	185	16068	228	2.697274	0.015669
pink	10028	GSE3982_DC_VS_TH2_UP	7	185	16068	228	2.697274	0.015669
pink	300	ESTABLISHMENT_OF_LOCALIZATION	18	735	15518	228	1.745757	0.015671
greenyello	5353	DARWICHE_PAPILLOMA_PROGRESSION_RISK	3	57	16196	150	5.702807	0.015676
brown	5394	PEREZ_TP63_TARGETS	53	294	15959	2190	1.337882	0.015682
blue	6055	TARTE_PLASMA_CELL_VS_B_LYMPHOCYTE_UP	22	76	16177	2978	1.579858	0.015687
red	8918	GSE17721_LPS_VS_POLYIC_4H_BMDM_UP	12	183	16070	524	2.033913	0.015688

red	9898	GSE36392_EOSINOPHIL_VS_NEUTROPHIL_IL25_TREATE	12	183	16070	524	2.033913	0.015688
red	10069	GSE3982_BCELL_VS_TH1_DN	12	183	16070	524	2.033913	0.015688
blue	9741	GSE31082_CD4_VS_CD8_SP_THYMOCYTE_DN	46	185	16068	2978	1.357047	0.015696
blue	9952	GSE3982_CTRL_VS_LPS_4H_MAC_UP	46	185	16068	2978	1.357047	0.015696
brown	1753	V$STAT1_02	42	224	16029	2190	1.391524	0.015711
brown	1381	TRANSFERASE_ACTIVITY_TRANSFERRING_PHOSPHORUS	69	398	15855	2190	1.286635	0.015715
purple	44	CONTRACTILE_FIBER	2	20	16233	157	10.35223	0.015718
purple	3995	PID_S1P_S1P1_PATHWAY	2	20	16233	157	10.35223	0.015718
purple	4508	REACTOME_TIGHT_JUNCTION_INTERACTIONS	2	20	16233	157	10.35223	0.015718
purple	5649	MURATA_VIRULENCE_OF_H_PILORI	2	20	16233	157	10.35223	0.015718
purple	6595	PAL_PRMT5_TARGETS_DN	2	20	16233	157	10.35223	0.015718
blue	9400	GSE22886_UNSTIM_VS_IL15_STIM_NKCELL_UP	43	171	16082	2978	1.372402	0.01573
greenyello	7978	AZARE_NEOPLASTIC_TRANSFORMATION_BY_STAT3_DN	4	104	16149	150	4.167436	0.01573
cyan	5283	WANG_ESOPHAGUS_CANCER_VS_NORMAL_UP	3	111	16142	77	5.704809	0.015732
green	7565	MIKKELSEN_MCV6_HCP_WITH_H3K27ME3	30	248	16005	1305	1.506581	0.015738
salmon	8420	GSE10239_MEMORY_VS_DAY4.5_EFF_CD8_TCELL_UP	5	187	16066	127	3.421828	0.01574
cyan	1722	V$STAT5A_01	4	203	16050	77	4.159171	0.015743
red	2872	KEGG_AMYOTROPHIC_LATERAL_SCLEROSIS_ALS	5	46	16207	524	3.371432	0.015783
red	6220	LEE_LIVER_CANCER_E2F1_DN	5	46	16207	524	3.371432	0.015783
blue	5031	RHEIN_ALL_GLUCOCORTICOID_THERAPY_DN	82	358	15895	2978	1.250085	0.015787
turquoise	8895	GSE17721_CTRL_VS_CPG_24H_BMDM_DN	58	175	16078	4169	1.292086	0.015794
salmon	3740	MODULE_279	4	123	16130	127	4.161833	0.015816
brown	5154	HAHTOLA_MYCOSIS_FUNGOIDES_CD4_UP	15	62	16191	2190	1.795515	0.015816
brown	6951	LIN_MELANOMA_COPY_NUMBER_UP	15	62	16191	2190	1.795515	0.015816
blue	584	ADAPTIVE_IMMUNE_RESPONSE_GO_0002460	9	23	16230	2978	2.135618	0.015819
greenyello	576	ANION_TRANSPORT	2	21	16232	150	10.31937	0.015839
greenyello	3178	chr5q21	2	21	16232	150	10.31937	0.015839
greenyello	3390	chr5q32	2	21	16232	150	10.31937	0.015839
greenyello	3978	PID_WNT_SIGNALING_PATHWAY	2	21	16232	150	10.31937	0.015839
greenyello	5734	HUI_MAPK14_TARGETS_UP	2	21	16232	150	10.31937	0.015839
greenyello	6272	HASLINGER_B_CLL_WITH_13Q14_DELETION	2	21	16232	150	10.31937	0.015839
greenyello	7590	ONO_FOXP3_TARGETS_UP	2	21	16232	150	10.31937	0.015839
green	2386	MORF_ERCC4	29	238	16015	1305	1.517554	0.015854
lightyellow	7175	BRUECKNER_TARGETS_OF_MIRLET7A3_UP	2	95	16158	33	10.36874	0.015868
black	8508	GSE12845_IGD_POS_BLOOD_VS_PRE_GC_TONSIL_BCEI	8	185	16068	283	2.483507	0.015904
black	9320	GSE22886_NAIVE_CD4_TCELL_VS_NKCELL_UP	8	185	16068	283	2.483507	0.015904
turquoise	1480	V$E2F_02	71	220	16033	4169	1.258164	0.015923
pink	725	TRANSPORT	17	682	15571	228	1.776901	0.015928
pink	34	INTERMEDIATE_FILAMENT_CYTOSKELETON	2	14	16239	228	10.18358	0.015956
pink	125	INTERMEDIATE_FILAMENT	2	14	16239	228	10.18358	0.015956
pink	673	RESPONSE_TO_TEMPERATURE_STIMULUS	2	14	16239	228	10.18358	0.015956
pink	869	PIGMENT_BIOSYNTHETIC_PROCESS	2	14	16239	228	10.18358	0.015956
pink	5378	MARKS_ACETYLATED_NON_HISTONE_PROTEINS	2	14	16239	228	10.18358	0.015956
pink	7343	RAY_TARGETS_OF_P210_BCR_ABL_FUSION_UP	2	14	16239	228	10.18358	0.015956
turquoise	7643	FOURNIER_ACINAR_DEVELOPMENT_LATE_2	85	269	15984	4169	1.23188	0.015959
green	10283	GSE9988_LPS_VS_CTRL_TREATED_MONOCYTE_DN	25	198	16055	1305	1.572526	0.015961
turquoise	301	REGULATION_OF_KINASE_ACTIVITY	48	141	16112	4169	1.327161	0.015969
turquoise	7824	WHITFIELD_CELL_CYCLE_M_G1	48	141	16112	4169	1.327161	0.015969
green	5262	LANDIS_ERBB2_BREAST_TUMORS_324_DN	18	130	16123	1305	1.724456	0.015989
magenta	1356	HORMONE_ACTIVITY	2	19	16234	167	10.24456	0.016002
magenta	6808	CLASPER_LYMPHATIC_VESSELS_DURING_METASTASIS_	2	19	16234	167	10.24456	0.016002
red	8222	MTOR_UP.V1_UP	10	141	16112	524	2.1998	0.016014
pink	949	NUCLEOTIDE_METABOLIC_PROCESS	3	38	16215	228	5.62777	0.016019
pink	5938	AMIT_EGF_RESPONSE_480_MCF10A	3	38	16215	228	5.62777	0.016019
pink	6529	ULE_SPLICING_VIA_NOVA2	3	38	16215	228	5.62777	0.016019
greenyello	8326	IL15_UP.V1_UP	5	159	16094	150	3.407338	0.016023
turquoise	1586	V$USF_Q6	75	234	16019	4169	1.249531	0.016025
lightgreen	1740	V$FOXO4_02	3	206	16047	42	5.635576	0.016027
blue	1628	V$NGFIC_01	54	223	16030	2978	1.321593	0.016028
magenta	8241	RELA_DN.V1_DN	4	94	16159	167	4.141419	0.016039

pink	4830	BERTUCCI_MEDULLARY_VS_DUCTAL_BREAST_CANCER_	6	144	16109	228	2.970212	0.016047
pink	6320	PETROVA_ENDOTHELIUM_LYMPHATIC_VS_BLOOD_DN	6	144	16109	228	2.970212	0.016047
brown	6626	MCLACHLAN_DENTAL_CARIES_DN	41	218	16035	2190	1.395779	0.016047
brown	4731	REACTOME_IMMUNE_SYSTEM	129	801	15452	2190	1.195217	0.016058
salmon	8921	GSE17721_LPS_VS_POLYIC_6H_BMDM_DN	5	188	16065	127	3.403627	0.016071
purple	4670	REACTOME_MEIOTIC_RECOMBINATION	3	55	16198	157	5.646671	0.016079
brown	8397	KAECH_DAY15_EFF_VS_MEMORY_CD8_TCELL_DN	37	193	16060	2190	1.422767	0.016082
brown	8606	GSE13738_TCR_VS_BYSTANDER_ACTIVATED_CD4_TCEL	37	193	16060	2190	1.422767	0.016082
brown	8975	GSE17721_CPG_VS_GARDIQUIMOD_6H_BMDM_DN	37	193	16060	2190	1.422767	0.016082
brown	9664	GSE29618_PDC_VS_MDC_UP	37	193	16060	2190	1.422767	0.016082
brown	9802	GSE360_CTRL_VS_M_TUBERCULOSIS_DC_UP	37	193	16060	2190	1.422767	0.016082
turquoise	3189	chr1p31	24	62	16191	4169	1.509111	0.016086
turquoise	3370	chr3q13	24	62	16191	4169	1.509111	0.016086
turquoise	6301	ZHAN_MULTIPLE_MYELOMA_UP	24	62	16191	4169	1.509111	0.016086
pink	9755	GSE32423_IL7_VS_IL7_IL4_MEMORY_CD8_TCELL_DN	7	186	16067	228	2.682772	0.016095
yellow	2561	GCM_TPT1	12	69	16184	1426	1.982194	0.016098
magenta	9631	GSE29614_CTRL_VS_TIV_FLU_VACCINE_PBMC_2007_D	5	143	16110	167	3.402914	0.016102
cyan	3247	chr12p12	2	41	16212	77	10.29648	0.016122
greenyello	136	INTEGRAL_TO_PLASMA_MEMBRANE	13	721	15532	150	1.953666	0.016127
lightyellow	883	ENDOTHELIAL_CELL_MIGRATION	1	8	16245	33	61.56439	0.016132
lightyellow	3594	MODULE_106	1	8	16245	33	61.56439	0.016132
lightyellow	5053	TONKS_TARGETS_OF_RUNX1_RUNX1T1_FUSION_SUST/	1	8	16245	33	61.56439	0.016132
lightyellow	8036	SCHMIDT_POR_TARGETS_IN_LIMB_BUD_DN	1	8	16245	33	61.56439	0.016132
grey60	3863	MODULE_454	1	6	16247	44	61.56439	0.016136
grey60	6473	INGRAM_SHH_TARGETS	1	6	16247	44	61.56439	0.016136
green	3625	MODULE_137	47	426	15827	1305	1.374078	0.016137
salmon	7737	YAO_TEMPORAL_RESPONSE_TO_PROGESTERONE_CLUS	3	68	16185	127	5.646017	0.016148
salmon	3761	MODULE_306	2	25	16228	127	10.23811	0.01616
salmon	4305	REACTOME_REGULATORY_RNA_PATHWAYS	2	25	16228	127	10.23811	0.01616
salmon	5688	YANG_BREAST_CANCER_ESR1_BULK_UP	2	25	16228	127	10.23811	0.01616
red	5047	TONKS_TARGETS_OF_RUNX1_RUNX1T1_FUSION_GRAN	3	17	16236	524	5.473619	0.016177
red	5408	MAHADEVAN_RESPONSE_TO_MP470_DN	3	17	16236	524	5.473619	0.016177
red	6450	NIELSEN_MALIGNAT_FIBROUS_HISTIOCYTOMA_UP	3	17	16236	524	5.473619	0.016177
red	7519	BOYAULT_LIVER_CANCER_SUBCLASS_G56_DN	3	17	16236	524	5.473619	0.016177
red	7750	MOOTHA_GLYCOGEN_METABOLISM	3	17	16236	524	5.473619	0.016177
lightcyan	5057	PAPASPYRIDONOS_UNSTABLE_ATEROSCLEROTIC_PLAQ	2	51	16202	62	10.2802	0.016191
lightcyan	6475	SESTO_RESPONSE_TO_UV_C2	2	51	16202	62	10.2802	0.016191
brown	3285	chr10q22	18	79	16174	2190	1.690966	0.01622
brown	4054	PID_CMYB_PATHWAY	18	79	16174	2190	1.690966	0.01622
magenta	8386	KAECH_NAIVE_VS_DAY8_EFF_CD8_TCELL_UP	6	197	16056	167	2.964163	0.016222
magenta	10151	GSE7460_TCONV_VS_TREG_LN_DN	6	197	16056	167	2.964163	0.016222
grey60	6847	LEIN_PONS_MARKERS	2	72	16181	44	10.26073	0.016238
brown	563	CELLULAR_CARBOHYDRATE_METABOLIC_PROCESS	23	108	16145	2190	1.580496	0.016239
turquoise	9011	GSE17721_POLYIC_VS_CPG_6H_BMDM_DN	62	189	16064	4169	1.278885	0.016245
turquoise	9028	GSE17721_PAM3CSK4_VS_GADIQUIMOD_6H_BMDM_	62	189	16064	4169	1.278885	0.016245
blue	9599	GSE27786_NKCELL_VS_NKTCELL_DN	44	176	16077	2978	1.364422	0.016251
brown	6173	FLECHNER_PBL_KIDNEY_TRANSPLANT_OK_VS_DONOR_	30	150	16103	2190	1.484292	0.016255
brown	8697	GSE1460_DP_THYMOCYTE_VS_NAIVE_CD4_TCELL_COR	30	150	16103	2190	1.484292	0.016255
black	3796	MODULE_350	4	56	16197	283	4.102221	0.016273
magenta	3185	chr18q21	3	52	16201	167	5.614809	0.016298
magenta	5700	HOWLIN_PUBERTAL_MAMMARY_GLAND	3	52	16201	167	5.614809	0.016298
magenta	8359	CORDENONSI_YAP_CONSERVED_SIGNATURE	3	52	16201	167	5.614809	0.016298
yellow	7741	YAO_TEMPORAL_RESPONSE_TO_PROGESTERONE_CLUS	20	137	16116	1426	1.663886	0.0163
red	10070	GSE3982_BCELL_VS_TH2_UP	12	184	16069	524	2.022859	0.016311
purple	2274	CCCNNGGGAR_V$OLF1_01	7	271	15982	157	2.674008	0.016313
turquoise	9925	GSE37416_CTRL_VS_3H_F_TULARENSIS_LVS_NEUTROP	55	165	16088	4169	1.299512	0.016341
red	1998	ATGTTAA,MIR-302C	13	206	16047	524	1.957395	0.01635
midnightb	5176	ENK_UV_RESPONSE_KERATINOCYTE_DN	6	468	15785	71	2.934814	0.016351
grey60	10072	GSE3982_BASOPHIL_VS_EFF_MEMORY_CD4_TCELL_UF	3	198	16055	44	5.596763	0.016352
greenyello	1461	AACTTT_UNKNOWN	22	1473	14780	150	1.618312	0.016352

yellow	1878	V$YY1_Q6	30	228	16025	1426	1.499686	0.016359
brown	8723	GSE14769_UNSTIM_VS_120MIN_LPS_BMDM_DN	36	187	16066	2190	1.42873	0.016359
brown	9023	GSE17721_PAM3CSK4_VS_GADIQUIMOD_1H_BMDM_	36	187	16066	2190	1.42873	0.016359
brown	9144	GSE17721_0.5H_VS_24H_GARDIQUIMOD_BMDM_UP	36	187	16066	2190	1.42873	0.016359
brown	9728	GSE30962_ACUTE_VS_CHRONIC_LCMV_SECONDARY_II	36	187	16066	2190	1.42873	0.016359
blue	3365	chr17q21	55	228	16025	2978	1.316548	0.016363
black	9479	GSE26669_CTRL_VS_COSTIM_BLOCK_MLR_CD4_TCELL_	8	186	16067	283	2.470155	0.016377
black	9755	GSE32423_IL7_VS_IL7_IL4_MEMORY_CD8_TCELL_DN	8	186	16067	283	2.470155	0.016377
black	5503	SCHLOSSER_MYC_AND_SERUM_RESPONSE_SYNERGY	3	31	16222	283	5.557848	0.016383
brown	2270	GGGNNTTTCC_V$NFKB_Q6_01	24	114	16139	2190	1.562413	0.016389
brown	5851	RICKMAN_TUMOR_DIFFERENTIATED_MODERATELY_VS	24	114	16139	2190	1.562413	0.016389
salmon	9028	GSE17721_PAM3CSK4_VS_GADIQUIMOD_6H_BMDM_	5	189	16064	127	3.385618	0.016408
salmon	9040	GSE17721_LPS_VS_CPG_1H_BMDM_UP	5	189	16064	127	3.385618	0.016408
salmon	9814	GSE360_CTRL_VS_M_TUBERCULOSIS_MAC_UP	5	189	16064	127	3.385618	0.016408
red	6930	STEARMAN_LUNG_CANCER_EARLY_VS_LATE_UP	9	121	16132	524	2.307063	0.016411
cyan	368	TRANSCRIPTION_DNA_DEPENDENT	7	558	15695	77	2.647931	0.016415
turquoise	6989	FUJII_YBX1_TARGETS_DN	64	196	16057	4169	1.272992	0.016418
turquoise	8480	GSE11924_TFH_VS_TH1_CD4_TCELL_UP	64	196	16057	4169	1.272992	0.016418
turquoise	8870	GSE17721_CTRL_VS_PAM3CSK4_4H_BMDM_UP	64	196	16057	4169	1.272992	0.016418
turquoise	9594	GSE27786_CD8_TCELL_VS_NEUTROPHIL_UP	64	196	16057	4169	1.272992	0.016418
turquoise	9596	GSE27786_CD8_TCELL_VS_MONO_MAC_UP	64	196	16057	4169	1.272992	0.016418
turquoise	9606	GSE27786_NKTCELL_VS_ERYTHROBLAST_UP	64	196	16057	4169	1.272992	0.016418
greenyello	2406	MORF_IL9	3	58	16195	150	5.604483	0.016421
greenyello	4464	REACTOME_CLASS_B_2_SECRETIN_FAMILY_RECEPTORS	3	58	16195	150	5.604483	0.016421
green	1288	3_5_EXONUCLEASE_ACTIVITY	4	13	16240	1305	3.832125	0.016426
green	5571	KORKOLA_EMBRYONAL_CARCINOMA	4	13	16240	1305	3.832125	0.016426
green	5823	GOUYER_TATI_TARGETS_DN	4	13	16240	1305	3.832125	0.016426
green	6505	MACLACHLAN_BRCA1_TARGETS_DN	4	13	16240	1305	3.832125	0.016426
green	8099	LIU_IL13_PRIMING_MODEL	4	13	16240	1305	3.832125	0.016426
turquoise	1226	SPECIFIC_TRANSCRIPTIONAL_REPRESSOR_ACTIVITY	7	12	16241	4169	2.274146	0.016428
turquoise	4165	REACTOME_TETRAHYDROBIOPTERIN_BH4_SYNTHESIS_	7	12	16241	4169	2.274146	0.016428
turquoise	4996	GARGALOVIC_RESPONSE_TO_OXIDIZED_PHOSPHOLIPID	7	12	16241	4169	2.274146	0.016428
turquoise	6138	CHEOK_RESPONSE_TO_MERCAPTOPURINE_UP	7	12	16241	4169	2.274146	0.016428
tan	4327	REACTOME_RNA_POL_I_TRANSCRIPTION	3	60	16193	145	5.604483	0.016432
turquoise	7938	BRUINS_UVC_RESPONSE_EARLY_LATE	92	294	15959	4169	1.21995	0.016444
lightgreen	2221	YGTCCTTGR_UNKNOWN	2	76	16177	42	10.18358	0.016465
lightgreen	6591	ZHU_CMV_24_HR_DN	2	76	16177	42	10.18358	0.016465
lightgreen	7360	ROSS_LEUKEMIA_WITH_MLL_FUSIONS	2	76	16177	42	10.18358	0.016465
yellow	9496	GSE26928_CENTR_MEMORY_VS_CXCR5_POS_CD4_TCE	21	146	16107	1426	1.639383	0.016469
purple	5312	MCBRYAN_PUBERTAL_TGFB1_TARGETS_UP	5	153	16100	157	3.383081	0.016477
purple	6855	GAVIN_FOXP3_TARGETS_CLUSTER_P3	5	153	16100	157	3.383081	0.016477
cyan	462	I_KAPPAB_KINASE_NF_KAPPAB_CASCADE	3	113	16140	77	5.603839	0.016495
cyan	2843	KEGG_JAK_STAT_SIGNALING_PATHWAY	3	113	16140	77	5.603839	0.016495
cyan	6315	GERY_CEBP_TARGETS	3	113	16140	77	5.603839	0.016495
brown	1596	V$SP1_Q6	43	231	16022	2190	1.381484	0.016501
midnightb	8134	ABRAMSON_INTERACT_WITH_AIRE	2	45	16208	71	10.17402	0.016503
red	4863	IGARASHI_ATF4_TARGETS_DN	7	82	16171	524	2.647808	0.016519
red	6249	GALINDO_IMMUNE_RESPONSE_TO_ENTEROTOXIN	7	82	16171	524	2.647808	0.016519
red	6336	NGUYEN_NOTCH1_TARGETS_DN	7	82	16171	524	2.647808	0.016519
pink	9482	GSE26669_CD4_VS_CD8_TCELL_IN_MLR_UP	7	187	16066	228	2.668426	0.016528
blue	6710	BACOLOD_RESISTANCE_TO_ALKYLATING_AGENTS_DN	17	55	16198	2978	1.686922	0.016539
yellow	2731	KEGG_PYRIMIDINE_METABOLISM	15	94	16159	1426	1.818768	0.016542
pink	8292	ESC_V6.5_UP_LATE.V1_UP	6	145	16108	228	2.949728	0.01655
green	8714	GSE14769_UNSTIM_VS_20MIN_LPS_BMDM_UP	19	140	16113	1305	1.690241	0.016552
turquoise	6742	SARTIPY_NORMAL_AT_INSULIN_RESISTANCE_DN	10	20	16233	4169	1.949268	0.016586
brown	2921	BIOCARTA_SPPA_PATHWAY	7	21	16232	2190	2.47382	0.016591
brown	2951	BIOCARTA_CXCR4_PATHWAY	7	21	16232	2190	2.47382	0.016591
brown	2993	BIOCARTA_IGF1_PATHWAY	7	21	16232	2190	2.47382	0.016591
brown	3009	BIOCARTA_INSULIN_PATHWAY	7	21	16232	2190	2.47382	0.016591
brown	4160	REACTOME_GROWTH_HORMONE_RECEPTOR_SIGNALI	7	21	16232	2190	2.47382	0.016591

brown	6605	MARIADASON_RESPONSE_TO_BUTYRATE_SULINDAC_4	7	21	16232	2190	2.47382	0.016591
magenta	77	GOLGI_APPARATUS_PART	4	95	16158	167	4.097825	0.016613
magenta	874	ION_HOMEOSTASIS	4	95	16158	167	4.097825	0.016613
salmon	7850	KIM_ALL_DISORDERS_CALB1_CORR_UP	9	500	15753	127	2.303575	0.01662
brown	8909	GSE17721_CTRL_VS_GARDIQUIMOD_12H_BMDM_DN	35	181	16072	2190	1.435089	0.016627
midnightb	5338	DARWICHE_SQUAMOUS_CELL_CARCINOMA_UP	3	123	16130	71	5.583305	0.016642
turquoise	4576	REACTOME_RNA_POL_II_PRE_TRANSCRIPTION_EVENTS	23	59	16194	4169	1.519769	0.016651
blue	2813	KEGG_UBIQUITIN_MEDIATED_PROTEOLYSIS	34	130	16123	2978	1.427396	0.016664
grey60	6333	KANG_IMMORTALIZED_BY_TERT_DN	2	73	16180	44	10.12017	0.016667
green	79	PORE_COMPLEX	7	34	16219	1305	2.564142	0.016668
red	4807	PICCALUGA_ANGIOIMMUNOBLASTIC_LYMPHOMA_UP	11	163	16090	524	2.093184	0.016671
blue	6855	GAVIN_FOXP3_TARGETS_CLUSTER_P3	39	153	16100	2978	1.391176	0.01668
greenyello	3519	MODULE_27	7	285	15968	150	2.66131	0.016681
brown	1965	CACTGTG,MIR-128A,MIR-128B	53	295	15958	2190	1.333347	0.016684
turquoise	7079	BONOME_OVARIAN_CANCER_SURVIVAL_SUBOPTIMAL_	137	455	15798	4169	1.173845	0.016703
cyan	510	RNA_BIOSYNTHETIC_PROCESS	7	560	15693	77	2.638474	0.016709
brown	7237	WANG_TUMOR_INVASIVENESS_DN	39	206	16047	2190	1.405034	0.016714
green	1815	V$ATF1_Q6	23	179	16074	1305	1.600287	0.016719
salmon	8668	GSE1432_6H_VS_24H_IFNG_MICROGLIA_UP	5	190	16063	127	3.367799	0.016749
salmon	8680	GSE1448_CTRL_VS_ANTI_VBETA5_DP_THYMOCYTE_UP	5	190	16063	127	3.367799	0.016749
greenyello	1886	V$CREB_Q3	6	221	16032	150	2.941719	0.01676
blue	1953	CACTGCC,MIR-34A,MIR-34C,MIR-449	61	257	15996	2978	1.295405	0.016779
blue	119	SPINDLE_MICROTUBULE	7	16	16237	2978	2.387739	0.016781
blue	6036	ONDER_CDH1_TARGETS_3_UP	7	16	16237	2978	2.387739	0.016781
yellow	5289	ROYLANCE_BREAST_CANCER_16Q_COPY_NUMBER_DN	5	18	16235	1426	3.166004	0.016787
salmon	6772	KYNG_RESPONSE_TO_H2O2	3	69	16184	127	5.56419	0.016788
turquoise	3551	MODULE_62	32	88	16165	4169	1.41765	0.016789
lightcyan	5313	MCBRYAN_PUBERTAL_TGFB1_TARGETS_DN	2	52	16201	62	10.08251	0.016798
pink	687	RESPONSE_TO_ABIOTIC_STIMULUS	4	70	16183	228	4.073434	0.016815
turquoise	3525	MODULE_36	52	155	16098	4169	1.307896	0.016856
turquoise	5504	SCHLOSSER_MYC_TARGETS_REPRESSED_BY_SERUM	52	155	16098	4169	1.307896	0.016856
black	10111	GSE39820_CTRL_VS_TGFBETA3_IL6_IL23A_CD4_TCELL_	8	187	16066	283	2.456945	0.01686
red	5940	AMIT_SERUM_RESPONSE_40_MCF10A	4	31	16222	524	4.002216	0.016861
turquoise	6820	CREIGHTON_ENDOCRINE_THERAPY_RESISTANCE_3	189	644	15609	4169	1.144136	0.016861
lightgreen	2132	AACTGGA,MIR-145	3	210	16043	42	5.528231	0.016864
red	2209	WGTTNNNNNAAA_UNKNOWN	23	442	15811	524	1.614016	0.016868
purple	3217	chr7q31	3	56	16197	157	5.545837	0.016871
purple	5290	JOHANSSON_GLIOMAGENESIS_BY_PDGFB_UP	3	56	16197	157	5.545837	0.016871
purple	6846	LEIN_MIDBRAIN_MARKERS	3	56	16197	157	5.545837	0.016871
purple	7612	FONTAINE_PAPILLARY_THYROID_CARCINOMA_UP	3	56	16197	157	5.545837	0.016871
purple	7708	NAKAYAMA_SOFT_TISSUE_TUMORS_PCA2_DN	3	56	16197	157	5.545837	0.016871
cyan	5054	TONKS_TARGETS_OF_RUNX1_RUNX1T1_FUSION_SUST/	2	42	16211	77	10.05133	0.016877
cyan	6067	SHIPP_DLBCL_CURED_VS_FATAL_DN	2	42	16211	77	10.05133	0.016877
cyan	7319	NUTT_GBM_VS_AO_GLIOMA_UP	2	42	16211	77	10.05133	0.016877
lightgreen	6287	HESS_TARGETS_OF_HOXA9_AND_MEIS1_DN	2	77	16176	42	10.05133	0.016877
lightgreen	6969	WALLACE_PROSTATE_CANCER_RACE_DN	2	77	16176	42	10.05133	0.016877
brown	10048	GSE3982_NEUTROPHIL_VS_BASOPHIL_UP	34	175	16078	2190	1.441884	0.016884
blue	2369	MORF_CSNK2B	67	286	15967	2978	1.27855	0.016895
purple	8352	KRAS.DF.V1_DN	5	154	16099	157	3.361113	0.016902
purple	8355	TBK1.DF_UP	7	273	15980	157	2.654418	0.016915
red	8925	GSE17721_LPS_VS_POLYIC_12H_BMDM_DN	12	185	16068	524	2.011925	0.016952
red	8938	GSE17721_POLYIC_VS_PAM3CSK4_6H_BMDM_UP	12	185	16068	524	2.011925	0.016952
red	10178	GSE7460_WT_VS_FOXP3_HET_ACT_TCONV_UP	12	185	16068	524	2.011925	0.016952
lightcyan	9352	GSE22886_NEUTROPHIL_VS_DC_UP	3	142	16111	62	5.538278	0.016968
pink	4478	REACTOME_SLC_MEDIATED_TRANSMEMBRANE_TRANS	7	188	16065	228	2.654232	0.016969
tan	8021	PURBEY_TARGETS_OF_CTBP1_NOT_SATB1_UP	7	296	15957	145	2.650769	0.016994
yellow	100	MITOCHONDRIAL_MATRIX	9	46	16207	1426	2.229968	0.016994
yellow	113	MITOCHONDRIAL_LUMEN	9	46	16207	1426	2.229968	0.016994
yellow	2564	GCM_VAV1	9	46	16207	1426	2.229968	0.016994
tan	8588	GSE13485_DAY3_VS_DAY7_YF17D_VACCINE_PBMC_UI	5	167	16086	145	3.355978	0.017

tan	9830	GSE360_L_DONOVANI_VS_L_MAJOR_DC_UP	5	167	16086	145	3.355978	0.017
blue	1894	V$PAX_Q6	49	200	16053	2978	1.337134	0.017031
blue	9388	GSE22886_CTRL_VS_LPS_24H_DC_UP	49	200	16053	2978	1.337134	0.017031
blue	2604	GNF2_SMC1L1	10	27	16226	2978	2.021367	0.017034
blue	3043	BIOCARTA_MYOSIN_PATHWAY	10	27	16226	2978	2.021367	0.017034
blue	3799	MODULE_355	10	27	16226	2978	2.021367	0.017034
blue	4696	REACTOME_NEP_NS2_INTERACTS_WITH_THE_CELLULA	10	27	16226	2978	2.021367	0.017034
blue	4698	REACTOME_TRANSPORT_OF_RIBONUCLEOPROTEINS_II	10	27	16226	2978	2.021367	0.017034
brown	474	REGULATION_OF_SIGNAL_TRANSDUCTION	38	200	16053	2190	1.410078	0.017044
blue	4072	PID_CASPASE_PATHWAY	16	51	16202	2978	1.712216	0.017073
lightgreen	5222	GRAESSMANN_APOPTOSIS_BY_SERUM_DEPRIVATION_	3	211	16042	42	5.502031	0.017077
salmon	8564	GSE13484_UNSTIM_VS_12H_YF17D_VACCINE_STIM_PI	5	191	16062	127	3.350167	0.017094
salmon	9504	GSE2706_UNSTIM_VS_8H_LPS_DC_UP	5	191	16062	127	3.350167	0.017094
green	2593	GNF2_MLH1	8	42	16211	1305	2.372268	0.017107
turquoise	2515	GCM_DFFA	44	128	16125	4169	1.340122	0.01712
magenta	926	LYMPHOCYTE_ACTIVATION	3	53	16200	167	5.508869	0.017148
magenta	5941	AMIT_SERUM_RESPONSE_60_MCF10A	3	53	16200	167	5.508869	0.017148
purple	8363	KRAS.300_UP.V1_UP	4	102	16151	157	4.059698	0.017151
greenyello	3813	MODULE_375	3	59	16194	150	5.509492	0.017186
greenyello	4173	REACTOME_IMMUNOREGULATORY_INTERACTIONS_BE	3	59	16194	150	5.509492	0.017186
turquoise	5193	CASTELLANO_HRAS_AND_NRAS_TARGETS_UP	4	5	16248	4169	3.118829	0.017188
turquoise	7107	JONES_TCOF1_TARGETS	4	5	16248	4169	3.118829	0.017188
magenta	7549	WOO_LIVER_CANCER_RECURRENCE_UP	4	96	16157	167	4.05514	0.017201
turquoise	2039	ATAACCT,MIR-154	22	56	16197	4169	1.531568	0.017204
midnightb	7735	YAO_TEMPORAL_RESPONSE_TO_PROGESTERONE_CLUS	2	46	16207	71	9.952848	0.017205
red	260	NUCLEOBASENUCLEOSIDE_AND_NUCLEOTIDE_METABC	5	47	16206	524	3.2997	0.017208
red	6151	LEE_LIVER_CANCER_MYC_E2F1_DN	5	47	16206	524	3.2997	0.017208
lightgreen	4824	LIU_PROSTATE_CANCER_DN	4	386	15867	42	4.010116	0.017228
red	6074	BYSTRYKH_HEMATOPOIESIS_STEM_CELL_QTL_CIS	9	122	16131	524	2.288152	0.017231
greenyello	1729	V$POU6F1_01	5	162	16091	150	3.344239	0.017235
greenyello	5016	TAKEDA_TARGETS_OF_NUP98_HOXA9_FUSION_16D_U	5	162	16091	150	3.344239	0.017235
greenyello	9766	GSE33513_TCF7_KO_VS_HET_EARLY_THYMIC_PROGEN	5	162	16091	150	3.344239	0.017235
grey60	960	CATABOLIC_PROCESS	3	202	16051	44	5.485936	0.017239
purple	53	TIGHT_JUNCTION	2	21	16232	157	9.859266	0.017263
purple	75	CYTOSOLIC_PART	2	21	16232	157	9.859266	0.017263
purple	1616	V$STAT3_01	2	21	16232	157	9.859266	0.017263
purple	2921	BIOCARTA_SPPA_PATHWAY	2	21	16232	157	9.859266	0.017263
purple	4395	REACTOME_G_PROTEIN_ACTIVATION	2	21	16232	157	9.859266	0.017263
purple	5118	GRAHAM_CML_QUIESCENT_VS_CML_DIVIDING_UP	2	21	16232	157	9.859266	0.017263
purple	6024	JI_METASTASIS_REPRESSED_BY_STK11	2	21	16232	157	9.859266	0.017263
purple	6108	IIZUKA_LIVER_CANCER_PROGRESSION_G2_G3_UP	2	21	16232	157	9.859266	0.017263
purple	6183	CHEOK_RESPONSE_TO_HD_MTX_UP	2	21	16232	157	9.859266	0.017263
purple	6534	KANG_FLUOROURACIL_RESISTANCE_UP	2	21	16232	157	9.859266	0.017263
purple	6734	BACOLOD_RESISTANCE_TO_ALKYLATING_AGENTS_UP	2	21	16232	157	9.859266	0.017263
purple	7529	CHIANG_LIVER_CANCER_SUBCLASS_POLYSOMY7_DN	2	21	16232	157	9.859266	0.017263
cyan	8043	SERVITJA_ISLET_HNF1A_TARGETS_UP	3	115	16138	77	5.506381	0.017278
brown	7352	RUTELLA_RESPONSE_TO_HGF_VS_CSF2RB_AND_IL4_DI	44	238	16015	2190	1.372035	0.017282
turquoise	9049	GSE17721_LPS_VS_CPG_8H_BMDM_DN	63	193	16060	4169	1.272579	0.01729
turquoise	9339	GSE22886_IGG_IGA_MEMORY_BCELL_VS_BLOOD_PLAS	63	193	16060	4169	1.272579	0.01729
turquoise	9542	GSE27786_LSK_VS_NEUTROPHIL_UP	63	193	16060	4169	1.272579	0.01729
lightgreen	7150	LABBE_TGFB1_TARGETS_DN	2	78	16175	42	9.922466	0.017293
blue	6218	PENG_RAPAMYCIN_RESPONSE_DN	58	243	16010	2978	1.302658	0.017303
yellow	4437	REACTOME_3_UTR_MEDIATED_TRANSLATIONAL_REGU	16	103	16150	1426	1.770503	0.017318
greenyello	3680	MODULE_200	2	22	16231	150	9.850303	0.017319
turquoise	375	REGULATION_OF_TRANSFERASE_ACTIVITY	49	145	16108	4169	1.317437	0.017323
green	5216	MARKEY_RB1_CHRONIC_LOF_DN	15	103	16150	1305	1.813748	0.017329
purple	1685	V$RSRFC4_Q2	5	155	16098	157	3.339429	0.017333
purple	5886	WU_CELL_MIGRATION	5	155	16098	157	3.339429	0.017333
yellow	8640	GSE14308_TH1_VS_NAIVE_CD4_TCELL_UP	26	192	16061	1426	1.543427	0.017343
yellow	9157	GSE17974_0H_VS_4H_IN_VITRO_ACT_CD4_TCELL_DN	26	192	16061	1426	1.543427	0.017343

brown	9553	GSE27786_LIN_NEG_VS_NKCELL_DN	37	194	16059	2190	1.415433	0.017369
brown	9900	GSE36392_MAC_VS_NEUTROPHIL_IL25_TREATED_LUN	37	194	16059	2190	1.415433	0.017369
salmon	5896	DEN_INTERACT_WITH_LCA5	2	26	16227	127	9.844337	0.017418
purple	5294	MCBRYAN_PUBERTAL_BREAST_4_5WK_UP	6	213	16040	157	2.916121	0.017431
purple	7650	HOSHIDA_LIVER_CANCER_SUBCLASS_S3	6	213	16040	157	2.916121	0.017431
purple	7875	DUTERTRE_ESTRADIOL_RESPONSE_6HR_UP	6	213	16040	157	2.916121	0.017431
salmon	2371	MORF_CUL1	3	70	16183	127	5.484702	0.017441
salmon	8463	GSE11864_UNTREATED_VS_CSF1_IFNG_IN_MAC_DN	5	192	16061	127	3.332718	0.017445
salmon	8556	GSE13411_IGM_MEMORY_BCELL_VS_PLASMA_CELL_U	5	192	16061	127	3.332718	0.017445
salmon	8858	GSE17721_CTRL_VS_POLYIC_8H_BMDM_UP	5	192	16061	127	3.332718	0.017445
salmon	9697	GSE29618_PRE_VS_DAY7_POST_TIV_FLU_VACCINE_PD	5	192	16061	127	3.332718	0.017445
salmon	10059	GSE3982_NEUTROPHIL_VS_TH2_DN	5	192	16061	127	3.332718	0.017445
blue	1917	V$SP1_Q6_01	54	224	16029	2978	1.315693	0.017451
grey60	635	IMMUNE_RESPONSE	3	203	16050	44	5.458912	0.017465
red	1216	ENZYME_REGULATOR_ACTIVITY	16	276	15977	524	1.798097	0.017472
greenyello	7069	SMID_BREAST_CANCER_BASAL_DN	11	574	15679	150	2.076458	0.017473
green	993	CELL_MIGRATION	12	76	16177	1305	1.966485	0.017497
lightgreen	7875	DUTERTRE_ESTRADIOL_RESPONSE_6HR_UP	3	213	16040	42	5.450369	0.017507
yellow	9775	GSE339_CD4POS_VS_CD8POS_DC_IN_CULTURE_DN	25	183	16070	1426	1.557051	0.017513
turquoise	2659	GNF2_KPNB1	27	72	16181	4169	1.461951	0.017513
turquoise	8658	GSE1432_CTRL_VS_IFNG_1H_MICROGLIA_UP	56	169	16084	4169	1.291823	0.017531
lightcyan	7172	RAY_TUMORIGENESIS_BY_ERBB2_CDC25A_DN	3	144	16109	62	5.461358	0.017606
cyan	7406	ZHANG_TLX_TARGETS_36HR_UP	4	210	16043	77	4.020532	0.017608
red	9719	GSE30083_SP2_VS_SP4_THYMOCYTE_DN	12	186	16067	524	2.001108	0.017613
brown	1652	V$NFY_01	40	213	16040	2190	1.393702	0.017622
cyan	1075	GTP_BINDING	2	43	16210	77	9.817578	0.017647
cyan	1297	GUANYL_NUCLEOTIDE_BINDING	2	43	16210	77	9.817578	0.017647
cyan	2654	GNF2_INPP5D	2	43	16210	77	9.817578	0.017647
cyan	5751	KHETCHOUMIAN_TRIM24_TARGETS_UP	2	43	16210	77	9.817578	0.017647
magenta	4586	REACTOME_INTERACTION_BETWEEN_L1_AND_ANKYRI	2	20	16233	167	9.732335	0.017661
magenta	5704	HATADA_METHYLATED_IN_LUNG_CANCER_DN	2	20	16233	167	9.732335	0.017661
magenta	6250	VERNELL_RETINOBLASTOMA_PATHWAY_DN	2	20	16233	167	9.732335	0.017661
magenta	6480	HEDENFALK_BREAST_CANCER_BRACX_UP	2	20	16233	167	9.732335	0.017661
purple	2254	TGTTTGY_V$HNF3_Q6	11	549	15704	157	2.074217	0.017662
blue	7865	WANG_RESPONSE_TO_GSK3_INHIBITOR_SB216763_DN	79	345	15908	2978	1.249732	0.017665
red	8110	PEDRIOLI_MIR31_TARGETS_DN	19	347	15906	524	1.698347	0.017681
purple	6631	WEIGEL_OXIDATIVE_STRESS_BY_HNE_AND_TBH	3	57	16196	157	5.448542	0.017685
brown	3215	chr1q21	36	188	16065	2190	1.421131	0.017687
brown	6637	WELCSH_BRCA1_TARGETS_UP	36	188	16065	2190	1.421131	0.017687
brown	8513	GSE12845_IGD_NEG_BLOOD_VS_NAIVE_TONSIL_BCELL	36	188	16065	2190	1.421131	0.017687
brown	8701	GSE1460_DP_THYMOCYTE_VS_THYMIC_STROMAL_CEL	36	188	16065	2190	1.421131	0.017687
brown	8921	GSE17721_LPS_VS_POLYIC_6H_BMDM_DN	36	188	16065	2190	1.421131	0.017687
brown	9012	GSE17721_POLYIC_VS_CPG_8H_BMDM_UP	36	188	16065	2190	1.421131	0.017687
brown	9649	GSE29617_CTRL_VS_DAY3_TIV_FLU_VACCINE_PBMC_2	36	188	16065	2190	1.421131	0.017687
brown	9756	GSE32423_IL7_VS_IL4_MEMORY_CD8_TCELL_UP	36	188	16065	2190	1.421131	0.017687
brown	10006	GSE3982_MAST_CELL_VS_NKCELL_UP	36	188	16065	2190	1.421131	0.017687
tan	3947	PID_GLYPICAN_1PATHWAY	2	23	16230	145	9.746927	0.01769
brown	511	DETECTION_OF_STIMULUS	8	26	16227	2190	2.283527	0.017691
brown	4235	REACTOME_SIGNALLING_TO_RAS	8	26	16227	2190	2.283527	0.017691
turquoise	1707	V$E2F1_Q6	71	221	16032	4169	1.252471	0.017701
turquoise	1886	V$CREB_Q3	71	221	16032	4169	1.252471	0.017701
blue	2290	GCCATNTTG_V$YY1_Q6	91	404	15849	2978	1.229331	0.017709
magenta	1196	SUBSTRATE_SPECIFIC_TRANSMEMBRANE_TRANSPORTE	7	259	15994	167	2.630361	0.017713
purple	8309	CRX_NRL_DN.V1_DN	4	103	16150	157	4.020283	0.017713
yellow	9056	GSE17721_POLYIC_VS_GARDIQUIMOD_0.5H_BMDM_U	23	165	16088	1426	1.588759	0.017716
blue	8549	GSE13411_NAIVE_VS_SWITCHED_MEMORY_BCELL_DN	47	191	16062	2978	1.342992	0.01772
blue	9073	GSE17721_POLYIC_VS_GARDIQUIMOD_24H_BMDM_D	47	191	16062	2978	1.342992	0.01772
blue	9222	GSE18791_CTRL_VS_NEWCASTLE_VIRUS_DC_4H_UP	47	191	16062	2978	1.342992	0.01772
blue	9560	GSE27786_LIN_NEG_VS_MONO_MAC_UP	47	191	16062	2978	1.342992	0.01772
blue	2595	GNF2_MSH6	11	31	16222	2978	1.9366	0.017733

magenta	7912	WIERENGA_STAT5A_TARGETS_UP	6	201	16052	167	2.905175	0.017733
brown	4129	REACTOME_APOPTOTIC_CLEAVAGE_OF_CELLULAR_PR(	10	36	16217	2190	2.061517	0.017734
turquoise	1811	V$TEL2_Q6	73	228	16025	4169	1.248216	0.017738
turquoise	2298	MORF_BRCA1	73	228	16025	4169	1.248216	0.017738
turquoise	2841	KEGG_RIG_I_LIKE_RECEPTOR_SIGNALING_PATHWAY	21	53	16200	4169	1.544703	0.017738
green	2809	KEGG_NEUROACTIVE_LIGAND_RECEPTOR_INTERACTIO	19	141	16112	1305	1.678253	0.017739
brown	1956	GGGYGTGNY_UNKNOWN	96	580	15673	2190	1.22838	0.017743
turquoise	1801	V$NFMUE1_Q6	75	235	16018	4169	1.244214	0.017753
green	3687	MODULE_207	23	180	16073	1305	1.591396	0.017763
green	8729	GSE14769_20MIN_VS_360MIN_LPS_BMDM_DN	23	180	16073	1305	1.591396	0.017763
purple	8336	TGFB_UP.V1_UP	5	156	16097	157	3.318022	0.017772
blue	1534	V$MAX_01	55	229	16024	2978	1.310799	0.017794
salmon	8501	GSE12366_PLASMA_CELL_VS_MEMORY_BCELL_DN	5	193	16060	127	3.31545	0.0178
salmon	9396	GSE22886_NAIVE_CD4_TCELL_VS_48H_ACT_TH2_UP	5	193	16060	127	3.31545	0.0178
salmon	10049	GSE3982_NEUTROPHIL_VS_BASOPHIL_DN	5	193	16060	127	3.31545	0.0178
blue	5041	MULLIGHAN_NPM1_SIGNATURE_3_DN	38	149	16104	2978	1.391894	0.017808
red	5600	DACOSTA_UV_RESPONSE_VIA_ERCC3_COMMON_DN	24	469	15784	524	1.587233	0.017826
greenyello	1626	CATRRAGC_UNKNOWN	4	108	16145	150	4.013086	0.017827
green	5948	SHEN_SMARCA2_TARGETS_DN	27	220	16033	1305	1.528495	0.017831
black	7014	HANN_RESISTANCE_TO_BCL2_INHIBITOR_DN	3	32	16221	283	5.384165	0.01785
brown	510	RNA_BIOSYNTHETIC_PROCESS	93	560	15693	2190	1.232493	0.017853
blue	4447	REACTOME_ACTIVATION_OF_CHAPERONE_GENES_BY_	14	43	16210	2978	1.776922	0.017874
pink	9270	GSE20366_TREG_VS_NAIVE_CD4_TCELL_DEC205_CON\	7	190	16063	228	2.626293	0.017878
brown	2118	GAGCTGG,MIR-337	29	145	16108	2190	1.484292	0.017907
brown	8070	HUANG_GATA2_TARGETS_UP	29	145	16108	2190	1.484292	0.017907
midnightb	497	MEMBRANE_LIPID_BIOSYNTHETIC_PROCESS	2	47	16206	71	9.741085	0.017919
midnightb	3250	chr6q21	2	47	16206	71	9.741085	0.017919
midnightb	6120	KENNY_CTNNB1_TARGETS_DN	2	47	16206	71	9.741085	0.017919
grey60	7913	WIERENGA_STAT5A_TARGETS_DN	3	205	16048	44	5.405654	0.017922
tan	3370	chr3q13	3	62	16191	145	5.423693	0.01793
turquoise	4382	REACTOME_DARPP_32_EVENTS	11	23	16230	4169	1.864518	0.017948
turquoise	4589	REACTOME_TRANSPORT_OF_VITAMINS_NUCLEOSIDES_	11	23	16230	4169	1.864518	0.017948
turquoise	5376	EGUCHI_CELL_CYCLE_RB1_TARGETS	11	23	16230	4169	1.864518	0.017948
lightgreen	899	ACTIN_FILAMENT_BASED_MOVEMENT	1	7	16246	42	55.28231	0.017953
lightgreen	1190	HORMONE_BINDING	1	7	16246	42	55.28231	0.017953
lightgreen	7010	GAUTSCHI_SRC_SIGNALING	1	7	16246	42	55.28231	0.017953
brown	269	REGULATION_OF_SECRETION	9	31	16222	2190	2.154618	0.017974
brown	3321	chr20p11	9	31	16222	2190	2.154618	0.017974
brown	3466	SIG_REGULATION_OF_THE_ACTIN_CYTOSKELETON_BY_	9	31	16222	2190	2.154618	0.017974
brown	7338	POTTI_CYTOXAN_SENSITIVITY	9	31	16222	2190	2.154618	0.017974
brown	5805	LOCKWOOD_AMPLIFIED_IN_LUNG_CANCER	39	207	16046	2190	1.398246	0.017996
brown	8674	GSE14350_IL2RB_KO_VS_WT_TREG_UP	35	182	16071	2190	1.427204	0.017998
yellow	2466	MORF_SART1	11	62	16191	1426	2.022158	0.018001
turquoise	872	CELL_CYCLE_GO_0007049	92	295	15958	4169	1.215815	0.018003
yellow	5449	HESSON_TUMOR_SUPPRESSOR_CLUSTER_3P21_3	3	7	16246	1426	4.884692	0.018015
yellow	5511	FARMER_BREAST_CANCER_CLUSTER_8	3	7	16246	1426	4.884692	0.018015
yellow	8154	HOLLEMAN_DAUNORUBICIN_ALL_UP	3	7	16246	1426	4.884692	0.018015
purple	5074	SENESE_HDAC3_TARGETS_DN	10	479	15774	157	2.161217	0.018022
blue	782	CELLULAR_MACROMOLECULE_CATABOLIC_PROCESS	27	99	16154	2978	1.488461	0.018023
magenta	1636	V$NRSF_01	3	54	16199	167	5.406853	0.018023
magenta	5049	TONKS_TARGETS_OF_RUNX1_RUNX1T1_FUSION_MON	3	54	16199	167	5.406853	0.018023
magenta	7661	GUTIERREZ_CHRONIC_LYMPHOCYTIC_LEUKEMIA_DN	3	54	16199	167	5.406853	0.018023
turquoise	9681	GSE29618_PRE_VS_DAY7_FLU_VACCINE_MONOCYTE_I	60	183	16070	4169	1.278209	0.018024
salmon	2351	MORF_AP3D1	4	128	16125	127	3.999262	0.018044
red	610	RESPONSE_TO_CHEMICAL_STIMULUS	15	254	15999	524	1.831723	0.018052
blue	2868	KEGG_VASOPRESSIN_REGULATED_WATER_REABSORPT	13	39	16214	2978	1.81923	0.018065
red	6703	BAELDE_DIABETIC_NEPHROPATHY_DN	21	396	15857	524	1.64485	0.018069
greenyello	1888	V$E2F_Q2	5	164	16089	150	3.303455	0.018076
greenyello	2107	AAAGACA,MIR-511	5	164	16089	150	3.303455	0.018076
greenyello	8226	VEGF_A_UP.V1_UP	5	164	16089	150	3.303455	0.018076

greenyello	10098	GSE3982_NKCELL_VS_TH2_UP	5	164	16089	150	3.303455	0.018076
turquoise	2259	CTTTGT_V$LEF1_Q2	448	1608	14645	4169	1.08616	0.018088
red	9638	GSE29615_CTRL_VS_DAY3_LAIV_IFLU_VACCINE_PBMC_	11	165	16088	524	2.067812	0.018093
magenta	1439	ION_TRANSMEMBRANE_TRANSPORTER_ACTIVITY	6	202	16051	167	2.890793	0.018126
magenta	8078	PLASARI_TGFB1_TARGETS_10HR_DN	6	202	16051	167	2.890793	0.018126
turquoise	7226	MUELLER_PLURINET	90	288	15965	4169	1.218293	0.01813
lightyellow	1227	METALLOEXOPEPTIDASE_ACTIVITY	1	9	16244	33	54.72391	0.01813
lightyellow	2697	GNF2_SERPINI2	1	9	16244	33	54.72391	0.01813
lightyellow	4137	REACTOME_SYNTHESIS_OF_BILE_ACIDS_AND_BILE_SAI	1	9	16244	33	54.72391	0.01813
lightyellow	4471	REACTOME_PROSTANOID_LIGAND_RECEPTORS	1	9	16244	33	54.72391	0.01813
lightgreen	5092	SABATES_COLORECTAL_ADENOMA_UP	2	80	16173	42	9.674405	0.018138
brown	3288	chr10q	2	2	16251	2190	7.421461	0.018149
grey60	1827	V$ZF5_01	3	206	16047	44	5.379413	0.018153
blue	6338	CHIARETTI_ACUTE_LYMPHOBLASTIC_LEUKEMIA_ZAP70	19	64	16189	2978	1.620252	0.018158
salmon	10030	GSE3982_MAC_VS_NEUTROPHIL_UP	5	194	16059	127	3.29836	0.01816
salmon	10053	GSE3982_NEUTROPHIL_VS_CENT_MEMORY_CD4_TCEL	5	194	16059	127	3.29836	0.01816
salmon	10224	GSE9006_HEALTHY_VS_TYPE_1_DIABETES_PBMC_AT_I	5	194	16059	127	3.29836	0.01816
cyan	7672	KYNG_WERNER_SYNDROM_AND_NORMAL_AGING_DN	4	212	16041	77	3.982602	0.018166
greenyello	2879	KEGG_PATHWAYS_IN_CANCER	7	290	15963	150	2.615425	0.018169
blue	10256	GSE9650_NAIVE_VS_EXHAUSTED_CD8_TCELL_UP	48	196	16057	2978	1.336577	0.018178
brown	8315	RB_P107_DN.V1_DN	25	121	16132	2190	1.53336	0.018191
red	1920	V$NFAT_Q4_01	13	209	16044	524	1.929298	0.018209
turquoise	8492	GSE12366_GC_BCELL_VS_PLASMA_CELL_UP	62	190	16063	4169	1.272154	0.018211
turquoise	9091	GSE17721_LPS_VS_GARDIQUIMOD_24H_BMDM_DN	62	190	16063	4169	1.272154	0.018211
turquoise	9109	GSE17721_0.5H_VS_24H_POLYIC_BMDM_DN	62	190	16063	4169	1.272154	0.018211
tan	8820	GSE16522_ANTI_CD3CD28_STIM_VS_UNSTIM_NAIVE_(	5	170	16083	145	3.296755	0.018217
tan	9054	GSE17721_LPS_VS_CPG_24H_BMDM_UP	5	170	16083	145	3.296755	0.018217
purple	8883	GSE17721_CTRL_VS_CPG_1H_BMDM_DN	5	157	16096	157	3.296888	0.018218
purple	9620	GSE28237_FOLLICULAR_VS_LATE_GC_BCELL_UP	5	157	16096	157	3.296888	0.018218
pink	448	CARBOHYDRATE_TRANSPORT	2	15	16238	228	9.504678	0.018242
pink	870	PIGMENT_METABOLIC_PROCESS	2	15	16238	228	9.504678	0.018242
pink	1154	SH3_DOMAIN_BINDING	2	15	16238	228	9.504678	0.018242
pink	1338	TRANSMEMBRANE_RECEPTOR_PROTEIN_PHOSPHATAS	2	15	16238	228	9.504678	0.018242
pink	4425	REACTOME_POST_CHAPERONIN_TUBULIN_FOLDING_P	2	15	16238	228	9.504678	0.018242
pink	4946	ROY_WOUND_BLOOD_VESSEL_DN	2	15	16238	228	9.504678	0.018242
pink	5051	TONKS_TARGETS_OF_RUNX1_RUNX1T1_FUSION_ERYTI	2	15	16238	228	9.504678	0.018242
pink	5965	SHIN_B_CELL_LYMPHOMA_CLUSTER_9	2	15	16238	228	9.504678	0.018242
pink	7711	MIKKELSEN_ES_HCP_WITH_H3K27ME3	2	15	16238	228	9.504678	0.018242
turquoise	2590	GNF2_HAT1	20	50	16203	4169	1.559415	0.018244
brown	4031	PID_AVB3_INTEGRIN_PATHWAY	15	63	16190	2190	1.767015	0.018247
brown	5174	ENK_UV_RESPONSE_EPIDERMIS_DN	79	467	15786	2190	1.255451	0.01825
purple	3572	MODULE_84	10	480	15773	157	2.156714	0.018257
blue	3970	PID_MYC_ACTIVPATHWAY	22	77	16176	2978	1.55934	0.018261
turquoise	6331	VERNELL_RETINOBLASTOMA_PATHWAY_UP	26	69	16184	4169	1.469014	0.018264
purple	3865	MODULE_456	4	104	16149	157	3.981627	0.018287
purple	7978	AZARE_NEOPLASTIC_TRANSFORMATION_BY_STAT3_DN	4	104	16149	157	3.981627	0.018287
red	7943	MIYAGAWA_TARGETS_OF_EWSR1_ETS_FUSIONS_DN	12	187	16066	524	1.990407	0.018292
red	8563	GSE13484_UNSTIM_VS_3H_YF17D_VACCINE_STIM_PB	12	187	16066	524	1.990407	0.018292
red	8571	GSE13484_12H_VS_3H_YF17D_VACCINE_STIM_PBMC_	12	187	16066	524	1.990407	0.018292
red	9300	GSE2197_IMMUNOSUPPRESSIVE_DNA_VS_UNTREATED	12	187	16066	524	1.990407	0.018292
red	9484	GSE26669_CD4_VS_CD8_TCELL_IN_MLR_COSTIM_BLO	12	187	16066	524	1.990407	0.018292
red	9829	GSE360_DC_VS_MAC_M_TUBERCULOSIS_DN	12	187	16066	524	1.990407	0.018292
red	10213	GSE8384_CTRL_VS_B_ABORTUS_4H_MAC_CELL_LINE_I	12	187	16066	524	1.990407	0.018292
green	5708	CALVET_IRINOTECAN_SENSITIVE_VS_REVERTED_DN	2	3	16250	1305	8.302937	0.018294
pink	1726	V$GATA6_01	7	191	16062	228	2.612542	0.018344
pink	10194	GSE7852_TREG_VS_TCONV_THYMUS_UP	7	191	16062	228	2.612542	0.018344
black	1476	V$CREBP1_01	7	154	16099	283	2.610504	0.01835
lightgreen	1798	V$MAF_Q6	3	217	16036	42	5.349901	0.018386
magenta	3383	chr20q13	5	148	16105	167	3.287951	0.018408
magenta	8800	GSE15930_STIM_VS_STIM_AND_TRICHOSTATINA_24H_	5	148	16105	167	3.287951	0.018408

blue	8674	GSE14350_IL2RB_KO_VS_WT_TREG_UP	45	182	16071	2978	1.349429	0.018413
greenyello	2233	TGANTCA_V$AP1_C	15	895	15358	150	1.815978	0.018418
yellow	8906	GSE17721_CTRL_VS_GARDIQUIMOD_8H_BMDM_UP	26	193	16060	1426	1.53543	0.018427
yellow	9339	GSE22886_IGG_IGA_MEMORY_BCELL_VS_BLOOD_PLAS	26	193	16060	1426	1.53543	0.018427
yellow	9360	GSE22886_NAIVE_TCELL_VS_DC_UP	26	193	16060	1426	1.53543	0.018427
cyan	4047	PID_IL6_7PATHWAY	2	44	16209	77	9.594451	0.018431
cyan	6310	ZHAN_MULTIPLE_MYELOMA_CD2_UP	2	44	16209	77	9.594451	0.018431
grey60	7935	BRUINS_UVC_RESPONSE_VIA_TP53_GROUP_C	2	77	16176	44	9.594451	0.018431
turquoise	3129	chr1p13	30	82	16171	4169	1.426294	0.018436
turquoise	6336	NGUYEN_NOTCH1_TARGETS_DN	30	82	16171	4169	1.426294	0.018436
lightcyan	7349	RUTELLA_RESPONSE_TO_HGF_UP	5	402	15851	62	3.260512	0.018456
green	538	NITROGEN_COMPOUND_METABOLIC_PROCESS	18	132	16121	1305	1.698328	0.018464
green	38	EXTRACELLULAR_REGION	33	282	15971	1305	1.457431	0.018467
turquoise	2153	RRAGTTGT_UNKNOWN	66	204	16049	4169	1.261291	0.01848
turquoise	7862	CHICAS_RB1_TARGETS_GROWING	66	204	16049	4169	1.261291	0.01848
purple	3583	MODULE_95	9	411	15842	157	2.266912	0.018486
greenyello	4868	DAVICIONI_TARGETS_OF_PAX_FOXO1_FUSIONS_UP	6	226	16027	150	2.876637	0.0185
greenyello	5392	EBAUER_TARGETS_OF_PAX3_FOXO1_FUSION_UP	5	165	16088	150	3.283434	0.018507
purple	4762	REACTOME_TELOMERE_MAINTENANCE	3	58	16195	157	5.354601	0.01852
salmon	8430	GSE10325_LUPUS_CD4_TCELL_VS_LUPUS_MYELOID_U	5	195	16058	127	3.281446	0.018525
salmon	8840	GSE17721_CTRL_VS_LPS_6H_BMDM_UP	5	195	16058	127	3.281446	0.018525
salmon	9096	GSE17721_0.5H_VS_8H_LPS_BMDM_UP	5	195	16058	127	3.281446	0.018525
blue	9307	GSE22886_CD8_VS_CD4_NAIVE_TCELL_DN	42	168	16085	2978	1.364422	0.018529
green	207	SYNAPSE	5	20	16233	1305	3.113602	0.018542
green	4465	REACTOME_GLUCAGON_TYPE_LIGAND_RECEPTORS	5	20	16233	1305	3.113602	0.018542
brown	998	ORGAN_DEVELOPMENT	67	388	15865	2190	1.281541	0.018542
lightcyan	9241	GSE18791_UNSTIM_VS_NEWCATSLE_VIRUS_DC_2H_DI	3	147	16106	62	5.349901	0.018587
lightcyan	9593	GSE27786_CD8_TCELL_VS_ERYTHROBLAST_DN	3	147	16106	62	5.349901	0.018587
yellow	8808	GSE15930_STIM_VS_STIM_AND_IL-12_72H_CD8_T_CE	25	184	16069	1426	1.548589	0.018635
yellow	9524	GSE2706_2H_VS_8H_LPS_STIM_DC_UP	25	184	16069	1426	1.548589	0.018635
red	1032	NUCLEOCYTOPLASMIC_TRANSPORT	7	84	16169	524	2.584765	0.01864
red	7806	SASSON_RESPONSE_TO_GONADOTROPHINS_UP	7	84	16169	524	2.584765	0.01864
yellow	5061	UDAYAKUMAR_MED1_TARGETS_UP	19	130	16123	1426	1.665805	0.018665
purple	773	PROTEIN_COMPLEX_ASSEMBLY	5	158	16095	157	3.276022	0.018671
turquoise	2275	CGTSACG_V$PAX3_B	45	132	16121	4169	1.329047	0.018673
lightcyan	5518	SCHEIDEREIT_IKK_INTERACTING_PROTEINS	2	55	16198	62	9.532551	0.018676
lightcyan	7915	WIERENGA_STAT5A_TARGETS_GROUP2	2	55	16198	62	9.532551	0.018676
turquoise	4153	REACTOME_CROSS_PRESENTATION_OF_SOLUBLE_EXO	19	47	16206	4169	1.576004	0.018708
red	4900	PRAMOONJAGO_SOX4_TARGETS_UP	5	48	16205	524	3.230956	0.018715
red	5351	BEGUM_TARGETS_OF_PAX3_FOXO1_FUSION_UP	5	48	16205	524	3.230956	0.018715
salmon	2719	KEGG_CITRATE_CYCLE_TCA_CYCLE	2	27	16226	127	9.479732	0.018717
brown	10255	GSE9650_NAIVE_VS_EFF_CD8_TCELL_DN	37	195	16058	2190	1.408175	0.018736
magenta	1315	RECEPTOR_BINDING	7	262	15991	167	2.600242	0.01874
purple	3647	MODULE_163	9	412	15841	157	2.261409	0.018747
purple	7931	JOHNSTONE_PARVB_TARGETS_3_UP	9	412	15841	157	2.261409	0.018747
greenyello	1468	V$E2F_01	3	61	16192	150	5.328852	0.018775
salmon	4159	REACTOME_ANTIGEN_PROCESSING_CROSS_PRESENTAT	3	72	16181	127	5.332349	0.018791
red	4661	REACTOME_ION_CHANNEL_TRANSPORT	4	32	16221	524	3.877147	0.018791
green	2108	ATAAGCT,MIR-21	15	104	16149	1305	1.796309	0.018798
grey60	4546	REACTOME_PURINE_CATABOLISM	1	7	16246	44	52.76948	0.018801
grey60	5043	TONKS_TARGETS_OF_RUNX1_RUNX1T1_FUSION_SUST	1	7	16246	44	52.76948	0.018801
green	4987	OSWALD_HEMATOPOIETIC_STEM_CELL_IN_COLLAGEN_	27	221	16032	1305	1.521579	0.018814
cyan	3014	BIOCARTA_LECTIN_PATHWAY	1	4	16249	77	52.76948	0.018818
cyan	5566	BEGUM_TARGETS_OF_PAX3_FOXO1_FUSION_AND_PA	1	4	16249	77	52.76948	0.018818
yellow	5129	GRAHAM_NORMAL_QUIESCENT_VS_NORMAL_DIVIDIN	14	87	16166	1426	1.834099	0.018825
red	9267	GSE20366_EX_VIVO_VS_DEC205_CONVERSION_NAIVE_	11	166	16087	524	2.055355	0.018836
blue	2690	GNF2_RAN	24	86	16167	2978	1.523076	0.018848
greenyello	4950	NEWMAN_ERCC6_TARGETS_DN	2	23	16230	150	9.422029	0.018855
green	9887	GSE360_HIGH_DOSE_B_MALAYI_VS_M_TUBERCULOSIS	23	181	16072	1305	1.582604	0.018857
brown	2107	AAAGACA,MIR-511	32	164	16089	2190	1.44809	0.018859

yellow	972	DNA_PACKAGING	7	32	16221	1426	2.493228	0.018862
brown	5145	RODRIGUES_THYROID_CARCINOMA_ANAPLASTIC_DN	81	481	15772	2190	1.249768	0.018863
turquoise	2947	BIOCARTA_HDAC_PATHWAY	12	26	16227	4169	1.799325	0.018864
turquoise	6866	MARSON_FOXP3_TARGETS_STIMULATED_UP	12	26	16227	4169	1.799325	0.018864
yellow	4429	REACTOME_METABOLISM_OF_PROTEINS	47	394	15859	1426	1.359614	0.01887
purple	1417	CALMODULIN_BINDING	2	22	16231	157	9.411118	0.018871
purple	2626	GNF2_CDH11	2	22	16231	157	9.411118	0.018871
purple	3050	BIOCARTA_RAC1_PATHWAY	2	22	16231	157	9.411118	0.018871
purple	4457	REACTOME_INHIBITION_OF_INSULIN_SECRETION_BY_A	2	22	16231	157	9.411118	0.018871
purple	7611	FONTAINE_THYROID_TUMOR_UNCERTAIN_MALIGNAN	2	22	16231	157	9.411118	0.018871
purple	7702	TIAN_TNF_SIGNALING_NOT_VIA_NFKB	2	22	16231	157	9.411118	0.018871
purple	2834	KEGG_TIGHT_JUNCTION	4	105	16148	157	3.943706	0.018873
salmon	8870	GSE17721_CTRL_VS_PAM3CSK4_4H_BMDM_UP	5	196	16057	127	3.264704	0.018894
salmon	9126	GSE17721_0.5H_VS_12H_CPG_BMDM_UP	5	196	16057	127	3.264704	0.018894
green	5222	GRAESSMANN_APOPTOSIS_BY_SERUM_DEPRIVATION_	26	211	16042	1305	1.534666	0.018902
blue	8426	GSE10325_BCELL_VS_MYELOID_UP	46	187	16066	2978	1.342533	0.018918
magenta	6924	ZHANG_GATA6_TARGETS_DN	3	55	16198	167	5.308547	0.018922
magenta	7915	WIERENGA_STAT5A_TARGETS_GROUP2	3	55	16198	167	5.308547	0.018922
greenyello	5167	KOKKINAKIS_METHIONINE_DEPRIVATION_96HR_UP	4	110	16143	150	3.940121	0.018937
brown	7034	ACEVEDO_LIVER_CANCER_UP	147	929	15324	2190	1.174332	0.018943
brown	613	REGULATION_OF_GENE_EXPRESSION	98	595	15658	2190	1.222358	0.018945
red	755	NUCLEOTIDE_BIOSYNTHETIC_PROCESS	3	18	16235	524	5.169529	0.018956
red	1389	CHANNEL_REGULATOR_ACTIVITY	3	18	16235	524	5.169529	0.018956
red	2962	BIOCARTA_CALCINEURIN_PATHWAY	3	18	16235	524	5.169529	0.018956
red	6081	FERRANDO_TAL1_NEIGHBORS	3	18	16235	524	5.169529	0.018956
red	6524	WU_HBX_TARGETS_3_UP	3	18	16235	524	5.169529	0.018956
red	7165	CLIMENT_BREAST_CANCER_COPY_NUMBER_UP	3	18	16235	524	5.169529	0.018956
red	7607	KUROZUMI_RESPONSE_TO_ONCOCYTIC_VIRUS_AND_C	3	18	16235	524	5.169529	0.018956
blue	7190	CADWELL_ATG16L1_TARGETS_DN	18	60	16193	2978	1.637307	0.018987
turquoise	8955	GSE17721_PAM3CSK4_VS_CPG_4H_BMDM_DN	59	180	16073	4169	1.277854	0.018988
turquoise	9506	GSE2706_UNSTIM_VS_2H_LPS_AND_R848_DC_UP	59	180	16073	4169	1.277854	0.018988
red	2268	TGGAAA_V$NFAT_Q4_01	63	1506	14747	524	1.297531	0.018988
turquoise	650	PROTEIN_CATABOLIC_PROCESS	25	66	16187	4169	1.476718	0.01903
turquoise	6027	TCGA_GLIOBLASTOMA_COPY_NUMBER_UP	25	66	16187	4169	1.476718	0.01903
magenta	663	SYNAPTIC_TRANSMISSION	4	99	16154	167	3.932257	0.019039
green	5230	LANDIS_ERBB2_BREAST_PRENEOPLASTIC_DN	9	51	16202	1305	2.197836	0.019058
grey60	1464	V$SP1_01	3	210	16043	44	5.276948	0.019094
brown	9814	GSE360_CTRL_VS_M_TUBERCULOSIS_MAC_UP	36	189	16064	2190	1.413612	0.0191
red	1444	ATP_BINDING	10	145	16108	524	2.139116	0.019107
red	6033	ONDER_CDH1_TARGETS_2_DN	20	374	15879	524	1.658672	0.019118
blue	7666	WINNEPENNINCKX_MELANOMA_METASTASIS_UP	40	159	16094	2978	1.373004	0.019152
red	6801	DURCHDEWALD_SKIN_CARCINOGENESIS_DN	14	233	16020	524	1.863693	0.019156
red	3516	MODULE_24	19	350	15903	524	1.68379	0.019158
blue	5457	LINDGREN_BLADDER_CANCER_WITH_LOH_IN_CHR9Q	30	113	16140	2978	1.448944	0.019172
red	3865	MODULE_456	8	104	16149	524	2.385937	0.019177
turquoise	9057	GSE17721_POLYIC_VS_GARDIQUIMOD_0.5H_BMDM_D	61	187	16066	4169	1.271715	0.019182
purple	3205	chr4q33	1	2	16251	157	51.76115	0.019227
purple	7752	LY_AGING_OLD_UP	1	2	16251	157	51.76115	0.019227
blue	4139	REACTOME_SIGNALING_BY_RHO_GTPASES	28	104	16149	2978	1.469378	0.01925
magenta	5863	SCHAEFFER_PROSTATE_DEVELOPMENT_48HR_DN	8	325	15928	167	2.395652	0.019252
lightcyan	8756	GSE15659_CD45RA_NEG_CD4_TCELL_VS_ACTIVATED_T	3	149	16104	62	5.27809	0.019259
red	7443	MEISSNER_BRAIN_HCP_WITH_H3K4ME3_AND_H3K27M	36	779	15474	524	1.4334	0.019261
salmon	9175	GSE17974_CTRL_VS_ACT_IL4_AND_ANTI_IL12_4H_CD4	5	197	16056	127	3.248131	0.019268
salmon	9226	GSE18791_CTRL_VS_NEWCASTLE_VIRUS_DC_8H_UP	5	197	16056	127	3.248131	0.019268
salmon	9392	GSE22886_NAIVE_CD4_TCELL_VS_48H_ACT_TH1_UP	5	197	16056	127	3.248131	0.019268
pink	9118	GSE17721_0.5H_VS_8H_PAM3CSK4_BMDM_UP	7	193	16060	228	2.58547	0.019303
pink	9132	GSE17721_0.5H_VS_24H_CPG_BMDM_UP	7	193	16060	228	2.58547	0.019303
green	8371	KRAS.BREAST_UP.V1_UP	13	86	16167	1305	1.882643	0.019313
cyan	1499	V$CP2_01	4	216	16037	77	3.90885	0.019313
cyan	7364	VANTVEER_BREAST_CANCER_ESR1_DN	4	216	16037	77	3.90885	0.019313

lightcyan	6241	NADLER_OBESITY_UP	2	56	16197	62	9.362327	0.01932
lightcyan	7708	NAKAYAMA_SOFT_TISSUE_TUMORS_PCA2_DN	2	56	16197	62	9.362327	0.01932
cyan	3750	MODULE_292	3	120	16133	77	5.276948	0.019327
brown	6195	LU_IL4_SIGNALING	20	92	16161	2190	1.613361	0.01933
turquoise	8570	GSE13484_12H_VS_3H_YF17D_VACCINE_STIM_PBMC_	63	194	16059	4169	1.26602	0.019337
turquoise	8585	GSE13485_DAY1_VS_DAY7_YF17D_VACCINE_PBMC_DI	63	194	16059	4169	1.26602	0.019337
turquoise	8811	GSE15930_STIM_VS_STIM_AND_IFNAB_72H_CD8_T_C	63	194	16059	4169	1.26602	0.019337
turquoise	8874	GSE17721_CTRL_VS_PAM3CSK4_8H_BMDM_UP	63	194	16059	4169	1.26602	0.019337
turquoise	1199	PROTEIN_KINASE_ACTIVITY	83	264	15989	4169	1.225676	0.01938
midnightb	6530	MARIADASON_REGULATED_BY_HISTONE_ACETYLATIOI	2	49	16204	71	9.34349	0.019386
black	5211	CREIGHTON_AKT1_SIGNALING_VIA_MTOR_UP	3	33	16220	283	5.221009	0.019388
greenyello	9793	GSE360_CTRL_VS_L_DONOVANI_DC_DN	5	167	16086	150	3.244112	0.019388
greenyello	10264	GSE9650_EXHAUSTED_VS_MEMORY_CD8_TCELL_UP	5	167	16086	150	3.244112	0.019388
magenta	7745	SENGUPTA_EBNA1_ANTICORRELATED	5	150	16103	167	3.244112	0.019388
magenta	268	NEGATIVE_REGULATION_OF_MULTICELLULAR_ORGANI	2	21	16232	167	9.268891	0.01939
magenta	672	REGULATION_OF_CYTOKINE_PRODUCTION	2	21	16232	167	9.268891	0.01939
magenta	747	POSITIVE_REGULATION_OF_LYMPHOCYTE_ACTIVATION	2	21	16232	167	9.268891	0.01939
magenta	3435	chr4q13	2	21	16232	167	9.268891	0.01939
magenta	5594	DACOSTA_ERCC3_ALLELE_XPCS_VS_TTD_UP	2	21	16232	167	9.268891	0.01939
magenta	6300	NUMATA_CSF3_SIGNALING_VIA_STAT3	2	21	16232	167	9.268891	0.01939
brown	6679	JAZAERI_BREAST_CANCER_BRCA1_VS_BRCA2_UP	12	47	16206	2190	1.894841	0.019398
pink	735	CELLULAR_HOMEOSTASIS	5	110	16143	228	3.240231	0.019398
blue	8466	GSE11864_UNTREATED_VS_CSF1_IFNG_PAM3CYS_IN_I	47	192	16061	2978	1.335997	0.019404
greenyello	5394	PEREZ_TP63_TARGETS	7	294	15959	150	2.579841	0.019426
turquoise	3451	ST_TUMOR_NECROSIS_FACTOR_PATHWAY	13	29	16224	4169	1.74762	0.019427
black	8924	GSE17721_LPS_VS_POLYIC_12H_BMDM_UP	8	192	16061	283	2.392962	0.019431
green	8131	BOSCO_EPITHELIAL_DIFFERENTIATION_MODULE	7	35	16218	1305	2.490881	0.019432
lightyellow	7443	MEISSNER_BRAIN_HCP_WITH_H3K4ME3_AND_H3K27I	5	779	15474	33	3.161201	0.019443
turquoise	6952	LIN_MELANOMA_COPY_NUMBER_DN	17	41	16212	4169	1.616466	0.019447
pink	3555	MODULE_66	12	433	15820	228	1.975568	0.019463
purple	3776	MODULE_324	4	106	16147	157	3.906502	0.019469
purple	8240	P53_DN.V2_UP	4	106	16147	157	3.906502	0.019469
salmon	2417	MORF_MAP2K2	4	131	16122	127	3.907676	0.01947
greenyello	3610	MODULE_122	4	111	16142	150	3.904625	0.019508
brown	1365	RHO_GTPASE_ACTIVATOR_ACTIVITY	6	17	16236	2190	2.619339	0.01955
brown	3834	MODULE_408	6	17	16236	2190	2.619339	0.01955
brown	3942	PID_NFKAPPABATYPICALPATHWAY	6	17	16236	2190	2.619339	0.01955
brown	6603	YIH_RESPONSE_TO_ARSENITE_C4	6	17	16236	2190	2.619339	0.01955
brown	7984	KASLER_HDAC7_TARGETS_1_DN	6	17	16236	2190	2.619339	0.01955
blue	1333	OXIDOREDUCTASE_ACTIVITY_ACTING_ON_THE_CH_CH	8	20	16233	2978	2.183076	0.019554
red	1332	PATTERN_RECOGNITION_RECEPTOR_ACTIVITY	2	7	16246	524	8.86205	0.019564
red	2701	GNF2_SPRR1B	2	7	16246	524	8.86205	0.019564
red	4194	REACTOME_DEFENSINS	2	7	16246	524	8.86205	0.019564
red	4277	REACTOME_ABACAVIR_TRANSPORT_AND_METABOLISI	2	7	16246	524	8.86205	0.019564
red	4546	REACTOME_PURINE_CATABOLISM	2	7	16246	524	8.86205	0.019564
red	7256	MOOTHA_ROS	2	7	16246	524	8.86205	0.019564
red	8013	RAFFEL_VEGFA_TARGETS_UP	2	7	16246	524	8.86205	0.019564
yellow	8448	GSE11057_NAIVE_CD4_VS_PBMC_CD4_TCELL_UP	26	194	16059	1426	1.527516	0.019565
yellow	8814	GSE16522_MEMORY_VS_NAIVE_CD8_TCELL_UP	26	194	16059	1426	1.527516	0.019565
yellow	8866	GSE17721_CTRL_VS_PAM3CSK4_1H_BMDM_UP	26	194	16059	1426	1.527516	0.019565
yellow	9116	GSE17721_0.5H_VS_4H_PAM3CSK4_BMDM_UP	26	194	16059	1426	1.527516	0.019565
lightcyan	8528	GSE13229_MATURE_VS_INTMATURE_NKCELL_UP	3	150	16103	62	5.242903	0.019599
purple	8261	RAF_UP.V1_DN	5	160	16093	157	3.235072	0.019599
purple	9716	GSE30083_SP2_VS_SP3_THYMOCYTE_UP	5	160	16093	157	3.235072	0.019599
greenyello	1661	V$MYCMAX_B	6	229	16024	150	2.838952	0.019601
red	8659	GSE1432_CTRL_VS_IFNG_1H_MICROGLIA_DN	11	167	16086	524	2.043047	0.019602
red	8839	GSE17721_CTRL_VS_LPS_4H_BMDM_DN	11	167	16086	524	2.043047	0.019602
red	9020	GSE17721_PAM3CSK4_VS_GADIQUIMOD_0.5H_BMDI	11	167	16086	524	2.043047	0.019602
pink	7563	MEISSNER_NPC_HCP_WITH_H3K4ME2	11	383	15870	228	2.047352	0.019618
salmon	8698	GSE1460_DP_THYMOCYTE_VS_NAIVE_CD4_TCELL_ADU	5	198	16055	127	3.231727	0.019648

salmon	9357	GSE22886_DC_VS_MONOCYTE_DN	5	198	16055	127	3.231727	0.019648
red	7414	HAN_SATB1_TARGETS_UP	19	351	15902	524	1.678992	0.019671
magenta	4366	REACTOME_PEPTIDE_LIGAND_BINDING_RECEPTORS	4	100	16153	167	3.892934	0.019678
magenta	8275	CTIP_DN.V1_DN	4	100	16153	167	3.892934	0.019678
turquoise	2491	GCM_HDAC1	16	38	16215	4169	1.641489	0.019679
lightgreen	4926	CHARAFE_BREAST_CANCER_LUMINAL_VS_BASAL_DN	4	402	15851	42	3.850509	0.01969
red	1403	PURINE_RIBONUCLEOTIDE_BINDING	12	189	16064	524	1.969344	0.019709
red	8717	GSE14769_UNSTIM_VS_40MIN_LPS_BMDM_DN	12	189	16064	524	1.969344	0.019709
red	8825	GSE17580_TREG_VS_TEFF_DN	12	189	16064	524	1.969344	0.019709
red	9160	GSE17974_0H_VS_12H_IN_VITRO_ACT_CD4_TCELL_UP	12	189	16064	524	1.969344	0.019709
red	10016	GSE3982_DC_VS_BCELL_UP	12	189	16064	524	1.969344	0.019709
turquoise	132	MICROTUBULE	14	32	16221	4169	1.70561	0.019712
turquoise	1431	GENERAL_RNA_POLYMERASE_II_TRANSCRIPTION_FACT	14	32	16221	4169	1.70561	0.019712
red	1335	TRANSCRIPTION_FACTOR_ACTIVITY	16	280	15973	524	1.77241	0.019715
purple	6207	FLECHNER_BIOPSY_KIDNEY_TRANSPLANT_REJECTED_V	10	486	15767	157	2.130088	0.019715
brown	578	LEUKOCYTE_ACTIVATION	14	58	16195	2190	1.791387	0.01972
brown	3964	PID_AR_PATHWAY	14	58	16195	2190	1.791387	0.01972
brown	380	INTRACELLULAR_PROTEIN_TRANSPORT	28	140	16113	2190	1.484292	0.019735
magenta	1825	V$TFIIA_Q6	6	206	16047	167	2.834661	0.019755
turquoise	1008	REGULATION_OF_CELL_CYCLE	56	170	16083	4169	1.284224	0.01976
turquoise	9220	GSE18791_CTRL_VS_NEWCASTLE_VIRUS_DC_2H_UP	56	170	16083	4169	1.284224	0.01976
brown	3429	chr3q	3	5	16248	2190	4.452877	0.019768
brown	4138	REACTOME_RECYCLING_OF_BILE_ACIDS_AND_SALTS	3	5	16248	2190	4.452877	0.019768
brown	6123	CHESLER_BRAIN_QTL_TRANS	3	5	16248	2190	4.452877	0.019768
red	409	NUCLEAR_TRANSPORT	7	85	16168	524	2.554356	0.019769
red	3417	chr3q21	7	85	16168	524	2.554356	0.019769
red	5080	TIEN_INTESTINE_PROBIOTICS_2HR_DN	7	85	16168	524	2.554356	0.019769
turquoise	482	TRANSCRIPTION_INITIATION	15	35	16218	4169	1.670801	0.01978
turquoise	6149	CHESLER_BRAIN_HIGHEST_EXPRESSION	15	35	16218	4169	1.670801	0.01978
turquoise	6611	GENTILE_UV_RESPONSE_CLUSTER_D6	15	35	16218	4169	1.670801	0.01978
turquoise	6659	MMS_MOUSE_LYMPH_HIGH_4HRS_UP	15	35	16218	4169	1.670801	0.01978
turquoise	7664	GUTIERREZ_MULTIPLE_MYELOMA_DN	15	35	16218	4169	1.670801	0.01978
pink	7144	BOQUEST_STEM_CELL_DN	7	194	16059	228	2.572142	0.019795
blue	2556	GCM_SMO	17	56	16197	2978	1.656799	0.0198
brown	8532	GSE13306_TREG_VS_TCONV_LAMINA_PROPRIA_UP	34	177	16076	2190	1.425591	0.019805
brown	8841	GSE17721_CTRL_VS_LPS_6H_BMDM_DN	34	177	16076	2190	1.425591	0.019805
yellow	8508	GSE12845_IGD_POS_BLOOD_VS_PRE_GC_TONSIL_BCEI	25	185	16068	1426	1.540218	0.019814
yellow	9320	GSE22886_NAIVE_CD4_TCELL_VS_NKCELL_UP	25	185	16068	1426	1.540218	0.019814
purple	164	MEMBRANE_FRACTION	7	282	15971	157	2.569702	0.019824
greenyello	9294	GSE20715_0H_VS_6H_OZONE_TLR4_KO_LUNG_UP	5	168	16085	150	3.224802	0.019839
magenta	5601	DIRMEIER_LMP1_RESPONSE_LATE_UP	3	56	16197	167	5.213751	0.019846
brown	1868	GGAANCGGAANY_UNKNOWN	22	104	16149	2190	1.569924	0.019853
red	5148	GOZGIT_ESR1_TARGETS_UP	9	125	16128	524	2.233237	0.019872
blue	9128	GSE17721_0.5H_VS_4H_CPG_BMDM_UP	48	197	16056	2978	1.329792	0.019874
blue	9401	GSE22886_UNSTIM_VS_IL15_STIM_NKCELL_DN	48	197	16056	2978	1.329792	0.019874
blue	9544	GSE27786_LSK_VS_MONO_MAC_UP	48	197	16056	2978	1.329792	0.019874
yellow	7703	WONG_EMBRYONIC_STEM_CELL_CORE	40	327	15926	1426	1.394204	0.01988
lightgreen	7372	SWEET_KRAS_ONCOGENIC_SIGNATURE	2	84	16169	42	9.213719	0.01988
pink	227	PLASMA_MEMBRANE	24	1095	15158	228	1.562413	0.019902
cyan	7942	MIYAGAWA_TARGETS_OF_EWSR1_ETS_FUSIONS_UP	4	218	16035	77	3.872989	0.019903
blue	4701	REACTOME_EARLY_PHASE_OF_HIV_LIFE_CYCLE	6	13	16240	2978	2.518934	0.019909
blue	8101	FU_INTERACT_WITH_ALKBH8	6	13	16240	2978	2.518934	0.019909
tan	8782	GSE15930_NAIVE_VS_48H_IN_VITRO_STIM_CD8_TCELI	5	174	16079	145	3.220967	0.019927
yellow	2381	MORF_EIF3S2	31	241	16012	1426	1.466083	0.019931
lightcyan	6705	DAZARD_UV_RESPONSE_CLUSTER_G1	2	57	16196	62	9.198076	0.019974
black	10122	GSE5463_CTRL_VS_DEXAMETHASONE_TREATED_THYM	8	193	16060	283	2.380564	0.019978
greenyello	8168	DURAND_STROMA_MAX_UP	6	230	16023	150	2.826609	0.019978
brown	2402	MORF_IKBKG	26	128	16125	2190	1.507484	0.019983
turquoise	239	RNA_METABOLIC_PROCESS	219	757	15496	4169	1.127846	0.019985
turquoise	9066	GSE17721_POLYIC_VS_GARDIQUIMOD_8H_BMDM_UP	58	177	16076	4169	1.277487	0.020005

yellow	8643	GSE14308_TH1_VS_INDUCED_TREG_DN	24	176	16077	1426	1.55422	0.020022
yellow	8647	GSE14308_TH17_VS_NAIVE_CD4_TCELL_DN	24	176	16077	1426	1.55422	0.020022
yellow	934	NEGATIVE_REGULATION_OF_CATALYTIC_ACTIVITY	10	55	16198	1426	2.072294	0.020026
yellow	5813	TONG_INTERACT_WITH_PTTG1	10	55	16198	1426	2.072294	0.020026
salmon	8702	GSE1460_CD4_THYMOCYTE_VS_NAIVE_CD4_TCELL_CO	5	199	16054	127	3.215487	0.020032
salmon	9442	GSE24634_TREG_VS_TCONV_POST_DAY5_IL4_CONVER	5	199	16054	127	3.215487	0.020032
salmon	9909	GSE36476_CTRL_VS_TSST_ACT_16H_MEMORY_CD4_T(	5	199	16054	127	3.215487	0.020032
brown	658	MEMBRANE_ORGANIZATION_AND_BIOGENESIS	25	122	16131	2190	1.520791	0.02004
midnightb	1981	TTGGAGA,MIR-515-5P,MIR-519E	3	132	16121	71	5.202625	0.020041
cyan	6560	APRELIKOVA_BRCA1_TARGETS	2	46	16207	77	9.177301	0.020043
salmon	3046	BIOCARTA_PROTEASOME_PATHWAY	2	28	16225	127	9.14117	0.020055
salmon	6777	JOSEPH_RESPONSE_TO_SODIUM_BUTYRATE_UP	2	28	16225	127	9.14117	0.020055
purple	9652	GSE29617_DAY3_VS_DAY7_TIV_FLU_VACCINE_PBMC_	5	161	16092	157	3.214978	0.020075
purple	1931	V$PR_02	4	107	16146	157	3.869992	0.020078
purple	7871	HOELZEL_NF1_TARGETS_UP	4	107	16146	157	3.869992	0.020078
brown	6166	ROSS_AML_WITH_MLL_FUSIONS	17	75	16178	2190	1.682198	0.020085
pink	2098	RAAGNYNNCTTY_UNKNOWN	5	111	16142	228	3.21104	0.020089
pink	3503	MODULE_11	12	435	15818	228	1.966485	0.020094
purple	2198	TGCTGAY_UNKNOWN	9	417	15836	157	2.234294	0.020095
lightyellow	1156	CARBOXY_LYASE_ACTIVITY	1	10	16243	33	49.25152	0.020125
lightyellow	2838	KEGG_RENIN_ANGIOTENSIN_SYSTEM	1	10	16243	33	49.25152	0.020125
lightyellow	2961	BIOCARTA_SKP2E2F_PATHWAY	1	10	16243	33	49.25152	0.020125
lightyellow	4357	REACTOME_ENDOGENOUS_STEROLS	1	10	16243	33	49.25152	0.020125
lightyellow	4504	REACTOME_FACILITATIVE_NA_INDEPENDENT_GLUCOSI	1	10	16243	33	49.25152	0.020125
lightyellow	5169	PASTURAL_RIZ1_TARGETS_UP	1	10	16243	33	49.25152	0.020125
yellow	5166	KOKKINAKIS_METHIONINE_DEPRIVATION_48HR_DN	11	63	16190	1426	1.99006	0.020136
midnightb	408	REGULATION_OF_GROWTH	2	50	16203	71	9.15662	0.020137
magenta	7439	MEISSNER_NPC_HCP_WITH_H3_UNMETHYLATED	9	392	15861	167	2.234465	0.020142
turquoise	312	CHROMATIN_ASSEMBLY	8	15	16238	4169	2.07922	0.020149
turquoise	339	PEROXISOME_ORGANIZATION_AND_BIOGENESIS	8	15	16238	4169	2.07922	0.020149
turquoise	3296	chr2q23	8	15	16238	4169	2.07922	0.020149
turquoise	4476	REACTOME_CYCLIN_A_B1_ASSOCIATED_EVENTS_DURI	8	15	16238	4169	2.07922	0.020149
turquoise	5210	KOINUMA_COLON_CANCER_MSI_DN	8	15	16238	4169	2.07922	0.020149
turquoise	8096	TIAN_BHLHA15_TARGETS	8	15	16238	4169	2.07922	0.020149
turquoise	1610	V$SREBP1_01	51	153	16100	4169	1.299512	0.020153
brown	9933	GSE37416_CTRL_VS_48H_F_TULARENSIS_LVS_NEUTRO	37	196	16057	2190	1.40099	0.020189
brown	10038	GSE3982_MAC_VS_CENT_MEMORY_CD4_TCELL_UP	37	196	16057	2190	1.40099	0.020189
brown	10209	GSE7852_THYMUS_VS_FAT_TCONV_DN	37	196	16057	2190	1.40099	0.020189
salmon	3715	MODULE_243	3	74	16179	127	5.188232	0.020197
blue	9681	GSE29618_PRE_VS_DAY7_FLU_VACCINE_MONOCYTE_I	45	183	16070	2978	1.342055	0.0202
turquoise	9781	GSE339_EX_VIVO_VS_IN_CULTURE_CD4POS_DC_DN	60	184	16069	4169	1.271262	0.020206
turquoise	9985	GSE3982_EOSINOPHIL_VS_CENT_MEMORY_CD4_TCELL	60	184	16069	4169	1.271262	0.020206
cyan	8189	ZWANG_TRANSIENTLY_UP_BY_1ST_EGF_PULSE_ONLY	13	1472	14781	77	1.864139	0.02021
turquoise	6790	BILD_CTNNB1_ONCOGENIC_SIGNATURE	28	76	16177	4169	1.436303	0.020217
purple	953	INTERPHASE_OF_MITOTIC_CELL_CYCLE	3	60	16193	157	5.176115	0.020255
purple	1151	LYASE_ACTIVITY	3	60	16193	157	5.176115	0.020255
purple	1615	CAGNYGKNAAA_UNKNOWN	3	60	16193	157	5.176115	0.020255
purple	4327	REACTOME_RNA_POL_I_TRANSCRIPTION	3	60	16193	157	5.176115	0.020255
purple	5224	VANHARANTA_UTERINE_FIBROID_DN	3	60	16193	157	5.176115	0.020255
purple	5716	SHETH_LIVER_CANCER_VS_TXNIP_LOSS_PAM3	3	60	16193	157	5.176115	0.020255
purple	6575	GAJATE_RESPONSE_TO_TRABECTEDIN_UP	3	60	16193	157	5.176115	0.020255
red	181	NUCLEAR_MEMBRANE	5	49	16204	524	3.165018	0.020307
grey60	459	DEFENSE_RESPONSE	3	215	16038	44	5.154228	0.020308
grey60	1460	V$AP4_01	3	215	16038	44	5.154228	0.020308
tan	2526	GCM_ING1	3	65	16188	145	5.173369	0.020313
tan	6720	SESTO_RESPONSE_TO_UV_C7	3	65	16188	145	5.173369	0.020313
magenta	1385	ION_CHANNEL_ACTIVITY	4	101	16152	167	3.85439	0.02033
blue	2612	GNF2_APEX1	25	91	16162	2978	1.499365	0.02036
turquoise	2550	GCM_RAN	62	191	16062	4169	1.265494	0.020366
turquoise	8888	GSE17721_CTRL_VS_CPG_6H_BMDM_UP	62	191	16062	4169	1.265494	0.020366

turquoise	9069	GSE17721_POLYIC_VS_GARDIQUIMOD_12H_BMDM_D	62	191	16062	4169	1.265494	0.020366
turquoise	9222	GSE18791_CTRL_VS_NEWCASTLE_VIRUS_DC_4H_UP	62	191	16062	4169	1.265494	0.020366
pink	8269	PRC2_SUZ12_UP.V1_DN	6	152	16101	228	2.813885	0.020379
red	8407	GSE10239_NAIVE_VS_MEMORY_CD8_TCELL_DN	11	168	16085	524	2.030886	0.020391
red	8871	GSE17721_CTRL_VS_PAM3CSK4_4H_BMDM_DN	11	168	16085	524	2.030886	0.020391
red	9872	GSE360_L_MAJOR_VS_B_MALAYI_HIGH_DOSE_MAC_U	11	168	16085	524	2.030886	0.020391
blue	8471	GSE11864_CSF1_VS_CSF1_PAM3CYS_IN_MAC_DN	42	169	16084	2978	1.356349	0.020406
blue	9833	GSE360_L_DONOVANI_VS_T_GONDII_DC_DN	42	169	16084	2978	1.356349	0.020406
salmon	6701	XU_GH1_AUTOCRINE_TARGETS_UP	5	200	16053	127	3.199409	0.020421
salmon	9336	GSE22886_NAIVE_BCELL_VS_BM_PLASMA_CELL_UP	5	200	16053	127	3.199409	0.020421
salmon	9435	GSE24634_NAIVE_CD4_TCELL_VS_DAY5_IL4_CONV_TR	5	200	16053	127	3.199409	0.020421
salmon	9911	GSE36476_CTRL_VS_TSST_ACT_40H_MEMORY_CD4_T(	5	200	16053	127	3.199409	0.020421
magenta	7415	HAN_SATB1_TARGETS_DN	9	393	15860	167	2.228779	0.020438
magenta	3695	MODULE_215	1	2	16251	167	48.66168	0.020445
magenta	7218	ALONSO_METASTASIS_EMT_DN	1	2	16251	167	48.66168	0.020445
red	1531	V$CEBPA_01	12	190	16063	524	1.95898	0.020448
red	8492	GSE12366_GC_BCELL_VS_PLASMA_CELL_UP	12	190	16063	524	1.95898	0.020448
red	9010	GSE17721_POLYIC_VS_CPG_6H_BMDM_UP	12	190	16063	524	1.95898	0.020448
red	9266	GSE20366_EX_VIVO_VS_DEC205_CONVERSION_NAIVE_	12	190	16063	524	1.95898	0.020448
red	9420	GSE24142_EARLY_THYMIC_PROGENITOR_VS_DN2_THY	12	190	16063	524	1.95898	0.020448
red	9908	GSE36476_CTRL_VS_TSST_ACT_16H_MEMORY_CD4_T(	12	190	16063	524	1.95898	0.020448
red	10274	GSE9988_ANTI_TREM1_VS_CTRL_TREATED_MONOCYT	12	190	16063	524	1.95898	0.020448
turquoise	10188	GSE7764_IL15_TREATED_VS_CTRL_NK_CELL_24H_UP	64	198	16055	4169	1.260133	0.020489
lightgreen	441	REGULATION_OF_INTERFERON_GAMMA_BIOSYNTHETI	1	8	16245	42	48.37202	0.020491
lightgreen	3447	ST_INTERFERON_GAMMA_PATHWAY	1	8	16245	42	48.37202	0.020491
lightgreen	4837	FOURNIER_ACINAR_DEVELOPMENT_LATE_UP	1	8	16245	42	48.37202	0.020491
lightgreen	4905	PIEPOLI_LGI1_TARGETS_DN	1	8	16245	42	48.37202	0.020491
lightgreen	6284	ROETH_TERT_TARGETS_DN	1	8	16245	42	48.37202	0.020491
lightgreen	7599	SEIKE_LUNG_CANCER_POOR_SURVIVAL	1	8	16245	42	48.37202	0.020491
lightgreen	7833	NOUSHMEHR_GBM_SOMATIC_MUTATED	1	8	16245	42	48.37202	0.020491
lightgreen	8112	PHONG_TNF_TARGETS_DN	1	8	16245	42	48.37202	0.020491
blue	6818	CREIGHTON_ENDOCRINE_THERAPY_RESISTANCE_1	102	461	15792	2978	1.207558	0.020494
brown	1409	SH3_SH2_ADAPTOR_ACTIVITY	11	42	16211	2190	1.943716	0.020504
brown	2511	GCM_DDX11	11	42	16211	2190	1.943716	0.020504
brown	7319	NUTT_GBM_VS_AO_GLIOMA_UP	11	42	16211	2190	1.943716	0.020504
black	9570	GSE27786_BCELL_VS_ERYTHROBLAST_UP	8	194	16059	283	2.368293	0.020534
black	10222	GSE8868_SPLEEN_VS_INTESTINE_CD11B_POS_CD11C_I	8	194	16059	283	2.368293	0.020534
purple	5541	HERNANDEZ_ABERRANT_MITOSIS_BY_DOCETACEL_2NI	2	23	16230	157	9.001939	0.020539
purple	5581	TERAMOTO_OPN_TARGETS_CLUSTER_6	2	23	16230	157	9.001939	0.020539
purple	8054	PEDERSEN_METASTASIS_BY_ERBB2_ISOFORM_6	2	23	16230	157	9.001939	0.020539
magenta	2279	CTAWWWATA_V$RSRFC4_Q2	7	267	15986	167	2.551549	0.020544
grey60	1499	V$CP2_01	3	216	16037	44	5.130366	0.020556
purple	1832	V$HNF3ALPHA_Q6	5	162	16091	157	3.195132	0.020558
purple	5016	TAKEDA_TARGETS_OF_NUP98_HOXA9_FUSION_16D_U	5	162	16091	157	3.195132	0.020558
blue	2757	KEGG_INOSITOL_PHOSPHATE_METABOLISM	16	52	16201	2978	1.679289	0.020576
turquoise	4728	REACTOME_SIGNALING_BY_TGF_BETA_RECEPTOR_COI	23	60	16193	4169	1.494439	0.020595
brown	8868	GSE17721_CTRL_VS_PAM3CSK4_2H_BMDM_UP	36	190	16063	2190	1.406172	0.020601
brown	9122	GSE17721_ALL_VS_24H_PAM3CSK4_BMDM_UP	36	190	16063	2190	1.406172	0.020601
brown	9835	GSE360_L_DONOVANI_VS_B_MALAYI_HIGH_DOSE_DC_	36	190	16063	2190	1.406172	0.020601
brown	10026	GSE3982_DC_VS_TH1_UP	36	190	16063	2190	1.406172	0.020601
magenta	2166	CTTTGCA,MIR-527	6	208	16045	167	2.807404	0.020606
cyan	6807	ZHONG_SECRETOME_OF_LUNG_CANCER_AND_FIBROB	3	123	16130	77	5.148242	0.020618
cyan	8317	RB_P130_DN.V1_DN	3	123	16130	77	5.148242	0.020618
turquoise	1459	V$CMYB_01	68	212	16041	4169	1.250474	0.020635
lightcyan	2622	GNF2_CD53	2	58	16195	62	9.039488	0.020638
turquoise	1535	V$USF_01	74	233	16020	4169	1.238162	0.020646
pink	798	PROTEIN_AMINO_ACID_O_LINKED_GLYCOSYLATION	2	16	16237	228	8.910636	0.020658
pink	3903	MODULE_530	2	16	16237	228	8.910636	0.020658
pink	4570	REACTOME_ENERGY_DEPENDENT_REGULATION_OF_M	2	16	16237	228	8.910636	0.020658
pink	6683	KYNG_RESPONSE_TO_H2O2_VIA_ERCC6	2	16	16237	228	8.910636	0.020658

pink	7631	STEIN_ESTROGEN_RESPONSE_NOT_VIA_ESRRA	2	16	16237	228	8.910636	0.020658
pink	8343	SINGH_KRAS_DEPENDENCY_SIGNATURE_	2	16	16237	228	8.910636	0.020658
black	5501	SCHLOSSER_SERUM_RESPONSE_UP	6	123	16130	283	2.801517	0.020677
lightgreen	3587	MODULE_99	3	227	16026	42	5.114223	0.02069
purple	2067	CCTGTGA,MIR-513	4	108	16145	157	3.834159	0.020698
purple	3773	MODULE_321	4	108	16145	157	3.834159	0.020698
tan	1009	CHROMATIN_ASSEMBLY_OR_DISASSEMBLY	2	25	16228	145	8.967172	0.020735
tan	3163	chr4q35	2	25	16228	145	8.967172	0.020735
tan	7261	BOYLAN_MULTIPLE_MYELOMA_D_CLUSTER_UP	2	25	16228	145	8.967172	0.020735
tan	8347	STK33_SKM_DN	6	240	16013	145	2.802241	0.020735
red	6770	MAHAJAN_RESPONSE_TO_IL1A_UP	6	67	16186	524	2.777658	0.020737
grey60	6186	SMITH_TERT_TARGETS_DN	2	82	16171	44	9.009424	0.020742
grey60	7917	ACOSTA_PROLIFERATION_INDEPENDENT_MYC_TARGET	2	82	16171	44	9.009424	0.020742
yellow	7397	LEE_DIFFERENTIATING_T_LYMPHOCYTE	26	195	16058	1426	1.519682	0.020756
greenyello	10128	GSE6269_HEALTHY_VS_E_COLI_INF_PBMC_UP	5	170	16083	150	3.186863	0.020761
brown	7043	MITSIADES_RESPONSE_TO_APLIDIN_UP	70	410	15843	2190	1.267079	0.02078
brown	3365	chr17q21	42	228	16025	2190	1.367111	0.020783
tan	10039	GSE3982_MAC_VS_CENT_MEMORY_CD4_TCELL_DN	5	176	16077	145	3.184365	0.02082
tan	10090	GSE3982_CENT_MEMORY_CD4_TCELL_VS_NKCELL_UP	5	176	16077	145	3.184365	0.02082
turquoise	2529	GCM_MAP4K4	55	167	16086	4169	1.283949	0.020824
midnightb	9188	GSE17974_IL4_AND_ANTI_IL12_VS_UNTREATED_1H_A	3	134	16119	71	5.124974	0.020845
red	1303	PHOSPHORIC_DIESTER_HYDROLASE_ACTIVITY	4	33	16220	524	3.759658	0.020853
red	2566	CAR_HPX	4	33	16220	524	3.759658	0.020853
red	4040	PID_UPA_UPAR_PATHWAY	4	33	16220	524	3.759658	0.020853
red	7284	SPIRA_SMOKERS_LUNG_CANCER_UP	4	33	16220	524	3.759658	0.020853
red	7482	VALK_AML_CLUSTER_5	4	33	16220	524	3.759658	0.020853
cyan	2647	GNF2_HLA-C	2	47	16206	77	8.982039	0.02087
cyan	7017	WORSCHECH_TUMOR_REJECTION_UP	2	47	16206	77	8.982039	0.02087
purple	1790	V$HNF4ALPHA_Q6	6	222	16031	157	2.7979	0.0209
green	3396	chr6p22	11	69	16184	1305	1.985485	0.020919
salmon	7739	YAO_TEMPORAL_RESPONSE_TO_PROGESTERONE_CLUS	3	75	16178	127	5.119055	0.020921
magenta	6855	GAVIN_FOXP3_TARGETS_CLUSTER_P3	5	153	16100	167	3.180502	0.020922
magenta	9698	GSE29618_PRE_VS_DAY7_POST_LAIV_FLU_VACCINE_M	5	153	16100	167	3.180502	0.020922
red	7349	RUTELLA_RESPONSE_TO_HGF_UP	21	402	15851	524	1.6203	0.020949
brown	2507	GCM_CRKL	15	64	16189	2190	1.739405	0.020952
brown	4074	PID_BETACATENIN_NUC_PATHWAY	15	64	16189	2190	1.739405	0.020952
brown	6091	BYSTRYKH_HEMATOPOIESIS_STEM_CELL_AND_BRAIN_	15	64	16189	2190	1.739405	0.020952
brown	7998	DUAN_PRDM5_TARGETS	15	64	16189	2190	1.739405	0.020952
pink	9002	GSE17721_POLYIC_VS_CPG_0.5H_BMDM_UP	6	153	16100	228	2.795494	0.020971
turquoise	6416	IVANOVA_HEMATOPOIESIS_MATURE_CELL	87	279	15974	4169	1.215673	0.020989
yellow	5936	AMIT_EGF_RESPONSE_120_MCF10A	8	40	16213	1426	2.279523	0.020995
lightcyan	5932	AMIT_EGF_RESPONSE_480_HELA	3	154	16099	62	5.106724	0.020995
lightcyan	8750	GSE15659_NAIVE_CD4_TCELL_VS_ACTIVATED_TREG_U	3	154	16099	62	5.106724	0.020995
magenta	7012	AMBROSINI_FLAVOPIRIDOL_TREATMENT_TP53	4	102	16151	167	3.816602	0.020996
black	3969	PID_FRA_PATHWAY	3	34	16219	283	5.06745	0.020996
salmon	2300	MORF_BUB3	6	275	15978	127	2.792212	0.020997
blue	8641	GSE14308_TH1_VS_NAIVE_CD4_TCELL_DN	43	174	16079	2978	1.348739	0.021
brown	8587	GSE13485_DAY1_VS_DAY21_YF17D_VACCINE_PBMC_D	35	184	16069	2190	1.411691	0.021009
brown	8907	GSE17721_CTRL_VS_GARDIQUIMOD_8H_BMDM_DN	35	184	16069	2190	1.411691	0.021009
brown	8944	GSE17721_POLYIC_VS_PAM3CSK4_16H_BMDM_UP	35	184	16069	2190	1.411691	0.021009
brown	8957	GSE17721_PAM3CSK4_VS_CPG_6H_BMDM_DN	35	184	16069	2190	1.411691	0.021009
brown	7934	BRUINS_UVC_RESPONSE_VIA_TP53_GROUP_B	79	470	15783	2190	1.247437	0.021028
purple	2023	GTATTAT,MIR-369-3P	5	163	16090	157	3.17553	0.021049
yellow	6223	PENG_RAPAMYCIN_RESPONSE_UP	25	186	16067	1426	1.531938	0.02105
yellow	8634	GSE14308_TH2_VS_INDUCED_TREG_UP	25	186	16067	1426	1.531938	0.02105
yellow	8649	GSE14308_TH17_VS_INDUCED_TREG_DN	25	186	16067	1426	1.531938	0.02105
pink	707	MACROMOLECULE_BIOSYNTHETIC_PROCESS	9	289	15964	228	2.219951	0.021077
turquoise	5259	OUELLET_OVARIAN_CANCER_INVASIVE_VS_LMP_UP	40	116	16137	4169	1.344323	0.02108
blue	6567	BROWNE_HCMV_INFECTION_14HR_UP	37	146	16107	2978	1.383113	0.021098
black	9893	GSE36392_TYPE_2_MYELOID_VS_MAC_IL25_TREATED_	8	195	16058	283	2.356148	0.021102

red	8348	STK33_SKM_UP	15	259	15994	524	1.796362	0.021108
purple	2901	KEGG_ARRHYTHMOGENIC_RIGHT_VENTRICULAR_CARD	3	61	16192	157	5.09126	0.021156
purple	6098	JECHLINGER_EPITHELIAL_TO_MESENCHYMAL_TRANSIT	3	61	16192	157	5.09126	0.021156
purple	6165	JECHLINGER_EPITHELIAL_TO_MESENCHYMAL_TRANSIT	3	61	16192	157	5.09126	0.021156
purple	6775	WESTON_VEGFA_TARGETS_3HR	3	61	16192	157	5.09126	0.021156
purple	7609	FONTAINE_FOLLICULAR_THYROID_ADENOMA_DN	3	61	16192	157	5.09126	0.021156
purple	7613	FONTAINE_PAPILLARY_THYROID_CARCINOMA_DN	3	61	16192	157	5.09126	0.021156
blue	5039	MULLIGHAN_MLL_SIGNATURE_2_DN	61	260	15993	2978	1.280458	0.021158
turquoise	2077	MCAATNNNNNGCG_UNKNOWN	27	73	16180	4169	1.441925	0.021158
turquoise	2400	MORF_HEAB	27	73	16180	4169	1.441925	0.021158
red	1726	V$GATA6_01	12	191	16062	524	1.948723	0.021207
red	9580	GSE27786_CD4_TCELL_VS_NKTCELL_UP	12	191	16062	524	1.948723	0.021207
red	10194	GSE7852_TREG_VS_TCONV_THYMUS_UP	12	191	16062	524	1.948723	0.021207
blue	7764	GERHOLD_RESPONSE_TO_TZD_UP	3	4	16249	2978	4.093267	0.02121
blue	9067	GSE17721_POLYIC_VS_GARDIQUIMOD_8H_BMDM_DN	47	193	16060	2978	1.329075	0.021216
blue	9542	GSE27786_LSK_VS_NEUTROPHIL_UP	47	193	16060	2978	1.329075	0.021216
blue	10049	GSE3982_NEUTROPHIL_VS_BASOPHIL_DN	47	193	16060	2978	1.329075	0.021216
grey60	6290	FERRANDO_T_ALL_WITH_MLL_ENL_FUSION_DN	2	83	16170	44	8.900876	0.021218
grey60	8351	KRAS.AMP.LUNG_UP.V1_UP	2	83	16170	44	8.900876	0.021218
turquoise	1122	LIGASE_ACTIVITY	34	96	16157	4169	1.380732	0.02123
turquoise	1193	TRANSFERASE_ACTIVITY_TRANSFERRING_GLYCOSYL_GI	34	96	16157	4169	1.380732	0.02123
greenyello	1809	V$TCF4_Q5	5	171	16082	150	3.168226	0.021232
greenyello	5395	PEREZ_TP53_AND_TP63_TARGETS	5	171	16082	150	3.168226	0.021232
greenyello	9400	GSE22886_UNSTIM_VS_IL15_STIM_NKCELL_UP	5	171	16082	150	3.168226	0.021232
salmon	5570	PATIL_LIVER_CANCER	11	700	15553	127	2.011057	0.021236
blue	7066	SMID_BREAST_CANCER_NORMAL_LIKE_UP	91	407	15846	2978	1.22027	0.021258
brown	1963	AAAGGGA,MIR-204,MIR-211	38	203	16050	2190	1.389239	0.021263
green	391	RESPONSE_TO_TOXIN	3	8	16245	1305	4.670402	0.021277
blue	105	OUTER_MEMBRANE	9	24	16229	2978	2.046634	0.021284
blue	290	CHROMATIN_REMODELING	9	24	16229	2978	2.046634	0.021284
blue	3483	ST_INTERLEUKIN_4_PATHWAY	9	24	16229	2978	2.046634	0.021284
blue	3640	MODULE_155	9	24	16229	2978	2.046634	0.021284
brown	1762	V$STAT6_02	41	222	16031	2190	1.37063	0.021291
greenyello	6844	LEIN_OLIGODENDROCYTE_MARKERS	3	64	16189	150	5.079063	0.021307
grey60	1819	V$HEB_Q6	3	219	16034	44	5.060087	0.021309
lightcyan	4038	PID_AP1_PATHWAY	2	59	16194	62	8.886277	0.02131
lightcyan	6111	SHEPARD_BMYB_TARGETS	2	59	16194	62	8.886277	0.02131
lightcyan	6339	PETROVA_PROX1_TARGETS_DN	2	59	16194	62	8.886277	0.02131
pink	8724	GSE14769_UNSTIM_VS_240MIN_LPS_BMDM_UP	7	197	16056	228	2.532973	0.021325
purple	4417	REACTOME_SIGNALING_BY_PDGF	4	109	16144	157	3.798983	0.021329
blue	3279	chr7p22	19	65	16188	2978	1.595325	0.021369
blue	2198	TGCTGAY_UNKNOWN	93	417	15836	2978	1.217183	0.02137
cyan	7158	WEST_ADRENOCORTICAL_TUMOR_DN	6	458	15795	77	2.765213	0.021381
turquoise	416	CELLULAR_PROTEIN_CATABOLIC_PROCESS	22	57	16196	4169	1.504698	0.021385
turquoise	2853	KEGG_LONG_TERM_POTENTIATION	22	57	16196	4169	1.504698	0.021385
turquoise	6129	PEART_HDAC_PROLIFERATION_CLUSTER_UP	22	57	16196	4169	1.504698	0.021385
yellow	6556	BURTON_ADIPOGENESIS_5	18	123	16130	1426	1.667944	0.021388
brown	7744	YAO_TEMPORAL_RESPONSE_TO_PROGESTERONE_CLUS	34	178	16075	2190	1.417582	0.021409
brown	8324	RPS14_DN.V1_UP	34	178	16075	2190	1.417582	0.021409
brown	8901	GSE17721_CTRL_VS_GARDIQUIMOD_2H_BMDM_DN	34	178	16075	2190	1.417582	0.021409
brown	9148	GSE17721_12H_VS_24H_GARDIQUIMOD_BMDM_UP	34	178	16075	2190	1.417582	0.021409
brown	9817	GSE360_DC_VS_MAC_DN	34	178	16075	2190	1.417582	0.021409
brown	10120	GSE39820_TGFBETA1_VS_TGFBETA3_IN_IL6_IL23A_TR	34	178	16075	2190	1.417582	0.021409
yellow	8010	VANLOO_SP3_TARGETS_DN	13	80	16173	1426	1.852113	0.021415
black	6725	BANDRES_RESPONSE_TO_CARMUSTIN_MGMT_48HR_I	6	124	16129	283	2.778924	0.021422
brown	2290	GCCATNTTG_V$YY1_Q6	69	404	15849	2190	1.267527	0.021427
cyan	7648	HOSHIDA_LIVER_CANCER_SUBCLASS_S1	4	223	16030	77	3.786151	0.021427
salmon	2791	KEGG_RNA_POLYMERASE	2	29	16224	127	8.825957	0.021431
salmon	4099	PID_BARD1PATHWAY	2	29	16224	127	8.825957	0.021431
black	8326	IL15_UP.V1_UP	7	159	16094	283	2.528413	0.021447

magenta	8352	KRAS.DF.V1_DN	5	154	16099	167	3.159849	0.02145
grey60	2998	BIOCARTA_IL5_PATHWAY	1	8	16245	44	46.1733	0.021458
grey60	4773	REACTOME_TRYPTOPHAN_CATABOLISM	1	8	16245	44	46.1733	0.021458
grey60	6061	LEI_HOXC8_TARGETS_UP	1	8	16245	44	46.1733	0.021458
grey60	6197	BYSTRYKH_HEMATOPOIESIS_STEM_CELL_FLI1	1	8	16245	44	46.1733	0.021458
grey60	7799	HOFFMAN_CLOCK_TARGETS_DN	1	8	16245	44	46.1733	0.021458
greenyello	1642	V$AML1_01	6	234	16019	150	2.778291	0.021535
greenyello	1854	V$AML1_Q6	6	234	16019	150	2.778291	0.021535
green	6766	URS_ADIPOCYTE_DIFFERENTIATION_DN	6	28	16225	1305	2.668801	0.021538
purple	3419	chr12p13	5	164	16089	157	3.156167	0.021547
purple	3791	MODULE_342	5	164	16089	157	3.156167	0.021547
purple	10098	GSE3982_NKCELL_VS_TH2_UP	5	164	16089	157	3.156167	0.021547
green	1404	AMINE_BINDING	4	14	16239	1305	3.558402	0.021563
green	3765	MODULE_311	4	14	16239	1305	3.558402	0.021563
green	4428	REACTOME_AMINE_LIGAND_BINDING_RECEPTORS	4	14	16239	1305	3.558402	0.021563
green	7841	STAMBOLSKY_BOUND_BY_MUTATED_TP53	4	14	16239	1305	3.558402	0.021563
brown	1417	CALMODULIN_BINDING	7	22	16231	2190	2.361374	0.021568
brown	3888	MODULE_500	7	22	16231	2190	2.361374	0.021568
brown	6150	MAGRANGEAS_MULTIPLE_MYELOMA_IGG_VS_IGA_DN	7	22	16231	2190	2.361374	0.021568
pink	5932	AMIT_EGF_RESPONSE_480_HELA	6	154	16099	228	2.777341	0.021575
turquoise	8512	GSE12845_IGD_NEG_BLOOD_VS_NAIVE_TONSIL_BCELL	63	195	16058	4169	1.259527	0.021576
turquoise	9397	GSE22886_NAIVE_CD4_TCELL_VS_48H_ACT_TH2_DN	63	195	16058	4169	1.259527	0.021576
purple	5089	KINSEY_TARGETS_OF_EWSR1_FLII_FUSION_DN	7	287	15966	157	2.524934	0.021583
purple	6282	LEI_MYB_TARGETS	7	287	15966	157	2.524934	0.021583
brown	6968	WALLACE_PROSTATE_CANCER_RACE_UP	48	267	15986	2190	1.334195	0.021622
turquoise	997	RESPONSE_TO_DNA_DAMAGE_STIMULUS	52	157	16096	4169	1.291235	0.021626
yellow	5237	GRAESSMANN_APOPTOSIS_BY_DOXORUBICIN_UP	113	1072	15181	1426	1.201428	0.02164
midnightb	5578	TERAMOTO_OPN_TARGETS_CLUSTER_3	1	5	16248	71	45.7831	0.021655
midnightb	5625	LI_WILMS_TUMOR_ANAPLASTIC_DN	1	5	16248	71	45.7831	0.021655
midnightb	7090	BASSO_HAIRY_CELL_LEUKEMIA_UP	1	5	16248	71	45.7831	0.021655
red	1898	V$CEBP_Q2_01	13	214	16039	524	1.884221	0.021657
salmon	6790	BILD_CTNNB1_ONCOGENIC_SIGNATURE	3	76	16177	127	5.051699	0.021659
magenta	8361	JAK2_DN.V1_UP	4	103	16150	167	3.779548	0.021674
midnightb	2620	GNF2_CD33	2	52	16201	71	8.804442	0.021676
lightgreen	7787	NIELSEN_GIST	2	88	16165	42	8.794913	0.02169
blue	8572	GSE13484_UNSTIM_VS_YF17D_VACCINE_STIM_PBMC_	48	198	16055	2978	1.323076	0.021695
grey60	5025	HOEBEKE_LYMPHOID_STEM_CELL_DN	2	84	16169	44	8.794913	0.021698
red	172	EXTRACELLULAR_SPACE	10	148	16105	524	2.095755	0.021698
cyan	2836	KEGG_COMPLEMENT_AND_COAGULATION_CASCADES	2	48	16205	77	8.794913	0.021712
cyan	6006	NIKOLSKY_BREAST_CANCER_16Q24_AMPLICON	2	48	16205	77	8.794913	0.021712
cyan	7282	VILIMAS_NOTCH1_TARGETS_UP	2	48	16205	77	8.794913	0.021712
brown	10265	GSE9650_EXHAUSTED_VS_MEMORY_CD8_TCELL_DN	37	197	16056	2190	1.393878	0.02173
brown	298	NEGATIVE_REGULATION_OF_APOPTOSIS	27	135	16118	2190	1.484292	0.021757
magenta	523	POSITIVE_REGULATION_OF_MULTICELLULAR_ORGANIS	3	58	16195	167	5.033967	0.021766
magenta	578	LEUKOCYTE_ACTIVATION	3	58	16195	167	5.033967	0.021766
magenta	8321	CAHOY_NEURONAL	3	58	16195	167	5.033967	0.021766
purple	6415	BLALOCK_ALZHEIMERS_DISEASE_INCIPIENT_UP	8	354	15899	157	2.339487	0.021786
red	7866	QI_HYPOXIA	9	127	16126	524	2.198068	0.021787
brown	1630	V$EGR2_01	33	172	16081	2190	1.423885	0.0218
brown	5385	LASTOWSKA_NEUROBLASTOMA_COPY_NUMBER_UP	33	172	16081	2190	1.423885	0.0218
purple	1322	RECEPTOR_ACTIVITY	9	423	15830	157	2.202602	0.021804
grey60	1836	V$SF1_Q6	3	221	16032	44	5.014295	0.02182
grey60	3343	chr11q13	3	221	16032	44	5.014295	0.02182
pink	8838	GSE17721_CTRL_VS_LPS_4H_BMDM_UP	7	198	16055	228	2.52018	0.021853
turquoise	8317	RB_P130_DN.V1_DN	42	123	16130	4169	1.331208	0.021856
brown	4197	REACTOME_SIGNALING_BY_EGFR_IN_CANCER	21	99	16154	2190	1.574249	0.021859
brown	2244	SCGGAAGY_V$ELK1_02	180	1162	15091	2190	1.149624	0.021864
red	854	REGULATION_OF_METABOLIC_PROCESS	33	708	15545	524	1.445716	0.021876
brown	368	TRANSCRIPTION_DNA_DEPENDENT	92	558	15695	2190	1.22361	0.02188
blue	6389	FAELT_B_CLL_WITH_VH3_21_UP	14	44	16209	2978	1.736538	0.02191

pink	6695	JIANG_HYPOXIA_NORMAL	9	291	15962	228	2.204693	0.021922
turquoise	2545	GCM_PTK2	47	140	16113	4169	1.308795	0.021923
salmon	64	ORGANELLE_LUMEN	8	439	15814	127	2.332144	0.021937
salmon	146	MEMBRANE_ENCLOSED_LUMEN	8	439	15814	127	2.332144	0.021937
brown	6446	RAMALHO_STEMNESS_DN	16	70	16183	2190	1.696334	0.021943
brown	7825	RAGHAVACHARI_PLATELET_SPECIFIC_GENES	16	70	16183	2190	1.696334	0.021943
turquoise	910	MRNA_METABOLIC_PROCESS	30	83	16170	4169	1.40911	0.021954
cyan	4024	PID_P53DOWNSTREAMPATHWAY	3	126	16127	77	5.025665	0.021956
purple	2008	AGGGCAG,MIR-18A	4	110	16143	157	3.764447	0.021973
purple	8045	SERVITJA_LIVER_HNF1A_TARGETS_UP	4	110	16143	157	3.764447	0.021973
red	2752	KEGG_GLYCOSAMINOGLYCAN_DEGRADATION	3	19	16234	524	4.897449	0.021982
red	5282	WANG_BARRETTS_ESOPHAGUS_DN	3	19	16234	524	4.897449	0.021982
red	5725	TOMLINS_METASTASIS_DN	3	19	16234	524	4.897449	0.021982
red	6700	SARTIPY_BLUNTED_BY_INSULIN_RESISTANCE_UP	3	19	16234	524	4.897449	0.021982
red	7389	HOFMANN_MYELODYSPLASTIC_SYNDROM_RISK_DN	3	19	16234	524	4.897449	0.021982
red	8666	GSE1432_1H_VS_24H_IFNG_MICROGLIA_UP	12	192	16061	524	1.938573	0.021987
red	8928	GSE17721_LPS_VS_POLYIC_24H_BMDM_UP	12	192	16061	524	1.938573	0.021987
red	9445	GSE24634_TREG_VS_TCONV_POST_DAY7_IL4_CONVER	12	192	16061	524	1.938573	0.021987
magenta	1685	V$RSRFC4_Q2	5	155	16098	167	3.139463	0.021987
magenta	9790	GSE34205_RSV_VS_FLU_INF_INFANT_PBMC_UP	5	155	16098	167	3.139463	0.021987
lightcyan	6575	GAJATE_RESPONSE_TO_TRABECTEDIN_UP	2	60	16193	62	8.738172	0.021991
salmon	4307	REACTOME_PROCESSING_OF_CAPPED_INTRON_CONT/	4	136	16117	127	3.764011	0.021998
yellow	9594	GSE27786_CD8_TCELL_VS_NEUTROPHIL_UP	26	196	16057	1426	1.511929	0.022004
yellow	10262	GSE9650_EFFECTOR_VS_MEMORY_CD8_TCELL_UP	26	196	16057	1426	1.511929	0.022004
red	5241	GAUSSMANN_MLL_AF4_FUSION_TARGETS_A_UP	11	170	16083	524	2.006994	0.022039
pink	5854	RICKMAN_METASTASIS_DN	8	244	16009	228	2.337216	0.022051
black	4984	GINESTIER_BREAST_CANCER_ZNF217_AMPLIFIED_DN	11	314	15939	283	2.011917	0.02206
purple	3608	MODULE_120	3	62	16191	157	5.009143	0.022077
purple	3713	MODULE_241	3	62	16191	157	5.009143	0.022077
greenyello	1273	PEPTIDE_RECEPTOR_ACTIVITY	2	25	16228	150	8.668267	0.022091
greenyello	3081	BIOCARTA_NKT_PATHWAY	2	25	16228	150	8.668267	0.022091
brown	3746	MODULE_287	9	32	16221	2190	2.087286	0.022097
brown	3757	MODULE_300	9	32	16221	2190	2.087286	0.022097
brown	3954	PID_HIF2PATHWAY	9	32	16221	2190	2.087286	0.022097
brown	4106	PID_MAPKTRKPATHWAY	9	32	16221	2190	2.087286	0.022097
brown	4113	PID_INTEGRIN_A4B1_PATHWAY	9	32	16221	2190	2.087286	0.022097
brown	5575	WANG_METHYLATED_IN_BREAST_CANCER	9	32	16221	2190	2.087286	0.022097
brown	5605	HEIDENBLAD_AMPLICON_12P11_12_UP	9	32	16221	2190	2.087286	0.022097
brown	6709	SEMENZA_HIF1_TARGETS	9	32	16221	2190	2.087286	0.022097
yellow	4900	PRAMOONJAGO_SOX4_TARGETS_UP	9	48	16205	1426	2.137053	0.022114
yellow	4901	PRAMOONJAGO_SOX4_TARGETS_DN	9	48	16205	1426	2.137053	0.022114
lightyellow	1170	CARBONATE_DEHYDRATASE_ACTIVITY	1	11	16242	33	44.7741	0.022116
lightyellow	1217	RNA_POLYMERASE_II_TRANSCRIPTION_FACTOR_ACTIV	1	11	16242	33	44.7741	0.022116
lightyellow	3114	BIOCARTA_TRKA_PATHWAY	1	11	16242	33	44.7741	0.022116
lightyellow	3506	MODULE_14	1	11	16242	33	44.7741	0.022116
lightyellow	4143	REACTOME_SYNTHESIS_OF_BILE_ACIDS_AND_BILE_SAI	1	11	16242	33	44.7741	0.022116
lightyellow	4301	REACTOME_RETROGRADE_NEUROTROPHIN_SIGNALLIN	1	11	16242	33	44.7741	0.022116
lightyellow	4904	PIEPOLI_LGI1_TARGETS_UP	1	11	16242	33	44.7741	0.022116
lightyellow	5119	GRAHAM_CML_QUIESCENT_VS_CML_DIVIDING_DN	1	11	16242	33	44.7741	0.022116
lightyellow	5463	MYLLYKANGAS_AMPLIFICATION_HOT_SPOT_24	1	11	16242	33	44.7741	0.022116
lightyellow	5921	PETRETTO_BLOOD_PRESSURE_UP	1	11	16242	33	44.7741	0.022116
blue	2403	MORF_IL13	45	184	16069	2978	1.334761	0.022122
turquoise	2506	GCM_CHUK	26	70	16183	4169	1.448028	0.022131
red	3529	MODULE_40	6	68	16185	524	2.73681	0.022147
red	3980	PID_CDC42_PATHWAY	6	68	16185	524	2.73681	0.022147
lightgreen	6765	BURTON_ADIPOGENESIS_9	2	89	16164	42	8.696094	0.022152
lightgreen	6769	BROWNE_HCMV_INFECTION_12HR_DN	2	89	16164	42	8.696094	0.022152
yellow	678	REGULATION_OF_ACTION_POTENTIAL	4	13	16240	1426	3.506959	0.022154
yellow	701	G1_PHASE_OF_MITOTIC_CELL_CYCLE	4	13	16240	1426	3.506959	0.022154
yellow	3517	MODULE_25	4	13	16240	1426	3.506959	0.022154

yellow	3539	MODULE_50	4	13	16240	1426	3.506959	0.022154
yellow	6146	DISTECHE_ESCAPED_FROM_X_INACTIVATION	4	13	16240	1426	3.506959	0.022154
red	1623	V$NKX25_01	7	87	16166	524	2.495635	0.022169
brown	6086	GOLDRATH_HOMEOSTATIC_PROLIFERATION	32	166	16087	2190	1.430643	0.022179
yellow	4352	REACTOME_RNA_POL_III_TRANSCRIPTION	7	33	16220	1426	2.417676	0.022179
black	6000	NIKOLSKY_BREAST_CANCER_11Q12_Q14_AMPLICON	6	125	16128	283	2.756693	0.022186
greenyello	709	RESPONSE_TO_OTHER_ORGANISM	3	65	16188	150	5.000923	0.02219
greenyello	6228	GOLDRATH_IMMUNE_MEMORY	3	65	16188	150	5.000923	0.02219
brown	8771	GSE15750_DAY6_VS_DAY10_EFF_CD8_TCELL_DN	36	191	16062	2190	1.398809	0.022195
brown	9408	GSE24142_EARLY_THYMIC_PROGENITOR_VS_DN2_THY	36	191	16062	2190	1.398809	0.022195
greenyello	9277	GSE20366_CD103_POS_VS_CD103_KLRG1_DP_TREG_D	5	173	16080	150	3.131599	0.022196
tan	9305	GSE22045_TREG_VS_TCONV_DN	5	179	16074	145	3.130996	0.022208
tan	10130	GSE6269_HEALTHY_VS_STREP_AUREUS_INF_PBMC_UP	5	179	16074	145	3.130996	0.022208
lightcyan	7418	CHEN_METABOLIC_SYNDROM_NETWORK	9	1094	15159	62	2.156587	0.022225
red	300	ESTABLISHMENT_OF_LOCALIZATION	34	735	15518	524	1.434808	0.022242
brown	2874	KEGG_PRION_DISEASES	8	27	16226	2190	2.198951	0.022246
brown	3900	MODULE_524	8	27	16226	2190	2.198951	0.022246
brown	4209	REACTOME_PRE_NOTCH_TRANSCRIPTION_AND_TRANS	8	27	16226	2190	2.198951	0.022246
brown	5028	AKL_HTLV1_INFECTION_UP	8	27	16226	2190	2.198951	0.022246
blue	1392	MONOVALENT_INORGANIC_CATION_TRANSMEMBRAN	10	28	16225	2978	1.949175	0.022248
purple	109	APICAL_JUNCTION_COMPLEX	2	24	16229	157	8.626858	0.022266
purple	198	APICOLATERAL_PLASMA_MEMBRANE	2	24	16229	157	8.626858	0.022266
purple	1099	OXIDOREDUCTASE_ACTIVITY_GO_0016705	2	24	16229	157	8.626858	0.022266
purple	5256	BERENJENO_TRANSFORMED_BY_RHOA_FOREVER_DN	2	24	16229	157	8.626858	0.022266
purple	6510	CUI_TCF21_TARGETS_DN	2	24	16229	157	8.626858	0.022266
purple	6674	SCHLINGEMANN_SKIN_CARCINOGENESIS_TPA_DN	2	24	16229	157	8.626858	0.022266
black	10285	GSE9988_LPS_VS_VEHICLE_TREATED_MONOCYTE_DN	8	197	16056	283	2.332227	0.022271
pink	5436	KLEIN_TARGETS_OF_BCR_ABL1_FUSION	3	43	16210	228	4.973378	0.022272
pink	7015	ENGELMANN_CANCER_PROGENITORS_UP	3	43	16210	228	4.973378	0.022272
brown	565	CELL_RECOGNITION	5	13	16240	2190	2.854408	0.022294
brown	678	REGULATION_OF_ACTION_POTENTIAL	5	13	16240	2190	2.854408	0.022294
brown	775	REGULATION_OF_CELL_MORPHOGENESIS	5	13	16240	2190	2.854408	0.022294
brown	3837	MODULE_412	5	13	16240	2190	2.854408	0.022294
brown	4014	PID_IL5_PATHWAY	5	13	16240	2190	2.854408	0.022294
brown	4156	REACTOME_SPRY_REGULATION_OF_FGF_SIGNALING	5	13	16240	2190	2.854408	0.022294
brown	7316	MARTINELLI_IMMATURE_NEUTROPHIL_DN	5	13	16240	2190	2.854408	0.022294
brown	7665	GUTIERREZ_WALDENSTROEMS_MACROGLOBULINEMIA	5	13	16240	2190	2.854408	0.022294
turquoise	1593	ATGGYGGA_UNKNOWN	33	93	16160	4169	1.383352	0.02233
turquoise	2305	MORF_ERCC2	33	93	16160	4169	1.383352	0.02233
yellow	10111	GSE39820_CTRL_VS_TGFBETA3_IL6_IL23A_CD4_TCELL_	25	187	16066	1426	1.523745	0.022346
green	1914	V$AP4_Q6_01	25	204	16049	1305	1.526275	0.022347
turquoise	101	VACUOLAR_PART	6	10	16243	4169	2.339122	0.022349
turquoise	123	LYSOSOMAL_MEMBRANE	6	10	16243	4169	2.339122	0.022349
turquoise	140	VACUOLAR_MEMBRANE	6	10	16243	4169	2.339122	0.022349
turquoise	1260	ACETYLGALACTOSAMINYLTRANSFERASE_ACTIVITY	6	10	16243	4169	2.339122	0.022349
turquoise	1296	PROTEIN_DEACETYLASE_ACTIVITY	6	10	16243	4169	2.339122	0.022349
turquoise	1323	DEACETYLASE_ACTIVITY	6	10	16243	4169	2.339122	0.022349
turquoise	3088	BIOCARTA_PTC1_PATHWAY	6	10	16243	4169	2.339122	0.022349
turquoise	3328	chr4q34	6	10	16243	4169	2.339122	0.022349
turquoise	5695	SEMBA_FHIT_TARGETS_DN	6	10	16243	4169	2.339122	0.022349
turquoise	7071	KUMAMOTO_RESPONSE_TO_NUTLIN_3A_DN	6	10	16243	4169	2.339122	0.022349
magenta	1204	SUBSTRATE_SPECIFIC_CHANNEL_ACTIVITY	4	104	16149	167	3.743206	0.022366
magenta	108	EXTRACELLULAR_REGION_PART	6	212	16041	167	2.754435	0.022382
magenta	1532	V$CEBPB_02	6	212	16041	167	2.754435	0.022382
blue	4463	REACTOME_UNFOLDED_PROTEIN_RESPONSE	21	74	16179	2978	1.548804	0.022386
blue	5492	LUI_THYROID_CANCER_CLUSTER_2	13	40	16213	2978	1.773749	0.022389
lightgreen	1642	V$AML1_01	3	234	16019	42	4.961233	0.022394
lightgreen	1854	V$AML1_Q6	3	234	16019	42	4.961233	0.022394
red	1964	AATGTGA,MIR-23A,MIR-23B	19	356	15897	524	1.655411	0.022397
red	3542	MODULE_53	19	356	15897	524	1.655411	0.022397

green	7854	VERHAAK_GLIOBLASTOMA_PRONEURAL	22	174	16079	1305	1.574695	0.022409
turquoise	9779	GSE339_CD8POS_VS_CD4CD8DN_DC_IN_CULTURE_DN	58	178	16075	4169	1.27031	0.022428
green	9113	GSE17721_12H_VS_24H_POLYIC_BMDM_DN	24	194	16059	1305	1.540751	0.022433
purple	1964	AATGTGA,MIR-23A,MIR-23B	8	356	15897	157	2.326344	0.02244
pink	3499	MODULE_6	10	341	15912	228	2.090472	0.022442
lightcyan	10144	GSE6269_STREP_AUREUS_VS_STREP_PNEUMO_INF_PE	3	158	16095	62	4.97744	0.022445
blue	1468	V$E2F_01	18	61	16192	2978	1.610466	0.022448
blue	2880	KEGG_COLORECTAL_CANCER	18	61	16192	2978	1.610466	0.022448
red	7331	YOSHIMURA_MAPK8_TARGETS_UP	42	947	15306	524	1.37563	0.022455
midnightb	8171	SMIRNOV_RESPONSE_TO_IR_2HR_DN	2	53	16200	71	8.63832	0.022463
green	3981	PID_RET_PATHWAY	7	36	16217	1305	2.42169	0.0225
greenyello	276	ENZYME_LINKED_RECEPTOR_PROTEIN_SIGNALING_PAT	4	116	16137	150	3.736322	0.022522
greenyello	7249	VART_KSHV_INFECTION_ANGIOGENIC_MARKERS_DN	4	116	16137	150	3.736322	0.022522
yellow	2584	GNF2_CCNB2	10	56	16197	1426	2.035289	0.022526
yellow	3796	MODULE_350	10	56	16197	1426	2.035289	0.022526
salmon	6383	NOUZOVA_TRETINOIN_AND_H4_ACETYLATION	4	137	16116	127	3.736537	0.022526
purple	1635	V$TATA_01	5	166	16087	157	3.118141	0.022566
cyan	4879	TURASHVILI_BREAST_DUCTAL_CARCINOMA_VS_LOBUL	2	49	16204	77	8.615425	0.022567
cyan	7873	DEMAGALHAES_AGING_UP	2	49	16204	77	8.615425	0.022567
purple	1830	V$CACCCBINDINGFACTOR_Q6	6	226	16027	157	2.748379	0.022582
yellow	7205	QI_PLASMACYTOMA_DN	14	89	16164	1426	1.792883	0.022583
turquoise	8911	GSE17721_CTRL_VS_GARDIQUIMOD_24H_BMDM_DN	60	185	16068	4169	1.26439	0.022596
turquoise	10123	GSE5463_CTRL_VS_DEXAMETHASONE_TREATED_THYM	60	185	16068	4169	1.26439	0.022596
purple	5283	WANG_ESOPHAGUS_CANCER_VS_NORMAL_UP	4	111	16142	157	3.730533	0.022628
lightgreen	4565	REACTOME_GPCR_LIGAND_BINDING	3	235	16018	42	4.940122	0.022644
yellow	8611	GSE14000_TRANSLATED_RNA_VS_MRNA_4H_LPS_DC_	24	178	16075	1426	1.536757	0.022648
yellow	9036	GSE17721_PAM3CSK4_VS_GADIQUIMOD_24H_BMDM_	24	178	16075	1426	1.536757	0.022648
blue	4710	REACTOME_INTERACTIONS_OF_VPR_WITH_HOST_CELI	11	32	16221	2978	1.876081	0.022656
blue	5021	BOGNI_TREATMENT_RELATED_MYELOID_LEUKEMIA_D	11	32	16221	2978	1.876081	0.022656
brown	10218	GSE8515_IL1_VS_IL6_4H_STIM_)MAC_UP	35	185	16068	2190	1.40406	0.022657
blue	3423	chr7q32	12	36	16217	2978	1.81923	0.022665
grey60	669	AMINO_ACID_AND_DERIVATIVE_METABOLIC_PROCESS	2	86	16167	44	8.590381	0.022673
lightcyan	3165	chr22q	1	6	16247	62	43.69086	0.022674
lightcyan	7286	LUDWICZEK_TREATING_IRON_OVERLOAD	1	6	16247	62	43.69086	0.022674
lightcyan	7293	SOUCEK_MYC_TARGETS	1	6	16247	62	43.69086	0.022674
black	7561	YOSHIOKA_LIVER_CANCER_EARLY_RECURRENCE_UP	3	35	16218	283	4.922665	0.022676
lightcyan	5022	BILBAN_B_CLL_LPL_UP	2	61	16192	62	8.594923	0.022682
greenyello	9563	GSE27786_BCELL_VS_CD4_TCELL_DN	5	174	16079	150	3.113602	0.022688
turquoise	8463	GSE11864_UNTREATED_VS_CSF1_IFNG_IN_MAC_DN	62	192	16061	4169	1.258903	0.022723
turquoise	8973	GSE17721_CPG_VS_GARDIQUIMOD_4H_BMDM_DN	62	192	16061	4169	1.258903	0.022723
brown	2903	KEGG_VIRAL_MYOCARDITIS	14	59	16194	2190	1.761025	0.022746
brown	4173	REACTOME_IMMUNOREGULATORY_INTERACTIONS_BE	14	59	16194	2190	1.761025	0.022746
brown	4499	REACTOME_SPHINGOLIPID_METABOLISM	14	59	16194	2190	1.761025	0.022746
brown	7964	BAKKER_FOXO3_TARGETS_UP	14	59	16194	2190	1.761025	0.022746
magenta	2903	KEGG_VIRAL_MYOCARDITIS	3	59	16194	167	4.948645	0.022763
magenta	5586	SEITZ_NEOPLASTIC_TRANSFORMATION_BY_8P_DELETI(	3	59	16194	167	4.948645	0.022763
turquoise	99	MEDIATOR_COMPLEX	9	18	16235	4169	1.949268	0.022764
turquoise	4528	REACTOME_METAL_ION_SLC_TRANSPORTERS	9	18	16235	4169	1.949268	0.022764
green	4759	REACTOME_INWARDLY_RECTIFYING_K_CHANNELS	5	21	16232	1305	2.965335	0.022767
purple	4957	BASAKI_YBX1_TARGETS_DN	8	357	15896	157	2.319827	0.022773
brown	1200	MOLECULAR_ADAPTOR_ACTIVITY	12	48	16205	2190	1.855365	0.022785
brown	3357	chrxq13	12	48	16205	2190	1.855365	0.022785
brown	4793	REACTOME_APOPTOTIC_EXECUTION_PHASE	12	48	16205	2190	1.855365	0.022785
red	8397	KAECH_DAY15_EFF_VS_MEMORY_CD8_TCELL_DN	12	193	16060	524	1.928529	0.022788
red	8457	GSE11057_NAIVE_VS_MEMORY_CD4_TCELL_DN	12	193	16060	524	1.928529	0.022788
red	8606	GSE13738_TCR_VS_BYSTANDER_ACTIVATED_CD4_TCEI	12	193	16060	524	1.928529	0.022788
red	9049	GSE17721_LPS_VS_CPG_8H_BMDM_DN	12	193	16060	524	1.928529	0.022788
red	9789	GSE34205_HEALTHY_VS_FLU_INF_INFANT_PBMC_DN	12	193	16060	524	1.928529	0.022788
red	10062	GSE3982_BCELL_VS_EFF_MEMORY_CD4_TCELL_UP	12	193	16060	524	1.928529	0.022788
red	10167	GSE7460_CTRL_VS_TGFB_TREATED_ACT_TCONV_DN	12	193	16060	524	1.928529	0.022788

red	10198	GSE7852_LN_VS_THYMUS_TREG_UP	12	193	16060	524	1.928529	0.022788
blue	7240	GRESHOCK_CANCER_COPY_NUMBER_UP	68	295	15958	2978	1.258044	0.022793
black	5400	DACOSTA_UV_RESPONSE_VIA_ERCC3_TTD_UP	4	62	16191	283	3.705232	0.022808
pink	8767	GSE15750_WT_VS_TRAF6KO_DAY6_EFF_CD8_TCELL_D	6	156	16097	228	2.741734	0.022817
red	1324	TRANSMEMBRANE_RECEPTOR_ACTIVITY	16	285	15968	524	1.741315	0.02282
red	3519	MODULE_27	16	285	15968	524	1.741315	0.02282
salmon	7997	KIM_TIAL1_TARGETS	2	30	16223	127	8.531759	0.022846
salmon	7237	WANG_TUMOR_INVASIVENESS_DN	5	206	16047	127	3.106223	0.022858
turquoise	1841	V$E2F1DP1_01	70	220	16033	4169	1.240444	0.022877
turquoise	1842	V$E2F1DP2_01	70	220	16033	4169	1.240444	0.022877
turquoise	1844	V$E2F4DP2_01	70	220	16033	4169	1.240444	0.022877
red	7401	SHEDDEN_LUNG_CANCER_GOOD_SURVIVAL_A4	11	171	16082	524	1.995257	0.022898
red	9853	GSE360_T_GONDII_VS_M_TUBERCULOSIS_DC_DN	11	171	16082	524	1.995257	0.022898
yellow	9565	GSE27786_BCELL_VS_CD8_TCELL_DN	23	169	16084	1426	1.551155	0.022902
blue	36	NUCLEAR_REPLICATION_FORK	5	10	16243	2978	2.728845	0.02291
blue	690	RNA_3END_PROCESSING	5	10	16243	2978	2.728845	0.02291
blue	5789	MANTOVANI_NFKB_TARGETS_DN	5	10	16243	2978	2.728845	0.02291
blue	7386	HOFMANN_MYELODYSPLASTIC_SYNDROM_HIGH_RISK_	5	10	16243	2978	2.728845	0.02291
pink	1569	V$SRY_02	7	200	16053	228	2.494978	0.022935
blue	5859	SCHAEFFER_PROSTATE_DEVELOPMENT_6HR_DN	105	478	15775	2978	1.198865	0.022941
salmon	6634	RODWELL_AGING_KIDNEY_UP	8	443	15810	127	2.311086	0.023007
pink	8043	SERVITJA_ISLET_HNF1A_TARGETS_UP	5	115	16138	228	3.099352	0.023012
purple	2120	RNCTGNYNRNCTGNY_UNKNOWN	3	63	16190	157	4.929633	0.023021
purple	3885	MODULE_493	3	63	16190	157	4.929633	0.023021
pink	7276	TOOKER_GEMCITABINE_RESISTANCE_UP	4	77	16176	228	3.703121	0.023021
pink	7935	BRUINS_UVC_RESPONSE_VIA_TP53_GROUP_C	4	77	16176	228	3.703121	0.023021
lightgreen	966	INTERFERON_GAMMA_BIOSYNTHETIC_PROCESS	1	9	16244	42	42.99735	0.023024
lightgreen	3923	MODULE_575	1	9	16244	42	42.99735	0.023024
lightgreen	4134	REACTOME_GAP_JUNCTION_DEGRADATION	1	9	16244	42	42.99735	0.023024
lightgreen	4341	REACTOME_APOPTOTIC_CLEAVAGE_OF_CELL_ADHESIO	1	9	16244	42	42.99735	0.023024
lightgreen	5665	SIMBULAN_UV_RESPONSE_NORMAL_UP	1	9	16244	42	42.99735	0.023024
lightgreen	5696	SHANK_TAL1_TARGETS_DN	1	9	16244	42	42.99735	0.023024
lightgreen	7021	MASRI_RESISTANCE_TO_TAMOXIFEN_AND_AROMATAS	1	9	16244	42	42.99735	0.023024
lightgreen	7952	ZHU_SKIL_TARGETS_DN	1	9	16244	42	42.99735	0.023024
green	5085	ZHOU_INFLAMMATORY_RESPONSE_FIMA_UP	45	414	15839	1305	1.35374	0.023032
blue	6238	SANSOM_APC_TARGETS_UP	29	110	16143	2978	1.438845	0.023032
cyan	7878	DUTERTRE_ESTRADIOL_RESPONSE_24HR_DN	6	466	15787	77	2.717741	0.023047
red	2573	GNF2_BNIP2	4	34	16219	524	3.649079	0.023048
red	3179	chr14q21	4	34	16219	524	3.649079	0.023048
magenta	1059	INTEGRIN_BINDING	2	23	16230	167	8.4629	0.02305
magenta	2949	BIOCARTA_CTCF_PATHWAY	2	23	16230	167	8.4629	0.02305
magenta	6048	CERVERA_SDHB_TARGETS_1_DN	2	23	16230	167	8.4629	0.02305
salmon	4691	REACTOME_APOPTOSIS	4	138	16115	127	3.70946	0.023062
salmon	7853	KIM_ALL_DISORDERS_DURATION_CORR_DN	4	138	16115	127	3.70946	0.023062
green	7457	LIU_VAV3_PROSTATE_CARCINOGENESIS_UP	11	70	16183	1305	1.957121	0.023081
lightgreen	5076	LEE_NEURAL_CREST_STEM_CELL_DN	2	91	16162	42	8.504971	0.02309
lightgreen	8052	PEDERSEN_METASTASIS_BY_ERBB2_ISOFORM_4	2	91	16162	42	8.504971	0.02309
yellow	531	CELL_CYCLE_PHASE	22	160	16093	1426	1.567172	0.023099
yellow	2427	MORF_NPM1	22	160	16093	1426	1.567172	0.023099
brown	8489	GSE11924_TH1_VS_TH17_CD4_TCELL_DN	34	179	16074	2190	1.409663	0.023114
turquoise	9652	GSE29617_DAY3_VS_DAY7_TIV_FLU_VACCINE_PBMC_	53	161	16092	4169	1.283369	0.023133
turquoise	3763	MODULE_308	25	67	16186	4169	1.454678	0.023136
blue	8515	GSE12845_IGD_NEG_BLOOD_VS_PRE_GC_TONSIL_BCE	47	194	16059	2978	1.322224	0.02316
green	8488	GSE11924_TH1_VS_TH17_CD4_TCELL_UP	19	145	16108	1305	1.631957	0.023162
lightyellow	4831	SCHUETZ_BREAST_CANCER_DUCTAL_INVASIVE_UP	3	303	15950	33	4.876388	0.023171
greenyello	6361	VERHAAK_AML_WITH_NPM1_MUTATED_DN	6	238	16015	150	2.731597	0.023172
salmon	2372	MORF_DAP	3	78	16175	127	4.922168	0.023178
salmon	4434	REACTOME_M_G1_TRANSITION	3	78	16175	127	4.922168	0.023178
salmon	6722	JIANG_HYPOXIA_CANCER	3	78	16175	127	4.922168	0.023178
salmon	7990	BILANGES_SERUM_SENSITIVE_GENES	3	78	16175	127	4.922168	0.023178

yellow	1834	V$USF2_Q6	29	225	16028	1426	1.469026	0.023187
greenyello	9382	GSE22886_TH1_VS_TH2_12H_ACT_UP	5	175	16078	150	3.09581	0.023188
pink	607	EXTRACELLULAR_STRUCTURE_ORGANIZATION_AND_BI	2	17	16236	228	8.386481	0.023199
pink	4280	REACTOME_SYNTHESIS_OF_PC	2	17	16236	228	8.386481	0.023199
pink	4426	REACTOME_STRIATED_MUSCLE_CONTRACTION	2	17	16236	228	8.386481	0.023199
pink	5939	AMIT_SERUM_RESPONSE_20_MCF10A	2	17	16236	228	8.386481	0.023199
pink	6277	ZHAN_MULTIPLE_MYELOMA_SPIKED	2	17	16236	228	8.386481	0.023199
pink	7564	MEISSNER_BRAIN_HCP_WITH_H3K4ME2	2	17	16236	228	8.386481	0.023199
pink	7789	NIELSEN_LEIOMYOSARCOMA_DN	2	17	16236	228	8.386481	0.023199
brown	834	CARBOHYDRATE_METABOLIC_PROCESS	29	148	16105	2190	1.454205	0.023211
yellow	6599	BROWNE_HCMV_INFECTION_18HR_DN	21	151	16102	1426	1.585099	0.023227
blue	2429	MORF_PAPSS1	27	101	16152	2978	1.458986	0.023228
blue	3336	chr22q12	25	92	16161	2978	1.483068	0.023228
pink	8012	KOINUMA_TARGETS_OF_SMAD2_OR_SMAD3	18	768	15485	228	1.670744	0.023232
red	5783	NUYTTEN_NIPP1_TARGETS_DN	36	790	15463	524	1.413441	0.02324
green	3588	MODULE_100	46	425	15828	1305	1.348006	0.02324
salmon	5708	CALVET_IRINOTECAN_SENSITIVE_VS_REVERTED_DN	1	3	16250	127	42.65879	0.023261
salmon	7616	WEBER_METHYLATED_LCP_IN_FIBROBLAST_DN	1	3	16250	127	42.65879	0.023261
yellow	6643	IVANOVA_HEMATOPOIESIS_INTERMEDIATE_PROGENIT	20	142	16111	1426	1.605298	0.023271
salmon	5208	LINDGREN_BLADDER_CANCER_CLUSTER_3_DN	5	207	16046	127	3.091217	0.023282
blue	3193	chr22q13	40	161	16092	2978	1.355948	0.023291
greenyello	6963	MASSARWEH_TAMOXIFEN_RESISTANCE_UP	10	523	15730	150	2.071765	0.023292
purple	5647	DAIRKEE_TERT_TARGETS_DN	4	112	16141	157	3.697225	0.023295
purple	8184	ZWANG_CLASS_1_TRANSIENTLY_INDUCED_BY_EGF	9	428	15825	157	2.176871	0.023307
yellow	3367	chr11p15	26	197	16056	1426	1.504254	0.02331
yellow	8398	GOLDRATH_NAIVE_VS_EFF_CD8_TCELL_UP	26	197	16056	1426	1.504254	0.02331
yellow	9183	GSE17974_CTRL_VS_ACT_IL4_AND_ANTI_IL12_48H_CD	26	197	16056	1426	1.504254	0.02331
yellow	9544	GSE27786_LSK_VS_MONO_MAC_UP	26	197	16056	1426	1.504254	0.02331
yellow	9615	GSE27786_ERYTHROBLAST_VS_MONO_MAC_DN	26	197	16056	1426	1.504254	0.02331
yellow	9730	GSE31082_DN_VS_DP_THYMOCYTE_UP	26	197	16056	1426	1.504254	0.02331
brown	7807	SASSON_RESPONSE_TO_GONADOTROPHINS_DN	18	82	16171	2190	1.629101	0.023324
grey60	50	MEMBRANE_PART	8	1316	14937	44	2.24551	0.02333
blue	8214	CSR_EARLY_UP.V1_UP	37	147	16106	2978	1.373704	0.023357
brown	1245	NUCLEOSIDE_TRIPHOSPHATASE_ACTIVITY	37	198	16055	2190	1.386839	0.023362
lightcyan	5891	GOTZMANN_EPITHELIAL_TO_MESENCHYMAL_TRANSIT	2	62	16191	62	8.456296	0.023381
purple	6958	MARTINEZ_RB1_AND_TP53_TARGETS_DN	10	500	15753	157	2.070446	0.023448
pink	1601	V$HNF1_C	6	157	16096	228	2.724271	0.023456
yellow	6579	BURTON_ADIPOGENESIS_3	15	98	16155	1426	1.744533	0.023462
cyan	904	TRIACYLGLYCEROL_METABOLIC_PROCESS	1	5	16248	77	42.21558	0.023467
cyan	5625	LI_WILMS_TUMOR_ANAPLASTIC_DN	1	5	16248	77	42.21558	0.023467
green	5504	SCHLOSSER_MYC_TARGETS_REPRESSED_BY_SERUM	20	155	16098	1305	1.60702	0.023471
yellow	6009	NIKOLSKY_BREAST_CANCER_17Q21_Q25_AMPLICON	36	292	15961	1426	1.405185	0.023473
black	3331	chr9q34	8	199	16054	283	2.308788	0.023485
turquoise	5680	KIM_MYCN_AMPLIFICATION_TARGETS_DN	32	90	16163	4169	1.386146	0.023486
pink	1509	V$MZF1_01	7	201	16052	228	2.482565	0.02349
pink	7925	BHAT_ESR1_TARGETS_VIA_AKT1_UP	8	247	16006	228	2.308829	0.023511
purple	2203	TTANTCA_UNKNOWN	13	725	15528	157	1.856262	0.023556
brown	8963	GSE17721_PAM3CSK4_VS_CPG_16H_BMDM_DN	33	173	16080	2190	1.415654	0.023563
lightgreen	8276	CTIP_DN.V1_UP	2	92	16161	42	8.412526	0.023565
lightgreen	8377	KRAS.LUNG.BREAST_UP.V1_UP	2	92	16161	42	8.412526	0.023565
lightcyan	731	HOMEOSTATIC_PROCESS	3	161	16092	62	4.884692	0.023567
lightcyan	8813	GSE15930_STIM_VS_STIM_AND_TRICHOSTATINA_72H_	3	161	16092	62	4.884692	0.023567
tan	7840	MARTENS_TRETINOIN_RESPONSE_DN	13	786	15467	145	1.8539	0.023577
brown	568	PEPTIDE_METABOLIC_PROCESS	4	9	16244	2190	3.298427	0.023593
brown	1358	PROTEIN_SERINE_THREONINE_TYROSINE_KINASE_ACTI	4	9	16244	2190	3.298427	0.023593
brown	2982	BIOCARTA_GLYCOLYSIS_PATHWAY	4	9	16244	2190	3.298427	0.023593
brown	3090	BIOCARTA_BARRESTIN_PATHWAY	4	9	16244	2190	3.298427	0.023593
brown	3829	MODULE_401	4	9	16244	2190	3.298427	0.023593
brown	4577	REACTOME_ROLE_OF_DCC_IN_REGULATING_APOPTOS	4	9	16244	2190	3.298427	0.023593
brown	4660	REACTOME_DSCAM_INTERACTIONS	4	9	16244	2190	3.298427	0.023593

brown	7551	YAMANAKA_GLIOBLASTOMA_SURVIVAL_UP	4	9	16244	2190	3.298427	0.023593
red	1138	PURINE_NUCLEOTIDE_BINDING	12	194	16059	524	1.918588	0.02361
red	2113	TTTGCAG,MIR-518A-2	12	194	16059	524	1.918588	0.02361
red	8408	GSE10239_NAIVE_VS_KLRG1INT_EFF_CD8_TCELL_UP	12	194	16059	524	1.918588	0.02361
red	9331	GSE22886_NAIVE_VS_IGM_MEMORY_BCELL_DN	12	194	16059	524	1.918588	0.02361
purple	1677	V$PAX4_04	5	168	16085	157	3.081021	0.023616
purple	8871	GSE17721_CTRL_VS_PAM3CSK4_4H_BMDM_DN	5	168	16085	157	3.081021	0.023616
purple	8894	GSE17721_CTRL_VS_CPG_24H_BMDM_UP	5	168	16085	157	3.081021	0.023616
purple	9271	GSE20366_TREG_VS_NAIVE_CD4_TCELL_DEC205_CONV	5	168	16085	157	3.081021	0.023616
blue	9546	GSE27786_LIN_NEG_VS_BCELL_UP	44	180	16073	2978	1.334102	0.023626
turquoise	8887	GSE17721_CTRL_VS_CPG_4H_BMDM_DN	57	175	16078	4169	1.269809	0.023632
turquoise	8916	GSE17721_LPS_VS_POLYIC_2H_BMDM_UP	57	175	16078	4169	1.269809	0.023632
blue	3331	chr9q34	48	199	16054	2978	1.316428	0.023648
blue	6877	SANSOM_APC_TARGETS_REQUIRE_MYC	48	199	16054	2978	1.316428	0.023648
blue	9572	GSE27786_BCELL_VS_NEUTROPHIL_UP	48	199	16054	2978	1.316428	0.023648
tan	9654	GSE29618_BCELL_VS_MONOCYTE_UP	5	182	16071	145	3.079386	0.023656
red	6681	DAZARD_RESPONSE_TO_UV_NHEK_DN	17	310	15943	524	1.700942	0.023656
pink	5727	LIANG_HEMATOPOIESIS_STEM_CELL_NUMBER_LARGE_	3	44	16209	228	4.860347	0.02366
pink	7771	KYNG_RESPONSE_TO_H2O2_VIA_ERCC6_DN	3	44	16209	228	4.860347	0.02366
pink	7960	CHANGOLKAR_H2AFY_TARGETS_UP	3	44	16209	228	4.860347	0.02366
magenta	5455	LINDGREN_BLADDER_CANCER_CLUSTER_2B	8	338	15915	167	2.303511	0.023676
turquoise	1206	CYSTEINE_TYPE_PEPTIDASE_ACTIVITY	19	48	16205	4169	1.543171	0.02369
turquoise	4250	REACTOME_P53_INDEPENDENT_G1_S_DNA_DAMAGE_	19	48	16205	4169	1.543171	0.02369
greenyello	1502	V$GATA3_01	5	176	16077	150	3.07822	0.023694
greenyello	8815	GSE16522_MEMORY_VS_NAIVE_CD8_TCELL_DN	5	176	16077	150	3.07822	0.023694
greenyello	9273	GSE20366_CD103_POS_VS_NEG_TREG_KLRG1NEG_DN	5	176	16077	150	3.07822	0.023694
tan	3396	chr6p22	3	69	16184	145	4.873463	0.023749
red	6475	SESTO_RESPONSE_TO_UV_C2	5	51	16202	524	3.0409	0.02375
turquoise	7868	GREGORY_SYNTHETIC_LETHAL_WITH_IMATINIB	43	127	16126	4169	1.319977	0.023757
green	3822	MODULE_389	22	175	16078	1305	1.565697	0.02376
brown	1681	CCCNNNNNNAAGWT_UNKNOWN	19	88	16165	2190	1.602361	0.023767
brown	2050	TTGGGAG,MIR-150	19	88	16165	2190	1.602361	0.023767
brown	2360	MORF_CASP2	19	88	16165	2190	1.602361	0.023767
brown	7537	HOSHIDA_LIVER_CANCER_SURVIVAL_DN	19	88	16165	2190	1.602361	0.023767
magenta	1594	V$NFKB_Q6	6	215	16038	167	2.716001	0.02378
red	8120	FOSTER_KDM1A_TARGETS_UP	11	172	16081	524	1.983657	0.023782
pink	853	CHEMICAL_HOMEOSTASIS	5	116	16137	228	3.072633	0.023783
pink	3349	chrxp11	5	116	16137	228	3.072633	0.023783
pink	4953	GAL_LEUKEMIC_STEM_CELL_UP	5	116	16137	228	3.072633	0.023783
magenta	6635	MCCLUNG_COCAIN_REWARD_4WK	3	60	16193	167	4.866168	0.023785
greenyello	2717	GNF2_ZAP70	2	26	16227	150	8.334872	0.023789
greenyello	3568	MODULE_80	2	26	16227	150	8.334872	0.023789
greenyello	4082	PID_TCRCALCIUMPATHWAY	2	26	16227	150	8.334872	0.023789
greenyello	5691	WANG_RESPONSE_TO_BEXAROTENE_DN	2	26	16227	150	8.334872	0.023789
magenta	8240	P53_DN.V2_UP	4	106	16147	167	3.672579	0.02379
greenyello	4450	REACTOME_G_ALPHA_Q_SIGNALLING_EVENTS	4	118	16135	150	3.672994	0.023803
turquoise	8565	GSE13484_UNSTIM_VS_12H_YF17D_VACCINE_STIM_PI	59	182	16071	4169	1.263811	0.023805
cyan	8235	WNT_UP.V1_DN	3	130	16123	77	4.871029	0.023811
red	2260	YTAATTAA_V$LHX3_01	9	129	16124	524	2.163989	0.023832
red	5001	GARGALOVIC_RESPONSE_TO_OXIDIZED_PHOSPHOLIPID	9	129	16124	524	2.163989	0.023832
yellow	1801	V$NFMUE1_Q6	30	235	16018	1426	1.455015	0.023875
brown	8466	GSE11864_UNTREATED_VS_CSF1_IFNG_PAM3CYS_IN_I	36	192	16061	2190	1.391524	0.023884
brown	8927	GSE17721_LPS_VS_POLYIC_16H_BMDM_DN	36	192	16061	2190	1.391524	0.023884
turquoise	9278	GSE20366_TREG_VS_NAIVE_CD4_TCELL_UP	61	189	16064	4169	1.258258	0.023933
lightcyan	8243	P53_DN.V1_DN	3	162	16091	62	4.85454	0.023948
lightcyan	9766	GSE33513_TCF7_KO_VS_HET_EARLY_THYMIC_PROGEN	3	162	16091	62	4.85454	0.023948
black	6655	WENG_POR_TARGETS_GLOBAL_UP	2	14	16239	283	8.204442	0.023949
brown	302	CELL_ACTIVATION	15	65	16188	2190	1.712645	0.023949
brown	2488	GCM_BECN1	15	65	16188	2190	1.712645	0.023949
brown	3929	PID_BCR_5PATHWAY	15	65	16188	2190	1.712645	0.023949

salmon	6181	SCHUHMACHER_MYC_TARGETS_UP	3	79	16174	127	4.859862	0.023959
salmon	6231	BHATTACHARYA_EMBRYONIC_STEM_CELL	3	79	16174	127	4.859862	0.023959
blue	10083	GSE3982_EFF_MEMORY_VS_CENT_MEMORY_CD4_TCE	41	166	16087	2978	1.347984	0.023966
purple	2832	KEGG_CELL_ADHESION_MOLECULES_CAMS	4	113	16140	157	3.664506	0.023973
purple	5277	MISSIAGLIA_REGULATED_BY_METHYLATION_UP	4	113	16140	157	3.664506	0.023973
tan	2503	GCM_CASP2	2	27	16226	145	8.302937	0.02398
pink	7150	LABBE_TGFB1_TARGETS_DN	4	78	16175	228	3.655646	0.024007
pink	7565	MIKKELSEN_MCV6_HCP_WITH_H3K27ME3	8	248	16005	228	2.299519	0.024013
greenyello	1443	CYTOKINE_ACTIVITY	3	67	16186	150	4.851642	0.024016
turquoise	8419	GSE10239_NAIVE_VS_DAY4.5_EFF_CD8_TCELL_DN	63	196	16057	4169	1.253101	0.024019
turquoise	8730	GSE14769_40MIN_VS_360MIN_LPS_BMDM_UP	63	196	16057	4169	1.253101	0.024019
turquoise	8856	GSE17721_CTRL_VS_POLYIC_6H_BMDM_UP	63	196	16057	4169	1.253101	0.024019
turquoise	9179	GSE17974_CTRL_VS_ACT_IL4_AND_ANTI_IL12_12H_CD	63	196	16057	4169	1.253101	0.024019
turquoise	10038	GSE3982_MAC_VS_CENT_MEMORY_CD4_TCELL_UP	63	196	16057	4169	1.253101	0.024019
turquoise	10087	GSE3982_EFF_MEMORY_CD4_TCELL_VS_TH1_DN	63	196	16057	4169	1.253101	0.024019
turquoise	10197	GSE7852_TREG_VS_TCONV_FAT_DN	63	196	16057	4169	1.253101	0.024019
turquoise	10200	GSE7852_LN_VS_FAT_TREG_UP	63	196	16057	4169	1.253101	0.024019
turquoise	10262	GSE9650_EFFECTOR_VS_MEMORY_CD8_TCELL_UP	63	196	16057	4169	1.253101	0.024019
lightgreen	5198	CHEBOTAEV_GR_TARGETS_DN	2	93	16160	42	8.322069	0.024044
purple	1273	PEPTIDE_RECEPTOR_ACTIVITY	2	25	16228	157	8.281783	0.02405
purple	1393	STRUCTURAL_CONSTITUENT_OF_MUSCLE	2	25	16228	157	8.281783	0.02405
purple	5252	GAUSSMANN_MLL_AF4_FUSION_TARGETS_F_DN	2	25	16228	157	8.281783	0.02405
purple	5951	SAKAI_TUMOR_INFILTRATING_MONOCYTES_UP	2	25	16228	157	8.281783	0.02405
purple	6441	GENTILE_UV_LOW_DOSE_UP	2	25	16228	157	8.281783	0.02405
purple	6900	JEPSEN_SMRT_TARGETS	2	25	16228	157	8.281783	0.02405
pink	8078	PLASARI_TGFB1_TARGETS_10HR_DN	7	202	16051	228	2.470275	0.024054
yellow	10185	GSE7460_CTRL_VS_FOXP3_OVEREXPR_TCONV_1_DN	24	179	16074	1426	1.528172	0.024057
green	4384	REACTOME_LOSS_OF_NLP_FROM_MITOTIC_CENTROSO	9	53	16200	1305	2.114899	0.024067
midnightb	2539	GCM_NUMA1	2	55	16198	71	8.3242	0.024073
lightcyan	2634	GNF2_DDX5	2	63	16190	62	8.322069	0.024089
lightcyan	6414	NATSUME_RESPONSE_TO_INTERFERON_BETA_UP	2	63	16190	62	8.322069	0.024089
lightcyan	7189	CADWELL_ATG16L1_TARGETS_UP	2	63	16190	62	8.322069	0.024089
blue	1732	V$AP2REP_01	35	138	16115	2978	1.384197	0.02409
blue	524	ACTIVATION_OF_NF_KAPPAB_TRANSCRIPTION_FACTOI	7	17	16236	2978	2.247284	0.024102
blue	3097	BIOCARTA_TEL_PATHWAY	7	17	16236	2978	2.247284	0.024102
lightyellow	2940	BIOCARTA_CELL2CELL_PATHWAY	1	12	16241	33	41.04293	0.024103
lightyellow	3491	SA_REG_CASCADE_OF_CYCLIN_EXPR	1	12	16241	33	41.04293	0.024103
lightyellow	3661	MODULE_178	1	12	16241	33	41.04293	0.024103
lightyellow	6258	HEDVAT_ELF4_TARGETS_UP	1	12	16241	33	41.04293	0.024103
lightyellow	6417	DAZARD_UV_RESPONSE_CLUSTER_G3	1	12	16241	33	41.04293	0.024103
lightyellow	7022	MASRI_RESISTANCE_TO_TAMOXIFEN_AND_AROMATAS	1	12	16241	33	41.04293	0.024103
lightyellow	7467	IRITANI_MAD1_TARGETS_UP	1	12	16241	33	41.04293	0.024103
lightyellow	7637	LOPEZ_MESOTHELIOMA_SURVIVAL_DN	1	12	16241	33	41.04293	0.024103
lightyellow	7790	NIELSEN_SYNOVIAL_SARCOMA_UP	1	12	16241	33	41.04293	0.024103
lightyellow	7887	FIGUEROA_AML_METHYLATION_CLUSTER_5_UP	1	12	16241	33	41.04293	0.024103
grey60	5905	BENPORATH_SUZ12_TARGETS	5	612	15641	44	3.017862	0.024108
grey60	889	PERIPHERAL_NERVOUS_SYSTEM_DEVELOPMENT	1	9	16244	44	41.04293	0.024108
grey60	3923	MODULE_575	1	9	16244	44	41.04293	0.024108
grey60	4471	REACTOME_PROSTANOID_LIGAND_RECEPTORS	1	9	16244	44	41.04293	0.024108
grey60	5344	SAENZ_DETOX_PATHWAY_AND_CARCINOGENESIS_DN	1	9	16244	44	41.04293	0.024108
grey60	5633	OXFORD_RALA_TARGETS_DN	1	9	16244	44	41.04293	0.024108
grey60	6007	NIKOLSKY_BREAST_CANCER_17P11_AMPLICON	1	9	16244	44	41.04293	0.024108
grey60	7517	BOYAULT_LIVER_CANCER_SUBCLASS_G23_DN	1	9	16244	44	41.04293	0.024108
grey60	7695	DORN_ADENOVIRUS_INFECTION_24HR_UP	1	9	16244	44	41.04293	0.024108
grey60	7765	GERHOLD_RESPONSE_TO_TZD_DN	1	9	16244	44	41.04293	0.024108
brown	809	ANATOMICAL_STRUCTURE_DEVELOPMENT	112	696	15557	2190	1.194258	0.024113
lightcyan	5486	FARMER_BREAST_CANCER_APOCRINE_VS_LUMINAL	4	288	15965	62	3.640905	0.024133
lightgreen	8184	ZWANG_CLASS_1_TRANSIENTLY_INDUCED_BY_EGF	4	428	15825	42	3.6166	0.024137
pink	1225	SUBSTRATE_SPECIFIC_TRANSPORTER_ACTIVITY	9	296	15957	228	2.167452	0.02414
purple	8732	GSE15215_CD2_POS_VS_NEG_PDC_UP	5	169	16084	157	3.06279	0.024153

purple	8903	GSE17721_CTRL_VS_GARDIQUIMOD_4H_BMDM_DN	5	169	16084	157	3.06279	0.024153
red	6282	LEI_MYB_TARGETS	16	287	15966	524	1.729181	0.02416
turquoise	148	MITOCHONDRIAL_INNER_MEMBRANE	24	64	16189	4169	1.461951	0.02417
turquoise	6826	PELLICCIOTTA_HDAC_IN_ANTIGEN_PRESENTATION_UP	24	64	16189	4169	1.461951	0.02417
black	8241	RELA_DN.V1_DN	5	94	16159	283	3.054846	0.024176
turquoise	5969	MORI_PRE_BI_LYMPHOCYTE_DN	28	77	16176	4169	1.41765	0.024182
blue	9015	GSE17721_POLYIC_VS_CPG_12H_BMDM_DN	45	185	16068	2978	1.327546	0.024187
blue	9460	GSE24634_IL4_VS_CTRL_TREATED_NAIVE_CD4_TCELL_	45	185	16068	2978	1.327546	0.024187
blue	9569	GSE27786_BCELL_VS_NKTCELL_DN	45	185	16068	2978	1.327546	0.024187
yellow	5622	MARTORIATI_MDM4_TARGETS_FETAL_LIVER_UP	27	207	16046	1426	1.486646	0.024199
greenyello	8532	GSE13306_TREG_VS_TCONV_LAMINA_PROPRIA_UP	5	177	16076	150	3.060829	0.024208
greenyello	9525	GSE2706_2H_VS_8H_LPS_STIM_DC_DN	5	177	16076	150	3.060829	0.024208
greenyello	10061	GSE3982_BCELL_VS_BASOPHIL_DN	5	177	16076	150	3.060829	0.024208
greenyello	10189	GSE7764_IL15_TREATED_VS_CTRL_NK_CELL_24H_DN	5	177	16076	150	3.060829	0.024208
purple	2046	TTGCCAA,MIR-182	7	294	15959	157	2.464816	0.024224
green	2199	TGGNNNNNNKCCAR_UNKNOWN	37	330	15923	1305	1.396403	0.024226
blue	5155	HAHTOLA_MYCOSIS_FUNGOIDES_CD4_DN	30	115	16138	2978	1.423745	0.024229
brown	8127	BOUDOUKHA_BOUND_BY_IGF2BP2	21	100	16153	2190	1.558507	0.024268
purple	2247	TTGTTT_V$FOXO4_01	24	1632	14621	157	1.522387	0.024274
magenta	1780	CTTTAAR_UNKNOWN	14	757	15496	167	1.799903	0.024302
lightcyan	4807	PICCALUGA_ANGIOIMMUNOBLASTIC_LYMPHOMA_UP	3	163	16090	62	4.824758	0.024332
yellow	10128	GSE6269_HEALTHY_VS_E_COLI_INF_PBMC_UP	23	170	16083	1426	1.54203	0.024366
turquoise	5082	TIEN_INTESTINE_PROBIOTICS_6HR_DN	52	158	16095	4169	1.283063	0.024386
yellow	7655	CAIRO_HEPATOBLASTOMA_CLASSES_UP	66	591	15662	1426	1.27283	0.024428
red	9008	GSE17721_POLYIC_VS_CPG_4H_BMDM_UP	12	195	16058	524	1.908749	0.024454
red	9449	GSE24634_TEFF_VS_TCONV_DAY3_IN_CULTURE_DN	12	195	16058	524	1.908749	0.024454
purple	7145	BOQUEST_STEM_CELL_CULTURED_VS_FRESH_UP	8	362	15891	157	2.287785	0.024488
brown	1932	V$ER_Q6_02	38	205	16048	2190	1.375685	0.024509
turquoise	3061	BIOCARTA_PGC1A_PATHWAY	10	21	16232	4169	1.856446	0.024514
turquoise	3233	chr4q28	10	21	16232	4169	1.856446	0.024514
turquoise	4243	REACTOME_DOWNREGULATION_OF_TGF_BETA_RECEF	10	21	16232	4169	1.856446	0.024514
green	4822	HOLLMANN_APOPTOSIS_VIA_CD40_DN	30	257	15996	1305	1.453822	0.024526
red	1201	ADENYL_RIBONUCLEOTIDE_BINDING	10	151	16102	524	2.054118	0.024537
purple	800	CELL_PROLIFERATION_GO_0008283	9	432	15821	157	2.156714	0.024561
blue	1535	V$USF_01	55	233	16020	2978	1.288296	0.024563
blue	5417	BAKER_HEMATOPOESIS_STAT5_TARGETS	4	7	16246	2978	3.11868	0.024584
blue	7947	MIKHAYLOVA_OXIDATIVE_STRESS_RESPONSE_VIA_VHL	4	7	16246	2978	3.11868	0.024584
salmon	2457	MORF_RAC1	5	210	16043	127	3.047057	0.024584
lightyellow	9632	GSE29614_CTRL_VS_DAY3_TIV_FLU_VACCINE_PBMC_U	2	120	16133	33	8.208586	0.02459
blue	930	CHROMATIN_MODIFICATION	16	53	16200	2978	1.647604	0.024598
blue	4384	REACTOME_LOSS_OF_NLP_FROM_MITOTIC_CENTROSC	16	53	16200	2978	1.647604	0.024598
blue	4748	REACTOME_MEIOTIC_SYNAPSIS	16	53	16200	2978	1.647604	0.024598
blue	7802	WU_APOPTOSIS_BY_CDKN1A_VIA_TP53	16	53	16200	2978	1.647604	0.024598
blue	10163	GSE7460_TREG_VS_TCONV_ACT_DN	42	171	16082	2978	1.340485	0.024614
yellow	8746	GSE15659_NAIVE_CD4_TCELL_VS_RESTING_TREG_UP	22	161	16092	1426	1.557438	0.024619
brown	1230	PEPTIDE_BINDING	13	54	16199	2190	1.786648	0.024633
brown	2309	MORF_GPX4	13	54	16199	2190	1.786648	0.024633
brown	4114	PID_RAC1_PATHWAY	13	54	16199	2190	1.786648	0.024633
tan	9204	GSE17974_0.5H_VS_72H_UNTREATED_IN_VITRO_CD4_	5	184	16069	145	3.045915	0.024654
tan	10055	GSE3982_NEUTROPHIL_VS_NKCELL_DN	5	184	16069	145	3.045915	0.024654
red	1975	ATGTAGC,MIR-221,MIR-222	8	109	16144	524	2.27649	0.024654
red	1992	GCACCTT,MIR-18A,MIR-18B	8	109	16144	524	2.27649	0.024654
purple	8296	BMI1_DN_MEL18_DN.V1_UP	4	114	16139	157	3.632361	0.024664
turquoise	8759	GSE15659_RESTING_TREG_VS_NONSUPPRESSIVE_TCEL	54	165	16088	4169	1.275885	0.024674
yellow	8521	GSE12845_NAIVE_VS_DARKZONE_GC_TONSIL_BCELL_D	26	198	16055	1426	1.496657	0.024674
lightcyan	5487	FARMER_BREAST_CANCER_APOCRINE_VS_BASAL	4	290	15963	62	3.615795	0.024676
red	8200	ERB2_UP.V1_UP	11	173	16080	524	1.97219	0.02469
red	9237	GSE18791_CTRL_VS_NEWCASTLE_VIRUS_DC_18H_DN	11	173	16080	524	1.97219	0.02469
red	9988	GSE3982_EOSINOPHIL_VS_TH1_UP	11	173	16080	524	1.97219	0.02469
purple	1540	V$GATA1_02	5	170	16083	157	3.044773	0.024697

green	1544	V$FOXD3_01	19	146	16107	1305	1.620779	0.024697
turquoise	5756	ALCALA_APOPTOSIS	31	87	16166	4169	1.389134	0.024701
green	6632	TSENG_IRS1_TARGETS_DN	16	117	16136	1305	1.703167	0.02471
salmon	7824	WHITFIELD_CELL_CYCLE_M_G1	4	141	16112	127	3.630536	0.024717
lightcyan	8849	GSE17721_CTRL_VS_POLYIC_0.5H_BMDM_DN	3	164	16089	62	4.795338	0.024719
blue	3391	chr20q11	24	88	16165	2978	1.488461	0.024723
blue	1840	V$CIZ_01	46	190	16063	2978	1.321335	0.024726
blue	8977	GSE17721_CPG_VS_GARDIQUIMOD_8H_BMDM_DN	46	190	16063	2978	1.321335	0.024726
blue	9481	GSE26669_CTRL_VS_COSTIM_BLOCK_MLR_CD8_TCELL_	46	190	16063	2978	1.321335	0.024726
greenyello	8615	GSE14000_UNSTIM_VS_4H_LPS_DC_TRANSLATED_RNA	5	178	16075	150	3.043633	0.024728
magenta	5037	MULLIGHAN_MLL_SIGNATURE_1_DN	6	217	16036	167	2.690968	0.024744
salmon	2292	MORF_ATOX1	3	80	16173	127	4.799114	0.024754
red	3351	chrxp22	7	89	16164	524	2.439553	0.024763
cyan	7254	LEE_LIVER_CANCER_SURVIVAL_UP	3	132	16121	77	4.797226	0.02477
blue	1019	POSITIVE_REGULATION_OF_TRANSCRIPTION	33	129	16124	2978	1.396153	0.024771
red	8022	PURBEY_TARGETS_OF_CTBP1_NOT_SATB1_DN	19	360	15893	524	1.637018	0.024779
brown	3525	MODULE_36	30	155	16098	2190	1.436412	0.024839
brown	6139	UEDA_PERIFERAL_CLOCK	30	155	16098	2190	1.436412	0.024839
purple	1015	SYSTEM_PROCESS	8	363	15890	157	2.281483	0.024842
blue	71	NON_MEMBRANE_BOUND_ORGANELLE	124	575	15678	2978	1.176963	0.024868
blue	177	INTRACELLULAR_NON_MEMBRANE_BOUND_ORGANEL	124	575	15678	2978	1.176963	0.024868
black	9690	GSE29618_PRE_VS_DAY7_POST_LAIV_FLU_VACCINE_M	7	164	16089	283	2.451327	0.024889
yellow	4694	REACTOME_INFLUENZA_LIFE_CYCLE	19	134	16119	1426	1.61608	0.024906
brown	8848	GSE17721_CTRL_VS_POLYIC_0.5H_BMDM_UP	34	180	16073	2190	1.401832	0.024924
greenyello	1270	RHODOPSIN_LIKE_RECEPTOR_ACTIVITY	3	68	16185	150	4.780294	0.024959
magenta	500	REGULATION_OF_T_CELL_ACTIVATION	2	24	16229	167	8.110279	0.024978
magenta	3747	MODULE_288	2	24	16229	167	8.110279	0.024978
magenta	7566	MIKKELSEN_MCV6_ICP_WITH_H3K4ME3_AND_H3K27M	2	24	16229	167	8.110279	0.024978
magenta	7627	WORSCHECH_TUMOR_EVASION_AND_TOLEROGENICIT	2	24	16229	167	8.110279	0.024978
blue	650	PROTEIN_CATABOLIC_PROCESS	19	66	16187	2978	1.571153	0.024999
green	7205	QI_PLASMACYTOMA_DN	13	89	16164	1305	1.819183	0.025021
green	4225	REACTOME_EXTRACELLULAR_MATRIX_ORGANIZATION	10	62	16191	1305	2.008775	0.025025
salmon	6580	DAZARD_RESPONSE_TO_UV_NHEK_UP	5	211	16042	127	3.032616	0.025028
green	9946	GSE37416_12H_VS_48H_F_TULARENSIS_LVS_NEUTROI	24	196	16057	1305	1.525029	0.025034
turquoise	8542	GSE13306_RA_VS_UNTREATED_TCONV_UP	58	179	16074	4169	1.263213	0.02508
turquoise	9561	GSE27786_LIN_NEG_VS_MONO_MAC_DN	58	179	16074	4169	1.263213	0.02508
brown	9214	GSE17974_2.5H_VS_72H_IL4_AND_ANTI_IL12_ACT_CD	37	199	16054	2190	1.37987	0.02509
brown	10228	GSE9006_HEALTHY_VS_TYPE_1_DIABETES_PBMC_4MC	37	199	16054	2190	1.37987	0.02509
pink	3766	MODULE_312	3	45	16208	228	4.752339	0.025094
lightcyan	8759	GSE15659_RESTING_TREG_VS_NONSUPPRESSIVE_TCEL	3	165	16088	62	4.766276	0.02511
yellow	4909	CASORELLI_ACUTE_PROMYELOCYTIC_LEUKEMIA_DN	72	653	15600	1426	1.256705	0.025121
yellow	8766	GSE15750_WT_VS_TRAF6KO_DAY6_EFF_CD8_TCELL_U	25	189	16064	1426	1.507621	0.025122
yellow	9427	GSE24142_ADULT_VS_FETAL_EARLY_THYMIC_PROGEN	25	189	16064	1426	1.507621	0.025122
greenyello	7342	POTTI_TOPOTECAN_SENSITIVITY	4	120	16133	150	3.611778	0.025127
yellow	5785	NUYTTEN_EZH2_TARGETS_DN	104	984	15269	1426	1.204626	0.025139
tan	8506	GSE12845_IGD_POS_BLOOD_VS_NAIVE_TONSIL_BCELL	5	185	16068	145	3.02945	0.025163
turquoise	7215	BLUM_RESPONSE_TO_SALIRASIB_DN	101	331	15922	4169	1.189584	0.025165
black	3242	chr5q35	5	95	16158	283	3.022689	0.025169
purple	6791	DOUGLAS_BMI1_TARGETS_UP	10	506	15747	157	2.045895	0.025195
turquoise	8992	GSE17721_LPS_VS_PAM3CSK4_6H_BMDM_UP	60	186	16067	4169	1.257593	0.025209
turquoise	9029	GSE17721_PAM3CSK4_VS_GADIQUIMOD_6H_BMDM_	60	186	16067	4169	1.257593	0.025209
turquoise	10271	GSE9988_ANTI_TREM1_VS_LOW_LPS_MONOCYTE_DN	60	186	16067	4169	1.257593	0.025209
cyan	3735	MODULE_273	2	52	16201	77	8.118382	0.025215
cyan	5626	BORLAK_LIVER_CANCER_EGF_UP	2	52	16201	77	8.118382	0.025215
cyan	5845	GARCIA_TARGETS_OF_FLI1_AND_DAX1_UP	2	52	16201	77	8.118382	0.025215
cyan	6100	KANNAN_TP53_TARGETS_UP	2	52	16201	77	8.118382	0.025215
turquoise	1813	GATGKMRGCG_UNKNOWN	23	61	16192	4169	1.46994	0.025229
blue	8991	GSE17721_LPS_VS_PAM3CSK4_4H_BMDM_DN	47	195	16058	2978	1.315443	0.025243
blue	9574	GSE27786_BCELL_VS_MONO_MAC_UP	47	195	16058	2978	1.315443	0.025243
blue	9991	GSE3982_EOSINOPHIL_VS_TH2_DN	47	195	16058	2978	1.315443	0.025243

purple	9523	GSE2706_2H_VS_8H_R848_STIM_DC_DN	5	171	16082	157	3.026968	0.02525
greenyello	1896	V$SRF_Q4	5	179	16074	150	3.026629	0.025256
greenyello	8417	GSE10239_KLRG1INT_VS_KLRG1HIGH_EFF_CD8_TCELL_	5	179	16074	150	3.026629	0.025256
red	1346	AUXILIARY_TRANSPORT_PROTEIN_ACTIVITY	3	20	16233	524	4.652576	0.025256
red	3808	MODULE_367	3	20	16233	524	4.652576	0.025256
red	5179	GESERICK_TERT_TARGETS_DN	3	20	16233	524	4.652576	0.025256
red	5731	SPIELMAN_LYMPHOBLAST_EUROPEAN_VS_ASIAN_2FC_	3	20	16233	524	4.652576	0.025256
red	7106	BUCKANOVICH_T_LYMPHOCYTE_HOMING_ON_TUMOI	3	20	16233	524	4.652576	0.025256
yellow	4853	GAZDA_DIAMOND_BLACKFAN_ANEMIA_ERYTHROID_D	53	461	15792	1426	1.310355	0.025265
green	5441	RASHI_RESPONSE_TO_IONIZING_RADIATION_3	8	45	16208	1305	2.214117	0.025279
salmon	2362	MORF_CDC10	4	142	16111	127	3.604968	0.025285
salmon	2380	MORF_EI24	4	142	16111	127	3.604968	0.025285
salmon	5988	KAUFFMANN_DNA_REPLICATION_GENES	4	142	16111	127	3.604968	0.025285
black	5748	SCHLESINGER_METHYLATED_DE_NOVO_IN_CANCER	4	64	16189	283	3.589443	0.025293
turquoise	9745	GSE32423_MEMORY_VS_NAIVE_CD8_TCELL_IL7_DN	62	193	16060	4169	1.25238	0.025294
turquoise	10097	GSE3982_NKCELL_VS_TH1_DN	62	193	16060	4169	1.25238	0.025294
turquoise	4585	REACTOME_METABOLISM_OF_LIPIDS_AND_LIPOPROTE	121	403	15850	4169	1.170528	0.025304
red	1654	V$FREAC2_01	12	196	16057	524	1.899011	0.02532
red	10209	GSE7852_THYMUS_VS_FAT_TCONV_DN	12	196	16057	524	1.899011	0.02532
red	10268	GSE9988_ANTI_TREM1_VS_LPS_MONOCYTE_UP	12	196	16057	524	1.899011	0.02532
turquoise	8402	GOLDRATH_EFF_VS_MEMORY_CD8_TCELL_UP	64	200	16053	4169	1.247532	0.02534
turquoise	9379	GSE22886_NAIVE_CD4_TCELL_VS_DC_DN	64	200	16053	4169	1.247532	0.02534
turquoise	9435	GSE24634_NAIVE_CD4_TCELL_VS_DAY5_IL4_CONV_TR	64	200	16053	4169	1.247532	0.02534
green	4099	PID_BARD1PATHWAY	6	29	16224	1305	2.576774	0.025366
green	4569	REACTOME_THROMBIN_SIGNALLING_THROUGH_PROT	6	29	16224	1305	2.576774	0.025366
green	6773	DAZARD_UV_RESPONSE_CLUSTER_G2	6	29	16224	1305	2.576774	0.025366
purple	8043	SERVITJA_ISLET_HNF1A_TARGETS_UP	4	115	16138	157	3.600775	0.025367
magenta	1060	CATION_TRANSMEMBRANE_TRANSPORTER_ACTIVITY	5	161	16092	167	3.022464	0.025393
magenta	9822	GSE360_DC_VS_MAC_T_GONDII_UP	5	161	16092	167	3.022464	0.025393
blue	2242	TTAYRTAA_V$E4BP4_01	52	219	16034	2978	1.29589	0.025409
brown	8782	GSE15930_NAIVE_VS_48H_IN_VITRO_STIM_CD8_TCELI	33	174	16079	2190	1.407519	0.025437
brown	9490	GSE26928_NAIVE_VS_CXCR5_POS_CD4_TCELL_UP	33	174	16079	2190	1.407519	0.025437
pink	4864	ZHONG_RESPONSE_TO_AZACITIDINE_AND_TSA_UP	6	160	16093	228	2.673191	0.025445
brown	2235	CAGGTA_V$AREB6_01	99	609	15644	2190	1.206444	0.025455
brown	2256	RGAANNTTC_V$HSF1_01	60	348	15905	2190	1.279562	0.025456
cyan	7371	SWEET_LUNG_CANCER_KRAS_DN	5	351	15902	77	3.006808	0.025459
yellow	3187	chr11q12	15	99	16154	1426	1.726911	0.02549
yellow	9341	GSE22886_IGG_IGA_MEMORY_BCELL_VS_BM_PLASMA	24	180	16073	1426	1.519682	0.025533
yellow	9588	GSE27786_CD8_TCELL_VS_NKCELL_UP	24	180	16073	1426	1.519682	0.025533
red	4593	REACTOME_ORGANIC_CATION_ANION_ZWITTERION_T	2	8	16245	524	7.754294	0.025535
red	5380	TAKAYAMA_BOUND_BY_AR	2	8	16245	524	7.754294	0.025535
red	5470	MYLLYKANGAS_AMPLIFICATION_HOT_SPOT_16	2	8	16245	524	7.754294	0.025535
red	6284	ROETH_TERT_TARGETS_DN	2	8	16245	524	7.754294	0.025535
red	6758	SASSON_FSH_RESPONSE	2	8	16245	524	7.754294	0.025535
red	7125	MATZUK_LUTEAL_GENES	2	8	16245	524	7.754294	0.025535
red	8036	SCHMIDT_POR_TARGETS_IN_LIMB_BUD_DN	2	8	16245	524	7.754294	0.025535
greenyello	1233	CHEMOKINE_ACTIVITY	2	27	16226	150	8.026173	0.025539
greenyello	2874	KEGG_PRION_DISEASES	2	27	16226	150	8.026173	0.025539
greenyello	7490	VALK_AML_CLUSTER_13	2	27	16226	150	8.026173	0.025539
lightgreen	711	LYSOSOMAL_TRANSPORT	1	10	16243	42	38.69762	0.02555
lightgreen	956	POSITIVE_REGULATION_OF_MAPKKK_CASCADE	1	10	16243	42	38.69762	0.02555
lightgreen	1084	HEMATOPOIETIN_INTERFERON_CLASSD200_DOMAIN_	1	10	16243	42	38.69762	0.02555
lightgreen	1231	ADP_BINDING	1	10	16243	42	38.69762	0.02555
lightgreen	2953	BIOCARTA_CFTR_PATHWAY	1	10	16243	42	38.69762	0.02555
lightgreen	4665	REACTOME_IKK_COMPLEX_RECRUITMENT_MEDIATED_	1	10	16243	42	38.69762	0.02555
lightgreen	5822	GOUYER_TATI_TARGETS_UP	1	10	16243	42	38.69762	0.02555
salmon	4634	REACTOME_REGULATION_OF_MRNA_STABILITY_BY_PI	3	81	16172	127	4.739866	0.025563
yellow	1567	V$COUP_01	27	208	16045	1426	1.479498	0.025569
grey60	826	RESPONSE_TO_EXTERNAL_STIMULUS	3	235	16018	44	4.715571	0.025582
turquoise	695	PROTEIN_AMINO_ACID_LIPIDATION	11	24	16229	4169	1.786829	0.025603

turquoise	739	PHOSPHOINOSITIDE_BIOSYNTHETIC_PROCESS	11	24	16229	4169	1.786829	0.025603
turquoise	4254	REACTOME_TGF_BETA_RECEPTOR_SIGNALING_ACTIVA	11	24	16229	4169	1.786829	0.025603
red	866	CELL_CYCLE_ARREST_GO_0007050	5	52	16201	524	2.982421	0.025604
red	5098	NAGASHIMA_EGF_SIGNALING_UP	5	52	16201	524	2.982421	0.025604
turquoise	2078	GCAAGAC,MIR-431	16	39	16214	4169	1.5994	0.025604
turquoise	7101	EHLERS_ANEUPLOIDY_UP	16	39	16214	4169	1.5994	0.025604
yellow	1525	V$NRF2_01	33	265	15988	1426	1.419326	0.025613
brown	6189	SANA_RESPONSE_TO_IFNG_DN	17	77	16176	2190	1.638504	0.025618
red	9869	GSE360_L_DONOVANI_VS_M_TUBERCULOSIS_MAC_DN	11	174	16079	524	1.960856	0.025623
red	9970	GSE3982_EOSINOPHIL_VS_MAST_CELL_UP	11	174	16079	524	1.960856	0.025623
blue	8357	TBK1.DN.48HRS_UP	15	49	16204	2978	1.670721	0.025629
brown	1420	PYROPHOSPHATASE_ACTIVITY	39	212	16041	2190	1.365269	0.025634
tan	6795	KRIGE_RESPONSE_TO_TOSEDOSTAT_6HR_DN	14	880	15373	145	1.783245	0.025645
brown	8568	GSE13484_12H_UNSTIM_VS_YF17D_VACCINE_STIM_PI	36	193	16060	2190	1.384314	0.025673
brown	8816	GSE16522_MEMORY_VS_NAIVE_ANTI_CD3CD28_STIM_	36	193	16060	2190	1.384314	0.025673
brown	9049	GSE17721_LPS_VS_CPG_8H_BMDM_DN	36	193	16060	2190	1.384314	0.025673
brown	9132	GSE17721_0.5H_VS_24H_CPG_BMDM_UP	36	193	16060	2190	1.384314	0.025673
brown	9329	GSE22886_NAIVE_VS_IGG_IGA_MEMORY_BCELL_DN	36	193	16060	2190	1.384314	0.025673
brown	9598	GSE27786_NKCELL_VS_NKTCELL_UP	36	193	16060	2190	1.384314	0.025673
tan	5247	GAUSSMANN_MLL_AF4_FUSION_TARGETS_D_UP	2	28	16225	145	8.006404	0.025676
tan	10132	GSE6269_HEALTHY_VS_STREP_PNEUMO_INF_PBMC_U	5	186	16067	145	3.013163	0.025678
pink	136	INTEGRAL_TO_PLASMA_MEMBRANE	17	721	15532	228	1.680786	0.025684
brown	3385	chr17q24	10	38	16215	2190	1.953016	0.025696
brown	4070	PID_ERBB1_INTERNALIZATION_PATHWAY	10	38	16215	2190	1.953016	0.025696
grey60	1504	V$EVI1_02	2	92	16161	44	8.030138	0.025704
cyan	7839	MARTENS_TRETINOIN_RESPONSE_UP	6	478	15775	77	2.649514	0.025707
blue	1665	V$ZF5_B	48	200	16053	2978	1.309846	0.025738
lightyellow	5159	PROVENZANI_METASTASIS_DN	2	123	16130	33	8.008376	0.025743
cyan	3607	MODULE_119	3	134	16119	77	4.725625	0.02575
cyan	6971	SARRIO_EPITHELIAL_MESENCHYMAL_TRANSITION_DN	3	134	16119	77	4.725625	0.02575
lightcyan	5394	PEREZ_TP63_TARGETS	4	294	15959	62	3.566601	0.025783
greenyello	1470	V$RSRFC4_01	5	180	16073	150	3.009815	0.025791
greenyello	8738	GSE15324_ELF4_KO_VS_WT_NAIVE_CD8_TCELL_UP	5	180	16073	150	3.009815	0.025791
blue	8266	PRC2_EDD_UP.V1_UP	44	181	16072	2978	1.326731	0.025832
blue	9182	GSE17974_CTRL_VS_ACT_IL4_AND_ANTI_IL12_48H_CD	44	181	16072	2978	1.326731	0.025832
salmon	3786	MODULE_334	4	143	16110	127	3.579759	0.02586
pink	1364	CARBOXYLESTERASE_ACTIVITY	2	18	16235	228	7.920565	0.025861
pink	3887	MODULE_497	2	18	16235	228	7.920565	0.025861
pink	4423	REACTOME_FORMATION_OF_TUBULIN_FOLDING_INTE	2	18	16235	228	7.920565	0.025861
red	8308	CRX_DN.V1_UP	8	110	16143	524	2.255795	0.025868
green	3302	chr2q21	7	37	16216	1305	2.356239	0.025888
purple	1412	GLYCOSAMINOGLYCAN_BINDING	2	26	16227	157	7.963253	0.025892
purple	4415	REACTOME_GLUCAGON_SIGNALING_IN_METABOLIC_R	2	26	16227	157	7.963253	0.025892
purple	5011	TAKEDA_TARGETS_OF_NUP98_HOXA9_FUSION_3D_DN	2	26	16227	157	7.963253	0.025892
purple	6528	SIMBULAN_PARP1_TARGETS_UP	2	26	16227	157	7.963253	0.025892
purple	6649	LIANG_SILENCED_BY_METHYLATION_UP	2	26	16227	157	7.963253	0.025892
purple	7479	VALK_AML_CLUSTER_2	2	26	16227	157	7.963253	0.025892
magenta	513	CYTOKINE_PRODUCTION	3	62	16191	167	4.709195	0.025902
magenta	4757	REACTOME_POTASSIUM_CHANNELS	3	62	16191	167	4.709195	0.025902
magenta	6301	ZHAN_MULTIPLE_MYELOMA_UP	3	62	16191	167	4.709195	0.025902
magenta	7274	GU_PDEF_TARGETS_UP	3	62	16191	167	4.709195	0.025902
turquoise	2311	MORF_HDAC1	79	253	16000	4169	1.21733	0.025915
greenyello	989	TRANSMEMBRANE_RECEPTOR_PROTEIN_TYROSINE_KII	3	69	16184	150	4.711014	0.025922
midnightb	2744	KEGG_TAURINE_AND_HYPOTAURINE_METABOLISM	1	6	16247	71	38.15258	0.02593
brown	62	CYTOPLASM	291	1950	14303	2190	1.10751	0.025941
green	7725	MIKKELSEN_MEF_HCP_WITH_H3K27ME3	34	300	15953	1305	1.411499	0.025947
turquoise	8181	ZHOU_CELL_CYCLE_GENES_IN_IR_RESPONSE_6HR	30	84	16169	4169	1.392335	0.025977
purple	443	INTERPHASE	3	66	16187	157	4.705559	0.025981
purple	7733	YAO_TEMPORAL_RESPONSE_TO_PROGESTERONE_CLUS	3	66	16187	157	4.705559	0.025981
turquoise	4035	PID_ERBB1_DOWNSTREAM_PATHWAY	36	104	16149	4169	1.349494	0.025995

magenta	1376	METAL_ION_TRANSMEMBRANE_TRANSPORTER_ACTIV	4	109	16144	167	3.571499	0.026026
midnightb	6754	BROWNE_HCMV_INFECTION_6HR_DN	3	146	16107	71	4.703743	0.026035
lightyellow	5207	LINDGREN_BLADDER_CANCER_CLUSTER_3_UP	3	317	15936	33	4.661027	0.026043
turquoise	6352	MA_MYELOID_DIFFERENTIATION_UP	15	36	16217	4169	1.62439	0.026048
turquoise	7341	POTTI_PACLITAXEL_SENSITIVITY	15	36	16217	4169	1.62439	0.026048
pink	2042	AGGCACT,MIR-515-3P	4	80	16173	228	3.564254	0.026054
pink	8010	VANLOO_SP3_TARGETS_DN	4	80	16173	228	3.564254	0.026054
brown	450	MUSCLE_CELL_DIFFERENTIATION	6	18	16235	2190	2.47382	0.026057
brown	606	B_CELL_ACTIVATION	6	18	16235	2190	2.47382	0.026057
brown	4273	REACTOME_SIGNALING_BY_FGFR1_FUSION_MUTANTS	6	18	16235	2190	2.47382	0.026057
brown	4484	REACTOME_ADHERENS_JUNCTIONS_INTERACTIONS	6	18	16235	2190	2.47382	0.026057
brown	4779	REACTOME_GAP_JUNCTION_TRAFFICKING	6	18	16235	2190	2.47382	0.026057
brown	5838	SUH_COEXPRESSED_WITH_ID1_AND_ID2_UP	6	18	16235	2190	2.47382	0.026057
lightyellow	1238	OXYGEN_BINDING	1	13	16240	33	37.88578	0.026085
lightyellow	3623	MODULE_135	1	13	16240	33	37.88578	0.026085
lightyellow	3841	MODULE_417	1	13	16240	33	37.88578	0.026085
lightyellow	3857	MODULE_444	1	13	16240	33	37.88578	0.026085
lightyellow	3896	MODULE_516	1	13	16240	33	37.88578	0.026085
lightyellow	4249	REACTOME_SIGNALLING_TO_P38_VIA_RIT_AND_RIN	1	13	16240	33	37.88578	0.026085
lightyellow	4795	NAKAMURA_CANCER_MICROENVIRONMENT_UP	1	13	16240	33	37.88578	0.026085
lightyellow	5196	CASTELLANO_NRAS_TARGETS_DN	1	13	16240	33	37.88578	0.026085
lightyellow	7011	VICENT_METASTASIS_UP	1	13	16240	33	37.88578	0.026085
lightyellow	7665	GUTIERREZ_WALDENSTROEMS_MACROGLOBULINEMIA	1	13	16240	33	37.88578	0.026085
yellow	9244	GSE18791_UNSTIM_VS_NEWCATSLE_VIRUS_DC_10H_U	26	199	16054	1426	1.489136	0.0261
brown	3927	PID_FCER1PATHWAY	14	60	16193	2190	1.731674	0.026105
yellow	6394	IVANOVA_HEMATOPOIESIS_EARLY_PROGENITOR	56	492	15761	1426	1.29729	0.026114
cyan	5442	RASHI_RESPONSE_TO_IONIZING_RADIATION_4	2	53	16200	77	7.965205	0.026125
lightyellow	6256	ALCALAY_AML_BY_NPM1_LOCALIZATION_UP	2	124	16129	33	7.943793	0.026132
pink	5898	PASQUALUCCI_LYMPHOMA_BY_GC_STAGE_DN	6	161	16092	228	2.656587	0.026132
red	1300	ENZYME_INHIBITOR_ACTIVITY	7	90	16163	524	2.412447	0.026135
salmon	69	NUCLEAR_LUMEN	7	370	15883	127	2.421175	0.02619
turquoise	3177	chr10p11	12	27	16226	4169	1.732683	0.026193
turquoise	3759	MODULE_303	12	27	16226	4169	1.732683	0.026193
turquoise	4682	REACTOME_PKB_MEDIATED_EVENTS	12	27	16226	4169	1.732683	0.026193
turquoise	4937	WANG_RESPONSE_TO_ANDROGEN_UP	12	27	16226	4169	1.732683	0.026193
turquoise	5263	BARRIER_CANCER_RELAPSE_NORMAL_SAMPLE_UP	12	27	16226	4169	1.732683	0.026193
turquoise	5533	AIYAR_COBRA1_TARGETS_DN	12	27	16226	4169	1.732683	0.026193
tan	10250	GSE9037_WT_VS_IRAK4_KO_LPS_1H_STIM_BMDM_UF	5	187	16066	145	2.99705	0.026201
red	1810	V$TEF_Q6	12	197	16056	524	1.889371	0.026208
red	8661	GSE1432_CTRL_VS_IFNG_6H_MICROGLIA_DN	12	197	16056	524	1.889371	0.026208
yellow	1884	V$MYC_Q2	22	162	16091	1426	1.547824	0.026213
grey60	785	SECOND_MESSENGER_MEDIATED_SIGNALING	2	93	16160	44	7.943793	0.026224
grey60	8293	ALK_DN.V1_DN	2	93	16160	44	7.943793	0.026224
magenta	1419	TRANSMEMBRANE_TRANSPORTER_ACTIVITY	7	281	15972	167	2.424425	0.026234
blue	3329	chr11q23	27	102	16151	2978	1.444683	0.026234
yellow	1212	S_ADENOSYLMETHIONINE_DEPENDENT_METHYLTRANS	5	20	16233	1426	2.849404	0.026245
yellow	5547	XU_HGF_SIGNALING_NOT_VIA_AKT1_48HR_DN	5	20	16233	1426	2.849404	0.026245
turquoise	8471	GSE11864_CSF1_VS_CSF1_PAM3CYS_IN_MAC_DN	55	169	16084	4169	1.268755	0.026246
lightcyan	4878	TURASHVILI_BREAST_DUCTAL_CARCINOMA_VS_LOBUL	2	66	16187	62	7.943793	0.026266
black	3174	chr16q22	6	130	16123	283	2.650666	0.026279
pink	7443	MEISSNER_BRAIN_HCP_WITH_H3K4ME3_AND_H3K27M	18	779	15474	228	1.647152	0.026291
lightgreen	7500	POOLA_INVASIVE_BREAST_CANCER_UP	3	249	16004	42	4.662364	0.026297
blue	8473	GSE11864_CSF1_VS_CSF1_IFNG_PAM3CYS_IN_MAC_D	41	167	16086	2978	1.339912	0.0263
blue	8588	GSE13485_DAY3_VS_DAY7_YF17D_VACCINE_PBMC_UI	41	167	16086	2978	1.339912	0.0263
blue	1032	NUCLEOCYTOPLASMIC_TRANSPORT	23	84	16169	2978	1.494367	0.026309
blue	2318	MORF_PRDX3	23	84	16169	2978	1.494367	0.026309
turquoise	1505	ACAWNRNSRCGG_UNKNOWN	22	58	16195	4169	1.478755	0.026311
turquoise	4166	REACTOME_ER_PHAGOSOME_PATHWAY	22	58	16195	4169	1.478755	0.026311
midnightb	6797	KRIGE_RESPONSE_TO_TOSEDOSTAT_24HR_DN	9	980	15273	71	2.102285	0.026313
red	6218	PENG_RAPAMYCIN_RESPONSE_DN	14	243	16010	524	1.786998	0.026326

turquoise	1927	V$AR_02	14	33	16220	4169	1.653925	0.026333
greenyello	10203	GSE7852_THYMUS_VS_FAT_TREG_DN	5	181	16072	150	2.993186	0.026333
blue	2084	GACAGGG,MIR-339	18	62	16191	2978	1.584491	0.026369
blue	2134	AGGAGTG,MIR-483	18	62	16191	2978	1.584491	0.026369
blue	5809	WEI_MYCN_TARGETS_WITH_E_BOX	159	754	15499	2978	1.150892	0.02637
purple	8708	GSE1460_CORD_VS_ADULT_BLOOD_NAIVE_CD4_TCELL	5	173	16080	157	2.991974	0.026378
purple	8788	GSE15930_NAIVE_VS_72H_IN_VITRO_STIM_CD8_TCELL	5	173	16080	157	2.991974	0.026378
purple	9327	GSE22886_CD4_TCELL_VS_BCELL_NAIVE_DN	5	173	16080	157	2.991974	0.026378
purple	2239	TAATTA_V$CHX10_01	11	584	15669	157	1.949906	0.02638
salmon	8019	PURBEY_TARGETS_OF_CTBP1_AND_SATB1_UP	3	82	16171	127	4.682063	0.026386
salmon	1756	V$BACH1_01	5	214	16039	127	2.990102	0.026392
blue	9714	GSE30083_SP1_VS_SP4_THYMOCYTE_UP	45	186	16067	2978	1.320409	0.026403
blue	10152	GSE7460_TCONV_VS_TREG_THYMUS_UP	45	186	16067	2978	1.320409	0.026403
lightcyan	377	SENSORY_PERCEPTION_OF_TASTE	1	7	16246	62	37.44931	0.026404
lightcyan	3377	chr1p	1	7	16246	62	37.44931	0.026404
lightcyan	6013	NIKOLSKY_BREAST_CANCER_20P13_AMPLICON	1	7	16246	62	37.44931	0.026404
lightcyan	7235	ISHIKAWA_STING_SIGNALING	1	7	16246	62	37.44931	0.026404
turquoise	905	PHOSPHOINOSITIDE_METABOLIC_PROCESS	13	30	16223	4169	1.689366	0.026405
turquoise	2974	BIOCARTA_FAS_PATHWAY	13	30	16223	4169	1.689366	0.026405
turquoise	4563	REACTOME_MAPK_TARGETS_NUCLEAR_EVENTS_MEDI	13	30	16223	4169	1.689366	0.026405
turquoise	4642	REACTOME_TRANSPORT_TO_THE_GOLGI_AND_SUBSE(	13	30	16223	4169	1.689366	0.026405
red	1590	V$ER_Q6	13	220	16033	524	1.832833	0.026411
green	8667	GSE1432_1H_VS_24H_IFNG_MICROGLIA_DN	24	197	16056	1305	1.517288	0.026419
turquoise	8974	GSE17721_CPG_VS_GARDIQUIMOD_6H_BMDM_UP	57	176	16077	4169	1.262594	0.026426
green	663	SYNAPTIC_TRANSMISSION	14	99	16154	1305	1.761229	0.026474
brown	4238	REACTOME_NGF_SIGNALLING_VIA_TRKA_FROM_THE_	25	125	16128	2190	1.484292	0.026479
greenyello	5165	KOKKINAKIS_METHIONINE_DEPRIVATION_48HR_UP	4	122	16131	150	3.552568	0.026494
tan	2818	KEGG_PEROXISOME	3	72	16181	145	4.670402	0.02652
tan	5850	RICKMAN_TUMOR_DIFFERENTIATED_WELL_VS_MODEI	3	72	16181	145	4.670402	0.02652
lightyellow	6550	RODWELL_AGING_KIDNEY_DN	2	125	16128	33	7.880242	0.026523
brown	7944	IVANOVSKA_MIR106B_TARGETS	19	89	16164	2190	1.584357	0.026524
green	8221	MTOR_UP.V1_DN	20	157	16096	1305	1.586549	0.026533
green	7726	MIKKELSEN_MEF_HCP_WITH_H3_UNMETHYLATED	16	118	16135	1305	1.688733	0.026535
cyan	239	RNA_METABOLIC_PROCESS	8	757	15496	77	2.230678	0.026548
turquoise	8829	GSE17580_UNINFECTED_VS_S_MANSONI_INF_TEFF_DI	59	183	16070	4169	1.256905	0.026555
green	2404	MORF_IL16	23	187	16066	1305	1.531825	0.026569
red	9627	GSE2826_WT_VS_BTK_KO_BCELL_DN	11	175	16078	524	1.949651	0.026582
red	9980	GSE3982_EOSINOPHIL_VS_BASOPHIL_UP	11	175	16078	524	1.949651	0.026582
red	10171	GSE7460_CTRL_VS_TGFB_TREATED_ACT_FOXP3_MUT_	11	175	16078	524	1.949651	0.026582
brown	4020	PID_PI3KCIPATHWAY	12	49	16204	2190	1.817501	0.026589
blue	1438	GUANYL_NUCLEOTIDE_EXCHANGE_FACTOR_ACTIVITY	14	45	16208	2978	1.697948	0.02659
blue	4276	REACTOME_PI_METABOLISM	14	45	16208	2978	1.697948	0.02659
blue	5426	MIDORIKAWA_AMPLIFIED_IN_LIVER_CANCER	14	45	16208	2978	1.697948	0.02659
yellow	9115	GSE17721_0.5H_VS_12H_PAM3CSK4_BMDM_DN	25	190	16063	1426	1.499686	0.026606
yellow	9154	GSE17974_0H_VS_2H_IN_VITRO_ACT_CD4_TCELL_UP	25	190	16063	1426	1.499686	0.026606
yellow	9260	GSE20366_EX_VIVO_VS_HOMEOSTATIC_CONVERSION_	25	190	16063	1426	1.499686	0.026606
turquoise	2171	AAGGGAT,MIR-188	26	71	16182	4169	1.427633	0.026608
salmon	2249	MGGAAGTG_V$GABP_B	11	725	15528	127	1.941711	0.026622
turquoise	8607	GSE13738_TCR_VS_BYSTANDER_ACTIVATED_CD4_TCEL	61	190	16063	4169	1.251636	0.026638
turquoise	9010	GSE17721_POLYIC_VS_CPG_6H_BMDM_UP	61	190	16063	4169	1.251636	0.026638
turquoise	9208	GSE17974_2H_VS_72H_UNTREATED_IN_VITRO_CD4_T	61	190	16063	4169	1.251636	0.026638
turquoise	9835	GSE360_L_DONOVANI_VS_B_MALAYI_HIGH_DOSE_DC_	61	190	16063	4169	1.251636	0.026638
green	9020	GSE17721_PAM3CSK4_VS_GADIQUIMOD_0.5H_BMDM	21	167	16086	1305	1.566123	0.02664
purple	1456	AAAYRNCTG_UNKNOWN	7	300	15953	157	2.41552	0.026659
purple	5877	KOYAMA_SEMA3B_TARGETS_DN	8	368	15885	157	2.250485	0.026664
blue	2457	MORF_RAC1	50	210	16043	2978	1.29945	0.026665
magenta	38	EXTRACELLULAR_REGION	7	282	15971	167	2.415828	0.026678
turquoise	9142	GSE17721_0.5H_VS_8H_GARDIQUIMOD_BMDM_UP	63	197	16056	4169	1.24674	0.026679
turquoise	9615	GSE27786_ERYTHROBLAST_VS_MONO_MAC_DN	63	197	16056	4169	1.24674	0.026679
turquoise	10191	GSE7764_IL15_NK_CELL_24H_VS_SPLENOCYTE_DN	63	197	16056	4169	1.24674	0.026679

turquoise	10226	GSE9006_HEALTHY_VS_TYPE_1_DIABETES_PBMC_1MC	63	197	16056	4169	1.24674	0.026679
lightcyan	10058	GSE3982_NEUTROPHIL_VS_TH2_UP	3	169	16084	62	4.653464	0.026707
brown	1199	PROTEIN_KINASE_ACTIVITY	47	264	15989	2190	1.321245	0.026717
midnightb	4824	LIU_PROSTATE_CANCER_DN	5	386	15867	71	2.965227	0.026718
grey60	2019	GTGTTGA,MIR-505	2	94	16159	44	7.859284	0.026749
grey60	5967	ZHANG_RESPONSE_TO_IKK_INHIBITOR_AND_TNF_DN	2	94	16159	44	7.859284	0.026749
grey60	914	AMYLOID_PRECURSOR_PROTEIN_METABOLIC_PROCES:	1	10	16243	44	36.93864	0.026752
grey60	1306	NUCLEOBASENUCLEOSIDENUCLEOTIDE_AND_NUCLEIC_	1	10	16243	44	36.93864	0.026752
grey60	7697	DORN_ADENOVIRUS_INFECTION_32HR_UP	1	10	16243	44	36.93864	0.026752
red	145	NUCLEAR_ENVELOPE	6	71	16182	524	2.62117	0.026758
brown	6344	MENSE_HYPOXIA_UP	20	95	16158	2190	1.562413	0.026758
yellow	1945	GGGNRMNNYCAT_UNKNOWN	12	74	16179	1426	1.848262	0.026823
pink	9421	GSE24142_EARLY_THYMIC_PROGENITOR_VS_DN2_THY	6	162	16091	228	2.640188	0.026831
brown	9158	GSE17974_0H_VS_6H_IN_VITRO_ACT_CD4_TCELL_UP	34	181	16072	2190	1.394087	0.026843
brown	9945	GSE37416_12H_VS_24H_F_TULARENSIS_LVS_NEUTROI	34	181	16072	2190	1.394087	0.026843
brown	4040	PID_UPA_UPAR_PATHWAY	9	33	16220	2190	2.024035	0.026856
yellow	7306	ICHIBA_GRAFT_VERSUS_HOST_DISEASE_D7_DN	6	27	16226	1426	2.532803	0.026877
brown	2147	GGCACTT,MIR-519E	22	107	16146	2190	1.525908	0.026891
grey60	5906	BENPORATH_EED_TARGETS	5	630	15623	44	2.931638	0.026904
greenyello	1286	ACTIN_BINDING	3	70	16183	150	4.643714	0.026904
greenyello	3631	MODULE_146	3	70	16183	150	4.643714	0.026904
lightyellow	3599	MODULE_111	2	126	16127	33	7.817701	0.026917
blue	9347	GSE22886_DAY0_VS_DAY1_MONOCYTE_IN_CULTURE_	46	191	16062	2978	1.314417	0.026949
blue	10175	GSE7460_FOXP3_MUT_VS_WT_ACT_WITH_TGFB_TCOI	46	191	16062	2978	1.314417	0.026949
purple	9136	GSE17721_12H_VS_24H_CPG_BMDM_UP	5	174	16079	157	2.974779	0.026954
purple	10119	GSE39820_TGFBETA1_VS_TGFBETA3_IN_IL6_TREATED_	5	174	16079	157	2.974779	0.026954
lightgreen	5100	KIM_WT1_TARGETS_DN	4	443	15810	42	3.494142	0.02696
magenta	5688	YANG_BREAST_CANCER_ESR1_BULK_UP	2	25	16228	167	7.785868	0.02697
magenta	6296	RORIE_TARGETS_OF_EWSR1_FLI1_FUSION_UP	2	25	16228	167	7.785868	0.02697
magenta	1208	SMALL_GTPASE_REGULATOR_ACTIVITY	3	63	16190	167	4.634445	0.026997
magenta	6348	HADDAD_T_LYMPHOCYTE_AND_NK_PROGENITOR_DN	3	63	16190	167	4.634445	0.026997
lightgreen	1697	V$MEIS1BHOXA9_01	2	99	16154	42	7.817701	0.027001
lightgreen	6143	WIELAND_UP_BY_HBV_INFECTION	2	99	16154	42	7.817701	0.027001
lightcyan	6770	MAHAJAN_RESPONSE_TO_IL1A_UP	2	67	16186	62	7.825229	0.027008
pink	3504	MODULE_12	9	302	15951	228	2.12439	0.027009
purple	3911	MODULE_544	3	67	16186	157	4.635327	0.027012
purple	4374	REACTOME_OPIOID_SIGNALLING	3	67	16186	157	4.635327	0.027012
pink	1612	V$HAND1E47_01	7	207	16046	228	2.410607	0.027015
pink	1829	V$CACBINDINGPROTEIN_Q6	7	207	16046	228	2.410607	0.027015
cyan	7661	GUTIERREZ_CHRONIC_LYMPHOCYTIC_LEUKEMIA_DN	2	54	16199	77	7.817701	0.027047
grey60	1322	RECEPTOR_ACTIVITY	4	423	15830	44	3.493015	0.027072
grey60	8124	RAO_BOUND_BY_SALL4_ISOFORM_B	4	423	15830	44	3.493015	0.027072
yellow	6058	SHEPARD_CRUSH_AND_BURN_MUTANT_UP	24	181	16072	1426	1.511286	0.027078
yellow	8266	PRC2_EDD_UP.V1_UP	24	181	16072	1426	1.511286	0.027078
lightcyan	10094	GSE3982_CENT_MEMORY_CD4_TCELL_VS_TH2_UP	3	170	16083	62	4.626091	0.027114
pink	7671	KYNG_WERNER_SYNDROM_AND_NORMAL_AGING_UP	4	81	16172	228	3.520251	0.027116
red	1853	V$HMGIY_Q6	12	198	16055	524	1.879829	0.027118
red	8439	GSE10325_MYELOID_VS_LUPUS_MYELOID_DN	12	198	16055	524	1.879829	0.027118
red	9321	GSE22886_NAIVE_CD4_TCELL_VS_NKCELL_DN	12	198	16055	524	1.879829	0.027118
blue	5033	MULLIGHAN_NPM1_MUTATED_SIGNATURE_1_DN	30	116	16137	2978	1.411471	0.027118
black	8248	E2F3_UP.V1_UP	7	167	16086	283	2.407291	0.027126
salmon	3532	MODULE_43	3	83	16170	127	4.625652	0.027223
tan	8768	GSE15750_WT_VS_TRAF6KO_DAY10_EFF_CD8_TCELL_	5	189	16064	145	2.965335	0.027266
grey60	874	ION_HOMEOSTASIS	2	95	16158	44	7.776555	0.027278
grey60	1221	OXIDOREDUCTASE_ACTIVITY	3	241	16012	44	4.598171	0.027295
lightyellow	4489	REACTOME_G_ALPHA_I_SIGNALLING_EVENTS	2	127	16126	33	7.756144	0.027314
salmon	3253	chr3q22	2	33	16220	127	7.756144	0.027314
turquoise	7671	KYNG_WERNER_SYNDROM_AND_NORMAL_AGING_UP	29	81	16172	4169	1.395772	0.027317
black	3856	MODULE_440	2	15	16238	283	7.657479	0.02732
black	7711	MIKKELSEN_ES_HCP_WITH_H3K27ME3	2	15	16238	283	7.657479	0.02732

salmon	1910	V$AP1_Q2_01	5	216	16037	127	2.962416	0.027327
greenyello	1268	CHEMOKINE_RECEPTOR_BINDING	2	28	16225	150	7.739524	0.027339
blue	773	PROTEIN_COMPLEX_ASSEMBLY	39	158	16095	2978	1.347151	0.027372
turquoise	7928	JOHNSTONE_PARVB_TARGETS_1_DN	21	55	16198	4169	1.488532	0.027409
tan	7749	MOOTHA_GLUCONEOGENESIS	2	29	16224	145	7.730321	0.027419
brown	9836	GSE360_L_DONOVANI_VS_B_MALAYI_LOW_DOSE_DC_	33	175	16078	2190	1.399476	0.027424
brown	10000	GSE3982_MAST_CELL_VS_BASOPHIL_UP	33	175	16078	2190	1.399476	0.027424
blue	5213	NADERI_BREAST_CANCER_PROGNOSIS_UP	13	41	16212	2978	1.730487	0.027436
blue	5304	LUI_THYROID_CANCER_PAX8_PPARG_UP	13	41	16212	2978	1.730487	0.027436
greenyello	1492	V$TAL1BETAE47_01	5	183	16070	150	2.960474	0.02744
greenyello	1708	V$TITF1_Q3	5	183	16070	150	2.960474	0.02744
greenyello	8677	GSE14350_IL2RB_KO_VS_WT_TEFF_DN	5	183	16070	150	2.960474	0.02744
greenyello	9402	GSE24026_PD1_LIGATION_VS_CTRL_IN_ACT_TCELL_LIN	5	183	16070	150	2.960474	0.02744
black	5854	RICKMAN_METASTASIS_DN	9	244	16009	283	2.11836	0.027455
cyan	6537	BLALOCK_ALZHEIMERS_DISEASE_UP	13	1535	14718	77	1.787631	0.027458
blue	8395	KAECH_DAY8_EFF_VS_MEMORY_CD8_TCELL_DN	47	196	16057	2978	1.308732	0.027471
blue	8400	GOLDRATH_NAIVE_VS_MEMORY_CD8_TCELL_UP	47	196	16057	2978	1.308732	0.027471
blue	9440	GSE24634_TREG_VS_TCONV_POST_DAY3_IL4_CONVER	47	196	16057	2978	1.308732	0.027471
brown	2720	KEGG_PENTOSE_PHOSPHATE_PATHWAY	7	23	16230	2190	2.258706	0.027492
brown	2723	KEGG_GALACTOSE_METABOLISM	7	23	16230	2190	2.258706	0.027492
brown	2968	BIOCARTA_ECM_PATHWAY	7	23	16230	2190	2.258706	0.027492
brown	4265	REACTOME_SIGNALING_BY_FGFR1_MUTANTS	7	23	16230	2190	2.258706	0.027492
brown	4530	REACTOME_SMOOTH_MUSCLE_CONTRACTION	7	23	16230	2190	2.258706	0.027492
brown	5342	TOMIDA_METASTASIS_UP	7	23	16230	2190	2.258706	0.027492
lightgreen	4366	REACTOME_PEPTIDE_LIGAND_BINDING_RECEPTORS	2	100	16153	42	7.739524	0.027508
yellow	9104	GSE17721_0.5H_VS_4H_POLYIC_BMDM_UP	23	172	16081	1426	1.5241	0.027508
lightcyan	8465	GSE11864_UNTREATED_VS_CSF1_PAM3CYS_IN_MAC_I	3	171	16082	62	4.599038	0.027525
lightcyan	8710	GSE1460_NAIVE_CD4_TCELL_CORD_BLOOD_VS_THYMI	3	171	16082	62	4.599038	0.027525
lightcyan	8761	GSE15659_RESTING_VS_ACTIVATED_TREG_DN	3	171	16082	62	4.599038	0.027525
lightcyan	9853	GSE360_T_GONDII_VS_M_TUBERCULOSIS_DC_DN	3	171	16082	62	4.599038	0.027525
purple	10000	GSE3982_MAST_CELL_VS_BASOPHIL_UP	5	175	16078	157	2.95778	0.027538
purple	6691	IVANOVA_HEMATOPOIESIS_STEM_CELL_AND_PROGEN	11	588	15665	157	1.936642	0.027545
brown	8484	GSE11924_TFH_VS_TH17_CD4_TCELL_UP	36	194	16059	2190	1.377178	0.027566
brown	8814	GSE16522_MEMORY_VS_NAIVE_CD8_TCELL_UP	36	194	16059	2190	1.377178	0.027566
brown	9470	GSE25087_TREG_VS_TCONV_ADULT_UP	36	194	16059	2190	1.377178	0.027566
red	9609	GSE27786_NKTCELL_VS_NEUTROPHIL_DN	11	176	16077	524	1.938573	0.027566
brown	7594	SCHOEN_NFKB_SIGNALING	8	28	16225	2190	2.120417	0.027568
green	556	AMINO_ACID_CATABOLIC_PROCESS	5	22	16231	1305	2.830547	0.027569
green	838	RESPONSE_TO_UV	5	22	16231	1305	2.830547	0.027569
green	4457	REACTOME_INHIBITION_OF_INSULIN_SECRETION_BY_/	5	22	16231	1305	2.830547	0.027569
green	163	DENDRITE	4	15	16238	1305	3.321175	0.027577
green	3906	MODULE_534	4	15	16238	1305	3.321175	0.027577
green	7546	LEE_LIVER_CANCER_HEPATOBLAST	4	15	16238	1305	3.321175	0.027577
magenta	999	TRANSMISSION_OF_NERVE_IMPULSE	4	111	16142	167	3.507148	0.027586
black	5906	BENPORATH_EED_TARGETS	18	630	15623	283	1.640888	0.027587
blue	10012	GSE3982_DC_VS_MAC_UP	43	177	16076	2978	1.325879	0.027593
green	1376	METAL_ION_TRANSMEMBRANE_TRANSPORTER_ACTIV	15	109	16144	1305	1.713909	0.027603
yellow	8179	WARTERS_IR_RESPONSE_5GY	8	42	16211	1426	2.170974	0.027605
salmon	9496	GSE26928_CENTR_MEMORY_VS_CXCR5_POS_CD4_TCE	4	146	16107	127	3.506202	0.027632
yellow	3173	chrxq28	11	66	16187	1426	1.899603	0.027657
turquoise	730	NEGATIVE_REGULATION_OF_TRANSCRIPTION	54	166	16087	4169	1.268199	0.027664
turquoise	6324	LI_WILMS_TUMOR_VS_FETAL_KIDNEY_1_UP	54	166	16087	4169	1.268199	0.027664
lightyellow	2114	AACATTC,MIR-409-3P	2	128	16125	33	7.695549	0.027713
tan	8314	RB_DN.V1_UP	4	128	16125	145	3.502802	0.027718
turquoise	5598	DACOSTA_UV_RESPONSE_VIA_ERCC3_UP	92	300	15953	4169	1.195551	0.027719
red	4892	GARY_CD5_TARGETS_UP	23	464	15789	524	1.537489	0.027739
turquoise	7304	GOLDRATH_ANTIGEN_RESPONSE	104	343	15910	4169	1.182064	0.027744
lightcyan	7176	BRUECKNER_TARGETS_OF_MIRLET7A3_DN	2	68	16185	62	7.710152	0.027759
lightcyan	7272	HELLEBREKERS_SILENCED_DURING_TUMOR_ANGIOGEI	2	68	16185	62	7.710152	0.027759
blue	1775	V$HTF_01	17	58	16195	2978	1.599668	0.027768

blue	1967	GKCGCNNNNNNNTGAYG_UNKNOWN	17	58	16195	2978	1.599668	0.027768
cyan	5683	TSAI_RESPONSE_TO_IONIZING_RADIATION	3	138	16115	77	4.58865	0.027771
cyan	8289	ESC_V6.5_UP_EARLY.V1_DN	3	138	16115	77	4.58865	0.027771
yellow	7368	AGUIRRE_PANCREATIC_CANCER_COPY_NUMBER_UP	35	286	15967	1426	1.394813	0.027772
purple	1188	POLYSACCHARIDE_BINDING	2	27	16226	157	7.668318	0.027789
purple	1233	CHEMOKINE_ACTIVITY	2	27	16226	157	7.668318	0.027789
purple	3382	chr4q25	2	27	16226	157	7.668318	0.027789
purple	4098	PID_EPHRINBREVPATHWAY	2	27	16226	157	7.668318	0.027789
tan	8884	GSE17721_CTRL_VS_CPG_2H_BMDM_UP	5	190	16063	145	2.949728	0.027809
tan	9474	GSE26495_NAIVE_VS_PD1LOW_CD8_TCELL_UP	5	190	16063	145	2.949728	0.027809
grey60	7962	PASINI_SUZ12_TARGETS_UP	2	96	16157	44	7.695549	0.027811
turquoise	3056	BIOCARTA_CK1_PATHWAY	7	13	16240	4169	2.099212	0.027815
turquoise	3060	BIOCARTA_P27_PATHWAY	7	13	16240	4169	2.099212	0.027815
turquoise	4163	REACTOME_ABCA_TRANSPORTERS_IN_LIPID_HOMEOS	7	13	16240	4169	2.099212	0.027815
turquoise	4555	REACTOME_REGULATION_OF_AMPK_ACTIVITY_VIA_LK	7	13	16240	4169	2.099212	0.027815
turquoise	6798	GHO_ATF5_TARGETS_UP	7	13	16240	4169	2.099212	0.027815
turquoise	7730	YAO_TEMPORAL_RESPONSE_TO_PROGESTERONE_CLUS	7	13	16240	4169	2.099212	0.027815
brown	2278	RGAGGAARY_V$PU1_Q6	73	436	15817	2190	1.242584	0.027816
red	6721	KAAB_FAILED_HEART_VENTRICLE_DN	4	36	16217	524	3.446353	0.027846
turquoise	9108	GSE17721_0.5H_VS_24H_POLYIC_BMDM_UP	56	173	16080	4169	1.261954	0.027847
tan	5853	RICKMAN_METASTASIS_UP	7	328	15925	145	2.392157	0.027848
pink	5162	LIU_CDX2_TARGETS_DN	1	2	16251	228	35.64254	0.02786
turquoise	1776	V$ARNT_02	70	222	16031	4169	1.229268	0.027872
yellow	111	ENVELOPE	22	163	16090	1426	1.538329	0.027886
yellow	134	ORGANELLE_ENVELOPE	22	163	16090	1426	1.538329	0.027886
green	1516	ACAWYAAAG_UNKNOWN	11	72	16181	1305	1.902756	0.027886
turquoise	7737	YAO_TEMPORAL_RESPONSE_TO_PROGESTERONE_CLUS	25	68	16185	4169	1.433286	0.027896
greenyello	488	LOCOMOTORY_BEHAVIOR	3	71	16182	150	4.57831	0.027906
blue	3721	MODULE_252	53	225	16028	2978	1.285589	0.027908
lightcyan	8120	FOSTER_KDM1A_TARGETS_UP	3	172	16081	62	4.572299	0.02794
lightcyan	8198	EGFR_UP.V1_UP	3	172	16081	62	4.572299	0.02794
turquoise	8729	GSE14769_20MIN_VS_360MIN_LPS_BMDM_DN	58	180	16073	4169	1.256195	0.027976
blue	3717	MODULE_245	9	25	16228	2978	1.964768	0.027982
blue	6085	IIZUKA_LIVER_CANCER_PROGRESSION_G1_G2_DN	9	25	16228	2978	1.964768	0.027982
blue	6202	COLLER_MYC_TARGETS_UP	9	25	16228	2978	1.964768	0.027982
cyan	5046	TONKS_TARGETS_OF_RUNX1_RUNX1T1_FUSION_GRAN	2	55	16198	77	7.675561	0.027983
lightyellow	6735	SATO_SILENCED_BY_METHYLATION_IN_PANCREATIC_C	3	326	15927	33	4.532348	0.027986
green	172	EXTRACELLULAR_SPACE	19	148	16105	1305	1.598876	0.027998
green	8800	GSE15930_STIM_VS_STIM_AND_TRICHOSTATINA_24H_	19	148	16105	1305	1.598876	0.027998
brown	9799	GSE360_CTRL_VS_B_MALAYI_HIGH_DOSE_DC_DN	32	169	16084	2190	1.405247	0.028001
brown	10113	GSE39820_IL1B_IL6_VS_IL1B_IL6_IL23A_TREATED_CD4	32	169	16084	2190	1.405247	0.028001
blue	2519	GCM_FANCC	26	98	16155	2978	1.447958	0.028003
greenyello	5070	SENESE_HDAC1_AND_HDAC2_TARGETS_DN	5	184	16069	150	2.944384	0.028004
greenyello	8824	GSE17580_TREG_VS_TEFF_UP	5	184	16069	150	2.944384	0.028004
greenyello	9268	GSE20366_TREG_VS_NAIVE_CD4_TCELL_HOMEOSTATI	5	184	16069	150	2.944384	0.028004
greenyello	10029	GSE3982_DC_VS_TH2_DN	5	184	16069	150	2.944384	0.028004
midnightb	4982	OSMAN_BLADDER_CANCER_DN	5	391	15862	71	2.927308	0.028035
turquoise	2166	CTTTGCA,MIR-527	66	208	16045	4169	1.237036	0.028047
red	1782	V$CEBPGAMMA_Q6	12	199	16054	524	1.870382	0.028051
red	8394	KAECH_DAY8_EFF_VS_MEMORY_CD8_TCELL_UP	12	199	16054	524	1.870382	0.028051
red	8603	GSE13738_RESTING_VS_TCR_ACTIVATED_CD4_TCELL_I	12	199	16054	524	1.870382	0.028051
turquoise	8946	GSE17721_POLYIC_VS_PAM3CSK4_24H_BMDM_UP	60	187	16066	4169	1.250867	0.028056
turquoise	10250	GSE9037_WT_VS_IRAK4_KO_LPS_1H_STIM_BMDM_UI	60	187	16066	4169	1.250867	0.028056
purple	1270	RHODOPSIN_LIKE_RECEPTOR_ACTIVITY	3	68	16185	157	4.56716	0.028063
purple	3877	MODULE_481	3	68	16185	157	4.56716	0.028063
purple	5197	CHEBOTAEV_GR_TARGETS_UP	3	68	16185	157	4.56716	0.028063
purple	7272	HELLEBREKERS_SILENCED_DURING_TUMOR_ANGIOGEI	3	68	16185	157	4.56716	0.028063
lightyellow	94	PEROXISOMAL_PART	1	14	16239	33	35.17965	0.028064
lightyellow	162	MICROBODY_PART	1	14	16239	33	35.17965	0.028064
lightyellow	484	PHAGOCYTOSIS	1	14	16239	33	35.17965	0.028064

lightyellow	806	REGULATION_OF_ENDOCYTOSIS	1	14	16239	33	35.17965	0.028064
lightyellow	1319	CARBON_CARBON_LYASE_ACTIVITY	1	14	16239	33	35.17965	0.028064
lightyellow	3798	MODULE_354	1	14	16239	33	35.17965	0.028064
lightyellow	4068	PID_INTEGRIN5_PATHWAY	1	14	16239	33	35.17965	0.028064
lightyellow	4428	REACTOME_AMINE_LIGAND_BINDING_RECEPTORS	1	14	16239	33	35.17965	0.028064
lightgreen	93	IMMUNOLOGICAL_SYNAPSE	1	11	16242	42	35.17965	0.02807
lightgreen	969	ADENYLATE_CYCLASE_ACTIVATION	1	11	16242	42	35.17965	0.02807
lightgreen	979	INTERFERON_GAMMA_PRODUCTION	1	11	16242	42	35.17965	0.02807
lightgreen	2954	BIOCARTA_CYTOKINE_PATHWAY	1	11	16242	42	35.17965	0.02807
lightgreen	2983	BIOCARTA_SET_PATHWAY	1	11	16242	42	35.17965	0.02807
lightgreen	3040	BIOCARTA_PLCE_PATHWAY	1	11	16242	42	35.17965	0.02807
lightgreen	3132	chr6q13	1	11	16242	42	35.17965	0.02807
lightgreen	3729	MODULE_262	1	11	16242	42	35.17965	0.02807
lightgreen	5958	SHIN_B_CELL_LYMPHOMA_CLUSTER_1	1	11	16242	42	35.17965	0.02807
salmon	2318	MORF_PRDX3	3	84	16169	127	4.570585	0.028075
salmon	7372	SWEET_KRAS_ONCOGENIC_SIGNATURE	3	84	16169	127	4.570585	0.028075
lightcyan	3504	MODULE_12	4	302	15951	62	3.472121	0.028088
turquoise	8775	GSE15767_MED_VS_SCS_MAC_LN_DN	62	194	16059	4169	1.245924	0.028092
turquoise	9532	GSE27786_LSK_VS_CD4_TCELL_UP	62	194	16059	4169	1.245924	0.028092
turquoise	9553	GSE27786_LIN_NEG_VS_NKCELL_DN	62	194	16059	4169	1.245924	0.028092
turquoise	10030	GSE3982_MAC_VS_NEUTROPHIL_UP	62	194	16059	4169	1.245924	0.028092
turquoise	10105	GSE39820_CTRL_VS_TGFBETA1_IL6_CD4_TCELL_DN	62	194	16059	4169	1.245924	0.028092
cyan	903	ACUTE_INFLAMMATORY_RESPONSE	1	6	16247	77	35.17965	0.028095
cyan	5630	DARWICHE_PAPILLOMA_RISK_HIGH_VS_LOW_UP	1	6	16247	77	35.17965	0.028095
cyan	6409	IYENGAR_RESPONSE_TO_ADIPOCYTE_FACTORS	1	6	16247	77	35.17965	0.028095
cyan	7047	NADELLA_PRKAR1A_TARGETS_DN	1	6	16247	77	35.17965	0.028095
cyan	7292	PALOMERO_GSI_SENSITIVITY_DN	1	6	16247	77	35.17965	0.028095
pink	260	NUCLEOBASENUCLEOSIDE_AND_NUCLEOTIDE_METABO	3	47	16206	228	4.550112	0.0281
pink	5505	SCHLOSSER_MYC_TARGETS_AND_SERUM_RESPONSE_[	3	47	16206	228	4.550112	0.0281
pink	5506	SCHLOSSER_MYC_TARGETS_AND_SERUM_RESPONSE_U	3	47	16206	228	4.550112	0.0281
pink	6667	LIU_SMARCA4_TARGETS	3	47	16206	228	4.550112	0.0281
pink	6673	ZHANG_ANTIVIRAL_RESPONSE_TO_RIBAVIRIN_DN	3	47	16206	228	4.550112	0.0281
blue	2493	GCM_RAD21	12	37	16216	2978	1.770062	0.028104
blue	4886	CHANDRAN_METASTASIS_TOP50_UP	12	37	16216	2978	1.770062	0.028104
blue	8144	HOLLEMAN_VINCRISTINE_RESISTANCE_B_ALL_UP	12	37	16216	2978	1.770062	0.028104
blue	111	ENVELOPE	40	163	16090	2978	1.33931	0.028106
blue	134	ORGANELLE_ENVELOPE	40	163	16090	2978	1.33931	0.028106
lightyellow	3675	MODULE_195	2	129	16124	33	7.635894	0.028114
lightyellow	8301	PTEN_DN.V1_DN	2	129	16124	33	7.635894	0.028114
magenta	5560	DIRMEIER_LMP1_RESPONSE_EARLY	3	64	16189	167	4.562032	0.028117
magenta	7998	DUAN_PRDM5_TARGETS	3	64	16189	167	4.562032	0.028117
yellow	8088	DELACROIX_RARG_BOUND_MEF	40	335	15918	1426	1.360909	0.028125
purple	8974	GSE17721_CPG_VS_GARDIQUIMOD_6H_BMDM_UP	5	176	16077	157	2.940974	0.028131
purple	9998	GSE3982_MAST_CELL_VS_BCELL_UP	5	176	16077	157	2.940974	0.028131
brown	1829	V$CACBINDINGPROTEIN_Q6	38	207	16046	2190	1.362394	0.028132
magenta	1949	TGAATGT,MIR-181A,MIR-181B,MIR-181C,MIR-181D	9	416	15837	167	2.105553	0.028153
yellow	8830	GSE17580_UNINFECTED_VS_S_MANSONI_INF_TREG_U	25	191	16062	1426	1.491835	0.028157
yellow	10045	GSE3982_MAC_VS_TH2_DN	25	191	16062	1426	1.491835	0.028157
green	9431	GSE24142_ADULT_VS_FETAL_DN3_THYMOCYTE_DN	22	178	16075	1305	1.539309	0.028187
turquoise	1991	ACACTGG,MIR-199A,MIR-199B	47	142	16111	4169	1.290361	0.028189
turquoise	2475	MORF_TPR	47	142	16111	4169	1.290361	0.028189
pink	4823	LIU_PROSTATE_CANCER_UP	4	82	16171	228	3.477321	0.028203
pink	5438	ROVERSI_GLIOMA_COPY_NUMBER_UP	4	82	16171	228	3.477321	0.028203
brown	8642	GSE14308_TH1_VS_INDUCED_TREG_UP	35	188	16065	2190	1.381655	0.028219
brown	9733	GSE31082_DN_VS_CD4_SP_THYMOCYTE_DN	35	188	16065	2190	1.381655	0.028219
lightgreen	8344	STK33_DN	3	256	15997	42	4.534877	0.028236
greenyello	7443	MEISSNER_BRAIN_HCP_WITH_H3K4ME3_AND_H3K27M	13	779	15474	150	1.808207	0.028242
red	5188	CHIARADONNA_NEOPLASTIC_TRANSFORMATION_KRAS	9	133	16120	524	2.098907	0.028324
lightcyan	8708	GSE1460_CORD_VS_ADULT_BLOOD_NAIVE_CD4_TCELL	3	173	16080	62	4.54587	0.028357
lightcyan	8837	GSE17721_CTRL_VS_LPS_2H_BMDM_DN	3	173	16080	62	4.54587	0.028357

lightcyan	8953	GSE17721_PAM3CSK4_VS_CPG_2H_BMDM_DN	3	173	16080	62	4.54587	0.028357
lightcyan	8963	GSE17721_PAM3CSK4_VS_CPG_16H_BMDM_DN	3	173	16080	62	4.54587	0.028357
brown	5797	LOPEZ_MBD_TARGETS	140	893	15360	2190	1.163499	0.028369
magenta	8299	MEL18_DN.V1_DN	4	112	16141	167	3.475834	0.028386
magenta	9218	GSE18791_CTRL_VS_NEWCASTLE_VIRUS_DC_1H_UP	4	112	16141	167	3.475834	0.028386
red	4293	REACTOME_GLYCEROPHOSPHOLIPID_BIOSYNTHESIS	6	72	16181	524	2.584765	0.028426
red	7945	KIM_GLIS2_TARGETS_UP	6	72	16181	524	2.584765	0.028426
tan	4463	REACTOME_UNFOLDED_PROTEIN_RESPONSE	3	74	16179	145	4.544175	0.02846
tan	6800	DURCHDEWALD_SKIN_CARCINOGENESIS_UP	3	74	16179	145	4.544175	0.02846
purple	2234	CAGCTG_V$AP4_Q5	19	1231	15022	157	1.597826	0.028463
magenta	9312	GSE22886_NAIVE_CD4_TCELL_VS_MEMORY_TCELL_UP	5	166	16087	167	2.931426	0.028476
magenta	9476	GSE26495_PD1HIGH_VS_PD1LOW_CD8_TCELL_UP	5	166	16087	167	2.931426	0.028476
brown	5623	MARTORIATI_MDM4_TARGETS_FETAL_LIVER_DN	78	470	15783	2190	1.231647	0.02849
blue	5410	APPIERTO_RESPONSE_TO_FENRETINIDE_UP	11	33	16220	2978	1.81923	0.028508
lightcyan	7377	HOFFMANN_PRE_BI_TO_LARGE_PRE_BII_LYMPHOCYTE	2	69	16184	62	7.59841	0.028519
blue	4081	PID_IL2_STAT5PATHWAY	10	29	16224	2978	1.881962	0.028526
turquoise	6467	WANG_SMARCE1_TARGETS_DN	100	329	15924	4169	1.184966	0.028535
brown	1563	V$SRF_01	11	44	16209	2190	1.855365	0.028573
brown	3476	ST_T_CELL_SIGNAL_TRANSDUCTION	11	44	16209	2190	1.855365	0.028573
brown	4047	PID_IL6_7PATHWAY	11	44	16209	2190	1.855365	0.028573
brown	5604	HEIDENBLAD_AMPLICON_8Q24_DN	11	44	16209	2190	1.855365	0.028573
brown	5618	TSUNODA_CISPLATIN_RESISTANCE_DN	11	44	16209	2190	1.855365	0.028573
brown	7413	MELLMAN_TUT1_TARGETS_DN	11	44	16209	2190	1.855365	0.028573
greenyello	1822	V$TEF1_Q6	5	185	16068	150	2.928468	0.028575
greenyello	9275	GSE20366_CD103_KLRG1_DP_VS_DN_TREG_DN	5	185	16068	150	2.928468	0.028575
greenyello	9987	GSE3982_EOSINOPHIL_VS_NKCELL_DN	5	185	16068	150	2.928468	0.028575
red	5914	STARK_PREFRONTAL_CORTEX_22Q11_DELETION_UP	11	177	16076	524	1.927621	0.028576
red	9499	GSE2706_UNSTIM_VS_2H_R848_DC_DN	11	177	16076	524	1.927621	0.028576
magenta	5393	PEREZ_TP53_TARGETS	17	1001	15252	167	1.652844	0.028577
turquoise	8182	ZHOU_CELL_CYCLE_GENES_IN_IR_RESPONSE_24HR	42	125	16128	4169	1.309908	0.028604
salmon	4183	REACTOME_CELL_CYCLE	7	377	15876	127	2.376219	0.028605
greenyello	1746	V$NCX_01	4	125	16128	150	3.467307	0.028628
pink	688	HOMEOSTASIS_OF_NUMBER_OF_CELLS	2	19	16234	228	7.503693	0.02864
pink	2708	GNF2_TM4SF2	2	19	16234	228	7.503693	0.02864
pink	4270	REACTOME_DOWNREGULATION_OF_SMAD2_3_SMAD	2	19	16234	228	7.503693	0.02864
pink	5525	LA_MEN1_TARGETS	2	19	16234	228	7.503693	0.02864
pink	5725	TOMLINS_METASTASIS_DN	2	19	16234	228	7.503693	0.02864
pink	7680	BYSTRYKH_HEMATOPOIESIS_STEM_CELL_SCP2_QTL_TF	2	19	16234	228	7.503693	0.02864
blue	1801	V$NFMUE1_Q6	55	235	16018	2978	1.277332	0.028645
blue	4968	WANG_CLIM2_TARGETS_UP	55	235	16018	2978	1.277332	0.028645
brown	5862	SCHAEFFER_PROSTATE_DEVELOPMENT_48HR_UP	66	390	15863	2190	1.25594	0.02865
black	8732	GSE15215_CD2_POS_VS_NEG_PDC_UP	7	169	16084	283	2.378803	0.028692
purple	3364	chr3p11	1	3	16250	157	34.50743	0.028702
purple	5574	KORKOLA_CHORIOCARCINOMA	1	3	16250	157	34.50743	0.028702
purple	5735	DIERICK_SEROTONIN_FUNCTION_GENES	1	3	16250	157	34.50743	0.028702
turquoise	4434	REACTOME_M_G1_TRANSITION	28	78	16175	4169	1.399475	0.028724
red	3525	MODULE_36	10	155	16098	524	2.001108	0.028725
purple	8684	GSE1460_INTRATHYMIC_T_PROGENITOR_VS_DP_THYM	5	177	16076	157	2.924359	0.028731
purple	8861	GSE17721_CTRL_VS_POLYIC_12H_BMDM_DN	5	177	16076	157	2.924359	0.028731
grey60	5032	MULLIGHAN_NPM1_MUTATED_SIGNATURE_1_UP	3	246	16007	44	4.504712	0.028769
magenta	7371	SWEET_LUNG_CANCER_KRAS_DN	8	351	15902	167	2.218196	0.028773
red	4160	REACTOME_GROWTH_HORMONE_RECEPTOR_SIGNALI	3	21	16232	524	4.431025	0.028777
red	4777	REACTOME_CHOLESTEROL_BIOSYNTHESIS	3	21	16232	524	4.431025	0.028777
red	7325	PARK_TRETINOIN_RESPONSE_AND_RARA_PLZF_FUSIOI	3	21	16232	524	4.431025	0.028777
lightcyan	8782	GSE15930_NAIVE_VS_48H_IN_VITRO_STIM_CD8_TCELI	3	174	16079	62	4.519744	0.028778
lightcyan	9136	GSE17721_12H_VS_24H_CPG_BMDM_UP	3	174	16079	62	4.519744	0.028778
brown	5972	MORI_SMALL_PRE_BII_LYMPHOCYTE_UP	17	78	16175	2190	1.617498	0.02878
black	1926	V$CP2_02	8	207	16046	283	2.219559	0.028807
blue	654	PROTEOLYSIS	41	168	16085	2978	1.331936	0.028808
blue	10147	GSE6566_STRONG_VS_WEAK_DC_STIMULATED_CD4_T	41	168	16085	2978	1.331936	0.028808

brown	9488	GSE26928_NAIVE_VS_CENT_MEMORY_CD4_TCELL_UP	34	182	16071	2190	1.386427	0.028875
brown	9875	GSE360_L_MAJOR_VS_B_MALAYI_LOW_DOSE_MAC_D	34	182	16071	2190	1.386427	0.028875
lightyellow	7008	RIGGI_EWING_SARCOMA_PROGENITOR_UP	3	330	15923	33	4.47741	0.028875
salmon	32	CHROMATIN	2	34	16219	127	7.528022	0.028875
grey60	4899	HOOI_ST7_TARGETS_DN	2	98	16155	44	7.538497	0.02889
pink	7406	ZHANG_TLX_TARGETS_36HR_UP	7	210	16043	228	2.37617	0.028906
yellow	63	NADH_DEHYDROGENASE_COMPLEX	4	14	16239	1426	3.256462	0.028909
yellow	126	MITOCHONDRIAL_RESPIRATORY_CHAIN_COMPLEX_I	4	14	16239	1426	3.256462	0.028909
yellow	180	PROTON_TRANSPORTING_TWO_SECTOR_ATPASE_COM	4	14	16239	1426	3.256462	0.028909
yellow	190	RESPIRATORY_CHAIN_COMPLEX_I	4	14	16239	1426	3.256462	0.028909
yellow	4707	REACTOME_VIRAL_MESSENGER_RNA_SYNTHESIS	4	14	16239	1426	3.256462	0.028909
tan	8444	GSE11057_NAIVE_VS_EFF_MEMORY_CD4_TCELL_UP	5	192	16061	145	2.919001	0.028915
tan	8625	GSE14000_4H_VS_16H_LPS_DC_DN	5	192	16061	145	2.919001	0.028915
tan	9149	GSE17721_12H_VS_24H_GARDIQUIMOD_BMDM_DN	5	192	16061	145	2.919001	0.028915
tan	9979	GSE3982_EOSINOPHIL_VS_BCELL_DN	5	192	16061	145	2.919001	0.028915
greenyello	5147	RODRIGUES_THYROID_CARCINOMA_DN	3	72	16181	150	4.514722	0.028927
greenyello	6854	GAVIN_FOXP3_TARGETS_CLUSTER_P2	3	72	16181	150	4.514722	0.028927
cyan	4897	SAMOLS_TARGETS_OF_KHSV_MIRNAS_DN	2	56	16197	77	7.538497	0.028932
cyan	5268	BARIS_THYROID_CANCER_DN	2	56	16197	77	7.538497	0.028932
salmon	7861	CHICAS_RB1_TARGETS_LOW_SERUM	3	85	16168	127	4.516813	0.02894
brown	1098	PHOSPHOTRANSFERASE_ACTIVITY_ALCOHOL_GROUP_A	54	311	15942	2190	1.288614	0.02895
blue	7164	BHATI_G2M_ARREST_BY_2METHOXYESTRADIOL_DN	29	112	16141	2978	1.413152	0.028986
pink	1792	V$HNF6_Q6	6	165	16088	228	2.592185	0.029004
pink	10131	GSE6269_HEALTHY_VS_STREP_AUREUS_INF_PBMC_DN	6	165	16088	228	2.592185	0.029004
pink	10133	GSE6269_HEALTHY_VS_STREP_PNEUMO_INF_PBMC_D	6	165	16088	228	2.592185	0.029004
red	2251	TGATTTRY_V$GFI1_01	12	200	16053	524	1.861031	0.029008
red	9437	GSE24634_NAIVE_CD4_TCELL_VS_DAY7_IL4_CONV_TR	12	200	16053	524	1.861031	0.029008
red	9911	GSE36476_CTRL_VS_TSST_ACT_40H_MEMORY_CD4_TC	12	200	16053	524	1.861031	0.029008
magenta	5881	RICKMAN_HEAD_AND_NECK_CANCER_B	2	26	16227	167	7.486412	0.029023
red	7370	SWEET_LUNG_CANCER_KRAS_UP	22	441	15812	524	1.547342	0.02904
lightgreen	5189	CHIARADONNA_NEOPLASTIC_TRANSFORMATION_CDC2	2	103	16150	42	7.514101	0.02905
lightgreen	8309	CRX_NRL_DN.V1_DN	2	103	16150	42	7.514101	0.02905
red	2054	GTACTGT,MIR-101	13	223	16030	524	1.808176	0.029057
blue	147	DNA_DIRECTED_RNA_POLYMERASEII_HOLOENZYME	19	67	16186	2978	1.547703	0.029078
green	9923	GSE37416_CTRL_VS_0H_F_TULARENSIS_LVS_NEUTROP	17	129	16124	1305	1.641278	0.029081
tan	1876	V$HNF3_Q6	4	130	16123	145	3.448912	0.029119
tan	5645	MOHANKUMAR_TLX1_TARGETS_DN	4	130	16123	145	3.448912	0.029119
magenta	1700	V$NKX61_01	5	167	16086	167	2.913873	0.02912
brown	5337	DARWICHE_PAPILLOMA_RISK_HIGH_DN	30	157	16096	2190	1.418114	0.029126
greenyello	1742	V$CDC5_01	5	186	16067	150	2.912724	0.029154
greenyello	1744	V$PITX2_Q2	5	186	16067	150	2.912724	0.029154
brown	821	TRANSCRIPTION	107	667	15586	2190	1.190549	0.02916
midnightb	641	GROWTH	2	61	16192	71	7.505426	0.029174
midnightb	3961	PID_IL12_2PATHWAY	2	61	16192	71	7.505426	0.029174
purple	7266	BOYLAN_MULTIPLE_MYELOMA_C_D_DN	6	240	16013	157	2.588057	0.029183
greenyello	3867	MODULE_458	2	29	16224	150	7.472644	0.02919
greenyello	7314	MARSHALL_VIRAL_INFECTION_RESPONSE_DN	2	29	16224	150	7.472644	0.02919
greenyello	7677	CHIARETTI_T_ALL_REFRACTORY_TO_THERAPY	2	29	16224	150	7.472644	0.02919
lightcyan	8887	GSE17721_CTRL_VS_CPG_4H_BMDM_DN	3	175	16078	62	4.493917	0.029202
lightcyan	8895	GSE17721_CTRL_VS_CPG_24H_BMDM_DN	3	175	16078	62	4.493917	0.029202
lightcyan	9753	GSE32423_CTRL_VS_IL7_IL4_MEMORY_CD8_TCELL_DN	3	175	16078	62	4.493917	0.029202
lightcyan	9844	GSE360_L_MAJOR_VS_B_MALAYI_LOW_DOSE_DC_UP	3	175	16078	62	4.493917	0.029202
tan	913	NEGATIVE_REGULATION_OF_TRANSFERASE_ACTIVITY	2	30	16223	145	7.472644	0.029209
tan	7298	PIONTEK_PKD1_TARGETS_UP	2	30	16223	145	7.472644	0.029209
brown	7140	GRADE_COLON_AND_RECTAL_CANCER_DN	18	84	16169	2190	1.590313	0.029231
brown	7342	POTTI_TOPOTECAN_SENSITIVITY	24	120	16133	2190	1.484292	0.029232
turquoise	3929	PID_BCR_5PATHWAY	24	65	16188	4169	1.43946	0.029234
magenta	302	CELL_ACTIVATION	3	65	16188	167	4.491847	0.029261
blue	10077	GSE3982_BASOPHIL_VS_NKCELL_DN	38	154	16099	2978	1.346703	0.029261
lightyellow	225	PLASMA_MEMBRANE_PART	5	867	15386	33	2.840341	0.029287

purple	6957	MARTINEZ_RB1_AND_TP53_TARGETS_UP	10	519	15734	157	1.994649	0.0293
pink	8351	KRAS.AMP.LUNG_UP.V1_UP	4	83	16170	228	3.435426	0.029316
blue	8388	KAECH_NAIVE_VS_DAY15_EFF_CD8_TCELL_UP	46	192	16061	2978	1.307571	0.029326
blue	8580	GSE13485_CTRL_VS_DAY21_YF17D_VACCINE_PBMC_U	46	192	16061	2978	1.307571	0.029326
blue	9149	GSE17721_12H_VS_24H_GARDIQUIMOD_BMDM_DN	46	192	16061	2978	1.307571	0.029326
lightyellow	3800	MODULE_356	2	132	16121	33	7.462351	0.029331
lightyellow	6472	RODWELL_AGING_KIDNEY_NO_BLOOD_DN	2	132	16121	33	7.462351	0.029331
greenyello	5174	ENK_UV_RESPONSE_EPIDERMIS_DN	9	467	15786	150	2.08818	0.029332
purple	5184	DELYS_THYROID_CANCER_DN	5	178	16075	157	2.90793	0.029339
purple	8195	E2F1_UP.V1_DN	5	178	16075	157	2.90793	0.029339
purple	9431	GSE24142_ADULT_VS_FETAL_DN3_THYMOCYTE_DN	5	178	16075	157	2.90793	0.029339
cyan	6094	SMITH_TERT_TARGETS_UP	3	141	16112	77	4.49102	0.029341
yellow	62	CYTOPLASM	194	1950	14303	1426	1.133917	0.02937
red	2203	TTANTCA_UNKNOWN	33	725	15528	524	1.411816	0.029371
grey60	440	GLUTAMINE_FAMILY_AMINO_ACID_METABOLIC_PROC	1	11	16242	44	33.58058	0.029388
grey60	969	ADENYLATE_CYCLASE_ACTIVATION	1	11	16242	44	33.58058	0.029388
grey60	3729	MODULE_262	1	11	16242	44	33.58058	0.029388
grey60	4451	REACTOME_P2Y_RECEPTORS	1	11	16242	44	33.58058	0.029388
grey60	6867	MARSON_FOXP3_TARGETS_STIMULATED_DN	1	11	16242	44	33.58058	0.029388
grey60	6939	CLAUS_PGR_POSITIVE_MENINGIOMA_DN	1	11	16242	44	33.58058	0.029388
turquoise	5064	ODONNELL_TFRC_TARGETS_DN	39	115	16138	4169	1.322112	0.0294
grey60	3187	chr11q12	2	99	16154	44	7.462351	0.029436
grey60	6049	LE_EGR2_TARGETS_DN	2	99	16154	44	7.462351	0.029436
pink	14	INTRINSIC_TO_PLASMA_MEMBRANE	17	733	15520	228	1.653269	0.029446
yellow	1934	GGCNRNWCTTYS_UNKNOWN	12	75	16178	1426	1.823619	0.029476
yellow	5168	KOKKINAKIS_METHIONINE_DEPRIVATION_96HR_DN	12	75	16178	1426	1.823619	0.029476
blue	9813	GSE360_CTRL_VS_B_MALAYI_LOW_DOSE_MAC_DN	42	173	16080	2978	1.324988	0.029479
blue	380	INTRACELLULAR_PROTEIN_TRANSPORT	35	140	16113	2978	1.364422	0.029497
black	9054	GSE17721_LPS_VS_CPG_24H_BMDM_UP	7	170	16083	283	2.36481	0.029497
brown	8974	GSE17721_CPG_VS_GARDIQUIMOD_6H_BMDM_UP	33	176	16077	2190	1.391524	0.02953
blue	122	HETEROGENEOUS_NUCLEAR_RIBONUCLEOPROTEIN_CC	6	14	16239	2978	2.33901	0.02954
blue	216	ENDOPLASMIC_RETICULUM_LUMEN	6	14	16239	2978	2.33901	0.02954
blue	1299	DNA_DIRECTED_DNA_POLYMERASE_ACTIVITY	6	14	16239	2978	2.33901	0.02954
blue	3340	chr11q25	6	14	16239	2978	2.33901	0.02954
blue	3803	MODULE_360	6	14	16239	2978	2.33901	0.02954
lightyellow	4859	CHEMNITZ_RESPONSE_TO_PROSTAGLANDIN_E2_DN	3	333	15920	33	4.437073	0.029551
turquoise	8539	GSE13306_TREG_VS_TCONV_DN	59	184	16069	4169	1.250074	0.029552
turquoise	9204	GSE17974_0.5H_VS_72H_UNTREATED_IN_VITRO_CD4_	59	184	16069	4169	1.250074	0.029552
pink	5222	GRAESSMANN_APOPTOSIS_BY_SERUM_DEPRIVATION_	7	211	16042	228	2.364908	0.029555
pink	6580	DAZARD_RESPONSE_TO_UV_NHEK_UP	7	211	16042	228	2.364908	0.029555
lightgreen	5073	SENESE_HDAC3_TARGETS_UP	4	456	15797	42	3.394528	0.029563
brown	8639	GSE14308_TH1_VS_TH17_DN	36	195	16058	2190	1.370116	0.029565
brown	9784	GSE339_EX_VIVO_VS_IN_CULTURE_CD4CD8DN_DC_UF	36	195	16058	2190	1.370116	0.029565
turquoise	8726	GSE14769_UNSTIM_VS_360MIN_LPS_BMDM_UP	63	198	16055	4169	1.240444	0.029568
turquoise	9228	GSE18791_CTRL_VS_NEWCASTLE_VIRUS_DC_10H_UP	63	198	16055	4169	1.240444	0.029568
turquoise	9255	GSE19825_NAIVE_VS_DAY3_EFF_CD8_TCELL_DN	63	198	16055	4169	1.240444	0.029568
turquoise	9391	GSE22886_NAIVE_CD4_TCELL_VS_12H_ACT_TH1_DN	63	198	16055	4169	1.240444	0.029568
lightgreen	1119	PROTEIN_HOMODIMERIZATION_ACTIVITY	2	104	16149	42	7.44185	0.029572
lightgreen	9240	GSE18791_UNSTIM_VS_NEWCATSLE_VIRUS_DC_2H_UI	2	104	16149	42	7.44185	0.029572
turquoise	9301	GSE2197_IMMUNOSUPPRESSIVE_DNA__VS_UNTREATE	61	191	16062	4169	1.245082	0.029581
turquoise	9703	GSE29618_LAIV_VS_TIV_FLU_VACCINE_DAY7_BCELL_D	61	191	16062	4169	1.245082	0.029581
red	1230	PEPTIDE_BINDING	5	54	16199	524	2.871961	0.029583
red	4114	PID_RAC1_PATHWAY	5	54	16199	524	2.871961	0.029583
green	6288	HALMOS_CEBPA_TARGETS_DN	7	38	16215	1305	2.294233	0.029608
red	1562	V$SRY_01	11	178	16075	524	1.916792	0.029613
red	9235	GSE18791_CTRL_VS_NEWCASTLE_VIRUS_DC_16H_DN	11	178	16075	524	1.916792	0.029613
red	10183	GSE7460_CTRL_VS_FOXP3_OVEREXPR_TCONV_DN	11	178	16075	524	1.916792	0.029613
brown	1634	V$XBP1_01	22	108	16145	2190	1.511779	0.029616
turquoise	3806	MODULE_363	19	49	16204	4169	1.511678	0.02962
turquoise	6295	NADLER_HYPERGLYCEMIA_AT_OBESITY	19	49	16204	4169	1.511678	0.02962

turquoise	6827	PELLICCIOTTA_HDAC_IN_ANTIGEN_PRESENTATION_DN	19	49	16204	4169	1.511678	0.02962
green	2857	KEGG_TASTE_TRANSDUCTION	6	30	16223	1305	2.490881	0.029621
purple	3534	MODULE_45	10	520	15733	157	1.990813	0.029635
yellow	8952	GSE17721_PAM3CSK4_VS_CPG_2H_BMDM_UP	22	164	16089	1426	1.528948	0.029638
pink	5159	PROVENZANI_METASTASIS_DN	5	123	16130	228	2.897768	0.029651
red	1668	V$ETS2_B	14	247	16006	524	1.758059	0.029691
turquoise	26	MITOCHONDRIAL_PART	46	139	16114	4169	1.290163	0.029736
purple	1268	CHEMOKINE_RECEPTOR_BINDING	2	28	16225	157	7.394449	0.02974
purple	4036	PID_SYNDECAN_4_PATHWAY	2	28	16225	157	7.394449	0.02974
purple	6019	WHITEHURST_PACLITAXEL_SENSITIVITY	2	28	16225	157	7.394449	0.02974
purple	6276	GILDEA_METASTASIS	2	28	16225	157	7.394449	0.02974
purple	8138	GENTLES_LEUKEMIC_STEM_CELL_UP	2	28	16225	157	7.394449	0.02974
greenyello	1550	V$OCT1_01	5	187	16066	150	2.897148	0.02974
greenyello	9768	GSE339_CD4POS_VS_CD8POS_DC_UP	5	187	16066	150	2.897148	0.02974
blue	1836	V$SF1_Q6	52	221	16032	2978	1.284162	0.029751
pink	1635	V$TATA_01	6	166	16087	228	2.576569	0.029753
pink	9603	GSE27786_NKCELL_VS_NEUTROPHIL_DN	6	166	16087	228	2.576569	0.029753
red	2843	KEGG_JAK_STAT_SIGNALING_PATHWAY	8	113	16140	524	2.195906	0.02976
red	4915	DIAZ_CHRONIC_MEYLOGENOUS_LEUKEMIA_DN	8	113	16140	524	2.195906	0.02976
lightcyan	5073	SENESE_HDAC3_TARGETS_UP	5	456	15797	62	2.874399	0.029773
magenta	9276	GSE20366_CD103_POS_VS_CD103_KLRG1_DP_TREG_U	5	168	16085	167	2.896528	0.029773
magenta	9294	GSE20715_0H_VS_6H_OZONE_TLR4_KO_LUNG_UP	5	168	16085	167	2.896528	0.029773
yellow	8655	GSE14308_NAIVE_CD4_TCELL_VS_NATURAL_TREG_DN	25	192	16061	1426	1.484065	0.029775
yellow	9554	GSE27786_LIN_NEG_VS_NKTCELL_UP	25	192	16061	1426	1.484065	0.029775
green	2450	MORF_PTPRB	22	179	16074	1305	1.530709	0.029794
green	4442	REACTOME_TRANSCRIPTION	22	179	16074	1305	1.530709	0.029794
turquoise	4549	REACTOME_METABOLISM_OF_RNA	78	251	16002	4169	1.211497	0.029806
grey60	2278	RGAGGAARY_V$PU1_Q6	4	436	15817	44	3.388866	0.02981
brown	6806	ZHONG_SECRETOME_OF_LUNG_CANCER_AND_ENDOT	14	61	16192	2190	1.703286	0.029817
salmon	1483	AACYNNNNTTCCS_UNKNOWN	3	86	16167	127	4.464292	0.029819
red	1205	ADENYL_NUCLEOTIDE_BINDING	10	156	16097	524	1.98828	0.029847
red	9272	GSE20366_CD103_POS_VS_NEG_TREG_KLRG1NEG_UP	10	156	16097	524	1.98828	0.029847
red	9901	GSE36392_MAC_VS_NEUTROPHIL_IL25_TREATED_LUN	10	156	16097	524	1.98828	0.029847
green	8443	GSE10856_CTRL_VS_TNFRSF6B_IN_MACROPHAGE_DN	21	169	16084	1305	1.547589	0.029891
blue	2731	KEGG_PYRIMIDINE_METABOLISM	25	94	16159	2978	1.451513	0.029893
cyan	6678	BURTON_ADIPOGENESIS_PEAK_AT_0HR	2	57	16196	77	7.406243	0.029894
lightcyan	4919	DEURIG_T_CELL_PROLYMPHOCYTIC_LEUKEMIA_DN	4	308	15945	62	3.404483	0.029895
yellow	6171	LE_EGR2_TARGETS_UP	15	101	16152	1426	1.692715	0.029931
green	4670	REACTOME_MEIOTIC_RECOMBINATION	9	55	16198	1305	2.037994	0.029948
purple	1514	V$IK3_01	5	179	16074	157	2.891684	0.029955
purple	8713	GSE1460_NAIVE_CD4_TCELL_ADULT_BLOOD_VS_THYM	5	179	16074	157	2.891684	0.029955
purple	8857	GSE17721_CTRL_VS_POLYIC_6H_BMDM_DN	5	179	16074	157	2.891684	0.029955
purple	8905	GSE17721_CTRL_VS_GARDIQUIMOD_6H_BMDM_DN	5	179	16074	157	2.891684	0.029955
purple	9009	GSE17721_POLYIC_VS_CPG_4H_BMDM_DN	5	179	16074	157	2.891684	0.029955
purple	9338	GSE22886_IGG_IGA_MEMORY_BCELL_VS_BLOOD_PLAS	5	179	16074	157	2.891684	0.029955
magenta	1850	V$ELF1_Q6	6	227	16026	167	2.572423	0.029957
lightgreen	1315	RECEPTOR_BINDING	3	262	15991	42	4.431025	0.029958
yellow	2585	GNF2_ESPL1	7	35	16218	1426	2.279523	0.029979
yellow	5993	NIKOLSKY_BREAST_CANCER_7P22_AMPLICON	7	35	16218	1426	2.279523	0.029979
yellow	6539	BURTON_ADIPOGENESIS_12	7	35	16218	1426	2.279523	0.029979
grey60	4583	REACTOME_SIGNALING_BY_ILS	2	100	16153	44	7.387727	0.029985
grey60	5642	WOOD_EBV_EBNA1_TARGETS_UP	2	100	16153	44	7.387727	0.029985
blue	409	NUCLEAR_TRANSPORT	23	85	16168	2978	1.476787	0.030002
red	601	REGULATION_OF_CELLULAR_METABOLIC_PROCESS	32	700	15553	524	1.417928	0.030021
green	608	INTERLEUKIN_8_PRODUCTION	3	9	16244	1305	4.151469	0.030039
green	683	ESTABLISHMENT_OF_VESICLE_LOCALIZATION	3	9	16244	1305	4.151469	0.030039
green	792	PROTEIN_AMINO_ACID_ADP_RIBOSYLATION	3	9	16244	1305	4.151469	0.030039
green	878	POSITIVE_REGULATION_OF_ANGIOGENESIS	3	9	16244	1305	4.151469	0.030039
lightyellow	2773	KEGG_RIBOFLAVIN_METABOLISM	1	15	16238	33	32.83434	0.03004
lightyellow	2775	KEGG_PANTOTHENATE_AND_COA_BIOSYNTHESIS	1	15	16238	33	32.83434	0.03004

lightyellow	3400	chr8q11	1	15	16238	33	32.83434	0.03004
lightyellow	3752	MODULE_294	1	15	16238	33	32.83434	0.03004
lightyellow	3805	MODULE_362	1	15	16238	33	32.83434	0.03004
lightyellow	4136	REACTOME_BILE_ACID_AND_BILE_SALT_METABOLISM	1	15	16238	33	32.83434	0.03004
lightyellow	4259	REACTOME_OXYGEN_DEPENDENT_PROLINE_HYDROXY	1	15	16238	33	32.83434	0.03004
lightyellow	4461	REACTOME_EICOSANOID_LIGAND_BINDING_RECEPTOF	1	15	16238	33	32.83434	0.03004
lightyellow	4476	REACTOME_CYCLIN_A_B1_ASSOCIATED_EVENTS_DURI	1	15	16238	33	32.83434	0.03004
lightyellow	5051	TONKS_TARGETS_OF_RUNX1_RUNX1T1_FUSION_ERYTI	1	15	16238	33	32.83434	0.03004
lightyellow	7548	WANG_RECURRENT_LIVER_CANCER_DN	1	15	16238	33	32.83434	0.03004
blue	6668	HEDENFALK_BREAST_CANCER_BRCA1_VS_BRCA2	39	159	16094	2978	1.338679	0.030043
yellow	1496	V$YY1_02	29	230	16023	1426	1.437091	0.03005
lightcyan	8841	GSE17721_CTRL_VS_LPS_6H_BMDM_DN	3	177	16076	62	4.443138	0.03006
lightcyan	8859	GSE17721_CTRL_VS_POLYIC_8H_BMDM_DN	3	177	16076	62	4.443138	0.03006
lightcyan	4161	REACTOME_TRIF_MEDIATED_TLR3_SIGNALING	2	71	16182	62	7.384371	0.030063
lightcyan	5299	MCBRYAN_PUBERTAL_BREAST_6_7WK_DN	2	71	16182	62	7.384371	0.030063
lightcyan	5855	FRIDMAN_SENESCENCE_UP	2	71	16182	62	7.384371	0.030063
lightcyan	6243	ADDYA_ERYTHROID_DIFFERENTIATION_BY_HEMIN	2	71	16182	62	7.384371	0.030063
lightcyan	7200	HUANG_DASATINIB_RESISTANCE_UP	2	71	16182	62	7.384371	0.030063
lightcyan	7646	BOYLAN_MULTIPLE_MYELOMA_PCA3_UP	2	71	16182	62	7.384371	0.030063
midnightb	7743	YAO_TEMPORAL_RESPONSE_TO_PROGESTERONE_CLUS	2	62	16191	71	7.384371	0.030063
cyan	4745	REACTOME_ADAPTIVE_IMMUNE_SYSTEM	6	496	15757	77	2.553362	0.030072
black	1124	HYDROLASE_ACTIVITY_ACTING_ON_CARBON_NITROGE	3	39	16214	283	4.417777	0.030104
black	7186	CHEN_HOXA5_TARGETS_9HR_DN	3	39	16214	283	4.417777	0.030104
salmon	6173	FLECHNER_PBL_KIDNEY_TRANSPLANT_OK_VS_DONOR_	4	150	16103	127	3.412703	0.030106
pink	5140	DODD_NASOPHARYNGEAL_CARCINOMA_UP	29	1439	14814	228	1.4366	0.030118
blue	1891	V$LEF1_Q2	43	178	16075	2978	1.318431	0.030119
blue	8656	GSE14308_INDUCED_VS_NATURAL_TREG_UP	43	178	16075	2978	1.318431	0.030119
blue	9038	GSE17721_LPS_VS_CPG_0.5H_BMDM_UP	43	178	16075	2978	1.318431	0.030119
lightcyan	5201	BERENJENO_TRANSFORMED_BY_RHOA_REVERSIBLY_U	1	8	16245	62	32.76815	0.030119
lightcyan	5553	XU_AKT1_TARGETS_48HR	1	8	16245	62	32.76815	0.030119
lightcyan	6061	LEI_HOXC8_TARGETS_UP	1	8	16245	62	32.76815	0.030119
lightcyan	6758	SASSON_FSH_RESPONSE	1	8	16245	62	32.76815	0.030119
red	6160	FRASOR_RESPONSE_TO_ESTRADIOL_DN	6	73	16180	524	2.549357	0.030162
purple	4894	ZHOU_INFLAMMATORY_RESPONSE_LIVE_UP	8	377	15876	157	2.19676	0.030175
brown	9220	GSE18791_CTRL_VS_NEWCASTLE_VIRUS_DC_2H_UP	32	170	16083	2190	1.396981	0.030184
midnightb	3814	MODULE_377	1	7	16246	71	32.70221	0.030187
midnightb	8033	KUWANO_RNA_STABILIZED_BY_NO	1	7	16246	71	32.70221	0.030187
purple	8159	ACEVEDO_FGFR1_TARGETS_IN_PROSTATE_CANCER_M	6	242	16011	157	2.566668	0.030219
purple	540	MONOCARBOXYLIC_ACID_METABOLIC_PROCESS	3	70	16183	157	4.43667	0.030231
purple	4455	REACTOME_REGULATION_OF_INSULIN_SECRETION	3	70	16183	157	4.43667	0.030231
salmon	1706	V$E2F1_Q4	5	222	16031	127	2.882351	0.030258
grey60	7416	SHEDDEN_LUNG_CANCER_GOOD_SURVIVAL_A12	3	251	16002	44	4.414976	0.030285
brown	8464	GSE11864_UNTREATED_VS_CSF1_PAM3CYS_IN_MAC_I	35	189	16064	2190	1.374345	0.030291
brown	8998	GSE17721_LPS_VS_PAM3CSK4_16H_BMDM_UP	35	189	16064	2190	1.374345	0.030291
brown	9031	GSE17721_PAM3CSK4_VS_GADIQUIMOD_8H_BMDM_	35	189	16064	2190	1.374345	0.030291
red	7415	HAN_SATB1_TARGETS_DN	20	393	15860	524	1.578482	0.030307
greenyello	10180	GSE7460_WT_VS_FOXP3_HET_ACT_WITH_TGFB_TCON	5	188	16065	150	2.881738	0.030334
midnightb	9494	GSE26928_EFF_MEMORY_VS_CXCR5_POS_CD4_TCELL_	3	155	16098	71	4.430622	0.030342
yellow	466	CELL_CYCLE_PROCESS	24	183	16070	1426	1.494769	0.030381
yellow	8900	GSE17721_CTRL_VS_GARDIQUIMOD_2H_BMDM_UP	24	183	16070	1426	1.494769	0.030381
yellow	9531	GSE27786_LSK_VS_BCELL_DN	24	183	16070	1426	1.494769	0.030381
turquoise	2169	CGTCTTA,MIR-208	5	8	16245	4169	2.436586	0.030385
turquoise	2776	KEGG_FOLATE_BIOSYNTHESIS	5	8	16245	4169	2.436586	0.030385
turquoise	4288	REACTOME_ACYL_CHAIN_REMODELLING_OF_PG	5	8	16245	4169	2.436586	0.030385
turquoise	4607	REACTOME_SYNTHESIS_SECRETION_AND_INACTIVATIC	5	8	16245	4169	2.436586	0.030385
turquoise	7273	REICHERT_G1S_REGULATORS_AS_PI3K_TARGETS	5	8	16245	4169	2.436586	0.030385
turquoise	8155	HOLLEMAN_DAUNORUBICIN_ALL_DN	5	8	16245	4169	2.436586	0.030385
cyan	3786	MODULE_334	3	143	16110	77	4.428208	0.030414
magenta	1769	CTGYNNCTYTAA_UNKNOWN	3	66	16187	167	4.423789	0.030429
magenta	2831	KEGG_ECM_RECEPTOR_INTERACTION	3	66	16187	167	4.423789	0.030429

magenta	6038	ONDER_CDH1_SIGNALING_VIA_CTNNB1	3	66	16187	167	4.423789	0.030429
magenta	8903	GSE17721_CTRL_VS_GARDIQUIMOD_4H_BMDM_DN	5	169	16084	167	2.879389	0.030435
turquoise	6344	MENSE_HYPOXIA_UP	33	95	16158	4169	1.354229	0.030452
pink	8069	GUO_TARGETS_OF_IRS1_AND_IRS2	4	84	16169	228	3.394528	0.030455
salmon	435	MICROTUBULE_CYTOSKELETON_ORGANIZATION_AND_	2	35	16218	127	7.312936	0.030471
salmon	4410	REACTOME_G1_PHASE	2	35	16218	127	7.312936	0.030471
salmon	5350	HU_ANGIOGENESIS_DN	2	35	16218	127	7.312936	0.030471
green	9634	GSE29614_CTRL_VS_DAY7_TIV_FLU_VACCINE_PBMC_U	16	120	16133	1305	1.660587	0.030484
cyan	7204	QI_PLASMACYTOMA_UP	4	249	16004	77	3.39081	0.030495
lightcyan	8195	E2F1_UP.V1_DN	3	178	16075	62	4.418177	0.030495
lightcyan	8324	RPS14_DN.V1_UP	3	178	16075	62	4.418177	0.030495
lightcyan	8889	GSE17721_CTRL_VS_CPG_6H_BMDM_DN	3	178	16075	62	4.418177	0.030495
lightcyan	8987	GSE17721_LPS_VS_PAM3CSK4_1H_BMDM_DN	3	178	16075	62	4.418177	0.030495
lightcyan	9817	GSE360_DC_VS_MAC_DN	3	178	16075	62	4.418177	0.030495
magenta	7723	MIKKELSEN_NPC_WITH_LCP_H3K27ME3	1	3	16250	167	32.44112	0.030511
brown	4032	PID_IFNGPATHWAY	10	39	16214	2190	1.902939	0.030514
brown	5722	TOMLINS_PROSTATE_CANCER_UP	10	39	16214	2190	1.902939	0.030514
brown	7279	BOHN_PRIMARY_IMMUNODEFICIENCY_SYNDROM_DN	10	39	16214	2190	1.902939	0.030514
pink	9793	GSE360_CTRL_VS_L_DONOVANI_DC_DN	6	167	16086	228	2.561141	0.030514
grey60	8129	BOSCO_TH1_CYTOTOXIC_MODULE	2	101	16152	44	7.314581	0.030539
yellow	6504	JIANG_VHL_TARGETS	19	137	16116	1426	1.580691	0.030542
turquoise	5080	TIEN_INTESTINE_PROBIOTICS_2HR_DN	30	85	16168	4169	1.375954	0.030548
turquoise	6175	MOOTHA_VOXPHOS	30	85	16168	4169	1.375954	0.030548
red	2043	ATAGGAA,MIR-202	7	93	16160	524	2.334626	0.03056
purple	8589	GSE13485_DAY3_VS_DAY7_YF17D_VACCINE_PBMC_DI	5	180	16073	157	2.875619	0.03058
lightgreen	92	ENDOCYTIC_VESICLE	1	12	16241	42	32.24802	0.030583
lightgreen	708	G_PROTEIN_SIGNALING_ADENYLATE_CYCLASE_ACTIVA	1	12	16241	42	32.24802	0.030583
lightgreen	948	DNA_DAMAGE_RESPONSESIGNAL_TRANSDUCTION_BY_	1	12	16241	42	32.24802	0.030583
lightgreen	2909	BIOCARTA_GRANULOCYTES_PATHWAY	1	12	16241	42	32.24802	0.030583
lightgreen	3672	MODULE_191	1	12	16241	42	32.24802	0.030583
lightgreen	4214	REACTOME_TRAFFICKING_AND_PROCESSING_OF_END(	1	12	16241	42	32.24802	0.030583
blue	4309	REACTOME_TCR_SIGNALING	15	50	16203	2978	1.637307	0.030593
blue	6396	MARTINEZ_RESPONSE_TO_TRABECTEDIN	15	50	16203	2978	1.637307	0.030593
brown	725	TRANSPORT	109	682	15571	2190	1.186128	0.030613
turquoise	6226	FLECHNER_PBL_KIDNEY_TRANSPLANT_REJECTED_VS_O	23	62	16191	4169	1.446231	0.030621
purple	6719	XU_GH1_AUTOCRINE_TARGETS_DN	4	122	16131	157	3.394174	0.030623
tan	6568	YAMAZAKI_TCEB3_TARGETS_DN	5	195	16058	145	2.874094	0.030626
red	9842	GSE360_L_MAJOR_VS_B_MALAYI_HIGH_DOSE_DC_UP	11	179	16074	524	1.906083	0.030676
red	10170	GSE7460_CTRL_VS_TGFB_TREATED_ACT_FOXP3_MUT_	11	179	16074	524	1.906083	0.030676
red	10204	GSE7852_LN_VS_THYMUS_TCONV_UP	11	179	16074	524	1.906083	0.030676
turquoise	1843	V$E2F4DP1_01	70	223	16030	4169	1.223756	0.030676
brown	5793	BUYTAERT_PHOTODYNAMIC_THERAPY_STRESS_DN	98	607	15646	2190	1.198193	0.030683
turquoise	6730	SESTO_RESPONSE_TO_UV_C5	18	46	16207	4169	1.525514	0.03071
blue	9651	GSE29617_CTRL_VS_DAY7_TIV_FLU_VACCINE_PBMC_2	44	183	16070	2978	1.312231	0.030729
blue	9845	GSE360_L_MAJOR_VS_B_MALAYI_LOW_DOSE_DC_DN	44	183	16070	2978	1.312231	0.030729
purple	6932	HELLER_SILENCED_BY_METHYLATION_UP	6	243	16010	157	2.556106	0.030746
brown	1642	V$AML1_01	42	234	16019	2190	1.332057	0.030753
brown	1854	V$AML1_Q6	42	234	16019	2190	1.332057	0.030753
blue	3455	SIG_PIP3_SIGNALING_IN_CARDIAC_MYOCTES	18	63	16190	2978	1.55934	0.030783
blue	5166	KOKKINAKIS_METHIONINE_DEPRIVATION_48HR_DN	18	63	16190	2978	1.55934	0.030783
blue	6401	LEE_AGING_NEOCORTEX_DN	18	63	16190	2978	1.55934	0.030783
blue	8604	GSE13738_RESTING_VS_BYSTANDER_ACTIVATED_CD4_	40	164	16089	2978	1.331144	0.030789
blue	9801	GSE360_CTRL_VS_B_MALAYI_LOW_DOSE_DC_DN	40	164	16089	2978	1.331144	0.030789
blue	9841	GSE360_L_MAJOR_VS_T_GONDII_DC_DN	40	164	16089	2978	1.331144	0.030789
blue	5805	LOCKWOOD_AMPLIFIED_IN_LUNG_CANCER	49	207	16046	2978	1.291917	0.030831
brown	3419	chr12p13	31	164	16089	2190	1.402837	0.030833
brown	8703	GSE1460_CD4_THYMOCYTE_VS_NAIVE_CD4_TCELL_CO	31	164	16089	2190	1.402837	0.030833
midnightb	8336	TGFB_UP.V1_UP	3	156	16097	71	4.402221	0.030843
midnightb	9639	GSE29615_CTRL_VS_DAY3_LAIV_IFLU_VACCINE_PBMC_	3	156	16097	71	4.402221	0.030843
lightcyan	7453	MILI_PSEUDOPODIA_CHEMOTAXIS_UP	2	72	16181	62	7.28181	0.030847

green	3555	MODULE_66	46	433	15820	1305	1.323101	0.030847
greenyello	4050	PID_PDGFRBPATHWAY	4	128	16125	150	3.386042	0.030862
cyan	4464	REACTOME_CLASS_B_2_SECRETIN_FAMILY_RECEPTORS	2	58	16195	77	7.278549	0.030868
cyan	4664	REACTOME_RIG_I_MDA5_MEDIATED_INDUCTION_OF_	2	58	16195	77	7.278549	0.030868
black	2726	KEGG_STEROID_BIOSYNTHESIS	2	16	16237	283	7.178887	0.03087
salmon	3292	chr11p	1	4	16249	127	31.99409	0.030894
salmon	4732	REACTOME_BINDING_AND_ENTRY_OF_HIV_VIRION	1	4	16249	127	31.99409	0.030894
salmon	4977	SMIRNOV_CIRCULATING_ENDOTHELIOCYTES_IN_CANC	1	4	16249	127	31.99409	0.030894
brown	484	PHAGOCYTOSIS	5	14	16239	2190	2.650522	0.030914
brown	772	OXYGEN_AND_REACTIVE_OXYGEN_SPECIES_METABOLI	5	14	16239	2190	2.650522	0.030914
brown	965	POSITIVE_REGULATION_OF_SECRETION	5	14	16239	2190	2.650522	0.030914
brown	3045	BIOCARTA_PS1_PATHWAY	5	14	16239	2190	2.650522	0.030914
brown	3869	MODULE_462	5	14	16239	2190	2.650522	0.030914
brown	4017	PID_THROMBIN_PAR4_PATHWAY	5	14	16239	2190	2.650522	0.030914
brown	4242	REACTOME_TGF_BETA_RECEPTOR_SIGNALING_IN_EMT	5	14	16239	2190	2.650522	0.030914
brown	4490	REACTOME_SEMA3A_PAK_DEPENDENT_AXON_REPULS	5	14	16239	2190	2.650522	0.030914
brown	4689	REACTOME_SOS_MEDIATED_SIGNALLING	5	14	16239	2190	2.650522	0.030914
brown	5139	WATANABE_ULCERATIVE_COLITIS_WITH_CANCER_DN	5	14	16239	2190	2.650522	0.030914
brown	6671	RAMPON_ENRICHED_LEARNING_ENVIRONMENT_EARL	5	14	16239	2190	2.650522	0.030914
magenta	3542	MODULE_53	8	356	15897	167	2.187042	0.030921
lightcyan	8510	GSE12845_IGD_POS_BLOOD_VS_DARKZONE_GC_TONS	3	179	16074	62	4.393494	0.030932
lightcyan	8857	GSE17721_CTRL_VS_POLYIC_6H_BMDM_DN	3	179	16074	62	4.393494	0.030932
lightcyan	8905	GSE17721_CTRL_VS_GARDIQUIMOD_6H_BMDM_DN	3	179	16074	62	4.393494	0.030932
lightcyan	9338	GSE22886_IGG_IGA_MEMORY_BCELL_VS_BLOOD_PLAS	3	179	16074	62	4.393494	0.030932
lightcyan	10204	GSE7852_LN_VS_THYMUS_TCONV_UP	3	179	16074	62	4.393494	0.030932
turquoise	9820	GSE360_DC_VS_MAC_L_MAJOR_UP	54	167	16086	4169	1.260605	0.030933
greenyello	9278	GSE20366_TREG_VS_NAIVE_CD4_TCELL_UP	5	189	16064	150	2.86649	0.030935
grey60	5907	BENPORATH_ES_WITH_H3K27ME3	5	654	15599	44	2.824055	0.030946
yellow	9490	GSE26928_NAIVE_VS_CXCR5_POS_CD4_TCELL_UP	23	174	16079	1426	1.506581	0.03095
yellow	9545	GSE27786_LSK_VS_MONO_MAC_DN	23	174	16079	1426	1.506581	0.03095
cyan	6320	PETROVA_ENDOTHELIUM_LYMPHATIC_VS_BLOOD_DN	3	144	16109	77	4.397457	0.030958
midnightb	2634	GNF2_DDX5	2	63	16190	71	7.267159	0.030962
midnightb	3303	chr3p25	2	63	16190	71	7.267159	0.030962
midnightb	3885	MODULE_493	2	63	16190	71	7.267159	0.030962
turquoise	5130	BIDUS_METASTASIS_UP	66	209	16044	4169	1.231117	0.030967
brown	8355	TBK1.DF_UP	48	273	15980	2190	1.304872	0.030976
lightyellow	8548	GSE13411_NAIVE_VS_SWITCHED_MEMORY_BCELL_UP	2	136	16117	33	7.24287	0.030987
red	1545	V$HNF3B_01	10	157	16096	524	1.975616	0.030999
red	9969	GSE3982_MAC_VS_NEUTROPHIL_LPS_STIM_DN	10	157	16096	524	1.975616	0.030999
red	4981	OSMAN_BLADDER_CANCER_UP	20	394	15859	524	1.574476	0.031009
brown	9051	GSE17721_LPS_VS_CPG_12H_BMDM_DN	34	183	16070	2190	1.378851	0.031024
tan	3236	chr13q34	2	31	16222	145	7.231591	0.031044
tan	3745	MODULE_286	2	31	16222	145	7.231591	0.031044
tan	4761	REACTOME_PACKAGING_OF_TELOMERE_ENDS	2	31	16222	145	7.231591	0.031044
tan	6974	YOKOE_CANCER_TESTIS_ANTIGENS	2	31	16222	145	7.231591	0.031044
cyan	7034	ACEVEDO_LIVER_CANCER_UP	9	929	15324	77	2.044888	0.031073
greenyello	3540	MODULE_51	2	30	16223	150	7.223556	0.031089
greenyello	3730	MODULE_263	2	30	16223	150	7.223556	0.031089
greenyello	6239	NAKAJIMA_EOSINOPHIL	2	30	16223	150	7.223556	0.031089
greenyello	8175	GHANDHI_DIRECT_IRRADIATION_DN	2	30	16223	150	7.223556	0.031089
magenta	7524	CHIANG_LIVER_CANCER_SUBCLASS_PROLIFERATION_U	5	170	16083	167	2.862452	0.031107
midnightb	1948	GTGCAAT,MIR-25,MIR-32,MIR-92,MIR-363,MIR-367	4	272	15981	71	3.366404	0.031123
turquoise	2349	MORF_ANP32B	62	195	16058	4169	1.239535	0.031129
turquoise	8971	GSE17721_CPG_VS_GARDIQUIMOD_2H_BMDM_DN	62	195	16058	4169	1.239535	0.031129
turquoise	9784	GSE339_EX_VIVO_VS_IN_CULTURE_CD4CD8DN_DC_UP	62	195	16058	4169	1.239535	0.031129
turquoise	8613	GSE14000_TRANSLATED_RNA_VS_MRNA_16H_LPS_DC	58	181	16072	4169	1.249255	0.031131
turquoise	9704	GSE29618_LAIV_VS_TIV_FLU_VACCINE_DAY7_MONOC	58	181	16072	4169	1.249255	0.031131
turquoise	10008	GSE3982_MAST_CELL_VS_TH1_UP	58	181	16072	4169	1.249255	0.031131
turquoise	10101	GSE39820_CTRL_VS_IL1B_IL6_CD4_TCELL_DN	58	181	16072	4169	1.249255	0.031131
magenta	135	LIPID_RAFT	2	27	16226	167	7.209137	0.031137

magenta	909	RESPONSE_TO_HORMONE_STIMULUS	2	27	16226	167	7.209137	0.031137
magenta	3380	chr2p16	2	27	16226	167	7.209137	0.031137
magenta	4744	REACTOME_VOLTAGE_GATED_POTASSIUM_CHANNELS	2	27	16226	167	7.209137	0.031137
magenta	5959	SHIN_B_CELL_LYMPHOMA_CLUSTER_2	2	27	16226	167	7.209137	0.031137
red	2227	YTAAYNGCT_UNKNOWN	8	114	16139	524	2.176644	0.031143
red	5851	RICKMAN_TUMOR_DIFFERENTIATED_MODERATELY_VS	8	114	16139	524	2.176644	0.031143
turquoise	8850	GSE17721_CTRL_VS_POLYIC_1H_BMDM_UP	60	188	16065	4169	1.244214	0.031152
brown	5188	CHIARADONNA_NEOPLASTIC_TRANSFORMATION_KRAS	26	133	16120	2190	1.450812	0.031154
black	5903	BENPORATH_SOX2_TARGETS	19	685	15568	283	1.592979	0.031198
tan	9230	GSE18791_CTRL_VS_NEWCASTLE_VIRUS_DC_12H_UP	5	196	16057	145	2.85943	0.03121
purple	9259	GSE20366_TREG_VS_TCONV_DN	5	181	16072	157	2.859732	0.031212
grey60	2273	YATGNWAAT_V$OCT_C	3	254	15999	44	4.362831	0.031214
green	9416	GSE24142_EARLY_THYMIC_PROGENITOR_VS_DN3_THY	23	190	16063	1305	1.507639	0.031232
brown	6242	SANSOM_APC_TARGETS_DN	55	319	15934	2190	1.279562	0.031273
greenyello	5863	SCHAEFFER_PROSTATE_DEVELOPMENT_48HR_DN	7	325	15928	150	2.333764	0.031282
blue	1919	V$SP1_Q2_01	50	212	16041	2978	1.287191	0.031286
pink	325	COFACTOR_METABOLIC_PROCESS	3	49	16204	228	4.364393	0.031288
pink	5978	MORI_PLASMA_CELL_UP	3	49	16204	228	4.364393	0.031288
pink	7873	DEMAGALHAES_AGING_UP	3	49	16204	228	4.364393	0.031288
yellow	7035	ACEVEDO_LIVER_CANCER_DN	52	457	15796	1426	1.296884	0.031289
tan	7060	SMID_BREAST_CANCER_LUMINAL_B_UP	4	133	16120	145	3.371117	0.031298
blue	9858	GSE360_LOW_DOSE_B_MALAYI_VS_M_TUBERCULOSIS	45	188	16065	2978	1.306362	0.03131
brown	459	DEFENSE_RESPONSE	39	215	16038	2190	1.346219	0.031315
purple	3926	PID_SMAD2_3NUCLEARPATHWAY	3	71	16182	157	4.374181	0.031348
purple	4884	TURASHVILI_BREAST_LOBULAR_CARCINOMA_VS_LOBU	3	71	16182	157	4.374181	0.031348
magenta	6652	CUI_TCF21_TARGETS_2_UP	8	357	15896	167	2.180915	0.031363
turquoise	1224	PHOSPHORIC_ESTER_HYDROLASE_ACTIVITY	45	136	16117	4169	1.289957	0.031372
turquoise	1289	TRANSCRIPTION_REPRESSOR_ACTIVITY	45	136	16117	4169	1.289957	0.031372
lightcyan	8738	GSE15324_ELF4_KO_VS_WT_NAIVE_CD8_TCELL_UP	3	180	16073	62	4.369086	0.031373
lightcyan	8955	GSE17721_PAM3CSK4_VS_CPG_4H_BMDM_DN	3	180	16073	62	4.369086	0.031373
lightcyan	9783	GSE339_EX_VIVO_VS_IN_CULTURE_CD8POS_DC_DN	3	180	16073	62	4.369086	0.031373
turquoise	1159	MICROTUBULE_MOTOR_ACTIVITY	8	16	16237	4169	1.949268	0.031391
turquoise	2082	CTACTAG,MIR-325	8	16	16237	4169	1.949268	0.031391
turquoise	4147	REACTOME_CTNNB1_PHOSPHORYLATION_CASCADE	8	16	16237	4169	1.949268	0.031391
turquoise	4567	REACTOME_JNK_C_JUN_KINASES_PHOSPHORYLATION_	8	16	16237	4169	1.949268	0.031391
turquoise	5513	FARMER_BREAST_CANCER_CLUSTER_6	8	16	16237	4169	1.949268	0.031391
turquoise	6756	CARDOSO_RESPONSE_TO_GAMMA_RADIATION_AND_	8	16	16237	4169	1.949268	0.031391
yellow	583	ELECTRON_TRANSPORT_GO_0006118	8	43	16210	1426	2.120487	0.031397
yellow	5275	KERLEY_RESPONSE_TO_CISPLATIN_UP	8	43	16210	1426	2.120487	0.031397
lightyellow	1384	PEPTIDASE_ACTIVITY	2	137	16116	33	7.190002	0.031407
lightyellow	9896	GSE36392_EOSINOPHIL_VS_MAC_IL25_TREATED_LUNG	2	137	16116	33	7.190002	0.031407
purple	6283	BROWN_MYELOID_CELL_DEVELOPMENT_DN	4	123	16130	157	3.366579	0.031422
purple	6807	ZHONG_SECRETOME_OF_LUNG_CANCER_AND_FIBROB	4	123	16130	157	3.366579	0.031422
yellow	9731	GSE31082_DN_VS_DP_THYMOCYTE_DN	25	193	16060	1426	1.476375	0.031464
yellow	10187	GSE7764_NKCELL_VS_SPLENOCYTE_DN	25	193	16060	1426	1.476375	0.031464
red	5175	ENK_UV_RESPONSE_KERATINOCYTE_UP	23	470	15783	524	1.517862	0.031475
midnightb	7139	GRADE_COLON_AND_RECTAL_CANCER_UP	4	273	15980	71	3.354073	0.031485
cyan	5773	GROSS_HYPOXIA_VIA_ELK3_DN	3	145	16108	77	4.367129	0.031507
cyan	7580	MIKKELSEN_IPS_LCP_WITH_H3K4ME3	3	145	16108	77	4.367129	0.031507
pink	1994	CGCTGCT,MIR-503	2	20	16233	228	7.128509	0.031533
pink	4586	REACTOME_INTERACTION_BETWEEN_L1_AND_ANKYRI	2	20	16233	228	7.128509	0.031533
pink	5704	HATADA_METHYLATED_IN_LUNG_CANCER_DN	2	20	16233	228	7.128509	0.031533
pink	6966	WALLACE_PROSTATE_CANCER_UP	2	20	16233	228	7.128509	0.031533
pink	7570	MIKKELSEN_MEF_ICP_WITH_H3K4ME3_AND_H3K27MI	2	20	16233	228	7.128509	0.031533
greenyello	1547	V$TST1_01	5	190	16063	150	2.851404	0.031543
greenyello	1781	V$CEBPDELTA_Q6	5	190	16063	150	2.851404	0.031543
greenyello	1840	V$CIZ_01	5	190	16063	150	2.851404	0.031543
greenyello	8544	GSE13306_RA_VS_UNTREATED_MEM_CD4_TCELL_UP	5	190	16063	150	2.851404	0.031543
turquoise	8355	TBK1.DF_UP	84	273	15980	4169	1.19955	0.031569
blue	2220	YGCGYRCGC_UNKNOWN	66	290	15963	2978	1.242095	0.031589

brown	1811	V$TEL2_Q6	41	228	16025	2190	1.334561	0.031619
pink	8174	GHANDHI_DIRECT_IRRADIATION_UP	4	85	16168	228	3.354592	0.03162
magenta	5718	SHETH_LIVER_CANCER_VS_TXNIP_LOSS_PAM5	3	67	16186	167	4.357762	0.031622
greenyello	8301	PTEN_DN.V1_DN	4	129	16124	150	3.359793	0.031629
greenyello	9354	GSE22886_NEUTROPHIL_VS_MONOCYTE_UP	4	129	16124	150	3.359793	0.031629
greenyello	9688	GSE29618_PRE_VS_DAY7_POST_TIV_FLU_VACCINE_BC	4	129	16124	150	3.359793	0.031629
lightcyan	6805	ZHONG_SECRETOME_OF_LUNG_CANCER_AND_MACRC	2	73	16180	62	7.182059	0.031639
blue	400	NEGATIVE_REGULATION_OF_CELL_CYCLE	20	72	16181	2978	1.516025	0.031651
blue	1611	V$SREBP1_02	20	72	16181	2978	1.516025	0.031651
blue	2659	GNF2_KPNB1	20	72	16181	2978	1.516025	0.031651
grey60	8282	PTEN_DN.V2_UP	2	103	16150	44	7.172551	0.031659
green	1575	V$AP1_Q6	26	221	16032	1305	1.465224	0.031661
brown	8623	GSE14000_4H_VS_16H_LPS_DC_TRANSLATED_RNA_DN	36	196	16057	2190	1.363126	0.031674
brown	9083	GSE17721_LPS_VS_GARDIQUIMOD_6H_BMDM_DN	36	196	16057	2190	1.363126	0.031674
brown	9936	GSE37416_0H_VS_6H_F_TULARENSIS_LVS_NEUTROPH	36	196	16057	2190	1.363126	0.031674
brown	5030	RHEIN_ALL_GLUCOCORTICOID_THERAPY_UP	16	73	16180	2190	1.626622	0.031676
red	7753	NAKAMURA_ADIPOGENESIS_EARLY_UP	5	55	16198	524	2.819743	0.031711
red	7915	WIERENGA_STAT5A_TARGETS_GROUP2	5	55	16198	524	2.819743	0.031711
red	7928	JOHNSTONE_PARVB_TARGETS_1_DN	5	55	16198	524	2.819743	0.031711
magenta	853	CHEMICAL_HOMEOSTASIS	4	116	16137	167	3.355978	0.031723
magenta	2175	RTTTNNNYTGGM_UNKNOWN	4	116	16137	167	3.355978	0.031723
magenta	7093	IZADPANAH_STEM_CELL_ADIPOSE_VS_BONE_UP	4	116	16137	167	3.355978	0.031723
yellow	6521	NATSUME_RESPONSE_TO_INTERFERON_BETA_DN	9	51	16202	1426	2.011344	0.031731
purple	3962	PID_S1P_S1P3_PATHWAY	2	29	16224	157	7.139468	0.031744
purple	4111	PID_ALPHASYNUCLEIN_PATHWAY	2	29	16224	157	7.139468	0.031744
purple	4569	REACTOME_THROMBIN_SIGNALLING_THROUGH_PROT	2	29	16224	157	7.139468	0.031744
purple	8122	YU_BAP1_TARGETS	2	29	16224	157	7.139468	0.031744
red	8355	TBK1.DF_UP	15	273	15980	524	1.70424	0.031746
yellow	8103	REICHERT_MITOSIS_LIN9_TARGETS	6	28	16225	1426	2.442346	0.031747
turquoise	706	MRNA_PROCESSING_GO_0006397	26	72	16181	4169	1.407805	0.031748
turquoise	7453	MILI_PSEUDOPODIA_CHEMOTAXIS_UP	26	72	16181	4169	1.407805	0.031748
brown	9503	GSE2706_UNSTIM_VS_2H_LPS_DC_DN	33	177	16076	2190	1.383662	0.031759
brown	9990	GSE3982_EOSINOPHIL_VS_TH2_UP	33	177	16076	2190	1.383662	0.031759
turquoise	5066	ODONNELL_TARGETS_OF_MYC_AND_TFRC_DN	17	43	16210	4169	1.541282	0.031766
turquoise	6273	HOFMANN_CELL_LYMPHOMA_UP	17	43	16210	4169	1.541282	0.031766
red	1589	V$CEBP_Q2	11	180	16073	524	1.895494	0.031766
red	9289	GSE20715_0H_VS_6H_OZONE_LUNG_DN	11	180	16073	524	1.895494	0.031766
red	10292	GSE9988_ANTI_TREM1_AND_LPS_VS_CTRL_TREATED_I	11	180	16073	524	1.895494	0.031766
yellow	707	MACROMOLECULE_BIOSYNTHETIC_PROCESS	35	289	15964	1426	1.380334	0.031771
magenta	4368	REACTOME_CLASS_A1_RHODOPSIN_LIKE_RECEPTORS	5	171	16082	167	2.845712	0.031788
magenta	10136	GSE6269_FLU_VS_STREP_AUREUS_INF_PBMC_UP	5	171	16082	167	2.845712	0.031788
tan	8398	GOLDRATH_NAIVE_VS_EFF_CD8_TCELL_UP	5	197	16056	145	2.844915	0.031802
brown	2245	TGACCTY_V$ERR1_Q2	135	862	15391	2190	1.162294	0.031806
lightcyan	8778	GSE15930_NAIVE_VS_24H_IN_VITRO_STIM_IL12_CD8_	3	181	16072	62	4.344947	0.031817
lightcyan	9080	GSE17721_LPS_VS_GARDIQUIMOD_4H_BMDM_UP	3	181	16072	62	4.344947	0.031817
blue	9290	GSE20715_0H_VS_24H_OZONE_LUNG_UP	46	193	16060	2978	1.300797	0.031864
blue	9464	GSE25087_FETAL_VS_ADULT_TREG_UP	46	193	16060	2978	1.300797	0.031864
blue	9676	GSE29618_PDC_VS_MDC_DAY7_FLU_VACCINE_UP	46	193	16060	2978	1.300797	0.031864
midnightb	4883	TURASHVILI_BREAST_LOBULAR_CARCINOMA_VS_DUCT	2	64	16189	71	7.153609	0.031872
lightgreen	5174	ENK_UV_RESPONSE_EPIDERMIS_DN	4	467	15786	42	3.314571	0.031879
red	3715	MODULE_243	6	74	16179	524	2.514906	0.031965
red	7264	BOYLAN_MULTIPLE_MYELOMA_D_DN	6	74	16179	524	2.514906	0.031965
blue	4141	REACTOME_METABOLISM_OF_NON_CODING_RNA	14	46	16207	2978	1.661036	0.031968
blue	7303	FRASOR_RESPONSE_TO_SERM_OR_FULVESTRANT_DN	14	46	16207	2978	1.661036	0.031968
blue	6069	UEDA_CENTRAL_CLOCK	22	81	16172	2978	1.482335	0.03198
lightyellow	221	INTEGRIN_COMPLEX	1	16	16237	33	30.7822	0.032011
lightyellow	3492	SA_TRKA_RECEPTOR	1	16	16237	33	30.7822	0.032011
lightyellow	3845	MODULE_424	1	16	16237	33	30.7822	0.032011
lightyellow	4233	REACTOME_ARMS_MEDIATED_ACTIVATION	1	16	16237	33	30.7822	0.032011
lightyellow	5320	ZIRN_TRETINOIN_RESPONSE_UP	1	16	16237	33	30.7822	0.032011

lightyellow	5462	MYLLYKANGAS_AMPLIFICATION_HOT_SPOT_17	1	16	16237	33	30.7822	0.032011
lightyellow	6607	DAZARD_UV_RESPONSE_CLUSTER_G4	1	16	16237	33	30.7822	0.032011
lightyellow	6718	NIELSEN_LEIOMYOSARCOMA_CNN1_UP	1	16	16237	33	30.7822	0.032011
grey60	271	BIOGENIC_AMINE_METABOLIC_PROCESS	1	12	16241	44	30.7822	0.032018
grey60	708	G_PROTEIN_SIGNALING_ADENYLATE_CYCLASE_ACTIVAT	1	12	16241	44	30.7822	0.032018
grey60	1161	OXIDOREDUCTASE_ACTIVITY_ACTING_ON_PEROXIDE_A	1	12	16241	44	30.7822	0.032018
grey60	3633	MODULE_148	1	12	16241	44	30.7822	0.032018
grey60	3672	MODULE_191	1	12	16241	44	30.7822	0.032018
grey60	3778	MODULE_326	1	12	16241	44	30.7822	0.032018
grey60	3850	MODULE_431	1	12	16241	44	30.7822	0.032018
grey60	3912	MODULE_545	1	12	16241	44	30.7822	0.032018
grey60	3916	MODULE_560	1	12	16241	44	30.7822	0.032018
grey60	6690	NIELSEN_SCHWANNOMA_UP	1	12	16241	44	30.7822	0.032018
grey60	7089	WEBER_METHYLATED_HCP_IN_SPERM_UP	1	12	16241	44	30.7822	0.032018
grey60	7323	PARK_TRETINOIN_RESPONSE	1	12	16241	44	30.7822	0.032018
grey60	7327	WU_ALZHEIMER_DISEASE_UP	1	12	16241	44	30.7822	0.032018
grey60	7830	WANG_METASTASIS_OF_BREAST_CANCER	1	12	16241	44	30.7822	0.032018
yellow	1092	DAMAGED_DNA_BINDING	5	21	16232	1426	2.713718	0.032029
yellow	5090	SABATES_COLORECTAL_ADENOMA_SIZE_UP	5	21	16232	1426	2.713718	0.032029
green	1089	PROTEIN_TYROSINE_PHOSPHATASE_ACTIVITY	8	47	16206	1305	2.119899	0.03204
green	4760	REACTOME_AMYLOIDS	8	47	16206	1305	2.119899	0.03204
pink	8853	GSE17721_CTRL_VS_POLYIC_2H_BMDM_DN	6	169	16084	228	2.530832	0.032076
pink	8903	GSE17721_CTRL_VS_GARDIQUIMOD_4H_BMDM_DN	6	169	16084	228	2.530832	0.032076
green	8159	ACEVEDO_FGFR1_TARGETS_IN_PROSTATE_CANCER_M	28	242	16011	1305	1.441006	0.03208
salmon	2793	KEGG_DNA_REPLICATION	2	36	16217	127	7.109799	0.032101
salmon	3634	MODULE_149	2	36	16217	127	7.109799	0.032101
salmon	6352	MA_MYELOID_DIFFERENTIATION_UP	2	36	16217	127	7.109799	0.032101
greenyello	6166	ROSS_AML_WITH_MLL_FUSIONS	3	75	16178	150	4.334133	0.032109
blue	1777	V$MYCMAX_03	52	222	16031	2978	1.278378	0.03213
red	2210	WTGAAAT_UNKNOWN	23	471	15782	524	1.514639	0.032133
black	7687	ZHAN_MULTIPLE_MYELOMA_PR_DN	3	40	16213	283	4.307332	0.032137
red	326	REGULATION_OF_HORMONE_SECRETION	2	9	16244	524	6.892706	0.032139
red	1222	DELAYED_RECTIFIER_POTASSIUM_CHANNEL_ACTIVITY	2	9	16244	524	6.892706	0.032139
red	1311	SIALYLTRANSFERASE_ACTIVITY	2	9	16244	524	6.892706	0.032139
red	4470	REACTOME_ACTIVATION_OF_CHAPERONE_GENES_BY_	2	9	16244	524	6.892706	0.032139
red	4860	ZERBINI_RESPONSE_TO_SULINDAC_UP	2	9	16244	524	6.892706	0.032139
red	5373	KONG_E2F1_TARGETS	2	9	16244	524	6.892706	0.032139
red	5870	SCHAEFFER_PROSTATE_DEVELOPMENT_AND_CANCER_	2	9	16244	524	6.892706	0.032139
yellow	8644	GSE14308_TH1_VS_NATURAL_TREG_UP	24	184	16069	1426	1.486646	0.032143
greenyello	9408	GSE24142_EARLY_THYMIC_PROGENITOR_VS_DN2_THY	5	191	16062	150	2.836475	0.032159
greenyello	10045	GSE3982_MAC_VS_TH2_DN	5	191	16062	150	2.836475	0.032159
blue	1750	V$NKX62_Q2	42	174	16079	2978	1.317373	0.032177
salmon	4371	REACTOME_CELL_CYCLE_MITOTIC	6	304	15949	127	2.52585	0.03218
red	8381	LEF1_UP.V1_UP	10	158	16095	524	1.963112	0.032183
red	9024	GSE17721_PAM3CSK4_VS_GADIQUIMOD_2H_BMDM_	10	158	16095	524	1.963112	0.032183
black	2228	YTCCCRNNAGGY_UNKNOWN	4	69	16184	283	3.329339	0.032196
brown	5065	ODONNELL_TARGETS_OF_MYC_AND_TFRC_UP	17	79	16174	2190	1.597023	0.032225
magenta	6746	WANG_SMARCE1_TARGETS_UP	6	231	16022	167	2.527879	0.032229
purple	1791	CYTAGCAAY_UNKNOWN	4	124	16129	157	3.339429	0.032234
lightgreen	6642	ZHU_CMV_ALL_DN	2	109	16144	42	7.100481	0.032237
turquoise	2378	MORF_DEK	80	259	15994	4169	1.204181	0.032253
lightcyan	7878	DUTERTRE_ESTRADIOL_RESPONSE_24HR_DN	5	466	15787	62	2.812716	0.032262
lightcyan	8776	GSE15930_NAIVE_VS_24H_IN_VITRO_STIM_CD8_TCELL	3	182	16071	62	4.321074	0.032265
lightcyan	9533	GSE27786_LSK_VS_CD4_TCELL_DN	3	182	16071	62	4.321074	0.032265
lightcyan	9999	GSE3982_MAST_CELL_VS_BCELL_DN	3	182	16071	62	4.321074	0.032265
brown	2699	GNF2_SPI1	9	34	16219	2190	1.964504	0.032292
brown	4010	PID_ERBB1_RECEPTOR_PROXIMAL_PATHWAY	9	34	16219	2190	1.964504	0.032292
brown	6426	WEIGEL_OXIDATIVE_STRESS_RESPONSE	9	34	16219	2190	1.964504	0.032292
brown	7112	HONMA_DOCETAXEL_RESISTANCE	9	34	16219	2190	1.964504	0.032292
lightcyan	7704	WONG_ADULT_TISSUE_STEM_MODULE	6	629	15624	62	2.50059	0.032295

yellow	8760	GSE15659_RESTING_VS_ACTIVATED_TREG_UP	20	147	16106	1426	1.550696	0.032323
lightgreen	2140	TGCTTTG,MIR-330	3	270	15983	42	4.299735	0.032339
blue	9228	GSE18791_CTRL_VS_NEWCASTLE_VIRUS_DC_10H_UP	47	198	16055	2978	1.295512	0.032391
blue	9311	GSE22886_UNSTIM_VS_STIM_MEMORY_TCELL_DN	47	198	16055	2978	1.295512	0.032391
blue	10148	GSE7400_CTRL_VS_CSF3_IN_VIVO_TREATED_PBMC_UI	47	198	16055	2978	1.295512	0.032391
greenyello	9248	GSE19825_NAIVE_VS_IL2RALOW_DAY3_EFF_CD8_TCEL	4	130	16123	150	3.333949	0.032406
red	7930	JOHNSTONE_PARVB_TARGETS_2_DN	17	322	15931	524	1.637553	0.032408
lightcyan	5433	HUMMERICH_SKIN_CANCER_PROGRESSION_UP	2	74	16179	62	7.085004	0.03244
lightcyan	5588	YORDY_RECIPROCAL_REGULATION_BY_ETS1_AND_SP1	2	74	16179	62	7.085004	0.03244
lightcyan	7264	BOYLAN_MULTIPLE_MYELOMA_D_DN	2	74	16179	62	7.085004	0.03244
red	4365	REACTOME_SIGNALING_BY_GPCR	21	421	15832	524	1.547175	0.032445
brown	8956	GSE17721_PAM3CSK4_VS_CPG_6H_BMDM_UP	35	190	16063	2190	1.367111	0.03248
brown	9035	GSE17721_PAM3CSK4_VS_GADIQUIMOD_16H_BMDM	35	190	16063	2190	1.367111	0.03248
brown	10239	GSE9006_TYPE_1_VS_TYPE_2_DIABETES_PBMC_AT_DX	35	190	16063	2190	1.367111	0.03248
purple	2285	CCAWWNAAGG_V$SRF_Q4	3	72	16181	157	4.313429	0.032485
purple	9501	GSE2706_UNSTIM_VS_8H_R848_DC_DN	5	183	16070	157	2.828478	0.032502
red	3029	BIOCARTA_NFKB_PATHWAY	3	22	16231	524	4.229615	0.032544
red	3190	chr10p13	3	22	16231	524	4.229615	0.032544
red	3562	MODULE_73	3	22	16231	524	4.229615	0.032544
red	4221	REACTOME_YAP1_AND_WWTR1_TAZ_STIMULATED_GI	3	22	16231	524	4.229615	0.032544
red	6859	GAVIN_PDE3B_TARGETS	3	22	16231	524	4.229615	0.032544
brown	4246	REACTOME_SIGNALING_BY_FGFR_IN_DISEASE	22	109	16144	2190	1.49791	0.032544
brown	7506	BOYAULT_LIVER_CANCER_SUBCLASS_G1_UP	22	109	16144	2190	1.49791	0.032544
brown	7649	HOSHIDA_LIVER_CANCER_SUBCLASS_S2	22	109	16144	2190	1.49791	0.032544
pink	7236	WANG_TUMOR_INVASIVENESS_UP	10	363	15890	228	1.963777	0.032549
blue	2469	MORF_SS18	17	59	16194	2978	1.572555	0.032551
red	880	CATION_TRANSPORT	8	115	16138	524	2.157717	0.032568
cyan	9197	GSE17974_IL4_AND_ANTI_IL12_VS_UNTREATED_12H_	3	147	16106	77	4.307713	0.03262
cyan	9221	GSE18791_CTRL_VS_NEWCASTLE_VIRUS_DC_2H_DN	3	147	16106	77	4.307713	0.03262
purple	5393	PEREZ_TP53_TARGETS	16	1001	15252	157	1.654702	0.032674
lightyellow	9198	GSE17974_IL4_AND_ANTI_IL12_VS_UNTREATED_24H_	2	140	16113	33	7.035931	0.03268
turquoise	9214	GSE17974_2.5H_VS_72H_IL4_AND_ANTI_IL12_ACT_CD	63	199	16054	4169	1.23421	0.032699
turquoise	9355	GSE22886_NEUTROPHIL_VS_MONOCYTE_DN	63	199	16054	4169	1.23421	0.032699
cyan	444	NEGATIVE_REGULATION_OF_ANGIOGENESIS	1	7	16246	77	30.15399	0.032701
cyan	512	SPERM_MOTILITY	1	7	16246	77	30.15399	0.032701
cyan	6075	SCHUHMACHER_MYC_TARGETS_DN	1	7	16246	77	30.15399	0.032701
salmon	4416	REACTOME_MRNA_PROCESSING	4	154	16099	127	3.324062	0.032707
salmon	7032	ACEVEDO_NORMAL_TISSUE_ADJACENT_TO_LIVER_TUI	4	154	16099	127	3.324062	0.032707
lightcyan	8721	GSE14769_UNSTIM_VS_80MIN_LPS_BMDM_DN	3	183	16070	62	4.297462	0.032715
lightcyan	8801	GSE15930_STIM_VS_STIM_AND_TRICHOSTATINA_24H_	3	183	16070	62	4.297462	0.032715
lightcyan	9141	GSE17721_0.5H_VS_4H_GARDIQUIMOD_BMDM_DN	3	183	16070	62	4.297462	0.032715
lightcyan	9827	GSE360_DC_VS_MAC_B_MALAYI_LOW_DOSE_DN	3	183	16070	62	4.297462	0.032715
lightcyan	10033	GSE3982_MAC_VS_BCELL_DN	3	183	16070	62	4.297462	0.032715
lightcyan	10243	GSE9037_CTRL_VS_LPS_4H_STIM_BMDM_DN	3	183	16070	62	4.297462	0.032715
purple	7079	BONOME_OVARIAN_CANCER_SURVIVAL_SUBOPTIMAL_	9	455	15798	157	2.047694	0.03272
turquoise	1975	ATGTAGC,MIR-221,MIR-222	37	109	16144	4169	1.323357	0.032737
turquoise	5980	MORI_EMU_MYC_LYMPHOMA_BY_ONSET_TIME_UP	37	109	16144	4169	1.323357	0.032737
green	1180	GATED_CHANNEL_ACTIVITY	12	83	16170	1305	1.800637	0.03276
turquoise	3121	chr5q23	16	40	16213	4169	1.559415	0.032766
turquoise	4414	REACTOME_RNA_POL_II_TRANSCRIPTION_PRE_INITIAT	16	40	16213	4169	1.559415	0.032766
turquoise	8728	GSE14769_20MIN_VS_360MIN_LPS_BMDM_UP	61	192	16061	4169	1.238598	0.032775
turquoise	8941	GSE17721_POLYIC_VS_PAM3CSK4_8H_BMDM_DN	61	192	16061	4169	1.238598	0.032775
turquoise	9537	GSE27786_LSK_VS_NKCELL_DN	61	192	16061	4169	1.238598	0.032775
turquoise	9589	GSE27786_CD8_TCELL_VS_NKCELL_DN	61	192	16061	4169	1.238598	0.032775
turquoise	10102	GSE39820_CTRL_VS_IL1B_IL6_IL23A_CD4_TCELL_UP	61	192	16061	4169	1.238598	0.032775
lightcyan	5176	ENK_UV_RESPONSE_KERATINOCYTE_DN	5	468	15785	62	2.800696	0.032775
greenyello	1591	V$GR_Q6	5	192	16061	150	2.821701	0.032782
greenyello	8687	GSE1460_INTRATHYMIC_T_PROGENITOR_VS_CD4_THY	5	192	16061	150	2.821701	0.032782
greenyello	8711	GSE1460_NAIVE_CD4_TCELL_CORD_BLOOD_VS_THYMI	5	192	16061	150	2.821701	0.032782
greenyello	8927	GSE17721_LPS_VS_POLYIC_16H_BMDM_DN	5	192	16061	150	2.821701	0.032782

greenyello	9729	GSE30962_ACUTE_VS_CHRONIC_LCMV_SECONDARY_II	5	192	16061	150	2.821701	0.032782
yellow	9751	GSE32423_CTRL_VS_IL4_MEMORY_CD8_TCELL_DN	23	175	16078	1426	1.497972	0.032789
midnightb	618	PROTEIN_RNA_COMPLEX_ASSEMBLY	2	65	16188	71	7.043554	0.032793
midnightb	3666	MODULE_183	2	65	16188	71	7.043554	0.032793
grey60	8348	STK33_SKM_UP	3	259	15994	44	4.278607	0.032796
turquoise	3593	MODULE_105	59	185	16068	4169	1.243317	0.032809
pink	4820	WATANABE_RECTAL_CANCER_RADIOTHERAPY_RESPON	4	86	16167	228	3.315585	0.03281
red	3606	MODULE_118	18	347	15906	524	1.60896	0.032837
yellow	1835	GCGNNANTTCC_UNKNOWN	17	120	16133	1426	1.614662	0.032838
magenta	4832	SCHUETZ_BREAST_CANCER_DUCTAL_INVASIVE_DN	3	68	16185	167	4.293677	0.032839
brown	1463	V$ELK1_01	44	248	16005	2190	1.316711	0.03284
cyan	5942	AMIT_SERUM_RESPONSE_120_MCF10A	2	60	16193	77	7.035931	0.032854
black	9563	GSE27786_BCELL_VS_CD4_TCELL_DN	7	174	16079	283	2.310446	0.032871
cyan	6388	KUMAR_TARGETS_OF_MLL_AF9_FUSION	5	376	15877	77	2.806887	0.032883
red	2191	SYATTGTG_UNKNOWN	11	181	16072	524	1.885022	0.032884
red	5892	GOTZMANN_EPITHELIAL_TO_MESENCHYMAL_TRANSIT	11	181	16072	524	1.885022	0.032884
red	8778	GSE15930_NAIVE_VS_24H_IN_VITRO_STIM_IL12_CD8_	11	181	16072	524	1.885022	0.032884
red	9158	GSE17974_0H_VS_6H_IN_VITRO_ACT_CD4_TCELL_UP	11	181	16072	524	1.885022	0.032884
red	9507	GSE2706_UNSTIM_VS_2H_LPS_AND_R848_DC_DN	11	181	16072	524	1.885022	0.032884
red	9710	GSE30083_SP1_VS_SP2_THYMOCYTE_UP	11	181	16072	524	1.885022	0.032884
midnightb	1929	V$GR_01	3	160	16093	71	4.292165	0.032888
blue	167	NUCLEOPLASM_PART	48	203	16050	2978	1.290488	0.032891
blue	7409	ZHANG_TLX_TARGETS_60HR_DN	62	271	15982	2978	1.248623	0.032906
magenta	5488	FARMER_BREAST_CANCER_BASAL_VS_LULMINAL	7	295	15958	167	2.309368	0.032918
magenta	7240	GRESHOCK_CANCER_COPY_NUMBER_UP	7	295	15958	167	2.309368	0.032918
tan	972	DNA_PACKAGING	2	32	16221	145	7.005603	0.032923
tan	4710	REACTOME_INTERACTIONS_OF_VPR_WITH_HOST_CELI	2	32	16221	145	7.005603	0.032923
tan	5824	CAFFAREL_RESPONSE_TO_THC_UP	2	32	16221	145	7.005603	0.032923
lightgreen	3605	MODULE_117	4	472	15781	42	3.279459	0.032966
green	1411	TRANSFERASE_ACTIVITY_TRANSFERRING_SULFUR_CON	5	23	16230	1305	2.70748	0.032968
tan	9244	GSE18791_UNSTIM_VS_NEWCATSLE_VIRUS_DC_10H_U	5	199	16054	145	2.816323	0.033005
tan	9572	GSE27786_BCELL_VS_NEUTROPHIL_UP	5	199	16054	145	2.816323	0.033005
pink	6955	MARTINEZ_TP53_TARGETS_UP	13	522	15731	228	1.775299	0.033015
greenyello	6103	FRASOR_RESPONSE_TO_ESTRADIOL_UP	2	31	16222	150	6.990538	0.033035
red	2281	TGACCTTG_V$SF1_Q6	12	204	16049	524	1.82454	0.03307
pink	3511	MODULE_19	8	264	15989	228	2.160154	0.033084
lightgreen	130	MYOSIN_COMPLEX	1	13	16240	42	29.7674	0.03309
lightgreen	623	VACUOLAR_TRANSPORT	1	13	16240	42	29.7674	0.03309
lightgreen	1034	POSITIVE_REGULATION_OF_CYTOKINE_PRODUCTION	1	13	16240	42	29.7674	0.03309
lightgreen	1143	ANION_CATION_SYMPORTER_ACTIVITY	1	13	16240	42	29.7674	0.03309
lightgreen	4632	REACTOME_REGULATION_OF_IFNG_SIGNALING	1	13	16240	42	29.7674	0.03309
lightgreen	4648	REACTOME_ACTIVATION_OF_IRF3_IRF7_MEDIATED_BY	1	13	16240	42	29.7674	0.03309
lightgreen	4663	REACTOME_TRAF6_MEDIATED_INDUCTION_OF_TAK1_	1	13	16240	42	29.7674	0.03309
lightgreen	5670	INAMURA_LUNG_CANCER_SCC_UP	1	13	16240	42	29.7674	0.03309
greenyello	8091	DELACROIX_RAR_BOUND_ES	8	402	15851	150	2.156285	0.033098
green	8264	PRC1_BMI_UP.V1_UP	17	131	16122	1305	1.616221	0.033117
purple	7923	BHAT_ESR1_TARGETS_NOT_VIA_AKT1_UP	5	184	16069	157	2.813106	0.033159
purple	9957	GSE3982_CTRL_VS_LPS_48H_DC_DN	5	184	16069	157	2.813106	0.033159
purple	9993	GSE3982_MAST_CELL_VS_DC_DN	5	184	16069	157	2.813106	0.033159
lightcyan	8808	GSE15930_STIM_VS_STIM_AND_IL-12_72H_CD8_T_CE	3	184	16069	62	4.274106	0.033169
lightcyan	8907	GSE17721_CTRL_VS_GARDIQUIMOD_8H_BMDM_DN	3	184	16069	62	4.274106	0.033169
lightcyan	9616	GSE27786_NEUTROPHIL_VS_MONO_MAC_UP	3	184	16069	62	4.274106	0.033169
lightcyan	9761	GSE3337_CTRL_VS_4H_IFNG_IN_CD8POS_DC_DN	3	184	16069	62	4.274106	0.033169
magenta	8262	RAF_UP.V1_UP	5	173	16080	167	2.812814	0.033177
magenta	8708	GSE1460_CORD_VS_ADULT_BLOOD_NAIVE_CD4_TCELL	5	173	16080	167	2.812814	0.033177
cyan	172	EXTRACELLULAR_SPACE	3	148	16105	77	4.278607	0.033185
cyan	8220	AKT_UP.V1_UP	3	148	16105	77	4.278607	0.033185
yellow	3522	MODULE_32	29	232	16021	1426	1.424702	0.033195
greenyello	8270	PRC2_SUZ12_UP.V1_UP	4	131	16122	150	3.308499	0.033195
red	525	BLOOD_COAGULATION	4	38	16215	524	3.264966	0.033196

red	835	COAGULATION	4	38	16215	524	3.264966	0.033196
red	4231	REACTOME_TRIGLYCERIDE_BIOSYNTHESIS	4	38	16215	524	3.264966	0.033196
red	7463	CROONQUIST_NRAS_SIGNALING_UP	4	38	16215	524	3.264966	0.033196
red	7589	ONO_AML1_TARGETS_DN	4	38	16215	524	3.264966	0.033196
red	7683	ZHAN_MULTIPLE_MYELOMA_LB_DN	4	38	16215	524	3.264966	0.033196
greenyello	993	CELL_MIGRATION	3	76	16177	150	4.277105	0.033209
greenyello	7360	ROSS_LEUKEMIA_WITH_MLL_FUSIONS	3	76	16177	150	4.277105	0.033209
yellow	1795	V$LBP1_Q6	25	194	16059	1426	1.468765	0.033224
brown	8885	GSE17721_CTRL_VS_CPG_2H_BMDM_DN	31	165	16088	2190	1.394335	0.033231
green	7188	LIN_NPAS4_TARGETS_DN	9	56	16197	1305	2.001601	0.033236
lightcyan	5168	KOKKINAKIS_METHIONINE_DEPRIVATION_96HR_DN	2	75	16178	62	6.990538	0.033248
lightcyan	6156	LIN_APC_TARGETS	2	75	16178	62	6.990538	0.033248
lightcyan	6166	ROSS_AML_WITH_MLL_FUSIONS	2	75	16178	62	6.990538	0.033248
lightcyan	6214	PEART_HDAC_PROLIFERATION_CLUSTER_DN	2	75	16178	62	6.990538	0.033248
lightgreen	4956	BASAKI_YBX1_TARGETS_UP	3	273	15980	42	4.252486	0.033256
cyan	8344	STK33_DN	4	256	15997	77	3.298093	0.03327
brown	8093	KRIEG_KDM3A_TARGETS_NOT_HYPOXIA	34	184	16069	2190	1.371357	0.033294
brown	9992	GSE3982_MAST_CELL_VS_DC_UP	34	184	16069	2190	1.371357	0.033294
magenta	4453	REACTOME_NCAM1_INTERACTIONS	2	28	16225	167	6.951668	0.03331
magenta	4958	NOJIMA_SFRP2_TARGETS_UP	2	28	16225	167	6.951668	0.03331
magenta	7701	TIAN_TNF_SIGNALING_VIA_NFKB	2	28	16225	167	6.951668	0.03331
greenyello	7839	MARTENS_TRETINOIN_RESPONSE_UP	9	478	15775	150	2.040126	0.033315
blue	3072	BIOCARTA_MEF2D_PATHWAY	7	18	16235	2978	2.122435	0.033346
blue	4423	REACTOME_FORMATION_OF_TUBULIN_FOLDING_INTE	7	18	16235	2978	2.122435	0.033346
blue	4522	REACTOME_DEADENYLATION_OF_MRNA	7	18	16235	2978	2.122435	0.033346
blue	7607	KUROZUMI_RESPONSE_TO_ONCOCYTIC_VIRUS_AND_C	7	18	16235	2978	2.122435	0.033346
brown	2825	KEGG_NOTCH_SIGNALING_PATHWAY	11	45	16208	2190	1.814135	0.033364
brown	4025	PID_AR_TF_PATHWAY	11	45	16208	2190	1.814135	0.033364
brown	4071	PID_HEDGEHOG_GLIPATHWAY	11	45	16208	2190	1.814135	0.033364
brown	6549	CHIBA_RESPONSE_TO_TSA	11	45	16208	2190	1.814135	0.033364
turquoise	6772	KYNG_RESPONSE_TO_H2O2	25	69	16184	4169	1.412513	0.033369
green	7445	TAVAZOIE_METASTASIS	11	74	16179	1305	1.851331	0.033371
salmon	6200	PENG_GLUCOSE_DEPRIVATION_DN	4	155	16098	127	3.302616	0.033377
red	4903	PUIFFE_INVASION_INHIBITED_BY_ASCITES_DN	9	137	16116	524	2.037625	0.033381
yellow	9438	GSE24634_NAIVE_CD4_TCELL_VS_DAY10_IL4_CONV_T	22	166	16087	1426	1.510527	0.033391
midnightb	5096	NAGASHIMA_NRG1_SIGNALING_UP	3	161	16092	71	4.265506	0.03341
midnightb	9643	GSE29615_DAY3_VS_DAY7_LAIV_FLU_VACCINE_PBMC_	3	161	16092	71	4.265506	0.03341
brown	7216	WINTER_HYPOXIA_METAGENE	39	216	16037	2190	1.339986	0.03341
greenyello	1788	V$GATA4_Q3	5	193	16060	150	2.807081	0.033413
blue	8491	GSE11924_TH2_VS_TH17_CD4_TCELL_DN	44	184	16069	2978	1.3051	0.033434
blue	9174	GSE17974_CTRL_VS_ACT_IL4_AND_ANTI_IL12_4H_CD4	44	184	16069	2978	1.3051	0.033434
blue	9204	GSE17974_0.5H_VS_72H_UNTREATED_IN_VITRO_CD4_	44	184	16069	2978	1.3051	0.033434
magenta	1225	SUBSTRATE_SPECIFIC_TRANSPORTER_ACTIVITY	7	296	15957	167	2.301566	0.033436
magenta	3582	MODULE_94	7	296	15957	167	2.301566	0.033436
turquoise	7858	HIRSCH_CELLULAR_TRANSFORMATION_SIGNATURE_UI	72	231	16022	4169	1.215128	0.033448
cyan	1433	DNA_BINDING	6	509	15744	77	2.488148	0.033513
turquoise	2790	KEGG_RNA_DEGRADATION	21	56	16197	4169	1.461951	0.033532
green	8935	GSE17721_POLYIC_VS_PAM3CSK4_2H_BMDM_DN	20	161	16092	1305	1.547131	0.033553
turquoise	29	RIBONUCLEOPROTEIN_COMPLEX	46	140	16113	4169	1.280948	0.033559
lightgreen	7963	PASINI_SUZ12_TARGETS_DN	3	274	15979	42	4.236966	0.033565
greenyello	2199	TGGNNNNNNKCCAR_UNKNOWN	7	330	15923	150	2.298404	0.033567
purple	5793	BUYTAERT_PHOTODYNAMIC_THERAPY_STRESS_DN	11	607	15646	157	1.876022	0.033595
turquoise	4814	KORKOLA_YOLK_SAC_TUMOR_UP	9	19	16234	4169	1.846675	0.033601
turquoise	5418	SEIDEN_MET_SIGNALING	9	19	16234	4169	1.846675	0.033601
turquoise	6916	KYNG_ENVIRONMENTAL_STRESS_RESPONSE_DN	9	19	16234	4169	1.846675	0.033601
turquoise	7547	WANG_RECURRENT_LIVER_CANCER_UP	9	19	16234	4169	1.846675	0.033601
lightcyan	9086	GSE17721_LPS_VS_GARDIQUIMOD_12H_BMDM_UP	3	185	16068	62	4.251003	0.033627
lightcyan	9765	GSE3337_4H_VS_16H_IFNG_IN_CD8POS_DC_DN	3	185	16068	62	4.251003	0.033627
lightcyan	9881	GSE360_T_GONDII_VS_B_MALAYI_LOW_DOSE_MAC_D	3	185	16068	62	4.251003	0.033627
blue	4006	PID_MTOR_4PATHWAY	19	68	16185	2978	1.524943	0.033636

salmon	7937	BRUINS_UVC_RESPONSE_LATE	14	1045	15208	127	1.714516	0.033637
purple	6160	FRASOR_RESPONSE_TO_ESTRADIOL_DN	3	73	16180	157	4.254341	0.033644
yellow	64	ORGANELLE_LUMEN	50	439	15814	1426	1.298134	0.033669
yellow	146	MEMBRANE_ENCLOSED_LUMEN	50	439	15814	1426	1.298134	0.033669
yellow	1538	ACTAYRNNNCCCR_UNKNOWN	50	439	15814	1426	1.298134	0.033669
green	2693	GNF2_RTN1	7	39	16214	1305	2.235406	0.033672
green	5861	SCHAEFFER_PROSTATE_DEVELOPMENT_12HR_DN	7	39	16214	1305	2.235406	0.033672
turquoise	628	REGULATION_OF_ORGANELLE_ORGANIZATION_AND_B	15	37	16216	4169	1.580488	0.033677
turquoise	3054	BIOCARTA_CHREBP2_PATHWAY	15	37	16216	4169	1.580488	0.033677
turquoise	4391	REACTOME_PLC_BETA_MEDIATED_EVENTS	15	37	16216	4169	1.580488	0.033677
turquoise	4601	REACTOME_IL1_SIGNALING	15	37	16216	4169	1.580488	0.033677
yellow	3394	chr1q22	11	68	16185	1426	1.843732	0.033679
yellow	7737	YAO_TEMPORAL_RESPONSE_TO_PROGESTERONE_CLUS	11	68	16185	1426	1.843732	0.033679
green	3511	MODULE_19	30	264	15989	1305	1.415273	0.033693
brown	2146	ACCAATC,MIR-509	8	29	16224	2190	2.0473	0.03371
brown	2912	BIOCARTA_AGR_PATHWAY	8	29	16224	2190	2.0473	0.03371
brown	4117	PID_HNF3APATHWAY	8	29	16224	2190	2.0473	0.03371
brown	4219	REACTOME_ANTIGEN_ACTIVATES_B_CELL_RECEPTOR_	8	29	16224	2190	2.0473	0.03371
black	9831	GSE360_L_DONOVANI_VS_L_MAJOR_DC_DN	7	175	16078	283	2.297244	0.033753
cyan	1455	AAANWWTGC_UNKNOWN	3	149	16104	77	4.249891	0.033754
pink	5175	ENK_UV_RESPONSE_KERATINOCYTE_UP	12	470	15783	228	1.820045	0.033756
salmon	3579	MODULE_91	2	37	16216	127	6.917642	0.033765
salmon	5453	LI_LUNG_CANCER	2	37	16216	127	6.917642	0.033765
salmon	6153	ZUCCHI_METASTASIS_UP	2	37	16216	127	6.917642	0.033765
salmon	6470	SESTO_RESPONSE_TO_UV_C6	2	37	16216	127	6.917642	0.033765
salmon	7366	VANTVEER_BREAST_CANCER_BRCA1_DN	2	37	16216	127	6.917642	0.033765
salmon	8017	TERAO_AOX4_TARGETS_SKIN_UP	2	37	16216	127	6.917642	0.033765
red	1715	V$AR_Q2	7	95	16158	524	2.285476	0.033774
red	2025	GCTTGAA,MIR-498	7	95	16158	524	2.285476	0.033774
red	6344	MENSE_HYPOXIA_UP	7	95	16158	524	2.285476	0.033774
red	6856	GAVIN_FOXP3_TARGETS_CLUSTER_P4	7	95	16158	524	2.285476	0.033774
purple	7353	SCHRAETS_MLL_TARGETS_UP	2	30	16223	157	6.901486	0.033799
lightcyan	361	DETECTION_OF_STIMULUS_INVOLVED_IN_SENSORY_PI	1	9	16244	62	29.12724	0.033821
lightcyan	4647	REACTOME_TRAF6_MEDIATED_IRF7_ACTIVATION_IN_	1	9	16244	62	29.12724	0.033821
lightcyan	5550	XU_HGF_TARGETS_REPRESSED_BY_AKT1_UP	1	9	16244	62	29.12724	0.033821
lightcyan	5613	SASAKI_TARGETS_OF_TP73_AND_TP63	1	9	16244	62	29.12724	0.033821
lightcyan	5634	OXFORD_RALB_TARGETS_DN	1	9	16244	62	29.12724	0.033821
lightcyan	6124	IIZUKA_LIVER_CANCER_PROGRESSION_G1_G2_UP	1	9	16244	62	29.12724	0.033821
lightcyan	6369	IGLESIAS_E2F_TARGETS_DN	1	9	16244	62	29.12724	0.033821
lightcyan	7746	YIH_RESPONSE_TO_ARSENITE_C5	1	9	16244	62	29.12724	0.033821
purple	8891	GSE17721_CTRL_VS_CPG_8H_BMDM_DN	5	185	16068	157	2.7979	0.033825
purple	9107	GSE17721_0.5H_VS_8H_POLYIC_BMDM_DN	5	185	16068	157	2.7979	0.033825
purple	10028	GSE3982_DC_VS_TH2_UP	5	185	16068	157	2.7979	0.033825
red	4902	PUIFFE_INVASION_INHIBITED_BY_ASCITES_UP	6	75	16178	524	2.481374	0.033837
red	5168	KOKKINAKIS_METHIONINE_DEPRIVATION_96HR_DN	6	75	16178	524	2.481374	0.033837
red	62	CYTOPLASM	77	1950	14303	524	1.224781	0.033848
brown	1275	PHOSPHOINOSITIDE_BINDING	6	19	16234	2190	2.343619	0.033856
brown	5282	WANG_BARRETTS_ESOPHAGUS_DN	6	19	16234	2190	2.343619	0.033856
cyan	3595	MODULE_107	2	61	16192	77	6.920588	0.033865
cyan	5006	GARGALOVIC_RESPONSE_TO_OXIDIZED_PHOSPHOLIPID	2	61	16192	77	6.920588	0.033865
cyan	5879	RICKMAN_HEAD_AND_NECK_CANCER_A	2	61	16192	77	6.920588	0.033865
cyan	6098	JECHLINGER_EPITHELIAL_TO_MESENCHYMAL_TRANSIT	2	61	16192	77	6.920588	0.033865
cyan	6775	WESTON_VEGFA_TARGETS_3HR	2	61	16192	77	6.920588	0.033865
lightgreen	6157	VANTVEER_BREAST_CANCER_METASTASIS_DN	2	112	16141	42	6.910289	0.03388
turquoise	6231	BHATTACHARYA_EMBRYONIC_STEM_CELL	28	79	16174	4169	1.38176	0.033893
purple	6304	LENAOUR_DENDRITIC_CELL_MATURATION_DN	4	126	16127	157	3.286422	0.033894
brown	8688	GSE1460_INTRATHYMIC_T_PROGENITOR_VS_NAIVE_CI	36	197	16056	2190	1.356206	0.033898
brown	9615	GSE27786_ERYTHROBLAST_VS_MONO_MAC_DN	36	197	16056	2190	1.356206	0.033898
brown	4026	PID_P75NTRPATHWAY	14	62	16191	2190	1.675814	0.0339
brown	7550	WOO_LIVER_CANCER_RECURRENCE_DN	14	62	16191	2190	1.675814	0.0339

red	2039	ATAACCT,MIR-154	5	56	16197	524	2.769391	0.033932
red	6842	LEIN_NEURON_MARKERS	5	56	16197	524	2.769391	0.033932
lightyellow	10219	GSE8515_IL1_VS_IL6_4H_STIM_)MAC_DN	2	143	16110	33	6.888324	0.033973
lightyellow	751	DIGESTION	1	17	16236	33	28.97148	0.033978
lightyellow	995	STEROID_BIOSYNTHETIC_PROCESS	1	17	16236	33	28.97148	0.033978
lightyellow	4725	REACTOME_G1_S_SPECIFIC_TRANSCRIPTION	1	17	16236	33	28.97148	0.033978
lightyellow	5535	LI_CISPLATIN_RESISTANCE_UP	1	17	16236	33	28.97148	0.033978
lightyellow	6029	DING_LUNG_CANCER_MUTATED_SIGNIFICANTLY	1	17	16236	33	28.97148	0.033978
lightyellow	8004	TORCHIA_TARGETS_OF_EWSR1_FLI1_FUSION_TOP20_U	1	17	16236	33	28.97148	0.033978
yellow	9106	GSE17721_0.5H_VS_8H_POLYIC_BMDM_UP	24	185	16068	1426	1.47861	0.033981
greenyello	4970	VECCHI_GASTRIC_CANCER_ADVANCED_VS_EARLY_UP	4	132	16121	150	3.283434	0.033996
greenyello	8247	E2F3_UP.V1_DN	4	132	16121	150	3.283434	0.033996
blue	8768	GSE15750_WT_VS_TRAF6KO_DAY10_EFF_CD8_TCELL_	45	189	16064	2978	1.29945	0.034016
blue	8825	GSE17580_TREG_VS_TEFF_DN	45	189	16064	2978	1.29945	0.034016
blue	9028	GSE17721_PAM3CSK4_VS_GADIQUIMOD_6H_BMDM_	45	189	16064	2978	1.29945	0.034016
blue	10079	GSE3982_BASOPHIL_VS_TH1_DN	45	189	16064	2978	1.29945	0.034016
pink	8376	KRAS.LUNG.BREAST_UP.V1_DN	4	87	16166	228	3.277475	0.034026
red	9509	GSE2706_UNSTIM_VS_8H_LPS_AND_R848_DC_DN	11	182	16071	524	1.874664	0.034029
red	853	CHEMICAL_HOMEOSTASIS	8	116	16137	524	2.139116	0.034037
red	2175	RTTTNNNYTGGM_UNKNOWN	8	116	16137	524	2.139116	0.034037
red	7093	IZADPANAH_STEM_CELL_ADIPOSE_VS_BONE_UP	8	116	16137	524	2.139116	0.034037
red	8312	NRL_DN.V1_UP	8	116	16137	524	2.139116	0.034037
salmon	5809	WEI_MYCN_TARGETS_WITH_E_BOX	11	754	15499	127	1.867029	0.034043
greenyello	9413	GSE24142_DN2_VS_DN3_THYMOCYTE_DN	5	194	16059	150	2.792612	0.034052
blue	1483	AACYNNNNTTCCS_UNKNOWN	23	86	16167	2978	1.459615	0.034074
magenta	3512	MODULE_20	3	69	16184	167	4.23145	0.03408
magenta	6982	CAMPS_COLON_CANCER_COPY_NUMBER_UP	3	69	16184	167	4.23145	0.03408
blue	6845	LEIN_CHOROID_PLEXUS_MARKERS	21	77	16176	2978	1.488461	0.034085
lightcyan	8651	GSE14308_TH17_VS_NATURAL_TREG_DN	3	186	16067	62	4.228148	0.034087
lightcyan	9133	GSE17721_0.5H_VS_24H_CPG_BMDM_DN	3	186	16067	62	4.228148	0.034087
lightcyan	9143	GSE17721_0.5H_VS_8H_GARDIQUIMOD_BMDM_DN	3	186	16067	62	4.228148	0.034087
lightcyan	9811	GSE360_CTRL_VS_B_MALAYI_HIGH_DOSE_MAC_DN	3	186	16067	62	4.228148	0.034087
brown	9891	GSE36392_TYPE_2_MYELOID_VS_EOSINOPHIL_IL25_TR	33	178	16075	2190	1.375889	0.034115
turquoise	2132	AACTGGA,MIR-145	66	210	16043	4169	1.225254	0.034122
turquoise	2457	MORF_RAC1	66	210	16043	4169	1.225254	0.034122
red	5608	AMUNDSON_RESPONSE_TO_ARSENITE	12	205	16048	524	1.81564	0.034146
lightgreen	4844	SENGUPTA_NASOPHARYNGEAL_CARCINOMA_UP	3	276	15977	42	4.206263	0.034188
green	1587	V$AP1_Q4	27	233	16020	1305	1.443214	0.034235
green	5940	AMIT_SERUM_RESPONSE_40_MCF10A	6	31	16222	1305	2.41053	0.034319
cyan	8697	GSE1460_DP_THYMOCYTE_VS_NAIVE_CD4_TCELL_COR	3	150	16103	77	4.221558	0.034329
greenyello	1318	RECEPTOR_SIGNALING_PROTEIN_ACTIVITY	3	77	16176	150	4.221558	0.034329
brown	1464	V$SP1_01	38	210	16043	2190	1.342931	0.034335
turquoise	1980	ACTACCT,MIR-196A,MIR-196B	41	123	16130	4169	1.299512	0.034342
turquoise	6556	BURTON_ADIPOGENESIS_5	41	123	16130	4169	1.299512	0.034342
tan	7820	WHITFIELD_CELL_CYCLE_G1_S	4	137	16116	145	3.272691	0.034348
turquoise	8985	GSE17721_LPS_VS_PAM3CSK4_0.5H_BMDM_DN	52	161	16092	4169	1.259155	0.034376
turquoise	2127	ACTGCCT,MIR-34B	62	196	16057	4169	1.233211	0.034419
turquoise	8791	GSE15930_NAIVE_VS_72H_IN_VITRO_STIM_IL12_CD8_	62	196	16057	4169	1.233211	0.034419
turquoise	9252	GSE19825_CD24LOW_VS_IL2RA_HIGH_DAY3_EFF_CD8	62	196	16057	4169	1.233211	0.034419
grey60	3511	MODULE_19	3	264	15989	44	4.197572	0.03442
midnightb	1426	TRANSITION_METAL_ION_TRANSMEMBRANE_TRANSP(	1	8	16245	71	28.61444	0.034425
midnightb	7183	CHEN_HOXA5_TARGETS_6HR_UP	1	8	16245	71	28.61444	0.034425
brown	346	NEGATIVE_REGULATION_OF_CELLULAR_BIOSYNTHETIC	7	24	16229	2190	2.164593	0.034428
brown	3028	BIOCARTA_NTHI_PATHWAY	7	24	16229	2190	2.164593	0.034428
brown	3452	SA_B_CELL_RECEPTOR_COMPLEXES	7	24	16229	2190	2.164593	0.034428
brown	4039	PID_INTEGRIN2_PATHWAY	7	24	16229	2190	2.164593	0.034428
brown	4356	REACTOME_AMINO_ACID_TRANSPORT_ACROSS_THE_I	7	24	16229	2190	2.164593	0.034428
brown	7584	OKAMOTO_LIVER_CANCER_MULTICENTRIC_OCCURREN	7	24	16229	2190	2.164593	0.034428
blue	564	REGULATION_OF_TRANSCRIPTION_FACTOR_ACTIVITY	12	38	16215	2978	1.723481	0.034428
blue	7589	ONO_AML1_TARGETS_DN	12	38	16215	2978	1.723481	0.034428

lightgreen	6315	GERY_CEBP_TARGETS	2	113	16140	42	6.849136	0.034435
brown	8059	WAKABAYASHI_ADIPOGENESIS_PPARG_RXRA_BOUND_	129	823	15430	2190	1.163267	0.03444
green	6514	BROWNE_HCMV_INFECTION_8HR_UP	13	93	16160	1305	1.740938	0.03445
turquoise	3944	PID_HDAC_CLASSII_PATHWAY	14	34	16219	4169	1.60528	0.034459
red	1972	CAGTGTT,MIR-141,MIR-200A	15	276	15977	524	1.685716	0.034462
green	897	POLYSACCHARIDE_METABOLIC_PROCESS	4	16	16237	1305	3.113602	0.034492
green	2924	BIOCARTA_BCELLSURVIVAL_PATHWAY	4	16	16237	1305	3.113602	0.034492
green	4620	REACTOME_PROSTACYCLIN_SIGNALLING_THROUGH_PI	4	16	16237	1305	3.113602	0.034492
green	7975	WANG_NFKB_TARGETS	4	16	16237	1305	3.113602	0.034492
yellow	3634	MODULE_149	7	36	16217	1426	2.216203	0.034494
purple	1585	V$SRF_Q6	5	186	16067	157	2.782857	0.034499
purple	1925	V$MEF2_Q6_01	5	186	16067	157	2.782857	0.034499
purple	9824	GSE360_DC_VS_MAC_B_MALAYI_HIGH_DOSE_UP	5	186	16067	157	2.782857	0.034499
purple	9964	GSE3982_DC_VS_MAC_LPS_STIM_UP	5	186	16067	157	2.782857	0.034499
greenyello	3535	MODULE_46	7	332	15921	150	2.284558	0.034512
cyan	6936	HELLER_HDAC_TARGETS_SILENCED_BY_METHYLATION_	5	381	15872	77	2.770051	0.034514
grey60	2828	KEGG_AXON_GUIDANCE	2	108	16145	44	6.840488	0.03453
grey60	9238	GSE18791_UNSTIM_VS_NEWCATSLE_VIRUS_DC_1H_UI	2	108	16145	44	6.840488	0.03453
pink	543	REGULATION_OF_NUCLEOCYTOPLASMIC_TRANSPORT	2	21	16232	228	6.789056	0.034536
pink	925	COFACTOR_BIOSYNTHETIC_PROCESS	2	21	16232	228	6.789056	0.034536
pink	3233	chr4q28	2	21	16232	228	6.789056	0.034536
pink	5594	DACOSTA_ERCC3_ALLELE_XPCS_VS_TTD_UP	2	21	16232	228	6.789056	0.034536
pink	6024	JI_METASTASIS_REPRESSED_BY_STK11	2	21	16232	228	6.789056	0.034536
pink	6108	IIZUKA_LIVER_CANCER_PROGRESSION_G2_G3_UP	2	21	16232	228	6.789056	0.034536
pink	7097	MISHRA_CARCINOMA_ASSOCIATED_FIBROBLAST_DN	2	21	16232	228	6.789056	0.034536
pink	7295	SEKI_INFLAMMATORY_RESPONSE_LPS_DN	2	21	16232	228	6.789056	0.034536
red	725	TRANSPORT	31	682	15571	524	1.409872	0.034545
lightcyan	8931	GSE17721_POLYIC_VS_PAM3CSK4_0.5H_BMDM_DN	3	187	16066	62	4.205537	0.034551
lightcyan	9171	GSE17974_CTRL_VS_ACT_IL4_AND_ANTI_IL12_1H_CD4	3	187	16066	62	4.205537	0.034551
lightcyan	9591	GSE27786_CD8_TCELL_VS_NKTCELL_DN	3	187	16066	62	4.205537	0.034551
blue	6791	DOUGLAS_BMI1_TARGETS_UP	109	506	15747	2978	1.175668	0.034553
turquoise	5152	HAHTOLA_MYCOSIS_FUNGOIDES_SKIN_UP	56	175	16078	4169	1.247532	0.034555
turquoise	9097	GSE17721_0.5H_VS_8H_LPS_BMDM_DN	56	175	16078	4169	1.247532	0.034555
turquoise	8863	GSE17721_CTRL_VS_POLYIC_24H_BMDM_DN	58	182	16071	4169	1.242391	0.034557
turquoise	9299	GSE20715_0H_VS_48H_OZONE_TLR4_KO_LUNG_DN	58	182	16071	4169	1.242391	0.034557
blue	2113	TTTGCAG,MIR-518A-2	46	194	16059	2978	1.294091	0.034569
blue	8484	GSE11924_TFH_VS_TH17_CD4_TCELL_UP	46	194	16059	2978	1.294091	0.034569
blue	8669	GSE1432_6H_VS_24H_IFNG_MICROGLIA_DN	46	194	16059	2978	1.294091	0.034569
blue	9135	GSE17721_4_VS_24H_CPG_BMDM_DN	46	194	16059	2978	1.294091	0.034569
grey60	356	CELL_SURFACE_RECEPTOR_LINKED_SIGNAL_TRANSDUC	4	457	15796	44	3.233141	0.034572
black	4626	REACTOME_DESTABILIZATION_OF_MRNA_BY_BRF1	2	17	16236	283	6.756599	0.034591
black	4641	REACTOME_DESTABILIZATION_OF_MRNA_BY_KSRP	2	17	16236	283	6.756599	0.034591
black	4644	REACTOME_DESTABILIZATION_OF_MRNA_BY_TRISTETI	2	17	16236	283	6.756599	0.034591
black	7776	ZEMBUTSU_SENSITIVITY_TO_DOXORUBICIN	2	17	16236	283	6.756599	0.034591
magenta	3617	MODULE_129	5	175	16078	167	2.780667	0.034604
magenta	4565	REACTOME_GPCR_LIGAND_BINDING	6	235	16018	167	2.484852	0.034611
magenta	7936	BRUINS_UVC_RESPONSE_VIA_TP53_GROUP_D	6	235	16018	167	2.484852	0.034611
brown	1628	V$NGFIC_01	40	223	16030	2190	1.331204	0.034627
blue	5980	MORI_EMU_MYC_LYMPHOMA_BY_ONSET_TIME_UP	28	109	16144	2978	1.401975	0.034636
blue	7506	BOYAULT_LIVER_CANCER_SUBCLASS_G1_UP	28	109	16144	2978	1.401975	0.034636
grey60	2762	KEGG_LINOLEIC_ACID_METABOLISM	1	13	16240	44	28.41434	0.03464
grey60	3103	BIOCARTA_LONGEVITY_PATHWAY	1	13	16240	44	28.41434	0.03464
grey60	4014	PID_IL5_PATHWAY	1	13	16240	44	28.41434	0.03464
grey60	4486	REACTOME_OTHER_SEMAPHORIN_INTERACTIONS	1	13	16240	44	28.41434	0.03464
grey60	6512	KANG_FLUOROURACIL_RESISTANCE_DN	1	13	16240	44	28.41434	0.03464
grey60	7699	DORN_ADENOVIRUS_INFECTION_48HR_UP	1	13	16240	44	28.41434	0.03464
green	6813	YANG_MUC2_TARGETS_COLON_3MO_DN	2	4	16249	1305	6.227203	0.034646
green	7086	WEBER_METHYLATED_HCP_IN_FIBROBLAST_UP	2	4	16249	1305	6.227203	0.034646
pink	6521	NATSUME_RESPONSE_TO_INTERFERON_BETA_DN	3	51	16202	228	4.19324	0.034658
midnightb	7688	DANG_REGULATED_BY_MYC_UP	2	67	16186	71	6.833298	0.034665

green	10102	GSE39820_CTRL_VS_IL1B_IL6_IL23A_CD4_TCELL_UP	23	192	16061	1305	1.491934	0.034667
greenyello	1608	V$TATA_C	5	195	16058	150	2.778291	0.034698
greenyello	1683	V$MMEF2_Q6	5	195	16058	150	2.778291	0.034698
greenyello	1864	V$CEBP_Q3	5	195	16058	150	2.778291	0.034698
greenyello	7143	BOQUEST_STEM_CELL_UP	5	195	16058	150	2.778291	0.034698
greenyello	9213	GSE17974_1.5H_VS_72H_IL4_AND_ANTI_IL12_ACT_CD	5	195	16058	150	2.778291	0.034698
greenyello	9389	GSE22886_CTRL_VS_LPS_24H_DC_DN	5	195	16058	150	2.778291	0.034698
pink	3605	MODULE_117	12	472	15781	228	1.812333	0.034698
yellow	8700	GSE1460_DP_THYMOCYTE_VS_THYMIC_STROMAL_CEL	23	176	16077	1426	1.489461	0.034708
yellow	9058	GSE17721_POLYIC_VS_GARDIQUIMOD_1H_BMDM_UP	23	176	16077	1426	1.489461	0.034708
tan	7263	BOYLAN_MULTIPLE_MYELOMA_D_UP	3	80	16173	145	4.203362	0.034718
brown	270	SYSTEM_DEVELOPMENT	94	583	15670	2190	1.196599	0.034724
red	9219	GSE18791_CTRL_VS_NEWCASTLE_VIRUS_DC_1H_DN	9	138	16115	524	2.022859	0.034737
purple	7866	QI_HYPOXIA	4	127	16126	157	3.260545	0.034742
purple	7868	GREGORY_SYNTHETIC_LETHAL_WITH_IMATINIB	4	127	16126	157	3.260545	0.034742
brown	8389	KAECH_NAIVE_VS_DAY15_EFF_CD8_TCELL_DN	35	191	16062	2190	1.359954	0.034787
brown	8549	GSE13411_NAIVE_VS_SWITCHED_MEMORY_BCELL_DN	35	191	16062	2190	1.359954	0.034787
brown	8828	GSE17580_UNINFECTED_VS_S_MANSONI_INF_TEFF_UI	35	191	16062	2190	1.359954	0.034787
brown	8831	GSE17580_UNINFECTED_VS_S_MANSONI_INF_TREG_D	35	191	16062	2190	1.359954	0.034787
brown	9065	GSE17721_POLYIC_VS_GARDIQUIMOD_6H_BMDM_DN	35	191	16062	2190	1.359954	0.034787
yellow	305	REGULATION_OF_PROTEIN_KINASE_ACTIVITY	19	139	16114	1426	1.557947	0.034796
greenyello	2122	CCCAGAG,MIR-326	4	133	16120	150	3.258747	0.034807
greenyello	3804	MODULE_361	4	133	16120	150	3.258747	0.034807
purple	2035	CAGGGTC,MIR-504	3	74	16179	157	4.19685	0.034825
turquoise	6011	NIKOLSKY_BREAST_CANCER_19Q13.1_AMPLICON	10	22	16231	4169	1.772062	0.034834
lightyellow	7580	MIKKELSEN_IPS_LCP_WITH_H3K4ME3	2	145	16108	33	6.793312	0.034846
tan	3392	chr6q24	2	33	16220	145	6.793312	0.034846
tan	6485	VARELA_ZMPSTE24_TARGETS_DN	2	33	16220	145	6.793312	0.034846
brown	2878	KEGG_LEISHMANIA_INFECTION	15	68	16185	2190	1.637087	0.034865
brown	3980	PID_CDC42_PATHWAY	15	68	16185	2190	1.637087	0.034865
lightcyan	6287	HESS_TARGETS_OF_HOXA9_AND_MEIS1_DN	2	77	16176	62	6.808965	0.034888
cyan	2451	MORF_PTPRR	2	62	16191	77	6.808965	0.034888
cyan	5154	HAHTOLA_MYCOSIS_FUNGOIDES_CD4_UP	2	62	16191	77	6.808965	0.034888
cyan	5400	DACOSTA_UV_RESPONSE_VIA_ERCC3_TTD_UP	2	62	16191	77	6.808965	0.034888
cyan	7550	WOO_LIVER_CANCER_RECURRENCE_DN	2	62	16191	77	6.808965	0.034888
cyan	5039	MULLIGHAN_MLL_SIGNATURE_2_DN	4	260	15993	77	3.247353	0.03492
lightgreen	7933	BRUINS_UVC_RESPONSE_VIA_TP53_GROUP_A	5	709	15544	42	2.729028	0.034965
blue	1	NUCLEOPLASM	61	267	15986	2978	1.246888	0.034984
midnightb	8604	GSE13738_RESTING_VS_BYSTANDER_ACTIVATED_CD4_	3	164	16089	71	4.187479	0.035003
midnightb	8849	GSE17721_CTRL_VS_POLYIC_0.5H_BMDM_DN	3	164	16089	71	4.187479	0.035003
lightcyan	8642	GSE14308_TH1_VS_INDUCED_TREG_UP	3	188	16065	62	4.183167	0.035019
lightcyan	8836	GSE17721_CTRL_VS_LPS_2H_BMDM_UP	3	188	16065	62	4.183167	0.035019
lightcyan	8929	GSE17721_LPS_VS_POLYIC_24H_BMDM_DN	3	188	16065	62	4.183167	0.035019
lightcyan	9064	GSE17721_POLYIC_VS_GARDIQUIMOD_6H_BMDM_UP	3	188	16065	62	4.183167	0.035019
lightcyan	9070	GSE17721_POLYIC_VS_GARDIQUIMOD_16H_BMDM_U	3	188	16065	62	4.183167	0.035019
lightcyan	9177	GSE17974_CTRL_VS_ACT_IL4_AND_ANTI_IL12_6H_CD4	3	188	16065	62	4.183167	0.035019
lightcyan	9819	GSE360_DC_VS_MAC_L_DONOVANI_DN	3	188	16065	62	4.183167	0.035019
greenyello	2657	GNF2_JAK1	2	32	16221	150	6.772083	0.035028
greenyello	2897	KEGG_ALLOGRAFT_REJECTION	2	32	16221	150	6.772083	0.035028
greenyello	5648	DAWSON_METHYLATED_IN_LYMPHOMA_TCL1	2	32	16221	150	6.772083	0.035028
turquoise	4118	PID_TGFBRPATHWAY	20	53	16200	4169	1.471146	0.035047
yellow	2349	MORF_ANP32B	25	195	16058	1426	1.461233	0.035058
yellow	9213	GSE17974_1.5H_VS_72H_IL4_AND_ANTI_IL12_ACT_CD	25	195	16058	1426	1.461233	0.035058
yellow	9224	GSE18791_CTRL_VS_NEWCASTLE_VIRUS_DC_6H_UP	25	195	16058	1426	1.461233	0.035058
yellow	9996	GSE3982_MAST_CELL_VS_NEUTROPHIL_UP	25	195	16058	1426	1.461233	0.035058
blue	9279	GSE20366_TREG_VS_NAIVE_CD4_TCELL_DN	42	175	16078	2978	1.309846	0.035062
purple	7943	MIYAGAWA_TARGETS_OF_EWSR1_ETS_FUSIONS_DN	5	187	16066	157	2.767976	0.035181
purple	9522	GSE2706_2H_VS_8H_R848_STIM_DC_UP	5	187	16066	157	2.767976	0.035181
cyan	5183	DELYS_THYROID_CANCER_UP	5	383	15870	77	2.755586	0.035181
blue	8578	GSE13485_CTRL_VS_DAY7_YF17D_VACCINE_PBMC_UP	38	156	16097	2978	1.329437	0.035189

brown	542	LEUKOCYTE_CHEMOTAXIS	4	10	16243	2190	2.968584	0.035201
brown	721	POSITIVE_REGULATION_OF_PROTEIN_SECRETION	4	10	16243	2190	2.968584	0.035201
brown	4272	REACTOME_REVERSIBLE_HYDRATION_OF_CARBON_DI(	4	10	16243	2190	2.968584	0.035201
brown	4621	REACTOME_GABA_SYNTHESIS_RELEASE_REUPTAKE_AN	4	10	16243	2190	2.968584	0.035201
brown	4672	REACTOME_IL_6_SIGNALING	4	10	16243	2190	2.968584	0.035201
brown	6043	ROZANOV_MMP14_CORRELATED	4	10	16243	2190	2.968584	0.035201
brown	7757	RAMPON_ENRICHED_LEARNING_ENVIRONMENT_EARL	4	10	16243	2190	2.968584	0.035201
brown	7772	VISALA_AGING_LYMPHOCYTE_UP	4	10	16243	2190	2.968584	0.035201
brown	8165	LIM_MAMMARY_LUMINAL_PROGENITOR_DN	4	10	16243	2190	2.968584	0.035201
red	1738	V$FOXO1_01	11	183	16070	524	1.86442	0.035202
red	8922	GSE17721_LPS_VS_POLYIC_8H_BMDM_UP	11	183	16070	524	1.86442	0.035202
red	9501	GSE2706_UNSTIM_VS_8H_R848_DC_DN	11	183	16070	524	1.86442	0.035202
red	9668	GSE29618_BCELL_VS_PDC_DAY7_FLU_VACCINE_UP	11	183	16070	524	1.86442	0.035202
tan	3196	chr19p	1	4	16249	145	28.02241	0.035214
red	7237	WANG_TUMOR_INVASIVENESS_DN	12	206	16047	524	1.806826	0.035247
brown	7958	LINSLEY_MIR16_TARGETS	37	204	16049	2190	1.346049	0.035278
lightyellow	6567	BROWNE_HCMV_INFECTION_14HR_UP	2	146	16107	33	6.746783	0.035285
blue	2920	BIOCARTA_CHEMICAL_PATHWAY	8	22	16231	2978	1.984614	0.035296
blue	6222	FLECHNER_BIOPSY_KIDNEY_TRANSPLANT_OK_VS_DON	8	22	16231	2978	1.984614	0.035296
green	3600	MODULE_112	21	172	16081	1305	1.520596	0.035316
magenta	1735	V$TBP_01	5	176	16077	167	2.764868	0.035331
magenta	6972	OUILLETTE_CLL_13Q14_DELETION_UP	3	70	16183	167	4.171001	0.035345
magenta	7294	SEKI_INFLAMMATORY_RESPONSE_LPS_UP	3	70	16183	167	4.171001	0.035345
pink	3675	MODULE_195	5	129	16124	228	2.762988	0.035349
greenyello	10192	GSE7852_TREG_VS_TCONV_LN_UP	5	196	16057	150	2.764116	0.035351
pink	8963	GSE17721_PAM3CSK4_VS_CPG_16H_BMDM_DN	6	173	16080	228	2.472315	0.035353
turquoise	469	SPHINGOLIPID_METABOLIC_PROCESS	11	25	16228	4169	1.715356	0.035365
turquoise	2665	GNF2_MBD4	11	25	16228	4169	1.715356	0.035365
turquoise	4669	REACTOME_ENDOSOMAL_SORTING_COMPLEX_REQUII	11	25	16228	4169	1.715356	0.035365
turquoise	7449	CHEOK_RESPONSE_TO_MERCAPTOPURINE_AND_HD_N	11	25	16228	4169	1.715356	0.035365
blue	233	NUCLEAR_CHROMOSOME_PART	11	34	16219	2978	1.765723	0.035369
blue	2768	KEGG_PYRUVATE_METABOLISM	11	34	16219	2978	1.765723	0.035369
blue	3983	PID_ATM_PATHWAY	11	34	16219	2978	1.765723	0.035369
blue	4474	REACTOME_MRNA_3_END_PROCESSING	11	34	16219	2978	1.765723	0.035369
blue	4547	REACTOME_GLUCOSE_TRANSPORT	11	34	16219	2978	1.765723	0.035369
yellow	8679	GSE1448_CTRL_VS_ANTI_VALPHA2_DP_THYMOCYTE_D	22	167	16086	1426	1.501482	0.035395
blue	4914	DIAZ_CHRONIC_MEYLOGENOUS_LEUKEMIA_UP	278	1378	14875	2978	1.101043	0.035411
salmon	7992	BILANGES_RAPAMYCIN_SENSITIVE_GENES	2	38	16215	127	6.735599	0.035462
greenyello	2804	KEGG_ERBB_SIGNALING_PATHWAY	3	78	16175	150	4.167436	0.035467
greenyello	5949	LIN_SILENCED_BY_TUMOR_MICROENVIRONMENT	3	78	16175	150	4.167436	0.035467
greenyello	6182	SWEET_KRAS_TARGETS_UP	3	78	16175	150	4.167436	0.035467
lightcyan	8514	GSE12845_IGD_NEG_BLOOD_VS_PRE_GC_TONSIL_BCE	3	189	16064	62	4.161034	0.035489
lightcyan	8717	GSE14769_UNSTIM_VS_40MIN_LPS_BMDM_DN	3	189	16064	62	4.161034	0.035489
yellow	6218	PENG_RAPAMYCIN_RESPONSE_DN	30	243	16010	1426	1.407113	0.035503
yellow	3476	ST_T_CELL_SIGNAL_TRANSDUCTION	8	44	16209	1426	2.072294	0.03553
yellow	7800	WHITFIELD_CELL_CYCLE_LITERATURE	8	44	16209	1426	2.072294	0.03553
brown	2847	KEGG_B_CELL_RECEPTOR_SIGNALING_PATHWAY	16	74	16179	2190	1.60464	0.035535
brown	5433	HUMMERICH_SKIN_CANCER_PROGRESSION_UP	16	74	16179	2190	1.60464	0.035535
magenta	882	REGULATION_OF_LYMPHOCYTE_ACTIVATION	2	29	16224	167	6.711955	0.03554
magenta	7677	CHIARETTI_T_ALL_REFRACTORY_TO_THERAPY	2	29	16224	167	6.711955	0.03554
midnightb	4933	DOANE_RESPONSE_TO_ANDROGEN_UP	3	165	16088	71	4.1621	0.035542
midnightb	5392	EBAUER_TARGETS_OF_PAX3_FOXO1_FUSION_UP	3	165	16088	71	4.1621	0.035542
brown	4629	REACTOME_CIRCADIAN_CLOCK	12	51	16202	2190	1.746226	0.035547
brown	5057	PAPASPYRIDONOS_UNSTABLE_ATEROSCLEROTIC_PLAQ	12	51	16202	2190	1.746226	0.035547
brown	6574	ZHANG_PROLIFERATING_VS_QUIESCENT	12	51	16202	2190	1.746226	0.035547
brown	6880	RIGGINS_TAMOXIFEN_RESISTANCE_UP	12	51	16202	2190	1.746226	0.035547
red	7277	TOOKER_GEMCITABINE_RESISTANCE_DN	8	117	16136	524	2.120833	0.035551
lightgreen	8242	RELA_DN.V1_UP	2	115	16138	42	6.730021	0.035556
lightgreen	8295	BMI1_DN_MEL18_DN.V1_DN	2	115	16138	42	6.730021	0.035556
lightgreen	900	NEGATIVE_REGULATION_OF_TRANSCRIPTION_FACTOR	1	14	16239	42	27.64116	0.03559

lightgreen	1404	AMINE_BINDING	1	14	16239	42	27.64116	0.03559
lightgreen	4428	REACTOME_AMINE_LIGAND_BINDING_RECEPTORS	1	14	16239	42	27.64116	0.03559
lightgreen	4679	REACTOME_SYNTHESIS_OF_VERY_LONG_CHAIN_FATTY	1	14	16239	42	27.64116	0.03559
lightgreen	5116	MOROSETTI_FACIOSCAPULOHUMERAL_MUSCULAR_DI!	1	14	16239	42	27.64116	0.03559
lightgreen	6460	ZHENG_RESPONSE_TO_ARSENITE_DN	1	14	16239	42	27.64116	0.03559
lightgreen	6942	AMUNDSON_GAMMA_RADIATION_RESISTANCE	1	14	16239	42	27.64116	0.03559
lightgreen	7321	PARK_APL_PATHOGENESIS_UP	1	14	16239	42	27.64116	0.03559
purple	8552	GSE13411_NAIVE_VS_MEMORY_BCELL_UP	4	128	16125	157	3.235072	0.035602
brown	5498	MYLLYKANGAS_AMPLIFICATION_HOT_SPOT_13	3	6	16247	2190	3.710731	0.035605
brown	5754	OHM_EMBRYONIC_CARCINOMA_UP	3	6	16247	2190	3.710731	0.035605
brown	7198	THUM_MIR21_TARGETS_HEART_DISEASE_DN	3	6	16247	2190	3.710731	0.035605
brown	7541	YAMASHITA_LIVER_CANCER_WITH_EPCAM_DN	3	6	16247	2190	3.710731	0.035605
brown	2261	GGGAGGRR_V$MAZ_Q6	279	1879	14374	2190	1.101963	0.035614
greenyello	8441	GSE10463_CD40L_AND_VA347_VS_CD40L_IN_DC_DN	4	134	16119	150	3.234428	0.03563
brown	5570	PATIL_LIVER_CANCER	111	700	15553	2190	1.176832	0.035644
brown	8938	GSE17721_POLYIC_VS_PAM3CSK4_6H_BMDM_UP	34	185	16068	2190	1.363944	0.035689
turquoise	3802	MODULE_358	27	76	16177	4169	1.385007	0.035701
turquoise	6155	NELSON_RESPONSE_TO_ANDROGEN_UP	27	76	16177	4169	1.385007	0.035701
grey60	735	CELLULAR_HOMEOSTASIS	2	110	16143	44	6.716116	0.035706
blue	9498	GSE2706_UNSTIM_VS_2H_R848_DC_UP	43	180	16073	2978	1.303781	0.035707
turquoise	1501	V$GATA2_01	30	86	16167	4169	1.359955	0.035711
turquoise	7152	LABBE_TARGETS_OF_TGFB1_AND_WNT3A_DN	30	86	16167	4169	1.359955	0.035711
lightcyan	6630	LEE_AGING_CEREBELLUM_UP	2	78	16175	62	6.721671	0.03572
lightcyan	7150	LABBE_TGFB1_TARGETS_DN	2	78	16175	62	6.721671	0.03572
lightyellow	8760	GSE15659_RESTING_VS_ACTIVATED_TREG_UP	2	147	16106	33	6.700886	0.035727
lightyellow	9920	GSE36476_YOUNG_VS_OLD_DONOR_MEMORY_CD4_T	2	147	16106	33	6.700886	0.035727
lightyellow	10217	GSE8515_CTRL_VS_IL6_4H_STIM_MAC_DN	2	147	16106	33	6.700886	0.035727
blue	1804	V$PEA3_Q6	55	238	16015	2978	1.261231	0.035747
purple	4805	NAKAMURA_TUMOR_ZONE_PERIPHERAL_VS_CENTRAL	6	252	16001	157	2.464816	0.035762
blue	8024	GOBERT_OLIGODENDROCYTE_DIFFERENTIATION_DN	202	983	15270	2978	1.121519	0.035766
blue	2918	BIOCARTA_ASBCELL_PATHWAY	5	11	16242	2978	2.480768	0.035772
blue	2954	BIOCARTA_CYTOKINE_PATHWAY	5	11	16242	2978	2.480768	0.035772
blue	4188	REACTOME_DOWNREGULATION_OF_ERBB2_ERBB3_SI	5	11	16242	2978	2.480768	0.035772
blue	4459	REACTOME_ACTIVATION_OF_CHAPERONES_BY_ATF6_	5	11	16242	2978	2.480768	0.035772
blue	4714	REACTOME_UNWINDING_OF_DNA	5	11	16242	2978	2.480768	0.035772
blue	5508	MATTIOLI_MULTIPLE_MYELOMA_SUBGROUPS	5	11	16242	2978	2.480768	0.035772
blue	5921	PETRETTO_BLOOD_PRESSURE_UP	5	11	16242	2978	2.480768	0.035772
blue	6080	IIZUKA_LIVER_CANCER_PROGRESSION_L1_G1_DN	5	11	16242	2978	2.480768	0.035772
blue	6263	ABBUD_LIF_SIGNALING_2_UP	5	11	16242	2978	2.480768	0.035772
blue	6684	KALMA_E2F1_TARGETS	5	11	16242	2978	2.480768	0.035772
blue	5988	KAUFFMANN_DNA_REPLICATION_GENES	35	142	16111	2978	1.345205	0.035797
green	4112	PID_FGF_PATHWAY	8	48	16205	1305	2.075734	0.035843
green	6627	BURTON_ADIPOGENESIS_PEAK_AT_2HR	8	48	16205	1305	2.075734	0.035843
purple	85	CYTOSOL	5	188	16065	157	2.753252	0.035871
purple	9412	GSE24142_DN2_VS_DN3_THYMOCYTE_UP	5	188	16065	157	2.753252	0.035871
purple	10196	GSE7852_TREG_VS_TCONV_FAT_UP	5	188	16065	157	2.753252	0.035871
purple	10201	GSE7852_LN_VS_FAT_TREG_DN	5	188	16065	157	2.753252	0.035871
greenyello	293	NERVOUS_SYSTEM_DEVELOPMENT	6	264	15989	150	2.462576	0.035872
turquoise	4907	THUM_SYSTOLIC_HEART_FAILURE_DN	65	207	16046	4169	1.224178	0.035896
brown	6932	HELLER_SILENCED_BY_METHYLATION_UP	43	243	16010	2190	1.313263	0.035896
purple	3065	BIOCARTA_RHO_PATHWAY	2	31	16222	157	6.678858	0.035905
purple	5160	PACHER_TARGETS_OF_IGF1_AND_IGF2_UP	2	31	16222	157	6.678858	0.035905
purple	7138	GRADE_COLON_CANCER_DN	2	31	16222	157	6.678858	0.035905
purple	7195	CONRAD_STEM_CELL	2	31	16222	157	6.678858	0.035905
purple	7338	POTTI_CYTOXAN_SENSITIVITY	2	31	16222	157	6.678858	0.035905
purple	7473	ZHAN_V1_LATE_DIFFERENTIATION_GENES_UP	2	31	16222	157	6.678858	0.035905
red	731	HOMEOSTATIC_PROCESS	10	161	16092	524	1.926533	0.035923
cyan	6414	NATSUME_RESPONSE_TO_INTERFERON_BETA_UP	2	63	16190	77	6.700886	0.035924
cyan	7247	BEIER_GLIOMA_STEM_CELL_DN	2	63	16190	77	6.700886	0.035924
brown	6164	SMITH_LIVER_CANCER	10	40	16213	2190	1.855365	0.035935

purple	2259	CTTTGT_V$LEF1_Q2	23	1608	14645	157	1.480729	0.035939
lightyellow	3070	BIOCARTA_ERK5_PATHWAY	1	18	16235	33	27.36195	0.035942
lightyellow	4234	REACTOME_PROLONGED_ERK_ACTIVATION_EVENTS	1	18	16235	33	27.36195	0.035942
lightyellow	5937	AMIT_EGF_RESPONSE_240_MCF10A	1	18	16235	33	27.36195	0.035942
lightyellow	6046	LANDEMAINE_LUNG_METASTASIS	1	18	16235	33	27.36195	0.035942
lightyellow	6184	MAGRANGEAS_MULTIPLE_MYELOMA_IGG_VS_IGA_UP	1	18	16235	33	27.36195	0.035942
lightyellow	6440	MARCHINI_TRABECTEDIN_RESISTANCE_UP	1	18	16235	33	27.36195	0.035942
blue	6546	DASU_IL6_SIGNALING_SCAR_UP	10	30	16223	2978	1.81923	0.035963
lightcyan	8476	GSE11864_CSF1_IFNG_VS_CSF1_IFNG_PAM3CYS_IN_M	3	190	16063	62	4.139134	0.035963
lightcyan	8497	GSE12366_GC_VS_MEMORY_BCELL_DN	3	190	16063	62	4.139134	0.035963
lightcyan	8607	GSE13738_TCR_VS_BYSTANDER_ACTIVATED_CD4_TCEL	3	190	16063	62	4.139134	0.035963
lightcyan	9035	GSE17721_PAM3CSK4_VS_GADIQUIMOD_16H_BMDM_	3	190	16063	62	4.139134	0.035963
lightcyan	9109	GSE17721_0.5H_VS_24H_POLYIC_BMDM_DN	3	190	16063	62	4.139134	0.035963
lightcyan	9260	GSE20366_EX_VIVO_VS_HOMEOSTATIC_CONVERSION_	3	190	16063	62	4.139134	0.035963
lightcyan	9430	GSE24142_ADULT_VS_FETAL_DN3_THYMOCYTE_UP	3	190	16063	62	4.139134	0.035963
lightcyan	10054	GSE3982_NEUTROPHIL_VS_NKCELL_UP	3	190	16063	62	4.139134	0.035963
lightcyan	10239	GSE9006_TYPE_1_VS_TYPE_2_DIABETES_PBMC_AT_DX	3	190	16063	62	4.139134	0.035963
lightcyan	10287	GSE9988_LOW_LPS_VS_ANTI_TREM1_AND_LPS_MONO	3	190	16063	62	4.139134	0.035963
blue	10086	GSE3982_EFF_MEMORY_CD4_TCELL_VS_TH1_UP	39	161	16092	2978	1.322049	0.035988
greenyello	1752	V$MAZR_01	5	197	16056	150	2.750085	0.036013
purple	6458	KAYO_CALORIE_RESTRICTION_MUSCLE_DN	3	75	16178	157	4.140892	0.036026
blue	2732	KEGG_ALANINE_ASPARTATE_AND_GLUTAMATE_META	9	26	16227	2978	1.8892	0.03603
blue	2771	KEGG_BUTANOATE_METABOLISM	9	26	16227	2978	1.8892	0.03603
blue	3277	chr20q12	9	26	16227	2978	1.8892	0.03603
blue	4418	REACTOME_ASSOCIATION_OF_TRIC_CCT_WITH_TARGE	9	26	16227	2978	1.8892	0.03603
blue	4961	WIKMAN_ASBESTOS_LUNG_CANCER_DN	9	26	16227	2978	1.8892	0.03603
blue	5136	MAYBURD_RESPONSE_TO_L663536_UP	9	26	16227	2978	1.8892	0.03603
yellow	8744	GSE15659_NAIVE_VS_PTPRC_NEG_CD4_TCELL_UP	21	158	16095	1426	1.514873	0.03603
magenta	8861	GSE17721_CTRL_VS_POLYIC_12H_BMDM_DN	5	177	16076	167	2.749247	0.036068
red	2756	KEGG_GLYCEROLIPID_METABOLISM	4	39	16214	524	3.181249	0.03608
red	6594	GENTILE_UV_RESPONSE_CLUSTER_D2	4	39	16214	524	3.181249	0.03608
turquoise	10238	GSE9006_TYPE_1_VS_TYPE_2_DIABETES_PBMC_AT_DX	63	200	16053	4169	1.228039	0.036083
cyan	5312	MCBRYAN_PUBERTAL_TGFB1_TARGETS_UP	3	153	16100	77	4.138783	0.036083
cyan	9150	GSE17974_0H_VS_0.5H_IN_VITRO_ACT_CD4_TCELL_UI	3	153	16100	77	4.138783	0.036083
midnightb	8490	GSE11924_TH2_VS_TH17_CD4_TCELL_UP	3	166	16087	71	4.137027	0.036086
midnightb	9848	GSE360_T_GONDII_VS_B_MALAYI_HIGH_DOSE_DC_UP	3	166	16087	71	4.137027	0.036086
midnightb	9883	GSE360_T_GONDII_VS_M_TUBERCULOSIS_MAC_DN	3	166	16087	71	4.137027	0.036086
lightgreen	276	ENZYME_LINKED_RECEPTOR_PROTEIN_SIGNALING_PAT	2	116	16137	42	6.672003	0.036121
lightgreen	7249	VART_KSHV_INFECTION_ANGIOGENIC_MARKERS_DN	2	116	16137	42	6.672003	0.036121
lightgreen	4828	ONKEN_UVEAL_MELANOMA_DN	4	486	15767	42	3.184989	0.036127
red	996	ION_TRANSPORT	9	139	16114	524	2.008306	0.03613
salmon	5620	MARTORIATI_MDM4_TARGETS_NEUROEPITHELIUM_UI	4	159	16094	127	3.219532	0.036138
lightyellow	3659	MODULE_176	2	148	16105	33	6.65561	0.036172
turquoise	8411	GSE10239_NAIVE_VS_KLRG1HIGH_EFF_CD8_TCELL_DN	61	193	16060	4169	1.23218	0.036232
turquoise	9731	GSE31082_DN_VS_DP_THYMOCYTE_DN	61	193	16060	4169	1.23218	0.036232
brown	4829	BERTUCCI_MEDULLARY_VS_DUCTAL_BREAST_CANCER_	36	198	16055	2190	1.349357	0.036239
brown	9475	GSE26495_NAIVE_VS_PD1LOW_CD8_TCELL_DN	36	198	16055	2190	1.349357	0.036239
red	4645	REACTOME_INTERFERON_GAMMA_SIGNALING	5	57	16196	524	2.720805	0.036248
red	5955	WANG_PROSTATE_CANCER_ANDROGEN_INDEPENDENT	5	57	16196	524	2.720805	0.036248
red	7268	BOYLAN_MULTIPLE_MYELOMA_C_DN	5	57	16196	524	2.720805	0.036248
brown	137	CELL_PROJECTION	19	92	16161	2190	1.532693	0.036248
brown	5113	ELVIDGE_HIF1A_AND_HIF2A_TARGETS_DN	19	92	16161	2190	1.532693	0.036248
grey60	1951	TGGTGCT,MIR-29A,MIR-29B,MIR-29C	4	464	15789	44	3.184365	0.036253
blue	9619	GSE28237_FOLLICULAR_VS_EARLY_GC_BCELL_DN	44	185	16068	2978	1.298045	0.036319
blue	10178	GSE7460_WT_VS_FOXP3_HET_ACT_TCONV_UP	44	185	16068	2978	1.298045	0.036319
cyan	6957	MARTINEZ_RB1_AND_TP53_TARGETS_UP	6	519	15734	77	2.440207	0.036327
pink	225	PLASMA_MEMBRANE_PART	19	867	15386	228	1.562188	0.036332
turquoise	1572	V$OCT1_06	59	186	16067	4169	1.236633	0.03634
turquoise	9004	GSE17721_POLYIC_VS_CPG_1H_BMDM_UP	59	186	16067	4169	1.236633	0.03634
turquoise	10244	GSE9037_CTRL_VS_LPS_1H_STIM_IRAK4_KO_BMDM_U	59	186	16067	4169	1.236633	0.03634

turquoise	9776	GSE339_CD4POS_VS_CD4CD8DN_DC_IN_CULTURE_UP	53	165	16088	4169	1.252257	0.036361
red	1969	TACTTGA,MIR-26A,MIR-26B	15	278	15975	524	1.673589	0.036364
red	5622	MARTORIATI_MDM4_TARGETS_FETAL_LIVER_UP	12	207	16046	524	1.798097	0.036373
green	2409	MORF_JAK3	11	75	16178	1305	1.826646	0.036381
green	2447	MORF_PTEN	11	75	16178	1305	1.826646	0.036381
blue	202	TRANSCRIPTION_FACTOR_COMPLEX	22	82	16171	2978	1.464258	0.036383
blue	4944	BORCZUK_MALIGNANT_MESOTHELIOMA_DN	22	82	16171	2978	1.464258	0.036383
red	8503	GSE12366_NAIVE_VS_MEMORY_BCELL_DN	11	184	16069	524	1.854288	0.036403
red	8944	GSE17721_POLYIC_VS_PAM3CSK4_16H_BMDM_UP	11	184	16069	524	1.854288	0.036403
red	9849	GSE360_T_GONDII_VS_B_MALAYI_HIGH_DOSE_DC_DN	11	184	16069	524	1.854288	0.036403
red	10158	GSE7460_CD8_TCELL_VS_TREG_ACT_UP	11	184	16069	524	1.854288	0.036403
turquoise	10273	GSE9988_ANTI_TREM1_VS_ANTI_TREM1_AND_LPS_M	55	172	16081	4169	1.246625	0.036411
greenyello	4732	REACTOME_BINDING_AND_ENTRY_OF_HIV_VIRION	1	4	16249	150	27.08833	0.036412
greenyello	5566	BEGUM_TARGETS_OF_PAX3_FOXO1_FUSION_AND_PA	1	4	16249	150	27.08833	0.036412
greenyello	7056	SMID_BREAST_CANCER_RELAPSE_IN_LIVER_UP	1	4	16249	150	27.08833	0.036412
greenyello	7834	NOUSHMEHR_GBM_GERMLINE_MUTATED	1	4	16249	150	27.08833	0.036412
black	8089	DELACROIX_RAR_TARGETS_UP	3	42	16211	283	4.102221	0.036414
magenta	2203	TTANTCA_UNKNOWN	13	725	15528	167	1.745108	0.036426
lightcyan	8803	GSE15930_STIM_VS_STIM_AND_IL-12_48H_CD8_T_CE	3	191	16062	62	4.117463	0.03644
lightcyan	8826	GSE17580_TREG_VS_TEFF_S_MANSONI_INF_UP	3	191	16062	62	4.117463	0.03644
lightcyan	9145	GSE17721_0.5H_VS_24H_GARDIQUIMOD_BMDM_DN	3	191	16062	62	4.117463	0.03644
turquoise	8007	TORCHIA_TARGETS_OF_EWSR1_FLI1_FUSION_DN	93	307	15946	4169	1.18099	0.036469
yellow	5120	GRAHAM_CML_QUIESCENT_VS_NORMAL_QUIESCENT_	13	86	16167	1426	1.722895	0.036487
green	1671	V$OCT1_B	23	193	16060	1305	1.484204	0.036487
green	9975	GSE3982_EOSINOPHIL_VS_MAC_DN	23	193	16060	1305	1.484204	0.036487
black	8987	GSE17721_LPS_VS_PAM3CSK4_1H_BMDM_DN	7	178	16075	283	2.258526	0.036493
green	2421	MORF_MLLT10	26	224	16029	1305	1.445601	0.036542
red	2933	BIOCARTA_CASPASE_PATHWAY	3	23	16230	524	4.045719	0.036554
lightcyan	5065	ODONNELL_TARGETS_OF_MYC_AND_TFRC_UP	2	79	16174	62	6.636586	0.03656
purple	8823	GSE16755_CTRL_VS_IFNA_TREATED_MAC_DN	5	189	16064	157	2.738685	0.03657
purple	8827	GSE17580_TREG_VS_TEFF_S_MANSONI_INF_DN	5	189	16064	157	2.738685	0.03657
purple	9427	GSE24142_ADULT_VS_FETAL_EARLY_THYMIC_PROGEN	5	189	16064	157	2.738685	0.03657
turquoise	2631	GNF2_CKS2	19	50	16203	4169	1.481444	0.036593
turquoise	4662	REACTOME_DESTABILIZATION_OF_MRNA_BY_AUF1_H	19	50	16203	4169	1.481444	0.036593
yellow	8756	GSE15659_CD45RA_NEG_CD4_TCELL_VS_ACTIVATED_T	20	149	16104	1426	1.529881	0.036598
brown	8525	GSE13229_IMM_VS_MATURE_NKCELL_DN	33	179	16074	2190	1.368202	0.036601
greenyello	5065	ODONNELL_TARGETS_OF_MYC_AND_TFRC_UP	3	79	16174	150	4.114684	0.036625
midnightb	1553	V$OCT1_04	3	167	16086	71	4.112254	0.036634
midnightb	9134	GSE17721_4_VS_24H_CPG_BMDM_UP	3	167	16086	71	4.112254	0.036634
cyan	10182	GSE7460_CTRL_VS_FOXP3_OVEREXPR_TCONV_UP	3	154	16099	77	4.111908	0.036678
greenyello	2007	CTATGCA,MIR-153	5	198	16055	150	2.736195	0.036681
magenta	4731	REACTOME_IMMUNE_SYSTEM	14	801	15452	167	1.701032	0.036683
yellow	10005	GSE3982_MAST_CELL_VS_CENT_MEMORY_CD4_TCELL_	23	177	16076	1426	1.481046	0.03671
yellow	616	NEGATIVE_REGULATION_OF_DNA_BINDING	4	15	16238	1426	3.039364	0.036752
yellow	765	NEGATIVE_REGULATION_OF_MAP_KINASE_ACTIVITY	4	15	16238	1426	3.039364	0.036752
yellow	816	G1_PHASE	4	15	16238	1426	3.039364	0.036752
yellow	970	BASE_EXCISION_REPAIR	4	15	16238	1426	3.039364	0.036752
yellow	2773	KEGG_RIBOFLAVIN_METABOLISM	4	15	16238	1426	3.039364	0.036752
yellow	4121	REACTOME_GLYCOGEN_BREAKDOWN_GLYCOGENOLYS	4	15	16238	1426	3.039364	0.036752
pink	6553	BROWNE_HCMV_INFECTION_48HR_DN	11	423	15830	228	1.853749	0.036754
tan	5608	AMUNDSON_RESPONSE_TO_ARSENITE	5	205	16048	145	2.733894	0.036784
tan	5003	GARGALOVIC_RESPONSE_TO_OXIDIZED_PHOSPHOLIPID	2	34	16219	145	6.593509	0.036811
magenta	1814	V$AP3_Q6	5	178	16075	167	2.733802	0.036814
magenta	9148	GSE17721_12H_VS_24H_GARDIQUIMOD_BMDM_UP	5	178	16075	167	2.733802	0.036814
turquoise	2634	GNF2_DDX5	23	63	16190	4169	1.423275	0.036839
green	2411	MORF_KDR	10	66	16187	1305	1.887031	0.036852
turquoise	5134	WAMUNYOKOLI_OVARIAN_CANCER_GRADES_1_2_UP	42	127	16126	4169	1.28928	0.036876
pink	8188	ZWANG_TRANSIENTLY_UP_BY_2ND_EGF_PULSE_ONLY	22	1044	15209	228	1.502176	0.036881
grey60	1508	V$EVI1_05	2	112	16141	44	6.596185	0.036897
blue	8544	GSE13306_RA_VS_UNTREATED_MEM_CD4_TCELL_UP	45	190	16063	2978	1.292611	0.036899

blue	8939	GSE17721_POLYIC_VS_PAM3CSK4_6H_BMDM_DN	45	190	16063	2978	1.292611	0.036899
lightcyan	8556	GSE13411_IGM_MEMORY_BCELL_VS_PLASMA_CELL_U	3	192	16061	62	4.096018	0.036921
lightcyan	9262	GSE20366_EX_VIVO_VS_DEC205_CONVERSION_UP	3	192	16061	62	4.096018	0.036921
lightcyan	10050	GSE3982_NEUTROPHIL_VS_EFF_MEMORY_CD4_TCELL_	3	192	16061	62	4.096018	0.036921
turquoise	3721	MODULE_252	70	225	16028	4169	1.212878	0.036949
greenyello	1943	CTACCTC,LET-7A,LET-7B,LET-7C,LET-7D,LET-7E,LET-7F,M	7	337	15916	150	2.250663	0.036951
blue	7703	WONG_EMBRYONIC_STEM_CELL_CORE	73	327	15926	2978	1.218383	0.036958
yellow	9440	GSE24634_TREG_VS_TCONV_POST_DAY3_IL4_CONVER	25	196	16057	1426	1.453778	0.036966
yellow	10200	GSE7852_LN_VS_FAT_TREG_UP	25	196	16057	1426	1.453778	0.036966
cyan	4189	REACTOME_ANTIVIRAL_MECHANISM_BY_IFN_STIMUL/	2	64	16189	77	6.596185	0.036971
turquoise	7846	KIM_ALL_DISORDERS_OLIGODENDROCYTE_NUMBER_C	205	717	15536	4169	1.114644	0.036973
cyan	8349	STK33_UP	4	265	15988	77	3.186082	0.037049
blue	280	POSITIVE_REGULATION_OF_TRANSCRIPTIONDNA_DEPE	27	105	16148	2978	1.403406	0.037066
greenyello	429	AXONOGENESIS	2	33	16220	150	6.566869	0.037066
greenyello	2895	KEGG_AUTOIMMUNE_THYROID_DISEASE	2	33	16220	150	6.566869	0.037066
greenyello	3200	chr16q13	2	33	16220	150	6.566869	0.037066
lightyellow	7745	SENGUPTA_EBNA1_ANTICORRELATED	2	150	16103	33	6.566869	0.037066
lightyellow	8758	GSE15659_RESTING_TREG_VS_NONSUPPRESSIVE_TCEL	2	150	16103	33	6.566869	0.037066
pink	8988	GSE17721_LPS_VS_PAM3CSK4_2H_BMDM_UP	6	175	16078	228	2.44406	0.037069
red	7111	BOCHKIS_FOXA2_TARGETS	19	377	15876	524	1.5632	0.037102
red	1566	V$RORA2_01	8	118	16135	524	2.102859	0.03711
magenta	9187	GSE17974_IL4_AND_ANTI_IL12_VS_UNTREATED_0.5H_	4	122	16131	167	3.19093	0.037145
yellow	420	REGULATION_OF_TRANSLATIONAL_INITIATION	6	29	16224	1426	2.358127	0.037157
purple	7415	HAN_SATB1_TARGETS_DN	8	393	15860	157	2.107324	0.037181
brown	4543	REACTOME_CELL_JUNCTION_ORGANIZATION	13	57	16196	2190	1.692614	0.037183
midnightb	9797	GSE360_CTRL_VS_T_GONDII_DC_DN	3	168	16085	71	4.087777	0.037187
midnightb	10007	GSE3982_MAST_CELL_VS_NKCELL_DN	3	168	16085	71	4.087777	0.037187
salmon	2683	GNF2_PPP6C	2	39	16214	127	6.562891	0.037192
salmon	7880	FIGUEROA_AML_METHYLATION_CLUSTER_1_DN	2	39	16214	127	6.562891	0.037192
cyan	6955	MARTINEZ_TP53_TARGETS_UP	6	522	15731	77	2.426183	0.037201
red	691	RESPONSE_TO_BIOTIC_STIMULUS	7	97	16156	524	2.238353	0.037206
red	3924	MODULE_576	7	97	16156	524	2.238353	0.037206
red	4674	REACTOME_SIGNALING_BY_NOTCH	7	97	16156	524	2.238353	0.037206
red	5284	WANG_ESOPHAGUS_CANCER_VS_NORMAL_DN	7	97	16156	524	2.238353	0.037206
red	5956	SUNG_METASTASIS_STROMA_UP	7	97	16156	524	2.238353	0.037206
magenta	4831	SCHUETZ_BREAST_CANCER_DUCTAL_INVASIVE_UP	7	303	15950	167	2.248394	0.037208
brown	8924	GSE17721_LPS_VS_POLYIC_12H_BMDM_UP	35	192	16061	2190	1.352871	0.037218
brown	9528	GSE27786_LSK_VS_LIN_NEG_CELL_UP	35	192	16061	2190	1.352871	0.037218
brown	9697	GSE29618_PRE_VS_DAY7_POST_TIV_FLU_VACCINE_PD	35	192	16061	2190	1.352871	0.037218
yellow	3239	chr7q22	14	95	16158	1426	1.679649	0.037221
purple	7878	DUTERTRE_ESTRADIOL_RESPONSE_24HR_DN	9	466	15787	157	1.999358	0.037222
purple	6155	NELSON_RESPONSE_TO_ANDROGEN_UP	3	76	16177	157	4.086406	0.037249
grey60	673	RESPONSE_TO_TEMPERATURE_STIMULUS	1	14	16239	44	26.38474	0.037256
grey60	1164	THIOLESTER_HYDROLASE_ACTIVITY	1	14	16239	44	26.38474	0.037256
grey60	1990	GCGCCTT,MIR-525,MIR-524	1	14	16239	44	26.38474	0.037256
grey60	3869	MODULE_462	1	14	16239	44	26.38474	0.037256
grey60	6812	YANG_MUC2_TARGETS_DUODENUM_6MO_DN	1	14	16239	44	26.38474	0.037256
lightgreen	4450	REACTOME_G_ALPHA_Q_SIGNALLING_EVENTS	2	118	16135	42	6.558918	0.037263
lightgreen	10278	GSE9988_LPS_VS_LOW_LPS_MONOCYTE_UP	2	118	16135	42	6.558918	0.037263
purple	9105	GSE17721_0.5H_VS_4H_POLYIC_BMDM_DN	5	190	16063	157	2.724271	0.037277
cyan	8212	CYCLIN_D1_UP.V1_UP	3	155	16098	77	4.085379	0.037278
cyan	2944	BIOCARTA_CLASSIC_PATHWAY	1	8	16245	77	26.38474	0.037286
cyan	3447	ST_INTERFERON_GAMMA_PATHWAY	1	8	16245	77	26.38474	0.037286
blue	1533	V$MYCMAX_01	52	224	16029	2978	1.266964	0.03733
greenyello	8702	GSE1460_CD4_THYMOCYTE_VS_NAIVE_CD4_TCELL_CO	5	199	16054	150	2.722446	0.037358
greenyello	9324	GSE22886_CD8_TCELL_VS_BCELL_NAIVE_UP	5	199	16054	150	2.722446	0.037358
purple	9248	GSE19825_NAIVE_VS_IL2RALOW_DAY3_EFF_CD8_TCEL	4	130	16123	157	3.185301	0.037361
brown	1019	POSITIVE_REGULATION_OF_TRANSCRIPTION	25	129	16124	2190	1.438268	0.037398
lightcyan	8411	GSE10239_NAIVE_VS_KLRG1HIGH_EFF_CD8_TCELL_DN	3	193	16060	62	4.074795	0.037404
lightcyan	9464	GSE25087_FETAL_VS_ADULT_TREG_UP	3	193	16060	62	4.074795	0.037404

lightcyan	9676	GSE29618_PDC_VS_MDC_DAY7_FLU_VACCINE_UP	3	193	16060	62	4.074795	0.037404
lightcyan	600	CELLULAR_CATION_HOMEOSTASIS	2	80	16173	62	6.553629	0.037407
lightcyan	3609	MODULE_121	2	80	16173	62	6.553629	0.037407
brown	5105	ELVIDGE_HYPOXIA_UP	29	154	16099	2190	1.397548	0.037421
blue	9344	GSE22886_IGM_MEMORY_BCELL_VS_BM_PLASMA_CE	46	195	16058	2978	1.287455	0.037448
blue	8465	GSE11864_UNTREATED_VS_CSF1_PAM3CYS_IN_MAC_I	41	171	16082	2978	1.308569	0.037462
lightcyan	101	VACUOLAR_PART	1	10	16243	62	26.21452	0.037509
lightcyan	123	LYSOSOMAL_MEMBRANE	1	10	16243	62	26.21452	0.037509
lightcyan	140	VACUOLAR_MEMBRANE	1	10	16243	62	26.21452	0.037509
lightcyan	588	REGULATION_OF_HOMEOSTATIC_PROCESS	1	10	16243	62	26.21452	0.037509
lightcyan	670	SYNAPSE_ORGANIZATION_AND_BIOGENESIS	1	10	16243	62	26.21452	0.037509
lightcyan	2959	BIOCARTA_RNA_PATHWAY	1	10	16243	62	26.21452	0.037509
lightcyan	3909	MODULE_540	1	10	16243	62	26.21452	0.037509
lightcyan	5354	SHIRAISHI_PLZF_TARGETS_UP	1	10	16243	62	26.21452	0.037509
lightcyan	5822	GOUYER_TATI_TARGETS_UP	1	10	16243	62	26.21452	0.037509
lightcyan	7018	WORSCHECH_TUMOR_REJECTION_DN	1	10	16243	62	26.21452	0.037509
lightcyan	7102	EHLERS_ANEUPLOIDY_DN	1	10	16243	62	26.21452	0.037509
lightcyan	7697	DORN_ADENOVIRUS_INFECTION_32HR_UP	1	10	16243	62	26.21452	0.037509
lightyellow	428	CARBOXYLIC_ACID_METABOLIC_PROCESS	2	151	16102	33	6.523379	0.037517
turquoise	6525	WANG_CISPLATIN_RESPONSE_AND_XPC_DN	66	211	16042	4169	1.219448	0.037522
red	2166	CTTTGCA,MIR-527	12	208	16045	524	1.789452	0.037524
midnightb	2692	GNF2_RBBP6	2	70	16183	71	6.540443	0.03755
salmon	2873	KEGG_HUNTINGTONS_DISEASE	4	161	16092	127	3.179537	0.037567
salmon	3193	chr22q13	4	161	16092	127	3.179537	0.037567
magenta	10155	GSE7460_CTRL_VS_TGFB_TREATED_ACT_CD8_TCELL_D	5	179	16074	167	2.718529	0.03757
turquoise	1098	PHOSPHOTRANSFERASE_ACTIVITY_ALCOHOL_GROUP_A	94	311	15942	4169	1.178336	0.037625
red	8280	MTOR_UP.N4.V1_UP	11	185	16068	524	1.844264	0.037633
red	8958	GSE17721_PAM3CSK4_VS_CPG_8H_BMDM_UP	11	185	16068	524	1.844264	0.037633
red	9086	GSE17721_LPS_VS_GARDIQUIMOD_12H_BMDM_UP	11	185	16068	524	1.844264	0.037633
red	9087	GSE17721_LPS_VS_GARDIQUIMOD_12H_BMDM_DN	11	185	16068	524	1.844264	0.037633
red	9176	GSE17974_CTRL_VS_ACT_IL4_AND_ANTI_IL12_6H_CD4	11	185	16068	524	1.844264	0.037633
red	9765	GSE3337_4H_VS_16H_IFNG_IN_CD8POS_DC_DN	11	185	16068	524	1.844264	0.037633
blue	2390	MORF_FANCG	37	152	16101	2978	1.328517	0.037633
blue	10140	GSE6269_E_COLI_VS_STREP_AUREUS_INF_PBMC_UP	37	152	16101	2978	1.328517	0.037633
pink	655	SECONDARY_METABOLIC_PROCESS	2	22	16231	228	6.480463	0.037645
pink	788	REGULATION_OF_INTRACELLULAR_TRANSPORT	2	22	16231	228	6.480463	0.037645
pink	1081	SYMPORTER_ACTIVITY	2	22	16231	228	6.480463	0.037645
pink	3238	chr4q22	2	22	16231	228	6.480463	0.037645
pink	4959	NOJIMA_SFRP2_TARGETS_DN	2	22	16231	228	6.480463	0.037645
pink	6150	MAGRANGEAS_MULTIPLE_MYELOMA_IGG_VS_IGA_DN	2	22	16231	228	6.480463	0.037645
pink	6198	MAGRANGEAS_MULTIPLE_MYELOMA_IGLL_VS_IGLK_D	2	22	16231	228	6.480463	0.037645
pink	3494	MODULE_1	9	321	15932	228	1.998647	0.037668
green	5339	DARWICHE_SQUAMOUS_CELL_CARCINOMA_DN	19	153	16100	1305	1.546626	0.037691
yellow	244	DNA_REPAIR	17	122	16131	1426	1.588192	0.037695
green	9216	GSE18148_CBFB_KO_VS_WT_TREG_UP	18	143	16110	1305	1.567687	0.037704
grey60	7934	BRUINS_UVC_RESPONSE_VIA_TP53_GROUP_B	4	470	15783	44	3.143714	0.037732
midnightb	10113	GSE39820_IL1B_IL6_VS_IL1B_IL6_IL23A_TREATED_CD4	3	169	16084	71	4.063589	0.037743
midnightb	10214	GSE8515_CTRL_VS_IL1_4H_STIM_MAC_UP	3	169	16084	71	4.063589	0.037743
yellow	6109	NING_CHRONIC_OBSTRUCTIVE_PULMONARY_DISEASE_	16	113	16140	1426	1.613822	0.037765
red	3684	MODULE_204	6	77	16176	524	2.416923	0.037791
greenyello	6402	REN_ALVEOLAR_RHABDOMYOSARCOMA_UP	3	80	16173	150	4.06325	0.037803
greenyello	6584	BURTON_ADIPOGENESIS_8	3	80	16173	150	4.06325	0.037803
pink	3588	MODULE_100	11	425	15828	228	1.845026	0.037821
pink	7061	SMID_BREAST_CANCER_LUMINAL_B_DN	11	425	15828	228	1.845026	0.037821
magenta	3235	chr6p25	2	30	16223	167	6.488224	0.037827
magenta	5447	AIGNER_ZEB1_TARGETS	2	30	16223	167	6.488224	0.037827
magenta	6546	DASU_IL6_SIGNALING_SCAR_UP	2	30	16223	167	6.488224	0.037827
magenta	7811	BAE_BRCA1_TARGETS_DN	2	30	16223	167	6.488224	0.037827
magenta	8175	GHANDHI_DIRECT_IRRADIATION_DN	2	30	16223	167	6.488224	0.037827
pink	341	MEMBRANE_LIPID_METABOLIC_PROCESS	4	90	16163	228	3.168226	0.037832

pink	5434	HUMMERICH_SKIN_CANCER_PROGRESSION_DN	4	90	16163	228	3.168226	0.037832
yellow	832	REGULATION_OF_NEUROGENESIS	3	9	16244	1426	3.799205	0.037857
yellow	1051	REGULATION_OF_ENDOTHELIAL_CELL_PROLIFERATION	3	9	16244	1426	3.799205	0.037857
yellow	4647	REACTOME_TRAF6_MEDIATED_IRF7_ACTIVATION_IN_T	3	9	16244	1426	3.799205	0.037857
yellow	4917	CASORELLI_APL_SECONDARY_VS_DE_NOVO_DN	3	9	16244	1426	3.799205	0.037857
yellow	5613	SASAKI_TARGETS_OF_TP73_AND_TP63	3	9	16244	1426	3.799205	0.037857
cyan	9239	GSE18791_UNSTIM_VS_NEWCATSLE_VIRUS_DC_1H_DI	3	156	16097	77	4.059191	0.037883
red	4585	REACTOME_METABOLISM_OF_LIPIDS_AND_LIPOPROTE	20	403	15850	524	1.539314	0.037885
lightcyan	8811	GSE15930_STIM_VS_STIM_AND_IFNAB_72H_CD8_T_C	3	194	16059	62	4.053791	0.037891
lightcyan	9006	GSE17721_POLYIC_VS_CPG_2H_BMDM_UP	3	194	16059	62	4.053791	0.037891
lightcyan	9116	GSE17721_0.5H_VS_4H_PAM3CSK4_BMDM_UP	3	194	16059	62	4.053791	0.037891
lightcyan	9334	GSE22886_NAIVE_BCELL_VS_BLOOD_PLASMA_CELL_UI	3	194	16059	62	4.053791	0.037891
lightcyan	9441	GSE24634_TREG_VS_TCONV_POST_DAY3_IL4_CONVER	3	194	16059	62	4.053791	0.037891
lightcyan	9968	GSE3982_MAC_VS_NEUTROPHIL_LPS_STIM_UP	3	194	16059	62	4.053791	0.037891
lightcyan	10270	GSE9988_ANTI_TREM1_VS_LOW_LPS_MONOCYTE_UP	3	194	16059	62	4.053791	0.037891
yellow	8420	GSE10239_MEMORY_VS_DAY4.5_EFF_CD8_TCELL_UP	24	187	16066	1426	1.462796	0.037892
yellow	9407	GSE24102_GRANULOCYSTIC_MDSC_VS_NEUTROPHIL_I	24	187	16066	1426	1.462796	0.037892
yellow	9417	GSE24142_EARLY_THYMIC_PROGENITOR_VS_DN3_THY	24	187	16066	1426	1.462796	0.037892
yellow	9566	GSE27786_BCELL_VS_NKCELL_UP	24	187	16066	1426	1.462796	0.037892
yellow	10213	GSE8384_CTRL_VS_B_ABORTUS_4H_MAC_CELL_LINE_I	24	187	16066	1426	1.462796	0.037892
lightyellow	481	POSITIVE_REGULATION_OF_TRANSPORT	1	19	16234	33	25.92185	0.037901
lightyellow	1339	EXONUCLEASE_ACTIVITY	1	19	16234	33	25.92185	0.037901
lightyellow	3120	BIOCARTA_ACTINY_PATHWAY	1	19	16234	33	25.92185	0.037901
lightyellow	3408	chr2p12	1	19	16234	33	25.92185	0.037901
lightyellow	5624	LI_WILMS_TUMOR_ANAPLASTIC_UP	1	19	16234	33	25.92185	0.037901
lightyellow	6362	RORIE_TARGETS_OF_EWSR1_FLI1_FUSION_DN	1	19	16234	33	25.92185	0.037901
brown	1690	V$AREB6_03	39	218	16035	2190	1.327693	0.037924
magenta	5147	RODRIGUES_THYROID_CARCINOMA_DN	3	72	16181	167	4.05514	0.037947
magenta	6847	LEIN_PONS_MARKERS	3	72	16181	167	4.05514	0.037947
magenta	6854	GAVIN_FOXP3_TARGETS_CLUSTER_P2	3	72	16181	167	4.05514	0.037947
turquoise	9349	GSE22886_DAY0_VS_DAY7_MONOCYTE_IN_CULTURE_	62	197	16056	4169	1.226951	0.037973
purple	1726	V$GATA6_01	5	191	16062	157	2.710008	0.037992
purple	5013	TAKEDA_TARGETS_OF_NUP98_HOXA9_FUSION_8D_DN	5	191	16062	157	2.710008	0.037992
purple	8828	GSE17580_UNINFECTED_VS_S_MANSONI_INF_TEFF_UI	5	191	16062	157	2.710008	0.037992
purple	10186	GSE7764_NKCELL_VS_SPLENOCYTE_UP	5	191	16062	157	2.710008	0.037992
brown	1824	GCCNNNWTAAR_UNKNOWN	24	123	16130	2190	1.44809	0.037999
cyan	7311	LINDSTEDT_DENDRITIC_CELL_MATURATION_C	2	65	16188	77	6.494705	0.03803
cyan	7705	NAKAYAMA_SOFT_TISSUE_TUMORS_PCA1_UP	2	65	16188	77	6.494705	0.03803
greenyello	7827	KARLSSON_TGFB1_TARGETS_DN	5	200	16053	150	2.708833	0.038042
purple	3037	BIOCARTA_PDGF_PATHWAY	2	32	16221	157	6.470143	0.038061
purple	4587	REACTOME_SIGNAL_TRANSDUCTION_BY_L1	2	32	16221	157	6.470143	0.038061
purple	5758	COWLING_MYCN_TARGETS	2	32	16221	157	6.470143	0.038061
purple	6709	SEMENZA_HIF1_TARGETS	2	32	16221	157	6.470143	0.038061
purple	7469	ZHAN_LATE_DIFFERENTIATION_GENES_UP	2	32	16221	157	6.470143	0.038061
greenyello	2261	GGGAGGRR_V$MAZ_Q6	25	1879	14374	150	1.441636	0.038065
lightgreen	141	ACTIN_FILAMENT	1	15	16238	42	25.79841	0.038085
lightgreen	616	NEGATIVE_REGULATION_OF_DNA_BINDING	1	15	16238	42	25.79841	0.038085
lightgreen	776	DEFENSE_RESPONSE_TO_BACTERIUM	1	15	16238	42	25.79841	0.038085
lightgreen	1070	INORGANIC_ANION_TRANSMEMBRANE_TRANSPORTER	1	15	16238	42	25.79841	0.038085
lightgreen	2957	BIOCARTA_DC_PATHWAY	1	15	16238	42	25.79841	0.038085
lightgreen	5365	MARKS_HDAC_TARGETS_DN	1	15	16238	42	25.79841	0.038085
lightgreen	5543	HERNANDEZ_MITOTIC_ARREST_BY_DOCETAXEL_2_DN	1	15	16238	42	25.79841	0.038085
lightgreen	6335	YAO_HOXA10_TARGETS_VIA_PROGESTERONE_DN	1	15	16238	42	25.79841	0.038085
lightgreen	7179	LU_TUMOR_ENDOTHELIAL_MARKERS_UP	1	15	16238	42	25.79841	0.038085
lightgreen	7548	WANG_RECURRENT_LIVER_CANCER_DN	1	15	16238	42	25.79841	0.038085
purple	1165	ORGANIC_ANION_TRANSMEMBRANE_TRANSPORTER_A	1	4	16249	157	25.88057	0.038086
purple	5458	NAKAMURA_ALVEOLAR_EPITHELIUM	1	4	16249	157	25.88057	0.038086
purple	6817	MCCOLLUM_GELDANAMYCIN_RESISTANCE_DN	1	4	16249	157	25.88057	0.038086
purple	6967	WALLACE_PROSTATE_CANCER_DN	1	4	16249	157	25.88057	0.038086
purple	7721	MIKKELSEN_NPC_ICP_WITH_H3K27ME3	1	4	16249	157	25.88057	0.038086

purple	8063	PARK_OSTEOBLAST_DIFFERENTIATION_BY_PHENYLAMI	1	4	16249	157	25.88057	0.038086
green	6312	ABBUD_LIF_SIGNALING_1_UP	7	40	16213	1305	2.179521	0.03809
blue	5985	LEE_TARGETS_OF_PTCH1_AND_SUFU_UP	14	47	16206	2978	1.625695	0.038094
magenta	3701	MODULE_223	4	123	16130	167	3.164987	0.038097
magenta	8329	IL21_UP.V1_DN	4	123	16130	167	3.164987	0.038097
magenta	9189	GSE17974_IL4_AND_ANTI_IL12_VS_UNTREATED_1H_A	4	123	16130	167	3.164987	0.038097
tan	1651	V$TCF11_01	5	207	16046	145	2.70748	0.038101
blue	10090	GSE3982_CENT_MEMORY_CD4_TCELL_VS_NKCELL_UP	42	176	16077	2978	1.302403	0.038141
turquoise	1547	V$TST1_01	60	190	16063	4169	1.231117	0.038144
turquoise	8904	GSE17721_CTRL_VS_GARDIQUIMOD_6H_BMDM_UP	60	190	16063	4169	1.231117	0.038144
turquoise	9125	GSE17721_12H_VS_24H_PAM3CSK4_BMDM_DN	60	190	16063	4169	1.231117	0.038144
red	7689	DANG_REGULATED_BY_MYC_DN	13	232	16021	524	1.738031	0.038151
turquoise	1020	UBIQUITIN_CYCLE	18	47	16206	4169	1.493057	0.038159
turquoise	2092	GGCACAT,MIR-455	18	47	16206	4169	1.493057	0.038159
turquoise	3250	chr6q21	18	47	16206	4169	1.493057	0.038159
turquoise	6120	KENNY_CTNNB1_TARGETS_DN	18	47	16206	4169	1.493057	0.038159
greenyello	4262	REACTOME_GASTRIN_CREB_SIGNALLING_PATHWAY_V	4	137	16116	150	3.163601	0.038165
pink	6439	ZHOU_TNF_SIGNALING_4HR	3	53	16200	228	4.035005	0.038209
brown	8629	GSE14308_TH2_VS_TH1_DN	34	186	16067	2190	1.356611	0.038213
brown	10001	GSE3982_MAST_CELL_VS_BASOPHIL_DN	34	186	16067	2190	1.356611	0.038213
turquoise	6200	PENG_GLUCOSE_DEPRIVATION_DN	50	155	16098	4169	1.257593	0.03822
brown	3383	chr20q13	28	148	16105	2190	1.40406	0.038238
purple	2805	KEGG_CALCIUM_SIGNALING_PATHWAY	4	131	16122	157	3.160986	0.038258
purple	8270	PRC2_SUZ12_UP.V1_UP	4	131	16122	157	3.160986	0.038258
turquoise	8812	GSE15930_STIM_VS_STIM_AND_TRICHOSTATINA_72H_	58	183	16070	4169	1.235602	0.03827
turquoise	9139	GSE17721_0.5H_VS_12H_GARDIQUIMOD_BMDM_DN	58	183	16070	4169	1.235602	0.03827
turquoise	9587	GSE27786_CD4_TCELL_VS_MONO_MAC_DN	58	183	16070	4169	1.235602	0.03827
turquoise	9866	GSE360_L_DONOVANI_VS_B_MALAYI_LOW_DOSE_MA	58	183	16070	4169	1.235602	0.03827
turquoise	5746	PUJANA_BRCA_CENTERED_NETWORK	39	117	16136	4169	1.299512	0.038278
pink	87	CYTOSKELETON	9	322	15931	228	1.99244	0.038299
midnightb	9039	GSE17721_LPS_VS_CPG_0.5H_BMDM_DN	3	170	16083	71	4.039685	0.038304
green	1204	SUBSTRATE_SPECIFIC_CHANNEL_ACTIVITY	14	104	16149	1305	1.676555	0.038334
green	7438	MEISSNER_NPC_HCP_WITH_H3K4ME3_AND_H3K27ME	14	104	16149	1305	1.676555	0.038334
green	9240	GSE18791_UNSTIM_VS_NEWCATSLE_VIRUS_DC_2H_UI	14	104	16149	1305	1.676555	0.038334
magenta	1470	V$RSRFC4_01	5	180	16073	167	2.703426	0.038335
magenta	1589	V$CEBP_Q2	5	180	16073	167	2.703426	0.038335
brown	6864	MARSON_BOUND_BY_FOXP3_UNSTIMULATED	177	1161	15092	2190	1.131437	0.03834
salmon	2455	MORF_RAB5A	3	95	16158	127	4.041359	0.038363
pink	3625	MODULE_137	11	426	15827	228	1.840695	0.038363
turquoise	8443	GSE10856_CTRL_VS_TNFRSF6B_IN_MACROPHAGE_DN	54	169	16084	4169	1.245686	0.038371
turquoise	8966	GSE17721_CPG_VS_GARDIQUIMOD_0.5H_BMDM_UP	54	169	16084	4169	1.245686	0.038371
turquoise	10092	GSE3982_CENT_MEMORY_CD4_TCELL_VS_TH1_UP	54	169	16084	4169	1.245686	0.038371
brown	1208	SMALL_GTPASE_REGULATOR_ACTIVITY	14	63	16190	2190	1.649214	0.038373
brown	3455	SIG_PIP3_SIGNALING_IN_CARDIAC_MYOCTES	14	63	16190	2190	1.649214	0.038373
lightcyan	8561	GSE13411_PLASMA_CELL_VS_MEMORY_BCELL_DN	3	195	16058	62	4.033002	0.038381
lightcyan	9895	GSE36392_TYPE_2_MYELOID_VS_NEUTROPHIL_IL25_TI	3	195	16058	62	4.033002	0.038381
tan	5335	DARWICHE_PAPILLOMA_RISK_LOW_DN	4	142	16111	145	3.157455	0.038397
lightgreen	3750	MODULE_292	2	120	16133	42	6.449603	0.038418
lightyellow	522	ORGANIC_ACID_METABOLIC_PROCESS	2	153	16100	33	6.438107	0.038425
lightyellow	5312	MCBRYAN_PUBERTAL_TGFB1_TARGETS_UP	2	153	16100	33	6.438107	0.038425
lightyellow	9002	GSE17721_POLYIC_VS_CPG_0.5H_BMDM_UP	2	153	16100	33	6.438107	0.038425
pink	3800	MODULE_356	5	132	16121	228	2.700193	0.038435
pink	5780	GROSS_HYPOXIA_VIA_ELK3_AND_HIF1A_UP	5	132	16121	228	2.700193	0.038435
brown	3105	BIOCARTA_PAR1_PATHWAY	9	35	16218	2190	1.908376	0.038445
brown	4158	REACTOME_BMAL1_CLOCK_NPAS2_ACTIVATES_CIRCAI	9	35	16218	2190	1.908376	0.038445
brown	6106	DORSAM_HOXA9_TARGETS_UP	9	35	16218	2190	1.908376	0.038445
brown	7900	SUBTIL_PROGESTIN_TARGETS	9	35	16218	2190	1.908376	0.038445
blue	7823	WHITFIELD_CELL_CYCLE_G2_M	48	205	16048	2978	1.277898	0.038459
greenyello	3563	MODULE_75	7	340	15913	150	2.230804	0.038467
salmon	4778	REACTOME_APOBEC3G_MEDIATED_RESISTANCE_TO_H	1	5	16248	127	25.59528	0.038469

salmon	5480	MYLLYKANGAS_AMPLIFICATION_HOT_SPOT_30	1	5	16248	127	25.59528	0.038469
yellow	2581	GNF2_CENPF	10	61	16192	1426	1.868462	0.038473
yellow	5006	GARGALOVIC_RESPONSE_TO_OXIDIZED_PHOSPHOLIPID	10	61	16192	1426	1.868462	0.038473
black	4737	REACTOME_COMPLEMENT_CASCADE	2	18	16235	283	6.381233	0.038476
black	6511	SU_THYMUS	2	18	16235	283	6.381233	0.038476
green	6723	ZAMORA_NOS2_TARGETS_DN	12	85	16168	1305	1.758269	0.038479
green	7872	HOELZEL_NF1_TARGETS_DN	12	85	16168	1305	1.758269	0.038479
cyan	997	RESPONSE_TO_DNA_DAMAGE_STIMULUS	3	157	16096	77	4.033336	0.038493
purple	863	POSITIVE_REGULATION_OF_TRANSFERASE_ACTIVITY	3	77	16176	157	4.033336	0.038493
purple	5249	GAUSSMANN_MLL_AF4_FUSION_TARGETS_E_UP	3	77	16176	157	4.033336	0.038493
brown	2879	KEGG_PATHWAYS_IN_CANCER	50	290	15963	2190	1.279562	0.038494
blue	3418	chr16p11	23	87	16166	2978	1.442838	0.038545
yellow	2484	GCM_ATM	5	22	16231	1426	2.590367	0.038547
yellow	3644	MODULE_159	12	78	16175	1426	1.753479	0.038565
turquoise	279	LYSOSOME_ORGANIZATION_AND_BIOGENESIS	6	11	16242	4169	2.126475	0.038639
turquoise	351	GLYCOPROTEIN_CATABOLIC_PROCESS	6	11	16242	4169	2.126475	0.038639
turquoise	620	VACUOLE_ORGANIZATION_AND_BIOGENESIS	6	11	16242	4169	2.126475	0.038639
turquoise	1293	CYCLIC_NUCLEOTIDE_PHOSPHODIESTERASE_ACTIVITY	6	11	16242	4169	2.126475	0.038639
turquoise	3132	chr6q13	6	11	16242	4169	2.126475	0.038639
turquoise	3342	chr21q11	6	11	16242	4169	2.126475	0.038639
turquoise	4451	REACTOME_P2Y_RECEPTORS	6	11	16242	4169	2.126475	0.038639
turquoise	4525	REACTOME_ZINC_TRANSPORTERS	6	11	16242	4169	2.126475	0.038639
turquoise	5004	GARGALOVIC_RESPONSE_TO_OXIDIZED_PHOSPHOLIPID	6	11	16242	4169	2.126475	0.038639
turquoise	6020	STEGMEIER_PRE-MITOTIC_CELL_CYCLE_REGULATORS	6	11	16242	4169	2.126475	0.038639
midnightb	4939	WANG_RESPONSE_TO_FORSKOLIN_DN	1	9	16244	71	25.43505	0.038645
red	8111	PHONG_TNF_TARGETS_UP	5	58	16195	524	2.673894	0.038658
turquoise	737	GLYCOPROTEIN_BIOSYNTHETIC_PROCESS	22	60	16193	4169	1.429464	0.03869
turquoise	2070	CTCAAGA,MIR-526B	22	60	16193	4169	1.429464	0.03869
brown	2872	KEGG_AMYOTROPHIC_LATERAL_SCLEROSIS_ALS	11	46	16207	2190	1.774697	0.038696
brown	6676	BRACHAT_RESPONSE_TO_CAMPTOTHECIN_DN	11	46	16207	2190	1.774697	0.038696
brown	9167	GSE17974_0H_VS_72H_IN_VITRO_ACT_CD4_TCELL_DN	36	199	16054	2190	1.342576	0.038702
brown	9909	GSE36476_CTRL_VS_TSST_ACT_16H_MEMORY_CD4_T(	36	199	16054	2190	1.342576	0.038702
blue	1239	RNA_BINDING	56	244	16009	2978	1.252585	0.03871
red	6359	YAGI_AML_SURVIVAL	8	119	16134	524	2.085188	0.038713
purple	8662	GSE1432_CTRL_VS_IFNG_24H_MICROGLIA_UP	5	192	16061	157	2.695893	0.038716
greenyello	1936	V$AR_Q6	5	201	16052	150	2.695357	0.038734
brown	5083	TIEN_INTESTINE_PROBIOTICS_24HR_UP	88	545	15708	2190	1.198328	0.038736
yellow	6865	MARSON_BOUND_BY_E2F4_UNSTIMULATED	75	699	15554	1426	1.22292	0.038737
blue	8520	GSE12845_NAIVE_VS_DARKZONE_GC_TONSIL_BCELL_U	43	181	16072	2978	1.296578	0.038784
yellow	8469	GSE11864_CSF1_VS_CSF1_IFNG_IN_MAC_DN	23	178	16075	1426	1.472726	0.038797
yellow	8951	GSE17721_PAM3CSK4_VS_CPG_1H_BMDM_DN	23	178	16075	1426	1.472726	0.038797
tan	4591	REACTOME_RNA_POL_I_PROMOTER_OPENING	2	35	16218	145	6.405123	0.038818
pink	9074	GSE17721_LPS_VS_GARDIQUIMOD_0.5H_BMDM_UP	6	177	16076	228	2.416444	0.038838
pink	9098	GSE17721_0.5H_VS_24H_LPS_BMDM_UP	6	177	16076	228	2.416444	0.038838
midnightb	8465	GSE11864_UNTREATED_VS_CSF1_PAM3CYS_IN_MAC_I	3	171	16082	71	4.016061	0.03887
midnightb	9078	GSE17721_LPS_VS_GARDIQUIMOD_2H_BMDM_UP	3	171	16082	71	4.016061	0.03887
midnightb	9713	GSE30083_SP1_VS_SP3_THYMOCYTE_DN	3	171	16082	71	4.016061	0.03887
midnightb	9852	GSE360_T_GONDII_VS_M_TUBERCULOSIS_DC_UP	3	171	16082	71	4.016061	0.03887
midnightb	9912	GSE36476_CTRL_VS_TSST_ACT_72H_MEMORY_CD4_T(	3	171	16082	71	4.016061	0.03887
lightcyan	8480	GSE11924_TFH_VS_TH1_CD4_TCELL_UP	3	196	16057	62	4.012426	0.038875
lightcyan	8870	GSE17721_CTRL_VS_PAM3CSK4_4H_BMDM_UP	3	196	16057	62	4.012426	0.038875
lightcyan	9942	GSE37416_0H_VS_48H_F_TULARENSIS_LVS_NEUTROPI	3	196	16057	62	4.012426	0.038875
lightcyan	9946	GSE37416_12H_VS_48H_F_TULARENSIS_LVS_NEUTROI	3	196	16057	62	4.012426	0.038875
lightyellow	8750	GSE15659_NAIVE_CD4_TCELL_VS_ACTIVATED_TREG_U	2	154	16099	33	6.396301	0.038881
green	9524	GSE2706_2H_VS_8H_LPS_STIM_DC_UP	22	184	16069	1305	1.489114	0.038885
red	2064	ATATGCA,MIR-448	11	186	16067	524	1.834349	0.038891
red	8429	GSE10325_LUPUS_CD4_TCELL_VS_LUPUS_BCELL_DN	11	186	16067	524	1.834349	0.038891
red	8629	GSE14308_TH2_VS_TH1_DN	11	186	16067	524	1.834349	0.038891
red	9027	GSE17721_PAM3CSK4_VS_GADIQUIMOD_4H_BMDM_	11	186	16067	524	1.834349	0.038891
red	9409	GSE24142_EARLY_THYMIC_PROGENITOR_VS_DN2_THY	11	186	16067	524	1.834349	0.038891

red	9778	GSE339_CD8POS_VS_CD4CD8DN_DC_IN_CULTURE_UP	11	186	16067	524	1.834349	0.038891
red	9811	GSE360_CTRL_VS_B_MALAYI_HIGH_DOSE_MAC_DN	11	186	16067	524	1.834349	0.038891
blue	1464	V$SP1_01	49	210	16043	2978	1.273461	0.038923
yellow	8618	GSE14000_UNSTIM_VS_4H_LPS_DC_UP	25	197	16056	1426	1.446398	0.038951
yellow	9161	GSE17974_0H_VS_12H_IN_VITRO_ACT_CD4_TCELL_DN	25	197	16056	1426	1.446398	0.038951
salmon	1406	UNFOLDED_PROTEIN_BINDING	2	40	16213	127	6.398819	0.038953
blue	2393	MORF_FOSL1	70	313	15940	2978	1.22057	0.038959
purple	5175	ENK_UV_RESPONSE_KERATINOCYTE_UP	9	470	15783	157	1.982342	0.038959
purple	7934	BRUINS_UVC_RESPONSE_VIA_TP53_GROUP_B	9	470	15783	157	1.982342	0.038959
green	631	AMINE_CATABOLIC_PROCESS	5	24	16229	1305	2.594668	0.038979
green	5720	LIEN_BREAST_CARCINOMA_METAPLASTIC	5	24	16229	1305	2.594668	0.038979
green	6563	BANDRES_RESPONSE_TO_CARMUSTIN_MGMT_24HR_I	5	24	16229	1305	2.594668	0.038979
green	6899	GAZIN_EPIGENETIC_SILENCING_BY_KRAS	5	24	16229	1305	2.594668	0.038979
greenyello	3748	MODULE_289	3	81	16172	150	4.013086	0.038999
red	2730	KEGG_PURINE_METABOLISM	9	141	16112	524	1.97982	0.03903
red	4858	CHEMNITZ_RESPONSE_TO_PROSTAGLANDIN_E2_UP	9	141	16112	524	1.97982	0.03903
red	9892	GSE36392_TYPE_2_MYELOID_VS_MAC_IL25_TREATED_	9	141	16112	524	1.97982	0.03903
greenyello	7853	KIM_ALL_DISORDERS_DURATION_CORR_DN	4	138	16115	150	3.140676	0.039033
brown	740	SECRETION	27	142	16111	2190	1.411123	0.039042
brown	9352	GSE22886_NEUTROPHIL_VS_DC_UP	27	142	16111	2190	1.411123	0.039042
magenta	8106	FORTSCHEGGER_PHF8_TARGETS_UP	6	242	16011	167	2.412976	0.039048
magenta	9386	GSE22886_IL2_VS_IL15_STIM_NKCELL_UP	4	124	16129	167	3.139463	0.039064
lightyellow	2131	RNGTGGGC_UNKNOWN	4	638	15615	33	3.087869	0.039083
pink	7848	KIM_BIPOLAR_DISORDER_OLIGODENDROCYTE_DENSIT	15	646	15607	228	1.655226	0.039088
cyan	6038	ONDER_CDH1_SIGNALING_VIA_CTNNB1	2	66	16187	77	6.396301	0.0391
cyan	7430	SHAFFER_IRF4_TARGETS_IN_PLASMA_CELL_VS_MATUI	2	66	16187	77	6.396301	0.0391
red	8029	IKEDA_MIR133_TARGETS_UP	4	40	16213	524	3.101718	0.039104
red	8164	LIM_MAMMARY_LUMINAL_PROGENITOR_UP	4	40	16213	524	3.101718	0.039104
magenta	8520	GSE12845_NAIVE_VS_DARKZONE_GC_TONSIL_BCELL_U	5	181	16072	167	2.68849	0.03911
magenta	9456	GSE24634_IL4_VS_CTRL_TREATED_NAIVE_CD4_TCELL_	5	181	16072	167	2.68849	0.03911
lightcyan	929	CATION_HOMEOSTASIS	2	82	16171	62	6.393784	0.039123
purple	6537	BLALOCK_ALZHEIMERS_DISEASE_UP	22	1535	14718	157	1.483707	0.039145
greenyello	1074	G_PROTEIN_COUPLED_RECEPTOR_BINDING	2	34	16219	150	6.373725	0.039149
greenyello	3932	PID_ERBB4_PATHWAY	2	34	16219	150	6.373725	0.039149
greenyello	5509	MATTIOLI_MULTIPLE_MYELOMA_WITH_14Q32_TRANS	2	34	16219	150	6.373725	0.039149
greenyello	5964	SHIN_B_CELL_LYMPHOMA_CLUSTER_8	2	34	16219	150	6.373725	0.039149
black	8290	ESC_V6.5_UP_EARLY.V1_UP	6	143	16110	283	2.409696	0.039178
brown	6077	CHESLER_BRAIN_QTL_CIS	15	69	16184	2190	1.613361	0.039198
brown	8981	GSE17721_CPG_VS_GARDIQUIMOD_16H_BMDM_DN	33	180	16073	2190	1.360601	0.039223
turquoise	4801	PARENT_MTOR_SIGNALING_UP	154	530	15723	4169	1.132782	0.039231
tan	599	RIBONUCLEOPROTEIN_COMPLEX_BIOGENESIS_AND_AS	3	84	16169	145	4.003202	0.039252
magenta	6353	RADMACHER_AML_PROGNOSIS	3	73	16180	167	3.99959	0.039284
turquoise	3526	MODULE_37	122	413	15840	4169	1.151626	0.039295
grey60	853	CHEMICAL_HOMEOSTASIS	2	116	16137	44	6.36873	0.039325
grey60	5187	CHIARADONNA_NEOPLASTIC_TRANSFORMATION_KRAS	2	116	16137	44	6.36873	0.039325
red	149	APICAL_PART_OF_CELL	2	10	16243	524	6.203435	0.03933
red	304	HORMONE_SECRETION	2	10	16243	524	6.203435	0.03933
red	1084	HEMATOPOIETIN_INTERFERON_CLASSD200_DOMAIN_(	2	10	16243	524	6.203435	0.03933
red	2838	KEGG_RENIN_ANGIOTENSIN_SYSTEM	2	10	16243	524	6.203435	0.03933
red	3077	BIOCARTA_RANMS_PATHWAY	2	10	16243	524	6.203435	0.03933
red	3323	chr4p13	2	10	16243	524	6.203435	0.03933
red	4220	REACTOME_REGULATION_OF_COMPLEMENT_CASCADI	2	10	16243	524	6.203435	0.03933
red	4257	REACTOME_CS_DS_DEGRADATION	2	10	16243	524	6.203435	0.03933
red	4332	REACTOME_REGULATED_PROTEOLYSIS_OF_P75NTR	2	10	16243	524	6.203435	0.03933
red	4466	REACTOME_REGULATION_OF_INSULIN_SECRETION_BY_	2	10	16243	524	6.203435	0.03933
red	5577	TERAMOTO_OPN_TARGETS_CLUSTER_1	2	10	16243	524	6.203435	0.03933
red	8143	HOLLEMAN_PREDNISOLONE_RESISTANCE_B_ALL_DN	2	10	16243	524	6.203435	0.03933
red	8150	HOLLEMAN_PREDNISOLONE_RESISTANCE_ALL_UP	2	10	16243	524	6.203435	0.03933
turquoise	2220	YGCGYRCGC_UNKNOWN	88	290	15963	4169	1.183004	0.039332
lightyellow	5858	SCHAEFFER_PROSTATE_DEVELOPMENT_6HR_UP	2	155	16098	33	6.355034	0.039341

red	4951	HORIUCHI_WTAP_TARGETS_UP	15	281	15972	524	1.655721	0.039358
lightcyan	3367	chr11p15	3	197	16056	62	3.992058	0.039372
lightcyan	9392	GSE22886_NAIVE_CD4_TCELL_VS_48H_ACT_TH1_UP	3	197	16056	62	3.992058	0.039372
lightcyan	9443	GSE24634_TREG_VS_TCONV_POST_DAY5_IL4_CONVER	3	197	16056	62	3.992058	0.039372
purple	6500	BROWNE_HCMV_INFECTION_14HR_DN	6	258	15995	157	2.407495	0.039383
brown	9949	GSE3982_CTRL_VS_PMA_STIM_EOSINOPHIL_DN	30	161	16092	2190	1.382881	0.039383
blue	8999	GSE17721_LPS_VS_PAM3CSK4_16H_BMDM_DN	44	186	16067	2978	1.291066	0.039392
blue	9166	GSE17974_0H_VS_72H_IN_VITRO_ACT_CD4_TCELL_UP	44	186	16067	2978	1.291066	0.039392
greenyello	1720	V$IRF7_01	5	202	16051	150	2.682013	0.039433
yellow	396	TRANSLATIONAL_INITIATION	7	37	16216	1426	2.156306	0.039439
yellow	2115	ACAACCT,MIR-453	7	37	16216	1426	2.156306	0.039439
yellow	2554	GCM_SMARCC1	7	37	16216	1426	2.156306	0.039439
yellow	5989	NIKOLSKY_BREAST_CANCER_1Q21_AMPLICON	7	37	16216	1426	2.156306	0.039439
yellow	6520	KEEN_RESPONSE_TO_ROSIGLITAZONE_UP	7	37	16216	1426	2.156306	0.039439
midnightb	9152	GSE17974_0H_VS_1H_IN_VITRO_ACT_CD4_TCELL_UP	3	172	16081	71	3.992712	0.039439
turquoise	2417	MORF_MAP2K2	43	131	16122	4169	1.279672	0.039446
purple	8411	GSE10239_NAIVE_VS_KLRG1HIGH_EFF_CD8_TCELL_DN	5	193	16060	157	2.681925	0.039448
brown	2502	GCM_CALM1	21	105	16148	2190	1.484292	0.039463
turquoise	4535	REACTOME_METABOLISM_OF_MRNA	65	208	16045	4169	1.218293	0.039464
green	4643	REACTOME_IRON_UPTAKE_AND_TRANSPORT	6	32	16221	1305	2.335201	0.039471
yellow	301	REGULATION_OF_KINASE_ACTIVITY	19	141	16112	1426	1.535849	0.039472
lightgreen	658	MEMBRANE_ORGANIZATION_AND_BIOGENESIS	2	122	16131	42	6.343872	0.039587
lightyellow	6033	ONDER_CDH1_TARGETS_2_DN	3	374	15879	33	3.950656	0.039633
green	6725	BANDRES_RESPONSE_TO_CARMUSTIN_MGMT_48HR_I	16	124	16129	1305	1.60702	0.039656
yellow	2435	MORF_PPP1CA	22	169	16084	1426	1.483713	0.039671
yellow	8531	GSE13306_LAMINA_PROPRIA_VS_SPLEEN_TREG_DN	22	169	16084	1426	1.483713	0.039671
blue	5849	RICKMAN_TUMOR_DIFFERENTIATED_WELL_VS_MODEI	26	101	16152	2978	1.40495	0.039678
cyan	8346	STK33_NOMO_UP	4	271	15982	77	3.115541	0.039702
brown	1993	GGCCAGT,MIR-193A,MIR-193B	16	75	16178	2190	1.583245	0.039725
brown	4581	REACTOME_L1CAM_INTERACTIONS	16	75	16178	2190	1.583245	0.039725
brown	4902	PUIFFE_INVASION_INHIBITED_BY_ASCITES_UP	16	75	16178	2190	1.583245	0.039725
brown	6156	LIN_APC_TARGETS	16	75	16178	2190	1.583245	0.039725
brown	7739	YAO_TEMPORAL_RESPONSE_TO_PROGESTERONE_CLUS	16	75	16178	2190	1.583245	0.039725
turquoise	196	MICROBODY	17	44	16209	4169	1.506253	0.039731
turquoise	228	PEROXISOME	17	44	16209	4169	1.506253	0.039731
turquoise	1432	TRANSFERASE_ACTIVITY_TRANSFERRING_GROUPS_OTH	17	44	16209	4169	1.506253	0.039731
black	5905	BENPORATH_SUZ12_TARGETS	17	612	15641	283	1.595308	0.039732
turquoise	5164	COLDREN_GEFITINIB_RESISTANCE_DN	63	201	16052	4169	1.221929	0.039732
turquoise	2011	GTTNYYNNGGTNA_UNKNOWN	28	80	16173	4169	1.364488	0.039735
turquoise	2044	KMCATNNWGGA_UNKNOWN	28	80	16173	4169	1.364488	0.039735
yellow	5793	BUYTAERT_PHOTODYNAMIC_THERAPY_STRESS_DN	66	607	15646	1426	1.239279	0.039739
pink	1891	V$LEF1_Q2	6	178	16075	228	2.402868	0.039743
pink	8195	E2F1_UP.V1_DN	6	178	16075	228	2.402868	0.039743
pink	9951	GSE3982_CTRL_VS_IGE_STIM_MAST_CELL_DN	6	178	16075	228	2.402868	0.039743
purple	7373	HINATA_NFKB_TARGETS_KERATINOCYTE_UP	3	78	16175	157	3.981627	0.039758
purple	8008	WINZEN_DEGRADED_VIA_KHSRP	3	78	16175	157	3.981627	0.039758
brown	617	POSITIVE_REGULATION_OF_DEVELOPMENTAL_PROCES	35	193	16060	2190	1.345861	0.039775
brown	8256	CAMP_UP.V1_UP	35	193	16060	2190	1.345861	0.039775
brown	10095	GSE3982_CENT_MEMORY_CD4_TCELL_VS_TH2_DN	35	193	16060	2190	1.345861	0.039775
brown	3890	MODULE_503	20	99	16154	2190	1.499285	0.039791
lightyellow	9272	GSE20366_CD103_POS_VS_NEG_TREG_KLRG1NEG_UP	2	156	16097	33	6.314297	0.039802
brown	2165	CAGCAGG,MIR-370	26	136	16117	2190	1.418809	0.039828
turquoise	7177	PODAR_RESPONSE_TO_ADAPHOSTIN_UP	45	138	16115	4169	1.271262	0.039837
lightyellow	2781	KEGG_NITROGEN_METABOLISM	1	20	16233	33	24.62576	0.039857
lightyellow	3753	MODULE_295	1	20	16233	33	24.62576	0.039857
lightyellow	3772	MODULE_320	1	20	16233	33	24.62576	0.039857
lightyellow	4400	REACTOME_STEROID_HORMONES	1	20	16233	33	24.62576	0.039857
lightyellow	4424	REACTOME_PEROXISOMAL_LIPID_METABOLISM	1	20	16233	33	24.62576	0.039857
lightyellow	5052	TONKS_TARGETS_OF_RUNX1_RUNX1T1_FUSION_SUST/	1	20	16233	33	24.62576	0.039857
lightyellow	6250	VERNELL_RETINOBLASTOMA_PATHWAY_DN	1	20	16233	33	24.62576	0.039857

lightyellow	6576	VERRECCHIA_RESPONSE_TO_TGFB1_C5	1	20	16233	33	24.62576	0.039857
lightyellow	6644	GENTILE_UV_HIGH_DOSE_UP	1	20	16233	33	24.62576	0.039857
grey60	4654	REACTOME_N_GLYCAN_ANTENNAE_ELONGATION_IN_	1	15	16238	44	24.62576	0.039864
grey60	6803	LI_CYTIDINE_ANALOG_PATHWAY	1	15	16238	44	24.62576	0.039864
grey60	6993	DE_YY1_TARGETS_UP	1	15	16238	44	24.62576	0.039864
blue	2335	MORF_STK17A	32	129	16124	2978	1.353846	0.039867
lightcyan	8399	GOLDRATH_NAIVE_VS_EFF_CD8_TCELL_DN	3	198	16055	62	3.971896	0.039872
lightcyan	8474	GSE11864_CSF1_IFNG_VS_CSF1_PAM3CYS_IN_MAC_U	3	198	16055	62	3.971896	0.039872
red	7373	HINATA_NFKB_TARGETS_KERATINOCYTE_UP	6	78	16175	524	2.385937	0.039873
green	10182	GSE7460_CTRL_VS_FOXP3_OVEREXPR_TCONV_UP	19	154	16099	1305	1.536583	0.039893
red	2250	GATAAGR_V$GATA_C	12	210	16043	524	1.77241	0.039903
red	3611	MODULE_123	12	210	16043	524	1.77241	0.039903
greenyello	2274	CCCNNGGGAR_V$OLF1_01	6	271	15982	150	2.398967	0.03993
greenyello	8346	STK33_NOMO_UP	6	271	15982	150	2.398967	0.03993
blue	1292	SEQUENCE_SPECIFIC_DNA_BINDING	13	43	16210	2978	1.649999	0.039936
yellow	2275	CGTSACG_V$PAX3_B	18	132	16121	1426	1.55422	0.039956
turquoise	8408	GSE10239_NAIVE_VS_KLRG1INT_EFF_CD8_TCELL_UP	61	194	16059	4169	1.225829	0.039966
turquoise	8562	GSE13484_UNSTIM_VS_3H_YF17D_VACCINE_STIM_PB	61	194	16059	4169	1.225829	0.039966
blue	9303	GSE2197_CPG_DNA_VS_UNTREATED_IN_DC_DN	45	191	16062	2978	1.285843	0.039966
yellow	8850	GSE17721_CTRL_VS_POLYIC_1H_BMDM_UP	24	188	16065	1426	1.455015	0.039968
yellow	9733	GSE31082_DN_VS_CD4_SP_THYMOCYTE_DN	24	188	16065	1426	1.455015	0.039968
yellow	2460	MORF_RAN	32	265	15988	1426	1.376316	0.040007
brown	6244	ZHANG_TARGETS_OF_EWSR1_FLI1_FUSION	17	81	16172	2190	1.557591	0.040008
midnightb	8262	RAF_UP.V1_UP	3	173	16080	71	3.969633	0.040013
midnightb	9277	GSE20366_CD103_POS_VS_CD103_KLRG1_DP_TREG_D	3	173	16080	71	3.969633	0.040013
midnightb	9298	GSE20715_0H_VS_48H_OZONE_TLR4_KO_LUNG_UP	3	173	16080	71	3.969633	0.040013
yellow	3744	MODULE_285	8	45	16208	1426	2.026243	0.040015
blue	10138	GSE6269_FLU_VS_STREP_PNEUMO_INF_PBMC_UP	40	167	16086	2978	1.307231	0.040034
turquoise	754	REGULATION_OF_CATALYTIC_ACTIVITY	72	233	16020	4169	1.204698	0.040035
blue	4157	REACTOME_TCA_CYCLE_AND_RESPIRATORY_ELECTRON	29	115	16138	2978	1.376287	0.040043
pink	4037	PID_ATF2_PATHWAY	3	54	16199	228	3.960283	0.040051
purple	5188	CHIARADONNA_NEOPLASTIC_TRANSFORMATION_KRAS	4	133	16120	157	3.113452	0.04009
purple	7060	SMID_BREAST_CANCER_LUMINAL_B_UP	4	133	16120	157	3.113452	0.04009
green	1177	HYDROLASE_ACTIVITY_ACTING_ON_ESTER_BONDS	26	226	16027	1305	1.432808	0.040095
greenyello	635	IMMUNE_RESPONSE	5	203	16050	150	2.668801	0.04014
cyan	5173	ENK_UV_RESPONSE_EPIDERMIS_UP	4	272	15981	77	3.104087	0.040154
green	3515	MODULE_23	44	419	15834	1305	1.307861	0.040156
turquoise	9032	GSE17721_PAM3CSK4_VS_GADIQUIMOD_12H_BMDM_	59	187	16066	4169	1.23002	0.040159
turquoise	9144	GSE17721_0.5H_VS_24H_GARDIQUIMOD_BMDM_UP	59	187	16066	4169	1.23002	0.040159
blue	1528	V$CREB_02	52	225	16028	2978	1.261333	0.040162
magenta	984	REGULATION_OF_CYTOKINE_BIOSYNTHETIC_PROCESS	2	31	16222	167	6.278926	0.040168
magenta	2687	GNF2_PTX3	2	31	16222	167	6.278926	0.040168
magenta	3307	chr12p11	2	31	16222	167	6.278926	0.040168
magenta	6748	HAN_JNK_SINGALING_DN	2	31	16222	167	6.278926	0.040168
red	1710	V$IPF1_Q4	11	187	16066	524	1.82454	0.040178
red	9522	GSE2706_2H_VS_8H_R848_STIM_DC_UP	11	187	16066	524	1.82454	0.040178
red	10111	GSE39820_CTRL_VS_TGFBETA3_IL6_IL23A_CD4_TCELL_	11	187	16066	524	1.82454	0.040178
cyan	3122	chr16q24	2	67	16186	77	6.300833	0.040182
cyan	3287	chr15q22	2	67	16186	77	6.300833	0.040182
brown	1968	CTCAGGG,MIR-125B,MIR-125A	52	304	15949	2190	1.26946	0.040182
purple	8694	GSE1460_DP_VS_CD4_THYMOCYTE_UP	5	194	16059	157	2.6681	0.040189
blue	2542	GCM_PPP1CC	16	56	16197	2978	1.55934	0.040199
blue	2586	GNF2_FEN1	16	56	16197	2978	1.55934	0.040199
blue	6326	GALE_APL_WITH_FLT3_MUTATED_UP	16	56	16197	2978	1.55934	0.040199
lightyellow	6820	CREIGHTON_ENDOCRINE_THERAPY_RESISTANCE_3	4	644	15609	33	3.0591	0.040236
brown	8950	GSE17721_PAM3CSK4_VS_CPG_1H_BMDM_UP	32	174	16079	2190	1.364866	0.040246
brown	9563	GSE27786_BCELL_VS_CD4_TCELL_DN	32	174	16079	2190	1.364866	0.040246
purple	3347	chr10p12	2	33	16220	157	6.274078	0.040265
purple	6381	GREENBAUM_E2A_TARGETS_UP	2	33	16220	157	6.274078	0.040265
purple	6526	MURAKAMI_UV_RESPONSE_6HR_UP	2	33	16220	157	6.274078	0.040265

lightyellow	8881	GSE17721_CTRL_VS_CPG_0.5H_BMDM_DN	2	157	16096	33	6.274078	0.040265
turquoise	8990	GSE17721_LPS_VS_PAM3CSK4_4H_BMDM_UP	57	180	16073	4169	1.234537	0.040306
turquoise	2425	MORF_NME2	49	152	16101	4169	1.256765	0.040308
yellow	9189	GSE17974_IL4_AND_ANTI_IL12_VS_UNTREATED_1H_A	17	123	16130	1426	1.57528	0.04031
green	3122	chr16q24	10	67	16186	1305	1.858867	0.040326
green	6921	KONDO_EZH2_TARGETS	23	195	16058	1305	1.468981	0.040339
cyan	8218	AKT_UP_MTOR_DN.V1_UP	3	160	16093	77	3.957711	0.040353
red	1128	GTPASE_REGULATOR_ACTIVITY	8	120	16133	524	2.067812	0.040363
red	3326	chr21q22	8	120	16133	524	2.067812	0.040363
black	9014	GSE17721_POLYIC_VS_CPG_12H_BMDM_UP	7	182	16071	283	2.208888	0.04037
black	10223	GSE8868_SPLEEN_VS_INTESTINE_CD11B_POS_CD11C_	7	182	16071	283	2.208888	0.04037
lightcyan	9324	GSE22886_CD8_TCELL_VS_BCELL_NAIVE_UP	3	199	16054	62	3.951937	0.040375
lightcyan	9665	GSE29618_PDC_VS_MDC_DN	3	199	16054	62	3.951937	0.040375
red	38	EXTRACELLULAR_REGION	15	282	15971	524	1.64985	0.040394
green	33	SYNAPSE_PART	3	10	16243	1305	3.736322	0.040402
green	760	VESICLE_LOCALIZATION	3	10	16243	1305	3.736322	0.040402
green	923	REGULATION_OF_NEURON_APOPTOSIS	3	10	16243	1305	3.736322	0.040402
green	1178	BETA_TUBULIN_BINDING	3	10	16243	1305	3.736322	0.040402
green	1337	PDZ_DOMAIN_BINDING	3	10	16243	1305	3.736322	0.040402
green	3261	chr11p12	3	10	16243	1305	3.736322	0.040402
green	4562	REACTOME_ACTIVATION_OF_THE_AP1_FAMILY_OF_TF	3	10	16243	1305	3.736322	0.040402
green	7257	DAVIES_MULTIPLE_MYELOMA_VS_MGUS_UP	3	10	16243	1305	3.736322	0.040402
tan	6268	MATSUDA_NATURAL_KILLER_DIFFERENTIATION	8	433	15820	145	2.070941	0.040407
black	7869	LU_EZH2_TARGETS_UP	9	262	15991	283	1.972824	0.040409
turquoise	4699	REACTOME_HIV_LIFE_CYCLE	38	114	16139	4169	1.299512	0.040424
lightyellow	4894	ZHOU_INFLAMMATORY_RESPONSE_LIVE_UP	3	377	15876	33	3.919219	0.040432
cyan	7351	RUTELLA_RESPONSE_TO_HGF_VS_CSF2RB_AND_IL4_U	5	398	15855	77	2.651733	0.040435
magenta	3344	chr9p	1	4	16249	167	24.33084	0.040475
magenta	4199	REACTOME_BETA_DEFENSINS	1	4	16249	167	24.33084	0.040475
magenta	4666	REACTOME_LIGAND_GATED_ION_CHANNEL_TRANSPO	1	4	16249	167	24.33084	0.040475
magenta	7721	MIKKELSEN_NPC_ICP_WITH_H3K27ME3	1	4	16249	167	24.33084	0.040475
yellow	8172	SMIRNOV_RESPONSE_TO_IR_6HR_UP	21	160	16093	1426	1.495937	0.040502
pink	1177	HYDROLASE_ACTIVITY_ACTING_ON_ESTER_BONDS	7	226	16027	228	2.207945	0.040506
blue	8733	GSE15215_CD2_POS_VS_NEG_PDC_DN	46	196	16057	2978	1.280886	0.040508
greenyellow	1948	GTGCAAT,MIR-25,MIR-32,MIR-92,MIR-363,MIR-367	6	272	15981	150	2.390147	0.040532
green	2406	MORF_IL9	9	58	16195	1305	1.93258	0.040541
red	6500	BROWNE_HCMV_INFECTION_14HR_DN	14	258	15995	524	1.683103	0.040554
grey60	4450	REACTOME_G_ALPHA_Q_SIGNALLING_EVENTS	2	118	16135	44	6.260786	0.040561
lightgreen	358	CELL_STRUCTURE_DISASSEMBLY_DURING_APOPTOSIS	1	16	16237	42	24.18601	0.040573
lightgreen	840	NEGATIVE_REGULATION_OF_BINDING	1	16	16237	42	24.18601	0.040573
lightgreen	1135	AMINE_RECEPTOR_ACTIVITY	1	16	16237	42	24.18601	0.040573
lightgreen	1312	DOUBLE_STRANDED_RNA_BINDING	1	16	16237	42	24.18601	0.040573
lightgreen	3031	BIOCARTA_NO2IL12_PATHWAY	1	16	16237	42	24.18601	0.040573
lightgreen	3100	BIOCARTA_41BB_PATHWAY	1	16	16237	42	24.18601	0.040573
lightgreen	3845	MODULE_424	1	16	16237	42	24.18601	0.040573
lightgreen	5214	NADERI_BREAST_CANCER_PROGNOSIS_DN	1	16	16237	42	24.18601	0.040573
lightgreen	5826	CAFFAREL_RESPONSE_TO_THC_8HR_5_UP	1	16	16237	42	24.18601	0.040573
lightgreen	7470	ZHAN_LATE_DIFFERENTIATION_GENES_DN	1	16	16237	42	24.18601	0.040573
lightgreen	7639	KORKOLA_EMBRYONIC_CARCINOMA_VS_SEMINOMA_	1	16	16237	42	24.18601	0.040573
lightgreen	7668	HAHTOLA_CTCL_PATHOGENESIS	1	16	16237	42	24.18601	0.040573
blue	1504	V$EVI1_02	24	92	16161	2978	1.423745	0.040579
pink	6491	BROWNE_HCMV_INFECTION_24HR_DN	5	134	16119	228	2.659891	0.040581
pink	9642	GSE29615_DAY3_VS_DAY7_LAIV_FLU_VACCINE_PBMC_	5	134	16119	228	2.659891	0.040581
pink	10146	GSE6566_STRONG_VS_WEAK_DC_STIMULATED_CD4_T	5	134	16119	228	2.659891	0.040581
lightyellow	7331	YOSHIMURA_MAPK8_TARGETS_UP	5	947	15306	33	2.600397	0.040618
purple	520	LIPID_METABOLIC_PROCESS	6	260	15993	157	2.388976	0.04064
purple	5039	MULLIGHAN_MLL_SIGNATURE_2_DN	6	260	15993	157	2.388976	0.04064
magenta	7148	LABBE_WNT3A_TARGETS_DN	3	74	16179	167	3.945541	0.040645
purple	7331	YOSHIMURA_MAPK8_TARGETS_UP	15	947	15306	157	1.639741	0.040656
magenta	1708	V$TITF1_Q3	5	183	16070	167	2.659108	0.040688

magenta	8918	GSE17721_LPS_VS_POLYIC_4H_BMDM_UP	5	183	16070	167	2.659108	0.040688
magenta	9425	GSE24142_DN2_VS_DN3_THYMOCYTE_FETAL_DN	5	183	16070	167	2.659108	0.040688
brown	142	RUFFLE	8	30	16223	2190	1.979056	0.040717
lightyellow	8381	LEF1_UP.V1_UP	2	158	16095	33	6.234369	0.04073
salmon	6170	ASTON_MAJOR_DEPRESSIVE_DISORDER_UP	2	41	16212	127	6.24275	0.040745
salmon	6375	KAMMINGA_EZH2_TARGETS	2	41	16212	127	6.24275	0.040745
salmon	8246	BCAT_BILD_ET_AL_UP	2	41	16212	127	6.24275	0.040745
brown	6581	LIAN_LIPA_TARGETS_3M	12	52	16201	2190	1.712645	0.040747
lightgreen	2370	MORF_CTSB	2	124	16129	42	6.241551	0.04077
lightgreen	2418	MORF_MAP2K7	2	124	16129	42	6.241551	0.04077
red	1114	RAS_GTPASE_BINDING	3	24	16229	524	3.877147	0.040806
red	1271	KINASE_INHIBITOR_ACTIVITY	3	24	16229	524	3.877147	0.040806
red	2615	GNF2_CASP4	3	24	16229	524	3.877147	0.040806
red	3792	MODULE_343	3	24	16229	524	3.877147	0.040806
red	4039	PID_INTEGRIN2_PATHWAY	3	24	16229	524	3.877147	0.040806
red	5256	BERENJENO_TRANSFORMED_BY_RHOA_FOREVER_DN	3	24	16229	524	3.877147	0.040806
black	5859	SCHAEFFER_PROSTATE_DEVELOPMENT_6HR_DN	14	478	15775	283	1.682082	0.040837
greenyello	1755	V$STAT6_01	5	204	16049	150	2.655719	0.040855
greenyello	3313	chr3p21	5	204	16049	150	2.655719	0.040855
pink	250	ACTIN_FILAMENT_ORGANIZATION	2	23	16230	228	6.198703	0.040857
pink	1360	OXIDOREDUCTASE_ACTIVITY_ACTING_ON_NADH_OR_I	2	23	16230	228	6.198703	0.040857
pink	4950	NEWMAN_ERCC6_TARGETS_DN	2	23	16230	228	6.198703	0.040857
pink	5257	CAIRO_PML_TARGETS_BOUND_BY_MYC_UP	2	23	16230	228	6.198703	0.040857
pink	5347	HWANG_PROSTATE_CANCER_MARKERS	2	23	16230	228	6.198703	0.040857
pink	7760	BAUS_TFF2_TARGETS_UP	2	23	16230	228	6.198703	0.040857
red	472	BEHAVIOR	7	99	16154	524	2.193134	0.040859
red	3890	MODULE_503	7	99	16154	524	2.193134	0.040859
red	7532	COULOUARN_TEMPORAL_TGFB1_SIGNATURE_UP	7	99	16154	524	2.193134	0.040859
red	7738	YAO_TEMPORAL_RESPONSE_TO_PROGESTERONE_CLUS	7	99	16154	524	2.193134	0.040859
brown	8567	GSE13484_3H_UNSTIM_VS_YF17D_VACCINE_STIM_PB	34	187	16066	2190	1.349357	0.040869
brown	8926	GSE17721_LPS_VS_POLYIC_16H_BMDM_UP	34	187	16066	2190	1.349357	0.040869
brown	9057	GSE17721_POLYIC_VS_GARDIQUIMOD_0.5H_BMDM_D	34	187	16066	2190	1.349357	0.040869
brown	9769	GSE339_CD4POS_VS_CD8POS_DC_DN	34	187	16066	2190	1.349357	0.040869
lightcyan	8374	KRAS.LUNG_UP.V1_DN	2	84	16169	62	6.241551	0.040869
black	2207	WCAANNNYCAG_UNKNOWN	8	222	16031	283	2.069589	0.040922
yellow	7317	NICK_RESPONSE_TO_PROC_TREATMENT_UP	2	4	16249	1426	5.698808	0.040942
black	4047	PID_IL6_7PATHWAY	3	44	16209	283	3.915757	0.040967
yellow	10060	GSE3982_BCELL_VS_BASOPHIL_UP	23	179	16074	1426	1.464498	0.04097
green	6500	BROWNE_HCMV_INFECTION_14HR_DN	29	258	15995	1305	1.399914	0.040971
cyan	9643	GSE29615_DAY3_VS_DAY7_LAIV_FLU_VACCINE_PBMC_	3	161	16092	77	3.933129	0.040983
turquoise	7043	MITSIADES_RESPONSE_TO_APLIDIN_UP	121	410	15843	4169	1.150544	0.040987
blue	2388	MORF_ESR1	33	134	16119	2978	1.344058	0.040999
blue	9642	GSE29615_DAY3_VS_DAY7_LAIV_FLU_VACCINE_PBMC_	33	134	16119	2978	1.344058	0.040999
yellow	1860	V$COUP_DR1_Q6	25	198	16055	1426	1.439093	0.041015
yellow	9159	GSE17974_0H_VS_6H_IN_VITRO_ACT_CD4_TCELL_DN	25	198	16055	1426	1.439093	0.041015
yellow	9732	GSE31082_DN_VS_CD4_SP_THYMOCYTE_UP	25	198	16055	1426	1.439093	0.041015
blue	2430	MORF_PAX7	47	201	16052	2978	1.276176	0.041019
purple	9511	GSE2706_R848_VS_LPS_2H_STIM_DC_DN	4	134	16119	157	3.090218	0.041025
grey60	7331	YOSHIMURA_MAPK8_TARGETS_UP	6	947	15306	44	2.340357	0.041033
purple	1518	V$PAX6_01	3	79	16174	157	3.931226	0.041043
purple	4331	REACTOME_BIOLOGICAL_OXIDATIONS	3	79	16174	157	3.931226	0.041043
brown	6890	FOSTER_TOLERANT_MACROPHAGE_DN	65	391	15862	2190	1.233747	0.041084
turquoise	5111	ELVIDGE_HIF2A_TARGETS_UP	4	6	16247	4169	2.599025	0.041104
turquoise	7003	YAMASHITA_SILENCED_BY_METHYLATION	4	6	16247	4169	2.599025	0.041104
turquoise	4224	REACTOME_SIGNALING_BY_THE_B_CELL_RECEPTOR_B	40	121	16132	4169	1.288773	0.041109
turquoise	854	REGULATION_OF_METABOLIC_PROCESS	202	708	15545	4169	1.112294	0.041109
turquoise	1239	RNA_BINDING	75	244	16009	4169	1.198321	0.041125
red	5717	SHETH_LIVER_CANCER_VS_TXNIP_LOSS_PAM4	12	211	16042	524	1.76401	0.041131
blue	9684	GSE29618_PRE_VS_DAY7_FLU_VACCINE_MDC_UP	37	153	16100	2978	1.319833	0.041151
red	3788	MODULE_337	5	59	16194	524	2.628574	0.041164

red	6339	PETROVA_PROX1_TARGETS_DN	5	59	16194	524	2.628574	0.041164
red	6986	RIZKI_TUMOR_INVASIVENESS_2D_UP	5	59	16194	524	2.628574	0.041164
midnightb	7499	JISON_SICKLE_CELL_DISEASE_DN	3	175	16078	71	3.924266	0.041173
lightcyan	552	DETECTION_OF_CHEMICAL_STIMULUS	1	11	16242	62	23.83138	0.041183
lightcyan	626	PROTEIN_TARGETING_TO_MEMBRANE	1	11	16242	62	23.83138	0.041183
lightcyan	698	SENSORY_PERCEPTION_OF_CHEMICAL_STIMULUS	1	11	16242	62	23.83138	0.041183
lightcyan	742	APOPTOTIC_MITOCHONDRIAL_CHANGES	1	11	16242	62	23.83138	0.041183
lightcyan	1052	REGULATION_OF_MEMBRANE_POTENTIAL	1	11	16242	62	23.83138	0.041183
lightcyan	3057	BIOCARTA_EIF2_PATHWAY	1	11	16242	62	23.83138	0.041183
lightcyan	3137	chr9p12	1	11	16242	62	23.83138	0.041183
lightcyan	4655	REACTOME_REGULATION_OF_IFNA_SIGNALING	1	11	16242	62	23.83138	0.041183
lightcyan	6211	IIZUKA_LIVER_CANCER_EARLY_RECURRENCE	1	11	16242	62	23.83138	0.041183
lightcyan	6433	WHITESIDE_CISPLATIN_RESISTANCE_UP	1	11	16242	62	23.83138	0.041183
blue	1072	PROTEIN_DOMAIN_SPECIFIC_BINDING	18	65	16188	2978	1.51136	0.041221
blue	3666	MODULE_183	18	65	16188	2978	1.51136	0.041221
blue	4427	REACTOME_METABOLISM_OF_NUCLEOTIDES	18	65	16188	2978	1.51136	0.041221
blue	5710	FURUKAWA_DUSP6_TARGETS_PCI35_DN	18	65	16188	2978	1.51136	0.041221
blue	6424	ZAMORA_NOS2_TARGETS_UP	18	65	16188	2978	1.51136	0.041221
cyan	3689	MODULE_209	2	68	16185	77	6.208174	0.041275
cyan	3707	MODULE_235	2	68	16185	77	6.208174	0.041275
greenyello	2865	KEGG_TYPE_I_DIABETES_MELLITUS	2	35	16218	150	6.191619	0.041276
salmon	7965	BAKKER_FOXO3_TARGETS_DN	4	166	16087	127	3.083768	0.04128
brown	8894	GSE17721_CTRL_VS_CPG_24H_BMDM_UP	31	168	16085	2190	1.369436	0.041281
turquoise	2599	GNF2_RFC3	16	41	16212	4169	1.52138	0.041289
cyan	348	NUCLEOBASENUCLEOSIDENUCLEOTIDE_AND_NUCLEIC_	10	1136	15117	77	1.85808	0.041312
brown	519	MESODERM_DEVELOPMENT	5	15	16238	2190	2.47382	0.041358
brown	2931	BIOCARTA_CDMAC_PATHWAY	5	15	16238	2190	2.47382	0.041358
brown	3794	MODULE_346	5	15	16238	2190	2.47382	0.041358
brown	4136	REACTOME_BILE_ACID_AND_BILE_SALT_METABOLISM	5	15	16238	2190	2.47382	0.041358
brown	5543	HERNANDEZ_MITOTIC_ARREST_BY_DOCETAXEL_2_DN	5	15	16238	2190	2.47382	0.041358
brown	8074	STEINER_ERYTHROCYTE_MEMBRANE_GENES	5	15	16238	2190	2.47382	0.041358
red	9925	GSE37416_CTRL_VS_3H_F_TULARENSIS_LVS_NEUTROP	10	165	16088	524	1.879829	0.041366
black	5045	TONKS_TARGETS_OF_RUNX1_RUNX1T1_FUSION_HSC_	7	183	16070	283	2.196818	0.041379
black	8699	GSE1460_DP_THYMOCYTE_VS_NAIVE_CD4_TCELL_ADU	7	183	16070	283	2.196818	0.041379
black	8736	GSE15324_NAIVE_VS_ACTIVATED_ELF4_KO_CD8_TCEL	7	183	16070	283	2.196818	0.041379
lightyellow	7988	LEE_BMP2_TARGETS_UP	4	650	15603	33	3.030862	0.041407
blue	939	ESTABLISHMENT_AND_OR_MAINTENANCE_OF_CHROM	20	74	16179	2978	1.475051	0.041486
blue	2035	CAGGGTC,MIR-504	20	74	16179	2978	1.475051	0.041486
blue	5804	LIU_COMMON_CANCER_GENES	20	74	16179	2978	1.475051	0.041486
yellow	5987	KAUFFMANN_DNA_REPAIR_GENES	28	227	16026	1426	1.405873	0.041487
magenta	8435	GSE10325_CD4_TCELL_VS_LUPUS_CD4_TCELL_DN	5	184	16069	167	2.644656	0.041491
magenta	8808	GSE15930_STIM_VS_STIM_AND_IL-12_72H_CD8_T_CE	5	184	16069	167	2.644656	0.041491
magenta	9527	GSE2706_2H_VS_8H_R848_AND_LPS_STIM_DC_DN	5	184	16069	167	2.644656	0.041491
magenta	9626	GSE2826_WT_VS_BTK_KO_BCELL_UP	5	184	16069	167	2.644656	0.041491
magenta	9818	GSE360_DC_VS_MAC_L_DONOVANI_UP	5	184	16069	167	2.644656	0.041491
red	8535	GSE13306_TREG_VS_TCONV_SPLEEN_DN	11	188	16065	524	1.814835	0.041495
red	8701	GSE1460_DP_THYMOCYTE_VS_THYMIC_STROMAL_CEL	11	188	16065	524	1.814835	0.041495
red	9412	GSE24142_DN2_VS_DN3_THYMOCYTE_UP	11	188	16065	524	1.814835	0.041495
red	9432	GSE24634_NAIVE_CD4_TCELL_VS_DAY3_IL4_CONV_TR	11	188	16065	524	1.814835	0.041495
red	10009	GSE3982_MAST_CELL_VS_TH1_DN	11	188	16065	524	1.814835	0.041495
red	10201	GSE7852_LN_VS_FAT_TREG_DN	11	188	16065	524	1.814835	0.041495
pink	5497	MYLLYKANGAS_AMPLIFICATION_HOT_SPOT_1	1	3	16250	228	23.7617	0.041499
lightyellow	6936	HELLER_HDAC_TARGETS_SILENCED_BY_METHYLATION_	3	381	15872	33	3.878072	0.04151
midnightb	5381	RIZ_ERYTHROID_DIFFERENTIATION	2	74	16179	71	6.186905	0.041533
brown	2350	MORF_AP2M1	38	213	16040	2190	1.324017	0.041536
purple	7349	RUTELLA_RESPONSE_TO_HGF_UP	8	402	15851	157	2.060145	0.041569
pink	7498	JISON_SICKLE_CELL_DISEASE_UP	6	180	16073	228	2.37617	0.041591
pink	8589	GSE13485_DAY3_VS_DAY7_YF17D_VACCINE_PBMC_DI	6	180	16073	228	2.37617	0.041591
pink	9341	GSE22886_IGG_IGA_MEMORY_BCELL_VS_BM_PLASMA	6	180	16073	228	2.37617	0.041591
cyan	8243	P53_DN.V1_DN	3	162	16091	77	3.90885	0.041618

cyan	8541	GSE13306_RA_VS_UNTREATED_TREG_DN	3	162	16091	77	3.90885	0.041618
lightyellow	9716	GSE30083_SP2_VS_SP3_THYMOCYTE_UP	2	160	16093	33	6.156439	0.041667
lightyellow	9720	GSE30083_SP3_VS_SP4_THYMOCYTE_UP	2	160	16093	33	6.156439	0.041667
pink	7265	BOYLAN_MULTIPLE_MYELOMA_C_D_UP	5	135	16118	228	2.640188	0.041681
grey60	1324	TRANSMEMBRANE_RECEPTOR_ACTIVITY	3	285	15968	44	3.888278	0.041691
purple	1599	V$GATA_C	5	196	16057	157	2.640875	0.041695
purple	5048	TONKS_TARGETS_OF_RUNX1_RUNX1T1_FUSION_MON	5	196	16057	157	2.640875	0.041695
purple	10038	GSE3982_MAC_VS_CENT_MEMORY_CD4_TCELL_UP	5	196	16057	157	2.640875	0.041695
purple	10209	GSE7852_THYMUS_VS_FAT_TCONV_DN	5	196	16057	157	2.640875	0.041695
greenyello	2809	KEGG_NEUROACTIVE_LIGAND_RECEPTOR_INTERACTIO	4	141	16112	150	3.073853	0.041703
greenyello	3816	MODULE_379	4	141	16112	150	3.073853	0.041703
greenyello	7523	CHIANG_LIVER_CANCER_SUBCLASS_CTNNB1_DN	4	141	16112	150	3.073853	0.041703
turquoise	4374	REACTOME_OPIOID_SIGNALLING	24	67	16186	4169	1.396491	0.041724
lightcyan	3642	MODULE_157	2	85	16168	62	6.168121	0.041754
lightcyan	7861	CHICAS_RB1_TARGETS_LOW_SERUM	2	85	16168	62	6.168121	0.041754
greenyello	7963	PASINI_SUZ12_TARGETS_DN	6	274	15979	150	2.372701	0.041755
midnightb	9834	GSE360_L_DONOVANI_VS_B_MALAYI_HIGH_DOSE_DC_	3	176	16077	71	3.901969	0.04176
blue	1977	TGCACGA,MIR-517A,MIR-517C	6	15	16238	2978	2.183076	0.04178
blue	2972	BIOCARTA_EIF_PATHWAY	6	15	16238	2978	2.183076	0.04178
blue	3115	BIOCARTA_ARF_PATHWAY	6	15	16238	2978	2.183076	0.04178
blue	4103	PID_ANTHRAXPATHWAY	6	15	16238	2978	2.183076	0.04178
blue	4253	REACTOME_SYNTHESIS_OF_PIPS_AT_THE_GOLGI_MEM	6	15	16238	2978	2.183076	0.04178
blue	4360	REACTOME_PROCESSIVE_SYNTHESIS_ON_THE_LAGGIN	6	15	16238	2978	2.183076	0.04178
blue	5852	RICKMAN_TUMOR_DIFFERENTIATED_MODERATELY_VS	6	15	16238	2978	2.183076	0.04178
blue	5983	COLLIS_PRKDC_REGULATORS	6	15	16238	2978	2.183076	0.04178
blue	6002	NIKOLSKY_BREAST_CANCER_12Q24_AMPLICON	6	15	16238	2978	2.183076	0.04178
blue	7344	RAY_TARGETS_OF_P210_BCR_ABL_FUSION_DN	6	15	16238	2978	2.183076	0.04178
turquoise	9212	GSE17974_1.5H_VS_72H_IL4_AND_ANTI_IL12_ACT_CD	62	198	16055	4169	1.220754	0.041805
turquoise	9732	GSE31082_DN_VS_CD4_SP_THYMOCYTE_UP	62	198	16055	4169	1.220754	0.041805
lightyellow	2774	KEGG_NICOTINATE_AND_NICOTINAMIDE_METABOLISM	1	21	16232	33	23.4531	0.041809
lightyellow	4144	REACTOME_METABOLISM_OF_STEROID_HORMONES_A	1	21	16232	33	23.4531	0.041809
lightyellow	4251	REACTOME_REGULATION_OF_HYPOXIA_INDUCIBLE_FA	1	21	16232	33	23.4531	0.041809
lightyellow	6024	JI_METASTASIS_REPRESSED_BY_STK11	1	21	16232	33	23.4531	0.041809
lightyellow	6585	DELLA_RESPONSE_TO_TSA_AND_BUTYRATE	1	21	16232	33	23.4531	0.041809
lightyellow	7890	FIGUEROA_AML_METHYLATION_CLUSTER_6_DN	1	21	16232	33	23.4531	0.041809
grey60	828	AMINE_METABOLIC_PROCESS	2	120	16133	44	6.156439	0.041813
grey60	8332	PDGF_ERK_DN.V1_UP	2	120	16133	44	6.156439	0.041813
green	2414	MORF_LTK	15	115	16138	1305	1.624488	0.041814
cyan	4767	REACTOME_INITIAL_TRIGGERING_OF_COMPLEMENT	1	9	16244	77	23.4531	0.041849
cyan	4860	ZERBINI_RESPONSE_TO_SULINDAC_UP	1	9	16244	77	23.4531	0.041849
cyan	5180	YEMELYANOV_GR_TARGETS_DN	1	9	16244	77	23.4531	0.041849
cyan	5634	OXFORD_RALB_TARGETS_DN	1	9	16244	77	23.4531	0.041849
cyan	7552	YAMANAKA_GLIOBLASTOMA_SURVIVAL_DN	1	9	16244	77	23.4531	0.041849
cyan	7746	YIH_RESPONSE_TO_ARSENITE_C5	1	9	16244	77	23.4531	0.041849
pink	1978	GTCNYYATGR_UNKNOWN	4	93	16160	228	3.066025	0.041872
pink	8082	PLASARI_TGFB1_SIGNALING_VIA_NFIC_1HR_DN	4	93	16160	228	3.066025	0.041872
cyan	7349	RUTELLA_RESPONSE_TO_HGF_UP	5	402	15851	77	2.625347	0.041913
cyan	8091	DELACROIX_RAR_BOUND_ES	5	402	15851	77	2.625347	0.041913
turquoise	3126	chr10q23	27	77	16176	4169	1.367019	0.041922
turquoise	6969	WALLACE_PROSTATE_CANCER_RACE_DN	27	77	16176	4169	1.367019	0.041922
pink	876	REGULATION_OF_TRANSPORT	3	55	16198	228	3.888278	0.041937
pink	3723	MODULE_254	3	55	16198	228	3.888278	0.041937
pink	6490	CHANG_IMMORTALIZED_BY_HPV31_DN	3	55	16198	228	3.888278	0.041937
pink	7420	MCMURRAY_TP53_HRAS_COOPERATION_RESPONSE_D	3	55	16198	228	3.888278	0.041937
yellow	4535	REACTOME_METABOLISM_OF_MRNA	26	208	16045	1426	1.424702	0.041945
yellow	9877	GSE360_L_MAJOR_VS_M_TUBERCULOSIS_MAC_DN	22	170	16083	1426	1.474986	0.041946
purple	395	POSITIVE_REGULATION_OF_CATALYTIC_ACTIVITY	4	135	16118	157	3.067327	0.041971
brown	10008	GSE3982_MAST_CELL_VS_TH1_UP	33	181	16072	2190	1.353084	0.041983
yellow	7433	HSIAO_HOUSEKEEPING_GENES	44	385	15868	1426	1.302585	0.041993
cyan	694	PROTEIN_KINASE_CASCADE	4	276	15977	77	3.0591	0.041993

brown	8158	ALTEMEIER_RESPONSE_TO_LPS_WITH_MECHANICAL_V	23	118	16135	2190	1.446556	0.042001
red	390	GAMETE_GENERATION	6	79	16174	524	2.355735	0.042027
red	2137	AGGTGCA,MIR-500	6	79	16174	524	2.355735	0.042027
magenta	6918	KONDO_PROSTATE_CANCER_WITH_H3K27ME3	3	75	16178	167	3.892934	0.042029
magenta	8178	WARTERS_RESPONSE_TO_IR_SKIN	3	75	16178	167	3.892934	0.042029
salmon	6218	PENG_RAPAMYCIN_RESPONSE_DN	5	243	16010	127	2.633259	0.042041
magenta	3198	chr22q11	4	127	16126	167	3.065302	0.042046
red	8310	CRX_NRL_DN.V1_UP	8	121	16132	524	2.050722	0.042059
turquoise	8882	GSE17721_CTRL_VS_CPG_1H_BMDM_UP	60	191	16062	4169	1.224671	0.042065
turquoise	8937	GSE17721_POLYIC_VS_PAM3CSK4_4H_BMDM_DN	60	191	16062	4169	1.224671	0.042065
red	6979	IWANAGA_CARCINOGENESIS_BY_KRAS_UP	9	143	16110	524	1.95213	0.042084
red	8272	JNK_DN.V1_UP	9	143	16110	524	1.95213	0.042084
blue	849	REGULATION_OF_CELL_CELL_ADHESION	4	8	16245	2978	2.728845	0.042127
blue	3101	BIOCARTA_KREB_PATHWAY	4	8	16245	2978	2.728845	0.042127
blue	4380	REACTOME_ADENYLATE_CYCLASE_ACTIVATING_PATHV	4	8	16245	2978	2.728845	0.042127
blue	5209	KOINUMA_COLON_CANCER_MSI_UP	4	8	16245	2978	2.728845	0.042127
blue	7425	MIKKELSEN_PARTIALLY_REPROGRAMMED_TO_PLURIP(	4	8	16245	2978	2.728845	0.042127
blue	7833	NOUSHMEHR_GBM_SOMATIC_MUTATED	4	8	16245	2978	2.728845	0.042127
yellow	8464	GSE11864_UNTREATED_VS_CSF1_PAM3CYS_IN_MAC_I	24	189	16064	1426	1.447316	0.042128
yellow	9211	GSE17974_0.5H_VS_72H_IL4_AND_ANTI_IL12_ACT_CD	24	189	16064	1426	1.447316	0.042128
yellow	9434	GSE24634_NAIVE_CD4_TCELL_VS_DAY5_IL4_CONV_TR	24	189	16064	1426	1.447316	0.042128
lightyellow	8935	GSE17721_POLYIC_VS_PAM3CSK4_2H_BMDM_DN	2	161	16092	33	6.118201	0.042138
lightyellow	8985	GSE17721_LPS_VS_PAM3CSK4_0.5H_BMDM_DN	2	161	16092	33	6.118201	0.042138
green	9687	GSE29618_PRE_VS_DAY7_POST_LAIV_FLU_VACCINE_B	19	155	16098	1305	1.526669	0.042188
brown	4059	PID_ERA_GENOMIC_PATHWAY	13	58	16195	2190	1.663431	0.042228
brown	6903	KYNG_DNA_DAMAGE_BY_UV	13	58	16195	2190	1.663431	0.042228
brown	5173	ENK_UV_RESPONSE_EPIDERMIS_UP	47	272	15981	2190	1.282385	0.042257
red	5423	GALLUZZI_PERMEABILIZE_MITOCHONDRIA	4	41	16212	524	3.026066	0.042268
red	5779	GROSS_HYPOXIA_VIA_ELK3_ONLY_DN	4	41	16212	524	3.026066	0.042268
red	6213	BECKER_TAMOXIFEN_RESISTANCE_DN	4	41	16212	524	3.026066	0.042268
turquoise	8644	GSE14308_TH1_VS_NATURAL_TREG_UP	58	184	16069	4169	1.228887	0.042284
turquoise	9263	GSE20366_EX_VIVO_VS_DEC205_CONVERSION_DN	58	184	16069	4169	1.228887	0.042284
magenta	8255	CAMP_UP.V1_DN	5	185	16068	167	2.630361	0.042304
magenta	8280	MTOR_UP.N4.V1_UP	5	185	16068	167	2.630361	0.042304
magenta	9868	GSE360_L_DONOVANI_VS_M_TUBERCULOSIS_MAC_UI	5	185	16068	167	2.630361	0.042304
greenyello	1867	V$MYB_Q3	5	206	16047	150	2.629935	0.042307
green	612	REGULATION_OF_NEUROTRANSMITTER_LEVELS	4	17	16236	1305	2.930449	0.042321
green	622	ESTABLISHMENT_OF_ORGANELLE_LOCALIZATION	4	17	16236	1305	2.930449	0.042321
green	751	DIGESTION	4	17	16236	1305	2.930449	0.042321
green	3097	BIOCARTA_TEL_PATHWAY	4	17	16236	1305	2.930449	0.042321
green	4212	REACTOME_SIGNALING_BY_HIPPO	4	17	16236	1305	2.930449	0.042321
green	4480	REACTOME_G_BETA_GAMMA_SIGNALLING_THROUGH	4	17	16236	1305	2.930449	0.042321
green	7785	RUAN_RESPONSE_TO_TROGLITAZONE_DN	4	17	16236	1305	2.930449	0.042321
turquoise	2362	MORF_CDC10	46	142	16111	4169	1.262906	0.042333
midnightb	10193	GSE7852_TREG_VS_TCONV_LN_DN	3	177	16076	71	3.879924	0.042351
greenyello	7850	KIM_ALL_DISORDERS_CALB1_CORR_UP	9	500	15753	150	1.95036	0.04238
magenta	5876	KOYAMA_SEMA3B_TARGETS_UP	6	247	16006	167	2.36413	0.042429
brown	1027	NEGATIVE_REGULATION_OF_BIOSYNTHETIC_PROCESS	7	25	16228	2190	2.078009	0.042433
brown	3111	BIOCARTA_CREB_PATHWAY	7	25	16228	2190	2.078009	0.042433
brown	4034	PID_LIS1PATHWAY	7	25	16228	2190	2.078009	0.042433
brown	6900	JEPSEN_SMRT_TARGETS	7	25	16228	2190	2.078009	0.042433
brown	7393	LEE_DOUBLE_POLAR_THYMOCYTE	7	25	16228	2190	2.078009	0.042433
brown	8157	HOLLEMAN_ASPARAGINASE_RESISTANCE_ALL_DN	7	25	16228	2190	2.078009	0.042433
turquoise	8861	GSE17721_CTRL_VS_POLYIC_12H_BMDM_DN	56	177	16076	4169	1.233435	0.042454
brown	8562	GSE13484_UNSTIM_VS_3H_YF17D_VACCINE_STIM_PB	35	194	16059	2190	1.338923	0.042462
brown	8574	GSE13485_CTRL_VS_DAY1_YF17D_VACCINE_PBMC_UP	35	194	16059	2190	1.338923	0.042462
brown	8694	GSE1460_DP_VS_CD4_THYMOCYTE_UP	35	194	16059	2190	1.338923	0.042462
brown	8993	GSE17721_LPS_VS_PAM3CSK4_6H_BMDM_DN	35	194	16059	2190	1.338923	0.042462
brown	9079	GSE17721_LPS_VS_GARDIQUIMOD_2H_BMDM_DN	35	194	16059	2190	1.338923	0.042462
brown	9763	GSE3337_CTRL_VS_16H_IFNG_IN_CD8POS_DC_DN	35	194	16059	2190	1.338923	0.042462

grey60	3903	MODULE_530	1	16	16237	44	23.08665	0.042466
grey60	5255	BERENJENO_TRANSFORMED_BY_RHOA_FOREVER_UP	1	16	16237	44	23.08665	0.042466
grey60	6054	NELSON_RESPONSE_TO_ANDROGEN_DN	1	16	16237	44	23.08665	0.042466
grey60	7203	MATTHEWS_AP1_TARGETS	1	16	16237	44	23.08665	0.042466
grey60	7219	ALONSO_METASTASIS_NEURAL_UP	1	16	16237	44	23.08665	0.042466
purple	1074	G_PROTEIN_COUPLED_RECEPTOR_BINDING	2	34	16219	157	6.089547	0.042516
purple	1367	PATTERN_BINDING	2	34	16219	157	6.089547	0.042516
purple	1467	V$HOX13_01	2	34	16219	157	6.089547	0.042516
purple	4448	REACTOME_REGULATION_OF_INSULIN_SECRETION_BY	2	34	16219	157	6.089547	0.042516
purple	4625	REACTOME_REGULATION_OF_WATER_BALANCE_BY_R	2	34	16219	157	6.089547	0.042516
purple	5539	HERNANDEZ_MITOTIC_ARREST_BY_DOCETAXEL_1_DN	2	34	16219	157	6.089547	0.042516
black	1356	HORMONE_ACTIVITY	2	19	16234	283	6.045378	0.042518
black	5250	GAUSSMANN_MLL_AF4_FUSION_TARGETS_E_DN	2	19	16234	283	6.045378	0.042518
black	5682	KIM_MYCL1_AMPLIFICATION_TARGETS_DN	2	19	16234	283	6.045378	0.042518
black	7388	HOFMANN_MYELODYSPLASTIC_SYNDROM_RISK_UP	2	19	16234	283	6.045378	0.042518
black	7784	DAZARD_UV_RESPONSE_CLUSTER_G28	2	19	16234	283	6.045378	0.042518
pink	1821	V$SMAD3_Q6	6	181	16072	228	2.363042	0.042535
pink	6364	YAGI_AML_FAB_MARKERS	6	181	16072	228	2.363042	0.042535
pink	9068	GSE17721_POLYIC_VS_GARDIQUIMOD_12H_BMDM_U	6	181	16072	228	2.363042	0.042535
pink	9075	GSE17721_LPS_VS_GARDIQUIMOD_0.5H_BMDM_DN	6	181	16072	228	2.363042	0.042535
pink	9415	GSE24142_EARLY_THYMIC_PROGENITOR_VS_DN2_THY	6	181	16072	228	2.363042	0.042535
midnightb	1934	GGCNRNWCTTYS_UNKNOWN	2	75	16178	71	6.104413	0.042553
midnightb	4902	PUIFFE_INVASION_INHIBITED_BY_ASCITES_UP	2	75	16178	71	6.104413	0.042553
midnightb	6166	ROSS_AML_WITH_MLL_FUSIONS	2	75	16178	71	6.104413	0.042553
salmon	6795	KRIGE_RESPONSE_TO_TOSEDOSTAT_6HR_DN	12	880	15373	127	1.745132	0.04256
magenta	266	POSITIVE_REGULATION_OF_RESPONSE_TO_STIMULUS	2	32	16221	167	6.08271	0.042563
magenta	5058	PAPASPYRIDONOS_UNSTABLE_ATEROSCLEROTIC_PLAQ	2	32	16221	167	6.08271	0.042563
lightgreen	8554	GSE13411_IGM_VS_SWITCHED_MEMORY_BCELL_UP	2	127	16126	42	6.094113	0.042569
salmon	553	POSITIVE_REGULATION_OF_HYDROLASE_ACTIVITY	2	42	16211	127	6.094113	0.042569
salmon	2549	GCM_RAF1	2	42	16211	127	6.094113	0.042569
blue	866	CELL_CYCLE_ARREST_GO_0007050	15	52	16201	2978	1.574334	0.042571
blue	5793	BUYTAERT_PHOTODYNAMIC_THERAPY_STRESS_DN	128	607	15646	2978	1.15088	0.042589
greenyello	6553	BROWNE_HCMV_INFECTION_48HR_DN	8	423	15830	150	2.049236	0.042598
turquoise	9239	GSE18791_UNSTIM_VS_NEWCATSLE_VIRUS_DC_1H_DI	50	156	16097	4169	1.249531	0.042611
lightyellow	5010	TAKEDA_TARGETS_OF_NUP98_HOXA9_FUSION_3D_UF	2	162	16091	33	6.080434	0.042612
greenyello	1748	V$NKX22_01	4	142	16111	150	3.052207	0.042615
greenyello	8330	IL21_UP.V1_UP	4	142	16111	150	3.052207	0.042615
turquoise	795	PEPTIDYL_AMINO_ACID_MODIFICATION	20	54	16199	4169	1.443903	0.042621
turquoise	6448	GENTILE_UV_RESPONSE_CLUSTER_D4	20	54	16199	4169	1.443903	0.042621
turquoise	2340	MORF_TERF2IP	37	111	16142	4169	1.299512	0.042698
greenyello	2885	KEGG_PROSTATE_CANCER	3	84	16169	150	3.869762	0.042704
tan	10217	GSE8515_CTRL_VS_IL6_4H_STIM_MAC_DN	4	147	16106	145	3.050059	0.042708
salmon	6797	KRIGE_RESPONSE_TO_TOSEDOSTAT_24HR_DN	13	980	15273	127	1.697646	0.04274
black	1917	V$SP1_Q6_01	8	224	16029	283	2.051111	0.042759
red	6377	BASSO_CD40_SIGNALING_UP	7	100	16153	524	2.171202	0.04277
pink	6373	IGLESIAS_E2F_TARGETS_UP	5	136	16117	228	2.620775	0.042799
red	6086	GOLDRATH_HOMEOSTATIC_PROLIFERATION	10	166	16087	524	1.868505	0.04281
red	8490	GSE11924_TH2_VS_TH17_CD4_TCELL_UP	10	166	16087	524	1.868505	0.04281
red	9883	GSE360_T_GONDII_VS_M_TUBERCULOSIS_MAC_DN	10	166	16087	524	1.868505	0.04281
salmon	605	RNA_PROCESSING	4	168	16085	127	3.047057	0.042821
salmon	5741	PUJANA_XPRSS_INT_NETWORK	4	168	16085	127	3.047057	0.042821
salmon	10147	GSE6566_STRONG_VS_WEAK_DC_STIMULATED_CD4_T	4	168	16085	127	3.047057	0.042821
red	8923	GSE17721_LPS_VS_POLYIC_8H_BMDM_DN	11	189	16064	524	1.805232	0.042841
red	10044	GSE3982_MAC_VS_TH2_UP	11	189	16064	524	1.805232	0.042841
midnightb	527	PURINE_RIBONUCLEOTIDE_METABOLIC_PROCESS	1	10	16243	71	22.89155	0.042847
midnightb	596	PROTEIN_STABILIZATION	1	10	16243	71	22.89155	0.042847
midnightb	4272	REACTOME_REVERSIBLE_HYDRATION_OF_CARBON_DI(	1	10	16243	71	22.89155	0.042847
midnightb	5406	TURJANSKI_MAPK14_TARGETS	1	10	16243	71	22.89155	0.042847
midnightb	5927	AMIT_EGF_RESPONSE_20_HELA	1	10	16243	71	22.89155	0.042847
green	5928	AMIT_EGF_RESPONSE_40_HELA	7	41	16212	1305	2.126362	0.042872

yellow	7537	HOSHIDA_LIVER_CANCER_SURVIVAL_DN	13	88	16165	1426	1.683739	0.042875
green	4365	REACTOME_SIGNALING_BY_GPCR	44	421	15832	1305	1.301648	0.042891
lightcyan	7068	SMID_BREAST_CANCER_BASAL_UP	5	504	15749	62	2.600646	0.042899
purple	8331	PDGF_ERK_DN.V1_DN	4	136	16117	157	3.044773	0.042931
green	1579	V$CREB_Q4	27	238	16015	1305	1.412895	0.042931
midnightb	9888	GSE360_LOW_DOSE_B_MALAYI_VS_M_TUBERCULOSIS	3	178	16075	71	3.858126	0.042946
brown	4835	FOURNIER_ACINAR_DEVELOPMENT_EARLY_UP	6	20	16233	2190	2.226438	0.043015
green	7962	PASINI_SUZ12_TARGETS_UP	13	96	16157	1305	1.686534	0.043024
green	9029	GSE17721_PAM3CSK4_VS_GADIQUIMOD_6H_BMDM_	22	186	16067	1305	1.473102	0.043043
green	9930	GSE37416_CTRL_VS_24H_F_TULARENSIS_LVS_NEUTRO	22	186	16067	1305	1.473102	0.043043
greenyello	1829	V$CACBINDINGPROTEIN_Q6	5	207	16046	150	2.61723	0.043045
lightgreen	431	REGULATION_OF_MAPKKK_CASCADE	1	17	16236	42	22.76331	0.043055
lightgreen	2943	BIOCARTA_TID_PATHWAY	1	17	16236	42	22.76331	0.043055
lightgreen	3099	BIOCARTA_TH1TH2_PATHWAY	1	17	16236	42	22.76331	0.043055
lightgreen	3875	MODULE_478	1	17	16236	42	22.76331	0.043055
lightgreen	4208	REACTOME_RIP_MEDIATED_NFKB_ACTIVATION_VIA_D	1	17	16236	42	22.76331	0.043055
lightgreen	6413	SUZUKI_RESPONSE_TO_TSA_AND_DECITABINE_1A	1	17	16236	42	22.76331	0.043055
lightgreen	7568	MIKKELSEN_MCV6_LCP_WITH_H3K27ME3	1	17	16236	42	22.76331	0.043055
magenta	8169	DURAND_STROMA_MAX_DN	4	128	16125	167	3.041355	0.043068
magenta	8552	GSE13411_NAIVE_VS_MEMORY_BCELL_UP	4	128	16125	167	3.041355	0.043068
brown	8683	GSE1448_ANTI_VALPHA2_VS_VBETA5_DP_THYMOCYTI	32	175	16078	2190	1.357067	0.043115
yellow	4105	PID_RAS_PATHWAY	6	30	16223	1426	2.279523	0.043122
yellow	7997	KIM_TIAL1_TARGETS	6	30	16223	1426	2.279523	0.043122
magenta	1916	V$OCT1_Q5_01	5	186	16067	167	2.616219	0.043127
magenta	1925	V$MEF2_Q6_01	5	186	16067	167	2.616219	0.043127
magenta	3536	MODULE_47	5	186	16067	167	2.616219	0.043127
magenta	9712	GSE30083_SP1_VS_SP3_THYMOCYTE_UP	5	186	16067	167	2.616219	0.043127
blue	1924	V$E2F1_Q6_01	52	226	16027	2978	1.255752	0.043156
yellow	9214	GSE17974_2.5H_VS_72H_IL4_AND_ANTI_IL12_ACT_CD	25	199	16054	1426	1.431861	0.043157
yellow	9343	GSE22886_IGM_MEMORY_BCELL_VS_BLOOD_PLASMA	25	199	16054	1426	1.431861	0.043157
grey60	807	IMMUNE_SYSTEM_PROCESS	3	289	15964	44	3.834461	0.043158
lightgreen	7522	CHIANG_LIVER_CANCER_SUBCLASS_CTNNB1_UP	2	128	16125	42	6.046503	0.043175
lightcyan	7455	MILI_PSEUDOPODIA_HAPTOTAXIS_UP	5	505	15748	62	2.595497	0.043204
blue	8556	GSE13411_IGM_MEMORY_BCELL_VS_PLASMA_CELL_U	45	192	16061	2978	1.279146	0.043223
purple	8698	GSE1460_DP_THYMOCYTE_VS_NAIVE_CD4_TCELL_ADU	5	198	16055	157	2.614199	0.043236
brown	5109	ELVIDGE_HIF1A_TARGETS_UP	14	64	16189	2190	1.623445	0.043251
brown	5560	DIRMEIER_LMP1_RESPONSE_EARLY	14	64	16189	2190	1.623445	0.043251
brown	7728	YAO_TEMPORAL_RESPONSE_TO_PROGESTERONE_CLUS	14	64	16189	2190	1.623445	0.043251
turquoise	1647	V$RFX1_01	65	209	16044	4169	1.212464	0.043299
blue	2644	GNF2_G22P1	11	35	16218	2978	1.715274	0.043309
blue	3105	BIOCARTA_PAR1_PATHWAY	11	35	16218	2978	1.715274	0.043309
blue	4056	PID_HIVNEFPATHWAY	11	35	16218	2978	1.715274	0.043309
blue	4591	REACTOME_RNA_POL_I_PROMOTER_OPENING	11	35	16218	2978	1.715274	0.043309
turquoise	5621	MARTORIATI_MDM4_TARGETS_NEUROEPITHELIUM_DI	39	118	16135	4169	1.288499	0.043417
greenyello	6404	TRAYNOR_RETT_SYNDROM_UP	2	36	16217	150	6.01963	0.043445
greenyello	7497	VALK_AML_WITH_FLT3_ITD	2	36	16217	150	6.01963	0.043445
black	10178	GSE7460_WT_VS_FOXP3_HET_ACT_TCONV_UP	7	185	16068	283	2.173068	0.043447
black	10246	GSE9037_CTRL_VS_LPS_4H_STIM_IRAK4_KO_BMDM_U	7	185	16068	283	2.173068	0.043447
blue	8001	FEVR_CTNNB1_TARGETS_DN	112	526	15727	2978	1.162094	0.043459
turquoise	439	POST_GOLGI_VESICLE_MEDIATED_TRANSPORT	7	14	16239	4169	1.949268	0.043541
turquoise	2779	KEGG_TERPENOID_BACKBONE_BIOSYNTHESIS	7	14	16239	4169	1.949268	0.043541
turquoise	3254	chr6q15	7	14	16239	4169	1.949268	0.043541
turquoise	3270	chr2p15	7	14	16239	4169	1.949268	0.043541
turquoise	5427	GILMORE_CORE_NFKB_PATHWAY	7	14	16239	4169	1.949268	0.043541
turquoise	5672	INAMURA_LUNG_CANCER_SCC_SUBTYPES_UP	7	14	16239	4169	1.949268	0.043541
blue	6945	AMUNDSON_POOR_SURVIVAL_AFTER_GAMMA_RADIA	40	168	16085	2978	1.29945	0.043543
lightcyan	5678	KIM_MYC_AMPLIFICATION_TARGETS_DN	2	87	16166	62	6.026326	0.043543
lightcyan	6285	NEMETH_INFLAMMATORY_RESPONSE_LPS_UP	2	87	16166	62	6.026326	0.043543
lightcyan	8376	KRAS.LUNG.BREAST_UP.V1_DN	2	87	16166	62	6.026326	0.043543
black	3316	chr10q26	4	76	16177	283	3.022689	0.043543

midnightb	9338	GSE22886_IGG_IGA_MEMORY_BCELL_VS_BLOOD_PLAS	3	179	16074	71	3.836573	0.043545
midnightb	10130	GSE6269_HEALTHY_VS_STREP_AUREUS_INF_PBMC_UP	3	179	16074	71	3.836573	0.043545
cyan	8759	GSE15659_RESTING_TREG_VS_NONSUPPRESSIVE_TCEL	3	165	16088	77	3.83778	0.043553
lightyellow	3552	MODULE_63	2	164	16089	33	6.006282	0.043565
lightyellow	6554	BROWNE_HCMV_INFECTION_48HR_UP	2	164	16089	33	6.006282	0.043565
lightyellow	9841	GSE360_L_MAJOR_VS_T_GONDII_DC_DN	2	164	16089	33	6.006282	0.043565
salmon	8536	GSE13306_TREG_RA_VS_TCONV_RA_UP	4	169	16084	127	3.029027	0.043604
salmon	10021	GSE3982_DC_VS_EFF_MEMORY_CD4_TCELL_DN	4	169	16084	127	3.029027	0.043604
turquoise	1720	V$IRF7_01	63	202	16051	4169	1.21588	0.04366
yellow	1	NUCLEOPLASM	32	267	15986	1426	1.366006	0.043661
brown	10042	GSE3982_MAC_VS_TH1_UP	34	188	16065	2190	1.342179	0.043662
red	6320	PETROVA_ENDOTHELIUM_LYMPHATIC_VS_BLOOD_DN	9	144	16109	524	1.938573	0.04367
brown	4631	REACTOME_FACTORS_INVOLVED_IN_MEGAKARYOCYTE	20	100	16153	2190	1.484292	0.043672
green	1935	V$VDR_Q6	24	207	16046	1305	1.443989	0.043678
blue	8672	GSE14350_TREG_VS_TEFF_IN_IL2RB_KO_UP	46	197	16056	2978	1.274384	0.043754
lightyellow	962	HORMONE_METABOLIC_PROCESS	1	22	16231	33	22.38705	0.043757
lightyellow	1175	LIGAND_DEPENDENT_NUCLEAR_RECEPTOR_ACTIVITY	1	22	16231	33	22.38705	0.043757
lightyellow	5585	SCHRAMM_INHBA_TARGETS_DN	1	22	16231	33	22.38705	0.043757
lightyellow	6350	LIAN_NEUTROPHIL_GRANULE_CONSTITUENTS	1	22	16231	33	22.38705	0.043757
lightyellow	7702	TIAN_TNF_SIGNALING_NOT_VIA_NFKB	1	22	16231	33	22.38705	0.043757
red	83	VACUOLE	5	60	16193	524	2.584765	0.043767
red	2070	CTCAAGA,MIR-526B	5	60	16193	524	2.584765	0.043767
red	5674	WATTEL_AUTONOMOUS_THYROID_ADENOMA_UP	5	60	16193	524	2.584765	0.043767
red	6025	GOTTWEIN_TARGETS_OF_KSHV_MIR_K12_11	5	60	16193	524	2.584765	0.043767
lightgreen	2379	MORF_DMPK	2	129	16124	42	5.999631	0.043784
lightgreen	5001	GARGALOVIC_RESPONSE_TO_OXIDIZED_PHOSPHOLIPID	2	129	16124	42	5.999631	0.043784
greenyello	1639	V$ISRE_01	5	208	16045	150	2.604647	0.043791
greenyello	1760	V$STAT4_01	5	208	16045	150	2.604647	0.043791
greenyello	2166	CTTTGCA,MIR-527	5	208	16045	150	2.604647	0.043791
red	658	MEMBRANE_ORGANIZATION_AND_BIOGENESIS	8	122	16131	524	2.033913	0.043801
red	5165	KOKKINAKIS_METHIONINE_DEPRIVATION_48HR_UP	8	122	16131	524	2.033913	0.043801
tan	397	PHOTOTRANSDUCTION	1	5	16248	145	22.41793	0.043824
tan	5844	FUJIWARA_PARK2_IN_LIVER_CANCER_DN	1	5	16248	145	22.41793	0.043824
tan	7795	DASU_IL6_SIGNALING_DN	1	5	16248	145	22.41793	0.043824
pink	7612	FONTAINE_PAPILLARY_THYROID_CARCINOMA_UP	3	56	16197	228	3.818844	0.043867
cyan	1335	TRANSCRIPTION_FACTOR_ACTIVITY	4	280	15973	77	3.015399	0.04388
blue	1813	GATGKMRGCG_UNKNOWN	17	61	16192	2978	1.520995	0.043889
blue	4505	REACTOME_COSTIMULATION_BY_THE_CD28_FAMILY	17	61	16192	2978	1.520995	0.043889
purple	8349	STK33_UP	6	265	15988	157	2.343901	0.043893
brown	1286	ACTIN_BINDING	15	70	16183	2190	1.590313	0.043904
brown	6390	SESTO_RESPONSE_TO_UV_C8	15	70	16183	2190	1.590313	0.043904
red	4853	GAZDA_DIAMOND_BLACKFAN_ANEMIA_ERYTHROID_D	22	461	15792	524	1.480212	0.043907
salmon	5785	NUYTTEN_EZH2_TARGETS_DN	13	984	15269	127	1.690745	0.043915
yellow	6552	KANG_DOXORUBICIN_RESISTANCE_UP	9	54	16199	1426	1.899603	0.043922
yellow	7661	GUTIERREZ_CHRONIC_LYMPHOCYTIC_LEUKEMIA_DN	9	54	16199	1426	1.899603	0.043922
magenta	1550	V$OCT1_01	5	187	16066	167	2.602229	0.043959
magenta	1710	V$IPF1_Q4	5	187	16066	167	2.602229	0.043959
magenta	8676	GSE14350_IL2RB_KO_VS_WT_TEFF_UP	5	187	16066	167	2.602229	0.043959
red	7043	MITSIADES_RESPONSE_TO_APLIDIN_UP	20	410	15843	524	1.513033	0.043964
greenyello	2125	AGCTCCT,MIR-28	3	85	16168	150	3.824235	0.043976
turquoise	9205	GSE17974_0.5H_VS_72H_UNTREATED_IN_VITRO_CD4_	61	195	16058	4169	1.219542	0.043988
turquoise	9740	GSE31082_CD4_VS_CD8_SP_THYMOCYTE_UP	61	195	16058	4169	1.219542	0.043988
green	4842	KIM_RESPONSE_TO_TSA_AND_DECITABINE_UP	14	106	16147	1305	1.644922	0.044002
green	13	PROTEINACEOUS_EXTRACELLULAR_MATRIX	10	68	16185	1305	1.83153	0.044019
tan	3438	chr14q24	3	88	16165	145	3.821238	0.044073
blue	2072	TCTGGAC,MIR-198	21	79	16174	2978	1.450778	0.044086
red	985	REGULATION_OF_APOPTOSIS	16	310	15943	524	1.600886	0.044123
yellow	1469	V$ELK1_02	29	238	16015	1426	1.388785	0.044133
midnightb	1660	V$NFAT_Q6	3	180	16073	71	3.815258	0.044148
midnightb	8738	GSE15324_ELF4_KO_VS_WT_NAIVE_CD8_TCELL_UP	3	180	16073	71	3.815258	0.044148

midnightb	8796	GSE15930_STIM_VS_STIM_AND_IL-12_24H_CD8_T_CE	3	180	16073	71	3.815258	0.044148
purple	7066	SMID_BREAST_CANCER_NORMAL_LIKE_UP	8	407	15846	157	2.034836	0.04415
turquoise	4708	REACTOME_LATE_PHASE_OF_HIV_LIFE_CYCLE	34	101	16152	4169	1.312379	0.044155
brown	7368	AGUIRRE_PANCREATIC_CANCER_COPY_NUMBER_UP	49	286	15967	2190	1.271509	0.044164
pink	901	HETEROCYCLE_METABOLIC_PROCESS	2	24	16229	228	5.940424	0.044168
pink	1340	ACTIN_FILAMENT_BINDING	2	24	16229	228	5.940424	0.044168
pink	3946	PID_HDAC_CLASSIII_PATHWAY	2	24	16229	228	5.940424	0.044168
pink	4356	REACTOME_AMINO_ACID_TRANSPORT_ACROSS_THE_I	2	24	16229	228	5.940424	0.044168
pink	4419	REACTOME_PREFOLDIN_MEDIATED_TRANSFER_OF_SU	2	24	16229	228	5.940424	0.044168
pink	6141	CHEN_LUNG_CANCER_SURVIVAL	2	24	16229	228	5.940424	0.044168
cyan	8162	LIM_MAMMARY_STEM_CELL_UP	5	408	15845	77	2.586739	0.044191
brown	1850	V$ELF1_Q6	40	227	16026	2190	1.307746	0.044195
pink	5183	DELYS_THYROID_CANCER_UP	10	383	15870	228	1.861229	0.044198
cyan	8835	GSE17721_CTRL_VS_LPS_1H_BMDM_DN	3	166	16087	77	3.814661	0.044208
cyan	9883	GSE360_T_GONDII_VS_M_TUBERCULOSIS_MAC_DN	3	166	16087	77	3.814661	0.044208
red	8544	GSE13306_RA_VS_UNTREATED_MEM_CD4_TCELL_UP	11	190	16063	524	1.795731	0.044218
red	8668	GSE1432_6H_VS_24H_IFNG_MICROGLIA_UP	11	190	16063	524	1.795731	0.044218
red	9122	GSE17721_ALL_VS_24H_PAM3CSK4_BMDM_UP	11	190	16063	524	1.795731	0.044218
red	10026	GSE3982_DC_VS_TH1_UP	11	190	16063	524	1.795731	0.044218
turquoise	5095	SCIBETTA_KDM5B_TARGETS_DN	26	74	16179	4169	1.369756	0.044234
turquoise	811	RNA_SPLICINGVIA_TRANSESTERIFICATION_REACTIONS	14	35	16218	4169	1.559415	0.044245
turquoise	2143	TAGAACC,MIR-182	14	35	16218	4169	1.559415	0.044245
turquoise	3245	chr3p24	14	35	16218	4169	1.559415	0.044245
blue	9462	GSE24634_IL4_VS_CTRL_TREATED_NAIVE_CD4_TCELL_	41	173	16080	2978	1.293441	0.044249
blue	9798	GSE360_CTRL_VS_B_MALAYI_HIGH_DOSE_DC_UP	41	173	16080	2978	1.293441	0.044249
blue	9839	GSE360_L_DONOVANI_VS_M_TUBERCULOSIS_DC_DN	41	173	16080	2978	1.293441	0.044249
red	600	CELLULAR_CATION_HOMEOSTASIS	6	80	16173	524	2.326288	0.044253
brown	6055	TARTE_PLASMA_CELL_VS_B_LYMPHOCYTE_UP	16	76	16177	2190	1.562413	0.044259
brown	8732	GSE15215_CD2_POS_VS_NEG_PDC_UP	31	169	16084	2190	1.361333	0.044265
lightyellow	6890	FOSTER_TOLERANT_MACROPHAGE_DN	3	391	15862	33	3.778889	0.044269
blue	2846	KEGG_T_CELL_RECEPTOR_SIGNALING_PATHWAY	26	102	16151	2978	1.391176	0.044277
blue	7012	AMBROSINI_FLAVOPIRIDOL_TREATMENT_TP53	26	102	16151	2978	1.391176	0.044277
turquoise	9092	GSE17721_0.5H_VS_12H_LPS_BMDM_UP	59	188	16065	4169	1.223477	0.044278
turquoise	9419	GSE24142_DN2_VS_DN3_THYMOCYTE_ADULT_DN	59	188	16065	4169	1.223477	0.044278
turquoise	10042	GSE3982_MAC_VS_TH1_UP	59	188	16065	4169	1.223477	0.044278
brown	2089	TCTCTCC,MIR-185	18	88	16165	2190	1.518026	0.044288
green	3308	chr9p13	8	50	16203	1305	1.992705	0.044335
blue	4953	GAL_LEUKEMIC_STEM_CELL_UP	29	116	16137	2978	1.364422	0.044352
grey60	9254	GSE19825_NAIVE_VS_DAY3_EFF_CD8_TCELL_UP	2	124	16129	44	5.957845	0.044358
grey60	9386	GSE22886_IL2_VS_IL15_STIM_NKCELL_UP	2	124	16129	44	5.957845	0.044358
yellow	1171	ELECTRON_CARRIER_ACTIVITY	11	71	16182	1426	1.765828	0.044361
brown	4823	LIU_PROSTATE_CANCER_UP	17	82	16171	2190	1.538596	0.044372
greenyello	7414	HAN_SATB1_TARGETS_UP	7	351	15902	150	2.160893	0.044377
salmon	7740	YAO_TEMPORAL_RESPONSE_TO_PROGESTERONE_CLUS	4	170	16083	127	3.011209	0.044395
lightgreen	4825	LIU_SOX4_TARGETS_UP	2	130	16123	42	5.95348	0.044397
lightgreen	5645	MOHANKUMAR_TLX1_TARGETS_DN	2	130	16123	42	5.95348	0.044397
salmon	3575	MODULE_87	2	43	16210	127	5.95239	0.044422
salmon	4226	REACTOME_NOTCH1_INTRACELLULAR_DOMAIN_REGU	2	43	16210	127	5.95239	0.044422
salmon	5367	CHOI_ATL_STAGE_PREDICTOR	2	43	16210	127	5.95239	0.044422
purple	4890	FULCHER_INFLAMMATORY_RESPONSE_LECTIN_VS_LPS	10	558	15695	157	1.855238	0.044438
lightcyan	7860	CHICAS_RB1_TARGETS_SENESCENT	5	509	15744	62	2.5751	0.044439
pink	1708	V$TITF1_Q3	6	183	16070	228	2.337216	0.044464
pink	8900	GSE17721_CTRL_VS_GARDIQUIMOD_2H_BMDM_UP	6	183	16070	228	2.337216	0.044464
pink	9297	GSE20715_0H_VS_24H_OZONE_TLR4_KO_LUNG_DN	6	183	16070	228	2.337216	0.044464
tan	1646	V$MIF1_01	4	149	16104	145	3.009118	0.044506
black	6223	PENG_RAPAMYCIN_RESPONSE_UP	7	186	16067	283	2.161385	0.044506
black	9285	GSE20715_WT_VS_TLR4_KO_24H_OZONE_LUNG_DN	7	186	16067	283	2.161385	0.044506
lightgreen	3534	MODULE_45	4	520	15733	42	2.97674	0.044519
magenta	5086	ZHOU_INFLAMMATORY_RESPONSE_FIMA_DN	6	250	16003	167	2.33576	0.044544
salmon	7455	MILI_PSEUDOPODIA_HAPTOTAXIS_UP	8	505	15748	127	2.027349	0.044581

cyan	3926	PID_SMAD2_3NUCLEARPATHWAY	2	71	16182	77	5.945857	0.044621
cyan	5299	MCBRYAN_PUBERTAL_BREAST_6_7WK_DN	2	71	16182	77	5.945857	0.044621
cyan	5855	FRIDMAN_SENESCENCE_UP	2	71	16182	77	5.945857	0.044621
cyan	7646	BOYLAN_MULTIPLE_MYELOMA_PCA3_UP	2	71	16182	77	5.945857	0.044621
midnightb	5969	MORI_PRE_BI_LYMPHOCYTE_DN	2	77	16176	71	5.945857	0.044621
blue	4477	REACTOME_ELONGATION_ARREST_AND_RECOVERY	10	31	16222	2978	1.760545	0.044644
blue	4481	REACTOME_CD28_CO_STIMULATION	10	31	16222	2978	1.760545	0.044644
blue	7209	HUPER_BREAST_BASAL_VS_LUMINAL_UP	10	31	16222	2978	1.760545	0.044644
blue	582	TRNA_METABOLIC_PROCESS	7	19	16234	2978	2.010728	0.044675
blue	4109	PID_EPHA2_FWDPATHWAY	7	19	16234	2978	2.010728	0.044675
blue	4564	REACTOME_SYNTHESIS_AND_INTERCONVERSION_OF_I	7	19	16234	2978	2.010728	0.044675
blue	6596	VISALA_AGING_LYMPHOCYTE_DN	7	19	16234	2978	2.010728	0.044675
blue	8135	MIZUSHIMA_AUTOPHAGOSOME_FORMATION	7	19	16234	2978	2.010728	0.044675
salmon	2429	MORF_PAPSS1	3	101	16152	127	3.801279	0.044677
salmon	4708	REACTOME_LATE_PHASE_OF_HIV_LIFE_CYCLE	3	101	16152	127	3.801279	0.044677
pink	874	ION_HOMEOSTASIS	4	95	16158	228	3.001477	0.044695
blue	1785	V$EFC_Q6	48	207	16046	2978	1.265551	0.044722
red	5849	RICKMAN_TUMOR_DIFFERENTIATED_WELL_VS_MODEI	7	101	16152	524	2.149705	0.044738
midnightb	2162	TGTGTGA,MIR-377	3	181	16072	71	3.794179	0.044756
lightgreen	6482	CUI_TCF21_TARGETS_2_DN	5	759	15494	42	2.54925	0.044792
magenta	1624	V$NKX25_02	5	188	16065	167	2.588387	0.0448
magenta	9419	GSE24142_DN2_VS_DN3_THYMOCYTE_ADULT_DN	5	188	16065	167	2.588387	0.0448
purple	692	G_PROTEIN_SIGNALING_COUPLED_TO_CAMP_NUCLEO	2	35	16218	157	5.91556	0.044813
purple	3162	chr2q32	2	35	16218	157	5.91556	0.044813
purple	4045	PID_BMPPATHWAY	2	35	16218	157	5.91556	0.044813
purple	4314	REACTOME_GAB1_SIGNALOSOME	2	35	16218	157	5.91556	0.044813
purple	5935	AMIT_EGF_RESPONSE_60_MCF10A	2	35	16218	157	5.91556	0.044813
purple	6260	STOSSI_RESPONSE_TO_ESTRADIOL	2	35	16218	157	5.91556	0.044813
purple	7486	VALK_AML_CLUSTER_9	2	35	16218	157	5.91556	0.044813
purple	7884	FIGUEROA_AML_METHYLATION_CLUSTER_3_DN	2	35	16218	157	5.91556	0.044813
yellow	1142	HYDROLASE_ACTIVITY_ACTING_ON_GLYCOSYL_BONDS	7	38	16215	1426	2.099561	0.044823
lightcyan	366	NEGATIVE_REGULATION_OF_HYDROLASE_ACTIVITY	1	12	16241	62	21.84543	0.044843
lightcyan	570	DEFENSE_RESPONSE_TO_VIRUS	1	12	16241	62	21.84543	0.044843
lightcyan	1440	PHOSPHOLIPID_TRANSPORTER_ACTIVITY	1	12	16241	62	21.84543	0.044843
lightcyan	4214	REACTOME_TRAFFICKING_AND_PROCESSING_OF_END(	1	12	16241	62	21.84543	0.044843
lightcyan	5831	CAFFAREL_RESPONSE_TO_THC_24HR_3_DN	1	12	16241	62	21.84543	0.044843
lightcyan	5887	TAGHAVI_NEOPLASTIC_TRANSFORMATION	1	12	16241	62	21.84543	0.044843
lightcyan	6168	KUROKAWA_LIVER_CANCER_CHEMOTHERAPY_UP	1	12	16241	62	21.84543	0.044843
lightcyan	6907	KYNG_ENVIRONMENTAL_STRESS_RESPONSE_NOT_BY_	1	12	16241	62	21.84543	0.044843
lightcyan	7907	PHESSE_TARGETS_OF_APC_AND_MBD2_DN	1	12	16241	62	21.84543	0.044843
lightcyan	8040	WANG_THOC1_TARGETS_DN	1	12	16241	62	21.84543	0.044843
lightcyan	7371	SWEET_LUNG_CANCER_KRAS_DN	4	351	15902	62	2.987409	0.044849
cyan	8679	GSE1448_CTRL_VS_ANTI_VALPHA2_DP_THYMOCYTE_D	3	167	16086	77	3.791819	0.044868
cyan	8741	GSE15324_ELF4_KO_VS_WT_ACTIVATED_CD8_TCELL_D	3	167	16086	77	3.791819	0.044868
cyan	8839	GSE17721_CTRL_VS_LPS_4H_BMDM_DN	3	167	16086	77	3.791819	0.044868
cyan	9517	GSE2706_R848_VS_R848_AND_LPS_8H_STIM_DC_DN	3	167	16086	77	3.791819	0.044868
magenta	2036	GCTCTTG,MIR-335	3	77	16176	167	3.791819	0.044868
purple	1732	V$AP2REP_01	4	138	16115	157	3.000646	0.044886
brown	8462	GSE11864_UNTREATED_VS_CSF1_IFNG_IN_MAC_UP	33	182	16071	2190	1.34565	0.044886
brown	8776	GSE15930_NAIVE_VS_24H_IN_VITRO_STIM_CD8_TCELI	33	182	16071	2190	1.34565	0.044886
brown	9100	GSE17721_12H_VS_24H_LPS_BMDM_UP	33	182	16071	2190	1.34565	0.044886
brown	9533	GSE27786_LSK_VS_CD4_TCELL_DN	33	182	16071	2190	1.34565	0.044886
brown	10035	GSE3982_MAC_VS_BASOPHIL_DN	33	182	16071	2190	1.34565	0.044886
brown	10078	GSE3982_BASOPHIL_VS_TH1_UP	33	182	16071	2190	1.34565	0.044886
blue	7363	VANTVEER_BREAST_CANCER_ESR1_UP	37	154	16099	2978	1.311263	0.044911
turquoise	9076	GSE17721_LPS_VS_GARDIQUIMOD_1H_BMDM_UP	51	160	16093	4169	1.242659	0.044935
red	1756	V$BACH1_01	12	214	16039	524	1.739281	0.044974
green	1224	PHOSPHORIC_ESTER_HYDROLASE_ACTIVITY	17	136	16117	1305	1.556801	0.044989
green	9168	GSE17974_CTRL_VS_ACT_IL4_AND_ANTI_IL12_0.5H_CI	17	136	16117	1305	1.556801	0.044989
grey60	8038	KATSANOU_ELAVL1_TARGETS_DN	2	125	16128	44	5.910182	0.045003

lightyellow	1400	ENZYME_BINDING	2	167	16086	33	5.898385	0.04501
magenta	3741	MODULE_280	2	33	16220	167	5.898385	0.04501
blue	1380	INORGANIC_CATION_TRANSMEMBRANE_TRANSPORTE	14	48	16205	2978	1.591826	0.045013
blue	2558	GCM_SUPT4H1	14	48	16205	2978	1.591826	0.045013
blue	3346	chr6q25	14	48	16205	2978	1.591826	0.045013
blue	5638	OXFORD_RALA_OR_RALB_TARGETS_UP	14	48	16205	2978	1.591826	0.045013
blue	6307	PARK_HSC_AND_MULTIPOTENT_PROGENITORS	14	48	16205	2978	1.591826	0.045013
blue	7300	CHAUHAN_RESPONSE_TO_METHOXYESTRADIOL_UP	14	48	16205	2978	1.591826	0.045013
purple	4167	REACTOME_MEIOSIS	3	82	16171	157	3.787401	0.045025
pink	1419	TRANSMEMBRANE_TRANSPORTER_ACTIVITY	8	281	15972	228	2.029469	0.045026
blue	7265	BOYLAN_MULTIPLE_MYELOMA_C_D_UP	33	135	16118	2978	1.334102	0.045031
black	3383	chr20q13	6	148	16105	283	2.328288	0.045048
grey60	1120	HEPARIN_BINDING	1	17	16236	44	21.72861	0.045061
grey60	1451	ANTIOXIDANT_ACTIVITY	1	17	16236	44	21.72861	0.045061
grey60	3259	chr3p26	1	17	16236	44	21.72861	0.045061
grey60	5047	TONKS_TARGETS_OF_RUNX1_RUNX1T1_FUSION_GRAN	1	17	16236	44	21.72861	0.045061
pink	8209	CYCLIN_D1_KE_.V1_DN	5	138	16115	228	2.582793	0.045088
turquoise	2205	TTCYRGAA_UNKNOWN	80	263	15990	4169	1.185867	0.045101
blue	7672	KYNG_WERNER_SYNDROM_AND_NORMAL_AGING_DN	49	212	16041	2978	1.261447	0.045161
red	991	REGULATION_OF_PROGRAMMED_CELL_DEATH	16	311	15942	524	1.595739	0.045198
greenyello	2839	KEGG_TOLL_LIKE_RECEPTOR_SIGNALING_PATHWAY	3	86	16167	150	3.779767	0.045268
greenyello	8371	KRAS.BREAST_UP.V1_UP	3	86	16167	150	3.779767	0.045268
brown	8840	GSE17721_CTRL_VS_LPS_6H_BMDM_UP	35	195	16058	2190	1.332057	0.045284
brown	9210	GSE17974_0.5H_VS_72H_IL4_AND_ANTI_IL12_ACT_CD	35	195	16058	2190	1.332057	0.045284
brown	9897	GSE36392_EOSINOPHIL_VS_MAC_IL25_TREATED_LUNG	35	195	16058	2190	1.332057	0.045284
brown	9996	GSE3982_MAST_CELL_VS_NEUTROPHIL_UP	35	195	16058	2190	1.332057	0.045284
brown	10040	GSE3982_MAC_VS_NKCELL_UP	35	195	16058	2190	1.332057	0.045284
red	1076	HYDRO_LYASE_ACTIVITY	3	25	16228	524	3.722061	0.045295
red	3081	BIOCARTA_NKT_PATHWAY	3	25	16228	524	3.722061	0.045295
red	6392	GENTILE_UV_RESPONSE_CLUSTER_D9	3	25	16228	524	3.722061	0.045295
greenyello	1754	V$STAT5A_03	5	210	16043	150	2.579841	0.045306
greenyello	591	NEUROPEPTIDE_SIGNALING_PATHWAY	1	5	16248	150	21.67067	0.045307
greenyello	3136	chr1p11	1	5	16248	150	21.67067	0.045307
greenyello	3280	chr5q	1	5	16248	150	21.67067	0.045307
greenyello	4778	REACTOME_APOBEC3G_MEDIATED_RESISTANCE_TO_H	1	5	16248	150	21.67067	0.045307
greenyello	5763	GALIE_TUMOR_STEMNESS_GENES	1	5	16248	150	21.67067	0.045307
greenyello	7288	JU_AGING_TERC_TARGETS_DN	1	5	16248	150	21.67067	0.045307
greenyello	7448	CHEOK_RESPONSE_TO_MERCAPTOPURINE_AND_HD_N	1	5	16248	150	21.67067	0.045307
brown	3423	chr7q32	9	36	16217	2190	1.855365	0.045349
brown	4431	REACTOME_MUSCLE_CONTRACTION	9	36	16217	2190	1.855365	0.045349
brown	4876	TURASHVILI_BREAST_DUCTAL_CARCINOMA_VS_DUCTA	9	36	16217	2190	1.855365	0.045349
brown	6657	GEISS_RESPONSE_TO_DSRNA_UP	9	36	16217	2190	1.855365	0.045349
brown	7155	NAKAMURA_METASTASIS_MODEL_DN	9	36	16217	2190	1.855365	0.045349
midnightb	8834	GSE17721_CTRL_VS_LPS_1H_BMDM_UP	3	182	16071	71	3.773332	0.045368
midnightb	9654	GSE29618_BCELL_VS_MONOCYTE_UP	3	182	16071	71	3.773332	0.045368
midnightb	9805	GSE360_CTRL_VS_L_DONOVANI_MAC_DN	3	182	16071	71	3.773332	0.045368
yellow	9437	GSE24634_NAIVE_CD4_TCELL_VS_DAY7_IL4_CONV_TR	25	200	16053	1426	1.424702	0.045381
red	8055	PEDERSEN_METASTASIS_BY_ERBB2_ISOFORM_7	18	361	15892	524	1.546563	0.045394
turquoise	2612	GNF2_APEX1	31	91	16162	4169	1.328073	0.045394
turquoise	4803	PYEON_HPV_POSITIVE_TUMORS_UP	31	91	16162	4169	1.328073	0.045394
lightyellow	3527	MODULE_38	3	395	15858	33	3.740621	0.045398
red	4482	REACTOME_GPCR_DOWNSTREAM_SIGNALING	17	336	15917	524	1.569321	0.045402
red	5657	GRUETZMANN_PANCREATIC_CANCER_UP	17	336	15917	524	1.569321	0.045402
greenyello	8769	GSE15750_WT_VS_TRAF6KO_DAY10_EFF_CD8_TCELL_	4	145	16108	150	2.989057	0.04542
brown	10096	GSE3982_NKCELL_VS_TH1_UP	30	163	16090	2190	1.365913	0.045429
pink	8491	GSE11924_TH2_VS_TH17_CD4_TCELL_DN	6	184	16069	228	2.324514	0.045449
lightyellow	654	PROTEOLYSIS	2	168	16085	33	5.863276	0.045495
lightyellow	6185	OKUMURA_INFLAMMATORY_RESPONSE_LPS	2	168	16085	33	5.863276	0.045495
lightyellow	9368	GSE22886_NAIVE_BCELL_VS_MONOCYTE_UP	2	168	16085	33	5.863276	0.045495
lightyellow	9725	GSE30962_PRIMARY_VS_SECONDARY_CHRONIC_LCMV	2	168	16085	33	5.863276	0.045495

lightyellow	9850	GSE360_T_GONDII_VS_B_MALAYI_LOW_DOSE_DC_UP	2	168	16085	33	5.863276	0.045495
pink	7433	HSIAO_HOUSEKEEPING_GENES	10	385	15868	228	1.851561	0.045501
blue	4566	REACTOME_RNA_POL_I_RNA_POL_III_AND_MITOCHOI	24	93	16160	2978	1.408436	0.04551
blue	3697	MODULE_219	9	27	16226	2978	1.81923	0.045527
blue	4155	REACTOME_DAG_AND_IP3_SIGNALING	9	27	16226	2978	1.81923	0.045527
blue	4768	REACTOME_EXTENSION_OF_TELOMERES	9	27	16226	2978	1.81923	0.045527
blue	7829	WANG_METASTASIS_OF_BREAST_CANCER_ESR1_DN	9	27	16226	2978	1.81923	0.045527
lightgreen	2948	BIOCARTA_GCR_PATHWAY	1	18	16235	42	21.49868	0.04553
lightgreen	3148	chr12q15	1	18	16235	42	21.49868	0.04553
lightgreen	4328	REACTOME_FATTY_ACYL_COA_BIOSYNTHESIS	1	18	16235	42	21.49868	0.04553
lightgreen	4779	REACTOME_GAP_JUNCTION_TRAFFICKING	1	18	16235	42	21.49868	0.04553
lightgreen	4834	DAVICIONI_PAX_FOXO1_SIGNATURE_IN_ARMS_DN	1	18	16235	42	21.49868	0.04553
lightgreen	5838	SUH_COEXPRESSED_WITH_ID1_AND_ID2_UP	1	18	16235	42	21.49868	0.04553
lightgreen	7607	KUROZUMI_RESPONSE_TO_ONCOCYTIC_VIRUS_AND_C	1	18	16235	42	21.49868	0.04553
lightgreen	7994	BILANGES_SERUM_SENSITIVE_VIA_TSC1	1	18	16235	42	21.49868	0.04553
cyan	9850	GSE360_T_GONDII_VS_B_MALAYI_LOW_DOSE_DC_UP	3	168	16085	77	3.769249	0.045533
blue	8677	GSE14350_IL2RB_KO_VS_WT_TEFF_DN	43	183	16070	2978	1.282408	0.045539
blue	8922	GSE17721_LPS_VS_POLYIC_8H_BMDM_UP	43	183	16070	2978	1.282408	0.045539
blue	9471	GSE25087_TREG_VS_TCONV_ADULT_DN	43	183	16070	2978	1.282408	0.045539
pink	1573	V$AP1FJ_Q2	7	232	16021	228	2.150843	0.04554
pink	7689	DANG_REGULATED_BY_MYC_DN	7	232	16021	228	2.150843	0.04554
turquoise	5516	FARMER_BREAST_CANCER_CLUSTER_2	13	32	16221	4169	1.583781	0.045554
turquoise	5832	CAFFAREL_RESPONSE_TO_THC_24HR_5_UP	13	32	16221	4169	1.583781	0.045554
red	1228	KINASE_REGULATOR_ACTIVITY	4	42	16211	524	2.954017	0.045573
red	3133	chr2p22	4	42	16211	524	2.954017	0.045573
red	3350	chr9q21	4	42	16211	524	2.954017	0.045573
red	3574	MODULE_86	4	42	16211	524	2.954017	0.045573
red	6559	BROWNE_HCMV_INFECTION_8HR_DN	4	42	16211	524	2.954017	0.045573
red	3701	MODULE_223	8	123	16130	524	2.017377	0.04559
red	5338	DARWICHE_SQUAMOUS_CELL_CARCINOMA_UP	8	123	16130	524	2.017377	0.04559
red	8317	RB_P130_DN.V1_DN	8	123	16130	524	2.017377	0.04559
red	8329	IL21_UP.V1_DN	8	123	16130	524	2.017377	0.04559
purple	5164	COLDREN_GEFITINIB_RESISTANCE_DN	5	201	16052	157	2.575181	0.04561
green	6392	GENTILE_UV_RESPONSE_CLUSTER_D9	5	25	16228	1305	2.490881	0.045615
red	9721	GSE30083_SP3_VS_SP4_THYMOCYTE_DN	11	191	16062	524	1.786329	0.045624
lightgreen	7345	RUIZ_TNC_TARGETS_UP	2	132	16121	42	5.863276	0.045632
magenta	1517	V$PBX1_01	5	189	16064	167	2.574692	0.045652
grey60	5034	MULLIGHAN_NPM1_MUTATED_SIGNATURE_2_UP	2	126	16127	44	5.863276	0.045652
greenyello	3054	BIOCARTA_CHREBP2_PATHWAY	2	37	16216	150	5.856937	0.045656
greenyello	6437	WEIGEL_OXIDATIVE_STRESS_BY_HNE_AND_H2O2	2	37	16216	150	5.856937	0.045656
greenyello	6988	FUJII_YBX1_TARGETS_UP	2	37	16216	150	5.856937	0.045656
pink	7846	KIM_ALL_DISORDERS_OLIGODENDROCYTE_NUMBER_C	16	717	15536	228	1.590741	0.045659
brown	5332	DARWICHE_SKIN_TUMOR_PROMOTER_UP	23	119	16134	2190	1.4344	0.045671
blue	133	ORGANELLE_OUTER_MEMBRANE	8	23	16230	2978	1.898327	0.045683
blue	645	POSITIVE_REGULATION_OF_TRANSCRIPTION_FACTOR_	8	23	16230	2978	1.898327	0.045683
blue	4990	GARGALOVIC_RESPONSE_TO_OXIDIZED_PHOSPHOLIPIE	8	23	16230	2978	1.898327	0.045683
yellow	840	NEGATIVE_REGULATION_OF_BINDING	4	16	16237	1426	2.849404	0.045699
yellow	1236	RNA_POLYMERASE_ACTIVITY	4	16	16237	1426	2.849404	0.045699
yellow	2726	KEGG_STEROID_BIOSYNTHESIS	4	16	16237	1426	2.849404	0.045699
yellow	3089	BIOCARTA_SPRY_PATHWAY	4	16	16237	1426	2.849404	0.045699
yellow	4233	REACTOME_ARMS_MEDIATED_ACTIVATION	4	16	16237	1426	2.849404	0.045699
lightyellow	2894	KEGG_ASTHMA	1	23	16230	33	21.4137	0.045701
lightyellow	2952	BIOCARTA_CELLCYCLE_PATHWAY	1	23	16230	33	21.4137	0.045701
lightyellow	3318	chr15q23	1	23	16230	33	21.4137	0.045701
lightyellow	3957	PID_INTEGRIN_CS_PATHWAY	1	23	16230	33	21.4137	0.045701
lightyellow	4169	REACTOME_G0_AND_EARLY_G1	1	23	16230	33	21.4137	0.045701
lightyellow	5318	ZIRN_TRETINOIN_RESPONSE_WT1_UP	1	23	16230	33	21.4137	0.045701
lightyellow	6919	KONDO_COLON_CANCER_HCP_WITH_H3K27ME1	1	23	16230	33	21.4137	0.045701
lightyellow	7783	DAZARD_UV_RESPONSE_CLUSTER_G24	1	23	16230	33	21.4137	0.045701
purple	3510	MODULE_18	8	410	15843	157	2.019947	0.045749

cyan	7945	KIM_GLIS2_TARGETS_UP	2	72	16181	77	5.863276	0.045758
black	3985	PID_LKB1_PATHWAY	3	46	16207	283	3.745506	0.045794
red	7253	LEE_LIVER_CANCER_SURVIVAL_DN	10	168	16085	524	1.84626	0.045801
red	8894	GSE17721_CTRL_VS_CPG_24H_BMDM_UP	10	168	16085	524	1.84626	0.045801
red	9797	GSE360_CTRL_VS_T_GONDII_DC_DN	10	168	16085	524	1.84626	0.045801
yellow	4124	REACTOME_RNA_POL_III_TRANSCRIPTION_INITIATION_	5	23	16230	1426	2.477743	0.045817
blue	3202	chr12p	3	5	16248	2978	3.274614	0.04582
blue	3294	chr4q	3	5	16248	2978	3.274614	0.04582
blue	5464	MYLLYKANGAS_AMPLIFICATION_HOT_SPOT_25	3	5	16248	2978	3.274614	0.04582
blue	5468	MYLLYKANGAS_AMPLIFICATION_HOT_SPOT_29	3	5	16248	2978	3.274614	0.04582
blue	7634	LOPEZ_MESOTHELIOMA_SURVIVAL_OVERALL_UP	3	5	16248	2978	3.274614	0.04582
blue	8068	IM_SREBF1A_TARGETS	3	5	16248	2978	3.274614	0.04582
pink	416	CELLULAR_PROTEIN_CATABOLIC_PROCESS	3	57	16196	228	3.751847	0.045841
pink	6631	WEIGEL_OXIDATIVE_STRESS_BY_HNE_AND_TBH	3	57	16196	228	3.751847	0.045841
grey60	2234	CAGCTG_V$AP4_Q5	7	1231	15022	44	2.100491	0.045851
turquoise	9600	GSE27786_NKCELL_VS_ERYTHROBLAST_UP	62	199	16054	4169	1.21462	0.045926
turquoise	10228	GSE9006_HEALTHY_VS_TYPE_1_DIABETES_PBMC_4MC	62	199	16054	4169	1.21462	0.045926
turquoise	3507	MODULE_15	101	339	15914	4169	1.161511	0.045951
blue	415	POSITIVE_REGULATION_OF_RNA_METABOLIC_PROCES	27	107	16146	2978	1.377174	0.045954
blue	1776	V$ARNT_02	51	222	16031	2978	1.253794	0.045957
lightyellow	1542	V$GATA1_04	2	169	16084	33	5.828582	0.045983
midnightb	8456	GSE11057_NAIVE_VS_MEMORY_CD4_TCELL_UP	3	183	16070	71	3.752713	0.045984
midnightb	8801	GSE15930_STIM_VS_STIM_AND_TRICHOSTATINA_24H_	3	183	16070	71	3.752713	0.045984
midnightb	9501	GSE2706_UNSTIM_VS_8H_R848_DC_DN	3	183	16070	71	3.752713	0.045984
midnightb	9668	GSE29618_BCELL_VS_PDC_DAY7_FLU_VACCINE_UP	3	183	16070	71	3.752713	0.045984
midnightb	9885	GSE360_HIGH_VS_LOW_DOSE_B_MALAYI_MAC_DN	3	183	16070	71	3.752713	0.045984
midnightb	9960	GSE3982_MEMORY_CD4_TCELL_VS_TH2_UP	3	183	16070	71	3.752713	0.045984
salmon	5192	CASTELLANO_HRAS_TARGETS_DN	1	6	16247	127	21.3294	0.045984
salmon	6051	BYSTRYKH_SCP2_QTL	1	6	16247	127	21.3294	0.045984
salmon	9034	GSE17721_PAM3CSK4_VS_GADIQUIMOD_16H_BMDM_	4	172	16081	127	2.976195	0.046
salmon	9104	GSE17721_0.5H_VS_4H_POLYIC_BMDM_UP	4	172	16081	127	2.976195	0.046
salmon	10073	GSE3982_BASOPHIL_VS_EFF_MEMORY_CD4_TCELL_DN	4	172	16081	127	2.976195	0.046
lightcyan	6415	BLALOCK_ALZHEIMERS_DISEASE_INCIPIENT_UP	4	354	15899	62	2.962092	0.046024
brown	2116	GTGCCAA,MIR-96	46	267	15986	2190	1.278604	0.046027
brown	5897	PASQUALUCCI_LYMPHOMA_BY_GC_STAGE_UP	46	267	15986	2190	1.278604	0.046027
brown	6937	HELLER_HDAC_TARGETS_SILENCED_BY_METHYLATION_	46	267	15986	2190	1.278604	0.046027
greenyello	1484	V$NFKAPPAB_01	5	211	16042	150	2.567615	0.046075
blue	6035	ONDER_CDH1_TARGETS_1_DN	34	140	16113	2978	1.325439	0.046098
blue	8522	GSE12845_PRE_GC_VS_DARKZONE_GC_TONSIL_BCELL_	44	188	16065	2978	1.277332	0.046126
blue	9428	GSE24142_ADULT_VS_FETAL_DN2_THYMOCYTE_UP	44	188	16065	2978	1.277332	0.046126
blue	9770	GSE339_CD4POS_VS_CD4CD8DN_DC_UP	44	188	16065	2978	1.277332	0.046126
pink	3632	MODULE_147	4	96	16157	228	2.970212	0.046146
pink	8166	LIM_MAMMARY_LUMINAL_MATURE_UP	4	96	16157	228	2.970212	0.046146
turquoise	1120	HEPARIN_BINDING	8	17	16236	4169	1.834606	0.046255
turquoise	4208	REACTOME_RIP_MEDIATED_NFKB_ACTIVATION_VIA_D	8	17	16236	4169	1.834606	0.046255
turquoise	4573	REACTOME_ACTIVATED_TAK1_MEDIATES_P38_MAPK_	8	17	16236	4169	1.834606	0.046255
turquoise	6551	YIH_RESPONSE_TO_ARSENITE_C2	8	17	16236	4169	1.834606	0.046255
lightcyan	6857	GAVIN_FOXP3_TARGETS_CLUSTER_P6	2	90	16163	62	5.825448	0.046281
turquoise	10059	GSE3982_NEUTROPHIL_VS_TH2_DN	60	192	16061	4169	1.218293	0.046288
red	3523	MODULE_33	16	312	15941	524	1.590624	0.046291
turquoise	2086	GGCAGTG,MIR-324-3P	28	81	16172	4169	1.347642	0.046295
cyan	3519	MODULE_27	4	285	15968	77	2.962497	0.046304
grey60	4489	REACTOME_G_ALPHA_I_SIGNALLING_EVENTS	2	127	16126	44	5.817108	0.046304
salmon	2799	KEGG_NUCLEOTIDE_EXCISION_REPAIR	2	44	16209	127	5.817108	0.046304
salmon	4413	REACTOME_TRANSCRIPTION_COUPLED_NER_TC_NER	2	44	16209	127	5.817108	0.046304
salmon	6771	JIANG_AGING_HYPOTHALAMUS_UP	2	44	16209	127	5.817108	0.046304
salmon	7136	GRADE_METASTASIS_DN	2	44	16209	127	5.817108	0.046304
red	1512	V$IK1_01	12	215	16038	524	1.731191	0.046308
red	1900	V$ER_Q6_01	12	215	16038	524	1.731191	0.046308
magenta	1680	V$EN1_01	3	78	16175	167	3.743206	0.046323

magenta	2892	KEGG_SMALL_CELL_LUNG_CANCER	3	78	16175	167	3.743206	0.046323
magenta	5972	MORI_SMALL_PRE_BII_LYMPHOCYTE_UP	3	78	16175	167	3.743206	0.046323
magenta	6115	SANA_TNF_SIGNALING_DN	3	78	16175	167	3.743206	0.046323
magenta	7576	MIKKELSEN_IPS_ICP_WITH_H3K4ME3_AND_H327ME3	3	78	16175	167	3.743206	0.046323
yellow	9186	GSE17974_IL4_AND_ANTI_IL12_VS_UNTREATED_0.5H_	20	153	16100	1426	1.489884	0.046376
greenyello	8265	PRC2_EDD_UP.V1_DN	4	146	16107	150	2.968584	0.046378
greenyello	8327	IL2_UP.V1_DN	4	146	16107	150	2.968584	0.046378
yellow	1620	V$AHRARNT_01	16	116	16137	1426	1.572085	0.046386
cyan	573	MYELOID_LEUKOCYTE_DIFFERENTIATION	1	10	16243	77	21.10779	0.046391
cyan	843	RESPONSE_TO_IONIZING_RADIATION	1	10	16243	77	21.10779	0.046391
cyan	2910	BIOCARTA_LYM_PATHWAY	1	10	16243	77	21.10779	0.046391
cyan	3021	BIOCARTA_MONOCYTE_PATHWAY	1	10	16243	77	21.10779	0.046391
cyan	3075	BIOCARTA_PARKIN_PATHWAY	1	10	16243	77	21.10779	0.046391
cyan	3207	chr2q34	1	10	16243	77	21.10779	0.046391
cyan	3873	MODULE_471	1	10	16243	77	21.10779	0.046391
cyan	4220	REACTOME_REGULATION_OF_COMPLEMENT_CASCADI	1	10	16243	77	21.10779	0.046391
cyan	4672	REACTOME_IL_6_SIGNALING	1	10	16243	77	21.10779	0.046391
purple	6614	LEE_AGING_NEOCORTEX_UP	3	83	16170	157	3.74177	0.046394
brown	1958	TGCACTT,MIR-519C,MIR-519B,MIR-519A	64	387	15866	2190	1.227322	0.046405
pink	9292	GSE20715_0H_VS_48H_OZONE_LUNG_UP	6	185	16068	228	2.311949	0.046447
pink	9765	GSE3337_4H_VS_16H_IFNG_IN_CD8POS_DC_DN	6	185	16068	228	2.311949	0.046447
brown	421	NEGATIVE_REGULATION_OF_TRANSCRIPTION_DNA_DE	22	113	16140	2190	1.444886	0.046454
brown	5277	MISSIAGLIA_REGULATED_BY_METHYLATION_UP	22	113	16140	2190	1.444886	0.046454
brown	926	LYMPHOCYTE_ACTIVATION	12	53	16200	2190	1.680331	0.046455
brown	3725	MODULE_256	12	53	16200	2190	1.680331	0.046455
purple	4828	ONKEN_UVEAL_MELANOMA_DN	9	486	15767	157	1.917079	0.046463
red	144	ENDOSOME	5	61	16192	524	2.542391	0.046465
red	1813	GATGKMRGCG_UNKNOWN	5	61	16192	524	2.542391	0.046465
lightyellow	9625	GSE2826_WT_VS_XID_BCELL_DN	2	170	16083	33	5.794296	0.046472
magenta	1547	V$TST1_01	5	190	16063	167	2.561141	0.046513
magenta	1581	V$MYB_Q6	5	190	16063	167	2.561141	0.046513
green	8500	GSE12366_PLASMA_CELL_VS_MEMORY_BCELL_UP	20	167	16086	1305	1.491546	0.046546
grey60	6978	IWANAGA_CARCINOGENESIS_BY_KRAS_PTEN_DN	3	298	15955	44	3.718655	0.046552
greenyello	1623	V$NKX25_01	3	87	16166	150	3.736322	0.046578
brown	8847	GSE17721_CTRL_VS_LPS_24H_BMDM_DN	34	189	16064	2190	1.335078	0.046595
brown	8923	GSE17721_LPS_VS_POLYIC_8H_BMDM_DN	34	189	16064	2190	1.335078	0.046595
brown	10153	GSE7460_TCONV_VS_TREG_THYMUS_DN	34	189	16064	2190	1.335078	0.046595
midnightb	8504	GSE12845_IGD_POS_VS_NEG_BLOOD_BCELL_UP	3	184	16069	71	3.732318	0.046604
midnightb	9174	GSE17974_CTRL_VS_ACT_IL4_AND_ANTI_IL12_4H_CD4	3	184	16069	71	3.732318	0.046604
midnightb	9318	GSE22886_NAIVE_CD8_TCELL_VS_NKCELL_UP	3	184	16069	71	3.732318	0.046604
midnightb	9666	GSE29618_BCELL_VS_MONOCYTE_DAY7_FLU_VACCINE	3	184	16069	71	3.732318	0.046604
midnightb	10055	GSE3982_NEUTROPHIL_VS_NKCELL_DN	3	184	16069	71	3.732318	0.046604
turquoise	9535	GSE27786_LSK_VS_CD8_TCELL_DN	58	185	16068	4169	1.222244	0.04661
purple	4845	SENGUPTA_NASOPHARYNGEAL_CARCINOMA_DN	6	269	15984	157	2.309047	0.046611
magenta	356	CELL_SURFACE_RECEPTOR_LINKED_SIGNAL_TRANSDUC	9	457	15796	167	1.916652	0.046623
yellow	8311	NRL_DN.V1_DN	15	107	16146	1426	1.597797	0.046647
blue	1767	V$ATF4_Q2	53	232	16021	2978	1.2468	0.046651
turquoise	1828	V$PAX8_01	12	29	16224	4169	1.613188	0.046661
turquoise	2915	BIOCARTA_ALK_PATHWAY	12	29	16224	4169	1.613188	0.046661
turquoise	5172	CHOW_RASSF1_TARGETS_DN	12	29	16224	4169	1.613188	0.046661
turquoise	7110	ZHENG_IL22_SIGNALING_DN	12	29	16224	4169	1.613188	0.046661
turquoise	8122	YU_BAP1_TARGETS	12	29	16224	4169	1.613188	0.046661
lightcyan	6580	DAZARD_RESPONSE_TO_UV_NHEK_UP	3	211	16042	62	3.727182	0.046665
black	8806	GSE15930_STIM_VS_STIM_AND_TRICHOSTATINA_48H_	7	188	16065	283	2.138392	0.046673
black	9406	GSE24102_GRANULOCYSTIC_MDSC_VS_NEUTROPHIL_U	7	188	16065	283	2.138392	0.046673
blue	2373	MORF_DAP3	45	193	16060	2978	1.272518	0.046677
blue	10108	GSE39820_CTRL_VS_TGFBETA3_IL6_CD4_TCELL_UP	45	193	16060	2978	1.272518	0.046677
yellow	1310	STRUCTURAL_MOLECULE_ACTIVITY	24	191	16062	1426	1.432161	0.046703
yellow	8910	GSE17721_CTRL_VS_GARDIQUIMOD_24H_BMDM_UP	24	191	16062	1426	1.432161	0.046703
yellow	9030	GSE17721_PAM3CSK4_VS_GADIQUIMOD_8H_BMDM_	24	191	16062	1426	1.432161	0.046703

yellow	9094	GSE17721_0.5H_VS_4H_LPS_BMDM_UP	24	191	16062	1426	1.432161	0.046703
yellow	9899	GSE36392_EOSINOPHIL_VS_NEUTROPHIL_IL25_TREATE	24	191	16062	1426	1.432161	0.046703
blue	416	CELLULAR_PROTEIN_CATABOLIC_PROCESS	16	57	16196	2978	1.531983	0.046704
black	214	RIBOSOMAL_SUBUNIT	2	20	16233	283	5.74311	0.046711
black	1445	METALLOENDOPEPTIDASE_ACTIVITY	2	20	16233	283	5.74311	0.046711
red	2846	KEGG_T_CELL_RECEPTOR_SIGNALING_PATHWAY	7	102	16151	524	2.12863	0.046764
cyan	8094	KRIEG_HYPOXIA_NOT_VIA_KDM3A	7	697	15556	77	2.119864	0.046798
salmon	9108	GSE17721_0.5H_VS_24H_POLYIC_BMDM_UP	4	173	16080	127	2.958991	0.046815
salmon	9954	GSE3982_CTRL_VS_LPS_1H_NEUTROPHIL_UP	4	173	16080	127	2.958991	0.046815
brown	307	BIOPOLYMER_METABOLIC_PROCESS	228	1529	14724	2190	1.106667	0.046839
magenta	5087	ZHOU_INFLAMMATORY_RESPONSE_LPS_UP	7	319	15934	167	2.135622	0.046859
turquoise	2379	MORF_DMPK	42	129	16124	4169	1.269291	0.046863
brown	1878	V$YY1_Q6	40	228	16025	2190	1.302011	0.046871
cyan	6217	SASAKI_ADULT_T_CELL_LEUKEMIA	3	170	16083	77	3.724905	0.046877
cyan	6327	AFFAR_YY1_TARGETS_UP	3	170	16083	77	3.724905	0.046877
cyan	7524	CHIANG_LIVER_CANCER_SUBCLASS_PROLIFERATION_U	3	170	16083	77	3.724905	0.046877
cyan	9513	GSE2706_R848_VS_LPS_8H_STIM_DC_DN	3	170	16083	77	3.724905	0.046877
lightgreen	8441	GSE10463_CD40L_AND_VA347_VS_CD40L_IN_DC_DN	2	134	16119	42	5.775764	0.04688
yellow	3549	MODULE_60	42	368	15885	1426	1.300815	0.046886
turquoise	9038	GSE17721_LPS_VS_CPG_0.5H_BMDM_UP	56	178	16075	4169	1.226506	0.046887
turquoise	9090	GSE17721_LPS_VS_GARDIQUIMOD_24H_BMDM_UP	56	178	16075	4169	1.226506	0.046887
green	1819	V$HEB_Q6	25	219	16034	1305	1.421736	0.046931
lightyellow	4368	REACTOME_CLASS_A1_RHODOPSIN_LIKE_RECEPTORS	2	171	16082	33	5.760411	0.046963
lightyellow	5044	TONKS_TARGETS_OF_RUNX1_RUNX1T1_FUSION_HSC_	2	171	16082	33	5.760411	0.046963
lightyellow	8224	PIGF_UP.V1_UP	2	171	16082	33	5.760411	0.046963
midnightb	846	TRANSITION_METAL_ION_TRANSPORT	1	11	16242	71	20.8105	0.047031
midnightb	1170	CARBONATE_DEHYDRATASE_ACTIVITY	1	11	16242	71	20.8105	0.047031
midnightb	1244	STEROID_HORMONE_RECEPTOR_ACTIVITY	1	11	16242	71	20.8105	0.047031
midnightb	1437	SINGLE_STRANDED_RNA_BINDING	1	11	16242	71	20.8105	0.047031
midnightb	2913	BIOCARTA_AKAP95_PATHWAY	1	11	16242	71	20.8105	0.047031
red	8662	GSE1432_CTRL_VS_IFNG_24H_MICROGLIA_UP	11	192	16061	524	1.777026	0.04706
red	1261	SULFURIC_ESTER_HYDROLASE_ACTIVITY	2	11	16242	524	5.639486	0.047063
red	1317	NF_KAPPAB_BINDING	2	11	16242	524	5.639486	0.047063
red	1347	POTASSIUM_CHANNEL_REGULATOR_ACTIVITY	2	11	16242	524	5.639486	0.047063
red	1437	SINGLE_STRANDED_RNA_BINDING	2	11	16242	524	5.639486	0.047063
red	2971	BIOCARTA_EPONFKB_PATHWAY	2	11	16242	524	5.639486	0.047063
red	4065	PID_P38GAMMADELTAPATHWAY	2	11	16242	524	5.639486	0.047063
red	4274	REACTOME_ALPHA_LINOLENIC_ACID_ALA_METABOLIS	2	11	16242	524	5.639486	0.047063
red	4459	REACTOME_ACTIVATION_OF_CHAPERONES_BY_ATF6_	2	11	16242	524	5.639486	0.047063
red	4487	REACTOME_ACTIVATION_OF_RAC	2	11	16242	524	5.639486	0.047063
red	4655	REACTOME_REGULATION_OF_IFNA_SIGNALING	2	11	16242	524	5.639486	0.047063
red	5508	MATTIOLI_MULTIPLE_MYELOMA_SUBGROUPS	2	11	16242	524	5.639486	0.047063
red	5769	WILLIAMS_ESR2_TARGETS_DN	2	11	16242	524	5.639486	0.047063
red	6432	RUAN_RESPONSE_TO_TNF_UP	2	11	16242	524	5.639486	0.047063
red	6939	CLAUS_PGR_POSITIVE_MENINGIOMA_DN	2	11	16242	524	5.639486	0.047063
blue	2118	GAGCTGG,MIR-337	35	145	16108	2978	1.317373	0.047099
purple	643	ANGIOGENESIS	2	36	16217	157	5.751238	0.047155
purple	1043	CAMP_MEDIATED_SIGNALING	2	36	16217	157	5.751238	0.047155
purple	6404	TRAYNOR_RETT_SYNDROM_UP	2	36	16217	157	5.751238	0.047155
purple	6486	SATO_SILENCED_BY_METHYLATION_IN_PANCREATIC_C	2	36	16217	157	5.751238	0.047155
purple	7155	NAKAMURA_METASTASIS_MODEL_DN	2	36	16217	157	5.751238	0.047155
purple	7835	NOUSHMEHR_GBM_SILENCED_BY_METHYLATION	2	36	16217	157	5.751238	0.047155
purple	8365	KRAS.50_UP.V1_UP	2	36	16217	157	5.751238	0.047155
blue	4571	REACTOME_MITOTIC_G2_G2_M_PHASES	20	75	16178	2978	1.455384	0.047161
salmon	1669	V$GABP_B	5	251	16002	127	2.54933	0.047167
brown	762	ANTI_APOPTOSIS	21	107	16146	2190	1.456548	0.047175
blue	967	PROTEIN_LOCALIZATION	46	198	16055	2978	1.267948	0.047193
blue	1245	NUCLEOSIDE_TRIPHOSPHATASE_ACTIVITY	46	198	16055	2978	1.267948	0.047193
midnightb	8508	GSE12845_IGD_POS_BLOOD_VS_PRE_GC_TONSIL_BCEI	3	185	16068	71	3.712143	0.047228
midnightb	8645	GSE14308_TH1_VS_NATURAL_TREG_DN	3	185	16068	71	3.712143	0.047228

midnightb	9483	GSE26669_CD4_VS_CD8_TCELL_IN_MLR_DN	3	185	16068	71	3.712143	0.047228
midnightb	9868	GSE360_L_DONOVANI_VS_M_TUBERCULOSIS_MAC_UI	3	185	16068	71	3.712143	0.047228
midnightb	9987	GSE3982_EOSINOPHIL_VS_NKCELL_DN	3	185	16068	71	3.712143	0.047228
tan	2495	GCM_TINF2	2	39	16214	145	5.748187	0.047246
turquoise	2522	GCM_GSPT1	52	164	16089	4169	1.236121	0.047277
magenta	4970	VECCHI_GASTRIC_CANCER_ADVANCED_VS_EARLY_UP	4	132	16121	167	2.949193	0.047296
magenta	8234	ATF2_UP.V1_UP	4	132	16121	167	2.949193	0.047296
greenyello	1344	CYTOSKELETAL_PROTEIN_BINDING	4	147	16106	150	2.94839	0.047346
greenyello	9197	GSE17974_IL4_AND_ANTI_IL12_VS_UNTREATED_12H_	4	147	16106	150	2.94839	0.047346
greenyello	9202	GSE17974_IL4_AND_ANTI_IL12_VS_UNTREATED_72H_	4	147	16106	150	2.94839	0.047346
red	8732	GSE15215_CD2_POS_VS_NEG_PDC_UP	10	169	16084	524	1.835336	0.047348
red	10241	GSE9037_CTRL_VS_LPS_1H_STIM_BMDM_DN	10	169	16084	524	1.835336	0.047348
red	10245	GSE9037_CTRL_VS_LPS_1H_STIM_IRAK4_KO_BMDM_D	10	169	16084	524	1.835336	0.047348
purple	4138	REACTOME_RECYCLING_OF_BILE_ACIDS_AND_SALTS	1	5	16248	157	20.70446	0.04738
purple	4804	PYEON_HPV_POSITIVE_TUMORS_DN	1	5	16248	157	20.70446	0.04738
purple	4881	TURASHVILI_BREAST_CARCINOMA_DUCTAL_VS_LOBUL	1	5	16248	157	20.70446	0.04738
purple	5346	OZANNE_AP1_TARGETS_DN	1	5	16248	157	20.70446	0.04738
purple	5763	GALIE_TUMOR_STEMNESS_GENES	1	5	16248	157	20.70446	0.04738
purple	6824	MILICIC_FAMILIAL_ADENOMATOUS_POLYPOSIS_DN	1	5	16248	157	20.70446	0.04738
purple	7167	SHARMA_ASTROCYTOMA_WITH_NF1_SYNDROM	1	5	16248	157	20.70446	0.04738
purple	7288	JU_AGING_TERC_TARGETS_DN	1	5	16248	157	20.70446	0.04738
purple	7602	TSAI_DNAJB4_TARGETS_DN	1	5	16248	157	20.70446	0.04738
purple	7927	JOHNSTONE_PARVB_TARGETS_1_UP	1	5	16248	157	20.70446	0.04738
magenta	1726	V$GATA6_01	5	191	16062	167	2.547732	0.047383
magenta	8455	GSE11057_CD4_CENT_MEM_VS_PBMC_DN	5	191	16062	167	2.547732	0.047383
magenta	9408	GSE24142_EARLY_THYMIC_PROGENITOR_VS_DN2_THY	5	191	16062	167	2.547732	0.047383
magenta	10169	GSE7460_CTRL_VS_TGFB_TREATED_ACT_FOXP3_HET_T	5	191	16062	167	2.547732	0.047383
magenta	10175	GSE7460_FOXP3_MUT_VS_WT_ACT_WITH_TGFB_TCOI	5	191	16062	167	2.547732	0.047383
brown	1823	V$TFIII_Q6	31	170	16083	2190	1.353325	0.047405
lightyellow	3600	MODULE_112	2	172	16081	33	5.72692	0.047457
pink	6223	PENG_RAPAMYCIN_RESPONSE_UP	6	186	16067	228	2.299519	0.047459
pink	8545	GSE13306_RA_VS_UNTREATED_MEM_CD4_TCELL_DN	6	186	16067	228	2.299519	0.047459
pink	9409	GSE24142_EARLY_THYMIC_PROGENITOR_VS_DN2_THY	6	186	16067	228	2.299519	0.047459
pink	9772	GSE339_CD8POS_VS_CD4CD8DN_DC_UP	6	186	16067	228	2.299519	0.047459
magenta	2273	YATGNWAAT_V$OCT_C	6	254	15999	167	2.298977	0.047465
turquoise	758	LIPOPROTEIN_BIOSYNTHETIC_PROCESS	11	26	16227	4169	1.649381	0.047465
turquoise	3702	MODULE_226	11	26	16227	4169	1.649381	0.047465
pink	998	ORGAN_DEVELOPMENT	10	388	15865	228	1.837245	0.047504
magenta	817	CYTOKINE_BIOSYNTHETIC_PROCESS	2	34	16219	167	5.724903	0.047507
magenta	2407	MORF_ITGA2	2	34	16219	167	5.724903	0.047507
magenta	6851	LEIN_LOCALIZED_TO_PROXIMAL_DENDRITES	2	34	16219	167	5.724903	0.047507
magenta	6990	OSADA_ASCL1_TARGETS_UP	2	34	16219	167	5.724903	0.047507
magenta	7428	SHAFFER_IRF4_MULTIPLE_MYELOMA_PROGRAM	2	34	16219	167	5.724903	0.047507
magenta	7698	DORN_ADENOVIRUS_INFECTION_32HR_DN	2	34	16219	167	5.724903	0.047507
magenta	7700	DORN_ADENOVIRUS_INFECTION_48HR_DN	2	34	16219	167	5.724903	0.047507
green	10201	GSE7852_LN_VS_FAT_TREG_DN	22	188	16065	1305	1.457431	0.047516
turquoise	4236	REACTOME_SIGNALING_BY_BMP	9	20	16233	4169	1.754342	0.047523
turquoise	4835	FOURNIER_ACINAR_DEVELOPMENT_EARLY_UP	9	20	16233	4169	1.754342	0.047523
turquoise	5731	SPIELMAN_LYMPHOBLAST_EUROPEAN_VS_ASIAN_2FC_	9	20	16233	4169	1.754342	0.047523
turquoise	7160	WEST_ADRENOCORTICAL_CARCINOMA_VS_ADENOMA	9	20	16233	4169	1.754342	0.047523
red	5455	LINDGREN_BLADDER_CANCER_CLUSTER_2B	17	338	15915	524	1.560035	0.047524
black	473	REGULATION_OF_PROTEIN_METABOLIC_PROCESS	6	150	16103	283	2.297244	0.047542
black	3726	MODULE_257	6	150	16103	283	2.297244	0.047542
brown	6415	BLALOCK_ALZHEIMERS_DISEASE_INCIPIENT_UP	59	354	15899	2190	1.23691	0.047553
cyan	9078	GSE17721_LPS_VS_GARDIQUIMOD_2H_BMDM_UP	3	171	16082	77	3.703121	0.047557
brown	239	RNA_METABOLIC_PROCESS	118	757	15496	2190	1.156846	0.047561
pink	3387	chr7q35	2	25	16228	228	5.702807	0.047574
pink	4245	REACTOME_SMAD2_SMAD3_SMAD4_HETEROTRIMER_	2	25	16228	228	5.702807	0.047574
pink	6604	ZHANG_ANTIVIRAL_RESPONSE_TO_RIBAVIRIN_UP	2	25	16228	228	5.702807	0.047574
pink	8084	PLASARI_TGFB1_SIGNALING_VIA_NFIC_10HR_DN	2	25	16228	228	5.702807	0.047574

grey60	1743	V$AR_01	2	129	16124	44	5.72692	0.047619
grey60	2260	YTAATTAA_V$LHX3_01	2	129	16124	44	5.72692	0.047619
grey60	9688	GSE29618_PRE_VS_DAY7_POST_TIV_FLU_VACCINE_BC	2	129	16124	44	5.72692	0.047619
pink	6047	CERVERA_SDHB_TARGETS_1_UP	4	97	16156	228	2.939591	0.047623
pink	7859	HIRSCH_CELLULAR_TRANSFORMATION_SIGNATURE_DN	4	97	16156	228	2.939591	0.047623
greenyello	1555	V$LYF1_01	5	213	16040	150	2.543505	0.047636
greenyello	7909	CHYLA_CBFA2T3_TARGETS_DN	5	213	16040	150	2.543505	0.047636
salmon	8481	GSE11924_TFH_VS_TH1_CD4_TCELL_DN	4	174	16079	127	2.941986	0.047638
salmon	9026	GSE17721_PAM3CSK4_VS_GADIQUIMOD_4H_BMDM_	4	174	16079	127	2.941986	0.047638
lightyellow	2567	CAR_IGFBP1	1	24	16229	33	20.52146	0.047641
lightyellow	2739	KEGG_HISTIDINE_METABOLISM	1	24	16229	33	20.52146	0.047641
lightyellow	3125	chr7p21	1	24	16229	33	20.52146	0.047641
lightyellow	3792	MODULE_343	1	24	16229	33	20.52146	0.047641
lightyellow	5256	BERENJENO_TRANSFORMED_BY_RHOA_FOREVER_DN	1	24	16229	33	20.52146	0.047641
lightyellow	6753	ABE_VEGFA_TARGETS_30MIN	1	24	16229	33	20.52146	0.047641
lightyellow	7367	WALLACE_JAK2_TARGETS_UP	1	24	16229	33	20.52146	0.047641
grey60	1368	INTERLEUKIN_RECEPTOR_ACTIVITY	1	18	16235	44	20.52146	0.047649
grey60	5272	TAKADA_GASTRIC_CANCER_COPY_NUMBER_DN	1	18	16235	44	20.52146	0.047649
grey60	6112	KANNAN_TP53_TARGETS_DN	1	18	16235	44	20.52146	0.047649
grey60	6810	YANG_MUC2_TARGETS_DUODENUM_3MO_DN	1	18	16235	44	20.52146	0.047649
grey60	7221	ALONSO_METASTASIS_DN	1	18	16235	44	20.52146	0.047649
grey60	7994	BILANGES_SERUM_SENSITIVE_VIA_TSC1	1	18	16235	44	20.52146	0.047649
red	1911	V$AP1_Q6_01	12	216	16037	524	1.723176	0.04767
red	7364	VANTVEER_BREAST_CANCER_ESR1_DN	12	216	16037	524	1.723176	0.04767
turquoise	1818	V$ETF_Q6	35	105	16148	4169	1.299512	0.04767
tan	2537	GCM_NF2	6	293	15960	145	2.295351	0.047672
lightcyan	7650	HOSHIDA_LIVER_CANCER_SUBCLASS_S3	3	213	16040	62	3.692185	0.047758
black	8514	GSE12845_IGD_NEG_BLOOD_VS_PRE_GC_TONSIL_BCE	7	189	16064	283	2.127078	0.047781
purple	8167	LIM_MAMMARY_LUMINAL_MATURE_DN	3	84	16169	157	3.697225	0.047783
turquoise	2796	KEGG_PROTEIN_EXPORT	10	23	16230	4169	1.695016	0.047823
turquoise	6724	YIH_RESPONSE_TO_ARSENITE_C1	10	23	16230	4169	1.695016	0.047823
midnightb	3500	MODULE_7	3	186	16067	71	3.692185	0.047857
midnightb	9004	GSE17721_POLYIC_VS_CPG_1H_BMDM_UP	3	186	16067	71	3.692185	0.047857
midnightb	9719	GSE30083_SP2_VS_SP4_THYMOCYTE_DN	3	186	16067	71	3.692185	0.047857
midnightb	10132	GSE6269_HEALTHY_VS_STREP_PNEUMO_INF_PBMC_U	3	186	16067	71	3.692185	0.047857
pink	6589	XU_GH1_EXOGENOUS_TARGETS_UP	3	58	16195	228	3.68716	0.047859
red	544	MULTICELLULAR_ORGANISMAL_DEVELOPMENT	32	729	15524	524	1.361522	0.047877
greenyello	2089	TCTCTCC,MIR-185	3	88	16165	150	3.693864	0.047907
greenyello	501	CELLULAR_MORPHOGENESIS_DURING_DIFFERENTIATIC	2	38	16215	150	5.702807	0.047907
greenyello	3244	chr12q12	2	38	16215	150	5.702807	0.047907
greenyello	3385	chr17q24	2	38	16215	150	5.702807	0.047907
greenyello	3990	PID_FASPATHWAY	2	38	16215	150	5.702807	0.047907
greenyello	4070	PID_ERBB1_INTERNALIZATION_PATHWAY	2	38	16215	150	5.702807	0.047907
greenyello	6288	HALMOS_CEBPA_TARGETS_DN	2	38	16215	150	5.702807	0.047907
greenyello	8185	ZWANG_CLASS_2_TRANSIENTLY_INDUCED_BY_EGF	2	38	16215	150	5.702807	0.047907
purple	2809	KEGG_NEUROACTIVE_LIGAND_RECEPTOR_INTERACTIO	4	141	16112	157	2.936803	0.047911
green	191	EXTRACELLULAR_MATRIX	10	69	16184	1305	1.804986	0.047934
brown	8624	GSE14000_4H_VS_16H_LPS_DC_UP	33	183	16070	2190	1.338296	0.047936
brown	8699	GSE1460_DP_THYMOCYTE_VS_NAIVE_CD4_TCELL_ADU	33	183	16070	2190	1.338296	0.047936
brown	9402	GSE24026_PD1_LIGATION_VS_CTRL_IN_ACT_TCELL_LIN	33	183	16070	2190	1.338296	0.047936
turquoise	601	REGULATION_OF_CELLULAR_METABOLIC_PROCESS	199	700	15553	4169	1.108298	0.047949
lightyellow	8262	RAF_UP.V1_UP	2	173	16080	33	5.693817	0.047952
lightyellow	9237	GSE18791_CTRL_VS_NEWCASTLE_VIRUS_DC_18H_DN	2	173	16080	33	5.693817	0.047952
green	2353	MORF_ARL3	26	230	16023	1305	1.407889	0.047961
blue	9026	GSE17721_PAM3CSK4_VS_GADIQUIMOD_4H_BMDM_	41	174	16079	2978	1.286007	0.047971
blue	9328	GSE22886_NAIVE_VS_IGG_IGA_MEMORY_BCELL_UP	41	174	16079	2978	1.286007	0.047971
blue	9490	GSE26928_NAIVE_VS_CXCR5_POS_CD4_TCELL_UP	41	174	16079	2978	1.286007	0.047971
blue	9491	GSE26928_NAIVE_VS_CXCR5_POS_CD4_TCELL_DN	41	174	16079	2978	1.286007	0.047971
blue	10233	GSE9006_TYPE_1_DIABETES_AT_DX_VS_1MONTH_POS	41	174	16079	2978	1.286007	0.047971
turquoise	7898	LI_DCP2_BOUND_MRNA	30	88	16165	4169	1.329047	0.047972

lightyellow	3553	MODULE_64	3	404	15849	33	3.657291	0.047991
lightgreen	2815	KEGG_REGULATION_OF_AUTOPHAGY	1	19	16234	42	20.36717	0.047999
lightgreen	2869	KEGG_PROXIMAL_TUBULE_BICARBONATE_RECLAMATI	1	19	16234	42	20.36717	0.047999
lightgreen	2955	BIOCARTA_INFLAM_PATHWAY	1	19	16234	42	20.36717	0.047999
lightgreen	3301	chr18q22	1	19	16234	42	20.36717	0.047999
lightgreen	5229	LANDIS_ERBB2_BREAST_PRENEOPLASTIC_UP	1	19	16234	42	20.36717	0.047999
lightgreen	6117	WILLERT_WNT_SIGNALING	1	19	16234	42	20.36717	0.047999
lightgreen	6828	LOPES_METHYLATED_IN_COLON_CANCER_UP	1	19	16234	42	20.36717	0.047999
lightgreen	6860	GAVIN_IL2_RESPONSIVE_FOXP3_TARGETS_UP	1	19	16234	42	20.36717	0.047999
lightgreen	7399	FINAK_BREAST_CANCER_SDPP_SIGNATURE	1	19	16234	42	20.36717	0.047999
lightgreen	7526	CHIANG_LIVER_CANCER_SUBCLASS_INTERFERON_UP	1	19	16234	42	20.36717	0.047999
salmon	1868	GGAANCGGAANY_UNKNOWN	3	104	16149	127	3.691626	0.048017
salmon	2871	KEGG_PARKINSONS_DISEASE	3	104	16149	127	3.691626	0.048017
salmon	4574	REACTOME_DNA_REPAIR	3	104	16149	127	3.691626	0.048017
purple	7862	CHICAS_RB1_TARGETS_GROWING	5	204	16049	157	2.537311	0.048061
cyan	3655	MODULE_172	2	74	16179	77	5.704809	0.048064
cyan	5588	YORDY_RECIPROCAL_REGULATION_BY_ETS1_AND_SP1	2	74	16179	77	5.704809	0.048064
cyan	7264	BOYLAN_MULTIPLE_MYELOMA_D_DN	2	74	16179	77	5.704809	0.048064
turquoise	707	MACROMOLECULE_BIOSYNTHETIC_PROCESS	87	289	15964	4169	1.173608	0.048094
blue	1800	V$MTF1_Q4	48	208	16045	2978	1.259467	0.048128
magenta	3495	MODULE_2	7	321	15932	167	2.122316	0.048169
pink	4968	WANG_CLIM2_TARGETS_UP	7	235	16018	228	2.123386	0.048201
magenta	1044	REGULATION_OF_CELL_PROLIFERATION	6	255	15998	167	2.289961	0.048213
salmon	3766	MODULE_312	2	45	16208	127	5.687839	0.048216
salmon	8134	ABRAMSON_INTERACT_WITH_AIRE	2	45	16208	127	5.687839	0.048216
blue	9694	GSE29618_PRE_VS_DAY7_POST_LAIV_FLU_VACCINE_P	32	131	16122	2978	1.333176	0.048219
brown	8418	GSE10239_NAIVE_VS_DAY4.5_EFF_CD8_TCELL_UP	35	196	16057	2190	1.325261	0.048243
brown	9252	GSE19825_CD24LOW_VS_IL2RA_HIGH_DAY3_EFF_CD8	35	196	16057	2190	1.325261	0.048243
brown	9385	GSE22886_TH1_VS_TH2_48H_ACT_DN	35	196	16057	2190	1.325261	0.048243
brown	9440	GSE24634_TREG_VS_TCONV_POST_DAY3_IL4_CONVER	35	196	16057	2190	1.325261	0.048243
brown	9782	GSE339_EX_VIVO_VS_IN_CULTURE_CD8POS_DC_UP	35	196	16057	2190	1.325261	0.048243
brown	10254	GSE9650_NAIVE_VS_EFF_CD8_TCELL_UP	35	196	16057	2190	1.325261	0.048243
magenta	1591	V$GR_Q6	5	192	16061	167	2.534462	0.048264
magenta	9155	GSE17974_0H_VS_2H_IN_VITRO_ACT_CD4_TCELL_DN	5	192	16061	167	2.534462	0.048264
magenta	9979	GSE3982_EOSINOPHIL_VS_BCELL_DN	5	192	16061	167	2.534462	0.048264
magenta	10020	GSE3982_DC_VS_EFF_MEMORY_CD4_TCELL_UP	5	192	16061	167	2.534462	0.048264
magenta	10103	GSE39820_CTRL_VS_IL1B_IL6_IL23A_CD4_TCELL_DN	5	192	16061	167	2.534462	0.048264
magenta	10210	GSE7852_TREG_VS_TCONV_UP	5	192	16061	167	2.534462	0.048264
green	9429	GSE24142_ADULT_VS_FETAL_DN2_THYMOCYTE_DN	21	178	16075	1305	1.46934	0.048306
turquoise	9939	GSE37416_0H_VS_12H_F_TULARENSIS_LVS_NEUTROPI	61	196	16057	4169	1.21332	0.048312
greenyello	3659	MODULE_176	4	148	16105	150	2.928468	0.048326
greenyello	3714	MODULE_242	4	148	16105	150	2.928468	0.048326
greenyello	10067	GSE3982_BCELL_VS_NKCELL_DN	4	148	16105	150	2.928468	0.048326
brown	7355	CHANG_CORE_SERUM_RESPONSE_UP	37	209	16044	2190	1.313847	0.048362
brown	5777	GROSS_HYPOXIA_VIA_HIF1A_DN	19	95	16158	2190	1.484292	0.048367
yellow	6522	BAELDE_DIABETIC_NEPHROPATHY_UP	11	72	16181	1426	1.741302	0.048385
yellow	7707	NAKAYAMA_SOFT_TISSUE_TUMORS_PCA2_UP	11	72	16181	1426	1.741302	0.048385
yellow	7844	STAMBOLSKY_RESPONSE_TO_VITAMIN_D3_UP	11	72	16181	1426	1.741302	0.048385
magenta	2122	CCCAGAG,MIR-326	4	133	16120	167	2.927018	0.048388
brown	2243	GTGACGY_V$E4F1_Q6	94	592	15661	2190	1.178408	0.048397
greenyello	1466	V$ATF_01	5	214	16039	150	2.53162	0.048428
greenyello	1526	V$CEBPB_01	5	214	16039	150	2.53162	0.048428
turquoise	4963	RODRIGUES_DCC_TARGETS_DN	37	112	16141	4169	1.287909	0.048459
pink	9417	GSE24142_EARLY_THYMIC_PROGENITOR_VS_DN3_THY	6	187	16066	228	2.287222	0.048484
lightcyan	1010	CELL_MATURATION	1	13	16240	62	20.16501	0.048489
lightcyan	2929	BIOCARTA_RANKL_PATHWAY	1	13	16240	62	20.16501	0.048489
lightcyan	4648	REACTOME_ACTIVATION_OF_IRF3_IRF7_MEDIATED_BY	1	13	16240	62	20.16501	0.048489
lightcyan	5368	WONG_ENDMETRIUM_CANCER_UP	1	13	16240	62	20.16501	0.048489
lightcyan	5571	KORKOLA_EMBRYONAL_CARCINOMA	1	13	16240	62	20.16501	0.048489
lightcyan	6831	TUOMISTO_TUMOR_SUPPRESSION_BY_COL13A1_UP	1	13	16240	62	20.16501	0.048489

lightcyan	7471	ZHAN_VARIABLE_EARLY_DIFFERENTIATION_GENES_UP	1	13	16240	62	20.16501	0.048489
lightcyan	7699	DORN_ADENOVIRUS_INFECTION_48HR_UP	1	13	16240	62	20.16501	0.048489
midnightb	8426	GSE10325_BCELL_VS_MYELOID_UP	3	187	16066	71	3.672441	0.048489
midnightb	8946	GSE17721_POLYIC_VS_PAM3CSK4_24H_BMDM_UP	3	187	16066	71	3.672441	0.048489
midnightb	9162	GSE17974_0H_VS_24H_IN_VITRO_ACT_CD4_TCELL_UP	3	187	16066	71	3.672441	0.048489
midnightb	9398	GSE22886_UNSTIM_VS_IL2_STIM_NKCELL_UP	3	187	16066	71	3.672441	0.048489
midnightb	9863	GSE360_L_DONOVANI_VS_T_GONDII_MAC_DN	3	187	16066	71	3.672441	0.048489
magenta	5862	SCHAEFFER_PROSTATE_DEVELOPMENT_48HR_UP	8	390	15863	167	1.996376	0.048491
red	617	POSITIVE_REGULATION_OF_DEVELOPMENTAL_PROCES	11	193	16060	524	1.767818	0.048527
red	8577	GSE13485_CTRL_VS_DAY3_YF17D_VACCINE_PBMC_DN	11	193	16060	524	1.767818	0.048527
red	9598	GSE27786_NKCELL_VS_NKTCELL_UP	11	193	16060	524	1.767818	0.048527
brown	7705	NAKAYAMA_SOFT_TISSUE_TUMORS_PCA1_UP	14	65	16188	2190	1.598469	0.048549
brown	4024	PID_P53DOWNSTREAMPATHWAY	24	126	16127	2190	1.413612	0.048582
yellow	7031	MOLENAAR_TARGETS_OF_CCND1_AND_CDK4_DN	9	55	16198	1426	1.865064	0.048597
red	3507	MODULE_15	17	339	15914	524	1.555434	0.048611
blue	10075	GSE3982_BASOPHIL_VS_CENT_MEMORY_CD4_TCELL_I	42	179	16074	2978	1.280575	0.048622
brown	3843	MODULE_419	8	31	16222	2190	1.915216	0.048627
brown	4087	PID_SYNDECAN_2_PATHWAY	8	31	16222	2190	1.915216	0.048627
brown	7473	ZHAN_V1_LATE_DIFFERENTIATION_GENES_UP	8	31	16222	2190	1.915216	0.048627
brown	7691	DANG_MYC_TARGETS_DN	8	31	16222	2190	1.915216	0.048627
brown	7888	FIGUEROA_AML_METHYLATION_CLUSTER_5_DN	8	31	16222	2190	1.915216	0.048627
pink	2730	KEGG_PURINE_METABOLISM	5	141	16112	228	2.52784	0.048657
pink	3816	MODULE_379	5	141	16112	228	2.52784	0.048657
brown	1169	PHOSPHOLIPID_BINDING	10	42	16211	2190	1.767015	0.048703
brown	3350	chr9q21	10	42	16211	2190	1.767015	0.048703
brown	3972	PID_RHOA_REG_PATHWAY	10	42	16211	2190	1.767015	0.048703
brown	4223	REACTOME_PRE_NOTCH_EXPRESSION_AND_PROCESSII	10	42	16211	2190	1.767015	0.048703
brown	6405	MCCLUNG_DELTA_FOSB_TARGETS_8WK	10	42	16211	2190	1.767015	0.048703
turquoise	9112	GSE17721_12H_VS_24H_POLYIC_BMDM_UP	59	189	16064	4169	1.217004	0.048711
lightgreen	4262	REACTOME_GASTRIN_CREB_SIGNALLING_PATHWAY_V	2	137	16116	42	5.649287	0.048775
greenyello	2240	CAGGTG_V$E12_Q6	26	2020	14233	150	1.394647	0.048795
black	6599	BROWNE_HCMV_INFECTION_18HR_DN	6	151	16102	283	2.28203	0.04882
black	8223	PIGF_UP.V1_DN	6	151	16102	283	2.28203	0.04882
green	3927	PID_FCER1PATHWAY	9	60	16193	1305	1.868161	0.048859
green	4375	REACTOME_RECRUITMENT_OF_MITOTIC_CENTROSOM	9	60	16193	1305	1.868161	0.048859
lightcyan	459	DEFENSE_RESPONSE	3	215	16038	62	3.657839	0.048863
purple	5069	SENESE_HDAC1_AND_HDAC2_TARGETS_UP	5	205	16048	157	2.524934	0.048895
black	8807	GSE15930_STIM_VS_STIM_AND_TRICHOSTATINA_48H_	7	190	16063	283	2.115882	0.048906
blue	8109	PEDRIOLI_MIR31_TARGETS_UP	37	155	16098	2978	1.302803	0.048921
red	202	TRANSCRIPTION_FACTOR_COMPLEX	6	82	16171	524	2.269549	0.048922
red	929	CATION_HOMEOSTASIS	6	82	16171	524	2.269549	0.048922
red	7131	MATZUK_SPERMATOZOA	6	82	16171	524	2.269549	0.048922
red	7807	SASSON_RESPONSE_TO_GONADOTROPHINS_DN	6	82	16171	524	2.269549	0.048922
green	10289	GSE9988_LOW_LPS_VS_CTRL_TREATED_MONOCYTE_D	23	199	16054	1305	1.439454	0.048929
cyan	8893	GSE17721_CTRL_VS_CPG_12H_BMDM_DN	3	173	16080	77	3.660311	0.048931
cyan	8914	GSE17721_LPS_VS_POLYIC_1H_BMDM_UP	3	173	16080	77	3.660311	0.048931
cyan	8953	GSE17721_PAM3CSK4_VS_CPG_2H_BMDM_DN	3	173	16080	77	3.660311	0.048931
cyan	9298	GSE20715_0H_VS_48H_OZONE_TLR4_KO_LUNG_UP	3	173	16080	77	3.660311	0.048931
cyan	9650	GSE29617_CTRL_VS_DAY7_TIV_FLU_VACCINE_PBMC_2	3	173	16080	77	3.660311	0.048931
cyan	9839	GSE360_L_DONOVANI_VS_M_TUBERCULOSIS_DC_DN	3	173	16080	77	3.660311	0.048931
lightyellow	3617	MODULE_129	2	175	16078	33	5.628745	0.048948
lightyellow	5152	HAHTOLA_MYCOSIS_FUNGOIDES_SKIN_UP	2	175	16078	33	5.628745	0.048948
lightyellow	9279	GSE20366_TREG_VS_NAIVE_CD4_TCELL_DN	2	175	16078	33	5.628745	0.048948
lightyellow	9330	GSE22886_NAIVE_VS_IGM_MEMORY_BCELL_UP	2	175	16078	33	5.628745	0.048948
brown	9840	GSE360_L_MAJOR_VS_T_GONDII_DC_UP	27	145	16108	2190	1.381927	0.048984
brown	6230	ROSS_AML_WITH_AML1_ETO_FUSION	15	71	16182	2190	1.567914	0.048996
brown	6243	ADDYA_ERYTHROID_DIFFERENTIATION_BY_HEMIN	15	71	16182	2190	1.567914	0.048996
black	4054	PID_CMYB_PATHWAY	4	79	16174	283	2.907904	0.049011
red	5305	LUI_THYROID_CANCER_PAX8_PPARG_DN	4	43	16210	524	2.885319	0.049018
red	5363	OLSSON_E2F3_TARGETS_DN	4	43	16210	524	2.885319	0.049018

red	6273	HOFMANN_CELL_LYMPHOMA_UP	4	43	16210	524	2.885319	0.049018
red	7238	JIANG_TIP30_TARGETS_UP	4	43	16210	524	2.885319	0.049018
red	7904	THILLAINADESAN_ZNF217_TARGETS_UP	4	43	16210	524	2.885319	0.049018
turquoise	2123	GCTGAGT,MIR-512-5P	17	45	16208	4169	1.472781	0.049042
turquoise	2150	CAGTCAC,MIR-134	17	45	16208	4169	1.472781	0.049042
turquoise	7520	BOYAULT_LIVER_CANCER_SUBCLASS_G123_UP	17	45	16208	4169	1.472781	0.049042
brown	6614	LEE_AGING_NEOCORTEX_UP	17	83	16170	2190	1.520058	0.049065
brown	7809	SASSON_RESPONSE_TO_FORSKOLIN_DN	17	83	16170	2190	1.520058	0.049065
turquoise	9100	GSE17721_12H_VS_24H_LPS_BMDM_UP	57	182	16071	4169	1.22097	0.049069
lightcyan	3652	MODULE_169	2	93	16160	62	5.63753	0.04908
lightcyan	6514	BROWNE_HCMV_INFECTION_8HR_UP	2	93	16160	62	5.63753	0.04908
magenta	4982	OSMAN_BLADDER_CANCER_DN	8	391	15862	167	1.991271	0.04909
yellow	8705	GSE1460_CD4_THYMOCYTE_VS_NAIVE_CD4_TCELL_AD	24	192	16061	1426	1.424702	0.049121
yellow	8718	GSE14769_UNSTIM_VS_60MIN_LPS_BMDM_UP	24	192	16061	1426	1.424702	0.049121
yellow	8728	GSE14769_20MIN_VS_360MIN_LPS_BMDM_UP	24	192	16061	1426	1.424702	0.049121
yellow	8858	GSE17721_CTRL_VS_POLYIC_8H_BMDM_UP	24	192	16061	1426	1.424702	0.049121
yellow	8932	GSE17721_POLYIC_VS_PAM3CSK4_1H_BMDM_UP	24	192	16061	1426	1.424702	0.049121
yellow	9394	GSE22886_NAIVE_CD4_TCELL_VS_12H_ACT_TH2_UP	24	192	16061	1426	1.424702	0.049121
yellow	10043	GSE3982_MAC_VS_TH1_DN	24	192	16061	1426	1.424702	0.049121
midnightb	8864	GSE17721_CTRL_VS_PAM3CSK4_0.5H_BMDM_UP	3	188	16065	71	3.652907	0.049126
midnightb	8920	GSE17721_LPS_VS_POLYIC_6H_BMDM_UP	3	188	16065	71	3.652907	0.049126
midnightb	9432	GSE24634_NAIVE_CD4_TCELL_VS_DAY3_IL4_CONV_TR	3	188	16065	71	3.652907	0.049126
midnightb	10281	GSE9988_LPS_VS_LPS_AND_ANTI_TREM1_MONOCYTE_	3	188	16065	71	3.652907	0.049126
pink	4899	HOOI_ST7_TARGETS_DN	4	98	16155	228	2.909595	0.049126
pink	6853	GAVIN_FOXP3_TARGETS_CLUSTER_T7	4	98	16155	228	2.909595	0.049126
brown	1629	V$EGR3_01	16	77	16176	2190	1.542122	0.049149
lightgreen	7443	MEISSNER_BRAIN_HCP_WITH_H3K4ME3_AND_H3K27N	5	779	15474	42	2.483801	0.04915
magenta	1788	V$GATA4_Q3	5	193	16060	167	2.52133	0.049154
magenta	8501	GSE12366_PLASMA_CELL_VS_MEMORY_BCELL_DN	5	193	16060	167	2.52133	0.049154
magenta	10097	GSE3982_NKCELL_VS_TH1_DN	5	193	16060	167	2.52133	0.049154
salmon	2729	KEGG_OXIDATIVE_PHOSPHORYLATION	3	105	16148	127	3.656468	0.049157
tan	1600	V$GRE_C	3	92	16161	145	3.655097	0.049174
tan	5251	GAUSSMANN_MLL_AF4_FUSION_TARGETS_F_UP	4	154	16099	145	2.91142	0.049184
purple	2145	CCAGGGG,MIR-331	3	85	16168	157	3.653728	0.049192
purple	5080	TIEN_INTESTINE_PROBIOTICS_2HR_DN	3	85	16168	157	3.653728	0.049192
red	1025	APOPTOSIS_GO	19	390	15863	524	1.511093	0.049199
greenyello	3664	MODULE_181	5	215	16038	150	2.519845	0.049228
blue	7923	BHAT_ESR1_TARGETS_NOT_VIA_AKT1_UP	43	184	16069	2978	1.275438	0.049231
blue	8948	GSE17721_PAM3CSK4_VS_CPG_0.5H_BMDM_UP	43	184	16069	2978	1.275438	0.049231
blue	9293	GSE20715_0H_VS_48H_OZONE_LUNG_DN	43	184	16069	2978	1.275438	0.049231
cyan	1095	LIPID_BINDING	2	75	16178	77	5.628745	0.049233
cyan	6651	LEE_CALORIE_RESTRICTION_NEOCORTEX_DN	2	75	16178	77	5.628745	0.049233
cyan	7375	HINATA_NFKB_TARGETS_FIBROBLAST_UP	2	75	16178	77	5.628745	0.049233
yellow	7937	BRUINS_UVC_RESPONSE_LATE	107	1045	15208	1426	1.167029	0.049253
greenyello	7094	IZADPANAH_STEM_CELL_ADIPOSE_VS_BONE_DN	3	89	16164	150	3.65236	0.049254
red	6274	GUO_HEX_TARGETS_DN	5	62	16191	524	2.501385	0.049261
red	6951	LIN_MELANOMA_COPY_NUMBER_UP	5	62	16191	524	2.501385	0.049261
red	7274	GU_PDEF_TARGETS_UP	5	62	16191	524	2.501385	0.049261
turquoise	1094	LIGASE_ACTIVITY_FORMING_CARBON_NITROGEN_BON	24	68	16185	4169	1.375954	0.049267
turquoise	6235	YAO_HOXA10_TARGETS_VIA_PROGESTERONE_UP	24	68	16185	4169	1.375954	0.049267
grey60	5040	MULLIGHAN_NPM1_SIGNATURE_3_UP	3	305	15948	44	3.633308	0.049281
magenta	447	TISSUE_DEVELOPMENT	3	80	16173	167	3.649626	0.049301
magenta	600	CELLULAR_CATION_HOMEOSTASIS	3	80	16173	167	3.649626	0.049301
purple	2240	CAGGTG_V$E12_Q6	27	2020	14233	157	1.383714	0.049303
yellow	5218	MARKEY_RB1_ACUTE_LOF_DN	27	221	16032	1426	1.392469	0.049303
salmon	8712	GSE1460_NAIVE_CD4_TCELL_ADULT_BLOOD_VS_THYM	4	176	16077	127	2.908554	0.049309
salmon	9685	GSE29618_PRE_VS_DAY7_FLU_VACCINE_MDC_DN	4	176	16077	127	2.908554	0.049309
red	1527	ACCTGTTG_UNKNOWN	8	125	16128	524	1.985099	0.049311
red	2090	AGCATTA,MIR-155	8	125	16128	524	1.985099	0.049311
red	2158	ACACTAC,MIR-142-3P	8	125	16128	524	1.985099	0.049311

greenyello	7720	MIKKELSEN_NPC_HCP_WITH_H3K4ME3_AND_H3K27M	4	149	16104	150	2.908814	0.049318
turquoise	8477	GSE11864_CSF1_IFNG_VS_CSF1_IFNG_PAM3CYS_IN_M	55	175	16078	4169	1.225254	0.04938
turquoise	8627	GSE14026_TH1_VS_TH17_DN	55	175	16078	4169	1.225254	0.04938
turquoise	9701	GSE29618_PRE_VS_DAY7_POST_TIV_FLU_VACCINE_MI	55	175	16078	4169	1.225254	0.04938
lightgreen	5683	TSAI_RESPONSE_TO_IONIZING_RADIATION	2	138	16115	42	5.608351	0.049413
lightgreen	7177	PODAR_RESPONSE_TO_ADAPHOSTIN_UP	2	138	16115	42	5.608351	0.049413
lightcyan	7364	VANTVEER_BREAST_CANCER_ESR1_DN	3	216	16037	62	3.640905	0.049421
tan	3860	MODULE_448	2	40	16213	145	5.604483	0.049449
lightyellow	9998	GSE3982_MAST_CELL_VS_BCELL_UP	2	176	16077	33	5.596763	0.049449
turquoise	2794	KEGG_SPLICEOSOME	41	126	16127	4169	1.268572	0.049488
green	1571	ARGGGTTAA_UNKNOWN	13	98	16155	1305	1.652115	0.049509
green	2188	RYTGCNWTGGNR_UNKNOWN	13	98	16155	1305	1.652115	0.049509
pink	85	CYTOSOL	6	188	16065	228	2.275056	0.049523
pink	8806	GSE15930_STIM_VS_STIM_AND_TRICHOSTATINA_48H_	6	188	16065	228	2.275056	0.049523
pink	9412	GSE24142_DN2_VS_DN3_THYMOCYTE_UP	6	188	16065	228	2.275056	0.049523
purple	494	ACTIVATION_OF_MAPK_ACTIVITY	2	37	16216	157	5.5958	0.049541
purple	4391	REACTOME_PLC_BETA_MEDIATED_EVENTS	2	37	16216	157	5.5958	0.049541
purple	5009	TAKEDA_TARGETS_OF_NUP98_HOXA9_FUSION_6HR_D	2	37	16216	157	5.5958	0.049541
purple	6564	GENTILE_UV_RESPONSE_CLUSTER_D5	2	37	16216	157	5.5958	0.049541
purple	6988	FUJII_YBX1_TARGETS_UP	2	37	16216	157	5.5958	0.049541
brown	351	GLYCOPROTEIN_CATABOLIC_PROCESS	4	11	16242	2190	2.698713	0.049551
brown	552	DETECTION_OF_CHEMICAL_STIMULUS	4	11	16242	2190	2.698713	0.049551
brown	908	CDC42_PROTEIN_SIGNAL_TRANSDUCTION	4	11	16242	2190	2.698713	0.049551
brown	1052	REGULATION_OF_MEMBRANE_POTENTIAL	4	11	16242	2190	2.698713	0.049551
brown	2958	BIOCARTA_P35ALZHEIMERS_PATHWAY	4	11	16242	2190	2.698713	0.049551
brown	3078	BIOCARTA_BARR_MAPK_PATHWAY	4	11	16242	2190	2.698713	0.049551
brown	3731	MODULE_264	4	11	16242	2190	2.698713	0.049551
brown	3870	MODULE_464	4	11	16242	2190	2.698713	0.049551
brown	4182	REACTOME_IL_7_SIGNALING	4	11	16242	2190	2.698713	0.049551
brown	4655	REACTOME_REGULATION_OF_IFNA_SIGNALING	4	11	16242	2190	2.698713	0.049551
brown	4750	REACTOME_THE_NLRP3_INFLAMMASOME	4	11	16242	2190	2.698713	0.049551
brown	5463	MYLLYKANGAS_AMPLIFICATION_HOT_SPOT_24	4	11	16242	2190	2.698713	0.049551
brown	6433	WHITESIDE_CISPLATIN_RESISTANCE_UP	4	11	16242	2190	2.698713	0.049551
brown	7967	VANDESLUIS_COMMD1_TARGETS_GROUP_2_UP	4	11	16242	2190	2.698713	0.049551
brown	4785	REACTOME_DIGESTION_OF_DIETARY_CARBOHYDRATE	2	3	16250	2190	4.947641	0.04956
brown	5497	MYLLYKANGAS_AMPLIFICATION_HOT_SPOT_1	2	3	16250	2190	4.947641	0.04956
brown	7169	SHARMA_PILOCYTIC_ASTROCYTOMA_LOCATION_DN	2	3	16250	2190	4.947641	0.04956
lightyellow	1076	HYDRO_LYASE_ACTIVITY	1	25	16228	33	19.70061	0.049578
lightyellow	1202	EXOPEPTIDASE_ACTIVITY	1	25	16228	33	19.70061	0.049578
lightyellow	1341	CALCIUM_CHANNEL_ACTIVITY	1	25	16228	33	19.70061	0.049578
lightyellow	5587	SEITZ_NEOPLASTIC_TRANSFORMATION_BY_8P_DELETI	1	25	16228	33	19.70061	0.049578
grey60	538	NITROGEN_COMPOUND_METABOLIC_PROCESS	2	132	16121	44	5.596763	0.049617
grey60	8247	E2F3_UP.V1_DN	2	132	16121	44	5.596763	0.049617
cyan	9136	GSE17721_12H_VS_24H_CPG_BMDM_UP	3	174	16079	77	3.639275	0.049625
turquoise	9815	GSE360_CTRL_VS_M_TUBERCULOSIS_MAC_DN	53	168	16085	4169	1.229896	0.049636
yellow	574	CHROMOSOME_SEGREGATION	6	31	16222	1426	2.20599	0.049654
yellow	5264	BARRIER_CANCER_RELAPSE_NORMAL_SAMPLE_DN	6	31	16222	1426	2.20599	0.049654
lightcyan	7864	WANG_RESPONSE_TO_GSK3_INHIBITOR_SB216763_UF	4	363	15890	62	2.888652	0.049654
blue	1023	REGULATION_OF_BINDING	15	53	16200	2978	1.544629	0.049666
brown	7983	KASLER_HDAC7_TARGETS_1_UP	34	190	16063	2190	1.328051	0.049671
brown	9109	GSE17721_0.5H_VS_24H_POLYIC_BMDM_DN	34	190	16063	2190	1.328051	0.049671
brown	9345	GSE22886_IGM_MEMORY_BCELL_VS_BM_PLASMA_CE	34	190	16063	2190	1.328051	0.049671
brown	9908	GSE36476_CTRL_VS_TSST_ACT_16H_MEMORY_CD4_T	34	190	16063	2190	1.328051	0.049671
red	6116	TARTE_PLASMA_CELL_VS_PLASMABLAST_UP	16	315	15938	524	1.575476	0.049682
purple	1867	V$MYB_Q3	5	206	16047	157	2.512677	0.049737
midnightb	8631	GSE14308_TH2_VS_TH17_DN	3	189	16064	71	3.633579	0.049767
midnightb	8766	GSE15750_WT_VS_TRAF6KO_DAY6_EFF_CD8_TCELL_U	3	189	16064	71	3.633579	0.049767
midnightb	8773	GSE15750_DAY6_VS_DAY10_TRAF6KO_EFF_CD8_TCELL	3	189	16064	71	3.633579	0.049767
midnightb	9011	GSE17721_POLYIC_VS_CPG_6H_BMDM_DN	3	189	16064	71	3.633579	0.049767
midnightb	9112	GSE17721_12H_VS_24H_POLYIC_BMDM_UP	3	189	16064	71	3.633579	0.049767

midnightb	9427	GSE24142_ADULT_VS_FETAL_EARLY_THYMIC_PROGEN	3	189	16064	71	3.633579	0.049767
turquoise	7657	CAIRO_LIVER_DEVELOPMENT_UP	43	133	16120	4169	1.260429	0.049775
blue	8773	GSE15750_DAY6_VS_DAY10_TRAF6KO_EFF_CD8_TCELI	44	189	16064	2978	1.270573	0.049801
brown	1911	V$AP1_Q6_01	38	216	16037	2190	1.305627	0.04982
purple	2248	RTAAACA_V$FREAC2_01	12	728	15525	157	1.706411	0.049824
brown	635	IMMUNE_RESPONSE	36	203	16050	2190	1.316121	0.049831
red	4806	NAKAMURA_TUMOR_ZONE_PERIPHERAL_VS_CENTRAL	26	572	15681	524	1.409872	0.049836
pink	6282	LEI_MYB_TARGETS	8	287	15966	228	1.987041	0.04984
green	7559	MEISSNER_NPC_HCP_WITH_H3K4ME2_AND_H3K27ME	22	189	16064	1305	1.449719	0.049874
green	9045	GSE17721_LPS_VS_CPG_4H_BMDM_DN	22	189	16064	1305	1.449719	0.049874
pink	5988	KAUFFMANN_DNA_REPLICATION_GENES	5	142	16111	228	2.510038	0.049883
pink	9250	GSE19825_NAIVE_VS_IL2RAHIGH_DAY3_EFF_CD8_TCEI	5	142	16111	228	2.510038	0.049883
midnightb	4942	LAIHO_COLORECTAL_CANCER_SERRATED_DN	2	82	16171	71	5.583305	0.049949
lightyellow	8695	GSE1460_DP_VS_CD4_THYMOCYTE_DN	2	177	16076	33	5.565143	0.049952
lightyellow	9525	GSE2706_2H_VS_8H_LPS_STIM_DC_DN	2	177	16076	33	5.565143	0.049952
lightyellow	10061	GSE3982_BCELL_VS_BASOPHIL_DN	2	177	16076	33	5.565143	0.049952
turquoise	7032	ACEVEDO_NORMAL_TISSUE_ADJACENT_TO_LIVER_TUI	49	154	16099	4169	1.240444	0.049953
blue	2678	GNF2_NS	12	40	16213	2978	1.637307	0.049978
blue	4559	REACTOME_PHOSPHOLIPASE_C_MEDIATED_CASCADE	12	40	16213	2978	1.637307	0.049978
lightcyan	5037	MULLIGHAN_MLL_SIGNATURE_1_DN	3	217	16036	62	3.624127	0.049982
lightcyan	7103	ZHENG_GLIOBLASTOMA_PLASTICITY_UP	3	217	16036	62	3.624127	0.049982
purple	1698	V$FOXJ2_01	4	143	16110	157	2.895728	0.049989
